# Supplementary material for: Fangs in the Ghats: Preclinical Insights into the Medical Importance of Pit Vipers from the Western Ghats
Source: Int J Mol Sci. 2023 May 30;24(11):9516. doi: 10.3390/ijms24119516 (PMC10253985; doi:10.3390/ijms24119516)
Supplement: Supplementary file 1 [file ijms-24-09516-s001.zip › Supplementary Figures_Fangs in the Ghats.pdf]

Supplementary Figures

Supplementary Figure S1. Fibrinogenolytic activities of pit viper venoms.

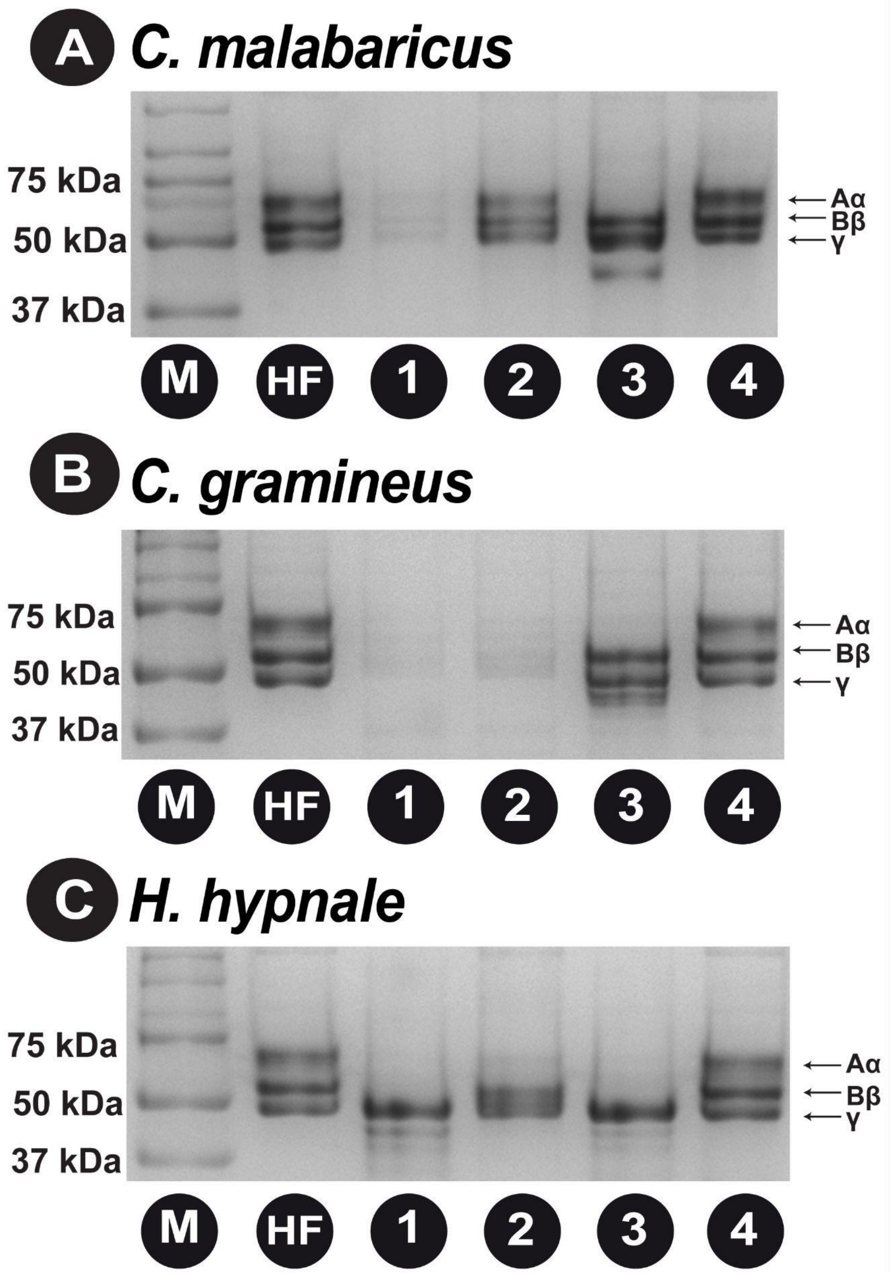

The figure highlights the fibrinogenolytic activity of pit viper venoms, namely (A) *C. malabaricus*, (B) *C. gramineus,* and (C) *H. hypnale*, respectively. The SDS-PAGE gel depicts the enzymatic cleavage of human fibrinogen Aα, Bβ and γ bands. In three gels (M): Prestained protein ladder; (HF): human fibrinogen only (negative control); (1): human fibrinogen mixed with venom; (2): human fibrinogen mixed with venom and EDTA; (3): human fibrinogen mixed with venom and PMSF; (4): human fibrinogen mixed with venom, EDTA and PMSF.

Supplementary Figure S2: Nephrotoxicity of pit viper venoms from the Western Ghats.

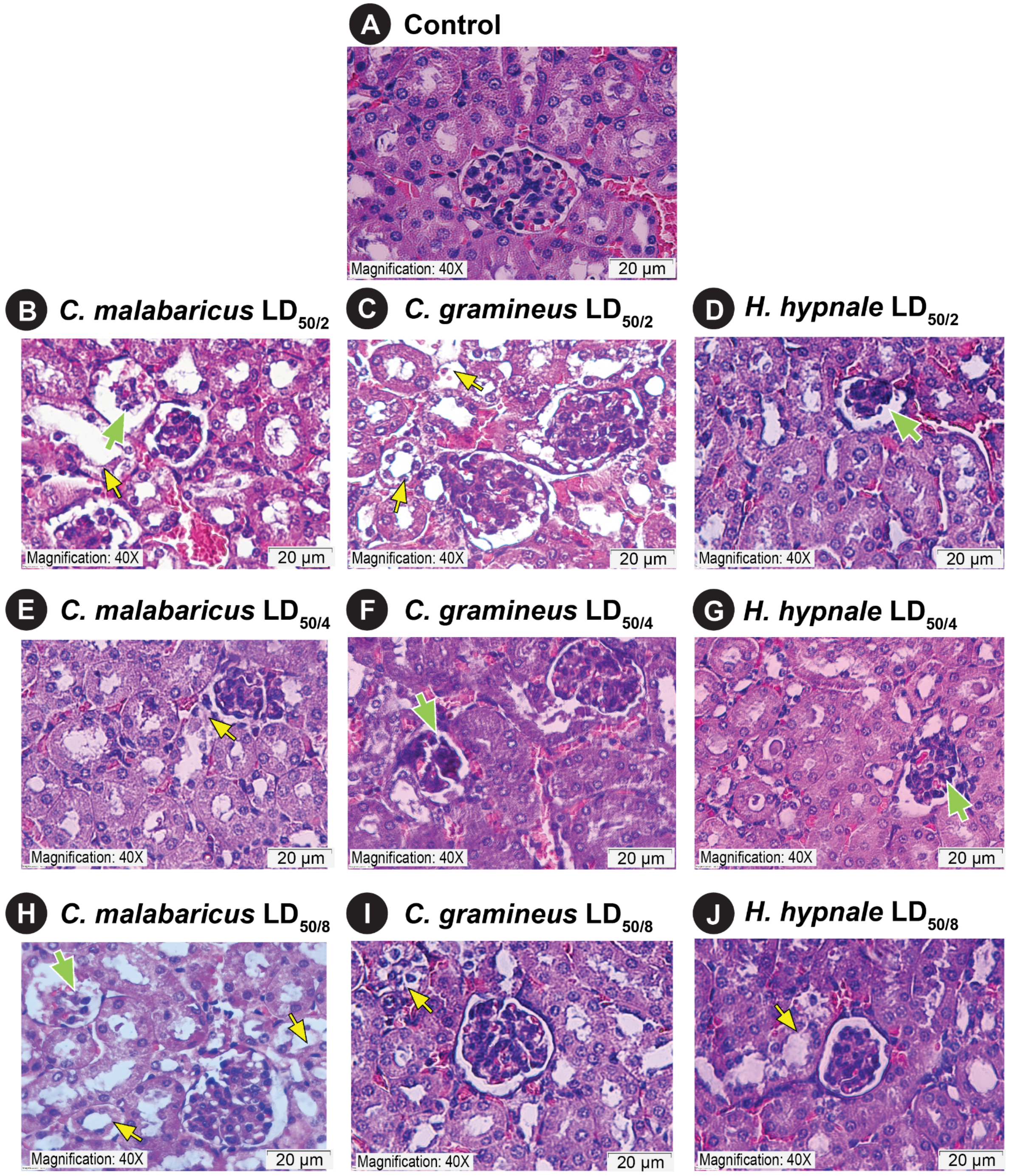

This figure shows the hematoxylin-eosin-stained kidney sections of *C. malabaricus, C. gramineus* and *H. hypnale* venoms (Intravenous). Panels (A) the control mice and (B to J) treatment groups: ½ LD_{50} (B-D), ¼ LD_{50} (E-G) and ⅛ LD_{50} (H-J) concentrations of *C. malabaricus, C. gramineus and H. hypnale* venoms, respectively. A scale bar of 20 µm is shown, along with green and yellow arrows that indicate glomerular degeneration and tubular injury, respectively.

Supplementary Figure S3: Nephrotic injury score of pit viper venoms from the Western Ghats.

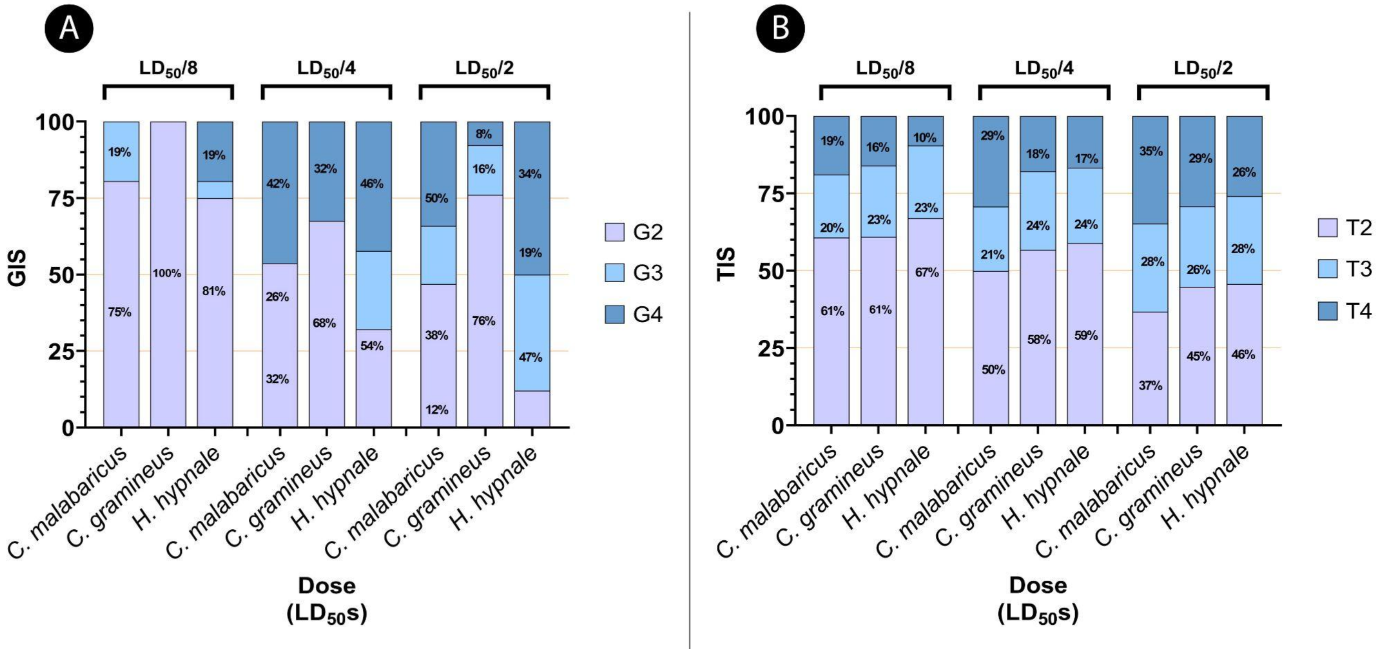

Panel A of this figure highlights the glomerular injury score (GIS), while panel B highlights the tubular injury score (TIS) of *C. malabaricus, C. gramineus* and *H. hypnale* venoms of the hematoxylin-eosin-stained kidney sections.

Supplementary Figure S4: Skeletal muscle injury of pit viper venoms from the Western Ghats.

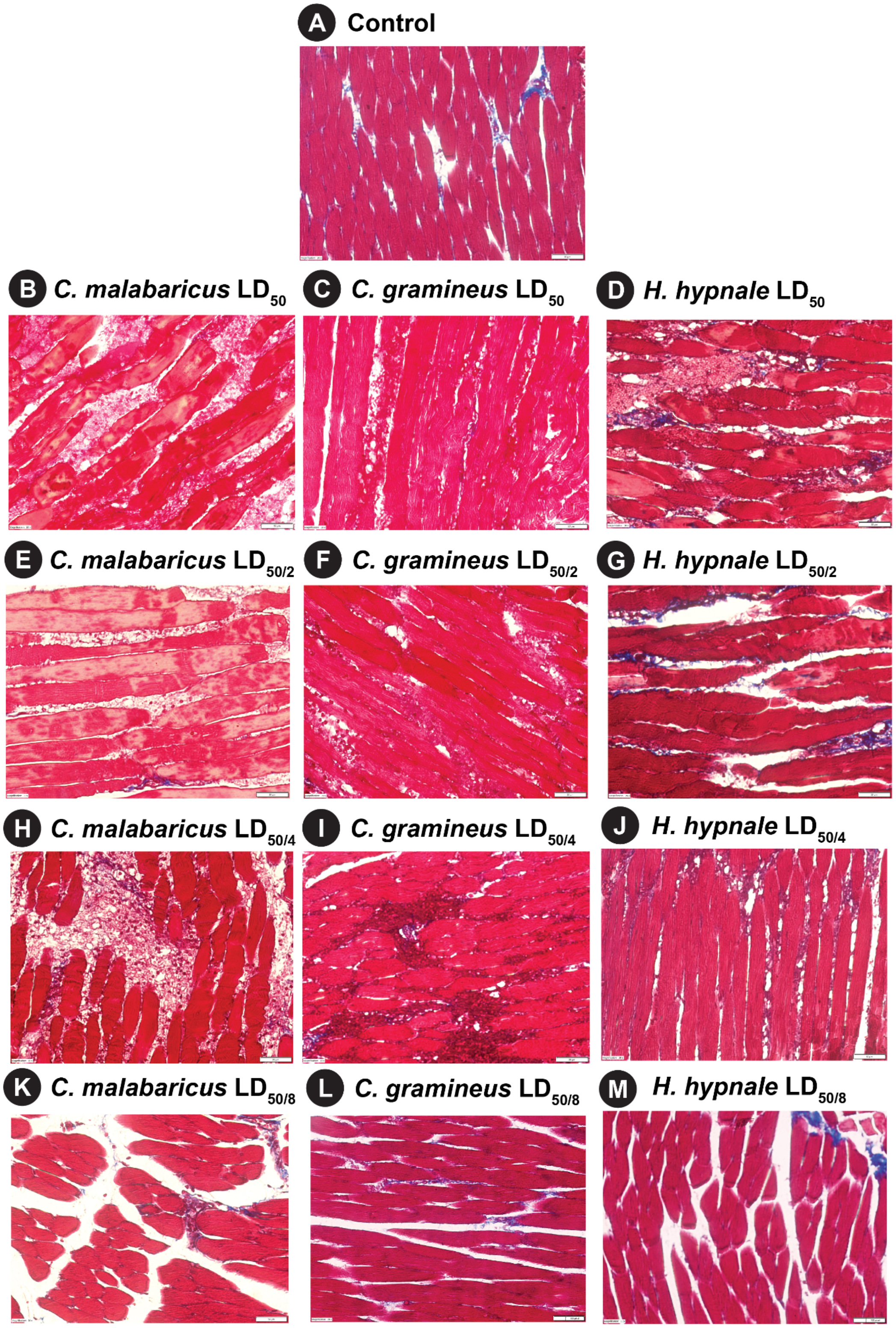

This figure highlights Masson Trichrome stained skeletal muscles of (A) the control mice and (B to M) treatment groups: LD_{50} (B-D), ½ LD_{50} (E-G), ¼ LD_{50} (H-J) and ⅛ LD_{50} (K-M) concentrations of *C. malabaricus, C. gramineus* and *H. hypnale* venoms, respectively. A scale bar of 50 µm is shown.
