# Supplementary material for: Fangs in the Ghats: Preclinical Insights into the Medical Importance of Pit Vipers from the Western Ghats
Source: Int J Mol Sci. 2023 May 30;24(11):9516. doi: 10.3390/ijms24119516 (PMC10253985; doi:10.3390/ijms24119516)
Supplement: Supplementary file 1 [file ijms-24-09516-s001.zip › Supplementary Tables _Fangs in the Ghats.pdf]

Supplementary Table S1A. Details of venom samples investigated in this study.

Common name	Species Name	Number of individuals	Protein concentration (mg/ml)	Sampling location
Malabar pit viper	*Craspedocephalus malabaricus*	2	0.191	Wayanad, Kerala
Bamboo pit viper	*Craspedocephalus gramineus*	Multiple	0.234	Pune, Maharashtra
Hump nosed pit viper	*Hypnale hypnale*	3	0.098	Hunsur, Karnataka

The table above shows source location and the number of individual snakes from which these venoms were collected and have been provided here with their respective protein concentrations.

Supplementary Table S1B. Details of antivenom samples investigated in this study.

Manufacturer	Batch	Manufacture (M) and expiry (E) dates	Protein content (mg/ml)	Marketed neutralizing efficacy (mg/ml)
**Indian polyvalent antivenoms**				
**Bharat Serums and Vaccines Ltd.**	A05318087	**M:** 10/2018<br>**E:** 09/2022	26.5 ± 0.77	*N. naja*: 0.60<br>*B. caeruleus*: 0.45<br>*D. russelii*: 0.60<br>*E. carinatus*: 0.45
**Haffkine BioPharmaceutical Corporation Ltd.**	AS180611	**M:** 06/2018<br>**E:** 11/2022	24.7± 0.5	
**Premium Serums & Vaccines Pvt. Ltd.**	ASVS(I)-Lyo013	**M:** 12/2018<br>**E:** 11/2022	26.2± 1.2	
**VINS Bioproducts Ltd.**	01AS18067	**M:** 08/2018<br>**E:** 07/2023	31.4 ± 0.54	
**Virchow Biotech Private Ltd. (Virchow)**	PAS00718	**M:** 09/2018<br>**E:** 08/2022	19.54±3.86	
**Sri Lankan polyvalent antivenom**				
**Premium Serums & Vaccines Pvt. Ltd.**	ASVS-SL/LY-001	**M:** 04/2019<br>**E:** 03/2023	25.9 ± 0.44	*N. naja*: 0.60<br>*B. caeruleus*: 0.45<br>*D. russelii*: 0.60<br>*E. carinatus*: 0.45<br>*H. hypnale*: 0.60

Details of the commercial Indian and Sri Lankan polyvalent antivenoms tested in this study are provided here. Batch numbers, manufacturing and expiry dates, protein concentrations and marketed neutralising potencies are shown.

Raw MS/MS spectra of each gel excised bands were searched against the National Center for Biotechnology Information's (NCBI) non-redundant (nr) database (Serpentes: 8570) using Peaks Studio X+ for the identification of toxin classes present in the venom. The key results of these searches, including the accession numbers, -10lgP values, coverage, number of high confidence peptides, unique peptides, percent abundance of each toxin hit, average molecular mass (kDa) and the toxin family of the matching NCBI entry was identified are listed here. The percentage indicated adjacent to the average mass column corresponds to its relative proportion in the venoms of *C. malabaricus*, **(Supplementary Table S2)**, *C. gramineus* **(Supplementary Table S3)**, and *H. hypnale* **(Supplementary Table S4)**.

Supplementary Table S2. The proteomic composition of *C. malabaricus* venom.

Sr. no.	Accession		-10lgP	Coverage	#Peptides	#Unique	Relative abundance of toxin hit (%)	Avg. Mass (kda)	Toxin Type
	**Snake venom serine protease (SVSP): 29.608%**								
1	JAA97975.1		240.74	29	17	2	1.1001	28.204	SVSP
2	JAV51280.1		233.43	18	17	1	0.0175	110.116	SVSP
3	XP_015676063.1		225.65	31	16	5	0.0515	77.032	SVSP
4	JAG45871.1		223.17	12	12	1	0.0043	110.767	SVSP
5	O13063.1		220.76	25	17	2	0.2942	28.034	SVSP
6	AAG10789.1		218.65	16	11	2	1.1253	28.104	SVSP
7	XP_026530513.1		198.18	19	12	1	0.0244	76.681	SVSP
8	XP_015687424.1		182.67	16	10	10	0.0446	106.458	SVSP
9	JAG65874.1		179.53	15	9	1	0.0012	76.736	SVSP
10	ASX97880.1		171.77	22	7	3	2.7258	28.515	SVSP
11	AUS82539.1		153.2	10	4	1	16.0256	28.39	SVSP
12	JAV01822.1		146.86	17	6	2	0.0721	28	SVSP
13	JAG47217.1		132.25	17	5	2	0.0019	43.509	SVSP
14	P0CJ41.1		130.89	15	4	1	0.0649	28.001	SVSP
15	P0DJF5.1		129.45	14	4	2	0.3514	28.428	SVSP
16	AUS82501.1		128.96	15	5	1	0.0155	28.553	SVSP
17	AUS82565.1		126.43	17	5	1	0.0008	28.164	SVSP
18	QBF53412.1		123.43	14	4	1	0.0018	28.317	SVSP
19	BAA20283.1		123.09	11	4	1	0.6148	27.894	SVSP
20	ETE66900.1		122.98	16	4	1	0.0020	30.492	SVSP
21	AUS82534.1		116.28	11	4	1	6.2474	29.104	SVSP
22	JAV01828.1		100.51	9	3	1	0.0003	28.523	SVSP
23	AUS82484.1		84.59	10	2	1	0.0174	27.907	SVSP
24	P26324.1		66.32	9	2	1	0.0111	26.57	SVSP
25	JAS03131.1		59.06	4	1	1	0.7102	28.382	SVSP
26	Q8AY81.1		111.91	16	4	1	0.0828	29.328	SVSP
	**Phospholipase A_2 (PLA_2): 27.490%**								
27	AHJ09587.1		170.76	17	4	3	23.7371	15.07	PLA2
28	AAF91498.1		117.95	24	4	3	0.0135	13.732	PLA2
29	Q2YHJ2.1		101.71	12	3	2	3.7295	15.742	PLA2
30	Q6H3C5.2		91.08	12	2	1	0.0102	13.819	PLA2

	**Cysteine-rich secretory proteins (CRISP): 14.322%**								
31	AMB36337.1		237.67	21	13	11	14.3038	26.517	CRISP
32	AAZ75600.1		100.89	8	3	1	0.0182	23.998	CRISP
	**Snake venom metalloproteinases (SVMP): 11.560%**								
33	BAP39913.1		122.33	9	3	2	5.4658	19.487	SVMP
34	P0DM87.1		121.44	3	2	2	0.3278	54.436	SVMP
35	XP_015683679.2		110.59	3	2	1	2.4467	73.339	SVMP
36	BAN89403.1		101.82	4	2	1	0.0123	67.822	SVMP
37	AEJ31991.1		86.06	6	2	2	3.1359	54.615	SVMP
38	Q9PVK7.1		80.81	4	2	2	0.0021	67.662	SVMP
39	XP_015685587.1		78.17	5	2	1	0.1013	39.726	SVMP
40	JAV51434.1		69.89	4	2	2	0.0058	68.93	SVMP
41	AEJ31985.1		69.82	1	1	1	0.0535	54.751	SVMP
42	XP_026534386.1		48.58	1	1	1	0.0084	106.048	SVMP
	**L-amino-acid oxidase (LAAO): 9.728%**								
43	BAN82013.1		311.27	36	37	2	0.0999	57.129	LAAO
44	XP_015673892.1		289.31	29	29	2	4.1383	57.159	LAAO
45	JAS05316.1		240.26	25	18	1	0.0082	58.784	LAAO
46	A0A024BTN9.1		237.53	20	19	1	0.0058	56.376	LAAO
47	AAY89681.1		231.83	17	11	1	3.9394	59.058	LAAO
48	JAS05152.1		214.4	15	10	1	0.0165	58.785	LAAO
49	P0C2D7.1		45.16	11	1	1	1.5205	10.295	LAAO
	**Lectins: 5.659%**								
50	JAS04797.1		62.63	5	1	1	0.2867	18.183	Lectin
51	ACZ34293.1		122.95	9	1	1	1.0436	18.146	Lectin
52	P0DJL3.1		102.8	15	2	2	4.3288	14.498	Lectin
53	XP_015685588.1		42.21	9	1	1	0.0001	18.348	Lectin
	**Phospholipase B (PLB): 0.707%**								
54	SMD28240.1		295.98	30	21	1	0.7018	64.104	PLB
55	QBF53421.1		249.48	21	14	1	0.0036	64.31	PLB
56	XP_007427768.1		229.67	16	10	1	0.0019	64.842	PLB
	**5’-nucleotidase (5’-NT): 0.629%**								
57	JAI10406.1		238.16	31	20	1	0.0021	64.682	5'-NT
58	BAP39972.1		237	30	20	1	0.2777	65.019	5'-NT
59	JAS04520.1		233.33	31	17	1	0.0028	64.602	5'-NT
60	JAG67188.1		169.92	10	6	1	0.3344	64.759	5'-NT
61	P0DJJ5.1		135	52	3	1	0.0120	6.012	5'-NT
	**Hyaluronidase (HYL): 0.243%**								
62	BAN89414.1		173.48	16	8	6	0.2433	49.636	HYL
	**Phosphodiesterase (PDE): 0.023%**								
63	BAN89426.1		335	29	37	1	0.0212	96.239	PDE
64	JAV01884.1		305.05	24	32	1	0.0014	91.859	PDE
	**Vascular endothelial growth factor (VEGF): 0.013%**								
65	JAS03130.1		69.98	5	1	1	0.0132	22.605	VEGF
	**Nerve growth factor (NGF): 0.009%**								
66	ACC85814.1		44.88	4	1	1	0.0086	21.685	NGF
	**Cystatin: 0.008%**								
67	E3P6N7.1		58.84	9	1	1	0.0075	15.886	Cystatin

Supplementary Table S3. The proteomic composition of *C. gramineus* venom.

Sr. no.	Accession	-10lgP	Coverage	#Peptides	#Unique	Relative abundance of toxin hit (%)	Avg. Mass (kda)	Toxin Type
**Phospholipase A_2 (PLA_2): 30.572%**								
1	AHJ09587.1	107.1	16	2	2	25.0034	15.07	PLA2
2	Q2YHJ5.1	77.93	13	2	2	5.5686	13.94	PLA2
**Snake venom metalloproteinases (SVMP): 20.747%**								
3	P0DM87.1	172.47	6	4	2	5.1245	54.44	SVMP
4	JAS05409.1	135.9	4	4	2	3.3809	67.04	SVMP
5	XP_029142320.1	128.98	3	4	3	0.1103	73.45	SVMP
6	P0C6E8.1	125.36	3	2	2	5.0676	48.20	SVMP
7	AFJ49231.1	118.22	4	3	1	0.1667	67.33	SVMP
8	AAP20639.1	115.58	2	2	2	0.9393	54.01	SVMP
9	XP_015685587.1	105.33	10	3	2	0.1036	39.73	SVMP
10	AFJ49243.1	62.59	3	1	1	5.4444	68.43	SVMP
11	Q7LZS9.1	54.7	5	1	1	0.4102	22.95	SVMP
**Cysteine-rich secretory proteins (CRISP): 17.623%**								
12	AMB36337.1	272.59	22	12	10	17.5681	26.52	CRISP
13	AXL95289.1	125.42	4	3	1	0.0551	26.94	CRISP
**Snake venom serine protease (SVSP): 13.998%**								
14	JAG47096.1	173.11	14	5	5	0.0739	55.48	SVSP
15	JAG45871.1	166.03	6	5	1	0.0075	110.77	SVSP
16	XP_015667388.1	162.62	6	5	1	0.0127	110.69	SVSP
17	JAS04411.1	159.14	14	6	2	1.2595	28.35	SVSP
18	JAS05258.1	140.11	13	6	1	6.5049	27.94	SVSP
19	AUS82539.1	123.75	10	4	1	1.0069	28.39	SVSP
20	JAV48393.1	123.73	13	4	1	3.7617	28.64	SVSP
21	ASX97880.1	122.38	12	3	2	0.9370	28.52	SVSP
22	BAN82028.1	122.18	13	2	1	0.0200	18.38	SVSP
23	XP_015675298.1	94.09	3	1	1	0.3028	53.90	SVSP
24	XP_015745242.1	89.49	1	1	1	0.0007	81.34	SVSP
25	P0CG03.1	62.9	4	1	1	0.1098	28.05	SVSP
**5'-nucleotidase (5'-NT): 5.952%**								
26	XP_015668350.1	219.05	15	9	1	5.8341	64.64	5'-NT
27	JAS04520.1	203.96	13	8	1	0.1175	64.60	5'-NT
**Phospholipase B (PLB): 5.060%**								
28	AFJ50957.1	248.96	27	13	1	5.0595	64.09	PLB
**Lectins: 3.797%**								
29	JAS04545.1	96.26	12	2	2	0.0581	18.66	Lectin
30	ACZ34293.1	113.7	9	1	1	1.0197	18.15	Lectin
31	P0DJL3.1	104.56	15	2	2	2.7188	14.50	Lectin
**L-amino-acid oxidase (LAAO): 1.668%**								
32	AAY89681.1	242.89	21	13	1	0.0425	59.06	LAAO
33	ACF70483.1	233.1	18	12	3	0.7513	56.89	LAAO
34	Q4JHE1.1	224.31	17	12	2	0.7325	58.74	LAAO
35	XP_026549343.1	165.82	18	4	2	0.1414	16.48	LAAO

**Phosphodiesterase (PDE): 0.582%**								
36	BAN89426.1	412.32	36	54	1	0.5818	96.24	PDE
**Hyaluronidase (HYL): 0.003%**								
37	ALB06111.1	64.06	3	1	1	0.0027	53.92	HYL

Supplementary Table S4. The proteomic composition of *H. hypnale* venom.

Sr. no.	Accession	-10lgP	Coverage	#Peptides	#Unique	Relative abundance of toxin hit (%)	Avg. Mass (kda)	Toxin Type
**Snake venom serine protease (SVSP): 44.369%**								
1	P82981.1	179.4	12	6	4	0.1485	25.41	SVSP
2	XP_015675298.1	167.87	22	8	8	0.1298	53.90	SVSP
3	JAG47218.1	135.24	11	3	3	0.0072	37.48	SVSP
4	JAV48393.1	116.63	12	4	1	0.8867	28.64	SVSP
5	P26324.1	116.46	13	3	2	19.2205	26.57	SVSP
6	AAQ02908.1	105.29	12	3	1	0.0763	28.51	SVSP
7	J3RYA3.1	100.31	8	3	1	0.0207	29.53	SVSP
8	XP_015687424.1	96.43	4	3	3	0.0018	106.46	SVSP
9	JAC94969.1	85.05	5	2	2	0.0022	43.34	SVSP
10	JAS03131.1	77.07	4	1	1	23.8725	28.38	SVSP
11	BAG82600.1	53.64	1	1	1	0.0004	106.11	SVSP
12	XP_013927550.1	50.36	6	1	1	0.0021	30.27	SVSP
**Cysteine-rich secretory proteins (CRISP): 23.133%**								
13	AMB36337.1	235.19	21	11	7	13.0263	26.52	CRISP
14	XP_007439730.1	124.09	5	4	4	10.0980	57.06	CRISP
15	ACN93671.1	67.61	7	1	1	0.0088	26.31	CRISP
**Lectins: 16.953%**								
16	JAS04545.1	156.01	13	5	5	11.1303	18.66	Lectin
17	JAG46154.1	133.59	16	3	3	1.4031	17.46	Lectin
18	BAP39929.1	86.88	8	1	1	0.0033	21.33	Lectin
19	P0DJL3.1	121.63	15	2	1	1.7306	14.50	Lectin
20	ACZ34293.1	115.29	9	1	1	0.9472	18.15	Lectin
21	P0DJL2.1	105.88	9	1	1	0.0439	15.61	Lectin
22	BAC77707.1	100.18	8	1	1	0.9287	17.76	Lectin
23	AAQ15159.1	97.81	16	2	1	0.1349	16.82	Lectin
24	D2YW39.2	74.99	8	1	1	0.6308	15.73	Lectin
**L-amino-acid oxidase (LAAO): 7.890%**								
25	Q90W54.1	311.92	30	28	1	0.2961	57.09	LAAO
26	2IID	282.58	32	24	2	7.4287	56.23	LAAO
27	AXL95287.1	174.25	9	7	1	0.1358	58.59	LAAO
**Snake venom metalloproteinases (SVMP): 4.686%**								
28	JAV51432.1	187.5	10	8	4	0.3049	68.11	SVMP
29	P0DM87.1	153.23	10	4	2	0.0698	54.44	SVMP
30	P0CB14.1	145.67	16	4	2	1.6481	47.45	SVMP
31	Q8JJ51.1	107.02	8	2	1	0.0368	46.91	SVMP
32	BAO23490.1	73.75	3	2	1	0.3721	67.77	SVMP
33	XP_026564825.1	58.54	1	1	1	2.2370	106.23	SVMP
34	QBF53415.1	55.16	2	1	1	0.0013	69.22	SVMP
35	P86802.1	43	8	1	1	0.0189	27.27	SVMP
**Phospholipase A_2 (PLA_2): 1.943%**								
36	AHJ09589.1	89.75	8	2	2	1.6823	15.86	PLA2
37	Q2YHJ2.1	59.65	6	1	1	0.1296	15.74	PLA2
38	AAZ53176.1	49.56	9	1	1	0.0084	15.59	PLA2

39	AAR14169.1	48.2	6	1	1	0.1229	13.94	PLA2
**Phospholipase B (PLB): 0.526%**								
40	AFJ50957.1	300.91	40	23	1	0.0204	64.09	PLB
41	XP_007427768.1	245.44	15	12	3	0.5058	64.84	PLB
**Nerve growth factor (NGF): 0.390%**								
42	ABA60128.1	97.89	8	2	2	0.3898	27.78	NGF
**Phosphodiesterase (PDE): 0.050%**								
43	BAN89426.1	95.06	4	3	3	0.0500	96.24	PDE
**Cystatin: 0.029%**								
44	XP_015672096.1	93.04	16	2	2	0.0285	15.69	Cystatin
**Hyaluronidase (HYL): 0.028%**								
45	BAP39986.1	77.71	4	2	2	0.0279	60.40	HYL

Supplementary Table S5A. Median lethal dose of *H. hypnale* venoms.

Name of sample	Venom Dose (µg)					Number of survivors					LD_{50} (µg/mouse)	LD_{50} (mg/kg)
H. Hypnale	15.56	23.34	35.01	52.21	78.77	4	4	1	0	0	26.48<br>21.41-32.75	1.32<br>1.07-1.63

The table indicates various dose groups, survival patterns and toxicities of hump nosed pit viper venom. Here, a group of five CD-1 mice were used per venom dose.

Supplementary Table S5B. Toxicity range estimates for *C. gramineus* and *C. malabaricus* venoms.

Name of sample	Venom Dose (µg)					Death pattern					Lethal range (µg/mouse)	Lethal range (mg/kg)
C. gramineus	10	20	40	80	100	L	L	L	L	D	80-100	4 mg/kg
C. malabaricus	40	60	80	100	120	L	L	L	L	L	≥120	≥6 mg/kg

The table indicates various *C. gramineus* and *C. malabaricus* venom concentrations used for toxicity range finding studies. Here, a single CD-1 mouse was used per venom dose. Survival status - L: Alive and D: Dead.

Supplementary Table S6. Neutralisation potencies of commercial Indian polyvalent and Sri Lankan polyvalent antivenoms against *H. hypnale*.

**Antivenom used:** Indian polyvalent antivenom manufactured by Premium Serums and Vaccines Pvt. Ltd. (Batch No. ASVS-I Lyo.013)					
**Challenge dose**	**Amount of antivenom injected in the venom-antivenom mixture** (µl)		$\mathbf{ED_{50}}$ (µl)	$\mathbf{ED_{50}}$ (µl antivenom/mg venom)	**Potency of antivenom** (mg/ml)
5X LD_{50}	166.67	111.12	NIL	NIL	NIL
3X LD_{50}	166.67	111.12	NIL	NIL	NIL
**Antivenom used:** Sri Lakan polyvalent antivenom manufactured by Premium Serums and Vaccines Pvt. Ltd. (Batch No. ASVS-SL/LY-001)					
**Challenge dose**	**Amount of antivenom injected in the venom-antivenom mixture** (µl)		$\mathbf{ED_{50}}$ (µl)	$\mathbf{ED_{50}}$ (µl antivenom/mg venom)	**Potency of antivenom** (mg/ml)
5X LD_{50}	166.67	111.12	NIL	NIL	NIL
3X LD_{50}	166.67	111.12	NIL	NIL	NIL

The neutralisation potencies of Premium Serums Indian and Sri Lankan polyvalent antivenoms are shown in the table above. The highest undiluted doses of Indian and Sri Lankan polyvalent antivenoms failed to neutralise at 3X and 5X challenge doses of the *H. hypnale* venom. (NIL - Neutralisation was not observed)
