# Supplementary material for: Fangs in the Ghats: Preclinical Insights into the Medical Importance of Pit Vipers from the Western Ghats
Source: Int J Mol Sci. 2023 May 30;24(11):9516. doi: 10.3390/ijms24119516 (PMC10253985; doi:10.3390/ijms24119516)
Supplement: Supplementary file 1 [file ijms-24-09516-s001.zip › Supplementary File S1.zip!/Supplementary File S1/CRGR/CRGR.html]

proteins


Summary

  

# 1. Notes

# 2. Result Statistics

**Figure 1.**
False discovery rate (FDR) curve. X axis is the number of peptide-spectrum matches (PSM) being kept. Y axis is the corresponding FDR.

  


  

**Figure 2.**
PSM score distribution.  **(a)**
Distribution of PEAKS peptide score;  **(b)**
Scatterplot of PEAKS peptide score versus precursor mass error.

**(a)**

**(b)**

**Table 1.**
Statistics of data.


|  |  |  |  |  |  |  |  |  |  |  |  |
| --- | --- | --- | --- | --- | --- | --- | --- | --- | --- | --- | --- |
|  | #Scans | | #Features | Identified | | | #Peptides | #Sequences | #Proteins^\*^ | | |
|  | MS1 | MS2 |  | #PSMs | #Scans | #Features |  |  | Groups | All | Top |
| Total | 142853 | 256534 | 303975 | 6064 | 5831 | 32284 | 891 | 797 | 113 | 1065 | 627 |
| F6 | 18941 | 19223 | 21864 | 496 | 439 | 1518 | 97 | 88 | 23 | 122 | 38 |
| F2 | 18631 | 24071 | 25190 | 257 | 254 | 2386 | 140 | 126 | 36 | 157 | 58 |
| F3 | 16973 | 47291 | 51442 | 926 | 898 | 8855 | 402 | 357 | 61 | 346 | 165 |
| F4 | 18071 | 43750 | 50423 | 499 | 491 | 5125 | 210 | 189 | 46 | 279 | 126 |
| F5 | 18597 | 27882 | 30165 | 445 | 439 | 3225 | 160 | 144 | 44 | 270 | 127 |
| F7 | 18138 | 27381 | 32816 | 652 | 644 | 2421 | 103 | 94 | 22 | 146 | 33 |
| F8 | 18240 | 26245 | 30193 | 448 | 441 | 2015 | 89 | 83 | 19 | 112 | 30 |
| F1 | 15262 | 40691 | 61882 | 2341 | 2225 | 6739 | 657 | 589 | 98 | 875 | 551 |

^\* proteins with significant peptides are used in counts.^

**Figure 3.**
Sample overlap for Proteins and Peptides (up to 8 samples).  **(a)**
All Proteins;  **(b)**
Top Proteins;  **(c)**
Peptides;

**(a)**

|  |  |  |  |  |  |  |  |  |  |  |  |  |  |  |  |  |  |  |  |  |  |  |  |  |  |  |  |  |  |  |  |  |  |  |  |  |  |  |  |  |  |  |  |  |  |  |  |  |  |  |  |  |  |  |  |  |  |  |  |  |  |  |  |  |  |  |  |  |  |  |  |  |  |  |  |  |  |  |  |  |  |  |  |  |  |  |  |  |  |  |
| --- | --- | --- | --- | --- | --- | --- | --- | --- | --- | --- | --- | --- | --- | --- | --- | --- | --- | --- | --- | --- | --- | --- | --- | --- | --- | --- | --- | --- | --- | --- | --- | --- | --- | --- | --- | --- | --- | --- | --- | --- | --- | --- | --- | --- | --- | --- | --- | --- | --- | --- | --- | --- | --- | --- | --- | --- | --- | --- | --- | --- | --- | --- | --- | --- | --- | --- | --- | --- | --- | --- | --- | --- | --- | --- | --- | --- | --- | --- | --- | --- | --- | --- | --- | --- | --- | --- | --- | --- | --- | --- |
|  | 1 | 2 | 3 | 4 | 5 | 6 | 7 | 8 | Unique | \|  \|  \|  \|  \|  \|  \|  \|  \|  \|  \| \| --- \| --- \| --- \| --- \| --- \| --- \| --- \| --- \| --- \| --- \| \| 1 \| 122 \| 92 \| 106 \| 105 \| 106 \| 89 \| 82 \| 107 \| 9 \| \| 2 \| 92 \| 157 \| 147 \| 144 \| 129 \| 105 \| 101 \| 145 \| 0 \| \| 3 \| 106 \| 147 \| 346 \| 222 \| 205 \| 108 \| 106 \| 250 \| 75 \| \| 4 \| 105 \| 144 \| 222 \| 279 \| 252 \| 96 \| 92 \| 205 \| 1 \| \| 5 \| 106 \| 129 \| 205 \| 252 \| 270 \| 108 \| 100 \| 201 \| 2 \| \| 6 \| 89 \| 105 \| 108 \| 96 \| 108 \| 146 \| 112 \| 117 \| 25 \| \| 7 \| 82 \| 101 \| 106 \| 92 \| 100 \| 112 \| 112 \| 112 \| 0 \| \| 8 \| 107 \| 145 \| 250 \| 205 \| 201 \| 117 \| 112 \| 875 \| 612 \| |

**(b)**

|  |  |  |  |  |  |  |  |  |  |  |  |  |  |  |  |  |  |  |  |  |  |  |  |  |  |  |  |  |  |  |  |  |  |  |  |  |  |  |  |  |  |  |  |  |  |  |  |  |  |  |  |  |  |  |  |  |  |  |  |  |  |  |  |  |  |  |  |  |  |  |  |  |  |  |  |  |  |  |  |  |  |  |  |  |  |  |  |  |  |  |
| --- | --- | --- | --- | --- | --- | --- | --- | --- | --- | --- | --- | --- | --- | --- | --- | --- | --- | --- | --- | --- | --- | --- | --- | --- | --- | --- | --- | --- | --- | --- | --- | --- | --- | --- | --- | --- | --- | --- | --- | --- | --- | --- | --- | --- | --- | --- | --- | --- | --- | --- | --- | --- | --- | --- | --- | --- | --- | --- | --- | --- | --- | --- | --- | --- | --- | --- | --- | --- | --- | --- | --- | --- | --- | --- | --- | --- | --- | --- | --- | --- | --- | --- | --- | --- | --- | --- | --- | --- | --- | --- |
|  | 1 | 2 | 3 | 4 | 5 | 6 | 7 | 8 | Unique | \|  \|  \|  \|  \|  \|  \|  \|  \|  \|  \| \| --- \| --- \| --- \| --- \| --- \| --- \| --- \| --- \| --- \| --- \| \| 1 \| 38 \| 25 \| 36 \| 34 \| 34 \| 20 \| 19 \| 34 \| 1 \| \| 2 \| 25 \| 58 \| 58 \| 52 \| 48 \| 25 \| 24 \| 54 \| 0 \| \| 3 \| 36 \| 58 \| 165 \| 95 \| 94 \| 32 \| 30 \| 123 \| 36 \| \| 4 \| 34 \| 52 \| 95 \| 126 \| 118 \| 22 \| 21 \| 90 \| 1 \| \| 5 \| 34 \| 48 \| 94 \| 118 \| 127 \| 28 \| 26 \| 92 \| 1 \| \| 6 \| 20 \| 25 \| 32 \| 22 \| 28 \| 33 \| 30 \| 32 \| 0 \| \| 7 \| 19 \| 24 \| 30 \| 21 \| 26 \| 30 \| 30 \| 30 \| 0 \| \| 8 \| 34 \| 54 \| 123 \| 90 \| 92 \| 32 \| 30 \| 551 \| 427 \| |

**(c)**

|  |  |  |  |  |  |  |  |  |  |  |  |  |  |  |  |  |  |  |  |  |  |  |  |  |  |  |  |  |  |  |  |  |  |  |  |  |  |  |  |  |  |  |  |  |  |  |  |  |  |  |  |  |  |  |  |  |  |  |  |  |  |  |  |  |  |  |  |  |  |  |  |  |  |  |  |  |  |  |  |  |  |  |  |  |  |  |  |  |  |  |
| --- | --- | --- | --- | --- | --- | --- | --- | --- | --- | --- | --- | --- | --- | --- | --- | --- | --- | --- | --- | --- | --- | --- | --- | --- | --- | --- | --- | --- | --- | --- | --- | --- | --- | --- | --- | --- | --- | --- | --- | --- | --- | --- | --- | --- | --- | --- | --- | --- | --- | --- | --- | --- | --- | --- | --- | --- | --- | --- | --- | --- | --- | --- | --- | --- | --- | --- | --- | --- | --- | --- | --- | --- | --- | --- | --- | --- | --- | --- | --- | --- | --- | --- | --- | --- | --- | --- | --- | --- | --- | --- |
|  | 1 | 2 | 3 | 4 | 5 | 6 | 7 | 8 | Unique | \|  \|  \|  \|  \|  \|  \|  \|  \|  \|  \| \| --- \| --- \| --- \| --- \| --- \| --- \| --- \| --- \| --- \| --- \| \| 1 \| 97 \| 64 \| 79 \| 74 \| 71 \| 65 \| 60 \| 80 \| 12 \| \| 2 \| 64 \| 140 \| 124 \| 106 \| 95 \| 73 \| 66 \| 112 \| 5 \| \| 3 \| 79 \| 124 \| 402 \| 159 \| 127 \| 90 \| 81 \| 251 \| 115 \| \| 4 \| 74 \| 106 \| 159 \| 210 \| 127 \| 84 \| 73 \| 141 \| 31 \| \| 5 \| 71 \| 95 \| 127 \| 127 \| 160 \| 86 \| 72 \| 123 \| 11 \| \| 6 \| 65 \| 73 \| 90 \| 84 \| 86 \| 103 \| 81 \| 93 \| 4 \| \| 7 \| 60 \| 66 \| 81 \| 73 \| 72 \| 81 \| 89 \| 80 \| 4 \| \| 8 \| 80 \| 112 \| 251 \| 141 \| 123 \| 93 \| 80 \| 657 \| 381 \| |

**Figure 4.**
Distribution of peptide feature detection.  **(a)**
Feature m/z distribution;  **(b)**
Feature RT distribution.

**(a)**

**(b)**

**Figure 5.**
Distribution of identified peptide features.  **(a)**
Feature abundance distribution;  **(b)**
 *De novo*
sequencing validation.

**(a)**

**(b)**

**Table 2.**
Result filtration parameters.

|  |  |
| --- | --- |
| Peptide -10lgP | ≥44.9 |
| PTM Ascore | ≥0 |
| Protein -10lgP | ≥20 |
| Proteins unique peptides | ≥1 |
| De novo score(%) | ≥50% |


**Table 3.**
Statistics of filtered result.

|  |  |
| --- | --- |
| FDR (Peptide-Spectrum Matches) | 0.1% |
| FDR (Peptide Sequences) | 0.4% |
| FDR (Protein Group) | 4.4% |
| De Novo Only Spectra | 57365 |

**Table 4.**
PTM profile.

| Name | ∆Mass | Position | #PSM | -10lgP | Abundance | AScore || Oxidation | 15.99 | M | 1005 | 100.04 | 1.31E7 | 1000.00 |
| Carbamidomethyl | 57.02 | C | 34 | 73.44 | 1.25E7 | 1000.00 |

# 3. Experiment Control

**Figure 6.**
Precursor mass error of peptide-spectrum matches (PSM) in filtered result.  **(a)**
Distribution of precursor mass error in ppm;  **(b)**
Scatterplot of precursor m/z versus precursor mass error in ppm.

**(a)**

**(b)**

**Table 5.**
Number of identified peptides in each sample by the number of missed cleavages.

|  |  |  |  |  |  |
| --- | --- | --- | --- | --- | --- |
| Missed Cleavages | 0 | 1 | 2 | 3 | 4+ |
| F6 | 91 | 6 | 0 | 0 | 0 |
| F2 | 136 | 4 | 0 | 0 | 0 |
| F3 | 298 | 95 | 9 | 0 | 0 |
| F4 | 182 | 27 | 1 | 0 | 0 |
| F5 | 146 | 12 | 2 | 0 | 0 |
| F7 | 99 | 4 | 0 | 0 | 0 |
| F8 | 85 | 4 | 0 | 0 | 0 |
| F1 | 523 | 122 | 11 | 1 | 0 |

# 4. Other Information

**Table 6.**
Search parameters.

Search Engine Name: PEAKS  
Parent Mass Error Tolerance: 10.0 ppm  
Fragment Mass Error Tolerance: 0.6 Da  
Precursor Mass Search Type: monoisotopic  
Enzyme: Trypsin  
Max Missed Cleavages: 3  
Digest Mode: Semispecific  
Fixed Modifications:   
  Carbamidomethylation: 57.02  
Variable Modifications:   
  Oxidation (M): 15.99  
Max Variable PTM Per Peptide: 3  
Database: NCBI\_Serpentes\_NR  
Taxon: All  
Contaminant Database: cRAP\_contaminants  
Searched Entry: 410049  
FDR Estimation: Enabled  
Merge Options: no merge  
Precursor Options: corrected  
Charge Options: no correction  
Filter Charge: 2 - 8  
Process: true  
Associate chimera: yes

**Table 7.**
Instrument parameters.

Fractions: 04012022\_RID\_3262\_6b.raw, 13012022\_RID\_3262\_2b.raw,   
13012022\_RID\_3262\_3b.raw, 13012022\_RID\_3262\_4b.raw, 13012022\_  
RID\_3262\_5b.raw, 17012022\_RID\_3262\_7b.raw, 17012022\_RID\_3262\_  
8b.raw, 31122021\_RID\_3262\_1b.raw  
Ion Source: ESI(nano-spray)  
Fragmentation Mode: CID, CAD(y and b ions)  
MS Scan Mode: FT-ICR/Orbitrap  
MS/MS Scan Mode: FT-ICR/Orbitrap

  

Protein List

  

Protein Accession Contains:

Protein Description Contains:

Protein Sample Area >=

Protein PTM Contains:

| Protein Group | Protein ID | Accession | -10lgP | Coverage (%) | Coverage (%) F6 | Coverage (%) F2 | Coverage (%) F3 | Coverage (%) F4 | Coverage (%) F5 | Coverage (%) F7 | Coverage (%) F8 | Coverage (%) F1 | Area F6 | Area F2 | Area F3 | Area F4 | Area F5 | Area F7 | Area F8 | Area F1 | #Peptides | #Unique | #Spec F6 | #Spec F2 | #Spec F3 | #Spec F4 | #Spec F5 | #Spec F7 | #Spec F8 | #Spec F1 | PTM | Avg. Mass | Description |
| --- | --- | --- | --- | --- | --- | --- | --- | --- | --- | --- | --- | --- | --- | --- | --- | --- | --- | --- | --- | --- | --- | --- | --- | --- | --- | --- | --- | --- | --- | --- | --- | --- | --- |
| 6 | 30 | BAN89426.1 | 412.32 | 36 | 0 | 10 | 15 | 8 | 2 | 0 | 0 | 35 |  |  |  |  |  |  |  | 1.0864E7 | 54 | 1 | 0 | 20 | 17 | 8 | 3 | 0 | 0 | 395 | Y | 96239 | phosphodiesterase [Ovophis okinavensis] |
| 6 | 31 | BAN89425.1 | 412.32 | 34 | 0 | 9 | 15 | 7 | 2 | 0 | 0 | 34 |  |  |  |  |  |  |  | 1.0864E7 | 54 | 1 | 0 | 20 | 17 | 8 | 3 | 0 | 0 | 395 | Y | 100879 | phosphodiesterase [Ovophis okinavensis] |
| 10 | 32 | JAV01884.1 | 384.38 | 33 | 0 | 7 | 15 | 7 | 3 | 0 | 0 | 33 |  |  |  |  |  |  |  | 0 | 42 | 1 | 0 | 12 | 14 | 7 | 3 | 0 | 0 | 306 | Y | 91859 | BATXPDE1 [Bothrops atrox] |
| 12 | 150 | ETE67131.1 | 275.54 | 4 | 1 | 2 | 4 | 3 | 3 | 2 | 2 | 4 |  | 4.71E5 | 1.087E6 | 4.8663E5 | 6.2675E5 | 8.4246E5 | 2.9874E5 | 3.4491E6 | 20 | 1 | 4 | 8 | 29 | 11 | 17 | 13 | 8 | 96 | Y | 240642 | Keratin, type II cytoskeletal 1, partial [Ophiophagus hannah] |
| 11 | 744 | AMB36337.1 | 272.59 | 22 | 22 | 9 | 11 | 9 | 9 | 15 | 11 | 16 | 1.0241E9 | 1.9462E6 | 6.7778E6 | 9.1431E6 | 2.3396E7 | 2.0591E7 | 2.3229E7 | 1.5753E7 | 12 | 10 | 217 | 3 | 3 | 2 | 7 | 40 | 72 | 9 | Y | 26517 | cysteine-rich secretory protein CRISP-1 [Vipera ammodytes ammodytes] |
| 120 | 177 | XP\_007422201.1 | 253.40 | 5 | 0 | 0 | 2 | 0 | 0 | 0 | 0 | 4 |  |  | 5.0256E5 |  |  |  |  |  | 14 | 2 | 0 | 0 | 6 | 1 | 0 | 0 | 0 | 16 | Y | 329911 | desmoplakin isoform X1 [Python bivittatus] |
| 60 | 91 | AFJ50957.1 | 248.96 | 27 | 3 | 0 | 6 | 27 | 5 | 0 | 0 | 7 | 2.8944E6 |  | 4.0146E5 | 2.8073E7 | 2.3975E6 |  |  | 4.8327E5 | 13 | 1 | 2 | 0 | 2 | 49 | 4 | 0 | 0 | 3 | Y | 64091 | Phospholipase B [Crotalus adamanteus] |
| 60 | 92 | AEJ31983.1 | 248.96 | 27 | 3 | 0 | 6 | 27 | 5 | 0 | 0 | 7 | 2.8944E6 |  | 4.0146E5 | 2.8073E7 | 2.3975E6 |  |  | 4.8327E5 | 13 | 1 | 2 | 0 | 2 | 49 | 4 | 0 | 0 | 3 | Y | 64049 | phospholipase B [Crotalus adamanteus] |
| 60 | 93 | JAI12031.1 | 248.96 | 27 | 3 | 0 | 6 | 27 | 5 | 0 | 0 | 7 | 2.8944E6 |  | 4.0146E5 | 2.8073E7 | 2.3975E6 |  |  | 4.8327E5 | 13 | 1 | 2 | 0 | 2 | 49 | 4 | 0 | 0 | 3 | Y | 64091 | Phospholipase B [Crotalus adamanteus] |
| 60 | 94 | SMD28240.1 | 248.96 | 27 | 3 | 0 | 6 | 27 | 5 | 0 | 0 | 7 | 2.8944E6 |  | 4.0146E5 | 2.8073E7 | 2.3975E6 |  |  | 4.8327E5 | 13 | 1 | 2 | 0 | 2 | 49 | 4 | 0 | 0 | 3 | Y | 64104 | phospholipase B CdtPLB1 [Crotalus durissus terrificus] |
| 60 | 95 | F8S101.1 | 248.96 | 27 | 3 | 0 | 6 | 27 | 5 | 0 | 0 | 7 | 2.8944E6 |  | 4.0146E5 | 2.8073E7 | 2.3975E6 |  |  | 4.8327E5 | 13 | 1 | 2 | 0 | 2 | 49 | 4 | 0 | 0 | 3 | Y | 64049 | RecName: Full=Phospholipase B; Short=PLB; Flags: Precursor |
| 60 | 96 | JAV49636.1 | 248.96 | 27 | 3 | 0 | 6 | 27 | 5 | 0 | 0 | 7 | 2.8944E6 |  | 4.0146E5 | 2.8073E7 | 2.3975E6 |  |  | 4.8327E5 | 13 | 1 | 2 | 0 | 2 | 49 | 4 | 0 | 0 | 3 | Y | 64129 | Phospholipase B [Agkistrodon contortrix contortrix] |
| 60 | 97 | JAV48419.1 | 248.96 | 27 | 3 | 0 | 6 | 27 | 5 | 0 | 0 | 7 | 2.8944E6 |  | 4.0146E5 | 2.8073E7 | 2.3975E6 |  |  | 4.8327E5 | 13 | 1 | 2 | 0 | 2 | 49 | 4 | 0 | 0 | 3 | Y | 64110 | Cadam10\_PLB-1 [Crotalus adamanteus] |
| 60 | 98 | BAN82026.1 | 248.96 | 27 | 3 | 0 | 6 | 27 | 5 | 0 | 0 | 7 | 2.8944E6 |  | 4.0146E5 | 2.8073E7 | 2.3975E6 |  |  | 4.8327E5 | 13 | 1 | 2 | 0 | 2 | 49 | 4 | 0 | 0 | 3 | Y | 64233 | phospholipase b [Protobothrops flavoviridis] |
| 60 | 99 | BAP39973.1 | 248.96 | 27 | 3 | 0 | 6 | 27 | 5 | 0 | 0 | 7 | 2.8944E6 |  | 4.0146E5 | 2.8073E7 | 2.3975E6 |  |  | 4.8327E5 | 13 | 1 | 2 | 0 | 2 | 49 | 4 | 0 | 0 | 3 | Y | 64233 | phospholipase B [Protobothrops flavoviridis] |
| 60 | 100 | BAP39922.1 | 248.96 | 27 | 3 | 0 | 6 | 27 | 5 | 0 | 0 | 7 | 2.8944E6 |  | 4.0146E5 | 2.8073E7 | 2.3975E6 |  |  | 4.8327E5 | 13 | 1 | 2 | 0 | 2 | 49 | 4 | 0 | 0 | 3 | Y | 64115 | phospholipase B [Protobothrops elegans] |
| 57 | 296 | AAY89681.1 | 242.89 | 21 | 2 | 4 | 21 | 4 | 4 | 2 | 2 | 4 |  |  | 3.2785E6 |  |  |  |  |  | 13 | 1 | 2 | 6 | 49 | 8 | 7 | 6 | 5 | 3 | Y | 59058 | L-amino acid oxidase precursor [Notechis scutatus] |
| 57 | 297 | Q4JHE2.1 | 242.89 | 21 | 2 | 4 | 21 | 4 | 4 | 2 | 2 | 4 |  |  | 3.2785E6 |  |  |  |  |  | 13 | 1 | 2 | 6 | 49 | 8 | 7 | 6 | 5 | 3 | Y | 59058 | RecName: Full=L-amino-acid oxidase; Short=LAAO; Short=LAO; Flags: Precursor |
| 57 | 299 | XP\_026538830.1 | 242.89 | 20 | 2 | 4 | 20 | 4 | 4 | 2 | 2 | 4 |  |  | 3.2785E6 |  |  |  |  |  | 13 | 1 | 2 | 6 | 49 | 8 | 7 | 6 | 5 | 3 | Y | 61914 | L-amino-acid oxidase [Notechis scutatus] |
| 58 | 288 | ACF70483.1 | 233.10 | 18 | 4 | 3 | 14 | 3 | 3 | 0 | 0 | 2 |  |  | 5.8013E7 |  |  |  |  |  | 12 | 3 | 2 | 3 | 40 | 18 | 12 | 0 | 0 | 2 | Y | 56888 | secreted L-amino acid oxidase precursor [Daboia russelii] |
| 36 | 237 | XP\_032067281.1 | 228.95 | 12 | 2 | 6 | 10 | 5 | 5 | 4 | 5 | 9 |  |  |  |  |  |  | 1.9473E7 |  | 12 | 1 | 2 | 6 | 19 | 5 | 7 | 8 | 7 | 54 | Y | 59706 | keratin, type II cytoskeletal 6A-like [Thamnophis elegans] |
| 36 | 238 | XP\_013915209.1 | 228.95 | 12 | 2 | 6 | 10 | 5 | 5 | 4 | 5 | 9 |  |  |  |  |  |  | 1.9473E7 |  | 12 | 1 | 2 | 6 | 19 | 5 | 7 | 8 | 7 | 54 | Y | 59764 | PREDICTED: keratin, type II cytoskeletal 6A-like [Thamnophis sirtalis] |
| 67 | 281 | Q4JHE1.1 | 224.31 | 17 | 6 | 2 | 13 | 3 | 2 | 0 | 0 | 0 |  |  | 5.656E7 |  |  |  |  |  | 12 | 2 | 3 | 1 | 42 | 7 | 1 | 0 | 0 | 0 | Y | 58744 | RecName: Full=L-amino-acid oxidase; Short=LAAO; Short=LAO; Flags: Precursor |
| 67 | 282 | AAY89682.1 | 224.31 | 17 | 6 | 2 | 13 | 3 | 2 | 0 | 0 | 0 |  |  | 5.656E7 |  |  |  |  |  | 12 | 2 | 3 | 1 | 42 | 7 | 1 | 0 | 0 | 0 | Y | 58744 | L-amino acid oxidase precursor [Pseudechis australis] |
| 99 | 219 | XP\_015668350.1 | 219.05 | 15 | 0 | 12 | 13 | 9 | 8 | 0 | 0 | 3 |  | 1.4421E7 |  | 6.0077E6 | 3.384E6 |  |  |  | 9 | 1 | 0 | 13 | 10 | 8 | 7 | 0 | 0 | 2 | Y | 64635 | 5'-nucleotidase [Protobothrops mucrosquamatus] |
| 99 | 220 | BAP39925.1 | 219.05 | 15 | 0 | 12 | 13 | 9 | 8 | 0 | 0 | 3 |  | 1.4421E7 |  | 6.0077E6 | 3.384E6 |  |  |  | 9 | 1 | 0 | 13 | 10 | 8 | 7 | 0 | 0 | 2 | Y | 64640 | 5-nucleotidase [Protobothrops elegans] |
| 61 | 227 | ETE70217.1 | 218.79 | 2 | 0 | 0 | 1 | 0 | 0 | 0 | 1 | 2 |  |  | 3.0328E6 |  |  |  |  |  | 12 | 1 | 3 | 2 | 9 | 2 | 2 | 4 | 3 | 12 | N | 436388 | Keratin, type I cytoskeletal 19, partial [Ophiophagus hannah] |
| 141 | 154 | XP\_025022318.1 | 216.77 | 14 | 0 | 0 | 9 | 1 | 1 | 0 | 0 | 13 |  |  | 2.1992E6 | 2.0478E5 | 2.1025E5 |  |  | 7.8432E6 | 11 | 11 | 0 | 0 | 6 | 1 | 2 | 0 | 0 | 17 | Y | 81985 | junction plakoglobin isoform X2 [Python bivittatus] |
| 141 | 155 | XP\_026576301.1 | 216.77 | 14 | 0 | 0 | 9 | 1 | 1 | 0 | 0 | 13 |  |  | 2.1992E6 | 2.0478E5 | 2.1025E5 |  |  | 7.8432E6 | 11 | 11 | 0 | 0 | 6 | 1 | 2 | 0 | 0 | 17 | Y | 81304 | junction plakoglobin isoform X2 [Pseudonaja textilis] |
| 141 | 156 | XP\_026525656.1 | 216.77 | 14 | 0 | 0 | 9 | 1 | 1 | 0 | 0 | 13 |  |  | 2.1992E6 | 2.0478E5 | 2.1025E5 |  |  | 7.8432E6 | 11 | 11 | 0 | 0 | 6 | 1 | 2 | 0 | 0 | 17 | Y | 81304 | junction plakoglobin isoform X2 [Notechis scutatus] |
| 141 | 157 | XP\_026576299.1 | 216.77 | 14 | 0 | 0 | 9 | 1 | 1 | 0 | 0 | 13 |  |  | 2.1992E6 | 2.0478E5 | 2.1025E5 |  |  | 7.8432E6 | 11 | 11 | 0 | 0 | 6 | 1 | 2 | 0 | 0 | 17 | Y | 81375 | junction plakoglobin isoform X1 [Pseudonaja textilis] |
| 141 | 158 | XP\_026525655.1 | 216.77 | 14 | 0 | 0 | 9 | 1 | 1 | 0 | 0 | 13 |  |  | 2.1992E6 | 2.0478E5 | 2.1025E5 |  |  | 7.8432E6 | 11 | 11 | 0 | 0 | 6 | 1 | 2 | 0 | 0 | 17 | Y | 81375 | junction plakoglobin isoform X1 [Notechis scutatus] |
| 141 | 159 | XP\_026525654.1 | 216.77 | 14 | 0 | 0 | 9 | 1 | 1 | 0 | 0 | 13 |  |  | 2.1992E6 | 2.0478E5 | 2.1025E5 |  |  | 7.8432E6 | 11 | 11 | 0 | 0 | 6 | 1 | 2 | 0 | 0 | 17 | Y | 81375 | junction plakoglobin isoform X1 [Notechis scutatus] |
| 141 | 160 | XP\_026576298.1 | 216.77 | 14 | 0 | 0 | 9 | 1 | 1 | 0 | 0 | 13 |  |  | 2.1992E6 | 2.0478E5 | 2.1025E5 |  |  | 7.8432E6 | 11 | 11 | 0 | 0 | 6 | 1 | 2 | 0 | 0 | 17 | Y | 81375 | junction plakoglobin isoform X1 [Pseudonaja textilis] |
| 141 | 161 | JAG44306.1 | 216.77 | 14 | 0 | 0 | 9 | 1 | 1 | 0 | 0 | 13 |  |  | 2.1992E6 | 2.0478E5 | 2.1025E5 |  |  | 7.8432E6 | 11 | 11 | 0 | 0 | 6 | 1 | 2 | 0 | 0 | 17 | Y | 81903 | junction plakoglobin [Crotalus horridus] |
| 141 | 162 | JAA96758.1 | 216.77 | 14 | 0 | 0 | 9 | 1 | 1 | 0 | 0 | 13 |  |  | 2.1992E6 | 2.0478E5 | 2.1025E5 |  |  | 7.8432E6 | 11 | 11 | 0 | 0 | 6 | 1 | 2 | 0 | 0 | 17 | Y | 81903 | junction plakoglobin [Crotalus horridus] |
| 141 | 163 | JAG46538.1 | 216.77 | 14 | 0 | 0 | 9 | 1 | 1 | 0 | 0 | 13 |  |  | 2.1992E6 | 2.0478E5 | 2.1025E5 |  |  | 7.8432E6 | 11 | 11 | 0 | 0 | 6 | 1 | 2 | 0 | 0 | 17 | Y | 81903 | junction plakoglobin [Crotalus horridus] |
| 141 | 164 | JAV50234.1 | 216.77 | 14 | 0 | 0 | 9 | 1 | 1 | 0 | 0 | 13 |  |  | 2.1992E6 | 2.0478E5 | 2.1025E5 |  |  | 7.8432E6 | 11 | 11 | 0 | 0 | 6 | 1 | 2 | 0 | 0 | 17 | Y | 81358 | JUP protein [Agkistrodon contortrix contortrix] |
| 141 | 165 | XP\_025022317.1 | 216.77 | 14 | 0 | 0 | 9 | 1 | 1 | 0 | 0 | 13 |  |  | 2.1992E6 | 2.0478E5 | 2.1025E5 |  |  | 7.8432E6 | 11 | 11 | 0 | 0 | 6 | 1 | 2 | 0 | 0 | 17 | Y | 82056 | junction plakoglobin isoform X1 [Python bivittatus] |
| 141 | 166 | XP\_007428111.1 | 216.77 | 14 | 0 | 0 | 9 | 1 | 1 | 0 | 0 | 13 |  |  | 2.1992E6 | 2.0478E5 | 2.1025E5 |  |  | 7.8432E6 | 11 | 11 | 0 | 0 | 6 | 1 | 2 | 0 | 0 | 17 | Y | 82056 | junction plakoglobin isoform X1 [Python bivittatus] |
| 141 | 167 | LAB43566.1 | 216.77 | 14 | 0 | 0 | 8 | 1 | 1 | 0 | 0 | 13 |  |  | 2.1992E6 | 2.0478E5 | 2.1025E5 |  |  | 7.8432E6 | 11 | 11 | 0 | 0 | 6 | 1 | 2 | 0 | 0 | 17 | Y | 83463 | hypothetical protein, partial [Micrurus spixii] |
| 141 | 168 | XP\_034271617.1 | 216.77 | 14 | 0 | 0 | 9 | 1 | 1 | 0 | 0 | 13 |  |  | 2.1992E6 | 2.0478E5 | 2.1025E5 |  |  | 7.8432E6 | 11 | 11 | 0 | 0 | 6 | 1 | 2 | 0 | 0 | 17 | Y | 81870 | junction plakoglobin isoform X2 [Pantherophis guttatus] |
| 141 | 169 | XP\_034271615.1 | 216.77 | 14 | 0 | 0 | 9 | 1 | 1 | 0 | 0 | 13 |  |  | 2.1992E6 | 2.0478E5 | 2.1025E5 |  |  | 7.8432E6 | 11 | 11 | 0 | 0 | 6 | 1 | 2 | 0 | 0 | 17 | Y | 81941 | junction plakoglobin isoform X1 [Pantherophis guttatus] |
| 141 | 170 | XP\_013919280.1 | 216.77 | 14 | 0 | 0 | 9 | 1 | 1 | 0 | 0 | 13 |  |  | 2.1992E6 | 2.0478E5 | 2.1025E5 |  |  | 7.8432E6 | 11 | 11 | 0 | 0 | 6 | 1 | 2 | 0 | 0 | 17 | Y | 81965 | PREDICTED: junction plakoglobin [Thamnophis sirtalis] |
| 141 | 171 | XP\_034271618.1 | 216.77 | 14 | 0 | 0 | 9 | 1 | 1 | 0 | 0 | 13 |  |  | 2.1992E6 | 2.0478E5 | 2.1025E5 |  |  | 7.8432E6 | 11 | 11 | 0 | 0 | 6 | 1 | 2 | 0 | 0 | 17 | Y | 81941 | junction plakoglobin isoform X1 [Pantherophis guttatus] |
| 141 | 172 | JAG67632.1 | 216.77 | 14 | 0 | 0 | 9 | 1 | 1 | 0 | 0 | 13 |  |  | 2.1992E6 | 2.0478E5 | 2.1025E5 |  |  | 7.8432E6 | 11 | 11 | 0 | 0 | 6 | 1 | 2 | 0 | 0 | 17 | Y | 81861 | junction plakoglobin [Boiga irregularis] |
| 141 | 173 | XP\_034271616.1 | 216.77 | 14 | 0 | 0 | 9 | 1 | 1 | 0 | 0 | 13 |  |  | 2.1992E6 | 2.0478E5 | 2.1025E5 |  |  | 7.8432E6 | 11 | 11 | 0 | 0 | 6 | 1 | 2 | 0 | 0 | 17 | Y | 81941 | junction plakoglobin isoform X1 [Pantherophis guttatus] |
| 141 | 174 | XP\_032091066.1 | 216.77 | 14 | 0 | 0 | 8 | 1 | 1 | 0 | 0 | 12 |  |  | 2.1992E6 | 2.0478E5 | 2.1025E5 |  |  | 7.8432E6 | 11 | 11 | 0 | 0 | 6 | 1 | 2 | 0 | 0 | 17 | Y | 86365 | junction plakoglobin [Thamnophis elegans] |
| 141 | 175 | XP\_032091065.1 | 216.77 | 14 | 0 | 0 | 8 | 1 | 1 | 0 | 0 | 12 |  |  | 2.1992E6 | 2.0478E5 | 2.1025E5 |  |  | 7.8432E6 | 11 | 11 | 0 | 0 | 6 | 1 | 2 | 0 | 0 | 17 | Y | 86365 | junction plakoglobin [Thamnophis elegans] |
| 141 | 188 | ETE68218.1 | 216.77 | 14 | 0 | 0 | 9 | 1 | 1 | 0 | 0 | 13 |  |  | 2.1992E6 | 2.0478E5 | 2.1025E5 |  |  | 7.8432E6 | 11 | 11 | 0 | 0 | 6 | 1 | 2 | 0 | 0 | 17 | Y | 83009 | Junction plakoglobin [Ophiophagus hannah] |
| 114 | 270 | JAS04520.1 | 203.96 | 13 | 0 | 10 | 12 | 6 | 5 | 0 | 0 | 2 |  | 3.7298E5 |  |  |  |  |  |  | 8 | 1 | 0 | 9 | 8 | 6 | 5 | 0 | 0 | 1 | Y | 64602 | Snake venom 5'-nucleotidase [Agkistrodon piscivorus conanti] |
| 134 | 340 | XP\_015674366.1 | 196.17 | 26 | 6 | 18 | 13 | 4 | 4 | 0 | 0 | 6 |  | 2.1103E6 |  | 6.0339E5 | 1.7716E5 |  |  |  | 7 | 2 | 3 | 8 | 4 | 2 | 1 | 0 | 0 | 2 | Y | 37677 | intestinal-type alkaline phosphatase-like [Protobothrops mucrosquamatus] |
| 160 | 243 | JAI10342.1 | 180.46 | 17 | 0 | 0 | 0 | 0 | 0 | 0 | 0 | 17 |  |  |  |  |  |  |  | 3.2529E6 | 5 | 5 | 0 | 0 | 0 | 0 | 0 | 0 | 0 | 8 | N | 41737 | Actin, cytoplasmic 1 [Micrurus fulvius] |
| 160 | 244 | JAB54771.1 | 180.46 | 17 | 0 | 0 | 0 | 0 | 0 | 0 | 0 | 17 |  |  |  |  |  |  |  | 3.2529E6 | 5 | 5 | 0 | 0 | 0 | 0 | 0 | 0 | 0 | 8 | N | 41737 | Actin [Micrurus fulvius] |
| 160 | 245 | JAA97901.1 | 180.46 | 17 | 0 | 0 | 0 | 0 | 0 | 0 | 0 | 17 |  |  |  |  |  |  |  | 3.2529E6 | 5 | 5 | 0 | 0 | 0 | 0 | 0 | 0 | 0 | 8 | N | 41737 | Actin, cytoplasmic 1 [Crotalus horridus] |
| 160 | 246 | JAG68844.1 | 180.46 | 17 | 0 | 0 | 0 | 0 | 0 | 0 | 0 | 17 |  |  |  |  |  |  |  | 3.2529E6 | 5 | 5 | 0 | 0 | 0 | 0 | 0 | 0 | 0 | 8 | N | 41737 | Actin, cytoplasmic 1 [Boiga irregularis] |
| 160 | 247 | JAV51374.1 | 180.46 | 17 | 0 | 0 | 0 | 0 | 0 | 0 | 0 | 17 |  |  |  |  |  |  |  | 3.2529E6 | 5 | 5 | 0 | 0 | 0 | 0 | 0 | 0 | 0 | 8 | N | 41737 | Actin, cytoplasmic 1 [Agkistrodon contortrix contortrix] |
| 160 | 248 | JAG47580.1 | 180.46 | 17 | 0 | 0 | 0 | 0 | 0 | 0 | 0 | 17 |  |  |  |  |  |  |  | 3.2529E6 | 5 | 5 | 0 | 0 | 0 | 0 | 0 | 0 | 0 | 8 | N | 41737 | Actin, cytoplasmic 1 [Crotalus horridus] |
| 160 | 249 | JAI14496.1 | 180.46 | 17 | 0 | 0 | 0 | 0 | 0 | 0 | 0 | 17 |  |  |  |  |  |  |  | 3.2529E6 | 5 | 5 | 0 | 0 | 0 | 0 | 0 | 0 | 0 | 8 | N | 41737 | Actin, cytoplasmic 1 [Crotalus adamanteus] |
| 160 | 250 | JAC96364.1 | 180.46 | 17 | 0 | 0 | 0 | 0 | 0 | 0 | 0 | 17 |  |  |  |  |  |  |  | 3.2529E6 | 5 | 5 | 0 | 0 | 0 | 0 | 0 | 0 | 0 | 8 | N | 41737 | Actin, cytoplasmic 1 [Hypsiglena sp. JMG-2014] |
| 160 | 251 | AFJ49302.1 | 180.46 | 17 | 0 | 0 | 0 | 0 | 0 | 0 | 0 | 17 |  |  |  |  |  |  |  | 3.2529E6 | 5 | 5 | 0 | 0 | 0 | 0 | 0 | 0 | 0 | 8 | N | 41737 | Actin, cytoplasmic 1 [Crotalus adamanteus] |
| 160 | 252 | BAQ22177.1 | 180.46 | 17 | 0 | 0 | 0 | 0 | 0 | 0 | 0 | 17 |  |  |  |  |  |  |  | 3.2529E6 | 5 | 5 | 0 | 0 | 0 | 0 | 0 | 0 | 0 | 8 | N | 41737 | beta-actin [Protobothrops flavoviridis] |
| 160 | 253 | JAG45999.1 | 180.46 | 17 | 0 | 0 | 0 | 0 | 0 | 0 | 0 | 17 |  |  |  |  |  |  |  | 3.2529E6 | 5 | 5 | 0 | 0 | 0 | 0 | 0 | 0 | 0 | 8 | N | 41737 | actin, cytoplasmic 1 [Crotalus horridus] |
| 160 | 254 | ETE57948.1 | 180.46 | 18 | 0 | 0 | 0 | 0 | 0 | 0 | 0 | 18 |  |  |  |  |  |  |  | 3.2529E6 | 5 | 5 | 0 | 0 | 0 | 0 | 0 | 0 | 0 | 8 | N | 40263 | hypothetical protein L345\_16333, partial [Ophiophagus hannah] |
| 160 | 255 | JAC96363.1 | 180.46 | 17 | 0 | 0 | 0 | 0 | 0 | 0 | 0 | 17 |  |  |  |  |  |  |  | 3.2529E6 | 5 | 5 | 0 | 0 | 0 | 0 | 0 | 0 | 0 | 8 | N | 41793 | Actin, cytoplasmic 2 [Hypsiglena sp. JMG-2014] |
| 160 | 256 | JAA97900.1 | 180.46 | 17 | 0 | 0 | 0 | 0 | 0 | 0 | 0 | 17 |  |  |  |  |  |  |  | 3.2529E6 | 5 | 5 | 0 | 0 | 0 | 0 | 0 | 0 | 0 | 8 | N | 41793 | Actin, cytoplasmic 2 [Crotalus horridus] |
| 160 | 257 | LAA21099.1 | 180.46 | 17 | 0 | 0 | 0 | 0 | 0 | 0 | 0 | 17 |  |  |  |  |  |  |  | 3.2529E6 | 5 | 5 | 0 | 0 | 0 | 0 | 0 | 0 | 0 | 8 | N | 41793 | hypothetical protein [Micrurus lemniscatus carvalhoi] |
| 160 | 258 | JAG68843.1 | 180.46 | 17 | 0 | 0 | 0 | 0 | 0 | 0 | 0 | 17 |  |  |  |  |  |  |  | 3.2529E6 | 5 | 5 | 0 | 0 | 0 | 0 | 0 | 0 | 0 | 8 | N | 41793 | Actin, cytoplasmic 2 [Boiga irregularis] |
| 160 | 259 | XP\_013921350.1 | 180.46 | 17 | 0 | 0 | 0 | 0 | 0 | 0 | 0 | 17 |  |  |  |  |  |  |  | 3.2529E6 | 5 | 5 | 0 | 0 | 0 | 0 | 0 | 0 | 0 | 8 | N | 41793 | PREDICTED: actin, cytoplasmic 2 [Thamnophis sirtalis] |
| 160 | 260 | LAA21098.1 | 180.46 | 17 | 0 | 0 | 0 | 0 | 0 | 0 | 0 | 17 |  |  |  |  |  |  |  | 3.2529E6 | 5 | 5 | 0 | 0 | 0 | 0 | 0 | 0 | 0 | 8 | N | 41793 | hypothetical protein [Micrurus lemniscatus carvalhoi] |
| 160 | 261 | AFJ49303.1 | 180.46 | 17 | 0 | 0 | 0 | 0 | 0 | 0 | 0 | 17 |  |  |  |  |  |  |  | 3.2529E6 | 5 | 5 | 0 | 0 | 0 | 0 | 0 | 0 | 0 | 8 | N | 41793 | Actin, cytoplasmic 2 [Crotalus adamanteus] |
| 160 | 262 | JAI10341.1 | 180.46 | 17 | 0 | 0 | 0 | 0 | 0 | 0 | 0 | 17 |  |  |  |  |  |  |  | 3.2529E6 | 5 | 5 | 0 | 0 | 0 | 0 | 0 | 0 | 0 | 8 | N | 41793 | Actin, cytoplasmic 2 [Micrurus fulvius] |
| 160 | 263 | JAI14495.1 | 180.46 | 17 | 0 | 0 | 0 | 0 | 0 | 0 | 0 | 17 |  |  |  |  |  |  |  | 3.2529E6 | 5 | 5 | 0 | 0 | 0 | 0 | 0 | 0 | 0 | 8 | N | 41793 | Actin, cytoplasmic 2 [Crotalus adamanteus] |
| 160 | 264 | JAG47579.1 | 180.46 | 17 | 0 | 0 | 0 | 0 | 0 | 0 | 0 | 17 |  |  |  |  |  |  |  | 3.2529E6 | 5 | 5 | 0 | 0 | 0 | 0 | 0 | 0 | 0 | 8 | N | 41793 | Actin, cytoplasmic 2 [Crotalus horridus] |
| 160 | 265 | JAV51372.1 | 180.46 | 17 | 0 | 0 | 0 | 0 | 0 | 0 | 0 | 17 |  |  |  |  |  |  |  | 3.2529E6 | 5 | 5 | 0 | 0 | 0 | 0 | 0 | 0 | 0 | 8 | N | 41793 | Actin, cytoplasmic 2 [Agkistrodon contortrix contortrix] |
| 160 | 266 | JAG45996.1 | 180.46 | 17 | 0 | 0 | 0 | 0 | 0 | 0 | 0 | 17 |  |  |  |  |  |  |  | 3.2529E6 | 5 | 5 | 0 | 0 | 0 | 0 | 0 | 0 | 0 | 8 | N | 41793 | Actin, cytoplasmic 2 [Crotalus horridus] |
| 160 | 267 | JAB54395.1 | 180.46 | 17 | 0 | 0 | 0 | 0 | 0 | 0 | 0 | 17 |  |  |  |  |  |  |  | 3.2529E6 | 5 | 5 | 0 | 0 | 0 | 0 | 0 | 0 | 0 | 8 | N | 41793 | cytoplasmic 2 [Micrurus fulvius] |
| 160 | 268 | ETE74025.1 | 180.46 | 17 | 0 | 0 | 0 | 0 | 0 | 0 | 0 | 17 |  |  |  |  |  |  |  | 3.2529E6 | 5 | 5 | 0 | 0 | 0 | 0 | 0 | 0 | 0 | 8 | N | 41793 | Actin, cytoplasmic 2 [Ophiophagus hannah] |
| 144 | 293 | AFE84764.1 | 174.05 | 26 | 0 | 0 | 0 | 23 | 10 | 0 | 0 | 0 |  |  |  | 9.5963E6 | 9.7108E5 |  |  |  | 8 | 8 | 0 | 0 | 0 | 15 | 3 | 0 | 0 | 0 | Y | 42350 | glutaminyl-peptide cyclotransferases [Protobothrops mucrosquamatus] |
| 144 | 294 | NP\_001310179.1 | 174.05 | 26 | 0 | 0 | 0 | 23 | 10 | 0 | 0 | 0 |  |  |  | 9.5963E6 | 9.7108E5 |  |  |  | 8 | 8 | 0 | 0 | 0 | 15 | 3 | 0 | 0 | 0 | Y | 42350 | glutaminyl-peptide cyclotransferase precursor [Protobothrops mucrosquamatus] |
| 144 | 298 | JAG46763.1 | 174.05 | 26 | 0 | 0 | 0 | 23 | 10 | 0 | 0 | 0 |  |  |  | 9.5963E6 | 9.7108E5 |  |  |  | 8 | 8 | 0 | 0 | 0 | 15 | 3 | 0 | 0 | 0 | Y | 42370 | glutaminyl-peptide cyclotransferases [Crotalus horridus] |
| 144 | 300 | BAN89376.1 | 174.05 | 32 | 0 | 0 | 0 | 28 | 12 | 0 | 0 | 0 |  |  |  | 9.5963E6 | 9.7108E5 |  |  |  | 8 | 8 | 0 | 0 | 0 | 15 | 3 | 0 | 0 | 0 | Y | 34563 | glutaminyl cyclase [Protobothrops flavoviridis] |
| 144 | 301 | BAN82005.1 | 174.05 | 29 | 0 | 0 | 0 | 25 | 11 | 0 | 0 | 0 |  |  |  | 9.5963E6 | 9.7108E5 |  |  |  | 8 | 8 | 0 | 0 | 0 | 15 | 3 | 0 | 0 | 0 | Y | 38114 | glutaminyl cyclase, partial [Protobothrops flavoviridis] |
| 144 | 302 | Q90YA8.1 | 174.05 | 26 | 0 | 0 | 0 | 23 | 10 | 0 | 0 | 0 |  |  |  | 9.5963E6 | 9.7108E5 |  |  |  | 8 | 8 | 0 | 0 | 0 | 15 | 3 | 0 | 0 | 0 | Y | 42267 | RecName: Full=Glutaminyl-peptide cyclotransferase; AltName: Full=Glutaminyl cyclase; Short=QC; AltName: Full=Glutaminyl-tRNA cyclotransferase; Flags: Precursor |
| 144 | 303 | BAP39934.1 | 174.05 | 26 | 0 | 0 | 0 | 23 | 10 | 0 | 0 | 0 |  |  |  | 9.5963E6 | 9.7108E5 |  |  |  | 8 | 8 | 0 | 0 | 0 | 15 | 3 | 0 | 0 | 0 | Y | 42305 | glutaminyl-peptide cyclotransferase [Protobothrops elegans] |
| 144 | 304 | AFE84763.1 | 174.05 | 26 | 0 | 0 | 0 | 23 | 10 | 0 | 0 | 0 |  |  |  | 9.5963E6 | 9.7108E5 |  |  |  | 8 | 8 | 0 | 0 | 0 | 15 | 3 | 0 | 0 | 0 | Y | 42295 | glutaminyl-peptide cyclotransferases [Trimeresurus gracilis] |
| 144 | 305 | BAP39982.1 | 174.05 | 26 | 0 | 0 | 0 | 23 | 10 | 0 | 0 | 0 |  |  |  | 9.5963E6 | 9.7108E5 |  |  |  | 8 | 8 | 0 | 0 | 0 | 15 | 3 | 0 | 0 | 0 | Y | 42219 | glutaminyl-peptide cyclotransferase [Protobothrops flavoviridis] |
| 144 | 306 | AFE84765.1 | 174.05 | 26 | 0 | 0 | 0 | 23 | 10 | 0 | 0 | 0 |  |  |  | 9.5963E6 | 9.7108E5 |  |  |  | 8 | 8 | 0 | 0 | 0 | 15 | 3 | 0 | 0 | 0 | Y | 42297 | glutaminyl-peptide cyclotransferases [Sistrurus tergeminus] |
| 144 | 307 | AFJ49187.1 | 174.05 | 26 | 0 | 0 | 0 | 23 | 10 | 0 | 0 | 0 |  |  |  | 9.5963E6 | 9.7108E5 |  |  |  | 8 | 8 | 0 | 0 | 0 | 15 | 3 | 0 | 0 | 0 | Y | 42354 | Glutaminyl-peptide cyclotransferase [Crotalus adamanteus] |
| 144 | 308 | JAA94895.1 | 174.05 | 26 | 0 | 0 | 0 | 23 | 10 | 0 | 0 | 0 |  |  |  | 9.5963E6 | 9.7108E5 |  |  |  | 8 | 8 | 0 | 0 | 0 | 15 | 3 | 0 | 0 | 0 | Y | 42369 | Glutaminyl-peptide cyclotransferase [Crotalus horridus] |
| 144 | 309 | JAI13216.1 | 174.05 | 26 | 0 | 0 | 0 | 23 | 10 | 0 | 0 | 0 |  |  |  | 9.5963E6 | 9.7108E5 |  |  |  | 8 | 8 | 0 | 0 | 0 | 15 | 3 | 0 | 0 | 0 | Y | 42354 | glutaminyl-peptide cyclotransferases [Crotalus adamanteus] |
| 144 | 310 | BAN82004.1 | 174.05 | 26 | 0 | 0 | 0 | 23 | 10 | 0 | 0 | 0 |  |  |  | 9.5963E6 | 9.7108E5 |  |  |  | 8 | 8 | 0 | 0 | 0 | 15 | 3 | 0 | 0 | 0 | Y | 42219 | glutaminyl cyclase [Protobothrops flavoviridis] |
| 144 | 311 | BAB69586.1 | 174.05 | 26 | 0 | 0 | 0 | 23 | 10 | 0 | 0 | 0 |  |  |  | 9.5963E6 | 9.7108E5 |  |  |  | 8 | 8 | 0 | 0 | 0 | 15 | 3 | 0 | 0 | 0 | Y | 42267 | glutaminyl cyclase [Gloydius blomhoffii] |
| 144 | 312 | JAG44660.1 | 174.05 | 26 | 0 | 0 | 0 | 23 | 10 | 0 | 0 | 0 |  |  |  | 9.5963E6 | 9.7108E5 |  |  |  | 8 | 8 | 0 | 0 | 0 | 15 | 3 | 0 | 0 | 0 | Y | 42369 | Glutaminyl-peptide cyclotransferase [Crotalus horridus] |
| 144 | 313 | BAN89375.1 | 174.05 | 26 | 0 | 0 | 0 | 23 | 10 | 0 | 0 | 0 |  |  |  | 9.5963E6 | 9.7108E5 |  |  |  | 8 | 8 | 0 | 0 | 0 | 15 | 3 | 0 | 0 | 0 | Y | 42219 | glutaminyl cyclase [Protobothrops flavoviridis] |
| 144 | 314 | BAN89450.1 | 174.05 | 27 | 0 | 0 | 0 | 24 | 10 | 0 | 0 | 0 |  |  |  | 9.5963E6 | 9.7108E5 |  |  |  | 8 | 8 | 0 | 0 | 0 | 15 | 3 | 0 | 0 | 0 | Y | 40742 | glutaminyl\_cyclase, partial [Ovophis okinavensis] |
| 144 | 315 | BAN89449.1 | 174.05 | 26 | 0 | 0 | 0 | 23 | 10 | 0 | 0 | 0 |  |  |  | 9.5963E6 | 9.7108E5 |  |  |  | 8 | 8 | 0 | 0 | 0 | 15 | 3 | 0 | 0 | 0 | Y | 42400 | glutaminyl\_cyclase, partial [Ovophis okinavensis] |
| 177 | 444 | JAG47096.1 | 173.11 | 14 | 0 | 0 | 14 | 0 | 0 | 0 | 0 | 0 |  |  | 5.7056E6 |  |  |  |  |  | 5 | 5 | 0 | 0 | 6 | 0 | 0 | 0 | 0 | 0 | N | 55483 | Putative serine carboxypeptidase CPVL-like protein [Crotalus horridus] |
| 177 | 449 | JAV50864.1 | 173.11 | 14 | 0 | 0 | 14 | 0 | 0 | 0 | 0 | 0 |  |  | 5.7056E6 |  |  |  |  |  | 5 | 5 | 0 | 0 | 6 | 0 | 0 | 0 | 0 | 0 | N | 55517 | putative serine carboxypeptidase CPVL-like protein [Agkistrodon contortrix contortrix] |
| 177 | 450 | XP\_015666085.1 | 173.11 | 14 | 0 | 0 | 14 | 0 | 0 | 0 | 0 | 0 |  |  | 5.7056E6 |  |  |  |  |  | 5 | 5 | 0 | 0 | 6 | 0 | 0 | 0 | 0 | 0 | N | 54741 | probable serine carboxypeptidase CPVL [Protobothrops mucrosquamatus] |
| 177 | 451 | XP\_015666086.1 | 173.11 | 14 | 0 | 0 | 14 | 0 | 0 | 0 | 0 | 0 |  |  | 5.7056E6 |  |  |  |  |  | 5 | 5 | 0 | 0 | 6 | 0 | 0 | 0 | 0 | 0 | N | 54741 | probable serine carboxypeptidase CPVL [Protobothrops mucrosquamatus] |
| 177 | 452 | JAG45243.1 | 173.11 | 14 | 0 | 0 | 14 | 0 | 0 | 0 | 0 | 0 |  |  | 5.7056E6 |  |  |  |  |  | 5 | 5 | 0 | 0 | 6 | 0 | 0 | 0 | 0 | 0 | N | 55505 | Putative serine carboxypeptidase CPVL-like protein [Crotalus horridus] |
| 177 | 453 | JAI13760.1 | 173.11 | 14 | 0 | 0 | 14 | 0 | 0 | 0 | 0 | 0 |  |  | 5.7056E6 |  |  |  |  |  | 5 | 5 | 0 | 0 | 6 | 0 | 0 | 0 | 0 | 0 | N | 55591 | putative serine carboxypeptidase CPVL-like protein [Crotalus adamanteus] |
| 177 | 454 | AFJ49766.1 | 173.11 | 14 | 0 | 0 | 14 | 0 | 0 | 0 | 0 | 0 |  |  | 5.7056E6 |  |  |  |  |  | 5 | 5 | 0 | 0 | 6 | 0 | 0 | 0 | 0 | 0 | N | 55591 | putative serine carboxypeptidase CPVL-like [Crotalus adamanteus] |
| 177 | 455 | JAA97361.1 | 173.11 | 14 | 0 | 0 | 14 | 0 | 0 | 0 | 0 | 0 |  |  | 5.7056E6 |  |  |  |  |  | 5 | 5 | 0 | 0 | 6 | 0 | 0 | 0 | 0 | 0 | N | 55505 | Putative serine carboxypeptidase CPVL-like protein [Crotalus horridus] |
| 177 | 459 | QHR82741.1 | 173.11 | 14 | 0 | 0 | 14 | 0 | 0 | 0 | 0 | 0 |  |  | 5.7056E6 |  |  |  |  |  | 5 | 5 | 0 | 0 | 6 | 0 | 0 | 0 | 0 | 0 | N | 55344 | putative serine carboxypeptidase 1 [Vipera anatolica senliki] |
| 112 | 460 | XP\_032067333.1 | 172.78 | 7 | 0 | 1 | 3 | 1 | 2 | 1 | 1 | 7 |  |  |  |  |  |  |  | 1.0192E5 | 7 | 1 | 0 | 1 | 6 | 1 | 3 | 4 | 2 | 9 | N | 62422 | keratin, type II cytoskeletal 6A-like [Thamnophis elegans] |
| 81 | 1557 | P0DM87.1 | 172.47 | 6 | 0 | 0 | 3 | 6 | 3 | 0 | 0 | 6 |  |  |  | 2.9504E7 |  |  |  | 8.2269E5 | 4 | 2 | 0 | 0 | 1 | 38 | 7 | 0 | 0 | 11 | Y | 54436 | RecName: Full=Zinc metalloproteinase-disintegrin stejnitin; AltName: Full=Snake venom metalloproteinase; Short=SVMP; Flags: Precursor |
| 136 | 521 | XP\_032080818.1 | 167.77 | 13 | 4 | 8 | 9 | 0 | 0 | 0 | 0 | 4 |  | 4.4338E6 |  |  |  |  |  |  | 6 | 1 | 3 | 8 | 4 | 0 | 0 | 0 | 0 | 2 | N | 57576 | intestinal-type alkaline phosphatase 1-like [Thamnophis elegans] |
| 97 | 604 | XP\_026529665.1 | 166.90 | 9 | 0 | 2 | 4 | 2 | 2 | 0 | 0 | 9 |  |  |  |  |  |  |  | 2.1862E5 | 6 | 1 | 0 | 3 | 5 | 1 | 3 | 0 | 0 | 32 | Y | 54010 | keratin, type II cytoskeletal 8 [Notechis scutatus] |
| 185 | 337 | JAG45871.1 | 166.03 | 6 | 0 | 0 | 0 | 0 | 0 | 0 | 0 | 6 |  |  |  |  |  |  |  | 1.404E5 | 5 | 1 | 0 | 0 | 0 | 0 | 0 | 0 | 0 | 5 | N | 110767 | Aminopeptidase N [Crotalus horridus] |
| 185 | 338 | JAA97801.1 | 166.03 | 6 | 0 | 0 | 0 | 0 | 0 | 0 | 0 | 6 |  |  |  |  |  |  |  | 1.404E5 | 5 | 1 | 0 | 0 | 0 | 0 | 0 | 0 | 0 | 5 | N | 110767 | Aminopeptidase N [Crotalus horridus] |
| 185 | 339 | JAG47501.1 | 166.03 | 6 | 0 | 0 | 0 | 0 | 0 | 0 | 0 | 6 |  |  |  |  |  |  |  | 1.404E5 | 5 | 1 | 0 | 0 | 0 | 0 | 0 | 0 | 0 | 5 | N | 110737 | Aminopeptidase N [Crotalus horridus] |
| 113 | 932 | XP\_026549343.1 | 165.82 | 18 | 7 | 0 | 18 | 7 | 0 | 0 | 0 | 8 |  |  | 1.0918E7 |  |  |  |  |  | 4 | 2 | 1 | 0 | 20 | 4 | 0 | 0 | 0 | 1 | Y | 16476 | L-amino-acid oxidase-like, partial [Notechis scutatus] |
| 180 | 357 | XP\_015667388.1 | 162.62 | 6 | 0 | 0 | 0 | 0 | 0 | 0 | 0 | 6 |  |  |  |  |  |  |  | 2.3746E5 | 5 | 1 | 0 | 0 | 0 | 0 | 0 | 0 | 0 | 5 | N | 110689 | aminopeptidase N [Protobothrops mucrosquamatus] |
| 66 | 724 | JAS04411.1 | 159.14 | 14 | 0 | 5 | 14 | 14 | 0 | 0 | 0 | 3 |  |  | 5.3201E6 | 6.9144E6 |  |  |  |  | 6 | 2 | 0 | 2 | 20 | 21 | 0 | 0 | 0 | 1 | Y | 28352 | Serine proteinase 2 [Agkistrodon piscivorus conanti] |
| 191 | 622 | XP\_032069201.1 | 157.52 | 10 | 0 | 0 | 0 | 0 | 0 | 0 | 0 | 10 |  |  |  |  |  |  |  | 2.1393E6 | 3 | 1 | 0 | 0 | 0 | 0 | 0 | 0 | 0 | 4 | N | 35787 | glyceraldehyde-3-phosphate dehydrogenase [Thamnophis elegans] |
| 181 | 491 | XP\_034265289.1 | 155.30 | 13 | 0 | 0 | 10 | 0 | 0 | 0 | 0 | 13 |  |  |  |  |  |  |  | 2.1385E5 | 4 | 1 | 0 | 0 | 4 | 0 | 0 | 0 | 0 | 5 | N | 38616 | annexin A2 [Pantherophis guttatus] |
| 181 | 553 | JAI10286.1 | 155.30 | 13 | 0 | 0 | 10 | 0 | 0 | 0 | 0 | 13 |  |  |  |  |  |  |  | 2.1385E5 | 4 | 1 | 0 | 0 | 4 | 0 | 0 | 0 | 0 | 5 | N | 38532 | Annexin A2-like protein [Micrurus fulvius] |
| 181 | 554 | LAB40267.1 | 155.30 | 13 | 0 | 0 | 10 | 0 | 0 | 0 | 0 | 13 |  |  |  |  |  |  |  | 2.1385E5 | 4 | 1 | 0 | 0 | 4 | 0 | 0 | 0 | 0 | 5 | N | 39471 | hypothetical protein, partial [Micrurus spixii] |
| 181 | 555 | XP\_032089545.1 | 155.30 | 13 | 0 | 0 | 10 | 0 | 0 | 0 | 0 | 13 |  |  |  |  |  |  |  | 2.1385E5 | 4 | 1 | 0 | 0 | 4 | 0 | 0 | 0 | 0 | 5 | N | 38475 | annexin A2 [Thamnophis elegans] |
| 181 | 556 | XP\_026579588.1 | 155.30 | 13 | 0 | 0 | 10 | 0 | 0 | 0 | 0 | 13 |  |  |  |  |  |  |  | 2.1385E5 | 4 | 1 | 0 | 0 | 4 | 0 | 0 | 0 | 0 | 5 | N | 38558 | annexin A2 [Pseudonaja textilis] |
| 181 | 557 | XP\_026579587.1 | 155.30 | 13 | 0 | 0 | 10 | 0 | 0 | 0 | 0 | 13 |  |  |  |  |  |  |  | 2.1385E5 | 4 | 1 | 0 | 0 | 4 | 0 | 0 | 0 | 0 | 5 | N | 38558 | annexin A2 [Pseudonaja textilis] |
| 181 | 558 | XP\_026579585.1 | 155.30 | 13 | 0 | 0 | 10 | 0 | 0 | 0 | 0 | 13 |  |  |  |  |  |  |  | 2.1385E5 | 4 | 1 | 0 | 0 | 4 | 0 | 0 | 0 | 0 | 5 | N | 38558 | annexin A2 [Pseudonaja textilis] |
| 181 | 559 | XP\_013930792.1 | 155.30 | 13 | 0 | 0 | 10 | 0 | 0 | 0 | 0 | 13 |  |  |  |  |  |  |  | 2.1385E5 | 4 | 1 | 0 | 0 | 4 | 0 | 0 | 0 | 0 | 5 | N | 40234 | PREDICTED: annexin A2 [Thamnophis sirtalis] |
| 186 | 561 | XP\_026537798.1 | 151.23 | 13 | 0 | 0 | 10 | 0 | 0 | 0 | 0 | 13 |  |  |  |  |  |  |  | 1.5203E4 | 4 | 1 | 0 | 0 | 4 | 0 | 0 | 0 | 0 | 5 | N | 40235 | annexin A2 [Notechis scutatus] |
| 77 | 727 | XP\_026570509.1 | 150.25 | 5 | 2 | 3 | 4 | 3 | 4 | 3 | 3 | 5 |  |  |  |  |  |  |  | 4.7863E5 | 4 | 1 | 4 | 3 | 8 | 3 | 5 | 9 | 6 | 15 | N | 52005 | keratin, type II cytoskeletal 4-like [Pseudonaja textilis] |
| 77 | 729 | XP\_026539658.1 | 150.25 | 5 | 2 | 3 | 4 | 3 | 4 | 3 | 3 | 5 |  |  |  |  |  |  |  | 4.7863E5 | 4 | 1 | 4 | 3 | 8 | 3 | 5 | 9 | 6 | 15 | N | 51886 | keratin, type II cytoskeletal 4-like [Notechis scutatus] |
| 195 | 467 | XP\_007439537.1 | 146.93 | 8 | 0 | 0 | 0 | 0 | 0 | 0 | 0 | 8 |  |  |  |  |  |  |  | 1.5629E6 | 5 | 5 | 0 | 0 | 0 | 0 | 0 | 0 | 0 | 5 | N | 73851 | histidine ammonia-lyase isoform X1 [Python bivittatus] |
| 124 | 591 | JAS05258.1 | 140.11 | 13 | 0 | 0 | 9 | 8 | 5 | 0 | 0 | 5 |  |  | 2.9418E8 |  | 3.5485E7 |  |  |  | 6 | 1 | 0 | 0 | 4 | 5 | 2 | 0 | 0 | 2 | Y | 27943 | Serine proteinase 14b [Sistrurus tergeminus] |
| 124 | 592 | JAS05257.1 | 140.11 | 13 | 0 | 0 | 9 | 8 | 5 | 0 | 0 | 5 |  |  | 2.9418E8 |  | 3.5485E7 |  |  |  | 6 | 1 | 0 | 0 | 4 | 5 | 2 | 0 | 0 | 2 | Y | 28013 | Serine proteinase 14c [Sistrurus tergeminus] |
| 124 | 593 | JAS05259.1 | 140.11 | 13 | 0 | 0 | 9 | 8 | 5 | 0 | 0 | 5 |  |  | 2.9418E8 |  | 3.5485E7 |  |  |  | 6 | 1 | 0 | 0 | 4 | 5 | 2 | 0 | 0 | 2 | Y | 28013 | Serine proteinase 14a [Sistrurus tergeminus] |
| 206 | 594 | JAG66915.1 | 139.54 | 19 | 0 | 0 | 0 | 0 | 0 | 0 | 0 | 19 |  |  |  |  |  |  |  | 4.2647E5 | 3 | 3 | 0 | 0 | 0 | 0 | 0 | 0 | 0 | 3 | N | 28000 | Proteasome subunit alpha type-7-like protein [Boiga irregularis] |
| 206 | 607 | JAB53603.1 | 139.54 | 19 | 0 | 0 | 0 | 0 | 0 | 0 | 0 | 19 |  |  |  |  |  |  |  | 4.2647E5 | 3 | 3 | 0 | 0 | 0 | 0 | 0 | 0 | 0 | 3 | N | 27972 | Proteasome subunit alpha type-7 [Micrurus fulvius] |
| 206 | 608 | JAA96031.1 | 139.54 | 19 | 0 | 0 | 0 | 0 | 0 | 0 | 0 | 19 |  |  |  |  |  |  |  | 4.2647E5 | 3 | 3 | 0 | 0 | 0 | 0 | 0 | 0 | 0 | 3 | N | 27972 | Proteasome subunit alpha type-7-like protein [Crotalus horridus] |
| 206 | 609 | AFJ51040.1 | 139.54 | 19 | 0 | 0 | 0 | 0 | 0 | 0 | 0 | 19 |  |  |  |  |  |  |  | 4.2647E5 | 3 | 3 | 0 | 0 | 0 | 0 | 0 | 0 | 0 | 3 | N | 27972 | Proteasome subunit alpha type-7-like [Crotalus adamanteus] |
| 206 | 610 | ETE69560.1 | 139.54 | 19 | 0 | 0 | 0 | 0 | 0 | 0 | 0 | 19 |  |  |  |  |  |  |  | 4.2647E5 | 3 | 3 | 0 | 0 | 0 | 0 | 0 | 0 | 0 | 3 | N | 27972 | Proteasome subunit alpha type-7 [Ophiophagus hannah] |
| 206 | 611 | XP\_015673624.1 | 139.54 | 19 | 0 | 0 | 0 | 0 | 0 | 0 | 0 | 19 |  |  |  |  |  |  |  | 4.2647E5 | 3 | 3 | 0 | 0 | 0 | 0 | 0 | 0 | 0 | 3 | N | 27945 | proteasome subunit alpha type-7 [Protobothrops mucrosquamatus] |
| 206 | 612 | JAI09615.1 | 139.54 | 19 | 0 | 0 | 0 | 0 | 0 | 0 | 0 | 19 |  |  |  |  |  |  |  | 4.2647E5 | 3 | 3 | 0 | 0 | 0 | 0 | 0 | 0 | 0 | 3 | N | 27972 | Proteasome subunit alpha type-7-like protein [Micrurus fulvius] |
| 206 | 613 | JAI11926.1 | 139.54 | 19 | 0 | 0 | 0 | 0 | 0 | 0 | 0 | 19 |  |  |  |  |  |  |  | 4.2647E5 | 3 | 3 | 0 | 0 | 0 | 0 | 0 | 0 | 0 | 3 | N | 27972 | Proteasome subunit alpha type-7-like protein [Crotalus adamanteus] |
| 206 | 614 | JAV49551.1 | 139.54 | 19 | 0 | 0 | 0 | 0 | 0 | 0 | 0 | 19 |  |  |  |  |  |  |  | 4.2647E5 | 3 | 3 | 0 | 0 | 0 | 0 | 0 | 0 | 0 | 3 | N | 27945 | Proteasome subunit alpha type-7-like protein [Agkistrodon contortrix contortrix] |
| 206 | 615 | XP\_026553199.1 | 139.54 | 19 | 0 | 0 | 0 | 0 | 0 | 0 | 0 | 19 |  |  |  |  |  |  |  | 4.2647E5 | 3 | 3 | 0 | 0 | 0 | 0 | 0 | 0 | 0 | 3 | N | 27972 | proteasome subunit alpha type-7 [Pseudonaja textilis] |
| 206 | 616 | XP\_034289033.1 | 139.54 | 19 | 0 | 0 | 0 | 0 | 0 | 0 | 0 | 19 |  |  |  |  |  |  |  | 4.2647E5 | 3 | 3 | 0 | 0 | 0 | 0 | 0 | 0 | 0 | 3 | N | 27972 | proteasome subunit alpha type-7 [Pantherophis guttatus] |
| 206 | 617 | XP\_032074995.1 | 139.54 | 19 | 0 | 0 | 0 | 0 | 0 | 0 | 0 | 19 |  |  |  |  |  |  |  | 4.2647E5 | 3 | 3 | 0 | 0 | 0 | 0 | 0 | 0 | 0 | 3 | N | 27988 | proteasome subunit alpha type-7 [Thamnophis elegans] |
| 206 | 618 | BAN82080.1 | 139.54 | 19 | 0 | 0 | 0 | 0 | 0 | 0 | 0 | 19 |  |  |  |  |  |  |  | 4.2647E5 | 3 | 3 | 0 | 0 | 0 | 0 | 0 | 0 | 0 | 3 | N | 27915 | hypothetical protein [Protobothrops flavoviridis] |
| 206 | 683 | XP\_013917261.1 | 139.54 | 19 | 0 | 0 | 0 | 0 | 0 | 0 | 0 | 19 |  |  |  |  |  |  |  | 4.2647E5 | 3 | 3 | 0 | 0 | 0 | 0 | 0 | 0 | 0 | 3 | N | 28045 | PREDICTED: proteasome subunit alpha type-7 [Thamnophis sirtalis] |
| 145 | 1564 | JAS05409.1 | 135.90 | 4 | 0 | 4 | 4 | 2 | 2 | 0 | 0 | 2 |  | 7.7874E6 | 7.1647E7 |  |  |  |  |  | 4 | 2 | 0 | 5 | 7 | 2 | 1 | 0 | 0 | 2 | Y | 67040 | Metalloproteinase type III 8b [Sistrurus miliarius barbouri] |
| 145 | 1566 | JAS05410.1 | 135.90 | 4 | 0 | 4 | 4 | 2 | 2 | 0 | 0 | 2 |  | 7.7874E6 | 7.1647E7 |  |  |  |  |  | 4 | 2 | 0 | 5 | 7 | 2 | 1 | 0 | 0 | 2 | Y | 66856 | Metalloproteinase type III 8a [Sistrurus miliarius barbouri] |
| 188 | 527 | AFJ49950.1 | 132.52 | 10 | 0 | 0 | 0 | 0 | 0 | 0 | 0 | 10 |  |  |  |  |  |  |  | 1.5256E6 | 4 | 4 | 0 | 0 | 0 | 0 | 0 | 0 | 0 | 6 | N | 50201 | Elongation factor 1-alpha 1-like [Crotalus adamanteus] |
| 188 | 528 | JAA97186.1 | 132.52 | 10 | 0 | 0 | 0 | 0 | 0 | 0 | 0 | 10 |  |  |  |  |  |  |  | 1.5256E6 | 4 | 4 | 0 | 0 | 0 | 0 | 0 | 0 | 0 | 6 | N | 50201 | Elongation factor 1-alpha 1 [Crotalus horridus] |
| 188 | 529 | JAI13488.1 | 132.52 | 10 | 0 | 0 | 0 | 0 | 0 | 0 | 0 | 10 |  |  |  |  |  |  |  | 1.5256E6 | 4 | 4 | 0 | 0 | 0 | 0 | 0 | 0 | 0 | 6 | N | 50201 | elongation factor 1-alpha 1 [Crotalus adamanteus] |
| 188 | 530 | XP\_007431170.1 | 132.52 | 10 | 0 | 0 | 0 | 0 | 0 | 0 | 0 | 10 |  |  |  |  |  |  |  | 1.5256E6 | 4 | 4 | 0 | 0 | 0 | 0 | 0 | 0 | 0 | 6 | N | 50155 | elongation factor 1-alpha 1 [Python bivittatus] |
| 188 | 531 | JAG46923.1 | 132.52 | 10 | 0 | 0 | 0 | 0 | 0 | 0 | 0 | 10 |  |  |  |  |  |  |  | 1.5256E6 | 4 | 4 | 0 | 0 | 0 | 0 | 0 | 0 | 0 | 6 | N | 50201 | Elongation factor 1-alpha 1 [Crotalus horridus] |
| 188 | 532 | XP\_015672964.1 | 132.52 | 10 | 0 | 0 | 0 | 0 | 0 | 0 | 0 | 10 |  |  |  |  |  |  |  | 1.5256E6 | 4 | 4 | 0 | 0 | 0 | 0 | 0 | 0 | 0 | 6 | N | 50155 | elongation factor 1-alpha 1 [Protobothrops mucrosquamatus] |
| 188 | 533 | JAG44960.1 | 132.52 | 10 | 0 | 0 | 0 | 0 | 0 | 0 | 0 | 10 |  |  |  |  |  |  |  | 1.5256E6 | 4 | 4 | 0 | 0 | 0 | 0 | 0 | 0 | 0 | 6 | N | 50201 | elongation factor 1-alpha 1 [Crotalus horridus] |
| 188 | 534 | JAV50678.1 | 132.52 | 10 | 0 | 0 | 0 | 0 | 0 | 0 | 0 | 10 |  |  |  |  |  |  |  | 1.5256E6 | 4 | 4 | 0 | 0 | 0 | 0 | 0 | 0 | 0 | 6 | N | 50201 | Elongation factor 1-alpha 1 [Agkistrodon contortrix contortrix] |
| 188 | 536 | ETE71213.1 | 132.52 | 10 | 0 | 0 | 0 | 0 | 0 | 0 | 0 | 10 |  |  |  |  |  |  |  | 1.5256E6 | 4 | 4 | 0 | 0 | 0 | 0 | 0 | 0 | 0 | 6 | N | 50095 | Elongation factor 1-alpha 1 [Ophiophagus hannah] |
| 188 | 537 | JAC95701.1 | 132.52 | 10 | 0 | 0 | 0 | 0 | 0 | 0 | 0 | 10 |  |  |  |  |  |  |  | 1.5256E6 | 4 | 4 | 0 | 0 | 0 | 0 | 0 | 0 | 0 | 6 | N | 50205 | elongation factor-1 alpha [Hypsiglena sp. JMG-2014] |
| 188 | 538 | JAC95700.1 | 132.52 | 10 | 0 | 0 | 0 | 0 | 0 | 0 | 0 | 10 |  |  |  |  |  |  |  | 1.5256E6 | 4 | 4 | 0 | 0 | 0 | 0 | 0 | 0 | 0 | 6 | N | 50205 | Elongation factor 1-alpha 1 [Hypsiglena sp. JMG-2014] |
| 188 | 539 | JAG68089.1 | 132.52 | 10 | 0 | 0 | 0 | 0 | 0 | 0 | 0 | 10 |  |  |  |  |  |  |  | 1.5256E6 | 4 | 4 | 0 | 0 | 0 | 0 | 0 | 0 | 0 | 6 | N | 50095 | elongation factor 1-alpha 1 [Boiga irregularis] |
| 188 | 540 | JAI10049.1 | 132.52 | 10 | 0 | 0 | 0 | 0 | 0 | 0 | 0 | 10 |  |  |  |  |  |  |  | 1.5256E6 | 4 | 4 | 0 | 0 | 0 | 0 | 0 | 0 | 0 | 6 | N | 50125 | elongation factor 1-alpha 1 [Micrurus fulvius] |
| 188 | 541 | XP\_034287789.1 | 132.52 | 10 | 0 | 0 | 0 | 0 | 0 | 0 | 0 | 10 |  |  |  |  |  |  |  | 1.5256E6 | 4 | 4 | 0 | 0 | 0 | 0 | 0 | 0 | 0 | 6 | N | 50095 | elongation factor 1-alpha 1 [Pantherophis guttatus] |
| 188 | 542 | XP\_026550930.1 | 132.52 | 10 | 0 | 0 | 0 | 0 | 0 | 0 | 0 | 10 |  |  |  |  |  |  |  | 1.5256E6 | 4 | 4 | 0 | 0 | 0 | 0 | 0 | 0 | 0 | 6 | N | 50095 | elongation factor 1-alpha 1 [Pseudonaja textilis] |
| 188 | 543 | JAB54325.1 | 132.52 | 10 | 0 | 0 | 0 | 0 | 0 | 0 | 0 | 10 |  |  |  |  |  |  |  | 1.5256E6 | 4 | 4 | 0 | 0 | 0 | 0 | 0 | 0 | 0 | 6 | N | 50125 | Elongation factor 1-alpha 1 [Micrurus fulvius] |
| 188 | 545 | XP\_013910443.1 | 132.52 | 10 | 0 | 0 | 0 | 0 | 0 | 0 | 0 | 10 |  |  |  |  |  |  |  | 1.5256E6 | 4 | 4 | 0 | 0 | 0 | 0 | 0 | 0 | 0 | 6 | N | 50109 | PREDICTED: elongation factor 1-alpha 1 [Thamnophis sirtalis] |
| 188 | 546 | XP\_026520989.1 | 132.52 | 10 | 0 | 0 | 0 | 0 | 0 | 0 | 0 | 10 |  |  |  |  |  |  |  | 1.5256E6 | 4 | 4 | 0 | 0 | 0 | 0 | 0 | 0 | 0 | 6 | N | 50109 | elongation factor 1-alpha 1 [Notechis scutatus] |
| 188 | 547 | XP\_032071033.1 | 132.52 | 10 | 0 | 0 | 0 | 0 | 0 | 0 | 0 | 10 |  |  |  |  |  |  |  | 1.5256E6 | 4 | 4 | 0 | 0 | 0 | 0 | 0 | 0 | 0 | 6 | N | 50109 | elongation factor 1-alpha 1 [Thamnophis elegans] |
| 188 | 560 | AEO96984.1 | 132.52 | 11 | 0 | 0 | 0 | 0 | 0 | 0 | 0 | 11 |  |  |  |  |  |  |  | 1.5256E6 | 4 | 4 | 0 | 0 | 0 | 0 | 0 | 0 | 0 | 6 | N | 44794 | eukaryotic translation elongation factor 1 alpha 1, partial [Elaphe taeniura] |
| 196 | 418 | LAA86151.1 | 130.52 | 10 | 0 | 0 | 0 | 0 | 0 | 0 | 0 | 10 |  |  |  |  |  |  |  | 1.0175E5 | 4 | 1 | 0 | 0 | 0 | 0 | 0 | 0 | 0 | 4 | N | 49952 | hypothetical protein [Micrurus lemniscatus lemniscatus] |
| 196 | 419 | JAI09230.1 | 130.52 | 10 | 0 | 0 | 0 | 0 | 0 | 0 | 0 | 10 |  |  |  |  |  |  |  | 1.0175E5 | 4 | 1 | 0 | 0 | 0 | 0 | 0 | 0 | 0 | 4 | N | 49981 | Tubulin alpha 6 [Micrurus fulvius] |
| 196 | 420 | AFJ51865.1 | 130.52 | 10 | 0 | 0 | 0 | 0 | 0 | 0 | 0 | 10 |  |  |  |  |  |  |  | 1.0175E5 | 4 | 1 | 0 | 0 | 0 | 0 | 0 | 0 | 0 | 4 | N | 49881 | Tubulin alpha-1C chain [Crotalus adamanteus] |
| 196 | 421 | JAA95197.1 | 130.52 | 10 | 0 | 0 | 0 | 0 | 0 | 0 | 0 | 10 |  |  |  |  |  |  |  | 1.0175E5 | 4 | 1 | 0 | 0 | 0 | 0 | 0 | 0 | 0 | 4 | N | 49895 | Tubulin alpha 6 [Crotalus horridus] |
| 196 | 422 | LAA55685.1 | 130.52 | 10 | 0 | 0 | 0 | 0 | 0 | 0 | 0 | 10 |  |  |  |  |  |  |  | 1.0175E5 | 4 | 1 | 0 | 0 | 0 | 0 | 0 | 0 | 0 | 4 | N | 49967 | hypothetical protein [Micrurus corallinus] |
| 196 | 423 | JAI10819.1 | 130.52 | 10 | 0 | 0 | 0 | 0 | 0 | 0 | 0 | 10 |  |  |  |  |  |  |  | 1.0175E5 | 4 | 1 | 0 | 0 | 0 | 0 | 0 | 0 | 0 | 4 | N | 49895 | Tubulin alpha 6 [Crotalus adamanteus] |
| 196 | 424 | JAG66080.1 | 130.52 | 10 | 0 | 0 | 0 | 0 | 0 | 0 | 0 | 10 |  |  |  |  |  |  |  | 1.0175E5 | 4 | 1 | 0 | 0 | 0 | 0 | 0 | 0 | 0 | 4 | N | 49881 | tubulin alpha-1B chain [Boiga irregularis] |
| 196 | 425 | JAG66077.1 | 130.52 | 10 | 0 | 0 | 0 | 0 | 0 | 0 | 0 | 10 |  |  |  |  |  |  |  | 1.0175E5 | 4 | 1 | 0 | 0 | 0 | 0 | 0 | 0 | 0 | 4 | N | 49951 | Tubulin alpha 6 [Boiga irregularis] |
| 196 | 426 | JAB53062.1 | 130.52 | 10 | 0 | 0 | 0 | 0 | 0 | 0 | 0 | 10 |  |  |  |  |  |  |  | 1.0175E5 | 4 | 1 | 0 | 0 | 0 | 0 | 0 | 0 | 0 | 4 | N | 50152 | Tubulin alpha-1B chain [Micrurus fulvius] |
| 196 | 427 | JAI10823.1 | 130.52 | 10 | 0 | 0 | 0 | 0 | 0 | 0 | 0 | 10 |  |  |  |  |  |  |  | 1.0175E5 | 4 | 1 | 0 | 0 | 0 | 0 | 0 | 0 | 0 | 4 | N | 50152 | Tubulin alpha-1B chain [Crotalus adamanteus] |
| 196 | 428 | LAA55681.1 | 130.52 | 10 | 0 | 0 | 0 | 0 | 0 | 0 | 0 | 10 |  |  |  |  |  |  |  | 1.0175E5 | 4 | 1 | 0 | 0 | 0 | 0 | 0 | 0 | 0 | 4 | N | 50152 | hypothetical protein [Micrurus corallinus] |
| 196 | 429 | AFJ51858.1 | 130.52 | 10 | 0 | 0 | 0 | 0 | 0 | 0 | 0 | 10 |  |  |  |  |  |  |  | 1.0175E5 | 4 | 1 | 0 | 0 | 0 | 0 | 0 | 0 | 0 | 4 | N | 50152 | Tubulin alpha-1B chain [Crotalus adamanteus] |
| 196 | 430 | JAA95201.1 | 130.52 | 10 | 0 | 0 | 0 | 0 | 0 | 0 | 0 | 10 |  |  |  |  |  |  |  | 1.0175E5 | 4 | 1 | 0 | 0 | 0 | 0 | 0 | 0 | 0 | 4 | N | 50152 | Tubulin alpha-1B chain [Crotalus horridus] |
| 196 | 431 | JAV48721.1 | 130.52 | 10 | 0 | 0 | 0 | 0 | 0 | 0 | 0 | 10 |  |  |  |  |  |  |  | 1.0175E5 | 4 | 1 | 0 | 0 | 0 | 0 | 0 | 0 | 0 | 4 | N | 50152 | Tubulin alpha-1B chain [Agkistrodon contortrix contortrix] |
| 196 | 432 | JAG66079.1 | 130.52 | 10 | 0 | 0 | 0 | 0 | 0 | 0 | 0 | 10 |  |  |  |  |  |  |  | 1.0175E5 | 4 | 1 | 0 | 0 | 0 | 0 | 0 | 0 | 0 | 4 | N | 50171 | Tubulin alpha-1B chain [Boiga irregularis] |
| 196 | 436 | XP\_013918982.1 | 130.52 | 10 | 0 | 0 | 0 | 0 | 0 | 0 | 0 | 10 |  |  |  |  |  |  |  | 1.0175E5 | 4 | 1 | 0 | 0 | 0 | 0 | 0 | 0 | 0 | 4 | N | 49865 | PREDICTED: tubulin alpha-1C chain [Thamnophis sirtalis] |
| 196 | 442 | JAV48718.1 | 130.52 | 10 | 0 | 0 | 0 | 0 | 0 | 0 | 0 | 10 |  |  |  |  |  |  |  | 1.0175E5 | 4 | 1 | 0 | 0 | 0 | 0 | 0 | 0 | 0 | 4 | N | 49865 | Tubulin alpha 6 [Agkistrodon contortrix contortrix] |
| 101 | 1351 | XP\_029142320.1 | 128.98 | 3 | 0 | 0 | 3 | 3 | 3 | 0 | 0 | 2 |  |  | 2.0399E6 | 3.2051E5 | 1.3197E5 |  |  | 3.4899E5 | 4 | 3 | 0 | 0 | 3 | 4 | 3 | 0 | 0 | 3 | Y | 73450 | LOW QUALITY PROTEIN: zinc metalloproteinase-disintegrin-like HV1 [Protobothrops mucrosquamatus] |
| 169 | 1543 | AXL95289.1 | 125.42 | 4 | 4 | 0 | 0 | 0 | 0 | 0 | 0 | 0 | 1.295E7 |  |  |  |  |  |  |  | 3 | 1 | 9 | 0 | 0 | 0 | 0 | 0 | 0 | 0 | N | 26943 | cysteine-rich secretory protein, partial [Spilotes sulphureus] |
| 111 | 958 | P0C6E8.1 | 125.36 | 3 | 0 | 0 | 0 | 0 | 3 | 0 | 0 | 3 |  |  |  |  | 6.6723E7 |  |  | 0 | 2 | 2 | 0 | 0 | 0 | 0 | 26 | 0 | 0 | 1 | Y | 48204 | RecName: Full=Zinc metalloproteinase/disintegrin; Contains: RecName: Full=Snake venom metalloproteinase graminelysin; Short=SVMP; AltName: Full=Graminelysin I; Contains: RecName: Full=Disintegrin-like; Flags: Precursor |
| 201 | 474 | LAA55683.1 | 124.03 | 10 | 0 | 0 | 0 | 0 | 0 | 0 | 0 | 10 |  |  |  |  |  |  |  | 0 | 4 | 1 | 0 | 0 | 0 | 0 | 0 | 0 | 0 | 4 | N | 49955 | hypothetical protein, partial [Micrurus corallinus] |
| 201 | 475 | JAB53061.1 | 124.03 | 11 | 0 | 0 | 0 | 0 | 0 | 0 | 0 | 11 |  |  |  |  |  |  |  | 0 | 4 | 1 | 0 | 0 | 0 | 0 | 0 | 0 | 0 | 4 | N | 48392 | Tubulin alpha-4A chain [Micrurus fulvius] |
| 201 | 476 | JAI09231.1 | 124.03 | 10 | 0 | 0 | 0 | 0 | 0 | 0 | 0 | 10 |  |  |  |  |  |  |  | 0 | 4 | 1 | 0 | 0 | 0 | 0 | 0 | 0 | 0 | 4 | N | 49988 | Tubulin alpha-4A chain-like protein [Micrurus fulvius] |
| 201 | 477 | LAA55679.1 | 124.03 | 10 | 0 | 0 | 0 | 0 | 0 | 0 | 0 | 10 |  |  |  |  |  |  |  | 0 | 4 | 1 | 0 | 0 | 0 | 0 | 0 | 0 | 0 | 4 | N | 50187 | hypothetical protein [Micrurus corallinus] |
| 95 | 769 | AUS82539.1 | 123.75 | 10 | 0 | 0 | 3 | 10 | 4 | 0 | 0 | 3 |  |  |  | 4.1625E6 | 3.8208E6 |  |  |  | 4 | 1 | 0 | 0 | 9 | 9 | 2 | 0 | 0 | 1 | Y | 28390 | serine endopeptidase [Crotalus scutulatus] |
| 84 | 898 | JAV48393.1 | 123.73 | 13 | 0 | 0 | 8 | 8 | 4 | 0 | 0 | 3 |  |  | 4.8766E6 | 2.1479E7 |  |  |  |  | 4 | 1 | 0 | 0 | 10 | 7 | 8 | 0 | 0 | 1 | Y | 28642 | Cadam10\_SVSP-11 [Crotalus adamanteus] |
| 152 | 808 | ASX97880.1 | 122.38 | 12 | 3 | 0 | 0 | 0 | 12 | 3 | 0 | 0 | 8.3095E5 |  |  |  | 1.053E7 | 1.0165E6 |  |  | 3 | 2 | 1 | 0 | 0 | 0 | 14 | 1 | 0 | 0 | Y | 28515 | venom plasminogen activator precursor 1 [Gloydius intermedius] |
| 200 | 1164 | BAN82028.1 | 122.18 | 13 | 0 | 0 | 6 | 0 | 7 | 0 | 0 | 0 |  |  | 1.5435E6 |  |  |  |  |  | 2 | 1 | 0 | 0 | 1 | 0 | 3 | 0 | 0 | 0 | N | 18376 | serine protease, partial [Protobothrops flavoviridis] |
| 163 | 1096 | AFJ49231.1 | 118.22 | 4 | 0 | 2 | 4 | 2 | 2 | 0 | 0 | 1 |  |  | 1.287E7 |  |  |  |  |  | 3 | 1 | 0 | 4 | 6 | 2 | 1 | 0 | 0 | 2 | N | 67330 | Snake venom metalloproteinase (type III) 1a [Crotalus adamanteus] |
| 163 | 1097 | JAI10389.1 | 118.22 | 4 | 0 | 2 | 4 | 2 | 2 | 0 | 0 | 1 |  |  | 1.287E7 |  |  |  |  |  | 3 | 1 | 0 | 4 | 6 | 2 | 1 | 0 | 0 | 2 | N | 67330 | Metalloproteinase (type III) 1a [Crotalus adamanteus] |
| 163 | 1098 | JAS04685.1 | 118.22 | 4 | 0 | 2 | 4 | 2 | 2 | 0 | 0 | 1 |  |  | 1.287E7 |  |  |  |  |  | 3 | 1 | 0 | 4 | 6 | 2 | 1 | 0 | 0 | 2 | N | 67330 | Metalloproteinase type III 1a [Crotalus adamanteus] |
| 163 | 1099 | JAI10388.1 | 118.22 | 4 | 0 | 2 | 4 | 2 | 2 | 0 | 0 | 1 |  |  | 1.287E7 |  |  |  |  |  | 3 | 1 | 0 | 4 | 6 | 2 | 1 | 0 | 0 | 2 | N | 67329 | Metalloproteinase (type III) 1b [Crotalus adamanteus] |
| 163 | 1100 | JAS04684.1 | 118.22 | 4 | 0 | 2 | 4 | 2 | 2 | 0 | 0 | 1 |  |  | 1.287E7 |  |  |  |  |  | 3 | 1 | 0 | 4 | 6 | 2 | 1 | 0 | 0 | 2 | N | 67329 | Metalloproteinase type III 1b [Crotalus adamanteus] |
| 163 | 1101 | AFJ49232.1 | 118.22 | 4 | 0 | 2 | 4 | 2 | 2 | 0 | 0 | 1 |  |  | 1.287E7 |  |  |  |  |  | 3 | 1 | 0 | 4 | 6 | 2 | 1 | 0 | 0 | 2 | N | 67329 | Snake venom metalloproteinase (type III) 1b [Crotalus adamanteus] |
| 163 | 1102 | JAV48411.1 | 118.22 | 4 | 0 | 2 | 4 | 2 | 2 | 0 | 0 | 1 |  |  | 1.287E7 |  |  |  |  |  | 3 | 1 | 0 | 4 | 6 | 2 | 1 | 0 | 0 | 2 | N | 67329 | Cadam10\_SVMPIII-3 [Crotalus adamanteus] |
| 163 | 1116 | QIV64921.1 | 118.22 | 4 | 0 | 2 | 4 | 2 | 2 | 0 | 0 | 1 |  |  | 1.287E7 |  |  |  |  |  | 3 | 1 | 0 | 4 | 6 | 2 | 1 | 0 | 0 | 2 | N | 67345 | MDC-6d [Crotalus scutulatus] |
| 163 | 1119 | QIV64948.1 | 118.22 | 4 | 0 | 2 | 4 | 2 | 2 | 0 | 0 | 1 |  |  | 1.287E7 |  |  |  |  |  | 3 | 1 | 0 | 4 | 6 | 2 | 1 | 0 | 0 | 2 | N | 67421 | MDC-6d [Crotalus atrox] |
| 178 | 944 | AAP20639.1 | 115.58 | 2 | 0 | 2 | 2 | 2 | 0 | 0 | 0 | 0 |  | 7.3064E5 | 3.2032E6 | 3.8774E6 |  |  |  |  | 2 | 2 | 0 | 1 | 1 | 2 | 0 | 0 | 0 | 0 | N | 54011 | metalloproteinase/disintegrin saxin precursor [Gloydius saxatilis] |
| 178 | 945 | Q7SZE0.1 | 115.58 | 2 | 0 | 2 | 2 | 2 | 0 | 0 | 0 | 0 |  | 7.3064E5 | 3.2032E6 | 3.8774E6 |  |  |  |  | 2 | 2 | 0 | 1 | 1 | 2 | 0 | 0 | 0 | 0 | N | 54011 | RecName: Full=Zinc metalloproteinase/disintegrin; Contains: RecName: Full=Snake venom metalloproteinase saxin; Short=SVMP; Contains: RecName: Full=Disintegrin; Flags: Precursor |
| 142 | 1768 | ACZ34293.1 | 113.70 | 9 | 9 | 9 | 9 | 9 | 9 | 9 | 9 | 9 | 1.7214E6 | 1.3028E6 | 1.0536E6 | 3.517E5 | 6.6503E5 | 1.194E6 | 3.8557E5 | 4.2422E6 | 1 | 1 | 3 | 2 | 1 | 2 | 2 | 6 | 2 | 2 | N | 18146 | TJ-GPIb-bp alpha chain [Protobothrops jerdonii] |
| 142 | 1769 | D1MGU0.1 | 113.70 | 9 | 9 | 9 | 9 | 9 | 9 | 9 | 9 | 9 | 1.7214E6 | 1.3028E6 | 1.0536E6 | 3.517E5 | 6.6503E5 | 1.194E6 | 3.8557E5 | 4.2422E6 | 1 | 1 | 3 | 2 | 1 | 2 | 2 | 6 | 2 | 2 | N | 18146 | RecName: Full=Snaclec jerdonibitin subunit alpha; AltName: Full=TJ-GPIb-bp subunit alpha; Flags: Precursor |
| 223 | 987 | ETE65153.1 | 113.51 | 4 | 0 | 0 | 0 | 0 | 0 | 0 | 0 | 4 |  |  |  |  |  |  |  | 4.2057E4 | 1 | 1 | 0 | 0 | 0 | 0 | 0 | 0 | 0 | 1 | N | 39446 | Malate dehydrogenase, mitochondrial, partial [Ophiophagus hannah] |
| 223 | 1003 | JAG67389.1 | 113.51 | 5 | 0 | 0 | 0 | 0 | 0 | 0 | 0 | 5 |  |  |  |  |  |  |  | 4.2057E4 | 1 | 1 | 0 | 0 | 0 | 0 | 0 | 0 | 0 | 1 | N | 35617 | Malate dehydrogenaseserine-threonine-like protein [Boiga irregularis] |
| 223 | 1004 | XP\_026528223.1 | 113.51 | 5 | 0 | 0 | 0 | 0 | 0 | 0 | 0 | 5 |  |  |  |  |  |  |  | 4.2057E4 | 1 | 1 | 0 | 0 | 0 | 0 | 0 | 0 | 0 | 1 | N | 35621 | malate dehydrogenase, mitochondrial [Notechis scutatus] |
| 223 | 1005 | XP\_026564338.1 | 113.51 | 5 | 0 | 0 | 0 | 0 | 0 | 0 | 0 | 5 |  |  |  |  |  |  |  | 4.2057E4 | 1 | 1 | 0 | 0 | 0 | 0 | 0 | 0 | 0 | 1 | N | 35667 | malate dehydrogenase, mitochondrial [Pseudonaja textilis] |
| 223 | 1006 | JAI09791.1 | 113.51 | 5 | 0 | 0 | 0 | 0 | 0 | 0 | 0 | 5 |  |  |  |  |  |  |  | 4.2057E4 | 1 | 1 | 0 | 0 | 0 | 0 | 0 | 0 | 0 | 1 | N | 35651 | Malate dehydrogenase, mitochondrial-like protein [Micrurus fulvius] |
| 223 | 1007 | JAB53935.1 | 113.51 | 5 | 0 | 0 | 0 | 0 | 0 | 0 | 0 | 5 |  |  |  |  |  |  |  | 4.2057E4 | 1 | 1 | 0 | 0 | 0 | 0 | 0 | 0 | 0 | 1 | N | 35651 | malate dehydrogenase [Micrurus fulvius] |
| 223 | 1010 | XP\_034277264.1 | 113.51 | 5 | 0 | 0 | 0 | 0 | 0 | 0 | 0 | 5 |  |  |  |  |  |  |  | 4.2057E4 | 1 | 1 | 0 | 0 | 0 | 0 | 0 | 0 | 0 | 1 | N | 35858 | malate dehydrogenase, mitochondrial [Pantherophis guttatus] |
| 223 | 1011 | JAC95102.1 | 113.51 | 5 | 0 | 0 | 0 | 0 | 0 | 0 | 0 | 5 |  |  |  |  |  |  |  | 4.2057E4 | 1 | 1 | 0 | 0 | 0 | 0 | 0 | 0 | 0 | 1 | N | 35709 | Malate dehydrogenase protein [Hypsiglena sp. JMG-2014] |
| 223 | 1012 | XP\_007433827.1 | 113.51 | 5 | 0 | 0 | 0 | 0 | 0 | 0 | 0 | 5 |  |  |  |  |  |  |  | 4.2057E4 | 1 | 1 | 0 | 0 | 0 | 0 | 0 | 0 | 0 | 1 | N | 35667 | malate dehydrogenase, mitochondrial [Python bivittatus] |
| 223 | 1015 | AFJ50538.1 | 113.51 | 5 | 0 | 0 | 0 | 0 | 0 | 0 | 0 | 5 |  |  |  |  |  |  |  | 4.2057E4 | 1 | 1 | 0 | 0 | 0 | 0 | 0 | 0 | 0 | 1 | N | 35870 | Malate dehydrogenase, mitochondrial-like [Crotalus adamanteus] |
| 223 | 1016 | JAV49984.1 | 113.51 | 5 | 0 | 0 | 0 | 0 | 0 | 0 | 0 | 5 |  |  |  |  |  |  |  | 4.2057E4 | 1 | 1 | 0 | 0 | 0 | 0 | 0 | 0 | 0 | 1 | N | 35857 | Malate dehydrogenase, mitochondrial-like protein [Agkistrodon contortrix contortrix] |
| 223 | 1017 | JAG46324.1 | 113.51 | 5 | 0 | 0 | 0 | 0 | 0 | 0 | 0 | 5 |  |  |  |  |  |  |  | 4.2057E4 | 1 | 1 | 0 | 0 | 0 | 0 | 0 | 0 | 0 | 1 | N | 35856 | Malate dehydrogenase, mitochondrial-like protein [Crotalus horridus] |
| 223 | 1018 | JAA96492.1 | 113.51 | 5 | 0 | 0 | 0 | 0 | 0 | 0 | 0 | 5 |  |  |  |  |  |  |  | 4.2057E4 | 1 | 1 | 0 | 0 | 0 | 0 | 0 | 0 | 0 | 1 | N | 35856 | Malate dehydrogenase, mitochondrial-like protein [Crotalus horridus] |
| 223 | 1019 | XP\_015674357.1 | 113.51 | 5 | 0 | 0 | 0 | 0 | 0 | 0 | 0 | 5 |  |  |  |  |  |  |  | 4.2057E4 | 1 | 1 | 0 | 0 | 0 | 0 | 0 | 0 | 0 | 1 | N | 35788 | malate dehydrogenase, mitochondrial [Protobothrops mucrosquamatus] |
| 223 | 1020 | JAI12538.1 | 113.51 | 5 | 0 | 0 | 0 | 0 | 0 | 0 | 0 | 5 |  |  |  |  |  |  |  | 4.2057E4 | 1 | 1 | 0 | 0 | 0 | 0 | 0 | 0 | 0 | 1 | N | 35870 | Malate dehydrogenase, mitochondrial-like protein [Crotalus adamanteus] |
| 223 | 1021 | JAG43926.1 | 113.51 | 5 | 0 | 0 | 0 | 0 | 0 | 0 | 0 | 5 |  |  |  |  |  |  |  | 4.2057E4 | 1 | 1 | 0 | 0 | 0 | 0 | 0 | 0 | 0 | 1 | N | 35856 | Malate dehydrogenase, mitochondrial-like protein [Crotalus horridus] |
| 223 | 1023 | XP\_032071513.1 | 113.51 | 5 | 0 | 0 | 0 | 0 | 0 | 0 | 0 | 5 |  |  |  |  |  |  |  | 4.2057E4 | 1 | 1 | 0 | 0 | 0 | 0 | 0 | 0 | 0 | 1 | N | 35651 | malate dehydrogenase, mitochondrial [Thamnophis elegans] |
| 223 | 1024 | XP\_013922804.1 | 113.51 | 5 | 0 | 0 | 0 | 0 | 0 | 0 | 0 | 5 |  |  |  |  |  |  |  | 4.2057E4 | 1 | 1 | 0 | 0 | 0 | 0 | 0 | 0 | 0 | 1 | N | 35651 | PREDICTED: malate dehydrogenase, mitochondrial [Thamnophis sirtalis] |
| 209 | 621 | XP\_015681610.1 | 111.78 | 5 | 0 | 0 | 0 | 0 | 0 | 0 | 0 | 5 |  |  |  |  |  |  |  | 2.4098E5 | 3 | 3 | 0 | 0 | 0 | 0 | 0 | 0 | 0 | 3 | N | 72155 | endoplasmic reticulum chaperone BiP [Protobothrops mucrosquamatus] |
| 209 | 623 | JAV50419.1 | 111.78 | 5 | 0 | 0 | 0 | 0 | 0 | 0 | 0 | 5 |  |  |  |  |  |  |  | 2.4098E5 | 3 | 3 | 0 | 0 | 0 | 0 | 0 | 0 | 0 | 3 | N | 72067 | 78 kDa glucose-regulated protein [Agkistrodon contortrix contortrix] |
| 209 | 624 | JAG44540.1 | 111.78 | 5 | 0 | 0 | 0 | 0 | 0 | 0 | 0 | 5 |  |  |  |  |  |  |  | 2.4098E5 | 3 | 3 | 0 | 0 | 0 | 0 | 0 | 0 | 0 | 3 | N | 72105 | 78 kDa glucose-regulated protein precursor [Crotalus horridus] |
| 209 | 625 | JAI13107.1 | 111.78 | 5 | 0 | 0 | 0 | 0 | 0 | 0 | 0 | 5 |  |  |  |  |  |  |  | 2.4098E5 | 3 | 3 | 0 | 0 | 0 | 0 | 0 | 0 | 0 | 3 | N | 72105 | 78 kDa glucose-regulated protein [Crotalus adamanteus] |
| 209 | 626 | JAG46692.1 | 111.78 | 5 | 0 | 0 | 0 | 0 | 0 | 0 | 0 | 5 |  |  |  |  |  |  |  | 2.4098E5 | 3 | 3 | 0 | 0 | 0 | 0 | 0 | 0 | 0 | 3 | N | 72105 | 78 kDa glucose-regulated protein [Crotalus horridus] |
| 209 | 627 | JAA96909.1 | 111.78 | 5 | 0 | 0 | 0 | 0 | 0 | 0 | 0 | 5 |  |  |  |  |  |  |  | 2.4098E5 | 3 | 3 | 0 | 0 | 0 | 0 | 0 | 0 | 0 | 3 | N | 72105 | 78 kDa glucose-regulated protein [Crotalus horridus] |
| 209 | 628 | AFJ50327.1 | 111.78 | 5 | 0 | 0 | 0 | 0 | 0 | 0 | 0 | 5 |  |  |  |  |  |  |  | 2.4098E5 | 3 | 3 | 0 | 0 | 0 | 0 | 0 | 0 | 0 | 3 | N | 72105 | Heat shock protein 5 [Crotalus adamanteus] |
| 209 | 629 | AFJ50239.1 | 111.78 | 5 | 0 | 0 | 0 | 0 | 0 | 0 | 0 | 5 |  |  |  |  |  |  |  | 2.4098E5 | 3 | 3 | 0 | 0 | 0 | 0 | 0 | 0 | 0 | 3 | N | 75480 | 78 kDa glucose-regulated protein precursor [Crotalus adamanteus] |
| 209 | 630 | XP\_026542482.1 | 111.78 | 5 | 0 | 0 | 0 | 0 | 0 | 0 | 0 | 5 |  |  |  |  |  |  |  | 2.4098E5 | 3 | 3 | 0 | 0 | 0 | 0 | 0 | 0 | 0 | 3 | N | 72286 | endoplasmic reticulum chaperone BiP [Notechis scutatus] |
| 209 | 631 | XP\_034262421.1 | 111.78 | 6 | 0 | 0 | 0 | 0 | 0 | 0 | 0 | 6 |  |  |  |  |  |  |  | 2.4098E5 | 3 | 3 | 0 | 0 | 0 | 0 | 0 | 0 | 0 | 3 | N | 68299 | endoplasmic reticulum chaperone BiP, partial [Pantherophis guttatus] |
| 209 | 632 | XP\_025022112.1 | 111.78 | 5 | 0 | 0 | 0 | 0 | 0 | 0 | 0 | 5 |  |  |  |  |  |  |  | 2.4098E5 | 3 | 3 | 0 | 0 | 0 | 0 | 0 | 0 | 0 | 3 | N | 71864 | endoplasmic reticulum chaperone BiP [Python bivittatus] |
| 209 | 633 | JAG67806.1 | 111.78 | 5 | 0 | 0 | 0 | 0 | 0 | 0 | 0 | 5 |  |  |  |  |  |  |  | 2.4098E5 | 3 | 3 | 0 | 0 | 0 | 0 | 0 | 0 | 0 | 3 | N | 71941 | 78 kDa glucose-regulated protein [Boiga irregularis] |
| 209 | 634 | XP\_032088936.1 | 111.78 | 5 | 0 | 0 | 0 | 0 | 0 | 0 | 0 | 5 |  |  |  |  |  |  |  | 2.4098E5 | 3 | 3 | 0 | 0 | 0 | 0 | 0 | 0 | 0 | 3 | N | 72006 | endoplasmic reticulum chaperone BiP [Thamnophis elegans] |
| 209 | 635 | XP\_013910585.1 | 111.78 | 5 | 0 | 0 | 0 | 0 | 0 | 0 | 0 | 5 |  |  |  |  |  |  |  | 2.4098E5 | 3 | 3 | 0 | 0 | 0 | 0 | 0 | 0 | 0 | 3 | N | 72006 | PREDICTED: 78 kDa glucose-regulated protein [Thamnophis sirtalis] |
| 209 | 636 | XP\_026568326.1 | 111.78 | 5 | 0 | 0 | 0 | 0 | 0 | 0 | 0 | 5 |  |  |  |  |  |  |  | 2.4098E5 | 3 | 3 | 0 | 0 | 0 | 0 | 0 | 0 | 0 | 3 | N | 72187 | endoplasmic reticulum chaperone BiP [Pseudonaja textilis] |
| 209 | 637 | JAC95528.1 | 111.78 | 5 | 0 | 0 | 0 | 0 | 0 | 0 | 0 | 5 |  |  |  |  |  |  |  | 2.4098E5 | 3 | 3 | 0 | 0 | 0 | 0 | 0 | 0 | 0 | 3 | N | 72299 | 78 kDa glucose-regulated protein-like [Hypsiglena sp. JMG-2014] |
| 209 | 642 | JAB54174.1 | 111.78 | 5 | 0 | 0 | 0 | 0 | 0 | 0 | 0 | 5 |  |  |  |  |  |  |  | 2.4098E5 | 3 | 3 | 0 | 0 | 0 | 0 | 0 | 0 | 0 | 3 | N | 72106 | glucose-regulated protein [Micrurus fulvius] |
| 26 | 1226 | AHJ09587.1 | 107.10 | 16 | 0 | 0 | 7 | 0 | 7 | 16 | 7 | 7 |  |  | 9.0994E6 |  | 0 | 1.0494E8 | 4.4489E7 | 6.4224E6 | 2 | 2 | 0 | 0 | 2 | 0 | 1 | 84 | 41 | 5 | Y | 15070 | phospholipase A2 [Trimeresurus cardamomensis] |
| 26 | 1762 | AHJ09589.1 | 107.10 | 16 | 0 | 0 | 7 | 0 | 7 | 16 | 7 | 7 |  |  | 9.0994E6 |  | 0 | 1.0494E8 | 4.4489E7 | 6.4224E6 | 2 | 2 | 0 | 0 | 2 | 0 | 1 | 84 | 41 | 5 | Y | 15864 | phospholipase A2 [Trimeresurus cardamomensis] |
| 26 | 1763 | AHJ09585.1 | 107.10 | 16 | 0 | 0 | 7 | 0 | 7 | 16 | 7 | 7 |  |  | 9.0994E6 |  | 0 | 1.0494E8 | 4.4489E7 | 6.4224E6 | 2 | 2 | 0 | 0 | 2 | 0 | 1 | 84 | 41 | 5 | Y | 15864 | phospholipase A2 [Trimeresurus cardamomensis] |
| 26 | 1764 | AHJ09594.1 | 107.10 | 16 | 0 | 0 | 7 | 0 | 7 | 16 | 7 | 7 |  |  | 9.0994E6 |  | 0 | 1.0494E8 | 4.4489E7 | 6.4224E6 | 2 | 2 | 0 | 0 | 2 | 0 | 1 | 84 | 41 | 5 | Y | 15864 | phospholipase A2 [Trimeresurus cardamomensis] |
| 26 | 1765 | AHJ09591.1 | 107.10 | 16 | 0 | 0 | 7 | 0 | 7 | 16 | 7 | 7 |  |  | 9.0994E6 |  | 0 | 1.0494E8 | 4.4489E7 | 6.4224E6 | 2 | 2 | 0 | 0 | 2 | 0 | 1 | 84 | 41 | 5 | Y | 15804 | phospholipase A2 [Trimeresurus cardamomensis] |
| 175 | 733 | XP\_015685587.1 | 105.33 | 10 | 0 | 7 | 5 | 3 | 0 | 0 | 0 | 7 |  | 2.5255E5 | 1.1101E6 |  |  |  |  | 1.8092E5 | 3 | 2 | 0 | 3 | 3 | 1 | 0 | 0 | 0 | 3 | N | 39726 | zinc metalloproteinase/disintegrin-like HR1a [Protobothrops mucrosquamatus] |
| 143 | 1123 | P0DJL3.1 | 104.56 | 15 | 0 | 8 | 7 | 0 | 0 | 8 | 15 | 8 |  | 5.9765E5 | 1.3707E6 |  |  | 2.2921E6 | 8.8993E6 | 1.1947E6 | 2 | 2 | 0 | 1 | 1 | 0 | 0 | 2 | 9 | 1 | N | 14498 | RecName: Full=Snaclec purpureotin subunit beta |
| 143 | 1124 | P81116.2 | 104.56 | 13 | 0 | 7 | 6 | 0 | 0 | 7 | 13 | 7 |  | 5.9765E5 | 1.3707E6 |  |  | 2.2921E6 | 8.8993E6 | 1.1947E6 | 2 | 2 | 0 | 1 | 1 | 0 | 0 | 2 | 9 | 1 | N | 16871 | RecName: Full=Snaclec alboaggregin-B subunit beta; Short=AL-B subunit beta; AltName: Full=25-kDa alboaggregin; Flags: Precursor |
| 143 | 1125 | ABS12077.1 | 104.56 | 13 | 0 | 7 | 6 | 0 | 0 | 7 | 13 | 7 |  | 5.9765E5 | 1.3707E6 |  |  | 2.2921E6 | 8.8993E6 | 1.1947E6 | 2 | 2 | 0 | 1 | 1 | 0 | 0 | 2 | 9 | 1 | N | 16871 | alboaggregin B beta subunit [Trimeresurus albolabris] |
| 216 | 811 | AFJ51037.1 | 102.58 | 4 | 0 | 0 | 0 | 0 | 0 | 0 | 0 | 4 |  |  |  |  |  |  |  | 1.7985E5 | 1 | 1 | 0 | 0 | 0 | 0 | 0 | 0 | 0 | 1 | N | 29453 | Proteasome subunit alpha type-4 [Crotalus adamanteus] |
| 216 | 812 | JAA96034.1 | 102.58 | 4 | 0 | 0 | 0 | 0 | 0 | 0 | 0 | 4 |  |  |  |  |  |  |  | 1.7985E5 | 1 | 1 | 0 | 0 | 0 | 0 | 0 | 0 | 0 | 1 | N | 29453 | Proteasome subunit alpha type-4 protein [Crotalus horridus] |
| 216 | 813 | JAV49554.1 | 102.58 | 4 | 0 | 0 | 0 | 0 | 0 | 0 | 0 | 4 |  |  |  |  |  |  |  | 1.7985E5 | 1 | 1 | 0 | 0 | 0 | 0 | 0 | 0 | 0 | 1 | N | 29453 | Proteasome subunit alpha type-4 protein [Agkistrodon contortrix contortrix] |
| 216 | 814 | JAI11929.1 | 102.58 | 4 | 0 | 0 | 0 | 0 | 0 | 0 | 0 | 4 |  |  |  |  |  |  |  | 1.7985E5 | 1 | 1 | 0 | 0 | 0 | 0 | 0 | 0 | 0 | 1 | N | 29453 | Proteasome subunit alpha type-4 protein [Crotalus adamanteus] |
| 212 | 645 | XP\_026544503.1 | 102.01 | 6 | 0 | 0 | 0 | 0 | 0 | 0 | 0 | 6 |  |  |  |  |  |  |  | 2.6262E5 | 2 | 2 | 0 | 0 | 0 | 0 | 0 | 0 | 0 | 2 | N | 47550 | alpha-enolase [Notechis scutatus] |
| 212 | 647 | XP\_032087801.1 | 102.01 | 6 | 0 | 0 | 0 | 0 | 0 | 0 | 0 | 6 |  |  |  |  |  |  |  | 2.6262E5 | 2 | 2 | 0 | 0 | 0 | 0 | 0 | 0 | 0 | 2 | N | 47512 | alpha-enolase [Thamnophis elegans] |
| 212 | 648 | JAG68757.1 | 102.01 | 6 | 0 | 0 | 0 | 0 | 0 | 0 | 0 | 6 |  |  |  |  |  |  |  | 2.6262E5 | 2 | 2 | 0 | 0 | 0 | 0 | 0 | 0 | 0 | 2 | N | 47546 | Alpha-enolase [Boiga irregularis] |
| 212 | 649 | XP\_034282306.1 | 102.01 | 6 | 0 | 0 | 0 | 0 | 0 | 0 | 0 | 6 |  |  |  |  |  |  |  | 2.6262E5 | 2 | 2 | 0 | 0 | 0 | 0 | 0 | 0 | 0 | 2 | N | 47546 | alpha-enolase [Pantherophis guttatus] |
| 212 | 650 | XP\_034282305.1 | 102.01 | 6 | 0 | 0 | 0 | 0 | 0 | 0 | 0 | 6 |  |  |  |  |  |  |  | 2.6262E5 | 2 | 2 | 0 | 0 | 0 | 0 | 0 | 0 | 0 | 2 | N | 47546 | alpha-enolase [Pantherophis guttatus] |
| 212 | 651 | XP\_034282304.1 | 102.01 | 6 | 0 | 0 | 0 | 0 | 0 | 0 | 0 | 6 |  |  |  |  |  |  |  | 2.6262E5 | 2 | 2 | 0 | 0 | 0 | 0 | 0 | 0 | 0 | 2 | N | 47546 | alpha-enolase [Pantherophis guttatus] |
| 212 | 652 | JAC96284.1 | 102.01 | 6 | 0 | 0 | 0 | 0 | 0 | 0 | 0 | 6 |  |  |  |  |  |  |  | 2.6262E5 | 2 | 2 | 0 | 0 | 0 | 0 | 0 | 0 | 0 | 2 | N | 47493 | alpha-enolase [Hypsiglena sp. JMG-2014] |
| 221 | 983 | XP\_015687424.1 | 101.80 | 1 | 0 | 0 | 1 | 0 | 0 | 0 | 0 | 0 |  |  | 5.7064E5 |  |  |  |  |  | 1 | 1 | 0 | 0 | 1 | 0 | 0 | 0 | 0 | 0 | N | 106458 | endoplasmic reticulum aminopeptidase 1 [Protobothrops mucrosquamatus] |
| 221 | 984 | JAI13418.1 | 101.80 | 1 | 0 | 0 | 1 | 0 | 0 | 0 | 0 | 0 |  |  | 5.7064E5 |  |  |  |  |  | 1 | 1 | 0 | 0 | 1 | 0 | 0 | 0 | 0 | 0 | N | 107364 | endoplasmic reticulum aminopeptidase 1-like protein [Crotalus adamanteus] |
| 238 | 1421 | LAB29750.1 | 101.58 | 6 | 0 | 0 | 0 | 0 | 0 | 0 | 0 | 6 |  |  |  |  |  |  |  | 1.3088E5 | 2 | 2 | 0 | 0 | 0 | 0 | 0 | 0 | 0 | 2 | N | 44334 | hypothetical protein, partial [Micrurus spixii] |
| 238 | 1422 | LAB29755.1 | 101.58 | 6 | 0 | 0 | 0 | 0 | 0 | 0 | 0 | 6 |  |  |  |  |  |  |  | 1.3088E5 | 2 | 2 | 0 | 0 | 0 | 0 | 0 | 0 | 0 | 2 | N | 47042 | hypothetical protein, partial [Micrurus spixii] |
| 238 | 1423 | XP\_025032359.1 | 101.58 | 6 | 0 | 0 | 0 | 0 | 0 | 0 | 0 | 6 |  |  |  |  |  |  |  | 1.3088E5 | 2 | 2 | 0 | 0 | 0 | 0 | 0 | 0 | 0 | 2 | N | 42156 | eukaryotic initiation factor 4A-I [Python bivittatus] |
| 238 | 1424 | JAB54310.1 | 101.58 | 6 | 0 | 0 | 0 | 0 | 0 | 0 | 0 | 6 |  |  |  |  |  |  |  | 1.3088E5 | 2 | 2 | 0 | 0 | 0 | 0 | 0 | 0 | 0 | 2 | N | 46097 | eukaryotic initiation factor 4A-1 [Micrurus fulvius] |
| 238 | 1425 | JAG66237.1 | 101.58 | 6 | 0 | 0 | 0 | 0 | 0 | 0 | 0 | 6 |  |  |  |  |  |  |  | 1.3088E5 | 2 | 2 | 0 | 0 | 0 | 0 | 0 | 0 | 0 | 2 | N | 46095 | eukaryotic initiation factor 4A-I [Boiga irregularis] |
| 238 | 1426 | XP\_034263139.1 | 101.58 | 6 | 0 | 0 | 0 | 0 | 0 | 0 | 0 | 6 |  |  |  |  |  |  |  | 1.3088E5 | 2 | 2 | 0 | 0 | 0 | 0 | 0 | 0 | 0 | 2 | N | 46111 | eukaryotic initiation factor 4A-I [Pantherophis guttatus] |
| 238 | 1445 | JAG46857.1 | 101.58 | 6 | 0 | 0 | 0 | 0 | 0 | 0 | 0 | 6 |  |  |  |  |  |  |  | 1.3088E5 | 2 | 2 | 0 | 0 | 0 | 0 | 0 | 0 | 0 | 2 | N | 46286 | eukaryotic initiation factor 4AII [Crotalus horridus] |
| 238 | 1446 | XP\_015677966.1 | 101.58 | 6 | 0 | 0 | 0 | 0 | 0 | 0 | 0 | 6 |  |  |  |  |  |  |  | 1.3088E5 | 2 | 2 | 0 | 0 | 0 | 0 | 0 | 0 | 0 | 2 | N | 46286 | eukaryotic initiation factor 4A-II isoform X1 [Protobothrops mucrosquamatus] |
| 238 | 1454 | XP\_032081049.1 | 101.58 | 6 | 0 | 0 | 0 | 0 | 0 | 0 | 0 | 6 |  |  |  |  |  |  |  | 1.3088E5 | 2 | 2 | 0 | 0 | 0 | 0 | 0 | 0 | 0 | 2 | N | 46286 | eukaryotic initiation factor 4A-II isoform X1 [Thamnophis elegans] |
| 238 | 1455 | XP\_026539620.1 | 101.58 | 6 | 0 | 0 | 0 | 0 | 0 | 0 | 0 | 6 |  |  |  |  |  |  |  | 1.3088E5 | 2 | 2 | 0 | 0 | 0 | 0 | 0 | 0 | 0 | 2 | N | 46286 | eukaryotic initiation factor 4A-II [Notechis scutatus] |
| 238 | 1456 | XP\_026561483.1 | 101.58 | 6 | 0 | 0 | 0 | 0 | 0 | 0 | 0 | 6 |  |  |  |  |  |  |  | 1.3088E5 | 2 | 2 | 0 | 0 | 0 | 0 | 0 | 0 | 0 | 2 | N | 46286 | eukaryotic initiation factor 4A-II [Pseudonaja textilis] |
| 238 | 1457 | XP\_013923864.1 | 101.58 | 6 | 0 | 0 | 0 | 0 | 0 | 0 | 0 | 6 |  |  |  |  |  |  |  | 1.3088E5 | 2 | 2 | 0 | 0 | 0 | 0 | 0 | 0 | 0 | 2 | N | 46286 | PREDICTED: eukaryotic initiation factor 4A-II isoform X1 [Thamnophis sirtalis] |
| 238 | 1458 | XP\_034294778.1 | 101.58 | 6 | 0 | 0 | 0 | 0 | 0 | 0 | 0 | 6 |  |  |  |  |  |  |  | 1.3088E5 | 2 | 2 | 0 | 0 | 0 | 0 | 0 | 0 | 0 | 2 | N | 46286 | eukaryotic initiation factor 4A-II [Pantherophis guttatus] |
| 238 | 1459 | XP\_007442965.1 | 101.58 | 6 | 0 | 0 | 0 | 0 | 0 | 0 | 0 | 6 |  |  |  |  |  |  |  | 1.3088E5 | 2 | 2 | 0 | 0 | 0 | 0 | 0 | 0 | 0 | 2 | N | 46286 | eukaryotic initiation factor 4A-II [Python bivittatus] |
| 238 | 1460 | JAB54309.1 | 101.58 | 7 | 0 | 0 | 0 | 0 | 0 | 0 | 0 | 7 |  |  |  |  |  |  |  | 1.3088E5 | 2 | 2 | 0 | 0 | 0 | 0 | 0 | 0 | 0 | 2 | N | 41204 | eukaryotic initiation factor 4A-2 [Micrurus fulvius] |
| 238 | 1461 | JAI12965.1 | 101.58 | 7 | 0 | 0 | 0 | 0 | 0 | 0 | 0 | 7 |  |  |  |  |  |  |  | 1.3088E5 | 2 | 2 | 0 | 0 | 0 | 0 | 0 | 0 | 0 | 2 | N | 41188 | eukaryotic initiation factor 4A-II-like [Crotalus adamanteus] |
| 238 | 1462 | XP\_015677967.1 | 101.58 | 7 | 0 | 0 | 0 | 0 | 0 | 0 | 0 | 7 |  |  |  |  |  |  |  | 1.3088E5 | 2 | 2 | 0 | 0 | 0 | 0 | 0 | 0 | 0 | 2 | N | 41188 | eukaryotic initiation factor 4A-II isoform X2 [Protobothrops mucrosquamatus] |
| 238 | 1466 | XP\_032081051.1 | 101.58 | 7 | 0 | 0 | 0 | 0 | 0 | 0 | 0 | 7 |  |  |  |  |  |  |  | 1.3088E5 | 2 | 2 | 0 | 0 | 0 | 0 | 0 | 0 | 0 | 2 | N | 41174 | eukaryotic initiation factor 4A-II isoform X3 [Thamnophis elegans] |
| 238 | 1467 | XP\_013923865.1 | 101.58 | 7 | 0 | 0 | 0 | 0 | 0 | 0 | 0 | 7 |  |  |  |  |  |  |  | 1.3088E5 | 2 | 2 | 0 | 0 | 0 | 0 | 0 | 0 | 0 | 2 | N | 41174 | PREDICTED: eukaryotic initiation factor 4A-II isoform X2 [Thamnophis sirtalis] |
| 238 | 1473 | ETE63526.1 | 101.58 | 7 | 0 | 0 | 0 | 0 | 0 | 0 | 0 | 7 |  |  |  |  |  |  |  | 1.3088E5 | 2 | 2 | 0 | 0 | 0 | 0 | 0 | 0 | 0 | 2 | N | 39213 | Eukaryotic initiation factor 4A-II [Ophiophagus hannah] |
| 171 | 1042 | JAS04545.1 | 96.26 | 12 | 0 | 0 | 0 | 0 | 0 | 0 | 0 | 12 |  |  |  |  |  |  |  | 1.0855E6 | 2 | 2 | 0 | 0 | 0 | 0 | 0 | 0 | 0 | 3 | N | 18656 | C-type lectin 2 [Agkistrodon piscivorus conanti] |
| 224 | 1041 | XP\_015675298.1 | 94.09 | 3 | 0 | 0 | 0 | 3 | 0 | 0 | 0 | 0 |  |  |  | 1.7583E6 |  |  |  |  | 1 | 1 | 0 | 0 | 0 | 1 | 0 | 0 | 0 | 0 | N | 53899 | carboxypeptidase E [Protobothrops mucrosquamatus] |
| 227 | 1058 | XP\_015745242.1 | 89.49 | 1 | 0 | 0 | 0 | 0 | 0 | 0 | 0 | 1 |  |  |  |  |  |  |  | 1.3303E4 | 1 | 1 | 0 | 0 | 0 | 0 | 0 | 0 | 0 | 1 | N | 81343 | calpain-1 catalytic subunit [Python bivittatus] |
| 176 | 1490 | ETE56723.1 | 88.20 | 9 | 0 | 0 | 0 | 9 | 9 | 0 | 0 | 0 |  |  |  | 9.9316E5 | 4.294E6 |  |  |  | 1 | 1 | 0 | 0 | 0 | 1 | 3 | 0 | 0 | 0 | N | 18083 | Endonuclease domain-containing 1 protein [Ophiophagus hannah] |
| 176 | 1500 | XP\_032076462.1 | 88.20 | 5 | 0 | 0 | 0 | 5 | 5 | 0 | 0 | 0 |  |  |  | 9.9316E5 | 4.294E6 |  |  |  | 1 | 1 | 0 | 0 | 0 | 1 | 3 | 0 | 0 | 0 | N | 31282 | endonuclease domain-containing 1 protein-like isoform X2 [Thamnophis elegans] |
| 176 | 1502 | XP\_026546198.1 | 88.20 | 5 | 0 | 0 | 0 | 5 | 5 | 0 | 0 | 0 |  |  |  | 9.9316E5 | 4.294E6 |  |  |  | 1 | 1 | 0 | 0 | 0 | 1 | 3 | 0 | 0 | 0 | N | 31377 | endonuclease domain-containing 1 protein-like isoform X2 [Notechis scutatus] |
| 176 | 1503 | XP\_026546199.1 | 88.20 | 5 | 0 | 0 | 0 | 5 | 5 | 0 | 0 | 0 |  |  |  | 9.9316E5 | 4.294E6 |  |  |  | 1 | 1 | 0 | 0 | 0 | 1 | 3 | 0 | 0 | 0 | N | 31377 | endonuclease domain-containing 1 protein-like isoform X2 [Notechis scutatus] |
| 176 | 1504 | XP\_026546197.1 | 88.20 | 5 | 0 | 0 | 0 | 5 | 5 | 0 | 0 | 0 |  |  |  | 9.9316E5 | 4.294E6 |  |  |  | 1 | 1 | 0 | 0 | 0 | 1 | 3 | 0 | 0 | 0 | N | 31377 | endonuclease domain-containing 1 protein-like isoform X2 [Notechis scutatus] |
| 176 | 1505 | XP\_026579206.1 | 88.20 | 5 | 0 | 0 | 0 | 5 | 5 | 0 | 0 | 0 |  |  |  | 9.9316E5 | 4.294E6 |  |  |  | 1 | 1 | 0 | 0 | 0 | 1 | 3 | 0 | 0 | 0 | N | 31175 | endonuclease domain-containing 1 protein-like [Pseudonaja textilis] |
| 176 | 1506 | XP\_026579205.1 | 88.20 | 5 | 0 | 0 | 0 | 5 | 5 | 0 | 0 | 0 |  |  |  | 9.9316E5 | 4.294E6 |  |  |  | 1 | 1 | 0 | 0 | 0 | 1 | 3 | 0 | 0 | 0 | N | 31175 | endonuclease domain-containing 1 protein-like [Pseudonaja textilis] |
| 176 | 1507 | XP\_026579207.1 | 88.20 | 5 | 0 | 0 | 0 | 5 | 5 | 0 | 0 | 0 |  |  |  | 9.9316E5 | 4.294E6 |  |  |  | 1 | 1 | 0 | 0 | 0 | 1 | 3 | 0 | 0 | 0 | N | 31175 | endonuclease domain-containing 1 protein-like [Pseudonaja textilis] |
| 176 | 1508 | XP\_032076461.1 | 88.20 | 5 | 0 | 0 | 0 | 5 | 5 | 0 | 0 | 0 |  |  |  | 9.9316E5 | 4.294E6 |  |  |  | 1 | 1 | 0 | 0 | 0 | 1 | 3 | 0 | 0 | 0 | N | 34799 | endonuclease domain-containing 1 protein-like isoform X1 [Thamnophis elegans] |
| 176 | 1509 | XP\_026546196.1 | 88.20 | 5 | 0 | 0 | 0 | 5 | 5 | 0 | 0 | 0 |  |  |  | 9.9316E5 | 4.294E6 |  |  |  | 1 | 1 | 0 | 0 | 0 | 1 | 3 | 0 | 0 | 0 | N | 33047 | endonuclease domain-containing 1 protein-like isoform X1 [Notechis scutatus] |
| 138 | 1992 | LAA93895.1 | 86.47 | 5 | 0 | 5 | 5 | 5 | 5 | 5 | 5 | 5 |  | 8.8509E5 | 6.3988E6 | 1.6611E6 | 2.3733E6 | 3.7532E6 | 9.5962E5 | 1.4812E7 | 1 | 1 | 0 | 2 | 2 | 1 | 3 | 6 | 1 | 8 | N | 19716 | hypothetical protein, partial [Micrurus lemniscatus lemniscatus] |
| 204 | 1128 | XP\_032085019.1 | 84.02 | 4 | 0 | 0 | 0 | 0 | 0 | 0 | 0 | 4 |  |  |  |  |  |  |  | 1.5034E6 | 1 | 1 | 0 | 0 | 0 | 0 | 0 | 0 | 0 | 1 | N | 51446 | polyubiquitin-C [Thamnophis elegans] |
| 242 | 1204 | JAI09617.1 | 80.17 | 5 | 0 | 0 | 0 | 0 | 0 | 0 | 0 | 5 |  |  |  |  |  |  |  | 1.3568E5 | 1 | 1 | 0 | 0 | 0 | 0 | 0 | 0 | 0 | 1 | N | 26469 | Proteasome subunit alpha type-5 protein [Micrurus fulvius] |
| 222 | 1691 | XP\_007433195.1 | 79.90 | 4 | 0 | 0 | 0 | 0 | 0 | 0 | 0 | 4 |  |  |  |  |  |  |  | 1.8537E5 | 1 | 1 | 0 | 0 | 0 | 0 | 0 | 0 | 0 | 1 | N | 32697 | tropomyosin alpha-1 chain isoform X2 [Python bivittatus] |
| 222 | 1698 | XP\_025025541.1 | 79.90 | 4 | 0 | 0 | 0 | 0 | 0 | 0 | 0 | 4 |  |  |  |  |  |  |  | 1.8537E5 | 1 | 1 | 0 | 0 | 0 | 0 | 0 | 0 | 0 | 1 | N | 28800 | tropomyosin alpha-4 chain isoform X4 [Python bivittatus] |
| 205 | 1560 | XP\_013914127.1 | 78.48 | 5 | 0 | 0 | 0 | 0 | 0 | 0 | 0 | 5 |  |  |  |  |  |  |  | 2.6626E5 | 1 | 1 | 0 | 0 | 0 | 0 | 0 | 0 | 0 | 1 | N | 23326 | PREDICTED: heat shock protein beta-1 [Thamnophis sirtalis] |
| 205 | 1561 | XP\_032072257.1 | 78.48 | 5 | 0 | 0 | 0 | 0 | 0 | 0 | 0 | 5 |  |  |  |  |  |  |  | 2.6626E5 | 1 | 1 | 0 | 0 | 0 | 0 | 0 | 0 | 0 | 1 | N | 23326 | heat shock protein beta-1 [Thamnophis elegans] |
| 192 | 1632 | BAP39929.1 | 78.33 | 8 | 0 | 0 | 0 | 0 | 0 | 0 | 0 | 8 |  |  |  |  |  |  |  | 0 | 1 | 1 | 0 | 0 | 0 | 0 | 0 | 0 | 0 | 1 | N | 21332 | C-type lectin B subunit, partial [Protobothrops elegans] |
| 192 | 1644 | XP\_015685588.1 | 78.33 | 9 | 0 | 0 | 0 | 0 | 0 | 0 | 0 | 9 |  |  |  |  |  |  |  | 0 | 1 | 1 | 0 | 0 | 0 | 0 | 0 | 0 | 0 | 1 | N | 18348 | snaclec jerdonibitin subunit alpha [Protobothrops mucrosquamatus] |
| 139 | 1565 | Q2YHJ5.1 | 77.93 | 13 | 0 | 0 | 6 | 0 | 0 | 13 | 6 | 6 |  |  | 1.8518E6 |  |  | 2.7028E7 | 7.6191E6 | 2.8034E6 | 2 | 2 | 0 | 0 | 2 | 0 | 0 | 12 | 7 | 1 | N | 13943 | RecName: Full=Acidic phospholipase A2 Tpu-E6b; Short=svPLA2; AltName: Full=Phosphatidylcholine 2-acylhydrolase |
| 234 | 905 | XP\_007437998.1 | 76.60 | 2 | 0 | 0 | 0 | 0 | 0 | 0 | 0 | 2 |  |  |  |  |  |  |  | 1.4855E5 | 2 | 2 | 0 | 0 | 0 | 0 | 0 | 0 | 0 | 2 | N | 95239 | elongation factor 2 [Python bivittatus] |
| 234 | 918 | XP\_013920852.1 | 76.60 | 2 | 0 | 0 | 0 | 0 | 0 | 0 | 0 | 2 |  |  |  |  |  |  |  | 1.4855E5 | 2 | 2 | 0 | 0 | 0 | 0 | 0 | 0 | 0 | 2 | N | 95399 | PREDICTED: elongation factor 2 [Thamnophis sirtalis] |
| 234 | 919 | XP\_032070431.1 | 76.60 | 2 | 0 | 0 | 0 | 0 | 0 | 0 | 0 | 2 |  |  |  |  |  |  |  | 1.4855E5 | 2 | 2 | 0 | 0 | 0 | 0 | 0 | 0 | 0 | 2 | N | 97430 | elongation factor 2 [Thamnophis elegans] |
| 234 | 920 | JAG46919.1 | 76.60 | 2 | 0 | 0 | 0 | 0 | 0 | 0 | 0 | 2 |  |  |  |  |  |  |  | 1.4855E5 | 2 | 2 | 0 | 0 | 0 | 0 | 0 | 0 | 0 | 2 | N | 95397 | Elongation factor 2 [Crotalus horridus] |
| 234 | 921 | JAI13483.1 | 76.60 | 2 | 0 | 0 | 0 | 0 | 0 | 0 | 0 | 2 |  |  |  |  |  |  |  | 1.4855E5 | 2 | 2 | 0 | 0 | 0 | 0 | 0 | 0 | 0 | 2 | N | 95410 | Elongation factor 2 [Crotalus adamanteus] |
| 234 | 922 | JAA97181.1 | 76.60 | 2 | 0 | 0 | 0 | 0 | 0 | 0 | 0 | 2 |  |  |  |  |  |  |  | 1.4855E5 | 2 | 2 | 0 | 0 | 0 | 0 | 0 | 0 | 0 | 2 | N | 95397 | Elongation factor 2 [Crotalus horridus] |
| 234 | 923 | JAV50654.1 | 76.60 | 2 | 0 | 0 | 0 | 0 | 0 | 0 | 0 | 2 |  |  |  |  |  |  |  | 1.4855E5 | 2 | 2 | 0 | 0 | 0 | 0 | 0 | 0 | 0 | 2 | N | 95424 | elongation factor 2 [Agkistrodon contortrix contortrix] |
| 234 | 924 | AFJ49955.1 | 76.60 | 2 | 0 | 0 | 0 | 0 | 0 | 0 | 0 | 2 |  |  |  |  |  |  |  | 1.4855E5 | 2 | 2 | 0 | 0 | 0 | 0 | 0 | 0 | 0 | 2 | N | 95410 | Eukaryotic translation elongation factor 2 [Crotalus adamanteus] |
| 234 | 925 | JAB54320.1 | 76.60 | 2 | 0 | 0 | 0 | 0 | 0 | 0 | 0 | 2 |  |  |  |  |  |  |  | 1.4855E5 | 2 | 2 | 0 | 0 | 0 | 0 | 0 | 0 | 0 | 2 | N | 95410 | Eukaryotic translation elongation factor 2 [Micrurus fulvius] |
| 234 | 926 | JAG44954.1 | 76.60 | 2 | 0 | 0 | 0 | 0 | 0 | 0 | 0 | 2 |  |  |  |  |  |  |  | 1.4855E5 | 2 | 2 | 0 | 0 | 0 | 0 | 0 | 0 | 0 | 2 | N | 95397 | Elongation factor 2 [Crotalus horridus] |
| 234 | 927 | JAC95695.1 | 76.60 | 2 | 0 | 0 | 0 | 0 | 0 | 0 | 0 | 2 |  |  |  |  |  |  |  | 1.4855E5 | 2 | 2 | 0 | 0 | 0 | 0 | 0 | 0 | 0 | 2 | N | 95327 | Elongation factor 2 [Hypsiglena sp. JMG-2014] |
| 234 | 928 | XP\_026543467.1 | 76.60 | 2 | 0 | 0 | 0 | 0 | 0 | 0 | 0 | 2 |  |  |  |  |  |  |  | 1.4855E5 | 2 | 2 | 0 | 0 | 0 | 0 | 0 | 0 | 0 | 2 | N | 95312 | elongation factor 2 [Notechis scutatus] |
| 234 | 929 | JAG68084.1 | 76.60 | 2 | 0 | 0 | 0 | 0 | 0 | 0 | 0 | 2 |  |  |  |  |  |  |  | 1.4855E5 | 2 | 2 | 0 | 0 | 0 | 0 | 0 | 0 | 0 | 2 | N | 95322 | Elongation factor 2 [Boiga irregularis] |
| 234 | 930 | XP\_034262248.1 | 76.60 | 2 | 0 | 0 | 0 | 0 | 0 | 0 | 0 | 2 |  |  |  |  |  |  |  | 1.4855E5 | 2 | 2 | 0 | 0 | 0 | 0 | 0 | 0 | 0 | 2 | N | 95300 | elongation factor 2 [Pantherophis guttatus] |
| 234 | 931 | ETE57963.1 | 76.60 | 2 | 0 | 0 | 0 | 0 | 0 | 0 | 0 | 2 |  |  |  |  |  |  |  | 1.4855E5 | 2 | 2 | 0 | 0 | 0 | 0 | 0 | 0 | 0 | 2 | N | 82409 | Elongation factor 2, partial [Ophiophagus hannah] |
| 234 | 933 | LAB65750.1 | 76.60 | 2 | 0 | 0 | 0 | 0 | 0 | 0 | 0 | 2 |  |  |  |  |  |  |  | 1.4855E5 | 2 | 2 | 0 | 0 | 0 | 0 | 0 | 0 | 0 | 2 | N | 85984 | hypothetical protein, partial [Micrurus surinamensis] |
| 234 | 934 | LAA97078.1 | 76.60 | 2 | 0 | 0 | 0 | 0 | 0 | 0 | 0 | 2 |  |  |  |  |  |  |  | 1.4855E5 | 2 | 2 | 0 | 0 | 0 | 0 | 0 | 0 | 0 | 2 | N | 85984 | hypothetical protein, partial [Micrurus lemniscatus lemniscatus] |
| 239 | 1271 | XP\_026562277.1 | 74.95 | 7 | 0 | 0 | 0 | 0 | 0 | 0 | 0 | 7 |  |  |  |  |  |  |  | 7.6495E4 | 2 | 2 | 0 | 0 | 0 | 0 | 0 | 0 | 0 | 2 | N | 40671 | proteasome subunit alpha type-1 [Pseudonaja textilis] |
| 239 | 1276 | XP\_007430978.1 | 74.95 | 10 | 0 | 0 | 0 | 0 | 0 | 0 | 0 | 10 |  |  |  |  |  |  |  | 7.6495E4 | 2 | 2 | 0 | 0 | 0 | 0 | 0 | 0 | 0 | 2 | N | 29352 | proteasome subunit alpha type-1 [Python bivittatus] |
| 239 | 1277 | XP\_026524124.1 | 74.95 | 10 | 0 | 0 | 0 | 0 | 0 | 0 | 0 | 10 |  |  |  |  |  |  |  | 7.6495E4 | 2 | 2 | 0 | 0 | 0 | 0 | 0 | 0 | 0 | 2 | N | 29366 | proteasome subunit alpha type-1 [Notechis scutatus] |
| 239 | 1278 | JAB53608.1 | 74.95 | 10 | 0 | 0 | 0 | 0 | 0 | 0 | 0 | 10 |  |  |  |  |  |  |  | 7.6495E4 | 2 | 2 | 0 | 0 | 0 | 0 | 0 | 0 | 0 | 2 | N | 29366 | proteasome subunit alpha type-1 [Micrurus fulvius] |
| 239 | 1279 | JAG66921.1 | 74.95 | 10 | 0 | 0 | 0 | 0 | 0 | 0 | 0 | 10 |  |  |  |  |  |  |  | 7.6495E4 | 2 | 2 | 0 | 0 | 0 | 0 | 0 | 0 | 0 | 2 | N | 29382 | Proteasome subunit alpha type-1-like protein [Boiga irregularis] |
| 239 | 1280 | XP\_032078732.1 | 74.95 | 10 | 0 | 0 | 0 | 0 | 0 | 0 | 0 | 10 |  |  |  |  |  |  |  | 7.6495E4 | 2 | 2 | 0 | 0 | 0 | 0 | 0 | 0 | 0 | 2 | N | 29368 | proteasome subunit alpha type-1 [Thamnophis elegans] |
| 239 | 1281 | XP\_034272893.1 | 74.95 | 10 | 0 | 0 | 0 | 0 | 0 | 0 | 0 | 10 |  |  |  |  |  |  |  | 7.6495E4 | 2 | 2 | 0 | 0 | 0 | 0 | 0 | 0 | 0 | 2 | N | 29382 | proteasome subunit alpha type-1 [Pantherophis guttatus] |
| 239 | 1282 | JAI09621.1 | 74.95 | 10 | 0 | 0 | 0 | 0 | 0 | 0 | 0 | 10 |  |  |  |  |  |  |  | 7.6495E4 | 2 | 2 | 0 | 0 | 0 | 0 | 0 | 0 | 0 | 2 | N | 29366 | Proteasome subunit alpha type-1-like protein [Micrurus fulvius] |
| 239 | 1283 | XP\_013919925.1 | 74.95 | 10 | 0 | 0 | 0 | 0 | 0 | 0 | 0 | 10 |  |  |  |  |  |  |  | 7.6495E4 | 2 | 2 | 0 | 0 | 0 | 0 | 0 | 0 | 0 | 2 | N | 29368 | PREDICTED: proteasome subunit alpha type-1 [Thamnophis sirtalis] |
| 239 | 1284 | LAA24276.1 | 74.95 | 8 | 0 | 0 | 0 | 0 | 0 | 0 | 0 | 8 |  |  |  |  |  |  |  | 7.6495E4 | 2 | 2 | 0 | 0 | 0 | 0 | 0 | 0 | 0 | 2 | N | 34135 | hypothetical protein, partial [Micrurus lemniscatus carvalhoi] |
| 239 | 1285 | ETE67788.1 | 74.95 | 8 | 0 | 0 | 0 | 0 | 0 | 0 | 0 | 8 |  |  |  |  |  |  |  | 7.6495E4 | 2 | 2 | 0 | 0 | 0 | 0 | 0 | 0 | 0 | 2 | N | 35050 | Proteasome subunit alpha type-1, partial [Ophiophagus hannah] |
| 239 | 1313 | JAI11932.1 | 74.95 | 10 | 0 | 0 | 0 | 0 | 0 | 0 | 0 | 10 |  |  |  |  |  |  |  | 7.6495E4 | 2 | 2 | 0 | 0 | 0 | 0 | 0 | 0 | 0 | 2 | N | 29322 | Proteasome subunit alpha type-1-like protein [Crotalus adamanteus] |
| 239 | 1314 | JAA96037.1 | 74.95 | 10 | 0 | 0 | 0 | 0 | 0 | 0 | 0 | 10 |  |  |  |  |  |  |  | 7.6495E4 | 2 | 2 | 0 | 0 | 0 | 0 | 0 | 0 | 0 | 2 | N | 29322 | Proteasome subunit alpha type-1-like protein [Crotalus horridus] |
| 239 | 1315 | JAV49557.1 | 74.95 | 10 | 0 | 0 | 0 | 0 | 0 | 0 | 0 | 10 |  |  |  |  |  |  |  | 7.6495E4 | 2 | 2 | 0 | 0 | 0 | 0 | 0 | 0 | 0 | 2 | N | 29322 | Proteasome subunit alpha type-1-like protein [Agkistrodon contortrix contortrix] |
| 239 | 1316 | AFJ51033.1 | 74.95 | 10 | 0 | 0 | 0 | 0 | 0 | 0 | 0 | 10 |  |  |  |  |  |  |  | 7.6495E4 | 2 | 2 | 0 | 0 | 0 | 0 | 0 | 0 | 0 | 2 | N | 29322 | Proteasome subunit alpha type-1 [Crotalus adamanteus] |
| 239 | 1317 | XP\_015679799.1 | 74.95 | 8 | 0 | 0 | 0 | 0 | 0 | 0 | 0 | 8 |  |  |  |  |  |  |  | 7.6495E4 | 2 | 2 | 0 | 0 | 0 | 0 | 0 | 0 | 0 | 2 | N | 33774 | proteasome subunit alpha type-1 [Protobothrops mucrosquamatus] |
| 305 | 1569 | AFJ51039.1 | 66.22 | 5 | 0 | 0 | 0 | 0 | 0 | 0 | 0 | 5 |  |  |  |  |  |  |  | 1.5738E5 | 1 | 1 | 0 | 0 | 0 | 0 | 0 | 0 | 0 | 1 | N | 27442 | Proteasome subunit alpha type-6-like [Crotalus adamanteus] |
| 305 | 1570 | XP\_026520282.1 | 66.22 | 5 | 0 | 0 | 0 | 0 | 0 | 0 | 0 | 5 |  |  |  |  |  |  |  | 1.5738E5 | 1 | 1 | 0 | 0 | 0 | 0 | 0 | 0 | 0 | 1 | N | 27455 | proteasome subunit alpha type-6 isoform X1 [Notechis scutatus] |
| 305 | 1571 | XP\_034273914.1 | 66.22 | 5 | 0 | 0 | 0 | 0 | 0 | 0 | 0 | 5 |  |  |  |  |  |  |  | 1.5738E5 | 1 | 1 | 0 | 0 | 0 | 0 | 0 | 0 | 0 | 1 | N | 27469 | proteasome subunit alpha type-6 [Pantherophis guttatus] |
| 305 | 1572 | XP\_026566911.1 | 66.22 | 5 | 0 | 0 | 0 | 0 | 0 | 0 | 0 | 5 |  |  |  |  |  |  |  | 1.5738E5 | 1 | 1 | 0 | 0 | 0 | 0 | 0 | 0 | 0 | 1 | N | 27455 | proteasome subunit alpha type-6 isoform X1 [Pseudonaja textilis] |
| 305 | 1573 | JAI11927.1 | 66.22 | 5 | 0 | 0 | 0 | 0 | 0 | 0 | 0 | 5 |  |  |  |  |  |  |  | 1.5738E5 | 1 | 1 | 0 | 0 | 0 | 0 | 0 | 0 | 0 | 1 | N | 27442 | Proteasome subunit alpha type-6-like protein [Crotalus adamanteus] |
| 305 | 1574 | XP\_032086764.1 | 66.22 | 5 | 0 | 0 | 0 | 0 | 0 | 0 | 0 | 5 |  |  |  |  |  |  |  | 1.5738E5 | 1 | 1 | 0 | 0 | 0 | 0 | 0 | 0 | 0 | 1 | N | 27510 | proteasome subunit alpha type-6 [Thamnophis elegans] |
| 305 | 1575 | JAI09616.1 | 66.22 | 5 | 0 | 0 | 0 | 0 | 0 | 0 | 0 | 5 |  |  |  |  |  |  |  | 1.5738E5 | 1 | 1 | 0 | 0 | 0 | 0 | 0 | 0 | 0 | 1 | N | 27455 | Proteasome subunit alpha type-6-like protein [Micrurus fulvius] |
| 305 | 1576 | XP\_015674567.1 | 66.22 | 5 | 0 | 0 | 0 | 0 | 0 | 0 | 0 | 5 |  |  |  |  |  |  |  | 1.5738E5 | 1 | 1 | 0 | 0 | 0 | 0 | 0 | 0 | 0 | 1 | N | 27410 | proteasome subunit alpha type-6 [Protobothrops mucrosquamatus] |
| 305 | 1577 | JAV49552.1 | 66.22 | 5 | 0 | 0 | 0 | 0 | 0 | 0 | 0 | 5 |  |  |  |  |  |  |  | 1.5738E5 | 1 | 1 | 0 | 0 | 0 | 0 | 0 | 0 | 0 | 1 | N | 27442 | Proteasome subunit alpha type-6-like protein [Agkistrodon contortrix contortrix] |
| 305 | 1578 | JAG66916.1 | 66.22 | 5 | 0 | 0 | 0 | 0 | 0 | 0 | 0 | 5 |  |  |  |  |  |  |  | 1.5738E5 | 1 | 1 | 0 | 0 | 0 | 0 | 0 | 0 | 0 | 1 | N | 27489 | Proteasome subunit alpha type-6-like protein [Boiga irregularis] |
| 305 | 1579 | JAB53610.1 | 66.22 | 5 | 0 | 0 | 0 | 0 | 0 | 0 | 0 | 5 |  |  |  |  |  |  |  | 1.5738E5 | 1 | 1 | 0 | 0 | 0 | 0 | 0 | 0 | 0 | 1 | N | 27455 | proteasome subunit alpha type-6 [Micrurus fulvius] |
| 305 | 1580 | XP\_013929267.1 | 66.22 | 5 | 0 | 0 | 0 | 0 | 0 | 0 | 0 | 5 |  |  |  |  |  |  |  | 1.5738E5 | 1 | 1 | 0 | 0 | 0 | 0 | 0 | 0 | 0 | 1 | N | 27483 | PREDICTED: proteasome subunit alpha type-6 isoform X1 [Thamnophis sirtalis] |
| 305 | 1581 | XP\_007436765.1 | 66.22 | 5 | 0 | 0 | 0 | 0 | 0 | 0 | 0 | 5 |  |  |  |  |  |  |  | 1.5738E5 | 1 | 1 | 0 | 0 | 0 | 0 | 0 | 0 | 0 | 1 | N | 27470 | proteasome subunit alpha type-6 [Python bivittatus] |
| 305 | 1582 | JAA96032.1 | 66.22 | 5 | 0 | 0 | 0 | 0 | 0 | 0 | 0 | 5 |  |  |  |  |  |  |  | 1.5738E5 | 1 | 1 | 0 | 0 | 0 | 0 | 0 | 0 | 0 | 1 | N | 27456 | Proteasome subunit alpha type-6-like protein [Crotalus horridus] |
| 305 | 1583 | ETE73021.1 | 66.22 | 5 | 0 | 0 | 0 | 0 | 0 | 0 | 0 | 5 |  |  |  |  |  |  |  | 1.5738E5 | 1 | 1 | 0 | 0 | 0 | 0 | 0 | 0 | 0 | 1 | N | 31077 | Proteasome subunit alpha type-6, partial [Ophiophagus hannah] |
| 306 | 2137 | ALB06111.1 | 64.06 | 3 | 0 | 0 | 3 | 0 | 0 | 0 | 0 | 0 |  |  | 2.0513E5 |  |  |  |  |  | 1 | 1 | 0 | 0 | 1 | 0 | 0 | 0 | 0 | 0 | N | 53920 | hyaluronoglucosaminidase [Gloydius intermedius] |
| 306 | 2486 | JAS05458.1 | 64.06 | 3 | 0 | 0 | 3 | 0 | 0 | 0 | 0 | 0 |  |  | 2.0513E5 |  |  |  |  |  | 1 | 1 | 0 | 0 | 1 | 0 | 0 | 0 | 0 | 0 | N | 52449 | Hyaluronidase [Sistrurus miliarius barbouri] |
| 306 | 2574 | JAS04525.1 | 64.06 | 3 | 0 | 0 | 3 | 0 | 0 | 0 | 0 | 0 |  |  | 2.0513E5 |  |  |  |  |  | 1 | 1 | 0 | 0 | 1 | 0 | 0 | 0 | 0 | 0 | N | 52303 | Hyaluronidase 1b [Agkistrodon piscivorus conanti] |
| 306 | 2575 | JAV50314.1 | 64.06 | 3 | 0 | 0 | 3 | 0 | 0 | 0 | 0 | 0 |  |  | 2.0513E5 |  |  |  |  |  | 1 | 1 | 0 | 0 | 1 | 0 | 0 | 0 | 0 | 0 | N | 52314 | Hyaluronidase [Agkistrodon contortrix contortrix] |
| 306 | 2576 | JAS04524.1 | 64.06 | 3 | 0 | 0 | 3 | 0 | 0 | 0 | 0 | 0 |  |  | 2.0513E5 |  |  |  |  |  | 1 | 1 | 0 | 0 | 1 | 0 | 0 | 0 | 0 | 0 | N | 52278 | Hyaluronidase 1c [Agkistrodon piscivorus conanti] |
| 306 | 2593 | JAS04526.1 | 64.06 | 3 | 0 | 0 | 3 | 0 | 0 | 0 | 0 | 0 |  |  | 2.0513E5 |  |  |  |  |  | 1 | 1 | 0 | 0 | 1 | 0 | 0 | 0 | 0 | 0 | N | 52280 | Hyaluronidase 1a [Agkistrodon piscivorus conanti] |
| 306 | 2872 | SMD28274.1 | 64.06 | 4 | 0 | 0 | 4 | 0 | 0 | 0 | 0 | 0 |  |  | 2.0513E5 |  |  |  |  |  | 1 | 1 | 0 | 0 | 1 | 0 | 0 | 0 | 0 | 0 | N | 39688 | truncated hyaluronidase CdtHya3 [Crotalus durissus terrificus] |
| 306 | 2873 | JAS04874.1 | 64.06 | 3 | 0 | 0 | 3 | 0 | 0 | 0 | 0 | 0 |  |  | 2.0513E5 |  |  |  |  |  | 1 | 1 | 0 | 0 | 1 | 0 | 0 | 0 | 0 | 0 | N | 52473 | Hyaluronidase 1b [Crotalus horridus] |
| 306 | 2874 | JAG46141.1 | 64.06 | 3 | 0 | 0 | 3 | 0 | 0 | 0 | 0 | 0 |  |  | 2.0513E5 |  |  |  |  |  | 1 | 1 | 0 | 0 | 1 | 0 | 0 | 0 | 0 | 0 | N | 52473 | Hyaluronidase 1a [Crotalus horridus] |
| 306 | 2875 | JAS04875.1 | 64.06 | 3 | 0 | 0 | 3 | 0 | 0 | 0 | 0 | 0 |  |  | 2.0513E5 |  |  |  |  |  | 1 | 1 | 0 | 0 | 1 | 0 | 0 | 0 | 0 | 0 | N | 52473 | Hyaluronidase 1a [Crotalus horridus] |
| 306 | 2876 | JAS04787.1 | 64.06 | 3 | 0 | 0 | 3 | 0 | 0 | 0 | 0 | 0 |  |  | 2.0513E5 |  |  |  |  |  | 1 | 1 | 0 | 0 | 1 | 0 | 0 | 0 | 0 | 0 | N | 52503 | Hyaluronidase 1 [Crotalus horridus] |
| 306 | 2877 | JAA94894.1 | 64.06 | 3 | 0 | 0 | 3 | 0 | 0 | 0 | 0 | 0 |  |  | 2.0513E5 |  |  |  |  |  | 1 | 1 | 0 | 0 | 1 | 0 | 0 | 0 | 0 | 0 | N | 52503 | Hyaluronidase [Crotalus horridus] |
| 306 | 2878 | SMD28273.1 | 64.06 | 3 | 0 | 0 | 3 | 0 | 0 | 0 | 0 | 0 |  |  | 2.0513E5 |  |  |  |  |  | 1 | 1 | 0 | 0 | 1 | 0 | 0 | 0 | 0 | 0 | N | 52458 | hyaluronidase CdtHya2 [Crotalus durissus terrificus] |
| 306 | 2879 | JAG46140.1 | 64.06 | 3 | 0 | 0 | 3 | 0 | 0 | 0 | 0 | 0 |  |  | 2.0513E5 |  |  |  |  |  | 1 | 1 | 0 | 0 | 1 | 0 | 0 | 0 | 0 | 0 | N | 52473 | Hyaluronidase 1b [Crotalus horridus] |
| 306 | 2880 | SMD28272.1 | 64.06 | 3 | 0 | 0 | 3 | 0 | 0 | 0 | 0 | 0 |  |  | 2.0513E5 |  |  |  |  |  | 1 | 1 | 0 | 0 | 1 | 0 | 0 | 0 | 0 | 0 | N | 52458 | hyaluronidase CdtHya1 [Crotalus durissus terrificus] |
| 306 | 2881 | JAG43657.1 | 64.06 | 3 | 0 | 0 | 3 | 0 | 0 | 0 | 0 | 0 |  |  | 2.0513E5 |  |  |  |  |  | 1 | 1 | 0 | 0 | 1 | 0 | 0 | 0 | 0 | 0 | N | 52503 | Hyaluronidase 1 [Crotalus horridus] |
| 306 | 3035 | JAV48430.1 | 64.06 | 3 | 0 | 0 | 3 | 0 | 0 | 0 | 0 | 0 |  |  | 2.0513E5 |  |  |  |  |  | 1 | 1 | 0 | 0 | 1 | 0 | 0 | 0 | 0 | 0 | N | 52484 | Cadam10\_HYAL-1 [Crotalus adamanteus] |
| 306 | 3036 | AFJ49188.1 | 64.06 | 3 | 0 | 0 | 3 | 0 | 0 | 0 | 0 | 0 |  |  | 2.0513E5 |  |  |  |  |  | 1 | 1 | 0 | 0 | 1 | 0 | 0 | 0 | 0 | 0 | N | 52484 | Hyaluronidase 1 [Crotalus adamanteus] |
| 306 | 3037 | JAS05321.1 | 64.06 | 3 | 0 | 0 | 3 | 0 | 0 | 0 | 0 | 0 |  |  | 2.0513E5 |  |  |  |  |  | 1 | 1 | 0 | 0 | 1 | 0 | 0 | 0 | 0 | 0 | N | 52431 | Hyaluronidase [Sistrurus tergeminus] |
| 306 | 3038 | JAI10412.1 | 64.06 | 3 | 0 | 0 | 3 | 0 | 0 | 0 | 0 | 0 |  |  | 2.0513E5 |  |  |  |  |  | 1 | 1 | 0 | 0 | 1 | 0 | 0 | 0 | 0 | 0 | N | 52484 | Hyaluronidase [Crotalus adamanteus] |
| 306 | 3039 | JAS04708.1 | 64.06 | 3 | 0 | 0 | 3 | 0 | 0 | 0 | 0 | 0 |  |  | 2.0513E5 |  |  |  |  |  | 1 | 1 | 0 | 0 | 1 | 0 | 0 | 0 | 0 | 0 | N | 52484 | Hyaluronidase [Crotalus adamanteus] |
| 306 | 3040 | J3S820.1 | 64.06 | 3 | 0 | 0 | 3 | 0 | 0 | 0 | 0 | 0 |  |  | 2.0513E5 |  |  |  |  |  | 1 | 1 | 0 | 0 | 1 | 0 | 0 | 0 | 0 | 0 | N | 52484 | RecName: Full=Hyaluronidase; AltName: Full=Hyaluronoglucosaminidase; AltName: Full=Venom spreading factor; Flags: Precursor |
| 306 | 3096 | JAV01892.1 | 64.06 | 3 | 0 | 0 | 3 | 0 | 0 | 0 | 0 | 0 |  |  | 2.0513E5 |  |  |  |  |  | 1 | 1 | 0 | 0 | 1 | 0 | 0 | 0 | 0 | 0 | N | 52550 | BATXHYAL1 [Bothrops atrox] |
| 306 | 3097 | ATU85542.1 | 64.06 | 3 | 0 | 0 | 3 | 0 | 0 | 0 | 0 | 0 |  |  | 2.0513E5 |  |  |  |  |  | 1 | 1 | 0 | 0 | 1 | 0 | 0 | 0 | 0 | 0 | N | 52550 | Hyaluronidase BmooHyal-1, partial [Bothrops moojeni] |
| 215 | 1327 | P0CG03.1 | 62.90 | 4 | 0 | 0 | 0 | 0 | 4 | 0 | 0 | 0 |  |  |  |  | 1.4462E6 |  |  |  | 1 | 1 | 0 | 0 | 0 | 0 | 1 | 0 | 0 | 0 | N | 28045 | RecName: Full=Thrombin-like enzyme bhalternin; Short=SVTLE; AltName: Full=Fibrinogen-clotting enzyme; AltName: Full=Snake venom serine protease; Short=SVSP; Flags: Precursor |
| 183 | 1626 | AFJ49243.1 | 62.59 | 3 | 0 | 3 | 3 | 3 | 3 | 0 | 0 | 3 |  | 9.91E6 | 4.837E7 | 7.8142E6 | 2.6837E6 |  |  | 2.7456E6 | 1 | 1 | 0 | 3 | 2 | 2 | 2 | 0 | 0 | 1 | Y | 68434 | Snake venom metalloproteinase (type III) 5 [Crotalus adamanteus] |
| 183 | 1778 | AEJ31991.1 | 62.59 | 4 | 0 | 4 | 4 | 4 | 4 | 0 | 0 | 4 |  | 9.91E6 | 4.837E7 | 7.8142E6 | 2.6837E6 |  |  | 2.7456E6 | 1 | 1 | 0 | 3 | 2 | 2 | 2 | 0 | 0 | 1 | Y | 54615 | metalloproteinase 8, partial [Crotalus adamanteus] |
| 183 | 2005 | JAV48412.1 | 62.59 | 3 | 0 | 3 | 3 | 3 | 3 | 0 | 0 | 3 |  | 9.91E6 | 4.837E7 | 7.8142E6 | 2.6837E6 |  |  | 2.7456E6 | 1 | 1 | 0 | 3 | 2 | 2 | 2 | 0 | 0 | 1 | Y | 68421 | Cadam10\_SVMPIII-2 [Crotalus adamanteus] |
| 213 | 1593 | XP\_015684827.1 | 62.54 | 2 | 0 | 0 | 0 | 2 | 2 | 0 | 0 | 0 |  |  |  | 0 | 6.927E4 |  |  |  | 1 | 1 | 0 | 0 | 0 | 1 | 1 | 0 | 0 | 0 | N | 50189 | beta-hexosaminidase subunit alpha [Protobothrops mucrosquamatus] |
| 237 | 1645 | XP\_026535627.1 | 62.20 | 1 | 0 | 0 | 0 | 0 | 0 | 0 | 0 | 1 |  |  |  |  |  |  |  | 6.1498E4 | 1 | 1 | 0 | 0 | 0 | 0 | 0 | 0 | 0 | 2 | N | 148584 | angiotensin-converting enzyme [Notechis scutatus] |
| 179 | 2181 | XP\_013924292.1 | 58.79 | 1 | 0 | 0 | 1 | 0 | 0 | 0 | 0 | 1 |  |  | 2.0994E6 |  |  |  |  | 7.9862E6 | 1 | 1 | 0 | 0 | 1 | 0 | 0 | 0 | 0 | 4 | Y | 119192 | PREDICTED: neurofilament medium polypeptide-like [Thamnophis sirtalis] |
| 179 | 2194 | XP\_034268135.1 | 58.79 | 1 | 0 | 0 | 1 | 0 | 0 | 0 | 0 | 1 |  |  | 2.0994E6 |  |  |  |  | 7.9862E6 | 1 | 1 | 0 | 0 | 1 | 0 | 0 | 0 | 0 | 4 | Y | 124073 | neurofilament medium polypeptide-like [Pantherophis guttatus] |
| 179 | 2350 | ETE71916.1 | 58.79 | 1 | 0 | 0 | 1 | 0 | 0 | 0 | 0 | 1 |  |  | 2.0994E6 |  |  |  |  | 7.9862E6 | 1 | 1 | 0 | 0 | 1 | 0 | 0 | 0 | 0 | 4 | Y | 82272 | Keratin, type II cytoskeletal 80 [Ophiophagus hannah] |
| 179 | 2571 | XP\_026573192.1 | 58.79 | 1 | 0 | 0 | 1 | 0 | 0 | 0 | 0 | 1 |  |  | 2.0994E6 |  |  |  |  | 7.9862E6 | 1 | 1 | 0 | 0 | 1 | 0 | 0 | 0 | 0 | 4 | Y | 118330 | neurofilament medium polypeptide-like [Pseudonaja textilis] |
| 179 | 2726 | XP\_032067334.1 | 58.79 | 2 | 0 | 0 | 2 | 0 | 0 | 0 | 0 | 2 |  |  | 2.0994E6 |  |  |  |  | 7.9862E6 | 1 | 1 | 0 | 0 | 1 | 0 | 0 | 0 | 0 | 4 | Y | 42280 | keratin, type II cytoskeletal 80 [Thamnophis elegans] |
| 179 | 3006 | XP\_026535749.1 | 58.79 | 1 | 0 | 0 | 1 | 0 | 0 | 0 | 0 | 1 |  |  | 2.0994E6 |  |  |  |  | 7.9862E6 | 1 | 1 | 0 | 0 | 1 | 0 | 0 | 0 | 0 | 4 | Y | 123281 | LOW QUALITY PROTEIN: neurofilament medium polypeptide-like [Notechis scutatus] |
| 179 | 3248 | XP\_029142905.1 | 58.79 | 4 | 0 | 0 | 4 | 0 | 0 | 0 | 0 | 4 |  |  | 2.0994E6 |  |  |  |  | 7.9862E6 | 1 | 1 | 0 | 0 | 1 | 0 | 0 | 0 | 0 | 4 | Y | 23545 | keratin, type II cytoskeletal 80-like, partial [Protobothrops mucrosquamatus] |
| 325 | 2059 | XP\_032066440.1 | 57.74 | 3 | 0 | 0 | 0 | 0 | 0 | 0 | 0 | 3 |  |  |  |  |  |  |  | 8.8493E4 | 1 | 1 | 0 | 0 | 0 | 0 | 0 | 0 | 0 | 1 | N | 34999 | voltage-dependent anion-selective channel protein 1 [Thamnophis elegans] |
| 325 | 2060 | XP\_013913912.1 | 57.74 | 3 | 0 | 0 | 0 | 0 | 0 | 0 | 0 | 3 |  |  |  |  |  |  |  | 8.8493E4 | 1 | 1 | 0 | 0 | 0 | 0 | 0 | 0 | 0 | 1 | N | 35013 | PREDICTED: voltage-dependent anion-selective channel protein 1 [Thamnophis sirtalis] |
| 294 | 1838 | JAG46544.1 | 56.43 | 3 | 0 | 0 | 0 | 0 | 0 | 0 | 0 | 3 |  |  |  |  |  |  |  | 2.9167E4 | 1 | 1 | 0 | 0 | 0 | 0 | 0 | 0 | 0 | 1 | N | 46713 | Isocitrate dehydrogenase (NADP) cytoplasmic-like protein [Crotalus horridus] |
| 294 | 1839 | JAA96763.1 | 56.43 | 3 | 0 | 0 | 0 | 0 | 0 | 0 | 0 | 3 |  |  |  |  |  |  |  | 2.9167E4 | 1 | 1 | 0 | 0 | 0 | 0 | 0 | 0 | 0 | 1 | N | 46713 | Isocitrate dehydrogenase (NADP) cytoplasmic-like protein [Crotalus horridus] |
| 294 | 1840 | JAG44318.1 | 56.43 | 3 | 0 | 0 | 0 | 0 | 0 | 0 | 0 | 3 |  |  |  |  |  |  |  | 2.9167E4 | 1 | 1 | 0 | 0 | 0 | 0 | 0 | 0 | 0 | 1 | N | 46713 | Isocitrate dehydrogenase (NADP) cytoplasmic-like protein [Crotalus horridus] |
| 294 | 1841 | XP\_026563554.1 | 56.43 | 3 | 0 | 0 | 0 | 0 | 0 | 0 | 0 | 3 |  |  |  |  |  |  |  | 2.9167E4 | 1 | 1 | 0 | 0 | 0 | 0 | 0 | 0 | 0 | 1 | N | 46817 | isocitrate dehydrogenase [NADP] cytoplasmic [Pseudonaja textilis] |
| 294 | 1842 | JAC95305.1 | 56.43 | 3 | 0 | 0 | 0 | 0 | 0 | 0 | 0 | 3 |  |  |  |  |  |  |  | 2.9167E4 | 1 | 1 | 0 | 0 | 0 | 0 | 0 | 0 | 0 | 1 | N | 46716 | Isocitrate dehydrogenase (NADP) cytoplasmic-like protein [Hypsiglena sp. JMG-2014] |
| 294 | 1843 | JAB54051.1 | 56.43 | 3 | 0 | 0 | 0 | 0 | 0 | 0 | 0 | 3 |  |  |  |  |  |  |  | 2.9167E4 | 1 | 1 | 0 | 0 | 0 | 0 | 0 | 0 | 0 | 1 | N | 46788 | Isocitrate dehydrogenase [Micrurus fulvius] |
| 294 | 1844 | XP\_026528963.1 | 56.43 | 3 | 0 | 0 | 0 | 0 | 0 | 0 | 0 | 3 |  |  |  |  |  |  |  | 2.9167E4 | 1 | 1 | 0 | 0 | 0 | 0 | 0 | 0 | 0 | 1 | N | 46717 | isocitrate dehydrogenase [NADP] cytoplasmic [Notechis scutatus] |
| 294 | 1845 | JAI09889.1 | 56.43 | 3 | 0 | 0 | 0 | 0 | 0 | 0 | 0 | 3 |  |  |  |  |  |  |  | 2.9167E4 | 1 | 1 | 0 | 0 | 0 | 0 | 0 | 0 | 0 | 1 | N | 46788 | Isocitrate dehydrogenase (NADP) cytoplasmic-like protein [Micrurus fulvius] |
| 294 | 1846 | ETE62592.1 | 56.43 | 3 | 0 | 0 | 0 | 0 | 0 | 0 | 0 | 3 |  |  |  |  |  |  |  | 2.9167E4 | 1 | 1 | 0 | 0 | 0 | 0 | 0 | 0 | 0 | 1 | N | 46686 | Isocitrate dehydrogenase [NADP] cytoplasmic [Ophiophagus hannah] |
| 294 | 1863 | JAI12893.1 | 56.43 | 3 | 0 | 0 | 0 | 0 | 0 | 0 | 0 | 3 |  |  |  |  |  |  |  | 2.9167E4 | 1 | 1 | 0 | 0 | 0 | 0 | 0 | 0 | 0 | 1 | N | 46698 | Isocitrate dehydrogenase (NADP) cytoplasmic-like protein [Crotalus adamanteus] |
| 294 | 1864 | AFJ50392.1 | 56.43 | 3 | 0 | 0 | 0 | 0 | 0 | 0 | 0 | 3 |  |  |  |  |  |  |  | 2.9167E4 | 1 | 1 | 0 | 0 | 0 | 0 | 0 | 0 | 0 | 1 | N | 46729 | Isocitrate dehydrogenase NADP cytoplasmic-like [Crotalus adamanteus] |
| 294 | 1865 | XP\_015669945.1 | 56.43 | 3 | 0 | 0 | 0 | 0 | 0 | 0 | 0 | 3 |  |  |  |  |  |  |  | 2.9167E4 | 1 | 1 | 0 | 0 | 0 | 0 | 0 | 0 | 0 | 1 | N | 46751 | isocitrate dehydrogenase [NADP] cytoplasmic [Protobothrops mucrosquamatus] |
| 294 | 2524 | XP\_032067252.1 | 56.43 | 3 | 0 | 0 | 0 | 0 | 0 | 0 | 0 | 3 |  |  |  |  |  |  |  | 2.9167E4 | 1 | 1 | 0 | 0 | 0 | 0 | 0 | 0 | 0 | 1 | N | 46736 | isocitrate dehydrogenase [NADP] cytoplasmic [Thamnophis elegans] |
| 294 | 2525 | JAG67637.1 | 56.43 | 3 | 0 | 0 | 0 | 0 | 0 | 0 | 0 | 3 |  |  |  |  |  |  |  | 2.9167E4 | 1 | 1 | 0 | 0 | 0 | 0 | 0 | 0 | 0 | 1 | N | 46729 | Isocitrate dehydrogenase (NADP) cytoplasmic-like protein [Boiga irregularis] |
| 294 | 2526 | XP\_034287847.1 | 56.43 | 3 | 0 | 0 | 0 | 0 | 0 | 0 | 0 | 3 |  |  |  |  |  |  |  | 2.9167E4 | 1 | 1 | 0 | 0 | 0 | 0 | 0 | 0 | 0 | 1 | N | 46785 | isocitrate dehydrogenase [NADP] cytoplasmic [Pantherophis guttatus] |
| 294 | 2554 | JAV50239.1 | 56.43 | 3 | 0 | 0 | 0 | 0 | 0 | 0 | 0 | 3 |  |  |  |  |  |  |  | 2.9167E4 | 1 | 1 | 0 | 0 | 0 | 0 | 0 | 0 | 0 | 1 | N | 46607 | Isocitrate dehydrogenase (NADP) cytoplasmic-like protein [Agkistrodon contortrix contortrix] |
| 287 | 1651 | XP\_032076181.1 | 55.88 | 1 | 0 | 0 | 0 | 0 | 0 | 0 | 0 | 1 |  |  |  |  |  |  |  | 5.0661E3 | 1 | 1 | 0 | 0 | 0 | 0 | 0 | 0 | 0 | 1 | N | 78293 | ATP-dependent RNA helicase DDX3X isoform X1 [Thamnophis elegans] |
| 287 | 1652 | XP\_013929400.1 | 55.88 | 1 | 0 | 0 | 0 | 0 | 0 | 0 | 0 | 1 |  |  |  |  |  |  |  | 5.0661E3 | 1 | 1 | 0 | 0 | 0 | 0 | 0 | 0 | 0 | 1 | N | 78351 | PREDICTED: ATP-dependent RNA helicase DDX3X isoform X1 [Thamnophis sirtalis] |
| 287 | 1656 | JAV50781.1 | 55.88 | 1 | 0 | 0 | 0 | 0 | 0 | 0 | 0 | 1 |  |  |  |  |  |  |  | 5.0661E3 | 1 | 1 | 0 | 0 | 0 | 0 | 0 | 0 | 0 | 1 | N | 78201 | ATP-dependent RNA helicase DDX3X-like [Agkistrodon contortrix contortrix] |
| 287 | 1672 | XP\_032076182.1 | 55.88 | 1 | 0 | 0 | 0 | 0 | 0 | 0 | 0 | 1 |  |  |  |  |  |  |  | 5.0661E3 | 1 | 1 | 0 | 0 | 0 | 0 | 0 | 0 | 0 | 1 | N | 73406 | ATP-dependent RNA helicase DDX3X isoform X2 [Thamnophis elegans] |
| 287 | 1673 | XP\_013929401.1 | 55.88 | 1 | 0 | 0 | 0 | 0 | 0 | 0 | 0 | 1 |  |  |  |  |  |  |  | 5.0661E3 | 1 | 1 | 0 | 0 | 0 | 0 | 0 | 0 | 0 | 1 | N | 73464 | PREDICTED: ATP-dependent RNA helicase DDX3X isoform X2 [Thamnophis sirtalis] |
| 287 | 1784 | XP\_007426567.1 | 55.88 | 1 | 0 | 0 | 0 | 0 | 0 | 0 | 0 | 1 |  |  |  |  |  |  |  | 5.0661E3 | 1 | 1 | 0 | 0 | 0 | 0 | 0 | 0 | 0 | 1 | N | 78101 | ATP-dependent RNA helicase DDX3X isoform X3 [Python bivittatus] |
| 287 | 1785 | XP\_007426565.1 | 55.88 | 1 | 0 | 0 | 0 | 0 | 0 | 0 | 0 | 1 |  |  |  |  |  |  |  | 5.0661E3 | 1 | 1 | 0 | 0 | 0 | 0 | 0 | 0 | 0 | 1 | N | 80547 | ATP-dependent RNA helicase DDX3X isoform X1 [Python bivittatus] |
| 287 | 1800 | XP\_007426569.1 | 55.88 | 2 | 0 | 0 | 0 | 0 | 0 | 0 | 0 | 2 |  |  |  |  |  |  |  | 5.0661E3 | 1 | 1 | 0 | 0 | 0 | 0 | 0 | 0 | 0 | 1 | N | 73214 | ATP-dependent RNA helicase DDX3X isoform X5 [Python bivittatus] |
| 287 | 1836 | XP\_034279735.1 | 55.88 | 1 | 0 | 0 | 0 | 0 | 0 | 0 | 0 | 1 |  |  |  |  |  |  |  | 5.0661E3 | 1 | 1 | 0 | 0 | 0 | 0 | 0 | 0 | 0 | 1 | N | 78093 | ATP-dependent RNA helicase DDX3X isoform X2 [Pantherophis guttatus] |
| 287 | 1837 | XP\_034279734.1 | 55.88 | 1 | 0 | 0 | 0 | 0 | 0 | 0 | 0 | 1 |  |  |  |  |  |  |  | 5.0661E3 | 1 | 1 | 0 | 0 | 0 | 0 | 0 | 0 | 0 | 1 | N | 80583 | ATP-dependent RNA helicase DDX3X isoform X1 [Pantherophis guttatus] |
| 287 | 1849 | AFJ51213.1 | 55.88 | 1 | 0 | 0 | 0 | 0 | 0 | 0 | 0 | 1 |  |  |  |  |  |  |  | 5.0661E3 | 1 | 1 | 0 | 0 | 0 | 0 | 0 | 0 | 0 | 1 | N | 77987 | ATP-dependent RNA helicase DDX3X-like isoform 3 [Crotalus adamanteus] |
| 287 | 1850 | JAI11687.1 | 55.88 | 1 | 0 | 0 | 0 | 0 | 0 | 0 | 0 | 1 |  |  |  |  |  |  |  | 5.0661E3 | 1 | 1 | 0 | 0 | 0 | 0 | 0 | 0 | 0 | 1 | N | 77987 | ATP-dependent RNA helicase DDX3X-like [Crotalus adamanteus] |
| 287 | 1866 | XP\_034279736.1 | 55.88 | 1 | 0 | 0 | 0 | 0 | 0 | 0 | 0 | 1 |  |  |  |  |  |  |  | 5.0661E3 | 1 | 1 | 0 | 0 | 0 | 0 | 0 | 0 | 0 | 1 | N | 73206 | ATP-dependent RNA helicase DDX3X isoform X3 [Pantherophis guttatus] |
| 287 | 1867 | JAG47014.1 | 55.88 | 2 | 0 | 0 | 0 | 0 | 0 | 0 | 0 | 2 |  |  |  |  |  |  |  | 5.0661E3 | 1 | 1 | 0 | 0 | 0 | 0 | 0 | 0 | 0 | 1 | N | 73068 | DEAD-box protein 3 [Crotalus horridus] |
| 287 | 1917 | XP\_015670389.1 | 55.88 | 1 | 0 | 0 | 0 | 0 | 0 | 0 | 0 | 1 |  |  |  |  |  |  |  | 5.0661E3 | 1 | 1 | 0 | 0 | 0 | 0 | 0 | 0 | 0 | 1 | N | 78156 | ATP-dependent RNA helicase DDX3X isoform X2 [Protobothrops mucrosquamatus] |
| 287 | 1918 | XP\_015670387.1 | 55.88 | 1 | 0 | 0 | 0 | 0 | 0 | 0 | 0 | 1 |  |  |  |  |  |  |  | 5.0661E3 | 1 | 1 | 0 | 0 | 0 | 0 | 0 | 0 | 0 | 1 | N | 80616 | ATP-dependent RNA helicase DDX3X isoform X1 [Protobothrops mucrosquamatus] |
| 287 | 1933 | XP\_015670390.1 | 55.88 | 1 | 0 | 0 | 0 | 0 | 0 | 0 | 0 | 1 |  |  |  |  |  |  |  | 5.0661E3 | 1 | 1 | 0 | 0 | 0 | 0 | 0 | 0 | 0 | 1 | N | 73211 | ATP-dependent RNA helicase DDX3X isoform X3 [Protobothrops mucrosquamatus] |
| 287 | 1953 | XP\_007426568.1 | 55.88 | 1 | 0 | 0 | 0 | 0 | 0 | 0 | 0 | 1 |  |  |  |  |  |  |  | 5.0661E3 | 1 | 1 | 0 | 0 | 0 | 0 | 0 | 0 | 0 | 1 | N | 77942 | ATP-dependent RNA helicase DDX3X isoform X4 [Python bivittatus] |
| 287 | 1954 | XP\_007426566.1 | 55.88 | 1 | 0 | 0 | 0 | 0 | 0 | 0 | 0 | 1 |  |  |  |  |  |  |  | 5.0661E3 | 1 | 1 | 0 | 0 | 0 | 0 | 0 | 0 | 0 | 1 | N | 80389 | ATP-dependent RNA helicase DDX3X isoform X2 [Python bivittatus] |
| 287 | 2021 | XP\_007426570.1 | 55.88 | 2 | 0 | 0 | 0 | 0 | 0 | 0 | 0 | 2 |  |  |  |  |  |  |  | 5.0661E3 | 1 | 1 | 0 | 0 | 0 | 0 | 0 | 0 | 0 | 1 | N | 73055 | ATP-dependent RNA helicase DDX3X isoform X6 [Python bivittatus] |
| 287 | 2124 | XP\_026569452.1 | 55.88 | 1 | 0 | 0 | 0 | 0 | 0 | 0 | 0 | 1 |  |  |  |  |  |  |  | 5.0661E3 | 1 | 1 | 0 | 0 | 0 | 0 | 0 | 0 | 0 | 1 | N | 80584 | ATP-dependent RNA helicase DDX3X isoform X4 [Pseudonaja textilis] |
| 287 | 2302 | XP\_026529838.1 | 55.88 | 1 | 0 | 0 | 0 | 0 | 0 | 0 | 0 | 1 |  |  |  |  |  |  |  | 5.0661E3 | 1 | 1 | 0 | 0 | 0 | 0 | 0 | 0 | 0 | 1 | N | 80760 | ATP-dependent RNA helicase DDX3X isoform X4 [Notechis scutatus] |
| 287 | 2367 | XP\_026569451.1 | 55.88 | 2 | 0 | 0 | 0 | 0 | 0 | 0 | 0 | 2 |  |  |  |  |  |  |  | 5.0661E3 | 1 | 1 | 0 | 0 | 0 | 0 | 0 | 0 | 0 | 1 | N | 73484 | ATP-dependent RNA helicase DDX3X isoform X3 [Pseudonaja textilis] |
| 287 | 2368 | XP\_026569449.1 | 55.88 | 1 | 0 | 0 | 0 | 0 | 0 | 0 | 0 | 1 |  |  |  |  |  |  |  | 5.0661E3 | 1 | 1 | 0 | 0 | 0 | 0 | 0 | 0 | 0 | 1 | N | 78102 | ATP-dependent RNA helicase DDX3X isoform X1 [Pseudonaja textilis] |
| 287 | 2413 | XP\_026569450.1 | 55.88 | 2 | 0 | 0 | 0 | 0 | 0 | 0 | 0 | 2 |  |  |  |  |  |  |  | 5.0661E3 | 1 | 1 | 0 | 0 | 0 | 0 | 0 | 0 | 0 | 1 | N | 73215 | ATP-dependent RNA helicase DDX3X isoform X2 [Pseudonaja textilis] |
| 287 | 2512 | ETE62077.1 | 55.88 | 2 | 0 | 0 | 0 | 0 | 0 | 0 | 0 | 2 |  |  |  |  |  |  |  | 5.0661E3 | 1 | 1 | 0 | 0 | 0 | 0 | 0 | 0 | 0 | 1 | N | 65647 | ATP-dependent RNA helicase DDX3X [Ophiophagus hannah] |
| 287 | 2558 | XP\_026529837.1 | 55.88 | 1 | 0 | 0 | 0 | 0 | 0 | 0 | 0 | 1 |  |  |  |  |  |  |  | 5.0661E3 | 1 | 1 | 0 | 0 | 0 | 0 | 0 | 0 | 0 | 1 | N | 73676 | ATP-dependent RNA helicase DDX3X isoform X3 [Notechis scutatus] |
| 287 | 2559 | XP\_026529835.1 | 55.88 | 1 | 0 | 0 | 0 | 0 | 0 | 0 | 0 | 1 |  |  |  |  |  |  |  | 5.0661E3 | 1 | 1 | 0 | 0 | 0 | 0 | 0 | 0 | 0 | 1 | N | 78278 | ATP-dependent RNA helicase DDX3X isoform X1 [Notechis scutatus] |
| 287 | 2578 | XP\_026529836.1 | 55.88 | 1 | 0 | 0 | 0 | 0 | 0 | 0 | 0 | 1 |  |  |  |  |  |  |  | 5.0661E3 | 1 | 1 | 0 | 0 | 0 | 0 | 0 | 0 | 0 | 1 | N | 73333 | ATP-dependent RNA helicase DDX3X isoform X2 [Notechis scutatus] |
| 287 | 3139 | JAB53490.1 | 55.88 | 2 | 0 | 0 | 0 | 0 | 0 | 0 | 0 | 2 |  |  |  |  |  |  |  | 5.0661E3 | 1 | 1 | 0 | 0 | 0 | 0 | 0 | 0 | 0 | 1 | N | 73081 | ATP-dependent RNA helicase DDX3X protein 2 [Micrurus fulvius] |
| 281 | 1967 | XP\_026539659.1 | 55.28 | 1 | 0 | 0 | 0 | 0 | 0 | 0 | 0 | 1 |  |  |  |  |  |  |  | 2.1449E5 | 1 | 1 | 0 | 0 | 0 | 0 | 0 | 0 | 0 | 1 | N | 70020 | keratin, type II cytoskeletal 7-like [Notechis scutatus] |
| 281 | 1983 | XP\_026570510.1 | 55.28 | 1 | 0 | 0 | 0 | 0 | 0 | 0 | 0 | 1 |  |  |  |  |  |  |  | 2.1449E5 | 1 | 1 | 0 | 0 | 0 | 0 | 0 | 0 | 0 | 1 | N | 70208 | keratin, type II cytoskeletal 7-like [Pseudonaja textilis] |
| 220 | 3252 | Q7LZS9.1 | 54.70 | 5 | 0 | 0 | 5 | 5 | 0 | 0 | 0 | 0 |  |  | 1.9595E7 | 9.0845E5 |  |  |  |  | 1 | 1 | 0 | 0 | 1 | 1 | 0 | 0 | 0 | 0 | Y | 22945 | RecName: Full=Snake venom metalloproteinase Ac1; Short=SVMP; AltName: Full=Ac1-proteinase |
| 328 | 2296 | XP\_007443339.1 | 53.35 | 2 | 0 | 0 | 0 | 0 | 0 | 0 | 0 | 2 |  |  |  |  |  |  |  | 3.8989E4 | 1 | 1 | 0 | 0 | 0 | 0 | 0 | 0 | 0 | 1 | N | 47560 | delta-aminolevulinic acid dehydratase-like [Python bivittatus] |
| 328 | 2358 | XP\_026568189.1 | 53.35 | 3 | 0 | 0 | 0 | 0 | 0 | 0 | 0 | 3 |  |  |  |  |  |  |  | 3.8989E4 | 1 | 1 | 0 | 0 | 0 | 0 | 0 | 0 | 0 | 1 | N | 36085 | delta-aminolevulinic acid dehydratase [Pseudonaja textilis] |
| 328 | 2359 | XP\_026542345.1 | 53.35 | 3 | 0 | 0 | 0 | 0 | 0 | 0 | 0 | 3 |  |  |  |  |  |  |  | 3.8989E4 | 1 | 1 | 0 | 0 | 0 | 0 | 0 | 0 | 0 | 1 | N | 35929 | delta-aminolevulinic acid dehydratase [Notechis scutatus] |
| 328 | 2674 | AFJ49864.1 | 53.35 | 3 | 0 | 0 | 0 | 0 | 0 | 0 | 0 | 3 |  |  |  |  |  |  |  | 3.8989E4 | 1 | 1 | 0 | 0 | 0 | 0 | 0 | 0 | 0 | 1 | N | 36044 | Delta-aminolevulinic acid dehydratase-like [Crotalus adamanteus] |
| 328 | 2675 | JAI12578.1 | 53.35 | 3 | 0 | 0 | 0 | 0 | 0 | 0 | 0 | 3 |  |  |  |  |  |  |  | 3.8989E4 | 1 | 1 | 0 | 0 | 0 | 0 | 0 | 0 | 0 | 1 | N | 36044 | MGC84775 protein [Crotalus adamanteus] |
| 328 | 2707 | XP\_032088448.1 | 53.35 | 3 | 0 | 0 | 0 | 0 | 0 | 0 | 0 | 3 |  |  |  |  |  |  |  | 3.8989E4 | 1 | 1 | 0 | 0 | 0 | 0 | 0 | 0 | 0 | 1 | N | 36154 | delta-aminolevulinic acid dehydratase [Thamnophis elegans] |
| 328 | 2992 | LAA91247.1 | 53.35 | 3 | 0 | 0 | 0 | 0 | 0 | 0 | 0 | 3 |  |  |  |  |  |  |  | 3.8989E4 | 1 | 1 | 0 | 0 | 0 | 0 | 0 | 0 | 0 | 1 | N | 31197 | hypothetical protein, partial [Micrurus lemniscatus lemniscatus] |
| 328 | 2993 | XP\_034292227.1 | 53.35 | 3 | 0 | 0 | 0 | 0 | 0 | 0 | 0 | 3 |  |  |  |  |  |  |  | 3.8989E4 | 1 | 1 | 0 | 0 | 0 | 0 | 0 | 0 | 0 | 1 | N | 36018 | delta-aminolevulinic acid dehydratase [Pantherophis guttatus] |
| 328 | 2994 | JAC96276.1 | 53.35 | 3 | 0 | 0 | 0 | 0 | 0 | 0 | 0 | 3 |  |  |  |  |  |  |  | 3.8989E4 | 1 | 1 | 0 | 0 | 0 | 0 | 0 | 0 | 0 | 1 | N | 36107 | Delta-aminolevulinic acid dehydratase-like [Hypsiglena sp. JMG-2014] |
| 328 | 2995 | XP\_015687137.1 | 53.35 | 3 | 0 | 0 | 0 | 0 | 0 | 0 | 0 | 3 |  |  |  |  |  |  |  | 3.8989E4 | 1 | 1 | 0 | 0 | 0 | 0 | 0 | 0 | 0 | 1 | N | 35914 | delta-aminolevulinic acid dehydratase [Protobothrops mucrosquamatus] |
| 328 | 2996 | JAV51283.1 | 53.35 | 3 | 0 | 0 | 0 | 0 | 0 | 0 | 0 | 3 |  |  |  |  |  |  |  | 3.8989E4 | 1 | 1 | 0 | 0 | 0 | 0 | 0 | 0 | 0 | 1 | N | 36224 | aminolevulinate dehydratase [Agkistrodon contortrix contortrix] |
| 328 | 2997 | JAG68750.1 | 53.35 | 3 | 0 | 0 | 0 | 0 | 0 | 0 | 0 | 3 |  |  |  |  |  |  |  | 3.8989E4 | 1 | 1 | 0 | 0 | 0 | 0 | 0 | 0 | 0 | 1 | N | 36017 | Delta-aminolevulinic acid dehydratase-like [Boiga irregularis] |
| 296 | 2028 | XP\_034263014.1 | 53.27 | 3 | 0 | 0 | 0 | 0 | 0 | 0 | 0 | 3 |  |  |  |  |  |  |  | 1.7509E5 | 1 | 1 | 0 | 0 | 0 | 0 | 0 | 0 | 0 | 1 | N | 53080 | calmodulin-like protein 12 [Pantherophis guttatus] |
| 296 | 2497 | LAA89550.1 | 53.27 | 8 | 0 | 0 | 0 | 0 | 0 | 0 | 0 | 8 |  |  |  |  |  |  |  | 1.7509E5 | 1 | 1 | 0 | 0 | 0 | 0 | 0 | 0 | 0 | 1 | N | 16284 | hypothetical protein, partial [Micrurus lemniscatus lemniscatus] |
| 296 | 2498 | LAA89553.1 | 53.27 | 7 | 0 | 0 | 0 | 0 | 0 | 0 | 0 | 7 |  |  |  |  |  |  |  | 1.7509E5 | 1 | 1 | 0 | 0 | 0 | 0 | 0 | 0 | 0 | 1 | N | 19887 | hypothetical protein, partial [Micrurus lemniscatus lemniscatus] |
| 296 | 2499 | XP\_026544519.1 | 53.27 | 4 | 0 | 0 | 0 | 0 | 0 | 0 | 0 | 4 |  |  |  |  |  |  |  | 1.7509E5 | 1 | 1 | 0 | 0 | 0 | 0 | 0 | 0 | 0 | 1 | N | 34439 | centrin-4-like [Notechis scutatus] |
| 296 | 2500 | XP\_026578673.1 | 53.27 | 3 | 0 | 0 | 0 | 0 | 0 | 0 | 0 | 3 |  |  |  |  |  |  |  | 1.7509E5 | 1 | 1 | 0 | 0 | 0 | 0 | 0 | 0 | 0 | 1 | N | 51802 | uncharacterized protein LOC113451503 [Pseudonaja textilis] |
| 296 | 3051 | XP\_013924398.1 | 53.27 | 8 | 0 | 0 | 0 | 0 | 0 | 0 | 0 | 8 |  |  |  |  |  |  |  | 1.7509E5 | 1 | 1 | 0 | 0 | 0 | 0 | 0 | 0 | 0 | 1 | N | 16943 | PREDICTED: calmodulin-beta-like [Thamnophis sirtalis] |
| 296 | 3052 | XP\_025025692.1 | 53.27 | 3 | 0 | 0 | 0 | 0 | 0 | 0 | 0 | 3 |  |  |  |  |  |  |  | 1.7509E5 | 1 | 1 | 0 | 0 | 0 | 0 | 0 | 0 | 0 | 1 | N | 43349 | calmodulin-like protein 12 [Python bivittatus] |
| 296 | 3053 | XP\_026578681.1 | 53.27 | 3 | 0 | 0 | 0 | 0 | 0 | 0 | 0 | 3 |  |  |  |  |  |  |  | 1.7509E5 | 1 | 1 | 0 | 0 | 0 | 0 | 0 | 0 | 0 | 1 | N | 52347 | uncharacterized protein LOC113451522 [Pseudonaja textilis] |
| 296 | 3384 | XP\_029142126.1 | 53.27 | 6 | 0 | 0 | 0 | 0 | 0 | 0 | 0 | 6 |  |  |  |  |  |  |  | 1.7509E5 | 1 | 1 | 0 | 0 | 0 | 0 | 0 | 0 | 0 | 1 | N | 22695 | calmodulin-A [Protobothrops mucrosquamatus] |
| 329 | 1919 | XP\_013923169.1 | 52.98 | 8 | 0 | 0 | 0 | 0 | 0 | 0 | 0 | 8 |  |  |  |  |  |  |  | 7.6993E4 | 1 | 1 | 0 | 0 | 0 | 0 | 0 | 0 | 0 | 1 | N | 14127 | PREDICTED: purine nucleoside phosphorylase-like [Thamnophis sirtalis] |
| 329 | 2702 | JAI11878.1 | 52.98 | 3 | 0 | 0 | 0 | 0 | 0 | 0 | 0 | 3 |  |  |  |  |  |  |  | 7.6993E4 | 1 | 1 | 0 | 0 | 0 | 0 | 0 | 0 | 0 | 1 | N | 32382 | Purine nucleoside phosphorylase-like protein [Crotalus adamanteus] |
| 329 | 2703 | AFJ50974.1 | 52.98 | 3 | 0 | 0 | 0 | 0 | 0 | 0 | 0 | 3 |  |  |  |  |  |  |  | 7.6993E4 | 1 | 1 | 0 | 0 | 0 | 0 | 0 | 0 | 0 | 1 | N | 32382 | Purine nucleoside phosphorylase-like [Crotalus adamanteus] |
| 329 | 2704 | JAA95993.1 | 52.98 | 3 | 0 | 0 | 0 | 0 | 0 | 0 | 0 | 3 |  |  |  |  |  |  |  | 7.6993E4 | 1 | 1 | 0 | 0 | 0 | 0 | 0 | 0 | 0 | 1 | N | 32352 | Purine nucleoside phosphorylase-like protein [Crotalus horridus] |
| 329 | 2794 | XP\_025032762.1 | 52.98 | 3 | 0 | 0 | 0 | 0 | 0 | 0 | 0 | 3 |  |  |  |  |  |  |  | 7.6993E4 | 1 | 1 | 0 | 0 | 0 | 0 | 0 | 0 | 0 | 1 | N | 33742 | purine nucleoside phosphorylase, partial [Python bivittatus] |
| 329 | 3054 | LAA42915.1 | 52.98 | 5 | 0 | 0 | 0 | 0 | 0 | 0 | 0 | 5 |  |  |  |  |  |  |  | 7.6993E4 | 1 | 1 | 0 | 0 | 0 | 0 | 0 | 0 | 0 | 1 | N | 23232 | hypothetical protein [Micrurus corallinus] |
| 329 | 3055 | LAB18974.1 | 52.98 | 4 | 0 | 0 | 0 | 0 | 0 | 0 | 0 | 4 |  |  |  |  |  |  |  | 7.6993E4 | 1 | 1 | 0 | 0 | 0 | 0 | 0 | 0 | 0 | 1 | N | 31361 | hypothetical protein, partial [Micrurus spixii] |
| 329 | 3056 | JAB53579.1 | 52.98 | 3 | 0 | 0 | 0 | 0 | 0 | 0 | 0 | 3 |  |  |  |  |  |  |  | 7.6993E4 | 1 | 1 | 0 | 0 | 0 | 0 | 0 | 0 | 0 | 1 | N | 32609 | purine nucleoside phosphorylase-like protein [Micrurus fulvius] |
| 329 | 3057 | XP\_026543188.1 | 52.98 | 3 | 0 | 0 | 0 | 0 | 0 | 0 | 0 | 3 |  |  |  |  |  |  |  | 7.6993E4 | 1 | 1 | 0 | 0 | 0 | 0 | 0 | 0 | 0 | 1 | N | 32551 | purine nucleoside phosphorylase [Notechis scutatus] |
| 329 | 3058 | XP\_015676887.1 | 52.98 | 3 | 0 | 0 | 0 | 0 | 0 | 0 | 0 | 3 |  |  |  |  |  |  |  | 7.6993E4 | 1 | 1 | 0 | 0 | 0 | 0 | 0 | 0 | 0 | 1 | N | 32362 | purine nucleoside phosphorylase [Protobothrops mucrosquamatus] |
| 329 | 3059 | JAV49515.1 | 52.98 | 3 | 0 | 0 | 0 | 0 | 0 | 0 | 0 | 3 |  |  |  |  |  |  |  | 7.6993E4 | 1 | 1 | 0 | 0 | 0 | 0 | 0 | 0 | 0 | 1 | N | 32369 | Purine nucleoside phosphorylase-like protein [Agkistrodon contortrix contortrix] |
| 329 | 3060 | XP\_026576404.1 | 52.98 | 3 | 0 | 0 | 0 | 0 | 0 | 0 | 0 | 3 |  |  |  |  |  |  |  | 7.6993E4 | 1 | 1 | 0 | 0 | 0 | 0 | 0 | 0 | 0 | 1 | N | 32625 | purine nucleoside phosphorylase [Pseudonaja textilis] |
| 329 | 3061 | JAG66882.1 | 52.98 | 3 | 0 | 0 | 0 | 0 | 0 | 0 | 0 | 3 |  |  |  |  |  |  |  | 7.6993E4 | 1 | 1 | 0 | 0 | 0 | 0 | 0 | 0 | 0 | 1 | N | 32375 | Purine nucleoside phosphorylase-like protein [Boiga irregularis] |
| 329 | 3062 | LAA42911.1 | 52.98 | 3 | 0 | 0 | 0 | 0 | 0 | 0 | 0 | 3 |  |  |  |  |  |  |  | 7.6993E4 | 1 | 1 | 0 | 0 | 0 | 0 | 0 | 0 | 0 | 1 | N | 33620 | hypothetical protein, partial [Micrurus corallinus] |
| 329 | 3063 | ETE69996.1 | 52.98 | 3 | 0 | 0 | 0 | 0 | 0 | 0 | 0 | 3 |  |  |  |  |  |  |  | 7.6993E4 | 1 | 1 | 0 | 0 | 0 | 0 | 0 | 0 | 0 | 1 | N | 36174 | Purine nucleoside phosphorylase, partial [Ophiophagus hannah] |
| 329 | 3064 | XP\_032091539.1 | 52.98 | 3 | 0 | 0 | 0 | 0 | 0 | 0 | 0 | 3 |  |  |  |  |  |  |  | 7.6993E4 | 1 | 1 | 0 | 0 | 0 | 0 | 0 | 0 | 0 | 1 | N | 33365 | LOW QUALITY PROTEIN: purine nucleoside phosphorylase [Thamnophis elegans] |
| 329 | 3386 | XP\_034267396.1 | 52.98 | 3 | 0 | 0 | 0 | 0 | 0 | 0 | 0 | 3 |  |  |  |  |  |  |  | 7.6993E4 | 1 | 1 | 0 | 0 | 0 | 0 | 0 | 0 | 0 | 1 | N | 32340 | purine nucleoside phosphorylase [Pantherophis guttatus] |
| 346 | 3140 | XP\_013908450.1 | 52.71 | 8 | 0 | 0 | 0 | 0 | 0 | 0 | 0 | 8 |  |  |  |  |  |  |  | 1.1916E4 | 1 | 1 | 0 | 0 | 0 | 0 | 0 | 0 | 0 | 1 | N | 14128 | PREDICTED: ADP-ribosylation factor 1 [Thamnophis sirtalis] |
| 346 | 3141 | JAG68790.1 | 52.71 | 6 | 0 | 0 | 0 | 0 | 0 | 0 | 0 | 6 |  |  |  |  |  |  |  | 1.1916E4 | 1 | 1 | 0 | 0 | 0 | 0 | 0 | 0 | 0 | 1 | N | 20697 | ADP-ribosylation factor 1 [Boiga irregularis] |
| 346 | 3142 | JAV51323.1 | 52.71 | 6 | 0 | 0 | 0 | 0 | 0 | 0 | 0 | 6 |  |  |  |  |  |  |  | 1.1916E4 | 1 | 1 | 0 | 0 | 0 | 0 | 0 | 0 | 0 | 1 | N | 20697 | ADP-ribosylation factor 1 [Agkistrodon contortrix contortrix] |
| 346 | 3143 | JAC96314.1 | 52.71 | 6 | 0 | 0 | 0 | 0 | 0 | 0 | 0 | 6 |  |  |  |  |  |  |  | 1.1916E4 | 1 | 1 | 0 | 0 | 0 | 0 | 0 | 0 | 0 | 1 | N | 20601 | ADP-ribosylation factor 3 [Hypsiglena sp. JMG-2014] |
| 346 | 3144 | JAI10317.1 | 52.71 | 6 | 0 | 0 | 0 | 0 | 0 | 0 | 0 | 6 |  |  |  |  |  |  |  | 1.1916E4 | 1 | 1 | 0 | 0 | 0 | 0 | 0 | 0 | 0 | 1 | N | 20697 | ADP-ribosylation factor 1 [Micrurus fulvius] |
| 346 | 3145 | JAB54735.1 | 52.71 | 6 | 0 | 0 | 0 | 0 | 0 | 0 | 0 | 6 |  |  |  |  |  |  |  | 1.1916E4 | 1 | 1 | 0 | 0 | 0 | 0 | 0 | 0 | 0 | 1 | N | 20697 | ADP-ribosylation factor 1 [Micrurus fulvius] |
| 346 | 3146 | JAG45931.1 | 52.71 | 6 | 0 | 0 | 0 | 0 | 0 | 0 | 0 | 6 |  |  |  |  |  |  |  | 1.1916E4 | 1 | 1 | 0 | 0 | 0 | 0 | 0 | 0 | 0 | 1 | N | 20697 | ADP-ribosylation factor 1 [Crotalus horridus] |
| 346 | 3147 | AFJ49358.1 | 52.71 | 6 | 0 | 0 | 0 | 0 | 0 | 0 | 0 | 6 |  |  |  |  |  |  |  | 1.1916E4 | 1 | 1 | 0 | 0 | 0 | 0 | 0 | 0 | 0 | 1 | N | 20697 | ADP-ribosylation factor 1 [Crotalus adamanteus] |
| 346 | 3148 | JAA97845.1 | 52.71 | 6 | 0 | 0 | 0 | 0 | 0 | 0 | 0 | 6 |  |  |  |  |  |  |  | 1.1916E4 | 1 | 1 | 0 | 0 | 0 | 0 | 0 | 0 | 0 | 1 | N | 20697 | ADP-ribosylation factor 1 [Crotalus horridus] |
| 346 | 3149 | JAC96307.1 | 52.71 | 6 | 0 | 0 | 0 | 0 | 0 | 0 | 0 | 6 |  |  |  |  |  |  |  | 1.1916E4 | 1 | 1 | 0 | 0 | 0 | 0 | 0 | 0 | 0 | 1 | N | 20697 | ADP-ribosylation factor 1 [Hypsiglena sp. JMG-2014] |
| 346 | 3150 | ETE72302.1 | 52.71 | 6 | 0 | 0 | 0 | 0 | 0 | 0 | 0 | 6 |  |  |  |  |  |  |  | 1.1916E4 | 1 | 1 | 0 | 0 | 0 | 0 | 0 | 0 | 0 | 1 | N | 20697 | ADP-ribosylation factor 1 [Ophiophagus hannah] |
| 346 | 3151 | AFJ49361.1 | 52.71 | 6 | 0 | 0 | 0 | 0 | 0 | 0 | 0 | 6 |  |  |  |  |  |  |  | 1.1916E4 | 1 | 1 | 0 | 0 | 0 | 0 | 0 | 0 | 0 | 1 | N | 20601 | ADP-ribosylation factor 3 [Crotalus adamanteus] |
| 346 | 3152 | XP\_013911895.1 | 52.71 | 6 | 0 | 0 | 0 | 0 | 0 | 0 | 0 | 6 |  |  |  |  |  |  |  | 1.1916E4 | 1 | 1 | 0 | 0 | 0 | 0 | 0 | 0 | 0 | 1 | N | 20729 | PREDICTED: ADP-ribosylation factor 1 [Thamnophis sirtalis] |
| 346 | 3153 | JAG68788.1 | 52.71 | 6 | 0 | 0 | 0 | 0 | 0 | 0 | 0 | 6 |  |  |  |  |  |  |  | 1.1916E4 | 1 | 1 | 0 | 0 | 0 | 0 | 0 | 0 | 0 | 1 | N | 20601 | ADP-ribosylation factor 3 [Boiga irregularis] |
| 346 | 3154 | JAG45930.1 | 52.71 | 6 | 0 | 0 | 0 | 0 | 0 | 0 | 0 | 6 |  |  |  |  |  |  |  | 1.1916E4 | 1 | 1 | 0 | 0 | 0 | 0 | 0 | 0 | 0 | 1 | N | 20697 | ADP-ribosylation factor 1 [Crotalus horridus] |
| 346 | 3155 | JAC96305.1 | 52.71 | 6 | 0 | 0 | 0 | 0 | 0 | 0 | 0 | 6 |  |  |  |  |  |  |  | 1.1916E4 | 1 | 1 | 0 | 0 | 0 | 0 | 0 | 0 | 0 | 1 | N | 20601 | ADP-ribosylation factor 3 [Hypsiglena sp. JMG-2014] |
| 346 | 3156 | JAA97840.1 | 52.71 | 6 | 0 | 0 | 0 | 0 | 0 | 0 | 0 | 6 |  |  |  |  |  |  |  | 1.1916E4 | 1 | 1 | 0 | 0 | 0 | 0 | 0 | 0 | 0 | 1 | N | 20601 | ADP-ribosylation factor 3 [Crotalus horridus] |
| 346 | 3157 | ETE74069.1 | 52.71 | 6 | 0 | 0 | 0 | 0 | 0 | 0 | 0 | 6 |  |  |  |  |  |  |  | 1.1916E4 | 1 | 1 | 0 | 0 | 0 | 0 | 0 | 0 | 0 | 1 | N | 20601 | ADP-ribosylation factor 3 [Ophiophagus hannah] |
| 346 | 3158 | JAG47534.1 | 52.71 | 6 | 0 | 0 | 0 | 0 | 0 | 0 | 0 | 6 |  |  |  |  |  |  |  | 1.1916E4 | 1 | 1 | 0 | 0 | 0 | 0 | 0 | 0 | 0 | 1 | N | 20601 | ADP-ribosylation factor 3 [Crotalus horridus] |
| 346 | 3159 | JAI14426.1 | 52.71 | 6 | 0 | 0 | 0 | 0 | 0 | 0 | 0 | 6 |  |  |  |  |  |  |  | 1.1916E4 | 1 | 1 | 0 | 0 | 0 | 0 | 0 | 0 | 0 | 1 | N | 20601 | ADP-ribosylation factor 3 [Crotalus adamanteus] |
| 346 | 3160 | JAB54737.1 | 52.71 | 6 | 0 | 0 | 0 | 0 | 0 | 0 | 0 | 6 |  |  |  |  |  |  |  | 1.1916E4 | 1 | 1 | 0 | 0 | 0 | 0 | 0 | 0 | 0 | 1 | N | 20601 | ADP-ribosylation factor 1 [Micrurus fulvius] |
| 346 | 3161 | JAA97844.1 | 52.71 | 6 | 0 | 0 | 0 | 0 | 0 | 0 | 0 | 6 |  |  |  |  |  |  |  | 1.1916E4 | 1 | 1 | 0 | 0 | 0 | 0 | 0 | 0 | 0 | 1 | N | 20697 | ADP-ribosylation factor 1 [Crotalus horridus] |
| 346 | 3162 | XP\_013922416.1 | 52.71 | 6 | 0 | 0 | 0 | 0 | 0 | 0 | 0 | 6 |  |  |  |  |  |  |  | 1.1916E4 | 1 | 1 | 0 | 0 | 0 | 0 | 0 | 0 | 0 | 1 | N | 20601 | PREDICTED: ADP-ribosylation factor 3 [Thamnophis sirtalis] |
| 346 | 3163 | JAI14429.1 | 52.71 | 6 | 0 | 0 | 0 | 0 | 0 | 0 | 0 | 6 |  |  |  |  |  |  |  | 1.1916E4 | 1 | 1 | 0 | 0 | 0 | 0 | 0 | 0 | 0 | 1 | N | 20697 | ADP-ribosylation factor 1 [Crotalus adamanteus] |
| 346 | 3164 | JAI10315.1 | 52.71 | 6 | 0 | 0 | 0 | 0 | 0 | 0 | 0 | 6 |  |  |  |  |  |  |  | 1.1916E4 | 1 | 1 | 0 | 0 | 0 | 0 | 0 | 0 | 0 | 1 | N | 20601 | ADP-ribosylation factor 3 [Micrurus fulvius] |
| 346 | 3165 | JAG45927.1 | 52.71 | 6 | 0 | 0 | 0 | 0 | 0 | 0 | 0 | 6 |  |  |  |  |  |  |  | 1.1916E4 | 1 | 1 | 0 | 0 | 0 | 0 | 0 | 0 | 0 | 1 | N | 20601 | ADP-ribosylation factor 3 [Crotalus horridus] |
| 346 | 3166 | JAV51321.1 | 52.71 | 6 | 0 | 0 | 0 | 0 | 0 | 0 | 0 | 6 |  |  |  |  |  |  |  | 1.1916E4 | 1 | 1 | 0 | 0 | 0 | 0 | 0 | 0 | 0 | 1 | N | 20601 | ADP-ribosylation factor 3 [Agkistrodon contortrix contortrix] |
| 346 | 3167 | AFJ49360.1 | 52.71 | 6 | 0 | 0 | 0 | 0 | 0 | 0 | 0 | 6 |  |  |  |  |  |  |  | 1.1916E4 | 1 | 1 | 0 | 0 | 0 | 0 | 0 | 0 | 0 | 1 | N | 20697 | ADP-ribosylation factor 1 [Crotalus adamanteus] |
| 346 | 3168 | JAA97843.1 | 52.71 | 6 | 0 | 0 | 0 | 0 | 0 | 0 | 0 | 6 |  |  |  |  |  |  |  | 1.1916E4 | 1 | 1 | 0 | 0 | 0 | 0 | 0 | 0 | 0 | 1 | N | 20697 | ADP-ribosylation factor 1 [Crotalus horridus] |
| 346 | 3169 | JAG47537.1 | 52.71 | 6 | 0 | 0 | 0 | 0 | 0 | 0 | 0 | 6 |  |  |  |  |  |  |  | 1.1916E4 | 1 | 1 | 0 | 0 | 0 | 0 | 0 | 0 | 0 | 1 | N | 20697 | ADP-ribosylation factor 1 [Crotalus horridus] |
| 324 | 2039 | ETE73522.1 | 51.77 | 4 | 0 | 0 | 0 | 0 | 0 | 0 | 0 | 4 |  |  |  |  |  |  |  | 4.84E4 | 1 | 1 | 0 | 0 | 0 | 0 | 0 | 0 | 0 | 1 | N | 27615 | Proteasome subunit alpha type-3 [Ophiophagus hannah] |
| 324 | 2040 | XP\_013912175.1 | 51.77 | 4 | 0 | 0 | 0 | 0 | 0 | 0 | 0 | 4 |  |  |  |  |  |  |  | 4.84E4 | 1 | 1 | 0 | 0 | 0 | 0 | 0 | 0 | 0 | 1 | N | 28424 | PREDICTED: proteasome subunit alpha type-3 [Thamnophis sirtalis] |
| 324 | 2041 | JAG66919.1 | 51.77 | 4 | 0 | 0 | 0 | 0 | 0 | 0 | 0 | 4 |  |  |  |  |  |  |  | 4.84E4 | 1 | 1 | 0 | 0 | 0 | 0 | 0 | 0 | 0 | 1 | N | 28410 | Proteasome subunit alpha type-3-like protein [Boiga irregularis] |
| 324 | 2042 | XP\_032092012.1 | 51.77 | 4 | 0 | 0 | 0 | 0 | 0 | 0 | 0 | 4 |  |  |  |  |  |  |  | 4.84E4 | 1 | 1 | 0 | 0 | 0 | 0 | 0 | 0 | 0 | 1 | N | 28424 | proteasome subunit alpha type-3 [Thamnophis elegans] |
| 324 | 2043 | XP\_034289368.1 | 51.77 | 4 | 0 | 0 | 0 | 0 | 0 | 0 | 0 | 4 |  |  |  |  |  |  |  | 4.84E4 | 1 | 1 | 0 | 0 | 0 | 0 | 0 | 0 | 0 | 1 | N | 28352 | proteasome subunit alpha type-3 [Pantherophis guttatus] |
| 324 | 2044 | XP\_026554461.1 | 51.77 | 4 | 0 | 0 | 0 | 0 | 0 | 0 | 0 | 4 |  |  |  |  |  |  |  | 4.84E4 | 1 | 1 | 0 | 0 | 0 | 0 | 0 | 0 | 0 | 1 | N | 28410 | proteasome subunit alpha type-3 [Pseudonaja textilis] |
| 324 | 2045 | JAI09619.1 | 51.77 | 4 | 0 | 0 | 0 | 0 | 0 | 0 | 0 | 4 |  |  |  |  |  |  |  | 4.84E4 | 1 | 1 | 0 | 0 | 0 | 0 | 0 | 0 | 0 | 1 | N | 28410 | Proteasome subunit alpha type-3-like protein [Micrurus fulvius] |
| 324 | 2046 | XP\_007422519.1 | 51.77 | 4 | 0 | 0 | 0 | 0 | 0 | 0 | 0 | 4 |  |  |  |  |  |  |  | 4.84E4 | 1 | 1 | 0 | 0 | 0 | 0 | 0 | 0 | 0 | 1 | N | 28410 | proteasome subunit alpha type-3 [Python bivittatus] |
| 324 | 2047 | XP\_026525981.1 | 51.77 | 4 | 0 | 0 | 0 | 0 | 0 | 0 | 0 | 4 |  |  |  |  |  |  |  | 4.84E4 | 1 | 1 | 0 | 0 | 0 | 0 | 0 | 0 | 0 | 1 | N | 28410 | proteasome subunit alpha type-3 [Notechis scutatus] |
| 324 | 2048 | JAG66904.1 | 51.77 | 4 | 0 | 0 | 0 | 0 | 0 | 0 | 0 | 4 |  |  |  |  |  |  |  | 4.84E4 | 1 | 1 | 0 | 0 | 0 | 0 | 0 | 0 | 0 | 1 | N | 28410 | Proteasome subunit alpha type-3-like [Boiga irregularis] |
| 324 | 2049 | JAB53606.1 | 51.77 | 3 | 0 | 0 | 0 | 0 | 0 | 0 | 0 | 3 |  |  |  |  |  |  |  | 4.84E4 | 1 | 1 | 0 | 0 | 0 | 0 | 0 | 0 | 0 | 1 | N | 30605 | Proteasome subunit alpha type-3 [Micrurus fulvius] |
| 324 | 2829 | JAA96035.1 | 51.77 | 4 | 0 | 0 | 0 | 0 | 0 | 0 | 0 | 4 |  |  |  |  |  |  |  | 4.84E4 | 1 | 1 | 0 | 0 | 0 | 0 | 0 | 0 | 0 | 1 | N | 28356 | Proteasome subunit alpha type-3-like protein [Crotalus horridus] |
| 324 | 2830 | XP\_015676739.1 | 51.77 | 4 | 0 | 0 | 0 | 0 | 0 | 0 | 0 | 4 |  |  |  |  |  |  |  | 4.84E4 | 1 | 1 | 0 | 0 | 0 | 0 | 0 | 0 | 0 | 1 | N | 28383 | proteasome subunit alpha type-3 [Protobothrops mucrosquamatus] |
| 324 | 2831 | JAV49555.1 | 51.77 | 4 | 0 | 0 | 0 | 0 | 0 | 0 | 0 | 4 |  |  |  |  |  |  |  | 4.84E4 | 1 | 1 | 0 | 0 | 0 | 0 | 0 | 0 | 0 | 1 | N | 28356 | Proteasome subunit alpha type-3-like protein [Agkistrodon contortrix contortrix] |
| 324 | 2832 | JAI11930.1 | 51.77 | 4 | 0 | 0 | 0 | 0 | 0 | 0 | 0 | 4 |  |  |  |  |  |  |  | 4.84E4 | 1 | 1 | 0 | 0 | 0 | 0 | 0 | 0 | 0 | 1 | N | 28356 | Proteasome subunit alpha type-3-like protein [Crotalus adamanteus] |
| 324 | 2833 | AFJ51036.1 | 51.77 | 3 | 0 | 0 | 0 | 0 | 0 | 0 | 0 | 3 |  |  |  |  |  |  |  | 4.84E4 | 1 | 1 | 0 | 0 | 0 | 0 | 0 | 0 | 0 | 1 | N | 30581 | Proteasome subunit alpha type-3-like [Crotalus adamanteus] |
| 310 | 1676 | LAA64694.1 | 48.00 | 3 | 0 | 0 | 0 | 0 | 0 | 0 | 0 | 3 |  |  |  |  |  |  |  | 8.4212E4 | 1 | 1 | 0 | 0 | 0 | 0 | 0 | 0 | 0 | 1 | N | 31963 | hypothetical protein [Micrurus corallinus] |
| 310 | 1677 | LAA64691.1 | 48.00 | 3 | 0 | 0 | 0 | 0 | 0 | 0 | 0 | 3 |  |  |  |  |  |  |  | 8.4212E4 | 1 | 1 | 0 | 0 | 0 | 0 | 0 | 0 | 0 | 1 | N | 31963 | hypothetical protein [Micrurus corallinus] |
| 310 | 1678 | JAB54700.1 | 48.00 | 3 | 0 | 0 | 0 | 0 | 0 | 0 | 0 | 3 |  |  |  |  |  |  |  | 8.4212E4 | 1 | 1 | 0 | 0 | 0 | 0 | 0 | 0 | 0 | 1 | N | 31963 | Voltage-dependent anion-selective channel protein 2-like protein [Micrurus fulvius] |
| 310 | 1701 | JAI10292.1 | 48.00 | 4 | 0 | 0 | 0 | 0 | 0 | 0 | 0 | 4 |  |  |  |  |  |  |  | 8.4212E4 | 1 | 1 | 0 | 0 | 0 | 0 | 0 | 0 | 0 | 1 | N | 30330 | Voltage-dependent anion-selective channel protein 2 [Micrurus fulvius] |
| 310 | 1702 | XP\_034291314.1 | 48.00 | 4 | 0 | 0 | 0 | 0 | 0 | 0 | 0 | 4 |  |  |  |  |  |  |  | 8.4212E4 | 1 | 1 | 0 | 0 | 0 | 0 | 0 | 0 | 0 | 1 | N | 30330 | voltage-dependent anion-selective channel protein 2 isoform X2 [Pantherophis guttatus] |
| 310 | 1703 | JAC96265.1 | 48.00 | 4 | 0 | 0 | 0 | 0 | 0 | 0 | 0 | 4 |  |  |  |  |  |  |  | 8.4212E4 | 1 | 1 | 0 | 0 | 0 | 0 | 0 | 0 | 0 | 1 | N | 30314 | Voltage-dependent anion-selective channel protein 2 [Hypsiglena sp. JMG-2014] |
| 310 | 1704 | LAA64698.1 | 48.00 | 4 | 0 | 0 | 0 | 0 | 0 | 0 | 0 | 4 |  |  |  |  |  |  |  | 8.4212E4 | 1 | 1 | 0 | 0 | 0 | 0 | 0 | 0 | 0 | 1 | N | 30330 | hypothetical protein [Micrurus corallinus] |
| 310 | 1705 | JAG45846.1 | 48.00 | 4 | 0 | 0 | 0 | 0 | 0 | 0 | 0 | 4 |  |  |  |  |  |  |  | 8.4212E4 | 1 | 1 | 0 | 0 | 0 | 0 | 0 | 0 | 0 | 1 | N | 30330 | Voltage-dependent anion-selective channel protein 2 [Crotalus horridus] |
| 310 | 1706 | JAI14353.1 | 48.00 | 4 | 0 | 0 | 0 | 0 | 0 | 0 | 0 | 4 |  |  |  |  |  |  |  | 8.4212E4 | 1 | 1 | 0 | 0 | 0 | 0 | 0 | 0 | 0 | 1 | N | 30330 | Voltage-dependent anion-selective channel protein 2 [Crotalus adamanteus] |
| 310 | 1707 | JAA97782.1 | 48.00 | 4 | 0 | 0 | 0 | 0 | 0 | 0 | 0 | 4 |  |  |  |  |  |  |  | 8.4212E4 | 1 | 1 | 0 | 0 | 0 | 0 | 0 | 0 | 0 | 1 | N | 30330 | Voltage-dependent anion-selective channel protein 2 [Crotalus horridus] |
| 310 | 1708 | JAV51267.1 | 48.00 | 4 | 0 | 0 | 0 | 0 | 0 | 0 | 0 | 4 |  |  |  |  |  |  |  | 8.4212E4 | 1 | 1 | 0 | 0 | 0 | 0 | 0 | 0 | 0 | 1 | N | 30330 | Voltage-dependent anion-selective channel protein 2 [Agkistrodon contortrix contortrix] |
| 310 | 1709 | JAG68733.1 | 48.00 | 4 | 0 | 0 | 0 | 0 | 0 | 0 | 0 | 4 |  |  |  |  |  |  |  | 8.4212E4 | 1 | 1 | 0 | 0 | 0 | 0 | 0 | 0 | 0 | 1 | N | 30330 | Voltage-dependent anion-selective channel protein 2 [Boiga irregularis] |
| 310 | 1710 | XP\_026579473.1 | 48.00 | 4 | 0 | 0 | 0 | 0 | 0 | 0 | 0 | 4 |  |  |  |  |  |  |  | 8.4212E4 | 1 | 1 | 0 | 0 | 0 | 0 | 0 | 0 | 0 | 1 | N | 30314 | voltage-dependent anion-selective channel protein 2 isoform X2 [Pseudonaja textilis] |
| 310 | 1711 | JAG47483.1 | 48.00 | 4 | 0 | 0 | 0 | 0 | 0 | 0 | 0 | 4 |  |  |  |  |  |  |  | 8.4212E4 | 1 | 1 | 0 | 0 | 0 | 0 | 0 | 0 | 0 | 1 | N | 30330 | Voltage-dependent anion-selective channel protein 2 [Crotalus horridus] |
| 310 | 1712 | XP\_034291315.1 | 48.00 | 4 | 0 | 0 | 0 | 0 | 0 | 0 | 0 | 4 |  |  |  |  |  |  |  | 8.4212E4 | 1 | 1 | 0 | 0 | 0 | 0 | 0 | 0 | 0 | 1 | N | 30330 | voltage-dependent anion-selective channel protein 2 isoform X2 [Pantherophis guttatus] |
| 310 | 1713 | XP\_026579477.1 | 48.00 | 4 | 0 | 0 | 0 | 0 | 0 | 0 | 0 | 4 |  |  |  |  |  |  |  | 8.4212E4 | 1 | 1 | 0 | 0 | 0 | 0 | 0 | 0 | 0 | 1 | N | 30314 | voltage-dependent anion-selective channel protein 2 isoform X2 [Pseudonaja textilis] |
| 310 | 1714 | XP\_032087627.1 | 48.00 | 3 | 0 | 0 | 0 | 0 | 0 | 0 | 0 | 3 |  |  |  |  |  |  |  | 8.4212E4 | 1 | 1 | 0 | 0 | 0 | 0 | 0 | 0 | 0 | 1 | N | 31895 | voltage-dependent anion-selective channel protein 2 [Thamnophis elegans] |
| 310 | 1715 | XP\_026579471.1 | 48.00 | 3 | 0 | 0 | 0 | 0 | 0 | 0 | 0 | 3 |  |  |  |  |  |  |  | 8.4212E4 | 1 | 1 | 0 | 0 | 0 | 0 | 0 | 0 | 0 | 1 | N | 31963 | voltage-dependent anion-selective channel protein 2 isoform X1 [Pseudonaja textilis] |
| 310 | 1716 | XP\_026579476.1 | 48.00 | 3 | 0 | 0 | 0 | 0 | 0 | 0 | 0 | 3 |  |  |  |  |  |  |  | 8.4212E4 | 1 | 1 | 0 | 0 | 0 | 0 | 0 | 0 | 0 | 1 | N | 31963 | voltage-dependent anion-selective channel protein 2 isoform X1 [Pseudonaja textilis] |
| 310 | 1717 | XP\_026579475.1 | 48.00 | 3 | 0 | 0 | 0 | 0 | 0 | 0 | 0 | 3 |  |  |  |  |  |  |  | 8.4212E4 | 1 | 1 | 0 | 0 | 0 | 0 | 0 | 0 | 0 | 1 | N | 31963 | voltage-dependent anion-selective channel protein 2 isoform X1 [Pseudonaja textilis] |
| 310 | 1718 | XP\_013907074.1 | 48.00 | 3 | 0 | 0 | 0 | 0 | 0 | 0 | 0 | 3 |  |  |  |  |  |  |  | 8.4212E4 | 1 | 1 | 0 | 0 | 0 | 0 | 0 | 0 | 0 | 1 | N | 31895 | PREDICTED: voltage-dependent anion-selective channel protein 2 [Thamnophis sirtalis] |
| 310 | 1719 | XP\_026579472.1 | 48.00 | 3 | 0 | 0 | 0 | 0 | 0 | 0 | 0 | 3 |  |  |  |  |  |  |  | 8.4212E4 | 1 | 1 | 0 | 0 | 0 | 0 | 0 | 0 | 0 | 1 | N | 31963 | voltage-dependent anion-selective channel protein 2 isoform X1 [Pseudonaja textilis] |
| 310 | 1720 | AFJ49407.1 | 48.00 | 3 | 0 | 0 | 0 | 0 | 0 | 0 | 0 | 3 |  |  |  |  |  |  |  | 8.4212E4 | 1 | 1 | 0 | 0 | 0 | 0 | 0 | 0 | 0 | 1 | N | 31937 | Voltage-dependent anion-selective channel protein 2-like [Crotalus adamanteus] |
| 310 | 1721 | XP\_026529972.1 | 48.00 | 3 | 0 | 0 | 0 | 0 | 0 | 0 | 0 | 3 |  |  |  |  |  |  |  | 8.4212E4 | 1 | 1 | 0 | 0 | 0 | 0 | 0 | 0 | 0 | 1 | N | 31963 | voltage-dependent anion-selective channel protein 2 [Notechis scutatus] |
| 310 | 1722 | XP\_034291313.1 | 48.00 | 3 | 0 | 0 | 0 | 0 | 0 | 0 | 0 | 3 |  |  |  |  |  |  |  | 8.4212E4 | 1 | 1 | 0 | 0 | 0 | 0 | 0 | 0 | 0 | 1 | N | 31994 | voltage-dependent anion-selective channel protein 2 isoform X1 [Pantherophis guttatus] |
| 310 | 1723 | XP\_029142427.1 | 48.00 | 3 | 0 | 0 | 0 | 0 | 0 | 0 | 0 | 3 |  |  |  |  |  |  |  | 8.4212E4 | 1 | 1 | 0 | 0 | 0 | 0 | 0 | 0 | 0 | 1 | N | 33947 | voltage-dependent anion-selective channel protein 2 [Protobothrops mucrosquamatus] |
| 279 | 1427 | JAI13875.1 | 47.92 | 1 | 0 | 0 | 0 | 0 | 0 | 0 | 0 | 1 |  |  |  |  |  |  |  | 0 | 1 | 1 | 0 | 0 | 0 | 0 | 0 | 0 | 0 | 1 | N | 117203 | Chain A, Crystal Structure Of A Cytoskeletal Protein [Crotalus adamanteus] |
| 279 | 1428 | AFJ52037.1 | 47.92 | 1 | 0 | 0 | 0 | 0 | 0 | 0 | 0 | 1 |  |  |  |  |  |  |  | 0 | 1 | 1 | 0 | 0 | 0 | 0 | 0 | 0 | 0 | 1 | N | 117203 | Vinculin-like [Crotalus adamanteus] |
| 279 | 1429 | JAG65919.1 | 47.92 | 1 | 0 | 0 | 0 | 0 | 0 | 0 | 0 | 1 |  |  |  |  |  |  |  | 0 | 1 | 1 | 0 | 0 | 0 | 0 | 0 | 0 | 0 | 1 | N | 117018 | Vinculin-like [Boiga irregularis] |
| 279 | 1430 | XP\_034291292.1 | 47.92 | 1 | 0 | 0 | 0 | 0 | 0 | 0 | 0 | 1 |  |  |  |  |  |  |  | 0 | 1 | 1 | 0 | 0 | 0 | 0 | 0 | 0 | 0 | 1 | N | 116939 | vinculin isoform X4 [Pantherophis guttatus] |
| 279 | 1431 | XP\_034291291.1 | 47.92 | 1 | 0 | 0 | 0 | 0 | 0 | 0 | 0 | 1 |  |  |  |  |  |  |  | 0 | 1 | 1 | 0 | 0 | 0 | 0 | 0 | 0 | 0 | 1 | N | 117067 | vinculin isoform X3 [Pantherophis guttatus] |
| 279 | 1432 | XP\_034291290.1 | 47.92 | 1 | 0 | 0 | 0 | 0 | 0 | 0 | 0 | 1 |  |  |  |  |  |  |  | 0 | 1 | 1 | 0 | 0 | 0 | 0 | 0 | 0 | 0 | 1 | N | 124885 | vinculin isoform X2 [Pantherophis guttatus] |
| 279 | 1433 | XP\_034291289.1 | 47.92 | 1 | 0 | 0 | 0 | 0 | 0 | 0 | 0 | 1 |  |  |  |  |  |  |  | 0 | 1 | 1 | 0 | 0 | 0 | 0 | 0 | 0 | 0 | 1 | N | 125014 | vinculin isoform X1 [Pantherophis guttatus] |
| 279 | 1434 | XP\_015671947.1 | 47.92 | 1 | 0 | 0 | 0 | 0 | 0 | 0 | 0 | 1 |  |  |  |  |  |  |  | 0 | 1 | 1 | 0 | 0 | 0 | 0 | 0 | 0 | 0 | 1 | N | 117418 | vinculin [Protobothrops mucrosquamatus] |
| 279 | 1439 | XP\_007424958.1 | 47.92 | 1 | 0 | 0 | 0 | 0 | 0 | 0 | 0 | 1 |  |  |  |  |  |  |  | 0 | 1 | 1 | 0 | 0 | 0 | 0 | 0 | 0 | 0 | 1 | N | 116824 | vinculin isoform X3 [Python bivittatus] |
| 279 | 1440 | XP\_007424957.1 | 47.92 | 1 | 0 | 0 | 0 | 0 | 0 | 0 | 0 | 1 |  |  |  |  |  |  |  | 0 | 1 | 1 | 0 | 0 | 0 | 0 | 0 | 0 | 0 | 1 | N | 116952 | vinculin isoform X2 [Python bivittatus] |
| 279 | 1441 | XP\_007424956.1 | 47.92 | 1 | 0 | 0 | 0 | 0 | 0 | 0 | 0 | 1 |  |  |  |  |  |  |  | 0 | 1 | 1 | 0 | 0 | 0 | 0 | 0 | 0 | 0 | 1 | N | 124940 | vinculin isoform X1 [Python bivittatus] |
| 279 | 1447 | XP\_026529964.1 | 47.92 | 1 | 0 | 0 | 0 | 0 | 0 | 0 | 0 | 1 |  |  |  |  |  |  |  | 0 | 1 | 1 | 0 | 0 | 0 | 0 | 0 | 0 | 0 | 1 | N | 114355 | vinculin [Notechis scutatus] |
| 279 | 1469 | XP\_032087779.1 | 47.92 | 1 | 0 | 0 | 0 | 0 | 0 | 0 | 0 | 1 |  |  |  |  |  |  |  | 0 | 1 | 1 | 0 | 0 | 0 | 0 | 0 | 0 | 0 | 1 | N | 116986 | vinculin isoform X1 [Thamnophis elegans] |
| 279 | 1470 | XP\_032087780.1 | 47.92 | 1 | 0 | 0 | 0 | 0 | 0 | 0 | 0 | 1 |  |  |  |  |  |  |  | 0 | 1 | 1 | 0 | 0 | 0 | 0 | 0 | 0 | 0 | 1 | N | 125252 | vinculin isoform X2 [Thamnophis elegans] |
| 279 | 2270 | LAA44292.1 | 47.92 | 3 | 0 | 0 | 0 | 0 | 0 | 0 | 0 | 3 |  |  |  |  |  |  |  | 0 | 1 | 1 | 0 | 0 | 0 | 0 | 0 | 0 | 0 | 1 | N | 41176 | hypothetical protein, partial [Micrurus corallinus] |
| 345 | 2254 | XP\_026569149.1 | 47.06 | 6 | 0 | 0 | 0 | 0 | 0 | 0 | 0 | 6 |  |  |  |  |  |  |  | 7.6533E4 | 1 | 1 | 0 | 0 | 0 | 0 | 0 | 0 | 0 | 1 | N | 26885 | triosephosphate isomerase [Pseudonaja textilis] |
| 345 | 2255 | JAI11012.1 | 47.06 | 6 | 0 | 0 | 0 | 0 | 0 | 0 | 0 | 6 |  |  |  |  |  |  |  | 7.6533E4 | 1 | 1 | 0 | 0 | 0 | 0 | 0 | 0 | 0 | 1 | N | 26938 | Triosephosphate isomerase [Crotalus adamanteus] |
| 345 | 2256 | XP\_032069541.1 | 47.06 | 6 | 0 | 0 | 0 | 0 | 0 | 0 | 0 | 6 |  |  |  |  |  |  |  | 7.6533E4 | 1 | 1 | 0 | 0 | 0 | 0 | 0 | 0 | 0 | 1 | N | 26856 | triosephosphate isomerase [Thamnophis elegans] |
| 345 | 2257 | XP\_015669508.1 | 47.06 | 6 | 0 | 0 | 0 | 0 | 0 | 0 | 0 | 6 |  |  |  |  |  |  |  | 7.6533E4 | 1 | 1 | 0 | 0 | 0 | 0 | 0 | 0 | 0 | 1 | N | 26910 | triosephosphate isomerase [Protobothrops mucrosquamatus] |
| 345 | 2258 | XP\_026542021.1 | 47.06 | 6 | 0 | 0 | 0 | 0 | 0 | 0 | 0 | 6 |  |  |  |  |  |  |  | 7.6533E4 | 1 | 1 | 0 | 0 | 0 | 0 | 0 | 0 | 0 | 1 | N | 26885 | triosephosphate isomerase [Notechis scutatus] |
| 345 | 2259 | JAG66211.1 | 47.06 | 6 | 0 | 0 | 0 | 0 | 0 | 0 | 0 | 6 |  |  |  |  |  |  |  | 7.6533E4 | 1 | 1 | 0 | 0 | 0 | 0 | 0 | 0 | 0 | 1 | N | 26881 | Triosephosphate isomerase [Boiga irregularis] |
| 345 | 2260 | XP\_013926209.1 | 47.06 | 6 | 0 | 0 | 0 | 0 | 0 | 0 | 0 | 6 |  |  |  |  |  |  |  | 7.6533E4 | 1 | 1 | 0 | 0 | 0 | 0 | 0 | 0 | 0 | 1 | N | 26829 | PREDICTED: triosephosphate isomerase [Thamnophis sirtalis] |
| 345 | 2261 | JAV48852.1 | 47.06 | 6 | 0 | 0 | 0 | 0 | 0 | 0 | 0 | 6 |  |  |  |  |  |  |  | 7.6533E4 | 1 | 1 | 0 | 0 | 0 | 0 | 0 | 0 | 0 | 1 | N | 26884 | Triosephosphate isomerase [Agkistrodon contortrix contortrix] |
| 345 | 2262 | JAA95339.1 | 47.06 | 6 | 0 | 0 | 0 | 0 | 0 | 0 | 0 | 6 |  |  |  |  |  |  |  | 7.6533E4 | 1 | 1 | 0 | 0 | 0 | 0 | 0 | 0 | 0 | 1 | N | 26910 | Triosephosphate isomerase [Crotalus horridus] |
| 345 | 2263 | JAI09292.1 | 47.06 | 6 | 0 | 0 | 0 | 0 | 0 | 0 | 0 | 6 |  |  |  |  |  |  |  | 7.6533E4 | 1 | 1 | 0 | 0 | 0 | 0 | 0 | 0 | 0 | 1 | N | 26899 | Triosephosphate isomerase [Micrurus fulvius] |
| 345 | 2264 | AFJ51829.1 | 47.06 | 6 | 0 | 0 | 0 | 0 | 0 | 0 | 0 | 6 |  |  |  |  |  |  |  | 7.6533E4 | 1 | 1 | 0 | 0 | 0 | 0 | 0 | 0 | 0 | 1 | N | 26938 | putative triosephosphate isomerase [Crotalus adamanteus] |
| 345 | 2265 | XP\_034266880.1 | 47.06 | 6 | 0 | 0 | 0 | 0 | 0 | 0 | 0 | 6 |  |  |  |  |  |  |  | 7.6533E4 | 1 | 1 | 0 | 0 | 0 | 0 | 0 | 0 | 0 | 1 | N | 26865 | triosephosphate isomerase [Pantherophis guttatus] |
| 345 | 2266 | ETE72092.1 | 47.06 | 6 | 0 | 0 | 0 | 0 | 0 | 0 | 0 | 6 |  |  |  |  |  |  |  | 7.6533E4 | 1 | 1 | 0 | 0 | 0 | 0 | 0 | 0 | 0 | 1 | N | 26899 | Triosephosphate isomerase [Ophiophagus hannah] |
| 345 | 2267 | JAB53077.1 | 47.06 | 6 | 0 | 0 | 0 | 0 | 0 | 0 | 0 | 6 |  |  |  |  |  |  |  | 7.6533E4 | 1 | 1 | 0 | 0 | 0 | 0 | 0 | 0 | 0 | 1 | N | 26899 | triosephosphate isomerase [Micrurus fulvius] |
| 345 | 2268 | XP\_007420484.1 | 47.06 | 6 | 0 | 0 | 0 | 0 | 0 | 0 | 0 | 6 |  |  |  |  |  |  |  | 7.6533E4 | 1 | 1 | 0 | 0 | 0 | 0 | 0 | 0 | 0 | 1 | N | 26830 | triosephosphate isomerase [Python bivittatus] |
| 345 | 3065 | ETE63728.1 | 47.06 | 9 | 0 | 0 | 0 | 0 | 0 | 0 | 0 | 9 |  |  |  |  |  |  |  | 7.6533E4 | 1 | 1 | 0 | 0 | 0 | 0 | 0 | 0 | 0 | 1 | N | 17675 | Triosephosphate isomerase, partial [Ophiophagus hannah] |
| 291 | 1887 | XP\_026555717.1 | 46.57 | 1 | 0 | 0 | 0 | 0 | 0 | 0 | 0 | 1 |  |  |  |  |  |  |  | 4.2276E4 | 1 | 1 | 0 | 0 | 0 | 0 | 0 | 0 | 0 | 1 | N | 120943 | uncharacterized protein LOC113435838 isoform X1 [Pseudonaja textilis] |
| 291 | 2156 | XP\_007421180.1 | 46.57 | 3 | 0 | 0 | 0 | 0 | 0 | 0 | 0 | 3 |  |  |  |  |  |  |  | 4.2276E4 | 1 | 1 | 0 | 0 | 0 | 0 | 0 | 0 | 0 | 1 | N | 47394 | multifunctional protein ADE2 isoform X2 [Python bivittatus] |
| 291 | 2158 | XP\_007421178.1 | 46.57 | 2 | 0 | 0 | 0 | 0 | 0 | 0 | 0 | 2 |  |  |  |  |  |  |  | 4.2276E4 | 1 | 1 | 0 | 0 | 0 | 0 | 0 | 0 | 0 | 1 | N | 76846 | multifunctional protein ADE2 isoform X1 [Python bivittatus] |
| 291 | 2188 | ETE65717.1 | 46.57 | 3 | 0 | 0 | 0 | 0 | 0 | 0 | 0 | 3 |  |  |  |  |  |  |  | 4.2276E4 | 1 | 1 | 0 | 0 | 0 | 0 | 0 | 0 | 0 | 1 | N | 45048 | Multifunctional protein ADE2, partial [Ophiophagus hannah] |
| 291 | 2189 | LAA41266.1 | 46.57 | 3 | 0 | 0 | 0 | 0 | 0 | 0 | 0 | 3 |  |  |  |  |  |  |  | 4.2276E4 | 1 | 1 | 0 | 0 | 0 | 0 | 0 | 0 | 0 | 1 | N | 47451 | hypothetical protein [Micrurus corallinus] |
| 291 | 2190 | LAA22516.1 | 46.57 | 3 | 0 | 0 | 0 | 0 | 0 | 0 | 0 | 3 |  |  |  |  |  |  |  | 4.2276E4 | 1 | 1 | 0 | 0 | 0 | 0 | 0 | 0 | 0 | 1 | N | 47511 | hypothetical protein [Micrurus lemniscatus carvalhoi] |
| 291 | 2191 | LAB38276.1 | 46.57 | 3 | 0 | 0 | 0 | 0 | 0 | 0 | 0 | 3 |  |  |  |  |  |  |  | 4.2276E4 | 1 | 1 | 0 | 0 | 0 | 0 | 0 | 0 | 0 | 1 | N | 47513 | hypothetical protein [Micrurus spixii] |
| 291 | 2222 | JAC96332.1 | 46.57 | 3 | 0 | 0 | 0 | 0 | 0 | 0 | 0 | 3 |  |  |  |  |  |  |  | 4.2276E4 | 1 | 1 | 0 | 0 | 0 | 0 | 0 | 0 | 0 | 1 | N | 47551 | Multifunctional protein ADE2-like [Hypsiglena sp. JMG-2014] |
| 291 | 2418 | XP\_013928441.1 | 46.57 | 4 | 0 | 0 | 0 | 0 | 0 | 0 | 0 | 4 |  |  |  |  |  |  |  | 4.2276E4 | 1 | 1 | 0 | 0 | 0 | 0 | 0 | 0 | 0 | 1 | N | 43862 | PREDICTED: multifunctional protein ADE2 [Thamnophis sirtalis] |
| 291 | 2510 | XP\_029142087.1 | 46.57 | 4 | 0 | 0 | 0 | 0 | 0 | 0 | 0 | 4 |  |  |  |  |  |  |  | 4.2276E4 | 1 | 1 | 0 | 0 | 0 | 0 | 0 | 0 | 0 | 1 | N | 37380 | multifunctional protein ADE2 [Protobothrops mucrosquamatus] |
| 291 | 2666 | JAI14454.1 | 46.57 | 3 | 0 | 0 | 0 | 0 | 0 | 0 | 0 | 3 |  |  |  |  |  |  |  | 4.2276E4 | 1 | 1 | 0 | 0 | 0 | 0 | 0 | 0 | 0 | 1 | N | 47557 | Multifunctional protein ADE2-like protein [Crotalus adamanteus] |
| 291 | 2667 | JAG47557.1 | 46.57 | 3 | 0 | 0 | 0 | 0 | 0 | 0 | 0 | 3 |  |  |  |  |  |  |  | 4.2276E4 | 1 | 1 | 0 | 0 | 0 | 0 | 0 | 0 | 0 | 1 | N | 47557 | Multifunctional protein ADE2-like protein [Crotalus horridus] |
| 291 | 2668 | JAA97867.1 | 46.57 | 3 | 0 | 0 | 0 | 0 | 0 | 0 | 0 | 3 |  |  |  |  |  |  |  | 4.2276E4 | 1 | 1 | 0 | 0 | 0 | 0 | 0 | 0 | 0 | 1 | N | 47557 | Multifunctional protein ADE2-like protein [Crotalus horridus] |
| 291 | 2669 | AFJ50690.1 | 46.57 | 3 | 0 | 0 | 0 | 0 | 0 | 0 | 0 | 3 |  |  |  |  |  |  |  | 4.2276E4 | 1 | 1 | 0 | 0 | 0 | 0 | 0 | 0 | 0 | 1 | N | 47557 | Multifunctional protein ADE2-like [Crotalus adamanteus] |
| 291 | 2670 | JAG45959.1 | 46.57 | 3 | 0 | 0 | 0 | 0 | 0 | 0 | 0 | 3 |  |  |  |  |  |  |  | 4.2276E4 | 1 | 1 | 0 | 0 | 0 | 0 | 0 | 0 | 0 | 1 | N | 47557 | Multifunctional protein ADE2-like protein [Crotalus horridus] |
| 291 | 2671 | XP\_034267284.1 | 46.57 | 3 | 0 | 0 | 0 | 0 | 0 | 0 | 0 | 3 |  |  |  |  |  |  |  | 4.2276E4 | 1 | 1 | 0 | 0 | 0 | 0 | 0 | 0 | 0 | 1 | N | 47536 | multifunctional protein ADE2 [Pantherophis guttatus] |
| 291 | 2760 | JAB54751.1 | 46.57 | 3 | 0 | 0 | 0 | 0 | 0 | 0 | 0 | 3 |  |  |  |  |  |  |  | 4.2276E4 | 1 | 1 | 0 | 0 | 0 | 0 | 0 | 0 | 0 | 1 | N | 47511 | protein ADE2 [Micrurus fulvius] |
| 291 | 2761 | LAA95407.1 | 46.57 | 3 | 0 | 0 | 0 | 0 | 0 | 0 | 0 | 3 |  |  |  |  |  |  |  | 4.2276E4 | 1 | 1 | 0 | 0 | 0 | 0 | 0 | 0 | 0 | 1 | N | 47527 | hypothetical protein [Micrurus lemniscatus lemniscatus] |
| 336 | 2735 | XP\_007443118.1 | 46.24 | 10 | 0 | 0 | 0 | 0 | 0 | 0 | 0 | 10 |  |  |  |  |  |  |  | 8.378E4 | 1 | 1 | 0 | 0 | 0 | 0 | 0 | 0 | 0 | 1 | N | 16348 | phosphatidylethanolamine-binding protein 1, partial [Python bivittatus] |
| total 605 proteins |  |  |  |  |  |  |  |  |  |  |  |  |  |  |  |  |  |  |  |  |  |  |  |  |  |  |  |  |  |  |  |  |  |
| --- | --- | --- | --- | --- | --- | --- | --- | --- | --- | --- | --- | --- | --- | --- | --- | --- | --- | --- | --- | --- | --- | --- | --- | --- | --- | --- | --- | --- | --- | --- | --- | --- | --- |

  

BAN89426.1

back to list

  

| Protein Coverage
| Supporting Peptides
|

Protein Coverage:

Supporting Peptides:

| Peptide | Uniq | -10lgP | Mass | Length | ppm | m/z | z | RT | Fraction | Scan | Source File | Area F6 | Area F2 | Area F3 | Area F4 | Area F5 | Area F7 | Area F8 | Area F1 | #Feature | #Feature F6 | #Feature F2 | #Feature F3 | #Feature F4 | #Feature F5 | #Feature F7 | #Feature F8 | #Feature F1 | Start | End | PTM | AScore | Found By |
| --- | --- | --- | --- | --- | --- | --- | --- | --- | --- | --- | --- | --- | --- | --- | --- | --- | --- | --- | --- | --- | --- | --- | --- | --- | --- | --- | --- | --- | --- | --- | --- | --- | --- |
| R.NPAWWGGQPIWHTATYQGLK.A | N | 103.11 | 2310.1333 | 20 | -0.2 | 1156.0737 | 2 | 50.36 | 8 | F8:28928 | 31122021\_RID\_3262\_1b.raw |  |  |  |  |  |  |  | 3.6924E7 | 2 | 0 | 0 | 0 | 0 | 0 | 0 | 0 | 2 | 223 | 242 |  |  | PEAKS DB |
| K.SMQAIFLAHGPGFNEK.N | N | 103.04 | 1745.8558 | 16 | -0.2 | 873.9351 | 2 | 42.38 | 8 | F8:22729 | 31122021\_RID\_3262\_1b.raw |  |  | 3.2331E5 |  |  |  |  | 8.2355E7 | 3 | 0 | 0 | 1 | 0 | 0 | 0 | 0 | 2 | 467 | 482 |  |  | PEAKS DB |
| N.VDFFMYEGPAPR.I | N | 96.79 | 1427.6543 | 12 | -0.1 | 714.8344 | 2 | 51.87 | 8 | F8:30116 | 31122021\_RID\_3262\_1b.raw |  | 8.9339E5 |  |  |  |  |  | 1.2101E8 | 4 | 0 | 1 | 0 | 0 | 0 | 0 | 0 | 3 | 370 | 381 |  |  | PEAKS DB |
| K.NVPKDFYTFDSEGIVK.N | N | 95.90 | 1857.9148 | 16 | 0.4 | 620.3124 | 3 | 43.93 | 8 | F8:24080 | 31122021\_RID\_3262\_1b.raw |  |  | 1.1786E6 |  |  |  |  | 3.437E7 | 4 | 0 | 0 | 2 | 0 | 0 | 0 | 0 | 2 | 386 | 401 |  |  | PEAKS DB |
| R.DVELLTGLNFYSGLK.Q | N | 94.09 | 1667.8770 | 15 | 0.8 | 834.9464 | 2 | 66.30 | 8 | F8:40834 | 31122021\_RID\_3262\_1b.raw |  |  |  |  |  |  |  | 3.1554E7 | 4 | 0 | 0 | 0 | 0 | 0 | 0 | 0 | 4 | 813 | 827 |  |  | PEAKS DB |
| K.YISAYSQDILMPLWSSYTIYR.S | N | 93.53 | 2569.2563 | 21 | 0.8 | 857.4268 | 3 | 70.86 | 8 | F8:42937 | 31122021\_RID\_3262\_1b.raw |  |  |  |  |  |  |  | 5.1443E6 | 2 | 0 | 0 | 0 | 0 | 0 | 0 | 0 | 2 | 603 | 623 |  |  | PEAKS DB |
| K.SM(+15.99)QAIFLAHGPGFNEK.N | N | 91.55 | 1761.8508 | 16 | 0.1 | 881.9327 | 2 | 36.86 | 8 | F8:18333 | 31122021\_RID\_3262\_1b.raw |  |  | 1.011E6 |  |  |  |  | 2.6382E7 | 8 | 0 | 0 | 1 | 0 | 0 | 0 | 0 | 7 | 467 | 482 | Oxidation (M) | M2:Oxidation (M):1000.00 | PEAKS DB |
| R.IDKVNLM(+15.99)VDQQWMAVR.D | N | 89.72 | 1960.9863 | 16 | 1.2 | 981.5016 | 2 | 45.32 | 8 | F8:25068 | 31122021\_RID\_3262\_1b.raw |  |  |  |  |  |  |  | 2.3151E7 | 4 | 0 | 0 | 0 | 0 | 0 | 0 | 0 | 4 | 432 | 447 | Oxidation (M) | M7:Oxidation (M):182.75 | PEAKS DB |
| R.IDKVNLMVDQQWMAVR.D | N | 88.44 | 1944.9913 | 16 | 0.4 | 973.5034 | 2 | 48.73 | 8 | F8:27572 | 31122021\_RID\_3262\_1b.raw |  |  |  |  |  |  |  | 9.0594E6 | 3 | 0 | 0 | 0 | 0 | 0 | 0 | 0 | 3 | 432 | 447 |  |  | PEAKS DB |
| R.IDKVNLMVDQQWM(+15.99)AVR.D | N | 86.82 | 1960.9863 | 16 | 0.3 | 654.6696 | 3 | 44.18 | 8 | F8:23999 | 31122021\_RID\_3262\_1b.raw |  |  |  |  |  |  |  | 6.7254E6 | 5 | 0 | 0 | 0 | 0 | 0 | 0 | 0 | 5 | 432 | 447 | Oxidation (M) | M13:Oxidation (M):156.85 | PEAKS DB |
| Y.SQDILMPLWSSYTIYR.S | N | 86.64 | 1971.9764 | 16 | 0.5 | 986.9960 | 2 | 65.42 | 8 | F8:39935 | 31122021\_RID\_3262\_1b.raw |  |  |  |  |  |  |  | 1.8734E6 | 1 | 0 | 0 | 0 | 0 | 0 | 0 | 0 | 1 | 608 | 623 |  |  | PEAKS DB |
| K.VNLM(+15.99)VDQQWMAVR.D | N | 85.00 | 1604.7803 | 13 | 0.8 | 803.3981 | 2 | 48.10 | 8 | F8:27251 | 31122021\_RID\_3262\_1b.raw |  |  | 2.977E5 |  |  |  |  | 8.7958E6 | 3 | 0 | 0 | 1 | 0 | 0 | 0 | 0 | 2 | 435 | 447 | Oxidation (M) | M4:Oxidation (M):203.88 | PEAKS DB |
| R.IDKVNLM(+15.99)VDQQWM(+15.99)AVR.D | N | 83.92 | 1976.9812 | 16 | 0.8 | 660.0015 | 3 | 40.70 | 8 | F8:21410 | 31122021\_RID\_3262\_1b.raw |  |  |  |  |  |  |  | 8.2962E6 | 5 | 0 | 0 | 0 | 0 | 0 | 0 | 0 | 5 | 432 | 447 | Oxidation (M) | M7:Oxidation (M):1000.00;M13:Oxidation (M):1000.00 | PEAKS DB |
| R.LWNYFHTTLIPK.Y | N | 82.18 | 1531.8187 | 12 | 0.3 | 766.9169 | 2 | 47.03 | 8 | F8:26408 | 31122021\_RID\_3262\_1b.raw |  |  |  |  |  |  |  | 8.903E7 | 2 | 0 | 0 | 0 | 0 | 0 | 0 | 0 | 2 | 696 | 707 |  |  | PEAKS DB |
| R.VRDVELLTGLNFYSGLK.Q | N | 82.16 | 1923.0465 | 17 | 0.6 | 642.0232 | 3 | 54.30 | 8 | F8:32508 | 31122021\_RID\_3262\_1b.raw |  |  |  |  |  |  |  | 2.8722E7 | 5 | 0 | 0 | 0 | 0 | 0 | 0 | 0 | 5 | 811 | 827 |  |  | PEAKS DB |
| K.YGPVSGEIIK.A | N | 81.32 | 1061.5757 | 10 | -0.7 | 531.7947 | 2 | 36.07 | 2 | F2:12468 | 13012022\_RID\_3262\_2b.raw |  | 6.2644E6 | 1.3043E7 | 5.1609E6 | 1.3681E6 |  |  | 2.2399E8 | 5 | 0 | 1 | 1 | 1 | 1 | 0 | 0 | 1 | 308 | 317 |  |  | PEAKS DB |
| K.DFYTFDSEGIVK.N | N | 80.91 | 1419.6558 | 12 | 0.0 | 710.8351 | 2 | 52.40 | 8 | F8:30658 | 31122021\_RID\_3262\_1b.raw |  | 2.3372E6 | 2.3227E6 | 6.1588E5 |  |  |  | 1.1376E8 | 7 | 0 | 1 | 1 | 1 | 0 | 0 | 0 | 4 | 390 | 401 |  |  | PEAKS DB |
| K.RPDFYTLYIEEPDTTGHK.Y | N | 80.16 | 2181.0378 | 18 | -0.2 | 1091.5260 | 2 | 40.97 | 8 | F8:21573 | 31122021\_RID\_3262\_1b.raw |  |  |  |  |  |  |  | 2.4259E8 | 3 | 0 | 0 | 0 | 0 | 0 | 0 | 0 | 3 | 290 | 307 |  |  | PEAKS DB |
| K.VNLMVDQQWMAVR.D | N | 80.00 | 1588.7854 | 13 | 0.8 | 795.4006 | 2 | 52.09 | 8 | F8:30422 | 31122021\_RID\_3262\_1b.raw |  |  |  |  |  |  |  | 4.1895E6 | 1 | 0 | 0 | 0 | 0 | 0 | 0 | 0 | 1 | 435 | 447 |  |  | PEAKS DB |
| K.YISAYSQDILM(+15.99)PLWSSYTIYR.S | N | 79.58 | 2585.2512 | 21 | 1.1 | 862.7587 | 3 | 66.89 | 8 | F8:40744 | 31122021\_RID\_3262\_1b.raw |  |  |  |  |  |  |  | 2.4259E6 | 2 | 0 | 0 | 0 | 0 | 0 | 0 | 0 | 2 | 603 | 623 | Oxidation (M) | M11:Oxidation (M):1000.00 | PEAKS DB |
| N.VDFFM(+15.99)YEGPAPR.I | N | 76.86 | 1443.6493 | 12 | 0.7 | 722.8325 | 2 | 49.36 | 8 | F8:30056 | 31122021\_RID\_3262\_1b.raw |  | 9.0306E5 | 6.2316E5 |  |  |  |  | 5.0987E7 | 6 | 0 | 2 | 1 | 0 | 0 | 0 | 0 | 3 | 370 | 381 | Oxidation (M) | M5:Oxidation (M):1000.00 | PEAKS DB |
| Y.SQDILM(+15.99)PLWSSYTIYR.S | N | 74.92 | 1987.9713 | 16 | 0.6 | 994.9935 | 2 | 61.25 | 8 | F8:37365 | 31122021\_RID\_3262\_1b.raw |  |  |  |  |  |  |  | 6.0219E5 | 1 | 0 | 0 | 0 | 0 | 0 | 0 | 0 | 1 | 608 | 623 | Oxidation (M) | M6:Oxidation (M):1000.00 | PEAKS DB |
| K.AATYFWPGSEVK.I | N | 73.56 | 1354.6558 | 12 | -0.3 | 678.3350 | 2 | 44.52 | 8 | F8:24361 | 31122021\_RID\_3262\_1b.raw |  | 1.7682E6 | 5.0699E6 | 1.4387E6 |  |  |  | 1.9551E8 | 4 | 0 | 1 | 1 | 1 | 0 | 0 | 0 | 1 | 243 | 254 |  |  | PEAKS DB |
| K.VNLM(+15.99)VDQQWM(+15.99)AVR.D | N | 72.26 | 1620.7753 | 13 | 0.9 | 811.3956 | 2 | 42.52 | 8 | F8:22889 | 31122021\_RID\_3262\_1b.raw |  |  |  |  |  |  |  | 3.4011E6 | 5 | 0 | 0 | 0 | 0 | 0 | 0 | 0 | 5 | 435 | 447 | Oxidation (M) | M4:Oxidation (M):1000.00;M10:Oxidation (M):1000.00 | PEAKS DB |
| R.TLGM(+15.99)LMEGLK.Q | N | 71.09 | 1107.5668 | 10 | 0.2 | 554.7908 | 2 | 43.40 | 8 | F8:23540 | 31122021\_RID\_3262\_1b.raw |  | 2.8854E6 | 5.0463E6 |  |  |  |  | 1.9938E8 | 8 | 0 | 1 | 2 | 0 | 0 | 0 | 0 | 5 | 325 | 334 | Oxidation (M) | M4:Oxidation (M):67.96 | PEAKS DB |
| K.NPFYTPSPAK.E | N | 70.36 | 1120.5553 | 10 | 0.1 | 561.2850 | 2 | 31.20 | 8 | F8:13940 | 31122021\_RID\_3262\_1b.raw |  | 3.4243E5 | 1.1099E6 | 5.0205E5 |  |  |  | 1.254E7 | 4 | 0 | 1 | 1 | 1 | 0 | 0 | 0 | 1 | 521 | 530 |  |  | PEAKS DB |
| K.TFLPIFVNPVN | N | 67.67 | 1259.6914 | 11 | 0.2 | 630.8531 | 2 | 72.02 | 8 | F8:43764 | 31122021\_RID\_3262\_1b.raw |  | 2.2902E6 | 3.7599E6 | 1.795E6 | 1.8129E6 |  |  | 1.0908E8 | 6 | 0 | 1 | 1 | 1 | 1 | 0 | 0 | 2 | 838 | 848 |  |  | PEAKS DB |
| I.FLAHGPGFNEK.N | N | 67.15 | 1215.6036 | 11 | 0.8 | 608.8096 | 2 | 25.74 | 8 | F8:9653 | 31122021\_RID\_3262\_1b.raw |  |  |  |  |  |  |  | 1.7745E5 | 1 | 0 | 0 | 0 | 0 | 0 | 0 | 0 | 1 | 472 | 482 |  |  | PEAKS DB |
| R.TLGMLMEGLK.Q | N | 67.03 | 1091.5719 | 10 | 0.1 | 546.7933 | 2 | 47.62 | 8 | F8:26617 | 31122021\_RID\_3262\_1b.raw |  |  |  |  |  |  |  | 1.2415E7 | 1 | 0 | 0 | 0 | 0 | 0 | 0 | 0 | 1 | 325 | 334 |  |  | PEAKS DB |
| K.VNLMVDQQWM(+15.99)AVR.D | N | 62.89 | 1604.7803 | 13 | -0.2 | 803.3972 | 2 | 52.11 | 8 | F8:30389 | 31122021\_RID\_3262\_1b.raw |  |  |  |  |  |  |  | 2.8558E6 | 3 | 0 | 0 | 0 | 0 | 0 | 0 | 0 | 3 | 435 | 447 | Oxidation (M) | M10:Oxidation (M):113.99 | PEAKS DB |
| R.NPAWWGGQPIWHTA.T | N | 61.48 | 1619.7633 | 14 | 0.5 | 810.8893 | 2 | 53.82 | 8 | F8:31774 | 31122021\_RID\_3262\_1b.raw |  |  |  |  |  |  |  | 8.4618E5 | 1 | 0 | 0 | 0 | 0 | 0 | 0 | 0 | 1 | 223 | 236 |  |  | PEAKS DB |
| R.NPAWWGGQPIWHT.A | N | 60.99 | 1548.7262 | 13 | 0.1 | 775.3704 | 2 | 55.72 | 8 | F8:33196 | 31122021\_RID\_3262\_1b.raw |  |  |  |  |  |  |  | 1.1047E6 | 1 | 0 | 0 | 0 | 0 | 0 | 0 | 0 | 1 | 223 | 235 |  |  | PEAKS DB |
| R.IDKVNLMVDQQWMAV.R | N | 60.10 | 1788.8903 | 15 | 0.4 | 895.4528 | 2 | 58.57 | 8 | F8:35442 | 31122021\_RID\_3262\_1b.raw |  |  |  |  |  |  |  | 6.3717E6 | 1 | 0 | 0 | 0 | 0 | 0 | 0 | 0 | 1 | 432 | 446 |  |  | PEAKS DB |
| R.NPAWWGGQPIWH.T | N | 59.14 | 1447.6786 | 12 | 0.5 | 724.8469 | 2 | 54.57 | 8 | F8:32349 | 31122021\_RID\_3262\_1b.raw |  |  |  |  |  |  |  | 1.8077E6 | 1 | 0 | 0 | 0 | 0 | 0 | 0 | 0 | 1 | 223 | 234 |  |  | PEAKS DB |
| R.TLGM(+15.99)LM(+15.99)EGLK.Q | N | 59.05 | 1123.5618 | 10 | 0.5 | 562.7885 | 2 | 41.42 | 8 | F8:23793 | 31122021\_RID\_3262\_1b.raw |  | 1.544E6 |  |  |  |  |  | 7.4572E7 | 8 | 0 | 1 | 0 | 0 | 0 | 0 | 0 | 7 | 325 | 334 | Oxidation (M) | M4:Oxidation (M):1000.00;M6:Oxidation (M):1000.00 | PEAKS DB |
| R.NPAWWGGQPIWHTAT.Y | N | 58.25 | 1720.8110 | 15 | 0.8 | 861.4135 | 2 | 53.71 | 8 | F8:31660 | 31122021\_RID\_3262\_1b.raw |  |  |  |  |  |  |  | 1.8953E6 | 1 | 0 | 0 | 0 | 0 | 0 | 0 | 0 | 1 | 223 | 237 |  |  | PEAKS DB |
| R.TLGMLM(+15.99)EGLK.Q | N | 58.20 | 1107.5668 | 10 | -0.4 | 554.7905 | 2 | 47.61 | 8 | F8:26867 | 31122021\_RID\_3262\_1b.raw |  |  |  |  |  |  |  | 6.9241E6 | 2 | 0 | 0 | 0 | 0 | 0 | 0 | 0 | 2 | 325 | 334 | Oxidation (M) | M6:Oxidation (M):60.92 | PEAKS DB |
| E.SPLILFSMDGFR.A | N | 57.71 | 1381.7064 | 12 | 0.3 | 691.8607 | 2 | 62.91 | 8 | F8:38370 | 31122021\_RID\_3262\_1b.raw |  |  |  |  |  |  |  | 2.2236E4 | 1 | 0 | 0 | 0 | 0 | 0 | 0 | 0 | 1 | 136 | 147 |  |  | PEAKS DB |
| R.IDKVNLM(+15.99)VDQQWMAV.R | N | 57.69 | 1804.8851 | 15 | 0.7 | 903.4505 | 2 | 54.37 | 8 | F8:32152 | 31122021\_RID\_3262\_1b.raw |  |  |  |  |  |  |  | 1.1309E7 | 1 | 0 | 0 | 0 | 0 | 0 | 0 | 0 | 1 | 432 | 446 | Oxidation (M) | M7:Oxidation (M):112.31 | PEAKS DB |
| R.IDKVNLM(+15.99)VDQQWM(+15.99)AV.R | N | 57.21 | 1820.8801 | 15 | 0.6 | 911.4479 | 2 | 53.49 | 8 | F8:32111 | 31122021\_RID\_3262\_1b.raw |  |  |  |  |  |  |  | 4.342E6 | 4 | 0 | 0 | 0 | 0 | 0 | 0 | 0 | 4 | 432 | 446 | Oxidation (M) | M7:Oxidation (M):1000.00;M13:Oxidation (M):1000.00 | PEAKS DB |
| K.QPLPETLQLK.T | N | 56.85 | 1165.6707 | 10 | -0.3 | 583.8424 | 2 | 39.53 | 8 | F8:20550 | 31122021\_RID\_3262\_1b.raw |  |  | 4.1963E6 | 1.1061E6 |  |  |  | 1.0052E8 | 3 | 0 | 0 | 1 | 1 | 0 | 0 | 0 | 1 | 828 | 837 |  |  | PEAKS DB |
| K.TESEAHNLPYGR.P | N | 56.82 | 1372.6371 | 12 | -0.1 | 687.3257 | 2 | 21.36 | 8 | F8:6439 | 31122021\_RID\_3262\_1b.raw |  |  |  |  |  |  |  | 7.9014E6 | 2 | 0 | 0 | 0 | 0 | 0 | 0 | 0 | 2 | 574 | 585 |  |  | PEAKS DB |
| N.LMVDQQWMAVR.D | N | 55.73 | 1375.6741 | 11 | 2.2 | 688.8458 | 2 | 47.23 | 8 | F8:26413 | 31122021\_RID\_3262\_1b.raw |  |  |  |  |  |  |  | 3.1228E4 | 1 | 0 | 0 | 0 | 0 | 0 | 0 | 0 | 1 | 437 | 447 |  |  | PEAKS DB |
| L.ILFSMDGFR.A | N | 54.86 | 1084.5375 | 9 | 0.3 | 543.2762 | 2 | 52.47 | 8 | F8:30540 | 31122021\_RID\_3262\_1b.raw |  |  |  |  |  |  |  | 7.1062E5 | 1 | 0 | 0 | 0 | 0 | 0 | 0 | 0 | 1 | 139 | 147 |  |  | PEAKS DB |
| R.NPAWWGGQPIWHTATY.Q | N | 54.41 | 1883.8744 | 16 | 1.0 | 942.9454 | 2 | 56.35 | 8 | F8:33653 | 31122021\_RID\_3262\_1b.raw |  |  |  |  |  |  |  | 2.4054E6 | 1 | 0 | 0 | 0 | 0 | 0 | 0 | 0 | 1 | 223 | 238 |  |  | PEAKS DB |
| K.YISAYSQDILMPL.W | N | 54.14 | 1512.7534 | 13 | 0.5 | 757.3843 | 2 | 68.83 | 8 | F8:41947 | 31122021\_RID\_3262\_1b.raw |  |  |  |  |  |  |  | 1.2569E6 | 1 | 0 | 0 | 0 | 0 | 0 | 0 | 0 | 1 | 603 | 615 |  |  | PEAKS DB |
| K.TESEAHNLPYGRPEVLQNHSK.Y | Y | 52.58 | 2405.1724 | 21 | 0.5 | 802.7318 | 3 | 25.25 | 8 | F8:9184 | 31122021\_RID\_3262\_1b.raw |  |  |  |  |  |  |  | 1.0864E7 | 2 | 0 | 0 | 0 | 0 | 0 | 0 | 0 | 2 | 574 | 594 |  |  | PEAKS DB |
| L.ITSNIVPMFK.G | N | 52.50 | 1148.6263 | 10 | 0.3 | 575.3206 | 2 | 44.67 | 8 | F8:24431 | 31122021\_RID\_3262\_1b.raw |  |  |  |  |  |  |  | 5.4274E5 | 1 | 0 | 0 | 0 | 0 | 0 | 0 | 0 | 1 | 682 | 691 |  |  | PEAKS DB |
| M.VDQQWMAVR.D | N | 52.40 | 1131.5496 | 9 | 0.2 | 566.7822 | 2 | 35.82 | 8 | F8:17412 | 31122021\_RID\_3262\_1b.raw |  |  |  |  |  |  |  | 2.5672E5 | 1 | 0 | 0 | 0 | 0 | 0 | 0 | 0 | 1 | 439 | 447 |  |  | PEAKS DB |
| F.MYEGPAPR.I | N | 52.23 | 919.4222 | 8 | -0.2 | 460.7183 | 2 | 22.38 | 8 | F8:7073 | 31122021\_RID\_3262\_1b.raw |  |  |  |  |  |  |  | 1.1389E6 | 1 | 0 | 0 | 0 | 0 | 0 | 0 | 0 | 1 | 374 | 381 |  |  | PEAKS DB |
| K.YISAYSQDILMPLWSSY.T | N | 51.82 | 2035.9601 | 17 | 0.6 | 1018.9880 | 2 | 78.75 | 8 | F8:46267 | 31122021\_RID\_3262\_1b.raw |  |  |  |  |  |  |  | 3.9027E6 | 1 | 0 | 0 | 0 | 0 | 0 | 0 | 0 | 1 | 603 | 619 |  |  | PEAKS DB |
| K.SMQAIFLAH.G | N | 50.78 | 1016.5114 | 9 | 0.6 | 509.2633 | 2 | 44.34 | 8 | F8:24102 | 31122021\_RID\_3262\_1b.raw |  |  |  |  |  |  |  | 2.2295E6 | 1 | 0 | 0 | 0 | 0 | 0 | 0 | 0 | 1 | 467 | 475 |  |  | PEAKS DB |
| K.IPIPTHYFV.V | N | 50.61 | 1085.5909 | 9 | -0.2 | 543.8026 | 2 | 48.99 | 8 | F8:27744 | 31122021\_RID\_3262\_1b.raw |  |  |  |  |  |  |  | 4.4642E5 | 1 | 0 | 0 | 0 | 0 | 0 | 0 | 0 | 1 | 746 | 754 |  |  | PEAKS DB |
| K.GETSWLKDK.C | N | 50.34 | 1062.5345 | 9 | 1.1 | 532.2751 | 2 | 22.35 | 8 | F8:7174 | 31122021\_RID\_3262\_1b.raw |  |  |  |  |  |  |  | 9.0657E5 | 1 | 0 | 0 | 0 | 0 | 0 | 0 | 0 | 1 | 112 | 120 |  |  | PEAKS DB |
| R.DVELLTGLNF.Y | N | 50.10 | 1119.5812 | 10 | 0.1 | 560.7979 | 2 | 77.66 | 8 | F8:45882 | 31122021\_RID\_3262\_1b.raw |  |  |  |  |  |  |  | 2.4718E5 | 1 | 0 | 0 | 0 | 0 | 0 | 0 | 0 | 1 | 813 | 822 |  |  | PEAKS DB |
| Y.TLYIEEPDTTGHK.Y | N | 50.08 | 1502.7252 | 13 | 0.6 | 752.3703 | 2 | 29.35 | 8 | F8:12577 | 31122021\_RID\_3262\_1b.raw |  |  |  |  |  |  |  | 1.6199E6 | 1 | 0 | 0 | 0 | 0 | 0 | 0 | 0 | 1 | 295 | 307 |  |  | PEAKS DB |
| N.LM(+15.99)VDQQWMAVR.D | N | 49.58 | 1391.6689 | 11 | -1.3 | 696.8408 | 2 | 43.90 | 8 | F8:23868 | 31122021\_RID\_3262\_1b.raw |  |  |  |  |  |  |  | 7.1676E4 | 1 | 0 | 0 | 0 | 0 | 0 | 0 | 0 | 1 | 437 | 447 | Oxidation (M) | M2:Oxidation (M):70.10 | PEAKS DB |
| K.TFLPIFVN.P | N | 49.12 | 949.5273 | 8 | -0.5 | 475.7707 | 2 | 69.23 | 8 | F8:42039 | 31122021\_RID\_3262\_1b.raw |  |  |  |  |  |  |  | 1.5864E5 | 1 | 0 | 0 | 0 | 0 | 0 | 0 | 0 | 1 | 838 | 845 |  |  | PEAKS DB |
| D.FYTLYIEEPDTTGHK.Y | N | 48.85 | 1812.8569 | 15 | -0.6 | 907.4352 | 2 | 42.38 | 8 | F8:22650 | 31122021\_RID\_3262\_1b.raw |  |  |  |  |  |  |  | 6.9586E4 | 1 | 0 | 0 | 0 | 0 | 0 | 0 | 0 | 1 | 293 | 307 |  |  | PEAKS DB |
| K.VLSFILPHRPDN.S | N | 48.71 | 1406.7670 | 12 | 0.8 | 704.3914 | 2 | 41.42 | 8 | F8:21857 | 31122021\_RID\_3262\_1b.raw |  |  |  |  |  |  |  | 1.8605E5 | 1 | 0 | 0 | 0 | 0 | 0 | 0 | 0 | 1 | 775 | 786 |  |  | PEAKS DB |
| L.YIEEPDTTGHK.Y | N | 48.56 | 1288.5935 | 11 | 0.1 | 645.3041 | 2 | 21.02 | 3 | F3:7745 | 13012022\_RID\_3262\_3b.raw |  |  | 4.5919E5 |  |  |  |  | 1.123E6 | 2 | 0 | 0 | 1 | 0 | 0 | 0 | 0 | 1 | 297 | 307 |  |  | PEAKS DB |
| K.YISAYSQDILMPLWSSYT.I | N | 48.45 | 2137.0078 | 18 | 0.5 | 1069.5117 | 2 | 78.53 | 8 | F8:46086 | 31122021\_RID\_3262\_1b.raw |  |  |  |  |  |  |  | 7.9829E5 | 1 | 0 | 0 | 0 | 0 | 0 | 0 | 0 | 1 | 603 | 620 |  |  | PEAKS DB |
| R.IDKVNLMVDQQWM(+15.99)AV.R | N | 48.31 | 1804.8851 | 15 | 0.7 | 903.4505 | 2 | 54.37 | 8 | F8:32568 | 31122021\_RID\_3262\_1b.raw |  |  |  |  |  |  |  | 1.1309E7 | 1 | 0 | 0 | 0 | 0 | 0 | 0 | 0 | 1 | 432 | 446 | Oxidation (M) | M13:Oxidation (M):95.35 | PEAKS DB |
| K.SIPFEAR.V | N | 48.00 | 818.4286 | 7 | 0.6 | 410.2218 | 2 | 32.78 | 2 | F2:10658 | 13012022\_RID\_3262\_2b.raw |  | 2.8129E6 |  |  |  |  |  |  | 1 | 0 | 1 | 0 | 0 | 0 | 0 | 0 | 0 | 269 | 275 |  |  | PEAKS DB |
| N.PAWWGGQPIWHTATYQGLK.A | N | 47.31 | 2196.0906 | 19 | 0.5 | 733.0378 | 3 | 50.42 | 8 | F8:28936 | 31122021\_RID\_3262\_1b.raw |  |  |  |  |  |  |  | 4.682E5 | 1 | 0 | 0 | 0 | 0 | 0 | 0 | 0 | 1 | 224 | 242 |  |  | PEAKS DB |
| R.DVELLTGLNFY.S | N | 47.24 | 1282.6445 | 11 | 0.3 | 642.3297 | 2 | 79.71 | 8 | F8:46647 | 31122021\_RID\_3262\_1b.raw |  |  |  |  |  |  |  | 2.9834E5 | 1 | 0 | 0 | 0 | 0 | 0 | 0 | 0 | 1 | 813 | 823 |  |  | PEAKS DB |
| D.FFMYEGPAPR.I | N | 46.65 | 1213.5590 | 10 | 0.7 | 607.7872 | 2 | 43.12 | 8 | F8:23185 | 31122021\_RID\_3262\_1b.raw |  |  |  |  |  |  |  | 2.7887E5 | 1 | 0 | 0 | 0 | 0 | 0 | 0 | 0 | 1 | 372 | 381 |  |  | PEAKS DB |
| F.YTFDSEGIVK.N | N | 46.54 | 1157.5604 | 10 | 1.2 | 579.7882 | 2 | 38.75 | 3 | F3:22609 | 13012022\_RID\_3262\_3b.raw |  |  | 1.1954E5 |  |  |  |  |  | 1 | 0 | 0 | 1 | 0 | 0 | 0 | 0 | 0 | 392 | 401 |  |  | PEAKS DB |
| R.NGLNVISGPIFDYN.S | N | 46.38 | 1521.7463 | 14 | 1.5 | 761.8816 | 2 | 65.30 | 8 | F8:39826 | 31122021\_RID\_3262\_1b.raw |  |  |  |  |  |  |  | 1.1708E4 | 1 | 0 | 0 | 0 | 0 | 0 | 0 | 0 | 1 | 713 | 726 |  |  | PEAKS DB |
| K.YISAYSQDILM(+15.99)PLWSSY.T | N | 46.31 | 2051.9551 | 17 | 0.5 | 1026.9854 | 2 | 73.46 | 8 | F8:44129 | 31122021\_RID\_3262\_1b.raw |  |  |  |  |  |  |  | 8.6735E5 | 1 | 0 | 0 | 0 | 0 | 0 | 0 | 0 | 1 | 603 | 619 | Oxidation (M) | M11:Oxidation (M):1000.00 | PEAKS DB |
| P.DFYTLYIEEPDTTGHK.Y | N | 46.00 | 1927.8839 | 16 | 0.7 | 964.9499 | 2 | 49.25 | 8 | F8:27984 | 31122021\_RID\_3262\_1b.raw |  |  |  |  |  |  |  | 1.6606E6 | 1 | 0 | 0 | 0 | 0 | 0 | 0 | 0 | 1 | 292 | 307 |  |  | PEAKS DB |
| K.DFYTFDSEGIV.K | N | 45.21 | 1291.5608 | 11 | 0.6 | 646.7881 | 2 | 70.18 | 8 | F8:42580 | 31122021\_RID\_3262\_1b.raw |  |  |  |  |  |  |  | 2.0262E5 | 1 | 0 | 0 | 0 | 0 | 0 | 0 | 0 | 1 | 390 | 400 |  |  | PEAKS DB |
| total 72 peptides |  |  |  |  |  |  |  |  |  |  |  |  |  |  |  |  |  |  |  |  |  |  |  |  |  |  |  |  |  |  |  |  |  |
| --- | --- | --- | --- | --- | --- | --- | --- | --- | --- | --- | --- | --- | --- | --- | --- | --- | --- | --- | --- | --- | --- | --- | --- | --- | --- | --- | --- | --- | --- | --- | --- | --- | --- |

BAN89425.1

back to list

  

| Protein Coverage
| Supporting Peptides
|

Protein Coverage:

Supporting Peptides:

| Peptide | Uniq | -10lgP | Mass | Length | ppm | m/z | z | RT | Fraction | Scan | Source File | Area F6 | Area F2 | Area F3 | Area F4 | Area F5 | Area F7 | Area F8 | Area F1 | #Feature | #Feature F6 | #Feature F2 | #Feature F3 | #Feature F4 | #Feature F5 | #Feature F7 | #Feature F8 | #Feature F1 | Start | End | PTM | AScore | Found By |
| --- | --- | --- | --- | --- | --- | --- | --- | --- | --- | --- | --- | --- | --- | --- | --- | --- | --- | --- | --- | --- | --- | --- | --- | --- | --- | --- | --- | --- | --- | --- | --- | --- | --- |
| R.NPAWWGGQPIWHTATYQGLK.A | N | 103.11 | 2310.1333 | 20 | -0.2 | 1156.0737 | 2 | 50.36 | 8 | F8:28928 | 31122021\_RID\_3262\_1b.raw |  |  |  |  |  |  |  | 3.6924E7 | 2 | 0 | 0 | 0 | 0 | 0 | 0 | 0 | 2 | 264 | 283 |  |  | PEAKS DB |
| K.SMQAIFLAHGPGFNEK.N | N | 103.04 | 1745.8558 | 16 | -0.2 | 873.9351 | 2 | 42.38 | 8 | F8:22729 | 31122021\_RID\_3262\_1b.raw |  |  | 3.2331E5 |  |  |  |  | 8.2355E7 | 3 | 0 | 0 | 1 | 0 | 0 | 0 | 0 | 2 | 508 | 523 |  |  | PEAKS DB |
| N.VDFFMYEGPAPR.I | N | 96.79 | 1427.6543 | 12 | -0.1 | 714.8344 | 2 | 51.87 | 8 | F8:30116 | 31122021\_RID\_3262\_1b.raw |  | 8.9339E5 |  |  |  |  |  | 1.2101E8 | 4 | 0 | 1 | 0 | 0 | 0 | 0 | 0 | 3 | 411 | 422 |  |  | PEAKS DB |
| K.NVPKDFYTFDSEGIVK.N | N | 95.90 | 1857.9148 | 16 | 0.4 | 620.3124 | 3 | 43.93 | 8 | F8:24080 | 31122021\_RID\_3262\_1b.raw |  |  | 1.1786E6 |  |  |  |  | 3.437E7 | 4 | 0 | 0 | 2 | 0 | 0 | 0 | 0 | 2 | 427 | 442 |  |  | PEAKS DB |
| R.DVELLTGLNFYSGLK.Q | N | 94.09 | 1667.8770 | 15 | 0.8 | 834.9464 | 2 | 66.30 | 8 | F8:40834 | 31122021\_RID\_3262\_1b.raw |  |  |  |  |  |  |  | 3.1554E7 | 4 | 0 | 0 | 0 | 0 | 0 | 0 | 0 | 4 | 854 | 868 |  |  | PEAKS DB |
| K.YISAYSQDILMPLWSSYTIYR.S | N | 93.53 | 2569.2563 | 21 | 0.8 | 857.4268 | 3 | 70.86 | 8 | F8:42937 | 31122021\_RID\_3262\_1b.raw |  |  |  |  |  |  |  | 5.1443E6 | 2 | 0 | 0 | 0 | 0 | 0 | 0 | 0 | 2 | 644 | 664 |  |  | PEAKS DB |
| K.SM(+15.99)QAIFLAHGPGFNEK.N | N | 91.55 | 1761.8508 | 16 | 0.1 | 881.9327 | 2 | 36.86 | 8 | F8:18333 | 31122021\_RID\_3262\_1b.raw |  |  | 1.011E6 |  |  |  |  | 2.6382E7 | 8 | 0 | 0 | 1 | 0 | 0 | 0 | 0 | 7 | 508 | 523 | Oxidation (M) | M2:Oxidation (M):1000.00 | PEAKS DB |
| R.IDKVNLM(+15.99)VDQQWMAVR.D | N | 89.72 | 1960.9863 | 16 | 1.2 | 981.5016 | 2 | 45.32 | 8 | F8:25068 | 31122021\_RID\_3262\_1b.raw |  |  |  |  |  |  |  | 2.3151E7 | 4 | 0 | 0 | 0 | 0 | 0 | 0 | 0 | 4 | 473 | 488 | Oxidation (M) | M7:Oxidation (M):182.75 | PEAKS DB |
| R.IDKVNLMVDQQWMAVR.D | N | 88.44 | 1944.9913 | 16 | 0.4 | 973.5034 | 2 | 48.73 | 8 | F8:27572 | 31122021\_RID\_3262\_1b.raw |  |  |  |  |  |  |  | 9.0594E6 | 3 | 0 | 0 | 0 | 0 | 0 | 0 | 0 | 3 | 473 | 488 |  |  | PEAKS DB |
| R.IDKVNLMVDQQWM(+15.99)AVR.D | N | 86.82 | 1960.9863 | 16 | 0.3 | 654.6696 | 3 | 44.18 | 8 | F8:23999 | 31122021\_RID\_3262\_1b.raw |  |  |  |  |  |  |  | 6.7254E6 | 5 | 0 | 0 | 0 | 0 | 0 | 0 | 0 | 5 | 473 | 488 | Oxidation (M) | M13:Oxidation (M):156.85 | PEAKS DB |
| Y.SQDILMPLWSSYTIYR.S | N | 86.64 | 1971.9764 | 16 | 0.5 | 986.9960 | 2 | 65.42 | 8 | F8:39935 | 31122021\_RID\_3262\_1b.raw |  |  |  |  |  |  |  | 1.8734E6 | 1 | 0 | 0 | 0 | 0 | 0 | 0 | 0 | 1 | 649 | 664 |  |  | PEAKS DB |
| K.VNLM(+15.99)VDQQWMAVR.D | N | 85.00 | 1604.7803 | 13 | 0.8 | 803.3981 | 2 | 48.10 | 8 | F8:27251 | 31122021\_RID\_3262\_1b.raw |  |  | 2.977E5 |  |  |  |  | 8.7958E6 | 3 | 0 | 0 | 1 | 0 | 0 | 0 | 0 | 2 | 476 | 488 | Oxidation (M) | M4:Oxidation (M):203.88 | PEAKS DB |
| R.IDKVNLM(+15.99)VDQQWM(+15.99)AVR.D | N | 83.92 | 1976.9812 | 16 | 0.8 | 660.0015 | 3 | 40.70 | 8 | F8:21410 | 31122021\_RID\_3262\_1b.raw |  |  |  |  |  |  |  | 8.2962E6 | 5 | 0 | 0 | 0 | 0 | 0 | 0 | 0 | 5 | 473 | 488 | Oxidation (M) | M7:Oxidation (M):1000.00;M13:Oxidation (M):1000.00 | PEAKS DB |
| R.LWNYFHTTLIPK.Y | N | 82.18 | 1531.8187 | 12 | 0.3 | 766.9169 | 2 | 47.03 | 8 | F8:26408 | 31122021\_RID\_3262\_1b.raw |  |  |  |  |  |  |  | 8.903E7 | 2 | 0 | 0 | 0 | 0 | 0 | 0 | 0 | 2 | 737 | 748 |  |  | PEAKS DB |
| R.VRDVELLTGLNFYSGLK.Q | N | 82.16 | 1923.0465 | 17 | 0.6 | 642.0232 | 3 | 54.30 | 8 | F8:32508 | 31122021\_RID\_3262\_1b.raw |  |  |  |  |  |  |  | 2.8722E7 | 5 | 0 | 0 | 0 | 0 | 0 | 0 | 0 | 5 | 852 | 868 |  |  | PEAKS DB |
| K.YGPVSGEIIK.A | N | 81.32 | 1061.5757 | 10 | -0.7 | 531.7947 | 2 | 36.07 | 2 | F2:12468 | 13012022\_RID\_3262\_2b.raw |  | 6.2644E6 | 1.3043E7 | 5.1609E6 | 1.3681E6 |  |  | 2.2399E8 | 5 | 0 | 1 | 1 | 1 | 1 | 0 | 0 | 1 | 349 | 358 |  |  | PEAKS DB |
| K.DFYTFDSEGIVK.N | N | 80.91 | 1419.6558 | 12 | 0.0 | 710.8351 | 2 | 52.40 | 8 | F8:30658 | 31122021\_RID\_3262\_1b.raw |  | 2.3372E6 | 2.3227E6 | 6.1588E5 |  |  |  | 1.1376E8 | 7 | 0 | 1 | 1 | 1 | 0 | 0 | 0 | 4 | 431 | 442 |  |  | PEAKS DB |
| K.RPDFYTLYIEEPDTTGHK.Y | N | 80.16 | 2181.0378 | 18 | -0.2 | 1091.5260 | 2 | 40.97 | 8 | F8:21573 | 31122021\_RID\_3262\_1b.raw |  |  |  |  |  |  |  | 2.4259E8 | 3 | 0 | 0 | 0 | 0 | 0 | 0 | 0 | 3 | 331 | 348 |  |  | PEAKS DB |
| K.VNLMVDQQWMAVR.D | N | 80.00 | 1588.7854 | 13 | 0.8 | 795.4006 | 2 | 52.09 | 8 | F8:30422 | 31122021\_RID\_3262\_1b.raw |  |  |  |  |  |  |  | 4.1895E6 | 1 | 0 | 0 | 0 | 0 | 0 | 0 | 0 | 1 | 476 | 488 |  |  | PEAKS DB |
| K.YISAYSQDILM(+15.99)PLWSSYTIYR.S | N | 79.58 | 2585.2512 | 21 | 1.1 | 862.7587 | 3 | 66.89 | 8 | F8:40744 | 31122021\_RID\_3262\_1b.raw |  |  |  |  |  |  |  | 2.4259E6 | 2 | 0 | 0 | 0 | 0 | 0 | 0 | 0 | 2 | 644 | 664 | Oxidation (M) | M11:Oxidation (M):1000.00 | PEAKS DB |
| N.VDFFM(+15.99)YEGPAPR.I | N | 76.86 | 1443.6493 | 12 | 0.7 | 722.8325 | 2 | 49.36 | 8 | F8:30056 | 31122021\_RID\_3262\_1b.raw |  | 9.0306E5 | 6.2316E5 |  |  |  |  | 5.0987E7 | 6 | 0 | 2 | 1 | 0 | 0 | 0 | 0 | 3 | 411 | 422 | Oxidation (M) | M5:Oxidation (M):1000.00 | PEAKS DB |
| Y.SQDILM(+15.99)PLWSSYTIYR.S | N | 74.92 | 1987.9713 | 16 | 0.6 | 994.9935 | 2 | 61.25 | 8 | F8:37365 | 31122021\_RID\_3262\_1b.raw |  |  |  |  |  |  |  | 6.0219E5 | 1 | 0 | 0 | 0 | 0 | 0 | 0 | 0 | 1 | 649 | 664 | Oxidation (M) | M6:Oxidation (M):1000.00 | PEAKS DB |
| K.AATYFWPGSEVK.I | N | 73.56 | 1354.6558 | 12 | -0.3 | 678.3350 | 2 | 44.52 | 8 | F8:24361 | 31122021\_RID\_3262\_1b.raw |  | 1.7682E6 | 5.0699E6 | 1.4387E6 |  |  |  | 1.9551E8 | 4 | 0 | 1 | 1 | 1 | 0 | 0 | 0 | 1 | 284 | 295 |  |  | PEAKS DB |
| K.VNLM(+15.99)VDQQWM(+15.99)AVR.D | N | 72.26 | 1620.7753 | 13 | 0.9 | 811.3956 | 2 | 42.52 | 8 | F8:22889 | 31122021\_RID\_3262\_1b.raw |  |  |  |  |  |  |  | 3.4011E6 | 5 | 0 | 0 | 0 | 0 | 0 | 0 | 0 | 5 | 476 | 488 | Oxidation (M) | M4:Oxidation (M):1000.00;M10:Oxidation (M):1000.00 | PEAKS DB |
| R.TLGM(+15.99)LMEGLK.Q | N | 71.09 | 1107.5668 | 10 | 0.2 | 554.7908 | 2 | 43.40 | 8 | F8:23540 | 31122021\_RID\_3262\_1b.raw |  | 2.8854E6 | 5.0463E6 |  |  |  |  | 1.9938E8 | 8 | 0 | 1 | 2 | 0 | 0 | 0 | 0 | 5 | 366 | 375 | Oxidation (M) | M4:Oxidation (M):67.96 | PEAKS DB |
| K.NPFYTPSPAK.E | N | 70.36 | 1120.5553 | 10 | 0.1 | 561.2850 | 2 | 31.20 | 8 | F8:13940 | 31122021\_RID\_3262\_1b.raw |  | 3.4243E5 | 1.1099E6 | 5.0205E5 |  |  |  | 1.254E7 | 4 | 0 | 1 | 1 | 1 | 0 | 0 | 0 | 1 | 562 | 571 |  |  | PEAKS DB |
| K.TFLPIFVNPVN | N | 67.67 | 1259.6914 | 11 | 0.2 | 630.8531 | 2 | 72.02 | 8 | F8:43764 | 31122021\_RID\_3262\_1b.raw |  | 2.2902E6 | 3.7599E6 | 1.795E6 | 1.8129E6 |  |  | 1.0908E8 | 6 | 0 | 1 | 1 | 1 | 1 | 0 | 0 | 2 | 879 | 889 |  |  | PEAKS DB |
| I.FLAHGPGFNEK.N | N | 67.15 | 1215.6036 | 11 | 0.8 | 608.8096 | 2 | 25.74 | 8 | F8:9653 | 31122021\_RID\_3262\_1b.raw |  |  |  |  |  |  |  | 1.7745E5 | 1 | 0 | 0 | 0 | 0 | 0 | 0 | 0 | 1 | 513 | 523 |  |  | PEAKS DB |
| R.TLGMLMEGLK.Q | N | 67.03 | 1091.5719 | 10 | 0.1 | 546.7933 | 2 | 47.62 | 8 | F8:26617 | 31122021\_RID\_3262\_1b.raw |  |  |  |  |  |  |  | 1.2415E7 | 1 | 0 | 0 | 0 | 0 | 0 | 0 | 0 | 1 | 366 | 375 |  |  | PEAKS DB |
| K.VNLMVDQQWM(+15.99)AVR.D | N | 62.89 | 1604.7803 | 13 | -0.2 | 803.3972 | 2 | 52.11 | 8 | F8:30389 | 31122021\_RID\_3262\_1b.raw |  |  |  |  |  |  |  | 2.8558E6 | 3 | 0 | 0 | 0 | 0 | 0 | 0 | 0 | 3 | 476 | 488 | Oxidation (M) | M10:Oxidation (M):113.99 | PEAKS DB |
| R.NPAWWGGQPIWHTA.T | N | 61.48 | 1619.7633 | 14 | 0.5 | 810.8893 | 2 | 53.82 | 8 | F8:31774 | 31122021\_RID\_3262\_1b.raw |  |  |  |  |  |  |  | 8.4618E5 | 1 | 0 | 0 | 0 | 0 | 0 | 0 | 0 | 1 | 264 | 277 |  |  | PEAKS DB |
| R.NPAWWGGQPIWHT.A | N | 60.99 | 1548.7262 | 13 | 0.1 | 775.3704 | 2 | 55.72 | 8 | F8:33196 | 31122021\_RID\_3262\_1b.raw |  |  |  |  |  |  |  | 1.1047E6 | 1 | 0 | 0 | 0 | 0 | 0 | 0 | 0 | 1 | 264 | 276 |  |  | PEAKS DB |
| R.IDKVNLMVDQQWMAV.R | N | 60.10 | 1788.8903 | 15 | 0.4 | 895.4528 | 2 | 58.57 | 8 | F8:35442 | 31122021\_RID\_3262\_1b.raw |  |  |  |  |  |  |  | 6.3717E6 | 1 | 0 | 0 | 0 | 0 | 0 | 0 | 0 | 1 | 473 | 487 |  |  | PEAKS DB |
| R.NPAWWGGQPIWH.T | N | 59.14 | 1447.6786 | 12 | 0.5 | 724.8469 | 2 | 54.57 | 8 | F8:32349 | 31122021\_RID\_3262\_1b.raw |  |  |  |  |  |  |  | 1.8077E6 | 1 | 0 | 0 | 0 | 0 | 0 | 0 | 0 | 1 | 264 | 275 |  |  | PEAKS DB |
| R.TLGM(+15.99)LM(+15.99)EGLK.Q | N | 59.05 | 1123.5618 | 10 | 0.5 | 562.7885 | 2 | 41.42 | 8 | F8:23793 | 31122021\_RID\_3262\_1b.raw |  | 1.544E6 |  |  |  |  |  | 7.4572E7 | 8 | 0 | 1 | 0 | 0 | 0 | 0 | 0 | 7 | 366 | 375 | Oxidation (M) | M4:Oxidation (M):1000.00;M6:Oxidation (M):1000.00 | PEAKS DB |
| R.NPAWWGGQPIWHTAT.Y | N | 58.25 | 1720.8110 | 15 | 0.8 | 861.4135 | 2 | 53.71 | 8 | F8:31660 | 31122021\_RID\_3262\_1b.raw |  |  |  |  |  |  |  | 1.8953E6 | 1 | 0 | 0 | 0 | 0 | 0 | 0 | 0 | 1 | 264 | 278 |  |  | PEAKS DB |
| R.TLGMLM(+15.99)EGLK.Q | N | 58.20 | 1107.5668 | 10 | -0.4 | 554.7905 | 2 | 47.61 | 8 | F8:26867 | 31122021\_RID\_3262\_1b.raw |  |  |  |  |  |  |  | 6.9241E6 | 2 | 0 | 0 | 0 | 0 | 0 | 0 | 0 | 2 | 366 | 375 | Oxidation (M) | M6:Oxidation (M):60.92 | PEAKS DB |
| E.SPLILFSMDGFR.A | N | 57.71 | 1381.7064 | 12 | 0.3 | 691.8607 | 2 | 62.91 | 8 | F8:38370 | 31122021\_RID\_3262\_1b.raw |  |  |  |  |  |  |  | 2.2236E4 | 1 | 0 | 0 | 0 | 0 | 0 | 0 | 0 | 1 | 177 | 188 |  |  | PEAKS DB |
| R.IDKVNLM(+15.99)VDQQWMAV.R | N | 57.69 | 1804.8851 | 15 | 0.7 | 903.4505 | 2 | 54.37 | 8 | F8:32152 | 31122021\_RID\_3262\_1b.raw |  |  |  |  |  |  |  | 1.1309E7 | 1 | 0 | 0 | 0 | 0 | 0 | 0 | 0 | 1 | 473 | 487 | Oxidation (M) | M7:Oxidation (M):112.31 | PEAKS DB |
| R.IDKVNLM(+15.99)VDQQWM(+15.99)AV.R | N | 57.21 | 1820.8801 | 15 | 0.6 | 911.4479 | 2 | 53.49 | 8 | F8:32111 | 31122021\_RID\_3262\_1b.raw |  |  |  |  |  |  |  | 4.342E6 | 4 | 0 | 0 | 0 | 0 | 0 | 0 | 0 | 4 | 473 | 487 | Oxidation (M) | M7:Oxidation (M):1000.00;M13:Oxidation (M):1000.00 | PEAKS DB |
| K.QPLPETLQLK.T | N | 56.85 | 1165.6707 | 10 | -0.3 | 583.8424 | 2 | 39.53 | 8 | F8:20550 | 31122021\_RID\_3262\_1b.raw |  |  | 4.1963E6 | 1.1061E6 |  |  |  | 1.0052E8 | 3 | 0 | 0 | 1 | 1 | 0 | 0 | 0 | 1 | 869 | 878 |  |  | PEAKS DB |
| K.TESEAHNLPYGR.P | N | 56.82 | 1372.6371 | 12 | -0.1 | 687.3257 | 2 | 21.36 | 8 | F8:6439 | 31122021\_RID\_3262\_1b.raw |  |  |  |  |  |  |  | 7.9014E6 | 2 | 0 | 0 | 0 | 0 | 0 | 0 | 0 | 2 | 615 | 626 |  |  | PEAKS DB |
| N.LMVDQQWMAVR.D | N | 55.73 | 1375.6741 | 11 | 2.2 | 688.8458 | 2 | 47.23 | 8 | F8:26413 | 31122021\_RID\_3262\_1b.raw |  |  |  |  |  |  |  | 3.1228E4 | 1 | 0 | 0 | 0 | 0 | 0 | 0 | 0 | 1 | 478 | 488 |  |  | PEAKS DB |
| L.ILFSMDGFR.A | N | 54.86 | 1084.5375 | 9 | 0.3 | 543.2762 | 2 | 52.47 | 8 | F8:30540 | 31122021\_RID\_3262\_1b.raw |  |  |  |  |  |  |  | 7.1062E5 | 1 | 0 | 0 | 0 | 0 | 0 | 0 | 0 | 1 | 180 | 188 |  |  | PEAKS DB |
| R.NPAWWGGQPIWHTATY.Q | N | 54.41 | 1883.8744 | 16 | 1.0 | 942.9454 | 2 | 56.35 | 8 | F8:33653 | 31122021\_RID\_3262\_1b.raw |  |  |  |  |  |  |  | 2.4054E6 | 1 | 0 | 0 | 0 | 0 | 0 | 0 | 0 | 1 | 264 | 279 |  |  | PEAKS DB |
| K.YISAYSQDILMPL.W | N | 54.14 | 1512.7534 | 13 | 0.5 | 757.3843 | 2 | 68.83 | 8 | F8:41947 | 31122021\_RID\_3262\_1b.raw |  |  |  |  |  |  |  | 1.2569E6 | 1 | 0 | 0 | 0 | 0 | 0 | 0 | 0 | 1 | 644 | 656 |  |  | PEAKS DB |
| K.TESEAHNLPYGRPEVLQNHSK.Y | Y | 52.58 | 2405.1724 | 21 | 0.5 | 802.7318 | 3 | 25.25 | 8 | F8:9184 | 31122021\_RID\_3262\_1b.raw |  |  |  |  |  |  |  | 1.0864E7 | 2 | 0 | 0 | 0 | 0 | 0 | 0 | 0 | 2 | 615 | 635 |  |  | PEAKS DB |
| L.ITSNIVPMFK.G | N | 52.50 | 1148.6263 | 10 | 0.3 | 575.3206 | 2 | 44.67 | 8 | F8:24431 | 31122021\_RID\_3262\_1b.raw |  |  |  |  |  |  |  | 5.4274E5 | 1 | 0 | 0 | 0 | 0 | 0 | 0 | 0 | 1 | 723 | 732 |  |  | PEAKS DB |
| M.VDQQWMAVR.D | N | 52.40 | 1131.5496 | 9 | 0.2 | 566.7822 | 2 | 35.82 | 8 | F8:17412 | 31122021\_RID\_3262\_1b.raw |  |  |  |  |  |  |  | 2.5672E5 | 1 | 0 | 0 | 0 | 0 | 0 | 0 | 0 | 1 | 480 | 488 |  |  | PEAKS DB |
| F.MYEGPAPR.I | N | 52.23 | 919.4222 | 8 | -0.2 | 460.7183 | 2 | 22.38 | 8 | F8:7073 | 31122021\_RID\_3262\_1b.raw |  |  |  |  |  |  |  | 1.1389E6 | 1 | 0 | 0 | 0 | 0 | 0 | 0 | 0 | 1 | 415 | 422 |  |  | PEAKS DB |
| K.YISAYSQDILMPLWSSY.T | N | 51.82 | 2035.9601 | 17 | 0.6 | 1018.9880 | 2 | 78.75 | 8 | F8:46267 | 31122021\_RID\_3262\_1b.raw |  |  |  |  |  |  |  | 3.9027E6 | 1 | 0 | 0 | 0 | 0 | 0 | 0 | 0 | 1 | 644 | 660 |  |  | PEAKS DB |
| K.SMQAIFLAH.G | N | 50.78 | 1016.5114 | 9 | 0.6 | 509.2633 | 2 | 44.34 | 8 | F8:24102 | 31122021\_RID\_3262\_1b.raw |  |  |  |  |  |  |  | 2.2295E6 | 1 | 0 | 0 | 0 | 0 | 0 | 0 | 0 | 1 | 508 | 516 |  |  | PEAKS DB |
| K.IPIPTHYFV.V | N | 50.61 | 1085.5909 | 9 | -0.2 | 543.8026 | 2 | 48.99 | 8 | F8:27744 | 31122021\_RID\_3262\_1b.raw |  |  |  |  |  |  |  | 4.4642E5 | 1 | 0 | 0 | 0 | 0 | 0 | 0 | 0 | 1 | 787 | 795 |  |  | PEAKS DB |
| K.GETSWLKDK.C | N | 50.34 | 1062.5345 | 9 | 1.1 | 532.2751 | 2 | 22.35 | 8 | F8:7174 | 31122021\_RID\_3262\_1b.raw |  |  |  |  |  |  |  | 9.0657E5 | 1 | 0 | 0 | 0 | 0 | 0 | 0 | 0 | 1 | 153 | 161 |  |  | PEAKS DB |
| R.DVELLTGLNF.Y | N | 50.10 | 1119.5812 | 10 | 0.1 | 560.7979 | 2 | 77.66 | 8 | F8:45882 | 31122021\_RID\_3262\_1b.raw |  |  |  |  |  |  |  | 2.4718E5 | 1 | 0 | 0 | 0 | 0 | 0 | 0 | 0 | 1 | 854 | 863 |  |  | PEAKS DB |
| Y.TLYIEEPDTTGHK.Y | N | 50.08 | 1502.7252 | 13 | 0.6 | 752.3703 | 2 | 29.35 | 8 | F8:12577 | 31122021\_RID\_3262\_1b.raw |  |  |  |  |  |  |  | 1.6199E6 | 1 | 0 | 0 | 0 | 0 | 0 | 0 | 0 | 1 | 336 | 348 |  |  | PEAKS DB |
| N.LM(+15.99)VDQQWMAVR.D | N | 49.58 | 1391.6689 | 11 | -1.3 | 696.8408 | 2 | 43.90 | 8 | F8:23868 | 31122021\_RID\_3262\_1b.raw |  |  |  |  |  |  |  | 7.1676E4 | 1 | 0 | 0 | 0 | 0 | 0 | 0 | 0 | 1 | 478 | 488 | Oxidation (M) | M2:Oxidation (M):70.10 | PEAKS DB |
| K.TFLPIFVN.P | N | 49.12 | 949.5273 | 8 | -0.5 | 475.7707 | 2 | 69.23 | 8 | F8:42039 | 31122021\_RID\_3262\_1b.raw |  |  |  |  |  |  |  | 1.5864E5 | 1 | 0 | 0 | 0 | 0 | 0 | 0 | 0 | 1 | 879 | 886 |  |  | PEAKS DB |
| D.FYTLYIEEPDTTGHK.Y | N | 48.85 | 1812.8569 | 15 | -0.6 | 907.4352 | 2 | 42.38 | 8 | F8:22650 | 31122021\_RID\_3262\_1b.raw |  |  |  |  |  |  |  | 6.9586E4 | 1 | 0 | 0 | 0 | 0 | 0 | 0 | 0 | 1 | 334 | 348 |  |  | PEAKS DB |
| K.VLSFILPHRPDN.S | N | 48.71 | 1406.7670 | 12 | 0.8 | 704.3914 | 2 | 41.42 | 8 | F8:21857 | 31122021\_RID\_3262\_1b.raw |  |  |  |  |  |  |  | 1.8605E5 | 1 | 0 | 0 | 0 | 0 | 0 | 0 | 0 | 1 | 816 | 827 |  |  | PEAKS DB |
| L.YIEEPDTTGHK.Y | N | 48.56 | 1288.5935 | 11 | 0.1 | 645.3041 | 2 | 21.02 | 3 | F3:7745 | 13012022\_RID\_3262\_3b.raw |  |  | 4.5919E5 |  |  |  |  | 1.123E6 | 2 | 0 | 0 | 1 | 0 | 0 | 0 | 0 | 1 | 338 | 348 |  |  | PEAKS DB |
| K.YISAYSQDILMPLWSSYT.I | N | 48.45 | 2137.0078 | 18 | 0.5 | 1069.5117 | 2 | 78.53 | 8 | F8:46086 | 31122021\_RID\_3262\_1b.raw |  |  |  |  |  |  |  | 7.9829E5 | 1 | 0 | 0 | 0 | 0 | 0 | 0 | 0 | 1 | 644 | 661 |  |  | PEAKS DB |
| R.IDKVNLMVDQQWM(+15.99)AV.R | N | 48.31 | 1804.8851 | 15 | 0.7 | 903.4505 | 2 | 54.37 | 8 | F8:32568 | 31122021\_RID\_3262\_1b.raw |  |  |  |  |  |  |  | 1.1309E7 | 1 | 0 | 0 | 0 | 0 | 0 | 0 | 0 | 1 | 473 | 487 | Oxidation (M) | M13:Oxidation (M):95.35 | PEAKS DB |
| K.SIPFEAR.V | N | 48.00 | 818.4286 | 7 | 0.6 | 410.2218 | 2 | 32.78 | 2 | F2:10658 | 13012022\_RID\_3262\_2b.raw |  | 2.8129E6 |  |  |  |  |  |  | 1 | 0 | 1 | 0 | 0 | 0 | 0 | 0 | 0 | 310 | 316 |  |  | PEAKS DB |
| N.PAWWGGQPIWHTATYQGLK.A | N | 47.31 | 2196.0906 | 19 | 0.5 | 733.0378 | 3 | 50.42 | 8 | F8:28936 | 31122021\_RID\_3262\_1b.raw |  |  |  |  |  |  |  | 4.682E5 | 1 | 0 | 0 | 0 | 0 | 0 | 0 | 0 | 1 | 265 | 283 |  |  | PEAKS DB |
| R.DVELLTGLNFY.S | N | 47.24 | 1282.6445 | 11 | 0.3 | 642.3297 | 2 | 79.71 | 8 | F8:46647 | 31122021\_RID\_3262\_1b.raw |  |  |  |  |  |  |  | 2.9834E5 | 1 | 0 | 0 | 0 | 0 | 0 | 0 | 0 | 1 | 854 | 864 |  |  | PEAKS DB |
| D.FFMYEGPAPR.I | N | 46.65 | 1213.5590 | 10 | 0.7 | 607.7872 | 2 | 43.12 | 8 | F8:23185 | 31122021\_RID\_3262\_1b.raw |  |  |  |  |  |  |  | 2.7887E5 | 1 | 0 | 0 | 0 | 0 | 0 | 0 | 0 | 1 | 413 | 422 |  |  | PEAKS DB |
| F.YTFDSEGIVK.N | N | 46.54 | 1157.5604 | 10 | 1.2 | 579.7882 | 2 | 38.75 | 3 | F3:22609 | 13012022\_RID\_3262\_3b.raw |  |  | 1.1954E5 |  |  |  |  |  | 1 | 0 | 0 | 1 | 0 | 0 | 0 | 0 | 0 | 433 | 442 |  |  | PEAKS DB |
| R.NGLNVISGPIFDYN.S | N | 46.38 | 1521.7463 | 14 | 1.5 | 761.8816 | 2 | 65.30 | 8 | F8:39826 | 31122021\_RID\_3262\_1b.raw |  |  |  |  |  |  |  | 1.1708E4 | 1 | 0 | 0 | 0 | 0 | 0 | 0 | 0 | 1 | 754 | 767 |  |  | PEAKS DB |
| K.YISAYSQDILM(+15.99)PLWSSY.T | N | 46.31 | 2051.9551 | 17 | 0.5 | 1026.9854 | 2 | 73.46 | 8 | F8:44129 | 31122021\_RID\_3262\_1b.raw |  |  |  |  |  |  |  | 8.6735E5 | 1 | 0 | 0 | 0 | 0 | 0 | 0 | 0 | 1 | 644 | 660 | Oxidation (M) | M11:Oxidation (M):1000.00 | PEAKS DB |
| P.DFYTLYIEEPDTTGHK.Y | N | 46.00 | 1927.8839 | 16 | 0.7 | 964.9499 | 2 | 49.25 | 8 | F8:27984 | 31122021\_RID\_3262\_1b.raw |  |  |  |  |  |  |  | 1.6606E6 | 1 | 0 | 0 | 0 | 0 | 0 | 0 | 0 | 1 | 333 | 348 |  |  | PEAKS DB |
| K.DFYTFDSEGIV.K | N | 45.21 | 1291.5608 | 11 | 0.6 | 646.7881 | 2 | 70.18 | 8 | F8:42580 | 31122021\_RID\_3262\_1b.raw |  |  |  |  |  |  |  | 2.0262E5 | 1 | 0 | 0 | 0 | 0 | 0 | 0 | 0 | 1 | 431 | 441 |  |  | PEAKS DB |
| total 72 peptides |  |  |  |  |  |  |  |  |  |  |  |  |  |  |  |  |  |  |  |  |  |  |  |  |  |  |  |  |  |  |  |  |  |
| --- | --- | --- | --- | --- | --- | --- | --- | --- | --- | --- | --- | --- | --- | --- | --- | --- | --- | --- | --- | --- | --- | --- | --- | --- | --- | --- | --- | --- | --- | --- | --- | --- | --- |

JAV01884.1

back to list

  

| Protein Coverage
| Supporting Peptides
|

Protein Coverage:

Supporting Peptides:

| Peptide | Uniq | -10lgP | Mass | Length | ppm | m/z | z | RT | Fraction | Scan | Source File | Area F6 | Area F2 | Area F3 | Area F4 | Area F5 | Area F7 | Area F8 | Area F1 | #Feature | #Feature F6 | #Feature F2 | #Feature F3 | #Feature F4 | #Feature F5 | #Feature F7 | #Feature F8 | #Feature F1 | Start | End | PTM | AScore | Found By |
| --- | --- | --- | --- | --- | --- | --- | --- | --- | --- | --- | --- | --- | --- | --- | --- | --- | --- | --- | --- | --- | --- | --- | --- | --- | --- | --- | --- | --- | --- | --- | --- | --- | --- |
| K.NPAWWGGQPIWHTATYQGLK.A | N | 103.11 | 2310.1333 | 20 | -0.2 | 1156.0737 | 2 | 50.36 | 8 | F8:28928 | 31122021\_RID\_3262\_1b.raw |  |  |  |  |  |  |  | 3.6924E7 | 2 | 0 | 0 | 0 | 0 | 0 | 0 | 0 | 2 | 185 | 204 |  |  | PEAKS DB |
| K.SMQAIFLAHGPGFNEK.K | N | 103.04 | 1745.8558 | 16 | -0.2 | 873.9351 | 2 | 42.38 | 8 | F8:22729 | 31122021\_RID\_3262\_1b.raw |  |  | 3.2331E5 |  |  |  |  | 8.2355E7 | 3 | 0 | 0 | 1 | 0 | 0 | 0 | 0 | 2 | 429 | 444 |  |  | PEAKS DB |
| R.DVELLTGLNFYSGLK.Q | N | 94.09 | 1667.8770 | 15 | 0.8 | 834.9464 | 2 | 66.30 | 8 | F8:40834 | 31122021\_RID\_3262\_1b.raw |  |  |  |  |  |  |  | 3.1554E7 | 4 | 0 | 0 | 0 | 0 | 0 | 0 | 0 | 4 | 775 | 789 |  |  | PEAKS DB |
| K.SM(+15.99)QAIFLAHGPGFNEK.K | N | 91.55 | 1761.8508 | 16 | 0.1 | 881.9327 | 2 | 36.86 | 8 | F8:18333 | 31122021\_RID\_3262\_1b.raw |  |  | 1.011E6 |  |  |  |  | 2.6382E7 | 8 | 0 | 0 | 1 | 0 | 0 | 0 | 0 | 7 | 429 | 444 | Oxidation (M) | M2:Oxidation (M):1000.00 | PEAKS DB |
| R.IDKVNLM(+15.99)VDQQWMAVR.N | N | 89.72 | 1960.9863 | 16 | 1.2 | 981.5016 | 2 | 45.32 | 8 | F8:25068 | 31122021\_RID\_3262\_1b.raw |  |  |  |  |  |  |  | 2.3151E7 | 4 | 0 | 0 | 0 | 0 | 0 | 0 | 0 | 4 | 394 | 409 | Oxidation (M) | M7:Oxidation (M):182.75 | PEAKS DB |
| R.IDKVNLMVDQQWMAVR.N | N | 88.44 | 1944.9913 | 16 | 0.4 | 973.5034 | 2 | 48.73 | 8 | F8:27572 | 31122021\_RID\_3262\_1b.raw |  |  |  |  |  |  |  | 9.0594E6 | 3 | 0 | 0 | 0 | 0 | 0 | 0 | 0 | 3 | 394 | 409 |  |  | PEAKS DB |
| R.IDKVNLMVDQQWM(+15.99)AVR.N | N | 86.82 | 1960.9863 | 16 | 0.3 | 654.6696 | 3 | 44.18 | 8 | F8:23999 | 31122021\_RID\_3262\_1b.raw |  |  |  |  |  |  |  | 6.7254E6 | 5 | 0 | 0 | 0 | 0 | 0 | 0 | 0 | 5 | 394 | 409 | Oxidation (M) | M13:Oxidation (M):156.85 | PEAKS DB |
| K.VNLM(+15.99)VDQQWMAVR.N | N | 85.00 | 1604.7803 | 13 | 0.8 | 803.3981 | 2 | 48.10 | 8 | F8:27251 | 31122021\_RID\_3262\_1b.raw |  |  | 2.977E5 |  |  |  |  | 8.7958E6 | 3 | 0 | 0 | 1 | 0 | 0 | 0 | 0 | 2 | 397 | 409 | Oxidation (M) | M4:Oxidation (M):203.88 | PEAKS DB |
| R.IDKVNLM(+15.99)VDQQWM(+15.99)AVR.N | N | 83.92 | 1976.9812 | 16 | 0.8 | 660.0015 | 3 | 40.70 | 8 | F8:21410 | 31122021\_RID\_3262\_1b.raw |  |  |  |  |  |  |  | 8.2962E6 | 5 | 0 | 0 | 0 | 0 | 0 | 0 | 0 | 5 | 394 | 409 | Oxidation (M) | M7:Oxidation (M):1000.00;M13:Oxidation (M):1000.00 | PEAKS DB |
| R.LWNYFHTTLIPK.Y | N | 82.18 | 1531.8187 | 12 | 0.3 | 766.9169 | 2 | 47.03 | 8 | F8:26408 | 31122021\_RID\_3262\_1b.raw |  |  |  |  |  |  |  | 8.903E7 | 2 | 0 | 0 | 0 | 0 | 0 | 0 | 0 | 2 | 658 | 669 |  |  | PEAKS DB |
| R.VRDVELLTGLNFYSGLK.Q | N | 82.16 | 1923.0465 | 17 | 0.6 | 642.0232 | 3 | 54.30 | 8 | F8:32508 | 31122021\_RID\_3262\_1b.raw |  |  |  |  |  |  |  | 2.8722E7 | 5 | 0 | 0 | 0 | 0 | 0 | 0 | 0 | 5 | 773 | 789 |  |  | PEAKS DB |
| N.YGPVSGEIIK.A | N | 81.32 | 1061.5757 | 10 | -0.7 | 531.7947 | 2 | 36.07 | 2 | F2:12468 | 13012022\_RID\_3262\_2b.raw |  | 6.2644E6 | 1.3043E7 | 5.1609E6 | 1.3681E6 |  |  | 2.2399E8 | 5 | 0 | 1 | 1 | 1 | 1 | 0 | 0 | 1 | 270 | 279 |  |  | PEAKS DB |
| K.VNLMVDQQWMAVR.N | N | 80.00 | 1588.7854 | 13 | 0.8 | 795.4006 | 2 | 52.09 | 8 | F8:30422 | 31122021\_RID\_3262\_1b.raw |  |  |  |  |  |  |  | 4.1895E6 | 1 | 0 | 0 | 0 | 0 | 0 | 0 | 0 | 1 | 397 | 409 |  |  | PEAKS DB |
| K.AATYFWPGSEVK.I | N | 73.56 | 1354.6558 | 12 | -0.3 | 678.3350 | 2 | 44.52 | 8 | F8:24361 | 31122021\_RID\_3262\_1b.raw |  | 1.7682E6 | 5.0699E6 | 1.4387E6 |  |  |  | 1.9551E8 | 4 | 0 | 1 | 1 | 1 | 0 | 0 | 0 | 1 | 205 | 216 |  |  | PEAKS DB |
| K.VNLM(+15.99)VDQQWM(+15.99)AVR.N | N | 72.26 | 1620.7753 | 13 | 0.9 | 811.3956 | 2 | 42.52 | 8 | F8:22889 | 31122021\_RID\_3262\_1b.raw |  |  |  |  |  |  |  | 3.4011E6 | 5 | 0 | 0 | 0 | 0 | 0 | 0 | 0 | 5 | 397 | 409 | Oxidation (M) | M4:Oxidation (M):1000.00;M10:Oxidation (M):1000.00 | PEAKS DB |
| R.TLGM(+15.99)LMEGLK.Q | N | 71.09 | 1107.5668 | 10 | 0.2 | 554.7908 | 2 | 43.40 | 8 | F8:23540 | 31122021\_RID\_3262\_1b.raw |  | 2.8854E6 | 5.0463E6 |  |  |  |  | 1.9938E8 | 8 | 0 | 1 | 2 | 0 | 0 | 0 | 0 | 5 | 287 | 296 | Oxidation (M) | M4:Oxidation (M):67.96 | PEAKS DB |
| K.RLHYVNNIR.I | N | 70.58 | 1183.6573 | 9 | -0.1 | 395.5597 | 3 | 17.86 | 8 | F8:4187 | 31122021\_RID\_3262\_1b.raw |  |  |  |  |  |  |  | 2.7601E7 | 3 | 0 | 0 | 0 | 0 | 0 | 0 | 0 | 3 | 385 | 393 |  |  | PEAKS DB |
| K.NPFYTPSPAK.E | N | 70.36 | 1120.5553 | 10 | 0.1 | 561.2850 | 2 | 31.20 | 8 | F8:13940 | 31122021\_RID\_3262\_1b.raw |  | 3.4243E5 | 1.1099E6 | 5.0205E5 |  |  |  | 1.254E7 | 4 | 0 | 1 | 1 | 1 | 0 | 0 | 0 | 1 | 483 | 492 |  |  | PEAKS DB |
| K.DYYTFDSEGIVK.N | Y | 70.06 | 1435.6506 | 12 | -1.2 | 718.8317 | 2 | 52.33 | 8 | F8:30492 | 31122021\_RID\_3262\_1b.raw |  |  |  |  |  |  |  | 0 | 0 | 0 | 0 | 0 | 0 | 0 | 0 | 0 | 0 | 352 | 363 |  |  | PEAKS DB |
| K.TFLPIFVNPVN | N | 67.67 | 1259.6914 | 11 | 0.2 | 630.8531 | 2 | 72.02 | 8 | F8:43764 | 31122021\_RID\_3262\_1b.raw |  | 2.2902E6 | 3.7599E6 | 1.795E6 | 1.8129E6 |  |  | 1.0908E8 | 6 | 0 | 1 | 1 | 1 | 1 | 0 | 0 | 2 | 800 | 810 |  |  | PEAKS DB |
| I.FLAHGPGFNEK.K | N | 67.15 | 1215.6036 | 11 | 0.8 | 608.8096 | 2 | 25.74 | 8 | F8:9653 | 31122021\_RID\_3262\_1b.raw |  |  |  |  |  |  |  | 1.7745E5 | 1 | 0 | 0 | 0 | 0 | 0 | 0 | 0 | 1 | 434 | 444 |  |  | PEAKS DB |
| R.TLGMLMEGLK.Q | N | 67.03 | 1091.5719 | 10 | 0.1 | 546.7933 | 2 | 47.62 | 8 | F8:26617 | 31122021\_RID\_3262\_1b.raw |  |  |  |  |  |  |  | 1.2415E7 | 1 | 0 | 0 | 0 | 0 | 0 | 0 | 0 | 1 | 287 | 296 |  |  | PEAKS DB |
| K.GGTHGYDNEFK.S | N | 64.95 | 1223.5206 | 11 | 0.5 | 612.7679 | 2 | 18.69 | 8 | F8:4517 | 31122021\_RID\_3262\_1b.raw |  |  | 1.0051E6 |  |  |  |  | 2.299E7 | 2 | 0 | 0 | 1 | 0 | 0 | 0 | 0 | 1 | 418 | 428 |  |  | PEAKS DB |
| K.VNLMVDQQWM(+15.99)AVR.N | N | 62.89 | 1604.7803 | 13 | -0.2 | 803.3972 | 2 | 52.11 | 8 | F8:30389 | 31122021\_RID\_3262\_1b.raw |  |  |  |  |  |  |  | 2.8558E6 | 3 | 0 | 0 | 0 | 0 | 0 | 0 | 0 | 3 | 397 | 409 | Oxidation (M) | M10:Oxidation (M):113.99 | PEAKS DB |
| K.NPAWWGGQPIWHTA.T | N | 61.48 | 1619.7633 | 14 | 0.5 | 810.8893 | 2 | 53.82 | 8 | F8:31774 | 31122021\_RID\_3262\_1b.raw |  |  |  |  |  |  |  | 8.4618E5 | 1 | 0 | 0 | 0 | 0 | 0 | 0 | 0 | 1 | 185 | 198 |  |  | PEAKS DB |
| K.NPAWWGGQPIWHT.A | N | 60.99 | 1548.7262 | 13 | 0.1 | 775.3704 | 2 | 55.72 | 8 | F8:33196 | 31122021\_RID\_3262\_1b.raw |  |  |  |  |  |  |  | 1.1047E6 | 1 | 0 | 0 | 0 | 0 | 0 | 0 | 0 | 1 | 185 | 197 |  |  | PEAKS DB |
| R.IDKVNLMVDQQWMAV.R | N | 60.10 | 1788.8903 | 15 | 0.4 | 895.4528 | 2 | 58.57 | 8 | F8:35442 | 31122021\_RID\_3262\_1b.raw |  |  |  |  |  |  |  | 6.3717E6 | 1 | 0 | 0 | 0 | 0 | 0 | 0 | 0 | 1 | 394 | 408 |  |  | PEAKS DB |
| K.NPAWWGGQPIWH.T | N | 59.14 | 1447.6786 | 12 | 0.5 | 724.8469 | 2 | 54.57 | 8 | F8:32349 | 31122021\_RID\_3262\_1b.raw |  |  |  |  |  |  |  | 1.8077E6 | 1 | 0 | 0 | 0 | 0 | 0 | 0 | 0 | 1 | 185 | 196 |  |  | PEAKS DB |
| R.TLGM(+15.99)LM(+15.99)EGLK.Q | N | 59.05 | 1123.5618 | 10 | 0.5 | 562.7885 | 2 | 41.42 | 8 | F8:23793 | 31122021\_RID\_3262\_1b.raw |  | 1.544E6 |  |  |  |  |  | 7.4572E7 | 8 | 0 | 1 | 0 | 0 | 0 | 0 | 0 | 7 | 287 | 296 | Oxidation (M) | M4:Oxidation (M):1000.00;M6:Oxidation (M):1000.00 | PEAKS DB |
| K.NPAWWGGQPIWHTAT.Y | N | 58.25 | 1720.8110 | 15 | 0.8 | 861.4135 | 2 | 53.71 | 8 | F8:31660 | 31122021\_RID\_3262\_1b.raw |  |  |  |  |  |  |  | 1.8953E6 | 1 | 0 | 0 | 0 | 0 | 0 | 0 | 0 | 1 | 185 | 199 |  |  | PEAKS DB |
| R.TLGMLM(+15.99)EGLK.Q | N | 58.20 | 1107.5668 | 10 | -0.4 | 554.7905 | 2 | 47.61 | 8 | F8:26867 | 31122021\_RID\_3262\_1b.raw |  |  |  |  |  |  |  | 6.9241E6 | 2 | 0 | 0 | 0 | 0 | 0 | 0 | 0 | 2 | 287 | 296 | Oxidation (M) | M6:Oxidation (M):60.92 | PEAKS DB |
| Q.SPLILFSMDGFR.A | N | 57.71 | 1381.7064 | 12 | 0.3 | 691.8607 | 2 | 62.91 | 8 | F8:38370 | 31122021\_RID\_3262\_1b.raw |  |  |  |  |  |  |  | 2.2236E4 | 1 | 0 | 0 | 0 | 0 | 0 | 0 | 0 | 1 | 98 | 109 |  |  | PEAKS DB |
| R.IDKVNLM(+15.99)VDQQWMAV.R | N | 57.69 | 1804.8851 | 15 | 0.7 | 903.4505 | 2 | 54.37 | 8 | F8:32152 | 31122021\_RID\_3262\_1b.raw |  |  |  |  |  |  |  | 1.1309E7 | 1 | 0 | 0 | 0 | 0 | 0 | 0 | 0 | 1 | 394 | 408 | Oxidation (M) | M7:Oxidation (M):112.31 | PEAKS DB |
| R.IDKVNLM(+15.99)VDQQWM(+15.99)AV.R | N | 57.21 | 1820.8801 | 15 | 0.6 | 911.4479 | 2 | 53.49 | 8 | F8:32111 | 31122021\_RID\_3262\_1b.raw |  |  |  |  |  |  |  | 4.342E6 | 4 | 0 | 0 | 0 | 0 | 0 | 0 | 0 | 4 | 394 | 408 | Oxidation (M) | M7:Oxidation (M):1000.00;M13:Oxidation (M):1000.00 | PEAKS DB |
| K.QPLPETLQLK.T | N | 56.85 | 1165.6707 | 10 | -0.3 | 583.8424 | 2 | 39.53 | 8 | F8:20550 | 31122021\_RID\_3262\_1b.raw |  |  | 4.1963E6 | 1.1061E6 |  |  |  | 1.0052E8 | 3 | 0 | 0 | 1 | 1 | 0 | 0 | 0 | 1 | 790 | 799 |  |  | PEAKS DB |
| N.LMVDQQWMAVR.N | N | 55.73 | 1375.6741 | 11 | 2.2 | 688.8458 | 2 | 47.23 | 8 | F8:26413 | 31122021\_RID\_3262\_1b.raw |  |  |  |  |  |  |  | 3.1228E4 | 1 | 0 | 0 | 0 | 0 | 0 | 0 | 0 | 1 | 399 | 409 |  |  | PEAKS DB |
| L.ILFSMDGFR.A | N | 54.86 | 1084.5375 | 9 | 0.3 | 543.2762 | 2 | 52.47 | 8 | F8:30540 | 31122021\_RID\_3262\_1b.raw |  |  |  |  |  |  |  | 7.1062E5 | 1 | 0 | 0 | 0 | 0 | 0 | 0 | 0 | 1 | 101 | 109 |  |  | PEAKS DB |
| K.NPAWWGGQPIWHTATY.Q | N | 54.41 | 1883.8744 | 16 | 1.0 | 942.9454 | 2 | 56.35 | 8 | F8:33653 | 31122021\_RID\_3262\_1b.raw |  |  |  |  |  |  |  | 2.4054E6 | 1 | 0 | 0 | 0 | 0 | 0 | 0 | 0 | 1 | 185 | 200 |  |  | PEAKS DB |
| K.YISAYSQDILMPL.W | N | 54.14 | 1512.7534 | 13 | 0.5 | 757.3843 | 2 | 68.83 | 8 | F8:41947 | 31122021\_RID\_3262\_1b.raw |  |  |  |  |  |  |  | 1.2569E6 | 1 | 0 | 0 | 0 | 0 | 0 | 0 | 0 | 1 | 565 | 577 |  |  | PEAKS DB |
| R.LHYVNNIR.I | N | 53.99 | 1027.5563 | 8 | 0.1 | 514.7855 | 2 | 20.71 | 8 | F8:5976 | 31122021\_RID\_3262\_1b.raw |  |  | 9.0646E5 |  |  |  |  | 1.2323E7 | 2 | 0 | 0 | 1 | 0 | 0 | 0 | 0 | 1 | 386 | 393 |  |  | PEAKS DB |
| L.ITSNIVPMFK.G | N | 52.50 | 1148.6263 | 10 | 0.3 | 575.3206 | 2 | 44.67 | 8 | F8:24431 | 31122021\_RID\_3262\_1b.raw |  |  |  |  |  |  |  | 5.4274E5 | 1 | 0 | 0 | 0 | 0 | 0 | 0 | 0 | 1 | 644 | 653 |  |  | PEAKS DB |
| M.VDQQWMAVR.N | N | 52.40 | 1131.5496 | 9 | 0.2 | 566.7822 | 2 | 35.82 | 8 | F8:17412 | 31122021\_RID\_3262\_1b.raw |  |  |  |  |  |  |  | 2.5672E5 | 1 | 0 | 0 | 0 | 0 | 0 | 0 | 0 | 1 | 401 | 409 |  |  | PEAKS DB |
| Y.MYEGPAPR.I | N | 52.23 | 919.4222 | 8 | -0.2 | 460.7183 | 2 | 22.38 | 8 | F8:7073 | 31122021\_RID\_3262\_1b.raw |  |  |  |  |  |  |  | 1.1389E6 | 1 | 0 | 0 | 0 | 0 | 0 | 0 | 0 | 1 | 336 | 343 |  |  | PEAKS DB |
| K.SMQAIFLAH.G | N | 50.78 | 1016.5114 | 9 | 0.6 | 509.2633 | 2 | 44.34 | 8 | F8:24102 | 31122021\_RID\_3262\_1b.raw |  |  |  |  |  |  |  | 2.2295E6 | 1 | 0 | 0 | 0 | 0 | 0 | 0 | 0 | 1 | 429 | 437 |  |  | PEAKS DB |
| K.IPIPTHYFV.V | N | 50.61 | 1085.5909 | 9 | -0.2 | 543.8026 | 2 | 48.99 | 8 | F8:27744 | 31122021\_RID\_3262\_1b.raw |  |  |  |  |  |  |  | 4.4642E5 | 1 | 0 | 0 | 0 | 0 | 0 | 0 | 0 | 1 | 708 | 716 |  |  | PEAKS DB |
| G.GTHGYDNEFK.S | N | 50.17 | 1166.4993 | 10 | 0.3 | 584.2571 | 2 | 17.71 | 8 | F8:4104 | 31122021\_RID\_3262\_1b.raw |  |  |  |  |  |  |  | 4.0494E4 | 1 | 0 | 0 | 0 | 0 | 0 | 0 | 0 | 1 | 419 | 428 |  |  | PEAKS DB |
| R.DVELLTGLNF.Y | N | 50.10 | 1119.5812 | 10 | 0.1 | 560.7979 | 2 | 77.66 | 8 | F8:45882 | 31122021\_RID\_3262\_1b.raw |  |  |  |  |  |  |  | 2.4718E5 | 1 | 0 | 0 | 0 | 0 | 0 | 0 | 0 | 1 | 775 | 784 |  |  | PEAKS DB |
| N.LM(+15.99)VDQQWMAVR.N | N | 49.58 | 1391.6689 | 11 | -1.3 | 696.8408 | 2 | 43.90 | 8 | F8:23868 | 31122021\_RID\_3262\_1b.raw |  |  |  |  |  |  |  | 7.1676E4 | 1 | 0 | 0 | 0 | 0 | 0 | 0 | 0 | 1 | 399 | 409 | Oxidation (M) | M2:Oxidation (M):70.10 | PEAKS DB |
| K.TFLPIFVN.P | N | 49.12 | 949.5273 | 8 | -0.5 | 475.7707 | 2 | 69.23 | 8 | F8:42039 | 31122021\_RID\_3262\_1b.raw |  |  |  |  |  |  |  | 1.5864E5 | 1 | 0 | 0 | 0 | 0 | 0 | 0 | 0 | 1 | 800 | 807 |  |  | PEAKS DB |
| K.VLSFILPHRPDN.S | N | 48.71 | 1406.7670 | 12 | 0.8 | 704.3914 | 2 | 41.42 | 8 | F8:21857 | 31122021\_RID\_3262\_1b.raw |  |  |  |  |  |  |  | 1.8605E5 | 1 | 0 | 0 | 0 | 0 | 0 | 0 | 0 | 1 | 737 | 748 |  |  | PEAKS DB |
| R.IDKVNLMVDQQWM(+15.99)AV.R | N | 48.31 | 1804.8851 | 15 | 0.7 | 903.4505 | 2 | 54.37 | 8 | F8:32568 | 31122021\_RID\_3262\_1b.raw |  |  |  |  |  |  |  | 1.1309E7 | 1 | 0 | 0 | 0 | 0 | 0 | 0 | 0 | 1 | 394 | 408 | Oxidation (M) | M13:Oxidation (M):95.35 | PEAKS DB |
| D.GHFDSYDTIK.Q | N | 47.87 | 1181.5353 | 10 | 0.8 | 591.7754 | 2 | 25.52 | 8 | F8:9466 | 31122021\_RID\_3262\_1b.raw |  |  |  |  |  |  |  | 2.1836E5 | 1 | 0 | 0 | 0 | 0 | 0 | 0 | 0 | 1 | 691 | 700 |  |  | PEAKS DB |
| N.PAWWGGQPIWHTATYQGLK.A | N | 47.31 | 2196.0906 | 19 | 0.5 | 733.0378 | 3 | 50.42 | 8 | F8:28936 | 31122021\_RID\_3262\_1b.raw |  |  |  |  |  |  |  | 4.682E5 | 1 | 0 | 0 | 0 | 0 | 0 | 0 | 0 | 1 | 186 | 204 |  |  | PEAKS DB |
| R.DVELLTGLNFY.S | N | 47.24 | 1282.6445 | 11 | 0.3 | 642.3297 | 2 | 79.71 | 8 | F8:46647 | 31122021\_RID\_3262\_1b.raw |  |  |  |  |  |  |  | 2.9834E5 | 1 | 0 | 0 | 0 | 0 | 0 | 0 | 0 | 1 | 775 | 785 |  |  | PEAKS DB |
| Y.YTFDSEGIVK.N | N | 46.54 | 1157.5604 | 10 | 1.2 | 579.7882 | 2 | 38.75 | 3 | F3:22609 | 13012022\_RID\_3262\_3b.raw |  |  | 1.1954E5 |  |  |  |  |  | 1 | 0 | 0 | 1 | 0 | 0 | 0 | 0 | 0 | 354 | 363 |  |  | PEAKS DB |
| R.NGLNVISGPIFDYN.S | N | 46.38 | 1521.7463 | 14 | 1.5 | 761.8816 | 2 | 65.30 | 8 | F8:39826 | 31122021\_RID\_3262\_1b.raw |  |  |  |  |  |  |  | 1.1708E4 | 1 | 0 | 0 | 0 | 0 | 0 | 0 | 0 | 1 | 675 | 688 |  |  | PEAKS DB |
| total 56 peptides |  |  |  |  |  |  |  |  |  |  |  |  |  |  |  |  |  |  |  |  |  |  |  |  |  |  |  |  |  |  |  |  |  |
| --- | --- | --- | --- | --- | --- | --- | --- | --- | --- | --- | --- | --- | --- | --- | --- | --- | --- | --- | --- | --- | --- | --- | --- | --- | --- | --- | --- | --- | --- | --- | --- | --- | --- |

ETE67131.1

back to list

  

| Protein Coverage
| Supporting Peptides
|

Protein Coverage:

Supporting Peptides:

| Peptide | Uniq | -10lgP | Mass | Length | ppm | m/z | z | RT | Fraction | Scan | Source File | Area F6 | Area F2 | Area F3 | Area F4 | Area F5 | Area F7 | Area F8 | Area F1 | #Feature | #Feature F6 | #Feature F2 | #Feature F3 | #Feature F4 | #Feature F5 | #Feature F7 | #Feature F8 | #Feature F1 | Start | End | PTM | AScore | Found By |
| --- | --- | --- | --- | --- | --- | --- | --- | --- | --- | --- | --- | --- | --- | --- | --- | --- | --- | --- | --- | --- | --- | --- | --- | --- | --- | --- | --- | --- | --- | --- | --- | --- | --- |
| R.DLDLDSIIAEVK.A | Y | 84.35 | 1329.7028 | 12 | 1.1 | 665.8594 | 2 | 66.05 | 8 | F8:40234 | 31122021\_RID\_3262\_1b.raw |  | 4.71E5 | 1.087E6 | 4.8663E5 | 6.2675E5 | 8.4246E5 | 2.9874E5 | 3.4491E6 | 13 | 0 | 1 | 2 | 2 | 2 | 2 | 1 | 3 | 1923 | 1934 |  |  | PEAKS DB |
| K.LALDIEIATYR.K | N | 78.30 | 1276.7026 | 11 | 0.6 | 639.3590 | 2 | 52.72 | 8 | F8:30857 | 31122021\_RID\_3262\_1b.raw |  | 1.2925E6 | 1.0345E7 | 2.8112E5 | 1.0579E6 |  |  | 1.4292E7 | 10 | 0 | 1 | 1 | 1 | 1 | 0 | 0 | 6 | 446 | 456 |  |  | PEAKS DB |
| K.LALDVEIATYR.T | N | 75.27 | 1262.6870 | 11 | 0.9 | 632.3513 | 2 | 48.83 | 8 | F8:27771 | 31122021\_RID\_3262\_1b.raw | 4.2901E5 | 4.1866E5 | 5.6979E6 | 8.6415E3 | 2.2794E5 |  |  | 3.6103E6 | 9 | 1 | 1 | 1 | 1 | 2 | 0 | 0 | 3 | 2052 | 2062 |  |  | PEAKS DB |
| K.AQYEDIAQK.S | N | 71.01 | 1064.5138 | 9 | 0.2 | 533.2643 | 2 | 22.69 | 8 | F8:7374 | 31122021\_RID\_3262\_1b.raw | 5.6213E6 | 4.6319E6 | 1.1417E8 | 3.7973E7 | 1.7343E7 | 3.8115E7 | 5.2156E7 | 1.9393E8 | 12 | 1 | 1 | 1 | 1 | 1 | 1 | 2 | 4 | 334 | 342 |  |  | PEAKS DB |
| R.DYQELMNVK.L | N | 70.10 | 1138.5328 | 9 | 0.6 | 570.2740 | 2 | 39.78 | 8 | F8:20760 | 31122021\_RID\_3262\_1b.raw | 8.5765E5 |  | 8.2376E6 | 2.4643E6 | 2.2846E6 | 2.0779E6 | 9.1069E5 | 1.8234E7 | 7 | 1 | 0 | 1 | 1 | 1 | 1 | 1 | 1 | 2043 | 2051 |  |  | PEAKS DB |
| K.FASFIDKVR.F | N | 65.83 | 1081.5920 | 9 | 1.0 | 541.8038 | 2 | 33.62 | 8 | F8:15859 | 31122021\_RID\_3262\_1b.raw |  |  | 2.2979E6 |  | 2.6797E5 |  |  | 2.3536E6 | 3 | 0 | 0 | 1 | 0 | 1 | 0 | 0 | 1 | 200 | 208 |  |  | PEAKS DB |
| K.NKYEDEINKR.T | N | 63.23 | 1307.6470 | 10 | -0.2 | 436.8895 | 3 | 19.88 | 3 | F3:6860 | 13012022\_RID\_3262\_3b.raw |  |  | 1.7338E7 |  | 7.4484E5 |  |  | 4.9777E7 | 4 | 0 | 0 | 1 | 0 | 1 | 0 | 0 | 2 | 1847 | 1856 |  |  | PEAKS DB |
| R.LLKEYQELM(+15.99)NVK.L | N | 63.16 | 1522.8065 | 12 | 0.0 | 508.6095 | 3 | 34.77 | 3 | F3:19359 | 13012022\_RID\_3262\_3b.raw |  |  | 2.7626E6 |  |  |  |  |  | 2 | 0 | 0 | 2 | 0 | 0 | 0 | 0 | 0 | 434 | 445 | Oxidation (M) | M9:Oxidation (M):1000.00 | PEAKS DB |
| D.LDLDSIIAEVK.A | N | 62.00 | 1214.6758 | 11 | 0.3 | 608.3453 | 2 | 60.72 | 8 | F8:36921 | 31122021\_RID\_3262\_1b.raw |  |  | 8.1093E5 |  |  |  |  | 3.4061E6 | 2 | 0 | 0 | 1 | 0 | 0 | 0 | 0 | 1 | 1924 | 1934 |  |  | PEAKS DB |
| R.DYQELM(+15.99)NVK.L | N | 61.98 | 1154.5277 | 9 | -0.1 | 578.2711 | 2 | 31.37 | 3 | F3:16348 | 13012022\_RID\_3262\_3b.raw |  |  | 6.5405E6 | 8.0585E5 | 1.0159E6 |  |  | 5.8533E6 | 9 | 0 | 0 | 1 | 1 | 2 | 0 | 0 | 5 | 2043 | 2051 | Oxidation (M) | M6:Oxidation (M):1000.00 | PEAKS DB |
| K.EYQELMNVK.L | N | 60.69 | 1152.5485 | 9 | 0.3 | 577.2817 | 2 | 33.98 | 8 | F8:15978 | 31122021\_RID\_3262\_1b.raw |  |  | 1.0316E6 |  |  |  |  | 1.0554E6 | 2 | 0 | 0 | 1 | 0 | 0 | 0 | 0 | 1 | 437 | 445 |  |  | PEAKS DB |
| L.DLDSIIAEVK.A | N | 58.84 | 1101.5917 | 10 | 0.6 | 551.8035 | 2 | 54.57 | 8 | F8:32317 | 31122021\_RID\_3262\_1b.raw |  |  | 1.0155E5 |  |  |  |  | 4.068E5 | 2 | 0 | 0 | 1 | 0 | 0 | 0 | 0 | 1 | 1925 | 1934 |  |  | PEAKS DB |
| R.LLRDYQELMNVK.L | N | 55.59 | 1520.8021 | 12 | 0.3 | 507.9415 | 3 | 39.68 | 8 | F8:20681 | 31122021\_RID\_3262\_1b.raw |  |  |  |  |  |  |  | 4.5587E6 | 2 | 0 | 0 | 0 | 0 | 0 | 0 | 0 | 2 | 2040 | 2051 |  |  | PEAKS DB |
| K.FASFIDK.V | N | 53.98 | 826.4225 | 7 | 0.0 | 414.2185 | 2 | 37.13 | 2 | F2:13215 | 13012022\_RID\_3262\_2b.raw |  | 3.8919E6 | 3.3996E7 | 9.4622E6 | 1.087E7 | 2.372E7 | 4.1967E6 | 3.8411E7 | 9 | 0 | 1 | 2 | 1 | 1 | 2 | 1 | 1 | 200 | 206 |  |  | PEAKS DB |
| K.NKYEDEINK.R | N | 53.89 | 1151.5458 | 9 | 0.4 | 576.7804 | 2 | 17.00 | 8 | F8:3573 | 31122021\_RID\_3262\_1b.raw |  |  | 3.1112E6 | 1.8214E5 |  |  |  | 6.6435E6 | 3 | 0 | 0 | 1 | 1 | 0 | 0 | 0 | 1 | 1847 | 1855 |  |  | PEAKS DB |
| R.LLKEYQELMNVK.L | N | 53.41 | 1506.8115 | 12 | 0.2 | 503.2779 | 3 | 39.69 | 8 | F8:20552 | 31122021\_RID\_3262\_1b.raw |  |  |  |  |  |  |  | 8.6831E5 | 2 | 0 | 0 | 0 | 0 | 0 | 0 | 0 | 2 | 434 | 445 |  |  | PEAKS DB |
| N.KYEDEINKR.T | N | 52.93 | 1193.6040 | 9 | 0.1 | 398.8753 | 3 | 17.04 | 3 | F3:4500 | 13012022\_RID\_3262\_3b.raw |  |  | 3.8499E6 |  |  |  |  |  | 1 | 0 | 0 | 1 | 0 | 0 | 0 | 0 | 0 | 1848 | 1856 |  |  | PEAKS DB |
| K.EYQELM(+15.99)NVK.L | N | 52.63 | 1168.5435 | 9 | 0.3 | 585.2792 | 2 | 28.16 | 3 | F3:13441 | 13012022\_RID\_3262\_3b.raw |  |  | 1.8851E6 |  |  |  |  | 1.7223E5 | 2 | 0 | 0 | 1 | 0 | 0 | 0 | 0 | 1 | 437 | 445 | Oxidation (M) | M6:Oxidation (M):1000.00 | PEAKS DB |
| K.AQYEDIAQKSR.A | N | 51.10 | 1307.6470 | 11 | -7.8 | 654.8257 | 2 | 27.24 | 8 | F8:10853 | 31122021\_RID\_3262\_1b.raw |  |  |  |  |  |  |  | 2.8701E4 | 1 | 0 | 0 | 0 | 0 | 0 | 0 | 0 | 1 | 334 | 344 |  |  | PEAKS DB |
| R.LLRDYQELM(+15.99)NVK.L | N | 50.21 | 1536.7970 | 12 | -0.1 | 513.2729 | 3 | 34.28 | 3 | F3:18689 | 13012022\_RID\_3262\_3b.raw |  |  | 1.9454E6 |  |  |  |  |  | 1 | 0 | 0 | 1 | 0 | 0 | 0 | 0 | 0 | 2040 | 2051 | Oxidation (M) | M9:Oxidation (M):1000.00 | PEAKS DB |
| N.NQFASFIDK.V | N | 49.40 | 1068.5239 | 9 | 1.1 | 535.2698 | 2 | 38.81 | 8 | F8:19909 | 31122021\_RID\_3262\_1b.raw |  |  |  |  |  |  |  | 9.2773E5 | 1 | 0 | 0 | 0 | 0 | 0 | 0 | 0 | 1 | 684 | 692 |  |  | PEAKS DB |
| L.ALDIEIATYR.K | N | 46.18 | 1163.6187 | 10 | -0.4 | 582.8163 | 2 | 45.58 | 8 | F8:25162 | 31122021\_RID\_3262\_1b.raw |  |  |  |  |  |  |  | 2.943E5 | 1 | 0 | 0 | 0 | 0 | 0 | 0 | 0 | 1 | 447 | 456 |  |  | PEAKS DB |
| A.LDVEIATYR.T | N | 46.13 | 1078.5658 | 9 | 0.8 | 540.2906 | 2 | 39.94 | 8 | F8:20761 | 31122021\_RID\_3262\_1b.raw |  |  |  |  |  |  |  | 7.8953E4 | 1 | 0 | 0 | 0 | 0 | 0 | 0 | 0 | 1 | 2054 | 2062 |  |  | PEAKS DB |
| A.LDIEIATYR.K | N | 45.35 | 1092.5815 | 9 | 0.0 | 547.2980 | 2 | 44.78 | 8 | F8:24515 | 31122021\_RID\_3262\_1b.raw |  |  |  |  |  |  |  | 3.5731E5 | 1 | 0 | 0 | 0 | 0 | 0 | 0 | 0 | 1 | 448 | 456 |  |  | PEAKS DB |
| total 24 peptides |  |  |  |  |  |  |  |  |  |  |  |  |  |  |  |  |  |  |  |  |  |  |  |  |  |  |  |  |  |  |  |  |  |
| --- | --- | --- | --- | --- | --- | --- | --- | --- | --- | --- | --- | --- | --- | --- | --- | --- | --- | --- | --- | --- | --- | --- | --- | --- | --- | --- | --- | --- | --- | --- | --- | --- | --- |

AMB36337.1

back to list

  

| Protein Coverage
| Supporting Peptides
|

Protein Coverage:

Supporting Peptides:

| Peptide | Uniq | -10lgP | Mass | Length | ppm | m/z | z | RT | Fraction | Scan | Source File | Area F6 | Area F2 | Area F3 | Area F4 | Area F5 | Area F7 | Area F8 | Area F1 | #Feature | #Feature F6 | #Feature F2 | #Feature F3 | #Feature F4 | #Feature F5 | #Feature F7 | #Feature F8 | #Feature F1 | Start | End | PTM | AScore | Found By |
| --- | --- | --- | --- | --- | --- | --- | --- | --- | --- | --- | --- | --- | --- | --- | --- | --- | --- | --- | --- | --- | --- | --- | --- | --- | --- | --- | --- | --- | --- | --- | --- | --- | --- |
| R.KPEIQNEIIDLHNSLR.R | Y | 110.33 | 1918.0272 | 16 | -0.1 | 640.3496 | 3 | 53.86 | 1 | F1:20064 | 04012022\_RID\_3262\_6b.raw | 1.4219E8 |  | 1.5534E6 |  |  | 6.3395E6 | 2.1411E7 | 5.8683E6 | 38 | 26 | 0 | 1 | 0 | 0 | 4 | 5 | 2 | 30 | 45 |  |  | PEAKS DB |
| R.SVNPTASNMLK.M | Y | 92.55 | 1160.5859 | 11 | -0.3 | 581.3000 | 2 | 31.48 | 1 | F1:11048 | 04012022\_RID\_3262\_6b.raw | 2.8341E8 |  |  | 4.3807E6 | 7.0746E6 | 4.1792E6 |  | 4.7676E6 | 5 | 1 | 0 | 0 | 1 | 1 | 1 | 0 | 1 | 47 | 57 |  |  | PEAKS DB |
| G.SVDFDSESPR.K | Y | 90.22 | 1137.4938 | 10 | -0.7 | 569.7538 | 2 | 29.53 | 1 | F1:9807 | 04012022\_RID\_3262\_6b.raw | 3.1216E8 | 1.5785E6 | 5.2244E6 | 4.7624E6 | 9.5313E6 | 4.8826E6 | 1.8175E6 | 4.9216E6 | 10 | 2 | 2 | 1 | 1 | 1 | 1 | 1 | 1 | 20 | 29 |  |  | PEAKS DB |
| R.SVNPTASNM(+15.99)LK.M | Y | 80.68 | 1176.5808 | 11 | -0.5 | 589.2974 | 2 | 22.90 | 1 | F1:6828 | 04012022\_RID\_3262\_6b.raw | 1.8362E8 | 3.6775E5 |  |  | 6.7902E6 | 5.1902E6 |  |  | 10 | 3 | 1 | 0 | 0 | 3 | 3 | 0 | 0 | 47 | 57 | Oxidation (M) | M9:Oxidation (M):1000.00 | PEAKS DB |
| S.VDFDSESPR.K | N | 69.49 | 1050.4618 | 9 | -0.5 | 526.2379 | 2 | 28.96 | 1 | F1:9510 | 04012022\_RID\_3262\_6b.raw | 1.3966E7 |  |  |  |  |  |  |  | 2 | 2 | 0 | 0 | 0 | 0 | 0 | 0 | 0 | 21 | 29 |  |  | PEAKS DB |
| K.MEWYPEAAANAER.W | Y | 65.44 | 1536.6667 | 13 | 0.4 | 769.3409 | 2 | 46.53 | 1 | F1:17587 | 04012022\_RID\_3262\_6b.raw | 2.3645E6 |  |  |  |  |  |  |  | 2 | 2 | 0 | 0 | 0 | 0 | 0 | 0 | 0 | 58 | 70 |  |  | PEAKS DB |
| R.KPEIQNEIIDLHNSL.R | Y | 63.70 | 1761.9260 | 15 | 0.5 | 881.9707 | 2 | 53.86 | 1 | F1:20281 | 04012022\_RID\_3262\_6b.raw | 6.4253E7 |  |  |  |  |  |  |  | 2 | 2 | 0 | 0 | 0 | 0 | 0 | 0 | 0 | 30 | 44 |  |  | PEAKS DB |
| E.WYPEAAANAER.W | Y | 62.94 | 1276.5836 | 11 | 0.4 | 639.2993 | 2 | 36.37 | 1 | F1:12817 | 04012022\_RID\_3262\_6b.raw | 1.7357E7 |  |  |  |  |  |  |  | 1 | 1 | 0 | 0 | 0 | 0 | 0 | 0 | 0 | 60 | 70 |  |  | PEAKS DB |
| R.SVNPTASNM(+15.99)L.K | Y | 61.90 | 1048.4860 | 10 | 0.4 | 525.2505 | 2 | 42.26 | 1 | F1:15475 | 04012022\_RID\_3262\_6b.raw | 1.228E6 |  |  |  |  |  |  |  | 1 | 1 | 0 | 0 | 0 | 0 | 0 | 0 | 0 | 47 | 56 | Oxidation (M) | M9:Oxidation (M):1000.00 | PEAKS DB |
| R.SVNPTASNML.K | Y | 56.63 | 1032.4910 | 10 | 0.7 | 517.2531 | 2 | 42.28 | 1 | F1:15395 | 04012022\_RID\_3262\_6b.raw | 5.6395E6 |  |  |  |  |  |  |  | 1 | 1 | 0 | 0 | 0 | 0 | 0 | 0 | 0 | 47 | 56 |  |  | PEAKS DB |
| S.GSVDFDSESPR.K | Y | 55.21 | 1194.5153 | 11 | 0.0 | 598.2649 | 2 | 29.71 | 1 | F1:9770 | 04012022\_RID\_3262\_6b.raw | 3.9696E5 |  |  |  |  |  |  |  | 1 | 1 | 0 | 0 | 0 | 0 | 0 | 0 | 0 | 19 | 29 |  |  | PEAKS DB |
| R.RSVNPTASNMLK.M | Y | 50.43 | 1316.6870 | 12 | 0.6 | 659.3512 | 2 | 37.90 | 1 | F1:13940 | 04012022\_RID\_3262\_6b.raw | 6.0711E6 |  |  |  |  |  |  | 1.953E5 | 2 | 1 | 0 | 0 | 0 | 0 | 0 | 0 | 1 | 46 | 57 |  |  | PEAKS DB |
| V.DFDSESPR.K | N | 50.10 | 951.3934 | 8 | -0.2 | 476.7039 | 2 | 24.61 | 1 | F1:7484 | 04012022\_RID\_3262\_6b.raw | 2.7217E6 |  |  |  |  |  |  |  | 1 | 1 | 0 | 0 | 0 | 0 | 0 | 0 | 0 | 22 | 29 |  |  | PEAKS DB |
| R.KPEIQNEIIDLHN.S | Y | 47.89 | 1561.8099 | 13 | -0.6 | 521.6103 | 3 | 46.70 | 1 | F1:18465 | 04012022\_RID\_3262\_6b.raw | 5.4069E6 |  |  |  |  |  |  |  | 3 | 3 | 0 | 0 | 0 | 0 | 0 | 0 | 0 | 30 | 42 |  |  | PEAKS DB |
| total 14 peptides |  |  |  |  |  |  |  |  |  |  |  |  |  |  |  |  |  |  |  |  |  |  |  |  |  |  |  |  |  |  |  |  |  |
| --- | --- | --- | --- | --- | --- | --- | --- | --- | --- | --- | --- | --- | --- | --- | --- | --- | --- | --- | --- | --- | --- | --- | --- | --- | --- | --- | --- | --- | --- | --- | --- | --- | --- |

XP\_007422201.1

back to list

  

| Protein Coverage
| Supporting Peptides
|

Protein Coverage:

Supporting Peptides:

| Peptide | Uniq | -10lgP | Mass | Length | ppm | m/z | z | RT | Fraction | Scan | Source File | Area F6 | Area F2 | Area F3 | Area F4 | Area F5 | Area F7 | Area F8 | Area F1 | #Feature | #Feature F6 | #Feature F2 | #Feature F3 | #Feature F4 | #Feature F5 | #Feature F7 | #Feature F8 | #Feature F1 | Start | End | PTM | AScore | Found By |
| --- | --- | --- | --- | --- | --- | --- | --- | --- | --- | --- | --- | --- | --- | --- | --- | --- | --- | --- | --- | --- | --- | --- | --- | --- | --- | --- | --- | --- | --- | --- | --- | --- | --- |
| K.GFFDPNTEENLTYLQLK.E | N | 95.00 | 2027.9840 | 17 | 1.2 | 1015.0005 | 2 | 59.85 | 8 | F8:36424 | 31122021\_RID\_3262\_1b.raw |  |  |  |  |  |  |  | 2.047E6 | 1 | 0 | 0 | 0 | 0 | 0 | 0 | 0 | 1 | 2407 | 2423 |  |  | PEAKS DB |
| R.LLEAQIATGGIIDPK.E | N | 81.77 | 1537.8715 | 15 | 1.1 | 769.9438 | 2 | 48.34 | 8 | F8:27443 | 31122021\_RID\_3262\_1b.raw |  |  | 8.6242E5 |  |  |  |  | 1.9093E6 | 2 | 0 | 0 | 1 | 0 | 0 | 0 | 0 | 1 | 2360 | 2374 |  |  | PEAKS DB |
| R.LTYEIEDEKR.K | N | 66.31 | 1294.6405 | 10 | 0.1 | 648.3276 | 2 | 25.34 | 8 | F8:9311 | 31122021\_RID\_3262\_1b.raw |  |  |  |  |  |  |  | 4.2477E5 | 1 | 0 | 0 | 0 | 0 | 0 | 0 | 0 | 1 | 1110 | 1119 |  |  | PEAKS DB |
| K.EHLMLEEELR.N | N | 60.78 | 1297.6335 | 10 | 0.9 | 649.8246 | 2 | 35.25 | 8 | F8:17002 | 31122021\_RID\_3262\_1b.raw |  |  |  |  |  |  |  | 3.3826E5 | 2 | 0 | 0 | 0 | 0 | 0 | 0 | 0 | 2 | 1712 | 1721 |  |  | PEAKS DB |
| R.AELIVQPELK.Y | N | 60.57 | 1138.6597 | 10 | -0.3 | 570.3369 | 2 | 40.19 | 8 | F8:20910 | 31122021\_RID\_3262\_1b.raw |  |  |  |  |  |  |  | 1.9131E6 | 1 | 0 | 0 | 0 | 0 | 0 | 0 | 0 | 1 | 94 | 103 |  |  | PEAKS DB |
| R.LTYEIEDEK.R | N | 59.28 | 1138.5393 | 9 | -0.1 | 570.2769 | 2 | 30.91 | 8 | F8:13546 | 31122021\_RID\_3262\_1b.raw |  |  |  |  |  |  |  | 5.7009E5 | 1 | 0 | 0 | 0 | 0 | 0 | 0 | 0 | 1 | 1110 | 1118 |  |  | PEAKS DB |
| R.QLQNIIQATSR.E | N | 58.54 | 1270.6993 | 11 | -0.2 | 636.3568 | 2 | 34.47 | 8 | F8:16560 | 31122021\_RID\_3262\_1b.raw |  |  | 7.1959E5 | 1.1986E5 |  |  |  | 9.9681E5 | 4 | 0 | 0 | 2 | 1 | 0 | 0 | 0 | 1 | 279 | 289 |  |  | PEAKS DB |
| R.EYENELAK.V | Y | 57.84 | 994.4607 | 8 | 1.0 | 498.2381 | 2 | 24.46 | 3 | F3:10543 | 13012022\_RID\_3262\_3b.raw |  |  | 5.0256E5 |  |  |  |  |  | 1 | 0 | 0 | 1 | 0 | 0 | 0 | 0 | 0 | 1183 | 1190 |  |  | PEAKS DB |
| R.GLVGIEFK.E | N | 53.09 | 861.4960 | 8 | 0.8 | 431.7556 | 2 | 41.72 | 8 | F8:22260 | 31122021\_RID\_3262\_1b.raw |  |  |  |  |  |  |  | 1.1302E6 | 1 | 0 | 0 | 0 | 0 | 0 | 0 | 0 | 1 | 2312 | 2319 |  |  | PEAKS DB |
| K.NKYETEINITK.T | N | 52.42 | 1351.6984 | 11 | 0.3 | 676.8567 | 2 | 25.79 | 8 | F8:9598 | 31122021\_RID\_3262\_1b.raw |  |  |  |  |  |  |  | 2.6043E5 | 1 | 0 | 0 | 0 | 0 | 0 | 0 | 0 | 1 | 1360 | 1370 |  |  | PEAKS DB |
| R.DLKDEIVR.L | N | 51.42 | 986.5396 | 8 | -0.2 | 494.2770 | 2 | 31.74 | 3 | F3:16512 | 13012022\_RID\_3262\_3b.raw |  |  | 0 |  |  |  |  |  | 0 | 0 | 0 | 0 | 0 | 0 | 0 | 0 | 0 | 1244 | 1251 |  |  | PEAKS DB |
| R.LLQLQEQM(+15.99)R.A | N | 49.44 | 1173.6176 | 9 | -0.2 | 587.8159 | 2 | 29.86 | 8 | F8:12793 | 31122021\_RID\_3262\_1b.raw |  |  |  |  |  |  |  | 2.7877E5 | 1 | 0 | 0 | 0 | 0 | 0 | 0 | 0 | 1 | 151 | 159 | Oxidation (M) | M8:Oxidation (M):1000.00 | PEAKS DB |
| R.NSQGSEMFGDDDK.R | N | 48.84 | 1428.5463 | 13 | 0.4 | 715.2807 | 2 | 26.02 | 8 | F8:9883 | 31122021\_RID\_3262\_1b.raw |  |  |  |  |  |  |  | 0 | 0 | 0 | 0 | 0 | 0 | 0 | 0 | 0 | 0 | 601 | 613 |  |  | PEAKS DB |
| R.LQSLTENLTK.E | Y | 45.35 | 1145.6292 | 10 | -0.2 | 573.8217 | 2 | 33.80 | 3 | F3:18198 | 13012022\_RID\_3262\_3b.raw |  |  | 0 |  |  |  |  |  | 0 | 0 | 0 | 0 | 0 | 0 | 0 | 0 | 0 | 1702 | 1711 |  |  | PEAKS DB |
| total 14 peptides |  |  |  |  |  |  |  |  |  |  |  |  |  |  |  |  |  |  |  |  |  |  |  |  |  |  |  |  |  |  |  |  |  |
| --- | --- | --- | --- | --- | --- | --- | --- | --- | --- | --- | --- | --- | --- | --- | --- | --- | --- | --- | --- | --- | --- | --- | --- | --- | --- | --- | --- | --- | --- | --- | --- | --- | --- |

AFJ50957.1

back to list

  

| Protein Coverage
| Supporting Peptides
|

Protein Coverage:

Supporting Peptides:

| Peptide | Uniq | -10lgP | Mass | Length | ppm | m/z | z | RT | Fraction | Scan | Source File | Area F6 | Area F2 | Area F3 | Area F4 | Area F5 | Area F7 | Area F8 | Area F1 | #Feature | #Feature F6 | #Feature F2 | #Feature F3 | #Feature F4 | #Feature F5 | #Feature F7 | #Feature F8 | #Feature F1 | Start | End | PTM | AScore | Found By |
| --- | --- | --- | --- | --- | --- | --- | --- | --- | --- | --- | --- | --- | --- | --- | --- | --- | --- | --- | --- | --- | --- | --- | --- | --- | --- | --- | --- | --- | --- | --- | --- | --- | --- |
| K.QNSGTYNNQYMILDTK.K | N | 86.08 | 1888.8625 | 16 | 0.4 | 945.4390 | 2 | 40.90 | 4 | F4:23353 | 13012022\_RID\_3262\_4b.raw |  |  | 0 | 3.4258E6 |  |  |  |  | 2 | 0 | 0 | 0 | 2 | 0 | 0 | 0 | 0 | 351 | 366 |  |  | PEAKS DB |
| K.FTAYAINGPPVEK.G | N | 84.12 | 1405.7241 | 13 | 0.8 | 703.8699 | 2 | 39.64 | 4 | F4:22495 | 13012022\_RID\_3262\_4b.raw |  |  |  | 6.1282E6 |  |  |  | 3.749E4 | 2 | 0 | 0 | 0 | 1 | 0 | 0 | 0 | 1 | 508 | 520 |  |  | PEAKS DB |
| K.VVPESLFAWER.V | N | 80.07 | 1331.6874 | 11 | 0.5 | 666.8513 | 2 | 54.58 | 4 | F4:31604 | 13012022\_RID\_3262\_4b.raw |  |  |  | 7.2111E6 | 1.2146E6 |  |  |  | 2 | 0 | 0 | 0 | 1 | 1 | 0 | 0 | 0 | 320 | 330 |  |  | PEAKS DB |
| R.SLEDGTLYIIEQVPK.L | Y | 79.29 | 1703.8981 | 15 | 0.1 | 852.9564 | 2 | 58.41 | 4 | F4:33371 | 13012022\_RID\_3262\_4b.raw | 2.8944E6 |  | 4.0146E5 | 2.8073E7 | 2.3975E6 |  |  | 4.8327E5 | 7 | 1 | 0 | 1 | 3 | 1 | 0 | 0 | 1 | 373 | 387 |  |  | PEAKS DB |
| R.YNNYKEDPYAK.H | N | 67.23 | 1403.6357 | 11 | 0.4 | 702.8254 | 2 | 21.90 | 4 | F4:8946 | 13012022\_RID\_3262\_4b.raw |  |  |  | 1.3653E6 |  |  |  |  | 1 | 0 | 0 | 0 | 1 | 0 | 0 | 0 | 0 | 461 | 471 |  |  | PEAKS DB |
| K.HQGLPESYNFDFVTM(+15.99)KPVL | N | 65.84 | 2237.0828 | 19 | 0.7 | 746.7020 | 3 | 55.00 | 4 | F4:31845 | 13012022\_RID\_3262\_4b.raw |  |  |  | 4.8499E6 |  |  |  |  | 1 | 0 | 0 | 0 | 1 | 0 | 0 | 0 | 0 | 535 | 553 | Oxidation (M) | M15:Oxidation (M):1000.00 | PEAKS DB |
| R.IANMMADSGK.T | N | 65.43 | 1036.4681 | 10 | 0.2 | 519.2415 | 2 | 26.71 | 4 | F4:12519 | 13012022\_RID\_3262\_4b.raw |  |  |  | 1.8735E6 |  |  |  |  | 1 | 0 | 0 | 0 | 1 | 0 | 0 | 0 | 0 | 333 | 342 |  |  | PEAKS DB |
| R.HGLEFSYEM(+15.99)APR.A | N | 61.72 | 1451.6503 | 12 | 0.3 | 484.8908 | 3 | 36.62 | 4 | F4:21922 | 13012022\_RID\_3262\_4b.raw |  |  |  | 5.3072E6 |  |  |  |  | 4 | 0 | 0 | 0 | 4 | 0 | 0 | 0 | 0 | 427 | 438 | Oxidation (M) | M9:Oxidation (M):1000.00 | PEAKS DB |
| K.HQGLPESYNFDFVTMKPVL | N | 59.98 | 2221.0876 | 19 | 0.1 | 741.3699 | 3 | 58.53 | 4 | F4:33351 | 13012022\_RID\_3262\_4b.raw |  |  |  | 1.4286E6 |  |  |  |  | 1 | 0 | 0 | 0 | 1 | 0 | 0 | 0 | 0 | 535 | 553 |  |  | PEAKS DB |
| K.VADISMAAK.F | N | 58.91 | 904.4688 | 9 | 0.2 | 453.2418 | 2 | 28.03 | 4 | F4:13402 | 13012022\_RID\_3262\_4b.raw |  |  |  | 5.4443E6 |  |  |  |  | 1 | 0 | 0 | 0 | 1 | 0 | 0 | 0 | 0 | 499 | 507 |  |  | PEAKS DB |
| K.TWAETFEK.Q | N | 55.83 | 1010.4709 | 8 | 0.3 | 506.2429 | 2 | 36.52 | 4 | F4:20193 | 13012022\_RID\_3262\_4b.raw |  |  |  | 1.3005E7 |  |  |  |  | 1 | 0 | 0 | 0 | 1 | 0 | 0 | 0 | 0 | 343 | 350 |  |  | PEAKS DB |
| K.VADISM(+15.99)AAK.F | N | 55.77 | 920.4637 | 9 | -0.1 | 461.2391 | 2 | 28.03 | 4 | F4:13483 | 13012022\_RID\_3262\_4b.raw |  |  |  | 2.148E6 |  |  |  |  | 1 | 0 | 0 | 0 | 1 | 0 | 0 | 0 | 0 | 499 | 507 | Oxidation (M) | M6:Oxidation (M):1000.00 | PEAKS DB |
| R.HGLEFSYEMAPR.A | N | 54.89 | 1435.6554 | 12 | 1.5 | 718.8361 | 2 | 44.96 | 4 | F4:26246 | 13012022\_RID\_3262\_4b.raw |  |  |  | 2.0563E5 |  |  |  | 3.225E4 | 3 | 0 | 0 | 0 | 2 | 0 | 0 | 0 | 1 | 427 | 438 |  |  | PEAKS DB |
| K.VTDMESMK.F | N | 51.87 | 939.4042 | 8 | -0.6 | 470.7091 | 2 | 23.58 | 4 | F4:10341 | 13012022\_RID\_3262\_4b.raw |  |  |  | 6.0853E6 |  |  |  |  | 1 | 0 | 0 | 0 | 1 | 0 | 0 | 0 | 0 | 449 | 456 |  |  | PEAKS DB |
| R.DQGKVTDMESMK.F | N | 48.72 | 1367.6061 | 12 | 0.4 | 684.8106 | 2 | 26.13 | 4 | F4:12043 | 13012022\_RID\_3262\_4b.raw |  |  |  | 2.3854E5 |  |  |  |  | 1 | 0 | 0 | 0 | 1 | 0 | 0 | 0 | 0 | 445 | 456 |  |  | PEAKS DB |
| R.NGYWPSYNIPFDK.E | N | 46.48 | 1599.7357 | 13 | 0.3 | 800.8754 | 2 | 53.87 | 4 | F4:31237 | 13012022\_RID\_3262\_4b.raw |  |  |  | 4.5781E5 |  |  |  |  | 1 | 0 | 0 | 0 | 1 | 0 | 0 | 0 | 0 | 400 | 412 |  |  | PEAKS DB |
| K.VTDM(+15.99)ESMK.F | N | 45.07 | 955.3990 | 8 | 1.9 | 478.7077 | 2 | 21.38 | 4 | F4:10176 | 13012022\_RID\_3262\_4b.raw |  |  |  | 2.4286E6 |  |  |  |  | 1 | 0 | 0 | 0 | 1 | 0 | 0 | 0 | 0 | 449 | 456 | Oxidation (M) | M4:Oxidation (M):21.57 | PEAKS DB |
| total 17 peptides |  |  |  |  |  |  |  |  |  |  |  |  |  |  |  |  |  |  |  |  |  |  |  |  |  |  |  |  |  |  |  |  |  |
| --- | --- | --- | --- | --- | --- | --- | --- | --- | --- | --- | --- | --- | --- | --- | --- | --- | --- | --- | --- | --- | --- | --- | --- | --- | --- | --- | --- | --- | --- | --- | --- | --- | --- |

AEJ31983.1

back to list

  

| Protein Coverage
| Supporting Peptides
|

Protein Coverage:

Supporting Peptides:

| Peptide | Uniq | -10lgP | Mass | Length | ppm | m/z | z | RT | Fraction | Scan | Source File | Area F6 | Area F2 | Area F3 | Area F4 | Area F5 | Area F7 | Area F8 | Area F1 | #Feature | #Feature F6 | #Feature F2 | #Feature F3 | #Feature F4 | #Feature F5 | #Feature F7 | #Feature F8 | #Feature F1 | Start | End | PTM | AScore | Found By |
| --- | --- | --- | --- | --- | --- | --- | --- | --- | --- | --- | --- | --- | --- | --- | --- | --- | --- | --- | --- | --- | --- | --- | --- | --- | --- | --- | --- | --- | --- | --- | --- | --- | --- |
| K.QNSGTYNNQYMILDTK.K | N | 86.08 | 1888.8625 | 16 | 0.4 | 945.4390 | 2 | 40.90 | 4 | F4:23353 | 13012022\_RID\_3262\_4b.raw |  |  | 0 | 3.4258E6 |  |  |  |  | 2 | 0 | 0 | 0 | 2 | 0 | 0 | 0 | 0 | 351 | 366 |  |  | PEAKS DB |
| K.FTAYAINGPPVEK.G | N | 84.12 | 1405.7241 | 13 | 0.8 | 703.8699 | 2 | 39.64 | 4 | F4:22495 | 13012022\_RID\_3262\_4b.raw |  |  |  | 6.1282E6 |  |  |  | 3.749E4 | 2 | 0 | 0 | 0 | 1 | 0 | 0 | 0 | 1 | 508 | 520 |  |  | PEAKS DB |
| K.VVPESLFAWER.V | N | 80.07 | 1331.6874 | 11 | 0.5 | 666.8513 | 2 | 54.58 | 4 | F4:31604 | 13012022\_RID\_3262\_4b.raw |  |  |  | 7.2111E6 | 1.2146E6 |  |  |  | 2 | 0 | 0 | 0 | 1 | 1 | 0 | 0 | 0 | 320 | 330 |  |  | PEAKS DB |
| R.SLEDGTLYIIEQVPK.L | Y | 79.29 | 1703.8981 | 15 | 0.1 | 852.9564 | 2 | 58.41 | 4 | F4:33371 | 13012022\_RID\_3262\_4b.raw | 2.8944E6 |  | 4.0146E5 | 2.8073E7 | 2.3975E6 |  |  | 4.8327E5 | 7 | 1 | 0 | 1 | 3 | 1 | 0 | 0 | 1 | 373 | 387 |  |  | PEAKS DB |
| R.YNNYKEDPYAK.H | N | 67.23 | 1403.6357 | 11 | 0.4 | 702.8254 | 2 | 21.90 | 4 | F4:8946 | 13012022\_RID\_3262\_4b.raw |  |  |  | 1.3653E6 |  |  |  |  | 1 | 0 | 0 | 0 | 1 | 0 | 0 | 0 | 0 | 461 | 471 |  |  | PEAKS DB |
| K.HQGLPESYNFDFVTM(+15.99)KPVL | N | 65.84 | 2237.0828 | 19 | 0.7 | 746.7020 | 3 | 55.00 | 4 | F4:31845 | 13012022\_RID\_3262\_4b.raw |  |  |  | 4.8499E6 |  |  |  |  | 1 | 0 | 0 | 0 | 1 | 0 | 0 | 0 | 0 | 535 | 553 | Oxidation (M) | M15:Oxidation (M):1000.00 | PEAKS DB |
| R.IANMMADSGK.T | N | 65.43 | 1036.4681 | 10 | 0.2 | 519.2415 | 2 | 26.71 | 4 | F4:12519 | 13012022\_RID\_3262\_4b.raw |  |  |  | 1.8735E6 |  |  |  |  | 1 | 0 | 0 | 0 | 1 | 0 | 0 | 0 | 0 | 333 | 342 |  |  | PEAKS DB |
| R.HGLEFSYEM(+15.99)APR.A | N | 61.72 | 1451.6503 | 12 | 0.3 | 484.8908 | 3 | 36.62 | 4 | F4:21922 | 13012022\_RID\_3262\_4b.raw |  |  |  | 5.3072E6 |  |  |  |  | 4 | 0 | 0 | 0 | 4 | 0 | 0 | 0 | 0 | 427 | 438 | Oxidation (M) | M9:Oxidation (M):1000.00 | PEAKS DB |
| K.HQGLPESYNFDFVTMKPVL | N | 59.98 | 2221.0876 | 19 | 0.1 | 741.3699 | 3 | 58.53 | 4 | F4:33351 | 13012022\_RID\_3262\_4b.raw |  |  |  | 1.4286E6 |  |  |  |  | 1 | 0 | 0 | 0 | 1 | 0 | 0 | 0 | 0 | 535 | 553 |  |  | PEAKS DB |
| K.VADISMAAK.F | N | 58.91 | 904.4688 | 9 | 0.2 | 453.2418 | 2 | 28.03 | 4 | F4:13402 | 13012022\_RID\_3262\_4b.raw |  |  |  | 5.4443E6 |  |  |  |  | 1 | 0 | 0 | 0 | 1 | 0 | 0 | 0 | 0 | 499 | 507 |  |  | PEAKS DB |
| K.TWAETFEK.Q | N | 55.83 | 1010.4709 | 8 | 0.3 | 506.2429 | 2 | 36.52 | 4 | F4:20193 | 13012022\_RID\_3262\_4b.raw |  |  |  | 1.3005E7 |  |  |  |  | 1 | 0 | 0 | 0 | 1 | 0 | 0 | 0 | 0 | 343 | 350 |  |  | PEAKS DB |
| K.VADISM(+15.99)AAK.F | N | 55.77 | 920.4637 | 9 | -0.1 | 461.2391 | 2 | 28.03 | 4 | F4:13483 | 13012022\_RID\_3262\_4b.raw |  |  |  | 2.148E6 |  |  |  |  | 1 | 0 | 0 | 0 | 1 | 0 | 0 | 0 | 0 | 499 | 507 | Oxidation (M) | M6:Oxidation (M):1000.00 | PEAKS DB |
| R.HGLEFSYEMAPR.A | N | 54.89 | 1435.6554 | 12 | 1.5 | 718.8361 | 2 | 44.96 | 4 | F4:26246 | 13012022\_RID\_3262\_4b.raw |  |  |  | 2.0563E5 |  |  |  | 3.225E4 | 3 | 0 | 0 | 0 | 2 | 0 | 0 | 0 | 1 | 427 | 438 |  |  | PEAKS DB |
| K.VTDMESMK.F | N | 51.87 | 939.4042 | 8 | -0.6 | 470.7091 | 2 | 23.58 | 4 | F4:10341 | 13012022\_RID\_3262\_4b.raw |  |  |  | 6.0853E6 |  |  |  |  | 1 | 0 | 0 | 0 | 1 | 0 | 0 | 0 | 0 | 449 | 456 |  |  | PEAKS DB |
| R.DQGKVTDMESMK.F | N | 48.72 | 1367.6061 | 12 | 0.4 | 684.8106 | 2 | 26.13 | 4 | F4:12043 | 13012022\_RID\_3262\_4b.raw |  |  |  | 2.3854E5 |  |  |  |  | 1 | 0 | 0 | 0 | 1 | 0 | 0 | 0 | 0 | 445 | 456 |  |  | PEAKS DB |
| R.NGYWPSYNIPFDK.E | N | 46.48 | 1599.7357 | 13 | 0.3 | 800.8754 | 2 | 53.87 | 4 | F4:31237 | 13012022\_RID\_3262\_4b.raw |  |  |  | 4.5781E5 |  |  |  |  | 1 | 0 | 0 | 0 | 1 | 0 | 0 | 0 | 0 | 400 | 412 |  |  | PEAKS DB |
| K.VTDM(+15.99)ESMK.F | N | 45.07 | 955.3990 | 8 | 1.9 | 478.7077 | 2 | 21.38 | 4 | F4:10176 | 13012022\_RID\_3262\_4b.raw |  |  |  | 2.4286E6 |  |  |  |  | 1 | 0 | 0 | 0 | 1 | 0 | 0 | 0 | 0 | 449 | 456 | Oxidation (M) | M4:Oxidation (M):21.57 | PEAKS DB |
| total 17 peptides |  |  |  |  |  |  |  |  |  |  |  |  |  |  |  |  |  |  |  |  |  |  |  |  |  |  |  |  |  |  |  |  |  |
| --- | --- | --- | --- | --- | --- | --- | --- | --- | --- | --- | --- | --- | --- | --- | --- | --- | --- | --- | --- | --- | --- | --- | --- | --- | --- | --- | --- | --- | --- | --- | --- | --- | --- |

JAI12031.1

back to list

  

| Protein Coverage
| Supporting Peptides
|

Protein Coverage:

Supporting Peptides:

| Peptide | Uniq | -10lgP | Mass | Length | ppm | m/z | z | RT | Fraction | Scan | Source File | Area F6 | Area F2 | Area F3 | Area F4 | Area F5 | Area F7 | Area F8 | Area F1 | #Feature | #Feature F6 | #Feature F2 | #Feature F3 | #Feature F4 | #Feature F5 | #Feature F7 | #Feature F8 | #Feature F1 | Start | End | PTM | AScore | Found By |
| --- | --- | --- | --- | --- | --- | --- | --- | --- | --- | --- | --- | --- | --- | --- | --- | --- | --- | --- | --- | --- | --- | --- | --- | --- | --- | --- | --- | --- | --- | --- | --- | --- | --- |
| K.QNSGTYNNQYMILDTK.K | N | 86.08 | 1888.8625 | 16 | 0.4 | 945.4390 | 2 | 40.90 | 4 | F4:23353 | 13012022\_RID\_3262\_4b.raw |  |  | 0 | 3.4258E6 |  |  |  |  | 2 | 0 | 0 | 0 | 2 | 0 | 0 | 0 | 0 | 351 | 366 |  |  | PEAKS DB |
| K.FTAYAINGPPVEK.G | N | 84.12 | 1405.7241 | 13 | 0.8 | 703.8699 | 2 | 39.64 | 4 | F4:22495 | 13012022\_RID\_3262\_4b.raw |  |  |  | 6.1282E6 |  |  |  | 3.749E4 | 2 | 0 | 0 | 0 | 1 | 0 | 0 | 0 | 1 | 508 | 520 |  |  | PEAKS DB |
| K.VVPESLFAWER.V | N | 80.07 | 1331.6874 | 11 | 0.5 | 666.8513 | 2 | 54.58 | 4 | F4:31604 | 13012022\_RID\_3262\_4b.raw |  |  |  | 7.2111E6 | 1.2146E6 |  |  |  | 2 | 0 | 0 | 0 | 1 | 1 | 0 | 0 | 0 | 320 | 330 |  |  | PEAKS DB |
| R.SLEDGTLYIIEQVPK.L | Y | 79.29 | 1703.8981 | 15 | 0.1 | 852.9564 | 2 | 58.41 | 4 | F4:33371 | 13012022\_RID\_3262\_4b.raw | 2.8944E6 |  | 4.0146E5 | 2.8073E7 | 2.3975E6 |  |  | 4.8327E5 | 7 | 1 | 0 | 1 | 3 | 1 | 0 | 0 | 1 | 373 | 387 |  |  | PEAKS DB |
| R.YNNYKEDPYAK.H | N | 67.23 | 1403.6357 | 11 | 0.4 | 702.8254 | 2 | 21.90 | 4 | F4:8946 | 13012022\_RID\_3262\_4b.raw |  |  |  | 1.3653E6 |  |  |  |  | 1 | 0 | 0 | 0 | 1 | 0 | 0 | 0 | 0 | 461 | 471 |  |  | PEAKS DB |
| K.HQGLPESYNFDFVTM(+15.99)KPVL | N | 65.84 | 2237.0828 | 19 | 0.7 | 746.7020 | 3 | 55.00 | 4 | F4:31845 | 13012022\_RID\_3262\_4b.raw |  |  |  | 4.8499E6 |  |  |  |  | 1 | 0 | 0 | 0 | 1 | 0 | 0 | 0 | 0 | 535 | 553 | Oxidation (M) | M15:Oxidation (M):1000.00 | PEAKS DB |
| R.IANMMADSGK.T | N | 65.43 | 1036.4681 | 10 | 0.2 | 519.2415 | 2 | 26.71 | 4 | F4:12519 | 13012022\_RID\_3262\_4b.raw |  |  |  | 1.8735E6 |  |  |  |  | 1 | 0 | 0 | 0 | 1 | 0 | 0 | 0 | 0 | 333 | 342 |  |  | PEAKS DB |
| R.HGLEFSYEM(+15.99)APR.A | N | 61.72 | 1451.6503 | 12 | 0.3 | 484.8908 | 3 | 36.62 | 4 | F4:21922 | 13012022\_RID\_3262\_4b.raw |  |  |  | 5.3072E6 |  |  |  |  | 4 | 0 | 0 | 0 | 4 | 0 | 0 | 0 | 0 | 427 | 438 | Oxidation (M) | M9:Oxidation (M):1000.00 | PEAKS DB |
| K.HQGLPESYNFDFVTMKPVL | N | 59.98 | 2221.0876 | 19 | 0.1 | 741.3699 | 3 | 58.53 | 4 | F4:33351 | 13012022\_RID\_3262\_4b.raw |  |  |  | 1.4286E6 |  |  |  |  | 1 | 0 | 0 | 0 | 1 | 0 | 0 | 0 | 0 | 535 | 553 |  |  | PEAKS DB |
| K.VADISMAAK.F | N | 58.91 | 904.4688 | 9 | 0.2 | 453.2418 | 2 | 28.03 | 4 | F4:13402 | 13012022\_RID\_3262\_4b.raw |  |  |  | 5.4443E6 |  |  |  |  | 1 | 0 | 0 | 0 | 1 | 0 | 0 | 0 | 0 | 499 | 507 |  |  | PEAKS DB |
| K.TWAETFEK.Q | N | 55.83 | 1010.4709 | 8 | 0.3 | 506.2429 | 2 | 36.52 | 4 | F4:20193 | 13012022\_RID\_3262\_4b.raw |  |  |  | 1.3005E7 |  |  |  |  | 1 | 0 | 0 | 0 | 1 | 0 | 0 | 0 | 0 | 343 | 350 |  |  | PEAKS DB |
| K.VADISM(+15.99)AAK.F | N | 55.77 | 920.4637 | 9 | -0.1 | 461.2391 | 2 | 28.03 | 4 | F4:13483 | 13012022\_RID\_3262\_4b.raw |  |  |  | 2.148E6 |  |  |  |  | 1 | 0 | 0 | 0 | 1 | 0 | 0 | 0 | 0 | 499 | 507 | Oxidation (M) | M6:Oxidation (M):1000.00 | PEAKS DB |
| R.HGLEFSYEMAPR.A | N | 54.89 | 1435.6554 | 12 | 1.5 | 718.8361 | 2 | 44.96 | 4 | F4:26246 | 13012022\_RID\_3262\_4b.raw |  |  |  | 2.0563E5 |  |  |  | 3.225E4 | 3 | 0 | 0 | 0 | 2 | 0 | 0 | 0 | 1 | 427 | 438 |  |  | PEAKS DB |
| K.VTDMESMK.F | N | 51.87 | 939.4042 | 8 | -0.6 | 470.7091 | 2 | 23.58 | 4 | F4:10341 | 13012022\_RID\_3262\_4b.raw |  |  |  | 6.0853E6 |  |  |  |  | 1 | 0 | 0 | 0 | 1 | 0 | 0 | 0 | 0 | 449 | 456 |  |  | PEAKS DB |
| R.DQGKVTDMESMK.F | N | 48.72 | 1367.6061 | 12 | 0.4 | 684.8106 | 2 | 26.13 | 4 | F4:12043 | 13012022\_RID\_3262\_4b.raw |  |  |  | 2.3854E5 |  |  |  |  | 1 | 0 | 0 | 0 | 1 | 0 | 0 | 0 | 0 | 445 | 456 |  |  | PEAKS DB |
| R.NGYWPSYNIPFDK.E | N | 46.48 | 1599.7357 | 13 | 0.3 | 800.8754 | 2 | 53.87 | 4 | F4:31237 | 13012022\_RID\_3262\_4b.raw |  |  |  | 4.5781E5 |  |  |  |  | 1 | 0 | 0 | 0 | 1 | 0 | 0 | 0 | 0 | 400 | 412 |  |  | PEAKS DB |
| K.VTDM(+15.99)ESMK.F | N | 45.07 | 955.3990 | 8 | 1.9 | 478.7077 | 2 | 21.38 | 4 | F4:10176 | 13012022\_RID\_3262\_4b.raw |  |  |  | 2.4286E6 |  |  |  |  | 1 | 0 | 0 | 0 | 1 | 0 | 0 | 0 | 0 | 449 | 456 | Oxidation (M) | M4:Oxidation (M):21.57 | PEAKS DB |
| total 17 peptides |  |  |  |  |  |  |  |  |  |  |  |  |  |  |  |  |  |  |  |  |  |  |  |  |  |  |  |  |  |  |  |  |  |
| --- | --- | --- | --- | --- | --- | --- | --- | --- | --- | --- | --- | --- | --- | --- | --- | --- | --- | --- | --- | --- | --- | --- | --- | --- | --- | --- | --- | --- | --- | --- | --- | --- | --- |

SMD28240.1

back to list

  

| Protein Coverage
| Supporting Peptides
|

Protein Coverage:

Supporting Peptides:

| Peptide | Uniq | -10lgP | Mass | Length | ppm | m/z | z | RT | Fraction | Scan | Source File | Area F6 | Area F2 | Area F3 | Area F4 | Area F5 | Area F7 | Area F8 | Area F1 | #Feature | #Feature F6 | #Feature F2 | #Feature F3 | #Feature F4 | #Feature F5 | #Feature F7 | #Feature F8 | #Feature F1 | Start | End | PTM | AScore | Found By |
| --- | --- | --- | --- | --- | --- | --- | --- | --- | --- | --- | --- | --- | --- | --- | --- | --- | --- | --- | --- | --- | --- | --- | --- | --- | --- | --- | --- | --- | --- | --- | --- | --- | --- |
| K.QNSGTYNNQYMILDTK.K | N | 86.08 | 1888.8625 | 16 | 0.4 | 945.4390 | 2 | 40.90 | 4 | F4:23353 | 13012022\_RID\_3262\_4b.raw |  |  | 0 | 3.4258E6 |  |  |  |  | 2 | 0 | 0 | 0 | 2 | 0 | 0 | 0 | 0 | 351 | 366 |  |  | PEAKS DB |
| K.FTAYAINGPPVEK.G | N | 84.12 | 1405.7241 | 13 | 0.8 | 703.8699 | 2 | 39.64 | 4 | F4:22495 | 13012022\_RID\_3262\_4b.raw |  |  |  | 6.1282E6 |  |  |  | 3.749E4 | 2 | 0 | 0 | 0 | 1 | 0 | 0 | 0 | 1 | 508 | 520 |  |  | PEAKS DB |
| K.VVPESLFAWER.V | N | 80.07 | 1331.6874 | 11 | 0.5 | 666.8513 | 2 | 54.58 | 4 | F4:31604 | 13012022\_RID\_3262\_4b.raw |  |  |  | 7.2111E6 | 1.2146E6 |  |  |  | 2 | 0 | 0 | 0 | 1 | 1 | 0 | 0 | 0 | 320 | 330 |  |  | PEAKS DB |
| R.SLEDGTLYIIEQVPK.L | Y | 79.29 | 1703.8981 | 15 | 0.1 | 852.9564 | 2 | 58.41 | 4 | F4:33371 | 13012022\_RID\_3262\_4b.raw | 2.8944E6 |  | 4.0146E5 | 2.8073E7 | 2.3975E6 |  |  | 4.8327E5 | 7 | 1 | 0 | 1 | 3 | 1 | 0 | 0 | 1 | 373 | 387 |  |  | PEAKS DB |
| R.YNNYKEDPYAK.R | N | 67.23 | 1403.6357 | 11 | 0.4 | 702.8254 | 2 | 21.90 | 4 | F4:8946 | 13012022\_RID\_3262\_4b.raw |  |  |  | 1.3653E6 |  |  |  |  | 1 | 0 | 0 | 0 | 1 | 0 | 0 | 0 | 0 | 461 | 471 |  |  | PEAKS DB |
| K.HQGLPESYNFDFVTM(+15.99)KPVL | N | 65.84 | 2237.0828 | 19 | 0.7 | 746.7020 | 3 | 55.00 | 4 | F4:31845 | 13012022\_RID\_3262\_4b.raw |  |  |  | 4.8499E6 |  |  |  |  | 1 | 0 | 0 | 0 | 1 | 0 | 0 | 0 | 0 | 535 | 553 | Oxidation (M) | M15:Oxidation (M):1000.00 | PEAKS DB |
| R.IANMMADSGK.T | N | 65.43 | 1036.4681 | 10 | 0.2 | 519.2415 | 2 | 26.71 | 4 | F4:12519 | 13012022\_RID\_3262\_4b.raw |  |  |  | 1.8735E6 |  |  |  |  | 1 | 0 | 0 | 0 | 1 | 0 | 0 | 0 | 0 | 333 | 342 |  |  | PEAKS DB |
| R.HGLEFSYEM(+15.99)APR.A | N | 61.72 | 1451.6503 | 12 | 0.3 | 484.8908 | 3 | 36.62 | 4 | F4:21922 | 13012022\_RID\_3262\_4b.raw |  |  |  | 5.3072E6 |  |  |  |  | 4 | 0 | 0 | 0 | 4 | 0 | 0 | 0 | 0 | 427 | 438 | Oxidation (M) | M9:Oxidation (M):1000.00 | PEAKS DB |
| K.HQGLPESYNFDFVTMKPVL | N | 59.98 | 2221.0876 | 19 | 0.1 | 741.3699 | 3 | 58.53 | 4 | F4:33351 | 13012022\_RID\_3262\_4b.raw |  |  |  | 1.4286E6 |  |  |  |  | 1 | 0 | 0 | 0 | 1 | 0 | 0 | 0 | 0 | 535 | 553 |  |  | PEAKS DB |
| K.VADISMAAK.F | N | 58.91 | 904.4688 | 9 | 0.2 | 453.2418 | 2 | 28.03 | 4 | F4:13402 | 13012022\_RID\_3262\_4b.raw |  |  |  | 5.4443E6 |  |  |  |  | 1 | 0 | 0 | 0 | 1 | 0 | 0 | 0 | 0 | 499 | 507 |  |  | PEAKS DB |
| K.TWAETFEK.Q | N | 55.83 | 1010.4709 | 8 | 0.3 | 506.2429 | 2 | 36.52 | 4 | F4:20193 | 13012022\_RID\_3262\_4b.raw |  |  |  | 1.3005E7 |  |  |  |  | 1 | 0 | 0 | 0 | 1 | 0 | 0 | 0 | 0 | 343 | 350 |  |  | PEAKS DB |
| K.VADISM(+15.99)AAK.F | N | 55.77 | 920.4637 | 9 | -0.1 | 461.2391 | 2 | 28.03 | 4 | F4:13483 | 13012022\_RID\_3262\_4b.raw |  |  |  | 2.148E6 |  |  |  |  | 1 | 0 | 0 | 0 | 1 | 0 | 0 | 0 | 0 | 499 | 507 | Oxidation (M) | M6:Oxidation (M):1000.00 | PEAKS DB |
| R.HGLEFSYEMAPR.A | N | 54.89 | 1435.6554 | 12 | 1.5 | 718.8361 | 2 | 44.96 | 4 | F4:26246 | 13012022\_RID\_3262\_4b.raw |  |  |  | 2.0563E5 |  |  |  | 3.225E4 | 3 | 0 | 0 | 0 | 2 | 0 | 0 | 0 | 1 | 427 | 438 |  |  | PEAKS DB |
| K.VTDMESMK.F | N | 51.87 | 939.4042 | 8 | -0.6 | 470.7091 | 2 | 23.58 | 4 | F4:10341 | 13012022\_RID\_3262\_4b.raw |  |  |  | 6.0853E6 |  |  |  |  | 1 | 0 | 0 | 0 | 1 | 0 | 0 | 0 | 0 | 449 | 456 |  |  | PEAKS DB |
| R.DQGKVTDMESMK.F | N | 48.72 | 1367.6061 | 12 | 0.4 | 684.8106 | 2 | 26.13 | 4 | F4:12043 | 13012022\_RID\_3262\_4b.raw |  |  |  | 2.3854E5 |  |  |  |  | 1 | 0 | 0 | 0 | 1 | 0 | 0 | 0 | 0 | 445 | 456 |  |  | PEAKS DB |
| R.NGYWPSYNIPFDK.E | N | 46.48 | 1599.7357 | 13 | 0.3 | 800.8754 | 2 | 53.87 | 4 | F4:31237 | 13012022\_RID\_3262\_4b.raw |  |  |  | 4.5781E5 |  |  |  |  | 1 | 0 | 0 | 0 | 1 | 0 | 0 | 0 | 0 | 400 | 412 |  |  | PEAKS DB |
| K.VTDM(+15.99)ESMK.F | N | 45.07 | 955.3990 | 8 | 1.9 | 478.7077 | 2 | 21.38 | 4 | F4:10176 | 13012022\_RID\_3262\_4b.raw |  |  |  | 2.4286E6 |  |  |  |  | 1 | 0 | 0 | 0 | 1 | 0 | 0 | 0 | 0 | 449 | 456 | Oxidation (M) | M4:Oxidation (M):21.57 | PEAKS DB |
| total 17 peptides |  |  |  |  |  |  |  |  |  |  |  |  |  |  |  |  |  |  |  |  |  |  |  |  |  |  |  |  |  |  |  |  |  |
| --- | --- | --- | --- | --- | --- | --- | --- | --- | --- | --- | --- | --- | --- | --- | --- | --- | --- | --- | --- | --- | --- | --- | --- | --- | --- | --- | --- | --- | --- | --- | --- | --- | --- |

F8S101.1

back to list

  

| Protein Coverage
| Supporting Peptides
|

Protein Coverage:

Supporting Peptides:

| Peptide | Uniq | -10lgP | Mass | Length | ppm | m/z | z | RT | Fraction | Scan | Source File | Area F6 | Area F2 | Area F3 | Area F4 | Area F5 | Area F7 | Area F8 | Area F1 | #Feature | #Feature F6 | #Feature F2 | #Feature F3 | #Feature F4 | #Feature F5 | #Feature F7 | #Feature F8 | #Feature F1 | Start | End | PTM | AScore | Found By |
| --- | --- | --- | --- | --- | --- | --- | --- | --- | --- | --- | --- | --- | --- | --- | --- | --- | --- | --- | --- | --- | --- | --- | --- | --- | --- | --- | --- | --- | --- | --- | --- | --- | --- |
| K.QNSGTYNNQYMILDTK.K | N | 86.08 | 1888.8625 | 16 | 0.4 | 945.4390 | 2 | 40.90 | 4 | F4:23353 | 13012022\_RID\_3262\_4b.raw |  |  | 0 | 3.4258E6 |  |  |  |  | 2 | 0 | 0 | 0 | 2 | 0 | 0 | 0 | 0 | 351 | 366 |  |  | PEAKS DB |
| K.FTAYAINGPPVEK.G | N | 84.12 | 1405.7241 | 13 | 0.8 | 703.8699 | 2 | 39.64 | 4 | F4:22495 | 13012022\_RID\_3262\_4b.raw |  |  |  | 6.1282E6 |  |  |  | 3.749E4 | 2 | 0 | 0 | 0 | 1 | 0 | 0 | 0 | 1 | 508 | 520 |  |  | PEAKS DB |
| K.VVPESLFAWER.V | N | 80.07 | 1331.6874 | 11 | 0.5 | 666.8513 | 2 | 54.58 | 4 | F4:31604 | 13012022\_RID\_3262\_4b.raw |  |  |  | 7.2111E6 | 1.2146E6 |  |  |  | 2 | 0 | 0 | 0 | 1 | 1 | 0 | 0 | 0 | 320 | 330 |  |  | PEAKS DB |
| R.SLEDGTLYIIEQVPK.L | Y | 79.29 | 1703.8981 | 15 | 0.1 | 852.9564 | 2 | 58.41 | 4 | F4:33371 | 13012022\_RID\_3262\_4b.raw | 2.8944E6 |  | 4.0146E5 | 2.8073E7 | 2.3975E6 |  |  | 4.8327E5 | 7 | 1 | 0 | 1 | 3 | 1 | 0 | 0 | 1 | 373 | 387 |  |  | PEAKS DB |
| R.YNNYKEDPYAK.H | N | 67.23 | 1403.6357 | 11 | 0.4 | 702.8254 | 2 | 21.90 | 4 | F4:8946 | 13012022\_RID\_3262\_4b.raw |  |  |  | 1.3653E6 |  |  |  |  | 1 | 0 | 0 | 0 | 1 | 0 | 0 | 0 | 0 | 461 | 471 |  |  | PEAKS DB |
| K.HQGLPESYNFDFVTM(+15.99)KPVL | N | 65.84 | 2237.0828 | 19 | 0.7 | 746.7020 | 3 | 55.00 | 4 | F4:31845 | 13012022\_RID\_3262\_4b.raw |  |  |  | 4.8499E6 |  |  |  |  | 1 | 0 | 0 | 0 | 1 | 0 | 0 | 0 | 0 | 535 | 553 | Oxidation (M) | M15:Oxidation (M):1000.00 | PEAKS DB |
| R.IANMMADSGK.T | N | 65.43 | 1036.4681 | 10 | 0.2 | 519.2415 | 2 | 26.71 | 4 | F4:12519 | 13012022\_RID\_3262\_4b.raw |  |  |  | 1.8735E6 |  |  |  |  | 1 | 0 | 0 | 0 | 1 | 0 | 0 | 0 | 0 | 333 | 342 |  |  | PEAKS DB |
| R.HGLEFSYEM(+15.99)APR.A | N | 61.72 | 1451.6503 | 12 | 0.3 | 484.8908 | 3 | 36.62 | 4 | F4:21922 | 13012022\_RID\_3262\_4b.raw |  |  |  | 5.3072E6 |  |  |  |  | 4 | 0 | 0 | 0 | 4 | 0 | 0 | 0 | 0 | 427 | 438 | Oxidation (M) | M9:Oxidation (M):1000.00 | PEAKS DB |
| K.HQGLPESYNFDFVTMKPVL | N | 59.98 | 2221.0876 | 19 | 0.1 | 741.3699 | 3 | 58.53 | 4 | F4:33351 | 13012022\_RID\_3262\_4b.raw |  |  |  | 1.4286E6 |  |  |  |  | 1 | 0 | 0 | 0 | 1 | 0 | 0 | 0 | 0 | 535 | 553 |  |  | PEAKS DB |
| K.VADISMAAK.F | N | 58.91 | 904.4688 | 9 | 0.2 | 453.2418 | 2 | 28.03 | 4 | F4:13402 | 13012022\_RID\_3262\_4b.raw |  |  |  | 5.4443E6 |  |  |  |  | 1 | 0 | 0 | 0 | 1 | 0 | 0 | 0 | 0 | 499 | 507 |  |  | PEAKS DB |
| K.TWAETFEK.Q | N | 55.83 | 1010.4709 | 8 | 0.3 | 506.2429 | 2 | 36.52 | 4 | F4:20193 | 13012022\_RID\_3262\_4b.raw |  |  |  | 1.3005E7 |  |  |  |  | 1 | 0 | 0 | 0 | 1 | 0 | 0 | 0 | 0 | 343 | 350 |  |  | PEAKS DB |
| K.VADISM(+15.99)AAK.F | N | 55.77 | 920.4637 | 9 | -0.1 | 461.2391 | 2 | 28.03 | 4 | F4:13483 | 13012022\_RID\_3262\_4b.raw |  |  |  | 2.148E6 |  |  |  |  | 1 | 0 | 0 | 0 | 1 | 0 | 0 | 0 | 0 | 499 | 507 | Oxidation (M) | M6:Oxidation (M):1000.00 | PEAKS DB |
| R.HGLEFSYEMAPR.A | N | 54.89 | 1435.6554 | 12 | 1.5 | 718.8361 | 2 | 44.96 | 4 | F4:26246 | 13012022\_RID\_3262\_4b.raw |  |  |  | 2.0563E5 |  |  |  | 3.225E4 | 3 | 0 | 0 | 0 | 2 | 0 | 0 | 0 | 1 | 427 | 438 |  |  | PEAKS DB |
| K.VTDMESMK.F | N | 51.87 | 939.4042 | 8 | -0.6 | 470.7091 | 2 | 23.58 | 4 | F4:10341 | 13012022\_RID\_3262\_4b.raw |  |  |  | 6.0853E6 |  |  |  |  | 1 | 0 | 0 | 0 | 1 | 0 | 0 | 0 | 0 | 449 | 456 |  |  | PEAKS DB |
| R.DQGKVTDMESMK.F | N | 48.72 | 1367.6061 | 12 | 0.4 | 684.8106 | 2 | 26.13 | 4 | F4:12043 | 13012022\_RID\_3262\_4b.raw |  |  |  | 2.3854E5 |  |  |  |  | 1 | 0 | 0 | 0 | 1 | 0 | 0 | 0 | 0 | 445 | 456 |  |  | PEAKS DB |
| R.NGYWPSYNIPFDK.E | N | 46.48 | 1599.7357 | 13 | 0.3 | 800.8754 | 2 | 53.87 | 4 | F4:31237 | 13012022\_RID\_3262\_4b.raw |  |  |  | 4.5781E5 |  |  |  |  | 1 | 0 | 0 | 0 | 1 | 0 | 0 | 0 | 0 | 400 | 412 |  |  | PEAKS DB |
| K.VTDM(+15.99)ESMK.F | N | 45.07 | 955.3990 | 8 | 1.9 | 478.7077 | 2 | 21.38 | 4 | F4:10176 | 13012022\_RID\_3262\_4b.raw |  |  |  | 2.4286E6 |  |  |  |  | 1 | 0 | 0 | 0 | 1 | 0 | 0 | 0 | 0 | 449 | 456 | Oxidation (M) | M4:Oxidation (M):21.57 | PEAKS DB |
| total 17 peptides |  |  |  |  |  |  |  |  |  |  |  |  |  |  |  |  |  |  |  |  |  |  |  |  |  |  |  |  |  |  |  |  |  |
| --- | --- | --- | --- | --- | --- | --- | --- | --- | --- | --- | --- | --- | --- | --- | --- | --- | --- | --- | --- | --- | --- | --- | --- | --- | --- | --- | --- | --- | --- | --- | --- | --- | --- |

JAV49636.1

back to list

  

| Protein Coverage
| Supporting Peptides
|

Protein Coverage:

Supporting Peptides:

| Peptide | Uniq | -10lgP | Mass | Length | ppm | m/z | z | RT | Fraction | Scan | Source File | Area F6 | Area F2 | Area F3 | Area F4 | Area F5 | Area F7 | Area F8 | Area F1 | #Feature | #Feature F6 | #Feature F2 | #Feature F3 | #Feature F4 | #Feature F5 | #Feature F7 | #Feature F8 | #Feature F1 | Start | End | PTM | AScore | Found By |
| --- | --- | --- | --- | --- | --- | --- | --- | --- | --- | --- | --- | --- | --- | --- | --- | --- | --- | --- | --- | --- | --- | --- | --- | --- | --- | --- | --- | --- | --- | --- | --- | --- | --- |
| K.QNSGTYNNQYMILDTK.K | N | 86.08 | 1888.8625 | 16 | 0.4 | 945.4390 | 2 | 40.90 | 4 | F4:23353 | 13012022\_RID\_3262\_4b.raw |  |  | 0 | 3.4258E6 |  |  |  |  | 2 | 0 | 0 | 0 | 2 | 0 | 0 | 0 | 0 | 351 | 366 |  |  | PEAKS DB |
| K.FTAYAINGPPVEK.G | N | 84.12 | 1405.7241 | 13 | 0.8 | 703.8699 | 2 | 39.64 | 4 | F4:22495 | 13012022\_RID\_3262\_4b.raw |  |  |  | 6.1282E6 |  |  |  | 3.749E4 | 2 | 0 | 0 | 0 | 1 | 0 | 0 | 0 | 1 | 508 | 520 |  |  | PEAKS DB |
| K.VVPESLFAWER.V | N | 80.07 | 1331.6874 | 11 | 0.5 | 666.8513 | 2 | 54.58 | 4 | F4:31604 | 13012022\_RID\_3262\_4b.raw |  |  |  | 7.2111E6 | 1.2146E6 |  |  |  | 2 | 0 | 0 | 0 | 1 | 1 | 0 | 0 | 0 | 320 | 330 |  |  | PEAKS DB |
| R.SLEDGTLYIIEQVPK.L | Y | 79.29 | 1703.8981 | 15 | 0.1 | 852.9564 | 2 | 58.41 | 4 | F4:33371 | 13012022\_RID\_3262\_4b.raw | 2.8944E6 |  | 4.0146E5 | 2.8073E7 | 2.3975E6 |  |  | 4.8327E5 | 7 | 1 | 0 | 1 | 3 | 1 | 0 | 0 | 1 | 373 | 387 |  |  | PEAKS DB |
| R.YNNYKEDPYAK.R | N | 67.23 | 1403.6357 | 11 | 0.4 | 702.8254 | 2 | 21.90 | 4 | F4:8946 | 13012022\_RID\_3262\_4b.raw |  |  |  | 1.3653E6 |  |  |  |  | 1 | 0 | 0 | 0 | 1 | 0 | 0 | 0 | 0 | 461 | 471 |  |  | PEAKS DB |
| K.HQGLPESYNFDFVTM(+15.99)KPVL | N | 65.84 | 2237.0828 | 19 | 0.7 | 746.7020 | 3 | 55.00 | 4 | F4:31845 | 13012022\_RID\_3262\_4b.raw |  |  |  | 4.8499E6 |  |  |  |  | 1 | 0 | 0 | 0 | 1 | 0 | 0 | 0 | 0 | 535 | 553 | Oxidation (M) | M15:Oxidation (M):1000.00 | PEAKS DB |
| R.IANMMADSGK.T | N | 65.43 | 1036.4681 | 10 | 0.2 | 519.2415 | 2 | 26.71 | 4 | F4:12519 | 13012022\_RID\_3262\_4b.raw |  |  |  | 1.8735E6 |  |  |  |  | 1 | 0 | 0 | 0 | 1 | 0 | 0 | 0 | 0 | 333 | 342 |  |  | PEAKS DB |
| R.HGLEFSYEM(+15.99)APR.A | N | 61.72 | 1451.6503 | 12 | 0.3 | 484.8908 | 3 | 36.62 | 4 | F4:21922 | 13012022\_RID\_3262\_4b.raw |  |  |  | 5.3072E6 |  |  |  |  | 4 | 0 | 0 | 0 | 4 | 0 | 0 | 0 | 0 | 427 | 438 | Oxidation (M) | M9:Oxidation (M):1000.00 | PEAKS DB |
| K.HQGLPESYNFDFVTMKPVL | N | 59.98 | 2221.0876 | 19 | 0.1 | 741.3699 | 3 | 58.53 | 4 | F4:33351 | 13012022\_RID\_3262\_4b.raw |  |  |  | 1.4286E6 |  |  |  |  | 1 | 0 | 0 | 0 | 1 | 0 | 0 | 0 | 0 | 535 | 553 |  |  | PEAKS DB |
| K.VADISMAAK.F | N | 58.91 | 904.4688 | 9 | 0.2 | 453.2418 | 2 | 28.03 | 4 | F4:13402 | 13012022\_RID\_3262\_4b.raw |  |  |  | 5.4443E6 |  |  |  |  | 1 | 0 | 0 | 0 | 1 | 0 | 0 | 0 | 0 | 499 | 507 |  |  | PEAKS DB |
| K.TWAETFEK.Q | N | 55.83 | 1010.4709 | 8 | 0.3 | 506.2429 | 2 | 36.52 | 4 | F4:20193 | 13012022\_RID\_3262\_4b.raw |  |  |  | 1.3005E7 |  |  |  |  | 1 | 0 | 0 | 0 | 1 | 0 | 0 | 0 | 0 | 343 | 350 |  |  | PEAKS DB |
| K.VADISM(+15.99)AAK.F | N | 55.77 | 920.4637 | 9 | -0.1 | 461.2391 | 2 | 28.03 | 4 | F4:13483 | 13012022\_RID\_3262\_4b.raw |  |  |  | 2.148E6 |  |  |  |  | 1 | 0 | 0 | 0 | 1 | 0 | 0 | 0 | 0 | 499 | 507 | Oxidation (M) | M6:Oxidation (M):1000.00 | PEAKS DB |
| R.HGLEFSYEMAPR.A | N | 54.89 | 1435.6554 | 12 | 1.5 | 718.8361 | 2 | 44.96 | 4 | F4:26246 | 13012022\_RID\_3262\_4b.raw |  |  |  | 2.0563E5 |  |  |  | 3.225E4 | 3 | 0 | 0 | 0 | 2 | 0 | 0 | 0 | 1 | 427 | 438 |  |  | PEAKS DB |
| K.VTDMESMK.F | N | 51.87 | 939.4042 | 8 | -0.6 | 470.7091 | 2 | 23.58 | 4 | F4:10341 | 13012022\_RID\_3262\_4b.raw |  |  |  | 6.0853E6 |  |  |  |  | 1 | 0 | 0 | 0 | 1 | 0 | 0 | 0 | 0 | 449 | 456 |  |  | PEAKS DB |
| R.DQGKVTDMESMK.F | N | 48.72 | 1367.6061 | 12 | 0.4 | 684.8106 | 2 | 26.13 | 4 | F4:12043 | 13012022\_RID\_3262\_4b.raw |  |  |  | 2.3854E5 |  |  |  |  | 1 | 0 | 0 | 0 | 1 | 0 | 0 | 0 | 0 | 445 | 456 |  |  | PEAKS DB |
| R.NGYWPSYNIPFDK.E | N | 46.48 | 1599.7357 | 13 | 0.3 | 800.8754 | 2 | 53.87 | 4 | F4:31237 | 13012022\_RID\_3262\_4b.raw |  |  |  | 4.5781E5 |  |  |  |  | 1 | 0 | 0 | 0 | 1 | 0 | 0 | 0 | 0 | 400 | 412 |  |  | PEAKS DB |
| K.VTDM(+15.99)ESMK.F | N | 45.07 | 955.3990 | 8 | 1.9 | 478.7077 | 2 | 21.38 | 4 | F4:10176 | 13012022\_RID\_3262\_4b.raw |  |  |  | 2.4286E6 |  |  |  |  | 1 | 0 | 0 | 0 | 1 | 0 | 0 | 0 | 0 | 449 | 456 | Oxidation (M) | M4:Oxidation (M):21.57 | PEAKS DB |
| total 17 peptides |  |  |  |  |  |  |  |  |  |  |  |  |  |  |  |  |  |  |  |  |  |  |  |  |  |  |  |  |  |  |  |  |  |
| --- | --- | --- | --- | --- | --- | --- | --- | --- | --- | --- | --- | --- | --- | --- | --- | --- | --- | --- | --- | --- | --- | --- | --- | --- | --- | --- | --- | --- | --- | --- | --- | --- | --- |

JAV48419.1

back to list

  

| Protein Coverage
| Supporting Peptides
|

Protein Coverage:

Supporting Peptides:

| Peptide | Uniq | -10lgP | Mass | Length | ppm | m/z | z | RT | Fraction | Scan | Source File | Area F6 | Area F2 | Area F3 | Area F4 | Area F5 | Area F7 | Area F8 | Area F1 | #Feature | #Feature F6 | #Feature F2 | #Feature F3 | #Feature F4 | #Feature F5 | #Feature F7 | #Feature F8 | #Feature F1 | Start | End | PTM | AScore | Found By |
| --- | --- | --- | --- | --- | --- | --- | --- | --- | --- | --- | --- | --- | --- | --- | --- | --- | --- | --- | --- | --- | --- | --- | --- | --- | --- | --- | --- | --- | --- | --- | --- | --- | --- |
| K.QNSGTYNNQYMILDTK.K | N | 86.08 | 1888.8625 | 16 | 0.4 | 945.4390 | 2 | 40.90 | 4 | F4:23353 | 13012022\_RID\_3262\_4b.raw |  |  | 0 | 3.4258E6 |  |  |  |  | 2 | 0 | 0 | 0 | 2 | 0 | 0 | 0 | 0 | 351 | 366 |  |  | PEAKS DB |
| K.FTAYAINGPPVEK.G | N | 84.12 | 1405.7241 | 13 | 0.8 | 703.8699 | 2 | 39.64 | 4 | F4:22495 | 13012022\_RID\_3262\_4b.raw |  |  |  | 6.1282E6 |  |  |  | 3.749E4 | 2 | 0 | 0 | 0 | 1 | 0 | 0 | 0 | 1 | 508 | 520 |  |  | PEAKS DB |
| K.VVPESLFAWER.V | N | 80.07 | 1331.6874 | 11 | 0.5 | 666.8513 | 2 | 54.58 | 4 | F4:31604 | 13012022\_RID\_3262\_4b.raw |  |  |  | 7.2111E6 | 1.2146E6 |  |  |  | 2 | 0 | 0 | 0 | 1 | 1 | 0 | 0 | 0 | 320 | 330 |  |  | PEAKS DB |
| R.SLEDGTLYIIEQVPK.L | Y | 79.29 | 1703.8981 | 15 | 0.1 | 852.9564 | 2 | 58.41 | 4 | F4:33371 | 13012022\_RID\_3262\_4b.raw | 2.8944E6 |  | 4.0146E5 | 2.8073E7 | 2.3975E6 |  |  | 4.8327E5 | 7 | 1 | 0 | 1 | 3 | 1 | 0 | 0 | 1 | 373 | 387 |  |  | PEAKS DB |
| R.YNNYKEDPYAK.R | N | 67.23 | 1403.6357 | 11 | 0.4 | 702.8254 | 2 | 21.90 | 4 | F4:8946 | 13012022\_RID\_3262\_4b.raw |  |  |  | 1.3653E6 |  |  |  |  | 1 | 0 | 0 | 0 | 1 | 0 | 0 | 0 | 0 | 461 | 471 |  |  | PEAKS DB |
| K.HQGLPESYNFDFVTM(+15.99)KPVL | N | 65.84 | 2237.0828 | 19 | 0.7 | 746.7020 | 3 | 55.00 | 4 | F4:31845 | 13012022\_RID\_3262\_4b.raw |  |  |  | 4.8499E6 |  |  |  |  | 1 | 0 | 0 | 0 | 1 | 0 | 0 | 0 | 0 | 535 | 553 | Oxidation (M) | M15:Oxidation (M):1000.00 | PEAKS DB |
| R.IANMMADSGK.T | N | 65.43 | 1036.4681 | 10 | 0.2 | 519.2415 | 2 | 26.71 | 4 | F4:12519 | 13012022\_RID\_3262\_4b.raw |  |  |  | 1.8735E6 |  |  |  |  | 1 | 0 | 0 | 0 | 1 | 0 | 0 | 0 | 0 | 333 | 342 |  |  | PEAKS DB |
| R.HGLEFSYEM(+15.99)APR.A | N | 61.72 | 1451.6503 | 12 | 0.3 | 484.8908 | 3 | 36.62 | 4 | F4:21922 | 13012022\_RID\_3262\_4b.raw |  |  |  | 5.3072E6 |  |  |  |  | 4 | 0 | 0 | 0 | 4 | 0 | 0 | 0 | 0 | 427 | 438 | Oxidation (M) | M9:Oxidation (M):1000.00 | PEAKS DB |
| K.HQGLPESYNFDFVTMKPVL | N | 59.98 | 2221.0876 | 19 | 0.1 | 741.3699 | 3 | 58.53 | 4 | F4:33351 | 13012022\_RID\_3262\_4b.raw |  |  |  | 1.4286E6 |  |  |  |  | 1 | 0 | 0 | 0 | 1 | 0 | 0 | 0 | 0 | 535 | 553 |  |  | PEAKS DB |
| K.VADISMAAK.F | N | 58.91 | 904.4688 | 9 | 0.2 | 453.2418 | 2 | 28.03 | 4 | F4:13402 | 13012022\_RID\_3262\_4b.raw |  |  |  | 5.4443E6 |  |  |  |  | 1 | 0 | 0 | 0 | 1 | 0 | 0 | 0 | 0 | 499 | 507 |  |  | PEAKS DB |
| K.TWAETFEK.Q | N | 55.83 | 1010.4709 | 8 | 0.3 | 506.2429 | 2 | 36.52 | 4 | F4:20193 | 13012022\_RID\_3262\_4b.raw |  |  |  | 1.3005E7 |  |  |  |  | 1 | 0 | 0 | 0 | 1 | 0 | 0 | 0 | 0 | 343 | 350 |  |  | PEAKS DB |
| K.VADISM(+15.99)AAK.F | N | 55.77 | 920.4637 | 9 | -0.1 | 461.2391 | 2 | 28.03 | 4 | F4:13483 | 13012022\_RID\_3262\_4b.raw |  |  |  | 2.148E6 |  |  |  |  | 1 | 0 | 0 | 0 | 1 | 0 | 0 | 0 | 0 | 499 | 507 | Oxidation (M) | M6:Oxidation (M):1000.00 | PEAKS DB |
| R.HGLEFSYEMAPR.A | N | 54.89 | 1435.6554 | 12 | 1.5 | 718.8361 | 2 | 44.96 | 4 | F4:26246 | 13012022\_RID\_3262\_4b.raw |  |  |  | 2.0563E5 |  |  |  | 3.225E4 | 3 | 0 | 0 | 0 | 2 | 0 | 0 | 0 | 1 | 427 | 438 |  |  | PEAKS DB |
| K.VTDMESMK.F | N | 51.87 | 939.4042 | 8 | -0.6 | 470.7091 | 2 | 23.58 | 4 | F4:10341 | 13012022\_RID\_3262\_4b.raw |  |  |  | 6.0853E6 |  |  |  |  | 1 | 0 | 0 | 0 | 1 | 0 | 0 | 0 | 0 | 449 | 456 |  |  | PEAKS DB |
| R.DQGKVTDMESMK.F | N | 48.72 | 1367.6061 | 12 | 0.4 | 684.8106 | 2 | 26.13 | 4 | F4:12043 | 13012022\_RID\_3262\_4b.raw |  |  |  | 2.3854E5 |  |  |  |  | 1 | 0 | 0 | 0 | 1 | 0 | 0 | 0 | 0 | 445 | 456 |  |  | PEAKS DB |
| R.NGYWPSYNIPFDK.E | N | 46.48 | 1599.7357 | 13 | 0.3 | 800.8754 | 2 | 53.87 | 4 | F4:31237 | 13012022\_RID\_3262\_4b.raw |  |  |  | 4.5781E5 |  |  |  |  | 1 | 0 | 0 | 0 | 1 | 0 | 0 | 0 | 0 | 400 | 412 |  |  | PEAKS DB |
| K.VTDM(+15.99)ESMK.F | N | 45.07 | 955.3990 | 8 | 1.9 | 478.7077 | 2 | 21.38 | 4 | F4:10176 | 13012022\_RID\_3262\_4b.raw |  |  |  | 2.4286E6 |  |  |  |  | 1 | 0 | 0 | 0 | 1 | 0 | 0 | 0 | 0 | 449 | 456 | Oxidation (M) | M4:Oxidation (M):21.57 | PEAKS DB |
| total 17 peptides |  |  |  |  |  |  |  |  |  |  |  |  |  |  |  |  |  |  |  |  |  |  |  |  |  |  |  |  |  |  |  |  |  |
| --- | --- | --- | --- | --- | --- | --- | --- | --- | --- | --- | --- | --- | --- | --- | --- | --- | --- | --- | --- | --- | --- | --- | --- | --- | --- | --- | --- | --- | --- | --- | --- | --- | --- |

BAN82026.1

back to list

  

| Protein Coverage
| Supporting Peptides
|

Protein Coverage:

Supporting Peptides:

| Peptide | Uniq | -10lgP | Mass | Length | ppm | m/z | z | RT | Fraction | Scan | Source File | Area F6 | Area F2 | Area F3 | Area F4 | Area F5 | Area F7 | Area F8 | Area F1 | #Feature | #Feature F6 | #Feature F2 | #Feature F3 | #Feature F4 | #Feature F5 | #Feature F7 | #Feature F8 | #Feature F1 | Start | End | PTM | AScore | Found By |
| --- | --- | --- | --- | --- | --- | --- | --- | --- | --- | --- | --- | --- | --- | --- | --- | --- | --- | --- | --- | --- | --- | --- | --- | --- | --- | --- | --- | --- | --- | --- | --- | --- | --- |
| K.QNSGTYNNQYMILDTK.K | N | 86.08 | 1888.8625 | 16 | 0.4 | 945.4390 | 2 | 40.90 | 4 | F4:23353 | 13012022\_RID\_3262\_4b.raw |  |  | 0 | 3.4258E6 |  |  |  |  | 2 | 0 | 0 | 0 | 2 | 0 | 0 | 0 | 0 | 351 | 366 |  |  | PEAKS DB |
| K.FTAYAINGPPVEK.G | N | 84.12 | 1405.7241 | 13 | 0.8 | 703.8699 | 2 | 39.64 | 4 | F4:22495 | 13012022\_RID\_3262\_4b.raw |  |  |  | 6.1282E6 |  |  |  | 3.749E4 | 2 | 0 | 0 | 0 | 1 | 0 | 0 | 0 | 1 | 508 | 520 |  |  | PEAKS DB |
| K.VVPESLFAWER.V | N | 80.07 | 1331.6874 | 11 | 0.5 | 666.8513 | 2 | 54.58 | 4 | F4:31604 | 13012022\_RID\_3262\_4b.raw |  |  |  | 7.2111E6 | 1.2146E6 |  |  |  | 2 | 0 | 0 | 0 | 1 | 1 | 0 | 0 | 0 | 320 | 330 |  |  | PEAKS DB |
| R.SLEDGTLYIIEQVPK.L | Y | 79.29 | 1703.8981 | 15 | 0.1 | 852.9564 | 2 | 58.41 | 4 | F4:33371 | 13012022\_RID\_3262\_4b.raw | 2.8944E6 |  | 4.0146E5 | 2.8073E7 | 2.3975E6 |  |  | 4.8327E5 | 7 | 1 | 0 | 1 | 3 | 1 | 0 | 0 | 1 | 373 | 387 |  |  | PEAKS DB |
| R.YNNYKEDPYAK.H | N | 67.23 | 1403.6357 | 11 | 0.4 | 702.8254 | 2 | 21.90 | 4 | F4:8946 | 13012022\_RID\_3262\_4b.raw |  |  |  | 1.3653E6 |  |  |  |  | 1 | 0 | 0 | 0 | 1 | 0 | 0 | 0 | 0 | 461 | 471 |  |  | PEAKS DB |
| K.HQGLPESYNFDFVTM(+15.99)KPVL | N | 65.84 | 2237.0828 | 19 | 0.7 | 746.7020 | 3 | 55.00 | 4 | F4:31845 | 13012022\_RID\_3262\_4b.raw |  |  |  | 4.8499E6 |  |  |  |  | 1 | 0 | 0 | 0 | 1 | 0 | 0 | 0 | 0 | 535 | 553 | Oxidation (M) | M15:Oxidation (M):1000.00 | PEAKS DB |
| R.IANMMADSGK.T | N | 65.43 | 1036.4681 | 10 | 0.2 | 519.2415 | 2 | 26.71 | 4 | F4:12519 | 13012022\_RID\_3262\_4b.raw |  |  |  | 1.8735E6 |  |  |  |  | 1 | 0 | 0 | 0 | 1 | 0 | 0 | 0 | 0 | 333 | 342 |  |  | PEAKS DB |
| R.HGLEFSYEM(+15.99)APR.A | N | 61.72 | 1451.6503 | 12 | 0.3 | 484.8908 | 3 | 36.62 | 4 | F4:21922 | 13012022\_RID\_3262\_4b.raw |  |  |  | 5.3072E6 |  |  |  |  | 4 | 0 | 0 | 0 | 4 | 0 | 0 | 0 | 0 | 427 | 438 | Oxidation (M) | M9:Oxidation (M):1000.00 | PEAKS DB |
| K.HQGLPESYNFDFVTMKPVL | N | 59.98 | 2221.0876 | 19 | 0.1 | 741.3699 | 3 | 58.53 | 4 | F4:33351 | 13012022\_RID\_3262\_4b.raw |  |  |  | 1.4286E6 |  |  |  |  | 1 | 0 | 0 | 0 | 1 | 0 | 0 | 0 | 0 | 535 | 553 |  |  | PEAKS DB |
| K.VADISMAAK.F | N | 58.91 | 904.4688 | 9 | 0.2 | 453.2418 | 2 | 28.03 | 4 | F4:13402 | 13012022\_RID\_3262\_4b.raw |  |  |  | 5.4443E6 |  |  |  |  | 1 | 0 | 0 | 0 | 1 | 0 | 0 | 0 | 0 | 499 | 507 |  |  | PEAKS DB |
| K.TWAETFEK.Q | N | 55.83 | 1010.4709 | 8 | 0.3 | 506.2429 | 2 | 36.52 | 4 | F4:20193 | 13012022\_RID\_3262\_4b.raw |  |  |  | 1.3005E7 |  |  |  |  | 1 | 0 | 0 | 0 | 1 | 0 | 0 | 0 | 0 | 343 | 350 |  |  | PEAKS DB |
| K.VADISM(+15.99)AAK.F | N | 55.77 | 920.4637 | 9 | -0.1 | 461.2391 | 2 | 28.03 | 4 | F4:13483 | 13012022\_RID\_3262\_4b.raw |  |  |  | 2.148E6 |  |  |  |  | 1 | 0 | 0 | 0 | 1 | 0 | 0 | 0 | 0 | 499 | 507 | Oxidation (M) | M6:Oxidation (M):1000.00 | PEAKS DB |
| R.HGLEFSYEMAPR.A | N | 54.89 | 1435.6554 | 12 | 1.5 | 718.8361 | 2 | 44.96 | 4 | F4:26246 | 13012022\_RID\_3262\_4b.raw |  |  |  | 2.0563E5 |  |  |  | 3.225E4 | 3 | 0 | 0 | 0 | 2 | 0 | 0 | 0 | 1 | 427 | 438 |  |  | PEAKS DB |
| K.VTDMESMK.S | N | 51.87 | 939.4042 | 8 | -0.6 | 470.7091 | 2 | 23.58 | 4 | F4:10341 | 13012022\_RID\_3262\_4b.raw |  |  |  | 6.0853E6 |  |  |  |  | 1 | 0 | 0 | 0 | 1 | 0 | 0 | 0 | 0 | 449 | 456 |  |  | PEAKS DB |
| R.DQGKVTDMESMK.S | N | 48.72 | 1367.6061 | 12 | 0.4 | 684.8106 | 2 | 26.13 | 4 | F4:12043 | 13012022\_RID\_3262\_4b.raw |  |  |  | 2.3854E5 |  |  |  |  | 1 | 0 | 0 | 0 | 1 | 0 | 0 | 0 | 0 | 445 | 456 |  |  | PEAKS DB |
| R.NGYWPSYNIPFDK.E | N | 46.48 | 1599.7357 | 13 | 0.3 | 800.8754 | 2 | 53.87 | 4 | F4:31237 | 13012022\_RID\_3262\_4b.raw |  |  |  | 4.5781E5 |  |  |  |  | 1 | 0 | 0 | 0 | 1 | 0 | 0 | 0 | 0 | 400 | 412 |  |  | PEAKS DB |
| K.VTDM(+15.99)ESMK.S | N | 45.07 | 955.3990 | 8 | 1.9 | 478.7077 | 2 | 21.38 | 4 | F4:10176 | 13012022\_RID\_3262\_4b.raw |  |  |  | 2.4286E6 |  |  |  |  | 1 | 0 | 0 | 0 | 1 | 0 | 0 | 0 | 0 | 449 | 456 | Oxidation (M) | M4:Oxidation (M):21.57 | PEAKS DB |
| total 17 peptides |  |  |  |  |  |  |  |  |  |  |  |  |  |  |  |  |  |  |  |  |  |  |  |  |  |  |  |  |  |  |  |  |  |
| --- | --- | --- | --- | --- | --- | --- | --- | --- | --- | --- | --- | --- | --- | --- | --- | --- | --- | --- | --- | --- | --- | --- | --- | --- | --- | --- | --- | --- | --- | --- | --- | --- | --- |

BAP39973.1

back to list

  

| Protein Coverage
| Supporting Peptides
|

Protein Coverage:

Supporting Peptides:

| Peptide | Uniq | -10lgP | Mass | Length | ppm | m/z | z | RT | Fraction | Scan | Source File | Area F6 | Area F2 | Area F3 | Area F4 | Area F5 | Area F7 | Area F8 | Area F1 | #Feature | #Feature F6 | #Feature F2 | #Feature F3 | #Feature F4 | #Feature F5 | #Feature F7 | #Feature F8 | #Feature F1 | Start | End | PTM | AScore | Found By |
| --- | --- | --- | --- | --- | --- | --- | --- | --- | --- | --- | --- | --- | --- | --- | --- | --- | --- | --- | --- | --- | --- | --- | --- | --- | --- | --- | --- | --- | --- | --- | --- | --- | --- |
| K.QNSGTYNNQYMILDTK.K | N | 86.08 | 1888.8625 | 16 | 0.4 | 945.4390 | 2 | 40.90 | 4 | F4:23353 | 13012022\_RID\_3262\_4b.raw |  |  | 0 | 3.4258E6 |  |  |  |  | 2 | 0 | 0 | 0 | 2 | 0 | 0 | 0 | 0 | 351 | 366 |  |  | PEAKS DB |
| K.FTAYAINGPPVEK.G | N | 84.12 | 1405.7241 | 13 | 0.8 | 703.8699 | 2 | 39.64 | 4 | F4:22495 | 13012022\_RID\_3262\_4b.raw |  |  |  | 6.1282E6 |  |  |  | 3.749E4 | 2 | 0 | 0 | 0 | 1 | 0 | 0 | 0 | 1 | 508 | 520 |  |  | PEAKS DB |
| K.VVPESLFAWER.V | N | 80.07 | 1331.6874 | 11 | 0.5 | 666.8513 | 2 | 54.58 | 4 | F4:31604 | 13012022\_RID\_3262\_4b.raw |  |  |  | 7.2111E6 | 1.2146E6 |  |  |  | 2 | 0 | 0 | 0 | 1 | 1 | 0 | 0 | 0 | 320 | 330 |  |  | PEAKS DB |
| R.SLEDGTLYIIEQVPK.L | Y | 79.29 | 1703.8981 | 15 | 0.1 | 852.9564 | 2 | 58.41 | 4 | F4:33371 | 13012022\_RID\_3262\_4b.raw | 2.8944E6 |  | 4.0146E5 | 2.8073E7 | 2.3975E6 |  |  | 4.8327E5 | 7 | 1 | 0 | 1 | 3 | 1 | 0 | 0 | 1 | 373 | 387 |  |  | PEAKS DB |
| R.YNNYKEDPYAK.H | N | 67.23 | 1403.6357 | 11 | 0.4 | 702.8254 | 2 | 21.90 | 4 | F4:8946 | 13012022\_RID\_3262\_4b.raw |  |  |  | 1.3653E6 |  |  |  |  | 1 | 0 | 0 | 0 | 1 | 0 | 0 | 0 | 0 | 461 | 471 |  |  | PEAKS DB |
| K.HQGLPESYNFDFVTM(+15.99)KPVL | N | 65.84 | 2237.0828 | 19 | 0.7 | 746.7020 | 3 | 55.00 | 4 | F4:31845 | 13012022\_RID\_3262\_4b.raw |  |  |  | 4.8499E6 |  |  |  |  | 1 | 0 | 0 | 0 | 1 | 0 | 0 | 0 | 0 | 535 | 553 | Oxidation (M) | M15:Oxidation (M):1000.00 | PEAKS DB |
| R.IANMMADSGK.T | N | 65.43 | 1036.4681 | 10 | 0.2 | 519.2415 | 2 | 26.71 | 4 | F4:12519 | 13012022\_RID\_3262\_4b.raw |  |  |  | 1.8735E6 |  |  |  |  | 1 | 0 | 0 | 0 | 1 | 0 | 0 | 0 | 0 | 333 | 342 |  |  | PEAKS DB |
| R.HGLEFSYEM(+15.99)APR.A | N | 61.72 | 1451.6503 | 12 | 0.3 | 484.8908 | 3 | 36.62 | 4 | F4:21922 | 13012022\_RID\_3262\_4b.raw |  |  |  | 5.3072E6 |  |  |  |  | 4 | 0 | 0 | 0 | 4 | 0 | 0 | 0 | 0 | 427 | 438 | Oxidation (M) | M9:Oxidation (M):1000.00 | PEAKS DB |
| K.HQGLPESYNFDFVTMKPVL | N | 59.98 | 2221.0876 | 19 | 0.1 | 741.3699 | 3 | 58.53 | 4 | F4:33351 | 13012022\_RID\_3262\_4b.raw |  |  |  | 1.4286E6 |  |  |  |  | 1 | 0 | 0 | 0 | 1 | 0 | 0 | 0 | 0 | 535 | 553 |  |  | PEAKS DB |
| K.VADISMAAK.F | N | 58.91 | 904.4688 | 9 | 0.2 | 453.2418 | 2 | 28.03 | 4 | F4:13402 | 13012022\_RID\_3262\_4b.raw |  |  |  | 5.4443E6 |  |  |  |  | 1 | 0 | 0 | 0 | 1 | 0 | 0 | 0 | 0 | 499 | 507 |  |  | PEAKS DB |
| K.TWAETFEK.Q | N | 55.83 | 1010.4709 | 8 | 0.3 | 506.2429 | 2 | 36.52 | 4 | F4:20193 | 13012022\_RID\_3262\_4b.raw |  |  |  | 1.3005E7 |  |  |  |  | 1 | 0 | 0 | 0 | 1 | 0 | 0 | 0 | 0 | 343 | 350 |  |  | PEAKS DB |
| K.VADISM(+15.99)AAK.F | N | 55.77 | 920.4637 | 9 | -0.1 | 461.2391 | 2 | 28.03 | 4 | F4:13483 | 13012022\_RID\_3262\_4b.raw |  |  |  | 2.148E6 |  |  |  |  | 1 | 0 | 0 | 0 | 1 | 0 | 0 | 0 | 0 | 499 | 507 | Oxidation (M) | M6:Oxidation (M):1000.00 | PEAKS DB |
| R.HGLEFSYEMAPR.A | N | 54.89 | 1435.6554 | 12 | 1.5 | 718.8361 | 2 | 44.96 | 4 | F4:26246 | 13012022\_RID\_3262\_4b.raw |  |  |  | 2.0563E5 |  |  |  | 3.225E4 | 3 | 0 | 0 | 0 | 2 | 0 | 0 | 0 | 1 | 427 | 438 |  |  | PEAKS DB |
| K.VTDMESMK.S | N | 51.87 | 939.4042 | 8 | -0.6 | 470.7091 | 2 | 23.58 | 4 | F4:10341 | 13012022\_RID\_3262\_4b.raw |  |  |  | 6.0853E6 |  |  |  |  | 1 | 0 | 0 | 0 | 1 | 0 | 0 | 0 | 0 | 449 | 456 |  |  | PEAKS DB |
| R.DQGKVTDMESMK.S | N | 48.72 | 1367.6061 | 12 | 0.4 | 684.8106 | 2 | 26.13 | 4 | F4:12043 | 13012022\_RID\_3262\_4b.raw |  |  |  | 2.3854E5 |  |  |  |  | 1 | 0 | 0 | 0 | 1 | 0 | 0 | 0 | 0 | 445 | 456 |  |  | PEAKS DB |
| R.NGYWPSYNIPFDK.E | N | 46.48 | 1599.7357 | 13 | 0.3 | 800.8754 | 2 | 53.87 | 4 | F4:31237 | 13012022\_RID\_3262\_4b.raw |  |  |  | 4.5781E5 |  |  |  |  | 1 | 0 | 0 | 0 | 1 | 0 | 0 | 0 | 0 | 400 | 412 |  |  | PEAKS DB |
| K.VTDM(+15.99)ESMK.S | N | 45.07 | 955.3990 | 8 | 1.9 | 478.7077 | 2 | 21.38 | 4 | F4:10176 | 13012022\_RID\_3262\_4b.raw |  |  |  | 2.4286E6 |  |  |  |  | 1 | 0 | 0 | 0 | 1 | 0 | 0 | 0 | 0 | 449 | 456 | Oxidation (M) | M4:Oxidation (M):21.57 | PEAKS DB |
| total 17 peptides |  |  |  |  |  |  |  |  |  |  |  |  |  |  |  |  |  |  |  |  |  |  |  |  |  |  |  |  |  |  |  |  |  |
| --- | --- | --- | --- | --- | --- | --- | --- | --- | --- | --- | --- | --- | --- | --- | --- | --- | --- | --- | --- | --- | --- | --- | --- | --- | --- | --- | --- | --- | --- | --- | --- | --- | --- |

BAP39922.1

back to list

  

| Protein Coverage
| Supporting Peptides
|

Protein Coverage:

Supporting Peptides:

| Peptide | Uniq | -10lgP | Mass | Length | ppm | m/z | z | RT | Fraction | Scan | Source File | Area F6 | Area F2 | Area F3 | Area F4 | Area F5 | Area F7 | Area F8 | Area F1 | #Feature | #Feature F6 | #Feature F2 | #Feature F3 | #Feature F4 | #Feature F5 | #Feature F7 | #Feature F8 | #Feature F1 | Start | End | PTM | AScore | Found By |
| --- | --- | --- | --- | --- | --- | --- | --- | --- | --- | --- | --- | --- | --- | --- | --- | --- | --- | --- | --- | --- | --- | --- | --- | --- | --- | --- | --- | --- | --- | --- | --- | --- | --- |
| K.QNSGTYNNQYMILDTK.K | N | 86.08 | 1888.8625 | 16 | 0.4 | 945.4390 | 2 | 40.90 | 4 | F4:23353 | 13012022\_RID\_3262\_4b.raw |  |  | 0 | 3.4258E6 |  |  |  |  | 2 | 0 | 0 | 0 | 2 | 0 | 0 | 0 | 0 | 351 | 366 |  |  | PEAKS DB |
| K.FTAYAINGPPVEK.G | N | 84.12 | 1405.7241 | 13 | 0.8 | 703.8699 | 2 | 39.64 | 4 | F4:22495 | 13012022\_RID\_3262\_4b.raw |  |  |  | 6.1282E6 |  |  |  | 3.749E4 | 2 | 0 | 0 | 0 | 1 | 0 | 0 | 0 | 1 | 508 | 520 |  |  | PEAKS DB |
| K.VVPESLFAWER.V | N | 80.07 | 1331.6874 | 11 | 0.5 | 666.8513 | 2 | 54.58 | 4 | F4:31604 | 13012022\_RID\_3262\_4b.raw |  |  |  | 7.2111E6 | 1.2146E6 |  |  |  | 2 | 0 | 0 | 0 | 1 | 1 | 0 | 0 | 0 | 320 | 330 |  |  | PEAKS DB |
| R.SLEDGTLYIIEQVPK.L | Y | 79.29 | 1703.8981 | 15 | 0.1 | 852.9564 | 2 | 58.41 | 4 | F4:33371 | 13012022\_RID\_3262\_4b.raw | 2.8944E6 |  | 4.0146E5 | 2.8073E7 | 2.3975E6 |  |  | 4.8327E5 | 7 | 1 | 0 | 1 | 3 | 1 | 0 | 0 | 1 | 373 | 387 |  |  | PEAKS DB |
| R.YNNYKEDPYAK.H | N | 67.23 | 1403.6357 | 11 | 0.4 | 702.8254 | 2 | 21.90 | 4 | F4:8946 | 13012022\_RID\_3262\_4b.raw |  |  |  | 1.3653E6 |  |  |  |  | 1 | 0 | 0 | 0 | 1 | 0 | 0 | 0 | 0 | 461 | 471 |  |  | PEAKS DB |
| K.HQGLPESYNFDFVTM(+15.99)KPVL | N | 65.84 | 2237.0828 | 19 | 0.7 | 746.7020 | 3 | 55.00 | 4 | F4:31845 | 13012022\_RID\_3262\_4b.raw |  |  |  | 4.8499E6 |  |  |  |  | 1 | 0 | 0 | 0 | 1 | 0 | 0 | 0 | 0 | 535 | 553 | Oxidation (M) | M15:Oxidation (M):1000.00 | PEAKS DB |
| R.IANMMADSGK.T | N | 65.43 | 1036.4681 | 10 | 0.2 | 519.2415 | 2 | 26.71 | 4 | F4:12519 | 13012022\_RID\_3262\_4b.raw |  |  |  | 1.8735E6 |  |  |  |  | 1 | 0 | 0 | 0 | 1 | 0 | 0 | 0 | 0 | 333 | 342 |  |  | PEAKS DB |
| K.HGLEFSYEM(+15.99)APR.A | N | 61.72 | 1451.6503 | 12 | 0.3 | 484.8908 | 3 | 36.62 | 4 | F4:21922 | 13012022\_RID\_3262\_4b.raw |  |  |  | 5.3072E6 |  |  |  |  | 4 | 0 | 0 | 0 | 4 | 0 | 0 | 0 | 0 | 427 | 438 | Oxidation (M) | M9:Oxidation (M):1000.00 | PEAKS DB |
| K.HQGLPESYNFDFVTMKPVL | N | 59.98 | 2221.0876 | 19 | 0.1 | 741.3699 | 3 | 58.53 | 4 | F4:33351 | 13012022\_RID\_3262\_4b.raw |  |  |  | 1.4286E6 |  |  |  |  | 1 | 0 | 0 | 0 | 1 | 0 | 0 | 0 | 0 | 535 | 553 |  |  | PEAKS DB |
| K.VADISMAAK.F | N | 58.91 | 904.4688 | 9 | 0.2 | 453.2418 | 2 | 28.03 | 4 | F4:13402 | 13012022\_RID\_3262\_4b.raw |  |  |  | 5.4443E6 |  |  |  |  | 1 | 0 | 0 | 0 | 1 | 0 | 0 | 0 | 0 | 499 | 507 |  |  | PEAKS DB |
| K.TWAETFEK.Q | N | 55.83 | 1010.4709 | 8 | 0.3 | 506.2429 | 2 | 36.52 | 4 | F4:20193 | 13012022\_RID\_3262\_4b.raw |  |  |  | 1.3005E7 |  |  |  |  | 1 | 0 | 0 | 0 | 1 | 0 | 0 | 0 | 0 | 343 | 350 |  |  | PEAKS DB |
| K.VADISM(+15.99)AAK.F | N | 55.77 | 920.4637 | 9 | -0.1 | 461.2391 | 2 | 28.03 | 4 | F4:13483 | 13012022\_RID\_3262\_4b.raw |  |  |  | 2.148E6 |  |  |  |  | 1 | 0 | 0 | 0 | 1 | 0 | 0 | 0 | 0 | 499 | 507 | Oxidation (M) | M6:Oxidation (M):1000.00 | PEAKS DB |
| K.HGLEFSYEMAPR.A | N | 54.89 | 1435.6554 | 12 | 1.5 | 718.8361 | 2 | 44.96 | 4 | F4:26246 | 13012022\_RID\_3262\_4b.raw |  |  |  | 2.0563E5 |  |  |  | 3.225E4 | 3 | 0 | 0 | 0 | 2 | 0 | 0 | 0 | 1 | 427 | 438 |  |  | PEAKS DB |
| K.VTDMESMK.S | N | 51.87 | 939.4042 | 8 | -0.6 | 470.7091 | 2 | 23.58 | 4 | F4:10341 | 13012022\_RID\_3262\_4b.raw |  |  |  | 6.0853E6 |  |  |  |  | 1 | 0 | 0 | 0 | 1 | 0 | 0 | 0 | 0 | 449 | 456 |  |  | PEAKS DB |
| R.DQGKVTDMESMK.S | N | 48.72 | 1367.6061 | 12 | 0.4 | 684.8106 | 2 | 26.13 | 4 | F4:12043 | 13012022\_RID\_3262\_4b.raw |  |  |  | 2.3854E5 |  |  |  |  | 1 | 0 | 0 | 0 | 1 | 0 | 0 | 0 | 0 | 445 | 456 |  |  | PEAKS DB |
| R.NGYWPSYNIPFDK.E | N | 46.48 | 1599.7357 | 13 | 0.3 | 800.8754 | 2 | 53.87 | 4 | F4:31237 | 13012022\_RID\_3262\_4b.raw |  |  |  | 4.5781E5 |  |  |  |  | 1 | 0 | 0 | 0 | 1 | 0 | 0 | 0 | 0 | 400 | 412 |  |  | PEAKS DB |
| K.VTDM(+15.99)ESMK.S | N | 45.07 | 955.3990 | 8 | 1.9 | 478.7077 | 2 | 21.38 | 4 | F4:10176 | 13012022\_RID\_3262\_4b.raw |  |  |  | 2.4286E6 |  |  |  |  | 1 | 0 | 0 | 0 | 1 | 0 | 0 | 0 | 0 | 449 | 456 | Oxidation (M) | M4:Oxidation (M):21.57 | PEAKS DB |
| total 17 peptides |  |  |  |  |  |  |  |  |  |  |  |  |  |  |  |  |  |  |  |  |  |  |  |  |  |  |  |  |  |  |  |  |  |
| --- | --- | --- | --- | --- | --- | --- | --- | --- | --- | --- | --- | --- | --- | --- | --- | --- | --- | --- | --- | --- | --- | --- | --- | --- | --- | --- | --- | --- | --- | --- | --- | --- | --- |

AAY89681.1

back to list

  

| Protein Coverage
| Supporting Peptides
|

Protein Coverage:

Supporting Peptides:

| Peptide | Uniq | -10lgP | Mass | Length | ppm | m/z | z | RT | Fraction | Scan | Source File | Area F6 | Area F2 | Area F3 | Area F4 | Area F5 | Area F7 | Area F8 | Area F1 | #Feature | #Feature F6 | #Feature F2 | #Feature F3 | #Feature F4 | #Feature F5 | #Feature F7 | #Feature F8 | #Feature F1 | Start | End | PTM | AScore | Found By |
| --- | --- | --- | --- | --- | --- | --- | --- | --- | --- | --- | --- | --- | --- | --- | --- | --- | --- | --- | --- | --- | --- | --- | --- | --- | --- | --- | --- | --- | --- | --- | --- | --- | --- |
| Q.EADYEEFLEIAR.N | N | 85.37 | 1483.6830 | 12 | 0.2 | 742.8489 | 2 | 55.72 | 2 | F2:24489 | 13012022\_RID\_3262\_2b.raw | 6.0602E6 | 1.2207E7 | 3.0247E8 | 1.2442E7 | 9.4429E6 | 5.4359E6 | 2.0471E6 | 3.6897E6 | 10 | 1 | 1 | 3 | 1 | 1 | 1 | 1 | 1 | 32 | 43 |  |  | PEAKS DB |
| T.EGWYVNLGPM(+15.99)R.L | N | 75.80 | 1336.6234 | 11 | 0.3 | 669.3192 | 2 | 45.61 | 3 | F3:28662 | 13012022\_RID\_3262\_3b.raw |  |  | 1.1921E7 |  |  |  |  |  | 2 | 0 | 0 | 2 | 0 | 0 | 0 | 0 | 0 | 99 | 109 | Oxidation (M) | M10:Oxidation (M):1000.00 | PEAKS DB |
| A.DIVINDLSLIHQLPK.E | N | 75.61 | 1716.9774 | 15 | 0.0 | 859.4960 | 2 | 54.14 | 3 | F3:35798 | 13012022\_RID\_3262\_3b.raw |  |  | 2.3604E6 |  |  |  |  |  | 2 | 0 | 0 | 2 | 0 | 0 | 0 | 0 | 0 | 410 | 424 |  |  | PEAKS DB |
| T.EGWYVNLGPMR.L | N | 70.02 | 1320.6284 | 11 | 0.4 | 661.3218 | 2 | 50.89 | 3 | F3:33016 | 13012022\_RID\_3262\_3b.raw |  |  | 1.2688E7 |  |  |  |  | 9.1762E4 | 2 | 0 | 0 | 1 | 0 | 0 | 0 | 0 | 1 | 99 | 109 |  |  | PEAKS DB |
| K.YDTYSTKEYLIK.E | N | 68.24 | 1522.7555 | 12 | 0.3 | 762.3853 | 2 | 34.64 | 3 | F3:18814 | 13012022\_RID\_3262\_3b.raw |  |  | 1.2832E6 |  |  |  |  |  | 2 | 0 | 0 | 2 | 0 | 0 | 0 | 0 | 0 | 199 | 210 |  |  | PEAKS DB |
| D.YEEFLEIAR.N | N | 64.34 | 1168.5764 | 9 | -0.1 | 585.2954 | 2 | 45.67 | 3 | F3:28690 | 13012022\_RID\_3262\_3b.raw |  |  | 1.8472E7 |  |  |  |  |  | 1 | 0 | 0 | 1 | 0 | 0 | 0 | 0 | 0 | 35 | 43 |  |  | PEAKS DB |
| D.IVINDLSLIHQLPK.E | N | 61.67 | 1601.9504 | 14 | 0.6 | 534.9911 | 3 | 48.31 | 3 | F3:30799 | 13012022\_RID\_3262\_3b.raw |  |  | 4.9507E6 |  |  |  |  |  | 2 | 0 | 0 | 2 | 0 | 0 | 0 | 0 | 0 | 411 | 424 |  |  | PEAKS DB |
| K.HVVVVGAGM(+15.99)AGLSAAYVLAGAGHNVTLLEASER.V | Y | 61.44 | 3234.6819 | 33 | 0.8 | 809.6784 | 4 | 56.50 | 3 | F3:37740 | 13012022\_RID\_3262\_3b.raw |  |  | 3.2785E6 |  |  |  |  |  | 3 | 0 | 0 | 3 | 0 | 0 | 0 | 0 | 0 | 54 | 86 | Oxidation (M) | M9:Oxidation (M):1000.00 | PEAKS DB |
| K.STTDLPSR.F | N | 58.41 | 875.4348 | 8 | 0.3 | 438.7248 | 2 | 21.23 | 4 | F4:8235 | 13012022\_RID\_3262\_4b.raw |  | 3.3314E6 | 1.6248E8 | 1.5408E7 | 9.3405E6 |  |  |  | 4 | 0 | 1 | 1 | 1 | 1 | 0 | 0 | 0 | 365 | 372 |  |  | PEAKS DB |
| N.DLSLIHQLPK.E | N | 58.16 | 1162.6710 | 10 | 0.6 | 582.3431 | 2 | 41.31 | 3 | F3:24879 | 13012022\_RID\_3262\_3b.raw |  |  | 4.4132E6 |  |  |  |  |  | 1 | 0 | 0 | 1 | 0 | 0 | 0 | 0 | 0 | 415 | 424 |  |  | PEAKS DB |
| Y.FEPPLPPKK.A | N | 54.07 | 1051.6066 | 9 | 0.2 | 526.8107 | 2 | 28.91 | 3 | F3:14056 | 13012022\_RID\_3262\_3b.raw |  |  | 1.498E7 |  |  |  |  |  | 1 | 0 | 0 | 1 | 0 | 0 | 0 | 0 | 0 | 323 | 331 |  |  | PEAKS DB |
| Y.FEPPLPPK.K | N | 50.89 | 923.5116 | 8 | 0.6 | 462.7633 | 2 | 36.44 | 3 | F3:20282 | 13012022\_RID\_3262\_3b.raw |  |  | 1.731E8 |  |  |  |  |  | 1 | 0 | 0 | 1 | 0 | 0 | 0 | 0 | 0 | 323 | 330 |  |  | PEAKS DB |
| D.LSLIHQLPK.E | N | 50.17 | 1047.6440 | 9 | -0.1 | 524.8292 | 2 | 34.27 | 3 | F3:18619 | 13012022\_RID\_3262\_3b.raw |  |  | 9.3032E5 |  |  |  |  |  | 1 | 0 | 0 | 1 | 0 | 0 | 0 | 0 | 0 | 416 | 424 |  |  | PEAKS DB |
| K.SGLTAARDVNR.A | N | 46.67 | 1158.6105 | 11 | 0.4 | 387.2109 | 3 | 20.31 | 3 | F3:7146 | 13012022\_RID\_3262\_3b.raw |  |  | 2.6229E5 |  |  |  |  |  | 1 | 0 | 0 | 1 | 0 | 0 | 0 | 0 | 0 | 491 | 501 |  |  | PEAKS DB |
| total 14 peptides |  |  |  |  |  |  |  |  |  |  |  |  |  |  |  |  |  |  |  |  |  |  |  |  |  |  |  |  |  |  |  |  |  |
| --- | --- | --- | --- | --- | --- | --- | --- | --- | --- | --- | --- | --- | --- | --- | --- | --- | --- | --- | --- | --- | --- | --- | --- | --- | --- | --- | --- | --- | --- | --- | --- | --- | --- |

Q4JHE2.1

back to list

  

| Protein Coverage
| Supporting Peptides
|

Protein Coverage:

Supporting Peptides:

| Peptide | Uniq | -10lgP | Mass | Length | ppm | m/z | z | RT | Fraction | Scan | Source File | Area F6 | Area F2 | Area F3 | Area F4 | Area F5 | Area F7 | Area F8 | Area F1 | #Feature | #Feature F6 | #Feature F2 | #Feature F3 | #Feature F4 | #Feature F5 | #Feature F7 | #Feature F8 | #Feature F1 | Start | End | PTM | AScore | Found By |
| --- | --- | --- | --- | --- | --- | --- | --- | --- | --- | --- | --- | --- | --- | --- | --- | --- | --- | --- | --- | --- | --- | --- | --- | --- | --- | --- | --- | --- | --- | --- | --- | --- | --- |
| Q.EADYEEFLEIAR.N | N | 85.37 | 1483.6830 | 12 | 0.2 | 742.8489 | 2 | 55.72 | 2 | F2:24489 | 13012022\_RID\_3262\_2b.raw | 6.0602E6 | 1.2207E7 | 3.0247E8 | 1.2442E7 | 9.4429E6 | 5.4359E6 | 2.0471E6 | 3.6897E6 | 10 | 1 | 1 | 3 | 1 | 1 | 1 | 1 | 1 | 32 | 43 |  |  | PEAKS DB |
| T.EGWYVNLGPM(+15.99)R.L | N | 75.80 | 1336.6234 | 11 | 0.3 | 669.3192 | 2 | 45.61 | 3 | F3:28662 | 13012022\_RID\_3262\_3b.raw |  |  | 1.1921E7 |  |  |  |  |  | 2 | 0 | 0 | 2 | 0 | 0 | 0 | 0 | 0 | 99 | 109 | Oxidation (M) | M10:Oxidation (M):1000.00 | PEAKS DB |
| A.DIVINDLSLIHQLPK.E | N | 75.61 | 1716.9774 | 15 | 0.0 | 859.4960 | 2 | 54.14 | 3 | F3:35798 | 13012022\_RID\_3262\_3b.raw |  |  | 2.3604E6 |  |  |  |  |  | 2 | 0 | 0 | 2 | 0 | 0 | 0 | 0 | 0 | 410 | 424 |  |  | PEAKS DB |
| T.EGWYVNLGPMR.L | N | 70.02 | 1320.6284 | 11 | 0.4 | 661.3218 | 2 | 50.89 | 3 | F3:33016 | 13012022\_RID\_3262\_3b.raw |  |  | 1.2688E7 |  |  |  |  | 9.1762E4 | 2 | 0 | 0 | 1 | 0 | 0 | 0 | 0 | 1 | 99 | 109 |  |  | PEAKS DB |
| K.YDTYSTKEYLIK.E | N | 68.24 | 1522.7555 | 12 | 0.3 | 762.3853 | 2 | 34.64 | 3 | F3:18814 | 13012022\_RID\_3262\_3b.raw |  |  | 1.2832E6 |  |  |  |  |  | 2 | 0 | 0 | 2 | 0 | 0 | 0 | 0 | 0 | 199 | 210 |  |  | PEAKS DB |
| D.YEEFLEIAR.N | N | 64.34 | 1168.5764 | 9 | -0.1 | 585.2954 | 2 | 45.67 | 3 | F3:28690 | 13012022\_RID\_3262\_3b.raw |  |  | 1.8472E7 |  |  |  |  |  | 1 | 0 | 0 | 1 | 0 | 0 | 0 | 0 | 0 | 35 | 43 |  |  | PEAKS DB |
| D.IVINDLSLIHQLPK.E | N | 61.67 | 1601.9504 | 14 | 0.6 | 534.9911 | 3 | 48.31 | 3 | F3:30799 | 13012022\_RID\_3262\_3b.raw |  |  | 4.9507E6 |  |  |  |  |  | 2 | 0 | 0 | 2 | 0 | 0 | 0 | 0 | 0 | 411 | 424 |  |  | PEAKS DB |
| K.HVVVVGAGM(+15.99)AGLSAAYVLAGAGHNVTLLEASER.V | Y | 61.44 | 3234.6819 | 33 | 0.8 | 809.6784 | 4 | 56.50 | 3 | F3:37740 | 13012022\_RID\_3262\_3b.raw |  |  | 3.2785E6 |  |  |  |  |  | 3 | 0 | 0 | 3 | 0 | 0 | 0 | 0 | 0 | 54 | 86 | Oxidation (M) | M9:Oxidation (M):1000.00 | PEAKS DB |
| K.STTDLPSR.F | N | 58.41 | 875.4348 | 8 | 0.3 | 438.7248 | 2 | 21.23 | 4 | F4:8235 | 13012022\_RID\_3262\_4b.raw |  | 3.3314E6 | 1.6248E8 | 1.5408E7 | 9.3405E6 |  |  |  | 4 | 0 | 1 | 1 | 1 | 1 | 0 | 0 | 0 | 365 | 372 |  |  | PEAKS DB |
| N.DLSLIHQLPK.E | N | 58.16 | 1162.6710 | 10 | 0.6 | 582.3431 | 2 | 41.31 | 3 | F3:24879 | 13012022\_RID\_3262\_3b.raw |  |  | 4.4132E6 |  |  |  |  |  | 1 | 0 | 0 | 1 | 0 | 0 | 0 | 0 | 0 | 415 | 424 |  |  | PEAKS DB |
| Y.FEPPLPPKK.A | N | 54.07 | 1051.6066 | 9 | 0.2 | 526.8107 | 2 | 28.91 | 3 | F3:14056 | 13012022\_RID\_3262\_3b.raw |  |  | 1.498E7 |  |  |  |  |  | 1 | 0 | 0 | 1 | 0 | 0 | 0 | 0 | 0 | 323 | 331 |  |  | PEAKS DB |
| Y.FEPPLPPK.K | N | 50.89 | 923.5116 | 8 | 0.6 | 462.7633 | 2 | 36.44 | 3 | F3:20282 | 13012022\_RID\_3262\_3b.raw |  |  | 1.731E8 |  |  |  |  |  | 1 | 0 | 0 | 1 | 0 | 0 | 0 | 0 | 0 | 323 | 330 |  |  | PEAKS DB |
| D.LSLIHQLPK.E | N | 50.17 | 1047.6440 | 9 | -0.1 | 524.8292 | 2 | 34.27 | 3 | F3:18619 | 13012022\_RID\_3262\_3b.raw |  |  | 9.3032E5 |  |  |  |  |  | 1 | 0 | 0 | 1 | 0 | 0 | 0 | 0 | 0 | 416 | 424 |  |  | PEAKS DB |
| K.SGLTAARDVNR.A | N | 46.67 | 1158.6105 | 11 | 0.4 | 387.2109 | 3 | 20.31 | 3 | F3:7146 | 13012022\_RID\_3262\_3b.raw |  |  | 2.6229E5 |  |  |  |  |  | 1 | 0 | 0 | 1 | 0 | 0 | 0 | 0 | 0 | 491 | 501 |  |  | PEAKS DB |
| total 14 peptides |  |  |  |  |  |  |  |  |  |  |  |  |  |  |  |  |  |  |  |  |  |  |  |  |  |  |  |  |  |  |  |  |  |
| --- | --- | --- | --- | --- | --- | --- | --- | --- | --- | --- | --- | --- | --- | --- | --- | --- | --- | --- | --- | --- | --- | --- | --- | --- | --- | --- | --- | --- | --- | --- | --- | --- | --- |

XP\_026538830.1

back to list

  

| Protein Coverage
| Supporting Peptides
|

Protein Coverage:

Supporting Peptides:

| Peptide | Uniq | -10lgP | Mass | Length | ppm | m/z | z | RT | Fraction | Scan | Source File | Area F6 | Area F2 | Area F3 | Area F4 | Area F5 | Area F7 | Area F8 | Area F1 | #Feature | #Feature F6 | #Feature F2 | #Feature F3 | #Feature F4 | #Feature F5 | #Feature F7 | #Feature F8 | #Feature F1 | Start | End | PTM | AScore | Found By |
| --- | --- | --- | --- | --- | --- | --- | --- | --- | --- | --- | --- | --- | --- | --- | --- | --- | --- | --- | --- | --- | --- | --- | --- | --- | --- | --- | --- | --- | --- | --- | --- | --- | --- |
| Q.EADYEEFLEIAR.N | N | 85.37 | 1483.6830 | 12 | 0.2 | 742.8489 | 2 | 55.72 | 2 | F2:24489 | 13012022\_RID\_3262\_2b.raw | 6.0602E6 | 1.2207E7 | 3.0247E8 | 1.2442E7 | 9.4429E6 | 5.4359E6 | 2.0471E6 | 3.6897E6 | 10 | 1 | 1 | 3 | 1 | 1 | 1 | 1 | 1 | 32 | 43 |  |  | PEAKS DB |
| T.EGWYVNLGPM(+15.99)R.L | N | 75.80 | 1336.6234 | 11 | 0.3 | 669.3192 | 2 | 45.61 | 3 | F3:28662 | 13012022\_RID\_3262\_3b.raw |  |  | 1.1921E7 |  |  |  |  |  | 2 | 0 | 0 | 2 | 0 | 0 | 0 | 0 | 0 | 99 | 109 | Oxidation (M) | M10:Oxidation (M):1000.00 | PEAKS DB |
| A.DIVINDLSLIHQLPK.E | N | 75.61 | 1716.9774 | 15 | 0.0 | 859.4960 | 2 | 54.14 | 3 | F3:35798 | 13012022\_RID\_3262\_3b.raw |  |  | 2.3604E6 |  |  |  |  |  | 2 | 0 | 0 | 2 | 0 | 0 | 0 | 0 | 0 | 410 | 424 |  |  | PEAKS DB |
| T.EGWYVNLGPMR.L | N | 70.02 | 1320.6284 | 11 | 0.4 | 661.3218 | 2 | 50.89 | 3 | F3:33016 | 13012022\_RID\_3262\_3b.raw |  |  | 1.2688E7 |  |  |  |  | 9.1762E4 | 2 | 0 | 0 | 1 | 0 | 0 | 0 | 0 | 1 | 99 | 109 |  |  | PEAKS DB |
| K.YDTYSTKEYLIK.E | N | 68.24 | 1522.7555 | 12 | 0.3 | 762.3853 | 2 | 34.64 | 3 | F3:18814 | 13012022\_RID\_3262\_3b.raw |  |  | 1.2832E6 |  |  |  |  |  | 2 | 0 | 0 | 2 | 0 | 0 | 0 | 0 | 0 | 199 | 210 |  |  | PEAKS DB |
| D.YEEFLEIAR.N | N | 64.34 | 1168.5764 | 9 | -0.1 | 585.2954 | 2 | 45.67 | 3 | F3:28690 | 13012022\_RID\_3262\_3b.raw |  |  | 1.8472E7 |  |  |  |  |  | 1 | 0 | 0 | 1 | 0 | 0 | 0 | 0 | 0 | 35 | 43 |  |  | PEAKS DB |
| D.IVINDLSLIHQLPK.E | N | 61.67 | 1601.9504 | 14 | 0.6 | 534.9911 | 3 | 48.31 | 3 | F3:30799 | 13012022\_RID\_3262\_3b.raw |  |  | 4.9507E6 |  |  |  |  |  | 2 | 0 | 0 | 2 | 0 | 0 | 0 | 0 | 0 | 411 | 424 |  |  | PEAKS DB |
| K.HVVVVGAGM(+15.99)AGLSAAYVLAGAGHNVTLLEASER.V | Y | 61.44 | 3234.6819 | 33 | 0.8 | 809.6784 | 4 | 56.50 | 3 | F3:37740 | 13012022\_RID\_3262\_3b.raw |  |  | 3.2785E6 |  |  |  |  |  | 3 | 0 | 0 | 3 | 0 | 0 | 0 | 0 | 0 | 54 | 86 | Oxidation (M) | M9:Oxidation (M):1000.00 | PEAKS DB |
| K.STTDLPSR.F | N | 58.41 | 875.4348 | 8 | 0.3 | 438.7248 | 2 | 21.23 | 4 | F4:8235 | 13012022\_RID\_3262\_4b.raw |  | 3.3314E6 | 1.6248E8 | 1.5408E7 | 9.3405E6 |  |  |  | 4 | 0 | 1 | 1 | 1 | 1 | 0 | 0 | 0 | 365 | 372 |  |  | PEAKS DB |
| N.DLSLIHQLPK.E | N | 58.16 | 1162.6710 | 10 | 0.6 | 582.3431 | 2 | 41.31 | 3 | F3:24879 | 13012022\_RID\_3262\_3b.raw |  |  | 4.4132E6 |  |  |  |  |  | 1 | 0 | 0 | 1 | 0 | 0 | 0 | 0 | 0 | 415 | 424 |  |  | PEAKS DB |
| Y.FEPPLPPKK.A | N | 54.07 | 1051.6066 | 9 | 0.2 | 526.8107 | 2 | 28.91 | 3 | F3:14056 | 13012022\_RID\_3262\_3b.raw |  |  | 1.498E7 |  |  |  |  |  | 1 | 0 | 0 | 1 | 0 | 0 | 0 | 0 | 0 | 323 | 331 |  |  | PEAKS DB |
| Y.FEPPLPPK.K | N | 50.89 | 923.5116 | 8 | 0.6 | 462.7633 | 2 | 36.44 | 3 | F3:20282 | 13012022\_RID\_3262\_3b.raw |  |  | 1.731E8 |  |  |  |  |  | 1 | 0 | 0 | 1 | 0 | 0 | 0 | 0 | 0 | 323 | 330 |  |  | PEAKS DB |
| D.LSLIHQLPK.E | N | 50.17 | 1047.6440 | 9 | -0.1 | 524.8292 | 2 | 34.27 | 3 | F3:18619 | 13012022\_RID\_3262\_3b.raw |  |  | 9.3032E5 |  |  |  |  |  | 1 | 0 | 0 | 1 | 0 | 0 | 0 | 0 | 0 | 416 | 424 |  |  | PEAKS DB |
| K.SGLTAARDVNR.A | N | 46.67 | 1158.6105 | 11 | 0.4 | 387.2109 | 3 | 20.31 | 3 | F3:7146 | 13012022\_RID\_3262\_3b.raw |  |  | 2.6229E5 |  |  |  |  |  | 1 | 0 | 0 | 1 | 0 | 0 | 0 | 0 | 0 | 491 | 501 |  |  | PEAKS DB |
| total 14 peptides |  |  |  |  |  |  |  |  |  |  |  |  |  |  |  |  |  |  |  |  |  |  |  |  |  |  |  |  |  |  |  |  |  |
| --- | --- | --- | --- | --- | --- | --- | --- | --- | --- | --- | --- | --- | --- | --- | --- | --- | --- | --- | --- | --- | --- | --- | --- | --- | --- | --- | --- | --- | --- | --- | --- | --- | --- |

ACF70483.1

back to list

  

| Protein Coverage
| Supporting Peptides
|

Protein Coverage:

Supporting Peptides:

| Peptide | Uniq | -10lgP | Mass | Length | ppm | m/z | z | RT | Fraction | Scan | Source File | Area F6 | Area F2 | Area F3 | Area F4 | Area F5 | Area F7 | Area F8 | Area F1 | #Feature | #Feature F6 | #Feature F2 | #Feature F3 | #Feature F4 | #Feature F5 | #Feature F7 | #Feature F8 | #Feature F1 | Start | End | PTM | AScore | Found By |
| --- | --- | --- | --- | --- | --- | --- | --- | --- | --- | --- | --- | --- | --- | --- | --- | --- | --- | --- | --- | --- | --- | --- | --- | --- | --- | --- | --- | --- | --- | --- | --- | --- | --- |
| K.HDDIFAYEK.R | N | 84.58 | 1136.5138 | 9 | -0.3 | 569.2640 | 2 | 32.40 | 3 | F3:17001 | 13012022\_RID\_3262\_3b.raw |  | 1.8575E6 | 2.7357E8 | 7.8264E6 | 4.7702E6 |  |  | 2.6433E6 | 10 | 0 | 1 | 4 | 2 | 2 | 0 | 0 | 1 | 241 | 249 |  |  | PEAKS DB |
| K.HIVIVGAGMSGLSAAY.V | Y | 74.04 | 1544.8021 | 16 | 0.6 | 773.4088 | 2 | 50.43 | 3 | F3:32609 | 13012022\_RID\_3262\_3b.raw |  |  | 6.0155E6 |  |  |  |  |  | 1 | 0 | 0 | 1 | 0 | 0 | 0 | 0 | 0 | 53 | 68 |  |  | PEAKS DB |
| K.YDTYSTKEYLIK.E | N | 68.24 | 1522.7555 | 12 | 0.3 | 762.3853 | 2 | 34.64 | 3 | F3:18814 | 13012022\_RID\_3262\_3b.raw |  |  | 1.2832E6 |  |  |  |  |  | 2 | 0 | 0 | 2 | 0 | 0 | 0 | 0 | 0 | 198 | 209 |  |  | PEAKS DB |
| K.HIVIVGAGMSGLSAA.Y | Y | 64.16 | 1381.7388 | 15 | 0.6 | 691.8771 | 2 | 46.04 | 3 | F3:29047 | 13012022\_RID\_3262\_3b.raw |  |  | 2.8561E7 |  |  |  |  |  | 1 | 0 | 0 | 1 | 0 | 0 | 0 | 0 | 0 | 53 | 67 |  |  | PEAKS DB |
| K.HDDIFAYEKR.F | N | 61.69 | 1292.6149 | 10 | 0.4 | 431.8791 | 3 | 28.28 | 3 | F3:13656 | 13012022\_RID\_3262\_3b.raw |  |  | 2.952E6 |  |  |  |  |  | 3 | 0 | 0 | 3 | 0 | 0 | 0 | 0 | 0 | 241 | 250 |  |  | PEAKS DB |
| K.STTDLPSR.F | N | 58.41 | 875.4348 | 8 | 0.3 | 438.7248 | 2 | 21.23 | 4 | F4:8235 | 13012022\_RID\_3262\_4b.raw |  | 3.3314E6 | 1.6248E8 | 1.5408E7 | 9.3405E6 |  |  |  | 4 | 0 | 1 | 1 | 1 | 1 | 0 | 0 | 0 | 364 | 371 |  |  | PEAKS DB |
| K.HIVIVGAGM(+15.99)SGLSAA.Y | Y | 58.27 | 1397.7336 | 15 | -0.5 | 699.8738 | 2 | 39.95 | 3 | F3:23668 | 13012022\_RID\_3262\_3b.raw |  |  | 1.7074E7 |  |  |  |  |  | 1 | 0 | 0 | 1 | 0 | 0 | 0 | 0 | 0 | 53 | 67 | Oxidation (M) | M9:Oxidation (M):1000.00 | PEAKS DB |
| K.RFDEIVGGMD.Q | N | 53.30 | 1137.5125 | 10 | -0.3 | 569.7633 | 2 | 42.41 | 3 | F3:25858 | 13012022\_RID\_3262\_3b.raw |  |  | 1.9491E6 |  |  |  |  |  | 1 | 0 | 0 | 1 | 0 | 0 | 0 | 0 | 0 | 250 | 259 |  |  | PEAKS DB |
| K.HIVIVGAGM(+15.99)SGL.S | Y | 51.90 | 1168.6274 | 12 | 0.2 | 585.3211 | 2 | 40.50 | 3 | F3:24231 | 13012022\_RID\_3262\_3b.raw |  |  | 3.2704E6 |  |  |  |  |  | 2 | 0 | 0 | 2 | 0 | 0 | 0 | 0 | 0 | 53 | 64 | Oxidation (M) | M9:Oxidation (M):1000.00 | PEAKS DB |
| K.HIVIVGAGM(+15.99)SGLSAAY.V | Y | 50.61 | 1560.7970 | 16 | 0.1 | 781.4059 | 2 | 44.51 | 3 | F3:27726 | 13012022\_RID\_3262\_3b.raw |  |  | 3.0914E6 |  |  |  |  |  | 1 | 0 | 0 | 1 | 0 | 0 | 0 | 0 | 0 | 53 | 68 | Oxidation (M) | M9:Oxidation (M):1000.00 | PEAKS DB |
| K.EGNLSPGAVDMIGDLLNED.S | N | 47.73 | 1957.8938 | 19 | -0.4 | 979.9537 | 2 | 76.70 | 1 | F1:26892 | 04012022\_RID\_3262\_6b.raw | 3.5828E5 |  |  |  |  |  |  |  | 2 | 2 | 0 | 0 | 0 | 0 | 0 | 0 | 0 | 210 | 228 |  |  | PEAKS DB |
| K.RFDEIVGGMDQLPTSM.Y | N | 46.82 | 1794.8280 | 16 | 0.3 | 898.4215 | 2 | 55.47 | 3 | F3:36763 | 13012022\_RID\_3262\_3b.raw |  |  | 2.0995E6 |  |  |  |  |  | 1 | 0 | 0 | 1 | 0 | 0 | 0 | 0 | 0 | 250 | 265 |  |  | PEAKS DB |
| K.SGLTAARDVNR.A | N | 46.67 | 1158.6105 | 11 | 0.4 | 387.2109 | 3 | 20.31 | 3 | F3:7146 | 13012022\_RID\_3262\_3b.raw |  |  | 2.6229E5 |  |  |  |  |  | 1 | 0 | 0 | 1 | 0 | 0 | 0 | 0 | 0 | 490 | 500 |  |  | PEAKS DB |
| K.RFDEIVGGMDQL.P | N | 45.79 | 1378.6550 | 12 | 1.0 | 690.3354 | 2 | 51.18 | 3 | F3:33282 | 13012022\_RID\_3262\_3b.raw |  |  | 3.4038E6 |  |  |  |  |  | 1 | 0 | 0 | 1 | 0 | 0 | 0 | 0 | 0 | 250 | 261 |  |  | PEAKS DB |
| total 14 peptides |  |  |  |  |  |  |  |  |  |  |  |  |  |  |  |  |  |  |  |  |  |  |  |  |  |  |  |  |  |  |  |  |  |
| --- | --- | --- | --- | --- | --- | --- | --- | --- | --- | --- | --- | --- | --- | --- | --- | --- | --- | --- | --- | --- | --- | --- | --- | --- | --- | --- | --- | --- | --- | --- | --- | --- | --- |

XP\_032067281.1

back to list

  

| Protein Coverage
| Supporting Peptides
|

Protein Coverage:

Supporting Peptides:

| Peptide | Uniq | -10lgP | Mass | Length | ppm | m/z | z | RT | Fraction | Scan | Source File | Area F6 | Area F2 | Area F3 | Area F4 | Area F5 | Area F7 | Area F8 | Area F1 | #Feature | #Feature F6 | #Feature F2 | #Feature F3 | #Feature F4 | #Feature F5 | #Feature F7 | #Feature F8 | #Feature F1 | Start | End | PTM | AScore | Found By |
| --- | --- | --- | --- | --- | --- | --- | --- | --- | --- | --- | --- | --- | --- | --- | --- | --- | --- | --- | --- | --- | --- | --- | --- | --- | --- | --- | --- | --- | --- | --- | --- | --- | --- |
| K.LALDIEIATYR.K | N | 78.30 | 1276.7026 | 11 | 0.6 | 639.3590 | 2 | 52.72 | 8 | F8:30857 | 31122021\_RID\_3262\_1b.raw |  | 1.2925E6 | 1.0345E7 | 2.8112E5 | 1.0579E6 |  |  | 1.4292E7 | 10 | 0 | 1 | 1 | 1 | 1 | 0 | 0 | 6 | 444 | 454 |  |  | PEAKS DB |
| K.AQYEDIAQK.S | N | 71.01 | 1064.5138 | 9 | 0.2 | 533.2643 | 2 | 22.69 | 8 | F8:7374 | 31122021\_RID\_3262\_1b.raw | 5.6213E6 | 4.6319E6 | 1.1417E8 | 3.7973E7 | 1.7343E7 | 3.8115E7 | 5.2156E7 | 1.9393E8 | 12 | 1 | 1 | 1 | 1 | 1 | 1 | 2 | 4 | 327 | 335 |  |  | PEAKS DB |
| K.FASFIDKVR.F | N | 65.83 | 1081.5920 | 9 | 1.0 | 541.8038 | 2 | 33.62 | 8 | F8:15859 | 31122021\_RID\_3262\_1b.raw |  |  | 2.2979E6 |  | 2.6797E5 |  |  | 2.3536E6 | 3 | 0 | 0 | 1 | 0 | 1 | 0 | 0 | 1 | 161 | 169 |  |  | PEAKS DB |
| K.DVDAAYMNKVELQAK.L | N | 63.82 | 1693.8345 | 15 | 1.5 | 847.9258 | 2 | 37.57 | 3 | F3:21522 | 13012022\_RID\_3262\_3b.raw |  |  | 1.7246E5 |  |  |  |  |  | 2 | 0 | 0 | 2 | 0 | 0 | 0 | 0 | 0 | 261 | 275 |  |  | PEAKS DB |
| R.LLKEYQELM(+15.99)NVK.L | N | 63.16 | 1522.8065 | 12 | 0.0 | 508.6095 | 3 | 34.77 | 3 | F3:19359 | 13012022\_RID\_3262\_3b.raw |  |  | 2.7626E6 |  |  |  |  |  | 2 | 0 | 0 | 2 | 0 | 0 | 0 | 0 | 0 | 432 | 443 | Oxidation (M) | M9:Oxidation (M):1000.00 | PEAKS DB |
| K.EYQELMNVK.L | N | 60.69 | 1152.5485 | 9 | 0.3 | 577.2817 | 2 | 33.98 | 8 | F8:15978 | 31122021\_RID\_3262\_1b.raw |  |  | 1.0316E6 |  |  |  |  | 1.0554E6 | 2 | 0 | 0 | 1 | 0 | 0 | 0 | 0 | 1 | 435 | 443 |  |  | PEAKS DB |
| K.DVDAAYM(+15.99)NKVELQAK.L | N | 57.31 | 1709.8293 | 15 | -0.8 | 570.9500 | 3 | 31.84 | 3 | F3:16567 | 13012022\_RID\_3262\_3b.raw |  |  | 0 |  |  |  |  |  | 0 | 0 | 0 | 0 | 0 | 0 | 0 | 0 | 0 | 261 | 275 | Oxidation (M) | M7:Oxidation (M):1000.00 | PEAKS DB |
| K.DVDAAYMNK.V | N | 55.69 | 1025.4487 | 9 | 0.8 | 513.7321 | 2 | 27.01 | 8 | F8:10605 | 31122021\_RID\_3262\_1b.raw |  | 8.0719E4 | 0 |  |  | 3.8382E5 |  | 6.5187E5 | 3 | 0 | 1 | 0 | 0 | 0 | 1 | 0 | 1 | 261 | 269 |  |  | PEAKS DB |
| K.FASFIDK.V | N | 53.98 | 826.4225 | 7 | 0.0 | 414.2185 | 2 | 37.13 | 2 | F2:13215 | 13012022\_RID\_3262\_2b.raw |  | 3.8919E6 | 3.3996E7 | 9.4622E6 | 1.087E7 | 2.372E7 | 4.1967E6 | 3.8411E7 | 9 | 0 | 1 | 2 | 1 | 1 | 2 | 1 | 1 | 161 | 167 |  |  | PEAKS DB |
| R.LLKEYQELMNVK.L | N | 53.41 | 1506.8115 | 12 | 0.2 | 503.2779 | 3 | 39.69 | 8 | F8:20552 | 31122021\_RID\_3262\_1b.raw |  |  |  |  |  |  |  | 8.6831E5 | 2 | 0 | 0 | 0 | 0 | 0 | 0 | 0 | 2 | 432 | 443 |  |  | PEAKS DB |
| K.EYQELM(+15.99)NVK.L | N | 52.63 | 1168.5435 | 9 | 0.3 | 585.2792 | 2 | 28.16 | 3 | F3:13441 | 13012022\_RID\_3262\_3b.raw |  |  | 1.8851E6 |  |  |  |  | 1.7223E5 | 2 | 0 | 0 | 1 | 0 | 0 | 0 | 0 | 1 | 435 | 443 | Oxidation (M) | M6:Oxidation (M):1000.00 | PEAKS DB |
| K.AQYEDIAQKSR.A | N | 51.10 | 1307.6470 | 11 | -7.8 | 654.8257 | 2 | 27.24 | 8 | F8:10853 | 31122021\_RID\_3262\_1b.raw |  |  |  |  |  |  |  | 2.8701E4 | 1 | 0 | 0 | 0 | 0 | 0 | 0 | 0 | 1 | 327 | 337 |  |  | PEAKS DB |
| K.YEELQVSVGR.H | Y | 49.14 | 1178.5931 | 10 | 0.4 | 590.3041 | 2 | 37.58 | 7 | F7:18127 | 17012022\_RID\_3262\_8b.raw |  |  |  |  |  |  | 1.9473E7 |  | 1 | 0 | 0 | 0 | 0 | 0 | 0 | 1 | 0 | 348 | 357 |  |  | PEAKS DB |
| L.ALDIEIATYR.K | N | 46.18 | 1163.6187 | 10 | -0.4 | 582.8163 | 2 | 45.58 | 8 | F8:25162 | 31122021\_RID\_3262\_1b.raw |  |  |  |  |  |  |  | 2.943E5 | 1 | 0 | 0 | 0 | 0 | 0 | 0 | 0 | 1 | 445 | 454 |  |  | PEAKS DB |
| A.LDIEIATYR.K | N | 45.35 | 1092.5815 | 9 | 0.0 | 547.2980 | 2 | 44.78 | 8 | F8:24515 | 31122021\_RID\_3262\_1b.raw |  |  |  |  |  |  |  | 3.5731E5 | 1 | 0 | 0 | 0 | 0 | 0 | 0 | 0 | 1 | 446 | 454 |  |  | PEAKS DB |
| total 15 peptides |  |  |  |  |  |  |  |  |  |  |  |  |  |  |  |  |  |  |  |  |  |  |  |  |  |  |  |  |  |  |  |  |  |
| --- | --- | --- | --- | --- | --- | --- | --- | --- | --- | --- | --- | --- | --- | --- | --- | --- | --- | --- | --- | --- | --- | --- | --- | --- | --- | --- | --- | --- | --- | --- | --- | --- | --- |

XP\_013915209.1

back to list

  

| Protein Coverage
| Supporting Peptides
|

Protein Coverage:

Supporting Peptides:

| Peptide | Uniq | -10lgP | Mass | Length | ppm | m/z | z | RT | Fraction | Scan | Source File | Area F6 | Area F2 | Area F3 | Area F4 | Area F5 | Area F7 | Area F8 | Area F1 | #Feature | #Feature F6 | #Feature F2 | #Feature F3 | #Feature F4 | #Feature F5 | #Feature F7 | #Feature F8 | #Feature F1 | Start | End | PTM | AScore | Found By |
| --- | --- | --- | --- | --- | --- | --- | --- | --- | --- | --- | --- | --- | --- | --- | --- | --- | --- | --- | --- | --- | --- | --- | --- | --- | --- | --- | --- | --- | --- | --- | --- | --- | --- |
| K.LALDIEIATYR.K | N | 78.30 | 1276.7026 | 11 | 0.6 | 639.3590 | 2 | 52.72 | 8 | F8:30857 | 31122021\_RID\_3262\_1b.raw |  | 1.2925E6 | 1.0345E7 | 2.8112E5 | 1.0579E6 |  |  | 1.4292E7 | 10 | 0 | 1 | 1 | 1 | 1 | 0 | 0 | 6 | 444 | 454 |  |  | PEAKS DB |
| K.AQYEDIAQK.S | N | 71.01 | 1064.5138 | 9 | 0.2 | 533.2643 | 2 | 22.69 | 8 | F8:7374 | 31122021\_RID\_3262\_1b.raw | 5.6213E6 | 4.6319E6 | 1.1417E8 | 3.7973E7 | 1.7343E7 | 3.8115E7 | 5.2156E7 | 1.9393E8 | 12 | 1 | 1 | 1 | 1 | 1 | 1 | 2 | 4 | 327 | 335 |  |  | PEAKS DB |
| K.FASFIDKVR.F | N | 65.83 | 1081.5920 | 9 | 1.0 | 541.8038 | 2 | 33.62 | 8 | F8:15859 | 31122021\_RID\_3262\_1b.raw |  |  | 2.2979E6 |  | 2.6797E5 |  |  | 2.3536E6 | 3 | 0 | 0 | 1 | 0 | 1 | 0 | 0 | 1 | 161 | 169 |  |  | PEAKS DB |
| K.DVDAAYMNKVELQAK.L | N | 63.82 | 1693.8345 | 15 | 1.5 | 847.9258 | 2 | 37.57 | 3 | F3:21522 | 13012022\_RID\_3262\_3b.raw |  |  | 1.7246E5 |  |  |  |  |  | 2 | 0 | 0 | 2 | 0 | 0 | 0 | 0 | 0 | 261 | 275 |  |  | PEAKS DB |
| R.LLKEYQELM(+15.99)NVK.L | N | 63.16 | 1522.8065 | 12 | 0.0 | 508.6095 | 3 | 34.77 | 3 | F3:19359 | 13012022\_RID\_3262\_3b.raw |  |  | 2.7626E6 |  |  |  |  |  | 2 | 0 | 0 | 2 | 0 | 0 | 0 | 0 | 0 | 432 | 443 | Oxidation (M) | M9:Oxidation (M):1000.00 | PEAKS DB |
| K.EYQELMNVK.L | N | 60.69 | 1152.5485 | 9 | 0.3 | 577.2817 | 2 | 33.98 | 8 | F8:15978 | 31122021\_RID\_3262\_1b.raw |  |  | 1.0316E6 |  |  |  |  | 1.0554E6 | 2 | 0 | 0 | 1 | 0 | 0 | 0 | 0 | 1 | 435 | 443 |  |  | PEAKS DB |
| K.DVDAAYM(+15.99)NKVELQAK.L | N | 57.31 | 1709.8293 | 15 | -0.8 | 570.9500 | 3 | 31.84 | 3 | F3:16567 | 13012022\_RID\_3262\_3b.raw |  |  | 0 |  |  |  |  |  | 0 | 0 | 0 | 0 | 0 | 0 | 0 | 0 | 0 | 261 | 275 | Oxidation (M) | M7:Oxidation (M):1000.00 | PEAKS DB |
| K.DVDAAYMNK.V | N | 55.69 | 1025.4487 | 9 | 0.8 | 513.7321 | 2 | 27.01 | 8 | F8:10605 | 31122021\_RID\_3262\_1b.raw |  | 8.0719E4 | 0 |  |  | 3.8382E5 |  | 6.5187E5 | 3 | 0 | 1 | 0 | 0 | 0 | 1 | 0 | 1 | 261 | 269 |  |  | PEAKS DB |
| K.FASFIDK.V | N | 53.98 | 826.4225 | 7 | 0.0 | 414.2185 | 2 | 37.13 | 2 | F2:13215 | 13012022\_RID\_3262\_2b.raw |  | 3.8919E6 | 3.3996E7 | 9.4622E6 | 1.087E7 | 2.372E7 | 4.1967E6 | 3.8411E7 | 9 | 0 | 1 | 2 | 1 | 1 | 2 | 1 | 1 | 161 | 167 |  |  | PEAKS DB |
| R.LLKEYQELMNVK.L | N | 53.41 | 1506.8115 | 12 | 0.2 | 503.2779 | 3 | 39.69 | 8 | F8:20552 | 31122021\_RID\_3262\_1b.raw |  |  |  |  |  |  |  | 8.6831E5 | 2 | 0 | 0 | 0 | 0 | 0 | 0 | 0 | 2 | 432 | 443 |  |  | PEAKS DB |
| K.EYQELM(+15.99)NVK.L | N | 52.63 | 1168.5435 | 9 | 0.3 | 585.2792 | 2 | 28.16 | 3 | F3:13441 | 13012022\_RID\_3262\_3b.raw |  |  | 1.8851E6 |  |  |  |  | 1.7223E5 | 2 | 0 | 0 | 1 | 0 | 0 | 0 | 0 | 1 | 435 | 443 | Oxidation (M) | M6:Oxidation (M):1000.00 | PEAKS DB |
| K.AQYEDIAQKSR.A | N | 51.10 | 1307.6470 | 11 | -7.8 | 654.8257 | 2 | 27.24 | 8 | F8:10853 | 31122021\_RID\_3262\_1b.raw |  |  |  |  |  |  |  | 2.8701E4 | 1 | 0 | 0 | 0 | 0 | 0 | 0 | 0 | 1 | 327 | 337 |  |  | PEAKS DB |
| K.YEELQVSVGR.H | Y | 49.14 | 1178.5931 | 10 | 0.4 | 590.3041 | 2 | 37.58 | 7 | F7:18127 | 17012022\_RID\_3262\_8b.raw |  |  |  |  |  |  | 1.9473E7 |  | 1 | 0 | 0 | 0 | 0 | 0 | 0 | 1 | 0 | 348 | 357 |  |  | PEAKS DB |
| L.ALDIEIATYR.K | N | 46.18 | 1163.6187 | 10 | -0.4 | 582.8163 | 2 | 45.58 | 8 | F8:25162 | 31122021\_RID\_3262\_1b.raw |  |  |  |  |  |  |  | 2.943E5 | 1 | 0 | 0 | 0 | 0 | 0 | 0 | 0 | 1 | 445 | 454 |  |  | PEAKS DB |
| A.LDIEIATYR.K | N | 45.35 | 1092.5815 | 9 | 0.0 | 547.2980 | 2 | 44.78 | 8 | F8:24515 | 31122021\_RID\_3262\_1b.raw |  |  |  |  |  |  |  | 3.5731E5 | 1 | 0 | 0 | 0 | 0 | 0 | 0 | 0 | 1 | 446 | 454 |  |  | PEAKS DB |
| total 15 peptides |  |  |  |  |  |  |  |  |  |  |  |  |  |  |  |  |  |  |  |  |  |  |  |  |  |  |  |  |  |  |  |  |  |
| --- | --- | --- | --- | --- | --- | --- | --- | --- | --- | --- | --- | --- | --- | --- | --- | --- | --- | --- | --- | --- | --- | --- | --- | --- | --- | --- | --- | --- | --- | --- | --- | --- | --- |

Q4JHE1.1

back to list

  

| Protein Coverage
| Supporting Peptides
|

Protein Coverage:

Supporting Peptides:

| Peptide | Uniq | -10lgP | Mass | Length | ppm | m/z | z | RT | Fraction | Scan | Source File | Area F6 | Area F2 | Area F3 | Area F4 | Area F5 | Area F7 | Area F8 | Area F1 | #Feature | #Feature F6 | #Feature F2 | #Feature F3 | #Feature F4 | #Feature F5 | #Feature F7 | #Feature F8 | #Feature F1 | Start | End | PTM | AScore | Found By |
| --- | --- | --- | --- | --- | --- | --- | --- | --- | --- | --- | --- | --- | --- | --- | --- | --- | --- | --- | --- | --- | --- | --- | --- | --- | --- | --- | --- | --- | --- | --- | --- | --- | --- |
| A.DIVINDLSLIHQLPK.E | N | 75.61 | 1716.9774 | 15 | 0.0 | 859.4960 | 2 | 54.14 | 3 | F3:35798 | 13012022\_RID\_3262\_3b.raw |  |  | 2.3604E6 |  |  |  |  |  | 2 | 0 | 0 | 2 | 0 | 0 | 0 | 0 | 0 | 410 | 424 |  |  | PEAKS DB |
| K.YPVKPSEEGK.S | N | 73.79 | 1132.5764 | 10 | 0.2 | 567.2956 | 2 | 18.85 | 3 | F3:5905 | 13012022\_RID\_3262\_3b.raw | 1.3691E4 |  | 4.5878E8 | 1.2567E7 |  |  |  |  | 5 | 1 | 0 | 2 | 2 | 0 | 0 | 0 | 0 | 161 | 170 |  |  | PEAKS DB |
| K.YDTYSTKEYLIK.E | N | 68.24 | 1522.7555 | 12 | 0.3 | 762.3853 | 2 | 34.64 | 3 | F3:18814 | 13012022\_RID\_3262\_3b.raw |  |  | 1.2832E6 |  |  |  |  |  | 2 | 0 | 0 | 2 | 0 | 0 | 0 | 0 | 0 | 199 | 210 |  |  | PEAKS DB |
| R.NEKDGWYVNLGPMR.L | Y | 62.39 | 1677.7933 | 14 | 0.6 | 839.9044 | 2 | 43.84 | 3 | F3:27118 | 13012022\_RID\_3262\_3b.raw |  |  | 2.8959E7 |  |  |  |  |  | 2 | 0 | 0 | 2 | 0 | 0 | 0 | 0 | 0 | 96 | 109 |  |  | PEAKS DB |
| D.IVINDLSLIHQLPK.E | N | 61.67 | 1601.9504 | 14 | 0.6 | 534.9911 | 3 | 48.31 | 3 | F3:30799 | 13012022\_RID\_3262\_3b.raw |  |  | 4.9507E6 |  |  |  |  |  | 2 | 0 | 0 | 2 | 0 | 0 | 0 | 0 | 0 | 411 | 424 |  |  | PEAKS DB |
| K.STTDLPSR.F | N | 58.41 | 875.4348 | 8 | 0.3 | 438.7248 | 2 | 21.23 | 4 | F4:8235 | 13012022\_RID\_3262\_4b.raw |  | 3.3314E6 | 1.6248E8 | 1.5408E7 | 9.3405E6 |  |  |  | 4 | 0 | 1 | 1 | 1 | 1 | 0 | 0 | 0 | 365 | 372 |  |  | PEAKS DB |
| N.DLSLIHQLPK.E | N | 58.16 | 1162.6710 | 10 | 0.6 | 582.3431 | 2 | 41.31 | 3 | F3:24879 | 13012022\_RID\_3262\_3b.raw |  |  | 4.4132E6 |  |  |  |  |  | 1 | 0 | 0 | 1 | 0 | 0 | 0 | 0 | 0 | 415 | 424 |  |  | PEAKS DB |
| R.NEKDGWYVNLGPM(+15.99)R.L | Y | 57.50 | 1693.7882 | 14 | 0.3 | 847.9017 | 2 | 38.99 | 3 | F3:22907 | 13012022\_RID\_3262\_3b.raw |  |  | 2.3129E7 |  |  |  |  |  | 2 | 0 | 0 | 2 | 0 | 0 | 0 | 0 | 0 | 96 | 109 | Oxidation (M) | M13:Oxidation (M):1000.00 | PEAKS DB |
| H.FEPPLPPKK.A | N | 54.07 | 1051.6066 | 9 | 0.2 | 526.8107 | 2 | 28.91 | 3 | F3:14056 | 13012022\_RID\_3262\_3b.raw |  |  | 1.498E7 |  |  |  |  |  | 1 | 0 | 0 | 1 | 0 | 0 | 0 | 0 | 0 | 323 | 331 |  |  | PEAKS DB |
| H.FEPPLPPK.K | N | 50.89 | 923.5116 | 8 | 0.6 | 462.7633 | 2 | 36.44 | 3 | F3:20282 | 13012022\_RID\_3262\_3b.raw |  |  | 1.731E8 |  |  |  |  |  | 1 | 0 | 0 | 1 | 0 | 0 | 0 | 0 | 0 | 323 | 330 |  |  | PEAKS DB |
| R.NEKDGWYVNLGPM(+15.99).R | Y | 50.39 | 1537.6871 | 13 | 0.5 | 769.8512 | 2 | 46.66 | 3 | F3:29582 | 13012022\_RID\_3262\_3b.raw |  |  | 4.4722E6 |  |  |  |  |  | 1 | 0 | 0 | 1 | 0 | 0 | 0 | 0 | 0 | 96 | 108 | Oxidation (M) | M13:Oxidation (M):1000.00 | PEAKS DB |
| D.LSLIHQLPK.E | N | 50.17 | 1047.6440 | 9 | -0.1 | 524.8292 | 2 | 34.27 | 3 | F3:18619 | 13012022\_RID\_3262\_3b.raw |  |  | 9.3032E5 |  |  |  |  |  | 1 | 0 | 0 | 1 | 0 | 0 | 0 | 0 | 0 | 416 | 424 |  |  | PEAKS DB |
| K.EGNLSPGAVDMIGDLLNED.S | N | 47.73 | 1957.8938 | 19 | -0.4 | 979.9537 | 2 | 76.70 | 1 | F1:26892 | 04012022\_RID\_3262\_6b.raw | 3.5828E5 |  |  |  |  |  |  |  | 2 | 2 | 0 | 0 | 0 | 0 | 0 | 0 | 0 | 211 | 229 |  |  | PEAKS DB |
| total 13 peptides |  |  |  |  |  |  |  |  |  |  |  |  |  |  |  |  |  |  |  |  |  |  |  |  |  |  |  |  |  |  |  |  |  |
| --- | --- | --- | --- | --- | --- | --- | --- | --- | --- | --- | --- | --- | --- | --- | --- | --- | --- | --- | --- | --- | --- | --- | --- | --- | --- | --- | --- | --- | --- | --- | --- | --- | --- |

AAY89682.1

back to list

  

| Protein Coverage
| Supporting Peptides
|

Protein Coverage:

Supporting Peptides:

| Peptide | Uniq | -10lgP | Mass | Length | ppm | m/z | z | RT | Fraction | Scan | Source File | Area F6 | Area F2 | Area F3 | Area F4 | Area F5 | Area F7 | Area F8 | Area F1 | #Feature | #Feature F6 | #Feature F2 | #Feature F3 | #Feature F4 | #Feature F5 | #Feature F7 | #Feature F8 | #Feature F1 | Start | End | PTM | AScore | Found By |
| --- | --- | --- | --- | --- | --- | --- | --- | --- | --- | --- | --- | --- | --- | --- | --- | --- | --- | --- | --- | --- | --- | --- | --- | --- | --- | --- | --- | --- | --- | --- | --- | --- | --- |
| A.DIVINDLSLIHQLPK.E | N | 75.61 | 1716.9774 | 15 | 0.0 | 859.4960 | 2 | 54.14 | 3 | F3:35798 | 13012022\_RID\_3262\_3b.raw |  |  | 2.3604E6 |  |  |  |  |  | 2 | 0 | 0 | 2 | 0 | 0 | 0 | 0 | 0 | 410 | 424 |  |  | PEAKS DB |
| K.YPVKPSEEGK.S | N | 73.79 | 1132.5764 | 10 | 0.2 | 567.2956 | 2 | 18.85 | 3 | F3:5905 | 13012022\_RID\_3262\_3b.raw | 1.3691E4 |  | 4.5878E8 | 1.2567E7 |  |  |  |  | 5 | 1 | 0 | 2 | 2 | 0 | 0 | 0 | 0 | 161 | 170 |  |  | PEAKS DB |
| K.YDTYSTKEYLIK.E | N | 68.24 | 1522.7555 | 12 | 0.3 | 762.3853 | 2 | 34.64 | 3 | F3:18814 | 13012022\_RID\_3262\_3b.raw |  |  | 1.2832E6 |  |  |  |  |  | 2 | 0 | 0 | 2 | 0 | 0 | 0 | 0 | 0 | 199 | 210 |  |  | PEAKS DB |
| R.NEKDGWYVNLGPMR.L | Y | 62.39 | 1677.7933 | 14 | 0.6 | 839.9044 | 2 | 43.84 | 3 | F3:27118 | 13012022\_RID\_3262\_3b.raw |  |  | 2.8959E7 |  |  |  |  |  | 2 | 0 | 0 | 2 | 0 | 0 | 0 | 0 | 0 | 96 | 109 |  |  | PEAKS DB |
| D.IVINDLSLIHQLPK.E | N | 61.67 | 1601.9504 | 14 | 0.6 | 534.9911 | 3 | 48.31 | 3 | F3:30799 | 13012022\_RID\_3262\_3b.raw |  |  | 4.9507E6 |  |  |  |  |  | 2 | 0 | 0 | 2 | 0 | 0 | 0 | 0 | 0 | 411 | 424 |  |  | PEAKS DB |
| K.STTDLPSR.F | N | 58.41 | 875.4348 | 8 | 0.3 | 438.7248 | 2 | 21.23 | 4 | F4:8235 | 13012022\_RID\_3262\_4b.raw |  | 3.3314E6 | 1.6248E8 | 1.5408E7 | 9.3405E6 |  |  |  | 4 | 0 | 1 | 1 | 1 | 1 | 0 | 0 | 0 | 365 | 372 |  |  | PEAKS DB |
| N.DLSLIHQLPK.E | N | 58.16 | 1162.6710 | 10 | 0.6 | 582.3431 | 2 | 41.31 | 3 | F3:24879 | 13012022\_RID\_3262\_3b.raw |  |  | 4.4132E6 |  |  |  |  |  | 1 | 0 | 0 | 1 | 0 | 0 | 0 | 0 | 0 | 415 | 424 |  |  | PEAKS DB |
| R.NEKDGWYVNLGPM(+15.99)R.L | Y | 57.50 | 1693.7882 | 14 | 0.3 | 847.9017 | 2 | 38.99 | 3 | F3:22907 | 13012022\_RID\_3262\_3b.raw |  |  | 2.3129E7 |  |  |  |  |  | 2 | 0 | 0 | 2 | 0 | 0 | 0 | 0 | 0 | 96 | 109 | Oxidation (M) | M13:Oxidation (M):1000.00 | PEAKS DB |
| H.FEPPLPPKK.A | N | 54.07 | 1051.6066 | 9 | 0.2 | 526.8107 | 2 | 28.91 | 3 | F3:14056 | 13012022\_RID\_3262\_3b.raw |  |  | 1.498E7 |  |  |  |  |  | 1 | 0 | 0 | 1 | 0 | 0 | 0 | 0 | 0 | 323 | 331 |  |  | PEAKS DB |
| H.FEPPLPPK.K | N | 50.89 | 923.5116 | 8 | 0.6 | 462.7633 | 2 | 36.44 | 3 | F3:20282 | 13012022\_RID\_3262\_3b.raw |  |  | 1.731E8 |  |  |  |  |  | 1 | 0 | 0 | 1 | 0 | 0 | 0 | 0 | 0 | 323 | 330 |  |  | PEAKS DB |
| R.NEKDGWYVNLGPM(+15.99).R | Y | 50.39 | 1537.6871 | 13 | 0.5 | 769.8512 | 2 | 46.66 | 3 | F3:29582 | 13012022\_RID\_3262\_3b.raw |  |  | 4.4722E6 |  |  |  |  |  | 1 | 0 | 0 | 1 | 0 | 0 | 0 | 0 | 0 | 96 | 108 | Oxidation (M) | M13:Oxidation (M):1000.00 | PEAKS DB |
| D.LSLIHQLPK.E | N | 50.17 | 1047.6440 | 9 | -0.1 | 524.8292 | 2 | 34.27 | 3 | F3:18619 | 13012022\_RID\_3262\_3b.raw |  |  | 9.3032E5 |  |  |  |  |  | 1 | 0 | 0 | 1 | 0 | 0 | 0 | 0 | 0 | 416 | 424 |  |  | PEAKS DB |
| K.EGNLSPGAVDMIGDLLNED.S | N | 47.73 | 1957.8938 | 19 | -0.4 | 979.9537 | 2 | 76.70 | 1 | F1:26892 | 04012022\_RID\_3262\_6b.raw | 3.5828E5 |  |  |  |  |  |  |  | 2 | 2 | 0 | 0 | 0 | 0 | 0 | 0 | 0 | 211 | 229 |  |  | PEAKS DB |
| total 13 peptides |  |  |  |  |  |  |  |  |  |  |  |  |  |  |  |  |  |  |  |  |  |  |  |  |  |  |  |  |  |  |  |  |  |
| --- | --- | --- | --- | --- | --- | --- | --- | --- | --- | --- | --- | --- | --- | --- | --- | --- | --- | --- | --- | --- | --- | --- | --- | --- | --- | --- | --- | --- | --- | --- | --- | --- | --- |

XP\_015668350.1

back to list

  

| Protein Coverage
| Supporting Peptides
|

Protein Coverage:

Supporting Peptides:

| Peptide | Uniq | -10lgP | Mass | Length | ppm | m/z | z | RT | Fraction | Scan | Source File | Area F6 | Area F2 | Area F3 | Area F4 | Area F5 | Area F7 | Area F8 | Area F1 | #Feature | #Feature F6 | #Feature F2 | #Feature F3 | #Feature F4 | #Feature F5 | #Feature F7 | #Feature F8 | #Feature F1 | Start | End | PTM | AScore | Found By |
| --- | --- | --- | --- | --- | --- | --- | --- | --- | --- | --- | --- | --- | --- | --- | --- | --- | --- | --- | --- | --- | --- | --- | --- | --- | --- | --- | --- | --- | --- | --- | --- | --- | --- |
| R.QVPVVQAYAFGK.Y | N | 86.85 | 1305.7081 | 12 | 0.9 | 653.8619 | 2 | 57.46 | 2 | F2:25189 | 13012022\_RID\_3262\_2b.raw |  | 4.6095E6 | 3.2149E6 | 2.7574E6 | 1.0716E6 |  |  |  | 7 | 0 | 2 | 2 | 1 | 2 | 0 | 0 | 0 | 288 | 299 |  |  | PEAKS DB |
| K.ASGNPILLNK.D | N | 81.59 | 1025.5869 | 10 | -0.1 | 513.8007 | 2 | 31.96 | 2 | F2:10230 | 13012022\_RID\_3262\_2b.raw |  | 8.476E6 | 6.1171E6 | 7.1882E6 | 1.6984E6 |  |  | 5.894E5 | 5 | 0 | 1 | 1 | 1 | 1 | 0 | 0 | 1 | 317 | 326 |  |  | PEAKS DB |
| K.QAFEHSVHR.H | N | 67.95 | 1109.5366 | 9 | 0.5 | 555.7759 | 2 | 15.68 | 3 | F3:3797 | 13012022\_RID\_3262\_3b.raw |  |  | 5.4735E4 |  |  |  |  |  | 1 | 0 | 0 | 1 | 0 | 0 | 0 | 0 | 0 | 448 | 456 |  |  | PEAKS DB |
| R.HGQGM(+15.99)GELLQVSGIK.V | N | 66.29 | 1568.7981 | 15 | 0.3 | 523.9401 | 3 | 34.38 | 3 | F3:18618 | 13012022\_RID\_3262\_3b.raw |  | 9.5404E5 | 1.0877E6 |  |  |  |  |  | 2 | 0 | 1 | 1 | 0 | 0 | 0 | 0 | 0 | 457 | 471 | Oxidation (M) | M5:Oxidation (M):1000.00 | PEAKS DB |
| K.VYPAVEGR.V | Y | 64.81 | 889.4657 | 8 | 0.4 | 445.7403 | 2 | 25.18 | 5 | F5:9121 | 13012022\_RID\_3262\_5b.raw |  | 1.4421E7 |  | 6.0077E6 | 3.384E6 |  |  |  | 3 | 0 | 1 | 0 | 1 | 1 | 0 | 0 | 0 | 553 | 560 |  |  | PEAKS DB |
| K.VGIIGYTTK.E | N | 55.69 | 950.5436 | 9 | -0.2 | 476.2790 | 2 | 35.28 | 3 | F3:19379 | 13012022\_RID\_3262\_3b.raw |  | 8.9517E6 | 6.0623E6 | 4.7337E6 | 2.7356E6 |  |  | 4.6716E5 | 5 | 0 | 1 | 1 | 1 | 1 | 0 | 0 | 1 | 185 | 193 |  |  | PEAKS DB |
| K.LTTLGVNK.I | N | 53.46 | 844.5018 | 8 | 0.4 | 423.2584 | 2 | 27.00 | 4 | F4:12784 | 13012022\_RID\_3262\_4b.raw |  | 7.1171E6 | 5.2677E6 | 5.2452E6 |  |  |  |  | 3 | 0 | 1 | 1 | 1 | 0 | 0 | 0 | 0 | 221 | 228 |  |  | PEAKS DB |
| K.VVYDLSR.K | N | 52.94 | 850.4548 | 7 | 0.5 | 426.2349 | 2 | 30.66 | 2 | F2:9588 | 13012022\_RID\_3262\_2b.raw |  | 4.2852E6 | 1.5807E6 | 1.8252E6 | 1.6963E6 |  |  |  | 4 | 0 | 1 | 1 | 1 | 1 | 0 | 0 | 0 | 472 | 478 |  |  | PEAKS DB |
| K.IINVGSEK.V | N | 47.29 | 858.4811 | 8 | -0.3 | 430.2477 | 2 | 23.68 | 3 | F3:9771 | 13012022\_RID\_3262\_3b.raw |  |  | 2.9001E6 |  |  |  |  |  | 1 | 0 | 0 | 1 | 0 | 0 | 0 | 0 | 0 | 177 | 184 |  |  | PEAKS DB |
| total 9 peptides |  |  |  |  |  |  |  |  |  |  |  |  |  |  |  |  |  |  |  |  |  |  |  |  |  |  |  |  |  |  |  |  |  |
| --- | --- | --- | --- | --- | --- | --- | --- | --- | --- | --- | --- | --- | --- | --- | --- | --- | --- | --- | --- | --- | --- | --- | --- | --- | --- | --- | --- | --- | --- | --- | --- | --- | --- |

BAP39925.1

back to list

  

| Protein Coverage
| Supporting Peptides
|

Protein Coverage:

Supporting Peptides:

| Peptide | Uniq | -10lgP | Mass | Length | ppm | m/z | z | RT | Fraction | Scan | Source File | Area F6 | Area F2 | Area F3 | Area F4 | Area F5 | Area F7 | Area F8 | Area F1 | #Feature | #Feature F6 | #Feature F2 | #Feature F3 | #Feature F4 | #Feature F5 | #Feature F7 | #Feature F8 | #Feature F1 | Start | End | PTM | AScore | Found By |
| --- | --- | --- | --- | --- | --- | --- | --- | --- | --- | --- | --- | --- | --- | --- | --- | --- | --- | --- | --- | --- | --- | --- | --- | --- | --- | --- | --- | --- | --- | --- | --- | --- | --- |
| R.QVPVVQAYAFGK.Y | N | 86.85 | 1305.7081 | 12 | 0.9 | 653.8619 | 2 | 57.46 | 2 | F2:25189 | 13012022\_RID\_3262\_2b.raw |  | 4.6095E6 | 3.2149E6 | 2.7574E6 | 1.0716E6 |  |  |  | 7 | 0 | 2 | 2 | 1 | 2 | 0 | 0 | 0 | 288 | 299 |  |  | PEAKS DB |
| K.ASGNPILLNK.D | N | 81.59 | 1025.5869 | 10 | -0.1 | 513.8007 | 2 | 31.96 | 2 | F2:10230 | 13012022\_RID\_3262\_2b.raw |  | 8.476E6 | 6.1171E6 | 7.1882E6 | 1.6984E6 |  |  | 5.894E5 | 5 | 0 | 1 | 1 | 1 | 1 | 0 | 0 | 1 | 317 | 326 |  |  | PEAKS DB |
| K.QAFEHSVHR.H | N | 67.95 | 1109.5366 | 9 | 0.5 | 555.7759 | 2 | 15.68 | 3 | F3:3797 | 13012022\_RID\_3262\_3b.raw |  |  | 5.4735E4 |  |  |  |  |  | 1 | 0 | 0 | 1 | 0 | 0 | 0 | 0 | 0 | 448 | 456 |  |  | PEAKS DB |
| R.HGQGM(+15.99)GELLQVSGIK.V | N | 66.29 | 1568.7981 | 15 | 0.3 | 523.9401 | 3 | 34.38 | 3 | F3:18618 | 13012022\_RID\_3262\_3b.raw |  | 9.5404E5 | 1.0877E6 |  |  |  |  |  | 2 | 0 | 1 | 1 | 0 | 0 | 0 | 0 | 0 | 457 | 471 | Oxidation (M) | M5:Oxidation (M):1000.00 | PEAKS DB |
| K.VYPAVEGR.V | Y | 64.81 | 889.4657 | 8 | 0.4 | 445.7403 | 2 | 25.18 | 5 | F5:9121 | 13012022\_RID\_3262\_5b.raw |  | 1.4421E7 |  | 6.0077E6 | 3.384E6 |  |  |  | 3 | 0 | 1 | 0 | 1 | 1 | 0 | 0 | 0 | 553 | 560 |  |  | PEAKS DB |
| K.VGIIGYTTK.E | N | 55.69 | 950.5436 | 9 | -0.2 | 476.2790 | 2 | 35.28 | 3 | F3:19379 | 13012022\_RID\_3262\_3b.raw |  | 8.9517E6 | 6.0623E6 | 4.7337E6 | 2.7356E6 |  |  | 4.6716E5 | 5 | 0 | 1 | 1 | 1 | 1 | 0 | 0 | 1 | 185 | 193 |  |  | PEAKS DB |
| K.LTTLGVNK.I | N | 53.46 | 844.5018 | 8 | 0.4 | 423.2584 | 2 | 27.00 | 4 | F4:12784 | 13012022\_RID\_3262\_4b.raw |  | 7.1171E6 | 5.2677E6 | 5.2452E6 |  |  |  |  | 3 | 0 | 1 | 1 | 1 | 0 | 0 | 0 | 0 | 221 | 228 |  |  | PEAKS DB |
| K.VVYDLSR.K | N | 52.94 | 850.4548 | 7 | 0.5 | 426.2349 | 2 | 30.66 | 2 | F2:9588 | 13012022\_RID\_3262\_2b.raw |  | 4.2852E6 | 1.5807E6 | 1.8252E6 | 1.6963E6 |  |  |  | 4 | 0 | 1 | 1 | 1 | 1 | 0 | 0 | 0 | 472 | 478 |  |  | PEAKS DB |
| K.IINVGSEK.V | N | 47.29 | 858.4811 | 8 | -0.3 | 430.2477 | 2 | 23.68 | 3 | F3:9771 | 13012022\_RID\_3262\_3b.raw |  |  | 2.9001E6 |  |  |  |  |  | 1 | 0 | 0 | 1 | 0 | 0 | 0 | 0 | 0 | 177 | 184 |  |  | PEAKS DB |
| total 9 peptides |  |  |  |  |  |  |  |  |  |  |  |  |  |  |  |  |  |  |  |  |  |  |  |  |  |  |  |  |  |  |  |  |  |
| --- | --- | --- | --- | --- | --- | --- | --- | --- | --- | --- | --- | --- | --- | --- | --- | --- | --- | --- | --- | --- | --- | --- | --- | --- | --- | --- | --- | --- | --- | --- | --- | --- | --- |

ETE70217.1

back to list

  

| Protein Coverage
| Supporting Peptides
|

Protein Coverage:

Supporting Peptides:

| Peptide | Uniq | -10lgP | Mass | Length | ppm | m/z | z | RT | Fraction | Scan | Source File | Area F6 | Area F2 | Area F3 | Area F4 | Area F5 | Area F7 | Area F8 | Area F1 | #Feature | #Feature F6 | #Feature F2 | #Feature F3 | #Feature F4 | #Feature F5 | #Feature F7 | #Feature F8 | #Feature F1 | Start | End | PTM | AScore | Found By |
| --- | --- | --- | --- | --- | --- | --- | --- | --- | --- | --- | --- | --- | --- | --- | --- | --- | --- | --- | --- | --- | --- | --- | --- | --- | --- | --- | --- | --- | --- | --- | --- | --- | --- |
| K.TRLEQEIATYR.N | N | 80.90 | 1378.7205 | 11 | -0.1 | 690.3674 | 2 | 32.96 | 3 | F3:17517 | 13012022\_RID\_3262\_3b.raw |  |  | 5.9915E6 |  |  |  |  | 4.7086E6 | 2 | 0 | 0 | 1 | 0 | 0 | 0 | 0 | 1 | 875 | 885 |  |  | PEAKS DB |
| R.ALEEANTELEVK.I | N | 67.00 | 1344.6772 | 12 | 0.3 | 673.3461 | 2 | 33.85 | 3 | F3:18186 | 13012022\_RID\_3262\_3b.raw |  |  | 3.5713E6 |  |  |  | 5.7938E5 | 1.6599E6 | 3 | 0 | 0 | 1 | 0 | 0 | 0 | 1 | 1 | 169 | 180 |  |  | PEAKS DB |
| R.LEQEIATYR.N | N | 60.03 | 1121.5717 | 9 | 0.6 | 561.7935 | 2 | 28.71 | 8 | F8:12050 | 31122021\_RID\_3262\_1b.raw |  |  | 5.6577E6 | 8.0603E5 | 1.2091E6 | 0 |  | 4.173E6 | 4 | 0 | 0 | 1 | 1 | 1 | 0 | 0 | 1 | 877 | 885 |  |  | PEAKS DB |
| R.MSVEADINGLR.R | N | 57.12 | 1203.5918 | 11 | -0.5 | 602.8029 | 2 | 39.13 | 8 | F8:20105 | 31122021\_RID\_3262\_1b.raw |  |  |  |  |  |  |  | 1.6814E5 | 1 | 0 | 0 | 0 | 0 | 0 | 0 | 0 | 1 | 246 | 256 |  |  | PEAKS DB |
| R.LAADDFRLK.Y | N | 52.16 | 1047.5713 | 9 | -0.8 | 524.7925 | 2 | 32.61 | 8 | F8:14898 | 31122021\_RID\_3262\_1b.raw |  |  |  |  |  |  |  | 3.4201E5 | 1 | 0 | 0 | 0 | 0 | 0 | 0 | 0 | 1 | 664 | 672 |  |  | PEAKS DB |
| R.LENEIETYR.H | N | 51.96 | 1165.5615 | 9 | 0.7 | 583.7885 | 2 | 29.02 | 8 | F8:12359 | 31122021\_RID\_3262\_1b.raw |  |  |  |  |  |  |  | 9.3203E5 | 1 | 0 | 0 | 0 | 0 | 0 | 0 | 0 | 1 | 2570 | 2578 |  |  | PEAKS DB |
| R.LAADDFR.L | N | 50.38 | 806.3922 | 7 | -0.2 | 404.2033 | 2 | 30.00 | 2 | F2:9232 | 13012022\_RID\_3262\_2b.raw | 9.4985E6 | 1.3018E7 | 9.3698E7 | 1.9576E7 | 2.294E7 | 4.1151E7 | 1.2883E7 | 4.5921E7 | 9 | 2 | 1 | 1 | 1 | 1 | 1 | 1 | 1 | 664 | 670 |  |  | PEAKS DB |
| E.SLTEELAYLK.K | N | 49.60 | 1165.6230 | 10 | 0.8 | 583.8193 | 2 | 46.33 | 8 | F8:25883 | 31122021\_RID\_3262\_1b.raw |  |  |  |  |  |  |  | 7.3841E5 | 1 | 0 | 0 | 0 | 0 | 0 | 0 | 0 | 1 | 3370 | 3379 |  |  | PEAKS DB |
| K.IRLENEIETYR.H | N | 48.57 | 1434.7467 | 11 | 0.1 | 718.3807 | 2 | 32.97 | 8 | F8:15318 | 31122021\_RID\_3262\_1b.raw |  |  |  |  |  |  |  | 1.3482E5 | 1 | 0 | 0 | 0 | 0 | 0 | 0 | 0 | 1 | 2568 | 2578 |  |  | PEAKS DB |
| K.NHEEEMK.N | N | 47.06 | 915.3756 | 7 | 0.4 | 458.6953 | 2 | 11.28 | 7 | F7:2022 | 17012022\_RID\_3262\_8b.raw |  |  |  |  |  |  | 5.7262E4 |  | 1 | 0 | 0 | 0 | 0 | 0 | 0 | 1 | 0 | 2414 | 2420 |  |  | PEAKS DB |
| K.SEVTELR.R | Y | 46.16 | 832.4290 | 7 | 0.0 | 417.2218 | 2 | 23.62 | 3 | F3:9790 | 13012022\_RID\_3262\_3b.raw |  |  | 3.0328E6 |  |  |  |  |  | 1 | 0 | 0 | 1 | 0 | 0 | 0 | 0 | 0 | 2991 | 2997 |  |  | PEAKS DB |
| K.TKFETEQALR.M | N | 45.37 | 1221.6353 | 10 | 0.4 | 611.8251 | 2 | 24.52 | 3 | F3:10522 | 13012022\_RID\_3262\_3b.raw |  |  | 5.4837E5 |  |  |  |  |  | 1 | 0 | 0 | 1 | 0 | 0 | 0 | 0 | 0 | 236 | 245 |  |  | PEAKS DB |
| total 12 peptides |  |  |  |  |  |  |  |  |  |  |  |  |  |  |  |  |  |  |  |  |  |  |  |  |  |  |  |  |  |  |  |  |  |
| --- | --- | --- | --- | --- | --- | --- | --- | --- | --- | --- | --- | --- | --- | --- | --- | --- | --- | --- | --- | --- | --- | --- | --- | --- | --- | --- | --- | --- | --- | --- | --- | --- | --- |

XP\_025022318.1

back to list

  

| Protein Coverage
| Supporting Peptides
|

Protein Coverage:

Supporting Peptides:

| Peptide | Uniq | -10lgP | Mass | Length | ppm | m/z | z | RT | Fraction | Scan | Source File | Area F6 | Area F2 | Area F3 | Area F4 | Area F5 | Area F7 | Area F8 | Area F1 | #Feature | #Feature F6 | #Feature F2 | #Feature F3 | #Feature F4 | #Feature F5 | #Feature F7 | #Feature F8 | #Feature F1 | Start | End | PTM | AScore | Found By |
| --- | --- | --- | --- | --- | --- | --- | --- | --- | --- | --- | --- | --- | --- | --- | --- | --- | --- | --- | --- | --- | --- | --- | --- | --- | --- | --- | --- | --- | --- | --- | --- | --- | --- |
| R.NEGTATYAAAVLFR.I | Y | 79.96 | 1482.7467 | 14 | 0.1 | 742.3807 | 2 | 48.35 | 8 | F8:27296 | 31122021\_RID\_3262\_1b.raw |  |  | 5.3493E5 |  |  |  |  | 7.7228E5 | 2 | 0 | 0 | 1 | 0 | 0 | 0 | 0 | 1 | 640 | 653 |  |  | PEAKS DB |
| R.LNYGIPAIVK.L | Y | 71.64 | 1086.6437 | 10 | 0.9 | 544.3296 | 2 | 44.80 | 8 | F8:24695 | 31122021\_RID\_3262\_1b.raw |  |  | 9.8473E5 | 2.0478E5 |  |  |  | 1.6368E6 | 3 | 0 | 0 | 1 | 1 | 0 | 0 | 0 | 1 | 480 | 489 |  |  | PEAKS DB |
| K.SAIVHLINYQDDAELATR.A | Y | 70.33 | 2028.0276 | 18 | 0.5 | 677.0168 | 3 | 43.83 | 3 | F3:27023 | 13012022\_RID\_3262\_3b.raw |  |  | 3.2757E5 |  |  |  |  | 1.1198E6 | 3 | 0 | 0 | 1 | 0 | 0 | 0 | 0 | 2 | 127 | 144 |  |  | PEAKS DB |
| R.VSVELTNSLFK.H | Y | 67.96 | 1235.6761 | 11 | -0.3 | 618.8452 | 2 | 47.06 | 8 | F8:26272 | 31122021\_RID\_3262\_1b.raw |  |  |  |  |  |  |  | 1.62E6 | 1 | 0 | 0 | 0 | 0 | 0 | 0 | 0 | 1 | 666 | 676 |  |  | PEAKS DB |
| R.TM(+15.99)QNTSDLDTAR.C | Y | 65.61 | 1367.5988 | 12 | 0.5 | 684.8070 | 2 | 18.97 | 8 | F8:4673 | 31122021\_RID\_3262\_1b.raw |  |  | 1.5244E5 |  |  |  |  | 1.2402E5 | 2 | 0 | 0 | 1 | 0 | 0 | 0 | 0 | 1 | 194 | 205 | Oxidation (M) | M2:Oxidation (M):1000.00 | PEAKS DB |
| R.TMQNTSDLDTAR.C | Y | 60.18 | 1351.6038 | 12 | 0.8 | 676.8097 | 2 | 24.85 | 3 | F3:10817 | 13012022\_RID\_3262\_3b.raw |  |  | 1.4351E5 |  |  |  |  | 4.1837E5 | 2 | 0 | 0 | 1 | 0 | 0 | 0 | 0 | 1 | 194 | 205 |  |  | PEAKS DB |
| K.AAMIVNQLSK.K | Y | 59.14 | 1073.5903 | 10 | 0.3 | 537.8026 | 2 | 32.46 | 8 | F8:14867 | 31122021\_RID\_3262\_1b.raw |  |  |  |  |  |  |  | 6.7476E5 | 1 | 0 | 0 | 0 | 0 | 0 | 0 | 0 | 1 | 164 | 173 |  |  | PEAKS DB |
| R.HPEAEMAQNSVR.L | Y | 57.43 | 1367.6251 | 12 | 0.4 | 456.8825 | 3 | 17.57 | 8 | F8:3951 | 31122021\_RID\_3262\_1b.raw |  |  |  |  |  |  |  | 4.095E5 | 2 | 0 | 0 | 0 | 0 | 0 | 0 | 0 | 2 | 468 | 479 |  |  | PEAKS DB |
| L.INYQDDAELATR.A | Y | 52.56 | 1407.6630 | 12 | 0.3 | 704.8390 | 2 | 32.97 | 8 | F8:15260 | 31122021\_RID\_3262\_1b.raw |  |  |  |  |  |  |  | 5.7938E4 | 1 | 0 | 0 | 0 | 0 | 0 | 0 | 0 | 1 | 133 | 144 |  |  | PEAKS DB |
| K.SGGIPALVR.M | Y | 51.41 | 868.5130 | 9 | 0.3 | 435.2639 | 2 | 34.23 | 8 | F8:16166 | 31122021\_RID\_3262\_1b.raw |  |  |  |  | 2.1025E5 |  |  | 9.0683E5 | 2 | 0 | 0 | 0 | 0 | 1 | 0 | 0 | 1 | 227 | 235 |  |  | PEAKS DB |
| R.ISEDKNPDYR.K | Y | 46.94 | 1235.5781 | 10 | 0.7 | 618.7968 | 2 | 18.42 | 3 | F3:5560 | 13012022\_RID\_3262\_3b.raw |  |  | 5.6021E4 |  |  |  |  |  | 1 | 0 | 0 | 1 | 0 | 0 | 0 | 0 | 0 | 654 | 663 |  |  | PEAKS DB |
| K.AAM(+15.99)IVNQLSK.K | Y | 45.42 | 1089.5852 | 10 | 1.1 | 545.8005 | 2 | 32.45 | 8 | F8:14843 | 31122021\_RID\_3262\_1b.raw |  |  |  |  |  |  |  | 6.6638E4 | 1 | 0 | 0 | 0 | 0 | 0 | 0 | 0 | 1 | 164 | 173 | Oxidation (M) | M3:Oxidation (M):1000.00 | PEAKS DB |
| K.RVSVELTNSLFK.H | Y | 45.25 | 1391.7772 | 12 | 0.9 | 696.8965 | 2 | 39.22 | 8 | F8:20209 | 31122021\_RID\_3262\_1b.raw |  |  |  |  |  |  |  | 3.6237E4 | 1 | 0 | 0 | 0 | 0 | 0 | 0 | 0 | 1 | 665 | 676 |  |  | PEAKS DB |
| total 13 peptides |  |  |  |  |  |  |  |  |  |  |  |  |  |  |  |  |  |  |  |  |  |  |  |  |  |  |  |  |  |  |  |  |  |
| --- | --- | --- | --- | --- | --- | --- | --- | --- | --- | --- | --- | --- | --- | --- | --- | --- | --- | --- | --- | --- | --- | --- | --- | --- | --- | --- | --- | --- | --- | --- | --- | --- | --- |

XP\_026576301.1

back to list

  

| Protein Coverage
| Supporting Peptides
|

Protein Coverage:

Supporting Peptides:

| Peptide | Uniq | -10lgP | Mass | Length | ppm | m/z | z | RT | Fraction | Scan | Source File | Area F6 | Area F2 | Area F3 | Area F4 | Area F5 | Area F7 | Area F8 | Area F1 | #Feature | #Feature F6 | #Feature F2 | #Feature F3 | #Feature F4 | #Feature F5 | #Feature F7 | #Feature F8 | #Feature F1 | Start | End | PTM | AScore | Found By |
| --- | --- | --- | --- | --- | --- | --- | --- | --- | --- | --- | --- | --- | --- | --- | --- | --- | --- | --- | --- | --- | --- | --- | --- | --- | --- | --- | --- | --- | --- | --- | --- | --- | --- |
| R.NEGTATYAAAVLFR.I | Y | 79.96 | 1482.7467 | 14 | 0.1 | 742.3807 | 2 | 48.35 | 8 | F8:27296 | 31122021\_RID\_3262\_1b.raw |  |  | 5.3493E5 |  |  |  |  | 7.7228E5 | 2 | 0 | 0 | 1 | 0 | 0 | 0 | 0 | 1 | 638 | 651 |  |  | PEAKS DB |
| R.LNYGIPAIVK.L | Y | 71.64 | 1086.6437 | 10 | 0.9 | 544.3296 | 2 | 44.80 | 8 | F8:24695 | 31122021\_RID\_3262\_1b.raw |  |  | 9.8473E5 | 2.0478E5 |  |  |  | 1.6368E6 | 3 | 0 | 0 | 1 | 1 | 0 | 0 | 0 | 1 | 478 | 487 |  |  | PEAKS DB |
| K.SAIVHLINYQDDAELATR.A | Y | 70.33 | 2028.0276 | 18 | 0.5 | 677.0168 | 3 | 43.83 | 3 | F3:27023 | 13012022\_RID\_3262\_3b.raw |  |  | 3.2757E5 |  |  |  |  | 1.1198E6 | 3 | 0 | 0 | 1 | 0 | 0 | 0 | 0 | 2 | 125 | 142 |  |  | PEAKS DB |
| R.VSVELTNSLFK.H | Y | 67.96 | 1235.6761 | 11 | -0.3 | 618.8452 | 2 | 47.06 | 8 | F8:26272 | 31122021\_RID\_3262\_1b.raw |  |  |  |  |  |  |  | 1.62E6 | 1 | 0 | 0 | 0 | 0 | 0 | 0 | 0 | 1 | 664 | 674 |  |  | PEAKS DB |
| R.TM(+15.99)QNTSDLDTAR.C | Y | 65.61 | 1367.5988 | 12 | 0.5 | 684.8070 | 2 | 18.97 | 8 | F8:4673 | 31122021\_RID\_3262\_1b.raw |  |  | 1.5244E5 |  |  |  |  | 1.2402E5 | 2 | 0 | 0 | 1 | 0 | 0 | 0 | 0 | 1 | 192 | 203 | Oxidation (M) | M2:Oxidation (M):1000.00 | PEAKS DB |
| R.TMQNTSDLDTAR.C | Y | 60.18 | 1351.6038 | 12 | 0.8 | 676.8097 | 2 | 24.85 | 3 | F3:10817 | 13012022\_RID\_3262\_3b.raw |  |  | 1.4351E5 |  |  |  |  | 4.1837E5 | 2 | 0 | 0 | 1 | 0 | 0 | 0 | 0 | 1 | 192 | 203 |  |  | PEAKS DB |
| K.AAMIVNQLSK.K | Y | 59.14 | 1073.5903 | 10 | 0.3 | 537.8026 | 2 | 32.46 | 8 | F8:14867 | 31122021\_RID\_3262\_1b.raw |  |  |  |  |  |  |  | 6.7476E5 | 1 | 0 | 0 | 0 | 0 | 0 | 0 | 0 | 1 | 162 | 171 |  |  | PEAKS DB |
| R.HPEAEMAQNSVR.L | Y | 57.43 | 1367.6251 | 12 | 0.4 | 456.8825 | 3 | 17.57 | 8 | F8:3951 | 31122021\_RID\_3262\_1b.raw |  |  |  |  |  |  |  | 4.095E5 | 2 | 0 | 0 | 0 | 0 | 0 | 0 | 0 | 2 | 466 | 477 |  |  | PEAKS DB |
| L.INYQDDAELATR.A | Y | 52.56 | 1407.6630 | 12 | 0.3 | 704.8390 | 2 | 32.97 | 8 | F8:15260 | 31122021\_RID\_3262\_1b.raw |  |  |  |  |  |  |  | 5.7938E4 | 1 | 0 | 0 | 0 | 0 | 0 | 0 | 0 | 1 | 131 | 142 |  |  | PEAKS DB |
| K.SGGIPALVR.M | Y | 51.41 | 868.5130 | 9 | 0.3 | 435.2639 | 2 | 34.23 | 8 | F8:16166 | 31122021\_RID\_3262\_1b.raw |  |  |  |  | 2.1025E5 |  |  | 9.0683E5 | 2 | 0 | 0 | 0 | 0 | 1 | 0 | 0 | 1 | 225 | 233 |  |  | PEAKS DB |
| R.ISEDKNPDYR.K | Y | 46.94 | 1235.5781 | 10 | 0.7 | 618.7968 | 2 | 18.42 | 3 | F3:5560 | 13012022\_RID\_3262\_3b.raw |  |  | 5.6021E4 |  |  |  |  |  | 1 | 0 | 0 | 1 | 0 | 0 | 0 | 0 | 0 | 652 | 661 |  |  | PEAKS DB |
| K.AAM(+15.99)IVNQLSK.K | Y | 45.42 | 1089.5852 | 10 | 1.1 | 545.8005 | 2 | 32.45 | 8 | F8:14843 | 31122021\_RID\_3262\_1b.raw |  |  |  |  |  |  |  | 6.6638E4 | 1 | 0 | 0 | 0 | 0 | 0 | 0 | 0 | 1 | 162 | 171 | Oxidation (M) | M3:Oxidation (M):1000.00 | PEAKS DB |
| K.RVSVELTNSLFK.H | Y | 45.25 | 1391.7772 | 12 | 0.9 | 696.8965 | 2 | 39.22 | 8 | F8:20209 | 31122021\_RID\_3262\_1b.raw |  |  |  |  |  |  |  | 3.6237E4 | 1 | 0 | 0 | 0 | 0 | 0 | 0 | 0 | 1 | 663 | 674 |  |  | PEAKS DB |
| total 13 peptides |  |  |  |  |  |  |  |  |  |  |  |  |  |  |  |  |  |  |  |  |  |  |  |  |  |  |  |  |  |  |  |  |  |
| --- | --- | --- | --- | --- | --- | --- | --- | --- | --- | --- | --- | --- | --- | --- | --- | --- | --- | --- | --- | --- | --- | --- | --- | --- | --- | --- | --- | --- | --- | --- | --- | --- | --- |

XP\_026525656.1

back to list

  

| Protein Coverage
| Supporting Peptides
|

Protein Coverage:

Supporting Peptides:

| Peptide | Uniq | -10lgP | Mass | Length | ppm | m/z | z | RT | Fraction | Scan | Source File | Area F6 | Area F2 | Area F3 | Area F4 | Area F5 | Area F7 | Area F8 | Area F1 | #Feature | #Feature F6 | #Feature F2 | #Feature F3 | #Feature F4 | #Feature F5 | #Feature F7 | #Feature F8 | #Feature F1 | Start | End | PTM | AScore | Found By |
| --- | --- | --- | --- | --- | --- | --- | --- | --- | --- | --- | --- | --- | --- | --- | --- | --- | --- | --- | --- | --- | --- | --- | --- | --- | --- | --- | --- | --- | --- | --- | --- | --- | --- |
| R.NEGTATYAAAVLFR.I | Y | 79.96 | 1482.7467 | 14 | 0.1 | 742.3807 | 2 | 48.35 | 8 | F8:27296 | 31122021\_RID\_3262\_1b.raw |  |  | 5.3493E5 |  |  |  |  | 7.7228E5 | 2 | 0 | 0 | 1 | 0 | 0 | 0 | 0 | 1 | 638 | 651 |  |  | PEAKS DB |
| R.LNYGIPAIVK.L | Y | 71.64 | 1086.6437 | 10 | 0.9 | 544.3296 | 2 | 44.80 | 8 | F8:24695 | 31122021\_RID\_3262\_1b.raw |  |  | 9.8473E5 | 2.0478E5 |  |  |  | 1.6368E6 | 3 | 0 | 0 | 1 | 1 | 0 | 0 | 0 | 1 | 478 | 487 |  |  | PEAKS DB |
| K.SAIVHLINYQDDAELATR.A | Y | 70.33 | 2028.0276 | 18 | 0.5 | 677.0168 | 3 | 43.83 | 3 | F3:27023 | 13012022\_RID\_3262\_3b.raw |  |  | 3.2757E5 |  |  |  |  | 1.1198E6 | 3 | 0 | 0 | 1 | 0 | 0 | 0 | 0 | 2 | 125 | 142 |  |  | PEAKS DB |
| R.VSVELTNSLFK.H | Y | 67.96 | 1235.6761 | 11 | -0.3 | 618.8452 | 2 | 47.06 | 8 | F8:26272 | 31122021\_RID\_3262\_1b.raw |  |  |  |  |  |  |  | 1.62E6 | 1 | 0 | 0 | 0 | 0 | 0 | 0 | 0 | 1 | 664 | 674 |  |  | PEAKS DB |
| R.TM(+15.99)QNTSDLDTAR.C | Y | 65.61 | 1367.5988 | 12 | 0.5 | 684.8070 | 2 | 18.97 | 8 | F8:4673 | 31122021\_RID\_3262\_1b.raw |  |  | 1.5244E5 |  |  |  |  | 1.2402E5 | 2 | 0 | 0 | 1 | 0 | 0 | 0 | 0 | 1 | 192 | 203 | Oxidation (M) | M2:Oxidation (M):1000.00 | PEAKS DB |
| R.TMQNTSDLDTAR.C | Y | 60.18 | 1351.6038 | 12 | 0.8 | 676.8097 | 2 | 24.85 | 3 | F3:10817 | 13012022\_RID\_3262\_3b.raw |  |  | 1.4351E5 |  |  |  |  | 4.1837E5 | 2 | 0 | 0 | 1 | 0 | 0 | 0 | 0 | 1 | 192 | 203 |  |  | PEAKS DB |
| K.AAMIVNQLSK.K | Y | 59.14 | 1073.5903 | 10 | 0.3 | 537.8026 | 2 | 32.46 | 8 | F8:14867 | 31122021\_RID\_3262\_1b.raw |  |  |  |  |  |  |  | 6.7476E5 | 1 | 0 | 0 | 0 | 0 | 0 | 0 | 0 | 1 | 162 | 171 |  |  | PEAKS DB |
| R.HPEAEMAQNSVR.L | Y | 57.43 | 1367.6251 | 12 | 0.4 | 456.8825 | 3 | 17.57 | 8 | F8:3951 | 31122021\_RID\_3262\_1b.raw |  |  |  |  |  |  |  | 4.095E5 | 2 | 0 | 0 | 0 | 0 | 0 | 0 | 0 | 2 | 466 | 477 |  |  | PEAKS DB |
| L.INYQDDAELATR.A | Y | 52.56 | 1407.6630 | 12 | 0.3 | 704.8390 | 2 | 32.97 | 8 | F8:15260 | 31122021\_RID\_3262\_1b.raw |  |  |  |  |  |  |  | 5.7938E4 | 1 | 0 | 0 | 0 | 0 | 0 | 0 | 0 | 1 | 131 | 142 |  |  | PEAKS DB |
| K.SGGIPALVR.M | Y | 51.41 | 868.5130 | 9 | 0.3 | 435.2639 | 2 | 34.23 | 8 | F8:16166 | 31122021\_RID\_3262\_1b.raw |  |  |  |  | 2.1025E5 |  |  | 9.0683E5 | 2 | 0 | 0 | 0 | 0 | 1 | 0 | 0 | 1 | 225 | 233 |  |  | PEAKS DB |
| R.ISEDKNPDYR.K | Y | 46.94 | 1235.5781 | 10 | 0.7 | 618.7968 | 2 | 18.42 | 3 | F3:5560 | 13012022\_RID\_3262\_3b.raw |  |  | 5.6021E4 |  |  |  |  |  | 1 | 0 | 0 | 1 | 0 | 0 | 0 | 0 | 0 | 652 | 661 |  |  | PEAKS DB |
| K.AAM(+15.99)IVNQLSK.K | Y | 45.42 | 1089.5852 | 10 | 1.1 | 545.8005 | 2 | 32.45 | 8 | F8:14843 | 31122021\_RID\_3262\_1b.raw |  |  |  |  |  |  |  | 6.6638E4 | 1 | 0 | 0 | 0 | 0 | 0 | 0 | 0 | 1 | 162 | 171 | Oxidation (M) | M3:Oxidation (M):1000.00 | PEAKS DB |
| K.RVSVELTNSLFK.H | Y | 45.25 | 1391.7772 | 12 | 0.9 | 696.8965 | 2 | 39.22 | 8 | F8:20209 | 31122021\_RID\_3262\_1b.raw |  |  |  |  |  |  |  | 3.6237E4 | 1 | 0 | 0 | 0 | 0 | 0 | 0 | 0 | 1 | 663 | 674 |  |  | PEAKS DB |
| total 13 peptides |  |  |  |  |  |  |  |  |  |  |  |  |  |  |  |  |  |  |  |  |  |  |  |  |  |  |  |  |  |  |  |  |  |
| --- | --- | --- | --- | --- | --- | --- | --- | --- | --- | --- | --- | --- | --- | --- | --- | --- | --- | --- | --- | --- | --- | --- | --- | --- | --- | --- | --- | --- | --- | --- | --- | --- | --- |

XP\_026576299.1

back to list

  

| Protein Coverage
| Supporting Peptides
|

Protein Coverage:

Supporting Peptides:

| Peptide | Uniq | -10lgP | Mass | Length | ppm | m/z | z | RT | Fraction | Scan | Source File | Area F6 | Area F2 | Area F3 | Area F4 | Area F5 | Area F7 | Area F8 | Area F1 | #Feature | #Feature F6 | #Feature F2 | #Feature F3 | #Feature F4 | #Feature F5 | #Feature F7 | #Feature F8 | #Feature F1 | Start | End | PTM | AScore | Found By |
| --- | --- | --- | --- | --- | --- | --- | --- | --- | --- | --- | --- | --- | --- | --- | --- | --- | --- | --- | --- | --- | --- | --- | --- | --- | --- | --- | --- | --- | --- | --- | --- | --- | --- |
| R.NEGTATYAAAVLFR.I | Y | 79.96 | 1482.7467 | 14 | 0.1 | 742.3807 | 2 | 48.35 | 8 | F8:27296 | 31122021\_RID\_3262\_1b.raw |  |  | 5.3493E5 |  |  |  |  | 7.7228E5 | 2 | 0 | 0 | 1 | 0 | 0 | 0 | 0 | 1 | 639 | 652 |  |  | PEAKS DB |
| R.LNYGIPAIVK.L | Y | 71.64 | 1086.6437 | 10 | 0.9 | 544.3296 | 2 | 44.80 | 8 | F8:24695 | 31122021\_RID\_3262\_1b.raw |  |  | 9.8473E5 | 2.0478E5 |  |  |  | 1.6368E6 | 3 | 0 | 0 | 1 | 1 | 0 | 0 | 0 | 1 | 479 | 488 |  |  | PEAKS DB |
| K.SAIVHLINYQDDAELATR.A | Y | 70.33 | 2028.0276 | 18 | 0.5 | 677.0168 | 3 | 43.83 | 3 | F3:27023 | 13012022\_RID\_3262\_3b.raw |  |  | 3.2757E5 |  |  |  |  | 1.1198E6 | 3 | 0 | 0 | 1 | 0 | 0 | 0 | 0 | 2 | 126 | 143 |  |  | PEAKS DB |
| R.VSVELTNSLFK.H | Y | 67.96 | 1235.6761 | 11 | -0.3 | 618.8452 | 2 | 47.06 | 8 | F8:26272 | 31122021\_RID\_3262\_1b.raw |  |  |  |  |  |  |  | 1.62E6 | 1 | 0 | 0 | 0 | 0 | 0 | 0 | 0 | 1 | 665 | 675 |  |  | PEAKS DB |
| R.TM(+15.99)QNTSDLDTAR.C | Y | 65.61 | 1367.5988 | 12 | 0.5 | 684.8070 | 2 | 18.97 | 8 | F8:4673 | 31122021\_RID\_3262\_1b.raw |  |  | 1.5244E5 |  |  |  |  | 1.2402E5 | 2 | 0 | 0 | 1 | 0 | 0 | 0 | 0 | 1 | 193 | 204 | Oxidation (M) | M2:Oxidation (M):1000.00 | PEAKS DB |
| R.TMQNTSDLDTAR.C | Y | 60.18 | 1351.6038 | 12 | 0.8 | 676.8097 | 2 | 24.85 | 3 | F3:10817 | 13012022\_RID\_3262\_3b.raw |  |  | 1.4351E5 |  |  |  |  | 4.1837E5 | 2 | 0 | 0 | 1 | 0 | 0 | 0 | 0 | 1 | 193 | 204 |  |  | PEAKS DB |
| K.AAMIVNQLSK.K | Y | 59.14 | 1073.5903 | 10 | 0.3 | 537.8026 | 2 | 32.46 | 8 | F8:14867 | 31122021\_RID\_3262\_1b.raw |  |  |  |  |  |  |  | 6.7476E5 | 1 | 0 | 0 | 0 | 0 | 0 | 0 | 0 | 1 | 163 | 172 |  |  | PEAKS DB |
| R.HPEAEMAQNSVR.L | Y | 57.43 | 1367.6251 | 12 | 0.4 | 456.8825 | 3 | 17.57 | 8 | F8:3951 | 31122021\_RID\_3262\_1b.raw |  |  |  |  |  |  |  | 4.095E5 | 2 | 0 | 0 | 0 | 0 | 0 | 0 | 0 | 2 | 467 | 478 |  |  | PEAKS DB |
| L.INYQDDAELATR.A | Y | 52.56 | 1407.6630 | 12 | 0.3 | 704.8390 | 2 | 32.97 | 8 | F8:15260 | 31122021\_RID\_3262\_1b.raw |  |  |  |  |  |  |  | 5.7938E4 | 1 | 0 | 0 | 0 | 0 | 0 | 0 | 0 | 1 | 132 | 143 |  |  | PEAKS DB |
| K.SGGIPALVR.M | Y | 51.41 | 868.5130 | 9 | 0.3 | 435.2639 | 2 | 34.23 | 8 | F8:16166 | 31122021\_RID\_3262\_1b.raw |  |  |  |  | 2.1025E5 |  |  | 9.0683E5 | 2 | 0 | 0 | 0 | 0 | 1 | 0 | 0 | 1 | 226 | 234 |  |  | PEAKS DB |
| R.ISEDKNPDYR.K | Y | 46.94 | 1235.5781 | 10 | 0.7 | 618.7968 | 2 | 18.42 | 3 | F3:5560 | 13012022\_RID\_3262\_3b.raw |  |  | 5.6021E4 |  |  |  |  |  | 1 | 0 | 0 | 1 | 0 | 0 | 0 | 0 | 0 | 653 | 662 |  |  | PEAKS DB |
| K.AAM(+15.99)IVNQLSK.K | Y | 45.42 | 1089.5852 | 10 | 1.1 | 545.8005 | 2 | 32.45 | 8 | F8:14843 | 31122021\_RID\_3262\_1b.raw |  |  |  |  |  |  |  | 6.6638E4 | 1 | 0 | 0 | 0 | 0 | 0 | 0 | 0 | 1 | 163 | 172 | Oxidation (M) | M3:Oxidation (M):1000.00 | PEAKS DB |
| K.RVSVELTNSLFK.H | Y | 45.25 | 1391.7772 | 12 | 0.9 | 696.8965 | 2 | 39.22 | 8 | F8:20209 | 31122021\_RID\_3262\_1b.raw |  |  |  |  |  |  |  | 3.6237E4 | 1 | 0 | 0 | 0 | 0 | 0 | 0 | 0 | 1 | 664 | 675 |  |  | PEAKS DB |
| total 13 peptides |  |  |  |  |  |  |  |  |  |  |  |  |  |  |  |  |  |  |  |  |  |  |  |  |  |  |  |  |  |  |  |  |  |
| --- | --- | --- | --- | --- | --- | --- | --- | --- | --- | --- | --- | --- | --- | --- | --- | --- | --- | --- | --- | --- | --- | --- | --- | --- | --- | --- | --- | --- | --- | --- | --- | --- | --- |

XP\_026525655.1

back to list

  

| Protein Coverage
| Supporting Peptides
|

Protein Coverage:

Supporting Peptides:

| Peptide | Uniq | -10lgP | Mass | Length | ppm | m/z | z | RT | Fraction | Scan | Source File | Area F6 | Area F2 | Area F3 | Area F4 | Area F5 | Area F7 | Area F8 | Area F1 | #Feature | #Feature F6 | #Feature F2 | #Feature F3 | #Feature F4 | #Feature F5 | #Feature F7 | #Feature F8 | #Feature F1 | Start | End | PTM | AScore | Found By |
| --- | --- | --- | --- | --- | --- | --- | --- | --- | --- | --- | --- | --- | --- | --- | --- | --- | --- | --- | --- | --- | --- | --- | --- | --- | --- | --- | --- | --- | --- | --- | --- | --- | --- |
| R.NEGTATYAAAVLFR.I | Y | 79.96 | 1482.7467 | 14 | 0.1 | 742.3807 | 2 | 48.35 | 8 | F8:27296 | 31122021\_RID\_3262\_1b.raw |  |  | 5.3493E5 |  |  |  |  | 7.7228E5 | 2 | 0 | 0 | 1 | 0 | 0 | 0 | 0 | 1 | 639 | 652 |  |  | PEAKS DB |
| R.LNYGIPAIVK.L | Y | 71.64 | 1086.6437 | 10 | 0.9 | 544.3296 | 2 | 44.80 | 8 | F8:24695 | 31122021\_RID\_3262\_1b.raw |  |  | 9.8473E5 | 2.0478E5 |  |  |  | 1.6368E6 | 3 | 0 | 0 | 1 | 1 | 0 | 0 | 0 | 1 | 479 | 488 |  |  | PEAKS DB |
| K.SAIVHLINYQDDAELATR.A | Y | 70.33 | 2028.0276 | 18 | 0.5 | 677.0168 | 3 | 43.83 | 3 | F3:27023 | 13012022\_RID\_3262\_3b.raw |  |  | 3.2757E5 |  |  |  |  | 1.1198E6 | 3 | 0 | 0 | 1 | 0 | 0 | 0 | 0 | 2 | 126 | 143 |  |  | PEAKS DB |
| R.VSVELTNSLFK.H | Y | 67.96 | 1235.6761 | 11 | -0.3 | 618.8452 | 2 | 47.06 | 8 | F8:26272 | 31122021\_RID\_3262\_1b.raw |  |  |  |  |  |  |  | 1.62E6 | 1 | 0 | 0 | 0 | 0 | 0 | 0 | 0 | 1 | 665 | 675 |  |  | PEAKS DB |
| R.TM(+15.99)QNTSDLDTAR.C | Y | 65.61 | 1367.5988 | 12 | 0.5 | 684.8070 | 2 | 18.97 | 8 | F8:4673 | 31122021\_RID\_3262\_1b.raw |  |  | 1.5244E5 |  |  |  |  | 1.2402E5 | 2 | 0 | 0 | 1 | 0 | 0 | 0 | 0 | 1 | 193 | 204 | Oxidation (M) | M2:Oxidation (M):1000.00 | PEAKS DB |
| R.TMQNTSDLDTAR.C | Y | 60.18 | 1351.6038 | 12 | 0.8 | 676.8097 | 2 | 24.85 | 3 | F3:10817 | 13012022\_RID\_3262\_3b.raw |  |  | 1.4351E5 |  |  |  |  | 4.1837E5 | 2 | 0 | 0 | 1 | 0 | 0 | 0 | 0 | 1 | 193 | 204 |  |  | PEAKS DB |
| K.AAMIVNQLSK.K | Y | 59.14 | 1073.5903 | 10 | 0.3 | 537.8026 | 2 | 32.46 | 8 | F8:14867 | 31122021\_RID\_3262\_1b.raw |  |  |  |  |  |  |  | 6.7476E5 | 1 | 0 | 0 | 0 | 0 | 0 | 0 | 0 | 1 | 163 | 172 |  |  | PEAKS DB |
| R.HPEAEMAQNSVR.L | Y | 57.43 | 1367.6251 | 12 | 0.4 | 456.8825 | 3 | 17.57 | 8 | F8:3951 | 31122021\_RID\_3262\_1b.raw |  |  |  |  |  |  |  | 4.095E5 | 2 | 0 | 0 | 0 | 0 | 0 | 0 | 0 | 2 | 467 | 478 |  |  | PEAKS DB |
| L.INYQDDAELATR.A | Y | 52.56 | 1407.6630 | 12 | 0.3 | 704.8390 | 2 | 32.97 | 8 | F8:15260 | 31122021\_RID\_3262\_1b.raw |  |  |  |  |  |  |  | 5.7938E4 | 1 | 0 | 0 | 0 | 0 | 0 | 0 | 0 | 1 | 132 | 143 |  |  | PEAKS DB |
| K.SGGIPALVR.M | Y | 51.41 | 868.5130 | 9 | 0.3 | 435.2639 | 2 | 34.23 | 8 | F8:16166 | 31122021\_RID\_3262\_1b.raw |  |  |  |  | 2.1025E5 |  |  | 9.0683E5 | 2 | 0 | 0 | 0 | 0 | 1 | 0 | 0 | 1 | 226 | 234 |  |  | PEAKS DB |
| R.ISEDKNPDYR.K | Y | 46.94 | 1235.5781 | 10 | 0.7 | 618.7968 | 2 | 18.42 | 3 | F3:5560 | 13012022\_RID\_3262\_3b.raw |  |  | 5.6021E4 |  |  |  |  |  | 1 | 0 | 0 | 1 | 0 | 0 | 0 | 0 | 0 | 653 | 662 |  |  | PEAKS DB |
| K.AAM(+15.99)IVNQLSK.K | Y | 45.42 | 1089.5852 | 10 | 1.1 | 545.8005 | 2 | 32.45 | 8 | F8:14843 | 31122021\_RID\_3262\_1b.raw |  |  |  |  |  |  |  | 6.6638E4 | 1 | 0 | 0 | 0 | 0 | 0 | 0 | 0 | 1 | 163 | 172 | Oxidation (M) | M3:Oxidation (M):1000.00 | PEAKS DB |
| K.RVSVELTNSLFK.H | Y | 45.25 | 1391.7772 | 12 | 0.9 | 696.8965 | 2 | 39.22 | 8 | F8:20209 | 31122021\_RID\_3262\_1b.raw |  |  |  |  |  |  |  | 3.6237E4 | 1 | 0 | 0 | 0 | 0 | 0 | 0 | 0 | 1 | 664 | 675 |  |  | PEAKS DB |
| total 13 peptides |  |  |  |  |  |  |  |  |  |  |  |  |  |  |  |  |  |  |  |  |  |  |  |  |  |  |  |  |  |  |  |  |  |
| --- | --- | --- | --- | --- | --- | --- | --- | --- | --- | --- | --- | --- | --- | --- | --- | --- | --- | --- | --- | --- | --- | --- | --- | --- | --- | --- | --- | --- | --- | --- | --- | --- | --- |

XP\_026525654.1

back to list

  

| Protein Coverage
| Supporting Peptides
|

Protein Coverage:

Supporting Peptides:

| Peptide | Uniq | -10lgP | Mass | Length | ppm | m/z | z | RT | Fraction | Scan | Source File | Area F6 | Area F2 | Area F3 | Area F4 | Area F5 | Area F7 | Area F8 | Area F1 | #Feature | #Feature F6 | #Feature F2 | #Feature F3 | #Feature F4 | #Feature F5 | #Feature F7 | #Feature F8 | #Feature F1 | Start | End | PTM | AScore | Found By |
| --- | --- | --- | --- | --- | --- | --- | --- | --- | --- | --- | --- | --- | --- | --- | --- | --- | --- | --- | --- | --- | --- | --- | --- | --- | --- | --- | --- | --- | --- | --- | --- | --- | --- |
| R.NEGTATYAAAVLFR.I | Y | 79.96 | 1482.7467 | 14 | 0.1 | 742.3807 | 2 | 48.35 | 8 | F8:27296 | 31122021\_RID\_3262\_1b.raw |  |  | 5.3493E5 |  |  |  |  | 7.7228E5 | 2 | 0 | 0 | 1 | 0 | 0 | 0 | 0 | 1 | 639 | 652 |  |  | PEAKS DB |
| R.LNYGIPAIVK.L | Y | 71.64 | 1086.6437 | 10 | 0.9 | 544.3296 | 2 | 44.80 | 8 | F8:24695 | 31122021\_RID\_3262\_1b.raw |  |  | 9.8473E5 | 2.0478E5 |  |  |  | 1.6368E6 | 3 | 0 | 0 | 1 | 1 | 0 | 0 | 0 | 1 | 479 | 488 |  |  | PEAKS DB |
| K.SAIVHLINYQDDAELATR.A | Y | 70.33 | 2028.0276 | 18 | 0.5 | 677.0168 | 3 | 43.83 | 3 | F3:27023 | 13012022\_RID\_3262\_3b.raw |  |  | 3.2757E5 |  |  |  |  | 1.1198E6 | 3 | 0 | 0 | 1 | 0 | 0 | 0 | 0 | 2 | 126 | 143 |  |  | PEAKS DB |
| R.VSVELTNSLFK.H | Y | 67.96 | 1235.6761 | 11 | -0.3 | 618.8452 | 2 | 47.06 | 8 | F8:26272 | 31122021\_RID\_3262\_1b.raw |  |  |  |  |  |  |  | 1.62E6 | 1 | 0 | 0 | 0 | 0 | 0 | 0 | 0 | 1 | 665 | 675 |  |  | PEAKS DB |
| R.TM(+15.99)QNTSDLDTAR.C | Y | 65.61 | 1367.5988 | 12 | 0.5 | 684.8070 | 2 | 18.97 | 8 | F8:4673 | 31122021\_RID\_3262\_1b.raw |  |  | 1.5244E5 |  |  |  |  | 1.2402E5 | 2 | 0 | 0 | 1 | 0 | 0 | 0 | 0 | 1 | 193 | 204 | Oxidation (M) | M2:Oxidation (M):1000.00 | PEAKS DB |
| R.TMQNTSDLDTAR.C | Y | 60.18 | 1351.6038 | 12 | 0.8 | 676.8097 | 2 | 24.85 | 3 | F3:10817 | 13012022\_RID\_3262\_3b.raw |  |  | 1.4351E5 |  |  |  |  | 4.1837E5 | 2 | 0 | 0 | 1 | 0 | 0 | 0 | 0 | 1 | 193 | 204 |  |  | PEAKS DB |
| K.AAMIVNQLSK.K | Y | 59.14 | 1073.5903 | 10 | 0.3 | 537.8026 | 2 | 32.46 | 8 | F8:14867 | 31122021\_RID\_3262\_1b.raw |  |  |  |  |  |  |  | 6.7476E5 | 1 | 0 | 0 | 0 | 0 | 0 | 0 | 0 | 1 | 163 | 172 |  |  | PEAKS DB |
| R.HPEAEMAQNSVR.L | Y | 57.43 | 1367.6251 | 12 | 0.4 | 456.8825 | 3 | 17.57 | 8 | F8:3951 | 31122021\_RID\_3262\_1b.raw |  |  |  |  |  |  |  | 4.095E5 | 2 | 0 | 0 | 0 | 0 | 0 | 0 | 0 | 2 | 467 | 478 |  |  | PEAKS DB |
| L.INYQDDAELATR.A | Y | 52.56 | 1407.6630 | 12 | 0.3 | 704.8390 | 2 | 32.97 | 8 | F8:15260 | 31122021\_RID\_3262\_1b.raw |  |  |  |  |  |  |  | 5.7938E4 | 1 | 0 | 0 | 0 | 0 | 0 | 0 | 0 | 1 | 132 | 143 |  |  | PEAKS DB |
| K.SGGIPALVR.M | Y | 51.41 | 868.5130 | 9 | 0.3 | 435.2639 | 2 | 34.23 | 8 | F8:16166 | 31122021\_RID\_3262\_1b.raw |  |  |  |  | 2.1025E5 |  |  | 9.0683E5 | 2 | 0 | 0 | 0 | 0 | 1 | 0 | 0 | 1 | 226 | 234 |  |  | PEAKS DB |
| R.ISEDKNPDYR.K | Y | 46.94 | 1235.5781 | 10 | 0.7 | 618.7968 | 2 | 18.42 | 3 | F3:5560 | 13012022\_RID\_3262\_3b.raw |  |  | 5.6021E4 |  |  |  |  |  | 1 | 0 | 0 | 1 | 0 | 0 | 0 | 0 | 0 | 653 | 662 |  |  | PEAKS DB |
| K.AAM(+15.99)IVNQLSK.K | Y | 45.42 | 1089.5852 | 10 | 1.1 | 545.8005 | 2 | 32.45 | 8 | F8:14843 | 31122021\_RID\_3262\_1b.raw |  |  |  |  |  |  |  | 6.6638E4 | 1 | 0 | 0 | 0 | 0 | 0 | 0 | 0 | 1 | 163 | 172 | Oxidation (M) | M3:Oxidation (M):1000.00 | PEAKS DB |
| K.RVSVELTNSLFK.H | Y | 45.25 | 1391.7772 | 12 | 0.9 | 696.8965 | 2 | 39.22 | 8 | F8:20209 | 31122021\_RID\_3262\_1b.raw |  |  |  |  |  |  |  | 3.6237E4 | 1 | 0 | 0 | 0 | 0 | 0 | 0 | 0 | 1 | 664 | 675 |  |  | PEAKS DB |
| total 13 peptides |  |  |  |  |  |  |  |  |  |  |  |  |  |  |  |  |  |  |  |  |  |  |  |  |  |  |  |  |  |  |  |  |  |
| --- | --- | --- | --- | --- | --- | --- | --- | --- | --- | --- | --- | --- | --- | --- | --- | --- | --- | --- | --- | --- | --- | --- | --- | --- | --- | --- | --- | --- | --- | --- | --- | --- | --- |

XP\_026576298.1

back to list

  

| Protein Coverage
| Supporting Peptides
|

Protein Coverage:

Supporting Peptides:

| Peptide | Uniq | -10lgP | Mass | Length | ppm | m/z | z | RT | Fraction | Scan | Source File | Area F6 | Area F2 | Area F3 | Area F4 | Area F5 | Area F7 | Area F8 | Area F1 | #Feature | #Feature F6 | #Feature F2 | #Feature F3 | #Feature F4 | #Feature F5 | #Feature F7 | #Feature F8 | #Feature F1 | Start | End | PTM | AScore | Found By |
| --- | --- | --- | --- | --- | --- | --- | --- | --- | --- | --- | --- | --- | --- | --- | --- | --- | --- | --- | --- | --- | --- | --- | --- | --- | --- | --- | --- | --- | --- | --- | --- | --- | --- |
| R.NEGTATYAAAVLFR.I | Y | 79.96 | 1482.7467 | 14 | 0.1 | 742.3807 | 2 | 48.35 | 8 | F8:27296 | 31122021\_RID\_3262\_1b.raw |  |  | 5.3493E5 |  |  |  |  | 7.7228E5 | 2 | 0 | 0 | 1 | 0 | 0 | 0 | 0 | 1 | 639 | 652 |  |  | PEAKS DB |
| R.LNYGIPAIVK.L | Y | 71.64 | 1086.6437 | 10 | 0.9 | 544.3296 | 2 | 44.80 | 8 | F8:24695 | 31122021\_RID\_3262\_1b.raw |  |  | 9.8473E5 | 2.0478E5 |  |  |  | 1.6368E6 | 3 | 0 | 0 | 1 | 1 | 0 | 0 | 0 | 1 | 479 | 488 |  |  | PEAKS DB |
| K.SAIVHLINYQDDAELATR.A | Y | 70.33 | 2028.0276 | 18 | 0.5 | 677.0168 | 3 | 43.83 | 3 | F3:27023 | 13012022\_RID\_3262\_3b.raw |  |  | 3.2757E5 |  |  |  |  | 1.1198E6 | 3 | 0 | 0 | 1 | 0 | 0 | 0 | 0 | 2 | 126 | 143 |  |  | PEAKS DB |
| R.VSVELTNSLFK.H | Y | 67.96 | 1235.6761 | 11 | -0.3 | 618.8452 | 2 | 47.06 | 8 | F8:26272 | 31122021\_RID\_3262\_1b.raw |  |  |  |  |  |  |  | 1.62E6 | 1 | 0 | 0 | 0 | 0 | 0 | 0 | 0 | 1 | 665 | 675 |  |  | PEAKS DB |
| R.TM(+15.99)QNTSDLDTAR.C | Y | 65.61 | 1367.5988 | 12 | 0.5 | 684.8070 | 2 | 18.97 | 8 | F8:4673 | 31122021\_RID\_3262\_1b.raw |  |  | 1.5244E5 |  |  |  |  | 1.2402E5 | 2 | 0 | 0 | 1 | 0 | 0 | 0 | 0 | 1 | 193 | 204 | Oxidation (M) | M2:Oxidation (M):1000.00 | PEAKS DB |
| R.TMQNTSDLDTAR.C | Y | 60.18 | 1351.6038 | 12 | 0.8 | 676.8097 | 2 | 24.85 | 3 | F3:10817 | 13012022\_RID\_3262\_3b.raw |  |  | 1.4351E5 |  |  |  |  | 4.1837E5 | 2 | 0 | 0 | 1 | 0 | 0 | 0 | 0 | 1 | 193 | 204 |  |  | PEAKS DB |
| K.AAMIVNQLSK.K | Y | 59.14 | 1073.5903 | 10 | 0.3 | 537.8026 | 2 | 32.46 | 8 | F8:14867 | 31122021\_RID\_3262\_1b.raw |  |  |  |  |  |  |  | 6.7476E5 | 1 | 0 | 0 | 0 | 0 | 0 | 0 | 0 | 1 | 163 | 172 |  |  | PEAKS DB |
| R.HPEAEMAQNSVR.L | Y | 57.43 | 1367.6251 | 12 | 0.4 | 456.8825 | 3 | 17.57 | 8 | F8:3951 | 31122021\_RID\_3262\_1b.raw |  |  |  |  |  |  |  | 4.095E5 | 2 | 0 | 0 | 0 | 0 | 0 | 0 | 0 | 2 | 467 | 478 |  |  | PEAKS DB |
| L.INYQDDAELATR.A | Y | 52.56 | 1407.6630 | 12 | 0.3 | 704.8390 | 2 | 32.97 | 8 | F8:15260 | 31122021\_RID\_3262\_1b.raw |  |  |  |  |  |  |  | 5.7938E4 | 1 | 0 | 0 | 0 | 0 | 0 | 0 | 0 | 1 | 132 | 143 |  |  | PEAKS DB |
| K.SGGIPALVR.M | Y | 51.41 | 868.5130 | 9 | 0.3 | 435.2639 | 2 | 34.23 | 8 | F8:16166 | 31122021\_RID\_3262\_1b.raw |  |  |  |  | 2.1025E5 |  |  | 9.0683E5 | 2 | 0 | 0 | 0 | 0 | 1 | 0 | 0 | 1 | 226 | 234 |  |  | PEAKS DB |
| R.ISEDKNPDYR.K | Y | 46.94 | 1235.5781 | 10 | 0.7 | 618.7968 | 2 | 18.42 | 3 | F3:5560 | 13012022\_RID\_3262\_3b.raw |  |  | 5.6021E4 |  |  |  |  |  | 1 | 0 | 0 | 1 | 0 | 0 | 0 | 0 | 0 | 653 | 662 |  |  | PEAKS DB |
| K.AAM(+15.99)IVNQLSK.K | Y | 45.42 | 1089.5852 | 10 | 1.1 | 545.8005 | 2 | 32.45 | 8 | F8:14843 | 31122021\_RID\_3262\_1b.raw |  |  |  |  |  |  |  | 6.6638E4 | 1 | 0 | 0 | 0 | 0 | 0 | 0 | 0 | 1 | 163 | 172 | Oxidation (M) | M3:Oxidation (M):1000.00 | PEAKS DB |
| K.RVSVELTNSLFK.H | Y | 45.25 | 1391.7772 | 12 | 0.9 | 696.8965 | 2 | 39.22 | 8 | F8:20209 | 31122021\_RID\_3262\_1b.raw |  |  |  |  |  |  |  | 3.6237E4 | 1 | 0 | 0 | 0 | 0 | 0 | 0 | 0 | 1 | 664 | 675 |  |  | PEAKS DB |
| total 13 peptides |  |  |  |  |  |  |  |  |  |  |  |  |  |  |  |  |  |  |  |  |  |  |  |  |  |  |  |  |  |  |  |  |  |
| --- | --- | --- | --- | --- | --- | --- | --- | --- | --- | --- | --- | --- | --- | --- | --- | --- | --- | --- | --- | --- | --- | --- | --- | --- | --- | --- | --- | --- | --- | --- | --- | --- | --- |

JAG44306.1

back to list

  

| Protein Coverage
| Supporting Peptides
|

Protein Coverage:

Supporting Peptides:

| Peptide | Uniq | -10lgP | Mass | Length | ppm | m/z | z | RT | Fraction | Scan | Source File | Area F6 | Area F2 | Area F3 | Area F4 | Area F5 | Area F7 | Area F8 | Area F1 | #Feature | #Feature F6 | #Feature F2 | #Feature F3 | #Feature F4 | #Feature F5 | #Feature F7 | #Feature F8 | #Feature F1 | Start | End | PTM | AScore | Found By |
| --- | --- | --- | --- | --- | --- | --- | --- | --- | --- | --- | --- | --- | --- | --- | --- | --- | --- | --- | --- | --- | --- | --- | --- | --- | --- | --- | --- | --- | --- | --- | --- | --- | --- |
| R.NEGTATYAAAVLFR.I | Y | 79.96 | 1482.7467 | 14 | 0.1 | 742.3807 | 2 | 48.35 | 8 | F8:27296 | 31122021\_RID\_3262\_1b.raw |  |  | 5.3493E5 |  |  |  |  | 7.7228E5 | 2 | 0 | 0 | 1 | 0 | 0 | 0 | 0 | 1 | 639 | 652 |  |  | PEAKS DB |
| R.LNYGIPAIVK.L | Y | 71.64 | 1086.6437 | 10 | 0.9 | 544.3296 | 2 | 44.80 | 8 | F8:24695 | 31122021\_RID\_3262\_1b.raw |  |  | 9.8473E5 | 2.0478E5 |  |  |  | 1.6368E6 | 3 | 0 | 0 | 1 | 1 | 0 | 0 | 0 | 1 | 479 | 488 |  |  | PEAKS DB |
| K.SAIVHLINYQDDAELATR.A | Y | 70.33 | 2028.0276 | 18 | 0.5 | 677.0168 | 3 | 43.83 | 3 | F3:27023 | 13012022\_RID\_3262\_3b.raw |  |  | 3.2757E5 |  |  |  |  | 1.1198E6 | 3 | 0 | 0 | 1 | 0 | 0 | 0 | 0 | 2 | 126 | 143 |  |  | PEAKS DB |
| R.VSVELTNSLFK.H | Y | 67.96 | 1235.6761 | 11 | -0.3 | 618.8452 | 2 | 47.06 | 8 | F8:26272 | 31122021\_RID\_3262\_1b.raw |  |  |  |  |  |  |  | 1.62E6 | 1 | 0 | 0 | 0 | 0 | 0 | 0 | 0 | 1 | 665 | 675 |  |  | PEAKS DB |
| R.TM(+15.99)QNTSDLDTAR.C | Y | 65.61 | 1367.5988 | 12 | 0.5 | 684.8070 | 2 | 18.97 | 8 | F8:4673 | 31122021\_RID\_3262\_1b.raw |  |  | 1.5244E5 |  |  |  |  | 1.2402E5 | 2 | 0 | 0 | 1 | 0 | 0 | 0 | 0 | 1 | 193 | 204 | Oxidation (M) | M2:Oxidation (M):1000.00 | PEAKS DB |
| R.TMQNTSDLDTAR.C | Y | 60.18 | 1351.6038 | 12 | 0.8 | 676.8097 | 2 | 24.85 | 3 | F3:10817 | 13012022\_RID\_3262\_3b.raw |  |  | 1.4351E5 |  |  |  |  | 4.1837E5 | 2 | 0 | 0 | 1 | 0 | 0 | 0 | 0 | 1 | 193 | 204 |  |  | PEAKS DB |
| K.AAMIVNQLSK.K | Y | 59.14 | 1073.5903 | 10 | 0.3 | 537.8026 | 2 | 32.46 | 8 | F8:14867 | 31122021\_RID\_3262\_1b.raw |  |  |  |  |  |  |  | 6.7476E5 | 1 | 0 | 0 | 0 | 0 | 0 | 0 | 0 | 1 | 163 | 172 |  |  | PEAKS DB |
| R.HPEAEMAQNSVR.L | Y | 57.43 | 1367.6251 | 12 | 0.4 | 456.8825 | 3 | 17.57 | 8 | F8:3951 | 31122021\_RID\_3262\_1b.raw |  |  |  |  |  |  |  | 4.095E5 | 2 | 0 | 0 | 0 | 0 | 0 | 0 | 0 | 2 | 467 | 478 |  |  | PEAKS DB |
| L.INYQDDAELATR.A | Y | 52.56 | 1407.6630 | 12 | 0.3 | 704.8390 | 2 | 32.97 | 8 | F8:15260 | 31122021\_RID\_3262\_1b.raw |  |  |  |  |  |  |  | 5.7938E4 | 1 | 0 | 0 | 0 | 0 | 0 | 0 | 0 | 1 | 132 | 143 |  |  | PEAKS DB |
| K.SGGIPALVR.M | Y | 51.41 | 868.5130 | 9 | 0.3 | 435.2639 | 2 | 34.23 | 8 | F8:16166 | 31122021\_RID\_3262\_1b.raw |  |  |  |  | 2.1025E5 |  |  | 9.0683E5 | 2 | 0 | 0 | 0 | 0 | 1 | 0 | 0 | 1 | 226 | 234 |  |  | PEAKS DB |
| R.ISEDKNPDYR.K | Y | 46.94 | 1235.5781 | 10 | 0.7 | 618.7968 | 2 | 18.42 | 3 | F3:5560 | 13012022\_RID\_3262\_3b.raw |  |  | 5.6021E4 |  |  |  |  |  | 1 | 0 | 0 | 1 | 0 | 0 | 0 | 0 | 0 | 653 | 662 |  |  | PEAKS DB |
| K.AAM(+15.99)IVNQLSK.K | Y | 45.42 | 1089.5852 | 10 | 1.1 | 545.8005 | 2 | 32.45 | 8 | F8:14843 | 31122021\_RID\_3262\_1b.raw |  |  |  |  |  |  |  | 6.6638E4 | 1 | 0 | 0 | 0 | 0 | 0 | 0 | 0 | 1 | 163 | 172 | Oxidation (M) | M3:Oxidation (M):1000.00 | PEAKS DB |
| K.RVSVELTNSLFK.H | Y | 45.25 | 1391.7772 | 12 | 0.9 | 696.8965 | 2 | 39.22 | 8 | F8:20209 | 31122021\_RID\_3262\_1b.raw |  |  |  |  |  |  |  | 3.6237E4 | 1 | 0 | 0 | 0 | 0 | 0 | 0 | 0 | 1 | 664 | 675 |  |  | PEAKS DB |
| total 13 peptides |  |  |  |  |  |  |  |  |  |  |  |  |  |  |  |  |  |  |  |  |  |  |  |  |  |  |  |  |  |  |  |  |  |
| --- | --- | --- | --- | --- | --- | --- | --- | --- | --- | --- | --- | --- | --- | --- | --- | --- | --- | --- | --- | --- | --- | --- | --- | --- | --- | --- | --- | --- | --- | --- | --- | --- | --- |

JAA96758.1

back to list

  

| Protein Coverage
| Supporting Peptides
|

Protein Coverage:

Supporting Peptides:

| Peptide | Uniq | -10lgP | Mass | Length | ppm | m/z | z | RT | Fraction | Scan | Source File | Area F6 | Area F2 | Area F3 | Area F4 | Area F5 | Area F7 | Area F8 | Area F1 | #Feature | #Feature F6 | #Feature F2 | #Feature F3 | #Feature F4 | #Feature F5 | #Feature F7 | #Feature F8 | #Feature F1 | Start | End | PTM | AScore | Found By |
| --- | --- | --- | --- | --- | --- | --- | --- | --- | --- | --- | --- | --- | --- | --- | --- | --- | --- | --- | --- | --- | --- | --- | --- | --- | --- | --- | --- | --- | --- | --- | --- | --- | --- |
| R.NEGTATYAAAVLFR.I | Y | 79.96 | 1482.7467 | 14 | 0.1 | 742.3807 | 2 | 48.35 | 8 | F8:27296 | 31122021\_RID\_3262\_1b.raw |  |  | 5.3493E5 |  |  |  |  | 7.7228E5 | 2 | 0 | 0 | 1 | 0 | 0 | 0 | 0 | 1 | 639 | 652 |  |  | PEAKS DB |
| R.LNYGIPAIVK.L | Y | 71.64 | 1086.6437 | 10 | 0.9 | 544.3296 | 2 | 44.80 | 8 | F8:24695 | 31122021\_RID\_3262\_1b.raw |  |  | 9.8473E5 | 2.0478E5 |  |  |  | 1.6368E6 | 3 | 0 | 0 | 1 | 1 | 0 | 0 | 0 | 1 | 479 | 488 |  |  | PEAKS DB |
| K.SAIVHLINYQDDAELATR.A | Y | 70.33 | 2028.0276 | 18 | 0.5 | 677.0168 | 3 | 43.83 | 3 | F3:27023 | 13012022\_RID\_3262\_3b.raw |  |  | 3.2757E5 |  |  |  |  | 1.1198E6 | 3 | 0 | 0 | 1 | 0 | 0 | 0 | 0 | 2 | 126 | 143 |  |  | PEAKS DB |
| R.VSVELTNSLFK.H | Y | 67.96 | 1235.6761 | 11 | -0.3 | 618.8452 | 2 | 47.06 | 8 | F8:26272 | 31122021\_RID\_3262\_1b.raw |  |  |  |  |  |  |  | 1.62E6 | 1 | 0 | 0 | 0 | 0 | 0 | 0 | 0 | 1 | 665 | 675 |  |  | PEAKS DB |
| R.TM(+15.99)QNTSDLDTAR.C | Y | 65.61 | 1367.5988 | 12 | 0.5 | 684.8070 | 2 | 18.97 | 8 | F8:4673 | 31122021\_RID\_3262\_1b.raw |  |  | 1.5244E5 |  |  |  |  | 1.2402E5 | 2 | 0 | 0 | 1 | 0 | 0 | 0 | 0 | 1 | 193 | 204 | Oxidation (M) | M2:Oxidation (M):1000.00 | PEAKS DB |
| R.TMQNTSDLDTAR.C | Y | 60.18 | 1351.6038 | 12 | 0.8 | 676.8097 | 2 | 24.85 | 3 | F3:10817 | 13012022\_RID\_3262\_3b.raw |  |  | 1.4351E5 |  |  |  |  | 4.1837E5 | 2 | 0 | 0 | 1 | 0 | 0 | 0 | 0 | 1 | 193 | 204 |  |  | PEAKS DB |
| K.AAMIVNQLSK.K | Y | 59.14 | 1073.5903 | 10 | 0.3 | 537.8026 | 2 | 32.46 | 8 | F8:14867 | 31122021\_RID\_3262\_1b.raw |  |  |  |  |  |  |  | 6.7476E5 | 1 | 0 | 0 | 0 | 0 | 0 | 0 | 0 | 1 | 163 | 172 |  |  | PEAKS DB |
| R.HPEAEMAQNSVR.L | Y | 57.43 | 1367.6251 | 12 | 0.4 | 456.8825 | 3 | 17.57 | 8 | F8:3951 | 31122021\_RID\_3262\_1b.raw |  |  |  |  |  |  |  | 4.095E5 | 2 | 0 | 0 | 0 | 0 | 0 | 0 | 0 | 2 | 467 | 478 |  |  | PEAKS DB |
| L.INYQDDAELATR.A | Y | 52.56 | 1407.6630 | 12 | 0.3 | 704.8390 | 2 | 32.97 | 8 | F8:15260 | 31122021\_RID\_3262\_1b.raw |  |  |  |  |  |  |  | 5.7938E4 | 1 | 0 | 0 | 0 | 0 | 0 | 0 | 0 | 1 | 132 | 143 |  |  | PEAKS DB |
| K.SGGIPALVR.M | Y | 51.41 | 868.5130 | 9 | 0.3 | 435.2639 | 2 | 34.23 | 8 | F8:16166 | 31122021\_RID\_3262\_1b.raw |  |  |  |  | 2.1025E5 |  |  | 9.0683E5 | 2 | 0 | 0 | 0 | 0 | 1 | 0 | 0 | 1 | 226 | 234 |  |  | PEAKS DB |
| R.ISEDKNPDYR.K | Y | 46.94 | 1235.5781 | 10 | 0.7 | 618.7968 | 2 | 18.42 | 3 | F3:5560 | 13012022\_RID\_3262\_3b.raw |  |  | 5.6021E4 |  |  |  |  |  | 1 | 0 | 0 | 1 | 0 | 0 | 0 | 0 | 0 | 653 | 662 |  |  | PEAKS DB |
| K.AAM(+15.99)IVNQLSK.K | Y | 45.42 | 1089.5852 | 10 | 1.1 | 545.8005 | 2 | 32.45 | 8 | F8:14843 | 31122021\_RID\_3262\_1b.raw |  |  |  |  |  |  |  | 6.6638E4 | 1 | 0 | 0 | 0 | 0 | 0 | 0 | 0 | 1 | 163 | 172 | Oxidation (M) | M3:Oxidation (M):1000.00 | PEAKS DB |
| K.RVSVELTNSLFK.H | Y | 45.25 | 1391.7772 | 12 | 0.9 | 696.8965 | 2 | 39.22 | 8 | F8:20209 | 31122021\_RID\_3262\_1b.raw |  |  |  |  |  |  |  | 3.6237E4 | 1 | 0 | 0 | 0 | 0 | 0 | 0 | 0 | 1 | 664 | 675 |  |  | PEAKS DB |
| total 13 peptides |  |  |  |  |  |  |  |  |  |  |  |  |  |  |  |  |  |  |  |  |  |  |  |  |  |  |  |  |  |  |  |  |  |
| --- | --- | --- | --- | --- | --- | --- | --- | --- | --- | --- | --- | --- | --- | --- | --- | --- | --- | --- | --- | --- | --- | --- | --- | --- | --- | --- | --- | --- | --- | --- | --- | --- | --- |

JAG46538.1

back to list

  

| Protein Coverage
| Supporting Peptides
|

Protein Coverage:

Supporting Peptides:

| Peptide | Uniq | -10lgP | Mass | Length | ppm | m/z | z | RT | Fraction | Scan | Source File | Area F6 | Area F2 | Area F3 | Area F4 | Area F5 | Area F7 | Area F8 | Area F1 | #Feature | #Feature F6 | #Feature F2 | #Feature F3 | #Feature F4 | #Feature F5 | #Feature F7 | #Feature F8 | #Feature F1 | Start | End | PTM | AScore | Found By |
| --- | --- | --- | --- | --- | --- | --- | --- | --- | --- | --- | --- | --- | --- | --- | --- | --- | --- | --- | --- | --- | --- | --- | --- | --- | --- | --- | --- | --- | --- | --- | --- | --- | --- |
| R.NEGTATYAAAVLFR.I | Y | 79.96 | 1482.7467 | 14 | 0.1 | 742.3807 | 2 | 48.35 | 8 | F8:27296 | 31122021\_RID\_3262\_1b.raw |  |  | 5.3493E5 |  |  |  |  | 7.7228E5 | 2 | 0 | 0 | 1 | 0 | 0 | 0 | 0 | 1 | 639 | 652 |  |  | PEAKS DB |
| R.LNYGIPAIVK.L | Y | 71.64 | 1086.6437 | 10 | 0.9 | 544.3296 | 2 | 44.80 | 8 | F8:24695 | 31122021\_RID\_3262\_1b.raw |  |  | 9.8473E5 | 2.0478E5 |  |  |  | 1.6368E6 | 3 | 0 | 0 | 1 | 1 | 0 | 0 | 0 | 1 | 479 | 488 |  |  | PEAKS DB |
| K.SAIVHLINYQDDAELATR.A | Y | 70.33 | 2028.0276 | 18 | 0.5 | 677.0168 | 3 | 43.83 | 3 | F3:27023 | 13012022\_RID\_3262\_3b.raw |  |  | 3.2757E5 |  |  |  |  | 1.1198E6 | 3 | 0 | 0 | 1 | 0 | 0 | 0 | 0 | 2 | 126 | 143 |  |  | PEAKS DB |
| R.VSVELTNSLFK.H | Y | 67.96 | 1235.6761 | 11 | -0.3 | 618.8452 | 2 | 47.06 | 8 | F8:26272 | 31122021\_RID\_3262\_1b.raw |  |  |  |  |  |  |  | 1.62E6 | 1 | 0 | 0 | 0 | 0 | 0 | 0 | 0 | 1 | 665 | 675 |  |  | PEAKS DB |
| R.TM(+15.99)QNTSDLDTAR.C | Y | 65.61 | 1367.5988 | 12 | 0.5 | 684.8070 | 2 | 18.97 | 8 | F8:4673 | 31122021\_RID\_3262\_1b.raw |  |  | 1.5244E5 |  |  |  |  | 1.2402E5 | 2 | 0 | 0 | 1 | 0 | 0 | 0 | 0 | 1 | 193 | 204 | Oxidation (M) | M2:Oxidation (M):1000.00 | PEAKS DB |
| R.TMQNTSDLDTAR.C | Y | 60.18 | 1351.6038 | 12 | 0.8 | 676.8097 | 2 | 24.85 | 3 | F3:10817 | 13012022\_RID\_3262\_3b.raw |  |  | 1.4351E5 |  |  |  |  | 4.1837E5 | 2 | 0 | 0 | 1 | 0 | 0 | 0 | 0 | 1 | 193 | 204 |  |  | PEAKS DB |
| K.AAMIVNQLSK.K | Y | 59.14 | 1073.5903 | 10 | 0.3 | 537.8026 | 2 | 32.46 | 8 | F8:14867 | 31122021\_RID\_3262\_1b.raw |  |  |  |  |  |  |  | 6.7476E5 | 1 | 0 | 0 | 0 | 0 | 0 | 0 | 0 | 1 | 163 | 172 |  |  | PEAKS DB |
| R.HPEAEMAQNSVR.L | Y | 57.43 | 1367.6251 | 12 | 0.4 | 456.8825 | 3 | 17.57 | 8 | F8:3951 | 31122021\_RID\_3262\_1b.raw |  |  |  |  |  |  |  | 4.095E5 | 2 | 0 | 0 | 0 | 0 | 0 | 0 | 0 | 2 | 467 | 478 |  |  | PEAKS DB |
| L.INYQDDAELATR.A | Y | 52.56 | 1407.6630 | 12 | 0.3 | 704.8390 | 2 | 32.97 | 8 | F8:15260 | 31122021\_RID\_3262\_1b.raw |  |  |  |  |  |  |  | 5.7938E4 | 1 | 0 | 0 | 0 | 0 | 0 | 0 | 0 | 1 | 132 | 143 |  |  | PEAKS DB |
| K.SGGIPALVR.M | Y | 51.41 | 868.5130 | 9 | 0.3 | 435.2639 | 2 | 34.23 | 8 | F8:16166 | 31122021\_RID\_3262\_1b.raw |  |  |  |  | 2.1025E5 |  |  | 9.0683E5 | 2 | 0 | 0 | 0 | 0 | 1 | 0 | 0 | 1 | 226 | 234 |  |  | PEAKS DB |
| R.ISEDKNPDYR.K | Y | 46.94 | 1235.5781 | 10 | 0.7 | 618.7968 | 2 | 18.42 | 3 | F3:5560 | 13012022\_RID\_3262\_3b.raw |  |  | 5.6021E4 |  |  |  |  |  | 1 | 0 | 0 | 1 | 0 | 0 | 0 | 0 | 0 | 653 | 662 |  |  | PEAKS DB |
| K.AAM(+15.99)IVNQLSK.K | Y | 45.42 | 1089.5852 | 10 | 1.1 | 545.8005 | 2 | 32.45 | 8 | F8:14843 | 31122021\_RID\_3262\_1b.raw |  |  |  |  |  |  |  | 6.6638E4 | 1 | 0 | 0 | 0 | 0 | 0 | 0 | 0 | 1 | 163 | 172 | Oxidation (M) | M3:Oxidation (M):1000.00 | PEAKS DB |
| K.RVSVELTNSLFK.H | Y | 45.25 | 1391.7772 | 12 | 0.9 | 696.8965 | 2 | 39.22 | 8 | F8:20209 | 31122021\_RID\_3262\_1b.raw |  |  |  |  |  |  |  | 3.6237E4 | 1 | 0 | 0 | 0 | 0 | 0 | 0 | 0 | 1 | 664 | 675 |  |  | PEAKS DB |
| total 13 peptides |  |  |  |  |  |  |  |  |  |  |  |  |  |  |  |  |  |  |  |  |  |  |  |  |  |  |  |  |  |  |  |  |  |
| --- | --- | --- | --- | --- | --- | --- | --- | --- | --- | --- | --- | --- | --- | --- | --- | --- | --- | --- | --- | --- | --- | --- | --- | --- | --- | --- | --- | --- | --- | --- | --- | --- | --- |

JAV50234.1

back to list

  

| Protein Coverage
| Supporting Peptides
|

Protein Coverage:

Supporting Peptides:

| Peptide | Uniq | -10lgP | Mass | Length | ppm | m/z | z | RT | Fraction | Scan | Source File | Area F6 | Area F2 | Area F3 | Area F4 | Area F5 | Area F7 | Area F8 | Area F1 | #Feature | #Feature F6 | #Feature F2 | #Feature F3 | #Feature F4 | #Feature F5 | #Feature F7 | #Feature F8 | #Feature F1 | Start | End | PTM | AScore | Found By |
| --- | --- | --- | --- | --- | --- | --- | --- | --- | --- | --- | --- | --- | --- | --- | --- | --- | --- | --- | --- | --- | --- | --- | --- | --- | --- | --- | --- | --- | --- | --- | --- | --- | --- |
| R.NEGTATYAAAVLFR.I | Y | 79.96 | 1482.7467 | 14 | 0.1 | 742.3807 | 2 | 48.35 | 8 | F8:27296 | 31122021\_RID\_3262\_1b.raw |  |  | 5.3493E5 |  |  |  |  | 7.7228E5 | 2 | 0 | 0 | 1 | 0 | 0 | 0 | 0 | 1 | 639 | 652 |  |  | PEAKS DB |
| R.LNYGIPAIVK.L | Y | 71.64 | 1086.6437 | 10 | 0.9 | 544.3296 | 2 | 44.80 | 8 | F8:24695 | 31122021\_RID\_3262\_1b.raw |  |  | 9.8473E5 | 2.0478E5 |  |  |  | 1.6368E6 | 3 | 0 | 0 | 1 | 1 | 0 | 0 | 0 | 1 | 479 | 488 |  |  | PEAKS DB |
| K.SAIVHLINYQDDAELATR.A | Y | 70.33 | 2028.0276 | 18 | 0.5 | 677.0168 | 3 | 43.83 | 3 | F3:27023 | 13012022\_RID\_3262\_3b.raw |  |  | 3.2757E5 |  |  |  |  | 1.1198E6 | 3 | 0 | 0 | 1 | 0 | 0 | 0 | 0 | 2 | 126 | 143 |  |  | PEAKS DB |
| R.VSVELTNSLFK.H | Y | 67.96 | 1235.6761 | 11 | -0.3 | 618.8452 | 2 | 47.06 | 8 | F8:26272 | 31122021\_RID\_3262\_1b.raw |  |  |  |  |  |  |  | 1.62E6 | 1 | 0 | 0 | 0 | 0 | 0 | 0 | 0 | 1 | 665 | 675 |  |  | PEAKS DB |
| R.TM(+15.99)QNTSDLDTAR.C | Y | 65.61 | 1367.5988 | 12 | 0.5 | 684.8070 | 2 | 18.97 | 8 | F8:4673 | 31122021\_RID\_3262\_1b.raw |  |  | 1.5244E5 |  |  |  |  | 1.2402E5 | 2 | 0 | 0 | 1 | 0 | 0 | 0 | 0 | 1 | 193 | 204 | Oxidation (M) | M2:Oxidation (M):1000.00 | PEAKS DB |
| R.TMQNTSDLDTAR.C | Y | 60.18 | 1351.6038 | 12 | 0.8 | 676.8097 | 2 | 24.85 | 3 | F3:10817 | 13012022\_RID\_3262\_3b.raw |  |  | 1.4351E5 |  |  |  |  | 4.1837E5 | 2 | 0 | 0 | 1 | 0 | 0 | 0 | 0 | 1 | 193 | 204 |  |  | PEAKS DB |
| K.AAMIVNQLSK.K | Y | 59.14 | 1073.5903 | 10 | 0.3 | 537.8026 | 2 | 32.46 | 8 | F8:14867 | 31122021\_RID\_3262\_1b.raw |  |  |  |  |  |  |  | 6.7476E5 | 1 | 0 | 0 | 0 | 0 | 0 | 0 | 0 | 1 | 163 | 172 |  |  | PEAKS DB |
| R.HPEAEMAQNSVR.L | Y | 57.43 | 1367.6251 | 12 | 0.4 | 456.8825 | 3 | 17.57 | 8 | F8:3951 | 31122021\_RID\_3262\_1b.raw |  |  |  |  |  |  |  | 4.095E5 | 2 | 0 | 0 | 0 | 0 | 0 | 0 | 0 | 2 | 467 | 478 |  |  | PEAKS DB |
| L.INYQDDAELATR.A | Y | 52.56 | 1407.6630 | 12 | 0.3 | 704.8390 | 2 | 32.97 | 8 | F8:15260 | 31122021\_RID\_3262\_1b.raw |  |  |  |  |  |  |  | 5.7938E4 | 1 | 0 | 0 | 0 | 0 | 0 | 0 | 0 | 1 | 132 | 143 |  |  | PEAKS DB |
| K.SGGIPALVR.M | Y | 51.41 | 868.5130 | 9 | 0.3 | 435.2639 | 2 | 34.23 | 8 | F8:16166 | 31122021\_RID\_3262\_1b.raw |  |  |  |  | 2.1025E5 |  |  | 9.0683E5 | 2 | 0 | 0 | 0 | 0 | 1 | 0 | 0 | 1 | 226 | 234 |  |  | PEAKS DB |
| R.ISEDKNPDYR.K | Y | 46.94 | 1235.5781 | 10 | 0.7 | 618.7968 | 2 | 18.42 | 3 | F3:5560 | 13012022\_RID\_3262\_3b.raw |  |  | 5.6021E4 |  |  |  |  |  | 1 | 0 | 0 | 1 | 0 | 0 | 0 | 0 | 0 | 653 | 662 |  |  | PEAKS DB |
| K.AAM(+15.99)IVNQLSK.K | Y | 45.42 | 1089.5852 | 10 | 1.1 | 545.8005 | 2 | 32.45 | 8 | F8:14843 | 31122021\_RID\_3262\_1b.raw |  |  |  |  |  |  |  | 6.6638E4 | 1 | 0 | 0 | 0 | 0 | 0 | 0 | 0 | 1 | 163 | 172 | Oxidation (M) | M3:Oxidation (M):1000.00 | PEAKS DB |
| K.RVSVELTNSLFK.H | Y | 45.25 | 1391.7772 | 12 | 0.9 | 696.8965 | 2 | 39.22 | 8 | F8:20209 | 31122021\_RID\_3262\_1b.raw |  |  |  |  |  |  |  | 3.6237E4 | 1 | 0 | 0 | 0 | 0 | 0 | 0 | 0 | 1 | 664 | 675 |  |  | PEAKS DB |
| total 13 peptides |  |  |  |  |  |  |  |  |  |  |  |  |  |  |  |  |  |  |  |  |  |  |  |  |  |  |  |  |  |  |  |  |  |
| --- | --- | --- | --- | --- | --- | --- | --- | --- | --- | --- | --- | --- | --- | --- | --- | --- | --- | --- | --- | --- | --- | --- | --- | --- | --- | --- | --- | --- | --- | --- | --- | --- | --- |

XP\_025022317.1

back to list

  

| Protein Coverage
| Supporting Peptides
|

Protein Coverage:

Supporting Peptides:

| Peptide | Uniq | -10lgP | Mass | Length | ppm | m/z | z | RT | Fraction | Scan | Source File | Area F6 | Area F2 | Area F3 | Area F4 | Area F5 | Area F7 | Area F8 | Area F1 | #Feature | #Feature F6 | #Feature F2 | #Feature F3 | #Feature F4 | #Feature F5 | #Feature F7 | #Feature F8 | #Feature F1 | Start | End | PTM | AScore | Found By |
| --- | --- | --- | --- | --- | --- | --- | --- | --- | --- | --- | --- | --- | --- | --- | --- | --- | --- | --- | --- | --- | --- | --- | --- | --- | --- | --- | --- | --- | --- | --- | --- | --- | --- |
| R.NEGTATYAAAVLFR.I | Y | 79.96 | 1482.7467 | 14 | 0.1 | 742.3807 | 2 | 48.35 | 8 | F8:27296 | 31122021\_RID\_3262\_1b.raw |  |  | 5.3493E5 |  |  |  |  | 7.7228E5 | 2 | 0 | 0 | 1 | 0 | 0 | 0 | 0 | 1 | 641 | 654 |  |  | PEAKS DB |
| R.LNYGIPAIVK.L | Y | 71.64 | 1086.6437 | 10 | 0.9 | 544.3296 | 2 | 44.80 | 8 | F8:24695 | 31122021\_RID\_3262\_1b.raw |  |  | 9.8473E5 | 2.0478E5 |  |  |  | 1.6368E6 | 3 | 0 | 0 | 1 | 1 | 0 | 0 | 0 | 1 | 481 | 490 |  |  | PEAKS DB |
| K.SAIVHLINYQDDAELATR.A | Y | 70.33 | 2028.0276 | 18 | 0.5 | 677.0168 | 3 | 43.83 | 3 | F3:27023 | 13012022\_RID\_3262\_3b.raw |  |  | 3.2757E5 |  |  |  |  | 1.1198E6 | 3 | 0 | 0 | 1 | 0 | 0 | 0 | 0 | 2 | 128 | 145 |  |  | PEAKS DB |
| R.VSVELTNSLFK.H | Y | 67.96 | 1235.6761 | 11 | -0.3 | 618.8452 | 2 | 47.06 | 8 | F8:26272 | 31122021\_RID\_3262\_1b.raw |  |  |  |  |  |  |  | 1.62E6 | 1 | 0 | 0 | 0 | 0 | 0 | 0 | 0 | 1 | 667 | 677 |  |  | PEAKS DB |
| R.TM(+15.99)QNTSDLDTAR.C | Y | 65.61 | 1367.5988 | 12 | 0.5 | 684.8070 | 2 | 18.97 | 8 | F8:4673 | 31122021\_RID\_3262\_1b.raw |  |  | 1.5244E5 |  |  |  |  | 1.2402E5 | 2 | 0 | 0 | 1 | 0 | 0 | 0 | 0 | 1 | 195 | 206 | Oxidation (M) | M2:Oxidation (M):1000.00 | PEAKS DB |
| R.TMQNTSDLDTAR.C | Y | 60.18 | 1351.6038 | 12 | 0.8 | 676.8097 | 2 | 24.85 | 3 | F3:10817 | 13012022\_RID\_3262\_3b.raw |  |  | 1.4351E5 |  |  |  |  | 4.1837E5 | 2 | 0 | 0 | 1 | 0 | 0 | 0 | 0 | 1 | 195 | 206 |  |  | PEAKS DB |
| K.AAMIVNQLSK.K | Y | 59.14 | 1073.5903 | 10 | 0.3 | 537.8026 | 2 | 32.46 | 8 | F8:14867 | 31122021\_RID\_3262\_1b.raw |  |  |  |  |  |  |  | 6.7476E5 | 1 | 0 | 0 | 0 | 0 | 0 | 0 | 0 | 1 | 165 | 174 |  |  | PEAKS DB |
| R.HPEAEMAQNSVR.L | Y | 57.43 | 1367.6251 | 12 | 0.4 | 456.8825 | 3 | 17.57 | 8 | F8:3951 | 31122021\_RID\_3262\_1b.raw |  |  |  |  |  |  |  | 4.095E5 | 2 | 0 | 0 | 0 | 0 | 0 | 0 | 0 | 2 | 469 | 480 |  |  | PEAKS DB |
| L.INYQDDAELATR.A | Y | 52.56 | 1407.6630 | 12 | 0.3 | 704.8390 | 2 | 32.97 | 8 | F8:15260 | 31122021\_RID\_3262\_1b.raw |  |  |  |  |  |  |  | 5.7938E4 | 1 | 0 | 0 | 0 | 0 | 0 | 0 | 0 | 1 | 134 | 145 |  |  | PEAKS DB |
| K.SGGIPALVR.M | Y | 51.41 | 868.5130 | 9 | 0.3 | 435.2639 | 2 | 34.23 | 8 | F8:16166 | 31122021\_RID\_3262\_1b.raw |  |  |  |  | 2.1025E5 |  |  | 9.0683E5 | 2 | 0 | 0 | 0 | 0 | 1 | 0 | 0 | 1 | 228 | 236 |  |  | PEAKS DB |
| R.ISEDKNPDYR.K | Y | 46.94 | 1235.5781 | 10 | 0.7 | 618.7968 | 2 | 18.42 | 3 | F3:5560 | 13012022\_RID\_3262\_3b.raw |  |  | 5.6021E4 |  |  |  |  |  | 1 | 0 | 0 | 1 | 0 | 0 | 0 | 0 | 0 | 655 | 664 |  |  | PEAKS DB |
| K.AAM(+15.99)IVNQLSK.K | Y | 45.42 | 1089.5852 | 10 | 1.1 | 545.8005 | 2 | 32.45 | 8 | F8:14843 | 31122021\_RID\_3262\_1b.raw |  |  |  |  |  |  |  | 6.6638E4 | 1 | 0 | 0 | 0 | 0 | 0 | 0 | 0 | 1 | 165 | 174 | Oxidation (M) | M3:Oxidation (M):1000.00 | PEAKS DB |
| K.RVSVELTNSLFK.H | Y | 45.25 | 1391.7772 | 12 | 0.9 | 696.8965 | 2 | 39.22 | 8 | F8:20209 | 31122021\_RID\_3262\_1b.raw |  |  |  |  |  |  |  | 3.6237E4 | 1 | 0 | 0 | 0 | 0 | 0 | 0 | 0 | 1 | 666 | 677 |  |  | PEAKS DB |
| total 13 peptides |  |  |  |  |  |  |  |  |  |  |  |  |  |  |  |  |  |  |  |  |  |  |  |  |  |  |  |  |  |  |  |  |  |
| --- | --- | --- | --- | --- | --- | --- | --- | --- | --- | --- | --- | --- | --- | --- | --- | --- | --- | --- | --- | --- | --- | --- | --- | --- | --- | --- | --- | --- | --- | --- | --- | --- | --- |

XP\_007428111.1

back to list

  

| Protein Coverage
| Supporting Peptides
|

Protein Coverage:

Supporting Peptides:

| Peptide | Uniq | -10lgP | Mass | Length | ppm | m/z | z | RT | Fraction | Scan | Source File | Area F6 | Area F2 | Area F3 | Area F4 | Area F5 | Area F7 | Area F8 | Area F1 | #Feature | #Feature F6 | #Feature F2 | #Feature F3 | #Feature F4 | #Feature F5 | #Feature F7 | #Feature F8 | #Feature F1 | Start | End | PTM | AScore | Found By |
| --- | --- | --- | --- | --- | --- | --- | --- | --- | --- | --- | --- | --- | --- | --- | --- | --- | --- | --- | --- | --- | --- | --- | --- | --- | --- | --- | --- | --- | --- | --- | --- | --- | --- |
| R.NEGTATYAAAVLFR.I | Y | 79.96 | 1482.7467 | 14 | 0.1 | 742.3807 | 2 | 48.35 | 8 | F8:27296 | 31122021\_RID\_3262\_1b.raw |  |  | 5.3493E5 |  |  |  |  | 7.7228E5 | 2 | 0 | 0 | 1 | 0 | 0 | 0 | 0 | 1 | 641 | 654 |  |  | PEAKS DB |
| R.LNYGIPAIVK.L | Y | 71.64 | 1086.6437 | 10 | 0.9 | 544.3296 | 2 | 44.80 | 8 | F8:24695 | 31122021\_RID\_3262\_1b.raw |  |  | 9.8473E5 | 2.0478E5 |  |  |  | 1.6368E6 | 3 | 0 | 0 | 1 | 1 | 0 | 0 | 0 | 1 | 481 | 490 |  |  | PEAKS DB |
| K.SAIVHLINYQDDAELATR.A | Y | 70.33 | 2028.0276 | 18 | 0.5 | 677.0168 | 3 | 43.83 | 3 | F3:27023 | 13012022\_RID\_3262\_3b.raw |  |  | 3.2757E5 |  |  |  |  | 1.1198E6 | 3 | 0 | 0 | 1 | 0 | 0 | 0 | 0 | 2 | 128 | 145 |  |  | PEAKS DB |
| R.VSVELTNSLFK.H | Y | 67.96 | 1235.6761 | 11 | -0.3 | 618.8452 | 2 | 47.06 | 8 | F8:26272 | 31122021\_RID\_3262\_1b.raw |  |  |  |  |  |  |  | 1.62E6 | 1 | 0 | 0 | 0 | 0 | 0 | 0 | 0 | 1 | 667 | 677 |  |  | PEAKS DB |
| R.TM(+15.99)QNTSDLDTAR.C | Y | 65.61 | 1367.5988 | 12 | 0.5 | 684.8070 | 2 | 18.97 | 8 | F8:4673 | 31122021\_RID\_3262\_1b.raw |  |  | 1.5244E5 |  |  |  |  | 1.2402E5 | 2 | 0 | 0 | 1 | 0 | 0 | 0 | 0 | 1 | 195 | 206 | Oxidation (M) | M2:Oxidation (M):1000.00 | PEAKS DB |
| R.TMQNTSDLDTAR.C | Y | 60.18 | 1351.6038 | 12 | 0.8 | 676.8097 | 2 | 24.85 | 3 | F3:10817 | 13012022\_RID\_3262\_3b.raw |  |  | 1.4351E5 |  |  |  |  | 4.1837E5 | 2 | 0 | 0 | 1 | 0 | 0 | 0 | 0 | 1 | 195 | 206 |  |  | PEAKS DB |
| K.AAMIVNQLSK.K | Y | 59.14 | 1073.5903 | 10 | 0.3 | 537.8026 | 2 | 32.46 | 8 | F8:14867 | 31122021\_RID\_3262\_1b.raw |  |  |  |  |  |  |  | 6.7476E5 | 1 | 0 | 0 | 0 | 0 | 0 | 0 | 0 | 1 | 165 | 174 |  |  | PEAKS DB |
| R.HPEAEMAQNSVR.L | Y | 57.43 | 1367.6251 | 12 | 0.4 | 456.8825 | 3 | 17.57 | 8 | F8:3951 | 31122021\_RID\_3262\_1b.raw |  |  |  |  |  |  |  | 4.095E5 | 2 | 0 | 0 | 0 | 0 | 0 | 0 | 0 | 2 | 469 | 480 |  |  | PEAKS DB |
| L.INYQDDAELATR.A | Y | 52.56 | 1407.6630 | 12 | 0.3 | 704.8390 | 2 | 32.97 | 8 | F8:15260 | 31122021\_RID\_3262\_1b.raw |  |  |  |  |  |  |  | 5.7938E4 | 1 | 0 | 0 | 0 | 0 | 0 | 0 | 0 | 1 | 134 | 145 |  |  | PEAKS DB |
| K.SGGIPALVR.M | Y | 51.41 | 868.5130 | 9 | 0.3 | 435.2639 | 2 | 34.23 | 8 | F8:16166 | 31122021\_RID\_3262\_1b.raw |  |  |  |  | 2.1025E5 |  |  | 9.0683E5 | 2 | 0 | 0 | 0 | 0 | 1 | 0 | 0 | 1 | 228 | 236 |  |  | PEAKS DB |
| R.ISEDKNPDYR.K | Y | 46.94 | 1235.5781 | 10 | 0.7 | 618.7968 | 2 | 18.42 | 3 | F3:5560 | 13012022\_RID\_3262\_3b.raw |  |  | 5.6021E4 |  |  |  |  |  | 1 | 0 | 0 | 1 | 0 | 0 | 0 | 0 | 0 | 655 | 664 |  |  | PEAKS DB |
| K.AAM(+15.99)IVNQLSK.K | Y | 45.42 | 1089.5852 | 10 | 1.1 | 545.8005 | 2 | 32.45 | 8 | F8:14843 | 31122021\_RID\_3262\_1b.raw |  |  |  |  |  |  |  | 6.6638E4 | 1 | 0 | 0 | 0 | 0 | 0 | 0 | 0 | 1 | 165 | 174 | Oxidation (M) | M3:Oxidation (M):1000.00 | PEAKS DB |
| K.RVSVELTNSLFK.H | Y | 45.25 | 1391.7772 | 12 | 0.9 | 696.8965 | 2 | 39.22 | 8 | F8:20209 | 31122021\_RID\_3262\_1b.raw |  |  |  |  |  |  |  | 3.6237E4 | 1 | 0 | 0 | 0 | 0 | 0 | 0 | 0 | 1 | 666 | 677 |  |  | PEAKS DB |
| total 13 peptides |  |  |  |  |  |  |  |  |  |  |  |  |  |  |  |  |  |  |  |  |  |  |  |  |  |  |  |  |  |  |  |  |  |
| --- | --- | --- | --- | --- | --- | --- | --- | --- | --- | --- | --- | --- | --- | --- | --- | --- | --- | --- | --- | --- | --- | --- | --- | --- | --- | --- | --- | --- | --- | --- | --- | --- | --- |

LAB43566.1

back to list

  

| Protein Coverage
| Supporting Peptides
|

Protein Coverage:

Supporting Peptides:

| Peptide | Uniq | -10lgP | Mass | Length | ppm | m/z | z | RT | Fraction | Scan | Source File | Area F6 | Area F2 | Area F3 | Area F4 | Area F5 | Area F7 | Area F8 | Area F1 | #Feature | #Feature F6 | #Feature F2 | #Feature F3 | #Feature F4 | #Feature F5 | #Feature F7 | #Feature F8 | #Feature F1 | Start | End | PTM | AScore | Found By |
| --- | --- | --- | --- | --- | --- | --- | --- | --- | --- | --- | --- | --- | --- | --- | --- | --- | --- | --- | --- | --- | --- | --- | --- | --- | --- | --- | --- | --- | --- | --- | --- | --- | --- |
| R.NEGTATYAAAVLFR.I | Y | 79.96 | 1482.7467 | 14 | 0.1 | 742.3807 | 2 | 48.35 | 8 | F8:27296 | 31122021\_RID\_3262\_1b.raw |  |  | 5.3493E5 |  |  |  |  | 7.7228E5 | 2 | 0 | 0 | 1 | 0 | 0 | 0 | 0 | 1 | 657 | 670 |  |  | PEAKS DB |
| R.LNYGIPAIVK.L | Y | 71.64 | 1086.6437 | 10 | 0.9 | 544.3296 | 2 | 44.80 | 8 | F8:24695 | 31122021\_RID\_3262\_1b.raw |  |  | 9.8473E5 | 2.0478E5 |  |  |  | 1.6368E6 | 3 | 0 | 0 | 1 | 1 | 0 | 0 | 0 | 1 | 503 | 512 |  |  | PEAKS DB |
| K.SAIVHLINYQDDAELATR.A | Y | 70.33 | 2028.0276 | 18 | 0.5 | 677.0168 | 3 | 43.83 | 3 | F3:27023 | 13012022\_RID\_3262\_3b.raw |  |  | 3.2757E5 |  |  |  |  | 1.1198E6 | 3 | 0 | 0 | 1 | 0 | 0 | 0 | 0 | 2 | 150 | 167 |  |  | PEAKS DB |
| R.VSVELTNSLFK.H | Y | 67.96 | 1235.6761 | 11 | -0.3 | 618.8452 | 2 | 47.06 | 8 | F8:26272 | 31122021\_RID\_3262\_1b.raw |  |  |  |  |  |  |  | 1.62E6 | 1 | 0 | 0 | 0 | 0 | 0 | 0 | 0 | 1 | 683 | 693 |  |  | PEAKS DB |
| R.TM(+15.99)QNTSDLDTAR.C | Y | 65.61 | 1367.5988 | 12 | 0.5 | 684.8070 | 2 | 18.97 | 8 | F8:4673 | 31122021\_RID\_3262\_1b.raw |  |  | 1.5244E5 |  |  |  |  | 1.2402E5 | 2 | 0 | 0 | 1 | 0 | 0 | 0 | 0 | 1 | 217 | 228 | Oxidation (M) | M2:Oxidation (M):1000.00 | PEAKS DB |
| R.TMQNTSDLDTAR.C | Y | 60.18 | 1351.6038 | 12 | 0.8 | 676.8097 | 2 | 24.85 | 3 | F3:10817 | 13012022\_RID\_3262\_3b.raw |  |  | 1.4351E5 |  |  |  |  | 4.1837E5 | 2 | 0 | 0 | 1 | 0 | 0 | 0 | 0 | 1 | 217 | 228 |  |  | PEAKS DB |
| K.AAMIVNQLSK.K | Y | 59.14 | 1073.5903 | 10 | 0.3 | 537.8026 | 2 | 32.46 | 8 | F8:14867 | 31122021\_RID\_3262\_1b.raw |  |  |  |  |  |  |  | 6.7476E5 | 1 | 0 | 0 | 0 | 0 | 0 | 0 | 0 | 1 | 187 | 196 |  |  | PEAKS DB |
| R.HPEAEMAQNSVR.L | Y | 57.43 | 1367.6251 | 12 | 0.4 | 456.8825 | 3 | 17.57 | 8 | F8:3951 | 31122021\_RID\_3262\_1b.raw |  |  |  |  |  |  |  | 4.095E5 | 2 | 0 | 0 | 0 | 0 | 0 | 0 | 0 | 2 | 491 | 502 |  |  | PEAKS DB |
| L.INYQDDAELATR.A | Y | 52.56 | 1407.6630 | 12 | 0.3 | 704.8390 | 2 | 32.97 | 8 | F8:15260 | 31122021\_RID\_3262\_1b.raw |  |  |  |  |  |  |  | 5.7938E4 | 1 | 0 | 0 | 0 | 0 | 0 | 0 | 0 | 1 | 156 | 167 |  |  | PEAKS DB |
| K.SGGIPALVR.M | Y | 51.41 | 868.5130 | 9 | 0.3 | 435.2639 | 2 | 34.23 | 8 | F8:16166 | 31122021\_RID\_3262\_1b.raw |  |  |  |  | 2.1025E5 |  |  | 9.0683E5 | 2 | 0 | 0 | 0 | 0 | 1 | 0 | 0 | 1 | 250 | 258 |  |  | PEAKS DB |
| R.ISEDKNPDYR.K | Y | 46.94 | 1235.5781 | 10 | 0.7 | 618.7968 | 2 | 18.42 | 3 | F3:5560 | 13012022\_RID\_3262\_3b.raw |  |  | 5.6021E4 |  |  |  |  |  | 1 | 0 | 0 | 1 | 0 | 0 | 0 | 0 | 0 | 671 | 680 |  |  | PEAKS DB |
| K.AAM(+15.99)IVNQLSK.K | Y | 45.42 | 1089.5852 | 10 | 1.1 | 545.8005 | 2 | 32.45 | 8 | F8:14843 | 31122021\_RID\_3262\_1b.raw |  |  |  |  |  |  |  | 6.6638E4 | 1 | 0 | 0 | 0 | 0 | 0 | 0 | 0 | 1 | 187 | 196 | Oxidation (M) | M3:Oxidation (M):1000.00 | PEAKS DB |
| K.RVSVELTNSLFK.H | Y | 45.25 | 1391.7772 | 12 | 0.9 | 696.8965 | 2 | 39.22 | 8 | F8:20209 | 31122021\_RID\_3262\_1b.raw |  |  |  |  |  |  |  | 3.6237E4 | 1 | 0 | 0 | 0 | 0 | 0 | 0 | 0 | 1 | 682 | 693 |  |  | PEAKS DB |
| total 13 peptides |  |  |  |  |  |  |  |  |  |  |  |  |  |  |  |  |  |  |  |  |  |  |  |  |  |  |  |  |  |  |  |  |  |
| --- | --- | --- | --- | --- | --- | --- | --- | --- | --- | --- | --- | --- | --- | --- | --- | --- | --- | --- | --- | --- | --- | --- | --- | --- | --- | --- | --- | --- | --- | --- | --- | --- | --- |

XP\_034271617.1

back to list

  

| Protein Coverage
| Supporting Peptides
|

Protein Coverage:

Supporting Peptides:

| Peptide | Uniq | -10lgP | Mass | Length | ppm | m/z | z | RT | Fraction | Scan | Source File | Area F6 | Area F2 | Area F3 | Area F4 | Area F5 | Area F7 | Area F8 | Area F1 | #Feature | #Feature F6 | #Feature F2 | #Feature F3 | #Feature F4 | #Feature F5 | #Feature F7 | #Feature F8 | #Feature F1 | Start | End | PTM | AScore | Found By |
| --- | --- | --- | --- | --- | --- | --- | --- | --- | --- | --- | --- | --- | --- | --- | --- | --- | --- | --- | --- | --- | --- | --- | --- | --- | --- | --- | --- | --- | --- | --- | --- | --- | --- |
| R.NEGTATYAAAVLFR.I | Y | 79.96 | 1482.7467 | 14 | 0.1 | 742.3807 | 2 | 48.35 | 8 | F8:27296 | 31122021\_RID\_3262\_1b.raw |  |  | 5.3493E5 |  |  |  |  | 7.7228E5 | 2 | 0 | 0 | 1 | 0 | 0 | 0 | 0 | 1 | 638 | 651 |  |  | PEAKS DB |
| R.LNYGIPAIVK.L | Y | 71.64 | 1086.6437 | 10 | 0.9 | 544.3296 | 2 | 44.80 | 8 | F8:24695 | 31122021\_RID\_3262\_1b.raw |  |  | 9.8473E5 | 2.0478E5 |  |  |  | 1.6368E6 | 3 | 0 | 0 | 1 | 1 | 0 | 0 | 0 | 1 | 478 | 487 |  |  | PEAKS DB |
| K.SAIVHLINYQDDAELATR.A | Y | 70.33 | 2028.0276 | 18 | 0.5 | 677.0168 | 3 | 43.83 | 3 | F3:27023 | 13012022\_RID\_3262\_3b.raw |  |  | 3.2757E5 |  |  |  |  | 1.1198E6 | 3 | 0 | 0 | 1 | 0 | 0 | 0 | 0 | 2 | 125 | 142 |  |  | PEAKS DB |
| R.VSVELTNSLFK.H | Y | 67.96 | 1235.6761 | 11 | -0.3 | 618.8452 | 2 | 47.06 | 8 | F8:26272 | 31122021\_RID\_3262\_1b.raw |  |  |  |  |  |  |  | 1.62E6 | 1 | 0 | 0 | 0 | 0 | 0 | 0 | 0 | 1 | 664 | 674 |  |  | PEAKS DB |
| R.TM(+15.99)QNTSDLDTAR.C | Y | 65.61 | 1367.5988 | 12 | 0.5 | 684.8070 | 2 | 18.97 | 8 | F8:4673 | 31122021\_RID\_3262\_1b.raw |  |  | 1.5244E5 |  |  |  |  | 1.2402E5 | 2 | 0 | 0 | 1 | 0 | 0 | 0 | 0 | 1 | 192 | 203 | Oxidation (M) | M2:Oxidation (M):1000.00 | PEAKS DB |
| R.TMQNTSDLDTAR.C | Y | 60.18 | 1351.6038 | 12 | 0.8 | 676.8097 | 2 | 24.85 | 3 | F3:10817 | 13012022\_RID\_3262\_3b.raw |  |  | 1.4351E5 |  |  |  |  | 4.1837E5 | 2 | 0 | 0 | 1 | 0 | 0 | 0 | 0 | 1 | 192 | 203 |  |  | PEAKS DB |
| K.AAMIVNQLSK.K | Y | 59.14 | 1073.5903 | 10 | 0.3 | 537.8026 | 2 | 32.46 | 8 | F8:14867 | 31122021\_RID\_3262\_1b.raw |  |  |  |  |  |  |  | 6.7476E5 | 1 | 0 | 0 | 0 | 0 | 0 | 0 | 0 | 1 | 162 | 171 |  |  | PEAKS DB |
| R.HPEAEMAQNSVR.L | Y | 57.43 | 1367.6251 | 12 | 0.4 | 456.8825 | 3 | 17.57 | 8 | F8:3951 | 31122021\_RID\_3262\_1b.raw |  |  |  |  |  |  |  | 4.095E5 | 2 | 0 | 0 | 0 | 0 | 0 | 0 | 0 | 2 | 466 | 477 |  |  | PEAKS DB |
| L.INYQDDAELATR.A | Y | 52.56 | 1407.6630 | 12 | 0.3 | 704.8390 | 2 | 32.97 | 8 | F8:15260 | 31122021\_RID\_3262\_1b.raw |  |  |  |  |  |  |  | 5.7938E4 | 1 | 0 | 0 | 0 | 0 | 0 | 0 | 0 | 1 | 131 | 142 |  |  | PEAKS DB |
| K.SGGIPALVR.M | Y | 51.41 | 868.5130 | 9 | 0.3 | 435.2639 | 2 | 34.23 | 8 | F8:16166 | 31122021\_RID\_3262\_1b.raw |  |  |  |  | 2.1025E5 |  |  | 9.0683E5 | 2 | 0 | 0 | 0 | 0 | 1 | 0 | 0 | 1 | 225 | 233 |  |  | PEAKS DB |
| R.ISEDKNPDYR.K | Y | 46.94 | 1235.5781 | 10 | 0.7 | 618.7968 | 2 | 18.42 | 3 | F3:5560 | 13012022\_RID\_3262\_3b.raw |  |  | 5.6021E4 |  |  |  |  |  | 1 | 0 | 0 | 1 | 0 | 0 | 0 | 0 | 0 | 652 | 661 |  |  | PEAKS DB |
| K.AAM(+15.99)IVNQLSK.K | Y | 45.42 | 1089.5852 | 10 | 1.1 | 545.8005 | 2 | 32.45 | 8 | F8:14843 | 31122021\_RID\_3262\_1b.raw |  |  |  |  |  |  |  | 6.6638E4 | 1 | 0 | 0 | 0 | 0 | 0 | 0 | 0 | 1 | 162 | 171 | Oxidation (M) | M3:Oxidation (M):1000.00 | PEAKS DB |
| K.RVSVELTNSLFK.H | Y | 45.25 | 1391.7772 | 12 | 0.9 | 696.8965 | 2 | 39.22 | 8 | F8:20209 | 31122021\_RID\_3262\_1b.raw |  |  |  |  |  |  |  | 3.6237E4 | 1 | 0 | 0 | 0 | 0 | 0 | 0 | 0 | 1 | 663 | 674 |  |  | PEAKS DB |
| total 13 peptides |  |  |  |  |  |  |  |  |  |  |  |  |  |  |  |  |  |  |  |  |  |  |  |  |  |  |  |  |  |  |  |  |  |
| --- | --- | --- | --- | --- | --- | --- | --- | --- | --- | --- | --- | --- | --- | --- | --- | --- | --- | --- | --- | --- | --- | --- | --- | --- | --- | --- | --- | --- | --- | --- | --- | --- | --- |

XP\_034271615.1

back to list

  

| Protein Coverage
| Supporting Peptides
|

Protein Coverage:

Supporting Peptides:

| Peptide | Uniq | -10lgP | Mass | Length | ppm | m/z | z | RT | Fraction | Scan | Source File | Area F6 | Area F2 | Area F3 | Area F4 | Area F5 | Area F7 | Area F8 | Area F1 | #Feature | #Feature F6 | #Feature F2 | #Feature F3 | #Feature F4 | #Feature F5 | #Feature F7 | #Feature F8 | #Feature F1 | Start | End | PTM | AScore | Found By |
| --- | --- | --- | --- | --- | --- | --- | --- | --- | --- | --- | --- | --- | --- | --- | --- | --- | --- | --- | --- | --- | --- | --- | --- | --- | --- | --- | --- | --- | --- | --- | --- | --- | --- |
| R.NEGTATYAAAVLFR.I | Y | 79.96 | 1482.7467 | 14 | 0.1 | 742.3807 | 2 | 48.35 | 8 | F8:27296 | 31122021\_RID\_3262\_1b.raw |  |  | 5.3493E5 |  |  |  |  | 7.7228E5 | 2 | 0 | 0 | 1 | 0 | 0 | 0 | 0 | 1 | 639 | 652 |  |  | PEAKS DB |
| R.LNYGIPAIVK.L | Y | 71.64 | 1086.6437 | 10 | 0.9 | 544.3296 | 2 | 44.80 | 8 | F8:24695 | 31122021\_RID\_3262\_1b.raw |  |  | 9.8473E5 | 2.0478E5 |  |  |  | 1.6368E6 | 3 | 0 | 0 | 1 | 1 | 0 | 0 | 0 | 1 | 479 | 488 |  |  | PEAKS DB |
| K.SAIVHLINYQDDAELATR.A | Y | 70.33 | 2028.0276 | 18 | 0.5 | 677.0168 | 3 | 43.83 | 3 | F3:27023 | 13012022\_RID\_3262\_3b.raw |  |  | 3.2757E5 |  |  |  |  | 1.1198E6 | 3 | 0 | 0 | 1 | 0 | 0 | 0 | 0 | 2 | 126 | 143 |  |  | PEAKS DB |
| R.VSVELTNSLFK.H | Y | 67.96 | 1235.6761 | 11 | -0.3 | 618.8452 | 2 | 47.06 | 8 | F8:26272 | 31122021\_RID\_3262\_1b.raw |  |  |  |  |  |  |  | 1.62E6 | 1 | 0 | 0 | 0 | 0 | 0 | 0 | 0 | 1 | 665 | 675 |  |  | PEAKS DB |
| R.TM(+15.99)QNTSDLDTAR.C | Y | 65.61 | 1367.5988 | 12 | 0.5 | 684.8070 | 2 | 18.97 | 8 | F8:4673 | 31122021\_RID\_3262\_1b.raw |  |  | 1.5244E5 |  |  |  |  | 1.2402E5 | 2 | 0 | 0 | 1 | 0 | 0 | 0 | 0 | 1 | 193 | 204 | Oxidation (M) | M2:Oxidation (M):1000.00 | PEAKS DB |
| R.TMQNTSDLDTAR.C | Y | 60.18 | 1351.6038 | 12 | 0.8 | 676.8097 | 2 | 24.85 | 3 | F3:10817 | 13012022\_RID\_3262\_3b.raw |  |  | 1.4351E5 |  |  |  |  | 4.1837E5 | 2 | 0 | 0 | 1 | 0 | 0 | 0 | 0 | 1 | 193 | 204 |  |  | PEAKS DB |
| K.AAMIVNQLSK.K | Y | 59.14 | 1073.5903 | 10 | 0.3 | 537.8026 | 2 | 32.46 | 8 | F8:14867 | 31122021\_RID\_3262\_1b.raw |  |  |  |  |  |  |  | 6.7476E5 | 1 | 0 | 0 | 0 | 0 | 0 | 0 | 0 | 1 | 163 | 172 |  |  | PEAKS DB |
| R.HPEAEMAQNSVR.L | Y | 57.43 | 1367.6251 | 12 | 0.4 | 456.8825 | 3 | 17.57 | 8 | F8:3951 | 31122021\_RID\_3262\_1b.raw |  |  |  |  |  |  |  | 4.095E5 | 2 | 0 | 0 | 0 | 0 | 0 | 0 | 0 | 2 | 467 | 478 |  |  | PEAKS DB |
| L.INYQDDAELATR.A | Y | 52.56 | 1407.6630 | 12 | 0.3 | 704.8390 | 2 | 32.97 | 8 | F8:15260 | 31122021\_RID\_3262\_1b.raw |  |  |  |  |  |  |  | 5.7938E4 | 1 | 0 | 0 | 0 | 0 | 0 | 0 | 0 | 1 | 132 | 143 |  |  | PEAKS DB |
| K.SGGIPALVR.M | Y | 51.41 | 868.5130 | 9 | 0.3 | 435.2639 | 2 | 34.23 | 8 | F8:16166 | 31122021\_RID\_3262\_1b.raw |  |  |  |  | 2.1025E5 |  |  | 9.0683E5 | 2 | 0 | 0 | 0 | 0 | 1 | 0 | 0 | 1 | 226 | 234 |  |  | PEAKS DB |
| R.ISEDKNPDYR.K | Y | 46.94 | 1235.5781 | 10 | 0.7 | 618.7968 | 2 | 18.42 | 3 | F3:5560 | 13012022\_RID\_3262\_3b.raw |  |  | 5.6021E4 |  |  |  |  |  | 1 | 0 | 0 | 1 | 0 | 0 | 0 | 0 | 0 | 653 | 662 |  |  | PEAKS DB |
| K.AAM(+15.99)IVNQLSK.K | Y | 45.42 | 1089.5852 | 10 | 1.1 | 545.8005 | 2 | 32.45 | 8 | F8:14843 | 31122021\_RID\_3262\_1b.raw |  |  |  |  |  |  |  | 6.6638E4 | 1 | 0 | 0 | 0 | 0 | 0 | 0 | 0 | 1 | 163 | 172 | Oxidation (M) | M3:Oxidation (M):1000.00 | PEAKS DB |
| K.RVSVELTNSLFK.H | Y | 45.25 | 1391.7772 | 12 | 0.9 | 696.8965 | 2 | 39.22 | 8 | F8:20209 | 31122021\_RID\_3262\_1b.raw |  |  |  |  |  |  |  | 3.6237E4 | 1 | 0 | 0 | 0 | 0 | 0 | 0 | 0 | 1 | 664 | 675 |  |  | PEAKS DB |
| total 13 peptides |  |  |  |  |  |  |  |  |  |  |  |  |  |  |  |  |  |  |  |  |  |  |  |  |  |  |  |  |  |  |  |  |  |
| --- | --- | --- | --- | --- | --- | --- | --- | --- | --- | --- | --- | --- | --- | --- | --- | --- | --- | --- | --- | --- | --- | --- | --- | --- | --- | --- | --- | --- | --- | --- | --- | --- | --- |

XP\_013919280.1

back to list

  

| Protein Coverage
| Supporting Peptides
|

Protein Coverage:

Supporting Peptides:

| Peptide | Uniq | -10lgP | Mass | Length | ppm | m/z | z | RT | Fraction | Scan | Source File | Area F6 | Area F2 | Area F3 | Area F4 | Area F5 | Area F7 | Area F8 | Area F1 | #Feature | #Feature F6 | #Feature F2 | #Feature F3 | #Feature F4 | #Feature F5 | #Feature F7 | #Feature F8 | #Feature F1 | Start | End | PTM | AScore | Found By |
| --- | --- | --- | --- | --- | --- | --- | --- | --- | --- | --- | --- | --- | --- | --- | --- | --- | --- | --- | --- | --- | --- | --- | --- | --- | --- | --- | --- | --- | --- | --- | --- | --- | --- |
| R.NEGTATYAAAVLFR.I | Y | 79.96 | 1482.7467 | 14 | 0.1 | 742.3807 | 2 | 48.35 | 8 | F8:27296 | 31122021\_RID\_3262\_1b.raw |  |  | 5.3493E5 |  |  |  |  | 7.7228E5 | 2 | 0 | 0 | 1 | 0 | 0 | 0 | 0 | 1 | 639 | 652 |  |  | PEAKS DB |
| R.LNYGIPAIVK.L | Y | 71.64 | 1086.6437 | 10 | 0.9 | 544.3296 | 2 | 44.80 | 8 | F8:24695 | 31122021\_RID\_3262\_1b.raw |  |  | 9.8473E5 | 2.0478E5 |  |  |  | 1.6368E6 | 3 | 0 | 0 | 1 | 1 | 0 | 0 | 0 | 1 | 479 | 488 |  |  | PEAKS DB |
| K.SAIVHLINYQDDAELATR.A | Y | 70.33 | 2028.0276 | 18 | 0.5 | 677.0168 | 3 | 43.83 | 3 | F3:27023 | 13012022\_RID\_3262\_3b.raw |  |  | 3.2757E5 |  |  |  |  | 1.1198E6 | 3 | 0 | 0 | 1 | 0 | 0 | 0 | 0 | 2 | 126 | 143 |  |  | PEAKS DB |
| R.VSVELTNSLFK.H | Y | 67.96 | 1235.6761 | 11 | -0.3 | 618.8452 | 2 | 47.06 | 8 | F8:26272 | 31122021\_RID\_3262\_1b.raw |  |  |  |  |  |  |  | 1.62E6 | 1 | 0 | 0 | 0 | 0 | 0 | 0 | 0 | 1 | 665 | 675 |  |  | PEAKS DB |
| R.TM(+15.99)QNTSDLDTAR.C | Y | 65.61 | 1367.5988 | 12 | 0.5 | 684.8070 | 2 | 18.97 | 8 | F8:4673 | 31122021\_RID\_3262\_1b.raw |  |  | 1.5244E5 |  |  |  |  | 1.2402E5 | 2 | 0 | 0 | 1 | 0 | 0 | 0 | 0 | 1 | 193 | 204 | Oxidation (M) | M2:Oxidation (M):1000.00 | PEAKS DB |
| R.TMQNTSDLDTAR.C | Y | 60.18 | 1351.6038 | 12 | 0.8 | 676.8097 | 2 | 24.85 | 3 | F3:10817 | 13012022\_RID\_3262\_3b.raw |  |  | 1.4351E5 |  |  |  |  | 4.1837E5 | 2 | 0 | 0 | 1 | 0 | 0 | 0 | 0 | 1 | 193 | 204 |  |  | PEAKS DB |
| K.AAMIVNQLSK.K | Y | 59.14 | 1073.5903 | 10 | 0.3 | 537.8026 | 2 | 32.46 | 8 | F8:14867 | 31122021\_RID\_3262\_1b.raw |  |  |  |  |  |  |  | 6.7476E5 | 1 | 0 | 0 | 0 | 0 | 0 | 0 | 0 | 1 | 163 | 172 |  |  | PEAKS DB |
| R.HPEAEMAQNSVR.L | Y | 57.43 | 1367.6251 | 12 | 0.4 | 456.8825 | 3 | 17.57 | 8 | F8:3951 | 31122021\_RID\_3262\_1b.raw |  |  |  |  |  |  |  | 4.095E5 | 2 | 0 | 0 | 0 | 0 | 0 | 0 | 0 | 2 | 467 | 478 |  |  | PEAKS DB |
| L.INYQDDAELATR.A | Y | 52.56 | 1407.6630 | 12 | 0.3 | 704.8390 | 2 | 32.97 | 8 | F8:15260 | 31122021\_RID\_3262\_1b.raw |  |  |  |  |  |  |  | 5.7938E4 | 1 | 0 | 0 | 0 | 0 | 0 | 0 | 0 | 1 | 132 | 143 |  |  | PEAKS DB |
| K.SGGIPALVR.M | Y | 51.41 | 868.5130 | 9 | 0.3 | 435.2639 | 2 | 34.23 | 8 | F8:16166 | 31122021\_RID\_3262\_1b.raw |  |  |  |  | 2.1025E5 |  |  | 9.0683E5 | 2 | 0 | 0 | 0 | 0 | 1 | 0 | 0 | 1 | 226 | 234 |  |  | PEAKS DB |
| R.ISEDKNPDYR.K | Y | 46.94 | 1235.5781 | 10 | 0.7 | 618.7968 | 2 | 18.42 | 3 | F3:5560 | 13012022\_RID\_3262\_3b.raw |  |  | 5.6021E4 |  |  |  |  |  | 1 | 0 | 0 | 1 | 0 | 0 | 0 | 0 | 0 | 653 | 662 |  |  | PEAKS DB |
| K.AAM(+15.99)IVNQLSK.K | Y | 45.42 | 1089.5852 | 10 | 1.1 | 545.8005 | 2 | 32.45 | 8 | F8:14843 | 31122021\_RID\_3262\_1b.raw |  |  |  |  |  |  |  | 6.6638E4 | 1 | 0 | 0 | 0 | 0 | 0 | 0 | 0 | 1 | 163 | 172 | Oxidation (M) | M3:Oxidation (M):1000.00 | PEAKS DB |
| K.RVSVELTNSLFK.H | Y | 45.25 | 1391.7772 | 12 | 0.9 | 696.8965 | 2 | 39.22 | 8 | F8:20209 | 31122021\_RID\_3262\_1b.raw |  |  |  |  |  |  |  | 3.6237E4 | 1 | 0 | 0 | 0 | 0 | 0 | 0 | 0 | 1 | 664 | 675 |  |  | PEAKS DB |
| total 13 peptides |  |  |  |  |  |  |  |  |  |  |  |  |  |  |  |  |  |  |  |  |  |  |  |  |  |  |  |  |  |  |  |  |  |
| --- | --- | --- | --- | --- | --- | --- | --- | --- | --- | --- | --- | --- | --- | --- | --- | --- | --- | --- | --- | --- | --- | --- | --- | --- | --- | --- | --- | --- | --- | --- | --- | --- | --- |

XP\_034271618.1

back to list

  

| Protein Coverage
| Supporting Peptides
|

Protein Coverage:

Supporting Peptides:

| Peptide | Uniq | -10lgP | Mass | Length | ppm | m/z | z | RT | Fraction | Scan | Source File | Area F6 | Area F2 | Area F3 | Area F4 | Area F5 | Area F7 | Area F8 | Area F1 | #Feature | #Feature F6 | #Feature F2 | #Feature F3 | #Feature F4 | #Feature F5 | #Feature F7 | #Feature F8 | #Feature F1 | Start | End | PTM | AScore | Found By |
| --- | --- | --- | --- | --- | --- | --- | --- | --- | --- | --- | --- | --- | --- | --- | --- | --- | --- | --- | --- | --- | --- | --- | --- | --- | --- | --- | --- | --- | --- | --- | --- | --- | --- |
| R.NEGTATYAAAVLFR.I | Y | 79.96 | 1482.7467 | 14 | 0.1 | 742.3807 | 2 | 48.35 | 8 | F8:27296 | 31122021\_RID\_3262\_1b.raw |  |  | 5.3493E5 |  |  |  |  | 7.7228E5 | 2 | 0 | 0 | 1 | 0 | 0 | 0 | 0 | 1 | 639 | 652 |  |  | PEAKS DB |
| R.LNYGIPAIVK.L | Y | 71.64 | 1086.6437 | 10 | 0.9 | 544.3296 | 2 | 44.80 | 8 | F8:24695 | 31122021\_RID\_3262\_1b.raw |  |  | 9.8473E5 | 2.0478E5 |  |  |  | 1.6368E6 | 3 | 0 | 0 | 1 | 1 | 0 | 0 | 0 | 1 | 479 | 488 |  |  | PEAKS DB |
| K.SAIVHLINYQDDAELATR.A | Y | 70.33 | 2028.0276 | 18 | 0.5 | 677.0168 | 3 | 43.83 | 3 | F3:27023 | 13012022\_RID\_3262\_3b.raw |  |  | 3.2757E5 |  |  |  |  | 1.1198E6 | 3 | 0 | 0 | 1 | 0 | 0 | 0 | 0 | 2 | 126 | 143 |  |  | PEAKS DB |
| R.VSVELTNSLFK.H | Y | 67.96 | 1235.6761 | 11 | -0.3 | 618.8452 | 2 | 47.06 | 8 | F8:26272 | 31122021\_RID\_3262\_1b.raw |  |  |  |  |  |  |  | 1.62E6 | 1 | 0 | 0 | 0 | 0 | 0 | 0 | 0 | 1 | 665 | 675 |  |  | PEAKS DB |
| R.TM(+15.99)QNTSDLDTAR.C | Y | 65.61 | 1367.5988 | 12 | 0.5 | 684.8070 | 2 | 18.97 | 8 | F8:4673 | 31122021\_RID\_3262\_1b.raw |  |  | 1.5244E5 |  |  |  |  | 1.2402E5 | 2 | 0 | 0 | 1 | 0 | 0 | 0 | 0 | 1 | 193 | 204 | Oxidation (M) | M2:Oxidation (M):1000.00 | PEAKS DB |
| R.TMQNTSDLDTAR.C | Y | 60.18 | 1351.6038 | 12 | 0.8 | 676.8097 | 2 | 24.85 | 3 | F3:10817 | 13012022\_RID\_3262\_3b.raw |  |  | 1.4351E5 |  |  |  |  | 4.1837E5 | 2 | 0 | 0 | 1 | 0 | 0 | 0 | 0 | 1 | 193 | 204 |  |  | PEAKS DB |
| K.AAMIVNQLSK.K | Y | 59.14 | 1073.5903 | 10 | 0.3 | 537.8026 | 2 | 32.46 | 8 | F8:14867 | 31122021\_RID\_3262\_1b.raw |  |  |  |  |  |  |  | 6.7476E5 | 1 | 0 | 0 | 0 | 0 | 0 | 0 | 0 | 1 | 163 | 172 |  |  | PEAKS DB |
| R.HPEAEMAQNSVR.L | Y | 57.43 | 1367.6251 | 12 | 0.4 | 456.8825 | 3 | 17.57 | 8 | F8:3951 | 31122021\_RID\_3262\_1b.raw |  |  |  |  |  |  |  | 4.095E5 | 2 | 0 | 0 | 0 | 0 | 0 | 0 | 0 | 2 | 467 | 478 |  |  | PEAKS DB |
| L.INYQDDAELATR.A | Y | 52.56 | 1407.6630 | 12 | 0.3 | 704.8390 | 2 | 32.97 | 8 | F8:15260 | 31122021\_RID\_3262\_1b.raw |  |  |  |  |  |  |  | 5.7938E4 | 1 | 0 | 0 | 0 | 0 | 0 | 0 | 0 | 1 | 132 | 143 |  |  | PEAKS DB |
| K.SGGIPALVR.M | Y | 51.41 | 868.5130 | 9 | 0.3 | 435.2639 | 2 | 34.23 | 8 | F8:16166 | 31122021\_RID\_3262\_1b.raw |  |  |  |  | 2.1025E5 |  |  | 9.0683E5 | 2 | 0 | 0 | 0 | 0 | 1 | 0 | 0 | 1 | 226 | 234 |  |  | PEAKS DB |
| R.ISEDKNPDYR.K | Y | 46.94 | 1235.5781 | 10 | 0.7 | 618.7968 | 2 | 18.42 | 3 | F3:5560 | 13012022\_RID\_3262\_3b.raw |  |  | 5.6021E4 |  |  |  |  |  | 1 | 0 | 0 | 1 | 0 | 0 | 0 | 0 | 0 | 653 | 662 |  |  | PEAKS DB |
| K.AAM(+15.99)IVNQLSK.K | Y | 45.42 | 1089.5852 | 10 | 1.1 | 545.8005 | 2 | 32.45 | 8 | F8:14843 | 31122021\_RID\_3262\_1b.raw |  |  |  |  |  |  |  | 6.6638E4 | 1 | 0 | 0 | 0 | 0 | 0 | 0 | 0 | 1 | 163 | 172 | Oxidation (M) | M3:Oxidation (M):1000.00 | PEAKS DB |
| K.RVSVELTNSLFK.H | Y | 45.25 | 1391.7772 | 12 | 0.9 | 696.8965 | 2 | 39.22 | 8 | F8:20209 | 31122021\_RID\_3262\_1b.raw |  |  |  |  |  |  |  | 3.6237E4 | 1 | 0 | 0 | 0 | 0 | 0 | 0 | 0 | 1 | 664 | 675 |  |  | PEAKS DB |
| total 13 peptides |  |  |  |  |  |  |  |  |  |  |  |  |  |  |  |  |  |  |  |  |  |  |  |  |  |  |  |  |  |  |  |  |  |
| --- | --- | --- | --- | --- | --- | --- | --- | --- | --- | --- | --- | --- | --- | --- | --- | --- | --- | --- | --- | --- | --- | --- | --- | --- | --- | --- | --- | --- | --- | --- | --- | --- | --- |

JAG67632.1

back to list

  

| Protein Coverage
| Supporting Peptides
|

Protein Coverage:

Supporting Peptides:

| Peptide | Uniq | -10lgP | Mass | Length | ppm | m/z | z | RT | Fraction | Scan | Source File | Area F6 | Area F2 | Area F3 | Area F4 | Area F5 | Area F7 | Area F8 | Area F1 | #Feature | #Feature F6 | #Feature F2 | #Feature F3 | #Feature F4 | #Feature F5 | #Feature F7 | #Feature F8 | #Feature F1 | Start | End | PTM | AScore | Found By |
| --- | --- | --- | --- | --- | --- | --- | --- | --- | --- | --- | --- | --- | --- | --- | --- | --- | --- | --- | --- | --- | --- | --- | --- | --- | --- | --- | --- | --- | --- | --- | --- | --- | --- |
| R.NEGTATYAAAVLFR.I | Y | 79.96 | 1482.7467 | 14 | 0.1 | 742.3807 | 2 | 48.35 | 8 | F8:27296 | 31122021\_RID\_3262\_1b.raw |  |  | 5.3493E5 |  |  |  |  | 7.7228E5 | 2 | 0 | 0 | 1 | 0 | 0 | 0 | 0 | 1 | 639 | 652 |  |  | PEAKS DB |
| R.LNYGIPAIVK.L | Y | 71.64 | 1086.6437 | 10 | 0.9 | 544.3296 | 2 | 44.80 | 8 | F8:24695 | 31122021\_RID\_3262\_1b.raw |  |  | 9.8473E5 | 2.0478E5 |  |  |  | 1.6368E6 | 3 | 0 | 0 | 1 | 1 | 0 | 0 | 0 | 1 | 479 | 488 |  |  | PEAKS DB |
| K.SAIVHLINYQDDAELATR.A | Y | 70.33 | 2028.0276 | 18 | 0.5 | 677.0168 | 3 | 43.83 | 3 | F3:27023 | 13012022\_RID\_3262\_3b.raw |  |  | 3.2757E5 |  |  |  |  | 1.1198E6 | 3 | 0 | 0 | 1 | 0 | 0 | 0 | 0 | 2 | 126 | 143 |  |  | PEAKS DB |
| R.VSVELTNSLFK.H | Y | 67.96 | 1235.6761 | 11 | -0.3 | 618.8452 | 2 | 47.06 | 8 | F8:26272 | 31122021\_RID\_3262\_1b.raw |  |  |  |  |  |  |  | 1.62E6 | 1 | 0 | 0 | 0 | 0 | 0 | 0 | 0 | 1 | 665 | 675 |  |  | PEAKS DB |
| R.TM(+15.99)QNTSDLDTAR.C | Y | 65.61 | 1367.5988 | 12 | 0.5 | 684.8070 | 2 | 18.97 | 8 | F8:4673 | 31122021\_RID\_3262\_1b.raw |  |  | 1.5244E5 |  |  |  |  | 1.2402E5 | 2 | 0 | 0 | 1 | 0 | 0 | 0 | 0 | 1 | 193 | 204 | Oxidation (M) | M2:Oxidation (M):1000.00 | PEAKS DB |
| R.TMQNTSDLDTAR.C | Y | 60.18 | 1351.6038 | 12 | 0.8 | 676.8097 | 2 | 24.85 | 3 | F3:10817 | 13012022\_RID\_3262\_3b.raw |  |  | 1.4351E5 |  |  |  |  | 4.1837E5 | 2 | 0 | 0 | 1 | 0 | 0 | 0 | 0 | 1 | 193 | 204 |  |  | PEAKS DB |
| K.AAMIVNQLSK.K | Y | 59.14 | 1073.5903 | 10 | 0.3 | 537.8026 | 2 | 32.46 | 8 | F8:14867 | 31122021\_RID\_3262\_1b.raw |  |  |  |  |  |  |  | 6.7476E5 | 1 | 0 | 0 | 0 | 0 | 0 | 0 | 0 | 1 | 163 | 172 |  |  | PEAKS DB |
| R.HPEAEMAQNSVR.L | Y | 57.43 | 1367.6251 | 12 | 0.4 | 456.8825 | 3 | 17.57 | 8 | F8:3951 | 31122021\_RID\_3262\_1b.raw |  |  |  |  |  |  |  | 4.095E5 | 2 | 0 | 0 | 0 | 0 | 0 | 0 | 0 | 2 | 467 | 478 |  |  | PEAKS DB |
| L.INYQDDAELATR.A | Y | 52.56 | 1407.6630 | 12 | 0.3 | 704.8390 | 2 | 32.97 | 8 | F8:15260 | 31122021\_RID\_3262\_1b.raw |  |  |  |  |  |  |  | 5.7938E4 | 1 | 0 | 0 | 0 | 0 | 0 | 0 | 0 | 1 | 132 | 143 |  |  | PEAKS DB |
| K.SGGIPALVR.M | Y | 51.41 | 868.5130 | 9 | 0.3 | 435.2639 | 2 | 34.23 | 8 | F8:16166 | 31122021\_RID\_3262\_1b.raw |  |  |  |  | 2.1025E5 |  |  | 9.0683E5 | 2 | 0 | 0 | 0 | 0 | 1 | 0 | 0 | 1 | 226 | 234 |  |  | PEAKS DB |
| R.ISEDKNPDYR.K | Y | 46.94 | 1235.5781 | 10 | 0.7 | 618.7968 | 2 | 18.42 | 3 | F3:5560 | 13012022\_RID\_3262\_3b.raw |  |  | 5.6021E4 |  |  |  |  |  | 1 | 0 | 0 | 1 | 0 | 0 | 0 | 0 | 0 | 653 | 662 |  |  | PEAKS DB |
| K.AAM(+15.99)IVNQLSK.K | Y | 45.42 | 1089.5852 | 10 | 1.1 | 545.8005 | 2 | 32.45 | 8 | F8:14843 | 31122021\_RID\_3262\_1b.raw |  |  |  |  |  |  |  | 6.6638E4 | 1 | 0 | 0 | 0 | 0 | 0 | 0 | 0 | 1 | 163 | 172 | Oxidation (M) | M3:Oxidation (M):1000.00 | PEAKS DB |
| K.RVSVELTNSLFK.H | Y | 45.25 | 1391.7772 | 12 | 0.9 | 696.8965 | 2 | 39.22 | 8 | F8:20209 | 31122021\_RID\_3262\_1b.raw |  |  |  |  |  |  |  | 3.6237E4 | 1 | 0 | 0 | 0 | 0 | 0 | 0 | 0 | 1 | 664 | 675 |  |  | PEAKS DB |
| total 13 peptides |  |  |  |  |  |  |  |  |  |  |  |  |  |  |  |  |  |  |  |  |  |  |  |  |  |  |  |  |  |  |  |  |  |
| --- | --- | --- | --- | --- | --- | --- | --- | --- | --- | --- | --- | --- | --- | --- | --- | --- | --- | --- | --- | --- | --- | --- | --- | --- | --- | --- | --- | --- | --- | --- | --- | --- | --- |

XP\_034271616.1

back to list

  

| Protein Coverage
| Supporting Peptides
|

Protein Coverage:

Supporting Peptides:

| Peptide | Uniq | -10lgP | Mass | Length | ppm | m/z | z | RT | Fraction | Scan | Source File | Area F6 | Area F2 | Area F3 | Area F4 | Area F5 | Area F7 | Area F8 | Area F1 | #Feature | #Feature F6 | #Feature F2 | #Feature F3 | #Feature F4 | #Feature F5 | #Feature F7 | #Feature F8 | #Feature F1 | Start | End | PTM | AScore | Found By |
| --- | --- | --- | --- | --- | --- | --- | --- | --- | --- | --- | --- | --- | --- | --- | --- | --- | --- | --- | --- | --- | --- | --- | --- | --- | --- | --- | --- | --- | --- | --- | --- | --- | --- |
| R.NEGTATYAAAVLFR.I | Y | 79.96 | 1482.7467 | 14 | 0.1 | 742.3807 | 2 | 48.35 | 8 | F8:27296 | 31122021\_RID\_3262\_1b.raw |  |  | 5.3493E5 |  |  |  |  | 7.7228E5 | 2 | 0 | 0 | 1 | 0 | 0 | 0 | 0 | 1 | 639 | 652 |  |  | PEAKS DB |
| R.LNYGIPAIVK.L | Y | 71.64 | 1086.6437 | 10 | 0.9 | 544.3296 | 2 | 44.80 | 8 | F8:24695 | 31122021\_RID\_3262\_1b.raw |  |  | 9.8473E5 | 2.0478E5 |  |  |  | 1.6368E6 | 3 | 0 | 0 | 1 | 1 | 0 | 0 | 0 | 1 | 479 | 488 |  |  | PEAKS DB |
| K.SAIVHLINYQDDAELATR.A | Y | 70.33 | 2028.0276 | 18 | 0.5 | 677.0168 | 3 | 43.83 | 3 | F3:27023 | 13012022\_RID\_3262\_3b.raw |  |  | 3.2757E5 |  |  |  |  | 1.1198E6 | 3 | 0 | 0 | 1 | 0 | 0 | 0 | 0 | 2 | 126 | 143 |  |  | PEAKS DB |
| R.VSVELTNSLFK.H | Y | 67.96 | 1235.6761 | 11 | -0.3 | 618.8452 | 2 | 47.06 | 8 | F8:26272 | 31122021\_RID\_3262\_1b.raw |  |  |  |  |  |  |  | 1.62E6 | 1 | 0 | 0 | 0 | 0 | 0 | 0 | 0 | 1 | 665 | 675 |  |  | PEAKS DB |
| R.TM(+15.99)QNTSDLDTAR.C | Y | 65.61 | 1367.5988 | 12 | 0.5 | 684.8070 | 2 | 18.97 | 8 | F8:4673 | 31122021\_RID\_3262\_1b.raw |  |  | 1.5244E5 |  |  |  |  | 1.2402E5 | 2 | 0 | 0 | 1 | 0 | 0 | 0 | 0 | 1 | 193 | 204 | Oxidation (M) | M2:Oxidation (M):1000.00 | PEAKS DB |
| R.TMQNTSDLDTAR.C | Y | 60.18 | 1351.6038 | 12 | 0.8 | 676.8097 | 2 | 24.85 | 3 | F3:10817 | 13012022\_RID\_3262\_3b.raw |  |  | 1.4351E5 |  |  |  |  | 4.1837E5 | 2 | 0 | 0 | 1 | 0 | 0 | 0 | 0 | 1 | 193 | 204 |  |  | PEAKS DB |
| K.AAMIVNQLSK.K | Y | 59.14 | 1073.5903 | 10 | 0.3 | 537.8026 | 2 | 32.46 | 8 | F8:14867 | 31122021\_RID\_3262\_1b.raw |  |  |  |  |  |  |  | 6.7476E5 | 1 | 0 | 0 | 0 | 0 | 0 | 0 | 0 | 1 | 163 | 172 |  |  | PEAKS DB |
| R.HPEAEMAQNSVR.L | Y | 57.43 | 1367.6251 | 12 | 0.4 | 456.8825 | 3 | 17.57 | 8 | F8:3951 | 31122021\_RID\_3262\_1b.raw |  |  |  |  |  |  |  | 4.095E5 | 2 | 0 | 0 | 0 | 0 | 0 | 0 | 0 | 2 | 467 | 478 |  |  | PEAKS DB |
| L.INYQDDAELATR.A | Y | 52.56 | 1407.6630 | 12 | 0.3 | 704.8390 | 2 | 32.97 | 8 | F8:15260 | 31122021\_RID\_3262\_1b.raw |  |  |  |  |  |  |  | 5.7938E4 | 1 | 0 | 0 | 0 | 0 | 0 | 0 | 0 | 1 | 132 | 143 |  |  | PEAKS DB |
| K.SGGIPALVR.M | Y | 51.41 | 868.5130 | 9 | 0.3 | 435.2639 | 2 | 34.23 | 8 | F8:16166 | 31122021\_RID\_3262\_1b.raw |  |  |  |  | 2.1025E5 |  |  | 9.0683E5 | 2 | 0 | 0 | 0 | 0 | 1 | 0 | 0 | 1 | 226 | 234 |  |  | PEAKS DB |
| R.ISEDKNPDYR.K | Y | 46.94 | 1235.5781 | 10 | 0.7 | 618.7968 | 2 | 18.42 | 3 | F3:5560 | 13012022\_RID\_3262\_3b.raw |  |  | 5.6021E4 |  |  |  |  |  | 1 | 0 | 0 | 1 | 0 | 0 | 0 | 0 | 0 | 653 | 662 |  |  | PEAKS DB |
| K.AAM(+15.99)IVNQLSK.K | Y | 45.42 | 1089.5852 | 10 | 1.1 | 545.8005 | 2 | 32.45 | 8 | F8:14843 | 31122021\_RID\_3262\_1b.raw |  |  |  |  |  |  |  | 6.6638E4 | 1 | 0 | 0 | 0 | 0 | 0 | 0 | 0 | 1 | 163 | 172 | Oxidation (M) | M3:Oxidation (M):1000.00 | PEAKS DB |
| K.RVSVELTNSLFK.H | Y | 45.25 | 1391.7772 | 12 | 0.9 | 696.8965 | 2 | 39.22 | 8 | F8:20209 | 31122021\_RID\_3262\_1b.raw |  |  |  |  |  |  |  | 3.6237E4 | 1 | 0 | 0 | 0 | 0 | 0 | 0 | 0 | 1 | 664 | 675 |  |  | PEAKS DB |
| total 13 peptides |  |  |  |  |  |  |  |  |  |  |  |  |  |  |  |  |  |  |  |  |  |  |  |  |  |  |  |  |  |  |  |  |  |
| --- | --- | --- | --- | --- | --- | --- | --- | --- | --- | --- | --- | --- | --- | --- | --- | --- | --- | --- | --- | --- | --- | --- | --- | --- | --- | --- | --- | --- | --- | --- | --- | --- | --- |

XP\_032091066.1

back to list

  

| Protein Coverage
| Supporting Peptides
|

Protein Coverage:

Supporting Peptides:

| Peptide | Uniq | -10lgP | Mass | Length | ppm | m/z | z | RT | Fraction | Scan | Source File | Area F6 | Area F2 | Area F3 | Area F4 | Area F5 | Area F7 | Area F8 | Area F1 | #Feature | #Feature F6 | #Feature F2 | #Feature F3 | #Feature F4 | #Feature F5 | #Feature F7 | #Feature F8 | #Feature F1 | Start | End | PTM | AScore | Found By |
| --- | --- | --- | --- | --- | --- | --- | --- | --- | --- | --- | --- | --- | --- | --- | --- | --- | --- | --- | --- | --- | --- | --- | --- | --- | --- | --- | --- | --- | --- | --- | --- | --- | --- |
| R.NEGTATYAAAVLFR.I | Y | 79.96 | 1482.7467 | 14 | 0.1 | 742.3807 | 2 | 48.35 | 8 | F8:27296 | 31122021\_RID\_3262\_1b.raw |  |  | 5.3493E5 |  |  |  |  | 7.7228E5 | 2 | 0 | 0 | 1 | 0 | 0 | 0 | 0 | 1 | 682 | 695 |  |  | PEAKS DB |
| R.LNYGIPAIVK.L | Y | 71.64 | 1086.6437 | 10 | 0.9 | 544.3296 | 2 | 44.80 | 8 | F8:24695 | 31122021\_RID\_3262\_1b.raw |  |  | 9.8473E5 | 2.0478E5 |  |  |  | 1.6368E6 | 3 | 0 | 0 | 1 | 1 | 0 | 0 | 0 | 1 | 522 | 531 |  |  | PEAKS DB |
| K.SAIVHLINYQDDAELATR.A | Y | 70.33 | 2028.0276 | 18 | 0.5 | 677.0168 | 3 | 43.83 | 3 | F3:27023 | 13012022\_RID\_3262\_3b.raw |  |  | 3.2757E5 |  |  |  |  | 1.1198E6 | 3 | 0 | 0 | 1 | 0 | 0 | 0 | 0 | 2 | 169 | 186 |  |  | PEAKS DB |
| R.VSVELTNSLFK.H | Y | 67.96 | 1235.6761 | 11 | -0.3 | 618.8452 | 2 | 47.06 | 8 | F8:26272 | 31122021\_RID\_3262\_1b.raw |  |  |  |  |  |  |  | 1.62E6 | 1 | 0 | 0 | 0 | 0 | 0 | 0 | 0 | 1 | 708 | 718 |  |  | PEAKS DB |
| R.TM(+15.99)QNTSDLDTAR.C | Y | 65.61 | 1367.5988 | 12 | 0.5 | 684.8070 | 2 | 18.97 | 8 | F8:4673 | 31122021\_RID\_3262\_1b.raw |  |  | 1.5244E5 |  |  |  |  | 1.2402E5 | 2 | 0 | 0 | 1 | 0 | 0 | 0 | 0 | 1 | 236 | 247 | Oxidation (M) | M2:Oxidation (M):1000.00 | PEAKS DB |
| R.TMQNTSDLDTAR.C | Y | 60.18 | 1351.6038 | 12 | 0.8 | 676.8097 | 2 | 24.85 | 3 | F3:10817 | 13012022\_RID\_3262\_3b.raw |  |  | 1.4351E5 |  |  |  |  | 4.1837E5 | 2 | 0 | 0 | 1 | 0 | 0 | 0 | 0 | 1 | 236 | 247 |  |  | PEAKS DB |
| K.AAMIVNQLSK.K | Y | 59.14 | 1073.5903 | 10 | 0.3 | 537.8026 | 2 | 32.46 | 8 | F8:14867 | 31122021\_RID\_3262\_1b.raw |  |  |  |  |  |  |  | 6.7476E5 | 1 | 0 | 0 | 0 | 0 | 0 | 0 | 0 | 1 | 206 | 215 |  |  | PEAKS DB |
| R.HPEAEMAQNSVR.L | Y | 57.43 | 1367.6251 | 12 | 0.4 | 456.8825 | 3 | 17.57 | 8 | F8:3951 | 31122021\_RID\_3262\_1b.raw |  |  |  |  |  |  |  | 4.095E5 | 2 | 0 | 0 | 0 | 0 | 0 | 0 | 0 | 2 | 510 | 521 |  |  | PEAKS DB |
| L.INYQDDAELATR.A | Y | 52.56 | 1407.6630 | 12 | 0.3 | 704.8390 | 2 | 32.97 | 8 | F8:15260 | 31122021\_RID\_3262\_1b.raw |  |  |  |  |  |  |  | 5.7938E4 | 1 | 0 | 0 | 0 | 0 | 0 | 0 | 0 | 1 | 175 | 186 |  |  | PEAKS DB |
| K.SGGIPALVR.M | Y | 51.41 | 868.5130 | 9 | 0.3 | 435.2639 | 2 | 34.23 | 8 | F8:16166 | 31122021\_RID\_3262\_1b.raw |  |  |  |  | 2.1025E5 |  |  | 9.0683E5 | 2 | 0 | 0 | 0 | 0 | 1 | 0 | 0 | 1 | 269 | 277 |  |  | PEAKS DB |
| R.ISEDKNPDYR.K | Y | 46.94 | 1235.5781 | 10 | 0.7 | 618.7968 | 2 | 18.42 | 3 | F3:5560 | 13012022\_RID\_3262\_3b.raw |  |  | 5.6021E4 |  |  |  |  |  | 1 | 0 | 0 | 1 | 0 | 0 | 0 | 0 | 0 | 696 | 705 |  |  | PEAKS DB |
| K.AAM(+15.99)IVNQLSK.K | Y | 45.42 | 1089.5852 | 10 | 1.1 | 545.8005 | 2 | 32.45 | 8 | F8:14843 | 31122021\_RID\_3262\_1b.raw |  |  |  |  |  |  |  | 6.6638E4 | 1 | 0 | 0 | 0 | 0 | 0 | 0 | 0 | 1 | 206 | 215 | Oxidation (M) | M3:Oxidation (M):1000.00 | PEAKS DB |
| K.RVSVELTNSLFK.H | Y | 45.25 | 1391.7772 | 12 | 0.9 | 696.8965 | 2 | 39.22 | 8 | F8:20209 | 31122021\_RID\_3262\_1b.raw |  |  |  |  |  |  |  | 3.6237E4 | 1 | 0 | 0 | 0 | 0 | 0 | 0 | 0 | 1 | 707 | 718 |  |  | PEAKS DB |
| total 13 peptides |  |  |  |  |  |  |  |  |  |  |  |  |  |  |  |  |  |  |  |  |  |  |  |  |  |  |  |  |  |  |  |  |  |
| --- | --- | --- | --- | --- | --- | --- | --- | --- | --- | --- | --- | --- | --- | --- | --- | --- | --- | --- | --- | --- | --- | --- | --- | --- | --- | --- | --- | --- | --- | --- | --- | --- | --- |

XP\_032091065.1

back to list

  

| Protein Coverage
| Supporting Peptides
|

Protein Coverage:

Supporting Peptides:

| Peptide | Uniq | -10lgP | Mass | Length | ppm | m/z | z | RT | Fraction | Scan | Source File | Area F6 | Area F2 | Area F3 | Area F4 | Area F5 | Area F7 | Area F8 | Area F1 | #Feature | #Feature F6 | #Feature F2 | #Feature F3 | #Feature F4 | #Feature F5 | #Feature F7 | #Feature F8 | #Feature F1 | Start | End | PTM | AScore | Found By |
| --- | --- | --- | --- | --- | --- | --- | --- | --- | --- | --- | --- | --- | --- | --- | --- | --- | --- | --- | --- | --- | --- | --- | --- | --- | --- | --- | --- | --- | --- | --- | --- | --- | --- |
| R.NEGTATYAAAVLFR.I | Y | 79.96 | 1482.7467 | 14 | 0.1 | 742.3807 | 2 | 48.35 | 8 | F8:27296 | 31122021\_RID\_3262\_1b.raw |  |  | 5.3493E5 |  |  |  |  | 7.7228E5 | 2 | 0 | 0 | 1 | 0 | 0 | 0 | 0 | 1 | 682 | 695 |  |  | PEAKS DB |
| R.LNYGIPAIVK.L | Y | 71.64 | 1086.6437 | 10 | 0.9 | 544.3296 | 2 | 44.80 | 8 | F8:24695 | 31122021\_RID\_3262\_1b.raw |  |  | 9.8473E5 | 2.0478E5 |  |  |  | 1.6368E6 | 3 | 0 | 0 | 1 | 1 | 0 | 0 | 0 | 1 | 522 | 531 |  |  | PEAKS DB |
| K.SAIVHLINYQDDAELATR.A | Y | 70.33 | 2028.0276 | 18 | 0.5 | 677.0168 | 3 | 43.83 | 3 | F3:27023 | 13012022\_RID\_3262\_3b.raw |  |  | 3.2757E5 |  |  |  |  | 1.1198E6 | 3 | 0 | 0 | 1 | 0 | 0 | 0 | 0 | 2 | 169 | 186 |  |  | PEAKS DB |
| R.VSVELTNSLFK.H | Y | 67.96 | 1235.6761 | 11 | -0.3 | 618.8452 | 2 | 47.06 | 8 | F8:26272 | 31122021\_RID\_3262\_1b.raw |  |  |  |  |  |  |  | 1.62E6 | 1 | 0 | 0 | 0 | 0 | 0 | 0 | 0 | 1 | 708 | 718 |  |  | PEAKS DB |
| R.TM(+15.99)QNTSDLDTAR.C | Y | 65.61 | 1367.5988 | 12 | 0.5 | 684.8070 | 2 | 18.97 | 8 | F8:4673 | 31122021\_RID\_3262\_1b.raw |  |  | 1.5244E5 |  |  |  |  | 1.2402E5 | 2 | 0 | 0 | 1 | 0 | 0 | 0 | 0 | 1 | 236 | 247 | Oxidation (M) | M2:Oxidation (M):1000.00 | PEAKS DB |
| R.TMQNTSDLDTAR.C | Y | 60.18 | 1351.6038 | 12 | 0.8 | 676.8097 | 2 | 24.85 | 3 | F3:10817 | 13012022\_RID\_3262\_3b.raw |  |  | 1.4351E5 |  |  |  |  | 4.1837E5 | 2 | 0 | 0 | 1 | 0 | 0 | 0 | 0 | 1 | 236 | 247 |  |  | PEAKS DB |
| K.AAMIVNQLSK.K | Y | 59.14 | 1073.5903 | 10 | 0.3 | 537.8026 | 2 | 32.46 | 8 | F8:14867 | 31122021\_RID\_3262\_1b.raw |  |  |  |  |  |  |  | 6.7476E5 | 1 | 0 | 0 | 0 | 0 | 0 | 0 | 0 | 1 | 206 | 215 |  |  | PEAKS DB |
| R.HPEAEMAQNSVR.L | Y | 57.43 | 1367.6251 | 12 | 0.4 | 456.8825 | 3 | 17.57 | 8 | F8:3951 | 31122021\_RID\_3262\_1b.raw |  |  |  |  |  |  |  | 4.095E5 | 2 | 0 | 0 | 0 | 0 | 0 | 0 | 0 | 2 | 510 | 521 |  |  | PEAKS DB |
| L.INYQDDAELATR.A | Y | 52.56 | 1407.6630 | 12 | 0.3 | 704.8390 | 2 | 32.97 | 8 | F8:15260 | 31122021\_RID\_3262\_1b.raw |  |  |  |  |  |  |  | 5.7938E4 | 1 | 0 | 0 | 0 | 0 | 0 | 0 | 0 | 1 | 175 | 186 |  |  | PEAKS DB |
| K.SGGIPALVR.M | Y | 51.41 | 868.5130 | 9 | 0.3 | 435.2639 | 2 | 34.23 | 8 | F8:16166 | 31122021\_RID\_3262\_1b.raw |  |  |  |  | 2.1025E5 |  |  | 9.0683E5 | 2 | 0 | 0 | 0 | 0 | 1 | 0 | 0 | 1 | 269 | 277 |  |  | PEAKS DB |
| R.ISEDKNPDYR.K | Y | 46.94 | 1235.5781 | 10 | 0.7 | 618.7968 | 2 | 18.42 | 3 | F3:5560 | 13012022\_RID\_3262\_3b.raw |  |  | 5.6021E4 |  |  |  |  |  | 1 | 0 | 0 | 1 | 0 | 0 | 0 | 0 | 0 | 696 | 705 |  |  | PEAKS DB |
| K.AAM(+15.99)IVNQLSK.K | Y | 45.42 | 1089.5852 | 10 | 1.1 | 545.8005 | 2 | 32.45 | 8 | F8:14843 | 31122021\_RID\_3262\_1b.raw |  |  |  |  |  |  |  | 6.6638E4 | 1 | 0 | 0 | 0 | 0 | 0 | 0 | 0 | 1 | 206 | 215 | Oxidation (M) | M3:Oxidation (M):1000.00 | PEAKS DB |
| K.RVSVELTNSLFK.H | Y | 45.25 | 1391.7772 | 12 | 0.9 | 696.8965 | 2 | 39.22 | 8 | F8:20209 | 31122021\_RID\_3262\_1b.raw |  |  |  |  |  |  |  | 3.6237E4 | 1 | 0 | 0 | 0 | 0 | 0 | 0 | 0 | 1 | 707 | 718 |  |  | PEAKS DB |
| total 13 peptides |  |  |  |  |  |  |  |  |  |  |  |  |  |  |  |  |  |  |  |  |  |  |  |  |  |  |  |  |  |  |  |  |  |
| --- | --- | --- | --- | --- | --- | --- | --- | --- | --- | --- | --- | --- | --- | --- | --- | --- | --- | --- | --- | --- | --- | --- | --- | --- | --- | --- | --- | --- | --- | --- | --- | --- | --- |

ETE68218.1

back to list

  

| Protein Coverage
| Supporting Peptides
|

Protein Coverage:

Supporting Peptides:

| Peptide | Uniq | -10lgP | Mass | Length | ppm | m/z | z | RT | Fraction | Scan | Source File | Area F6 | Area F2 | Area F3 | Area F4 | Area F5 | Area F7 | Area F8 | Area F1 | #Feature | #Feature F6 | #Feature F2 | #Feature F3 | #Feature F4 | #Feature F5 | #Feature F7 | #Feature F8 | #Feature F1 | Start | End | PTM | AScore | Found By |
| --- | --- | --- | --- | --- | --- | --- | --- | --- | --- | --- | --- | --- | --- | --- | --- | --- | --- | --- | --- | --- | --- | --- | --- | --- | --- | --- | --- | --- | --- | --- | --- | --- | --- |
| R.NEGTATYAAAVLFR.I | Y | 79.96 | 1482.7467 | 14 | 0.1 | 742.3807 | 2 | 48.35 | 8 | F8:27296 | 31122021\_RID\_3262\_1b.raw |  |  | 5.3493E5 |  |  |  |  | 7.7228E5 | 2 | 0 | 0 | 1 | 0 | 0 | 0 | 0 | 1 | 647 | 660 |  |  | PEAKS DB |
| R.LNYGIPAIVK.L | Y | 71.64 | 1086.6437 | 10 | 0.9 | 544.3296 | 2 | 44.80 | 8 | F8:24695 | 31122021\_RID\_3262\_1b.raw |  |  | 9.8473E5 | 2.0478E5 |  |  |  | 1.6368E6 | 3 | 0 | 0 | 1 | 1 | 0 | 0 | 0 | 1 | 478 | 487 |  |  | PEAKS DB |
| K.SAIVHLINYQDDAELATR.A | Y | 70.33 | 2028.0276 | 18 | 0.5 | 677.0168 | 3 | 43.83 | 3 | F3:27023 | 13012022\_RID\_3262\_3b.raw |  |  | 3.2757E5 |  |  |  |  | 1.1198E6 | 3 | 0 | 0 | 1 | 0 | 0 | 0 | 0 | 2 | 125 | 142 |  |  | PEAKS DB |
| R.VSVELTNSLFK.H | Y | 67.96 | 1235.6761 | 11 | -0.3 | 618.8452 | 2 | 47.06 | 8 | F8:26272 | 31122021\_RID\_3262\_1b.raw |  |  |  |  |  |  |  | 1.62E6 | 1 | 0 | 0 | 0 | 0 | 0 | 0 | 0 | 1 | 673 | 683 |  |  | PEAKS DB |
| R.TM(+15.99)QNTSDLDTAR.C | Y | 65.61 | 1367.5988 | 12 | 0.5 | 684.8070 | 2 | 18.97 | 8 | F8:4673 | 31122021\_RID\_3262\_1b.raw |  |  | 1.5244E5 |  |  |  |  | 1.2402E5 | 2 | 0 | 0 | 1 | 0 | 0 | 0 | 0 | 1 | 192 | 203 | Oxidation (M) | M2:Oxidation (M):1000.00 | PEAKS DB |
| R.TMQNTSDLDTAR.C | Y | 60.18 | 1351.6038 | 12 | 0.8 | 676.8097 | 2 | 24.85 | 3 | F3:10817 | 13012022\_RID\_3262\_3b.raw |  |  | 1.4351E5 |  |  |  |  | 4.1837E5 | 2 | 0 | 0 | 1 | 0 | 0 | 0 | 0 | 1 | 192 | 203 |  |  | PEAKS DB |
| K.AAMIVNQLSK.K | Y | 59.14 | 1073.5903 | 10 | 0.3 | 537.8026 | 2 | 32.46 | 8 | F8:14867 | 31122021\_RID\_3262\_1b.raw |  |  |  |  |  |  |  | 6.7476E5 | 1 | 0 | 0 | 0 | 0 | 0 | 0 | 0 | 1 | 162 | 171 |  |  | PEAKS DB |
| R.HPEAEMAQNSVR.L | Y | 57.43 | 1367.6251 | 12 | 0.4 | 456.8825 | 3 | 17.57 | 8 | F8:3951 | 31122021\_RID\_3262\_1b.raw |  |  |  |  |  |  |  | 4.095E5 | 2 | 0 | 0 | 0 | 0 | 0 | 0 | 0 | 2 | 466 | 477 |  |  | PEAKS DB |
| L.INYQDDAELATR.A | Y | 52.56 | 1407.6630 | 12 | 0.3 | 704.8390 | 2 | 32.97 | 8 | F8:15260 | 31122021\_RID\_3262\_1b.raw |  |  |  |  |  |  |  | 5.7938E4 | 1 | 0 | 0 | 0 | 0 | 0 | 0 | 0 | 1 | 131 | 142 |  |  | PEAKS DB |
| K.SGGIPALVR.M | Y | 51.41 | 868.5130 | 9 | 0.3 | 435.2639 | 2 | 34.23 | 8 | F8:16166 | 31122021\_RID\_3262\_1b.raw |  |  |  |  | 2.1025E5 |  |  | 9.0683E5 | 2 | 0 | 0 | 0 | 0 | 1 | 0 | 0 | 1 | 225 | 233 |  |  | PEAKS DB |
| R.ISEDKNPDYR.K | Y | 46.94 | 1235.5781 | 10 | 0.7 | 618.7968 | 2 | 18.42 | 3 | F3:5560 | 13012022\_RID\_3262\_3b.raw |  |  | 5.6021E4 |  |  |  |  |  | 1 | 0 | 0 | 1 | 0 | 0 | 0 | 0 | 0 | 661 | 670 |  |  | PEAKS DB |
| K.AAM(+15.99)IVNQLSK.K | Y | 45.42 | 1089.5852 | 10 | 1.1 | 545.8005 | 2 | 32.45 | 8 | F8:14843 | 31122021\_RID\_3262\_1b.raw |  |  |  |  |  |  |  | 6.6638E4 | 1 | 0 | 0 | 0 | 0 | 0 | 0 | 0 | 1 | 162 | 171 | Oxidation (M) | M3:Oxidation (M):1000.00 | PEAKS DB |
| K.RVSVELTNSLFK.H | Y | 45.25 | 1391.7772 | 12 | 0.9 | 696.8965 | 2 | 39.22 | 8 | F8:20209 | 31122021\_RID\_3262\_1b.raw |  |  |  |  |  |  |  | 3.6237E4 | 1 | 0 | 0 | 0 | 0 | 0 | 0 | 0 | 1 | 672 | 683 |  |  | PEAKS DB |
| total 13 peptides |  |  |  |  |  |  |  |  |  |  |  |  |  |  |  |  |  |  |  |  |  |  |  |  |  |  |  |  |  |  |  |  |  |
| --- | --- | --- | --- | --- | --- | --- | --- | --- | --- | --- | --- | --- | --- | --- | --- | --- | --- | --- | --- | --- | --- | --- | --- | --- | --- | --- | --- | --- | --- | --- | --- | --- | --- |

JAS04520.1

back to list

  

| Protein Coverage
| Supporting Peptides
|

Protein Coverage:

Supporting Peptides:

| Peptide | Uniq | -10lgP | Mass | Length | ppm | m/z | z | RT | Fraction | Scan | Source File | Area F6 | Area F2 | Area F3 | Area F4 | Area F5 | Area F7 | Area F8 | Area F1 | #Feature | #Feature F6 | #Feature F2 | #Feature F3 | #Feature F4 | #Feature F5 | #Feature F7 | #Feature F8 | #Feature F1 | Start | End | PTM | AScore | Found By |
| --- | --- | --- | --- | --- | --- | --- | --- | --- | --- | --- | --- | --- | --- | --- | --- | --- | --- | --- | --- | --- | --- | --- | --- | --- | --- | --- | --- | --- | --- | --- | --- | --- | --- |
| R.QVPVVQAYAFGK.Y | N | 86.85 | 1305.7081 | 12 | 0.9 | 653.8619 | 2 | 57.46 | 2 | F2:25189 | 13012022\_RID\_3262\_2b.raw |  | 4.6095E6 | 3.2149E6 | 2.7574E6 | 1.0716E6 |  |  |  | 7 | 0 | 2 | 2 | 1 | 2 | 0 | 0 | 0 | 288 | 299 |  |  | PEAKS DB |
| K.QAFEHSVHR.H | N | 67.95 | 1109.5366 | 9 | 0.5 | 555.7759 | 2 | 15.68 | 3 | F3:3797 | 13012022\_RID\_3262\_3b.raw |  |  | 5.4735E4 |  |  |  |  |  | 1 | 0 | 0 | 1 | 0 | 0 | 0 | 0 | 0 | 448 | 456 |  |  | PEAKS DB |
| R.HGQGM(+15.99)GELLQVSGIK.V | N | 66.29 | 1568.7981 | 15 | 0.3 | 523.9401 | 3 | 34.38 | 3 | F3:18618 | 13012022\_RID\_3262\_3b.raw |  | 9.5404E5 | 1.0877E6 |  |  |  |  |  | 2 | 0 | 1 | 1 | 0 | 0 | 0 | 0 | 0 | 457 | 471 | Oxidation (M) | M5:Oxidation (M):1000.00 | PEAKS DB |
| K.VGIIGYTTK.E | N | 55.69 | 950.5436 | 9 | -0.2 | 476.2790 | 2 | 35.28 | 3 | F3:19379 | 13012022\_RID\_3262\_3b.raw |  | 8.9517E6 | 6.0623E6 | 4.7337E6 | 2.7356E6 |  |  | 4.6716E5 | 5 | 0 | 1 | 1 | 1 | 1 | 0 | 0 | 1 | 185 | 193 |  |  | PEAKS DB |
| K.LTTLGVNK.I | N | 53.46 | 844.5018 | 8 | 0.4 | 423.2584 | 2 | 27.00 | 4 | F4:12784 | 13012022\_RID\_3262\_4b.raw |  | 7.1171E6 | 5.2677E6 | 5.2452E6 |  |  |  |  | 3 | 0 | 1 | 1 | 1 | 0 | 0 | 0 | 0 | 221 | 228 |  |  | PEAKS DB |
| K.VVYDLSR.K | N | 52.94 | 850.4548 | 7 | 0.5 | 426.2349 | 2 | 30.66 | 2 | F2:9588 | 13012022\_RID\_3262\_2b.raw |  | 4.2852E6 | 1.5807E6 | 1.8252E6 | 1.6963E6 |  |  |  | 4 | 0 | 1 | 1 | 1 | 1 | 0 | 0 | 0 | 472 | 478 |  |  | PEAKS DB |
| K.DISEDQDMK.A | Y | 47.65 | 1079.4441 | 9 | 0.7 | 540.7297 | 2 | 24.08 | 2 | F2:6754 | 13012022\_RID\_3262\_2b.raw |  | 3.7298E5 |  |  |  |  |  |  | 1 | 0 | 1 | 0 | 0 | 0 | 0 | 0 | 0 | 327 | 335 |  |  | PEAKS DB |
| K.IINVGSEK.V | N | 47.29 | 858.4811 | 8 | -0.3 | 430.2477 | 2 | 23.68 | 3 | F3:9771 | 13012022\_RID\_3262\_3b.raw |  |  | 2.9001E6 |  |  |  |  |  | 1 | 0 | 0 | 1 | 0 | 0 | 0 | 0 | 0 | 177 | 184 |  |  | PEAKS DB |
| total 8 peptides |  |  |  |  |  |  |  |  |  |  |  |  |  |  |  |  |  |  |  |  |  |  |  |  |  |  |  |  |  |  |  |  |  |
| --- | --- | --- | --- | --- | --- | --- | --- | --- | --- | --- | --- | --- | --- | --- | --- | --- | --- | --- | --- | --- | --- | --- | --- | --- | --- | --- | --- | --- | --- | --- | --- | --- | --- |

XP\_015674366.1

back to list

  

| Protein Coverage
| Supporting Peptides
|

Protein Coverage:

Supporting Peptides:

| Peptide | Uniq | -10lgP | Mass | Length | ppm | m/z | z | RT | Fraction | Scan | Source File | Area F6 | Area F2 | Area F3 | Area F4 | Area F5 | Area F7 | Area F8 | Area F1 | #Feature | #Feature F6 | #Feature F2 | #Feature F3 | #Feature F4 | #Feature F5 | #Feature F7 | #Feature F8 | #Feature F1 | Start | End | PTM | AScore | Found By |
| --- | --- | --- | --- | --- | --- | --- | --- | --- | --- | --- | --- | --- | --- | --- | --- | --- | --- | --- | --- | --- | --- | --- | --- | --- | --- | --- | --- | --- | --- | --- | --- | --- | --- |
| K.GNYGTIGLSAAAR.Y | Y | 87.22 | 1249.6415 | 13 | 0.8 | 625.8285 | 2 | 35.91 | 2 | F2:12353 | 13012022\_RID\_3262\_2b.raw |  | 1.7457E6 |  | 6.0339E5 | 1.7716E5 |  |  |  | 3 | 0 | 1 | 0 | 1 | 1 | 0 | 0 | 0 | 113 | 125 |  |  | PEAKS DB |
| R.LMGLFEPSDMK.Y | N | 72.85 | 1266.5988 | 11 | 0.3 | 634.3068 | 2 | 51.05 | 2 | F2:22048 | 13012022\_RID\_3262\_2b.raw |  | 1.7026E6 | 7.4096E5 |  |  |  |  |  | 2 | 0 | 1 | 1 | 0 | 0 | 0 | 0 | 0 | 228 | 238 |  |  | PEAKS DB |
| R.ALTEAIEFDK.A | N | 66.72 | 1135.5760 | 10 | 0.2 | 568.7954 | 2 | 40.70 | 2 | F2:15380 | 13012022\_RID\_3262\_2b.raw | 1.082E6 | 7.046E6 | 2.1709E6 |  |  |  |  |  | 3 | 1 | 1 | 1 | 0 | 0 | 0 | 0 | 0 | 290 | 299 |  |  | PEAKS DB |
| K.GFYLFVEGGR.I | N | 64.89 | 1143.5713 | 10 | 0.7 | 572.7933 | 2 | 50.12 | 8 | F8:28714 | 31122021\_RID\_3262\_1b.raw |  |  |  |  |  |  |  | 1.0902E5 | 1 | 0 | 0 | 0 | 0 | 0 | 0 | 0 | 1 | 268 | 277 |  |  | PEAKS DB |
| K.NLILFLGDGM(+15.99)GVPTITATR.I | Y | 63.48 | 2004.0714 | 19 | 0.2 | 1003.0432 | 2 | 62.12 | 2 | F2:27027 | 13012022\_RID\_3262\_2b.raw |  | 3.6464E5 |  |  |  |  |  |  | 1 | 0 | 1 | 0 | 0 | 0 | 0 | 0 | 0 | 43 | 61 | Oxidation (M) | M10:Oxidation (M):1000.00 | PEAKS DB |
| K.SVGIVTTTR.V | N | 59.29 | 932.5291 | 9 | -0.6 | 467.2715 | 2 | 27.35 | 2 | F2:8028 | 13012022\_RID\_3262\_2b.raw | 1.3812E4 | 2.0541E6 | 1.4792E6 |  |  |  |  | 1.5073E5 | 4 | 1 | 1 | 1 | 0 | 0 | 0 | 0 | 1 | 150 | 158 |  |  | PEAKS DB |
| R.VQHASPAGNYAHVVNR.N | N | 50.35 | 1718.8601 | 16 | 0.5 | 573.9609 | 3 | 22.34 | 3 | F3:8686 | 13012022\_RID\_3262\_3b.raw |  |  | 3.414E5 |  |  |  |  |  | 1 | 0 | 0 | 1 | 0 | 0 | 0 | 0 | 0 | 159 | 174 |  |  | PEAKS DB |
| total 7 peptides |  |  |  |  |  |  |  |  |  |  |  |  |  |  |  |  |  |  |  |  |  |  |  |  |  |  |  |  |  |  |  |  |  |
| --- | --- | --- | --- | --- | --- | --- | --- | --- | --- | --- | --- | --- | --- | --- | --- | --- | --- | --- | --- | --- | --- | --- | --- | --- | --- | --- | --- | --- | --- | --- | --- | --- | --- |

JAI10342.1

back to list

  

| Protein Coverage
| Supporting Peptides
|

Protein Coverage:

Supporting Peptides:

| Peptide | Uniq | -10lgP | Mass | Length | ppm | m/z | z | RT | Fraction | Scan | Source File | Area F6 | Area F2 | Area F3 | Area F4 | Area F5 | Area F7 | Area F8 | Area F1 | #Feature | #Feature F6 | #Feature F2 | #Feature F3 | #Feature F4 | #Feature F5 | #Feature F7 | #Feature F8 | #Feature F1 | Start | End | PTM | AScore | Found By |
| --- | --- | --- | --- | --- | --- | --- | --- | --- | --- | --- | --- | --- | --- | --- | --- | --- | --- | --- | --- | --- | --- | --- | --- | --- | --- | --- | --- | --- | --- | --- | --- | --- | --- |
| K.SYELPDGQVITIGNER.F | Y | 80.13 | 1789.8846 | 16 | 1.1 | 895.9506 | 2 | 49.57 | 8 | F8:28417 | 31122021\_RID\_3262\_1b.raw |  |  |  |  |  |  |  | 1.1005E6 | 1 | 0 | 0 | 0 | 0 | 0 | 0 | 0 | 1 | 239 | 254 |  |  | PEAKS DB |
| R.VAPEEHPVLLTEAPLNPK.A | Y | 63.63 | 1953.0570 | 18 | 0.2 | 652.0264 | 3 | 40.19 | 8 | F8:20941 | 31122021\_RID\_3262\_1b.raw |  |  |  |  |  |  |  | 7.9964E5 | 2 | 0 | 0 | 0 | 0 | 0 | 0 | 0 | 2 | 96 | 113 |  |  | PEAKS DB |
| R.GYSFTTTAER.E | Y | 63.40 | 1131.5197 | 10 | 0.3 | 566.7673 | 2 | 28.58 | 8 | F8:11995 | 31122021\_RID\_3262\_1b.raw |  |  |  |  |  |  |  | 7.8615E5 | 1 | 0 | 0 | 0 | 0 | 0 | 0 | 0 | 1 | 197 | 206 |  |  | PEAKS DB |
| K.EITALAPSTMK.I | Y | 50.44 | 1160.6111 | 11 | 1.2 | 581.3135 | 2 | 35.42 | 8 | F8:17136 | 31122021\_RID\_3262\_1b.raw |  |  |  |  |  |  |  | 4.2069E5 | 1 | 0 | 0 | 0 | 0 | 0 | 0 | 0 | 1 | 316 | 326 |  |  | PEAKS DB |
| R.AVFPSIVGR.P | Y | 45.16 | 944.5443 | 9 | -0.4 | 473.2792 | 2 | 41.75 | 8 | F8:22126 | 31122021\_RID\_3262\_1b.raw |  |  |  |  |  |  |  | 1.4594E5 | 1 | 0 | 0 | 0 | 0 | 0 | 0 | 0 | 1 | 29 | 37 |  |  | PEAKS DB |
| total 5 peptides |  |  |  |  |  |  |  |  |  |  |  |  |  |  |  |  |  |  |  |  |  |  |  |  |  |  |  |  |  |  |  |  |  |
| --- | --- | --- | --- | --- | --- | --- | --- | --- | --- | --- | --- | --- | --- | --- | --- | --- | --- | --- | --- | --- | --- | --- | --- | --- | --- | --- | --- | --- | --- | --- | --- | --- | --- |

JAB54771.1

back to list

  

| Protein Coverage
| Supporting Peptides
|

Protein Coverage:

Supporting Peptides:

| Peptide | Uniq | -10lgP | Mass | Length | ppm | m/z | z | RT | Fraction | Scan | Source File | Area F6 | Area F2 | Area F3 | Area F4 | Area F5 | Area F7 | Area F8 | Area F1 | #Feature | #Feature F6 | #Feature F2 | #Feature F3 | #Feature F4 | #Feature F5 | #Feature F7 | #Feature F8 | #Feature F1 | Start | End | PTM | AScore | Found By |
| --- | --- | --- | --- | --- | --- | --- | --- | --- | --- | --- | --- | --- | --- | --- | --- | --- | --- | --- | --- | --- | --- | --- | --- | --- | --- | --- | --- | --- | --- | --- | --- | --- | --- |
| K.SYELPDGQVITIGNER.F | Y | 80.13 | 1789.8846 | 16 | 1.1 | 895.9506 | 2 | 49.57 | 8 | F8:28417 | 31122021\_RID\_3262\_1b.raw |  |  |  |  |  |  |  | 1.1005E6 | 1 | 0 | 0 | 0 | 0 | 0 | 0 | 0 | 1 | 239 | 254 |  |  | PEAKS DB |
| R.VAPEEHPVLLTEAPLNPK.A | Y | 63.63 | 1953.0570 | 18 | 0.2 | 652.0264 | 3 | 40.19 | 8 | F8:20941 | 31122021\_RID\_3262\_1b.raw |  |  |  |  |  |  |  | 7.9964E5 | 2 | 0 | 0 | 0 | 0 | 0 | 0 | 0 | 2 | 96 | 113 |  |  | PEAKS DB |
| R.GYSFTTTAER.E | Y | 63.40 | 1131.5197 | 10 | 0.3 | 566.7673 | 2 | 28.58 | 8 | F8:11995 | 31122021\_RID\_3262\_1b.raw |  |  |  |  |  |  |  | 7.8615E5 | 1 | 0 | 0 | 0 | 0 | 0 | 0 | 0 | 1 | 197 | 206 |  |  | PEAKS DB |
| K.EITALAPSTMK.I | Y | 50.44 | 1160.6111 | 11 | 1.2 | 581.3135 | 2 | 35.42 | 8 | F8:17136 | 31122021\_RID\_3262\_1b.raw |  |  |  |  |  |  |  | 4.2069E5 | 1 | 0 | 0 | 0 | 0 | 0 | 0 | 0 | 1 | 316 | 326 |  |  | PEAKS DB |
| R.AVFPSIVGR.P | Y | 45.16 | 944.5443 | 9 | -0.4 | 473.2792 | 2 | 41.75 | 8 | F8:22126 | 31122021\_RID\_3262\_1b.raw |  |  |  |  |  |  |  | 1.4594E5 | 1 | 0 | 0 | 0 | 0 | 0 | 0 | 0 | 1 | 29 | 37 |  |  | PEAKS DB |
| total 5 peptides |  |  |  |  |  |  |  |  |  |  |  |  |  |  |  |  |  |  |  |  |  |  |  |  |  |  |  |  |  |  |  |  |  |
| --- | --- | --- | --- | --- | --- | --- | --- | --- | --- | --- | --- | --- | --- | --- | --- | --- | --- | --- | --- | --- | --- | --- | --- | --- | --- | --- | --- | --- | --- | --- | --- | --- | --- |

JAA97901.1

back to list

  

| Protein Coverage
| Supporting Peptides
|

Protein Coverage:

Supporting Peptides:

| Peptide | Uniq | -10lgP | Mass | Length | ppm | m/z | z | RT | Fraction | Scan | Source File | Area F6 | Area F2 | Area F3 | Area F4 | Area F5 | Area F7 | Area F8 | Area F1 | #Feature | #Feature F6 | #Feature F2 | #Feature F3 | #Feature F4 | #Feature F5 | #Feature F7 | #Feature F8 | #Feature F1 | Start | End | PTM | AScore | Found By |
| --- | --- | --- | --- | --- | --- | --- | --- | --- | --- | --- | --- | --- | --- | --- | --- | --- | --- | --- | --- | --- | --- | --- | --- | --- | --- | --- | --- | --- | --- | --- | --- | --- | --- |
| K.SYELPDGQVITIGNER.F | Y | 80.13 | 1789.8846 | 16 | 1.1 | 895.9506 | 2 | 49.57 | 8 | F8:28417 | 31122021\_RID\_3262\_1b.raw |  |  |  |  |  |  |  | 1.1005E6 | 1 | 0 | 0 | 0 | 0 | 0 | 0 | 0 | 1 | 239 | 254 |  |  | PEAKS DB |
| R.VAPEEHPVLLTEAPLNPK.A | Y | 63.63 | 1953.0570 | 18 | 0.2 | 652.0264 | 3 | 40.19 | 8 | F8:20941 | 31122021\_RID\_3262\_1b.raw |  |  |  |  |  |  |  | 7.9964E5 | 2 | 0 | 0 | 0 | 0 | 0 | 0 | 0 | 2 | 96 | 113 |  |  | PEAKS DB |
| R.GYSFTTTAER.E | Y | 63.40 | 1131.5197 | 10 | 0.3 | 566.7673 | 2 | 28.58 | 8 | F8:11995 | 31122021\_RID\_3262\_1b.raw |  |  |  |  |  |  |  | 7.8615E5 | 1 | 0 | 0 | 0 | 0 | 0 | 0 | 0 | 1 | 197 | 206 |  |  | PEAKS DB |
| K.EITALAPSTMK.I | Y | 50.44 | 1160.6111 | 11 | 1.2 | 581.3135 | 2 | 35.42 | 8 | F8:17136 | 31122021\_RID\_3262\_1b.raw |  |  |  |  |  |  |  | 4.2069E5 | 1 | 0 | 0 | 0 | 0 | 0 | 0 | 0 | 1 | 316 | 326 |  |  | PEAKS DB |
| R.AVFPSIVGR.P | Y | 45.16 | 944.5443 | 9 | -0.4 | 473.2792 | 2 | 41.75 | 8 | F8:22126 | 31122021\_RID\_3262\_1b.raw |  |  |  |  |  |  |  | 1.4594E5 | 1 | 0 | 0 | 0 | 0 | 0 | 0 | 0 | 1 | 29 | 37 |  |  | PEAKS DB |
| total 5 peptides |  |  |  |  |  |  |  |  |  |  |  |  |  |  |  |  |  |  |  |  |  |  |  |  |  |  |  |  |  |  |  |  |  |
| --- | --- | --- | --- | --- | --- | --- | --- | --- | --- | --- | --- | --- | --- | --- | --- | --- | --- | --- | --- | --- | --- | --- | --- | --- | --- | --- | --- | --- | --- | --- | --- | --- | --- |

JAG68844.1

back to list

  

| Protein Coverage
| Supporting Peptides
|

Protein Coverage:

Supporting Peptides:

| Peptide | Uniq | -10lgP | Mass | Length | ppm | m/z | z | RT | Fraction | Scan | Source File | Area F6 | Area F2 | Area F3 | Area F4 | Area F5 | Area F7 | Area F8 | Area F1 | #Feature | #Feature F6 | #Feature F2 | #Feature F3 | #Feature F4 | #Feature F5 | #Feature F7 | #Feature F8 | #Feature F1 | Start | End | PTM | AScore | Found By |
| --- | --- | --- | --- | --- | --- | --- | --- | --- | --- | --- | --- | --- | --- | --- | --- | --- | --- | --- | --- | --- | --- | --- | --- | --- | --- | --- | --- | --- | --- | --- | --- | --- | --- |
| K.SYELPDGQVITIGNER.F | Y | 80.13 | 1789.8846 | 16 | 1.1 | 895.9506 | 2 | 49.57 | 8 | F8:28417 | 31122021\_RID\_3262\_1b.raw |  |  |  |  |  |  |  | 1.1005E6 | 1 | 0 | 0 | 0 | 0 | 0 | 0 | 0 | 1 | 239 | 254 |  |  | PEAKS DB |
| R.VAPEEHPVLLTEAPLNPK.A | Y | 63.63 | 1953.0570 | 18 | 0.2 | 652.0264 | 3 | 40.19 | 8 | F8:20941 | 31122021\_RID\_3262\_1b.raw |  |  |  |  |  |  |  | 7.9964E5 | 2 | 0 | 0 | 0 | 0 | 0 | 0 | 0 | 2 | 96 | 113 |  |  | PEAKS DB |
| R.GYSFTTTAER.E | Y | 63.40 | 1131.5197 | 10 | 0.3 | 566.7673 | 2 | 28.58 | 8 | F8:11995 | 31122021\_RID\_3262\_1b.raw |  |  |  |  |  |  |  | 7.8615E5 | 1 | 0 | 0 | 0 | 0 | 0 | 0 | 0 | 1 | 197 | 206 |  |  | PEAKS DB |
| K.EITALAPSTMK.I | Y | 50.44 | 1160.6111 | 11 | 1.2 | 581.3135 | 2 | 35.42 | 8 | F8:17136 | 31122021\_RID\_3262\_1b.raw |  |  |  |  |  |  |  | 4.2069E5 | 1 | 0 | 0 | 0 | 0 | 0 | 0 | 0 | 1 | 316 | 326 |  |  | PEAKS DB |
| R.AVFPSIVGR.P | Y | 45.16 | 944.5443 | 9 | -0.4 | 473.2792 | 2 | 41.75 | 8 | F8:22126 | 31122021\_RID\_3262\_1b.raw |  |  |  |  |  |  |  | 1.4594E5 | 1 | 0 | 0 | 0 | 0 | 0 | 0 | 0 | 1 | 29 | 37 |  |  | PEAKS DB |
| total 5 peptides |  |  |  |  |  |  |  |  |  |  |  |  |  |  |  |  |  |  |  |  |  |  |  |  |  |  |  |  |  |  |  |  |  |
| --- | --- | --- | --- | --- | --- | --- | --- | --- | --- | --- | --- | --- | --- | --- | --- | --- | --- | --- | --- | --- | --- | --- | --- | --- | --- | --- | --- | --- | --- | --- | --- | --- | --- |

JAV51374.1

back to list

  

| Protein Coverage
| Supporting Peptides
|

Protein Coverage:

Supporting Peptides:

| Peptide | Uniq | -10lgP | Mass | Length | ppm | m/z | z | RT | Fraction | Scan | Source File | Area F6 | Area F2 | Area F3 | Area F4 | Area F5 | Area F7 | Area F8 | Area F1 | #Feature | #Feature F6 | #Feature F2 | #Feature F3 | #Feature F4 | #Feature F5 | #Feature F7 | #Feature F8 | #Feature F1 | Start | End | PTM | AScore | Found By |
| --- | --- | --- | --- | --- | --- | --- | --- | --- | --- | --- | --- | --- | --- | --- | --- | --- | --- | --- | --- | --- | --- | --- | --- | --- | --- | --- | --- | --- | --- | --- | --- | --- | --- |
| K.SYELPDGQVITIGNER.F | Y | 80.13 | 1789.8846 | 16 | 1.1 | 895.9506 | 2 | 49.57 | 8 | F8:28417 | 31122021\_RID\_3262\_1b.raw |  |  |  |  |  |  |  | 1.1005E6 | 1 | 0 | 0 | 0 | 0 | 0 | 0 | 0 | 1 | 239 | 254 |  |  | PEAKS DB |
| R.VAPEEHPVLLTEAPLNPK.A | Y | 63.63 | 1953.0570 | 18 | 0.2 | 652.0264 | 3 | 40.19 | 8 | F8:20941 | 31122021\_RID\_3262\_1b.raw |  |  |  |  |  |  |  | 7.9964E5 | 2 | 0 | 0 | 0 | 0 | 0 | 0 | 0 | 2 | 96 | 113 |  |  | PEAKS DB |
| R.GYSFTTTAER.E | Y | 63.40 | 1131.5197 | 10 | 0.3 | 566.7673 | 2 | 28.58 | 8 | F8:11995 | 31122021\_RID\_3262\_1b.raw |  |  |  |  |  |  |  | 7.8615E5 | 1 | 0 | 0 | 0 | 0 | 0 | 0 | 0 | 1 | 197 | 206 |  |  | PEAKS DB |
| K.EITALAPSTMK.I | Y | 50.44 | 1160.6111 | 11 | 1.2 | 581.3135 | 2 | 35.42 | 8 | F8:17136 | 31122021\_RID\_3262\_1b.raw |  |  |  |  |  |  |  | 4.2069E5 | 1 | 0 | 0 | 0 | 0 | 0 | 0 | 0 | 1 | 316 | 326 |  |  | PEAKS DB |
| R.AVFPSIVGR.P | Y | 45.16 | 944.5443 | 9 | -0.4 | 473.2792 | 2 | 41.75 | 8 | F8:22126 | 31122021\_RID\_3262\_1b.raw |  |  |  |  |  |  |  | 1.4594E5 | 1 | 0 | 0 | 0 | 0 | 0 | 0 | 0 | 1 | 29 | 37 |  |  | PEAKS DB |
| total 5 peptides |  |  |  |  |  |  |  |  |  |  |  |  |  |  |  |  |  |  |  |  |  |  |  |  |  |  |  |  |  |  |  |  |  |
| --- | --- | --- | --- | --- | --- | --- | --- | --- | --- | --- | --- | --- | --- | --- | --- | --- | --- | --- | --- | --- | --- | --- | --- | --- | --- | --- | --- | --- | --- | --- | --- | --- | --- |

JAG47580.1

back to list

  

| Protein Coverage
| Supporting Peptides
|

Protein Coverage:

Supporting Peptides:

| Peptide | Uniq | -10lgP | Mass | Length | ppm | m/z | z | RT | Fraction | Scan | Source File | Area F6 | Area F2 | Area F3 | Area F4 | Area F5 | Area F7 | Area F8 | Area F1 | #Feature | #Feature F6 | #Feature F2 | #Feature F3 | #Feature F4 | #Feature F5 | #Feature F7 | #Feature F8 | #Feature F1 | Start | End | PTM | AScore | Found By |
| --- | --- | --- | --- | --- | --- | --- | --- | --- | --- | --- | --- | --- | --- | --- | --- | --- | --- | --- | --- | --- | --- | --- | --- | --- | --- | --- | --- | --- | --- | --- | --- | --- | --- |
| K.SYELPDGQVITIGNER.F | Y | 80.13 | 1789.8846 | 16 | 1.1 | 895.9506 | 2 | 49.57 | 8 | F8:28417 | 31122021\_RID\_3262\_1b.raw |  |  |  |  |  |  |  | 1.1005E6 | 1 | 0 | 0 | 0 | 0 | 0 | 0 | 0 | 1 | 239 | 254 |  |  | PEAKS DB |
| R.VAPEEHPVLLTEAPLNPK.A | Y | 63.63 | 1953.0570 | 18 | 0.2 | 652.0264 | 3 | 40.19 | 8 | F8:20941 | 31122021\_RID\_3262\_1b.raw |  |  |  |  |  |  |  | 7.9964E5 | 2 | 0 | 0 | 0 | 0 | 0 | 0 | 0 | 2 | 96 | 113 |  |  | PEAKS DB |
| R.GYSFTTTAER.E | Y | 63.40 | 1131.5197 | 10 | 0.3 | 566.7673 | 2 | 28.58 | 8 | F8:11995 | 31122021\_RID\_3262\_1b.raw |  |  |  |  |  |  |  | 7.8615E5 | 1 | 0 | 0 | 0 | 0 | 0 | 0 | 0 | 1 | 197 | 206 |  |  | PEAKS DB |
| K.EITALAPSTMK.I | Y | 50.44 | 1160.6111 | 11 | 1.2 | 581.3135 | 2 | 35.42 | 8 | F8:17136 | 31122021\_RID\_3262\_1b.raw |  |  |  |  |  |  |  | 4.2069E5 | 1 | 0 | 0 | 0 | 0 | 0 | 0 | 0 | 1 | 316 | 326 |  |  | PEAKS DB |
| R.AVFPSIVGR.P | Y | 45.16 | 944.5443 | 9 | -0.4 | 473.2792 | 2 | 41.75 | 8 | F8:22126 | 31122021\_RID\_3262\_1b.raw |  |  |  |  |  |  |  | 1.4594E5 | 1 | 0 | 0 | 0 | 0 | 0 | 0 | 0 | 1 | 29 | 37 |  |  | PEAKS DB |
| total 5 peptides |  |  |  |  |  |  |  |  |  |  |  |  |  |  |  |  |  |  |  |  |  |  |  |  |  |  |  |  |  |  |  |  |  |
| --- | --- | --- | --- | --- | --- | --- | --- | --- | --- | --- | --- | --- | --- | --- | --- | --- | --- | --- | --- | --- | --- | --- | --- | --- | --- | --- | --- | --- | --- | --- | --- | --- | --- |

JAI14496.1

back to list

  

| Protein Coverage
| Supporting Peptides
|

Protein Coverage:

Supporting Peptides:

| Peptide | Uniq | -10lgP | Mass | Length | ppm | m/z | z | RT | Fraction | Scan | Source File | Area F6 | Area F2 | Area F3 | Area F4 | Area F5 | Area F7 | Area F8 | Area F1 | #Feature | #Feature F6 | #Feature F2 | #Feature F3 | #Feature F4 | #Feature F5 | #Feature F7 | #Feature F8 | #Feature F1 | Start | End | PTM | AScore | Found By |
| --- | --- | --- | --- | --- | --- | --- | --- | --- | --- | --- | --- | --- | --- | --- | --- | --- | --- | --- | --- | --- | --- | --- | --- | --- | --- | --- | --- | --- | --- | --- | --- | --- | --- |
| K.SYELPDGQVITIGNER.F | Y | 80.13 | 1789.8846 | 16 | 1.1 | 895.9506 | 2 | 49.57 | 8 | F8:28417 | 31122021\_RID\_3262\_1b.raw |  |  |  |  |  |  |  | 1.1005E6 | 1 | 0 | 0 | 0 | 0 | 0 | 0 | 0 | 1 | 239 | 254 |  |  | PEAKS DB |
| R.VAPEEHPVLLTEAPLNPK.A | Y | 63.63 | 1953.0570 | 18 | 0.2 | 652.0264 | 3 | 40.19 | 8 | F8:20941 | 31122021\_RID\_3262\_1b.raw |  |  |  |  |  |  |  | 7.9964E5 | 2 | 0 | 0 | 0 | 0 | 0 | 0 | 0 | 2 | 96 | 113 |  |  | PEAKS DB |
| R.GYSFTTTAER.E | Y | 63.40 | 1131.5197 | 10 | 0.3 | 566.7673 | 2 | 28.58 | 8 | F8:11995 | 31122021\_RID\_3262\_1b.raw |  |  |  |  |  |  |  | 7.8615E5 | 1 | 0 | 0 | 0 | 0 | 0 | 0 | 0 | 1 | 197 | 206 |  |  | PEAKS DB |
| K.EITALAPSTMK.I | Y | 50.44 | 1160.6111 | 11 | 1.2 | 581.3135 | 2 | 35.42 | 8 | F8:17136 | 31122021\_RID\_3262\_1b.raw |  |  |  |  |  |  |  | 4.2069E5 | 1 | 0 | 0 | 0 | 0 | 0 | 0 | 0 | 1 | 316 | 326 |  |  | PEAKS DB |
| R.AVFPSIVGR.P | Y | 45.16 | 944.5443 | 9 | -0.4 | 473.2792 | 2 | 41.75 | 8 | F8:22126 | 31122021\_RID\_3262\_1b.raw |  |  |  |  |  |  |  | 1.4594E5 | 1 | 0 | 0 | 0 | 0 | 0 | 0 | 0 | 1 | 29 | 37 |  |  | PEAKS DB |
| total 5 peptides |  |  |  |  |  |  |  |  |  |  |  |  |  |  |  |  |  |  |  |  |  |  |  |  |  |  |  |  |  |  |  |  |  |
| --- | --- | --- | --- | --- | --- | --- | --- | --- | --- | --- | --- | --- | --- | --- | --- | --- | --- | --- | --- | --- | --- | --- | --- | --- | --- | --- | --- | --- | --- | --- | --- | --- | --- |

JAC96364.1

back to list

  

| Protein Coverage
| Supporting Peptides
|

Protein Coverage:

Supporting Peptides:

| Peptide | Uniq | -10lgP | Mass | Length | ppm | m/z | z | RT | Fraction | Scan | Source File | Area F6 | Area F2 | Area F3 | Area F4 | Area F5 | Area F7 | Area F8 | Area F1 | #Feature | #Feature F6 | #Feature F2 | #Feature F3 | #Feature F4 | #Feature F5 | #Feature F7 | #Feature F8 | #Feature F1 | Start | End | PTM | AScore | Found By |
| --- | --- | --- | --- | --- | --- | --- | --- | --- | --- | --- | --- | --- | --- | --- | --- | --- | --- | --- | --- | --- | --- | --- | --- | --- | --- | --- | --- | --- | --- | --- | --- | --- | --- |
| K.SYELPDGQVITIGNER.F | Y | 80.13 | 1789.8846 | 16 | 1.1 | 895.9506 | 2 | 49.57 | 8 | F8:28417 | 31122021\_RID\_3262\_1b.raw |  |  |  |  |  |  |  | 1.1005E6 | 1 | 0 | 0 | 0 | 0 | 0 | 0 | 0 | 1 | 239 | 254 |  |  | PEAKS DB |
| R.VAPEEHPVLLTEAPLNPK.A | Y | 63.63 | 1953.0570 | 18 | 0.2 | 652.0264 | 3 | 40.19 | 8 | F8:20941 | 31122021\_RID\_3262\_1b.raw |  |  |  |  |  |  |  | 7.9964E5 | 2 | 0 | 0 | 0 | 0 | 0 | 0 | 0 | 2 | 96 | 113 |  |  | PEAKS DB |
| R.GYSFTTTAER.E | Y | 63.40 | 1131.5197 | 10 | 0.3 | 566.7673 | 2 | 28.58 | 8 | F8:11995 | 31122021\_RID\_3262\_1b.raw |  |  |  |  |  |  |  | 7.8615E5 | 1 | 0 | 0 | 0 | 0 | 0 | 0 | 0 | 1 | 197 | 206 |  |  | PEAKS DB |
| K.EITALAPSTMK.I | Y | 50.44 | 1160.6111 | 11 | 1.2 | 581.3135 | 2 | 35.42 | 8 | F8:17136 | 31122021\_RID\_3262\_1b.raw |  |  |  |  |  |  |  | 4.2069E5 | 1 | 0 | 0 | 0 | 0 | 0 | 0 | 0 | 1 | 316 | 326 |  |  | PEAKS DB |
| R.AVFPSIVGR.P | Y | 45.16 | 944.5443 | 9 | -0.4 | 473.2792 | 2 | 41.75 | 8 | F8:22126 | 31122021\_RID\_3262\_1b.raw |  |  |  |  |  |  |  | 1.4594E5 | 1 | 0 | 0 | 0 | 0 | 0 | 0 | 0 | 1 | 29 | 37 |  |  | PEAKS DB |
| total 5 peptides |  |  |  |  |  |  |  |  |  |  |  |  |  |  |  |  |  |  |  |  |  |  |  |  |  |  |  |  |  |  |  |  |  |
| --- | --- | --- | --- | --- | --- | --- | --- | --- | --- | --- | --- | --- | --- | --- | --- | --- | --- | --- | --- | --- | --- | --- | --- | --- | --- | --- | --- | --- | --- | --- | --- | --- | --- |

AFJ49302.1

back to list

  

| Protein Coverage
| Supporting Peptides
|

Protein Coverage:

Supporting Peptides:

| Peptide | Uniq | -10lgP | Mass | Length | ppm | m/z | z | RT | Fraction | Scan | Source File | Area F6 | Area F2 | Area F3 | Area F4 | Area F5 | Area F7 | Area F8 | Area F1 | #Feature | #Feature F6 | #Feature F2 | #Feature F3 | #Feature F4 | #Feature F5 | #Feature F7 | #Feature F8 | #Feature F1 | Start | End | PTM | AScore | Found By |
| --- | --- | --- | --- | --- | --- | --- | --- | --- | --- | --- | --- | --- | --- | --- | --- | --- | --- | --- | --- | --- | --- | --- | --- | --- | --- | --- | --- | --- | --- | --- | --- | --- | --- |
| K.SYELPDGQVITIGNER.F | Y | 80.13 | 1789.8846 | 16 | 1.1 | 895.9506 | 2 | 49.57 | 8 | F8:28417 | 31122021\_RID\_3262\_1b.raw |  |  |  |  |  |  |  | 1.1005E6 | 1 | 0 | 0 | 0 | 0 | 0 | 0 | 0 | 1 | 239 | 254 |  |  | PEAKS DB |
| R.VAPEEHPVLLTEAPLNPK.A | Y | 63.63 | 1953.0570 | 18 | 0.2 | 652.0264 | 3 | 40.19 | 8 | F8:20941 | 31122021\_RID\_3262\_1b.raw |  |  |  |  |  |  |  | 7.9964E5 | 2 | 0 | 0 | 0 | 0 | 0 | 0 | 0 | 2 | 96 | 113 |  |  | PEAKS DB |
| R.GYSFTTTAER.E | Y | 63.40 | 1131.5197 | 10 | 0.3 | 566.7673 | 2 | 28.58 | 8 | F8:11995 | 31122021\_RID\_3262\_1b.raw |  |  |  |  |  |  |  | 7.8615E5 | 1 | 0 | 0 | 0 | 0 | 0 | 0 | 0 | 1 | 197 | 206 |  |  | PEAKS DB |
| K.EITALAPSTMK.I | Y | 50.44 | 1160.6111 | 11 | 1.2 | 581.3135 | 2 | 35.42 | 8 | F8:17136 | 31122021\_RID\_3262\_1b.raw |  |  |  |  |  |  |  | 4.2069E5 | 1 | 0 | 0 | 0 | 0 | 0 | 0 | 0 | 1 | 316 | 326 |  |  | PEAKS DB |
| R.AVFPSIVGR.P | Y | 45.16 | 944.5443 | 9 | -0.4 | 473.2792 | 2 | 41.75 | 8 | F8:22126 | 31122021\_RID\_3262\_1b.raw |  |  |  |  |  |  |  | 1.4594E5 | 1 | 0 | 0 | 0 | 0 | 0 | 0 | 0 | 1 | 29 | 37 |  |  | PEAKS DB |
| total 5 peptides |  |  |  |  |  |  |  |  |  |  |  |  |  |  |  |  |  |  |  |  |  |  |  |  |  |  |  |  |  |  |  |  |  |
| --- | --- | --- | --- | --- | --- | --- | --- | --- | --- | --- | --- | --- | --- | --- | --- | --- | --- | --- | --- | --- | --- | --- | --- | --- | --- | --- | --- | --- | --- | --- | --- | --- | --- |

BAQ22177.1

back to list

  

| Protein Coverage
| Supporting Peptides
|

Protein Coverage:

Supporting Peptides:

| Peptide | Uniq | -10lgP | Mass | Length | ppm | m/z | z | RT | Fraction | Scan | Source File | Area F6 | Area F2 | Area F3 | Area F4 | Area F5 | Area F7 | Area F8 | Area F1 | #Feature | #Feature F6 | #Feature F2 | #Feature F3 | #Feature F4 | #Feature F5 | #Feature F7 | #Feature F8 | #Feature F1 | Start | End | PTM | AScore | Found By |
| --- | --- | --- | --- | --- | --- | --- | --- | --- | --- | --- | --- | --- | --- | --- | --- | --- | --- | --- | --- | --- | --- | --- | --- | --- | --- | --- | --- | --- | --- | --- | --- | --- | --- |
| K.SYELPDGQVITIGNER.F | Y | 80.13 | 1789.8846 | 16 | 1.1 | 895.9506 | 2 | 49.57 | 8 | F8:28417 | 31122021\_RID\_3262\_1b.raw |  |  |  |  |  |  |  | 1.1005E6 | 1 | 0 | 0 | 0 | 0 | 0 | 0 | 0 | 1 | 239 | 254 |  |  | PEAKS DB |
| R.VAPEEHPVLLTEAPLNPK.A | Y | 63.63 | 1953.0570 | 18 | 0.2 | 652.0264 | 3 | 40.19 | 8 | F8:20941 | 31122021\_RID\_3262\_1b.raw |  |  |  |  |  |  |  | 7.9964E5 | 2 | 0 | 0 | 0 | 0 | 0 | 0 | 0 | 2 | 96 | 113 |  |  | PEAKS DB |
| R.GYSFTTTAER.E | Y | 63.40 | 1131.5197 | 10 | 0.3 | 566.7673 | 2 | 28.58 | 8 | F8:11995 | 31122021\_RID\_3262\_1b.raw |  |  |  |  |  |  |  | 7.8615E5 | 1 | 0 | 0 | 0 | 0 | 0 | 0 | 0 | 1 | 197 | 206 |  |  | PEAKS DB |
| K.EITALAPSTMK.I | Y | 50.44 | 1160.6111 | 11 | 1.2 | 581.3135 | 2 | 35.42 | 8 | F8:17136 | 31122021\_RID\_3262\_1b.raw |  |  |  |  |  |  |  | 4.2069E5 | 1 | 0 | 0 | 0 | 0 | 0 | 0 | 0 | 1 | 316 | 326 |  |  | PEAKS DB |
| R.AVFPSIVGR.P | Y | 45.16 | 944.5443 | 9 | -0.4 | 473.2792 | 2 | 41.75 | 8 | F8:22126 | 31122021\_RID\_3262\_1b.raw |  |  |  |  |  |  |  | 1.4594E5 | 1 | 0 | 0 | 0 | 0 | 0 | 0 | 0 | 1 | 29 | 37 |  |  | PEAKS DB |
| total 5 peptides |  |  |  |  |  |  |  |  |  |  |  |  |  |  |  |  |  |  |  |  |  |  |  |  |  |  |  |  |  |  |  |  |  |
| --- | --- | --- | --- | --- | --- | --- | --- | --- | --- | --- | --- | --- | --- | --- | --- | --- | --- | --- | --- | --- | --- | --- | --- | --- | --- | --- | --- | --- | --- | --- | --- | --- | --- |

JAG45999.1

back to list

  

| Protein Coverage
| Supporting Peptides
|

Protein Coverage:

Supporting Peptides:

| Peptide | Uniq | -10lgP | Mass | Length | ppm | m/z | z | RT | Fraction | Scan | Source File | Area F6 | Area F2 | Area F3 | Area F4 | Area F5 | Area F7 | Area F8 | Area F1 | #Feature | #Feature F6 | #Feature F2 | #Feature F3 | #Feature F4 | #Feature F5 | #Feature F7 | #Feature F8 | #Feature F1 | Start | End | PTM | AScore | Found By |
| --- | --- | --- | --- | --- | --- | --- | --- | --- | --- | --- | --- | --- | --- | --- | --- | --- | --- | --- | --- | --- | --- | --- | --- | --- | --- | --- | --- | --- | --- | --- | --- | --- | --- |
| K.SYELPDGQVITIGNER.F | Y | 80.13 | 1789.8846 | 16 | 1.1 | 895.9506 | 2 | 49.57 | 8 | F8:28417 | 31122021\_RID\_3262\_1b.raw |  |  |  |  |  |  |  | 1.1005E6 | 1 | 0 | 0 | 0 | 0 | 0 | 0 | 0 | 1 | 239 | 254 |  |  | PEAKS DB |
| R.VAPEEHPVLLTEAPLNPK.A | Y | 63.63 | 1953.0570 | 18 | 0.2 | 652.0264 | 3 | 40.19 | 8 | F8:20941 | 31122021\_RID\_3262\_1b.raw |  |  |  |  |  |  |  | 7.9964E5 | 2 | 0 | 0 | 0 | 0 | 0 | 0 | 0 | 2 | 96 | 113 |  |  | PEAKS DB |
| R.GYSFTTTAER.E | Y | 63.40 | 1131.5197 | 10 | 0.3 | 566.7673 | 2 | 28.58 | 8 | F8:11995 | 31122021\_RID\_3262\_1b.raw |  |  |  |  |  |  |  | 7.8615E5 | 1 | 0 | 0 | 0 | 0 | 0 | 0 | 0 | 1 | 197 | 206 |  |  | PEAKS DB |
| K.EITALAPSTMK.I | Y | 50.44 | 1160.6111 | 11 | 1.2 | 581.3135 | 2 | 35.42 | 8 | F8:17136 | 31122021\_RID\_3262\_1b.raw |  |  |  |  |  |  |  | 4.2069E5 | 1 | 0 | 0 | 0 | 0 | 0 | 0 | 0 | 1 | 316 | 326 |  |  | PEAKS DB |
| R.AVFPSIVGR.P | Y | 45.16 | 944.5443 | 9 | -0.4 | 473.2792 | 2 | 41.75 | 8 | F8:22126 | 31122021\_RID\_3262\_1b.raw |  |  |  |  |  |  |  | 1.4594E5 | 1 | 0 | 0 | 0 | 0 | 0 | 0 | 0 | 1 | 29 | 37 |  |  | PEAKS DB |
| total 5 peptides |  |  |  |  |  |  |  |  |  |  |  |  |  |  |  |  |  |  |  |  |  |  |  |  |  |  |  |  |  |  |  |  |  |
| --- | --- | --- | --- | --- | --- | --- | --- | --- | --- | --- | --- | --- | --- | --- | --- | --- | --- | --- | --- | --- | --- | --- | --- | --- | --- | --- | --- | --- | --- | --- | --- | --- | --- |

ETE57948.1

back to list

  

| Protein Coverage
| Supporting Peptides
|

Protein Coverage:

Supporting Peptides:

| Peptide | Uniq | -10lgP | Mass | Length | ppm | m/z | z | RT | Fraction | Scan | Source File | Area F6 | Area F2 | Area F3 | Area F4 | Area F5 | Area F7 | Area F8 | Area F1 | #Feature | #Feature F6 | #Feature F2 | #Feature F3 | #Feature F4 | #Feature F5 | #Feature F7 | #Feature F8 | #Feature F1 | Start | End | PTM | AScore | Found By |
| --- | --- | --- | --- | --- | --- | --- | --- | --- | --- | --- | --- | --- | --- | --- | --- | --- | --- | --- | --- | --- | --- | --- | --- | --- | --- | --- | --- | --- | --- | --- | --- | --- | --- |
| K.SYELPDGQVITIGNER.F | Y | 80.13 | 1789.8846 | 16 | 1.1 | 895.9506 | 2 | 49.57 | 8 | F8:28417 | 31122021\_RID\_3262\_1b.raw |  |  |  |  |  |  |  | 1.1005E6 | 1 | 0 | 0 | 0 | 0 | 0 | 0 | 0 | 1 | 224 | 239 |  |  | PEAKS DB |
| R.VAPEEHPVLLTEAPLNPK.A | Y | 63.63 | 1953.0570 | 18 | 0.2 | 652.0264 | 3 | 40.19 | 8 | F8:20941 | 31122021\_RID\_3262\_1b.raw |  |  |  |  |  |  |  | 7.9964E5 | 2 | 0 | 0 | 0 | 0 | 0 | 0 | 0 | 2 | 81 | 98 |  |  | PEAKS DB |
| R.GYSFTTTAER.E | Y | 63.40 | 1131.5197 | 10 | 0.3 | 566.7673 | 2 | 28.58 | 8 | F8:11995 | 31122021\_RID\_3262\_1b.raw |  |  |  |  |  |  |  | 7.8615E5 | 1 | 0 | 0 | 0 | 0 | 0 | 0 | 0 | 1 | 182 | 191 |  |  | PEAKS DB |
| K.EITALAPSTMK.I | Y | 50.44 | 1160.6111 | 11 | 1.2 | 581.3135 | 2 | 35.42 | 8 | F8:17136 | 31122021\_RID\_3262\_1b.raw |  |  |  |  |  |  |  | 4.2069E5 | 1 | 0 | 0 | 0 | 0 | 0 | 0 | 0 | 1 | 301 | 311 |  |  | PEAKS DB |
| R.AVFPSIVGR.P | Y | 45.16 | 944.5443 | 9 | -0.4 | 473.2792 | 2 | 41.75 | 8 | F8:22126 | 31122021\_RID\_3262\_1b.raw |  |  |  |  |  |  |  | 1.4594E5 | 1 | 0 | 0 | 0 | 0 | 0 | 0 | 0 | 1 | 14 | 22 |  |  | PEAKS DB |
| total 5 peptides |  |  |  |  |  |  |  |  |  |  |  |  |  |  |  |  |  |  |  |  |  |  |  |  |  |  |  |  |  |  |  |  |  |
| --- | --- | --- | --- | --- | --- | --- | --- | --- | --- | --- | --- | --- | --- | --- | --- | --- | --- | --- | --- | --- | --- | --- | --- | --- | --- | --- | --- | --- | --- | --- | --- | --- | --- |

JAC96363.1

back to list

  

| Protein Coverage
| Supporting Peptides
|

Protein Coverage:

Supporting Peptides:

| Peptide | Uniq | -10lgP | Mass | Length | ppm | m/z | z | RT | Fraction | Scan | Source File | Area F6 | Area F2 | Area F3 | Area F4 | Area F5 | Area F7 | Area F8 | Area F1 | #Feature | #Feature F6 | #Feature F2 | #Feature F3 | #Feature F4 | #Feature F5 | #Feature F7 | #Feature F8 | #Feature F1 | Start | End | PTM | AScore | Found By |
| --- | --- | --- | --- | --- | --- | --- | --- | --- | --- | --- | --- | --- | --- | --- | --- | --- | --- | --- | --- | --- | --- | --- | --- | --- | --- | --- | --- | --- | --- | --- | --- | --- | --- |
| K.SYELPDGQVITIGNER.F | Y | 80.13 | 1789.8846 | 16 | 1.1 | 895.9506 | 2 | 49.57 | 8 | F8:28417 | 31122021\_RID\_3262\_1b.raw |  |  |  |  |  |  |  | 1.1005E6 | 1 | 0 | 0 | 0 | 0 | 0 | 0 | 0 | 1 | 239 | 254 |  |  | PEAKS DB |
| R.VAPEEHPVLLTEAPLNPK.A | Y | 63.63 | 1953.0570 | 18 | 0.2 | 652.0264 | 3 | 40.19 | 8 | F8:20941 | 31122021\_RID\_3262\_1b.raw |  |  |  |  |  |  |  | 7.9964E5 | 2 | 0 | 0 | 0 | 0 | 0 | 0 | 0 | 2 | 96 | 113 |  |  | PEAKS DB |
| R.GYSFTTTAER.E | Y | 63.40 | 1131.5197 | 10 | 0.3 | 566.7673 | 2 | 28.58 | 8 | F8:11995 | 31122021\_RID\_3262\_1b.raw |  |  |  |  |  |  |  | 7.8615E5 | 1 | 0 | 0 | 0 | 0 | 0 | 0 | 0 | 1 | 197 | 206 |  |  | PEAKS DB |
| K.EITALAPSTMK.I | Y | 50.44 | 1160.6111 | 11 | 1.2 | 581.3135 | 2 | 35.42 | 8 | F8:17136 | 31122021\_RID\_3262\_1b.raw |  |  |  |  |  |  |  | 4.2069E5 | 1 | 0 | 0 | 0 | 0 | 0 | 0 | 0 | 1 | 316 | 326 |  |  | PEAKS DB |
| R.AVFPSIVGR.P | Y | 45.16 | 944.5443 | 9 | -0.4 | 473.2792 | 2 | 41.75 | 8 | F8:22126 | 31122021\_RID\_3262\_1b.raw |  |  |  |  |  |  |  | 1.4594E5 | 1 | 0 | 0 | 0 | 0 | 0 | 0 | 0 | 1 | 29 | 37 |  |  | PEAKS DB |
| total 5 peptides |  |  |  |  |  |  |  |  |  |  |  |  |  |  |  |  |  |  |  |  |  |  |  |  |  |  |  |  |  |  |  |  |  |
| --- | --- | --- | --- | --- | --- | --- | --- | --- | --- | --- | --- | --- | --- | --- | --- | --- | --- | --- | --- | --- | --- | --- | --- | --- | --- | --- | --- | --- | --- | --- | --- | --- | --- |

JAA97900.1

back to list

  

| Protein Coverage
| Supporting Peptides
|

Protein Coverage:

Supporting Peptides:

| Peptide | Uniq | -10lgP | Mass | Length | ppm | m/z | z | RT | Fraction | Scan | Source File | Area F6 | Area F2 | Area F3 | Area F4 | Area F5 | Area F7 | Area F8 | Area F1 | #Feature | #Feature F6 | #Feature F2 | #Feature F3 | #Feature F4 | #Feature F5 | #Feature F7 | #Feature F8 | #Feature F1 | Start | End | PTM | AScore | Found By |
| --- | --- | --- | --- | --- | --- | --- | --- | --- | --- | --- | --- | --- | --- | --- | --- | --- | --- | --- | --- | --- | --- | --- | --- | --- | --- | --- | --- | --- | --- | --- | --- | --- | --- |
| K.SYELPDGQVITIGNER.F | Y | 80.13 | 1789.8846 | 16 | 1.1 | 895.9506 | 2 | 49.57 | 8 | F8:28417 | 31122021\_RID\_3262\_1b.raw |  |  |  |  |  |  |  | 1.1005E6 | 1 | 0 | 0 | 0 | 0 | 0 | 0 | 0 | 1 | 239 | 254 |  |  | PEAKS DB |
| R.VAPEEHPVLLTEAPLNPK.A | Y | 63.63 | 1953.0570 | 18 | 0.2 | 652.0264 | 3 | 40.19 | 8 | F8:20941 | 31122021\_RID\_3262\_1b.raw |  |  |  |  |  |  |  | 7.9964E5 | 2 | 0 | 0 | 0 | 0 | 0 | 0 | 0 | 2 | 96 | 113 |  |  | PEAKS DB |
| R.GYSFTTTAER.E | Y | 63.40 | 1131.5197 | 10 | 0.3 | 566.7673 | 2 | 28.58 | 8 | F8:11995 | 31122021\_RID\_3262\_1b.raw |  |  |  |  |  |  |  | 7.8615E5 | 1 | 0 | 0 | 0 | 0 | 0 | 0 | 0 | 1 | 197 | 206 |  |  | PEAKS DB |
| K.EITALAPSTMK.I | Y | 50.44 | 1160.6111 | 11 | 1.2 | 581.3135 | 2 | 35.42 | 8 | F8:17136 | 31122021\_RID\_3262\_1b.raw |  |  |  |  |  |  |  | 4.2069E5 | 1 | 0 | 0 | 0 | 0 | 0 | 0 | 0 | 1 | 316 | 326 |  |  | PEAKS DB |
| R.AVFPSIVGR.P | Y | 45.16 | 944.5443 | 9 | -0.4 | 473.2792 | 2 | 41.75 | 8 | F8:22126 | 31122021\_RID\_3262\_1b.raw |  |  |  |  |  |  |  | 1.4594E5 | 1 | 0 | 0 | 0 | 0 | 0 | 0 | 0 | 1 | 29 | 37 |  |  | PEAKS DB |
| total 5 peptides |  |  |  |  |  |  |  |  |  |  |  |  |  |  |  |  |  |  |  |  |  |  |  |  |  |  |  |  |  |  |  |  |  |
| --- | --- | --- | --- | --- | --- | --- | --- | --- | --- | --- | --- | --- | --- | --- | --- | --- | --- | --- | --- | --- | --- | --- | --- | --- | --- | --- | --- | --- | --- | --- | --- | --- | --- |

LAA21099.1

back to list

  

| Protein Coverage
| Supporting Peptides
|

Protein Coverage:

Supporting Peptides:

| Peptide | Uniq | -10lgP | Mass | Length | ppm | m/z | z | RT | Fraction | Scan | Source File | Area F6 | Area F2 | Area F3 | Area F4 | Area F5 | Area F7 | Area F8 | Area F1 | #Feature | #Feature F6 | #Feature F2 | #Feature F3 | #Feature F4 | #Feature F5 | #Feature F7 | #Feature F8 | #Feature F1 | Start | End | PTM | AScore | Found By |
| --- | --- | --- | --- | --- | --- | --- | --- | --- | --- | --- | --- | --- | --- | --- | --- | --- | --- | --- | --- | --- | --- | --- | --- | --- | --- | --- | --- | --- | --- | --- | --- | --- | --- |
| K.SYELPDGQVITIGNER.F | Y | 80.13 | 1789.8846 | 16 | 1.1 | 895.9506 | 2 | 49.57 | 8 | F8:28417 | 31122021\_RID\_3262\_1b.raw |  |  |  |  |  |  |  | 1.1005E6 | 1 | 0 | 0 | 0 | 0 | 0 | 0 | 0 | 1 | 239 | 254 |  |  | PEAKS DB |
| R.VAPEEHPVLLTEAPLNPK.A | Y | 63.63 | 1953.0570 | 18 | 0.2 | 652.0264 | 3 | 40.19 | 8 | F8:20941 | 31122021\_RID\_3262\_1b.raw |  |  |  |  |  |  |  | 7.9964E5 | 2 | 0 | 0 | 0 | 0 | 0 | 0 | 0 | 2 | 96 | 113 |  |  | PEAKS DB |
| R.GYSFTTTAER.E | Y | 63.40 | 1131.5197 | 10 | 0.3 | 566.7673 | 2 | 28.58 | 8 | F8:11995 | 31122021\_RID\_3262\_1b.raw |  |  |  |  |  |  |  | 7.8615E5 | 1 | 0 | 0 | 0 | 0 | 0 | 0 | 0 | 1 | 197 | 206 |  |  | PEAKS DB |
| K.EITALAPSTMK.I | Y | 50.44 | 1160.6111 | 11 | 1.2 | 581.3135 | 2 | 35.42 | 8 | F8:17136 | 31122021\_RID\_3262\_1b.raw |  |  |  |  |  |  |  | 4.2069E5 | 1 | 0 | 0 | 0 | 0 | 0 | 0 | 0 | 1 | 316 | 326 |  |  | PEAKS DB |
| R.AVFPSIVGR.P | Y | 45.16 | 944.5443 | 9 | -0.4 | 473.2792 | 2 | 41.75 | 8 | F8:22126 | 31122021\_RID\_3262\_1b.raw |  |  |  |  |  |  |  | 1.4594E5 | 1 | 0 | 0 | 0 | 0 | 0 | 0 | 0 | 1 | 29 | 37 |  |  | PEAKS DB |
| total 5 peptides |  |  |  |  |  |  |  |  |  |  |  |  |  |  |  |  |  |  |  |  |  |  |  |  |  |  |  |  |  |  |  |  |  |
| --- | --- | --- | --- | --- | --- | --- | --- | --- | --- | --- | --- | --- | --- | --- | --- | --- | --- | --- | --- | --- | --- | --- | --- | --- | --- | --- | --- | --- | --- | --- | --- | --- | --- |

JAG68843.1

back to list

  

| Protein Coverage
| Supporting Peptides
|

Protein Coverage:

Supporting Peptides:

| Peptide | Uniq | -10lgP | Mass | Length | ppm | m/z | z | RT | Fraction | Scan | Source File | Area F6 | Area F2 | Area F3 | Area F4 | Area F5 | Area F7 | Area F8 | Area F1 | #Feature | #Feature F6 | #Feature F2 | #Feature F3 | #Feature F4 | #Feature F5 | #Feature F7 | #Feature F8 | #Feature F1 | Start | End | PTM | AScore | Found By |
| --- | --- | --- | --- | --- | --- | --- | --- | --- | --- | --- | --- | --- | --- | --- | --- | --- | --- | --- | --- | --- | --- | --- | --- | --- | --- | --- | --- | --- | --- | --- | --- | --- | --- |
| K.SYELPDGQVITIGNER.F | Y | 80.13 | 1789.8846 | 16 | 1.1 | 895.9506 | 2 | 49.57 | 8 | F8:28417 | 31122021\_RID\_3262\_1b.raw |  |  |  |  |  |  |  | 1.1005E6 | 1 | 0 | 0 | 0 | 0 | 0 | 0 | 0 | 1 | 239 | 254 |  |  | PEAKS DB |
| R.VAPEEHPVLLTEAPLNPK.A | Y | 63.63 | 1953.0570 | 18 | 0.2 | 652.0264 | 3 | 40.19 | 8 | F8:20941 | 31122021\_RID\_3262\_1b.raw |  |  |  |  |  |  |  | 7.9964E5 | 2 | 0 | 0 | 0 | 0 | 0 | 0 | 0 | 2 | 96 | 113 |  |  | PEAKS DB |
| R.GYSFTTTAER.E | Y | 63.40 | 1131.5197 | 10 | 0.3 | 566.7673 | 2 | 28.58 | 8 | F8:11995 | 31122021\_RID\_3262\_1b.raw |  |  |  |  |  |  |  | 7.8615E5 | 1 | 0 | 0 | 0 | 0 | 0 | 0 | 0 | 1 | 197 | 206 |  |  | PEAKS DB |
| K.EITALAPSTMK.I | Y | 50.44 | 1160.6111 | 11 | 1.2 | 581.3135 | 2 | 35.42 | 8 | F8:17136 | 31122021\_RID\_3262\_1b.raw |  |  |  |  |  |  |  | 4.2069E5 | 1 | 0 | 0 | 0 | 0 | 0 | 0 | 0 | 1 | 316 | 326 |  |  | PEAKS DB |
| R.AVFPSIVGR.P | Y | 45.16 | 944.5443 | 9 | -0.4 | 473.2792 | 2 | 41.75 | 8 | F8:22126 | 31122021\_RID\_3262\_1b.raw |  |  |  |  |  |  |  | 1.4594E5 | 1 | 0 | 0 | 0 | 0 | 0 | 0 | 0 | 1 | 29 | 37 |  |  | PEAKS DB |
| total 5 peptides |  |  |  |  |  |  |  |  |  |  |  |  |  |  |  |  |  |  |  |  |  |  |  |  |  |  |  |  |  |  |  |  |  |
| --- | --- | --- | --- | --- | --- | --- | --- | --- | --- | --- | --- | --- | --- | --- | --- | --- | --- | --- | --- | --- | --- | --- | --- | --- | --- | --- | --- | --- | --- | --- | --- | --- | --- |

XP\_013921350.1

back to list

  

| Protein Coverage
| Supporting Peptides
|

Protein Coverage:

Supporting Peptides:

| Peptide | Uniq | -10lgP | Mass | Length | ppm | m/z | z | RT | Fraction | Scan | Source File | Area F6 | Area F2 | Area F3 | Area F4 | Area F5 | Area F7 | Area F8 | Area F1 | #Feature | #Feature F6 | #Feature F2 | #Feature F3 | #Feature F4 | #Feature F5 | #Feature F7 | #Feature F8 | #Feature F1 | Start | End | PTM | AScore | Found By |
| --- | --- | --- | --- | --- | --- | --- | --- | --- | --- | --- | --- | --- | --- | --- | --- | --- | --- | --- | --- | --- | --- | --- | --- | --- | --- | --- | --- | --- | --- | --- | --- | --- | --- |
| K.SYELPDGQVITIGNER.F | Y | 80.13 | 1789.8846 | 16 | 1.1 | 895.9506 | 2 | 49.57 | 8 | F8:28417 | 31122021\_RID\_3262\_1b.raw |  |  |  |  |  |  |  | 1.1005E6 | 1 | 0 | 0 | 0 | 0 | 0 | 0 | 0 | 1 | 239 | 254 |  |  | PEAKS DB |
| R.VAPEEHPVLLTEAPLNPK.A | Y | 63.63 | 1953.0570 | 18 | 0.2 | 652.0264 | 3 | 40.19 | 8 | F8:20941 | 31122021\_RID\_3262\_1b.raw |  |  |  |  |  |  |  | 7.9964E5 | 2 | 0 | 0 | 0 | 0 | 0 | 0 | 0 | 2 | 96 | 113 |  |  | PEAKS DB |
| R.GYSFTTTAER.E | Y | 63.40 | 1131.5197 | 10 | 0.3 | 566.7673 | 2 | 28.58 | 8 | F8:11995 | 31122021\_RID\_3262\_1b.raw |  |  |  |  |  |  |  | 7.8615E5 | 1 | 0 | 0 | 0 | 0 | 0 | 0 | 0 | 1 | 197 | 206 |  |  | PEAKS DB |
| K.EITALAPSTMK.I | Y | 50.44 | 1160.6111 | 11 | 1.2 | 581.3135 | 2 | 35.42 | 8 | F8:17136 | 31122021\_RID\_3262\_1b.raw |  |  |  |  |  |  |  | 4.2069E5 | 1 | 0 | 0 | 0 | 0 | 0 | 0 | 0 | 1 | 316 | 326 |  |  | PEAKS DB |
| R.AVFPSIVGR.P | Y | 45.16 | 944.5443 | 9 | -0.4 | 473.2792 | 2 | 41.75 | 8 | F8:22126 | 31122021\_RID\_3262\_1b.raw |  |  |  |  |  |  |  | 1.4594E5 | 1 | 0 | 0 | 0 | 0 | 0 | 0 | 0 | 1 | 29 | 37 |  |  | PEAKS DB |
| total 5 peptides |  |  |  |  |  |  |  |  |  |  |  |  |  |  |  |  |  |  |  |  |  |  |  |  |  |  |  |  |  |  |  |  |  |
| --- | --- | --- | --- | --- | --- | --- | --- | --- | --- | --- | --- | --- | --- | --- | --- | --- | --- | --- | --- | --- | --- | --- | --- | --- | --- | --- | --- | --- | --- | --- | --- | --- | --- |

LAA21098.1

back to list

  

| Protein Coverage
| Supporting Peptides
|

Protein Coverage:

Supporting Peptides:

| Peptide | Uniq | -10lgP | Mass | Length | ppm | m/z | z | RT | Fraction | Scan | Source File | Area F6 | Area F2 | Area F3 | Area F4 | Area F5 | Area F7 | Area F8 | Area F1 | #Feature | #Feature F6 | #Feature F2 | #Feature F3 | #Feature F4 | #Feature F5 | #Feature F7 | #Feature F8 | #Feature F1 | Start | End | PTM | AScore | Found By |
| --- | --- | --- | --- | --- | --- | --- | --- | --- | --- | --- | --- | --- | --- | --- | --- | --- | --- | --- | --- | --- | --- | --- | --- | --- | --- | --- | --- | --- | --- | --- | --- | --- | --- |
| K.SYELPDGQVITIGNER.F | Y | 80.13 | 1789.8846 | 16 | 1.1 | 895.9506 | 2 | 49.57 | 8 | F8:28417 | 31122021\_RID\_3262\_1b.raw |  |  |  |  |  |  |  | 1.1005E6 | 1 | 0 | 0 | 0 | 0 | 0 | 0 | 0 | 1 | 239 | 254 |  |  | PEAKS DB |
| R.VAPEEHPVLLTEAPLNPK.A | Y | 63.63 | 1953.0570 | 18 | 0.2 | 652.0264 | 3 | 40.19 | 8 | F8:20941 | 31122021\_RID\_3262\_1b.raw |  |  |  |  |  |  |  | 7.9964E5 | 2 | 0 | 0 | 0 | 0 | 0 | 0 | 0 | 2 | 96 | 113 |  |  | PEAKS DB |
| R.GYSFTTTAER.E | Y | 63.40 | 1131.5197 | 10 | 0.3 | 566.7673 | 2 | 28.58 | 8 | F8:11995 | 31122021\_RID\_3262\_1b.raw |  |  |  |  |  |  |  | 7.8615E5 | 1 | 0 | 0 | 0 | 0 | 0 | 0 | 0 | 1 | 197 | 206 |  |  | PEAKS DB |
| K.EITALAPSTMK.I | Y | 50.44 | 1160.6111 | 11 | 1.2 | 581.3135 | 2 | 35.42 | 8 | F8:17136 | 31122021\_RID\_3262\_1b.raw |  |  |  |  |  |  |  | 4.2069E5 | 1 | 0 | 0 | 0 | 0 | 0 | 0 | 0 | 1 | 316 | 326 |  |  | PEAKS DB |
| R.AVFPSIVGR.P | Y | 45.16 | 944.5443 | 9 | -0.4 | 473.2792 | 2 | 41.75 | 8 | F8:22126 | 31122021\_RID\_3262\_1b.raw |  |  |  |  |  |  |  | 1.4594E5 | 1 | 0 | 0 | 0 | 0 | 0 | 0 | 0 | 1 | 29 | 37 |  |  | PEAKS DB |
| total 5 peptides |  |  |  |  |  |  |  |  |  |  |  |  |  |  |  |  |  |  |  |  |  |  |  |  |  |  |  |  |  |  |  |  |  |
| --- | --- | --- | --- | --- | --- | --- | --- | --- | --- | --- | --- | --- | --- | --- | --- | --- | --- | --- | --- | --- | --- | --- | --- | --- | --- | --- | --- | --- | --- | --- | --- | --- | --- |

AFJ49303.1

back to list

  

| Protein Coverage
| Supporting Peptides
|

Protein Coverage:

Supporting Peptides:

| Peptide | Uniq | -10lgP | Mass | Length | ppm | m/z | z | RT | Fraction | Scan | Source File | Area F6 | Area F2 | Area F3 | Area F4 | Area F5 | Area F7 | Area F8 | Area F1 | #Feature | #Feature F6 | #Feature F2 | #Feature F3 | #Feature F4 | #Feature F5 | #Feature F7 | #Feature F8 | #Feature F1 | Start | End | PTM | AScore | Found By |
| --- | --- | --- | --- | --- | --- | --- | --- | --- | --- | --- | --- | --- | --- | --- | --- | --- | --- | --- | --- | --- | --- | --- | --- | --- | --- | --- | --- | --- | --- | --- | --- | --- | --- |
| K.SYELPDGQVITIGNER.F | Y | 80.13 | 1789.8846 | 16 | 1.1 | 895.9506 | 2 | 49.57 | 8 | F8:28417 | 31122021\_RID\_3262\_1b.raw |  |  |  |  |  |  |  | 1.1005E6 | 1 | 0 | 0 | 0 | 0 | 0 | 0 | 0 | 1 | 239 | 254 |  |  | PEAKS DB |
| R.VAPEEHPVLLTEAPLNPK.A | Y | 63.63 | 1953.0570 | 18 | 0.2 | 652.0264 | 3 | 40.19 | 8 | F8:20941 | 31122021\_RID\_3262\_1b.raw |  |  |  |  |  |  |  | 7.9964E5 | 2 | 0 | 0 | 0 | 0 | 0 | 0 | 0 | 2 | 96 | 113 |  |  | PEAKS DB |
| R.GYSFTTTAER.E | Y | 63.40 | 1131.5197 | 10 | 0.3 | 566.7673 | 2 | 28.58 | 8 | F8:11995 | 31122021\_RID\_3262\_1b.raw |  |  |  |  |  |  |  | 7.8615E5 | 1 | 0 | 0 | 0 | 0 | 0 | 0 | 0 | 1 | 197 | 206 |  |  | PEAKS DB |
| K.EITALAPSTMK.I | Y | 50.44 | 1160.6111 | 11 | 1.2 | 581.3135 | 2 | 35.42 | 8 | F8:17136 | 31122021\_RID\_3262\_1b.raw |  |  |  |  |  |  |  | 4.2069E5 | 1 | 0 | 0 | 0 | 0 | 0 | 0 | 0 | 1 | 316 | 326 |  |  | PEAKS DB |
| R.AVFPSIVGR.P | Y | 45.16 | 944.5443 | 9 | -0.4 | 473.2792 | 2 | 41.75 | 8 | F8:22126 | 31122021\_RID\_3262\_1b.raw |  |  |  |  |  |  |  | 1.4594E5 | 1 | 0 | 0 | 0 | 0 | 0 | 0 | 0 | 1 | 29 | 37 |  |  | PEAKS DB |
| total 5 peptides |  |  |  |  |  |  |  |  |  |  |  |  |  |  |  |  |  |  |  |  |  |  |  |  |  |  |  |  |  |  |  |  |  |
| --- | --- | --- | --- | --- | --- | --- | --- | --- | --- | --- | --- | --- | --- | --- | --- | --- | --- | --- | --- | --- | --- | --- | --- | --- | --- | --- | --- | --- | --- | --- | --- | --- | --- |

JAI10341.1

back to list

  

| Protein Coverage
| Supporting Peptides
|

Protein Coverage:

Supporting Peptides:

| Peptide | Uniq | -10lgP | Mass | Length | ppm | m/z | z | RT | Fraction | Scan | Source File | Area F6 | Area F2 | Area F3 | Area F4 | Area F5 | Area F7 | Area F8 | Area F1 | #Feature | #Feature F6 | #Feature F2 | #Feature F3 | #Feature F4 | #Feature F5 | #Feature F7 | #Feature F8 | #Feature F1 | Start | End | PTM | AScore | Found By |
| --- | --- | --- | --- | --- | --- | --- | --- | --- | --- | --- | --- | --- | --- | --- | --- | --- | --- | --- | --- | --- | --- | --- | --- | --- | --- | --- | --- | --- | --- | --- | --- | --- | --- |
| K.SYELPDGQVITIGNER.F | Y | 80.13 | 1789.8846 | 16 | 1.1 | 895.9506 | 2 | 49.57 | 8 | F8:28417 | 31122021\_RID\_3262\_1b.raw |  |  |  |  |  |  |  | 1.1005E6 | 1 | 0 | 0 | 0 | 0 | 0 | 0 | 0 | 1 | 239 | 254 |  |  | PEAKS DB |
| R.VAPEEHPVLLTEAPLNPK.A | Y | 63.63 | 1953.0570 | 18 | 0.2 | 652.0264 | 3 | 40.19 | 8 | F8:20941 | 31122021\_RID\_3262\_1b.raw |  |  |  |  |  |  |  | 7.9964E5 | 2 | 0 | 0 | 0 | 0 | 0 | 0 | 0 | 2 | 96 | 113 |  |  | PEAKS DB |
| R.GYSFTTTAER.E | Y | 63.40 | 1131.5197 | 10 | 0.3 | 566.7673 | 2 | 28.58 | 8 | F8:11995 | 31122021\_RID\_3262\_1b.raw |  |  |  |  |  |  |  | 7.8615E5 | 1 | 0 | 0 | 0 | 0 | 0 | 0 | 0 | 1 | 197 | 206 |  |  | PEAKS DB |
| K.EITALAPSTMK.I | Y | 50.44 | 1160.6111 | 11 | 1.2 | 581.3135 | 2 | 35.42 | 8 | F8:17136 | 31122021\_RID\_3262\_1b.raw |  |  |  |  |  |  |  | 4.2069E5 | 1 | 0 | 0 | 0 | 0 | 0 | 0 | 0 | 1 | 316 | 326 |  |  | PEAKS DB |
| R.AVFPSIVGR.P | Y | 45.16 | 944.5443 | 9 | -0.4 | 473.2792 | 2 | 41.75 | 8 | F8:22126 | 31122021\_RID\_3262\_1b.raw |  |  |  |  |  |  |  | 1.4594E5 | 1 | 0 | 0 | 0 | 0 | 0 | 0 | 0 | 1 | 29 | 37 |  |  | PEAKS DB |
| total 5 peptides |  |  |  |  |  |  |  |  |  |  |  |  |  |  |  |  |  |  |  |  |  |  |  |  |  |  |  |  |  |  |  |  |  |
| --- | --- | --- | --- | --- | --- | --- | --- | --- | --- | --- | --- | --- | --- | --- | --- | --- | --- | --- | --- | --- | --- | --- | --- | --- | --- | --- | --- | --- | --- | --- | --- | --- | --- |

JAI14495.1

back to list

  

| Protein Coverage
| Supporting Peptides
|

Protein Coverage:

Supporting Peptides:

| Peptide | Uniq | -10lgP | Mass | Length | ppm | m/z | z | RT | Fraction | Scan | Source File | Area F6 | Area F2 | Area F3 | Area F4 | Area F5 | Area F7 | Area F8 | Area F1 | #Feature | #Feature F6 | #Feature F2 | #Feature F3 | #Feature F4 | #Feature F5 | #Feature F7 | #Feature F8 | #Feature F1 | Start | End | PTM | AScore | Found By |
| --- | --- | --- | --- | --- | --- | --- | --- | --- | --- | --- | --- | --- | --- | --- | --- | --- | --- | --- | --- | --- | --- | --- | --- | --- | --- | --- | --- | --- | --- | --- | --- | --- | --- |
| K.SYELPDGQVITIGNER.F | Y | 80.13 | 1789.8846 | 16 | 1.1 | 895.9506 | 2 | 49.57 | 8 | F8:28417 | 31122021\_RID\_3262\_1b.raw |  |  |  |  |  |  |  | 1.1005E6 | 1 | 0 | 0 | 0 | 0 | 0 | 0 | 0 | 1 | 239 | 254 |  |  | PEAKS DB |
| R.VAPEEHPVLLTEAPLNPK.A | Y | 63.63 | 1953.0570 | 18 | 0.2 | 652.0264 | 3 | 40.19 | 8 | F8:20941 | 31122021\_RID\_3262\_1b.raw |  |  |  |  |  |  |  | 7.9964E5 | 2 | 0 | 0 | 0 | 0 | 0 | 0 | 0 | 2 | 96 | 113 |  |  | PEAKS DB |
| R.GYSFTTTAER.E | Y | 63.40 | 1131.5197 | 10 | 0.3 | 566.7673 | 2 | 28.58 | 8 | F8:11995 | 31122021\_RID\_3262\_1b.raw |  |  |  |  |  |  |  | 7.8615E5 | 1 | 0 | 0 | 0 | 0 | 0 | 0 | 0 | 1 | 197 | 206 |  |  | PEAKS DB |
| K.EITALAPSTMK.I | Y | 50.44 | 1160.6111 | 11 | 1.2 | 581.3135 | 2 | 35.42 | 8 | F8:17136 | 31122021\_RID\_3262\_1b.raw |  |  |  |  |  |  |  | 4.2069E5 | 1 | 0 | 0 | 0 | 0 | 0 | 0 | 0 | 1 | 316 | 326 |  |  | PEAKS DB |
| R.AVFPSIVGR.P | Y | 45.16 | 944.5443 | 9 | -0.4 | 473.2792 | 2 | 41.75 | 8 | F8:22126 | 31122021\_RID\_3262\_1b.raw |  |  |  |  |  |  |  | 1.4594E5 | 1 | 0 | 0 | 0 | 0 | 0 | 0 | 0 | 1 | 29 | 37 |  |  | PEAKS DB |
| total 5 peptides |  |  |  |  |  |  |  |  |  |  |  |  |  |  |  |  |  |  |  |  |  |  |  |  |  |  |  |  |  |  |  |  |  |
| --- | --- | --- | --- | --- | --- | --- | --- | --- | --- | --- | --- | --- | --- | --- | --- | --- | --- | --- | --- | --- | --- | --- | --- | --- | --- | --- | --- | --- | --- | --- | --- | --- | --- |

JAG47579.1

back to list

  

| Protein Coverage
| Supporting Peptides
|

Protein Coverage:

Supporting Peptides:

| Peptide | Uniq | -10lgP | Mass | Length | ppm | m/z | z | RT | Fraction | Scan | Source File | Area F6 | Area F2 | Area F3 | Area F4 | Area F5 | Area F7 | Area F8 | Area F1 | #Feature | #Feature F6 | #Feature F2 | #Feature F3 | #Feature F4 | #Feature F5 | #Feature F7 | #Feature F8 | #Feature F1 | Start | End | PTM | AScore | Found By |
| --- | --- | --- | --- | --- | --- | --- | --- | --- | --- | --- | --- | --- | --- | --- | --- | --- | --- | --- | --- | --- | --- | --- | --- | --- | --- | --- | --- | --- | --- | --- | --- | --- | --- |
| K.SYELPDGQVITIGNER.F | Y | 80.13 | 1789.8846 | 16 | 1.1 | 895.9506 | 2 | 49.57 | 8 | F8:28417 | 31122021\_RID\_3262\_1b.raw |  |  |  |  |  |  |  | 1.1005E6 | 1 | 0 | 0 | 0 | 0 | 0 | 0 | 0 | 1 | 239 | 254 |  |  | PEAKS DB |
| R.VAPEEHPVLLTEAPLNPK.A | Y | 63.63 | 1953.0570 | 18 | 0.2 | 652.0264 | 3 | 40.19 | 8 | F8:20941 | 31122021\_RID\_3262\_1b.raw |  |  |  |  |  |  |  | 7.9964E5 | 2 | 0 | 0 | 0 | 0 | 0 | 0 | 0 | 2 | 96 | 113 |  |  | PEAKS DB |
| R.GYSFTTTAER.E | Y | 63.40 | 1131.5197 | 10 | 0.3 | 566.7673 | 2 | 28.58 | 8 | F8:11995 | 31122021\_RID\_3262\_1b.raw |  |  |  |  |  |  |  | 7.8615E5 | 1 | 0 | 0 | 0 | 0 | 0 | 0 | 0 | 1 | 197 | 206 |  |  | PEAKS DB |
| K.EITALAPSTMK.I | Y | 50.44 | 1160.6111 | 11 | 1.2 | 581.3135 | 2 | 35.42 | 8 | F8:17136 | 31122021\_RID\_3262\_1b.raw |  |  |  |  |  |  |  | 4.2069E5 | 1 | 0 | 0 | 0 | 0 | 0 | 0 | 0 | 1 | 316 | 326 |  |  | PEAKS DB |
| R.AVFPSIVGR.P | Y | 45.16 | 944.5443 | 9 | -0.4 | 473.2792 | 2 | 41.75 | 8 | F8:22126 | 31122021\_RID\_3262\_1b.raw |  |  |  |  |  |  |  | 1.4594E5 | 1 | 0 | 0 | 0 | 0 | 0 | 0 | 0 | 1 | 29 | 37 |  |  | PEAKS DB |
| total 5 peptides |  |  |  |  |  |  |  |  |  |  |  |  |  |  |  |  |  |  |  |  |  |  |  |  |  |  |  |  |  |  |  |  |  |
| --- | --- | --- | --- | --- | --- | --- | --- | --- | --- | --- | --- | --- | --- | --- | --- | --- | --- | --- | --- | --- | --- | --- | --- | --- | --- | --- | --- | --- | --- | --- | --- | --- | --- |

JAV51372.1

back to list

  

| Protein Coverage
| Supporting Peptides
|

Protein Coverage:

Supporting Peptides:

| Peptide | Uniq | -10lgP | Mass | Length | ppm | m/z | z | RT | Fraction | Scan | Source File | Area F6 | Area F2 | Area F3 | Area F4 | Area F5 | Area F7 | Area F8 | Area F1 | #Feature | #Feature F6 | #Feature F2 | #Feature F3 | #Feature F4 | #Feature F5 | #Feature F7 | #Feature F8 | #Feature F1 | Start | End | PTM | AScore | Found By |
| --- | --- | --- | --- | --- | --- | --- | --- | --- | --- | --- | --- | --- | --- | --- | --- | --- | --- | --- | --- | --- | --- | --- | --- | --- | --- | --- | --- | --- | --- | --- | --- | --- | --- |
| K.SYELPDGQVITIGNER.F | Y | 80.13 | 1789.8846 | 16 | 1.1 | 895.9506 | 2 | 49.57 | 8 | F8:28417 | 31122021\_RID\_3262\_1b.raw |  |  |  |  |  |  |  | 1.1005E6 | 1 | 0 | 0 | 0 | 0 | 0 | 0 | 0 | 1 | 239 | 254 |  |  | PEAKS DB |
| R.VAPEEHPVLLTEAPLNPK.A | Y | 63.63 | 1953.0570 | 18 | 0.2 | 652.0264 | 3 | 40.19 | 8 | F8:20941 | 31122021\_RID\_3262\_1b.raw |  |  |  |  |  |  |  | 7.9964E5 | 2 | 0 | 0 | 0 | 0 | 0 | 0 | 0 | 2 | 96 | 113 |  |  | PEAKS DB |
| R.GYSFTTTAER.E | Y | 63.40 | 1131.5197 | 10 | 0.3 | 566.7673 | 2 | 28.58 | 8 | F8:11995 | 31122021\_RID\_3262\_1b.raw |  |  |  |  |  |  |  | 7.8615E5 | 1 | 0 | 0 | 0 | 0 | 0 | 0 | 0 | 1 | 197 | 206 |  |  | PEAKS DB |
| K.EITALAPSTMK.I | Y | 50.44 | 1160.6111 | 11 | 1.2 | 581.3135 | 2 | 35.42 | 8 | F8:17136 | 31122021\_RID\_3262\_1b.raw |  |  |  |  |  |  |  | 4.2069E5 | 1 | 0 | 0 | 0 | 0 | 0 | 0 | 0 | 1 | 316 | 326 |  |  | PEAKS DB |
| R.AVFPSIVGR.P | Y | 45.16 | 944.5443 | 9 | -0.4 | 473.2792 | 2 | 41.75 | 8 | F8:22126 | 31122021\_RID\_3262\_1b.raw |  |  |  |  |  |  |  | 1.4594E5 | 1 | 0 | 0 | 0 | 0 | 0 | 0 | 0 | 1 | 29 | 37 |  |  | PEAKS DB |
| total 5 peptides |  |  |  |  |  |  |  |  |  |  |  |  |  |  |  |  |  |  |  |  |  |  |  |  |  |  |  |  |  |  |  |  |  |
| --- | --- | --- | --- | --- | --- | --- | --- | --- | --- | --- | --- | --- | --- | --- | --- | --- | --- | --- | --- | --- | --- | --- | --- | --- | --- | --- | --- | --- | --- | --- | --- | --- | --- |

JAG45996.1

back to list

  

| Protein Coverage
| Supporting Peptides
|

Protein Coverage:

Supporting Peptides:

| Peptide | Uniq | -10lgP | Mass | Length | ppm | m/z | z | RT | Fraction | Scan | Source File | Area F6 | Area F2 | Area F3 | Area F4 | Area F5 | Area F7 | Area F8 | Area F1 | #Feature | #Feature F6 | #Feature F2 | #Feature F3 | #Feature F4 | #Feature F5 | #Feature F7 | #Feature F8 | #Feature F1 | Start | End | PTM | AScore | Found By |
| --- | --- | --- | --- | --- | --- | --- | --- | --- | --- | --- | --- | --- | --- | --- | --- | --- | --- | --- | --- | --- | --- | --- | --- | --- | --- | --- | --- | --- | --- | --- | --- | --- | --- |
| K.SYELPDGQVITIGNER.F | Y | 80.13 | 1789.8846 | 16 | 1.1 | 895.9506 | 2 | 49.57 | 8 | F8:28417 | 31122021\_RID\_3262\_1b.raw |  |  |  |  |  |  |  | 1.1005E6 | 1 | 0 | 0 | 0 | 0 | 0 | 0 | 0 | 1 | 239 | 254 |  |  | PEAKS DB |
| R.VAPEEHPVLLTEAPLNPK.A | Y | 63.63 | 1953.0570 | 18 | 0.2 | 652.0264 | 3 | 40.19 | 8 | F8:20941 | 31122021\_RID\_3262\_1b.raw |  |  |  |  |  |  |  | 7.9964E5 | 2 | 0 | 0 | 0 | 0 | 0 | 0 | 0 | 2 | 96 | 113 |  |  | PEAKS DB |
| R.GYSFTTTAER.E | Y | 63.40 | 1131.5197 | 10 | 0.3 | 566.7673 | 2 | 28.58 | 8 | F8:11995 | 31122021\_RID\_3262\_1b.raw |  |  |  |  |  |  |  | 7.8615E5 | 1 | 0 | 0 | 0 | 0 | 0 | 0 | 0 | 1 | 197 | 206 |  |  | PEAKS DB |
| K.EITALAPSTMK.I | Y | 50.44 | 1160.6111 | 11 | 1.2 | 581.3135 | 2 | 35.42 | 8 | F8:17136 | 31122021\_RID\_3262\_1b.raw |  |  |  |  |  |  |  | 4.2069E5 | 1 | 0 | 0 | 0 | 0 | 0 | 0 | 0 | 1 | 316 | 326 |  |  | PEAKS DB |
| R.AVFPSIVGR.P | Y | 45.16 | 944.5443 | 9 | -0.4 | 473.2792 | 2 | 41.75 | 8 | F8:22126 | 31122021\_RID\_3262\_1b.raw |  |  |  |  |  |  |  | 1.4594E5 | 1 | 0 | 0 | 0 | 0 | 0 | 0 | 0 | 1 | 29 | 37 |  |  | PEAKS DB |
| total 5 peptides |  |  |  |  |  |  |  |  |  |  |  |  |  |  |  |  |  |  |  |  |  |  |  |  |  |  |  |  |  |  |  |  |  |
| --- | --- | --- | --- | --- | --- | --- | --- | --- | --- | --- | --- | --- | --- | --- | --- | --- | --- | --- | --- | --- | --- | --- | --- | --- | --- | --- | --- | --- | --- | --- | --- | --- | --- |

JAB54395.1

back to list

  

| Protein Coverage
| Supporting Peptides
|

Protein Coverage:

Supporting Peptides:

| Peptide | Uniq | -10lgP | Mass | Length | ppm | m/z | z | RT | Fraction | Scan | Source File | Area F6 | Area F2 | Area F3 | Area F4 | Area F5 | Area F7 | Area F8 | Area F1 | #Feature | #Feature F6 | #Feature F2 | #Feature F3 | #Feature F4 | #Feature F5 | #Feature F7 | #Feature F8 | #Feature F1 | Start | End | PTM | AScore | Found By |
| --- | --- | --- | --- | --- | --- | --- | --- | --- | --- | --- | --- | --- | --- | --- | --- | --- | --- | --- | --- | --- | --- | --- | --- | --- | --- | --- | --- | --- | --- | --- | --- | --- | --- |
| K.SYELPDGQVITIGNER.F | Y | 80.13 | 1789.8846 | 16 | 1.1 | 895.9506 | 2 | 49.57 | 8 | F8:28417 | 31122021\_RID\_3262\_1b.raw |  |  |  |  |  |  |  | 1.1005E6 | 1 | 0 | 0 | 0 | 0 | 0 | 0 | 0 | 1 | 239 | 254 |  |  | PEAKS DB |
| R.VAPEEHPVLLTEAPLNPK.A | Y | 63.63 | 1953.0570 | 18 | 0.2 | 652.0264 | 3 | 40.19 | 8 | F8:20941 | 31122021\_RID\_3262\_1b.raw |  |  |  |  |  |  |  | 7.9964E5 | 2 | 0 | 0 | 0 | 0 | 0 | 0 | 0 | 2 | 96 | 113 |  |  | PEAKS DB |
| R.GYSFTTTAER.E | Y | 63.40 | 1131.5197 | 10 | 0.3 | 566.7673 | 2 | 28.58 | 8 | F8:11995 | 31122021\_RID\_3262\_1b.raw |  |  |  |  |  |  |  | 7.8615E5 | 1 | 0 | 0 | 0 | 0 | 0 | 0 | 0 | 1 | 197 | 206 |  |  | PEAKS DB |
| K.EITALAPSTMK.I | Y | 50.44 | 1160.6111 | 11 | 1.2 | 581.3135 | 2 | 35.42 | 8 | F8:17136 | 31122021\_RID\_3262\_1b.raw |  |  |  |  |  |  |  | 4.2069E5 | 1 | 0 | 0 | 0 | 0 | 0 | 0 | 0 | 1 | 316 | 326 |  |  | PEAKS DB |
| R.AVFPSIVGR.P | Y | 45.16 | 944.5443 | 9 | -0.4 | 473.2792 | 2 | 41.75 | 8 | F8:22126 | 31122021\_RID\_3262\_1b.raw |  |  |  |  |  |  |  | 1.4594E5 | 1 | 0 | 0 | 0 | 0 | 0 | 0 | 0 | 1 | 29 | 37 |  |  | PEAKS DB |
| total 5 peptides |  |  |  |  |  |  |  |  |  |  |  |  |  |  |  |  |  |  |  |  |  |  |  |  |  |  |  |  |  |  |  |  |  |
| --- | --- | --- | --- | --- | --- | --- | --- | --- | --- | --- | --- | --- | --- | --- | --- | --- | --- | --- | --- | --- | --- | --- | --- | --- | --- | --- | --- | --- | --- | --- | --- | --- | --- |

ETE74025.1

back to list

  

| Protein Coverage
| Supporting Peptides
|

Protein Coverage:

Supporting Peptides:

| Peptide | Uniq | -10lgP | Mass | Length | ppm | m/z | z | RT | Fraction | Scan | Source File | Area F6 | Area F2 | Area F3 | Area F4 | Area F5 | Area F7 | Area F8 | Area F1 | #Feature | #Feature F6 | #Feature F2 | #Feature F3 | #Feature F4 | #Feature F5 | #Feature F7 | #Feature F8 | #Feature F1 | Start | End | PTM | AScore | Found By |
| --- | --- | --- | --- | --- | --- | --- | --- | --- | --- | --- | --- | --- | --- | --- | --- | --- | --- | --- | --- | --- | --- | --- | --- | --- | --- | --- | --- | --- | --- | --- | --- | --- | --- |
| K.SYELPDGQVITIGNER.F | Y | 80.13 | 1789.8846 | 16 | 1.1 | 895.9506 | 2 | 49.57 | 8 | F8:28417 | 31122021\_RID\_3262\_1b.raw |  |  |  |  |  |  |  | 1.1005E6 | 1 | 0 | 0 | 0 | 0 | 0 | 0 | 0 | 1 | 239 | 254 |  |  | PEAKS DB |
| R.VAPEEHPVLLTEAPLNPK.A | Y | 63.63 | 1953.0570 | 18 | 0.2 | 652.0264 | 3 | 40.19 | 8 | F8:20941 | 31122021\_RID\_3262\_1b.raw |  |  |  |  |  |  |  | 7.9964E5 | 2 | 0 | 0 | 0 | 0 | 0 | 0 | 0 | 2 | 96 | 113 |  |  | PEAKS DB |
| R.GYSFTTTAER.E | Y | 63.40 | 1131.5197 | 10 | 0.3 | 566.7673 | 2 | 28.58 | 8 | F8:11995 | 31122021\_RID\_3262\_1b.raw |  |  |  |  |  |  |  | 7.8615E5 | 1 | 0 | 0 | 0 | 0 | 0 | 0 | 0 | 1 | 197 | 206 |  |  | PEAKS DB |
| K.EITALAPSTMK.I | Y | 50.44 | 1160.6111 | 11 | 1.2 | 581.3135 | 2 | 35.42 | 8 | F8:17136 | 31122021\_RID\_3262\_1b.raw |  |  |  |  |  |  |  | 4.2069E5 | 1 | 0 | 0 | 0 | 0 | 0 | 0 | 0 | 1 | 316 | 326 |  |  | PEAKS DB |
| R.AVFPSIVGR.P | Y | 45.16 | 944.5443 | 9 | -0.4 | 473.2792 | 2 | 41.75 | 8 | F8:22126 | 31122021\_RID\_3262\_1b.raw |  |  |  |  |  |  |  | 1.4594E5 | 1 | 0 | 0 | 0 | 0 | 0 | 0 | 0 | 1 | 29 | 37 |  |  | PEAKS DB |
| total 5 peptides |  |  |  |  |  |  |  |  |  |  |  |  |  |  |  |  |  |  |  |  |  |  |  |  |  |  |  |  |  |  |  |  |  |
| --- | --- | --- | --- | --- | --- | --- | --- | --- | --- | --- | --- | --- | --- | --- | --- | --- | --- | --- | --- | --- | --- | --- | --- | --- | --- | --- | --- | --- | --- | --- | --- | --- | --- |

AFE84764.1

back to list

  

| Protein Coverage
| Supporting Peptides
|

Protein Coverage:

Supporting Peptides:

| Peptide | Uniq | -10lgP | Mass | Length | ppm | m/z | z | RT | Fraction | Scan | Source File | Area F6 | Area F2 | Area F3 | Area F4 | Area F5 | Area F7 | Area F8 | Area F1 | #Feature | #Feature F6 | #Feature F2 | #Feature F3 | #Feature F4 | #Feature F5 | #Feature F7 | #Feature F8 | #Feature F1 | Start | End | PTM | AScore | Found By |
| --- | --- | --- | --- | --- | --- | --- | --- | --- | --- | --- | --- | --- | --- | --- | --- | --- | --- | --- | --- | --- | --- | --- | --- | --- | --- | --- | --- | --- | --- | --- | --- | --- | --- |
| R.TFSNIISTLNPLAK.R | Y | 67.62 | 1517.8453 | 14 | 0.6 | 759.9304 | 2 | 58.92 | 4 | F4:33771 | 13012022\_RID\_3262\_4b.raw |  |  |  | 1.3808E6 |  |  |  |  | 1 | 0 | 0 | 0 | 1 | 0 | 0 | 0 | 0 | 123 | 136 |  |  | PEAKS DB |
| R.WSPSDSLYGSR.S | Y | 65.59 | 1253.5676 | 11 | -0.3 | 627.7909 | 2 | 39.85 | 4 | F4:22700 | 13012022\_RID\_3262\_4b.raw |  |  |  | 1.1335E6 | 3.8712E5 |  |  |  | 2 | 0 | 0 | 0 | 1 | 1 | 0 | 0 | 0 | 213 | 223 |  |  | PEAKS DB |
| R.YPGSPGSYAVR.Q | Y | 61.64 | 1152.5563 | 11 | 0.4 | 577.2856 | 2 | 30.09 | 4 | F4:15330 | 13012022\_RID\_3262\_4b.raw |  |  |  | 3.0986E6 |  |  |  |  | 1 | 0 | 0 | 0 | 1 | 0 | 0 | 0 | 0 | 82 | 92 |  |  | PEAKS DB |
| R.NLYDLGLLNNYSSER.Q | Y | 57.61 | 1769.8584 | 15 | 0.3 | 885.9368 | 2 | 54.90 | 4 | F4:31914 | 13012022\_RID\_3262\_4b.raw |  |  |  | 4.1197E5 |  |  |  |  | 1 | 0 | 0 | 0 | 1 | 0 | 0 | 0 | 0 | 283 | 297 |  |  | PEAKS DB |
| R.NPVFPVYFLNTAR.W | Y | 57.19 | 1536.8088 | 13 | 0.2 | 769.4119 | 2 | 58.74 | 4 | F4:33587 | 13012022\_RID\_3262\_4b.raw |  |  |  | 1.2502E6 | 3.6499E5 |  |  |  | 2 | 0 | 0 | 0 | 1 | 1 | 0 | 0 | 0 | 260 | 272 |  |  | PEAKS DB |
| K.LIFFDGEEAFVR.W | Y | 56.69 | 1441.7241 | 12 | -0.1 | 721.8693 | 2 | 59.83 | 5 | F5:29308 | 13012022\_RID\_3262\_5b.raw |  |  |  |  | 2.1896E5 |  |  |  | 1 | 0 | 0 | 0 | 0 | 1 | 0 | 0 | 0 | 201 | 212 |  |  | PEAKS DB |
| K.MASTPHPPGAR.N | Y | 50.10 | 1120.5447 | 11 | 0.5 | 561.2799 | 2 | 19.93 | 4 | F4:7339 | 13012022\_RID\_3262\_4b.raw |  |  |  | 1.6545E5 |  |  |  |  | 1 | 0 | 0 | 0 | 1 | 0 | 0 | 0 | 0 | 229 | 239 |  |  | PEAKS DB |
| K.M(+15.99)ASTPHPPGAR.N | Y | 49.55 | 1136.5397 | 11 | 0.8 | 569.2776 | 2 | 17.65 | 4 | F4:5444 | 13012022\_RID\_3262\_4b.raw |  |  |  | 1.1854E5 |  |  |  |  | 1 | 0 | 0 | 0 | 1 | 0 | 0 | 0 | 0 | 229 | 239 | Oxidation (M) | M1:Oxidation (M):1000.00 | PEAKS DB |
| K.YFPPQLDGK.V | Y | 46.92 | 1063.5338 | 9 | -0.3 | 532.7740 | 2 | 37.67 | 4 | F4:21235 | 13012022\_RID\_3262\_4b.raw |  |  |  | 2.0372E6 |  |  |  |  | 1 | 0 | 0 | 0 | 1 | 0 | 0 | 0 | 0 | 149 | 157 |  |  | PEAKS DB |
| total 9 peptides |  |  |  |  |  |  |  |  |  |  |  |  |  |  |  |  |  |  |  |  |  |  |  |  |  |  |  |  |  |  |  |  |  |
| --- | --- | --- | --- | --- | --- | --- | --- | --- | --- | --- | --- | --- | --- | --- | --- | --- | --- | --- | --- | --- | --- | --- | --- | --- | --- | --- | --- | --- | --- | --- | --- | --- | --- |

NP\_001310179.1

back to list

  

| Protein Coverage
| Supporting Peptides
|

Protein Coverage:

Supporting Peptides:

| Peptide | Uniq | -10lgP | Mass | Length | ppm | m/z | z | RT | Fraction | Scan | Source File | Area F6 | Area F2 | Area F3 | Area F4 | Area F5 | Area F7 | Area F8 | Area F1 | #Feature | #Feature F6 | #Feature F2 | #Feature F3 | #Feature F4 | #Feature F5 | #Feature F7 | #Feature F8 | #Feature F1 | Start | End | PTM | AScore | Found By |
| --- | --- | --- | --- | --- | --- | --- | --- | --- | --- | --- | --- | --- | --- | --- | --- | --- | --- | --- | --- | --- | --- | --- | --- | --- | --- | --- | --- | --- | --- | --- | --- | --- | --- |
| R.TFSNIISTLNPLAK.R | Y | 67.62 | 1517.8453 | 14 | 0.6 | 759.9304 | 2 | 58.92 | 4 | F4:33771 | 13012022\_RID\_3262\_4b.raw |  |  |  | 1.3808E6 |  |  |  |  | 1 | 0 | 0 | 0 | 1 | 0 | 0 | 0 | 0 | 123 | 136 |  |  | PEAKS DB |
| R.WSPSDSLYGSR.S | Y | 65.59 | 1253.5676 | 11 | -0.3 | 627.7909 | 2 | 39.85 | 4 | F4:22700 | 13012022\_RID\_3262\_4b.raw |  |  |  | 1.1335E6 | 3.8712E5 |  |  |  | 2 | 0 | 0 | 0 | 1 | 1 | 0 | 0 | 0 | 213 | 223 |  |  | PEAKS DB |
| R.YPGSPGSYAVR.Q | Y | 61.64 | 1152.5563 | 11 | 0.4 | 577.2856 | 2 | 30.09 | 4 | F4:15330 | 13012022\_RID\_3262\_4b.raw |  |  |  | 3.0986E6 |  |  |  |  | 1 | 0 | 0 | 0 | 1 | 0 | 0 | 0 | 0 | 82 | 92 |  |  | PEAKS DB |
| R.NLYDLGLLNNYSSER.Q | Y | 57.61 | 1769.8584 | 15 | 0.3 | 885.9368 | 2 | 54.90 | 4 | F4:31914 | 13012022\_RID\_3262\_4b.raw |  |  |  | 4.1197E5 |  |  |  |  | 1 | 0 | 0 | 0 | 1 | 0 | 0 | 0 | 0 | 283 | 297 |  |  | PEAKS DB |
| R.NPVFPVYFLNTAR.W | Y | 57.19 | 1536.8088 | 13 | 0.2 | 769.4119 | 2 | 58.74 | 4 | F4:33587 | 13012022\_RID\_3262\_4b.raw |  |  |  | 1.2502E6 | 3.6499E5 |  |  |  | 2 | 0 | 0 | 0 | 1 | 1 | 0 | 0 | 0 | 260 | 272 |  |  | PEAKS DB |
| K.LIFFDGEEAFVR.W | Y | 56.69 | 1441.7241 | 12 | -0.1 | 721.8693 | 2 | 59.83 | 5 | F5:29308 | 13012022\_RID\_3262\_5b.raw |  |  |  |  | 2.1896E5 |  |  |  | 1 | 0 | 0 | 0 | 0 | 1 | 0 | 0 | 0 | 201 | 212 |  |  | PEAKS DB |
| K.MASTPHPPGAR.N | Y | 50.10 | 1120.5447 | 11 | 0.5 | 561.2799 | 2 | 19.93 | 4 | F4:7339 | 13012022\_RID\_3262\_4b.raw |  |  |  | 1.6545E5 |  |  |  |  | 1 | 0 | 0 | 0 | 1 | 0 | 0 | 0 | 0 | 229 | 239 |  |  | PEAKS DB |
| K.M(+15.99)ASTPHPPGAR.N | Y | 49.55 | 1136.5397 | 11 | 0.8 | 569.2776 | 2 | 17.65 | 4 | F4:5444 | 13012022\_RID\_3262\_4b.raw |  |  |  | 1.1854E5 |  |  |  |  | 1 | 0 | 0 | 0 | 1 | 0 | 0 | 0 | 0 | 229 | 239 | Oxidation (M) | M1:Oxidation (M):1000.00 | PEAKS DB |
| K.YFPPQLDGK.V | Y | 46.92 | 1063.5338 | 9 | -0.3 | 532.7740 | 2 | 37.67 | 4 | F4:21235 | 13012022\_RID\_3262\_4b.raw |  |  |  | 2.0372E6 |  |  |  |  | 1 | 0 | 0 | 0 | 1 | 0 | 0 | 0 | 0 | 149 | 157 |  |  | PEAKS DB |
| total 9 peptides |  |  |  |  |  |  |  |  |  |  |  |  |  |  |  |  |  |  |  |  |  |  |  |  |  |  |  |  |  |  |  |  |  |
| --- | --- | --- | --- | --- | --- | --- | --- | --- | --- | --- | --- | --- | --- | --- | --- | --- | --- | --- | --- | --- | --- | --- | --- | --- | --- | --- | --- | --- | --- | --- | --- | --- | --- |

JAG46763.1

back to list

  

| Protein Coverage
| Supporting Peptides
|

Protein Coverage:

Supporting Peptides:

| Peptide | Uniq | -10lgP | Mass | Length | ppm | m/z | z | RT | Fraction | Scan | Source File | Area F6 | Area F2 | Area F3 | Area F4 | Area F5 | Area F7 | Area F8 | Area F1 | #Feature | #Feature F6 | #Feature F2 | #Feature F3 | #Feature F4 | #Feature F5 | #Feature F7 | #Feature F8 | #Feature F1 | Start | End | PTM | AScore | Found By |
| --- | --- | --- | --- | --- | --- | --- | --- | --- | --- | --- | --- | --- | --- | --- | --- | --- | --- | --- | --- | --- | --- | --- | --- | --- | --- | --- | --- | --- | --- | --- | --- | --- | --- |
| R.TFSNIISTLNPLAK.R | Y | 67.62 | 1517.8453 | 14 | 0.6 | 759.9304 | 2 | 58.92 | 4 | F4:33771 | 13012022\_RID\_3262\_4b.raw |  |  |  | 1.3808E6 |  |  |  |  | 1 | 0 | 0 | 0 | 1 | 0 | 0 | 0 | 0 | 123 | 136 |  |  | PEAKS DB |
| R.WSPSDSLYGSR.S | Y | 65.59 | 1253.5676 | 11 | -0.3 | 627.7909 | 2 | 39.85 | 4 | F4:22700 | 13012022\_RID\_3262\_4b.raw |  |  |  | 1.1335E6 | 3.8712E5 |  |  |  | 2 | 0 | 0 | 0 | 1 | 1 | 0 | 0 | 0 | 213 | 223 |  |  | PEAKS DB |
| R.YPGSPGSYAVR.Q | Y | 61.64 | 1152.5563 | 11 | 0.4 | 577.2856 | 2 | 30.09 | 4 | F4:15330 | 13012022\_RID\_3262\_4b.raw |  |  |  | 3.0986E6 |  |  |  |  | 1 | 0 | 0 | 0 | 1 | 0 | 0 | 0 | 0 | 82 | 92 |  |  | PEAKS DB |
| R.NLYDLGLLNNYSSER.Q | Y | 57.61 | 1769.8584 | 15 | 0.3 | 885.9368 | 2 | 54.90 | 4 | F4:31914 | 13012022\_RID\_3262\_4b.raw |  |  |  | 4.1197E5 |  |  |  |  | 1 | 0 | 0 | 0 | 1 | 0 | 0 | 0 | 0 | 283 | 297 |  |  | PEAKS DB |
| R.NPVFPVYFLNTAR.W | Y | 57.19 | 1536.8088 | 13 | 0.2 | 769.4119 | 2 | 58.74 | 4 | F4:33587 | 13012022\_RID\_3262\_4b.raw |  |  |  | 1.2502E6 | 3.6499E5 |  |  |  | 2 | 0 | 0 | 0 | 1 | 1 | 0 | 0 | 0 | 260 | 272 |  |  | PEAKS DB |
| K.LIFFDGEEAFVR.W | Y | 56.69 | 1441.7241 | 12 | -0.1 | 721.8693 | 2 | 59.83 | 5 | F5:29308 | 13012022\_RID\_3262\_5b.raw |  |  |  |  | 2.1896E5 |  |  |  | 1 | 0 | 0 | 0 | 0 | 1 | 0 | 0 | 0 | 201 | 212 |  |  | PEAKS DB |
| K.MASTPHPPGAR.N | Y | 50.10 | 1120.5447 | 11 | 0.5 | 561.2799 | 2 | 19.93 | 4 | F4:7339 | 13012022\_RID\_3262\_4b.raw |  |  |  | 1.6545E5 |  |  |  |  | 1 | 0 | 0 | 0 | 1 | 0 | 0 | 0 | 0 | 229 | 239 |  |  | PEAKS DB |
| K.M(+15.99)ASTPHPPGAR.N | Y | 49.55 | 1136.5397 | 11 | 0.8 | 569.2776 | 2 | 17.65 | 4 | F4:5444 | 13012022\_RID\_3262\_4b.raw |  |  |  | 1.1854E5 |  |  |  |  | 1 | 0 | 0 | 0 | 1 | 0 | 0 | 0 | 0 | 229 | 239 | Oxidation (M) | M1:Oxidation (M):1000.00 | PEAKS DB |
| K.YFPPQLDGK.V | Y | 46.92 | 1063.5338 | 9 | -0.3 | 532.7740 | 2 | 37.67 | 4 | F4:21235 | 13012022\_RID\_3262\_4b.raw |  |  |  | 2.0372E6 |  |  |  |  | 1 | 0 | 0 | 0 | 1 | 0 | 0 | 0 | 0 | 149 | 157 |  |  | PEAKS DB |
| total 9 peptides |  |  |  |  |  |  |  |  |  |  |  |  |  |  |  |  |  |  |  |  |  |  |  |  |  |  |  |  |  |  |  |  |  |
| --- | --- | --- | --- | --- | --- | --- | --- | --- | --- | --- | --- | --- | --- | --- | --- | --- | --- | --- | --- | --- | --- | --- | --- | --- | --- | --- | --- | --- | --- | --- | --- | --- | --- |

BAN89376.1

back to list

  

| Protein Coverage
| Supporting Peptides
|

Protein Coverage:

Supporting Peptides:

| Peptide | Uniq | -10lgP | Mass | Length | ppm | m/z | z | RT | Fraction | Scan | Source File | Area F6 | Area F2 | Area F3 | Area F4 | Area F5 | Area F7 | Area F8 | Area F1 | #Feature | #Feature F6 | #Feature F2 | #Feature F3 | #Feature F4 | #Feature F5 | #Feature F7 | #Feature F8 | #Feature F1 | Start | End | PTM | AScore | Found By |
| --- | --- | --- | --- | --- | --- | --- | --- | --- | --- | --- | --- | --- | --- | --- | --- | --- | --- | --- | --- | --- | --- | --- | --- | --- | --- | --- | --- | --- | --- | --- | --- | --- | --- |
| R.TFSNIISTLNPLAK.R | Y | 67.62 | 1517.8453 | 14 | 0.6 | 759.9304 | 2 | 58.92 | 4 | F4:33771 | 13012022\_RID\_3262\_4b.raw |  |  |  | 1.3808E6 |  |  |  |  | 1 | 0 | 0 | 0 | 1 | 0 | 0 | 0 | 0 | 55 | 68 |  |  | PEAKS DB |
| R.WSPSDSLYGSR.S | Y | 65.59 | 1253.5676 | 11 | -0.3 | 627.7909 | 2 | 39.85 | 4 | F4:22700 | 13012022\_RID\_3262\_4b.raw |  |  |  | 1.1335E6 | 3.8712E5 |  |  |  | 2 | 0 | 0 | 0 | 1 | 1 | 0 | 0 | 0 | 145 | 155 |  |  | PEAKS DB |
| R.YPGSPGSYAVR.Q | Y | 61.64 | 1152.5563 | 11 | 0.4 | 577.2856 | 2 | 30.09 | 4 | F4:15330 | 13012022\_RID\_3262\_4b.raw |  |  |  | 3.0986E6 |  |  |  |  | 1 | 0 | 0 | 0 | 1 | 0 | 0 | 0 | 0 | 14 | 24 |  |  | PEAKS DB |
| R.NLYDLGLLNNYSSER.Q | Y | 57.61 | 1769.8584 | 15 | 0.3 | 885.9368 | 2 | 54.90 | 4 | F4:31914 | 13012022\_RID\_3262\_4b.raw |  |  |  | 4.1197E5 |  |  |  |  | 1 | 0 | 0 | 0 | 1 | 0 | 0 | 0 | 0 | 215 | 229 |  |  | PEAKS DB |
| R.NPVFPVYFLNTAR.W | Y | 57.19 | 1536.8088 | 13 | 0.2 | 769.4119 | 2 | 58.74 | 4 | F4:33587 | 13012022\_RID\_3262\_4b.raw |  |  |  | 1.2502E6 | 3.6499E5 |  |  |  | 2 | 0 | 0 | 0 | 1 | 1 | 0 | 0 | 0 | 192 | 204 |  |  | PEAKS DB |
| K.LIFFDGEEAFVR.W | Y | 56.69 | 1441.7241 | 12 | -0.1 | 721.8693 | 2 | 59.83 | 5 | F5:29308 | 13012022\_RID\_3262\_5b.raw |  |  |  |  | 2.1896E5 |  |  |  | 1 | 0 | 0 | 0 | 0 | 1 | 0 | 0 | 0 | 133 | 144 |  |  | PEAKS DB |
| K.MASTPHPPGAR.N | Y | 50.10 | 1120.5447 | 11 | 0.5 | 561.2799 | 2 | 19.93 | 4 | F4:7339 | 13012022\_RID\_3262\_4b.raw |  |  |  | 1.6545E5 |  |  |  |  | 1 | 0 | 0 | 0 | 1 | 0 | 0 | 0 | 0 | 161 | 171 |  |  | PEAKS DB |
| K.M(+15.99)ASTPHPPGAR.N | Y | 49.55 | 1136.5397 | 11 | 0.8 | 569.2776 | 2 | 17.65 | 4 | F4:5444 | 13012022\_RID\_3262\_4b.raw |  |  |  | 1.1854E5 |  |  |  |  | 1 | 0 | 0 | 0 | 1 | 0 | 0 | 0 | 0 | 161 | 171 | Oxidation (M) | M1:Oxidation (M):1000.00 | PEAKS DB |
| K.YFPPQLDGK.V | Y | 46.92 | 1063.5338 | 9 | -0.3 | 532.7740 | 2 | 37.67 | 4 | F4:21235 | 13012022\_RID\_3262\_4b.raw |  |  |  | 2.0372E6 |  |  |  |  | 1 | 0 | 0 | 0 | 1 | 0 | 0 | 0 | 0 | 81 | 89 |  |  | PEAKS DB |
| total 9 peptides |  |  |  |  |  |  |  |  |  |  |  |  |  |  |  |  |  |  |  |  |  |  |  |  |  |  |  |  |  |  |  |  |  |
| --- | --- | --- | --- | --- | --- | --- | --- | --- | --- | --- | --- | --- | --- | --- | --- | --- | --- | --- | --- | --- | --- | --- | --- | --- | --- | --- | --- | --- | --- | --- | --- | --- | --- |

BAN82005.1

back to list

  

| Protein Coverage
| Supporting Peptides
|

Protein Coverage:

Supporting Peptides:

| Peptide | Uniq | -10lgP | Mass | Length | ppm | m/z | z | RT | Fraction | Scan | Source File | Area F6 | Area F2 | Area F3 | Area F4 | Area F5 | Area F7 | Area F8 | Area F1 | #Feature | #Feature F6 | #Feature F2 | #Feature F3 | #Feature F4 | #Feature F5 | #Feature F7 | #Feature F8 | #Feature F1 | Start | End | PTM | AScore | Found By |
| --- | --- | --- | --- | --- | --- | --- | --- | --- | --- | --- | --- | --- | --- | --- | --- | --- | --- | --- | --- | --- | --- | --- | --- | --- | --- | --- | --- | --- | --- | --- | --- | --- | --- |
| R.TFSNIISTLNPLAK.R | Y | 67.62 | 1517.8453 | 14 | 0.6 | 759.9304 | 2 | 58.92 | 4 | F4:33771 | 13012022\_RID\_3262\_4b.raw |  |  |  | 1.3808E6 |  |  |  |  | 1 | 0 | 0 | 0 | 1 | 0 | 0 | 0 | 0 | 87 | 100 |  |  | PEAKS DB |
| R.WSPSDSLYGSR.S | Y | 65.59 | 1253.5676 | 11 | -0.3 | 627.7909 | 2 | 39.85 | 4 | F4:22700 | 13012022\_RID\_3262\_4b.raw |  |  |  | 1.1335E6 | 3.8712E5 |  |  |  | 2 | 0 | 0 | 0 | 1 | 1 | 0 | 0 | 0 | 177 | 187 |  |  | PEAKS DB |
| R.YPGSPGSYAVR.Q | Y | 61.64 | 1152.5563 | 11 | 0.4 | 577.2856 | 2 | 30.09 | 4 | F4:15330 | 13012022\_RID\_3262\_4b.raw |  |  |  | 3.0986E6 |  |  |  |  | 1 | 0 | 0 | 0 | 1 | 0 | 0 | 0 | 0 | 46 | 56 |  |  | PEAKS DB |
| R.NLYDLGLLNNYSSER.Q | Y | 57.61 | 1769.8584 | 15 | 0.3 | 885.9368 | 2 | 54.90 | 4 | F4:31914 | 13012022\_RID\_3262\_4b.raw |  |  |  | 4.1197E5 |  |  |  |  | 1 | 0 | 0 | 0 | 1 | 0 | 0 | 0 | 0 | 247 | 261 |  |  | PEAKS DB |
| R.NPVFPVYFLNTAR.W | Y | 57.19 | 1536.8088 | 13 | 0.2 | 769.4119 | 2 | 58.74 | 4 | F4:33587 | 13012022\_RID\_3262\_4b.raw |  |  |  | 1.2502E6 | 3.6499E5 |  |  |  | 2 | 0 | 0 | 0 | 1 | 1 | 0 | 0 | 0 | 224 | 236 |  |  | PEAKS DB |
| K.LIFFDGEEAFVR.W | Y | 56.69 | 1441.7241 | 12 | -0.1 | 721.8693 | 2 | 59.83 | 5 | F5:29308 | 13012022\_RID\_3262\_5b.raw |  |  |  |  | 2.1896E5 |  |  |  | 1 | 0 | 0 | 0 | 0 | 1 | 0 | 0 | 0 | 165 | 176 |  |  | PEAKS DB |
| K.MASTPHPPGAR.N | Y | 50.10 | 1120.5447 | 11 | 0.5 | 561.2799 | 2 | 19.93 | 4 | F4:7339 | 13012022\_RID\_3262\_4b.raw |  |  |  | 1.6545E5 |  |  |  |  | 1 | 0 | 0 | 0 | 1 | 0 | 0 | 0 | 0 | 193 | 203 |  |  | PEAKS DB |
| K.M(+15.99)ASTPHPPGAR.N | Y | 49.55 | 1136.5397 | 11 | 0.8 | 569.2776 | 2 | 17.65 | 4 | F4:5444 | 13012022\_RID\_3262\_4b.raw |  |  |  | 1.1854E5 |  |  |  |  | 1 | 0 | 0 | 0 | 1 | 0 | 0 | 0 | 0 | 193 | 203 | Oxidation (M) | M1:Oxidation (M):1000.00 | PEAKS DB |
| K.YFPPQLDGK.V | Y | 46.92 | 1063.5338 | 9 | -0.3 | 532.7740 | 2 | 37.67 | 4 | F4:21235 | 13012022\_RID\_3262\_4b.raw |  |  |  | 2.0372E6 |  |  |  |  | 1 | 0 | 0 | 0 | 1 | 0 | 0 | 0 | 0 | 113 | 121 |  |  | PEAKS DB |
| total 9 peptides |  |  |  |  |  |  |  |  |  |  |  |  |  |  |  |  |  |  |  |  |  |  |  |  |  |  |  |  |  |  |  |  |  |
| --- | --- | --- | --- | --- | --- | --- | --- | --- | --- | --- | --- | --- | --- | --- | --- | --- | --- | --- | --- | --- | --- | --- | --- | --- | --- | --- | --- | --- | --- | --- | --- | --- | --- |

Q90YA8.1

back to list

  

| Protein Coverage
| Supporting Peptides
|

Protein Coverage:

Supporting Peptides:

| Peptide | Uniq | -10lgP | Mass | Length | ppm | m/z | z | RT | Fraction | Scan | Source File | Area F6 | Area F2 | Area F3 | Area F4 | Area F5 | Area F7 | Area F8 | Area F1 | #Feature | #Feature F6 | #Feature F2 | #Feature F3 | #Feature F4 | #Feature F5 | #Feature F7 | #Feature F8 | #Feature F1 | Start | End | PTM | AScore | Found By |
| --- | --- | --- | --- | --- | --- | --- | --- | --- | --- | --- | --- | --- | --- | --- | --- | --- | --- | --- | --- | --- | --- | --- | --- | --- | --- | --- | --- | --- | --- | --- | --- | --- | --- |
| R.TFSNIISTLNPLAK.R | Y | 67.62 | 1517.8453 | 14 | 0.6 | 759.9304 | 2 | 58.92 | 4 | F4:33771 | 13012022\_RID\_3262\_4b.raw |  |  |  | 1.3808E6 |  |  |  |  | 1 | 0 | 0 | 0 | 1 | 0 | 0 | 0 | 0 | 123 | 136 |  |  | PEAKS DB |
| R.WSPSDSLYGSR.S | Y | 65.59 | 1253.5676 | 11 | -0.3 | 627.7909 | 2 | 39.85 | 4 | F4:22700 | 13012022\_RID\_3262\_4b.raw |  |  |  | 1.1335E6 | 3.8712E5 |  |  |  | 2 | 0 | 0 | 0 | 1 | 1 | 0 | 0 | 0 | 213 | 223 |  |  | PEAKS DB |
| R.YPGSPGSYAVR.Q | Y | 61.64 | 1152.5563 | 11 | 0.4 | 577.2856 | 2 | 30.09 | 4 | F4:15330 | 13012022\_RID\_3262\_4b.raw |  |  |  | 3.0986E6 |  |  |  |  | 1 | 0 | 0 | 0 | 1 | 0 | 0 | 0 | 0 | 82 | 92 |  |  | PEAKS DB |
| Q.NLYDLGLLNNYSSER.Q | Y | 57.61 | 1769.8584 | 15 | 0.3 | 885.9368 | 2 | 54.90 | 4 | F4:31914 | 13012022\_RID\_3262\_4b.raw |  |  |  | 4.1197E5 |  |  |  |  | 1 | 0 | 0 | 0 | 1 | 0 | 0 | 0 | 0 | 283 | 297 |  |  | PEAKS DB |
| R.NPVFPVYFLNTAR.W | Y | 57.19 | 1536.8088 | 13 | 0.2 | 769.4119 | 2 | 58.74 | 4 | F4:33587 | 13012022\_RID\_3262\_4b.raw |  |  |  | 1.2502E6 | 3.6499E5 |  |  |  | 2 | 0 | 0 | 0 | 1 | 1 | 0 | 0 | 0 | 260 | 272 |  |  | PEAKS DB |
| K.LIFFDGEEAFVR.W | Y | 56.69 | 1441.7241 | 12 | -0.1 | 721.8693 | 2 | 59.83 | 5 | F5:29308 | 13012022\_RID\_3262\_5b.raw |  |  |  |  | 2.1896E5 |  |  |  | 1 | 0 | 0 | 0 | 0 | 1 | 0 | 0 | 0 | 201 | 212 |  |  | PEAKS DB |
| K.MASTPHPPGAR.N | Y | 50.10 | 1120.5447 | 11 | 0.5 | 561.2799 | 2 | 19.93 | 4 | F4:7339 | 13012022\_RID\_3262\_4b.raw |  |  |  | 1.6545E5 |  |  |  |  | 1 | 0 | 0 | 0 | 1 | 0 | 0 | 0 | 0 | 229 | 239 |  |  | PEAKS DB |
| K.M(+15.99)ASTPHPPGAR.N | Y | 49.55 | 1136.5397 | 11 | 0.8 | 569.2776 | 2 | 17.65 | 4 | F4:5444 | 13012022\_RID\_3262\_4b.raw |  |  |  | 1.1854E5 |  |  |  |  | 1 | 0 | 0 | 0 | 1 | 0 | 0 | 0 | 0 | 229 | 239 | Oxidation (M) | M1:Oxidation (M):1000.00 | PEAKS DB |
| K.YFPPQLDGK.V | Y | 46.92 | 1063.5338 | 9 | -0.3 | 532.7740 | 2 | 37.67 | 4 | F4:21235 | 13012022\_RID\_3262\_4b.raw |  |  |  | 2.0372E6 |  |  |  |  | 1 | 0 | 0 | 0 | 1 | 0 | 0 | 0 | 0 | 149 | 157 |  |  | PEAKS DB |
| total 9 peptides |  |  |  |  |  |  |  |  |  |  |  |  |  |  |  |  |  |  |  |  |  |  |  |  |  |  |  |  |  |  |  |  |  |
| --- | --- | --- | --- | --- | --- | --- | --- | --- | --- | --- | --- | --- | --- | --- | --- | --- | --- | --- | --- | --- | --- | --- | --- | --- | --- | --- | --- | --- | --- | --- | --- | --- | --- |

BAP39934.1

back to list

  

| Protein Coverage
| Supporting Peptides
|

Protein Coverage:

Supporting Peptides:

| Peptide | Uniq | -10lgP | Mass | Length | ppm | m/z | z | RT | Fraction | Scan | Source File | Area F6 | Area F2 | Area F3 | Area F4 | Area F5 | Area F7 | Area F8 | Area F1 | #Feature | #Feature F6 | #Feature F2 | #Feature F3 | #Feature F4 | #Feature F5 | #Feature F7 | #Feature F8 | #Feature F1 | Start | End | PTM | AScore | Found By |
| --- | --- | --- | --- | --- | --- | --- | --- | --- | --- | --- | --- | --- | --- | --- | --- | --- | --- | --- | --- | --- | --- | --- | --- | --- | --- | --- | --- | --- | --- | --- | --- | --- | --- |
| R.TFSNIISTLNPLAK.R | Y | 67.62 | 1517.8453 | 14 | 0.6 | 759.9304 | 2 | 58.92 | 4 | F4:33771 | 13012022\_RID\_3262\_4b.raw |  |  |  | 1.3808E6 |  |  |  |  | 1 | 0 | 0 | 0 | 1 | 0 | 0 | 0 | 0 | 123 | 136 |  |  | PEAKS DB |
| R.WSPSDSLYGSR.S | Y | 65.59 | 1253.5676 | 11 | -0.3 | 627.7909 | 2 | 39.85 | 4 | F4:22700 | 13012022\_RID\_3262\_4b.raw |  |  |  | 1.1335E6 | 3.8712E5 |  |  |  | 2 | 0 | 0 | 0 | 1 | 1 | 0 | 0 | 0 | 213 | 223 |  |  | PEAKS DB |
| R.YPGSPGSYAVR.Q | Y | 61.64 | 1152.5563 | 11 | 0.4 | 577.2856 | 2 | 30.09 | 4 | F4:15330 | 13012022\_RID\_3262\_4b.raw |  |  |  | 3.0986E6 |  |  |  |  | 1 | 0 | 0 | 0 | 1 | 0 | 0 | 0 | 0 | 82 | 92 |  |  | PEAKS DB |
| R.NLYDLGLLNNYSSER.Q | Y | 57.61 | 1769.8584 | 15 | 0.3 | 885.9368 | 2 | 54.90 | 4 | F4:31914 | 13012022\_RID\_3262\_4b.raw |  |  |  | 4.1197E5 |  |  |  |  | 1 | 0 | 0 | 0 | 1 | 0 | 0 | 0 | 0 | 283 | 297 |  |  | PEAKS DB |
| R.NPVFPVYFLNTAR.W | Y | 57.19 | 1536.8088 | 13 | 0.2 | 769.4119 | 2 | 58.74 | 4 | F4:33587 | 13012022\_RID\_3262\_4b.raw |  |  |  | 1.2502E6 | 3.6499E5 |  |  |  | 2 | 0 | 0 | 0 | 1 | 1 | 0 | 0 | 0 | 260 | 272 |  |  | PEAKS DB |
| K.LIFFDGEEAFVR.W | Y | 56.69 | 1441.7241 | 12 | -0.1 | 721.8693 | 2 | 59.83 | 5 | F5:29308 | 13012022\_RID\_3262\_5b.raw |  |  |  |  | 2.1896E5 |  |  |  | 1 | 0 | 0 | 0 | 0 | 1 | 0 | 0 | 0 | 201 | 212 |  |  | PEAKS DB |
| K.MASTPHPPGAR.N | Y | 50.10 | 1120.5447 | 11 | 0.5 | 561.2799 | 2 | 19.93 | 4 | F4:7339 | 13012022\_RID\_3262\_4b.raw |  |  |  | 1.6545E5 |  |  |  |  | 1 | 0 | 0 | 0 | 1 | 0 | 0 | 0 | 0 | 229 | 239 |  |  | PEAKS DB |
| K.M(+15.99)ASTPHPPGAR.N | Y | 49.55 | 1136.5397 | 11 | 0.8 | 569.2776 | 2 | 17.65 | 4 | F4:5444 | 13012022\_RID\_3262\_4b.raw |  |  |  | 1.1854E5 |  |  |  |  | 1 | 0 | 0 | 0 | 1 | 0 | 0 | 0 | 0 | 229 | 239 | Oxidation (M) | M1:Oxidation (M):1000.00 | PEAKS DB |
| K.YFPPQLDGK.V | Y | 46.92 | 1063.5338 | 9 | -0.3 | 532.7740 | 2 | 37.67 | 4 | F4:21235 | 13012022\_RID\_3262\_4b.raw |  |  |  | 2.0372E6 |  |  |  |  | 1 | 0 | 0 | 0 | 1 | 0 | 0 | 0 | 0 | 149 | 157 |  |  | PEAKS DB |
| total 9 peptides |  |  |  |  |  |  |  |  |  |  |  |  |  |  |  |  |  |  |  |  |  |  |  |  |  |  |  |  |  |  |  |  |  |
| --- | --- | --- | --- | --- | --- | --- | --- | --- | --- | --- | --- | --- | --- | --- | --- | --- | --- | --- | --- | --- | --- | --- | --- | --- | --- | --- | --- | --- | --- | --- | --- | --- | --- |

AFE84763.1

back to list

  

| Protein Coverage
| Supporting Peptides
|

Protein Coverage:

Supporting Peptides:

| Peptide | Uniq | -10lgP | Mass | Length | ppm | m/z | z | RT | Fraction | Scan | Source File | Area F6 | Area F2 | Area F3 | Area F4 | Area F5 | Area F7 | Area F8 | Area F1 | #Feature | #Feature F6 | #Feature F2 | #Feature F3 | #Feature F4 | #Feature F5 | #Feature F7 | #Feature F8 | #Feature F1 | Start | End | PTM | AScore | Found By |
| --- | --- | --- | --- | --- | --- | --- | --- | --- | --- | --- | --- | --- | --- | --- | --- | --- | --- | --- | --- | --- | --- | --- | --- | --- | --- | --- | --- | --- | --- | --- | --- | --- | --- |
| R.TFSNIISTLNPLAK.R | Y | 67.62 | 1517.8453 | 14 | 0.6 | 759.9304 | 2 | 58.92 | 4 | F4:33771 | 13012022\_RID\_3262\_4b.raw |  |  |  | 1.3808E6 |  |  |  |  | 1 | 0 | 0 | 0 | 1 | 0 | 0 | 0 | 0 | 123 | 136 |  |  | PEAKS DB |
| R.WSPSDSLYGSR.S | Y | 65.59 | 1253.5676 | 11 | -0.3 | 627.7909 | 2 | 39.85 | 4 | F4:22700 | 13012022\_RID\_3262\_4b.raw |  |  |  | 1.1335E6 | 3.8712E5 |  |  |  | 2 | 0 | 0 | 0 | 1 | 1 | 0 | 0 | 0 | 213 | 223 |  |  | PEAKS DB |
| R.YPGSPGSYAVR.Q | Y | 61.64 | 1152.5563 | 11 | 0.4 | 577.2856 | 2 | 30.09 | 4 | F4:15330 | 13012022\_RID\_3262\_4b.raw |  |  |  | 3.0986E6 |  |  |  |  | 1 | 0 | 0 | 0 | 1 | 0 | 0 | 0 | 0 | 82 | 92 |  |  | PEAKS DB |
| R.NLYDLGLLNNYSSER.Q | Y | 57.61 | 1769.8584 | 15 | 0.3 | 885.9368 | 2 | 54.90 | 4 | F4:31914 | 13012022\_RID\_3262\_4b.raw |  |  |  | 4.1197E5 |  |  |  |  | 1 | 0 | 0 | 0 | 1 | 0 | 0 | 0 | 0 | 283 | 297 |  |  | PEAKS DB |
| R.NPVFPVYFLNTAR.W | Y | 57.19 | 1536.8088 | 13 | 0.2 | 769.4119 | 2 | 58.74 | 4 | F4:33587 | 13012022\_RID\_3262\_4b.raw |  |  |  | 1.2502E6 | 3.6499E5 |  |  |  | 2 | 0 | 0 | 0 | 1 | 1 | 0 | 0 | 0 | 260 | 272 |  |  | PEAKS DB |
| K.LIFFDGEEAFVR.W | Y | 56.69 | 1441.7241 | 12 | -0.1 | 721.8693 | 2 | 59.83 | 5 | F5:29308 | 13012022\_RID\_3262\_5b.raw |  |  |  |  | 2.1896E5 |  |  |  | 1 | 0 | 0 | 0 | 0 | 1 | 0 | 0 | 0 | 201 | 212 |  |  | PEAKS DB |
| K.MASTPHPPGAR.N | Y | 50.10 | 1120.5447 | 11 | 0.5 | 561.2799 | 2 | 19.93 | 4 | F4:7339 | 13012022\_RID\_3262\_4b.raw |  |  |  | 1.6545E5 |  |  |  |  | 1 | 0 | 0 | 0 | 1 | 0 | 0 | 0 | 0 | 229 | 239 |  |  | PEAKS DB |
| K.M(+15.99)ASTPHPPGAR.N | Y | 49.55 | 1136.5397 | 11 | 0.8 | 569.2776 | 2 | 17.65 | 4 | F4:5444 | 13012022\_RID\_3262\_4b.raw |  |  |  | 1.1854E5 |  |  |  |  | 1 | 0 | 0 | 0 | 1 | 0 | 0 | 0 | 0 | 229 | 239 | Oxidation (M) | M1:Oxidation (M):1000.00 | PEAKS DB |
| K.YFPPQLDGK.V | Y | 46.92 | 1063.5338 | 9 | -0.3 | 532.7740 | 2 | 37.67 | 4 | F4:21235 | 13012022\_RID\_3262\_4b.raw |  |  |  | 2.0372E6 |  |  |  |  | 1 | 0 | 0 | 0 | 1 | 0 | 0 | 0 | 0 | 149 | 157 |  |  | PEAKS DB |
| total 9 peptides |  |  |  |  |  |  |  |  |  |  |  |  |  |  |  |  |  |  |  |  |  |  |  |  |  |  |  |  |  |  |  |  |  |
| --- | --- | --- | --- | --- | --- | --- | --- | --- | --- | --- | --- | --- | --- | --- | --- | --- | --- | --- | --- | --- | --- | --- | --- | --- | --- | --- | --- | --- | --- | --- | --- | --- | --- |

BAP39982.1

back to list

  

| Protein Coverage
| Supporting Peptides
|

Protein Coverage:

Supporting Peptides:

| Peptide | Uniq | -10lgP | Mass | Length | ppm | m/z | z | RT | Fraction | Scan | Source File | Area F6 | Area F2 | Area F3 | Area F4 | Area F5 | Area F7 | Area F8 | Area F1 | #Feature | #Feature F6 | #Feature F2 | #Feature F3 | #Feature F4 | #Feature F5 | #Feature F7 | #Feature F8 | #Feature F1 | Start | End | PTM | AScore | Found By |
| --- | --- | --- | --- | --- | --- | --- | --- | --- | --- | --- | --- | --- | --- | --- | --- | --- | --- | --- | --- | --- | --- | --- | --- | --- | --- | --- | --- | --- | --- | --- | --- | --- | --- |
| R.TFSNIISTLNPLAK.R | Y | 67.62 | 1517.8453 | 14 | 0.6 | 759.9304 | 2 | 58.92 | 4 | F4:33771 | 13012022\_RID\_3262\_4b.raw |  |  |  | 1.3808E6 |  |  |  |  | 1 | 0 | 0 | 0 | 1 | 0 | 0 | 0 | 0 | 123 | 136 |  |  | PEAKS DB |
| R.WSPSDSLYGSR.S | Y | 65.59 | 1253.5676 | 11 | -0.3 | 627.7909 | 2 | 39.85 | 4 | F4:22700 | 13012022\_RID\_3262\_4b.raw |  |  |  | 1.1335E6 | 3.8712E5 |  |  |  | 2 | 0 | 0 | 0 | 1 | 1 | 0 | 0 | 0 | 213 | 223 |  |  | PEAKS DB |
| R.YPGSPGSYAVR.Q | Y | 61.64 | 1152.5563 | 11 | 0.4 | 577.2856 | 2 | 30.09 | 4 | F4:15330 | 13012022\_RID\_3262\_4b.raw |  |  |  | 3.0986E6 |  |  |  |  | 1 | 0 | 0 | 0 | 1 | 0 | 0 | 0 | 0 | 82 | 92 |  |  | PEAKS DB |
| R.NLYDLGLLNNYSSER.Q | Y | 57.61 | 1769.8584 | 15 | 0.3 | 885.9368 | 2 | 54.90 | 4 | F4:31914 | 13012022\_RID\_3262\_4b.raw |  |  |  | 4.1197E5 |  |  |  |  | 1 | 0 | 0 | 0 | 1 | 0 | 0 | 0 | 0 | 283 | 297 |  |  | PEAKS DB |
| R.NPVFPVYFLNTAR.W | Y | 57.19 | 1536.8088 | 13 | 0.2 | 769.4119 | 2 | 58.74 | 4 | F4:33587 | 13012022\_RID\_3262\_4b.raw |  |  |  | 1.2502E6 | 3.6499E5 |  |  |  | 2 | 0 | 0 | 0 | 1 | 1 | 0 | 0 | 0 | 260 | 272 |  |  | PEAKS DB |
| K.LIFFDGEEAFVR.W | Y | 56.69 | 1441.7241 | 12 | -0.1 | 721.8693 | 2 | 59.83 | 5 | F5:29308 | 13012022\_RID\_3262\_5b.raw |  |  |  |  | 2.1896E5 |  |  |  | 1 | 0 | 0 | 0 | 0 | 1 | 0 | 0 | 0 | 201 | 212 |  |  | PEAKS DB |
| K.MASTPHPPGAR.N | Y | 50.10 | 1120.5447 | 11 | 0.5 | 561.2799 | 2 | 19.93 | 4 | F4:7339 | 13012022\_RID\_3262\_4b.raw |  |  |  | 1.6545E5 |  |  |  |  | 1 | 0 | 0 | 0 | 1 | 0 | 0 | 0 | 0 | 229 | 239 |  |  | PEAKS DB |
| K.M(+15.99)ASTPHPPGAR.N | Y | 49.55 | 1136.5397 | 11 | 0.8 | 569.2776 | 2 | 17.65 | 4 | F4:5444 | 13012022\_RID\_3262\_4b.raw |  |  |  | 1.1854E5 |  |  |  |  | 1 | 0 | 0 | 0 | 1 | 0 | 0 | 0 | 0 | 229 | 239 | Oxidation (M) | M1:Oxidation (M):1000.00 | PEAKS DB |
| K.YFPPQLDGK.V | Y | 46.92 | 1063.5338 | 9 | -0.3 | 532.7740 | 2 | 37.67 | 4 | F4:21235 | 13012022\_RID\_3262\_4b.raw |  |  |  | 2.0372E6 |  |  |  |  | 1 | 0 | 0 | 0 | 1 | 0 | 0 | 0 | 0 | 149 | 157 |  |  | PEAKS DB |
| total 9 peptides |  |  |  |  |  |  |  |  |  |  |  |  |  |  |  |  |  |  |  |  |  |  |  |  |  |  |  |  |  |  |  |  |  |
| --- | --- | --- | --- | --- | --- | --- | --- | --- | --- | --- | --- | --- | --- | --- | --- | --- | --- | --- | --- | --- | --- | --- | --- | --- | --- | --- | --- | --- | --- | --- | --- | --- | --- |

AFE84765.1

back to list

  

| Protein Coverage
| Supporting Peptides
|

Protein Coverage:

Supporting Peptides:

| Peptide | Uniq | -10lgP | Mass | Length | ppm | m/z | z | RT | Fraction | Scan | Source File | Area F6 | Area F2 | Area F3 | Area F4 | Area F5 | Area F7 | Area F8 | Area F1 | #Feature | #Feature F6 | #Feature F2 | #Feature F3 | #Feature F4 | #Feature F5 | #Feature F7 | #Feature F8 | #Feature F1 | Start | End | PTM | AScore | Found By |
| --- | --- | --- | --- | --- | --- | --- | --- | --- | --- | --- | --- | --- | --- | --- | --- | --- | --- | --- | --- | --- | --- | --- | --- | --- | --- | --- | --- | --- | --- | --- | --- | --- | --- |
| R.TFSNIISTLNPLAK.R | Y | 67.62 | 1517.8453 | 14 | 0.6 | 759.9304 | 2 | 58.92 | 4 | F4:33771 | 13012022\_RID\_3262\_4b.raw |  |  |  | 1.3808E6 |  |  |  |  | 1 | 0 | 0 | 0 | 1 | 0 | 0 | 0 | 0 | 123 | 136 |  |  | PEAKS DB |
| R.WSPSDSLYGSR.S | Y | 65.59 | 1253.5676 | 11 | -0.3 | 627.7909 | 2 | 39.85 | 4 | F4:22700 | 13012022\_RID\_3262\_4b.raw |  |  |  | 1.1335E6 | 3.8712E5 |  |  |  | 2 | 0 | 0 | 0 | 1 | 1 | 0 | 0 | 0 | 213 | 223 |  |  | PEAKS DB |
| R.YPGSPGSYAVR.Q | Y | 61.64 | 1152.5563 | 11 | 0.4 | 577.2856 | 2 | 30.09 | 4 | F4:15330 | 13012022\_RID\_3262\_4b.raw |  |  |  | 3.0986E6 |  |  |  |  | 1 | 0 | 0 | 0 | 1 | 0 | 0 | 0 | 0 | 82 | 92 |  |  | PEAKS DB |
| R.NLYDLGLLNNYSSER.Q | Y | 57.61 | 1769.8584 | 15 | 0.3 | 885.9368 | 2 | 54.90 | 4 | F4:31914 | 13012022\_RID\_3262\_4b.raw |  |  |  | 4.1197E5 |  |  |  |  | 1 | 0 | 0 | 0 | 1 | 0 | 0 | 0 | 0 | 283 | 297 |  |  | PEAKS DB |
| R.NPVFPVYFLNTAR.W | Y | 57.19 | 1536.8088 | 13 | 0.2 | 769.4119 | 2 | 58.74 | 4 | F4:33587 | 13012022\_RID\_3262\_4b.raw |  |  |  | 1.2502E6 | 3.6499E5 |  |  |  | 2 | 0 | 0 | 0 | 1 | 1 | 0 | 0 | 0 | 260 | 272 |  |  | PEAKS DB |
| K.LIFFDGEEAFVR.W | Y | 56.69 | 1441.7241 | 12 | -0.1 | 721.8693 | 2 | 59.83 | 5 | F5:29308 | 13012022\_RID\_3262\_5b.raw |  |  |  |  | 2.1896E5 |  |  |  | 1 | 0 | 0 | 0 | 0 | 1 | 0 | 0 | 0 | 201 | 212 |  |  | PEAKS DB |
| K.MASTPHPPGAR.N | Y | 50.10 | 1120.5447 | 11 | 0.5 | 561.2799 | 2 | 19.93 | 4 | F4:7339 | 13012022\_RID\_3262\_4b.raw |  |  |  | 1.6545E5 |  |  |  |  | 1 | 0 | 0 | 0 | 1 | 0 | 0 | 0 | 0 | 229 | 239 |  |  | PEAKS DB |
| K.M(+15.99)ASTPHPPGAR.N | Y | 49.55 | 1136.5397 | 11 | 0.8 | 569.2776 | 2 | 17.65 | 4 | F4:5444 | 13012022\_RID\_3262\_4b.raw |  |  |  | 1.1854E5 |  |  |  |  | 1 | 0 | 0 | 0 | 1 | 0 | 0 | 0 | 0 | 229 | 239 | Oxidation (M) | M1:Oxidation (M):1000.00 | PEAKS DB |
| K.YFPPQLDGK.V | Y | 46.92 | 1063.5338 | 9 | -0.3 | 532.7740 | 2 | 37.67 | 4 | F4:21235 | 13012022\_RID\_3262\_4b.raw |  |  |  | 2.0372E6 |  |  |  |  | 1 | 0 | 0 | 0 | 1 | 0 | 0 | 0 | 0 | 149 | 157 |  |  | PEAKS DB |
| total 9 peptides |  |  |  |  |  |  |  |  |  |  |  |  |  |  |  |  |  |  |  |  |  |  |  |  |  |  |  |  |  |  |  |  |  |
| --- | --- | --- | --- | --- | --- | --- | --- | --- | --- | --- | --- | --- | --- | --- | --- | --- | --- | --- | --- | --- | --- | --- | --- | --- | --- | --- | --- | --- | --- | --- | --- | --- | --- |

AFJ49187.1

back to list

  

| Protein Coverage
| Supporting Peptides
|

Protein Coverage:

Supporting Peptides:

| Peptide | Uniq | -10lgP | Mass | Length | ppm | m/z | z | RT | Fraction | Scan | Source File | Area F6 | Area F2 | Area F3 | Area F4 | Area F5 | Area F7 | Area F8 | Area F1 | #Feature | #Feature F6 | #Feature F2 | #Feature F3 | #Feature F4 | #Feature F5 | #Feature F7 | #Feature F8 | #Feature F1 | Start | End | PTM | AScore | Found By |
| --- | --- | --- | --- | --- | --- | --- | --- | --- | --- | --- | --- | --- | --- | --- | --- | --- | --- | --- | --- | --- | --- | --- | --- | --- | --- | --- | --- | --- | --- | --- | --- | --- | --- |
| R.TFSNIISTLNPLAK.R | Y | 67.62 | 1517.8453 | 14 | 0.6 | 759.9304 | 2 | 58.92 | 4 | F4:33771 | 13012022\_RID\_3262\_4b.raw |  |  |  | 1.3808E6 |  |  |  |  | 1 | 0 | 0 | 0 | 1 | 0 | 0 | 0 | 0 | 123 | 136 |  |  | PEAKS DB |
| R.WSPSDSLYGSR.S | Y | 65.59 | 1253.5676 | 11 | -0.3 | 627.7909 | 2 | 39.85 | 4 | F4:22700 | 13012022\_RID\_3262\_4b.raw |  |  |  | 1.1335E6 | 3.8712E5 |  |  |  | 2 | 0 | 0 | 0 | 1 | 1 | 0 | 0 | 0 | 213 | 223 |  |  | PEAKS DB |
| R.YPGSPGSYAVR.Q | Y | 61.64 | 1152.5563 | 11 | 0.4 | 577.2856 | 2 | 30.09 | 4 | F4:15330 | 13012022\_RID\_3262\_4b.raw |  |  |  | 3.0986E6 |  |  |  |  | 1 | 0 | 0 | 0 | 1 | 0 | 0 | 0 | 0 | 82 | 92 |  |  | PEAKS DB |
| R.NLYDLGLLNNYSSER.Q | Y | 57.61 | 1769.8584 | 15 | 0.3 | 885.9368 | 2 | 54.90 | 4 | F4:31914 | 13012022\_RID\_3262\_4b.raw |  |  |  | 4.1197E5 |  |  |  |  | 1 | 0 | 0 | 0 | 1 | 0 | 0 | 0 | 0 | 283 | 297 |  |  | PEAKS DB |
| R.NPVFPVYFLNTAR.W | Y | 57.19 | 1536.8088 | 13 | 0.2 | 769.4119 | 2 | 58.74 | 4 | F4:33587 | 13012022\_RID\_3262\_4b.raw |  |  |  | 1.2502E6 | 3.6499E5 |  |  |  | 2 | 0 | 0 | 0 | 1 | 1 | 0 | 0 | 0 | 260 | 272 |  |  | PEAKS DB |
| K.LIFFDGEEAFVR.W | Y | 56.69 | 1441.7241 | 12 | -0.1 | 721.8693 | 2 | 59.83 | 5 | F5:29308 | 13012022\_RID\_3262\_5b.raw |  |  |  |  | 2.1896E5 |  |  |  | 1 | 0 | 0 | 0 | 0 | 1 | 0 | 0 | 0 | 201 | 212 |  |  | PEAKS DB |
| K.MASTPHPPGAR.N | Y | 50.10 | 1120.5447 | 11 | 0.5 | 561.2799 | 2 | 19.93 | 4 | F4:7339 | 13012022\_RID\_3262\_4b.raw |  |  |  | 1.6545E5 |  |  |  |  | 1 | 0 | 0 | 0 | 1 | 0 | 0 | 0 | 0 | 229 | 239 |  |  | PEAKS DB |
| K.M(+15.99)ASTPHPPGAR.N | Y | 49.55 | 1136.5397 | 11 | 0.8 | 569.2776 | 2 | 17.65 | 4 | F4:5444 | 13012022\_RID\_3262\_4b.raw |  |  |  | 1.1854E5 |  |  |  |  | 1 | 0 | 0 | 0 | 1 | 0 | 0 | 0 | 0 | 229 | 239 | Oxidation (M) | M1:Oxidation (M):1000.00 | PEAKS DB |
| K.YFPPQLDGK.V | Y | 46.92 | 1063.5338 | 9 | -0.3 | 532.7740 | 2 | 37.67 | 4 | F4:21235 | 13012022\_RID\_3262\_4b.raw |  |  |  | 2.0372E6 |  |  |  |  | 1 | 0 | 0 | 0 | 1 | 0 | 0 | 0 | 0 | 149 | 157 |  |  | PEAKS DB |
| total 9 peptides |  |  |  |  |  |  |  |  |  |  |  |  |  |  |  |  |  |  |  |  |  |  |  |  |  |  |  |  |  |  |  |  |  |
| --- | --- | --- | --- | --- | --- | --- | --- | --- | --- | --- | --- | --- | --- | --- | --- | --- | --- | --- | --- | --- | --- | --- | --- | --- | --- | --- | --- | --- | --- | --- | --- | --- | --- |

JAA94895.1

back to list

  

| Protein Coverage
| Supporting Peptides
|

Protein Coverage:

Supporting Peptides:

| Peptide | Uniq | -10lgP | Mass | Length | ppm | m/z | z | RT | Fraction | Scan | Source File | Area F6 | Area F2 | Area F3 | Area F4 | Area F5 | Area F7 | Area F8 | Area F1 | #Feature | #Feature F6 | #Feature F2 | #Feature F3 | #Feature F4 | #Feature F5 | #Feature F7 | #Feature F8 | #Feature F1 | Start | End | PTM | AScore | Found By |
| --- | --- | --- | --- | --- | --- | --- | --- | --- | --- | --- | --- | --- | --- | --- | --- | --- | --- | --- | --- | --- | --- | --- | --- | --- | --- | --- | --- | --- | --- | --- | --- | --- | --- |
| R.TFSNIISTLNPLAK.R | Y | 67.62 | 1517.8453 | 14 | 0.6 | 759.9304 | 2 | 58.92 | 4 | F4:33771 | 13012022\_RID\_3262\_4b.raw |  |  |  | 1.3808E6 |  |  |  |  | 1 | 0 | 0 | 0 | 1 | 0 | 0 | 0 | 0 | 123 | 136 |  |  | PEAKS DB |
| R.WSPSDSLYGSR.S | Y | 65.59 | 1253.5676 | 11 | -0.3 | 627.7909 | 2 | 39.85 | 4 | F4:22700 | 13012022\_RID\_3262\_4b.raw |  |  |  | 1.1335E6 | 3.8712E5 |  |  |  | 2 | 0 | 0 | 0 | 1 | 1 | 0 | 0 | 0 | 213 | 223 |  |  | PEAKS DB |
| R.YPGSPGSYAVR.Q | Y | 61.64 | 1152.5563 | 11 | 0.4 | 577.2856 | 2 | 30.09 | 4 | F4:15330 | 13012022\_RID\_3262\_4b.raw |  |  |  | 3.0986E6 |  |  |  |  | 1 | 0 | 0 | 0 | 1 | 0 | 0 | 0 | 0 | 82 | 92 |  |  | PEAKS DB |
| R.NLYDLGLLNNYSSER.Q | Y | 57.61 | 1769.8584 | 15 | 0.3 | 885.9368 | 2 | 54.90 | 4 | F4:31914 | 13012022\_RID\_3262\_4b.raw |  |  |  | 4.1197E5 |  |  |  |  | 1 | 0 | 0 | 0 | 1 | 0 | 0 | 0 | 0 | 283 | 297 |  |  | PEAKS DB |
| R.NPVFPVYFLNTAR.W | Y | 57.19 | 1536.8088 | 13 | 0.2 | 769.4119 | 2 | 58.74 | 4 | F4:33587 | 13012022\_RID\_3262\_4b.raw |  |  |  | 1.2502E6 | 3.6499E5 |  |  |  | 2 | 0 | 0 | 0 | 1 | 1 | 0 | 0 | 0 | 260 | 272 |  |  | PEAKS DB |
| K.LIFFDGEEAFVR.W | Y | 56.69 | 1441.7241 | 12 | -0.1 | 721.8693 | 2 | 59.83 | 5 | F5:29308 | 13012022\_RID\_3262\_5b.raw |  |  |  |  | 2.1896E5 |  |  |  | 1 | 0 | 0 | 0 | 0 | 1 | 0 | 0 | 0 | 201 | 212 |  |  | PEAKS DB |
| K.MASTPHPPGAR.N | Y | 50.10 | 1120.5447 | 11 | 0.5 | 561.2799 | 2 | 19.93 | 4 | F4:7339 | 13012022\_RID\_3262\_4b.raw |  |  |  | 1.6545E5 |  |  |  |  | 1 | 0 | 0 | 0 | 1 | 0 | 0 | 0 | 0 | 229 | 239 |  |  | PEAKS DB |
| K.M(+15.99)ASTPHPPGAR.N | Y | 49.55 | 1136.5397 | 11 | 0.8 | 569.2776 | 2 | 17.65 | 4 | F4:5444 | 13012022\_RID\_3262\_4b.raw |  |  |  | 1.1854E5 |  |  |  |  | 1 | 0 | 0 | 0 | 1 | 0 | 0 | 0 | 0 | 229 | 239 | Oxidation (M) | M1:Oxidation (M):1000.00 | PEAKS DB |
| K.YFPPQLDGK.V | Y | 46.92 | 1063.5338 | 9 | -0.3 | 532.7740 | 2 | 37.67 | 4 | F4:21235 | 13012022\_RID\_3262\_4b.raw |  |  |  | 2.0372E6 |  |  |  |  | 1 | 0 | 0 | 0 | 1 | 0 | 0 | 0 | 0 | 149 | 157 |  |  | PEAKS DB |
| total 9 peptides |  |  |  |  |  |  |  |  |  |  |  |  |  |  |  |  |  |  |  |  |  |  |  |  |  |  |  |  |  |  |  |  |  |
| --- | --- | --- | --- | --- | --- | --- | --- | --- | --- | --- | --- | --- | --- | --- | --- | --- | --- | --- | --- | --- | --- | --- | --- | --- | --- | --- | --- | --- | --- | --- | --- | --- | --- |

JAI13216.1

back to list

  

| Protein Coverage
| Supporting Peptides
|

Protein Coverage:

Supporting Peptides:

| Peptide | Uniq | -10lgP | Mass | Length | ppm | m/z | z | RT | Fraction | Scan | Source File | Area F6 | Area F2 | Area F3 | Area F4 | Area F5 | Area F7 | Area F8 | Area F1 | #Feature | #Feature F6 | #Feature F2 | #Feature F3 | #Feature F4 | #Feature F5 | #Feature F7 | #Feature F8 | #Feature F1 | Start | End | PTM | AScore | Found By |
| --- | --- | --- | --- | --- | --- | --- | --- | --- | --- | --- | --- | --- | --- | --- | --- | --- | --- | --- | --- | --- | --- | --- | --- | --- | --- | --- | --- | --- | --- | --- | --- | --- | --- |
| R.TFSNIISTLNPLAK.R | Y | 67.62 | 1517.8453 | 14 | 0.6 | 759.9304 | 2 | 58.92 | 4 | F4:33771 | 13012022\_RID\_3262\_4b.raw |  |  |  | 1.3808E6 |  |  |  |  | 1 | 0 | 0 | 0 | 1 | 0 | 0 | 0 | 0 | 123 | 136 |  |  | PEAKS DB |
| R.WSPSDSLYGSR.S | Y | 65.59 | 1253.5676 | 11 | -0.3 | 627.7909 | 2 | 39.85 | 4 | F4:22700 | 13012022\_RID\_3262\_4b.raw |  |  |  | 1.1335E6 | 3.8712E5 |  |  |  | 2 | 0 | 0 | 0 | 1 | 1 | 0 | 0 | 0 | 213 | 223 |  |  | PEAKS DB |
| R.YPGSPGSYAVR.Q | Y | 61.64 | 1152.5563 | 11 | 0.4 | 577.2856 | 2 | 30.09 | 4 | F4:15330 | 13012022\_RID\_3262\_4b.raw |  |  |  | 3.0986E6 |  |  |  |  | 1 | 0 | 0 | 0 | 1 | 0 | 0 | 0 | 0 | 82 | 92 |  |  | PEAKS DB |
| R.NLYDLGLLNNYSSER.Q | Y | 57.61 | 1769.8584 | 15 | 0.3 | 885.9368 | 2 | 54.90 | 4 | F4:31914 | 13012022\_RID\_3262\_4b.raw |  |  |  | 4.1197E5 |  |  |  |  | 1 | 0 | 0 | 0 | 1 | 0 | 0 | 0 | 0 | 283 | 297 |  |  | PEAKS DB |
| R.NPVFPVYFLNTAR.W | Y | 57.19 | 1536.8088 | 13 | 0.2 | 769.4119 | 2 | 58.74 | 4 | F4:33587 | 13012022\_RID\_3262\_4b.raw |  |  |  | 1.2502E6 | 3.6499E5 |  |  |  | 2 | 0 | 0 | 0 | 1 | 1 | 0 | 0 | 0 | 260 | 272 |  |  | PEAKS DB |
| K.LIFFDGEEAFVR.W | Y | 56.69 | 1441.7241 | 12 | -0.1 | 721.8693 | 2 | 59.83 | 5 | F5:29308 | 13012022\_RID\_3262\_5b.raw |  |  |  |  | 2.1896E5 |  |  |  | 1 | 0 | 0 | 0 | 0 | 1 | 0 | 0 | 0 | 201 | 212 |  |  | PEAKS DB |
| K.MASTPHPPGAR.N | Y | 50.10 | 1120.5447 | 11 | 0.5 | 561.2799 | 2 | 19.93 | 4 | F4:7339 | 13012022\_RID\_3262\_4b.raw |  |  |  | 1.6545E5 |  |  |  |  | 1 | 0 | 0 | 0 | 1 | 0 | 0 | 0 | 0 | 229 | 239 |  |  | PEAKS DB |
| K.M(+15.99)ASTPHPPGAR.N | Y | 49.55 | 1136.5397 | 11 | 0.8 | 569.2776 | 2 | 17.65 | 4 | F4:5444 | 13012022\_RID\_3262\_4b.raw |  |  |  | 1.1854E5 |  |  |  |  | 1 | 0 | 0 | 0 | 1 | 0 | 0 | 0 | 0 | 229 | 239 | Oxidation (M) | M1:Oxidation (M):1000.00 | PEAKS DB |
| K.YFPPQLDGK.V | Y | 46.92 | 1063.5338 | 9 | -0.3 | 532.7740 | 2 | 37.67 | 4 | F4:21235 | 13012022\_RID\_3262\_4b.raw |  |  |  | 2.0372E6 |  |  |  |  | 1 | 0 | 0 | 0 | 1 | 0 | 0 | 0 | 0 | 149 | 157 |  |  | PEAKS DB |
| total 9 peptides |  |  |  |  |  |  |  |  |  |  |  |  |  |  |  |  |  |  |  |  |  |  |  |  |  |  |  |  |  |  |  |  |  |
| --- | --- | --- | --- | --- | --- | --- | --- | --- | --- | --- | --- | --- | --- | --- | --- | --- | --- | --- | --- | --- | --- | --- | --- | --- | --- | --- | --- | --- | --- | --- | --- | --- | --- |

BAN82004.1

back to list

  

| Protein Coverage
| Supporting Peptides
|

Protein Coverage:

Supporting Peptides:

| Peptide | Uniq | -10lgP | Mass | Length | ppm | m/z | z | RT | Fraction | Scan | Source File | Area F6 | Area F2 | Area F3 | Area F4 | Area F5 | Area F7 | Area F8 | Area F1 | #Feature | #Feature F6 | #Feature F2 | #Feature F3 | #Feature F4 | #Feature F5 | #Feature F7 | #Feature F8 | #Feature F1 | Start | End | PTM | AScore | Found By |
| --- | --- | --- | --- | --- | --- | --- | --- | --- | --- | --- | --- | --- | --- | --- | --- | --- | --- | --- | --- | --- | --- | --- | --- | --- | --- | --- | --- | --- | --- | --- | --- | --- | --- |
| R.TFSNIISTLNPLAK.R | Y | 67.62 | 1517.8453 | 14 | 0.6 | 759.9304 | 2 | 58.92 | 4 | F4:33771 | 13012022\_RID\_3262\_4b.raw |  |  |  | 1.3808E6 |  |  |  |  | 1 | 0 | 0 | 0 | 1 | 0 | 0 | 0 | 0 | 123 | 136 |  |  | PEAKS DB |
| R.WSPSDSLYGSR.S | Y | 65.59 | 1253.5676 | 11 | -0.3 | 627.7909 | 2 | 39.85 | 4 | F4:22700 | 13012022\_RID\_3262\_4b.raw |  |  |  | 1.1335E6 | 3.8712E5 |  |  |  | 2 | 0 | 0 | 0 | 1 | 1 | 0 | 0 | 0 | 213 | 223 |  |  | PEAKS DB |
| R.YPGSPGSYAVR.Q | Y | 61.64 | 1152.5563 | 11 | 0.4 | 577.2856 | 2 | 30.09 | 4 | F4:15330 | 13012022\_RID\_3262\_4b.raw |  |  |  | 3.0986E6 |  |  |  |  | 1 | 0 | 0 | 0 | 1 | 0 | 0 | 0 | 0 | 82 | 92 |  |  | PEAKS DB |
| R.NLYDLGLLNNYSSER.Q | Y | 57.61 | 1769.8584 | 15 | 0.3 | 885.9368 | 2 | 54.90 | 4 | F4:31914 | 13012022\_RID\_3262\_4b.raw |  |  |  | 4.1197E5 |  |  |  |  | 1 | 0 | 0 | 0 | 1 | 0 | 0 | 0 | 0 | 283 | 297 |  |  | PEAKS DB |
| R.NPVFPVYFLNTAR.W | Y | 57.19 | 1536.8088 | 13 | 0.2 | 769.4119 | 2 | 58.74 | 4 | F4:33587 | 13012022\_RID\_3262\_4b.raw |  |  |  | 1.2502E6 | 3.6499E5 |  |  |  | 2 | 0 | 0 | 0 | 1 | 1 | 0 | 0 | 0 | 260 | 272 |  |  | PEAKS DB |
| K.LIFFDGEEAFVR.W | Y | 56.69 | 1441.7241 | 12 | -0.1 | 721.8693 | 2 | 59.83 | 5 | F5:29308 | 13012022\_RID\_3262\_5b.raw |  |  |  |  | 2.1896E5 |  |  |  | 1 | 0 | 0 | 0 | 0 | 1 | 0 | 0 | 0 | 201 | 212 |  |  | PEAKS DB |
| K.MASTPHPPGAR.N | Y | 50.10 | 1120.5447 | 11 | 0.5 | 561.2799 | 2 | 19.93 | 4 | F4:7339 | 13012022\_RID\_3262\_4b.raw |  |  |  | 1.6545E5 |  |  |  |  | 1 | 0 | 0 | 0 | 1 | 0 | 0 | 0 | 0 | 229 | 239 |  |  | PEAKS DB |
| K.M(+15.99)ASTPHPPGAR.N | Y | 49.55 | 1136.5397 | 11 | 0.8 | 569.2776 | 2 | 17.65 | 4 | F4:5444 | 13012022\_RID\_3262\_4b.raw |  |  |  | 1.1854E5 |  |  |  |  | 1 | 0 | 0 | 0 | 1 | 0 | 0 | 0 | 0 | 229 | 239 | Oxidation (M) | M1:Oxidation (M):1000.00 | PEAKS DB |
| K.YFPPQLDGK.V | Y | 46.92 | 1063.5338 | 9 | -0.3 | 532.7740 | 2 | 37.67 | 4 | F4:21235 | 13012022\_RID\_3262\_4b.raw |  |  |  | 2.0372E6 |  |  |  |  | 1 | 0 | 0 | 0 | 1 | 0 | 0 | 0 | 0 | 149 | 157 |  |  | PEAKS DB |
| total 9 peptides |  |  |  |  |  |  |  |  |  |  |  |  |  |  |  |  |  |  |  |  |  |  |  |  |  |  |  |  |  |  |  |  |  |
| --- | --- | --- | --- | --- | --- | --- | --- | --- | --- | --- | --- | --- | --- | --- | --- | --- | --- | --- | --- | --- | --- | --- | --- | --- | --- | --- | --- | --- | --- | --- | --- | --- | --- |

BAB69586.1

back to list

  

| Protein Coverage
| Supporting Peptides
|

Protein Coverage:

Supporting Peptides:

| Peptide | Uniq | -10lgP | Mass | Length | ppm | m/z | z | RT | Fraction | Scan | Source File | Area F6 | Area F2 | Area F3 | Area F4 | Area F5 | Area F7 | Area F8 | Area F1 | #Feature | #Feature F6 | #Feature F2 | #Feature F3 | #Feature F4 | #Feature F5 | #Feature F7 | #Feature F8 | #Feature F1 | Start | End | PTM | AScore | Found By |
| --- | --- | --- | --- | --- | --- | --- | --- | --- | --- | --- | --- | --- | --- | --- | --- | --- | --- | --- | --- | --- | --- | --- | --- | --- | --- | --- | --- | --- | --- | --- | --- | --- | --- |
| R.TFSNIISTLNPLAK.R | Y | 67.62 | 1517.8453 | 14 | 0.6 | 759.9304 | 2 | 58.92 | 4 | F4:33771 | 13012022\_RID\_3262\_4b.raw |  |  |  | 1.3808E6 |  |  |  |  | 1 | 0 | 0 | 0 | 1 | 0 | 0 | 0 | 0 | 123 | 136 |  |  | PEAKS DB |
| R.WSPSDSLYGSR.S | Y | 65.59 | 1253.5676 | 11 | -0.3 | 627.7909 | 2 | 39.85 | 4 | F4:22700 | 13012022\_RID\_3262\_4b.raw |  |  |  | 1.1335E6 | 3.8712E5 |  |  |  | 2 | 0 | 0 | 0 | 1 | 1 | 0 | 0 | 0 | 213 | 223 |  |  | PEAKS DB |
| R.YPGSPGSYAVR.Q | Y | 61.64 | 1152.5563 | 11 | 0.4 | 577.2856 | 2 | 30.09 | 4 | F4:15330 | 13012022\_RID\_3262\_4b.raw |  |  |  | 3.0986E6 |  |  |  |  | 1 | 0 | 0 | 0 | 1 | 0 | 0 | 0 | 0 | 82 | 92 |  |  | PEAKS DB |
| Q.NLYDLGLLNNYSSER.Q | Y | 57.61 | 1769.8584 | 15 | 0.3 | 885.9368 | 2 | 54.90 | 4 | F4:31914 | 13012022\_RID\_3262\_4b.raw |  |  |  | 4.1197E5 |  |  |  |  | 1 | 0 | 0 | 0 | 1 | 0 | 0 | 0 | 0 | 283 | 297 |  |  | PEAKS DB |
| R.NPVFPVYFLNTAR.W | Y | 57.19 | 1536.8088 | 13 | 0.2 | 769.4119 | 2 | 58.74 | 4 | F4:33587 | 13012022\_RID\_3262\_4b.raw |  |  |  | 1.2502E6 | 3.6499E5 |  |  |  | 2 | 0 | 0 | 0 | 1 | 1 | 0 | 0 | 0 | 260 | 272 |  |  | PEAKS DB |
| K.LIFFDGEEAFVR.W | Y | 56.69 | 1441.7241 | 12 | -0.1 | 721.8693 | 2 | 59.83 | 5 | F5:29308 | 13012022\_RID\_3262\_5b.raw |  |  |  |  | 2.1896E5 |  |  |  | 1 | 0 | 0 | 0 | 0 | 1 | 0 | 0 | 0 | 201 | 212 |  |  | PEAKS DB |
| K.MASTPHPPGAR.N | Y | 50.10 | 1120.5447 | 11 | 0.5 | 561.2799 | 2 | 19.93 | 4 | F4:7339 | 13012022\_RID\_3262\_4b.raw |  |  |  | 1.6545E5 |  |  |  |  | 1 | 0 | 0 | 0 | 1 | 0 | 0 | 0 | 0 | 229 | 239 |  |  | PEAKS DB |
| K.M(+15.99)ASTPHPPGAR.N | Y | 49.55 | 1136.5397 | 11 | 0.8 | 569.2776 | 2 | 17.65 | 4 | F4:5444 | 13012022\_RID\_3262\_4b.raw |  |  |  | 1.1854E5 |  |  |  |  | 1 | 0 | 0 | 0 | 1 | 0 | 0 | 0 | 0 | 229 | 239 | Oxidation (M) | M1:Oxidation (M):1000.00 | PEAKS DB |
| K.YFPPQLDGK.V | Y | 46.92 | 1063.5338 | 9 | -0.3 | 532.7740 | 2 | 37.67 | 4 | F4:21235 | 13012022\_RID\_3262\_4b.raw |  |  |  | 2.0372E6 |  |  |  |  | 1 | 0 | 0 | 0 | 1 | 0 | 0 | 0 | 0 | 149 | 157 |  |  | PEAKS DB |
| total 9 peptides |  |  |  |  |  |  |  |  |  |  |  |  |  |  |  |  |  |  |  |  |  |  |  |  |  |  |  |  |  |  |  |  |  |
| --- | --- | --- | --- | --- | --- | --- | --- | --- | --- | --- | --- | --- | --- | --- | --- | --- | --- | --- | --- | --- | --- | --- | --- | --- | --- | --- | --- | --- | --- | --- | --- | --- | --- |

JAG44660.1

back to list

  

| Protein Coverage
| Supporting Peptides
|

Protein Coverage:

Supporting Peptides:

| Peptide | Uniq | -10lgP | Mass | Length | ppm | m/z | z | RT | Fraction | Scan | Source File | Area F6 | Area F2 | Area F3 | Area F4 | Area F5 | Area F7 | Area F8 | Area F1 | #Feature | #Feature F6 | #Feature F2 | #Feature F3 | #Feature F4 | #Feature F5 | #Feature F7 | #Feature F8 | #Feature F1 | Start | End | PTM | AScore | Found By |
| --- | --- | --- | --- | --- | --- | --- | --- | --- | --- | --- | --- | --- | --- | --- | --- | --- | --- | --- | --- | --- | --- | --- | --- | --- | --- | --- | --- | --- | --- | --- | --- | --- | --- |
| R.TFSNIISTLNPLAK.R | Y | 67.62 | 1517.8453 | 14 | 0.6 | 759.9304 | 2 | 58.92 | 4 | F4:33771 | 13012022\_RID\_3262\_4b.raw |  |  |  | 1.3808E6 |  |  |  |  | 1 | 0 | 0 | 0 | 1 | 0 | 0 | 0 | 0 | 123 | 136 |  |  | PEAKS DB |
| R.WSPSDSLYGSR.S | Y | 65.59 | 1253.5676 | 11 | -0.3 | 627.7909 | 2 | 39.85 | 4 | F4:22700 | 13012022\_RID\_3262\_4b.raw |  |  |  | 1.1335E6 | 3.8712E5 |  |  |  | 2 | 0 | 0 | 0 | 1 | 1 | 0 | 0 | 0 | 213 | 223 |  |  | PEAKS DB |
| R.YPGSPGSYAVR.Q | Y | 61.64 | 1152.5563 | 11 | 0.4 | 577.2856 | 2 | 30.09 | 4 | F4:15330 | 13012022\_RID\_3262\_4b.raw |  |  |  | 3.0986E6 |  |  |  |  | 1 | 0 | 0 | 0 | 1 | 0 | 0 | 0 | 0 | 82 | 92 |  |  | PEAKS DB |
| R.NLYDLGLLNNYSSER.Q | Y | 57.61 | 1769.8584 | 15 | 0.3 | 885.9368 | 2 | 54.90 | 4 | F4:31914 | 13012022\_RID\_3262\_4b.raw |  |  |  | 4.1197E5 |  |  |  |  | 1 | 0 | 0 | 0 | 1 | 0 | 0 | 0 | 0 | 283 | 297 |  |  | PEAKS DB |
| R.NPVFPVYFLNTAR.W | Y | 57.19 | 1536.8088 | 13 | 0.2 | 769.4119 | 2 | 58.74 | 4 | F4:33587 | 13012022\_RID\_3262\_4b.raw |  |  |  | 1.2502E6 | 3.6499E5 |  |  |  | 2 | 0 | 0 | 0 | 1 | 1 | 0 | 0 | 0 | 260 | 272 |  |  | PEAKS DB |
| K.LIFFDGEEAFVR.W | Y | 56.69 | 1441.7241 | 12 | -0.1 | 721.8693 | 2 | 59.83 | 5 | F5:29308 | 13012022\_RID\_3262\_5b.raw |  |  |  |  | 2.1896E5 |  |  |  | 1 | 0 | 0 | 0 | 0 | 1 | 0 | 0 | 0 | 201 | 212 |  |  | PEAKS DB |
| K.MASTPHPPGAR.N | Y | 50.10 | 1120.5447 | 11 | 0.5 | 561.2799 | 2 | 19.93 | 4 | F4:7339 | 13012022\_RID\_3262\_4b.raw |  |  |  | 1.6545E5 |  |  |  |  | 1 | 0 | 0 | 0 | 1 | 0 | 0 | 0 | 0 | 229 | 239 |  |  | PEAKS DB |
| K.M(+15.99)ASTPHPPGAR.N | Y | 49.55 | 1136.5397 | 11 | 0.8 | 569.2776 | 2 | 17.65 | 4 | F4:5444 | 13012022\_RID\_3262\_4b.raw |  |  |  | 1.1854E5 |  |  |  |  | 1 | 0 | 0 | 0 | 1 | 0 | 0 | 0 | 0 | 229 | 239 | Oxidation (M) | M1:Oxidation (M):1000.00 | PEAKS DB |
| K.YFPPQLDGK.V | Y | 46.92 | 1063.5338 | 9 | -0.3 | 532.7740 | 2 | 37.67 | 4 | F4:21235 | 13012022\_RID\_3262\_4b.raw |  |  |  | 2.0372E6 |  |  |  |  | 1 | 0 | 0 | 0 | 1 | 0 | 0 | 0 | 0 | 149 | 157 |  |  | PEAKS DB |
| total 9 peptides |  |  |  |  |  |  |  |  |  |  |  |  |  |  |  |  |  |  |  |  |  |  |  |  |  |  |  |  |  |  |  |  |  |
| --- | --- | --- | --- | --- | --- | --- | --- | --- | --- | --- | --- | --- | --- | --- | --- | --- | --- | --- | --- | --- | --- | --- | --- | --- | --- | --- | --- | --- | --- | --- | --- | --- | --- |

BAN89375.1

back to list

  

| Protein Coverage
| Supporting Peptides
|

Protein Coverage:

Supporting Peptides:

| Peptide | Uniq | -10lgP | Mass | Length | ppm | m/z | z | RT | Fraction | Scan | Source File | Area F6 | Area F2 | Area F3 | Area F4 | Area F5 | Area F7 | Area F8 | Area F1 | #Feature | #Feature F6 | #Feature F2 | #Feature F3 | #Feature F4 | #Feature F5 | #Feature F7 | #Feature F8 | #Feature F1 | Start | End | PTM | AScore | Found By |
| --- | --- | --- | --- | --- | --- | --- | --- | --- | --- | --- | --- | --- | --- | --- | --- | --- | --- | --- | --- | --- | --- | --- | --- | --- | --- | --- | --- | --- | --- | --- | --- | --- | --- |
| R.TFSNIISTLNPLAK.R | Y | 67.62 | 1517.8453 | 14 | 0.6 | 759.9304 | 2 | 58.92 | 4 | F4:33771 | 13012022\_RID\_3262\_4b.raw |  |  |  | 1.3808E6 |  |  |  |  | 1 | 0 | 0 | 0 | 1 | 0 | 0 | 0 | 0 | 123 | 136 |  |  | PEAKS DB |
| R.WSPSDSLYGSR.S | Y | 65.59 | 1253.5676 | 11 | -0.3 | 627.7909 | 2 | 39.85 | 4 | F4:22700 | 13012022\_RID\_3262\_4b.raw |  |  |  | 1.1335E6 | 3.8712E5 |  |  |  | 2 | 0 | 0 | 0 | 1 | 1 | 0 | 0 | 0 | 213 | 223 |  |  | PEAKS DB |
| R.YPGSPGSYAVR.Q | Y | 61.64 | 1152.5563 | 11 | 0.4 | 577.2856 | 2 | 30.09 | 4 | F4:15330 | 13012022\_RID\_3262\_4b.raw |  |  |  | 3.0986E6 |  |  |  |  | 1 | 0 | 0 | 0 | 1 | 0 | 0 | 0 | 0 | 82 | 92 |  |  | PEAKS DB |
| R.NLYDLGLLNNYSSER.Q | Y | 57.61 | 1769.8584 | 15 | 0.3 | 885.9368 | 2 | 54.90 | 4 | F4:31914 | 13012022\_RID\_3262\_4b.raw |  |  |  | 4.1197E5 |  |  |  |  | 1 | 0 | 0 | 0 | 1 | 0 | 0 | 0 | 0 | 283 | 297 |  |  | PEAKS DB |
| R.NPVFPVYFLNTAR.W | Y | 57.19 | 1536.8088 | 13 | 0.2 | 769.4119 | 2 | 58.74 | 4 | F4:33587 | 13012022\_RID\_3262\_4b.raw |  |  |  | 1.2502E6 | 3.6499E5 |  |  |  | 2 | 0 | 0 | 0 | 1 | 1 | 0 | 0 | 0 | 260 | 272 |  |  | PEAKS DB |
| K.LIFFDGEEAFVR.W | Y | 56.69 | 1441.7241 | 12 | -0.1 | 721.8693 | 2 | 59.83 | 5 | F5:29308 | 13012022\_RID\_3262\_5b.raw |  |  |  |  | 2.1896E5 |  |  |  | 1 | 0 | 0 | 0 | 0 | 1 | 0 | 0 | 0 | 201 | 212 |  |  | PEAKS DB |
| K.MASTPHPPGAR.N | Y | 50.10 | 1120.5447 | 11 | 0.5 | 561.2799 | 2 | 19.93 | 4 | F4:7339 | 13012022\_RID\_3262\_4b.raw |  |  |  | 1.6545E5 |  |  |  |  | 1 | 0 | 0 | 0 | 1 | 0 | 0 | 0 | 0 | 229 | 239 |  |  | PEAKS DB |
| K.M(+15.99)ASTPHPPGAR.N | Y | 49.55 | 1136.5397 | 11 | 0.8 | 569.2776 | 2 | 17.65 | 4 | F4:5444 | 13012022\_RID\_3262\_4b.raw |  |  |  | 1.1854E5 |  |  |  |  | 1 | 0 | 0 | 0 | 1 | 0 | 0 | 0 | 0 | 229 | 239 | Oxidation (M) | M1:Oxidation (M):1000.00 | PEAKS DB |
| K.YFPPQLDGK.V | Y | 46.92 | 1063.5338 | 9 | -0.3 | 532.7740 | 2 | 37.67 | 4 | F4:21235 | 13012022\_RID\_3262\_4b.raw |  |  |  | 2.0372E6 |  |  |  |  | 1 | 0 | 0 | 0 | 1 | 0 | 0 | 0 | 0 | 149 | 157 |  |  | PEAKS DB |
| total 9 peptides |  |  |  |  |  |  |  |  |  |  |  |  |  |  |  |  |  |  |  |  |  |  |  |  |  |  |  |  |  |  |  |  |  |
| --- | --- | --- | --- | --- | --- | --- | --- | --- | --- | --- | --- | --- | --- | --- | --- | --- | --- | --- | --- | --- | --- | --- | --- | --- | --- | --- | --- | --- | --- | --- | --- | --- | --- |

BAN89450.1

back to list

  

| Protein Coverage
| Supporting Peptides
|

Protein Coverage:

Supporting Peptides:

| Peptide | Uniq | -10lgP | Mass | Length | ppm | m/z | z | RT | Fraction | Scan | Source File | Area F6 | Area F2 | Area F3 | Area F4 | Area F5 | Area F7 | Area F8 | Area F1 | #Feature | #Feature F6 | #Feature F2 | #Feature F3 | #Feature F4 | #Feature F5 | #Feature F7 | #Feature F8 | #Feature F1 | Start | End | PTM | AScore | Found By |
| --- | --- | --- | --- | --- | --- | --- | --- | --- | --- | --- | --- | --- | --- | --- | --- | --- | --- | --- | --- | --- | --- | --- | --- | --- | --- | --- | --- | --- | --- | --- | --- | --- | --- |
| R.TFSNIISTLNPLAK.R | Y | 67.62 | 1517.8453 | 14 | 0.6 | 759.9304 | 2 | 58.92 | 4 | F4:33771 | 13012022\_RID\_3262\_4b.raw |  |  |  | 1.3808E6 |  |  |  |  | 1 | 0 | 0 | 0 | 1 | 0 | 0 | 0 | 0 | 110 | 123 |  |  | PEAKS DB |
| R.WSPSDSLYGSR.S | Y | 65.59 | 1253.5676 | 11 | -0.3 | 627.7909 | 2 | 39.85 | 4 | F4:22700 | 13012022\_RID\_3262\_4b.raw |  |  |  | 1.1335E6 | 3.8712E5 |  |  |  | 2 | 0 | 0 | 0 | 1 | 1 | 0 | 0 | 0 | 200 | 210 |  |  | PEAKS DB |
| R.YPGSPGSYAVR.Q | Y | 61.64 | 1152.5563 | 11 | 0.4 | 577.2856 | 2 | 30.09 | 4 | F4:15330 | 13012022\_RID\_3262\_4b.raw |  |  |  | 3.0986E6 |  |  |  |  | 1 | 0 | 0 | 0 | 1 | 0 | 0 | 0 | 0 | 69 | 79 |  |  | PEAKS DB |
| R.NLYDLGLLNNYSSER.Q | Y | 57.61 | 1769.8584 | 15 | 0.3 | 885.9368 | 2 | 54.90 | 4 | F4:31914 | 13012022\_RID\_3262\_4b.raw |  |  |  | 4.1197E5 |  |  |  |  | 1 | 0 | 0 | 0 | 1 | 0 | 0 | 0 | 0 | 270 | 284 |  |  | PEAKS DB |
| R.NPVFPVYFLNTAR.W | Y | 57.19 | 1536.8088 | 13 | 0.2 | 769.4119 | 2 | 58.74 | 4 | F4:33587 | 13012022\_RID\_3262\_4b.raw |  |  |  | 1.2502E6 | 3.6499E5 |  |  |  | 2 | 0 | 0 | 0 | 1 | 1 | 0 | 0 | 0 | 247 | 259 |  |  | PEAKS DB |
| K.LIFFDGEEAFVR.W | Y | 56.69 | 1441.7241 | 12 | -0.1 | 721.8693 | 2 | 59.83 | 5 | F5:29308 | 13012022\_RID\_3262\_5b.raw |  |  |  |  | 2.1896E5 |  |  |  | 1 | 0 | 0 | 0 | 0 | 1 | 0 | 0 | 0 | 188 | 199 |  |  | PEAKS DB |
| K.MASTPHPPGAR.N | Y | 50.10 | 1120.5447 | 11 | 0.5 | 561.2799 | 2 | 19.93 | 4 | F4:7339 | 13012022\_RID\_3262\_4b.raw |  |  |  | 1.6545E5 |  |  |  |  | 1 | 0 | 0 | 0 | 1 | 0 | 0 | 0 | 0 | 216 | 226 |  |  | PEAKS DB |
| K.M(+15.99)ASTPHPPGAR.N | Y | 49.55 | 1136.5397 | 11 | 0.8 | 569.2776 | 2 | 17.65 | 4 | F4:5444 | 13012022\_RID\_3262\_4b.raw |  |  |  | 1.1854E5 |  |  |  |  | 1 | 0 | 0 | 0 | 1 | 0 | 0 | 0 | 0 | 216 | 226 | Oxidation (M) | M1:Oxidation (M):1000.00 | PEAKS DB |
| K.YFPPQLDGK.V | Y | 46.92 | 1063.5338 | 9 | -0.3 | 532.7740 | 2 | 37.67 | 4 | F4:21235 | 13012022\_RID\_3262\_4b.raw |  |  |  | 2.0372E6 |  |  |  |  | 1 | 0 | 0 | 0 | 1 | 0 | 0 | 0 | 0 | 136 | 144 |  |  | PEAKS DB |
| total 9 peptides |  |  |  |  |  |  |  |  |  |  |  |  |  |  |  |  |  |  |  |  |  |  |  |  |  |  |  |  |  |  |  |  |  |
| --- | --- | --- | --- | --- | --- | --- | --- | --- | --- | --- | --- | --- | --- | --- | --- | --- | --- | --- | --- | --- | --- | --- | --- | --- | --- | --- | --- | --- | --- | --- | --- | --- | --- |

BAN89449.1

back to list

  

| Protein Coverage
| Supporting Peptides
|

Protein Coverage:

Supporting Peptides:

| Peptide | Uniq | -10lgP | Mass | Length | ppm | m/z | z | RT | Fraction | Scan | Source File | Area F6 | Area F2 | Area F3 | Area F4 | Area F5 | Area F7 | Area F8 | Area F1 | #Feature | #Feature F6 | #Feature F2 | #Feature F3 | #Feature F4 | #Feature F5 | #Feature F7 | #Feature F8 | #Feature F1 | Start | End | PTM | AScore | Found By |
| --- | --- | --- | --- | --- | --- | --- | --- | --- | --- | --- | --- | --- | --- | --- | --- | --- | --- | --- | --- | --- | --- | --- | --- | --- | --- | --- | --- | --- | --- | --- | --- | --- | --- |
| R.TFSNIISTLNPLAK.R | Y | 67.62 | 1517.8453 | 14 | 0.6 | 759.9304 | 2 | 58.92 | 4 | F4:33771 | 13012022\_RID\_3262\_4b.raw |  |  |  | 1.3808E6 |  |  |  |  | 1 | 0 | 0 | 0 | 1 | 0 | 0 | 0 | 0 | 124 | 137 |  |  | PEAKS DB |
| R.WSPSDSLYGSR.S | Y | 65.59 | 1253.5676 | 11 | -0.3 | 627.7909 | 2 | 39.85 | 4 | F4:22700 | 13012022\_RID\_3262\_4b.raw |  |  |  | 1.1335E6 | 3.8712E5 |  |  |  | 2 | 0 | 0 | 0 | 1 | 1 | 0 | 0 | 0 | 214 | 224 |  |  | PEAKS DB |
| R.YPGSPGSYAVR.Q | Y | 61.64 | 1152.5563 | 11 | 0.4 | 577.2856 | 2 | 30.09 | 4 | F4:15330 | 13012022\_RID\_3262\_4b.raw |  |  |  | 3.0986E6 |  |  |  |  | 1 | 0 | 0 | 0 | 1 | 0 | 0 | 0 | 0 | 83 | 93 |  |  | PEAKS DB |
| R.NLYDLGLLNNYSSER.Q | Y | 57.61 | 1769.8584 | 15 | 0.3 | 885.9368 | 2 | 54.90 | 4 | F4:31914 | 13012022\_RID\_3262\_4b.raw |  |  |  | 4.1197E5 |  |  |  |  | 1 | 0 | 0 | 0 | 1 | 0 | 0 | 0 | 0 | 284 | 298 |  |  | PEAKS DB |
| R.NPVFPVYFLNTAR.W | Y | 57.19 | 1536.8088 | 13 | 0.2 | 769.4119 | 2 | 58.74 | 4 | F4:33587 | 13012022\_RID\_3262\_4b.raw |  |  |  | 1.2502E6 | 3.6499E5 |  |  |  | 2 | 0 | 0 | 0 | 1 | 1 | 0 | 0 | 0 | 261 | 273 |  |  | PEAKS DB |
| K.LIFFDGEEAFVR.W | Y | 56.69 | 1441.7241 | 12 | -0.1 | 721.8693 | 2 | 59.83 | 5 | F5:29308 | 13012022\_RID\_3262\_5b.raw |  |  |  |  | 2.1896E5 |  |  |  | 1 | 0 | 0 | 0 | 0 | 1 | 0 | 0 | 0 | 202 | 213 |  |  | PEAKS DB |
| K.MASTPHPPGAR.N | Y | 50.10 | 1120.5447 | 11 | 0.5 | 561.2799 | 2 | 19.93 | 4 | F4:7339 | 13012022\_RID\_3262\_4b.raw |  |  |  | 1.6545E5 |  |  |  |  | 1 | 0 | 0 | 0 | 1 | 0 | 0 | 0 | 0 | 230 | 240 |  |  | PEAKS DB |
| K.M(+15.99)ASTPHPPGAR.N | Y | 49.55 | 1136.5397 | 11 | 0.8 | 569.2776 | 2 | 17.65 | 4 | F4:5444 | 13012022\_RID\_3262\_4b.raw |  |  |  | 1.1854E5 |  |  |  |  | 1 | 0 | 0 | 0 | 1 | 0 | 0 | 0 | 0 | 230 | 240 | Oxidation (M) | M1:Oxidation (M):1000.00 | PEAKS DB |
| K.YFPPQLDGK.V | Y | 46.92 | 1063.5338 | 9 | -0.3 | 532.7740 | 2 | 37.67 | 4 | F4:21235 | 13012022\_RID\_3262\_4b.raw |  |  |  | 2.0372E6 |  |  |  |  | 1 | 0 | 0 | 0 | 1 | 0 | 0 | 0 | 0 | 150 | 158 |  |  | PEAKS DB |
| total 9 peptides |  |  |  |  |  |  |  |  |  |  |  |  |  |  |  |  |  |  |  |  |  |  |  |  |  |  |  |  |  |  |  |  |  |
| --- | --- | --- | --- | --- | --- | --- | --- | --- | --- | --- | --- | --- | --- | --- | --- | --- | --- | --- | --- | --- | --- | --- | --- | --- | --- | --- | --- | --- | --- | --- | --- | --- | --- |

JAG47096.1

back to list

  

| Protein Coverage
| Supporting Peptides
|

Protein Coverage:

Supporting Peptides:

| Peptide | Uniq | -10lgP | Mass | Length | ppm | m/z | z | RT | Fraction | Scan | Source File | Area F6 | Area F2 | Area F3 | Area F4 | Area F5 | Area F7 | Area F8 | Area F1 | #Feature | #Feature F6 | #Feature F2 | #Feature F3 | #Feature F4 | #Feature F5 | #Feature F7 | #Feature F8 | #Feature F1 | Start | End | PTM | AScore | Found By |
| --- | --- | --- | --- | --- | --- | --- | --- | --- | --- | --- | --- | --- | --- | --- | --- | --- | --- | --- | --- | --- | --- | --- | --- | --- | --- | --- | --- | --- | --- | --- | --- | --- | --- |
| K.YVPAIGYYIHSNNPTAK.I | Y | 73.32 | 1906.9578 | 17 | 0.5 | 636.6602 | 3 | 40.16 | 3 | F3:23742 | 13012022\_RID\_3262\_3b.raw |  |  | 1.9334E6 |  |  |  |  |  | 1 | 0 | 0 | 1 | 0 | 0 | 0 | 0 | 0 | 217 | 233 |  |  | PEAKS DB |
| R.GGGHILPYDQPER.A | Y | 71.97 | 1437.7001 | 13 | 0.6 | 480.2409 | 3 | 29.58 | 3 | F3:14679 | 13012022\_RID\_3262\_3b.raw |  |  | 1.9362E6 |  |  |  |  |  | 1 | 0 | 0 | 1 | 0 | 0 | 0 | 0 | 0 | 453 | 465 |  |  | PEAKS DB |
| K.NDFYATGESYAGK.Y | Y | 64.79 | 1421.6099 | 13 | 0.8 | 711.8127 | 2 | 34.61 | 3 | F3:18781 | 13012022\_RID\_3262\_3b.raw |  |  | 1.2949E6 |  |  |  |  |  | 1 | 0 | 0 | 1 | 0 | 0 | 0 | 0 | 0 | 204 | 216 |  |  | PEAKS DB |
| D.PGQPLFLTPYIESGK.I | Y | 51.55 | 1645.8715 | 15 | 0.9 | 823.9437 | 2 | 52.63 | 3 | F3:34355 | 13012022\_RID\_3262\_3b.raw |  |  | 4.0593E5 |  |  |  |  |  | 1 | 0 | 0 | 1 | 0 | 0 | 0 | 0 | 0 | 47 | 61 |  |  | PEAKS DB |
| P.SQGDPGQPLFLTPYIESGKIEEGR.Q | Y | 46.52 | 2617.3022 | 24 | 0.6 | 873.4419 | 3 | 51.19 | 3 | F3:33289 | 13012022\_RID\_3262\_3b.raw |  |  | 1.351E5 |  |  |  |  |  | 1 | 0 | 0 | 1 | 0 | 0 | 0 | 0 | 0 | 43 | 66 |  |  | PEAKS DB |
| total 5 peptides |  |  |  |  |  |  |  |  |  |  |  |  |  |  |  |  |  |  |  |  |  |  |  |  |  |  |  |  |  |  |  |  |  |
| --- | --- | --- | --- | --- | --- | --- | --- | --- | --- | --- | --- | --- | --- | --- | --- | --- | --- | --- | --- | --- | --- | --- | --- | --- | --- | --- | --- | --- | --- | --- | --- | --- | --- |

JAV50864.1

back to list

  

| Protein Coverage
| Supporting Peptides
|

Protein Coverage:

Supporting Peptides:

| Peptide | Uniq | -10lgP | Mass | Length | ppm | m/z | z | RT | Fraction | Scan | Source File | Area F6 | Area F2 | Area F3 | Area F4 | Area F5 | Area F7 | Area F8 | Area F1 | #Feature | #Feature F6 | #Feature F2 | #Feature F3 | #Feature F4 | #Feature F5 | #Feature F7 | #Feature F8 | #Feature F1 | Start | End | PTM | AScore | Found By |
| --- | --- | --- | --- | --- | --- | --- | --- | --- | --- | --- | --- | --- | --- | --- | --- | --- | --- | --- | --- | --- | --- | --- | --- | --- | --- | --- | --- | --- | --- | --- | --- | --- | --- |
| K.YVPAIGYYIHSNNPTAK.I | Y | 73.32 | 1906.9578 | 17 | 0.5 | 636.6602 | 3 | 40.16 | 3 | F3:23742 | 13012022\_RID\_3262\_3b.raw |  |  | 1.9334E6 |  |  |  |  |  | 1 | 0 | 0 | 1 | 0 | 0 | 0 | 0 | 0 | 217 | 233 |  |  | PEAKS DB |
| R.GGGHILPYDQPER.A | Y | 71.97 | 1437.7001 | 13 | 0.6 | 480.2409 | 3 | 29.58 | 3 | F3:14679 | 13012022\_RID\_3262\_3b.raw |  |  | 1.9362E6 |  |  |  |  |  | 1 | 0 | 0 | 1 | 0 | 0 | 0 | 0 | 0 | 453 | 465 |  |  | PEAKS DB |
| K.NDFYATGESYAGK.Y | Y | 64.79 | 1421.6099 | 13 | 0.8 | 711.8127 | 2 | 34.61 | 3 | F3:18781 | 13012022\_RID\_3262\_3b.raw |  |  | 1.2949E6 |  |  |  |  |  | 1 | 0 | 0 | 1 | 0 | 0 | 0 | 0 | 0 | 204 | 216 |  |  | PEAKS DB |
| D.PGQPLFLTPYIESGK.I | Y | 51.55 | 1645.8715 | 15 | 0.9 | 823.9437 | 2 | 52.63 | 3 | F3:34355 | 13012022\_RID\_3262\_3b.raw |  |  | 4.0593E5 |  |  |  |  |  | 1 | 0 | 0 | 1 | 0 | 0 | 0 | 0 | 0 | 47 | 61 |  |  | PEAKS DB |
| P.SQGDPGQPLFLTPYIESGKIEEGR.Q | Y | 46.52 | 2617.3022 | 24 | 0.6 | 873.4419 | 3 | 51.19 | 3 | F3:33289 | 13012022\_RID\_3262\_3b.raw |  |  | 1.351E5 |  |  |  |  |  | 1 | 0 | 0 | 1 | 0 | 0 | 0 | 0 | 0 | 43 | 66 |  |  | PEAKS DB |
| total 5 peptides |  |  |  |  |  |  |  |  |  |  |  |  |  |  |  |  |  |  |  |  |  |  |  |  |  |  |  |  |  |  |  |  |  |
| --- | --- | --- | --- | --- | --- | --- | --- | --- | --- | --- | --- | --- | --- | --- | --- | --- | --- | --- | --- | --- | --- | --- | --- | --- | --- | --- | --- | --- | --- | --- | --- | --- | --- |

XP\_015666085.1

back to list

  

| Protein Coverage
| Supporting Peptides
|

Protein Coverage:

Supporting Peptides:

| Peptide | Uniq | -10lgP | Mass | Length | ppm | m/z | z | RT | Fraction | Scan | Source File | Area F6 | Area F2 | Area F3 | Area F4 | Area F5 | Area F7 | Area F8 | Area F1 | #Feature | #Feature F6 | #Feature F2 | #Feature F3 | #Feature F4 | #Feature F5 | #Feature F7 | #Feature F8 | #Feature F1 | Start | End | PTM | AScore | Found By |
| --- | --- | --- | --- | --- | --- | --- | --- | --- | --- | --- | --- | --- | --- | --- | --- | --- | --- | --- | --- | --- | --- | --- | --- | --- | --- | --- | --- | --- | --- | --- | --- | --- | --- |
| K.YVPAIGYYIHSNNPTAK.I | Y | 73.32 | 1906.9578 | 17 | 0.5 | 636.6602 | 3 | 40.16 | 3 | F3:23742 | 13012022\_RID\_3262\_3b.raw |  |  | 1.9334E6 |  |  |  |  |  | 1 | 0 | 0 | 1 | 0 | 0 | 0 | 0 | 0 | 217 | 233 |  |  | PEAKS DB |
| R.GGGHILPYDQPER.A | Y | 71.97 | 1437.7001 | 13 | 0.6 | 480.2409 | 3 | 29.58 | 3 | F3:14679 | 13012022\_RID\_3262\_3b.raw |  |  | 1.9362E6 |  |  |  |  |  | 1 | 0 | 0 | 1 | 0 | 0 | 0 | 0 | 0 | 453 | 465 |  |  | PEAKS DB |
| K.NDFYATGESYAGK.Y | Y | 64.79 | 1421.6099 | 13 | 0.8 | 711.8127 | 2 | 34.61 | 3 | F3:18781 | 13012022\_RID\_3262\_3b.raw |  |  | 1.2949E6 |  |  |  |  |  | 1 | 0 | 0 | 1 | 0 | 0 | 0 | 0 | 0 | 204 | 216 |  |  | PEAKS DB |
| D.PGQPLFLTPYIESGK.I | Y | 51.55 | 1645.8715 | 15 | 0.9 | 823.9437 | 2 | 52.63 | 3 | F3:34355 | 13012022\_RID\_3262\_3b.raw |  |  | 4.0593E5 |  |  |  |  |  | 1 | 0 | 0 | 1 | 0 | 0 | 0 | 0 | 0 | 47 | 61 |  |  | PEAKS DB |
| P.SQGDPGQPLFLTPYIESGKIEEGR.Q | Y | 46.52 | 2617.3022 | 24 | 0.6 | 873.4419 | 3 | 51.19 | 3 | F3:33289 | 13012022\_RID\_3262\_3b.raw |  |  | 1.351E5 |  |  |  |  |  | 1 | 0 | 0 | 1 | 0 | 0 | 0 | 0 | 0 | 43 | 66 |  |  | PEAKS DB |
| total 5 peptides |  |  |  |  |  |  |  |  |  |  |  |  |  |  |  |  |  |  |  |  |  |  |  |  |  |  |  |  |  |  |  |  |  |
| --- | --- | --- | --- | --- | --- | --- | --- | --- | --- | --- | --- | --- | --- | --- | --- | --- | --- | --- | --- | --- | --- | --- | --- | --- | --- | --- | --- | --- | --- | --- | --- | --- | --- |

XP\_015666086.1

back to list

  

| Protein Coverage
| Supporting Peptides
|

Protein Coverage:

Supporting Peptides:

| Peptide | Uniq | -10lgP | Mass | Length | ppm | m/z | z | RT | Fraction | Scan | Source File | Area F6 | Area F2 | Area F3 | Area F4 | Area F5 | Area F7 | Area F8 | Area F1 | #Feature | #Feature F6 | #Feature F2 | #Feature F3 | #Feature F4 | #Feature F5 | #Feature F7 | #Feature F8 | #Feature F1 | Start | End | PTM | AScore | Found By |
| --- | --- | --- | --- | --- | --- | --- | --- | --- | --- | --- | --- | --- | --- | --- | --- | --- | --- | --- | --- | --- | --- | --- | --- | --- | --- | --- | --- | --- | --- | --- | --- | --- | --- |
| K.YVPAIGYYIHSNNPTAK.I | Y | 73.32 | 1906.9578 | 17 | 0.5 | 636.6602 | 3 | 40.16 | 3 | F3:23742 | 13012022\_RID\_3262\_3b.raw |  |  | 1.9334E6 |  |  |  |  |  | 1 | 0 | 0 | 1 | 0 | 0 | 0 | 0 | 0 | 217 | 233 |  |  | PEAKS DB |
| R.GGGHILPYDQPER.A | Y | 71.97 | 1437.7001 | 13 | 0.6 | 480.2409 | 3 | 29.58 | 3 | F3:14679 | 13012022\_RID\_3262\_3b.raw |  |  | 1.9362E6 |  |  |  |  |  | 1 | 0 | 0 | 1 | 0 | 0 | 0 | 0 | 0 | 453 | 465 |  |  | PEAKS DB |
| K.NDFYATGESYAGK.Y | Y | 64.79 | 1421.6099 | 13 | 0.8 | 711.8127 | 2 | 34.61 | 3 | F3:18781 | 13012022\_RID\_3262\_3b.raw |  |  | 1.2949E6 |  |  |  |  |  | 1 | 0 | 0 | 1 | 0 | 0 | 0 | 0 | 0 | 204 | 216 |  |  | PEAKS DB |
| D.PGQPLFLTPYIESGK.I | Y | 51.55 | 1645.8715 | 15 | 0.9 | 823.9437 | 2 | 52.63 | 3 | F3:34355 | 13012022\_RID\_3262\_3b.raw |  |  | 4.0593E5 |  |  |  |  |  | 1 | 0 | 0 | 1 | 0 | 0 | 0 | 0 | 0 | 47 | 61 |  |  | PEAKS DB |
| P.SQGDPGQPLFLTPYIESGKIEEGR.Q | Y | 46.52 | 2617.3022 | 24 | 0.6 | 873.4419 | 3 | 51.19 | 3 | F3:33289 | 13012022\_RID\_3262\_3b.raw |  |  | 1.351E5 |  |  |  |  |  | 1 | 0 | 0 | 1 | 0 | 0 | 0 | 0 | 0 | 43 | 66 |  |  | PEAKS DB |
| total 5 peptides |  |  |  |  |  |  |  |  |  |  |  |  |  |  |  |  |  |  |  |  |  |  |  |  |  |  |  |  |  |  |  |  |  |
| --- | --- | --- | --- | --- | --- | --- | --- | --- | --- | --- | --- | --- | --- | --- | --- | --- | --- | --- | --- | --- | --- | --- | --- | --- | --- | --- | --- | --- | --- | --- | --- | --- | --- |

JAG45243.1

back to list

  

| Protein Coverage
| Supporting Peptides
|

Protein Coverage:

Supporting Peptides:

| Peptide | Uniq | -10lgP | Mass | Length | ppm | m/z | z | RT | Fraction | Scan | Source File | Area F6 | Area F2 | Area F3 | Area F4 | Area F5 | Area F7 | Area F8 | Area F1 | #Feature | #Feature F6 | #Feature F2 | #Feature F3 | #Feature F4 | #Feature F5 | #Feature F7 | #Feature F8 | #Feature F1 | Start | End | PTM | AScore | Found By |
| --- | --- | --- | --- | --- | --- | --- | --- | --- | --- | --- | --- | --- | --- | --- | --- | --- | --- | --- | --- | --- | --- | --- | --- | --- | --- | --- | --- | --- | --- | --- | --- | --- | --- |
| K.YVPAIGYYIHSNNPTAK.I | Y | 73.32 | 1906.9578 | 17 | 0.5 | 636.6602 | 3 | 40.16 | 3 | F3:23742 | 13012022\_RID\_3262\_3b.raw |  |  | 1.9334E6 |  |  |  |  |  | 1 | 0 | 0 | 1 | 0 | 0 | 0 | 0 | 0 | 217 | 233 |  |  | PEAKS DB |
| R.GGGHILPYDQPER.A | Y | 71.97 | 1437.7001 | 13 | 0.6 | 480.2409 | 3 | 29.58 | 3 | F3:14679 | 13012022\_RID\_3262\_3b.raw |  |  | 1.9362E6 |  |  |  |  |  | 1 | 0 | 0 | 1 | 0 | 0 | 0 | 0 | 0 | 453 | 465 |  |  | PEAKS DB |
| K.NDFYATGESYAGK.Y | Y | 64.79 | 1421.6099 | 13 | 0.8 | 711.8127 | 2 | 34.61 | 3 | F3:18781 | 13012022\_RID\_3262\_3b.raw |  |  | 1.2949E6 |  |  |  |  |  | 1 | 0 | 0 | 1 | 0 | 0 | 0 | 0 | 0 | 204 | 216 |  |  | PEAKS DB |
| D.PGQPLFLTPYIESGK.I | Y | 51.55 | 1645.8715 | 15 | 0.9 | 823.9437 | 2 | 52.63 | 3 | F3:34355 | 13012022\_RID\_3262\_3b.raw |  |  | 4.0593E5 |  |  |  |  |  | 1 | 0 | 0 | 1 | 0 | 0 | 0 | 0 | 0 | 47 | 61 |  |  | PEAKS DB |
| P.SQGDPGQPLFLTPYIESGKIEEGR.Q | Y | 46.52 | 2617.3022 | 24 | 0.6 | 873.4419 | 3 | 51.19 | 3 | F3:33289 | 13012022\_RID\_3262\_3b.raw |  |  | 1.351E5 |  |  |  |  |  | 1 | 0 | 0 | 1 | 0 | 0 | 0 | 0 | 0 | 43 | 66 |  |  | PEAKS DB |
| total 5 peptides |  |  |  |  |  |  |  |  |  |  |  |  |  |  |  |  |  |  |  |  |  |  |  |  |  |  |  |  |  |  |  |  |  |
| --- | --- | --- | --- | --- | --- | --- | --- | --- | --- | --- | --- | --- | --- | --- | --- | --- | --- | --- | --- | --- | --- | --- | --- | --- | --- | --- | --- | --- | --- | --- | --- | --- | --- |

JAI13760.1

back to list

  

| Protein Coverage
| Supporting Peptides
|

Protein Coverage:

Supporting Peptides:

| Peptide | Uniq | -10lgP | Mass | Length | ppm | m/z | z | RT | Fraction | Scan | Source File | Area F6 | Area F2 | Area F3 | Area F4 | Area F5 | Area F7 | Area F8 | Area F1 | #Feature | #Feature F6 | #Feature F2 | #Feature F3 | #Feature F4 | #Feature F5 | #Feature F7 | #Feature F8 | #Feature F1 | Start | End | PTM | AScore | Found By |
| --- | --- | --- | --- | --- | --- | --- | --- | --- | --- | --- | --- | --- | --- | --- | --- | --- | --- | --- | --- | --- | --- | --- | --- | --- | --- | --- | --- | --- | --- | --- | --- | --- | --- |
| K.YVPAIGYYIHSNNPTAK.I | Y | 73.32 | 1906.9578 | 17 | 0.5 | 636.6602 | 3 | 40.16 | 3 | F3:23742 | 13012022\_RID\_3262\_3b.raw |  |  | 1.9334E6 |  |  |  |  |  | 1 | 0 | 0 | 1 | 0 | 0 | 0 | 0 | 0 | 217 | 233 |  |  | PEAKS DB |
| R.GGGHILPYDQPER.A | Y | 71.97 | 1437.7001 | 13 | 0.6 | 480.2409 | 3 | 29.58 | 3 | F3:14679 | 13012022\_RID\_3262\_3b.raw |  |  | 1.9362E6 |  |  |  |  |  | 1 | 0 | 0 | 1 | 0 | 0 | 0 | 0 | 0 | 453 | 465 |  |  | PEAKS DB |
| K.NDFYATGESYAGK.Y | Y | 64.79 | 1421.6099 | 13 | 0.8 | 711.8127 | 2 | 34.61 | 3 | F3:18781 | 13012022\_RID\_3262\_3b.raw |  |  | 1.2949E6 |  |  |  |  |  | 1 | 0 | 0 | 1 | 0 | 0 | 0 | 0 | 0 | 204 | 216 |  |  | PEAKS DB |
| D.PGQPLFLTPYIESGK.I | Y | 51.55 | 1645.8715 | 15 | 0.9 | 823.9437 | 2 | 52.63 | 3 | F3:34355 | 13012022\_RID\_3262\_3b.raw |  |  | 4.0593E5 |  |  |  |  |  | 1 | 0 | 0 | 1 | 0 | 0 | 0 | 0 | 0 | 47 | 61 |  |  | PEAKS DB |
| P.SQGDPGQPLFLTPYIESGKIEEGR.Q | Y | 46.52 | 2617.3022 | 24 | 0.6 | 873.4419 | 3 | 51.19 | 3 | F3:33289 | 13012022\_RID\_3262\_3b.raw |  |  | 1.351E5 |  |  |  |  |  | 1 | 0 | 0 | 1 | 0 | 0 | 0 | 0 | 0 | 43 | 66 |  |  | PEAKS DB |
| total 5 peptides |  |  |  |  |  |  |  |  |  |  |  |  |  |  |  |  |  |  |  |  |  |  |  |  |  |  |  |  |  |  |  |  |  |
| --- | --- | --- | --- | --- | --- | --- | --- | --- | --- | --- | --- | --- | --- | --- | --- | --- | --- | --- | --- | --- | --- | --- | --- | --- | --- | --- | --- | --- | --- | --- | --- | --- | --- |

AFJ49766.1

back to list

  

| Protein Coverage
| Supporting Peptides
|

Protein Coverage:

Supporting Peptides:

| Peptide | Uniq | -10lgP | Mass | Length | ppm | m/z | z | RT | Fraction | Scan | Source File | Area F6 | Area F2 | Area F3 | Area F4 | Area F5 | Area F7 | Area F8 | Area F1 | #Feature | #Feature F6 | #Feature F2 | #Feature F3 | #Feature F4 | #Feature F5 | #Feature F7 | #Feature F8 | #Feature F1 | Start | End | PTM | AScore | Found By |
| --- | --- | --- | --- | --- | --- | --- | --- | --- | --- | --- | --- | --- | --- | --- | --- | --- | --- | --- | --- | --- | --- | --- | --- | --- | --- | --- | --- | --- | --- | --- | --- | --- | --- |
| K.YVPAIGYYIHSNNPTAK.I | Y | 73.32 | 1906.9578 | 17 | 0.5 | 636.6602 | 3 | 40.16 | 3 | F3:23742 | 13012022\_RID\_3262\_3b.raw |  |  | 1.9334E6 |  |  |  |  |  | 1 | 0 | 0 | 1 | 0 | 0 | 0 | 0 | 0 | 217 | 233 |  |  | PEAKS DB |
| R.GGGHILPYDQPER.A | Y | 71.97 | 1437.7001 | 13 | 0.6 | 480.2409 | 3 | 29.58 | 3 | F3:14679 | 13012022\_RID\_3262\_3b.raw |  |  | 1.9362E6 |  |  |  |  |  | 1 | 0 | 0 | 1 | 0 | 0 | 0 | 0 | 0 | 453 | 465 |  |  | PEAKS DB |
| K.NDFYATGESYAGK.Y | Y | 64.79 | 1421.6099 | 13 | 0.8 | 711.8127 | 2 | 34.61 | 3 | F3:18781 | 13012022\_RID\_3262\_3b.raw |  |  | 1.2949E6 |  |  |  |  |  | 1 | 0 | 0 | 1 | 0 | 0 | 0 | 0 | 0 | 204 | 216 |  |  | PEAKS DB |
| D.PGQPLFLTPYIESGK.I | Y | 51.55 | 1645.8715 | 15 | 0.9 | 823.9437 | 2 | 52.63 | 3 | F3:34355 | 13012022\_RID\_3262\_3b.raw |  |  | 4.0593E5 |  |  |  |  |  | 1 | 0 | 0 | 1 | 0 | 0 | 0 | 0 | 0 | 47 | 61 |  |  | PEAKS DB |
| P.SQGDPGQPLFLTPYIESGKIEEGR.Q | Y | 46.52 | 2617.3022 | 24 | 0.6 | 873.4419 | 3 | 51.19 | 3 | F3:33289 | 13012022\_RID\_3262\_3b.raw |  |  | 1.351E5 |  |  |  |  |  | 1 | 0 | 0 | 1 | 0 | 0 | 0 | 0 | 0 | 43 | 66 |  |  | PEAKS DB |
| total 5 peptides |  |  |  |  |  |  |  |  |  |  |  |  |  |  |  |  |  |  |  |  |  |  |  |  |  |  |  |  |  |  |  |  |  |
| --- | --- | --- | --- | --- | --- | --- | --- | --- | --- | --- | --- | --- | --- | --- | --- | --- | --- | --- | --- | --- | --- | --- | --- | --- | --- | --- | --- | --- | --- | --- | --- | --- | --- |

JAA97361.1

back to list

  

| Protein Coverage
| Supporting Peptides
|

Protein Coverage:

Supporting Peptides:

| Peptide | Uniq | -10lgP | Mass | Length | ppm | m/z | z | RT | Fraction | Scan | Source File | Area F6 | Area F2 | Area F3 | Area F4 | Area F5 | Area F7 | Area F8 | Area F1 | #Feature | #Feature F6 | #Feature F2 | #Feature F3 | #Feature F4 | #Feature F5 | #Feature F7 | #Feature F8 | #Feature F1 | Start | End | PTM | AScore | Found By |
| --- | --- | --- | --- | --- | --- | --- | --- | --- | --- | --- | --- | --- | --- | --- | --- | --- | --- | --- | --- | --- | --- | --- | --- | --- | --- | --- | --- | --- | --- | --- | --- | --- | --- |
| K.YVPAIGYYIHSNNPTAK.I | Y | 73.32 | 1906.9578 | 17 | 0.5 | 636.6602 | 3 | 40.16 | 3 | F3:23742 | 13012022\_RID\_3262\_3b.raw |  |  | 1.9334E6 |  |  |  |  |  | 1 | 0 | 0 | 1 | 0 | 0 | 0 | 0 | 0 | 217 | 233 |  |  | PEAKS DB |
| R.GGGHILPYDQPER.A | Y | 71.97 | 1437.7001 | 13 | 0.6 | 480.2409 | 3 | 29.58 | 3 | F3:14679 | 13012022\_RID\_3262\_3b.raw |  |  | 1.9362E6 |  |  |  |  |  | 1 | 0 | 0 | 1 | 0 | 0 | 0 | 0 | 0 | 453 | 465 |  |  | PEAKS DB |
| K.NDFYATGESYAGK.Y | Y | 64.79 | 1421.6099 | 13 | 0.8 | 711.8127 | 2 | 34.61 | 3 | F3:18781 | 13012022\_RID\_3262\_3b.raw |  |  | 1.2949E6 |  |  |  |  |  | 1 | 0 | 0 | 1 | 0 | 0 | 0 | 0 | 0 | 204 | 216 |  |  | PEAKS DB |
| D.PGQPLFLTPYIESGK.I | Y | 51.55 | 1645.8715 | 15 | 0.9 | 823.9437 | 2 | 52.63 | 3 | F3:34355 | 13012022\_RID\_3262\_3b.raw |  |  | 4.0593E5 |  |  |  |  |  | 1 | 0 | 0 | 1 | 0 | 0 | 0 | 0 | 0 | 47 | 61 |  |  | PEAKS DB |
| P.SQGDPGQPLFLTPYIESGKIEEGR.Q | Y | 46.52 | 2617.3022 | 24 | 0.6 | 873.4419 | 3 | 51.19 | 3 | F3:33289 | 13012022\_RID\_3262\_3b.raw |  |  | 1.351E5 |  |  |  |  |  | 1 | 0 | 0 | 1 | 0 | 0 | 0 | 0 | 0 | 43 | 66 |  |  | PEAKS DB |
| total 5 peptides |  |  |  |  |  |  |  |  |  |  |  |  |  |  |  |  |  |  |  |  |  |  |  |  |  |  |  |  |  |  |  |  |  |
| --- | --- | --- | --- | --- | --- | --- | --- | --- | --- | --- | --- | --- | --- | --- | --- | --- | --- | --- | --- | --- | --- | --- | --- | --- | --- | --- | --- | --- | --- | --- | --- | --- | --- |

QHR82741.1

back to list

  

| Protein Coverage
| Supporting Peptides
|

Protein Coverage:

Supporting Peptides:

| Peptide | Uniq | -10lgP | Mass | Length | ppm | m/z | z | RT | Fraction | Scan | Source File | Area F6 | Area F2 | Area F3 | Area F4 | Area F5 | Area F7 | Area F8 | Area F1 | #Feature | #Feature F6 | #Feature F2 | #Feature F3 | #Feature F4 | #Feature F5 | #Feature F7 | #Feature F8 | #Feature F1 | Start | End | PTM | AScore | Found By |
| --- | --- | --- | --- | --- | --- | --- | --- | --- | --- | --- | --- | --- | --- | --- | --- | --- | --- | --- | --- | --- | --- | --- | --- | --- | --- | --- | --- | --- | --- | --- | --- | --- | --- |
| K.YVPAIGYYIHSNNPTAK.I | Y | 73.32 | 1906.9578 | 17 | 0.5 | 636.6602 | 3 | 40.16 | 3 | F3:23742 | 13012022\_RID\_3262\_3b.raw |  |  | 1.9334E6 |  |  |  |  |  | 1 | 0 | 0 | 1 | 0 | 0 | 0 | 0 | 0 | 217 | 233 |  |  | PEAKS DB |
| R.GGGHILPYDQPER.A | Y | 71.97 | 1437.7001 | 13 | 0.6 | 480.2409 | 3 | 29.58 | 3 | F3:14679 | 13012022\_RID\_3262\_3b.raw |  |  | 1.9362E6 |  |  |  |  |  | 1 | 0 | 0 | 1 | 0 | 0 | 0 | 0 | 0 | 453 | 465 |  |  | PEAKS DB |
| K.NDFYATGESYAGK.Y | Y | 64.79 | 1421.6099 | 13 | 0.8 | 711.8127 | 2 | 34.61 | 3 | F3:18781 | 13012022\_RID\_3262\_3b.raw |  |  | 1.2949E6 |  |  |  |  |  | 1 | 0 | 0 | 1 | 0 | 0 | 0 | 0 | 0 | 204 | 216 |  |  | PEAKS DB |
| D.PGQPLFLTPYIESGK.I | Y | 51.55 | 1645.8715 | 15 | 0.9 | 823.9437 | 2 | 52.63 | 3 | F3:34355 | 13012022\_RID\_3262\_3b.raw |  |  | 4.0593E5 |  |  |  |  |  | 1 | 0 | 0 | 1 | 0 | 0 | 0 | 0 | 0 | 47 | 61 |  |  | PEAKS DB |
| P.SQGDPGQPLFLTPYIESGKIEEGR.Q | Y | 46.52 | 2617.3022 | 24 | 0.6 | 873.4419 | 3 | 51.19 | 3 | F3:33289 | 13012022\_RID\_3262\_3b.raw |  |  | 1.351E5 |  |  |  |  |  | 1 | 0 | 0 | 1 | 0 | 0 | 0 | 0 | 0 | 43 | 66 |  |  | PEAKS DB |
| total 5 peptides |  |  |  |  |  |  |  |  |  |  |  |  |  |  |  |  |  |  |  |  |  |  |  |  |  |  |  |  |  |  |  |  |  |
| --- | --- | --- | --- | --- | --- | --- | --- | --- | --- | --- | --- | --- | --- | --- | --- | --- | --- | --- | --- | --- | --- | --- | --- | --- | --- | --- | --- | --- | --- | --- | --- | --- | --- |

XP\_032067333.1

back to list

  

| Protein Coverage
| Supporting Peptides
|

Protein Coverage:

Supporting Peptides:

| Peptide | Uniq | -10lgP | Mass | Length | ppm | m/z | z | RT | Fraction | Scan | Source File | Area F6 | Area F2 | Area F3 | Area F4 | Area F5 | Area F7 | Area F8 | Area F1 | #Feature | #Feature F6 | #Feature F2 | #Feature F3 | #Feature F4 | #Feature F5 | #Feature F7 | #Feature F8 | #Feature F1 | Start | End | PTM | AScore | Found By |
| --- | --- | --- | --- | --- | --- | --- | --- | --- | --- | --- | --- | --- | --- | --- | --- | --- | --- | --- | --- | --- | --- | --- | --- | --- | --- | --- | --- | --- | --- | --- | --- | --- | --- |
| K.FASFIDKVR.F | N | 65.83 | 1081.5920 | 9 | 1.0 | 541.8038 | 2 | 33.62 | 8 | F8:15859 | 31122021\_RID\_3262\_1b.raw |  |  | 2.2979E6 |  | 2.6797E5 |  |  | 2.3536E6 | 3 | 0 | 0 | 1 | 0 | 1 | 0 | 0 | 1 | 166 | 174 |  |  | PEAKS DB |
| S.VILSMDNNR.Y | N | 56.96 | 1060.5336 | 9 | 0.4 | 531.2743 | 2 | 30.70 | 8 | F8:13454 | 31122021\_RID\_3262\_1b.raw |  |  |  |  |  |  |  | 5.6605E5 | 1 | 0 | 0 | 0 | 0 | 0 | 0 | 0 | 1 | 310 | 318 |  |  | PEAKS DB |
| K.LSLDVEIATYR.K | Y | 56.11 | 1278.6819 | 11 | -0.6 | 640.3478 | 2 | 48.88 | 8 | F8:28016 | 31122021\_RID\_3262\_1b.raw |  |  |  |  |  |  |  | 1.0192E5 | 1 | 0 | 0 | 0 | 0 | 0 | 0 | 0 | 1 | 448 | 458 |  |  | PEAKS DB |
| K.FASFIDK.V | N | 53.98 | 826.4225 | 7 | 0.0 | 414.2185 | 2 | 37.13 | 2 | F2:13215 | 13012022\_RID\_3262\_2b.raw |  | 3.8919E6 | 3.3996E7 | 9.4622E6 | 1.087E7 | 2.372E7 | 4.1967E6 | 3.8411E7 | 9 | 0 | 1 | 2 | 1 | 1 | 2 | 1 | 1 | 166 | 172 |  |  | PEAKS DB |
| S.KYEDEINKR.T | N | 52.93 | 1193.6040 | 9 | 0.1 | 398.8753 | 3 | 17.04 | 3 | F3:4500 | 13012022\_RID\_3262\_3b.raw |  |  | 3.8499E6 |  |  |  |  |  | 1 | 0 | 0 | 1 | 0 | 0 | 0 | 0 | 0 | 244 | 252 |  |  | PEAKS DB |
| R.SKYEDEINKR.T | N | 51.82 | 1280.6360 | 10 | -0.2 | 427.8859 | 3 | 14.63 | 8 | F8:2574 | 31122021\_RID\_3262\_1b.raw |  |  |  |  |  |  |  | 1.6755E5 | 1 | 0 | 0 | 0 | 0 | 0 | 0 | 0 | 1 | 243 | 252 |  |  | PEAKS DB |
| S.LDVEIATYR.K | N | 46.13 | 1078.5658 | 9 | 0.8 | 540.2906 | 2 | 39.94 | 8 | F8:20761 | 31122021\_RID\_3262\_1b.raw |  |  |  |  |  |  |  | 7.8953E4 | 1 | 0 | 0 | 0 | 0 | 0 | 0 | 0 | 1 | 450 | 458 |  |  | PEAKS DB |
| total 7 peptides |  |  |  |  |  |  |  |  |  |  |  |  |  |  |  |  |  |  |  |  |  |  |  |  |  |  |  |  |  |  |  |  |  |
| --- | --- | --- | --- | --- | --- | --- | --- | --- | --- | --- | --- | --- | --- | --- | --- | --- | --- | --- | --- | --- | --- | --- | --- | --- | --- | --- | --- | --- | --- | --- | --- | --- | --- |

P0DM87.1

back to list

  

| Protein Coverage
| Supporting Peptides
|

Protein Coverage:

Supporting Peptides:

| Peptide | Uniq | -10lgP | Mass | Length | ppm | m/z | z | RT | Fraction | Scan | Source File | Area F6 | Area F2 | Area F3 | Area F4 | Area F5 | Area F7 | Area F8 | Area F1 | #Feature | #Feature F6 | #Feature F2 | #Feature F3 | #Feature F4 | #Feature F5 | #Feature F7 | #Feature F8 | #Feature F1 | Start | End | PTM | AScore | Found By |
| --- | --- | --- | --- | --- | --- | --- | --- | --- | --- | --- | --- | --- | --- | --- | --- | --- | --- | --- | --- | --- | --- | --- | --- | --- | --- | --- | --- | --- | --- | --- | --- | --- | --- |
| R.HDNAQLLTGM(+15.99)IFNEK.I | N | 85.98 | 1745.8407 | 15 | -0.3 | 873.9274 | 2 | 46.88 | 4 | F4:27120 | 13012022\_RID\_3262\_4b.raw |  |  | 1.345E6 | 1.2628E7 | 5.8866E6 |  |  | 1.3649E6 | 8 | 0 | 0 | 1 | 2 | 2 | 0 | 0 | 3 | 280 | 294 | Oxidation (M) | M10:Oxidation (M):1000.00 | PEAKS DB |
| R.MYTKYDGDETEISSK.I | Y | 85.42 | 1765.7716 | 15 | 0.4 | 883.8934 | 2 | 28.30 | 4 | F4:13730 | 13012022\_RID\_3262\_4b.raw |  |  |  | 1.1005E7 |  |  |  | 8.5935E4 | 3 | 0 | 0 | 0 | 2 | 0 | 0 | 0 | 1 | 206 | 220 |  |  | PEAKS DB |
| R.M(+15.99)YTKYDGDETEISSK.I | Y | 80.15 | 1781.7665 | 15 | 0.9 | 594.9300 | 3 | 26.34 | 4 | F4:12126 | 13012022\_RID\_3262\_4b.raw |  |  |  | 4.0292E6 |  |  |  |  | 2 | 0 | 0 | 0 | 2 | 0 | 0 | 0 | 0 | 206 | 220 | Oxidation (M) | M1:Oxidation (M):1000.00 | PEAKS DB |
| K.YDGDETEISSK.I | Y | 72.15 | 1242.5251 | 11 | -0.3 | 622.2697 | 2 | 23.95 | 4 | F4:10576 | 13012022\_RID\_3262\_4b.raw |  |  |  | 1.447E7 |  |  |  | 7.3675E5 | 3 | 0 | 0 | 0 | 2 | 0 | 0 | 0 | 1 | 210 | 220 |  |  | PEAKS DB |
| R.HDNAQLLTGMIFNEK.I | N | 53.03 | 1729.8457 | 15 | 0.3 | 577.6227 | 3 | 47.54 | 8 | F8:26712 | 31122021\_RID\_3262\_1b.raw |  |  |  |  |  |  |  | 0 | 0 | 0 | 0 | 0 | 0 | 0 | 0 | 0 | 0 | 280 | 294 |  |  | PEAKS DB |
| K.RHDNAQLLTGM(+15.99)IFNEK.I | N | 51.26 | 1901.9418 | 16 | 0.3 | 634.9880 | 3 | 35.09 | 8 | F8:16937 | 31122021\_RID\_3262\_1b.raw |  |  |  |  |  |  |  | 1.5341E5 | 1 | 0 | 0 | 0 | 0 | 0 | 0 | 0 | 1 | 279 | 294 | Oxidation (M) | M11:Oxidation (M):1000.00 | PEAKS DB |
| total 6 peptides |  |  |  |  |  |  |  |  |  |  |  |  |  |  |  |  |  |  |  |  |  |  |  |  |  |  |  |  |  |  |  |  |  |
| --- | --- | --- | --- | --- | --- | --- | --- | --- | --- | --- | --- | --- | --- | --- | --- | --- | --- | --- | --- | --- | --- | --- | --- | --- | --- | --- | --- | --- | --- | --- | --- | --- | --- |

XP\_032080818.1

back to list

  

| Protein Coverage
| Supporting Peptides
|

Protein Coverage:

Supporting Peptides:

| Peptide | Uniq | -10lgP | Mass | Length | ppm | m/z | z | RT | Fraction | Scan | Source File | Area F6 | Area F2 | Area F3 | Area F4 | Area F5 | Area F7 | Area F8 | Area F1 | #Feature | #Feature F6 | #Feature F2 | #Feature F3 | #Feature F4 | #Feature F5 | #Feature F7 | #Feature F8 | #Feature F1 | Start | End | PTM | AScore | Found By |
| --- | --- | --- | --- | --- | --- | --- | --- | --- | --- | --- | --- | --- | --- | --- | --- | --- | --- | --- | --- | --- | --- | --- | --- | --- | --- | --- | --- | --- | --- | --- | --- | --- | --- |
| R.LMGLFEPSDMK.Y | N | 72.85 | 1266.5988 | 11 | 0.3 | 634.3068 | 2 | 51.05 | 2 | F2:22048 | 13012022\_RID\_3262\_2b.raw |  | 1.7026E6 | 7.4096E5 |  |  |  |  |  | 2 | 0 | 1 | 1 | 0 | 0 | 0 | 0 | 0 | 290 | 300 |  |  | PEAKS DB |
| H.ALTEAIEFDK.A | N | 66.72 | 1135.5760 | 10 | 0.2 | 568.7954 | 2 | 40.70 | 2 | F2:15380 | 13012022\_RID\_3262\_2b.raw | 1.082E6 | 7.046E6 | 2.1709E6 |  |  |  |  |  | 3 | 1 | 1 | 1 | 0 | 0 | 0 | 0 | 0 | 352 | 361 |  |  | PEAKS DB |
| K.GFYLFVEGGR.I | N | 64.89 | 1143.5713 | 10 | 0.7 | 572.7933 | 2 | 50.12 | 8 | F8:28714 | 31122021\_RID\_3262\_1b.raw |  |  |  |  |  |  |  | 1.0902E5 | 1 | 0 | 0 | 0 | 0 | 0 | 0 | 0 | 1 | 330 | 339 |  |  | PEAKS DB |
| K.SVGIVTTTR.V | N | 59.29 | 932.5291 | 9 | -0.6 | 467.2715 | 2 | 27.35 | 2 | F2:8028 | 13012022\_RID\_3262\_2b.raw | 1.3812E4 | 2.0541E6 | 1.4792E6 |  |  |  |  | 1.5073E5 | 4 | 1 | 1 | 1 | 0 | 0 | 0 | 0 | 1 | 167 | 175 |  |  | PEAKS DB |
| R.GTSIFGLAPEK.A | Y | 59.08 | 1118.5972 | 11 | -0.3 | 560.3057 | 2 | 44.12 | 2 | F2:17426 | 13012022\_RID\_3262\_2b.raw |  | 4.4338E6 |  |  |  |  |  |  | 1 | 0 | 1 | 0 | 0 | 0 | 0 | 0 | 0 | 396 | 406 |  |  | PEAKS DB |
| R.VQHASPAGNYAHVVNR.D | N | 50.35 | 1718.8601 | 16 | 0.5 | 573.9609 | 3 | 22.34 | 3 | F3:8686 | 13012022\_RID\_3262\_3b.raw |  |  | 3.414E5 |  |  |  |  |  | 1 | 0 | 0 | 1 | 0 | 0 | 0 | 0 | 0 | 176 | 191 |  |  | PEAKS DB |
| total 6 peptides |  |  |  |  |  |  |  |  |  |  |  |  |  |  |  |  |  |  |  |  |  |  |  |  |  |  |  |  |  |  |  |  |  |
| --- | --- | --- | --- | --- | --- | --- | --- | --- | --- | --- | --- | --- | --- | --- | --- | --- | --- | --- | --- | --- | --- | --- | --- | --- | --- | --- | --- | --- | --- | --- | --- | --- | --- |

XP\_026529665.1

back to list

  

| Protein Coverage
| Supporting Peptides
|

Protein Coverage:

Supporting Peptides:

| Peptide | Uniq | -10lgP | Mass | Length | ppm | m/z | z | RT | Fraction | Scan | Source File | Area F6 | Area F2 | Area F3 | Area F4 | Area F5 | Area F7 | Area F8 | Area F1 | #Feature | #Feature F6 | #Feature F2 | #Feature F3 | #Feature F4 | #Feature F5 | #Feature F7 | #Feature F8 | #Feature F1 | Start | End | PTM | AScore | Found By |
| --- | --- | --- | --- | --- | --- | --- | --- | --- | --- | --- | --- | --- | --- | --- | --- | --- | --- | --- | --- | --- | --- | --- | --- | --- | --- | --- | --- | --- | --- | --- | --- | --- | --- |
| K.LALDIEIATYR.K | N | 78.30 | 1276.7026 | 11 | 0.6 | 639.3590 | 2 | 52.72 | 8 | F8:30857 | 31122021\_RID\_3262\_1b.raw |  | 1.2925E6 | 1.0345E7 | 2.8112E5 | 1.0579E6 |  |  | 1.4292E7 | 10 | 0 | 1 | 1 | 1 | 1 | 0 | 0 | 6 | 382 | 392 |  |  | PEAKS DB |
| R.EYQELMNVK.L | N | 60.69 | 1152.5485 | 9 | 0.3 | 577.2817 | 2 | 33.98 | 8 | F8:15978 | 31122021\_RID\_3262\_1b.raw |  |  | 1.0316E6 |  |  |  |  | 1.0554E6 | 2 | 0 | 0 | 1 | 0 | 0 | 0 | 0 | 1 | 373 | 381 |  |  | PEAKS DB |
| S.VILSMDNNR.S | N | 56.96 | 1060.5336 | 9 | 0.4 | 531.2743 | 2 | 30.70 | 8 | F8:13454 | 31122021\_RID\_3262\_1b.raw |  |  |  |  |  |  |  | 5.6605E5 | 1 | 0 | 0 | 0 | 0 | 0 | 0 | 0 | 1 | 244 | 252 |  |  | PEAKS DB |
| R.EYQELM(+15.99)NVK.L | N | 52.63 | 1168.5435 | 9 | 0.3 | 585.2792 | 2 | 28.16 | 3 | F3:13441 | 13012022\_RID\_3262\_3b.raw |  |  | 1.8851E6 |  |  |  |  | 1.7223E5 | 2 | 0 | 0 | 1 | 0 | 0 | 0 | 0 | 1 | 373 | 381 | Oxidation (M) | M6:Oxidation (M):1000.00 | PEAKS DB |
| R.SLDLDGIIAEVK.A | Y | 48.24 | 1271.6973 | 12 | -0.1 | 636.8558 | 2 | 60.54 | 8 | F8:36788 | 31122021\_RID\_3262\_1b.raw |  |  |  |  |  |  |  | 2.1862E5 | 1 | 0 | 0 | 0 | 0 | 0 | 0 | 0 | 1 | 253 | 264 |  |  | PEAKS DB |
| L.ALDIEIATYR.K | N | 46.18 | 1163.6187 | 10 | -0.4 | 582.8163 | 2 | 45.58 | 8 | F8:25162 | 31122021\_RID\_3262\_1b.raw |  |  |  |  |  |  |  | 2.943E5 | 1 | 0 | 0 | 0 | 0 | 0 | 0 | 0 | 1 | 383 | 392 |  |  | PEAKS DB |
| A.LDIEIATYR.K | N | 45.35 | 1092.5815 | 9 | 0.0 | 547.2980 | 2 | 44.78 | 8 | F8:24515 | 31122021\_RID\_3262\_1b.raw |  |  |  |  |  |  |  | 3.5731E5 | 1 | 0 | 0 | 0 | 0 | 0 | 0 | 0 | 1 | 384 | 392 |  |  | PEAKS DB |
| total 7 peptides |  |  |  |  |  |  |  |  |  |  |  |  |  |  |  |  |  |  |  |  |  |  |  |  |  |  |  |  |  |  |  |  |  |
| --- | --- | --- | --- | --- | --- | --- | --- | --- | --- | --- | --- | --- | --- | --- | --- | --- | --- | --- | --- | --- | --- | --- | --- | --- | --- | --- | --- | --- | --- | --- | --- | --- | --- |

JAG45871.1

back to list

  

| Protein Coverage
| Supporting Peptides
|

Protein Coverage:

Supporting Peptides:

| Peptide | Uniq | -10lgP | Mass | Length | ppm | m/z | z | RT | Fraction | Scan | Source File | Area F6 | Area F2 | Area F3 | Area F4 | Area F5 | Area F7 | Area F8 | Area F1 | #Feature | #Feature F6 | #Feature F2 | #Feature F3 | #Feature F4 | #Feature F5 | #Feature F7 | #Feature F8 | #Feature F1 | Start | End | PTM | AScore | Found By |
| --- | --- | --- | --- | --- | --- | --- | --- | --- | --- | --- | --- | --- | --- | --- | --- | --- | --- | --- | --- | --- | --- | --- | --- | --- | --- | --- | --- | --- | --- | --- | --- | --- | --- |
| K.LLATTQMQAADAR.K | N | 76.07 | 1388.7081 | 13 | -1.1 | 695.3606 | 2 | 31.19 | 8 | F8:13875 | 31122021\_RID\_3262\_1b.raw |  |  |  |  |  |  |  | 1.964E5 | 1 | 0 | 0 | 0 | 0 | 0 | 0 | 0 | 1 | 219 | 231 |  |  | PEAKS DB |
| R.DNADVGFGSATR.A | N | 73.48 | 1208.5421 | 12 | 0.6 | 605.2787 | 2 | 30.11 | 8 | F8:13029 | 31122021\_RID\_3262\_1b.raw |  |  |  |  |  |  |  | 9.9736E4 | 1 | 0 | 0 | 0 | 0 | 0 | 0 | 0 | 1 | 938 | 949 |  |  | PEAKS DB |
| R.YLQYTLDPSK.I | Y | 57.83 | 1226.6183 | 10 | 0.1 | 614.3165 | 2 | 37.73 | 8 | F8:18976 | 31122021\_RID\_3262\_1b.raw |  |  |  |  |  |  |  | 1.404E5 | 1 | 0 | 0 | 0 | 0 | 0 | 0 | 0 | 1 | 858 | 867 |  |  | PEAKS DB |
| R.AQIIDDAFNLAR.A | N | 49.61 | 1345.6990 | 12 | 0.1 | 673.8568 | 2 | 49.86 | 8 | F8:28464 | 31122021\_RID\_3262\_1b.raw |  |  |  |  |  |  |  | 3.4019E5 | 1 | 0 | 0 | 0 | 0 | 0 | 0 | 0 | 1 | 673 | 684 |  |  | PEAKS DB |
| K.TLFQQFGR.S | N | 45.92 | 995.5189 | 8 | 0.5 | 498.7670 | 2 | 39.36 | 8 | F8:20462 | 31122021\_RID\_3262\_1b.raw |  |  |  |  |  |  |  | 9.541E4 | 1 | 0 | 0 | 0 | 0 | 0 | 0 | 0 | 1 | 900 | 907 |  |  | PEAKS DB |
| total 5 peptides |  |  |  |  |  |  |  |  |  |  |  |  |  |  |  |  |  |  |  |  |  |  |  |  |  |  |  |  |  |  |  |  |  |
| --- | --- | --- | --- | --- | --- | --- | --- | --- | --- | --- | --- | --- | --- | --- | --- | --- | --- | --- | --- | --- | --- | --- | --- | --- | --- | --- | --- | --- | --- | --- | --- | --- | --- |

JAA97801.1

back to list

  

| Protein Coverage
| Supporting Peptides
|

Protein Coverage:

Supporting Peptides:

| Peptide | Uniq | -10lgP | Mass | Length | ppm | m/z | z | RT | Fraction | Scan | Source File | Area F6 | Area F2 | Area F3 | Area F4 | Area F5 | Area F7 | Area F8 | Area F1 | #Feature | #Feature F6 | #Feature F2 | #Feature F3 | #Feature F4 | #Feature F5 | #Feature F7 | #Feature F8 | #Feature F1 | Start | End | PTM | AScore | Found By |
| --- | --- | --- | --- | --- | --- | --- | --- | --- | --- | --- | --- | --- | --- | --- | --- | --- | --- | --- | --- | --- | --- | --- | --- | --- | --- | --- | --- | --- | --- | --- | --- | --- | --- |
| K.LLATTQMQAADAR.K | N | 76.07 | 1388.7081 | 13 | -1.1 | 695.3606 | 2 | 31.19 | 8 | F8:13875 | 31122021\_RID\_3262\_1b.raw |  |  |  |  |  |  |  | 1.964E5 | 1 | 0 | 0 | 0 | 0 | 0 | 0 | 0 | 1 | 219 | 231 |  |  | PEAKS DB |
| R.DNADVGFGSATR.A | N | 73.48 | 1208.5421 | 12 | 0.6 | 605.2787 | 2 | 30.11 | 8 | F8:13029 | 31122021\_RID\_3262\_1b.raw |  |  |  |  |  |  |  | 9.9736E4 | 1 | 0 | 0 | 0 | 0 | 0 | 0 | 0 | 1 | 938 | 949 |  |  | PEAKS DB |
| R.YLQYTLDPSK.I | Y | 57.83 | 1226.6183 | 10 | 0.1 | 614.3165 | 2 | 37.73 | 8 | F8:18976 | 31122021\_RID\_3262\_1b.raw |  |  |  |  |  |  |  | 1.404E5 | 1 | 0 | 0 | 0 | 0 | 0 | 0 | 0 | 1 | 858 | 867 |  |  | PEAKS DB |
| R.AQIIDDAFNLAR.A | N | 49.61 | 1345.6990 | 12 | 0.1 | 673.8568 | 2 | 49.86 | 8 | F8:28464 | 31122021\_RID\_3262\_1b.raw |  |  |  |  |  |  |  | 3.4019E5 | 1 | 0 | 0 | 0 | 0 | 0 | 0 | 0 | 1 | 673 | 684 |  |  | PEAKS DB |
| K.TLFQQFGR.S | N | 45.92 | 995.5189 | 8 | 0.5 | 498.7670 | 2 | 39.36 | 8 | F8:20462 | 31122021\_RID\_3262\_1b.raw |  |  |  |  |  |  |  | 9.541E4 | 1 | 0 | 0 | 0 | 0 | 0 | 0 | 0 | 1 | 900 | 907 |  |  | PEAKS DB |
| total 5 peptides |  |  |  |  |  |  |  |  |  |  |  |  |  |  |  |  |  |  |  |  |  |  |  |  |  |  |  |  |  |  |  |  |  |
| --- | --- | --- | --- | --- | --- | --- | --- | --- | --- | --- | --- | --- | --- | --- | --- | --- | --- | --- | --- | --- | --- | --- | --- | --- | --- | --- | --- | --- | --- | --- | --- | --- | --- |

JAG47501.1

back to list

  

| Protein Coverage
| Supporting Peptides
|

Protein Coverage:

Supporting Peptides:

| Peptide | Uniq | -10lgP | Mass | Length | ppm | m/z | z | RT | Fraction | Scan | Source File | Area F6 | Area F2 | Area F3 | Area F4 | Area F5 | Area F7 | Area F8 | Area F1 | #Feature | #Feature F6 | #Feature F2 | #Feature F3 | #Feature F4 | #Feature F5 | #Feature F7 | #Feature F8 | #Feature F1 | Start | End | PTM | AScore | Found By |
| --- | --- | --- | --- | --- | --- | --- | --- | --- | --- | --- | --- | --- | --- | --- | --- | --- | --- | --- | --- | --- | --- | --- | --- | --- | --- | --- | --- | --- | --- | --- | --- | --- | --- |
| K.LLATTQMQAADAR.K | N | 76.07 | 1388.7081 | 13 | -1.1 | 695.3606 | 2 | 31.19 | 8 | F8:13875 | 31122021\_RID\_3262\_1b.raw |  |  |  |  |  |  |  | 1.964E5 | 1 | 0 | 0 | 0 | 0 | 0 | 0 | 0 | 1 | 219 | 231 |  |  | PEAKS DB |
| R.DNADVGFGSATR.A | N | 73.48 | 1208.5421 | 12 | 0.6 | 605.2787 | 2 | 30.11 | 8 | F8:13029 | 31122021\_RID\_3262\_1b.raw |  |  |  |  |  |  |  | 9.9736E4 | 1 | 0 | 0 | 0 | 0 | 0 | 0 | 0 | 1 | 938 | 949 |  |  | PEAKS DB |
| R.YLQYTLDPSK.I | Y | 57.83 | 1226.6183 | 10 | 0.1 | 614.3165 | 2 | 37.73 | 8 | F8:18976 | 31122021\_RID\_3262\_1b.raw |  |  |  |  |  |  |  | 1.404E5 | 1 | 0 | 0 | 0 | 0 | 0 | 0 | 0 | 1 | 858 | 867 |  |  | PEAKS DB |
| R.AQIIDDAFNLAR.A | N | 49.61 | 1345.6990 | 12 | 0.1 | 673.8568 | 2 | 49.86 | 8 | F8:28464 | 31122021\_RID\_3262\_1b.raw |  |  |  |  |  |  |  | 3.4019E5 | 1 | 0 | 0 | 0 | 0 | 0 | 0 | 0 | 1 | 673 | 684 |  |  | PEAKS DB |
| K.TLFQQFGR.S | N | 45.92 | 995.5189 | 8 | 0.5 | 498.7670 | 2 | 39.36 | 8 | F8:20462 | 31122021\_RID\_3262\_1b.raw |  |  |  |  |  |  |  | 9.541E4 | 1 | 0 | 0 | 0 | 0 | 0 | 0 | 0 | 1 | 900 | 907 |  |  | PEAKS DB |
| total 5 peptides |  |  |  |  |  |  |  |  |  |  |  |  |  |  |  |  |  |  |  |  |  |  |  |  |  |  |  |  |  |  |  |  |  |
| --- | --- | --- | --- | --- | --- | --- | --- | --- | --- | --- | --- | --- | --- | --- | --- | --- | --- | --- | --- | --- | --- | --- | --- | --- | --- | --- | --- | --- | --- | --- | --- | --- | --- |

XP\_026549343.1

back to list

  

| Protein Coverage
| Supporting Peptides
|

Protein Coverage:

Supporting Peptides:

| Peptide | Uniq | -10lgP | Mass | Length | ppm | m/z | z | RT | Fraction | Scan | Source File | Area F6 | Area F2 | Area F3 | Area F4 | Area F5 | Area F7 | Area F8 | Area F1 | #Feature | #Feature F6 | #Feature F2 | #Feature F3 | #Feature F4 | #Feature F5 | #Feature F7 | #Feature F8 | #Feature F1 | Start | End | PTM | AScore | Found By |
| --- | --- | --- | --- | --- | --- | --- | --- | --- | --- | --- | --- | --- | --- | --- | --- | --- | --- | --- | --- | --- | --- | --- | --- | --- | --- | --- | --- | --- | --- | --- | --- | --- | --- |
| K.EGWYVNLGPM(+15.99)R.L | N | 75.80 | 1336.6234 | 11 | 0.3 | 669.3192 | 2 | 45.61 | 3 | F3:28662 | 13012022\_RID\_3262\_3b.raw |  |  | 1.1921E7 |  |  |  |  |  | 2 | 0 | 0 | 2 | 0 | 0 | 0 | 0 | 0 | 6 | 16 | Oxidation (M) | M10:Oxidation (M):1000.00 | PEAKS DB |
| K.YPVKPSEEGK.S | N | 73.79 | 1132.5764 | 10 | 0.2 | 567.2956 | 2 | 18.85 | 3 | F3:5905 | 13012022\_RID\_3262\_3b.raw | 1.3691E4 |  | 4.5878E8 | 1.2567E7 |  |  |  |  | 5 | 1 | 0 | 2 | 2 | 0 | 0 | 0 | 0 | 68 | 77 |  |  | PEAKS DB |
| K.EGWYVNLGPMR.L | N | 70.02 | 1320.6284 | 11 | 0.4 | 661.3218 | 2 | 50.89 | 3 | F3:33016 | 13012022\_RID\_3262\_3b.raw |  |  | 1.2688E7 |  |  |  |  | 9.1762E4 | 2 | 0 | 0 | 1 | 0 | 0 | 0 | 0 | 1 | 6 | 16 |  |  | PEAKS DB |
| K.EGWYVNLGPM(+15.99)RLPEK.H | Y | 60.25 | 1803.8977 | 15 | 0.8 | 602.3070 | 3 | 43.72 | 3 | F3:27022 | 13012022\_RID\_3262\_3b.raw |  |  | 8.1831E6 |  |  |  |  |  | 2 | 0 | 0 | 2 | 0 | 0 | 0 | 0 | 0 | 6 | 20 | Oxidation (M) | M10:Oxidation (M):1000.00 | PEAKS DB |
| W.YVNLGPM(+15.99)RLPEK.H | Y | 51.77 | 1431.7544 | 12 | 0.4 | 716.8848 | 2 | 30.93 | 3 | F3:16035 | 13012022\_RID\_3262\_3b.raw |  |  | 2.7353E6 |  |  |  |  |  | 1 | 0 | 0 | 1 | 0 | 0 | 0 | 0 | 0 | 9 | 20 | Oxidation (M) | M7:Oxidation (M):1000.00 | PEAKS DB |
| total 5 peptides |  |  |  |  |  |  |  |  |  |  |  |  |  |  |  |  |  |  |  |  |  |  |  |  |  |  |  |  |  |  |  |  |  |
| --- | --- | --- | --- | --- | --- | --- | --- | --- | --- | --- | --- | --- | --- | --- | --- | --- | --- | --- | --- | --- | --- | --- | --- | --- | --- | --- | --- | --- | --- | --- | --- | --- | --- |

XP\_015667388.1

back to list

  

| Protein Coverage
| Supporting Peptides
|

Protein Coverage:

Supporting Peptides:

| Peptide | Uniq | -10lgP | Mass | Length | ppm | m/z | z | RT | Fraction | Scan | Source File | Area F6 | Area F2 | Area F3 | Area F4 | Area F5 | Area F7 | Area F8 | Area F1 | #Feature | #Feature F6 | #Feature F2 | #Feature F3 | #Feature F4 | #Feature F5 | #Feature F7 | #Feature F8 | #Feature F1 | Start | End | PTM | AScore | Found By |
| --- | --- | --- | --- | --- | --- | --- | --- | --- | --- | --- | --- | --- | --- | --- | --- | --- | --- | --- | --- | --- | --- | --- | --- | --- | --- | --- | --- | --- | --- | --- | --- | --- | --- |
| K.LLATTQMQAADAR.K | N | 76.07 | 1388.7081 | 13 | -1.1 | 695.3606 | 2 | 31.19 | 8 | F8:13875 | 31122021\_RID\_3262\_1b.raw |  |  |  |  |  |  |  | 1.964E5 | 1 | 0 | 0 | 0 | 0 | 0 | 0 | 0 | 1 | 215 | 227 |  |  | PEAKS DB |
| K.DNADVGFGSATR.A | N | 73.48 | 1208.5421 | 12 | 0.6 | 605.2787 | 2 | 30.11 | 8 | F8:13029 | 31122021\_RID\_3262\_1b.raw |  |  |  |  |  |  |  | 9.9736E4 | 1 | 0 | 0 | 0 | 0 | 0 | 0 | 0 | 1 | 934 | 945 |  |  | PEAKS DB |
| R.AQIIDDAFNLAR.A | N | 49.61 | 1345.6990 | 12 | 0.1 | 673.8568 | 2 | 49.86 | 8 | F8:28464 | 31122021\_RID\_3262\_1b.raw |  |  |  |  |  |  |  | 3.4019E5 | 1 | 0 | 0 | 0 | 0 | 0 | 0 | 0 | 1 | 669 | 680 |  |  | PEAKS DB |
| R.ESALLFDQEYSSIGNK.E | Y | 46.90 | 1799.8577 | 16 | 1.1 | 900.9371 | 2 | 48.18 | 8 | F8:27159 | 31122021\_RID\_3262\_1b.raw |  |  |  |  |  |  |  | 2.3746E5 | 1 | 0 | 0 | 0 | 0 | 0 | 0 | 0 | 1 | 373 | 388 |  |  | PEAKS DB |
| K.TLFQQFGR.S | N | 45.92 | 995.5189 | 8 | 0.5 | 498.7670 | 2 | 39.36 | 8 | F8:20462 | 31122021\_RID\_3262\_1b.raw |  |  |  |  |  |  |  | 9.541E4 | 1 | 0 | 0 | 0 | 0 | 0 | 0 | 0 | 1 | 896 | 903 |  |  | PEAKS DB |
| total 5 peptides |  |  |  |  |  |  |  |  |  |  |  |  |  |  |  |  |  |  |  |  |  |  |  |  |  |  |  |  |  |  |  |  |  |
| --- | --- | --- | --- | --- | --- | --- | --- | --- | --- | --- | --- | --- | --- | --- | --- | --- | --- | --- | --- | --- | --- | --- | --- | --- | --- | --- | --- | --- | --- | --- | --- | --- | --- |

JAS04411.1

back to list

  

| Protein Coverage
| Supporting Peptides
|

Protein Coverage:

Supporting Peptides:

| Peptide | Uniq | -10lgP | Mass | Length | ppm | m/z | z | RT | Fraction | Scan | Source File | Area F6 | Area F2 | Area F3 | Area F4 | Area F5 | Area F7 | Area F8 | Area F1 | #Feature | #Feature F6 | #Feature F2 | #Feature F3 | #Feature F4 | #Feature F5 | #Feature F7 | #Feature F8 | #Feature F1 | Start | End | PTM | AScore | Found By |
| --- | --- | --- | --- | --- | --- | --- | --- | --- | --- | --- | --- | --- | --- | --- | --- | --- | --- | --- | --- | --- | --- | --- | --- | --- | --- | --- | --- | --- | --- | --- | --- | --- | --- |
| K.NVPNEDEQTR.V | Y | 72.09 | 1200.5371 | 10 | 0.3 | 601.2760 | 2 | 17.92 | 4 | F4:5401 | 13012022\_RID\_3262\_4b.raw |  |  | 2.8599E5 | 1.6185E6 |  |  |  |  | 3 | 0 | 0 | 1 | 2 | 0 | 0 | 0 | 0 | 81 | 90 |  |  | PEAKS DB |
| K.NVPNEDEQTRVPK.E | Y | 70.53 | 1524.7532 | 13 | 0.5 | 763.3842 | 2 | 21.59 | 4 | F4:8692 | 13012022\_RID\_3262\_4b.raw |  |  | 5.0341E6 | 5.2959E6 |  |  |  |  | 4 | 0 | 0 | 2 | 2 | 0 | 0 | 0 | 0 | 81 | 93 |  |  | PEAKS DB |
| K.WDKDIMLIR.L | N | 59.44 | 1188.6324 | 9 | -0.6 | 595.3231 | 2 | 42.59 | 3 | F3:26121 | 13012022\_RID\_3262\_3b.raw |  |  | 7.7454E7 | 4.9887E6 |  |  |  | 2.131E6 | 5 | 0 | 0 | 2 | 2 | 0 | 0 | 0 | 1 | 107 | 115 |  |  | PEAKS DB |
| R.EPGLYTK.V | N | 57.24 | 806.4174 | 7 | 0.7 | 404.2162 | 2 | 23.91 | 4 | F4:10666 | 13012022\_RID\_3262\_4b.raw |  | 1.5277E6 | 1.3483E7 | 4.9704E7 |  |  |  |  | 3 | 0 | 1 | 1 | 1 | 0 | 0 | 0 | 0 | 230 | 236 |  |  | PEAKS DB |
| K.WDKDIM(+15.99)LIR.L | N | 53.70 | 1204.6274 | 9 | 0.1 | 603.3210 | 2 | 37.60 | 3 | F3:21664 | 13012022\_RID\_3262\_3b.raw |  |  | 1.4633E8 | 3.3538E6 |  |  |  |  | 3 | 0 | 0 | 2 | 1 | 0 | 0 | 0 | 0 | 107 | 115 | Oxidation (M) | M6:Oxidation (M):1000.00 | PEAKS DB |
| H.FLVALYK.S | N | 50.45 | 852.5109 | 7 | 0.7 | 427.2630 | 2 | 45.19 | 4 | F4:25862 | 13012022\_RID\_3262\_4b.raw |  | 1.9685E6 | 2.3483E7 | 4.3958E7 |  |  |  |  | 3 | 0 | 1 | 1 | 1 | 0 | 0 | 0 | 0 | 38 | 44 |  |  | PEAKS DB |
| W.DKDIMLIR.L | N | 45.33 | 1002.5532 | 8 | 0.8 | 502.2842 | 2 | 34.98 | 4 | F4:18968 | 13012022\_RID\_3262\_4b.raw |  |  |  | 7.1188E5 |  |  |  |  | 1 | 0 | 0 | 0 | 1 | 0 | 0 | 0 | 0 | 108 | 115 |  |  | PEAKS DB |
| total 7 peptides |  |  |  |  |  |  |  |  |  |  |  |  |  |  |  |  |  |  |  |  |  |  |  |  |  |  |  |  |  |  |  |  |  |
| --- | --- | --- | --- | --- | --- | --- | --- | --- | --- | --- | --- | --- | --- | --- | --- | --- | --- | --- | --- | --- | --- | --- | --- | --- | --- | --- | --- | --- | --- | --- | --- | --- | --- |

XP\_032069201.1

back to list

  

| Protein Coverage
| Supporting Peptides
|

Protein Coverage:

Supporting Peptides:

| Peptide | Uniq | -10lgP | Mass | Length | ppm | m/z | z | RT | Fraction | Scan | Source File | Area F6 | Area F2 | Area F3 | Area F4 | Area F5 | Area F7 | Area F8 | Area F1 | #Feature | #Feature F6 | #Feature F2 | #Feature F3 | #Feature F4 | #Feature F5 | #Feature F7 | #Feature F8 | #Feature F1 | Start | End | PTM | AScore | Found By |
| --- | --- | --- | --- | --- | --- | --- | --- | --- | --- | --- | --- | --- | --- | --- | --- | --- | --- | --- | --- | --- | --- | --- | --- | --- | --- | --- | --- | --- | --- | --- | --- | --- | --- |
| K.LISWYDNEFGYSNR.V | Y | 87.25 | 1762.7950 | 14 | 1.5 | 882.4061 | 2 | 51.55 | 8 | F8:29892 | 31122021\_RID\_3262\_1b.raw |  |  |  |  |  |  |  | 2.1393E6 | 1 | 0 | 0 | 0 | 0 | 0 | 0 | 0 | 1 | 308 | 321 |  |  | PEAKS DB |
| R.LEKPAKYDDIKK.V | N | 58.76 | 1446.8081 | 12 | 0.0 | 483.2766 | 3 | 15.77 | 8 | F8:2947 | 31122021\_RID\_3262\_1b.raw |  |  |  |  |  |  |  | 1.4047E5 | 1 | 0 | 0 | 0 | 0 | 0 | 0 | 0 | 1 | 247 | 258 |  |  | PEAKS DB |
| K.LTGMAFR.V | N | 49.28 | 794.4109 | 7 | -0.3 | 398.2126 | 2 | 30.53 | 8 | F8:13455 | 31122021\_RID\_3262\_1b.raw |  |  |  |  |  |  |  | 1.1461E6 | 1 | 0 | 0 | 0 | 0 | 0 | 0 | 0 | 1 | 226 | 232 |  |  | PEAKS DB |
| total 3 peptides |  |  |  |  |  |  |  |  |  |  |  |  |  |  |  |  |  |  |  |  |  |  |  |  |  |  |  |  |  |  |  |  |  |
| --- | --- | --- | --- | --- | --- | --- | --- | --- | --- | --- | --- | --- | --- | --- | --- | --- | --- | --- | --- | --- | --- | --- | --- | --- | --- | --- | --- | --- | --- | --- | --- | --- | --- |

XP\_034265289.1

back to list

  

| Protein Coverage
| Supporting Peptides
|

Protein Coverage:

Supporting Peptides:

| Peptide | Uniq | -10lgP | Mass | Length | ppm | m/z | z | RT | Fraction | Scan | Source File | Area F6 | Area F2 | Area F3 | Area F4 | Area F5 | Area F7 | Area F8 | Area F1 | #Feature | #Feature F6 | #Feature F2 | #Feature F3 | #Feature F4 | #Feature F5 | #Feature F7 | #Feature F8 | #Feature F1 | Start | End | PTM | AScore | Found By |
| --- | --- | --- | --- | --- | --- | --- | --- | --- | --- | --- | --- | --- | --- | --- | --- | --- | --- | --- | --- | --- | --- | --- | --- | --- | --- | --- | --- | --- | --- | --- | --- | --- | --- |
| K.TPAQYDASELK.A | N | 72.43 | 1221.5876 | 11 | -0.5 | 611.8008 | 2 | 27.03 | 8 | F8:10630 | 31122021\_RID\_3262\_1b.raw |  |  | 2.9074E5 |  |  |  |  | 7.6887E5 | 3 | 0 | 0 | 2 | 0 | 0 | 0 | 0 | 1 | 105 | 115 |  |  | PEAKS DB |
| K.GVDEVTIVNLLTNR.S | N | 69.50 | 1541.8413 | 14 | 0.2 | 771.9281 | 2 | 59.12 | 8 | F8:35905 | 31122021\_RID\_3262\_1b.raw |  |  | 1.6481E5 |  |  |  |  | 1.3768E6 | 2 | 0 | 0 | 1 | 0 | 0 | 0 | 0 | 1 | 50 | 63 |  |  | PEAKS DB |
| K.DIISDTSGDFR.K | Y | 62.86 | 1224.5623 | 11 | -0.5 | 613.2881 | 2 | 42.92 | 8 | F8:23088 | 31122021\_RID\_3262\_1b.raw |  |  |  |  |  |  |  | 2.1385E5 | 1 | 0 | 0 | 0 | 0 | 0 | 0 | 0 | 1 | 158 | 168 |  |  | PEAKS DB |
| R.QDIAFAYQR.R | N | 55.17 | 1110.5458 | 9 | 0.6 | 556.2805 | 2 | 34.91 | 8 | F8:16733 | 31122021\_RID\_3262\_1b.raw |  |  | 1.0819E5 |  |  |  |  | 3.9407E5 | 2 | 0 | 0 | 1 | 0 | 0 | 0 | 0 | 1 | 69 | 77 |  |  | PEAKS DB |
| total 4 peptides |  |  |  |  |  |  |  |  |  |  |  |  |  |  |  |  |  |  |  |  |  |  |  |  |  |  |  |  |  |  |  |  |  |
| --- | --- | --- | --- | --- | --- | --- | --- | --- | --- | --- | --- | --- | --- | --- | --- | --- | --- | --- | --- | --- | --- | --- | --- | --- | --- | --- | --- | --- | --- | --- | --- | --- | --- |

JAI10286.1

back to list

  

| Protein Coverage
| Supporting Peptides
|

Protein Coverage:

Supporting Peptides:

| Peptide | Uniq | -10lgP | Mass | Length | ppm | m/z | z | RT | Fraction | Scan | Source File | Area F6 | Area F2 | Area F3 | Area F4 | Area F5 | Area F7 | Area F8 | Area F1 | #Feature | #Feature F6 | #Feature F2 | #Feature F3 | #Feature F4 | #Feature F5 | #Feature F7 | #Feature F8 | #Feature F1 | Start | End | PTM | AScore | Found By |
| --- | --- | --- | --- | --- | --- | --- | --- | --- | --- | --- | --- | --- | --- | --- | --- | --- | --- | --- | --- | --- | --- | --- | --- | --- | --- | --- | --- | --- | --- | --- | --- | --- | --- |
| K.TPAQYDASELK.A | N | 72.43 | 1221.5876 | 11 | -0.5 | 611.8008 | 2 | 27.03 | 8 | F8:10630 | 31122021\_RID\_3262\_1b.raw |  |  | 2.9074E5 |  |  |  |  | 7.6887E5 | 3 | 0 | 0 | 2 | 0 | 0 | 0 | 0 | 1 | 105 | 115 |  |  | PEAKS DB |
| K.GVDEVTIVNLLTNR.S | N | 69.50 | 1541.8413 | 14 | 0.2 | 771.9281 | 2 | 59.12 | 8 | F8:35905 | 31122021\_RID\_3262\_1b.raw |  |  | 1.6481E5 |  |  |  |  | 1.3768E6 | 2 | 0 | 0 | 1 | 0 | 0 | 0 | 0 | 1 | 50 | 63 |  |  | PEAKS DB |
| K.DIISDTSGDFR.K | Y | 62.86 | 1224.5623 | 11 | -0.5 | 613.2881 | 2 | 42.92 | 8 | F8:23088 | 31122021\_RID\_3262\_1b.raw |  |  |  |  |  |  |  | 2.1385E5 | 1 | 0 | 0 | 0 | 0 | 0 | 0 | 0 | 1 | 158 | 168 |  |  | PEAKS DB |
| R.QDIAFAYQR.R | N | 55.17 | 1110.5458 | 9 | 0.6 | 556.2805 | 2 | 34.91 | 8 | F8:16733 | 31122021\_RID\_3262\_1b.raw |  |  | 1.0819E5 |  |  |  |  | 3.9407E5 | 2 | 0 | 0 | 1 | 0 | 0 | 0 | 0 | 1 | 69 | 77 |  |  | PEAKS DB |
| total 4 peptides |  |  |  |  |  |  |  |  |  |  |  |  |  |  |  |  |  |  |  |  |  |  |  |  |  |  |  |  |  |  |  |  |  |
| --- | --- | --- | --- | --- | --- | --- | --- | --- | --- | --- | --- | --- | --- | --- | --- | --- | --- | --- | --- | --- | --- | --- | --- | --- | --- | --- | --- | --- | --- | --- | --- | --- | --- |

LAB40267.1

back to list

  

| Protein Coverage
| Supporting Peptides
|

Protein Coverage:

Supporting Peptides:

| Peptide | Uniq | -10lgP | Mass | Length | ppm | m/z | z | RT | Fraction | Scan | Source File | Area F6 | Area F2 | Area F3 | Area F4 | Area F5 | Area F7 | Area F8 | Area F1 | #Feature | #Feature F6 | #Feature F2 | #Feature F3 | #Feature F4 | #Feature F5 | #Feature F7 | #Feature F8 | #Feature F1 | Start | End | PTM | AScore | Found By |
| --- | --- | --- | --- | --- | --- | --- | --- | --- | --- | --- | --- | --- | --- | --- | --- | --- | --- | --- | --- | --- | --- | --- | --- | --- | --- | --- | --- | --- | --- | --- | --- | --- | --- |
| K.TPAQYDASELK.A | N | 72.43 | 1221.5876 | 11 | -0.5 | 611.8008 | 2 | 27.03 | 8 | F8:10630 | 31122021\_RID\_3262\_1b.raw |  |  | 2.9074E5 |  |  |  |  | 7.6887E5 | 3 | 0 | 0 | 2 | 0 | 0 | 0 | 0 | 1 | 113 | 123 |  |  | PEAKS DB |
| K.GVDEVTIVNLLTNR.S | N | 69.50 | 1541.8413 | 14 | 0.2 | 771.9281 | 2 | 59.12 | 8 | F8:35905 | 31122021\_RID\_3262\_1b.raw |  |  | 1.6481E5 |  |  |  |  | 1.3768E6 | 2 | 0 | 0 | 1 | 0 | 0 | 0 | 0 | 1 | 58 | 71 |  |  | PEAKS DB |
| K.DIISDTSGDFR.K | Y | 62.86 | 1224.5623 | 11 | -0.5 | 613.2881 | 2 | 42.92 | 8 | F8:23088 | 31122021\_RID\_3262\_1b.raw |  |  |  |  |  |  |  | 2.1385E5 | 1 | 0 | 0 | 0 | 0 | 0 | 0 | 0 | 1 | 166 | 176 |  |  | PEAKS DB |
| R.QDIAFAYQR.R | N | 55.17 | 1110.5458 | 9 | 0.6 | 556.2805 | 2 | 34.91 | 8 | F8:16733 | 31122021\_RID\_3262\_1b.raw |  |  | 1.0819E5 |  |  |  |  | 3.9407E5 | 2 | 0 | 0 | 1 | 0 | 0 | 0 | 0 | 1 | 77 | 85 |  |  | PEAKS DB |
| total 4 peptides |  |  |  |  |  |  |  |  |  |  |  |  |  |  |  |  |  |  |  |  |  |  |  |  |  |  |  |  |  |  |  |  |  |
| --- | --- | --- | --- | --- | --- | --- | --- | --- | --- | --- | --- | --- | --- | --- | --- | --- | --- | --- | --- | --- | --- | --- | --- | --- | --- | --- | --- | --- | --- | --- | --- | --- | --- |

XP\_032089545.1

back to list

  

| Protein Coverage
| Supporting Peptides
|

Protein Coverage:

Supporting Peptides:

| Peptide | Uniq | -10lgP | Mass | Length | ppm | m/z | z | RT | Fraction | Scan | Source File | Area F6 | Area F2 | Area F3 | Area F4 | Area F5 | Area F7 | Area F8 | Area F1 | #Feature | #Feature F6 | #Feature F2 | #Feature F3 | #Feature F4 | #Feature F5 | #Feature F7 | #Feature F8 | #Feature F1 | Start | End | PTM | AScore | Found By |
| --- | --- | --- | --- | --- | --- | --- | --- | --- | --- | --- | --- | --- | --- | --- | --- | --- | --- | --- | --- | --- | --- | --- | --- | --- | --- | --- | --- | --- | --- | --- | --- | --- | --- |
| K.TPAQYDASELK.G | N | 72.43 | 1221.5876 | 11 | -0.5 | 611.8008 | 2 | 27.03 | 8 | F8:10630 | 31122021\_RID\_3262\_1b.raw |  |  | 2.9074E5 |  |  |  |  | 7.6887E5 | 3 | 0 | 0 | 2 | 0 | 0 | 0 | 0 | 1 | 105 | 115 |  |  | PEAKS DB |
| K.GVDEVTIVNLLTNR.S | N | 69.50 | 1541.8413 | 14 | 0.2 | 771.9281 | 2 | 59.12 | 8 | F8:35905 | 31122021\_RID\_3262\_1b.raw |  |  | 1.6481E5 |  |  |  |  | 1.3768E6 | 2 | 0 | 0 | 1 | 0 | 0 | 0 | 0 | 1 | 50 | 63 |  |  | PEAKS DB |
| K.DIISDTSGDFR.K | Y | 62.86 | 1224.5623 | 11 | -0.5 | 613.2881 | 2 | 42.92 | 8 | F8:23088 | 31122021\_RID\_3262\_1b.raw |  |  |  |  |  |  |  | 2.1385E5 | 1 | 0 | 0 | 0 | 0 | 0 | 0 | 0 | 1 | 158 | 168 |  |  | PEAKS DB |
| R.QDIAFAYQR.R | N | 55.17 | 1110.5458 | 9 | 0.6 | 556.2805 | 2 | 34.91 | 8 | F8:16733 | 31122021\_RID\_3262\_1b.raw |  |  | 1.0819E5 |  |  |  |  | 3.9407E5 | 2 | 0 | 0 | 1 | 0 | 0 | 0 | 0 | 1 | 69 | 77 |  |  | PEAKS DB |
| total 4 peptides |  |  |  |  |  |  |  |  |  |  |  |  |  |  |  |  |  |  |  |  |  |  |  |  |  |  |  |  |  |  |  |  |  |
| --- | --- | --- | --- | --- | --- | --- | --- | --- | --- | --- | --- | --- | --- | --- | --- | --- | --- | --- | --- | --- | --- | --- | --- | --- | --- | --- | --- | --- | --- | --- | --- | --- | --- |

XP\_026579588.1

back to list

  

| Protein Coverage
| Supporting Peptides
|

Protein Coverage:

Supporting Peptides:

| Peptide | Uniq | -10lgP | Mass | Length | ppm | m/z | z | RT | Fraction | Scan | Source File | Area F6 | Area F2 | Area F3 | Area F4 | Area F5 | Area F7 | Area F8 | Area F1 | #Feature | #Feature F6 | #Feature F2 | #Feature F3 | #Feature F4 | #Feature F5 | #Feature F7 | #Feature F8 | #Feature F1 | Start | End | PTM | AScore | Found By |
| --- | --- | --- | --- | --- | --- | --- | --- | --- | --- | --- | --- | --- | --- | --- | --- | --- | --- | --- | --- | --- | --- | --- | --- | --- | --- | --- | --- | --- | --- | --- | --- | --- | --- |
| K.TPAQYDASELK.A | N | 72.43 | 1221.5876 | 11 | -0.5 | 611.8008 | 2 | 27.03 | 8 | F8:10630 | 31122021\_RID\_3262\_1b.raw |  |  | 2.9074E5 |  |  |  |  | 7.6887E5 | 3 | 0 | 0 | 2 | 0 | 0 | 0 | 0 | 1 | 105 | 115 |  |  | PEAKS DB |
| K.GVDEVTIVNLLTNR.S | N | 69.50 | 1541.8413 | 14 | 0.2 | 771.9281 | 2 | 59.12 | 8 | F8:35905 | 31122021\_RID\_3262\_1b.raw |  |  | 1.6481E5 |  |  |  |  | 1.3768E6 | 2 | 0 | 0 | 1 | 0 | 0 | 0 | 0 | 1 | 50 | 63 |  |  | PEAKS DB |
| K.DIISDTSGDFR.K | Y | 62.86 | 1224.5623 | 11 | -0.5 | 613.2881 | 2 | 42.92 | 8 | F8:23088 | 31122021\_RID\_3262\_1b.raw |  |  |  |  |  |  |  | 2.1385E5 | 1 | 0 | 0 | 0 | 0 | 0 | 0 | 0 | 1 | 158 | 168 |  |  | PEAKS DB |
| R.QDIAFAYQR.R | N | 55.17 | 1110.5458 | 9 | 0.6 | 556.2805 | 2 | 34.91 | 8 | F8:16733 | 31122021\_RID\_3262\_1b.raw |  |  | 1.0819E5 |  |  |  |  | 3.9407E5 | 2 | 0 | 0 | 1 | 0 | 0 | 0 | 0 | 1 | 69 | 77 |  |  | PEAKS DB |
| total 4 peptides |  |  |  |  |  |  |  |  |  |  |  |  |  |  |  |  |  |  |  |  |  |  |  |  |  |  |  |  |  |  |  |  |  |
| --- | --- | --- | --- | --- | --- | --- | --- | --- | --- | --- | --- | --- | --- | --- | --- | --- | --- | --- | --- | --- | --- | --- | --- | --- | --- | --- | --- | --- | --- | --- | --- | --- | --- |

XP\_026579587.1

back to list

  

| Protein Coverage
| Supporting Peptides
|

Protein Coverage:

Supporting Peptides:

| Peptide | Uniq | -10lgP | Mass | Length | ppm | m/z | z | RT | Fraction | Scan | Source File | Area F6 | Area F2 | Area F3 | Area F4 | Area F5 | Area F7 | Area F8 | Area F1 | #Feature | #Feature F6 | #Feature F2 | #Feature F3 | #Feature F4 | #Feature F5 | #Feature F7 | #Feature F8 | #Feature F1 | Start | End | PTM | AScore | Found By |
| --- | --- | --- | --- | --- | --- | --- | --- | --- | --- | --- | --- | --- | --- | --- | --- | --- | --- | --- | --- | --- | --- | --- | --- | --- | --- | --- | --- | --- | --- | --- | --- | --- | --- |
| K.TPAQYDASELK.A | N | 72.43 | 1221.5876 | 11 | -0.5 | 611.8008 | 2 | 27.03 | 8 | F8:10630 | 31122021\_RID\_3262\_1b.raw |  |  | 2.9074E5 |  |  |  |  | 7.6887E5 | 3 | 0 | 0 | 2 | 0 | 0 | 0 | 0 | 1 | 105 | 115 |  |  | PEAKS DB |
| K.GVDEVTIVNLLTNR.S | N | 69.50 | 1541.8413 | 14 | 0.2 | 771.9281 | 2 | 59.12 | 8 | F8:35905 | 31122021\_RID\_3262\_1b.raw |  |  | 1.6481E5 |  |  |  |  | 1.3768E6 | 2 | 0 | 0 | 1 | 0 | 0 | 0 | 0 | 1 | 50 | 63 |  |  | PEAKS DB |
| K.DIISDTSGDFR.K | Y | 62.86 | 1224.5623 | 11 | -0.5 | 613.2881 | 2 | 42.92 | 8 | F8:23088 | 31122021\_RID\_3262\_1b.raw |  |  |  |  |  |  |  | 2.1385E5 | 1 | 0 | 0 | 0 | 0 | 0 | 0 | 0 | 1 | 158 | 168 |  |  | PEAKS DB |
| R.QDIAFAYQR.R | N | 55.17 | 1110.5458 | 9 | 0.6 | 556.2805 | 2 | 34.91 | 8 | F8:16733 | 31122021\_RID\_3262\_1b.raw |  |  | 1.0819E5 |  |  |  |  | 3.9407E5 | 2 | 0 | 0 | 1 | 0 | 0 | 0 | 0 | 1 | 69 | 77 |  |  | PEAKS DB |
| total 4 peptides |  |  |  |  |  |  |  |  |  |  |  |  |  |  |  |  |  |  |  |  |  |  |  |  |  |  |  |  |  |  |  |  |  |
| --- | --- | --- | --- | --- | --- | --- | --- | --- | --- | --- | --- | --- | --- | --- | --- | --- | --- | --- | --- | --- | --- | --- | --- | --- | --- | --- | --- | --- | --- | --- | --- | --- | --- |

XP\_026579585.1

back to list

  

| Protein Coverage
| Supporting Peptides
|

Protein Coverage:

Supporting Peptides:

| Peptide | Uniq | -10lgP | Mass | Length | ppm | m/z | z | RT | Fraction | Scan | Source File | Area F6 | Area F2 | Area F3 | Area F4 | Area F5 | Area F7 | Area F8 | Area F1 | #Feature | #Feature F6 | #Feature F2 | #Feature F3 | #Feature F4 | #Feature F5 | #Feature F7 | #Feature F8 | #Feature F1 | Start | End | PTM | AScore | Found By |
| --- | --- | --- | --- | --- | --- | --- | --- | --- | --- | --- | --- | --- | --- | --- | --- | --- | --- | --- | --- | --- | --- | --- | --- | --- | --- | --- | --- | --- | --- | --- | --- | --- | --- |
| K.TPAQYDASELK.A | N | 72.43 | 1221.5876 | 11 | -0.5 | 611.8008 | 2 | 27.03 | 8 | F8:10630 | 31122021\_RID\_3262\_1b.raw |  |  | 2.9074E5 |  |  |  |  | 7.6887E5 | 3 | 0 | 0 | 2 | 0 | 0 | 0 | 0 | 1 | 105 | 115 |  |  | PEAKS DB |
| K.GVDEVTIVNLLTNR.S | N | 69.50 | 1541.8413 | 14 | 0.2 | 771.9281 | 2 | 59.12 | 8 | F8:35905 | 31122021\_RID\_3262\_1b.raw |  |  | 1.6481E5 |  |  |  |  | 1.3768E6 | 2 | 0 | 0 | 1 | 0 | 0 | 0 | 0 | 1 | 50 | 63 |  |  | PEAKS DB |
| K.DIISDTSGDFR.K | Y | 62.86 | 1224.5623 | 11 | -0.5 | 613.2881 | 2 | 42.92 | 8 | F8:23088 | 31122021\_RID\_3262\_1b.raw |  |  |  |  |  |  |  | 2.1385E5 | 1 | 0 | 0 | 0 | 0 | 0 | 0 | 0 | 1 | 158 | 168 |  |  | PEAKS DB |
| R.QDIAFAYQR.R | N | 55.17 | 1110.5458 | 9 | 0.6 | 556.2805 | 2 | 34.91 | 8 | F8:16733 | 31122021\_RID\_3262\_1b.raw |  |  | 1.0819E5 |  |  |  |  | 3.9407E5 | 2 | 0 | 0 | 1 | 0 | 0 | 0 | 0 | 1 | 69 | 77 |  |  | PEAKS DB |
| total 4 peptides |  |  |  |  |  |  |  |  |  |  |  |  |  |  |  |  |  |  |  |  |  |  |  |  |  |  |  |  |  |  |  |  |  |
| --- | --- | --- | --- | --- | --- | --- | --- | --- | --- | --- | --- | --- | --- | --- | --- | --- | --- | --- | --- | --- | --- | --- | --- | --- | --- | --- | --- | --- | --- | --- | --- | --- | --- |

XP\_013930792.1

back to list

  

| Protein Coverage
| Supporting Peptides
|

Protein Coverage:

Supporting Peptides:

| Peptide | Uniq | -10lgP | Mass | Length | ppm | m/z | z | RT | Fraction | Scan | Source File | Area F6 | Area F2 | Area F3 | Area F4 | Area F5 | Area F7 | Area F8 | Area F1 | #Feature | #Feature F6 | #Feature F2 | #Feature F3 | #Feature F4 | #Feature F5 | #Feature F7 | #Feature F8 | #Feature F1 | Start | End | PTM | AScore | Found By |
| --- | --- | --- | --- | --- | --- | --- | --- | --- | --- | --- | --- | --- | --- | --- | --- | --- | --- | --- | --- | --- | --- | --- | --- | --- | --- | --- | --- | --- | --- | --- | --- | --- | --- |
| K.TPAQYDASELK.G | N | 72.43 | 1221.5876 | 11 | -0.5 | 611.8008 | 2 | 27.03 | 8 | F8:10630 | 31122021\_RID\_3262\_1b.raw |  |  | 2.9074E5 |  |  |  |  | 7.6887E5 | 3 | 0 | 0 | 2 | 0 | 0 | 0 | 0 | 1 | 120 | 130 |  |  | PEAKS DB |
| K.GVDEVTIVNLLTNR.S | N | 69.50 | 1541.8413 | 14 | 0.2 | 771.9281 | 2 | 59.12 | 8 | F8:35905 | 31122021\_RID\_3262\_1b.raw |  |  | 1.6481E5 |  |  |  |  | 1.3768E6 | 2 | 0 | 0 | 1 | 0 | 0 | 0 | 0 | 1 | 65 | 78 |  |  | PEAKS DB |
| K.DIISDTSGDFR.K | Y | 62.86 | 1224.5623 | 11 | -0.5 | 613.2881 | 2 | 42.92 | 8 | F8:23088 | 31122021\_RID\_3262\_1b.raw |  |  |  |  |  |  |  | 2.1385E5 | 1 | 0 | 0 | 0 | 0 | 0 | 0 | 0 | 1 | 173 | 183 |  |  | PEAKS DB |
| R.QDIAFAYQR.R | N | 55.17 | 1110.5458 | 9 | 0.6 | 556.2805 | 2 | 34.91 | 8 | F8:16733 | 31122021\_RID\_3262\_1b.raw |  |  | 1.0819E5 |  |  |  |  | 3.9407E5 | 2 | 0 | 0 | 1 | 0 | 0 | 0 | 0 | 1 | 84 | 92 |  |  | PEAKS DB |
| total 4 peptides |  |  |  |  |  |  |  |  |  |  |  |  |  |  |  |  |  |  |  |  |  |  |  |  |  |  |  |  |  |  |  |  |  |
| --- | --- | --- | --- | --- | --- | --- | --- | --- | --- | --- | --- | --- | --- | --- | --- | --- | --- | --- | --- | --- | --- | --- | --- | --- | --- | --- | --- | --- | --- | --- | --- | --- | --- |

XP\_026537798.1

back to list

  

| Protein Coverage
| Supporting Peptides
|

Protein Coverage:

Supporting Peptides:

| Peptide | Uniq | -10lgP | Mass | Length | ppm | m/z | z | RT | Fraction | Scan | Source File | Area F6 | Area F2 | Area F3 | Area F4 | Area F5 | Area F7 | Area F8 | Area F1 | #Feature | #Feature F6 | #Feature F2 | #Feature F3 | #Feature F4 | #Feature F5 | #Feature F7 | #Feature F8 | #Feature F1 | Start | End | PTM | AScore | Found By |
| --- | --- | --- | --- | --- | --- | --- | --- | --- | --- | --- | --- | --- | --- | --- | --- | --- | --- | --- | --- | --- | --- | --- | --- | --- | --- | --- | --- | --- | --- | --- | --- | --- | --- |
| K.TPAQYDASELK.A | N | 72.43 | 1221.5876 | 11 | -0.5 | 611.8008 | 2 | 27.03 | 8 | F8:10630 | 31122021\_RID\_3262\_1b.raw |  |  | 2.9074E5 |  |  |  |  | 7.6887E5 | 3 | 0 | 0 | 2 | 0 | 0 | 0 | 0 | 1 | 120 | 130 |  |  | PEAKS DB |
| K.GVDEVTIVNLLTNR.S | N | 69.50 | 1541.8413 | 14 | 0.2 | 771.9281 | 2 | 59.12 | 8 | F8:35905 | 31122021\_RID\_3262\_1b.raw |  |  | 1.6481E5 |  |  |  |  | 1.3768E6 | 2 | 0 | 0 | 1 | 0 | 0 | 0 | 0 | 1 | 65 | 78 |  |  | PEAKS DB |
| R.QDIAFAYQR.R | N | 55.17 | 1110.5458 | 9 | 0.6 | 556.2805 | 2 | 34.91 | 8 | F8:16733 | 31122021\_RID\_3262\_1b.raw |  |  | 1.0819E5 |  |  |  |  | 3.9407E5 | 2 | 0 | 0 | 1 | 0 | 0 | 0 | 0 | 1 | 84 | 92 |  |  | PEAKS DB |
| K.DITSDTSGDFR.K | Y | 49.17 | 1212.5259 | 11 | 1.5 | 607.2711 | 2 | 33.29 | 8 | F8:15542 | 31122021\_RID\_3262\_1b.raw |  |  |  |  |  |  |  | 1.5203E4 | 1 | 0 | 0 | 0 | 0 | 0 | 0 | 0 | 1 | 173 | 183 |  |  | PEAKS DB |
| total 4 peptides |  |  |  |  |  |  |  |  |  |  |  |  |  |  |  |  |  |  |  |  |  |  |  |  |  |  |  |  |  |  |  |  |  |
| --- | --- | --- | --- | --- | --- | --- | --- | --- | --- | --- | --- | --- | --- | --- | --- | --- | --- | --- | --- | --- | --- | --- | --- | --- | --- | --- | --- | --- | --- | --- | --- | --- | --- |

XP\_026570509.1

back to list

  

| Protein Coverage
| Supporting Peptides
|

Protein Coverage:

Supporting Peptides:

| Peptide | Uniq | -10lgP | Mass | Length | ppm | m/z | z | RT | Fraction | Scan | Source File | Area F6 | Area F2 | Area F3 | Area F4 | Area F5 | Area F7 | Area F8 | Area F1 | #Feature | #Feature F6 | #Feature F2 | #Feature F3 | #Feature F4 | #Feature F5 | #Feature F7 | #Feature F8 | #Feature F1 | Start | End | PTM | AScore | Found By |
| --- | --- | --- | --- | --- | --- | --- | --- | --- | --- | --- | --- | --- | --- | --- | --- | --- | --- | --- | --- | --- | --- | --- | --- | --- | --- | --- | --- | --- | --- | --- | --- | --- | --- |
| E.FASFIDKVR.N | N | 65.83 | 1081.5920 | 9 | 1.0 | 541.8038 | 2 | 33.62 | 8 | F8:15859 | 31122021\_RID\_3262\_1b.raw |  |  | 2.2979E6 |  | 2.6797E5 |  |  | 2.3536E6 | 3 | 0 | 0 | 1 | 0 | 1 | 0 | 0 | 1 | 160 | 168 |  |  | PEAKS DB |
| K.TLLEGEESR | N | 65.37 | 1032.5087 | 9 | 0.2 | 517.2617 | 2 | 27.61 | 3 | F3:13050 | 13012022\_RID\_3262\_3b.raw | 1.1643E7 | 1.435E7 | 1.7632E8 | 5.1183E7 | 4.4484E7 | 7.5538E7 | 3.5338E7 | 2.3285E8 | 8 | 1 | 1 | 1 | 1 | 1 | 1 | 1 | 1 | 452 | 460 |  |  | PEAKS DB |
| E.FASFIDK.V | N | 53.98 | 826.4225 | 7 | 0.0 | 414.2185 | 2 | 37.13 | 2 | F2:13215 | 13012022\_RID\_3262\_2b.raw |  | 3.8919E6 | 3.3996E7 | 9.4622E6 | 1.087E7 | 2.372E7 | 4.1967E6 | 3.8411E7 | 9 | 0 | 1 | 2 | 1 | 1 | 2 | 1 | 1 | 160 | 166 |  |  | PEAKS DB |
| K.NLNNEFASFIDK.V | Y | 52.64 | 1410.6779 | 12 | 1.2 | 706.3470 | 2 | 60.91 | 8 | F8:36943 | 31122021\_RID\_3262\_1b.raw |  |  |  |  |  |  |  | 4.7863E5 | 1 | 0 | 0 | 0 | 0 | 0 | 0 | 0 | 1 | 155 | 166 |  |  | PEAKS DB |
| total 4 peptides |  |  |  |  |  |  |  |  |  |  |  |  |  |  |  |  |  |  |  |  |  |  |  |  |  |  |  |  |  |  |  |  |  |
| --- | --- | --- | --- | --- | --- | --- | --- | --- | --- | --- | --- | --- | --- | --- | --- | --- | --- | --- | --- | --- | --- | --- | --- | --- | --- | --- | --- | --- | --- | --- | --- | --- | --- |

XP\_026539658.1

back to list

  

| Protein Coverage
| Supporting Peptides
|

Protein Coverage:

Supporting Peptides:

| Peptide | Uniq | -10lgP | Mass | Length | ppm | m/z | z | RT | Fraction | Scan | Source File | Area F6 | Area F2 | Area F3 | Area F4 | Area F5 | Area F7 | Area F8 | Area F1 | #Feature | #Feature F6 | #Feature F2 | #Feature F3 | #Feature F4 | #Feature F5 | #Feature F7 | #Feature F8 | #Feature F1 | Start | End | PTM | AScore | Found By |
| --- | --- | --- | --- | --- | --- | --- | --- | --- | --- | --- | --- | --- | --- | --- | --- | --- | --- | --- | --- | --- | --- | --- | --- | --- | --- | --- | --- | --- | --- | --- | --- | --- | --- |
| E.FASFIDKVR.N | N | 65.83 | 1081.5920 | 9 | 1.0 | 541.8038 | 2 | 33.62 | 8 | F8:15859 | 31122021\_RID\_3262\_1b.raw |  |  | 2.2979E6 |  | 2.6797E5 |  |  | 2.3536E6 | 3 | 0 | 0 | 1 | 0 | 1 | 0 | 0 | 1 | 160 | 168 |  |  | PEAKS DB |
| K.TLLEGEESR | N | 65.37 | 1032.5087 | 9 | 0.2 | 517.2617 | 2 | 27.61 | 3 | F3:13050 | 13012022\_RID\_3262\_3b.raw | 1.1643E7 | 1.435E7 | 1.7632E8 | 5.1183E7 | 4.4484E7 | 7.5538E7 | 3.5338E7 | 2.3285E8 | 8 | 1 | 1 | 1 | 1 | 1 | 1 | 1 | 1 | 452 | 460 |  |  | PEAKS DB |
| E.FASFIDK.V | N | 53.98 | 826.4225 | 7 | 0.0 | 414.2185 | 2 | 37.13 | 2 | F2:13215 | 13012022\_RID\_3262\_2b.raw |  | 3.8919E6 | 3.3996E7 | 9.4622E6 | 1.087E7 | 2.372E7 | 4.1967E6 | 3.8411E7 | 9 | 0 | 1 | 2 | 1 | 1 | 2 | 1 | 1 | 160 | 166 |  |  | PEAKS DB |
| K.NLNNEFASFIDK.V | Y | 52.64 | 1410.6779 | 12 | 1.2 | 706.3470 | 2 | 60.91 | 8 | F8:36943 | 31122021\_RID\_3262\_1b.raw |  |  |  |  |  |  |  | 4.7863E5 | 1 | 0 | 0 | 0 | 0 | 0 | 0 | 0 | 1 | 155 | 166 |  |  | PEAKS DB |
| total 4 peptides |  |  |  |  |  |  |  |  |  |  |  |  |  |  |  |  |  |  |  |  |  |  |  |  |  |  |  |  |  |  |  |  |  |
| --- | --- | --- | --- | --- | --- | --- | --- | --- | --- | --- | --- | --- | --- | --- | --- | --- | --- | --- | --- | --- | --- | --- | --- | --- | --- | --- | --- | --- | --- | --- | --- | --- | --- |

XP\_007439537.1

back to list

  

| Protein Coverage
| Supporting Peptides
|

Protein Coverage:

Supporting Peptides:

| Peptide | Uniq | -10lgP | Mass | Length | ppm | m/z | z | RT | Fraction | Scan | Source File | Area F6 | Area F2 | Area F3 | Area F4 | Area F5 | Area F7 | Area F8 | Area F1 | #Feature | #Feature F6 | #Feature F2 | #Feature F3 | #Feature F4 | #Feature F5 | #Feature F7 | #Feature F8 | #Feature F1 | Start | End | PTM | AScore | Found By |
| --- | --- | --- | --- | --- | --- | --- | --- | --- | --- | --- | --- | --- | --- | --- | --- | --- | --- | --- | --- | --- | --- | --- | --- | --- | --- | --- | --- | --- | --- | --- | --- | --- | --- |
| Q.TVVYGITTGFGK.F | Y | 72.09 | 1241.6655 | 12 | 0.9 | 621.8406 | 2 | 42.64 | 8 | F8:22872 | 31122021\_RID\_3262\_1b.raw |  |  |  |  |  |  |  | 4.643E5 | 1 | 0 | 0 | 0 | 0 | 0 | 0 | 0 | 1 | 161 | 172 |  |  | PEAKS DB |
| K.LQELQVNLVR.S | Y | 57.33 | 1210.7034 | 10 | 0.7 | 606.3594 | 2 | 41.27 | 8 | F8:21772 | 31122021\_RID\_3262\_1b.raw |  |  |  |  |  |  |  | 4.3424E5 | 1 | 0 | 0 | 0 | 0 | 0 | 0 | 0 | 1 | 183 | 192 |  |  | PEAKS DB |
| R.VQDAYTLR.C | Y | 52.95 | 964.4978 | 8 | 0.1 | 483.2562 | 2 | 25.74 | 8 | F8:9621 | 31122021\_RID\_3262\_1b.raw |  |  |  |  |  |  |  | 2.2781E5 | 1 | 0 | 0 | 0 | 0 | 0 | 0 | 0 | 1 | 391 | 398 |  |  | PEAKS DB |
| S.LTTEDLVNLGK.G | Y | 51.80 | 1201.6554 | 11 | 0.7 | 601.8354 | 2 | 44.71 | 8 | F8:24472 | 31122021\_RID\_3262\_1b.raw |  |  |  |  |  |  |  | 3.007E5 | 1 | 0 | 0 | 0 | 0 | 0 | 0 | 0 | 1 | 121 | 131 |  |  | PEAKS DB |
| R.FMAPDIEAAHR.L | Y | 49.62 | 1256.5972 | 11 | 1.0 | 419.8734 | 3 | 32.22 | 8 | F8:14639 | 31122021\_RID\_3262\_1b.raw |  |  |  |  |  |  |  | 1.3581E5 | 1 | 0 | 0 | 0 | 0 | 0 | 0 | 0 | 1 | 595 | 605 |  |  | PEAKS DB |
| total 5 peptides |  |  |  |  |  |  |  |  |  |  |  |  |  |  |  |  |  |  |  |  |  |  |  |  |  |  |  |  |  |  |  |  |  |
| --- | --- | --- | --- | --- | --- | --- | --- | --- | --- | --- | --- | --- | --- | --- | --- | --- | --- | --- | --- | --- | --- | --- | --- | --- | --- | --- | --- | --- | --- | --- | --- | --- | --- |

JAS05258.1

back to list

  

| Protein Coverage
| Supporting Peptides
|

Protein Coverage:

Supporting Peptides:

| Peptide | Uniq | -10lgP | Mass | Length | ppm | m/z | z | RT | Fraction | Scan | Source File | Area F6 | Area F2 | Area F3 | Area F4 | Area F5 | Area F7 | Area F8 | Area F1 | #Feature | #Feature F6 | #Feature F2 | #Feature F3 | #Feature F4 | #Feature F5 | #Feature F7 | #Feature F8 | #Feature F1 | Start | End | PTM | AScore | Found By |
| --- | --- | --- | --- | --- | --- | --- | --- | --- | --- | --- | --- | --- | --- | --- | --- | --- | --- | --- | --- | --- | --- | --- | --- | --- | --- | --- | --- | --- | --- | --- | --- | --- | --- |
| K.ILNEDEQTR.D | N | 57.57 | 1116.5411 | 9 | 0.0 | 559.2778 | 2 | 22.02 | 4 | F4:9107 | 13012022\_RID\_3262\_4b.raw |  |  | 2.9744E5 | 8.5819E6 |  |  |  | 2.6155E5 | 3 | 0 | 0 | 1 | 1 | 0 | 0 | 0 | 1 | 82 | 90 |  |  | PEAKS DB |
| K.KILNEDEQTR.D | N | 54.25 | 1244.6360 | 10 | 0.7 | 623.3257 | 2 | 19.43 | 4 | F4:6795 | 13012022\_RID\_3262\_4b.raw |  |  |  | 7.7829E5 |  |  |  |  | 1 | 0 | 0 | 0 | 1 | 0 | 0 | 0 | 0 | 81 | 90 |  |  | PEAKS DB |
| K.ILNEDEQTRDPK.E | N | 49.32 | 1456.7157 | 12 | 1.0 | 729.3658 | 2 | 18.03 | 8 | F8:4481 | 31122021\_RID\_3262\_1b.raw |  |  |  | 3.4529E6 |  |  |  | 1.2735E5 | 2 | 0 | 0 | 0 | 1 | 0 | 0 | 0 | 1 | 82 | 93 |  |  | PEAKS DB |
| L.VVGGEEC(+57.02)NINEHR.S | Y | 48.53 | 1511.6787 | 13 | 0.5 | 504.9005 | 3 | 21.42 | 3 | F3:8085 | 13012022\_RID\_3262\_3b.raw |  |  | 2.9418E8 |  | 3.5485E7 |  |  |  | 5 | 0 | 0 | 3 | 0 | 2 | 0 | 0 | 0 | 25 | 37 | Carbamidomethylation | C7:Carbamidomethylation:1000.00 | PEAKS DB |
| K.DKDIMLIR.L | N | 45.33 | 1002.5532 | 8 | 0.8 | 502.2842 | 2 | 34.98 | 4 | F4:18968 | 13012022\_RID\_3262\_4b.raw |  |  |  | 7.1188E5 |  |  |  |  | 1 | 0 | 0 | 0 | 1 | 0 | 0 | 0 | 0 | 108 | 115 |  |  | PEAKS DB |
| K.KILNEDEQTRDPK.E | N | 44.92 | 1584.8107 | 13 | -0.1 | 529.2775 | 3 | 18.98 | 4 | F4:6613 | 13012022\_RID\_3262\_4b.raw |  |  |  | 3.347E6 |  |  |  |  | 1 | 0 | 0 | 0 | 1 | 0 | 0 | 0 | 0 | 81 | 93 |  |  | PEAKS DB |
| total 6 peptides |  |  |  |  |  |  |  |  |  |  |  |  |  |  |  |  |  |  |  |  |  |  |  |  |  |  |  |  |  |  |  |  |  |
| --- | --- | --- | --- | --- | --- | --- | --- | --- | --- | --- | --- | --- | --- | --- | --- | --- | --- | --- | --- | --- | --- | --- | --- | --- | --- | --- | --- | --- | --- | --- | --- | --- | --- |

JAS05257.1

back to list

  

| Protein Coverage
| Supporting Peptides
|

Protein Coverage:

Supporting Peptides:

| Peptide | Uniq | -10lgP | Mass | Length | ppm | m/z | z | RT | Fraction | Scan | Source File | Area F6 | Area F2 | Area F3 | Area F4 | Area F5 | Area F7 | Area F8 | Area F1 | #Feature | #Feature F6 | #Feature F2 | #Feature F3 | #Feature F4 | #Feature F5 | #Feature F7 | #Feature F8 | #Feature F1 | Start | End | PTM | AScore | Found By |
| --- | --- | --- | --- | --- | --- | --- | --- | --- | --- | --- | --- | --- | --- | --- | --- | --- | --- | --- | --- | --- | --- | --- | --- | --- | --- | --- | --- | --- | --- | --- | --- | --- | --- |
| K.ILNEDEQTR.D | N | 57.57 | 1116.5411 | 9 | 0.0 | 559.2778 | 2 | 22.02 | 4 | F4:9107 | 13012022\_RID\_3262\_4b.raw |  |  | 2.9744E5 | 8.5819E6 |  |  |  | 2.6155E5 | 3 | 0 | 0 | 1 | 1 | 0 | 0 | 0 | 1 | 82 | 90 |  |  | PEAKS DB |
| K.KILNEDEQTR.D | N | 54.25 | 1244.6360 | 10 | 0.7 | 623.3257 | 2 | 19.43 | 4 | F4:6795 | 13012022\_RID\_3262\_4b.raw |  |  |  | 7.7829E5 |  |  |  |  | 1 | 0 | 0 | 0 | 1 | 0 | 0 | 0 | 0 | 81 | 90 |  |  | PEAKS DB |
| K.ILNEDEQTRDPK.E | N | 49.32 | 1456.7157 | 12 | 1.0 | 729.3658 | 2 | 18.03 | 8 | F8:4481 | 31122021\_RID\_3262\_1b.raw |  |  |  | 3.4529E6 |  |  |  | 1.2735E5 | 2 | 0 | 0 | 0 | 1 | 0 | 0 | 0 | 1 | 82 | 93 |  |  | PEAKS DB |
| L.VVGGEEC(+57.02)NINEHR.S | Y | 48.53 | 1511.6787 | 13 | 0.5 | 504.9005 | 3 | 21.42 | 3 | F3:8085 | 13012022\_RID\_3262\_3b.raw |  |  | 2.9418E8 |  | 3.5485E7 |  |  |  | 5 | 0 | 0 | 3 | 0 | 2 | 0 | 0 | 0 | 25 | 37 | Carbamidomethylation | C7:Carbamidomethylation:1000.00 | PEAKS DB |
| K.DKDIMLIR.L | N | 45.33 | 1002.5532 | 8 | 0.8 | 502.2842 | 2 | 34.98 | 4 | F4:18968 | 13012022\_RID\_3262\_4b.raw |  |  |  | 7.1188E5 |  |  |  |  | 1 | 0 | 0 | 0 | 1 | 0 | 0 | 0 | 0 | 108 | 115 |  |  | PEAKS DB |
| K.KILNEDEQTRDPK.E | N | 44.92 | 1584.8107 | 13 | -0.1 | 529.2775 | 3 | 18.98 | 4 | F4:6613 | 13012022\_RID\_3262\_4b.raw |  |  |  | 3.347E6 |  |  |  |  | 1 | 0 | 0 | 0 | 1 | 0 | 0 | 0 | 0 | 81 | 93 |  |  | PEAKS DB |
| total 6 peptides |  |  |  |  |  |  |  |  |  |  |  |  |  |  |  |  |  |  |  |  |  |  |  |  |  |  |  |  |  |  |  |  |  |
| --- | --- | --- | --- | --- | --- | --- | --- | --- | --- | --- | --- | --- | --- | --- | --- | --- | --- | --- | --- | --- | --- | --- | --- | --- | --- | --- | --- | --- | --- | --- | --- | --- | --- |

JAS05259.1

back to list

  

| Protein Coverage
| Supporting Peptides
|

Protein Coverage:

Supporting Peptides:

| Peptide | Uniq | -10lgP | Mass | Length | ppm | m/z | z | RT | Fraction | Scan | Source File | Area F6 | Area F2 | Area F3 | Area F4 | Area F5 | Area F7 | Area F8 | Area F1 | #Feature | #Feature F6 | #Feature F2 | #Feature F3 | #Feature F4 | #Feature F5 | #Feature F7 | #Feature F8 | #Feature F1 | Start | End | PTM | AScore | Found By |
| --- | --- | --- | --- | --- | --- | --- | --- | --- | --- | --- | --- | --- | --- | --- | --- | --- | --- | --- | --- | --- | --- | --- | --- | --- | --- | --- | --- | --- | --- | --- | --- | --- | --- |
| K.ILNEDEQTR.D | N | 57.57 | 1116.5411 | 9 | 0.0 | 559.2778 | 2 | 22.02 | 4 | F4:9107 | 13012022\_RID\_3262\_4b.raw |  |  | 2.9744E5 | 8.5819E6 |  |  |  | 2.6155E5 | 3 | 0 | 0 | 1 | 1 | 0 | 0 | 0 | 1 | 82 | 90 |  |  | PEAKS DB |
| K.KILNEDEQTR.D | N | 54.25 | 1244.6360 | 10 | 0.7 | 623.3257 | 2 | 19.43 | 4 | F4:6795 | 13012022\_RID\_3262\_4b.raw |  |  |  | 7.7829E5 |  |  |  |  | 1 | 0 | 0 | 0 | 1 | 0 | 0 | 0 | 0 | 81 | 90 |  |  | PEAKS DB |
| K.ILNEDEQTRDPK.E | N | 49.32 | 1456.7157 | 12 | 1.0 | 729.3658 | 2 | 18.03 | 8 | F8:4481 | 31122021\_RID\_3262\_1b.raw |  |  |  | 3.4529E6 |  |  |  | 1.2735E5 | 2 | 0 | 0 | 0 | 1 | 0 | 0 | 0 | 1 | 82 | 93 |  |  | PEAKS DB |
| L.VVGGEEC(+57.02)NINEHR.S | Y | 48.53 | 1511.6787 | 13 | 0.5 | 504.9005 | 3 | 21.42 | 3 | F3:8085 | 13012022\_RID\_3262\_3b.raw |  |  | 2.9418E8 |  | 3.5485E7 |  |  |  | 5 | 0 | 0 | 3 | 0 | 2 | 0 | 0 | 0 | 25 | 37 | Carbamidomethylation | C7:Carbamidomethylation:1000.00 | PEAKS DB |
| K.DKDIMLIR.L | N | 45.33 | 1002.5532 | 8 | 0.8 | 502.2842 | 2 | 34.98 | 4 | F4:18968 | 13012022\_RID\_3262\_4b.raw |  |  |  | 7.1188E5 |  |  |  |  | 1 | 0 | 0 | 0 | 1 | 0 | 0 | 0 | 0 | 108 | 115 |  |  | PEAKS DB |
| K.KILNEDEQTRDPK.E | N | 44.92 | 1584.8107 | 13 | -0.1 | 529.2775 | 3 | 18.98 | 4 | F4:6613 | 13012022\_RID\_3262\_4b.raw |  |  |  | 3.347E6 |  |  |  |  | 1 | 0 | 0 | 0 | 1 | 0 | 0 | 0 | 0 | 81 | 93 |  |  | PEAKS DB |
| total 6 peptides |  |  |  |  |  |  |  |  |  |  |  |  |  |  |  |  |  |  |  |  |  |  |  |  |  |  |  |  |  |  |  |  |  |
| --- | --- | --- | --- | --- | --- | --- | --- | --- | --- | --- | --- | --- | --- | --- | --- | --- | --- | --- | --- | --- | --- | --- | --- | --- | --- | --- | --- | --- | --- | --- | --- | --- | --- |

JAG66915.1

back to list

  

| Protein Coverage
| Supporting Peptides
|

Protein Coverage:

Supporting Peptides:

| Peptide | Uniq | -10lgP | Mass | Length | ppm | m/z | z | RT | Fraction | Scan | Source File | Area F6 | Area F2 | Area F3 | Area F4 | Area F5 | Area F7 | Area F8 | Area F1 | #Feature | #Feature F6 | #Feature F2 | #Feature F3 | #Feature F4 | #Feature F5 | #Feature F7 | #Feature F8 | #Feature F1 | Start | End | PTM | AScore | Found By |
| --- | --- | --- | --- | --- | --- | --- | --- | --- | --- | --- | --- | --- | --- | --- | --- | --- | --- | --- | --- | --- | --- | --- | --- | --- | --- | --- | --- | --- | --- | --- | --- | --- | --- |
| R.LTVEDPVTVEYITR.Y | Y | 74.58 | 1633.8563 | 14 | 1.1 | 817.9363 | 2 | 49.19 | 8 | F8:27990 | 31122021\_RID\_3262\_1b.raw |  |  |  |  |  |  |  | 1.2989E5 | 1 | 0 | 0 | 0 | 0 | 0 | 0 | 0 | 1 | 96 | 109 |  |  | PEAKS DB |
| K.ALLEVVQSGGK.N | Y | 65.78 | 1099.6237 | 11 | 0.8 | 550.8195 | 2 | 36.13 | 8 | F8:17692 | 31122021\_RID\_3262\_1b.raw |  |  |  |  |  |  |  | 2.1171E5 | 1 | 0 | 0 | 0 | 0 | 0 | 0 | 0 | 1 | 194 | 204 |  |  | PEAKS DB |
| R.AITVFSPDGHLFQVEYAQEAVK.K | Y | 46.30 | 2448.2324 | 22 | 0.6 | 817.0852 | 3 | 53.64 | 8 | F8:31471 | 31122021\_RID\_3262\_1b.raw |  |  |  |  |  |  |  | 8.4864E4 | 1 | 0 | 0 | 0 | 0 | 0 | 0 | 0 | 1 | 6 | 27 |  |  | PEAKS DB |
| total 3 peptides |  |  |  |  |  |  |  |  |  |  |  |  |  |  |  |  |  |  |  |  |  |  |  |  |  |  |  |  |  |  |  |  |  |
| --- | --- | --- | --- | --- | --- | --- | --- | --- | --- | --- | --- | --- | --- | --- | --- | --- | --- | --- | --- | --- | --- | --- | --- | --- | --- | --- | --- | --- | --- | --- | --- | --- | --- |

JAB53603.1

back to list

  

| Protein Coverage
| Supporting Peptides
|

Protein Coverage:

Supporting Peptides:

| Peptide | Uniq | -10lgP | Mass | Length | ppm | m/z | z | RT | Fraction | Scan | Source File | Area F6 | Area F2 | Area F3 | Area F4 | Area F5 | Area F7 | Area F8 | Area F1 | #Feature | #Feature F6 | #Feature F2 | #Feature F3 | #Feature F4 | #Feature F5 | #Feature F7 | #Feature F8 | #Feature F1 | Start | End | PTM | AScore | Found By |
| --- | --- | --- | --- | --- | --- | --- | --- | --- | --- | --- | --- | --- | --- | --- | --- | --- | --- | --- | --- | --- | --- | --- | --- | --- | --- | --- | --- | --- | --- | --- | --- | --- | --- |
| R.LTVEDPVTVEYITR.Y | Y | 74.58 | 1633.8563 | 14 | 1.1 | 817.9363 | 2 | 49.19 | 8 | F8:27990 | 31122021\_RID\_3262\_1b.raw |  |  |  |  |  |  |  | 1.2989E5 | 1 | 0 | 0 | 0 | 0 | 0 | 0 | 0 | 1 | 96 | 109 |  |  | PEAKS DB |
| K.ALLEVVQSGGK.N | Y | 65.78 | 1099.6237 | 11 | 0.8 | 550.8195 | 2 | 36.13 | 8 | F8:17692 | 31122021\_RID\_3262\_1b.raw |  |  |  |  |  |  |  | 2.1171E5 | 1 | 0 | 0 | 0 | 0 | 0 | 0 | 0 | 1 | 194 | 204 |  |  | PEAKS DB |
| R.AITVFSPDGHLFQVEYAQEAVK.K | Y | 46.30 | 2448.2324 | 22 | 0.6 | 817.0852 | 3 | 53.64 | 8 | F8:31471 | 31122021\_RID\_3262\_1b.raw |  |  |  |  |  |  |  | 8.4864E4 | 1 | 0 | 0 | 0 | 0 | 0 | 0 | 0 | 1 | 6 | 27 |  |  | PEAKS DB |
| total 3 peptides |  |  |  |  |  |  |  |  |  |  |  |  |  |  |  |  |  |  |  |  |  |  |  |  |  |  |  |  |  |  |  |  |  |
| --- | --- | --- | --- | --- | --- | --- | --- | --- | --- | --- | --- | --- | --- | --- | --- | --- | --- | --- | --- | --- | --- | --- | --- | --- | --- | --- | --- | --- | --- | --- | --- | --- | --- |

JAA96031.1

back to list

  

| Protein Coverage
| Supporting Peptides
|

Protein Coverage:

Supporting Peptides:

| Peptide | Uniq | -10lgP | Mass | Length | ppm | m/z | z | RT | Fraction | Scan | Source File | Area F6 | Area F2 | Area F3 | Area F4 | Area F5 | Area F7 | Area F8 | Area F1 | #Feature | #Feature F6 | #Feature F2 | #Feature F3 | #Feature F4 | #Feature F5 | #Feature F7 | #Feature F8 | #Feature F1 | Start | End | PTM | AScore | Found By |
| --- | --- | --- | --- | --- | --- | --- | --- | --- | --- | --- | --- | --- | --- | --- | --- | --- | --- | --- | --- | --- | --- | --- | --- | --- | --- | --- | --- | --- | --- | --- | --- | --- | --- |
| R.LTVEDPVTVEYITR.Y | Y | 74.58 | 1633.8563 | 14 | 1.1 | 817.9363 | 2 | 49.19 | 8 | F8:27990 | 31122021\_RID\_3262\_1b.raw |  |  |  |  |  |  |  | 1.2989E5 | 1 | 0 | 0 | 0 | 0 | 0 | 0 | 0 | 1 | 96 | 109 |  |  | PEAKS DB |
| K.ALLEVVQSGGK.N | Y | 65.78 | 1099.6237 | 11 | 0.8 | 550.8195 | 2 | 36.13 | 8 | F8:17692 | 31122021\_RID\_3262\_1b.raw |  |  |  |  |  |  |  | 2.1171E5 | 1 | 0 | 0 | 0 | 0 | 0 | 0 | 0 | 1 | 194 | 204 |  |  | PEAKS DB |
| R.AITVFSPDGHLFQVEYAQEAVK.K | Y | 46.30 | 2448.2324 | 22 | 0.6 | 817.0852 | 3 | 53.64 | 8 | F8:31471 | 31122021\_RID\_3262\_1b.raw |  |  |  |  |  |  |  | 8.4864E4 | 1 | 0 | 0 | 0 | 0 | 0 | 0 | 0 | 1 | 6 | 27 |  |  | PEAKS DB |
| total 3 peptides |  |  |  |  |  |  |  |  |  |  |  |  |  |  |  |  |  |  |  |  |  |  |  |  |  |  |  |  |  |  |  |  |  |
| --- | --- | --- | --- | --- | --- | --- | --- | --- | --- | --- | --- | --- | --- | --- | --- | --- | --- | --- | --- | --- | --- | --- | --- | --- | --- | --- | --- | --- | --- | --- | --- | --- | --- |

AFJ51040.1

back to list

  

| Protein Coverage
| Supporting Peptides
|

Protein Coverage:

Supporting Peptides:

| Peptide | Uniq | -10lgP | Mass | Length | ppm | m/z | z | RT | Fraction | Scan | Source File | Area F6 | Area F2 | Area F3 | Area F4 | Area F5 | Area F7 | Area F8 | Area F1 | #Feature | #Feature F6 | #Feature F2 | #Feature F3 | #Feature F4 | #Feature F5 | #Feature F7 | #Feature F8 | #Feature F1 | Start | End | PTM | AScore | Found By |
| --- | --- | --- | --- | --- | --- | --- | --- | --- | --- | --- | --- | --- | --- | --- | --- | --- | --- | --- | --- | --- | --- | --- | --- | --- | --- | --- | --- | --- | --- | --- | --- | --- | --- |
| R.LTVEDPVTVEYITR.Y | Y | 74.58 | 1633.8563 | 14 | 1.1 | 817.9363 | 2 | 49.19 | 8 | F8:27990 | 31122021\_RID\_3262\_1b.raw |  |  |  |  |  |  |  | 1.2989E5 | 1 | 0 | 0 | 0 | 0 | 0 | 0 | 0 | 1 | 96 | 109 |  |  | PEAKS DB |
| K.ALLEVVQSGGK.N | Y | 65.78 | 1099.6237 | 11 | 0.8 | 550.8195 | 2 | 36.13 | 8 | F8:17692 | 31122021\_RID\_3262\_1b.raw |  |  |  |  |  |  |  | 2.1171E5 | 1 | 0 | 0 | 0 | 0 | 0 | 0 | 0 | 1 | 194 | 204 |  |  | PEAKS DB |
| R.AITVFSPDGHLFQVEYAQEAVK.K | Y | 46.30 | 2448.2324 | 22 | 0.6 | 817.0852 | 3 | 53.64 | 8 | F8:31471 | 31122021\_RID\_3262\_1b.raw |  |  |  |  |  |  |  | 8.4864E4 | 1 | 0 | 0 | 0 | 0 | 0 | 0 | 0 | 1 | 6 | 27 |  |  | PEAKS DB |
| total 3 peptides |  |  |  |  |  |  |  |  |  |  |  |  |  |  |  |  |  |  |  |  |  |  |  |  |  |  |  |  |  |  |  |  |  |
| --- | --- | --- | --- | --- | --- | --- | --- | --- | --- | --- | --- | --- | --- | --- | --- | --- | --- | --- | --- | --- | --- | --- | --- | --- | --- | --- | --- | --- | --- | --- | --- | --- | --- |

ETE69560.1

back to list

  

| Protein Coverage
| Supporting Peptides
|

Protein Coverage:

Supporting Peptides:

| Peptide | Uniq | -10lgP | Mass | Length | ppm | m/z | z | RT | Fraction | Scan | Source File | Area F6 | Area F2 | Area F3 | Area F4 | Area F5 | Area F7 | Area F8 | Area F1 | #Feature | #Feature F6 | #Feature F2 | #Feature F3 | #Feature F4 | #Feature F5 | #Feature F7 | #Feature F8 | #Feature F1 | Start | End | PTM | AScore | Found By |
| --- | --- | --- | --- | --- | --- | --- | --- | --- | --- | --- | --- | --- | --- | --- | --- | --- | --- | --- | --- | --- | --- | --- | --- | --- | --- | --- | --- | --- | --- | --- | --- | --- | --- |
| R.LTVEDPVTVEYITR.Y | Y | 74.58 | 1633.8563 | 14 | 1.1 | 817.9363 | 2 | 49.19 | 8 | F8:27990 | 31122021\_RID\_3262\_1b.raw |  |  |  |  |  |  |  | 1.2989E5 | 1 | 0 | 0 | 0 | 0 | 0 | 0 | 0 | 1 | 96 | 109 |  |  | PEAKS DB |
| K.ALLEVVQSGGK.N | Y | 65.78 | 1099.6237 | 11 | 0.8 | 550.8195 | 2 | 36.13 | 8 | F8:17692 | 31122021\_RID\_3262\_1b.raw |  |  |  |  |  |  |  | 2.1171E5 | 1 | 0 | 0 | 0 | 0 | 0 | 0 | 0 | 1 | 194 | 204 |  |  | PEAKS DB |
| R.AITVFSPDGHLFQVEYAQEAVK.K | Y | 46.30 | 2448.2324 | 22 | 0.6 | 817.0852 | 3 | 53.64 | 8 | F8:31471 | 31122021\_RID\_3262\_1b.raw |  |  |  |  |  |  |  | 8.4864E4 | 1 | 0 | 0 | 0 | 0 | 0 | 0 | 0 | 1 | 6 | 27 |  |  | PEAKS DB |
| total 3 peptides |  |  |  |  |  |  |  |  |  |  |  |  |  |  |  |  |  |  |  |  |  |  |  |  |  |  |  |  |  |  |  |  |  |
| --- | --- | --- | --- | --- | --- | --- | --- | --- | --- | --- | --- | --- | --- | --- | --- | --- | --- | --- | --- | --- | --- | --- | --- | --- | --- | --- | --- | --- | --- | --- | --- | --- | --- |

XP\_015673624.1

back to list

  

| Protein Coverage
| Supporting Peptides
|

Protein Coverage:

Supporting Peptides:

| Peptide | Uniq | -10lgP | Mass | Length | ppm | m/z | z | RT | Fraction | Scan | Source File | Area F6 | Area F2 | Area F3 | Area F4 | Area F5 | Area F7 | Area F8 | Area F1 | #Feature | #Feature F6 | #Feature F2 | #Feature F3 | #Feature F4 | #Feature F5 | #Feature F7 | #Feature F8 | #Feature F1 | Start | End | PTM | AScore | Found By |
| --- | --- | --- | --- | --- | --- | --- | --- | --- | --- | --- | --- | --- | --- | --- | --- | --- | --- | --- | --- | --- | --- | --- | --- | --- | --- | --- | --- | --- | --- | --- | --- | --- | --- |
| R.LTVEDPVTVEYITR.Y | Y | 74.58 | 1633.8563 | 14 | 1.1 | 817.9363 | 2 | 49.19 | 8 | F8:27990 | 31122021\_RID\_3262\_1b.raw |  |  |  |  |  |  |  | 1.2989E5 | 1 | 0 | 0 | 0 | 0 | 0 | 0 | 0 | 1 | 96 | 109 |  |  | PEAKS DB |
| K.ALLEVVQSGGK.N | Y | 65.78 | 1099.6237 | 11 | 0.8 | 550.8195 | 2 | 36.13 | 8 | F8:17692 | 31122021\_RID\_3262\_1b.raw |  |  |  |  |  |  |  | 2.1171E5 | 1 | 0 | 0 | 0 | 0 | 0 | 0 | 0 | 1 | 194 | 204 |  |  | PEAKS DB |
| R.AITVFSPDGHLFQVEYAQEAVK.K | Y | 46.30 | 2448.2324 | 22 | 0.6 | 817.0852 | 3 | 53.64 | 8 | F8:31471 | 31122021\_RID\_3262\_1b.raw |  |  |  |  |  |  |  | 8.4864E4 | 1 | 0 | 0 | 0 | 0 | 0 | 0 | 0 | 1 | 6 | 27 |  |  | PEAKS DB |
| total 3 peptides |  |  |  |  |  |  |  |  |  |  |  |  |  |  |  |  |  |  |  |  |  |  |  |  |  |  |  |  |  |  |  |  |  |
| --- | --- | --- | --- | --- | --- | --- | --- | --- | --- | --- | --- | --- | --- | --- | --- | --- | --- | --- | --- | --- | --- | --- | --- | --- | --- | --- | --- | --- | --- | --- | --- | --- | --- |

JAI09615.1

back to list

  

| Protein Coverage
| Supporting Peptides
|

Protein Coverage:

Supporting Peptides:

| Peptide | Uniq | -10lgP | Mass | Length | ppm | m/z | z | RT | Fraction | Scan | Source File | Area F6 | Area F2 | Area F3 | Area F4 | Area F5 | Area F7 | Area F8 | Area F1 | #Feature | #Feature F6 | #Feature F2 | #Feature F3 | #Feature F4 | #Feature F5 | #Feature F7 | #Feature F8 | #Feature F1 | Start | End | PTM | AScore | Found By |
| --- | --- | --- | --- | --- | --- | --- | --- | --- | --- | --- | --- | --- | --- | --- | --- | --- | --- | --- | --- | --- | --- | --- | --- | --- | --- | --- | --- | --- | --- | --- | --- | --- | --- |
| R.LTVEDPVTVEYITR.Y | Y | 74.58 | 1633.8563 | 14 | 1.1 | 817.9363 | 2 | 49.19 | 8 | F8:27990 | 31122021\_RID\_3262\_1b.raw |  |  |  |  |  |  |  | 1.2989E5 | 1 | 0 | 0 | 0 | 0 | 0 | 0 | 0 | 1 | 96 | 109 |  |  | PEAKS DB |
| K.ALLEVVQSGGK.N | Y | 65.78 | 1099.6237 | 11 | 0.8 | 550.8195 | 2 | 36.13 | 8 | F8:17692 | 31122021\_RID\_3262\_1b.raw |  |  |  |  |  |  |  | 2.1171E5 | 1 | 0 | 0 | 0 | 0 | 0 | 0 | 0 | 1 | 194 | 204 |  |  | PEAKS DB |
| R.AITVFSPDGHLFQVEYAQEAVK.K | Y | 46.30 | 2448.2324 | 22 | 0.6 | 817.0852 | 3 | 53.64 | 8 | F8:31471 | 31122021\_RID\_3262\_1b.raw |  |  |  |  |  |  |  | 8.4864E4 | 1 | 0 | 0 | 0 | 0 | 0 | 0 | 0 | 1 | 6 | 27 |  |  | PEAKS DB |
| total 3 peptides |  |  |  |  |  |  |  |  |  |  |  |  |  |  |  |  |  |  |  |  |  |  |  |  |  |  |  |  |  |  |  |  |  |
| --- | --- | --- | --- | --- | --- | --- | --- | --- | --- | --- | --- | --- | --- | --- | --- | --- | --- | --- | --- | --- | --- | --- | --- | --- | --- | --- | --- | --- | --- | --- | --- | --- | --- |

JAI11926.1

back to list

  

| Protein Coverage
| Supporting Peptides
|

Protein Coverage:

Supporting Peptides:

| Peptide | Uniq | -10lgP | Mass | Length | ppm | m/z | z | RT | Fraction | Scan | Source File | Area F6 | Area F2 | Area F3 | Area F4 | Area F5 | Area F7 | Area F8 | Area F1 | #Feature | #Feature F6 | #Feature F2 | #Feature F3 | #Feature F4 | #Feature F5 | #Feature F7 | #Feature F8 | #Feature F1 | Start | End | PTM | AScore | Found By |
| --- | --- | --- | --- | --- | --- | --- | --- | --- | --- | --- | --- | --- | --- | --- | --- | --- | --- | --- | --- | --- | --- | --- | --- | --- | --- | --- | --- | --- | --- | --- | --- | --- | --- |
| R.LTVEDPVTVEYITR.Y | Y | 74.58 | 1633.8563 | 14 | 1.1 | 817.9363 | 2 | 49.19 | 8 | F8:27990 | 31122021\_RID\_3262\_1b.raw |  |  |  |  |  |  |  | 1.2989E5 | 1 | 0 | 0 | 0 | 0 | 0 | 0 | 0 | 1 | 96 | 109 |  |  | PEAKS DB |
| K.ALLEVVQSGGK.N | Y | 65.78 | 1099.6237 | 11 | 0.8 | 550.8195 | 2 | 36.13 | 8 | F8:17692 | 31122021\_RID\_3262\_1b.raw |  |  |  |  |  |  |  | 2.1171E5 | 1 | 0 | 0 | 0 | 0 | 0 | 0 | 0 | 1 | 194 | 204 |  |  | PEAKS DB |
| R.AITVFSPDGHLFQVEYAQEAVK.K | Y | 46.30 | 2448.2324 | 22 | 0.6 | 817.0852 | 3 | 53.64 | 8 | F8:31471 | 31122021\_RID\_3262\_1b.raw |  |  |  |  |  |  |  | 8.4864E4 | 1 | 0 | 0 | 0 | 0 | 0 | 0 | 0 | 1 | 6 | 27 |  |  | PEAKS DB |
| total 3 peptides |  |  |  |  |  |  |  |  |  |  |  |  |  |  |  |  |  |  |  |  |  |  |  |  |  |  |  |  |  |  |  |  |  |
| --- | --- | --- | --- | --- | --- | --- | --- | --- | --- | --- | --- | --- | --- | --- | --- | --- | --- | --- | --- | --- | --- | --- | --- | --- | --- | --- | --- | --- | --- | --- | --- | --- | --- |

JAV49551.1

back to list

  

| Protein Coverage
| Supporting Peptides
|

Protein Coverage:

Supporting Peptides:

| Peptide | Uniq | -10lgP | Mass | Length | ppm | m/z | z | RT | Fraction | Scan | Source File | Area F6 | Area F2 | Area F3 | Area F4 | Area F5 | Area F7 | Area F8 | Area F1 | #Feature | #Feature F6 | #Feature F2 | #Feature F3 | #Feature F4 | #Feature F5 | #Feature F7 | #Feature F8 | #Feature F1 | Start | End | PTM | AScore | Found By |
| --- | --- | --- | --- | --- | --- | --- | --- | --- | --- | --- | --- | --- | --- | --- | --- | --- | --- | --- | --- | --- | --- | --- | --- | --- | --- | --- | --- | --- | --- | --- | --- | --- | --- |
| R.LTVEDPVTVEYITR.Y | Y | 74.58 | 1633.8563 | 14 | 1.1 | 817.9363 | 2 | 49.19 | 8 | F8:27990 | 31122021\_RID\_3262\_1b.raw |  |  |  |  |  |  |  | 1.2989E5 | 1 | 0 | 0 | 0 | 0 | 0 | 0 | 0 | 1 | 96 | 109 |  |  | PEAKS DB |
| K.ALLEVVQSGGK.N | Y | 65.78 | 1099.6237 | 11 | 0.8 | 550.8195 | 2 | 36.13 | 8 | F8:17692 | 31122021\_RID\_3262\_1b.raw |  |  |  |  |  |  |  | 2.1171E5 | 1 | 0 | 0 | 0 | 0 | 0 | 0 | 0 | 1 | 194 | 204 |  |  | PEAKS DB |
| R.AITVFSPDGHLFQVEYAQEAVK.K | Y | 46.30 | 2448.2324 | 22 | 0.6 | 817.0852 | 3 | 53.64 | 8 | F8:31471 | 31122021\_RID\_3262\_1b.raw |  |  |  |  |  |  |  | 8.4864E4 | 1 | 0 | 0 | 0 | 0 | 0 | 0 | 0 | 1 | 6 | 27 |  |  | PEAKS DB |
| total 3 peptides |  |  |  |  |  |  |  |  |  |  |  |  |  |  |  |  |  |  |  |  |  |  |  |  |  |  |  |  |  |  |  |  |  |
| --- | --- | --- | --- | --- | --- | --- | --- | --- | --- | --- | --- | --- | --- | --- | --- | --- | --- | --- | --- | --- | --- | --- | --- | --- | --- | --- | --- | --- | --- | --- | --- | --- | --- |

XP\_026553199.1

back to list

  

| Protein Coverage
| Supporting Peptides
|

Protein Coverage:

Supporting Peptides:

| Peptide | Uniq | -10lgP | Mass | Length | ppm | m/z | z | RT | Fraction | Scan | Source File | Area F6 | Area F2 | Area F3 | Area F4 | Area F5 | Area F7 | Area F8 | Area F1 | #Feature | #Feature F6 | #Feature F2 | #Feature F3 | #Feature F4 | #Feature F5 | #Feature F7 | #Feature F8 | #Feature F1 | Start | End | PTM | AScore | Found By |
| --- | --- | --- | --- | --- | --- | --- | --- | --- | --- | --- | --- | --- | --- | --- | --- | --- | --- | --- | --- | --- | --- | --- | --- | --- | --- | --- | --- | --- | --- | --- | --- | --- | --- |
| R.LTVEDPVTVEYITR.Y | Y | 74.58 | 1633.8563 | 14 | 1.1 | 817.9363 | 2 | 49.19 | 8 | F8:27990 | 31122021\_RID\_3262\_1b.raw |  |  |  |  |  |  |  | 1.2989E5 | 1 | 0 | 0 | 0 | 0 | 0 | 0 | 0 | 1 | 96 | 109 |  |  | PEAKS DB |
| K.ALLEVVQSGGK.N | Y | 65.78 | 1099.6237 | 11 | 0.8 | 550.8195 | 2 | 36.13 | 8 | F8:17692 | 31122021\_RID\_3262\_1b.raw |  |  |  |  |  |  |  | 2.1171E5 | 1 | 0 | 0 | 0 | 0 | 0 | 0 | 0 | 1 | 194 | 204 |  |  | PEAKS DB |
| R.AITVFSPDGHLFQVEYAQEAVK.K | Y | 46.30 | 2448.2324 | 22 | 0.6 | 817.0852 | 3 | 53.64 | 8 | F8:31471 | 31122021\_RID\_3262\_1b.raw |  |  |  |  |  |  |  | 8.4864E4 | 1 | 0 | 0 | 0 | 0 | 0 | 0 | 0 | 1 | 6 | 27 |  |  | PEAKS DB |
| total 3 peptides |  |  |  |  |  |  |  |  |  |  |  |  |  |  |  |  |  |  |  |  |  |  |  |  |  |  |  |  |  |  |  |  |  |
| --- | --- | --- | --- | --- | --- | --- | --- | --- | --- | --- | --- | --- | --- | --- | --- | --- | --- | --- | --- | --- | --- | --- | --- | --- | --- | --- | --- | --- | --- | --- | --- | --- | --- |

XP\_034289033.1

back to list

  

| Protein Coverage
| Supporting Peptides
|

Protein Coverage:

Supporting Peptides:

| Peptide | Uniq | -10lgP | Mass | Length | ppm | m/z | z | RT | Fraction | Scan | Source File | Area F6 | Area F2 | Area F3 | Area F4 | Area F5 | Area F7 | Area F8 | Area F1 | #Feature | #Feature F6 | #Feature F2 | #Feature F3 | #Feature F4 | #Feature F5 | #Feature F7 | #Feature F8 | #Feature F1 | Start | End | PTM | AScore | Found By |
| --- | --- | --- | --- | --- | --- | --- | --- | --- | --- | --- | --- | --- | --- | --- | --- | --- | --- | --- | --- | --- | --- | --- | --- | --- | --- | --- | --- | --- | --- | --- | --- | --- | --- |
| R.LTVEDPVTVEYITR.Y | Y | 74.58 | 1633.8563 | 14 | 1.1 | 817.9363 | 2 | 49.19 | 8 | F8:27990 | 31122021\_RID\_3262\_1b.raw |  |  |  |  |  |  |  | 1.2989E5 | 1 | 0 | 0 | 0 | 0 | 0 | 0 | 0 | 1 | 96 | 109 |  |  | PEAKS DB |
| K.ALLEVVQSGGK.N | Y | 65.78 | 1099.6237 | 11 | 0.8 | 550.8195 | 2 | 36.13 | 8 | F8:17692 | 31122021\_RID\_3262\_1b.raw |  |  |  |  |  |  |  | 2.1171E5 | 1 | 0 | 0 | 0 | 0 | 0 | 0 | 0 | 1 | 194 | 204 |  |  | PEAKS DB |
| R.AITVFSPDGHLFQVEYAQEAVK.K | Y | 46.30 | 2448.2324 | 22 | 0.6 | 817.0852 | 3 | 53.64 | 8 | F8:31471 | 31122021\_RID\_3262\_1b.raw |  |  |  |  |  |  |  | 8.4864E4 | 1 | 0 | 0 | 0 | 0 | 0 | 0 | 0 | 1 | 6 | 27 |  |  | PEAKS DB |
| total 3 peptides |  |  |  |  |  |  |  |  |  |  |  |  |  |  |  |  |  |  |  |  |  |  |  |  |  |  |  |  |  |  |  |  |  |
| --- | --- | --- | --- | --- | --- | --- | --- | --- | --- | --- | --- | --- | --- | --- | --- | --- | --- | --- | --- | --- | --- | --- | --- | --- | --- | --- | --- | --- | --- | --- | --- | --- | --- |

XP\_032074995.1

back to list

  

| Protein Coverage
| Supporting Peptides
|

Protein Coverage:

Supporting Peptides:

| Peptide | Uniq | -10lgP | Mass | Length | ppm | m/z | z | RT | Fraction | Scan | Source File | Area F6 | Area F2 | Area F3 | Area F4 | Area F5 | Area F7 | Area F8 | Area F1 | #Feature | #Feature F6 | #Feature F2 | #Feature F3 | #Feature F4 | #Feature F5 | #Feature F7 | #Feature F8 | #Feature F1 | Start | End | PTM | AScore | Found By |
| --- | --- | --- | --- | --- | --- | --- | --- | --- | --- | --- | --- | --- | --- | --- | --- | --- | --- | --- | --- | --- | --- | --- | --- | --- | --- | --- | --- | --- | --- | --- | --- | --- | --- |
| R.LTVEDPVTVEYITR.Y | Y | 74.58 | 1633.8563 | 14 | 1.1 | 817.9363 | 2 | 49.19 | 8 | F8:27990 | 31122021\_RID\_3262\_1b.raw |  |  |  |  |  |  |  | 1.2989E5 | 1 | 0 | 0 | 0 | 0 | 0 | 0 | 0 | 1 | 96 | 109 |  |  | PEAKS DB |
| K.ALLEVVQSGGK.N | Y | 65.78 | 1099.6237 | 11 | 0.8 | 550.8195 | 2 | 36.13 | 8 | F8:17692 | 31122021\_RID\_3262\_1b.raw |  |  |  |  |  |  |  | 2.1171E5 | 1 | 0 | 0 | 0 | 0 | 0 | 0 | 0 | 1 | 194 | 204 |  |  | PEAKS DB |
| R.AITVFSPDGHLFQVEYAQEAVK.K | Y | 46.30 | 2448.2324 | 22 | 0.6 | 817.0852 | 3 | 53.64 | 8 | F8:31471 | 31122021\_RID\_3262\_1b.raw |  |  |  |  |  |  |  | 8.4864E4 | 1 | 0 | 0 | 0 | 0 | 0 | 0 | 0 | 1 | 6 | 27 |  |  | PEAKS DB |
| total 3 peptides |  |  |  |  |  |  |  |  |  |  |  |  |  |  |  |  |  |  |  |  |  |  |  |  |  |  |  |  |  |  |  |  |  |
| --- | --- | --- | --- | --- | --- | --- | --- | --- | --- | --- | --- | --- | --- | --- | --- | --- | --- | --- | --- | --- | --- | --- | --- | --- | --- | --- | --- | --- | --- | --- | --- | --- | --- |

BAN82080.1

back to list

  

| Protein Coverage
| Supporting Peptides
|

Protein Coverage:

Supporting Peptides:

| Peptide | Uniq | -10lgP | Mass | Length | ppm | m/z | z | RT | Fraction | Scan | Source File | Area F6 | Area F2 | Area F3 | Area F4 | Area F5 | Area F7 | Area F8 | Area F1 | #Feature | #Feature F6 | #Feature F2 | #Feature F3 | #Feature F4 | #Feature F5 | #Feature F7 | #Feature F8 | #Feature F1 | Start | End | PTM | AScore | Found By |
| --- | --- | --- | --- | --- | --- | --- | --- | --- | --- | --- | --- | --- | --- | --- | --- | --- | --- | --- | --- | --- | --- | --- | --- | --- | --- | --- | --- | --- | --- | --- | --- | --- | --- |
| R.LTVEDPVTVEYITR.Y | Y | 74.58 | 1633.8563 | 14 | 1.1 | 817.9363 | 2 | 49.19 | 8 | F8:27990 | 31122021\_RID\_3262\_1b.raw |  |  |  |  |  |  |  | 1.2989E5 | 1 | 0 | 0 | 0 | 0 | 0 | 0 | 0 | 1 | 96 | 109 |  |  | PEAKS DB |
| K.ALLEVVQSGGK.N | Y | 65.78 | 1099.6237 | 11 | 0.8 | 550.8195 | 2 | 36.13 | 8 | F8:17692 | 31122021\_RID\_3262\_1b.raw |  |  |  |  |  |  |  | 2.1171E5 | 1 | 0 | 0 | 0 | 0 | 0 | 0 | 0 | 1 | 194 | 204 |  |  | PEAKS DB |
| R.AITVFSPDGHLFQVEYAQEAVK.K | Y | 46.30 | 2448.2324 | 22 | 0.6 | 817.0852 | 3 | 53.64 | 8 | F8:31471 | 31122021\_RID\_3262\_1b.raw |  |  |  |  |  |  |  | 8.4864E4 | 1 | 0 | 0 | 0 | 0 | 0 | 0 | 0 | 1 | 6 | 27 |  |  | PEAKS DB |
| total 3 peptides |  |  |  |  |  |  |  |  |  |  |  |  |  |  |  |  |  |  |  |  |  |  |  |  |  |  |  |  |  |  |  |  |  |
| --- | --- | --- | --- | --- | --- | --- | --- | --- | --- | --- | --- | --- | --- | --- | --- | --- | --- | --- | --- | --- | --- | --- | --- | --- | --- | --- | --- | --- | --- | --- | --- | --- | --- |

XP\_013917261.1

back to list

  

| Protein Coverage
| Supporting Peptides
|

Protein Coverage:

Supporting Peptides:

| Peptide | Uniq | -10lgP | Mass | Length | ppm | m/z | z | RT | Fraction | Scan | Source File | Area F6 | Area F2 | Area F3 | Area F4 | Area F5 | Area F7 | Area F8 | Area F1 | #Feature | #Feature F6 | #Feature F2 | #Feature F3 | #Feature F4 | #Feature F5 | #Feature F7 | #Feature F8 | #Feature F1 | Start | End | PTM | AScore | Found By |
| --- | --- | --- | --- | --- | --- | --- | --- | --- | --- | --- | --- | --- | --- | --- | --- | --- | --- | --- | --- | --- | --- | --- | --- | --- | --- | --- | --- | --- | --- | --- | --- | --- | --- |
| R.LTVEDPVTVEYITR.Y | Y | 74.58 | 1633.8563 | 14 | 1.1 | 817.9363 | 2 | 49.19 | 8 | F8:27990 | 31122021\_RID\_3262\_1b.raw |  |  |  |  |  |  |  | 1.2989E5 | 1 | 0 | 0 | 0 | 0 | 0 | 0 | 0 | 1 | 96 | 109 |  |  | PEAKS DB |
| K.ALLEVVQSGGK.N | Y | 65.78 | 1099.6237 | 11 | 0.8 | 550.8195 | 2 | 36.13 | 8 | F8:17692 | 31122021\_RID\_3262\_1b.raw |  |  |  |  |  |  |  | 2.1171E5 | 1 | 0 | 0 | 0 | 0 | 0 | 0 | 0 | 1 | 194 | 204 |  |  | PEAKS DB |
| R.AITVFSPDGHLFQVEYAQEAVK.K | Y | 46.30 | 2448.2324 | 22 | 0.6 | 817.0852 | 3 | 53.64 | 8 | F8:31471 | 31122021\_RID\_3262\_1b.raw |  |  |  |  |  |  |  | 8.4864E4 | 1 | 0 | 0 | 0 | 0 | 0 | 0 | 0 | 1 | 6 | 27 |  |  | PEAKS DB |
| total 3 peptides |  |  |  |  |  |  |  |  |  |  |  |  |  |  |  |  |  |  |  |  |  |  |  |  |  |  |  |  |  |  |  |  |  |
| --- | --- | --- | --- | --- | --- | --- | --- | --- | --- | --- | --- | --- | --- | --- | --- | --- | --- | --- | --- | --- | --- | --- | --- | --- | --- | --- | --- | --- | --- | --- | --- | --- | --- |

JAS05409.1

back to list

  

| Protein Coverage
| Supporting Peptides
|

Protein Coverage:

Supporting Peptides:

| Peptide | Uniq | -10lgP | Mass | Length | ppm | m/z | z | RT | Fraction | Scan | Source File | Area F6 | Area F2 | Area F3 | Area F4 | Area F5 | Area F7 | Area F8 | Area F1 | #Feature | #Feature F6 | #Feature F2 | #Feature F3 | #Feature F4 | #Feature F5 | #Feature F7 | #Feature F8 | #Feature F1 | Start | End | PTM | AScore | Found By |
| --- | --- | --- | --- | --- | --- | --- | --- | --- | --- | --- | --- | --- | --- | --- | --- | --- | --- | --- | --- | --- | --- | --- | --- | --- | --- | --- | --- | --- | --- | --- | --- | --- | --- |
| R.FTSAGNIC(+57.02)RPAR.S | Y | 64.44 | 1348.6670 | 12 | -0.7 | 675.3403 | 2 | 21.05 | 3 | F3:7830 | 13012022\_RID\_3262\_3b.raw |  |  | 2.3638E7 |  |  |  |  |  | 1 | 0 | 0 | 1 | 0 | 0 | 0 | 0 | 0 | 450 | 461 | Carbamidomethylation | C8:Carbamidomethylation:1000.00 | PEAKS DB |
| S.QLVVTAEQQR.L | N | 61.06 | 1170.6356 | 10 | -0.6 | 586.3247 | 2 | 41.64 | 2 | F2:15936 | 13012022\_RID\_3262\_2b.raw |  | 1.1474E6 | 0 | 3.4155E5 | 8.5962E4 |  |  |  | 3 | 0 | 1 | 0 | 1 | 1 | 0 | 0 | 0 | 185 | 194 |  |  | PEAKS DB |
| Q.LVVTAEQQR.L | N | 59.96 | 1042.5771 | 9 | -0.3 | 522.2957 | 2 | 24.54 | 2 | F2:6838 | 13012022\_RID\_3262\_2b.raw |  | 4.3551E6 | 7.7997E6 | 4.2093E6 |  |  |  | 4.824E5 | 4 | 0 | 1 | 1 | 1 | 0 | 0 | 0 | 1 | 186 | 194 |  |  | PEAKS DB |
| R.FTSAGNIC(+57.02)RPA.R | Y | 53.80 | 1192.5659 | 11 | 0.7 | 597.2906 | 2 | 25.94 | 2 | F2:7462 | 13012022\_RID\_3262\_2b.raw |  | 7.7874E6 | 4.8009E7 |  |  |  |  |  | 2 | 0 | 1 | 1 | 0 | 0 | 0 | 0 | 0 | 450 | 460 | Carbamidomethylation | C8:Carbamidomethylation:1000.00 | PEAKS DB |
| total 4 peptides |  |  |  |  |  |  |  |  |  |  |  |  |  |  |  |  |  |  |  |  |  |  |  |  |  |  |  |  |  |  |  |  |  |
| --- | --- | --- | --- | --- | --- | --- | --- | --- | --- | --- | --- | --- | --- | --- | --- | --- | --- | --- | --- | --- | --- | --- | --- | --- | --- | --- | --- | --- | --- | --- | --- | --- | --- |

JAS05410.1

back to list

  

| Protein Coverage
| Supporting Peptides
|

Protein Coverage:

Supporting Peptides:

| Peptide | Uniq | -10lgP | Mass | Length | ppm | m/z | z | RT | Fraction | Scan | Source File | Area F6 | Area F2 | Area F3 | Area F4 | Area F5 | Area F7 | Area F8 | Area F1 | #Feature | #Feature F6 | #Feature F2 | #Feature F3 | #Feature F4 | #Feature F5 | #Feature F7 | #Feature F8 | #Feature F1 | Start | End | PTM | AScore | Found By |
| --- | --- | --- | --- | --- | --- | --- | --- | --- | --- | --- | --- | --- | --- | --- | --- | --- | --- | --- | --- | --- | --- | --- | --- | --- | --- | --- | --- | --- | --- | --- | --- | --- | --- |
| R.FTSAGNIC(+57.02)RPAR.S | Y | 64.44 | 1348.6670 | 12 | -0.7 | 675.3403 | 2 | 21.05 | 3 | F3:7830 | 13012022\_RID\_3262\_3b.raw |  |  | 2.3638E7 |  |  |  |  |  | 1 | 0 | 0 | 1 | 0 | 0 | 0 | 0 | 0 | 450 | 461 | Carbamidomethylation | C8:Carbamidomethylation:1000.00 | PEAKS DB |
| S.QLVVTAEQQR.L | N | 61.06 | 1170.6356 | 10 | -0.6 | 586.3247 | 2 | 41.64 | 2 | F2:15936 | 13012022\_RID\_3262\_2b.raw |  | 1.1474E6 | 0 | 3.4155E5 | 8.5962E4 |  |  |  | 3 | 0 | 1 | 0 | 1 | 1 | 0 | 0 | 0 | 185 | 194 |  |  | PEAKS DB |
| Q.LVVTAEQQR.L | N | 59.96 | 1042.5771 | 9 | -0.3 | 522.2957 | 2 | 24.54 | 2 | F2:6838 | 13012022\_RID\_3262\_2b.raw |  | 4.3551E6 | 7.7997E6 | 4.2093E6 |  |  |  | 4.824E5 | 4 | 0 | 1 | 1 | 1 | 0 | 0 | 0 | 1 | 186 | 194 |  |  | PEAKS DB |
| R.FTSAGNIC(+57.02)RPA.R | Y | 53.80 | 1192.5659 | 11 | 0.7 | 597.2906 | 2 | 25.94 | 2 | F2:7462 | 13012022\_RID\_3262\_2b.raw |  | 7.7874E6 | 4.8009E7 |  |  |  |  |  | 2 | 0 | 1 | 1 | 0 | 0 | 0 | 0 | 0 | 450 | 460 | Carbamidomethylation | C8:Carbamidomethylation:1000.00 | PEAKS DB |
| total 4 peptides |  |  |  |  |  |  |  |  |  |  |  |  |  |  |  |  |  |  |  |  |  |  |  |  |  |  |  |  |  |  |  |  |  |
| --- | --- | --- | --- | --- | --- | --- | --- | --- | --- | --- | --- | --- | --- | --- | --- | --- | --- | --- | --- | --- | --- | --- | --- | --- | --- | --- | --- | --- | --- | --- | --- | --- | --- |

AFJ49950.1

back to list

  

| Protein Coverage
| Supporting Peptides
|

Protein Coverage:

Supporting Peptides:

| Peptide | Uniq | -10lgP | Mass | Length | ppm | m/z | z | RT | Fraction | Scan | Source File | Area F6 | Area F2 | Area F3 | Area F4 | Area F5 | Area F7 | Area F8 | Area F1 | #Feature | #Feature F6 | #Feature F2 | #Feature F3 | #Feature F4 | #Feature F5 | #Feature F7 | #Feature F8 | #Feature F1 | Start | End | PTM | AScore | Found By |
| --- | --- | --- | --- | --- | --- | --- | --- | --- | --- | --- | --- | --- | --- | --- | --- | --- | --- | --- | --- | --- | --- | --- | --- | --- | --- | --- | --- | --- | --- | --- | --- | --- | --- |
| R.EHALLAYTLGVK.Q | Y | 64.19 | 1313.7343 | 12 | -0.3 | 657.8742 | 2 | 39.94 | 8 | F8:20708 | 31122021\_RID\_3262\_1b.raw |  |  |  |  |  |  |  | 1.7594E5 | 1 | 0 | 0 | 0 | 0 | 0 | 0 | 0 | 1 | 135 | 146 |  |  | PEAKS DB |
| K.STTTGHLIYK.C | Y | 60.44 | 1119.5924 | 10 | 0.8 | 560.8040 | 2 | 19.24 | 8 | F8:4903 | 31122021\_RID\_3262\_1b.raw |  |  |  |  |  |  |  | 4.9695E5 | 1 | 0 | 0 | 0 | 0 | 0 | 0 | 0 | 1 | 21 | 30 |  |  | PEAKS DB |
| K.YYVTIIDAPGHR.D | Y | 57.50 | 1403.7197 | 12 | 0.7 | 468.9142 | 3 | 36.23 | 8 | F8:17780 | 31122021\_RID\_3262\_1b.raw |  |  |  |  |  |  |  | 5.8111E5 | 2 | 0 | 0 | 0 | 0 | 0 | 0 | 0 | 2 | 85 | 96 |  |  | PEAKS DB |
| K.IGGIGTVPVGR.V | Y | 45.56 | 1024.6029 | 11 | 0.3 | 513.3089 | 2 | 34.95 | 8 | F8:16761 | 31122021\_RID\_3262\_1b.raw |  |  |  |  |  |  |  | 2.7161E5 | 1 | 0 | 0 | 0 | 0 | 0 | 0 | 0 | 1 | 256 | 266 |  |  | PEAKS DB |
| total 4 peptides |  |  |  |  |  |  |  |  |  |  |  |  |  |  |  |  |  |  |  |  |  |  |  |  |  |  |  |  |  |  |  |  |  |
| --- | --- | --- | --- | --- | --- | --- | --- | --- | --- | --- | --- | --- | --- | --- | --- | --- | --- | --- | --- | --- | --- | --- | --- | --- | --- | --- | --- | --- | --- | --- | --- | --- | --- |

JAA97186.1

back to list

  

| Protein Coverage
| Supporting Peptides
|

Protein Coverage:

Supporting Peptides:

| Peptide | Uniq | -10lgP | Mass | Length | ppm | m/z | z | RT | Fraction | Scan | Source File | Area F6 | Area F2 | Area F3 | Area F4 | Area F5 | Area F7 | Area F8 | Area F1 | #Feature | #Feature F6 | #Feature F2 | #Feature F3 | #Feature F4 | #Feature F5 | #Feature F7 | #Feature F8 | #Feature F1 | Start | End | PTM | AScore | Found By |
| --- | --- | --- | --- | --- | --- | --- | --- | --- | --- | --- | --- | --- | --- | --- | --- | --- | --- | --- | --- | --- | --- | --- | --- | --- | --- | --- | --- | --- | --- | --- | --- | --- | --- |
| R.EHALLAYTLGVK.Q | Y | 64.19 | 1313.7343 | 12 | -0.3 | 657.8742 | 2 | 39.94 | 8 | F8:20708 | 31122021\_RID\_3262\_1b.raw |  |  |  |  |  |  |  | 1.7594E5 | 1 | 0 | 0 | 0 | 0 | 0 | 0 | 0 | 1 | 135 | 146 |  |  | PEAKS DB |
| K.STTTGHLIYK.C | Y | 60.44 | 1119.5924 | 10 | 0.8 | 560.8040 | 2 | 19.24 | 8 | F8:4903 | 31122021\_RID\_3262\_1b.raw |  |  |  |  |  |  |  | 4.9695E5 | 1 | 0 | 0 | 0 | 0 | 0 | 0 | 0 | 1 | 21 | 30 |  |  | PEAKS DB |
| K.YYVTIIDAPGHR.D | Y | 57.50 | 1403.7197 | 12 | 0.7 | 468.9142 | 3 | 36.23 | 8 | F8:17780 | 31122021\_RID\_3262\_1b.raw |  |  |  |  |  |  |  | 5.8111E5 | 2 | 0 | 0 | 0 | 0 | 0 | 0 | 0 | 2 | 85 | 96 |  |  | PEAKS DB |
| K.IGGIGTVPVGR.V | Y | 45.56 | 1024.6029 | 11 | 0.3 | 513.3089 | 2 | 34.95 | 8 | F8:16761 | 31122021\_RID\_3262\_1b.raw |  |  |  |  |  |  |  | 2.7161E5 | 1 | 0 | 0 | 0 | 0 | 0 | 0 | 0 | 1 | 256 | 266 |  |  | PEAKS DB |
| total 4 peptides |  |  |  |  |  |  |  |  |  |  |  |  |  |  |  |  |  |  |  |  |  |  |  |  |  |  |  |  |  |  |  |  |  |
| --- | --- | --- | --- | --- | --- | --- | --- | --- | --- | --- | --- | --- | --- | --- | --- | --- | --- | --- | --- | --- | --- | --- | --- | --- | --- | --- | --- | --- | --- | --- | --- | --- | --- |

JAI13488.1

back to list

  

| Protein Coverage
| Supporting Peptides
|

Protein Coverage:

Supporting Peptides:

| Peptide | Uniq | -10lgP | Mass | Length | ppm | m/z | z | RT | Fraction | Scan | Source File | Area F6 | Area F2 | Area F3 | Area F4 | Area F5 | Area F7 | Area F8 | Area F1 | #Feature | #Feature F6 | #Feature F2 | #Feature F3 | #Feature F4 | #Feature F5 | #Feature F7 | #Feature F8 | #Feature F1 | Start | End | PTM | AScore | Found By |
| --- | --- | --- | --- | --- | --- | --- | --- | --- | --- | --- | --- | --- | --- | --- | --- | --- | --- | --- | --- | --- | --- | --- | --- | --- | --- | --- | --- | --- | --- | --- | --- | --- | --- |
| R.EHALLAYTLGVK.Q | Y | 64.19 | 1313.7343 | 12 | -0.3 | 657.8742 | 2 | 39.94 | 8 | F8:20708 | 31122021\_RID\_3262\_1b.raw |  |  |  |  |  |  |  | 1.7594E5 | 1 | 0 | 0 | 0 | 0 | 0 | 0 | 0 | 1 | 135 | 146 |  |  | PEAKS DB |
| K.STTTGHLIYK.C | Y | 60.44 | 1119.5924 | 10 | 0.8 | 560.8040 | 2 | 19.24 | 8 | F8:4903 | 31122021\_RID\_3262\_1b.raw |  |  |  |  |  |  |  | 4.9695E5 | 1 | 0 | 0 | 0 | 0 | 0 | 0 | 0 | 1 | 21 | 30 |  |  | PEAKS DB |
| K.YYVTIIDAPGHR.D | Y | 57.50 | 1403.7197 | 12 | 0.7 | 468.9142 | 3 | 36.23 | 8 | F8:17780 | 31122021\_RID\_3262\_1b.raw |  |  |  |  |  |  |  | 5.8111E5 | 2 | 0 | 0 | 0 | 0 | 0 | 0 | 0 | 2 | 85 | 96 |  |  | PEAKS DB |
| K.IGGIGTVPVGR.V | Y | 45.56 | 1024.6029 | 11 | 0.3 | 513.3089 | 2 | 34.95 | 8 | F8:16761 | 31122021\_RID\_3262\_1b.raw |  |  |  |  |  |  |  | 2.7161E5 | 1 | 0 | 0 | 0 | 0 | 0 | 0 | 0 | 1 | 256 | 266 |  |  | PEAKS DB |
| total 4 peptides |  |  |  |  |  |  |  |  |  |  |  |  |  |  |  |  |  |  |  |  |  |  |  |  |  |  |  |  |  |  |  |  |  |
| --- | --- | --- | --- | --- | --- | --- | --- | --- | --- | --- | --- | --- | --- | --- | --- | --- | --- | --- | --- | --- | --- | --- | --- | --- | --- | --- | --- | --- | --- | --- | --- | --- | --- |

XP\_007431170.1

back to list

  

| Protein Coverage
| Supporting Peptides
|

Protein Coverage:

Supporting Peptides:

| Peptide | Uniq | -10lgP | Mass | Length | ppm | m/z | z | RT | Fraction | Scan | Source File | Area F6 | Area F2 | Area F3 | Area F4 | Area F5 | Area F7 | Area F8 | Area F1 | #Feature | #Feature F6 | #Feature F2 | #Feature F3 | #Feature F4 | #Feature F5 | #Feature F7 | #Feature F8 | #Feature F1 | Start | End | PTM | AScore | Found By |
| --- | --- | --- | --- | --- | --- | --- | --- | --- | --- | --- | --- | --- | --- | --- | --- | --- | --- | --- | --- | --- | --- | --- | --- | --- | --- | --- | --- | --- | --- | --- | --- | --- | --- |
| R.EHALLAYTLGVK.Q | Y | 64.19 | 1313.7343 | 12 | -0.3 | 657.8742 | 2 | 39.94 | 8 | F8:20708 | 31122021\_RID\_3262\_1b.raw |  |  |  |  |  |  |  | 1.7594E5 | 1 | 0 | 0 | 0 | 0 | 0 | 0 | 0 | 1 | 135 | 146 |  |  | PEAKS DB |
| K.STTTGHLIYK.C | Y | 60.44 | 1119.5924 | 10 | 0.8 | 560.8040 | 2 | 19.24 | 8 | F8:4903 | 31122021\_RID\_3262\_1b.raw |  |  |  |  |  |  |  | 4.9695E5 | 1 | 0 | 0 | 0 | 0 | 0 | 0 | 0 | 1 | 21 | 30 |  |  | PEAKS DB |
| K.YYVTIIDAPGHR.D | Y | 57.50 | 1403.7197 | 12 | 0.7 | 468.9142 | 3 | 36.23 | 8 | F8:17780 | 31122021\_RID\_3262\_1b.raw |  |  |  |  |  |  |  | 5.8111E5 | 2 | 0 | 0 | 0 | 0 | 0 | 0 | 0 | 2 | 85 | 96 |  |  | PEAKS DB |
| K.IGGIGTVPVGR.V | Y | 45.56 | 1024.6029 | 11 | 0.3 | 513.3089 | 2 | 34.95 | 8 | F8:16761 | 31122021\_RID\_3262\_1b.raw |  |  |  |  |  |  |  | 2.7161E5 | 1 | 0 | 0 | 0 | 0 | 0 | 0 | 0 | 1 | 256 | 266 |  |  | PEAKS DB |
| total 4 peptides |  |  |  |  |  |  |  |  |  |  |  |  |  |  |  |  |  |  |  |  |  |  |  |  |  |  |  |  |  |  |  |  |  |
| --- | --- | --- | --- | --- | --- | --- | --- | --- | --- | --- | --- | --- | --- | --- | --- | --- | --- | --- | --- | --- | --- | --- | --- | --- | --- | --- | --- | --- | --- | --- | --- | --- | --- |

JAG46923.1

back to list

  

| Protein Coverage
| Supporting Peptides
|

Protein Coverage:

Supporting Peptides:

| Peptide | Uniq | -10lgP | Mass | Length | ppm | m/z | z | RT | Fraction | Scan | Source File | Area F6 | Area F2 | Area F3 | Area F4 | Area F5 | Area F7 | Area F8 | Area F1 | #Feature | #Feature F6 | #Feature F2 | #Feature F3 | #Feature F4 | #Feature F5 | #Feature F7 | #Feature F8 | #Feature F1 | Start | End | PTM | AScore | Found By |
| --- | --- | --- | --- | --- | --- | --- | --- | --- | --- | --- | --- | --- | --- | --- | --- | --- | --- | --- | --- | --- | --- | --- | --- | --- | --- | --- | --- | --- | --- | --- | --- | --- | --- |
| R.EHALLAYTLGVK.Q | Y | 64.19 | 1313.7343 | 12 | -0.3 | 657.8742 | 2 | 39.94 | 8 | F8:20708 | 31122021\_RID\_3262\_1b.raw |  |  |  |  |  |  |  | 1.7594E5 | 1 | 0 | 0 | 0 | 0 | 0 | 0 | 0 | 1 | 135 | 146 |  |  | PEAKS DB |
| K.STTTGHLIYK.C | Y | 60.44 | 1119.5924 | 10 | 0.8 | 560.8040 | 2 | 19.24 | 8 | F8:4903 | 31122021\_RID\_3262\_1b.raw |  |  |  |  |  |  |  | 4.9695E5 | 1 | 0 | 0 | 0 | 0 | 0 | 0 | 0 | 1 | 21 | 30 |  |  | PEAKS DB |
| K.YYVTIIDAPGHR.D | Y | 57.50 | 1403.7197 | 12 | 0.7 | 468.9142 | 3 | 36.23 | 8 | F8:17780 | 31122021\_RID\_3262\_1b.raw |  |  |  |  |  |  |  | 5.8111E5 | 2 | 0 | 0 | 0 | 0 | 0 | 0 | 0 | 2 | 85 | 96 |  |  | PEAKS DB |
| K.IGGIGTVPVGR.V | Y | 45.56 | 1024.6029 | 11 | 0.3 | 513.3089 | 2 | 34.95 | 8 | F8:16761 | 31122021\_RID\_3262\_1b.raw |  |  |  |  |  |  |  | 2.7161E5 | 1 | 0 | 0 | 0 | 0 | 0 | 0 | 0 | 1 | 256 | 266 |  |  | PEAKS DB |
| total 4 peptides |  |  |  |  |  |  |  |  |  |  |  |  |  |  |  |  |  |  |  |  |  |  |  |  |  |  |  |  |  |  |  |  |  |
| --- | --- | --- | --- | --- | --- | --- | --- | --- | --- | --- | --- | --- | --- | --- | --- | --- | --- | --- | --- | --- | --- | --- | --- | --- | --- | --- | --- | --- | --- | --- | --- | --- | --- |

XP\_015672964.1

back to list

  

| Protein Coverage
| Supporting Peptides
|

Protein Coverage:

Supporting Peptides:

| Peptide | Uniq | -10lgP | Mass | Length | ppm | m/z | z | RT | Fraction | Scan | Source File | Area F6 | Area F2 | Area F3 | Area F4 | Area F5 | Area F7 | Area F8 | Area F1 | #Feature | #Feature F6 | #Feature F2 | #Feature F3 | #Feature F4 | #Feature F5 | #Feature F7 | #Feature F8 | #Feature F1 | Start | End | PTM | AScore | Found By |
| --- | --- | --- | --- | --- | --- | --- | --- | --- | --- | --- | --- | --- | --- | --- | --- | --- | --- | --- | --- | --- | --- | --- | --- | --- | --- | --- | --- | --- | --- | --- | --- | --- | --- |
| R.EHALLAYTLGVK.Q | Y | 64.19 | 1313.7343 | 12 | -0.3 | 657.8742 | 2 | 39.94 | 8 | F8:20708 | 31122021\_RID\_3262\_1b.raw |  |  |  |  |  |  |  | 1.7594E5 | 1 | 0 | 0 | 0 | 0 | 0 | 0 | 0 | 1 | 135 | 146 |  |  | PEAKS DB |
| K.STTTGHLIYK.C | Y | 60.44 | 1119.5924 | 10 | 0.8 | 560.8040 | 2 | 19.24 | 8 | F8:4903 | 31122021\_RID\_3262\_1b.raw |  |  |  |  |  |  |  | 4.9695E5 | 1 | 0 | 0 | 0 | 0 | 0 | 0 | 0 | 1 | 21 | 30 |  |  | PEAKS DB |
| K.YYVTIIDAPGHR.D | Y | 57.50 | 1403.7197 | 12 | 0.7 | 468.9142 | 3 | 36.23 | 8 | F8:17780 | 31122021\_RID\_3262\_1b.raw |  |  |  |  |  |  |  | 5.8111E5 | 2 | 0 | 0 | 0 | 0 | 0 | 0 | 0 | 2 | 85 | 96 |  |  | PEAKS DB |
| K.IGGIGTVPVGR.V | Y | 45.56 | 1024.6029 | 11 | 0.3 | 513.3089 | 2 | 34.95 | 8 | F8:16761 | 31122021\_RID\_3262\_1b.raw |  |  |  |  |  |  |  | 2.7161E5 | 1 | 0 | 0 | 0 | 0 | 0 | 0 | 0 | 1 | 256 | 266 |  |  | PEAKS DB |
| total 4 peptides |  |  |  |  |  |  |  |  |  |  |  |  |  |  |  |  |  |  |  |  |  |  |  |  |  |  |  |  |  |  |  |  |  |
| --- | --- | --- | --- | --- | --- | --- | --- | --- | --- | --- | --- | --- | --- | --- | --- | --- | --- | --- | --- | --- | --- | --- | --- | --- | --- | --- | --- | --- | --- | --- | --- | --- | --- |

JAG44960.1

back to list

  

| Protein Coverage
| Supporting Peptides
|

Protein Coverage:

Supporting Peptides:

| Peptide | Uniq | -10lgP | Mass | Length | ppm | m/z | z | RT | Fraction | Scan | Source File | Area F6 | Area F2 | Area F3 | Area F4 | Area F5 | Area F7 | Area F8 | Area F1 | #Feature | #Feature F6 | #Feature F2 | #Feature F3 | #Feature F4 | #Feature F5 | #Feature F7 | #Feature F8 | #Feature F1 | Start | End | PTM | AScore | Found By |
| --- | --- | --- | --- | --- | --- | --- | --- | --- | --- | --- | --- | --- | --- | --- | --- | --- | --- | --- | --- | --- | --- | --- | --- | --- | --- | --- | --- | --- | --- | --- | --- | --- | --- |
| R.EHALLAYTLGVK.Q | Y | 64.19 | 1313.7343 | 12 | -0.3 | 657.8742 | 2 | 39.94 | 8 | F8:20708 | 31122021\_RID\_3262\_1b.raw |  |  |  |  |  |  |  | 1.7594E5 | 1 | 0 | 0 | 0 | 0 | 0 | 0 | 0 | 1 | 135 | 146 |  |  | PEAKS DB |
| K.STTTGHLIYK.C | Y | 60.44 | 1119.5924 | 10 | 0.8 | 560.8040 | 2 | 19.24 | 8 | F8:4903 | 31122021\_RID\_3262\_1b.raw |  |  |  |  |  |  |  | 4.9695E5 | 1 | 0 | 0 | 0 | 0 | 0 | 0 | 0 | 1 | 21 | 30 |  |  | PEAKS DB |
| K.YYVTIIDAPGHR.D | Y | 57.50 | 1403.7197 | 12 | 0.7 | 468.9142 | 3 | 36.23 | 8 | F8:17780 | 31122021\_RID\_3262\_1b.raw |  |  |  |  |  |  |  | 5.8111E5 | 2 | 0 | 0 | 0 | 0 | 0 | 0 | 0 | 2 | 85 | 96 |  |  | PEAKS DB |
| K.IGGIGTVPVGR.V | Y | 45.56 | 1024.6029 | 11 | 0.3 | 513.3089 | 2 | 34.95 | 8 | F8:16761 | 31122021\_RID\_3262\_1b.raw |  |  |  |  |  |  |  | 2.7161E5 | 1 | 0 | 0 | 0 | 0 | 0 | 0 | 0 | 1 | 256 | 266 |  |  | PEAKS DB |
| total 4 peptides |  |  |  |  |  |  |  |  |  |  |  |  |  |  |  |  |  |  |  |  |  |  |  |  |  |  |  |  |  |  |  |  |  |
| --- | --- | --- | --- | --- | --- | --- | --- | --- | --- | --- | --- | --- | --- | --- | --- | --- | --- | --- | --- | --- | --- | --- | --- | --- | --- | --- | --- | --- | --- | --- | --- | --- | --- |

JAV50678.1

back to list

  

| Protein Coverage
| Supporting Peptides
|

Protein Coverage:

Supporting Peptides:

| Peptide | Uniq | -10lgP | Mass | Length | ppm | m/z | z | RT | Fraction | Scan | Source File | Area F6 | Area F2 | Area F3 | Area F4 | Area F5 | Area F7 | Area F8 | Area F1 | #Feature | #Feature F6 | #Feature F2 | #Feature F3 | #Feature F4 | #Feature F5 | #Feature F7 | #Feature F8 | #Feature F1 | Start | End | PTM | AScore | Found By |
| --- | --- | --- | --- | --- | --- | --- | --- | --- | --- | --- | --- | --- | --- | --- | --- | --- | --- | --- | --- | --- | --- | --- | --- | --- | --- | --- | --- | --- | --- | --- | --- | --- | --- |
| R.EHALLAYTLGVK.Q | Y | 64.19 | 1313.7343 | 12 | -0.3 | 657.8742 | 2 | 39.94 | 8 | F8:20708 | 31122021\_RID\_3262\_1b.raw |  |  |  |  |  |  |  | 1.7594E5 | 1 | 0 | 0 | 0 | 0 | 0 | 0 | 0 | 1 | 135 | 146 |  |  | PEAKS DB |
| K.STTTGHLIYK.C | Y | 60.44 | 1119.5924 | 10 | 0.8 | 560.8040 | 2 | 19.24 | 8 | F8:4903 | 31122021\_RID\_3262\_1b.raw |  |  |  |  |  |  |  | 4.9695E5 | 1 | 0 | 0 | 0 | 0 | 0 | 0 | 0 | 1 | 21 | 30 |  |  | PEAKS DB |
| K.YYVTIIDAPGHR.D | Y | 57.50 | 1403.7197 | 12 | 0.7 | 468.9142 | 3 | 36.23 | 8 | F8:17780 | 31122021\_RID\_3262\_1b.raw |  |  |  |  |  |  |  | 5.8111E5 | 2 | 0 | 0 | 0 | 0 | 0 | 0 | 0 | 2 | 85 | 96 |  |  | PEAKS DB |
| K.IGGIGTVPVGR.V | Y | 45.56 | 1024.6029 | 11 | 0.3 | 513.3089 | 2 | 34.95 | 8 | F8:16761 | 31122021\_RID\_3262\_1b.raw |  |  |  |  |  |  |  | 2.7161E5 | 1 | 0 | 0 | 0 | 0 | 0 | 0 | 0 | 1 | 256 | 266 |  |  | PEAKS DB |
| total 4 peptides |  |  |  |  |  |  |  |  |  |  |  |  |  |  |  |  |  |  |  |  |  |  |  |  |  |  |  |  |  |  |  |  |  |
| --- | --- | --- | --- | --- | --- | --- | --- | --- | --- | --- | --- | --- | --- | --- | --- | --- | --- | --- | --- | --- | --- | --- | --- | --- | --- | --- | --- | --- | --- | --- | --- | --- | --- |

ETE71213.1

back to list

  

| Protein Coverage
| Supporting Peptides
|

Protein Coverage:

Supporting Peptides:

| Peptide | Uniq | -10lgP | Mass | Length | ppm | m/z | z | RT | Fraction | Scan | Source File | Area F6 | Area F2 | Area F3 | Area F4 | Area F5 | Area F7 | Area F8 | Area F1 | #Feature | #Feature F6 | #Feature F2 | #Feature F3 | #Feature F4 | #Feature F5 | #Feature F7 | #Feature F8 | #Feature F1 | Start | End | PTM | AScore | Found By |
| --- | --- | --- | --- | --- | --- | --- | --- | --- | --- | --- | --- | --- | --- | --- | --- | --- | --- | --- | --- | --- | --- | --- | --- | --- | --- | --- | --- | --- | --- | --- | --- | --- | --- |
| R.EHALLAYTLGVK.Q | Y | 64.19 | 1313.7343 | 12 | -0.3 | 657.8742 | 2 | 39.94 | 8 | F8:20708 | 31122021\_RID\_3262\_1b.raw |  |  |  |  |  |  |  | 1.7594E5 | 1 | 0 | 0 | 0 | 0 | 0 | 0 | 0 | 1 | 135 | 146 |  |  | PEAKS DB |
| K.STTTGHLIYK.C | Y | 60.44 | 1119.5924 | 10 | 0.8 | 560.8040 | 2 | 19.24 | 8 | F8:4903 | 31122021\_RID\_3262\_1b.raw |  |  |  |  |  |  |  | 4.9695E5 | 1 | 0 | 0 | 0 | 0 | 0 | 0 | 0 | 1 | 21 | 30 |  |  | PEAKS DB |
| K.YYVTIIDAPGHR.D | Y | 57.50 | 1403.7197 | 12 | 0.7 | 468.9142 | 3 | 36.23 | 8 | F8:17780 | 31122021\_RID\_3262\_1b.raw |  |  |  |  |  |  |  | 5.8111E5 | 2 | 0 | 0 | 0 | 0 | 0 | 0 | 0 | 2 | 85 | 96 |  |  | PEAKS DB |
| K.IGGIGTVPVGR.V | Y | 45.56 | 1024.6029 | 11 | 0.3 | 513.3089 | 2 | 34.95 | 8 | F8:16761 | 31122021\_RID\_3262\_1b.raw |  |  |  |  |  |  |  | 2.7161E5 | 1 | 0 | 0 | 0 | 0 | 0 | 0 | 0 | 1 | 256 | 266 |  |  | PEAKS DB |
| total 4 peptides |  |  |  |  |  |  |  |  |  |  |  |  |  |  |  |  |  |  |  |  |  |  |  |  |  |  |  |  |  |  |  |  |  |
| --- | --- | --- | --- | --- | --- | --- | --- | --- | --- | --- | --- | --- | --- | --- | --- | --- | --- | --- | --- | --- | --- | --- | --- | --- | --- | --- | --- | --- | --- | --- | --- | --- | --- |

JAC95701.1

back to list

  

| Protein Coverage
| Supporting Peptides
|

Protein Coverage:

Supporting Peptides:

| Peptide | Uniq | -10lgP | Mass | Length | ppm | m/z | z | RT | Fraction | Scan | Source File | Area F6 | Area F2 | Area F3 | Area F4 | Area F5 | Area F7 | Area F8 | Area F1 | #Feature | #Feature F6 | #Feature F2 | #Feature F3 | #Feature F4 | #Feature F5 | #Feature F7 | #Feature F8 | #Feature F1 | Start | End | PTM | AScore | Found By |
| --- | --- | --- | --- | --- | --- | --- | --- | --- | --- | --- | --- | --- | --- | --- | --- | --- | --- | --- | --- | --- | --- | --- | --- | --- | --- | --- | --- | --- | --- | --- | --- | --- | --- |
| R.EHALLAYTLGVK.Q | Y | 64.19 | 1313.7343 | 12 | -0.3 | 657.8742 | 2 | 39.94 | 8 | F8:20708 | 31122021\_RID\_3262\_1b.raw |  |  |  |  |  |  |  | 1.7594E5 | 1 | 0 | 0 | 0 | 0 | 0 | 0 | 0 | 1 | 135 | 146 |  |  | PEAKS DB |
| K.STTTGHLIYK.C | Y | 60.44 | 1119.5924 | 10 | 0.8 | 560.8040 | 2 | 19.24 | 8 | F8:4903 | 31122021\_RID\_3262\_1b.raw |  |  |  |  |  |  |  | 4.9695E5 | 1 | 0 | 0 | 0 | 0 | 0 | 0 | 0 | 1 | 21 | 30 |  |  | PEAKS DB |
| K.YYVTIIDAPGHR.D | Y | 57.50 | 1403.7197 | 12 | 0.7 | 468.9142 | 3 | 36.23 | 8 | F8:17780 | 31122021\_RID\_3262\_1b.raw |  |  |  |  |  |  |  | 5.8111E5 | 2 | 0 | 0 | 0 | 0 | 0 | 0 | 0 | 2 | 85 | 96 |  |  | PEAKS DB |
| K.IGGIGTVPVGR.V | Y | 45.56 | 1024.6029 | 11 | 0.3 | 513.3089 | 2 | 34.95 | 8 | F8:16761 | 31122021\_RID\_3262\_1b.raw |  |  |  |  |  |  |  | 2.7161E5 | 1 | 0 | 0 | 0 | 0 | 0 | 0 | 0 | 1 | 256 | 266 |  |  | PEAKS DB |
| total 4 peptides |  |  |  |  |  |  |  |  |  |  |  |  |  |  |  |  |  |  |  |  |  |  |  |  |  |  |  |  |  |  |  |  |  |
| --- | --- | --- | --- | --- | --- | --- | --- | --- | --- | --- | --- | --- | --- | --- | --- | --- | --- | --- | --- | --- | --- | --- | --- | --- | --- | --- | --- | --- | --- | --- | --- | --- | --- |

JAC95700.1

back to list

  

| Protein Coverage
| Supporting Peptides
|

Protein Coverage:

Supporting Peptides:

| Peptide | Uniq | -10lgP | Mass | Length | ppm | m/z | z | RT | Fraction | Scan | Source File | Area F6 | Area F2 | Area F3 | Area F4 | Area F5 | Area F7 | Area F8 | Area F1 | #Feature | #Feature F6 | #Feature F2 | #Feature F3 | #Feature F4 | #Feature F5 | #Feature F7 | #Feature F8 | #Feature F1 | Start | End | PTM | AScore | Found By |
| --- | --- | --- | --- | --- | --- | --- | --- | --- | --- | --- | --- | --- | --- | --- | --- | --- | --- | --- | --- | --- | --- | --- | --- | --- | --- | --- | --- | --- | --- | --- | --- | --- | --- |
| R.EHALLAYTLGVK.Q | Y | 64.19 | 1313.7343 | 12 | -0.3 | 657.8742 | 2 | 39.94 | 8 | F8:20708 | 31122021\_RID\_3262\_1b.raw |  |  |  |  |  |  |  | 1.7594E5 | 1 | 0 | 0 | 0 | 0 | 0 | 0 | 0 | 1 | 135 | 146 |  |  | PEAKS DB |
| K.STTTGHLIYK.C | Y | 60.44 | 1119.5924 | 10 | 0.8 | 560.8040 | 2 | 19.24 | 8 | F8:4903 | 31122021\_RID\_3262\_1b.raw |  |  |  |  |  |  |  | 4.9695E5 | 1 | 0 | 0 | 0 | 0 | 0 | 0 | 0 | 1 | 21 | 30 |  |  | PEAKS DB |
| K.YYVTIIDAPGHR.D | Y | 57.50 | 1403.7197 | 12 | 0.7 | 468.9142 | 3 | 36.23 | 8 | F8:17780 | 31122021\_RID\_3262\_1b.raw |  |  |  |  |  |  |  | 5.8111E5 | 2 | 0 | 0 | 0 | 0 | 0 | 0 | 0 | 2 | 85 | 96 |  |  | PEAKS DB |
| K.IGGIGTVPVGR.V | Y | 45.56 | 1024.6029 | 11 | 0.3 | 513.3089 | 2 | 34.95 | 8 | F8:16761 | 31122021\_RID\_3262\_1b.raw |  |  |  |  |  |  |  | 2.7161E5 | 1 | 0 | 0 | 0 | 0 | 0 | 0 | 0 | 1 | 256 | 266 |  |  | PEAKS DB |
| total 4 peptides |  |  |  |  |  |  |  |  |  |  |  |  |  |  |  |  |  |  |  |  |  |  |  |  |  |  |  |  |  |  |  |  |  |
| --- | --- | --- | --- | --- | --- | --- | --- | --- | --- | --- | --- | --- | --- | --- | --- | --- | --- | --- | --- | --- | --- | --- | --- | --- | --- | --- | --- | --- | --- | --- | --- | --- | --- |

JAG68089.1

back to list

  

| Protein Coverage
| Supporting Peptides
|

Protein Coverage:

Supporting Peptides:

| Peptide | Uniq | -10lgP | Mass | Length | ppm | m/z | z | RT | Fraction | Scan | Source File | Area F6 | Area F2 | Area F3 | Area F4 | Area F5 | Area F7 | Area F8 | Area F1 | #Feature | #Feature F6 | #Feature F2 | #Feature F3 | #Feature F4 | #Feature F5 | #Feature F7 | #Feature F8 | #Feature F1 | Start | End | PTM | AScore | Found By |
| --- | --- | --- | --- | --- | --- | --- | --- | --- | --- | --- | --- | --- | --- | --- | --- | --- | --- | --- | --- | --- | --- | --- | --- | --- | --- | --- | --- | --- | --- | --- | --- | --- | --- |
| R.EHALLAYTLGVK.Q | Y | 64.19 | 1313.7343 | 12 | -0.3 | 657.8742 | 2 | 39.94 | 8 | F8:20708 | 31122021\_RID\_3262\_1b.raw |  |  |  |  |  |  |  | 1.7594E5 | 1 | 0 | 0 | 0 | 0 | 0 | 0 | 0 | 1 | 135 | 146 |  |  | PEAKS DB |
| K.STTTGHLIYK.C | Y | 60.44 | 1119.5924 | 10 | 0.8 | 560.8040 | 2 | 19.24 | 8 | F8:4903 | 31122021\_RID\_3262\_1b.raw |  |  |  |  |  |  |  | 4.9695E5 | 1 | 0 | 0 | 0 | 0 | 0 | 0 | 0 | 1 | 21 | 30 |  |  | PEAKS DB |
| K.YYVTIIDAPGHR.D | Y | 57.50 | 1403.7197 | 12 | 0.7 | 468.9142 | 3 | 36.23 | 8 | F8:17780 | 31122021\_RID\_3262\_1b.raw |  |  |  |  |  |  |  | 5.8111E5 | 2 | 0 | 0 | 0 | 0 | 0 | 0 | 0 | 2 | 85 | 96 |  |  | PEAKS DB |
| K.IGGIGTVPVGR.V | Y | 45.56 | 1024.6029 | 11 | 0.3 | 513.3089 | 2 | 34.95 | 8 | F8:16761 | 31122021\_RID\_3262\_1b.raw |  |  |  |  |  |  |  | 2.7161E5 | 1 | 0 | 0 | 0 | 0 | 0 | 0 | 0 | 1 | 256 | 266 |  |  | PEAKS DB |
| total 4 peptides |  |  |  |  |  |  |  |  |  |  |  |  |  |  |  |  |  |  |  |  |  |  |  |  |  |  |  |  |  |  |  |  |  |
| --- | --- | --- | --- | --- | --- | --- | --- | --- | --- | --- | --- | --- | --- | --- | --- | --- | --- | --- | --- | --- | --- | --- | --- | --- | --- | --- | --- | --- | --- | --- | --- | --- | --- |

JAI10049.1

back to list

  

| Protein Coverage
| Supporting Peptides
|

Protein Coverage:

Supporting Peptides:

| Peptide | Uniq | -10lgP | Mass | Length | ppm | m/z | z | RT | Fraction | Scan | Source File | Area F6 | Area F2 | Area F3 | Area F4 | Area F5 | Area F7 | Area F8 | Area F1 | #Feature | #Feature F6 | #Feature F2 | #Feature F3 | #Feature F4 | #Feature F5 | #Feature F7 | #Feature F8 | #Feature F1 | Start | End | PTM | AScore | Found By |
| --- | --- | --- | --- | --- | --- | --- | --- | --- | --- | --- | --- | --- | --- | --- | --- | --- | --- | --- | --- | --- | --- | --- | --- | --- | --- | --- | --- | --- | --- | --- | --- | --- | --- |
| R.EHALLAYTLGVK.Q | Y | 64.19 | 1313.7343 | 12 | -0.3 | 657.8742 | 2 | 39.94 | 8 | F8:20708 | 31122021\_RID\_3262\_1b.raw |  |  |  |  |  |  |  | 1.7594E5 | 1 | 0 | 0 | 0 | 0 | 0 | 0 | 0 | 1 | 135 | 146 |  |  | PEAKS DB |
| K.STTTGHLIYK.C | Y | 60.44 | 1119.5924 | 10 | 0.8 | 560.8040 | 2 | 19.24 | 8 | F8:4903 | 31122021\_RID\_3262\_1b.raw |  |  |  |  |  |  |  | 4.9695E5 | 1 | 0 | 0 | 0 | 0 | 0 | 0 | 0 | 1 | 21 | 30 |  |  | PEAKS DB |
| K.YYVTIIDAPGHR.D | Y | 57.50 | 1403.7197 | 12 | 0.7 | 468.9142 | 3 | 36.23 | 8 | F8:17780 | 31122021\_RID\_3262\_1b.raw |  |  |  |  |  |  |  | 5.8111E5 | 2 | 0 | 0 | 0 | 0 | 0 | 0 | 0 | 2 | 85 | 96 |  |  | PEAKS DB |
| K.IGGIGTVPVGR.V | Y | 45.56 | 1024.6029 | 11 | 0.3 | 513.3089 | 2 | 34.95 | 8 | F8:16761 | 31122021\_RID\_3262\_1b.raw |  |  |  |  |  |  |  | 2.7161E5 | 1 | 0 | 0 | 0 | 0 | 0 | 0 | 0 | 1 | 256 | 266 |  |  | PEAKS DB |
| total 4 peptides |  |  |  |  |  |  |  |  |  |  |  |  |  |  |  |  |  |  |  |  |  |  |  |  |  |  |  |  |  |  |  |  |  |
| --- | --- | --- | --- | --- | --- | --- | --- | --- | --- | --- | --- | --- | --- | --- | --- | --- | --- | --- | --- | --- | --- | --- | --- | --- | --- | --- | --- | --- | --- | --- | --- | --- | --- |

XP\_034287789.1

back to list

  

| Protein Coverage
| Supporting Peptides
|

Protein Coverage:

Supporting Peptides:

| Peptide | Uniq | -10lgP | Mass | Length | ppm | m/z | z | RT | Fraction | Scan | Source File | Area F6 | Area F2 | Area F3 | Area F4 | Area F5 | Area F7 | Area F8 | Area F1 | #Feature | #Feature F6 | #Feature F2 | #Feature F3 | #Feature F4 | #Feature F5 | #Feature F7 | #Feature F8 | #Feature F1 | Start | End | PTM | AScore | Found By |
| --- | --- | --- | --- | --- | --- | --- | --- | --- | --- | --- | --- | --- | --- | --- | --- | --- | --- | --- | --- | --- | --- | --- | --- | --- | --- | --- | --- | --- | --- | --- | --- | --- | --- |
| R.EHALLAYTLGVK.Q | Y | 64.19 | 1313.7343 | 12 | -0.3 | 657.8742 | 2 | 39.94 | 8 | F8:20708 | 31122021\_RID\_3262\_1b.raw |  |  |  |  |  |  |  | 1.7594E5 | 1 | 0 | 0 | 0 | 0 | 0 | 0 | 0 | 1 | 135 | 146 |  |  | PEAKS DB |
| K.STTTGHLIYK.C | Y | 60.44 | 1119.5924 | 10 | 0.8 | 560.8040 | 2 | 19.24 | 8 | F8:4903 | 31122021\_RID\_3262\_1b.raw |  |  |  |  |  |  |  | 4.9695E5 | 1 | 0 | 0 | 0 | 0 | 0 | 0 | 0 | 1 | 21 | 30 |  |  | PEAKS DB |
| K.YYVTIIDAPGHR.D | Y | 57.50 | 1403.7197 | 12 | 0.7 | 468.9142 | 3 | 36.23 | 8 | F8:17780 | 31122021\_RID\_3262\_1b.raw |  |  |  |  |  |  |  | 5.8111E5 | 2 | 0 | 0 | 0 | 0 | 0 | 0 | 0 | 2 | 85 | 96 |  |  | PEAKS DB |
| K.IGGIGTVPVGR.V | Y | 45.56 | 1024.6029 | 11 | 0.3 | 513.3089 | 2 | 34.95 | 8 | F8:16761 | 31122021\_RID\_3262\_1b.raw |  |  |  |  |  |  |  | 2.7161E5 | 1 | 0 | 0 | 0 | 0 | 0 | 0 | 0 | 1 | 256 | 266 |  |  | PEAKS DB |
| total 4 peptides |  |  |  |  |  |  |  |  |  |  |  |  |  |  |  |  |  |  |  |  |  |  |  |  |  |  |  |  |  |  |  |  |  |
| --- | --- | --- | --- | --- | --- | --- | --- | --- | --- | --- | --- | --- | --- | --- | --- | --- | --- | --- | --- | --- | --- | --- | --- | --- | --- | --- | --- | --- | --- | --- | --- | --- | --- |

XP\_026550930.1

back to list

  

| Protein Coverage
| Supporting Peptides
|

Protein Coverage:

Supporting Peptides:

| Peptide | Uniq | -10lgP | Mass | Length | ppm | m/z | z | RT | Fraction | Scan | Source File | Area F6 | Area F2 | Area F3 | Area F4 | Area F5 | Area F7 | Area F8 | Area F1 | #Feature | #Feature F6 | #Feature F2 | #Feature F3 | #Feature F4 | #Feature F5 | #Feature F7 | #Feature F8 | #Feature F1 | Start | End | PTM | AScore | Found By |
| --- | --- | --- | --- | --- | --- | --- | --- | --- | --- | --- | --- | --- | --- | --- | --- | --- | --- | --- | --- | --- | --- | --- | --- | --- | --- | --- | --- | --- | --- | --- | --- | --- | --- |
| R.EHALLAYTLGVK.Q | Y | 64.19 | 1313.7343 | 12 | -0.3 | 657.8742 | 2 | 39.94 | 8 | F8:20708 | 31122021\_RID\_3262\_1b.raw |  |  |  |  |  |  |  | 1.7594E5 | 1 | 0 | 0 | 0 | 0 | 0 | 0 | 0 | 1 | 135 | 146 |  |  | PEAKS DB |
| K.STTTGHLIYK.C | Y | 60.44 | 1119.5924 | 10 | 0.8 | 560.8040 | 2 | 19.24 | 8 | F8:4903 | 31122021\_RID\_3262\_1b.raw |  |  |  |  |  |  |  | 4.9695E5 | 1 | 0 | 0 | 0 | 0 | 0 | 0 | 0 | 1 | 21 | 30 |  |  | PEAKS DB |
| K.YYVTIIDAPGHR.D | Y | 57.50 | 1403.7197 | 12 | 0.7 | 468.9142 | 3 | 36.23 | 8 | F8:17780 | 31122021\_RID\_3262\_1b.raw |  |  |  |  |  |  |  | 5.8111E5 | 2 | 0 | 0 | 0 | 0 | 0 | 0 | 0 | 2 | 85 | 96 |  |  | PEAKS DB |
| K.IGGIGTVPVGR.V | Y | 45.56 | 1024.6029 | 11 | 0.3 | 513.3089 | 2 | 34.95 | 8 | F8:16761 | 31122021\_RID\_3262\_1b.raw |  |  |  |  |  |  |  | 2.7161E5 | 1 | 0 | 0 | 0 | 0 | 0 | 0 | 0 | 1 | 256 | 266 |  |  | PEAKS DB |
| total 4 peptides |  |  |  |  |  |  |  |  |  |  |  |  |  |  |  |  |  |  |  |  |  |  |  |  |  |  |  |  |  |  |  |  |  |
| --- | --- | --- | --- | --- | --- | --- | --- | --- | --- | --- | --- | --- | --- | --- | --- | --- | --- | --- | --- | --- | --- | --- | --- | --- | --- | --- | --- | --- | --- | --- | --- | --- | --- |

JAB54325.1

back to list

  

| Protein Coverage
| Supporting Peptides
|

Protein Coverage:

Supporting Peptides:

| Peptide | Uniq | -10lgP | Mass | Length | ppm | m/z | z | RT | Fraction | Scan | Source File | Area F6 | Area F2 | Area F3 | Area F4 | Area F5 | Area F7 | Area F8 | Area F1 | #Feature | #Feature F6 | #Feature F2 | #Feature F3 | #Feature F4 | #Feature F5 | #Feature F7 | #Feature F8 | #Feature F1 | Start | End | PTM | AScore | Found By |
| --- | --- | --- | --- | --- | --- | --- | --- | --- | --- | --- | --- | --- | --- | --- | --- | --- | --- | --- | --- | --- | --- | --- | --- | --- | --- | --- | --- | --- | --- | --- | --- | --- | --- |
| R.EHALLAYTLGVK.Q | Y | 64.19 | 1313.7343 | 12 | -0.3 | 657.8742 | 2 | 39.94 | 8 | F8:20708 | 31122021\_RID\_3262\_1b.raw |  |  |  |  |  |  |  | 1.7594E5 | 1 | 0 | 0 | 0 | 0 | 0 | 0 | 0 | 1 | 135 | 146 |  |  | PEAKS DB |
| K.STTTGHLIYK.C | Y | 60.44 | 1119.5924 | 10 | 0.8 | 560.8040 | 2 | 19.24 | 8 | F8:4903 | 31122021\_RID\_3262\_1b.raw |  |  |  |  |  |  |  | 4.9695E5 | 1 | 0 | 0 | 0 | 0 | 0 | 0 | 0 | 1 | 21 | 30 |  |  | PEAKS DB |
| K.YYVTIIDAPGHR.D | Y | 57.50 | 1403.7197 | 12 | 0.7 | 468.9142 | 3 | 36.23 | 8 | F8:17780 | 31122021\_RID\_3262\_1b.raw |  |  |  |  |  |  |  | 5.8111E5 | 2 | 0 | 0 | 0 | 0 | 0 | 0 | 0 | 2 | 85 | 96 |  |  | PEAKS DB |
| K.IGGIGTVPVGR.V | Y | 45.56 | 1024.6029 | 11 | 0.3 | 513.3089 | 2 | 34.95 | 8 | F8:16761 | 31122021\_RID\_3262\_1b.raw |  |  |  |  |  |  |  | 2.7161E5 | 1 | 0 | 0 | 0 | 0 | 0 | 0 | 0 | 1 | 256 | 266 |  |  | PEAKS DB |
| total 4 peptides |  |  |  |  |  |  |  |  |  |  |  |  |  |  |  |  |  |  |  |  |  |  |  |  |  |  |  |  |  |  |  |  |  |
| --- | --- | --- | --- | --- | --- | --- | --- | --- | --- | --- | --- | --- | --- | --- | --- | --- | --- | --- | --- | --- | --- | --- | --- | --- | --- | --- | --- | --- | --- | --- | --- | --- | --- |

XP\_013910443.1

back to list

  

| Protein Coverage
| Supporting Peptides
|

Protein Coverage:

Supporting Peptides:

| Peptide | Uniq | -10lgP | Mass | Length | ppm | m/z | z | RT | Fraction | Scan | Source File | Area F6 | Area F2 | Area F3 | Area F4 | Area F5 | Area F7 | Area F8 | Area F1 | #Feature | #Feature F6 | #Feature F2 | #Feature F3 | #Feature F4 | #Feature F5 | #Feature F7 | #Feature F8 | #Feature F1 | Start | End | PTM | AScore | Found By |
| --- | --- | --- | --- | --- | --- | --- | --- | --- | --- | --- | --- | --- | --- | --- | --- | --- | --- | --- | --- | --- | --- | --- | --- | --- | --- | --- | --- | --- | --- | --- | --- | --- | --- |
| R.EHALLAYTLGVK.Q | Y | 64.19 | 1313.7343 | 12 | -0.3 | 657.8742 | 2 | 39.94 | 8 | F8:20708 | 31122021\_RID\_3262\_1b.raw |  |  |  |  |  |  |  | 1.7594E5 | 1 | 0 | 0 | 0 | 0 | 0 | 0 | 0 | 1 | 135 | 146 |  |  | PEAKS DB |
| K.STTTGHLIYK.C | Y | 60.44 | 1119.5924 | 10 | 0.8 | 560.8040 | 2 | 19.24 | 8 | F8:4903 | 31122021\_RID\_3262\_1b.raw |  |  |  |  |  |  |  | 4.9695E5 | 1 | 0 | 0 | 0 | 0 | 0 | 0 | 0 | 1 | 21 | 30 |  |  | PEAKS DB |
| K.YYVTIIDAPGHR.D | Y | 57.50 | 1403.7197 | 12 | 0.7 | 468.9142 | 3 | 36.23 | 8 | F8:17780 | 31122021\_RID\_3262\_1b.raw |  |  |  |  |  |  |  | 5.8111E5 | 2 | 0 | 0 | 0 | 0 | 0 | 0 | 0 | 2 | 85 | 96 |  |  | PEAKS DB |
| K.IGGIGTVPVGR.V | Y | 45.56 | 1024.6029 | 11 | 0.3 | 513.3089 | 2 | 34.95 | 8 | F8:16761 | 31122021\_RID\_3262\_1b.raw |  |  |  |  |  |  |  | 2.7161E5 | 1 | 0 | 0 | 0 | 0 | 0 | 0 | 0 | 1 | 256 | 266 |  |  | PEAKS DB |
| total 4 peptides |  |  |  |  |  |  |  |  |  |  |  |  |  |  |  |  |  |  |  |  |  |  |  |  |  |  |  |  |  |  |  |  |  |
| --- | --- | --- | --- | --- | --- | --- | --- | --- | --- | --- | --- | --- | --- | --- | --- | --- | --- | --- | --- | --- | --- | --- | --- | --- | --- | --- | --- | --- | --- | --- | --- | --- | --- |

XP\_026520989.1

back to list

  

| Protein Coverage
| Supporting Peptides
|

Protein Coverage:

Supporting Peptides:

| Peptide | Uniq | -10lgP | Mass | Length | ppm | m/z | z | RT | Fraction | Scan | Source File | Area F6 | Area F2 | Area F3 | Area F4 | Area F5 | Area F7 | Area F8 | Area F1 | #Feature | #Feature F6 | #Feature F2 | #Feature F3 | #Feature F4 | #Feature F5 | #Feature F7 | #Feature F8 | #Feature F1 | Start | End | PTM | AScore | Found By |
| --- | --- | --- | --- | --- | --- | --- | --- | --- | --- | --- | --- | --- | --- | --- | --- | --- | --- | --- | --- | --- | --- | --- | --- | --- | --- | --- | --- | --- | --- | --- | --- | --- | --- |
| R.EHALLAYTLGVK.Q | Y | 64.19 | 1313.7343 | 12 | -0.3 | 657.8742 | 2 | 39.94 | 8 | F8:20708 | 31122021\_RID\_3262\_1b.raw |  |  |  |  |  |  |  | 1.7594E5 | 1 | 0 | 0 | 0 | 0 | 0 | 0 | 0 | 1 | 135 | 146 |  |  | PEAKS DB |
| K.STTTGHLIYK.C | Y | 60.44 | 1119.5924 | 10 | 0.8 | 560.8040 | 2 | 19.24 | 8 | F8:4903 | 31122021\_RID\_3262\_1b.raw |  |  |  |  |  |  |  | 4.9695E5 | 1 | 0 | 0 | 0 | 0 | 0 | 0 | 0 | 1 | 21 | 30 |  |  | PEAKS DB |
| K.YYVTIIDAPGHR.D | Y | 57.50 | 1403.7197 | 12 | 0.7 | 468.9142 | 3 | 36.23 | 8 | F8:17780 | 31122021\_RID\_3262\_1b.raw |  |  |  |  |  |  |  | 5.8111E5 | 2 | 0 | 0 | 0 | 0 | 0 | 0 | 0 | 2 | 85 | 96 |  |  | PEAKS DB |
| K.IGGIGTVPVGR.V | Y | 45.56 | 1024.6029 | 11 | 0.3 | 513.3089 | 2 | 34.95 | 8 | F8:16761 | 31122021\_RID\_3262\_1b.raw |  |  |  |  |  |  |  | 2.7161E5 | 1 | 0 | 0 | 0 | 0 | 0 | 0 | 0 | 1 | 256 | 266 |  |  | PEAKS DB |
| total 4 peptides |  |  |  |  |  |  |  |  |  |  |  |  |  |  |  |  |  |  |  |  |  |  |  |  |  |  |  |  |  |  |  |  |  |
| --- | --- | --- | --- | --- | --- | --- | --- | --- | --- | --- | --- | --- | --- | --- | --- | --- | --- | --- | --- | --- | --- | --- | --- | --- | --- | --- | --- | --- | --- | --- | --- | --- | --- |

XP\_032071033.1

back to list

  

| Protein Coverage
| Supporting Peptides
|

Protein Coverage:

Supporting Peptides:

| Peptide | Uniq | -10lgP | Mass | Length | ppm | m/z | z | RT | Fraction | Scan | Source File | Area F6 | Area F2 | Area F3 | Area F4 | Area F5 | Area F7 | Area F8 | Area F1 | #Feature | #Feature F6 | #Feature F2 | #Feature F3 | #Feature F4 | #Feature F5 | #Feature F7 | #Feature F8 | #Feature F1 | Start | End | PTM | AScore | Found By |
| --- | --- | --- | --- | --- | --- | --- | --- | --- | --- | --- | --- | --- | --- | --- | --- | --- | --- | --- | --- | --- | --- | --- | --- | --- | --- | --- | --- | --- | --- | --- | --- | --- | --- |
| R.EHALLAYTLGVK.Q | Y | 64.19 | 1313.7343 | 12 | -0.3 | 657.8742 | 2 | 39.94 | 8 | F8:20708 | 31122021\_RID\_3262\_1b.raw |  |  |  |  |  |  |  | 1.7594E5 | 1 | 0 | 0 | 0 | 0 | 0 | 0 | 0 | 1 | 135 | 146 |  |  | PEAKS DB |
| K.STTTGHLIYK.C | Y | 60.44 | 1119.5924 | 10 | 0.8 | 560.8040 | 2 | 19.24 | 8 | F8:4903 | 31122021\_RID\_3262\_1b.raw |  |  |  |  |  |  |  | 4.9695E5 | 1 | 0 | 0 | 0 | 0 | 0 | 0 | 0 | 1 | 21 | 30 |  |  | PEAKS DB |
| K.YYVTIIDAPGHR.D | Y | 57.50 | 1403.7197 | 12 | 0.7 | 468.9142 | 3 | 36.23 | 8 | F8:17780 | 31122021\_RID\_3262\_1b.raw |  |  |  |  |  |  |  | 5.8111E5 | 2 | 0 | 0 | 0 | 0 | 0 | 0 | 0 | 2 | 85 | 96 |  |  | PEAKS DB |
| K.IGGIGTVPVGR.V | Y | 45.56 | 1024.6029 | 11 | 0.3 | 513.3089 | 2 | 34.95 | 8 | F8:16761 | 31122021\_RID\_3262\_1b.raw |  |  |  |  |  |  |  | 2.7161E5 | 1 | 0 | 0 | 0 | 0 | 0 | 0 | 0 | 1 | 256 | 266 |  |  | PEAKS DB |
| total 4 peptides |  |  |  |  |  |  |  |  |  |  |  |  |  |  |  |  |  |  |  |  |  |  |  |  |  |  |  |  |  |  |  |  |  |
| --- | --- | --- | --- | --- | --- | --- | --- | --- | --- | --- | --- | --- | --- | --- | --- | --- | --- | --- | --- | --- | --- | --- | --- | --- | --- | --- | --- | --- | --- | --- | --- | --- | --- |

AEO96984.1

back to list

  

| Protein Coverage
| Supporting Peptides
|

Protein Coverage:

Supporting Peptides:

| Peptide | Uniq | -10lgP | Mass | Length | ppm | m/z | z | RT | Fraction | Scan | Source File | Area F6 | Area F2 | Area F3 | Area F4 | Area F5 | Area F7 | Area F8 | Area F1 | #Feature | #Feature F6 | #Feature F2 | #Feature F3 | #Feature F4 | #Feature F5 | #Feature F7 | #Feature F8 | #Feature F1 | Start | End | PTM | AScore | Found By |
| --- | --- | --- | --- | --- | --- | --- | --- | --- | --- | --- | --- | --- | --- | --- | --- | --- | --- | --- | --- | --- | --- | --- | --- | --- | --- | --- | --- | --- | --- | --- | --- | --- | --- |
| R.EHALLAYTLGVK.Q | Y | 64.19 | 1313.7343 | 12 | -0.3 | 657.8742 | 2 | 39.94 | 8 | F8:20708 | 31122021\_RID\_3262\_1b.raw |  |  |  |  |  |  |  | 1.7594E5 | 1 | 0 | 0 | 0 | 0 | 0 | 0 | 0 | 1 | 131 | 142 |  |  | PEAKS DB |
| K.STTTGHLIYK.C | Y | 60.44 | 1119.5924 | 10 | 0.8 | 560.8040 | 2 | 19.24 | 8 | F8:4903 | 31122021\_RID\_3262\_1b.raw |  |  |  |  |  |  |  | 4.9695E5 | 1 | 0 | 0 | 0 | 0 | 0 | 0 | 0 | 1 | 17 | 26 |  |  | PEAKS DB |
| K.YYVTIIDAPGHR.D | Y | 57.50 | 1403.7197 | 12 | 0.7 | 468.9142 | 3 | 36.23 | 8 | F8:17780 | 31122021\_RID\_3262\_1b.raw |  |  |  |  |  |  |  | 5.8111E5 | 2 | 0 | 0 | 0 | 0 | 0 | 0 | 0 | 2 | 81 | 92 |  |  | PEAKS DB |
| K.IGGIGTVPVGR.V | Y | 45.56 | 1024.6029 | 11 | 0.3 | 513.3089 | 2 | 34.95 | 8 | F8:16761 | 31122021\_RID\_3262\_1b.raw |  |  |  |  |  |  |  | 2.7161E5 | 1 | 0 | 0 | 0 | 0 | 0 | 0 | 0 | 1 | 252 | 262 |  |  | PEAKS DB |
| total 4 peptides |  |  |  |  |  |  |  |  |  |  |  |  |  |  |  |  |  |  |  |  |  |  |  |  |  |  |  |  |  |  |  |  |  |
| --- | --- | --- | --- | --- | --- | --- | --- | --- | --- | --- | --- | --- | --- | --- | --- | --- | --- | --- | --- | --- | --- | --- | --- | --- | --- | --- | --- | --- | --- | --- | --- | --- | --- |

LAA86151.1

back to list

  

| Protein Coverage
| Supporting Peptides
|

Protein Coverage:

Supporting Peptides:

| Peptide | Uniq | -10lgP | Mass | Length | ppm | m/z | z | RT | Fraction | Scan | Source File | Area F6 | Area F2 | Area F3 | Area F4 | Area F5 | Area F7 | Area F8 | Area F1 | #Feature | #Feature F6 | #Feature F2 | #Feature F3 | #Feature F4 | #Feature F5 | #Feature F7 | #Feature F8 | #Feature F1 | Start | End | PTM | AScore | Found By |
| --- | --- | --- | --- | --- | --- | --- | --- | --- | --- | --- | --- | --- | --- | --- | --- | --- | --- | --- | --- | --- | --- | --- | --- | --- | --- | --- | --- | --- | --- | --- | --- | --- | --- |
| R.LISQIVSSITASLR.F | N | 56.73 | 1486.8718 | 14 | 0.8 | 744.4438 | 2 | 61.97 | 8 | F8:37801 | 31122021\_RID\_3262\_1b.raw |  |  |  |  |  |  |  | 9.8349E4 | 1 | 0 | 0 | 0 | 0 | 0 | 0 | 0 | 1 | 230 | 243 |  |  | PEAKS DB |
| R.NLDIERPTYTNLN.R | N | 52.43 | 1561.7736 | 13 | 0.5 | 781.8944 | 2 | 41.10 | 8 | F8:21657 | 31122021\_RID\_3262\_1b.raw |  |  |  |  |  |  |  | 1.1027E4 | 1 | 0 | 0 | 0 | 0 | 0 | 0 | 0 | 1 | 216 | 228 |  |  | PEAKS DB |
| K.DVNAAIATIK.T | Y | 49.86 | 1014.5709 | 10 | 0.0 | 508.2927 | 2 | 37.55 | 8 | F8:19028 | 31122021\_RID\_3262\_1b.raw |  |  |  |  |  |  |  | 1.0175E5 | 1 | 0 | 0 | 0 | 0 | 0 | 0 | 0 | 1 | 327 | 336 |  |  | PEAKS DB |
| K.EIIDLVLDR.I | N | 45.89 | 1084.6128 | 9 | -0.1 | 543.3136 | 2 | 54.37 | 8 | F8:32070 | 31122021\_RID\_3262\_1b.raw |  |  |  |  |  |  |  | 1.6656E5 | 1 | 0 | 0 | 0 | 0 | 0 | 0 | 0 | 1 | 113 | 121 |  |  | PEAKS DB |
| total 4 peptides |  |  |  |  |  |  |  |  |  |  |  |  |  |  |  |  |  |  |  |  |  |  |  |  |  |  |  |  |  |  |  |  |  |
| --- | --- | --- | --- | --- | --- | --- | --- | --- | --- | --- | --- | --- | --- | --- | --- | --- | --- | --- | --- | --- | --- | --- | --- | --- | --- | --- | --- | --- | --- | --- | --- | --- | --- |

JAI09230.1

back to list

  

| Protein Coverage
| Supporting Peptides
|

Protein Coverage:

Supporting Peptides:

| Peptide | Uniq | -10lgP | Mass | Length | ppm | m/z | z | RT | Fraction | Scan | Source File | Area F6 | Area F2 | Area F3 | Area F4 | Area F5 | Area F7 | Area F8 | Area F1 | #Feature | #Feature F6 | #Feature F2 | #Feature F3 | #Feature F4 | #Feature F5 | #Feature F7 | #Feature F8 | #Feature F1 | Start | End | PTM | AScore | Found By |
| --- | --- | --- | --- | --- | --- | --- | --- | --- | --- | --- | --- | --- | --- | --- | --- | --- | --- | --- | --- | --- | --- | --- | --- | --- | --- | --- | --- | --- | --- | --- | --- | --- | --- |
| R.LISQIVSSITASLR.F | N | 56.73 | 1486.8718 | 14 | 0.8 | 744.4438 | 2 | 61.97 | 8 | F8:37801 | 31122021\_RID\_3262\_1b.raw |  |  |  |  |  |  |  | 9.8349E4 | 1 | 0 | 0 | 0 | 0 | 0 | 0 | 0 | 1 | 230 | 243 |  |  | PEAKS DB |
| R.NLDIERPTYTNLN.R | N | 52.43 | 1561.7736 | 13 | 0.5 | 781.8944 | 2 | 41.10 | 8 | F8:21657 | 31122021\_RID\_3262\_1b.raw |  |  |  |  |  |  |  | 1.1027E4 | 1 | 0 | 0 | 0 | 0 | 0 | 0 | 0 | 1 | 216 | 228 |  |  | PEAKS DB |
| K.DVNAAIATIK.T | Y | 49.86 | 1014.5709 | 10 | 0.0 | 508.2927 | 2 | 37.55 | 8 | F8:19028 | 31122021\_RID\_3262\_1b.raw |  |  |  |  |  |  |  | 1.0175E5 | 1 | 0 | 0 | 0 | 0 | 0 | 0 | 0 | 1 | 327 | 336 |  |  | PEAKS DB |
| K.EIIDLVLDR.I | N | 45.89 | 1084.6128 | 9 | -0.1 | 543.3136 | 2 | 54.37 | 8 | F8:32070 | 31122021\_RID\_3262\_1b.raw |  |  |  |  |  |  |  | 1.6656E5 | 1 | 0 | 0 | 0 | 0 | 0 | 0 | 0 | 1 | 113 | 121 |  |  | PEAKS DB |
| total 4 peptides |  |  |  |  |  |  |  |  |  |  |  |  |  |  |  |  |  |  |  |  |  |  |  |  |  |  |  |  |  |  |  |  |  |
| --- | --- | --- | --- | --- | --- | --- | --- | --- | --- | --- | --- | --- | --- | --- | --- | --- | --- | --- | --- | --- | --- | --- | --- | --- | --- | --- | --- | --- | --- | --- | --- | --- | --- |

AFJ51865.1

back to list

  

| Protein Coverage
| Supporting Peptides
|

Protein Coverage:

Supporting Peptides:

| Peptide | Uniq | -10lgP | Mass | Length | ppm | m/z | z | RT | Fraction | Scan | Source File | Area F6 | Area F2 | Area F3 | Area F4 | Area F5 | Area F7 | Area F8 | Area F1 | #Feature | #Feature F6 | #Feature F2 | #Feature F3 | #Feature F4 | #Feature F5 | #Feature F7 | #Feature F8 | #Feature F1 | Start | End | PTM | AScore | Found By |
| --- | --- | --- | --- | --- | --- | --- | --- | --- | --- | --- | --- | --- | --- | --- | --- | --- | --- | --- | --- | --- | --- | --- | --- | --- | --- | --- | --- | --- | --- | --- | --- | --- | --- |
| R.LISQIVSSITASLR.F | N | 56.73 | 1486.8718 | 14 | 0.8 | 744.4438 | 2 | 61.97 | 8 | F8:37801 | 31122021\_RID\_3262\_1b.raw |  |  |  |  |  |  |  | 9.8349E4 | 1 | 0 | 0 | 0 | 0 | 0 | 0 | 0 | 1 | 230 | 243 |  |  | PEAKS DB |
| R.NLDIERPTYTNLN.R | N | 52.43 | 1561.7736 | 13 | 0.5 | 781.8944 | 2 | 41.10 | 8 | F8:21657 | 31122021\_RID\_3262\_1b.raw |  |  |  |  |  |  |  | 1.1027E4 | 1 | 0 | 0 | 0 | 0 | 0 | 0 | 0 | 1 | 216 | 228 |  |  | PEAKS DB |
| K.DVNAAIATIK.T | Y | 49.86 | 1014.5709 | 10 | 0.0 | 508.2927 | 2 | 37.55 | 8 | F8:19028 | 31122021\_RID\_3262\_1b.raw |  |  |  |  |  |  |  | 1.0175E5 | 1 | 0 | 0 | 0 | 0 | 0 | 0 | 0 | 1 | 327 | 336 |  |  | PEAKS DB |
| K.EIIDLVLDR.I | N | 45.89 | 1084.6128 | 9 | -0.1 | 543.3136 | 2 | 54.37 | 8 | F8:32070 | 31122021\_RID\_3262\_1b.raw |  |  |  |  |  |  |  | 1.6656E5 | 1 | 0 | 0 | 0 | 0 | 0 | 0 | 0 | 1 | 113 | 121 |  |  | PEAKS DB |
| total 4 peptides |  |  |  |  |  |  |  |  |  |  |  |  |  |  |  |  |  |  |  |  |  |  |  |  |  |  |  |  |  |  |  |  |  |
| --- | --- | --- | --- | --- | --- | --- | --- | --- | --- | --- | --- | --- | --- | --- | --- | --- | --- | --- | --- | --- | --- | --- | --- | --- | --- | --- | --- | --- | --- | --- | --- | --- | --- |

JAA95197.1

back to list

  

| Protein Coverage
| Supporting Peptides
|

Protein Coverage:

Supporting Peptides:

| Peptide | Uniq | -10lgP | Mass | Length | ppm | m/z | z | RT | Fraction | Scan | Source File | Area F6 | Area F2 | Area F3 | Area F4 | Area F5 | Area F7 | Area F8 | Area F1 | #Feature | #Feature F6 | #Feature F2 | #Feature F3 | #Feature F4 | #Feature F5 | #Feature F7 | #Feature F8 | #Feature F1 | Start | End | PTM | AScore | Found By |
| --- | --- | --- | --- | --- | --- | --- | --- | --- | --- | --- | --- | --- | --- | --- | --- | --- | --- | --- | --- | --- | --- | --- | --- | --- | --- | --- | --- | --- | --- | --- | --- | --- | --- |
| R.LISQIVSSITASLR.F | N | 56.73 | 1486.8718 | 14 | 0.8 | 744.4438 | 2 | 61.97 | 8 | F8:37801 | 31122021\_RID\_3262\_1b.raw |  |  |  |  |  |  |  | 9.8349E4 | 1 | 0 | 0 | 0 | 0 | 0 | 0 | 0 | 1 | 230 | 243 |  |  | PEAKS DB |
| R.NLDIERPTYTNLN.R | N | 52.43 | 1561.7736 | 13 | 0.5 | 781.8944 | 2 | 41.10 | 8 | F8:21657 | 31122021\_RID\_3262\_1b.raw |  |  |  |  |  |  |  | 1.1027E4 | 1 | 0 | 0 | 0 | 0 | 0 | 0 | 0 | 1 | 216 | 228 |  |  | PEAKS DB |
| K.DVNAAIATIK.T | Y | 49.86 | 1014.5709 | 10 | 0.0 | 508.2927 | 2 | 37.55 | 8 | F8:19028 | 31122021\_RID\_3262\_1b.raw |  |  |  |  |  |  |  | 1.0175E5 | 1 | 0 | 0 | 0 | 0 | 0 | 0 | 0 | 1 | 327 | 336 |  |  | PEAKS DB |
| K.EIIDLVLDR.I | N | 45.89 | 1084.6128 | 9 | -0.1 | 543.3136 | 2 | 54.37 | 8 | F8:32070 | 31122021\_RID\_3262\_1b.raw |  |  |  |  |  |  |  | 1.6656E5 | 1 | 0 | 0 | 0 | 0 | 0 | 0 | 0 | 1 | 113 | 121 |  |  | PEAKS DB |
| total 4 peptides |  |  |  |  |  |  |  |  |  |  |  |  |  |  |  |  |  |  |  |  |  |  |  |  |  |  |  |  |  |  |  |  |  |
| --- | --- | --- | --- | --- | --- | --- | --- | --- | --- | --- | --- | --- | --- | --- | --- | --- | --- | --- | --- | --- | --- | --- | --- | --- | --- | --- | --- | --- | --- | --- | --- | --- | --- |

LAA55685.1

back to list

  

| Protein Coverage
| Supporting Peptides
|

Protein Coverage:

Supporting Peptides:

| Peptide | Uniq | -10lgP | Mass | Length | ppm | m/z | z | RT | Fraction | Scan | Source File | Area F6 | Area F2 | Area F3 | Area F4 | Area F5 | Area F7 | Area F8 | Area F1 | #Feature | #Feature F6 | #Feature F2 | #Feature F3 | #Feature F4 | #Feature F5 | #Feature F7 | #Feature F8 | #Feature F1 | Start | End | PTM | AScore | Found By |
| --- | --- | --- | --- | --- | --- | --- | --- | --- | --- | --- | --- | --- | --- | --- | --- | --- | --- | --- | --- | --- | --- | --- | --- | --- | --- | --- | --- | --- | --- | --- | --- | --- | --- |
| R.LISQIVSSITASLR.F | N | 56.73 | 1486.8718 | 14 | 0.8 | 744.4438 | 2 | 61.97 | 8 | F8:37801 | 31122021\_RID\_3262\_1b.raw |  |  |  |  |  |  |  | 9.8349E4 | 1 | 0 | 0 | 0 | 0 | 0 | 0 | 0 | 1 | 230 | 243 |  |  | PEAKS DB |
| R.NLDIERPTYTNLN.R | N | 52.43 | 1561.7736 | 13 | 0.5 | 781.8944 | 2 | 41.10 | 8 | F8:21657 | 31122021\_RID\_3262\_1b.raw |  |  |  |  |  |  |  | 1.1027E4 | 1 | 0 | 0 | 0 | 0 | 0 | 0 | 0 | 1 | 216 | 228 |  |  | PEAKS DB |
| K.DVNAAIATIK.T | Y | 49.86 | 1014.5709 | 10 | 0.0 | 508.2927 | 2 | 37.55 | 8 | F8:19028 | 31122021\_RID\_3262\_1b.raw |  |  |  |  |  |  |  | 1.0175E5 | 1 | 0 | 0 | 0 | 0 | 0 | 0 | 0 | 1 | 327 | 336 |  |  | PEAKS DB |
| K.EIIDLVLDR.I | N | 45.89 | 1084.6128 | 9 | -0.1 | 543.3136 | 2 | 54.37 | 8 | F8:32070 | 31122021\_RID\_3262\_1b.raw |  |  |  |  |  |  |  | 1.6656E5 | 1 | 0 | 0 | 0 | 0 | 0 | 0 | 0 | 1 | 113 | 121 |  |  | PEAKS DB |
| total 4 peptides |  |  |  |  |  |  |  |  |  |  |  |  |  |  |  |  |  |  |  |  |  |  |  |  |  |  |  |  |  |  |  |  |  |
| --- | --- | --- | --- | --- | --- | --- | --- | --- | --- | --- | --- | --- | --- | --- | --- | --- | --- | --- | --- | --- | --- | --- | --- | --- | --- | --- | --- | --- | --- | --- | --- | --- | --- |

JAI10819.1

back to list

  

| Protein Coverage
| Supporting Peptides
|

Protein Coverage:

Supporting Peptides:

| Peptide | Uniq | -10lgP | Mass | Length | ppm | m/z | z | RT | Fraction | Scan | Source File | Area F6 | Area F2 | Area F3 | Area F4 | Area F5 | Area F7 | Area F8 | Area F1 | #Feature | #Feature F6 | #Feature F2 | #Feature F3 | #Feature F4 | #Feature F5 | #Feature F7 | #Feature F8 | #Feature F1 | Start | End | PTM | AScore | Found By |
| --- | --- | --- | --- | --- | --- | --- | --- | --- | --- | --- | --- | --- | --- | --- | --- | --- | --- | --- | --- | --- | --- | --- | --- | --- | --- | --- | --- | --- | --- | --- | --- | --- | --- |
| R.LISQIVSSITASLR.F | N | 56.73 | 1486.8718 | 14 | 0.8 | 744.4438 | 2 | 61.97 | 8 | F8:37801 | 31122021\_RID\_3262\_1b.raw |  |  |  |  |  |  |  | 9.8349E4 | 1 | 0 | 0 | 0 | 0 | 0 | 0 | 0 | 1 | 230 | 243 |  |  | PEAKS DB |
| R.NLDIERPTYTNLN.R | N | 52.43 | 1561.7736 | 13 | 0.5 | 781.8944 | 2 | 41.10 | 8 | F8:21657 | 31122021\_RID\_3262\_1b.raw |  |  |  |  |  |  |  | 1.1027E4 | 1 | 0 | 0 | 0 | 0 | 0 | 0 | 0 | 1 | 216 | 228 |  |  | PEAKS DB |
| K.DVNAAIATIK.T | Y | 49.86 | 1014.5709 | 10 | 0.0 | 508.2927 | 2 | 37.55 | 8 | F8:19028 | 31122021\_RID\_3262\_1b.raw |  |  |  |  |  |  |  | 1.0175E5 | 1 | 0 | 0 | 0 | 0 | 0 | 0 | 0 | 1 | 327 | 336 |  |  | PEAKS DB |
| K.EIIDLVLDR.I | N | 45.89 | 1084.6128 | 9 | -0.1 | 543.3136 | 2 | 54.37 | 8 | F8:32070 | 31122021\_RID\_3262\_1b.raw |  |  |  |  |  |  |  | 1.6656E5 | 1 | 0 | 0 | 0 | 0 | 0 | 0 | 0 | 1 | 113 | 121 |  |  | PEAKS DB |
| total 4 peptides |  |  |  |  |  |  |  |  |  |  |  |  |  |  |  |  |  |  |  |  |  |  |  |  |  |  |  |  |  |  |  |  |  |
| --- | --- | --- | --- | --- | --- | --- | --- | --- | --- | --- | --- | --- | --- | --- | --- | --- | --- | --- | --- | --- | --- | --- | --- | --- | --- | --- | --- | --- | --- | --- | --- | --- | --- |

JAG66080.1

back to list

  

| Protein Coverage
| Supporting Peptides
|

Protein Coverage:

Supporting Peptides:

| Peptide | Uniq | -10lgP | Mass | Length | ppm | m/z | z | RT | Fraction | Scan | Source File | Area F6 | Area F2 | Area F3 | Area F4 | Area F5 | Area F7 | Area F8 | Area F1 | #Feature | #Feature F6 | #Feature F2 | #Feature F3 | #Feature F4 | #Feature F5 | #Feature F7 | #Feature F8 | #Feature F1 | Start | End | PTM | AScore | Found By |
| --- | --- | --- | --- | --- | --- | --- | --- | --- | --- | --- | --- | --- | --- | --- | --- | --- | --- | --- | --- | --- | --- | --- | --- | --- | --- | --- | --- | --- | --- | --- | --- | --- | --- |
| R.LISQIVSSITASLR.F | N | 56.73 | 1486.8718 | 14 | 0.8 | 744.4438 | 2 | 61.97 | 8 | F8:37801 | 31122021\_RID\_3262\_1b.raw |  |  |  |  |  |  |  | 9.8349E4 | 1 | 0 | 0 | 0 | 0 | 0 | 0 | 0 | 1 | 230 | 243 |  |  | PEAKS DB |
| R.NLDIERPTYTNLN.R | N | 52.43 | 1561.7736 | 13 | 0.5 | 781.8944 | 2 | 41.10 | 8 | F8:21657 | 31122021\_RID\_3262\_1b.raw |  |  |  |  |  |  |  | 1.1027E4 | 1 | 0 | 0 | 0 | 0 | 0 | 0 | 0 | 1 | 216 | 228 |  |  | PEAKS DB |
| K.DVNAAIATIK.T | Y | 49.86 | 1014.5709 | 10 | 0.0 | 508.2927 | 2 | 37.55 | 8 | F8:19028 | 31122021\_RID\_3262\_1b.raw |  |  |  |  |  |  |  | 1.0175E5 | 1 | 0 | 0 | 0 | 0 | 0 | 0 | 0 | 1 | 327 | 336 |  |  | PEAKS DB |
| K.EIIDLVLDR.I | N | 45.89 | 1084.6128 | 9 | -0.1 | 543.3136 | 2 | 54.37 | 8 | F8:32070 | 31122021\_RID\_3262\_1b.raw |  |  |  |  |  |  |  | 1.6656E5 | 1 | 0 | 0 | 0 | 0 | 0 | 0 | 0 | 1 | 113 | 121 |  |  | PEAKS DB |
| total 4 peptides |  |  |  |  |  |  |  |  |  |  |  |  |  |  |  |  |  |  |  |  |  |  |  |  |  |  |  |  |  |  |  |  |  |
| --- | --- | --- | --- | --- | --- | --- | --- | --- | --- | --- | --- | --- | --- | --- | --- | --- | --- | --- | --- | --- | --- | --- | --- | --- | --- | --- | --- | --- | --- | --- | --- | --- | --- |

JAG66077.1

back to list

  

| Protein Coverage
| Supporting Peptides
|

Protein Coverage:

Supporting Peptides:

| Peptide | Uniq | -10lgP | Mass | Length | ppm | m/z | z | RT | Fraction | Scan | Source File | Area F6 | Area F2 | Area F3 | Area F4 | Area F5 | Area F7 | Area F8 | Area F1 | #Feature | #Feature F6 | #Feature F2 | #Feature F3 | #Feature F4 | #Feature F5 | #Feature F7 | #Feature F8 | #Feature F1 | Start | End | PTM | AScore | Found By |
| --- | --- | --- | --- | --- | --- | --- | --- | --- | --- | --- | --- | --- | --- | --- | --- | --- | --- | --- | --- | --- | --- | --- | --- | --- | --- | --- | --- | --- | --- | --- | --- | --- | --- |
| R.LISQIVSSITASLR.F | N | 56.73 | 1486.8718 | 14 | 0.8 | 744.4438 | 2 | 61.97 | 8 | F8:37801 | 31122021\_RID\_3262\_1b.raw |  |  |  |  |  |  |  | 9.8349E4 | 1 | 0 | 0 | 0 | 0 | 0 | 0 | 0 | 1 | 230 | 243 |  |  | PEAKS DB |
| R.NLDIERPTYTNLN.R | N | 52.43 | 1561.7736 | 13 | 0.5 | 781.8944 | 2 | 41.10 | 8 | F8:21657 | 31122021\_RID\_3262\_1b.raw |  |  |  |  |  |  |  | 1.1027E4 | 1 | 0 | 0 | 0 | 0 | 0 | 0 | 0 | 1 | 216 | 228 |  |  | PEAKS DB |
| K.DVNAAIATIK.T | Y | 49.86 | 1014.5709 | 10 | 0.0 | 508.2927 | 2 | 37.55 | 8 | F8:19028 | 31122021\_RID\_3262\_1b.raw |  |  |  |  |  |  |  | 1.0175E5 | 1 | 0 | 0 | 0 | 0 | 0 | 0 | 0 | 1 | 327 | 336 |  |  | PEAKS DB |
| K.EIIDLVLDR.I | N | 45.89 | 1084.6128 | 9 | -0.1 | 543.3136 | 2 | 54.37 | 8 | F8:32070 | 31122021\_RID\_3262\_1b.raw |  |  |  |  |  |  |  | 1.6656E5 | 1 | 0 | 0 | 0 | 0 | 0 | 0 | 0 | 1 | 113 | 121 |  |  | PEAKS DB |
| total 4 peptides |  |  |  |  |  |  |  |  |  |  |  |  |  |  |  |  |  |  |  |  |  |  |  |  |  |  |  |  |  |  |  |  |  |
| --- | --- | --- | --- | --- | --- | --- | --- | --- | --- | --- | --- | --- | --- | --- | --- | --- | --- | --- | --- | --- | --- | --- | --- | --- | --- | --- | --- | --- | --- | --- | --- | --- | --- |

JAB53062.1

back to list

  

| Protein Coverage
| Supporting Peptides
|

Protein Coverage:

Supporting Peptides:

| Peptide | Uniq | -10lgP | Mass | Length | ppm | m/z | z | RT | Fraction | Scan | Source File | Area F6 | Area F2 | Area F3 | Area F4 | Area F5 | Area F7 | Area F8 | Area F1 | #Feature | #Feature F6 | #Feature F2 | #Feature F3 | #Feature F4 | #Feature F5 | #Feature F7 | #Feature F8 | #Feature F1 | Start | End | PTM | AScore | Found By |
| --- | --- | --- | --- | --- | --- | --- | --- | --- | --- | --- | --- | --- | --- | --- | --- | --- | --- | --- | --- | --- | --- | --- | --- | --- | --- | --- | --- | --- | --- | --- | --- | --- | --- |
| R.LISQIVSSITASLR.F | N | 56.73 | 1486.8718 | 14 | 0.8 | 744.4438 | 2 | 61.97 | 8 | F8:37801 | 31122021\_RID\_3262\_1b.raw |  |  |  |  |  |  |  | 9.8349E4 | 1 | 0 | 0 | 0 | 0 | 0 | 0 | 0 | 1 | 230 | 243 |  |  | PEAKS DB |
| R.NLDIERPTYTNLN.R | N | 52.43 | 1561.7736 | 13 | 0.5 | 781.8944 | 2 | 41.10 | 8 | F8:21657 | 31122021\_RID\_3262\_1b.raw |  |  |  |  |  |  |  | 1.1027E4 | 1 | 0 | 0 | 0 | 0 | 0 | 0 | 0 | 1 | 216 | 228 |  |  | PEAKS DB |
| K.DVNAAIATIK.T | Y | 49.86 | 1014.5709 | 10 | 0.0 | 508.2927 | 2 | 37.55 | 8 | F8:19028 | 31122021\_RID\_3262\_1b.raw |  |  |  |  |  |  |  | 1.0175E5 | 1 | 0 | 0 | 0 | 0 | 0 | 0 | 0 | 1 | 327 | 336 |  |  | PEAKS DB |
| K.EIIDLVLDR.I | N | 45.89 | 1084.6128 | 9 | -0.1 | 543.3136 | 2 | 54.37 | 8 | F8:32070 | 31122021\_RID\_3262\_1b.raw |  |  |  |  |  |  |  | 1.6656E5 | 1 | 0 | 0 | 0 | 0 | 0 | 0 | 0 | 1 | 113 | 121 |  |  | PEAKS DB |
| total 4 peptides |  |  |  |  |  |  |  |  |  |  |  |  |  |  |  |  |  |  |  |  |  |  |  |  |  |  |  |  |  |  |  |  |  |
| --- | --- | --- | --- | --- | --- | --- | --- | --- | --- | --- | --- | --- | --- | --- | --- | --- | --- | --- | --- | --- | --- | --- | --- | --- | --- | --- | --- | --- | --- | --- | --- | --- | --- |

JAI10823.1

back to list

  

| Protein Coverage
| Supporting Peptides
|

Protein Coverage:

Supporting Peptides:

| Peptide | Uniq | -10lgP | Mass | Length | ppm | m/z | z | RT | Fraction | Scan | Source File | Area F6 | Area F2 | Area F3 | Area F4 | Area F5 | Area F7 | Area F8 | Area F1 | #Feature | #Feature F6 | #Feature F2 | #Feature F3 | #Feature F4 | #Feature F5 | #Feature F7 | #Feature F8 | #Feature F1 | Start | End | PTM | AScore | Found By |
| --- | --- | --- | --- | --- | --- | --- | --- | --- | --- | --- | --- | --- | --- | --- | --- | --- | --- | --- | --- | --- | --- | --- | --- | --- | --- | --- | --- | --- | --- | --- | --- | --- | --- |
| R.LISQIVSSITASLR.F | N | 56.73 | 1486.8718 | 14 | 0.8 | 744.4438 | 2 | 61.97 | 8 | F8:37801 | 31122021\_RID\_3262\_1b.raw |  |  |  |  |  |  |  | 9.8349E4 | 1 | 0 | 0 | 0 | 0 | 0 | 0 | 0 | 1 | 230 | 243 |  |  | PEAKS DB |
| R.NLDIERPTYTNLN.R | N | 52.43 | 1561.7736 | 13 | 0.5 | 781.8944 | 2 | 41.10 | 8 | F8:21657 | 31122021\_RID\_3262\_1b.raw |  |  |  |  |  |  |  | 1.1027E4 | 1 | 0 | 0 | 0 | 0 | 0 | 0 | 0 | 1 | 216 | 228 |  |  | PEAKS DB |
| K.DVNAAIATIK.T | Y | 49.86 | 1014.5709 | 10 | 0.0 | 508.2927 | 2 | 37.55 | 8 | F8:19028 | 31122021\_RID\_3262\_1b.raw |  |  |  |  |  |  |  | 1.0175E5 | 1 | 0 | 0 | 0 | 0 | 0 | 0 | 0 | 1 | 327 | 336 |  |  | PEAKS DB |
| K.EIIDLVLDR.I | N | 45.89 | 1084.6128 | 9 | -0.1 | 543.3136 | 2 | 54.37 | 8 | F8:32070 | 31122021\_RID\_3262\_1b.raw |  |  |  |  |  |  |  | 1.6656E5 | 1 | 0 | 0 | 0 | 0 | 0 | 0 | 0 | 1 | 113 | 121 |  |  | PEAKS DB |
| total 4 peptides |  |  |  |  |  |  |  |  |  |  |  |  |  |  |  |  |  |  |  |  |  |  |  |  |  |  |  |  |  |  |  |  |  |
| --- | --- | --- | --- | --- | --- | --- | --- | --- | --- | --- | --- | --- | --- | --- | --- | --- | --- | --- | --- | --- | --- | --- | --- | --- | --- | --- | --- | --- | --- | --- | --- | --- | --- |

LAA55681.1

back to list

  

| Protein Coverage
| Supporting Peptides
|

Protein Coverage:

Supporting Peptides:

| Peptide | Uniq | -10lgP | Mass | Length | ppm | m/z | z | RT | Fraction | Scan | Source File | Area F6 | Area F2 | Area F3 | Area F4 | Area F5 | Area F7 | Area F8 | Area F1 | #Feature | #Feature F6 | #Feature F2 | #Feature F3 | #Feature F4 | #Feature F5 | #Feature F7 | #Feature F8 | #Feature F1 | Start | End | PTM | AScore | Found By |
| --- | --- | --- | --- | --- | --- | --- | --- | --- | --- | --- | --- | --- | --- | --- | --- | --- | --- | --- | --- | --- | --- | --- | --- | --- | --- | --- | --- | --- | --- | --- | --- | --- | --- |
| R.LISQIVSSITASLR.F | N | 56.73 | 1486.8718 | 14 | 0.8 | 744.4438 | 2 | 61.97 | 8 | F8:37801 | 31122021\_RID\_3262\_1b.raw |  |  |  |  |  |  |  | 9.8349E4 | 1 | 0 | 0 | 0 | 0 | 0 | 0 | 0 | 1 | 230 | 243 |  |  | PEAKS DB |
| R.NLDIERPTYTNLN.R | N | 52.43 | 1561.7736 | 13 | 0.5 | 781.8944 | 2 | 41.10 | 8 | F8:21657 | 31122021\_RID\_3262\_1b.raw |  |  |  |  |  |  |  | 1.1027E4 | 1 | 0 | 0 | 0 | 0 | 0 | 0 | 0 | 1 | 216 | 228 |  |  | PEAKS DB |
| K.DVNAAIATIK.T | Y | 49.86 | 1014.5709 | 10 | 0.0 | 508.2927 | 2 | 37.55 | 8 | F8:19028 | 31122021\_RID\_3262\_1b.raw |  |  |  |  |  |  |  | 1.0175E5 | 1 | 0 | 0 | 0 | 0 | 0 | 0 | 0 | 1 | 327 | 336 |  |  | PEAKS DB |
| K.EIIDLVLDR.I | N | 45.89 | 1084.6128 | 9 | -0.1 | 543.3136 | 2 | 54.37 | 8 | F8:32070 | 31122021\_RID\_3262\_1b.raw |  |  |  |  |  |  |  | 1.6656E5 | 1 | 0 | 0 | 0 | 0 | 0 | 0 | 0 | 1 | 113 | 121 |  |  | PEAKS DB |
| total 4 peptides |  |  |  |  |  |  |  |  |  |  |  |  |  |  |  |  |  |  |  |  |  |  |  |  |  |  |  |  |  |  |  |  |  |
| --- | --- | --- | --- | --- | --- | --- | --- | --- | --- | --- | --- | --- | --- | --- | --- | --- | --- | --- | --- | --- | --- | --- | --- | --- | --- | --- | --- | --- | --- | --- | --- | --- | --- |

AFJ51858.1

back to list

  

| Protein Coverage
| Supporting Peptides
|

Protein Coverage:

Supporting Peptides:

| Peptide | Uniq | -10lgP | Mass | Length | ppm | m/z | z | RT | Fraction | Scan | Source File | Area F6 | Area F2 | Area F3 | Area F4 | Area F5 | Area F7 | Area F8 | Area F1 | #Feature | #Feature F6 | #Feature F2 | #Feature F3 | #Feature F4 | #Feature F5 | #Feature F7 | #Feature F8 | #Feature F1 | Start | End | PTM | AScore | Found By |
| --- | --- | --- | --- | --- | --- | --- | --- | --- | --- | --- | --- | --- | --- | --- | --- | --- | --- | --- | --- | --- | --- | --- | --- | --- | --- | --- | --- | --- | --- | --- | --- | --- | --- |
| R.LISQIVSSITASLR.F | N | 56.73 | 1486.8718 | 14 | 0.8 | 744.4438 | 2 | 61.97 | 8 | F8:37801 | 31122021\_RID\_3262\_1b.raw |  |  |  |  |  |  |  | 9.8349E4 | 1 | 0 | 0 | 0 | 0 | 0 | 0 | 0 | 1 | 230 | 243 |  |  | PEAKS DB |
| R.NLDIERPTYTNLN.R | N | 52.43 | 1561.7736 | 13 | 0.5 | 781.8944 | 2 | 41.10 | 8 | F8:21657 | 31122021\_RID\_3262\_1b.raw |  |  |  |  |  |  |  | 1.1027E4 | 1 | 0 | 0 | 0 | 0 | 0 | 0 | 0 | 1 | 216 | 228 |  |  | PEAKS DB |
| K.DVNAAIATIK.T | Y | 49.86 | 1014.5709 | 10 | 0.0 | 508.2927 | 2 | 37.55 | 8 | F8:19028 | 31122021\_RID\_3262\_1b.raw |  |  |  |  |  |  |  | 1.0175E5 | 1 | 0 | 0 | 0 | 0 | 0 | 0 | 0 | 1 | 327 | 336 |  |  | PEAKS DB |
| K.EIIDLVLDR.I | N | 45.89 | 1084.6128 | 9 | -0.1 | 543.3136 | 2 | 54.37 | 8 | F8:32070 | 31122021\_RID\_3262\_1b.raw |  |  |  |  |  |  |  | 1.6656E5 | 1 | 0 | 0 | 0 | 0 | 0 | 0 | 0 | 1 | 113 | 121 |  |  | PEAKS DB |
| total 4 peptides |  |  |  |  |  |  |  |  |  |  |  |  |  |  |  |  |  |  |  |  |  |  |  |  |  |  |  |  |  |  |  |  |  |
| --- | --- | --- | --- | --- | --- | --- | --- | --- | --- | --- | --- | --- | --- | --- | --- | --- | --- | --- | --- | --- | --- | --- | --- | --- | --- | --- | --- | --- | --- | --- | --- | --- | --- |

JAA95201.1

back to list

  

| Protein Coverage
| Supporting Peptides
|

Protein Coverage:

Supporting Peptides:

| Peptide | Uniq | -10lgP | Mass | Length | ppm | m/z | z | RT | Fraction | Scan | Source File | Area F6 | Area F2 | Area F3 | Area F4 | Area F5 | Area F7 | Area F8 | Area F1 | #Feature | #Feature F6 | #Feature F2 | #Feature F3 | #Feature F4 | #Feature F5 | #Feature F7 | #Feature F8 | #Feature F1 | Start | End | PTM | AScore | Found By |
| --- | --- | --- | --- | --- | --- | --- | --- | --- | --- | --- | --- | --- | --- | --- | --- | --- | --- | --- | --- | --- | --- | --- | --- | --- | --- | --- | --- | --- | --- | --- | --- | --- | --- |
| R.LISQIVSSITASLR.F | N | 56.73 | 1486.8718 | 14 | 0.8 | 744.4438 | 2 | 61.97 | 8 | F8:37801 | 31122021\_RID\_3262\_1b.raw |  |  |  |  |  |  |  | 9.8349E4 | 1 | 0 | 0 | 0 | 0 | 0 | 0 | 0 | 1 | 230 | 243 |  |  | PEAKS DB |
| R.NLDIERPTYTNLN.R | N | 52.43 | 1561.7736 | 13 | 0.5 | 781.8944 | 2 | 41.10 | 8 | F8:21657 | 31122021\_RID\_3262\_1b.raw |  |  |  |  |  |  |  | 1.1027E4 | 1 | 0 | 0 | 0 | 0 | 0 | 0 | 0 | 1 | 216 | 228 |  |  | PEAKS DB |
| K.DVNAAIATIK.T | Y | 49.86 | 1014.5709 | 10 | 0.0 | 508.2927 | 2 | 37.55 | 8 | F8:19028 | 31122021\_RID\_3262\_1b.raw |  |  |  |  |  |  |  | 1.0175E5 | 1 | 0 | 0 | 0 | 0 | 0 | 0 | 0 | 1 | 327 | 336 |  |  | PEAKS DB |
| K.EIIDLVLDR.I | N | 45.89 | 1084.6128 | 9 | -0.1 | 543.3136 | 2 | 54.37 | 8 | F8:32070 | 31122021\_RID\_3262\_1b.raw |  |  |  |  |  |  |  | 1.6656E5 | 1 | 0 | 0 | 0 | 0 | 0 | 0 | 0 | 1 | 113 | 121 |  |  | PEAKS DB |
| total 4 peptides |  |  |  |  |  |  |  |  |  |  |  |  |  |  |  |  |  |  |  |  |  |  |  |  |  |  |  |  |  |  |  |  |  |
| --- | --- | --- | --- | --- | --- | --- | --- | --- | --- | --- | --- | --- | --- | --- | --- | --- | --- | --- | --- | --- | --- | --- | --- | --- | --- | --- | --- | --- | --- | --- | --- | --- | --- |

JAV48721.1

back to list

  

| Protein Coverage
| Supporting Peptides
|

Protein Coverage:

Supporting Peptides:

| Peptide | Uniq | -10lgP | Mass | Length | ppm | m/z | z | RT | Fraction | Scan | Source File | Area F6 | Area F2 | Area F3 | Area F4 | Area F5 | Area F7 | Area F8 | Area F1 | #Feature | #Feature F6 | #Feature F2 | #Feature F3 | #Feature F4 | #Feature F5 | #Feature F7 | #Feature F8 | #Feature F1 | Start | End | PTM | AScore | Found By |
| --- | --- | --- | --- | --- | --- | --- | --- | --- | --- | --- | --- | --- | --- | --- | --- | --- | --- | --- | --- | --- | --- | --- | --- | --- | --- | --- | --- | --- | --- | --- | --- | --- | --- |
| R.LISQIVSSITASLR.F | N | 56.73 | 1486.8718 | 14 | 0.8 | 744.4438 | 2 | 61.97 | 8 | F8:37801 | 31122021\_RID\_3262\_1b.raw |  |  |  |  |  |  |  | 9.8349E4 | 1 | 0 | 0 | 0 | 0 | 0 | 0 | 0 | 1 | 230 | 243 |  |  | PEAKS DB |
| R.NLDIERPTYTNLN.R | N | 52.43 | 1561.7736 | 13 | 0.5 | 781.8944 | 2 | 41.10 | 8 | F8:21657 | 31122021\_RID\_3262\_1b.raw |  |  |  |  |  |  |  | 1.1027E4 | 1 | 0 | 0 | 0 | 0 | 0 | 0 | 0 | 1 | 216 | 228 |  |  | PEAKS DB |
| K.DVNAAIATIK.T | Y | 49.86 | 1014.5709 | 10 | 0.0 | 508.2927 | 2 | 37.55 | 8 | F8:19028 | 31122021\_RID\_3262\_1b.raw |  |  |  |  |  |  |  | 1.0175E5 | 1 | 0 | 0 | 0 | 0 | 0 | 0 | 0 | 1 | 327 | 336 |  |  | PEAKS DB |
| K.EIIDLVLDR.I | N | 45.89 | 1084.6128 | 9 | -0.1 | 543.3136 | 2 | 54.37 | 8 | F8:32070 | 31122021\_RID\_3262\_1b.raw |  |  |  |  |  |  |  | 1.6656E5 | 1 | 0 | 0 | 0 | 0 | 0 | 0 | 0 | 1 | 113 | 121 |  |  | PEAKS DB |
| total 4 peptides |  |  |  |  |  |  |  |  |  |  |  |  |  |  |  |  |  |  |  |  |  |  |  |  |  |  |  |  |  |  |  |  |  |
| --- | --- | --- | --- | --- | --- | --- | --- | --- | --- | --- | --- | --- | --- | --- | --- | --- | --- | --- | --- | --- | --- | --- | --- | --- | --- | --- | --- | --- | --- | --- | --- | --- | --- |

JAG66079.1

back to list

  

| Protein Coverage
| Supporting Peptides
|

Protein Coverage:

Supporting Peptides:

| Peptide | Uniq | -10lgP | Mass | Length | ppm | m/z | z | RT | Fraction | Scan | Source File | Area F6 | Area F2 | Area F3 | Area F4 | Area F5 | Area F7 | Area F8 | Area F1 | #Feature | #Feature F6 | #Feature F2 | #Feature F3 | #Feature F4 | #Feature F5 | #Feature F7 | #Feature F8 | #Feature F1 | Start | End | PTM | AScore | Found By |
| --- | --- | --- | --- | --- | --- | --- | --- | --- | --- | --- | --- | --- | --- | --- | --- | --- | --- | --- | --- | --- | --- | --- | --- | --- | --- | --- | --- | --- | --- | --- | --- | --- | --- |
| R.LISQIVSSITASLR.F | N | 56.73 | 1486.8718 | 14 | 0.8 | 744.4438 | 2 | 61.97 | 8 | F8:37801 | 31122021\_RID\_3262\_1b.raw |  |  |  |  |  |  |  | 9.8349E4 | 1 | 0 | 0 | 0 | 0 | 0 | 0 | 0 | 1 | 230 | 243 |  |  | PEAKS DB |
| R.NLDIERPTYTNLN.R | N | 52.43 | 1561.7736 | 13 | 0.5 | 781.8944 | 2 | 41.10 | 8 | F8:21657 | 31122021\_RID\_3262\_1b.raw |  |  |  |  |  |  |  | 1.1027E4 | 1 | 0 | 0 | 0 | 0 | 0 | 0 | 0 | 1 | 216 | 228 |  |  | PEAKS DB |
| K.DVNAAIATIK.T | Y | 49.86 | 1014.5709 | 10 | 0.0 | 508.2927 | 2 | 37.55 | 8 | F8:19028 | 31122021\_RID\_3262\_1b.raw |  |  |  |  |  |  |  | 1.0175E5 | 1 | 0 | 0 | 0 | 0 | 0 | 0 | 0 | 1 | 327 | 336 |  |  | PEAKS DB |
| K.EIIDLVLDR.I | N | 45.89 | 1084.6128 | 9 | -0.1 | 543.3136 | 2 | 54.37 | 8 | F8:32070 | 31122021\_RID\_3262\_1b.raw |  |  |  |  |  |  |  | 1.6656E5 | 1 | 0 | 0 | 0 | 0 | 0 | 0 | 0 | 1 | 113 | 121 |  |  | PEAKS DB |
| total 4 peptides |  |  |  |  |  |  |  |  |  |  |  |  |  |  |  |  |  |  |  |  |  |  |  |  |  |  |  |  |  |  |  |  |  |
| --- | --- | --- | --- | --- | --- | --- | --- | --- | --- | --- | --- | --- | --- | --- | --- | --- | --- | --- | --- | --- | --- | --- | --- | --- | --- | --- | --- | --- | --- | --- | --- | --- | --- |

XP\_013918982.1

back to list

  

| Protein Coverage
| Supporting Peptides
|

Protein Coverage:

Supporting Peptides:

| Peptide | Uniq | -10lgP | Mass | Length | ppm | m/z | z | RT | Fraction | Scan | Source File | Area F6 | Area F2 | Area F3 | Area F4 | Area F5 | Area F7 | Area F8 | Area F1 | #Feature | #Feature F6 | #Feature F2 | #Feature F3 | #Feature F4 | #Feature F5 | #Feature F7 | #Feature F8 | #Feature F1 | Start | End | PTM | AScore | Found By |
| --- | --- | --- | --- | --- | --- | --- | --- | --- | --- | --- | --- | --- | --- | --- | --- | --- | --- | --- | --- | --- | --- | --- | --- | --- | --- | --- | --- | --- | --- | --- | --- | --- | --- |
| R.LISQIVSSITASLR.F | N | 56.73 | 1486.8718 | 14 | 0.8 | 744.4438 | 2 | 61.97 | 8 | F8:37801 | 31122021\_RID\_3262\_1b.raw |  |  |  |  |  |  |  | 9.8349E4 | 1 | 0 | 0 | 0 | 0 | 0 | 0 | 0 | 1 | 230 | 243 |  |  | PEAKS DB |
| R.NLDIERPTYTNLN.R | N | 52.43 | 1561.7736 | 13 | 0.5 | 781.8944 | 2 | 41.10 | 8 | F8:21657 | 31122021\_RID\_3262\_1b.raw |  |  |  |  |  |  |  | 1.1027E4 | 1 | 0 | 0 | 0 | 0 | 0 | 0 | 0 | 1 | 216 | 228 |  |  | PEAKS DB |
| K.DVNAAIATIK.T | Y | 49.86 | 1014.5709 | 10 | 0.0 | 508.2927 | 2 | 37.55 | 8 | F8:19028 | 31122021\_RID\_3262\_1b.raw |  |  |  |  |  |  |  | 1.0175E5 | 1 | 0 | 0 | 0 | 0 | 0 | 0 | 0 | 1 | 327 | 336 |  |  | PEAKS DB |
| K.EIIDLVLDR.I | N | 45.89 | 1084.6128 | 9 | -0.1 | 543.3136 | 2 | 54.37 | 8 | F8:32070 | 31122021\_RID\_3262\_1b.raw |  |  |  |  |  |  |  | 1.6656E5 | 1 | 0 | 0 | 0 | 0 | 0 | 0 | 0 | 1 | 113 | 121 |  |  | PEAKS DB |
| total 4 peptides |  |  |  |  |  |  |  |  |  |  |  |  |  |  |  |  |  |  |  |  |  |  |  |  |  |  |  |  |  |  |  |  |  |
| --- | --- | --- | --- | --- | --- | --- | --- | --- | --- | --- | --- | --- | --- | --- | --- | --- | --- | --- | --- | --- | --- | --- | --- | --- | --- | --- | --- | --- | --- | --- | --- | --- | --- |

JAV48718.1

back to list

  

| Protein Coverage
| Supporting Peptides
|

Protein Coverage:

Supporting Peptides:

| Peptide | Uniq | -10lgP | Mass | Length | ppm | m/z | z | RT | Fraction | Scan | Source File | Area F6 | Area F2 | Area F3 | Area F4 | Area F5 | Area F7 | Area F8 | Area F1 | #Feature | #Feature F6 | #Feature F2 | #Feature F3 | #Feature F4 | #Feature F5 | #Feature F7 | #Feature F8 | #Feature F1 | Start | End | PTM | AScore | Found By |
| --- | --- | --- | --- | --- | --- | --- | --- | --- | --- | --- | --- | --- | --- | --- | --- | --- | --- | --- | --- | --- | --- | --- | --- | --- | --- | --- | --- | --- | --- | --- | --- | --- | --- |
| R.LISQIVSSITASLR.F | N | 56.73 | 1486.8718 | 14 | 0.8 | 744.4438 | 2 | 61.97 | 8 | F8:37801 | 31122021\_RID\_3262\_1b.raw |  |  |  |  |  |  |  | 9.8349E4 | 1 | 0 | 0 | 0 | 0 | 0 | 0 | 0 | 1 | 230 | 243 |  |  | PEAKS DB |
| R.NLDIERPTYTNLN.R | N | 52.43 | 1561.7736 | 13 | 0.5 | 781.8944 | 2 | 41.10 | 8 | F8:21657 | 31122021\_RID\_3262\_1b.raw |  |  |  |  |  |  |  | 1.1027E4 | 1 | 0 | 0 | 0 | 0 | 0 | 0 | 0 | 1 | 216 | 228 |  |  | PEAKS DB |
| K.DVNAAIATIK.T | Y | 49.86 | 1014.5709 | 10 | 0.0 | 508.2927 | 2 | 37.55 | 8 | F8:19028 | 31122021\_RID\_3262\_1b.raw |  |  |  |  |  |  |  | 1.0175E5 | 1 | 0 | 0 | 0 | 0 | 0 | 0 | 0 | 1 | 327 | 336 |  |  | PEAKS DB |
| K.EIIDLVLDR.I | N | 45.89 | 1084.6128 | 9 | -0.1 | 543.3136 | 2 | 54.37 | 8 | F8:32070 | 31122021\_RID\_3262\_1b.raw |  |  |  |  |  |  |  | 1.6656E5 | 1 | 0 | 0 | 0 | 0 | 0 | 0 | 0 | 1 | 113 | 121 |  |  | PEAKS DB |
| total 4 peptides |  |  |  |  |  |  |  |  |  |  |  |  |  |  |  |  |  |  |  |  |  |  |  |  |  |  |  |  |  |  |  |  |  |
| --- | --- | --- | --- | --- | --- | --- | --- | --- | --- | --- | --- | --- | --- | --- | --- | --- | --- | --- | --- | --- | --- | --- | --- | --- | --- | --- | --- | --- | --- | --- | --- | --- | --- |

XP\_029142320.1

back to list

  

| Protein Coverage
| Supporting Peptides
|

Protein Coverage:

Supporting Peptides:

| Peptide | Uniq | -10lgP | Mass | Length | ppm | m/z | z | RT | Fraction | Scan | Source File | Area F6 | Area F2 | Area F3 | Area F4 | Area F5 | Area F7 | Area F8 | Area F1 | #Feature | #Feature F6 | #Feature F2 | #Feature F3 | #Feature F4 | #Feature F5 | #Feature F7 | #Feature F8 | #Feature F1 | Start | End | PTM | AScore | Found By |
| --- | --- | --- | --- | --- | --- | --- | --- | --- | --- | --- | --- | --- | --- | --- | --- | --- | --- | --- | --- | --- | --- | --- | --- | --- | --- | --- | --- | --- | --- | --- | --- | --- | --- |
| R.M(+15.99)FDTVNIVNLILQR.I | Y | 72.40 | 1690.9076 | 14 | 0.6 | 846.4615 | 2 | 68.24 | 4 | F4:37429 | 13012022\_RID\_3262\_4b.raw |  |  |  | 3.2051E5 | 1.3197E5 |  |  | 1.1308E5 | 3 | 0 | 0 | 0 | 1 | 1 | 0 | 0 | 1 | 266 | 279 | Oxidation (M) | M1:Oxidation (M):1000.00 | PEAKS DB |
| R.MFDTVNIVNLILQR.I | Y | 61.33 | 1674.9127 | 14 | 0.8 | 838.4643 | 2 | 72.87 | 8 | F8:43942 | 31122021\_RID\_3262\_1b.raw |  |  |  |  |  |  |  | 1.597E5 | 1 | 0 | 0 | 0 | 0 | 0 | 0 | 0 | 1 | 266 | 279 |  |  | PEAKS DB |
| R.M(+15.99)FDTVNIVNLILQ.R | Y | 52.85 | 1534.8065 | 13 | 0.6 | 768.4110 | 2 | 81.71 | 8 | F8:47178 | 31122021\_RID\_3262\_1b.raw |  |  |  |  |  |  |  | 7.6205E4 | 1 | 0 | 0 | 0 | 0 | 0 | 0 | 0 | 1 | 266 | 278 | Oxidation (M) | M1:Oxidation (M):1000.00 | PEAKS DB |
| K.GMVLPGTK.C | N | 52.52 | 801.4418 | 8 | 0.5 | 401.7284 | 2 | 30.84 | 3 | F3:15989 | 13012022\_RID\_3262\_3b.raw |  |  | 6.6025E6 | 3.2965E7 | 4.1669E6 |  |  |  | 3 | 0 | 0 | 1 | 1 | 1 | 0 | 0 | 0 | 624 | 631 |  |  | PEAKS DB |
| D.TVNIVNLILQR.I | Y | 50.56 | 1281.7769 | 11 | 1.0 | 641.8964 | 2 | 59.27 | 3 | F3:39711 | 13012022\_RID\_3262\_3b.raw |  |  | 2.0399E6 |  |  |  |  |  | 1 | 0 | 0 | 1 | 0 | 0 | 0 | 0 | 0 | 269 | 279 |  |  | PEAKS DB |
| K.GM(+15.99)VLPGTK.C | N | 47.60 | 817.4368 | 8 | 0.3 | 409.7258 | 2 | 27.43 | 3 | F3:12884 | 13012022\_RID\_3262\_3b.raw |  |  | 0 | 1.679E7 | 3.8436E6 |  |  |  | 2 | 0 | 0 | 0 | 1 | 1 | 0 | 0 | 0 | 624 | 631 | Oxidation (M) | M2:Oxidation (M):1000.00 | PEAKS DB |
| total 6 peptides |  |  |  |  |  |  |  |  |  |  |  |  |  |  |  |  |  |  |  |  |  |  |  |  |  |  |  |  |  |  |  |  |  |
| --- | --- | --- | --- | --- | --- | --- | --- | --- | --- | --- | --- | --- | --- | --- | --- | --- | --- | --- | --- | --- | --- | --- | --- | --- | --- | --- | --- | --- | --- | --- | --- | --- | --- |

AXL95289.1

back to list

  

| Protein Coverage
| Supporting Peptides
|

Protein Coverage:

Supporting Peptides:

| Peptide | Uniq | -10lgP | Mass | Length | ppm | m/z | z | RT | Fraction | Scan | Source File | Area F6 | Area F2 | Area F3 | Area F4 | Area F5 | Area F7 | Area F8 | Area F1 | #Feature | #Feature F6 | #Feature F2 | #Feature F3 | #Feature F4 | #Feature F5 | #Feature F7 | #Feature F8 | #Feature F1 | Start | End | PTM | AScore | Found By |
| --- | --- | --- | --- | --- | --- | --- | --- | --- | --- | --- | --- | --- | --- | --- | --- | --- | --- | --- | --- | --- | --- | --- | --- | --- | --- | --- | --- | --- | --- | --- | --- | --- | --- |
| D.VDFDSESPR.R | N | 69.49 | 1050.4618 | 9 | -0.5 | 526.2379 | 2 | 28.96 | 1 | F1:9510 | 04012022\_RID\_3262\_6b.raw | 1.3966E7 |  |  |  |  |  |  |  | 2 | 2 | 0 | 0 | 0 | 0 | 0 | 0 | 0 | 20 | 28 |  |  | PEAKS DB |
| G.DVDFDSESPR.R | Y | 62.61 | 1165.4886 | 10 | 0.8 | 583.7521 | 2 | 42.29 | 1 | F1:15487 | 04012022\_RID\_3262\_6b.raw | 1.295E7 |  |  |  |  |  |  |  | 1 | 1 | 0 | 0 | 0 | 0 | 0 | 0 | 0 | 19 | 28 |  |  | PEAKS DB |
| V.DFDSESPR.R | N | 50.10 | 951.3934 | 8 | -0.2 | 476.7039 | 2 | 24.61 | 1 | F1:7484 | 04012022\_RID\_3262\_6b.raw | 2.7217E6 |  |  |  |  |  |  |  | 1 | 1 | 0 | 0 | 0 | 0 | 0 | 0 | 0 | 21 | 28 |  |  | PEAKS DB |
| total 3 peptides |  |  |  |  |  |  |  |  |  |  |  |  |  |  |  |  |  |  |  |  |  |  |  |  |  |  |  |  |  |  |  |  |  |
| --- | --- | --- | --- | --- | --- | --- | --- | --- | --- | --- | --- | --- | --- | --- | --- | --- | --- | --- | --- | --- | --- | --- | --- | --- | --- | --- | --- | --- | --- | --- | --- | --- | --- |

P0C6E8.1

back to list

  

| Protein Coverage
| Supporting Peptides
|

Protein Coverage:

Supporting Peptides:

| Peptide | Uniq | -10lgP | Mass | Length | ppm | m/z | z | RT | Fraction | Scan | Source File | Area F6 | Area F2 | Area F3 | Area F4 | Area F5 | Area F7 | Area F8 | Area F1 | #Feature | #Feature F6 | #Feature F2 | #Feature F3 | #Feature F4 | #Feature F5 | #Feature F7 | #Feature F8 | #Feature F1 | Start | End | PTM | AScore | Found By |
| --- | --- | --- | --- | --- | --- | --- | --- | --- | --- | --- | --- | --- | --- | --- | --- | --- | --- | --- | --- | --- | --- | --- | --- | --- | --- | --- | --- | --- | --- | --- | --- | --- | --- |
| K.LGIFVDHGMYTK.Y | Y | 79.59 | 1379.6907 | 12 | 0.0 | 690.8526 | 2 | 50.22 | 5 | F5:25013 | 13012022\_RID\_3262\_5b.raw |  |  |  |  | 8.7013E6 |  |  | 0 | 1 | 0 | 0 | 0 | 0 | 1 | 0 | 0 | 0 | 37 | 48 |  |  | PEAKS DB |
| K.LGIFVDHGM(+15.99)YTK.Y | Y | 70.86 | 1395.6857 | 12 | 0.5 | 698.8505 | 2 | 46.12 | 5 | F5:21981 | 13012022\_RID\_3262\_5b.raw |  |  |  |  | 5.3051E7 |  |  |  | 2 | 0 | 0 | 0 | 0 | 2 | 0 | 0 | 0 | 37 | 48 | Oxidation (M) | M9:Oxidation (M):1000.00 | PEAKS DB |
| K.LGIFVDHGMYT.K | Y | 46.72 | 1251.5958 | 11 | 0.7 | 626.8056 | 2 | 51.32 | 5 | F5:25449 | 13012022\_RID\_3262\_5b.raw |  |  |  |  | 4.97E6 |  |  |  | 1 | 0 | 0 | 0 | 0 | 1 | 0 | 0 | 0 | 37 | 47 |  |  | PEAKS DB |
| total 3 peptides |  |  |  |  |  |  |  |  |  |  |  |  |  |  |  |  |  |  |  |  |  |  |  |  |  |  |  |  |  |  |  |  |  |
| --- | --- | --- | --- | --- | --- | --- | --- | --- | --- | --- | --- | --- | --- | --- | --- | --- | --- | --- | --- | --- | --- | --- | --- | --- | --- | --- | --- | --- | --- | --- | --- | --- | --- |

LAA55683.1

back to list

  

| Protein Coverage
| Supporting Peptides
|

Protein Coverage:

Supporting Peptides:

| Peptide | Uniq | -10lgP | Mass | Length | ppm | m/z | z | RT | Fraction | Scan | Source File | Area F6 | Area F2 | Area F3 | Area F4 | Area F5 | Area F7 | Area F8 | Area F1 | #Feature | #Feature F6 | #Feature F2 | #Feature F3 | #Feature F4 | #Feature F5 | #Feature F7 | #Feature F8 | #Feature F1 | Start | End | PTM | AScore | Found By |
| --- | --- | --- | --- | --- | --- | --- | --- | --- | --- | --- | --- | --- | --- | --- | --- | --- | --- | --- | --- | --- | --- | --- | --- | --- | --- | --- | --- | --- | --- | --- | --- | --- | --- |
| R.LISQIVSSITASLR.F | N | 56.73 | 1486.8718 | 14 | 0.8 | 744.4438 | 2 | 61.97 | 8 | F8:37801 | 31122021\_RID\_3262\_1b.raw |  |  |  |  |  |  |  | 9.8349E4 | 1 | 0 | 0 | 0 | 0 | 0 | 0 | 0 | 1 | 248 | 261 |  |  | PEAKS DB |
| R.NLDIERPTYTNLN.R | N | 52.43 | 1561.7736 | 13 | 0.5 | 781.8944 | 2 | 41.10 | 8 | F8:21657 | 31122021\_RID\_3262\_1b.raw |  |  |  |  |  |  |  | 1.1027E4 | 1 | 0 | 0 | 0 | 0 | 0 | 0 | 0 | 1 | 234 | 246 |  |  | PEAKS DB |
| K.DVNAAIAAIK.T | Y | 50.98 | 984.5604 | 10 | 0.3 | 493.2876 | 2 | 39.88 | 8 | F8:20755 | 31122021\_RID\_3262\_1b.raw |  |  |  |  |  |  |  | 0 | 0 | 0 | 0 | 0 | 0 | 0 | 0 | 0 | 0 | 345 | 354 |  |  | PEAKS DB |
| K.EIIDLVLDR.I | N | 45.89 | 1084.6128 | 9 | -0.1 | 543.3136 | 2 | 54.37 | 8 | F8:32070 | 31122021\_RID\_3262\_1b.raw |  |  |  |  |  |  |  | 1.6656E5 | 1 | 0 | 0 | 0 | 0 | 0 | 0 | 0 | 1 | 131 | 139 |  |  | PEAKS DB |
| total 4 peptides |  |  |  |  |  |  |  |  |  |  |  |  |  |  |  |  |  |  |  |  |  |  |  |  |  |  |  |  |  |  |  |  |  |
| --- | --- | --- | --- | --- | --- | --- | --- | --- | --- | --- | --- | --- | --- | --- | --- | --- | --- | --- | --- | --- | --- | --- | --- | --- | --- | --- | --- | --- | --- | --- | --- | --- | --- |

JAB53061.1

back to list

  

| Protein Coverage
| Supporting Peptides
|

Protein Coverage:

Supporting Peptides:

| Peptide | Uniq | -10lgP | Mass | Length | ppm | m/z | z | RT | Fraction | Scan | Source File | Area F6 | Area F2 | Area F3 | Area F4 | Area F5 | Area F7 | Area F8 | Area F1 | #Feature | #Feature F6 | #Feature F2 | #Feature F3 | #Feature F4 | #Feature F5 | #Feature F7 | #Feature F8 | #Feature F1 | Start | End | PTM | AScore | Found By |
| --- | --- | --- | --- | --- | --- | --- | --- | --- | --- | --- | --- | --- | --- | --- | --- | --- | --- | --- | --- | --- | --- | --- | --- | --- | --- | --- | --- | --- | --- | --- | --- | --- | --- |
| R.LISQIVSSITASLR.F | N | 56.73 | 1486.8718 | 14 | 0.8 | 744.4438 | 2 | 61.97 | 8 | F8:37801 | 31122021\_RID\_3262\_1b.raw |  |  |  |  |  |  |  | 9.8349E4 | 1 | 0 | 0 | 0 | 0 | 0 | 0 | 0 | 1 | 215 | 228 |  |  | PEAKS DB |
| R.NLDIERPTYTNLN.R | N | 52.43 | 1561.7736 | 13 | 0.5 | 781.8944 | 2 | 41.10 | 8 | F8:21657 | 31122021\_RID\_3262\_1b.raw |  |  |  |  |  |  |  | 1.1027E4 | 1 | 0 | 0 | 0 | 0 | 0 | 0 | 0 | 1 | 201 | 213 |  |  | PEAKS DB |
| K.DVNAAIAAIK.T | Y | 50.98 | 984.5604 | 10 | 0.3 | 493.2876 | 2 | 39.88 | 8 | F8:20755 | 31122021\_RID\_3262\_1b.raw |  |  |  |  |  |  |  | 0 | 0 | 0 | 0 | 0 | 0 | 0 | 0 | 0 | 0 | 312 | 321 |  |  | PEAKS DB |
| K.EIIDLVLDR.I | N | 45.89 | 1084.6128 | 9 | -0.1 | 543.3136 | 2 | 54.37 | 8 | F8:32070 | 31122021\_RID\_3262\_1b.raw |  |  |  |  |  |  |  | 1.6656E5 | 1 | 0 | 0 | 0 | 0 | 0 | 0 | 0 | 1 | 98 | 106 |  |  | PEAKS DB |
| total 4 peptides |  |  |  |  |  |  |  |  |  |  |  |  |  |  |  |  |  |  |  |  |  |  |  |  |  |  |  |  |  |  |  |  |  |
| --- | --- | --- | --- | --- | --- | --- | --- | --- | --- | --- | --- | --- | --- | --- | --- | --- | --- | --- | --- | --- | --- | --- | --- | --- | --- | --- | --- | --- | --- | --- | --- | --- | --- |

JAI09231.1

back to list

  

| Protein Coverage
| Supporting Peptides
|

Protein Coverage:

Supporting Peptides:

| Peptide | Uniq | -10lgP | Mass | Length | ppm | m/z | z | RT | Fraction | Scan | Source File | Area F6 | Area F2 | Area F3 | Area F4 | Area F5 | Area F7 | Area F8 | Area F1 | #Feature | #Feature F6 | #Feature F2 | #Feature F3 | #Feature F4 | #Feature F5 | #Feature F7 | #Feature F8 | #Feature F1 | Start | End | PTM | AScore | Found By |
| --- | --- | --- | --- | --- | --- | --- | --- | --- | --- | --- | --- | --- | --- | --- | --- | --- | --- | --- | --- | --- | --- | --- | --- | --- | --- | --- | --- | --- | --- | --- | --- | --- | --- |
| R.LISQIVSSITASLR.F | N | 56.73 | 1486.8718 | 14 | 0.8 | 744.4438 | 2 | 61.97 | 8 | F8:37801 | 31122021\_RID\_3262\_1b.raw |  |  |  |  |  |  |  | 9.8349E4 | 1 | 0 | 0 | 0 | 0 | 0 | 0 | 0 | 1 | 230 | 243 |  |  | PEAKS DB |
| R.NLDIERPTYTNLN.R | N | 52.43 | 1561.7736 | 13 | 0.5 | 781.8944 | 2 | 41.10 | 8 | F8:21657 | 31122021\_RID\_3262\_1b.raw |  |  |  |  |  |  |  | 1.1027E4 | 1 | 0 | 0 | 0 | 0 | 0 | 0 | 0 | 1 | 216 | 228 |  |  | PEAKS DB |
| K.DVNAAIAAIK.T | Y | 50.98 | 984.5604 | 10 | 0.3 | 493.2876 | 2 | 39.88 | 8 | F8:20755 | 31122021\_RID\_3262\_1b.raw |  |  |  |  |  |  |  | 0 | 0 | 0 | 0 | 0 | 0 | 0 | 0 | 0 | 0 | 327 | 336 |  |  | PEAKS DB |
| K.EIIDLVLDR.I | N | 45.89 | 1084.6128 | 9 | -0.1 | 543.3136 | 2 | 54.37 | 8 | F8:32070 | 31122021\_RID\_3262\_1b.raw |  |  |  |  |  |  |  | 1.6656E5 | 1 | 0 | 0 | 0 | 0 | 0 | 0 | 0 | 1 | 113 | 121 |  |  | PEAKS DB |
| total 4 peptides |  |  |  |  |  |  |  |  |  |  |  |  |  |  |  |  |  |  |  |  |  |  |  |  |  |  |  |  |  |  |  |  |  |
| --- | --- | --- | --- | --- | --- | --- | --- | --- | --- | --- | --- | --- | --- | --- | --- | --- | --- | --- | --- | --- | --- | --- | --- | --- | --- | --- | --- | --- | --- | --- | --- | --- | --- |

LAA55679.1

back to list

  

| Protein Coverage
| Supporting Peptides
|

Protein Coverage:

Supporting Peptides:

| Peptide | Uniq | -10lgP | Mass | Length | ppm | m/z | z | RT | Fraction | Scan | Source File | Area F6 | Area F2 | Area F3 | Area F4 | Area F5 | Area F7 | Area F8 | Area F1 | #Feature | #Feature F6 | #Feature F2 | #Feature F3 | #Feature F4 | #Feature F5 | #Feature F7 | #Feature F8 | #Feature F1 | Start | End | PTM | AScore | Found By |
| --- | --- | --- | --- | --- | --- | --- | --- | --- | --- | --- | --- | --- | --- | --- | --- | --- | --- | --- | --- | --- | --- | --- | --- | --- | --- | --- | --- | --- | --- | --- | --- | --- | --- |
| R.LISQIVSSITASLR.F | N | 56.73 | 1486.8718 | 14 | 0.8 | 744.4438 | 2 | 61.97 | 8 | F8:37801 | 31122021\_RID\_3262\_1b.raw |  |  |  |  |  |  |  | 9.8349E4 | 1 | 0 | 0 | 0 | 0 | 0 | 0 | 0 | 1 | 230 | 243 |  |  | PEAKS DB |
| R.NLDIERPTYTNLN.R | N | 52.43 | 1561.7736 | 13 | 0.5 | 781.8944 | 2 | 41.10 | 8 | F8:21657 | 31122021\_RID\_3262\_1b.raw |  |  |  |  |  |  |  | 1.1027E4 | 1 | 0 | 0 | 0 | 0 | 0 | 0 | 0 | 1 | 216 | 228 |  |  | PEAKS DB |
| K.DVNAAIAAIK.T | Y | 50.98 | 984.5604 | 10 | 0.3 | 493.2876 | 2 | 39.88 | 8 | F8:20755 | 31122021\_RID\_3262\_1b.raw |  |  |  |  |  |  |  | 0 | 0 | 0 | 0 | 0 | 0 | 0 | 0 | 0 | 0 | 327 | 336 |  |  | PEAKS DB |
| K.EIIDLVLDR.I | N | 45.89 | 1084.6128 | 9 | -0.1 | 543.3136 | 2 | 54.37 | 8 | F8:32070 | 31122021\_RID\_3262\_1b.raw |  |  |  |  |  |  |  | 1.6656E5 | 1 | 0 | 0 | 0 | 0 | 0 | 0 | 0 | 1 | 113 | 121 |  |  | PEAKS DB |
| total 4 peptides |  |  |  |  |  |  |  |  |  |  |  |  |  |  |  |  |  |  |  |  |  |  |  |  |  |  |  |  |  |  |  |  |  |
| --- | --- | --- | --- | --- | --- | --- | --- | --- | --- | --- | --- | --- | --- | --- | --- | --- | --- | --- | --- | --- | --- | --- | --- | --- | --- | --- | --- | --- | --- | --- | --- | --- | --- |

AUS82539.1

back to list

  

| Protein Coverage
| Supporting Peptides
|

Protein Coverage:

Supporting Peptides:

| Peptide | Uniq | -10lgP | Mass | Length | ppm | m/z | z | RT | Fraction | Scan | Source File | Area F6 | Area F2 | Area F3 | Area F4 | Area F5 | Area F7 | Area F8 | Area F1 | #Feature | #Feature F6 | #Feature F2 | #Feature F3 | #Feature F4 | #Feature F5 | #Feature F7 | #Feature F8 | #Feature F1 | Start | End | PTM | AScore | Found By |
| --- | --- | --- | --- | --- | --- | --- | --- | --- | --- | --- | --- | --- | --- | --- | --- | --- | --- | --- | --- | --- | --- | --- | --- | --- | --- | --- | --- | --- | --- | --- | --- | --- | --- |
| R.IMGWGTISDTK.V | Y | 64.96 | 1207.5907 | 11 | -0.3 | 604.8024 | 2 | 44.77 | 4 | F4:25775 | 13012022\_RID\_3262\_4b.raw |  |  |  | 4.1625E6 | 3.8208E6 |  |  |  | 2 | 0 | 0 | 0 | 1 | 1 | 0 | 0 | 0 | 143 | 153 |  |  | PEAKS DB |
| F.WDKDIMLIR.L | N | 59.44 | 1188.6324 | 9 | -0.6 | 595.3231 | 2 | 42.59 | 3 | F3:26121 | 13012022\_RID\_3262\_3b.raw |  |  | 7.7454E7 | 4.9887E6 |  |  |  | 2.131E6 | 5 | 0 | 0 | 2 | 2 | 0 | 0 | 0 | 1 | 106 | 114 |  |  | PEAKS DB |
| F.WDKDIM(+15.99)LIR.L | N | 53.70 | 1204.6274 | 9 | 0.1 | 603.3210 | 2 | 37.60 | 3 | F3:21664 | 13012022\_RID\_3262\_3b.raw |  |  | 1.4633E8 | 3.3538E6 |  |  |  |  | 3 | 0 | 0 | 2 | 1 | 0 | 0 | 0 | 0 | 106 | 114 | Oxidation (M) | M6:Oxidation (M):1000.00 | PEAKS DB |
| R.EPALYTK.V | N | 53.18 | 820.4330 | 7 | 0.6 | 411.2240 | 2 | 24.56 | 4 | F4:11031 | 13012022\_RID\_3262\_4b.raw |  |  |  | 8.5115E5 |  |  |  |  | 1 | 0 | 0 | 0 | 1 | 0 | 0 | 0 | 0 | 231 | 237 |  |  | PEAKS DB |
| W.DKDIMLIR.L | N | 45.33 | 1002.5532 | 8 | 0.8 | 502.2842 | 2 | 34.98 | 4 | F4:18968 | 13012022\_RID\_3262\_4b.raw |  |  |  | 7.1188E5 |  |  |  |  | 1 | 0 | 0 | 0 | 1 | 0 | 0 | 0 | 0 | 107 | 114 |  |  | PEAKS DB |
| total 5 peptides |  |  |  |  |  |  |  |  |  |  |  |  |  |  |  |  |  |  |  |  |  |  |  |  |  |  |  |  |  |  |  |  |  |
| --- | --- | --- | --- | --- | --- | --- | --- | --- | --- | --- | --- | --- | --- | --- | --- | --- | --- | --- | --- | --- | --- | --- | --- | --- | --- | --- | --- | --- | --- | --- | --- | --- | --- |

JAV48393.1

back to list

  

| Protein Coverage
| Supporting Peptides
|

Protein Coverage:

Supporting Peptides:

| Peptide | Uniq | -10lgP | Mass | Length | ppm | m/z | z | RT | Fraction | Scan | Source File | Area F6 | Area F2 | Area F3 | Area F4 | Area F5 | Area F7 | Area F8 | Area F1 | #Feature | #Feature F6 | #Feature F2 | #Feature F3 | #Feature F4 | #Feature F5 | #Feature F7 | #Feature F8 | #Feature F1 | Start | End | PTM | AScore | Found By |
| --- | --- | --- | --- | --- | --- | --- | --- | --- | --- | --- | --- | --- | --- | --- | --- | --- | --- | --- | --- | --- | --- | --- | --- | --- | --- | --- | --- | --- | --- | --- | --- | --- | --- |
| R.IMGWGTISSTK.E | N | 63.66 | 1179.5958 | 11 | -0.2 | 590.8051 | 2 | 43.40 | 5 | F5:20913 | 13012022\_RID\_3262\_5b.raw |  |  |  |  | 6.9453E6 |  |  |  | 2 | 0 | 0 | 0 | 0 | 2 | 0 | 0 | 0 | 146 | 156 |  |  | PEAKS DB |
| K.WDKDIMLIR.L | N | 59.44 | 1188.6324 | 9 | -0.6 | 595.3231 | 2 | 42.59 | 3 | F3:26121 | 13012022\_RID\_3262\_3b.raw |  |  | 7.7454E7 | 4.9887E6 |  |  |  | 2.131E6 | 5 | 0 | 0 | 2 | 2 | 0 | 0 | 0 | 1 | 109 | 117 |  |  | PEAKS DB |
| R.IM(+15.99)GWGTISSTK.E | N | 59.44 | 1195.5907 | 11 | 0.1 | 598.8027 | 2 | 43.49 | 5 | F5:20273 | 13012022\_RID\_3262\_5b.raw |  |  |  |  | 3.9703E6 |  |  |  | 2 | 0 | 0 | 0 | 0 | 2 | 0 | 0 | 0 | 146 | 156 | Oxidation (M) | M2:Oxidation (M):1000.00 | PEAKS DB |
| L.VIGGEEC(+57.02)NINEHR.F | Y | 57.03 | 1525.6943 | 13 | 0.5 | 763.8549 | 2 | 24.02 | 4 | F4:10498 | 13012022\_RID\_3262\_4b.raw |  |  | 4.8766E6 | 2.1479E7 |  |  |  |  | 3 | 0 | 0 | 1 | 2 | 0 | 0 | 0 | 0 | 25 | 37 | Carbamidomethylation | C7:Carbamidomethylation:1000.00 | PEAKS DB |
| K.WDKDIM(+15.99)LIR.L | N | 53.70 | 1204.6274 | 9 | 0.1 | 603.3210 | 2 | 37.60 | 3 | F3:21664 | 13012022\_RID\_3262\_3b.raw |  |  | 1.4633E8 | 3.3538E6 |  |  |  |  | 3 | 0 | 0 | 2 | 1 | 0 | 0 | 0 | 0 | 109 | 117 | Oxidation (M) | M6:Oxidation (M):1000.00 | PEAKS DB |
| W.DKDIMLIR.L | N | 45.33 | 1002.5532 | 8 | 0.8 | 502.2842 | 2 | 34.98 | 4 | F4:18968 | 13012022\_RID\_3262\_4b.raw |  |  |  | 7.1188E5 |  |  |  |  | 1 | 0 | 0 | 0 | 1 | 0 | 0 | 0 | 0 | 110 | 117 |  |  | PEAKS DB |
| total 6 peptides |  |  |  |  |  |  |  |  |  |  |  |  |  |  |  |  |  |  |  |  |  |  |  |  |  |  |  |  |  |  |  |  |  |
| --- | --- | --- | --- | --- | --- | --- | --- | --- | --- | --- | --- | --- | --- | --- | --- | --- | --- | --- | --- | --- | --- | --- | --- | --- | --- | --- | --- | --- | --- | --- | --- | --- | --- |

ASX97880.1

back to list

  

| Protein Coverage
| Supporting Peptides
|

Protein Coverage:

Supporting Peptides:

| Peptide | Uniq | -10lgP | Mass | Length | ppm | m/z | z | RT | Fraction | Scan | Source File | Area F6 | Area F2 | Area F3 | Area F4 | Area F5 | Area F7 | Area F8 | Area F1 | #Feature | #Feature F6 | #Feature F2 | #Feature F3 | #Feature F4 | #Feature F5 | #Feature F7 | #Feature F8 | #Feature F1 | Start | End | PTM | AScore | Found By |
| --- | --- | --- | --- | --- | --- | --- | --- | --- | --- | --- | --- | --- | --- | --- | --- | --- | --- | --- | --- | --- | --- | --- | --- | --- | --- | --- | --- | --- | --- | --- | --- | --- | --- |
| R.AAYPWWPATTR.T | Y | 71.65 | 1318.6459 | 11 | 0.6 | 660.3306 | 2 | 51.99 | 5 | F5:25699 | 13012022\_RID\_3262\_5b.raw |  |  |  |  | 5.8582E6 |  |  |  | 1 | 0 | 0 | 0 | 0 | 1 | 0 | 0 | 0 | 176 | 186 |  |  | PEAKS DB |
| R.IMGWGTISSTK.E | N | 63.66 | 1179.5958 | 11 | -0.2 | 590.8051 | 2 | 43.40 | 5 | F5:20913 | 13012022\_RID\_3262\_5b.raw |  |  |  |  | 6.9453E6 |  |  |  | 2 | 0 | 0 | 0 | 0 | 2 | 0 | 0 | 0 | 144 | 154 |  |  | PEAKS DB |
| R.IM(+15.99)GWGTISSTK.E | N | 59.44 | 1195.5907 | 11 | 0.1 | 598.8027 | 2 | 43.49 | 5 | F5:20273 | 13012022\_RID\_3262\_5b.raw |  |  |  |  | 3.9703E6 |  |  |  | 2 | 0 | 0 | 0 | 0 | 2 | 0 | 0 | 0 | 144 | 154 | Oxidation (M) | M2:Oxidation (M):1000.00 | PEAKS DB |
| K.ETYPNVPR.C | Y | 56.69 | 974.4821 | 8 | 0.7 | 488.2487 | 2 | 27.83 | 5 | F5:10860 | 13012022\_RID\_3262\_5b.raw | 8.3095E5 |  |  |  | 4.6723E6 | 1.0165E6 |  |  | 3 | 1 | 0 | 0 | 0 | 1 | 1 | 0 | 0 | 155 | 162 |  |  | PEAKS DB |
| total 4 peptides |  |  |  |  |  |  |  |  |  |  |  |  |  |  |  |  |  |  |  |  |  |  |  |  |  |  |  |  |  |  |  |  |  |
| --- | --- | --- | --- | --- | --- | --- | --- | --- | --- | --- | --- | --- | --- | --- | --- | --- | --- | --- | --- | --- | --- | --- | --- | --- | --- | --- | --- | --- | --- | --- | --- | --- | --- |

BAN82028.1

back to list

  

| Protein Coverage
| Supporting Peptides
|

Protein Coverage:

Supporting Peptides:

| Peptide | Uniq | -10lgP | Mass | Length | ppm | m/z | z | RT | Fraction | Scan | Source File | Area F6 | Area F2 | Area F3 | Area F4 | Area F5 | Area F7 | Area F8 | Area F1 | #Feature | #Feature F6 | #Feature F2 | #Feature F3 | #Feature F4 | #Feature F5 | #Feature F7 | #Feature F8 | #Feature F1 | Start | End | PTM | AScore | Found By |
| --- | --- | --- | --- | --- | --- | --- | --- | --- | --- | --- | --- | --- | --- | --- | --- | --- | --- | --- | --- | --- | --- | --- | --- | --- | --- | --- | --- | --- | --- | --- | --- | --- | --- |
| K.PGYPELLPEYR.T | N | 70.94 | 1332.6714 | 11 | 0.4 | 667.3433 | 2 | 48.15 | 5 | F5:23263 | 13012022\_RID\_3262\_5b.raw |  |  |  |  | 6.1183E6 |  |  |  | 1 | 0 | 0 | 0 | 0 | 1 | 0 | 0 | 0 | 150 | 160 |  |  | PEAKS DB |
| E.NVKFDDEQGR.S | Y | 54.01 | 1206.5629 | 10 | 0.8 | 604.2892 | 2 | 20.67 | 3 | F3:7545 | 13012022\_RID\_3262\_3b.raw |  |  | 1.5435E6 |  |  |  |  |  | 1 | 0 | 0 | 1 | 0 | 0 | 0 | 0 | 0 | 55 | 64 |  |  | PEAKS DB |
| total 2 peptides |  |  |  |  |  |  |  |  |  |  |  |  |  |  |  |  |  |  |  |  |  |  |  |  |  |  |  |  |  |  |  |  |  |
| --- | --- | --- | --- | --- | --- | --- | --- | --- | --- | --- | --- | --- | --- | --- | --- | --- | --- | --- | --- | --- | --- | --- | --- | --- | --- | --- | --- | --- | --- | --- | --- | --- | --- |

AFJ49231.1

back to list

  

| Protein Coverage
| Supporting Peptides
|

Protein Coverage:

Supporting Peptides:

| Peptide | Uniq | -10lgP | Mass | Length | ppm | m/z | z | RT | Fraction | Scan | Source File | Area F6 | Area F2 | Area F3 | Area F4 | Area F5 | Area F7 | Area F8 | Area F1 | #Feature | #Feature F6 | #Feature F2 | #Feature F3 | #Feature F4 | #Feature F5 | #Feature F7 | #Feature F8 | #Feature F1 | Start | End | PTM | AScore | Found By |
| --- | --- | --- | --- | --- | --- | --- | --- | --- | --- | --- | --- | --- | --- | --- | --- | --- | --- | --- | --- | --- | --- | --- | --- | --- | --- | --- | --- | --- | --- | --- | --- | --- | --- |
| S.QLVVTAEQQR.L | N | 61.06 | 1170.6356 | 10 | -0.6 | 586.3247 | 2 | 41.64 | 2 | F2:15936 | 13012022\_RID\_3262\_2b.raw |  | 1.1474E6 | 0 | 3.4155E5 | 8.5962E4 |  |  |  | 3 | 0 | 1 | 0 | 1 | 1 | 0 | 0 | 0 | 184 | 193 |  |  | PEAKS DB |
| Q.LVVTAEQQR.L | N | 59.96 | 1042.5771 | 9 | -0.3 | 522.2957 | 2 | 24.54 | 2 | F2:6838 | 13012022\_RID\_3262\_2b.raw |  | 4.3551E6 | 7.7997E6 | 4.2093E6 |  |  |  | 4.824E5 | 4 | 0 | 1 | 1 | 1 | 0 | 0 | 0 | 1 | 185 | 193 |  |  | PEAKS DB |
| R.HDNAQLLTAIDFDGR.T | Y | 55.73 | 1684.8169 | 15 | -0.2 | 843.4155 | 2 | 45.97 | 3 | F3:28890 | 13012022\_RID\_3262\_3b.raw |  |  | 1.287E7 |  |  |  |  |  | 2 | 0 | 0 | 2 | 0 | 0 | 0 | 0 | 0 | 280 | 294 |  |  | PEAKS DB |
| total 3 peptides |  |  |  |  |  |  |  |  |  |  |  |  |  |  |  |  |  |  |  |  |  |  |  |  |  |  |  |  |  |  |  |  |  |
| --- | --- | --- | --- | --- | --- | --- | --- | --- | --- | --- | --- | --- | --- | --- | --- | --- | --- | --- | --- | --- | --- | --- | --- | --- | --- | --- | --- | --- | --- | --- | --- | --- | --- |

JAI10389.1

back to list

  

| Protein Coverage
| Supporting Peptides
|

Protein Coverage:

Supporting Peptides:

| Peptide | Uniq | -10lgP | Mass | Length | ppm | m/z | z | RT | Fraction | Scan | Source File | Area F6 | Area F2 | Area F3 | Area F4 | Area F5 | Area F7 | Area F8 | Area F1 | #Feature | #Feature F6 | #Feature F2 | #Feature F3 | #Feature F4 | #Feature F5 | #Feature F7 | #Feature F8 | #Feature F1 | Start | End | PTM | AScore | Found By |
| --- | --- | --- | --- | --- | --- | --- | --- | --- | --- | --- | --- | --- | --- | --- | --- | --- | --- | --- | --- | --- | --- | --- | --- | --- | --- | --- | --- | --- | --- | --- | --- | --- | --- |
| S.QLVVTAEQQR.L | N | 61.06 | 1170.6356 | 10 | -0.6 | 586.3247 | 2 | 41.64 | 2 | F2:15936 | 13012022\_RID\_3262\_2b.raw |  | 1.1474E6 | 0 | 3.4155E5 | 8.5962E4 |  |  |  | 3 | 0 | 1 | 0 | 1 | 1 | 0 | 0 | 0 | 184 | 193 |  |  | PEAKS DB |
| Q.LVVTAEQQR.L | N | 59.96 | 1042.5771 | 9 | -0.3 | 522.2957 | 2 | 24.54 | 2 | F2:6838 | 13012022\_RID\_3262\_2b.raw |  | 4.3551E6 | 7.7997E6 | 4.2093E6 |  |  |  | 4.824E5 | 4 | 0 | 1 | 1 | 1 | 0 | 0 | 0 | 1 | 185 | 193 |  |  | PEAKS DB |
| R.HDNAQLLTAIDFDGR.T | Y | 55.73 | 1684.8169 | 15 | -0.2 | 843.4155 | 2 | 45.97 | 3 | F3:28890 | 13012022\_RID\_3262\_3b.raw |  |  | 1.287E7 |  |  |  |  |  | 2 | 0 | 0 | 2 | 0 | 0 | 0 | 0 | 0 | 280 | 294 |  |  | PEAKS DB |
| total 3 peptides |  |  |  |  |  |  |  |  |  |  |  |  |  |  |  |  |  |  |  |  |  |  |  |  |  |  |  |  |  |  |  |  |  |
| --- | --- | --- | --- | --- | --- | --- | --- | --- | --- | --- | --- | --- | --- | --- | --- | --- | --- | --- | --- | --- | --- | --- | --- | --- | --- | --- | --- | --- | --- | --- | --- | --- | --- |

JAS04685.1

back to list

  

| Protein Coverage
| Supporting Peptides
|

Protein Coverage:

Supporting Peptides:

| Peptide | Uniq | -10lgP | Mass | Length | ppm | m/z | z | RT | Fraction | Scan | Source File | Area F6 | Area F2 | Area F3 | Area F4 | Area F5 | Area F7 | Area F8 | Area F1 | #Feature | #Feature F6 | #Feature F2 | #Feature F3 | #Feature F4 | #Feature F5 | #Feature F7 | #Feature F8 | #Feature F1 | Start | End | PTM | AScore | Found By |
| --- | --- | --- | --- | --- | --- | --- | --- | --- | --- | --- | --- | --- | --- | --- | --- | --- | --- | --- | --- | --- | --- | --- | --- | --- | --- | --- | --- | --- | --- | --- | --- | --- | --- |
| S.QLVVTAEQQR.L | N | 61.06 | 1170.6356 | 10 | -0.6 | 586.3247 | 2 | 41.64 | 2 | F2:15936 | 13012022\_RID\_3262\_2b.raw |  | 1.1474E6 | 0 | 3.4155E5 | 8.5962E4 |  |  |  | 3 | 0 | 1 | 0 | 1 | 1 | 0 | 0 | 0 | 184 | 193 |  |  | PEAKS DB |
| Q.LVVTAEQQR.L | N | 59.96 | 1042.5771 | 9 | -0.3 | 522.2957 | 2 | 24.54 | 2 | F2:6838 | 13012022\_RID\_3262\_2b.raw |  | 4.3551E6 | 7.7997E6 | 4.2093E6 |  |  |  | 4.824E5 | 4 | 0 | 1 | 1 | 1 | 0 | 0 | 0 | 1 | 185 | 193 |  |  | PEAKS DB |
| R.HDNAQLLTAIDFDGR.T | Y | 55.73 | 1684.8169 | 15 | -0.2 | 843.4155 | 2 | 45.97 | 3 | F3:28890 | 13012022\_RID\_3262\_3b.raw |  |  | 1.287E7 |  |  |  |  |  | 2 | 0 | 0 | 2 | 0 | 0 | 0 | 0 | 0 | 280 | 294 |  |  | PEAKS DB |
| total 3 peptides |  |  |  |  |  |  |  |  |  |  |  |  |  |  |  |  |  |  |  |  |  |  |  |  |  |  |  |  |  |  |  |  |  |
| --- | --- | --- | --- | --- | --- | --- | --- | --- | --- | --- | --- | --- | --- | --- | --- | --- | --- | --- | --- | --- | --- | --- | --- | --- | --- | --- | --- | --- | --- | --- | --- | --- | --- |

JAI10388.1

back to list

  

| Protein Coverage
| Supporting Peptides
|

Protein Coverage:

Supporting Peptides:

| Peptide | Uniq | -10lgP | Mass | Length | ppm | m/z | z | RT | Fraction | Scan | Source File | Area F6 | Area F2 | Area F3 | Area F4 | Area F5 | Area F7 | Area F8 | Area F1 | #Feature | #Feature F6 | #Feature F2 | #Feature F3 | #Feature F4 | #Feature F5 | #Feature F7 | #Feature F8 | #Feature F1 | Start | End | PTM | AScore | Found By |
| --- | --- | --- | --- | --- | --- | --- | --- | --- | --- | --- | --- | --- | --- | --- | --- | --- | --- | --- | --- | --- | --- | --- | --- | --- | --- | --- | --- | --- | --- | --- | --- | --- | --- |
| S.QLVVTAEQQR.L | N | 61.06 | 1170.6356 | 10 | -0.6 | 586.3247 | 2 | 41.64 | 2 | F2:15936 | 13012022\_RID\_3262\_2b.raw |  | 1.1474E6 | 0 | 3.4155E5 | 8.5962E4 |  |  |  | 3 | 0 | 1 | 0 | 1 | 1 | 0 | 0 | 0 | 184 | 193 |  |  | PEAKS DB |
| Q.LVVTAEQQR.L | N | 59.96 | 1042.5771 | 9 | -0.3 | 522.2957 | 2 | 24.54 | 2 | F2:6838 | 13012022\_RID\_3262\_2b.raw |  | 4.3551E6 | 7.7997E6 | 4.2093E6 |  |  |  | 4.824E5 | 4 | 0 | 1 | 1 | 1 | 0 | 0 | 0 | 1 | 185 | 193 |  |  | PEAKS DB |
| R.HDNAQLLTAIDFDGR.T | Y | 55.73 | 1684.8169 | 15 | -0.2 | 843.4155 | 2 | 45.97 | 3 | F3:28890 | 13012022\_RID\_3262\_3b.raw |  |  | 1.287E7 |  |  |  |  |  | 2 | 0 | 0 | 2 | 0 | 0 | 0 | 0 | 0 | 280 | 294 |  |  | PEAKS DB |
| total 3 peptides |  |  |  |  |  |  |  |  |  |  |  |  |  |  |  |  |  |  |  |  |  |  |  |  |  |  |  |  |  |  |  |  |  |
| --- | --- | --- | --- | --- | --- | --- | --- | --- | --- | --- | --- | --- | --- | --- | --- | --- | --- | --- | --- | --- | --- | --- | --- | --- | --- | --- | --- | --- | --- | --- | --- | --- | --- |

JAS04684.1

back to list

  

| Protein Coverage
| Supporting Peptides
|

Protein Coverage:

Supporting Peptides:

| Peptide | Uniq | -10lgP | Mass | Length | ppm | m/z | z | RT | Fraction | Scan | Source File | Area F6 | Area F2 | Area F3 | Area F4 | Area F5 | Area F7 | Area F8 | Area F1 | #Feature | #Feature F6 | #Feature F2 | #Feature F3 | #Feature F4 | #Feature F5 | #Feature F7 | #Feature F8 | #Feature F1 | Start | End | PTM | AScore | Found By |
| --- | --- | --- | --- | --- | --- | --- | --- | --- | --- | --- | --- | --- | --- | --- | --- | --- | --- | --- | --- | --- | --- | --- | --- | --- | --- | --- | --- | --- | --- | --- | --- | --- | --- |
| S.QLVVTAEQQR.L | N | 61.06 | 1170.6356 | 10 | -0.6 | 586.3247 | 2 | 41.64 | 2 | F2:15936 | 13012022\_RID\_3262\_2b.raw |  | 1.1474E6 | 0 | 3.4155E5 | 8.5962E4 |  |  |  | 3 | 0 | 1 | 0 | 1 | 1 | 0 | 0 | 0 | 184 | 193 |  |  | PEAKS DB |
| Q.LVVTAEQQR.L | N | 59.96 | 1042.5771 | 9 | -0.3 | 522.2957 | 2 | 24.54 | 2 | F2:6838 | 13012022\_RID\_3262\_2b.raw |  | 4.3551E6 | 7.7997E6 | 4.2093E6 |  |  |  | 4.824E5 | 4 | 0 | 1 | 1 | 1 | 0 | 0 | 0 | 1 | 185 | 193 |  |  | PEAKS DB |
| R.HDNAQLLTAIDFDGR.T | Y | 55.73 | 1684.8169 | 15 | -0.2 | 843.4155 | 2 | 45.97 | 3 | F3:28890 | 13012022\_RID\_3262\_3b.raw |  |  | 1.287E7 |  |  |  |  |  | 2 | 0 | 0 | 2 | 0 | 0 | 0 | 0 | 0 | 280 | 294 |  |  | PEAKS DB |
| total 3 peptides |  |  |  |  |  |  |  |  |  |  |  |  |  |  |  |  |  |  |  |  |  |  |  |  |  |  |  |  |  |  |  |  |  |
| --- | --- | --- | --- | --- | --- | --- | --- | --- | --- | --- | --- | --- | --- | --- | --- | --- | --- | --- | --- | --- | --- | --- | --- | --- | --- | --- | --- | --- | --- | --- | --- | --- | --- |

AFJ49232.1

back to list

  

| Protein Coverage
| Supporting Peptides
|

Protein Coverage:

Supporting Peptides:

| Peptide | Uniq | -10lgP | Mass | Length | ppm | m/z | z | RT | Fraction | Scan | Source File | Area F6 | Area F2 | Area F3 | Area F4 | Area F5 | Area F7 | Area F8 | Area F1 | #Feature | #Feature F6 | #Feature F2 | #Feature F3 | #Feature F4 | #Feature F5 | #Feature F7 | #Feature F8 | #Feature F1 | Start | End | PTM | AScore | Found By |
| --- | --- | --- | --- | --- | --- | --- | --- | --- | --- | --- | --- | --- | --- | --- | --- | --- | --- | --- | --- | --- | --- | --- | --- | --- | --- | --- | --- | --- | --- | --- | --- | --- | --- |
| S.QLVVTAEQQR.L | N | 61.06 | 1170.6356 | 10 | -0.6 | 586.3247 | 2 | 41.64 | 2 | F2:15936 | 13012022\_RID\_3262\_2b.raw |  | 1.1474E6 | 0 | 3.4155E5 | 8.5962E4 |  |  |  | 3 | 0 | 1 | 0 | 1 | 1 | 0 | 0 | 0 | 184 | 193 |  |  | PEAKS DB |
| Q.LVVTAEQQR.L | N | 59.96 | 1042.5771 | 9 | -0.3 | 522.2957 | 2 | 24.54 | 2 | F2:6838 | 13012022\_RID\_3262\_2b.raw |  | 4.3551E6 | 7.7997E6 | 4.2093E6 |  |  |  | 4.824E5 | 4 | 0 | 1 | 1 | 1 | 0 | 0 | 0 | 1 | 185 | 193 |  |  | PEAKS DB |
| R.HDNAQLLTAIDFDGR.T | Y | 55.73 | 1684.8169 | 15 | -0.2 | 843.4155 | 2 | 45.97 | 3 | F3:28890 | 13012022\_RID\_3262\_3b.raw |  |  | 1.287E7 |  |  |  |  |  | 2 | 0 | 0 | 2 | 0 | 0 | 0 | 0 | 0 | 280 | 294 |  |  | PEAKS DB |
| total 3 peptides |  |  |  |  |  |  |  |  |  |  |  |  |  |  |  |  |  |  |  |  |  |  |  |  |  |  |  |  |  |  |  |  |  |
| --- | --- | --- | --- | --- | --- | --- | --- | --- | --- | --- | --- | --- | --- | --- | --- | --- | --- | --- | --- | --- | --- | --- | --- | --- | --- | --- | --- | --- | --- | --- | --- | --- | --- |

JAV48411.1

back to list

  

| Protein Coverage
| Supporting Peptides
|

Protein Coverage:

Supporting Peptides:

| Peptide | Uniq | -10lgP | Mass | Length | ppm | m/z | z | RT | Fraction | Scan | Source File | Area F6 | Area F2 | Area F3 | Area F4 | Area F5 | Area F7 | Area F8 | Area F1 | #Feature | #Feature F6 | #Feature F2 | #Feature F3 | #Feature F4 | #Feature F5 | #Feature F7 | #Feature F8 | #Feature F1 | Start | End | PTM | AScore | Found By |
| --- | --- | --- | --- | --- | --- | --- | --- | --- | --- | --- | --- | --- | --- | --- | --- | --- | --- | --- | --- | --- | --- | --- | --- | --- | --- | --- | --- | --- | --- | --- | --- | --- | --- |
| S.QLVVTAEQQR.L | N | 61.06 | 1170.6356 | 10 | -0.6 | 586.3247 | 2 | 41.64 | 2 | F2:15936 | 13012022\_RID\_3262\_2b.raw |  | 1.1474E6 | 0 | 3.4155E5 | 8.5962E4 |  |  |  | 3 | 0 | 1 | 0 | 1 | 1 | 0 | 0 | 0 | 184 | 193 |  |  | PEAKS DB |
| Q.LVVTAEQQR.L | N | 59.96 | 1042.5771 | 9 | -0.3 | 522.2957 | 2 | 24.54 | 2 | F2:6838 | 13012022\_RID\_3262\_2b.raw |  | 4.3551E6 | 7.7997E6 | 4.2093E6 |  |  |  | 4.824E5 | 4 | 0 | 1 | 1 | 1 | 0 | 0 | 0 | 1 | 185 | 193 |  |  | PEAKS DB |
| R.HDNAQLLTAIDFDGR.T | Y | 55.73 | 1684.8169 | 15 | -0.2 | 843.4155 | 2 | 45.97 | 3 | F3:28890 | 13012022\_RID\_3262\_3b.raw |  |  | 1.287E7 |  |  |  |  |  | 2 | 0 | 0 | 2 | 0 | 0 | 0 | 0 | 0 | 280 | 294 |  |  | PEAKS DB |
| total 3 peptides |  |  |  |  |  |  |  |  |  |  |  |  |  |  |  |  |  |  |  |  |  |  |  |  |  |  |  |  |  |  |  |  |  |
| --- | --- | --- | --- | --- | --- | --- | --- | --- | --- | --- | --- | --- | --- | --- | --- | --- | --- | --- | --- | --- | --- | --- | --- | --- | --- | --- | --- | --- | --- | --- | --- | --- | --- |

QIV64921.1

back to list

  

| Protein Coverage
| Supporting Peptides
|

Protein Coverage:

Supporting Peptides:

| Peptide | Uniq | -10lgP | Mass | Length | ppm | m/z | z | RT | Fraction | Scan | Source File | Area F6 | Area F2 | Area F3 | Area F4 | Area F5 | Area F7 | Area F8 | Area F1 | #Feature | #Feature F6 | #Feature F2 | #Feature F3 | #Feature F4 | #Feature F5 | #Feature F7 | #Feature F8 | #Feature F1 | Start | End | PTM | AScore | Found By |
| --- | --- | --- | --- | --- | --- | --- | --- | --- | --- | --- | --- | --- | --- | --- | --- | --- | --- | --- | --- | --- | --- | --- | --- | --- | --- | --- | --- | --- | --- | --- | --- | --- | --- |
| S.QLVVTAEQQR.L | N | 61.06 | 1170.6356 | 10 | -0.6 | 586.3247 | 2 | 41.64 | 2 | F2:15936 | 13012022\_RID\_3262\_2b.raw |  | 1.1474E6 | 0 | 3.4155E5 | 8.5962E4 |  |  |  | 3 | 0 | 1 | 0 | 1 | 1 | 0 | 0 | 0 | 184 | 193 |  |  | PEAKS DB |
| Q.LVVTAEQQR.L | N | 59.96 | 1042.5771 | 9 | -0.3 | 522.2957 | 2 | 24.54 | 2 | F2:6838 | 13012022\_RID\_3262\_2b.raw |  | 4.3551E6 | 7.7997E6 | 4.2093E6 |  |  |  | 4.824E5 | 4 | 0 | 1 | 1 | 1 | 0 | 0 | 0 | 1 | 185 | 193 |  |  | PEAKS DB |
| R.HDNAQLLTAIDFDGR.T | Y | 55.73 | 1684.8169 | 15 | -0.2 | 843.4155 | 2 | 45.97 | 3 | F3:28890 | 13012022\_RID\_3262\_3b.raw |  |  | 1.287E7 |  |  |  |  |  | 2 | 0 | 0 | 2 | 0 | 0 | 0 | 0 | 0 | 280 | 294 |  |  | PEAKS DB |
| total 3 peptides |  |  |  |  |  |  |  |  |  |  |  |  |  |  |  |  |  |  |  |  |  |  |  |  |  |  |  |  |  |  |  |  |  |
| --- | --- | --- | --- | --- | --- | --- | --- | --- | --- | --- | --- | --- | --- | --- | --- | --- | --- | --- | --- | --- | --- | --- | --- | --- | --- | --- | --- | --- | --- | --- | --- | --- | --- |

QIV64948.1

back to list

  

| Protein Coverage
| Supporting Peptides
|

Protein Coverage:

Supporting Peptides:

| Peptide | Uniq | -10lgP | Mass | Length | ppm | m/z | z | RT | Fraction | Scan | Source File | Area F6 | Area F2 | Area F3 | Area F4 | Area F5 | Area F7 | Area F8 | Area F1 | #Feature | #Feature F6 | #Feature F2 | #Feature F3 | #Feature F4 | #Feature F5 | #Feature F7 | #Feature F8 | #Feature F1 | Start | End | PTM | AScore | Found By |
| --- | --- | --- | --- | --- | --- | --- | --- | --- | --- | --- | --- | --- | --- | --- | --- | --- | --- | --- | --- | --- | --- | --- | --- | --- | --- | --- | --- | --- | --- | --- | --- | --- | --- |
| S.QLVVTAEQQR.L | N | 61.06 | 1170.6356 | 10 | -0.6 | 586.3247 | 2 | 41.64 | 2 | F2:15936 | 13012022\_RID\_3262\_2b.raw |  | 1.1474E6 | 0 | 3.4155E5 | 8.5962E4 |  |  |  | 3 | 0 | 1 | 0 | 1 | 1 | 0 | 0 | 0 | 184 | 193 |  |  | PEAKS DB |
| Q.LVVTAEQQR.L | N | 59.96 | 1042.5771 | 9 | -0.3 | 522.2957 | 2 | 24.54 | 2 | F2:6838 | 13012022\_RID\_3262\_2b.raw |  | 4.3551E6 | 7.7997E6 | 4.2093E6 |  |  |  | 4.824E5 | 4 | 0 | 1 | 1 | 1 | 0 | 0 | 0 | 1 | 185 | 193 |  |  | PEAKS DB |
| R.HDNAQLLTAIDFDGR.T | Y | 55.73 | 1684.8169 | 15 | -0.2 | 843.4155 | 2 | 45.97 | 3 | F3:28890 | 13012022\_RID\_3262\_3b.raw |  |  | 1.287E7 |  |  |  |  |  | 2 | 0 | 0 | 2 | 0 | 0 | 0 | 0 | 0 | 280 | 294 |  |  | PEAKS DB |
| total 3 peptides |  |  |  |  |  |  |  |  |  |  |  |  |  |  |  |  |  |  |  |  |  |  |  |  |  |  |  |  |  |  |  |  |  |
| --- | --- | --- | --- | --- | --- | --- | --- | --- | --- | --- | --- | --- | --- | --- | --- | --- | --- | --- | --- | --- | --- | --- | --- | --- | --- | --- | --- | --- | --- | --- | --- | --- | --- |

AAP20639.1

back to list

  

| Protein Coverage
| Supporting Peptides
|

Protein Coverage:

Supporting Peptides:

| Peptide | Uniq | -10lgP | Mass | Length | ppm | m/z | z | RT | Fraction | Scan | Source File | Area F6 | Area F2 | Area F3 | Area F4 | Area F5 | Area F7 | Area F8 | Area F1 | #Feature | #Feature F6 | #Feature F2 | #Feature F3 | #Feature F4 | #Feature F5 | #Feature F7 | #Feature F8 | #Feature F1 | Start | End | PTM | AScore | Found By |
| --- | --- | --- | --- | --- | --- | --- | --- | --- | --- | --- | --- | --- | --- | --- | --- | --- | --- | --- | --- | --- | --- | --- | --- | --- | --- | --- | --- | --- | --- | --- | --- | --- | --- |
| Q.LVVTPEQQR.F | Y | 50.68 | 1068.5928 | 9 | 0.7 | 535.3040 | 2 | 26.40 | 3 | F3:12023 | 13012022\_RID\_3262\_3b.raw |  |  | 3.2032E6 | 2.9924E6 |  |  |  |  | 2 | 0 | 0 | 1 | 1 | 0 | 0 | 0 | 0 | 186 | 194 |  |  | PEAKS DB |
| S.QLVVTPEQQR.F | Y | 50.06 | 1196.6514 | 10 | 0.0 | 599.3329 | 2 | 43.05 | 2 | F2:16664 | 13012022\_RID\_3262\_2b.raw |  | 7.3064E5 |  | 8.8499E5 |  |  |  |  | 2 | 0 | 1 | 0 | 1 | 0 | 0 | 0 | 0 | 185 | 194 |  |  | PEAKS DB |
| total 2 peptides |  |  |  |  |  |  |  |  |  |  |  |  |  |  |  |  |  |  |  |  |  |  |  |  |  |  |  |  |  |  |  |  |  |
| --- | --- | --- | --- | --- | --- | --- | --- | --- | --- | --- | --- | --- | --- | --- | --- | --- | --- | --- | --- | --- | --- | --- | --- | --- | --- | --- | --- | --- | --- | --- | --- | --- | --- |

Q7SZE0.1

back to list

  

| Protein Coverage
| Supporting Peptides
|

Protein Coverage:

Supporting Peptides:

| Peptide | Uniq | -10lgP | Mass | Length | ppm | m/z | z | RT | Fraction | Scan | Source File | Area F6 | Area F2 | Area F3 | Area F4 | Area F5 | Area F7 | Area F8 | Area F1 | #Feature | #Feature F6 | #Feature F2 | #Feature F3 | #Feature F4 | #Feature F5 | #Feature F7 | #Feature F8 | #Feature F1 | Start | End | PTM | AScore | Found By |
| --- | --- | --- | --- | --- | --- | --- | --- | --- | --- | --- | --- | --- | --- | --- | --- | --- | --- | --- | --- | --- | --- | --- | --- | --- | --- | --- | --- | --- | --- | --- | --- | --- | --- |
| Q.LVVTPEQQR.F | Y | 50.68 | 1068.5928 | 9 | 0.7 | 535.3040 | 2 | 26.40 | 3 | F3:12023 | 13012022\_RID\_3262\_3b.raw |  |  | 3.2032E6 | 2.9924E6 |  |  |  |  | 2 | 0 | 0 | 1 | 1 | 0 | 0 | 0 | 0 | 186 | 194 |  |  | PEAKS DB |
| S.QLVVTPEQQR.F | Y | 50.06 | 1196.6514 | 10 | 0.0 | 599.3329 | 2 | 43.05 | 2 | F2:16664 | 13012022\_RID\_3262\_2b.raw |  | 7.3064E5 |  | 8.8499E5 |  |  |  |  | 2 | 0 | 1 | 0 | 1 | 0 | 0 | 0 | 0 | 185 | 194 |  |  | PEAKS DB |
| total 2 peptides |  |  |  |  |  |  |  |  |  |  |  |  |  |  |  |  |  |  |  |  |  |  |  |  |  |  |  |  |  |  |  |  |  |
| --- | --- | --- | --- | --- | --- | --- | --- | --- | --- | --- | --- | --- | --- | --- | --- | --- | --- | --- | --- | --- | --- | --- | --- | --- | --- | --- | --- | --- | --- | --- | --- | --- | --- |

ACZ34293.1

back to list

  

| Protein Coverage
| Supporting Peptides
|

Protein Coverage:

Supporting Peptides:

| Peptide | Uniq | -10lgP | Mass | Length | ppm | m/z | z | RT | Fraction | Scan | Source File | Area F6 | Area F2 | Area F3 | Area F4 | Area F5 | Area F7 | Area F8 | Area F1 | #Feature | #Feature F6 | #Feature F2 | #Feature F3 | #Feature F4 | #Feature F5 | #Feature F7 | #Feature F8 | #Feature F1 | Start | End | PTM | AScore | Found By |
| --- | --- | --- | --- | --- | --- | --- | --- | --- | --- | --- | --- | --- | --- | --- | --- | --- | --- | --- | --- | --- | --- | --- | --- | --- | --- | --- | --- | --- | --- | --- | --- | --- | --- |
| R.EAVFVAQLLSENIK.A | Y | 81.03 | 1559.8558 | 14 | 0.8 | 780.9359 | 2 | 58.86 | 8 | F8:35652 | 31122021\_RID\_3262\_1b.raw | 1.7214E6 | 1.3028E6 | 1.0536E6 | 3.517E5 | 6.6503E5 | 1.194E6 | 3.8557E5 | 4.2422E6 | 11 | 3 | 1 | 1 | 1 | 1 | 1 | 2 | 1 | 70 | 83 |  |  | PEAKS DB |
| total 1 peptides |  |  |  |  |  |  |  |  |  |  |  |  |  |  |  |  |  |  |  |  |  |  |  |  |  |  |  |  |  |  |  |  |  |
| --- | --- | --- | --- | --- | --- | --- | --- | --- | --- | --- | --- | --- | --- | --- | --- | --- | --- | --- | --- | --- | --- | --- | --- | --- | --- | --- | --- | --- | --- | --- | --- | --- | --- |

D1MGU0.1

back to list

  

| Protein Coverage
| Supporting Peptides
|

Protein Coverage:

Supporting Peptides:

| Peptide | Uniq | -10lgP | Mass | Length | ppm | m/z | z | RT | Fraction | Scan | Source File | Area F6 | Area F2 | Area F3 | Area F4 | Area F5 | Area F7 | Area F8 | Area F1 | #Feature | #Feature F6 | #Feature F2 | #Feature F3 | #Feature F4 | #Feature F5 | #Feature F7 | #Feature F8 | #Feature F1 | Start | End | PTM | AScore | Found By |
| --- | --- | --- | --- | --- | --- | --- | --- | --- | --- | --- | --- | --- | --- | --- | --- | --- | --- | --- | --- | --- | --- | --- | --- | --- | --- | --- | --- | --- | --- | --- | --- | --- | --- |
| R.EAVFVAQLLSENIK.A | Y | 81.03 | 1559.8558 | 14 | 0.8 | 780.9359 | 2 | 58.86 | 8 | F8:35652 | 31122021\_RID\_3262\_1b.raw | 1.7214E6 | 1.3028E6 | 1.0536E6 | 3.517E5 | 6.6503E5 | 1.194E6 | 3.8557E5 | 4.2422E6 | 11 | 3 | 1 | 1 | 1 | 1 | 1 | 2 | 1 | 70 | 83 |  |  | PEAKS DB |
| total 1 peptides |  |  |  |  |  |  |  |  |  |  |  |  |  |  |  |  |  |  |  |  |  |  |  |  |  |  |  |  |  |  |  |  |  |
| --- | --- | --- | --- | --- | --- | --- | --- | --- | --- | --- | --- | --- | --- | --- | --- | --- | --- | --- | --- | --- | --- | --- | --- | --- | --- | --- | --- | --- | --- | --- | --- | --- | --- |

ETE65153.1

back to list

  

| Protein Coverage
| Supporting Peptides
|

Protein Coverage:

Supporting Peptides:

| Peptide | Uniq | -10lgP | Mass | Length | ppm | m/z | z | RT | Fraction | Scan | Source File | Area F6 | Area F2 | Area F3 | Area F4 | Area F5 | Area F7 | Area F8 | Area F1 | #Feature | #Feature F6 | #Feature F2 | #Feature F3 | #Feature F4 | #Feature F5 | #Feature F7 | #Feature F8 | #Feature F1 | Start | End | PTM | AScore | Found By |
| --- | --- | --- | --- | --- | --- | --- | --- | --- | --- | --- | --- | --- | --- | --- | --- | --- | --- | --- | --- | --- | --- | --- | --- | --- | --- | --- | --- | --- | --- | --- | --- | --- | --- |
| K.AGAGSATLSMAYAGAR.F | Y | 78.98 | 1453.6984 | 16 | 0.2 | 727.8566 | 2 | 35.09 | 8 | F8:16915 | 31122021\_RID\_3262\_1b.raw |  |  |  |  |  |  |  | 4.2057E4 | 1 | 0 | 0 | 0 | 0 | 0 | 0 | 0 | 1 | 276 | 291 |  |  | PEAKS DB |
| total 1 peptides |  |  |  |  |  |  |  |  |  |  |  |  |  |  |  |  |  |  |  |  |  |  |  |  |  |  |  |  |  |  |  |  |  |
| --- | --- | --- | --- | --- | --- | --- | --- | --- | --- | --- | --- | --- | --- | --- | --- | --- | --- | --- | --- | --- | --- | --- | --- | --- | --- | --- | --- | --- | --- | --- | --- | --- | --- |

JAG67389.1

back to list

  

| Protein Coverage
| Supporting Peptides
|

Protein Coverage:

Supporting Peptides:

| Peptide | Uniq | -10lgP | Mass | Length | ppm | m/z | z | RT | Fraction | Scan | Source File | Area F6 | Area F2 | Area F3 | Area F4 | Area F5 | Area F7 | Area F8 | Area F1 | #Feature | #Feature F6 | #Feature F2 | #Feature F3 | #Feature F4 | #Feature F5 | #Feature F7 | #Feature F8 | #Feature F1 | Start | End | PTM | AScore | Found By |
| --- | --- | --- | --- | --- | --- | --- | --- | --- | --- | --- | --- | --- | --- | --- | --- | --- | --- | --- | --- | --- | --- | --- | --- | --- | --- | --- | --- | --- | --- | --- | --- | --- | --- |
| K.AGAGSATLSMAYAGAR.F | Y | 78.98 | 1453.6984 | 16 | 0.2 | 727.8566 | 2 | 35.09 | 8 | F8:16915 | 31122021\_RID\_3262\_1b.raw |  |  |  |  |  |  |  | 4.2057E4 | 1 | 0 | 0 | 0 | 0 | 0 | 0 | 0 | 1 | 242 | 257 |  |  | PEAKS DB |
| total 1 peptides |  |  |  |  |  |  |  |  |  |  |  |  |  |  |  |  |  |  |  |  |  |  |  |  |  |  |  |  |  |  |  |  |  |
| --- | --- | --- | --- | --- | --- | --- | --- | --- | --- | --- | --- | --- | --- | --- | --- | --- | --- | --- | --- | --- | --- | --- | --- | --- | --- | --- | --- | --- | --- | --- | --- | --- | --- |

XP\_026528223.1

back to list

  

| Protein Coverage
| Supporting Peptides
|

Protein Coverage:

Supporting Peptides:

| Peptide | Uniq | -10lgP | Mass | Length | ppm | m/z | z | RT | Fraction | Scan | Source File | Area F6 | Area F2 | Area F3 | Area F4 | Area F5 | Area F7 | Area F8 | Area F1 | #Feature | #Feature F6 | #Feature F2 | #Feature F3 | #Feature F4 | #Feature F5 | #Feature F7 | #Feature F8 | #Feature F1 | Start | End | PTM | AScore | Found By |
| --- | --- | --- | --- | --- | --- | --- | --- | --- | --- | --- | --- | --- | --- | --- | --- | --- | --- | --- | --- | --- | --- | --- | --- | --- | --- | --- | --- | --- | --- | --- | --- | --- | --- |
| K.AGAGSATLSMAYAGAR.F | Y | 78.98 | 1453.6984 | 16 | 0.2 | 727.8566 | 2 | 35.09 | 8 | F8:16915 | 31122021\_RID\_3262\_1b.raw |  |  |  |  |  |  |  | 4.2057E4 | 1 | 0 | 0 | 0 | 0 | 0 | 0 | 0 | 1 | 242 | 257 |  |  | PEAKS DB |
| total 1 peptides |  |  |  |  |  |  |  |  |  |  |  |  |  |  |  |  |  |  |  |  |  |  |  |  |  |  |  |  |  |  |  |  |  |
| --- | --- | --- | --- | --- | --- | --- | --- | --- | --- | --- | --- | --- | --- | --- | --- | --- | --- | --- | --- | --- | --- | --- | --- | --- | --- | --- | --- | --- | --- | --- | --- | --- | --- |

XP\_026564338.1

back to list

  

| Protein Coverage
| Supporting Peptides
|

Protein Coverage:

Supporting Peptides:

| Peptide | Uniq | -10lgP | Mass | Length | ppm | m/z | z | RT | Fraction | Scan | Source File | Area F6 | Area F2 | Area F3 | Area F4 | Area F5 | Area F7 | Area F8 | Area F1 | #Feature | #Feature F6 | #Feature F2 | #Feature F3 | #Feature F4 | #Feature F5 | #Feature F7 | #Feature F8 | #Feature F1 | Start | End | PTM | AScore | Found By |
| --- | --- | --- | --- | --- | --- | --- | --- | --- | --- | --- | --- | --- | --- | --- | --- | --- | --- | --- | --- | --- | --- | --- | --- | --- | --- | --- | --- | --- | --- | --- | --- | --- | --- |
| K.AGAGSATLSMAYAGAR.F | Y | 78.98 | 1453.6984 | 16 | 0.2 | 727.8566 | 2 | 35.09 | 8 | F8:16915 | 31122021\_RID\_3262\_1b.raw |  |  |  |  |  |  |  | 4.2057E4 | 1 | 0 | 0 | 0 | 0 | 0 | 0 | 0 | 1 | 242 | 257 |  |  | PEAKS DB |
| total 1 peptides |  |  |  |  |  |  |  |  |  |  |  |  |  |  |  |  |  |  |  |  |  |  |  |  |  |  |  |  |  |  |  |  |  |
| --- | --- | --- | --- | --- | --- | --- | --- | --- | --- | --- | --- | --- | --- | --- | --- | --- | --- | --- | --- | --- | --- | --- | --- | --- | --- | --- | --- | --- | --- | --- | --- | --- | --- |

JAI09791.1

back to list

  

| Protein Coverage
| Supporting Peptides
|

Protein Coverage:

Supporting Peptides:

| Peptide | Uniq | -10lgP | Mass | Length | ppm | m/z | z | RT | Fraction | Scan | Source File | Area F6 | Area F2 | Area F3 | Area F4 | Area F5 | Area F7 | Area F8 | Area F1 | #Feature | #Feature F6 | #Feature F2 | #Feature F3 | #Feature F4 | #Feature F5 | #Feature F7 | #Feature F8 | #Feature F1 | Start | End | PTM | AScore | Found By |
| --- | --- | --- | --- | --- | --- | --- | --- | --- | --- | --- | --- | --- | --- | --- | --- | --- | --- | --- | --- | --- | --- | --- | --- | --- | --- | --- | --- | --- | --- | --- | --- | --- | --- |
| K.AGAGSATLSMAYAGAR.F | Y | 78.98 | 1453.6984 | 16 | 0.2 | 727.8566 | 2 | 35.09 | 8 | F8:16915 | 31122021\_RID\_3262\_1b.raw |  |  |  |  |  |  |  | 4.2057E4 | 1 | 0 | 0 | 0 | 0 | 0 | 0 | 0 | 1 | 242 | 257 |  |  | PEAKS DB |
| total 1 peptides |  |  |  |  |  |  |  |  |  |  |  |  |  |  |  |  |  |  |  |  |  |  |  |  |  |  |  |  |  |  |  |  |  |
| --- | --- | --- | --- | --- | --- | --- | --- | --- | --- | --- | --- | --- | --- | --- | --- | --- | --- | --- | --- | --- | --- | --- | --- | --- | --- | --- | --- | --- | --- | --- | --- | --- | --- |

JAB53935.1

back to list

  

| Protein Coverage
| Supporting Peptides
|

Protein Coverage:

Supporting Peptides:

| Peptide | Uniq | -10lgP | Mass | Length | ppm | m/z | z | RT | Fraction | Scan | Source File | Area F6 | Area F2 | Area F3 | Area F4 | Area F5 | Area F7 | Area F8 | Area F1 | #Feature | #Feature F6 | #Feature F2 | #Feature F3 | #Feature F4 | #Feature F5 | #Feature F7 | #Feature F8 | #Feature F1 | Start | End | PTM | AScore | Found By |
| --- | --- | --- | --- | --- | --- | --- | --- | --- | --- | --- | --- | --- | --- | --- | --- | --- | --- | --- | --- | --- | --- | --- | --- | --- | --- | --- | --- | --- | --- | --- | --- | --- | --- |
| K.AGAGSATLSMAYAGAR.F | Y | 78.98 | 1453.6984 | 16 | 0.2 | 727.8566 | 2 | 35.09 | 8 | F8:16915 | 31122021\_RID\_3262\_1b.raw |  |  |  |  |  |  |  | 4.2057E4 | 1 | 0 | 0 | 0 | 0 | 0 | 0 | 0 | 1 | 242 | 257 |  |  | PEAKS DB |
| total 1 peptides |  |  |  |  |  |  |  |  |  |  |  |  |  |  |  |  |  |  |  |  |  |  |  |  |  |  |  |  |  |  |  |  |  |
| --- | --- | --- | --- | --- | --- | --- | --- | --- | --- | --- | --- | --- | --- | --- | --- | --- | --- | --- | --- | --- | --- | --- | --- | --- | --- | --- | --- | --- | --- | --- | --- | --- | --- |

XP\_034277264.1

back to list

  

| Protein Coverage
| Supporting Peptides
|

Protein Coverage:

Supporting Peptides:

| Peptide | Uniq | -10lgP | Mass | Length | ppm | m/z | z | RT | Fraction | Scan | Source File | Area F6 | Area F2 | Area F3 | Area F4 | Area F5 | Area F7 | Area F8 | Area F1 | #Feature | #Feature F6 | #Feature F2 | #Feature F3 | #Feature F4 | #Feature F5 | #Feature F7 | #Feature F8 | #Feature F1 | Start | End | PTM | AScore | Found By |
| --- | --- | --- | --- | --- | --- | --- | --- | --- | --- | --- | --- | --- | --- | --- | --- | --- | --- | --- | --- | --- | --- | --- | --- | --- | --- | --- | --- | --- | --- | --- | --- | --- | --- |
| K.AGAGSATLSMAYAGAR.F | Y | 78.98 | 1453.6984 | 16 | 0.2 | 727.8566 | 2 | 35.09 | 8 | F8:16915 | 31122021\_RID\_3262\_1b.raw |  |  |  |  |  |  |  | 4.2057E4 | 1 | 0 | 0 | 0 | 0 | 0 | 0 | 0 | 1 | 242 | 257 |  |  | PEAKS DB |
| total 1 peptides |  |  |  |  |  |  |  |  |  |  |  |  |  |  |  |  |  |  |  |  |  |  |  |  |  |  |  |  |  |  |  |  |  |
| --- | --- | --- | --- | --- | --- | --- | --- | --- | --- | --- | --- | --- | --- | --- | --- | --- | --- | --- | --- | --- | --- | --- | --- | --- | --- | --- | --- | --- | --- | --- | --- | --- | --- |

JAC95102.1

back to list

  

| Protein Coverage
| Supporting Peptides
|

Protein Coverage:

Supporting Peptides:

| Peptide | Uniq | -10lgP | Mass | Length | ppm | m/z | z | RT | Fraction | Scan | Source File | Area F6 | Area F2 | Area F3 | Area F4 | Area F5 | Area F7 | Area F8 | Area F1 | #Feature | #Feature F6 | #Feature F2 | #Feature F3 | #Feature F4 | #Feature F5 | #Feature F7 | #Feature F8 | #Feature F1 | Start | End | PTM | AScore | Found By |
| --- | --- | --- | --- | --- | --- | --- | --- | --- | --- | --- | --- | --- | --- | --- | --- | --- | --- | --- | --- | --- | --- | --- | --- | --- | --- | --- | --- | --- | --- | --- | --- | --- | --- |
| K.AGAGSATLSMAYAGAR.F | Y | 78.98 | 1453.6984 | 16 | 0.2 | 727.8566 | 2 | 35.09 | 8 | F8:16915 | 31122021\_RID\_3262\_1b.raw |  |  |  |  |  |  |  | 4.2057E4 | 1 | 0 | 0 | 0 | 0 | 0 | 0 | 0 | 1 | 242 | 257 |  |  | PEAKS DB |
| total 1 peptides |  |  |  |  |  |  |  |  |  |  |  |  |  |  |  |  |  |  |  |  |  |  |  |  |  |  |  |  |  |  |  |  |  |
| --- | --- | --- | --- | --- | --- | --- | --- | --- | --- | --- | --- | --- | --- | --- | --- | --- | --- | --- | --- | --- | --- | --- | --- | --- | --- | --- | --- | --- | --- | --- | --- | --- | --- |

XP\_007433827.1

back to list

  

| Protein Coverage
| Supporting Peptides
|

Protein Coverage:

Supporting Peptides:

| Peptide | Uniq | -10lgP | Mass | Length | ppm | m/z | z | RT | Fraction | Scan | Source File | Area F6 | Area F2 | Area F3 | Area F4 | Area F5 | Area F7 | Area F8 | Area F1 | #Feature | #Feature F6 | #Feature F2 | #Feature F3 | #Feature F4 | #Feature F5 | #Feature F7 | #Feature F8 | #Feature F1 | Start | End | PTM | AScore | Found By |
| --- | --- | --- | --- | --- | --- | --- | --- | --- | --- | --- | --- | --- | --- | --- | --- | --- | --- | --- | --- | --- | --- | --- | --- | --- | --- | --- | --- | --- | --- | --- | --- | --- | --- |
| K.AGAGSATLSMAYAGAR.F | Y | 78.98 | 1453.6984 | 16 | 0.2 | 727.8566 | 2 | 35.09 | 8 | F8:16915 | 31122021\_RID\_3262\_1b.raw |  |  |  |  |  |  |  | 4.2057E4 | 1 | 0 | 0 | 0 | 0 | 0 | 0 | 0 | 1 | 242 | 257 |  |  | PEAKS DB |
| total 1 peptides |  |  |  |  |  |  |  |  |  |  |  |  |  |  |  |  |  |  |  |  |  |  |  |  |  |  |  |  |  |  |  |  |  |
| --- | --- | --- | --- | --- | --- | --- | --- | --- | --- | --- | --- | --- | --- | --- | --- | --- | --- | --- | --- | --- | --- | --- | --- | --- | --- | --- | --- | --- | --- | --- | --- | --- | --- |

AFJ50538.1

back to list

  

| Protein Coverage
| Supporting Peptides
|

Protein Coverage:

Supporting Peptides:

| Peptide | Uniq | -10lgP | Mass | Length | ppm | m/z | z | RT | Fraction | Scan | Source File | Area F6 | Area F2 | Area F3 | Area F4 | Area F5 | Area F7 | Area F8 | Area F1 | #Feature | #Feature F6 | #Feature F2 | #Feature F3 | #Feature F4 | #Feature F5 | #Feature F7 | #Feature F8 | #Feature F1 | Start | End | PTM | AScore | Found By |
| --- | --- | --- | --- | --- | --- | --- | --- | --- | --- | --- | --- | --- | --- | --- | --- | --- | --- | --- | --- | --- | --- | --- | --- | --- | --- | --- | --- | --- | --- | --- | --- | --- | --- |
| K.AGAGSATLSMAYAGAR.F | Y | 78.98 | 1453.6984 | 16 | 0.2 | 727.8566 | 2 | 35.09 | 8 | F8:16915 | 31122021\_RID\_3262\_1b.raw |  |  |  |  |  |  |  | 4.2057E4 | 1 | 0 | 0 | 0 | 0 | 0 | 0 | 0 | 1 | 242 | 257 |  |  | PEAKS DB |
| total 1 peptides |  |  |  |  |  |  |  |  |  |  |  |  |  |  |  |  |  |  |  |  |  |  |  |  |  |  |  |  |  |  |  |  |  |
| --- | --- | --- | --- | --- | --- | --- | --- | --- | --- | --- | --- | --- | --- | --- | --- | --- | --- | --- | --- | --- | --- | --- | --- | --- | --- | --- | --- | --- | --- | --- | --- | --- | --- |

JAV49984.1

back to list

  

| Protein Coverage
| Supporting Peptides
|

Protein Coverage:

Supporting Peptides:

| Peptide | Uniq | -10lgP | Mass | Length | ppm | m/z | z | RT | Fraction | Scan | Source File | Area F6 | Area F2 | Area F3 | Area F4 | Area F5 | Area F7 | Area F8 | Area F1 | #Feature | #Feature F6 | #Feature F2 | #Feature F3 | #Feature F4 | #Feature F5 | #Feature F7 | #Feature F8 | #Feature F1 | Start | End | PTM | AScore | Found By |
| --- | --- | --- | --- | --- | --- | --- | --- | --- | --- | --- | --- | --- | --- | --- | --- | --- | --- | --- | --- | --- | --- | --- | --- | --- | --- | --- | --- | --- | --- | --- | --- | --- | --- |
| K.AGAGSATLSMAYAGAR.F | Y | 78.98 | 1453.6984 | 16 | 0.2 | 727.8566 | 2 | 35.09 | 8 | F8:16915 | 31122021\_RID\_3262\_1b.raw |  |  |  |  |  |  |  | 4.2057E4 | 1 | 0 | 0 | 0 | 0 | 0 | 0 | 0 | 1 | 242 | 257 |  |  | PEAKS DB |
| total 1 peptides |  |  |  |  |  |  |  |  |  |  |  |  |  |  |  |  |  |  |  |  |  |  |  |  |  |  |  |  |  |  |  |  |  |
| --- | --- | --- | --- | --- | --- | --- | --- | --- | --- | --- | --- | --- | --- | --- | --- | --- | --- | --- | --- | --- | --- | --- | --- | --- | --- | --- | --- | --- | --- | --- | --- | --- | --- |

JAG46324.1

back to list

  

| Protein Coverage
| Supporting Peptides
|

Protein Coverage:

Supporting Peptides:

| Peptide | Uniq | -10lgP | Mass | Length | ppm | m/z | z | RT | Fraction | Scan | Source File | Area F6 | Area F2 | Area F3 | Area F4 | Area F5 | Area F7 | Area F8 | Area F1 | #Feature | #Feature F6 | #Feature F2 | #Feature F3 | #Feature F4 | #Feature F5 | #Feature F7 | #Feature F8 | #Feature F1 | Start | End | PTM | AScore | Found By |
| --- | --- | --- | --- | --- | --- | --- | --- | --- | --- | --- | --- | --- | --- | --- | --- | --- | --- | --- | --- | --- | --- | --- | --- | --- | --- | --- | --- | --- | --- | --- | --- | --- | --- |
| K.AGAGSATLSMAYAGAR.F | Y | 78.98 | 1453.6984 | 16 | 0.2 | 727.8566 | 2 | 35.09 | 8 | F8:16915 | 31122021\_RID\_3262\_1b.raw |  |  |  |  |  |  |  | 4.2057E4 | 1 | 0 | 0 | 0 | 0 | 0 | 0 | 0 | 1 | 242 | 257 |  |  | PEAKS DB |
| total 1 peptides |  |  |  |  |  |  |  |  |  |  |  |  |  |  |  |  |  |  |  |  |  |  |  |  |  |  |  |  |  |  |  |  |  |
| --- | --- | --- | --- | --- | --- | --- | --- | --- | --- | --- | --- | --- | --- | --- | --- | --- | --- | --- | --- | --- | --- | --- | --- | --- | --- | --- | --- | --- | --- | --- | --- | --- | --- |

JAA96492.1

back to list

  

| Protein Coverage
| Supporting Peptides
|

Protein Coverage:

Supporting Peptides:

| Peptide | Uniq | -10lgP | Mass | Length | ppm | m/z | z | RT | Fraction | Scan | Source File | Area F6 | Area F2 | Area F3 | Area F4 | Area F5 | Area F7 | Area F8 | Area F1 | #Feature | #Feature F6 | #Feature F2 | #Feature F3 | #Feature F4 | #Feature F5 | #Feature F7 | #Feature F8 | #Feature F1 | Start | End | PTM | AScore | Found By |
| --- | --- | --- | --- | --- | --- | --- | --- | --- | --- | --- | --- | --- | --- | --- | --- | --- | --- | --- | --- | --- | --- | --- | --- | --- | --- | --- | --- | --- | --- | --- | --- | --- | --- |
| K.AGAGSATLSMAYAGAR.F | Y | 78.98 | 1453.6984 | 16 | 0.2 | 727.8566 | 2 | 35.09 | 8 | F8:16915 | 31122021\_RID\_3262\_1b.raw |  |  |  |  |  |  |  | 4.2057E4 | 1 | 0 | 0 | 0 | 0 | 0 | 0 | 0 | 1 | 242 | 257 |  |  | PEAKS DB |
| total 1 peptides |  |  |  |  |  |  |  |  |  |  |  |  |  |  |  |  |  |  |  |  |  |  |  |  |  |  |  |  |  |  |  |  |  |
| --- | --- | --- | --- | --- | --- | --- | --- | --- | --- | --- | --- | --- | --- | --- | --- | --- | --- | --- | --- | --- | --- | --- | --- | --- | --- | --- | --- | --- | --- | --- | --- | --- | --- |

XP\_015674357.1

back to list

  

| Protein Coverage
| Supporting Peptides
|

Protein Coverage:

Supporting Peptides:

| Peptide | Uniq | -10lgP | Mass | Length | ppm | m/z | z | RT | Fraction | Scan | Source File | Area F6 | Area F2 | Area F3 | Area F4 | Area F5 | Area F7 | Area F8 | Area F1 | #Feature | #Feature F6 | #Feature F2 | #Feature F3 | #Feature F4 | #Feature F5 | #Feature F7 | #Feature F8 | #Feature F1 | Start | End | PTM | AScore | Found By |
| --- | --- | --- | --- | --- | --- | --- | --- | --- | --- | --- | --- | --- | --- | --- | --- | --- | --- | --- | --- | --- | --- | --- | --- | --- | --- | --- | --- | --- | --- | --- | --- | --- | --- |
| K.AGAGSATLSMAYAGAR.F | Y | 78.98 | 1453.6984 | 16 | 0.2 | 727.8566 | 2 | 35.09 | 8 | F8:16915 | 31122021\_RID\_3262\_1b.raw |  |  |  |  |  |  |  | 4.2057E4 | 1 | 0 | 0 | 0 | 0 | 0 | 0 | 0 | 1 | 242 | 257 |  |  | PEAKS DB |
| total 1 peptides |  |  |  |  |  |  |  |  |  |  |  |  |  |  |  |  |  |  |  |  |  |  |  |  |  |  |  |  |  |  |  |  |  |
| --- | --- | --- | --- | --- | --- | --- | --- | --- | --- | --- | --- | --- | --- | --- | --- | --- | --- | --- | --- | --- | --- | --- | --- | --- | --- | --- | --- | --- | --- | --- | --- | --- | --- |

JAI12538.1

back to list

  

| Protein Coverage
| Supporting Peptides
|

Protein Coverage:

Supporting Peptides:

| Peptide | Uniq | -10lgP | Mass | Length | ppm | m/z | z | RT | Fraction | Scan | Source File | Area F6 | Area F2 | Area F3 | Area F4 | Area F5 | Area F7 | Area F8 | Area F1 | #Feature | #Feature F6 | #Feature F2 | #Feature F3 | #Feature F4 | #Feature F5 | #Feature F7 | #Feature F8 | #Feature F1 | Start | End | PTM | AScore | Found By |
| --- | --- | --- | --- | --- | --- | --- | --- | --- | --- | --- | --- | --- | --- | --- | --- | --- | --- | --- | --- | --- | --- | --- | --- | --- | --- | --- | --- | --- | --- | --- | --- | --- | --- |
| K.AGAGSATLSMAYAGAR.F | Y | 78.98 | 1453.6984 | 16 | 0.2 | 727.8566 | 2 | 35.09 | 8 | F8:16915 | 31122021\_RID\_3262\_1b.raw |  |  |  |  |  |  |  | 4.2057E4 | 1 | 0 | 0 | 0 | 0 | 0 | 0 | 0 | 1 | 242 | 257 |  |  | PEAKS DB |
| total 1 peptides |  |  |  |  |  |  |  |  |  |  |  |  |  |  |  |  |  |  |  |  |  |  |  |  |  |  |  |  |  |  |  |  |  |
| --- | --- | --- | --- | --- | --- | --- | --- | --- | --- | --- | --- | --- | --- | --- | --- | --- | --- | --- | --- | --- | --- | --- | --- | --- | --- | --- | --- | --- | --- | --- | --- | --- | --- |

JAG43926.1

back to list

  

| Protein Coverage
| Supporting Peptides
|

Protein Coverage:

Supporting Peptides:

| Peptide | Uniq | -10lgP | Mass | Length | ppm | m/z | z | RT | Fraction | Scan | Source File | Area F6 | Area F2 | Area F3 | Area F4 | Area F5 | Area F7 | Area F8 | Area F1 | #Feature | #Feature F6 | #Feature F2 | #Feature F3 | #Feature F4 | #Feature F5 | #Feature F7 | #Feature F8 | #Feature F1 | Start | End | PTM | AScore | Found By |
| --- | --- | --- | --- | --- | --- | --- | --- | --- | --- | --- | --- | --- | --- | --- | --- | --- | --- | --- | --- | --- | --- | --- | --- | --- | --- | --- | --- | --- | --- | --- | --- | --- | --- |
| K.AGAGSATLSMAYAGAR.F | Y | 78.98 | 1453.6984 | 16 | 0.2 | 727.8566 | 2 | 35.09 | 8 | F8:16915 | 31122021\_RID\_3262\_1b.raw |  |  |  |  |  |  |  | 4.2057E4 | 1 | 0 | 0 | 0 | 0 | 0 | 0 | 0 | 1 | 242 | 257 |  |  | PEAKS DB |
| total 1 peptides |  |  |  |  |  |  |  |  |  |  |  |  |  |  |  |  |  |  |  |  |  |  |  |  |  |  |  |  |  |  |  |  |  |
| --- | --- | --- | --- | --- | --- | --- | --- | --- | --- | --- | --- | --- | --- | --- | --- | --- | --- | --- | --- | --- | --- | --- | --- | --- | --- | --- | --- | --- | --- | --- | --- | --- | --- |

XP\_032071513.1

back to list

  

| Protein Coverage
| Supporting Peptides
|

Protein Coverage:

Supporting Peptides:

| Peptide | Uniq | -10lgP | Mass | Length | ppm | m/z | z | RT | Fraction | Scan | Source File | Area F6 | Area F2 | Area F3 | Area F4 | Area F5 | Area F7 | Area F8 | Area F1 | #Feature | #Feature F6 | #Feature F2 | #Feature F3 | #Feature F4 | #Feature F5 | #Feature F7 | #Feature F8 | #Feature F1 | Start | End | PTM | AScore | Found By |
| --- | --- | --- | --- | --- | --- | --- | --- | --- | --- | --- | --- | --- | --- | --- | --- | --- | --- | --- | --- | --- | --- | --- | --- | --- | --- | --- | --- | --- | --- | --- | --- | --- | --- |
| K.AGAGSATLSMAYAGAR.F | Y | 78.98 | 1453.6984 | 16 | 0.2 | 727.8566 | 2 | 35.09 | 8 | F8:16915 | 31122021\_RID\_3262\_1b.raw |  |  |  |  |  |  |  | 4.2057E4 | 1 | 0 | 0 | 0 | 0 | 0 | 0 | 0 | 1 | 242 | 257 |  |  | PEAKS DB |
| total 1 peptides |  |  |  |  |  |  |  |  |  |  |  |  |  |  |  |  |  |  |  |  |  |  |  |  |  |  |  |  |  |  |  |  |  |
| --- | --- | --- | --- | --- | --- | --- | --- | --- | --- | --- | --- | --- | --- | --- | --- | --- | --- | --- | --- | --- | --- | --- | --- | --- | --- | --- | --- | --- | --- | --- | --- | --- | --- |

XP\_013922804.1

back to list

  

| Protein Coverage
| Supporting Peptides
|

Protein Coverage:

Supporting Peptides:

| Peptide | Uniq | -10lgP | Mass | Length | ppm | m/z | z | RT | Fraction | Scan | Source File | Area F6 | Area F2 | Area F3 | Area F4 | Area F5 | Area F7 | Area F8 | Area F1 | #Feature | #Feature F6 | #Feature F2 | #Feature F3 | #Feature F4 | #Feature F5 | #Feature F7 | #Feature F8 | #Feature F1 | Start | End | PTM | AScore | Found By |
| --- | --- | --- | --- | --- | --- | --- | --- | --- | --- | --- | --- | --- | --- | --- | --- | --- | --- | --- | --- | --- | --- | --- | --- | --- | --- | --- | --- | --- | --- | --- | --- | --- | --- |
| K.AGAGSATLSMAYAGAR.F | Y | 78.98 | 1453.6984 | 16 | 0.2 | 727.8566 | 2 | 35.09 | 8 | F8:16915 | 31122021\_RID\_3262\_1b.raw |  |  |  |  |  |  |  | 4.2057E4 | 1 | 0 | 0 | 0 | 0 | 0 | 0 | 0 | 1 | 242 | 257 |  |  | PEAKS DB |
| total 1 peptides |  |  |  |  |  |  |  |  |  |  |  |  |  |  |  |  |  |  |  |  |  |  |  |  |  |  |  |  |  |  |  |  |  |
| --- | --- | --- | --- | --- | --- | --- | --- | --- | --- | --- | --- | --- | --- | --- | --- | --- | --- | --- | --- | --- | --- | --- | --- | --- | --- | --- | --- | --- | --- | --- | --- | --- | --- |

XP\_015681610.1

back to list

  

| Protein Coverage
| Supporting Peptides
|

Protein Coverage:

Supporting Peptides:

| Peptide | Uniq | -10lgP | Mass | Length | ppm | m/z | z | RT | Fraction | Scan | Source File | Area F6 | Area F2 | Area F3 | Area F4 | Area F5 | Area F7 | Area F8 | Area F1 | #Feature | #Feature F6 | #Feature F2 | #Feature F3 | #Feature F4 | #Feature F5 | #Feature F7 | #Feature F8 | #Feature F1 | Start | End | PTM | AScore | Found By |
| --- | --- | --- | --- | --- | --- | --- | --- | --- | --- | --- | --- | --- | --- | --- | --- | --- | --- | --- | --- | --- | --- | --- | --- | --- | --- | --- | --- | --- | --- | --- | --- | --- | --- |
| K.VYEGERPLTK.D | Y | 61.30 | 1190.6295 | 10 | 0.8 | 596.3225 | 2 | 18.85 | 8 | F8:4637 | 31122021\_RID\_3262\_1b.raw |  |  |  |  |  |  |  | 7.869E4 | 1 | 0 | 0 | 0 | 0 | 0 | 0 | 0 | 1 | 464 | 473 |  |  | PEAKS DB |
| R.VEIIANDQGNR.I | Y | 53.00 | 1227.6207 | 11 | 0.0 | 614.8176 | 2 | 26.25 | 8 | F8:10120 | 31122021\_RID\_3262\_1b.raw |  |  |  |  |  |  |  | 1.5054E5 | 1 | 0 | 0 | 0 | 0 | 0 | 0 | 0 | 1 | 49 | 59 |  |  | PEAKS DB |
| R.ITPSYVAFTPEGER.L | Y | 46.56 | 1565.7725 | 14 | 1.3 | 783.8945 | 2 | 42.21 | 8 | F8:22560 | 31122021\_RID\_3262\_1b.raw |  |  |  |  |  |  |  | 1.1743E4 | 1 | 0 | 0 | 0 | 0 | 0 | 0 | 0 | 1 | 60 | 73 |  |  | PEAKS DB |
| total 3 peptides |  |  |  |  |  |  |  |  |  |  |  |  |  |  |  |  |  |  |  |  |  |  |  |  |  |  |  |  |  |  |  |  |  |
| --- | --- | --- | --- | --- | --- | --- | --- | --- | --- | --- | --- | --- | --- | --- | --- | --- | --- | --- | --- | --- | --- | --- | --- | --- | --- | --- | --- | --- | --- | --- | --- | --- | --- |

JAV50419.1

back to list

  

| Protein Coverage
| Supporting Peptides
|

Protein Coverage:

Supporting Peptides:

| Peptide | Uniq | -10lgP | Mass | Length | ppm | m/z | z | RT | Fraction | Scan | Source File | Area F6 | Area F2 | Area F3 | Area F4 | Area F5 | Area F7 | Area F8 | Area F1 | #Feature | #Feature F6 | #Feature F2 | #Feature F3 | #Feature F4 | #Feature F5 | #Feature F7 | #Feature F8 | #Feature F1 | Start | End | PTM | AScore | Found By |
| --- | --- | --- | --- | --- | --- | --- | --- | --- | --- | --- | --- | --- | --- | --- | --- | --- | --- | --- | --- | --- | --- | --- | --- | --- | --- | --- | --- | --- | --- | --- | --- | --- | --- |
| K.VYEGERPLTK.D | Y | 61.30 | 1190.6295 | 10 | 0.8 | 596.3225 | 2 | 18.85 | 8 | F8:4637 | 31122021\_RID\_3262\_1b.raw |  |  |  |  |  |  |  | 7.869E4 | 1 | 0 | 0 | 0 | 0 | 0 | 0 | 0 | 1 | 464 | 473 |  |  | PEAKS DB |
| R.VEIIANDQGNR.I | Y | 53.00 | 1227.6207 | 11 | 0.0 | 614.8176 | 2 | 26.25 | 8 | F8:10120 | 31122021\_RID\_3262\_1b.raw |  |  |  |  |  |  |  | 1.5054E5 | 1 | 0 | 0 | 0 | 0 | 0 | 0 | 0 | 1 | 49 | 59 |  |  | PEAKS DB |
| R.ITPSYVAFTPEGER.L | Y | 46.56 | 1565.7725 | 14 | 1.3 | 783.8945 | 2 | 42.21 | 8 | F8:22560 | 31122021\_RID\_3262\_1b.raw |  |  |  |  |  |  |  | 1.1743E4 | 1 | 0 | 0 | 0 | 0 | 0 | 0 | 0 | 1 | 60 | 73 |  |  | PEAKS DB |
| total 3 peptides |  |  |  |  |  |  |  |  |  |  |  |  |  |  |  |  |  |  |  |  |  |  |  |  |  |  |  |  |  |  |  |  |  |
| --- | --- | --- | --- | --- | --- | --- | --- | --- | --- | --- | --- | --- | --- | --- | --- | --- | --- | --- | --- | --- | --- | --- | --- | --- | --- | --- | --- | --- | --- | --- | --- | --- | --- |

JAG44540.1

back to list

  

| Protein Coverage
| Supporting Peptides
|

Protein Coverage:

Supporting Peptides:

| Peptide | Uniq | -10lgP | Mass | Length | ppm | m/z | z | RT | Fraction | Scan | Source File | Area F6 | Area F2 | Area F3 | Area F4 | Area F5 | Area F7 | Area F8 | Area F1 | #Feature | #Feature F6 | #Feature F2 | #Feature F3 | #Feature F4 | #Feature F5 | #Feature F7 | #Feature F8 | #Feature F1 | Start | End | PTM | AScore | Found By |
| --- | --- | --- | --- | --- | --- | --- | --- | --- | --- | --- | --- | --- | --- | --- | --- | --- | --- | --- | --- | --- | --- | --- | --- | --- | --- | --- | --- | --- | --- | --- | --- | --- | --- |
| K.VYEGERPLTK.D | Y | 61.30 | 1190.6295 | 10 | 0.8 | 596.3225 | 2 | 18.85 | 8 | F8:4637 | 31122021\_RID\_3262\_1b.raw |  |  |  |  |  |  |  | 7.869E4 | 1 | 0 | 0 | 0 | 0 | 0 | 0 | 0 | 1 | 464 | 473 |  |  | PEAKS DB |
| R.VEIIANDQGNR.I | Y | 53.00 | 1227.6207 | 11 | 0.0 | 614.8176 | 2 | 26.25 | 8 | F8:10120 | 31122021\_RID\_3262\_1b.raw |  |  |  |  |  |  |  | 1.5054E5 | 1 | 0 | 0 | 0 | 0 | 0 | 0 | 0 | 1 | 49 | 59 |  |  | PEAKS DB |
| R.ITPSYVAFTPEGER.L | Y | 46.56 | 1565.7725 | 14 | 1.3 | 783.8945 | 2 | 42.21 | 8 | F8:22560 | 31122021\_RID\_3262\_1b.raw |  |  |  |  |  |  |  | 1.1743E4 | 1 | 0 | 0 | 0 | 0 | 0 | 0 | 0 | 1 | 60 | 73 |  |  | PEAKS DB |
| total 3 peptides |  |  |  |  |  |  |  |  |  |  |  |  |  |  |  |  |  |  |  |  |  |  |  |  |  |  |  |  |  |  |  |  |  |
| --- | --- | --- | --- | --- | --- | --- | --- | --- | --- | --- | --- | --- | --- | --- | --- | --- | --- | --- | --- | --- | --- | --- | --- | --- | --- | --- | --- | --- | --- | --- | --- | --- | --- |

JAI13107.1

back to list

  

| Protein Coverage
| Supporting Peptides
|

Protein Coverage:

Supporting Peptides:

| Peptide | Uniq | -10lgP | Mass | Length | ppm | m/z | z | RT | Fraction | Scan | Source File | Area F6 | Area F2 | Area F3 | Area F4 | Area F5 | Area F7 | Area F8 | Area F1 | #Feature | #Feature F6 | #Feature F2 | #Feature F3 | #Feature F4 | #Feature F5 | #Feature F7 | #Feature F8 | #Feature F1 | Start | End | PTM | AScore | Found By |
| --- | --- | --- | --- | --- | --- | --- | --- | --- | --- | --- | --- | --- | --- | --- | --- | --- | --- | --- | --- | --- | --- | --- | --- | --- | --- | --- | --- | --- | --- | --- | --- | --- | --- |
| K.VYEGERPLTK.D | Y | 61.30 | 1190.6295 | 10 | 0.8 | 596.3225 | 2 | 18.85 | 8 | F8:4637 | 31122021\_RID\_3262\_1b.raw |  |  |  |  |  |  |  | 7.869E4 | 1 | 0 | 0 | 0 | 0 | 0 | 0 | 0 | 1 | 464 | 473 |  |  | PEAKS DB |
| R.VEIIANDQGNR.I | Y | 53.00 | 1227.6207 | 11 | 0.0 | 614.8176 | 2 | 26.25 | 8 | F8:10120 | 31122021\_RID\_3262\_1b.raw |  |  |  |  |  |  |  | 1.5054E5 | 1 | 0 | 0 | 0 | 0 | 0 | 0 | 0 | 1 | 49 | 59 |  |  | PEAKS DB |
| R.ITPSYVAFTPEGER.L | Y | 46.56 | 1565.7725 | 14 | 1.3 | 783.8945 | 2 | 42.21 | 8 | F8:22560 | 31122021\_RID\_3262\_1b.raw |  |  |  |  |  |  |  | 1.1743E4 | 1 | 0 | 0 | 0 | 0 | 0 | 0 | 0 | 1 | 60 | 73 |  |  | PEAKS DB |
| total 3 peptides |  |  |  |  |  |  |  |  |  |  |  |  |  |  |  |  |  |  |  |  |  |  |  |  |  |  |  |  |  |  |  |  |  |
| --- | --- | --- | --- | --- | --- | --- | --- | --- | --- | --- | --- | --- | --- | --- | --- | --- | --- | --- | --- | --- | --- | --- | --- | --- | --- | --- | --- | --- | --- | --- | --- | --- | --- |

JAG46692.1

back to list

  

| Protein Coverage
| Supporting Peptides
|

Protein Coverage:

Supporting Peptides:

| Peptide | Uniq | -10lgP | Mass | Length | ppm | m/z | z | RT | Fraction | Scan | Source File | Area F6 | Area F2 | Area F3 | Area F4 | Area F5 | Area F7 | Area F8 | Area F1 | #Feature | #Feature F6 | #Feature F2 | #Feature F3 | #Feature F4 | #Feature F5 | #Feature F7 | #Feature F8 | #Feature F1 | Start | End | PTM | AScore | Found By |
| --- | --- | --- | --- | --- | --- | --- | --- | --- | --- | --- | --- | --- | --- | --- | --- | --- | --- | --- | --- | --- | --- | --- | --- | --- | --- | --- | --- | --- | --- | --- | --- | --- | --- |
| K.VYEGERPLTK.D | Y | 61.30 | 1190.6295 | 10 | 0.8 | 596.3225 | 2 | 18.85 | 8 | F8:4637 | 31122021\_RID\_3262\_1b.raw |  |  |  |  |  |  |  | 7.869E4 | 1 | 0 | 0 | 0 | 0 | 0 | 0 | 0 | 1 | 464 | 473 |  |  | PEAKS DB |
| R.VEIIANDQGNR.I | Y | 53.00 | 1227.6207 | 11 | 0.0 | 614.8176 | 2 | 26.25 | 8 | F8:10120 | 31122021\_RID\_3262\_1b.raw |  |  |  |  |  |  |  | 1.5054E5 | 1 | 0 | 0 | 0 | 0 | 0 | 0 | 0 | 1 | 49 | 59 |  |  | PEAKS DB |
| R.ITPSYVAFTPEGER.L | Y | 46.56 | 1565.7725 | 14 | 1.3 | 783.8945 | 2 | 42.21 | 8 | F8:22560 | 31122021\_RID\_3262\_1b.raw |  |  |  |  |  |  |  | 1.1743E4 | 1 | 0 | 0 | 0 | 0 | 0 | 0 | 0 | 1 | 60 | 73 |  |  | PEAKS DB |
| total 3 peptides |  |  |  |  |  |  |  |  |  |  |  |  |  |  |  |  |  |  |  |  |  |  |  |  |  |  |  |  |  |  |  |  |  |
| --- | --- | --- | --- | --- | --- | --- | --- | --- | --- | --- | --- | --- | --- | --- | --- | --- | --- | --- | --- | --- | --- | --- | --- | --- | --- | --- | --- | --- | --- | --- | --- | --- | --- |

JAA96909.1

back to list

  

| Protein Coverage
| Supporting Peptides
|

Protein Coverage:

Supporting Peptides:

| Peptide | Uniq | -10lgP | Mass | Length | ppm | m/z | z | RT | Fraction | Scan | Source File | Area F6 | Area F2 | Area F3 | Area F4 | Area F5 | Area F7 | Area F8 | Area F1 | #Feature | #Feature F6 | #Feature F2 | #Feature F3 | #Feature F4 | #Feature F5 | #Feature F7 | #Feature F8 | #Feature F1 | Start | End | PTM | AScore | Found By |
| --- | --- | --- | --- | --- | --- | --- | --- | --- | --- | --- | --- | --- | --- | --- | --- | --- | --- | --- | --- | --- | --- | --- | --- | --- | --- | --- | --- | --- | --- | --- | --- | --- | --- |
| K.VYEGERPLTK.D | Y | 61.30 | 1190.6295 | 10 | 0.8 | 596.3225 | 2 | 18.85 | 8 | F8:4637 | 31122021\_RID\_3262\_1b.raw |  |  |  |  |  |  |  | 7.869E4 | 1 | 0 | 0 | 0 | 0 | 0 | 0 | 0 | 1 | 464 | 473 |  |  | PEAKS DB |
| R.VEIIANDQGNR.I | Y | 53.00 | 1227.6207 | 11 | 0.0 | 614.8176 | 2 | 26.25 | 8 | F8:10120 | 31122021\_RID\_3262\_1b.raw |  |  |  |  |  |  |  | 1.5054E5 | 1 | 0 | 0 | 0 | 0 | 0 | 0 | 0 | 1 | 49 | 59 |  |  | PEAKS DB |
| R.ITPSYVAFTPEGER.L | Y | 46.56 | 1565.7725 | 14 | 1.3 | 783.8945 | 2 | 42.21 | 8 | F8:22560 | 31122021\_RID\_3262\_1b.raw |  |  |  |  |  |  |  | 1.1743E4 | 1 | 0 | 0 | 0 | 0 | 0 | 0 | 0 | 1 | 60 | 73 |  |  | PEAKS DB |
| total 3 peptides |  |  |  |  |  |  |  |  |  |  |  |  |  |  |  |  |  |  |  |  |  |  |  |  |  |  |  |  |  |  |  |  |  |
| --- | --- | --- | --- | --- | --- | --- | --- | --- | --- | --- | --- | --- | --- | --- | --- | --- | --- | --- | --- | --- | --- | --- | --- | --- | --- | --- | --- | --- | --- | --- | --- | --- | --- |

AFJ50327.1

back to list

  

| Protein Coverage
| Supporting Peptides
|

Protein Coverage:

Supporting Peptides:

| Peptide | Uniq | -10lgP | Mass | Length | ppm | m/z | z | RT | Fraction | Scan | Source File | Area F6 | Area F2 | Area F3 | Area F4 | Area F5 | Area F7 | Area F8 | Area F1 | #Feature | #Feature F6 | #Feature F2 | #Feature F3 | #Feature F4 | #Feature F5 | #Feature F7 | #Feature F8 | #Feature F1 | Start | End | PTM | AScore | Found By |
| --- | --- | --- | --- | --- | --- | --- | --- | --- | --- | --- | --- | --- | --- | --- | --- | --- | --- | --- | --- | --- | --- | --- | --- | --- | --- | --- | --- | --- | --- | --- | --- | --- | --- |
| K.VYEGERPLTK.D | Y | 61.30 | 1190.6295 | 10 | 0.8 | 596.3225 | 2 | 18.85 | 8 | F8:4637 | 31122021\_RID\_3262\_1b.raw |  |  |  |  |  |  |  | 7.869E4 | 1 | 0 | 0 | 0 | 0 | 0 | 0 | 0 | 1 | 464 | 473 |  |  | PEAKS DB |
| R.VEIIANDQGNR.I | Y | 53.00 | 1227.6207 | 11 | 0.0 | 614.8176 | 2 | 26.25 | 8 | F8:10120 | 31122021\_RID\_3262\_1b.raw |  |  |  |  |  |  |  | 1.5054E5 | 1 | 0 | 0 | 0 | 0 | 0 | 0 | 0 | 1 | 49 | 59 |  |  | PEAKS DB |
| R.ITPSYVAFTPEGER.L | Y | 46.56 | 1565.7725 | 14 | 1.3 | 783.8945 | 2 | 42.21 | 8 | F8:22560 | 31122021\_RID\_3262\_1b.raw |  |  |  |  |  |  |  | 1.1743E4 | 1 | 0 | 0 | 0 | 0 | 0 | 0 | 0 | 1 | 60 | 73 |  |  | PEAKS DB |
| total 3 peptides |  |  |  |  |  |  |  |  |  |  |  |  |  |  |  |  |  |  |  |  |  |  |  |  |  |  |  |  |  |  |  |  |  |
| --- | --- | --- | --- | --- | --- | --- | --- | --- | --- | --- | --- | --- | --- | --- | --- | --- | --- | --- | --- | --- | --- | --- | --- | --- | --- | --- | --- | --- | --- | --- | --- | --- | --- |

AFJ50239.1

back to list

  

| Protein Coverage
| Supporting Peptides
|

Protein Coverage:

Supporting Peptides:

| Peptide | Uniq | -10lgP | Mass | Length | ppm | m/z | z | RT | Fraction | Scan | Source File | Area F6 | Area F2 | Area F3 | Area F4 | Area F5 | Area F7 | Area F8 | Area F1 | #Feature | #Feature F6 | #Feature F2 | #Feature F3 | #Feature F4 | #Feature F5 | #Feature F7 | #Feature F8 | #Feature F1 | Start | End | PTM | AScore | Found By |
| --- | --- | --- | --- | --- | --- | --- | --- | --- | --- | --- | --- | --- | --- | --- | --- | --- | --- | --- | --- | --- | --- | --- | --- | --- | --- | --- | --- | --- | --- | --- | --- | --- | --- |
| K.VYEGERPLTK.D | Y | 61.30 | 1190.6295 | 10 | 0.8 | 596.3225 | 2 | 18.85 | 8 | F8:4637 | 31122021\_RID\_3262\_1b.raw |  |  |  |  |  |  |  | 7.869E4 | 1 | 0 | 0 | 0 | 0 | 0 | 0 | 0 | 1 | 464 | 473 |  |  | PEAKS DB |
| R.VEIIANDQGNR.I | Y | 53.00 | 1227.6207 | 11 | 0.0 | 614.8176 | 2 | 26.25 | 8 | F8:10120 | 31122021\_RID\_3262\_1b.raw |  |  |  |  |  |  |  | 1.5054E5 | 1 | 0 | 0 | 0 | 0 | 0 | 0 | 0 | 1 | 49 | 59 |  |  | PEAKS DB |
| R.ITPSYVAFTPEGER.L | Y | 46.56 | 1565.7725 | 14 | 1.3 | 783.8945 | 2 | 42.21 | 8 | F8:22560 | 31122021\_RID\_3262\_1b.raw |  |  |  |  |  |  |  | 1.1743E4 | 1 | 0 | 0 | 0 | 0 | 0 | 0 | 0 | 1 | 60 | 73 |  |  | PEAKS DB |
| total 3 peptides |  |  |  |  |  |  |  |  |  |  |  |  |  |  |  |  |  |  |  |  |  |  |  |  |  |  |  |  |  |  |  |  |  |
| --- | --- | --- | --- | --- | --- | --- | --- | --- | --- | --- | --- | --- | --- | --- | --- | --- | --- | --- | --- | --- | --- | --- | --- | --- | --- | --- | --- | --- | --- | --- | --- | --- | --- |

XP\_026542482.1

back to list

  

| Protein Coverage
| Supporting Peptides
|

Protein Coverage:

Supporting Peptides:

| Peptide | Uniq | -10lgP | Mass | Length | ppm | m/z | z | RT | Fraction | Scan | Source File | Area F6 | Area F2 | Area F3 | Area F4 | Area F5 | Area F7 | Area F8 | Area F1 | #Feature | #Feature F6 | #Feature F2 | #Feature F3 | #Feature F4 | #Feature F5 | #Feature F7 | #Feature F8 | #Feature F1 | Start | End | PTM | AScore | Found By |
| --- | --- | --- | --- | --- | --- | --- | --- | --- | --- | --- | --- | --- | --- | --- | --- | --- | --- | --- | --- | --- | --- | --- | --- | --- | --- | --- | --- | --- | --- | --- | --- | --- | --- |
| K.VYEGERPLTK.D | Y | 61.30 | 1190.6295 | 10 | 0.8 | 596.3225 | 2 | 18.85 | 8 | F8:4637 | 31122021\_RID\_3262\_1b.raw |  |  |  |  |  |  |  | 7.869E4 | 1 | 0 | 0 | 0 | 0 | 0 | 0 | 0 | 1 | 465 | 474 |  |  | PEAKS DB |
| R.VEIIANDQGNR.I | Y | 53.00 | 1227.6207 | 11 | 0.0 | 614.8176 | 2 | 26.25 | 8 | F8:10120 | 31122021\_RID\_3262\_1b.raw |  |  |  |  |  |  |  | 1.5054E5 | 1 | 0 | 0 | 0 | 0 | 0 | 0 | 0 | 1 | 50 | 60 |  |  | PEAKS DB |
| R.ITPSYVAFTPEGER.L | Y | 46.56 | 1565.7725 | 14 | 1.3 | 783.8945 | 2 | 42.21 | 8 | F8:22560 | 31122021\_RID\_3262\_1b.raw |  |  |  |  |  |  |  | 1.1743E4 | 1 | 0 | 0 | 0 | 0 | 0 | 0 | 0 | 1 | 61 | 74 |  |  | PEAKS DB |
| total 3 peptides |  |  |  |  |  |  |  |  |  |  |  |  |  |  |  |  |  |  |  |  |  |  |  |  |  |  |  |  |  |  |  |  |  |
| --- | --- | --- | --- | --- | --- | --- | --- | --- | --- | --- | --- | --- | --- | --- | --- | --- | --- | --- | --- | --- | --- | --- | --- | --- | --- | --- | --- | --- | --- | --- | --- | --- | --- |

XP\_034262421.1

back to list

  

| Protein Coverage
| Supporting Peptides
|

Protein Coverage:

Supporting Peptides:

| Peptide | Uniq | -10lgP | Mass | Length | ppm | m/z | z | RT | Fraction | Scan | Source File | Area F6 | Area F2 | Area F3 | Area F4 | Area F5 | Area F7 | Area F8 | Area F1 | #Feature | #Feature F6 | #Feature F2 | #Feature F3 | #Feature F4 | #Feature F5 | #Feature F7 | #Feature F8 | #Feature F1 | Start | End | PTM | AScore | Found By |
| --- | --- | --- | --- | --- | --- | --- | --- | --- | --- | --- | --- | --- | --- | --- | --- | --- | --- | --- | --- | --- | --- | --- | --- | --- | --- | --- | --- | --- | --- | --- | --- | --- | --- |
| K.VYEGERPLTK.D | Y | 61.30 | 1190.6295 | 10 | 0.8 | 596.3225 | 2 | 18.85 | 8 | F8:4637 | 31122021\_RID\_3262\_1b.raw |  |  |  |  |  |  |  | 7.869E4 | 1 | 0 | 0 | 0 | 0 | 0 | 0 | 0 | 1 | 430 | 439 |  |  | PEAKS DB |
| R.VEIIANDQGNR.I | Y | 53.00 | 1227.6207 | 11 | 0.0 | 614.8176 | 2 | 26.25 | 8 | F8:10120 | 31122021\_RID\_3262\_1b.raw |  |  |  |  |  |  |  | 1.5054E5 | 1 | 0 | 0 | 0 | 0 | 0 | 0 | 0 | 1 | 15 | 25 |  |  | PEAKS DB |
| R.ITPSYVAFTPEGER.L | Y | 46.56 | 1565.7725 | 14 | 1.3 | 783.8945 | 2 | 42.21 | 8 | F8:22560 | 31122021\_RID\_3262\_1b.raw |  |  |  |  |  |  |  | 1.1743E4 | 1 | 0 | 0 | 0 | 0 | 0 | 0 | 0 | 1 | 26 | 39 |  |  | PEAKS DB |
| total 3 peptides |  |  |  |  |  |  |  |  |  |  |  |  |  |  |  |  |  |  |  |  |  |  |  |  |  |  |  |  |  |  |  |  |  |
| --- | --- | --- | --- | --- | --- | --- | --- | --- | --- | --- | --- | --- | --- | --- | --- | --- | --- | --- | --- | --- | --- | --- | --- | --- | --- | --- | --- | --- | --- | --- | --- | --- | --- |

XP\_025022112.1

back to list

  

| Protein Coverage
| Supporting Peptides
|

Protein Coverage:

Supporting Peptides:

| Peptide | Uniq | -10lgP | Mass | Length | ppm | m/z | z | RT | Fraction | Scan | Source File | Area F6 | Area F2 | Area F3 | Area F4 | Area F5 | Area F7 | Area F8 | Area F1 | #Feature | #Feature F6 | #Feature F2 | #Feature F3 | #Feature F4 | #Feature F5 | #Feature F7 | #Feature F8 | #Feature F1 | Start | End | PTM | AScore | Found By |
| --- | --- | --- | --- | --- | --- | --- | --- | --- | --- | --- | --- | --- | --- | --- | --- | --- | --- | --- | --- | --- | --- | --- | --- | --- | --- | --- | --- | --- | --- | --- | --- | --- | --- |
| K.VYEGERPLTK.D | Y | 61.30 | 1190.6295 | 10 | 0.8 | 596.3225 | 2 | 18.85 | 8 | F8:4637 | 31122021\_RID\_3262\_1b.raw |  |  |  |  |  |  |  | 7.869E4 | 1 | 0 | 0 | 0 | 0 | 0 | 0 | 0 | 1 | 463 | 472 |  |  | PEAKS DB |
| R.VEIIANDQGNR.I | Y | 53.00 | 1227.6207 | 11 | 0.0 | 614.8176 | 2 | 26.25 | 8 | F8:10120 | 31122021\_RID\_3262\_1b.raw |  |  |  |  |  |  |  | 1.5054E5 | 1 | 0 | 0 | 0 | 0 | 0 | 0 | 0 | 1 | 48 | 58 |  |  | PEAKS DB |
| R.ITPSYVAFTPEGER.L | Y | 46.56 | 1565.7725 | 14 | 1.3 | 783.8945 | 2 | 42.21 | 8 | F8:22560 | 31122021\_RID\_3262\_1b.raw |  |  |  |  |  |  |  | 1.1743E4 | 1 | 0 | 0 | 0 | 0 | 0 | 0 | 0 | 1 | 59 | 72 |  |  | PEAKS DB |
| total 3 peptides |  |  |  |  |  |  |  |  |  |  |  |  |  |  |  |  |  |  |  |  |  |  |  |  |  |  |  |  |  |  |  |  |  |
| --- | --- | --- | --- | --- | --- | --- | --- | --- | --- | --- | --- | --- | --- | --- | --- | --- | --- | --- | --- | --- | --- | --- | --- | --- | --- | --- | --- | --- | --- | --- | --- | --- | --- |

JAG67806.1

back to list

  

| Protein Coverage
| Supporting Peptides
|

Protein Coverage:

Supporting Peptides:

| Peptide | Uniq | -10lgP | Mass | Length | ppm | m/z | z | RT | Fraction | Scan | Source File | Area F6 | Area F2 | Area F3 | Area F4 | Area F5 | Area F7 | Area F8 | Area F1 | #Feature | #Feature F6 | #Feature F2 | #Feature F3 | #Feature F4 | #Feature F5 | #Feature F7 | #Feature F8 | #Feature F1 | Start | End | PTM | AScore | Found By |
| --- | --- | --- | --- | --- | --- | --- | --- | --- | --- | --- | --- | --- | --- | --- | --- | --- | --- | --- | --- | --- | --- | --- | --- | --- | --- | --- | --- | --- | --- | --- | --- | --- | --- |
| K.VYEGERPLTK.D | Y | 61.30 | 1190.6295 | 10 | 0.8 | 596.3225 | 2 | 18.85 | 8 | F8:4637 | 31122021\_RID\_3262\_1b.raw |  |  |  |  |  |  |  | 7.869E4 | 1 | 0 | 0 | 0 | 0 | 0 | 0 | 0 | 1 | 464 | 473 |  |  | PEAKS DB |
| R.VEIIANDQGNR.I | Y | 53.00 | 1227.6207 | 11 | 0.0 | 614.8176 | 2 | 26.25 | 8 | F8:10120 | 31122021\_RID\_3262\_1b.raw |  |  |  |  |  |  |  | 1.5054E5 | 1 | 0 | 0 | 0 | 0 | 0 | 0 | 0 | 1 | 49 | 59 |  |  | PEAKS DB |
| R.ITPSYVAFTPEGER.L | Y | 46.56 | 1565.7725 | 14 | 1.3 | 783.8945 | 2 | 42.21 | 8 | F8:22560 | 31122021\_RID\_3262\_1b.raw |  |  |  |  |  |  |  | 1.1743E4 | 1 | 0 | 0 | 0 | 0 | 0 | 0 | 0 | 1 | 60 | 73 |  |  | PEAKS DB |
| total 3 peptides |  |  |  |  |  |  |  |  |  |  |  |  |  |  |  |  |  |  |  |  |  |  |  |  |  |  |  |  |  |  |  |  |  |
| --- | --- | --- | --- | --- | --- | --- | --- | --- | --- | --- | --- | --- | --- | --- | --- | --- | --- | --- | --- | --- | --- | --- | --- | --- | --- | --- | --- | --- | --- | --- | --- | --- | --- |

XP\_032088936.1

back to list

  

| Protein Coverage
| Supporting Peptides
|

Protein Coverage:

Supporting Peptides:

| Peptide | Uniq | -10lgP | Mass | Length | ppm | m/z | z | RT | Fraction | Scan | Source File | Area F6 | Area F2 | Area F3 | Area F4 | Area F5 | Area F7 | Area F8 | Area F1 | #Feature | #Feature F6 | #Feature F2 | #Feature F3 | #Feature F4 | #Feature F5 | #Feature F7 | #Feature F8 | #Feature F1 | Start | End | PTM | AScore | Found By |
| --- | --- | --- | --- | --- | --- | --- | --- | --- | --- | --- | --- | --- | --- | --- | --- | --- | --- | --- | --- | --- | --- | --- | --- | --- | --- | --- | --- | --- | --- | --- | --- | --- | --- |
| K.VYEGERPLTK.D | Y | 61.30 | 1190.6295 | 10 | 0.8 | 596.3225 | 2 | 18.85 | 8 | F8:4637 | 31122021\_RID\_3262\_1b.raw |  |  |  |  |  |  |  | 7.869E4 | 1 | 0 | 0 | 0 | 0 | 0 | 0 | 0 | 1 | 464 | 473 |  |  | PEAKS DB |
| R.VEIIANDQGNR.I | Y | 53.00 | 1227.6207 | 11 | 0.0 | 614.8176 | 2 | 26.25 | 8 | F8:10120 | 31122021\_RID\_3262\_1b.raw |  |  |  |  |  |  |  | 1.5054E5 | 1 | 0 | 0 | 0 | 0 | 0 | 0 | 0 | 1 | 49 | 59 |  |  | PEAKS DB |
| R.ITPSYVAFTPEGER.L | Y | 46.56 | 1565.7725 | 14 | 1.3 | 783.8945 | 2 | 42.21 | 8 | F8:22560 | 31122021\_RID\_3262\_1b.raw |  |  |  |  |  |  |  | 1.1743E4 | 1 | 0 | 0 | 0 | 0 | 0 | 0 | 0 | 1 | 60 | 73 |  |  | PEAKS DB |
| total 3 peptides |  |  |  |  |  |  |  |  |  |  |  |  |  |  |  |  |  |  |  |  |  |  |  |  |  |  |  |  |  |  |  |  |  |
| --- | --- | --- | --- | --- | --- | --- | --- | --- | --- | --- | --- | --- | --- | --- | --- | --- | --- | --- | --- | --- | --- | --- | --- | --- | --- | --- | --- | --- | --- | --- | --- | --- | --- |

XP\_013910585.1

back to list

  

| Protein Coverage
| Supporting Peptides
|

Protein Coverage:

Supporting Peptides:

| Peptide | Uniq | -10lgP | Mass | Length | ppm | m/z | z | RT | Fraction | Scan | Source File | Area F6 | Area F2 | Area F3 | Area F4 | Area F5 | Area F7 | Area F8 | Area F1 | #Feature | #Feature F6 | #Feature F2 | #Feature F3 | #Feature F4 | #Feature F5 | #Feature F7 | #Feature F8 | #Feature F1 | Start | End | PTM | AScore | Found By |
| --- | --- | --- | --- | --- | --- | --- | --- | --- | --- | --- | --- | --- | --- | --- | --- | --- | --- | --- | --- | --- | --- | --- | --- | --- | --- | --- | --- | --- | --- | --- | --- | --- | --- |
| K.VYEGERPLTK.D | Y | 61.30 | 1190.6295 | 10 | 0.8 | 596.3225 | 2 | 18.85 | 8 | F8:4637 | 31122021\_RID\_3262\_1b.raw |  |  |  |  |  |  |  | 7.869E4 | 1 | 0 | 0 | 0 | 0 | 0 | 0 | 0 | 1 | 464 | 473 |  |  | PEAKS DB |
| R.VEIIANDQGNR.I | Y | 53.00 | 1227.6207 | 11 | 0.0 | 614.8176 | 2 | 26.25 | 8 | F8:10120 | 31122021\_RID\_3262\_1b.raw |  |  |  |  |  |  |  | 1.5054E5 | 1 | 0 | 0 | 0 | 0 | 0 | 0 | 0 | 1 | 49 | 59 |  |  | PEAKS DB |
| R.ITPSYVAFTPEGER.L | Y | 46.56 | 1565.7725 | 14 | 1.3 | 783.8945 | 2 | 42.21 | 8 | F8:22560 | 31122021\_RID\_3262\_1b.raw |  |  |  |  |  |  |  | 1.1743E4 | 1 | 0 | 0 | 0 | 0 | 0 | 0 | 0 | 1 | 60 | 73 |  |  | PEAKS DB |
| total 3 peptides |  |  |  |  |  |  |  |  |  |  |  |  |  |  |  |  |  |  |  |  |  |  |  |  |  |  |  |  |  |  |  |  |  |
| --- | --- | --- | --- | --- | --- | --- | --- | --- | --- | --- | --- | --- | --- | --- | --- | --- | --- | --- | --- | --- | --- | --- | --- | --- | --- | --- | --- | --- | --- | --- | --- | --- | --- |

XP\_026568326.1

back to list

  

| Protein Coverage
| Supporting Peptides
|

Protein Coverage:

Supporting Peptides:

| Peptide | Uniq | -10lgP | Mass | Length | ppm | m/z | z | RT | Fraction | Scan | Source File | Area F6 | Area F2 | Area F3 | Area F4 | Area F5 | Area F7 | Area F8 | Area F1 | #Feature | #Feature F6 | #Feature F2 | #Feature F3 | #Feature F4 | #Feature F5 | #Feature F7 | #Feature F8 | #Feature F1 | Start | End | PTM | AScore | Found By |
| --- | --- | --- | --- | --- | --- | --- | --- | --- | --- | --- | --- | --- | --- | --- | --- | --- | --- | --- | --- | --- | --- | --- | --- | --- | --- | --- | --- | --- | --- | --- | --- | --- | --- |
| K.VYEGERPLTK.D | Y | 61.30 | 1190.6295 | 10 | 0.8 | 596.3225 | 2 | 18.85 | 8 | F8:4637 | 31122021\_RID\_3262\_1b.raw |  |  |  |  |  |  |  | 7.869E4 | 1 | 0 | 0 | 0 | 0 | 0 | 0 | 0 | 1 | 465 | 474 |  |  | PEAKS DB |
| R.VEIIANDQGNR.I | Y | 53.00 | 1227.6207 | 11 | 0.0 | 614.8176 | 2 | 26.25 | 8 | F8:10120 | 31122021\_RID\_3262\_1b.raw |  |  |  |  |  |  |  | 1.5054E5 | 1 | 0 | 0 | 0 | 0 | 0 | 0 | 0 | 1 | 50 | 60 |  |  | PEAKS DB |
| R.ITPSYVAFTPEGER.L | Y | 46.56 | 1565.7725 | 14 | 1.3 | 783.8945 | 2 | 42.21 | 8 | F8:22560 | 31122021\_RID\_3262\_1b.raw |  |  |  |  |  |  |  | 1.1743E4 | 1 | 0 | 0 | 0 | 0 | 0 | 0 | 0 | 1 | 61 | 74 |  |  | PEAKS DB |
| total 3 peptides |  |  |  |  |  |  |  |  |  |  |  |  |  |  |  |  |  |  |  |  |  |  |  |  |  |  |  |  |  |  |  |  |  |
| --- | --- | --- | --- | --- | --- | --- | --- | --- | --- | --- | --- | --- | --- | --- | --- | --- | --- | --- | --- | --- | --- | --- | --- | --- | --- | --- | --- | --- | --- | --- | --- | --- | --- |

JAC95528.1

back to list

  

| Protein Coverage
| Supporting Peptides
|

Protein Coverage:

Supporting Peptides:

| Peptide | Uniq | -10lgP | Mass | Length | ppm | m/z | z | RT | Fraction | Scan | Source File | Area F6 | Area F2 | Area F3 | Area F4 | Area F5 | Area F7 | Area F8 | Area F1 | #Feature | #Feature F6 | #Feature F2 | #Feature F3 | #Feature F4 | #Feature F5 | #Feature F7 | #Feature F8 | #Feature F1 | Start | End | PTM | AScore | Found By |
| --- | --- | --- | --- | --- | --- | --- | --- | --- | --- | --- | --- | --- | --- | --- | --- | --- | --- | --- | --- | --- | --- | --- | --- | --- | --- | --- | --- | --- | --- | --- | --- | --- | --- |
| K.VYEGERPLTK.D | Y | 61.30 | 1190.6295 | 10 | 0.8 | 596.3225 | 2 | 18.85 | 8 | F8:4637 | 31122021\_RID\_3262\_1b.raw |  |  |  |  |  |  |  | 7.869E4 | 1 | 0 | 0 | 0 | 0 | 0 | 0 | 0 | 1 | 465 | 474 |  |  | PEAKS DB |
| R.VEIIANDQGNR.I | Y | 53.00 | 1227.6207 | 11 | 0.0 | 614.8176 | 2 | 26.25 | 8 | F8:10120 | 31122021\_RID\_3262\_1b.raw |  |  |  |  |  |  |  | 1.5054E5 | 1 | 0 | 0 | 0 | 0 | 0 | 0 | 0 | 1 | 50 | 60 |  |  | PEAKS DB |
| R.ITPSYVAFTPEGER.L | Y | 46.56 | 1565.7725 | 14 | 1.3 | 783.8945 | 2 | 42.21 | 8 | F8:22560 | 31122021\_RID\_3262\_1b.raw |  |  |  |  |  |  |  | 1.1743E4 | 1 | 0 | 0 | 0 | 0 | 0 | 0 | 0 | 1 | 61 | 74 |  |  | PEAKS DB |
| total 3 peptides |  |  |  |  |  |  |  |  |  |  |  |  |  |  |  |  |  |  |  |  |  |  |  |  |  |  |  |  |  |  |  |  |  |
| --- | --- | --- | --- | --- | --- | --- | --- | --- | --- | --- | --- | --- | --- | --- | --- | --- | --- | --- | --- | --- | --- | --- | --- | --- | --- | --- | --- | --- | --- | --- | --- | --- | --- |

JAB54174.1

back to list

  

| Protein Coverage
| Supporting Peptides
|

Protein Coverage:

Supporting Peptides:

| Peptide | Uniq | -10lgP | Mass | Length | ppm | m/z | z | RT | Fraction | Scan | Source File | Area F6 | Area F2 | Area F3 | Area F4 | Area F5 | Area F7 | Area F8 | Area F1 | #Feature | #Feature F6 | #Feature F2 | #Feature F3 | #Feature F4 | #Feature F5 | #Feature F7 | #Feature F8 | #Feature F1 | Start | End | PTM | AScore | Found By |
| --- | --- | --- | --- | --- | --- | --- | --- | --- | --- | --- | --- | --- | --- | --- | --- | --- | --- | --- | --- | --- | --- | --- | --- | --- | --- | --- | --- | --- | --- | --- | --- | --- | --- |
| K.VYEGERPLTK.D | Y | 61.30 | 1190.6295 | 10 | 0.8 | 596.3225 | 2 | 18.85 | 8 | F8:4637 | 31122021\_RID\_3262\_1b.raw |  |  |  |  |  |  |  | 7.869E4 | 1 | 0 | 0 | 0 | 0 | 0 | 0 | 0 | 1 | 465 | 474 |  |  | PEAKS DB |
| R.VEIIANDQGNR.I | Y | 53.00 | 1227.6207 | 11 | 0.0 | 614.8176 | 2 | 26.25 | 8 | F8:10120 | 31122021\_RID\_3262\_1b.raw |  |  |  |  |  |  |  | 1.5054E5 | 1 | 0 | 0 | 0 | 0 | 0 | 0 | 0 | 1 | 50 | 60 |  |  | PEAKS DB |
| R.ITPSYVAFTPEGER.L | Y | 46.56 | 1565.7725 | 14 | 1.3 | 783.8945 | 2 | 42.21 | 8 | F8:22560 | 31122021\_RID\_3262\_1b.raw |  |  |  |  |  |  |  | 1.1743E4 | 1 | 0 | 0 | 0 | 0 | 0 | 0 | 0 | 1 | 61 | 74 |  |  | PEAKS DB |
| total 3 peptides |  |  |  |  |  |  |  |  |  |  |  |  |  |  |  |  |  |  |  |  |  |  |  |  |  |  |  |  |  |  |  |  |  |
| --- | --- | --- | --- | --- | --- | --- | --- | --- | --- | --- | --- | --- | --- | --- | --- | --- | --- | --- | --- | --- | --- | --- | --- | --- | --- | --- | --- | --- | --- | --- | --- | --- | --- |

AHJ09587.1

back to list

  

| Protein Coverage
| Supporting Peptides
|

Protein Coverage:

Supporting Peptides:

| Peptide | Uniq | -10lgP | Mass | Length | ppm | m/z | z | RT | Fraction | Scan | Source File | Area F6 | Area F2 | Area F3 | Area F4 | Area F5 | Area F7 | Area F8 | Area F1 | #Feature | #Feature F6 | #Feature F2 | #Feature F3 | #Feature F4 | #Feature F5 | #Feature F7 | #Feature F8 | #Feature F1 | Start | End | PTM | AScore | Found By |
| --- | --- | --- | --- | --- | --- | --- | --- | --- | --- | --- | --- | --- | --- | --- | --- | --- | --- | --- | --- | --- | --- | --- | --- | --- | --- | --- | --- | --- | --- | --- | --- | --- | --- |
| K.GGQGRPQDASDR.C | Y | 72.59 | 1242.5701 | 12 | -0.3 | 622.2921 | 2 | 11.65 | 6 | F6:2020 | 17012022\_RID\_3262\_7b.raw |  |  |  |  |  | 1.9138E6 |  |  | 2 | 0 | 0 | 0 | 0 | 0 | 2 | 0 | 0 | 47 | 58 |  |  | PEAKS DB |
| G.HLM(+15.99)QFETMIK.K | Y | 69.00 | 1292.6257 | 10 | -0.3 | 647.3199 | 2 | 35.21 | 3 | F3:19455 | 13012022\_RID\_3262\_3b.raw |  |  | 6.4304E6 |  | 0 | 4.6583E7 | 2.0638E7 | 5.6241E6 | 5 | 0 | 0 | 1 | 0 | 0 | 2 | 1 | 1 | 17 | 26 | Oxidation (M) | M3:Oxidation (M):130.10 | PEAKS DB |
| G.HLM(+15.99)QFETM(+15.99)IK.K | Y | 59.37 | 1308.6206 | 10 | 0.4 | 655.3179 | 2 | 62.98 | 6 | F6:28458 | 17012022\_RID\_3262\_7b.raw |  |  | 2.669E6 |  |  | 5.6444E7 | 2.385E7 |  | 4 | 0 | 0 | 1 | 0 | 0 | 1 | 2 | 0 | 17 | 26 | Oxidation (M) | M3:Oxidation (M):1000.00;M8:Oxidation (M):1000.00 | PEAKS DB |
| G.HLMQFETMIK.K | Y | 49.22 | 1276.6309 | 10 | 0.1 | 639.3228 | 2 | 38.95 | 8 | F8:20142 | 31122021\_RID\_3262\_1b.raw |  |  |  |  |  |  |  | 7.984E5 | 1 | 0 | 0 | 0 | 0 | 0 | 0 | 0 | 1 | 17 | 26 |  |  | PEAKS DB |
| total 4 peptides |  |  |  |  |  |  |  |  |  |  |  |  |  |  |  |  |  |  |  |  |  |  |  |  |  |  |  |  |  |  |  |  |  |
| --- | --- | --- | --- | --- | --- | --- | --- | --- | --- | --- | --- | --- | --- | --- | --- | --- | --- | --- | --- | --- | --- | --- | --- | --- | --- | --- | --- | --- | --- | --- | --- | --- | --- |

AHJ09589.1

back to list

  

| Protein Coverage
| Supporting Peptides
|

Protein Coverage:

Supporting Peptides:

| Peptide | Uniq | -10lgP | Mass | Length | ppm | m/z | z | RT | Fraction | Scan | Source File | Area F6 | Area F2 | Area F3 | Area F4 | Area F5 | Area F7 | Area F8 | Area F1 | #Feature | #Feature F6 | #Feature F2 | #Feature F3 | #Feature F4 | #Feature F5 | #Feature F7 | #Feature F8 | #Feature F1 | Start | End | PTM | AScore | Found By |
| --- | --- | --- | --- | --- | --- | --- | --- | --- | --- | --- | --- | --- | --- | --- | --- | --- | --- | --- | --- | --- | --- | --- | --- | --- | --- | --- | --- | --- | --- | --- | --- | --- | --- |
| K.GGQGRPQDASDR.C | Y | 72.59 | 1242.5701 | 12 | -0.3 | 622.2921 | 2 | 11.65 | 6 | F6:2020 | 17012022\_RID\_3262\_7b.raw |  |  |  |  |  | 1.9138E6 |  |  | 2 | 0 | 0 | 0 | 0 | 0 | 2 | 0 | 0 | 47 | 58 |  |  | PEAKS DB |
| G.HLM(+15.99)QFETMIK.K | Y | 69.00 | 1292.6257 | 10 | -0.3 | 647.3199 | 2 | 35.21 | 3 | F3:19455 | 13012022\_RID\_3262\_3b.raw |  |  | 6.4304E6 |  | 0 | 4.6583E7 | 2.0638E7 | 5.6241E6 | 5 | 0 | 0 | 1 | 0 | 0 | 2 | 1 | 1 | 17 | 26 | Oxidation (M) | M3:Oxidation (M):130.10 | PEAKS DB |
| G.HLM(+15.99)QFETM(+15.99)IK.K | Y | 59.37 | 1308.6206 | 10 | 0.4 | 655.3179 | 2 | 62.98 | 6 | F6:28458 | 17012022\_RID\_3262\_7b.raw |  |  | 2.669E6 |  |  | 5.6444E7 | 2.385E7 |  | 4 | 0 | 0 | 1 | 0 | 0 | 1 | 2 | 0 | 17 | 26 | Oxidation (M) | M3:Oxidation (M):1000.00;M8:Oxidation (M):1000.00 | PEAKS DB |
| G.HLMQFETMIK.K | Y | 49.22 | 1276.6309 | 10 | 0.1 | 639.3228 | 2 | 38.95 | 8 | F8:20142 | 31122021\_RID\_3262\_1b.raw |  |  |  |  |  |  |  | 7.984E5 | 1 | 0 | 0 | 0 | 0 | 0 | 0 | 0 | 1 | 17 | 26 |  |  | PEAKS DB |
| total 4 peptides |  |  |  |  |  |  |  |  |  |  |  |  |  |  |  |  |  |  |  |  |  |  |  |  |  |  |  |  |  |  |  |  |  |
| --- | --- | --- | --- | --- | --- | --- | --- | --- | --- | --- | --- | --- | --- | --- | --- | --- | --- | --- | --- | --- | --- | --- | --- | --- | --- | --- | --- | --- | --- | --- | --- | --- | --- |

AHJ09585.1

back to list

  

| Protein Coverage
| Supporting Peptides
|

Protein Coverage:

Supporting Peptides:

| Peptide | Uniq | -10lgP | Mass | Length | ppm | m/z | z | RT | Fraction | Scan | Source File | Area F6 | Area F2 | Area F3 | Area F4 | Area F5 | Area F7 | Area F8 | Area F1 | #Feature | #Feature F6 | #Feature F2 | #Feature F3 | #Feature F4 | #Feature F5 | #Feature F7 | #Feature F8 | #Feature F1 | Start | End | PTM | AScore | Found By |
| --- | --- | --- | --- | --- | --- | --- | --- | --- | --- | --- | --- | --- | --- | --- | --- | --- | --- | --- | --- | --- | --- | --- | --- | --- | --- | --- | --- | --- | --- | --- | --- | --- | --- |
| K.GGQGRPQDASDR.C | Y | 72.59 | 1242.5701 | 12 | -0.3 | 622.2921 | 2 | 11.65 | 6 | F6:2020 | 17012022\_RID\_3262\_7b.raw |  |  |  |  |  | 1.9138E6 |  |  | 2 | 0 | 0 | 0 | 0 | 0 | 2 | 0 | 0 | 47 | 58 |  |  | PEAKS DB |
| G.HLM(+15.99)QFETMIK.K | Y | 69.00 | 1292.6257 | 10 | -0.3 | 647.3199 | 2 | 35.21 | 3 | F3:19455 | 13012022\_RID\_3262\_3b.raw |  |  | 6.4304E6 |  | 0 | 4.6583E7 | 2.0638E7 | 5.6241E6 | 5 | 0 | 0 | 1 | 0 | 0 | 2 | 1 | 1 | 17 | 26 | Oxidation (M) | M3:Oxidation (M):130.10 | PEAKS DB |
| G.HLM(+15.99)QFETM(+15.99)IK.K | Y | 59.37 | 1308.6206 | 10 | 0.4 | 655.3179 | 2 | 62.98 | 6 | F6:28458 | 17012022\_RID\_3262\_7b.raw |  |  | 2.669E6 |  |  | 5.6444E7 | 2.385E7 |  | 4 | 0 | 0 | 1 | 0 | 0 | 1 | 2 | 0 | 17 | 26 | Oxidation (M) | M3:Oxidation (M):1000.00;M8:Oxidation (M):1000.00 | PEAKS DB |
| G.HLMQFETMIK.K | Y | 49.22 | 1276.6309 | 10 | 0.1 | 639.3228 | 2 | 38.95 | 8 | F8:20142 | 31122021\_RID\_3262\_1b.raw |  |  |  |  |  |  |  | 7.984E5 | 1 | 0 | 0 | 0 | 0 | 0 | 0 | 0 | 1 | 17 | 26 |  |  | PEAKS DB |
| total 4 peptides |  |  |  |  |  |  |  |  |  |  |  |  |  |  |  |  |  |  |  |  |  |  |  |  |  |  |  |  |  |  |  |  |  |
| --- | --- | --- | --- | --- | --- | --- | --- | --- | --- | --- | --- | --- | --- | --- | --- | --- | --- | --- | --- | --- | --- | --- | --- | --- | --- | --- | --- | --- | --- | --- | --- | --- | --- |

AHJ09594.1

back to list

  

| Protein Coverage
| Supporting Peptides
|

Protein Coverage:

Supporting Peptides:

| Peptide | Uniq | -10lgP | Mass | Length | ppm | m/z | z | RT | Fraction | Scan | Source File | Area F6 | Area F2 | Area F3 | Area F4 | Area F5 | Area F7 | Area F8 | Area F1 | #Feature | #Feature F6 | #Feature F2 | #Feature F3 | #Feature F4 | #Feature F5 | #Feature F7 | #Feature F8 | #Feature F1 | Start | End | PTM | AScore | Found By |
| --- | --- | --- | --- | --- | --- | --- | --- | --- | --- | --- | --- | --- | --- | --- | --- | --- | --- | --- | --- | --- | --- | --- | --- | --- | --- | --- | --- | --- | --- | --- | --- | --- | --- |
| K.GGQGRPQDASDR.C | Y | 72.59 | 1242.5701 | 12 | -0.3 | 622.2921 | 2 | 11.65 | 6 | F6:2020 | 17012022\_RID\_3262\_7b.raw |  |  |  |  |  | 1.9138E6 |  |  | 2 | 0 | 0 | 0 | 0 | 0 | 2 | 0 | 0 | 47 | 58 |  |  | PEAKS DB |
| G.HLM(+15.99)QFETMIK.K | Y | 69.00 | 1292.6257 | 10 | -0.3 | 647.3199 | 2 | 35.21 | 3 | F3:19455 | 13012022\_RID\_3262\_3b.raw |  |  | 6.4304E6 |  | 0 | 4.6583E7 | 2.0638E7 | 5.6241E6 | 5 | 0 | 0 | 1 | 0 | 0 | 2 | 1 | 1 | 17 | 26 | Oxidation (M) | M3:Oxidation (M):130.10 | PEAKS DB |
| G.HLM(+15.99)QFETM(+15.99)IK.K | Y | 59.37 | 1308.6206 | 10 | 0.4 | 655.3179 | 2 | 62.98 | 6 | F6:28458 | 17012022\_RID\_3262\_7b.raw |  |  | 2.669E6 |  |  | 5.6444E7 | 2.385E7 |  | 4 | 0 | 0 | 1 | 0 | 0 | 1 | 2 | 0 | 17 | 26 | Oxidation (M) | M3:Oxidation (M):1000.00;M8:Oxidation (M):1000.00 | PEAKS DB |
| G.HLMQFETMIK.K | Y | 49.22 | 1276.6309 | 10 | 0.1 | 639.3228 | 2 | 38.95 | 8 | F8:20142 | 31122021\_RID\_3262\_1b.raw |  |  |  |  |  |  |  | 7.984E5 | 1 | 0 | 0 | 0 | 0 | 0 | 0 | 0 | 1 | 17 | 26 |  |  | PEAKS DB |
| total 4 peptides |  |  |  |  |  |  |  |  |  |  |  |  |  |  |  |  |  |  |  |  |  |  |  |  |  |  |  |  |  |  |  |  |  |
| --- | --- | --- | --- | --- | --- | --- | --- | --- | --- | --- | --- | --- | --- | --- | --- | --- | --- | --- | --- | --- | --- | --- | --- | --- | --- | --- | --- | --- | --- | --- | --- | --- | --- |

AHJ09591.1

back to list

  

| Protein Coverage
| Supporting Peptides
|

Protein Coverage:

Supporting Peptides:

| Peptide | Uniq | -10lgP | Mass | Length | ppm | m/z | z | RT | Fraction | Scan | Source File | Area F6 | Area F2 | Area F3 | Area F4 | Area F5 | Area F7 | Area F8 | Area F1 | #Feature | #Feature F6 | #Feature F2 | #Feature F3 | #Feature F4 | #Feature F5 | #Feature F7 | #Feature F8 | #Feature F1 | Start | End | PTM | AScore | Found By |
| --- | --- | --- | --- | --- | --- | --- | --- | --- | --- | --- | --- | --- | --- | --- | --- | --- | --- | --- | --- | --- | --- | --- | --- | --- | --- | --- | --- | --- | --- | --- | --- | --- | --- |
| K.GGQGRPQDASDR.C | Y | 72.59 | 1242.5701 | 12 | -0.3 | 622.2921 | 2 | 11.65 | 6 | F6:2020 | 17012022\_RID\_3262\_7b.raw |  |  |  |  |  | 1.9138E6 |  |  | 2 | 0 | 0 | 0 | 0 | 0 | 2 | 0 | 0 | 47 | 58 |  |  | PEAKS DB |
| G.HLM(+15.99)QFETMIK.K | Y | 69.00 | 1292.6257 | 10 | -0.3 | 647.3199 | 2 | 35.21 | 3 | F3:19455 | 13012022\_RID\_3262\_3b.raw |  |  | 6.4304E6 |  | 0 | 4.6583E7 | 2.0638E7 | 5.6241E6 | 5 | 0 | 0 | 1 | 0 | 0 | 2 | 1 | 1 | 17 | 26 | Oxidation (M) | M3:Oxidation (M):130.10 | PEAKS DB |
| G.HLM(+15.99)QFETM(+15.99)IK.K | Y | 59.37 | 1308.6206 | 10 | 0.4 | 655.3179 | 2 | 62.98 | 6 | F6:28458 | 17012022\_RID\_3262\_7b.raw |  |  | 2.669E6 |  |  | 5.6444E7 | 2.385E7 |  | 4 | 0 | 0 | 1 | 0 | 0 | 1 | 2 | 0 | 17 | 26 | Oxidation (M) | M3:Oxidation (M):1000.00;M8:Oxidation (M):1000.00 | PEAKS DB |
| G.HLMQFETMIK.K | Y | 49.22 | 1276.6309 | 10 | 0.1 | 639.3228 | 2 | 38.95 | 8 | F8:20142 | 31122021\_RID\_3262\_1b.raw |  |  |  |  |  |  |  | 7.984E5 | 1 | 0 | 0 | 0 | 0 | 0 | 0 | 0 | 1 | 17 | 26 |  |  | PEAKS DB |
| total 4 peptides |  |  |  |  |  |  |  |  |  |  |  |  |  |  |  |  |  |  |  |  |  |  |  |  |  |  |  |  |  |  |  |  |  |
| --- | --- | --- | --- | --- | --- | --- | --- | --- | --- | --- | --- | --- | --- | --- | --- | --- | --- | --- | --- | --- | --- | --- | --- | --- | --- | --- | --- | --- | --- | --- | --- | --- | --- |

XP\_015685587.1

back to list

  

| Protein Coverage
| Supporting Peptides
|

Protein Coverage:

Supporting Peptides:

| Peptide | Uniq | -10lgP | Mass | Length | ppm | m/z | z | RT | Fraction | Scan | Source File | Area F6 | Area F2 | Area F3 | Area F4 | Area F5 | Area F7 | Area F8 | Area F1 | #Feature | #Feature F6 | #Feature F2 | #Feature F3 | #Feature F4 | #Feature F5 | #Feature F7 | #Feature F8 | #Feature F1 | Start | End | PTM | AScore | Found By |
| --- | --- | --- | --- | --- | --- | --- | --- | --- | --- | --- | --- | --- | --- | --- | --- | --- | --- | --- | --- | --- | --- | --- | --- | --- | --- | --- | --- | --- | --- | --- | --- | --- | --- |
| K.LVVTAEQQR.Y | N | 59.96 | 1042.5771 | 9 | -0.3 | 522.2957 | 2 | 24.54 | 2 | F2:6838 | 13012022\_RID\_3262\_2b.raw |  | 4.3551E6 | 7.7997E6 | 4.2093E6 |  |  |  | 4.824E5 | 4 | 0 | 1 | 1 | 1 | 0 | 0 | 0 | 1 | 223 | 231 |  |  | PEAKS DB |
| K.TWGYEIVNTLNEIYR.Y | Y | 58.99 | 1869.9260 | 15 | 0.8 | 935.9711 | 2 | 63.72 | 8 | F8:38888 | 31122021\_RID\_3262\_1b.raw |  | 2.5255E5 |  |  |  |  |  | 1.8092E5 | 2 | 0 | 1 | 0 | 0 | 0 | 0 | 0 | 1 | 262 | 276 |  |  | PEAKS DB |
| K.FNSNLTEVK.T | Y | 47.62 | 1050.5345 | 9 | 0.6 | 526.2748 | 2 | 30.12 | 3 | F3:15153 | 13012022\_RID\_3262\_3b.raw |  |  | 1.1101E6 |  |  |  |  |  | 1 | 0 | 0 | 1 | 0 | 0 | 0 | 0 | 0 | 253 | 261 |  |  | PEAKS DB |
| total 3 peptides |  |  |  |  |  |  |  |  |  |  |  |  |  |  |  |  |  |  |  |  |  |  |  |  |  |  |  |  |  |  |  |  |  |
| --- | --- | --- | --- | --- | --- | --- | --- | --- | --- | --- | --- | --- | --- | --- | --- | --- | --- | --- | --- | --- | --- | --- | --- | --- | --- | --- | --- | --- | --- | --- | --- | --- | --- |

P0DJL3.1

back to list

  

| Protein Coverage
| Supporting Peptides
|

Protein Coverage:

Supporting Peptides:

| Peptide | Uniq | -10lgP | Mass | Length | ppm | m/z | z | RT | Fraction | Scan | Source File | Area F6 | Area F2 | Area F3 | Area F4 | Area F5 | Area F7 | Area F8 | Area F1 | #Feature | #Feature F6 | #Feature F2 | #Feature F3 | #Feature F4 | #Feature F5 | #Feature F7 | #Feature F8 | #Feature F1 | Start | End | PTM | AScore | Found By |
| --- | --- | --- | --- | --- | --- | --- | --- | --- | --- | --- | --- | --- | --- | --- | --- | --- | --- | --- | --- | --- | --- | --- | --- | --- | --- | --- | --- | --- | --- | --- | --- | --- | --- |
| K.TTDNQWWTR.S | Y | 61.25 | 1206.5417 | 9 | -0.3 | 604.2780 | 2 | 41.71 | 7 | F7:18569 | 17012022\_RID\_3262\_8b.raw |  |  | 1.3707E6 |  |  |  | 6.1973E6 |  | 3 | 0 | 0 | 1 | 0 | 0 | 0 | 2 | 0 | 101 | 109 |  |  | PEAKS DB |
| R.LQWSDGTELK.Y | Y | 58.58 | 1175.5823 | 10 | 0.3 | 588.7986 | 2 | 40.53 | 7 | F7:17181 | 17012022\_RID\_3262\_8b.raw |  | 5.9765E5 |  |  |  | 2.2921E6 | 2.702E6 | 1.1947E6 | 4 | 0 | 1 | 0 | 0 | 0 | 1 | 1 | 1 | 77 | 86 |  |  | PEAKS DB |
| total 2 peptides |  |  |  |  |  |  |  |  |  |  |  |  |  |  |  |  |  |  |  |  |  |  |  |  |  |  |  |  |  |  |  |  |  |
| --- | --- | --- | --- | --- | --- | --- | --- | --- | --- | --- | --- | --- | --- | --- | --- | --- | --- | --- | --- | --- | --- | --- | --- | --- | --- | --- | --- | --- | --- | --- | --- | --- | --- |

P81116.2

back to list

  

| Protein Coverage
| Supporting Peptides
|

Protein Coverage:

Supporting Peptides:

| Peptide | Uniq | -10lgP | Mass | Length | ppm | m/z | z | RT | Fraction | Scan | Source File | Area F6 | Area F2 | Area F3 | Area F4 | Area F5 | Area F7 | Area F8 | Area F1 | #Feature | #Feature F6 | #Feature F2 | #Feature F3 | #Feature F4 | #Feature F5 | #Feature F7 | #Feature F8 | #Feature F1 | Start | End | PTM | AScore | Found By |
| --- | --- | --- | --- | --- | --- | --- | --- | --- | --- | --- | --- | --- | --- | --- | --- | --- | --- | --- | --- | --- | --- | --- | --- | --- | --- | --- | --- | --- | --- | --- | --- | --- | --- |
| K.TTDNQWWTR.S | Y | 61.25 | 1206.5417 | 9 | -0.3 | 604.2780 | 2 | 41.71 | 7 | F7:18569 | 17012022\_RID\_3262\_8b.raw |  |  | 1.3707E6 |  |  |  | 6.1973E6 |  | 3 | 0 | 0 | 1 | 0 | 0 | 0 | 2 | 0 | 124 | 132 |  |  | PEAKS DB |
| R.LQWSDGTELK.Y | Y | 58.58 | 1175.5823 | 10 | 0.3 | 588.7986 | 2 | 40.53 | 7 | F7:17181 | 17012022\_RID\_3262\_8b.raw |  | 5.9765E5 |  |  |  | 2.2921E6 | 2.702E6 | 1.1947E6 | 4 | 0 | 1 | 0 | 0 | 0 | 1 | 1 | 1 | 100 | 109 |  |  | PEAKS DB |
| total 2 peptides |  |  |  |  |  |  |  |  |  |  |  |  |  |  |  |  |  |  |  |  |  |  |  |  |  |  |  |  |  |  |  |  |  |
| --- | --- | --- | --- | --- | --- | --- | --- | --- | --- | --- | --- | --- | --- | --- | --- | --- | --- | --- | --- | --- | --- | --- | --- | --- | --- | --- | --- | --- | --- | --- | --- | --- | --- |

ABS12077.1

back to list

  

| Protein Coverage
| Supporting Peptides
|

Protein Coverage:

Supporting Peptides:

| Peptide | Uniq | -10lgP | Mass | Length | ppm | m/z | z | RT | Fraction | Scan | Source File | Area F6 | Area F2 | Area F3 | Area F4 | Area F5 | Area F7 | Area F8 | Area F1 | #Feature | #Feature F6 | #Feature F2 | #Feature F3 | #Feature F4 | #Feature F5 | #Feature F7 | #Feature F8 | #Feature F1 | Start | End | PTM | AScore | Found By |
| --- | --- | --- | --- | --- | --- | --- | --- | --- | --- | --- | --- | --- | --- | --- | --- | --- | --- | --- | --- | --- | --- | --- | --- | --- | --- | --- | --- | --- | --- | --- | --- | --- | --- |
| K.TTDNQWWTR.S | Y | 61.25 | 1206.5417 | 9 | -0.3 | 604.2780 | 2 | 41.71 | 7 | F7:18569 | 17012022\_RID\_3262\_8b.raw |  |  | 1.3707E6 |  |  |  | 6.1973E6 |  | 3 | 0 | 0 | 1 | 0 | 0 | 0 | 2 | 0 | 124 | 132 |  |  | PEAKS DB |
| R.LQWSDGTELK.Y | Y | 58.58 | 1175.5823 | 10 | 0.3 | 588.7986 | 2 | 40.53 | 7 | F7:17181 | 17012022\_RID\_3262\_8b.raw |  | 5.9765E5 |  |  |  | 2.2921E6 | 2.702E6 | 1.1947E6 | 4 | 0 | 1 | 0 | 0 | 0 | 1 | 1 | 1 | 100 | 109 |  |  | PEAKS DB |
| total 2 peptides |  |  |  |  |  |  |  |  |  |  |  |  |  |  |  |  |  |  |  |  |  |  |  |  |  |  |  |  |  |  |  |  |  |
| --- | --- | --- | --- | --- | --- | --- | --- | --- | --- | --- | --- | --- | --- | --- | --- | --- | --- | --- | --- | --- | --- | --- | --- | --- | --- | --- | --- | --- | --- | --- | --- | --- | --- |

AFJ51037.1

back to list

  

| Protein Coverage
| Supporting Peptides
|

Protein Coverage:

Supporting Peptides:

| Peptide | Uniq | -10lgP | Mass | Length | ppm | m/z | z | RT | Fraction | Scan | Source File | Area F6 | Area F2 | Area F3 | Area F4 | Area F5 | Area F7 | Area F8 | Area F1 | #Feature | #Feature F6 | #Feature F2 | #Feature F3 | #Feature F4 | #Feature F5 | #Feature F7 | #Feature F8 | #Feature F1 | Start | End | PTM | AScore | Found By |
| --- | --- | --- | --- | --- | --- | --- | --- | --- | --- | --- | --- | --- | --- | --- | --- | --- | --- | --- | --- | --- | --- | --- | --- | --- | --- | --- | --- | --- | --- | --- | --- | --- | --- |
| K.LLDEVFFSEK.I | Y | 60.40 | 1225.6230 | 10 | 0.1 | 613.8188 | 2 | 51.78 | 8 | F8:29981 | 31122021\_RID\_3262\_1b.raw |  |  |  |  |  |  |  | 1.7985E5 | 1 | 0 | 0 | 0 | 0 | 0 | 0 | 0 | 1 | 55 | 64 |  |  | PEAKS DB |
| total 1 peptides |  |  |  |  |  |  |  |  |  |  |  |  |  |  |  |  |  |  |  |  |  |  |  |  |  |  |  |  |  |  |  |  |  |
| --- | --- | --- | --- | --- | --- | --- | --- | --- | --- | --- | --- | --- | --- | --- | --- | --- | --- | --- | --- | --- | --- | --- | --- | --- | --- | --- | --- | --- | --- | --- | --- | --- | --- |

JAA96034.1

back to list

  

| Protein Coverage
| Supporting Peptides
|

Protein Coverage:

Supporting Peptides:

| Peptide | Uniq | -10lgP | Mass | Length | ppm | m/z | z | RT | Fraction | Scan | Source File | Area F6 | Area F2 | Area F3 | Area F4 | Area F5 | Area F7 | Area F8 | Area F1 | #Feature | #Feature F6 | #Feature F2 | #Feature F3 | #Feature F4 | #Feature F5 | #Feature F7 | #Feature F8 | #Feature F1 | Start | End | PTM | AScore | Found By |
| --- | --- | --- | --- | --- | --- | --- | --- | --- | --- | --- | --- | --- | --- | --- | --- | --- | --- | --- | --- | --- | --- | --- | --- | --- | --- | --- | --- | --- | --- | --- | --- | --- | --- |
| K.LLDEVFFSEK.I | Y | 60.40 | 1225.6230 | 10 | 0.1 | 613.8188 | 2 | 51.78 | 8 | F8:29981 | 31122021\_RID\_3262\_1b.raw |  |  |  |  |  |  |  | 1.7985E5 | 1 | 0 | 0 | 0 | 0 | 0 | 0 | 0 | 1 | 55 | 64 |  |  | PEAKS DB |
| total 1 peptides |  |  |  |  |  |  |  |  |  |  |  |  |  |  |  |  |  |  |  |  |  |  |  |  |  |  |  |  |  |  |  |  |  |
| --- | --- | --- | --- | --- | --- | --- | --- | --- | --- | --- | --- | --- | --- | --- | --- | --- | --- | --- | --- | --- | --- | --- | --- | --- | --- | --- | --- | --- | --- | --- | --- | --- | --- |

JAV49554.1

back to list

  

| Protein Coverage
| Supporting Peptides
|

Protein Coverage:

Supporting Peptides:

| Peptide | Uniq | -10lgP | Mass | Length | ppm | m/z | z | RT | Fraction | Scan | Source File | Area F6 | Area F2 | Area F3 | Area F4 | Area F5 | Area F7 | Area F8 | Area F1 | #Feature | #Feature F6 | #Feature F2 | #Feature F3 | #Feature F4 | #Feature F5 | #Feature F7 | #Feature F8 | #Feature F1 | Start | End | PTM | AScore | Found By |
| --- | --- | --- | --- | --- | --- | --- | --- | --- | --- | --- | --- | --- | --- | --- | --- | --- | --- | --- | --- | --- | --- | --- | --- | --- | --- | --- | --- | --- | --- | --- | --- | --- | --- |
| K.LLDEVFFSEK.I | Y | 60.40 | 1225.6230 | 10 | 0.1 | 613.8188 | 2 | 51.78 | 8 | F8:29981 | 31122021\_RID\_3262\_1b.raw |  |  |  |  |  |  |  | 1.7985E5 | 1 | 0 | 0 | 0 | 0 | 0 | 0 | 0 | 1 | 55 | 64 |  |  | PEAKS DB |
| total 1 peptides |  |  |  |  |  |  |  |  |  |  |  |  |  |  |  |  |  |  |  |  |  |  |  |  |  |  |  |  |  |  |  |  |  |
| --- | --- | --- | --- | --- | --- | --- | --- | --- | --- | --- | --- | --- | --- | --- | --- | --- | --- | --- | --- | --- | --- | --- | --- | --- | --- | --- | --- | --- | --- | --- | --- | --- | --- |

JAI11929.1

back to list

  

| Protein Coverage
| Supporting Peptides
|

Protein Coverage:

Supporting Peptides:

| Peptide | Uniq | -10lgP | Mass | Length | ppm | m/z | z | RT | Fraction | Scan | Source File | Area F6 | Area F2 | Area F3 | Area F4 | Area F5 | Area F7 | Area F8 | Area F1 | #Feature | #Feature F6 | #Feature F2 | #Feature F3 | #Feature F4 | #Feature F5 | #Feature F7 | #Feature F8 | #Feature F1 | Start | End | PTM | AScore | Found By |
| --- | --- | --- | --- | --- | --- | --- | --- | --- | --- | --- | --- | --- | --- | --- | --- | --- | --- | --- | --- | --- | --- | --- | --- | --- | --- | --- | --- | --- | --- | --- | --- | --- | --- |
| K.LLDEVFFSEK.I | Y | 60.40 | 1225.6230 | 10 | 0.1 | 613.8188 | 2 | 51.78 | 8 | F8:29981 | 31122021\_RID\_3262\_1b.raw |  |  |  |  |  |  |  | 1.7985E5 | 1 | 0 | 0 | 0 | 0 | 0 | 0 | 0 | 1 | 55 | 64 |  |  | PEAKS DB |
| total 1 peptides |  |  |  |  |  |  |  |  |  |  |  |  |  |  |  |  |  |  |  |  |  |  |  |  |  |  |  |  |  |  |  |  |  |
| --- | --- | --- | --- | --- | --- | --- | --- | --- | --- | --- | --- | --- | --- | --- | --- | --- | --- | --- | --- | --- | --- | --- | --- | --- | --- | --- | --- | --- | --- | --- | --- | --- | --- |

XP\_026544503.1

back to list

  

| Protein Coverage
| Supporting Peptides
|

Protein Coverage:

Supporting Peptides:

| Peptide | Uniq | -10lgP | Mass | Length | ppm | m/z | z | RT | Fraction | Scan | Source File | Area F6 | Area F2 | Area F3 | Area F4 | Area F5 | Area F7 | Area F8 | Area F1 | #Feature | #Feature F6 | #Feature F2 | #Feature F3 | #Feature F4 | #Feature F5 | #Feature F7 | #Feature F8 | #Feature F1 | Start | End | PTM | AScore | Found By |
| --- | --- | --- | --- | --- | --- | --- | --- | --- | --- | --- | --- | --- | --- | --- | --- | --- | --- | --- | --- | --- | --- | --- | --- | --- | --- | --- | --- | --- | --- | --- | --- | --- | --- |
| R.AAVPSGASTGIYEALELR.D | Y | 53.50 | 1803.9366 | 18 | 0.3 | 902.9759 | 2 | 51.87 | 8 | F8:30047 | 31122021\_RID\_3262\_1b.raw |  |  |  |  |  |  |  | 2.2092E5 | 1 | 0 | 0 | 0 | 0 | 0 | 0 | 0 | 1 | 33 | 50 |  |  | PEAKS DB |
| R.IGAEVYHNLK.N | Y | 50.54 | 1142.6084 | 10 | 0.2 | 572.3116 | 2 | 24.70 | 8 | F8:8831 | 31122021\_RID\_3262\_1b.raw |  |  |  |  |  |  |  | 4.1709E4 | 1 | 0 | 0 | 0 | 0 | 0 | 0 | 0 | 1 | 184 | 193 |  |  | PEAKS DB |
| total 2 peptides |  |  |  |  |  |  |  |  |  |  |  |  |  |  |  |  |  |  |  |  |  |  |  |  |  |  |  |  |  |  |  |  |  |
| --- | --- | --- | --- | --- | --- | --- | --- | --- | --- | --- | --- | --- | --- | --- | --- | --- | --- | --- | --- | --- | --- | --- | --- | --- | --- | --- | --- | --- | --- | --- | --- | --- | --- |

XP\_032087801.1

back to list

  

| Protein Coverage
| Supporting Peptides
|

Protein Coverage:

Supporting Peptides:

| Peptide | Uniq | -10lgP | Mass | Length | ppm | m/z | z | RT | Fraction | Scan | Source File | Area F6 | Area F2 | Area F3 | Area F4 | Area F5 | Area F7 | Area F8 | Area F1 | #Feature | #Feature F6 | #Feature F2 | #Feature F3 | #Feature F4 | #Feature F5 | #Feature F7 | #Feature F8 | #Feature F1 | Start | End | PTM | AScore | Found By |
| --- | --- | --- | --- | --- | --- | --- | --- | --- | --- | --- | --- | --- | --- | --- | --- | --- | --- | --- | --- | --- | --- | --- | --- | --- | --- | --- | --- | --- | --- | --- | --- | --- | --- |
| R.AAVPSGASTGIYEALELR.D | Y | 53.50 | 1803.9366 | 18 | 0.3 | 902.9759 | 2 | 51.87 | 8 | F8:30047 | 31122021\_RID\_3262\_1b.raw |  |  |  |  |  |  |  | 2.2092E5 | 1 | 0 | 0 | 0 | 0 | 0 | 0 | 0 | 1 | 33 | 50 |  |  | PEAKS DB |
| R.IGAEVYHNLK.N | Y | 50.54 | 1142.6084 | 10 | 0.2 | 572.3116 | 2 | 24.70 | 8 | F8:8831 | 31122021\_RID\_3262\_1b.raw |  |  |  |  |  |  |  | 4.1709E4 | 1 | 0 | 0 | 0 | 0 | 0 | 0 | 0 | 1 | 184 | 193 |  |  | PEAKS DB |
| total 2 peptides |  |  |  |  |  |  |  |  |  |  |  |  |  |  |  |  |  |  |  |  |  |  |  |  |  |  |  |  |  |  |  |  |  |
| --- | --- | --- | --- | --- | --- | --- | --- | --- | --- | --- | --- | --- | --- | --- | --- | --- | --- | --- | --- | --- | --- | --- | --- | --- | --- | --- | --- | --- | --- | --- | --- | --- | --- |

JAG68757.1

back to list

  

| Protein Coverage
| Supporting Peptides
|

Protein Coverage:

Supporting Peptides:

| Peptide | Uniq | -10lgP | Mass | Length | ppm | m/z | z | RT | Fraction | Scan | Source File | Area F6 | Area F2 | Area F3 | Area F4 | Area F5 | Area F7 | Area F8 | Area F1 | #Feature | #Feature F6 | #Feature F2 | #Feature F3 | #Feature F4 | #Feature F5 | #Feature F7 | #Feature F8 | #Feature F1 | Start | End | PTM | AScore | Found By |
| --- | --- | --- | --- | --- | --- | --- | --- | --- | --- | --- | --- | --- | --- | --- | --- | --- | --- | --- | --- | --- | --- | --- | --- | --- | --- | --- | --- | --- | --- | --- | --- | --- | --- |
| R.AAVPSGASTGIYEALELR.D | Y | 53.50 | 1803.9366 | 18 | 0.3 | 902.9759 | 2 | 51.87 | 8 | F8:30047 | 31122021\_RID\_3262\_1b.raw |  |  |  |  |  |  |  | 2.2092E5 | 1 | 0 | 0 | 0 | 0 | 0 | 0 | 0 | 1 | 33 | 50 |  |  | PEAKS DB |
| R.IGAEVYHNLK.N | Y | 50.54 | 1142.6084 | 10 | 0.2 | 572.3116 | 2 | 24.70 | 8 | F8:8831 | 31122021\_RID\_3262\_1b.raw |  |  |  |  |  |  |  | 4.1709E4 | 1 | 0 | 0 | 0 | 0 | 0 | 0 | 0 | 1 | 184 | 193 |  |  | PEAKS DB |
| total 2 peptides |  |  |  |  |  |  |  |  |  |  |  |  |  |  |  |  |  |  |  |  |  |  |  |  |  |  |  |  |  |  |  |  |  |
| --- | --- | --- | --- | --- | --- | --- | --- | --- | --- | --- | --- | --- | --- | --- | --- | --- | --- | --- | --- | --- | --- | --- | --- | --- | --- | --- | --- | --- | --- | --- | --- | --- | --- |

XP\_034282306.1

back to list

  

| Protein Coverage
| Supporting Peptides
|

Protein Coverage:

Supporting Peptides:

| Peptide | Uniq | -10lgP | Mass | Length | ppm | m/z | z | RT | Fraction | Scan | Source File | Area F6 | Area F2 | Area F3 | Area F4 | Area F5 | Area F7 | Area F8 | Area F1 | #Feature | #Feature F6 | #Feature F2 | #Feature F3 | #Feature F4 | #Feature F5 | #Feature F7 | #Feature F8 | #Feature F1 | Start | End | PTM | AScore | Found By |
| --- | --- | --- | --- | --- | --- | --- | --- | --- | --- | --- | --- | --- | --- | --- | --- | --- | --- | --- | --- | --- | --- | --- | --- | --- | --- | --- | --- | --- | --- | --- | --- | --- | --- |
| R.AAVPSGASTGIYEALELR.D | Y | 53.50 | 1803.9366 | 18 | 0.3 | 902.9759 | 2 | 51.87 | 8 | F8:30047 | 31122021\_RID\_3262\_1b.raw |  |  |  |  |  |  |  | 2.2092E5 | 1 | 0 | 0 | 0 | 0 | 0 | 0 | 0 | 1 | 33 | 50 |  |  | PEAKS DB |
| R.IGAEVYHNLK.N | Y | 50.54 | 1142.6084 | 10 | 0.2 | 572.3116 | 2 | 24.70 | 8 | F8:8831 | 31122021\_RID\_3262\_1b.raw |  |  |  |  |  |  |  | 4.1709E4 | 1 | 0 | 0 | 0 | 0 | 0 | 0 | 0 | 1 | 184 | 193 |  |  | PEAKS DB |
| total 2 peptides |  |  |  |  |  |  |  |  |  |  |  |  |  |  |  |  |  |  |  |  |  |  |  |  |  |  |  |  |  |  |  |  |  |
| --- | --- | --- | --- | --- | --- | --- | --- | --- | --- | --- | --- | --- | --- | --- | --- | --- | --- | --- | --- | --- | --- | --- | --- | --- | --- | --- | --- | --- | --- | --- | --- | --- | --- |

XP\_034282305.1

back to list

  

| Protein Coverage
| Supporting Peptides
|

Protein Coverage:

Supporting Peptides:

| Peptide | Uniq | -10lgP | Mass | Length | ppm | m/z | z | RT | Fraction | Scan | Source File | Area F6 | Area F2 | Area F3 | Area F4 | Area F5 | Area F7 | Area F8 | Area F1 | #Feature | #Feature F6 | #Feature F2 | #Feature F3 | #Feature F4 | #Feature F5 | #Feature F7 | #Feature F8 | #Feature F1 | Start | End | PTM | AScore | Found By |
| --- | --- | --- | --- | --- | --- | --- | --- | --- | --- | --- | --- | --- | --- | --- | --- | --- | --- | --- | --- | --- | --- | --- | --- | --- | --- | --- | --- | --- | --- | --- | --- | --- | --- |
| R.AAVPSGASTGIYEALELR.D | Y | 53.50 | 1803.9366 | 18 | 0.3 | 902.9759 | 2 | 51.87 | 8 | F8:30047 | 31122021\_RID\_3262\_1b.raw |  |  |  |  |  |  |  | 2.2092E5 | 1 | 0 | 0 | 0 | 0 | 0 | 0 | 0 | 1 | 33 | 50 |  |  | PEAKS DB |
| R.IGAEVYHNLK.N | Y | 50.54 | 1142.6084 | 10 | 0.2 | 572.3116 | 2 | 24.70 | 8 | F8:8831 | 31122021\_RID\_3262\_1b.raw |  |  |  |  |  |  |  | 4.1709E4 | 1 | 0 | 0 | 0 | 0 | 0 | 0 | 0 | 1 | 184 | 193 |  |  | PEAKS DB |
| total 2 peptides |  |  |  |  |  |  |  |  |  |  |  |  |  |  |  |  |  |  |  |  |  |  |  |  |  |  |  |  |  |  |  |  |  |
| --- | --- | --- | --- | --- | --- | --- | --- | --- | --- | --- | --- | --- | --- | --- | --- | --- | --- | --- | --- | --- | --- | --- | --- | --- | --- | --- | --- | --- | --- | --- | --- | --- | --- |

XP\_034282304.1

back to list

  

| Protein Coverage
| Supporting Peptides
|

Protein Coverage:

Supporting Peptides:

| Peptide | Uniq | -10lgP | Mass | Length | ppm | m/z | z | RT | Fraction | Scan | Source File | Area F6 | Area F2 | Area F3 | Area F4 | Area F5 | Area F7 | Area F8 | Area F1 | #Feature | #Feature F6 | #Feature F2 | #Feature F3 | #Feature F4 | #Feature F5 | #Feature F7 | #Feature F8 | #Feature F1 | Start | End | PTM | AScore | Found By |
| --- | --- | --- | --- | --- | --- | --- | --- | --- | --- | --- | --- | --- | --- | --- | --- | --- | --- | --- | --- | --- | --- | --- | --- | --- | --- | --- | --- | --- | --- | --- | --- | --- | --- |
| R.AAVPSGASTGIYEALELR.D | Y | 53.50 | 1803.9366 | 18 | 0.3 | 902.9759 | 2 | 51.87 | 8 | F8:30047 | 31122021\_RID\_3262\_1b.raw |  |  |  |  |  |  |  | 2.2092E5 | 1 | 0 | 0 | 0 | 0 | 0 | 0 | 0 | 1 | 33 | 50 |  |  | PEAKS DB |
| R.IGAEVYHNLK.N | Y | 50.54 | 1142.6084 | 10 | 0.2 | 572.3116 | 2 | 24.70 | 8 | F8:8831 | 31122021\_RID\_3262\_1b.raw |  |  |  |  |  |  |  | 4.1709E4 | 1 | 0 | 0 | 0 | 0 | 0 | 0 | 0 | 1 | 184 | 193 |  |  | PEAKS DB |
| total 2 peptides |  |  |  |  |  |  |  |  |  |  |  |  |  |  |  |  |  |  |  |  |  |  |  |  |  |  |  |  |  |  |  |  |  |
| --- | --- | --- | --- | --- | --- | --- | --- | --- | --- | --- | --- | --- | --- | --- | --- | --- | --- | --- | --- | --- | --- | --- | --- | --- | --- | --- | --- | --- | --- | --- | --- | --- | --- |

JAC96284.1

back to list

  

| Protein Coverage
| Supporting Peptides
|

Protein Coverage:

Supporting Peptides:

| Peptide | Uniq | -10lgP | Mass | Length | ppm | m/z | z | RT | Fraction | Scan | Source File | Area F6 | Area F2 | Area F3 | Area F4 | Area F5 | Area F7 | Area F8 | Area F1 | #Feature | #Feature F6 | #Feature F2 | #Feature F3 | #Feature F4 | #Feature F5 | #Feature F7 | #Feature F8 | #Feature F1 | Start | End | PTM | AScore | Found By |
| --- | --- | --- | --- | --- | --- | --- | --- | --- | --- | --- | --- | --- | --- | --- | --- | --- | --- | --- | --- | --- | --- | --- | --- | --- | --- | --- | --- | --- | --- | --- | --- | --- | --- |
| R.AAVPSGASTGIYEALELR.D | Y | 53.50 | 1803.9366 | 18 | 0.3 | 902.9759 | 2 | 51.87 | 8 | F8:30047 | 31122021\_RID\_3262\_1b.raw |  |  |  |  |  |  |  | 2.2092E5 | 1 | 0 | 0 | 0 | 0 | 0 | 0 | 0 | 1 | 33 | 50 |  |  | PEAKS DB |
| R.IGAEVYHNLK.N | Y | 50.54 | 1142.6084 | 10 | 0.2 | 572.3116 | 2 | 24.70 | 8 | F8:8831 | 31122021\_RID\_3262\_1b.raw |  |  |  |  |  |  |  | 4.1709E4 | 1 | 0 | 0 | 0 | 0 | 0 | 0 | 0 | 1 | 184 | 193 |  |  | PEAKS DB |
| total 2 peptides |  |  |  |  |  |  |  |  |  |  |  |  |  |  |  |  |  |  |  |  |  |  |  |  |  |  |  |  |  |  |  |  |  |
| --- | --- | --- | --- | --- | --- | --- | --- | --- | --- | --- | --- | --- | --- | --- | --- | --- | --- | --- | --- | --- | --- | --- | --- | --- | --- | --- | --- | --- | --- | --- | --- | --- | --- |

XP\_015687424.1

back to list

  

| Protein Coverage
| Supporting Peptides
|

Protein Coverage:

Supporting Peptides:

| Peptide | Uniq | -10lgP | Mass | Length | ppm | m/z | z | RT | Fraction | Scan | Source File | Area F6 | Area F2 | Area F3 | Area F4 | Area F5 | Area F7 | Area F8 | Area F1 | #Feature | #Feature F6 | #Feature F2 | #Feature F3 | #Feature F4 | #Feature F5 | #Feature F7 | #Feature F8 | #Feature F1 | Start | End | PTM | AScore | Found By |
| --- | --- | --- | --- | --- | --- | --- | --- | --- | --- | --- | --- | --- | --- | --- | --- | --- | --- | --- | --- | --- | --- | --- | --- | --- | --- | --- | --- | --- | --- | --- | --- | --- | --- |
| R.VLASTQFEATAAR.M | Y | 68.35 | 1363.7096 | 13 | 0.1 | 682.8621 | 2 | 34.02 | 3 | F3:18312 | 13012022\_RID\_3262\_3b.raw |  |  | 5.7064E5 |  |  |  |  |  | 1 | 0 | 0 | 1 | 0 | 0 | 0 | 0 | 0 | 161 | 173 |  |  | PEAKS DB |
| total 1 peptides |  |  |  |  |  |  |  |  |  |  |  |  |  |  |  |  |  |  |  |  |  |  |  |  |  |  |  |  |  |  |  |  |  |
| --- | --- | --- | --- | --- | --- | --- | --- | --- | --- | --- | --- | --- | --- | --- | --- | --- | --- | --- | --- | --- | --- | --- | --- | --- | --- | --- | --- | --- | --- | --- | --- | --- | --- |

JAI13418.1

back to list

  

| Protein Coverage
| Supporting Peptides
|

Protein Coverage:

Supporting Peptides:

| Peptide | Uniq | -10lgP | Mass | Length | ppm | m/z | z | RT | Fraction | Scan | Source File | Area F6 | Area F2 | Area F3 | Area F4 | Area F5 | Area F7 | Area F8 | Area F1 | #Feature | #Feature F6 | #Feature F2 | #Feature F3 | #Feature F4 | #Feature F5 | #Feature F7 | #Feature F8 | #Feature F1 | Start | End | PTM | AScore | Found By |
| --- | --- | --- | --- | --- | --- | --- | --- | --- | --- | --- | --- | --- | --- | --- | --- | --- | --- | --- | --- | --- | --- | --- | --- | --- | --- | --- | --- | --- | --- | --- | --- | --- | --- |
| R.VLASTQFEATAAR.M | Y | 68.35 | 1363.7096 | 13 | 0.1 | 682.8621 | 2 | 34.02 | 3 | F3:18312 | 13012022\_RID\_3262\_3b.raw |  |  | 5.7064E5 |  |  |  |  |  | 1 | 0 | 0 | 1 | 0 | 0 | 0 | 0 | 0 | 169 | 181 |  |  | PEAKS DB |
| total 1 peptides |  |  |  |  |  |  |  |  |  |  |  |  |  |  |  |  |  |  |  |  |  |  |  |  |  |  |  |  |  |  |  |  |  |
| --- | --- | --- | --- | --- | --- | --- | --- | --- | --- | --- | --- | --- | --- | --- | --- | --- | --- | --- | --- | --- | --- | --- | --- | --- | --- | --- | --- | --- | --- | --- | --- | --- | --- |

LAB29750.1

back to list

  

| Protein Coverage
| Supporting Peptides
|

Protein Coverage:

Supporting Peptides:

| Peptide | Uniq | -10lgP | Mass | Length | ppm | m/z | z | RT | Fraction | Scan | Source File | Area F6 | Area F2 | Area F3 | Area F4 | Area F5 | Area F7 | Area F8 | Area F1 | #Feature | #Feature F6 | #Feature F2 | #Feature F3 | #Feature F4 | #Feature F5 | #Feature F7 | #Feature F8 | #Feature F1 | Start | End | PTM | AScore | Found By |
| --- | --- | --- | --- | --- | --- | --- | --- | --- | --- | --- | --- | --- | --- | --- | --- | --- | --- | --- | --- | --- | --- | --- | --- | --- | --- | --- | --- | --- | --- | --- | --- | --- | --- |
| K.GYDVIAQAQSGTGK.T | Y | 75.27 | 1393.6837 | 14 | 0.9 | 697.8497 | 2 | 32.05 | 8 | F8:14545 | 31122021\_RID\_3262\_1b.raw |  |  |  |  |  |  |  | 1.5589E4 | 1 | 0 | 0 | 0 | 0 | 0 | 0 | 0 | 1 | 52 | 65 |  |  | PEAKS DB |
| R.VLITTDLLAR.G | Y | 52.62 | 1113.6758 | 10 | 0.4 | 557.8454 | 2 | 48.17 | 8 | F8:27172 | 31122021\_RID\_3262\_1b.raw |  |  |  |  |  |  |  | 1.1529E5 | 1 | 0 | 0 | 0 | 0 | 0 | 0 | 0 | 1 | 308 | 317 |  |  | PEAKS DB |
| total 2 peptides |  |  |  |  |  |  |  |  |  |  |  |  |  |  |  |  |  |  |  |  |  |  |  |  |  |  |  |  |  |  |  |  |  |
| --- | --- | --- | --- | --- | --- | --- | --- | --- | --- | --- | --- | --- | --- | --- | --- | --- | --- | --- | --- | --- | --- | --- | --- | --- | --- | --- | --- | --- | --- | --- | --- | --- | --- |

LAB29755.1

back to list

  

| Protein Coverage
| Supporting Peptides
|

Protein Coverage:

Supporting Peptides:

| Peptide | Uniq | -10lgP | Mass | Length | ppm | m/z | z | RT | Fraction | Scan | Source File | Area F6 | Area F2 | Area F3 | Area F4 | Area F5 | Area F7 | Area F8 | Area F1 | #Feature | #Feature F6 | #Feature F2 | #Feature F3 | #Feature F4 | #Feature F5 | #Feature F7 | #Feature F8 | #Feature F1 | Start | End | PTM | AScore | Found By |
| --- | --- | --- | --- | --- | --- | --- | --- | --- | --- | --- | --- | --- | --- | --- | --- | --- | --- | --- | --- | --- | --- | --- | --- | --- | --- | --- | --- | --- | --- | --- | --- | --- | --- |
| K.GYDVIAQAQSGTGK.T | Y | 75.27 | 1393.6837 | 14 | 0.9 | 697.8497 | 2 | 32.05 | 8 | F8:14545 | 31122021\_RID\_3262\_1b.raw |  |  |  |  |  |  |  | 1.5589E4 | 1 | 0 | 0 | 0 | 0 | 0 | 0 | 0 | 1 | 52 | 65 |  |  | PEAKS DB |
| R.VLITTDLLAR.G | Y | 52.62 | 1113.6758 | 10 | 0.4 | 557.8454 | 2 | 48.17 | 8 | F8:27172 | 31122021\_RID\_3262\_1b.raw |  |  |  |  |  |  |  | 1.1529E5 | 1 | 0 | 0 | 0 | 0 | 0 | 0 | 0 | 1 | 308 | 317 |  |  | PEAKS DB |
| total 2 peptides |  |  |  |  |  |  |  |  |  |  |  |  |  |  |  |  |  |  |  |  |  |  |  |  |  |  |  |  |  |  |  |  |  |
| --- | --- | --- | --- | --- | --- | --- | --- | --- | --- | --- | --- | --- | --- | --- | --- | --- | --- | --- | --- | --- | --- | --- | --- | --- | --- | --- | --- | --- | --- | --- | --- | --- | --- |

XP\_025032359.1

back to list

  

| Protein Coverage
| Supporting Peptides
|

Protein Coverage:

Supporting Peptides:

| Peptide | Uniq | -10lgP | Mass | Length | ppm | m/z | z | RT | Fraction | Scan | Source File | Area F6 | Area F2 | Area F3 | Area F4 | Area F5 | Area F7 | Area F8 | Area F1 | #Feature | #Feature F6 | #Feature F2 | #Feature F3 | #Feature F4 | #Feature F5 | #Feature F7 | #Feature F8 | #Feature F1 | Start | End | PTM | AScore | Found By |
| --- | --- | --- | --- | --- | --- | --- | --- | --- | --- | --- | --- | --- | --- | --- | --- | --- | --- | --- | --- | --- | --- | --- | --- | --- | --- | --- | --- | --- | --- | --- | --- | --- | --- |
| K.GYDVIAQAQSGTGK.T | Y | 75.27 | 1393.6837 | 14 | 0.9 | 697.8497 | 2 | 32.05 | 8 | F8:14545 | 31122021\_RID\_3262\_1b.raw |  |  |  |  |  |  |  | 1.5589E4 | 1 | 0 | 0 | 0 | 0 | 0 | 0 | 0 | 1 | 33 | 46 |  |  | PEAKS DB |
| R.VLITTDLLAR.G | Y | 52.62 | 1113.6758 | 10 | 0.4 | 557.8454 | 2 | 48.17 | 8 | F8:27172 | 31122021\_RID\_3262\_1b.raw |  |  |  |  |  |  |  | 1.1529E5 | 1 | 0 | 0 | 0 | 0 | 0 | 0 | 0 | 1 | 289 | 298 |  |  | PEAKS DB |
| total 2 peptides |  |  |  |  |  |  |  |  |  |  |  |  |  |  |  |  |  |  |  |  |  |  |  |  |  |  |  |  |  |  |  |  |  |
| --- | --- | --- | --- | --- | --- | --- | --- | --- | --- | --- | --- | --- | --- | --- | --- | --- | --- | --- | --- | --- | --- | --- | --- | --- | --- | --- | --- | --- | --- | --- | --- | --- | --- |

JAB54310.1

back to list

  

| Protein Coverage
| Supporting Peptides
|

Protein Coverage:

Supporting Peptides:

| Peptide | Uniq | -10lgP | Mass | Length | ppm | m/z | z | RT | Fraction | Scan | Source File | Area F6 | Area F2 | Area F3 | Area F4 | Area F5 | Area F7 | Area F8 | Area F1 | #Feature | #Feature F6 | #Feature F2 | #Feature F3 | #Feature F4 | #Feature F5 | #Feature F7 | #Feature F8 | #Feature F1 | Start | End | PTM | AScore | Found By |
| --- | --- | --- | --- | --- | --- | --- | --- | --- | --- | --- | --- | --- | --- | --- | --- | --- | --- | --- | --- | --- | --- | --- | --- | --- | --- | --- | --- | --- | --- | --- | --- | --- | --- |
| K.GYDVIAQAQSGTGK.T | Y | 75.27 | 1393.6837 | 14 | 0.9 | 697.8497 | 2 | 32.05 | 8 | F8:14545 | 31122021\_RID\_3262\_1b.raw |  |  |  |  |  |  |  | 1.5589E4 | 1 | 0 | 0 | 0 | 0 | 0 | 0 | 0 | 1 | 69 | 82 |  |  | PEAKS DB |
| R.VLITTDLLAR.G | Y | 52.62 | 1113.6758 | 10 | 0.4 | 557.8454 | 2 | 48.17 | 8 | F8:27172 | 31122021\_RID\_3262\_1b.raw |  |  |  |  |  |  |  | 1.1529E5 | 1 | 0 | 0 | 0 | 0 | 0 | 0 | 0 | 1 | 325 | 334 |  |  | PEAKS DB |
| total 2 peptides |  |  |  |  |  |  |  |  |  |  |  |  |  |  |  |  |  |  |  |  |  |  |  |  |  |  |  |  |  |  |  |  |  |
| --- | --- | --- | --- | --- | --- | --- | --- | --- | --- | --- | --- | --- | --- | --- | --- | --- | --- | --- | --- | --- | --- | --- | --- | --- | --- | --- | --- | --- | --- | --- | --- | --- | --- |

JAG66237.1

back to list

  

| Protein Coverage
| Supporting Peptides
|

Protein Coverage:

Supporting Peptides:

| Peptide | Uniq | -10lgP | Mass | Length | ppm | m/z | z | RT | Fraction | Scan | Source File | Area F6 | Area F2 | Area F3 | Area F4 | Area F5 | Area F7 | Area F8 | Area F1 | #Feature | #Feature F6 | #Feature F2 | #Feature F3 | #Feature F4 | #Feature F5 | #Feature F7 | #Feature F8 | #Feature F1 | Start | End | PTM | AScore | Found By |
| --- | --- | --- | --- | --- | --- | --- | --- | --- | --- | --- | --- | --- | --- | --- | --- | --- | --- | --- | --- | --- | --- | --- | --- | --- | --- | --- | --- | --- | --- | --- | --- | --- | --- |
| K.GYDVIAQAQSGTGK.T | Y | 75.27 | 1393.6837 | 14 | 0.9 | 697.8497 | 2 | 32.05 | 8 | F8:14545 | 31122021\_RID\_3262\_1b.raw |  |  |  |  |  |  |  | 1.5589E4 | 1 | 0 | 0 | 0 | 0 | 0 | 0 | 0 | 1 | 69 | 82 |  |  | PEAKS DB |
| R.VLITTDLLAR.G | Y | 52.62 | 1113.6758 | 10 | 0.4 | 557.8454 | 2 | 48.17 | 8 | F8:27172 | 31122021\_RID\_3262\_1b.raw |  |  |  |  |  |  |  | 1.1529E5 | 1 | 0 | 0 | 0 | 0 | 0 | 0 | 0 | 1 | 325 | 334 |  |  | PEAKS DB |
| total 2 peptides |  |  |  |  |  |  |  |  |  |  |  |  |  |  |  |  |  |  |  |  |  |  |  |  |  |  |  |  |  |  |  |  |  |
| --- | --- | --- | --- | --- | --- | --- | --- | --- | --- | --- | --- | --- | --- | --- | --- | --- | --- | --- | --- | --- | --- | --- | --- | --- | --- | --- | --- | --- | --- | --- | --- | --- | --- |

XP\_034263139.1

back to list

  

| Protein Coverage
| Supporting Peptides
|

Protein Coverage:

Supporting Peptides:

| Peptide | Uniq | -10lgP | Mass | Length | ppm | m/z | z | RT | Fraction | Scan | Source File | Area F6 | Area F2 | Area F3 | Area F4 | Area F5 | Area F7 | Area F8 | Area F1 | #Feature | #Feature F6 | #Feature F2 | #Feature F3 | #Feature F4 | #Feature F5 | #Feature F7 | #Feature F8 | #Feature F1 | Start | End | PTM | AScore | Found By |
| --- | --- | --- | --- | --- | --- | --- | --- | --- | --- | --- | --- | --- | --- | --- | --- | --- | --- | --- | --- | --- | --- | --- | --- | --- | --- | --- | --- | --- | --- | --- | --- | --- | --- |
| K.GYDVIAQAQSGTGK.T | Y | 75.27 | 1393.6837 | 14 | 0.9 | 697.8497 | 2 | 32.05 | 8 | F8:14545 | 31122021\_RID\_3262\_1b.raw |  |  |  |  |  |  |  | 1.5589E4 | 1 | 0 | 0 | 0 | 0 | 0 | 0 | 0 | 1 | 69 | 82 |  |  | PEAKS DB |
| R.VLITTDLLAR.G | Y | 52.62 | 1113.6758 | 10 | 0.4 | 557.8454 | 2 | 48.17 | 8 | F8:27172 | 31122021\_RID\_3262\_1b.raw |  |  |  |  |  |  |  | 1.1529E5 | 1 | 0 | 0 | 0 | 0 | 0 | 0 | 0 | 1 | 325 | 334 |  |  | PEAKS DB |
| total 2 peptides |  |  |  |  |  |  |  |  |  |  |  |  |  |  |  |  |  |  |  |  |  |  |  |  |  |  |  |  |  |  |  |  |  |
| --- | --- | --- | --- | --- | --- | --- | --- | --- | --- | --- | --- | --- | --- | --- | --- | --- | --- | --- | --- | --- | --- | --- | --- | --- | --- | --- | --- | --- | --- | --- | --- | --- | --- |

JAG46857.1

back to list

  

| Protein Coverage
| Supporting Peptides
|

Protein Coverage:

Supporting Peptides:

| Peptide | Uniq | -10lgP | Mass | Length | ppm | m/z | z | RT | Fraction | Scan | Source File | Area F6 | Area F2 | Area F3 | Area F4 | Area F5 | Area F7 | Area F8 | Area F1 | #Feature | #Feature F6 | #Feature F2 | #Feature F3 | #Feature F4 | #Feature F5 | #Feature F7 | #Feature F8 | #Feature F1 | Start | End | PTM | AScore | Found By |
| --- | --- | --- | --- | --- | --- | --- | --- | --- | --- | --- | --- | --- | --- | --- | --- | --- | --- | --- | --- | --- | --- | --- | --- | --- | --- | --- | --- | --- | --- | --- | --- | --- | --- |
| K.GYDVIAQAQSGTGK.T | Y | 75.27 | 1393.6837 | 14 | 0.9 | 697.8497 | 2 | 32.05 | 8 | F8:14545 | 31122021\_RID\_3262\_1b.raw |  |  |  |  |  |  |  | 1.5589E4 | 1 | 0 | 0 | 0 | 0 | 0 | 0 | 0 | 1 | 69 | 82 |  |  | PEAKS DB |
| R.VLITTDLLAR.G | Y | 52.62 | 1113.6758 | 10 | 0.4 | 557.8454 | 2 | 48.17 | 8 | F8:27172 | 31122021\_RID\_3262\_1b.raw |  |  |  |  |  |  |  | 1.1529E5 | 1 | 0 | 0 | 0 | 0 | 0 | 0 | 0 | 1 | 325 | 334 |  |  | PEAKS DB |
| total 2 peptides |  |  |  |  |  |  |  |  |  |  |  |  |  |  |  |  |  |  |  |  |  |  |  |  |  |  |  |  |  |  |  |  |  |
| --- | --- | --- | --- | --- | --- | --- | --- | --- | --- | --- | --- | --- | --- | --- | --- | --- | --- | --- | --- | --- | --- | --- | --- | --- | --- | --- | --- | --- | --- | --- | --- | --- | --- |

XP\_015677966.1

back to list

  

| Protein Coverage
| Supporting Peptides
|

Protein Coverage:

Supporting Peptides:

| Peptide | Uniq | -10lgP | Mass | Length | ppm | m/z | z | RT | Fraction | Scan | Source File | Area F6 | Area F2 | Area F3 | Area F4 | Area F5 | Area F7 | Area F8 | Area F1 | #Feature | #Feature F6 | #Feature F2 | #Feature F3 | #Feature F4 | #Feature F5 | #Feature F7 | #Feature F8 | #Feature F1 | Start | End | PTM | AScore | Found By |
| --- | --- | --- | --- | --- | --- | --- | --- | --- | --- | --- | --- | --- | --- | --- | --- | --- | --- | --- | --- | --- | --- | --- | --- | --- | --- | --- | --- | --- | --- | --- | --- | --- | --- |
| K.GYDVIAQAQSGTGK.T | Y | 75.27 | 1393.6837 | 14 | 0.9 | 697.8497 | 2 | 32.05 | 8 | F8:14545 | 31122021\_RID\_3262\_1b.raw |  |  |  |  |  |  |  | 1.5589E4 | 1 | 0 | 0 | 0 | 0 | 0 | 0 | 0 | 1 | 69 | 82 |  |  | PEAKS DB |
| R.VLITTDLLAR.G | Y | 52.62 | 1113.6758 | 10 | 0.4 | 557.8454 | 2 | 48.17 | 8 | F8:27172 | 31122021\_RID\_3262\_1b.raw |  |  |  |  |  |  |  | 1.1529E5 | 1 | 0 | 0 | 0 | 0 | 0 | 0 | 0 | 1 | 325 | 334 |  |  | PEAKS DB |
| total 2 peptides |  |  |  |  |  |  |  |  |  |  |  |  |  |  |  |  |  |  |  |  |  |  |  |  |  |  |  |  |  |  |  |  |  |
| --- | --- | --- | --- | --- | --- | --- | --- | --- | --- | --- | --- | --- | --- | --- | --- | --- | --- | --- | --- | --- | --- | --- | --- | --- | --- | --- | --- | --- | --- | --- | --- | --- | --- |

XP\_032081049.1

back to list

  

| Protein Coverage
| Supporting Peptides
|

Protein Coverage:

Supporting Peptides:

| Peptide | Uniq | -10lgP | Mass | Length | ppm | m/z | z | RT | Fraction | Scan | Source File | Area F6 | Area F2 | Area F3 | Area F4 | Area F5 | Area F7 | Area F8 | Area F1 | #Feature | #Feature F6 | #Feature F2 | #Feature F3 | #Feature F4 | #Feature F5 | #Feature F7 | #Feature F8 | #Feature F1 | Start | End | PTM | AScore | Found By |
| --- | --- | --- | --- | --- | --- | --- | --- | --- | --- | --- | --- | --- | --- | --- | --- | --- | --- | --- | --- | --- | --- | --- | --- | --- | --- | --- | --- | --- | --- | --- | --- | --- | --- |
| K.GYDVIAQAQSGTGK.T | Y | 75.27 | 1393.6837 | 14 | 0.9 | 697.8497 | 2 | 32.05 | 8 | F8:14545 | 31122021\_RID\_3262\_1b.raw |  |  |  |  |  |  |  | 1.5589E4 | 1 | 0 | 0 | 0 | 0 | 0 | 0 | 0 | 1 | 69 | 82 |  |  | PEAKS DB |
| R.VLITTDLLAR.G | Y | 52.62 | 1113.6758 | 10 | 0.4 | 557.8454 | 2 | 48.17 | 8 | F8:27172 | 31122021\_RID\_3262\_1b.raw |  |  |  |  |  |  |  | 1.1529E5 | 1 | 0 | 0 | 0 | 0 | 0 | 0 | 0 | 1 | 325 | 334 |  |  | PEAKS DB |
| total 2 peptides |  |  |  |  |  |  |  |  |  |  |  |  |  |  |  |  |  |  |  |  |  |  |  |  |  |  |  |  |  |  |  |  |  |
| --- | --- | --- | --- | --- | --- | --- | --- | --- | --- | --- | --- | --- | --- | --- | --- | --- | --- | --- | --- | --- | --- | --- | --- | --- | --- | --- | --- | --- | --- | --- | --- | --- | --- |

XP\_026539620.1

back to list

  

| Protein Coverage
| Supporting Peptides
|

Protein Coverage:

Supporting Peptides:

| Peptide | Uniq | -10lgP | Mass | Length | ppm | m/z | z | RT | Fraction | Scan | Source File | Area F6 | Area F2 | Area F3 | Area F4 | Area F5 | Area F7 | Area F8 | Area F1 | #Feature | #Feature F6 | #Feature F2 | #Feature F3 | #Feature F4 | #Feature F5 | #Feature F7 | #Feature F8 | #Feature F1 | Start | End | PTM | AScore | Found By |
| --- | --- | --- | --- | --- | --- | --- | --- | --- | --- | --- | --- | --- | --- | --- | --- | --- | --- | --- | --- | --- | --- | --- | --- | --- | --- | --- | --- | --- | --- | --- | --- | --- | --- |
| K.GYDVIAQAQSGTGK.T | Y | 75.27 | 1393.6837 | 14 | 0.9 | 697.8497 | 2 | 32.05 | 8 | F8:14545 | 31122021\_RID\_3262\_1b.raw |  |  |  |  |  |  |  | 1.5589E4 | 1 | 0 | 0 | 0 | 0 | 0 | 0 | 0 | 1 | 69 | 82 |  |  | PEAKS DB |
| R.VLITTDLLAR.G | Y | 52.62 | 1113.6758 | 10 | 0.4 | 557.8454 | 2 | 48.17 | 8 | F8:27172 | 31122021\_RID\_3262\_1b.raw |  |  |  |  |  |  |  | 1.1529E5 | 1 | 0 | 0 | 0 | 0 | 0 | 0 | 0 | 1 | 325 | 334 |  |  | PEAKS DB |
| total 2 peptides |  |  |  |  |  |  |  |  |  |  |  |  |  |  |  |  |  |  |  |  |  |  |  |  |  |  |  |  |  |  |  |  |  |
| --- | --- | --- | --- | --- | --- | --- | --- | --- | --- | --- | --- | --- | --- | --- | --- | --- | --- | --- | --- | --- | --- | --- | --- | --- | --- | --- | --- | --- | --- | --- | --- | --- | --- |

XP\_026561483.1

back to list

  

| Protein Coverage
| Supporting Peptides
|

Protein Coverage:

Supporting Peptides:

| Peptide | Uniq | -10lgP | Mass | Length | ppm | m/z | z | RT | Fraction | Scan | Source File | Area F6 | Area F2 | Area F3 | Area F4 | Area F5 | Area F7 | Area F8 | Area F1 | #Feature | #Feature F6 | #Feature F2 | #Feature F3 | #Feature F4 | #Feature F5 | #Feature F7 | #Feature F8 | #Feature F1 | Start | End | PTM | AScore | Found By |
| --- | --- | --- | --- | --- | --- | --- | --- | --- | --- | --- | --- | --- | --- | --- | --- | --- | --- | --- | --- | --- | --- | --- | --- | --- | --- | --- | --- | --- | --- | --- | --- | --- | --- |
| K.GYDVIAQAQSGTGK.T | Y | 75.27 | 1393.6837 | 14 | 0.9 | 697.8497 | 2 | 32.05 | 8 | F8:14545 | 31122021\_RID\_3262\_1b.raw |  |  |  |  |  |  |  | 1.5589E4 | 1 | 0 | 0 | 0 | 0 | 0 | 0 | 0 | 1 | 69 | 82 |  |  | PEAKS DB |
| R.VLITTDLLAR.G | Y | 52.62 | 1113.6758 | 10 | 0.4 | 557.8454 | 2 | 48.17 | 8 | F8:27172 | 31122021\_RID\_3262\_1b.raw |  |  |  |  |  |  |  | 1.1529E5 | 1 | 0 | 0 | 0 | 0 | 0 | 0 | 0 | 1 | 325 | 334 |  |  | PEAKS DB |
| total 2 peptides |  |  |  |  |  |  |  |  |  |  |  |  |  |  |  |  |  |  |  |  |  |  |  |  |  |  |  |  |  |  |  |  |  |
| --- | --- | --- | --- | --- | --- | --- | --- | --- | --- | --- | --- | --- | --- | --- | --- | --- | --- | --- | --- | --- | --- | --- | --- | --- | --- | --- | --- | --- | --- | --- | --- | --- | --- |

XP\_013923864.1

back to list

  

| Protein Coverage
| Supporting Peptides
|

Protein Coverage:

Supporting Peptides:

| Peptide | Uniq | -10lgP | Mass | Length | ppm | m/z | z | RT | Fraction | Scan | Source File | Area F6 | Area F2 | Area F3 | Area F4 | Area F5 | Area F7 | Area F8 | Area F1 | #Feature | #Feature F6 | #Feature F2 | #Feature F3 | #Feature F4 | #Feature F5 | #Feature F7 | #Feature F8 | #Feature F1 | Start | End | PTM | AScore | Found By |
| --- | --- | --- | --- | --- | --- | --- | --- | --- | --- | --- | --- | --- | --- | --- | --- | --- | --- | --- | --- | --- | --- | --- | --- | --- | --- | --- | --- | --- | --- | --- | --- | --- | --- |
| K.GYDVIAQAQSGTGK.T | Y | 75.27 | 1393.6837 | 14 | 0.9 | 697.8497 | 2 | 32.05 | 8 | F8:14545 | 31122021\_RID\_3262\_1b.raw |  |  |  |  |  |  |  | 1.5589E4 | 1 | 0 | 0 | 0 | 0 | 0 | 0 | 0 | 1 | 69 | 82 |  |  | PEAKS DB |
| R.VLITTDLLAR.G | Y | 52.62 | 1113.6758 | 10 | 0.4 | 557.8454 | 2 | 48.17 | 8 | F8:27172 | 31122021\_RID\_3262\_1b.raw |  |  |  |  |  |  |  | 1.1529E5 | 1 | 0 | 0 | 0 | 0 | 0 | 0 | 0 | 1 | 325 | 334 |  |  | PEAKS DB |
| total 2 peptides |  |  |  |  |  |  |  |  |  |  |  |  |  |  |  |  |  |  |  |  |  |  |  |  |  |  |  |  |  |  |  |  |  |
| --- | --- | --- | --- | --- | --- | --- | --- | --- | --- | --- | --- | --- | --- | --- | --- | --- | --- | --- | --- | --- | --- | --- | --- | --- | --- | --- | --- | --- | --- | --- | --- | --- | --- |

XP\_034294778.1

back to list

  

| Protein Coverage
| Supporting Peptides
|

Protein Coverage:

Supporting Peptides:

| Peptide | Uniq | -10lgP | Mass | Length | ppm | m/z | z | RT | Fraction | Scan | Source File | Area F6 | Area F2 | Area F3 | Area F4 | Area F5 | Area F7 | Area F8 | Area F1 | #Feature | #Feature F6 | #Feature F2 | #Feature F3 | #Feature F4 | #Feature F5 | #Feature F7 | #Feature F8 | #Feature F1 | Start | End | PTM | AScore | Found By |
| --- | --- | --- | --- | --- | --- | --- | --- | --- | --- | --- | --- | --- | --- | --- | --- | --- | --- | --- | --- | --- | --- | --- | --- | --- | --- | --- | --- | --- | --- | --- | --- | --- | --- |
| K.GYDVIAQAQSGTGK.T | Y | 75.27 | 1393.6837 | 14 | 0.9 | 697.8497 | 2 | 32.05 | 8 | F8:14545 | 31122021\_RID\_3262\_1b.raw |  |  |  |  |  |  |  | 1.5589E4 | 1 | 0 | 0 | 0 | 0 | 0 | 0 | 0 | 1 | 69 | 82 |  |  | PEAKS DB |
| R.VLITTDLLAR.G | Y | 52.62 | 1113.6758 | 10 | 0.4 | 557.8454 | 2 | 48.17 | 8 | F8:27172 | 31122021\_RID\_3262\_1b.raw |  |  |  |  |  |  |  | 1.1529E5 | 1 | 0 | 0 | 0 | 0 | 0 | 0 | 0 | 1 | 325 | 334 |  |  | PEAKS DB |
| total 2 peptides |  |  |  |  |  |  |  |  |  |  |  |  |  |  |  |  |  |  |  |  |  |  |  |  |  |  |  |  |  |  |  |  |  |
| --- | --- | --- | --- | --- | --- | --- | --- | --- | --- | --- | --- | --- | --- | --- | --- | --- | --- | --- | --- | --- | --- | --- | --- | --- | --- | --- | --- | --- | --- | --- | --- | --- | --- |

XP\_007442965.1

back to list

  

| Protein Coverage
| Supporting Peptides
|

Protein Coverage:

Supporting Peptides:

| Peptide | Uniq | -10lgP | Mass | Length | ppm | m/z | z | RT | Fraction | Scan | Source File | Area F6 | Area F2 | Area F3 | Area F4 | Area F5 | Area F7 | Area F8 | Area F1 | #Feature | #Feature F6 | #Feature F2 | #Feature F3 | #Feature F4 | #Feature F5 | #Feature F7 | #Feature F8 | #Feature F1 | Start | End | PTM | AScore | Found By |
| --- | --- | --- | --- | --- | --- | --- | --- | --- | --- | --- | --- | --- | --- | --- | --- | --- | --- | --- | --- | --- | --- | --- | --- | --- | --- | --- | --- | --- | --- | --- | --- | --- | --- |
| K.GYDVIAQAQSGTGK.T | Y | 75.27 | 1393.6837 | 14 | 0.9 | 697.8497 | 2 | 32.05 | 8 | F8:14545 | 31122021\_RID\_3262\_1b.raw |  |  |  |  |  |  |  | 1.5589E4 | 1 | 0 | 0 | 0 | 0 | 0 | 0 | 0 | 1 | 69 | 82 |  |  | PEAKS DB |
| R.VLITTDLLAR.G | Y | 52.62 | 1113.6758 | 10 | 0.4 | 557.8454 | 2 | 48.17 | 8 | F8:27172 | 31122021\_RID\_3262\_1b.raw |  |  |  |  |  |  |  | 1.1529E5 | 1 | 0 | 0 | 0 | 0 | 0 | 0 | 0 | 1 | 325 | 334 |  |  | PEAKS DB |
| total 2 peptides |  |  |  |  |  |  |  |  |  |  |  |  |  |  |  |  |  |  |  |  |  |  |  |  |  |  |  |  |  |  |  |  |  |
| --- | --- | --- | --- | --- | --- | --- | --- | --- | --- | --- | --- | --- | --- | --- | --- | --- | --- | --- | --- | --- | --- | --- | --- | --- | --- | --- | --- | --- | --- | --- | --- | --- | --- |

JAB54309.1

back to list

  

| Protein Coverage
| Supporting Peptides
|

Protein Coverage:

Supporting Peptides:

| Peptide | Uniq | -10lgP | Mass | Length | ppm | m/z | z | RT | Fraction | Scan | Source File | Area F6 | Area F2 | Area F3 | Area F4 | Area F5 | Area F7 | Area F8 | Area F1 | #Feature | #Feature F6 | #Feature F2 | #Feature F3 | #Feature F4 | #Feature F5 | #Feature F7 | #Feature F8 | #Feature F1 | Start | End | PTM | AScore | Found By |
| --- | --- | --- | --- | --- | --- | --- | --- | --- | --- | --- | --- | --- | --- | --- | --- | --- | --- | --- | --- | --- | --- | --- | --- | --- | --- | --- | --- | --- | --- | --- | --- | --- | --- |
| K.GYDVIAQAQSGTGK.T | Y | 75.27 | 1393.6837 | 14 | 0.9 | 697.8497 | 2 | 32.05 | 8 | F8:14545 | 31122021\_RID\_3262\_1b.raw |  |  |  |  |  |  |  | 1.5589E4 | 1 | 0 | 0 | 0 | 0 | 0 | 0 | 0 | 1 | 69 | 82 |  |  | PEAKS DB |
| R.VLITTDLLAR.G | Y | 52.62 | 1113.6758 | 10 | 0.4 | 557.8454 | 2 | 48.17 | 8 | F8:27172 | 31122021\_RID\_3262\_1b.raw |  |  |  |  |  |  |  | 1.1529E5 | 1 | 0 | 0 | 0 | 0 | 0 | 0 | 0 | 1 | 325 | 334 |  |  | PEAKS DB |
| total 2 peptides |  |  |  |  |  |  |  |  |  |  |  |  |  |  |  |  |  |  |  |  |  |  |  |  |  |  |  |  |  |  |  |  |  |
| --- | --- | --- | --- | --- | --- | --- | --- | --- | --- | --- | --- | --- | --- | --- | --- | --- | --- | --- | --- | --- | --- | --- | --- | --- | --- | --- | --- | --- | --- | --- | --- | --- | --- |

JAI12965.1

back to list

  

| Protein Coverage
| Supporting Peptides
|

Protein Coverage:

Supporting Peptides:

| Peptide | Uniq | -10lgP | Mass | Length | ppm | m/z | z | RT | Fraction | Scan | Source File | Area F6 | Area F2 | Area F3 | Area F4 | Area F5 | Area F7 | Area F8 | Area F1 | #Feature | #Feature F6 | #Feature F2 | #Feature F3 | #Feature F4 | #Feature F5 | #Feature F7 | #Feature F8 | #Feature F1 | Start | End | PTM | AScore | Found By |
| --- | --- | --- | --- | --- | --- | --- | --- | --- | --- | --- | --- | --- | --- | --- | --- | --- | --- | --- | --- | --- | --- | --- | --- | --- | --- | --- | --- | --- | --- | --- | --- | --- | --- |
| K.GYDVIAQAQSGTGK.T | Y | 75.27 | 1393.6837 | 14 | 0.9 | 697.8497 | 2 | 32.05 | 8 | F8:14545 | 31122021\_RID\_3262\_1b.raw |  |  |  |  |  |  |  | 1.5589E4 | 1 | 0 | 0 | 0 | 0 | 0 | 0 | 0 | 1 | 69 | 82 |  |  | PEAKS DB |
| R.VLITTDLLAR.G | Y | 52.62 | 1113.6758 | 10 | 0.4 | 557.8454 | 2 | 48.17 | 8 | F8:27172 | 31122021\_RID\_3262\_1b.raw |  |  |  |  |  |  |  | 1.1529E5 | 1 | 0 | 0 | 0 | 0 | 0 | 0 | 0 | 1 | 325 | 334 |  |  | PEAKS DB |
| total 2 peptides |  |  |  |  |  |  |  |  |  |  |  |  |  |  |  |  |  |  |  |  |  |  |  |  |  |  |  |  |  |  |  |  |  |
| --- | --- | --- | --- | --- | --- | --- | --- | --- | --- | --- | --- | --- | --- | --- | --- | --- | --- | --- | --- | --- | --- | --- | --- | --- | --- | --- | --- | --- | --- | --- | --- | --- | --- |

XP\_015677967.1

back to list

  

| Protein Coverage
| Supporting Peptides
|

Protein Coverage:

Supporting Peptides:

| Peptide | Uniq | -10lgP | Mass | Length | ppm | m/z | z | RT | Fraction | Scan | Source File | Area F6 | Area F2 | Area F3 | Area F4 | Area F5 | Area F7 | Area F8 | Area F1 | #Feature | #Feature F6 | #Feature F2 | #Feature F3 | #Feature F4 | #Feature F5 | #Feature F7 | #Feature F8 | #Feature F1 | Start | End | PTM | AScore | Found By |
| --- | --- | --- | --- | --- | --- | --- | --- | --- | --- | --- | --- | --- | --- | --- | --- | --- | --- | --- | --- | --- | --- | --- | --- | --- | --- | --- | --- | --- | --- | --- | --- | --- | --- |
| K.GYDVIAQAQSGTGK.T | Y | 75.27 | 1393.6837 | 14 | 0.9 | 697.8497 | 2 | 32.05 | 8 | F8:14545 | 31122021\_RID\_3262\_1b.raw |  |  |  |  |  |  |  | 1.5589E4 | 1 | 0 | 0 | 0 | 0 | 0 | 0 | 0 | 1 | 69 | 82 |  |  | PEAKS DB |
| R.VLITTDLLAR.G | Y | 52.62 | 1113.6758 | 10 | 0.4 | 557.8454 | 2 | 48.17 | 8 | F8:27172 | 31122021\_RID\_3262\_1b.raw |  |  |  |  |  |  |  | 1.1529E5 | 1 | 0 | 0 | 0 | 0 | 0 | 0 | 0 | 1 | 325 | 334 |  |  | PEAKS DB |
| total 2 peptides |  |  |  |  |  |  |  |  |  |  |  |  |  |  |  |  |  |  |  |  |  |  |  |  |  |  |  |  |  |  |  |  |  |
| --- | --- | --- | --- | --- | --- | --- | --- | --- | --- | --- | --- | --- | --- | --- | --- | --- | --- | --- | --- | --- | --- | --- | --- | --- | --- | --- | --- | --- | --- | --- | --- | --- | --- |

XP\_032081051.1

back to list

  

| Protein Coverage
| Supporting Peptides
|

Protein Coverage:

Supporting Peptides:

| Peptide | Uniq | -10lgP | Mass | Length | ppm | m/z | z | RT | Fraction | Scan | Source File | Area F6 | Area F2 | Area F3 | Area F4 | Area F5 | Area F7 | Area F8 | Area F1 | #Feature | #Feature F6 | #Feature F2 | #Feature F3 | #Feature F4 | #Feature F5 | #Feature F7 | #Feature F8 | #Feature F1 | Start | End | PTM | AScore | Found By |
| --- | --- | --- | --- | --- | --- | --- | --- | --- | --- | --- | --- | --- | --- | --- | --- | --- | --- | --- | --- | --- | --- | --- | --- | --- | --- | --- | --- | --- | --- | --- | --- | --- | --- |
| K.GYDVIAQAQSGTGK.T | Y | 75.27 | 1393.6837 | 14 | 0.9 | 697.8497 | 2 | 32.05 | 8 | F8:14545 | 31122021\_RID\_3262\_1b.raw |  |  |  |  |  |  |  | 1.5589E4 | 1 | 0 | 0 | 0 | 0 | 0 | 0 | 0 | 1 | 69 | 82 |  |  | PEAKS DB |
| R.VLITTDLLAR.G | Y | 52.62 | 1113.6758 | 10 | 0.4 | 557.8454 | 2 | 48.17 | 8 | F8:27172 | 31122021\_RID\_3262\_1b.raw |  |  |  |  |  |  |  | 1.1529E5 | 1 | 0 | 0 | 0 | 0 | 0 | 0 | 0 | 1 | 325 | 334 |  |  | PEAKS DB |
| total 2 peptides |  |  |  |  |  |  |  |  |  |  |  |  |  |  |  |  |  |  |  |  |  |  |  |  |  |  |  |  |  |  |  |  |  |
| --- | --- | --- | --- | --- | --- | --- | --- | --- | --- | --- | --- | --- | --- | --- | --- | --- | --- | --- | --- | --- | --- | --- | --- | --- | --- | --- | --- | --- | --- | --- | --- | --- | --- |

XP\_013923865.1

back to list

  

| Protein Coverage
| Supporting Peptides
|

Protein Coverage:

Supporting Peptides:

| Peptide | Uniq | -10lgP | Mass | Length | ppm | m/z | z | RT | Fraction | Scan | Source File | Area F6 | Area F2 | Area F3 | Area F4 | Area F5 | Area F7 | Area F8 | Area F1 | #Feature | #Feature F6 | #Feature F2 | #Feature F3 | #Feature F4 | #Feature F5 | #Feature F7 | #Feature F8 | #Feature F1 | Start | End | PTM | AScore | Found By |
| --- | --- | --- | --- | --- | --- | --- | --- | --- | --- | --- | --- | --- | --- | --- | --- | --- | --- | --- | --- | --- | --- | --- | --- | --- | --- | --- | --- | --- | --- | --- | --- | --- | --- |
| K.GYDVIAQAQSGTGK.T | Y | 75.27 | 1393.6837 | 14 | 0.9 | 697.8497 | 2 | 32.05 | 8 | F8:14545 | 31122021\_RID\_3262\_1b.raw |  |  |  |  |  |  |  | 1.5589E4 | 1 | 0 | 0 | 0 | 0 | 0 | 0 | 0 | 1 | 69 | 82 |  |  | PEAKS DB |
| R.VLITTDLLAR.G | Y | 52.62 | 1113.6758 | 10 | 0.4 | 557.8454 | 2 | 48.17 | 8 | F8:27172 | 31122021\_RID\_3262\_1b.raw |  |  |  |  |  |  |  | 1.1529E5 | 1 | 0 | 0 | 0 | 0 | 0 | 0 | 0 | 1 | 325 | 334 |  |  | PEAKS DB |
| total 2 peptides |  |  |  |  |  |  |  |  |  |  |  |  |  |  |  |  |  |  |  |  |  |  |  |  |  |  |  |  |  |  |  |  |  |
| --- | --- | --- | --- | --- | --- | --- | --- | --- | --- | --- | --- | --- | --- | --- | --- | --- | --- | --- | --- | --- | --- | --- | --- | --- | --- | --- | --- | --- | --- | --- | --- | --- | --- |

ETE63526.1

back to list

  

| Protein Coverage
| Supporting Peptides
|

Protein Coverage:

Supporting Peptides:

| Peptide | Uniq | -10lgP | Mass | Length | ppm | m/z | z | RT | Fraction | Scan | Source File | Area F6 | Area F2 | Area F3 | Area F4 | Area F5 | Area F7 | Area F8 | Area F1 | #Feature | #Feature F6 | #Feature F2 | #Feature F3 | #Feature F4 | #Feature F5 | #Feature F7 | #Feature F8 | #Feature F1 | Start | End | PTM | AScore | Found By |
| --- | --- | --- | --- | --- | --- | --- | --- | --- | --- | --- | --- | --- | --- | --- | --- | --- | --- | --- | --- | --- | --- | --- | --- | --- | --- | --- | --- | --- | --- | --- | --- | --- | --- |
| K.GYDVIAQAQSGTGK.T | Y | 75.27 | 1393.6837 | 14 | 0.9 | 697.8497 | 2 | 32.05 | 8 | F8:14545 | 31122021\_RID\_3262\_1b.raw |  |  |  |  |  |  |  | 1.5589E4 | 1 | 0 | 0 | 0 | 0 | 0 | 0 | 0 | 1 | 53 | 66 |  |  | PEAKS DB |
| R.VLITTDLLAR.G | Y | 52.62 | 1113.6758 | 10 | 0.4 | 557.8454 | 2 | 48.17 | 8 | F8:27172 | 31122021\_RID\_3262\_1b.raw |  |  |  |  |  |  |  | 1.1529E5 | 1 | 0 | 0 | 0 | 0 | 0 | 0 | 0 | 1 | 263 | 272 |  |  | PEAKS DB |
| total 2 peptides |  |  |  |  |  |  |  |  |  |  |  |  |  |  |  |  |  |  |  |  |  |  |  |  |  |  |  |  |  |  |  |  |  |
| --- | --- | --- | --- | --- | --- | --- | --- | --- | --- | --- | --- | --- | --- | --- | --- | --- | --- | --- | --- | --- | --- | --- | --- | --- | --- | --- | --- | --- | --- | --- | --- | --- | --- |

JAS04545.1

back to list

  

| Protein Coverage
| Supporting Peptides
|

Protein Coverage:

Supporting Peptides:

| Peptide | Uniq | -10lgP | Mass | Length | ppm | m/z | z | RT | Fraction | Scan | Source File | Area F6 | Area F2 | Area F3 | Area F4 | Area F5 | Area F7 | Area F8 | Area F1 | #Feature | #Feature F6 | #Feature F2 | #Feature F3 | #Feature F4 | #Feature F5 | #Feature F7 | #Feature F8 | #Feature F1 | Start | End | PTM | AScore | Found By |
| --- | --- | --- | --- | --- | --- | --- | --- | --- | --- | --- | --- | --- | --- | --- | --- | --- | --- | --- | --- | --- | --- | --- | --- | --- | --- | --- | --- | --- | --- | --- | --- | --- | --- |
| K.DFSWEWTDR.S | Y | 57.56 | 1240.5149 | 9 | 0.8 | 621.2652 | 2 | 53.56 | 8 | F8:31342 | 31122021\_RID\_3262\_1b.raw |  |  |  |  |  |  |  | 8.3802E5 | 1 | 0 | 0 | 0 | 0 | 0 | 0 | 0 | 1 | 99 | 107 |  |  | PEAKS DB |
| Q.AEVWIGLWDK.K | Y | 48.48 | 1215.6288 | 10 | -0.2 | 608.8215 | 2 | 55.10 | 8 | F8:32713 | 31122021\_RID\_3262\_1b.raw |  |  |  |  |  |  |  | 2.4749E5 | 1 | 0 | 0 | 0 | 0 | 0 | 0 | 0 | 1 | 87 | 96 |  |  | PEAKS DB |
| total 2 peptides |  |  |  |  |  |  |  |  |  |  |  |  |  |  |  |  |  |  |  |  |  |  |  |  |  |  |  |  |  |  |  |  |  |
| --- | --- | --- | --- | --- | --- | --- | --- | --- | --- | --- | --- | --- | --- | --- | --- | --- | --- | --- | --- | --- | --- | --- | --- | --- | --- | --- | --- | --- | --- | --- | --- | --- | --- |

XP\_015675298.1

back to list

  

| Protein Coverage
| Supporting Peptides
|

Protein Coverage:

Supporting Peptides:

| Peptide | Uniq | -10lgP | Mass | Length | ppm | m/z | z | RT | Fraction | Scan | Source File | Area F6 | Area F2 | Area F3 | Area F4 | Area F5 | Area F7 | Area F8 | Area F1 | #Feature | #Feature F6 | #Feature F2 | #Feature F3 | #Feature F4 | #Feature F5 | #Feature F7 | #Feature F8 | #Feature F1 | Start | End | PTM | AScore | Found By |
| --- | --- | --- | --- | --- | --- | --- | --- | --- | --- | --- | --- | --- | --- | --- | --- | --- | --- | --- | --- | --- | --- | --- | --- | --- | --- | --- | --- | --- | --- | --- | --- | --- | --- |
| K.VTASAPDYLAITK.K | Y | 63.68 | 1348.7238 | 13 | 0.8 | 675.3697 | 2 | 41.56 | 4 | F4:23779 | 13012022\_RID\_3262\_4b.raw |  |  |  | 1.7583E6 |  |  |  |  | 1 | 0 | 0 | 0 | 1 | 0 | 0 | 0 | 0 | 425 | 437 |  |  | PEAKS DB |
| total 1 peptides |  |  |  |  |  |  |  |  |  |  |  |  |  |  |  |  |  |  |  |  |  |  |  |  |  |  |  |  |  |  |  |  |  |
| --- | --- | --- | --- | --- | --- | --- | --- | --- | --- | --- | --- | --- | --- | --- | --- | --- | --- | --- | --- | --- | --- | --- | --- | --- | --- | --- | --- | --- | --- | --- | --- | --- | --- |

XP\_015745242.1

back to list

  

| Protein Coverage
| Supporting Peptides
|

Protein Coverage:

Supporting Peptides:

| Peptide | Uniq | -10lgP | Mass | Length | ppm | m/z | z | RT | Fraction | Scan | Source File | Area F6 | Area F2 | Area F3 | Area F4 | Area F5 | Area F7 | Area F8 | Area F1 | #Feature | #Feature F6 | #Feature F2 | #Feature F3 | #Feature F4 | #Feature F5 | #Feature F7 | #Feature F8 | #Feature F1 | Start | End | PTM | AScore | Found By |
| --- | --- | --- | --- | --- | --- | --- | --- | --- | --- | --- | --- | --- | --- | --- | --- | --- | --- | --- | --- | --- | --- | --- | --- | --- | --- | --- | --- | --- | --- | --- | --- | --- | --- |
| K.SGSMSAYEMR.M | Y | 54.95 | 1117.4532 | 10 | 1.1 | 559.7345 | 2 | 25.88 | 8 | F8:9763 | 31122021\_RID\_3262\_1b.raw |  |  |  |  |  |  |  | 1.3303E4 | 1 | 0 | 0 | 0 | 0 | 0 | 0 | 0 | 1 | 632 | 641 |  |  | PEAKS DB |
| total 1 peptides |  |  |  |  |  |  |  |  |  |  |  |  |  |  |  |  |  |  |  |  |  |  |  |  |  |  |  |  |  |  |  |  |  |
| --- | --- | --- | --- | --- | --- | --- | --- | --- | --- | --- | --- | --- | --- | --- | --- | --- | --- | --- | --- | --- | --- | --- | --- | --- | --- | --- | --- | --- | --- | --- | --- | --- | --- |

ETE56723.1

back to list

  

| Protein Coverage
| Supporting Peptides
|

Protein Coverage:

Supporting Peptides:

| Peptide | Uniq | -10lgP | Mass | Length | ppm | m/z | z | RT | Fraction | Scan | Source File | Area F6 | Area F2 | Area F3 | Area F4 | Area F5 | Area F7 | Area F8 | Area F1 | #Feature | #Feature F6 | #Feature F2 | #Feature F3 | #Feature F4 | #Feature F5 | #Feature F7 | #Feature F8 | #Feature F1 | Start | End | PTM | AScore | Found By |
| --- | --- | --- | --- | --- | --- | --- | --- | --- | --- | --- | --- | --- | --- | --- | --- | --- | --- | --- | --- | --- | --- | --- | --- | --- | --- | --- | --- | --- | --- | --- | --- | --- | --- |
| K.SSTFTLTNIVPQFIK.L | Y | 69.04 | 1694.9243 | 15 | 0.6 | 848.4699 | 2 | 62.06 | 5 | F5:30027 | 13012022\_RID\_3262\_5b.raw |  |  |  | 9.9316E5 | 4.294E6 |  |  |  | 2 | 0 | 0 | 0 | 1 | 1 | 0 | 0 | 0 | 51 | 65 |  |  | PEAKS DB |
| total 1 peptides |  |  |  |  |  |  |  |  |  |  |  |  |  |  |  |  |  |  |  |  |  |  |  |  |  |  |  |  |  |  |  |  |  |
| --- | --- | --- | --- | --- | --- | --- | --- | --- | --- | --- | --- | --- | --- | --- | --- | --- | --- | --- | --- | --- | --- | --- | --- | --- | --- | --- | --- | --- | --- | --- | --- | --- | --- |

XP\_032076462.1

back to list

  

| Protein Coverage
| Supporting Peptides
|

Protein Coverage:

Supporting Peptides:

| Peptide | Uniq | -10lgP | Mass | Length | ppm | m/z | z | RT | Fraction | Scan | Source File | Area F6 | Area F2 | Area F3 | Area F4 | Area F5 | Area F7 | Area F8 | Area F1 | #Feature | #Feature F6 | #Feature F2 | #Feature F3 | #Feature F4 | #Feature F5 | #Feature F7 | #Feature F8 | #Feature F1 | Start | End | PTM | AScore | Found By |
| --- | --- | --- | --- | --- | --- | --- | --- | --- | --- | --- | --- | --- | --- | --- | --- | --- | --- | --- | --- | --- | --- | --- | --- | --- | --- | --- | --- | --- | --- | --- | --- | --- | --- |
| K.SSTFTLTNIVPQFIK.L | Y | 69.04 | 1694.9243 | 15 | 0.6 | 848.4699 | 2 | 62.06 | 5 | F5:30027 | 13012022\_RID\_3262\_5b.raw |  |  |  | 9.9316E5 | 4.294E6 |  |  |  | 2 | 0 | 0 | 0 | 1 | 1 | 0 | 0 | 0 | 154 | 168 |  |  | PEAKS DB |
| total 1 peptides |  |  |  |  |  |  |  |  |  |  |  |  |  |  |  |  |  |  |  |  |  |  |  |  |  |  |  |  |  |  |  |  |  |
| --- | --- | --- | --- | --- | --- | --- | --- | --- | --- | --- | --- | --- | --- | --- | --- | --- | --- | --- | --- | --- | --- | --- | --- | --- | --- | --- | --- | --- | --- | --- | --- | --- | --- |

XP\_026546198.1

back to list

  

| Protein Coverage
| Supporting Peptides
|

Protein Coverage:

Supporting Peptides:

| Peptide | Uniq | -10lgP | Mass | Length | ppm | m/z | z | RT | Fraction | Scan | Source File | Area F6 | Area F2 | Area F3 | Area F4 | Area F5 | Area F7 | Area F8 | Area F1 | #Feature | #Feature F6 | #Feature F2 | #Feature F3 | #Feature F4 | #Feature F5 | #Feature F7 | #Feature F8 | #Feature F1 | Start | End | PTM | AScore | Found By |
| --- | --- | --- | --- | --- | --- | --- | --- | --- | --- | --- | --- | --- | --- | --- | --- | --- | --- | --- | --- | --- | --- | --- | --- | --- | --- | --- | --- | --- | --- | --- | --- | --- | --- |
| K.SSTFTLTNIVPQFIK.L | Y | 69.04 | 1694.9243 | 15 | 0.6 | 848.4699 | 2 | 62.06 | 5 | F5:30027 | 13012022\_RID\_3262\_5b.raw |  |  |  | 9.9316E5 | 4.294E6 |  |  |  | 2 | 0 | 0 | 0 | 1 | 1 | 0 | 0 | 0 | 154 | 168 |  |  | PEAKS DB |
| total 1 peptides |  |  |  |  |  |  |  |  |  |  |  |  |  |  |  |  |  |  |  |  |  |  |  |  |  |  |  |  |  |  |  |  |  |
| --- | --- | --- | --- | --- | --- | --- | --- | --- | --- | --- | --- | --- | --- | --- | --- | --- | --- | --- | --- | --- | --- | --- | --- | --- | --- | --- | --- | --- | --- | --- | --- | --- | --- |

XP\_026546199.1

back to list

  

| Protein Coverage
| Supporting Peptides
|

Protein Coverage:

Supporting Peptides:

| Peptide | Uniq | -10lgP | Mass | Length | ppm | m/z | z | RT | Fraction | Scan | Source File | Area F6 | Area F2 | Area F3 | Area F4 | Area F5 | Area F7 | Area F8 | Area F1 | #Feature | #Feature F6 | #Feature F2 | #Feature F3 | #Feature F4 | #Feature F5 | #Feature F7 | #Feature F8 | #Feature F1 | Start | End | PTM | AScore | Found By |
| --- | --- | --- | --- | --- | --- | --- | --- | --- | --- | --- | --- | --- | --- | --- | --- | --- | --- | --- | --- | --- | --- | --- | --- | --- | --- | --- | --- | --- | --- | --- | --- | --- | --- |
| K.SSTFTLTNIVPQFIK.L | Y | 69.04 | 1694.9243 | 15 | 0.6 | 848.4699 | 2 | 62.06 | 5 | F5:30027 | 13012022\_RID\_3262\_5b.raw |  |  |  | 9.9316E5 | 4.294E6 |  |  |  | 2 | 0 | 0 | 0 | 1 | 1 | 0 | 0 | 0 | 154 | 168 |  |  | PEAKS DB |
| total 1 peptides |  |  |  |  |  |  |  |  |  |  |  |  |  |  |  |  |  |  |  |  |  |  |  |  |  |  |  |  |  |  |  |  |  |
| --- | --- | --- | --- | --- | --- | --- | --- | --- | --- | --- | --- | --- | --- | --- | --- | --- | --- | --- | --- | --- | --- | --- | --- | --- | --- | --- | --- | --- | --- | --- | --- | --- | --- |

XP\_026546197.1

back to list

  

| Protein Coverage
| Supporting Peptides
|

Protein Coverage:

Supporting Peptides:

| Peptide | Uniq | -10lgP | Mass | Length | ppm | m/z | z | RT | Fraction | Scan | Source File | Area F6 | Area F2 | Area F3 | Area F4 | Area F5 | Area F7 | Area F8 | Area F1 | #Feature | #Feature F6 | #Feature F2 | #Feature F3 | #Feature F4 | #Feature F5 | #Feature F7 | #Feature F8 | #Feature F1 | Start | End | PTM | AScore | Found By |
| --- | --- | --- | --- | --- | --- | --- | --- | --- | --- | --- | --- | --- | --- | --- | --- | --- | --- | --- | --- | --- | --- | --- | --- | --- | --- | --- | --- | --- | --- | --- | --- | --- | --- |
| K.SSTFTLTNIVPQFIK.L | Y | 69.04 | 1694.9243 | 15 | 0.6 | 848.4699 | 2 | 62.06 | 5 | F5:30027 | 13012022\_RID\_3262\_5b.raw |  |  |  | 9.9316E5 | 4.294E6 |  |  |  | 2 | 0 | 0 | 0 | 1 | 1 | 0 | 0 | 0 | 154 | 168 |  |  | PEAKS DB |
| total 1 peptides |  |  |  |  |  |  |  |  |  |  |  |  |  |  |  |  |  |  |  |  |  |  |  |  |  |  |  |  |  |  |  |  |  |
| --- | --- | --- | --- | --- | --- | --- | --- | --- | --- | --- | --- | --- | --- | --- | --- | --- | --- | --- | --- | --- | --- | --- | --- | --- | --- | --- | --- | --- | --- | --- | --- | --- | --- |

XP\_026579206.1

back to list

  

| Protein Coverage
| Supporting Peptides
|

Protein Coverage:

Supporting Peptides:

| Peptide | Uniq | -10lgP | Mass | Length | ppm | m/z | z | RT | Fraction | Scan | Source File | Area F6 | Area F2 | Area F3 | Area F4 | Area F5 | Area F7 | Area F8 | Area F1 | #Feature | #Feature F6 | #Feature F2 | #Feature F3 | #Feature F4 | #Feature F5 | #Feature F7 | #Feature F8 | #Feature F1 | Start | End | PTM | AScore | Found By |
| --- | --- | --- | --- | --- | --- | --- | --- | --- | --- | --- | --- | --- | --- | --- | --- | --- | --- | --- | --- | --- | --- | --- | --- | --- | --- | --- | --- | --- | --- | --- | --- | --- | --- |
| K.SSTFTLTNIVPQFIK.L | Y | 69.04 | 1694.9243 | 15 | 0.6 | 848.4699 | 2 | 62.06 | 5 | F5:30027 | 13012022\_RID\_3262\_5b.raw |  |  |  | 9.9316E5 | 4.294E6 |  |  |  | 2 | 0 | 0 | 0 | 1 | 1 | 0 | 0 | 0 | 154 | 168 |  |  | PEAKS DB |
| total 1 peptides |  |  |  |  |  |  |  |  |  |  |  |  |  |  |  |  |  |  |  |  |  |  |  |  |  |  |  |  |  |  |  |  |  |
| --- | --- | --- | --- | --- | --- | --- | --- | --- | --- | --- | --- | --- | --- | --- | --- | --- | --- | --- | --- | --- | --- | --- | --- | --- | --- | --- | --- | --- | --- | --- | --- | --- | --- |

XP\_026579205.1

back to list

  

| Protein Coverage
| Supporting Peptides
|

Protein Coverage:

Supporting Peptides:

| Peptide | Uniq | -10lgP | Mass | Length | ppm | m/z | z | RT | Fraction | Scan | Source File | Area F6 | Area F2 | Area F3 | Area F4 | Area F5 | Area F7 | Area F8 | Area F1 | #Feature | #Feature F6 | #Feature F2 | #Feature F3 | #Feature F4 | #Feature F5 | #Feature F7 | #Feature F8 | #Feature F1 | Start | End | PTM | AScore | Found By |
| --- | --- | --- | --- | --- | --- | --- | --- | --- | --- | --- | --- | --- | --- | --- | --- | --- | --- | --- | --- | --- | --- | --- | --- | --- | --- | --- | --- | --- | --- | --- | --- | --- | --- |
| K.SSTFTLTNIVPQFIK.L | Y | 69.04 | 1694.9243 | 15 | 0.6 | 848.4699 | 2 | 62.06 | 5 | F5:30027 | 13012022\_RID\_3262\_5b.raw |  |  |  | 9.9316E5 | 4.294E6 |  |  |  | 2 | 0 | 0 | 0 | 1 | 1 | 0 | 0 | 0 | 154 | 168 |  |  | PEAKS DB |
| total 1 peptides |  |  |  |  |  |  |  |  |  |  |  |  |  |  |  |  |  |  |  |  |  |  |  |  |  |  |  |  |  |  |  |  |  |
| --- | --- | --- | --- | --- | --- | --- | --- | --- | --- | --- | --- | --- | --- | --- | --- | --- | --- | --- | --- | --- | --- | --- | --- | --- | --- | --- | --- | --- | --- | --- | --- | --- | --- |

XP\_026579207.1

back to list

  

| Protein Coverage
| Supporting Peptides
|

Protein Coverage:

Supporting Peptides:

| Peptide | Uniq | -10lgP | Mass | Length | ppm | m/z | z | RT | Fraction | Scan | Source File | Area F6 | Area F2 | Area F3 | Area F4 | Area F5 | Area F7 | Area F8 | Area F1 | #Feature | #Feature F6 | #Feature F2 | #Feature F3 | #Feature F4 | #Feature F5 | #Feature F7 | #Feature F8 | #Feature F1 | Start | End | PTM | AScore | Found By |
| --- | --- | --- | --- | --- | --- | --- | --- | --- | --- | --- | --- | --- | --- | --- | --- | --- | --- | --- | --- | --- | --- | --- | --- | --- | --- | --- | --- | --- | --- | --- | --- | --- | --- |
| K.SSTFTLTNIVPQFIK.L | Y | 69.04 | 1694.9243 | 15 | 0.6 | 848.4699 | 2 | 62.06 | 5 | F5:30027 | 13012022\_RID\_3262\_5b.raw |  |  |  | 9.9316E5 | 4.294E6 |  |  |  | 2 | 0 | 0 | 0 | 1 | 1 | 0 | 0 | 0 | 154 | 168 |  |  | PEAKS DB |
| total 1 peptides |  |  |  |  |  |  |  |  |  |  |  |  |  |  |  |  |  |  |  |  |  |  |  |  |  |  |  |  |  |  |  |  |  |
| --- | --- | --- | --- | --- | --- | --- | --- | --- | --- | --- | --- | --- | --- | --- | --- | --- | --- | --- | --- | --- | --- | --- | --- | --- | --- | --- | --- | --- | --- | --- | --- | --- | --- |

XP\_032076461.1

back to list

  

| Protein Coverage
| Supporting Peptides
|

Protein Coverage:

Supporting Peptides:

| Peptide | Uniq | -10lgP | Mass | Length | ppm | m/z | z | RT | Fraction | Scan | Source File | Area F6 | Area F2 | Area F3 | Area F4 | Area F5 | Area F7 | Area F8 | Area F1 | #Feature | #Feature F6 | #Feature F2 | #Feature F3 | #Feature F4 | #Feature F5 | #Feature F7 | #Feature F8 | #Feature F1 | Start | End | PTM | AScore | Found By |
| --- | --- | --- | --- | --- | --- | --- | --- | --- | --- | --- | --- | --- | --- | --- | --- | --- | --- | --- | --- | --- | --- | --- | --- | --- | --- | --- | --- | --- | --- | --- | --- | --- | --- |
| K.SSTFTLTNIVPQFIK.L | Y | 69.04 | 1694.9243 | 15 | 0.6 | 848.4699 | 2 | 62.06 | 5 | F5:30027 | 13012022\_RID\_3262\_5b.raw |  |  |  | 9.9316E5 | 4.294E6 |  |  |  | 2 | 0 | 0 | 0 | 1 | 1 | 0 | 0 | 0 | 183 | 197 |  |  | PEAKS DB |
| total 1 peptides |  |  |  |  |  |  |  |  |  |  |  |  |  |  |  |  |  |  |  |  |  |  |  |  |  |  |  |  |  |  |  |  |  |
| --- | --- | --- | --- | --- | --- | --- | --- | --- | --- | --- | --- | --- | --- | --- | --- | --- | --- | --- | --- | --- | --- | --- | --- | --- | --- | --- | --- | --- | --- | --- | --- | --- | --- |

XP\_026546196.1

back to list

  

| Protein Coverage
| Supporting Peptides
|

Protein Coverage:

Supporting Peptides:

| Peptide | Uniq | -10lgP | Mass | Length | ppm | m/z | z | RT | Fraction | Scan | Source File | Area F6 | Area F2 | Area F3 | Area F4 | Area F5 | Area F7 | Area F8 | Area F1 | #Feature | #Feature F6 | #Feature F2 | #Feature F3 | #Feature F4 | #Feature F5 | #Feature F7 | #Feature F8 | #Feature F1 | Start | End | PTM | AScore | Found By |
| --- | --- | --- | --- | --- | --- | --- | --- | --- | --- | --- | --- | --- | --- | --- | --- | --- | --- | --- | --- | --- | --- | --- | --- | --- | --- | --- | --- | --- | --- | --- | --- | --- | --- |
| K.SSTFTLTNIVPQFIK.L | Y | 69.04 | 1694.9243 | 15 | 0.6 | 848.4699 | 2 | 62.06 | 5 | F5:30027 | 13012022\_RID\_3262\_5b.raw |  |  |  | 9.9316E5 | 4.294E6 |  |  |  | 2 | 0 | 0 | 0 | 1 | 1 | 0 | 0 | 0 | 168 | 182 |  |  | PEAKS DB |
| total 1 peptides |  |  |  |  |  |  |  |  |  |  |  |  |  |  |  |  |  |  |  |  |  |  |  |  |  |  |  |  |  |  |  |  |  |
| --- | --- | --- | --- | --- | --- | --- | --- | --- | --- | --- | --- | --- | --- | --- | --- | --- | --- | --- | --- | --- | --- | --- | --- | --- | --- | --- | --- | --- | --- | --- | --- | --- | --- |

LAA93895.1

back to list

  

| Protein Coverage
| Supporting Peptides
|

Protein Coverage:

Supporting Peptides:

| Peptide | Uniq | -10lgP | Mass | Length | ppm | m/z | z | RT | Fraction | Scan | Source File | Area F6 | Area F2 | Area F3 | Area F4 | Area F5 | Area F7 | Area F8 | Area F1 | #Feature | #Feature F6 | #Feature F2 | #Feature F3 | #Feature F4 | #Feature F5 | #Feature F7 | #Feature F8 | #Feature F1 | Start | End | PTM | AScore | Found By |
| --- | --- | --- | --- | --- | --- | --- | --- | --- | --- | --- | --- | --- | --- | --- | --- | --- | --- | --- | --- | --- | --- | --- | --- | --- | --- | --- | --- | --- | --- | --- | --- | --- | --- |
| K.LAELEEALQK.A | Y | 65.50 | 1142.6183 | 10 | -0.4 | 572.3162 | 2 | 38.95 | 2 | F2:14354 | 13012022\_RID\_3262\_2b.raw |  | 8.8509E5 | 6.3988E6 | 1.6611E6 | 2.3733E6 | 3.7532E6 | 9.5962E5 | 1.4812E7 | 8 | 0 | 1 | 1 | 1 | 1 | 1 | 1 | 2 | 68 | 77 |  |  | PEAKS DB |
| total 1 peptides |  |  |  |  |  |  |  |  |  |  |  |  |  |  |  |  |  |  |  |  |  |  |  |  |  |  |  |  |  |  |  |  |  |
| --- | --- | --- | --- | --- | --- | --- | --- | --- | --- | --- | --- | --- | --- | --- | --- | --- | --- | --- | --- | --- | --- | --- | --- | --- | --- | --- | --- | --- | --- | --- | --- | --- | --- |

XP\_032085019.1

back to list

  

| Protein Coverage
| Supporting Peptides
|

Protein Coverage:

Supporting Peptides:

| Peptide | Uniq | -10lgP | Mass | Length | ppm | m/z | z | RT | Fraction | Scan | Source File | Area F6 | Area F2 | Area F3 | Area F4 | Area F5 | Area F7 | Area F8 | Area F1 | #Feature | #Feature F6 | #Feature F2 | #Feature F3 | #Feature F4 | #Feature F5 | #Feature F7 | #Feature F8 | #Feature F1 | Start | End | PTM | AScore | Found By |
| --- | --- | --- | --- | --- | --- | --- | --- | --- | --- | --- | --- | --- | --- | --- | --- | --- | --- | --- | --- | --- | --- | --- | --- | --- | --- | --- | --- | --- | --- | --- | --- | --- | --- |
| K.TITLEVEPSDTIENVK.A | Y | 52.64 | 1786.9200 | 16 | 0.5 | 894.4678 | 2 | 46.41 | 8 | F8:25780 | 31122021\_RID\_3262\_1b.raw |  |  |  |  |  |  |  | 1.5034E6 | 1 | 0 | 0 | 0 | 0 | 0 | 0 | 0 | 1 | 12 | 27 |  |  | PEAKS DB |
| total 1 peptides |  |  |  |  |  |  |  |  |  |  |  |  |  |  |  |  |  |  |  |  |  |  |  |  |  |  |  |  |  |  |  |  |  |
| --- | --- | --- | --- | --- | --- | --- | --- | --- | --- | --- | --- | --- | --- | --- | --- | --- | --- | --- | --- | --- | --- | --- | --- | --- | --- | --- | --- | --- | --- | --- | --- | --- | --- |

JAI09617.1

back to list

  

| Protein Coverage
| Supporting Peptides
|

Protein Coverage:

Supporting Peptides:

| Peptide | Uniq | -10lgP | Mass | Length | ppm | m/z | z | RT | Fraction | Scan | Source File | Area F6 | Area F2 | Area F3 | Area F4 | Area F5 | Area F7 | Area F8 | Area F1 | #Feature | #Feature F6 | #Feature F2 | #Feature F3 | #Feature F4 | #Feature F5 | #Feature F7 | #Feature F8 | #Feature F1 | Start | End | PTM | AScore | Found By |
| --- | --- | --- | --- | --- | --- | --- | --- | --- | --- | --- | --- | --- | --- | --- | --- | --- | --- | --- | --- | --- | --- | --- | --- | --- | --- | --- | --- | --- | --- | --- | --- | --- | --- |
| R.LFQVEYAIEAIK.L | Y | 59.98 | 1422.7758 | 12 | 0.6 | 712.3956 | 2 | 55.39 | 8 | F8:32856 | 31122021\_RID\_3262\_1b.raw |  |  |  |  |  |  |  | 1.3568E5 | 1 | 0 | 0 | 0 | 0 | 0 | 0 | 0 | 1 | 21 | 32 |  |  | PEAKS DB |
| total 1 peptides |  |  |  |  |  |  |  |  |  |  |  |  |  |  |  |  |  |  |  |  |  |  |  |  |  |  |  |  |  |  |  |  |  |
| --- | --- | --- | --- | --- | --- | --- | --- | --- | --- | --- | --- | --- | --- | --- | --- | --- | --- | --- | --- | --- | --- | --- | --- | --- | --- | --- | --- | --- | --- | --- | --- | --- | --- |

XP\_007433195.1

back to list

  

| Protein Coverage
| Supporting Peptides
|

Protein Coverage:

Supporting Peptides:

| Peptide | Uniq | -10lgP | Mass | Length | ppm | m/z | z | RT | Fraction | Scan | Source File | Area F6 | Area F2 | Area F3 | Area F4 | Area F5 | Area F7 | Area F8 | Area F1 | #Feature | #Feature F6 | #Feature F2 | #Feature F3 | #Feature F4 | #Feature F5 | #Feature F7 | #Feature F8 | #Feature F1 | Start | End | PTM | AScore | Found By |
| --- | --- | --- | --- | --- | --- | --- | --- | --- | --- | --- | --- | --- | --- | --- | --- | --- | --- | --- | --- | --- | --- | --- | --- | --- | --- | --- | --- | --- | --- | --- | --- | --- | --- |
| R.IQLVEEELDR.A | Y | 52.21 | 1242.6455 | 10 | -0.1 | 622.3300 | 2 | 39.82 | 8 | F8:20647 | 31122021\_RID\_3262\_1b.raw |  |  |  |  |  |  |  | 1.8537E5 | 1 | 0 | 0 | 0 | 0 | 0 | 0 | 0 | 1 | 92 | 101 |  |  | PEAKS DB |
| total 1 peptides |  |  |  |  |  |  |  |  |  |  |  |  |  |  |  |  |  |  |  |  |  |  |  |  |  |  |  |  |  |  |  |  |  |
| --- | --- | --- | --- | --- | --- | --- | --- | --- | --- | --- | --- | --- | --- | --- | --- | --- | --- | --- | --- | --- | --- | --- | --- | --- | --- | --- | --- | --- | --- | --- | --- | --- | --- |

XP\_025025541.1

back to list

  

| Protein Coverage
| Supporting Peptides
|

Protein Coverage:

Supporting Peptides:

| Peptide | Uniq | -10lgP | Mass | Length | ppm | m/z | z | RT | Fraction | Scan | Source File | Area F6 | Area F2 | Area F3 | Area F4 | Area F5 | Area F7 | Area F8 | Area F1 | #Feature | #Feature F6 | #Feature F2 | #Feature F3 | #Feature F4 | #Feature F5 | #Feature F7 | #Feature F8 | #Feature F1 | Start | End | PTM | AScore | Found By |
| --- | --- | --- | --- | --- | --- | --- | --- | --- | --- | --- | --- | --- | --- | --- | --- | --- | --- | --- | --- | --- | --- | --- | --- | --- | --- | --- | --- | --- | --- | --- | --- | --- | --- |
| R.IQLVEEELDR.A | Y | 52.21 | 1242.6455 | 10 | -0.1 | 622.3300 | 2 | 39.82 | 8 | F8:20647 | 31122021\_RID\_3262\_1b.raw |  |  |  |  |  |  |  | 1.8537E5 | 1 | 0 | 0 | 0 | 0 | 0 | 0 | 0 | 1 | 57 | 66 |  |  | PEAKS DB |
| total 1 peptides |  |  |  |  |  |  |  |  |  |  |  |  |  |  |  |  |  |  |  |  |  |  |  |  |  |  |  |  |  |  |  |  |  |
| --- | --- | --- | --- | --- | --- | --- | --- | --- | --- | --- | --- | --- | --- | --- | --- | --- | --- | --- | --- | --- | --- | --- | --- | --- | --- | --- | --- | --- | --- | --- | --- | --- | --- |

XP\_013914127.1

back to list

  

| Protein Coverage
| Supporting Peptides
|

Protein Coverage:

Supporting Peptides:

| Peptide | Uniq | -10lgP | Mass | Length | ppm | m/z | z | RT | Fraction | Scan | Source File | Area F6 | Area F2 | Area F3 | Area F4 | Area F5 | Area F7 | Area F8 | Area F1 | #Feature | #Feature F6 | #Feature F2 | #Feature F3 | #Feature F4 | #Feature F5 | #Feature F7 | #Feature F8 | #Feature F1 | Start | End | PTM | AScore | Found By |
| --- | --- | --- | --- | --- | --- | --- | --- | --- | --- | --- | --- | --- | --- | --- | --- | --- | --- | --- | --- | --- | --- | --- | --- | --- | --- | --- | --- | --- | --- | --- | --- | --- | --- |
| K.TKDGVVEITGK.H | Y | 60.09 | 1145.6292 | 11 | 1.2 | 573.8225 | 2 | 20.98 | 8 | F8:6018 | 31122021\_RID\_3262\_1b.raw |  |  |  |  |  |  |  | 2.6626E5 | 1 | 0 | 0 | 0 | 0 | 0 | 0 | 0 | 1 | 118 | 128 |  |  | PEAKS DB |
| total 1 peptides |  |  |  |  |  |  |  |  |  |  |  |  |  |  |  |  |  |  |  |  |  |  |  |  |  |  |  |  |  |  |  |  |  |
| --- | --- | --- | --- | --- | --- | --- | --- | --- | --- | --- | --- | --- | --- | --- | --- | --- | --- | --- | --- | --- | --- | --- | --- | --- | --- | --- | --- | --- | --- | --- | --- | --- | --- |

XP\_032072257.1

back to list

  

| Protein Coverage
| Supporting Peptides
|

Protein Coverage:

Supporting Peptides:

| Peptide | Uniq | -10lgP | Mass | Length | ppm | m/z | z | RT | Fraction | Scan | Source File | Area F6 | Area F2 | Area F3 | Area F4 | Area F5 | Area F7 | Area F8 | Area F1 | #Feature | #Feature F6 | #Feature F2 | #Feature F3 | #Feature F4 | #Feature F5 | #Feature F7 | #Feature F8 | #Feature F1 | Start | End | PTM | AScore | Found By |
| --- | --- | --- | --- | --- | --- | --- | --- | --- | --- | --- | --- | --- | --- | --- | --- | --- | --- | --- | --- | --- | --- | --- | --- | --- | --- | --- | --- | --- | --- | --- | --- | --- | --- |
| K.TKDGVVEITGK.H | Y | 60.09 | 1145.6292 | 11 | 1.2 | 573.8225 | 2 | 20.98 | 8 | F8:6018 | 31122021\_RID\_3262\_1b.raw |  |  |  |  |  |  |  | 2.6626E5 | 1 | 0 | 0 | 0 | 0 | 0 | 0 | 0 | 1 | 118 | 128 |  |  | PEAKS DB |
| total 1 peptides |  |  |  |  |  |  |  |  |  |  |  |  |  |  |  |  |  |  |  |  |  |  |  |  |  |  |  |  |  |  |  |  |  |
| --- | --- | --- | --- | --- | --- | --- | --- | --- | --- | --- | --- | --- | --- | --- | --- | --- | --- | --- | --- | --- | --- | --- | --- | --- | --- | --- | --- | --- | --- | --- | --- | --- | --- |

BAP39929.1

back to list

  

| Protein Coverage
| Supporting Peptides
|

Protein Coverage:

Supporting Peptides:

| Peptide | Uniq | -10lgP | Mass | Length | ppm | m/z | z | RT | Fraction | Scan | Source File | Area F6 | Area F2 | Area F3 | Area F4 | Area F5 | Area F7 | Area F8 | Area F1 | #Feature | #Feature F6 | #Feature F2 | #Feature F3 | #Feature F4 | #Feature F5 | #Feature F7 | #Feature F8 | #Feature F1 | Start | End | PTM | AScore | Found By |
| --- | --- | --- | --- | --- | --- | --- | --- | --- | --- | --- | --- | --- | --- | --- | --- | --- | --- | --- | --- | --- | --- | --- | --- | --- | --- | --- | --- | --- | --- | --- | --- | --- | --- |
| R.EAVFVAQLLSEDIK.T | Y | 56.64 | 1560.8398 | 14 | 3.7 | 781.4301 | 2 | 60.22 | 8 | F8:36604 | 31122021\_RID\_3262\_1b.raw |  |  |  |  |  |  |  | 0 | 0 | 0 | 0 | 0 | 0 | 0 | 0 | 0 | 0 | 98 | 111 |  |  | PEAKS DB |
| total 1 peptides |  |  |  |  |  |  |  |  |  |  |  |  |  |  |  |  |  |  |  |  |  |  |  |  |  |  |  |  |  |  |  |  |  |
| --- | --- | --- | --- | --- | --- | --- | --- | --- | --- | --- | --- | --- | --- | --- | --- | --- | --- | --- | --- | --- | --- | --- | --- | --- | --- | --- | --- | --- | --- | --- | --- | --- | --- |

XP\_015685588.1

back to list

  

| Protein Coverage
| Supporting Peptides
|

Protein Coverage:

Supporting Peptides:

| Peptide | Uniq | -10lgP | Mass | Length | ppm | m/z | z | RT | Fraction | Scan | Source File | Area F6 | Area F2 | Area F3 | Area F4 | Area F5 | Area F7 | Area F8 | Area F1 | #Feature | #Feature F6 | #Feature F2 | #Feature F3 | #Feature F4 | #Feature F5 | #Feature F7 | #Feature F8 | #Feature F1 | Start | End | PTM | AScore | Found By |
| --- | --- | --- | --- | --- | --- | --- | --- | --- | --- | --- | --- | --- | --- | --- | --- | --- | --- | --- | --- | --- | --- | --- | --- | --- | --- | --- | --- | --- | --- | --- | --- | --- | --- |
| R.EAVFVAQLLSEDIK.T | Y | 56.64 | 1560.8398 | 14 | 3.7 | 781.4301 | 2 | 60.22 | 8 | F8:36604 | 31122021\_RID\_3262\_1b.raw |  |  |  |  |  |  |  | 0 | 0 | 0 | 0 | 0 | 0 | 0 | 0 | 0 | 0 | 70 | 83 |  |  | PEAKS DB |
| total 1 peptides |  |  |  |  |  |  |  |  |  |  |  |  |  |  |  |  |  |  |  |  |  |  |  |  |  |  |  |  |  |  |  |  |  |
| --- | --- | --- | --- | --- | --- | --- | --- | --- | --- | --- | --- | --- | --- | --- | --- | --- | --- | --- | --- | --- | --- | --- | --- | --- | --- | --- | --- | --- | --- | --- | --- | --- | --- |

Q2YHJ5.1

back to list

  

| Protein Coverage
| Supporting Peptides
|

Protein Coverage:

Supporting Peptides:

| Peptide | Uniq | -10lgP | Mass | Length | ppm | m/z | z | RT | Fraction | Scan | Source File | Area F6 | Area F2 | Area F3 | Area F4 | Area F5 | Area F7 | Area F8 | Area F1 | #Feature | #Feature F6 | #Feature F2 | #Feature F3 | #Feature F4 | #Feature F5 | #Feature F7 | #Feature F8 | #Feature F1 | Start | End | PTM | AScore | Found By |
| --- | --- | --- | --- | --- | --- | --- | --- | --- | --- | --- | --- | --- | --- | --- | --- | --- | --- | --- | --- | --- | --- | --- | --- | --- | --- | --- | --- | --- | --- | --- | --- | --- | --- |
| K.YWFYPTK.Y | Y | 55.16 | 1003.4803 | 7 | -0.1 | 502.7474 | 2 | 54.34 | 6 | F6:25654 | 17012022\_RID\_3262\_7b.raw |  |  | 1.8518E6 |  |  | 2.6308E7 | 7.6191E6 | 2.8034E6 | 4 | 0 | 0 | 1 | 0 | 0 | 1 | 1 | 1 | 107 | 113 |  |  | PEAKS DB |
| R.DNMDTYQNK.Y | Y | 45.55 | 1127.4553 | 9 | 0.0 | 564.7349 | 2 | 21.38 | 6 | F6:6440 | 17012022\_RID\_3262\_7b.raw |  |  |  |  |  | 7.1938E5 |  |  | 1 | 0 | 0 | 0 | 0 | 0 | 1 | 0 | 0 | 98 | 106 |  |  | PEAKS DB |
| total 2 peptides |  |  |  |  |  |  |  |  |  |  |  |  |  |  |  |  |  |  |  |  |  |  |  |  |  |  |  |  |  |  |  |  |  |
| --- | --- | --- | --- | --- | --- | --- | --- | --- | --- | --- | --- | --- | --- | --- | --- | --- | --- | --- | --- | --- | --- | --- | --- | --- | --- | --- | --- | --- | --- | --- | --- | --- | --- |

XP\_007437998.1

back to list

  

| Protein Coverage
| Supporting Peptides
|

Protein Coverage:

Supporting Peptides:

| Peptide | Uniq | -10lgP | Mass | Length | ppm | m/z | z | RT | Fraction | Scan | Source File | Area F6 | Area F2 | Area F3 | Area F4 | Area F5 | Area F7 | Area F8 | Area F1 | #Feature | #Feature F6 | #Feature F2 | #Feature F3 | #Feature F4 | #Feature F5 | #Feature F7 | #Feature F8 | #Feature F1 | Start | End | PTM | AScore | Found By |
| --- | --- | --- | --- | --- | --- | --- | --- | --- | --- | --- | --- | --- | --- | --- | --- | --- | --- | --- | --- | --- | --- | --- | --- | --- | --- | --- | --- | --- | --- | --- | --- | --- | --- |
| K.YEWDVAEAR.K | Y | 53.01 | 1137.5090 | 9 | 0.8 | 569.7623 | 2 | 37.47 | 8 | F8:18812 | 31122021\_RID\_3262\_1b.raw |  |  |  |  |  |  |  | 1.0724E5 | 1 | 0 | 0 | 0 | 0 | 0 | 0 | 0 | 1 | 639 | 647 |  |  | PEAKS DB |
| M.VNFTVDQIR.A | Y | 47.18 | 1090.5771 | 9 | -0.6 | 546.2955 | 2 | 37.58 | 8 | F8:18942 | 31122021\_RID\_3262\_1b.raw |  |  |  |  |  |  |  | 4.1315E4 | 1 | 0 | 0 | 0 | 0 | 0 | 0 | 0 | 1 | 2 | 10 |  |  | PEAKS DB |
| total 2 peptides |  |  |  |  |  |  |  |  |  |  |  |  |  |  |  |  |  |  |  |  |  |  |  |  |  |  |  |  |  |  |  |  |  |
| --- | --- | --- | --- | --- | --- | --- | --- | --- | --- | --- | --- | --- | --- | --- | --- | --- | --- | --- | --- | --- | --- | --- | --- | --- | --- | --- | --- | --- | --- | --- | --- | --- | --- |

XP\_013920852.1

back to list

  

| Protein Coverage
| Supporting Peptides
|

Protein Coverage:

Supporting Peptides:

| Peptide | Uniq | -10lgP | Mass | Length | ppm | m/z | z | RT | Fraction | Scan | Source File | Area F6 | Area F2 | Area F3 | Area F4 | Area F5 | Area F7 | Area F8 | Area F1 | #Feature | #Feature F6 | #Feature F2 | #Feature F3 | #Feature F4 | #Feature F5 | #Feature F7 | #Feature F8 | #Feature F1 | Start | End | PTM | AScore | Found By |
| --- | --- | --- | --- | --- | --- | --- | --- | --- | --- | --- | --- | --- | --- | --- | --- | --- | --- | --- | --- | --- | --- | --- | --- | --- | --- | --- | --- | --- | --- | --- | --- | --- | --- |
| K.YEWDVAEAR.K | Y | 53.01 | 1137.5090 | 9 | 0.8 | 569.7623 | 2 | 37.47 | 8 | F8:18812 | 31122021\_RID\_3262\_1b.raw |  |  |  |  |  |  |  | 1.0724E5 | 1 | 0 | 0 | 0 | 0 | 0 | 0 | 0 | 1 | 639 | 647 |  |  | PEAKS DB |
| M.VNFTVDQIR.A | Y | 47.18 | 1090.5771 | 9 | -0.6 | 546.2955 | 2 | 37.58 | 8 | F8:18942 | 31122021\_RID\_3262\_1b.raw |  |  |  |  |  |  |  | 4.1315E4 | 1 | 0 | 0 | 0 | 0 | 0 | 0 | 0 | 1 | 2 | 10 |  |  | PEAKS DB |
| total 2 peptides |  |  |  |  |  |  |  |  |  |  |  |  |  |  |  |  |  |  |  |  |  |  |  |  |  |  |  |  |  |  |  |  |  |
| --- | --- | --- | --- | --- | --- | --- | --- | --- | --- | --- | --- | --- | --- | --- | --- | --- | --- | --- | --- | --- | --- | --- | --- | --- | --- | --- | --- | --- | --- | --- | --- | --- | --- |

XP\_032070431.1

back to list

  

| Protein Coverage
| Supporting Peptides
|

Protein Coverage:

Supporting Peptides:

| Peptide | Uniq | -10lgP | Mass | Length | ppm | m/z | z | RT | Fraction | Scan | Source File | Area F6 | Area F2 | Area F3 | Area F4 | Area F5 | Area F7 | Area F8 | Area F1 | #Feature | #Feature F6 | #Feature F2 | #Feature F3 | #Feature F4 | #Feature F5 | #Feature F7 | #Feature F8 | #Feature F1 | Start | End | PTM | AScore | Found By |
| --- | --- | --- | --- | --- | --- | --- | --- | --- | --- | --- | --- | --- | --- | --- | --- | --- | --- | --- | --- | --- | --- | --- | --- | --- | --- | --- | --- | --- | --- | --- | --- | --- | --- |
| K.YEWDVAEAR.K | Y | 53.01 | 1137.5090 | 9 | 0.8 | 569.7623 | 2 | 37.47 | 8 | F8:18812 | 31122021\_RID\_3262\_1b.raw |  |  |  |  |  |  |  | 1.0724E5 | 1 | 0 | 0 | 0 | 0 | 0 | 0 | 0 | 1 | 658 | 666 |  |  | PEAKS DB |
| K.VNFTVDQIR.A | Y | 47.18 | 1090.5771 | 9 | -0.6 | 546.2955 | 2 | 37.58 | 8 | F8:18942 | 31122021\_RID\_3262\_1b.raw |  |  |  |  |  |  |  | 4.1315E4 | 1 | 0 | 0 | 0 | 0 | 0 | 0 | 0 | 1 | 21 | 29 |  |  | PEAKS DB |
| total 2 peptides |  |  |  |  |  |  |  |  |  |  |  |  |  |  |  |  |  |  |  |  |  |  |  |  |  |  |  |  |  |  |  |  |  |
| --- | --- | --- | --- | --- | --- | --- | --- | --- | --- | --- | --- | --- | --- | --- | --- | --- | --- | --- | --- | --- | --- | --- | --- | --- | --- | --- | --- | --- | --- | --- | --- | --- | --- |

JAG46919.1

back to list

  

| Protein Coverage
| Supporting Peptides
|

Protein Coverage:

Supporting Peptides:

| Peptide | Uniq | -10lgP | Mass | Length | ppm | m/z | z | RT | Fraction | Scan | Source File | Area F6 | Area F2 | Area F3 | Area F4 | Area F5 | Area F7 | Area F8 | Area F1 | #Feature | #Feature F6 | #Feature F2 | #Feature F3 | #Feature F4 | #Feature F5 | #Feature F7 | #Feature F8 | #Feature F1 | Start | End | PTM | AScore | Found By |
| --- | --- | --- | --- | --- | --- | --- | --- | --- | --- | --- | --- | --- | --- | --- | --- | --- | --- | --- | --- | --- | --- | --- | --- | --- | --- | --- | --- | --- | --- | --- | --- | --- | --- |
| K.YEWDVAEAR.K | Y | 53.01 | 1137.5090 | 9 | 0.8 | 569.7623 | 2 | 37.47 | 8 | F8:18812 | 31122021\_RID\_3262\_1b.raw |  |  |  |  |  |  |  | 1.0724E5 | 1 | 0 | 0 | 0 | 0 | 0 | 0 | 0 | 1 | 639 | 647 |  |  | PEAKS DB |
| M.VNFTVDQIR.A | Y | 47.18 | 1090.5771 | 9 | -0.6 | 546.2955 | 2 | 37.58 | 8 | F8:18942 | 31122021\_RID\_3262\_1b.raw |  |  |  |  |  |  |  | 4.1315E4 | 1 | 0 | 0 | 0 | 0 | 0 | 0 | 0 | 1 | 2 | 10 |  |  | PEAKS DB |
| total 2 peptides |  |  |  |  |  |  |  |  |  |  |  |  |  |  |  |  |  |  |  |  |  |  |  |  |  |  |  |  |  |  |  |  |  |
| --- | --- | --- | --- | --- | --- | --- | --- | --- | --- | --- | --- | --- | --- | --- | --- | --- | --- | --- | --- | --- | --- | --- | --- | --- | --- | --- | --- | --- | --- | --- | --- | --- | --- |

JAI13483.1

back to list

  

| Protein Coverage
| Supporting Peptides
|

Protein Coverage:

Supporting Peptides:

| Peptide | Uniq | -10lgP | Mass | Length | ppm | m/z | z | RT | Fraction | Scan | Source File | Area F6 | Area F2 | Area F3 | Area F4 | Area F5 | Area F7 | Area F8 | Area F1 | #Feature | #Feature F6 | #Feature F2 | #Feature F3 | #Feature F4 | #Feature F5 | #Feature F7 | #Feature F8 | #Feature F1 | Start | End | PTM | AScore | Found By |
| --- | --- | --- | --- | --- | --- | --- | --- | --- | --- | --- | --- | --- | --- | --- | --- | --- | --- | --- | --- | --- | --- | --- | --- | --- | --- | --- | --- | --- | --- | --- | --- | --- | --- |
| K.YEWDVAEAR.K | Y | 53.01 | 1137.5090 | 9 | 0.8 | 569.7623 | 2 | 37.47 | 8 | F8:18812 | 31122021\_RID\_3262\_1b.raw |  |  |  |  |  |  |  | 1.0724E5 | 1 | 0 | 0 | 0 | 0 | 0 | 0 | 0 | 1 | 639 | 647 |  |  | PEAKS DB |
| M.VNFTVDQIR.A | Y | 47.18 | 1090.5771 | 9 | -0.6 | 546.2955 | 2 | 37.58 | 8 | F8:18942 | 31122021\_RID\_3262\_1b.raw |  |  |  |  |  |  |  | 4.1315E4 | 1 | 0 | 0 | 0 | 0 | 0 | 0 | 0 | 1 | 2 | 10 |  |  | PEAKS DB |
| total 2 peptides |  |  |  |  |  |  |  |  |  |  |  |  |  |  |  |  |  |  |  |  |  |  |  |  |  |  |  |  |  |  |  |  |  |
| --- | --- | --- | --- | --- | --- | --- | --- | --- | --- | --- | --- | --- | --- | --- | --- | --- | --- | --- | --- | --- | --- | --- | --- | --- | --- | --- | --- | --- | --- | --- | --- | --- | --- |

JAA97181.1

back to list

  

| Protein Coverage
| Supporting Peptides
|

Protein Coverage:

Supporting Peptides:

| Peptide | Uniq | -10lgP | Mass | Length | ppm | m/z | z | RT | Fraction | Scan | Source File | Area F6 | Area F2 | Area F3 | Area F4 | Area F5 | Area F7 | Area F8 | Area F1 | #Feature | #Feature F6 | #Feature F2 | #Feature F3 | #Feature F4 | #Feature F5 | #Feature F7 | #Feature F8 | #Feature F1 | Start | End | PTM | AScore | Found By |
| --- | --- | --- | --- | --- | --- | --- | --- | --- | --- | --- | --- | --- | --- | --- | --- | --- | --- | --- | --- | --- | --- | --- | --- | --- | --- | --- | --- | --- | --- | --- | --- | --- | --- |
| K.YEWDVAEAR.K | Y | 53.01 | 1137.5090 | 9 | 0.8 | 569.7623 | 2 | 37.47 | 8 | F8:18812 | 31122021\_RID\_3262\_1b.raw |  |  |  |  |  |  |  | 1.0724E5 | 1 | 0 | 0 | 0 | 0 | 0 | 0 | 0 | 1 | 639 | 647 |  |  | PEAKS DB |
| M.VNFTVDQIR.A | Y | 47.18 | 1090.5771 | 9 | -0.6 | 546.2955 | 2 | 37.58 | 8 | F8:18942 | 31122021\_RID\_3262\_1b.raw |  |  |  |  |  |  |  | 4.1315E4 | 1 | 0 | 0 | 0 | 0 | 0 | 0 | 0 | 1 | 2 | 10 |  |  | PEAKS DB |
| total 2 peptides |  |  |  |  |  |  |  |  |  |  |  |  |  |  |  |  |  |  |  |  |  |  |  |  |  |  |  |  |  |  |  |  |  |
| --- | --- | --- | --- | --- | --- | --- | --- | --- | --- | --- | --- | --- | --- | --- | --- | --- | --- | --- | --- | --- | --- | --- | --- | --- | --- | --- | --- | --- | --- | --- | --- | --- | --- |

JAV50654.1

back to list

  

| Protein Coverage
| Supporting Peptides
|

Protein Coverage:

Supporting Peptides:

| Peptide | Uniq | -10lgP | Mass | Length | ppm | m/z | z | RT | Fraction | Scan | Source File | Area F6 | Area F2 | Area F3 | Area F4 | Area F5 | Area F7 | Area F8 | Area F1 | #Feature | #Feature F6 | #Feature F2 | #Feature F3 | #Feature F4 | #Feature F5 | #Feature F7 | #Feature F8 | #Feature F1 | Start | End | PTM | AScore | Found By |
| --- | --- | --- | --- | --- | --- | --- | --- | --- | --- | --- | --- | --- | --- | --- | --- | --- | --- | --- | --- | --- | --- | --- | --- | --- | --- | --- | --- | --- | --- | --- | --- | --- | --- |
| K.YEWDVAEAR.K | Y | 53.01 | 1137.5090 | 9 | 0.8 | 569.7623 | 2 | 37.47 | 8 | F8:18812 | 31122021\_RID\_3262\_1b.raw |  |  |  |  |  |  |  | 1.0724E5 | 1 | 0 | 0 | 0 | 0 | 0 | 0 | 0 | 1 | 639 | 647 |  |  | PEAKS DB |
| M.VNFTVDQIR.A | Y | 47.18 | 1090.5771 | 9 | -0.6 | 546.2955 | 2 | 37.58 | 8 | F8:18942 | 31122021\_RID\_3262\_1b.raw |  |  |  |  |  |  |  | 4.1315E4 | 1 | 0 | 0 | 0 | 0 | 0 | 0 | 0 | 1 | 2 | 10 |  |  | PEAKS DB |
| total 2 peptides |  |  |  |  |  |  |  |  |  |  |  |  |  |  |  |  |  |  |  |  |  |  |  |  |  |  |  |  |  |  |  |  |  |
| --- | --- | --- | --- | --- | --- | --- | --- | --- | --- | --- | --- | --- | --- | --- | --- | --- | --- | --- | --- | --- | --- | --- | --- | --- | --- | --- | --- | --- | --- | --- | --- | --- | --- |

AFJ49955.1

back to list

  

| Protein Coverage
| Supporting Peptides
|

Protein Coverage:

Supporting Peptides:

| Peptide | Uniq | -10lgP | Mass | Length | ppm | m/z | z | RT | Fraction | Scan | Source File | Area F6 | Area F2 | Area F3 | Area F4 | Area F5 | Area F7 | Area F8 | Area F1 | #Feature | #Feature F6 | #Feature F2 | #Feature F3 | #Feature F4 | #Feature F5 | #Feature F7 | #Feature F8 | #Feature F1 | Start | End | PTM | AScore | Found By |
| --- | --- | --- | --- | --- | --- | --- | --- | --- | --- | --- | --- | --- | --- | --- | --- | --- | --- | --- | --- | --- | --- | --- | --- | --- | --- | --- | --- | --- | --- | --- | --- | --- | --- |
| K.YEWDVAEAR.K | Y | 53.01 | 1137.5090 | 9 | 0.8 | 569.7623 | 2 | 37.47 | 8 | F8:18812 | 31122021\_RID\_3262\_1b.raw |  |  |  |  |  |  |  | 1.0724E5 | 1 | 0 | 0 | 0 | 0 | 0 | 0 | 0 | 1 | 639 | 647 |  |  | PEAKS DB |
| M.VNFTVDQIR.A | Y | 47.18 | 1090.5771 | 9 | -0.6 | 546.2955 | 2 | 37.58 | 8 | F8:18942 | 31122021\_RID\_3262\_1b.raw |  |  |  |  |  |  |  | 4.1315E4 | 1 | 0 | 0 | 0 | 0 | 0 | 0 | 0 | 1 | 2 | 10 |  |  | PEAKS DB |
| total 2 peptides |  |  |  |  |  |  |  |  |  |  |  |  |  |  |  |  |  |  |  |  |  |  |  |  |  |  |  |  |  |  |  |  |  |
| --- | --- | --- | --- | --- | --- | --- | --- | --- | --- | --- | --- | --- | --- | --- | --- | --- | --- | --- | --- | --- | --- | --- | --- | --- | --- | --- | --- | --- | --- | --- | --- | --- | --- |

JAB54320.1

back to list

  

| Protein Coverage
| Supporting Peptides
|

Protein Coverage:

Supporting Peptides:

| Peptide | Uniq | -10lgP | Mass | Length | ppm | m/z | z | RT | Fraction | Scan | Source File | Area F6 | Area F2 | Area F3 | Area F4 | Area F5 | Area F7 | Area F8 | Area F1 | #Feature | #Feature F6 | #Feature F2 | #Feature F3 | #Feature F4 | #Feature F5 | #Feature F7 | #Feature F8 | #Feature F1 | Start | End | PTM | AScore | Found By |
| --- | --- | --- | --- | --- | --- | --- | --- | --- | --- | --- | --- | --- | --- | --- | --- | --- | --- | --- | --- | --- | --- | --- | --- | --- | --- | --- | --- | --- | --- | --- | --- | --- | --- |
| K.YEWDVAEAR.K | Y | 53.01 | 1137.5090 | 9 | 0.8 | 569.7623 | 2 | 37.47 | 8 | F8:18812 | 31122021\_RID\_3262\_1b.raw |  |  |  |  |  |  |  | 1.0724E5 | 1 | 0 | 0 | 0 | 0 | 0 | 0 | 0 | 1 | 639 | 647 |  |  | PEAKS DB |
| M.VNFTVDQIR.A | Y | 47.18 | 1090.5771 | 9 | -0.6 | 546.2955 | 2 | 37.58 | 8 | F8:18942 | 31122021\_RID\_3262\_1b.raw |  |  |  |  |  |  |  | 4.1315E4 | 1 | 0 | 0 | 0 | 0 | 0 | 0 | 0 | 1 | 2 | 10 |  |  | PEAKS DB |
| total 2 peptides |  |  |  |  |  |  |  |  |  |  |  |  |  |  |  |  |  |  |  |  |  |  |  |  |  |  |  |  |  |  |  |  |  |
| --- | --- | --- | --- | --- | --- | --- | --- | --- | --- | --- | --- | --- | --- | --- | --- | --- | --- | --- | --- | --- | --- | --- | --- | --- | --- | --- | --- | --- | --- | --- | --- | --- | --- |

JAG44954.1

back to list

  

| Protein Coverage
| Supporting Peptides
|

Protein Coverage:

Supporting Peptides:

| Peptide | Uniq | -10lgP | Mass | Length | ppm | m/z | z | RT | Fraction | Scan | Source File | Area F6 | Area F2 | Area F3 | Area F4 | Area F5 | Area F7 | Area F8 | Area F1 | #Feature | #Feature F6 | #Feature F2 | #Feature F3 | #Feature F4 | #Feature F5 | #Feature F7 | #Feature F8 | #Feature F1 | Start | End | PTM | AScore | Found By |
| --- | --- | --- | --- | --- | --- | --- | --- | --- | --- | --- | --- | --- | --- | --- | --- | --- | --- | --- | --- | --- | --- | --- | --- | --- | --- | --- | --- | --- | --- | --- | --- | --- | --- |
| K.YEWDVAEAR.K | Y | 53.01 | 1137.5090 | 9 | 0.8 | 569.7623 | 2 | 37.47 | 8 | F8:18812 | 31122021\_RID\_3262\_1b.raw |  |  |  |  |  |  |  | 1.0724E5 | 1 | 0 | 0 | 0 | 0 | 0 | 0 | 0 | 1 | 639 | 647 |  |  | PEAKS DB |
| M.VNFTVDQIR.A | Y | 47.18 | 1090.5771 | 9 | -0.6 | 546.2955 | 2 | 37.58 | 8 | F8:18942 | 31122021\_RID\_3262\_1b.raw |  |  |  |  |  |  |  | 4.1315E4 | 1 | 0 | 0 | 0 | 0 | 0 | 0 | 0 | 1 | 2 | 10 |  |  | PEAKS DB |
| total 2 peptides |  |  |  |  |  |  |  |  |  |  |  |  |  |  |  |  |  |  |  |  |  |  |  |  |  |  |  |  |  |  |  |  |  |
| --- | --- | --- | --- | --- | --- | --- | --- | --- | --- | --- | --- | --- | --- | --- | --- | --- | --- | --- | --- | --- | --- | --- | --- | --- | --- | --- | --- | --- | --- | --- | --- | --- | --- |

JAC95695.1

back to list

  

| Protein Coverage
| Supporting Peptides
|

Protein Coverage:

Supporting Peptides:

| Peptide | Uniq | -10lgP | Mass | Length | ppm | m/z | z | RT | Fraction | Scan | Source File | Area F6 | Area F2 | Area F3 | Area F4 | Area F5 | Area F7 | Area F8 | Area F1 | #Feature | #Feature F6 | #Feature F2 | #Feature F3 | #Feature F4 | #Feature F5 | #Feature F7 | #Feature F8 | #Feature F1 | Start | End | PTM | AScore | Found By |
| --- | --- | --- | --- | --- | --- | --- | --- | --- | --- | --- | --- | --- | --- | --- | --- | --- | --- | --- | --- | --- | --- | --- | --- | --- | --- | --- | --- | --- | --- | --- | --- | --- | --- |
| K.YEWDVAEAR.K | Y | 53.01 | 1137.5090 | 9 | 0.8 | 569.7623 | 2 | 37.47 | 8 | F8:18812 | 31122021\_RID\_3262\_1b.raw |  |  |  |  |  |  |  | 1.0724E5 | 1 | 0 | 0 | 0 | 0 | 0 | 0 | 0 | 1 | 639 | 647 |  |  | PEAKS DB |
| M.VNFTVDQIR.A | Y | 47.18 | 1090.5771 | 9 | -0.6 | 546.2955 | 2 | 37.58 | 8 | F8:18942 | 31122021\_RID\_3262\_1b.raw |  |  |  |  |  |  |  | 4.1315E4 | 1 | 0 | 0 | 0 | 0 | 0 | 0 | 0 | 1 | 2 | 10 |  |  | PEAKS DB |
| total 2 peptides |  |  |  |  |  |  |  |  |  |  |  |  |  |  |  |  |  |  |  |  |  |  |  |  |  |  |  |  |  |  |  |  |  |
| --- | --- | --- | --- | --- | --- | --- | --- | --- | --- | --- | --- | --- | --- | --- | --- | --- | --- | --- | --- | --- | --- | --- | --- | --- | --- | --- | --- | --- | --- | --- | --- | --- | --- |

XP\_026543467.1

back to list

  

| Protein Coverage
| Supporting Peptides
|

Protein Coverage:

Supporting Peptides:

| Peptide | Uniq | -10lgP | Mass | Length | ppm | m/z | z | RT | Fraction | Scan | Source File | Area F6 | Area F2 | Area F3 | Area F4 | Area F5 | Area F7 | Area F8 | Area F1 | #Feature | #Feature F6 | #Feature F2 | #Feature F3 | #Feature F4 | #Feature F5 | #Feature F7 | #Feature F8 | #Feature F1 | Start | End | PTM | AScore | Found By |
| --- | --- | --- | --- | --- | --- | --- | --- | --- | --- | --- | --- | --- | --- | --- | --- | --- | --- | --- | --- | --- | --- | --- | --- | --- | --- | --- | --- | --- | --- | --- | --- | --- | --- |
| K.YEWDVAEAR.K | Y | 53.01 | 1137.5090 | 9 | 0.8 | 569.7623 | 2 | 37.47 | 8 | F8:18812 | 31122021\_RID\_3262\_1b.raw |  |  |  |  |  |  |  | 1.0724E5 | 1 | 0 | 0 | 0 | 0 | 0 | 0 | 0 | 1 | 639 | 647 |  |  | PEAKS DB |
| M.VNFTVDQIR.A | Y | 47.18 | 1090.5771 | 9 | -0.6 | 546.2955 | 2 | 37.58 | 8 | F8:18942 | 31122021\_RID\_3262\_1b.raw |  |  |  |  |  |  |  | 4.1315E4 | 1 | 0 | 0 | 0 | 0 | 0 | 0 | 0 | 1 | 2 | 10 |  |  | PEAKS DB |
| total 2 peptides |  |  |  |  |  |  |  |  |  |  |  |  |  |  |  |  |  |  |  |  |  |  |  |  |  |  |  |  |  |  |  |  |  |
| --- | --- | --- | --- | --- | --- | --- | --- | --- | --- | --- | --- | --- | --- | --- | --- | --- | --- | --- | --- | --- | --- | --- | --- | --- | --- | --- | --- | --- | --- | --- | --- | --- | --- |

JAG68084.1

back to list

  

| Protein Coverage
| Supporting Peptides
|

Protein Coverage:

Supporting Peptides:

| Peptide | Uniq | -10lgP | Mass | Length | ppm | m/z | z | RT | Fraction | Scan | Source File | Area F6 | Area F2 | Area F3 | Area F4 | Area F5 | Area F7 | Area F8 | Area F1 | #Feature | #Feature F6 | #Feature F2 | #Feature F3 | #Feature F4 | #Feature F5 | #Feature F7 | #Feature F8 | #Feature F1 | Start | End | PTM | AScore | Found By |
| --- | --- | --- | --- | --- | --- | --- | --- | --- | --- | --- | --- | --- | --- | --- | --- | --- | --- | --- | --- | --- | --- | --- | --- | --- | --- | --- | --- | --- | --- | --- | --- | --- | --- |
| K.YEWDVAEAR.K | Y | 53.01 | 1137.5090 | 9 | 0.8 | 569.7623 | 2 | 37.47 | 8 | F8:18812 | 31122021\_RID\_3262\_1b.raw |  |  |  |  |  |  |  | 1.0724E5 | 1 | 0 | 0 | 0 | 0 | 0 | 0 | 0 | 1 | 639 | 647 |  |  | PEAKS DB |
| M.VNFTVDQIR.A | Y | 47.18 | 1090.5771 | 9 | -0.6 | 546.2955 | 2 | 37.58 | 8 | F8:18942 | 31122021\_RID\_3262\_1b.raw |  |  |  |  |  |  |  | 4.1315E4 | 1 | 0 | 0 | 0 | 0 | 0 | 0 | 0 | 1 | 2 | 10 |  |  | PEAKS DB |
| total 2 peptides |  |  |  |  |  |  |  |  |  |  |  |  |  |  |  |  |  |  |  |  |  |  |  |  |  |  |  |  |  |  |  |  |  |
| --- | --- | --- | --- | --- | --- | --- | --- | --- | --- | --- | --- | --- | --- | --- | --- | --- | --- | --- | --- | --- | --- | --- | --- | --- | --- | --- | --- | --- | --- | --- | --- | --- | --- |

XP\_034262248.1

back to list

  

| Protein Coverage
| Supporting Peptides
|

Protein Coverage:

Supporting Peptides:

| Peptide | Uniq | -10lgP | Mass | Length | ppm | m/z | z | RT | Fraction | Scan | Source File | Area F6 | Area F2 | Area F3 | Area F4 | Area F5 | Area F7 | Area F8 | Area F1 | #Feature | #Feature F6 | #Feature F2 | #Feature F3 | #Feature F4 | #Feature F5 | #Feature F7 | #Feature F8 | #Feature F1 | Start | End | PTM | AScore | Found By |
| --- | --- | --- | --- | --- | --- | --- | --- | --- | --- | --- | --- | --- | --- | --- | --- | --- | --- | --- | --- | --- | --- | --- | --- | --- | --- | --- | --- | --- | --- | --- | --- | --- | --- |
| K.YEWDVAEAR.K | Y | 53.01 | 1137.5090 | 9 | 0.8 | 569.7623 | 2 | 37.47 | 8 | F8:18812 | 31122021\_RID\_3262\_1b.raw |  |  |  |  |  |  |  | 1.0724E5 | 1 | 0 | 0 | 0 | 0 | 0 | 0 | 0 | 1 | 639 | 647 |  |  | PEAKS DB |
| M.VNFTVDQIR.A | Y | 47.18 | 1090.5771 | 9 | -0.6 | 546.2955 | 2 | 37.58 | 8 | F8:18942 | 31122021\_RID\_3262\_1b.raw |  |  |  |  |  |  |  | 4.1315E4 | 1 | 0 | 0 | 0 | 0 | 0 | 0 | 0 | 1 | 2 | 10 |  |  | PEAKS DB |
| total 2 peptides |  |  |  |  |  |  |  |  |  |  |  |  |  |  |  |  |  |  |  |  |  |  |  |  |  |  |  |  |  |  |  |  |  |
| --- | --- | --- | --- | --- | --- | --- | --- | --- | --- | --- | --- | --- | --- | --- | --- | --- | --- | --- | --- | --- | --- | --- | --- | --- | --- | --- | --- | --- | --- | --- | --- | --- | --- |

ETE57963.1

back to list

  

| Protein Coverage
| Supporting Peptides
|

Protein Coverage:

Supporting Peptides:

| Peptide | Uniq | -10lgP | Mass | Length | ppm | m/z | z | RT | Fraction | Scan | Source File | Area F6 | Area F2 | Area F3 | Area F4 | Area F5 | Area F7 | Area F8 | Area F1 | #Feature | #Feature F6 | #Feature F2 | #Feature F3 | #Feature F4 | #Feature F5 | #Feature F7 | #Feature F8 | #Feature F1 | Start | End | PTM | AScore | Found By |
| --- | --- | --- | --- | --- | --- | --- | --- | --- | --- | --- | --- | --- | --- | --- | --- | --- | --- | --- | --- | --- | --- | --- | --- | --- | --- | --- | --- | --- | --- | --- | --- | --- | --- |
| K.YEWDVAEAR.K | Y | 53.01 | 1137.5090 | 9 | 0.8 | 569.7623 | 2 | 37.47 | 8 | F8:18812 | 31122021\_RID\_3262\_1b.raw |  |  |  |  |  |  |  | 1.0724E5 | 1 | 0 | 0 | 0 | 0 | 0 | 0 | 0 | 1 | 562 | 570 |  |  | PEAKS DB |
| L.VNFTVDQIR.A | Y | 47.18 | 1090.5771 | 9 | -0.6 | 546.2955 | 2 | 37.58 | 8 | F8:18942 | 31122021\_RID\_3262\_1b.raw |  |  |  |  |  |  |  | 4.1315E4 | 1 | 0 | 0 | 0 | 0 | 0 | 0 | 0 | 1 | 20 | 28 |  |  | PEAKS DB |
| total 2 peptides |  |  |  |  |  |  |  |  |  |  |  |  |  |  |  |  |  |  |  |  |  |  |  |  |  |  |  |  |  |  |  |  |  |
| --- | --- | --- | --- | --- | --- | --- | --- | --- | --- | --- | --- | --- | --- | --- | --- | --- | --- | --- | --- | --- | --- | --- | --- | --- | --- | --- | --- | --- | --- | --- | --- | --- | --- |

LAB65750.1

back to list

  

| Protein Coverage
| Supporting Peptides
|

Protein Coverage:

Supporting Peptides:

| Peptide | Uniq | -10lgP | Mass | Length | ppm | m/z | z | RT | Fraction | Scan | Source File | Area F6 | Area F2 | Area F3 | Area F4 | Area F5 | Area F7 | Area F8 | Area F1 | #Feature | #Feature F6 | #Feature F2 | #Feature F3 | #Feature F4 | #Feature F5 | #Feature F7 | #Feature F8 | #Feature F1 | Start | End | PTM | AScore | Found By |
| --- | --- | --- | --- | --- | --- | --- | --- | --- | --- | --- | --- | --- | --- | --- | --- | --- | --- | --- | --- | --- | --- | --- | --- | --- | --- | --- | --- | --- | --- | --- | --- | --- | --- |
| K.YEWDVAEAR.K | Y | 53.01 | 1137.5090 | 9 | 0.8 | 569.7623 | 2 | 37.47 | 8 | F8:18812 | 31122021\_RID\_3262\_1b.raw |  |  |  |  |  |  |  | 1.0724E5 | 1 | 0 | 0 | 0 | 0 | 0 | 0 | 0 | 1 | 677 | 685 |  |  | PEAKS DB |
| M.VNFTVDQIR.A | Y | 47.18 | 1090.5771 | 9 | -0.6 | 546.2955 | 2 | 37.58 | 8 | F8:18942 | 31122021\_RID\_3262\_1b.raw |  |  |  |  |  |  |  | 4.1315E4 | 1 | 0 | 0 | 0 | 0 | 0 | 0 | 0 | 1 | 40 | 48 |  |  | PEAKS DB |
| total 2 peptides |  |  |  |  |  |  |  |  |  |  |  |  |  |  |  |  |  |  |  |  |  |  |  |  |  |  |  |  |  |  |  |  |  |
| --- | --- | --- | --- | --- | --- | --- | --- | --- | --- | --- | --- | --- | --- | --- | --- | --- | --- | --- | --- | --- | --- | --- | --- | --- | --- | --- | --- | --- | --- | --- | --- | --- | --- |

LAA97078.1

back to list

  

| Protein Coverage
| Supporting Peptides
|

Protein Coverage:

Supporting Peptides:

| Peptide | Uniq | -10lgP | Mass | Length | ppm | m/z | z | RT | Fraction | Scan | Source File | Area F6 | Area F2 | Area F3 | Area F4 | Area F5 | Area F7 | Area F8 | Area F1 | #Feature | #Feature F6 | #Feature F2 | #Feature F3 | #Feature F4 | #Feature F5 | #Feature F7 | #Feature F8 | #Feature F1 | Start | End | PTM | AScore | Found By |
| --- | --- | --- | --- | --- | --- | --- | --- | --- | --- | --- | --- | --- | --- | --- | --- | --- | --- | --- | --- | --- | --- | --- | --- | --- | --- | --- | --- | --- | --- | --- | --- | --- | --- |
| K.YEWDVAEAR.K | Y | 53.01 | 1137.5090 | 9 | 0.8 | 569.7623 | 2 | 37.47 | 8 | F8:18812 | 31122021\_RID\_3262\_1b.raw |  |  |  |  |  |  |  | 1.0724E5 | 1 | 0 | 0 | 0 | 0 | 0 | 0 | 0 | 1 | 677 | 685 |  |  | PEAKS DB |
| M.VNFTVDQIR.A | Y | 47.18 | 1090.5771 | 9 | -0.6 | 546.2955 | 2 | 37.58 | 8 | F8:18942 | 31122021\_RID\_3262\_1b.raw |  |  |  |  |  |  |  | 4.1315E4 | 1 | 0 | 0 | 0 | 0 | 0 | 0 | 0 | 1 | 40 | 48 |  |  | PEAKS DB |
| total 2 peptides |  |  |  |  |  |  |  |  |  |  |  |  |  |  |  |  |  |  |  |  |  |  |  |  |  |  |  |  |  |  |  |  |  |
| --- | --- | --- | --- | --- | --- | --- | --- | --- | --- | --- | --- | --- | --- | --- | --- | --- | --- | --- | --- | --- | --- | --- | --- | --- | --- | --- | --- | --- | --- | --- | --- | --- | --- |

XP\_026562277.1

back to list

  

| Protein Coverage
| Supporting Peptides
|

Protein Coverage:

Supporting Peptides:

| Peptide | Uniq | -10lgP | Mass | Length | ppm | m/z | z | RT | Fraction | Scan | Source File | Area F6 | Area F2 | Area F3 | Area F4 | Area F5 | Area F7 | Area F8 | Area F1 | #Feature | #Feature F6 | #Feature F2 | #Feature F3 | #Feature F4 | #Feature F5 | #Feature F7 | #Feature F8 | #Feature F1 | Start | End | PTM | AScore | Found By |
| --- | --- | --- | --- | --- | --- | --- | --- | --- | --- | --- | --- | --- | --- | --- | --- | --- | --- | --- | --- | --- | --- | --- | --- | --- | --- | --- | --- | --- | --- | --- | --- | --- | --- |
| R.NQYDNDVTVWSPQGR.I | Y | 51.68 | 1777.8020 | 15 | 0.5 | 889.9087 | 2 | 40.39 | 8 | F8:21153 | 31122021\_RID\_3262\_1b.raw |  |  |  |  |  |  |  | 8.2996E3 | 1 | 0 | 0 | 0 | 0 | 0 | 0 | 0 | 1 | 108 | 122 |  |  | PEAKS DB |
| R.FVFDRPLPVS.R | Y | 46.54 | 1175.6338 | 10 | 1.1 | 588.8248 | 2 | 49.32 | 8 | F8:28075 | 31122021\_RID\_3262\_1b.raw |  |  |  |  |  |  |  | 6.8195E4 | 1 | 0 | 0 | 0 | 0 | 0 | 0 | 0 | 1 | 201 | 210 |  |  | PEAKS DB |
| total 2 peptides |  |  |  |  |  |  |  |  |  |  |  |  |  |  |  |  |  |  |  |  |  |  |  |  |  |  |  |  |  |  |  |  |  |
| --- | --- | --- | --- | --- | --- | --- | --- | --- | --- | --- | --- | --- | --- | --- | --- | --- | --- | --- | --- | --- | --- | --- | --- | --- | --- | --- | --- | --- | --- | --- | --- | --- | --- |

XP\_007430978.1

back to list

  

| Protein Coverage
| Supporting Peptides
|

Protein Coverage:

Supporting Peptides:

| Peptide | Uniq | -10lgP | Mass | Length | ppm | m/z | z | RT | Fraction | Scan | Source File | Area F6 | Area F2 | Area F3 | Area F4 | Area F5 | Area F7 | Area F8 | Area F1 | #Feature | #Feature F6 | #Feature F2 | #Feature F3 | #Feature F4 | #Feature F5 | #Feature F7 | #Feature F8 | #Feature F1 | Start | End | PTM | AScore | Found By |
| --- | --- | --- | --- | --- | --- | --- | --- | --- | --- | --- | --- | --- | --- | --- | --- | --- | --- | --- | --- | --- | --- | --- | --- | --- | --- | --- | --- | --- | --- | --- | --- | --- | --- |
| R.NQYDNDVTVWSPQGR.I | Y | 51.68 | 1777.8020 | 15 | 0.5 | 889.9087 | 2 | 40.39 | 8 | F8:21153 | 31122021\_RID\_3262\_1b.raw |  |  |  |  |  |  |  | 8.2996E3 | 1 | 0 | 0 | 0 | 0 | 0 | 0 | 0 | 1 | 4 | 18 |  |  | PEAKS DB |
| R.FVFDRPLPVS.R | Y | 46.54 | 1175.6338 | 10 | 1.1 | 588.8248 | 2 | 49.32 | 8 | F8:28075 | 31122021\_RID\_3262\_1b.raw |  |  |  |  |  |  |  | 6.8195E4 | 1 | 0 | 0 | 0 | 0 | 0 | 0 | 0 | 1 | 97 | 106 |  |  | PEAKS DB |
| total 2 peptides |  |  |  |  |  |  |  |  |  |  |  |  |  |  |  |  |  |  |  |  |  |  |  |  |  |  |  |  |  |  |  |  |  |
| --- | --- | --- | --- | --- | --- | --- | --- | --- | --- | --- | --- | --- | --- | --- | --- | --- | --- | --- | --- | --- | --- | --- | --- | --- | --- | --- | --- | --- | --- | --- | --- | --- | --- |

XP\_026524124.1

back to list

  

| Protein Coverage
| Supporting Peptides
|

Protein Coverage:

Supporting Peptides:

| Peptide | Uniq | -10lgP | Mass | Length | ppm | m/z | z | RT | Fraction | Scan | Source File | Area F6 | Area F2 | Area F3 | Area F4 | Area F5 | Area F7 | Area F8 | Area F1 | #Feature | #Feature F6 | #Feature F2 | #Feature F3 | #Feature F4 | #Feature F5 | #Feature F7 | #Feature F8 | #Feature F1 | Start | End | PTM | AScore | Found By |
| --- | --- | --- | --- | --- | --- | --- | --- | --- | --- | --- | --- | --- | --- | --- | --- | --- | --- | --- | --- | --- | --- | --- | --- | --- | --- | --- | --- | --- | --- | --- | --- | --- | --- |
| R.NQYDNDVTVWSPQGR.I | Y | 51.68 | 1777.8020 | 15 | 0.5 | 889.9087 | 2 | 40.39 | 8 | F8:21153 | 31122021\_RID\_3262\_1b.raw |  |  |  |  |  |  |  | 8.2996E3 | 1 | 0 | 0 | 0 | 0 | 0 | 0 | 0 | 1 | 4 | 18 |  |  | PEAKS DB |
| R.FVFDRPLPVS.R | Y | 46.54 | 1175.6338 | 10 | 1.1 | 588.8248 | 2 | 49.32 | 8 | F8:28075 | 31122021\_RID\_3262\_1b.raw |  |  |  |  |  |  |  | 6.8195E4 | 1 | 0 | 0 | 0 | 0 | 0 | 0 | 0 | 1 | 97 | 106 |  |  | PEAKS DB |
| total 2 peptides |  |  |  |  |  |  |  |  |  |  |  |  |  |  |  |  |  |  |  |  |  |  |  |  |  |  |  |  |  |  |  |  |  |
| --- | --- | --- | --- | --- | --- | --- | --- | --- | --- | --- | --- | --- | --- | --- | --- | --- | --- | --- | --- | --- | --- | --- | --- | --- | --- | --- | --- | --- | --- | --- | --- | --- | --- |

JAB53608.1

back to list

  

| Protein Coverage
| Supporting Peptides
|

Protein Coverage:

Supporting Peptides:

| Peptide | Uniq | -10lgP | Mass | Length | ppm | m/z | z | RT | Fraction | Scan | Source File | Area F6 | Area F2 | Area F3 | Area F4 | Area F5 | Area F7 | Area F8 | Area F1 | #Feature | #Feature F6 | #Feature F2 | #Feature F3 | #Feature F4 | #Feature F5 | #Feature F7 | #Feature F8 | #Feature F1 | Start | End | PTM | AScore | Found By |
| --- | --- | --- | --- | --- | --- | --- | --- | --- | --- | --- | --- | --- | --- | --- | --- | --- | --- | --- | --- | --- | --- | --- | --- | --- | --- | --- | --- | --- | --- | --- | --- | --- | --- |
| R.NQYDNDVTVWSPQGR.I | Y | 51.68 | 1777.8020 | 15 | 0.5 | 889.9087 | 2 | 40.39 | 8 | F8:21153 | 31122021\_RID\_3262\_1b.raw |  |  |  |  |  |  |  | 8.2996E3 | 1 | 0 | 0 | 0 | 0 | 0 | 0 | 0 | 1 | 4 | 18 |  |  | PEAKS DB |
| R.FVFDRPLPVS.R | Y | 46.54 | 1175.6338 | 10 | 1.1 | 588.8248 | 2 | 49.32 | 8 | F8:28075 | 31122021\_RID\_3262\_1b.raw |  |  |  |  |  |  |  | 6.8195E4 | 1 | 0 | 0 | 0 | 0 | 0 | 0 | 0 | 1 | 97 | 106 |  |  | PEAKS DB |
| total 2 peptides |  |  |  |  |  |  |  |  |  |  |  |  |  |  |  |  |  |  |  |  |  |  |  |  |  |  |  |  |  |  |  |  |  |
| --- | --- | --- | --- | --- | --- | --- | --- | --- | --- | --- | --- | --- | --- | --- | --- | --- | --- | --- | --- | --- | --- | --- | --- | --- | --- | --- | --- | --- | --- | --- | --- | --- | --- |

JAG66921.1

back to list

  

| Protein Coverage
| Supporting Peptides
|

Protein Coverage:

Supporting Peptides:

| Peptide | Uniq | -10lgP | Mass | Length | ppm | m/z | z | RT | Fraction | Scan | Source File | Area F6 | Area F2 | Area F3 | Area F4 | Area F5 | Area F7 | Area F8 | Area F1 | #Feature | #Feature F6 | #Feature F2 | #Feature F3 | #Feature F4 | #Feature F5 | #Feature F7 | #Feature F8 | #Feature F1 | Start | End | PTM | AScore | Found By |
| --- | --- | --- | --- | --- | --- | --- | --- | --- | --- | --- | --- | --- | --- | --- | --- | --- | --- | --- | --- | --- | --- | --- | --- | --- | --- | --- | --- | --- | --- | --- | --- | --- | --- |
| R.NQYDNDVTVWSPQGR.I | Y | 51.68 | 1777.8020 | 15 | 0.5 | 889.9087 | 2 | 40.39 | 8 | F8:21153 | 31122021\_RID\_3262\_1b.raw |  |  |  |  |  |  |  | 8.2996E3 | 1 | 0 | 0 | 0 | 0 | 0 | 0 | 0 | 1 | 4 | 18 |  |  | PEAKS DB |
| R.FVFDRPLPVS.R | Y | 46.54 | 1175.6338 | 10 | 1.1 | 588.8248 | 2 | 49.32 | 8 | F8:28075 | 31122021\_RID\_3262\_1b.raw |  |  |  |  |  |  |  | 6.8195E4 | 1 | 0 | 0 | 0 | 0 | 0 | 0 | 0 | 1 | 97 | 106 |  |  | PEAKS DB |
| total 2 peptides |  |  |  |  |  |  |  |  |  |  |  |  |  |  |  |  |  |  |  |  |  |  |  |  |  |  |  |  |  |  |  |  |  |
| --- | --- | --- | --- | --- | --- | --- | --- | --- | --- | --- | --- | --- | --- | --- | --- | --- | --- | --- | --- | --- | --- | --- | --- | --- | --- | --- | --- | --- | --- | --- | --- | --- | --- |

XP\_032078732.1

back to list

  

| Protein Coverage
| Supporting Peptides
|

Protein Coverage:

Supporting Peptides:

| Peptide | Uniq | -10lgP | Mass | Length | ppm | m/z | z | RT | Fraction | Scan | Source File | Area F6 | Area F2 | Area F3 | Area F4 | Area F5 | Area F7 | Area F8 | Area F1 | #Feature | #Feature F6 | #Feature F2 | #Feature F3 | #Feature F4 | #Feature F5 | #Feature F7 | #Feature F8 | #Feature F1 | Start | End | PTM | AScore | Found By |
| --- | --- | --- | --- | --- | --- | --- | --- | --- | --- | --- | --- | --- | --- | --- | --- | --- | --- | --- | --- | --- | --- | --- | --- | --- | --- | --- | --- | --- | --- | --- | --- | --- | --- |
| R.NQYDNDVTVWSPQGR.I | Y | 51.68 | 1777.8020 | 15 | 0.5 | 889.9087 | 2 | 40.39 | 8 | F8:21153 | 31122021\_RID\_3262\_1b.raw |  |  |  |  |  |  |  | 8.2996E3 | 1 | 0 | 0 | 0 | 0 | 0 | 0 | 0 | 1 | 4 | 18 |  |  | PEAKS DB |
| R.FVFDRPLPVS.R | Y | 46.54 | 1175.6338 | 10 | 1.1 | 588.8248 | 2 | 49.32 | 8 | F8:28075 | 31122021\_RID\_3262\_1b.raw |  |  |  |  |  |  |  | 6.8195E4 | 1 | 0 | 0 | 0 | 0 | 0 | 0 | 0 | 1 | 97 | 106 |  |  | PEAKS DB |
| total 2 peptides |  |  |  |  |  |  |  |  |  |  |  |  |  |  |  |  |  |  |  |  |  |  |  |  |  |  |  |  |  |  |  |  |  |
| --- | --- | --- | --- | --- | --- | --- | --- | --- | --- | --- | --- | --- | --- | --- | --- | --- | --- | --- | --- | --- | --- | --- | --- | --- | --- | --- | --- | --- | --- | --- | --- | --- | --- |

XP\_034272893.1

back to list

  

| Protein Coverage
| Supporting Peptides
|

Protein Coverage:

Supporting Peptides:

| Peptide | Uniq | -10lgP | Mass | Length | ppm | m/z | z | RT | Fraction | Scan | Source File | Area F6 | Area F2 | Area F3 | Area F4 | Area F5 | Area F7 | Area F8 | Area F1 | #Feature | #Feature F6 | #Feature F2 | #Feature F3 | #Feature F4 | #Feature F5 | #Feature F7 | #Feature F8 | #Feature F1 | Start | End | PTM | AScore | Found By |
| --- | --- | --- | --- | --- | --- | --- | --- | --- | --- | --- | --- | --- | --- | --- | --- | --- | --- | --- | --- | --- | --- | --- | --- | --- | --- | --- | --- | --- | --- | --- | --- | --- | --- |
| R.NQYDNDVTVWSPQGR.I | Y | 51.68 | 1777.8020 | 15 | 0.5 | 889.9087 | 2 | 40.39 | 8 | F8:21153 | 31122021\_RID\_3262\_1b.raw |  |  |  |  |  |  |  | 8.2996E3 | 1 | 0 | 0 | 0 | 0 | 0 | 0 | 0 | 1 | 4 | 18 |  |  | PEAKS DB |
| R.FVFDRPLPVS.R | Y | 46.54 | 1175.6338 | 10 | 1.1 | 588.8248 | 2 | 49.32 | 8 | F8:28075 | 31122021\_RID\_3262\_1b.raw |  |  |  |  |  |  |  | 6.8195E4 | 1 | 0 | 0 | 0 | 0 | 0 | 0 | 0 | 1 | 97 | 106 |  |  | PEAKS DB |
| total 2 peptides |  |  |  |  |  |  |  |  |  |  |  |  |  |  |  |  |  |  |  |  |  |  |  |  |  |  |  |  |  |  |  |  |  |
| --- | --- | --- | --- | --- | --- | --- | --- | --- | --- | --- | --- | --- | --- | --- | --- | --- | --- | --- | --- | --- | --- | --- | --- | --- | --- | --- | --- | --- | --- | --- | --- | --- | --- |

JAI09621.1

back to list

  

| Protein Coverage
| Supporting Peptides
|

Protein Coverage:

Supporting Peptides:

| Peptide | Uniq | -10lgP | Mass | Length | ppm | m/z | z | RT | Fraction | Scan | Source File | Area F6 | Area F2 | Area F3 | Area F4 | Area F5 | Area F7 | Area F8 | Area F1 | #Feature | #Feature F6 | #Feature F2 | #Feature F3 | #Feature F4 | #Feature F5 | #Feature F7 | #Feature F8 | #Feature F1 | Start | End | PTM | AScore | Found By |
| --- | --- | --- | --- | --- | --- | --- | --- | --- | --- | --- | --- | --- | --- | --- | --- | --- | --- | --- | --- | --- | --- | --- | --- | --- | --- | --- | --- | --- | --- | --- | --- | --- | --- |
| R.NQYDNDVTVWSPQGR.I | Y | 51.68 | 1777.8020 | 15 | 0.5 | 889.9087 | 2 | 40.39 | 8 | F8:21153 | 31122021\_RID\_3262\_1b.raw |  |  |  |  |  |  |  | 8.2996E3 | 1 | 0 | 0 | 0 | 0 | 0 | 0 | 0 | 1 | 4 | 18 |  |  | PEAKS DB |
| R.FVFDRPLPVS.R | Y | 46.54 | 1175.6338 | 10 | 1.1 | 588.8248 | 2 | 49.32 | 8 | F8:28075 | 31122021\_RID\_3262\_1b.raw |  |  |  |  |  |  |  | 6.8195E4 | 1 | 0 | 0 | 0 | 0 | 0 | 0 | 0 | 1 | 97 | 106 |  |  | PEAKS DB |
| total 2 peptides |  |  |  |  |  |  |  |  |  |  |  |  |  |  |  |  |  |  |  |  |  |  |  |  |  |  |  |  |  |  |  |  |  |
| --- | --- | --- | --- | --- | --- | --- | --- | --- | --- | --- | --- | --- | --- | --- | --- | --- | --- | --- | --- | --- | --- | --- | --- | --- | --- | --- | --- | --- | --- | --- | --- | --- | --- |

XP\_013919925.1

back to list

  

| Protein Coverage
| Supporting Peptides
|

Protein Coverage:

Supporting Peptides:

| Peptide | Uniq | -10lgP | Mass | Length | ppm | m/z | z | RT | Fraction | Scan | Source File | Area F6 | Area F2 | Area F3 | Area F4 | Area F5 | Area F7 | Area F8 | Area F1 | #Feature | #Feature F6 | #Feature F2 | #Feature F3 | #Feature F4 | #Feature F5 | #Feature F7 | #Feature F8 | #Feature F1 | Start | End | PTM | AScore | Found By |
| --- | --- | --- | --- | --- | --- | --- | --- | --- | --- | --- | --- | --- | --- | --- | --- | --- | --- | --- | --- | --- | --- | --- | --- | --- | --- | --- | --- | --- | --- | --- | --- | --- | --- |
| R.NQYDNDVTVWSPQGR.I | Y | 51.68 | 1777.8020 | 15 | 0.5 | 889.9087 | 2 | 40.39 | 8 | F8:21153 | 31122021\_RID\_3262\_1b.raw |  |  |  |  |  |  |  | 8.2996E3 | 1 | 0 | 0 | 0 | 0 | 0 | 0 | 0 | 1 | 4 | 18 |  |  | PEAKS DB |
| R.FVFDRPLPVS.R | Y | 46.54 | 1175.6338 | 10 | 1.1 | 588.8248 | 2 | 49.32 | 8 | F8:28075 | 31122021\_RID\_3262\_1b.raw |  |  |  |  |  |  |  | 6.8195E4 | 1 | 0 | 0 | 0 | 0 | 0 | 0 | 0 | 1 | 97 | 106 |  |  | PEAKS DB |
| total 2 peptides |  |  |  |  |  |  |  |  |  |  |  |  |  |  |  |  |  |  |  |  |  |  |  |  |  |  |  |  |  |  |  |  |  |
| --- | --- | --- | --- | --- | --- | --- | --- | --- | --- | --- | --- | --- | --- | --- | --- | --- | --- | --- | --- | --- | --- | --- | --- | --- | --- | --- | --- | --- | --- | --- | --- | --- | --- |

LAA24276.1

back to list

  

| Protein Coverage
| Supporting Peptides
|

Protein Coverage:

Supporting Peptides:

| Peptide | Uniq | -10lgP | Mass | Length | ppm | m/z | z | RT | Fraction | Scan | Source File | Area F6 | Area F2 | Area F3 | Area F4 | Area F5 | Area F7 | Area F8 | Area F1 | #Feature | #Feature F6 | #Feature F2 | #Feature F3 | #Feature F4 | #Feature F5 | #Feature F7 | #Feature F8 | #Feature F1 | Start | End | PTM | AScore | Found By |
| --- | --- | --- | --- | --- | --- | --- | --- | --- | --- | --- | --- | --- | --- | --- | --- | --- | --- | --- | --- | --- | --- | --- | --- | --- | --- | --- | --- | --- | --- | --- | --- | --- | --- |
| R.NQYDNDVTVWSPQGR.I | Y | 51.68 | 1777.8020 | 15 | 0.5 | 889.9087 | 2 | 40.39 | 8 | F8:21153 | 31122021\_RID\_3262\_1b.raw |  |  |  |  |  |  |  | 8.2996E3 | 1 | 0 | 0 | 0 | 0 | 0 | 0 | 0 | 1 | 49 | 63 |  |  | PEAKS DB |
| R.FVFDRPLPVS.R | Y | 46.54 | 1175.6338 | 10 | 1.1 | 588.8248 | 2 | 49.32 | 8 | F8:28075 | 31122021\_RID\_3262\_1b.raw |  |  |  |  |  |  |  | 6.8195E4 | 1 | 0 | 0 | 0 | 0 | 0 | 0 | 0 | 1 | 142 | 151 |  |  | PEAKS DB |
| total 2 peptides |  |  |  |  |  |  |  |  |  |  |  |  |  |  |  |  |  |  |  |  |  |  |  |  |  |  |  |  |  |  |  |  |  |
| --- | --- | --- | --- | --- | --- | --- | --- | --- | --- | --- | --- | --- | --- | --- | --- | --- | --- | --- | --- | --- | --- | --- | --- | --- | --- | --- | --- | --- | --- | --- | --- | --- | --- |

ETE67788.1

back to list

  

| Protein Coverage
| Supporting Peptides
|

Protein Coverage:

Supporting Peptides:

| Peptide | Uniq | -10lgP | Mass | Length | ppm | m/z | z | RT | Fraction | Scan | Source File | Area F6 | Area F2 | Area F3 | Area F4 | Area F5 | Area F7 | Area F8 | Area F1 | #Feature | #Feature F6 | #Feature F2 | #Feature F3 | #Feature F4 | #Feature F5 | #Feature F7 | #Feature F8 | #Feature F1 | Start | End | PTM | AScore | Found By |
| --- | --- | --- | --- | --- | --- | --- | --- | --- | --- | --- | --- | --- | --- | --- | --- | --- | --- | --- | --- | --- | --- | --- | --- | --- | --- | --- | --- | --- | --- | --- | --- | --- | --- |
| R.NQYDNDVTVWSPQGR.I | Y | 51.68 | 1777.8020 | 15 | 0.5 | 889.9087 | 2 | 40.39 | 8 | F8:21153 | 31122021\_RID\_3262\_1b.raw |  |  |  |  |  |  |  | 8.2996E3 | 1 | 0 | 0 | 0 | 0 | 0 | 0 | 0 | 1 | 78 | 92 |  |  | PEAKS DB |
| R.FVFDRPLPVS.R | Y | 46.54 | 1175.6338 | 10 | 1.1 | 588.8248 | 2 | 49.32 | 8 | F8:28075 | 31122021\_RID\_3262\_1b.raw |  |  |  |  |  |  |  | 6.8195E4 | 1 | 0 | 0 | 0 | 0 | 0 | 0 | 0 | 1 | 171 | 180 |  |  | PEAKS DB |
| total 2 peptides |  |  |  |  |  |  |  |  |  |  |  |  |  |  |  |  |  |  |  |  |  |  |  |  |  |  |  |  |  |  |  |  |  |
| --- | --- | --- | --- | --- | --- | --- | --- | --- | --- | --- | --- | --- | --- | --- | --- | --- | --- | --- | --- | --- | --- | --- | --- | --- | --- | --- | --- | --- | --- | --- | --- | --- | --- |

JAI11932.1

back to list

  

| Protein Coverage
| Supporting Peptides
|

Protein Coverage:

Supporting Peptides:

| Peptide | Uniq | -10lgP | Mass | Length | ppm | m/z | z | RT | Fraction | Scan | Source File | Area F6 | Area F2 | Area F3 | Area F4 | Area F5 | Area F7 | Area F8 | Area F1 | #Feature | #Feature F6 | #Feature F2 | #Feature F3 | #Feature F4 | #Feature F5 | #Feature F7 | #Feature F8 | #Feature F1 | Start | End | PTM | AScore | Found By |
| --- | --- | --- | --- | --- | --- | --- | --- | --- | --- | --- | --- | --- | --- | --- | --- | --- | --- | --- | --- | --- | --- | --- | --- | --- | --- | --- | --- | --- | --- | --- | --- | --- | --- |
| R.NQYDNDVTVWSPQGR.I | Y | 51.68 | 1777.8020 | 15 | 0.5 | 889.9087 | 2 | 40.39 | 8 | F8:21153 | 31122021\_RID\_3262\_1b.raw |  |  |  |  |  |  |  | 8.2996E3 | 1 | 0 | 0 | 0 | 0 | 0 | 0 | 0 | 1 | 4 | 18 |  |  | PEAKS DB |
| R.FVFDRPLPVS.R | Y | 46.54 | 1175.6338 | 10 | 1.1 | 588.8248 | 2 | 49.32 | 8 | F8:28075 | 31122021\_RID\_3262\_1b.raw |  |  |  |  |  |  |  | 6.8195E4 | 1 | 0 | 0 | 0 | 0 | 0 | 0 | 0 | 1 | 97 | 106 |  |  | PEAKS DB |
| total 2 peptides |  |  |  |  |  |  |  |  |  |  |  |  |  |  |  |  |  |  |  |  |  |  |  |  |  |  |  |  |  |  |  |  |  |
| --- | --- | --- | --- | --- | --- | --- | --- | --- | --- | --- | --- | --- | --- | --- | --- | --- | --- | --- | --- | --- | --- | --- | --- | --- | --- | --- | --- | --- | --- | --- | --- | --- | --- |

JAA96037.1

back to list

  

| Protein Coverage
| Supporting Peptides
|

Protein Coverage:

Supporting Peptides:

| Peptide | Uniq | -10lgP | Mass | Length | ppm | m/z | z | RT | Fraction | Scan | Source File | Area F6 | Area F2 | Area F3 | Area F4 | Area F5 | Area F7 | Area F8 | Area F1 | #Feature | #Feature F6 | #Feature F2 | #Feature F3 | #Feature F4 | #Feature F5 | #Feature F7 | #Feature F8 | #Feature F1 | Start | End | PTM | AScore | Found By |
| --- | --- | --- | --- | --- | --- | --- | --- | --- | --- | --- | --- | --- | --- | --- | --- | --- | --- | --- | --- | --- | --- | --- | --- | --- | --- | --- | --- | --- | --- | --- | --- | --- | --- |
| R.NQYDNDVTVWSPQGR.I | Y | 51.68 | 1777.8020 | 15 | 0.5 | 889.9087 | 2 | 40.39 | 8 | F8:21153 | 31122021\_RID\_3262\_1b.raw |  |  |  |  |  |  |  | 8.2996E3 | 1 | 0 | 0 | 0 | 0 | 0 | 0 | 0 | 1 | 4 | 18 |  |  | PEAKS DB |
| R.FVFDRPLPVS.R | Y | 46.54 | 1175.6338 | 10 | 1.1 | 588.8248 | 2 | 49.32 | 8 | F8:28075 | 31122021\_RID\_3262\_1b.raw |  |  |  |  |  |  |  | 6.8195E4 | 1 | 0 | 0 | 0 | 0 | 0 | 0 | 0 | 1 | 97 | 106 |  |  | PEAKS DB |
| total 2 peptides |  |  |  |  |  |  |  |  |  |  |  |  |  |  |  |  |  |  |  |  |  |  |  |  |  |  |  |  |  |  |  |  |  |
| --- | --- | --- | --- | --- | --- | --- | --- | --- | --- | --- | --- | --- | --- | --- | --- | --- | --- | --- | --- | --- | --- | --- | --- | --- | --- | --- | --- | --- | --- | --- | --- | --- | --- |

JAV49557.1

back to list

  

| Protein Coverage
| Supporting Peptides
|

Protein Coverage:

Supporting Peptides:

| Peptide | Uniq | -10lgP | Mass | Length | ppm | m/z | z | RT | Fraction | Scan | Source File | Area F6 | Area F2 | Area F3 | Area F4 | Area F5 | Area F7 | Area F8 | Area F1 | #Feature | #Feature F6 | #Feature F2 | #Feature F3 | #Feature F4 | #Feature F5 | #Feature F7 | #Feature F8 | #Feature F1 | Start | End | PTM | AScore | Found By |
| --- | --- | --- | --- | --- | --- | --- | --- | --- | --- | --- | --- | --- | --- | --- | --- | --- | --- | --- | --- | --- | --- | --- | --- | --- | --- | --- | --- | --- | --- | --- | --- | --- | --- |
| R.NQYDNDVTVWSPQGR.I | Y | 51.68 | 1777.8020 | 15 | 0.5 | 889.9087 | 2 | 40.39 | 8 | F8:21153 | 31122021\_RID\_3262\_1b.raw |  |  |  |  |  |  |  | 8.2996E3 | 1 | 0 | 0 | 0 | 0 | 0 | 0 | 0 | 1 | 4 | 18 |  |  | PEAKS DB |
| R.FVFDRPLPVS.R | Y | 46.54 | 1175.6338 | 10 | 1.1 | 588.8248 | 2 | 49.32 | 8 | F8:28075 | 31122021\_RID\_3262\_1b.raw |  |  |  |  |  |  |  | 6.8195E4 | 1 | 0 | 0 | 0 | 0 | 0 | 0 | 0 | 1 | 97 | 106 |  |  | PEAKS DB |
| total 2 peptides |  |  |  |  |  |  |  |  |  |  |  |  |  |  |  |  |  |  |  |  |  |  |  |  |  |  |  |  |  |  |  |  |  |
| --- | --- | --- | --- | --- | --- | --- | --- | --- | --- | --- | --- | --- | --- | --- | --- | --- | --- | --- | --- | --- | --- | --- | --- | --- | --- | --- | --- | --- | --- | --- | --- | --- | --- |

AFJ51033.1

back to list

  

| Protein Coverage
| Supporting Peptides
|

Protein Coverage:

Supporting Peptides:

| Peptide | Uniq | -10lgP | Mass | Length | ppm | m/z | z | RT | Fraction | Scan | Source File | Area F6 | Area F2 | Area F3 | Area F4 | Area F5 | Area F7 | Area F8 | Area F1 | #Feature | #Feature F6 | #Feature F2 | #Feature F3 | #Feature F4 | #Feature F5 | #Feature F7 | #Feature F8 | #Feature F1 | Start | End | PTM | AScore | Found By |
| --- | --- | --- | --- | --- | --- | --- | --- | --- | --- | --- | --- | --- | --- | --- | --- | --- | --- | --- | --- | --- | --- | --- | --- | --- | --- | --- | --- | --- | --- | --- | --- | --- | --- |
| R.NQYDNDVTVWSPQGR.I | Y | 51.68 | 1777.8020 | 15 | 0.5 | 889.9087 | 2 | 40.39 | 8 | F8:21153 | 31122021\_RID\_3262\_1b.raw |  |  |  |  |  |  |  | 8.2996E3 | 1 | 0 | 0 | 0 | 0 | 0 | 0 | 0 | 1 | 4 | 18 |  |  | PEAKS DB |
| R.FVFDRPLPVS.R | Y | 46.54 | 1175.6338 | 10 | 1.1 | 588.8248 | 2 | 49.32 | 8 | F8:28075 | 31122021\_RID\_3262\_1b.raw |  |  |  |  |  |  |  | 6.8195E4 | 1 | 0 | 0 | 0 | 0 | 0 | 0 | 0 | 1 | 97 | 106 |  |  | PEAKS DB |
| total 2 peptides |  |  |  |  |  |  |  |  |  |  |  |  |  |  |  |  |  |  |  |  |  |  |  |  |  |  |  |  |  |  |  |  |  |
| --- | --- | --- | --- | --- | --- | --- | --- | --- | --- | --- | --- | --- | --- | --- | --- | --- | --- | --- | --- | --- | --- | --- | --- | --- | --- | --- | --- | --- | --- | --- | --- | --- | --- |

XP\_015679799.1

back to list

  

| Protein Coverage
| Supporting Peptides
|

Protein Coverage:

Supporting Peptides:

| Peptide | Uniq | -10lgP | Mass | Length | ppm | m/z | z | RT | Fraction | Scan | Source File | Area F6 | Area F2 | Area F3 | Area F4 | Area F5 | Area F7 | Area F8 | Area F1 | #Feature | #Feature F6 | #Feature F2 | #Feature F3 | #Feature F4 | #Feature F5 | #Feature F7 | #Feature F8 | #Feature F1 | Start | End | PTM | AScore | Found By |
| --- | --- | --- | --- | --- | --- | --- | --- | --- | --- | --- | --- | --- | --- | --- | --- | --- | --- | --- | --- | --- | --- | --- | --- | --- | --- | --- | --- | --- | --- | --- | --- | --- | --- |
| R.NQYDNDVTVWSPQGR.I | Y | 51.68 | 1777.8020 | 15 | 0.5 | 889.9087 | 2 | 40.39 | 8 | F8:21153 | 31122021\_RID\_3262\_1b.raw |  |  |  |  |  |  |  | 8.2996E3 | 1 | 0 | 0 | 0 | 0 | 0 | 0 | 0 | 1 | 42 | 56 |  |  | PEAKS DB |
| R.FVFDRPLPVS.R | Y | 46.54 | 1175.6338 | 10 | 1.1 | 588.8248 | 2 | 49.32 | 8 | F8:28075 | 31122021\_RID\_3262\_1b.raw |  |  |  |  |  |  |  | 6.8195E4 | 1 | 0 | 0 | 0 | 0 | 0 | 0 | 0 | 1 | 135 | 144 |  |  | PEAKS DB |
| total 2 peptides |  |  |  |  |  |  |  |  |  |  |  |  |  |  |  |  |  |  |  |  |  |  |  |  |  |  |  |  |  |  |  |  |  |
| --- | --- | --- | --- | --- | --- | --- | --- | --- | --- | --- | --- | --- | --- | --- | --- | --- | --- | --- | --- | --- | --- | --- | --- | --- | --- | --- | --- | --- | --- | --- | --- | --- | --- |

AFJ51039.1

back to list

  

| Protein Coverage
| Supporting Peptides
|

Protein Coverage:

Supporting Peptides:

| Peptide | Uniq | -10lgP | Mass | Length | ppm | m/z | z | RT | Fraction | Scan | Source File | Area F6 | Area F2 | Area F3 | Area F4 | Area F5 | Area F7 | Area F8 | Area F1 | #Feature | #Feature F6 | #Feature F2 | #Feature F3 | #Feature F4 | #Feature F5 | #Feature F7 | #Feature F8 | #Feature F1 | Start | End | PTM | AScore | Found By |
| --- | --- | --- | --- | --- | --- | --- | --- | --- | --- | --- | --- | --- | --- | --- | --- | --- | --- | --- | --- | --- | --- | --- | --- | --- | --- | --- | --- | --- | --- | --- | --- | --- | --- |
| K.AINQGGLTSVAVR.G | Y | 66.22 | 1284.7150 | 13 | 0.6 | 643.3651 | 2 | 33.23 | 8 | F8:15490 | 31122021\_RID\_3262\_1b.raw |  |  |  |  |  |  |  | 1.5738E5 | 1 | 0 | 0 | 0 | 0 | 0 | 0 | 0 | 1 | 31 | 43 |  |  | PEAKS DB |
| total 1 peptides |  |  |  |  |  |  |  |  |  |  |  |  |  |  |  |  |  |  |  |  |  |  |  |  |  |  |  |  |  |  |  |  |  |
| --- | --- | --- | --- | --- | --- | --- | --- | --- | --- | --- | --- | --- | --- | --- | --- | --- | --- | --- | --- | --- | --- | --- | --- | --- | --- | --- | --- | --- | --- | --- | --- | --- | --- |

XP\_026520282.1

back to list

  

| Protein Coverage
| Supporting Peptides
|

Protein Coverage:

Supporting Peptides:

| Peptide | Uniq | -10lgP | Mass | Length | ppm | m/z | z | RT | Fraction | Scan | Source File | Area F6 | Area F2 | Area F3 | Area F4 | Area F5 | Area F7 | Area F8 | Area F1 | #Feature | #Feature F6 | #Feature F2 | #Feature F3 | #Feature F4 | #Feature F5 | #Feature F7 | #Feature F8 | #Feature F1 | Start | End | PTM | AScore | Found By |
| --- | --- | --- | --- | --- | --- | --- | --- | --- | --- | --- | --- | --- | --- | --- | --- | --- | --- | --- | --- | --- | --- | --- | --- | --- | --- | --- | --- | --- | --- | --- | --- | --- | --- |
| K.AINQGGLTSVAVR.G | Y | 66.22 | 1284.7150 | 13 | 0.6 | 643.3651 | 2 | 33.23 | 8 | F8:15490 | 31122021\_RID\_3262\_1b.raw |  |  |  |  |  |  |  | 1.5738E5 | 1 | 0 | 0 | 0 | 0 | 0 | 0 | 0 | 1 | 31 | 43 |  |  | PEAKS DB |
| total 1 peptides |  |  |  |  |  |  |  |  |  |  |  |  |  |  |  |  |  |  |  |  |  |  |  |  |  |  |  |  |  |  |  |  |  |
| --- | --- | --- | --- | --- | --- | --- | --- | --- | --- | --- | --- | --- | --- | --- | --- | --- | --- | --- | --- | --- | --- | --- | --- | --- | --- | --- | --- | --- | --- | --- | --- | --- | --- |

XP\_034273914.1

back to list

  

| Protein Coverage
| Supporting Peptides
|

Protein Coverage:

Supporting Peptides:

| Peptide | Uniq | -10lgP | Mass | Length | ppm | m/z | z | RT | Fraction | Scan | Source File | Area F6 | Area F2 | Area F3 | Area F4 | Area F5 | Area F7 | Area F8 | Area F1 | #Feature | #Feature F6 | #Feature F2 | #Feature F3 | #Feature F4 | #Feature F5 | #Feature F7 | #Feature F8 | #Feature F1 | Start | End | PTM | AScore | Found By |
| --- | --- | --- | --- | --- | --- | --- | --- | --- | --- | --- | --- | --- | --- | --- | --- | --- | --- | --- | --- | --- | --- | --- | --- | --- | --- | --- | --- | --- | --- | --- | --- | --- | --- |
| K.AINQGGLTSVAVR.G | Y | 66.22 | 1284.7150 | 13 | 0.6 | 643.3651 | 2 | 33.23 | 8 | F8:15490 | 31122021\_RID\_3262\_1b.raw |  |  |  |  |  |  |  | 1.5738E5 | 1 | 0 | 0 | 0 | 0 | 0 | 0 | 0 | 1 | 31 | 43 |  |  | PEAKS DB |
| total 1 peptides |  |  |  |  |  |  |  |  |  |  |  |  |  |  |  |  |  |  |  |  |  |  |  |  |  |  |  |  |  |  |  |  |  |
| --- | --- | --- | --- | --- | --- | --- | --- | --- | --- | --- | --- | --- | --- | --- | --- | --- | --- | --- | --- | --- | --- | --- | --- | --- | --- | --- | --- | --- | --- | --- | --- | --- | --- |

XP\_026566911.1

back to list

  

| Protein Coverage
| Supporting Peptides
|

Protein Coverage:

Supporting Peptides:

| Peptide | Uniq | -10lgP | Mass | Length | ppm | m/z | z | RT | Fraction | Scan | Source File | Area F6 | Area F2 | Area F3 | Area F4 | Area F5 | Area F7 | Area F8 | Area F1 | #Feature | #Feature F6 | #Feature F2 | #Feature F3 | #Feature F4 | #Feature F5 | #Feature F7 | #Feature F8 | #Feature F1 | Start | End | PTM | AScore | Found By |
| --- | --- | --- | --- | --- | --- | --- | --- | --- | --- | --- | --- | --- | --- | --- | --- | --- | --- | --- | --- | --- | --- | --- | --- | --- | --- | --- | --- | --- | --- | --- | --- | --- | --- |
| K.AINQGGLTSVAVR.G | Y | 66.22 | 1284.7150 | 13 | 0.6 | 643.3651 | 2 | 33.23 | 8 | F8:15490 | 31122021\_RID\_3262\_1b.raw |  |  |  |  |  |  |  | 1.5738E5 | 1 | 0 | 0 | 0 | 0 | 0 | 0 | 0 | 1 | 31 | 43 |  |  | PEAKS DB |
| total 1 peptides |  |  |  |  |  |  |  |  |  |  |  |  |  |  |  |  |  |  |  |  |  |  |  |  |  |  |  |  |  |  |  |  |  |
| --- | --- | --- | --- | --- | --- | --- | --- | --- | --- | --- | --- | --- | --- | --- | --- | --- | --- | --- | --- | --- | --- | --- | --- | --- | --- | --- | --- | --- | --- | --- | --- | --- | --- |

JAI11927.1

back to list

  

| Protein Coverage
| Supporting Peptides
|

Protein Coverage:

Supporting Peptides:

| Peptide | Uniq | -10lgP | Mass | Length | ppm | m/z | z | RT | Fraction | Scan | Source File | Area F6 | Area F2 | Area F3 | Area F4 | Area F5 | Area F7 | Area F8 | Area F1 | #Feature | #Feature F6 | #Feature F2 | #Feature F3 | #Feature F4 | #Feature F5 | #Feature F7 | #Feature F8 | #Feature F1 | Start | End | PTM | AScore | Found By |
| --- | --- | --- | --- | --- | --- | --- | --- | --- | --- | --- | --- | --- | --- | --- | --- | --- | --- | --- | --- | --- | --- | --- | --- | --- | --- | --- | --- | --- | --- | --- | --- | --- | --- |
| K.AINQGGLTSVAVR.G | Y | 66.22 | 1284.7150 | 13 | 0.6 | 643.3651 | 2 | 33.23 | 8 | F8:15490 | 31122021\_RID\_3262\_1b.raw |  |  |  |  |  |  |  | 1.5738E5 | 1 | 0 | 0 | 0 | 0 | 0 | 0 | 0 | 1 | 31 | 43 |  |  | PEAKS DB |
| total 1 peptides |  |  |  |  |  |  |  |  |  |  |  |  |  |  |  |  |  |  |  |  |  |  |  |  |  |  |  |  |  |  |  |  |  |
| --- | --- | --- | --- | --- | --- | --- | --- | --- | --- | --- | --- | --- | --- | --- | --- | --- | --- | --- | --- | --- | --- | --- | --- | --- | --- | --- | --- | --- | --- | --- | --- | --- | --- |

XP\_032086764.1

back to list

  

| Protein Coverage
| Supporting Peptides
|

Protein Coverage:

Supporting Peptides:

| Peptide | Uniq | -10lgP | Mass | Length | ppm | m/z | z | RT | Fraction | Scan | Source File | Area F6 | Area F2 | Area F3 | Area F4 | Area F5 | Area F7 | Area F8 | Area F1 | #Feature | #Feature F6 | #Feature F2 | #Feature F3 | #Feature F4 | #Feature F5 | #Feature F7 | #Feature F8 | #Feature F1 | Start | End | PTM | AScore | Found By |
| --- | --- | --- | --- | --- | --- | --- | --- | --- | --- | --- | --- | --- | --- | --- | --- | --- | --- | --- | --- | --- | --- | --- | --- | --- | --- | --- | --- | --- | --- | --- | --- | --- | --- |
| K.AINQGGLTSVAVR.G | Y | 66.22 | 1284.7150 | 13 | 0.6 | 643.3651 | 2 | 33.23 | 8 | F8:15490 | 31122021\_RID\_3262\_1b.raw |  |  |  |  |  |  |  | 1.5738E5 | 1 | 0 | 0 | 0 | 0 | 0 | 0 | 0 | 1 | 31 | 43 |  |  | PEAKS DB |
| total 1 peptides |  |  |  |  |  |  |  |  |  |  |  |  |  |  |  |  |  |  |  |  |  |  |  |  |  |  |  |  |  |  |  |  |  |
| --- | --- | --- | --- | --- | --- | --- | --- | --- | --- | --- | --- | --- | --- | --- | --- | --- | --- | --- | --- | --- | --- | --- | --- | --- | --- | --- | --- | --- | --- | --- | --- | --- | --- |

JAI09616.1

back to list

  

| Protein Coverage
| Supporting Peptides
|

Protein Coverage:

Supporting Peptides:

| Peptide | Uniq | -10lgP | Mass | Length | ppm | m/z | z | RT | Fraction | Scan | Source File | Area F6 | Area F2 | Area F3 | Area F4 | Area F5 | Area F7 | Area F8 | Area F1 | #Feature | #Feature F6 | #Feature F2 | #Feature F3 | #Feature F4 | #Feature F5 | #Feature F7 | #Feature F8 | #Feature F1 | Start | End | PTM | AScore | Found By |
| --- | --- | --- | --- | --- | --- | --- | --- | --- | --- | --- | --- | --- | --- | --- | --- | --- | --- | --- | --- | --- | --- | --- | --- | --- | --- | --- | --- | --- | --- | --- | --- | --- | --- |
| K.AINQGGLTSVAVR.G | Y | 66.22 | 1284.7150 | 13 | 0.6 | 643.3651 | 2 | 33.23 | 8 | F8:15490 | 31122021\_RID\_3262\_1b.raw |  |  |  |  |  |  |  | 1.5738E5 | 1 | 0 | 0 | 0 | 0 | 0 | 0 | 0 | 1 | 31 | 43 |  |  | PEAKS DB |
| total 1 peptides |  |  |  |  |  |  |  |  |  |  |  |  |  |  |  |  |  |  |  |  |  |  |  |  |  |  |  |  |  |  |  |  |  |
| --- | --- | --- | --- | --- | --- | --- | --- | --- | --- | --- | --- | --- | --- | --- | --- | --- | --- | --- | --- | --- | --- | --- | --- | --- | --- | --- | --- | --- | --- | --- | --- | --- | --- |

XP\_015674567.1

back to list

  

| Protein Coverage
| Supporting Peptides
|

Protein Coverage:

Supporting Peptides:

| Peptide | Uniq | -10lgP | Mass | Length | ppm | m/z | z | RT | Fraction | Scan | Source File | Area F6 | Area F2 | Area F3 | Area F4 | Area F5 | Area F7 | Area F8 | Area F1 | #Feature | #Feature F6 | #Feature F2 | #Feature F3 | #Feature F4 | #Feature F5 | #Feature F7 | #Feature F8 | #Feature F1 | Start | End | PTM | AScore | Found By |
| --- | --- | --- | --- | --- | --- | --- | --- | --- | --- | --- | --- | --- | --- | --- | --- | --- | --- | --- | --- | --- | --- | --- | --- | --- | --- | --- | --- | --- | --- | --- | --- | --- | --- |
| K.AINQGGLTSVAVR.G | Y | 66.22 | 1284.7150 | 13 | 0.6 | 643.3651 | 2 | 33.23 | 8 | F8:15490 | 31122021\_RID\_3262\_1b.raw |  |  |  |  |  |  |  | 1.5738E5 | 1 | 0 | 0 | 0 | 0 | 0 | 0 | 0 | 1 | 31 | 43 |  |  | PEAKS DB |
| total 1 peptides |  |  |  |  |  |  |  |  |  |  |  |  |  |  |  |  |  |  |  |  |  |  |  |  |  |  |  |  |  |  |  |  |  |
| --- | --- | --- | --- | --- | --- | --- | --- | --- | --- | --- | --- | --- | --- | --- | --- | --- | --- | --- | --- | --- | --- | --- | --- | --- | --- | --- | --- | --- | --- | --- | --- | --- | --- |

JAV49552.1

back to list

  

| Protein Coverage
| Supporting Peptides
|

Protein Coverage:

Supporting Peptides:

| Peptide | Uniq | -10lgP | Mass | Length | ppm | m/z | z | RT | Fraction | Scan | Source File | Area F6 | Area F2 | Area F3 | Area F4 | Area F5 | Area F7 | Area F8 | Area F1 | #Feature | #Feature F6 | #Feature F2 | #Feature F3 | #Feature F4 | #Feature F5 | #Feature F7 | #Feature F8 | #Feature F1 | Start | End | PTM | AScore | Found By |
| --- | --- | --- | --- | --- | --- | --- | --- | --- | --- | --- | --- | --- | --- | --- | --- | --- | --- | --- | --- | --- | --- | --- | --- | --- | --- | --- | --- | --- | --- | --- | --- | --- | --- |
| K.AINQGGLTSVAVR.G | Y | 66.22 | 1284.7150 | 13 | 0.6 | 643.3651 | 2 | 33.23 | 8 | F8:15490 | 31122021\_RID\_3262\_1b.raw |  |  |  |  |  |  |  | 1.5738E5 | 1 | 0 | 0 | 0 | 0 | 0 | 0 | 0 | 1 | 31 | 43 |  |  | PEAKS DB |
| total 1 peptides |  |  |  |  |  |  |  |  |  |  |  |  |  |  |  |  |  |  |  |  |  |  |  |  |  |  |  |  |  |  |  |  |  |
| --- | --- | --- | --- | --- | --- | --- | --- | --- | --- | --- | --- | --- | --- | --- | --- | --- | --- | --- | --- | --- | --- | --- | --- | --- | --- | --- | --- | --- | --- | --- | --- | --- | --- |

JAG66916.1

back to list

  

| Protein Coverage
| Supporting Peptides
|

Protein Coverage:

Supporting Peptides:

| Peptide | Uniq | -10lgP | Mass | Length | ppm | m/z | z | RT | Fraction | Scan | Source File | Area F6 | Area F2 | Area F3 | Area F4 | Area F5 | Area F7 | Area F8 | Area F1 | #Feature | #Feature F6 | #Feature F2 | #Feature F3 | #Feature F4 | #Feature F5 | #Feature F7 | #Feature F8 | #Feature F1 | Start | End | PTM | AScore | Found By |
| --- | --- | --- | --- | --- | --- | --- | --- | --- | --- | --- | --- | --- | --- | --- | --- | --- | --- | --- | --- | --- | --- | --- | --- | --- | --- | --- | --- | --- | --- | --- | --- | --- | --- |
| K.AINQGGLTSVAVR.G | Y | 66.22 | 1284.7150 | 13 | 0.6 | 643.3651 | 2 | 33.23 | 8 | F8:15490 | 31122021\_RID\_3262\_1b.raw |  |  |  |  |  |  |  | 1.5738E5 | 1 | 0 | 0 | 0 | 0 | 0 | 0 | 0 | 1 | 31 | 43 |  |  | PEAKS DB |
| total 1 peptides |  |  |  |  |  |  |  |  |  |  |  |  |  |  |  |  |  |  |  |  |  |  |  |  |  |  |  |  |  |  |  |  |  |
| --- | --- | --- | --- | --- | --- | --- | --- | --- | --- | --- | --- | --- | --- | --- | --- | --- | --- | --- | --- | --- | --- | --- | --- | --- | --- | --- | --- | --- | --- | --- | --- | --- | --- |

JAB53610.1

back to list

  

| Protein Coverage
| Supporting Peptides
|

Protein Coverage:

Supporting Peptides:

| Peptide | Uniq | -10lgP | Mass | Length | ppm | m/z | z | RT | Fraction | Scan | Source File | Area F6 | Area F2 | Area F3 | Area F4 | Area F5 | Area F7 | Area F8 | Area F1 | #Feature | #Feature F6 | #Feature F2 | #Feature F3 | #Feature F4 | #Feature F5 | #Feature F7 | #Feature F8 | #Feature F1 | Start | End | PTM | AScore | Found By |
| --- | --- | --- | --- | --- | --- | --- | --- | --- | --- | --- | --- | --- | --- | --- | --- | --- | --- | --- | --- | --- | --- | --- | --- | --- | --- | --- | --- | --- | --- | --- | --- | --- | --- |
| K.AINQGGLTSVAVR.G | Y | 66.22 | 1284.7150 | 13 | 0.6 | 643.3651 | 2 | 33.23 | 8 | F8:15490 | 31122021\_RID\_3262\_1b.raw |  |  |  |  |  |  |  | 1.5738E5 | 1 | 0 | 0 | 0 | 0 | 0 | 0 | 0 | 1 | 31 | 43 |  |  | PEAKS DB |
| total 1 peptides |  |  |  |  |  |  |  |  |  |  |  |  |  |  |  |  |  |  |  |  |  |  |  |  |  |  |  |  |  |  |  |  |  |
| --- | --- | --- | --- | --- | --- | --- | --- | --- | --- | --- | --- | --- | --- | --- | --- | --- | --- | --- | --- | --- | --- | --- | --- | --- | --- | --- | --- | --- | --- | --- | --- | --- | --- |

XP\_013929267.1

back to list

  

| Protein Coverage
| Supporting Peptides
|

Protein Coverage:

Supporting Peptides:

| Peptide | Uniq | -10lgP | Mass | Length | ppm | m/z | z | RT | Fraction | Scan | Source File | Area F6 | Area F2 | Area F3 | Area F4 | Area F5 | Area F7 | Area F8 | Area F1 | #Feature | #Feature F6 | #Feature F2 | #Feature F3 | #Feature F4 | #Feature F5 | #Feature F7 | #Feature F8 | #Feature F1 | Start | End | PTM | AScore | Found By |
| --- | --- | --- | --- | --- | --- | --- | --- | --- | --- | --- | --- | --- | --- | --- | --- | --- | --- | --- | --- | --- | --- | --- | --- | --- | --- | --- | --- | --- | --- | --- | --- | --- | --- |
| K.AINQGGLTSVAVR.G | Y | 66.22 | 1284.7150 | 13 | 0.6 | 643.3651 | 2 | 33.23 | 8 | F8:15490 | 31122021\_RID\_3262\_1b.raw |  |  |  |  |  |  |  | 1.5738E5 | 1 | 0 | 0 | 0 | 0 | 0 | 0 | 0 | 1 | 31 | 43 |  |  | PEAKS DB |
| total 1 peptides |  |  |  |  |  |  |  |  |  |  |  |  |  |  |  |  |  |  |  |  |  |  |  |  |  |  |  |  |  |  |  |  |  |
| --- | --- | --- | --- | --- | --- | --- | --- | --- | --- | --- | --- | --- | --- | --- | --- | --- | --- | --- | --- | --- | --- | --- | --- | --- | --- | --- | --- | --- | --- | --- | --- | --- | --- |

XP\_007436765.1

back to list

  

| Protein Coverage
| Supporting Peptides
|

Protein Coverage:

Supporting Peptides:

| Peptide | Uniq | -10lgP | Mass | Length | ppm | m/z | z | RT | Fraction | Scan | Source File | Area F6 | Area F2 | Area F3 | Area F4 | Area F5 | Area F7 | Area F8 | Area F1 | #Feature | #Feature F6 | #Feature F2 | #Feature F3 | #Feature F4 | #Feature F5 | #Feature F7 | #Feature F8 | #Feature F1 | Start | End | PTM | AScore | Found By |
| --- | --- | --- | --- | --- | --- | --- | --- | --- | --- | --- | --- | --- | --- | --- | --- | --- | --- | --- | --- | --- | --- | --- | --- | --- | --- | --- | --- | --- | --- | --- | --- | --- | --- |
| K.AINQGGLTSVAVR.G | Y | 66.22 | 1284.7150 | 13 | 0.6 | 643.3651 | 2 | 33.23 | 8 | F8:15490 | 31122021\_RID\_3262\_1b.raw |  |  |  |  |  |  |  | 1.5738E5 | 1 | 0 | 0 | 0 | 0 | 0 | 0 | 0 | 1 | 31 | 43 |  |  | PEAKS DB |
| total 1 peptides |  |  |  |  |  |  |  |  |  |  |  |  |  |  |  |  |  |  |  |  |  |  |  |  |  |  |  |  |  |  |  |  |  |
| --- | --- | --- | --- | --- | --- | --- | --- | --- | --- | --- | --- | --- | --- | --- | --- | --- | --- | --- | --- | --- | --- | --- | --- | --- | --- | --- | --- | --- | --- | --- | --- | --- | --- |

JAA96032.1

back to list

  

| Protein Coverage
| Supporting Peptides
|

Protein Coverage:

Supporting Peptides:

| Peptide | Uniq | -10lgP | Mass | Length | ppm | m/z | z | RT | Fraction | Scan | Source File | Area F6 | Area F2 | Area F3 | Area F4 | Area F5 | Area F7 | Area F8 | Area F1 | #Feature | #Feature F6 | #Feature F2 | #Feature F3 | #Feature F4 | #Feature F5 | #Feature F7 | #Feature F8 | #Feature F1 | Start | End | PTM | AScore | Found By |
| --- | --- | --- | --- | --- | --- | --- | --- | --- | --- | --- | --- | --- | --- | --- | --- | --- | --- | --- | --- | --- | --- | --- | --- | --- | --- | --- | --- | --- | --- | --- | --- | --- | --- |
| K.AINQGGLTSVAVR.G | Y | 66.22 | 1284.7150 | 13 | 0.6 | 643.3651 | 2 | 33.23 | 8 | F8:15490 | 31122021\_RID\_3262\_1b.raw |  |  |  |  |  |  |  | 1.5738E5 | 1 | 0 | 0 | 0 | 0 | 0 | 0 | 0 | 1 | 31 | 43 |  |  | PEAKS DB |
| total 1 peptides |  |  |  |  |  |  |  |  |  |  |  |  |  |  |  |  |  |  |  |  |  |  |  |  |  |  |  |  |  |  |  |  |  |
| --- | --- | --- | --- | --- | --- | --- | --- | --- | --- | --- | --- | --- | --- | --- | --- | --- | --- | --- | --- | --- | --- | --- | --- | --- | --- | --- | --- | --- | --- | --- | --- | --- | --- |

ETE73021.1

back to list

  

| Protein Coverage
| Supporting Peptides
|

Protein Coverage:

Supporting Peptides:

| Peptide | Uniq | -10lgP | Mass | Length | ppm | m/z | z | RT | Fraction | Scan | Source File | Area F6 | Area F2 | Area F3 | Area F4 | Area F5 | Area F7 | Area F8 | Area F1 | #Feature | #Feature F6 | #Feature F2 | #Feature F3 | #Feature F4 | #Feature F5 | #Feature F7 | #Feature F8 | #Feature F1 | Start | End | PTM | AScore | Found By |
| --- | --- | --- | --- | --- | --- | --- | --- | --- | --- | --- | --- | --- | --- | --- | --- | --- | --- | --- | --- | --- | --- | --- | --- | --- | --- | --- | --- | --- | --- | --- | --- | --- | --- |
| K.AINQGGLTSVAVR.G | Y | 66.22 | 1284.7150 | 13 | 0.6 | 643.3651 | 2 | 33.23 | 8 | F8:15490 | 31122021\_RID\_3262\_1b.raw |  |  |  |  |  |  |  | 1.5738E5 | 1 | 0 | 0 | 0 | 0 | 0 | 0 | 0 | 1 | 64 | 76 |  |  | PEAKS DB |
| total 1 peptides |  |  |  |  |  |  |  |  |  |  |  |  |  |  |  |  |  |  |  |  |  |  |  |  |  |  |  |  |  |  |  |  |  |
| --- | --- | --- | --- | --- | --- | --- | --- | --- | --- | --- | --- | --- | --- | --- | --- | --- | --- | --- | --- | --- | --- | --- | --- | --- | --- | --- | --- | --- | --- | --- | --- | --- | --- |

ALB06111.1

back to list

  

| Protein Coverage
| Supporting Peptides
|

Protein Coverage:

Supporting Peptides:

| Peptide | Uniq | -10lgP | Mass | Length | ppm | m/z | z | RT | Fraction | Scan | Source File | Area F6 | Area F2 | Area F3 | Area F4 | Area F5 | Area F7 | Area F8 | Area F1 | #Feature | #Feature F6 | #Feature F2 | #Feature F3 | #Feature F4 | #Feature F5 | #Feature F7 | #Feature F8 | #Feature F1 | Start | End | PTM | AScore | Found By |
| --- | --- | --- | --- | --- | --- | --- | --- | --- | --- | --- | --- | --- | --- | --- | --- | --- | --- | --- | --- | --- | --- | --- | --- | --- | --- | --- | --- | --- | --- | --- | --- | --- | --- |
| R.DLHPELSEDEIKR.L | Y | 64.06 | 1579.7842 | 13 | 0.6 | 527.6023 | 3 | 30.55 | 3 | F3:15585 | 13012022\_RID\_3262\_3b.raw |  |  | 2.0513E5 |  |  |  |  |  | 1 | 0 | 0 | 1 | 0 | 0 | 0 | 0 | 0 | 161 | 173 |  |  | PEAKS DB |
| total 1 peptides |  |  |  |  |  |  |  |  |  |  |  |  |  |  |  |  |  |  |  |  |  |  |  |  |  |  |  |  |  |  |  |  |  |
| --- | --- | --- | --- | --- | --- | --- | --- | --- | --- | --- | --- | --- | --- | --- | --- | --- | --- | --- | --- | --- | --- | --- | --- | --- | --- | --- | --- | --- | --- | --- | --- | --- | --- |

JAS05458.1

back to list

  

| Protein Coverage
| Supporting Peptides
|

Protein Coverage:

Supporting Peptides:

| Peptide | Uniq | -10lgP | Mass | Length | ppm | m/z | z | RT | Fraction | Scan | Source File | Area F6 | Area F2 | Area F3 | Area F4 | Area F5 | Area F7 | Area F8 | Area F1 | #Feature | #Feature F6 | #Feature F2 | #Feature F3 | #Feature F4 | #Feature F5 | #Feature F7 | #Feature F8 | #Feature F1 | Start | End | PTM | AScore | Found By |
| --- | --- | --- | --- | --- | --- | --- | --- | --- | --- | --- | --- | --- | --- | --- | --- | --- | --- | --- | --- | --- | --- | --- | --- | --- | --- | --- | --- | --- | --- | --- | --- | --- | --- |
| R.DLHPELSEDEIKR.L | Y | 64.06 | 1579.7842 | 13 | 0.6 | 527.6023 | 3 | 30.55 | 3 | F3:15585 | 13012022\_RID\_3262\_3b.raw |  |  | 2.0513E5 |  |  |  |  |  | 1 | 0 | 0 | 1 | 0 | 0 | 0 | 0 | 0 | 161 | 173 |  |  | PEAKS DB |
| total 1 peptides |  |  |  |  |  |  |  |  |  |  |  |  |  |  |  |  |  |  |  |  |  |  |  |  |  |  |  |  |  |  |  |  |  |
| --- | --- | --- | --- | --- | --- | --- | --- | --- | --- | --- | --- | --- | --- | --- | --- | --- | --- | --- | --- | --- | --- | --- | --- | --- | --- | --- | --- | --- | --- | --- | --- | --- | --- |

JAS04525.1

back to list

  

| Protein Coverage
| Supporting Peptides
|

Protein Coverage:

Supporting Peptides:

| Peptide | Uniq | -10lgP | Mass | Length | ppm | m/z | z | RT | Fraction | Scan | Source File | Area F6 | Area F2 | Area F3 | Area F4 | Area F5 | Area F7 | Area F8 | Area F1 | #Feature | #Feature F6 | #Feature F2 | #Feature F3 | #Feature F4 | #Feature F5 | #Feature F7 | #Feature F8 | #Feature F1 | Start | End | PTM | AScore | Found By |
| --- | --- | --- | --- | --- | --- | --- | --- | --- | --- | --- | --- | --- | --- | --- | --- | --- | --- | --- | --- | --- | --- | --- | --- | --- | --- | --- | --- | --- | --- | --- | --- | --- | --- |
| R.DLHPELSEDEIKR.L | Y | 64.06 | 1579.7842 | 13 | 0.6 | 527.6023 | 3 | 30.55 | 3 | F3:15585 | 13012022\_RID\_3262\_3b.raw |  |  | 2.0513E5 |  |  |  |  |  | 1 | 0 | 0 | 1 | 0 | 0 | 0 | 0 | 0 | 161 | 173 |  |  | PEAKS DB |
| total 1 peptides |  |  |  |  |  |  |  |  |  |  |  |  |  |  |  |  |  |  |  |  |  |  |  |  |  |  |  |  |  |  |  |  |  |
| --- | --- | --- | --- | --- | --- | --- | --- | --- | --- | --- | --- | --- | --- | --- | --- | --- | --- | --- | --- | --- | --- | --- | --- | --- | --- | --- | --- | --- | --- | --- | --- | --- | --- |

JAV50314.1

back to list

  

| Protein Coverage
| Supporting Peptides
|

Protein Coverage:

Supporting Peptides:

| Peptide | Uniq | -10lgP | Mass | Length | ppm | m/z | z | RT | Fraction | Scan | Source File | Area F6 | Area F2 | Area F3 | Area F4 | Area F5 | Area F7 | Area F8 | Area F1 | #Feature | #Feature F6 | #Feature F2 | #Feature F3 | #Feature F4 | #Feature F5 | #Feature F7 | #Feature F8 | #Feature F1 | Start | End | PTM | AScore | Found By |
| --- | --- | --- | --- | --- | --- | --- | --- | --- | --- | --- | --- | --- | --- | --- | --- | --- | --- | --- | --- | --- | --- | --- | --- | --- | --- | --- | --- | --- | --- | --- | --- | --- | --- |
| R.DLHPELSEDEIKR.L | Y | 64.06 | 1579.7842 | 13 | 0.6 | 527.6023 | 3 | 30.55 | 3 | F3:15585 | 13012022\_RID\_3262\_3b.raw |  |  | 2.0513E5 |  |  |  |  |  | 1 | 0 | 0 | 1 | 0 | 0 | 0 | 0 | 0 | 161 | 173 |  |  | PEAKS DB |
| total 1 peptides |  |  |  |  |  |  |  |  |  |  |  |  |  |  |  |  |  |  |  |  |  |  |  |  |  |  |  |  |  |  |  |  |  |
| --- | --- | --- | --- | --- | --- | --- | --- | --- | --- | --- | --- | --- | --- | --- | --- | --- | --- | --- | --- | --- | --- | --- | --- | --- | --- | --- | --- | --- | --- | --- | --- | --- | --- |

JAS04524.1

back to list

  

| Protein Coverage
| Supporting Peptides
|

Protein Coverage:

Supporting Peptides:

| Peptide | Uniq | -10lgP | Mass | Length | ppm | m/z | z | RT | Fraction | Scan | Source File | Area F6 | Area F2 | Area F3 | Area F4 | Area F5 | Area F7 | Area F8 | Area F1 | #Feature | #Feature F6 | #Feature F2 | #Feature F3 | #Feature F4 | #Feature F5 | #Feature F7 | #Feature F8 | #Feature F1 | Start | End | PTM | AScore | Found By |
| --- | --- | --- | --- | --- | --- | --- | --- | --- | --- | --- | --- | --- | --- | --- | --- | --- | --- | --- | --- | --- | --- | --- | --- | --- | --- | --- | --- | --- | --- | --- | --- | --- | --- |
| R.DLHPELSEDEIKR.L | Y | 64.06 | 1579.7842 | 13 | 0.6 | 527.6023 | 3 | 30.55 | 3 | F3:15585 | 13012022\_RID\_3262\_3b.raw |  |  | 2.0513E5 |  |  |  |  |  | 1 | 0 | 0 | 1 | 0 | 0 | 0 | 0 | 0 | 161 | 173 |  |  | PEAKS DB |
| total 1 peptides |  |  |  |  |  |  |  |  |  |  |  |  |  |  |  |  |  |  |  |  |  |  |  |  |  |  |  |  |  |  |  |  |  |
| --- | --- | --- | --- | --- | --- | --- | --- | --- | --- | --- | --- | --- | --- | --- | --- | --- | --- | --- | --- | --- | --- | --- | --- | --- | --- | --- | --- | --- | --- | --- | --- | --- | --- |

JAS04526.1

back to list

  

| Protein Coverage
| Supporting Peptides
|

Protein Coverage:

Supporting Peptides:

| Peptide | Uniq | -10lgP | Mass | Length | ppm | m/z | z | RT | Fraction | Scan | Source File | Area F6 | Area F2 | Area F3 | Area F4 | Area F5 | Area F7 | Area F8 | Area F1 | #Feature | #Feature F6 | #Feature F2 | #Feature F3 | #Feature F4 | #Feature F5 | #Feature F7 | #Feature F8 | #Feature F1 | Start | End | PTM | AScore | Found By |
| --- | --- | --- | --- | --- | --- | --- | --- | --- | --- | --- | --- | --- | --- | --- | --- | --- | --- | --- | --- | --- | --- | --- | --- | --- | --- | --- | --- | --- | --- | --- | --- | --- | --- |
| R.DLHPELSEDEIKR.L | Y | 64.06 | 1579.7842 | 13 | 0.6 | 527.6023 | 3 | 30.55 | 3 | F3:15585 | 13012022\_RID\_3262\_3b.raw |  |  | 2.0513E5 |  |  |  |  |  | 1 | 0 | 0 | 1 | 0 | 0 | 0 | 0 | 0 | 161 | 173 |  |  | PEAKS DB |
| total 1 peptides |  |  |  |  |  |  |  |  |  |  |  |  |  |  |  |  |  |  |  |  |  |  |  |  |  |  |  |  |  |  |  |  |  |
| --- | --- | --- | --- | --- | --- | --- | --- | --- | --- | --- | --- | --- | --- | --- | --- | --- | --- | --- | --- | --- | --- | --- | --- | --- | --- | --- | --- | --- | --- | --- | --- | --- | --- |

SMD28274.1

back to list

  

| Protein Coverage
| Supporting Peptides
|

Protein Coverage:

Supporting Peptides:

| Peptide | Uniq | -10lgP | Mass | Length | ppm | m/z | z | RT | Fraction | Scan | Source File | Area F6 | Area F2 | Area F3 | Area F4 | Area F5 | Area F7 | Area F8 | Area F1 | #Feature | #Feature F6 | #Feature F2 | #Feature F3 | #Feature F4 | #Feature F5 | #Feature F7 | #Feature F8 | #Feature F1 | Start | End | PTM | AScore | Found By |
| --- | --- | --- | --- | --- | --- | --- | --- | --- | --- | --- | --- | --- | --- | --- | --- | --- | --- | --- | --- | --- | --- | --- | --- | --- | --- | --- | --- | --- | --- | --- | --- | --- | --- |
| R.DLHPELSEDEIKR.L | Y | 64.06 | 1579.7842 | 13 | 0.6 | 527.6023 | 3 | 30.55 | 3 | F3:15585 | 13012022\_RID\_3262\_3b.raw |  |  | 2.0513E5 |  |  |  |  |  | 1 | 0 | 0 | 1 | 0 | 0 | 0 | 0 | 0 | 161 | 173 |  |  | PEAKS DB |
| total 1 peptides |  |  |  |  |  |  |  |  |  |  |  |  |  |  |  |  |  |  |  |  |  |  |  |  |  |  |  |  |  |  |  |  |  |
| --- | --- | --- | --- | --- | --- | --- | --- | --- | --- | --- | --- | --- | --- | --- | --- | --- | --- | --- | --- | --- | --- | --- | --- | --- | --- | --- | --- | --- | --- | --- | --- | --- | --- |

JAS04874.1

back to list

  

| Protein Coverage
| Supporting Peptides
|

Protein Coverage:

Supporting Peptides:

| Peptide | Uniq | -10lgP | Mass | Length | ppm | m/z | z | RT | Fraction | Scan | Source File | Area F6 | Area F2 | Area F3 | Area F4 | Area F5 | Area F7 | Area F8 | Area F1 | #Feature | #Feature F6 | #Feature F2 | #Feature F3 | #Feature F4 | #Feature F5 | #Feature F7 | #Feature F8 | #Feature F1 | Start | End | PTM | AScore | Found By |
| --- | --- | --- | --- | --- | --- | --- | --- | --- | --- | --- | --- | --- | --- | --- | --- | --- | --- | --- | --- | --- | --- | --- | --- | --- | --- | --- | --- | --- | --- | --- | --- | --- | --- |
| R.DLHPELSEDEIKR.L | Y | 64.06 | 1579.7842 | 13 | 0.6 | 527.6023 | 3 | 30.55 | 3 | F3:15585 | 13012022\_RID\_3262\_3b.raw |  |  | 2.0513E5 |  |  |  |  |  | 1 | 0 | 0 | 1 | 0 | 0 | 0 | 0 | 0 | 161 | 173 |  |  | PEAKS DB |
| total 1 peptides |  |  |  |  |  |  |  |  |  |  |  |  |  |  |  |  |  |  |  |  |  |  |  |  |  |  |  |  |  |  |  |  |  |
| --- | --- | --- | --- | --- | --- | --- | --- | --- | --- | --- | --- | --- | --- | --- | --- | --- | --- | --- | --- | --- | --- | --- | --- | --- | --- | --- | --- | --- | --- | --- | --- | --- | --- |

JAG46141.1

back to list

  

| Protein Coverage
| Supporting Peptides
|

Protein Coverage:

Supporting Peptides:

| Peptide | Uniq | -10lgP | Mass | Length | ppm | m/z | z | RT | Fraction | Scan | Source File | Area F6 | Area F2 | Area F3 | Area F4 | Area F5 | Area F7 | Area F8 | Area F1 | #Feature | #Feature F6 | #Feature F2 | #Feature F3 | #Feature F4 | #Feature F5 | #Feature F7 | #Feature F8 | #Feature F1 | Start | End | PTM | AScore | Found By |
| --- | --- | --- | --- | --- | --- | --- | --- | --- | --- | --- | --- | --- | --- | --- | --- | --- | --- | --- | --- | --- | --- | --- | --- | --- | --- | --- | --- | --- | --- | --- | --- | --- | --- |
| R.DLHPELSEDEIKR.L | Y | 64.06 | 1579.7842 | 13 | 0.6 | 527.6023 | 3 | 30.55 | 3 | F3:15585 | 13012022\_RID\_3262\_3b.raw |  |  | 2.0513E5 |  |  |  |  |  | 1 | 0 | 0 | 1 | 0 | 0 | 0 | 0 | 0 | 161 | 173 |  |  | PEAKS DB |
| total 1 peptides |  |  |  |  |  |  |  |  |  |  |  |  |  |  |  |  |  |  |  |  |  |  |  |  |  |  |  |  |  |  |  |  |  |
| --- | --- | --- | --- | --- | --- | --- | --- | --- | --- | --- | --- | --- | --- | --- | --- | --- | --- | --- | --- | --- | --- | --- | --- | --- | --- | --- | --- | --- | --- | --- | --- | --- | --- |

JAS04875.1

back to list

  

| Protein Coverage
| Supporting Peptides
|

Protein Coverage:

Supporting Peptides:

| Peptide | Uniq | -10lgP | Mass | Length | ppm | m/z | z | RT | Fraction | Scan | Source File | Area F6 | Area F2 | Area F3 | Area F4 | Area F5 | Area F7 | Area F8 | Area F1 | #Feature | #Feature F6 | #Feature F2 | #Feature F3 | #Feature F4 | #Feature F5 | #Feature F7 | #Feature F8 | #Feature F1 | Start | End | PTM | AScore | Found By |
| --- | --- | --- | --- | --- | --- | --- | --- | --- | --- | --- | --- | --- | --- | --- | --- | --- | --- | --- | --- | --- | --- | --- | --- | --- | --- | --- | --- | --- | --- | --- | --- | --- | --- |
| R.DLHPELSEDEIKR.L | Y | 64.06 | 1579.7842 | 13 | 0.6 | 527.6023 | 3 | 30.55 | 3 | F3:15585 | 13012022\_RID\_3262\_3b.raw |  |  | 2.0513E5 |  |  |  |  |  | 1 | 0 | 0 | 1 | 0 | 0 | 0 | 0 | 0 | 161 | 173 |  |  | PEAKS DB |
| total 1 peptides |  |  |  |  |  |  |  |  |  |  |  |  |  |  |  |  |  |  |  |  |  |  |  |  |  |  |  |  |  |  |  |  |  |
| --- | --- | --- | --- | --- | --- | --- | --- | --- | --- | --- | --- | --- | --- | --- | --- | --- | --- | --- | --- | --- | --- | --- | --- | --- | --- | --- | --- | --- | --- | --- | --- | --- | --- |

JAS04787.1

back to list

  

| Protein Coverage
| Supporting Peptides
|

Protein Coverage:

Supporting Peptides:

| Peptide | Uniq | -10lgP | Mass | Length | ppm | m/z | z | RT | Fraction | Scan | Source File | Area F6 | Area F2 | Area F3 | Area F4 | Area F5 | Area F7 | Area F8 | Area F1 | #Feature | #Feature F6 | #Feature F2 | #Feature F3 | #Feature F4 | #Feature F5 | #Feature F7 | #Feature F8 | #Feature F1 | Start | End | PTM | AScore | Found By |
| --- | --- | --- | --- | --- | --- | --- | --- | --- | --- | --- | --- | --- | --- | --- | --- | --- | --- | --- | --- | --- | --- | --- | --- | --- | --- | --- | --- | --- | --- | --- | --- | --- | --- |
| R.DLHPELSEDEIKR.L | Y | 64.06 | 1579.7842 | 13 | 0.6 | 527.6023 | 3 | 30.55 | 3 | F3:15585 | 13012022\_RID\_3262\_3b.raw |  |  | 2.0513E5 |  |  |  |  |  | 1 | 0 | 0 | 1 | 0 | 0 | 0 | 0 | 0 | 161 | 173 |  |  | PEAKS DB |
| total 1 peptides |  |  |  |  |  |  |  |  |  |  |  |  |  |  |  |  |  |  |  |  |  |  |  |  |  |  |  |  |  |  |  |  |  |
| --- | --- | --- | --- | --- | --- | --- | --- | --- | --- | --- | --- | --- | --- | --- | --- | --- | --- | --- | --- | --- | --- | --- | --- | --- | --- | --- | --- | --- | --- | --- | --- | --- | --- |

JAA94894.1

back to list

  

| Protein Coverage
| Supporting Peptides
|

Protein Coverage:

Supporting Peptides:

| Peptide | Uniq | -10lgP | Mass | Length | ppm | m/z | z | RT | Fraction | Scan | Source File | Area F6 | Area F2 | Area F3 | Area F4 | Area F5 | Area F7 | Area F8 | Area F1 | #Feature | #Feature F6 | #Feature F2 | #Feature F3 | #Feature F4 | #Feature F5 | #Feature F7 | #Feature F8 | #Feature F1 | Start | End | PTM | AScore | Found By |
| --- | --- | --- | --- | --- | --- | --- | --- | --- | --- | --- | --- | --- | --- | --- | --- | --- | --- | --- | --- | --- | --- | --- | --- | --- | --- | --- | --- | --- | --- | --- | --- | --- | --- |
| R.DLHPELSEDEIKR.L | Y | 64.06 | 1579.7842 | 13 | 0.6 | 527.6023 | 3 | 30.55 | 3 | F3:15585 | 13012022\_RID\_3262\_3b.raw |  |  | 2.0513E5 |  |  |  |  |  | 1 | 0 | 0 | 1 | 0 | 0 | 0 | 0 | 0 | 161 | 173 |  |  | PEAKS DB |
| total 1 peptides |  |  |  |  |  |  |  |  |  |  |  |  |  |  |  |  |  |  |  |  |  |  |  |  |  |  |  |  |  |  |  |  |  |
| --- | --- | --- | --- | --- | --- | --- | --- | --- | --- | --- | --- | --- | --- | --- | --- | --- | --- | --- | --- | --- | --- | --- | --- | --- | --- | --- | --- | --- | --- | --- | --- | --- | --- |

SMD28273.1

back to list

  

| Protein Coverage
| Supporting Peptides
|

Protein Coverage:

Supporting Peptides:

| Peptide | Uniq | -10lgP | Mass | Length | ppm | m/z | z | RT | Fraction | Scan | Source File | Area F6 | Area F2 | Area F3 | Area F4 | Area F5 | Area F7 | Area F8 | Area F1 | #Feature | #Feature F6 | #Feature F2 | #Feature F3 | #Feature F4 | #Feature F5 | #Feature F7 | #Feature F8 | #Feature F1 | Start | End | PTM | AScore | Found By |
| --- | --- | --- | --- | --- | --- | --- | --- | --- | --- | --- | --- | --- | --- | --- | --- | --- | --- | --- | --- | --- | --- | --- | --- | --- | --- | --- | --- | --- | --- | --- | --- | --- | --- |
| R.DLHPELSEDEIKR.L | Y | 64.06 | 1579.7842 | 13 | 0.6 | 527.6023 | 3 | 30.55 | 3 | F3:15585 | 13012022\_RID\_3262\_3b.raw |  |  | 2.0513E5 |  |  |  |  |  | 1 | 0 | 0 | 1 | 0 | 0 | 0 | 0 | 0 | 161 | 173 |  |  | PEAKS DB |
| total 1 peptides |  |  |  |  |  |  |  |  |  |  |  |  |  |  |  |  |  |  |  |  |  |  |  |  |  |  |  |  |  |  |  |  |  |
| --- | --- | --- | --- | --- | --- | --- | --- | --- | --- | --- | --- | --- | --- | --- | --- | --- | --- | --- | --- | --- | --- | --- | --- | --- | --- | --- | --- | --- | --- | --- | --- | --- | --- |

JAG46140.1

back to list

  

| Protein Coverage
| Supporting Peptides
|

Protein Coverage:

Supporting Peptides:

| Peptide | Uniq | -10lgP | Mass | Length | ppm | m/z | z | RT | Fraction | Scan | Source File | Area F6 | Area F2 | Area F3 | Area F4 | Area F5 | Area F7 | Area F8 | Area F1 | #Feature | #Feature F6 | #Feature F2 | #Feature F3 | #Feature F4 | #Feature F5 | #Feature F7 | #Feature F8 | #Feature F1 | Start | End | PTM | AScore | Found By |
| --- | --- | --- | --- | --- | --- | --- | --- | --- | --- | --- | --- | --- | --- | --- | --- | --- | --- | --- | --- | --- | --- | --- | --- | --- | --- | --- | --- | --- | --- | --- | --- | --- | --- |
| R.DLHPELSEDEIKR.L | Y | 64.06 | 1579.7842 | 13 | 0.6 | 527.6023 | 3 | 30.55 | 3 | F3:15585 | 13012022\_RID\_3262\_3b.raw |  |  | 2.0513E5 |  |  |  |  |  | 1 | 0 | 0 | 1 | 0 | 0 | 0 | 0 | 0 | 161 | 173 |  |  | PEAKS DB |
| total 1 peptides |  |  |  |  |  |  |  |  |  |  |  |  |  |  |  |  |  |  |  |  |  |  |  |  |  |  |  |  |  |  |  |  |  |
| --- | --- | --- | --- | --- | --- | --- | --- | --- | --- | --- | --- | --- | --- | --- | --- | --- | --- | --- | --- | --- | --- | --- | --- | --- | --- | --- | --- | --- | --- | --- | --- | --- | --- |

SMD28272.1

back to list

  

| Protein Coverage
| Supporting Peptides
|

Protein Coverage:

Supporting Peptides:

| Peptide | Uniq | -10lgP | Mass | Length | ppm | m/z | z | RT | Fraction | Scan | Source File | Area F6 | Area F2 | Area F3 | Area F4 | Area F5 | Area F7 | Area F8 | Area F1 | #Feature | #Feature F6 | #Feature F2 | #Feature F3 | #Feature F4 | #Feature F5 | #Feature F7 | #Feature F8 | #Feature F1 | Start | End | PTM | AScore | Found By |
| --- | --- | --- | --- | --- | --- | --- | --- | --- | --- | --- | --- | --- | --- | --- | --- | --- | --- | --- | --- | --- | --- | --- | --- | --- | --- | --- | --- | --- | --- | --- | --- | --- | --- |
| R.DLHPELSEDEIKR.L | Y | 64.06 | 1579.7842 | 13 | 0.6 | 527.6023 | 3 | 30.55 | 3 | F3:15585 | 13012022\_RID\_3262\_3b.raw |  |  | 2.0513E5 |  |  |  |  |  | 1 | 0 | 0 | 1 | 0 | 0 | 0 | 0 | 0 | 161 | 173 |  |  | PEAKS DB |
| total 1 peptides |  |  |  |  |  |  |  |  |  |  |  |  |  |  |  |  |  |  |  |  |  |  |  |  |  |  |  |  |  |  |  |  |  |
| --- | --- | --- | --- | --- | --- | --- | --- | --- | --- | --- | --- | --- | --- | --- | --- | --- | --- | --- | --- | --- | --- | --- | --- | --- | --- | --- | --- | --- | --- | --- | --- | --- | --- |

JAG43657.1

back to list

  

| Protein Coverage
| Supporting Peptides
|

Protein Coverage:

Supporting Peptides:

| Peptide | Uniq | -10lgP | Mass | Length | ppm | m/z | z | RT | Fraction | Scan | Source File | Area F6 | Area F2 | Area F3 | Area F4 | Area F5 | Area F7 | Area F8 | Area F1 | #Feature | #Feature F6 | #Feature F2 | #Feature F3 | #Feature F4 | #Feature F5 | #Feature F7 | #Feature F8 | #Feature F1 | Start | End | PTM | AScore | Found By |
| --- | --- | --- | --- | --- | --- | --- | --- | --- | --- | --- | --- | --- | --- | --- | --- | --- | --- | --- | --- | --- | --- | --- | --- | --- | --- | --- | --- | --- | --- | --- | --- | --- | --- |
| R.DLHPELSEDEIKR.L | Y | 64.06 | 1579.7842 | 13 | 0.6 | 527.6023 | 3 | 30.55 | 3 | F3:15585 | 13012022\_RID\_3262\_3b.raw |  |  | 2.0513E5 |  |  |  |  |  | 1 | 0 | 0 | 1 | 0 | 0 | 0 | 0 | 0 | 161 | 173 |  |  | PEAKS DB |
| total 1 peptides |  |  |  |  |  |  |  |  |  |  |  |  |  |  |  |  |  |  |  |  |  |  |  |  |  |  |  |  |  |  |  |  |  |
| --- | --- | --- | --- | --- | --- | --- | --- | --- | --- | --- | --- | --- | --- | --- | --- | --- | --- | --- | --- | --- | --- | --- | --- | --- | --- | --- | --- | --- | --- | --- | --- | --- | --- |

JAV48430.1

back to list

  

| Protein Coverage
| Supporting Peptides
|

Protein Coverage:

Supporting Peptides:

| Peptide | Uniq | -10lgP | Mass | Length | ppm | m/z | z | RT | Fraction | Scan | Source File | Area F6 | Area F2 | Area F3 | Area F4 | Area F5 | Area F7 | Area F8 | Area F1 | #Feature | #Feature F6 | #Feature F2 | #Feature F3 | #Feature F4 | #Feature F5 | #Feature F7 | #Feature F8 | #Feature F1 | Start | End | PTM | AScore | Found By |
| --- | --- | --- | --- | --- | --- | --- | --- | --- | --- | --- | --- | --- | --- | --- | --- | --- | --- | --- | --- | --- | --- | --- | --- | --- | --- | --- | --- | --- | --- | --- | --- | --- | --- |
| R.DLHPELSEDEIKR.L | Y | 64.06 | 1579.7842 | 13 | 0.6 | 527.6023 | 3 | 30.55 | 3 | F3:15585 | 13012022\_RID\_3262\_3b.raw |  |  | 2.0513E5 |  |  |  |  |  | 1 | 0 | 0 | 1 | 0 | 0 | 0 | 0 | 0 | 161 | 173 |  |  | PEAKS DB |
| total 1 peptides |  |  |  |  |  |  |  |  |  |  |  |  |  |  |  |  |  |  |  |  |  |  |  |  |  |  |  |  |  |  |  |  |  |
| --- | --- | --- | --- | --- | --- | --- | --- | --- | --- | --- | --- | --- | --- | --- | --- | --- | --- | --- | --- | --- | --- | --- | --- | --- | --- | --- | --- | --- | --- | --- | --- | --- | --- |

AFJ49188.1

back to list

  

| Protein Coverage
| Supporting Peptides
|

Protein Coverage:

Supporting Peptides:

| Peptide | Uniq | -10lgP | Mass | Length | ppm | m/z | z | RT | Fraction | Scan | Source File | Area F6 | Area F2 | Area F3 | Area F4 | Area F5 | Area F7 | Area F8 | Area F1 | #Feature | #Feature F6 | #Feature F2 | #Feature F3 | #Feature F4 | #Feature F5 | #Feature F7 | #Feature F8 | #Feature F1 | Start | End | PTM | AScore | Found By |
| --- | --- | --- | --- | --- | --- | --- | --- | --- | --- | --- | --- | --- | --- | --- | --- | --- | --- | --- | --- | --- | --- | --- | --- | --- | --- | --- | --- | --- | --- | --- | --- | --- | --- |
| R.DLHPELSEDEIKR.L | Y | 64.06 | 1579.7842 | 13 | 0.6 | 527.6023 | 3 | 30.55 | 3 | F3:15585 | 13012022\_RID\_3262\_3b.raw |  |  | 2.0513E5 |  |  |  |  |  | 1 | 0 | 0 | 1 | 0 | 0 | 0 | 0 | 0 | 161 | 173 |  |  | PEAKS DB |
| total 1 peptides |  |  |  |  |  |  |  |  |  |  |  |  |  |  |  |  |  |  |  |  |  |  |  |  |  |  |  |  |  |  |  |  |  |
| --- | --- | --- | --- | --- | --- | --- | --- | --- | --- | --- | --- | --- | --- | --- | --- | --- | --- | --- | --- | --- | --- | --- | --- | --- | --- | --- | --- | --- | --- | --- | --- | --- | --- |

JAS05321.1

back to list

  

| Protein Coverage
| Supporting Peptides
|

Protein Coverage:

Supporting Peptides:

| Peptide | Uniq | -10lgP | Mass | Length | ppm | m/z | z | RT | Fraction | Scan | Source File | Area F6 | Area F2 | Area F3 | Area F4 | Area F5 | Area F7 | Area F8 | Area F1 | #Feature | #Feature F6 | #Feature F2 | #Feature F3 | #Feature F4 | #Feature F5 | #Feature F7 | #Feature F8 | #Feature F1 | Start | End | PTM | AScore | Found By |
| --- | --- | --- | --- | --- | --- | --- | --- | --- | --- | --- | --- | --- | --- | --- | --- | --- | --- | --- | --- | --- | --- | --- | --- | --- | --- | --- | --- | --- | --- | --- | --- | --- | --- |
| R.DLHPELSEDEIKR.L | Y | 64.06 | 1579.7842 | 13 | 0.6 | 527.6023 | 3 | 30.55 | 3 | F3:15585 | 13012022\_RID\_3262\_3b.raw |  |  | 2.0513E5 |  |  |  |  |  | 1 | 0 | 0 | 1 | 0 | 0 | 0 | 0 | 0 | 161 | 173 |  |  | PEAKS DB |
| total 1 peptides |  |  |  |  |  |  |  |  |  |  |  |  |  |  |  |  |  |  |  |  |  |  |  |  |  |  |  |  |  |  |  |  |  |
| --- | --- | --- | --- | --- | --- | --- | --- | --- | --- | --- | --- | --- | --- | --- | --- | --- | --- | --- | --- | --- | --- | --- | --- | --- | --- | --- | --- | --- | --- | --- | --- | --- | --- |

JAI10412.1

back to list

  

| Protein Coverage
| Supporting Peptides
|

Protein Coverage:

Supporting Peptides:

| Peptide | Uniq | -10lgP | Mass | Length | ppm | m/z | z | RT | Fraction | Scan | Source File | Area F6 | Area F2 | Area F3 | Area F4 | Area F5 | Area F7 | Area F8 | Area F1 | #Feature | #Feature F6 | #Feature F2 | #Feature F3 | #Feature F4 | #Feature F5 | #Feature F7 | #Feature F8 | #Feature F1 | Start | End | PTM | AScore | Found By |
| --- | --- | --- | --- | --- | --- | --- | --- | --- | --- | --- | --- | --- | --- | --- | --- | --- | --- | --- | --- | --- | --- | --- | --- | --- | --- | --- | --- | --- | --- | --- | --- | --- | --- |
| R.DLHPELSEDEIKR.L | Y | 64.06 | 1579.7842 | 13 | 0.6 | 527.6023 | 3 | 30.55 | 3 | F3:15585 | 13012022\_RID\_3262\_3b.raw |  |  | 2.0513E5 |  |  |  |  |  | 1 | 0 | 0 | 1 | 0 | 0 | 0 | 0 | 0 | 161 | 173 |  |  | PEAKS DB |
| total 1 peptides |  |  |  |  |  |  |  |  |  |  |  |  |  |  |  |  |  |  |  |  |  |  |  |  |  |  |  |  |  |  |  |  |  |
| --- | --- | --- | --- | --- | --- | --- | --- | --- | --- | --- | --- | --- | --- | --- | --- | --- | --- | --- | --- | --- | --- | --- | --- | --- | --- | --- | --- | --- | --- | --- | --- | --- | --- |

JAS04708.1

back to list

  

| Protein Coverage
| Supporting Peptides
|

Protein Coverage:

Supporting Peptides:

| Peptide | Uniq | -10lgP | Mass | Length | ppm | m/z | z | RT | Fraction | Scan | Source File | Area F6 | Area F2 | Area F3 | Area F4 | Area F5 | Area F7 | Area F8 | Area F1 | #Feature | #Feature F6 | #Feature F2 | #Feature F3 | #Feature F4 | #Feature F5 | #Feature F7 | #Feature F8 | #Feature F1 | Start | End | PTM | AScore | Found By |
| --- | --- | --- | --- | --- | --- | --- | --- | --- | --- | --- | --- | --- | --- | --- | --- | --- | --- | --- | --- | --- | --- | --- | --- | --- | --- | --- | --- | --- | --- | --- | --- | --- | --- |
| R.DLHPELSEDEIKR.L | Y | 64.06 | 1579.7842 | 13 | 0.6 | 527.6023 | 3 | 30.55 | 3 | F3:15585 | 13012022\_RID\_3262\_3b.raw |  |  | 2.0513E5 |  |  |  |  |  | 1 | 0 | 0 | 1 | 0 | 0 | 0 | 0 | 0 | 161 | 173 |  |  | PEAKS DB |
| total 1 peptides |  |  |  |  |  |  |  |  |  |  |  |  |  |  |  |  |  |  |  |  |  |  |  |  |  |  |  |  |  |  |  |  |  |
| --- | --- | --- | --- | --- | --- | --- | --- | --- | --- | --- | --- | --- | --- | --- | --- | --- | --- | --- | --- | --- | --- | --- | --- | --- | --- | --- | --- | --- | --- | --- | --- | --- | --- |

J3S820.1

back to list

  

| Protein Coverage
| Supporting Peptides
|

Protein Coverage:

Supporting Peptides:

| Peptide | Uniq | -10lgP | Mass | Length | ppm | m/z | z | RT | Fraction | Scan | Source File | Area F6 | Area F2 | Area F3 | Area F4 | Area F5 | Area F7 | Area F8 | Area F1 | #Feature | #Feature F6 | #Feature F2 | #Feature F3 | #Feature F4 | #Feature F5 | #Feature F7 | #Feature F8 | #Feature F1 | Start | End | PTM | AScore | Found By |
| --- | --- | --- | --- | --- | --- | --- | --- | --- | --- | --- | --- | --- | --- | --- | --- | --- | --- | --- | --- | --- | --- | --- | --- | --- | --- | --- | --- | --- | --- | --- | --- | --- | --- |
| R.DLHPELSEDEIKR.L | Y | 64.06 | 1579.7842 | 13 | 0.6 | 527.6023 | 3 | 30.55 | 3 | F3:15585 | 13012022\_RID\_3262\_3b.raw |  |  | 2.0513E5 |  |  |  |  |  | 1 | 0 | 0 | 1 | 0 | 0 | 0 | 0 | 0 | 161 | 173 |  |  | PEAKS DB |
| total 1 peptides |  |  |  |  |  |  |  |  |  |  |  |  |  |  |  |  |  |  |  |  |  |  |  |  |  |  |  |  |  |  |  |  |  |
| --- | --- | --- | --- | --- | --- | --- | --- | --- | --- | --- | --- | --- | --- | --- | --- | --- | --- | --- | --- | --- | --- | --- | --- | --- | --- | --- | --- | --- | --- | --- | --- | --- | --- |

JAV01892.1

back to list

  

| Protein Coverage
| Supporting Peptides
|

Protein Coverage:

Supporting Peptides:

| Peptide | Uniq | -10lgP | Mass | Length | ppm | m/z | z | RT | Fraction | Scan | Source File | Area F6 | Area F2 | Area F3 | Area F4 | Area F5 | Area F7 | Area F8 | Area F1 | #Feature | #Feature F6 | #Feature F2 | #Feature F3 | #Feature F4 | #Feature F5 | #Feature F7 | #Feature F8 | #Feature F1 | Start | End | PTM | AScore | Found By |
| --- | --- | --- | --- | --- | --- | --- | --- | --- | --- | --- | --- | --- | --- | --- | --- | --- | --- | --- | --- | --- | --- | --- | --- | --- | --- | --- | --- | --- | --- | --- | --- | --- | --- |
| R.DLHPELSEDEIKR.L | Y | 64.06 | 1579.7842 | 13 | 0.6 | 527.6023 | 3 | 30.55 | 3 | F3:15585 | 13012022\_RID\_3262\_3b.raw |  |  | 2.0513E5 |  |  |  |  |  | 1 | 0 | 0 | 1 | 0 | 0 | 0 | 0 | 0 | 161 | 173 |  |  | PEAKS DB |
| total 1 peptides |  |  |  |  |  |  |  |  |  |  |  |  |  |  |  |  |  |  |  |  |  |  |  |  |  |  |  |  |  |  |  |  |  |
| --- | --- | --- | --- | --- | --- | --- | --- | --- | --- | --- | --- | --- | --- | --- | --- | --- | --- | --- | --- | --- | --- | --- | --- | --- | --- | --- | --- | --- | --- | --- | --- | --- | --- |

ATU85542.1

back to list

  

| Protein Coverage
| Supporting Peptides
|

Protein Coverage:

Supporting Peptides:

| Peptide | Uniq | -10lgP | Mass | Length | ppm | m/z | z | RT | Fraction | Scan | Source File | Area F6 | Area F2 | Area F3 | Area F4 | Area F5 | Area F7 | Area F8 | Area F1 | #Feature | #Feature F6 | #Feature F2 | #Feature F3 | #Feature F4 | #Feature F5 | #Feature F7 | #Feature F8 | #Feature F1 | Start | End | PTM | AScore | Found By |
| --- | --- | --- | --- | --- | --- | --- | --- | --- | --- | --- | --- | --- | --- | --- | --- | --- | --- | --- | --- | --- | --- | --- | --- | --- | --- | --- | --- | --- | --- | --- | --- | --- | --- |
| R.DLHPELSEDEIKR.L | Y | 64.06 | 1579.7842 | 13 | 0.6 | 527.6023 | 3 | 30.55 | 3 | F3:15585 | 13012022\_RID\_3262\_3b.raw |  |  | 2.0513E5 |  |  |  |  |  | 1 | 0 | 0 | 1 | 0 | 0 | 0 | 0 | 0 | 161 | 173 |  |  | PEAKS DB |
| total 1 peptides |  |  |  |  |  |  |  |  |  |  |  |  |  |  |  |  |  |  |  |  |  |  |  |  |  |  |  |  |  |  |  |  |  |
| --- | --- | --- | --- | --- | --- | --- | --- | --- | --- | --- | --- | --- | --- | --- | --- | --- | --- | --- | --- | --- | --- | --- | --- | --- | --- | --- | --- | --- | --- | --- | --- | --- | --- |

P0CG03.1

back to list

  

| Protein Coverage
| Supporting Peptides
|

Protein Coverage:

Supporting Peptides:

| Peptide | Uniq | -10lgP | Mass | Length | ppm | m/z | z | RT | Fraction | Scan | Source File | Area F6 | Area F2 | Area F3 | Area F4 | Area F5 | Area F7 | Area F8 | Area F1 | #Feature | #Feature F6 | #Feature F2 | #Feature F3 | #Feature F4 | #Feature F5 | #Feature F7 | #Feature F8 | #Feature F1 | Start | End | PTM | AScore | Found By |
| --- | --- | --- | --- | --- | --- | --- | --- | --- | --- | --- | --- | --- | --- | --- | --- | --- | --- | --- | --- | --- | --- | --- | --- | --- | --- | --- | --- | --- | --- | --- | --- | --- | --- |
| R.VMGWGTISPTK.V | Y | 46.12 | 1175.6008 | 11 | 0.4 | 588.8079 | 2 | 42.67 | 5 | F5:20179 | 13012022\_RID\_3262\_5b.raw |  |  |  |  | 1.4462E6 |  |  |  | 1 | 0 | 0 | 0 | 0 | 1 | 0 | 0 | 0 | 146 | 156 |  |  | PEAKS DB |
| total 1 peptides |  |  |  |  |  |  |  |  |  |  |  |  |  |  |  |  |  |  |  |  |  |  |  |  |  |  |  |  |  |  |  |  |  |
| --- | --- | --- | --- | --- | --- | --- | --- | --- | --- | --- | --- | --- | --- | --- | --- | --- | --- | --- | --- | --- | --- | --- | --- | --- | --- | --- | --- | --- | --- | --- | --- | --- | --- |

AFJ49243.1

back to list

  

| Protein Coverage
| Supporting Peptides
|

Protein Coverage:

Supporting Peptides:

| Peptide | Uniq | -10lgP | Mass | Length | ppm | m/z | z | RT | Fraction | Scan | Source File | Area F6 | Area F2 | Area F3 | Area F4 | Area F5 | Area F7 | Area F8 | Area F1 | #Feature | #Feature F6 | #Feature F2 | #Feature F3 | #Feature F4 | #Feature F5 | #Feature F7 | #Feature F8 | #Feature F1 | Start | End | PTM | AScore | Found By |
| --- | --- | --- | --- | --- | --- | --- | --- | --- | --- | --- | --- | --- | --- | --- | --- | --- | --- | --- | --- | --- | --- | --- | --- | --- | --- | --- | --- | --- | --- | --- | --- | --- | --- |
| R.TDIVSPPIC(+57.02)GNELLEVG.E | Y | 62.59 | 1811.8975 | 17 | 7.2 | 906.9625 | 2 | 47.87 | 8 | F8:27025 | 31122021\_RID\_3262\_1b.raw |  | 9.91E6 | 4.837E7 | 7.8142E6 | 2.6837E6 |  |  | 2.7456E6 | 5 | 0 | 1 | 1 | 1 | 1 | 0 | 0 | 1 | 399 | 415 | Carbamidomethylation | C9:Carbamidomethylation:1000.00 | PEAKS DB |
| total 1 peptides |  |  |  |  |  |  |  |  |  |  |  |  |  |  |  |  |  |  |  |  |  |  |  |  |  |  |  |  |  |  |  |  |  |
| --- | --- | --- | --- | --- | --- | --- | --- | --- | --- | --- | --- | --- | --- | --- | --- | --- | --- | --- | --- | --- | --- | --- | --- | --- | --- | --- | --- | --- | --- | --- | --- | --- | --- |

AEJ31991.1

back to list

  

| Protein Coverage
| Supporting Peptides
|

Protein Coverage:

Supporting Peptides:

| Peptide | Uniq | -10lgP | Mass | Length | ppm | m/z | z | RT | Fraction | Scan | Source File | Area F6 | Area F2 | Area F3 | Area F4 | Area F5 | Area F7 | Area F8 | Area F1 | #Feature | #Feature F6 | #Feature F2 | #Feature F3 | #Feature F4 | #Feature F5 | #Feature F7 | #Feature F8 | #Feature F1 | Start | End | PTM | AScore | Found By |
| --- | --- | --- | --- | --- | --- | --- | --- | --- | --- | --- | --- | --- | --- | --- | --- | --- | --- | --- | --- | --- | --- | --- | --- | --- | --- | --- | --- | --- | --- | --- | --- | --- | --- |
| R.TDIVSPPIC(+57.02)GNELLEVG.E | Y | 62.59 | 1811.8975 | 17 | 7.2 | 906.9625 | 2 | 47.87 | 8 | F8:27025 | 31122021\_RID\_3262\_1b.raw |  | 9.91E6 | 4.837E7 | 7.8142E6 | 2.6837E6 |  |  | 2.7456E6 | 5 | 0 | 1 | 1 | 1 | 1 | 0 | 0 | 1 | 275 | 291 | Carbamidomethylation | C9:Carbamidomethylation:1000.00 | PEAKS DB |
| total 1 peptides |  |  |  |  |  |  |  |  |  |  |  |  |  |  |  |  |  |  |  |  |  |  |  |  |  |  |  |  |  |  |  |  |  |
| --- | --- | --- | --- | --- | --- | --- | --- | --- | --- | --- | --- | --- | --- | --- | --- | --- | --- | --- | --- | --- | --- | --- | --- | --- | --- | --- | --- | --- | --- | --- | --- | --- | --- |

JAV48412.1

back to list

  

| Protein Coverage
| Supporting Peptides
|

Protein Coverage:

Supporting Peptides:

| Peptide | Uniq | -10lgP | Mass | Length | ppm | m/z | z | RT | Fraction | Scan | Source File | Area F6 | Area F2 | Area F3 | Area F4 | Area F5 | Area F7 | Area F8 | Area F1 | #Feature | #Feature F6 | #Feature F2 | #Feature F3 | #Feature F4 | #Feature F5 | #Feature F7 | #Feature F8 | #Feature F1 | Start | End | PTM | AScore | Found By |
| --- | --- | --- | --- | --- | --- | --- | --- | --- | --- | --- | --- | --- | --- | --- | --- | --- | --- | --- | --- | --- | --- | --- | --- | --- | --- | --- | --- | --- | --- | --- | --- | --- | --- |
| R.TDIVSPPIC(+57.02)GNELLEVG.E | Y | 62.59 | 1811.8975 | 17 | 7.2 | 906.9625 | 2 | 47.87 | 8 | F8:27025 | 31122021\_RID\_3262\_1b.raw |  | 9.91E6 | 4.837E7 | 7.8142E6 | 2.6837E6 |  |  | 2.7456E6 | 5 | 0 | 1 | 1 | 1 | 1 | 0 | 0 | 1 | 399 | 415 | Carbamidomethylation | C9:Carbamidomethylation:1000.00 | PEAKS DB |
| total 1 peptides |  |  |  |  |  |  |  |  |  |  |  |  |  |  |  |  |  |  |  |  |  |  |  |  |  |  |  |  |  |  |  |  |  |
| --- | --- | --- | --- | --- | --- | --- | --- | --- | --- | --- | --- | --- | --- | --- | --- | --- | --- | --- | --- | --- | --- | --- | --- | --- | --- | --- | --- | --- | --- | --- | --- | --- | --- |

XP\_015684827.1

back to list

  

| Protein Coverage
| Supporting Peptides
|

Protein Coverage:

Supporting Peptides:

| Peptide | Uniq | -10lgP | Mass | Length | ppm | m/z | z | RT | Fraction | Scan | Source File | Area F6 | Area F2 | Area F3 | Area F4 | Area F5 | Area F7 | Area F8 | Area F1 | #Feature | #Feature F6 | #Feature F2 | #Feature F3 | #Feature F4 | #Feature F5 | #Feature F7 | #Feature F8 | #Feature F1 | Start | End | PTM | AScore | Found By |
| --- | --- | --- | --- | --- | --- | --- | --- | --- | --- | --- | --- | --- | --- | --- | --- | --- | --- | --- | --- | --- | --- | --- | --- | --- | --- | --- | --- | --- | --- | --- | --- | --- | --- |
| K.TDVVDFPR.F | Y | 46.04 | 947.4712 | 8 | -0.5 | 474.7426 | 2 | 37.60 | 4 | F4:21020 | 13012022\_RID\_3262\_4b.raw |  |  |  | 0 | 6.927E4 |  |  |  | 1 | 0 | 0 | 0 | 0 | 1 | 0 | 0 | 0 | 72 | 79 |  |  | PEAKS DB |
| total 1 peptides |  |  |  |  |  |  |  |  |  |  |  |  |  |  |  |  |  |  |  |  |  |  |  |  |  |  |  |  |  |  |  |  |  |
| --- | --- | --- | --- | --- | --- | --- | --- | --- | --- | --- | --- | --- | --- | --- | --- | --- | --- | --- | --- | --- | --- | --- | --- | --- | --- | --- | --- | --- | --- | --- | --- | --- | --- |

XP\_026535627.1

back to list

  

| Protein Coverage
| Supporting Peptides
|

Protein Coverage:

Supporting Peptides:

| Peptide | Uniq | -10lgP | Mass | Length | ppm | m/z | z | RT | Fraction | Scan | Source File | Area F6 | Area F2 | Area F3 | Area F4 | Area F5 | Area F7 | Area F8 | Area F1 | #Feature | #Feature F6 | #Feature F2 | #Feature F3 | #Feature F4 | #Feature F5 | #Feature F7 | #Feature F8 | #Feature F1 | Start | End | PTM | AScore | Found By |
| --- | --- | --- | --- | --- | --- | --- | --- | --- | --- | --- | --- | --- | --- | --- | --- | --- | --- | --- | --- | --- | --- | --- | --- | --- | --- | --- | --- | --- | --- | --- | --- | --- | --- |
| R.VSEEFFTSLGMFK.M | Y | 62.20 | 1520.7220 | 13 | 0.2 | 761.3685 | 2 | 59.64 | 8 | F8:36218 | 31122021\_RID\_3262\_1b.raw |  |  |  |  |  |  |  | 6.1498E4 | 1 | 0 | 0 | 0 | 0 | 0 | 0 | 0 | 1 | 314 | 326 |  |  | PEAKS DB |
| total 1 peptides |  |  |  |  |  |  |  |  |  |  |  |  |  |  |  |  |  |  |  |  |  |  |  |  |  |  |  |  |  |  |  |  |  |
| --- | --- | --- | --- | --- | --- | --- | --- | --- | --- | --- | --- | --- | --- | --- | --- | --- | --- | --- | --- | --- | --- | --- | --- | --- | --- | --- | --- | --- | --- | --- | --- | --- | --- |

XP\_013924292.1

back to list

  

| Protein Coverage
| Supporting Peptides
|

Protein Coverage:

Supporting Peptides:

| Peptide | Uniq | -10lgP | Mass | Length | ppm | m/z | z | RT | Fraction | Scan | Source File | Area F6 | Area F2 | Area F3 | Area F4 | Area F5 | Area F7 | Area F8 | Area F1 | #Feature | #Feature F6 | #Feature F2 | #Feature F3 | #Feature F4 | #Feature F5 | #Feature F7 | #Feature F8 | #Feature F1 | Start | End | PTM | AScore | Found By |
| --- | --- | --- | --- | --- | --- | --- | --- | --- | --- | --- | --- | --- | --- | --- | --- | --- | --- | --- | --- | --- | --- | --- | --- | --- | --- | --- | --- | --- | --- | --- | --- | --- | --- |
| K.EYQELMNTK.L | Y | 58.79 | 1154.5277 | 9 | 0.6 | 578.2715 | 2 | 28.66 | 8 | F8:12011 | 31122021\_RID\_3262\_1b.raw |  |  | 2.0994E6 |  |  |  |  | 6.7955E6 | 2 | 0 | 0 | 1 | 0 | 0 | 0 | 0 | 1 | 380 | 388 |  |  | PEAKS DB |
| K.EYQELM(+15.99)NTK.L | Y | 48.14 | 1170.5227 | 9 | 1.0 | 586.2692 | 2 | 28.65 | 8 | F8:11928 | 31122021\_RID\_3262\_1b.raw |  |  |  |  |  |  |  | 1.1907E6 | 2 | 0 | 0 | 0 | 0 | 0 | 0 | 0 | 2 | 380 | 388 | Oxidation (M) | M6:Oxidation (M):1000.00 | PEAKS DB |
| total 2 peptides |  |  |  |  |  |  |  |  |  |  |  |  |  |  |  |  |  |  |  |  |  |  |  |  |  |  |  |  |  |  |  |  |  |
| --- | --- | --- | --- | --- | --- | --- | --- | --- | --- | --- | --- | --- | --- | --- | --- | --- | --- | --- | --- | --- | --- | --- | --- | --- | --- | --- | --- | --- | --- | --- | --- | --- | --- |

XP\_034268135.1

back to list

  

| Protein Coverage
| Supporting Peptides
|

Protein Coverage:

Supporting Peptides:

| Peptide | Uniq | -10lgP | Mass | Length | ppm | m/z | z | RT | Fraction | Scan | Source File | Area F6 | Area F2 | Area F3 | Area F4 | Area F5 | Area F7 | Area F8 | Area F1 | #Feature | #Feature F6 | #Feature F2 | #Feature F3 | #Feature F4 | #Feature F5 | #Feature F7 | #Feature F8 | #Feature F1 | Start | End | PTM | AScore | Found By |
| --- | --- | --- | --- | --- | --- | --- | --- | --- | --- | --- | --- | --- | --- | --- | --- | --- | --- | --- | --- | --- | --- | --- | --- | --- | --- | --- | --- | --- | --- | --- | --- | --- | --- |
| K.EYQELMNTK.L | Y | 58.79 | 1154.5277 | 9 | 0.6 | 578.2715 | 2 | 28.66 | 8 | F8:12011 | 31122021\_RID\_3262\_1b.raw |  |  | 2.0994E6 |  |  |  |  | 6.7955E6 | 2 | 0 | 0 | 1 | 0 | 0 | 0 | 0 | 1 | 380 | 388 |  |  | PEAKS DB |
| K.EYQELM(+15.99)NTK.L | Y | 48.14 | 1170.5227 | 9 | 1.0 | 586.2692 | 2 | 28.65 | 8 | F8:11928 | 31122021\_RID\_3262\_1b.raw |  |  |  |  |  |  |  | 1.1907E6 | 2 | 0 | 0 | 0 | 0 | 0 | 0 | 0 | 2 | 380 | 388 | Oxidation (M) | M6:Oxidation (M):1000.00 | PEAKS DB |
| total 2 peptides |  |  |  |  |  |  |  |  |  |  |  |  |  |  |  |  |  |  |  |  |  |  |  |  |  |  |  |  |  |  |  |  |  |
| --- | --- | --- | --- | --- | --- | --- | --- | --- | --- | --- | --- | --- | --- | --- | --- | --- | --- | --- | --- | --- | --- | --- | --- | --- | --- | --- | --- | --- | --- | --- | --- | --- | --- |

ETE71916.1

back to list

  

| Protein Coverage
| Supporting Peptides
|

Protein Coverage:

Supporting Peptides:

| Peptide | Uniq | -10lgP | Mass | Length | ppm | m/z | z | RT | Fraction | Scan | Source File | Area F6 | Area F2 | Area F3 | Area F4 | Area F5 | Area F7 | Area F8 | Area F1 | #Feature | #Feature F6 | #Feature F2 | #Feature F3 | #Feature F4 | #Feature F5 | #Feature F7 | #Feature F8 | #Feature F1 | Start | End | PTM | AScore | Found By |
| --- | --- | --- | --- | --- | --- | --- | --- | --- | --- | --- | --- | --- | --- | --- | --- | --- | --- | --- | --- | --- | --- | --- | --- | --- | --- | --- | --- | --- | --- | --- | --- | --- | --- |
| K.EYQELMNTK.L | Y | 58.79 | 1154.5277 | 9 | 0.6 | 578.2715 | 2 | 28.66 | 8 | F8:12011 | 31122021\_RID\_3262\_1b.raw |  |  | 2.0994E6 |  |  |  |  | 6.7955E6 | 2 | 0 | 0 | 1 | 0 | 0 | 0 | 0 | 1 | 68 | 76 |  |  | PEAKS DB |
| K.EYQELM(+15.99)NTK.L | Y | 48.14 | 1170.5227 | 9 | 1.0 | 586.2692 | 2 | 28.65 | 8 | F8:11928 | 31122021\_RID\_3262\_1b.raw |  |  |  |  |  |  |  | 1.1907E6 | 2 | 0 | 0 | 0 | 0 | 0 | 0 | 0 | 2 | 68 | 76 | Oxidation (M) | M6:Oxidation (M):1000.00 | PEAKS DB |
| total 2 peptides |  |  |  |  |  |  |  |  |  |  |  |  |  |  |  |  |  |  |  |  |  |  |  |  |  |  |  |  |  |  |  |  |  |
| --- | --- | --- | --- | --- | --- | --- | --- | --- | --- | --- | --- | --- | --- | --- | --- | --- | --- | --- | --- | --- | --- | --- | --- | --- | --- | --- | --- | --- | --- | --- | --- | --- | --- |

XP\_026573192.1

back to list

  

| Protein Coverage
| Supporting Peptides
|

Protein Coverage:

Supporting Peptides:

| Peptide | Uniq | -10lgP | Mass | Length | ppm | m/z | z | RT | Fraction | Scan | Source File | Area F6 | Area F2 | Area F3 | Area F4 | Area F5 | Area F7 | Area F8 | Area F1 | #Feature | #Feature F6 | #Feature F2 | #Feature F3 | #Feature F4 | #Feature F5 | #Feature F7 | #Feature F8 | #Feature F1 | Start | End | PTM | AScore | Found By |
| --- | --- | --- | --- | --- | --- | --- | --- | --- | --- | --- | --- | --- | --- | --- | --- | --- | --- | --- | --- | --- | --- | --- | --- | --- | --- | --- | --- | --- | --- | --- | --- | --- | --- |
| K.EYQELMNTK.L | Y | 58.79 | 1154.5277 | 9 | 0.6 | 578.2715 | 2 | 28.66 | 8 | F8:12011 | 31122021\_RID\_3262\_1b.raw |  |  | 2.0994E6 |  |  |  |  | 6.7955E6 | 2 | 0 | 0 | 1 | 0 | 0 | 0 | 0 | 1 | 379 | 387 |  |  | PEAKS DB |
| K.EYQELM(+15.99)NTK.L | Y | 48.14 | 1170.5227 | 9 | 1.0 | 586.2692 | 2 | 28.65 | 8 | F8:11928 | 31122021\_RID\_3262\_1b.raw |  |  |  |  |  |  |  | 1.1907E6 | 2 | 0 | 0 | 0 | 0 | 0 | 0 | 0 | 2 | 379 | 387 | Oxidation (M) | M6:Oxidation (M):1000.00 | PEAKS DB |
| total 2 peptides |  |  |  |  |  |  |  |  |  |  |  |  |  |  |  |  |  |  |  |  |  |  |  |  |  |  |  |  |  |  |  |  |  |
| --- | --- | --- | --- | --- | --- | --- | --- | --- | --- | --- | --- | --- | --- | --- | --- | --- | --- | --- | --- | --- | --- | --- | --- | --- | --- | --- | --- | --- | --- | --- | --- | --- | --- |

XP\_032067334.1

back to list

  

| Protein Coverage
| Supporting Peptides
|

Protein Coverage:

Supporting Peptides:

| Peptide | Uniq | -10lgP | Mass | Length | ppm | m/z | z | RT | Fraction | Scan | Source File | Area F6 | Area F2 | Area F3 | Area F4 | Area F5 | Area F7 | Area F8 | Area F1 | #Feature | #Feature F6 | #Feature F2 | #Feature F3 | #Feature F4 | #Feature F5 | #Feature F7 | #Feature F8 | #Feature F1 | Start | End | PTM | AScore | Found By |
| --- | --- | --- | --- | --- | --- | --- | --- | --- | --- | --- | --- | --- | --- | --- | --- | --- | --- | --- | --- | --- | --- | --- | --- | --- | --- | --- | --- | --- | --- | --- | --- | --- | --- |
| K.EYQELMNTK.L | Y | 58.79 | 1154.5277 | 9 | 0.6 | 578.2715 | 2 | 28.66 | 8 | F8:12011 | 31122021\_RID\_3262\_1b.raw |  |  | 2.0994E6 |  |  |  |  | 6.7955E6 | 2 | 0 | 0 | 1 | 0 | 0 | 0 | 0 | 1 | 336 | 344 |  |  | PEAKS DB |
| K.EYQELM(+15.99)NTK.L | Y | 48.14 | 1170.5227 | 9 | 1.0 | 586.2692 | 2 | 28.65 | 8 | F8:11928 | 31122021\_RID\_3262\_1b.raw |  |  |  |  |  |  |  | 1.1907E6 | 2 | 0 | 0 | 0 | 0 | 0 | 0 | 0 | 2 | 336 | 344 | Oxidation (M) | M6:Oxidation (M):1000.00 | PEAKS DB |
| total 2 peptides |  |  |  |  |  |  |  |  |  |  |  |  |  |  |  |  |  |  |  |  |  |  |  |  |  |  |  |  |  |  |  |  |  |
| --- | --- | --- | --- | --- | --- | --- | --- | --- | --- | --- | --- | --- | --- | --- | --- | --- | --- | --- | --- | --- | --- | --- | --- | --- | --- | --- | --- | --- | --- | --- | --- | --- | --- |

XP\_026535749.1

back to list

  

| Protein Coverage
| Supporting Peptides
|

Protein Coverage:

Supporting Peptides:

| Peptide | Uniq | -10lgP | Mass | Length | ppm | m/z | z | RT | Fraction | Scan | Source File | Area F6 | Area F2 | Area F3 | Area F4 | Area F5 | Area F7 | Area F8 | Area F1 | #Feature | #Feature F6 | #Feature F2 | #Feature F3 | #Feature F4 | #Feature F5 | #Feature F7 | #Feature F8 | #Feature F1 | Start | End | PTM | AScore | Found By |
| --- | --- | --- | --- | --- | --- | --- | --- | --- | --- | --- | --- | --- | --- | --- | --- | --- | --- | --- | --- | --- | --- | --- | --- | --- | --- | --- | --- | --- | --- | --- | --- | --- | --- |
| K.EYQELMNTK.L | Y | 58.79 | 1154.5277 | 9 | 0.6 | 578.2715 | 2 | 28.66 | 8 | F8:12011 | 31122021\_RID\_3262\_1b.raw |  |  | 2.0994E6 |  |  |  |  | 6.7955E6 | 2 | 0 | 0 | 1 | 0 | 0 | 0 | 0 | 1 | 379 | 387 |  |  | PEAKS DB |
| K.EYQELM(+15.99)NTK.L | Y | 48.14 | 1170.5227 | 9 | 1.0 | 586.2692 | 2 | 28.65 | 8 | F8:11928 | 31122021\_RID\_3262\_1b.raw |  |  |  |  |  |  |  | 1.1907E6 | 2 | 0 | 0 | 0 | 0 | 0 | 0 | 0 | 2 | 379 | 387 | Oxidation (M) | M6:Oxidation (M):1000.00 | PEAKS DB |
| total 2 peptides |  |  |  |  |  |  |  |  |  |  |  |  |  |  |  |  |  |  |  |  |  |  |  |  |  |  |  |  |  |  |  |  |  |
| --- | --- | --- | --- | --- | --- | --- | --- | --- | --- | --- | --- | --- | --- | --- | --- | --- | --- | --- | --- | --- | --- | --- | --- | --- | --- | --- | --- | --- | --- | --- | --- | --- | --- |

XP\_029142905.1

back to list

  

| Protein Coverage
| Supporting Peptides
|

Protein Coverage:

Supporting Peptides:

| Peptide | Uniq | -10lgP | Mass | Length | ppm | m/z | z | RT | Fraction | Scan | Source File | Area F6 | Area F2 | Area F3 | Area F4 | Area F5 | Area F7 | Area F8 | Area F1 | #Feature | #Feature F6 | #Feature F2 | #Feature F3 | #Feature F4 | #Feature F5 | #Feature F7 | #Feature F8 | #Feature F1 | Start | End | PTM | AScore | Found By |
| --- | --- | --- | --- | --- | --- | --- | --- | --- | --- | --- | --- | --- | --- | --- | --- | --- | --- | --- | --- | --- | --- | --- | --- | --- | --- | --- | --- | --- | --- | --- | --- | --- | --- |
| K.EYQELMNTK.L | Y | 58.79 | 1154.5277 | 9 | 0.6 | 578.2715 | 2 | 28.66 | 8 | F8:12011 | 31122021\_RID\_3262\_1b.raw |  |  | 2.0994E6 |  |  |  |  | 6.7955E6 | 2 | 0 | 0 | 1 | 0 | 0 | 0 | 0 | 1 | 176 | 184 |  |  | PEAKS DB |
| K.EYQELM(+15.99)NTK.L | Y | 48.14 | 1170.5227 | 9 | 1.0 | 586.2692 | 2 | 28.65 | 8 | F8:11928 | 31122021\_RID\_3262\_1b.raw |  |  |  |  |  |  |  | 1.1907E6 | 2 | 0 | 0 | 0 | 0 | 0 | 0 | 0 | 2 | 176 | 184 | Oxidation (M) | M6:Oxidation (M):1000.00 | PEAKS DB |
| total 2 peptides |  |  |  |  |  |  |  |  |  |  |  |  |  |  |  |  |  |  |  |  |  |  |  |  |  |  |  |  |  |  |  |  |  |
| --- | --- | --- | --- | --- | --- | --- | --- | --- | --- | --- | --- | --- | --- | --- | --- | --- | --- | --- | --- | --- | --- | --- | --- | --- | --- | --- | --- | --- | --- | --- | --- | --- | --- |

XP\_032066440.1

back to list

  

| Protein Coverage
| Supporting Peptides
|

Protein Coverage:

Supporting Peptides:

| Peptide | Uniq | -10lgP | Mass | Length | ppm | m/z | z | RT | Fraction | Scan | Source File | Area F6 | Area F2 | Area F3 | Area F4 | Area F5 | Area F7 | Area F8 | Area F1 | #Feature | #Feature F6 | #Feature F2 | #Feature F3 | #Feature F4 | #Feature F5 | #Feature F7 | #Feature F8 | #Feature F1 | Start | End | PTM | AScore | Found By |
| --- | --- | --- | --- | --- | --- | --- | --- | --- | --- | --- | --- | --- | --- | --- | --- | --- | --- | --- | --- | --- | --- | --- | --- | --- | --- | --- | --- | --- | --- | --- | --- | --- | --- |
| R.VTQSNFAVGYK.T | Y | 57.74 | 1212.6139 | 11 | 0.1 | 607.3143 | 2 | 30.03 | 8 | F8:12938 | 31122021\_RID\_3262\_1b.raw |  |  |  |  |  |  |  | 8.8493E4 | 1 | 0 | 0 | 0 | 0 | 0 | 0 | 0 | 1 | 206 | 216 |  |  | PEAKS DB |
| total 1 peptides |  |  |  |  |  |  |  |  |  |  |  |  |  |  |  |  |  |  |  |  |  |  |  |  |  |  |  |  |  |  |  |  |  |
| --- | --- | --- | --- | --- | --- | --- | --- | --- | --- | --- | --- | --- | --- | --- | --- | --- | --- | --- | --- | --- | --- | --- | --- | --- | --- | --- | --- | --- | --- | --- | --- | --- | --- |

XP\_013913912.1

back to list

  

| Protein Coverage
| Supporting Peptides
|

Protein Coverage:

Supporting Peptides:

| Peptide | Uniq | -10lgP | Mass | Length | ppm | m/z | z | RT | Fraction | Scan | Source File | Area F6 | Area F2 | Area F3 | Area F4 | Area F5 | Area F7 | Area F8 | Area F1 | #Feature | #Feature F6 | #Feature F2 | #Feature F3 | #Feature F4 | #Feature F5 | #Feature F7 | #Feature F8 | #Feature F1 | Start | End | PTM | AScore | Found By |
| --- | --- | --- | --- | --- | --- | --- | --- | --- | --- | --- | --- | --- | --- | --- | --- | --- | --- | --- | --- | --- | --- | --- | --- | --- | --- | --- | --- | --- | --- | --- | --- | --- | --- |
| R.VTQSNFAVGYK.T | Y | 57.74 | 1212.6139 | 11 | 0.1 | 607.3143 | 2 | 30.03 | 8 | F8:12938 | 31122021\_RID\_3262\_1b.raw |  |  |  |  |  |  |  | 8.8493E4 | 1 | 0 | 0 | 0 | 0 | 0 | 0 | 0 | 1 | 206 | 216 |  |  | PEAKS DB |
| total 1 peptides |  |  |  |  |  |  |  |  |  |  |  |  |  |  |  |  |  |  |  |  |  |  |  |  |  |  |  |  |  |  |  |  |  |
| --- | --- | --- | --- | --- | --- | --- | --- | --- | --- | --- | --- | --- | --- | --- | --- | --- | --- | --- | --- | --- | --- | --- | --- | --- | --- | --- | --- | --- | --- | --- | --- | --- | --- |

JAG46544.1

back to list

  

| Protein Coverage
| Supporting Peptides
|

Protein Coverage:

Supporting Peptides:

| Peptide | Uniq | -10lgP | Mass | Length | ppm | m/z | z | RT | Fraction | Scan | Source File | Area F6 | Area F2 | Area F3 | Area F4 | Area F5 | Area F7 | Area F8 | Area F1 | #Feature | #Feature F6 | #Feature F2 | #Feature F3 | #Feature F4 | #Feature F5 | #Feature F7 | #Feature F8 | #Feature F1 | Start | End | PTM | AScore | Found By |
| --- | --- | --- | --- | --- | --- | --- | --- | --- | --- | --- | --- | --- | --- | --- | --- | --- | --- | --- | --- | --- | --- | --- | --- | --- | --- | --- | --- | --- | --- | --- | --- | --- | --- |
| R.ATDFVVPGPGK.V | Y | 56.43 | 1086.5709 | 11 | 1.4 | 544.2935 | 2 | 35.18 | 8 | F8:17067 | 31122021\_RID\_3262\_1b.raw |  |  |  |  |  |  |  | 2.9167E4 | 1 | 0 | 0 | 0 | 0 | 0 | 0 | 0 | 1 | 141 | 151 |  |  | PEAKS DB |
| total 1 peptides |  |  |  |  |  |  |  |  |  |  |  |  |  |  |  |  |  |  |  |  |  |  |  |  |  |  |  |  |  |  |  |  |  |
| --- | --- | --- | --- | --- | --- | --- | --- | --- | --- | --- | --- | --- | --- | --- | --- | --- | --- | --- | --- | --- | --- | --- | --- | --- | --- | --- | --- | --- | --- | --- | --- | --- | --- |

JAA96763.1

back to list

  

| Protein Coverage
| Supporting Peptides
|

Protein Coverage:

Supporting Peptides:

| Peptide | Uniq | -10lgP | Mass | Length | ppm | m/z | z | RT | Fraction | Scan | Source File | Area F6 | Area F2 | Area F3 | Area F4 | Area F5 | Area F7 | Area F8 | Area F1 | #Feature | #Feature F6 | #Feature F2 | #Feature F3 | #Feature F4 | #Feature F5 | #Feature F7 | #Feature F8 | #Feature F1 | Start | End | PTM | AScore | Found By |
| --- | --- | --- | --- | --- | --- | --- | --- | --- | --- | --- | --- | --- | --- | --- | --- | --- | --- | --- | --- | --- | --- | --- | --- | --- | --- | --- | --- | --- | --- | --- | --- | --- | --- |
| R.ATDFVVPGPGK.V | Y | 56.43 | 1086.5709 | 11 | 1.4 | 544.2935 | 2 | 35.18 | 8 | F8:17067 | 31122021\_RID\_3262\_1b.raw |  |  |  |  |  |  |  | 2.9167E4 | 1 | 0 | 0 | 0 | 0 | 0 | 0 | 0 | 1 | 141 | 151 |  |  | PEAKS DB |
| total 1 peptides |  |  |  |  |  |  |  |  |  |  |  |  |  |  |  |  |  |  |  |  |  |  |  |  |  |  |  |  |  |  |  |  |  |
| --- | --- | --- | --- | --- | --- | --- | --- | --- | --- | --- | --- | --- | --- | --- | --- | --- | --- | --- | --- | --- | --- | --- | --- | --- | --- | --- | --- | --- | --- | --- | --- | --- | --- |

JAG44318.1

back to list

  

| Protein Coverage
| Supporting Peptides
|

Protein Coverage:

Supporting Peptides:

| Peptide | Uniq | -10lgP | Mass | Length | ppm | m/z | z | RT | Fraction | Scan | Source File | Area F6 | Area F2 | Area F3 | Area F4 | Area F5 | Area F7 | Area F8 | Area F1 | #Feature | #Feature F6 | #Feature F2 | #Feature F3 | #Feature F4 | #Feature F5 | #Feature F7 | #Feature F8 | #Feature F1 | Start | End | PTM | AScore | Found By |
| --- | --- | --- | --- | --- | --- | --- | --- | --- | --- | --- | --- | --- | --- | --- | --- | --- | --- | --- | --- | --- | --- | --- | --- | --- | --- | --- | --- | --- | --- | --- | --- | --- | --- |
| R.ATDFVVPGPGK.V | Y | 56.43 | 1086.5709 | 11 | 1.4 | 544.2935 | 2 | 35.18 | 8 | F8:17067 | 31122021\_RID\_3262\_1b.raw |  |  |  |  |  |  |  | 2.9167E4 | 1 | 0 | 0 | 0 | 0 | 0 | 0 | 0 | 1 | 141 | 151 |  |  | PEAKS DB |
| total 1 peptides |  |  |  |  |  |  |  |  |  |  |  |  |  |  |  |  |  |  |  |  |  |  |  |  |  |  |  |  |  |  |  |  |  |
| --- | --- | --- | --- | --- | --- | --- | --- | --- | --- | --- | --- | --- | --- | --- | --- | --- | --- | --- | --- | --- | --- | --- | --- | --- | --- | --- | --- | --- | --- | --- | --- | --- | --- |

XP\_026563554.1

back to list

  

| Protein Coverage
| Supporting Peptides
|

Protein Coverage:

Supporting Peptides:

| Peptide | Uniq | -10lgP | Mass | Length | ppm | m/z | z | RT | Fraction | Scan | Source File | Area F6 | Area F2 | Area F3 | Area F4 | Area F5 | Area F7 | Area F8 | Area F1 | #Feature | #Feature F6 | #Feature F2 | #Feature F3 | #Feature F4 | #Feature F5 | #Feature F7 | #Feature F8 | #Feature F1 | Start | End | PTM | AScore | Found By |
| --- | --- | --- | --- | --- | --- | --- | --- | --- | --- | --- | --- | --- | --- | --- | --- | --- | --- | --- | --- | --- | --- | --- | --- | --- | --- | --- | --- | --- | --- | --- | --- | --- | --- |
| R.ATDFVVPGPGK.V | Y | 56.43 | 1086.5709 | 11 | 1.4 | 544.2935 | 2 | 35.18 | 8 | F8:17067 | 31122021\_RID\_3262\_1b.raw |  |  |  |  |  |  |  | 2.9167E4 | 1 | 0 | 0 | 0 | 0 | 0 | 0 | 0 | 1 | 141 | 151 |  |  | PEAKS DB |
| total 1 peptides |  |  |  |  |  |  |  |  |  |  |  |  |  |  |  |  |  |  |  |  |  |  |  |  |  |  |  |  |  |  |  |  |  |
| --- | --- | --- | --- | --- | --- | --- | --- | --- | --- | --- | --- | --- | --- | --- | --- | --- | --- | --- | --- | --- | --- | --- | --- | --- | --- | --- | --- | --- | --- | --- | --- | --- | --- |

JAC95305.1

back to list

  

| Protein Coverage
| Supporting Peptides
|

Protein Coverage:

Supporting Peptides:

| Peptide | Uniq | -10lgP | Mass | Length | ppm | m/z | z | RT | Fraction | Scan | Source File | Area F6 | Area F2 | Area F3 | Area F4 | Area F5 | Area F7 | Area F8 | Area F1 | #Feature | #Feature F6 | #Feature F2 | #Feature F3 | #Feature F4 | #Feature F5 | #Feature F7 | #Feature F8 | #Feature F1 | Start | End | PTM | AScore | Found By |
| --- | --- | --- | --- | --- | --- | --- | --- | --- | --- | --- | --- | --- | --- | --- | --- | --- | --- | --- | --- | --- | --- | --- | --- | --- | --- | --- | --- | --- | --- | --- | --- | --- | --- |
| R.ATDFVVPGPGK.V | Y | 56.43 | 1086.5709 | 11 | 1.4 | 544.2935 | 2 | 35.18 | 8 | F8:17067 | 31122021\_RID\_3262\_1b.raw |  |  |  |  |  |  |  | 2.9167E4 | 1 | 0 | 0 | 0 | 0 | 0 | 0 | 0 | 1 | 141 | 151 |  |  | PEAKS DB |
| total 1 peptides |  |  |  |  |  |  |  |  |  |  |  |  |  |  |  |  |  |  |  |  |  |  |  |  |  |  |  |  |  |  |  |  |  |
| --- | --- | --- | --- | --- | --- | --- | --- | --- | --- | --- | --- | --- | --- | --- | --- | --- | --- | --- | --- | --- | --- | --- | --- | --- | --- | --- | --- | --- | --- | --- | --- | --- | --- |

JAB54051.1

back to list

  

| Protein Coverage
| Supporting Peptides
|

Protein Coverage:

Supporting Peptides:

| Peptide | Uniq | -10lgP | Mass | Length | ppm | m/z | z | RT | Fraction | Scan | Source File | Area F6 | Area F2 | Area F3 | Area F4 | Area F5 | Area F7 | Area F8 | Area F1 | #Feature | #Feature F6 | #Feature F2 | #Feature F3 | #Feature F4 | #Feature F5 | #Feature F7 | #Feature F8 | #Feature F1 | Start | End | PTM | AScore | Found By |
| --- | --- | --- | --- | --- | --- | --- | --- | --- | --- | --- | --- | --- | --- | --- | --- | --- | --- | --- | --- | --- | --- | --- | --- | --- | --- | --- | --- | --- | --- | --- | --- | --- | --- |
| R.ATDFVVPGPGK.V | Y | 56.43 | 1086.5709 | 11 | 1.4 | 544.2935 | 2 | 35.18 | 8 | F8:17067 | 31122021\_RID\_3262\_1b.raw |  |  |  |  |  |  |  | 2.9167E4 | 1 | 0 | 0 | 0 | 0 | 0 | 0 | 0 | 1 | 141 | 151 |  |  | PEAKS DB |
| total 1 peptides |  |  |  |  |  |  |  |  |  |  |  |  |  |  |  |  |  |  |  |  |  |  |  |  |  |  |  |  |  |  |  |  |  |
| --- | --- | --- | --- | --- | --- | --- | --- | --- | --- | --- | --- | --- | --- | --- | --- | --- | --- | --- | --- | --- | --- | --- | --- | --- | --- | --- | --- | --- | --- | --- | --- | --- | --- |

XP\_026528963.1

back to list

  

| Protein Coverage
| Supporting Peptides
|

Protein Coverage:

Supporting Peptides:

| Peptide | Uniq | -10lgP | Mass | Length | ppm | m/z | z | RT | Fraction | Scan | Source File | Area F6 | Area F2 | Area F3 | Area F4 | Area F5 | Area F7 | Area F8 | Area F1 | #Feature | #Feature F6 | #Feature F2 | #Feature F3 | #Feature F4 | #Feature F5 | #Feature F7 | #Feature F8 | #Feature F1 | Start | End | PTM | AScore | Found By |
| --- | --- | --- | --- | --- | --- | --- | --- | --- | --- | --- | --- | --- | --- | --- | --- | --- | --- | --- | --- | --- | --- | --- | --- | --- | --- | --- | --- | --- | --- | --- | --- | --- | --- |
| R.ATDFVVPGPGK.V | Y | 56.43 | 1086.5709 | 11 | 1.4 | 544.2935 | 2 | 35.18 | 8 | F8:17067 | 31122021\_RID\_3262\_1b.raw |  |  |  |  |  |  |  | 2.9167E4 | 1 | 0 | 0 | 0 | 0 | 0 | 0 | 0 | 1 | 141 | 151 |  |  | PEAKS DB |
| total 1 peptides |  |  |  |  |  |  |  |  |  |  |  |  |  |  |  |  |  |  |  |  |  |  |  |  |  |  |  |  |  |  |  |  |  |
| --- | --- | --- | --- | --- | --- | --- | --- | --- | --- | --- | --- | --- | --- | --- | --- | --- | --- | --- | --- | --- | --- | --- | --- | --- | --- | --- | --- | --- | --- | --- | --- | --- | --- |

JAI09889.1

back to list

  

| Protein Coverage
| Supporting Peptides
|

Protein Coverage:

Supporting Peptides:

| Peptide | Uniq | -10lgP | Mass | Length | ppm | m/z | z | RT | Fraction | Scan | Source File | Area F6 | Area F2 | Area F3 | Area F4 | Area F5 | Area F7 | Area F8 | Area F1 | #Feature | #Feature F6 | #Feature F2 | #Feature F3 | #Feature F4 | #Feature F5 | #Feature F7 | #Feature F8 | #Feature F1 | Start | End | PTM | AScore | Found By |
| --- | --- | --- | --- | --- | --- | --- | --- | --- | --- | --- | --- | --- | --- | --- | --- | --- | --- | --- | --- | --- | --- | --- | --- | --- | --- | --- | --- | --- | --- | --- | --- | --- | --- |
| R.ATDFVVPGPGK.V | Y | 56.43 | 1086.5709 | 11 | 1.4 | 544.2935 | 2 | 35.18 | 8 | F8:17067 | 31122021\_RID\_3262\_1b.raw |  |  |  |  |  |  |  | 2.9167E4 | 1 | 0 | 0 | 0 | 0 | 0 | 0 | 0 | 1 | 141 | 151 |  |  | PEAKS DB |
| total 1 peptides |  |  |  |  |  |  |  |  |  |  |  |  |  |  |  |  |  |  |  |  |  |  |  |  |  |  |  |  |  |  |  |  |  |
| --- | --- | --- | --- | --- | --- | --- | --- | --- | --- | --- | --- | --- | --- | --- | --- | --- | --- | --- | --- | --- | --- | --- | --- | --- | --- | --- | --- | --- | --- | --- | --- | --- | --- |

ETE62592.1

back to list

  

| Protein Coverage
| Supporting Peptides
|

Protein Coverage:

Supporting Peptides:

| Peptide | Uniq | -10lgP | Mass | Length | ppm | m/z | z | RT | Fraction | Scan | Source File | Area F6 | Area F2 | Area F3 | Area F4 | Area F5 | Area F7 | Area F8 | Area F1 | #Feature | #Feature F6 | #Feature F2 | #Feature F3 | #Feature F4 | #Feature F5 | #Feature F7 | #Feature F8 | #Feature F1 | Start | End | PTM | AScore | Found By |
| --- | --- | --- | --- | --- | --- | --- | --- | --- | --- | --- | --- | --- | --- | --- | --- | --- | --- | --- | --- | --- | --- | --- | --- | --- | --- | --- | --- | --- | --- | --- | --- | --- | --- |
| R.ATDFVVPGPGK.V | Y | 56.43 | 1086.5709 | 11 | 1.4 | 544.2935 | 2 | 35.18 | 8 | F8:17067 | 31122021\_RID\_3262\_1b.raw |  |  |  |  |  |  |  | 2.9167E4 | 1 | 0 | 0 | 0 | 0 | 0 | 0 | 0 | 1 | 141 | 151 |  |  | PEAKS DB |
| total 1 peptides |  |  |  |  |  |  |  |  |  |  |  |  |  |  |  |  |  |  |  |  |  |  |  |  |  |  |  |  |  |  |  |  |  |
| --- | --- | --- | --- | --- | --- | --- | --- | --- | --- | --- | --- | --- | --- | --- | --- | --- | --- | --- | --- | --- | --- | --- | --- | --- | --- | --- | --- | --- | --- | --- | --- | --- | --- |

JAI12893.1

back to list

  

| Protein Coverage
| Supporting Peptides
|

Protein Coverage:

Supporting Peptides:

| Peptide | Uniq | -10lgP | Mass | Length | ppm | m/z | z | RT | Fraction | Scan | Source File | Area F6 | Area F2 | Area F3 | Area F4 | Area F5 | Area F7 | Area F8 | Area F1 | #Feature | #Feature F6 | #Feature F2 | #Feature F3 | #Feature F4 | #Feature F5 | #Feature F7 | #Feature F8 | #Feature F1 | Start | End | PTM | AScore | Found By |
| --- | --- | --- | --- | --- | --- | --- | --- | --- | --- | --- | --- | --- | --- | --- | --- | --- | --- | --- | --- | --- | --- | --- | --- | --- | --- | --- | --- | --- | --- | --- | --- | --- | --- |
| R.ATDFVVPGPGK.V | Y | 56.43 | 1086.5709 | 11 | 1.4 | 544.2935 | 2 | 35.18 | 8 | F8:17067 | 31122021\_RID\_3262\_1b.raw |  |  |  |  |  |  |  | 2.9167E4 | 1 | 0 | 0 | 0 | 0 | 0 | 0 | 0 | 1 | 141 | 151 |  |  | PEAKS DB |
| total 1 peptides |  |  |  |  |  |  |  |  |  |  |  |  |  |  |  |  |  |  |  |  |  |  |  |  |  |  |  |  |  |  |  |  |  |
| --- | --- | --- | --- | --- | --- | --- | --- | --- | --- | --- | --- | --- | --- | --- | --- | --- | --- | --- | --- | --- | --- | --- | --- | --- | --- | --- | --- | --- | --- | --- | --- | --- | --- |

AFJ50392.1

back to list

  

| Protein Coverage
| Supporting Peptides
|

Protein Coverage:

Supporting Peptides:

| Peptide | Uniq | -10lgP | Mass | Length | ppm | m/z | z | RT | Fraction | Scan | Source File | Area F6 | Area F2 | Area F3 | Area F4 | Area F5 | Area F7 | Area F8 | Area F1 | #Feature | #Feature F6 | #Feature F2 | #Feature F3 | #Feature F4 | #Feature F5 | #Feature F7 | #Feature F8 | #Feature F1 | Start | End | PTM | AScore | Found By |
| --- | --- | --- | --- | --- | --- | --- | --- | --- | --- | --- | --- | --- | --- | --- | --- | --- | --- | --- | --- | --- | --- | --- | --- | --- | --- | --- | --- | --- | --- | --- | --- | --- | --- |
| R.ATDFVVPGPGK.V | Y | 56.43 | 1086.5709 | 11 | 1.4 | 544.2935 | 2 | 35.18 | 8 | F8:17067 | 31122021\_RID\_3262\_1b.raw |  |  |  |  |  |  |  | 2.9167E4 | 1 | 0 | 0 | 0 | 0 | 0 | 0 | 0 | 1 | 141 | 151 |  |  | PEAKS DB |
| total 1 peptides |  |  |  |  |  |  |  |  |  |  |  |  |  |  |  |  |  |  |  |  |  |  |  |  |  |  |  |  |  |  |  |  |  |
| --- | --- | --- | --- | --- | --- | --- | --- | --- | --- | --- | --- | --- | --- | --- | --- | --- | --- | --- | --- | --- | --- | --- | --- | --- | --- | --- | --- | --- | --- | --- | --- | --- | --- |

XP\_015669945.1

back to list

  

| Protein Coverage
| Supporting Peptides
|

Protein Coverage:

Supporting Peptides:

| Peptide | Uniq | -10lgP | Mass | Length | ppm | m/z | z | RT | Fraction | Scan | Source File | Area F6 | Area F2 | Area F3 | Area F4 | Area F5 | Area F7 | Area F8 | Area F1 | #Feature | #Feature F6 | #Feature F2 | #Feature F3 | #Feature F4 | #Feature F5 | #Feature F7 | #Feature F8 | #Feature F1 | Start | End | PTM | AScore | Found By |
| --- | --- | --- | --- | --- | --- | --- | --- | --- | --- | --- | --- | --- | --- | --- | --- | --- | --- | --- | --- | --- | --- | --- | --- | --- | --- | --- | --- | --- | --- | --- | --- | --- | --- |
| R.ATDFVVPGPGK.V | Y | 56.43 | 1086.5709 | 11 | 1.4 | 544.2935 | 2 | 35.18 | 8 | F8:17067 | 31122021\_RID\_3262\_1b.raw |  |  |  |  |  |  |  | 2.9167E4 | 1 | 0 | 0 | 0 | 0 | 0 | 0 | 0 | 1 | 141 | 151 |  |  | PEAKS DB |
| total 1 peptides |  |  |  |  |  |  |  |  |  |  |  |  |  |  |  |  |  |  |  |  |  |  |  |  |  |  |  |  |  |  |  |  |  |
| --- | --- | --- | --- | --- | --- | --- | --- | --- | --- | --- | --- | --- | --- | --- | --- | --- | --- | --- | --- | --- | --- | --- | --- | --- | --- | --- | --- | --- | --- | --- | --- | --- | --- |

XP\_032067252.1

back to list

  

| Protein Coverage
| Supporting Peptides
|

Protein Coverage:

Supporting Peptides:

| Peptide | Uniq | -10lgP | Mass | Length | ppm | m/z | z | RT | Fraction | Scan | Source File | Area F6 | Area F2 | Area F3 | Area F4 | Area F5 | Area F7 | Area F8 | Area F1 | #Feature | #Feature F6 | #Feature F2 | #Feature F3 | #Feature F4 | #Feature F5 | #Feature F7 | #Feature F8 | #Feature F1 | Start | End | PTM | AScore | Found By |
| --- | --- | --- | --- | --- | --- | --- | --- | --- | --- | --- | --- | --- | --- | --- | --- | --- | --- | --- | --- | --- | --- | --- | --- | --- | --- | --- | --- | --- | --- | --- | --- | --- | --- |
| R.ATDFVVPGPGK.V | Y | 56.43 | 1086.5709 | 11 | 1.4 | 544.2935 | 2 | 35.18 | 8 | F8:17067 | 31122021\_RID\_3262\_1b.raw |  |  |  |  |  |  |  | 2.9167E4 | 1 | 0 | 0 | 0 | 0 | 0 | 0 | 0 | 1 | 141 | 151 |  |  | PEAKS DB |
| total 1 peptides |  |  |  |  |  |  |  |  |  |  |  |  |  |  |  |  |  |  |  |  |  |  |  |  |  |  |  |  |  |  |  |  |  |
| --- | --- | --- | --- | --- | --- | --- | --- | --- | --- | --- | --- | --- | --- | --- | --- | --- | --- | --- | --- | --- | --- | --- | --- | --- | --- | --- | --- | --- | --- | --- | --- | --- | --- |

JAG67637.1

back to list

  

| Protein Coverage
| Supporting Peptides
|

Protein Coverage:

Supporting Peptides:

| Peptide | Uniq | -10lgP | Mass | Length | ppm | m/z | z | RT | Fraction | Scan | Source File | Area F6 | Area F2 | Area F3 | Area F4 | Area F5 | Area F7 | Area F8 | Area F1 | #Feature | #Feature F6 | #Feature F2 | #Feature F3 | #Feature F4 | #Feature F5 | #Feature F7 | #Feature F8 | #Feature F1 | Start | End | PTM | AScore | Found By |
| --- | --- | --- | --- | --- | --- | --- | --- | --- | --- | --- | --- | --- | --- | --- | --- | --- | --- | --- | --- | --- | --- | --- | --- | --- | --- | --- | --- | --- | --- | --- | --- | --- | --- |
| R.ATDFVVPGPGK.V | Y | 56.43 | 1086.5709 | 11 | 1.4 | 544.2935 | 2 | 35.18 | 8 | F8:17067 | 31122021\_RID\_3262\_1b.raw |  |  |  |  |  |  |  | 2.9167E4 | 1 | 0 | 0 | 0 | 0 | 0 | 0 | 0 | 1 | 141 | 151 |  |  | PEAKS DB |
| total 1 peptides |  |  |  |  |  |  |  |  |  |  |  |  |  |  |  |  |  |  |  |  |  |  |  |  |  |  |  |  |  |  |  |  |  |
| --- | --- | --- | --- | --- | --- | --- | --- | --- | --- | --- | --- | --- | --- | --- | --- | --- | --- | --- | --- | --- | --- | --- | --- | --- | --- | --- | --- | --- | --- | --- | --- | --- | --- |

XP\_034287847.1

back to list

  

| Protein Coverage
| Supporting Peptides
|

Protein Coverage:

Supporting Peptides:

| Peptide | Uniq | -10lgP | Mass | Length | ppm | m/z | z | RT | Fraction | Scan | Source File | Area F6 | Area F2 | Area F3 | Area F4 | Area F5 | Area F7 | Area F8 | Area F1 | #Feature | #Feature F6 | #Feature F2 | #Feature F3 | #Feature F4 | #Feature F5 | #Feature F7 | #Feature F8 | #Feature F1 | Start | End | PTM | AScore | Found By |
| --- | --- | --- | --- | --- | --- | --- | --- | --- | --- | --- | --- | --- | --- | --- | --- | --- | --- | --- | --- | --- | --- | --- | --- | --- | --- | --- | --- | --- | --- | --- | --- | --- | --- |
| R.ATDFVVPGPGK.V | Y | 56.43 | 1086.5709 | 11 | 1.4 | 544.2935 | 2 | 35.18 | 8 | F8:17067 | 31122021\_RID\_3262\_1b.raw |  |  |  |  |  |  |  | 2.9167E4 | 1 | 0 | 0 | 0 | 0 | 0 | 0 | 0 | 1 | 141 | 151 |  |  | PEAKS DB |
| total 1 peptides |  |  |  |  |  |  |  |  |  |  |  |  |  |  |  |  |  |  |  |  |  |  |  |  |  |  |  |  |  |  |  |  |  |
| --- | --- | --- | --- | --- | --- | --- | --- | --- | --- | --- | --- | --- | --- | --- | --- | --- | --- | --- | --- | --- | --- | --- | --- | --- | --- | --- | --- | --- | --- | --- | --- | --- | --- |

JAV50239.1

back to list

  

| Protein Coverage
| Supporting Peptides
|

Protein Coverage:

Supporting Peptides:

| Peptide | Uniq | -10lgP | Mass | Length | ppm | m/z | z | RT | Fraction | Scan | Source File | Area F6 | Area F2 | Area F3 | Area F4 | Area F5 | Area F7 | Area F8 | Area F1 | #Feature | #Feature F6 | #Feature F2 | #Feature F3 | #Feature F4 | #Feature F5 | #Feature F7 | #Feature F8 | #Feature F1 | Start | End | PTM | AScore | Found By |
| --- | --- | --- | --- | --- | --- | --- | --- | --- | --- | --- | --- | --- | --- | --- | --- | --- | --- | --- | --- | --- | --- | --- | --- | --- | --- | --- | --- | --- | --- | --- | --- | --- | --- |
| R.ATDFVVPGPGK.V | Y | 56.43 | 1086.5709 | 11 | 1.4 | 544.2935 | 2 | 35.18 | 8 | F8:17067 | 31122021\_RID\_3262\_1b.raw |  |  |  |  |  |  |  | 2.9167E4 | 1 | 0 | 0 | 0 | 0 | 0 | 0 | 0 | 1 | 141 | 151 |  |  | PEAKS DB |
| total 1 peptides |  |  |  |  |  |  |  |  |  |  |  |  |  |  |  |  |  |  |  |  |  |  |  |  |  |  |  |  |  |  |  |  |  |
| --- | --- | --- | --- | --- | --- | --- | --- | --- | --- | --- | --- | --- | --- | --- | --- | --- | --- | --- | --- | --- | --- | --- | --- | --- | --- | --- | --- | --- | --- | --- | --- | --- | --- |

XP\_032076181.1

back to list

  

| Protein Coverage
| Supporting Peptides
|

Protein Coverage:

Supporting Peptides:

| Peptide | Uniq | -10lgP | Mass | Length | ppm | m/z | z | RT | Fraction | Scan | Source File | Area F6 | Area F2 | Area F3 | Area F4 | Area F5 | Area F7 | Area F8 | Area F1 | #Feature | #Feature F6 | #Feature F2 | #Feature F3 | #Feature F4 | #Feature F5 | #Feature F7 | #Feature F8 | #Feature F1 | Start | End | PTM | AScore | Found By |
| --- | --- | --- | --- | --- | --- | --- | --- | --- | --- | --- | --- | --- | --- | --- | --- | --- | --- | --- | --- | --- | --- | --- | --- | --- | --- | --- | --- | --- | --- | --- | --- | --- | --- |
| K.DLLDLLVEAK.Q | Y | 55.88 | 1127.6438 | 10 | 0.2 | 564.8293 | 2 | 68.78 | 8 | F8:41830 | 31122021\_RID\_3262\_1b.raw |  |  |  |  |  |  |  | 5.0661E3 | 1 | 0 | 0 | 0 | 0 | 0 | 0 | 0 | 1 | 600 | 609 |  |  | PEAKS DB |
| total 1 peptides |  |  |  |  |  |  |  |  |  |  |  |  |  |  |  |  |  |  |  |  |  |  |  |  |  |  |  |  |  |  |  |  |  |
| --- | --- | --- | --- | --- | --- | --- | --- | --- | --- | --- | --- | --- | --- | --- | --- | --- | --- | --- | --- | --- | --- | --- | --- | --- | --- | --- | --- | --- | --- | --- | --- | --- | --- |

XP\_013929400.1

back to list

  

| Protein Coverage
| Supporting Peptides
|

Protein Coverage:

Supporting Peptides:

| Peptide | Uniq | -10lgP | Mass | Length | ppm | m/z | z | RT | Fraction | Scan | Source File | Area F6 | Area F2 | Area F3 | Area F4 | Area F5 | Area F7 | Area F8 | Area F1 | #Feature | #Feature F6 | #Feature F2 | #Feature F3 | #Feature F4 | #Feature F5 | #Feature F7 | #Feature F8 | #Feature F1 | Start | End | PTM | AScore | Found By |
| --- | --- | --- | --- | --- | --- | --- | --- | --- | --- | --- | --- | --- | --- | --- | --- | --- | --- | --- | --- | --- | --- | --- | --- | --- | --- | --- | --- | --- | --- | --- | --- | --- | --- |
| K.DLLDLLVEAK.Q | Y | 55.88 | 1127.6438 | 10 | 0.2 | 564.8293 | 2 | 68.78 | 8 | F8:41830 | 31122021\_RID\_3262\_1b.raw |  |  |  |  |  |  |  | 5.0661E3 | 1 | 0 | 0 | 0 | 0 | 0 | 0 | 0 | 1 | 600 | 609 |  |  | PEAKS DB |
| total 1 peptides |  |  |  |  |  |  |  |  |  |  |  |  |  |  |  |  |  |  |  |  |  |  |  |  |  |  |  |  |  |  |  |  |  |
| --- | --- | --- | --- | --- | --- | --- | --- | --- | --- | --- | --- | --- | --- | --- | --- | --- | --- | --- | --- | --- | --- | --- | --- | --- | --- | --- | --- | --- | --- | --- | --- | --- | --- |

JAV50781.1

back to list

  

| Protein Coverage
| Supporting Peptides
|

Protein Coverage:

Supporting Peptides:

| Peptide | Uniq | -10lgP | Mass | Length | ppm | m/z | z | RT | Fraction | Scan | Source File | Area F6 | Area F2 | Area F3 | Area F4 | Area F5 | Area F7 | Area F8 | Area F1 | #Feature | #Feature F6 | #Feature F2 | #Feature F3 | #Feature F4 | #Feature F5 | #Feature F7 | #Feature F8 | #Feature F1 | Start | End | PTM | AScore | Found By |
| --- | --- | --- | --- | --- | --- | --- | --- | --- | --- | --- | --- | --- | --- | --- | --- | --- | --- | --- | --- | --- | --- | --- | --- | --- | --- | --- | --- | --- | --- | --- | --- | --- | --- |
| K.DLLDLLVEAK.Q | Y | 55.88 | 1127.6438 | 10 | 0.2 | 564.8293 | 2 | 68.78 | 8 | F8:41830 | 31122021\_RID\_3262\_1b.raw |  |  |  |  |  |  |  | 5.0661E3 | 1 | 0 | 0 | 0 | 0 | 0 | 0 | 0 | 1 | 599 | 608 |  |  | PEAKS DB |
| total 1 peptides |  |  |  |  |  |  |  |  |  |  |  |  |  |  |  |  |  |  |  |  |  |  |  |  |  |  |  |  |  |  |  |  |  |
| --- | --- | --- | --- | --- | --- | --- | --- | --- | --- | --- | --- | --- | --- | --- | --- | --- | --- | --- | --- | --- | --- | --- | --- | --- | --- | --- | --- | --- | --- | --- | --- | --- | --- |

XP\_032076182.1

back to list

  

| Protein Coverage
| Supporting Peptides
|

Protein Coverage:

Supporting Peptides:

| Peptide | Uniq | -10lgP | Mass | Length | ppm | m/z | z | RT | Fraction | Scan | Source File | Area F6 | Area F2 | Area F3 | Area F4 | Area F5 | Area F7 | Area F8 | Area F1 | #Feature | #Feature F6 | #Feature F2 | #Feature F3 | #Feature F4 | #Feature F5 | #Feature F7 | #Feature F8 | #Feature F1 | Start | End | PTM | AScore | Found By |
| --- | --- | --- | --- | --- | --- | --- | --- | --- | --- | --- | --- | --- | --- | --- | --- | --- | --- | --- | --- | --- | --- | --- | --- | --- | --- | --- | --- | --- | --- | --- | --- | --- | --- |
| K.DLLDLLVEAK.Q | Y | 55.88 | 1127.6438 | 10 | 0.2 | 564.8293 | 2 | 68.78 | 8 | F8:41830 | 31122021\_RID\_3262\_1b.raw |  |  |  |  |  |  |  | 5.0661E3 | 1 | 0 | 0 | 0 | 0 | 0 | 0 | 0 | 1 | 556 | 565 |  |  | PEAKS DB |
| total 1 peptides |  |  |  |  |  |  |  |  |  |  |  |  |  |  |  |  |  |  |  |  |  |  |  |  |  |  |  |  |  |  |  |  |  |
| --- | --- | --- | --- | --- | --- | --- | --- | --- | --- | --- | --- | --- | --- | --- | --- | --- | --- | --- | --- | --- | --- | --- | --- | --- | --- | --- | --- | --- | --- | --- | --- | --- | --- |

XP\_013929401.1

back to list

  

| Protein Coverage
| Supporting Peptides
|

Protein Coverage:

Supporting Peptides:

| Peptide | Uniq | -10lgP | Mass | Length | ppm | m/z | z | RT | Fraction | Scan | Source File | Area F6 | Area F2 | Area F3 | Area F4 | Area F5 | Area F7 | Area F8 | Area F1 | #Feature | #Feature F6 | #Feature F2 | #Feature F3 | #Feature F4 | #Feature F5 | #Feature F7 | #Feature F8 | #Feature F1 | Start | End | PTM | AScore | Found By |
| --- | --- | --- | --- | --- | --- | --- | --- | --- | --- | --- | --- | --- | --- | --- | --- | --- | --- | --- | --- | --- | --- | --- | --- | --- | --- | --- | --- | --- | --- | --- | --- | --- | --- |
| K.DLLDLLVEAK.Q | Y | 55.88 | 1127.6438 | 10 | 0.2 | 564.8293 | 2 | 68.78 | 8 | F8:41830 | 31122021\_RID\_3262\_1b.raw |  |  |  |  |  |  |  | 5.0661E3 | 1 | 0 | 0 | 0 | 0 | 0 | 0 | 0 | 1 | 556 | 565 |  |  | PEAKS DB |
| total 1 peptides |  |  |  |  |  |  |  |  |  |  |  |  |  |  |  |  |  |  |  |  |  |  |  |  |  |  |  |  |  |  |  |  |  |
| --- | --- | --- | --- | --- | --- | --- | --- | --- | --- | --- | --- | --- | --- | --- | --- | --- | --- | --- | --- | --- | --- | --- | --- | --- | --- | --- | --- | --- | --- | --- | --- | --- | --- |

XP\_007426567.1

back to list

  

| Protein Coverage
| Supporting Peptides
|

Protein Coverage:

Supporting Peptides:

| Peptide | Uniq | -10lgP | Mass | Length | ppm | m/z | z | RT | Fraction | Scan | Source File | Area F6 | Area F2 | Area F3 | Area F4 | Area F5 | Area F7 | Area F8 | Area F1 | #Feature | #Feature F6 | #Feature F2 | #Feature F3 | #Feature F4 | #Feature F5 | #Feature F7 | #Feature F8 | #Feature F1 | Start | End | PTM | AScore | Found By |
| --- | --- | --- | --- | --- | --- | --- | --- | --- | --- | --- | --- | --- | --- | --- | --- | --- | --- | --- | --- | --- | --- | --- | --- | --- | --- | --- | --- | --- | --- | --- | --- | --- | --- |
| K.DLLDLLVEAK.Q | Y | 55.88 | 1127.6438 | 10 | 0.2 | 564.8293 | 2 | 68.78 | 8 | F8:41830 | 31122021\_RID\_3262\_1b.raw |  |  |  |  |  |  |  | 5.0661E3 | 1 | 0 | 0 | 0 | 0 | 0 | 0 | 0 | 1 | 601 | 610 |  |  | PEAKS DB |
| total 1 peptides |  |  |  |  |  |  |  |  |  |  |  |  |  |  |  |  |  |  |  |  |  |  |  |  |  |  |  |  |  |  |  |  |  |
| --- | --- | --- | --- | --- | --- | --- | --- | --- | --- | --- | --- | --- | --- | --- | --- | --- | --- | --- | --- | --- | --- | --- | --- | --- | --- | --- | --- | --- | --- | --- | --- | --- | --- |

XP\_007426565.1

back to list

  

| Protein Coverage
| Supporting Peptides
|

Protein Coverage:

Supporting Peptides:

| Peptide | Uniq | -10lgP | Mass | Length | ppm | m/z | z | RT | Fraction | Scan | Source File | Area F6 | Area F2 | Area F3 | Area F4 | Area F5 | Area F7 | Area F8 | Area F1 | #Feature | #Feature F6 | #Feature F2 | #Feature F3 | #Feature F4 | #Feature F5 | #Feature F7 | #Feature F8 | #Feature F1 | Start | End | PTM | AScore | Found By |
| --- | --- | --- | --- | --- | --- | --- | --- | --- | --- | --- | --- | --- | --- | --- | --- | --- | --- | --- | --- | --- | --- | --- | --- | --- | --- | --- | --- | --- | --- | --- | --- | --- | --- |
| K.DLLDLLVEAK.Q | Y | 55.88 | 1127.6438 | 10 | 0.2 | 564.8293 | 2 | 68.78 | 8 | F8:41830 | 31122021\_RID\_3262\_1b.raw |  |  |  |  |  |  |  | 5.0661E3 | 1 | 0 | 0 | 0 | 0 | 0 | 0 | 0 | 1 | 622 | 631 |  |  | PEAKS DB |
| total 1 peptides |  |  |  |  |  |  |  |  |  |  |  |  |  |  |  |  |  |  |  |  |  |  |  |  |  |  |  |  |  |  |  |  |  |
| --- | --- | --- | --- | --- | --- | --- | --- | --- | --- | --- | --- | --- | --- | --- | --- | --- | --- | --- | --- | --- | --- | --- | --- | --- | --- | --- | --- | --- | --- | --- | --- | --- | --- |

XP\_007426569.1

back to list

  

| Protein Coverage
| Supporting Peptides
|

Protein Coverage:

Supporting Peptides:

| Peptide | Uniq | -10lgP | Mass | Length | ppm | m/z | z | RT | Fraction | Scan | Source File | Area F6 | Area F2 | Area F3 | Area F4 | Area F5 | Area F7 | Area F8 | Area F1 | #Feature | #Feature F6 | #Feature F2 | #Feature F3 | #Feature F4 | #Feature F5 | #Feature F7 | #Feature F8 | #Feature F1 | Start | End | PTM | AScore | Found By |
| --- | --- | --- | --- | --- | --- | --- | --- | --- | --- | --- | --- | --- | --- | --- | --- | --- | --- | --- | --- | --- | --- | --- | --- | --- | --- | --- | --- | --- | --- | --- | --- | --- | --- |
| K.DLLDLLVEAK.Q | Y | 55.88 | 1127.6438 | 10 | 0.2 | 564.8293 | 2 | 68.78 | 8 | F8:41830 | 31122021\_RID\_3262\_1b.raw |  |  |  |  |  |  |  | 5.0661E3 | 1 | 0 | 0 | 0 | 0 | 0 | 0 | 0 | 1 | 557 | 566 |  |  | PEAKS DB |
| total 1 peptides |  |  |  |  |  |  |  |  |  |  |  |  |  |  |  |  |  |  |  |  |  |  |  |  |  |  |  |  |  |  |  |  |  |
| --- | --- | --- | --- | --- | --- | --- | --- | --- | --- | --- | --- | --- | --- | --- | --- | --- | --- | --- | --- | --- | --- | --- | --- | --- | --- | --- | --- | --- | --- | --- | --- | --- | --- |

XP\_034279735.1

back to list

  

| Protein Coverage
| Supporting Peptides
|

Protein Coverage:

Supporting Peptides:

| Peptide | Uniq | -10lgP | Mass | Length | ppm | m/z | z | RT | Fraction | Scan | Source File | Area F6 | Area F2 | Area F3 | Area F4 | Area F5 | Area F7 | Area F8 | Area F1 | #Feature | #Feature F6 | #Feature F2 | #Feature F3 | #Feature F4 | #Feature F5 | #Feature F7 | #Feature F8 | #Feature F1 | Start | End | PTM | AScore | Found By |
| --- | --- | --- | --- | --- | --- | --- | --- | --- | --- | --- | --- | --- | --- | --- | --- | --- | --- | --- | --- | --- | --- | --- | --- | --- | --- | --- | --- | --- | --- | --- | --- | --- | --- |
| K.DLLDLLVEAK.Q | Y | 55.88 | 1127.6438 | 10 | 0.2 | 564.8293 | 2 | 68.78 | 8 | F8:41830 | 31122021\_RID\_3262\_1b.raw |  |  |  |  |  |  |  | 5.0661E3 | 1 | 0 | 0 | 0 | 0 | 0 | 0 | 0 | 1 | 600 | 609 |  |  | PEAKS DB |
| total 1 peptides |  |  |  |  |  |  |  |  |  |  |  |  |  |  |  |  |  |  |  |  |  |  |  |  |  |  |  |  |  |  |  |  |  |
| --- | --- | --- | --- | --- | --- | --- | --- | --- | --- | --- | --- | --- | --- | --- | --- | --- | --- | --- | --- | --- | --- | --- | --- | --- | --- | --- | --- | --- | --- | --- | --- | --- | --- |

XP\_034279734.1

back to list

  

| Protein Coverage
| Supporting Peptides
|

Protein Coverage:

Supporting Peptides:

| Peptide | Uniq | -10lgP | Mass | Length | ppm | m/z | z | RT | Fraction | Scan | Source File | Area F6 | Area F2 | Area F3 | Area F4 | Area F5 | Area F7 | Area F8 | Area F1 | #Feature | #Feature F6 | #Feature F2 | #Feature F3 | #Feature F4 | #Feature F5 | #Feature F7 | #Feature F8 | #Feature F1 | Start | End | PTM | AScore | Found By |
| --- | --- | --- | --- | --- | --- | --- | --- | --- | --- | --- | --- | --- | --- | --- | --- | --- | --- | --- | --- | --- | --- | --- | --- | --- | --- | --- | --- | --- | --- | --- | --- | --- | --- |
| K.DLLDLLVEAK.Q | Y | 55.88 | 1127.6438 | 10 | 0.2 | 564.8293 | 2 | 68.78 | 8 | F8:41830 | 31122021\_RID\_3262\_1b.raw |  |  |  |  |  |  |  | 5.0661E3 | 1 | 0 | 0 | 0 | 0 | 0 | 0 | 0 | 1 | 621 | 630 |  |  | PEAKS DB |
| total 1 peptides |  |  |  |  |  |  |  |  |  |  |  |  |  |  |  |  |  |  |  |  |  |  |  |  |  |  |  |  |  |  |  |  |  |
| --- | --- | --- | --- | --- | --- | --- | --- | --- | --- | --- | --- | --- | --- | --- | --- | --- | --- | --- | --- | --- | --- | --- | --- | --- | --- | --- | --- | --- | --- | --- | --- | --- | --- |

AFJ51213.1

back to list

  

| Protein Coverage
| Supporting Peptides
|

Protein Coverage:

Supporting Peptides:

| Peptide | Uniq | -10lgP | Mass | Length | ppm | m/z | z | RT | Fraction | Scan | Source File | Area F6 | Area F2 | Area F3 | Area F4 | Area F5 | Area F7 | Area F8 | Area F1 | #Feature | #Feature F6 | #Feature F2 | #Feature F3 | #Feature F4 | #Feature F5 | #Feature F7 | #Feature F8 | #Feature F1 | Start | End | PTM | AScore | Found By |
| --- | --- | --- | --- | --- | --- | --- | --- | --- | --- | --- | --- | --- | --- | --- | --- | --- | --- | --- | --- | --- | --- | --- | --- | --- | --- | --- | --- | --- | --- | --- | --- | --- | --- |
| K.DLLDLLVEAK.Q | Y | 55.88 | 1127.6438 | 10 | 0.2 | 564.8293 | 2 | 68.78 | 8 | F8:41830 | 31122021\_RID\_3262\_1b.raw |  |  |  |  |  |  |  | 5.0661E3 | 1 | 0 | 0 | 0 | 0 | 0 | 0 | 0 | 1 | 599 | 608 |  |  | PEAKS DB |
| total 1 peptides |  |  |  |  |  |  |  |  |  |  |  |  |  |  |  |  |  |  |  |  |  |  |  |  |  |  |  |  |  |  |  |  |  |
| --- | --- | --- | --- | --- | --- | --- | --- | --- | --- | --- | --- | --- | --- | --- | --- | --- | --- | --- | --- | --- | --- | --- | --- | --- | --- | --- | --- | --- | --- | --- | --- | --- | --- |

JAI11687.1

back to list

  

| Protein Coverage
| Supporting Peptides
|

Protein Coverage:

Supporting Peptides:

| Peptide | Uniq | -10lgP | Mass | Length | ppm | m/z | z | RT | Fraction | Scan | Source File | Area F6 | Area F2 | Area F3 | Area F4 | Area F5 | Area F7 | Area F8 | Area F1 | #Feature | #Feature F6 | #Feature F2 | #Feature F3 | #Feature F4 | #Feature F5 | #Feature F7 | #Feature F8 | #Feature F1 | Start | End | PTM | AScore | Found By |
| --- | --- | --- | --- | --- | --- | --- | --- | --- | --- | --- | --- | --- | --- | --- | --- | --- | --- | --- | --- | --- | --- | --- | --- | --- | --- | --- | --- | --- | --- | --- | --- | --- | --- |
| K.DLLDLLVEAK.Q | Y | 55.88 | 1127.6438 | 10 | 0.2 | 564.8293 | 2 | 68.78 | 8 | F8:41830 | 31122021\_RID\_3262\_1b.raw |  |  |  |  |  |  |  | 5.0661E3 | 1 | 0 | 0 | 0 | 0 | 0 | 0 | 0 | 1 | 599 | 608 |  |  | PEAKS DB |
| total 1 peptides |  |  |  |  |  |  |  |  |  |  |  |  |  |  |  |  |  |  |  |  |  |  |  |  |  |  |  |  |  |  |  |  |  |
| --- | --- | --- | --- | --- | --- | --- | --- | --- | --- | --- | --- | --- | --- | --- | --- | --- | --- | --- | --- | --- | --- | --- | --- | --- | --- | --- | --- | --- | --- | --- | --- | --- | --- |

XP\_034279736.1

back to list

  

| Protein Coverage
| Supporting Peptides
|

Protein Coverage:

Supporting Peptides:

| Peptide | Uniq | -10lgP | Mass | Length | ppm | m/z | z | RT | Fraction | Scan | Source File | Area F6 | Area F2 | Area F3 | Area F4 | Area F5 | Area F7 | Area F8 | Area F1 | #Feature | #Feature F6 | #Feature F2 | #Feature F3 | #Feature F4 | #Feature F5 | #Feature F7 | #Feature F8 | #Feature F1 | Start | End | PTM | AScore | Found By |
| --- | --- | --- | --- | --- | --- | --- | --- | --- | --- | --- | --- | --- | --- | --- | --- | --- | --- | --- | --- | --- | --- | --- | --- | --- | --- | --- | --- | --- | --- | --- | --- | --- | --- |
| K.DLLDLLVEAK.Q | Y | 55.88 | 1127.6438 | 10 | 0.2 | 564.8293 | 2 | 68.78 | 8 | F8:41830 | 31122021\_RID\_3262\_1b.raw |  |  |  |  |  |  |  | 5.0661E3 | 1 | 0 | 0 | 0 | 0 | 0 | 0 | 0 | 1 | 556 | 565 |  |  | PEAKS DB |
| total 1 peptides |  |  |  |  |  |  |  |  |  |  |  |  |  |  |  |  |  |  |  |  |  |  |  |  |  |  |  |  |  |  |  |  |  |
| --- | --- | --- | --- | --- | --- | --- | --- | --- | --- | --- | --- | --- | --- | --- | --- | --- | --- | --- | --- | --- | --- | --- | --- | --- | --- | --- | --- | --- | --- | --- | --- | --- | --- |

JAG47014.1

back to list

  

| Protein Coverage
| Supporting Peptides
|

Protein Coverage:

Supporting Peptides:

| Peptide | Uniq | -10lgP | Mass | Length | ppm | m/z | z | RT | Fraction | Scan | Source File | Area F6 | Area F2 | Area F3 | Area F4 | Area F5 | Area F7 | Area F8 | Area F1 | #Feature | #Feature F6 | #Feature F2 | #Feature F3 | #Feature F4 | #Feature F5 | #Feature F7 | #Feature F8 | #Feature F1 | Start | End | PTM | AScore | Found By |
| --- | --- | --- | --- | --- | --- | --- | --- | --- | --- | --- | --- | --- | --- | --- | --- | --- | --- | --- | --- | --- | --- | --- | --- | --- | --- | --- | --- | --- | --- | --- | --- | --- | --- |
| K.DLLDLLVEAK.Q | Y | 55.88 | 1127.6438 | 10 | 0.2 | 564.8293 | 2 | 68.78 | 8 | F8:41830 | 31122021\_RID\_3262\_1b.raw |  |  |  |  |  |  |  | 5.0661E3 | 1 | 0 | 0 | 0 | 0 | 0 | 0 | 0 | 1 | 555 | 564 |  |  | PEAKS DB |
| total 1 peptides |  |  |  |  |  |  |  |  |  |  |  |  |  |  |  |  |  |  |  |  |  |  |  |  |  |  |  |  |  |  |  |  |  |
| --- | --- | --- | --- | --- | --- | --- | --- | --- | --- | --- | --- | --- | --- | --- | --- | --- | --- | --- | --- | --- | --- | --- | --- | --- | --- | --- | --- | --- | --- | --- | --- | --- | --- |

XP\_015670389.1

back to list

  

| Protein Coverage
| Supporting Peptides
|

Protein Coverage:

Supporting Peptides:

| Peptide | Uniq | -10lgP | Mass | Length | ppm | m/z | z | RT | Fraction | Scan | Source File | Area F6 | Area F2 | Area F3 | Area F4 | Area F5 | Area F7 | Area F8 | Area F1 | #Feature | #Feature F6 | #Feature F2 | #Feature F3 | #Feature F4 | #Feature F5 | #Feature F7 | #Feature F8 | #Feature F1 | Start | End | PTM | AScore | Found By |
| --- | --- | --- | --- | --- | --- | --- | --- | --- | --- | --- | --- | --- | --- | --- | --- | --- | --- | --- | --- | --- | --- | --- | --- | --- | --- | --- | --- | --- | --- | --- | --- | --- | --- |
| K.DLLDLLVEAK.Q | Y | 55.88 | 1127.6438 | 10 | 0.2 | 564.8293 | 2 | 68.78 | 8 | F8:41830 | 31122021\_RID\_3262\_1b.raw |  |  |  |  |  |  |  | 5.0661E3 | 1 | 0 | 0 | 0 | 0 | 0 | 0 | 0 | 1 | 599 | 608 |  |  | PEAKS DB |
| total 1 peptides |  |  |  |  |  |  |  |  |  |  |  |  |  |  |  |  |  |  |  |  |  |  |  |  |  |  |  |  |  |  |  |  |  |
| --- | --- | --- | --- | --- | --- | --- | --- | --- | --- | --- | --- | --- | --- | --- | --- | --- | --- | --- | --- | --- | --- | --- | --- | --- | --- | --- | --- | --- | --- | --- | --- | --- | --- |

XP\_015670387.1

back to list

  

| Protein Coverage
| Supporting Peptides
|

Protein Coverage:

Supporting Peptides:

| Peptide | Uniq | -10lgP | Mass | Length | ppm | m/z | z | RT | Fraction | Scan | Source File | Area F6 | Area F2 | Area F3 | Area F4 | Area F5 | Area F7 | Area F8 | Area F1 | #Feature | #Feature F6 | #Feature F2 | #Feature F3 | #Feature F4 | #Feature F5 | #Feature F7 | #Feature F8 | #Feature F1 | Start | End | PTM | AScore | Found By |
| --- | --- | --- | --- | --- | --- | --- | --- | --- | --- | --- | --- | --- | --- | --- | --- | --- | --- | --- | --- | --- | --- | --- | --- | --- | --- | --- | --- | --- | --- | --- | --- | --- | --- |
| K.DLLDLLVEAK.Q | Y | 55.88 | 1127.6438 | 10 | 0.2 | 564.8293 | 2 | 68.78 | 8 | F8:41830 | 31122021\_RID\_3262\_1b.raw |  |  |  |  |  |  |  | 5.0661E3 | 1 | 0 | 0 | 0 | 0 | 0 | 0 | 0 | 1 | 620 | 629 |  |  | PEAKS DB |
| total 1 peptides |  |  |  |  |  |  |  |  |  |  |  |  |  |  |  |  |  |  |  |  |  |  |  |  |  |  |  |  |  |  |  |  |  |
| --- | --- | --- | --- | --- | --- | --- | --- | --- | --- | --- | --- | --- | --- | --- | --- | --- | --- | --- | --- | --- | --- | --- | --- | --- | --- | --- | --- | --- | --- | --- | --- | --- | --- |

XP\_015670390.1

back to list

  

| Protein Coverage
| Supporting Peptides
|

Protein Coverage:

Supporting Peptides:

| Peptide | Uniq | -10lgP | Mass | Length | ppm | m/z | z | RT | Fraction | Scan | Source File | Area F6 | Area F2 | Area F3 | Area F4 | Area F5 | Area F7 | Area F8 | Area F1 | #Feature | #Feature F6 | #Feature F2 | #Feature F3 | #Feature F4 | #Feature F5 | #Feature F7 | #Feature F8 | #Feature F1 | Start | End | PTM | AScore | Found By |
| --- | --- | --- | --- | --- | --- | --- | --- | --- | --- | --- | --- | --- | --- | --- | --- | --- | --- | --- | --- | --- | --- | --- | --- | --- | --- | --- | --- | --- | --- | --- | --- | --- | --- |
| K.DLLDLLVEAK.Q | Y | 55.88 | 1127.6438 | 10 | 0.2 | 564.8293 | 2 | 68.78 | 8 | F8:41830 | 31122021\_RID\_3262\_1b.raw |  |  |  |  |  |  |  | 5.0661E3 | 1 | 0 | 0 | 0 | 0 | 0 | 0 | 0 | 1 | 555 | 564 |  |  | PEAKS DB |
| total 1 peptides |  |  |  |  |  |  |  |  |  |  |  |  |  |  |  |  |  |  |  |  |  |  |  |  |  |  |  |  |  |  |  |  |  |
| --- | --- | --- | --- | --- | --- | --- | --- | --- | --- | --- | --- | --- | --- | --- | --- | --- | --- | --- | --- | --- | --- | --- | --- | --- | --- | --- | --- | --- | --- | --- | --- | --- | --- |

XP\_007426568.1

back to list

  

| Protein Coverage
| Supporting Peptides
|

Protein Coverage:

Supporting Peptides:

| Peptide | Uniq | -10lgP | Mass | Length | ppm | m/z | z | RT | Fraction | Scan | Source File | Area F6 | Area F2 | Area F3 | Area F4 | Area F5 | Area F7 | Area F8 | Area F1 | #Feature | #Feature F6 | #Feature F2 | #Feature F3 | #Feature F4 | #Feature F5 | #Feature F7 | #Feature F8 | #Feature F1 | Start | End | PTM | AScore | Found By |
| --- | --- | --- | --- | --- | --- | --- | --- | --- | --- | --- | --- | --- | --- | --- | --- | --- | --- | --- | --- | --- | --- | --- | --- | --- | --- | --- | --- | --- | --- | --- | --- | --- | --- |
| K.DLLDLLVEAK.Q | Y | 55.88 | 1127.6438 | 10 | 0.2 | 564.8293 | 2 | 68.78 | 8 | F8:41830 | 31122021\_RID\_3262\_1b.raw |  |  |  |  |  |  |  | 5.0661E3 | 1 | 0 | 0 | 0 | 0 | 0 | 0 | 0 | 1 | 599 | 608 |  |  | PEAKS DB |
| total 1 peptides |  |  |  |  |  |  |  |  |  |  |  |  |  |  |  |  |  |  |  |  |  |  |  |  |  |  |  |  |  |  |  |  |  |
| --- | --- | --- | --- | --- | --- | --- | --- | --- | --- | --- | --- | --- | --- | --- | --- | --- | --- | --- | --- | --- | --- | --- | --- | --- | --- | --- | --- | --- | --- | --- | --- | --- | --- |

XP\_007426566.1

back to list

  

| Protein Coverage
| Supporting Peptides
|

Protein Coverage:

Supporting Peptides:

| Peptide | Uniq | -10lgP | Mass | Length | ppm | m/z | z | RT | Fraction | Scan | Source File | Area F6 | Area F2 | Area F3 | Area F4 | Area F5 | Area F7 | Area F8 | Area F1 | #Feature | #Feature F6 | #Feature F2 | #Feature F3 | #Feature F4 | #Feature F5 | #Feature F7 | #Feature F8 | #Feature F1 | Start | End | PTM | AScore | Found By |
| --- | --- | --- | --- | --- | --- | --- | --- | --- | --- | --- | --- | --- | --- | --- | --- | --- | --- | --- | --- | --- | --- | --- | --- | --- | --- | --- | --- | --- | --- | --- | --- | --- | --- |
| K.DLLDLLVEAK.Q | Y | 55.88 | 1127.6438 | 10 | 0.2 | 564.8293 | 2 | 68.78 | 8 | F8:41830 | 31122021\_RID\_3262\_1b.raw |  |  |  |  |  |  |  | 5.0661E3 | 1 | 0 | 0 | 0 | 0 | 0 | 0 | 0 | 1 | 620 | 629 |  |  | PEAKS DB |
| total 1 peptides |  |  |  |  |  |  |  |  |  |  |  |  |  |  |  |  |  |  |  |  |  |  |  |  |  |  |  |  |  |  |  |  |  |
| --- | --- | --- | --- | --- | --- | --- | --- | --- | --- | --- | --- | --- | --- | --- | --- | --- | --- | --- | --- | --- | --- | --- | --- | --- | --- | --- | --- | --- | --- | --- | --- | --- | --- |

XP\_007426570.1

back to list

  

| Protein Coverage
| Supporting Peptides
|

Protein Coverage:

Supporting Peptides:

| Peptide | Uniq | -10lgP | Mass | Length | ppm | m/z | z | RT | Fraction | Scan | Source File | Area F6 | Area F2 | Area F3 | Area F4 | Area F5 | Area F7 | Area F8 | Area F1 | #Feature | #Feature F6 | #Feature F2 | #Feature F3 | #Feature F4 | #Feature F5 | #Feature F7 | #Feature F8 | #Feature F1 | Start | End | PTM | AScore | Found By |
| --- | --- | --- | --- | --- | --- | --- | --- | --- | --- | --- | --- | --- | --- | --- | --- | --- | --- | --- | --- | --- | --- | --- | --- | --- | --- | --- | --- | --- | --- | --- | --- | --- | --- |
| K.DLLDLLVEAK.Q | Y | 55.88 | 1127.6438 | 10 | 0.2 | 564.8293 | 2 | 68.78 | 8 | F8:41830 | 31122021\_RID\_3262\_1b.raw |  |  |  |  |  |  |  | 5.0661E3 | 1 | 0 | 0 | 0 | 0 | 0 | 0 | 0 | 1 | 555 | 564 |  |  | PEAKS DB |
| total 1 peptides |  |  |  |  |  |  |  |  |  |  |  |  |  |  |  |  |  |  |  |  |  |  |  |  |  |  |  |  |  |  |  |  |  |
| --- | --- | --- | --- | --- | --- | --- | --- | --- | --- | --- | --- | --- | --- | --- | --- | --- | --- | --- | --- | --- | --- | --- | --- | --- | --- | --- | --- | --- | --- | --- | --- | --- | --- |

XP\_026569452.1

back to list

  

| Protein Coverage
| Supporting Peptides
|

Protein Coverage:

Supporting Peptides:

| Peptide | Uniq | -10lgP | Mass | Length | ppm | m/z | z | RT | Fraction | Scan | Source File | Area F6 | Area F2 | Area F3 | Area F4 | Area F5 | Area F7 | Area F8 | Area F1 | #Feature | #Feature F6 | #Feature F2 | #Feature F3 | #Feature F4 | #Feature F5 | #Feature F7 | #Feature F8 | #Feature F1 | Start | End | PTM | AScore | Found By |
| --- | --- | --- | --- | --- | --- | --- | --- | --- | --- | --- | --- | --- | --- | --- | --- | --- | --- | --- | --- | --- | --- | --- | --- | --- | --- | --- | --- | --- | --- | --- | --- | --- | --- |
| K.DLLDLLVEAK.Q | Y | 55.88 | 1127.6438 | 10 | 0.2 | 564.8293 | 2 | 68.78 | 8 | F8:41830 | 31122021\_RID\_3262\_1b.raw |  |  |  |  |  |  |  | 5.0661E3 | 1 | 0 | 0 | 0 | 0 | 0 | 0 | 0 | 1 | 620 | 629 |  |  | PEAKS DB |
| total 1 peptides |  |  |  |  |  |  |  |  |  |  |  |  |  |  |  |  |  |  |  |  |  |  |  |  |  |  |  |  |  |  |  |  |  |
| --- | --- | --- | --- | --- | --- | --- | --- | --- | --- | --- | --- | --- | --- | --- | --- | --- | --- | --- | --- | --- | --- | --- | --- | --- | --- | --- | --- | --- | --- | --- | --- | --- | --- |

XP\_026529838.1

back to list

  

| Protein Coverage
| Supporting Peptides
|

Protein Coverage:

Supporting Peptides:

| Peptide | Uniq | -10lgP | Mass | Length | ppm | m/z | z | RT | Fraction | Scan | Source File | Area F6 | Area F2 | Area F3 | Area F4 | Area F5 | Area F7 | Area F8 | Area F1 | #Feature | #Feature F6 | #Feature F2 | #Feature F3 | #Feature F4 | #Feature F5 | #Feature F7 | #Feature F8 | #Feature F1 | Start | End | PTM | AScore | Found By |
| --- | --- | --- | --- | --- | --- | --- | --- | --- | --- | --- | --- | --- | --- | --- | --- | --- | --- | --- | --- | --- | --- | --- | --- | --- | --- | --- | --- | --- | --- | --- | --- | --- | --- |
| K.DLLDLLVEAK.Q | Y | 55.88 | 1127.6438 | 10 | 0.2 | 564.8293 | 2 | 68.78 | 8 | F8:41830 | 31122021\_RID\_3262\_1b.raw |  |  |  |  |  |  |  | 5.0661E3 | 1 | 0 | 0 | 0 | 0 | 0 | 0 | 0 | 1 | 620 | 629 |  |  | PEAKS DB |
| total 1 peptides |  |  |  |  |  |  |  |  |  |  |  |  |  |  |  |  |  |  |  |  |  |  |  |  |  |  |  |  |  |  |  |  |  |
| --- | --- | --- | --- | --- | --- | --- | --- | --- | --- | --- | --- | --- | --- | --- | --- | --- | --- | --- | --- | --- | --- | --- | --- | --- | --- | --- | --- | --- | --- | --- | --- | --- | --- |

XP\_026569451.1

back to list

  

| Protein Coverage
| Supporting Peptides
|

Protein Coverage:

Supporting Peptides:

| Peptide | Uniq | -10lgP | Mass | Length | ppm | m/z | z | RT | Fraction | Scan | Source File | Area F6 | Area F2 | Area F3 | Area F4 | Area F5 | Area F7 | Area F8 | Area F1 | #Feature | #Feature F6 | #Feature F2 | #Feature F3 | #Feature F4 | #Feature F5 | #Feature F7 | #Feature F8 | #Feature F1 | Start | End | PTM | AScore | Found By |
| --- | --- | --- | --- | --- | --- | --- | --- | --- | --- | --- | --- | --- | --- | --- | --- | --- | --- | --- | --- | --- | --- | --- | --- | --- | --- | --- | --- | --- | --- | --- | --- | --- | --- |
| K.DLLDLLVEAK.Q | Y | 55.88 | 1127.6438 | 10 | 0.2 | 564.8293 | 2 | 68.78 | 8 | F8:41830 | 31122021\_RID\_3262\_1b.raw |  |  |  |  |  |  |  | 5.0661E3 | 1 | 0 | 0 | 0 | 0 | 0 | 0 | 0 | 1 | 554 | 563 |  |  | PEAKS DB |
| total 1 peptides |  |  |  |  |  |  |  |  |  |  |  |  |  |  |  |  |  |  |  |  |  |  |  |  |  |  |  |  |  |  |  |  |  |
| --- | --- | --- | --- | --- | --- | --- | --- | --- | --- | --- | --- | --- | --- | --- | --- | --- | --- | --- | --- | --- | --- | --- | --- | --- | --- | --- | --- | --- | --- | --- | --- | --- | --- |

XP\_026569449.1

back to list

  

| Protein Coverage
| Supporting Peptides
|

Protein Coverage:

Supporting Peptides:

| Peptide | Uniq | -10lgP | Mass | Length | ppm | m/z | z | RT | Fraction | Scan | Source File | Area F6 | Area F2 | Area F3 | Area F4 | Area F5 | Area F7 | Area F8 | Area F1 | #Feature | #Feature F6 | #Feature F2 | #Feature F3 | #Feature F4 | #Feature F5 | #Feature F7 | #Feature F8 | #Feature F1 | Start | End | PTM | AScore | Found By |
| --- | --- | --- | --- | --- | --- | --- | --- | --- | --- | --- | --- | --- | --- | --- | --- | --- | --- | --- | --- | --- | --- | --- | --- | --- | --- | --- | --- | --- | --- | --- | --- | --- | --- |
| K.DLLDLLVEAK.Q | Y | 55.88 | 1127.6438 | 10 | 0.2 | 564.8293 | 2 | 68.78 | 8 | F8:41830 | 31122021\_RID\_3262\_1b.raw |  |  |  |  |  |  |  | 5.0661E3 | 1 | 0 | 0 | 0 | 0 | 0 | 0 | 0 | 1 | 599 | 608 |  |  | PEAKS DB |
| total 1 peptides |  |  |  |  |  |  |  |  |  |  |  |  |  |  |  |  |  |  |  |  |  |  |  |  |  |  |  |  |  |  |  |  |  |
| --- | --- | --- | --- | --- | --- | --- | --- | --- | --- | --- | --- | --- | --- | --- | --- | --- | --- | --- | --- | --- | --- | --- | --- | --- | --- | --- | --- | --- | --- | --- | --- | --- | --- |

XP\_026569450.1

back to list

  

| Protein Coverage
| Supporting Peptides
|

Protein Coverage:

Supporting Peptides:

| Peptide | Uniq | -10lgP | Mass | Length | ppm | m/z | z | RT | Fraction | Scan | Source File | Area F6 | Area F2 | Area F3 | Area F4 | Area F5 | Area F7 | Area F8 | Area F1 | #Feature | #Feature F6 | #Feature F2 | #Feature F3 | #Feature F4 | #Feature F5 | #Feature F7 | #Feature F8 | #Feature F1 | Start | End | PTM | AScore | Found By |
| --- | --- | --- | --- | --- | --- | --- | --- | --- | --- | --- | --- | --- | --- | --- | --- | --- | --- | --- | --- | --- | --- | --- | --- | --- | --- | --- | --- | --- | --- | --- | --- | --- | --- |
| K.DLLDLLVEAK.Q | Y | 55.88 | 1127.6438 | 10 | 0.2 | 564.8293 | 2 | 68.78 | 8 | F8:41830 | 31122021\_RID\_3262\_1b.raw |  |  |  |  |  |  |  | 5.0661E3 | 1 | 0 | 0 | 0 | 0 | 0 | 0 | 0 | 1 | 555 | 564 |  |  | PEAKS DB |
| total 1 peptides |  |  |  |  |  |  |  |  |  |  |  |  |  |  |  |  |  |  |  |  |  |  |  |  |  |  |  |  |  |  |  |  |  |
| --- | --- | --- | --- | --- | --- | --- | --- | --- | --- | --- | --- | --- | --- | --- | --- | --- | --- | --- | --- | --- | --- | --- | --- | --- | --- | --- | --- | --- | --- | --- | --- | --- | --- |

ETE62077.1

back to list

  

| Protein Coverage
| Supporting Peptides
|

Protein Coverage:

Supporting Peptides:

| Peptide | Uniq | -10lgP | Mass | Length | ppm | m/z | z | RT | Fraction | Scan | Source File | Area F6 | Area F2 | Area F3 | Area F4 | Area F5 | Area F7 | Area F8 | Area F1 | #Feature | #Feature F6 | #Feature F2 | #Feature F3 | #Feature F4 | #Feature F5 | #Feature F7 | #Feature F8 | #Feature F1 | Start | End | PTM | AScore | Found By |
| --- | --- | --- | --- | --- | --- | --- | --- | --- | --- | --- | --- | --- | --- | --- | --- | --- | --- | --- | --- | --- | --- | --- | --- | --- | --- | --- | --- | --- | --- | --- | --- | --- | --- |
| K.DLLDLLVEAK.Q | Y | 55.88 | 1127.6438 | 10 | 0.2 | 564.8293 | 2 | 68.78 | 8 | F8:41830 | 31122021\_RID\_3262\_1b.raw |  |  |  |  |  |  |  | 5.0661E3 | 1 | 0 | 0 | 0 | 0 | 0 | 0 | 0 | 1 | 531 | 540 |  |  | PEAKS DB |
| total 1 peptides |  |  |  |  |  |  |  |  |  |  |  |  |  |  |  |  |  |  |  |  |  |  |  |  |  |  |  |  |  |  |  |  |  |
| --- | --- | --- | --- | --- | --- | --- | --- | --- | --- | --- | --- | --- | --- | --- | --- | --- | --- | --- | --- | --- | --- | --- | --- | --- | --- | --- | --- | --- | --- | --- | --- | --- | --- |

XP\_026529837.1

back to list

  

| Protein Coverage
| Supporting Peptides
|

Protein Coverage:

Supporting Peptides:

| Peptide | Uniq | -10lgP | Mass | Length | ppm | m/z | z | RT | Fraction | Scan | Source File | Area F6 | Area F2 | Area F3 | Area F4 | Area F5 | Area F7 | Area F8 | Area F1 | #Feature | #Feature F6 | #Feature F2 | #Feature F3 | #Feature F4 | #Feature F5 | #Feature F7 | #Feature F8 | #Feature F1 | Start | End | PTM | AScore | Found By |
| --- | --- | --- | --- | --- | --- | --- | --- | --- | --- | --- | --- | --- | --- | --- | --- | --- | --- | --- | --- | --- | --- | --- | --- | --- | --- | --- | --- | --- | --- | --- | --- | --- | --- |
| K.DLLDLLVEAK.Q | Y | 55.88 | 1127.6438 | 10 | 0.2 | 564.8293 | 2 | 68.78 | 8 | F8:41830 | 31122021\_RID\_3262\_1b.raw |  |  |  |  |  |  |  | 5.0661E3 | 1 | 0 | 0 | 0 | 0 | 0 | 0 | 0 | 1 | 554 | 563 |  |  | PEAKS DB |
| total 1 peptides |  |  |  |  |  |  |  |  |  |  |  |  |  |  |  |  |  |  |  |  |  |  |  |  |  |  |  |  |  |  |  |  |  |
| --- | --- | --- | --- | --- | --- | --- | --- | --- | --- | --- | --- | --- | --- | --- | --- | --- | --- | --- | --- | --- | --- | --- | --- | --- | --- | --- | --- | --- | --- | --- | --- | --- | --- |

XP\_026529835.1

back to list

  

| Protein Coverage
| Supporting Peptides
|

Protein Coverage:

Supporting Peptides:

| Peptide | Uniq | -10lgP | Mass | Length | ppm | m/z | z | RT | Fraction | Scan | Source File | Area F6 | Area F2 | Area F3 | Area F4 | Area F5 | Area F7 | Area F8 | Area F1 | #Feature | #Feature F6 | #Feature F2 | #Feature F3 | #Feature F4 | #Feature F5 | #Feature F7 | #Feature F8 | #Feature F1 | Start | End | PTM | AScore | Found By |
| --- | --- | --- | --- | --- | --- | --- | --- | --- | --- | --- | --- | --- | --- | --- | --- | --- | --- | --- | --- | --- | --- | --- | --- | --- | --- | --- | --- | --- | --- | --- | --- | --- | --- |
| K.DLLDLLVEAK.Q | Y | 55.88 | 1127.6438 | 10 | 0.2 | 564.8293 | 2 | 68.78 | 8 | F8:41830 | 31122021\_RID\_3262\_1b.raw |  |  |  |  |  |  |  | 5.0661E3 | 1 | 0 | 0 | 0 | 0 | 0 | 0 | 0 | 1 | 599 | 608 |  |  | PEAKS DB |
| total 1 peptides |  |  |  |  |  |  |  |  |  |  |  |  |  |  |  |  |  |  |  |  |  |  |  |  |  |  |  |  |  |  |  |  |  |
| --- | --- | --- | --- | --- | --- | --- | --- | --- | --- | --- | --- | --- | --- | --- | --- | --- | --- | --- | --- | --- | --- | --- | --- | --- | --- | --- | --- | --- | --- | --- | --- | --- | --- |

XP\_026529836.1

back to list

  

| Protein Coverage
| Supporting Peptides
|

Protein Coverage:

Supporting Peptides:

| Peptide | Uniq | -10lgP | Mass | Length | ppm | m/z | z | RT | Fraction | Scan | Source File | Area F6 | Area F2 | Area F3 | Area F4 | Area F5 | Area F7 | Area F8 | Area F1 | #Feature | #Feature F6 | #Feature F2 | #Feature F3 | #Feature F4 | #Feature F5 | #Feature F7 | #Feature F8 | #Feature F1 | Start | End | PTM | AScore | Found By |
| --- | --- | --- | --- | --- | --- | --- | --- | --- | --- | --- | --- | --- | --- | --- | --- | --- | --- | --- | --- | --- | --- | --- | --- | --- | --- | --- | --- | --- | --- | --- | --- | --- | --- |
| K.DLLDLLVEAK.Q | Y | 55.88 | 1127.6438 | 10 | 0.2 | 564.8293 | 2 | 68.78 | 8 | F8:41830 | 31122021\_RID\_3262\_1b.raw |  |  |  |  |  |  |  | 5.0661E3 | 1 | 0 | 0 | 0 | 0 | 0 | 0 | 0 | 1 | 555 | 564 |  |  | PEAKS DB |
| total 1 peptides |  |  |  |  |  |  |  |  |  |  |  |  |  |  |  |  |  |  |  |  |  |  |  |  |  |  |  |  |  |  |  |  |  |
| --- | --- | --- | --- | --- | --- | --- | --- | --- | --- | --- | --- | --- | --- | --- | --- | --- | --- | --- | --- | --- | --- | --- | --- | --- | --- | --- | --- | --- | --- | --- | --- | --- | --- |

JAB53490.1

back to list

  

| Protein Coverage
| Supporting Peptides
|

Protein Coverage:

Supporting Peptides:

| Peptide | Uniq | -10lgP | Mass | Length | ppm | m/z | z | RT | Fraction | Scan | Source File | Area F6 | Area F2 | Area F3 | Area F4 | Area F5 | Area F7 | Area F8 | Area F1 | #Feature | #Feature F6 | #Feature F2 | #Feature F3 | #Feature F4 | #Feature F5 | #Feature F7 | #Feature F8 | #Feature F1 | Start | End | PTM | AScore | Found By |
| --- | --- | --- | --- | --- | --- | --- | --- | --- | --- | --- | --- | --- | --- | --- | --- | --- | --- | --- | --- | --- | --- | --- | --- | --- | --- | --- | --- | --- | --- | --- | --- | --- | --- |
| K.DLLDLLVEAK.Q | Y | 55.88 | 1127.6438 | 10 | 0.2 | 564.8293 | 2 | 68.78 | 8 | F8:41830 | 31122021\_RID\_3262\_1b.raw |  |  |  |  |  |  |  | 5.0661E3 | 1 | 0 | 0 | 0 | 0 | 0 | 0 | 0 | 1 | 555 | 564 |  |  | PEAKS DB |
| total 1 peptides |  |  |  |  |  |  |  |  |  |  |  |  |  |  |  |  |  |  |  |  |  |  |  |  |  |  |  |  |  |  |  |  |  |
| --- | --- | --- | --- | --- | --- | --- | --- | --- | --- | --- | --- | --- | --- | --- | --- | --- | --- | --- | --- | --- | --- | --- | --- | --- | --- | --- | --- | --- | --- | --- | --- | --- | --- |

XP\_026539659.1

back to list

  

| Protein Coverage
| Supporting Peptides
|

Protein Coverage:

Supporting Peptides:

| Peptide | Uniq | -10lgP | Mass | Length | ppm | m/z | z | RT | Fraction | Scan | Source File | Area F6 | Area F2 | Area F3 | Area F4 | Area F5 | Area F7 | Area F8 | Area F1 | #Feature | #Feature F6 | #Feature F2 | #Feature F3 | #Feature F4 | #Feature F5 | #Feature F7 | #Feature F8 | #Feature F1 | Start | End | PTM | AScore | Found By |
| --- | --- | --- | --- | --- | --- | --- | --- | --- | --- | --- | --- | --- | --- | --- | --- | --- | --- | --- | --- | --- | --- | --- | --- | --- | --- | --- | --- | --- | --- | --- | --- | --- | --- |
| R.SQYEEIAQR.S | Y | 55.28 | 1122.5305 | 9 | -0.5 | 562.2723 | 2 | 24.04 | 8 | F8:8318 | 31122021\_RID\_3262\_1b.raw |  |  |  |  |  |  |  | 2.1449E5 | 1 | 0 | 0 | 0 | 0 | 0 | 0 | 0 | 1 | 353 | 361 |  |  | PEAKS DB |
| total 1 peptides |  |  |  |  |  |  |  |  |  |  |  |  |  |  |  |  |  |  |  |  |  |  |  |  |  |  |  |  |  |  |  |  |  |
| --- | --- | --- | --- | --- | --- | --- | --- | --- | --- | --- | --- | --- | --- | --- | --- | --- | --- | --- | --- | --- | --- | --- | --- | --- | --- | --- | --- | --- | --- | --- | --- | --- | --- |

XP\_026570510.1

back to list

  

| Protein Coverage
| Supporting Peptides
|

Protein Coverage:

Supporting Peptides:

| Peptide | Uniq | -10lgP | Mass | Length | ppm | m/z | z | RT | Fraction | Scan | Source File | Area F6 | Area F2 | Area F3 | Area F4 | Area F5 | Area F7 | Area F8 | Area F1 | #Feature | #Feature F6 | #Feature F2 | #Feature F3 | #Feature F4 | #Feature F5 | #Feature F7 | #Feature F8 | #Feature F1 | Start | End | PTM | AScore | Found By |
| --- | --- | --- | --- | --- | --- | --- | --- | --- | --- | --- | --- | --- | --- | --- | --- | --- | --- | --- | --- | --- | --- | --- | --- | --- | --- | --- | --- | --- | --- | --- | --- | --- | --- |
| R.SQYEEIAQR.S | Y | 55.28 | 1122.5305 | 9 | -0.5 | 562.2723 | 2 | 24.04 | 8 | F8:8318 | 31122021\_RID\_3262\_1b.raw |  |  |  |  |  |  |  | 2.1449E5 | 1 | 0 | 0 | 0 | 0 | 0 | 0 | 0 | 1 | 353 | 361 |  |  | PEAKS DB |
| total 1 peptides |  |  |  |  |  |  |  |  |  |  |  |  |  |  |  |  |  |  |  |  |  |  |  |  |  |  |  |  |  |  |  |  |  |
| --- | --- | --- | --- | --- | --- | --- | --- | --- | --- | --- | --- | --- | --- | --- | --- | --- | --- | --- | --- | --- | --- | --- | --- | --- | --- | --- | --- | --- | --- | --- | --- | --- | --- |

Q7LZS9.1

back to list

  

| Protein Coverage
| Supporting Peptides
|

Protein Coverage:

Supporting Peptides:

| Peptide | Uniq | -10lgP | Mass | Length | ppm | m/z | z | RT | Fraction | Scan | Source File | Area F6 | Area F2 | Area F3 | Area F4 | Area F5 | Area F7 | Area F8 | Area F1 | #Feature | #Feature F6 | #Feature F2 | #Feature F3 | #Feature F4 | #Feature F5 | #Feature F7 | #Feature F8 | #Feature F1 | Start | End | PTM | AScore | Found By |
| --- | --- | --- | --- | --- | --- | --- | --- | --- | --- | --- | --- | --- | --- | --- | --- | --- | --- | --- | --- | --- | --- | --- | --- | --- | --- | --- | --- | --- | --- | --- | --- | --- | --- |
| L.AYVASMC(+57.02)EPK.L | Y | 54.70 | 1154.5100 | 10 | 0.5 | 578.2626 | 2 | 28.84 | 3 | F3:14028 | 13012022\_RID\_3262\_3b.raw |  |  | 1.9595E7 | 9.0845E5 |  |  |  |  | 2 | 0 | 0 | 1 | 1 | 0 | 0 | 0 | 0 | 111 | 120 | Carbamidomethylation | C7:Carbamidomethylation:1000.00 | PEAKS DB |
| total 1 peptides |  |  |  |  |  |  |  |  |  |  |  |  |  |  |  |  |  |  |  |  |  |  |  |  |  |  |  |  |  |  |  |  |  |
| --- | --- | --- | --- | --- | --- | --- | --- | --- | --- | --- | --- | --- | --- | --- | --- | --- | --- | --- | --- | --- | --- | --- | --- | --- | --- | --- | --- | --- | --- | --- | --- | --- | --- |

XP\_007443339.1

back to list

  

| Protein Coverage
| Supporting Peptides
|

Protein Coverage:

Supporting Peptides:

| Peptide | Uniq | -10lgP | Mass | Length | ppm | m/z | z | RT | Fraction | Scan | Source File | Area F6 | Area F2 | Area F3 | Area F4 | Area F5 | Area F7 | Area F8 | Area F1 | #Feature | #Feature F6 | #Feature F2 | #Feature F3 | #Feature F4 | #Feature F5 | #Feature F7 | #Feature F8 | #Feature F1 | Start | End | PTM | AScore | Found By |
| --- | --- | --- | --- | --- | --- | --- | --- | --- | --- | --- | --- | --- | --- | --- | --- | --- | --- | --- | --- | --- | --- | --- | --- | --- | --- | --- | --- | --- | --- | --- | --- | --- | --- |
| R.LAEVALAYAK.A | Y | 53.35 | 1047.5964 | 10 | 0.9 | 524.8060 | 2 | 37.49 | 8 | F8:18857 | 31122021\_RID\_3262\_1b.raw |  |  |  |  |  |  |  | 3.8989E4 | 1 | 0 | 0 | 0 | 0 | 0 | 0 | 0 | 1 | 256 | 265 |  |  | PEAKS DB |
| total 1 peptides |  |  |  |  |  |  |  |  |  |  |  |  |  |  |  |  |  |  |  |  |  |  |  |  |  |  |  |  |  |  |  |  |  |
| --- | --- | --- | --- | --- | --- | --- | --- | --- | --- | --- | --- | --- | --- | --- | --- | --- | --- | --- | --- | --- | --- | --- | --- | --- | --- | --- | --- | --- | --- | --- | --- | --- | --- |

XP\_026568189.1

back to list

  

| Protein Coverage
| Supporting Peptides
|

Protein Coverage:

Supporting Peptides:

| Peptide | Uniq | -10lgP | Mass | Length | ppm | m/z | z | RT | Fraction | Scan | Source File | Area F6 | Area F2 | Area F3 | Area F4 | Area F5 | Area F7 | Area F8 | Area F1 | #Feature | #Feature F6 | #Feature F2 | #Feature F3 | #Feature F4 | #Feature F5 | #Feature F7 | #Feature F8 | #Feature F1 | Start | End | PTM | AScore | Found By |
| --- | --- | --- | --- | --- | --- | --- | --- | --- | --- | --- | --- | --- | --- | --- | --- | --- | --- | --- | --- | --- | --- | --- | --- | --- | --- | --- | --- | --- | --- | --- | --- | --- | --- |
| R.LAEVALAYAK.A | Y | 53.35 | 1047.5964 | 10 | 0.9 | 524.8060 | 2 | 37.49 | 8 | F8:18857 | 31122021\_RID\_3262\_1b.raw |  |  |  |  |  |  |  | 3.8989E4 | 1 | 0 | 0 | 0 | 0 | 0 | 0 | 0 | 1 | 150 | 159 |  |  | PEAKS DB |
| total 1 peptides |  |  |  |  |  |  |  |  |  |  |  |  |  |  |  |  |  |  |  |  |  |  |  |  |  |  |  |  |  |  |  |  |  |
| --- | --- | --- | --- | --- | --- | --- | --- | --- | --- | --- | --- | --- | --- | --- | --- | --- | --- | --- | --- | --- | --- | --- | --- | --- | --- | --- | --- | --- | --- | --- | --- | --- | --- |

XP\_026542345.1

back to list

  

| Protein Coverage
| Supporting Peptides
|

Protein Coverage:

Supporting Peptides:

| Peptide | Uniq | -10lgP | Mass | Length | ppm | m/z | z | RT | Fraction | Scan | Source File | Area F6 | Area F2 | Area F3 | Area F4 | Area F5 | Area F7 | Area F8 | Area F1 | #Feature | #Feature F6 | #Feature F2 | #Feature F3 | #Feature F4 | #Feature F5 | #Feature F7 | #Feature F8 | #Feature F1 | Start | End | PTM | AScore | Found By |
| --- | --- | --- | --- | --- | --- | --- | --- | --- | --- | --- | --- | --- | --- | --- | --- | --- | --- | --- | --- | --- | --- | --- | --- | --- | --- | --- | --- | --- | --- | --- | --- | --- | --- |
| R.LAEVALAYAK.A | Y | 53.35 | 1047.5964 | 10 | 0.9 | 524.8060 | 2 | 37.49 | 8 | F8:18857 | 31122021\_RID\_3262\_1b.raw |  |  |  |  |  |  |  | 3.8989E4 | 1 | 0 | 0 | 0 | 0 | 0 | 0 | 0 | 1 | 150 | 159 |  |  | PEAKS DB |
| total 1 peptides |  |  |  |  |  |  |  |  |  |  |  |  |  |  |  |  |  |  |  |  |  |  |  |  |  |  |  |  |  |  |  |  |  |
| --- | --- | --- | --- | --- | --- | --- | --- | --- | --- | --- | --- | --- | --- | --- | --- | --- | --- | --- | --- | --- | --- | --- | --- | --- | --- | --- | --- | --- | --- | --- | --- | --- | --- |

AFJ49864.1

back to list

  

| Protein Coverage
| Supporting Peptides
|

Protein Coverage:

Supporting Peptides:

| Peptide | Uniq | -10lgP | Mass | Length | ppm | m/z | z | RT | Fraction | Scan | Source File | Area F6 | Area F2 | Area F3 | Area F4 | Area F5 | Area F7 | Area F8 | Area F1 | #Feature | #Feature F6 | #Feature F2 | #Feature F3 | #Feature F4 | #Feature F5 | #Feature F7 | #Feature F8 | #Feature F1 | Start | End | PTM | AScore | Found By |
| --- | --- | --- | --- | --- | --- | --- | --- | --- | --- | --- | --- | --- | --- | --- | --- | --- | --- | --- | --- | --- | --- | --- | --- | --- | --- | --- | --- | --- | --- | --- | --- | --- | --- |
| R.LAEVALAYAK.A | Y | 53.35 | 1047.5964 | 10 | 0.9 | 524.8060 | 2 | 37.49 | 8 | F8:18857 | 31122021\_RID\_3262\_1b.raw |  |  |  |  |  |  |  | 3.8989E4 | 1 | 0 | 0 | 0 | 0 | 0 | 0 | 0 | 1 | 150 | 159 |  |  | PEAKS DB |
| total 1 peptides |  |  |  |  |  |  |  |  |  |  |  |  |  |  |  |  |  |  |  |  |  |  |  |  |  |  |  |  |  |  |  |  |  |
| --- | --- | --- | --- | --- | --- | --- | --- | --- | --- | --- | --- | --- | --- | --- | --- | --- | --- | --- | --- | --- | --- | --- | --- | --- | --- | --- | --- | --- | --- | --- | --- | --- | --- |

JAI12578.1

back to list

  

| Protein Coverage
| Supporting Peptides
|

Protein Coverage:

Supporting Peptides:

| Peptide | Uniq | -10lgP | Mass | Length | ppm | m/z | z | RT | Fraction | Scan | Source File | Area F6 | Area F2 | Area F3 | Area F4 | Area F5 | Area F7 | Area F8 | Area F1 | #Feature | #Feature F6 | #Feature F2 | #Feature F3 | #Feature F4 | #Feature F5 | #Feature F7 | #Feature F8 | #Feature F1 | Start | End | PTM | AScore | Found By |
| --- | --- | --- | --- | --- | --- | --- | --- | --- | --- | --- | --- | --- | --- | --- | --- | --- | --- | --- | --- | --- | --- | --- | --- | --- | --- | --- | --- | --- | --- | --- | --- | --- | --- |
| R.LAEVALAYAK.A | Y | 53.35 | 1047.5964 | 10 | 0.9 | 524.8060 | 2 | 37.49 | 8 | F8:18857 | 31122021\_RID\_3262\_1b.raw |  |  |  |  |  |  |  | 3.8989E4 | 1 | 0 | 0 | 0 | 0 | 0 | 0 | 0 | 1 | 150 | 159 |  |  | PEAKS DB |
| total 1 peptides |  |  |  |  |  |  |  |  |  |  |  |  |  |  |  |  |  |  |  |  |  |  |  |  |  |  |  |  |  |  |  |  |  |
| --- | --- | --- | --- | --- | --- | --- | --- | --- | --- | --- | --- | --- | --- | --- | --- | --- | --- | --- | --- | --- | --- | --- | --- | --- | --- | --- | --- | --- | --- | --- | --- | --- | --- |

XP\_032088448.1

back to list

  

| Protein Coverage
| Supporting Peptides
|

Protein Coverage:

Supporting Peptides:

| Peptide | Uniq | -10lgP | Mass | Length | ppm | m/z | z | RT | Fraction | Scan | Source File | Area F6 | Area F2 | Area F3 | Area F4 | Area F5 | Area F7 | Area F8 | Area F1 | #Feature | #Feature F6 | #Feature F2 | #Feature F3 | #Feature F4 | #Feature F5 | #Feature F7 | #Feature F8 | #Feature F1 | Start | End | PTM | AScore | Found By |
| --- | --- | --- | --- | --- | --- | --- | --- | --- | --- | --- | --- | --- | --- | --- | --- | --- | --- | --- | --- | --- | --- | --- | --- | --- | --- | --- | --- | --- | --- | --- | --- | --- | --- |
| R.LAEVALAYAK.A | Y | 53.35 | 1047.5964 | 10 | 0.9 | 524.8060 | 2 | 37.49 | 8 | F8:18857 | 31122021\_RID\_3262\_1b.raw |  |  |  |  |  |  |  | 3.8989E4 | 1 | 0 | 0 | 0 | 0 | 0 | 0 | 0 | 1 | 150 | 159 |  |  | PEAKS DB |
| total 1 peptides |  |  |  |  |  |  |  |  |  |  |  |  |  |  |  |  |  |  |  |  |  |  |  |  |  |  |  |  |  |  |  |  |  |
| --- | --- | --- | --- | --- | --- | --- | --- | --- | --- | --- | --- | --- | --- | --- | --- | --- | --- | --- | --- | --- | --- | --- | --- | --- | --- | --- | --- | --- | --- | --- | --- | --- | --- |

LAA91247.1

back to list

  

| Protein Coverage
| Supporting Peptides
|

Protein Coverage:

Supporting Peptides:

| Peptide | Uniq | -10lgP | Mass | Length | ppm | m/z | z | RT | Fraction | Scan | Source File | Area F6 | Area F2 | Area F3 | Area F4 | Area F5 | Area F7 | Area F8 | Area F1 | #Feature | #Feature F6 | #Feature F2 | #Feature F3 | #Feature F4 | #Feature F5 | #Feature F7 | #Feature F8 | #Feature F1 | Start | End | PTM | AScore | Found By |
| --- | --- | --- | --- | --- | --- | --- | --- | --- | --- | --- | --- | --- | --- | --- | --- | --- | --- | --- | --- | --- | --- | --- | --- | --- | --- | --- | --- | --- | --- | --- | --- | --- | --- |
| R.LAEVALAYAK.A | Y | 53.35 | 1047.5964 | 10 | 0.9 | 524.8060 | 2 | 37.49 | 8 | F8:18857 | 31122021\_RID\_3262\_1b.raw |  |  |  |  |  |  |  | 3.8989E4 | 1 | 0 | 0 | 0 | 0 | 0 | 0 | 0 | 1 | 119 | 128 |  |  | PEAKS DB |
| total 1 peptides |  |  |  |  |  |  |  |  |  |  |  |  |  |  |  |  |  |  |  |  |  |  |  |  |  |  |  |  |  |  |  |  |  |
| --- | --- | --- | --- | --- | --- | --- | --- | --- | --- | --- | --- | --- | --- | --- | --- | --- | --- | --- | --- | --- | --- | --- | --- | --- | --- | --- | --- | --- | --- | --- | --- | --- | --- |

XP\_034292227.1

back to list

  

| Protein Coverage
| Supporting Peptides
|

Protein Coverage:

Supporting Peptides:

| Peptide | Uniq | -10lgP | Mass | Length | ppm | m/z | z | RT | Fraction | Scan | Source File | Area F6 | Area F2 | Area F3 | Area F4 | Area F5 | Area F7 | Area F8 | Area F1 | #Feature | #Feature F6 | #Feature F2 | #Feature F3 | #Feature F4 | #Feature F5 | #Feature F7 | #Feature F8 | #Feature F1 | Start | End | PTM | AScore | Found By |
| --- | --- | --- | --- | --- | --- | --- | --- | --- | --- | --- | --- | --- | --- | --- | --- | --- | --- | --- | --- | --- | --- | --- | --- | --- | --- | --- | --- | --- | --- | --- | --- | --- | --- |
| R.LAEVALAYAK.A | Y | 53.35 | 1047.5964 | 10 | 0.9 | 524.8060 | 2 | 37.49 | 8 | F8:18857 | 31122021\_RID\_3262\_1b.raw |  |  |  |  |  |  |  | 3.8989E4 | 1 | 0 | 0 | 0 | 0 | 0 | 0 | 0 | 1 | 150 | 159 |  |  | PEAKS DB |
| total 1 peptides |  |  |  |  |  |  |  |  |  |  |  |  |  |  |  |  |  |  |  |  |  |  |  |  |  |  |  |  |  |  |  |  |  |
| --- | --- | --- | --- | --- | --- | --- | --- | --- | --- | --- | --- | --- | --- | --- | --- | --- | --- | --- | --- | --- | --- | --- | --- | --- | --- | --- | --- | --- | --- | --- | --- | --- | --- |

JAC96276.1

back to list

  

| Protein Coverage
| Supporting Peptides
|

Protein Coverage:

Supporting Peptides:

| Peptide | Uniq | -10lgP | Mass | Length | ppm | m/z | z | RT | Fraction | Scan | Source File | Area F6 | Area F2 | Area F3 | Area F4 | Area F5 | Area F7 | Area F8 | Area F1 | #Feature | #Feature F6 | #Feature F2 | #Feature F3 | #Feature F4 | #Feature F5 | #Feature F7 | #Feature F8 | #Feature F1 | Start | End | PTM | AScore | Found By |
| --- | --- | --- | --- | --- | --- | --- | --- | --- | --- | --- | --- | --- | --- | --- | --- | --- | --- | --- | --- | --- | --- | --- | --- | --- | --- | --- | --- | --- | --- | --- | --- | --- | --- |
| R.LAEVALAYAK.A | Y | 53.35 | 1047.5964 | 10 | 0.9 | 524.8060 | 2 | 37.49 | 8 | F8:18857 | 31122021\_RID\_3262\_1b.raw |  |  |  |  |  |  |  | 3.8989E4 | 1 | 0 | 0 | 0 | 0 | 0 | 0 | 0 | 1 | 150 | 159 |  |  | PEAKS DB |
| total 1 peptides |  |  |  |  |  |  |  |  |  |  |  |  |  |  |  |  |  |  |  |  |  |  |  |  |  |  |  |  |  |  |  |  |  |
| --- | --- | --- | --- | --- | --- | --- | --- | --- | --- | --- | --- | --- | --- | --- | --- | --- | --- | --- | --- | --- | --- | --- | --- | --- | --- | --- | --- | --- | --- | --- | --- | --- | --- |

XP\_015687137.1

back to list

  

| Protein Coverage
| Supporting Peptides
|

Protein Coverage:

Supporting Peptides:

| Peptide | Uniq | -10lgP | Mass | Length | ppm | m/z | z | RT | Fraction | Scan | Source File | Area F6 | Area F2 | Area F3 | Area F4 | Area F5 | Area F7 | Area F8 | Area F1 | #Feature | #Feature F6 | #Feature F2 | #Feature F3 | #Feature F4 | #Feature F5 | #Feature F7 | #Feature F8 | #Feature F1 | Start | End | PTM | AScore | Found By |
| --- | --- | --- | --- | --- | --- | --- | --- | --- | --- | --- | --- | --- | --- | --- | --- | --- | --- | --- | --- | --- | --- | --- | --- | --- | --- | --- | --- | --- | --- | --- | --- | --- | --- |
| R.LAEVALAYAK.A | Y | 53.35 | 1047.5964 | 10 | 0.9 | 524.8060 | 2 | 37.49 | 8 | F8:18857 | 31122021\_RID\_3262\_1b.raw |  |  |  |  |  |  |  | 3.8989E4 | 1 | 0 | 0 | 0 | 0 | 0 | 0 | 0 | 1 | 150 | 159 |  |  | PEAKS DB |
| total 1 peptides |  |  |  |  |  |  |  |  |  |  |  |  |  |  |  |  |  |  |  |  |  |  |  |  |  |  |  |  |  |  |  |  |  |
| --- | --- | --- | --- | --- | --- | --- | --- | --- | --- | --- | --- | --- | --- | --- | --- | --- | --- | --- | --- | --- | --- | --- | --- | --- | --- | --- | --- | --- | --- | --- | --- | --- | --- |

JAV51283.1

back to list

  

| Protein Coverage
| Supporting Peptides
|

Protein Coverage:

Supporting Peptides:

| Peptide | Uniq | -10lgP | Mass | Length | ppm | m/z | z | RT | Fraction | Scan | Source File | Area F6 | Area F2 | Area F3 | Area F4 | Area F5 | Area F7 | Area F8 | Area F1 | #Feature | #Feature F6 | #Feature F2 | #Feature F3 | #Feature F4 | #Feature F5 | #Feature F7 | #Feature F8 | #Feature F1 | Start | End | PTM | AScore | Found By |
| --- | --- | --- | --- | --- | --- | --- | --- | --- | --- | --- | --- | --- | --- | --- | --- | --- | --- | --- | --- | --- | --- | --- | --- | --- | --- | --- | --- | --- | --- | --- | --- | --- | --- |
| R.LAEVALAYAK.A | Y | 53.35 | 1047.5964 | 10 | 0.9 | 524.8060 | 2 | 37.49 | 8 | F8:18857 | 31122021\_RID\_3262\_1b.raw |  |  |  |  |  |  |  | 3.8989E4 | 1 | 0 | 0 | 0 | 0 | 0 | 0 | 0 | 1 | 150 | 159 |  |  | PEAKS DB |
| total 1 peptides |  |  |  |  |  |  |  |  |  |  |  |  |  |  |  |  |  |  |  |  |  |  |  |  |  |  |  |  |  |  |  |  |  |
| --- | --- | --- | --- | --- | --- | --- | --- | --- | --- | --- | --- | --- | --- | --- | --- | --- | --- | --- | --- | --- | --- | --- | --- | --- | --- | --- | --- | --- | --- | --- | --- | --- | --- |

JAG68750.1

back to list

  

| Protein Coverage
| Supporting Peptides
|

Protein Coverage:

Supporting Peptides:

| Peptide | Uniq | -10lgP | Mass | Length | ppm | m/z | z | RT | Fraction | Scan | Source File | Area F6 | Area F2 | Area F3 | Area F4 | Area F5 | Area F7 | Area F8 | Area F1 | #Feature | #Feature F6 | #Feature F2 | #Feature F3 | #Feature F4 | #Feature F5 | #Feature F7 | #Feature F8 | #Feature F1 | Start | End | PTM | AScore | Found By |
| --- | --- | --- | --- | --- | --- | --- | --- | --- | --- | --- | --- | --- | --- | --- | --- | --- | --- | --- | --- | --- | --- | --- | --- | --- | --- | --- | --- | --- | --- | --- | --- | --- | --- |
| R.LAEVALAYAK.A | Y | 53.35 | 1047.5964 | 10 | 0.9 | 524.8060 | 2 | 37.49 | 8 | F8:18857 | 31122021\_RID\_3262\_1b.raw |  |  |  |  |  |  |  | 3.8989E4 | 1 | 0 | 0 | 0 | 0 | 0 | 0 | 0 | 1 | 150 | 159 |  |  | PEAKS DB |
| total 1 peptides |  |  |  |  |  |  |  |  |  |  |  |  |  |  |  |  |  |  |  |  |  |  |  |  |  |  |  |  |  |  |  |  |  |
| --- | --- | --- | --- | --- | --- | --- | --- | --- | --- | --- | --- | --- | --- | --- | --- | --- | --- | --- | --- | --- | --- | --- | --- | --- | --- | --- | --- | --- | --- | --- | --- | --- | --- |

XP\_034263014.1

back to list

  

| Protein Coverage
| Supporting Peptides
|

Protein Coverage:

Supporting Peptides:

| Peptide | Uniq | -10lgP | Mass | Length | ppm | m/z | z | RT | Fraction | Scan | Source File | Area F6 | Area F2 | Area F3 | Area F4 | Area F5 | Area F7 | Area F8 | Area F1 | #Feature | #Feature F6 | #Feature F2 | #Feature F3 | #Feature F4 | #Feature F5 | #Feature F7 | #Feature F8 | #Feature F1 | Start | End | PTM | AScore | Found By |
| --- | --- | --- | --- | --- | --- | --- | --- | --- | --- | --- | --- | --- | --- | --- | --- | --- | --- | --- | --- | --- | --- | --- | --- | --- | --- | --- | --- | --- | --- | --- | --- | --- | --- |
| R.SLGQNPTEAELR.E | Y | 53.27 | 1313.6575 | 12 | 0.9 | 657.8366 | 2 | 29.27 | 8 | F8:12361 | 31122021\_RID\_3262\_1b.raw |  |  |  |  |  |  |  | 1.7509E5 | 1 | 0 | 0 | 0 | 0 | 0 | 0 | 0 | 1 | 354 | 365 |  |  | PEAKS DB |
| total 1 peptides |  |  |  |  |  |  |  |  |  |  |  |  |  |  |  |  |  |  |  |  |  |  |  |  |  |  |  |  |  |  |  |  |  |
| --- | --- | --- | --- | --- | --- | --- | --- | --- | --- | --- | --- | --- | --- | --- | --- | --- | --- | --- | --- | --- | --- | --- | --- | --- | --- | --- | --- | --- | --- | --- | --- | --- | --- |

LAA89550.1

back to list

  

| Protein Coverage
| Supporting Peptides
|

Protein Coverage:

Supporting Peptides:

| Peptide | Uniq | -10lgP | Mass | Length | ppm | m/z | z | RT | Fraction | Scan | Source File | Area F6 | Area F2 | Area F3 | Area F4 | Area F5 | Area F7 | Area F8 | Area F1 | #Feature | #Feature F6 | #Feature F2 | #Feature F3 | #Feature F4 | #Feature F5 | #Feature F7 | #Feature F8 | #Feature F1 | Start | End | PTM | AScore | Found By |
| --- | --- | --- | --- | --- | --- | --- | --- | --- | --- | --- | --- | --- | --- | --- | --- | --- | --- | --- | --- | --- | --- | --- | --- | --- | --- | --- | --- | --- | --- | --- | --- | --- | --- |
| R.SLGQNPTEAELR.E | Y | 53.27 | 1313.6575 | 12 | 0.9 | 657.8366 | 2 | 29.27 | 8 | F8:12361 | 31122021\_RID\_3262\_1b.raw |  |  |  |  |  |  |  | 1.7509E5 | 1 | 0 | 0 | 0 | 0 | 0 | 0 | 0 | 1 | 33 | 44 |  |  | PEAKS DB |
| total 1 peptides |  |  |  |  |  |  |  |  |  |  |  |  |  |  |  |  |  |  |  |  |  |  |  |  |  |  |  |  |  |  |  |  |  |
| --- | --- | --- | --- | --- | --- | --- | --- | --- | --- | --- | --- | --- | --- | --- | --- | --- | --- | --- | --- | --- | --- | --- | --- | --- | --- | --- | --- | --- | --- | --- | --- | --- | --- |

LAA89553.1

back to list

  

| Protein Coverage
| Supporting Peptides
|

Protein Coverage:

Supporting Peptides:

| Peptide | Uniq | -10lgP | Mass | Length | ppm | m/z | z | RT | Fraction | Scan | Source File | Area F6 | Area F2 | Area F3 | Area F4 | Area F5 | Area F7 | Area F8 | Area F1 | #Feature | #Feature F6 | #Feature F2 | #Feature F3 | #Feature F4 | #Feature F5 | #Feature F7 | #Feature F8 | #Feature F1 | Start | End | PTM | AScore | Found By |
| --- | --- | --- | --- | --- | --- | --- | --- | --- | --- | --- | --- | --- | --- | --- | --- | --- | --- | --- | --- | --- | --- | --- | --- | --- | --- | --- | --- | --- | --- | --- | --- | --- | --- |
| R.SLGQNPTEAELR.E | Y | 53.27 | 1313.6575 | 12 | 0.9 | 657.8366 | 2 | 29.27 | 8 | F8:12361 | 31122021\_RID\_3262\_1b.raw |  |  |  |  |  |  |  | 1.7509E5 | 1 | 0 | 0 | 0 | 0 | 0 | 0 | 0 | 1 | 67 | 78 |  |  | PEAKS DB |
| total 1 peptides |  |  |  |  |  |  |  |  |  |  |  |  |  |  |  |  |  |  |  |  |  |  |  |  |  |  |  |  |  |  |  |  |  |
| --- | --- | --- | --- | --- | --- | --- | --- | --- | --- | --- | --- | --- | --- | --- | --- | --- | --- | --- | --- | --- | --- | --- | --- | --- | --- | --- | --- | --- | --- | --- | --- | --- | --- |

XP\_026544519.1

back to list

  

| Protein Coverage
| Supporting Peptides
|

Protein Coverage:

Supporting Peptides:

| Peptide | Uniq | -10lgP | Mass | Length | ppm | m/z | z | RT | Fraction | Scan | Source File | Area F6 | Area F2 | Area F3 | Area F4 | Area F5 | Area F7 | Area F8 | Area F1 | #Feature | #Feature F6 | #Feature F2 | #Feature F3 | #Feature F4 | #Feature F5 | #Feature F7 | #Feature F8 | #Feature F1 | Start | End | PTM | AScore | Found By |
| --- | --- | --- | --- | --- | --- | --- | --- | --- | --- | --- | --- | --- | --- | --- | --- | --- | --- | --- | --- | --- | --- | --- | --- | --- | --- | --- | --- | --- | --- | --- | --- | --- | --- |
| R.SLGQNPTEAELR.E | Y | 53.27 | 1313.6575 | 12 | 0.9 | 657.8366 | 2 | 29.27 | 8 | F8:12361 | 31122021\_RID\_3262\_1b.raw |  |  |  |  |  |  |  | 1.7509E5 | 1 | 0 | 0 | 0 | 0 | 0 | 0 | 0 | 1 | 194 | 205 |  |  | PEAKS DB |
| total 1 peptides |  |  |  |  |  |  |  |  |  |  |  |  |  |  |  |  |  |  |  |  |  |  |  |  |  |  |  |  |  |  |  |  |  |
| --- | --- | --- | --- | --- | --- | --- | --- | --- | --- | --- | --- | --- | --- | --- | --- | --- | --- | --- | --- | --- | --- | --- | --- | --- | --- | --- | --- | --- | --- | --- | --- | --- | --- |

XP\_026578673.1

back to list

  

| Protein Coverage
| Supporting Peptides
|

Protein Coverage:

Supporting Peptides:

| Peptide | Uniq | -10lgP | Mass | Length | ppm | m/z | z | RT | Fraction | Scan | Source File | Area F6 | Area F2 | Area F3 | Area F4 | Area F5 | Area F7 | Area F8 | Area F1 | #Feature | #Feature F6 | #Feature F2 | #Feature F3 | #Feature F4 | #Feature F5 | #Feature F7 | #Feature F8 | #Feature F1 | Start | End | PTM | AScore | Found By |
| --- | --- | --- | --- | --- | --- | --- | --- | --- | --- | --- | --- | --- | --- | --- | --- | --- | --- | --- | --- | --- | --- | --- | --- | --- | --- | --- | --- | --- | --- | --- | --- | --- | --- |
| R.SLGQNPTEAELR.E | Y | 53.27 | 1313.6575 | 12 | 0.9 | 657.8366 | 2 | 29.27 | 8 | F8:12361 | 31122021\_RID\_3262\_1b.raw |  |  |  |  |  |  |  | 1.7509E5 | 1 | 0 | 0 | 0 | 0 | 0 | 0 | 0 | 1 | 347 | 358 |  |  | PEAKS DB |
| total 1 peptides |  |  |  |  |  |  |  |  |  |  |  |  |  |  |  |  |  |  |  |  |  |  |  |  |  |  |  |  |  |  |  |  |  |
| --- | --- | --- | --- | --- | --- | --- | --- | --- | --- | --- | --- | --- | --- | --- | --- | --- | --- | --- | --- | --- | --- | --- | --- | --- | --- | --- | --- | --- | --- | --- | --- | --- | --- |

XP\_013924398.1

back to list

  

| Protein Coverage
| Supporting Peptides
|

Protein Coverage:

Supporting Peptides:

| Peptide | Uniq | -10lgP | Mass | Length | ppm | m/z | z | RT | Fraction | Scan | Source File | Area F6 | Area F2 | Area F3 | Area F4 | Area F5 | Area F7 | Area F8 | Area F1 | #Feature | #Feature F6 | #Feature F2 | #Feature F3 | #Feature F4 | #Feature F5 | #Feature F7 | #Feature F8 | #Feature F1 | Start | End | PTM | AScore | Found By |
| --- | --- | --- | --- | --- | --- | --- | --- | --- | --- | --- | --- | --- | --- | --- | --- | --- | --- | --- | --- | --- | --- | --- | --- | --- | --- | --- | --- | --- | --- | --- | --- | --- | --- |
| R.SLGQNPTEAELR.E | Y | 53.27 | 1313.6575 | 12 | 0.9 | 657.8366 | 2 | 29.27 | 8 | F8:12361 | 31122021\_RID\_3262\_1b.raw |  |  |  |  |  |  |  | 1.7509E5 | 1 | 0 | 0 | 0 | 0 | 0 | 0 | 0 | 1 | 38 | 49 |  |  | PEAKS DB |
| total 1 peptides |  |  |  |  |  |  |  |  |  |  |  |  |  |  |  |  |  |  |  |  |  |  |  |  |  |  |  |  |  |  |  |  |  |
| --- | --- | --- | --- | --- | --- | --- | --- | --- | --- | --- | --- | --- | --- | --- | --- | --- | --- | --- | --- | --- | --- | --- | --- | --- | --- | --- | --- | --- | --- | --- | --- | --- | --- |

XP\_025025692.1

back to list

  

| Protein Coverage
| Supporting Peptides
|

Protein Coverage:

Supporting Peptides:

| Peptide | Uniq | -10lgP | Mass | Length | ppm | m/z | z | RT | Fraction | Scan | Source File | Area F6 | Area F2 | Area F3 | Area F4 | Area F5 | Area F7 | Area F8 | Area F1 | #Feature | #Feature F6 | #Feature F2 | #Feature F3 | #Feature F4 | #Feature F5 | #Feature F7 | #Feature F8 | #Feature F1 | Start | End | PTM | AScore | Found By |
| --- | --- | --- | --- | --- | --- | --- | --- | --- | --- | --- | --- | --- | --- | --- | --- | --- | --- | --- | --- | --- | --- | --- | --- | --- | --- | --- | --- | --- | --- | --- | --- | --- | --- |
| R.SLGQNPTEAELR.E | Y | 53.27 | 1313.6575 | 12 | 0.9 | 657.8366 | 2 | 29.27 | 8 | F8:12361 | 31122021\_RID\_3262\_1b.raw |  |  |  |  |  |  |  | 1.7509E5 | 1 | 0 | 0 | 0 | 0 | 0 | 0 | 0 | 1 | 274 | 285 |  |  | PEAKS DB |
| total 1 peptides |  |  |  |  |  |  |  |  |  |  |  |  |  |  |  |  |  |  |  |  |  |  |  |  |  |  |  |  |  |  |  |  |  |
| --- | --- | --- | --- | --- | --- | --- | --- | --- | --- | --- | --- | --- | --- | --- | --- | --- | --- | --- | --- | --- | --- | --- | --- | --- | --- | --- | --- | --- | --- | --- | --- | --- | --- |

XP\_026578681.1

back to list

  

| Protein Coverage
| Supporting Peptides
|

Protein Coverage:

Supporting Peptides:

| Peptide | Uniq | -10lgP | Mass | Length | ppm | m/z | z | RT | Fraction | Scan | Source File | Area F6 | Area F2 | Area F3 | Area F4 | Area F5 | Area F7 | Area F8 | Area F1 | #Feature | #Feature F6 | #Feature F2 | #Feature F3 | #Feature F4 | #Feature F5 | #Feature F7 | #Feature F8 | #Feature F1 | Start | End | PTM | AScore | Found By |
| --- | --- | --- | --- | --- | --- | --- | --- | --- | --- | --- | --- | --- | --- | --- | --- | --- | --- | --- | --- | --- | --- | --- | --- | --- | --- | --- | --- | --- | --- | --- | --- | --- | --- |
| R.SLGQNPTEAELR.E | Y | 53.27 | 1313.6575 | 12 | 0.9 | 657.8366 | 2 | 29.27 | 8 | F8:12361 | 31122021\_RID\_3262\_1b.raw |  |  |  |  |  |  |  | 1.7509E5 | 1 | 0 | 0 | 0 | 0 | 0 | 0 | 0 | 1 | 352 | 363 |  |  | PEAKS DB |
| total 1 peptides |  |  |  |  |  |  |  |  |  |  |  |  |  |  |  |  |  |  |  |  |  |  |  |  |  |  |  |  |  |  |  |  |  |
| --- | --- | --- | --- | --- | --- | --- | --- | --- | --- | --- | --- | --- | --- | --- | --- | --- | --- | --- | --- | --- | --- | --- | --- | --- | --- | --- | --- | --- | --- | --- | --- | --- | --- |

XP\_029142126.1

back to list

  

| Protein Coverage
| Supporting Peptides
|

Protein Coverage:

Supporting Peptides:

| Peptide | Uniq | -10lgP | Mass | Length | ppm | m/z | z | RT | Fraction | Scan | Source File | Area F6 | Area F2 | Area F3 | Area F4 | Area F5 | Area F7 | Area F8 | Area F1 | #Feature | #Feature F6 | #Feature F2 | #Feature F3 | #Feature F4 | #Feature F5 | #Feature F7 | #Feature F8 | #Feature F1 | Start | End | PTM | AScore | Found By |
| --- | --- | --- | --- | --- | --- | --- | --- | --- | --- | --- | --- | --- | --- | --- | --- | --- | --- | --- | --- | --- | --- | --- | --- | --- | --- | --- | --- | --- | --- | --- | --- | --- | --- |
| R.SLGQNPTEAELR.E | Y | 53.27 | 1313.6575 | 12 | 0.9 | 657.8366 | 2 | 29.27 | 8 | F8:12361 | 31122021\_RID\_3262\_1b.raw |  |  |  |  |  |  |  | 1.7509E5 | 1 | 0 | 0 | 0 | 0 | 0 | 0 | 0 | 1 | 88 | 99 |  |  | PEAKS DB |
| total 1 peptides |  |  |  |  |  |  |  |  |  |  |  |  |  |  |  |  |  |  |  |  |  |  |  |  |  |  |  |  |  |  |  |  |  |
| --- | --- | --- | --- | --- | --- | --- | --- | --- | --- | --- | --- | --- | --- | --- | --- | --- | --- | --- | --- | --- | --- | --- | --- | --- | --- | --- | --- | --- | --- | --- | --- | --- | --- |

XP\_013923169.1

back to list

  

| Protein Coverage
| Supporting Peptides
|

Protein Coverage:

Supporting Peptides:

| Peptide | Uniq | -10lgP | Mass | Length | ppm | m/z | z | RT | Fraction | Scan | Source File | Area F6 | Area F2 | Area F3 | Area F4 | Area F5 | Area F7 | Area F8 | Area F1 | #Feature | #Feature F6 | #Feature F2 | #Feature F3 | #Feature F4 | #Feature F5 | #Feature F7 | #Feature F8 | #Feature F1 | Start | End | PTM | AScore | Found By |
| --- | --- | --- | --- | --- | --- | --- | --- | --- | --- | --- | --- | --- | --- | --- | --- | --- | --- | --- | --- | --- | --- | --- | --- | --- | --- | --- | --- | --- | --- | --- | --- | --- | --- |
| R.VFGFSLITNK.A | Y | 52.98 | 1124.6229 | 10 | 0.1 | 563.3188 | 2 | 51.56 | 8 | F8:29894 | 31122021\_RID\_3262\_1b.raw |  |  |  |  |  |  |  | 7.6993E4 | 1 | 0 | 0 | 0 | 0 | 0 | 0 | 0 | 1 | 74 | 83 |  |  | PEAKS DB |
| total 1 peptides |  |  |  |  |  |  |  |  |  |  |  |  |  |  |  |  |  |  |  |  |  |  |  |  |  |  |  |  |  |  |  |  |  |
| --- | --- | --- | --- | --- | --- | --- | --- | --- | --- | --- | --- | --- | --- | --- | --- | --- | --- | --- | --- | --- | --- | --- | --- | --- | --- | --- | --- | --- | --- | --- | --- | --- | --- |

JAI11878.1

back to list

  

| Protein Coverage
| Supporting Peptides
|

Protein Coverage:

Supporting Peptides:

| Peptide | Uniq | -10lgP | Mass | Length | ppm | m/z | z | RT | Fraction | Scan | Source File | Area F6 | Area F2 | Area F3 | Area F4 | Area F5 | Area F7 | Area F8 | Area F1 | #Feature | #Feature F6 | #Feature F2 | #Feature F3 | #Feature F4 | #Feature F5 | #Feature F7 | #Feature F8 | #Feature F1 | Start | End | PTM | AScore | Found By |
| --- | --- | --- | --- | --- | --- | --- | --- | --- | --- | --- | --- | --- | --- | --- | --- | --- | --- | --- | --- | --- | --- | --- | --- | --- | --- | --- | --- | --- | --- | --- | --- | --- | --- |
| R.VFGFSLITNK.A | Y | 52.98 | 1124.6229 | 10 | 0.1 | 563.3188 | 2 | 51.56 | 8 | F8:29894 | 31122021\_RID\_3262\_1b.raw |  |  |  |  |  |  |  | 7.6993E4 | 1 | 0 | 0 | 0 | 0 | 0 | 0 | 0 | 1 | 239 | 248 |  |  | PEAKS DB |
| total 1 peptides |  |  |  |  |  |  |  |  |  |  |  |  |  |  |  |  |  |  |  |  |  |  |  |  |  |  |  |  |  |  |  |  |  |
| --- | --- | --- | --- | --- | --- | --- | --- | --- | --- | --- | --- | --- | --- | --- | --- | --- | --- | --- | --- | --- | --- | --- | --- | --- | --- | --- | --- | --- | --- | --- | --- | --- | --- |

AFJ50974.1

back to list

  

| Protein Coverage
| Supporting Peptides
|

Protein Coverage:

Supporting Peptides:

| Peptide | Uniq | -10lgP | Mass | Length | ppm | m/z | z | RT | Fraction | Scan | Source File | Area F6 | Area F2 | Area F3 | Area F4 | Area F5 | Area F7 | Area F8 | Area F1 | #Feature | #Feature F6 | #Feature F2 | #Feature F3 | #Feature F4 | #Feature F5 | #Feature F7 | #Feature F8 | #Feature F1 | Start | End | PTM | AScore | Found By |
| --- | --- | --- | --- | --- | --- | --- | --- | --- | --- | --- | --- | --- | --- | --- | --- | --- | --- | --- | --- | --- | --- | --- | --- | --- | --- | --- | --- | --- | --- | --- | --- | --- | --- |
| R.VFGFSLITNK.A | Y | 52.98 | 1124.6229 | 10 | 0.1 | 563.3188 | 2 | 51.56 | 8 | F8:29894 | 31122021\_RID\_3262\_1b.raw |  |  |  |  |  |  |  | 7.6993E4 | 1 | 0 | 0 | 0 | 0 | 0 | 0 | 0 | 1 | 239 | 248 |  |  | PEAKS DB |
| total 1 peptides |  |  |  |  |  |  |  |  |  |  |  |  |  |  |  |  |  |  |  |  |  |  |  |  |  |  |  |  |  |  |  |  |  |
| --- | --- | --- | --- | --- | --- | --- | --- | --- | --- | --- | --- | --- | --- | --- | --- | --- | --- | --- | --- | --- | --- | --- | --- | --- | --- | --- | --- | --- | --- | --- | --- | --- | --- |

JAA95993.1

back to list

  

| Protein Coverage
| Supporting Peptides
|

Protein Coverage:

Supporting Peptides:

| Peptide | Uniq | -10lgP | Mass | Length | ppm | m/z | z | RT | Fraction | Scan | Source File | Area F6 | Area F2 | Area F3 | Area F4 | Area F5 | Area F7 | Area F8 | Area F1 | #Feature | #Feature F6 | #Feature F2 | #Feature F3 | #Feature F4 | #Feature F5 | #Feature F7 | #Feature F8 | #Feature F1 | Start | End | PTM | AScore | Found By |
| --- | --- | --- | --- | --- | --- | --- | --- | --- | --- | --- | --- | --- | --- | --- | --- | --- | --- | --- | --- | --- | --- | --- | --- | --- | --- | --- | --- | --- | --- | --- | --- | --- | --- |
| R.VFGFSLITNK.A | Y | 52.98 | 1124.6229 | 10 | 0.1 | 563.3188 | 2 | 51.56 | 8 | F8:29894 | 31122021\_RID\_3262\_1b.raw |  |  |  |  |  |  |  | 7.6993E4 | 1 | 0 | 0 | 0 | 0 | 0 | 0 | 0 | 1 | 239 | 248 |  |  | PEAKS DB |
| total 1 peptides |  |  |  |  |  |  |  |  |  |  |  |  |  |  |  |  |  |  |  |  |  |  |  |  |  |  |  |  |  |  |  |  |  |
| --- | --- | --- | --- | --- | --- | --- | --- | --- | --- | --- | --- | --- | --- | --- | --- | --- | --- | --- | --- | --- | --- | --- | --- | --- | --- | --- | --- | --- | --- | --- | --- | --- | --- |

XP\_025032762.1

back to list

  

| Protein Coverage
| Supporting Peptides
|

Protein Coverage:

Supporting Peptides:

| Peptide | Uniq | -10lgP | Mass | Length | ppm | m/z | z | RT | Fraction | Scan | Source File | Area F6 | Area F2 | Area F3 | Area F4 | Area F5 | Area F7 | Area F8 | Area F1 | #Feature | #Feature F6 | #Feature F2 | #Feature F3 | #Feature F4 | #Feature F5 | #Feature F7 | #Feature F8 | #Feature F1 | Start | End | PTM | AScore | Found By |
| --- | --- | --- | --- | --- | --- | --- | --- | --- | --- | --- | --- | --- | --- | --- | --- | --- | --- | --- | --- | --- | --- | --- | --- | --- | --- | --- | --- | --- | --- | --- | --- | --- | --- |
| R.VFGFSLITNK.A | Y | 52.98 | 1124.6229 | 10 | 0.1 | 563.3188 | 2 | 51.56 | 8 | F8:29894 | 31122021\_RID\_3262\_1b.raw |  |  |  |  |  |  |  | 7.6993E4 | 1 | 0 | 0 | 0 | 0 | 0 | 0 | 0 | 1 | 252 | 261 |  |  | PEAKS DB |
| total 1 peptides |  |  |  |  |  |  |  |  |  |  |  |  |  |  |  |  |  |  |  |  |  |  |  |  |  |  |  |  |  |  |  |  |  |
| --- | --- | --- | --- | --- | --- | --- | --- | --- | --- | --- | --- | --- | --- | --- | --- | --- | --- | --- | --- | --- | --- | --- | --- | --- | --- | --- | --- | --- | --- | --- | --- | --- | --- |

LAA42915.1

back to list

  

| Protein Coverage
| Supporting Peptides
|

Protein Coverage:

Supporting Peptides:

| Peptide | Uniq | -10lgP | Mass | Length | ppm | m/z | z | RT | Fraction | Scan | Source File | Area F6 | Area F2 | Area F3 | Area F4 | Area F5 | Area F7 | Area F8 | Area F1 | #Feature | #Feature F6 | #Feature F2 | #Feature F3 | #Feature F4 | #Feature F5 | #Feature F7 | #Feature F8 | #Feature F1 | Start | End | PTM | AScore | Found By |
| --- | --- | --- | --- | --- | --- | --- | --- | --- | --- | --- | --- | --- | --- | --- | --- | --- | --- | --- | --- | --- | --- | --- | --- | --- | --- | --- | --- | --- | --- | --- | --- | --- | --- |
| R.VFGFSLITNK.V | Y | 52.98 | 1124.6229 | 10 | 0.1 | 563.3188 | 2 | 51.56 | 8 | F8:29894 | 31122021\_RID\_3262\_1b.raw |  |  |  |  |  |  |  | 7.6993E4 | 1 | 0 | 0 | 0 | 0 | 0 | 0 | 0 | 1 | 155 | 164 |  |  | PEAKS DB |
| total 1 peptides |  |  |  |  |  |  |  |  |  |  |  |  |  |  |  |  |  |  |  |  |  |  |  |  |  |  |  |  |  |  |  |  |  |
| --- | --- | --- | --- | --- | --- | --- | --- | --- | --- | --- | --- | --- | --- | --- | --- | --- | --- | --- | --- | --- | --- | --- | --- | --- | --- | --- | --- | --- | --- | --- | --- | --- | --- |

LAB18974.1

back to list

  

| Protein Coverage
| Supporting Peptides
|

Protein Coverage:

Supporting Peptides:

| Peptide | Uniq | -10lgP | Mass | Length | ppm | m/z | z | RT | Fraction | Scan | Source File | Area F6 | Area F2 | Area F3 | Area F4 | Area F5 | Area F7 | Area F8 | Area F1 | #Feature | #Feature F6 | #Feature F2 | #Feature F3 | #Feature F4 | #Feature F5 | #Feature F7 | #Feature F8 | #Feature F1 | Start | End | PTM | AScore | Found By |
| --- | --- | --- | --- | --- | --- | --- | --- | --- | --- | --- | --- | --- | --- | --- | --- | --- | --- | --- | --- | --- | --- | --- | --- | --- | --- | --- | --- | --- | --- | --- | --- | --- | --- |
| R.VFGFSLITNK.A | Y | 52.98 | 1124.6229 | 10 | 0.1 | 563.3188 | 2 | 51.56 | 8 | F8:29894 | 31122021\_RID\_3262\_1b.raw |  |  |  |  |  |  |  | 7.6993E4 | 1 | 0 | 0 | 0 | 0 | 0 | 0 | 0 | 1 | 230 | 239 |  |  | PEAKS DB |
| total 1 peptides |  |  |  |  |  |  |  |  |  |  |  |  |  |  |  |  |  |  |  |  |  |  |  |  |  |  |  |  |  |  |  |  |  |
| --- | --- | --- | --- | --- | --- | --- | --- | --- | --- | --- | --- | --- | --- | --- | --- | --- | --- | --- | --- | --- | --- | --- | --- | --- | --- | --- | --- | --- | --- | --- | --- | --- | --- |

JAB53579.1

back to list

  

| Protein Coverage
| Supporting Peptides
|

Protein Coverage:

Supporting Peptides:

| Peptide | Uniq | -10lgP | Mass | Length | ppm | m/z | z | RT | Fraction | Scan | Source File | Area F6 | Area F2 | Area F3 | Area F4 | Area F5 | Area F7 | Area F8 | Area F1 | #Feature | #Feature F6 | #Feature F2 | #Feature F3 | #Feature F4 | #Feature F5 | #Feature F7 | #Feature F8 | #Feature F1 | Start | End | PTM | AScore | Found By |
| --- | --- | --- | --- | --- | --- | --- | --- | --- | --- | --- | --- | --- | --- | --- | --- | --- | --- | --- | --- | --- | --- | --- | --- | --- | --- | --- | --- | --- | --- | --- | --- | --- | --- |
| R.VFGFSLITNK.V | Y | 52.98 | 1124.6229 | 10 | 0.1 | 563.3188 | 2 | 51.56 | 8 | F8:29894 | 31122021\_RID\_3262\_1b.raw |  |  |  |  |  |  |  | 7.6993E4 | 1 | 0 | 0 | 0 | 0 | 0 | 0 | 0 | 1 | 239 | 248 |  |  | PEAKS DB |
| total 1 peptides |  |  |  |  |  |  |  |  |  |  |  |  |  |  |  |  |  |  |  |  |  |  |  |  |  |  |  |  |  |  |  |  |  |
| --- | --- | --- | --- | --- | --- | --- | --- | --- | --- | --- | --- | --- | --- | --- | --- | --- | --- | --- | --- | --- | --- | --- | --- | --- | --- | --- | --- | --- | --- | --- | --- | --- | --- |

XP\_026543188.1

back to list

  

| Protein Coverage
| Supporting Peptides
|

Protein Coverage:

Supporting Peptides:

| Peptide | Uniq | -10lgP | Mass | Length | ppm | m/z | z | RT | Fraction | Scan | Source File | Area F6 | Area F2 | Area F3 | Area F4 | Area F5 | Area F7 | Area F8 | Area F1 | #Feature | #Feature F6 | #Feature F2 | #Feature F3 | #Feature F4 | #Feature F5 | #Feature F7 | #Feature F8 | #Feature F1 | Start | End | PTM | AScore | Found By |
| --- | --- | --- | --- | --- | --- | --- | --- | --- | --- | --- | --- | --- | --- | --- | --- | --- | --- | --- | --- | --- | --- | --- | --- | --- | --- | --- | --- | --- | --- | --- | --- | --- | --- |
| R.VFGFSLITNK.A | Y | 52.98 | 1124.6229 | 10 | 0.1 | 563.3188 | 2 | 51.56 | 8 | F8:29894 | 31122021\_RID\_3262\_1b.raw |  |  |  |  |  |  |  | 7.6993E4 | 1 | 0 | 0 | 0 | 0 | 0 | 0 | 0 | 1 | 239 | 248 |  |  | PEAKS DB |
| total 1 peptides |  |  |  |  |  |  |  |  |  |  |  |  |  |  |  |  |  |  |  |  |  |  |  |  |  |  |  |  |  |  |  |  |  |
| --- | --- | --- | --- | --- | --- | --- | --- | --- | --- | --- | --- | --- | --- | --- | --- | --- | --- | --- | --- | --- | --- | --- | --- | --- | --- | --- | --- | --- | --- | --- | --- | --- | --- |

XP\_015676887.1

back to list

  

| Protein Coverage
| Supporting Peptides
|

Protein Coverage:

Supporting Peptides:

| Peptide | Uniq | -10lgP | Mass | Length | ppm | m/z | z | RT | Fraction | Scan | Source File | Area F6 | Area F2 | Area F3 | Area F4 | Area F5 | Area F7 | Area F8 | Area F1 | #Feature | #Feature F6 | #Feature F2 | #Feature F3 | #Feature F4 | #Feature F5 | #Feature F7 | #Feature F8 | #Feature F1 | Start | End | PTM | AScore | Found By |
| --- | --- | --- | --- | --- | --- | --- | --- | --- | --- | --- | --- | --- | --- | --- | --- | --- | --- | --- | --- | --- | --- | --- | --- | --- | --- | --- | --- | --- | --- | --- | --- | --- | --- |
| R.VFGFSLITNK.A | Y | 52.98 | 1124.6229 | 10 | 0.1 | 563.3188 | 2 | 51.56 | 8 | F8:29894 | 31122021\_RID\_3262\_1b.raw |  |  |  |  |  |  |  | 7.6993E4 | 1 | 0 | 0 | 0 | 0 | 0 | 0 | 0 | 1 | 239 | 248 |  |  | PEAKS DB |
| total 1 peptides |  |  |  |  |  |  |  |  |  |  |  |  |  |  |  |  |  |  |  |  |  |  |  |  |  |  |  |  |  |  |  |  |  |
| --- | --- | --- | --- | --- | --- | --- | --- | --- | --- | --- | --- | --- | --- | --- | --- | --- | --- | --- | --- | --- | --- | --- | --- | --- | --- | --- | --- | --- | --- | --- | --- | --- | --- |

JAV49515.1

back to list

  

| Protein Coverage
| Supporting Peptides
|

Protein Coverage:

Supporting Peptides:

| Peptide | Uniq | -10lgP | Mass | Length | ppm | m/z | z | RT | Fraction | Scan | Source File | Area F6 | Area F2 | Area F3 | Area F4 | Area F5 | Area F7 | Area F8 | Area F1 | #Feature | #Feature F6 | #Feature F2 | #Feature F3 | #Feature F4 | #Feature F5 | #Feature F7 | #Feature F8 | #Feature F1 | Start | End | PTM | AScore | Found By |
| --- | --- | --- | --- | --- | --- | --- | --- | --- | --- | --- | --- | --- | --- | --- | --- | --- | --- | --- | --- | --- | --- | --- | --- | --- | --- | --- | --- | --- | --- | --- | --- | --- | --- |
| R.VFGFSLITNK.A | Y | 52.98 | 1124.6229 | 10 | 0.1 | 563.3188 | 2 | 51.56 | 8 | F8:29894 | 31122021\_RID\_3262\_1b.raw |  |  |  |  |  |  |  | 7.6993E4 | 1 | 0 | 0 | 0 | 0 | 0 | 0 | 0 | 1 | 239 | 248 |  |  | PEAKS DB |
| total 1 peptides |  |  |  |  |  |  |  |  |  |  |  |  |  |  |  |  |  |  |  |  |  |  |  |  |  |  |  |  |  |  |  |  |  |
| --- | --- | --- | --- | --- | --- | --- | --- | --- | --- | --- | --- | --- | --- | --- | --- | --- | --- | --- | --- | --- | --- | --- | --- | --- | --- | --- | --- | --- | --- | --- | --- | --- | --- |

XP\_026576404.1

back to list

  

| Protein Coverage
| Supporting Peptides
|

Protein Coverage:

Supporting Peptides:

| Peptide | Uniq | -10lgP | Mass | Length | ppm | m/z | z | RT | Fraction | Scan | Source File | Area F6 | Area F2 | Area F3 | Area F4 | Area F5 | Area F7 | Area F8 | Area F1 | #Feature | #Feature F6 | #Feature F2 | #Feature F3 | #Feature F4 | #Feature F5 | #Feature F7 | #Feature F8 | #Feature F1 | Start | End | PTM | AScore | Found By |
| --- | --- | --- | --- | --- | --- | --- | --- | --- | --- | --- | --- | --- | --- | --- | --- | --- | --- | --- | --- | --- | --- | --- | --- | --- | --- | --- | --- | --- | --- | --- | --- | --- | --- |
| R.VFGFSLITNK.A | Y | 52.98 | 1124.6229 | 10 | 0.1 | 563.3188 | 2 | 51.56 | 8 | F8:29894 | 31122021\_RID\_3262\_1b.raw |  |  |  |  |  |  |  | 7.6993E4 | 1 | 0 | 0 | 0 | 0 | 0 | 0 | 0 | 1 | 239 | 248 |  |  | PEAKS DB |
| total 1 peptides |  |  |  |  |  |  |  |  |  |  |  |  |  |  |  |  |  |  |  |  |  |  |  |  |  |  |  |  |  |  |  |  |  |
| --- | --- | --- | --- | --- | --- | --- | --- | --- | --- | --- | --- | --- | --- | --- | --- | --- | --- | --- | --- | --- | --- | --- | --- | --- | --- | --- | --- | --- | --- | --- | --- | --- | --- |

JAG66882.1

back to list

  

| Protein Coverage
| Supporting Peptides
|

Protein Coverage:

Supporting Peptides:

| Peptide | Uniq | -10lgP | Mass | Length | ppm | m/z | z | RT | Fraction | Scan | Source File | Area F6 | Area F2 | Area F3 | Area F4 | Area F5 | Area F7 | Area F8 | Area F1 | #Feature | #Feature F6 | #Feature F2 | #Feature F3 | #Feature F4 | #Feature F5 | #Feature F7 | #Feature F8 | #Feature F1 | Start | End | PTM | AScore | Found By |
| --- | --- | --- | --- | --- | --- | --- | --- | --- | --- | --- | --- | --- | --- | --- | --- | --- | --- | --- | --- | --- | --- | --- | --- | --- | --- | --- | --- | --- | --- | --- | --- | --- | --- |
| R.VFGFSLITNK.A | Y | 52.98 | 1124.6229 | 10 | 0.1 | 563.3188 | 2 | 51.56 | 8 | F8:29894 | 31122021\_RID\_3262\_1b.raw |  |  |  |  |  |  |  | 7.6993E4 | 1 | 0 | 0 | 0 | 0 | 0 | 0 | 0 | 1 | 239 | 248 |  |  | PEAKS DB |
| total 1 peptides |  |  |  |  |  |  |  |  |  |  |  |  |  |  |  |  |  |  |  |  |  |  |  |  |  |  |  |  |  |  |  |  |  |
| --- | --- | --- | --- | --- | --- | --- | --- | --- | --- | --- | --- | --- | --- | --- | --- | --- | --- | --- | --- | --- | --- | --- | --- | --- | --- | --- | --- | --- | --- | --- | --- | --- | --- |

LAA42911.1

back to list

  

| Protein Coverage
| Supporting Peptides
|

Protein Coverage:

Supporting Peptides:

| Peptide | Uniq | -10lgP | Mass | Length | ppm | m/z | z | RT | Fraction | Scan | Source File | Area F6 | Area F2 | Area F3 | Area F4 | Area F5 | Area F7 | Area F8 | Area F1 | #Feature | #Feature F6 | #Feature F2 | #Feature F3 | #Feature F4 | #Feature F5 | #Feature F7 | #Feature F8 | #Feature F1 | Start | End | PTM | AScore | Found By |
| --- | --- | --- | --- | --- | --- | --- | --- | --- | --- | --- | --- | --- | --- | --- | --- | --- | --- | --- | --- | --- | --- | --- | --- | --- | --- | --- | --- | --- | --- | --- | --- | --- | --- |
| R.VFGFSLITNK.V | Y | 52.98 | 1124.6229 | 10 | 0.1 | 563.3188 | 2 | 51.56 | 8 | F8:29894 | 31122021\_RID\_3262\_1b.raw |  |  |  |  |  |  |  | 7.6993E4 | 1 | 0 | 0 | 0 | 0 | 0 | 0 | 0 | 1 | 248 | 257 |  |  | PEAKS DB |
| total 1 peptides |  |  |  |  |  |  |  |  |  |  |  |  |  |  |  |  |  |  |  |  |  |  |  |  |  |  |  |  |  |  |  |  |  |
| --- | --- | --- | --- | --- | --- | --- | --- | --- | --- | --- | --- | --- | --- | --- | --- | --- | --- | --- | --- | --- | --- | --- | --- | --- | --- | --- | --- | --- | --- | --- | --- | --- | --- |

ETE69996.1

back to list

  

| Protein Coverage
| Supporting Peptides
|

Protein Coverage:

Supporting Peptides:

| Peptide | Uniq | -10lgP | Mass | Length | ppm | m/z | z | RT | Fraction | Scan | Source File | Area F6 | Area F2 | Area F3 | Area F4 | Area F5 | Area F7 | Area F8 | Area F1 | #Feature | #Feature F6 | #Feature F2 | #Feature F3 | #Feature F4 | #Feature F5 | #Feature F7 | #Feature F8 | #Feature F1 | Start | End | PTM | AScore | Found By |
| --- | --- | --- | --- | --- | --- | --- | --- | --- | --- | --- | --- | --- | --- | --- | --- | --- | --- | --- | --- | --- | --- | --- | --- | --- | --- | --- | --- | --- | --- | --- | --- | --- | --- |
| R.VFGFSLITNK.A | Y | 52.98 | 1124.6229 | 10 | 0.1 | 563.3188 | 2 | 51.56 | 8 | F8:29894 | 31122021\_RID\_3262\_1b.raw |  |  |  |  |  |  |  | 7.6993E4 | 1 | 0 | 0 | 0 | 0 | 0 | 0 | 0 | 1 | 271 | 280 |  |  | PEAKS DB |
| total 1 peptides |  |  |  |  |  |  |  |  |  |  |  |  |  |  |  |  |  |  |  |  |  |  |  |  |  |  |  |  |  |  |  |  |  |
| --- | --- | --- | --- | --- | --- | --- | --- | --- | --- | --- | --- | --- | --- | --- | --- | --- | --- | --- | --- | --- | --- | --- | --- | --- | --- | --- | --- | --- | --- | --- | --- | --- | --- |

XP\_032091539.1

back to list

  

| Protein Coverage
| Supporting Peptides
|

Protein Coverage:

Supporting Peptides:

| Peptide | Uniq | -10lgP | Mass | Length | ppm | m/z | z | RT | Fraction | Scan | Source File | Area F6 | Area F2 | Area F3 | Area F4 | Area F5 | Area F7 | Area F8 | Area F1 | #Feature | #Feature F6 | #Feature F2 | #Feature F3 | #Feature F4 | #Feature F5 | #Feature F7 | #Feature F8 | #Feature F1 | Start | End | PTM | AScore | Found By |
| --- | --- | --- | --- | --- | --- | --- | --- | --- | --- | --- | --- | --- | --- | --- | --- | --- | --- | --- | --- | --- | --- | --- | --- | --- | --- | --- | --- | --- | --- | --- | --- | --- | --- |
| R.VFGFSLITNK.A | Y | 52.98 | 1124.6229 | 10 | 0.1 | 563.3188 | 2 | 51.56 | 8 | F8:29894 | 31122021\_RID\_3262\_1b.raw |  |  |  |  |  |  |  | 7.6993E4 | 1 | 0 | 0 | 0 | 0 | 0 | 0 | 0 | 1 | 239 | 248 |  |  | PEAKS DB |
| total 1 peptides |  |  |  |  |  |  |  |  |  |  |  |  |  |  |  |  |  |  |  |  |  |  |  |  |  |  |  |  |  |  |  |  |  |
| --- | --- | --- | --- | --- | --- | --- | --- | --- | --- | --- | --- | --- | --- | --- | --- | --- | --- | --- | --- | --- | --- | --- | --- | --- | --- | --- | --- | --- | --- | --- | --- | --- | --- |

XP\_034267396.1

back to list

  

| Protein Coverage
| Supporting Peptides
|

Protein Coverage:

Supporting Peptides:

| Peptide | Uniq | -10lgP | Mass | Length | ppm | m/z | z | RT | Fraction | Scan | Source File | Area F6 | Area F2 | Area F3 | Area F4 | Area F5 | Area F7 | Area F8 | Area F1 | #Feature | #Feature F6 | #Feature F2 | #Feature F3 | #Feature F4 | #Feature F5 | #Feature F7 | #Feature F8 | #Feature F1 | Start | End | PTM | AScore | Found By |
| --- | --- | --- | --- | --- | --- | --- | --- | --- | --- | --- | --- | --- | --- | --- | --- | --- | --- | --- | --- | --- | --- | --- | --- | --- | --- | --- | --- | --- | --- | --- | --- | --- | --- |
| R.VFGFSLITNK.A | Y | 52.98 | 1124.6229 | 10 | 0.1 | 563.3188 | 2 | 51.56 | 8 | F8:29894 | 31122021\_RID\_3262\_1b.raw |  |  |  |  |  |  |  | 7.6993E4 | 1 | 0 | 0 | 0 | 0 | 0 | 0 | 0 | 1 | 239 | 248 |  |  | PEAKS DB |
| total 1 peptides |  |  |  |  |  |  |  |  |  |  |  |  |  |  |  |  |  |  |  |  |  |  |  |  |  |  |  |  |  |  |  |  |  |
| --- | --- | --- | --- | --- | --- | --- | --- | --- | --- | --- | --- | --- | --- | --- | --- | --- | --- | --- | --- | --- | --- | --- | --- | --- | --- | --- | --- | --- | --- | --- | --- | --- | --- |

XP\_013908450.1

back to list

  

| Protein Coverage
| Supporting Peptides
|

Protein Coverage:

Supporting Peptides:

| Peptide | Uniq | -10lgP | Mass | Length | ppm | m/z | z | RT | Fraction | Scan | Source File | Area F6 | Area F2 | Area F3 | Area F4 | Area F5 | Area F7 | Area F8 | Area F1 | #Feature | #Feature F6 | #Feature F2 | #Feature F3 | #Feature F4 | #Feature F5 | #Feature F7 | #Feature F8 | #Feature F1 | Start | End | PTM | AScore | Found By |
| --- | --- | --- | --- | --- | --- | --- | --- | --- | --- | --- | --- | --- | --- | --- | --- | --- | --- | --- | --- | --- | --- | --- | --- | --- | --- | --- | --- | --- | --- | --- | --- | --- | --- |
| R.DAVLLVFANK.Q | Y | 52.71 | 1088.6229 | 10 | 0.3 | 545.3189 | 2 | 53.49 | 8 | F8:31406 | 31122021\_RID\_3262\_1b.raw |  |  |  |  |  |  |  | 1.1916E4 | 1 | 0 | 0 | 0 | 0 | 0 | 0 | 0 | 1 | 61 | 70 |  |  | PEAKS DB |
| total 1 peptides |  |  |  |  |  |  |  |  |  |  |  |  |  |  |  |  |  |  |  |  |  |  |  |  |  |  |  |  |  |  |  |  |  |
| --- | --- | --- | --- | --- | --- | --- | --- | --- | --- | --- | --- | --- | --- | --- | --- | --- | --- | --- | --- | --- | --- | --- | --- | --- | --- | --- | --- | --- | --- | --- | --- | --- | --- |

JAG68790.1

back to list

  

| Protein Coverage
| Supporting Peptides
|

Protein Coverage:

Supporting Peptides:

| Peptide | Uniq | -10lgP | Mass | Length | ppm | m/z | z | RT | Fraction | Scan | Source File | Area F6 | Area F2 | Area F3 | Area F4 | Area F5 | Area F7 | Area F8 | Area F1 | #Feature | #Feature F6 | #Feature F2 | #Feature F3 | #Feature F4 | #Feature F5 | #Feature F7 | #Feature F8 | #Feature F1 | Start | End | PTM | AScore | Found By |
| --- | --- | --- | --- | --- | --- | --- | --- | --- | --- | --- | --- | --- | --- | --- | --- | --- | --- | --- | --- | --- | --- | --- | --- | --- | --- | --- | --- | --- | --- | --- | --- | --- | --- |
| R.DAVLLVFANK.Q | Y | 52.71 | 1088.6229 | 10 | 0.3 | 545.3189 | 2 | 53.49 | 8 | F8:31406 | 31122021\_RID\_3262\_1b.raw |  |  |  |  |  |  |  | 1.1916E4 | 1 | 0 | 0 | 0 | 0 | 0 | 0 | 0 | 1 | 118 | 127 |  |  | PEAKS DB |
| total 1 peptides |  |  |  |  |  |  |  |  |  |  |  |  |  |  |  |  |  |  |  |  |  |  |  |  |  |  |  |  |  |  |  |  |  |
| --- | --- | --- | --- | --- | --- | --- | --- | --- | --- | --- | --- | --- | --- | --- | --- | --- | --- | --- | --- | --- | --- | --- | --- | --- | --- | --- | --- | --- | --- | --- | --- | --- | --- |

JAV51323.1

back to list

  

| Protein Coverage
| Supporting Peptides
|

Protein Coverage:

Supporting Peptides:

| Peptide | Uniq | -10lgP | Mass | Length | ppm | m/z | z | RT | Fraction | Scan | Source File | Area F6 | Area F2 | Area F3 | Area F4 | Area F5 | Area F7 | Area F8 | Area F1 | #Feature | #Feature F6 | #Feature F2 | #Feature F3 | #Feature F4 | #Feature F5 | #Feature F7 | #Feature F8 | #Feature F1 | Start | End | PTM | AScore | Found By |
| --- | --- | --- | --- | --- | --- | --- | --- | --- | --- | --- | --- | --- | --- | --- | --- | --- | --- | --- | --- | --- | --- | --- | --- | --- | --- | --- | --- | --- | --- | --- | --- | --- | --- |
| R.DAVLLVFANK.Q | Y | 52.71 | 1088.6229 | 10 | 0.3 | 545.3189 | 2 | 53.49 | 8 | F8:31406 | 31122021\_RID\_3262\_1b.raw |  |  |  |  |  |  |  | 1.1916E4 | 1 | 0 | 0 | 0 | 0 | 0 | 0 | 0 | 1 | 118 | 127 |  |  | PEAKS DB |
| total 1 peptides |  |  |  |  |  |  |  |  |  |  |  |  |  |  |  |  |  |  |  |  |  |  |  |  |  |  |  |  |  |  |  |  |  |
| --- | --- | --- | --- | --- | --- | --- | --- | --- | --- | --- | --- | --- | --- | --- | --- | --- | --- | --- | --- | --- | --- | --- | --- | --- | --- | --- | --- | --- | --- | --- | --- | --- | --- |

JAC96314.1

back to list

  

| Protein Coverage
| Supporting Peptides
|

Protein Coverage:

Supporting Peptides:

| Peptide | Uniq | -10lgP | Mass | Length | ppm | m/z | z | RT | Fraction | Scan | Source File | Area F6 | Area F2 | Area F3 | Area F4 | Area F5 | Area F7 | Area F8 | Area F1 | #Feature | #Feature F6 | #Feature F2 | #Feature F3 | #Feature F4 | #Feature F5 | #Feature F7 | #Feature F8 | #Feature F1 | Start | End | PTM | AScore | Found By |
| --- | --- | --- | --- | --- | --- | --- | --- | --- | --- | --- | --- | --- | --- | --- | --- | --- | --- | --- | --- | --- | --- | --- | --- | --- | --- | --- | --- | --- | --- | --- | --- | --- | --- |
| R.DAVLLVFANK.Q | Y | 52.71 | 1088.6229 | 10 | 0.3 | 545.3189 | 2 | 53.49 | 8 | F8:31406 | 31122021\_RID\_3262\_1b.raw |  |  |  |  |  |  |  | 1.1916E4 | 1 | 0 | 0 | 0 | 0 | 0 | 0 | 0 | 1 | 118 | 127 |  |  | PEAKS DB |
| total 1 peptides |  |  |  |  |  |  |  |  |  |  |  |  |  |  |  |  |  |  |  |  |  |  |  |  |  |  |  |  |  |  |  |  |  |
| --- | --- | --- | --- | --- | --- | --- | --- | --- | --- | --- | --- | --- | --- | --- | --- | --- | --- | --- | --- | --- | --- | --- | --- | --- | --- | --- | --- | --- | --- | --- | --- | --- | --- |

JAI10317.1

back to list

  

| Protein Coverage
| Supporting Peptides
|

Protein Coverage:

Supporting Peptides:

| Peptide | Uniq | -10lgP | Mass | Length | ppm | m/z | z | RT | Fraction | Scan | Source File | Area F6 | Area F2 | Area F3 | Area F4 | Area F5 | Area F7 | Area F8 | Area F1 | #Feature | #Feature F6 | #Feature F2 | #Feature F3 | #Feature F4 | #Feature F5 | #Feature F7 | #Feature F8 | #Feature F1 | Start | End | PTM | AScore | Found By |
| --- | --- | --- | --- | --- | --- | --- | --- | --- | --- | --- | --- | --- | --- | --- | --- | --- | --- | --- | --- | --- | --- | --- | --- | --- | --- | --- | --- | --- | --- | --- | --- | --- | --- |
| R.DAVLLVFANK.Q | Y | 52.71 | 1088.6229 | 10 | 0.3 | 545.3189 | 2 | 53.49 | 8 | F8:31406 | 31122021\_RID\_3262\_1b.raw |  |  |  |  |  |  |  | 1.1916E4 | 1 | 0 | 0 | 0 | 0 | 0 | 0 | 0 | 1 | 118 | 127 |  |  | PEAKS DB |
| total 1 peptides |  |  |  |  |  |  |  |  |  |  |  |  |  |  |  |  |  |  |  |  |  |  |  |  |  |  |  |  |  |  |  |  |  |
| --- | --- | --- | --- | --- | --- | --- | --- | --- | --- | --- | --- | --- | --- | --- | --- | --- | --- | --- | --- | --- | --- | --- | --- | --- | --- | --- | --- | --- | --- | --- | --- | --- | --- |

JAB54735.1

back to list

  

| Protein Coverage
| Supporting Peptides
|

Protein Coverage:

Supporting Peptides:

| Peptide | Uniq | -10lgP | Mass | Length | ppm | m/z | z | RT | Fraction | Scan | Source File | Area F6 | Area F2 | Area F3 | Area F4 | Area F5 | Area F7 | Area F8 | Area F1 | #Feature | #Feature F6 | #Feature F2 | #Feature F3 | #Feature F4 | #Feature F5 | #Feature F7 | #Feature F8 | #Feature F1 | Start | End | PTM | AScore | Found By |
| --- | --- | --- | --- | --- | --- | --- | --- | --- | --- | --- | --- | --- | --- | --- | --- | --- | --- | --- | --- | --- | --- | --- | --- | --- | --- | --- | --- | --- | --- | --- | --- | --- | --- |
| R.DAVLLVFANK.Q | Y | 52.71 | 1088.6229 | 10 | 0.3 | 545.3189 | 2 | 53.49 | 8 | F8:31406 | 31122021\_RID\_3262\_1b.raw |  |  |  |  |  |  |  | 1.1916E4 | 1 | 0 | 0 | 0 | 0 | 0 | 0 | 0 | 1 | 118 | 127 |  |  | PEAKS DB |
| total 1 peptides |  |  |  |  |  |  |  |  |  |  |  |  |  |  |  |  |  |  |  |  |  |  |  |  |  |  |  |  |  |  |  |  |  |
| --- | --- | --- | --- | --- | --- | --- | --- | --- | --- | --- | --- | --- | --- | --- | --- | --- | --- | --- | --- | --- | --- | --- | --- | --- | --- | --- | --- | --- | --- | --- | --- | --- | --- |

JAG45931.1

back to list

  

| Protein Coverage
| Supporting Peptides
|

Protein Coverage:

Supporting Peptides:

| Peptide | Uniq | -10lgP | Mass | Length | ppm | m/z | z | RT | Fraction | Scan | Source File | Area F6 | Area F2 | Area F3 | Area F4 | Area F5 | Area F7 | Area F8 | Area F1 | #Feature | #Feature F6 | #Feature F2 | #Feature F3 | #Feature F4 | #Feature F5 | #Feature F7 | #Feature F8 | #Feature F1 | Start | End | PTM | AScore | Found By |
| --- | --- | --- | --- | --- | --- | --- | --- | --- | --- | --- | --- | --- | --- | --- | --- | --- | --- | --- | --- | --- | --- | --- | --- | --- | --- | --- | --- | --- | --- | --- | --- | --- | --- |
| R.DAVLLVFANK.Q | Y | 52.71 | 1088.6229 | 10 | 0.3 | 545.3189 | 2 | 53.49 | 8 | F8:31406 | 31122021\_RID\_3262\_1b.raw |  |  |  |  |  |  |  | 1.1916E4 | 1 | 0 | 0 | 0 | 0 | 0 | 0 | 0 | 1 | 118 | 127 |  |  | PEAKS DB |
| total 1 peptides |  |  |  |  |  |  |  |  |  |  |  |  |  |  |  |  |  |  |  |  |  |  |  |  |  |  |  |  |  |  |  |  |  |
| --- | --- | --- | --- | --- | --- | --- | --- | --- | --- | --- | --- | --- | --- | --- | --- | --- | --- | --- | --- | --- | --- | --- | --- | --- | --- | --- | --- | --- | --- | --- | --- | --- | --- |

AFJ49358.1

back to list

  

| Protein Coverage
| Supporting Peptides
|

Protein Coverage:

Supporting Peptides:

| Peptide | Uniq | -10lgP | Mass | Length | ppm | m/z | z | RT | Fraction | Scan | Source File | Area F6 | Area F2 | Area F3 | Area F4 | Area F5 | Area F7 | Area F8 | Area F1 | #Feature | #Feature F6 | #Feature F2 | #Feature F3 | #Feature F4 | #Feature F5 | #Feature F7 | #Feature F8 | #Feature F1 | Start | End | PTM | AScore | Found By |
| --- | --- | --- | --- | --- | --- | --- | --- | --- | --- | --- | --- | --- | --- | --- | --- | --- | --- | --- | --- | --- | --- | --- | --- | --- | --- | --- | --- | --- | --- | --- | --- | --- | --- |
| R.DAVLLVFANK.Q | Y | 52.71 | 1088.6229 | 10 | 0.3 | 545.3189 | 2 | 53.49 | 8 | F8:31406 | 31122021\_RID\_3262\_1b.raw |  |  |  |  |  |  |  | 1.1916E4 | 1 | 0 | 0 | 0 | 0 | 0 | 0 | 0 | 1 | 118 | 127 |  |  | PEAKS DB |
| total 1 peptides |  |  |  |  |  |  |  |  |  |  |  |  |  |  |  |  |  |  |  |  |  |  |  |  |  |  |  |  |  |  |  |  |  |
| --- | --- | --- | --- | --- | --- | --- | --- | --- | --- | --- | --- | --- | --- | --- | --- | --- | --- | --- | --- | --- | --- | --- | --- | --- | --- | --- | --- | --- | --- | --- | --- | --- | --- |

JAA97845.1

back to list

  

| Protein Coverage
| Supporting Peptides
|

Protein Coverage:

Supporting Peptides:

| Peptide | Uniq | -10lgP | Mass | Length | ppm | m/z | z | RT | Fraction | Scan | Source File | Area F6 | Area F2 | Area F3 | Area F4 | Area F5 | Area F7 | Area F8 | Area F1 | #Feature | #Feature F6 | #Feature F2 | #Feature F3 | #Feature F4 | #Feature F5 | #Feature F7 | #Feature F8 | #Feature F1 | Start | End | PTM | AScore | Found By |
| --- | --- | --- | --- | --- | --- | --- | --- | --- | --- | --- | --- | --- | --- | --- | --- | --- | --- | --- | --- | --- | --- | --- | --- | --- | --- | --- | --- | --- | --- | --- | --- | --- | --- |
| R.DAVLLVFANK.Q | Y | 52.71 | 1088.6229 | 10 | 0.3 | 545.3189 | 2 | 53.49 | 8 | F8:31406 | 31122021\_RID\_3262\_1b.raw |  |  |  |  |  |  |  | 1.1916E4 | 1 | 0 | 0 | 0 | 0 | 0 | 0 | 0 | 1 | 118 | 127 |  |  | PEAKS DB |
| total 1 peptides |  |  |  |  |  |  |  |  |  |  |  |  |  |  |  |  |  |  |  |  |  |  |  |  |  |  |  |  |  |  |  |  |  |
| --- | --- | --- | --- | --- | --- | --- | --- | --- | --- | --- | --- | --- | --- | --- | --- | --- | --- | --- | --- | --- | --- | --- | --- | --- | --- | --- | --- | --- | --- | --- | --- | --- | --- |

JAC96307.1

back to list

  

| Protein Coverage
| Supporting Peptides
|

Protein Coverage:

Supporting Peptides:

| Peptide | Uniq | -10lgP | Mass | Length | ppm | m/z | z | RT | Fraction | Scan | Source File | Area F6 | Area F2 | Area F3 | Area F4 | Area F5 | Area F7 | Area F8 | Area F1 | #Feature | #Feature F6 | #Feature F2 | #Feature F3 | #Feature F4 | #Feature F5 | #Feature F7 | #Feature F8 | #Feature F1 | Start | End | PTM | AScore | Found By |
| --- | --- | --- | --- | --- | --- | --- | --- | --- | --- | --- | --- | --- | --- | --- | --- | --- | --- | --- | --- | --- | --- | --- | --- | --- | --- | --- | --- | --- | --- | --- | --- | --- | --- |
| R.DAVLLVFANK.Q | Y | 52.71 | 1088.6229 | 10 | 0.3 | 545.3189 | 2 | 53.49 | 8 | F8:31406 | 31122021\_RID\_3262\_1b.raw |  |  |  |  |  |  |  | 1.1916E4 | 1 | 0 | 0 | 0 | 0 | 0 | 0 | 0 | 1 | 118 | 127 |  |  | PEAKS DB |
| total 1 peptides |  |  |  |  |  |  |  |  |  |  |  |  |  |  |  |  |  |  |  |  |  |  |  |  |  |  |  |  |  |  |  |  |  |
| --- | --- | --- | --- | --- | --- | --- | --- | --- | --- | --- | --- | --- | --- | --- | --- | --- | --- | --- | --- | --- | --- | --- | --- | --- | --- | --- | --- | --- | --- | --- | --- | --- | --- |

ETE72302.1

back to list

  

| Protein Coverage
| Supporting Peptides
|

Protein Coverage:

Supporting Peptides:

| Peptide | Uniq | -10lgP | Mass | Length | ppm | m/z | z | RT | Fraction | Scan | Source File | Area F6 | Area F2 | Area F3 | Area F4 | Area F5 | Area F7 | Area F8 | Area F1 | #Feature | #Feature F6 | #Feature F2 | #Feature F3 | #Feature F4 | #Feature F5 | #Feature F7 | #Feature F8 | #Feature F1 | Start | End | PTM | AScore | Found By |
| --- | --- | --- | --- | --- | --- | --- | --- | --- | --- | --- | --- | --- | --- | --- | --- | --- | --- | --- | --- | --- | --- | --- | --- | --- | --- | --- | --- | --- | --- | --- | --- | --- | --- |
| R.DAVLLVFANK.Q | Y | 52.71 | 1088.6229 | 10 | 0.3 | 545.3189 | 2 | 53.49 | 8 | F8:31406 | 31122021\_RID\_3262\_1b.raw |  |  |  |  |  |  |  | 1.1916E4 | 1 | 0 | 0 | 0 | 0 | 0 | 0 | 0 | 1 | 118 | 127 |  |  | PEAKS DB |
| total 1 peptides |  |  |  |  |  |  |  |  |  |  |  |  |  |  |  |  |  |  |  |  |  |  |  |  |  |  |  |  |  |  |  |  |  |
| --- | --- | --- | --- | --- | --- | --- | --- | --- | --- | --- | --- | --- | --- | --- | --- | --- | --- | --- | --- | --- | --- | --- | --- | --- | --- | --- | --- | --- | --- | --- | --- | --- | --- |

AFJ49361.1

back to list

  

| Protein Coverage
| Supporting Peptides
|

Protein Coverage:

Supporting Peptides:

| Peptide | Uniq | -10lgP | Mass | Length | ppm | m/z | z | RT | Fraction | Scan | Source File | Area F6 | Area F2 | Area F3 | Area F4 | Area F5 | Area F7 | Area F8 | Area F1 | #Feature | #Feature F6 | #Feature F2 | #Feature F3 | #Feature F4 | #Feature F5 | #Feature F7 | #Feature F8 | #Feature F1 | Start | End | PTM | AScore | Found By |
| --- | --- | --- | --- | --- | --- | --- | --- | --- | --- | --- | --- | --- | --- | --- | --- | --- | --- | --- | --- | --- | --- | --- | --- | --- | --- | --- | --- | --- | --- | --- | --- | --- | --- |
| R.DAVLLVFANK.Q | Y | 52.71 | 1088.6229 | 10 | 0.3 | 545.3189 | 2 | 53.49 | 8 | F8:31406 | 31122021\_RID\_3262\_1b.raw |  |  |  |  |  |  |  | 1.1916E4 | 1 | 0 | 0 | 0 | 0 | 0 | 0 | 0 | 1 | 118 | 127 |  |  | PEAKS DB |
| total 1 peptides |  |  |  |  |  |  |  |  |  |  |  |  |  |  |  |  |  |  |  |  |  |  |  |  |  |  |  |  |  |  |  |  |  |
| --- | --- | --- | --- | --- | --- | --- | --- | --- | --- | --- | --- | --- | --- | --- | --- | --- | --- | --- | --- | --- | --- | --- | --- | --- | --- | --- | --- | --- | --- | --- | --- | --- | --- |

XP\_013911895.1

back to list

  

| Protein Coverage
| Supporting Peptides
|

Protein Coverage:

Supporting Peptides:

| Peptide | Uniq | -10lgP | Mass | Length | ppm | m/z | z | RT | Fraction | Scan | Source File | Area F6 | Area F2 | Area F3 | Area F4 | Area F5 | Area F7 | Area F8 | Area F1 | #Feature | #Feature F6 | #Feature F2 | #Feature F3 | #Feature F4 | #Feature F5 | #Feature F7 | #Feature F8 | #Feature F1 | Start | End | PTM | AScore | Found By |
| --- | --- | --- | --- | --- | --- | --- | --- | --- | --- | --- | --- | --- | --- | --- | --- | --- | --- | --- | --- | --- | --- | --- | --- | --- | --- | --- | --- | --- | --- | --- | --- | --- | --- |
| R.DAVLLVFANK.Q | Y | 52.71 | 1088.6229 | 10 | 0.3 | 545.3189 | 2 | 53.49 | 8 | F8:31406 | 31122021\_RID\_3262\_1b.raw |  |  |  |  |  |  |  | 1.1916E4 | 1 | 0 | 0 | 0 | 0 | 0 | 0 | 0 | 1 | 118 | 127 |  |  | PEAKS DB |
| total 1 peptides |  |  |  |  |  |  |  |  |  |  |  |  |  |  |  |  |  |  |  |  |  |  |  |  |  |  |  |  |  |  |  |  |  |
| --- | --- | --- | --- | --- | --- | --- | --- | --- | --- | --- | --- | --- | --- | --- | --- | --- | --- | --- | --- | --- | --- | --- | --- | --- | --- | --- | --- | --- | --- | --- | --- | --- | --- |

JAG68788.1

back to list

  

| Protein Coverage
| Supporting Peptides
|

Protein Coverage:

Supporting Peptides:

| Peptide | Uniq | -10lgP | Mass | Length | ppm | m/z | z | RT | Fraction | Scan | Source File | Area F6 | Area F2 | Area F3 | Area F4 | Area F5 | Area F7 | Area F8 | Area F1 | #Feature | #Feature F6 | #Feature F2 | #Feature F3 | #Feature F4 | #Feature F5 | #Feature F7 | #Feature F8 | #Feature F1 | Start | End | PTM | AScore | Found By |
| --- | --- | --- | --- | --- | --- | --- | --- | --- | --- | --- | --- | --- | --- | --- | --- | --- | --- | --- | --- | --- | --- | --- | --- | --- | --- | --- | --- | --- | --- | --- | --- | --- | --- |
| R.DAVLLVFANK.Q | Y | 52.71 | 1088.6229 | 10 | 0.3 | 545.3189 | 2 | 53.49 | 8 | F8:31406 | 31122021\_RID\_3262\_1b.raw |  |  |  |  |  |  |  | 1.1916E4 | 1 | 0 | 0 | 0 | 0 | 0 | 0 | 0 | 1 | 118 | 127 |  |  | PEAKS DB |
| total 1 peptides |  |  |  |  |  |  |  |  |  |  |  |  |  |  |  |  |  |  |  |  |  |  |  |  |  |  |  |  |  |  |  |  |  |
| --- | --- | --- | --- | --- | --- | --- | --- | --- | --- | --- | --- | --- | --- | --- | --- | --- | --- | --- | --- | --- | --- | --- | --- | --- | --- | --- | --- | --- | --- | --- | --- | --- | --- |

JAG45930.1

back to list

  

| Protein Coverage
| Supporting Peptides
|

Protein Coverage:

Supporting Peptides:

| Peptide | Uniq | -10lgP | Mass | Length | ppm | m/z | z | RT | Fraction | Scan | Source File | Area F6 | Area F2 | Area F3 | Area F4 | Area F5 | Area F7 | Area F8 | Area F1 | #Feature | #Feature F6 | #Feature F2 | #Feature F3 | #Feature F4 | #Feature F5 | #Feature F7 | #Feature F8 | #Feature F1 | Start | End | PTM | AScore | Found By |
| --- | --- | --- | --- | --- | --- | --- | --- | --- | --- | --- | --- | --- | --- | --- | --- | --- | --- | --- | --- | --- | --- | --- | --- | --- | --- | --- | --- | --- | --- | --- | --- | --- | --- |
| R.DAVLLVFANK.Q | Y | 52.71 | 1088.6229 | 10 | 0.3 | 545.3189 | 2 | 53.49 | 8 | F8:31406 | 31122021\_RID\_3262\_1b.raw |  |  |  |  |  |  |  | 1.1916E4 | 1 | 0 | 0 | 0 | 0 | 0 | 0 | 0 | 1 | 118 | 127 |  |  | PEAKS DB |
| total 1 peptides |  |  |  |  |  |  |  |  |  |  |  |  |  |  |  |  |  |  |  |  |  |  |  |  |  |  |  |  |  |  |  |  |  |
| --- | --- | --- | --- | --- | --- | --- | --- | --- | --- | --- | --- | --- | --- | --- | --- | --- | --- | --- | --- | --- | --- | --- | --- | --- | --- | --- | --- | --- | --- | --- | --- | --- | --- |

JAC96305.1

back to list

  

| Protein Coverage
| Supporting Peptides
|

Protein Coverage:

Supporting Peptides:

| Peptide | Uniq | -10lgP | Mass | Length | ppm | m/z | z | RT | Fraction | Scan | Source File | Area F6 | Area F2 | Area F3 | Area F4 | Area F5 | Area F7 | Area F8 | Area F1 | #Feature | #Feature F6 | #Feature F2 | #Feature F3 | #Feature F4 | #Feature F5 | #Feature F7 | #Feature F8 | #Feature F1 | Start | End | PTM | AScore | Found By |
| --- | --- | --- | --- | --- | --- | --- | --- | --- | --- | --- | --- | --- | --- | --- | --- | --- | --- | --- | --- | --- | --- | --- | --- | --- | --- | --- | --- | --- | --- | --- | --- | --- | --- |
| R.DAVLLVFANK.Q | Y | 52.71 | 1088.6229 | 10 | 0.3 | 545.3189 | 2 | 53.49 | 8 | F8:31406 | 31122021\_RID\_3262\_1b.raw |  |  |  |  |  |  |  | 1.1916E4 | 1 | 0 | 0 | 0 | 0 | 0 | 0 | 0 | 1 | 118 | 127 |  |  | PEAKS DB |
| total 1 peptides |  |  |  |  |  |  |  |  |  |  |  |  |  |  |  |  |  |  |  |  |  |  |  |  |  |  |  |  |  |  |  |  |  |
| --- | --- | --- | --- | --- | --- | --- | --- | --- | --- | --- | --- | --- | --- | --- | --- | --- | --- | --- | --- | --- | --- | --- | --- | --- | --- | --- | --- | --- | --- | --- | --- | --- | --- |

JAA97840.1

back to list

  

| Protein Coverage
| Supporting Peptides
|

Protein Coverage:

Supporting Peptides:

| Peptide | Uniq | -10lgP | Mass | Length | ppm | m/z | z | RT | Fraction | Scan | Source File | Area F6 | Area F2 | Area F3 | Area F4 | Area F5 | Area F7 | Area F8 | Area F1 | #Feature | #Feature F6 | #Feature F2 | #Feature F3 | #Feature F4 | #Feature F5 | #Feature F7 | #Feature F8 | #Feature F1 | Start | End | PTM | AScore | Found By |
| --- | --- | --- | --- | --- | --- | --- | --- | --- | --- | --- | --- | --- | --- | --- | --- | --- | --- | --- | --- | --- | --- | --- | --- | --- | --- | --- | --- | --- | --- | --- | --- | --- | --- |
| R.DAVLLVFANK.Q | Y | 52.71 | 1088.6229 | 10 | 0.3 | 545.3189 | 2 | 53.49 | 8 | F8:31406 | 31122021\_RID\_3262\_1b.raw |  |  |  |  |  |  |  | 1.1916E4 | 1 | 0 | 0 | 0 | 0 | 0 | 0 | 0 | 1 | 118 | 127 |  |  | PEAKS DB |
| total 1 peptides |  |  |  |  |  |  |  |  |  |  |  |  |  |  |  |  |  |  |  |  |  |  |  |  |  |  |  |  |  |  |  |  |  |
| --- | --- | --- | --- | --- | --- | --- | --- | --- | --- | --- | --- | --- | --- | --- | --- | --- | --- | --- | --- | --- | --- | --- | --- | --- | --- | --- | --- | --- | --- | --- | --- | --- | --- |

ETE74069.1

back to list

  

| Protein Coverage
| Supporting Peptides
|

Protein Coverage:

Supporting Peptides:

| Peptide | Uniq | -10lgP | Mass | Length | ppm | m/z | z | RT | Fraction | Scan | Source File | Area F6 | Area F2 | Area F3 | Area F4 | Area F5 | Area F7 | Area F8 | Area F1 | #Feature | #Feature F6 | #Feature F2 | #Feature F3 | #Feature F4 | #Feature F5 | #Feature F7 | #Feature F8 | #Feature F1 | Start | End | PTM | AScore | Found By |
| --- | --- | --- | --- | --- | --- | --- | --- | --- | --- | --- | --- | --- | --- | --- | --- | --- | --- | --- | --- | --- | --- | --- | --- | --- | --- | --- | --- | --- | --- | --- | --- | --- | --- |
| R.DAVLLVFANK.Q | Y | 52.71 | 1088.6229 | 10 | 0.3 | 545.3189 | 2 | 53.49 | 8 | F8:31406 | 31122021\_RID\_3262\_1b.raw |  |  |  |  |  |  |  | 1.1916E4 | 1 | 0 | 0 | 0 | 0 | 0 | 0 | 0 | 1 | 118 | 127 |  |  | PEAKS DB |
| total 1 peptides |  |  |  |  |  |  |  |  |  |  |  |  |  |  |  |  |  |  |  |  |  |  |  |  |  |  |  |  |  |  |  |  |  |
| --- | --- | --- | --- | --- | --- | --- | --- | --- | --- | --- | --- | --- | --- | --- | --- | --- | --- | --- | --- | --- | --- | --- | --- | --- | --- | --- | --- | --- | --- | --- | --- | --- | --- |

JAG47534.1

back to list

  

| Protein Coverage
| Supporting Peptides
|

Protein Coverage:

Supporting Peptides:

| Peptide | Uniq | -10lgP | Mass | Length | ppm | m/z | z | RT | Fraction | Scan | Source File | Area F6 | Area F2 | Area F3 | Area F4 | Area F5 | Area F7 | Area F8 | Area F1 | #Feature | #Feature F6 | #Feature F2 | #Feature F3 | #Feature F4 | #Feature F5 | #Feature F7 | #Feature F8 | #Feature F1 | Start | End | PTM | AScore | Found By |
| --- | --- | --- | --- | --- | --- | --- | --- | --- | --- | --- | --- | --- | --- | --- | --- | --- | --- | --- | --- | --- | --- | --- | --- | --- | --- | --- | --- | --- | --- | --- | --- | --- | --- |
| R.DAVLLVFANK.Q | Y | 52.71 | 1088.6229 | 10 | 0.3 | 545.3189 | 2 | 53.49 | 8 | F8:31406 | 31122021\_RID\_3262\_1b.raw |  |  |  |  |  |  |  | 1.1916E4 | 1 | 0 | 0 | 0 | 0 | 0 | 0 | 0 | 1 | 118 | 127 |  |  | PEAKS DB |
| total 1 peptides |  |  |  |  |  |  |  |  |  |  |  |  |  |  |  |  |  |  |  |  |  |  |  |  |  |  |  |  |  |  |  |  |  |
| --- | --- | --- | --- | --- | --- | --- | --- | --- | --- | --- | --- | --- | --- | --- | --- | --- | --- | --- | --- | --- | --- | --- | --- | --- | --- | --- | --- | --- | --- | --- | --- | --- | --- |

JAI14426.1

back to list

  

| Protein Coverage
| Supporting Peptides
|

Protein Coverage:

Supporting Peptides:

| Peptide | Uniq | -10lgP | Mass | Length | ppm | m/z | z | RT | Fraction | Scan | Source File | Area F6 | Area F2 | Area F3 | Area F4 | Area F5 | Area F7 | Area F8 | Area F1 | #Feature | #Feature F6 | #Feature F2 | #Feature F3 | #Feature F4 | #Feature F5 | #Feature F7 | #Feature F8 | #Feature F1 | Start | End | PTM | AScore | Found By |
| --- | --- | --- | --- | --- | --- | --- | --- | --- | --- | --- | --- | --- | --- | --- | --- | --- | --- | --- | --- | --- | --- | --- | --- | --- | --- | --- | --- | --- | --- | --- | --- | --- | --- |
| R.DAVLLVFANK.Q | Y | 52.71 | 1088.6229 | 10 | 0.3 | 545.3189 | 2 | 53.49 | 8 | F8:31406 | 31122021\_RID\_3262\_1b.raw |  |  |  |  |  |  |  | 1.1916E4 | 1 | 0 | 0 | 0 | 0 | 0 | 0 | 0 | 1 | 118 | 127 |  |  | PEAKS DB |
| total 1 peptides |  |  |  |  |  |  |  |  |  |  |  |  |  |  |  |  |  |  |  |  |  |  |  |  |  |  |  |  |  |  |  |  |  |
| --- | --- | --- | --- | --- | --- | --- | --- | --- | --- | --- | --- | --- | --- | --- | --- | --- | --- | --- | --- | --- | --- | --- | --- | --- | --- | --- | --- | --- | --- | --- | --- | --- | --- |

JAB54737.1

back to list

  

| Protein Coverage
| Supporting Peptides
|

Protein Coverage:

Supporting Peptides:

| Peptide | Uniq | -10lgP | Mass | Length | ppm | m/z | z | RT | Fraction | Scan | Source File | Area F6 | Area F2 | Area F3 | Area F4 | Area F5 | Area F7 | Area F8 | Area F1 | #Feature | #Feature F6 | #Feature F2 | #Feature F3 | #Feature F4 | #Feature F5 | #Feature F7 | #Feature F8 | #Feature F1 | Start | End | PTM | AScore | Found By |
| --- | --- | --- | --- | --- | --- | --- | --- | --- | --- | --- | --- | --- | --- | --- | --- | --- | --- | --- | --- | --- | --- | --- | --- | --- | --- | --- | --- | --- | --- | --- | --- | --- | --- |
| R.DAVLLVFANK.Q | Y | 52.71 | 1088.6229 | 10 | 0.3 | 545.3189 | 2 | 53.49 | 8 | F8:31406 | 31122021\_RID\_3262\_1b.raw |  |  |  |  |  |  |  | 1.1916E4 | 1 | 0 | 0 | 0 | 0 | 0 | 0 | 0 | 1 | 118 | 127 |  |  | PEAKS DB |
| total 1 peptides |  |  |  |  |  |  |  |  |  |  |  |  |  |  |  |  |  |  |  |  |  |  |  |  |  |  |  |  |  |  |  |  |  |
| --- | --- | --- | --- | --- | --- | --- | --- | --- | --- | --- | --- | --- | --- | --- | --- | --- | --- | --- | --- | --- | --- | --- | --- | --- | --- | --- | --- | --- | --- | --- | --- | --- | --- |

JAA97844.1

back to list

  

| Protein Coverage
| Supporting Peptides
|

Protein Coverage:

Supporting Peptides:

| Peptide | Uniq | -10lgP | Mass | Length | ppm | m/z | z | RT | Fraction | Scan | Source File | Area F6 | Area F2 | Area F3 | Area F4 | Area F5 | Area F7 | Area F8 | Area F1 | #Feature | #Feature F6 | #Feature F2 | #Feature F3 | #Feature F4 | #Feature F5 | #Feature F7 | #Feature F8 | #Feature F1 | Start | End | PTM | AScore | Found By |
| --- | --- | --- | --- | --- | --- | --- | --- | --- | --- | --- | --- | --- | --- | --- | --- | --- | --- | --- | --- | --- | --- | --- | --- | --- | --- | --- | --- | --- | --- | --- | --- | --- | --- |
| R.DAVLLVFANK.Q | Y | 52.71 | 1088.6229 | 10 | 0.3 | 545.3189 | 2 | 53.49 | 8 | F8:31406 | 31122021\_RID\_3262\_1b.raw |  |  |  |  |  |  |  | 1.1916E4 | 1 | 0 | 0 | 0 | 0 | 0 | 0 | 0 | 1 | 118 | 127 |  |  | PEAKS DB |
| total 1 peptides |  |  |  |  |  |  |  |  |  |  |  |  |  |  |  |  |  |  |  |  |  |  |  |  |  |  |  |  |  |  |  |  |  |
| --- | --- | --- | --- | --- | --- | --- | --- | --- | --- | --- | --- | --- | --- | --- | --- | --- | --- | --- | --- | --- | --- | --- | --- | --- | --- | --- | --- | --- | --- | --- | --- | --- | --- |

XP\_013922416.1

back to list

  

| Protein Coverage
| Supporting Peptides
|

Protein Coverage:

Supporting Peptides:

| Peptide | Uniq | -10lgP | Mass | Length | ppm | m/z | z | RT | Fraction | Scan | Source File | Area F6 | Area F2 | Area F3 | Area F4 | Area F5 | Area F7 | Area F8 | Area F1 | #Feature | #Feature F6 | #Feature F2 | #Feature F3 | #Feature F4 | #Feature F5 | #Feature F7 | #Feature F8 | #Feature F1 | Start | End | PTM | AScore | Found By |
| --- | --- | --- | --- | --- | --- | --- | --- | --- | --- | --- | --- | --- | --- | --- | --- | --- | --- | --- | --- | --- | --- | --- | --- | --- | --- | --- | --- | --- | --- | --- | --- | --- | --- |
| R.DAVLLVFANK.Q | Y | 52.71 | 1088.6229 | 10 | 0.3 | 545.3189 | 2 | 53.49 | 8 | F8:31406 | 31122021\_RID\_3262\_1b.raw |  |  |  |  |  |  |  | 1.1916E4 | 1 | 0 | 0 | 0 | 0 | 0 | 0 | 0 | 1 | 118 | 127 |  |  | PEAKS DB |
| total 1 peptides |  |  |  |  |  |  |  |  |  |  |  |  |  |  |  |  |  |  |  |  |  |  |  |  |  |  |  |  |  |  |  |  |  |
| --- | --- | --- | --- | --- | --- | --- | --- | --- | --- | --- | --- | --- | --- | --- | --- | --- | --- | --- | --- | --- | --- | --- | --- | --- | --- | --- | --- | --- | --- | --- | --- | --- | --- |

JAI14429.1

back to list

  

| Protein Coverage
| Supporting Peptides
|

Protein Coverage:

Supporting Peptides:

| Peptide | Uniq | -10lgP | Mass | Length | ppm | m/z | z | RT | Fraction | Scan | Source File | Area F6 | Area F2 | Area F3 | Area F4 | Area F5 | Area F7 | Area F8 | Area F1 | #Feature | #Feature F6 | #Feature F2 | #Feature F3 | #Feature F4 | #Feature F5 | #Feature F7 | #Feature F8 | #Feature F1 | Start | End | PTM | AScore | Found By |
| --- | --- | --- | --- | --- | --- | --- | --- | --- | --- | --- | --- | --- | --- | --- | --- | --- | --- | --- | --- | --- | --- | --- | --- | --- | --- | --- | --- | --- | --- | --- | --- | --- | --- |
| R.DAVLLVFANK.Q | Y | 52.71 | 1088.6229 | 10 | 0.3 | 545.3189 | 2 | 53.49 | 8 | F8:31406 | 31122021\_RID\_3262\_1b.raw |  |  |  |  |  |  |  | 1.1916E4 | 1 | 0 | 0 | 0 | 0 | 0 | 0 | 0 | 1 | 118 | 127 |  |  | PEAKS DB |
| total 1 peptides |  |  |  |  |  |  |  |  |  |  |  |  |  |  |  |  |  |  |  |  |  |  |  |  |  |  |  |  |  |  |  |  |  |
| --- | --- | --- | --- | --- | --- | --- | --- | --- | --- | --- | --- | --- | --- | --- | --- | --- | --- | --- | --- | --- | --- | --- | --- | --- | --- | --- | --- | --- | --- | --- | --- | --- | --- |

JAI10315.1

back to list

  

| Protein Coverage
| Supporting Peptides
|

Protein Coverage:

Supporting Peptides:

| Peptide | Uniq | -10lgP | Mass | Length | ppm | m/z | z | RT | Fraction | Scan | Source File | Area F6 | Area F2 | Area F3 | Area F4 | Area F5 | Area F7 | Area F8 | Area F1 | #Feature | #Feature F6 | #Feature F2 | #Feature F3 | #Feature F4 | #Feature F5 | #Feature F7 | #Feature F8 | #Feature F1 | Start | End | PTM | AScore | Found By |
| --- | --- | --- | --- | --- | --- | --- | --- | --- | --- | --- | --- | --- | --- | --- | --- | --- | --- | --- | --- | --- | --- | --- | --- | --- | --- | --- | --- | --- | --- | --- | --- | --- | --- |
| R.DAVLLVFANK.Q | Y | 52.71 | 1088.6229 | 10 | 0.3 | 545.3189 | 2 | 53.49 | 8 | F8:31406 | 31122021\_RID\_3262\_1b.raw |  |  |  |  |  |  |  | 1.1916E4 | 1 | 0 | 0 | 0 | 0 | 0 | 0 | 0 | 1 | 118 | 127 |  |  | PEAKS DB |
| total 1 peptides |  |  |  |  |  |  |  |  |  |  |  |  |  |  |  |  |  |  |  |  |  |  |  |  |  |  |  |  |  |  |  |  |  |
| --- | --- | --- | --- | --- | --- | --- | --- | --- | --- | --- | --- | --- | --- | --- | --- | --- | --- | --- | --- | --- | --- | --- | --- | --- | --- | --- | --- | --- | --- | --- | --- | --- | --- |

JAG45927.1

back to list

  

| Protein Coverage
| Supporting Peptides
|

Protein Coverage:

Supporting Peptides:

| Peptide | Uniq | -10lgP | Mass | Length | ppm | m/z | z | RT | Fraction | Scan | Source File | Area F6 | Area F2 | Area F3 | Area F4 | Area F5 | Area F7 | Area F8 | Area F1 | #Feature | #Feature F6 | #Feature F2 | #Feature F3 | #Feature F4 | #Feature F5 | #Feature F7 | #Feature F8 | #Feature F1 | Start | End | PTM | AScore | Found By |
| --- | --- | --- | --- | --- | --- | --- | --- | --- | --- | --- | --- | --- | --- | --- | --- | --- | --- | --- | --- | --- | --- | --- | --- | --- | --- | --- | --- | --- | --- | --- | --- | --- | --- |
| R.DAVLLVFANK.Q | Y | 52.71 | 1088.6229 | 10 | 0.3 | 545.3189 | 2 | 53.49 | 8 | F8:31406 | 31122021\_RID\_3262\_1b.raw |  |  |  |  |  |  |  | 1.1916E4 | 1 | 0 | 0 | 0 | 0 | 0 | 0 | 0 | 1 | 118 | 127 |  |  | PEAKS DB |
| total 1 peptides |  |  |  |  |  |  |  |  |  |  |  |  |  |  |  |  |  |  |  |  |  |  |  |  |  |  |  |  |  |  |  |  |  |
| --- | --- | --- | --- | --- | --- | --- | --- | --- | --- | --- | --- | --- | --- | --- | --- | --- | --- | --- | --- | --- | --- | --- | --- | --- | --- | --- | --- | --- | --- | --- | --- | --- | --- |

JAV51321.1

back to list

  

| Protein Coverage
| Supporting Peptides
|

Protein Coverage:

Supporting Peptides:

| Peptide | Uniq | -10lgP | Mass | Length | ppm | m/z | z | RT | Fraction | Scan | Source File | Area F6 | Area F2 | Area F3 | Area F4 | Area F5 | Area F7 | Area F8 | Area F1 | #Feature | #Feature F6 | #Feature F2 | #Feature F3 | #Feature F4 | #Feature F5 | #Feature F7 | #Feature F8 | #Feature F1 | Start | End | PTM | AScore | Found By |
| --- | --- | --- | --- | --- | --- | --- | --- | --- | --- | --- | --- | --- | --- | --- | --- | --- | --- | --- | --- | --- | --- | --- | --- | --- | --- | --- | --- | --- | --- | --- | --- | --- | --- |
| R.DAVLLVFANK.Q | Y | 52.71 | 1088.6229 | 10 | 0.3 | 545.3189 | 2 | 53.49 | 8 | F8:31406 | 31122021\_RID\_3262\_1b.raw |  |  |  |  |  |  |  | 1.1916E4 | 1 | 0 | 0 | 0 | 0 | 0 | 0 | 0 | 1 | 118 | 127 |  |  | PEAKS DB |
| total 1 peptides |  |  |  |  |  |  |  |  |  |  |  |  |  |  |  |  |  |  |  |  |  |  |  |  |  |  |  |  |  |  |  |  |  |
| --- | --- | --- | --- | --- | --- | --- | --- | --- | --- | --- | --- | --- | --- | --- | --- | --- | --- | --- | --- | --- | --- | --- | --- | --- | --- | --- | --- | --- | --- | --- | --- | --- | --- |

AFJ49360.1

back to list

  

| Protein Coverage
| Supporting Peptides
|

Protein Coverage:

Supporting Peptides:

| Peptide | Uniq | -10lgP | Mass | Length | ppm | m/z | z | RT | Fraction | Scan | Source File | Area F6 | Area F2 | Area F3 | Area F4 | Area F5 | Area F7 | Area F8 | Area F1 | #Feature | #Feature F6 | #Feature F2 | #Feature F3 | #Feature F4 | #Feature F5 | #Feature F7 | #Feature F8 | #Feature F1 | Start | End | PTM | AScore | Found By |
| --- | --- | --- | --- | --- | --- | --- | --- | --- | --- | --- | --- | --- | --- | --- | --- | --- | --- | --- | --- | --- | --- | --- | --- | --- | --- | --- | --- | --- | --- | --- | --- | --- | --- |
| R.DAVLLVFANK.Q | Y | 52.71 | 1088.6229 | 10 | 0.3 | 545.3189 | 2 | 53.49 | 8 | F8:31406 | 31122021\_RID\_3262\_1b.raw |  |  |  |  |  |  |  | 1.1916E4 | 1 | 0 | 0 | 0 | 0 | 0 | 0 | 0 | 1 | 118 | 127 |  |  | PEAKS DB |
| total 1 peptides |  |  |  |  |  |  |  |  |  |  |  |  |  |  |  |  |  |  |  |  |  |  |  |  |  |  |  |  |  |  |  |  |  |
| --- | --- | --- | --- | --- | --- | --- | --- | --- | --- | --- | --- | --- | --- | --- | --- | --- | --- | --- | --- | --- | --- | --- | --- | --- | --- | --- | --- | --- | --- | --- | --- | --- | --- |

JAA97843.1

back to list

  

| Protein Coverage
| Supporting Peptides
|

Protein Coverage:

Supporting Peptides:

| Peptide | Uniq | -10lgP | Mass | Length | ppm | m/z | z | RT | Fraction | Scan | Source File | Area F6 | Area F2 | Area F3 | Area F4 | Area F5 | Area F7 | Area F8 | Area F1 | #Feature | #Feature F6 | #Feature F2 | #Feature F3 | #Feature F4 | #Feature F5 | #Feature F7 | #Feature F8 | #Feature F1 | Start | End | PTM | AScore | Found By |
| --- | --- | --- | --- | --- | --- | --- | --- | --- | --- | --- | --- | --- | --- | --- | --- | --- | --- | --- | --- | --- | --- | --- | --- | --- | --- | --- | --- | --- | --- | --- | --- | --- | --- |
| R.DAVLLVFANK.Q | Y | 52.71 | 1088.6229 | 10 | 0.3 | 545.3189 | 2 | 53.49 | 8 | F8:31406 | 31122021\_RID\_3262\_1b.raw |  |  |  |  |  |  |  | 1.1916E4 | 1 | 0 | 0 | 0 | 0 | 0 | 0 | 0 | 1 | 118 | 127 |  |  | PEAKS DB |
| total 1 peptides |  |  |  |  |  |  |  |  |  |  |  |  |  |  |  |  |  |  |  |  |  |  |  |  |  |  |  |  |  |  |  |  |  |
| --- | --- | --- | --- | --- | --- | --- | --- | --- | --- | --- | --- | --- | --- | --- | --- | --- | --- | --- | --- | --- | --- | --- | --- | --- | --- | --- | --- | --- | --- | --- | --- | --- | --- |

JAG47537.1

back to list

  

| Protein Coverage
| Supporting Peptides
|

Protein Coverage:

Supporting Peptides:

| Peptide | Uniq | -10lgP | Mass | Length | ppm | m/z | z | RT | Fraction | Scan | Source File | Area F6 | Area F2 | Area F3 | Area F4 | Area F5 | Area F7 | Area F8 | Area F1 | #Feature | #Feature F6 | #Feature F2 | #Feature F3 | #Feature F4 | #Feature F5 | #Feature F7 | #Feature F8 | #Feature F1 | Start | End | PTM | AScore | Found By |
| --- | --- | --- | --- | --- | --- | --- | --- | --- | --- | --- | --- | --- | --- | --- | --- | --- | --- | --- | --- | --- | --- | --- | --- | --- | --- | --- | --- | --- | --- | --- | --- | --- | --- |
| R.DAVLLVFANK.Q | Y | 52.71 | 1088.6229 | 10 | 0.3 | 545.3189 | 2 | 53.49 | 8 | F8:31406 | 31122021\_RID\_3262\_1b.raw |  |  |  |  |  |  |  | 1.1916E4 | 1 | 0 | 0 | 0 | 0 | 0 | 0 | 0 | 1 | 118 | 127 |  |  | PEAKS DB |
| total 1 peptides |  |  |  |  |  |  |  |  |  |  |  |  |  |  |  |  |  |  |  |  |  |  |  |  |  |  |  |  |  |  |  |  |  |
| --- | --- | --- | --- | --- | --- | --- | --- | --- | --- | --- | --- | --- | --- | --- | --- | --- | --- | --- | --- | --- | --- | --- | --- | --- | --- | --- | --- | --- | --- | --- | --- | --- | --- |

ETE73522.1

back to list

  

| Protein Coverage
| Supporting Peptides
|

Protein Coverage:

Supporting Peptides:

| Peptide | Uniq | -10lgP | Mass | Length | ppm | m/z | z | RT | Fraction | Scan | Source File | Area F6 | Area F2 | Area F3 | Area F4 | Area F5 | Area F7 | Area F8 | Area F1 | #Feature | #Feature F6 | #Feature F2 | #Feature F3 | #Feature F4 | #Feature F5 | #Feature F7 | #Feature F8 | #Feature F1 | Start | End | PTM | AScore | Found By |
| --- | --- | --- | --- | --- | --- | --- | --- | --- | --- | --- | --- | --- | --- | --- | --- | --- | --- | --- | --- | --- | --- | --- | --- | --- | --- | --- | --- | --- | --- | --- | --- | --- | --- |
| R.VFQVEYAMK.A | Y | 51.77 | 1113.5529 | 9 | 0.2 | 557.7838 | 2 | 38.51 | 8 | F8:19620 | 31122021\_RID\_3262\_1b.raw |  |  |  |  |  |  |  | 4.84E4 | 1 | 0 | 0 | 0 | 0 | 0 | 0 | 0 | 1 | 21 | 29 |  |  | PEAKS DB |
| total 1 peptides |  |  |  |  |  |  |  |  |  |  |  |  |  |  |  |  |  |  |  |  |  |  |  |  |  |  |  |  |  |  |  |  |  |
| --- | --- | --- | --- | --- | --- | --- | --- | --- | --- | --- | --- | --- | --- | --- | --- | --- | --- | --- | --- | --- | --- | --- | --- | --- | --- | --- | --- | --- | --- | --- | --- | --- | --- |

XP\_013912175.1

back to list

  

| Protein Coverage
| Supporting Peptides
|

Protein Coverage:

Supporting Peptides:

| Peptide | Uniq | -10lgP | Mass | Length | ppm | m/z | z | RT | Fraction | Scan | Source File | Area F6 | Area F2 | Area F3 | Area F4 | Area F5 | Area F7 | Area F8 | Area F1 | #Feature | #Feature F6 | #Feature F2 | #Feature F3 | #Feature F4 | #Feature F5 | #Feature F7 | #Feature F8 | #Feature F1 | Start | End | PTM | AScore | Found By |
| --- | --- | --- | --- | --- | --- | --- | --- | --- | --- | --- | --- | --- | --- | --- | --- | --- | --- | --- | --- | --- | --- | --- | --- | --- | --- | --- | --- | --- | --- | --- | --- | --- | --- |
| R.VFQVEYAMK.A | Y | 51.77 | 1113.5529 | 9 | 0.2 | 557.7838 | 2 | 38.51 | 8 | F8:19620 | 31122021\_RID\_3262\_1b.raw |  |  |  |  |  |  |  | 4.84E4 | 1 | 0 | 0 | 0 | 0 | 0 | 0 | 0 | 1 | 21 | 29 |  |  | PEAKS DB |
| total 1 peptides |  |  |  |  |  |  |  |  |  |  |  |  |  |  |  |  |  |  |  |  |  |  |  |  |  |  |  |  |  |  |  |  |  |
| --- | --- | --- | --- | --- | --- | --- | --- | --- | --- | --- | --- | --- | --- | --- | --- | --- | --- | --- | --- | --- | --- | --- | --- | --- | --- | --- | --- | --- | --- | --- | --- | --- | --- |

JAG66919.1

back to list

  

| Protein Coverage
| Supporting Peptides
|

Protein Coverage:

Supporting Peptides:

| Peptide | Uniq | -10lgP | Mass | Length | ppm | m/z | z | RT | Fraction | Scan | Source File | Area F6 | Area F2 | Area F3 | Area F4 | Area F5 | Area F7 | Area F8 | Area F1 | #Feature | #Feature F6 | #Feature F2 | #Feature F3 | #Feature F4 | #Feature F5 | #Feature F7 | #Feature F8 | #Feature F1 | Start | End | PTM | AScore | Found By |
| --- | --- | --- | --- | --- | --- | --- | --- | --- | --- | --- | --- | --- | --- | --- | --- | --- | --- | --- | --- | --- | --- | --- | --- | --- | --- | --- | --- | --- | --- | --- | --- | --- | --- |
| R.VFQVEYAMK.A | Y | 51.77 | 1113.5529 | 9 | 0.2 | 557.7838 | 2 | 38.51 | 8 | F8:19620 | 31122021\_RID\_3262\_1b.raw |  |  |  |  |  |  |  | 4.84E4 | 1 | 0 | 0 | 0 | 0 | 0 | 0 | 0 | 1 | 21 | 29 |  |  | PEAKS DB |
| total 1 peptides |  |  |  |  |  |  |  |  |  |  |  |  |  |  |  |  |  |  |  |  |  |  |  |  |  |  |  |  |  |  |  |  |  |
| --- | --- | --- | --- | --- | --- | --- | --- | --- | --- | --- | --- | --- | --- | --- | --- | --- | --- | --- | --- | --- | --- | --- | --- | --- | --- | --- | --- | --- | --- | --- | --- | --- | --- |

XP\_032092012.1

back to list

  

| Protein Coverage
| Supporting Peptides
|

Protein Coverage:

Supporting Peptides:

| Peptide | Uniq | -10lgP | Mass | Length | ppm | m/z | z | RT | Fraction | Scan | Source File | Area F6 | Area F2 | Area F3 | Area F4 | Area F5 | Area F7 | Area F8 | Area F1 | #Feature | #Feature F6 | #Feature F2 | #Feature F3 | #Feature F4 | #Feature F5 | #Feature F7 | #Feature F8 | #Feature F1 | Start | End | PTM | AScore | Found By |
| --- | --- | --- | --- | --- | --- | --- | --- | --- | --- | --- | --- | --- | --- | --- | --- | --- | --- | --- | --- | --- | --- | --- | --- | --- | --- | --- | --- | --- | --- | --- | --- | --- | --- |
| R.VFQVEYAMK.A | Y | 51.77 | 1113.5529 | 9 | 0.2 | 557.7838 | 2 | 38.51 | 8 | F8:19620 | 31122021\_RID\_3262\_1b.raw |  |  |  |  |  |  |  | 4.84E4 | 1 | 0 | 0 | 0 | 0 | 0 | 0 | 0 | 1 | 21 | 29 |  |  | PEAKS DB |
| total 1 peptides |  |  |  |  |  |  |  |  |  |  |  |  |  |  |  |  |  |  |  |  |  |  |  |  |  |  |  |  |  |  |  |  |  |
| --- | --- | --- | --- | --- | --- | --- | --- | --- | --- | --- | --- | --- | --- | --- | --- | --- | --- | --- | --- | --- | --- | --- | --- | --- | --- | --- | --- | --- | --- | --- | --- | --- | --- |

XP\_034289368.1

back to list

  

| Protein Coverage
| Supporting Peptides
|

Protein Coverage:

Supporting Peptides:

| Peptide | Uniq | -10lgP | Mass | Length | ppm | m/z | z | RT | Fraction | Scan | Source File | Area F6 | Area F2 | Area F3 | Area F4 | Area F5 | Area F7 | Area F8 | Area F1 | #Feature | #Feature F6 | #Feature F2 | #Feature F3 | #Feature F4 | #Feature F5 | #Feature F7 | #Feature F8 | #Feature F1 | Start | End | PTM | AScore | Found By |
| --- | --- | --- | --- | --- | --- | --- | --- | --- | --- | --- | --- | --- | --- | --- | --- | --- | --- | --- | --- | --- | --- | --- | --- | --- | --- | --- | --- | --- | --- | --- | --- | --- | --- |
| R.VFQVEYAMK.A | Y | 51.77 | 1113.5529 | 9 | 0.2 | 557.7838 | 2 | 38.51 | 8 | F8:19620 | 31122021\_RID\_3262\_1b.raw |  |  |  |  |  |  |  | 4.84E4 | 1 | 0 | 0 | 0 | 0 | 0 | 0 | 0 | 1 | 21 | 29 |  |  | PEAKS DB |
| total 1 peptides |  |  |  |  |  |  |  |  |  |  |  |  |  |  |  |  |  |  |  |  |  |  |  |  |  |  |  |  |  |  |  |  |  |
| --- | --- | --- | --- | --- | --- | --- | --- | --- | --- | --- | --- | --- | --- | --- | --- | --- | --- | --- | --- | --- | --- | --- | --- | --- | --- | --- | --- | --- | --- | --- | --- | --- | --- |

XP\_026554461.1

back to list

  

| Protein Coverage
| Supporting Peptides
|

Protein Coverage:

Supporting Peptides:

| Peptide | Uniq | -10lgP | Mass | Length | ppm | m/z | z | RT | Fraction | Scan | Source File | Area F6 | Area F2 | Area F3 | Area F4 | Area F5 | Area F7 | Area F8 | Area F1 | #Feature | #Feature F6 | #Feature F2 | #Feature F3 | #Feature F4 | #Feature F5 | #Feature F7 | #Feature F8 | #Feature F1 | Start | End | PTM | AScore | Found By |
| --- | --- | --- | --- | --- | --- | --- | --- | --- | --- | --- | --- | --- | --- | --- | --- | --- | --- | --- | --- | --- | --- | --- | --- | --- | --- | --- | --- | --- | --- | --- | --- | --- | --- |
| R.VFQVEYAMK.A | Y | 51.77 | 1113.5529 | 9 | 0.2 | 557.7838 | 2 | 38.51 | 8 | F8:19620 | 31122021\_RID\_3262\_1b.raw |  |  |  |  |  |  |  | 4.84E4 | 1 | 0 | 0 | 0 | 0 | 0 | 0 | 0 | 1 | 21 | 29 |  |  | PEAKS DB |
| total 1 peptides |  |  |  |  |  |  |  |  |  |  |  |  |  |  |  |  |  |  |  |  |  |  |  |  |  |  |  |  |  |  |  |  |  |
| --- | --- | --- | --- | --- | --- | --- | --- | --- | --- | --- | --- | --- | --- | --- | --- | --- | --- | --- | --- | --- | --- | --- | --- | --- | --- | --- | --- | --- | --- | --- | --- | --- | --- |

JAI09619.1

back to list

  

| Protein Coverage
| Supporting Peptides
|

Protein Coverage:

Supporting Peptides:

| Peptide | Uniq | -10lgP | Mass | Length | ppm | m/z | z | RT | Fraction | Scan | Source File | Area F6 | Area F2 | Area F3 | Area F4 | Area F5 | Area F7 | Area F8 | Area F1 | #Feature | #Feature F6 | #Feature F2 | #Feature F3 | #Feature F4 | #Feature F5 | #Feature F7 | #Feature F8 | #Feature F1 | Start | End | PTM | AScore | Found By |
| --- | --- | --- | --- | --- | --- | --- | --- | --- | --- | --- | --- | --- | --- | --- | --- | --- | --- | --- | --- | --- | --- | --- | --- | --- | --- | --- | --- | --- | --- | --- | --- | --- | --- |
| R.VFQVEYAMK.A | Y | 51.77 | 1113.5529 | 9 | 0.2 | 557.7838 | 2 | 38.51 | 8 | F8:19620 | 31122021\_RID\_3262\_1b.raw |  |  |  |  |  |  |  | 4.84E4 | 1 | 0 | 0 | 0 | 0 | 0 | 0 | 0 | 1 | 21 | 29 |  |  | PEAKS DB |
| total 1 peptides |  |  |  |  |  |  |  |  |  |  |  |  |  |  |  |  |  |  |  |  |  |  |  |  |  |  |  |  |  |  |  |  |  |
| --- | --- | --- | --- | --- | --- | --- | --- | --- | --- | --- | --- | --- | --- | --- | --- | --- | --- | --- | --- | --- | --- | --- | --- | --- | --- | --- | --- | --- | --- | --- | --- | --- | --- |

XP\_007422519.1

back to list

  

| Protein Coverage
| Supporting Peptides
|

Protein Coverage:

Supporting Peptides:

| Peptide | Uniq | -10lgP | Mass | Length | ppm | m/z | z | RT | Fraction | Scan | Source File | Area F6 | Area F2 | Area F3 | Area F4 | Area F5 | Area F7 | Area F8 | Area F1 | #Feature | #Feature F6 | #Feature F2 | #Feature F3 | #Feature F4 | #Feature F5 | #Feature F7 | #Feature F8 | #Feature F1 | Start | End | PTM | AScore | Found By |
| --- | --- | --- | --- | --- | --- | --- | --- | --- | --- | --- | --- | --- | --- | --- | --- | --- | --- | --- | --- | --- | --- | --- | --- | --- | --- | --- | --- | --- | --- | --- | --- | --- | --- |
| R.VFQVEYAMK.A | Y | 51.77 | 1113.5529 | 9 | 0.2 | 557.7838 | 2 | 38.51 | 8 | F8:19620 | 31122021\_RID\_3262\_1b.raw |  |  |  |  |  |  |  | 4.84E4 | 1 | 0 | 0 | 0 | 0 | 0 | 0 | 0 | 1 | 21 | 29 |  |  | PEAKS DB |
| total 1 peptides |  |  |  |  |  |  |  |  |  |  |  |  |  |  |  |  |  |  |  |  |  |  |  |  |  |  |  |  |  |  |  |  |  |
| --- | --- | --- | --- | --- | --- | --- | --- | --- | --- | --- | --- | --- | --- | --- | --- | --- | --- | --- | --- | --- | --- | --- | --- | --- | --- | --- | --- | --- | --- | --- | --- | --- | --- |

XP\_026525981.1

back to list

  

| Protein Coverage
| Supporting Peptides
|

Protein Coverage:

Supporting Peptides:

| Peptide | Uniq | -10lgP | Mass | Length | ppm | m/z | z | RT | Fraction | Scan | Source File | Area F6 | Area F2 | Area F3 | Area F4 | Area F5 | Area F7 | Area F8 | Area F1 | #Feature | #Feature F6 | #Feature F2 | #Feature F3 | #Feature F4 | #Feature F5 | #Feature F7 | #Feature F8 | #Feature F1 | Start | End | PTM | AScore | Found By |
| --- | --- | --- | --- | --- | --- | --- | --- | --- | --- | --- | --- | --- | --- | --- | --- | --- | --- | --- | --- | --- | --- | --- | --- | --- | --- | --- | --- | --- | --- | --- | --- | --- | --- |
| R.VFQVEYAMK.A | Y | 51.77 | 1113.5529 | 9 | 0.2 | 557.7838 | 2 | 38.51 | 8 | F8:19620 | 31122021\_RID\_3262\_1b.raw |  |  |  |  |  |  |  | 4.84E4 | 1 | 0 | 0 | 0 | 0 | 0 | 0 | 0 | 1 | 21 | 29 |  |  | PEAKS DB |
| total 1 peptides |  |  |  |  |  |  |  |  |  |  |  |  |  |  |  |  |  |  |  |  |  |  |  |  |  |  |  |  |  |  |  |  |  |
| --- | --- | --- | --- | --- | --- | --- | --- | --- | --- | --- | --- | --- | --- | --- | --- | --- | --- | --- | --- | --- | --- | --- | --- | --- | --- | --- | --- | --- | --- | --- | --- | --- | --- |

JAG66904.1

back to list

  

| Protein Coverage
| Supporting Peptides
|

Protein Coverage:

Supporting Peptides:

| Peptide | Uniq | -10lgP | Mass | Length | ppm | m/z | z | RT | Fraction | Scan | Source File | Area F6 | Area F2 | Area F3 | Area F4 | Area F5 | Area F7 | Area F8 | Area F1 | #Feature | #Feature F6 | #Feature F2 | #Feature F3 | #Feature F4 | #Feature F5 | #Feature F7 | #Feature F8 | #Feature F1 | Start | End | PTM | AScore | Found By |
| --- | --- | --- | --- | --- | --- | --- | --- | --- | --- | --- | --- | --- | --- | --- | --- | --- | --- | --- | --- | --- | --- | --- | --- | --- | --- | --- | --- | --- | --- | --- | --- | --- | --- |
| R.VFQVEYAMK.A | Y | 51.77 | 1113.5529 | 9 | 0.2 | 557.7838 | 2 | 38.51 | 8 | F8:19620 | 31122021\_RID\_3262\_1b.raw |  |  |  |  |  |  |  | 4.84E4 | 1 | 0 | 0 | 0 | 0 | 0 | 0 | 0 | 1 | 21 | 29 |  |  | PEAKS DB |
| total 1 peptides |  |  |  |  |  |  |  |  |  |  |  |  |  |  |  |  |  |  |  |  |  |  |  |  |  |  |  |  |  |  |  |  |  |
| --- | --- | --- | --- | --- | --- | --- | --- | --- | --- | --- | --- | --- | --- | --- | --- | --- | --- | --- | --- | --- | --- | --- | --- | --- | --- | --- | --- | --- | --- | --- | --- | --- | --- |

JAB53606.1

back to list

  

| Protein Coverage
| Supporting Peptides
|

Protein Coverage:

Supporting Peptides:

| Peptide | Uniq | -10lgP | Mass | Length | ppm | m/z | z | RT | Fraction | Scan | Source File | Area F6 | Area F2 | Area F3 | Area F4 | Area F5 | Area F7 | Area F8 | Area F1 | #Feature | #Feature F6 | #Feature F2 | #Feature F3 | #Feature F4 | #Feature F5 | #Feature F7 | #Feature F8 | #Feature F1 | Start | End | PTM | AScore | Found By |
| --- | --- | --- | --- | --- | --- | --- | --- | --- | --- | --- | --- | --- | --- | --- | --- | --- | --- | --- | --- | --- | --- | --- | --- | --- | --- | --- | --- | --- | --- | --- | --- | --- | --- |
| R.VFQVEYAMK.A | Y | 51.77 | 1113.5529 | 9 | 0.2 | 557.7838 | 2 | 38.51 | 8 | F8:19620 | 31122021\_RID\_3262\_1b.raw |  |  |  |  |  |  |  | 4.84E4 | 1 | 0 | 0 | 0 | 0 | 0 | 0 | 0 | 1 | 21 | 29 |  |  | PEAKS DB |
| total 1 peptides |  |  |  |  |  |  |  |  |  |  |  |  |  |  |  |  |  |  |  |  |  |  |  |  |  |  |  |  |  |  |  |  |  |
| --- | --- | --- | --- | --- | --- | --- | --- | --- | --- | --- | --- | --- | --- | --- | --- | --- | --- | --- | --- | --- | --- | --- | --- | --- | --- | --- | --- | --- | --- | --- | --- | --- | --- |

JAA96035.1

back to list

  

| Protein Coverage
| Supporting Peptides
|

Protein Coverage:

Supporting Peptides:

| Peptide | Uniq | -10lgP | Mass | Length | ppm | m/z | z | RT | Fraction | Scan | Source File | Area F6 | Area F2 | Area F3 | Area F4 | Area F5 | Area F7 | Area F8 | Area F1 | #Feature | #Feature F6 | #Feature F2 | #Feature F3 | #Feature F4 | #Feature F5 | #Feature F7 | #Feature F8 | #Feature F1 | Start | End | PTM | AScore | Found By |
| --- | --- | --- | --- | --- | --- | --- | --- | --- | --- | --- | --- | --- | --- | --- | --- | --- | --- | --- | --- | --- | --- | --- | --- | --- | --- | --- | --- | --- | --- | --- | --- | --- | --- |
| R.VFQVEYAMK.A | Y | 51.77 | 1113.5529 | 9 | 0.2 | 557.7838 | 2 | 38.51 | 8 | F8:19620 | 31122021\_RID\_3262\_1b.raw |  |  |  |  |  |  |  | 4.84E4 | 1 | 0 | 0 | 0 | 0 | 0 | 0 | 0 | 1 | 21 | 29 |  |  | PEAKS DB |
| total 1 peptides |  |  |  |  |  |  |  |  |  |  |  |  |  |  |  |  |  |  |  |  |  |  |  |  |  |  |  |  |  |  |  |  |  |
| --- | --- | --- | --- | --- | --- | --- | --- | --- | --- | --- | --- | --- | --- | --- | --- | --- | --- | --- | --- | --- | --- | --- | --- | --- | --- | --- | --- | --- | --- | --- | --- | --- | --- |

XP\_015676739.1

back to list

  

| Protein Coverage
| Supporting Peptides
|

Protein Coverage:

Supporting Peptides:

| Peptide | Uniq | -10lgP | Mass | Length | ppm | m/z | z | RT | Fraction | Scan | Source File | Area F6 | Area F2 | Area F3 | Area F4 | Area F5 | Area F7 | Area F8 | Area F1 | #Feature | #Feature F6 | #Feature F2 | #Feature F3 | #Feature F4 | #Feature F5 | #Feature F7 | #Feature F8 | #Feature F1 | Start | End | PTM | AScore | Found By |
| --- | --- | --- | --- | --- | --- | --- | --- | --- | --- | --- | --- | --- | --- | --- | --- | --- | --- | --- | --- | --- | --- | --- | --- | --- | --- | --- | --- | --- | --- | --- | --- | --- | --- |
| R.VFQVEYAMK.A | Y | 51.77 | 1113.5529 | 9 | 0.2 | 557.7838 | 2 | 38.51 | 8 | F8:19620 | 31122021\_RID\_3262\_1b.raw |  |  |  |  |  |  |  | 4.84E4 | 1 | 0 | 0 | 0 | 0 | 0 | 0 | 0 | 1 | 21 | 29 |  |  | PEAKS DB |
| total 1 peptides |  |  |  |  |  |  |  |  |  |  |  |  |  |  |  |  |  |  |  |  |  |  |  |  |  |  |  |  |  |  |  |  |  |
| --- | --- | --- | --- | --- | --- | --- | --- | --- | --- | --- | --- | --- | --- | --- | --- | --- | --- | --- | --- | --- | --- | --- | --- | --- | --- | --- | --- | --- | --- | --- | --- | --- | --- |

JAV49555.1

back to list

  

| Protein Coverage
| Supporting Peptides
|

Protein Coverage:

Supporting Peptides:

| Peptide | Uniq | -10lgP | Mass | Length | ppm | m/z | z | RT | Fraction | Scan | Source File | Area F6 | Area F2 | Area F3 | Area F4 | Area F5 | Area F7 | Area F8 | Area F1 | #Feature | #Feature F6 | #Feature F2 | #Feature F3 | #Feature F4 | #Feature F5 | #Feature F7 | #Feature F8 | #Feature F1 | Start | End | PTM | AScore | Found By |
| --- | --- | --- | --- | --- | --- | --- | --- | --- | --- | --- | --- | --- | --- | --- | --- | --- | --- | --- | --- | --- | --- | --- | --- | --- | --- | --- | --- | --- | --- | --- | --- | --- | --- |
| R.VFQVEYAMK.A | Y | 51.77 | 1113.5529 | 9 | 0.2 | 557.7838 | 2 | 38.51 | 8 | F8:19620 | 31122021\_RID\_3262\_1b.raw |  |  |  |  |  |  |  | 4.84E4 | 1 | 0 | 0 | 0 | 0 | 0 | 0 | 0 | 1 | 21 | 29 |  |  | PEAKS DB |
| total 1 peptides |  |  |  |  |  |  |  |  |  |  |  |  |  |  |  |  |  |  |  |  |  |  |  |  |  |  |  |  |  |  |  |  |  |
| --- | --- | --- | --- | --- | --- | --- | --- | --- | --- | --- | --- | --- | --- | --- | --- | --- | --- | --- | --- | --- | --- | --- | --- | --- | --- | --- | --- | --- | --- | --- | --- | --- | --- |

JAI11930.1

back to list

  

| Protein Coverage
| Supporting Peptides
|

Protein Coverage:

Supporting Peptides:

| Peptide | Uniq | -10lgP | Mass | Length | ppm | m/z | z | RT | Fraction | Scan | Source File | Area F6 | Area F2 | Area F3 | Area F4 | Area F5 | Area F7 | Area F8 | Area F1 | #Feature | #Feature F6 | #Feature F2 | #Feature F3 | #Feature F4 | #Feature F5 | #Feature F7 | #Feature F8 | #Feature F1 | Start | End | PTM | AScore | Found By |
| --- | --- | --- | --- | --- | --- | --- | --- | --- | --- | --- | --- | --- | --- | --- | --- | --- | --- | --- | --- | --- | --- | --- | --- | --- | --- | --- | --- | --- | --- | --- | --- | --- | --- |
| R.VFQVEYAMK.A | Y | 51.77 | 1113.5529 | 9 | 0.2 | 557.7838 | 2 | 38.51 | 8 | F8:19620 | 31122021\_RID\_3262\_1b.raw |  |  |  |  |  |  |  | 4.84E4 | 1 | 0 | 0 | 0 | 0 | 0 | 0 | 0 | 1 | 21 | 29 |  |  | PEAKS DB |
| total 1 peptides |  |  |  |  |  |  |  |  |  |  |  |  |  |  |  |  |  |  |  |  |  |  |  |  |  |  |  |  |  |  |  |  |  |
| --- | --- | --- | --- | --- | --- | --- | --- | --- | --- | --- | --- | --- | --- | --- | --- | --- | --- | --- | --- | --- | --- | --- | --- | --- | --- | --- | --- | --- | --- | --- | --- | --- | --- |

AFJ51036.1

back to list

  

| Protein Coverage
| Supporting Peptides
|

Protein Coverage:

Supporting Peptides:

| Peptide | Uniq | -10lgP | Mass | Length | ppm | m/z | z | RT | Fraction | Scan | Source File | Area F6 | Area F2 | Area F3 | Area F4 | Area F5 | Area F7 | Area F8 | Area F1 | #Feature | #Feature F6 | #Feature F2 | #Feature F3 | #Feature F4 | #Feature F5 | #Feature F7 | #Feature F8 | #Feature F1 | Start | End | PTM | AScore | Found By |
| --- | --- | --- | --- | --- | --- | --- | --- | --- | --- | --- | --- | --- | --- | --- | --- | --- | --- | --- | --- | --- | --- | --- | --- | --- | --- | --- | --- | --- | --- | --- | --- | --- | --- |
| R.VFQVEYAMK.A | Y | 51.77 | 1113.5529 | 9 | 0.2 | 557.7838 | 2 | 38.51 | 8 | F8:19620 | 31122021\_RID\_3262\_1b.raw |  |  |  |  |  |  |  | 4.84E4 | 1 | 0 | 0 | 0 | 0 | 0 | 0 | 0 | 1 | 21 | 29 |  |  | PEAKS DB |
| total 1 peptides |  |  |  |  |  |  |  |  |  |  |  |  |  |  |  |  |  |  |  |  |  |  |  |  |  |  |  |  |  |  |  |  |  |
| --- | --- | --- | --- | --- | --- | --- | --- | --- | --- | --- | --- | --- | --- | --- | --- | --- | --- | --- | --- | --- | --- | --- | --- | --- | --- | --- | --- | --- | --- | --- | --- | --- | --- |

LAA64694.1

back to list

  

| Protein Coverage
| Supporting Peptides
|

Protein Coverage:

Supporting Peptides:

| Peptide | Uniq | -10lgP | Mass | Length | ppm | m/z | z | RT | Fraction | Scan | Source File | Area F6 | Area F2 | Area F3 | Area F4 | Area F5 | Area F7 | Area F8 | Area F1 | #Feature | #Feature F6 | #Feature F2 | #Feature F3 | #Feature F4 | #Feature F5 | #Feature F7 | #Feature F8 | #Feature F1 | Start | End | PTM | AScore | Found By |
| --- | --- | --- | --- | --- | --- | --- | --- | --- | --- | --- | --- | --- | --- | --- | --- | --- | --- | --- | --- | --- | --- | --- | --- | --- | --- | --- | --- | --- | --- | --- | --- | --- | --- |
| K.LTLSALVDGK.N | Y | 48.00 | 1015.5913 | 10 | 1.1 | 508.8035 | 2 | 44.95 | 8 | F8:24669 | 31122021\_RID\_3262\_1b.raw |  |  |  |  |  |  |  | 8.4212E4 | 1 | 0 | 0 | 0 | 0 | 0 | 0 | 0 | 1 | 271 | 280 |  |  | PEAKS DB |
| total 1 peptides |  |  |  |  |  |  |  |  |  |  |  |  |  |  |  |  |  |  |  |  |  |  |  |  |  |  |  |  |  |  |  |  |  |
| --- | --- | --- | --- | --- | --- | --- | --- | --- | --- | --- | --- | --- | --- | --- | --- | --- | --- | --- | --- | --- | --- | --- | --- | --- | --- | --- | --- | --- | --- | --- | --- | --- | --- |

LAA64691.1

back to list

  

| Protein Coverage
| Supporting Peptides
|

Protein Coverage:

Supporting Peptides:

| Peptide | Uniq | -10lgP | Mass | Length | ppm | m/z | z | RT | Fraction | Scan | Source File | Area F6 | Area F2 | Area F3 | Area F4 | Area F5 | Area F7 | Area F8 | Area F1 | #Feature | #Feature F6 | #Feature F2 | #Feature F3 | #Feature F4 | #Feature F5 | #Feature F7 | #Feature F8 | #Feature F1 | Start | End | PTM | AScore | Found By |
| --- | --- | --- | --- | --- | --- | --- | --- | --- | --- | --- | --- | --- | --- | --- | --- | --- | --- | --- | --- | --- | --- | --- | --- | --- | --- | --- | --- | --- | --- | --- | --- | --- | --- |
| K.LTLSALVDGK.N | Y | 48.00 | 1015.5913 | 10 | 1.1 | 508.8035 | 2 | 44.95 | 8 | F8:24669 | 31122021\_RID\_3262\_1b.raw |  |  |  |  |  |  |  | 8.4212E4 | 1 | 0 | 0 | 0 | 0 | 0 | 0 | 0 | 1 | 271 | 280 |  |  | PEAKS DB |
| total 1 peptides |  |  |  |  |  |  |  |  |  |  |  |  |  |  |  |  |  |  |  |  |  |  |  |  |  |  |  |  |  |  |  |  |  |
| --- | --- | --- | --- | --- | --- | --- | --- | --- | --- | --- | --- | --- | --- | --- | --- | --- | --- | --- | --- | --- | --- | --- | --- | --- | --- | --- | --- | --- | --- | --- | --- | --- | --- |

JAB54700.1

back to list

  

| Protein Coverage
| Supporting Peptides
|

Protein Coverage:

Supporting Peptides:

| Peptide | Uniq | -10lgP | Mass | Length | ppm | m/z | z | RT | Fraction | Scan | Source File | Area F6 | Area F2 | Area F3 | Area F4 | Area F5 | Area F7 | Area F8 | Area F1 | #Feature | #Feature F6 | #Feature F2 | #Feature F3 | #Feature F4 | #Feature F5 | #Feature F7 | #Feature F8 | #Feature F1 | Start | End | PTM | AScore | Found By |
| --- | --- | --- | --- | --- | --- | --- | --- | --- | --- | --- | --- | --- | --- | --- | --- | --- | --- | --- | --- | --- | --- | --- | --- | --- | --- | --- | --- | --- | --- | --- | --- | --- | --- |
| K.LTLSALVDGK.N | Y | 48.00 | 1015.5913 | 10 | 1.1 | 508.8035 | 2 | 44.95 | 8 | F8:24669 | 31122021\_RID\_3262\_1b.raw |  |  |  |  |  |  |  | 8.4212E4 | 1 | 0 | 0 | 0 | 0 | 0 | 0 | 0 | 1 | 271 | 280 |  |  | PEAKS DB |
| total 1 peptides |  |  |  |  |  |  |  |  |  |  |  |  |  |  |  |  |  |  |  |  |  |  |  |  |  |  |  |  |  |  |  |  |  |
| --- | --- | --- | --- | --- | --- | --- | --- | --- | --- | --- | --- | --- | --- | --- | --- | --- | --- | --- | --- | --- | --- | --- | --- | --- | --- | --- | --- | --- | --- | --- | --- | --- | --- |

JAI10292.1

back to list

  

| Protein Coverage
| Supporting Peptides
|

Protein Coverage:

Supporting Peptides:

| Peptide | Uniq | -10lgP | Mass | Length | ppm | m/z | z | RT | Fraction | Scan | Source File | Area F6 | Area F2 | Area F3 | Area F4 | Area F5 | Area F7 | Area F8 | Area F1 | #Feature | #Feature F6 | #Feature F2 | #Feature F3 | #Feature F4 | #Feature F5 | #Feature F7 | #Feature F8 | #Feature F1 | Start | End | PTM | AScore | Found By |
| --- | --- | --- | --- | --- | --- | --- | --- | --- | --- | --- | --- | --- | --- | --- | --- | --- | --- | --- | --- | --- | --- | --- | --- | --- | --- | --- | --- | --- | --- | --- | --- | --- | --- |
| K.LTLSALVDGK.N | Y | 48.00 | 1015.5913 | 10 | 1.1 | 508.8035 | 2 | 44.95 | 8 | F8:24669 | 31122021\_RID\_3262\_1b.raw |  |  |  |  |  |  |  | 8.4212E4 | 1 | 0 | 0 | 0 | 0 | 0 | 0 | 0 | 1 | 257 | 266 |  |  | PEAKS DB |
| total 1 peptides |  |  |  |  |  |  |  |  |  |  |  |  |  |  |  |  |  |  |  |  |  |  |  |  |  |  |  |  |  |  |  |  |  |
| --- | --- | --- | --- | --- | --- | --- | --- | --- | --- | --- | --- | --- | --- | --- | --- | --- | --- | --- | --- | --- | --- | --- | --- | --- | --- | --- | --- | --- | --- | --- | --- | --- | --- |

XP\_034291314.1

back to list

  

| Protein Coverage
| Supporting Peptides
|

Protein Coverage:

Supporting Peptides:

| Peptide | Uniq | -10lgP | Mass | Length | ppm | m/z | z | RT | Fraction | Scan | Source File | Area F6 | Area F2 | Area F3 | Area F4 | Area F5 | Area F7 | Area F8 | Area F1 | #Feature | #Feature F6 | #Feature F2 | #Feature F3 | #Feature F4 | #Feature F5 | #Feature F7 | #Feature F8 | #Feature F1 | Start | End | PTM | AScore | Found By |
| --- | --- | --- | --- | --- | --- | --- | --- | --- | --- | --- | --- | --- | --- | --- | --- | --- | --- | --- | --- | --- | --- | --- | --- | --- | --- | --- | --- | --- | --- | --- | --- | --- | --- |
| K.LTLSALVDGK.N | Y | 48.00 | 1015.5913 | 10 | 1.1 | 508.8035 | 2 | 44.95 | 8 | F8:24669 | 31122021\_RID\_3262\_1b.raw |  |  |  |  |  |  |  | 8.4212E4 | 1 | 0 | 0 | 0 | 0 | 0 | 0 | 0 | 1 | 257 | 266 |  |  | PEAKS DB |
| total 1 peptides |  |  |  |  |  |  |  |  |  |  |  |  |  |  |  |  |  |  |  |  |  |  |  |  |  |  |  |  |  |  |  |  |  |
| --- | --- | --- | --- | --- | --- | --- | --- | --- | --- | --- | --- | --- | --- | --- | --- | --- | --- | --- | --- | --- | --- | --- | --- | --- | --- | --- | --- | --- | --- | --- | --- | --- | --- |

JAC96265.1

back to list

  

| Protein Coverage
| Supporting Peptides
|

Protein Coverage:

Supporting Peptides:

| Peptide | Uniq | -10lgP | Mass | Length | ppm | m/z | z | RT | Fraction | Scan | Source File | Area F6 | Area F2 | Area F3 | Area F4 | Area F5 | Area F7 | Area F8 | Area F1 | #Feature | #Feature F6 | #Feature F2 | #Feature F3 | #Feature F4 | #Feature F5 | #Feature F7 | #Feature F8 | #Feature F1 | Start | End | PTM | AScore | Found By |
| --- | --- | --- | --- | --- | --- | --- | --- | --- | --- | --- | --- | --- | --- | --- | --- | --- | --- | --- | --- | --- | --- | --- | --- | --- | --- | --- | --- | --- | --- | --- | --- | --- | --- |
| K.LTLSALVDGK.N | Y | 48.00 | 1015.5913 | 10 | 1.1 | 508.8035 | 2 | 44.95 | 8 | F8:24669 | 31122021\_RID\_3262\_1b.raw |  |  |  |  |  |  |  | 8.4212E4 | 1 | 0 | 0 | 0 | 0 | 0 | 0 | 0 | 1 | 257 | 266 |  |  | PEAKS DB |
| total 1 peptides |  |  |  |  |  |  |  |  |  |  |  |  |  |  |  |  |  |  |  |  |  |  |  |  |  |  |  |  |  |  |  |  |  |
| --- | --- | --- | --- | --- | --- | --- | --- | --- | --- | --- | --- | --- | --- | --- | --- | --- | --- | --- | --- | --- | --- | --- | --- | --- | --- | --- | --- | --- | --- | --- | --- | --- | --- |

LAA64698.1

back to list

  

| Protein Coverage
| Supporting Peptides
|

Protein Coverage:

Supporting Peptides:

| Peptide | Uniq | -10lgP | Mass | Length | ppm | m/z | z | RT | Fraction | Scan | Source File | Area F6 | Area F2 | Area F3 | Area F4 | Area F5 | Area F7 | Area F8 | Area F1 | #Feature | #Feature F6 | #Feature F2 | #Feature F3 | #Feature F4 | #Feature F5 | #Feature F7 | #Feature F8 | #Feature F1 | Start | End | PTM | AScore | Found By |
| --- | --- | --- | --- | --- | --- | --- | --- | --- | --- | --- | --- | --- | --- | --- | --- | --- | --- | --- | --- | --- | --- | --- | --- | --- | --- | --- | --- | --- | --- | --- | --- | --- | --- |
| K.LTLSALVDGK.N | Y | 48.00 | 1015.5913 | 10 | 1.1 | 508.8035 | 2 | 44.95 | 8 | F8:24669 | 31122021\_RID\_3262\_1b.raw |  |  |  |  |  |  |  | 8.4212E4 | 1 | 0 | 0 | 0 | 0 | 0 | 0 | 0 | 1 | 257 | 266 |  |  | PEAKS DB |
| total 1 peptides |  |  |  |  |  |  |  |  |  |  |  |  |  |  |  |  |  |  |  |  |  |  |  |  |  |  |  |  |  |  |  |  |  |
| --- | --- | --- | --- | --- | --- | --- | --- | --- | --- | --- | --- | --- | --- | --- | --- | --- | --- | --- | --- | --- | --- | --- | --- | --- | --- | --- | --- | --- | --- | --- | --- | --- | --- |

JAG45846.1

back to list

  

| Protein Coverage
| Supporting Peptides
|

Protein Coverage:

Supporting Peptides:

| Peptide | Uniq | -10lgP | Mass | Length | ppm | m/z | z | RT | Fraction | Scan | Source File | Area F6 | Area F2 | Area F3 | Area F4 | Area F5 | Area F7 | Area F8 | Area F1 | #Feature | #Feature F6 | #Feature F2 | #Feature F3 | #Feature F4 | #Feature F5 | #Feature F7 | #Feature F8 | #Feature F1 | Start | End | PTM | AScore | Found By |
| --- | --- | --- | --- | --- | --- | --- | --- | --- | --- | --- | --- | --- | --- | --- | --- | --- | --- | --- | --- | --- | --- | --- | --- | --- | --- | --- | --- | --- | --- | --- | --- | --- | --- |
| K.LTLSALVDGK.N | Y | 48.00 | 1015.5913 | 10 | 1.1 | 508.8035 | 2 | 44.95 | 8 | F8:24669 | 31122021\_RID\_3262\_1b.raw |  |  |  |  |  |  |  | 8.4212E4 | 1 | 0 | 0 | 0 | 0 | 0 | 0 | 0 | 1 | 257 | 266 |  |  | PEAKS DB |
| total 1 peptides |  |  |  |  |  |  |  |  |  |  |  |  |  |  |  |  |  |  |  |  |  |  |  |  |  |  |  |  |  |  |  |  |  |
| --- | --- | --- | --- | --- | --- | --- | --- | --- | --- | --- | --- | --- | --- | --- | --- | --- | --- | --- | --- | --- | --- | --- | --- | --- | --- | --- | --- | --- | --- | --- | --- | --- | --- |

JAI14353.1

back to list

  

| Protein Coverage
| Supporting Peptides
|

Protein Coverage:

Supporting Peptides:

| Peptide | Uniq | -10lgP | Mass | Length | ppm | m/z | z | RT | Fraction | Scan | Source File | Area F6 | Area F2 | Area F3 | Area F4 | Area F5 | Area F7 | Area F8 | Area F1 | #Feature | #Feature F6 | #Feature F2 | #Feature F3 | #Feature F4 | #Feature F5 | #Feature F7 | #Feature F8 | #Feature F1 | Start | End | PTM | AScore | Found By |
| --- | --- | --- | --- | --- | --- | --- | --- | --- | --- | --- | --- | --- | --- | --- | --- | --- | --- | --- | --- | --- | --- | --- | --- | --- | --- | --- | --- | --- | --- | --- | --- | --- | --- |
| K.LTLSALVDGK.N | Y | 48.00 | 1015.5913 | 10 | 1.1 | 508.8035 | 2 | 44.95 | 8 | F8:24669 | 31122021\_RID\_3262\_1b.raw |  |  |  |  |  |  |  | 8.4212E4 | 1 | 0 | 0 | 0 | 0 | 0 | 0 | 0 | 1 | 257 | 266 |  |  | PEAKS DB |
| total 1 peptides |  |  |  |  |  |  |  |  |  |  |  |  |  |  |  |  |  |  |  |  |  |  |  |  |  |  |  |  |  |  |  |  |  |
| --- | --- | --- | --- | --- | --- | --- | --- | --- | --- | --- | --- | --- | --- | --- | --- | --- | --- | --- | --- | --- | --- | --- | --- | --- | --- | --- | --- | --- | --- | --- | --- | --- | --- |

JAA97782.1

back to list

  

| Protein Coverage
| Supporting Peptides
|

Protein Coverage:

Supporting Peptides:

| Peptide | Uniq | -10lgP | Mass | Length | ppm | m/z | z | RT | Fraction | Scan | Source File | Area F6 | Area F2 | Area F3 | Area F4 | Area F5 | Area F7 | Area F8 | Area F1 | #Feature | #Feature F6 | #Feature F2 | #Feature F3 | #Feature F4 | #Feature F5 | #Feature F7 | #Feature F8 | #Feature F1 | Start | End | PTM | AScore | Found By |
| --- | --- | --- | --- | --- | --- | --- | --- | --- | --- | --- | --- | --- | --- | --- | --- | --- | --- | --- | --- | --- | --- | --- | --- | --- | --- | --- | --- | --- | --- | --- | --- | --- | --- |
| K.LTLSALVDGK.N | Y | 48.00 | 1015.5913 | 10 | 1.1 | 508.8035 | 2 | 44.95 | 8 | F8:24669 | 31122021\_RID\_3262\_1b.raw |  |  |  |  |  |  |  | 8.4212E4 | 1 | 0 | 0 | 0 | 0 | 0 | 0 | 0 | 1 | 257 | 266 |  |  | PEAKS DB |
| total 1 peptides |  |  |  |  |  |  |  |  |  |  |  |  |  |  |  |  |  |  |  |  |  |  |  |  |  |  |  |  |  |  |  |  |  |
| --- | --- | --- | --- | --- | --- | --- | --- | --- | --- | --- | --- | --- | --- | --- | --- | --- | --- | --- | --- | --- | --- | --- | --- | --- | --- | --- | --- | --- | --- | --- | --- | --- | --- |

JAV51267.1

back to list

  

| Protein Coverage
| Supporting Peptides
|

Protein Coverage:

Supporting Peptides:

| Peptide | Uniq | -10lgP | Mass | Length | ppm | m/z | z | RT | Fraction | Scan | Source File | Area F6 | Area F2 | Area F3 | Area F4 | Area F5 | Area F7 | Area F8 | Area F1 | #Feature | #Feature F6 | #Feature F2 | #Feature F3 | #Feature F4 | #Feature F5 | #Feature F7 | #Feature F8 | #Feature F1 | Start | End | PTM | AScore | Found By |
| --- | --- | --- | --- | --- | --- | --- | --- | --- | --- | --- | --- | --- | --- | --- | --- | --- | --- | --- | --- | --- | --- | --- | --- | --- | --- | --- | --- | --- | --- | --- | --- | --- | --- |
| K.LTLSALVDGK.N | Y | 48.00 | 1015.5913 | 10 | 1.1 | 508.8035 | 2 | 44.95 | 8 | F8:24669 | 31122021\_RID\_3262\_1b.raw |  |  |  |  |  |  |  | 8.4212E4 | 1 | 0 | 0 | 0 | 0 | 0 | 0 | 0 | 1 | 257 | 266 |  |  | PEAKS DB |
| total 1 peptides |  |  |  |  |  |  |  |  |  |  |  |  |  |  |  |  |  |  |  |  |  |  |  |  |  |  |  |  |  |  |  |  |  |
| --- | --- | --- | --- | --- | --- | --- | --- | --- | --- | --- | --- | --- | --- | --- | --- | --- | --- | --- | --- | --- | --- | --- | --- | --- | --- | --- | --- | --- | --- | --- | --- | --- | --- |

JAG68733.1

back to list

  

| Protein Coverage
| Supporting Peptides
|

Protein Coverage:

Supporting Peptides:

| Peptide | Uniq | -10lgP | Mass | Length | ppm | m/z | z | RT | Fraction | Scan | Source File | Area F6 | Area F2 | Area F3 | Area F4 | Area F5 | Area F7 | Area F8 | Area F1 | #Feature | #Feature F6 | #Feature F2 | #Feature F3 | #Feature F4 | #Feature F5 | #Feature F7 | #Feature F8 | #Feature F1 | Start | End | PTM | AScore | Found By |
| --- | --- | --- | --- | --- | --- | --- | --- | --- | --- | --- | --- | --- | --- | --- | --- | --- | --- | --- | --- | --- | --- | --- | --- | --- | --- | --- | --- | --- | --- | --- | --- | --- | --- |
| K.LTLSALVDGK.N | Y | 48.00 | 1015.5913 | 10 | 1.1 | 508.8035 | 2 | 44.95 | 8 | F8:24669 | 31122021\_RID\_3262\_1b.raw |  |  |  |  |  |  |  | 8.4212E4 | 1 | 0 | 0 | 0 | 0 | 0 | 0 | 0 | 1 | 257 | 266 |  |  | PEAKS DB |
| total 1 peptides |  |  |  |  |  |  |  |  |  |  |  |  |  |  |  |  |  |  |  |  |  |  |  |  |  |  |  |  |  |  |  |  |  |
| --- | --- | --- | --- | --- | --- | --- | --- | --- | --- | --- | --- | --- | --- | --- | --- | --- | --- | --- | --- | --- | --- | --- | --- | --- | --- | --- | --- | --- | --- | --- | --- | --- | --- |

XP\_026579473.1

back to list

  

| Protein Coverage
| Supporting Peptides
|

Protein Coverage:

Supporting Peptides:

| Peptide | Uniq | -10lgP | Mass | Length | ppm | m/z | z | RT | Fraction | Scan | Source File | Area F6 | Area F2 | Area F3 | Area F4 | Area F5 | Area F7 | Area F8 | Area F1 | #Feature | #Feature F6 | #Feature F2 | #Feature F3 | #Feature F4 | #Feature F5 | #Feature F7 | #Feature F8 | #Feature F1 | Start | End | PTM | AScore | Found By |
| --- | --- | --- | --- | --- | --- | --- | --- | --- | --- | --- | --- | --- | --- | --- | --- | --- | --- | --- | --- | --- | --- | --- | --- | --- | --- | --- | --- | --- | --- | --- | --- | --- | --- |
| K.LTLSALVDGK.N | Y | 48.00 | 1015.5913 | 10 | 1.1 | 508.8035 | 2 | 44.95 | 8 | F8:24669 | 31122021\_RID\_3262\_1b.raw |  |  |  |  |  |  |  | 8.4212E4 | 1 | 0 | 0 | 0 | 0 | 0 | 0 | 0 | 1 | 257 | 266 |  |  | PEAKS DB |
| total 1 peptides |  |  |  |  |  |  |  |  |  |  |  |  |  |  |  |  |  |  |  |  |  |  |  |  |  |  |  |  |  |  |  |  |  |
| --- | --- | --- | --- | --- | --- | --- | --- | --- | --- | --- | --- | --- | --- | --- | --- | --- | --- | --- | --- | --- | --- | --- | --- | --- | --- | --- | --- | --- | --- | --- | --- | --- | --- |

JAG47483.1

back to list

  

| Protein Coverage
| Supporting Peptides
|

Protein Coverage:

Supporting Peptides:

| Peptide | Uniq | -10lgP | Mass | Length | ppm | m/z | z | RT | Fraction | Scan | Source File | Area F6 | Area F2 | Area F3 | Area F4 | Area F5 | Area F7 | Area F8 | Area F1 | #Feature | #Feature F6 | #Feature F2 | #Feature F3 | #Feature F4 | #Feature F5 | #Feature F7 | #Feature F8 | #Feature F1 | Start | End | PTM | AScore | Found By |
| --- | --- | --- | --- | --- | --- | --- | --- | --- | --- | --- | --- | --- | --- | --- | --- | --- | --- | --- | --- | --- | --- | --- | --- | --- | --- | --- | --- | --- | --- | --- | --- | --- | --- |
| K.LTLSALVDGK.N | Y | 48.00 | 1015.5913 | 10 | 1.1 | 508.8035 | 2 | 44.95 | 8 | F8:24669 | 31122021\_RID\_3262\_1b.raw |  |  |  |  |  |  |  | 8.4212E4 | 1 | 0 | 0 | 0 | 0 | 0 | 0 | 0 | 1 | 257 | 266 |  |  | PEAKS DB |
| total 1 peptides |  |  |  |  |  |  |  |  |  |  |  |  |  |  |  |  |  |  |  |  |  |  |  |  |  |  |  |  |  |  |  |  |  |
| --- | --- | --- | --- | --- | --- | --- | --- | --- | --- | --- | --- | --- | --- | --- | --- | --- | --- | --- | --- | --- | --- | --- | --- | --- | --- | --- | --- | --- | --- | --- | --- | --- | --- |

XP\_034291315.1

back to list

  

| Protein Coverage
| Supporting Peptides
|

Protein Coverage:

Supporting Peptides:

| Peptide | Uniq | -10lgP | Mass | Length | ppm | m/z | z | RT | Fraction | Scan | Source File | Area F6 | Area F2 | Area F3 | Area F4 | Area F5 | Area F7 | Area F8 | Area F1 | #Feature | #Feature F6 | #Feature F2 | #Feature F3 | #Feature F4 | #Feature F5 | #Feature F7 | #Feature F8 | #Feature F1 | Start | End | PTM | AScore | Found By |
| --- | --- | --- | --- | --- | --- | --- | --- | --- | --- | --- | --- | --- | --- | --- | --- | --- | --- | --- | --- | --- | --- | --- | --- | --- | --- | --- | --- | --- | --- | --- | --- | --- | --- |
| K.LTLSALVDGK.N | Y | 48.00 | 1015.5913 | 10 | 1.1 | 508.8035 | 2 | 44.95 | 8 | F8:24669 | 31122021\_RID\_3262\_1b.raw |  |  |  |  |  |  |  | 8.4212E4 | 1 | 0 | 0 | 0 | 0 | 0 | 0 | 0 | 1 | 257 | 266 |  |  | PEAKS DB |
| total 1 peptides |  |  |  |  |  |  |  |  |  |  |  |  |  |  |  |  |  |  |  |  |  |  |  |  |  |  |  |  |  |  |  |  |  |
| --- | --- | --- | --- | --- | --- | --- | --- | --- | --- | --- | --- | --- | --- | --- | --- | --- | --- | --- | --- | --- | --- | --- | --- | --- | --- | --- | --- | --- | --- | --- | --- | --- | --- |

XP\_026579477.1

back to list

  

| Protein Coverage
| Supporting Peptides
|

Protein Coverage:

Supporting Peptides:

| Peptide | Uniq | -10lgP | Mass | Length | ppm | m/z | z | RT | Fraction | Scan | Source File | Area F6 | Area F2 | Area F3 | Area F4 | Area F5 | Area F7 | Area F8 | Area F1 | #Feature | #Feature F6 | #Feature F2 | #Feature F3 | #Feature F4 | #Feature F5 | #Feature F7 | #Feature F8 | #Feature F1 | Start | End | PTM | AScore | Found By |
| --- | --- | --- | --- | --- | --- | --- | --- | --- | --- | --- | --- | --- | --- | --- | --- | --- | --- | --- | --- | --- | --- | --- | --- | --- | --- | --- | --- | --- | --- | --- | --- | --- | --- |
| K.LTLSALVDGK.N | Y | 48.00 | 1015.5913 | 10 | 1.1 | 508.8035 | 2 | 44.95 | 8 | F8:24669 | 31122021\_RID\_3262\_1b.raw |  |  |  |  |  |  |  | 8.4212E4 | 1 | 0 | 0 | 0 | 0 | 0 | 0 | 0 | 1 | 257 | 266 |  |  | PEAKS DB |
| total 1 peptides |  |  |  |  |  |  |  |  |  |  |  |  |  |  |  |  |  |  |  |  |  |  |  |  |  |  |  |  |  |  |  |  |  |
| --- | --- | --- | --- | --- | --- | --- | --- | --- | --- | --- | --- | --- | --- | --- | --- | --- | --- | --- | --- | --- | --- | --- | --- | --- | --- | --- | --- | --- | --- | --- | --- | --- | --- |

XP\_032087627.1

back to list

  

| Protein Coverage
| Supporting Peptides
|

Protein Coverage:

Supporting Peptides:

| Peptide | Uniq | -10lgP | Mass | Length | ppm | m/z | z | RT | Fraction | Scan | Source File | Area F6 | Area F2 | Area F3 | Area F4 | Area F5 | Area F7 | Area F8 | Area F1 | #Feature | #Feature F6 | #Feature F2 | #Feature F3 | #Feature F4 | #Feature F5 | #Feature F7 | #Feature F8 | #Feature F1 | Start | End | PTM | AScore | Found By |
| --- | --- | --- | --- | --- | --- | --- | --- | --- | --- | --- | --- | --- | --- | --- | --- | --- | --- | --- | --- | --- | --- | --- | --- | --- | --- | --- | --- | --- | --- | --- | --- | --- | --- |
| K.LTLSALVDGK.N | Y | 48.00 | 1015.5913 | 10 | 1.1 | 508.8035 | 2 | 44.95 | 8 | F8:24669 | 31122021\_RID\_3262\_1b.raw |  |  |  |  |  |  |  | 8.4212E4 | 1 | 0 | 0 | 0 | 0 | 0 | 0 | 0 | 1 | 271 | 280 |  |  | PEAKS DB |
| total 1 peptides |  |  |  |  |  |  |  |  |  |  |  |  |  |  |  |  |  |  |  |  |  |  |  |  |  |  |  |  |  |  |  |  |  |
| --- | --- | --- | --- | --- | --- | --- | --- | --- | --- | --- | --- | --- | --- | --- | --- | --- | --- | --- | --- | --- | --- | --- | --- | --- | --- | --- | --- | --- | --- | --- | --- | --- | --- |

XP\_026579471.1

back to list

  

| Protein Coverage
| Supporting Peptides
|

Protein Coverage:

Supporting Peptides:

| Peptide | Uniq | -10lgP | Mass | Length | ppm | m/z | z | RT | Fraction | Scan | Source File | Area F6 | Area F2 | Area F3 | Area F4 | Area F5 | Area F7 | Area F8 | Area F1 | #Feature | #Feature F6 | #Feature F2 | #Feature F3 | #Feature F4 | #Feature F5 | #Feature F7 | #Feature F8 | #Feature F1 | Start | End | PTM | AScore | Found By |
| --- | --- | --- | --- | --- | --- | --- | --- | --- | --- | --- | --- | --- | --- | --- | --- | --- | --- | --- | --- | --- | --- | --- | --- | --- | --- | --- | --- | --- | --- | --- | --- | --- | --- |
| K.LTLSALVDGK.N | Y | 48.00 | 1015.5913 | 10 | 1.1 | 508.8035 | 2 | 44.95 | 8 | F8:24669 | 31122021\_RID\_3262\_1b.raw |  |  |  |  |  |  |  | 8.4212E4 | 1 | 0 | 0 | 0 | 0 | 0 | 0 | 0 | 1 | 271 | 280 |  |  | PEAKS DB |
| total 1 peptides |  |  |  |  |  |  |  |  |  |  |  |  |  |  |  |  |  |  |  |  |  |  |  |  |  |  |  |  |  |  |  |  |  |
| --- | --- | --- | --- | --- | --- | --- | --- | --- | --- | --- | --- | --- | --- | --- | --- | --- | --- | --- | --- | --- | --- | --- | --- | --- | --- | --- | --- | --- | --- | --- | --- | --- | --- |

XP\_026579476.1

back to list

  

| Protein Coverage
| Supporting Peptides
|

Protein Coverage:

Supporting Peptides:

| Peptide | Uniq | -10lgP | Mass | Length | ppm | m/z | z | RT | Fraction | Scan | Source File | Area F6 | Area F2 | Area F3 | Area F4 | Area F5 | Area F7 | Area F8 | Area F1 | #Feature | #Feature F6 | #Feature F2 | #Feature F3 | #Feature F4 | #Feature F5 | #Feature F7 | #Feature F8 | #Feature F1 | Start | End | PTM | AScore | Found By |
| --- | --- | --- | --- | --- | --- | --- | --- | --- | --- | --- | --- | --- | --- | --- | --- | --- | --- | --- | --- | --- | --- | --- | --- | --- | --- | --- | --- | --- | --- | --- | --- | --- | --- |
| K.LTLSALVDGK.N | Y | 48.00 | 1015.5913 | 10 | 1.1 | 508.8035 | 2 | 44.95 | 8 | F8:24669 | 31122021\_RID\_3262\_1b.raw |  |  |  |  |  |  |  | 8.4212E4 | 1 | 0 | 0 | 0 | 0 | 0 | 0 | 0 | 1 | 271 | 280 |  |  | PEAKS DB |
| total 1 peptides |  |  |  |  |  |  |  |  |  |  |  |  |  |  |  |  |  |  |  |  |  |  |  |  |  |  |  |  |  |  |  |  |  |
| --- | --- | --- | --- | --- | --- | --- | --- | --- | --- | --- | --- | --- | --- | --- | --- | --- | --- | --- | --- | --- | --- | --- | --- | --- | --- | --- | --- | --- | --- | --- | --- | --- | --- |

XP\_026579475.1

back to list

  

| Protein Coverage
| Supporting Peptides
|

Protein Coverage:

Supporting Peptides:

| Peptide | Uniq | -10lgP | Mass | Length | ppm | m/z | z | RT | Fraction | Scan | Source File | Area F6 | Area F2 | Area F3 | Area F4 | Area F5 | Area F7 | Area F8 | Area F1 | #Feature | #Feature F6 | #Feature F2 | #Feature F3 | #Feature F4 | #Feature F5 | #Feature F7 | #Feature F8 | #Feature F1 | Start | End | PTM | AScore | Found By |
| --- | --- | --- | --- | --- | --- | --- | --- | --- | --- | --- | --- | --- | --- | --- | --- | --- | --- | --- | --- | --- | --- | --- | --- | --- | --- | --- | --- | --- | --- | --- | --- | --- | --- |
| K.LTLSALVDGK.N | Y | 48.00 | 1015.5913 | 10 | 1.1 | 508.8035 | 2 | 44.95 | 8 | F8:24669 | 31122021\_RID\_3262\_1b.raw |  |  |  |  |  |  |  | 8.4212E4 | 1 | 0 | 0 | 0 | 0 | 0 | 0 | 0 | 1 | 271 | 280 |  |  | PEAKS DB |
| total 1 peptides |  |  |  |  |  |  |  |  |  |  |  |  |  |  |  |  |  |  |  |  |  |  |  |  |  |  |  |  |  |  |  |  |  |
| --- | --- | --- | --- | --- | --- | --- | --- | --- | --- | --- | --- | --- | --- | --- | --- | --- | --- | --- | --- | --- | --- | --- | --- | --- | --- | --- | --- | --- | --- | --- | --- | --- | --- |

XP\_013907074.1

back to list

  

| Protein Coverage
| Supporting Peptides
|

Protein Coverage:

Supporting Peptides:

| Peptide | Uniq | -10lgP | Mass | Length | ppm | m/z | z | RT | Fraction | Scan | Source File | Area F6 | Area F2 | Area F3 | Area F4 | Area F5 | Area F7 | Area F8 | Area F1 | #Feature | #Feature F6 | #Feature F2 | #Feature F3 | #Feature F4 | #Feature F5 | #Feature F7 | #Feature F8 | #Feature F1 | Start | End | PTM | AScore | Found By |
| --- | --- | --- | --- | --- | --- | --- | --- | --- | --- | --- | --- | --- | --- | --- | --- | --- | --- | --- | --- | --- | --- | --- | --- | --- | --- | --- | --- | --- | --- | --- | --- | --- | --- |
| K.LTLSALVDGK.N | Y | 48.00 | 1015.5913 | 10 | 1.1 | 508.8035 | 2 | 44.95 | 8 | F8:24669 | 31122021\_RID\_3262\_1b.raw |  |  |  |  |  |  |  | 8.4212E4 | 1 | 0 | 0 | 0 | 0 | 0 | 0 | 0 | 1 | 271 | 280 |  |  | PEAKS DB |
| total 1 peptides |  |  |  |  |  |  |  |  |  |  |  |  |  |  |  |  |  |  |  |  |  |  |  |  |  |  |  |  |  |  |  |  |  |
| --- | --- | --- | --- | --- | --- | --- | --- | --- | --- | --- | --- | --- | --- | --- | --- | --- | --- | --- | --- | --- | --- | --- | --- | --- | --- | --- | --- | --- | --- | --- | --- | --- | --- |

XP\_026579472.1

back to list

  

| Protein Coverage
| Supporting Peptides
|

Protein Coverage:

Supporting Peptides:

| Peptide | Uniq | -10lgP | Mass | Length | ppm | m/z | z | RT | Fraction | Scan | Source File | Area F6 | Area F2 | Area F3 | Area F4 | Area F5 | Area F7 | Area F8 | Area F1 | #Feature | #Feature F6 | #Feature F2 | #Feature F3 | #Feature F4 | #Feature F5 | #Feature F7 | #Feature F8 | #Feature F1 | Start | End | PTM | AScore | Found By |
| --- | --- | --- | --- | --- | --- | --- | --- | --- | --- | --- | --- | --- | --- | --- | --- | --- | --- | --- | --- | --- | --- | --- | --- | --- | --- | --- | --- | --- | --- | --- | --- | --- | --- |
| K.LTLSALVDGK.N | Y | 48.00 | 1015.5913 | 10 | 1.1 | 508.8035 | 2 | 44.95 | 8 | F8:24669 | 31122021\_RID\_3262\_1b.raw |  |  |  |  |  |  |  | 8.4212E4 | 1 | 0 | 0 | 0 | 0 | 0 | 0 | 0 | 1 | 271 | 280 |  |  | PEAKS DB |
| total 1 peptides |  |  |  |  |  |  |  |  |  |  |  |  |  |  |  |  |  |  |  |  |  |  |  |  |  |  |  |  |  |  |  |  |  |
| --- | --- | --- | --- | --- | --- | --- | --- | --- | --- | --- | --- | --- | --- | --- | --- | --- | --- | --- | --- | --- | --- | --- | --- | --- | --- | --- | --- | --- | --- | --- | --- | --- | --- |

AFJ49407.1

back to list

  

| Protein Coverage
| Supporting Peptides
|

Protein Coverage:

Supporting Peptides:

| Peptide | Uniq | -10lgP | Mass | Length | ppm | m/z | z | RT | Fraction | Scan | Source File | Area F6 | Area F2 | Area F3 | Area F4 | Area F5 | Area F7 | Area F8 | Area F1 | #Feature | #Feature F6 | #Feature F2 | #Feature F3 | #Feature F4 | #Feature F5 | #Feature F7 | #Feature F8 | #Feature F1 | Start | End | PTM | AScore | Found By |
| --- | --- | --- | --- | --- | --- | --- | --- | --- | --- | --- | --- | --- | --- | --- | --- | --- | --- | --- | --- | --- | --- | --- | --- | --- | --- | --- | --- | --- | --- | --- | --- | --- | --- |
| K.LTLSALVDGK.N | Y | 48.00 | 1015.5913 | 10 | 1.1 | 508.8035 | 2 | 44.95 | 8 | F8:24669 | 31122021\_RID\_3262\_1b.raw |  |  |  |  |  |  |  | 8.4212E4 | 1 | 0 | 0 | 0 | 0 | 0 | 0 | 0 | 1 | 271 | 280 |  |  | PEAKS DB |
| total 1 peptides |  |  |  |  |  |  |  |  |  |  |  |  |  |  |  |  |  |  |  |  |  |  |  |  |  |  |  |  |  |  |  |  |  |
| --- | --- | --- | --- | --- | --- | --- | --- | --- | --- | --- | --- | --- | --- | --- | --- | --- | --- | --- | --- | --- | --- | --- | --- | --- | --- | --- | --- | --- | --- | --- | --- | --- | --- |

XP\_026529972.1

back to list

  

| Protein Coverage
| Supporting Peptides
|

Protein Coverage:

Supporting Peptides:

| Peptide | Uniq | -10lgP | Mass | Length | ppm | m/z | z | RT | Fraction | Scan | Source File | Area F6 | Area F2 | Area F3 | Area F4 | Area F5 | Area F7 | Area F8 | Area F1 | #Feature | #Feature F6 | #Feature F2 | #Feature F3 | #Feature F4 | #Feature F5 | #Feature F7 | #Feature F8 | #Feature F1 | Start | End | PTM | AScore | Found By |
| --- | --- | --- | --- | --- | --- | --- | --- | --- | --- | --- | --- | --- | --- | --- | --- | --- | --- | --- | --- | --- | --- | --- | --- | --- | --- | --- | --- | --- | --- | --- | --- | --- | --- |
| K.LTLSALVDGK.N | Y | 48.00 | 1015.5913 | 10 | 1.1 | 508.8035 | 2 | 44.95 | 8 | F8:24669 | 31122021\_RID\_3262\_1b.raw |  |  |  |  |  |  |  | 8.4212E4 | 1 | 0 | 0 | 0 | 0 | 0 | 0 | 0 | 1 | 271 | 280 |  |  | PEAKS DB |
| total 1 peptides |  |  |  |  |  |  |  |  |  |  |  |  |  |  |  |  |  |  |  |  |  |  |  |  |  |  |  |  |  |  |  |  |  |
| --- | --- | --- | --- | --- | --- | --- | --- | --- | --- | --- | --- | --- | --- | --- | --- | --- | --- | --- | --- | --- | --- | --- | --- | --- | --- | --- | --- | --- | --- | --- | --- | --- | --- |

XP\_034291313.1

back to list

  

| Protein Coverage
| Supporting Peptides
|

Protein Coverage:

Supporting Peptides:

| Peptide | Uniq | -10lgP | Mass | Length | ppm | m/z | z | RT | Fraction | Scan | Source File | Area F6 | Area F2 | Area F3 | Area F4 | Area F5 | Area F7 | Area F8 | Area F1 | #Feature | #Feature F6 | #Feature F2 | #Feature F3 | #Feature F4 | #Feature F5 | #Feature F7 | #Feature F8 | #Feature F1 | Start | End | PTM | AScore | Found By |
| --- | --- | --- | --- | --- | --- | --- | --- | --- | --- | --- | --- | --- | --- | --- | --- | --- | --- | --- | --- | --- | --- | --- | --- | --- | --- | --- | --- | --- | --- | --- | --- | --- | --- |
| K.LTLSALVDGK.N | Y | 48.00 | 1015.5913 | 10 | 1.1 | 508.8035 | 2 | 44.95 | 8 | F8:24669 | 31122021\_RID\_3262\_1b.raw |  |  |  |  |  |  |  | 8.4212E4 | 1 | 0 | 0 | 0 | 0 | 0 | 0 | 0 | 1 | 271 | 280 |  |  | PEAKS DB |
| total 1 peptides |  |  |  |  |  |  |  |  |  |  |  |  |  |  |  |  |  |  |  |  |  |  |  |  |  |  |  |  |  |  |  |  |  |
| --- | --- | --- | --- | --- | --- | --- | --- | --- | --- | --- | --- | --- | --- | --- | --- | --- | --- | --- | --- | --- | --- | --- | --- | --- | --- | --- | --- | --- | --- | --- | --- | --- | --- |

XP\_029142427.1

back to list

  

| Protein Coverage
| Supporting Peptides
|

Protein Coverage:

Supporting Peptides:

| Peptide | Uniq | -10lgP | Mass | Length | ppm | m/z | z | RT | Fraction | Scan | Source File | Area F6 | Area F2 | Area F3 | Area F4 | Area F5 | Area F7 | Area F8 | Area F1 | #Feature | #Feature F6 | #Feature F2 | #Feature F3 | #Feature F4 | #Feature F5 | #Feature F7 | #Feature F8 | #Feature F1 | Start | End | PTM | AScore | Found By |
| --- | --- | --- | --- | --- | --- | --- | --- | --- | --- | --- | --- | --- | --- | --- | --- | --- | --- | --- | --- | --- | --- | --- | --- | --- | --- | --- | --- | --- | --- | --- | --- | --- | --- |
| K.LTLSALVDGK.N | Y | 48.00 | 1015.5913 | 10 | 1.1 | 508.8035 | 2 | 44.95 | 8 | F8:24669 | 31122021\_RID\_3262\_1b.raw |  |  |  |  |  |  |  | 8.4212E4 | 1 | 0 | 0 | 0 | 0 | 0 | 0 | 0 | 1 | 287 | 296 |  |  | PEAKS DB |
| total 1 peptides |  |  |  |  |  |  |  |  |  |  |  |  |  |  |  |  |  |  |  |  |  |  |  |  |  |  |  |  |  |  |  |  |  |
| --- | --- | --- | --- | --- | --- | --- | --- | --- | --- | --- | --- | --- | --- | --- | --- | --- | --- | --- | --- | --- | --- | --- | --- | --- | --- | --- | --- | --- | --- | --- | --- | --- | --- |

JAI13875.1

back to list

  

| Protein Coverage
| Supporting Peptides
|

Protein Coverage:

Supporting Peptides:

| Peptide | Uniq | -10lgP | Mass | Length | ppm | m/z | z | RT | Fraction | Scan | Source File | Area F6 | Area F2 | Area F3 | Area F4 | Area F5 | Area F7 | Area F8 | Area F1 | #Feature | #Feature F6 | #Feature F2 | #Feature F3 | #Feature F4 | #Feature F5 | #Feature F7 | #Feature F8 | #Feature F1 | Start | End | PTM | AScore | Found By |
| --- | --- | --- | --- | --- | --- | --- | --- | --- | --- | --- | --- | --- | --- | --- | --- | --- | --- | --- | --- | --- | --- | --- | --- | --- | --- | --- | --- | --- | --- | --- | --- | --- | --- |
| R.ELTPQVVSAAR.I | Y | 47.92 | 1169.6404 | 11 | 0.5 | 585.8278 | 2 | 32.80 | 8 | F8:15165 | 31122021\_RID\_3262\_1b.raw |  |  |  |  |  |  |  | 0 | 0 | 0 | 0 | 0 | 0 | 0 | 0 | 0 | 0 | 670 | 680 |  |  | PEAKS DB |
| total 1 peptides |  |  |  |  |  |  |  |  |  |  |  |  |  |  |  |  |  |  |  |  |  |  |  |  |  |  |  |  |  |  |  |  |  |
| --- | --- | --- | --- | --- | --- | --- | --- | --- | --- | --- | --- | --- | --- | --- | --- | --- | --- | --- | --- | --- | --- | --- | --- | --- | --- | --- | --- | --- | --- | --- | --- | --- | --- |

AFJ52037.1

back to list

  

| Protein Coverage
| Supporting Peptides
|

Protein Coverage:

Supporting Peptides:

| Peptide | Uniq | -10lgP | Mass | Length | ppm | m/z | z | RT | Fraction | Scan | Source File | Area F6 | Area F2 | Area F3 | Area F4 | Area F5 | Area F7 | Area F8 | Area F1 | #Feature | #Feature F6 | #Feature F2 | #Feature F3 | #Feature F4 | #Feature F5 | #Feature F7 | #Feature F8 | #Feature F1 | Start | End | PTM | AScore | Found By |
| --- | --- | --- | --- | --- | --- | --- | --- | --- | --- | --- | --- | --- | --- | --- | --- | --- | --- | --- | --- | --- | --- | --- | --- | --- | --- | --- | --- | --- | --- | --- | --- | --- | --- |
| R.ELTPQVVSAAR.I | Y | 47.92 | 1169.6404 | 11 | 0.5 | 585.8278 | 2 | 32.80 | 8 | F8:15165 | 31122021\_RID\_3262\_1b.raw |  |  |  |  |  |  |  | 0 | 0 | 0 | 0 | 0 | 0 | 0 | 0 | 0 | 0 | 670 | 680 |  |  | PEAKS DB |
| total 1 peptides |  |  |  |  |  |  |  |  |  |  |  |  |  |  |  |  |  |  |  |  |  |  |  |  |  |  |  |  |  |  |  |  |  |
| --- | --- | --- | --- | --- | --- | --- | --- | --- | --- | --- | --- | --- | --- | --- | --- | --- | --- | --- | --- | --- | --- | --- | --- | --- | --- | --- | --- | --- | --- | --- | --- | --- | --- |

JAG65919.1

back to list

  

| Protein Coverage
| Supporting Peptides
|

Protein Coverage:

Supporting Peptides:

| Peptide | Uniq | -10lgP | Mass | Length | ppm | m/z | z | RT | Fraction | Scan | Source File | Area F6 | Area F2 | Area F3 | Area F4 | Area F5 | Area F7 | Area F8 | Area F1 | #Feature | #Feature F6 | #Feature F2 | #Feature F3 | #Feature F4 | #Feature F5 | #Feature F7 | #Feature F8 | #Feature F1 | Start | End | PTM | AScore | Found By |
| --- | --- | --- | --- | --- | --- | --- | --- | --- | --- | --- | --- | --- | --- | --- | --- | --- | --- | --- | --- | --- | --- | --- | --- | --- | --- | --- | --- | --- | --- | --- | --- | --- | --- |
| R.ELTPQVVSAAR.I | Y | 47.92 | 1169.6404 | 11 | 0.5 | 585.8278 | 2 | 32.80 | 8 | F8:15165 | 31122021\_RID\_3262\_1b.raw |  |  |  |  |  |  |  | 0 | 0 | 0 | 0 | 0 | 0 | 0 | 0 | 0 | 0 | 670 | 680 |  |  | PEAKS DB |
| total 1 peptides |  |  |  |  |  |  |  |  |  |  |  |  |  |  |  |  |  |  |  |  |  |  |  |  |  |  |  |  |  |  |  |  |  |
| --- | --- | --- | --- | --- | --- | --- | --- | --- | --- | --- | --- | --- | --- | --- | --- | --- | --- | --- | --- | --- | --- | --- | --- | --- | --- | --- | --- | --- | --- | --- | --- | --- | --- |

XP\_034291292.1

back to list

  

| Protein Coverage
| Supporting Peptides
|

Protein Coverage:

Supporting Peptides:

| Peptide | Uniq | -10lgP | Mass | Length | ppm | m/z | z | RT | Fraction | Scan | Source File | Area F6 | Area F2 | Area F3 | Area F4 | Area F5 | Area F7 | Area F8 | Area F1 | #Feature | #Feature F6 | #Feature F2 | #Feature F3 | #Feature F4 | #Feature F5 | #Feature F7 | #Feature F8 | #Feature F1 | Start | End | PTM | AScore | Found By |
| --- | --- | --- | --- | --- | --- | --- | --- | --- | --- | --- | --- | --- | --- | --- | --- | --- | --- | --- | --- | --- | --- | --- | --- | --- | --- | --- | --- | --- | --- | --- | --- | --- | --- |
| R.ELTPQVVSAAR.I | Y | 47.92 | 1169.6404 | 11 | 0.5 | 585.8278 | 2 | 32.80 | 8 | F8:15165 | 31122021\_RID\_3262\_1b.raw |  |  |  |  |  |  |  | 0 | 0 | 0 | 0 | 0 | 0 | 0 | 0 | 0 | 0 | 670 | 680 |  |  | PEAKS DB |
| total 1 peptides |  |  |  |  |  |  |  |  |  |  |  |  |  |  |  |  |  |  |  |  |  |  |  |  |  |  |  |  |  |  |  |  |  |
| --- | --- | --- | --- | --- | --- | --- | --- | --- | --- | --- | --- | --- | --- | --- | --- | --- | --- | --- | --- | --- | --- | --- | --- | --- | --- | --- | --- | --- | --- | --- | --- | --- | --- |

XP\_034291291.1

back to list

  

| Protein Coverage
| Supporting Peptides
|

Protein Coverage:

Supporting Peptides:

| Peptide | Uniq | -10lgP | Mass | Length | ppm | m/z | z | RT | Fraction | Scan | Source File | Area F6 | Area F2 | Area F3 | Area F4 | Area F5 | Area F7 | Area F8 | Area F1 | #Feature | #Feature F6 | #Feature F2 | #Feature F3 | #Feature F4 | #Feature F5 | #Feature F7 | #Feature F8 | #Feature F1 | Start | End | PTM | AScore | Found By |
| --- | --- | --- | --- | --- | --- | --- | --- | --- | --- | --- | --- | --- | --- | --- | --- | --- | --- | --- | --- | --- | --- | --- | --- | --- | --- | --- | --- | --- | --- | --- | --- | --- | --- |
| R.ELTPQVVSAAR.I | Y | 47.92 | 1169.6404 | 11 | 0.5 | 585.8278 | 2 | 32.80 | 8 | F8:15165 | 31122021\_RID\_3262\_1b.raw |  |  |  |  |  |  |  | 0 | 0 | 0 | 0 | 0 | 0 | 0 | 0 | 0 | 0 | 671 | 681 |  |  | PEAKS DB |
| total 1 peptides |  |  |  |  |  |  |  |  |  |  |  |  |  |  |  |  |  |  |  |  |  |  |  |  |  |  |  |  |  |  |  |  |  |
| --- | --- | --- | --- | --- | --- | --- | --- | --- | --- | --- | --- | --- | --- | --- | --- | --- | --- | --- | --- | --- | --- | --- | --- | --- | --- | --- | --- | --- | --- | --- | --- | --- | --- |

XP\_034291290.1

back to list

  

| Protein Coverage
| Supporting Peptides
|

Protein Coverage:

Supporting Peptides:

| Peptide | Uniq | -10lgP | Mass | Length | ppm | m/z | z | RT | Fraction | Scan | Source File | Area F6 | Area F2 | Area F3 | Area F4 | Area F5 | Area F7 | Area F8 | Area F1 | #Feature | #Feature F6 | #Feature F2 | #Feature F3 | #Feature F4 | #Feature F5 | #Feature F7 | #Feature F8 | #Feature F1 | Start | End | PTM | AScore | Found By |
| --- | --- | --- | --- | --- | --- | --- | --- | --- | --- | --- | --- | --- | --- | --- | --- | --- | --- | --- | --- | --- | --- | --- | --- | --- | --- | --- | --- | --- | --- | --- | --- | --- | --- |
| R.ELTPQVVSAAR.I | Y | 47.92 | 1169.6404 | 11 | 0.5 | 585.8278 | 2 | 32.80 | 8 | F8:15165 | 31122021\_RID\_3262\_1b.raw |  |  |  |  |  |  |  | 0 | 0 | 0 | 0 | 0 | 0 | 0 | 0 | 0 | 0 | 670 | 680 |  |  | PEAKS DB |
| total 1 peptides |  |  |  |  |  |  |  |  |  |  |  |  |  |  |  |  |  |  |  |  |  |  |  |  |  |  |  |  |  |  |  |  |  |
| --- | --- | --- | --- | --- | --- | --- | --- | --- | --- | --- | --- | --- | --- | --- | --- | --- | --- | --- | --- | --- | --- | --- | --- | --- | --- | --- | --- | --- | --- | --- | --- | --- | --- |

XP\_034291289.1

back to list

  

| Protein Coverage
| Supporting Peptides
|

Protein Coverage:

Supporting Peptides:

| Peptide | Uniq | -10lgP | Mass | Length | ppm | m/z | z | RT | Fraction | Scan | Source File | Area F6 | Area F2 | Area F3 | Area F4 | Area F5 | Area F7 | Area F8 | Area F1 | #Feature | #Feature F6 | #Feature F2 | #Feature F3 | #Feature F4 | #Feature F5 | #Feature F7 | #Feature F8 | #Feature F1 | Start | End | PTM | AScore | Found By |
| --- | --- | --- | --- | --- | --- | --- | --- | --- | --- | --- | --- | --- | --- | --- | --- | --- | --- | --- | --- | --- | --- | --- | --- | --- | --- | --- | --- | --- | --- | --- | --- | --- | --- |
| R.ELTPQVVSAAR.I | Y | 47.92 | 1169.6404 | 11 | 0.5 | 585.8278 | 2 | 32.80 | 8 | F8:15165 | 31122021\_RID\_3262\_1b.raw |  |  |  |  |  |  |  | 0 | 0 | 0 | 0 | 0 | 0 | 0 | 0 | 0 | 0 | 671 | 681 |  |  | PEAKS DB |
| total 1 peptides |  |  |  |  |  |  |  |  |  |  |  |  |  |  |  |  |  |  |  |  |  |  |  |  |  |  |  |  |  |  |  |  |  |
| --- | --- | --- | --- | --- | --- | --- | --- | --- | --- | --- | --- | --- | --- | --- | --- | --- | --- | --- | --- | --- | --- | --- | --- | --- | --- | --- | --- | --- | --- | --- | --- | --- | --- |

XP\_015671947.1

back to list

  

| Protein Coverage
| Supporting Peptides
|

Protein Coverage:

Supporting Peptides:

| Peptide | Uniq | -10lgP | Mass | Length | ppm | m/z | z | RT | Fraction | Scan | Source File | Area F6 | Area F2 | Area F3 | Area F4 | Area F5 | Area F7 | Area F8 | Area F1 | #Feature | #Feature F6 | #Feature F2 | #Feature F3 | #Feature F4 | #Feature F5 | #Feature F7 | #Feature F8 | #Feature F1 | Start | End | PTM | AScore | Found By |
| --- | --- | --- | --- | --- | --- | --- | --- | --- | --- | --- | --- | --- | --- | --- | --- | --- | --- | --- | --- | --- | --- | --- | --- | --- | --- | --- | --- | --- | --- | --- | --- | --- | --- |
| R.ELTPQVVSAAR.I | Y | 47.92 | 1169.6404 | 11 | 0.5 | 585.8278 | 2 | 32.80 | 8 | F8:15165 | 31122021\_RID\_3262\_1b.raw |  |  |  |  |  |  |  | 0 | 0 | 0 | 0 | 0 | 0 | 0 | 0 | 0 | 0 | 595 | 605 |  |  | PEAKS DB |
| total 1 peptides |  |  |  |  |  |  |  |  |  |  |  |  |  |  |  |  |  |  |  |  |  |  |  |  |  |  |  |  |  |  |  |  |  |
| --- | --- | --- | --- | --- | --- | --- | --- | --- | --- | --- | --- | --- | --- | --- | --- | --- | --- | --- | --- | --- | --- | --- | --- | --- | --- | --- | --- | --- | --- | --- | --- | --- | --- |

XP\_007424958.1

back to list

  

| Protein Coverage
| Supporting Peptides
|

Protein Coverage:

Supporting Peptides:

| Peptide | Uniq | -10lgP | Mass | Length | ppm | m/z | z | RT | Fraction | Scan | Source File | Area F6 | Area F2 | Area F3 | Area F4 | Area F5 | Area F7 | Area F8 | Area F1 | #Feature | #Feature F6 | #Feature F2 | #Feature F3 | #Feature F4 | #Feature F5 | #Feature F7 | #Feature F8 | #Feature F1 | Start | End | PTM | AScore | Found By |
| --- | --- | --- | --- | --- | --- | --- | --- | --- | --- | --- | --- | --- | --- | --- | --- | --- | --- | --- | --- | --- | --- | --- | --- | --- | --- | --- | --- | --- | --- | --- | --- | --- | --- |
| R.ELTPQVVSAAR.I | Y | 47.92 | 1169.6404 | 11 | 0.5 | 585.8278 | 2 | 32.80 | 8 | F8:15165 | 31122021\_RID\_3262\_1b.raw |  |  |  |  |  |  |  | 0 | 0 | 0 | 0 | 0 | 0 | 0 | 0 | 0 | 0 | 670 | 680 |  |  | PEAKS DB |
| total 1 peptides |  |  |  |  |  |  |  |  |  |  |  |  |  |  |  |  |  |  |  |  |  |  |  |  |  |  |  |  |  |  |  |  |  |
| --- | --- | --- | --- | --- | --- | --- | --- | --- | --- | --- | --- | --- | --- | --- | --- | --- | --- | --- | --- | --- | --- | --- | --- | --- | --- | --- | --- | --- | --- | --- | --- | --- | --- |

XP\_007424957.1

back to list

  

| Protein Coverage
| Supporting Peptides
|

Protein Coverage:

Supporting Peptides:

| Peptide | Uniq | -10lgP | Mass | Length | ppm | m/z | z | RT | Fraction | Scan | Source File | Area F6 | Area F2 | Area F3 | Area F4 | Area F5 | Area F7 | Area F8 | Area F1 | #Feature | #Feature F6 | #Feature F2 | #Feature F3 | #Feature F4 | #Feature F5 | #Feature F7 | #Feature F8 | #Feature F1 | Start | End | PTM | AScore | Found By |
| --- | --- | --- | --- | --- | --- | --- | --- | --- | --- | --- | --- | --- | --- | --- | --- | --- | --- | --- | --- | --- | --- | --- | --- | --- | --- | --- | --- | --- | --- | --- | --- | --- | --- |
| R.ELTPQVVSAAR.I | Y | 47.92 | 1169.6404 | 11 | 0.5 | 585.8278 | 2 | 32.80 | 8 | F8:15165 | 31122021\_RID\_3262\_1b.raw |  |  |  |  |  |  |  | 0 | 0 | 0 | 0 | 0 | 0 | 0 | 0 | 0 | 0 | 671 | 681 |  |  | PEAKS DB |
| total 1 peptides |  |  |  |  |  |  |  |  |  |  |  |  |  |  |  |  |  |  |  |  |  |  |  |  |  |  |  |  |  |  |  |  |  |
| --- | --- | --- | --- | --- | --- | --- | --- | --- | --- | --- | --- | --- | --- | --- | --- | --- | --- | --- | --- | --- | --- | --- | --- | --- | --- | --- | --- | --- | --- | --- | --- | --- | --- |

XP\_007424956.1

back to list

  

| Protein Coverage
| Supporting Peptides
|

Protein Coverage:

Supporting Peptides:

| Peptide | Uniq | -10lgP | Mass | Length | ppm | m/z | z | RT | Fraction | Scan | Source File | Area F6 | Area F2 | Area F3 | Area F4 | Area F5 | Area F7 | Area F8 | Area F1 | #Feature | #Feature F6 | #Feature F2 | #Feature F3 | #Feature F4 | #Feature F5 | #Feature F7 | #Feature F8 | #Feature F1 | Start | End | PTM | AScore | Found By |
| --- | --- | --- | --- | --- | --- | --- | --- | --- | --- | --- | --- | --- | --- | --- | --- | --- | --- | --- | --- | --- | --- | --- | --- | --- | --- | --- | --- | --- | --- | --- | --- | --- | --- |
| R.ELTPQVVSAAR.I | Y | 47.92 | 1169.6404 | 11 | 0.5 | 585.8278 | 2 | 32.80 | 8 | F8:15165 | 31122021\_RID\_3262\_1b.raw |  |  |  |  |  |  |  | 0 | 0 | 0 | 0 | 0 | 0 | 0 | 0 | 0 | 0 | 670 | 680 |  |  | PEAKS DB |
| total 1 peptides |  |  |  |  |  |  |  |  |  |  |  |  |  |  |  |  |  |  |  |  |  |  |  |  |  |  |  |  |  |  |  |  |  |
| --- | --- | --- | --- | --- | --- | --- | --- | --- | --- | --- | --- | --- | --- | --- | --- | --- | --- | --- | --- | --- | --- | --- | --- | --- | --- | --- | --- | --- | --- | --- | --- | --- | --- |

XP\_026529964.1

back to list

  

| Protein Coverage
| Supporting Peptides
|

Protein Coverage:

Supporting Peptides:

| Peptide | Uniq | -10lgP | Mass | Length | ppm | m/z | z | RT | Fraction | Scan | Source File | Area F6 | Area F2 | Area F3 | Area F4 | Area F5 | Area F7 | Area F8 | Area F1 | #Feature | #Feature F6 | #Feature F2 | #Feature F3 | #Feature F4 | #Feature F5 | #Feature F7 | #Feature F8 | #Feature F1 | Start | End | PTM | AScore | Found By |
| --- | --- | --- | --- | --- | --- | --- | --- | --- | --- | --- | --- | --- | --- | --- | --- | --- | --- | --- | --- | --- | --- | --- | --- | --- | --- | --- | --- | --- | --- | --- | --- | --- | --- |
| R.ELTPQVVSAAR.I | Y | 47.92 | 1169.6404 | 11 | 0.5 | 585.8278 | 2 | 32.80 | 8 | F8:15165 | 31122021\_RID\_3262\_1b.raw |  |  |  |  |  |  |  | 0 | 0 | 0 | 0 | 0 | 0 | 0 | 0 | 0 | 0 | 571 | 581 |  |  | PEAKS DB |
| total 1 peptides |  |  |  |  |  |  |  |  |  |  |  |  |  |  |  |  |  |  |  |  |  |  |  |  |  |  |  |  |  |  |  |  |  |
| --- | --- | --- | --- | --- | --- | --- | --- | --- | --- | --- | --- | --- | --- | --- | --- | --- | --- | --- | --- | --- | --- | --- | --- | --- | --- | --- | --- | --- | --- | --- | --- | --- | --- |

XP\_032087779.1

back to list

  

| Protein Coverage
| Supporting Peptides
|

Protein Coverage:

Supporting Peptides:

| Peptide | Uniq | -10lgP | Mass | Length | ppm | m/z | z | RT | Fraction | Scan | Source File | Area F6 | Area F2 | Area F3 | Area F4 | Area F5 | Area F7 | Area F8 | Area F1 | #Feature | #Feature F6 | #Feature F2 | #Feature F3 | #Feature F4 | #Feature F5 | #Feature F7 | #Feature F8 | #Feature F1 | Start | End | PTM | AScore | Found By |
| --- | --- | --- | --- | --- | --- | --- | --- | --- | --- | --- | --- | --- | --- | --- | --- | --- | --- | --- | --- | --- | --- | --- | --- | --- | --- | --- | --- | --- | --- | --- | --- | --- | --- |
| R.ELTPQVVSAAR.I | Y | 47.92 | 1169.6404 | 11 | 0.5 | 585.8278 | 2 | 32.80 | 8 | F8:15165 | 31122021\_RID\_3262\_1b.raw |  |  |  |  |  |  |  | 0 | 0 | 0 | 0 | 0 | 0 | 0 | 0 | 0 | 0 | 670 | 680 |  |  | PEAKS DB |
| total 1 peptides |  |  |  |  |  |  |  |  |  |  |  |  |  |  |  |  |  |  |  |  |  |  |  |  |  |  |  |  |  |  |  |  |  |
| --- | --- | --- | --- | --- | --- | --- | --- | --- | --- | --- | --- | --- | --- | --- | --- | --- | --- | --- | --- | --- | --- | --- | --- | --- | --- | --- | --- | --- | --- | --- | --- | --- | --- |

XP\_032087780.1

back to list

  

| Protein Coverage
| Supporting Peptides
|

Protein Coverage:

Supporting Peptides:

| Peptide | Uniq | -10lgP | Mass | Length | ppm | m/z | z | RT | Fraction | Scan | Source File | Area F6 | Area F2 | Area F3 | Area F4 | Area F5 | Area F7 | Area F8 | Area F1 | #Feature | #Feature F6 | #Feature F2 | #Feature F3 | #Feature F4 | #Feature F5 | #Feature F7 | #Feature F8 | #Feature F1 | Start | End | PTM | AScore | Found By |
| --- | --- | --- | --- | --- | --- | --- | --- | --- | --- | --- | --- | --- | --- | --- | --- | --- | --- | --- | --- | --- | --- | --- | --- | --- | --- | --- | --- | --- | --- | --- | --- | --- | --- |
| R.ELTPQVVSAAR.I | Y | 47.92 | 1169.6404 | 11 | 0.5 | 585.8278 | 2 | 32.80 | 8 | F8:15165 | 31122021\_RID\_3262\_1b.raw |  |  |  |  |  |  |  | 0 | 0 | 0 | 0 | 0 | 0 | 0 | 0 | 0 | 0 | 670 | 680 |  |  | PEAKS DB |
| total 1 peptides |  |  |  |  |  |  |  |  |  |  |  |  |  |  |  |  |  |  |  |  |  |  |  |  |  |  |  |  |  |  |  |  |  |
| --- | --- | --- | --- | --- | --- | --- | --- | --- | --- | --- | --- | --- | --- | --- | --- | --- | --- | --- | --- | --- | --- | --- | --- | --- | --- | --- | --- | --- | --- | --- | --- | --- | --- |

LAA44292.1

back to list

  

| Protein Coverage
| Supporting Peptides
|

Protein Coverage:

Supporting Peptides:

| Peptide | Uniq | -10lgP | Mass | Length | ppm | m/z | z | RT | Fraction | Scan | Source File | Area F6 | Area F2 | Area F3 | Area F4 | Area F5 | Area F7 | Area F8 | Area F1 | #Feature | #Feature F6 | #Feature F2 | #Feature F3 | #Feature F4 | #Feature F5 | #Feature F7 | #Feature F8 | #Feature F1 | Start | End | PTM | AScore | Found By |
| --- | --- | --- | --- | --- | --- | --- | --- | --- | --- | --- | --- | --- | --- | --- | --- | --- | --- | --- | --- | --- | --- | --- | --- | --- | --- | --- | --- | --- | --- | --- | --- | --- | --- |
| R.ELTPQVVSAAR.I | Y | 47.92 | 1169.6404 | 11 | 0.5 | 585.8278 | 2 | 32.80 | 8 | F8:15165 | 31122021\_RID\_3262\_1b.raw |  |  |  |  |  |  |  | 0 | 0 | 0 | 0 | 0 | 0 | 0 | 0 | 0 | 0 | 367 | 377 |  |  | PEAKS DB |
| total 1 peptides |  |  |  |  |  |  |  |  |  |  |  |  |  |  |  |  |  |  |  |  |  |  |  |  |  |  |  |  |  |  |  |  |  |
| --- | --- | --- | --- | --- | --- | --- | --- | --- | --- | --- | --- | --- | --- | --- | --- | --- | --- | --- | --- | --- | --- | --- | --- | --- | --- | --- | --- | --- | --- | --- | --- | --- | --- |

XP\_026569149.1

back to list

  

| Protein Coverage
| Supporting Peptides
|

Protein Coverage:

Supporting Peptides:

| Peptide | Uniq | -10lgP | Mass | Length | ppm | m/z | z | RT | Fraction | Scan | Source File | Area F6 | Area F2 | Area F3 | Area F4 | Area F5 | Area F7 | Area F8 | Area F1 | #Feature | #Feature F6 | #Feature F2 | #Feature F3 | #Feature F4 | #Feature F5 | #Feature F7 | #Feature F8 | #Feature F1 | Start | End | PTM | AScore | Found By |
| --- | --- | --- | --- | --- | --- | --- | --- | --- | --- | --- | --- | --- | --- | --- | --- | --- | --- | --- | --- | --- | --- | --- | --- | --- | --- | --- | --- | --- | --- | --- | --- | --- | --- |
| K.VVLAYEPVWAIGTGK.T | Y | 47.06 | 1601.8817 | 15 | 0.7 | 801.9487 | 2 | 56.04 | 8 | F8:33405 | 31122021\_RID\_3262\_1b.raw |  |  |  |  |  |  |  | 7.6533E4 | 1 | 0 | 0 | 0 | 0 | 0 | 0 | 0 | 1 | 160 | 174 |  |  | PEAKS DB |
| total 1 peptides |  |  |  |  |  |  |  |  |  |  |  |  |  |  |  |  |  |  |  |  |  |  |  |  |  |  |  |  |  |  |  |  |  |
| --- | --- | --- | --- | --- | --- | --- | --- | --- | --- | --- | --- | --- | --- | --- | --- | --- | --- | --- | --- | --- | --- | --- | --- | --- | --- | --- | --- | --- | --- | --- | --- | --- | --- |

JAI11012.1

back to list

  

| Protein Coverage
| Supporting Peptides
|

Protein Coverage:

Supporting Peptides:

| Peptide | Uniq | -10lgP | Mass | Length | ppm | m/z | z | RT | Fraction | Scan | Source File | Area F6 | Area F2 | Area F3 | Area F4 | Area F5 | Area F7 | Area F8 | Area F1 | #Feature | #Feature F6 | #Feature F2 | #Feature F3 | #Feature F4 | #Feature F5 | #Feature F7 | #Feature F8 | #Feature F1 | Start | End | PTM | AScore | Found By |
| --- | --- | --- | --- | --- | --- | --- | --- | --- | --- | --- | --- | --- | --- | --- | --- | --- | --- | --- | --- | --- | --- | --- | --- | --- | --- | --- | --- | --- | --- | --- | --- | --- | --- |
| K.VVLAYEPVWAIGTGK.T | Y | 47.06 | 1601.8817 | 15 | 0.7 | 801.9487 | 2 | 56.04 | 8 | F8:33405 | 31122021\_RID\_3262\_1b.raw |  |  |  |  |  |  |  | 7.6533E4 | 1 | 0 | 0 | 0 | 0 | 0 | 0 | 0 | 1 | 160 | 174 |  |  | PEAKS DB |
| total 1 peptides |  |  |  |  |  |  |  |  |  |  |  |  |  |  |  |  |  |  |  |  |  |  |  |  |  |  |  |  |  |  |  |  |  |
| --- | --- | --- | --- | --- | --- | --- | --- | --- | --- | --- | --- | --- | --- | --- | --- | --- | --- | --- | --- | --- | --- | --- | --- | --- | --- | --- | --- | --- | --- | --- | --- | --- | --- |

XP\_032069541.1

back to list

  

| Protein Coverage
| Supporting Peptides
|

Protein Coverage:

Supporting Peptides:

| Peptide | Uniq | -10lgP | Mass | Length | ppm | m/z | z | RT | Fraction | Scan | Source File | Area F6 | Area F2 | Area F3 | Area F4 | Area F5 | Area F7 | Area F8 | Area F1 | #Feature | #Feature F6 | #Feature F2 | #Feature F3 | #Feature F4 | #Feature F5 | #Feature F7 | #Feature F8 | #Feature F1 | Start | End | PTM | AScore | Found By |
| --- | --- | --- | --- | --- | --- | --- | --- | --- | --- | --- | --- | --- | --- | --- | --- | --- | --- | --- | --- | --- | --- | --- | --- | --- | --- | --- | --- | --- | --- | --- | --- | --- | --- |
| K.VVLAYEPVWAIGTGK.T | Y | 47.06 | 1601.8817 | 15 | 0.7 | 801.9487 | 2 | 56.04 | 8 | F8:33405 | 31122021\_RID\_3262\_1b.raw |  |  |  |  |  |  |  | 7.6533E4 | 1 | 0 | 0 | 0 | 0 | 0 | 0 | 0 | 1 | 160 | 174 |  |  | PEAKS DB |
| total 1 peptides |  |  |  |  |  |  |  |  |  |  |  |  |  |  |  |  |  |  |  |  |  |  |  |  |  |  |  |  |  |  |  |  |  |
| --- | --- | --- | --- | --- | --- | --- | --- | --- | --- | --- | --- | --- | --- | --- | --- | --- | --- | --- | --- | --- | --- | --- | --- | --- | --- | --- | --- | --- | --- | --- | --- | --- | --- |

XP\_015669508.1

back to list

  

| Protein Coverage
| Supporting Peptides
|

Protein Coverage:

Supporting Peptides:

| Peptide | Uniq | -10lgP | Mass | Length | ppm | m/z | z | RT | Fraction | Scan | Source File | Area F6 | Area F2 | Area F3 | Area F4 | Area F5 | Area F7 | Area F8 | Area F1 | #Feature | #Feature F6 | #Feature F2 | #Feature F3 | #Feature F4 | #Feature F5 | #Feature F7 | #Feature F8 | #Feature F1 | Start | End | PTM | AScore | Found By |
| --- | --- | --- | --- | --- | --- | --- | --- | --- | --- | --- | --- | --- | --- | --- | --- | --- | --- | --- | --- | --- | --- | --- | --- | --- | --- | --- | --- | --- | --- | --- | --- | --- | --- |
| K.VVLAYEPVWAIGTGK.T | Y | 47.06 | 1601.8817 | 15 | 0.7 | 801.9487 | 2 | 56.04 | 8 | F8:33405 | 31122021\_RID\_3262\_1b.raw |  |  |  |  |  |  |  | 7.6533E4 | 1 | 0 | 0 | 0 | 0 | 0 | 0 | 0 | 1 | 160 | 174 |  |  | PEAKS DB |
| total 1 peptides |  |  |  |  |  |  |  |  |  |  |  |  |  |  |  |  |  |  |  |  |  |  |  |  |  |  |  |  |  |  |  |  |  |
| --- | --- | --- | --- | --- | --- | --- | --- | --- | --- | --- | --- | --- | --- | --- | --- | --- | --- | --- | --- | --- | --- | --- | --- | --- | --- | --- | --- | --- | --- | --- | --- | --- | --- |

XP\_026542021.1

back to list

  

| Protein Coverage
| Supporting Peptides
|

Protein Coverage:

Supporting Peptides:

| Peptide | Uniq | -10lgP | Mass | Length | ppm | m/z | z | RT | Fraction | Scan | Source File | Area F6 | Area F2 | Area F3 | Area F4 | Area F5 | Area F7 | Area F8 | Area F1 | #Feature | #Feature F6 | #Feature F2 | #Feature F3 | #Feature F4 | #Feature F5 | #Feature F7 | #Feature F8 | #Feature F1 | Start | End | PTM | AScore | Found By |
| --- | --- | --- | --- | --- | --- | --- | --- | --- | --- | --- | --- | --- | --- | --- | --- | --- | --- | --- | --- | --- | --- | --- | --- | --- | --- | --- | --- | --- | --- | --- | --- | --- | --- |
| K.VVLAYEPVWAIGTGK.T | Y | 47.06 | 1601.8817 | 15 | 0.7 | 801.9487 | 2 | 56.04 | 8 | F8:33405 | 31122021\_RID\_3262\_1b.raw |  |  |  |  |  |  |  | 7.6533E4 | 1 | 0 | 0 | 0 | 0 | 0 | 0 | 0 | 1 | 160 | 174 |  |  | PEAKS DB |
| total 1 peptides |  |  |  |  |  |  |  |  |  |  |  |  |  |  |  |  |  |  |  |  |  |  |  |  |  |  |  |  |  |  |  |  |  |
| --- | --- | --- | --- | --- | --- | --- | --- | --- | --- | --- | --- | --- | --- | --- | --- | --- | --- | --- | --- | --- | --- | --- | --- | --- | --- | --- | --- | --- | --- | --- | --- | --- | --- |

JAG66211.1

back to list

  

| Protein Coverage
| Supporting Peptides
|

Protein Coverage:

Supporting Peptides:

| Peptide | Uniq | -10lgP | Mass | Length | ppm | m/z | z | RT | Fraction | Scan | Source File | Area F6 | Area F2 | Area F3 | Area F4 | Area F5 | Area F7 | Area F8 | Area F1 | #Feature | #Feature F6 | #Feature F2 | #Feature F3 | #Feature F4 | #Feature F5 | #Feature F7 | #Feature F8 | #Feature F1 | Start | End | PTM | AScore | Found By |
| --- | --- | --- | --- | --- | --- | --- | --- | --- | --- | --- | --- | --- | --- | --- | --- | --- | --- | --- | --- | --- | --- | --- | --- | --- | --- | --- | --- | --- | --- | --- | --- | --- | --- |
| K.VVLAYEPVWAIGTGK.T | Y | 47.06 | 1601.8817 | 15 | 0.7 | 801.9487 | 2 | 56.04 | 8 | F8:33405 | 31122021\_RID\_3262\_1b.raw |  |  |  |  |  |  |  | 7.6533E4 | 1 | 0 | 0 | 0 | 0 | 0 | 0 | 0 | 1 | 160 | 174 |  |  | PEAKS DB |
| total 1 peptides |  |  |  |  |  |  |  |  |  |  |  |  |  |  |  |  |  |  |  |  |  |  |  |  |  |  |  |  |  |  |  |  |  |
| --- | --- | --- | --- | --- | --- | --- | --- | --- | --- | --- | --- | --- | --- | --- | --- | --- | --- | --- | --- | --- | --- | --- | --- | --- | --- | --- | --- | --- | --- | --- | --- | --- | --- |

XP\_013926209.1

back to list

  

| Protein Coverage
| Supporting Peptides
|

Protein Coverage:

Supporting Peptides:

| Peptide | Uniq | -10lgP | Mass | Length | ppm | m/z | z | RT | Fraction | Scan | Source File | Area F6 | Area F2 | Area F3 | Area F4 | Area F5 | Area F7 | Area F8 | Area F1 | #Feature | #Feature F6 | #Feature F2 | #Feature F3 | #Feature F4 | #Feature F5 | #Feature F7 | #Feature F8 | #Feature F1 | Start | End | PTM | AScore | Found By |
| --- | --- | --- | --- | --- | --- | --- | --- | --- | --- | --- | --- | --- | --- | --- | --- | --- | --- | --- | --- | --- | --- | --- | --- | --- | --- | --- | --- | --- | --- | --- | --- | --- | --- |
| K.VVLAYEPVWAIGTGK.T | Y | 47.06 | 1601.8817 | 15 | 0.7 | 801.9487 | 2 | 56.04 | 8 | F8:33405 | 31122021\_RID\_3262\_1b.raw |  |  |  |  |  |  |  | 7.6533E4 | 1 | 0 | 0 | 0 | 0 | 0 | 0 | 0 | 1 | 160 | 174 |  |  | PEAKS DB |
| total 1 peptides |  |  |  |  |  |  |  |  |  |  |  |  |  |  |  |  |  |  |  |  |  |  |  |  |  |  |  |  |  |  |  |  |  |
| --- | --- | --- | --- | --- | --- | --- | --- | --- | --- | --- | --- | --- | --- | --- | --- | --- | --- | --- | --- | --- | --- | --- | --- | --- | --- | --- | --- | --- | --- | --- | --- | --- | --- |

JAV48852.1

back to list

  

| Protein Coverage
| Supporting Peptides
|

Protein Coverage:

Supporting Peptides:

| Peptide | Uniq | -10lgP | Mass | Length | ppm | m/z | z | RT | Fraction | Scan | Source File | Area F6 | Area F2 | Area F3 | Area F4 | Area F5 | Area F7 | Area F8 | Area F1 | #Feature | #Feature F6 | #Feature F2 | #Feature F3 | #Feature F4 | #Feature F5 | #Feature F7 | #Feature F8 | #Feature F1 | Start | End | PTM | AScore | Found By |
| --- | --- | --- | --- | --- | --- | --- | --- | --- | --- | --- | --- | --- | --- | --- | --- | --- | --- | --- | --- | --- | --- | --- | --- | --- | --- | --- | --- | --- | --- | --- | --- | --- | --- |
| K.VVLAYEPVWAIGTGK.T | Y | 47.06 | 1601.8817 | 15 | 0.7 | 801.9487 | 2 | 56.04 | 8 | F8:33405 | 31122021\_RID\_3262\_1b.raw |  |  |  |  |  |  |  | 7.6533E4 | 1 | 0 | 0 | 0 | 0 | 0 | 0 | 0 | 1 | 160 | 174 |  |  | PEAKS DB |
| total 1 peptides |  |  |  |  |  |  |  |  |  |  |  |  |  |  |  |  |  |  |  |  |  |  |  |  |  |  |  |  |  |  |  |  |  |
| --- | --- | --- | --- | --- | --- | --- | --- | --- | --- | --- | --- | --- | --- | --- | --- | --- | --- | --- | --- | --- | --- | --- | --- | --- | --- | --- | --- | --- | --- | --- | --- | --- | --- |

JAA95339.1

back to list

  

| Protein Coverage
| Supporting Peptides
|

Protein Coverage:

Supporting Peptides:

| Peptide | Uniq | -10lgP | Mass | Length | ppm | m/z | z | RT | Fraction | Scan | Source File | Area F6 | Area F2 | Area F3 | Area F4 | Area F5 | Area F7 | Area F8 | Area F1 | #Feature | #Feature F6 | #Feature F2 | #Feature F3 | #Feature F4 | #Feature F5 | #Feature F7 | #Feature F8 | #Feature F1 | Start | End | PTM | AScore | Found By |
| --- | --- | --- | --- | --- | --- | --- | --- | --- | --- | --- | --- | --- | --- | --- | --- | --- | --- | --- | --- | --- | --- | --- | --- | --- | --- | --- | --- | --- | --- | --- | --- | --- | --- |
| K.VVLAYEPVWAIGTGK.T | Y | 47.06 | 1601.8817 | 15 | 0.7 | 801.9487 | 2 | 56.04 | 8 | F8:33405 | 31122021\_RID\_3262\_1b.raw |  |  |  |  |  |  |  | 7.6533E4 | 1 | 0 | 0 | 0 | 0 | 0 | 0 | 0 | 1 | 160 | 174 |  |  | PEAKS DB |
| total 1 peptides |  |  |  |  |  |  |  |  |  |  |  |  |  |  |  |  |  |  |  |  |  |  |  |  |  |  |  |  |  |  |  |  |  |
| --- | --- | --- | --- | --- | --- | --- | --- | --- | --- | --- | --- | --- | --- | --- | --- | --- | --- | --- | --- | --- | --- | --- | --- | --- | --- | --- | --- | --- | --- | --- | --- | --- | --- |

JAI09292.1

back to list

  

| Protein Coverage
| Supporting Peptides
|

Protein Coverage:

Supporting Peptides:

| Peptide | Uniq | -10lgP | Mass | Length | ppm | m/z | z | RT | Fraction | Scan | Source File | Area F6 | Area F2 | Area F3 | Area F4 | Area F5 | Area F7 | Area F8 | Area F1 | #Feature | #Feature F6 | #Feature F2 | #Feature F3 | #Feature F4 | #Feature F5 | #Feature F7 | #Feature F8 | #Feature F1 | Start | End | PTM | AScore | Found By |
| --- | --- | --- | --- | --- | --- | --- | --- | --- | --- | --- | --- | --- | --- | --- | --- | --- | --- | --- | --- | --- | --- | --- | --- | --- | --- | --- | --- | --- | --- | --- | --- | --- | --- |
| K.VVLAYEPVWAIGTGK.T | Y | 47.06 | 1601.8817 | 15 | 0.7 | 801.9487 | 2 | 56.04 | 8 | F8:33405 | 31122021\_RID\_3262\_1b.raw |  |  |  |  |  |  |  | 7.6533E4 | 1 | 0 | 0 | 0 | 0 | 0 | 0 | 0 | 1 | 160 | 174 |  |  | PEAKS DB |
| total 1 peptides |  |  |  |  |  |  |  |  |  |  |  |  |  |  |  |  |  |  |  |  |  |  |  |  |  |  |  |  |  |  |  |  |  |
| --- | --- | --- | --- | --- | --- | --- | --- | --- | --- | --- | --- | --- | --- | --- | --- | --- | --- | --- | --- | --- | --- | --- | --- | --- | --- | --- | --- | --- | --- | --- | --- | --- | --- |

AFJ51829.1

back to list

  

| Protein Coverage
| Supporting Peptides
|

Protein Coverage:

Supporting Peptides:

| Peptide | Uniq | -10lgP | Mass | Length | ppm | m/z | z | RT | Fraction | Scan | Source File | Area F6 | Area F2 | Area F3 | Area F4 | Area F5 | Area F7 | Area F8 | Area F1 | #Feature | #Feature F6 | #Feature F2 | #Feature F3 | #Feature F4 | #Feature F5 | #Feature F7 | #Feature F8 | #Feature F1 | Start | End | PTM | AScore | Found By |
| --- | --- | --- | --- | --- | --- | --- | --- | --- | --- | --- | --- | --- | --- | --- | --- | --- | --- | --- | --- | --- | --- | --- | --- | --- | --- | --- | --- | --- | --- | --- | --- | --- | --- |
| K.VVLAYEPVWAIGTGK.T | Y | 47.06 | 1601.8817 | 15 | 0.7 | 801.9487 | 2 | 56.04 | 8 | F8:33405 | 31122021\_RID\_3262\_1b.raw |  |  |  |  |  |  |  | 7.6533E4 | 1 | 0 | 0 | 0 | 0 | 0 | 0 | 0 | 1 | 160 | 174 |  |  | PEAKS DB |
| total 1 peptides |  |  |  |  |  |  |  |  |  |  |  |  |  |  |  |  |  |  |  |  |  |  |  |  |  |  |  |  |  |  |  |  |  |
| --- | --- | --- | --- | --- | --- | --- | --- | --- | --- | --- | --- | --- | --- | --- | --- | --- | --- | --- | --- | --- | --- | --- | --- | --- | --- | --- | --- | --- | --- | --- | --- | --- | --- |

XP\_034266880.1

back to list

  

| Protein Coverage
| Supporting Peptides
|

Protein Coverage:

Supporting Peptides:

| Peptide | Uniq | -10lgP | Mass | Length | ppm | m/z | z | RT | Fraction | Scan | Source File | Area F6 | Area F2 | Area F3 | Area F4 | Area F5 | Area F7 | Area F8 | Area F1 | #Feature | #Feature F6 | #Feature F2 | #Feature F3 | #Feature F4 | #Feature F5 | #Feature F7 | #Feature F8 | #Feature F1 | Start | End | PTM | AScore | Found By |
| --- | --- | --- | --- | --- | --- | --- | --- | --- | --- | --- | --- | --- | --- | --- | --- | --- | --- | --- | --- | --- | --- | --- | --- | --- | --- | --- | --- | --- | --- | --- | --- | --- | --- |
| K.VVLAYEPVWAIGTGK.T | Y | 47.06 | 1601.8817 | 15 | 0.7 | 801.9487 | 2 | 56.04 | 8 | F8:33405 | 31122021\_RID\_3262\_1b.raw |  |  |  |  |  |  |  | 7.6533E4 | 1 | 0 | 0 | 0 | 0 | 0 | 0 | 0 | 1 | 160 | 174 |  |  | PEAKS DB |
| total 1 peptides |  |  |  |  |  |  |  |  |  |  |  |  |  |  |  |  |  |  |  |  |  |  |  |  |  |  |  |  |  |  |  |  |  |
| --- | --- | --- | --- | --- | --- | --- | --- | --- | --- | --- | --- | --- | --- | --- | --- | --- | --- | --- | --- | --- | --- | --- | --- | --- | --- | --- | --- | --- | --- | --- | --- | --- | --- |

ETE72092.1

back to list

  

| Protein Coverage
| Supporting Peptides
|

Protein Coverage:

Supporting Peptides:

| Peptide | Uniq | -10lgP | Mass | Length | ppm | m/z | z | RT | Fraction | Scan | Source File | Area F6 | Area F2 | Area F3 | Area F4 | Area F5 | Area F7 | Area F8 | Area F1 | #Feature | #Feature F6 | #Feature F2 | #Feature F3 | #Feature F4 | #Feature F5 | #Feature F7 | #Feature F8 | #Feature F1 | Start | End | PTM | AScore | Found By |
| --- | --- | --- | --- | --- | --- | --- | --- | --- | --- | --- | --- | --- | --- | --- | --- | --- | --- | --- | --- | --- | --- | --- | --- | --- | --- | --- | --- | --- | --- | --- | --- | --- | --- |
| K.VVLAYEPVWAIGTGK.T | Y | 47.06 | 1601.8817 | 15 | 0.7 | 801.9487 | 2 | 56.04 | 8 | F8:33405 | 31122021\_RID\_3262\_1b.raw |  |  |  |  |  |  |  | 7.6533E4 | 1 | 0 | 0 | 0 | 0 | 0 | 0 | 0 | 1 | 160 | 174 |  |  | PEAKS DB |
| total 1 peptides |  |  |  |  |  |  |  |  |  |  |  |  |  |  |  |  |  |  |  |  |  |  |  |  |  |  |  |  |  |  |  |  |  |
| --- | --- | --- | --- | --- | --- | --- | --- | --- | --- | --- | --- | --- | --- | --- | --- | --- | --- | --- | --- | --- | --- | --- | --- | --- | --- | --- | --- | --- | --- | --- | --- | --- | --- |

JAB53077.1

back to list

  

| Protein Coverage
| Supporting Peptides
|

Protein Coverage:

Supporting Peptides:

| Peptide | Uniq | -10lgP | Mass | Length | ppm | m/z | z | RT | Fraction | Scan | Source File | Area F6 | Area F2 | Area F3 | Area F4 | Area F5 | Area F7 | Area F8 | Area F1 | #Feature | #Feature F6 | #Feature F2 | #Feature F3 | #Feature F4 | #Feature F5 | #Feature F7 | #Feature F8 | #Feature F1 | Start | End | PTM | AScore | Found By |
| --- | --- | --- | --- | --- | --- | --- | --- | --- | --- | --- | --- | --- | --- | --- | --- | --- | --- | --- | --- | --- | --- | --- | --- | --- | --- | --- | --- | --- | --- | --- | --- | --- | --- |
| K.VVLAYEPVWAIGTGK.T | Y | 47.06 | 1601.8817 | 15 | 0.7 | 801.9487 | 2 | 56.04 | 8 | F8:33405 | 31122021\_RID\_3262\_1b.raw |  |  |  |  |  |  |  | 7.6533E4 | 1 | 0 | 0 | 0 | 0 | 0 | 0 | 0 | 1 | 160 | 174 |  |  | PEAKS DB |
| total 1 peptides |  |  |  |  |  |  |  |  |  |  |  |  |  |  |  |  |  |  |  |  |  |  |  |  |  |  |  |  |  |  |  |  |  |
| --- | --- | --- | --- | --- | --- | --- | --- | --- | --- | --- | --- | --- | --- | --- | --- | --- | --- | --- | --- | --- | --- | --- | --- | --- | --- | --- | --- | --- | --- | --- | --- | --- | --- |

XP\_007420484.1

back to list

  

| Protein Coverage
| Supporting Peptides
|

Protein Coverage:

Supporting Peptides:

| Peptide | Uniq | -10lgP | Mass | Length | ppm | m/z | z | RT | Fraction | Scan | Source File | Area F6 | Area F2 | Area F3 | Area F4 | Area F5 | Area F7 | Area F8 | Area F1 | #Feature | #Feature F6 | #Feature F2 | #Feature F3 | #Feature F4 | #Feature F5 | #Feature F7 | #Feature F8 | #Feature F1 | Start | End | PTM | AScore | Found By |
| --- | --- | --- | --- | --- | --- | --- | --- | --- | --- | --- | --- | --- | --- | --- | --- | --- | --- | --- | --- | --- | --- | --- | --- | --- | --- | --- | --- | --- | --- | --- | --- | --- | --- |
| K.VVLAYEPVWAIGTGK.T | Y | 47.06 | 1601.8817 | 15 | 0.7 | 801.9487 | 2 | 56.04 | 8 | F8:33405 | 31122021\_RID\_3262\_1b.raw |  |  |  |  |  |  |  | 7.6533E4 | 1 | 0 | 0 | 0 | 0 | 0 | 0 | 0 | 1 | 160 | 174 |  |  | PEAKS DB |
| total 1 peptides |  |  |  |  |  |  |  |  |  |  |  |  |  |  |  |  |  |  |  |  |  |  |  |  |  |  |  |  |  |  |  |  |  |
| --- | --- | --- | --- | --- | --- | --- | --- | --- | --- | --- | --- | --- | --- | --- | --- | --- | --- | --- | --- | --- | --- | --- | --- | --- | --- | --- | --- | --- | --- | --- | --- | --- | --- |

ETE63728.1

back to list

  

| Protein Coverage
| Supporting Peptides
|

Protein Coverage:

Supporting Peptides:

| Peptide | Uniq | -10lgP | Mass | Length | ppm | m/z | z | RT | Fraction | Scan | Source File | Area F6 | Area F2 | Area F3 | Area F4 | Area F5 | Area F7 | Area F8 | Area F1 | #Feature | #Feature F6 | #Feature F2 | #Feature F3 | #Feature F4 | #Feature F5 | #Feature F7 | #Feature F8 | #Feature F1 | Start | End | PTM | AScore | Found By |
| --- | --- | --- | --- | --- | --- | --- | --- | --- | --- | --- | --- | --- | --- | --- | --- | --- | --- | --- | --- | --- | --- | --- | --- | --- | --- | --- | --- | --- | --- | --- | --- | --- | --- |
| K.VVLAYEPVWAIGTGK.T | Y | 47.06 | 1601.8817 | 15 | 0.7 | 801.9487 | 2 | 56.04 | 8 | F8:33405 | 31122021\_RID\_3262\_1b.raw |  |  |  |  |  |  |  | 7.6533E4 | 1 | 0 | 0 | 0 | 0 | 0 | 0 | 0 | 1 | 95 | 109 |  |  | PEAKS DB |
| total 1 peptides |  |  |  |  |  |  |  |  |  |  |  |  |  |  |  |  |  |  |  |  |  |  |  |  |  |  |  |  |  |  |  |  |  |
| --- | --- | --- | --- | --- | --- | --- | --- | --- | --- | --- | --- | --- | --- | --- | --- | --- | --- | --- | --- | --- | --- | --- | --- | --- | --- | --- | --- | --- | --- | --- | --- | --- | --- |

XP\_026555717.1

back to list

  

| Protein Coverage
| Supporting Peptides
|

Protein Coverage:

Supporting Peptides:

| Peptide | Uniq | -10lgP | Mass | Length | ppm | m/z | z | RT | Fraction | Scan | Source File | Area F6 | Area F2 | Area F3 | Area F4 | Area F5 | Area F7 | Area F8 | Area F1 | #Feature | #Feature F6 | #Feature F2 | #Feature F3 | #Feature F4 | #Feature F5 | #Feature F7 | #Feature F8 | #Feature F1 | Start | End | PTM | AScore | Found By |
| --- | --- | --- | --- | --- | --- | --- | --- | --- | --- | --- | --- | --- | --- | --- | --- | --- | --- | --- | --- | --- | --- | --- | --- | --- | --- | --- | --- | --- | --- | --- | --- | --- | --- |
| K.EIVLADVIDNDSWR.L | Y | 46.57 | 1643.8154 | 14 | 0.7 | 822.9155 | 2 | 59.21 | 8 | F8:35877 | 31122021\_RID\_3262\_1b.raw |  |  |  |  |  |  |  | 4.2276E4 | 1 | 0 | 0 | 0 | 0 | 0 | 0 | 0 | 1 | 840 | 853 |  |  | PEAKS DB |
| total 1 peptides |  |  |  |  |  |  |  |  |  |  |  |  |  |  |  |  |  |  |  |  |  |  |  |  |  |  |  |  |  |  |  |  |  |
| --- | --- | --- | --- | --- | --- | --- | --- | --- | --- | --- | --- | --- | --- | --- | --- | --- | --- | --- | --- | --- | --- | --- | --- | --- | --- | --- | --- | --- | --- | --- | --- | --- | --- |

XP\_007421180.1

back to list

  

| Protein Coverage
| Supporting Peptides
|

Protein Coverage:

Supporting Peptides:

| Peptide | Uniq | -10lgP | Mass | Length | ppm | m/z | z | RT | Fraction | Scan | Source File | Area F6 | Area F2 | Area F3 | Area F4 | Area F5 | Area F7 | Area F8 | Area F1 | #Feature | #Feature F6 | #Feature F2 | #Feature F3 | #Feature F4 | #Feature F5 | #Feature F7 | #Feature F8 | #Feature F1 | Start | End | PTM | AScore | Found By |
| --- | --- | --- | --- | --- | --- | --- | --- | --- | --- | --- | --- | --- | --- | --- | --- | --- | --- | --- | --- | --- | --- | --- | --- | --- | --- | --- | --- | --- | --- | --- | --- | --- | --- |
| K.EIVLADVIDNDSWR.L | Y | 46.57 | 1643.8154 | 14 | 0.7 | 822.9155 | 2 | 59.21 | 8 | F8:35877 | 31122021\_RID\_3262\_1b.raw |  |  |  |  |  |  |  | 4.2276E4 | 1 | 0 | 0 | 0 | 0 | 0 | 0 | 0 | 1 | 203 | 216 |  |  | PEAKS DB |
| total 1 peptides |  |  |  |  |  |  |  |  |  |  |  |  |  |  |  |  |  |  |  |  |  |  |  |  |  |  |  |  |  |  |  |  |  |
| --- | --- | --- | --- | --- | --- | --- | --- | --- | --- | --- | --- | --- | --- | --- | --- | --- | --- | --- | --- | --- | --- | --- | --- | --- | --- | --- | --- | --- | --- | --- | --- | --- | --- |

XP\_007421178.1

back to list

  

| Protein Coverage
| Supporting Peptides
|

Protein Coverage:

Supporting Peptides:

| Peptide | Uniq | -10lgP | Mass | Length | ppm | m/z | z | RT | Fraction | Scan | Source File | Area F6 | Area F2 | Area F3 | Area F4 | Area F5 | Area F7 | Area F8 | Area F1 | #Feature | #Feature F6 | #Feature F2 | #Feature F3 | #Feature F4 | #Feature F5 | #Feature F7 | #Feature F8 | #Feature F1 | Start | End | PTM | AScore | Found By |
| --- | --- | --- | --- | --- | --- | --- | --- | --- | --- | --- | --- | --- | --- | --- | --- | --- | --- | --- | --- | --- | --- | --- | --- | --- | --- | --- | --- | --- | --- | --- | --- | --- | --- |
| K.EIVLADVIDNDSWR.L | Y | 46.57 | 1643.8154 | 14 | 0.7 | 822.9155 | 2 | 59.21 | 8 | F8:35877 | 31122021\_RID\_3262\_1b.raw |  |  |  |  |  |  |  | 4.2276E4 | 1 | 0 | 0 | 0 | 0 | 0 | 0 | 0 | 1 | 458 | 471 |  |  | PEAKS DB |
| total 1 peptides |  |  |  |  |  |  |  |  |  |  |  |  |  |  |  |  |  |  |  |  |  |  |  |  |  |  |  |  |  |  |  |  |  |
| --- | --- | --- | --- | --- | --- | --- | --- | --- | --- | --- | --- | --- | --- | --- | --- | --- | --- | --- | --- | --- | --- | --- | --- | --- | --- | --- | --- | --- | --- | --- | --- | --- | --- |

ETE65717.1

back to list

  

| Protein Coverage
| Supporting Peptides
|

Protein Coverage:

Supporting Peptides:

| Peptide | Uniq | -10lgP | Mass | Length | ppm | m/z | z | RT | Fraction | Scan | Source File | Area F6 | Area F2 | Area F3 | Area F4 | Area F5 | Area F7 | Area F8 | Area F1 | #Feature | #Feature F6 | #Feature F2 | #Feature F3 | #Feature F4 | #Feature F5 | #Feature F7 | #Feature F8 | #Feature F1 | Start | End | PTM | AScore | Found By |
| --- | --- | --- | --- | --- | --- | --- | --- | --- | --- | --- | --- | --- | --- | --- | --- | --- | --- | --- | --- | --- | --- | --- | --- | --- | --- | --- | --- | --- | --- | --- | --- | --- | --- |
| K.EIVLADVIDNDSWR.L | Y | 46.57 | 1643.8154 | 14 | 0.7 | 822.9155 | 2 | 59.21 | 8 | F8:35877 | 31122021\_RID\_3262\_1b.raw |  |  |  |  |  |  |  | 4.2276E4 | 1 | 0 | 0 | 0 | 0 | 0 | 0 | 0 | 1 | 194 | 207 |  |  | PEAKS DB |
| total 1 peptides |  |  |  |  |  |  |  |  |  |  |  |  |  |  |  |  |  |  |  |  |  |  |  |  |  |  |  |  |  |  |  |  |  |
| --- | --- | --- | --- | --- | --- | --- | --- | --- | --- | --- | --- | --- | --- | --- | --- | --- | --- | --- | --- | --- | --- | --- | --- | --- | --- | --- | --- | --- | --- | --- | --- | --- | --- |

LAA41266.1

back to list

  

| Protein Coverage
| Supporting Peptides
|

Protein Coverage:

Supporting Peptides:

| Peptide | Uniq | -10lgP | Mass | Length | ppm | m/z | z | RT | Fraction | Scan | Source File | Area F6 | Area F2 | Area F3 | Area F4 | Area F5 | Area F7 | Area F8 | Area F1 | #Feature | #Feature F6 | #Feature F2 | #Feature F3 | #Feature F4 | #Feature F5 | #Feature F7 | #Feature F8 | #Feature F1 | Start | End | PTM | AScore | Found By |
| --- | --- | --- | --- | --- | --- | --- | --- | --- | --- | --- | --- | --- | --- | --- | --- | --- | --- | --- | --- | --- | --- | --- | --- | --- | --- | --- | --- | --- | --- | --- | --- | --- | --- |
| K.EIVLADVIDNDSWR.L | Y | 46.57 | 1643.8154 | 14 | 0.7 | 822.9155 | 2 | 59.21 | 8 | F8:35877 | 31122021\_RID\_3262\_1b.raw |  |  |  |  |  |  |  | 4.2276E4 | 1 | 0 | 0 | 0 | 0 | 0 | 0 | 0 | 1 | 203 | 216 |  |  | PEAKS DB |
| total 1 peptides |  |  |  |  |  |  |  |  |  |  |  |  |  |  |  |  |  |  |  |  |  |  |  |  |  |  |  |  |  |  |  |  |  |
| --- | --- | --- | --- | --- | --- | --- | --- | --- | --- | --- | --- | --- | --- | --- | --- | --- | --- | --- | --- | --- | --- | --- | --- | --- | --- | --- | --- | --- | --- | --- | --- | --- | --- |

LAA22516.1

back to list

  

| Protein Coverage
| Supporting Peptides
|

Protein Coverage:

Supporting Peptides:

| Peptide | Uniq | -10lgP | Mass | Length | ppm | m/z | z | RT | Fraction | Scan | Source File | Area F6 | Area F2 | Area F3 | Area F4 | Area F5 | Area F7 | Area F8 | Area F1 | #Feature | #Feature F6 | #Feature F2 | #Feature F3 | #Feature F4 | #Feature F5 | #Feature F7 | #Feature F8 | #Feature F1 | Start | End | PTM | AScore | Found By |
| --- | --- | --- | --- | --- | --- | --- | --- | --- | --- | --- | --- | --- | --- | --- | --- | --- | --- | --- | --- | --- | --- | --- | --- | --- | --- | --- | --- | --- | --- | --- | --- | --- | --- |
| K.EIVLADVIDNDSWR.L | Y | 46.57 | 1643.8154 | 14 | 0.7 | 822.9155 | 2 | 59.21 | 8 | F8:35877 | 31122021\_RID\_3262\_1b.raw |  |  |  |  |  |  |  | 4.2276E4 | 1 | 0 | 0 | 0 | 0 | 0 | 0 | 0 | 1 | 203 | 216 |  |  | PEAKS DB |
| total 1 peptides |  |  |  |  |  |  |  |  |  |  |  |  |  |  |  |  |  |  |  |  |  |  |  |  |  |  |  |  |  |  |  |  |  |
| --- | --- | --- | --- | --- | --- | --- | --- | --- | --- | --- | --- | --- | --- | --- | --- | --- | --- | --- | --- | --- | --- | --- | --- | --- | --- | --- | --- | --- | --- | --- | --- | --- | --- |

LAB38276.1

back to list

  

| Protein Coverage
| Supporting Peptides
|

Protein Coverage:

Supporting Peptides:

| Peptide | Uniq | -10lgP | Mass | Length | ppm | m/z | z | RT | Fraction | Scan | Source File | Area F6 | Area F2 | Area F3 | Area F4 | Area F5 | Area F7 | Area F8 | Area F1 | #Feature | #Feature F6 | #Feature F2 | #Feature F3 | #Feature F4 | #Feature F5 | #Feature F7 | #Feature F8 | #Feature F1 | Start | End | PTM | AScore | Found By |
| --- | --- | --- | --- | --- | --- | --- | --- | --- | --- | --- | --- | --- | --- | --- | --- | --- | --- | --- | --- | --- | --- | --- | --- | --- | --- | --- | --- | --- | --- | --- | --- | --- | --- |
| K.EIVLADVIDNDSWR.L | Y | 46.57 | 1643.8154 | 14 | 0.7 | 822.9155 | 2 | 59.21 | 8 | F8:35877 | 31122021\_RID\_3262\_1b.raw |  |  |  |  |  |  |  | 4.2276E4 | 1 | 0 | 0 | 0 | 0 | 0 | 0 | 0 | 1 | 203 | 216 |  |  | PEAKS DB |
| total 1 peptides |  |  |  |  |  |  |  |  |  |  |  |  |  |  |  |  |  |  |  |  |  |  |  |  |  |  |  |  |  |  |  |  |  |
| --- | --- | --- | --- | --- | --- | --- | --- | --- | --- | --- | --- | --- | --- | --- | --- | --- | --- | --- | --- | --- | --- | --- | --- | --- | --- | --- | --- | --- | --- | --- | --- | --- | --- |

JAC96332.1

back to list

  

| Protein Coverage
| Supporting Peptides
|

Protein Coverage:

Supporting Peptides:

| Peptide | Uniq | -10lgP | Mass | Length | ppm | m/z | z | RT | Fraction | Scan | Source File | Area F6 | Area F2 | Area F3 | Area F4 | Area F5 | Area F7 | Area F8 | Area F1 | #Feature | #Feature F6 | #Feature F2 | #Feature F3 | #Feature F4 | #Feature F5 | #Feature F7 | #Feature F8 | #Feature F1 | Start | End | PTM | AScore | Found By |
| --- | --- | --- | --- | --- | --- | --- | --- | --- | --- | --- | --- | --- | --- | --- | --- | --- | --- | --- | --- | --- | --- | --- | --- | --- | --- | --- | --- | --- | --- | --- | --- | --- | --- |
| K.EIVLADVIDNDSWR.L | Y | 46.57 | 1643.8154 | 14 | 0.7 | 822.9155 | 2 | 59.21 | 8 | F8:35877 | 31122021\_RID\_3262\_1b.raw |  |  |  |  |  |  |  | 4.2276E4 | 1 | 0 | 0 | 0 | 0 | 0 | 0 | 0 | 1 | 203 | 216 |  |  | PEAKS DB |
| total 1 peptides |  |  |  |  |  |  |  |  |  |  |  |  |  |  |  |  |  |  |  |  |  |  |  |  |  |  |  |  |  |  |  |  |  |
| --- | --- | --- | --- | --- | --- | --- | --- | --- | --- | --- | --- | --- | --- | --- | --- | --- | --- | --- | --- | --- | --- | --- | --- | --- | --- | --- | --- | --- | --- | --- | --- | --- | --- |

XP\_013928441.1

back to list

  

| Protein Coverage
| Supporting Peptides
|

Protein Coverage:

Supporting Peptides:

| Peptide | Uniq | -10lgP | Mass | Length | ppm | m/z | z | RT | Fraction | Scan | Source File | Area F6 | Area F2 | Area F3 | Area F4 | Area F5 | Area F7 | Area F8 | Area F1 | #Feature | #Feature F6 | #Feature F2 | #Feature F3 | #Feature F4 | #Feature F5 | #Feature F7 | #Feature F8 | #Feature F1 | Start | End | PTM | AScore | Found By |
| --- | --- | --- | --- | --- | --- | --- | --- | --- | --- | --- | --- | --- | --- | --- | --- | --- | --- | --- | --- | --- | --- | --- | --- | --- | --- | --- | --- | --- | --- | --- | --- | --- | --- |
| K.EIVLADVIDNDSWR.L | Y | 46.57 | 1643.8154 | 14 | 0.7 | 822.9155 | 2 | 59.21 | 8 | F8:35877 | 31122021\_RID\_3262\_1b.raw |  |  |  |  |  |  |  | 4.2276E4 | 1 | 0 | 0 | 0 | 0 | 0 | 0 | 0 | 1 | 170 | 183 |  |  | PEAKS DB |
| total 1 peptides |  |  |  |  |  |  |  |  |  |  |  |  |  |  |  |  |  |  |  |  |  |  |  |  |  |  |  |  |  |  |  |  |  |
| --- | --- | --- | --- | --- | --- | --- | --- | --- | --- | --- | --- | --- | --- | --- | --- | --- | --- | --- | --- | --- | --- | --- | --- | --- | --- | --- | --- | --- | --- | --- | --- | --- | --- |

XP\_029142087.1

back to list

  

| Protein Coverage
| Supporting Peptides
|

Protein Coverage:

Supporting Peptides:

| Peptide | Uniq | -10lgP | Mass | Length | ppm | m/z | z | RT | Fraction | Scan | Source File | Area F6 | Area F2 | Area F3 | Area F4 | Area F5 | Area F7 | Area F8 | Area F1 | #Feature | #Feature F6 | #Feature F2 | #Feature F3 | #Feature F4 | #Feature F5 | #Feature F7 | #Feature F8 | #Feature F1 | Start | End | PTM | AScore | Found By |
| --- | --- | --- | --- | --- | --- | --- | --- | --- | --- | --- | --- | --- | --- | --- | --- | --- | --- | --- | --- | --- | --- | --- | --- | --- | --- | --- | --- | --- | --- | --- | --- | --- | --- |
| K.EIVLADVIDNDSWR.L | Y | 46.57 | 1643.8154 | 14 | 0.7 | 822.9155 | 2 | 59.21 | 8 | F8:35877 | 31122021\_RID\_3262\_1b.raw |  |  |  |  |  |  |  | 4.2276E4 | 1 | 0 | 0 | 0 | 0 | 0 | 0 | 0 | 1 | 110 | 123 |  |  | PEAKS DB |
| total 1 peptides |  |  |  |  |  |  |  |  |  |  |  |  |  |  |  |  |  |  |  |  |  |  |  |  |  |  |  |  |  |  |  |  |  |
| --- | --- | --- | --- | --- | --- | --- | --- | --- | --- | --- | --- | --- | --- | --- | --- | --- | --- | --- | --- | --- | --- | --- | --- | --- | --- | --- | --- | --- | --- | --- | --- | --- | --- |

JAI14454.1

back to list

  

| Protein Coverage
| Supporting Peptides
|

Protein Coverage:

Supporting Peptides:

| Peptide | Uniq | -10lgP | Mass | Length | ppm | m/z | z | RT | Fraction | Scan | Source File | Area F6 | Area F2 | Area F3 | Area F4 | Area F5 | Area F7 | Area F8 | Area F1 | #Feature | #Feature F6 | #Feature F2 | #Feature F3 | #Feature F4 | #Feature F5 | #Feature F7 | #Feature F8 | #Feature F1 | Start | End | PTM | AScore | Found By |
| --- | --- | --- | --- | --- | --- | --- | --- | --- | --- | --- | --- | --- | --- | --- | --- | --- | --- | --- | --- | --- | --- | --- | --- | --- | --- | --- | --- | --- | --- | --- | --- | --- | --- |
| K.EIVLADVIDNDSWR.L | Y | 46.57 | 1643.8154 | 14 | 0.7 | 822.9155 | 2 | 59.21 | 8 | F8:35877 | 31122021\_RID\_3262\_1b.raw |  |  |  |  |  |  |  | 4.2276E4 | 1 | 0 | 0 | 0 | 0 | 0 | 0 | 0 | 1 | 203 | 216 |  |  | PEAKS DB |
| total 1 peptides |  |  |  |  |  |  |  |  |  |  |  |  |  |  |  |  |  |  |  |  |  |  |  |  |  |  |  |  |  |  |  |  |  |
| --- | --- | --- | --- | --- | --- | --- | --- | --- | --- | --- | --- | --- | --- | --- | --- | --- | --- | --- | --- | --- | --- | --- | --- | --- | --- | --- | --- | --- | --- | --- | --- | --- | --- |

JAG47557.1

back to list

  

| Protein Coverage
| Supporting Peptides
|

Protein Coverage:

Supporting Peptides:

| Peptide | Uniq | -10lgP | Mass | Length | ppm | m/z | z | RT | Fraction | Scan | Source File | Area F6 | Area F2 | Area F3 | Area F4 | Area F5 | Area F7 | Area F8 | Area F1 | #Feature | #Feature F6 | #Feature F2 | #Feature F3 | #Feature F4 | #Feature F5 | #Feature F7 | #Feature F8 | #Feature F1 | Start | End | PTM | AScore | Found By |
| --- | --- | --- | --- | --- | --- | --- | --- | --- | --- | --- | --- | --- | --- | --- | --- | --- | --- | --- | --- | --- | --- | --- | --- | --- | --- | --- | --- | --- | --- | --- | --- | --- | --- |
| K.EIVLADVIDNDSWR.L | Y | 46.57 | 1643.8154 | 14 | 0.7 | 822.9155 | 2 | 59.21 | 8 | F8:35877 | 31122021\_RID\_3262\_1b.raw |  |  |  |  |  |  |  | 4.2276E4 | 1 | 0 | 0 | 0 | 0 | 0 | 0 | 0 | 1 | 203 | 216 |  |  | PEAKS DB |
| total 1 peptides |  |  |  |  |  |  |  |  |  |  |  |  |  |  |  |  |  |  |  |  |  |  |  |  |  |  |  |  |  |  |  |  |  |
| --- | --- | --- | --- | --- | --- | --- | --- | --- | --- | --- | --- | --- | --- | --- | --- | --- | --- | --- | --- | --- | --- | --- | --- | --- | --- | --- | --- | --- | --- | --- | --- | --- | --- |

JAA97867.1

back to list

  

| Protein Coverage
| Supporting Peptides
|

Protein Coverage:

Supporting Peptides:

| Peptide | Uniq | -10lgP | Mass | Length | ppm | m/z | z | RT | Fraction | Scan | Source File | Area F6 | Area F2 | Area F3 | Area F4 | Area F5 | Area F7 | Area F8 | Area F1 | #Feature | #Feature F6 | #Feature F2 | #Feature F3 | #Feature F4 | #Feature F5 | #Feature F7 | #Feature F8 | #Feature F1 | Start | End | PTM | AScore | Found By |
| --- | --- | --- | --- | --- | --- | --- | --- | --- | --- | --- | --- | --- | --- | --- | --- | --- | --- | --- | --- | --- | --- | --- | --- | --- | --- | --- | --- | --- | --- | --- | --- | --- | --- |
| K.EIVLADVIDNDSWR.L | Y | 46.57 | 1643.8154 | 14 | 0.7 | 822.9155 | 2 | 59.21 | 8 | F8:35877 | 31122021\_RID\_3262\_1b.raw |  |  |  |  |  |  |  | 4.2276E4 | 1 | 0 | 0 | 0 | 0 | 0 | 0 | 0 | 1 | 203 | 216 |  |  | PEAKS DB |
| total 1 peptides |  |  |  |  |  |  |  |  |  |  |  |  |  |  |  |  |  |  |  |  |  |  |  |  |  |  |  |  |  |  |  |  |  |
| --- | --- | --- | --- | --- | --- | --- | --- | --- | --- | --- | --- | --- | --- | --- | --- | --- | --- | --- | --- | --- | --- | --- | --- | --- | --- | --- | --- | --- | --- | --- | --- | --- | --- |

AFJ50690.1

back to list

  

| Protein Coverage
| Supporting Peptides
|

Protein Coverage:

Supporting Peptides:

| Peptide | Uniq | -10lgP | Mass | Length | ppm | m/z | z | RT | Fraction | Scan | Source File | Area F6 | Area F2 | Area F3 | Area F4 | Area F5 | Area F7 | Area F8 | Area F1 | #Feature | #Feature F6 | #Feature F2 | #Feature F3 | #Feature F4 | #Feature F5 | #Feature F7 | #Feature F8 | #Feature F1 | Start | End | PTM | AScore | Found By |
| --- | --- | --- | --- | --- | --- | --- | --- | --- | --- | --- | --- | --- | --- | --- | --- | --- | --- | --- | --- | --- | --- | --- | --- | --- | --- | --- | --- | --- | --- | --- | --- | --- | --- |
| K.EIVLADVIDNDSWR.L | Y | 46.57 | 1643.8154 | 14 | 0.7 | 822.9155 | 2 | 59.21 | 8 | F8:35877 | 31122021\_RID\_3262\_1b.raw |  |  |  |  |  |  |  | 4.2276E4 | 1 | 0 | 0 | 0 | 0 | 0 | 0 | 0 | 1 | 203 | 216 |  |  | PEAKS DB |
| total 1 peptides |  |  |  |  |  |  |  |  |  |  |  |  |  |  |  |  |  |  |  |  |  |  |  |  |  |  |  |  |  |  |  |  |  |
| --- | --- | --- | --- | --- | --- | --- | --- | --- | --- | --- | --- | --- | --- | --- | --- | --- | --- | --- | --- | --- | --- | --- | --- | --- | --- | --- | --- | --- | --- | --- | --- | --- | --- |

JAG45959.1

back to list

  

| Protein Coverage
| Supporting Peptides
|

Protein Coverage:

Supporting Peptides:

| Peptide | Uniq | -10lgP | Mass | Length | ppm | m/z | z | RT | Fraction | Scan | Source File | Area F6 | Area F2 | Area F3 | Area F4 | Area F5 | Area F7 | Area F8 | Area F1 | #Feature | #Feature F6 | #Feature F2 | #Feature F3 | #Feature F4 | #Feature F5 | #Feature F7 | #Feature F8 | #Feature F1 | Start | End | PTM | AScore | Found By |
| --- | --- | --- | --- | --- | --- | --- | --- | --- | --- | --- | --- | --- | --- | --- | --- | --- | --- | --- | --- | --- | --- | --- | --- | --- | --- | --- | --- | --- | --- | --- | --- | --- | --- |
| K.EIVLADVIDNDSWR.L | Y | 46.57 | 1643.8154 | 14 | 0.7 | 822.9155 | 2 | 59.21 | 8 | F8:35877 | 31122021\_RID\_3262\_1b.raw |  |  |  |  |  |  |  | 4.2276E4 | 1 | 0 | 0 | 0 | 0 | 0 | 0 | 0 | 1 | 203 | 216 |  |  | PEAKS DB |
| total 1 peptides |  |  |  |  |  |  |  |  |  |  |  |  |  |  |  |  |  |  |  |  |  |  |  |  |  |  |  |  |  |  |  |  |  |
| --- | --- | --- | --- | --- | --- | --- | --- | --- | --- | --- | --- | --- | --- | --- | --- | --- | --- | --- | --- | --- | --- | --- | --- | --- | --- | --- | --- | --- | --- | --- | --- | --- | --- |

XP\_034267284.1

back to list

  

| Protein Coverage
| Supporting Peptides
|

Protein Coverage:

Supporting Peptides:

| Peptide | Uniq | -10lgP | Mass | Length | ppm | m/z | z | RT | Fraction | Scan | Source File | Area F6 | Area F2 | Area F3 | Area F4 | Area F5 | Area F7 | Area F8 | Area F1 | #Feature | #Feature F6 | #Feature F2 | #Feature F3 | #Feature F4 | #Feature F5 | #Feature F7 | #Feature F8 | #Feature F1 | Start | End | PTM | AScore | Found By |
| --- | --- | --- | --- | --- | --- | --- | --- | --- | --- | --- | --- | --- | --- | --- | --- | --- | --- | --- | --- | --- | --- | --- | --- | --- | --- | --- | --- | --- | --- | --- | --- | --- | --- |
| K.EIVLADVIDNDSWR.L | Y | 46.57 | 1643.8154 | 14 | 0.7 | 822.9155 | 2 | 59.21 | 8 | F8:35877 | 31122021\_RID\_3262\_1b.raw |  |  |  |  |  |  |  | 4.2276E4 | 1 | 0 | 0 | 0 | 0 | 0 | 0 | 0 | 1 | 203 | 216 |  |  | PEAKS DB |
| total 1 peptides |  |  |  |  |  |  |  |  |  |  |  |  |  |  |  |  |  |  |  |  |  |  |  |  |  |  |  |  |  |  |  |  |  |
| --- | --- | --- | --- | --- | --- | --- | --- | --- | --- | --- | --- | --- | --- | --- | --- | --- | --- | --- | --- | --- | --- | --- | --- | --- | --- | --- | --- | --- | --- | --- | --- | --- | --- |

JAB54751.1

back to list

  

| Protein Coverage
| Supporting Peptides
|

Protein Coverage:

Supporting Peptides:

| Peptide | Uniq | -10lgP | Mass | Length | ppm | m/z | z | RT | Fraction | Scan | Source File | Area F6 | Area F2 | Area F3 | Area F4 | Area F5 | Area F7 | Area F8 | Area F1 | #Feature | #Feature F6 | #Feature F2 | #Feature F3 | #Feature F4 | #Feature F5 | #Feature F7 | #Feature F8 | #Feature F1 | Start | End | PTM | AScore | Found By |
| --- | --- | --- | --- | --- | --- | --- | --- | --- | --- | --- | --- | --- | --- | --- | --- | --- | --- | --- | --- | --- | --- | --- | --- | --- | --- | --- | --- | --- | --- | --- | --- | --- | --- |
| K.EIVLADVIDNDSWR.L | Y | 46.57 | 1643.8154 | 14 | 0.7 | 822.9155 | 2 | 59.21 | 8 | F8:35877 | 31122021\_RID\_3262\_1b.raw |  |  |  |  |  |  |  | 4.2276E4 | 1 | 0 | 0 | 0 | 0 | 0 | 0 | 0 | 1 | 203 | 216 |  |  | PEAKS DB |
| total 1 peptides |  |  |  |  |  |  |  |  |  |  |  |  |  |  |  |  |  |  |  |  |  |  |  |  |  |  |  |  |  |  |  |  |  |
| --- | --- | --- | --- | --- | --- | --- | --- | --- | --- | --- | --- | --- | --- | --- | --- | --- | --- | --- | --- | --- | --- | --- | --- | --- | --- | --- | --- | --- | --- | --- | --- | --- | --- |

LAA95407.1

back to list

  

| Protein Coverage
| Supporting Peptides
|

Protein Coverage:

Supporting Peptides:

| Peptide | Uniq | -10lgP | Mass | Length | ppm | m/z | z | RT | Fraction | Scan | Source File | Area F6 | Area F2 | Area F3 | Area F4 | Area F5 | Area F7 | Area F8 | Area F1 | #Feature | #Feature F6 | #Feature F2 | #Feature F3 | #Feature F4 | #Feature F5 | #Feature F7 | #Feature F8 | #Feature F1 | Start | End | PTM | AScore | Found By |
| --- | --- | --- | --- | --- | --- | --- | --- | --- | --- | --- | --- | --- | --- | --- | --- | --- | --- | --- | --- | --- | --- | --- | --- | --- | --- | --- | --- | --- | --- | --- | --- | --- | --- |
| K.EIVLADVIDNDSWR.L | Y | 46.57 | 1643.8154 | 14 | 0.7 | 822.9155 | 2 | 59.21 | 8 | F8:35877 | 31122021\_RID\_3262\_1b.raw |  |  |  |  |  |  |  | 4.2276E4 | 1 | 0 | 0 | 0 | 0 | 0 | 0 | 0 | 1 | 203 | 216 |  |  | PEAKS DB |
| total 1 peptides |  |  |  |  |  |  |  |  |  |  |  |  |  |  |  |  |  |  |  |  |  |  |  |  |  |  |  |  |  |  |  |  |  |
| --- | --- | --- | --- | --- | --- | --- | --- | --- | --- | --- | --- | --- | --- | --- | --- | --- | --- | --- | --- | --- | --- | --- | --- | --- | --- | --- | --- | --- | --- | --- | --- | --- | --- |

XP\_007443118.1

back to list

  

| Protein Coverage
| Supporting Peptides
|

Protein Coverage:

Supporting Peptides:

| Peptide | Uniq | -10lgP | Mass | Length | ppm | m/z | z | RT | Fraction | Scan | Source File | Area F6 | Area F2 | Area F3 | Area F4 | Area F5 | Area F7 | Area F8 | Area F1 | #Feature | #Feature F6 | #Feature F2 | #Feature F3 | #Feature F4 | #Feature F5 | #Feature F7 | #Feature F8 | #Feature F1 | Start | End | PTM | AScore | Found By |
| --- | --- | --- | --- | --- | --- | --- | --- | --- | --- | --- | --- | --- | --- | --- | --- | --- | --- | --- | --- | --- | --- | --- | --- | --- | --- | --- | --- | --- | --- | --- | --- | --- | --- |
| K.LYTLVLTDPDAPSR.K | Y | 46.24 | 1559.8195 | 14 | 1.0 | 780.9178 | 2 | 49.13 | 8 | F8:27933 | 31122021\_RID\_3262\_1b.raw |  |  |  |  |  |  |  | 8.378E4 | 1 | 0 | 0 | 0 | 0 | 0 | 0 | 0 | 1 | 18 | 31 |  |  | PEAKS DB |
| total 1 peptides |  |  |  |  |  |  |  |  |  |  |  |  |  |  |  |  |  |  |  |  |  |  |  |  |  |  |  |  |  |  |  |  |  |
| --- | --- | --- | --- | --- | --- | --- | --- | --- | --- | --- | --- | --- | --- | --- | --- | --- | --- | --- | --- | --- | --- | --- | --- | --- | --- | --- | --- | --- | --- | --- | --- | --- | --- |

Peptide List

  
  

---

Prepared with PEAKS ™ (bioinfor.com)
