# Supplementary material for: Fangs in the Ghats: Preclinical Insights into the Medical Importance of Pit Vipers from the Western Ghats
Source: Int J Mol Sci. 2023 May 30;24(11):9516. doi: 10.3390/ijms24119516 (PMC10253985; doi:10.3390/ijms24119516)
Supplement: Supplementary file 1 [file ijms-24-09516-s001.zip › Supplementary File S1.zip!/Supplementary File S1/CRML/CRML.html]

proteins


Summary

  

# 1. Notes

# 2. Result Statistics

**Figure 1.**
False discovery rate (FDR) curve. X axis is the number of peptide-spectrum matches (PSM) being kept. Y axis is the corresponding FDR.

  


  

**Figure 2.**
PSM score distribution.  **(a)**
Distribution of PEAKS peptide score;  **(b)**
Scatterplot of PEAKS peptide score versus precursor mass error.

**(a)**

**(b)**

**Table 1.**
Statistics of data.


|  |  |  |  |  |  |  |  |  |  |  |  |
| --- | --- | --- | --- | --- | --- | --- | --- | --- | --- | --- | --- |
|  | #Scans | | #Features | Identified | | | #Peptides | #Sequences | #Proteins^\*^ | | |
|  | MS1 | MS2 |  | #PSMs | #Scans | #Features |  |  | Groups | All | Top |
| Total | 168471 | 387156 | 484323 | 13298 | 12914 | 52995 | 1389 | 1223 | 148 | 1089 | 510 |
| F4 | 14814 | 64914 | 63382 | 1682 | 1646 | 792 | 518 | 447 | 87 | 458 | 198 |
| F5 | 17652 | 48434 | 53144 | 957 | 910 | 7338 | 298 | 260 | 78 | 364 | 187 |
| F6 | 18396 | 34879 | 40372 | 350 | 339 | 3760 | 159 | 141 | 56 | 216 | 99 |
| F10 | 16421 | 54046 | 61338 | 2567 | 2517 | 9683 | 409 | 366 | 67 | 471 | 223 |
| F8 | 17993 | 12974 | 22494 | 208 | 207 | 468 | 34 | 33 | 12 | 39 | 14 |
| F9 | 16124 | 54002 | 62072 | 2230 | 2193 | 10909 | 435 | 386 | 71 | 454 | 225 |
| F7 | 17727 | 27830 | 42651 | 479 | 465 | 3892 | 200 | 179 | 60 | 392 | 180 |
| F1 | 14746 | 41923 | 61439 | 2891 | 2777 | 8184 | 857 | 762 | 112 | 794 | 371 |
| F2 | 14790 | 39487 | 71389 | 1559 | 1515 | 6847 | 571 | 501 | 104 | 655 | 251 |
| F3 | 19808 | 8667 | 6042 | 375 | 345 | 1122 | 174 | 157 | 41 | 157 | 81 |

^\* proteins with significant peptides are used in counts.^

**Figure 3.**
Sample overlap for Proteins and Peptides (up to 8 samples).  **(a)**
All Proteins;  **(b)**
Top Proteins;  **(c)**
Peptides;

**(a)**

**Do not support more than 8 samples**

**(b)**

**Do not support more than 8 samples**

**(c)**

**Do not support more than 8 samples**

**Figure 4.**
Distribution of peptide feature detection.  **(a)**
Feature m/z distribution;  **(b)**
Feature RT distribution.

**(a)**

**(b)**

**Figure 5.**
Distribution of identified peptide features.  **(a)**
Feature abundance distribution;  **(b)**
 *De novo*
sequencing validation.

**(a)**

**(b)**

**Table 2.**
Result filtration parameters.

|  |  |
| --- | --- |
| Peptide -10lgP | ≥40.9 |
| PTM Ascore | ≥0 |
| Protein -10lgP | ≥20 |
| Proteins unique peptides | ≥1 |
| De novo score(%) | ≥50% |


**Table 3.**
Statistics of filtered result.

|  |  |
| --- | --- |
| FDR (Peptide-Spectrum Matches) | 0.1% |
| FDR (Peptide Sequences) | 0.4% |
| FDR (Protein Group) | 4.9% |
| De Novo Only Spectra | 82230 |

**Table 4.**
PTM profile.

| Name | ∆Mass | Position | #PSM | -10lgP | Abundance | AScore || Oxidation | 15.99 | M | 2282 | 93.86 | 4.76E6 | 1000.00 |
| Carbamidomethyl | 57.02 | C | 175 | 84.75 | 1.81E7 | 1000.00 |

# 3. Experiment Control

**Figure 6.**
Precursor mass error of peptide-spectrum matches (PSM) in filtered result.  **(a)**
Distribution of precursor mass error in ppm;  **(b)**
Scatterplot of precursor m/z versus precursor mass error in ppm.

**(a)**

**(b)**

**Table 5.**
Number of identified peptides in each sample by the number of missed cleavages.

|  |  |  |  |  |  |
| --- | --- | --- | --- | --- | --- |
| Missed Cleavages | 0 | 1 | 2 | 3 | 4+ |
| F4 | 312 | 174 | 27 | 5 | 0 |
| F5 | 228 | 60 | 10 | 0 | 0 |
| F6 | 145 | 14 | 0 | 0 | 0 |
| F10 | 306 | 93 | 10 | 0 | 0 |
| F8 | 34 | 0 | 0 | 0 | 0 |
| F9 | 319 | 101 | 14 | 1 | 0 |
| F7 | 143 | 48 | 9 | 0 | 0 |
| F1 | 674 | 162 | 20 | 1 | 0 |
| F2 | 460 | 104 | 7 | 0 | 0 |
| F3 | 131 | 39 | 4 | 0 | 0 |

# 4. Other Information

**Table 6.**
Search parameters.

Search Engine Name: PEAKS  
Parent Mass Error Tolerance: 10.0 ppm  
Fragment Mass Error Tolerance: 0.6 Da  
Precursor Mass Search Type: monoisotopic  
Enzyme: Trypsin  
Max Missed Cleavages: 3  
Digest Mode: Semispecific  
Fixed Modifications:   
  Carbamidomethylation: 57.02  
Variable Modifications:   
  Oxidation (M): 15.99  
Max Variable PTM Per Peptide: 3  
Database: NCBI\_Serpentes\_NR  
Taxon: All  
Contaminant Database: cRAP\_contaminants  
Searched Entry: 410049  
FDR Estimation: Enabled  
Merge Options: no merge  
Precursor Options: corrected  
Charge Options: no correction  
Filter Charge: 2 - 8  
Process: true  
Associate chimera: yes

**Table 7.**
Instrument parameters.

Fractions: 13012022\_RID\_3262\_4a.raw, 13012022\_RID\_3262\_5a.raw,   
13012022\_RID\_3262\_6a.raw, 17012022\_RID\_3262\_10a .raw, 1701202  
2\_RID\_3262\_8a.raw, 17012022\_RID\_3262\_9a.raw, 21012022\_RID\_326  
2\_7a.raw, 31122021\_RID\_3262\_1a.raw, 31122021\_RID\_3262\_2a.raw,  
 31122021\_RID\_3262\_3a.raw  
Ion Source: ESI(nano-spray)  
Fragmentation Mode: CID, CAD(y and b ions)  
MS Scan Mode: FT-ICR/Orbitrap  
MS/MS Scan Mode: FT-ICR/Orbitrap

  

Protein List

  

Protein Accession Contains:

Protein Description Contains:

Protein Sample Area >=

Protein PTM Contains:

| Protein Group | Protein ID | Accession | -10lgP | Coverage (%) | Coverage (%) F4 | Coverage (%) F5 | Coverage (%) F6 | Coverage (%) F10 | Coverage (%) F8 | Coverage (%) F9 | Coverage (%) F7 | Coverage (%) F1 | Coverage (%) F2 | Coverage (%) F3 | Area F4 | Area F5 | Area F6 | Area F10 | Area F8 | Area F9 | Area F7 | Area F1 | Area F2 | Area F3 | #Peptides | #Unique | #Spec F4 | #Spec F5 | #Spec F6 | #Spec F10 | #Spec F8 | #Spec F9 | #Spec F7 | #Spec F1 | #Spec F2 | #Spec F3 | PTM | Avg. Mass | Description |
| --- | --- | --- | --- | --- | --- | --- | --- | --- | --- | --- | --- | --- | --- | --- | --- | --- | --- | --- | --- | --- | --- | --- | --- | --- | --- | --- | --- | --- | --- | --- | --- | --- | --- | --- | --- | --- | --- | --- | --- |
| 21 | 26 | BAN89426.1 | 335.00 | 29 | 15 | 1 | 0 | 0 | 0 | 0 | 0 | 29 | 17 | 1 |  |  |  |  |  |  |  | 3.5345E6 |  |  | 37 | 1 | 12 | 1 | 0 | 0 | 0 | 0 | 0 | 245 | 44 | 1 | Y | 96239 | phosphodiesterase [Ovophis okinavensis] |
| 21 | 27 | BAN89425.1 | 335.00 | 28 | 14 | 1 | 0 | 0 | 0 | 0 | 0 | 28 | 16 | 1 |  |  |  |  |  |  |  | 3.5345E6 |  |  | 37 | 1 | 12 | 1 | 0 | 0 | 0 | 0 | 0 | 245 | 44 | 1 | Y | 100879 | phosphodiesterase [Ovophis okinavensis] |
| 10 | 41 | BAN82013.1 | 311.27 | 36 | 27 | 18 | 6 | 14 | 0 | 18 | 13 | 27 | 21 | 30 | 5.3703E7 |  |  |  |  |  |  | 6.7671E5 |  | 0 | 37 | 2 | 86 | 25 | 10 | 18 | 0 | 27 | 24 | 40 | 43 | 60 | Y | 57129 | l-amino acid oxidase [Protobothrops flavoviridis] |
| 10 | 42 | BAP39950.1 | 311.27 | 36 | 27 | 18 | 6 | 14 | 0 | 18 | 13 | 27 | 21 | 30 | 5.3703E7 |  |  |  |  |  |  | 6.7671E5 |  | 0 | 37 | 2 | 86 | 25 | 10 | 18 | 0 | 27 | 24 | 40 | 43 | 60 | Y | 57129 | L-amino acid oxidase [Protobothrops flavoviridis] |
| 44 | 34 | JAV01884.1 | 305.05 | 24 | 10 | 1 | 0 | 0 | 0 | 0 | 0 | 24 | 14 | 0 |  |  |  |  |  |  |  | 2.2913E5 |  |  | 32 | 1 | 8 | 1 | 0 | 0 | 0 | 0 | 0 | 183 | 35 | 0 | Y | 91859 | BATXPDE1 [Bothrops atrox] |
| 46 | 35 | SMD28240.1 | 295.98 | 30 | 30 | 16 | 5 | 0 | 0 | 0 | 2 | 16 | 7 | 0 | 3.0719E8 | 5.1718E6 | 1.8973E6 |  |  |  |  | 3.1069E6 | 1.3137E6 |  | 21 | 1 | 187 | 14 | 3 | 0 | 0 | 0 | 1 | 12 | 3 | 0 | Y | 64104 | phospholipase B CdtPLB1 [Crotalus durissus terrificus] |
| 46 | 36 | JAV49636.1 | 295.98 | 30 | 30 | 16 | 5 | 0 | 0 | 0 | 2 | 16 | 7 | 0 | 3.0719E8 | 5.1718E6 | 1.8973E6 |  |  |  |  | 3.1069E6 | 1.3137E6 |  | 21 | 1 | 187 | 14 | 3 | 0 | 0 | 0 | 1 | 12 | 3 | 0 | Y | 64129 | Phospholipase B [Agkistrodon contortrix contortrix] |
| 30 | 97 | XP\_015673892.1 | 289.31 | 29 | 19 | 15 | 4 | 14 | 0 | 15 | 11 | 19 | 18 | 24 |  |  |  | 8.9187E5 |  | 1.0903E6 | 8.4446E5 |  |  | 2.2953E8 | 29 | 2 | 70 | 20 | 5 | 19 | 0 | 22 | 19 | 34 | 36 | 53 | Y | 57159 | L-amino-acid oxidase isoform X1 [Protobothrops mucrosquamatus] |
| 79 | 38 | JAG46763.1 | 275.05 | 53 | 53 | 10 | 0 | 0 | 0 | 0 | 3 | 3 | 3 | 0 | 1.8304E8 |  |  |  |  |  |  |  |  |  | 18 | 7 | 118 | 4 | 0 | 0 | 0 | 0 | 1 | 1 | 1 | 0 | Y | 42370 | glutaminyl-peptide cyclotransferases [Crotalus horridus] |
| 79 | 39 | JAA94895.1 | 275.05 | 53 | 53 | 10 | 0 | 0 | 0 | 0 | 3 | 3 | 3 | 0 | 1.8304E8 |  |  |  |  |  |  |  |  |  | 18 | 7 | 118 | 4 | 0 | 0 | 0 | 0 | 1 | 1 | 1 | 0 | Y | 42369 | Glutaminyl-peptide cyclotransferase [Crotalus horridus] |
| 79 | 40 | JAG44660.1 | 275.05 | 53 | 53 | 10 | 0 | 0 | 0 | 0 | 3 | 3 | 3 | 0 | 1.8304E8 |  |  |  |  |  |  |  |  |  | 18 | 7 | 118 | 4 | 0 | 0 | 0 | 0 | 1 | 1 | 1 | 0 | Y | 42369 | Glutaminyl-peptide cyclotransferase [Crotalus horridus] |
| 79 | 43 | AFE84765.1 | 275.05 | 53 | 53 | 10 | 0 | 0 | 0 | 0 | 3 | 3 | 3 | 0 | 1.8304E8 |  |  |  |  |  |  |  |  |  | 18 | 7 | 118 | 4 | 0 | 0 | 0 | 0 | 1 | 1 | 1 | 0 | Y | 42297 | glutaminyl-peptide cyclotransferases [Sistrurus tergeminus] |
| 79 | 44 | AFE84763.1 | 275.05 | 53 | 53 | 10 | 0 | 0 | 0 | 0 | 3 | 3 | 3 | 0 | 1.8304E8 |  |  |  |  |  |  |  |  |  | 18 | 7 | 118 | 4 | 0 | 0 | 0 | 0 | 1 | 1 | 1 | 0 | Y | 42295 | glutaminyl-peptide cyclotransferases [Trimeresurus gracilis] |
| 79 | 45 | BAB69586.1 | 275.05 | 53 | 53 | 10 | 0 | 0 | 0 | 0 | 3 | 3 | 3 | 0 | 1.8304E8 |  |  |  |  |  |  |  |  |  | 18 | 7 | 118 | 4 | 0 | 0 | 0 | 0 | 1 | 1 | 1 | 0 | Y | 42267 | glutaminyl cyclase [Gloydius blomhoffii] |
| 71 | 179 | XP\_015674366.1 | 270.56 | 36 | 29 | 6 | 0 | 0 | 0 | 0 | 0 | 23 | 36 | 10 | 2.3625E7 | 4.9792E5 |  |  |  |  |  | 2.761E6 | 1.4018E8 | 1.8999E7 | 17 | 7 | 14 | 2 | 0 | 0 | 0 | 0 | 0 | 10 | 98 | 4 | Y | 37677 | intestinal-type alkaline phosphatase-like [Protobothrops mucrosquamatus] |
| 6 | 164 | ETE67131.1 | 265.67 | 5 | 3 | 3 | 2 | 4 | 0 | 4 | 2 | 4 | 4 | 3 | 2.6752E6 | 8.2242E5 | 3.2474E5 | 1.6764E6 |  | 5.6646E6 |  | 9.4427E6 | 4.808E6 |  | 27 | 1 | 30 | 21 | 9 | 122 | 4 | 57 | 18 | 131 | 57 | 15 | Y | 240642 | Keratin, type II cytoskeletal 1, partial [Ophiophagus hannah] |
| 76 | 188 | QBF53421.1 | 249.48 | 21 | 21 | 11 | 2 | 0 | 0 | 0 | 0 | 9 | 2 | 0 | 2.0162E6 |  |  |  |  |  |  |  |  |  | 14 | 1 | 101 | 8 | 2 | 0 | 0 | 0 | 0 | 4 | 1 | 0 | Y | 64310 | phospholipase B [Vipera ammodytes ammodytes] |
| 109 | 187 | XP\_007422201.1 | 248.32 | 5 | 1 | 1 | 0 | 4 | 0 | 4 | 0 | 4 | 3 | 1 |  | 4.0563E5 |  | 4.4156E6 |  | 4.5002E6 |  | 1.3029E6 |  |  | 16 | 2 | 2 | 2 | 0 | 13 | 0 | 18 | 0 | 13 | 7 | 1 | Y | 329911 | desmoplakin isoform X1 [Python bivittatus] |
| 56 | 458 | JAA97975.1 | 240.74 | 29 | 17 | 21 | 20 | 5 | 0 | 5 | 24 | 16 | 12 | 3 | 1.6154E7 | 3.702E6 | 1.8052E7 |  |  |  | 2.7529E6 | 3.7427E6 |  |  | 17 | 2 | 17 | 93 | 35 | 7 | 0 | 4 | 16 | 8 | 2 | 1 | Y | 28204 | Kallikrein-CohID-4 [Crotalus oreganus helleri] |
| 42 | 275 | JAS05316.1 | 240.26 | 25 | 12 | 9 | 4 | 6 | 0 | 6 | 8 | 10 | 10 | 19 |  |  |  |  |  |  |  |  |  | 4.5567E5 | 18 | 1 | 25 | 22 | 11 | 29 | 0 | 12 | 13 | 17 | 18 | 25 | Y | 58784 | L-amino acid oxidase 1d [Sistrurus tergeminus] |
| 42 | 276 | JAS05318.1 | 240.26 | 25 | 12 | 9 | 4 | 6 | 0 | 6 | 8 | 10 | 10 | 19 |  |  |  |  |  |  |  |  |  | 4.5567E5 | 18 | 1 | 25 | 22 | 11 | 29 | 0 | 12 | 13 | 17 | 18 | 25 | Y | 58840 | L-amino acid oxidase 1b [Sistrurus tergeminus] |
| 42 | 277 | JAS05319.1 | 240.26 | 25 | 12 | 9 | 4 | 6 | 0 | 6 | 8 | 10 | 10 | 19 |  |  |  |  |  |  |  |  |  | 4.5567E5 | 18 | 1 | 25 | 22 | 11 | 29 | 0 | 12 | 13 | 17 | 18 | 25 | Y | 58840 | L-amino acid oxidase 1a [Sistrurus tergeminus] |
| 210 | 66 | JAV50419.1 | 239.41 | 31 | 30 | 0 | 0 | 2 | 0 | 2 | 0 | 0 | 2 | 0 | 5.9769E7 |  |  | 2.9969E5 |  |  |  |  |  |  | 18 | 16 | 28 | 0 | 0 | 1 | 0 | 1 | 0 | 0 | 1 | 0 | Y | 72067 | 78 kDa glucose-regulated protein [Agkistrodon contortrix contortrix] |
| 210 | 67 | JAG44540.1 | 239.41 | 31 | 30 | 0 | 0 | 2 | 0 | 2 | 0 | 0 | 2 | 0 | 5.9769E7 |  |  | 2.9969E5 |  |  |  |  |  |  | 18 | 16 | 28 | 0 | 0 | 1 | 0 | 1 | 0 | 0 | 1 | 0 | Y | 72105 | 78 kDa glucose-regulated protein precursor [Crotalus horridus] |
| 210 | 68 | JAI13107.1 | 239.41 | 31 | 30 | 0 | 0 | 2 | 0 | 2 | 0 | 0 | 2 | 0 | 5.9769E7 |  |  | 2.9969E5 |  |  |  |  |  |  | 18 | 16 | 28 | 0 | 0 | 1 | 0 | 1 | 0 | 0 | 1 | 0 | Y | 72105 | 78 kDa glucose-regulated protein [Crotalus adamanteus] |
| 210 | 69 | JAG46692.1 | 239.41 | 31 | 30 | 0 | 0 | 2 | 0 | 2 | 0 | 0 | 2 | 0 | 5.9769E7 |  |  | 2.9969E5 |  |  |  |  |  |  | 18 | 16 | 28 | 0 | 0 | 1 | 0 | 1 | 0 | 0 | 1 | 0 | Y | 72105 | 78 kDa glucose-regulated protein [Crotalus horridus] |
| 210 | 70 | JAA96909.1 | 239.41 | 31 | 30 | 0 | 0 | 2 | 0 | 2 | 0 | 0 | 2 | 0 | 5.9769E7 |  |  | 2.9969E5 |  |  |  |  |  |  | 18 | 16 | 28 | 0 | 0 | 1 | 0 | 1 | 0 | 0 | 1 | 0 | Y | 72105 | 78 kDa glucose-regulated protein [Crotalus horridus] |
| 210 | 71 | XP\_015681610.1 | 239.41 | 31 | 30 | 0 | 0 | 2 | 0 | 2 | 0 | 0 | 2 | 0 | 5.9769E7 |  |  | 2.9969E5 |  |  |  |  |  |  | 18 | 16 | 28 | 0 | 0 | 1 | 0 | 1 | 0 | 0 | 1 | 0 | Y | 72155 | endoplasmic reticulum chaperone BiP [Protobothrops mucrosquamatus] |
| 210 | 72 | AFJ50327.1 | 239.41 | 31 | 30 | 0 | 0 | 2 | 0 | 2 | 0 | 0 | 2 | 0 | 5.9769E7 |  |  | 2.9969E5 |  |  |  |  |  |  | 18 | 16 | 28 | 0 | 0 | 1 | 0 | 1 | 0 | 0 | 1 | 0 | Y | 72105 | Heat shock protein 5 [Crotalus adamanteus] |
| 210 | 73 | AFJ50239.1 | 239.41 | 30 | 28 | 0 | 0 | 1 | 0 | 2 | 0 | 0 | 2 | 0 | 5.9769E7 |  |  | 2.9969E5 |  |  |  |  |  |  | 18 | 16 | 28 | 0 | 0 | 1 | 0 | 1 | 0 | 0 | 1 | 0 | Y | 75480 | 78 kDa glucose-regulated protein precursor [Crotalus adamanteus] |
| 183 | 103 | JAI10406.1 | 238.16 | 31 | 9 | 7 | 2 | 0 | 0 | 0 | 2 | 4 | 31 | 0 |  |  |  |  |  |  |  |  | 1.2697E5 |  | 20 | 1 | 6 | 5 | 1 | 0 | 0 | 0 | 1 | 3 | 35 | 0 | Y | 64682 | ecto-5'-nucleotidase [Crotalus adamanteus] |
| 183 | 104 | JAS04702.1 | 238.16 | 31 | 9 | 7 | 2 | 0 | 0 | 0 | 2 | 4 | 31 | 0 |  |  |  |  |  |  |  |  | 1.2697E5 |  | 20 | 1 | 6 | 5 | 1 | 0 | 0 | 0 | 1 | 3 | 35 | 0 | Y | 64682 | ecto-5'-nucleotidase [Crotalus adamanteus] |
| 183 | 105 | AFJ49197.1 | 238.16 | 31 | 9 | 7 | 2 | 0 | 0 | 0 | 2 | 4 | 31 | 0 |  |  |  |  |  |  |  |  | 1.2697E5 |  | 20 | 1 | 6 | 5 | 1 | 0 | 0 | 0 | 1 | 3 | 35 | 0 | Y | 64682 | Ecto-5'-nucleotidase [Crotalus adamanteus] |
| 183 | 106 | F8S0Z7.2 | 238.16 | 31 | 9 | 7 | 2 | 0 | 0 | 0 | 2 | 4 | 31 | 0 |  |  |  |  |  |  |  |  | 1.2697E5 |  | 20 | 1 | 6 | 5 | 1 | 0 | 0 | 0 | 1 | 3 | 35 | 0 | Y | 64682 | RecName: Full=Snake venom 5'-nucleotidase; Short=5'-NT; AltName: Full=Ecto-5'-nucleotidase; Flags: Precursor |
| 183 | 107 | JAV48423.1 | 238.16 | 31 | 9 | 7 | 2 | 0 | 0 | 0 | 2 | 4 | 31 | 0 |  |  |  |  |  |  |  |  | 1.2697E5 |  | 20 | 1 | 6 | 5 | 1 | 0 | 0 | 0 | 1 | 3 | 35 | 0 | Y | 64682 | Cadam10\_NUC-1 [Crotalus adamanteus] |
| 183 | 108 | SMD28261.1 | 238.16 | 31 | 9 | 7 | 2 | 0 | 0 | 0 | 2 | 4 | 31 | 0 |  |  |  |  |  |  |  |  | 1.2697E5 |  | 20 | 1 | 6 | 5 | 1 | 0 | 0 | 0 | 1 | 3 | 35 | 0 | Y | 64697 | nucleotidase Cdt5'-NT1 [Crotalus durissus terrificus] |
| 183 | 109 | JAS05314.1 | 238.16 | 31 | 9 | 7 | 2 | 0 | 0 | 0 | 2 | 4 | 31 | 0 |  |  |  |  |  |  |  |  | 1.2697E5 |  | 20 | 1 | 6 | 5 | 1 | 0 | 0 | 0 | 1 | 3 | 35 | 0 | Y | 64651 | ecto-5'-nucleotidase [Sistrurus tergeminus] |
| 183 | 110 | JAS04863.1 | 238.16 | 31 | 9 | 7 | 2 | 0 | 0 | 0 | 2 | 4 | 31 | 0 |  |  |  |  |  |  |  |  | 1.2697E5 |  | 20 | 1 | 6 | 5 | 1 | 0 | 0 | 0 | 1 | 3 | 35 | 0 | Y | 64817 | Ecto-5'-nucleotidase [Crotalus horridus] |
| 183 | 111 | JAG46129.1 | 238.16 | 31 | 9 | 7 | 2 | 0 | 0 | 0 | 2 | 4 | 31 | 0 |  |  |  |  |  |  |  |  | 1.2697E5 |  | 20 | 1 | 6 | 5 | 1 | 0 | 0 | 0 | 1 | 3 | 35 | 0 | Y | 64817 | Ecto-5'-nucleotidase [Crotalus horridus] |
| 183 | 112 | JAG43650.1 | 238.16 | 31 | 9 | 7 | 2 | 0 | 0 | 0 | 2 | 4 | 31 | 0 |  |  |  |  |  |  |  |  | 1.2697E5 |  | 20 | 1 | 6 | 5 | 1 | 0 | 0 | 0 | 1 | 3 | 35 | 0 | Y | 64817 | ecto-5'-nucleotidase [Crotalus horridus] |
| 183 | 113 | JAA94887.1 | 238.16 | 31 | 9 | 7 | 2 | 0 | 0 | 0 | 2 | 4 | 31 | 0 |  |  |  |  |  |  |  |  | 1.2697E5 |  | 20 | 1 | 6 | 5 | 1 | 0 | 0 | 0 | 1 | 3 | 35 | 0 | Y | 64817 | ecto-5'-nucleotidase [Crotalus horridus] |
| 183 | 114 | JAS04780.1 | 238.16 | 31 | 9 | 7 | 2 | 0 | 0 | 0 | 2 | 4 | 31 | 0 |  |  |  |  |  |  |  |  | 1.2697E5 |  | 20 | 1 | 6 | 5 | 1 | 0 | 0 | 0 | 1 | 3 | 35 | 0 | Y | 64817 | ecto-5'-nucleotidase [Crotalus horridus] |
| 7 | 642 | AMB36337.1 | 237.67 | 21 | 21 | 21 | 14 | 21 | 7 | 21 | 21 | 21 | 21 | 7 | 3.7999E8 | 8.8041E7 | 2.0461E7 | 8.6982E7 | 8.7525E6 | 1.4658E8 | 3.2308E8 | 7.5427E7 | 5.6345E7 | 2.2549E7 | 13 | 11 | 41 | 16 | 11 | 111 | 81 | 128 | 48 | 26 | 20 | 3 | Y | 26517 | cysteine-rich secretory protein CRISP-1 [Vipera ammodytes ammodytes] |
| 62 | 288 | A0A024BTN9.1 | 237.53 | 20 | 16 | 13 | 2 | 11 | 0 | 9 | 5 | 12 | 10 | 8 | 3.2487E6 |  |  |  |  |  |  |  |  |  | 19 | 1 | 30 | 14 | 1 | 10 | 0 | 9 | 8 | 19 | 19 | 28 | N | 56376 | RecName: Full=L-amino acid oxidase Bs29; AltName: Full=BsLAAO; Short=LAO; Flags: Precursor |
| 62 | 289 | AHZ20792.1 | 237.53 | 20 | 16 | 13 | 2 | 11 | 0 | 9 | 5 | 12 | 10 | 8 | 3.2487E6 |  |  |  |  |  |  |  |  |  | 19 | 1 | 30 | 14 | 1 | 10 | 0 | 9 | 8 | 19 | 19 | 28 | N | 56376 | L-amino acid oxidase, partial [Bothriechis schlegelii] |
| 176 | 117 | BAP39972.1 | 237.00 | 30 | 13 | 5 | 2 | 0 | 0 | 0 | 2 | 4 | 30 | 0 | 1.2181E7 |  |  |  |  |  |  |  | 1.5359E7 |  | 20 | 1 | 9 | 4 | 1 | 0 | 0 | 0 | 1 | 3 | 36 | 0 | Y | 65019 | 5-nucleotidase [Protobothrops flavoviridis] |
| 176 | 118 | XP\_015668350.1 | 237.00 | 31 | 13 | 5 | 2 | 0 | 0 | 0 | 2 | 4 | 31 | 0 | 1.2181E7 |  |  |  |  |  |  |  | 1.5359E7 |  | 20 | 1 | 9 | 4 | 1 | 0 | 0 | 0 | 1 | 3 | 36 | 0 | Y | 64635 | 5'-nucleotidase [Protobothrops mucrosquamatus] |
| 176 | 119 | BAP39925.1 | 237.00 | 31 | 13 | 5 | 2 | 0 | 0 | 0 | 2 | 4 | 31 | 0 | 1.2181E7 |  |  |  |  |  |  |  | 1.5359E7 |  | 20 | 1 | 9 | 4 | 1 | 0 | 0 | 0 | 1 | 3 | 36 | 0 | Y | 64640 | 5-nucleotidase [Protobothrops elegans] |
| 197 | 61 | JAV51280.1 | 233.43 | 18 | 1 | 2 | 0 | 0 | 0 | 0 | 0 | 18 | 6 | 0 |  |  |  |  |  |  |  | 2.9127E6 |  |  | 17 | 1 | 1 | 2 | 0 | 0 | 0 | 0 | 0 | 28 | 5 | 0 | N | 110116 | Aminopeptidase A [Agkistrodon contortrix contortrix] |
| 177 | 96 | JAS04520.1 | 233.33 | 31 | 11 | 9 | 2 | 0 | 0 | 0 | 2 | 4 | 30 | 0 | 1.5655E6 |  |  |  |  |  |  |  |  |  | 17 | 1 | 10 | 6 | 1 | 0 | 0 | 0 | 1 | 3 | 32 | 0 | Y | 64602 | Snake venom 5'-nucleotidase [Agkistrodon piscivorus conanti] |
| 60 | 450 | AAY89681.1 | 231.83 | 17 | 13 | 5 | 2 | 6 | 0 | 4 | 4 | 13 | 10 | 11 | 7.2329E7 |  |  |  |  |  |  | 1.8957E6 |  | 2.1198E8 | 11 | 1 | 62 | 9 | 2 | 16 | 0 | 8 | 2 | 13 | 11 | 27 | Y | 59058 | L-amino acid oxidase precursor [Notechis scutatus] |
| 60 | 451 | Q4JHE2.1 | 231.83 | 17 | 13 | 5 | 2 | 6 | 0 | 4 | 4 | 13 | 10 | 11 | 7.2329E7 |  |  |  |  |  |  | 1.8957E6 |  | 2.1198E8 | 11 | 1 | 62 | 9 | 2 | 16 | 0 | 8 | 2 | 13 | 11 | 27 | Y | 59058 | RecName: Full=L-amino-acid oxidase; Short=LAAO; Short=LAO; Flags: Precursor |
| 60 | 453 | XP\_026538830.1 | 231.83 | 16 | 12 | 5 | 2 | 5 | 0 | 4 | 4 | 13 | 9 | 10 | 7.2329E7 |  |  |  |  |  |  | 1.8957E6 |  | 2.1198E8 | 11 | 1 | 62 | 9 | 2 | 16 | 0 | 8 | 2 | 13 | 11 | 27 | Y | 61914 | L-amino-acid oxidase [Notechis scutatus] |
| 102 | 411 | XP\_007427768.1 | 229.67 | 16 | 13 | 5 | 0 | 0 | 0 | 0 | 0 | 5 | 0 | 0 |  | 1.2067E6 |  |  |  |  |  |  |  |  | 10 | 1 | 93 | 3 | 0 | 0 | 0 | 0 | 0 | 2 | 0 | 0 | Y | 64842 | phospholipase B-like 1 [Python bivittatus] |
| 63 | 208 | ETE70217.1 | 227.14 | 3 | 1 | 0 | 0 | 2 | 0 | 2 | 1 | 3 | 2 | 0 |  |  |  | 4.869E6 |  | 3.3371E6 |  | 5.9702E5 | 1.1633E6 |  | 16 | 3 | 4 | 5 | 3 | 21 | 3 | 16 | 3 | 26 | 25 | 4 | Y | 436388 | Keratin, type I cytoskeletal 19, partial [Ophiophagus hannah] |
| 187 | 59 | XP\_015676063.1 | 225.65 | 31 | 1 | 0 | 0 | 0 | 0 | 0 | 0 | 30 | 16 | 0 |  |  |  |  |  |  |  | 6.8534E6 | 6.1906E5 |  | 16 | 5 | 1 | 0 | 0 | 0 | 0 | 0 | 0 | 34 | 13 | 0 | Y | 77032 | xaa-Pro aminopeptidase 2 [Protobothrops mucrosquamatus] |
| 228 | 62 | JAG45871.1 | 223.17 | 12 | 0 | 0 | 0 | 0 | 0 | 0 | 0 | 12 | 4 | 0 |  |  |  |  |  |  |  | 7.1673E5 |  |  | 12 | 1 | 0 | 0 | 0 | 0 | 0 | 0 | 0 | 21 | 3 | 0 | Y | 110767 | Aminopeptidase N [Crotalus horridus] |
| 228 | 63 | JAA97801.1 | 223.17 | 12 | 0 | 0 | 0 | 0 | 0 | 0 | 0 | 12 | 4 | 0 |  |  |  |  |  |  |  | 7.1673E5 |  |  | 12 | 1 | 0 | 0 | 0 | 0 | 0 | 0 | 0 | 21 | 3 | 0 | Y | 110767 | Aminopeptidase N [Crotalus horridus] |
| 228 | 64 | JAG47501.1 | 223.17 | 12 | 0 | 0 | 0 | 0 | 0 | 0 | 0 | 12 | 4 | 0 |  |  |  |  |  |  |  | 7.1673E5 |  |  | 12 | 1 | 0 | 0 | 0 | 0 | 0 | 0 | 0 | 21 | 3 | 0 | Y | 110737 | Aminopeptidase N [Crotalus horridus] |
| 34 | 304 | XP\_032067281.1 | 220.93 | 14 | 5 | 5 | 5 | 8 | 2 | 9 | 6 | 7 | 10 | 4 |  |  | 0 |  |  |  |  |  |  |  | 15 | 1 | 13 | 8 | 5 | 58 | 4 | 21 | 6 | 76 | 31 | 5 | Y | 59706 | keratin, type II cytoskeletal 6A-like [Thamnophis elegans] |
| 34 | 305 | XP\_013915209.1 | 220.93 | 14 | 5 | 5 | 5 | 8 | 2 | 9 | 6 | 7 | 10 | 4 |  |  | 0 |  |  |  |  |  |  |  | 15 | 1 | 13 | 8 | 5 | 58 | 4 | 21 | 6 | 76 | 31 | 5 | Y | 59764 | PREDICTED: keratin, type II cytoskeletal 6A-like [Thamnophis sirtalis] |
| 106 | 686 | O13063.1 | 220.76 | 25 | 20 | 9 | 0 | 0 | 0 | 0 | 3 | 3 | 3 | 0 | 1.6489E8 |  |  |  |  |  |  |  |  |  | 17 | 2 | 41 | 6 | 0 | 0 | 0 | 0 | 2 | 4 | 1 | 0 | Y | 28034 | RecName: Full=Snake venom serine protease 3; Short=SVSP 3; Flags: Precursor |
| 106 | 687 | BAA19983.1 | 220.76 | 25 | 20 | 9 | 0 | 0 | 0 | 0 | 3 | 3 | 3 | 0 | 1.6489E8 |  |  |  |  |  |  |  |  |  | 17 | 2 | 41 | 6 | 0 | 0 | 0 | 0 | 2 | 4 | 1 | 0 | Y | 28034 | serine protease [Trimeresurus gramineus] |
| 57 | 633 | AAG10789.1 | 218.65 | 16 | 16 | 16 | 14 | 5 | 0 | 10 | 10 | 15 | 13 | 3 | 3.6357E7 | 8.5971E7 | 1.569E7 |  |  | 6.3517E6 | 1.0946E6 | 3.8961E6 | 5.7101E5 |  | 11 | 2 | 20 | 44 | 31 | 7 | 0 | 10 | 12 | 12 | 4 | 1 | Y | 28104 | serine proteinase 2 precursor [Protobothrops jerdonii] |
| 57 | 634 | Q9DF67.1 | 218.65 | 16 | 16 | 16 | 14 | 5 | 0 | 10 | 10 | 15 | 13 | 3 | 3.6357E7 | 8.5971E7 | 1.569E7 |  |  | 6.3517E6 | 1.0946E6 | 3.8961E6 | 5.7101E5 |  | 11 | 2 | 20 | 44 | 31 | 7 | 0 | 10 | 12 | 12 | 4 | 1 | Y | 28104 | RecName: Full=Snake venom serine protease 2; Short=SP2; Short=SVSP; AltName: Full=Jerdonobin-II; Flags: Precursor |
| 90 | 516 | JAS05152.1 | 214.40 | 15 | 9 | 6 | 4 | 6 | 0 | 6 | 6 | 8 | 8 | 9 |  |  |  |  |  |  |  |  |  | 9.2051E5 | 10 | 1 | 18 | 11 | 3 | 17 | 0 | 12 | 8 | 10 | 9 | 16 | N | 58785 | L-amino acid oxidase 1b [Micrurus tener] |
| 17 | 467 | XP\_026539627.1 | 214.06 | 11 | 7 | 6 | 3 | 8 | 0 | 9 | 6 | 9 | 9 | 5 |  |  |  |  |  |  |  | 8.2015E5 | 2.3951E5 |  | 15 | 1 | 18 | 14 | 5 | 81 | 0 | 42 | 16 | 51 | 22 | 9 | Y | 63000 | keratin, type II cytoskeletal 6A-like [Notechis scutatus] |
| 131 | 387 | XP\_032080818.1 | 209.91 | 15 | 10 | 2 | 2 | 0 | 0 | 0 | 0 | 9 | 15 | 2 |  |  | 4.641E5 |  |  |  |  | 1.1313E6 | 3.604E7 |  | 10 | 1 | 9 | 1 | 1 | 0 | 0 | 0 | 0 | 6 | 55 | 1 | Y | 57576 | intestinal-type alkaline phosphatase 1-like [Thamnophis elegans] |
| 208 | 239 | ETE57948.1 | 205.49 | 35 | 18 | 3 | 0 | 14 | 0 | 16 | 9 | 15 | 13 | 0 | 1.4154E6 |  |  | 1.3304E6 |  |  | 1.3673E6 | 2.9994E5 | 2.4553E5 |  | 10 | 3 | 5 | 1 | 0 | 4 | 0 | 5 | 4 | 4 | 5 | 0 | Y | 40263 | hypothetical protein L345\_16333, partial [Ophiophagus hannah] |
| 208 | 240 | LAA58652.1 | 205.49 | 34 | 17 | 3 | 0 | 13 | 0 | 15 | 9 | 15 | 13 | 0 | 1.4154E6 |  |  | 1.3304E6 |  |  | 1.3673E6 | 2.9994E5 | 2.4553E5 |  | 10 | 3 | 5 | 1 | 0 | 4 | 0 | 5 | 4 | 4 | 5 | 0 | Y | 41737 | hypothetical protein [Micrurus corallinus] |
| 208 | 241 | JAI10342.1 | 205.49 | 34 | 17 | 3 | 0 | 13 | 0 | 15 | 9 | 15 | 13 | 0 | 1.4154E6 |  |  | 1.3304E6 |  |  | 1.3673E6 | 2.9994E5 | 2.4553E5 |  | 10 | 3 | 5 | 1 | 0 | 4 | 0 | 5 | 4 | 4 | 5 | 0 | Y | 41737 | Actin, cytoplasmic 1 [Micrurus fulvius] |
| 208 | 242 | JAB54771.1 | 205.49 | 34 | 17 | 3 | 0 | 13 | 0 | 15 | 9 | 15 | 13 | 0 | 1.4154E6 |  |  | 1.3304E6 |  |  | 1.3673E6 | 2.9994E5 | 2.4553E5 |  | 10 | 3 | 5 | 1 | 0 | 4 | 0 | 5 | 4 | 4 | 5 | 0 | Y | 41737 | Actin [Micrurus fulvius] |
| 208 | 243 | JAA97901.1 | 205.49 | 34 | 17 | 3 | 0 | 13 | 0 | 15 | 9 | 15 | 13 | 0 | 1.4154E6 |  |  | 1.3304E6 |  |  | 1.3673E6 | 2.9994E5 | 2.4553E5 |  | 10 | 3 | 5 | 1 | 0 | 4 | 0 | 5 | 4 | 4 | 5 | 0 | Y | 41737 | Actin, cytoplasmic 1 [Crotalus horridus] |
| 208 | 244 | JAG68844.1 | 205.49 | 34 | 17 | 3 | 0 | 13 | 0 | 15 | 9 | 15 | 13 | 0 | 1.4154E6 |  |  | 1.3304E6 |  |  | 1.3673E6 | 2.9994E5 | 2.4553E5 |  | 10 | 3 | 5 | 1 | 0 | 4 | 0 | 5 | 4 | 4 | 5 | 0 | Y | 41737 | Actin, cytoplasmic 1 [Boiga irregularis] |
| 208 | 245 | JAV51374.1 | 205.49 | 34 | 17 | 3 | 0 | 13 | 0 | 15 | 9 | 15 | 13 | 0 | 1.4154E6 |  |  | 1.3304E6 |  |  | 1.3673E6 | 2.9994E5 | 2.4553E5 |  | 10 | 3 | 5 | 1 | 0 | 4 | 0 | 5 | 4 | 4 | 5 | 0 | Y | 41737 | Actin, cytoplasmic 1 [Agkistrodon contortrix contortrix] |
| 208 | 246 | JAG47580.1 | 205.49 | 34 | 17 | 3 | 0 | 13 | 0 | 15 | 9 | 15 | 13 | 0 | 1.4154E6 |  |  | 1.3304E6 |  |  | 1.3673E6 | 2.9994E5 | 2.4553E5 |  | 10 | 3 | 5 | 1 | 0 | 4 | 0 | 5 | 4 | 4 | 5 | 0 | Y | 41737 | Actin, cytoplasmic 1 [Crotalus horridus] |
| 208 | 247 | JAI14496.1 | 205.49 | 34 | 17 | 3 | 0 | 13 | 0 | 15 | 9 | 15 | 13 | 0 | 1.4154E6 |  |  | 1.3304E6 |  |  | 1.3673E6 | 2.9994E5 | 2.4553E5 |  | 10 | 3 | 5 | 1 | 0 | 4 | 0 | 5 | 4 | 4 | 5 | 0 | Y | 41737 | Actin, cytoplasmic 1 [Crotalus adamanteus] |
| 208 | 248 | XP\_007436312.1 | 205.49 | 34 | 17 | 3 | 0 | 13 | 0 | 15 | 9 | 15 | 13 | 0 | 1.4154E6 |  |  | 1.3304E6 |  |  | 1.3673E6 | 2.9994E5 | 2.4553E5 |  | 10 | 3 | 5 | 1 | 0 | 4 | 0 | 5 | 4 | 4 | 5 | 0 | Y | 41737 | actin-like protein 8 [Python bivittatus] |
| 208 | 249 | JAC96364.1 | 205.49 | 34 | 17 | 3 | 0 | 13 | 0 | 15 | 9 | 15 | 13 | 0 | 1.4154E6 |  |  | 1.3304E6 |  |  | 1.3673E6 | 2.9994E5 | 2.4553E5 |  | 10 | 3 | 5 | 1 | 0 | 4 | 0 | 5 | 4 | 4 | 5 | 0 | Y | 41737 | Actin, cytoplasmic 1 [Hypsiglena sp. JMG-2014] |
| 208 | 250 | AFJ49302.1 | 205.49 | 34 | 17 | 3 | 0 | 13 | 0 | 15 | 9 | 15 | 13 | 0 | 1.4154E6 |  |  | 1.3304E6 |  |  | 1.3673E6 | 2.9994E5 | 2.4553E5 |  | 10 | 3 | 5 | 1 | 0 | 4 | 0 | 5 | 4 | 4 | 5 | 0 | Y | 41737 | Actin, cytoplasmic 1 [Crotalus adamanteus] |
| 208 | 251 | BAQ22177.1 | 205.49 | 34 | 17 | 3 | 0 | 13 | 0 | 15 | 9 | 15 | 13 | 0 | 1.4154E6 |  |  | 1.3304E6 |  |  | 1.3673E6 | 2.9994E5 | 2.4553E5 |  | 10 | 3 | 5 | 1 | 0 | 4 | 0 | 5 | 4 | 4 | 5 | 0 | Y | 41737 | beta-actin [Protobothrops flavoviridis] |
| 208 | 252 | JAG45999.1 | 205.49 | 34 | 17 | 3 | 0 | 13 | 0 | 15 | 9 | 15 | 13 | 0 | 1.4154E6 |  |  | 1.3304E6 |  |  | 1.3673E6 | 2.9994E5 | 2.4553E5 |  | 10 | 3 | 5 | 1 | 0 | 4 | 0 | 5 | 4 | 4 | 5 | 0 | Y | 41737 | actin, cytoplasmic 1 [Crotalus horridus] |
| 208 | 253 | JAC96363.1 | 205.49 | 34 | 17 | 3 | 0 | 13 | 0 | 15 | 9 | 15 | 13 | 0 | 1.4154E6 |  |  | 1.3304E6 |  |  | 1.3673E6 | 2.9994E5 | 2.4553E5 |  | 10 | 3 | 5 | 1 | 0 | 4 | 0 | 5 | 4 | 4 | 5 | 0 | Y | 41793 | Actin, cytoplasmic 2 [Hypsiglena sp. JMG-2014] |
| 208 | 254 | JAA97900.1 | 205.49 | 34 | 17 | 3 | 0 | 13 | 0 | 15 | 9 | 15 | 13 | 0 | 1.4154E6 |  |  | 1.3304E6 |  |  | 1.3673E6 | 2.9994E5 | 2.4553E5 |  | 10 | 3 | 5 | 1 | 0 | 4 | 0 | 5 | 4 | 4 | 5 | 0 | Y | 41793 | Actin, cytoplasmic 2 [Crotalus horridus] |
| 208 | 255 | LAA21099.1 | 205.49 | 34 | 17 | 3 | 0 | 13 | 0 | 15 | 9 | 15 | 13 | 0 | 1.4154E6 |  |  | 1.3304E6 |  |  | 1.3673E6 | 2.9994E5 | 2.4553E5 |  | 10 | 3 | 5 | 1 | 0 | 4 | 0 | 5 | 4 | 4 | 5 | 0 | Y | 41793 | hypothetical protein [Micrurus lemniscatus carvalhoi] |
| 208 | 256 | JAG68843.1 | 205.49 | 34 | 17 | 3 | 0 | 13 | 0 | 15 | 9 | 15 | 13 | 0 | 1.4154E6 |  |  | 1.3304E6 |  |  | 1.3673E6 | 2.9994E5 | 2.4553E5 |  | 10 | 3 | 5 | 1 | 0 | 4 | 0 | 5 | 4 | 4 | 5 | 0 | Y | 41793 | Actin, cytoplasmic 2 [Boiga irregularis] |
| 208 | 257 | XP\_013921350.1 | 205.49 | 34 | 17 | 3 | 0 | 13 | 0 | 15 | 9 | 15 | 13 | 0 | 1.4154E6 |  |  | 1.3304E6 |  |  | 1.3673E6 | 2.9994E5 | 2.4553E5 |  | 10 | 3 | 5 | 1 | 0 | 4 | 0 | 5 | 4 | 4 | 5 | 0 | Y | 41793 | PREDICTED: actin, cytoplasmic 2 [Thamnophis sirtalis] |
| 208 | 258 | XP\_026531551.1 | 205.49 | 34 | 17 | 3 | 0 | 13 | 0 | 15 | 9 | 15 | 13 | 0 | 1.4154E6 |  |  | 1.3304E6 |  |  | 1.3673E6 | 2.9994E5 | 2.4553E5 |  | 10 | 3 | 5 | 1 | 0 | 4 | 0 | 5 | 4 | 4 | 5 | 0 | Y | 41793 | actin, cytoplasmic 2 [Notechis scutatus] |
| 208 | 259 | LAA21098.1 | 205.49 | 34 | 17 | 3 | 0 | 13 | 0 | 15 | 9 | 15 | 13 | 0 | 1.4154E6 |  |  | 1.3304E6 |  |  | 1.3673E6 | 2.9994E5 | 2.4553E5 |  | 10 | 3 | 5 | 1 | 0 | 4 | 0 | 5 | 4 | 4 | 5 | 0 | Y | 41793 | hypothetical protein [Micrurus lemniscatus carvalhoi] |
| 208 | 260 | AFJ49303.1 | 205.49 | 34 | 17 | 3 | 0 | 13 | 0 | 15 | 9 | 15 | 13 | 0 | 1.4154E6 |  |  | 1.3304E6 |  |  | 1.3673E6 | 2.9994E5 | 2.4553E5 |  | 10 | 3 | 5 | 1 | 0 | 4 | 0 | 5 | 4 | 4 | 5 | 0 | Y | 41793 | Actin, cytoplasmic 2 [Crotalus adamanteus] |
| 208 | 261 | JAI10341.1 | 205.49 | 34 | 17 | 3 | 0 | 13 | 0 | 15 | 9 | 15 | 13 | 0 | 1.4154E6 |  |  | 1.3304E6 |  |  | 1.3673E6 | 2.9994E5 | 2.4553E5 |  | 10 | 3 | 5 | 1 | 0 | 4 | 0 | 5 | 4 | 4 | 5 | 0 | Y | 41793 | Actin, cytoplasmic 2 [Micrurus fulvius] |
| 208 | 262 | JAI14495.1 | 205.49 | 34 | 17 | 3 | 0 | 13 | 0 | 15 | 9 | 15 | 13 | 0 | 1.4154E6 |  |  | 1.3304E6 |  |  | 1.3673E6 | 2.9994E5 | 2.4553E5 |  | 10 | 3 | 5 | 1 | 0 | 4 | 0 | 5 | 4 | 4 | 5 | 0 | Y | 41793 | Actin, cytoplasmic 2 [Crotalus adamanteus] |
| 208 | 263 | XP\_007419915.2 | 205.49 | 34 | 17 | 3 | 0 | 13 | 0 | 15 | 9 | 15 | 13 | 0 | 1.4154E6 |  |  | 1.3304E6 |  |  | 1.3673E6 | 2.9994E5 | 2.4553E5 |  | 10 | 3 | 5 | 1 | 0 | 4 | 0 | 5 | 4 | 4 | 5 | 0 | Y | 41793 | actin, cytoplasmic 2 [Python bivittatus] |
| 208 | 264 | JAG47579.1 | 205.49 | 34 | 17 | 3 | 0 | 13 | 0 | 15 | 9 | 15 | 13 | 0 | 1.4154E6 |  |  | 1.3304E6 |  |  | 1.3673E6 | 2.9994E5 | 2.4553E5 |  | 10 | 3 | 5 | 1 | 0 | 4 | 0 | 5 | 4 | 4 | 5 | 0 | Y | 41793 | Actin, cytoplasmic 2 [Crotalus horridus] |
| 208 | 265 | JAV51372.1 | 205.49 | 34 | 17 | 3 | 0 | 13 | 0 | 15 | 9 | 15 | 13 | 0 | 1.4154E6 |  |  | 1.3304E6 |  |  | 1.3673E6 | 2.9994E5 | 2.4553E5 |  | 10 | 3 | 5 | 1 | 0 | 4 | 0 | 5 | 4 | 4 | 5 | 0 | Y | 41793 | Actin, cytoplasmic 2 [Agkistrodon contortrix contortrix] |
| 208 | 266 | JAG45996.1 | 205.49 | 34 | 17 | 3 | 0 | 13 | 0 | 15 | 9 | 15 | 13 | 0 | 1.4154E6 |  |  | 1.3304E6 |  |  | 1.3673E6 | 2.9994E5 | 2.4553E5 |  | 10 | 3 | 5 | 1 | 0 | 4 | 0 | 5 | 4 | 4 | 5 | 0 | Y | 41793 | Actin, cytoplasmic 2 [Crotalus horridus] |
| 208 | 267 | JAB54395.1 | 205.49 | 34 | 17 | 3 | 0 | 13 | 0 | 15 | 9 | 15 | 13 | 0 | 1.4154E6 |  |  | 1.3304E6 |  |  | 1.3673E6 | 2.9994E5 | 2.4553E5 |  | 10 | 3 | 5 | 1 | 0 | 4 | 0 | 5 | 4 | 4 | 5 | 0 | Y | 41793 | cytoplasmic 2 [Micrurus fulvius] |
| 208 | 268 | ETE74025.1 | 205.49 | 34 | 17 | 3 | 0 | 13 | 0 | 15 | 9 | 15 | 13 | 0 | 1.4154E6 |  |  | 1.3304E6 |  |  | 1.3673E6 | 2.9994E5 | 2.4553E5 |  | 10 | 3 | 5 | 1 | 0 | 4 | 0 | 5 | 4 | 4 | 5 | 0 | Y | 41793 | Actin, cytoplasmic 2 [Ophiophagus hannah] |
| 205 | 189 | XP\_026530513.1 | 198.18 | 19 | 1 | 0 | 0 | 0 | 0 | 0 | 0 | 18 | 13 | 0 |  |  |  |  |  |  |  | 2.1251E6 | 6.9519E5 |  | 12 | 1 | 1 | 0 | 0 | 0 | 0 | 0 | 0 | 23 | 12 | 0 | Y | 76681 | xaa-Pro aminopeptidase 2 [Notechis scutatus] |
| 78 | 375 | XP\_032067333.1 | 197.06 | 14 | 3 | 3 | 1 | 8 | 0 | 8 | 4 | 7 | 3 | 0 |  |  |  |  |  |  |  | 7.118E5 |  |  | 15 | 1 | 6 | 4 | 1 | 35 | 0 | 22 | 8 | 15 | 4 | 0 | N | 62422 | keratin, type II cytoskeletal 6A-like [Thamnophis elegans] |
| 191 | 220 | XP\_026576301.1 | 194.90 | 14 | 6 | 4 | 0 | 10 | 0 | 10 | 3 | 12 | 12 | 1 | 1.081E6 | 6.5459E5 |  | 1.1006E7 |  | 1.1046E7 | 1.3158E5 | 7.6933E6 | 2.903E6 | 2.6941E6 | 10 | 9 | 3 | 5 | 0 | 11 | 0 | 9 | 2 | 12 | 9 | 1 | Y | 81304 | junction plakoglobin isoform X2 [Pseudonaja textilis] |
| 191 | 221 | XP\_026525656.1 | 194.90 | 14 | 6 | 4 | 0 | 10 | 0 | 10 | 3 | 12 | 12 | 1 | 1.081E6 | 6.5459E5 |  | 1.1006E7 |  | 1.1046E7 | 1.3158E5 | 7.6933E6 | 2.903E6 | 2.6941E6 | 10 | 9 | 3 | 5 | 0 | 11 | 0 | 9 | 2 | 12 | 9 | 1 | Y | 81304 | junction plakoglobin isoform X2 [Notechis scutatus] |
| 191 | 222 | XP\_026576299.1 | 194.90 | 14 | 6 | 4 | 0 | 10 | 0 | 10 | 3 | 12 | 11 | 1 | 1.081E6 | 6.5459E5 |  | 1.1006E7 |  | 1.1046E7 | 1.3158E5 | 7.6933E6 | 2.903E6 | 2.6941E6 | 10 | 9 | 3 | 5 | 0 | 11 | 0 | 9 | 2 | 12 | 9 | 1 | Y | 81375 | junction plakoglobin isoform X1 [Pseudonaja textilis] |
| 191 | 223 | XP\_026525655.1 | 194.90 | 14 | 6 | 4 | 0 | 10 | 0 | 10 | 3 | 12 | 11 | 1 | 1.081E6 | 6.5459E5 |  | 1.1006E7 |  | 1.1046E7 | 1.3158E5 | 7.6933E6 | 2.903E6 | 2.6941E6 | 10 | 9 | 3 | 5 | 0 | 11 | 0 | 9 | 2 | 12 | 9 | 1 | Y | 81375 | junction plakoglobin isoform X1 [Notechis scutatus] |
| 191 | 224 | XP\_026525654.1 | 194.90 | 14 | 6 | 4 | 0 | 10 | 0 | 10 | 3 | 12 | 11 | 1 | 1.081E6 | 6.5459E5 |  | 1.1006E7 |  | 1.1046E7 | 1.3158E5 | 7.6933E6 | 2.903E6 | 2.6941E6 | 10 | 9 | 3 | 5 | 0 | 11 | 0 | 9 | 2 | 12 | 9 | 1 | Y | 81375 | junction plakoglobin isoform X1 [Notechis scutatus] |
| 191 | 225 | XP\_026576298.1 | 194.90 | 14 | 6 | 4 | 0 | 10 | 0 | 10 | 3 | 12 | 11 | 1 | 1.081E6 | 6.5459E5 |  | 1.1006E7 |  | 1.1046E7 | 1.3158E5 | 7.6933E6 | 2.903E6 | 2.6941E6 | 10 | 9 | 3 | 5 | 0 | 11 | 0 | 9 | 2 | 12 | 9 | 1 | Y | 81375 | junction plakoglobin isoform X1 [Pseudonaja textilis] |
| 191 | 226 | JAG67632.1 | 194.90 | 14 | 6 | 4 | 0 | 10 | 0 | 10 | 3 | 12 | 11 | 1 | 1.081E6 | 6.5459E5 |  | 1.1006E7 |  | 1.1046E7 | 1.3158E5 | 7.6933E6 | 2.903E6 | 2.6941E6 | 10 | 9 | 3 | 5 | 0 | 11 | 0 | 9 | 2 | 12 | 9 | 1 | Y | 81861 | junction plakoglobin [Boiga irregularis] |
| 191 | 227 | XP\_025022317.1 | 194.90 | 14 | 6 | 4 | 0 | 10 | 0 | 10 | 3 | 12 | 11 | 1 | 1.081E6 | 6.5459E5 |  | 1.1006E7 |  | 1.1046E7 | 1.3158E5 | 7.6933E6 | 2.903E6 | 2.6941E6 | 10 | 9 | 3 | 5 | 0 | 11 | 0 | 9 | 2 | 12 | 9 | 1 | Y | 82056 | junction plakoglobin isoform X1 [Python bivittatus] |
| 191 | 228 | XP\_007428111.1 | 194.90 | 14 | 6 | 4 | 0 | 10 | 0 | 10 | 3 | 12 | 11 | 1 | 1.081E6 | 6.5459E5 |  | 1.1006E7 |  | 1.1046E7 | 1.3158E5 | 7.6933E6 | 2.903E6 | 2.6941E6 | 10 | 9 | 3 | 5 | 0 | 11 | 0 | 9 | 2 | 12 | 9 | 1 | Y | 82056 | junction plakoglobin isoform X1 [Python bivittatus] |
| 191 | 229 | JAG44306.1 | 194.90 | 14 | 6 | 4 | 0 | 10 | 0 | 10 | 3 | 12 | 11 | 1 | 1.081E6 | 6.5459E5 |  | 1.1006E7 |  | 1.1046E7 | 1.3158E5 | 7.6933E6 | 2.903E6 | 2.6941E6 | 10 | 9 | 3 | 5 | 0 | 11 | 0 | 9 | 2 | 12 | 9 | 1 | Y | 81903 | junction plakoglobin [Crotalus horridus] |
| 191 | 230 | JAA96758.1 | 194.90 | 14 | 6 | 4 | 0 | 10 | 0 | 10 | 3 | 12 | 11 | 1 | 1.081E6 | 6.5459E5 |  | 1.1006E7 |  | 1.1046E7 | 1.3158E5 | 7.6933E6 | 2.903E6 | 2.6941E6 | 10 | 9 | 3 | 5 | 0 | 11 | 0 | 9 | 2 | 12 | 9 | 1 | Y | 81903 | junction plakoglobin [Crotalus horridus] |
| 191 | 231 | JAG46538.1 | 194.90 | 14 | 6 | 4 | 0 | 10 | 0 | 10 | 3 | 12 | 11 | 1 | 1.081E6 | 6.5459E5 |  | 1.1006E7 |  | 1.1046E7 | 1.3158E5 | 7.6933E6 | 2.903E6 | 2.6941E6 | 10 | 9 | 3 | 5 | 0 | 11 | 0 | 9 | 2 | 12 | 9 | 1 | Y | 81903 | junction plakoglobin [Crotalus horridus] |
| 191 | 232 | JAV50234.1 | 194.90 | 14 | 6 | 4 | 0 | 10 | 0 | 10 | 3 | 12 | 11 | 1 | 1.081E6 | 6.5459E5 |  | 1.1006E7 |  | 1.1046E7 | 1.3158E5 | 7.6933E6 | 2.903E6 | 2.6941E6 | 10 | 9 | 3 | 5 | 0 | 11 | 0 | 9 | 2 | 12 | 9 | 1 | Y | 81358 | JUP protein [Agkistrodon contortrix contortrix] |
| 191 | 233 | XP\_025022318.1 | 194.90 | 14 | 6 | 4 | 0 | 10 | 0 | 10 | 3 | 12 | 11 | 1 | 1.081E6 | 6.5459E5 |  | 1.1006E7 |  | 1.1046E7 | 1.3158E5 | 7.6933E6 | 2.903E6 | 2.6941E6 | 10 | 9 | 3 | 5 | 0 | 11 | 0 | 9 | 2 | 12 | 9 | 1 | Y | 81985 | junction plakoglobin isoform X2 [Python bivittatus] |
| 191 | 234 | XP\_034271617.1 | 194.90 | 14 | 6 | 4 | 0 | 10 | 0 | 10 | 3 | 12 | 11 | 1 | 1.081E6 | 6.5459E5 |  | 1.1006E7 |  | 1.1046E7 | 1.3158E5 | 7.6933E6 | 2.903E6 | 2.6941E6 | 10 | 9 | 3 | 5 | 0 | 11 | 0 | 9 | 2 | 12 | 9 | 1 | Y | 81870 | junction plakoglobin isoform X2 [Pantherophis guttatus] |
| 191 | 235 | XP\_013919280.1 | 194.90 | 14 | 6 | 4 | 0 | 10 | 0 | 10 | 3 | 12 | 11 | 1 | 1.081E6 | 6.5459E5 |  | 1.1006E7 |  | 1.1046E7 | 1.3158E5 | 7.6933E6 | 2.903E6 | 2.6941E6 | 10 | 9 | 3 | 5 | 0 | 11 | 0 | 9 | 2 | 12 | 9 | 1 | Y | 81965 | PREDICTED: junction plakoglobin [Thamnophis sirtalis] |
| 191 | 236 | XP\_034271615.1 | 194.90 | 14 | 6 | 4 | 0 | 10 | 0 | 10 | 3 | 12 | 11 | 1 | 1.081E6 | 6.5459E5 |  | 1.1006E7 |  | 1.1046E7 | 1.3158E5 | 7.6933E6 | 2.903E6 | 2.6941E6 | 10 | 9 | 3 | 5 | 0 | 11 | 0 | 9 | 2 | 12 | 9 | 1 | Y | 81941 | junction plakoglobin isoform X1 [Pantherophis guttatus] |
| 191 | 237 | XP\_034271618.1 | 194.90 | 14 | 6 | 4 | 0 | 10 | 0 | 10 | 3 | 12 | 11 | 1 | 1.081E6 | 6.5459E5 |  | 1.1006E7 |  | 1.1046E7 | 1.3158E5 | 7.6933E6 | 2.903E6 | 2.6941E6 | 10 | 9 | 3 | 5 | 0 | 11 | 0 | 9 | 2 | 12 | 9 | 1 | Y | 81941 | junction plakoglobin isoform X1 [Pantherophis guttatus] |
| 191 | 238 | XP\_034271616.1 | 194.90 | 14 | 6 | 4 | 0 | 10 | 0 | 10 | 3 | 12 | 11 | 1 | 1.081E6 | 6.5459E5 |  | 1.1006E7 |  | 1.1046E7 | 1.3158E5 | 7.6933E6 | 2.903E6 | 2.6941E6 | 10 | 9 | 3 | 5 | 0 | 11 | 0 | 9 | 2 | 12 | 9 | 1 | Y | 81941 | junction plakoglobin isoform X1 [Pantherophis guttatus] |
| 191 | 269 | LAB43566.1 | 194.90 | 14 | 6 | 4 | 0 | 10 | 0 | 10 | 2 | 11 | 11 | 1 | 1.081E6 | 6.5459E5 |  | 1.1006E7 |  | 1.1046E7 | 1.3158E5 | 7.6933E6 | 2.903E6 | 2.6941E6 | 10 | 9 | 3 | 5 | 0 | 11 | 0 | 9 | 2 | 12 | 9 | 1 | Y | 83463 | hypothetical protein, partial [Micrurus spixii] |
| 191 | 272 | XP\_032091066.1 | 194.90 | 13 | 5 | 4 | 0 | 9 | 0 | 10 | 2 | 11 | 11 | 1 | 1.081E6 | 6.5459E5 |  | 1.1006E7 |  | 1.1046E7 | 1.3158E5 | 7.6933E6 | 2.903E6 | 2.6941E6 | 10 | 9 | 3 | 5 | 0 | 11 | 0 | 9 | 2 | 12 | 9 | 1 | Y | 86365 | junction plakoglobin [Thamnophis elegans] |
| 191 | 273 | XP\_032091065.1 | 194.90 | 13 | 5 | 4 | 0 | 9 | 0 | 10 | 2 | 11 | 11 | 1 | 1.081E6 | 6.5459E5 |  | 1.1006E7 |  | 1.1046E7 | 1.3158E5 | 7.6933E6 | 2.903E6 | 2.6941E6 | 10 | 9 | 3 | 5 | 0 | 11 | 0 | 9 | 2 | 12 | 9 | 1 | Y | 86365 | junction plakoglobin [Thamnophis elegans] |
| 191 | 278 | ETE68218.1 | 194.90 | 14 | 6 | 4 | 0 | 10 | 0 | 10 | 3 | 11 | 11 | 1 | 1.081E6 | 6.5459E5 |  | 1.1006E7 |  | 1.1046E7 | 1.3158E5 | 7.6933E6 | 2.903E6 | 2.6941E6 | 10 | 9 | 3 | 5 | 0 | 11 | 0 | 9 | 2 | 12 | 9 | 1 | Y | 83009 | Junction plakoglobin [Ophiophagus hannah] |
| 231 | 344 | XP\_015687424.1 | 182.67 | 16 | 16 | 0 | 0 | 0 | 0 | 0 | 0 | 1 | 0 | 0 | 2.4742E7 |  |  |  |  |  |  | 7.325E4 |  |  | 10 | 10 | 17 | 0 | 0 | 0 | 0 | 0 | 0 | 1 | 0 | 0 | Y | 106458 | endoplasmic reticulum aminopeptidase 1 [Protobothrops mucrosquamatus] |
| 224 | 328 | JAG65874.1 | 179.53 | 15 | 1 | 0 | 0 | 0 | 0 | 0 | 0 | 14 | 9 | 0 |  |  |  |  |  |  |  | 2.0806E5 |  |  | 9 | 1 | 1 | 0 | 0 | 0 | 0 | 0 | 0 | 14 | 8 | 0 | Y | 76736 | Xaa-Pro aminopeptidase 2 [Boiga irregularis] |
| 219 | 592 | XP\_015743663.1 | 178.38 | 11 | 7 | 2 | 0 | 0 | 0 | 0 | 0 | 5 | 11 | 2 |  |  |  |  |  |  |  |  | 0 |  | 7 | 1 | 6 | 1 | 0 | 0 | 0 | 0 | 0 | 3 | 22 | 1 | N | 56333 | intestinal-type alkaline phosphatase isoform X2 [Python bivittatus] |
| 219 | 599 | XP\_025022776.1 | 178.38 | 12 | 7 | 2 | 0 | 0 | 0 | 0 | 0 | 5 | 12 | 2 |  |  |  |  |  |  |  |  | 0 |  | 7 | 1 | 6 | 1 | 0 | 0 | 0 | 0 | 0 | 3 | 22 | 1 | N | 50994 | intestinal-type alkaline phosphatase isoform X3 [Python bivittatus] |
| 219 | 600 | XP\_025022775.1 | 178.38 | 10 | 6 | 2 | 0 | 0 | 0 | 0 | 0 | 4 | 10 | 2 |  |  |  |  |  |  |  |  | 0 |  | 7 | 1 | 6 | 1 | 0 | 0 | 0 | 0 | 0 | 3 | 22 | 1 | N | 60659 | intestinal-type alkaline phosphatase isoform X1 [Python bivittatus] |
| 226 | 597 | BAN89414.1 | 173.48 | 16 | 6 | 0 | 0 | 0 | 0 | 0 | 0 | 4 | 14 | 2 |  |  |  |  |  |  |  |  | 4.0106E6 | 9.8456E6 | 8 | 6 | 3 | 0 | 0 | 0 | 0 | 0 | 0 | 1 | 13 | 1 | Y | 49636 | hyaluronidase [Ovophis okinavensis] |
| 226 | 598 | BAN89413.1 | 173.48 | 15 | 5 | 0 | 0 | 0 | 0 | 0 | 0 | 4 | 13 | 2 |  |  |  |  |  |  |  |  | 4.0106E6 | 9.8456E6 | 8 | 6 | 3 | 0 | 0 | 0 | 0 | 0 | 0 | 1 | 13 | 1 | Y | 52464 | hyaluronidase [Ovophis okinavensis] |
| 137 | 463 | ASX97880.1 | 171.77 | 22 | 12 | 15 | 15 | 3 | 0 | 12 | 20 | 9 | 4 | 0 | 3.4114E6 | 3.5572E6 | 4.5094E6 | 3.0855E6 |  | 4.2246E7 | 1.5268E8 | 1.16E6 | 3.9737E5 |  | 7 | 3 | 3 | 16 | 16 | 1 | 0 | 5 | 19 | 2 | 1 | 0 | Y | 28515 | venom plasminogen activator precursor 1 [Gloydius intermedius] |
| 69 | 702 | XP\_007435739.1 | 171.70 | 8 | 2 | 4 | 2 | 6 | 0 | 6 | 2 | 8 | 8 | 2 |  | 5.7112E6 | 8.6592E5 | 5.2178E7 |  | 4.4375E7 | 2.6971E6 | 1.0894E7 | 5.7894E6 |  | 6 | 1 | 8 | 3 | 1 | 27 | 0 | 4 | 2 | 59 | 23 | 4 | Y | 53751 | keratin, type II cytoskeletal 8 [Python bivittatus] |
| 5 | 1480 | AHJ09587.1 | 170.76 | 17 | 8 | 7 | 0 | 8 | 0 | 17 | 7 | 8 | 8 | 0 | 2.4834E8 | 1.7728E7 |  | 3.259E8 |  | 1.9288E10 | 3.5643E6 | 5.5212E7 | 4.0776E7 |  | 4 | 3 | 13 | 6 | 0 | 115 | 0 | 647 | 3 | 18 | 13 | 0 | Y | 15070 | phospholipase A2 [Trimeresurus cardamomensis] |
| 5 | 1525 | AHJ09591.1 | 170.76 | 17 | 8 | 7 | 0 | 8 | 0 | 17 | 7 | 8 | 8 | 0 | 2.4834E8 | 1.7728E7 |  | 3.259E8 |  | 1.9288E10 | 3.5643E6 | 5.5212E7 | 4.0776E7 |  | 4 | 3 | 13 | 6 | 0 | 115 | 0 | 647 | 3 | 18 | 13 | 0 | Y | 15804 | phospholipase A2 [Trimeresurus cardamomensis] |
| 5 | 1567 | AHJ09589.1 | 170.76 | 17 | 8 | 7 | 0 | 8 | 0 | 17 | 7 | 8 | 8 | 0 | 2.4834E8 | 1.7728E7 |  | 3.259E8 |  | 1.9288E10 | 3.5643E6 | 5.5212E7 | 4.0776E7 |  | 4 | 3 | 13 | 6 | 0 | 115 | 0 | 647 | 3 | 18 | 13 | 0 | Y | 15864 | phospholipase A2 [Trimeresurus cardamomensis] |
| 5 | 1568 | AHJ09585.1 | 170.76 | 17 | 8 | 7 | 0 | 8 | 0 | 17 | 7 | 8 | 8 | 0 | 2.4834E8 | 1.7728E7 |  | 3.259E8 |  | 1.9288E10 | 3.5643E6 | 5.5212E7 | 4.0776E7 |  | 4 | 3 | 13 | 6 | 0 | 115 | 0 | 647 | 3 | 18 | 13 | 0 | Y | 15864 | phospholipase A2 [Trimeresurus cardamomensis] |
| 5 | 1569 | AHJ09594.1 | 170.76 | 17 | 8 | 7 | 0 | 8 | 0 | 17 | 7 | 8 | 8 | 0 | 2.4834E8 | 1.7728E7 |  | 3.259E8 |  | 1.9288E10 | 3.5643E6 | 5.5212E7 | 4.0776E7 |  | 4 | 3 | 13 | 6 | 0 | 115 | 0 | 647 | 3 | 18 | 13 | 0 | Y | 15864 | phospholipase A2 [Trimeresurus cardamomensis] |
| 223 | 424 | JAG67188.1 | 169.92 | 10 | 5 | 6 | 3 | 0 | 0 | 0 | 2 | 4 | 10 | 0 | 7.609E6 | 1.8482E6 | 2.445E5 |  |  |  |  | 5.4762E5 | 1.8065E7 |  | 6 | 1 | 4 | 5 | 2 | 0 | 0 | 0 | 1 | 3 | 14 | 0 | N | 64759 | Snake venom 5'-nucleotidase [Boiga irregularis] |
| 246 | 486 | XP\_034265289.1 | 167.87 | 13 | 4 | 0 | 0 | 6 | 0 | 5 | 3 | 7 | 7 | 0 |  |  |  | 1.5803E5 |  | 5.945E5 |  |  |  |  | 4 | 2 | 1 | 0 | 0 | 2 | 0 | 2 | 1 | 6 | 2 | 0 | N | 38616 | annexin A2 [Pantherophis guttatus] |
| 73 | 845 | XP\_026529665.1 | 166.05 | 9 | 2 | 2 | 0 | 4 | 0 | 4 | 0 | 9 | 9 | 2 |  |  |  |  |  |  |  | 3.1816E6 | 3.5252E6 |  | 6 | 1 | 8 | 2 | 0 | 25 | 0 | 2 | 0 | 58 | 22 | 4 | Y | 54010 | keratin, type II cytoskeletal 8 [Notechis scutatus] |
| 274 | 315 | XP\_029143063.1 | 163.11 | 8 | 0 | 0 | 0 | 0 | 0 | 0 | 0 | 8 | 0 | 0 |  |  |  |  |  |  |  | 2.4395E6 |  |  | 8 | 8 | 0 | 0 | 0 | 0 | 0 | 0 | 0 | 9 | 0 | 0 | Y | 151426 | angiotensin-converting enzyme [Protobothrops mucrosquamatus] |
| 248 | 635 | XP\_026537798.1 | 158.70 | 10 | 4 | 0 | 0 | 6 | 0 | 3 | 3 | 10 | 10 | 0 |  |  |  | 3.226E5 |  |  |  | 4.7166E4 | 2.2543E4 |  | 3 | 1 | 1 | 0 | 0 | 2 | 0 | 1 | 1 | 7 | 3 | 0 | N | 40235 | annexin A2 [Notechis scutatus] |
| 299 | 570 | XP\_007424908.1 | 153.41 | 4 | 0 | 0 | 0 | 0 | 0 | 0 | 0 | 0 | 4 | 0 |  |  |  |  |  |  |  |  | 7.6213E5 |  | 4 | 2 | 0 | 0 | 0 | 0 | 0 | 0 | 0 | 0 | 4 | 0 | Y | 137669 | collagen alpha-1(I) chain [Python bivittatus] |
| 43 | 746 | AUS82539.1 | 153.20 | 10 | 10 | 8 | 4 | 4 | 0 | 7 | 8 | 8 | 8 | 4 | 2.543E9 | 5.1576E9 | 3.907E7 | 9.4019E5 |  | 1.227E6 | 2.7278E7 | 1.9579E7 | 1.1888E7 | 2.5806E7 | 4 | 1 | 63 | 87 | 17 | 1 | 0 | 3 | 11 | 28 | 5 | 1 | Y | 28390 | serine endopeptidase [Crotalus scutulatus] |
| 201 | 1354 | JAV01822.1 | 146.86 | 17 | 13 | 14 | 5 | 5 | 0 | 5 | 8 | 3 | 3 | 3 | 2.1078E7 | 2.1926E7 |  |  |  |  |  |  |  |  | 6 | 2 | 7 | 13 | 6 | 4 | 0 | 2 | 3 | 2 | 1 | 1 | Y | 28000 | BATXSVSP14 [Bothrops atrox] |
| 268 | 490 | AFJ49950.1 | 135.50 | 11 | 0 | 0 | 0 | 6 | 0 | 7 | 0 | 7 | 3 | 0 |  |  |  | 2.9659E6 |  | 6.5617E5 |  | 3.5252E5 | 1.3607E4 |  | 5 | 5 | 0 | 0 | 0 | 3 | 0 | 3 | 0 | 3 | 1 | 0 | N | 50201 | Elongation factor 1-alpha 1-like [Crotalus adamanteus] |
| 268 | 491 | JAA97186.1 | 135.50 | 11 | 0 | 0 | 0 | 6 | 0 | 7 | 0 | 7 | 3 | 0 |  |  |  | 2.9659E6 |  | 6.5617E5 |  | 3.5252E5 | 1.3607E4 |  | 5 | 5 | 0 | 0 | 0 | 3 | 0 | 3 | 0 | 3 | 1 | 0 | N | 50201 | Elongation factor 1-alpha 1 [Crotalus horridus] |
| 268 | 492 | JAI13488.1 | 135.50 | 11 | 0 | 0 | 0 | 6 | 0 | 7 | 0 | 7 | 3 | 0 |  |  |  | 2.9659E6 |  | 6.5617E5 |  | 3.5252E5 | 1.3607E4 |  | 5 | 5 | 0 | 0 | 0 | 3 | 0 | 3 | 0 | 3 | 1 | 0 | N | 50201 | elongation factor 1-alpha 1 [Crotalus adamanteus] |
| 268 | 493 | XP\_007431170.1 | 135.50 | 11 | 0 | 0 | 0 | 6 | 0 | 7 | 0 | 7 | 3 | 0 |  |  |  | 2.9659E6 |  | 6.5617E5 |  | 3.5252E5 | 1.3607E4 |  | 5 | 5 | 0 | 0 | 0 | 3 | 0 | 3 | 0 | 3 | 1 | 0 | N | 50155 | elongation factor 1-alpha 1 [Python bivittatus] |
| 268 | 494 | JAG46923.1 | 135.50 | 11 | 0 | 0 | 0 | 6 | 0 | 7 | 0 | 7 | 3 | 0 |  |  |  | 2.9659E6 |  | 6.5617E5 |  | 3.5252E5 | 1.3607E4 |  | 5 | 5 | 0 | 0 | 0 | 3 | 0 | 3 | 0 | 3 | 1 | 0 | N | 50201 | Elongation factor 1-alpha 1 [Crotalus horridus] |
| 268 | 495 | XP\_015672964.1 | 135.50 | 11 | 0 | 0 | 0 | 6 | 0 | 7 | 0 | 7 | 3 | 0 |  |  |  | 2.9659E6 |  | 6.5617E5 |  | 3.5252E5 | 1.3607E4 |  | 5 | 5 | 0 | 0 | 0 | 3 | 0 | 3 | 0 | 3 | 1 | 0 | N | 50155 | elongation factor 1-alpha 1 [Protobothrops mucrosquamatus] |
| 268 | 496 | JAG44960.1 | 135.50 | 11 | 0 | 0 | 0 | 6 | 0 | 7 | 0 | 7 | 3 | 0 |  |  |  | 2.9659E6 |  | 6.5617E5 |  | 3.5252E5 | 1.3607E4 |  | 5 | 5 | 0 | 0 | 0 | 3 | 0 | 3 | 0 | 3 | 1 | 0 | N | 50201 | elongation factor 1-alpha 1 [Crotalus horridus] |
| 268 | 497 | JAV50678.1 | 135.50 | 11 | 0 | 0 | 0 | 6 | 0 | 7 | 0 | 7 | 3 | 0 |  |  |  | 2.9659E6 |  | 6.5617E5 |  | 3.5252E5 | 1.3607E4 |  | 5 | 5 | 0 | 0 | 0 | 3 | 0 | 3 | 0 | 3 | 1 | 0 | N | 50201 | Elongation factor 1-alpha 1 [Agkistrodon contortrix contortrix] |
| 81 | 946 | XP\_026570509.1 | 135.11 | 5 | 4 | 3 | 3 | 4 | 2 | 4 | 3 | 5 | 5 | 2 |  |  |  |  |  |  |  | 5.4786E5 | 1.3457E5 |  | 4 | 1 | 7 | 6 | 3 | 30 | 3 | 16 | 6 | 18 | 8 | 2 | N | 52005 | keratin, type II cytoskeletal 4-like [Pseudonaja textilis] |
| 81 | 954 | XP\_026539658.1 | 135.11 | 5 | 4 | 3 | 3 | 4 | 2 | 4 | 3 | 5 | 5 | 2 |  |  |  |  |  |  |  | 5.4786E5 | 1.3457E5 |  | 4 | 1 | 7 | 6 | 3 | 30 | 3 | 16 | 6 | 18 | 8 | 2 | N | 51886 | keratin, type II cytoskeletal 4-like [Notechis scutatus] |
| 282 | 1280 | P0DJJ5.1 | 135.00 | 52 | 0 | 24 | 0 | 0 | 0 | 0 | 0 | 0 | 52 | 0 |  |  |  |  |  |  |  |  | 7.189E5 |  | 3 | 1 | 0 | 1 | 0 | 0 | 0 | 0 | 0 | 0 | 5 | 0 | N | 6012 | RecName: Full=Snake venom 5'-nucleotidase; Short=5'-NT; AltName: Full=Ecto-5'-nucleotidase |
| 311 | 768 | JAG47217.1 | 132.25 | 17 | 17 | 0 | 0 | 0 | 0 | 0 | 0 | 0 | 0 | 0 | 1.0397E6 |  |  |  |  |  |  |  |  |  | 5 | 2 | 5 | 0 | 0 | 0 | 0 | 0 | 0 | 0 | 0 | 0 | Y | 43509 | Cathepsin D [Crotalus horridus] |
| 217 | 658 | P0CJ41.1 | 130.89 | 15 | 5 | 0 | 0 | 0 | 0 | 5 | 15 | 5 | 0 | 0 | 1.0071E7 |  |  |  |  | 1.611E6 | 1.8767E6 | 2.6993E6 |  |  | 4 | 1 | 1 | 0 | 0 | 0 | 0 | 1 | 5 | 2 | 0 | 0 | Y | 28001 | RecName: Full=Alpha-fibrinogenase albofibrase; AltName: Full=Snake venom serine protease; Short=SVSP; Flags: Precursor |
| 236 | 1062 | P0DJF5.1 | 129.45 | 14 | 9 | 14 | 9 | 0 | 0 | 0 | 0 | 4 | 4 | 0 |  | 1.0159E6 | 6.3134E6 |  |  |  |  |  |  |  | 4 | 2 | 3 | 4 | 6 | 0 | 0 | 0 | 0 | 3 | 2 | 0 | N | 28428 | RecName: Full=Venom plasminogen activator GPV-PA; AltName: Full=Green pit viper plasminogen activator; Short=GPV-PA; AltName: Full=Snake venom serine protease; Short=SVSP; Flags: Precursor |
| 136 | 823 | AUS82501.1 | 128.96 | 15 | 6 | 10 | 7 | 0 | 0 | 0 | 8 | 6 | 3 | 0 |  |  |  |  |  |  | 9.9886E5 |  |  |  | 5 | 1 | 21 | 11 | 2 | 0 | 0 | 0 | 3 | 5 | 1 | 0 | Y | 28553 | serine endopeptidase [Crotalus lepidus] |
| 233 | 755 | AUS82565.1 | 126.43 | 17 | 4 | 9 | 9 | 0 | 0 | 0 | 8 | 4 | 4 | 0 |  |  |  |  |  |  |  | 1.3612E5 |  |  | 5 | 1 | 2 | 5 | 5 | 0 | 0 | 0 | 2 | 4 | 2 | 0 | Y | 28164 | serine endopeptidase [Crotalus tigris] |
| 281 | 799 | XP\_015676332.1 | 126.08 | 4 | 0 | 0 | 0 | 0 | 0 | 0 | 0 | 4 | 2 | 0 |  |  |  |  |  |  |  | 3.8152E6 | 6.2212E4 |  | 3 | 3 | 0 | 0 | 0 | 0 | 0 | 0 | 0 | 3 | 1 | 0 | N | 61048 | N-acetylglucosamine-6-sulfatase isoform X1 [Protobothrops mucrosquamatus] |
| 281 | 801 | LAA85609.1 | 126.08 | 4 | 0 | 0 | 0 | 0 | 0 | 0 | 0 | 4 | 2 | 0 |  |  |  |  |  |  |  | 3.8152E6 | 6.2212E4 |  | 3 | 3 | 0 | 0 | 0 | 0 | 0 | 0 | 0 | 3 | 1 | 0 | N | 61053 | hypothetical protein [Micrurus lemniscatus lemniscatus] |
| 281 | 802 | LAA85607.1 | 126.08 | 4 | 0 | 0 | 0 | 0 | 0 | 0 | 0 | 4 | 2 | 0 |  |  |  |  |  |  |  | 3.8152E6 | 6.2212E4 |  | 3 | 3 | 0 | 0 | 0 | 0 | 0 | 0 | 0 | 3 | 1 | 0 | N | 61053 | hypothetical protein [Micrurus lemniscatus lemniscatus] |
| 280 | 519 | XP\_007439537.1 | 123.74 | 7 | 0 | 0 | 0 | 2 | 0 | 2 | 0 | 7 | 0 | 0 |  |  |  | 4.172E4 |  | 5.1027E5 |  | 1.2075E6 |  |  | 4 | 4 | 0 | 0 | 0 | 1 | 0 | 1 | 0 | 4 | 0 | 0 | Y | 73851 | histidine ammonia-lyase isoform X1 [Python bivittatus] |
| 123 | 1443 | QBF53412.1 | 123.43 | 14 | 10 | 8 | 4 | 0 | 0 | 4 | 8 | 8 | 4 | 0 | 1.0189E6 |  |  |  |  |  |  |  |  |  | 4 | 1 | 19 | 7 | 3 | 0 | 0 | 2 | 13 | 5 | 1 | 0 | Y | 28317 | serine proteinase SP-6 [Vipera ammodytes ammodytes] |
| 149 | 1006 | BAA20283.1 | 123.09 | 11 | 6 | 4 | 4 | 0 | 0 | 3 | 8 | 8 | 8 | 0 |  |  | 3.8883E6 |  |  |  | 2.5403E7 | 0 | 2.6616E5 |  | 4 | 1 | 20 | 5 | 1 | 0 | 0 | 2 | 4 | 5 | 2 | 0 | Y | 27894 | KN-BJ2 [Bothrops jararaca] |
| 149 | 1007 | O13069.1 | 123.09 | 11 | 6 | 4 | 4 | 0 | 0 | 3 | 8 | 8 | 8 | 0 |  |  | 3.8883E6 |  |  |  | 2.5403E7 | 0 | 2.6616E5 |  | 4 | 1 | 20 | 5 | 1 | 0 | 0 | 2 | 4 | 5 | 2 | 0 | Y | 27894 | RecName: Full=Thrombin-like enzyme KN-BJ 2; Short=SVTLE; AltName: Full=Kinin-releasing and fibrinogen-clotting serine protease 2; Flags: Precursor |
| 334 | 1087 | ETE66900.1 | 122.98 | 16 | 16 | 0 | 0 | 0 | 0 | 0 | 0 | 0 | 0 | 0 | 1.0944E6 |  |  |  |  |  |  |  |  |  | 4 | 1 | 4 | 0 | 0 | 0 | 0 | 0 | 0 | 0 | 0 | 0 | Y | 30492 | Cathepsin D, partial [Ophiophagus hannah] |
| 178 | 1813 | ACZ34293.1 | 122.95 | 9 | 9 | 9 | 9 | 9 | 0 | 9 | 0 | 9 | 9 | 0 | 2.5025E6 | 4.3527E5 | 2.1003E5 | 7.7427E7 |  | 3.7591E7 |  | 4.8101E6 | 2.1619E6 |  | 1 | 1 | 2 | 1 | 1 | 31 | 0 | 19 | 0 | 3 | 2 | 0 | N | 18146 | TJ-GPIb-bp alpha chain [Protobothrops jerdonii] |
| 178 | 1814 | D1MGU0.1 | 122.95 | 9 | 9 | 9 | 9 | 9 | 0 | 9 | 0 | 9 | 9 | 0 | 2.5025E6 | 4.3527E5 | 2.1003E5 | 7.7427E7 |  | 3.7591E7 |  | 4.8101E6 | 2.1619E6 |  | 1 | 1 | 2 | 1 | 1 | 31 | 0 | 19 | 0 | 3 | 2 | 0 | N | 18146 | RecName: Full=Snaclec jerdonibitin subunit alpha; AltName: Full=TJ-GPIb-bp subunit alpha; Flags: Precursor |
| 119 | 2441 | BAP39913.1 | 122.33 | 9 | 9 | 9 | 9 | 9 | 0 | 0 | 9 | 9 | 9 | 9 | 2.7643E9 | 2.0814E7 | 1.8361E6 | 2.5601E6 |  |  | 8.5889E6 | 1.1085E7 | 2.5945E6 | 7.0215E6 | 3 | 2 | 30 | 7 | 4 | 1 | 0 | 0 | 6 | 6 | 2 | 1 | Y | 19487 | metalloprotease P-IIIc, partial [Protobothrops elegans] |
| 213 | 2091 | P0DM87.1 | 121.44 | 3 | 3 | 3 | 2 | 0 | 0 | 0 | 3 | 3 | 2 | 0 | 1.3193E8 | 4.8825E6 | 9.5314E5 |  |  |  | 1.8633E6 | 4.3807E5 | 2.0418E4 |  | 2 | 2 | 18 | 4 | 2 | 0 | 0 | 0 | 3 | 3 | 1 | 0 | Y | 54436 | RecName: Full=Zinc metalloproteinase-disintegrin stejnitin; AltName: Full=Snake venom metalloproteinase; Short=SVMP; Flags: Precursor |
| 322 | 2021 | AAF91498.1 | 117.95 | 24 | 0 | 0 | 0 | 0 | 0 | 24 | 0 | 0 | 0 | 0 |  |  |  |  |  | 1.4266E7 |  |  |  |  | 4 | 3 | 0 | 0 | 0 | 0 | 0 | 4 | 0 | 0 | 0 | 0 | Y | 13732 | phospholipase A2, partial [Bothrops pictus] |
| 322 | 2022 | Q9I8F8.1 | 117.95 | 24 | 0 | 0 | 0 | 0 | 0 | 24 | 0 | 0 | 0 | 0 |  |  |  |  |  | 1.4266E7 |  |  |  |  | 4 | 3 | 0 | 0 | 0 | 0 | 0 | 4 | 0 | 0 | 0 | 0 | Y | 13732 | RecName: Full=Acidic phospholipase A2; Short=svPLA2; AltName: Full=Phosphatidylcholine 2-acylhydrolase |
| 303 | 1842 | XP\_015680726.1 | 117.94 | 21 | 0 | 0 | 0 | 0 | 0 | 0 | 0 | 0 | 21 | 0 |  |  |  |  |  |  |  |  | 2.5161E6 |  | 3 | 2 | 0 | 0 | 0 | 0 | 0 | 0 | 0 | 0 | 4 | 0 | N | 13512 | alkaline phosphatase, germ cell type-like, partial [Protobothrops mucrosquamatus] |
| 260 | 1150 | XP\_032069201.1 | 117.41 | 6 | 4 | 0 | 2 | 0 | 0 | 0 | 0 | 4 | 6 | 0 | 3.7581E5 |  |  |  |  |  |  | 1.3561E6 | 2.7915E5 |  | 2 | 1 | 1 | 0 | 1 | 0 | 0 | 0 | 0 | 2 | 2 | 0 | N | 35787 | glyceraldehyde-3-phosphate dehydrogenase [Thamnophis elegans] |
| 122 | 979 | AUS82534.1 | 116.28 | 11 | 6 | 11 | 3 | 3 | 0 | 3 | 3 | 6 | 6 | 0 | 3.0178E9 | 8.3757E7 | 7.6285E6 | 7.8221E5 |  | 5.6121E6 |  | 1.0754E7 | 1.38E7 |  | 4 | 1 | 24 | 8 | 1 | 1 | 0 | 1 | 2 | 5 | 2 | 0 | Y | 29104 | serine endopeptidase [Crotalus molossus] |
| 294 | 580 | XP\_026566978.1 | 114.79 | 8 | 3 | 0 | 0 | 0 | 0 | 0 | 3 | 2 | 0 | 0 |  |  |  |  |  |  | 6.1041E4 |  |  |  | 3 | 1 | 1 | 0 | 0 | 0 | 0 | 0 | 1 | 1 | 0 | 0 | N | 46297 | tubulin alpha-1A chain [Pseudonaja textilis] |
| 294 | 581 | XP\_026521383.1 | 114.79 | 8 | 3 | 0 | 0 | 0 | 0 | 0 | 3 | 2 | 0 | 0 |  |  |  |  |  |  | 6.1041E4 |  |  |  | 3 | 1 | 1 | 0 | 0 | 0 | 0 | 0 | 1 | 1 | 0 | 0 | N | 46456 | tubulin alpha-1D chain [Notechis scutatus] |
| 294 | 582 | XP\_025021189.1 | 114.79 | 8 | 3 | 0 | 0 | 0 | 0 | 0 | 3 | 2 | 0 | 0 |  |  |  |  |  |  | 6.1041E4 |  |  |  | 3 | 1 | 1 | 0 | 0 | 0 | 0 | 0 | 1 | 1 | 0 | 0 | N | 50184 | tubulin alpha-1A chain isoform X3 [Python bivittatus] |
| 294 | 583 | XP\_007426281.1 | 114.79 | 8 | 3 | 0 | 0 | 0 | 0 | 0 | 3 | 2 | 0 | 0 |  |  |  |  |  |  | 6.1041E4 |  |  |  | 3 | 1 | 1 | 0 | 0 | 0 | 0 | 0 | 1 | 1 | 0 | 0 | N | 50184 | tubulin alpha-1A chain isoform X2 [Python bivittatus] |
| 294 | 584 | XP\_026563732.1 | 114.79 | 8 | 3 | 0 | 0 | 0 | 0 | 0 | 3 | 2 | 0 | 0 |  |  |  |  |  |  | 6.1041E4 |  |  |  | 3 | 1 | 1 | 0 | 0 | 0 | 0 | 0 | 1 | 1 | 0 | 0 | N | 51412 | tubulin alpha-1D chain [Pseudonaja textilis] |
| 294 | 585 | XP\_007426279.2 | 114.79 | 7 | 2 | 0 | 0 | 0 | 0 | 0 | 3 | 2 | 0 | 0 |  |  |  |  |  |  | 6.1041E4 |  |  |  | 3 | 1 | 1 | 0 | 0 | 0 | 0 | 0 | 1 | 1 | 0 | 0 | N | 53653 | tubulin alpha-1A chain isoform X1 [Python bivittatus] |
| 163 | 980 | Q8AY81.1 | 111.91 | 16 | 3 | 8 | 7 | 0 | 0 | 0 | 3 | 3 | 3 | 0 |  |  | 1.4947E6 |  |  |  |  |  |  |  | 4 | 1 | 18 | 6 | 1 | 0 | 0 | 0 | 2 | 4 | 1 | 0 | Y | 29328 | RecName: Full=Thrombin-like enzyme stejnobin; Short=SVTLE; AltName: Full=Fibrinogen-clotting enzyme; AltName: Full=Snake venom serine protease; Short=SVSP; Flags: Precursor |
| 163 | 981 | AAN52347.1 | 111.91 | 16 | 3 | 8 | 7 | 0 | 0 | 0 | 3 | 3 | 3 | 0 |  |  | 1.4947E6 |  |  |  |  |  |  |  | 4 | 1 | 18 | 6 | 1 | 0 | 0 | 0 | 2 | 4 | 1 | 0 | Y | 29328 | stejnobin [Trimeresurus stejnegeri] |
| 273 | 826 | XP\_015746202.2 | 111.49 | 0 | 0 | 0 | 0 | 0 | 0 | 0 | 0 | 0 | 0 | 0 |  |  |  |  |  | 1.9801E6 |  |  |  |  | 2 | 1 | 0 | 0 | 0 | 1 | 0 | 1 | 0 | 0 | 0 | 0 | N | 523685 | plectin isoform X4 [Python bivittatus] |
| 273 | 827 | XP\_015746201.2 | 111.49 | 0 | 0 | 0 | 0 | 0 | 0 | 0 | 0 | 0 | 0 | 0 |  |  |  |  |  | 1.9801E6 |  |  |  |  | 2 | 1 | 0 | 0 | 0 | 1 | 0 | 1 | 0 | 0 | 0 | 0 | N | 524365 | plectin isoform X3 [Python bivittatus] |
| 273 | 828 | XP\_015746200.2 | 111.49 | 0 | 0 | 0 | 0 | 0 | 0 | 0 | 0 | 0 | 0 | 0 |  |  |  |  |  | 1.9801E6 |  |  |  |  | 2 | 1 | 0 | 0 | 0 | 1 | 0 | 1 | 0 | 0 | 0 | 0 | N | 525204 | plectin isoform X2 [Python bivittatus] |
| 273 | 829 | XP\_015746199.2 | 111.49 | 0 | 0 | 0 | 0 | 0 | 0 | 0 | 0 | 0 | 0 | 0 |  |  |  |  |  | 1.9801E6 |  |  |  |  | 2 | 1 | 0 | 0 | 0 | 1 | 0 | 1 | 0 | 0 | 0 | 0 | N | 525884 | plectin isoform X1 [Python bivittatus] |
| 329 | 873 | XP\_032091650.1 | 111.24 | 4 | 0 | 0 | 0 | 0 | 0 | 0 | 0 | 0 | 4 | 0 |  |  |  |  |  |  |  |  | 8.1161E6 |  | 3 | 1 | 0 | 0 | 0 | 0 | 0 | 0 | 0 | 0 | 3 | 0 | Y | 130571 | collagen alpha-1(I) chain isoform X2 [Thamnophis elegans] |
| 329 | 876 | XP\_032091649.1 | 111.24 | 4 | 0 | 0 | 0 | 0 | 0 | 0 | 0 | 0 | 4 | 0 |  |  |  |  |  |  |  |  | 8.1161E6 |  | 3 | 1 | 0 | 0 | 0 | 0 | 0 | 0 | 0 | 0 | 3 | 0 | Y | 137363 | collagen alpha-1(I) chain isoform X1 [Thamnophis elegans] |
| 329 | 906 | XP\_013916526.1 | 111.24 | 4 | 0 | 0 | 0 | 0 | 0 | 0 | 0 | 0 | 4 | 0 |  |  |  |  |  |  |  |  | 8.1161E6 |  | 3 | 1 | 0 | 0 | 0 | 0 | 0 | 0 | 0 | 0 | 3 | 0 | Y | 137592 | PREDICTED: collagen alpha-1(I) chain [Thamnophis sirtalis] |
| 192 | 1444 | XP\_015683679.2 | 110.59 | 3 | 0 | 2 | 2 | 0 | 0 | 2 | 2 | 2 | 3 | 2 |  | 4.8914E6 | 1.3339E6 |  |  | 1.4908E6 | 2.0807E6 | 7.4737E6 | 1.201E8 | 1.5891E7 | 2 | 1 | 0 | 3 | 1 | 0 | 0 | 2 | 3 | 6 | 41 | 2 | Y | 73339 | zinc metalloproteinase/disintegrin-like HR1a [Protobothrops mucrosquamatus] |
| 283 | 865 | XP\_026542921.1 | 106.87 | 0 | 0 | 0 | 0 | 0 | 0 | 0 | 0 | 0 | 0 | 0 |  |  |  |  |  |  |  | 7.3629E4 |  |  | 2 | 1 | 0 | 0 | 0 | 1 | 0 | 0 | 0 | 1 | 0 | 0 | N | 533554 | plectin [Notechis scutatus] |
| 283 | 877 | XP\_026577682.1 | 106.87 | 0 | 0 | 0 | 0 | 0 | 0 | 0 | 0 | 0 | 0 | 0 |  |  |  |  |  |  |  | 7.3629E4 |  |  | 2 | 1 | 0 | 0 | 0 | 1 | 0 | 0 | 0 | 1 | 0 | 0 | N | 512854 | plectin isoform X14 [Pseudonaja textilis] |
| 283 | 878 | XP\_026577681.1 | 106.87 | 0 | 0 | 0 | 0 | 0 | 0 | 0 | 0 | 0 | 0 | 0 |  |  |  |  |  |  |  | 7.3629E4 |  |  | 2 | 1 | 0 | 0 | 0 | 1 | 0 | 0 | 0 | 1 | 0 | 0 | N | 520222 | plectin isoform X13 [Pseudonaja textilis] |
| 283 | 879 | XP\_026577680.1 | 106.87 | 0 | 0 | 0 | 0 | 0 | 0 | 0 | 0 | 0 | 0 | 0 |  |  |  |  |  |  |  | 7.3629E4 |  |  | 2 | 1 | 0 | 0 | 0 | 1 | 0 | 0 | 0 | 1 | 0 | 0 | N | 520902 | plectin isoform X12 [Pseudonaja textilis] |
| 283 | 880 | XP\_029140206.1 | 106.87 | 0 | 0 | 0 | 0 | 0 | 0 | 0 | 0 | 0 | 0 | 0 |  |  |  |  |  |  |  | 7.3629E4 |  |  | 2 | 1 | 0 | 0 | 0 | 1 | 0 | 0 | 0 | 1 | 0 | 0 | N | 513681 | plectin [Protobothrops mucrosquamatus] |
| 283 | 881 | XP\_026577679.1 | 106.87 | 0 | 0 | 0 | 0 | 0 | 0 | 0 | 0 | 0 | 0 | 0 |  |  |  |  |  |  |  | 7.3629E4 |  |  | 2 | 1 | 0 | 0 | 0 | 1 | 0 | 0 | 0 | 1 | 0 | 0 | N | 523074 | plectin isoform X11 [Pseudonaja textilis] |
| 283 | 882 | XP\_026577678.1 | 106.87 | 0 | 0 | 0 | 0 | 0 | 0 | 0 | 0 | 0 | 0 | 0 |  |  |  |  |  |  |  | 7.3629E4 |  |  | 2 | 1 | 0 | 0 | 0 | 1 | 0 | 0 | 0 | 1 | 0 | 0 | N | 523819 | plectin isoform X10 [Pseudonaja textilis] |
| 283 | 883 | XP\_026577677.1 | 106.87 | 0 | 0 | 0 | 0 | 0 | 0 | 0 | 0 | 0 | 0 | 0 |  |  |  |  |  |  |  | 7.3629E4 |  |  | 2 | 1 | 0 | 0 | 0 | 1 | 0 | 0 | 0 | 1 | 0 | 0 | N | 524433 | plectin isoform X9 [Pseudonaja textilis] |
| 283 | 884 | XP\_026577675.1 | 106.87 | 0 | 0 | 0 | 0 | 0 | 0 | 0 | 0 | 0 | 0 | 0 |  |  |  |  |  |  |  | 7.3629E4 |  |  | 2 | 1 | 0 | 0 | 0 | 1 | 0 | 0 | 0 | 1 | 0 | 0 | N | 524367 | plectin isoform X8 [Pseudonaja textilis] |
| 283 | 885 | XP\_026577674.1 | 106.87 | 0 | 0 | 0 | 0 | 0 | 0 | 0 | 0 | 0 | 0 | 0 |  |  |  |  |  |  |  | 7.3629E4 |  |  | 2 | 1 | 0 | 0 | 0 | 1 | 0 | 0 | 0 | 1 | 0 | 0 | N | 524499 | plectin isoform X7 [Pseudonaja textilis] |
| 283 | 886 | XP\_026577673.1 | 106.87 | 0 | 0 | 0 | 0 | 0 | 0 | 0 | 0 | 0 | 0 | 0 |  |  |  |  |  |  |  | 7.3629E4 |  |  | 2 | 1 | 0 | 0 | 0 | 1 | 0 | 0 | 0 | 1 | 0 | 0 | N | 525113 | plectin isoform X6 [Pseudonaja textilis] |
| 283 | 887 | XP\_026577672.1 | 106.87 | 0 | 0 | 0 | 0 | 0 | 0 | 0 | 0 | 0 | 0 | 0 |  |  |  |  |  |  |  | 7.3629E4 |  |  | 2 | 1 | 0 | 0 | 0 | 1 | 0 | 0 | 0 | 1 | 0 | 0 | N | 525046 | plectin isoform X5 [Pseudonaja textilis] |
| 283 | 890 | XP\_026577671.1 | 106.87 | 0 | 0 | 0 | 0 | 0 | 0 | 0 | 0 | 0 | 0 | 0 |  |  |  |  |  |  |  | 7.3629E4 |  |  | 2 | 1 | 0 | 0 | 0 | 1 | 0 | 0 | 0 | 1 | 0 | 0 | N | 526447 | plectin isoform X4 [Pseudonaja textilis] |
| 283 | 891 | XP\_026577670.1 | 106.87 | 0 | 0 | 0 | 0 | 0 | 0 | 0 | 0 | 0 | 0 | 0 |  |  |  |  |  |  |  | 7.3629E4 |  |  | 2 | 1 | 0 | 0 | 0 | 1 | 0 | 0 | 0 | 1 | 0 | 0 | N | 527127 | plectin isoform X3 [Pseudonaja textilis] |
| 283 | 892 | XP\_034275868.1 | 106.87 | 0 | 0 | 0 | 0 | 0 | 0 | 0 | 0 | 0 | 0 | 0 |  |  |  |  |  |  |  | 7.3629E4 |  |  | 2 | 1 | 0 | 0 | 0 | 1 | 0 | 0 | 0 | 1 | 0 | 0 | N | 522418 | plectin isoform X9 [Pantherophis guttatus] |
| 283 | 893 | XP\_034275867.1 | 106.87 | 0 | 0 | 0 | 0 | 0 | 0 | 0 | 0 | 0 | 0 | 0 |  |  |  |  |  |  |  | 7.3629E4 |  |  | 2 | 1 | 0 | 0 | 0 | 1 | 0 | 0 | 0 | 1 | 0 | 0 | N | 523098 | plectin isoform X8 [Pantherophis guttatus] |
| 283 | 894 | XP\_034275879.1 | 106.87 | 0 | 0 | 0 | 0 | 0 | 0 | 0 | 0 | 0 | 0 | 0 |  |  |  |  |  |  |  | 7.3629E4 |  |  | 2 | 1 | 0 | 0 | 0 | 1 | 0 | 0 | 0 | 1 | 0 | 0 | N | 524699 | plectin isoform X19 [Pantherophis guttatus] |
| 283 | 895 | XP\_026577669.1 | 106.87 | 0 | 0 | 0 | 0 | 0 | 0 | 0 | 0 | 0 | 0 | 0 |  |  |  |  |  |  |  | 7.3629E4 |  |  | 2 | 1 | 0 | 0 | 0 | 1 | 0 | 0 | 0 | 1 | 0 | 0 | N | 538013 | plectin isoform X2 [Pseudonaja textilis] |
| 283 | 896 | XP\_026577668.1 | 106.87 | 0 | 0 | 0 | 0 | 0 | 0 | 0 | 0 | 0 | 0 | 0 |  |  |  |  |  |  |  | 7.3629E4 |  |  | 2 | 1 | 0 | 0 | 0 | 1 | 0 | 0 | 0 | 1 | 0 | 0 | N | 538693 | plectin isoform X1 [Pseudonaja textilis] |
| 283 | 897 | XP\_034275875.1 | 106.87 | 0 | 0 | 0 | 0 | 0 | 0 | 0 | 0 | 0 | 0 | 0 |  |  |  |  |  |  |  | 7.3629E4 |  |  | 2 | 1 | 0 | 0 | 0 | 1 | 0 | 0 | 0 | 1 | 0 | 0 | N | 526218 | plectin isoform X15 [Pantherophis guttatus] |
| 283 | 898 | XP\_034275865.1 | 106.87 | 0 | 0 | 0 | 0 | 0 | 0 | 0 | 0 | 0 | 0 | 0 |  |  |  |  |  |  |  | 7.3629E4 |  |  | 2 | 1 | 0 | 0 | 0 | 1 | 0 | 0 | 0 | 1 | 0 | 0 | N | 538854 | plectin isoform X6 [Pantherophis guttatus] |
| 283 | 899 | XP\_034275864.1 | 106.87 | 0 | 0 | 0 | 0 | 0 | 0 | 0 | 0 | 0 | 0 | 0 |  |  |  |  |  |  |  | 7.3629E4 |  |  | 2 | 1 | 0 | 0 | 0 | 1 | 0 | 0 | 0 | 1 | 0 | 0 | N | 539534 | plectin isoform X5 [Pantherophis guttatus] |
| 283 | 900 | XP\_034275862.1 | 106.87 | 0 | 0 | 0 | 0 | 0 | 0 | 0 | 0 | 0 | 0 | 0 |  |  |  |  |  |  |  | 7.3629E4 |  |  | 2 | 1 | 0 | 0 | 0 | 1 | 0 | 0 | 0 | 1 | 0 | 0 | N | 540373 | plectin isoform X3 [Pantherophis guttatus] |
| 283 | 901 | XP\_034275860.1 | 106.87 | 0 | 0 | 0 | 0 | 0 | 0 | 0 | 0 | 0 | 0 | 0 |  |  |  |  |  |  |  | 7.3629E4 |  |  | 2 | 1 | 0 | 0 | 0 | 1 | 0 | 0 | 0 | 1 | 0 | 0 | N | 541053 | plectin isoform X1 [Pantherophis guttatus] |
| 283 | 907 | XP\_034275866.1 | 106.87 | 0 | 0 | 0 | 0 | 0 | 0 | 0 | 0 | 0 | 0 | 0 |  |  |  |  |  |  |  | 7.3629E4 |  |  | 2 | 1 | 0 | 0 | 0 | 1 | 0 | 0 | 0 | 1 | 0 | 0 | N | 537428 | plectin isoform X7 [Pantherophis guttatus] |
| 283 | 909 | XP\_034275863.1 | 106.87 | 0 | 0 | 0 | 0 | 0 | 0 | 0 | 0 | 0 | 0 | 0 |  |  |  |  |  |  |  | 7.3629E4 |  |  | 2 | 1 | 0 | 0 | 0 | 1 | 0 | 0 | 0 | 1 | 0 | 0 | N | 538947 | plectin isoform X4 [Pantherophis guttatus] |
| 283 | 910 | XP\_034275861.1 | 106.87 | 0 | 0 | 0 | 0 | 0 | 0 | 0 | 0 | 0 | 0 | 0 |  |  |  |  |  |  |  | 7.3629E4 |  |  | 2 | 1 | 0 | 0 | 0 | 1 | 0 | 0 | 0 | 1 | 0 | 0 | N | 539627 | plectin isoform X2 [Pantherophis guttatus] |
| 278 | 1330 | XP\_015682690.1 | 105.53 | 4 | 2 | 0 | 0 | 0 | 0 | 0 | 0 | 3 | 3 | 0 | 1.1731E5 |  |  |  |  |  |  | 2.4656E5 | 1.5382E6 |  | 2 | 2 | 1 | 0 | 0 | 0 | 0 | 0 | 0 | 1 | 3 | 0 | Y | 57849 | pantetheinase-like [Protobothrops mucrosquamatus] |
| 301 | 1196 | JAG66642.1 | 104.93 | 22 | 0 | 0 | 0 | 0 | 0 | 12 | 0 | 16 | 0 | 0 |  |  |  |  |  | 1.5106E6 |  | 1.755E6 |  |  | 3 | 3 | 0 | 0 | 0 | 0 | 0 | 2 | 0 | 2 | 0 | 0 | N | 17979 | ubiquitin-40S ribosomal protein S27a [Boiga irregularis] |
| 301 | 1197 | JAA95751.1 | 104.93 | 22 | 0 | 0 | 0 | 0 | 0 | 12 | 0 | 16 | 0 | 0 |  |  |  |  |  | 1.5106E6 |  | 1.755E6 |  |  | 3 | 3 | 0 | 0 | 0 | 0 | 0 | 2 | 0 | 2 | 0 | 0 | N | 17979 | ubiquitin-40S ribosomal protein S27a [Crotalus horridus] |
| 301 | 1198 | JAB53407.1 | 104.93 | 22 | 0 | 0 | 0 | 0 | 0 | 12 | 0 | 16 | 0 | 0 |  |  |  |  |  | 1.5106E6 |  | 1.755E6 |  |  | 3 | 3 | 0 | 0 | 0 | 0 | 0 | 2 | 0 | 2 | 0 | 0 | N | 17979 | Ubiquitin-40S ribosomal protein S27a [Micrurus fulvius] |
| 301 | 1199 | JAI11580.1 | 104.93 | 22 | 0 | 0 | 0 | 0 | 0 | 12 | 0 | 16 | 0 | 0 |  |  |  |  |  | 1.5106E6 |  | 1.755E6 |  |  | 3 | 3 | 0 | 0 | 0 | 0 | 0 | 2 | 0 | 2 | 0 | 0 | N | 17979 | ubiquitin-40S ribosomal protein S27a [Crotalus adamanteus] |
| 301 | 1201 | JAI09470.1 | 104.93 | 22 | 0 | 0 | 0 | 0 | 0 | 12 | 0 | 16 | 0 | 0 |  |  |  |  |  | 1.5106E6 |  | 1.755E6 |  |  | 3 | 3 | 0 | 0 | 0 | 0 | 0 | 2 | 0 | 2 | 0 | 0 | N | 17979 | ubiquitin-40S ribosomal protein S27a [Micrurus fulvius] |
| 301 | 1202 | AFJ51304.1 | 104.93 | 22 | 0 | 0 | 0 | 0 | 0 | 12 | 0 | 16 | 0 | 0 |  |  |  |  |  | 1.5106E6 |  | 1.755E6 |  |  | 3 | 3 | 0 | 0 | 0 | 0 | 0 | 2 | 0 | 2 | 0 | 0 | N | 17979 | Ubiquitin-40S ribosomal protein S27a-like [Crotalus adamanteus] |
| 301 | 1219 | JAI10738.1 | 104.93 | 35 | 0 | 0 | 0 | 0 | 0 | 18 | 0 | 26 | 0 | 0 |  |  |  |  |  | 1.5106E6 |  | 1.755E6 |  |  | 3 | 3 | 0 | 0 | 0 | 0 | 0 | 2 | 0 | 2 | 0 | 0 | N | 10986 | ubiquitin C [Crotalus adamanteus] |
| 301 | 1220 | JAA95781.1 | 104.93 | 27 | 0 | 0 | 0 | 0 | 0 | 14 | 0 | 20 | 0 | 0 |  |  |  |  |  | 1.5106E6 |  | 1.755E6 |  |  | 3 | 3 | 0 | 0 | 0 | 0 | 0 | 2 | 0 | 2 | 0 | 0 | N | 14728 | 60S ribosomal protein L40 [Crotalus horridus] |
| 301 | 1221 | XP\_013929631.1 | 104.93 | 27 | 0 | 0 | 0 | 0 | 0 | 14 | 0 | 20 | 0 | 0 |  |  |  |  |  | 1.5106E6 |  | 1.755E6 |  |  | 3 | 3 | 0 | 0 | 0 | 0 | 0 | 2 | 0 | 2 | 0 | 0 | N | 14674 | PREDICTED: ubiquitin-60S ribosomal protein L40 [Thamnophis sirtalis] |
| 301 | 1222 | JAB53440.1 | 104.93 | 27 | 0 | 0 | 0 | 0 | 0 | 14 | 0 | 20 | 0 | 0 |  |  |  |  |  | 1.5106E6 |  | 1.755E6 |  |  | 3 | 3 | 0 | 0 | 0 | 0 | 0 | 2 | 0 | 2 | 0 | 0 | N | 14716 | ubiquitin-60S ribosomal protein L40 [Micrurus fulvius] |
| 301 | 1223 | JAI09497.1 | 104.93 | 27 | 0 | 0 | 0 | 0 | 0 | 14 | 0 | 20 | 0 | 0 |  |  |  |  |  | 1.5106E6 |  | 1.755E6 |  |  | 3 | 3 | 0 | 0 | 0 | 0 | 0 | 2 | 0 | 2 | 0 | 0 | N | 14728 | 60S ribosomal protein L40 [Micrurus fulvius] |
| 301 | 1224 | JAB53439.1 | 104.93 | 27 | 0 | 0 | 0 | 0 | 0 | 14 | 0 | 20 | 0 | 0 |  |  |  |  |  | 1.5106E6 |  | 1.755E6 |  |  | 3 | 3 | 0 | 0 | 0 | 0 | 0 | 2 | 0 | 2 | 0 | 0 | N | 14728 | Ubiquitin-60S ribosomal protein L40 [Micrurus fulvius] |
| 301 | 1225 | JAI11613.1 | 104.93 | 27 | 0 | 0 | 0 | 0 | 0 | 14 | 0 | 20 | 0 | 0 |  |  |  |  |  | 1.5106E6 |  | 1.755E6 |  |  | 3 | 3 | 0 | 0 | 0 | 0 | 0 | 2 | 0 | 2 | 0 | 0 | N | 14728 | 60S ribosomal protein L40 [Crotalus adamanteus] |
| 301 | 1226 | XP\_013929630.1 | 104.93 | 27 | 0 | 0 | 0 | 0 | 0 | 14 | 0 | 20 | 0 | 0 |  |  |  |  |  | 1.5106E6 |  | 1.755E6 |  |  | 3 | 3 | 0 | 0 | 0 | 0 | 0 | 2 | 0 | 2 | 0 | 0 | N | 14674 | PREDICTED: ubiquitin-60S ribosomal protein L40 [Thamnophis sirtalis] |
| 301 | 1227 | AFJ51273.1 | 104.93 | 27 | 0 | 0 | 0 | 0 | 0 | 14 | 0 | 20 | 0 | 0 |  |  |  |  |  | 1.5106E6 |  | 1.755E6 |  |  | 3 | 3 | 0 | 0 | 0 | 0 | 0 | 2 | 0 | 2 | 0 | 0 | N | 14728 | Ubiquitin-60S ribosomal protein L40 [Crotalus adamanteus] |
| 301 | 1228 | JAG66673.1 | 104.93 | 27 | 0 | 0 | 0 | 0 | 0 | 14 | 0 | 20 | 0 | 0 |  |  |  |  |  | 1.5106E6 |  | 1.755E6 |  |  | 3 | 3 | 0 | 0 | 0 | 0 | 0 | 2 | 0 | 2 | 0 | 0 | N | 14728 | 60S ribosomal protein L40 [Boiga irregularis] |
| 301 | 1229 | AAG17445.1 | 104.93 | 27 | 0 | 0 | 0 | 0 | 0 | 14 | 0 | 20 | 0 | 0 |  |  |  |  |  | 1.5106E6 |  | 1.755E6 |  |  | 3 | 3 | 0 | 0 | 0 | 0 | 0 | 2 | 0 | 2 | 0 | 0 | N | 14728 | ubiquitin fusion protein [Ophiophagus hannah] |
| 301 | 1231 | AFJ51941.1 | 104.93 | 15 | 0 | 0 | 0 | 0 | 0 | 8 | 0 | 11 | 0 | 0 |  |  |  |  |  | 1.5106E6 |  | 1.755E6 |  |  | 3 | 3 | 0 | 0 | 0 | 0 | 0 | 2 | 0 | 2 | 0 | 0 | N | 25806 | putative ubiquitin B [Crotalus adamanteus] |
| 301 | 1232 | XP\_013928637.1 | 104.93 | 15 | 0 | 0 | 0 | 0 | 0 | 8 | 0 | 11 | 0 | 0 |  |  |  |  |  | 1.5106E6 |  | 1.755E6 |  |  | 3 | 3 | 0 | 0 | 0 | 0 | 0 | 2 | 0 | 2 | 0 | 0 | N | 25822 | PREDICTED: polyubiquitin-B [Thamnophis sirtalis] |
| 301 | 1236 | JAB53023.1 | 104.93 | 9 | 0 | 0 | 0 | 0 | 0 | 5 | 0 | 7 | 0 | 0 |  |  |  |  |  | 1.5106E6 |  | 1.755E6 |  |  | 3 | 3 | 0 | 0 | 0 | 0 | 0 | 2 | 0 | 2 | 0 | 0 | N | 42865 | polyubiquitin-C isoform 2 [Micrurus fulvius] |
| 259 | 1284 | XP\_032091816.1 | 103.24 | 4 | 2 | 0 | 0 | 2 | 0 | 2 | 0 | 4 | 2 | 0 |  |  |  |  |  |  |  | 2.8262E5 |  |  | 3 | 1 | 1 | 0 | 0 | 1 | 0 | 1 | 0 | 4 | 3 | 0 | N | 55277 | keratin, type I cytoskeletal 12-like, partial [Thamnophis elegans] |
| 259 | 1572 | XP\_013917303.1 | 103.24 | 4 | 2 | 0 | 0 | 2 | 0 | 2 | 0 | 4 | 3 | 0 |  |  |  |  |  |  |  | 2.8262E5 |  |  | 3 | 1 | 1 | 0 | 0 | 1 | 0 | 1 | 0 | 4 | 3 | 0 | N | 46619 | PREDICTED: keratin, type I cytoskeletal 10-like [Thamnophis sirtalis] |
| 259 | 1576 | XP\_034271639.1 | 103.24 | 4 | 2 | 0 | 0 | 2 | 0 | 2 | 0 | 4 | 3 | 0 |  |  |  |  |  |  |  | 2.8262E5 |  |  | 3 | 1 | 1 | 0 | 0 | 1 | 0 | 1 | 0 | 4 | 3 | 0 | N | 46209 | keratin, type I cytoskeletal 12-like [Pantherophis guttatus] |
| 263 | 1586 | P0DJL3.1 | 102.80 | 15 | 0 | 8 | 0 | 15 | 0 | 7 | 0 | 0 | 0 | 0 |  | 0 |  | 3.6137E8 |  | 6.4193E6 |  |  |  |  | 2 | 2 | 0 | 1 | 0 | 10 | 0 | 3 | 0 | 0 | 0 | 0 | N | 14498 | RecName: Full=Snaclec purpureotin subunit beta |
| 263 | 1587 | P81116.2 | 102.80 | 13 | 0 | 7 | 0 | 13 | 0 | 6 | 0 | 0 | 0 | 0 |  | 0 |  | 3.6137E8 |  | 6.4193E6 |  |  |  |  | 2 | 2 | 0 | 1 | 0 | 10 | 0 | 3 | 0 | 0 | 0 | 0 | N | 16871 | RecName: Full=Snaclec alboaggregin-B subunit beta; Short=AL-B subunit beta; AltName: Full=25-kDa alboaggregin; Flags: Precursor |
| 263 | 1588 | ABS12077.1 | 102.80 | 13 | 0 | 7 | 0 | 13 | 0 | 6 | 0 | 0 | 0 | 0 |  | 0 |  | 3.6137E8 |  | 6.4193E6 |  |  |  |  | 2 | 2 | 0 | 1 | 0 | 10 | 0 | 3 | 0 | 0 | 0 | 0 | N | 16871 | alboaggregin B beta subunit [Trimeresurus albolabris] |
| 207 | 1625 | BAN89403.1 | 101.82 | 4 | 4 | 1 | 1 | 0 | 0 | 0 | 1 | 1 | 0 | 0 | 6.8904E6 |  |  |  |  |  |  |  |  |  | 2 | 1 | 10 | 3 | 3 | 0 | 0 | 0 | 2 | 1 | 0 | 0 | Y | 67822 | p-iii\_metalloprotease [Ovophis okinavensis] |
| 216 | 1286 | LAA93895.1 | 101.75 | 15 | 5 | 5 | 5 | 15 | 0 | 10 | 10 | 5 | 5 | 0 | 7.2289E6 | 5.7848E6 | 2.9885E6 | 2.9771E7 |  | 3.548E7 | 6.434E5 | 2.3478E7 | 7.6904E6 |  | 3 | 1 | 2 | 1 | 1 | 5 | 0 | 3 | 2 | 9 | 7 | 0 | N | 19716 | hypothetical protein, partial [Micrurus lemniscatus lemniscatus] |
| 89 | 1331 | Q2YHJ2.1 | 101.71 | 12 | 6 | 6 | 6 | 11 | 0 | 12 | 0 | 6 | 11 | 6 | 1.7238E7 | 1.1289E6 | 2.5258E5 | 2.8026E8 |  | 7.8998E7 |  | 9.5005E6 | 4.0119E6 | 7.3435E6 | 3 | 2 | 2 | 1 | 1 | 22 | 0 | 4 | 0 | 4 | 4 | 2 | N | 15742 | RecName: Full=Basic phospholipase A2 Tbo-G6D49; Short=svPLA2; AltName: Full=Phosphatidylcholine 2-acylhydrolase; Flags: Precursor |
| 89 | 1332 | AAR14173.1 | 101.71 | 12 | 6 | 6 | 6 | 11 | 0 | 12 | 0 | 6 | 11 | 6 | 1.7238E7 | 1.1289E6 | 2.5258E5 | 2.8026E8 |  | 7.8998E7 |  | 9.5005E6 | 4.0119E6 | 7.3435E6 | 3 | 2 | 2 | 1 | 1 | 22 | 0 | 4 | 0 | 4 | 4 | 2 | N | 15742 | G6D49 phospholipase A2 [Trimeresurus borneensis] |
| 89 | 1500 | Q2YHJ7.1 | 101.71 | 12 | 6 | 6 | 6 | 11 | 0 | 12 | 0 | 6 | 11 | 6 | 1.7238E7 | 1.1289E6 | 2.5258E5 | 2.8026E8 |  | 7.8998E7 |  | 9.5005E6 | 4.0119E6 | 7.3435E6 | 3 | 2 | 2 | 1 | 1 | 22 | 0 | 4 | 0 | 4 | 4 | 2 | N | 15699 | RecName: Full=Basic phospholipase A2 Tpu-G6D49; Short=svPLA2; AltName: Full=Phosphatidylcholine 2-acylhydrolase; Flags: Precursor |
| 89 | 1501 | AAR14167.1 | 101.71 | 12 | 6 | 6 | 6 | 11 | 0 | 12 | 0 | 6 | 11 | 6 | 1.7238E7 | 1.1289E6 | 2.5258E5 | 2.8026E8 |  | 7.8998E7 |  | 9.5005E6 | 4.0119E6 | 7.3435E6 | 3 | 2 | 2 | 1 | 1 | 22 | 0 | 4 | 0 | 4 | 4 | 2 | N | 15699 | G6D49 phospholipase A2 [Trimeresurus puniceus] |
| 296 | 1152 | JAB53718.1 | 101.62 | 15 | 0 | 0 | 0 | 0 | 0 | 10 | 0 | 5 | 0 | 0 |  |  |  |  |  | 5.7206E5 |  |  |  |  | 3 | 1 | 0 | 0 | 0 | 0 | 0 | 2 | 0 | 1 | 0 | 0 | N | 22304 | peroxiredoxin-1 [Micrurus fulvius] |
| 296 | 1153 | XP\_026521545.1 | 101.62 | 15 | 0 | 0 | 0 | 0 | 0 | 10 | 0 | 5 | 0 | 0 |  |  |  |  |  | 5.7206E5 |  |  |  |  | 3 | 1 | 0 | 0 | 0 | 0 | 0 | 2 | 0 | 1 | 0 | 0 | N | 22288 | peroxiredoxin-1 [Notechis scutatus] |
| 296 | 1154 | JAI09680.1 | 101.62 | 15 | 0 | 0 | 0 | 0 | 0 | 10 | 0 | 5 | 0 | 0 |  |  |  |  |  | 5.7206E5 |  |  |  |  | 3 | 1 | 0 | 0 | 0 | 0 | 0 | 2 | 0 | 1 | 0 | 0 | N | 22304 | peroxiredoxin-1-like protein [Micrurus fulvius] |
| 296 | 1155 | XP\_026562803.1 | 101.62 | 15 | 0 | 0 | 0 | 0 | 0 | 10 | 0 | 5 | 0 | 0 |  |  |  |  |  | 5.7206E5 |  |  |  |  | 3 | 1 | 0 | 0 | 0 | 0 | 0 | 2 | 0 | 1 | 0 | 0 | N | 22311 | peroxiredoxin-1 [Pseudonaja textilis] |
| 296 | 1156 | XP\_026521546.1 | 101.62 | 15 | 0 | 0 | 0 | 0 | 0 | 10 | 0 | 5 | 0 | 0 |  |  |  |  |  | 5.7206E5 |  |  |  |  | 3 | 1 | 0 | 0 | 0 | 0 | 0 | 2 | 0 | 1 | 0 | 0 | N | 22288 | peroxiredoxin-1 [Notechis scutatus] |
| 296 | 1206 | XP\_007420267.1 | 101.62 | 15 | 0 | 0 | 0 | 0 | 0 | 10 | 0 | 5 | 0 | 0 |  |  |  |  |  | 5.7206E5 |  |  |  |  | 3 | 1 | 0 | 0 | 0 | 0 | 0 | 2 | 0 | 1 | 0 | 0 | N | 22316 | peroxiredoxin-1 [Python bivittatus] |
| 296 | 1207 | JAV49689.1 | 101.62 | 15 | 0 | 0 | 0 | 0 | 0 | 10 | 0 | 5 | 0 | 0 |  |  |  |  |  | 5.7206E5 |  |  |  |  | 3 | 1 | 0 | 0 | 0 | 0 | 0 | 2 | 0 | 1 | 0 | 0 | N | 22327 | peroxiredoxin-1-like protein [Agkistrodon contortrix contortrix] |
| 296 | 1208 | XP\_015666821.1 | 101.62 | 15 | 0 | 0 | 0 | 0 | 0 | 10 | 0 | 5 | 0 | 0 |  |  |  |  |  | 5.7206E5 |  |  |  |  | 3 | 1 | 0 | 0 | 0 | 0 | 0 | 2 | 0 | 1 | 0 | 0 | N | 22327 | peroxiredoxin-1 [Protobothrops mucrosquamatus] |
| 296 | 1209 | JAI12123.1 | 101.62 | 15 | 0 | 0 | 0 | 0 | 0 | 10 | 0 | 5 | 0 | 0 |  |  |  |  |  | 5.7206E5 |  |  |  |  | 3 | 1 | 0 | 0 | 0 | 0 | 0 | 2 | 0 | 1 | 0 | 0 | N | 22327 | peroxiredoxin-1-like protein [Crotalus adamanteus] |
| 296 | 1210 | AFJ50897.1 | 101.62 | 15 | 0 | 0 | 0 | 0 | 0 | 10 | 0 | 5 | 0 | 0 |  |  |  |  |  | 5.7206E5 |  |  |  |  | 3 | 1 | 0 | 0 | 0 | 0 | 0 | 2 | 0 | 1 | 0 | 0 | N | 22327 | Peroxiredoxin-1-like [Crotalus adamanteus] |
| 296 | 1211 | JAA96190.1 | 101.62 | 15 | 0 | 0 | 0 | 0 | 0 | 10 | 0 | 5 | 0 | 0 |  |  |  |  |  | 5.7206E5 |  |  |  |  | 3 | 1 | 0 | 0 | 0 | 0 | 0 | 2 | 0 | 1 | 0 | 0 | N | 22327 | peroxiredoxin-1-like protein [Crotalus horridus] |
| 296 | 1212 | XP\_007420268.1 | 101.62 | 15 | 0 | 0 | 0 | 0 | 0 | 10 | 0 | 5 | 0 | 0 |  |  |  |  |  | 5.7206E5 |  |  |  |  | 3 | 1 | 0 | 0 | 0 | 0 | 0 | 2 | 0 | 1 | 0 | 0 | N | 22316 | peroxiredoxin-1 [Python bivittatus] |
| 296 | 1213 | XP\_015666820.1 | 101.62 | 15 | 0 | 0 | 0 | 0 | 0 | 10 | 0 | 5 | 0 | 0 |  |  |  |  |  | 5.7206E5 |  |  |  |  | 3 | 1 | 0 | 0 | 0 | 0 | 0 | 2 | 0 | 1 | 0 | 0 | N | 22327 | peroxiredoxin-1 [Protobothrops mucrosquamatus] |
| 272 | 1615 | AAZ75600.1 | 100.89 | 8 | 0 | 5 | 4 | 0 | 0 | 0 | 8 | 4 | 4 | 0 |  | 2.0776E5 |  |  |  |  | 1.1494E6 |  |  |  | 3 | 1 | 0 | 1 | 1 | 0 | 0 | 0 | 6 | 1 | 1 | 0 | N | 23998 | CRISP-LEI1, partial [Leioheterodon madagascariensis] |
| 272 | 1616 | Q2XXQ1.1 | 100.89 | 8 | 0 | 5 | 4 | 0 | 0 | 0 | 8 | 4 | 4 | 0 |  | 2.0776E5 |  |  |  |  | 1.1494E6 |  |  |  | 3 | 1 | 0 | 1 | 1 | 0 | 0 | 0 | 6 | 1 | 1 | 0 | N | 23998 | RecName: Full=Cysteine-rich venom protein LEI1; Short=CRVP; AltName: Full=Cysteine-rich secretory protein LEI1; Short=CRISP-LEI1; Flags: Precursor |
| 279 | 908 | JAV01828.1 | 100.51 | 9 | 4 | 0 | 0 | 0 | 0 | 0 | 6 | 0 | 0 | 0 | 1.7494E5 |  |  |  |  |  |  |  |  |  | 3 | 1 | 1 | 0 | 0 | 0 | 0 | 0 | 2 | 0 | 0 | 0 | N | 28523 | BATXSVSP8 [Bothrops atrox] |
| 302 | 1170 | JAB53603.1 | 96.67 | 10 | 0 | 0 | 0 | 0 | 0 | 4 | 0 | 10 | 0 | 0 |  |  |  |  |  | 3.0807E5 |  | 3.3954E5 |  |  | 2 | 2 | 0 | 0 | 0 | 0 | 0 | 1 | 0 | 2 | 0 | 0 | N | 27972 | Proteasome subunit alpha type-7 [Micrurus fulvius] |
| 302 | 1171 | JAA96031.1 | 96.67 | 10 | 0 | 0 | 0 | 0 | 0 | 4 | 0 | 10 | 0 | 0 |  |  |  |  |  | 3.0807E5 |  | 3.3954E5 |  |  | 2 | 2 | 0 | 0 | 0 | 0 | 0 | 1 | 0 | 2 | 0 | 0 | N | 27972 | Proteasome subunit alpha type-7-like protein [Crotalus horridus] |
| 302 | 1172 | AFJ51040.1 | 96.67 | 10 | 0 | 0 | 0 | 0 | 0 | 4 | 0 | 10 | 0 | 0 |  |  |  |  |  | 3.0807E5 |  | 3.3954E5 |  |  | 2 | 2 | 0 | 0 | 0 | 0 | 0 | 1 | 0 | 2 | 0 | 0 | N | 27972 | Proteasome subunit alpha type-7-like [Crotalus adamanteus] |
| 302 | 1173 | ETE69560.1 | 96.67 | 10 | 0 | 0 | 0 | 0 | 0 | 4 | 0 | 10 | 0 | 0 |  |  |  |  |  | 3.0807E5 |  | 3.3954E5 |  |  | 2 | 2 | 0 | 0 | 0 | 0 | 0 | 1 | 0 | 2 | 0 | 0 | N | 27972 | Proteasome subunit alpha type-7 [Ophiophagus hannah] |
| 302 | 1174 | XP\_015673624.1 | 96.67 | 10 | 0 | 0 | 0 | 0 | 0 | 4 | 0 | 10 | 0 | 0 |  |  |  |  |  | 3.0807E5 |  | 3.3954E5 |  |  | 2 | 2 | 0 | 0 | 0 | 0 | 0 | 1 | 0 | 2 | 0 | 0 | N | 27945 | proteasome subunit alpha type-7 [Protobothrops mucrosquamatus] |
| 302 | 1175 | JAI09615.1 | 96.67 | 10 | 0 | 0 | 0 | 0 | 0 | 4 | 0 | 10 | 0 | 0 |  |  |  |  |  | 3.0807E5 |  | 3.3954E5 |  |  | 2 | 2 | 0 | 0 | 0 | 0 | 0 | 1 | 0 | 2 | 0 | 0 | N | 27972 | Proteasome subunit alpha type-7-like protein [Micrurus fulvius] |
| 302 | 1176 | JAI11926.1 | 96.67 | 10 | 0 | 0 | 0 | 0 | 0 | 4 | 0 | 10 | 0 | 0 |  |  |  |  |  | 3.0807E5 |  | 3.3954E5 |  |  | 2 | 2 | 0 | 0 | 0 | 0 | 0 | 1 | 0 | 2 | 0 | 0 | N | 27972 | Proteasome subunit alpha type-7-like protein [Crotalus adamanteus] |
| 302 | 1177 | JAG66915.1 | 96.67 | 10 | 0 | 0 | 0 | 0 | 0 | 4 | 0 | 10 | 0 | 0 |  |  |  |  |  | 3.0807E5 |  | 3.3954E5 |  |  | 2 | 2 | 0 | 0 | 0 | 0 | 0 | 1 | 0 | 2 | 0 | 0 | N | 28000 | Proteasome subunit alpha type-7-like protein [Boiga irregularis] |
| 302 | 1178 | JAV49551.1 | 96.67 | 10 | 0 | 0 | 0 | 0 | 0 | 4 | 0 | 10 | 0 | 0 |  |  |  |  |  | 3.0807E5 |  | 3.3954E5 |  |  | 2 | 2 | 0 | 0 | 0 | 0 | 0 | 1 | 0 | 2 | 0 | 0 | N | 27945 | Proteasome subunit alpha type-7-like protein [Agkistrodon contortrix contortrix] |
| 302 | 1179 | XP\_026553199.1 | 96.67 | 10 | 0 | 0 | 0 | 0 | 0 | 4 | 0 | 10 | 0 | 0 |  |  |  |  |  | 3.0807E5 |  | 3.3954E5 |  |  | 2 | 2 | 0 | 0 | 0 | 0 | 0 | 1 | 0 | 2 | 0 | 0 | N | 27972 | proteasome subunit alpha type-7 [Pseudonaja textilis] |
| 302 | 1180 | XP\_034289033.1 | 96.67 | 10 | 0 | 0 | 0 | 0 | 0 | 4 | 0 | 10 | 0 | 0 |  |  |  |  |  | 3.0807E5 |  | 3.3954E5 |  |  | 2 | 2 | 0 | 0 | 0 | 0 | 0 | 1 | 0 | 2 | 0 | 0 | N | 27972 | proteasome subunit alpha type-7 [Pantherophis guttatus] |
| 302 | 1181 | XP\_032074995.1 | 96.67 | 10 | 0 | 0 | 0 | 0 | 0 | 4 | 0 | 10 | 0 | 0 |  |  |  |  |  | 3.0807E5 |  | 3.3954E5 |  |  | 2 | 2 | 0 | 0 | 0 | 0 | 0 | 1 | 0 | 2 | 0 | 0 | N | 27988 | proteasome subunit alpha type-7 [Thamnophis elegans] |
| 302 | 1182 | BAN82080.1 | 96.67 | 10 | 0 | 0 | 0 | 0 | 0 | 4 | 0 | 10 | 0 | 0 |  |  |  |  |  | 3.0807E5 |  | 3.3954E5 |  |  | 2 | 2 | 0 | 0 | 0 | 0 | 0 | 1 | 0 | 2 | 0 | 0 | N | 27915 | hypothetical protein [Protobothrops flavoviridis] |
| 302 | 1184 | XP\_013917261.1 | 96.67 | 10 | 0 | 0 | 0 | 0 | 0 | 4 | 0 | 10 | 0 | 0 |  |  |  |  |  | 3.0807E5 |  | 3.3954E5 |  |  | 2 | 2 | 0 | 0 | 0 | 0 | 0 | 1 | 0 | 2 | 0 | 0 | N | 28045 | PREDICTED: proteasome subunit alpha type-7 [Thamnophis sirtalis] |
| 184 | 1069 | Q6H3C5.2 | 91.08 | 12 | 0 | 0 | 0 | 12 | 0 | 6 | 0 | 0 | 6 | 0 |  |  |  | 8.5341E5 |  |  |  |  |  |  | 2 | 1 | 0 | 0 | 0 | 2 | 0 | 2 | 0 | 0 | 1 | 0 | N | 13819 | RecName: Full=Basic phospholipase A2 Ts-G6D49; Short=svPLA2; AltName: Full=Phosphatidylcholine 2-acylhydrolase |
| 345 | 2099 | AFJ51714.1 | 90.41 | 7 | 0 | 0 | 0 | 0 | 0 | 0 | 0 | 7 | 0 | 0 |  |  |  |  |  |  |  | 1.1516E5 |  |  | 1 | 1 | 0 | 0 | 0 | 0 | 0 | 0 | 0 | 2 | 0 | 0 | N | 26451 | Eukaryotic translation initiation factor 6-like [Crotalus adamanteus] |
| 345 | 2100 | JAI11019.1 | 90.41 | 7 | 0 | 0 | 0 | 0 | 0 | 0 | 0 | 7 | 0 | 0 |  |  |  |  |  |  |  | 1.1516E5 |  |  | 1 | 1 | 0 | 0 | 0 | 0 | 0 | 0 | 0 | 2 | 0 | 0 | N | 26451 | Eukaryotic translation initiation factor 6-like protein [Crotalus adamanteus] |
| 345 | 2101 | JAA95343.1 | 90.41 | 7 | 0 | 0 | 0 | 0 | 0 | 0 | 0 | 7 | 0 | 0 |  |  |  |  |  |  |  | 1.1516E5 |  |  | 1 | 1 | 0 | 0 | 0 | 0 | 0 | 0 | 0 | 2 | 0 | 0 | N | 26451 | Eukaryotic translation initiation factor 6-like protein [Crotalus horridus] |
| 345 | 2491 | LAB21681.1 | 90.41 | 8 | 0 | 0 | 0 | 0 | 0 | 0 | 0 | 8 | 0 | 0 |  |  |  |  |  |  |  | 1.1516E5 |  |  | 1 | 1 | 0 | 0 | 0 | 0 | 0 | 0 | 0 | 2 | 0 | 0 | N | 25129 | hypothetical protein [Micrurus spixii] |
| 345 | 2492 | JAV48857.1 | 90.41 | 7 | 0 | 0 | 0 | 0 | 0 | 0 | 0 | 7 | 0 | 0 |  |  |  |  |  |  |  | 1.1516E5 |  |  | 1 | 1 | 0 | 0 | 0 | 0 | 0 | 0 | 0 | 2 | 0 | 0 | N | 26481 | Eukaryotic translation initiation factor 6-like protein [Agkistrodon contortrix contortrix] |
| 345 | 2493 | XP\_026553507.1 | 90.41 | 7 | 0 | 0 | 0 | 0 | 0 | 0 | 0 | 7 | 0 | 0 |  |  |  |  |  |  |  | 1.1516E5 |  |  | 1 | 1 | 0 | 0 | 0 | 0 | 0 | 0 | 0 | 2 | 0 | 0 | N | 26525 | eukaryotic translation initiation factor 6 [Pseudonaja textilis] |
| 345 | 2494 | XP\_026540970.1 | 90.41 | 7 | 0 | 0 | 0 | 0 | 0 | 0 | 0 | 7 | 0 | 0 |  |  |  |  |  |  |  | 1.1516E5 |  |  | 1 | 1 | 0 | 0 | 0 | 0 | 0 | 0 | 0 | 2 | 0 | 0 | N | 26525 | eukaryotic translation initiation factor 6 [Notechis scutatus] |
| 345 | 2495 | XP\_015671062.1 | 90.41 | 7 | 0 | 0 | 0 | 0 | 0 | 0 | 0 | 7 | 0 | 0 |  |  |  |  |  |  |  | 1.1516E5 |  |  | 1 | 1 | 0 | 0 | 0 | 0 | 0 | 0 | 0 | 2 | 0 | 0 | N | 26497 | eukaryotic translation initiation factor 6 [Protobothrops mucrosquamatus] |
| 345 | 2496 | XP\_007439387.1 | 90.41 | 7 | 0 | 0 | 0 | 0 | 0 | 0 | 0 | 7 | 0 | 0 |  |  |  |  |  |  |  | 1.1516E5 |  |  | 1 | 1 | 0 | 0 | 0 | 0 | 0 | 0 | 0 | 2 | 0 | 0 | N | 26683 | eukaryotic translation initiation factor 6 [Python bivittatus] |
| 345 | 2497 | LAA26046.1 | 90.41 | 6 | 0 | 0 | 0 | 0 | 0 | 0 | 0 | 6 | 0 | 0 |  |  |  |  |  |  |  | 1.1516E5 |  |  | 1 | 1 | 0 | 0 | 0 | 0 | 0 | 0 | 0 | 2 | 0 | 0 | N | 29964 | hypothetical protein, partial [Micrurus lemniscatus carvalhoi] |
| 319 | 1187 | AFJ51037.1 | 88.13 | 7 | 0 | 0 | 0 | 0 | 0 | 3 | 0 | 4 | 0 | 0 |  |  |  |  |  | 1.4105E5 |  | 1.9782E5 |  |  | 2 | 2 | 0 | 0 | 0 | 0 | 0 | 1 | 0 | 1 | 0 | 0 | N | 29453 | Proteasome subunit alpha type-4 [Crotalus adamanteus] |
| 319 | 1188 | JAA96034.1 | 88.13 | 7 | 0 | 0 | 0 | 0 | 0 | 3 | 0 | 4 | 0 | 0 |  |  |  |  |  | 1.4105E5 |  | 1.9782E5 |  |  | 2 | 2 | 0 | 0 | 0 | 0 | 0 | 1 | 0 | 1 | 0 | 0 | N | 29453 | Proteasome subunit alpha type-4 protein [Crotalus horridus] |
| 319 | 1189 | JAV49554.1 | 88.13 | 7 | 0 | 0 | 0 | 0 | 0 | 3 | 0 | 4 | 0 | 0 |  |  |  |  |  | 1.4105E5 |  | 1.9782E5 |  |  | 2 | 2 | 0 | 0 | 0 | 0 | 0 | 1 | 0 | 1 | 0 | 0 | N | 29453 | Proteasome subunit alpha type-4 protein [Agkistrodon contortrix contortrix] |
| 319 | 1190 | JAI11929.1 | 88.13 | 7 | 0 | 0 | 0 | 0 | 0 | 3 | 0 | 4 | 0 | 0 |  |  |  |  |  | 1.4105E5 |  | 1.9782E5 |  |  | 2 | 2 | 0 | 0 | 0 | 0 | 0 | 1 | 0 | 1 | 0 | 0 | N | 29453 | Proteasome subunit alpha type-4 protein [Crotalus adamanteus] |
| 252 | 1832 | AEJ31991.1 | 86.06 | 6 | 4 | 0 | 0 | 2 | 0 | 0 | 2 | 6 | 6 | 4 | 6.6124E6 |  |  | 2.2861E5 |  |  | 4.066E5 | 1.3187E7 | 1.0571E8 | 7.1088E7 | 2 | 2 | 1 | 0 | 0 | 1 | 0 | 0 | 1 | 3 | 6 | 1 | Y | 54615 | metalloproteinase 8, partial [Crotalus adamanteus] |
| 252 | 1839 | AFJ49243.1 | 86.06 | 5 | 3 | 0 | 0 | 2 | 0 | 0 | 2 | 5 | 5 | 3 | 6.6124E6 |  |  | 2.2861E5 |  |  | 4.066E5 | 1.3187E7 | 1.0571E8 | 7.1088E7 | 2 | 2 | 1 | 0 | 0 | 1 | 0 | 0 | 1 | 3 | 6 | 1 | Y | 68434 | Snake venom metalloproteinase (type III) 5 [Crotalus adamanteus] |
| 252 | 1905 | JAV48412.1 | 86.06 | 5 | 3 | 0 | 0 | 2 | 0 | 0 | 2 | 5 | 5 | 3 | 6.6124E6 |  |  | 2.2861E5 |  |  | 4.066E5 | 1.3187E7 | 1.0571E8 | 7.1088E7 | 2 | 2 | 1 | 0 | 0 | 1 | 0 | 0 | 1 | 3 | 6 | 1 | Y | 68421 | Cadam10\_SVMPIII-2 [Crotalus adamanteus] |
| 289 | 1489 | AUS82484.1 | 84.59 | 10 | 0 | 5 | 0 | 0 | 0 | 0 | 5 | 0 | 0 | 0 |  | 1.1075E7 |  |  |  |  |  |  |  |  | 2 | 1 | 0 | 3 | 0 | 0 | 0 | 0 | 1 | 0 | 0 | 0 | Y | 27907 | serine endopeptidase [Crotalus atrox] |
| 342 | 2061 | Q9PVK7.1 | 80.81 | 4 | 0 | 0 | 0 | 0 | 0 | 0 | 0 | 4 | 2 | 0 |  |  |  |  |  |  |  | 1.4703E5 | 7.0812E4 |  | 2 | 2 | 0 | 0 | 0 | 0 | 0 | 0 | 0 | 2 | 1 | 0 | N | 67662 | RecName: Full=Zinc metalloproteinase-disintegrin-like cobrin; AltName: Full=Snake venom metalloproteinase; Short=SVMP; Flags: Precursor |
| 342 | 2062 | AAF00693.1 | 80.81 | 4 | 0 | 0 | 0 | 0 | 0 | 0 | 0 | 4 | 2 | 0 |  |  |  |  |  |  |  | 1.4703E5 | 7.0812E4 |  | 2 | 2 | 0 | 0 | 0 | 0 | 0 | 0 | 0 | 2 | 1 | 0 | N | 67662 | cobrin precursor [Naja naja] |
| 337 | 1066 | XP\_026544503.1 | 80.22 | 6 | 0 | 0 | 0 | 0 | 0 | 0 | 0 | 2 | 4 | 0 |  |  |  |  |  |  |  | 2.1124E4 | 9.5933E4 |  | 2 | 2 | 0 | 0 | 0 | 0 | 0 | 0 | 0 | 1 | 1 | 0 | N | 47550 | alpha-enolase [Notechis scutatus] |
| 337 | 1117 | JAG68757.1 | 80.22 | 6 | 0 | 0 | 0 | 0 | 0 | 0 | 0 | 2 | 4 | 0 |  |  |  |  |  |  |  | 2.1124E4 | 9.5933E4 |  | 2 | 2 | 0 | 0 | 0 | 0 | 0 | 0 | 0 | 1 | 1 | 0 | N | 47546 | Alpha-enolase [Boiga irregularis] |
| 337 | 1122 | JAC96284.1 | 80.22 | 6 | 0 | 0 | 0 | 0 | 0 | 0 | 0 | 2 | 4 | 0 |  |  |  |  |  |  |  | 2.1124E4 | 9.5933E4 |  | 2 | 2 | 0 | 0 | 0 | 0 | 0 | 0 | 0 | 1 | 1 | 0 | N | 47493 | alpha-enolase [Hypsiglena sp. JMG-2014] |
| 315 | 1445 | XP\_015685587.1 | 78.17 | 5 | 0 | 0 | 0 | 0 | 0 | 0 | 0 | 0 | 5 | 0 |  |  |  |  |  |  |  |  | 6.0805E6 |  | 2 | 1 | 0 | 0 | 0 | 0 | 0 | 0 | 0 | 0 | 4 | 0 | N | 39726 | zinc metalloproteinase/disintegrin-like HR1a [Protobothrops mucrosquamatus] |
| 355 | 1620 | BAD82814.1 | 76.08 | 2 | 0 | 0 | 0 | 0 | 0 | 0 | 0 | 0 | 2 | 0 |  |  |  |  |  |  |  |  | 6.9991E4 |  | 1 | 1 | 0 | 0 | 0 | 0 | 0 | 0 | 0 | 0 | 1 | 0 | N | 69705 | serum albumin [Protobothrops flavoviridis] |
| 346 | 1693 | XP\_026551425.1 | 75.98 | 2 | 0 | 0 | 0 | 0 | 0 | 0 | 0 | 2 | 0 | 0 |  |  |  |  |  |  |  | 5.8122E4 |  |  | 2 | 2 | 0 | 0 | 0 | 0 | 0 | 0 | 0 | 2 | 0 | 0 | N | 227171 | myosin-9 isoform X2 [Pseudonaja textilis] |
| 346 | 1694 | XP\_026551417.1 | 75.98 | 2 | 0 | 0 | 0 | 0 | 0 | 0 | 0 | 2 | 0 | 0 |  |  |  |  |  |  |  | 5.8122E4 |  |  | 2 | 2 | 0 | 0 | 0 | 0 | 0 | 0 | 0 | 2 | 0 | 0 | N | 229768 | myosin-9 isoform X1 [Pseudonaja textilis] |
| 346 | 1697 | BAN82211.1 | 75.98 | 2 | 0 | 0 | 0 | 0 | 0 | 0 | 0 | 2 | 0 | 0 |  |  |  |  |  |  |  | 5.8122E4 |  |  | 2 | 2 | 0 | 0 | 0 | 0 | 0 | 0 | 0 | 2 | 0 | 0 | N | 227345 | hypothetical protein [Ovophis okinavensis] |
| 346 | 1698 | JAI12437.1 | 75.98 | 2 | 0 | 0 | 0 | 0 | 0 | 0 | 0 | 2 | 0 | 0 |  |  |  |  |  |  |  | 5.8122E4 |  |  | 2 | 2 | 0 | 0 | 0 | 0 | 0 | 0 | 0 | 2 | 0 | 0 | N | 227373 | myosin-9-like [Crotalus adamanteus] |
| 346 | 1699 | XP\_015687322.1 | 75.98 | 2 | 0 | 0 | 0 | 0 | 0 | 0 | 0 | 2 | 0 | 0 |  |  |  |  |  |  |  | 5.8122E4 |  |  | 2 | 2 | 0 | 0 | 0 | 0 | 0 | 0 | 0 | 2 | 0 | 0 | N | 227364 | myosin-9 isoform X2 [Protobothrops mucrosquamatus] |
| 346 | 1700 | JAV49917.1 | 75.98 | 2 | 0 | 0 | 0 | 0 | 0 | 0 | 0 | 2 | 0 | 0 |  |  |  |  |  |  |  | 5.8122E4 |  |  | 2 | 2 | 0 | 0 | 0 | 0 | 0 | 0 | 0 | 2 | 0 | 0 | N | 227431 | myosin-9 isoform 1 [Agkistrodon contortrix contortrix] |
| 346 | 1701 | JAG46253.1 | 75.98 | 2 | 0 | 0 | 0 | 0 | 0 | 0 | 0 | 2 | 0 | 0 |  |  |  |  |  |  |  | 5.8122E4 |  |  | 2 | 2 | 0 | 0 | 0 | 0 | 0 | 0 | 0 | 2 | 0 | 0 | N | 227374 | myosin-9 [Crotalus horridus] |
| 346 | 1702 | XP\_015687313.1 | 75.98 | 2 | 0 | 0 | 0 | 0 | 0 | 0 | 0 | 2 | 0 | 0 |  |  |  |  |  |  |  | 5.8122E4 |  |  | 2 | 2 | 0 | 0 | 0 | 0 | 0 | 0 | 0 | 2 | 0 | 0 | N | 229979 | myosin-9 isoform X1 [Protobothrops mucrosquamatus] |
| 346 | 1703 | JAB53856.1 | 75.98 | 2 | 0 | 0 | 0 | 0 | 0 | 0 | 0 | 2 | 0 | 0 |  |  |  |  |  |  |  | 5.8122E4 |  |  | 2 | 2 | 0 | 0 | 0 | 0 | 0 | 0 | 0 | 2 | 0 | 0 | N | 227239 | myosin-9 [Micrurus fulvius] |
| 346 | 1704 | LAB26995.1 | 75.98 | 2 | 0 | 0 | 0 | 0 | 0 | 0 | 0 | 2 | 0 | 0 |  |  |  |  |  |  |  | 5.8122E4 |  |  | 2 | 2 | 0 | 0 | 0 | 0 | 0 | 0 | 0 | 2 | 0 | 0 | N | 227253 | hypothetical protein [Micrurus spixii] |
| 346 | 1705 | LAB26993.1 | 75.98 | 2 | 0 | 0 | 0 | 0 | 0 | 0 | 0 | 2 | 0 | 0 |  |  |  |  |  |  |  | 5.8122E4 |  |  | 2 | 2 | 0 | 0 | 0 | 0 | 0 | 0 | 0 | 2 | 0 | 0 | N | 227253 | hypothetical protein [Micrurus spixii] |
| 346 | 1706 | LAA85344.1 | 75.98 | 2 | 0 | 0 | 0 | 0 | 0 | 0 | 0 | 2 | 0 | 0 |  |  |  |  |  |  |  | 5.8122E4 |  |  | 2 | 2 | 0 | 0 | 0 | 0 | 0 | 0 | 0 | 2 | 0 | 0 | N | 227250 | hypothetical protein [Micrurus lemniscatus lemniscatus] |
| 346 | 1707 | XP\_026550725.1 | 75.98 | 2 | 0 | 0 | 0 | 0 | 0 | 0 | 0 | 2 | 0 | 0 |  |  |  |  |  |  |  | 5.8122E4 |  |  | 2 | 2 | 0 | 0 | 0 | 0 | 0 | 0 | 0 | 2 | 0 | 0 | N | 227143 | myosin-9 isoform X2 [Notechis scutatus] |
| 346 | 1708 | XP\_026550724.1 | 75.98 | 2 | 0 | 0 | 0 | 0 | 0 | 0 | 0 | 2 | 0 | 0 |  |  |  |  |  |  |  | 5.8122E4 |  |  | 2 | 2 | 0 | 0 | 0 | 0 | 0 | 0 | 0 | 2 | 0 | 0 | N | 229740 | myosin-9 isoform X1 [Notechis scutatus] |
| 346 | 1709 | LAB55089.1 | 75.98 | 3 | 0 | 0 | 0 | 0 | 0 | 0 | 0 | 3 | 0 | 0 |  |  |  |  |  |  |  | 5.8122E4 |  |  | 2 | 2 | 0 | 0 | 0 | 0 | 0 | 0 | 0 | 2 | 0 | 0 | N | 139932 | hypothetical protein, partial [Micrurus surinamensis] |
| 346 | 1747 | XP\_007430787.1 | 75.98 | 2 | 0 | 0 | 0 | 0 | 0 | 0 | 0 | 2 | 0 | 0 |  |  |  |  |  |  |  | 5.8122E4 |  |  | 2 | 2 | 0 | 0 | 0 | 0 | 0 | 0 | 0 | 2 | 0 | 0 | N | 227016 | myosin-9 [Python bivittatus] |
| 346 | 1748 | XP\_015744014.1 | 75.98 | 2 | 0 | 0 | 0 | 0 | 0 | 0 | 0 | 2 | 0 | 0 |  |  |  |  |  |  |  | 5.8122E4 |  |  | 2 | 2 | 0 | 0 | 0 | 0 | 0 | 0 | 0 | 2 | 0 | 0 | N | 227016 | myosin-9 [Python bivittatus] |
| 346 | 1749 | XP\_015744015.1 | 75.98 | 2 | 0 | 0 | 0 | 0 | 0 | 0 | 0 | 2 | 0 | 0 |  |  |  |  |  |  |  | 5.8122E4 |  |  | 2 | 2 | 0 | 0 | 0 | 0 | 0 | 0 | 0 | 2 | 0 | 0 | N | 227016 | myosin-9 [Python bivittatus] |
| 346 | 1750 | ETE73299.1 | 75.98 | 2 | 0 | 0 | 0 | 0 | 0 | 0 | 0 | 2 | 0 | 0 |  |  |  |  |  |  |  | 5.8122E4 |  |  | 2 | 2 | 0 | 0 | 0 | 0 | 0 | 0 | 0 | 2 | 0 | 0 | N | 221001 | Myosin-9, partial [Ophiophagus hannah] |
| 255 | 1949 | XP\_034268135.1 | 70.00 | 1 | 0 | 0 | 1 | 1 | 0 | 0 | 0 | 1 | 1 | 0 |  |  | 5.9763E5 | 4.2669E6 |  |  |  | 6.5049E6 | 3.1299E6 |  | 1 | 1 | 0 | 0 | 1 | 1 | 0 | 0 | 0 | 2 | 2 | 0 | Y | 124073 | neurofilament medium polypeptide-like [Pantherophis guttatus] |
| 255 | 1952 | XP\_013924292.1 | 70.00 | 1 | 0 | 0 | 1 | 1 | 0 | 0 | 0 | 1 | 1 | 0 |  |  | 5.9763E5 | 4.2669E6 |  |  |  | 6.5049E6 | 3.1299E6 |  | 1 | 1 | 0 | 0 | 1 | 1 | 0 | 0 | 0 | 2 | 2 | 0 | Y | 119192 | PREDICTED: neurofilament medium polypeptide-like [Thamnophis sirtalis] |
| 255 | 1953 | XP\_032067334.1 | 70.00 | 2 | 0 | 0 | 2 | 2 | 0 | 0 | 0 | 2 | 2 | 0 |  |  | 5.9763E5 | 4.2669E6 |  |  |  | 6.5049E6 | 3.1299E6 |  | 1 | 1 | 0 | 0 | 1 | 1 | 0 | 0 | 0 | 2 | 2 | 0 | Y | 42280 | keratin, type II cytoskeletal 80 [Thamnophis elegans] |
| 255 | 1960 | XP\_026573192.1 | 70.00 | 1 | 0 | 0 | 1 | 1 | 0 | 0 | 0 | 1 | 1 | 0 |  |  | 5.9763E5 | 4.2669E6 |  |  |  | 6.5049E6 | 3.1299E6 |  | 1 | 1 | 0 | 0 | 1 | 1 | 0 | 0 | 0 | 2 | 2 | 0 | Y | 118330 | neurofilament medium polypeptide-like [Pseudonaja textilis] |
| 255 | 1963 | XP\_026535749.1 | 70.00 | 1 | 0 | 0 | 1 | 1 | 0 | 0 | 0 | 1 | 1 | 0 |  |  | 5.9763E5 | 4.2669E6 |  |  |  | 6.5049E6 | 3.1299E6 |  | 1 | 1 | 0 | 0 | 1 | 1 | 0 | 0 | 0 | 2 | 2 | 0 | Y | 123281 | LOW QUALITY PROTEIN: neurofilament medium polypeptide-like [Notechis scutatus] |
| 255 | 2272 | ETE71916.1 | 70.00 | 1 | 0 | 0 | 1 | 1 | 0 | 0 | 0 | 1 | 1 | 0 |  |  | 5.9763E5 | 4.2669E6 |  |  |  | 6.5049E6 | 3.1299E6 |  | 1 | 1 | 0 | 0 | 1 | 1 | 0 | 0 | 0 | 2 | 2 | 0 | Y | 82272 | Keratin, type II cytoskeletal 80 [Ophiophagus hannah] |
| 255 | 2971 | XP\_029142905.1 | 70.00 | 4 | 0 | 0 | 4 | 4 | 0 | 0 | 0 | 4 | 4 | 0 |  |  | 5.9763E5 | 4.2669E6 |  |  |  | 6.5049E6 | 3.1299E6 |  | 1 | 1 | 0 | 0 | 1 | 1 | 0 | 0 | 0 | 2 | 2 | 0 | Y | 23545 | keratin, type II cytoskeletal 80-like, partial [Protobothrops mucrosquamatus] |
| 304 | 3471 | JAS03130.1 | 69.98 | 5 | 0 | 0 | 0 | 5 | 0 | 0 | 0 | 0 | 0 | 0 |  |  |  | 1.1042E6 |  |  |  |  |  |  | 1 | 1 | 0 | 0 | 0 | 1 | 0 | 0 | 0 | 0 | 0 | 0 | N | 22605 | Vascular Endothelial Growth Factor [Phalotris mertensi] |
| 369 | 1877 | JAV51434.1 | 69.89 | 4 | 0 | 0 | 0 | 2 | 0 | 0 | 0 | 0 | 2 | 0 |  |  |  | 1.8544E5 |  |  |  |  | 2.1629E5 |  | 2 | 2 | 0 | 0 | 0 | 1 | 0 | 0 | 0 | 0 | 1 | 0 | N | 68930 | Metalloproteinase (type III) 4 [Agkistrodon contortrix contortrix] |
| 369 | 1888 | JAS04446.1 | 69.89 | 4 | 0 | 0 | 0 | 2 | 0 | 0 | 0 | 0 | 2 | 0 |  |  |  | 1.8544E5 |  |  |  |  | 2.1629E5 |  | 2 | 2 | 0 | 0 | 0 | 1 | 0 | 0 | 0 | 0 | 1 | 0 | N | 68904 | Metalloproteinase type III 8 [Agkistrodon piscivorus conanti] |
| 320 | 1649 | AEJ31985.1 | 69.82 | 1 | 0 | 0 | 0 | 0 | 0 | 0 | 1 | 0 | 0 | 0 |  |  |  |  |  |  | 3.442E6 |  |  |  | 1 | 1 | 0 | 0 | 0 | 0 | 0 | 0 | 4 | 0 | 0 | 0 | N | 54751 | metalloproteinase 2 [Crotalus adamanteus] |
| 320 | 1654 | AFJ49219.1 | 69.82 | 1 | 0 | 0 | 0 | 0 | 0 | 0 | 1 | 0 | 0 | 0 |  |  |  |  |  |  | 3.442E6 |  |  |  | 1 | 1 | 0 | 0 | 0 | 0 | 0 | 0 | 4 | 0 | 0 | 0 | N | 54232 | Snake venom metalloproteinase (type II) 4 [Crotalus adamanteus] |
| 320 | 1846 | JAS05303.1 | 69.82 | 1 | 0 | 0 | 0 | 0 | 0 | 0 | 1 | 0 | 0 | 0 |  |  |  |  |  |  | 3.442E6 |  |  |  | 1 | 1 | 0 | 0 | 0 | 0 | 0 | 0 | 4 | 0 | 0 | 0 | N | 58338 | Metalloproteinase type II 4 [Sistrurus tergeminus] |
| 320 | 1847 | AFS49715.1 | 69.82 | 1 | 0 | 0 | 0 | 0 | 0 | 0 | 1 | 0 | 0 | 0 |  |  |  |  |  |  | 3.442E6 |  |  |  | 1 | 1 | 0 | 0 | 0 | 0 | 0 | 0 | 4 | 0 | 0 | 0 | N | 54823 | VMPII [Crotalus adamanteus] |
| 320 | 1850 | AFJ49230.1 | 69.82 | 1 | 0 | 0 | 0 | 0 | 0 | 0 | 1 | 0 | 0 | 0 |  |  |  |  |  |  | 3.442E6 |  |  |  | 1 | 1 | 0 | 0 | 0 | 0 | 0 | 0 | 4 | 0 | 0 | 0 | N | 54827 | Snake venom metalloproteinase (type II) 8 [Crotalus adamanteus] |
| 320 | 1853 | JAI10398.1 | 69.82 | 1 | 0 | 0 | 0 | 0 | 0 | 0 | 1 | 0 | 0 | 0 |  |  |  |  |  |  | 3.442E6 |  |  |  | 1 | 1 | 0 | 0 | 0 | 0 | 0 | 0 | 4 | 0 | 0 | 0 | N | 54879 | Metalloproteinase (type II) 1a [Crotalus adamanteus] |
| 320 | 1854 | JAI10397.1 | 69.82 | 1 | 0 | 0 | 0 | 0 | 0 | 0 | 1 | 0 | 0 | 0 |  |  |  |  |  |  | 3.442E6 |  |  |  | 1 | 1 | 0 | 0 | 0 | 0 | 0 | 0 | 4 | 0 | 0 | 0 | N | 54865 | Metalloproteinase (type II) 1b [Crotalus adamanteus] |
| 320 | 1855 | JAI10396.1 | 69.82 | 1 | 0 | 0 | 0 | 0 | 0 | 0 | 1 | 0 | 0 | 0 |  |  |  |  |  |  | 3.442E6 |  |  |  | 1 | 1 | 0 | 0 | 0 | 0 | 0 | 0 | 4 | 0 | 0 | 0 | N | 54849 | Metalloproteinase (type II) 1c [Crotalus adamanteus] |
| 320 | 1856 | JAS04694.1 | 69.82 | 1 | 0 | 0 | 0 | 0 | 0 | 0 | 1 | 0 | 0 | 0 |  |  |  |  |  |  | 3.442E6 |  |  |  | 1 | 1 | 0 | 0 | 0 | 0 | 0 | 0 | 4 | 0 | 0 | 0 | N | 54879 | Metalloproteinase type II 1a [Crotalus adamanteus] |
| 320 | 1857 | JAI10395.1 | 69.82 | 1 | 0 | 0 | 0 | 0 | 0 | 0 | 1 | 0 | 0 | 0 |  |  |  |  |  |  | 3.442E6 |  |  |  | 1 | 1 | 0 | 0 | 0 | 0 | 0 | 0 | 4 | 0 | 0 | 0 | N | 54835 | Metalloproteinase (type II) 1d [Crotalus adamanteus] |
| 320 | 1858 | AFJ49217.1 | 69.82 | 1 | 0 | 0 | 0 | 0 | 0 | 0 | 1 | 0 | 0 | 0 |  |  |  |  |  |  | 3.442E6 |  |  |  | 1 | 1 | 0 | 0 | 0 | 0 | 0 | 0 | 4 | 0 | 0 | 0 | N | 54865 | Snake venom metalloproteinase (type II) 3c [Crotalus adamanteus] |
| 320 | 1859 | JAS04693.1 | 69.82 | 1 | 0 | 0 | 0 | 0 | 0 | 0 | 1 | 0 | 0 | 0 |  |  |  |  |  |  | 3.442E6 |  |  |  | 1 | 1 | 0 | 0 | 0 | 0 | 0 | 0 | 4 | 0 | 0 | 0 | N | 54865 | Metalloproteinase type II 1b [Crotalus adamanteus] |
| 320 | 1860 | AEJ31984.1 | 69.82 | 1 | 0 | 0 | 0 | 0 | 0 | 0 | 1 | 0 | 0 | 0 |  |  |  |  |  |  | 3.442E6 |  |  |  | 1 | 1 | 0 | 0 | 0 | 0 | 0 | 0 | 4 | 0 | 0 | 0 | N | 54835 | metalloproteinase 1 [Crotalus adamanteus] |
| 320 | 1861 | JAS04691.1 | 69.82 | 1 | 0 | 0 | 0 | 0 | 0 | 0 | 1 | 0 | 0 | 0 |  |  |  |  |  |  | 3.442E6 |  |  |  | 1 | 1 | 0 | 0 | 0 | 0 | 0 | 0 | 4 | 0 | 0 | 0 | N | 54835 | Metalloproteinase type II 1d [Crotalus adamanteus] |
| 320 | 1862 | AFJ49216.1 | 69.82 | 1 | 0 | 0 | 0 | 0 | 0 | 0 | 1 | 0 | 0 | 0 |  |  |  |  |  |  | 3.442E6 |  |  |  | 1 | 1 | 0 | 0 | 0 | 0 | 0 | 0 | 4 | 0 | 0 | 0 | N | 54835 | Snake venom metalloproteinase (type II) 3b [Crotalus adamanteus] |
| 320 | 1863 | AFJ49218.1 | 69.82 | 1 | 0 | 0 | 0 | 0 | 0 | 0 | 1 | 0 | 0 | 0 |  |  |  |  |  |  | 3.442E6 |  |  |  | 1 | 1 | 0 | 0 | 0 | 0 | 0 | 0 | 4 | 0 | 0 | 0 | N | 54849 | Snake venom metalloproteinase (type II) 3d [Crotalus adamanteus] |
| 320 | 1864 | AFJ49215.1 | 69.82 | 1 | 0 | 0 | 0 | 0 | 0 | 0 | 1 | 0 | 0 | 0 |  |  |  |  |  |  | 3.442E6 |  |  |  | 1 | 1 | 0 | 0 | 0 | 0 | 0 | 0 | 4 | 0 | 0 | 0 | N | 54879 | Snake venom metalloproteinase (type II) 3a [Crotalus adamanteus] |
| 320 | 1865 | JAS04692.1 | 69.82 | 1 | 0 | 0 | 0 | 0 | 0 | 0 | 1 | 0 | 0 | 0 |  |  |  |  |  |  | 3.442E6 |  |  |  | 1 | 1 | 0 | 0 | 0 | 0 | 0 | 0 | 4 | 0 | 0 | 0 | N | 54849 | Metalloproteinase type II 1c [Crotalus adamanteus] |
| 320 | 1869 | AFJ49211.1 | 69.82 | 1 | 0 | 0 | 0 | 0 | 0 | 0 | 1 | 0 | 0 | 0 |  |  |  |  |  |  | 3.442E6 |  |  |  | 1 | 1 | 0 | 0 | 0 | 0 | 0 | 0 | 4 | 0 | 0 | 0 | N | 54175 | Snake venom metalloproteinase (type II) 1d [Crotalus adamanteus] |
| 320 | 1870 | AFJ49208.1 | 69.82 | 1 | 0 | 0 | 0 | 0 | 0 | 0 | 1 | 0 | 0 | 0 |  |  |  |  |  |  | 3.442E6 |  |  |  | 1 | 1 | 0 | 0 | 0 | 0 | 0 | 0 | 4 | 0 | 0 | 0 | N | 54308 | Snake venom metalloproteinase (type II) 1a [Crotalus adamanteus] |
| 320 | 1871 | AFJ49209.1 | 69.82 | 1 | 0 | 0 | 0 | 0 | 0 | 0 | 1 | 0 | 0 | 0 |  |  |  |  |  |  | 3.442E6 |  |  |  | 1 | 1 | 0 | 0 | 0 | 0 | 0 | 0 | 4 | 0 | 0 | 0 | N | 54278 | Snake venom metalloproteinase (type II) 1b [Crotalus adamanteus] |
| 320 | 1872 | AFJ49210.1 | 69.82 | 1 | 0 | 0 | 0 | 0 | 0 | 0 | 1 | 0 | 0 | 0 |  |  |  |  |  |  | 3.442E6 |  |  |  | 1 | 1 | 0 | 0 | 0 | 0 | 0 | 0 | 4 | 0 | 0 | 0 | N | 54308 | Snake venom metalloproteinase (type II) 1c [Crotalus adamanteus] |
| 320 | 1873 | AFJ49212.1 | 69.82 | 1 | 0 | 0 | 0 | 0 | 0 | 0 | 1 | 0 | 0 | 0 |  |  |  |  |  |  | 3.442E6 |  |  |  | 1 | 1 | 0 | 0 | 0 | 0 | 0 | 0 | 4 | 0 | 0 | 0 | N | 54308 | Snake venom metalloproteinase (type II) 1e [Crotalus adamanteus] |
| 320 | 1880 | JAS05427.1 | 69.82 | 1 | 0 | 0 | 0 | 0 | 0 | 0 | 1 | 0 | 0 | 0 |  |  |  |  |  |  | 3.442E6 |  |  |  | 1 | 1 | 0 | 0 | 0 | 0 | 0 | 0 | 4 | 0 | 0 | 0 | N | 58394 | Metalloproteinase type II 6b [Sistrurus miliarius barbouri] |
| 320 | 1881 | JAS05426.1 | 69.82 | 1 | 0 | 0 | 0 | 0 | 0 | 0 | 1 | 0 | 0 | 0 |  |  |  |  |  |  | 3.442E6 |  |  |  | 1 | 1 | 0 | 0 | 0 | 0 | 0 | 0 | 4 | 0 | 0 | 0 | N | 58394 | Metalloproteinase type II 6c [Sistrurus miliarius barbouri] |
| 320 | 1882 | JAS05428.1 | 69.82 | 1 | 0 | 0 | 0 | 0 | 0 | 0 | 1 | 0 | 0 | 0 |  |  |  |  |  |  | 3.442E6 |  |  |  | 1 | 1 | 0 | 0 | 0 | 0 | 0 | 0 | 4 | 0 | 0 | 0 | N | 58521 | Metalloproteinase type II 6a [Sistrurus miliarius barbouri] |
| 352 | 2261 | XP\_026539659.1 | 66.33 | 1 | 0 | 0 | 0 | 0 | 0 | 0 | 0 | 0 | 1 | 0 |  |  |  |  |  |  |  |  | 2.213E5 |  | 1 | 1 | 0 | 0 | 0 | 0 | 0 | 0 | 0 | 0 | 1 | 0 | N | 70020 | keratin, type II cytoskeletal 7-like [Notechis scutatus] |
| 352 | 2271 | XP\_026570510.1 | 66.33 | 1 | 0 | 0 | 0 | 0 | 0 | 0 | 0 | 0 | 1 | 0 |  |  |  |  |  |  |  |  | 2.213E5 |  | 1 | 1 | 0 | 0 | 0 | 0 | 0 | 0 | 0 | 0 | 1 | 0 | N | 70208 | keratin, type II cytoskeletal 7-like [Pseudonaja textilis] |
| 318 | 1678 | P26324.1 | 66.32 | 9 | 0 | 9 | 0 | 0 | 0 | 0 | 0 | 0 | 0 | 0 |  | 7.0834E6 |  |  |  |  |  |  |  |  | 2 | 1 | 0 | 2 | 0 | 0 | 0 | 0 | 0 | 0 | 0 | 0 | Y | 26570 | RecName: Full=Thrombin-like enzyme ancrod; Short=SVTLE; AltName: Full=Fibrinogen-clotting enzyme; AltName: Full=Snake venom serine protease; Short=SVSP; AltName: Full=Venombin A |
| 318 | 1679 | AAA03254.1 | 66.32 | 9 | 0 | 9 | 0 | 0 | 0 | 0 | 0 | 0 | 0 | 0 |  | 7.0834E6 |  |  |  |  |  |  |  |  | 2 | 1 | 0 | 2 | 0 | 0 | 0 | 0 | 0 | 0 | 0 | 0 | Y | 26570 | Ancrod=thrombin-like alpha-fibrinogenase [Akistrodon rhodostoma, venom, Peptide, 234 aa] |
| 377 | 2072 | XP\_013919189.1 | 64.35 | 7 | 0 | 0 | 0 | 0 | 0 | 0 | 0 | 0 | 7 | 0 |  |  |  |  |  |  |  |  | 2.2355E5 |  | 1 | 1 | 0 | 0 | 0 | 0 | 0 | 0 | 0 | 0 | 1 | 0 | N | 13507 | PREDICTED: histone H2A type 2-B-like [Thamnophis sirtalis] |
| 377 | 2073 | XP\_013919188.1 | 64.35 | 7 | 0 | 0 | 0 | 0 | 0 | 0 | 0 | 0 | 7 | 0 |  |  |  |  |  |  |  |  | 2.2355E5 |  | 1 | 1 | 0 | 0 | 0 | 0 | 0 | 0 | 0 | 0 | 1 | 0 | N | 13507 | PREDICTED: histone H2A type 2-B-like [Thamnophis sirtalis] |
| 377 | 2074 | ETE65301.1 | 64.35 | 7 | 0 | 0 | 0 | 0 | 0 | 0 | 0 | 0 | 7 | 0 |  |  |  |  |  |  |  |  | 2.2355E5 |  | 1 | 1 | 0 | 0 | 0 | 0 | 0 | 0 | 0 | 0 | 1 | 0 | N | 13507 | Histone H2A type 2-B, partial [Ophiophagus hannah] |
| 377 | 2076 | XP\_013931116.1 | 64.35 | 7 | 0 | 0 | 0 | 0 | 0 | 0 | 0 | 0 | 7 | 0 |  |  |  |  |  |  |  |  | 2.2355E5 |  | 1 | 1 | 0 | 0 | 0 | 0 | 0 | 0 | 0 | 0 | 1 | 0 | N | 13940 | PREDICTED: histone H2A type 2-C [Thamnophis sirtalis] |
| 377 | 2077 | XP\_013908942.1 | 64.35 | 7 | 0 | 0 | 0 | 0 | 0 | 0 | 0 | 0 | 7 | 0 |  |  |  |  |  |  |  |  | 2.2355E5 |  | 1 | 1 | 0 | 0 | 0 | 0 | 0 | 0 | 0 | 0 | 1 | 0 | N | 14026 | PREDICTED: histone H2A-IV-like [Thamnophis sirtalis] |
| 377 | 2114 | ETE65060.1 | 64.35 | 7 | 0 | 0 | 0 | 0 | 0 | 0 | 0 | 0 | 7 | 0 |  |  |  |  |  |  |  |  | 2.2355E5 |  | 1 | 1 | 0 | 0 | 0 | 0 | 0 | 0 | 0 | 0 | 1 | 0 | N | 13855 | hypothetical protein L345\_09168, partial [Ophiophagus hannah] |
| 377 | 2115 | XP\_013915314.1 | 64.35 | 7 | 0 | 0 | 0 | 0 | 0 | 0 | 0 | 0 | 7 | 0 |  |  |  |  |  |  |  |  | 2.2355E5 |  | 1 | 1 | 0 | 0 | 0 | 0 | 0 | 0 | 0 | 0 | 1 | 0 | N | 13855 | PREDICTED: histone H2A type 2-C [Thamnophis sirtalis] |
| 286 | 4375 | JAS04797.1 | 62.63 | 5 | 0 | 0 | 0 | 5 | 0 | 5 | 0 | 0 | 0 | 0 |  |  |  | 1.7937E7 |  | 7.5976E7 |  |  |  |  | 1 | 1 | 0 | 0 | 0 | 3 | 0 | 3 | 0 | 0 | 0 | 0 | N | 18183 | C-type lectin 11 [Crotalus horridus] |
| 286 | 4376 | JAA94903.1 | 62.63 | 5 | 0 | 0 | 0 | 5 | 0 | 5 | 0 | 0 | 0 | 0 |  |  |  | 1.7937E7 |  | 7.5976E7 |  |  |  |  | 1 | 1 | 0 | 0 | 0 | 3 | 0 | 3 | 0 | 0 | 0 | 0 | N | 18183 | C-type lectin 2b [Crotalus horridus] |
| 286 | 4377 | JAG43667.1 | 62.63 | 5 | 0 | 0 | 0 | 5 | 0 | 5 | 0 | 0 | 0 | 0 |  |  |  | 1.7937E7 |  | 7.5976E7 |  |  |  |  | 1 | 1 | 0 | 0 | 0 | 3 | 0 | 3 | 0 | 0 | 0 | 0 | N | 18183 | C-type lectin 11 [Crotalus horridus] |
| 286 | 4378 | JAG46149.1 | 62.63 | 5 | 0 | 0 | 0 | 5 | 0 | 5 | 0 | 0 | 0 | 0 |  |  |  | 1.7937E7 |  | 7.5976E7 |  |  |  |  | 1 | 1 | 0 | 0 | 0 | 3 | 0 | 3 | 0 | 0 | 0 | 0 | N | 18183 | C-type lectin 7 [Crotalus horridus] |
| 286 | 4379 | JAS04883.1 | 62.63 | 5 | 0 | 0 | 0 | 5 | 0 | 5 | 0 | 0 | 0 | 0 |  |  |  | 1.7937E7 |  | 7.5976E7 |  |  |  |  | 1 | 1 | 0 | 0 | 0 | 3 | 0 | 3 | 0 | 0 | 0 | 0 | N | 18183 | C-type lectin 7 [Crotalus horridus] |
| 286 | 4380 | JAA94904.1 | 62.63 | 5 | 0 | 0 | 0 | 5 | 0 | 5 | 0 | 0 | 0 | 0 |  |  |  | 1.7937E7 |  | 7.5976E7 |  |  |  |  | 1 | 1 | 0 | 0 | 0 | 3 | 0 | 3 | 0 | 0 | 0 | 0 | N | 18183 | C-type lectin 2a [Crotalus horridus] |
| 324 | 2409 | XP\_032091818.1 | 62.51 | 2 | 0 | 0 | 0 | 2 | 0 | 2 | 0 | 0 | 0 | 0 |  |  |  | 2.1836E6 |  | 2.8352E6 |  |  |  |  | 1 | 1 | 0 | 0 | 0 | 1 | 0 | 1 | 0 | 0 | 0 | 0 | N | 54697 | keratin, type I cytoskeletal 10-like [Thamnophis elegans] |
| 324 | 2413 | XP\_026541829.1 | 62.51 | 2 | 0 | 0 | 0 | 2 | 0 | 2 | 0 | 0 | 0 | 0 |  |  |  | 2.1836E6 |  | 2.8352E6 |  |  |  |  | 1 | 1 | 0 | 0 | 0 | 1 | 0 | 1 | 0 | 0 | 0 | 0 | N | 57476 | keratin, type I cytoskeletal 10-like [Notechis scutatus] |
| 324 | 2427 | XP\_032091819.1 | 62.51 | 2 | 0 | 0 | 0 | 2 | 0 | 2 | 0 | 0 | 0 | 0 |  |  |  | 2.1836E6 |  | 2.8352E6 |  |  |  |  | 1 | 1 | 0 | 0 | 0 | 1 | 0 | 1 | 0 | 0 | 0 | 0 | N | 50559 | keratin, type I cytoskeletal 10-like [Thamnophis elegans] |
| 324 | 2438 | XP\_013921793.1 | 62.51 | 2 | 0 | 0 | 0 | 2 | 0 | 2 | 0 | 0 | 0 | 0 |  |  |  | 2.1836E6 |  | 2.8352E6 |  |  |  |  | 1 | 1 | 0 | 0 | 0 | 1 | 0 | 1 | 0 | 0 | 0 | 0 | N | 63178 | PREDICTED: keratin, type I cytoskeletal 10-like [Thamnophis sirtalis] |
| 324 | 2514 | XP\_034263292.1 | 62.51 | 2 | 0 | 0 | 0 | 2 | 0 | 2 | 0 | 0 | 0 | 0 |  |  |  | 2.1836E6 |  | 2.8352E6 |  |  |  |  | 1 | 1 | 0 | 0 | 0 | 1 | 0 | 1 | 0 | 0 | 0 | 0 | N | 48789 | keratin, type I cytoskeletal 10-like [Pantherophis guttatus] |
| 324 | 3474 | LAB46984.1 | 62.51 | 3 | 0 | 0 | 0 | 3 | 0 | 3 | 0 | 0 | 0 | 0 |  |  |  | 2.1836E6 |  | 2.8352E6 |  |  |  |  | 1 | 1 | 0 | 0 | 0 | 1 | 0 | 1 | 0 | 0 | 0 | 0 | N | 33276 | hypothetical protein, partial [Micrurus surinamensis] |
| 324 | 3622 | XP\_025025771.1 | 62.51 | 2 | 0 | 0 | 0 | 2 | 0 | 2 | 0 | 0 | 0 | 0 |  |  |  | 2.1836E6 |  | 2.8352E6 |  |  |  |  | 1 | 1 | 0 | 0 | 0 | 1 | 0 | 1 | 0 | 0 | 0 | 0 | N | 52570 | keratin, type I cytoskeletal 17-like [Python bivittatus] |
| 324 | 4266 | LAA25601.1 | 62.51 | 5 | 0 | 0 | 0 | 5 | 0 | 5 | 0 | 0 | 0 | 0 |  |  |  | 2.1836E6 |  | 2.8352E6 |  |  |  |  | 1 | 1 | 0 | 0 | 0 | 1 | 0 | 1 | 0 | 0 | 0 | 0 | N | 16958 | hypothetical protein, partial [Micrurus lemniscatus carvalhoi] |
| 467 | 2014 | JAV50411.1 | 59.65 | 2 | 0 | 0 | 0 | 0 | 0 | 0 | 0 | 0 | 2 | 0 |  |  |  |  |  |  |  |  | 1.1072E5 |  | 1 | 1 | 0 | 0 | 0 | 0 | 0 | 0 | 0 | 0 | 1 | 0 | N | 57430 | glucosamine (N-acetyl)-6-sulfatase [Agkistrodon contortrix contortrix] |
| 220 | 2453 | JAS03131.1 | 59.06 | 4 | 0 | 4 | 0 | 0 | 0 | 0 | 0 | 0 | 0 | 0 |  | 4.5167E8 |  |  |  |  |  |  |  |  | 1 | 1 | 0 | 25 | 0 | 0 | 0 | 0 | 0 | 0 | 0 | 0 | Y | 28382 | Snake Venom Serine Proteinase [Phalotris mertensi] |
| 220 | 2571 | ABN72545.1 | 59.06 | 4 | 0 | 4 | 0 | 0 | 0 | 0 | 0 | 0 | 0 | 0 |  | 4.5167E8 |  |  |  |  |  |  |  |  | 1 | 1 | 0 | 25 | 0 | 0 | 0 | 0 | 0 | 0 | 0 | 0 | Y | 31010 | putative serine protease, partial [Bungarus multicinctus] |
| 220 | 2572 | A8QL57.1 | 59.06 | 4 | 0 | 4 | 0 | 0 | 0 | 0 | 0 | 0 | 0 | 0 |  | 4.5167E8 |  |  |  |  |  |  |  |  | 1 | 1 | 0 | 25 | 0 | 0 | 0 | 0 | 0 | 0 | 0 | 0 | Y | 31010 | RecName: Full=Snake venom serine protease BmSP; Short=SVSP; Flags: Precursor |
| 220 | 2581 | ABN72541.1 | 59.06 | 4 | 0 | 4 | 0 | 0 | 0 | 0 | 0 | 0 | 0 | 0 |  | 4.5167E8 |  |  |  |  |  |  |  |  | 1 | 1 | 0 | 25 | 0 | 0 | 0 | 0 | 0 | 0 | 0 | 0 | Y | 31137 | putative serine protease, partial [Naja atra] |
| 220 | 2582 | A8QL53.1 | 59.06 | 4 | 0 | 4 | 0 | 0 | 0 | 0 | 0 | 0 | 0 | 0 |  | 4.5167E8 |  |  |  |  |  |  |  |  | 1 | 1 | 0 | 25 | 0 | 0 | 0 | 0 | 0 | 0 | 0 | 0 | Y | 31137 | RecName: Full=Snake venom serine protease NaSP; Short=SVSP; Flags: Precursor |
| 516 | 2403 | E3P6N7.1 | 58.84 | 9 | 0 | 0 | 0 | 9 | 0 | 0 | 0 | 0 | 0 | 0 |  |  |  | 6.295E5 |  |  |  |  |  |  | 1 | 1 | 0 | 0 | 0 | 1 | 0 | 0 | 0 | 0 | 0 | 0 | N | 15886 | RecName: Full=Cystatin; Flags: Precursor |
| 516 | 2404 | ACR83843.1 | 58.84 | 9 | 0 | 0 | 0 | 9 | 0 | 0 | 0 | 0 | 0 | 0 |  |  |  | 6.295E5 |  |  |  |  |  |  | 1 | 1 | 0 | 0 | 0 | 1 | 0 | 0 | 0 | 0 | 0 | 0 | N | 15886 | cystatin precursor [Pseudechis porphyriacus] |
| 516 | 2446 | E3P6P3.1 | 58.84 | 9 | 0 | 0 | 0 | 9 | 0 | 0 | 0 | 0 | 0 | 0 |  |  |  | 6.295E5 |  |  |  |  |  |  | 1 | 1 | 0 | 0 | 0 | 1 | 0 | 0 | 0 | 0 | 0 | 0 | N | 15846 | RecName: Full=Cystatin; Flags: Precursor |
| 516 | 2447 | ACR83849.1 | 58.84 | 9 | 0 | 0 | 0 | 9 | 0 | 0 | 0 | 0 | 0 | 0 |  |  |  | 6.295E5 |  |  |  |  |  |  | 1 | 1 | 0 | 0 | 0 | 1 | 0 | 0 | 0 | 0 | 0 | 0 | N | 15846 | cystatin precursor [Micropechis ikaheca] |
| 516 | 2459 | ACR83844.1 | 58.84 | 9 | 0 | 0 | 0 | 9 | 0 | 0 | 0 | 0 | 0 | 0 |  |  |  | 6.295E5 |  |  |  |  |  |  | 1 | 1 | 0 | 0 | 0 | 1 | 0 | 0 | 0 | 0 | 0 | 0 | N | 15763 | cystatin precursor [Pseudechis australis] |
| 516 | 2460 | E3P6N8.1 | 58.84 | 9 | 0 | 0 | 0 | 9 | 0 | 0 | 0 | 0 | 0 | 0 |  |  |  | 6.295E5 |  |  |  |  |  |  | 1 | 1 | 0 | 0 | 0 | 1 | 0 | 0 | 0 | 0 | 0 | 0 | N | 15763 | RecName: Full=Cystatin; Flags: Precursor |
| 371 | 1475 | JAA97708.1 | 58.69 | 2 | 0 | 0 | 0 | 2 | 0 | 0 | 0 | 0 | 0 | 0 |  |  |  | 5.6349E4 |  |  |  |  |  |  | 1 | 1 | 0 | 0 | 0 | 1 | 0 | 0 | 0 | 0 | 0 | 0 | N | 59979 | ATP synthase subunit alpha, mitochondrial-like protein [Crotalus horridus] |
| 434 | 2193 | XP\_013918352.1 | 56.49 | 3 | 0 | 0 | 0 | 0 | 0 | 0 | 3 | 0 | 0 | 0 |  |  |  |  |  |  | 9.1107E4 |  |  |  | 1 | 1 | 0 | 0 | 0 | 0 | 0 | 0 | 1 | 0 | 0 | 0 | N | 35830 | PREDICTED: annexin A1 [Thamnophis sirtalis] |
| 434 | 2194 | XP\_032070448.1 | 56.49 | 3 | 0 | 0 | 0 | 0 | 0 | 0 | 3 | 0 | 0 | 0 |  |  |  |  |  |  | 9.1107E4 |  |  |  | 1 | 1 | 0 | 0 | 0 | 0 | 0 | 0 | 1 | 0 | 0 | 0 | N | 38296 | annexin A1 [Thamnophis elegans] |
| 512 | 2978 | XP\_007430670.1 | 54.85 | 3 | 0 | 0 | 0 | 0 | 0 | 0 | 0 | 3 | 0 | 0 |  |  |  |  |  |  |  | 7.3889E4 |  |  | 1 | 1 | 0 | 0 | 0 | 0 | 0 | 0 | 0 | 1 | 0 | 0 | N | 35266 | voltage-dependent anion-selective channel protein 1 [Python bivittatus] |
| 512 | 3453 | XP\_026543118.1 | 54.85 | 4 | 0 | 0 | 0 | 0 | 0 | 0 | 0 | 4 | 0 | 0 |  |  |  |  |  |  |  | 7.3889E4 |  |  | 1 | 1 | 0 | 0 | 0 | 0 | 0 | 0 | 0 | 1 | 0 | 0 | N | 30834 | voltage-dependent anion-selective channel protein 1 [Notechis scutatus] |
| 512 | 3454 | JAI09157.1 | 54.85 | 4 | 0 | 0 | 0 | 0 | 0 | 0 | 0 | 4 | 0 | 0 |  |  |  |  |  |  |  | 7.3889E4 |  |  | 1 | 1 | 0 | 0 | 0 | 0 | 0 | 0 | 0 | 1 | 0 | 0 | N | 30836 | Voltage-dependent anion-selective channel protein 1 -like protein [Micrurus fulvius] |
| 512 | 3455 | JAB52928.1 | 54.85 | 4 | 0 | 0 | 0 | 0 | 0 | 0 | 0 | 4 | 0 | 0 |  |  |  |  |  |  |  | 7.3889E4 |  |  | 1 | 1 | 0 | 0 | 0 | 0 | 0 | 0 | 0 | 1 | 0 | 0 | N | 30836 | voltage-dependent anion-selective channel protein [Micrurus fulvius] |
| 512 | 3456 | XP\_026552260.1 | 54.85 | 3 | 0 | 0 | 0 | 0 | 0 | 0 | 0 | 3 | 0 | 0 |  |  |  |  |  |  |  | 7.3889E4 |  |  | 1 | 1 | 0 | 0 | 0 | 0 | 0 | 0 | 0 | 1 | 0 | 0 | N | 35191 | voltage-dependent anion-selective channel protein 1 [Pseudonaja textilis] |
| 512 | 3457 | LAA28449.1 | 54.85 | 3 | 0 | 0 | 0 | 0 | 0 | 0 | 0 | 3 | 0 | 0 |  |  |  |  |  |  |  | 7.3889E4 |  |  | 1 | 1 | 0 | 0 | 0 | 0 | 0 | 0 | 0 | 1 | 0 | 0 | N | 39467 | hypothetical protein, partial [Micrurus lemniscatus carvalhoi] |
| 512 | 3935 | JAI10615.1 | 54.85 | 4 | 0 | 0 | 0 | 0 | 0 | 0 | 0 | 4 | 0 | 0 |  |  |  |  |  |  |  | 7.3889E4 |  |  | 1 | 1 | 0 | 0 | 0 | 0 | 0 | 0 | 0 | 1 | 0 | 0 | N | 30806 | Voltage-dependent anion-selective channel protein 1 -like protein [Crotalus adamanteus] |
| 512 | 3936 | JAG65932.1 | 54.85 | 4 | 0 | 0 | 0 | 0 | 0 | 0 | 0 | 4 | 0 | 0 |  |  |  |  |  |  |  | 7.3889E4 |  |  | 1 | 1 | 0 | 0 | 0 | 0 | 0 | 0 | 0 | 1 | 0 | 0 | N | 30864 | Voltage-dependent anion-selective channel protein 1 -like protein [Boiga irregularis] |
| 512 | 3937 | JAV48559.1 | 54.85 | 4 | 0 | 0 | 0 | 0 | 0 | 0 | 0 | 4 | 0 | 0 |  |  |  |  |  |  |  | 7.3889E4 |  |  | 1 | 1 | 0 | 0 | 0 | 0 | 0 | 0 | 0 | 1 | 0 | 0 | N | 30820 | Voltage-dependent anion-selective channel protein 1 -like protein [Agkistrodon contortrix contortrix] |
| 512 | 3938 | XP\_034260052.1 | 54.85 | 4 | 0 | 0 | 0 | 0 | 0 | 0 | 0 | 4 | 0 | 0 |  |  |  |  |  |  |  | 7.3889E4 |  |  | 1 | 1 | 0 | 0 | 0 | 0 | 0 | 0 | 0 | 1 | 0 | 0 | N | 30830 | voltage-dependent anion-selective channel protein 1 isoform X2 [Pantherophis guttatus] |
| 512 | 3939 | XP\_034260044.1 | 54.85 | 4 | 0 | 0 | 0 | 0 | 0 | 0 | 0 | 4 | 0 | 0 |  |  |  |  |  |  |  | 7.3889E4 |  |  | 1 | 1 | 0 | 0 | 0 | 0 | 0 | 0 | 0 | 1 | 0 | 0 | N | 30830 | voltage-dependent anion-selective channel protein 1 isoform X2 [Pantherophis guttatus] |
| 512 | 3940 | JAA95034.1 | 54.85 | 4 | 0 | 0 | 0 | 0 | 0 | 0 | 0 | 4 | 0 | 0 |  |  |  |  |  |  |  | 7.3889E4 |  |  | 1 | 1 | 0 | 0 | 0 | 0 | 0 | 0 | 0 | 1 | 0 | 0 | N | 30806 | Voltage-dependent anion-selective channel protein 1-like protein [Crotalus horridus] |
| 512 | 3941 | BAN82166.1 | 54.85 | 3 | 0 | 0 | 0 | 0 | 0 | 0 | 0 | 3 | 0 | 0 |  |  |  |  |  |  |  | 7.3889E4 |  |  | 1 | 1 | 0 | 0 | 0 | 0 | 0 | 0 | 0 | 1 | 0 | 0 | N | 35102 | hypothetical protein [Ovophis okinavensis] |
| 512 | 3942 | XP\_032066440.1 | 54.85 | 3 | 0 | 0 | 0 | 0 | 0 | 0 | 0 | 3 | 0 | 0 |  |  |  |  |  |  |  | 7.3889E4 |  |  | 1 | 1 | 0 | 0 | 0 | 0 | 0 | 0 | 0 | 1 | 0 | 0 | N | 34999 | voltage-dependent anion-selective channel protein 1 [Thamnophis elegans] |
| 512 | 3943 | XP\_015679656.1 | 54.85 | 3 | 0 | 0 | 0 | 0 | 0 | 0 | 0 | 3 | 0 | 0 |  |  |  |  |  |  |  | 7.3889E4 |  |  | 1 | 1 | 0 | 0 | 0 | 0 | 0 | 0 | 0 | 1 | 0 | 0 | N | 35116 | voltage-dependent anion-selective channel protein 1 [Protobothrops mucrosquamatus] |
| 512 | 3944 | XP\_034260037.1 | 54.85 | 3 | 0 | 0 | 0 | 0 | 0 | 0 | 0 | 3 | 0 | 0 |  |  |  |  |  |  |  | 7.3889E4 |  |  | 1 | 1 | 0 | 0 | 0 | 0 | 0 | 0 | 0 | 1 | 0 | 0 | N | 35065 | voltage-dependent anion-selective channel protein 1 isoform X1 [Pantherophis guttatus] |
| 512 | 3945 | XP\_013913912.1 | 54.85 | 3 | 0 | 0 | 0 | 0 | 0 | 0 | 0 | 3 | 0 | 0 |  |  |  |  |  |  |  | 7.3889E4 |  |  | 1 | 1 | 0 | 0 | 0 | 0 | 0 | 0 | 0 | 1 | 0 | 0 | N | 35013 | PREDICTED: voltage-dependent anion-selective channel protein 1 [Thamnophis sirtalis] |
| 514 | 1775 | XP\_026577138.1 | 53.19 | 3 | 3 | 0 | 0 | 0 | 0 | 0 | 0 | 0 | 0 | 0 | 3.9013E5 |  |  |  |  |  |  |  |  |  | 1 | 1 | 1 | 0 | 0 | 0 | 0 | 0 | 0 | 0 | 0 | 0 | N | 44958 | gastric intrinsic factor-like [Pseudonaja textilis] |
| 514 | 2693 | XP\_029140647.1 | 53.19 | 3 | 3 | 0 | 0 | 0 | 0 | 0 | 0 | 0 | 0 | 0 | 3.9013E5 |  |  |  |  |  |  |  |  |  | 1 | 1 | 1 | 0 | 0 | 0 | 0 | 0 | 0 | 0 | 0 | 0 | N | 45199 | cobalamin binding intrinsic factor-like [Protobothrops mucrosquamatus] |
| 486 | 2536 | XP\_026534386.1 | 48.58 | 1 | 0 | 1 | 0 | 0 | 0 | 0 | 0 | 0 | 0 | 0 |  | 5.3478E6 |  |  |  |  |  |  |  |  | 1 | 1 | 0 | 1 | 0 | 0 | 0 | 0 | 0 | 0 | 0 | 0 | N | 106048 | A disintegrin and metalloproteinase with thrombospondin motifs 15 isoform X2 [Notechis scutatus] |
| 486 | 2537 | XP\_026534385.1 | 48.58 | 1 | 0 | 1 | 0 | 0 | 0 | 0 | 0 | 0 | 0 | 0 |  | 5.3478E6 |  |  |  |  |  |  |  |  | 1 | 1 | 0 | 1 | 0 | 0 | 0 | 0 | 0 | 0 | 0 | 0 | N | 106245 | A disintegrin and metalloproteinase with thrombospondin motifs 15 isoform X1 [Notechis scutatus] |
| 486 | 2890 | XP\_015673523.1 | 48.58 | 1 | 0 | 1 | 0 | 0 | 0 | 0 | 0 | 0 | 0 | 0 |  | 5.3478E6 |  |  |  |  |  |  |  |  | 1 | 1 | 0 | 1 | 0 | 0 | 0 | 0 | 0 | 0 | 0 | 0 | N | 105960 | A disintegrin and metalloproteinase with thrombospondin motifs 15 [Protobothrops mucrosquamatus] |
| 486 | 3087 | XP\_026564825.1 | 48.58 | 1 | 0 | 1 | 0 | 0 | 0 | 0 | 0 | 0 | 0 | 0 |  | 5.3478E6 |  |  |  |  |  |  |  |  | 1 | 1 | 0 | 1 | 0 | 0 | 0 | 0 | 0 | 0 | 0 | 0 | N | 106234 | A disintegrin and metalloproteinase with thrombospondin motifs 15 isoform X2 [Pseudonaja textilis] |
| 486 | 3088 | XP\_026564824.1 | 48.58 | 1 | 0 | 1 | 0 | 0 | 0 | 0 | 0 | 0 | 0 | 0 |  | 5.3478E6 |  |  |  |  |  |  |  |  | 1 | 1 | 0 | 1 | 0 | 0 | 0 | 0 | 0 | 0 | 0 | 0 | N | 106430 | A disintegrin and metalloproteinase with thrombospondin motifs 15 isoform X1 [Pseudonaja textilis] |
| 486 | 3745 | XP\_034277870.1 | 48.58 | 1 | 0 | 1 | 0 | 0 | 0 | 0 | 0 | 0 | 0 | 0 |  | 5.3478E6 |  |  |  |  |  |  |  |  | 1 | 1 | 0 | 1 | 0 | 0 | 0 | 0 | 0 | 0 | 0 | 0 | N | 105888 | A disintegrin and metalloproteinase with thrombospondin motifs 15 [Pantherophis guttatus] |
| 486 | 4180 | XP\_007438168.1 | 48.58 | 1 | 0 | 1 | 0 | 0 | 0 | 0 | 0 | 0 | 0 | 0 |  | 5.3478E6 |  |  |  |  |  |  |  |  | 1 | 1 | 0 | 1 | 0 | 0 | 0 | 0 | 0 | 0 | 0 | 0 | N | 98239 | A disintegrin and metalloproteinase with thrombospondin motifs 15 [Python bivittatus] |
| 486 | 4314 | LAA53077.1 | 48.58 | 9 | 0 | 9 | 0 | 0 | 0 | 0 | 0 | 0 | 0 | 0 |  | 5.3478E6 |  |  |  |  |  |  |  |  | 1 | 1 | 0 | 1 | 0 | 0 | 0 | 0 | 0 | 0 | 0 | 0 | N | 12463 | hypothetical protein, partial [Micrurus corallinus] |
| 486 | 4316 | XP\_032084777.1 | 48.58 | 4 | 0 | 4 | 0 | 0 | 0 | 0 | 0 | 0 | 0 | 0 |  | 5.3478E6 |  |  |  |  |  |  |  |  | 1 | 1 | 0 | 1 | 0 | 0 | 0 | 0 | 0 | 0 | 0 | 0 | N | 31212 | A disintegrin and metalloproteinase with thrombospondin motifs 15-like [Thamnophis elegans] |
| 357 | 2681 | XP\_034292227.1 | 46.75 | 3 | 0 | 0 | 0 | 0 | 0 | 0 | 0 | 3 | 3 | 0 |  |  |  |  |  |  |  | 6.0544E4 | 1.1426E4 |  | 1 | 1 | 0 | 0 | 0 | 0 | 0 | 0 | 0 | 1 | 1 | 0 | N | 36018 | delta-aminolevulinic acid dehydratase [Pantherophis guttatus] |
| 357 | 2682 | XP\_015687137.1 | 46.75 | 3 | 0 | 0 | 0 | 0 | 0 | 0 | 0 | 3 | 3 | 0 |  |  |  |  |  |  |  | 6.0544E4 | 1.1426E4 |  | 1 | 1 | 0 | 0 | 0 | 0 | 0 | 0 | 0 | 1 | 1 | 0 | N | 35914 | delta-aminolevulinic acid dehydratase [Protobothrops mucrosquamatus] |
| 357 | 2683 | JAG68750.1 | 46.75 | 3 | 0 | 0 | 0 | 0 | 0 | 0 | 0 | 3 | 3 | 0 |  |  |  |  |  |  |  | 6.0544E4 | 1.1426E4 |  | 1 | 1 | 0 | 0 | 0 | 0 | 0 | 0 | 0 | 1 | 1 | 0 | N | 36017 | Delta-aminolevulinic acid dehydratase-like [Boiga irregularis] |
| 357 | 2900 | LAA91247.1 | 46.75 | 3 | 0 | 0 | 0 | 0 | 0 | 0 | 0 | 3 | 3 | 0 |  |  |  |  |  |  |  | 6.0544E4 | 1.1426E4 |  | 1 | 1 | 0 | 0 | 0 | 0 | 0 | 0 | 0 | 1 | 1 | 0 | N | 31197 | hypothetical protein, partial [Micrurus lemniscatus lemniscatus] |
| 357 | 3086 | XP\_007443339.1 | 46.75 | 2 | 0 | 0 | 0 | 0 | 0 | 0 | 0 | 2 | 2 | 0 |  |  |  |  |  |  |  | 6.0544E4 | 1.1426E4 |  | 1 | 1 | 0 | 0 | 0 | 0 | 0 | 0 | 0 | 1 | 1 | 0 | N | 47560 | delta-aminolevulinic acid dehydratase-like [Python bivittatus] |
| 357 | 3133 | JAV51283.1 | 46.75 | 3 | 0 | 0 | 0 | 0 | 0 | 0 | 0 | 3 | 3 | 0 |  |  |  |  |  |  |  | 6.0544E4 | 1.1426E4 |  | 1 | 1 | 0 | 0 | 0 | 0 | 0 | 0 | 0 | 1 | 1 | 0 | N | 36224 | aminolevulinate dehydratase [Agkistrodon contortrix contortrix] |
| 357 | 3588 | JAC96276.1 | 46.75 | 3 | 0 | 0 | 0 | 0 | 0 | 0 | 0 | 3 | 3 | 0 |  |  |  |  |  |  |  | 6.0544E4 | 1.1426E4 |  | 1 | 1 | 0 | 0 | 0 | 0 | 0 | 0 | 0 | 1 | 1 | 0 | N | 36107 | Delta-aminolevulinic acid dehydratase-like [Hypsiglena sp. JMG-2014] |
| 357 | 3671 | XP\_032088448.1 | 46.75 | 3 | 0 | 0 | 0 | 0 | 0 | 0 | 0 | 3 | 3 | 0 |  |  |  |  |  |  |  | 6.0544E4 | 1.1426E4 |  | 1 | 1 | 0 | 0 | 0 | 0 | 0 | 0 | 0 | 1 | 1 | 0 | N | 36154 | delta-aminolevulinic acid dehydratase [Thamnophis elegans] |
| 357 | 4285 | AFJ49864.1 | 46.75 | 3 | 0 | 0 | 0 | 0 | 0 | 0 | 0 | 3 | 3 | 0 |  |  |  |  |  |  |  | 6.0544E4 | 1.1426E4 |  | 1 | 1 | 0 | 0 | 0 | 0 | 0 | 0 | 0 | 1 | 1 | 0 | N | 36044 | Delta-aminolevulinic acid dehydratase-like [Crotalus adamanteus] |
| 357 | 4286 | XP\_026568189.1 | 46.75 | 3 | 0 | 0 | 0 | 0 | 0 | 0 | 0 | 3 | 3 | 0 |  |  |  |  |  |  |  | 6.0544E4 | 1.1426E4 |  | 1 | 1 | 0 | 0 | 0 | 0 | 0 | 0 | 0 | 1 | 1 | 0 | N | 36085 | delta-aminolevulinic acid dehydratase [Pseudonaja textilis] |
| 357 | 4287 | JAI12578.1 | 46.75 | 3 | 0 | 0 | 0 | 0 | 0 | 0 | 0 | 3 | 3 | 0 |  |  |  |  |  |  |  | 6.0544E4 | 1.1426E4 |  | 1 | 1 | 0 | 0 | 0 | 0 | 0 | 0 | 0 | 1 | 1 | 0 | N | 36044 | MGC84775 protein [Crotalus adamanteus] |
| 357 | 4288 | XP\_026542345.1 | 46.75 | 3 | 0 | 0 | 0 | 0 | 0 | 0 | 0 | 3 | 3 | 0 |  |  |  |  |  |  |  | 6.0544E4 | 1.1426E4 |  | 1 | 1 | 0 | 0 | 0 | 0 | 0 | 0 | 0 | 1 | 1 | 0 | N | 35929 | delta-aminolevulinic acid dehydratase [Notechis scutatus] |
| 373 | 2375 | XP\_034287864.1 | 46.22 | 1 | 0 | 0 | 0 | 0 | 0 | 0 | 0 | 0 | 1 | 0 |  |  |  |  |  |  |  |  | 5.6673E5 |  | 1 | 1 | 0 | 0 | 0 | 0 | 0 | 0 | 0 | 0 | 1 | 0 | N | 91779 | collagen alpha-1(IX) chain [Pantherophis guttatus] |
| 325 | 2326 | P0C2D7.1 | 45.16 | 11 | 0 | 0 | 0 | 0 | 0 | 0 | 0 | 0 | 0 | 11 |  |  |  |  |  |  |  |  |  | 8.4843E7 | 1 | 1 | 0 | 0 | 0 | 0 | 0 | 0 | 0 | 0 | 0 | 1 | N | 10295 | RecName: Full=L-amino-acid oxidase; Short=LAAO; Short=LAO |
| 354 | 4398 | ACC85814.1 | 44.88 | 4 | 0 | 0 | 0 | 0 | 0 | 4 | 0 | 0 | 0 | 0 |  |  |  |  |  | 9.0689E6 |  |  |  |  | 1 | 1 | 0 | 0 | 0 | 0 | 0 | 1 | 0 | 0 | 0 | 0 | N | 21685 | nerve growth factor beta polypeptide, partial [Diadophis punctatus] |
| 354 | 4420 | JAG46130.1 | 44.88 | 3 | 0 | 0 | 0 | 0 | 0 | 3 | 0 | 0 | 0 | 0 |  |  |  |  |  | 9.0689E6 |  |  |  |  | 1 | 1 | 0 | 0 | 0 | 0 | 0 | 1 | 0 | 0 | 0 | 0 | N | 27141 | Venom nerve growth factor [Crotalus horridus] |
| 468 | 2135 | XP\_015686279.2 | 43.78 | 4 | 0 | 0 | 4 | 0 | 0 | 0 | 0 | 0 | 0 | 0 |  |  | 1.2301E5 |  |  |  |  |  |  |  | 1 | 1 | 0 | 0 | 1 | 0 | 0 | 0 | 0 | 0 | 0 | 0 | N | 31521 | palmitoyl-protein thioesterase 1 [Protobothrops mucrosquamatus] |
| 468 | 2268 | XP\_026536429.1 | 43.78 | 3 | 0 | 0 | 3 | 0 | 0 | 0 | 0 | 0 | 0 | 0 |  |  | 1.2301E5 |  |  |  |  |  |  |  | 1 | 1 | 0 | 0 | 1 | 0 | 0 | 0 | 0 | 0 | 0 | 0 | N | 34292 | palmitoyl-protein thioesterase 1 [Notechis scutatus] |
| 468 | 2276 | XP\_026558426.1 | 43.78 | 3 | 0 | 0 | 3 | 0 | 0 | 0 | 0 | 0 | 0 | 0 |  |  | 1.2301E5 |  |  |  |  |  |  |  | 1 | 1 | 0 | 0 | 1 | 0 | 0 | 0 | 0 | 0 | 0 | 0 | N | 34281 | palmitoyl-protein thioesterase 1 [Pseudonaja textilis] |
| 468 | 2420 | XP\_034269326.1 | 43.78 | 3 | 0 | 0 | 3 | 0 | 0 | 0 | 0 | 0 | 0 | 0 |  |  | 1.2301E5 |  |  |  |  |  |  |  | 1 | 1 | 0 | 0 | 1 | 0 | 0 | 0 | 0 | 0 | 0 | 0 | N | 35574 | palmitoyl-protein thioesterase 1 isoform X1 [Pantherophis guttatus] |
| 468 | 2421 | XP\_034269327.1 | 43.78 | 4 | 0 | 0 | 4 | 0 | 0 | 0 | 0 | 0 | 0 | 0 |  |  | 1.2301E5 |  |  |  |  |  |  |  | 1 | 1 | 0 | 0 | 1 | 0 | 0 | 0 | 0 | 0 | 0 | 0 | N | 30735 | palmitoyl-protein thioesterase 1 isoform X2 [Pantherophis guttatus] |
| 468 | 2467 | XP\_032084406.1 | 43.78 | 3 | 0 | 0 | 3 | 0 | 0 | 0 | 0 | 0 | 0 | 0 |  |  | 1.2301E5 |  |  |  |  |  |  |  | 1 | 1 | 0 | 0 | 1 | 0 | 0 | 0 | 0 | 0 | 0 | 0 | N | 35338 | palmitoyl-protein thioesterase 1 [Thamnophis elegans] |
| 468 | 2475 | XP\_013915921.1 | 43.78 | 4 | 0 | 0 | 4 | 0 | 0 | 0 | 0 | 0 | 0 | 0 |  |  | 1.2301E5 |  |  |  |  |  |  |  | 1 | 1 | 0 | 0 | 1 | 0 | 0 | 0 | 0 | 0 | 0 | 0 | N | 31606 | PREDICTED: palmitoyl-protein thioesterase 1 isoform X2 [Thamnophis sirtalis] |
| 468 | 2547 | XP\_007430564.1 | 43.78 | 3 | 0 | 0 | 3 | 0 | 0 | 0 | 0 | 0 | 0 | 0 |  |  | 1.2301E5 |  |  |  |  |  |  |  | 1 | 1 | 0 | 0 | 1 | 0 | 0 | 0 | 0 | 0 | 0 | 0 | N | 34760 | palmitoyl-protein thioesterase 1 [Python bivittatus] |
| 326 | 4492 | XP\_026556752.1 | 42.85 | 2 | 0 | 0 | 0 | 0 | 0 | 2 | 0 | 0 | 0 | 0 |  |  |  |  |  | 1.3731E6 |  |  |  |  | 1 | 1 | 0 | 0 | 0 | 0 | 0 | 1 | 0 | 0 | 0 | 0 | N | 48527 | transcriptional adapter 2-beta [Pseudonaja textilis] |
| 532 | 2972 | XP\_034291152.1 | 42.76 | 2 | 0 | 0 | 0 | 0 | 0 | 0 | 0 | 2 | 0 | 0 |  |  |  |  |  |  |  | 0 |  |  | 1 | 1 | 0 | 0 | 0 | 0 | 0 | 0 | 0 | 1 | 0 | 0 | N | 41114 | annexin A7 isoform X4 [Pantherophis guttatus] |
| 532 | 2974 | XP\_034291151.1 | 42.76 | 2 | 0 | 0 | 0 | 0 | 0 | 0 | 0 | 2 | 0 | 0 |  |  |  |  |  |  |  | 0 |  |  | 1 | 1 | 0 | 0 | 0 | 0 | 0 | 0 | 0 | 1 | 0 | 0 | N | 49708 | annexin A7 isoform X3 [Pantherophis guttatus] |
| 532 | 2975 | XP\_034291150.1 | 42.76 | 2 | 0 | 0 | 0 | 0 | 0 | 0 | 0 | 2 | 0 | 0 |  |  |  |  |  |  |  | 0 |  |  | 1 | 1 | 0 | 0 | 0 | 0 | 0 | 0 | 0 | 1 | 0 | 0 | N | 49708 | annexin A7 isoform X3 [Pantherophis guttatus] |
| 532 | 2976 | XP\_034291149.1 | 42.76 | 2 | 0 | 0 | 0 | 0 | 0 | 0 | 0 | 2 | 0 | 0 |  |  |  |  |  |  |  | 0 |  |  | 1 | 1 | 0 | 0 | 0 | 0 | 0 | 0 | 0 | 1 | 0 | 0 | N | 51451 | annexin A7 isoform X2 [Pantherophis guttatus] |
| 532 | 2977 | XP\_034291148.1 | 42.76 | 2 | 0 | 0 | 0 | 0 | 0 | 0 | 0 | 2 | 0 | 0 |  |  |  |  |  |  |  | 0 |  |  | 1 | 1 | 0 | 0 | 0 | 0 | 0 | 0 | 0 | 1 | 0 | 0 | N | 51897 | annexin A7 isoform X1 [Pantherophis guttatus] |
| 376 | 4187 | XP\_013914127.1 | 42.34 | 5 | 0 | 0 | 0 | 0 | 0 | 5 | 0 | 5 | 0 | 0 |  |  |  |  |  | 1.3494E5 |  | 2.5834E4 |  |  | 1 | 1 | 0 | 0 | 0 | 0 | 0 | 1 | 0 | 1 | 0 | 0 | N | 23326 | PREDICTED: heat shock protein beta-1 [Thamnophis sirtalis] |
| 376 | 4188 | XP\_032072257.1 | 42.34 | 5 | 0 | 0 | 0 | 0 | 0 | 5 | 0 | 5 | 0 | 0 |  |  |  |  |  | 1.3494E5 |  | 2.5834E4 |  |  | 1 | 1 | 0 | 0 | 0 | 0 | 0 | 1 | 0 | 1 | 0 | 0 | N | 23326 | heat shock protein beta-1 [Thamnophis elegans] |
| 376 | 4189 | XP\_015679331.1 | 42.34 | 5 | 0 | 0 | 0 | 0 | 0 | 5 | 0 | 5 | 0 | 0 |  |  |  |  |  | 1.3494E5 |  | 2.5834E4 |  |  | 1 | 1 | 0 | 0 | 0 | 0 | 0 | 1 | 0 | 1 | 0 | 0 | N | 23517 | heat shock protein beta-1 [Protobothrops mucrosquamatus] |
| 349 | 1821 | XP\_015685588.1 | 42.21 | 9 | 0 | 0 | 0 | 0 | 0 | 0 | 0 | 9 | 0 | 0 |  |  |  |  |  |  |  | 1.3789E4 |  |  | 1 | 1 | 0 | 0 | 0 | 0 | 0 | 0 | 0 | 1 | 0 | 0 | N | 18348 | snaclec jerdonibitin subunit alpha [Protobothrops mucrosquamatus] |
| 349 | 2156 | BAP39929.1 | 42.21 | 8 | 0 | 0 | 0 | 0 | 0 | 0 | 0 | 8 | 0 | 0 |  |  |  |  |  |  |  | 1.3789E4 |  |  | 1 | 1 | 0 | 0 | 0 | 0 | 0 | 0 | 0 | 1 | 0 | 0 | N | 21332 | C-type lectin B subunit, partial [Protobothrops elegans] |
| 380 | 2780 | ETE73522.1 | 41.53 | 5 | 0 | 0 | 0 | 5 | 0 | 0 | 0 | 0 | 0 | 0 |  |  |  | 2.1242E5 |  |  |  |  |  |  | 1 | 1 | 0 | 0 | 0 | 1 | 0 | 0 | 0 | 0 | 0 | 0 | N | 27615 | Proteasome subunit alpha type-3 [Ophiophagus hannah] |
| 380 | 2781 | XP\_013912175.1 | 41.53 | 5 | 0 | 0 | 0 | 5 | 0 | 0 | 0 | 0 | 0 | 0 |  |  |  | 2.1242E5 |  |  |  |  |  |  | 1 | 1 | 0 | 0 | 0 | 1 | 0 | 0 | 0 | 0 | 0 | 0 | N | 28424 | PREDICTED: proteasome subunit alpha type-3 [Thamnophis sirtalis] |
| 380 | 2782 | JAG66919.1 | 41.53 | 5 | 0 | 0 | 0 | 5 | 0 | 0 | 0 | 0 | 0 | 0 |  |  |  | 2.1242E5 |  |  |  |  |  |  | 1 | 1 | 0 | 0 | 0 | 1 | 0 | 0 | 0 | 0 | 0 | 0 | N | 28410 | Proteasome subunit alpha type-3-like protein [Boiga irregularis] |
| 380 | 2783 | XP\_032092012.1 | 41.53 | 5 | 0 | 0 | 0 | 5 | 0 | 0 | 0 | 0 | 0 | 0 |  |  |  | 2.1242E5 |  |  |  |  |  |  | 1 | 1 | 0 | 0 | 0 | 1 | 0 | 0 | 0 | 0 | 0 | 0 | N | 28424 | proteasome subunit alpha type-3 [Thamnophis elegans] |
| 380 | 2784 | XP\_034289368.1 | 41.53 | 5 | 0 | 0 | 0 | 5 | 0 | 0 | 0 | 0 | 0 | 0 |  |  |  | 2.1242E5 |  |  |  |  |  |  | 1 | 1 | 0 | 0 | 0 | 1 | 0 | 0 | 0 | 0 | 0 | 0 | N | 28352 | proteasome subunit alpha type-3 [Pantherophis guttatus] |
| 380 | 2785 | XP\_026554461.1 | 41.53 | 5 | 0 | 0 | 0 | 5 | 0 | 0 | 0 | 0 | 0 | 0 |  |  |  | 2.1242E5 |  |  |  |  |  |  | 1 | 1 | 0 | 0 | 0 | 1 | 0 | 0 | 0 | 0 | 0 | 0 | N | 28410 | proteasome subunit alpha type-3 [Pseudonaja textilis] |
| 380 | 2786 | JAI09619.1 | 41.53 | 5 | 0 | 0 | 0 | 5 | 0 | 0 | 0 | 0 | 0 | 0 |  |  |  | 2.1242E5 |  |  |  |  |  |  | 1 | 1 | 0 | 0 | 0 | 1 | 0 | 0 | 0 | 0 | 0 | 0 | N | 28410 | Proteasome subunit alpha type-3-like protein [Micrurus fulvius] |
| 380 | 2787 | XP\_007422519.1 | 41.53 | 5 | 0 | 0 | 0 | 5 | 0 | 0 | 0 | 0 | 0 | 0 |  |  |  | 2.1242E5 |  |  |  |  |  |  | 1 | 1 | 0 | 0 | 0 | 1 | 0 | 0 | 0 | 0 | 0 | 0 | N | 28410 | proteasome subunit alpha type-3 [Python bivittatus] |
| 380 | 2788 | XP\_026525981.1 | 41.53 | 5 | 0 | 0 | 0 | 5 | 0 | 0 | 0 | 0 | 0 | 0 |  |  |  | 2.1242E5 |  |  |  |  |  |  | 1 | 1 | 0 | 0 | 0 | 1 | 0 | 0 | 0 | 0 | 0 | 0 | N | 28410 | proteasome subunit alpha type-3 [Notechis scutatus] |
| 380 | 2789 | JAG66904.1 | 41.53 | 5 | 0 | 0 | 0 | 5 | 0 | 0 | 0 | 0 | 0 | 0 |  |  |  | 2.1242E5 |  |  |  |  |  |  | 1 | 1 | 0 | 0 | 0 | 1 | 0 | 0 | 0 | 0 | 0 | 0 | N | 28410 | Proteasome subunit alpha type-3-like [Boiga irregularis] |
| 380 | 2790 | JAB53606.1 | 41.53 | 4 | 0 | 0 | 0 | 4 | 0 | 0 | 0 | 0 | 0 | 0 |  |  |  | 2.1242E5 |  |  |  |  |  |  | 1 | 1 | 0 | 0 | 0 | 1 | 0 | 0 | 0 | 0 | 0 | 0 | N | 30605 | Proteasome subunit alpha type-3 [Micrurus fulvius] |
| 380 | 4385 | JAA96035.1 | 41.53 | 5 | 0 | 0 | 0 | 5 | 0 | 0 | 0 | 0 | 0 | 0 |  |  |  | 2.1242E5 |  |  |  |  |  |  | 1 | 1 | 0 | 0 | 0 | 1 | 0 | 0 | 0 | 0 | 0 | 0 | N | 28356 | Proteasome subunit alpha type-3-like protein [Crotalus horridus] |
| 380 | 4386 | XP\_015676739.1 | 41.53 | 5 | 0 | 0 | 0 | 5 | 0 | 0 | 0 | 0 | 0 | 0 |  |  |  | 2.1242E5 |  |  |  |  |  |  | 1 | 1 | 0 | 0 | 0 | 1 | 0 | 0 | 0 | 0 | 0 | 0 | N | 28383 | proteasome subunit alpha type-3 [Protobothrops mucrosquamatus] |
| 380 | 4387 | JAV49555.1 | 41.53 | 5 | 0 | 0 | 0 | 5 | 0 | 0 | 0 | 0 | 0 | 0 |  |  |  | 2.1242E5 |  |  |  |  |  |  | 1 | 1 | 0 | 0 | 0 | 1 | 0 | 0 | 0 | 0 | 0 | 0 | N | 28356 | Proteasome subunit alpha type-3-like protein [Agkistrodon contortrix contortrix] |
| 380 | 4388 | JAI11930.1 | 41.53 | 5 | 0 | 0 | 0 | 5 | 0 | 0 | 0 | 0 | 0 | 0 |  |  |  | 2.1242E5 |  |  |  |  |  |  | 1 | 1 | 0 | 0 | 0 | 1 | 0 | 0 | 0 | 0 | 0 | 0 | N | 28356 | Proteasome subunit alpha type-3-like protein [Crotalus adamanteus] |
| 380 | 4389 | AFJ51036.1 | 41.53 | 4 | 0 | 0 | 0 | 4 | 0 | 0 | 0 | 0 | 0 | 0 |  |  |  | 2.1242E5 |  |  |  |  |  |  | 1 | 1 | 0 | 0 | 0 | 1 | 0 | 0 | 0 | 0 | 0 | 0 | N | 30581 | Proteasome subunit alpha type-3-like [Crotalus adamanteus] |
| total 484 proteins |  |  |  |  |  |  |  |  |  |  |  |  |  |  |  |  |  |  |  |  |  |  |  |  |  |  |  |  |  |  |  |  |  |  |  |  |  |  |  |
| --- | --- | --- | --- | --- | --- | --- | --- | --- | --- | --- | --- | --- | --- | --- | --- | --- | --- | --- | --- | --- | --- | --- | --- | --- | --- | --- | --- | --- | --- | --- | --- | --- | --- | --- | --- | --- | --- | --- | --- |

  

BAN89426.1

back to list

  

| Protein Coverage
| Supporting Peptides
|

Protein Coverage:

Supporting Peptides:

| Peptide | Uniq | -10lgP | Mass | Length | ppm | m/z | z | RT | Fraction | Scan | Source File | Area F4 | Area F5 | Area F6 | Area F10 | Area F8 | Area F9 | Area F7 | Area F1 | Area F2 | Area F3 | #Feature | #Feature F4 | #Feature F5 | #Feature F6 | #Feature F10 | #Feature F8 | #Feature F9 | #Feature F7 | #Feature F1 | #Feature F2 | #Feature F3 | Start | End | PTM | AScore | Found By |
| --- | --- | --- | --- | --- | --- | --- | --- | --- | --- | --- | --- | --- | --- | --- | --- | --- | --- | --- | --- | --- | --- | --- | --- | --- | --- | --- | --- | --- | --- | --- | --- | --- | --- | --- | --- | --- | --- |
| R.IDKVNLMVDQQWMAVR.D | N | 89.89 | 1944.9913 | 16 | 0.6 | 649.3381 | 3 | 49.05 | 8 | F8:27583 | 31122021\_RID\_3262\_1a.raw |  |  |  |  |  |  |  | 1.2194E7 | 3.5597E5 |  | 3 | 0 | 0 | 0 | 0 | 0 | 0 | 0 | 2 | 1 | 0 | 432 | 447 |  |  | PEAKS DB |
| K.YISAYSQDILM(+15.99)PLWSSYTIYR.S | N | 86.70 | 2585.2512 | 21 | 1.0 | 862.7585 | 3 | 67.22 | 8 | F8:41605 | 31122021\_RID\_3262\_1a.raw |  |  |  |  |  |  |  | 4.0338E6 |  |  | 3 | 0 | 0 | 0 | 0 | 0 | 0 | 0 | 3 | 0 | 0 | 603 | 623 | Oxidation (M) | M11:Oxidation (M):1000.00 | PEAKS DB |
| N.VDFFMYEGPAPR.I | N | 84.54 | 1427.6543 | 12 | 0.4 | 714.8347 | 2 | 52.03 | 8 | F8:29943 | 31122021\_RID\_3262\_1a.raw | 3.8891E5 |  |  |  |  |  |  | 3.2833E7 | 1.1005E6 | 4.0735E5 | 4 | 1 | 0 | 0 | 0 | 0 | 0 | 0 | 1 | 1 | 1 | 370 | 381 |  |  | PEAKS DB |
| K.YISAYSQDILMPLWSSYTIYR.S | N | 83.99 | 2569.2563 | 21 | 0.7 | 857.4267 | 3 | 71.20 | 8 | F8:44628 | 31122021\_RID\_3262\_1a.raw |  |  |  |  |  |  |  | 7.9234E6 |  |  | 2 | 0 | 0 | 0 | 0 | 0 | 0 | 0 | 2 | 0 | 0 | 603 | 623 |  |  | PEAKS DB |
| K.VNLMVDQQWMAVR.D | N | 80.06 | 1588.7854 | 13 | 0.8 | 795.4006 | 2 | 52.32 | 8 | F8:30174 | 31122021\_RID\_3262\_1a.raw |  |  |  |  |  |  |  | 3.4969E6 |  |  | 1 | 0 | 0 | 0 | 0 | 0 | 0 | 0 | 1 | 0 | 0 | 435 | 447 |  |  | PEAKS DB |
| R.DVELLTGLNFYSGLK.Q | N | 79.71 | 1667.8770 | 15 | 0.4 | 834.9461 | 2 | 67.85 | 8 | F8:42145 | 31122021\_RID\_3262\_1a.raw |  |  |  |  |  |  |  | 1.5005E7 | 4.8402E5 |  | 5 | 0 | 0 | 0 | 0 | 0 | 0 | 0 | 4 | 1 | 0 | 813 | 827 |  |  | PEAKS DB |
| K.DFYTFDSEGIVK.N | N | 78.54 | 1419.6558 | 12 | 0.4 | 710.8354 | 2 | 52.61 | 8 | F8:30396 | 31122021\_RID\_3262\_1a.raw | 3.7096E5 |  |  |  |  |  |  | 2.8875E7 | 1.731E6 |  | 5 | 1 | 0 | 0 | 0 | 0 | 0 | 0 | 3 | 1 | 0 | 390 | 401 |  |  | PEAKS DB |
| R.NPAWWGGQPIWHTATYQGLK.A | N | 77.54 | 2310.1333 | 20 | 1.0 | 771.0525 | 3 | 50.58 | 8 | F8:28794 | 31122021\_RID\_3262\_1a.raw |  |  |  |  |  |  |  | 2.7775E7 |  |  | 2 | 0 | 0 | 0 | 0 | 0 | 0 | 0 | 2 | 0 | 0 | 223 | 242 |  |  | PEAKS DB |
| K.NVPKDFYTFDSEGIVK.N | N | 76.77 | 1857.9148 | 16 | 0.6 | 929.9653 | 2 | 44.01 | 8 | F8:23615 | 31122021\_RID\_3262\_1a.raw |  |  |  |  |  |  |  | 1.8075E7 | 7.4834E5 |  | 3 | 0 | 0 | 0 | 0 | 0 | 0 | 0 | 2 | 1 | 0 | 386 | 401 |  |  | PEAKS DB |
| Y.SQDILMPLWSSYTIYR.S | N | 74.09 | 1971.9764 | 16 | 0.5 | 986.9960 | 2 | 65.69 | 8 | F8:40533 | 31122021\_RID\_3262\_1a.raw |  |  |  |  |  |  |  | 2.1206E6 |  |  | 2 | 0 | 0 | 0 | 0 | 0 | 0 | 0 | 2 | 0 | 0 | 608 | 623 |  |  | PEAKS DB |
| R.IDKVNLM(+15.99)VDQQWM(+15.99)AVR.D | N | 73.40 | 1976.9812 | 16 | 0.5 | 660.0013 | 3 | 40.77 | 8 | F8:21007 | 31122021\_RID\_3262\_1a.raw |  |  |  |  |  |  |  | 1.5146E6 |  |  | 1 | 0 | 0 | 0 | 0 | 0 | 0 | 0 | 1 | 0 | 0 | 432 | 447 | Oxidation (M) | M7:Oxidation (M):1000.00;M13:Oxidation (M):1000.00 | PEAKS DB |
| R.LWNYFHTTLIPK.Y | N | 73.37 | 1531.8187 | 12 | 0.5 | 766.9170 | 2 | 47.15 | 8 | F8:26107 | 31122021\_RID\_3262\_1a.raw | 8.3337E5 |  |  |  |  |  |  | 5.0587E7 |  |  | 3 | 1 | 0 | 0 | 0 | 0 | 0 | 0 | 2 | 0 | 0 | 696 | 707 |  |  | PEAKS DB |
| K.RPDFYTLYIEEPDTTGHK.Y | N | 73.22 | 2181.0378 | 18 | 0.2 | 1091.5264 | 2 | 41.13 | 8 | F8:21378 | 31122021\_RID\_3262\_1a.raw | 2.7863E6 |  |  |  |  |  |  | 9.8043E7 | 4.47E6 |  | 7 | 1 | 0 | 0 | 0 | 0 | 0 | 0 | 4 | 2 | 0 | 290 | 307 |  |  | PEAKS DB |
| K.VNLM(+15.99)VDQQWMAVR.D | N | 70.81 | 1604.7803 | 13 | 0.4 | 803.3977 | 2 | 48.28 | 8 | F8:27028 | 31122021\_RID\_3262\_1a.raw |  |  |  |  |  |  |  | 2.7138E6 | 8.2065E4 |  | 2 | 0 | 0 | 0 | 0 | 0 | 0 | 0 | 1 | 1 | 0 | 435 | 447 | Oxidation (M) | M4:Oxidation (M):176.18 | PEAKS DB |
| K.NPFYTPSPAK.E | N | 68.72 | 1120.5553 | 10 | -0.2 | 561.2848 | 2 | 31.38 | 8 | F8:13580 | 31122021\_RID\_3262\_1a.raw | 2.0471E6 |  |  |  |  |  |  | 3.8456E7 | 4.8509E6 |  | 4 | 1 | 0 | 0 | 0 | 0 | 0 | 0 | 2 | 1 | 0 | 521 | 530 |  |  | PEAKS DB |
| R.VRDVELLTGLNFYSGLK.Q | N | 68.06 | 1923.0465 | 17 | 0.9 | 642.0234 | 3 | 54.52 | 8 | F8:31944 | 31122021\_RID\_3262\_1a.raw | 5.1091E5 |  |  |  |  |  |  | 2.4463E7 | 3.6342E5 |  | 5 | 1 | 0 | 0 | 0 | 0 | 0 | 0 | 3 | 1 | 0 | 811 | 827 |  |  | PEAKS DB |
| K.YGPVSGEIIK.A | N | 66.88 | 1061.5757 | 10 | -0.3 | 531.7950 | 2 | 34.11 | 8 | F8:15575 | 31122021\_RID\_3262\_1a.raw | 5.5409E6 | 5.2937E5 |  |  |  |  |  | 8.5643E7 | 1.0829E7 |  | 4 | 1 | 1 | 0 | 0 | 0 | 0 | 0 | 1 | 1 | 0 | 308 | 317 |  |  | PEAKS DB |
| N.VDFFM(+15.99)YEGPAPR.I | N | 66.39 | 1443.6493 | 12 | 0.5 | 722.8323 | 2 | 48.28 | 8 | F8:26949 | 31122021\_RID\_3262\_1a.raw |  |  |  |  |  |  |  | 1.8808E7 | 1.7049E5 |  | 5 | 0 | 0 | 0 | 0 | 0 | 0 | 0 | 4 | 1 | 0 | 370 | 381 | Oxidation (M) | M5:Oxidation (M):1000.00 | PEAKS DB |
| K.AATYFWPGSEVK.I | N | 65.68 | 1354.6558 | 12 | 0.6 | 678.3356 | 2 | 44.70 | 8 | F8:24117 | 31122021\_RID\_3262\_1a.raw | 4.0357E6 |  |  |  |  |  |  | 5.8345E7 | 2.2909E6 |  | 3 | 1 | 0 | 0 | 0 | 0 | 0 | 0 | 1 | 1 | 0 | 243 | 254 |  |  | PEAKS DB |
| R.IDKVNLM(+15.99)VDQQWMAVR.D | N | 65.60 | 1960.9863 | 16 | 0.2 | 654.6695 | 3 | 45.45 | 8 | F8:24763 | 31122021\_RID\_3262\_1a.raw |  |  |  |  |  |  |  | 1.034E7 | 1.8006E5 |  | 5 | 0 | 0 | 0 | 0 | 0 | 0 | 0 | 4 | 1 | 0 | 432 | 447 | Oxidation (M) | M7:Oxidation (M):156.85 | PEAKS DB |
| R.TLGMLMEGLK.Q | N | 65.55 | 1091.5719 | 10 | 0.0 | 546.7932 | 2 | 47.83 | 8 | F8:26797 | 31122021\_RID\_3262\_1a.raw |  |  |  |  |  |  |  | 2.0277E7 | 9.0693E5 |  | 2 | 0 | 0 | 0 | 0 | 0 | 0 | 0 | 1 | 1 | 0 | 325 | 334 |  |  | PEAKS DB |
| R.TLGM(+15.99)LMEGLK.Q | N | 65.37 | 1107.5668 | 10 | 0.5 | 554.7910 | 2 | 43.66 | 8 | F8:23669 | 31122021\_RID\_3262\_1a.raw |  |  |  |  |  |  |  | 1.1063E8 | 4.1807E6 |  | 5 | 0 | 0 | 0 | 0 | 0 | 0 | 0 | 3 | 2 | 0 | 325 | 334 | Oxidation (M) | M4:Oxidation (M):67.96 | PEAKS DB |
| K.TFLPIFVNPVN | N | 64.77 | 1259.6914 | 11 | 0.6 | 630.8533 | 2 | 72.29 | 8 | F8:45470 | 31122021\_RID\_3262\_1a.raw | 3.3682E6 |  |  |  |  |  |  | 3.0009E7 | 1.5173E6 |  | 3 | 1 | 0 | 0 | 0 | 0 | 0 | 0 | 1 | 1 | 0 | 838 | 848 |  |  | PEAKS DB |
| R.TLGMLM(+15.99)EGLK.Q | N | 58.46 | 1107.5668 | 10 | 0.5 | 554.7910 | 2 | 44.17 | 8 | F8:26515 | 31122021\_RID\_3262\_1a.raw |  |  |  |  |  |  |  | 4.7543E7 |  |  | 2 | 0 | 0 | 0 | 0 | 0 | 0 | 0 | 2 | 0 | 0 | 325 | 334 | Oxidation (M) | M6:Oxidation (M):67.96 | PEAKS DB |
| R.TLGM(+15.99)LM(+15.99)EGLK.Q | N | 53.04 | 1123.5618 | 10 | -0.5 | 562.7879 | 2 | 44.08 | 8 | F8:23974 | 31122021\_RID\_3262\_1a.raw |  |  |  |  |  |  |  | 2.6054E7 | 1.1517E6 |  | 12 | 0 | 0 | 0 | 0 | 0 | 0 | 0 | 10 | 2 | 0 | 325 | 334 | Oxidation (M) | M4:Oxidation (M):1000.00;M6:Oxidation (M):1000.00 | PEAKS DB |
| K.QPLPETLQLK.T | N | 52.54 | 1165.6707 | 10 | 1.0 | 583.8432 | 2 | 39.62 | 8 | F8:20222 | 31122021\_RID\_3262\_1a.raw | 2.6906E6 |  |  |  |  |  |  | 4.8465E7 | 1.0101E6 |  | 3 | 1 | 0 | 0 | 0 | 0 | 0 | 0 | 1 | 1 | 0 | 828 | 837 |  |  | PEAKS DB |
| R.IDKVNLMVDQQWM(+15.99)AVR.D | N | 52.50 | 1960.9863 | 16 | 0.6 | 654.6698 | 3 | 44.30 | 8 | F8:23687 | 31122021\_RID\_3262\_1a.raw |  |  |  |  |  |  |  | 4.3693E6 |  |  | 4 | 0 | 0 | 0 | 0 | 0 | 0 | 0 | 4 | 0 | 0 | 432 | 447 | Oxidation (M) | M13:Oxidation (M):108.85 | PEAKS DB |
| K.VNLMVDQQWM(+15.99)AVR.D | N | 52.36 | 1604.7803 | 13 | -1.8 | 803.3960 | 2 | 46.44 | 8 | F8:25468 | 31122021\_RID\_3262\_1a.raw |  |  |  |  |  |  |  | 0 |  |  | 0 | 0 | 0 | 0 | 0 | 0 | 0 | 0 | 0 | 0 | 0 | 435 | 447 | Oxidation (M) | M10:Oxidation (M):109.28 | PEAKS DB |
| R.IDKVNLMVDQQWMAV.R | N | 51.38 | 1788.8903 | 15 | 0.6 | 895.4529 | 2 | 58.81 | 8 | F8:35116 | 31122021\_RID\_3262\_1a.raw |  |  |  |  |  |  |  | 2.4944E6 |  |  | 1 | 0 | 0 | 0 | 0 | 0 | 0 | 0 | 1 | 0 | 0 | 432 | 446 |  |  | PEAKS DB |
| K.TESEAHNLPYGRPEVLQNHSK.Y | Y | 51.36 | 2405.1724 | 21 | 0.4 | 802.7317 | 3 | 25.30 | 8 | F8:8903 | 31122021\_RID\_3262\_1a.raw |  |  |  |  |  |  |  | 3.5345E6 |  |  | 2 | 0 | 0 | 0 | 0 | 0 | 0 | 0 | 2 | 0 | 0 | 574 | 594 |  |  | PEAKS DB |
| K.TFLPIFVNPV.N | N | 50.01 | 1145.6484 | 10 | 0.9 | 573.8320 | 2 | 74.94 | 8 | F8:46923 | 31122021\_RID\_3262\_1a.raw |  |  |  |  |  |  |  | 2.2916E6 | 1.7438E5 |  | 2 | 0 | 0 | 0 | 0 | 0 | 0 | 0 | 1 | 1 | 0 | 838 | 847 |  |  | PEAKS DB |
| R.DVELLTGLNFYSGLKQPLPETLQLK.T | N | 49.64 | 2815.5371 | 25 | 1.2 | 939.5208 | 3 | 67.16 | 8 | F8:41552 | 31122021\_RID\_3262\_1a.raw |  |  |  |  |  |  |  | 2.4935E5 |  |  | 1 | 0 | 0 | 0 | 0 | 0 | 0 | 0 | 1 | 0 | 0 | 813 | 837 |  |  | PEAKS DB |
| L.ILFSMDGFR.A | N | 49.34 | 1084.5375 | 9 | 0.2 | 543.2761 | 2 | 52.66 | 8 | F8:30315 | 31122021\_RID\_3262\_1a.raw |  |  |  |  |  |  |  | 1.6418E5 |  |  | 1 | 0 | 0 | 0 | 0 | 0 | 0 | 0 | 1 | 0 | 0 | 139 | 147 |  |  | PEAKS DB |
| M.VDQQWMAVR.D | N | 48.37 | 1131.5496 | 9 | 0.7 | 566.7825 | 2 | 36.04 | 8 | F8:17176 | 31122021\_RID\_3262\_1a.raw |  |  |  |  |  |  |  | 1.0158E5 |  |  | 1 | 0 | 0 | 0 | 0 | 0 | 0 | 0 | 1 | 0 | 0 | 439 | 447 |  |  | PEAKS DB |
| R.NPAWWGGQPIWHTATYQGL.K | N | 48.24 | 2182.0383 | 19 | 0.2 | 1092.0266 | 2 | 59.92 | 8 | F8:36107 | 31122021\_RID\_3262\_1a.raw |  |  |  |  |  |  |  | 8.5153E5 |  |  | 1 | 0 | 0 | 0 | 0 | 0 | 0 | 0 | 1 | 0 | 0 | 223 | 241 |  |  | PEAKS DB |
| Y.DALITSNIVPMFK.G | N | 48.12 | 1447.7744 | 13 | 1.0 | 724.8952 | 2 | 60.81 | 8 | F8:36680 | 31122021\_RID\_3262\_1a.raw |  |  |  |  |  |  |  | 4.3711E4 |  |  | 1 | 0 | 0 | 0 | 0 | 0 | 0 | 0 | 1 | 0 | 0 | 679 | 691 |  |  | PEAKS DB |
| R.NPAWWGGQPIWH.T | N | 48.02 | 1447.6786 | 12 | 8.8 | 724.8529 | 2 | 54.69 | 8 | F8:31999 | 31122021\_RID\_3262\_1a.raw |  |  |  |  |  |  |  | 3.0315E5 |  |  | 2 | 0 | 0 | 0 | 0 | 0 | 0 | 0 | 2 | 0 | 0 | 223 | 234 |  |  | PEAKS DB |
| K.VNLM(+15.99)VDQQWM(+15.99)AVR.D | N | 47.24 | 1620.7753 | 13 | 0.4 | 811.3953 | 2 | 42.79 | 8 | F8:22684 | 31122021\_RID\_3262\_1a.raw |  |  |  |  |  |  |  | 6.7039E5 |  |  | 2 | 0 | 0 | 0 | 0 | 0 | 0 | 0 | 2 | 0 | 0 | 435 | 447 | Oxidation (M) | M4:Oxidation (M):1000.00;M10:Oxidation (M):1000.00 | PEAKS DB |
| R.VRDVELLTGLNFYSGL.K | N | 47.12 | 1794.9515 | 16 | 1.1 | 898.4840 | 2 | 65.47 | 8 | F8:40254 | 31122021\_RID\_3262\_1a.raw |  |  |  |  |  |  |  | 2.3332E5 |  |  | 1 | 0 | 0 | 0 | 0 | 0 | 0 | 0 | 1 | 0 | 0 | 811 | 826 |  |  | PEAKS DB |
| R.VRDVELLTGLNF.Y | N | 47.05 | 1374.7507 | 12 | 0.6 | 688.3831 | 2 | 57.99 | 8 | F8:34552 | 31122021\_RID\_3262\_1a.raw |  |  |  |  |  |  |  | 8.975E4 |  |  | 1 | 0 | 0 | 0 | 0 | 0 | 0 | 0 | 1 | 0 | 0 | 811 | 822 |  |  | PEAKS DB |
| K.YISAYSQDILMPLWSSY.T | N | 46.18 | 2035.9601 | 17 | 0.5 | 1018.9878 | 2 | 79.09 | 8 | F8:48729 | 31122021\_RID\_3262\_1a.raw |  |  |  |  |  |  |  | 1.08E6 |  |  | 1 | 0 | 0 | 0 | 0 | 0 | 0 | 0 | 1 | 0 | 0 | 603 | 619 |  |  | PEAKS DB |
| L.YIEEPDTTGHK.Y | N | 46.15 | 1288.5935 | 11 | 0.5 | 645.3043 | 2 | 18.75 | 8 | F8:4441 | 31122021\_RID\_3262\_1a.raw |  |  |  |  |  |  |  | 1.0358E6 |  |  | 2 | 0 | 0 | 0 | 0 | 0 | 0 | 0 | 2 | 0 | 0 | 297 | 307 |  |  | PEAKS DB |
| K.YISAYSQDILM(+15.99)PLWSSY.T | N | 45.47 | 2051.9551 | 17 | 0.3 | 1026.9851 | 2 | 73.72 | 8 | F8:46274 | 31122021\_RID\_3262\_1a.raw |  |  |  |  |  |  |  | 2.6762E5 |  |  | 1 | 0 | 0 | 0 | 0 | 0 | 0 | 0 | 1 | 0 | 0 | 603 | 619 | Oxidation (M) | M11:Oxidation (M):1000.00 | PEAKS DB |
| R.NPAWWGGQPIWHT.A | N | 45.39 | 1548.7262 | 13 | 1.0 | 775.3712 | 2 | 55.91 | 8 | F8:32881 | 31122021\_RID\_3262\_1a.raw |  |  |  |  |  |  |  | 1.6497E5 |  |  | 1 | 0 | 0 | 0 | 0 | 0 | 0 | 0 | 1 | 0 | 0 | 223 | 235 |  |  | PEAKS DB |
| K.SMQAIFLAH.G | N | 44.69 | 1016.5114 | 9 | 0.0 | 509.2629 | 2 | 44.53 | 8 | F8:23867 | 31122021\_RID\_3262\_1a.raw |  |  |  |  |  |  |  | 9.9535E5 |  |  | 1 | 0 | 0 | 0 | 0 | 0 | 0 | 0 | 1 | 0 | 0 | 467 | 475 |  |  | PEAKS DB |
| N.PFYTPSPAK.E | N | 42.64 | 1006.5123 | 9 | 0.3 | 504.2636 | 2 | 29.12 | 8 | F8:11767 | 31122021\_RID\_3262\_1a.raw |  |  |  |  |  |  |  | 8.451E5 |  |  | 1 | 0 | 0 | 0 | 0 | 0 | 0 | 0 | 1 | 0 | 0 | 522 | 530 |  |  | PEAKS DB |
| K.VNLMVDQQWMAV.R | N | 41.30 | 1432.6843 | 12 | 0.5 | 717.3498 | 2 | 66.02 | 8 | F8:40783 | 31122021\_RID\_3262\_1a.raw |  |  |  |  |  |  |  | 6.3792E5 |  |  | 1 | 0 | 0 | 0 | 0 | 0 | 0 | 0 | 1 | 0 | 0 | 435 | 446 |  |  | PEAKS DB |
| E.SPLILFSMDGFR.A | N | 41.10 | 1381.7064 | 12 | -0.3 | 691.8603 | 2 | 63.15 | 8 | F8:38506 | 31122021\_RID\_3262\_1a.raw |  |  |  |  |  |  |  | 2.0761E4 |  |  | 1 | 0 | 0 | 0 | 0 | 0 | 0 | 0 | 1 | 0 | 0 | 136 | 147 |  |  | PEAKS DB |
| R.VRDVELLTGLNFY.S | N | 40.90 | 1537.8140 | 13 | 0.5 | 769.9147 | 2 | 60.07 | 8 | F8:36272 | 31122021\_RID\_3262\_1a.raw |  |  |  |  |  |  |  | 7.5704E5 |  |  | 1 | 0 | 0 | 0 | 0 | 0 | 0 | 0 | 1 | 0 | 0 | 811 | 823 |  |  | PEAKS DB |
| total 49 peptides |  |  |  |  |  |  |  |  |  |  |  |  |  |  |  |  |  |  |  |  |  |  |  |  |  |  |  |  |  |  |  |  |  |  |  |  |  |
| --- | --- | --- | --- | --- | --- | --- | --- | --- | --- | --- | --- | --- | --- | --- | --- | --- | --- | --- | --- | --- | --- | --- | --- | --- | --- | --- | --- | --- | --- | --- | --- | --- | --- | --- | --- | --- | --- |

BAN89425.1

back to list

  

| Protein Coverage
| Supporting Peptides
|

Protein Coverage:

Supporting Peptides:

| Peptide | Uniq | -10lgP | Mass | Length | ppm | m/z | z | RT | Fraction | Scan | Source File | Area F4 | Area F5 | Area F6 | Area F10 | Area F8 | Area F9 | Area F7 | Area F1 | Area F2 | Area F3 | #Feature | #Feature F4 | #Feature F5 | #Feature F6 | #Feature F10 | #Feature F8 | #Feature F9 | #Feature F7 | #Feature F1 | #Feature F2 | #Feature F3 | Start | End | PTM | AScore | Found By |
| --- | --- | --- | --- | --- | --- | --- | --- | --- | --- | --- | --- | --- | --- | --- | --- | --- | --- | --- | --- | --- | --- | --- | --- | --- | --- | --- | --- | --- | --- | --- | --- | --- | --- | --- | --- | --- | --- |
| R.IDKVNLMVDQQWMAVR.D | N | 89.89 | 1944.9913 | 16 | 0.6 | 649.3381 | 3 | 49.05 | 8 | F8:27583 | 31122021\_RID\_3262\_1a.raw |  |  |  |  |  |  |  | 1.2194E7 | 3.5597E5 |  | 3 | 0 | 0 | 0 | 0 | 0 | 0 | 0 | 2 | 1 | 0 | 473 | 488 |  |  | PEAKS DB |
| K.YISAYSQDILM(+15.99)PLWSSYTIYR.S | N | 86.70 | 2585.2512 | 21 | 1.0 | 862.7585 | 3 | 67.22 | 8 | F8:41605 | 31122021\_RID\_3262\_1a.raw |  |  |  |  |  |  |  | 4.0338E6 |  |  | 3 | 0 | 0 | 0 | 0 | 0 | 0 | 0 | 3 | 0 | 0 | 644 | 664 | Oxidation (M) | M11:Oxidation (M):1000.00 | PEAKS DB |
| N.VDFFMYEGPAPR.I | N | 84.54 | 1427.6543 | 12 | 0.4 | 714.8347 | 2 | 52.03 | 8 | F8:29943 | 31122021\_RID\_3262\_1a.raw | 3.8891E5 |  |  |  |  |  |  | 3.2833E7 | 1.1005E6 | 4.0735E5 | 4 | 1 | 0 | 0 | 0 | 0 | 0 | 0 | 1 | 1 | 1 | 411 | 422 |  |  | PEAKS DB |
| K.YISAYSQDILMPLWSSYTIYR.S | N | 83.99 | 2569.2563 | 21 | 0.7 | 857.4267 | 3 | 71.20 | 8 | F8:44628 | 31122021\_RID\_3262\_1a.raw |  |  |  |  |  |  |  | 7.9234E6 |  |  | 2 | 0 | 0 | 0 | 0 | 0 | 0 | 0 | 2 | 0 | 0 | 644 | 664 |  |  | PEAKS DB |
| K.VNLMVDQQWMAVR.D | N | 80.06 | 1588.7854 | 13 | 0.8 | 795.4006 | 2 | 52.32 | 8 | F8:30174 | 31122021\_RID\_3262\_1a.raw |  |  |  |  |  |  |  | 3.4969E6 |  |  | 1 | 0 | 0 | 0 | 0 | 0 | 0 | 0 | 1 | 0 | 0 | 476 | 488 |  |  | PEAKS DB |
| R.DVELLTGLNFYSGLK.Q | N | 79.71 | 1667.8770 | 15 | 0.4 | 834.9461 | 2 | 67.85 | 8 | F8:42145 | 31122021\_RID\_3262\_1a.raw |  |  |  |  |  |  |  | 1.5005E7 | 4.8402E5 |  | 5 | 0 | 0 | 0 | 0 | 0 | 0 | 0 | 4 | 1 | 0 | 854 | 868 |  |  | PEAKS DB |
| K.DFYTFDSEGIVK.N | N | 78.54 | 1419.6558 | 12 | 0.4 | 710.8354 | 2 | 52.61 | 8 | F8:30396 | 31122021\_RID\_3262\_1a.raw | 3.7096E5 |  |  |  |  |  |  | 2.8875E7 | 1.731E6 |  | 5 | 1 | 0 | 0 | 0 | 0 | 0 | 0 | 3 | 1 | 0 | 431 | 442 |  |  | PEAKS DB |
| R.NPAWWGGQPIWHTATYQGLK.A | N | 77.54 | 2310.1333 | 20 | 1.0 | 771.0525 | 3 | 50.58 | 8 | F8:28794 | 31122021\_RID\_3262\_1a.raw |  |  |  |  |  |  |  | 2.7775E7 |  |  | 2 | 0 | 0 | 0 | 0 | 0 | 0 | 0 | 2 | 0 | 0 | 264 | 283 |  |  | PEAKS DB |
| K.NVPKDFYTFDSEGIVK.N | N | 76.77 | 1857.9148 | 16 | 0.6 | 929.9653 | 2 | 44.01 | 8 | F8:23615 | 31122021\_RID\_3262\_1a.raw |  |  |  |  |  |  |  | 1.8075E7 | 7.4834E5 |  | 3 | 0 | 0 | 0 | 0 | 0 | 0 | 0 | 2 | 1 | 0 | 427 | 442 |  |  | PEAKS DB |
| Y.SQDILMPLWSSYTIYR.S | N | 74.09 | 1971.9764 | 16 | 0.5 | 986.9960 | 2 | 65.69 | 8 | F8:40533 | 31122021\_RID\_3262\_1a.raw |  |  |  |  |  |  |  | 2.1206E6 |  |  | 2 | 0 | 0 | 0 | 0 | 0 | 0 | 0 | 2 | 0 | 0 | 649 | 664 |  |  | PEAKS DB |
| R.IDKVNLM(+15.99)VDQQWM(+15.99)AVR.D | N | 73.40 | 1976.9812 | 16 | 0.5 | 660.0013 | 3 | 40.77 | 8 | F8:21007 | 31122021\_RID\_3262\_1a.raw |  |  |  |  |  |  |  | 1.5146E6 |  |  | 1 | 0 | 0 | 0 | 0 | 0 | 0 | 0 | 1 | 0 | 0 | 473 | 488 | Oxidation (M) | M7:Oxidation (M):1000.00;M13:Oxidation (M):1000.00 | PEAKS DB |
| R.LWNYFHTTLIPK.Y | N | 73.37 | 1531.8187 | 12 | 0.5 | 766.9170 | 2 | 47.15 | 8 | F8:26107 | 31122021\_RID\_3262\_1a.raw | 8.3337E5 |  |  |  |  |  |  | 5.0587E7 |  |  | 3 | 1 | 0 | 0 | 0 | 0 | 0 | 0 | 2 | 0 | 0 | 737 | 748 |  |  | PEAKS DB |
| K.RPDFYTLYIEEPDTTGHK.Y | N | 73.22 | 2181.0378 | 18 | 0.2 | 1091.5264 | 2 | 41.13 | 8 | F8:21378 | 31122021\_RID\_3262\_1a.raw | 2.7863E6 |  |  |  |  |  |  | 9.8043E7 | 4.47E6 |  | 7 | 1 | 0 | 0 | 0 | 0 | 0 | 0 | 4 | 2 | 0 | 331 | 348 |  |  | PEAKS DB |
| K.VNLM(+15.99)VDQQWMAVR.D | N | 70.81 | 1604.7803 | 13 | 0.4 | 803.3977 | 2 | 48.28 | 8 | F8:27028 | 31122021\_RID\_3262\_1a.raw |  |  |  |  |  |  |  | 2.7138E6 | 8.2065E4 |  | 2 | 0 | 0 | 0 | 0 | 0 | 0 | 0 | 1 | 1 | 0 | 476 | 488 | Oxidation (M) | M4:Oxidation (M):176.18 | PEAKS DB |
| K.NPFYTPSPAK.E | N | 68.72 | 1120.5553 | 10 | -0.2 | 561.2848 | 2 | 31.38 | 8 | F8:13580 | 31122021\_RID\_3262\_1a.raw | 2.0471E6 |  |  |  |  |  |  | 3.8456E7 | 4.8509E6 |  | 4 | 1 | 0 | 0 | 0 | 0 | 0 | 0 | 2 | 1 | 0 | 562 | 571 |  |  | PEAKS DB |
| R.VRDVELLTGLNFYSGLK.Q | N | 68.06 | 1923.0465 | 17 | 0.9 | 642.0234 | 3 | 54.52 | 8 | F8:31944 | 31122021\_RID\_3262\_1a.raw | 5.1091E5 |  |  |  |  |  |  | 2.4463E7 | 3.6342E5 |  | 5 | 1 | 0 | 0 | 0 | 0 | 0 | 0 | 3 | 1 | 0 | 852 | 868 |  |  | PEAKS DB |
| K.YGPVSGEIIK.A | N | 66.88 | 1061.5757 | 10 | -0.3 | 531.7950 | 2 | 34.11 | 8 | F8:15575 | 31122021\_RID\_3262\_1a.raw | 5.5409E6 | 5.2937E5 |  |  |  |  |  | 8.5643E7 | 1.0829E7 |  | 4 | 1 | 1 | 0 | 0 | 0 | 0 | 0 | 1 | 1 | 0 | 349 | 358 |  |  | PEAKS DB |
| N.VDFFM(+15.99)YEGPAPR.I | N | 66.39 | 1443.6493 | 12 | 0.5 | 722.8323 | 2 | 48.28 | 8 | F8:26949 | 31122021\_RID\_3262\_1a.raw |  |  |  |  |  |  |  | 1.8808E7 | 1.7049E5 |  | 5 | 0 | 0 | 0 | 0 | 0 | 0 | 0 | 4 | 1 | 0 | 411 | 422 | Oxidation (M) | M5:Oxidation (M):1000.00 | PEAKS DB |
| K.AATYFWPGSEVK.I | N | 65.68 | 1354.6558 | 12 | 0.6 | 678.3356 | 2 | 44.70 | 8 | F8:24117 | 31122021\_RID\_3262\_1a.raw | 4.0357E6 |  |  |  |  |  |  | 5.8345E7 | 2.2909E6 |  | 3 | 1 | 0 | 0 | 0 | 0 | 0 | 0 | 1 | 1 | 0 | 284 | 295 |  |  | PEAKS DB |
| R.IDKVNLM(+15.99)VDQQWMAVR.D | N | 65.60 | 1960.9863 | 16 | 0.2 | 654.6695 | 3 | 45.45 | 8 | F8:24763 | 31122021\_RID\_3262\_1a.raw |  |  |  |  |  |  |  | 1.034E7 | 1.8006E5 |  | 5 | 0 | 0 | 0 | 0 | 0 | 0 | 0 | 4 | 1 | 0 | 473 | 488 | Oxidation (M) | M7:Oxidation (M):156.85 | PEAKS DB |
| R.TLGMLMEGLK.Q | N | 65.55 | 1091.5719 | 10 | 0.0 | 546.7932 | 2 | 47.83 | 8 | F8:26797 | 31122021\_RID\_3262\_1a.raw |  |  |  |  |  |  |  | 2.0277E7 | 9.0693E5 |  | 2 | 0 | 0 | 0 | 0 | 0 | 0 | 0 | 1 | 1 | 0 | 366 | 375 |  |  | PEAKS DB |
| R.TLGM(+15.99)LMEGLK.Q | N | 65.37 | 1107.5668 | 10 | 0.5 | 554.7910 | 2 | 43.66 | 8 | F8:23669 | 31122021\_RID\_3262\_1a.raw |  |  |  |  |  |  |  | 1.1063E8 | 4.1807E6 |  | 5 | 0 | 0 | 0 | 0 | 0 | 0 | 0 | 3 | 2 | 0 | 366 | 375 | Oxidation (M) | M4:Oxidation (M):67.96 | PEAKS DB |
| K.TFLPIFVNPVN | N | 64.77 | 1259.6914 | 11 | 0.6 | 630.8533 | 2 | 72.29 | 8 | F8:45470 | 31122021\_RID\_3262\_1a.raw | 3.3682E6 |  |  |  |  |  |  | 3.0009E7 | 1.5173E6 |  | 3 | 1 | 0 | 0 | 0 | 0 | 0 | 0 | 1 | 1 | 0 | 879 | 889 |  |  | PEAKS DB |
| R.TLGMLM(+15.99)EGLK.Q | N | 58.46 | 1107.5668 | 10 | 0.5 | 554.7910 | 2 | 44.17 | 8 | F8:26515 | 31122021\_RID\_3262\_1a.raw |  |  |  |  |  |  |  | 4.7543E7 |  |  | 2 | 0 | 0 | 0 | 0 | 0 | 0 | 0 | 2 | 0 | 0 | 366 | 375 | Oxidation (M) | M6:Oxidation (M):67.96 | PEAKS DB |
| R.TLGM(+15.99)LM(+15.99)EGLK.Q | N | 53.04 | 1123.5618 | 10 | -0.5 | 562.7879 | 2 | 44.08 | 8 | F8:23974 | 31122021\_RID\_3262\_1a.raw |  |  |  |  |  |  |  | 2.6054E7 | 1.1517E6 |  | 12 | 0 | 0 | 0 | 0 | 0 | 0 | 0 | 10 | 2 | 0 | 366 | 375 | Oxidation (M) | M4:Oxidation (M):1000.00;M6:Oxidation (M):1000.00 | PEAKS DB |
| K.QPLPETLQLK.T | N | 52.54 | 1165.6707 | 10 | 1.0 | 583.8432 | 2 | 39.62 | 8 | F8:20222 | 31122021\_RID\_3262\_1a.raw | 2.6906E6 |  |  |  |  |  |  | 4.8465E7 | 1.0101E6 |  | 3 | 1 | 0 | 0 | 0 | 0 | 0 | 0 | 1 | 1 | 0 | 869 | 878 |  |  | PEAKS DB |
| R.IDKVNLMVDQQWM(+15.99)AVR.D | N | 52.50 | 1960.9863 | 16 | 0.6 | 654.6698 | 3 | 44.30 | 8 | F8:23687 | 31122021\_RID\_3262\_1a.raw |  |  |  |  |  |  |  | 4.3693E6 |  |  | 4 | 0 | 0 | 0 | 0 | 0 | 0 | 0 | 4 | 0 | 0 | 473 | 488 | Oxidation (M) | M13:Oxidation (M):108.85 | PEAKS DB |
| K.VNLMVDQQWM(+15.99)AVR.D | N | 52.36 | 1604.7803 | 13 | -1.8 | 803.3960 | 2 | 46.44 | 8 | F8:25468 | 31122021\_RID\_3262\_1a.raw |  |  |  |  |  |  |  | 0 |  |  | 0 | 0 | 0 | 0 | 0 | 0 | 0 | 0 | 0 | 0 | 0 | 476 | 488 | Oxidation (M) | M10:Oxidation (M):109.28 | PEAKS DB |
| R.IDKVNLMVDQQWMAV.R | N | 51.38 | 1788.8903 | 15 | 0.6 | 895.4529 | 2 | 58.81 | 8 | F8:35116 | 31122021\_RID\_3262\_1a.raw |  |  |  |  |  |  |  | 2.4944E6 |  |  | 1 | 0 | 0 | 0 | 0 | 0 | 0 | 0 | 1 | 0 | 0 | 473 | 487 |  |  | PEAKS DB |
| K.TESEAHNLPYGRPEVLQNHSK.Y | Y | 51.36 | 2405.1724 | 21 | 0.4 | 802.7317 | 3 | 25.30 | 8 | F8:8903 | 31122021\_RID\_3262\_1a.raw |  |  |  |  |  |  |  | 3.5345E6 |  |  | 2 | 0 | 0 | 0 | 0 | 0 | 0 | 0 | 2 | 0 | 0 | 615 | 635 |  |  | PEAKS DB |
| K.TFLPIFVNPV.N | N | 50.01 | 1145.6484 | 10 | 0.9 | 573.8320 | 2 | 74.94 | 8 | F8:46923 | 31122021\_RID\_3262\_1a.raw |  |  |  |  |  |  |  | 2.2916E6 | 1.7438E5 |  | 2 | 0 | 0 | 0 | 0 | 0 | 0 | 0 | 1 | 1 | 0 | 879 | 888 |  |  | PEAKS DB |
| R.DVELLTGLNFYSGLKQPLPETLQLK.T | N | 49.64 | 2815.5371 | 25 | 1.2 | 939.5208 | 3 | 67.16 | 8 | F8:41552 | 31122021\_RID\_3262\_1a.raw |  |  |  |  |  |  |  | 2.4935E5 |  |  | 1 | 0 | 0 | 0 | 0 | 0 | 0 | 0 | 1 | 0 | 0 | 854 | 878 |  |  | PEAKS DB |
| L.ILFSMDGFR.A | N | 49.34 | 1084.5375 | 9 | 0.2 | 543.2761 | 2 | 52.66 | 8 | F8:30315 | 31122021\_RID\_3262\_1a.raw |  |  |  |  |  |  |  | 1.6418E5 |  |  | 1 | 0 | 0 | 0 | 0 | 0 | 0 | 0 | 1 | 0 | 0 | 180 | 188 |  |  | PEAKS DB |
| M.VDQQWMAVR.D | N | 48.37 | 1131.5496 | 9 | 0.7 | 566.7825 | 2 | 36.04 | 8 | F8:17176 | 31122021\_RID\_3262\_1a.raw |  |  |  |  |  |  |  | 1.0158E5 |  |  | 1 | 0 | 0 | 0 | 0 | 0 | 0 | 0 | 1 | 0 | 0 | 480 | 488 |  |  | PEAKS DB |
| R.NPAWWGGQPIWHTATYQGL.K | N | 48.24 | 2182.0383 | 19 | 0.2 | 1092.0266 | 2 | 59.92 | 8 | F8:36107 | 31122021\_RID\_3262\_1a.raw |  |  |  |  |  |  |  | 8.5153E5 |  |  | 1 | 0 | 0 | 0 | 0 | 0 | 0 | 0 | 1 | 0 | 0 | 264 | 282 |  |  | PEAKS DB |
| Y.DALITSNIVPMFK.G | N | 48.12 | 1447.7744 | 13 | 1.0 | 724.8952 | 2 | 60.81 | 8 | F8:36680 | 31122021\_RID\_3262\_1a.raw |  |  |  |  |  |  |  | 4.3711E4 |  |  | 1 | 0 | 0 | 0 | 0 | 0 | 0 | 0 | 1 | 0 | 0 | 720 | 732 |  |  | PEAKS DB |
| R.NPAWWGGQPIWH.T | N | 48.02 | 1447.6786 | 12 | 8.8 | 724.8529 | 2 | 54.69 | 8 | F8:31999 | 31122021\_RID\_3262\_1a.raw |  |  |  |  |  |  |  | 3.0315E5 |  |  | 2 | 0 | 0 | 0 | 0 | 0 | 0 | 0 | 2 | 0 | 0 | 264 | 275 |  |  | PEAKS DB |
| K.VNLM(+15.99)VDQQWM(+15.99)AVR.D | N | 47.24 | 1620.7753 | 13 | 0.4 | 811.3953 | 2 | 42.79 | 8 | F8:22684 | 31122021\_RID\_3262\_1a.raw |  |  |  |  |  |  |  | 6.7039E5 |  |  | 2 | 0 | 0 | 0 | 0 | 0 | 0 | 0 | 2 | 0 | 0 | 476 | 488 | Oxidation (M) | M4:Oxidation (M):1000.00;M10:Oxidation (M):1000.00 | PEAKS DB |
| R.VRDVELLTGLNFYSGL.K | N | 47.12 | 1794.9515 | 16 | 1.1 | 898.4840 | 2 | 65.47 | 8 | F8:40254 | 31122021\_RID\_3262\_1a.raw |  |  |  |  |  |  |  | 2.3332E5 |  |  | 1 | 0 | 0 | 0 | 0 | 0 | 0 | 0 | 1 | 0 | 0 | 852 | 867 |  |  | PEAKS DB |
| R.VRDVELLTGLNF.Y | N | 47.05 | 1374.7507 | 12 | 0.6 | 688.3831 | 2 | 57.99 | 8 | F8:34552 | 31122021\_RID\_3262\_1a.raw |  |  |  |  |  |  |  | 8.975E4 |  |  | 1 | 0 | 0 | 0 | 0 | 0 | 0 | 0 | 1 | 0 | 0 | 852 | 863 |  |  | PEAKS DB |
| K.YISAYSQDILMPLWSSY.T | N | 46.18 | 2035.9601 | 17 | 0.5 | 1018.9878 | 2 | 79.09 | 8 | F8:48729 | 31122021\_RID\_3262\_1a.raw |  |  |  |  |  |  |  | 1.08E6 |  |  | 1 | 0 | 0 | 0 | 0 | 0 | 0 | 0 | 1 | 0 | 0 | 644 | 660 |  |  | PEAKS DB |
| L.YIEEPDTTGHK.Y | N | 46.15 | 1288.5935 | 11 | 0.5 | 645.3043 | 2 | 18.75 | 8 | F8:4441 | 31122021\_RID\_3262\_1a.raw |  |  |  |  |  |  |  | 1.0358E6 |  |  | 2 | 0 | 0 | 0 | 0 | 0 | 0 | 0 | 2 | 0 | 0 | 338 | 348 |  |  | PEAKS DB |
| K.YISAYSQDILM(+15.99)PLWSSY.T | N | 45.47 | 2051.9551 | 17 | 0.3 | 1026.9851 | 2 | 73.72 | 8 | F8:46274 | 31122021\_RID\_3262\_1a.raw |  |  |  |  |  |  |  | 2.6762E5 |  |  | 1 | 0 | 0 | 0 | 0 | 0 | 0 | 0 | 1 | 0 | 0 | 644 | 660 | Oxidation (M) | M11:Oxidation (M):1000.00 | PEAKS DB |
| R.NPAWWGGQPIWHT.A | N | 45.39 | 1548.7262 | 13 | 1.0 | 775.3712 | 2 | 55.91 | 8 | F8:32881 | 31122021\_RID\_3262\_1a.raw |  |  |  |  |  |  |  | 1.6497E5 |  |  | 1 | 0 | 0 | 0 | 0 | 0 | 0 | 0 | 1 | 0 | 0 | 264 | 276 |  |  | PEAKS DB |
| K.SMQAIFLAH.G | N | 44.69 | 1016.5114 | 9 | 0.0 | 509.2629 | 2 | 44.53 | 8 | F8:23867 | 31122021\_RID\_3262\_1a.raw |  |  |  |  |  |  |  | 9.9535E5 |  |  | 1 | 0 | 0 | 0 | 0 | 0 | 0 | 0 | 1 | 0 | 0 | 508 | 516 |  |  | PEAKS DB |
| N.PFYTPSPAK.E | N | 42.64 | 1006.5123 | 9 | 0.3 | 504.2636 | 2 | 29.12 | 8 | F8:11767 | 31122021\_RID\_3262\_1a.raw |  |  |  |  |  |  |  | 8.451E5 |  |  | 1 | 0 | 0 | 0 | 0 | 0 | 0 | 0 | 1 | 0 | 0 | 563 | 571 |  |  | PEAKS DB |
| K.VNLMVDQQWMAV.R | N | 41.30 | 1432.6843 | 12 | 0.5 | 717.3498 | 2 | 66.02 | 8 | F8:40783 | 31122021\_RID\_3262\_1a.raw |  |  |  |  |  |  |  | 6.3792E5 |  |  | 1 | 0 | 0 | 0 | 0 | 0 | 0 | 0 | 1 | 0 | 0 | 476 | 487 |  |  | PEAKS DB |
| E.SPLILFSMDGFR.A | N | 41.10 | 1381.7064 | 12 | -0.3 | 691.8603 | 2 | 63.15 | 8 | F8:38506 | 31122021\_RID\_3262\_1a.raw |  |  |  |  |  |  |  | 2.0761E4 |  |  | 1 | 0 | 0 | 0 | 0 | 0 | 0 | 0 | 1 | 0 | 0 | 177 | 188 |  |  | PEAKS DB |
| R.VRDVELLTGLNFY.S | N | 40.90 | 1537.8140 | 13 | 0.5 | 769.9147 | 2 | 60.07 | 8 | F8:36272 | 31122021\_RID\_3262\_1a.raw |  |  |  |  |  |  |  | 7.5704E5 |  |  | 1 | 0 | 0 | 0 | 0 | 0 | 0 | 0 | 1 | 0 | 0 | 852 | 864 |  |  | PEAKS DB |
| total 49 peptides |  |  |  |  |  |  |  |  |  |  |  |  |  |  |  |  |  |  |  |  |  |  |  |  |  |  |  |  |  |  |  |  |  |  |  |  |  |
| --- | --- | --- | --- | --- | --- | --- | --- | --- | --- | --- | --- | --- | --- | --- | --- | --- | --- | --- | --- | --- | --- | --- | --- | --- | --- | --- | --- | --- | --- | --- | --- | --- | --- | --- | --- | --- | --- |

BAN82013.1

back to list

  

| Protein Coverage
| Supporting Peptides
|

Protein Coverage:

Supporting Peptides:

| Peptide | Uniq | -10lgP | Mass | Length | ppm | m/z | z | RT | Fraction | Scan | Source File | Area F4 | Area F5 | Area F6 | Area F10 | Area F8 | Area F9 | Area F7 | Area F1 | Area F2 | Area F3 | #Feature | #Feature F4 | #Feature F5 | #Feature F6 | #Feature F10 | #Feature F8 | #Feature F9 | #Feature F7 | #Feature F1 | #Feature F2 | #Feature F3 | Start | End | PTM | AScore | Found By |
| --- | --- | --- | --- | --- | --- | --- | --- | --- | --- | --- | --- | --- | --- | --- | --- | --- | --- | --- | --- | --- | --- | --- | --- | --- | --- | --- | --- | --- | --- | --- | --- | --- | --- | --- | --- | --- | --- |
| K.KDPGVLKYPVKPSEEGK.S | N | 79.57 | 1870.0199 | 17 | -0.7 | 468.5119 | 4 | 24.47 | 1 | F1:11529 | 13012022\_RID\_3262\_4a.raw | 3.7125E8 | 1.4174E6 |  |  |  | 8.1047E6 | 1.2066E6 | 5.7794E6 | 6.6831E6 | 5.2885E7 | 16 | 4 | 2 | 0 | 0 | 0 | 2 | 2 | 2 | 2 | 2 | 153 | 169 |  |  | PEAKS DB |
| K.DPGVLKYPVKPSEEGK.S | N | 78.91 | 1741.9249 | 16 | -0.1 | 581.6489 | 3 | 29.60 | 1 | F1:16075 | 13012022\_RID\_3262\_4a.raw | 3.1808E7 | 7.5919E5 |  |  |  |  | 2.7752E5 | 4.9105E5 | 7.4531E5 | 1.0783E7 | 8 | 2 | 1 | 0 | 0 | 0 | 0 | 1 | 1 | 2 | 1 | 154 | 169 |  |  | PEAKS DB |
| K.YPVKPSEEGKSAGQLYEESLR.K | N | 72.83 | 2366.1753 | 21 | 0.7 | 592.5515 | 4 | 32.87 | 1 | F1:19085 | 13012022\_RID\_3262\_4a.raw | 3.4004E6 |  |  |  |  |  |  |  |  |  | 2 | 2 | 0 | 0 | 0 | 0 | 0 | 0 | 0 | 0 | 0 | 160 | 180 |  |  | PEAKS DB |
| K.SAGQLYEESLR.K | N | 72.77 | 1251.6095 | 11 | 0.4 | 626.8123 | 2 | 32.12 | 8 | F8:14092 | 31122021\_RID\_3262\_1a.raw | 1.2366E9 | 8.6285E7 | 3.6644E7 | 6.5882E5 |  | 6.8121E6 | 2.7435E7 | 6.1833E7 | 1.0188E8 | 2.1008E9 | 11 | 2 | 1 | 1 | 1 | 0 | 1 | 1 | 1 | 1 | 2 | 170 | 180 |  |  | PEAKS DB |
| K.HDDIFAYEK.R | N | 71.92 | 1136.5138 | 9 | 0.2 | 569.2643 | 2 | 33.07 | 1 | F1:19276 | 13012022\_RID\_3262\_4a.raw | 2.205E8 | 5.0368E7 | 1.6541E6 | 5.2373E7 |  | 6.378E6 | 6.4081E6 | 1.8941E7 | 3.5546E7 | 1.9591E9 | 33 | 5 | 2 | 2 | 4 | 0 | 2 | 2 | 4 | 4 | 8 | 241 | 249 |  |  | PEAKS DB |
| K.KFWEDDGIHGGK.S | N | 70.84 | 1387.6520 | 12 | 1.0 | 694.8340 | 2 | 27.52 | 1 | F1:14353 | 13012022\_RID\_3262\_4a.raw | 2.2688E8 | 4.7295E4 |  |  |  |  |  | 1.1825E7 | 1.8101E7 | 8.0079E8 | 17 | 4 | 1 | 0 | 0 | 0 | 0 | 0 | 3 | 3 | 6 | 352 | 363 |  |  | PEAKS DB |
| Y.GIGDDANFFQALDFK.S | N | 70.77 | 1656.7783 | 15 | 0.6 | 829.3970 | 2 | 63.80 | 9 | F9:39957 | 31122021\_RID\_3262\_2a.raw |  | 4.1343E5 |  |  |  |  |  | 2.2577E6 | 1.7889E6 | 7.0811E7 | 6 | 0 | 1 | 0 | 0 | 0 | 0 | 0 | 2 | 1 | 2 | 391 | 405 |  |  | PEAKS DB |
| D.PGVLKYPVKPSEEGK.S | N | 70.72 | 1626.8981 | 15 | 0.0 | 543.3066 | 3 | 25.59 | 1 | F1:12547 | 13012022\_RID\_3262\_4a.raw | 1.4062E8 |  |  | 4.7646E5 |  | 4.3107E6 |  | 4.013E5 |  | 5.7619E6 | 8 | 3 | 0 | 0 | 1 | 0 | 2 | 0 | 1 | 0 | 1 | 155 | 169 |  |  | PEAKS DB |
| K.YPVKPSEEGK.S | N | 69.75 | 1132.5764 | 10 | 0.7 | 567.2959 | 2 | 17.88 | 6 | F6:5952 | 17012022\_RID\_3262\_9a.raw | 4.2299E8 | 5.2685E7 | 2.5077E5 | 3.2771E7 |  | 6.9462E7 | 2.576E7 |  |  |  | 12 | 2 | 2 | 1 | 3 | 0 | 2 | 2 | 0 | 0 | 0 | 160 | 169 |  |  | PEAKS DB |
| I.AYGIGDDANFFQALDFK.S | N | 69.60 | 1890.8788 | 17 | 0.2 | 946.4468 | 2 | 65.58 | 8 | F8:40325 | 31122021\_RID\_3262\_1a.raw |  |  |  |  |  |  |  | 1.5459E5 |  |  | 1 | 0 | 0 | 0 | 0 | 0 | 0 | 0 | 1 | 0 | 0 | 389 | 405 |  |  | PEAKS DB |
| L.SAAYVLAGAGHQVTVLEASER.A | N | 67.91 | 2128.0913 | 21 | 0.0 | 1065.0530 | 2 | 41.69 | 10 | F10:10487 | 31122021\_RID\_3262\_3a.raw | 1.4728E6 |  |  |  |  |  |  | 6.4802E5 | 6.599E5 | 2.6305E7 | 6 | 1 | 0 | 0 | 0 | 0 | 0 | 0 | 2 | 1 | 2 | 65 | 85 |  |  | PEAKS DB |
| K.FWEDDGIHGGK.S | N | 65.97 | 1259.5570 | 11 | 0.4 | 630.7860 | 2 | 31.13 | 1 | F1:17598 | 13012022\_RID\_3262\_4a.raw | 7.5921E7 | 9.0996E6 |  |  |  | 1.9943E6 |  | 6.128E6 | 1.0893E7 | 5.5749E8 | 17 | 2 | 2 | 0 | 0 | 0 | 1 | 0 | 3 | 3 | 6 | 353 | 363 |  |  | PEAKS DB |
| K.EGNLSPGAVDMIGDLLNEDSGYYVSFIESMK.H | Y | 65.38 | 3349.5369 | 31 | 0.7 | 1117.5204 | 3 | 81.20 | 1 | F1:59522 | 13012022\_RID\_3262\_4a.raw | 7.7147E6 |  |  |  |  |  |  |  |  |  | 1 | 1 | 0 | 0 | 0 | 0 | 0 | 0 | 0 | 0 | 0 | 210 | 240 |  |  | PEAKS DB |
| A.YVLAGAGHQVTVLEASER.A | N | 64.94 | 1898.9850 | 18 | 0.8 | 950.5005 | 2 | 37.54 | 10 | F10:8957 | 31122021\_RID\_3262\_3a.raw | 9.515E5 |  |  |  |  |  |  |  |  | 6.9519E6 | 3 | 1 | 0 | 0 | 0 | 0 | 0 | 0 | 0 | 0 | 2 | 68 | 85 |  |  | PEAKS DB |
| K.EGNLSPGAVDM(+15.99)IGDLLNEDSGYYVSFIESMK.H | Y | 61.82 | 3365.5320 | 31 | 0.2 | 1122.8514 | 3 | 75.85 | 1 | F1:55688 | 13012022\_RID\_3262\_4a.raw | 1.9966E7 |  |  |  |  |  |  | 6.7671E5 |  |  | 2 | 1 | 0 | 0 | 0 | 0 | 0 | 0 | 1 | 0 | 0 | 210 | 240 | Oxidation (M) | M11:Oxidation (M):117.45 | PEAKS DB |
| K.HDDIFAYEKR.F | N | 61.79 | 1292.6149 | 10 | 0.7 | 431.8792 | 3 | 27.51 | 1 | F1:14388 | 13012022\_RID\_3262\_4a.raw | 1.0662E7 |  |  | 1.628E5 |  |  |  |  |  |  | 3 | 2 | 0 | 0 | 1 | 0 | 0 | 0 | 0 | 0 | 0 | 241 | 250 |  |  | PEAKS DB |
| F.IESM(+15.99)KHDDIFAYEK.R | N | 61.58 | 1740.8029 | 14 | 0.9 | 581.2754 | 3 | 29.10 | 6 | F6:15895 | 17012022\_RID\_3262\_9a.raw |  |  |  |  |  | 3.2962E6 |  |  |  |  | 1 | 0 | 0 | 0 | 0 | 0 | 1 | 0 | 0 | 0 | 0 | 236 | 249 | Oxidation (M) | M4:Oxidation (M):1000.00 | PEAKS DB |
| Y.VLAGAGHQVTVLEASER.A | N | 61.46 | 1735.9216 | 17 | 0.9 | 579.6483 | 3 | 33.06 | 6 | F6:19337 | 17012022\_RID\_3262\_9a.raw | 2.8948E5 | 1.1934E6 |  | 6.6625E5 |  | 3.8459E6 |  | 1.4002E6 | 1.9495E6 | 3.1893E7 | 10 | 1 | 1 | 0 | 1 | 0 | 1 | 0 | 2 | 2 | 2 | 69 | 85 |  |  | PEAKS DB |
| K.VTVTYQTPAK.D | N | 59.36 | 1106.5972 | 10 | -0.1 | 554.3058 | 2 | 27.24 | 7 | F7:10262 | 21012022\_RID\_3262\_7a.raw |  |  |  |  |  | 1.7478E6 | 1.5831E6 |  |  |  | 2 | 0 | 0 | 0 | 0 | 0 | 1 | 1 | 0 | 0 | 0 | 289 | 298 |  |  | PEAKS DB |
| S.MKHDDIFAYEK.R | N | 57.56 | 1395.6493 | 11 | 0.9 | 466.2241 | 3 | 28.76 | 6 | F6:15683 | 17012022\_RID\_3262\_9a.raw |  |  |  |  |  | 1.5918E6 |  |  |  |  | 2 | 0 | 0 | 0 | 0 | 0 | 2 | 0 | 0 | 0 | 0 | 239 | 249 |  |  | PEAKS DB |
| P.GVLKYPVKPSEEGK.S | N | 54.97 | 1529.8452 | 14 | -0.7 | 510.9553 | 3 | 24.59 | 1 | F1:11477 | 13012022\_RID\_3262\_4a.raw | 2.1231E6 |  |  |  |  |  |  |  |  |  | 1 | 1 | 0 | 0 | 0 | 0 | 0 | 0 | 0 | 0 | 0 | 156 | 169 |  |  | PEAKS DB |
| D.YEEFLEIAR.N | N | 54.84 | 1168.5764 | 9 | 0.3 | 585.2957 | 2 | 46.57 | 4 | F4:30508 | 17012022\_RID\_3262\_10a .raw | 2.2869E6 |  |  | 1.3254E6 |  |  |  | 2.3858E5 | 1.5579E5 | 1.3646E7 | 5 | 1 | 0 | 0 | 1 | 0 | 0 | 0 | 1 | 1 | 1 | 34 | 42 |  |  | PEAKS DB |
| F.IESMKHDDIFAYEK.R | N | 54.51 | 1724.8079 | 14 | 1.1 | 575.9438 | 3 | 33.93 | 6 | F6:19920 | 17012022\_RID\_3262\_9a.raw |  |  |  |  |  | 3.2417E6 |  |  |  |  | 1 | 0 | 0 | 0 | 0 | 0 | 1 | 0 | 0 | 0 | 0 | 236 | 249 |  |  | PEAKS DB |
| K.EGNLSPGAVDM(+15.99)IGDLLNEDSGYYVSFIESM(+15.99)KHDDIFAYEK.R | Y | 53.18 | 4500.0298 | 40 | -0.3 | 1126.0144 | 4 | 68.67 | 1 | F1:49993 | 13012022\_RID\_3262\_4a.raw | 1.464E7 |  |  |  |  |  |  |  |  |  | 1 | 1 | 0 | 0 | 0 | 0 | 0 | 0 | 0 | 0 | 0 | 210 | 249 | Oxidation (M) | M11:Oxidation (M):1000.00;M30:Oxidation (M):1000.00 | PEAKS DB |
| K.YDTYSTKEYLIK.E | N | 53.18 | 1522.7555 | 12 | 0.9 | 762.3857 | 2 | 34.13 | 1 | F1:20150 | 13012022\_RID\_3262\_4a.raw | 4.6273E6 |  |  |  |  |  |  |  |  |  | 2 | 2 | 0 | 0 | 0 | 0 | 0 | 0 | 0 | 0 | 0 | 198 | 209 |  |  | PEAKS DB |
| K.SAGQLYEESLRK.V | N | 52.72 | 1379.7045 | 12 | 0.5 | 690.8599 | 2 | 24.65 | 9 | F9:9094 | 31122021\_RID\_3262\_2a.raw | 4.3022E7 | 1.5347E6 |  |  |  | 1.5382E4 | 5.71E5 |  | 1.2745E6 | 3.1589E7 | 7 | 2 | 1 | 0 | 0 | 0 | 1 | 1 | 0 | 1 | 1 | 170 | 181 |  |  | PEAKS DB |
| K.STTDLPSR.F | N | 52.16 | 875.4348 | 8 | 0.4 | 438.7249 | 2 | 21.40 | 6 | F6:8926 | 17012022\_RID\_3262\_9a.raw |  | 2.9854E7 |  | 4.8043E6 |  | 1.4473E7 | 1.9071E6 |  |  |  | 4 | 0 | 1 | 0 | 1 | 0 | 1 | 1 | 0 | 0 | 0 | 364 | 371 |  |  | PEAKS DB |
| N.DLSLIHQLPK.K | N | 51.65 | 1162.6710 | 10 | -0.7 | 582.3424 | 2 | 40.68 | 10 | F10:10098 | 31122021\_RID\_3262\_3a.raw |  |  |  |  |  |  |  | 1.197E6 | 2.7589E5 | 1.8174E7 | 3 | 0 | 0 | 0 | 0 | 0 | 0 | 0 | 1 | 1 | 1 | 414 | 423 |  |  | PEAKS DB |
| S.AAYVLAGAGHQVTVLEASER.A | N | 50.51 | 2041.0592 | 20 | 1.3 | 681.3612 | 3 | 40.58 | 1 | F1:26109 | 13012022\_RID\_3262\_4a.raw | 5.4454E5 |  |  |  |  |  |  |  |  |  | 1 | 1 | 0 | 0 | 0 | 0 | 0 | 0 | 0 | 0 | 0 | 66 | 85 |  |  | PEAKS DB |
| A.GAGHQVTVLEASER.A | N | 49.80 | 1452.7321 | 14 | 0.7 | 727.3738 | 2 | 23.21 | 9 | F9:7765 | 31122021\_RID\_3262\_2a.raw | 4.3064E5 |  |  |  |  | 6.0642E5 |  | 6.5701E4 | 8.2054E4 |  | 4 | 1 | 0 | 0 | 0 | 0 | 1 | 0 | 1 | 1 | 0 | 72 | 85 |  |  | PEAKS DB |
| K.RFDEIVGGMDQLPTSM.Y | N | 48.45 | 1794.8280 | 16 | -0.6 | 898.4208 | 2 | 56.96 | 10 | F10:16573 | 31122021\_RID\_3262\_3a.raw |  |  |  |  |  |  |  |  |  | 1.5934E7 | 1 | 0 | 0 | 0 | 0 | 0 | 0 | 0 | 0 | 0 | 1 | 250 | 265 |  |  | PEAKS DB |
| K.EGNLSPGAVDM(+15.99)IGDLLNEDSGYYVSFIESMKHDDIFAYEK.R | Y | 46.38 | 4484.0352 | 40 | 0.4 | 1122.0165 | 4 | 72.19 | 1 | F1:53209 | 13012022\_RID\_3262\_4a.raw | 5.4604E6 |  |  |  |  |  |  |  |  |  | 1 | 1 | 0 | 0 | 0 | 0 | 0 | 0 | 0 | 0 | 0 | 210 | 249 | Oxidation (M) | M11:Oxidation (M):59.55 | PEAKS DB |
| K.KDPGVLKYPVKPSEEGKSAGQLYEESLR.K | N | 42.88 | 3103.6189 | 28 | 1.3 | 776.9130 | 4 | 34.91 | 1 | F1:20825 | 13012022\_RID\_3262\_4a.raw | 7.3864E5 |  |  |  |  |  |  |  |  |  | 1 | 1 | 0 | 0 | 0 | 0 | 0 | 0 | 0 | 0 | 0 | 153 | 180 |  |  | PEAKS DB |
| K.RFDEIVGGM(+15.99)DQLPTSM(+15.99).Y | N | 42.69 | 1826.8179 | 16 | -0.4 | 914.4158 | 2 | 56.93 | 10 | F10:16509 | 31122021\_RID\_3262\_3a.raw |  |  |  |  |  |  |  |  |  | 0 | 0 | 0 | 0 | 0 | 0 | 0 | 0 | 0 | 0 | 0 | 0 | 250 | 265 | Oxidation (M) | M9:Oxidation (M):1000.00;M16:Oxidation (M):1000.00 | PEAKS DB |
| Q.VTVLEASER.A | N | 42.68 | 1002.5345 | 9 | -0.1 | 502.2745 | 2 | 28.85 | 7 | F7:11280 | 21012022\_RID\_3262\_7a.raw |  |  |  |  |  |  | 1.7683E5 |  |  |  | 1 | 0 | 0 | 0 | 0 | 0 | 0 | 1 | 0 | 0 | 0 | 77 | 85 |  |  | PEAKS DB |
| S.FIESMKHDDIFAYEK.R | N | 42.11 | 1871.8763 | 15 | -0.8 | 624.9656 | 3 | 39.08 | 1 | F1:24705 | 13012022\_RID\_3262\_4a.raw | 7.5077E5 |  |  |  |  |  |  |  |  |  | 1 | 1 | 0 | 0 | 0 | 0 | 0 | 0 | 0 | 0 | 0 | 235 | 249 |  |  | PEAKS DB |
| A.GHQVTVLEASER.A | N | 42.02 | 1324.6735 | 12 | 1.8 | 663.3452 | 2 | 22.45 | 8 | F8:6784 | 31122021\_RID\_3262\_1a.raw |  |  |  |  |  |  |  | 1.4851E4 |  |  | 1 | 0 | 0 | 0 | 0 | 0 | 0 | 0 | 1 | 0 | 0 | 74 | 85 |  |  | PEAKS DB |
| N.EDSGYYVSFIESMK.H | N | 41.95 | 1653.7231 | 14 | 1.1 | 827.8698 | 2 | 57.32 | 8 | F8:33993 | 31122021\_RID\_3262\_1a.raw |  |  |  |  |  |  |  | 7.6176E4 |  |  | 1 | 0 | 0 | 0 | 0 | 0 | 0 | 0 | 1 | 0 | 0 | 227 | 240 |  |  | PEAKS DB |
| H.QVTVLEASER.A | N | 41.88 | 1130.5931 | 10 | -0.3 | 566.3036 | 2 | 28.32 | 9 | F9:11956 | 31122021\_RID\_3262\_2a.raw |  |  |  |  |  |  |  |  | 3.805E5 |  | 1 | 0 | 0 | 0 | 0 | 0 | 0 | 0 | 0 | 1 | 0 | 76 | 85 |  |  | PEAKS DB |
| K.EGNLSPGAVDM(+15.99)IGDLLNEDSGYYVSFIESM(+15.99)K.H | Y | 41.64 | 3381.5269 | 31 | 0.0 | 1128.1829 | 3 | 70.66 | 1 | F1:51623 | 13012022\_RID\_3262\_4a.raw | 5.9214E6 |  |  |  |  |  |  |  |  | 0 | 1 | 1 | 0 | 0 | 0 | 0 | 0 | 0 | 0 | 0 | 0 | 210 | 240 | Oxidation (M) | M11:Oxidation (M):1000.00;M30:Oxidation (M):1000.00 | PEAKS DB |
| A.AYVLAGAGHQVTVLEASER.A | N | 41.51 | 1970.0221 | 19 | -0.2 | 657.6812 | 3 | 38.48 | 10 | F10:9343 | 31122021\_RID\_3262\_3a.raw |  |  |  |  |  |  |  |  |  | 1.157E6 | 1 | 0 | 0 | 0 | 0 | 0 | 0 | 0 | 0 | 0 | 1 | 67 | 85 |  |  | PEAKS DB |
| K.RFDEIVGGMDQLPT.S | N | 41.44 | 1576.7555 | 14 | -0.6 | 789.3846 | 2 | 51.27 | 1 | F1:35302 | 13012022\_RID\_3262\_4a.raw | 1.0726E6 |  |  |  |  |  |  |  |  |  | 1 | 1 | 0 | 0 | 0 | 0 | 0 | 0 | 0 | 0 | 0 | 250 | 263 |  |  | PEAKS DB |
| total 42 peptides |  |  |  |  |  |  |  |  |  |  |  |  |  |  |  |  |  |  |  |  |  |  |  |  |  |  |  |  |  |  |  |  |  |  |  |  |  |
| --- | --- | --- | --- | --- | --- | --- | --- | --- | --- | --- | --- | --- | --- | --- | --- | --- | --- | --- | --- | --- | --- | --- | --- | --- | --- | --- | --- | --- | --- | --- | --- | --- | --- | --- | --- | --- | --- |

BAP39950.1

back to list

  

| Protein Coverage
| Supporting Peptides
|

Protein Coverage:

Supporting Peptides:

| Peptide | Uniq | -10lgP | Mass | Length | ppm | m/z | z | RT | Fraction | Scan | Source File | Area F4 | Area F5 | Area F6 | Area F10 | Area F8 | Area F9 | Area F7 | Area F1 | Area F2 | Area F3 | #Feature | #Feature F4 | #Feature F5 | #Feature F6 | #Feature F10 | #Feature F8 | #Feature F9 | #Feature F7 | #Feature F1 | #Feature F2 | #Feature F3 | Start | End | PTM | AScore | Found By |
| --- | --- | --- | --- | --- | --- | --- | --- | --- | --- | --- | --- | --- | --- | --- | --- | --- | --- | --- | --- | --- | --- | --- | --- | --- | --- | --- | --- | --- | --- | --- | --- | --- | --- | --- | --- | --- | --- |
| K.KDPGVLKYPVKPSEEGK.S | N | 79.57 | 1870.0199 | 17 | -0.7 | 468.5119 | 4 | 24.47 | 1 | F1:11529 | 13012022\_RID\_3262\_4a.raw | 3.7125E8 | 1.4174E6 |  |  |  | 8.1047E6 | 1.2066E6 | 5.7794E6 | 6.6831E6 | 5.2885E7 | 16 | 4 | 2 | 0 | 0 | 0 | 2 | 2 | 2 | 2 | 2 | 153 | 169 |  |  | PEAKS DB |
| K.DPGVLKYPVKPSEEGK.S | N | 78.91 | 1741.9249 | 16 | -0.1 | 581.6489 | 3 | 29.60 | 1 | F1:16075 | 13012022\_RID\_3262\_4a.raw | 3.1808E7 | 7.5919E5 |  |  |  |  | 2.7752E5 | 4.9105E5 | 7.4531E5 | 1.0783E7 | 8 | 2 | 1 | 0 | 0 | 0 | 0 | 1 | 1 | 2 | 1 | 154 | 169 |  |  | PEAKS DB |
| K.YPVKPSEEGKSAGQLYEESLR.K | N | 72.83 | 2366.1753 | 21 | 0.7 | 592.5515 | 4 | 32.87 | 1 | F1:19085 | 13012022\_RID\_3262\_4a.raw | 3.4004E6 |  |  |  |  |  |  |  |  |  | 2 | 2 | 0 | 0 | 0 | 0 | 0 | 0 | 0 | 0 | 0 | 160 | 180 |  |  | PEAKS DB |
| K.SAGQLYEESLR.K | N | 72.77 | 1251.6095 | 11 | 0.4 | 626.8123 | 2 | 32.12 | 8 | F8:14092 | 31122021\_RID\_3262\_1a.raw | 1.2366E9 | 8.6285E7 | 3.6644E7 | 6.5882E5 |  | 6.8121E6 | 2.7435E7 | 6.1833E7 | 1.0188E8 | 2.1008E9 | 11 | 2 | 1 | 1 | 1 | 0 | 1 | 1 | 1 | 1 | 2 | 170 | 180 |  |  | PEAKS DB |
| K.HDDIFAYEK.R | N | 71.92 | 1136.5138 | 9 | 0.2 | 569.2643 | 2 | 33.07 | 1 | F1:19276 | 13012022\_RID\_3262\_4a.raw | 2.205E8 | 5.0368E7 | 1.6541E6 | 5.2373E7 |  | 6.378E6 | 6.4081E6 | 1.8941E7 | 3.5546E7 | 1.9591E9 | 33 | 5 | 2 | 2 | 4 | 0 | 2 | 2 | 4 | 4 | 8 | 241 | 249 |  |  | PEAKS DB |
| K.KFWEDDGIHGGK.S | N | 70.84 | 1387.6520 | 12 | 1.0 | 694.8340 | 2 | 27.52 | 1 | F1:14353 | 13012022\_RID\_3262\_4a.raw | 2.2688E8 | 4.7295E4 |  |  |  |  |  | 1.1825E7 | 1.8101E7 | 8.0079E8 | 17 | 4 | 1 | 0 | 0 | 0 | 0 | 0 | 3 | 3 | 6 | 352 | 363 |  |  | PEAKS DB |
| Y.GIGDDANFFQALDFK.S | N | 70.77 | 1656.7783 | 15 | 0.6 | 829.3970 | 2 | 63.80 | 9 | F9:39957 | 31122021\_RID\_3262\_2a.raw |  | 4.1343E5 |  |  |  |  |  | 2.2577E6 | 1.7889E6 | 7.0811E7 | 6 | 0 | 1 | 0 | 0 | 0 | 0 | 0 | 2 | 1 | 2 | 391 | 405 |  |  | PEAKS DB |
| D.PGVLKYPVKPSEEGK.S | N | 70.72 | 1626.8981 | 15 | 0.0 | 543.3066 | 3 | 25.59 | 1 | F1:12547 | 13012022\_RID\_3262\_4a.raw | 1.4062E8 |  |  | 4.7646E5 |  | 4.3107E6 |  | 4.013E5 |  | 5.7619E6 | 8 | 3 | 0 | 0 | 1 | 0 | 2 | 0 | 1 | 0 | 1 | 155 | 169 |  |  | PEAKS DB |
| K.YPVKPSEEGK.S | N | 69.75 | 1132.5764 | 10 | 0.7 | 567.2959 | 2 | 17.88 | 6 | F6:5952 | 17012022\_RID\_3262\_9a.raw | 4.2299E8 | 5.2685E7 | 2.5077E5 | 3.2771E7 |  | 6.9462E7 | 2.576E7 |  |  |  | 12 | 2 | 2 | 1 | 3 | 0 | 2 | 2 | 0 | 0 | 0 | 160 | 169 |  |  | PEAKS DB |
| I.AYGIGDDANFFQALDFK.S | N | 69.60 | 1890.8788 | 17 | 0.2 | 946.4468 | 2 | 65.58 | 8 | F8:40325 | 31122021\_RID\_3262\_1a.raw |  |  |  |  |  |  |  | 1.5459E5 |  |  | 1 | 0 | 0 | 0 | 0 | 0 | 0 | 0 | 1 | 0 | 0 | 389 | 405 |  |  | PEAKS DB |
| L.SAAYVLAGAGHQVTVLEASER.A | N | 67.91 | 2128.0913 | 21 | 0.0 | 1065.0530 | 2 | 41.69 | 10 | F10:10487 | 31122021\_RID\_3262\_3a.raw | 1.4728E6 |  |  |  |  |  |  | 6.4802E5 | 6.599E5 | 2.6305E7 | 6 | 1 | 0 | 0 | 0 | 0 | 0 | 0 | 2 | 1 | 2 | 65 | 85 |  |  | PEAKS DB |
| K.FWEDDGIHGGK.S | N | 65.97 | 1259.5570 | 11 | 0.4 | 630.7860 | 2 | 31.13 | 1 | F1:17598 | 13012022\_RID\_3262\_4a.raw | 7.5921E7 | 9.0996E6 |  |  |  | 1.9943E6 |  | 6.128E6 | 1.0893E7 | 5.5749E8 | 17 | 2 | 2 | 0 | 0 | 0 | 1 | 0 | 3 | 3 | 6 | 353 | 363 |  |  | PEAKS DB |
| K.EGNLSPGAVDMIGDLLNEDSGYYVSFIESMK.H | Y | 65.38 | 3349.5369 | 31 | 0.7 | 1117.5204 | 3 | 81.20 | 1 | F1:59522 | 13012022\_RID\_3262\_4a.raw | 7.7147E6 |  |  |  |  |  |  |  |  |  | 1 | 1 | 0 | 0 | 0 | 0 | 0 | 0 | 0 | 0 | 0 | 210 | 240 |  |  | PEAKS DB |
| A.YVLAGAGHQVTVLEASER.A | N | 64.94 | 1898.9850 | 18 | 0.8 | 950.5005 | 2 | 37.54 | 10 | F10:8957 | 31122021\_RID\_3262\_3a.raw | 9.515E5 |  |  |  |  |  |  |  |  | 6.9519E6 | 3 | 1 | 0 | 0 | 0 | 0 | 0 | 0 | 0 | 0 | 2 | 68 | 85 |  |  | PEAKS DB |
| K.EGNLSPGAVDM(+15.99)IGDLLNEDSGYYVSFIESMK.H | Y | 61.82 | 3365.5320 | 31 | 0.2 | 1122.8514 | 3 | 75.85 | 1 | F1:55688 | 13012022\_RID\_3262\_4a.raw | 1.9966E7 |  |  |  |  |  |  | 6.7671E5 |  |  | 2 | 1 | 0 | 0 | 0 | 0 | 0 | 0 | 1 | 0 | 0 | 210 | 240 | Oxidation (M) | M11:Oxidation (M):117.45 | PEAKS DB |
| K.HDDIFAYEKR.F | N | 61.79 | 1292.6149 | 10 | 0.7 | 431.8792 | 3 | 27.51 | 1 | F1:14388 | 13012022\_RID\_3262\_4a.raw | 1.0662E7 |  |  | 1.628E5 |  |  |  |  |  |  | 3 | 2 | 0 | 0 | 1 | 0 | 0 | 0 | 0 | 0 | 0 | 241 | 250 |  |  | PEAKS DB |
| F.IESM(+15.99)KHDDIFAYEK.R | N | 61.58 | 1740.8029 | 14 | 0.9 | 581.2754 | 3 | 29.10 | 6 | F6:15895 | 17012022\_RID\_3262\_9a.raw |  |  |  |  |  | 3.2962E6 |  |  |  |  | 1 | 0 | 0 | 0 | 0 | 0 | 1 | 0 | 0 | 0 | 0 | 236 | 249 | Oxidation (M) | M4:Oxidation (M):1000.00 | PEAKS DB |
| Y.VLAGAGHQVTVLEASER.A | N | 61.46 | 1735.9216 | 17 | 0.9 | 579.6483 | 3 | 33.06 | 6 | F6:19337 | 17012022\_RID\_3262\_9a.raw | 2.8948E5 | 1.1934E6 |  | 6.6625E5 |  | 3.8459E6 |  | 1.4002E6 | 1.9495E6 | 3.1893E7 | 10 | 1 | 1 | 0 | 1 | 0 | 1 | 0 | 2 | 2 | 2 | 69 | 85 |  |  | PEAKS DB |
| K.VTVTYQTPAK.D | N | 59.36 | 1106.5972 | 10 | -0.1 | 554.3058 | 2 | 27.24 | 7 | F7:10262 | 21012022\_RID\_3262\_7a.raw |  |  |  |  |  | 1.7478E6 | 1.5831E6 |  |  |  | 2 | 0 | 0 | 0 | 0 | 0 | 1 | 1 | 0 | 0 | 0 | 289 | 298 |  |  | PEAKS DB |
| S.MKHDDIFAYEK.R | N | 57.56 | 1395.6493 | 11 | 0.9 | 466.2241 | 3 | 28.76 | 6 | F6:15683 | 17012022\_RID\_3262\_9a.raw |  |  |  |  |  | 1.5918E6 |  |  |  |  | 2 | 0 | 0 | 0 | 0 | 0 | 2 | 0 | 0 | 0 | 0 | 239 | 249 |  |  | PEAKS DB |
| P.GVLKYPVKPSEEGK.S | N | 54.97 | 1529.8452 | 14 | -0.7 | 510.9553 | 3 | 24.59 | 1 | F1:11477 | 13012022\_RID\_3262\_4a.raw | 2.1231E6 |  |  |  |  |  |  |  |  |  | 1 | 1 | 0 | 0 | 0 | 0 | 0 | 0 | 0 | 0 | 0 | 156 | 169 |  |  | PEAKS DB |
| D.YEEFLEIAR.N | N | 54.84 | 1168.5764 | 9 | 0.3 | 585.2957 | 2 | 46.57 | 4 | F4:30508 | 17012022\_RID\_3262\_10a .raw | 2.2869E6 |  |  | 1.3254E6 |  |  |  | 2.3858E5 | 1.5579E5 | 1.3646E7 | 5 | 1 | 0 | 0 | 1 | 0 | 0 | 0 | 1 | 1 | 1 | 34 | 42 |  |  | PEAKS DB |
| F.IESMKHDDIFAYEK.R | N | 54.51 | 1724.8079 | 14 | 1.1 | 575.9438 | 3 | 33.93 | 6 | F6:19920 | 17012022\_RID\_3262\_9a.raw |  |  |  |  |  | 3.2417E6 |  |  |  |  | 1 | 0 | 0 | 0 | 0 | 0 | 1 | 0 | 0 | 0 | 0 | 236 | 249 |  |  | PEAKS DB |
| K.EGNLSPGAVDM(+15.99)IGDLLNEDSGYYVSFIESM(+15.99)KHDDIFAYEK.R | Y | 53.18 | 4500.0298 | 40 | -0.3 | 1126.0144 | 4 | 68.67 | 1 | F1:49993 | 13012022\_RID\_3262\_4a.raw | 1.464E7 |  |  |  |  |  |  |  |  |  | 1 | 1 | 0 | 0 | 0 | 0 | 0 | 0 | 0 | 0 | 0 | 210 | 249 | Oxidation (M) | M11:Oxidation (M):1000.00;M30:Oxidation (M):1000.00 | PEAKS DB |
| K.YDTYSTKEYLIK.E | N | 53.18 | 1522.7555 | 12 | 0.9 | 762.3857 | 2 | 34.13 | 1 | F1:20150 | 13012022\_RID\_3262\_4a.raw | 4.6273E6 |  |  |  |  |  |  |  |  |  | 2 | 2 | 0 | 0 | 0 | 0 | 0 | 0 | 0 | 0 | 0 | 198 | 209 |  |  | PEAKS DB |
| K.SAGQLYEESLRK.V | N | 52.72 | 1379.7045 | 12 | 0.5 | 690.8599 | 2 | 24.65 | 9 | F9:9094 | 31122021\_RID\_3262\_2a.raw | 4.3022E7 | 1.5347E6 |  |  |  | 1.5382E4 | 5.71E5 |  | 1.2745E6 | 3.1589E7 | 7 | 2 | 1 | 0 | 0 | 0 | 1 | 1 | 0 | 1 | 1 | 170 | 181 |  |  | PEAKS DB |
| K.STTDLPSR.F | N | 52.16 | 875.4348 | 8 | 0.4 | 438.7249 | 2 | 21.40 | 6 | F6:8926 | 17012022\_RID\_3262\_9a.raw |  | 2.9854E7 |  | 4.8043E6 |  | 1.4473E7 | 1.9071E6 |  |  |  | 4 | 0 | 1 | 0 | 1 | 0 | 1 | 1 | 0 | 0 | 0 | 364 | 371 |  |  | PEAKS DB |
| N.DLSLIHQLPK.K | N | 51.65 | 1162.6710 | 10 | -0.7 | 582.3424 | 2 | 40.68 | 10 | F10:10098 | 31122021\_RID\_3262\_3a.raw |  |  |  |  |  |  |  | 1.197E6 | 2.7589E5 | 1.8174E7 | 3 | 0 | 0 | 0 | 0 | 0 | 0 | 0 | 1 | 1 | 1 | 414 | 423 |  |  | PEAKS DB |
| S.AAYVLAGAGHQVTVLEASER.A | N | 50.51 | 2041.0592 | 20 | 1.3 | 681.3612 | 3 | 40.58 | 1 | F1:26109 | 13012022\_RID\_3262\_4a.raw | 5.4454E5 |  |  |  |  |  |  |  |  |  | 1 | 1 | 0 | 0 | 0 | 0 | 0 | 0 | 0 | 0 | 0 | 66 | 85 |  |  | PEAKS DB |
| A.GAGHQVTVLEASER.A | N | 49.80 | 1452.7321 | 14 | 0.7 | 727.3738 | 2 | 23.21 | 9 | F9:7765 | 31122021\_RID\_3262\_2a.raw | 4.3064E5 |  |  |  |  | 6.0642E5 |  | 6.5701E4 | 8.2054E4 |  | 4 | 1 | 0 | 0 | 0 | 0 | 1 | 0 | 1 | 1 | 0 | 72 | 85 |  |  | PEAKS DB |
| K.RFDEIVGGMDQLPTSM.Y | N | 48.45 | 1794.8280 | 16 | -0.6 | 898.4208 | 2 | 56.96 | 10 | F10:16573 | 31122021\_RID\_3262\_3a.raw |  |  |  |  |  |  |  |  |  | 1.5934E7 | 1 | 0 | 0 | 0 | 0 | 0 | 0 | 0 | 0 | 0 | 1 | 250 | 265 |  |  | PEAKS DB |
| K.EGNLSPGAVDM(+15.99)IGDLLNEDSGYYVSFIESMKHDDIFAYEK.R | Y | 46.38 | 4484.0352 | 40 | 0.4 | 1122.0165 | 4 | 72.19 | 1 | F1:53209 | 13012022\_RID\_3262\_4a.raw | 5.4604E6 |  |  |  |  |  |  |  |  |  | 1 | 1 | 0 | 0 | 0 | 0 | 0 | 0 | 0 | 0 | 0 | 210 | 249 | Oxidation (M) | M11:Oxidation (M):59.55 | PEAKS DB |
| K.KDPGVLKYPVKPSEEGKSAGQLYEESLR.K | N | 42.88 | 3103.6189 | 28 | 1.3 | 776.9130 | 4 | 34.91 | 1 | F1:20825 | 13012022\_RID\_3262\_4a.raw | 7.3864E5 |  |  |  |  |  |  |  |  |  | 1 | 1 | 0 | 0 | 0 | 0 | 0 | 0 | 0 | 0 | 0 | 153 | 180 |  |  | PEAKS DB |
| K.RFDEIVGGM(+15.99)DQLPTSM(+15.99).Y | N | 42.69 | 1826.8179 | 16 | -0.4 | 914.4158 | 2 | 56.93 | 10 | F10:16509 | 31122021\_RID\_3262\_3a.raw |  |  |  |  |  |  |  |  |  | 0 | 0 | 0 | 0 | 0 | 0 | 0 | 0 | 0 | 0 | 0 | 0 | 250 | 265 | Oxidation (M) | M9:Oxidation (M):1000.00;M16:Oxidation (M):1000.00 | PEAKS DB |
| Q.VTVLEASER.A | N | 42.68 | 1002.5345 | 9 | -0.1 | 502.2745 | 2 | 28.85 | 7 | F7:11280 | 21012022\_RID\_3262\_7a.raw |  |  |  |  |  |  | 1.7683E5 |  |  |  | 1 | 0 | 0 | 0 | 0 | 0 | 0 | 1 | 0 | 0 | 0 | 77 | 85 |  |  | PEAKS DB |
| S.FIESMKHDDIFAYEK.R | N | 42.11 | 1871.8763 | 15 | -0.8 | 624.9656 | 3 | 39.08 | 1 | F1:24705 | 13012022\_RID\_3262\_4a.raw | 7.5077E5 |  |  |  |  |  |  |  |  |  | 1 | 1 | 0 | 0 | 0 | 0 | 0 | 0 | 0 | 0 | 0 | 235 | 249 |  |  | PEAKS DB |
| A.GHQVTVLEASER.A | N | 42.02 | 1324.6735 | 12 | 1.8 | 663.3452 | 2 | 22.45 | 8 | F8:6784 | 31122021\_RID\_3262\_1a.raw |  |  |  |  |  |  |  | 1.4851E4 |  |  | 1 | 0 | 0 | 0 | 0 | 0 | 0 | 0 | 1 | 0 | 0 | 74 | 85 |  |  | PEAKS DB |
| N.EDSGYYVSFIESMK.H | N | 41.95 | 1653.7231 | 14 | 1.1 | 827.8698 | 2 | 57.32 | 8 | F8:33993 | 31122021\_RID\_3262\_1a.raw |  |  |  |  |  |  |  | 7.6176E4 |  |  | 1 | 0 | 0 | 0 | 0 | 0 | 0 | 0 | 1 | 0 | 0 | 227 | 240 |  |  | PEAKS DB |
| H.QVTVLEASER.A | N | 41.88 | 1130.5931 | 10 | -0.3 | 566.3036 | 2 | 28.32 | 9 | F9:11956 | 31122021\_RID\_3262\_2a.raw |  |  |  |  |  |  |  |  | 3.805E5 |  | 1 | 0 | 0 | 0 | 0 | 0 | 0 | 0 | 0 | 1 | 0 | 76 | 85 |  |  | PEAKS DB |
| K.EGNLSPGAVDM(+15.99)IGDLLNEDSGYYVSFIESM(+15.99)K.H | Y | 41.64 | 3381.5269 | 31 | 0.0 | 1128.1829 | 3 | 70.66 | 1 | F1:51623 | 13012022\_RID\_3262\_4a.raw | 5.9214E6 |  |  |  |  |  |  |  |  | 0 | 1 | 1 | 0 | 0 | 0 | 0 | 0 | 0 | 0 | 0 | 0 | 210 | 240 | Oxidation (M) | M11:Oxidation (M):1000.00;M30:Oxidation (M):1000.00 | PEAKS DB |
| A.AYVLAGAGHQVTVLEASER.A | N | 41.51 | 1970.0221 | 19 | -0.2 | 657.6812 | 3 | 38.48 | 10 | F10:9343 | 31122021\_RID\_3262\_3a.raw |  |  |  |  |  |  |  |  |  | 1.157E6 | 1 | 0 | 0 | 0 | 0 | 0 | 0 | 0 | 0 | 0 | 1 | 67 | 85 |  |  | PEAKS DB |
| K.RFDEIVGGMDQLPT.S | N | 41.44 | 1576.7555 | 14 | -0.6 | 789.3846 | 2 | 51.27 | 1 | F1:35302 | 13012022\_RID\_3262\_4a.raw | 1.0726E6 |  |  |  |  |  |  |  |  |  | 1 | 1 | 0 | 0 | 0 | 0 | 0 | 0 | 0 | 0 | 0 | 250 | 263 |  |  | PEAKS DB |
| total 42 peptides |  |  |  |  |  |  |  |  |  |  |  |  |  |  |  |  |  |  |  |  |  |  |  |  |  |  |  |  |  |  |  |  |  |  |  |  |  |
| --- | --- | --- | --- | --- | --- | --- | --- | --- | --- | --- | --- | --- | --- | --- | --- | --- | --- | --- | --- | --- | --- | --- | --- | --- | --- | --- | --- | --- | --- | --- | --- | --- | --- | --- | --- | --- | --- |

JAV01884.1

back to list

  

| Protein Coverage
| Supporting Peptides
|

Protein Coverage:

Supporting Peptides:

| Peptide | Uniq | -10lgP | Mass | Length | ppm | m/z | z | RT | Fraction | Scan | Source File | Area F4 | Area F5 | Area F6 | Area F10 | Area F8 | Area F9 | Area F7 | Area F1 | Area F2 | Area F3 | #Feature | #Feature F4 | #Feature F5 | #Feature F6 | #Feature F10 | #Feature F8 | #Feature F9 | #Feature F7 | #Feature F1 | #Feature F2 | #Feature F3 | Start | End | PTM | AScore | Found By |
| --- | --- | --- | --- | --- | --- | --- | --- | --- | --- | --- | --- | --- | --- | --- | --- | --- | --- | --- | --- | --- | --- | --- | --- | --- | --- | --- | --- | --- | --- | --- | --- | --- | --- | --- | --- | --- | --- |
| R.IDKVNLMVDQQWMAVR.N | N | 89.89 | 1944.9913 | 16 | 0.6 | 649.3381 | 3 | 49.05 | 8 | F8:27583 | 31122021\_RID\_3262\_1a.raw |  |  |  |  |  |  |  | 1.2194E7 | 3.5597E5 |  | 3 | 0 | 0 | 0 | 0 | 0 | 0 | 0 | 2 | 1 | 0 | 394 | 409 |  |  | PEAKS DB |
| K.VNLMVDQQWMAVR.N | N | 80.06 | 1588.7854 | 13 | 0.8 | 795.4006 | 2 | 52.32 | 8 | F8:30174 | 31122021\_RID\_3262\_1a.raw |  |  |  |  |  |  |  | 3.4969E6 |  |  | 1 | 0 | 0 | 0 | 0 | 0 | 0 | 0 | 1 | 0 | 0 | 397 | 409 |  |  | PEAKS DB |
| R.DVELLTGLNFYSGLK.Q | N | 79.71 | 1667.8770 | 15 | 0.4 | 834.9461 | 2 | 67.85 | 8 | F8:42145 | 31122021\_RID\_3262\_1a.raw |  |  |  |  |  |  |  | 1.5005E7 | 4.8402E5 |  | 5 | 0 | 0 | 0 | 0 | 0 | 0 | 0 | 4 | 1 | 0 | 775 | 789 |  |  | PEAKS DB |
| K.NPAWWGGQPIWHTATYQGLK.A | N | 77.54 | 2310.1333 | 20 | 1.0 | 771.0525 | 3 | 50.58 | 8 | F8:28794 | 31122021\_RID\_3262\_1a.raw |  |  |  |  |  |  |  | 2.7775E7 |  |  | 2 | 0 | 0 | 0 | 0 | 0 | 0 | 0 | 2 | 0 | 0 | 185 | 204 |  |  | PEAKS DB |
| R.IDKVNLM(+15.99)VDQQWM(+15.99)AVR.N | N | 73.40 | 1976.9812 | 16 | 0.5 | 660.0013 | 3 | 40.77 | 8 | F8:21007 | 31122021\_RID\_3262\_1a.raw |  |  |  |  |  |  |  | 1.5146E6 |  |  | 1 | 0 | 0 | 0 | 0 | 0 | 0 | 0 | 1 | 0 | 0 | 394 | 409 | Oxidation (M) | M7:Oxidation (M):1000.00;M13:Oxidation (M):1000.00 | PEAKS DB |
| R.LWNYFHTTLIPK.Y | N | 73.37 | 1531.8187 | 12 | 0.5 | 766.9170 | 2 | 47.15 | 8 | F8:26107 | 31122021\_RID\_3262\_1a.raw | 8.3337E5 |  |  |  |  |  |  | 5.0587E7 |  |  | 3 | 1 | 0 | 0 | 0 | 0 | 0 | 0 | 2 | 0 | 0 | 658 | 669 |  |  | PEAKS DB |
| K.VNLM(+15.99)VDQQWMAVR.N | N | 70.81 | 1604.7803 | 13 | 0.4 | 803.3977 | 2 | 48.28 | 8 | F8:27028 | 31122021\_RID\_3262\_1a.raw |  |  |  |  |  |  |  | 2.7138E6 | 8.2065E4 |  | 2 | 0 | 0 | 0 | 0 | 0 | 0 | 0 | 1 | 1 | 0 | 397 | 409 | Oxidation (M) | M4:Oxidation (M):176.18 | PEAKS DB |
| K.NPFYTPSPAK.E | N | 68.72 | 1120.5553 | 10 | -0.2 | 561.2848 | 2 | 31.38 | 8 | F8:13580 | 31122021\_RID\_3262\_1a.raw | 2.0471E6 |  |  |  |  |  |  | 3.8456E7 | 4.8509E6 |  | 4 | 1 | 0 | 0 | 0 | 0 | 0 | 0 | 2 | 1 | 0 | 483 | 492 |  |  | PEAKS DB |
| R.VRDVELLTGLNFYSGLK.Q | N | 68.06 | 1923.0465 | 17 | 0.9 | 642.0234 | 3 | 54.52 | 8 | F8:31944 | 31122021\_RID\_3262\_1a.raw | 5.1091E5 |  |  |  |  |  |  | 2.4463E7 | 3.6342E5 |  | 5 | 1 | 0 | 0 | 0 | 0 | 0 | 0 | 3 | 1 | 0 | 773 | 789 |  |  | PEAKS DB |
| N.YGPVSGEIIK.A | N | 66.88 | 1061.5757 | 10 | -0.3 | 531.7950 | 2 | 34.11 | 8 | F8:15575 | 31122021\_RID\_3262\_1a.raw | 5.5409E6 | 5.2937E5 |  |  |  |  |  | 8.5643E7 | 1.0829E7 |  | 4 | 1 | 1 | 0 | 0 | 0 | 0 | 0 | 1 | 1 | 0 | 270 | 279 |  |  | PEAKS DB |
| K.AATYFWPGSEVK.I | N | 65.68 | 1354.6558 | 12 | 0.6 | 678.3356 | 2 | 44.70 | 8 | F8:24117 | 31122021\_RID\_3262\_1a.raw | 4.0357E6 |  |  |  |  |  |  | 5.8345E7 | 2.2909E6 |  | 3 | 1 | 0 | 0 | 0 | 0 | 0 | 0 | 1 | 1 | 0 | 205 | 216 |  |  | PEAKS DB |
| R.IDKVNLM(+15.99)VDQQWMAVR.N | N | 65.60 | 1960.9863 | 16 | 0.2 | 654.6695 | 3 | 45.45 | 8 | F8:24763 | 31122021\_RID\_3262\_1a.raw |  |  |  |  |  |  |  | 1.034E7 | 1.8006E5 |  | 5 | 0 | 0 | 0 | 0 | 0 | 0 | 0 | 4 | 1 | 0 | 394 | 409 | Oxidation (M) | M7:Oxidation (M):156.85 | PEAKS DB |
| R.TLGMLMEGLK.Q | N | 65.55 | 1091.5719 | 10 | 0.0 | 546.7932 | 2 | 47.83 | 8 | F8:26797 | 31122021\_RID\_3262\_1a.raw |  |  |  |  |  |  |  | 2.0277E7 | 9.0693E5 |  | 2 | 0 | 0 | 0 | 0 | 0 | 0 | 0 | 1 | 1 | 0 | 287 | 296 |  |  | PEAKS DB |
| R.TLGM(+15.99)LMEGLK.Q | N | 65.37 | 1107.5668 | 10 | 0.5 | 554.7910 | 2 | 43.66 | 8 | F8:23669 | 31122021\_RID\_3262\_1a.raw |  |  |  |  |  |  |  | 1.1063E8 | 4.1807E6 |  | 5 | 0 | 0 | 0 | 0 | 0 | 0 | 0 | 3 | 2 | 0 | 287 | 296 | Oxidation (M) | M4:Oxidation (M):67.96 | PEAKS DB |
| K.GGTHGYDNEFK.S | N | 64.84 | 1223.5206 | 11 | 0.4 | 612.7678 | 2 | 18.14 | 8 | F8:4090 | 31122021\_RID\_3262\_1a.raw |  |  |  |  |  |  |  | 1.1563E7 | 3.8742E5 |  | 3 | 0 | 0 | 0 | 0 | 0 | 0 | 0 | 2 | 1 | 0 | 418 | 428 |  |  | PEAKS DB |
| K.TFLPIFVNPVN | N | 64.77 | 1259.6914 | 11 | 0.6 | 630.8533 | 2 | 72.29 | 8 | F8:45470 | 31122021\_RID\_3262\_1a.raw | 3.3682E6 |  |  |  |  |  |  | 3.0009E7 | 1.5173E6 |  | 3 | 1 | 0 | 0 | 0 | 0 | 0 | 0 | 1 | 1 | 0 | 800 | 810 |  |  | PEAKS DB |
| R.TLGMLM(+15.99)EGLK.Q | N | 58.46 | 1107.5668 | 10 | 0.5 | 554.7910 | 2 | 44.17 | 8 | F8:26515 | 31122021\_RID\_3262\_1a.raw |  |  |  |  |  |  |  | 4.7543E7 |  |  | 2 | 0 | 0 | 0 | 0 | 0 | 0 | 0 | 2 | 0 | 0 | 287 | 296 | Oxidation (M) | M6:Oxidation (M):67.96 | PEAKS DB |
| K.RLHYVNNIR.I | N | 55.97 | 1183.6573 | 9 | -0.5 | 395.5595 | 3 | 17.72 | 8 | F8:3970 | 31122021\_RID\_3262\_1a.raw |  |  |  |  |  |  |  | 7.9803E6 | 1.085E6 |  | 2 | 0 | 0 | 0 | 0 | 0 | 0 | 0 | 1 | 1 | 0 | 385 | 393 |  |  | PEAKS DB |
| R.TLGM(+15.99)LM(+15.99)EGLK.Q | N | 53.04 | 1123.5618 | 10 | -0.5 | 562.7879 | 2 | 44.08 | 8 | F8:23974 | 31122021\_RID\_3262\_1a.raw |  |  |  |  |  |  |  | 2.6054E7 | 1.1517E6 |  | 12 | 0 | 0 | 0 | 0 | 0 | 0 | 0 | 10 | 2 | 0 | 287 | 296 | Oxidation (M) | M4:Oxidation (M):1000.00;M6:Oxidation (M):1000.00 | PEAKS DB |
| K.QPLPETLQLK.T | N | 52.54 | 1165.6707 | 10 | 1.0 | 583.8432 | 2 | 39.62 | 8 | F8:20222 | 31122021\_RID\_3262\_1a.raw | 2.6906E6 |  |  |  |  |  |  | 4.8465E7 | 1.0101E6 |  | 3 | 1 | 0 | 0 | 0 | 0 | 0 | 0 | 1 | 1 | 0 | 790 | 799 |  |  | PEAKS DB |
| R.IDKVNLMVDQQWM(+15.99)AVR.N | N | 52.50 | 1960.9863 | 16 | 0.6 | 654.6698 | 3 | 44.30 | 8 | F8:23687 | 31122021\_RID\_3262\_1a.raw |  |  |  |  |  |  |  | 4.3693E6 |  |  | 4 | 0 | 0 | 0 | 0 | 0 | 0 | 0 | 4 | 0 | 0 | 394 | 409 | Oxidation (M) | M13:Oxidation (M):108.85 | PEAKS DB |
| K.VNLMVDQQWM(+15.99)AVR.N | N | 52.36 | 1604.7803 | 13 | -1.8 | 803.3960 | 2 | 46.44 | 8 | F8:25468 | 31122021\_RID\_3262\_1a.raw |  |  |  |  |  |  |  | 0 |  |  | 0 | 0 | 0 | 0 | 0 | 0 | 0 | 0 | 0 | 0 | 0 | 397 | 409 | Oxidation (M) | M10:Oxidation (M):109.28 | PEAKS DB |
| R.IDKVNLMVDQQWMAV.R | N | 51.38 | 1788.8903 | 15 | 0.6 | 895.4529 | 2 | 58.81 | 8 | F8:35116 | 31122021\_RID\_3262\_1a.raw |  |  |  |  |  |  |  | 2.4944E6 |  |  | 1 | 0 | 0 | 0 | 0 | 0 | 0 | 0 | 1 | 0 | 0 | 394 | 408 |  |  | PEAKS DB |
| K.TFLPIFVNPV.N | N | 50.01 | 1145.6484 | 10 | 0.9 | 573.8320 | 2 | 74.94 | 8 | F8:46923 | 31122021\_RID\_3262\_1a.raw |  |  |  |  |  |  |  | 2.2916E6 | 1.7438E5 |  | 2 | 0 | 0 | 0 | 0 | 0 | 0 | 0 | 1 | 1 | 0 | 800 | 809 |  |  | PEAKS DB |
| R.DVELLTGLNFYSGLKQPLPETLQLK.T | N | 49.64 | 2815.5371 | 25 | 1.2 | 939.5208 | 3 | 67.16 | 8 | F8:41552 | 31122021\_RID\_3262\_1a.raw |  |  |  |  |  |  |  | 2.4935E5 |  |  | 1 | 0 | 0 | 0 | 0 | 0 | 0 | 0 | 1 | 0 | 0 | 775 | 799 |  |  | PEAKS DB |
| L.ILFSMDGFR.A | N | 49.34 | 1084.5375 | 9 | 0.2 | 543.2761 | 2 | 52.66 | 8 | F8:30315 | 31122021\_RID\_3262\_1a.raw |  |  |  |  |  |  |  | 1.6418E5 |  |  | 1 | 0 | 0 | 0 | 0 | 0 | 0 | 0 | 1 | 0 | 0 | 101 | 109 |  |  | PEAKS DB |
| M.VDQQWMAVR.N | N | 48.37 | 1131.5496 | 9 | 0.7 | 566.7825 | 2 | 36.04 | 8 | F8:17176 | 31122021\_RID\_3262\_1a.raw |  |  |  |  |  |  |  | 1.0158E5 |  |  | 1 | 0 | 0 | 0 | 0 | 0 | 0 | 0 | 1 | 0 | 0 | 401 | 409 |  |  | PEAKS DB |
| K.DYYTFDSEGIVK.N | Y | 48.36 | 1435.6506 | 12 | -1.6 | 718.8314 | 2 | 52.63 | 8 | F8:30301 | 31122021\_RID\_3262\_1a.raw |  |  |  |  |  |  |  | 2.2913E5 |  |  | 1 | 0 | 0 | 0 | 0 | 0 | 0 | 0 | 1 | 0 | 0 | 352 | 363 |  |  | PEAKS DB |
| K.NPAWWGGQPIWHTATYQGL.K | N | 48.24 | 2182.0383 | 19 | 0.2 | 1092.0266 | 2 | 59.92 | 8 | F8:36107 | 31122021\_RID\_3262\_1a.raw |  |  |  |  |  |  |  | 8.5153E5 |  |  | 1 | 0 | 0 | 0 | 0 | 0 | 0 | 0 | 1 | 0 | 0 | 185 | 203 |  |  | PEAKS DB |
| Y.DALITSNIVPMFK.G | N | 48.12 | 1447.7744 | 13 | 1.0 | 724.8952 | 2 | 60.81 | 8 | F8:36680 | 31122021\_RID\_3262\_1a.raw |  |  |  |  |  |  |  | 4.3711E4 |  |  | 1 | 0 | 0 | 0 | 0 | 0 | 0 | 0 | 1 | 0 | 0 | 641 | 653 |  |  | PEAKS DB |
| K.NPAWWGGQPIWH.T | N | 48.02 | 1447.6786 | 12 | 8.8 | 724.8529 | 2 | 54.69 | 8 | F8:31999 | 31122021\_RID\_3262\_1a.raw |  |  |  |  |  |  |  | 3.0315E5 |  |  | 2 | 0 | 0 | 0 | 0 | 0 | 0 | 0 | 2 | 0 | 0 | 185 | 196 |  |  | PEAKS DB |
| K.VNLM(+15.99)VDQQWM(+15.99)AVR.N | N | 47.24 | 1620.7753 | 13 | 0.4 | 811.3953 | 2 | 42.79 | 8 | F8:22684 | 31122021\_RID\_3262\_1a.raw |  |  |  |  |  |  |  | 6.7039E5 |  |  | 2 | 0 | 0 | 0 | 0 | 0 | 0 | 0 | 2 | 0 | 0 | 397 | 409 | Oxidation (M) | M4:Oxidation (M):1000.00;M10:Oxidation (M):1000.00 | PEAKS DB |
| R.VRDVELLTGLNFYSGL.K | N | 47.12 | 1794.9515 | 16 | 1.1 | 898.4840 | 2 | 65.47 | 8 | F8:40254 | 31122021\_RID\_3262\_1a.raw |  |  |  |  |  |  |  | 2.3332E5 |  |  | 1 | 0 | 0 | 0 | 0 | 0 | 0 | 0 | 1 | 0 | 0 | 773 | 788 |  |  | PEAKS DB |
| R.VRDVELLTGLNF.Y | N | 47.05 | 1374.7507 | 12 | 0.6 | 688.3831 | 2 | 57.99 | 8 | F8:34552 | 31122021\_RID\_3262\_1a.raw |  |  |  |  |  |  |  | 8.975E4 |  |  | 1 | 0 | 0 | 0 | 0 | 0 | 0 | 0 | 1 | 0 | 0 | 773 | 784 |  |  | PEAKS DB |
| R.LHYVNNIR.I | N | 46.24 | 1027.5563 | 8 | -0.3 | 514.7853 | 2 | 20.66 | 8 | F8:5659 | 31122021\_RID\_3262\_1a.raw |  |  |  |  |  |  |  | 3.7778E6 |  |  | 1 | 0 | 0 | 0 | 0 | 0 | 0 | 0 | 1 | 0 | 0 | 386 | 393 |  |  | PEAKS DB |
| K.NPAWWGGQPIWHT.A | N | 45.39 | 1548.7262 | 13 | 1.0 | 775.3712 | 2 | 55.91 | 8 | F8:32881 | 31122021\_RID\_3262\_1a.raw |  |  |  |  |  |  |  | 1.6497E5 |  |  | 1 | 0 | 0 | 0 | 0 | 0 | 0 | 0 | 1 | 0 | 0 | 185 | 197 |  |  | PEAKS DB |
| K.SMQAIFLAH.G | N | 44.69 | 1016.5114 | 9 | 0.0 | 509.2629 | 2 | 44.53 | 8 | F8:23867 | 31122021\_RID\_3262\_1a.raw |  |  |  |  |  |  |  | 9.9535E5 |  |  | 1 | 0 | 0 | 0 | 0 | 0 | 0 | 0 | 1 | 0 | 0 | 429 | 437 |  |  | PEAKS DB |
| N.PFYTPSPAK.E | N | 42.64 | 1006.5123 | 9 | 0.3 | 504.2636 | 2 | 29.12 | 8 | F8:11767 | 31122021\_RID\_3262\_1a.raw |  |  |  |  |  |  |  | 8.451E5 |  |  | 1 | 0 | 0 | 0 | 0 | 0 | 0 | 0 | 1 | 0 | 0 | 484 | 492 |  |  | PEAKS DB |
| K.VNLMVDQQWMAV.R | N | 41.30 | 1432.6843 | 12 | 0.5 | 717.3498 | 2 | 66.02 | 8 | F8:40783 | 31122021\_RID\_3262\_1a.raw |  |  |  |  |  |  |  | 6.3792E5 |  |  | 1 | 0 | 0 | 0 | 0 | 0 | 0 | 0 | 1 | 0 | 0 | 397 | 408 |  |  | PEAKS DB |
| Q.SPLILFSMDGFR.A | N | 41.10 | 1381.7064 | 12 | -0.3 | 691.8603 | 2 | 63.15 | 8 | F8:38506 | 31122021\_RID\_3262\_1a.raw |  |  |  |  |  |  |  | 2.0761E4 |  |  | 1 | 0 | 0 | 0 | 0 | 0 | 0 | 0 | 1 | 0 | 0 | 98 | 109 |  |  | PEAKS DB |
| R.VRDVELLTGLNFY.S | N | 40.90 | 1537.8140 | 13 | 0.5 | 769.9147 | 2 | 60.07 | 8 | F8:36272 | 31122021\_RID\_3262\_1a.raw |  |  |  |  |  |  |  | 7.5704E5 |  |  | 1 | 0 | 0 | 0 | 0 | 0 | 0 | 0 | 1 | 0 | 0 | 773 | 785 |  |  | PEAKS DB |
| total 41 peptides |  |  |  |  |  |  |  |  |  |  |  |  |  |  |  |  |  |  |  |  |  |  |  |  |  |  |  |  |  |  |  |  |  |  |  |  |  |
| --- | --- | --- | --- | --- | --- | --- | --- | --- | --- | --- | --- | --- | --- | --- | --- | --- | --- | --- | --- | --- | --- | --- | --- | --- | --- | --- | --- | --- | --- | --- | --- | --- | --- | --- | --- | --- | --- |

SMD28240.1

back to list

  

| Protein Coverage
| Supporting Peptides
|

Protein Coverage:

Supporting Peptides:

| Peptide | Uniq | -10lgP | Mass | Length | ppm | m/z | z | RT | Fraction | Scan | Source File | Area F4 | Area F5 | Area F6 | Area F10 | Area F8 | Area F9 | Area F7 | Area F1 | Area F2 | Area F3 | #Feature | #Feature F4 | #Feature F5 | #Feature F6 | #Feature F10 | #Feature F8 | #Feature F9 | #Feature F7 | #Feature F1 | #Feature F2 | #Feature F3 | Start | End | PTM | AScore | Found By |
| --- | --- | --- | --- | --- | --- | --- | --- | --- | --- | --- | --- | --- | --- | --- | --- | --- | --- | --- | --- | --- | --- | --- | --- | --- | --- | --- | --- | --- | --- | --- | --- | --- | --- | --- | --- | --- | --- |
| R.HGLEFSYEMAPR.A | N | 89.78 | 1435.6554 | 12 | 0.2 | 718.8351 | 2 | 38.88 | 1 | F1:24671 | 13012022\_RID\_3262\_4a.raw | 1.3085E8 |  |  |  |  |  |  | 4.3045E5 | 5.2978E4 |  | 6 | 3 | 0 | 0 | 0 | 0 | 0 | 0 | 2 | 1 | 0 | 427 | 438 |  |  | PEAKS DB |
| K.HQGLPESYNFDFVTMKPVL | N | 88.30 | 2221.0876 | 19 | 1.0 | 741.3705 | 3 | 60.75 | 1 | F1:42994 | 13012022\_RID\_3262\_4a.raw | 1.5758E8 |  |  |  |  |  |  | 9.1193E5 |  |  | 5 | 4 | 0 | 0 | 0 | 0 | 0 | 0 | 1 | 0 | 0 | 535 | 553 |  |  | PEAKS DB |
| K.HQGLPESYNFDFVTM(+15.99)KPVL | N | 82.55 | 2237.0828 | 19 | 0.9 | 746.7022 | 3 | 54.75 | 1 | F1:37685 | 13012022\_RID\_3262\_4a.raw | 8.3546E7 |  |  |  |  |  |  |  |  |  | 4 | 4 | 0 | 0 | 0 | 0 | 0 | 0 | 0 | 0 | 0 | 535 | 553 | Oxidation (M) | M15:Oxidation (M):1000.00 | PEAKS DB |
| K.FTAYAINGPPVEK.G | N | 79.96 | 1405.7241 | 13 | 0.8 | 703.8699 | 2 | 39.56 | 1 | F1:25211 | 13012022\_RID\_3262\_4a.raw | 6.1472E7 | 2.7303E6 | 5.4329E5 |  |  |  |  | 1.3216E5 |  |  | 4 | 1 | 1 | 1 | 0 | 0 | 0 | 0 | 1 | 0 | 0 | 508 | 520 |  |  | PEAKS DB |
| K.QNSGTYNNQYMILDTK.K | N | 76.97 | 1888.8625 | 16 | -0.2 | 945.4384 | 2 | 40.58 | 1 | F1:26159 | 13012022\_RID\_3262\_4a.raw | 4.9824E7 |  |  |  |  |  |  |  |  |  | 3 | 3 | 0 | 0 | 0 | 0 | 0 | 0 | 0 | 0 | 0 | 351 | 366 |  |  | PEAKS DB |
| R.YNNYKEDPYAK.R | N | 76.33 | 1403.6357 | 11 | 0.4 | 702.8254 | 2 | 21.66 | 1 | F1:9212 | 13012022\_RID\_3262\_4a.raw | 1.0942E8 | 9.3729E5 |  |  |  |  | 9.4335E4 | 1.4673E5 |  |  | 6 | 3 | 1 | 0 | 0 | 0 | 0 | 1 | 1 | 0 | 0 | 461 | 471 |  |  | PEAKS DB |
| R.HGLEFSYEM(+15.99)APR.A | N | 75.94 | 1451.6503 | 12 | 0.5 | 726.8328 | 2 | 33.69 | 1 | F1:19718 | 13012022\_RID\_3262\_4a.raw | 7.8446E7 | 8.9934E5 |  |  |  |  |  |  |  |  | 7 | 5 | 2 | 0 | 0 | 0 | 0 | 0 | 0 | 0 | 0 | 427 | 438 | Oxidation (M) | M9:Oxidation (M):1000.00 | PEAKS DB |
| R.YNNYKEDPYAKR.N | N | 75.65 | 1559.7368 | 12 | -0.7 | 520.9192 | 3 | 18.70 | 1 | F1:6501 | 13012022\_RID\_3262\_4a.raw | 1.0783E7 |  |  |  |  |  |  |  |  |  | 2 | 2 | 0 | 0 | 0 | 0 | 0 | 0 | 0 | 0 | 0 | 461 | 472 |  |  | PEAKS DB |
| R.SLEDGTLYIIEQVPK.L | Y | 74.84 | 1703.8981 | 15 | 0.9 | 852.9571 | 2 | 58.14 | 3 | F3:28167 | 13012022\_RID\_3262\_6a.raw | 3.0719E8 | 5.1718E6 | 1.8973E6 |  |  |  |  | 3.1069E6 | 1.3137E6 |  | 10 | 4 | 1 | 1 | 0 | 0 | 0 | 0 | 3 | 1 | 0 | 373 | 387 |  |  | PEAKS DB |
| K.VVPESLFAWER.V | N | 74.17 | 1331.6874 | 11 | 0.3 | 666.8512 | 2 | 53.43 | 1 | F1:37684 | 13012022\_RID\_3262\_4a.raw | 1.6375E8 | 3.8989E6 |  |  |  |  |  | 1.2567E6 |  |  | 3 | 1 | 1 | 0 | 0 | 0 | 0 | 0 | 1 | 0 | 0 | 320 | 330 |  |  | PEAKS DB |
| K.TKHQGLPESYNFDFVTMKPVL | N | 73.21 | 2450.2305 | 21 | 0.9 | 613.5654 | 4 | 61.28 | 1 | F1:43650 | 13012022\_RID\_3262\_4a.raw | 2.0337E7 |  |  |  |  |  |  |  |  |  | 3 | 3 | 0 | 0 | 0 | 0 | 0 | 0 | 0 | 0 | 0 | 533 | 553 |  |  | PEAKS DB |
| K.QNSGTYNNQYM(+15.99)ILDTK.K | N | 70.11 | 1904.8574 | 16 | 0.2 | 953.4362 | 2 | 36.64 | 1 | F1:22592 | 13012022\_RID\_3262\_4a.raw | 7.9472E6 |  |  |  |  |  |  |  |  |  | 2 | 2 | 0 | 0 | 0 | 0 | 0 | 0 | 0 | 0 | 0 | 351 | 366 | Oxidation (M) | M11:Oxidation (M):1000.00 | PEAKS DB |
| R.IANM(+15.99)M(+15.99)ADSGKTWAETFEK.Q | N | 69.13 | 2060.9182 | 18 | 0.8 | 687.9805 | 3 | 35.55 | 1 | F1:21622 | 13012022\_RID\_3262\_4a.raw | 5.8045E6 |  |  |  |  |  |  |  |  |  | 1 | 1 | 0 | 0 | 0 | 0 | 0 | 0 | 0 | 0 | 0 | 333 | 350 | Oxidation (M) | M4:Oxidation (M):1000.00;M5:Oxidation (M):1000.00 | PEAKS DB |
| K.KVVPESLFAWER.V | N | 68.16 | 1459.7823 | 12 | 0.2 | 730.8986 | 2 | 45.80 | 1 | F1:30481 | 13012022\_RID\_3262\_4a.raw | 6.9146E7 |  |  |  |  |  |  | 2.9993E5 |  |  | 9 | 8 | 0 | 0 | 0 | 0 | 0 | 0 | 1 | 0 | 0 | 319 | 330 |  |  | PEAKS DB |
| R.NGYWPSYNIPFDK.E | N | 67.37 | 1599.7357 | 13 | 0.3 | 800.8754 | 2 | 52.81 | 1 | F1:36697 | 13012022\_RID\_3262\_4a.raw | 1.6428E7 |  |  |  |  |  |  |  |  |  | 1 | 1 | 0 | 0 | 0 | 0 | 0 | 0 | 0 | 0 | 0 | 400 | 412 |  |  | PEAKS DB |
| K.QNSGTYNNQYMILDTKK.I | N | 66.25 | 2016.9575 | 17 | 0.0 | 673.3265 | 3 | 33.86 | 1 | F1:19896 | 13012022\_RID\_3262\_4a.raw | 2.1321E7 |  |  |  |  |  |  |  |  |  | 2 | 2 | 0 | 0 | 0 | 0 | 0 | 0 | 0 | 0 | 0 | 351 | 367 |  |  | PEAKS DB |
| R.IANM(+15.99)MADSGKTWAETFEK.Q | N | 66.09 | 2044.9233 | 18 | 0.8 | 682.6489 | 3 | 40.44 | 1 | F1:25809 | 13012022\_RID\_3262\_4a.raw | 8.9561E6 |  |  |  |  |  |  |  |  |  | 2 | 2 | 0 | 0 | 0 | 0 | 0 | 0 | 0 | 0 | 0 | 333 | 350 | Oxidation (M) | M4:Oxidation (M):20.00 | PEAKS DB |
| R.DQGKVTDMESMK.F | N | 65.74 | 1367.6061 | 12 | 0.0 | 684.8103 | 2 | 25.62 | 1 | F1:12494 | 13012022\_RID\_3262\_4a.raw | 3.691E7 | 1.0003E5 |  |  |  |  |  |  |  |  | 3 | 2 | 1 | 0 | 0 | 0 | 0 | 0 | 0 | 0 | 0 | 445 | 456 |  |  | PEAKS DB |
| R.IANMMADSGK.T | N | 60.65 | 1036.4681 | 10 | 0.3 | 519.2415 | 2 | 26.29 | 1 | F1:13060 | 13012022\_RID\_3262\_4a.raw | 1.1087E7 |  |  |  |  |  |  |  |  |  | 1 | 1 | 0 | 0 | 0 | 0 | 0 | 0 | 0 | 0 | 0 | 333 | 342 |  |  | PEAKS DB |
| K.VADISMAAK.F | N | 59.57 | 904.4688 | 9 | 0.6 | 453.2419 | 2 | 26.16 | 8 | F8:9517 | 31122021\_RID\_3262\_1a.raw | 6.1959E7 | 2.0025E6 |  |  |  |  |  | 2.035E5 | 1.4637E5 |  | 4 | 1 | 1 | 0 | 0 | 0 | 0 | 0 | 1 | 1 | 0 | 499 | 507 |  |  | PEAKS DB |
| R.IANMM(+15.99)ADSGK.T | N | 57.25 | 1052.4630 | 10 | -0.7 | 527.2384 | 2 | 26.29 | 1 | F1:13130 | 13012022\_RID\_3262\_4a.raw | 1.8568E6 |  |  |  |  |  |  |  |  |  | 1 | 1 | 0 | 0 | 0 | 0 | 0 | 0 | 0 | 0 | 0 | 333 | 342 | Oxidation (M) | M5:Oxidation (M):18.17 | PEAKS DB |
| K.TKHQGLPESYNFDFVTM(+15.99)KPVL | N | 52.45 | 2466.2253 | 21 | 0.6 | 823.0829 | 3 | 46.88 | 1 | F1:31507 | 13012022\_RID\_3262\_4a.raw | 3.3945E6 |  |  |  |  |  |  |  |  |  | 2 | 2 | 0 | 0 | 0 | 0 | 0 | 0 | 0 | 0 | 0 | 533 | 553 | Oxidation (M) | M17:Oxidation (M):1000.00 | PEAKS DB |
| K.QNSGTYNNQYM(+15.99)ILDTKK.I | N | 51.05 | 2032.9524 | 17 | 0.1 | 678.6581 | 3 | 33.88 | 1 | F1:19963 | 13012022\_RID\_3262\_4a.raw | 1.3281E6 |  |  |  |  |  |  |  |  |  | 1 | 1 | 0 | 0 | 0 | 0 | 0 | 0 | 0 | 0 | 0 | 351 | 367 | Oxidation (M) | M11:Oxidation (M):1000.00 | PEAKS DB |
| K.HQGLPESYNFDFVTMKPV.L | N | 51.01 | 2108.0037 | 18 | 0.6 | 703.6756 | 3 | 51.54 | 1 | F1:35496 | 13012022\_RID\_3262\_4a.raw | 1.7469E6 |  |  |  |  |  |  |  |  |  | 1 | 1 | 0 | 0 | 0 | 0 | 0 | 0 | 0 | 0 | 0 | 535 | 552 |  |  | PEAKS DB |
| R.DQGKVTDMESM(+15.99)K.F | N | 50.79 | 1383.6010 | 12 | 0.2 | 692.8079 | 2 | 25.63 | 1 | F1:12244 | 13012022\_RID\_3262\_4a.raw | 3.9723E6 |  |  |  |  |  |  |  |  |  | 1 | 1 | 0 | 0 | 0 | 0 | 0 | 0 | 0 | 0 | 0 | 445 | 456 | Oxidation (M) | M11:Oxidation (M):30.72 | PEAKS DB |
| R.IANMMADSGKTWAETFEK.Q | N | 49.19 | 2028.9285 | 18 | 0.7 | 677.3173 | 3 | 45.14 | 1 | F1:30139 | 13012022\_RID\_3262\_4a.raw | 7.7306E5 |  |  |  |  |  |  |  |  |  | 1 | 1 | 0 | 0 | 0 | 0 | 0 | 0 | 0 | 0 | 0 | 333 | 350 |  |  | PEAKS DB |
| K.TWAETFEK.Q | N | 48.61 | 1010.4709 | 8 | -0.3 | 506.2426 | 2 | 35.90 | 2 | F2:19245 | 13012022\_RID\_3262\_5a.raw | 1.3699E8 | 4.4157E6 |  |  |  |  |  |  |  |  | 2 | 1 | 1 | 0 | 0 | 0 | 0 | 0 | 0 | 0 | 0 | 343 | 350 |  |  | PEAKS DB |
| K.VLPGYENIYFAH.S | N | 46.73 | 1421.6979 | 12 | 0.8 | 711.8568 | 2 | 49.22 | 1 | F1:33555 | 13012022\_RID\_3262\_4a.raw | 3.4702E6 |  |  |  |  |  |  |  |  |  | 1 | 1 | 0 | 0 | 0 | 0 | 0 | 0 | 0 | 0 | 0 | 239 | 250 |  |  | PEAKS DB |
| R.DQGKVTDM(+15.99)ESMK.F | N | 46.37 | 1383.6010 | 12 | 0.2 | 692.8079 | 2 | 25.63 | 1 | F1:12495 | 13012022\_RID\_3262\_4a.raw | 5.5334E6 |  |  |  |  |  |  |  |  |  | 2 | 2 | 0 | 0 | 0 | 0 | 0 | 0 | 0 | 0 | 0 | 445 | 456 | Oxidation (M) | M8:Oxidation (M):11.67 | PEAKS DB |
| K.HQGLPESYNFDFVTM(+15.99)KPV.L | N | 44.49 | 2123.9985 | 18 | 0.3 | 709.0070 | 3 | 47.31 | 1 | F1:31932 | 13012022\_RID\_3262\_4a.raw | 9.0568E5 |  |  |  |  |  |  |  |  |  | 1 | 1 | 0 | 0 | 0 | 0 | 0 | 0 | 0 | 0 | 0 | 535 | 552 | Oxidation (M) | M15:Oxidation (M):1000.00 | PEAKS DB |
| N.FDFVTMKPVL | N | 43.97 | 1195.6311 | 10 | 0.3 | 598.8230 | 2 | 56.81 | 1 | F1:39982 | 13012022\_RID\_3262\_4a.raw | 8.4752E5 |  |  |  |  |  |  |  |  |  | 1 | 1 | 0 | 0 | 0 | 0 | 0 | 0 | 0 | 0 | 0 | 544 | 553 |  |  | PEAKS DB |
| K.VADISM(+15.99)AAK.F | N | 42.64 | 920.4637 | 9 | -0.1 | 461.2391 | 2 | 27.69 | 1 | F1:14400 | 13012022\_RID\_3262\_4a.raw | 4.2128E6 |  |  |  |  |  |  |  |  |  | 1 | 1 | 0 | 0 | 0 | 0 | 0 | 0 | 0 | 0 | 0 | 499 | 507 | Oxidation (M) | M6:Oxidation (M):1000.00 | PEAKS DB |
| K.VTDMESM(+15.99)K.F | N | 41.24 | 955.3990 | 8 | 0.5 | 478.7070 | 2 | 18.80 | 2 | F2:5395 | 13012022\_RID\_3262\_5a.raw |  | 4.526E5 |  |  |  |  |  |  |  |  | 1 | 0 | 1 | 0 | 0 | 0 | 0 | 0 | 0 | 0 | 0 | 449 | 456 | Oxidation (M) | M7:Oxidation (M):30.72 | PEAKS DB |
| total 33 peptides |  |  |  |  |  |  |  |  |  |  |  |  |  |  |  |  |  |  |  |  |  |  |  |  |  |  |  |  |  |  |  |  |  |  |  |  |  |
| --- | --- | --- | --- | --- | --- | --- | --- | --- | --- | --- | --- | --- | --- | --- | --- | --- | --- | --- | --- | --- | --- | --- | --- | --- | --- | --- | --- | --- | --- | --- | --- | --- | --- | --- | --- | --- | --- |

JAV49636.1

back to list

  

| Protein Coverage
| Supporting Peptides
|

Protein Coverage:

Supporting Peptides:

| Peptide | Uniq | -10lgP | Mass | Length | ppm | m/z | z | RT | Fraction | Scan | Source File | Area F4 | Area F5 | Area F6 | Area F10 | Area F8 | Area F9 | Area F7 | Area F1 | Area F2 | Area F3 | #Feature | #Feature F4 | #Feature F5 | #Feature F6 | #Feature F10 | #Feature F8 | #Feature F9 | #Feature F7 | #Feature F1 | #Feature F2 | #Feature F3 | Start | End | PTM | AScore | Found By |
| --- | --- | --- | --- | --- | --- | --- | --- | --- | --- | --- | --- | --- | --- | --- | --- | --- | --- | --- | --- | --- | --- | --- | --- | --- | --- | --- | --- | --- | --- | --- | --- | --- | --- | --- | --- | --- | --- |
| R.HGLEFSYEMAPR.A | N | 89.78 | 1435.6554 | 12 | 0.2 | 718.8351 | 2 | 38.88 | 1 | F1:24671 | 13012022\_RID\_3262\_4a.raw | 1.3085E8 |  |  |  |  |  |  | 4.3045E5 | 5.2978E4 |  | 6 | 3 | 0 | 0 | 0 | 0 | 0 | 0 | 2 | 1 | 0 | 427 | 438 |  |  | PEAKS DB |
| K.HQGLPESYNFDFVTMKPVL | N | 88.30 | 2221.0876 | 19 | 1.0 | 741.3705 | 3 | 60.75 | 1 | F1:42994 | 13012022\_RID\_3262\_4a.raw | 1.5758E8 |  |  |  |  |  |  | 9.1193E5 |  |  | 5 | 4 | 0 | 0 | 0 | 0 | 0 | 0 | 1 | 0 | 0 | 535 | 553 |  |  | PEAKS DB |
| K.HQGLPESYNFDFVTM(+15.99)KPVL | N | 82.55 | 2237.0828 | 19 | 0.9 | 746.7022 | 3 | 54.75 | 1 | F1:37685 | 13012022\_RID\_3262\_4a.raw | 8.3546E7 |  |  |  |  |  |  |  |  |  | 4 | 4 | 0 | 0 | 0 | 0 | 0 | 0 | 0 | 0 | 0 | 535 | 553 | Oxidation (M) | M15:Oxidation (M):1000.00 | PEAKS DB |
| K.FTAYAINGPPVEK.G | N | 79.96 | 1405.7241 | 13 | 0.8 | 703.8699 | 2 | 39.56 | 1 | F1:25211 | 13012022\_RID\_3262\_4a.raw | 6.1472E7 | 2.7303E6 | 5.4329E5 |  |  |  |  | 1.3216E5 |  |  | 4 | 1 | 1 | 1 | 0 | 0 | 0 | 0 | 1 | 0 | 0 | 508 | 520 |  |  | PEAKS DB |
| K.QNSGTYNNQYMILDTK.K | N | 76.97 | 1888.8625 | 16 | -0.2 | 945.4384 | 2 | 40.58 | 1 | F1:26159 | 13012022\_RID\_3262\_4a.raw | 4.9824E7 |  |  |  |  |  |  |  |  |  | 3 | 3 | 0 | 0 | 0 | 0 | 0 | 0 | 0 | 0 | 0 | 351 | 366 |  |  | PEAKS DB |
| R.YNNYKEDPYAK.R | N | 76.33 | 1403.6357 | 11 | 0.4 | 702.8254 | 2 | 21.66 | 1 | F1:9212 | 13012022\_RID\_3262\_4a.raw | 1.0942E8 | 9.3729E5 |  |  |  |  | 9.4335E4 | 1.4673E5 |  |  | 6 | 3 | 1 | 0 | 0 | 0 | 0 | 1 | 1 | 0 | 0 | 461 | 471 |  |  | PEAKS DB |
| R.HGLEFSYEM(+15.99)APR.A | N | 75.94 | 1451.6503 | 12 | 0.5 | 726.8328 | 2 | 33.69 | 1 | F1:19718 | 13012022\_RID\_3262\_4a.raw | 7.8446E7 | 8.9934E5 |  |  |  |  |  |  |  |  | 7 | 5 | 2 | 0 | 0 | 0 | 0 | 0 | 0 | 0 | 0 | 427 | 438 | Oxidation (M) | M9:Oxidation (M):1000.00 | PEAKS DB |
| R.YNNYKEDPYAKR.N | N | 75.65 | 1559.7368 | 12 | -0.7 | 520.9192 | 3 | 18.70 | 1 | F1:6501 | 13012022\_RID\_3262\_4a.raw | 1.0783E7 |  |  |  |  |  |  |  |  |  | 2 | 2 | 0 | 0 | 0 | 0 | 0 | 0 | 0 | 0 | 0 | 461 | 472 |  |  | PEAKS DB |
| R.SLEDGTLYIIEQVPK.L | Y | 74.84 | 1703.8981 | 15 | 0.9 | 852.9571 | 2 | 58.14 | 3 | F3:28167 | 13012022\_RID\_3262\_6a.raw | 3.0719E8 | 5.1718E6 | 1.8973E6 |  |  |  |  | 3.1069E6 | 1.3137E6 |  | 10 | 4 | 1 | 1 | 0 | 0 | 0 | 0 | 3 | 1 | 0 | 373 | 387 |  |  | PEAKS DB |
| K.VVPESLFAWER.V | N | 74.17 | 1331.6874 | 11 | 0.3 | 666.8512 | 2 | 53.43 | 1 | F1:37684 | 13012022\_RID\_3262\_4a.raw | 1.6375E8 | 3.8989E6 |  |  |  |  |  | 1.2567E6 |  |  | 3 | 1 | 1 | 0 | 0 | 0 | 0 | 0 | 1 | 0 | 0 | 320 | 330 |  |  | PEAKS DB |
| K.TKHQGLPESYNFDFVTMKPVL | N | 73.21 | 2450.2305 | 21 | 0.9 | 613.5654 | 4 | 61.28 | 1 | F1:43650 | 13012022\_RID\_3262\_4a.raw | 2.0337E7 |  |  |  |  |  |  |  |  |  | 3 | 3 | 0 | 0 | 0 | 0 | 0 | 0 | 0 | 0 | 0 | 533 | 553 |  |  | PEAKS DB |
| K.QNSGTYNNQYM(+15.99)ILDTK.K | N | 70.11 | 1904.8574 | 16 | 0.2 | 953.4362 | 2 | 36.64 | 1 | F1:22592 | 13012022\_RID\_3262\_4a.raw | 7.9472E6 |  |  |  |  |  |  |  |  |  | 2 | 2 | 0 | 0 | 0 | 0 | 0 | 0 | 0 | 0 | 0 | 351 | 366 | Oxidation (M) | M11:Oxidation (M):1000.00 | PEAKS DB |
| R.IANM(+15.99)M(+15.99)ADSGKTWAETFEK.Q | N | 69.13 | 2060.9182 | 18 | 0.8 | 687.9805 | 3 | 35.55 | 1 | F1:21622 | 13012022\_RID\_3262\_4a.raw | 5.8045E6 |  |  |  |  |  |  |  |  |  | 1 | 1 | 0 | 0 | 0 | 0 | 0 | 0 | 0 | 0 | 0 | 333 | 350 | Oxidation (M) | M4:Oxidation (M):1000.00;M5:Oxidation (M):1000.00 | PEAKS DB |
| K.KVVPESLFAWER.V | N | 68.16 | 1459.7823 | 12 | 0.2 | 730.8986 | 2 | 45.80 | 1 | F1:30481 | 13012022\_RID\_3262\_4a.raw | 6.9146E7 |  |  |  |  |  |  | 2.9993E5 |  |  | 9 | 8 | 0 | 0 | 0 | 0 | 0 | 0 | 1 | 0 | 0 | 319 | 330 |  |  | PEAKS DB |
| R.NGYWPSYNIPFDK.E | N | 67.37 | 1599.7357 | 13 | 0.3 | 800.8754 | 2 | 52.81 | 1 | F1:36697 | 13012022\_RID\_3262\_4a.raw | 1.6428E7 |  |  |  |  |  |  |  |  |  | 1 | 1 | 0 | 0 | 0 | 0 | 0 | 0 | 0 | 0 | 0 | 400 | 412 |  |  | PEAKS DB |
| K.QNSGTYNNQYMILDTKK.I | N | 66.25 | 2016.9575 | 17 | 0.0 | 673.3265 | 3 | 33.86 | 1 | F1:19896 | 13012022\_RID\_3262\_4a.raw | 2.1321E7 |  |  |  |  |  |  |  |  |  | 2 | 2 | 0 | 0 | 0 | 0 | 0 | 0 | 0 | 0 | 0 | 351 | 367 |  |  | PEAKS DB |
| R.IANM(+15.99)MADSGKTWAETFEK.Q | N | 66.09 | 2044.9233 | 18 | 0.8 | 682.6489 | 3 | 40.44 | 1 | F1:25809 | 13012022\_RID\_3262\_4a.raw | 8.9561E6 |  |  |  |  |  |  |  |  |  | 2 | 2 | 0 | 0 | 0 | 0 | 0 | 0 | 0 | 0 | 0 | 333 | 350 | Oxidation (M) | M4:Oxidation (M):20.00 | PEAKS DB |
| R.DQGKVTDMESMK.F | N | 65.74 | 1367.6061 | 12 | 0.0 | 684.8103 | 2 | 25.62 | 1 | F1:12494 | 13012022\_RID\_3262\_4a.raw | 3.691E7 | 1.0003E5 |  |  |  |  |  |  |  |  | 3 | 2 | 1 | 0 | 0 | 0 | 0 | 0 | 0 | 0 | 0 | 445 | 456 |  |  | PEAKS DB |
| R.IANMMADSGK.T | N | 60.65 | 1036.4681 | 10 | 0.3 | 519.2415 | 2 | 26.29 | 1 | F1:13060 | 13012022\_RID\_3262\_4a.raw | 1.1087E7 |  |  |  |  |  |  |  |  |  | 1 | 1 | 0 | 0 | 0 | 0 | 0 | 0 | 0 | 0 | 0 | 333 | 342 |  |  | PEAKS DB |
| K.VADISMAAK.F | N | 59.57 | 904.4688 | 9 | 0.6 | 453.2419 | 2 | 26.16 | 8 | F8:9517 | 31122021\_RID\_3262\_1a.raw | 6.1959E7 | 2.0025E6 |  |  |  |  |  | 2.035E5 | 1.4637E5 |  | 4 | 1 | 1 | 0 | 0 | 0 | 0 | 0 | 1 | 1 | 0 | 499 | 507 |  |  | PEAKS DB |
| R.IANMM(+15.99)ADSGK.T | N | 57.25 | 1052.4630 | 10 | -0.7 | 527.2384 | 2 | 26.29 | 1 | F1:13130 | 13012022\_RID\_3262\_4a.raw | 1.8568E6 |  |  |  |  |  |  |  |  |  | 1 | 1 | 0 | 0 | 0 | 0 | 0 | 0 | 0 | 0 | 0 | 333 | 342 | Oxidation (M) | M5:Oxidation (M):18.17 | PEAKS DB |
| K.TKHQGLPESYNFDFVTM(+15.99)KPVL | N | 52.45 | 2466.2253 | 21 | 0.6 | 823.0829 | 3 | 46.88 | 1 | F1:31507 | 13012022\_RID\_3262\_4a.raw | 3.3945E6 |  |  |  |  |  |  |  |  |  | 2 | 2 | 0 | 0 | 0 | 0 | 0 | 0 | 0 | 0 | 0 | 533 | 553 | Oxidation (M) | M17:Oxidation (M):1000.00 | PEAKS DB |
| K.QNSGTYNNQYM(+15.99)ILDTKK.I | N | 51.05 | 2032.9524 | 17 | 0.1 | 678.6581 | 3 | 33.88 | 1 | F1:19963 | 13012022\_RID\_3262\_4a.raw | 1.3281E6 |  |  |  |  |  |  |  |  |  | 1 | 1 | 0 | 0 | 0 | 0 | 0 | 0 | 0 | 0 | 0 | 351 | 367 | Oxidation (M) | M11:Oxidation (M):1000.00 | PEAKS DB |
| K.HQGLPESYNFDFVTMKPV.L | N | 51.01 | 2108.0037 | 18 | 0.6 | 703.6756 | 3 | 51.54 | 1 | F1:35496 | 13012022\_RID\_3262\_4a.raw | 1.7469E6 |  |  |  |  |  |  |  |  |  | 1 | 1 | 0 | 0 | 0 | 0 | 0 | 0 | 0 | 0 | 0 | 535 | 552 |  |  | PEAKS DB |
| R.DQGKVTDMESM(+15.99)K.F | N | 50.79 | 1383.6010 | 12 | 0.2 | 692.8079 | 2 | 25.63 | 1 | F1:12244 | 13012022\_RID\_3262\_4a.raw | 3.9723E6 |  |  |  |  |  |  |  |  |  | 1 | 1 | 0 | 0 | 0 | 0 | 0 | 0 | 0 | 0 | 0 | 445 | 456 | Oxidation (M) | M11:Oxidation (M):30.72 | PEAKS DB |
| R.IANMMADSGKTWAETFEK.Q | N | 49.19 | 2028.9285 | 18 | 0.7 | 677.3173 | 3 | 45.14 | 1 | F1:30139 | 13012022\_RID\_3262\_4a.raw | 7.7306E5 |  |  |  |  |  |  |  |  |  | 1 | 1 | 0 | 0 | 0 | 0 | 0 | 0 | 0 | 0 | 0 | 333 | 350 |  |  | PEAKS DB |
| K.TWAETFEK.Q | N | 48.61 | 1010.4709 | 8 | -0.3 | 506.2426 | 2 | 35.90 | 2 | F2:19245 | 13012022\_RID\_3262\_5a.raw | 1.3699E8 | 4.4157E6 |  |  |  |  |  |  |  |  | 2 | 1 | 1 | 0 | 0 | 0 | 0 | 0 | 0 | 0 | 0 | 343 | 350 |  |  | PEAKS DB |
| K.VLPGYENIYFAH.S | N | 46.73 | 1421.6979 | 12 | 0.8 | 711.8568 | 2 | 49.22 | 1 | F1:33555 | 13012022\_RID\_3262\_4a.raw | 3.4702E6 |  |  |  |  |  |  |  |  |  | 1 | 1 | 0 | 0 | 0 | 0 | 0 | 0 | 0 | 0 | 0 | 239 | 250 |  |  | PEAKS DB |
| R.DQGKVTDM(+15.99)ESMK.F | N | 46.37 | 1383.6010 | 12 | 0.2 | 692.8079 | 2 | 25.63 | 1 | F1:12495 | 13012022\_RID\_3262\_4a.raw | 5.5334E6 |  |  |  |  |  |  |  |  |  | 2 | 2 | 0 | 0 | 0 | 0 | 0 | 0 | 0 | 0 | 0 | 445 | 456 | Oxidation (M) | M8:Oxidation (M):11.67 | PEAKS DB |
| K.HQGLPESYNFDFVTM(+15.99)KPV.L | N | 44.49 | 2123.9985 | 18 | 0.3 | 709.0070 | 3 | 47.31 | 1 | F1:31932 | 13012022\_RID\_3262\_4a.raw | 9.0568E5 |  |  |  |  |  |  |  |  |  | 1 | 1 | 0 | 0 | 0 | 0 | 0 | 0 | 0 | 0 | 0 | 535 | 552 | Oxidation (M) | M15:Oxidation (M):1000.00 | PEAKS DB |
| N.FDFVTMKPVL | N | 43.97 | 1195.6311 | 10 | 0.3 | 598.8230 | 2 | 56.81 | 1 | F1:39982 | 13012022\_RID\_3262\_4a.raw | 8.4752E5 |  |  |  |  |  |  |  |  |  | 1 | 1 | 0 | 0 | 0 | 0 | 0 | 0 | 0 | 0 | 0 | 544 | 553 |  |  | PEAKS DB |
| K.VADISM(+15.99)AAK.F | N | 42.64 | 920.4637 | 9 | -0.1 | 461.2391 | 2 | 27.69 | 1 | F1:14400 | 13012022\_RID\_3262\_4a.raw | 4.2128E6 |  |  |  |  |  |  |  |  |  | 1 | 1 | 0 | 0 | 0 | 0 | 0 | 0 | 0 | 0 | 0 | 499 | 507 | Oxidation (M) | M6:Oxidation (M):1000.00 | PEAKS DB |
| K.VTDMESM(+15.99)K.F | N | 41.24 | 955.3990 | 8 | 0.5 | 478.7070 | 2 | 18.80 | 2 | F2:5395 | 13012022\_RID\_3262\_5a.raw |  | 4.526E5 |  |  |  |  |  |  |  |  | 1 | 0 | 1 | 0 | 0 | 0 | 0 | 0 | 0 | 0 | 0 | 449 | 456 | Oxidation (M) | M7:Oxidation (M):30.72 | PEAKS DB |
| total 33 peptides |  |  |  |  |  |  |  |  |  |  |  |  |  |  |  |  |  |  |  |  |  |  |  |  |  |  |  |  |  |  |  |  |  |  |  |  |  |
| --- | --- | --- | --- | --- | --- | --- | --- | --- | --- | --- | --- | --- | --- | --- | --- | --- | --- | --- | --- | --- | --- | --- | --- | --- | --- | --- | --- | --- | --- | --- | --- | --- | --- | --- | --- | --- | --- |

XP\_015673892.1

back to list

  

| Protein Coverage
| Supporting Peptides
|

Protein Coverage:

Supporting Peptides:

| Peptide | Uniq | -10lgP | Mass | Length | ppm | m/z | z | RT | Fraction | Scan | Source File | Area F4 | Area F5 | Area F6 | Area F10 | Area F8 | Area F9 | Area F7 | Area F1 | Area F2 | Area F3 | #Feature | #Feature F4 | #Feature F5 | #Feature F6 | #Feature F10 | #Feature F8 | #Feature F9 | #Feature F7 | #Feature F1 | #Feature F2 | #Feature F3 | Start | End | PTM | AScore | Found By |
| --- | --- | --- | --- | --- | --- | --- | --- | --- | --- | --- | --- | --- | --- | --- | --- | --- | --- | --- | --- | --- | --- | --- | --- | --- | --- | --- | --- | --- | --- | --- | --- | --- | --- | --- | --- | --- | --- |
| K.KDPGVLKYPVKPSEEGK.S | N | 79.57 | 1870.0199 | 17 | -0.7 | 468.5119 | 4 | 24.47 | 1 | F1:11529 | 13012022\_RID\_3262\_4a.raw | 3.7125E8 | 1.4174E6 |  |  |  | 8.1047E6 | 1.2066E6 | 5.7794E6 | 6.6831E6 | 5.2885E7 | 16 | 4 | 2 | 0 | 0 | 0 | 2 | 2 | 2 | 2 | 2 | 153 | 169 |  |  | PEAKS DB |
| K.DPGVLKYPVKPSEEGK.S | N | 78.91 | 1741.9249 | 16 | -0.1 | 581.6489 | 3 | 29.60 | 1 | F1:16075 | 13012022\_RID\_3262\_4a.raw | 3.1808E7 | 7.5919E5 |  |  |  |  | 2.7752E5 | 4.9105E5 | 7.4531E5 | 1.0783E7 | 8 | 2 | 1 | 0 | 0 | 0 | 0 | 1 | 1 | 2 | 1 | 154 | 169 |  |  | PEAKS DB |
| K.HDDIFAYEK.R | N | 71.92 | 1136.5138 | 9 | 0.2 | 569.2643 | 2 | 33.07 | 1 | F1:19276 | 13012022\_RID\_3262\_4a.raw | 2.205E8 | 5.0368E7 | 1.6541E6 | 5.2373E7 |  | 6.378E6 | 6.4081E6 | 1.8941E7 | 3.5546E7 | 1.9591E9 | 33 | 5 | 2 | 2 | 4 | 0 | 2 | 2 | 4 | 4 | 8 | 241 | 249 |  |  | PEAKS DB |
| K.KFWEDDGIHGGK.S | N | 70.84 | 1387.6520 | 12 | 1.0 | 694.8340 | 2 | 27.52 | 1 | F1:14353 | 13012022\_RID\_3262\_4a.raw | 2.2688E8 | 4.7295E4 |  |  |  |  |  | 1.1825E7 | 1.8101E7 | 8.0079E8 | 17 | 4 | 1 | 0 | 0 | 0 | 0 | 0 | 3 | 3 | 6 | 352 | 363 |  |  | PEAKS DB |
| Y.GIGDDANFFQALDFK.S | N | 70.77 | 1656.7783 | 15 | 0.6 | 829.3970 | 2 | 63.80 | 9 | F9:39957 | 31122021\_RID\_3262\_2a.raw |  | 4.1343E5 |  |  |  |  |  | 2.2577E6 | 1.7889E6 | 7.0811E7 | 6 | 0 | 1 | 0 | 0 | 0 | 0 | 0 | 2 | 1 | 2 | 391 | 405 |  |  | PEAKS DB |
| D.PGVLKYPVKPSEEGK.S | N | 70.72 | 1626.8981 | 15 | 0.0 | 543.3066 | 3 | 25.59 | 1 | F1:12547 | 13012022\_RID\_3262\_4a.raw | 1.4062E8 |  |  | 4.7646E5 |  | 4.3107E6 |  | 4.013E5 |  | 5.7619E6 | 8 | 3 | 0 | 0 | 1 | 0 | 2 | 0 | 1 | 0 | 1 | 155 | 169 |  |  | PEAKS DB |
| K.YPVKPSEEGK.S | N | 69.75 | 1132.5764 | 10 | 0.7 | 567.2959 | 2 | 17.88 | 6 | F6:5952 | 17012022\_RID\_3262\_9a.raw | 4.2299E8 | 5.2685E7 | 2.5077E5 | 3.2771E7 |  | 6.9462E7 | 2.576E7 |  |  |  | 12 | 2 | 2 | 1 | 3 | 0 | 2 | 2 | 0 | 0 | 0 | 160 | 169 |  |  | PEAKS DB |
| I.AYGIGDDANFFQALDFK.S | N | 69.60 | 1890.8788 | 17 | 0.2 | 946.4468 | 2 | 65.58 | 8 | F8:40325 | 31122021\_RID\_3262\_1a.raw |  |  |  |  |  |  |  | 1.5459E5 |  |  | 1 | 0 | 0 | 0 | 0 | 0 | 0 | 0 | 1 | 0 | 0 | 389 | 405 |  |  | PEAKS DB |
| L.SAAYVLAGAGHQVTVLEASER.A | N | 67.91 | 2128.0913 | 21 | 0.0 | 1065.0530 | 2 | 41.69 | 10 | F10:10487 | 31122021\_RID\_3262\_3a.raw | 1.4728E6 |  |  |  |  |  |  | 6.4802E5 | 6.599E5 | 2.6305E7 | 6 | 1 | 0 | 0 | 0 | 0 | 0 | 0 | 2 | 1 | 2 | 65 | 85 |  |  | PEAKS DB |
| K.FWEDDGIHGGK.S | N | 65.97 | 1259.5570 | 11 | 0.4 | 630.7860 | 2 | 31.13 | 1 | F1:17598 | 13012022\_RID\_3262\_4a.raw | 7.5921E7 | 9.0996E6 |  |  |  | 1.9943E6 |  | 6.128E6 | 1.0893E7 | 5.5749E8 | 17 | 2 | 2 | 0 | 0 | 0 | 1 | 0 | 3 | 3 | 6 | 353 | 363 |  |  | PEAKS DB |
| A.YVLAGAGHQVTVLEASER.A | N | 64.94 | 1898.9850 | 18 | 0.8 | 950.5005 | 2 | 37.54 | 10 | F10:8957 | 31122021\_RID\_3262\_3a.raw | 9.515E5 |  |  |  |  |  |  |  |  | 6.9519E6 | 3 | 1 | 0 | 0 | 0 | 0 | 0 | 0 | 0 | 0 | 2 | 68 | 85 |  |  | PEAKS DB |
| K.HDDIFAYEKR.F | N | 61.79 | 1292.6149 | 10 | 0.7 | 431.8792 | 3 | 27.51 | 1 | F1:14388 | 13012022\_RID\_3262\_4a.raw | 1.0662E7 |  |  | 1.628E5 |  |  |  |  |  |  | 3 | 2 | 0 | 0 | 1 | 0 | 0 | 0 | 0 | 0 | 0 | 241 | 250 |  |  | PEAKS DB |
| Y.VLAGAGHQVTVLEASER.A | N | 61.46 | 1735.9216 | 17 | 0.9 | 579.6483 | 3 | 33.06 | 6 | F6:19337 | 17012022\_RID\_3262\_9a.raw | 2.8948E5 | 1.1934E6 |  | 6.6625E5 |  | 3.8459E6 |  | 1.4002E6 | 1.9495E6 | 3.1893E7 | 10 | 1 | 1 | 0 | 1 | 0 | 1 | 0 | 2 | 2 | 2 | 69 | 85 |  |  | PEAKS DB |
| S.MKHDDIFAYEK.R | N | 57.56 | 1395.6493 | 11 | 0.9 | 466.2241 | 3 | 28.76 | 6 | F6:15683 | 17012022\_RID\_3262\_9a.raw |  |  |  |  |  | 1.5918E6 |  |  |  |  | 2 | 0 | 0 | 0 | 0 | 0 | 2 | 0 | 0 | 0 | 0 | 239 | 249 |  |  | PEAKS DB |
| P.GVLKYPVKPSEEGK.S | N | 54.97 | 1529.8452 | 14 | -0.7 | 510.9553 | 3 | 24.59 | 1 | F1:11477 | 13012022\_RID\_3262\_4a.raw | 2.1231E6 |  |  |  |  |  |  |  |  |  | 1 | 1 | 0 | 0 | 0 | 0 | 0 | 0 | 0 | 0 | 0 | 156 | 169 |  |  | PEAKS DB |
| D.YEEFLEIAR.N | N | 54.84 | 1168.5764 | 9 | 0.3 | 585.2957 | 2 | 46.57 | 4 | F4:30508 | 17012022\_RID\_3262\_10a .raw | 2.2869E6 |  |  | 1.3254E6 |  |  |  | 2.3858E5 | 1.5579E5 | 1.3646E7 | 5 | 1 | 0 | 0 | 1 | 0 | 0 | 0 | 1 | 1 | 1 | 34 | 42 |  |  | PEAKS DB |
| K.YDTYSTKEYLIK.E | N | 53.18 | 1522.7555 | 12 | 0.9 | 762.3857 | 2 | 34.13 | 1 | F1:20150 | 13012022\_RID\_3262\_4a.raw | 4.6273E6 |  |  |  |  |  |  |  |  |  | 2 | 2 | 0 | 0 | 0 | 0 | 0 | 0 | 0 | 0 | 0 | 198 | 209 |  |  | PEAKS DB |
| K.STTDLPSR.F | N | 52.16 | 875.4348 | 8 | 0.4 | 438.7249 | 2 | 21.40 | 6 | F6:8926 | 17012022\_RID\_3262\_9a.raw |  | 2.9854E7 |  | 4.8043E6 |  | 1.4473E7 | 1.9071E6 |  |  |  | 4 | 0 | 1 | 0 | 1 | 0 | 1 | 1 | 0 | 0 | 0 | 364 | 371 |  |  | PEAKS DB |
| N.DLSLIHQLPK.K | N | 51.65 | 1162.6710 | 10 | -0.7 | 582.3424 | 2 | 40.68 | 10 | F10:10098 | 31122021\_RID\_3262\_3a.raw |  |  |  |  |  |  |  | 1.197E6 | 2.7589E5 | 1.8174E7 | 3 | 0 | 0 | 0 | 0 | 0 | 0 | 0 | 1 | 1 | 1 | 414 | 423 |  |  | PEAKS DB |
| S.AAYVLAGAGHQVTVLEASER.A | N | 50.51 | 2041.0592 | 20 | 1.3 | 681.3612 | 3 | 40.58 | 1 | F1:26109 | 13012022\_RID\_3262\_4a.raw | 5.4454E5 |  |  |  |  |  |  |  |  |  | 1 | 1 | 0 | 0 | 0 | 0 | 0 | 0 | 0 | 0 | 0 | 66 | 85 |  |  | PEAKS DB |
| A.GAGHQVTVLEASER.A | N | 49.80 | 1452.7321 | 14 | 0.7 | 727.3738 | 2 | 23.21 | 9 | F9:7765 | 31122021\_RID\_3262\_2a.raw | 4.3064E5 |  |  |  |  | 6.0642E5 |  | 6.5701E4 | 8.2054E4 |  | 4 | 1 | 0 | 0 | 0 | 0 | 1 | 0 | 1 | 1 | 0 | 72 | 85 |  |  | PEAKS DB |
| R.ITFRPPLPPK.K | Y | 48.71 | 1164.7019 | 10 | -9.0 | 389.2377 | 3 | 35.71 | 10 | F10:8130 | 31122021\_RID\_3262\_3a.raw |  |  |  |  |  |  |  |  |  | 2.2953E8 | 1 | 0 | 0 | 0 | 0 | 0 | 0 | 0 | 0 | 0 | 1 | 320 | 329 |  |  | PEAKS DB |
| K.RFDEIVGGMDQLPTSM.Y | N | 48.45 | 1794.8280 | 16 | -0.6 | 898.4208 | 2 | 56.96 | 10 | F10:16573 | 31122021\_RID\_3262\_3a.raw |  |  |  |  |  |  |  |  |  | 1.5934E7 | 1 | 0 | 0 | 0 | 0 | 0 | 0 | 0 | 0 | 0 | 1 | 250 | 265 |  |  | PEAKS DB |
| R.ITFRPPLPPKK.A | Y | 43.33 | 1292.7969 | 11 | -9.0 | 431.9357 | 3 | 38.92 | 4 | F4:24149 | 17012022\_RID\_3262\_10a .raw |  |  |  | 8.9187E5 |  | 1.0903E6 | 8.4446E5 |  |  |  | 4 | 0 | 0 | 0 | 2 | 0 | 1 | 1 | 0 | 0 | 0 | 320 | 330 |  |  | PEAKS DB |
| K.RFDEIVGGM(+15.99)DQLPTSM(+15.99).Y | N | 42.69 | 1826.8179 | 16 | -0.4 | 914.4158 | 2 | 56.93 | 10 | F10:16509 | 31122021\_RID\_3262\_3a.raw |  |  |  |  |  |  |  |  |  | 0 | 0 | 0 | 0 | 0 | 0 | 0 | 0 | 0 | 0 | 0 | 0 | 250 | 265 | Oxidation (M) | M9:Oxidation (M):1000.00;M16:Oxidation (M):1000.00 | PEAKS DB |
| Q.VTVLEASER.A | N | 42.68 | 1002.5345 | 9 | -0.1 | 502.2745 | 2 | 28.85 | 7 | F7:11280 | 21012022\_RID\_3262\_7a.raw |  |  |  |  |  |  | 1.7683E5 |  |  |  | 1 | 0 | 0 | 0 | 0 | 0 | 0 | 1 | 0 | 0 | 0 | 77 | 85 |  |  | PEAKS DB |
| A.GHQVTVLEASER.A | N | 42.02 | 1324.6735 | 12 | 1.8 | 663.3452 | 2 | 22.45 | 8 | F8:6784 | 31122021\_RID\_3262\_1a.raw |  |  |  |  |  |  |  | 1.4851E4 |  |  | 1 | 0 | 0 | 0 | 0 | 0 | 0 | 0 | 1 | 0 | 0 | 74 | 85 |  |  | PEAKS DB |
| H.QVTVLEASER.A | N | 41.88 | 1130.5931 | 10 | -0.3 | 566.3036 | 2 | 28.32 | 9 | F9:11956 | 31122021\_RID\_3262\_2a.raw |  |  |  |  |  |  |  |  | 3.805E5 |  | 1 | 0 | 0 | 0 | 0 | 0 | 0 | 0 | 0 | 1 | 0 | 76 | 85 |  |  | PEAKS DB |
| A.AYVLAGAGHQVTVLEASER.A | N | 41.51 | 1970.0221 | 19 | -0.2 | 657.6812 | 3 | 38.48 | 10 | F10:9343 | 31122021\_RID\_3262\_3a.raw |  |  |  |  |  |  |  |  |  | 1.157E6 | 1 | 0 | 0 | 0 | 0 | 0 | 0 | 0 | 0 | 0 | 1 | 67 | 85 |  |  | PEAKS DB |
| K.RFDEIVGGMDQLPT.S | N | 41.44 | 1576.7555 | 14 | -0.6 | 789.3846 | 2 | 51.27 | 1 | F1:35302 | 13012022\_RID\_3262\_4a.raw | 1.0726E6 |  |  |  |  |  |  |  |  |  | 1 | 1 | 0 | 0 | 0 | 0 | 0 | 0 | 0 | 0 | 0 | 250 | 263 |  |  | PEAKS DB |
| total 30 peptides |  |  |  |  |  |  |  |  |  |  |  |  |  |  |  |  |  |  |  |  |  |  |  |  |  |  |  |  |  |  |  |  |  |  |  |  |  |
| --- | --- | --- | --- | --- | --- | --- | --- | --- | --- | --- | --- | --- | --- | --- | --- | --- | --- | --- | --- | --- | --- | --- | --- | --- | --- | --- | --- | --- | --- | --- | --- | --- | --- | --- | --- | --- | --- |

JAG46763.1

back to list

  

| Protein Coverage
| Supporting Peptides
|

Protein Coverage:

Supporting Peptides:

| Peptide | Uniq | -10lgP | Mass | Length | ppm | m/z | z | RT | Fraction | Scan | Source File | Area F4 | Area F5 | Area F6 | Area F10 | Area F8 | Area F9 | Area F7 | Area F1 | Area F2 | Area F3 | #Feature | #Feature F4 | #Feature F5 | #Feature F6 | #Feature F10 | #Feature F8 | #Feature F9 | #Feature F7 | #Feature F1 | #Feature F2 | #Feature F3 | Start | End | PTM | AScore | Found By |
| --- | --- | --- | --- | --- | --- | --- | --- | --- | --- | --- | --- | --- | --- | --- | --- | --- | --- | --- | --- | --- | --- | --- | --- | --- | --- | --- | --- | --- | --- | --- | --- | --- | --- | --- | --- | --- | --- |
| R.HPVEDDHIPFLR.R | N | 87.60 | 1473.7365 | 12 | 1.0 | 492.2532 | 3 | 36.85 | 1 | F1:22768 | 13012022\_RID\_3262\_4a.raw | 1.9829E7 |  |  |  |  |  |  |  |  |  | 2 | 2 | 0 | 0 | 0 | 0 | 0 | 0 | 0 | 0 | 0 | 307 | 318 |  |  | PEAKS DB |
| R.RHPVEDDHIPFLR.R | Y | 82.46 | 1629.8375 | 13 | 0.5 | 408.4669 | 4 | 33.21 | 1 | F1:19452 | 13012022\_RID\_3262\_4a.raw | 1.4012E7 |  |  |  |  |  |  |  |  |  | 2 | 2 | 0 | 0 | 0 | 0 | 0 | 0 | 0 | 0 | 0 | 306 | 318 |  |  | PEAKS DB |
| R.VWHTMEDNEENLDKPTIDNLSK.I | Y | 79.12 | 2627.2173 | 22 | 0.9 | 876.7471 | 3 | 38.10 | 1 | F1:23706 | 13012022\_RID\_3262\_4a.raw | 4.0097E7 |  |  |  |  |  |  |  |  |  | 2 | 2 | 0 | 0 | 0 | 0 | 0 | 0 | 0 | 0 | 0 | 334 | 355 |  |  | PEAKS DB |
| R.MWQNDLHPILIER.Y | Y | 78.74 | 1663.8505 | 13 | 0.6 | 832.9330 | 2 | 45.36 | 1 | F1:30222 | 13012022\_RID\_3262\_4a.raw | 3.0808E7 |  |  |  |  |  |  |  |  |  | 4 | 4 | 0 | 0 | 0 | 0 | 0 | 0 | 0 | 0 | 0 | 69 | 81 |  |  | PEAKS DB |
| R.LQGLQAGWLVEEDTFQSHTPYGYR.T | N | 78.47 | 2794.3350 | 24 | -0.1 | 932.4522 | 3 | 53.43 | 1 | F1:37300 | 13012022\_RID\_3262\_4a.raw | 9.3842E6 |  |  |  |  |  |  |  |  |  | 2 | 2 | 0 | 0 | 0 | 0 | 0 | 0 | 0 | 0 | 0 | 99 | 122 |  |  | PEAKS DB |
| R.VWHTM(+15.99)EDNEENLDKPTIDNLSK.I | Y | 76.44 | 2643.2122 | 22 | 0.8 | 882.0787 | 3 | 34.34 | 1 | F1:20323 | 13012022\_RID\_3262\_4a.raw | 5.0954E7 |  |  |  |  |  |  |  |  |  | 3 | 3 | 0 | 0 | 0 | 0 | 0 | 0 | 0 | 0 | 0 | 334 | 355 | Oxidation (M) | M5:Oxidation (M):1000.00 | PEAKS DB |
| R.NPVFPVYFLNTAR.W | N | 73.70 | 1536.8088 | 13 | 0.4 | 769.4120 | 2 | 57.72 | 1 | F1:40818 | 13012022\_RID\_3262\_4a.raw | 3.1417E7 |  |  |  |  |  |  |  |  |  | 1 | 1 | 0 | 0 | 0 | 0 | 0 | 0 | 0 | 0 | 0 | 260 | 272 |  |  | PEAKS DB |
| R.TFSNIISTLNPLAK.R | N | 72.28 | 1517.8453 | 14 | 0.6 | 759.9304 | 2 | 57.62 | 1 | F1:41207 | 13012022\_RID\_3262\_4a.raw | 3.6427E7 | 7.9354E5 |  |  |  |  |  |  |  |  | 3 | 2 | 1 | 0 | 0 | 0 | 0 | 0 | 0 | 0 | 0 | 123 | 136 |  |  | PEAKS DB |
| R.NLYDLGLLNNYSSER.Q | Y | 69.68 | 1769.8584 | 15 | 0.2 | 885.9366 | 2 | 54.24 | 1 | F1:37925 | 13012022\_RID\_3262\_4a.raw | 1.6508E7 |  |  |  |  |  |  |  |  |  | 1 | 1 | 0 | 0 | 0 | 0 | 0 | 0 | 0 | 0 | 0 | 283 | 297 |  |  | PEAKS DB |
| K.LIFFDGEEAFVR.W | N | 68.80 | 1441.7241 | 12 | 0.4 | 721.8696 | 2 | 58.43 | 1 | F1:41693 | 13012022\_RID\_3262\_4a.raw | 3.2977E7 |  |  |  |  |  |  | 1.1946E5 | 6.5911E4 |  | 5 | 3 | 0 | 0 | 0 | 0 | 0 | 0 | 1 | 1 | 0 | 201 | 212 |  |  | PEAKS DB |
| K.MASTPHPPGAR.N | Y | 67.88 | 1120.5447 | 11 | -0.5 | 561.2794 | 2 | 19.51 | 1 | F1:7129 | 13012022\_RID\_3262\_4a.raw | 1.4622E7 |  |  |  |  |  |  |  |  |  | 1 | 1 | 0 | 0 | 0 | 0 | 0 | 0 | 0 | 0 | 0 | 229 | 239 |  |  | PEAKS DB |
| R.GVPILHLIPSPFPR.V | N | 62.06 | 1541.9082 | 14 | 0.7 | 771.9619 | 2 | 55.02 | 1 | F1:38548 | 13012022\_RID\_3262\_4a.raw | 6.1636E7 |  |  |  |  |  |  |  |  |  | 7 | 7 | 0 | 0 | 0 | 0 | 0 | 0 | 0 | 0 | 0 | 320 | 333 |  |  | PEAKS DB |
| R.YPGSPGSYAVR.Q | N | 60.61 | 1152.5563 | 11 | 0.8 | 577.2859 | 2 | 30.03 | 1 | F1:16317 | 13012022\_RID\_3262\_4a.raw | 6.9462E7 | 1.6839E6 |  |  |  |  | 0 |  |  |  | 2 | 1 | 1 | 0 | 0 | 0 | 0 | 0 | 0 | 0 | 0 | 82 | 92 |  |  | PEAKS DB |
| R.WSPSDSLYGSR.S | N | 58.77 | 1253.5676 | 11 | 1.1 | 627.7917 | 2 | 39.64 | 2 | F2:21914 | 13012022\_RID\_3262\_5a.raw | 4.1144E7 | 1.4027E6 |  |  |  |  |  |  |  |  | 3 | 2 | 1 | 0 | 0 | 0 | 0 | 0 | 0 | 0 | 0 | 213 | 223 |  |  | PEAKS DB |
| K.YFPPQLDGK.V | N | 58.52 | 1063.5338 | 9 | 0.4 | 532.7744 | 2 | 36.88 | 1 | F1:22880 | 13012022\_RID\_3262\_4a.raw | 2.5246E7 |  |  |  |  |  |  |  |  |  | 1 | 1 | 0 | 0 | 0 | 0 | 0 | 0 | 0 | 0 | 0 | 149 | 157 |  |  | PEAKS DB |
| R.M(+15.99)WQNDLHPILIER.Y | Y | 54.75 | 1679.8453 | 13 | 0.1 | 840.9300 | 2 | 43.35 | 1 | F1:28468 | 13012022\_RID\_3262\_4a.raw | 8.2051E6 |  |  |  |  |  |  |  |  |  | 2 | 2 | 0 | 0 | 0 | 0 | 0 | 0 | 0 | 0 | 0 | 69 | 81 | Oxidation (M) | M1:Oxidation (M):1000.00 | PEAKS DB |
| K.M(+15.99)ASTPHPPGAR.N | Y | 52.07 | 1136.5397 | 11 | -0.2 | 569.2770 | 2 | 19.47 | 1 | F1:7161 | 13012022\_RID\_3262\_4a.raw | 3.6399E6 |  |  |  |  |  |  |  |  |  | 1 | 1 | 0 | 0 | 0 | 0 | 0 | 0 | 0 | 0 | 0 | 229 | 239 | Oxidation (M) | M1:Oxidation (M):1000.00 | PEAKS DB |
| R.RGVPILHLIPSPFPR.V | N | 46.62 | 1698.0093 | 15 | -0.5 | 567.0101 | 3 | 62.24 | 1 | F1:45802 | 13012022\_RID\_3262\_4a.raw | 2.9364E6 |  |  |  |  |  |  |  |  |  | 2 | 2 | 0 | 0 | 0 | 0 | 0 | 0 | 0 | 0 | 0 | 319 | 333 |  |  | PEAKS DB |
| R.HPVEDDHIPFL.R | Y | 46.43 | 1317.6353 | 11 | 0.4 | 659.8252 | 2 | 44.87 | 1 | F1:29872 | 13012022\_RID\_3262\_4a.raw | 3.2746E6 |  |  |  |  |  |  |  |  |  | 1 | 1 | 0 | 0 | 0 | 0 | 0 | 0 | 0 | 0 | 0 | 307 | 317 |  |  | PEAKS DB |
| K.QSSLPPKADLSLK.L | Y | 44.08 | 1382.7769 | 13 | 1.5 | 692.3967 | 2 | 32.06 | 1 | F1:18359 | 13012022\_RID\_3262\_4a.raw | 9.2166E5 |  |  |  |  |  |  |  |  |  | 1 | 1 | 0 | 0 | 0 | 0 | 0 | 0 | 0 | 0 | 0 | 188 | 200 |  |  | PEAKS DB |
| N.PVFPVYFLNTAR.W | N | 42.68 | 1422.7659 | 12 | 0.1 | 712.3903 | 2 | 57.74 | 1 | F1:40783 | 13012022\_RID\_3262\_4a.raw | 3.2461E5 |  |  |  |  |  |  |  |  |  | 1 | 1 | 0 | 0 | 0 | 0 | 0 | 0 | 0 | 0 | 0 | 261 | 272 |  |  | PEAKS DB |
| total 21 peptides |  |  |  |  |  |  |  |  |  |  |  |  |  |  |  |  |  |  |  |  |  |  |  |  |  |  |  |  |  |  |  |  |  |  |  |  |  |
| --- | --- | --- | --- | --- | --- | --- | --- | --- | --- | --- | --- | --- | --- | --- | --- | --- | --- | --- | --- | --- | --- | --- | --- | --- | --- | --- | --- | --- | --- | --- | --- | --- | --- | --- | --- | --- | --- |

JAA94895.1

back to list

  

| Protein Coverage
| Supporting Peptides
|

Protein Coverage:

Supporting Peptides:

| Peptide | Uniq | -10lgP | Mass | Length | ppm | m/z | z | RT | Fraction | Scan | Source File | Area F4 | Area F5 | Area F6 | Area F10 | Area F8 | Area F9 | Area F7 | Area F1 | Area F2 | Area F3 | #Feature | #Feature F4 | #Feature F5 | #Feature F6 | #Feature F10 | #Feature F8 | #Feature F9 | #Feature F7 | #Feature F1 | #Feature F2 | #Feature F3 | Start | End | PTM | AScore | Found By |
| --- | --- | --- | --- | --- | --- | --- | --- | --- | --- | --- | --- | --- | --- | --- | --- | --- | --- | --- | --- | --- | --- | --- | --- | --- | --- | --- | --- | --- | --- | --- | --- | --- | --- | --- | --- | --- | --- |
| R.HPVEDDHIPFLR.R | N | 87.60 | 1473.7365 | 12 | 1.0 | 492.2532 | 3 | 36.85 | 1 | F1:22768 | 13012022\_RID\_3262\_4a.raw | 1.9829E7 |  |  |  |  |  |  |  |  |  | 2 | 2 | 0 | 0 | 0 | 0 | 0 | 0 | 0 | 0 | 0 | 307 | 318 |  |  | PEAKS DB |
| R.RHPVEDDHIPFLR.R | Y | 82.46 | 1629.8375 | 13 | 0.5 | 408.4669 | 4 | 33.21 | 1 | F1:19452 | 13012022\_RID\_3262\_4a.raw | 1.4012E7 |  |  |  |  |  |  |  |  |  | 2 | 2 | 0 | 0 | 0 | 0 | 0 | 0 | 0 | 0 | 0 | 306 | 318 |  |  | PEAKS DB |
| R.VWHTMEDNEENLDKPTIDNLSK.I | Y | 79.12 | 2627.2173 | 22 | 0.9 | 876.7471 | 3 | 38.10 | 1 | F1:23706 | 13012022\_RID\_3262\_4a.raw | 4.0097E7 |  |  |  |  |  |  |  |  |  | 2 | 2 | 0 | 0 | 0 | 0 | 0 | 0 | 0 | 0 | 0 | 334 | 355 |  |  | PEAKS DB |
| R.MWQNDLHPILIER.Y | Y | 78.74 | 1663.8505 | 13 | 0.6 | 832.9330 | 2 | 45.36 | 1 | F1:30222 | 13012022\_RID\_3262\_4a.raw | 3.0808E7 |  |  |  |  |  |  |  |  |  | 4 | 4 | 0 | 0 | 0 | 0 | 0 | 0 | 0 | 0 | 0 | 69 | 81 |  |  | PEAKS DB |
| R.LQGLQAGWLVEEDTFQSHTPYGYR.T | N | 78.47 | 2794.3350 | 24 | -0.1 | 932.4522 | 3 | 53.43 | 1 | F1:37300 | 13012022\_RID\_3262\_4a.raw | 9.3842E6 |  |  |  |  |  |  |  |  |  | 2 | 2 | 0 | 0 | 0 | 0 | 0 | 0 | 0 | 0 | 0 | 99 | 122 |  |  | PEAKS DB |
| R.VWHTM(+15.99)EDNEENLDKPTIDNLSK.I | Y | 76.44 | 2643.2122 | 22 | 0.8 | 882.0787 | 3 | 34.34 | 1 | F1:20323 | 13012022\_RID\_3262\_4a.raw | 5.0954E7 |  |  |  |  |  |  |  |  |  | 3 | 3 | 0 | 0 | 0 | 0 | 0 | 0 | 0 | 0 | 0 | 334 | 355 | Oxidation (M) | M5:Oxidation (M):1000.00 | PEAKS DB |
| R.NPVFPVYFLNTAR.W | N | 73.70 | 1536.8088 | 13 | 0.4 | 769.4120 | 2 | 57.72 | 1 | F1:40818 | 13012022\_RID\_3262\_4a.raw | 3.1417E7 |  |  |  |  |  |  |  |  |  | 1 | 1 | 0 | 0 | 0 | 0 | 0 | 0 | 0 | 0 | 0 | 260 | 272 |  |  | PEAKS DB |
| R.TFSNIISTLNPLAK.R | N | 72.28 | 1517.8453 | 14 | 0.6 | 759.9304 | 2 | 57.62 | 1 | F1:41207 | 13012022\_RID\_3262\_4a.raw | 3.6427E7 | 7.9354E5 |  |  |  |  |  |  |  |  | 3 | 2 | 1 | 0 | 0 | 0 | 0 | 0 | 0 | 0 | 0 | 123 | 136 |  |  | PEAKS DB |
| R.NLYDLGLLNNYSSER.Q | Y | 69.68 | 1769.8584 | 15 | 0.2 | 885.9366 | 2 | 54.24 | 1 | F1:37925 | 13012022\_RID\_3262\_4a.raw | 1.6508E7 |  |  |  |  |  |  |  |  |  | 1 | 1 | 0 | 0 | 0 | 0 | 0 | 0 | 0 | 0 | 0 | 283 | 297 |  |  | PEAKS DB |
| K.LIFFDGEEAFVR.W | N | 68.80 | 1441.7241 | 12 | 0.4 | 721.8696 | 2 | 58.43 | 1 | F1:41693 | 13012022\_RID\_3262\_4a.raw | 3.2977E7 |  |  |  |  |  |  | 1.1946E5 | 6.5911E4 |  | 5 | 3 | 0 | 0 | 0 | 0 | 0 | 0 | 1 | 1 | 0 | 201 | 212 |  |  | PEAKS DB |
| K.MASTPHPPGAR.N | Y | 67.88 | 1120.5447 | 11 | -0.5 | 561.2794 | 2 | 19.51 | 1 | F1:7129 | 13012022\_RID\_3262\_4a.raw | 1.4622E7 |  |  |  |  |  |  |  |  |  | 1 | 1 | 0 | 0 | 0 | 0 | 0 | 0 | 0 | 0 | 0 | 229 | 239 |  |  | PEAKS DB |
| R.GVPILHLIPSPFPR.V | N | 62.06 | 1541.9082 | 14 | 0.7 | 771.9619 | 2 | 55.02 | 1 | F1:38548 | 13012022\_RID\_3262\_4a.raw | 6.1636E7 |  |  |  |  |  |  |  |  |  | 7 | 7 | 0 | 0 | 0 | 0 | 0 | 0 | 0 | 0 | 0 | 320 | 333 |  |  | PEAKS DB |
| R.YPGSPGSYAVR.Q | N | 60.61 | 1152.5563 | 11 | 0.8 | 577.2859 | 2 | 30.03 | 1 | F1:16317 | 13012022\_RID\_3262\_4a.raw | 6.9462E7 | 1.6839E6 |  |  |  |  | 0 |  |  |  | 2 | 1 | 1 | 0 | 0 | 0 | 0 | 0 | 0 | 0 | 0 | 82 | 92 |  |  | PEAKS DB |
| R.WSPSDSLYGSR.S | N | 58.77 | 1253.5676 | 11 | 1.1 | 627.7917 | 2 | 39.64 | 2 | F2:21914 | 13012022\_RID\_3262\_5a.raw | 4.1144E7 | 1.4027E6 |  |  |  |  |  |  |  |  | 3 | 2 | 1 | 0 | 0 | 0 | 0 | 0 | 0 | 0 | 0 | 213 | 223 |  |  | PEAKS DB |
| K.YFPPQLDGK.V | N | 58.52 | 1063.5338 | 9 | 0.4 | 532.7744 | 2 | 36.88 | 1 | F1:22880 | 13012022\_RID\_3262\_4a.raw | 2.5246E7 |  |  |  |  |  |  |  |  |  | 1 | 1 | 0 | 0 | 0 | 0 | 0 | 0 | 0 | 0 | 0 | 149 | 157 |  |  | PEAKS DB |
| R.M(+15.99)WQNDLHPILIER.Y | Y | 54.75 | 1679.8453 | 13 | 0.1 | 840.9300 | 2 | 43.35 | 1 | F1:28468 | 13012022\_RID\_3262\_4a.raw | 8.2051E6 |  |  |  |  |  |  |  |  |  | 2 | 2 | 0 | 0 | 0 | 0 | 0 | 0 | 0 | 0 | 0 | 69 | 81 | Oxidation (M) | M1:Oxidation (M):1000.00 | PEAKS DB |
| K.M(+15.99)ASTPHPPGAR.N | Y | 52.07 | 1136.5397 | 11 | -0.2 | 569.2770 | 2 | 19.47 | 1 | F1:7161 | 13012022\_RID\_3262\_4a.raw | 3.6399E6 |  |  |  |  |  |  |  |  |  | 1 | 1 | 0 | 0 | 0 | 0 | 0 | 0 | 0 | 0 | 0 | 229 | 239 | Oxidation (M) | M1:Oxidation (M):1000.00 | PEAKS DB |
| R.RGVPILHLIPSPFPR.V | N | 46.62 | 1698.0093 | 15 | -0.5 | 567.0101 | 3 | 62.24 | 1 | F1:45802 | 13012022\_RID\_3262\_4a.raw | 2.9364E6 |  |  |  |  |  |  |  |  |  | 2 | 2 | 0 | 0 | 0 | 0 | 0 | 0 | 0 | 0 | 0 | 319 | 333 |  |  | PEAKS DB |
| R.HPVEDDHIPFL.R | Y | 46.43 | 1317.6353 | 11 | 0.4 | 659.8252 | 2 | 44.87 | 1 | F1:29872 | 13012022\_RID\_3262\_4a.raw | 3.2746E6 |  |  |  |  |  |  |  |  |  | 1 | 1 | 0 | 0 | 0 | 0 | 0 | 0 | 0 | 0 | 0 | 307 | 317 |  |  | PEAKS DB |
| K.QSSLPPKADLSLK.L | Y | 44.08 | 1382.7769 | 13 | 1.5 | 692.3967 | 2 | 32.06 | 1 | F1:18359 | 13012022\_RID\_3262\_4a.raw | 9.2166E5 |  |  |  |  |  |  |  |  |  | 1 | 1 | 0 | 0 | 0 | 0 | 0 | 0 | 0 | 0 | 0 | 188 | 200 |  |  | PEAKS DB |
| N.PVFPVYFLNTAR.W | N | 42.68 | 1422.7659 | 12 | 0.1 | 712.3903 | 2 | 57.74 | 1 | F1:40783 | 13012022\_RID\_3262\_4a.raw | 3.2461E5 |  |  |  |  |  |  |  |  |  | 1 | 1 | 0 | 0 | 0 | 0 | 0 | 0 | 0 | 0 | 0 | 261 | 272 |  |  | PEAKS DB |
| total 21 peptides |  |  |  |  |  |  |  |  |  |  |  |  |  |  |  |  |  |  |  |  |  |  |  |  |  |  |  |  |  |  |  |  |  |  |  |  |  |
| --- | --- | --- | --- | --- | --- | --- | --- | --- | --- | --- | --- | --- | --- | --- | --- | --- | --- | --- | --- | --- | --- | --- | --- | --- | --- | --- | --- | --- | --- | --- | --- | --- | --- | --- | --- | --- | --- |

JAG44660.1

back to list

  

| Protein Coverage
| Supporting Peptides
|

Protein Coverage:

Supporting Peptides:

| Peptide | Uniq | -10lgP | Mass | Length | ppm | m/z | z | RT | Fraction | Scan | Source File | Area F4 | Area F5 | Area F6 | Area F10 | Area F8 | Area F9 | Area F7 | Area F1 | Area F2 | Area F3 | #Feature | #Feature F4 | #Feature F5 | #Feature F6 | #Feature F10 | #Feature F8 | #Feature F9 | #Feature F7 | #Feature F1 | #Feature F2 | #Feature F3 | Start | End | PTM | AScore | Found By |
| --- | --- | --- | --- | --- | --- | --- | --- | --- | --- | --- | --- | --- | --- | --- | --- | --- | --- | --- | --- | --- | --- | --- | --- | --- | --- | --- | --- | --- | --- | --- | --- | --- | --- | --- | --- | --- | --- |
| R.HPVEDDHIPFLR.R | N | 87.60 | 1473.7365 | 12 | 1.0 | 492.2532 | 3 | 36.85 | 1 | F1:22768 | 13012022\_RID\_3262\_4a.raw | 1.9829E7 |  |  |  |  |  |  |  |  |  | 2 | 2 | 0 | 0 | 0 | 0 | 0 | 0 | 0 | 0 | 0 | 307 | 318 |  |  | PEAKS DB |
| R.RHPVEDDHIPFLR.R | Y | 82.46 | 1629.8375 | 13 | 0.5 | 408.4669 | 4 | 33.21 | 1 | F1:19452 | 13012022\_RID\_3262\_4a.raw | 1.4012E7 |  |  |  |  |  |  |  |  |  | 2 | 2 | 0 | 0 | 0 | 0 | 0 | 0 | 0 | 0 | 0 | 306 | 318 |  |  | PEAKS DB |
| R.VWHTMEDNEENLDKPTIDNLSK.I | Y | 79.12 | 2627.2173 | 22 | 0.9 | 876.7471 | 3 | 38.10 | 1 | F1:23706 | 13012022\_RID\_3262\_4a.raw | 4.0097E7 |  |  |  |  |  |  |  |  |  | 2 | 2 | 0 | 0 | 0 | 0 | 0 | 0 | 0 | 0 | 0 | 334 | 355 |  |  | PEAKS DB |
| R.MWQNDLHPILIER.Y | Y | 78.74 | 1663.8505 | 13 | 0.6 | 832.9330 | 2 | 45.36 | 1 | F1:30222 | 13012022\_RID\_3262\_4a.raw | 3.0808E7 |  |  |  |  |  |  |  |  |  | 4 | 4 | 0 | 0 | 0 | 0 | 0 | 0 | 0 | 0 | 0 | 69 | 81 |  |  | PEAKS DB |
| R.LQGLQAGWLVEEDTFQSHTPYGYR.T | N | 78.47 | 2794.3350 | 24 | -0.1 | 932.4522 | 3 | 53.43 | 1 | F1:37300 | 13012022\_RID\_3262\_4a.raw | 9.3842E6 |  |  |  |  |  |  |  |  |  | 2 | 2 | 0 | 0 | 0 | 0 | 0 | 0 | 0 | 0 | 0 | 99 | 122 |  |  | PEAKS DB |
| R.VWHTM(+15.99)EDNEENLDKPTIDNLSK.I | Y | 76.44 | 2643.2122 | 22 | 0.8 | 882.0787 | 3 | 34.34 | 1 | F1:20323 | 13012022\_RID\_3262\_4a.raw | 5.0954E7 |  |  |  |  |  |  |  |  |  | 3 | 3 | 0 | 0 | 0 | 0 | 0 | 0 | 0 | 0 | 0 | 334 | 355 | Oxidation (M) | M5:Oxidation (M):1000.00 | PEAKS DB |
| R.NPVFPVYFLNTAR.W | N | 73.70 | 1536.8088 | 13 | 0.4 | 769.4120 | 2 | 57.72 | 1 | F1:40818 | 13012022\_RID\_3262\_4a.raw | 3.1417E7 |  |  |  |  |  |  |  |  |  | 1 | 1 | 0 | 0 | 0 | 0 | 0 | 0 | 0 | 0 | 0 | 260 | 272 |  |  | PEAKS DB |
| R.TFSNIISTLNPLAK.R | N | 72.28 | 1517.8453 | 14 | 0.6 | 759.9304 | 2 | 57.62 | 1 | F1:41207 | 13012022\_RID\_3262\_4a.raw | 3.6427E7 | 7.9354E5 |  |  |  |  |  |  |  |  | 3 | 2 | 1 | 0 | 0 | 0 | 0 | 0 | 0 | 0 | 0 | 123 | 136 |  |  | PEAKS DB |
| R.NLYDLGLLNNYSSER.Q | Y | 69.68 | 1769.8584 | 15 | 0.2 | 885.9366 | 2 | 54.24 | 1 | F1:37925 | 13012022\_RID\_3262\_4a.raw | 1.6508E7 |  |  |  |  |  |  |  |  |  | 1 | 1 | 0 | 0 | 0 | 0 | 0 | 0 | 0 | 0 | 0 | 283 | 297 |  |  | PEAKS DB |
| K.LIFFDGEEAFVR.W | N | 68.80 | 1441.7241 | 12 | 0.4 | 721.8696 | 2 | 58.43 | 1 | F1:41693 | 13012022\_RID\_3262\_4a.raw | 3.2977E7 |  |  |  |  |  |  | 1.1946E5 | 6.5911E4 |  | 5 | 3 | 0 | 0 | 0 | 0 | 0 | 0 | 1 | 1 | 0 | 201 | 212 |  |  | PEAKS DB |
| K.MASTPHPPGAR.N | Y | 67.88 | 1120.5447 | 11 | -0.5 | 561.2794 | 2 | 19.51 | 1 | F1:7129 | 13012022\_RID\_3262\_4a.raw | 1.4622E7 |  |  |  |  |  |  |  |  |  | 1 | 1 | 0 | 0 | 0 | 0 | 0 | 0 | 0 | 0 | 0 | 229 | 239 |  |  | PEAKS DB |
| R.GVPILHLIPSPFPR.V | N | 62.06 | 1541.9082 | 14 | 0.7 | 771.9619 | 2 | 55.02 | 1 | F1:38548 | 13012022\_RID\_3262\_4a.raw | 6.1636E7 |  |  |  |  |  |  |  |  |  | 7 | 7 | 0 | 0 | 0 | 0 | 0 | 0 | 0 | 0 | 0 | 320 | 333 |  |  | PEAKS DB |
| R.YPGSPGSYAVR.Q | N | 60.61 | 1152.5563 | 11 | 0.8 | 577.2859 | 2 | 30.03 | 1 | F1:16317 | 13012022\_RID\_3262\_4a.raw | 6.9462E7 | 1.6839E6 |  |  |  |  | 0 |  |  |  | 2 | 1 | 1 | 0 | 0 | 0 | 0 | 0 | 0 | 0 | 0 | 82 | 92 |  |  | PEAKS DB |
| R.WSPSDSLYGSR.S | N | 58.77 | 1253.5676 | 11 | 1.1 | 627.7917 | 2 | 39.64 | 2 | F2:21914 | 13012022\_RID\_3262\_5a.raw | 4.1144E7 | 1.4027E6 |  |  |  |  |  |  |  |  | 3 | 2 | 1 | 0 | 0 | 0 | 0 | 0 | 0 | 0 | 0 | 213 | 223 |  |  | PEAKS DB |
| K.YFPPQLDGK.V | N | 58.52 | 1063.5338 | 9 | 0.4 | 532.7744 | 2 | 36.88 | 1 | F1:22880 | 13012022\_RID\_3262\_4a.raw | 2.5246E7 |  |  |  |  |  |  |  |  |  | 1 | 1 | 0 | 0 | 0 | 0 | 0 | 0 | 0 | 0 | 0 | 149 | 157 |  |  | PEAKS DB |
| R.M(+15.99)WQNDLHPILIER.Y | Y | 54.75 | 1679.8453 | 13 | 0.1 | 840.9300 | 2 | 43.35 | 1 | F1:28468 | 13012022\_RID\_3262\_4a.raw | 8.2051E6 |  |  |  |  |  |  |  |  |  | 2 | 2 | 0 | 0 | 0 | 0 | 0 | 0 | 0 | 0 | 0 | 69 | 81 | Oxidation (M) | M1:Oxidation (M):1000.00 | PEAKS DB |
| K.M(+15.99)ASTPHPPGAR.N | Y | 52.07 | 1136.5397 | 11 | -0.2 | 569.2770 | 2 | 19.47 | 1 | F1:7161 | 13012022\_RID\_3262\_4a.raw | 3.6399E6 |  |  |  |  |  |  |  |  |  | 1 | 1 | 0 | 0 | 0 | 0 | 0 | 0 | 0 | 0 | 0 | 229 | 239 | Oxidation (M) | M1:Oxidation (M):1000.00 | PEAKS DB |
| R.RGVPILHLIPSPFPR.V | N | 46.62 | 1698.0093 | 15 | -0.5 | 567.0101 | 3 | 62.24 | 1 | F1:45802 | 13012022\_RID\_3262\_4a.raw | 2.9364E6 |  |  |  |  |  |  |  |  |  | 2 | 2 | 0 | 0 | 0 | 0 | 0 | 0 | 0 | 0 | 0 | 319 | 333 |  |  | PEAKS DB |
| R.HPVEDDHIPFL.R | Y | 46.43 | 1317.6353 | 11 | 0.4 | 659.8252 | 2 | 44.87 | 1 | F1:29872 | 13012022\_RID\_3262\_4a.raw | 3.2746E6 |  |  |  |  |  |  |  |  |  | 1 | 1 | 0 | 0 | 0 | 0 | 0 | 0 | 0 | 0 | 0 | 307 | 317 |  |  | PEAKS DB |
| K.QSSLPPKADLSLK.L | Y | 44.08 | 1382.7769 | 13 | 1.5 | 692.3967 | 2 | 32.06 | 1 | F1:18359 | 13012022\_RID\_3262\_4a.raw | 9.2166E5 |  |  |  |  |  |  |  |  |  | 1 | 1 | 0 | 0 | 0 | 0 | 0 | 0 | 0 | 0 | 0 | 188 | 200 |  |  | PEAKS DB |
| N.PVFPVYFLNTAR.W | N | 42.68 | 1422.7659 | 12 | 0.1 | 712.3903 | 2 | 57.74 | 1 | F1:40783 | 13012022\_RID\_3262\_4a.raw | 3.2461E5 |  |  |  |  |  |  |  |  |  | 1 | 1 | 0 | 0 | 0 | 0 | 0 | 0 | 0 | 0 | 0 | 261 | 272 |  |  | PEAKS DB |
| total 21 peptides |  |  |  |  |  |  |  |  |  |  |  |  |  |  |  |  |  |  |  |  |  |  |  |  |  |  |  |  |  |  |  |  |  |  |  |  |  |
| --- | --- | --- | --- | --- | --- | --- | --- | --- | --- | --- | --- | --- | --- | --- | --- | --- | --- | --- | --- | --- | --- | --- | --- | --- | --- | --- | --- | --- | --- | --- | --- | --- | --- | --- | --- | --- | --- |

AFE84765.1

back to list

  

| Protein Coverage
| Supporting Peptides
|

Protein Coverage:

Supporting Peptides:

| Peptide | Uniq | -10lgP | Mass | Length | ppm | m/z | z | RT | Fraction | Scan | Source File | Area F4 | Area F5 | Area F6 | Area F10 | Area F8 | Area F9 | Area F7 | Area F1 | Area F2 | Area F3 | #Feature | #Feature F4 | #Feature F5 | #Feature F6 | #Feature F10 | #Feature F8 | #Feature F9 | #Feature F7 | #Feature F1 | #Feature F2 | #Feature F3 | Start | End | PTM | AScore | Found By |
| --- | --- | --- | --- | --- | --- | --- | --- | --- | --- | --- | --- | --- | --- | --- | --- | --- | --- | --- | --- | --- | --- | --- | --- | --- | --- | --- | --- | --- | --- | --- | --- | --- | --- | --- | --- | --- | --- |
| R.HPVEDDHIPFLR.R | N | 87.60 | 1473.7365 | 12 | 1.0 | 492.2532 | 3 | 36.85 | 1 | F1:22768 | 13012022\_RID\_3262\_4a.raw | 1.9829E7 |  |  |  |  |  |  |  |  |  | 2 | 2 | 0 | 0 | 0 | 0 | 0 | 0 | 0 | 0 | 0 | 307 | 318 |  |  | PEAKS DB |
| R.RHPVEDDHIPFLR.R | Y | 82.46 | 1629.8375 | 13 | 0.5 | 408.4669 | 4 | 33.21 | 1 | F1:19452 | 13012022\_RID\_3262\_4a.raw | 1.4012E7 |  |  |  |  |  |  |  |  |  | 2 | 2 | 0 | 0 | 0 | 0 | 0 | 0 | 0 | 0 | 0 | 306 | 318 |  |  | PEAKS DB |
| R.VWHTMEDNEENLDKPTIDNLSK.I | Y | 79.12 | 2627.2173 | 22 | 0.9 | 876.7471 | 3 | 38.10 | 1 | F1:23706 | 13012022\_RID\_3262\_4a.raw | 4.0097E7 |  |  |  |  |  |  |  |  |  | 2 | 2 | 0 | 0 | 0 | 0 | 0 | 0 | 0 | 0 | 0 | 334 | 355 |  |  | PEAKS DB |
| R.MWQNDLHPILIER.Y | Y | 78.74 | 1663.8505 | 13 | 0.6 | 832.9330 | 2 | 45.36 | 1 | F1:30222 | 13012022\_RID\_3262\_4a.raw | 3.0808E7 |  |  |  |  |  |  |  |  |  | 4 | 4 | 0 | 0 | 0 | 0 | 0 | 0 | 0 | 0 | 0 | 69 | 81 |  |  | PEAKS DB |
| R.LQGLQAGWLVEEDTFQSHTPYGYR.T | N | 78.47 | 2794.3350 | 24 | -0.1 | 932.4522 | 3 | 53.43 | 1 | F1:37300 | 13012022\_RID\_3262\_4a.raw | 9.3842E6 |  |  |  |  |  |  |  |  |  | 2 | 2 | 0 | 0 | 0 | 0 | 0 | 0 | 0 | 0 | 0 | 99 | 122 |  |  | PEAKS DB |
| R.VWHTM(+15.99)EDNEENLDKPTIDNLSK.I | Y | 76.44 | 2643.2122 | 22 | 0.8 | 882.0787 | 3 | 34.34 | 1 | F1:20323 | 13012022\_RID\_3262\_4a.raw | 5.0954E7 |  |  |  |  |  |  |  |  |  | 3 | 3 | 0 | 0 | 0 | 0 | 0 | 0 | 0 | 0 | 0 | 334 | 355 | Oxidation (M) | M5:Oxidation (M):1000.00 | PEAKS DB |
| R.NPVFPVYFLNTAR.W | N | 73.70 | 1536.8088 | 13 | 0.4 | 769.4120 | 2 | 57.72 | 1 | F1:40818 | 13012022\_RID\_3262\_4a.raw | 3.1417E7 |  |  |  |  |  |  |  |  |  | 1 | 1 | 0 | 0 | 0 | 0 | 0 | 0 | 0 | 0 | 0 | 260 | 272 |  |  | PEAKS DB |
| R.TFSNIISTLNPLAK.R | N | 72.28 | 1517.8453 | 14 | 0.6 | 759.9304 | 2 | 57.62 | 1 | F1:41207 | 13012022\_RID\_3262\_4a.raw | 3.6427E7 | 7.9354E5 |  |  |  |  |  |  |  |  | 3 | 2 | 1 | 0 | 0 | 0 | 0 | 0 | 0 | 0 | 0 | 123 | 136 |  |  | PEAKS DB |
| R.NLYDLGLLNNYSSER.Q | Y | 69.68 | 1769.8584 | 15 | 0.2 | 885.9366 | 2 | 54.24 | 1 | F1:37925 | 13012022\_RID\_3262\_4a.raw | 1.6508E7 |  |  |  |  |  |  |  |  |  | 1 | 1 | 0 | 0 | 0 | 0 | 0 | 0 | 0 | 0 | 0 | 283 | 297 |  |  | PEAKS DB |
| K.LIFFDGEEAFVR.W | N | 68.80 | 1441.7241 | 12 | 0.4 | 721.8696 | 2 | 58.43 | 1 | F1:41693 | 13012022\_RID\_3262\_4a.raw | 3.2977E7 |  |  |  |  |  |  | 1.1946E5 | 6.5911E4 |  | 5 | 3 | 0 | 0 | 0 | 0 | 0 | 0 | 1 | 1 | 0 | 201 | 212 |  |  | PEAKS DB |
| K.MASTPHPPGAR.N | Y | 67.88 | 1120.5447 | 11 | -0.5 | 561.2794 | 2 | 19.51 | 1 | F1:7129 | 13012022\_RID\_3262\_4a.raw | 1.4622E7 |  |  |  |  |  |  |  |  |  | 1 | 1 | 0 | 0 | 0 | 0 | 0 | 0 | 0 | 0 | 0 | 229 | 239 |  |  | PEAKS DB |
| R.GVPILHLIPSPFPR.V | N | 62.06 | 1541.9082 | 14 | 0.7 | 771.9619 | 2 | 55.02 | 1 | F1:38548 | 13012022\_RID\_3262\_4a.raw | 6.1636E7 |  |  |  |  |  |  |  |  |  | 7 | 7 | 0 | 0 | 0 | 0 | 0 | 0 | 0 | 0 | 0 | 320 | 333 |  |  | PEAKS DB |
| R.YPGSPGSYAVR.Q | N | 60.61 | 1152.5563 | 11 | 0.8 | 577.2859 | 2 | 30.03 | 1 | F1:16317 | 13012022\_RID\_3262\_4a.raw | 6.9462E7 | 1.6839E6 |  |  |  |  | 0 |  |  |  | 2 | 1 | 1 | 0 | 0 | 0 | 0 | 0 | 0 | 0 | 0 | 82 | 92 |  |  | PEAKS DB |
| R.WSPSDSLYGSR.S | N | 58.77 | 1253.5676 | 11 | 1.1 | 627.7917 | 2 | 39.64 | 2 | F2:21914 | 13012022\_RID\_3262\_5a.raw | 4.1144E7 | 1.4027E6 |  |  |  |  |  |  |  |  | 3 | 2 | 1 | 0 | 0 | 0 | 0 | 0 | 0 | 0 | 0 | 213 | 223 |  |  | PEAKS DB |
| K.YFPPQLDGK.V | N | 58.52 | 1063.5338 | 9 | 0.4 | 532.7744 | 2 | 36.88 | 1 | F1:22880 | 13012022\_RID\_3262\_4a.raw | 2.5246E7 |  |  |  |  |  |  |  |  |  | 1 | 1 | 0 | 0 | 0 | 0 | 0 | 0 | 0 | 0 | 0 | 149 | 157 |  |  | PEAKS DB |
| R.M(+15.99)WQNDLHPILIER.Y | Y | 54.75 | 1679.8453 | 13 | 0.1 | 840.9300 | 2 | 43.35 | 1 | F1:28468 | 13012022\_RID\_3262\_4a.raw | 8.2051E6 |  |  |  |  |  |  |  |  |  | 2 | 2 | 0 | 0 | 0 | 0 | 0 | 0 | 0 | 0 | 0 | 69 | 81 | Oxidation (M) | M1:Oxidation (M):1000.00 | PEAKS DB |
| K.M(+15.99)ASTPHPPGAR.N | Y | 52.07 | 1136.5397 | 11 | -0.2 | 569.2770 | 2 | 19.47 | 1 | F1:7161 | 13012022\_RID\_3262\_4a.raw | 3.6399E6 |  |  |  |  |  |  |  |  |  | 1 | 1 | 0 | 0 | 0 | 0 | 0 | 0 | 0 | 0 | 0 | 229 | 239 | Oxidation (M) | M1:Oxidation (M):1000.00 | PEAKS DB |
| R.RGVPILHLIPSPFPR.V | N | 46.62 | 1698.0093 | 15 | -0.5 | 567.0101 | 3 | 62.24 | 1 | F1:45802 | 13012022\_RID\_3262\_4a.raw | 2.9364E6 |  |  |  |  |  |  |  |  |  | 2 | 2 | 0 | 0 | 0 | 0 | 0 | 0 | 0 | 0 | 0 | 319 | 333 |  |  | PEAKS DB |
| R.HPVEDDHIPFL.R | Y | 46.43 | 1317.6353 | 11 | 0.4 | 659.8252 | 2 | 44.87 | 1 | F1:29872 | 13012022\_RID\_3262\_4a.raw | 3.2746E6 |  |  |  |  |  |  |  |  |  | 1 | 1 | 0 | 0 | 0 | 0 | 0 | 0 | 0 | 0 | 0 | 307 | 317 |  |  | PEAKS DB |
| K.QSSLPPKADLSLK.L | Y | 44.08 | 1382.7769 | 13 | 1.5 | 692.3967 | 2 | 32.06 | 1 | F1:18359 | 13012022\_RID\_3262\_4a.raw | 9.2166E5 |  |  |  |  |  |  |  |  |  | 1 | 1 | 0 | 0 | 0 | 0 | 0 | 0 | 0 | 0 | 0 | 188 | 200 |  |  | PEAKS DB |
| N.PVFPVYFLNTAR.W | N | 42.68 | 1422.7659 | 12 | 0.1 | 712.3903 | 2 | 57.74 | 1 | F1:40783 | 13012022\_RID\_3262\_4a.raw | 3.2461E5 |  |  |  |  |  |  |  |  |  | 1 | 1 | 0 | 0 | 0 | 0 | 0 | 0 | 0 | 0 | 0 | 261 | 272 |  |  | PEAKS DB |
| total 21 peptides |  |  |  |  |  |  |  |  |  |  |  |  |  |  |  |  |  |  |  |  |  |  |  |  |  |  |  |  |  |  |  |  |  |  |  |  |  |
| --- | --- | --- | --- | --- | --- | --- | --- | --- | --- | --- | --- | --- | --- | --- | --- | --- | --- | --- | --- | --- | --- | --- | --- | --- | --- | --- | --- | --- | --- | --- | --- | --- | --- | --- | --- | --- | --- |

AFE84763.1

back to list

  

| Protein Coverage
| Supporting Peptides
|

Protein Coverage:

Supporting Peptides:

| Peptide | Uniq | -10lgP | Mass | Length | ppm | m/z | z | RT | Fraction | Scan | Source File | Area F4 | Area F5 | Area F6 | Area F10 | Area F8 | Area F9 | Area F7 | Area F1 | Area F2 | Area F3 | #Feature | #Feature F4 | #Feature F5 | #Feature F6 | #Feature F10 | #Feature F8 | #Feature F9 | #Feature F7 | #Feature F1 | #Feature F2 | #Feature F3 | Start | End | PTM | AScore | Found By |
| --- | --- | --- | --- | --- | --- | --- | --- | --- | --- | --- | --- | --- | --- | --- | --- | --- | --- | --- | --- | --- | --- | --- | --- | --- | --- | --- | --- | --- | --- | --- | --- | --- | --- | --- | --- | --- | --- |
| R.HPVEDDHIPFLR.R | N | 87.60 | 1473.7365 | 12 | 1.0 | 492.2532 | 3 | 36.85 | 1 | F1:22768 | 13012022\_RID\_3262\_4a.raw | 1.9829E7 |  |  |  |  |  |  |  |  |  | 2 | 2 | 0 | 0 | 0 | 0 | 0 | 0 | 0 | 0 | 0 | 307 | 318 |  |  | PEAKS DB |
| R.RHPVEDDHIPFLR.R | Y | 82.46 | 1629.8375 | 13 | 0.5 | 408.4669 | 4 | 33.21 | 1 | F1:19452 | 13012022\_RID\_3262\_4a.raw | 1.4012E7 |  |  |  |  |  |  |  |  |  | 2 | 2 | 0 | 0 | 0 | 0 | 0 | 0 | 0 | 0 | 0 | 306 | 318 |  |  | PEAKS DB |
| R.VWHTMEDNEENLDKPTIDNLSK.I | Y | 79.12 | 2627.2173 | 22 | 0.9 | 876.7471 | 3 | 38.10 | 1 | F1:23706 | 13012022\_RID\_3262\_4a.raw | 4.0097E7 |  |  |  |  |  |  |  |  |  | 2 | 2 | 0 | 0 | 0 | 0 | 0 | 0 | 0 | 0 | 0 | 334 | 355 |  |  | PEAKS DB |
| R.MWQNDLHPILIER.Y | Y | 78.74 | 1663.8505 | 13 | 0.6 | 832.9330 | 2 | 45.36 | 1 | F1:30222 | 13012022\_RID\_3262\_4a.raw | 3.0808E7 |  |  |  |  |  |  |  |  |  | 4 | 4 | 0 | 0 | 0 | 0 | 0 | 0 | 0 | 0 | 0 | 69 | 81 |  |  | PEAKS DB |
| R.LQGLQAGWLVEEDTFQSHTPYGYR.T | N | 78.47 | 2794.3350 | 24 | -0.1 | 932.4522 | 3 | 53.43 | 1 | F1:37300 | 13012022\_RID\_3262\_4a.raw | 9.3842E6 |  |  |  |  |  |  |  |  |  | 2 | 2 | 0 | 0 | 0 | 0 | 0 | 0 | 0 | 0 | 0 | 99 | 122 |  |  | PEAKS DB |
| R.VWHTM(+15.99)EDNEENLDKPTIDNLSK.I | Y | 76.44 | 2643.2122 | 22 | 0.8 | 882.0787 | 3 | 34.34 | 1 | F1:20323 | 13012022\_RID\_3262\_4a.raw | 5.0954E7 |  |  |  |  |  |  |  |  |  | 3 | 3 | 0 | 0 | 0 | 0 | 0 | 0 | 0 | 0 | 0 | 334 | 355 | Oxidation (M) | M5:Oxidation (M):1000.00 | PEAKS DB |
| R.NPVFPVYFLNTAR.W | N | 73.70 | 1536.8088 | 13 | 0.4 | 769.4120 | 2 | 57.72 | 1 | F1:40818 | 13012022\_RID\_3262\_4a.raw | 3.1417E7 |  |  |  |  |  |  |  |  |  | 1 | 1 | 0 | 0 | 0 | 0 | 0 | 0 | 0 | 0 | 0 | 260 | 272 |  |  | PEAKS DB |
| R.TFSNIISTLNPLAK.R | N | 72.28 | 1517.8453 | 14 | 0.6 | 759.9304 | 2 | 57.62 | 1 | F1:41207 | 13012022\_RID\_3262\_4a.raw | 3.6427E7 | 7.9354E5 |  |  |  |  |  |  |  |  | 3 | 2 | 1 | 0 | 0 | 0 | 0 | 0 | 0 | 0 | 0 | 123 | 136 |  |  | PEAKS DB |
| R.NLYDLGLLNNYSSER.Q | Y | 69.68 | 1769.8584 | 15 | 0.2 | 885.9366 | 2 | 54.24 | 1 | F1:37925 | 13012022\_RID\_3262\_4a.raw | 1.6508E7 |  |  |  |  |  |  |  |  |  | 1 | 1 | 0 | 0 | 0 | 0 | 0 | 0 | 0 | 0 | 0 | 283 | 297 |  |  | PEAKS DB |
| K.LIFFDGEEAFVR.W | N | 68.80 | 1441.7241 | 12 | 0.4 | 721.8696 | 2 | 58.43 | 1 | F1:41693 | 13012022\_RID\_3262\_4a.raw | 3.2977E7 |  |  |  |  |  |  | 1.1946E5 | 6.5911E4 |  | 5 | 3 | 0 | 0 | 0 | 0 | 0 | 0 | 1 | 1 | 0 | 201 | 212 |  |  | PEAKS DB |
| K.MASTPHPPGAR.N | Y | 67.88 | 1120.5447 | 11 | -0.5 | 561.2794 | 2 | 19.51 | 1 | F1:7129 | 13012022\_RID\_3262\_4a.raw | 1.4622E7 |  |  |  |  |  |  |  |  |  | 1 | 1 | 0 | 0 | 0 | 0 | 0 | 0 | 0 | 0 | 0 | 229 | 239 |  |  | PEAKS DB |
| R.GVPILHLIPSPFPR.V | N | 62.06 | 1541.9082 | 14 | 0.7 | 771.9619 | 2 | 55.02 | 1 | F1:38548 | 13012022\_RID\_3262\_4a.raw | 6.1636E7 |  |  |  |  |  |  |  |  |  | 7 | 7 | 0 | 0 | 0 | 0 | 0 | 0 | 0 | 0 | 0 | 320 | 333 |  |  | PEAKS DB |
| R.YPGSPGSYAVR.Q | N | 60.61 | 1152.5563 | 11 | 0.8 | 577.2859 | 2 | 30.03 | 1 | F1:16317 | 13012022\_RID\_3262\_4a.raw | 6.9462E7 | 1.6839E6 |  |  |  |  | 0 |  |  |  | 2 | 1 | 1 | 0 | 0 | 0 | 0 | 0 | 0 | 0 | 0 | 82 | 92 |  |  | PEAKS DB |
| R.WSPSDSLYGSR.S | N | 58.77 | 1253.5676 | 11 | 1.1 | 627.7917 | 2 | 39.64 | 2 | F2:21914 | 13012022\_RID\_3262\_5a.raw | 4.1144E7 | 1.4027E6 |  |  |  |  |  |  |  |  | 3 | 2 | 1 | 0 | 0 | 0 | 0 | 0 | 0 | 0 | 0 | 213 | 223 |  |  | PEAKS DB |
| K.YFPPQLDGK.V | N | 58.52 | 1063.5338 | 9 | 0.4 | 532.7744 | 2 | 36.88 | 1 | F1:22880 | 13012022\_RID\_3262\_4a.raw | 2.5246E7 |  |  |  |  |  |  |  |  |  | 1 | 1 | 0 | 0 | 0 | 0 | 0 | 0 | 0 | 0 | 0 | 149 | 157 |  |  | PEAKS DB |
| R.M(+15.99)WQNDLHPILIER.Y | Y | 54.75 | 1679.8453 | 13 | 0.1 | 840.9300 | 2 | 43.35 | 1 | F1:28468 | 13012022\_RID\_3262\_4a.raw | 8.2051E6 |  |  |  |  |  |  |  |  |  | 2 | 2 | 0 | 0 | 0 | 0 | 0 | 0 | 0 | 0 | 0 | 69 | 81 | Oxidation (M) | M1:Oxidation (M):1000.00 | PEAKS DB |
| K.M(+15.99)ASTPHPPGAR.N | Y | 52.07 | 1136.5397 | 11 | -0.2 | 569.2770 | 2 | 19.47 | 1 | F1:7161 | 13012022\_RID\_3262\_4a.raw | 3.6399E6 |  |  |  |  |  |  |  |  |  | 1 | 1 | 0 | 0 | 0 | 0 | 0 | 0 | 0 | 0 | 0 | 229 | 239 | Oxidation (M) | M1:Oxidation (M):1000.00 | PEAKS DB |
| R.RGVPILHLIPSPFPR.V | N | 46.62 | 1698.0093 | 15 | -0.5 | 567.0101 | 3 | 62.24 | 1 | F1:45802 | 13012022\_RID\_3262\_4a.raw | 2.9364E6 |  |  |  |  |  |  |  |  |  | 2 | 2 | 0 | 0 | 0 | 0 | 0 | 0 | 0 | 0 | 0 | 319 | 333 |  |  | PEAKS DB |
| R.HPVEDDHIPFL.R | Y | 46.43 | 1317.6353 | 11 | 0.4 | 659.8252 | 2 | 44.87 | 1 | F1:29872 | 13012022\_RID\_3262\_4a.raw | 3.2746E6 |  |  |  |  |  |  |  |  |  | 1 | 1 | 0 | 0 | 0 | 0 | 0 | 0 | 0 | 0 | 0 | 307 | 317 |  |  | PEAKS DB |
| K.QSSLPPKADLSLK.L | Y | 44.08 | 1382.7769 | 13 | 1.5 | 692.3967 | 2 | 32.06 | 1 | F1:18359 | 13012022\_RID\_3262\_4a.raw | 9.2166E5 |  |  |  |  |  |  |  |  |  | 1 | 1 | 0 | 0 | 0 | 0 | 0 | 0 | 0 | 0 | 0 | 188 | 200 |  |  | PEAKS DB |
| N.PVFPVYFLNTAR.W | N | 42.68 | 1422.7659 | 12 | 0.1 | 712.3903 | 2 | 57.74 | 1 | F1:40783 | 13012022\_RID\_3262\_4a.raw | 3.2461E5 |  |  |  |  |  |  |  |  |  | 1 | 1 | 0 | 0 | 0 | 0 | 0 | 0 | 0 | 0 | 0 | 261 | 272 |  |  | PEAKS DB |
| total 21 peptides |  |  |  |  |  |  |  |  |  |  |  |  |  |  |  |  |  |  |  |  |  |  |  |  |  |  |  |  |  |  |  |  |  |  |  |  |  |
| --- | --- | --- | --- | --- | --- | --- | --- | --- | --- | --- | --- | --- | --- | --- | --- | --- | --- | --- | --- | --- | --- | --- | --- | --- | --- | --- | --- | --- | --- | --- | --- | --- | --- | --- | --- | --- | --- |

BAB69586.1

back to list

  

| Protein Coverage
| Supporting Peptides
|

Protein Coverage:

Supporting Peptides:

| Peptide | Uniq | -10lgP | Mass | Length | ppm | m/z | z | RT | Fraction | Scan | Source File | Area F4 | Area F5 | Area F6 | Area F10 | Area F8 | Area F9 | Area F7 | Area F1 | Area F2 | Area F3 | #Feature | #Feature F4 | #Feature F5 | #Feature F6 | #Feature F10 | #Feature F8 | #Feature F9 | #Feature F7 | #Feature F1 | #Feature F2 | #Feature F3 | Start | End | PTM | AScore | Found By |
| --- | --- | --- | --- | --- | --- | --- | --- | --- | --- | --- | --- | --- | --- | --- | --- | --- | --- | --- | --- | --- | --- | --- | --- | --- | --- | --- | --- | --- | --- | --- | --- | --- | --- | --- | --- | --- | --- |
| R.HPVEDDHIPFLR.R | N | 87.60 | 1473.7365 | 12 | 1.0 | 492.2532 | 3 | 36.85 | 1 | F1:22768 | 13012022\_RID\_3262\_4a.raw | 1.9829E7 |  |  |  |  |  |  |  |  |  | 2 | 2 | 0 | 0 | 0 | 0 | 0 | 0 | 0 | 0 | 0 | 307 | 318 |  |  | PEAKS DB |
| R.RHPVEDDHIPFLR.R | Y | 82.46 | 1629.8375 | 13 | 0.5 | 408.4669 | 4 | 33.21 | 1 | F1:19452 | 13012022\_RID\_3262\_4a.raw | 1.4012E7 |  |  |  |  |  |  |  |  |  | 2 | 2 | 0 | 0 | 0 | 0 | 0 | 0 | 0 | 0 | 0 | 306 | 318 |  |  | PEAKS DB |
| R.VWHTMEDNEENLDKPTIDNLSK.I | Y | 79.12 | 2627.2173 | 22 | 0.9 | 876.7471 | 3 | 38.10 | 1 | F1:23706 | 13012022\_RID\_3262\_4a.raw | 4.0097E7 |  |  |  |  |  |  |  |  |  | 2 | 2 | 0 | 0 | 0 | 0 | 0 | 0 | 0 | 0 | 0 | 334 | 355 |  |  | PEAKS DB |
| R.MWQNDLHPILIER.Y | Y | 78.74 | 1663.8505 | 13 | 0.6 | 832.9330 | 2 | 45.36 | 1 | F1:30222 | 13012022\_RID\_3262\_4a.raw | 3.0808E7 |  |  |  |  |  |  |  |  |  | 4 | 4 | 0 | 0 | 0 | 0 | 0 | 0 | 0 | 0 | 0 | 69 | 81 |  |  | PEAKS DB |
| R.LQGLQAGWLVEEDTFQSHTPYGYR.T | N | 78.47 | 2794.3350 | 24 | -0.1 | 932.4522 | 3 | 53.43 | 1 | F1:37300 | 13012022\_RID\_3262\_4a.raw | 9.3842E6 |  |  |  |  |  |  |  |  |  | 2 | 2 | 0 | 0 | 0 | 0 | 0 | 0 | 0 | 0 | 0 | 99 | 122 |  |  | PEAKS DB |
| R.VWHTM(+15.99)EDNEENLDKPTIDNLSK.I | Y | 76.44 | 2643.2122 | 22 | 0.8 | 882.0787 | 3 | 34.34 | 1 | F1:20323 | 13012022\_RID\_3262\_4a.raw | 5.0954E7 |  |  |  |  |  |  |  |  |  | 3 | 3 | 0 | 0 | 0 | 0 | 0 | 0 | 0 | 0 | 0 | 334 | 355 | Oxidation (M) | M5:Oxidation (M):1000.00 | PEAKS DB |
| R.NPVFPVYFLNTAR.W | N | 73.70 | 1536.8088 | 13 | 0.4 | 769.4120 | 2 | 57.72 | 1 | F1:40818 | 13012022\_RID\_3262\_4a.raw | 3.1417E7 |  |  |  |  |  |  |  |  |  | 1 | 1 | 0 | 0 | 0 | 0 | 0 | 0 | 0 | 0 | 0 | 260 | 272 |  |  | PEAKS DB |
| R.TFSNIISTLNPLAK.R | N | 72.28 | 1517.8453 | 14 | 0.6 | 759.9304 | 2 | 57.62 | 1 | F1:41207 | 13012022\_RID\_3262\_4a.raw | 3.6427E7 | 7.9354E5 |  |  |  |  |  |  |  |  | 3 | 2 | 1 | 0 | 0 | 0 | 0 | 0 | 0 | 0 | 0 | 123 | 136 |  |  | PEAKS DB |
| Q.NLYDLGLLNNYSSER.Q | Y | 69.68 | 1769.8584 | 15 | 0.2 | 885.9366 | 2 | 54.24 | 1 | F1:37925 | 13012022\_RID\_3262\_4a.raw | 1.6508E7 |  |  |  |  |  |  |  |  |  | 1 | 1 | 0 | 0 | 0 | 0 | 0 | 0 | 0 | 0 | 0 | 283 | 297 |  |  | PEAKS DB |
| K.LIFFDGEEAFVR.W | N | 68.80 | 1441.7241 | 12 | 0.4 | 721.8696 | 2 | 58.43 | 1 | F1:41693 | 13012022\_RID\_3262\_4a.raw | 3.2977E7 |  |  |  |  |  |  | 1.1946E5 | 6.5911E4 |  | 5 | 3 | 0 | 0 | 0 | 0 | 0 | 0 | 1 | 1 | 0 | 201 | 212 |  |  | PEAKS DB |
| K.MASTPHPPGAR.N | Y | 67.88 | 1120.5447 | 11 | -0.5 | 561.2794 | 2 | 19.51 | 1 | F1:7129 | 13012022\_RID\_3262\_4a.raw | 1.4622E7 |  |  |  |  |  |  |  |  |  | 1 | 1 | 0 | 0 | 0 | 0 | 0 | 0 | 0 | 0 | 0 | 229 | 239 |  |  | PEAKS DB |
| R.GVPILHLIPSPFPR.V | N | 62.06 | 1541.9082 | 14 | 0.7 | 771.9619 | 2 | 55.02 | 1 | F1:38548 | 13012022\_RID\_3262\_4a.raw | 6.1636E7 |  |  |  |  |  |  |  |  |  | 7 | 7 | 0 | 0 | 0 | 0 | 0 | 0 | 0 | 0 | 0 | 320 | 333 |  |  | PEAKS DB |
| R.YPGSPGSYAVR.Q | N | 60.61 | 1152.5563 | 11 | 0.8 | 577.2859 | 2 | 30.03 | 1 | F1:16317 | 13012022\_RID\_3262\_4a.raw | 6.9462E7 | 1.6839E6 |  |  |  |  | 0 |  |  |  | 2 | 1 | 1 | 0 | 0 | 0 | 0 | 0 | 0 | 0 | 0 | 82 | 92 |  |  | PEAKS DB |
| R.WSPSDSLYGSR.S | N | 58.77 | 1253.5676 | 11 | 1.1 | 627.7917 | 2 | 39.64 | 2 | F2:21914 | 13012022\_RID\_3262\_5a.raw | 4.1144E7 | 1.4027E6 |  |  |  |  |  |  |  |  | 3 | 2 | 1 | 0 | 0 | 0 | 0 | 0 | 0 | 0 | 0 | 213 | 223 |  |  | PEAKS DB |
| K.YFPPQLDGK.V | N | 58.52 | 1063.5338 | 9 | 0.4 | 532.7744 | 2 | 36.88 | 1 | F1:22880 | 13012022\_RID\_3262\_4a.raw | 2.5246E7 |  |  |  |  |  |  |  |  |  | 1 | 1 | 0 | 0 | 0 | 0 | 0 | 0 | 0 | 0 | 0 | 149 | 157 |  |  | PEAKS DB |
| R.M(+15.99)WQNDLHPILIER.Y | Y | 54.75 | 1679.8453 | 13 | 0.1 | 840.9300 | 2 | 43.35 | 1 | F1:28468 | 13012022\_RID\_3262\_4a.raw | 8.2051E6 |  |  |  |  |  |  |  |  |  | 2 | 2 | 0 | 0 | 0 | 0 | 0 | 0 | 0 | 0 | 0 | 69 | 81 | Oxidation (M) | M1:Oxidation (M):1000.00 | PEAKS DB |
| K.M(+15.99)ASTPHPPGAR.N | Y | 52.07 | 1136.5397 | 11 | -0.2 | 569.2770 | 2 | 19.47 | 1 | F1:7161 | 13012022\_RID\_3262\_4a.raw | 3.6399E6 |  |  |  |  |  |  |  |  |  | 1 | 1 | 0 | 0 | 0 | 0 | 0 | 0 | 0 | 0 | 0 | 229 | 239 | Oxidation (M) | M1:Oxidation (M):1000.00 | PEAKS DB |
| R.RGVPILHLIPSPFPR.V | N | 46.62 | 1698.0093 | 15 | -0.5 | 567.0101 | 3 | 62.24 | 1 | F1:45802 | 13012022\_RID\_3262\_4a.raw | 2.9364E6 |  |  |  |  |  |  |  |  |  | 2 | 2 | 0 | 0 | 0 | 0 | 0 | 0 | 0 | 0 | 0 | 319 | 333 |  |  | PEAKS DB |
| R.HPVEDDHIPFL.R | Y | 46.43 | 1317.6353 | 11 | 0.4 | 659.8252 | 2 | 44.87 | 1 | F1:29872 | 13012022\_RID\_3262\_4a.raw | 3.2746E6 |  |  |  |  |  |  |  |  |  | 1 | 1 | 0 | 0 | 0 | 0 | 0 | 0 | 0 | 0 | 0 | 307 | 317 |  |  | PEAKS DB |
| K.QSSLPPKADLSLK.L | Y | 44.08 | 1382.7769 | 13 | 1.5 | 692.3967 | 2 | 32.06 | 1 | F1:18359 | 13012022\_RID\_3262\_4a.raw | 9.2166E5 |  |  |  |  |  |  |  |  |  | 1 | 1 | 0 | 0 | 0 | 0 | 0 | 0 | 0 | 0 | 0 | 188 | 200 |  |  | PEAKS DB |
| N.PVFPVYFLNTAR.W | N | 42.68 | 1422.7659 | 12 | 0.1 | 712.3903 | 2 | 57.74 | 1 | F1:40783 | 13012022\_RID\_3262\_4a.raw | 3.2461E5 |  |  |  |  |  |  |  |  |  | 1 | 1 | 0 | 0 | 0 | 0 | 0 | 0 | 0 | 0 | 0 | 261 | 272 |  |  | PEAKS DB |
| total 21 peptides |  |  |  |  |  |  |  |  |  |  |  |  |  |  |  |  |  |  |  |  |  |  |  |  |  |  |  |  |  |  |  |  |  |  |  |  |  |
| --- | --- | --- | --- | --- | --- | --- | --- | --- | --- | --- | --- | --- | --- | --- | --- | --- | --- | --- | --- | --- | --- | --- | --- | --- | --- | --- | --- | --- | --- | --- | --- | --- | --- | --- | --- | --- | --- |

XP\_015674366.1

back to list

  

| Protein Coverage
| Supporting Peptides
|

Protein Coverage:

Supporting Peptides:

| Peptide | Uniq | -10lgP | Mass | Length | ppm | m/z | z | RT | Fraction | Scan | Source File | Area F4 | Area F5 | Area F6 | Area F10 | Area F8 | Area F9 | Area F7 | Area F1 | Area F2 | Area F3 | #Feature | #Feature F4 | #Feature F5 | #Feature F6 | #Feature F10 | #Feature F8 | #Feature F9 | #Feature F7 | #Feature F1 | #Feature F2 | #Feature F3 | Start | End | PTM | AScore | Found By |
| --- | --- | --- | --- | --- | --- | --- | --- | --- | --- | --- | --- | --- | --- | --- | --- | --- | --- | --- | --- | --- | --- | --- | --- | --- | --- | --- | --- | --- | --- | --- | --- | --- | --- | --- | --- | --- | --- |
| K.NLILFLGDGMGVPTITATR.I | Y | 96.73 | 1988.0764 | 19 | 0.2 | 995.0457 | 2 | 68.14 | 9 | F9:42700 | 31122021\_RID\_3262\_2a.raw | 1.3449E6 |  |  |  |  |  |  | 4.1158E5 | 1.3673E7 |  | 4 | 1 | 0 | 0 | 0 | 0 | 0 | 0 | 1 | 2 | 0 | 43 | 61 |  |  | PEAKS DB |
| K.NLILFLGDGM(+15.99)GVPTITATR.I | Y | 93.86 | 2004.0714 | 19 | 0.0 | 1003.0430 | 2 | 63.20 | 9 | F9:39512 | 31122021\_RID\_3262\_2a.raw |  |  |  |  |  |  |  |  | 7.0787E6 |  | 3 | 0 | 0 | 0 | 0 | 0 | 0 | 0 | 0 | 3 | 0 | 43 | 61 | Oxidation (M) | M10:Oxidation (M):1000.00 | PEAKS DB |
| K.GNYGTIGLSAAAR.Y | Y | 80.94 | 1249.6415 | 13 | 0.8 | 625.8285 | 2 | 33.58 | 9 | F9:16148 | 31122021\_RID\_3262\_2a.raw |  |  |  |  |  |  |  | 3.3202E5 | 1.2892E7 | 2.1309E6 | 3 | 0 | 0 | 0 | 0 | 0 | 0 | 0 | 1 | 1 | 1 | 113 | 125 |  |  | PEAKS DB |
| R.VQHASPAGNYAHVVNR.N | N | 80.89 | 1718.8601 | 16 | 0.0 | 573.9606 | 3 | 19.73 | 9 | F9:5169 | 31122021\_RID\_3262\_2a.raw | 5.9402E6 |  |  |  |  |  |  | 2.0488E5 | 2.9449E7 |  | 6 | 2 | 0 | 0 | 0 | 0 | 0 | 0 | 1 | 3 | 0 | 159 | 174 |  |  | PEAKS DB |
| R.ALTEAIEFDKAIER.A | N | 73.25 | 1604.8409 | 14 | 0.1 | 803.4279 | 2 | 41.60 | 9 | F9:22846 | 31122021\_RID\_3262\_2a.raw | 1.0763E6 |  |  |  |  |  |  |  | 3.2856E6 |  | 3 | 1 | 0 | 0 | 0 | 0 | 0 | 0 | 0 | 2 | 0 | 290 | 303 |  |  | PEAKS DB |
| G.VIPVEEEKPSFWNELADK.A | Y | 67.40 | 2129.0681 | 18 | 0.4 | 1065.5417 | 2 | 47.40 | 9 | F9:27417 | 31122021\_RID\_3262\_2a.raw | 6.5033E6 |  |  |  |  |  |  |  | 2.8604E6 |  | 2 | 1 | 0 | 0 | 0 | 0 | 0 | 0 | 0 | 1 | 0 | 9 | 26 |  |  | PEAKS DB |
| R.IDLLQAVSDPK.V | Y | 66.67 | 1197.6604 | 11 | 0.6 | 599.8378 | 2 | 47.81 | 9 | F9:27734 | 31122021\_RID\_3262\_2a.raw | 1.5776E7 | 4.9792E5 |  |  |  |  |  | 2.0174E6 | 9.7775E7 | 1.6868E7 | 6 | 1 | 1 | 0 | 0 | 0 | 0 | 0 | 1 | 2 | 1 | 214 | 224 |  |  | PEAKS DB |
| R.ALTEAIEFDK.A | N | 66.55 | 1135.5760 | 10 | 0.3 | 568.7955 | 2 | 39.48 | 9 | F9:21119 | 31122021\_RID\_3262\_2a.raw | 4.5481E6 | 5.9828E5 |  |  |  |  |  |  | 3.4018E7 | 6.8465E6 | 5 | 1 | 1 | 0 | 0 | 0 | 0 | 0 | 0 | 2 | 1 | 290 | 299 |  |  | PEAKS DB |
| R.LMGLFEPSDMK.Y | N | 63.72 | 1266.5988 | 11 | 0.9 | 634.3073 | 2 | 49.66 | 9 | F9:29211 | 31122021\_RID\_3262\_2a.raw | 4.9848E6 |  |  |  |  |  |  |  | 1.3471E7 |  | 5 | 1 | 0 | 0 | 0 | 0 | 0 | 0 | 0 | 4 | 0 | 228 | 238 |  |  | PEAKS DB |
| R.LM(+15.99)GLFEPSDMK.Y | N | 63.14 | 1282.5938 | 11 | 0.7 | 642.3046 | 2 | 42.96 | 9 | F9:23996 | 31122021\_RID\_3262\_2a.raw |  |  |  |  |  |  |  |  | 4.4808E6 |  | 2 | 0 | 0 | 0 | 0 | 0 | 0 | 0 | 0 | 2 | 0 | 228 | 238 | Oxidation (M) | M2:Oxidation (M):192.66 | PEAKS DB |
| R.LMGLFEPSDM(+15.99)K.Y | N | 62.01 | 1282.5938 | 11 | 0.6 | 642.3046 | 2 | 45.18 | 9 | F9:25650 | 31122021\_RID\_3262\_2a.raw | 7.7041E5 |  |  |  |  |  |  |  | 1.3371E7 |  | 5 | 1 | 0 | 0 | 0 | 0 | 0 | 0 | 0 | 4 | 0 | 228 | 238 | Oxidation (M) | M10:Oxidation (M):139.69 | PEAKS DB |
| K.GFYLFVEGGR.I | N | 61.76 | 1143.5713 | 10 | 0.8 | 572.7934 | 2 | 50.09 | 9 | F9:29523 | 31122021\_RID\_3262\_2a.raw |  |  |  |  |  |  |  | 4.3408E5 | 1.1514E7 |  | 2 | 0 | 0 | 0 | 0 | 0 | 0 | 0 | 1 | 1 | 0 | 268 | 277 |  |  | PEAKS DB |
| R.LM(+15.99)GLFEPSDM(+15.99)K.Y | N | 57.65 | 1298.5886 | 11 | 0.4 | 650.3018 | 2 | 49.67 | 9 | F9:29202 | 31122021\_RID\_3262\_2a.raw |  |  |  |  |  |  |  |  | 6.5419E6 |  | 4 | 0 | 0 | 0 | 0 | 0 | 0 | 0 | 0 | 4 | 0 | 228 | 238 | Oxidation (M) | M2:Oxidation (M):1000.00;M10:Oxidation (M):1000.00 | PEAKS DB |
| K.NLILFLGDGMGVPTITAT.R | Y | 55.84 | 1831.9753 | 18 | 0.1 | 916.9950 | 2 | 81.27 | 9 | F9:47217 | 31122021\_RID\_3262\_2a.raw |  |  |  |  |  |  |  |  | 1.9989E6 |  | 1 | 0 | 0 | 0 | 0 | 0 | 0 | 0 | 0 | 1 | 0 | 43 | 60 |  |  | PEAKS DB |
| K.SVGIVTTTR.V | N | 55.56 | 932.5291 | 9 | -0.6 | 467.2715 | 2 | 24.85 | 9 | F9:9035 | 31122021\_RID\_3262\_2a.raw | 4.7751E6 |  |  |  |  |  |  | 3.9398E5 | 2.2112E7 |  | 3 | 1 | 0 | 0 | 0 | 0 | 0 | 0 | 1 | 1 | 0 | 150 | 158 |  |  | PEAKS DB |
| K.RALTEAIEFDK.A | N | 53.46 | 1291.6771 | 11 | -0.5 | 431.5661 | 3 | 32.85 | 9 | F9:15657 | 31122021\_RID\_3262\_2a.raw |  |  |  |  |  |  |  |  | 2.1679E5 |  | 1 | 0 | 0 | 0 | 0 | 0 | 0 | 0 | 0 | 1 | 0 | 289 | 299 |  |  | PEAKS DB |
| K.NLILFLGDGM(+15.99)GVPTITAT.R | Y | 53.29 | 1847.9702 | 18 | 1.0 | 924.9933 | 2 | 75.62 | 9 | F9:45983 | 31122021\_RID\_3262\_2a.raw |  |  |  |  |  |  |  |  | 1.8995E6 |  | 3 | 0 | 0 | 0 | 0 | 0 | 0 | 0 | 0 | 3 | 0 | 43 | 60 | Oxidation (M) | M10:Oxidation (M):1000.00 | PEAKS DB |
| R.RLMGLFEPSDMK.Y | N | 52.96 | 1422.7000 | 12 | -0.1 | 712.3572 | 2 | 41.16 | 9 | F9:22395 | 31122021\_RID\_3262\_2a.raw |  |  |  |  |  |  |  |  | 2.0343E6 |  | 1 | 0 | 0 | 0 | 0 | 0 | 0 | 0 | 0 | 1 | 0 | 227 | 238 |  |  | PEAKS DB |
| R.IDLLQAVSDPKV.R | Y | 49.86 | 1296.7289 | 12 | 0.7 | 649.3722 | 2 | 53.33 | 9 | F9:31991 | 31122021\_RID\_3262\_2a.raw |  |  |  |  |  |  |  |  | 2.3902E5 |  | 1 | 0 | 0 | 0 | 0 | 0 | 0 | 0 | 0 | 1 | 0 | 214 | 225 |  |  | PEAKS DB |
| R.VQHASPAGNYAHVVN.R | N | 47.90 | 1562.7589 | 15 | 0.5 | 782.3871 | 2 | 22.55 | 9 | F9:7231 | 31122021\_RID\_3262\_2a.raw |  |  |  |  |  |  |  |  | 4.0973E6 |  | 2 | 0 | 0 | 0 | 0 | 0 | 0 | 0 | 0 | 2 | 0 | 159 | 173 |  |  | PEAKS DB |
| K.RALTEAIEFDKAIER.A | N | 46.92 | 1760.9420 | 15 | 0.3 | 587.9881 | 3 | 38.36 | 1 | F1:24057 | 13012022\_RID\_3262\_4a.raw | 1.1573E5 |  |  |  |  |  |  |  |  |  | 1 | 1 | 0 | 0 | 0 | 0 | 0 | 0 | 0 | 0 | 0 | 289 | 303 |  |  | PEAKS DB |
| K.GNYGTIGLSAAA.R | Y | 44.39 | 1093.5404 | 12 | -0.4 | 547.7773 | 2 | 43.81 | 9 | F9:24460 | 31122021\_RID\_3262\_2a.raw |  |  |  |  |  |  |  |  | 1.7625E6 |  | 1 | 0 | 0 | 0 | 0 | 0 | 0 | 0 | 0 | 1 | 0 | 113 | 124 |  |  | PEAKS DB |
| total 22 peptides |  |  |  |  |  |  |  |  |  |  |  |  |  |  |  |  |  |  |  |  |  |  |  |  |  |  |  |  |  |  |  |  |  |  |  |  |  |
| --- | --- | --- | --- | --- | --- | --- | --- | --- | --- | --- | --- | --- | --- | --- | --- | --- | --- | --- | --- | --- | --- | --- | --- | --- | --- | --- | --- | --- | --- | --- | --- | --- | --- | --- | --- | --- | --- |

ETE67131.1

back to list

  

| Protein Coverage
| Supporting Peptides
|

Protein Coverage:

Supporting Peptides:

| Peptide | Uniq | -10lgP | Mass | Length | ppm | m/z | z | RT | Fraction | Scan | Source File | Area F4 | Area F5 | Area F6 | Area F10 | Area F8 | Area F9 | Area F7 | Area F1 | Area F2 | Area F3 | #Feature | #Feature F4 | #Feature F5 | #Feature F6 | #Feature F10 | #Feature F8 | #Feature F9 | #Feature F7 | #Feature F1 | #Feature F2 | #Feature F3 | Start | End | PTM | AScore | Found By |
| --- | --- | --- | --- | --- | --- | --- | --- | --- | --- | --- | --- | --- | --- | --- | --- | --- | --- | --- | --- | --- | --- | --- | --- | --- | --- | --- | --- | --- | --- | --- | --- | --- | --- | --- | --- | --- | --- |
| R.DLDLDSIIAEVK.A | Y | 73.51 | 1329.7028 | 12 | 0.3 | 665.8588 | 2 | 69.53 | 8 | F8:43454 | 31122021\_RID\_3262\_1a.raw | 2.6752E6 | 8.2242E5 | 3.2474E5 | 1.6764E6 |  | 5.6646E6 |  | 9.4427E6 | 4.808E6 |  | 16 | 2 | 2 | 1 | 2 | 0 | 2 | 0 | 4 | 3 | 0 | 1923 | 1934 |  |  | PEAKS DB |
| K.LALDIEIATYR.K | N | 65.96 | 1276.7026 | 11 | 1.4 | 639.3595 | 2 | 52.91 | 8 | F8:30667 | 31122021\_RID\_3262\_1a.raw | 2.7385E7 | 1.0818E6 |  | 1.3337E7 |  |  |  | 4.2941E7 | 2.4275E7 | 6.0353E7 | 24 | 1 | 1 | 0 | 2 | 0 | 0 | 0 | 13 | 6 | 1 | 446 | 456 |  |  | PEAKS DB |
| R.DYQELMNVK.L | N | 65.21 | 1138.5328 | 9 | 0.4 | 570.2739 | 2 | 41.02 | 4 | F4:25980 | 17012022\_RID\_3262\_10a .raw | 1.1187E7 | 8.8848E6 | 2.6711E6 | 4.9346E7 |  | 7.1078E7 | 1.1383E6 | 2.5131E7 | 5.6427E6 | 4.6395E7 | 9 | 1 | 1 | 1 | 1 | 0 | 1 | 1 | 1 | 1 | 1 | 2043 | 2051 |  |  | PEAKS DB |
| R.LLKEYQELMNVK.L | N | 64.24 | 1506.8115 | 12 | 0.0 | 754.4130 | 2 | 39.68 | 8 | F8:20154 | 31122021\_RID\_3262\_1a.raw |  |  |  |  |  |  |  | 3.2141E6 | 1.0992E5 |  | 3 | 0 | 0 | 0 | 0 | 0 | 0 | 0 | 2 | 1 | 0 | 434 | 445 |  |  | PEAKS DB |
| K.LALDVEIATYR.T | N | 63.88 | 1262.6870 | 11 | 0.8 | 632.3513 | 2 | 49.15 | 8 | F8:27481 | 31122021\_RID\_3262\_1a.raw | 1.1555E7 | 4.2265E5 |  | 6.3798E6 |  | 4.7079E5 |  | 1.6274E7 | 6.9194E6 | 1.6595E7 | 16 | 1 | 2 | 0 | 6 | 0 | 2 | 0 | 2 | 2 | 1 | 2052 | 2062 |  |  | PEAKS DB |
| K.EYQELMNVK.L | N | 62.87 | 1152.5485 | 9 | 0.7 | 577.2819 | 2 | 35.46 | 6 | F6:21360 | 17012022\_RID\_3262\_9a.raw |  |  |  | 3.3062E6 |  | 5.5812E6 |  | 1.6845E6 | 1.1435E6 |  | 4 | 0 | 0 | 0 | 1 | 0 | 1 | 0 | 1 | 1 | 0 | 437 | 445 |  |  | PEAKS DB |
| K.AQYEDIAQK.S | N | 62.68 | 1064.5138 | 9 | -0.5 | 533.2639 | 2 | 24.47 | 4 | F4:11436 | 17012022\_RID\_3262\_10a .raw | 2.7369E8 | 8.02E7 | 1.3508E7 | 8.8805E8 | 3.7376E6 | 7.4491E8 | 3.6578E7 | 1.9002E8 | 1.1631E8 | 2.661E8 | 15 | 1 | 1 | 1 | 4 | 1 | 2 | 1 | 2 | 1 | 1 | 334 | 342 |  |  | PEAKS DB |
| K.NKYEDEINKR.T | N | 62.23 | 1307.6470 | 10 | -0.2 | 436.8895 | 3 | 19.57 | 6 | F6:7264 | 17012022\_RID\_3262\_9a.raw | 1.0196E8 | 9.8399E6 |  | 2.0043E8 |  | 2.2923E8 | 1.0038E7 | 4.397E7 | 3.6098E7 | 6.8711E7 | 17 | 3 | 1 | 0 | 3 | 0 | 2 | 2 | 2 | 2 | 2 | 1847 | 1856 |  |  | PEAKS DB |
| R.DYQELM(+15.99)NVK.L | N | 60.86 | 1154.5277 | 9 | -0.1 | 578.2711 | 2 | 31.71 | 4 | F4:18149 | 17012022\_RID\_3262\_10a .raw | 4.0442E6 | 2.0104E6 | 1.8647E6 | 1.8465E7 |  | 1.5647E7 | 9.5906E5 | 6.5695E6 | 4.2189E6 | 3.5004E6 | 15 | 1 | 1 | 2 | 1 | 0 | 2 | 1 | 4 | 2 | 1 | 2043 | 2051 | Oxidation (M) | M6:Oxidation (M):1000.00 | PEAKS DB |
| R.LLRDYQELMNVK.L | N | 60.01 | 1520.8021 | 12 | -0.1 | 507.9413 | 3 | 41.03 | 1 | F1:26617 | 13012022\_RID\_3262\_4a.raw | 7.7543E6 |  |  |  |  |  |  | 1.4523E7 |  |  | 3 | 1 | 0 | 0 | 0 | 0 | 0 | 0 | 2 | 0 | 0 | 2040 | 2051 |  |  | PEAKS DB |
| N.KYEDEINK.R | N | 59.49 | 1037.5029 | 8 | 0.1 | 519.7588 | 2 | 17.64 | 6 | F6:5589 | 17012022\_RID\_3262\_9a.raw |  |  |  | 7.1505E6 |  | 8.6198E6 |  |  |  |  | 2 | 0 | 0 | 0 | 1 | 0 | 1 | 0 | 0 | 0 | 0 | 1848 | 1855 |  |  | PEAKS DB |
| K.FASFIDKVR.F | N | 59.23 | 1081.5920 | 9 | 0.3 | 541.8035 | 2 | 35.45 | 6 | F6:21540 | 17012022\_RID\_3262\_9a.raw | 1.5335E7 |  |  | 9.5108E6 |  | 1.2654E7 |  | 1.0527E7 | 1.5026E6 |  | 9 | 1 | 0 | 0 | 3 | 0 | 1 | 0 | 3 | 1 | 0 | 200 | 208 |  |  | PEAKS DB |
| D.LDLDSIIAEVK.A | N | 58.53 | 1214.6758 | 11 | 1.0 | 608.3458 | 2 | 60.66 | 9 | F9:37537 | 31122021\_RID\_3262\_2a.raw | 2.0183E6 |  |  | 1.8764E6 |  | 7.295E5 |  | 1.0288E7 | 2.275E6 | 2.6971E5 | 7 | 1 | 0 | 0 | 1 | 0 | 1 | 0 | 1 | 2 | 1 | 1924 | 1934 |  |  | PEAKS DB |
| K.NKYEDEINK.R | N | 58.15 | 1151.5458 | 9 | -0.4 | 576.7799 | 2 | 19.99 | 6 | F6:7851 | 17012022\_RID\_3262\_9a.raw |  |  |  | 3.8328E7 |  | 2.7795E7 | 1.8307E6 | 3.6735E6 | 3.5275E6 | 3.3686E6 | 8 | 0 | 0 | 0 | 2 | 0 | 2 | 1 | 1 | 1 | 1 | 1847 | 1855 |  |  | PEAKS DB |
| L.ALDIEIATYR.K | N | 57.72 | 1163.6187 | 10 | 0.7 | 582.8170 | 2 | 45.74 | 8 | F8:24835 | 31122021\_RID\_3262\_1a.raw |  |  |  |  |  |  |  | 2.3394E6 | 1.9465E5 |  | 2 | 0 | 0 | 0 | 0 | 0 | 0 | 0 | 1 | 1 | 0 | 447 | 456 |  |  | PEAKS DB |
| N.KYEDEINKR.T | N | 54.63 | 1193.6040 | 9 | -0.3 | 597.8091 | 2 | 16.55 | 6 | F6:4717 | 17012022\_RID\_3262\_9a.raw | 2.4731E7 | 1.7851E6 |  | 5.25E7 |  | 7.1702E7 | 3.2062E6 |  |  |  | 9 | 1 | 1 | 0 | 2 | 0 | 3 | 2 | 0 | 0 | 0 | 1848 | 1856 |  |  | PEAKS DB |
| K.YEDEINKR.T | N | 54.27 | 1065.5090 | 8 | 0.2 | 533.7619 | 2 | 17.06 | 6 | F6:5136 | 17012022\_RID\_3262\_9a.raw |  |  |  | 3.7263E6 |  | 5.4143E6 | 1.8172E5 |  |  |  | 3 | 0 | 0 | 0 | 1 | 0 | 1 | 1 | 0 | 0 | 0 | 1849 | 1856 |  |  | PEAKS DB |
| V.LNNQFASFIDK.V | N | 53.71 | 1295.6510 | 11 | 2.4 | 648.8344 | 2 | 44.17 | 8 | F8:23553 | 31122021\_RID\_3262\_1a.raw |  |  |  |  |  |  |  | 4.0037E5 |  |  | 1 | 0 | 0 | 0 | 0 | 0 | 0 | 0 | 1 | 0 | 0 | 682 | 692 |  |  | PEAKS DB |
| R.LLKEYQELM(+15.99)NVK.L | N | 53.34 | 1522.8065 | 12 | -0.1 | 762.4105 | 2 | 33.16 | 8 | F8:14908 | 31122021\_RID\_3262\_1a.raw |  |  |  |  |  |  |  | 1.5111E5 |  |  | 1 | 0 | 0 | 0 | 0 | 0 | 0 | 0 | 1 | 0 | 0 | 434 | 445 | Oxidation (M) | M9:Oxidation (M):1000.00 | PEAKS DB |
| K.FASFIDK.V | N | 52.67 | 826.4225 | 7 | 0.7 | 414.2188 | 2 | 36.24 | 6 | F6:22289 | 17012022\_RID\_3262\_9a.raw | 2.5611E7 | 1.8348E7 | 6.6379E6 | 1.2204E8 |  | 1.3051E8 | 4.5017E6 | 4.1444E7 | 1.2872E7 |  | 15 | 2 | 2 | 1 | 4 | 0 | 3 | 1 | 1 | 1 | 0 | 200 | 206 |  |  | PEAKS DB |
| A.LDVEIATYR.T | N | 51.71 | 1078.5658 | 9 | 5.1 | 540.2929 | 2 | 39.98 | 8 | F8:20433 | 31122021\_RID\_3262\_1a.raw |  |  |  |  |  |  |  | 2.3839E5 |  |  | 1 | 0 | 0 | 0 | 0 | 0 | 0 | 0 | 1 | 0 | 0 | 2054 | 2062 |  |  | PEAKS DB |
| L.DLDSIIAEVK.A | N | 50.24 | 1101.5917 | 10 | -0.8 | 551.8027 | 2 | 60.53 | 8 | F8:36544 | 31122021\_RID\_3262\_1a.raw |  |  |  | 1.0752E6 |  |  |  | 3.1086E6 | 8.419E5 |  | 5 | 0 | 0 | 0 | 1 | 0 | 0 | 0 | 2 | 2 | 0 | 1925 | 1934 |  |  | PEAKS DB |
| L.ALDVEIATYR.T | N | 49.94 | 1149.6029 | 10 | 1.0 | 575.8093 | 2 | 40.83 | 9 | F9:22185 | 31122021\_RID\_3262\_2a.raw |  |  |  |  |  |  |  |  | 4.3303E4 |  | 1 | 0 | 0 | 0 | 0 | 0 | 0 | 0 | 0 | 1 | 0 | 2053 | 2062 |  |  | PEAKS DB |
| R.GELALKDAR.D | N | 49.06 | 971.5399 | 9 | -0.6 | 486.7769 | 2 | 22.10 | 6 | F6:9646 | 17012022\_RID\_3262\_9a.raw |  |  |  | 1.6772E6 |  | 1.879E6 | 8.5932E4 |  |  |  | 3 | 0 | 0 | 0 | 1 | 0 | 1 | 1 | 0 | 0 | 0 | 406 | 414 |  |  | PEAKS DB |
| R.LLRDYQELM(+15.99)NVK.L | N | 48.43 | 1536.7970 | 12 | 0.1 | 513.2730 | 3 | 34.30 | 6 | F6:20494 | 17012022\_RID\_3262\_9a.raw |  |  |  |  |  | 3.6586E6 |  | 2.0582E6 |  |  | 2 | 0 | 0 | 0 | 0 | 0 | 1 | 0 | 1 | 0 | 0 | 2040 | 2051 | Oxidation (M) | M9:Oxidation (M):1000.00 | PEAKS DB |
| K.EYQELM(+15.99)NVK.L | N | 46.90 | 1168.5435 | 9 | -0.5 | 585.2787 | 2 | 27.89 | 4 | F4:14727 | 17012022\_RID\_3262\_10a .raw |  |  |  | 1.3738E6 |  |  |  | 6.2988E5 | 4.4468E5 |  | 4 | 0 | 0 | 0 | 1 | 0 | 0 | 0 | 2 | 1 | 0 | 437 | 445 | Oxidation (M) | M6:Oxidation (M):1000.00 | PEAKS DB |
| L.NNQFASFIDK.V | N | 45.05 | 1182.5669 | 10 | -0.2 | 592.2906 | 2 | 41.14 | 6 | F6:26593 | 17012022\_RID\_3262\_9a.raw |  |  |  |  |  | 5.6121E5 |  |  |  |  | 1 | 0 | 0 | 0 | 0 | 0 | 1 | 0 | 0 | 0 | 0 | 683 | 692 |  |  | PEAKS DB |
| A.LDIEIATYR.K | N | 43.99 | 1092.5815 | 9 | 0.1 | 547.2981 | 2 | 45.00 | 8 | F8:24235 | 31122021\_RID\_3262\_1a.raw |  |  |  |  |  |  |  | 8.2061E5 |  |  | 1 | 0 | 0 | 0 | 0 | 0 | 0 | 0 | 1 | 0 | 0 | 448 | 456 |  |  | PEAKS DB |
| K.FASFIDKV.R | N | 43.37 | 925.4908 | 8 | 0.2 | 463.7528 | 2 | 44.26 | 4 | F4:28348 | 17012022\_RID\_3262\_10a .raw |  |  |  | 1.1458E7 |  |  |  | 5.4178E6 | 1.9415E6 |  | 3 | 0 | 0 | 0 | 1 | 0 | 0 | 0 | 1 | 1 | 0 | 200 | 207 |  |  | PEAKS DB |
| N.NQFASFIDK.V | N | 42.09 | 1068.5239 | 9 | 0.0 | 535.2692 | 2 | 40.31 | 6 | F6:25830 | 17012022\_RID\_3262\_9a.raw |  |  |  |  |  | 2.5162E6 |  |  |  |  | 1 | 0 | 0 | 0 | 0 | 0 | 1 | 0 | 0 | 0 | 0 | 684 | 692 |  |  | PEAKS DB |
| V.KAQYEDIAQK.S | N | 41.09 | 1192.6088 | 10 | 0.6 | 597.3120 | 2 | 20.16 | 3 | F3:6968 | 13012022\_RID\_3262\_6a.raw |  |  | 3.7665E4 |  |  |  |  |  |  |  | 1 | 0 | 0 | 1 | 0 | 0 | 0 | 0 | 0 | 0 | 0 | 333 | 342 |  |  | PEAKS DB |
| total 31 peptides |  |  |  |  |  |  |  |  |  |  |  |  |  |  |  |  |  |  |  |  |  |  |  |  |  |  |  |  |  |  |  |  |  |  |  |  |  |
| --- | --- | --- | --- | --- | --- | --- | --- | --- | --- | --- | --- | --- | --- | --- | --- | --- | --- | --- | --- | --- | --- | --- | --- | --- | --- | --- | --- | --- | --- | --- | --- | --- | --- | --- | --- | --- | --- |

QBF53421.1

back to list

  

| Protein Coverage
| Supporting Peptides
|

Protein Coverage:

Supporting Peptides:

| Peptide | Uniq | -10lgP | Mass | Length | ppm | m/z | z | RT | Fraction | Scan | Source File | Area F4 | Area F5 | Area F6 | Area F10 | Area F8 | Area F9 | Area F7 | Area F1 | Area F2 | Area F3 | #Feature | #Feature F4 | #Feature F5 | #Feature F6 | #Feature F10 | #Feature F8 | #Feature F9 | #Feature F7 | #Feature F1 | #Feature F2 | #Feature F3 | Start | End | PTM | AScore | Found By |
| --- | --- | --- | --- | --- | --- | --- | --- | --- | --- | --- | --- | --- | --- | --- | --- | --- | --- | --- | --- | --- | --- | --- | --- | --- | --- | --- | --- | --- | --- | --- | --- | --- | --- | --- | --- | --- | --- |
| K.HQGLPESYNFDFVTMKPVL | N | 88.30 | 2221.0876 | 19 | 1.0 | 741.3705 | 3 | 60.75 | 1 | F1:42994 | 13012022\_RID\_3262\_4a.raw | 1.5758E8 |  |  |  |  |  |  | 9.1193E5 |  |  | 5 | 4 | 0 | 0 | 0 | 0 | 0 | 0 | 1 | 0 | 0 | 535 | 553 |  |  | PEAKS DB |
| K.HQGLPESYNFDFVTM(+15.99)KPVL | N | 82.55 | 2237.0828 | 19 | 0.9 | 746.7022 | 3 | 54.75 | 1 | F1:37685 | 13012022\_RID\_3262\_4a.raw | 8.3546E7 |  |  |  |  |  |  |  |  |  | 4 | 4 | 0 | 0 | 0 | 0 | 0 | 0 | 0 | 0 | 0 | 535 | 553 | Oxidation (M) | M15:Oxidation (M):1000.00 | PEAKS DB |
| K.FTAYAINGPPVEK.D | N | 79.96 | 1405.7241 | 13 | 0.8 | 703.8699 | 2 | 39.56 | 1 | F1:25211 | 13012022\_RID\_3262\_4a.raw | 6.1472E7 | 2.7303E6 | 5.4329E5 |  |  |  |  | 1.3216E5 |  |  | 4 | 1 | 1 | 1 | 0 | 0 | 0 | 0 | 1 | 0 | 0 | 508 | 520 |  |  | PEAKS DB |
| Q.VVPESLFAWER.V | N | 74.17 | 1331.6874 | 11 | 0.3 | 666.8512 | 2 | 53.43 | 1 | F1:37684 | 13012022\_RID\_3262\_4a.raw | 1.6375E8 | 3.8989E6 |  |  |  |  |  | 1.2567E6 |  |  | 3 | 1 | 1 | 0 | 0 | 0 | 0 | 0 | 1 | 0 | 0 | 320 | 330 |  |  | PEAKS DB |
| Q.TKHQGLPESYNFDFVTMKPVL | N | 73.21 | 2450.2305 | 21 | 0.9 | 613.5654 | 4 | 61.28 | 1 | F1:43650 | 13012022\_RID\_3262\_4a.raw | 2.0337E7 |  |  |  |  |  |  |  |  |  | 3 | 3 | 0 | 0 | 0 | 0 | 0 | 0 | 0 | 0 | 0 | 533 | 553 |  |  | PEAKS DB |
| R.YGLEFSYEMAPR.A | Y | 72.14 | 1461.6598 | 12 | 8.8 | 731.8436 | 2 | 46.42 | 1 | F1:31261 | 13012022\_RID\_3262\_4a.raw | 1.6804E6 |  |  |  |  |  |  |  |  |  | 3 | 3 | 0 | 0 | 0 | 0 | 0 | 0 | 0 | 0 | 0 | 427 | 438 |  |  | PEAKS DB |
| R.DQGKVTDMESMK.H | N | 65.74 | 1367.6061 | 12 | 0.0 | 684.8103 | 2 | 25.62 | 1 | F1:12494 | 13012022\_RID\_3262\_4a.raw | 3.691E7 | 1.0003E5 |  |  |  |  |  |  |  |  | 3 | 2 | 1 | 0 | 0 | 0 | 0 | 0 | 0 | 0 | 0 | 445 | 456 |  |  | PEAKS DB |
| K.ENSGTYNNQYMILDTK.K | N | 63.72 | 1889.8466 | 16 | 0.3 | 945.9308 | 2 | 44.50 | 1 | F1:29592 | 13012022\_RID\_3262\_4a.raw | 2.1739E7 | 1.2764E5 |  |  |  |  |  |  |  |  | 5 | 4 | 1 | 0 | 0 | 0 | 0 | 0 | 0 | 0 | 0 | 351 | 366 |  |  | PEAKS DB |
| R.IANMMADSGK.T | N | 60.65 | 1036.4681 | 10 | 0.3 | 519.2415 | 2 | 26.29 | 1 | F1:13060 | 13012022\_RID\_3262\_4a.raw | 1.1087E7 |  |  |  |  |  |  |  |  |  | 1 | 1 | 0 | 0 | 0 | 0 | 0 | 0 | 0 | 0 | 0 | 333 | 342 |  |  | PEAKS DB |
| K.VADISMAAK.F | N | 59.57 | 904.4688 | 9 | 0.6 | 453.2419 | 2 | 26.16 | 8 | F8:9517 | 31122021\_RID\_3262\_1a.raw | 6.1959E7 | 2.0025E6 |  |  |  |  |  | 2.035E5 | 1.4637E5 |  | 4 | 1 | 1 | 0 | 0 | 0 | 0 | 0 | 1 | 1 | 0 | 499 | 507 |  |  | PEAKS DB |
| R.IANMM(+15.99)ADSGK.T | N | 57.25 | 1052.4630 | 10 | -0.7 | 527.2384 | 2 | 26.29 | 1 | F1:13130 | 13012022\_RID\_3262\_4a.raw | 1.8568E6 |  |  |  |  |  |  |  |  |  | 1 | 1 | 0 | 0 | 0 | 0 | 0 | 0 | 0 | 0 | 0 | 333 | 342 | Oxidation (M) | M5:Oxidation (M):18.17 | PEAKS DB |
| K.ENSGTYNNQYMILDTKK.I | N | 52.85 | 2017.9415 | 17 | 0.1 | 673.6545 | 3 | 37.13 | 1 | F1:22888 | 13012022\_RID\_3262\_4a.raw | 3.8253E6 |  |  |  |  |  |  |  |  |  | 1 | 1 | 0 | 0 | 0 | 0 | 0 | 0 | 0 | 0 | 0 | 351 | 367 |  |  | PEAKS DB |
| Q.TKHQGLPESYNFDFVTM(+15.99)KPVL | N | 52.45 | 2466.2253 | 21 | 0.6 | 823.0829 | 3 | 46.88 | 1 | F1:31507 | 13012022\_RID\_3262\_4a.raw | 3.3945E6 |  |  |  |  |  |  |  |  |  | 2 | 2 | 0 | 0 | 0 | 0 | 0 | 0 | 0 | 0 | 0 | 533 | 553 | Oxidation (M) | M17:Oxidation (M):1000.00 | PEAKS DB |
| K.HQGLPESYNFDFVTMKPV.L | N | 51.01 | 2108.0037 | 18 | 0.6 | 703.6756 | 3 | 51.54 | 1 | F1:35496 | 13012022\_RID\_3262\_4a.raw | 1.7469E6 |  |  |  |  |  |  |  |  |  | 1 | 1 | 0 | 0 | 0 | 0 | 0 | 0 | 0 | 0 | 0 | 535 | 552 |  |  | PEAKS DB |
| R.DQGKVTDMESM(+15.99)K.H | N | 50.79 | 1383.6010 | 12 | 0.2 | 692.8079 | 2 | 25.63 | 1 | F1:12244 | 13012022\_RID\_3262\_4a.raw | 3.9723E6 |  |  |  |  |  |  |  |  |  | 1 | 1 | 0 | 0 | 0 | 0 | 0 | 0 | 0 | 0 | 0 | 445 | 456 | Oxidation (M) | M11:Oxidation (M):30.72 | PEAKS DB |
| R.YGLEFSYEM(+15.99)APR.A | Y | 50.28 | 1477.6547 | 12 | 8.5 | 739.8409 | 2 | 41.19 | 1 | F1:26588 | 13012022\_RID\_3262\_4a.raw | 3.3585E5 |  |  |  |  |  |  |  |  |  | 1 | 1 | 0 | 0 | 0 | 0 | 0 | 0 | 0 | 0 | 0 | 427 | 438 | Oxidation (M) | M9:Oxidation (M):1000.00 | PEAKS DB |
| K.ENSGTYNNQYM(+15.99)ILDTK.K | N | 49.57 | 1905.8414 | 16 | 0.4 | 953.9283 | 2 | 40.31 | 1 | F1:25777 | 13012022\_RID\_3262\_4a.raw | 1.8961E6 |  |  |  |  |  |  |  |  |  | 1 | 1 | 0 | 0 | 0 | 0 | 0 | 0 | 0 | 0 | 0 | 351 | 366 | Oxidation (M) | M11:Oxidation (M):1000.00 | PEAKS DB |
| K.VLPGYENIYFAH.S | N | 46.73 | 1421.6979 | 12 | 0.8 | 711.8568 | 2 | 49.22 | 1 | F1:33555 | 13012022\_RID\_3262\_4a.raw | 3.4702E6 |  |  |  |  |  |  |  |  |  | 1 | 1 | 0 | 0 | 0 | 0 | 0 | 0 | 0 | 0 | 0 | 239 | 250 |  |  | PEAKS DB |
| R.DQGKVTDM(+15.99)ESMK.H | N | 46.37 | 1383.6010 | 12 | 0.2 | 692.8079 | 2 | 25.63 | 1 | F1:12495 | 13012022\_RID\_3262\_4a.raw | 5.5334E6 |  |  |  |  |  |  |  |  |  | 2 | 2 | 0 | 0 | 0 | 0 | 0 | 0 | 0 | 0 | 0 | 445 | 456 | Oxidation (M) | M8:Oxidation (M):11.67 | PEAKS DB |
| K.HQGLPESYNFDFVTM(+15.99)KPV.L | N | 44.49 | 2123.9985 | 18 | 0.3 | 709.0070 | 3 | 47.31 | 1 | F1:31932 | 13012022\_RID\_3262\_4a.raw | 9.0568E5 |  |  |  |  |  |  |  |  |  | 1 | 1 | 0 | 0 | 0 | 0 | 0 | 0 | 0 | 0 | 0 | 535 | 552 | Oxidation (M) | M15:Oxidation (M):1000.00 | PEAKS DB |
| N.FDFVTMKPVL | N | 43.97 | 1195.6311 | 10 | 0.3 | 598.8230 | 2 | 56.81 | 1 | F1:39982 | 13012022\_RID\_3262\_4a.raw | 8.4752E5 |  |  |  |  |  |  |  |  |  | 1 | 1 | 0 | 0 | 0 | 0 | 0 | 0 | 0 | 0 | 0 | 544 | 553 |  |  | PEAKS DB |
| K.VADISM(+15.99)AAK.F | N | 42.64 | 920.4637 | 9 | -0.1 | 461.2391 | 2 | 27.69 | 1 | F1:14400 | 13012022\_RID\_3262\_4a.raw | 4.2128E6 |  |  |  |  |  |  |  |  |  | 1 | 1 | 0 | 0 | 0 | 0 | 0 | 0 | 0 | 0 | 0 | 499 | 507 | Oxidation (M) | M6:Oxidation (M):1000.00 | PEAKS DB |
| K.VTDMESM(+15.99)K.H | N | 41.24 | 955.3990 | 8 | 0.5 | 478.7070 | 2 | 18.80 | 2 | F2:5395 | 13012022\_RID\_3262\_5a.raw |  | 4.526E5 |  |  |  |  |  |  |  |  | 1 | 0 | 1 | 0 | 0 | 0 | 0 | 0 | 0 | 0 | 0 | 449 | 456 | Oxidation (M) | M7:Oxidation (M):30.72 | PEAKS DB |
| total 23 peptides |  |  |  |  |  |  |  |  |  |  |  |  |  |  |  |  |  |  |  |  |  |  |  |  |  |  |  |  |  |  |  |  |  |  |  |  |  |
| --- | --- | --- | --- | --- | --- | --- | --- | --- | --- | --- | --- | --- | --- | --- | --- | --- | --- | --- | --- | --- | --- | --- | --- | --- | --- | --- | --- | --- | --- | --- | --- | --- | --- | --- | --- | --- | --- |

XP\_007422201.1

back to list

  

| Protein Coverage
| Supporting Peptides
|

Protein Coverage:

Supporting Peptides:

| Peptide | Uniq | -10lgP | Mass | Length | ppm | m/z | z | RT | Fraction | Scan | Source File | Area F4 | Area F5 | Area F6 | Area F10 | Area F8 | Area F9 | Area F7 | Area F1 | Area F2 | Area F3 | #Feature | #Feature F4 | #Feature F5 | #Feature F6 | #Feature F10 | #Feature F8 | #Feature F9 | #Feature F7 | #Feature F1 | #Feature F2 | #Feature F3 | Start | End | PTM | AScore | Found By |
| --- | --- | --- | --- | --- | --- | --- | --- | --- | --- | --- | --- | --- | --- | --- | --- | --- | --- | --- | --- | --- | --- | --- | --- | --- | --- | --- | --- | --- | --- | --- | --- | --- | --- | --- | --- | --- | --- |
| K.GFFDPNTEENLTYLQLK.E | N | 89.09 | 2027.9840 | 17 | 0.6 | 1014.9999 | 2 | 60.08 | 8 | F8:36271 | 31122021\_RID\_3262\_1a.raw |  |  |  |  |  | 3.8909E5 |  | 2.3132E6 | 6.8517E5 |  | 3 | 0 | 0 | 0 | 0 | 0 | 1 | 0 | 1 | 1 | 0 | 2407 | 2423 |  |  | PEAKS DB |
| R.LLEAQIATGGIIDPK.E | N | 71.52 | 1537.8715 | 15 | 0.8 | 769.9436 | 2 | 48.54 | 8 | F8:27038 | 31122021\_RID\_3262\_1a.raw | 1.1992E6 |  |  | 8.0905E5 |  | 1.0643E6 |  | 2.9774E6 | 9.1227E5 | 2.1458E6 | 6 | 1 | 0 | 0 | 1 | 0 | 1 | 0 | 1 | 1 | 1 | 2360 | 2374 |  |  | PEAKS DB |
| K.NKYETEINITK.T | N | 65.77 | 1351.6984 | 11 | 0.4 | 676.8568 | 2 | 27.99 | 6 | F6:15025 | 17012022\_RID\_3262\_9a.raw | 0 |  |  |  |  | 3.758E5 |  |  |  |  | 1 | 0 | 0 | 0 | 0 | 0 | 1 | 0 | 0 | 0 | 0 | 1360 | 1370 |  |  | PEAKS DB |
| R.LTYEIEDEK.R | N | 59.56 | 1138.5393 | 9 | 1.2 | 570.2776 | 2 | 32.56 | 4 | F4:18668 | 17012022\_RID\_3262\_10a .raw |  |  |  | 1.2576E6 |  | 1.2039E6 |  | 3.6182E5 | 2.3301E5 |  | 4 | 0 | 0 | 0 | 1 | 0 | 1 | 0 | 1 | 1 | 0 | 1110 | 1118 |  |  | PEAKS DB |
| K.EHLMLEEELR.N | N | 58.57 | 1297.6335 | 10 | 0.1 | 433.5518 | 3 | 37.12 | 6 | F6:22970 | 17012022\_RID\_3262\_9a.raw |  |  |  |  |  | 0 |  |  |  |  | 0 | 0 | 0 | 0 | 0 | 0 | 0 | 0 | 0 | 0 | 0 | 1712 | 1721 |  |  | PEAKS DB |
| R.EYENELAK.V | Y | 57.89 | 994.4607 | 8 | -0.1 | 498.2375 | 2 | 24.37 | 6 | F6:11703 | 17012022\_RID\_3262\_9a.raw |  |  |  | 1.6134E6 |  | 1.5483E6 |  |  |  |  | 2 | 0 | 0 | 0 | 1 | 0 | 1 | 0 | 0 | 0 | 0 | 1183 | 1190 |  |  | PEAKS DB |
| R.AELIVQPELK.Y | N | 55.25 | 1138.6597 | 10 | -0.3 | 570.3369 | 2 | 40.26 | 8 | F8:20618 | 31122021\_RID\_3262\_1a.raw |  |  |  | 3.1244E6 |  | 3.183E6 |  | 1.8084E6 | 8.7092E5 |  | 4 | 0 | 0 | 0 | 1 | 0 | 1 | 0 | 1 | 1 | 0 | 94 | 103 |  |  | PEAKS DB |
| R.LTYEIEDEKR.K | N | 53.23 | 1294.6405 | 10 | -0.1 | 432.5541 | 3 | 27.67 | 6 | F6:14693 | 17012022\_RID\_3262\_9a.raw |  |  |  | 2.6098E6 |  | 2.3333E6 |  | 3.2982E5 |  |  | 5 | 0 | 0 | 0 | 2 | 0 | 2 | 0 | 1 | 0 | 0 | 1110 | 1119 |  |  | PEAKS DB |
| R.LQSLTENLTK.E | Y | 52.69 | 1145.6292 | 10 | 0.5 | 573.8221 | 2 | 33.91 | 6 | F6:20185 | 17012022\_RID\_3262\_9a.raw |  | 4.0563E5 |  | 2.8023E6 |  | 2.9519E6 |  | 1.3029E6 |  |  | 4 | 0 | 1 | 0 | 1 | 0 | 1 | 0 | 1 | 0 | 0 | 1702 | 1711 |  |  | PEAKS DB |
| R.QLQNIIQATSR.E | N | 52.19 | 1270.6993 | 11 | -0.2 | 636.3568 | 2 | 35.86 | 6 | F6:21789 | 17012022\_RID\_3262\_9a.raw |  | 3.2783E5 |  | 1.3901E6 |  | 1.2113E6 |  | 1.1624E6 | 2.8151E5 |  | 7 | 0 | 1 | 0 | 2 | 0 | 2 | 0 | 1 | 1 | 0 | 279 | 289 |  |  | PEAKS DB |
| R.GLVGIEFK.E | N | 49.93 | 861.4960 | 8 | -0.5 | 431.7551 | 2 | 42.92 | 4 | F4:27239 | 17012022\_RID\_3262\_10a .raw |  |  |  | 2.3936E6 |  |  |  | 1.2886E6 | 5.6242E5 |  | 3 | 0 | 0 | 0 | 1 | 0 | 0 | 0 | 1 | 1 | 0 | 2312 | 2319 |  |  | PEAKS DB |
| R.DLKDEIVR.L | N | 49.83 | 986.5396 | 8 | -0.6 | 494.2768 | 2 | 31.53 | 6 | F6:18021 | 17012022\_RID\_3262\_9a.raw |  |  |  | 1.6467E6 |  | 1.0864E6 |  |  | 2.7225E5 |  | 3 | 0 | 0 | 0 | 1 | 0 | 1 | 0 | 0 | 1 | 0 | 1244 | 1251 |  |  | PEAKS DB |
| R.LLQLQEQM(+15.99)R.A | N | 46.98 | 1173.6176 | 9 | -0.4 | 587.8158 | 2 | 30.05 | 8 | F8:12398 | 31122021\_RID\_3262\_1a.raw |  |  |  |  |  |  |  | 5.7625E5 |  |  | 1 | 0 | 0 | 0 | 0 | 0 | 0 | 0 | 1 | 0 | 0 | 151 | 159 | Oxidation (M) | M8:Oxidation (M):1000.00 | PEAKS DB |
| R.LLQLQEQMR.A | N | 46.22 | 1157.6227 | 9 | 0.1 | 579.8187 | 2 | 36.87 | 6 | F6:22725 | 17012022\_RID\_3262\_9a.raw |  |  |  |  |  | 2.3411E6 |  |  |  |  | 1 | 0 | 0 | 0 | 0 | 0 | 1 | 0 | 0 | 0 | 0 | 151 | 159 |  |  | PEAKS DB |
| D.PNTEENLTYLQLK.E | N | 44.07 | 1561.7987 | 13 | 1.2 | 781.9076 | 2 | 43.35 | 6 | F6:28598 | 17012022\_RID\_3262\_9a.raw |  |  |  | 5.0937E5 |  | 4.3471E5 |  |  |  |  | 2 | 0 | 0 | 0 | 1 | 0 | 1 | 0 | 0 | 0 | 0 | 2411 | 2423 |  |  | PEAKS DB |
| K.EHLM(+15.99)LEEELR.N | N | 43.92 | 1313.6285 | 10 | 1.1 | 657.8223 | 2 | 31.95 | 8 | F8:13931 | 31122021\_RID\_3262\_1a.raw |  |  |  |  |  |  |  | 9.575E4 |  |  | 1 | 0 | 0 | 0 | 0 | 0 | 0 | 0 | 1 | 0 | 0 | 1712 | 1721 | Oxidation (M) | M4:Oxidation (M):1000.00 | PEAKS DB |
| R.YIELLTR.S | N | 43.13 | 906.5175 | 7 | 0.2 | 454.2661 | 2 | 40.36 | 6 | F6:26058 | 17012022\_RID\_3262\_9a.raw |  |  |  |  |  | 2.4176E6 |  |  |  |  | 1 | 0 | 0 | 0 | 0 | 0 | 1 | 0 | 0 | 0 | 0 | 999 | 1005 |  |  | PEAKS DB |
| R.QVQNLVNK.S | N | 42.51 | 941.5294 | 8 | 0.6 | 471.7722 | 2 | 22.50 | 4 | F4:9622 | 17012022\_RID\_3262\_10a .raw |  |  |  | 9.779E5 |  |  |  |  |  |  | 1 | 0 | 0 | 0 | 1 | 0 | 0 | 0 | 0 | 0 | 0 | 442 | 449 |  |  | PEAKS DB |
| total 18 peptides |  |  |  |  |  |  |  |  |  |  |  |  |  |  |  |  |  |  |  |  |  |  |  |  |  |  |  |  |  |  |  |  |  |  |  |  |  |
| --- | --- | --- | --- | --- | --- | --- | --- | --- | --- | --- | --- | --- | --- | --- | --- | --- | --- | --- | --- | --- | --- | --- | --- | --- | --- | --- | --- | --- | --- | --- | --- | --- | --- | --- | --- | --- | --- |

JAA97975.1

back to list

  

| Protein Coverage
| Supporting Peptides
|

Protein Coverage:

Supporting Peptides:

| Peptide | Uniq | -10lgP | Mass | Length | ppm | m/z | z | RT | Fraction | Scan | Source File | Area F4 | Area F5 | Area F6 | Area F10 | Area F8 | Area F9 | Area F7 | Area F1 | Area F2 | Area F3 | #Feature | #Feature F4 | #Feature F5 | #Feature F6 | #Feature F10 | #Feature F8 | #Feature F9 | #Feature F7 | #Feature F1 | #Feature F2 | #Feature F3 | Start | End | PTM | AScore | Found By |
| --- | --- | --- | --- | --- | --- | --- | --- | --- | --- | --- | --- | --- | --- | --- | --- | --- | --- | --- | --- | --- | --- | --- | --- | --- | --- | --- | --- | --- | --- | --- | --- | --- | --- | --- | --- | --- | --- |
| R.LDSSVSNSEHIAPLSLPSSPPSVGSIC(+57.02)R.I | Y | 84.75 | 2892.4287 | 28 | 0.1 | 965.1503 | 3 | 48.05 | 3 | F3:23636 | 13012022\_RID\_3262\_6a.raw | 1.6154E7 | 3.702E6 | 1.8052E7 |  |  |  | 2.7529E6 | 3.6348E6 |  |  | 5 | 1 | 1 | 1 | 0 | 0 | 0 | 1 | 1 | 0 | 0 | 116 | 143 | Carbamidomethylation | C27:Carbamidomethylation:1000.00 | PEAKS DB |
| S.NSEHIAPLSLPSSPPSVGSIC(+57.02)R.I | N | 77.01 | 2304.1531 | 22 | 0.9 | 769.0590 | 3 | 46.87 | 2 | F2:28483 | 13012022\_RID\_3262\_5a.raw | 2.0078E8 | 8.7551E8 | 5.3784E6 |  |  |  |  | 4.4386E6 | 1.4584E6 |  | 8 | 2 | 3 | 1 | 0 | 0 | 0 | 0 | 1 | 1 | 0 | 122 | 143 | Carbamidomethylation | C21:Carbamidomethylation:1000.00 | PEAKS DB |
| N.SEHIAPLSLPSSPPSVGSIC(+57.02)R.I | N | 64.27 | 2190.1104 | 21 | 0.7 | 731.0446 | 3 | 47.21 | 2 | F2:27952 | 13012022\_RID\_3262\_5a.raw | 7.6183E6 | 1.4583E7 |  |  |  |  |  |  |  |  | 2 | 1 | 1 | 0 | 0 | 0 | 0 | 0 | 0 | 0 | 0 | 123 | 143 | Carbamidomethylation | C20:Carbamidomethylation:1000.00 | PEAKS DB |
| K.ILNEDEQTRDPK.E | N | 63.25 | 1456.7157 | 12 | 0.2 | 729.3652 | 2 | 20.40 | 2 | F2:7523 | 13012022\_RID\_3262\_5a.raw | 2.5611E7 | 5.5662E7 | 3.8171E6 | 7.1395E6 |  | 5.2046E6 | 7.5202E6 |  |  |  | 12 | 2 | 3 | 2 | 2 | 0 | 1 | 2 | 0 | 0 | 0 | 82 | 93 |  |  | PEAKS DB |
| K.ILNEDEQTR.D | N | 62.86 | 1116.5411 | 9 | -0.7 | 559.2775 | 2 | 22.53 | 4 | F4:9650 | 17012022\_RID\_3262\_10a .raw |  | 2.4557E7 | 3.3128E7 | 8.9808E6 |  | 5.8113E6 |  | 6.3735E5 | 9.79E5 | 0 | 7 | 0 | 1 | 2 | 1 | 0 | 1 | 0 | 1 | 1 | 0 | 82 | 90 |  |  | PEAKS DB |
| K.KILNEDEQTR.D | N | 60.21 | 1244.6360 | 10 | -0.2 | 623.3251 | 2 | 18.75 | 2 | F2:6250 | 13012022\_RID\_3262\_5a.raw |  | 1.5363E7 |  | 8.4896E4 |  | 1.9432E6 | 2.5564E6 | 1.5673E4 |  |  | 8 | 0 | 3 | 0 | 1 | 0 | 1 | 2 | 1 | 0 | 0 | 81 | 90 |  |  | PEAKS DB |
| E.HIAPLSLPSSPPSVGSIC(+57.02)R.I | N | 57.25 | 1974.0356 | 19 | -0.2 | 659.0190 | 3 | 45.79 | 3 | F3:22025 | 13012022\_RID\_3262\_6a.raw | 1.1698E7 | 2.4569E7 | 3.1622E7 |  |  |  |  |  |  |  | 5 | 2 | 1 | 2 | 0 | 0 | 0 | 0 | 0 | 0 | 0 | 125 | 143 | Carbamidomethylation | C18:Carbamidomethylation:1000.00 | PEAKS DB |
| K.KILNEDEQTRDPK.E | N | 56.63 | 1584.8107 | 13 | 0.3 | 793.4128 | 2 | 19.17 | 7 | F7:4895 | 21012022\_RID\_3262\_7a.raw | 2.769E6 | 3.177E7 |  | 5.4009E6 |  | 7.2018E6 | 6.457E6 | 6.1213E4 |  |  | 8 | 1 | 2 | 0 | 1 | 0 | 1 | 2 | 1 | 0 | 0 | 81 | 93 |  |  | PEAKS DB |
| K.KILNEDEQTRD.P | N | 55.83 | 1359.6630 | 11 | 0.3 | 680.8390 | 2 | 20.13 | 7 | F7:5537 | 21012022\_RID\_3262\_7a.raw |  | 2.0037E6 |  | 4.1763E6 |  |  | 2.6434E6 |  |  |  | 4 | 0 | 1 | 0 | 1 | 0 | 0 | 2 | 0 | 0 | 0 | 81 | 91 |  |  | PEAKS DB |
| L.SLPSSPPSVGSIC(+57.02)R.I | N | 55.06 | 1442.7188 | 14 | 0.4 | 722.3669 | 2 | 34.98 | 3 | F3:15674 | 13012022\_RID\_3262\_6a.raw |  | 1.1398E7 | 2.4327E6 |  |  |  | 2.4086E6 |  |  |  | 3 | 0 | 1 | 1 | 0 | 0 | 0 | 1 | 0 | 0 | 0 | 130 | 143 | Carbamidomethylation | C13:Carbamidomethylation:1000.00 | PEAKS DB |
| K.KILNEDEQTRDPKEK.F | N | 52.44 | 1841.9482 | 15 | 0.0 | 461.4943 | 4 | 16.95 | 1 | F1:5225 | 13012022\_RID\_3262\_4a.raw | 8.7274E6 |  |  |  |  |  |  |  |  |  | 2 | 2 | 0 | 0 | 0 | 0 | 0 | 0 | 0 | 0 | 0 | 81 | 95 |  |  | PEAKS DB |
| K.ILNEDEQTRDPKEK.F | N | 47.76 | 1713.8533 | 14 | 0.4 | 572.2919 | 3 | 18.15 | 2 | F2:5610 | 13012022\_RID\_3262\_5a.raw |  | 1.6162E6 |  |  |  |  |  |  |  |  | 1 | 0 | 1 | 0 | 0 | 0 | 0 | 0 | 0 | 0 | 0 | 82 | 95 |  |  | PEAKS DB |
| R.IMGWGTISPTK.E | N | 47.73 | 1189.6166 | 11 | 1.3 | 595.8163 | 2 | 44.01 | 3 | F3:20987 | 13012022\_RID\_3262\_6a.raw |  | 2.3901E7 | 3.6021E5 |  |  |  |  |  |  |  | 2 | 0 | 1 | 1 | 0 | 0 | 0 | 0 | 0 | 0 | 0 | 144 | 154 |  |  | PEAKS DB |
| R.IM(+15.99)GWGTISPTK.E | N | 46.88 | 1205.6115 | 11 | 1.2 | 603.8137 | 2 | 37.85 | 2 | F2:20821 | 13012022\_RID\_3262\_5a.raw |  | 5.8537E6 |  |  |  |  |  |  |  |  | 1 | 0 | 1 | 0 | 0 | 0 | 0 | 0 | 0 | 0 | 0 | 144 | 154 | Oxidation (M) | M2:Oxidation (M):1000.00 | PEAKS DB |
| R.LDSSVSNSEHIAPL.S | N | 46.11 | 1467.7205 | 14 | 0.9 | 734.8682 | 2 | 40.20 | 7 | F7:19377 | 21012022\_RID\_3262\_7a.raw |  |  | 2.3044E7 |  |  |  | 2.7606E7 | 1.6388E6 |  |  | 4 | 0 | 0 | 2 | 0 | 0 | 0 | 1 | 1 | 0 | 0 | 116 | 129 |  |  | PEAKS DB |
| L.VVGGDEC(+57.02)NINEHR.S | N | 43.47 | 1497.6630 | 13 | 1.7 | 749.8400 | 2 | 21.08 | 7 | F7:6186 | 21012022\_RID\_3262\_7a.raw |  |  |  |  |  |  | 0 |  |  |  | 0 | 0 | 0 | 0 | 0 | 0 | 0 | 0 | 0 | 0 | 0 | 25 | 37 | Carbamidomethylation | C7:Carbamidomethylation:1000.00 | PEAKS DB |
| R.LDSSVSNSEHIAPLSLPSSPPSVGSIC(+57.02).R | Y | 42.61 | 2736.3276 | 27 | 0.7 | 1369.1721 | 2 | 55.26 | 8 | F8:32402 | 31122021\_RID\_3262\_1a.raw |  |  |  |  |  |  |  | 1.0795E5 |  |  | 1 | 0 | 0 | 0 | 0 | 0 | 0 | 0 | 1 | 0 | 0 | 116 | 142 | Carbamidomethylation | C27:Carbamidomethylation:1000.00 | PEAKS DB |
| K.DKDIMLIR.L | N | 41.99 | 1002.5532 | 8 | 0.7 | 502.2842 | 2 | 34.59 | 7 | F7:15514 | 21012022\_RID\_3262\_7a.raw |  |  |  |  |  |  | 2.6461E6 |  |  |  | 1 | 0 | 0 | 0 | 0 | 0 | 0 | 1 | 0 | 0 | 0 | 108 | 115 |  |  | PEAKS DB |
| total 18 peptides |  |  |  |  |  |  |  |  |  |  |  |  |  |  |  |  |  |  |  |  |  |  |  |  |  |  |  |  |  |  |  |  |  |  |  |  |  |
| --- | --- | --- | --- | --- | --- | --- | --- | --- | --- | --- | --- | --- | --- | --- | --- | --- | --- | --- | --- | --- | --- | --- | --- | --- | --- | --- | --- | --- | --- | --- | --- | --- | --- | --- | --- | --- | --- |

JAS05316.1

back to list

  

| Protein Coverage
| Supporting Peptides
|

Protein Coverage:

Supporting Peptides:

| Peptide | Uniq | -10lgP | Mass | Length | ppm | m/z | z | RT | Fraction | Scan | Source File | Area F4 | Area F5 | Area F6 | Area F10 | Area F8 | Area F9 | Area F7 | Area F1 | Area F2 | Area F3 | #Feature | #Feature F4 | #Feature F5 | #Feature F6 | #Feature F10 | #Feature F8 | #Feature F9 | #Feature F7 | #Feature F1 | #Feature F2 | #Feature F3 | Start | End | PTM | AScore | Found By |
| --- | --- | --- | --- | --- | --- | --- | --- | --- | --- | --- | --- | --- | --- | --- | --- | --- | --- | --- | --- | --- | --- | --- | --- | --- | --- | --- | --- | --- | --- | --- | --- | --- | --- | --- | --- | --- | --- |
| K.SAGQLYEESLR.K | N | 72.77 | 1251.6095 | 11 | 0.4 | 626.8123 | 2 | 32.12 | 8 | F8:14092 | 31122021\_RID\_3262\_1a.raw | 1.2366E9 | 8.6285E7 | 3.6644E7 | 6.5882E5 |  | 6.8121E6 | 2.7435E7 | 6.1833E7 | 1.0188E8 | 2.1008E9 | 11 | 2 | 1 | 1 | 1 | 0 | 1 | 1 | 1 | 1 | 2 | 170 | 180 |  |  | PEAKS DB |
| Y.GIGDDANFFQALDFK.D | N | 70.77 | 1656.7783 | 15 | 0.6 | 829.3970 | 2 | 63.80 | 9 | F9:39957 | 31122021\_RID\_3262\_2a.raw |  | 4.1343E5 |  |  |  |  |  | 2.2577E6 | 1.7889E6 | 7.0811E7 | 6 | 0 | 1 | 0 | 0 | 0 | 0 | 0 | 2 | 1 | 2 | 393 | 407 |  |  | PEAKS DB |
| I.AYGIGDDANFFQALDFK.D | N | 69.60 | 1890.8788 | 17 | 0.2 | 946.4468 | 2 | 65.58 | 8 | F8:40325 | 31122021\_RID\_3262\_1a.raw |  |  |  |  |  |  |  | 1.5459E5 |  |  | 1 | 0 | 0 | 0 | 0 | 0 | 0 | 0 | 1 | 0 | 0 | 391 | 407 |  |  | PEAKS DB |
| R.IKFEPPLPPK.K | N | 57.33 | 1164.6907 | 10 | 0.6 | 583.3530 | 2 | 38.07 | 4 | F4:23645 | 17012022\_RID\_3262\_10a .raw | 2.0942E8 | 5.2488E7 | 3.8344E6 | 8.8895E7 |  | 2.1537E7 | 4.1213E6 | 1.9315E7 | 1.4243E7 | 9.9326E8 | 18 | 1 | 2 | 1 | 4 | 0 | 2 | 1 | 2 | 2 | 3 | 322 | 331 |  |  | PEAKS DB |
| K.YDTYSTKEYLLK.E | N | 53.18 | 1522.7555 | 12 | 0.9 | 762.3857 | 2 | 34.13 | 1 | F1:20150 | 13012022\_RID\_3262\_4a.raw | 4.6273E6 |  |  |  |  |  |  |  |  |  | 2 | 2 | 0 | 0 | 0 | 0 | 0 | 0 | 0 | 0 | 0 | 198 | 209 |  |  | PEAKS DB |
| K.SAGQLYEESLRK.V | N | 52.72 | 1379.7045 | 12 | 0.5 | 690.8599 | 2 | 24.65 | 9 | F9:9094 | 31122021\_RID\_3262\_2a.raw | 4.3022E7 | 1.5347E6 |  |  |  | 1.5382E4 | 5.71E5 |  | 1.2745E6 | 3.1589E7 | 7 | 2 | 1 | 0 | 0 | 0 | 1 | 1 | 0 | 1 | 1 | 170 | 181 |  |  | PEAKS DB |
| K.KFWEDEGIHGGK.S | N | 52.53 | 1401.6677 | 12 | 0.0 | 468.2299 | 3 | 28.13 | 1 | F1:14816 | 13012022\_RID\_3262\_4a.raw | 4.3552E6 |  |  |  |  |  |  |  |  | 1.3068E7 | 4 | 2 | 0 | 0 | 0 | 0 | 0 | 0 | 0 | 0 | 2 | 354 | 365 |  |  | PEAKS DB |
| K.STTDLPSR.F | N | 52.16 | 875.4348 | 8 | 0.4 | 438.7249 | 2 | 21.40 | 6 | F6:8926 | 17012022\_RID\_3262\_9a.raw |  | 2.9854E7 |  | 4.8043E6 |  | 1.4473E7 | 1.9071E6 |  |  |  | 4 | 0 | 1 | 0 | 1 | 0 | 1 | 1 | 0 | 0 | 0 | 366 | 373 |  |  | PEAKS DB |
| N.DLSLIHQLPK.K | N | 51.65 | 1162.6710 | 10 | -0.7 | 582.3424 | 2 | 40.68 | 10 | F10:10098 | 31122021\_RID\_3262\_3a.raw |  |  |  |  |  |  |  | 1.197E6 | 2.7589E5 | 1.8174E7 | 3 | 0 | 0 | 0 | 0 | 0 | 0 | 0 | 1 | 1 | 1 | 416 | 425 |  |  | PEAKS DB |
| A.DIVINDLSLIHQLPK.K | N | 49.33 | 1716.9774 | 15 | 0.0 | 859.4960 | 2 | 55.52 | 8 | F8:32578 | 31122021\_RID\_3262\_1a.raw |  |  |  |  |  |  |  | 1.2793E5 | 1.1614E5 |  | 2 | 0 | 0 | 0 | 0 | 0 | 0 | 0 | 1 | 1 | 0 | 411 | 425 |  |  | PEAKS DB |
| K.RFDEIVGGMDQLPTSM.Y | N | 48.45 | 1794.8280 | 16 | -0.6 | 898.4208 | 2 | 56.96 | 10 | F10:16573 | 31122021\_RID\_3262\_3a.raw |  |  |  |  |  |  |  |  |  | 1.5934E7 | 1 | 0 | 0 | 0 | 0 | 0 | 0 | 0 | 0 | 0 | 1 | 250 | 265 |  |  | PEAKS DB |
| R.IKFEPPLPPKK.A | N | 48.17 | 1292.7855 | 11 | -0.6 | 431.9355 | 3 | 28.73 | 10 | F10:6015 | 31122021\_RID\_3262\_3a.raw |  |  |  | 2.6401E6 |  |  |  | 2.4685E6 |  | 9.6849E7 | 4 | 0 | 0 | 0 | 2 | 0 | 0 | 0 | 1 | 0 | 1 | 322 | 332 |  |  | PEAKS DB |
| R.RIKFEPPLPPK.K | N | 47.70 | 1320.7917 | 11 | -0.2 | 441.2711 | 3 | 31.51 | 6 | F6:18014 | 17012022\_RID\_3262\_9a.raw | 9.1946E7 |  |  |  |  | 4.9304E5 |  |  |  |  | 2 | 1 | 0 | 0 | 0 | 0 | 1 | 0 | 0 | 0 | 0 | 321 | 331 |  |  | PEAKS DB |
| K.FEPPLPPK.K | N | 46.74 | 923.5116 | 8 | -0.6 | 462.7628 | 2 | 36.61 | 4 | F4:21855 | 17012022\_RID\_3262\_10a .raw | 8.6451E7 | 2.7491E7 |  | 4.4878E7 |  |  |  | 7.8215E6 | 8.5594E6 |  | 5 | 1 | 1 | 0 | 1 | 0 | 0 | 0 | 1 | 1 | 0 | 324 | 331 |  |  | PEAKS DB |
| M.AGLSAAYVLAGAGHEVTVLEASER.A | Y | 46.58 | 2370.2178 | 24 | 7.8 | 791.0860 | 3 | 51.01 | 10 | F10:14383 | 31122021\_RID\_3262\_3a.raw |  |  |  |  |  |  |  |  |  | 4.5567E5 | 1 | 0 | 0 | 0 | 0 | 0 | 0 | 0 | 0 | 0 | 1 | 62 | 85 |  |  | PEAKS DB |
| K.FEPPLPPKK.A | N | 43.61 | 1051.6066 | 9 | 0.7 | 526.8109 | 2 | 29.21 | 4 | F4:15245 | 17012022\_RID\_3262\_10a .raw |  |  |  | 4.2511E6 |  |  |  |  |  |  | 1 | 0 | 0 | 0 | 1 | 0 | 0 | 0 | 0 | 0 | 0 | 324 | 332 |  |  | PEAKS DB |
| K.RFDEIVGGM(+15.99)DQLPTSM(+15.99).Y | N | 42.69 | 1826.8179 | 16 | -0.4 | 914.4158 | 2 | 56.93 | 10 | F10:16509 | 31122021\_RID\_3262\_3a.raw |  |  |  |  |  |  |  |  |  | 0 | 0 | 0 | 0 | 0 | 0 | 0 | 0 | 0 | 0 | 0 | 0 | 250 | 265 | Oxidation (M) | M9:Oxidation (M):1000.00;M16:Oxidation (M):1000.00 | PEAKS DB |
| E.VTVLEASER.A | N | 42.68 | 1002.5345 | 9 | -0.1 | 502.2745 | 2 | 28.85 | 7 | F7:11280 | 21012022\_RID\_3262\_7a.raw |  |  |  |  |  |  | 1.7683E5 |  |  |  | 1 | 0 | 0 | 0 | 0 | 0 | 0 | 1 | 0 | 0 | 0 | 77 | 85 |  |  | PEAKS DB |
| K.RFDEIVGGMDQLPT.S | N | 41.44 | 1576.7555 | 14 | -0.6 | 789.3846 | 2 | 51.27 | 1 | F1:35302 | 13012022\_RID\_3262\_4a.raw | 1.0726E6 |  |  |  |  |  |  |  |  |  | 1 | 1 | 0 | 0 | 0 | 0 | 0 | 0 | 0 | 0 | 0 | 250 | 263 |  |  | PEAKS DB |
| total 19 peptides |  |  |  |  |  |  |  |  |  |  |  |  |  |  |  |  |  |  |  |  |  |  |  |  |  |  |  |  |  |  |  |  |  |  |  |  |  |
| --- | --- | --- | --- | --- | --- | --- | --- | --- | --- | --- | --- | --- | --- | --- | --- | --- | --- | --- | --- | --- | --- | --- | --- | --- | --- | --- | --- | --- | --- | --- | --- | --- | --- | --- | --- | --- | --- |

JAS05318.1

back to list

  

| Protein Coverage
| Supporting Peptides
|

Protein Coverage:

Supporting Peptides:

| Peptide | Uniq | -10lgP | Mass | Length | ppm | m/z | z | RT | Fraction | Scan | Source File | Area F4 | Area F5 | Area F6 | Area F10 | Area F8 | Area F9 | Area F7 | Area F1 | Area F2 | Area F3 | #Feature | #Feature F4 | #Feature F5 | #Feature F6 | #Feature F10 | #Feature F8 | #Feature F9 | #Feature F7 | #Feature F1 | #Feature F2 | #Feature F3 | Start | End | PTM | AScore | Found By |
| --- | --- | --- | --- | --- | --- | --- | --- | --- | --- | --- | --- | --- | --- | --- | --- | --- | --- | --- | --- | --- | --- | --- | --- | --- | --- | --- | --- | --- | --- | --- | --- | --- | --- | --- | --- | --- | --- |
| K.SAGQLYEESLR.K | N | 72.77 | 1251.6095 | 11 | 0.4 | 626.8123 | 2 | 32.12 | 8 | F8:14092 | 31122021\_RID\_3262\_1a.raw | 1.2366E9 | 8.6285E7 | 3.6644E7 | 6.5882E5 |  | 6.8121E6 | 2.7435E7 | 6.1833E7 | 1.0188E8 | 2.1008E9 | 11 | 2 | 1 | 1 | 1 | 0 | 1 | 1 | 1 | 1 | 2 | 170 | 180 |  |  | PEAKS DB |
| Y.GIGDDANFFQALDFK.D | N | 70.77 | 1656.7783 | 15 | 0.6 | 829.3970 | 2 | 63.80 | 9 | F9:39957 | 31122021\_RID\_3262\_2a.raw |  | 4.1343E5 |  |  |  |  |  | 2.2577E6 | 1.7889E6 | 7.0811E7 | 6 | 0 | 1 | 0 | 0 | 0 | 0 | 0 | 2 | 1 | 2 | 393 | 407 |  |  | PEAKS DB |
| I.AYGIGDDANFFQALDFK.D | N | 69.60 | 1890.8788 | 17 | 0.2 | 946.4468 | 2 | 65.58 | 8 | F8:40325 | 31122021\_RID\_3262\_1a.raw |  |  |  |  |  |  |  | 1.5459E5 |  |  | 1 | 0 | 0 | 0 | 0 | 0 | 0 | 0 | 1 | 0 | 0 | 391 | 407 |  |  | PEAKS DB |
| R.IKFEPPLPPK.K | N | 57.33 | 1164.6907 | 10 | 0.6 | 583.3530 | 2 | 38.07 | 4 | F4:23645 | 17012022\_RID\_3262\_10a .raw | 2.0942E8 | 5.2488E7 | 3.8344E6 | 8.8895E7 |  | 2.1537E7 | 4.1213E6 | 1.9315E7 | 1.4243E7 | 9.9326E8 | 18 | 1 | 2 | 1 | 4 | 0 | 2 | 1 | 2 | 2 | 3 | 322 | 331 |  |  | PEAKS DB |
| K.YDTYSTKEYLLK.E | N | 53.18 | 1522.7555 | 12 | 0.9 | 762.3857 | 2 | 34.13 | 1 | F1:20150 | 13012022\_RID\_3262\_4a.raw | 4.6273E6 |  |  |  |  |  |  |  |  |  | 2 | 2 | 0 | 0 | 0 | 0 | 0 | 0 | 0 | 0 | 0 | 198 | 209 |  |  | PEAKS DB |
| K.SAGQLYEESLRK.V | N | 52.72 | 1379.7045 | 12 | 0.5 | 690.8599 | 2 | 24.65 | 9 | F9:9094 | 31122021\_RID\_3262\_2a.raw | 4.3022E7 | 1.5347E6 |  |  |  | 1.5382E4 | 5.71E5 |  | 1.2745E6 | 3.1589E7 | 7 | 2 | 1 | 0 | 0 | 0 | 1 | 1 | 0 | 1 | 1 | 170 | 181 |  |  | PEAKS DB |
| K.KFWEDEGIHGGK.S | N | 52.53 | 1401.6677 | 12 | 0.0 | 468.2299 | 3 | 28.13 | 1 | F1:14816 | 13012022\_RID\_3262\_4a.raw | 4.3552E6 |  |  |  |  |  |  |  |  | 1.3068E7 | 4 | 2 | 0 | 0 | 0 | 0 | 0 | 0 | 0 | 0 | 2 | 354 | 365 |  |  | PEAKS DB |
| K.STTDLPSR.F | N | 52.16 | 875.4348 | 8 | 0.4 | 438.7249 | 2 | 21.40 | 6 | F6:8926 | 17012022\_RID\_3262\_9a.raw |  | 2.9854E7 |  | 4.8043E6 |  | 1.4473E7 | 1.9071E6 |  |  |  | 4 | 0 | 1 | 0 | 1 | 0 | 1 | 1 | 0 | 0 | 0 | 366 | 373 |  |  | PEAKS DB |
| N.DLSLIHQLPK.K | N | 51.65 | 1162.6710 | 10 | -0.7 | 582.3424 | 2 | 40.68 | 10 | F10:10098 | 31122021\_RID\_3262\_3a.raw |  |  |  |  |  |  |  | 1.197E6 | 2.7589E5 | 1.8174E7 | 3 | 0 | 0 | 0 | 0 | 0 | 0 | 0 | 1 | 1 | 1 | 416 | 425 |  |  | PEAKS DB |
| A.DIVINDLSLIHQLPK.K | N | 49.33 | 1716.9774 | 15 | 0.0 | 859.4960 | 2 | 55.52 | 8 | F8:32578 | 31122021\_RID\_3262\_1a.raw |  |  |  |  |  |  |  | 1.2793E5 | 1.1614E5 |  | 2 | 0 | 0 | 0 | 0 | 0 | 0 | 0 | 1 | 1 | 0 | 411 | 425 |  |  | PEAKS DB |
| K.RFDEIVGGMDQLPTSM.Y | N | 48.45 | 1794.8280 | 16 | -0.6 | 898.4208 | 2 | 56.96 | 10 | F10:16573 | 31122021\_RID\_3262\_3a.raw |  |  |  |  |  |  |  |  |  | 1.5934E7 | 1 | 0 | 0 | 0 | 0 | 0 | 0 | 0 | 0 | 0 | 1 | 250 | 265 |  |  | PEAKS DB |
| R.IKFEPPLPPKK.A | N | 48.17 | 1292.7855 | 11 | -0.6 | 431.9355 | 3 | 28.73 | 10 | F10:6015 | 31122021\_RID\_3262\_3a.raw |  |  |  | 2.6401E6 |  |  |  | 2.4685E6 |  | 9.6849E7 | 4 | 0 | 0 | 0 | 2 | 0 | 0 | 0 | 1 | 0 | 1 | 322 | 332 |  |  | PEAKS DB |
| R.RIKFEPPLPPK.K | N | 47.70 | 1320.7917 | 11 | -0.2 | 441.2711 | 3 | 31.51 | 6 | F6:18014 | 17012022\_RID\_3262\_9a.raw | 9.1946E7 |  |  |  |  | 4.9304E5 |  |  |  |  | 2 | 1 | 0 | 0 | 0 | 0 | 1 | 0 | 0 | 0 | 0 | 321 | 331 |  |  | PEAKS DB |
| K.FEPPLPPK.K | N | 46.74 | 923.5116 | 8 | -0.6 | 462.7628 | 2 | 36.61 | 4 | F4:21855 | 17012022\_RID\_3262\_10a .raw | 8.6451E7 | 2.7491E7 |  | 4.4878E7 |  |  |  | 7.8215E6 | 8.5594E6 |  | 5 | 1 | 1 | 0 | 1 | 0 | 0 | 0 | 1 | 1 | 0 | 324 | 331 |  |  | PEAKS DB |
| M.AGLSAAYVLAGAGHEVTVLEASER.A | Y | 46.58 | 2370.2178 | 24 | 7.8 | 791.0860 | 3 | 51.01 | 10 | F10:14383 | 31122021\_RID\_3262\_3a.raw |  |  |  |  |  |  |  |  |  | 4.5567E5 | 1 | 0 | 0 | 0 | 0 | 0 | 0 | 0 | 0 | 0 | 1 | 62 | 85 |  |  | PEAKS DB |
| K.FEPPLPPKK.A | N | 43.61 | 1051.6066 | 9 | 0.7 | 526.8109 | 2 | 29.21 | 4 | F4:15245 | 17012022\_RID\_3262\_10a .raw |  |  |  | 4.2511E6 |  |  |  |  |  |  | 1 | 0 | 0 | 0 | 1 | 0 | 0 | 0 | 0 | 0 | 0 | 324 | 332 |  |  | PEAKS DB |
| K.RFDEIVGGM(+15.99)DQLPTSM(+15.99).Y | N | 42.69 | 1826.8179 | 16 | -0.4 | 914.4158 | 2 | 56.93 | 10 | F10:16509 | 31122021\_RID\_3262\_3a.raw |  |  |  |  |  |  |  |  |  | 0 | 0 | 0 | 0 | 0 | 0 | 0 | 0 | 0 | 0 | 0 | 0 | 250 | 265 | Oxidation (M) | M9:Oxidation (M):1000.00;M16:Oxidation (M):1000.00 | PEAKS DB |
| E.VTVLEASER.A | N | 42.68 | 1002.5345 | 9 | -0.1 | 502.2745 | 2 | 28.85 | 7 | F7:11280 | 21012022\_RID\_3262\_7a.raw |  |  |  |  |  |  | 1.7683E5 |  |  |  | 1 | 0 | 0 | 0 | 0 | 0 | 0 | 1 | 0 | 0 | 0 | 77 | 85 |  |  | PEAKS DB |
| K.RFDEIVGGMDQLPT.S | N | 41.44 | 1576.7555 | 14 | -0.6 | 789.3846 | 2 | 51.27 | 1 | F1:35302 | 13012022\_RID\_3262\_4a.raw | 1.0726E6 |  |  |  |  |  |  |  |  |  | 1 | 1 | 0 | 0 | 0 | 0 | 0 | 0 | 0 | 0 | 0 | 250 | 263 |  |  | PEAKS DB |
| total 19 peptides |  |  |  |  |  |  |  |  |  |  |  |  |  |  |  |  |  |  |  |  |  |  |  |  |  |  |  |  |  |  |  |  |  |  |  |  |  |
| --- | --- | --- | --- | --- | --- | --- | --- | --- | --- | --- | --- | --- | --- | --- | --- | --- | --- | --- | --- | --- | --- | --- | --- | --- | --- | --- | --- | --- | --- | --- | --- | --- | --- | --- | --- | --- | --- |

JAS05319.1

back to list

  

| Protein Coverage
| Supporting Peptides
|

Protein Coverage:

Supporting Peptides:

| Peptide | Uniq | -10lgP | Mass | Length | ppm | m/z | z | RT | Fraction | Scan | Source File | Area F4 | Area F5 | Area F6 | Area F10 | Area F8 | Area F9 | Area F7 | Area F1 | Area F2 | Area F3 | #Feature | #Feature F4 | #Feature F5 | #Feature F6 | #Feature F10 | #Feature F8 | #Feature F9 | #Feature F7 | #Feature F1 | #Feature F2 | #Feature F3 | Start | End | PTM | AScore | Found By |
| --- | --- | --- | --- | --- | --- | --- | --- | --- | --- | --- | --- | --- | --- | --- | --- | --- | --- | --- | --- | --- | --- | --- | --- | --- | --- | --- | --- | --- | --- | --- | --- | --- | --- | --- | --- | --- | --- |
| K.SAGQLYEESLR.K | N | 72.77 | 1251.6095 | 11 | 0.4 | 626.8123 | 2 | 32.12 | 8 | F8:14092 | 31122021\_RID\_3262\_1a.raw | 1.2366E9 | 8.6285E7 | 3.6644E7 | 6.5882E5 |  | 6.8121E6 | 2.7435E7 | 6.1833E7 | 1.0188E8 | 2.1008E9 | 11 | 2 | 1 | 1 | 1 | 0 | 1 | 1 | 1 | 1 | 2 | 170 | 180 |  |  | PEAKS DB |
| Y.GIGDDANFFQALDFK.D | N | 70.77 | 1656.7783 | 15 | 0.6 | 829.3970 | 2 | 63.80 | 9 | F9:39957 | 31122021\_RID\_3262\_2a.raw |  | 4.1343E5 |  |  |  |  |  | 2.2577E6 | 1.7889E6 | 7.0811E7 | 6 | 0 | 1 | 0 | 0 | 0 | 0 | 0 | 2 | 1 | 2 | 393 | 407 |  |  | PEAKS DB |
| I.AYGIGDDANFFQALDFK.D | N | 69.60 | 1890.8788 | 17 | 0.2 | 946.4468 | 2 | 65.58 | 8 | F8:40325 | 31122021\_RID\_3262\_1a.raw |  |  |  |  |  |  |  | 1.5459E5 |  |  | 1 | 0 | 0 | 0 | 0 | 0 | 0 | 0 | 1 | 0 | 0 | 391 | 407 |  |  | PEAKS DB |
| R.IKFEPPLPPK.K | N | 57.33 | 1164.6907 | 10 | 0.6 | 583.3530 | 2 | 38.07 | 4 | F4:23645 | 17012022\_RID\_3262\_10a .raw | 2.0942E8 | 5.2488E7 | 3.8344E6 | 8.8895E7 |  | 2.1537E7 | 4.1213E6 | 1.9315E7 | 1.4243E7 | 9.9326E8 | 18 | 1 | 2 | 1 | 4 | 0 | 2 | 1 | 2 | 2 | 3 | 322 | 331 |  |  | PEAKS DB |
| K.YDTYSTKEYLLK.E | N | 53.18 | 1522.7555 | 12 | 0.9 | 762.3857 | 2 | 34.13 | 1 | F1:20150 | 13012022\_RID\_3262\_4a.raw | 4.6273E6 |  |  |  |  |  |  |  |  |  | 2 | 2 | 0 | 0 | 0 | 0 | 0 | 0 | 0 | 0 | 0 | 198 | 209 |  |  | PEAKS DB |
| K.SAGQLYEESLRK.V | N | 52.72 | 1379.7045 | 12 | 0.5 | 690.8599 | 2 | 24.65 | 9 | F9:9094 | 31122021\_RID\_3262\_2a.raw | 4.3022E7 | 1.5347E6 |  |  |  | 1.5382E4 | 5.71E5 |  | 1.2745E6 | 3.1589E7 | 7 | 2 | 1 | 0 | 0 | 0 | 1 | 1 | 0 | 1 | 1 | 170 | 181 |  |  | PEAKS DB |
| K.KFWEDEGIHGGK.S | N | 52.53 | 1401.6677 | 12 | 0.0 | 468.2299 | 3 | 28.13 | 1 | F1:14816 | 13012022\_RID\_3262\_4a.raw | 4.3552E6 |  |  |  |  |  |  |  |  | 1.3068E7 | 4 | 2 | 0 | 0 | 0 | 0 | 0 | 0 | 0 | 0 | 2 | 354 | 365 |  |  | PEAKS DB |
| K.STTDLPSR.F | N | 52.16 | 875.4348 | 8 | 0.4 | 438.7249 | 2 | 21.40 | 6 | F6:8926 | 17012022\_RID\_3262\_9a.raw |  | 2.9854E7 |  | 4.8043E6 |  | 1.4473E7 | 1.9071E6 |  |  |  | 4 | 0 | 1 | 0 | 1 | 0 | 1 | 1 | 0 | 0 | 0 | 366 | 373 |  |  | PEAKS DB |
| N.DLSLIHQLPK.K | N | 51.65 | 1162.6710 | 10 | -0.7 | 582.3424 | 2 | 40.68 | 10 | F10:10098 | 31122021\_RID\_3262\_3a.raw |  |  |  |  |  |  |  | 1.197E6 | 2.7589E5 | 1.8174E7 | 3 | 0 | 0 | 0 | 0 | 0 | 0 | 0 | 1 | 1 | 1 | 416 | 425 |  |  | PEAKS DB |
| A.DIVINDLSLIHQLPK.K | N | 49.33 | 1716.9774 | 15 | 0.0 | 859.4960 | 2 | 55.52 | 8 | F8:32578 | 31122021\_RID\_3262\_1a.raw |  |  |  |  |  |  |  | 1.2793E5 | 1.1614E5 |  | 2 | 0 | 0 | 0 | 0 | 0 | 0 | 0 | 1 | 1 | 0 | 411 | 425 |  |  | PEAKS DB |
| K.RFDEIVGGMDQLPTSM.Y | N | 48.45 | 1794.8280 | 16 | -0.6 | 898.4208 | 2 | 56.96 | 10 | F10:16573 | 31122021\_RID\_3262\_3a.raw |  |  |  |  |  |  |  |  |  | 1.5934E7 | 1 | 0 | 0 | 0 | 0 | 0 | 0 | 0 | 0 | 0 | 1 | 250 | 265 |  |  | PEAKS DB |
| R.IKFEPPLPPKK.A | N | 48.17 | 1292.7855 | 11 | -0.6 | 431.9355 | 3 | 28.73 | 10 | F10:6015 | 31122021\_RID\_3262\_3a.raw |  |  |  | 2.6401E6 |  |  |  | 2.4685E6 |  | 9.6849E7 | 4 | 0 | 0 | 0 | 2 | 0 | 0 | 0 | 1 | 0 | 1 | 322 | 332 |  |  | PEAKS DB |
| R.RIKFEPPLPPK.K | N | 47.70 | 1320.7917 | 11 | -0.2 | 441.2711 | 3 | 31.51 | 6 | F6:18014 | 17012022\_RID\_3262\_9a.raw | 9.1946E7 |  |  |  |  | 4.9304E5 |  |  |  |  | 2 | 1 | 0 | 0 | 0 | 0 | 1 | 0 | 0 | 0 | 0 | 321 | 331 |  |  | PEAKS DB |
| K.FEPPLPPK.K | N | 46.74 | 923.5116 | 8 | -0.6 | 462.7628 | 2 | 36.61 | 4 | F4:21855 | 17012022\_RID\_3262\_10a .raw | 8.6451E7 | 2.7491E7 |  | 4.4878E7 |  |  |  | 7.8215E6 | 8.5594E6 |  | 5 | 1 | 1 | 0 | 1 | 0 | 0 | 0 | 1 | 1 | 0 | 324 | 331 |  |  | PEAKS DB |
| M.AGLSAAYVLAGAGHEVTVLEASER.A | Y | 46.58 | 2370.2178 | 24 | 7.8 | 791.0860 | 3 | 51.01 | 10 | F10:14383 | 31122021\_RID\_3262\_3a.raw |  |  |  |  |  |  |  |  |  | 4.5567E5 | 1 | 0 | 0 | 0 | 0 | 0 | 0 | 0 | 0 | 0 | 1 | 62 | 85 |  |  | PEAKS DB |
| K.FEPPLPPKK.A | N | 43.61 | 1051.6066 | 9 | 0.7 | 526.8109 | 2 | 29.21 | 4 | F4:15245 | 17012022\_RID\_3262\_10a .raw |  |  |  | 4.2511E6 |  |  |  |  |  |  | 1 | 0 | 0 | 0 | 1 | 0 | 0 | 0 | 0 | 0 | 0 | 324 | 332 |  |  | PEAKS DB |
| K.RFDEIVGGM(+15.99)DQLPTSM(+15.99).Y | N | 42.69 | 1826.8179 | 16 | -0.4 | 914.4158 | 2 | 56.93 | 10 | F10:16509 | 31122021\_RID\_3262\_3a.raw |  |  |  |  |  |  |  |  |  | 0 | 0 | 0 | 0 | 0 | 0 | 0 | 0 | 0 | 0 | 0 | 0 | 250 | 265 | Oxidation (M) | M9:Oxidation (M):1000.00;M16:Oxidation (M):1000.00 | PEAKS DB |
| E.VTVLEASER.A | N | 42.68 | 1002.5345 | 9 | -0.1 | 502.2745 | 2 | 28.85 | 7 | F7:11280 | 21012022\_RID\_3262\_7a.raw |  |  |  |  |  |  | 1.7683E5 |  |  |  | 1 | 0 | 0 | 0 | 0 | 0 | 0 | 1 | 0 | 0 | 0 | 77 | 85 |  |  | PEAKS DB |
| K.RFDEIVGGMDQLPT.S | N | 41.44 | 1576.7555 | 14 | -0.6 | 789.3846 | 2 | 51.27 | 1 | F1:35302 | 13012022\_RID\_3262\_4a.raw | 1.0726E6 |  |  |  |  |  |  |  |  |  | 1 | 1 | 0 | 0 | 0 | 0 | 0 | 0 | 0 | 0 | 0 | 250 | 263 |  |  | PEAKS DB |
| total 19 peptides |  |  |  |  |  |  |  |  |  |  |  |  |  |  |  |  |  |  |  |  |  |  |  |  |  |  |  |  |  |  |  |  |  |  |  |  |  |
| --- | --- | --- | --- | --- | --- | --- | --- | --- | --- | --- | --- | --- | --- | --- | --- | --- | --- | --- | --- | --- | --- | --- | --- | --- | --- | --- | --- | --- | --- | --- | --- | --- | --- | --- | --- | --- | --- |

JAV50419.1

back to list

  

| Protein Coverage
| Supporting Peptides
|

Protein Coverage:

Supporting Peptides:

| Peptide | Uniq | -10lgP | Mass | Length | ppm | m/z | z | RT | Fraction | Scan | Source File | Area F4 | Area F5 | Area F6 | Area F10 | Area F8 | Area F9 | Area F7 | Area F1 | Area F2 | Area F3 | #Feature | #Feature F4 | #Feature F5 | #Feature F6 | #Feature F10 | #Feature F8 | #Feature F9 | #Feature F7 | #Feature F1 | #Feature F2 | #Feature F3 | Start | End | PTM | AScore | Found By |
| --- | --- | --- | --- | --- | --- | --- | --- | --- | --- | --- | --- | --- | --- | --- | --- | --- | --- | --- | --- | --- | --- | --- | --- | --- | --- | --- | --- | --- | --- | --- | --- | --- | --- | --- | --- | --- | --- |
| K.TFAPEEISAMVLTK.M | Y | 76.75 | 1535.7905 | 14 | 0.4 | 768.9028 | 2 | 54.76 | 1 | F1:38401 | 13012022\_RID\_3262\_4a.raw | 3.7208E6 |  |  |  |  |  |  |  |  |  | 1 | 1 | 0 | 0 | 0 | 0 | 0 | 0 | 0 | 0 | 0 | 138 | 151 |  |  | PEAKS DB |
| K.VLEDADLKKPDIDEIVLVGGSTR.I | Y | 72.81 | 2481.3325 | 23 | 0.8 | 828.1188 | 3 | 47.19 | 1 | F1:31786 | 13012022\_RID\_3262\_4a.raw | 4.5067E6 |  |  |  |  |  |  |  |  |  | 2 | 2 | 0 | 0 | 0 | 0 | 0 | 0 | 0 | 0 | 0 | 344 | 366 |  |  | PEAKS DB |
| R.IINEPTAAAIAYGLDKR.E | Y | 69.41 | 1814.9890 | 17 | 0.4 | 606.0038 | 3 | 43.28 | 1 | F1:28563 | 13012022\_RID\_3262\_4a.raw | 5.6277E6 |  |  |  |  |  |  |  |  |  | 2 | 2 | 0 | 0 | 0 | 0 | 0 | 0 | 0 | 0 | 0 | 197 | 213 |  |  | PEAKS DB |
| K.VTHAVVTVPAYFNDAQR.Q | Y | 69.06 | 1886.9639 | 17 | -0.2 | 629.9951 | 3 | 39.80 | 1 | F1:25353 | 13012022\_RID\_3262\_4a.raw | 4.8131E6 |  |  |  |  |  |  |  |  |  | 2 | 2 | 0 | 0 | 0 | 0 | 0 | 0 | 0 | 0 | 0 | 164 | 180 |  |  | PEAKS DB |
| R.IEIESFFEGEDFSETLTR.A | Y | 64.45 | 2147.9897 | 18 | 1.2 | 1075.0034 | 2 | 64.33 | 1 | F1:46230 | 13012022\_RID\_3262\_4a.raw | 6.9522E5 |  |  |  |  |  |  |  |  |  | 1 | 1 | 0 | 0 | 0 | 0 | 0 | 0 | 0 | 0 | 0 | 306 | 323 |  |  | PEAKS DB |
| K.NQLTSNPENTVFDAKR.L | Y | 59.96 | 1832.9016 | 16 | -0.7 | 611.9741 | 3 | 32.88 | 1 | F1:19052 | 13012022\_RID\_3262\_4a.raw | 1.4316E6 |  |  |  |  |  |  |  |  |  | 1 | 1 | 0 | 0 | 0 | 0 | 0 | 0 | 0 | 0 | 0 | 81 | 96 |  |  | PEAKS DB |
| R.TWNDPSVQQDIK.Y | Y | 58.15 | 1429.6837 | 12 | 0.3 | 715.8494 | 2 | 34.73 | 1 | F1:20927 | 13012022\_RID\_3262\_4a.raw | 4.7471E6 |  |  |  |  |  |  |  |  |  | 1 | 1 | 0 | 0 | 0 | 0 | 0 | 0 | 0 | 0 | 0 | 101 | 112 |  |  | PEAKS DB |
| K.KVTHAVVTVPAYFNDAQR.Q | Y | 57.12 | 2015.0588 | 18 | 0.0 | 504.7720 | 4 | 34.90 | 1 | F1:20930 | 13012022\_RID\_3262\_4a.raw | 2.9277E5 |  |  |  |  |  |  |  |  |  | 1 | 1 | 0 | 0 | 0 | 0 | 0 | 0 | 0 | 0 | 0 | 163 | 180 |  |  | PEAKS DB |
| R.IINEPTAAAIAYGLDK.R | N | 55.26 | 1658.8879 | 16 | 0.4 | 830.4515 | 2 | 48.16 | 1 | F1:32643 | 13012022\_RID\_3262\_4a.raw | 8.615E5 |  |  |  |  |  |  |  |  |  | 1 | 1 | 0 | 0 | 0 | 0 | 0 | 0 | 0 | 0 | 0 | 197 | 212 |  |  | PEAKS DB |
| R.AKFEELNM(+15.99)DLFR.S | Y | 54.16 | 1527.7391 | 12 | 0.5 | 764.8773 | 2 | 42.07 | 1 | F1:27411 | 13012022\_RID\_3262\_4a.raw | 1.0234E6 |  |  |  |  |  |  |  |  |  | 1 | 1 | 0 | 0 | 0 | 0 | 0 | 0 | 0 | 0 | 0 | 324 | 335 | Oxidation (M) | M8:Oxidation (M):1000.00 | PEAKS DB |
| K.NQLTSNPENTVFDAK.R | Y | 50.60 | 1676.8005 | 15 | 0.1 | 839.4076 | 2 | 37.73 | 1 | F1:23391 | 13012022\_RID\_3262\_4a.raw | 6.3806E6 |  |  |  |  |  |  |  |  |  | 1 | 1 | 0 | 0 | 0 | 0 | 0 | 0 | 0 | 0 | 0 | 81 | 95 |  |  | PEAKS DB |
| K.VYEGERPLTK.D | Y | 50.32 | 1190.6295 | 10 | 0.6 | 596.3224 | 2 | 21.87 | 4 | F4:9030 | 17012022\_RID\_3262\_10a .raw |  |  |  | 2.9969E5 |  |  |  |  |  |  | 1 | 0 | 0 | 0 | 1 | 0 | 0 | 0 | 0 | 0 | 0 | 464 | 473 |  |  | PEAKS DB |
| R.ITPSYVAFTPEGER.L | Y | 49.55 | 1565.7725 | 14 | 0.6 | 783.8940 | 2 | 42.54 | 1 | F1:27752 | 13012022\_RID\_3262\_4a.raw | 8.2712E6 |  |  |  |  |  |  |  |  |  | 1 | 1 | 0 | 0 | 0 | 0 | 0 | 0 | 0 | 0 | 0 | 60 | 73 |  |  | PEAKS DB |
| R.VEIIANDQGNR.I | N | 48.68 | 1227.6207 | 11 | -0.1 | 614.8176 | 2 | 28.00 | 6 | F6:15050 | 17012022\_RID\_3262\_9a.raw | 9.9798E5 |  |  |  |  | 1.3198E5 |  |  | 7.0664E4 |  | 3 | 1 | 0 | 0 | 0 | 0 | 1 | 0 | 0 | 1 | 0 | 49 | 59 |  |  | PEAKS DB |
| K.TKPYIQVDIGGGQTK.T | Y | 48.32 | 1603.8569 | 15 | 0.9 | 535.6267 | 3 | 33.09 | 1 | F1:19277 | 13012022\_RID\_3262\_4a.raw | 3.2398E6 |  |  |  |  |  |  |  |  |  | 1 | 1 | 0 | 0 | 0 | 0 | 0 | 0 | 0 | 0 | 0 | 123 | 137 |  |  | PEAKS DB |
| K.MKETAEAYLGKK.V | Y | 47.88 | 1367.7118 | 12 | 0.4 | 456.9114 | 3 | 22.11 | 1 | F1:9440 | 13012022\_RID\_3262\_4a.raw | 1.7366E6 |  |  |  |  |  |  |  |  |  | 1 | 1 | 0 | 0 | 0 | 0 | 0 | 0 | 0 | 0 | 0 | 152 | 163 |  |  | PEAKS DB |
| K.DAGTIAGLNVMR.I | Y | 43.20 | 1216.6234 | 12 | 2.6 | 609.3206 | 2 | 44.39 | 1 | F1:30149 | 13012022\_RID\_3262\_4a.raw | 1.2625E7 |  |  |  |  |  |  |  |  |  | 2 | 2 | 0 | 0 | 0 | 0 | 0 | 0 | 0 | 0 | 0 | 185 | 196 |  |  | PEAKS DB |
| K.KTKPYIQVDIGGGQTK.T | Y | 40.93 | 1731.9519 | 16 | 1.5 | 578.3254 | 3 | 27.89 | 1 | F1:14640 | 13012022\_RID\_3262\_4a.raw | 6.5794E5 |  |  |  |  |  |  |  |  |  | 1 | 1 | 0 | 0 | 0 | 0 | 0 | 0 | 0 | 0 | 0 | 122 | 137 |  |  | PEAKS DB |
| total 18 peptides |  |  |  |  |  |  |  |  |  |  |  |  |  |  |  |  |  |  |  |  |  |  |  |  |  |  |  |  |  |  |  |  |  |  |  |  |  |
| --- | --- | --- | --- | --- | --- | --- | --- | --- | --- | --- | --- | --- | --- | --- | --- | --- | --- | --- | --- | --- | --- | --- | --- | --- | --- | --- | --- | --- | --- | --- | --- | --- | --- | --- | --- | --- | --- |

JAG44540.1

back to list

  

| Protein Coverage
| Supporting Peptides
|

Protein Coverage:

Supporting Peptides:

| Peptide | Uniq | -10lgP | Mass | Length | ppm | m/z | z | RT | Fraction | Scan | Source File | Area F4 | Area F5 | Area F6 | Area F10 | Area F8 | Area F9 | Area F7 | Area F1 | Area F2 | Area F3 | #Feature | #Feature F4 | #Feature F5 | #Feature F6 | #Feature F10 | #Feature F8 | #Feature F9 | #Feature F7 | #Feature F1 | #Feature F2 | #Feature F3 | Start | End | PTM | AScore | Found By |
| --- | --- | --- | --- | --- | --- | --- | --- | --- | --- | --- | --- | --- | --- | --- | --- | --- | --- | --- | --- | --- | --- | --- | --- | --- | --- | --- | --- | --- | --- | --- | --- | --- | --- | --- | --- | --- | --- |
| K.TFAPEEISAMVLTK.M | Y | 76.75 | 1535.7905 | 14 | 0.4 | 768.9028 | 2 | 54.76 | 1 | F1:38401 | 13012022\_RID\_3262\_4a.raw | 3.7208E6 |  |  |  |  |  |  |  |  |  | 1 | 1 | 0 | 0 | 0 | 0 | 0 | 0 | 0 | 0 | 0 | 138 | 151 |  |  | PEAKS DB |
| K.VLEDADLKKPDIDEIVLVGGSTR.I | Y | 72.81 | 2481.3325 | 23 | 0.8 | 828.1188 | 3 | 47.19 | 1 | F1:31786 | 13012022\_RID\_3262\_4a.raw | 4.5067E6 |  |  |  |  |  |  |  |  |  | 2 | 2 | 0 | 0 | 0 | 0 | 0 | 0 | 0 | 0 | 0 | 344 | 366 |  |  | PEAKS DB |
| R.IINEPTAAAIAYGLDKR.E | Y | 69.41 | 1814.9890 | 17 | 0.4 | 606.0038 | 3 | 43.28 | 1 | F1:28563 | 13012022\_RID\_3262\_4a.raw | 5.6277E6 |  |  |  |  |  |  |  |  |  | 2 | 2 | 0 | 0 | 0 | 0 | 0 | 0 | 0 | 0 | 0 | 197 | 213 |  |  | PEAKS DB |
| K.VTHAVVTVPAYFNDAQR.Q | Y | 69.06 | 1886.9639 | 17 | -0.2 | 629.9951 | 3 | 39.80 | 1 | F1:25353 | 13012022\_RID\_3262\_4a.raw | 4.8131E6 |  |  |  |  |  |  |  |  |  | 2 | 2 | 0 | 0 | 0 | 0 | 0 | 0 | 0 | 0 | 0 | 164 | 180 |  |  | PEAKS DB |
| R.IEIESFFEGEDFSETLTR.A | Y | 64.45 | 2147.9897 | 18 | 1.2 | 1075.0034 | 2 | 64.33 | 1 | F1:46230 | 13012022\_RID\_3262\_4a.raw | 6.9522E5 |  |  |  |  |  |  |  |  |  | 1 | 1 | 0 | 0 | 0 | 0 | 0 | 0 | 0 | 0 | 0 | 306 | 323 |  |  | PEAKS DB |
| K.NQLTSNPENTVFDAKR.L | Y | 59.96 | 1832.9016 | 16 | -0.7 | 611.9741 | 3 | 32.88 | 1 | F1:19052 | 13012022\_RID\_3262\_4a.raw | 1.4316E6 |  |  |  |  |  |  |  |  |  | 1 | 1 | 0 | 0 | 0 | 0 | 0 | 0 | 0 | 0 | 0 | 81 | 96 |  |  | PEAKS DB |
| R.TWNDPSVQQDIK.Y | Y | 58.15 | 1429.6837 | 12 | 0.3 | 715.8494 | 2 | 34.73 | 1 | F1:20927 | 13012022\_RID\_3262\_4a.raw | 4.7471E6 |  |  |  |  |  |  |  |  |  | 1 | 1 | 0 | 0 | 0 | 0 | 0 | 0 | 0 | 0 | 0 | 101 | 112 |  |  | PEAKS DB |
| K.KVTHAVVTVPAYFNDAQR.Q | Y | 57.12 | 2015.0588 | 18 | 0.0 | 504.7720 | 4 | 34.90 | 1 | F1:20930 | 13012022\_RID\_3262\_4a.raw | 2.9277E5 |  |  |  |  |  |  |  |  |  | 1 | 1 | 0 | 0 | 0 | 0 | 0 | 0 | 0 | 0 | 0 | 163 | 180 |  |  | PEAKS DB |
| R.IINEPTAAAIAYGLDK.R | N | 55.26 | 1658.8879 | 16 | 0.4 | 830.4515 | 2 | 48.16 | 1 | F1:32643 | 13012022\_RID\_3262\_4a.raw | 8.615E5 |  |  |  |  |  |  |  |  |  | 1 | 1 | 0 | 0 | 0 | 0 | 0 | 0 | 0 | 0 | 0 | 197 | 212 |  |  | PEAKS DB |
| R.AKFEELNM(+15.99)DLFR.S | Y | 54.16 | 1527.7391 | 12 | 0.5 | 764.8773 | 2 | 42.07 | 1 | F1:27411 | 13012022\_RID\_3262\_4a.raw | 1.0234E6 |  |  |  |  |  |  |  |  |  | 1 | 1 | 0 | 0 | 0 | 0 | 0 | 0 | 0 | 0 | 0 | 324 | 335 | Oxidation (M) | M8:Oxidation (M):1000.00 | PEAKS DB |
| K.NQLTSNPENTVFDAK.R | Y | 50.60 | 1676.8005 | 15 | 0.1 | 839.4076 | 2 | 37.73 | 1 | F1:23391 | 13012022\_RID\_3262\_4a.raw | 6.3806E6 |  |  |  |  |  |  |  |  |  | 1 | 1 | 0 | 0 | 0 | 0 | 0 | 0 | 0 | 0 | 0 | 81 | 95 |  |  | PEAKS DB |
| K.VYEGERPLTK.D | Y | 50.32 | 1190.6295 | 10 | 0.6 | 596.3224 | 2 | 21.87 | 4 | F4:9030 | 17012022\_RID\_3262\_10a .raw |  |  |  | 2.9969E5 |  |  |  |  |  |  | 1 | 0 | 0 | 0 | 1 | 0 | 0 | 0 | 0 | 0 | 0 | 464 | 473 |  |  | PEAKS DB |
| R.ITPSYVAFTPEGER.L | Y | 49.55 | 1565.7725 | 14 | 0.6 | 783.8940 | 2 | 42.54 | 1 | F1:27752 | 13012022\_RID\_3262\_4a.raw | 8.2712E6 |  |  |  |  |  |  |  |  |  | 1 | 1 | 0 | 0 | 0 | 0 | 0 | 0 | 0 | 0 | 0 | 60 | 73 |  |  | PEAKS DB |
| R.VEIIANDQGNR.I | N | 48.68 | 1227.6207 | 11 | -0.1 | 614.8176 | 2 | 28.00 | 6 | F6:15050 | 17012022\_RID\_3262\_9a.raw | 9.9798E5 |  |  |  |  | 1.3198E5 |  |  | 7.0664E4 |  | 3 | 1 | 0 | 0 | 0 | 0 | 1 | 0 | 0 | 1 | 0 | 49 | 59 |  |  | PEAKS DB |
| K.TKPYIQVDIGGGQTK.T | Y | 48.32 | 1603.8569 | 15 | 0.9 | 535.6267 | 3 | 33.09 | 1 | F1:19277 | 13012022\_RID\_3262\_4a.raw | 3.2398E6 |  |  |  |  |  |  |  |  |  | 1 | 1 | 0 | 0 | 0 | 0 | 0 | 0 | 0 | 0 | 0 | 123 | 137 |  |  | PEAKS DB |
| K.MKETAEAYLGKK.V | Y | 47.88 | 1367.7118 | 12 | 0.4 | 456.9114 | 3 | 22.11 | 1 | F1:9440 | 13012022\_RID\_3262\_4a.raw | 1.7366E6 |  |  |  |  |  |  |  |  |  | 1 | 1 | 0 | 0 | 0 | 0 | 0 | 0 | 0 | 0 | 0 | 152 | 163 |  |  | PEAKS DB |
| K.DAGTIAGLNVMR.I | Y | 43.20 | 1216.6234 | 12 | 2.6 | 609.3206 | 2 | 44.39 | 1 | F1:30149 | 13012022\_RID\_3262\_4a.raw | 1.2625E7 |  |  |  |  |  |  |  |  |  | 2 | 2 | 0 | 0 | 0 | 0 | 0 | 0 | 0 | 0 | 0 | 185 | 196 |  |  | PEAKS DB |
| K.KTKPYIQVDIGGGQTK.T | Y | 40.93 | 1731.9519 | 16 | 1.5 | 578.3254 | 3 | 27.89 | 1 | F1:14640 | 13012022\_RID\_3262\_4a.raw | 6.5794E5 |  |  |  |  |  |  |  |  |  | 1 | 1 | 0 | 0 | 0 | 0 | 0 | 0 | 0 | 0 | 0 | 122 | 137 |  |  | PEAKS DB |
| total 18 peptides |  |  |  |  |  |  |  |  |  |  |  |  |  |  |  |  |  |  |  |  |  |  |  |  |  |  |  |  |  |  |  |  |  |  |  |  |  |
| --- | --- | --- | --- | --- | --- | --- | --- | --- | --- | --- | --- | --- | --- | --- | --- | --- | --- | --- | --- | --- | --- | --- | --- | --- | --- | --- | --- | --- | --- | --- | --- | --- | --- | --- | --- | --- | --- |

JAI13107.1

back to list

  

| Protein Coverage
| Supporting Peptides
|

Protein Coverage:

Supporting Peptides:

| Peptide | Uniq | -10lgP | Mass | Length | ppm | m/z | z | RT | Fraction | Scan | Source File | Area F4 | Area F5 | Area F6 | Area F10 | Area F8 | Area F9 | Area F7 | Area F1 | Area F2 | Area F3 | #Feature | #Feature F4 | #Feature F5 | #Feature F6 | #Feature F10 | #Feature F8 | #Feature F9 | #Feature F7 | #Feature F1 | #Feature F2 | #Feature F3 | Start | End | PTM | AScore | Found By |
| --- | --- | --- | --- | --- | --- | --- | --- | --- | --- | --- | --- | --- | --- | --- | --- | --- | --- | --- | --- | --- | --- | --- | --- | --- | --- | --- | --- | --- | --- | --- | --- | --- | --- | --- | --- | --- | --- |
| K.TFAPEEISAMVLTK.M | Y | 76.75 | 1535.7905 | 14 | 0.4 | 768.9028 | 2 | 54.76 | 1 | F1:38401 | 13012022\_RID\_3262\_4a.raw | 3.7208E6 |  |  |  |  |  |  |  |  |  | 1 | 1 | 0 | 0 | 0 | 0 | 0 | 0 | 0 | 0 | 0 | 138 | 151 |  |  | PEAKS DB |
| K.VLEDADLKKPDIDEIVLVGGSTR.I | Y | 72.81 | 2481.3325 | 23 | 0.8 | 828.1188 | 3 | 47.19 | 1 | F1:31786 | 13012022\_RID\_3262\_4a.raw | 4.5067E6 |  |  |  |  |  |  |  |  |  | 2 | 2 | 0 | 0 | 0 | 0 | 0 | 0 | 0 | 0 | 0 | 344 | 366 |  |  | PEAKS DB |
| R.IINEPTAAAIAYGLDKR.E | Y | 69.41 | 1814.9890 | 17 | 0.4 | 606.0038 | 3 | 43.28 | 1 | F1:28563 | 13012022\_RID\_3262\_4a.raw | 5.6277E6 |  |  |  |  |  |  |  |  |  | 2 | 2 | 0 | 0 | 0 | 0 | 0 | 0 | 0 | 0 | 0 | 197 | 213 |  |  | PEAKS DB |
| K.VTHAVVTVPAYFNDAQR.Q | Y | 69.06 | 1886.9639 | 17 | -0.2 | 629.9951 | 3 | 39.80 | 1 | F1:25353 | 13012022\_RID\_3262\_4a.raw | 4.8131E6 |  |  |  |  |  |  |  |  |  | 2 | 2 | 0 | 0 | 0 | 0 | 0 | 0 | 0 | 0 | 0 | 164 | 180 |  |  | PEAKS DB |
| R.IEIESFFEGEDFSETLTR.A | Y | 64.45 | 2147.9897 | 18 | 1.2 | 1075.0034 | 2 | 64.33 | 1 | F1:46230 | 13012022\_RID\_3262\_4a.raw | 6.9522E5 |  |  |  |  |  |  |  |  |  | 1 | 1 | 0 | 0 | 0 | 0 | 0 | 0 | 0 | 0 | 0 | 306 | 323 |  |  | PEAKS DB |
| K.NQLTSNPENTVFDAKR.L | Y | 59.96 | 1832.9016 | 16 | -0.7 | 611.9741 | 3 | 32.88 | 1 | F1:19052 | 13012022\_RID\_3262\_4a.raw | 1.4316E6 |  |  |  |  |  |  |  |  |  | 1 | 1 | 0 | 0 | 0 | 0 | 0 | 0 | 0 | 0 | 0 | 81 | 96 |  |  | PEAKS DB |
| R.TWNDPSVQQDIK.Y | Y | 58.15 | 1429.6837 | 12 | 0.3 | 715.8494 | 2 | 34.73 | 1 | F1:20927 | 13012022\_RID\_3262\_4a.raw | 4.7471E6 |  |  |  |  |  |  |  |  |  | 1 | 1 | 0 | 0 | 0 | 0 | 0 | 0 | 0 | 0 | 0 | 101 | 112 |  |  | PEAKS DB |
| K.KVTHAVVTVPAYFNDAQR.Q | Y | 57.12 | 2015.0588 | 18 | 0.0 | 504.7720 | 4 | 34.90 | 1 | F1:20930 | 13012022\_RID\_3262\_4a.raw | 2.9277E5 |  |  |  |  |  |  |  |  |  | 1 | 1 | 0 | 0 | 0 | 0 | 0 | 0 | 0 | 0 | 0 | 163 | 180 |  |  | PEAKS DB |
| R.IINEPTAAAIAYGLDK.R | N | 55.26 | 1658.8879 | 16 | 0.4 | 830.4515 | 2 | 48.16 | 1 | F1:32643 | 13012022\_RID\_3262\_4a.raw | 8.615E5 |  |  |  |  |  |  |  |  |  | 1 | 1 | 0 | 0 | 0 | 0 | 0 | 0 | 0 | 0 | 0 | 197 | 212 |  |  | PEAKS DB |
| R.AKFEELNM(+15.99)DLFR.S | Y | 54.16 | 1527.7391 | 12 | 0.5 | 764.8773 | 2 | 42.07 | 1 | F1:27411 | 13012022\_RID\_3262\_4a.raw | 1.0234E6 |  |  |  |  |  |  |  |  |  | 1 | 1 | 0 | 0 | 0 | 0 | 0 | 0 | 0 | 0 | 0 | 324 | 335 | Oxidation (M) | M8:Oxidation (M):1000.00 | PEAKS DB |
| K.NQLTSNPENTVFDAK.R | Y | 50.60 | 1676.8005 | 15 | 0.1 | 839.4076 | 2 | 37.73 | 1 | F1:23391 | 13012022\_RID\_3262\_4a.raw | 6.3806E6 |  |  |  |  |  |  |  |  |  | 1 | 1 | 0 | 0 | 0 | 0 | 0 | 0 | 0 | 0 | 0 | 81 | 95 |  |  | PEAKS DB |
| K.VYEGERPLTK.D | Y | 50.32 | 1190.6295 | 10 | 0.6 | 596.3224 | 2 | 21.87 | 4 | F4:9030 | 17012022\_RID\_3262\_10a .raw |  |  |  | 2.9969E5 |  |  |  |  |  |  | 1 | 0 | 0 | 0 | 1 | 0 | 0 | 0 | 0 | 0 | 0 | 464 | 473 |  |  | PEAKS DB |
| R.ITPSYVAFTPEGER.L | Y | 49.55 | 1565.7725 | 14 | 0.6 | 783.8940 | 2 | 42.54 | 1 | F1:27752 | 13012022\_RID\_3262\_4a.raw | 8.2712E6 |  |  |  |  |  |  |  |  |  | 1 | 1 | 0 | 0 | 0 | 0 | 0 | 0 | 0 | 0 | 0 | 60 | 73 |  |  | PEAKS DB |
| R.VEIIANDQGNR.I | N | 48.68 | 1227.6207 | 11 | -0.1 | 614.8176 | 2 | 28.00 | 6 | F6:15050 | 17012022\_RID\_3262\_9a.raw | 9.9798E5 |  |  |  |  | 1.3198E5 |  |  | 7.0664E4 |  | 3 | 1 | 0 | 0 | 0 | 0 | 1 | 0 | 0 | 1 | 0 | 49 | 59 |  |  | PEAKS DB |
| K.TKPYIQVDIGGGQTK.T | Y | 48.32 | 1603.8569 | 15 | 0.9 | 535.6267 | 3 | 33.09 | 1 | F1:19277 | 13012022\_RID\_3262\_4a.raw | 3.2398E6 |  |  |  |  |  |  |  |  |  | 1 | 1 | 0 | 0 | 0 | 0 | 0 | 0 | 0 | 0 | 0 | 123 | 137 |  |  | PEAKS DB |
| K.MKETAEAYLGKK.V | Y | 47.88 | 1367.7118 | 12 | 0.4 | 456.9114 | 3 | 22.11 | 1 | F1:9440 | 13012022\_RID\_3262\_4a.raw | 1.7366E6 |  |  |  |  |  |  |  |  |  | 1 | 1 | 0 | 0 | 0 | 0 | 0 | 0 | 0 | 0 | 0 | 152 | 163 |  |  | PEAKS DB |
| K.DAGTIAGLNVMR.I | Y | 43.20 | 1216.6234 | 12 | 2.6 | 609.3206 | 2 | 44.39 | 1 | F1:30149 | 13012022\_RID\_3262\_4a.raw | 1.2625E7 |  |  |  |  |  |  |  |  |  | 2 | 2 | 0 | 0 | 0 | 0 | 0 | 0 | 0 | 0 | 0 | 185 | 196 |  |  | PEAKS DB |
| K.KTKPYIQVDIGGGQTK.T | Y | 40.93 | 1731.9519 | 16 | 1.5 | 578.3254 | 3 | 27.89 | 1 | F1:14640 | 13012022\_RID\_3262\_4a.raw | 6.5794E5 |  |  |  |  |  |  |  |  |  | 1 | 1 | 0 | 0 | 0 | 0 | 0 | 0 | 0 | 0 | 0 | 122 | 137 |  |  | PEAKS DB |
| total 18 peptides |  |  |  |  |  |  |  |  |  |  |  |  |  |  |  |  |  |  |  |  |  |  |  |  |  |  |  |  |  |  |  |  |  |  |  |  |  |
| --- | --- | --- | --- | --- | --- | --- | --- | --- | --- | --- | --- | --- | --- | --- | --- | --- | --- | --- | --- | --- | --- | --- | --- | --- | --- | --- | --- | --- | --- | --- | --- | --- | --- | --- | --- | --- | --- |

JAG46692.1

back to list

  

| Protein Coverage
| Supporting Peptides
|

Protein Coverage:

Supporting Peptides:

| Peptide | Uniq | -10lgP | Mass | Length | ppm | m/z | z | RT | Fraction | Scan | Source File | Area F4 | Area F5 | Area F6 | Area F10 | Area F8 | Area F9 | Area F7 | Area F1 | Area F2 | Area F3 | #Feature | #Feature F4 | #Feature F5 | #Feature F6 | #Feature F10 | #Feature F8 | #Feature F9 | #Feature F7 | #Feature F1 | #Feature F2 | #Feature F3 | Start | End | PTM | AScore | Found By |
| --- | --- | --- | --- | --- | --- | --- | --- | --- | --- | --- | --- | --- | --- | --- | --- | --- | --- | --- | --- | --- | --- | --- | --- | --- | --- | --- | --- | --- | --- | --- | --- | --- | --- | --- | --- | --- | --- |
| K.TFAPEEISAMVLTK.M | Y | 76.75 | 1535.7905 | 14 | 0.4 | 768.9028 | 2 | 54.76 | 1 | F1:38401 | 13012022\_RID\_3262\_4a.raw | 3.7208E6 |  |  |  |  |  |  |  |  |  | 1 | 1 | 0 | 0 | 0 | 0 | 0 | 0 | 0 | 0 | 0 | 138 | 151 |  |  | PEAKS DB |
| K.VLEDADLKKPDIDEIVLVGGSTR.I | Y | 72.81 | 2481.3325 | 23 | 0.8 | 828.1188 | 3 | 47.19 | 1 | F1:31786 | 13012022\_RID\_3262\_4a.raw | 4.5067E6 |  |  |  |  |  |  |  |  |  | 2 | 2 | 0 | 0 | 0 | 0 | 0 | 0 | 0 | 0 | 0 | 344 | 366 |  |  | PEAKS DB |
| R.IINEPTAAAIAYGLDKR.E | Y | 69.41 | 1814.9890 | 17 | 0.4 | 606.0038 | 3 | 43.28 | 1 | F1:28563 | 13012022\_RID\_3262\_4a.raw | 5.6277E6 |  |  |  |  |  |  |  |  |  | 2 | 2 | 0 | 0 | 0 | 0 | 0 | 0 | 0 | 0 | 0 | 197 | 213 |  |  | PEAKS DB |
| K.VTHAVVTVPAYFNDAQR.Q | Y | 69.06 | 1886.9639 | 17 | -0.2 | 629.9951 | 3 | 39.80 | 1 | F1:25353 | 13012022\_RID\_3262\_4a.raw | 4.8131E6 |  |  |  |  |  |  |  |  |  | 2 | 2 | 0 | 0 | 0 | 0 | 0 | 0 | 0 | 0 | 0 | 164 | 180 |  |  | PEAKS DB |
| R.IEIESFFEGEDFSETLTR.A | Y | 64.45 | 2147.9897 | 18 | 1.2 | 1075.0034 | 2 | 64.33 | 1 | F1:46230 | 13012022\_RID\_3262\_4a.raw | 6.9522E5 |  |  |  |  |  |  |  |  |  | 1 | 1 | 0 | 0 | 0 | 0 | 0 | 0 | 0 | 0 | 0 | 306 | 323 |  |  | PEAKS DB |
| K.NQLTSNPENTVFDAKR.L | Y | 59.96 | 1832.9016 | 16 | -0.7 | 611.9741 | 3 | 32.88 | 1 | F1:19052 | 13012022\_RID\_3262\_4a.raw | 1.4316E6 |  |  |  |  |  |  |  |  |  | 1 | 1 | 0 | 0 | 0 | 0 | 0 | 0 | 0 | 0 | 0 | 81 | 96 |  |  | PEAKS DB |
| R.TWNDPSVQQDIK.Y | Y | 58.15 | 1429.6837 | 12 | 0.3 | 715.8494 | 2 | 34.73 | 1 | F1:20927 | 13012022\_RID\_3262\_4a.raw | 4.7471E6 |  |  |  |  |  |  |  |  |  | 1 | 1 | 0 | 0 | 0 | 0 | 0 | 0 | 0 | 0 | 0 | 101 | 112 |  |  | PEAKS DB |
| K.KVTHAVVTVPAYFNDAQR.Q | Y | 57.12 | 2015.0588 | 18 | 0.0 | 504.7720 | 4 | 34.90 | 1 | F1:20930 | 13012022\_RID\_3262\_4a.raw | 2.9277E5 |  |  |  |  |  |  |  |  |  | 1 | 1 | 0 | 0 | 0 | 0 | 0 | 0 | 0 | 0 | 0 | 163 | 180 |  |  | PEAKS DB |
| R.IINEPTAAAIAYGLDK.R | N | 55.26 | 1658.8879 | 16 | 0.4 | 830.4515 | 2 | 48.16 | 1 | F1:32643 | 13012022\_RID\_3262\_4a.raw | 8.615E5 |  |  |  |  |  |  |  |  |  | 1 | 1 | 0 | 0 | 0 | 0 | 0 | 0 | 0 | 0 | 0 | 197 | 212 |  |  | PEAKS DB |
| R.AKFEELNM(+15.99)DLFR.S | Y | 54.16 | 1527.7391 | 12 | 0.5 | 764.8773 | 2 | 42.07 | 1 | F1:27411 | 13012022\_RID\_3262\_4a.raw | 1.0234E6 |  |  |  |  |  |  |  |  |  | 1 | 1 | 0 | 0 | 0 | 0 | 0 | 0 | 0 | 0 | 0 | 324 | 335 | Oxidation (M) | M8:Oxidation (M):1000.00 | PEAKS DB |
| K.NQLTSNPENTVFDAK.R | Y | 50.60 | 1676.8005 | 15 | 0.1 | 839.4076 | 2 | 37.73 | 1 | F1:23391 | 13012022\_RID\_3262\_4a.raw | 6.3806E6 |  |  |  |  |  |  |  |  |  | 1 | 1 | 0 | 0 | 0 | 0 | 0 | 0 | 0 | 0 | 0 | 81 | 95 |  |  | PEAKS DB |
| K.VYEGERPLTK.D | Y | 50.32 | 1190.6295 | 10 | 0.6 | 596.3224 | 2 | 21.87 | 4 | F4:9030 | 17012022\_RID\_3262\_10a .raw |  |  |  | 2.9969E5 |  |  |  |  |  |  | 1 | 0 | 0 | 0 | 1 | 0 | 0 | 0 | 0 | 0 | 0 | 464 | 473 |  |  | PEAKS DB |
| R.ITPSYVAFTPEGER.L | Y | 49.55 | 1565.7725 | 14 | 0.6 | 783.8940 | 2 | 42.54 | 1 | F1:27752 | 13012022\_RID\_3262\_4a.raw | 8.2712E6 |  |  |  |  |  |  |  |  |  | 1 | 1 | 0 | 0 | 0 | 0 | 0 | 0 | 0 | 0 | 0 | 60 | 73 |  |  | PEAKS DB |
| R.VEIIANDQGNR.I | N | 48.68 | 1227.6207 | 11 | -0.1 | 614.8176 | 2 | 28.00 | 6 | F6:15050 | 17012022\_RID\_3262\_9a.raw | 9.9798E5 |  |  |  |  | 1.3198E5 |  |  | 7.0664E4 |  | 3 | 1 | 0 | 0 | 0 | 0 | 1 | 0 | 0 | 1 | 0 | 49 | 59 |  |  | PEAKS DB |
| K.TKPYIQVDIGGGQTK.T | Y | 48.32 | 1603.8569 | 15 | 0.9 | 535.6267 | 3 | 33.09 | 1 | F1:19277 | 13012022\_RID\_3262\_4a.raw | 3.2398E6 |  |  |  |  |  |  |  |  |  | 1 | 1 | 0 | 0 | 0 | 0 | 0 | 0 | 0 | 0 | 0 | 123 | 137 |  |  | PEAKS DB |
| K.MKETAEAYLGKK.V | Y | 47.88 | 1367.7118 | 12 | 0.4 | 456.9114 | 3 | 22.11 | 1 | F1:9440 | 13012022\_RID\_3262\_4a.raw | 1.7366E6 |  |  |  |  |  |  |  |  |  | 1 | 1 | 0 | 0 | 0 | 0 | 0 | 0 | 0 | 0 | 0 | 152 | 163 |  |  | PEAKS DB |
| K.DAGTIAGLNVMR.I | Y | 43.20 | 1216.6234 | 12 | 2.6 | 609.3206 | 2 | 44.39 | 1 | F1:30149 | 13012022\_RID\_3262\_4a.raw | 1.2625E7 |  |  |  |  |  |  |  |  |  | 2 | 2 | 0 | 0 | 0 | 0 | 0 | 0 | 0 | 0 | 0 | 185 | 196 |  |  | PEAKS DB |
| K.KTKPYIQVDIGGGQTK.T | Y | 40.93 | 1731.9519 | 16 | 1.5 | 578.3254 | 3 | 27.89 | 1 | F1:14640 | 13012022\_RID\_3262\_4a.raw | 6.5794E5 |  |  |  |  |  |  |  |  |  | 1 | 1 | 0 | 0 | 0 | 0 | 0 | 0 | 0 | 0 | 0 | 122 | 137 |  |  | PEAKS DB |
| total 18 peptides |  |  |  |  |  |  |  |  |  |  |  |  |  |  |  |  |  |  |  |  |  |  |  |  |  |  |  |  |  |  |  |  |  |  |  |  |  |
| --- | --- | --- | --- | --- | --- | --- | --- | --- | --- | --- | --- | --- | --- | --- | --- | --- | --- | --- | --- | --- | --- | --- | --- | --- | --- | --- | --- | --- | --- | --- | --- | --- | --- | --- | --- | --- | --- |

JAA96909.1

back to list

  

| Protein Coverage
| Supporting Peptides
|

Protein Coverage:

Supporting Peptides:

| Peptide | Uniq | -10lgP | Mass | Length | ppm | m/z | z | RT | Fraction | Scan | Source File | Area F4 | Area F5 | Area F6 | Area F10 | Area F8 | Area F9 | Area F7 | Area F1 | Area F2 | Area F3 | #Feature | #Feature F4 | #Feature F5 | #Feature F6 | #Feature F10 | #Feature F8 | #Feature F9 | #Feature F7 | #Feature F1 | #Feature F2 | #Feature F3 | Start | End | PTM | AScore | Found By |
| --- | --- | --- | --- | --- | --- | --- | --- | --- | --- | --- | --- | --- | --- | --- | --- | --- | --- | --- | --- | --- | --- | --- | --- | --- | --- | --- | --- | --- | --- | --- | --- | --- | --- | --- | --- | --- | --- |
| K.TFAPEEISAMVLTK.M | Y | 76.75 | 1535.7905 | 14 | 0.4 | 768.9028 | 2 | 54.76 | 1 | F1:38401 | 13012022\_RID\_3262\_4a.raw | 3.7208E6 |  |  |  |  |  |  |  |  |  | 1 | 1 | 0 | 0 | 0 | 0 | 0 | 0 | 0 | 0 | 0 | 138 | 151 |  |  | PEAKS DB |
| K.VLEDADLKKPDIDEIVLVGGSTR.I | Y | 72.81 | 2481.3325 | 23 | 0.8 | 828.1188 | 3 | 47.19 | 1 | F1:31786 | 13012022\_RID\_3262\_4a.raw | 4.5067E6 |  |  |  |  |  |  |  |  |  | 2 | 2 | 0 | 0 | 0 | 0 | 0 | 0 | 0 | 0 | 0 | 344 | 366 |  |  | PEAKS DB |
| R.IINEPTAAAIAYGLDKR.E | Y | 69.41 | 1814.9890 | 17 | 0.4 | 606.0038 | 3 | 43.28 | 1 | F1:28563 | 13012022\_RID\_3262\_4a.raw | 5.6277E6 |  |  |  |  |  |  |  |  |  | 2 | 2 | 0 | 0 | 0 | 0 | 0 | 0 | 0 | 0 | 0 | 197 | 213 |  |  | PEAKS DB |
| K.VTHAVVTVPAYFNDAQR.Q | Y | 69.06 | 1886.9639 | 17 | -0.2 | 629.9951 | 3 | 39.80 | 1 | F1:25353 | 13012022\_RID\_3262\_4a.raw | 4.8131E6 |  |  |  |  |  |  |  |  |  | 2 | 2 | 0 | 0 | 0 | 0 | 0 | 0 | 0 | 0 | 0 | 164 | 180 |  |  | PEAKS DB |
| R.IEIESFFEGEDFSETLTR.A | Y | 64.45 | 2147.9897 | 18 | 1.2 | 1075.0034 | 2 | 64.33 | 1 | F1:46230 | 13012022\_RID\_3262\_4a.raw | 6.9522E5 |  |  |  |  |  |  |  |  |  | 1 | 1 | 0 | 0 | 0 | 0 | 0 | 0 | 0 | 0 | 0 | 306 | 323 |  |  | PEAKS DB |
| K.NQLTSNPENTVFDAKR.L | Y | 59.96 | 1832.9016 | 16 | -0.7 | 611.9741 | 3 | 32.88 | 1 | F1:19052 | 13012022\_RID\_3262\_4a.raw | 1.4316E6 |  |  |  |  |  |  |  |  |  | 1 | 1 | 0 | 0 | 0 | 0 | 0 | 0 | 0 | 0 | 0 | 81 | 96 |  |  | PEAKS DB |
| R.TWNDPSVQQDIK.Y | Y | 58.15 | 1429.6837 | 12 | 0.3 | 715.8494 | 2 | 34.73 | 1 | F1:20927 | 13012022\_RID\_3262\_4a.raw | 4.7471E6 |  |  |  |  |  |  |  |  |  | 1 | 1 | 0 | 0 | 0 | 0 | 0 | 0 | 0 | 0 | 0 | 101 | 112 |  |  | PEAKS DB |
| K.KVTHAVVTVPAYFNDAQR.Q | Y | 57.12 | 2015.0588 | 18 | 0.0 | 504.7720 | 4 | 34.90 | 1 | F1:20930 | 13012022\_RID\_3262\_4a.raw | 2.9277E5 |  |  |  |  |  |  |  |  |  | 1 | 1 | 0 | 0 | 0 | 0 | 0 | 0 | 0 | 0 | 0 | 163 | 180 |  |  | PEAKS DB |
| R.IINEPTAAAIAYGLDK.R | N | 55.26 | 1658.8879 | 16 | 0.4 | 830.4515 | 2 | 48.16 | 1 | F1:32643 | 13012022\_RID\_3262\_4a.raw | 8.615E5 |  |  |  |  |  |  |  |  |  | 1 | 1 | 0 | 0 | 0 | 0 | 0 | 0 | 0 | 0 | 0 | 197 | 212 |  |  | PEAKS DB |
| R.AKFEELNM(+15.99)DLFR.S | Y | 54.16 | 1527.7391 | 12 | 0.5 | 764.8773 | 2 | 42.07 | 1 | F1:27411 | 13012022\_RID\_3262\_4a.raw | 1.0234E6 |  |  |  |  |  |  |  |  |  | 1 | 1 | 0 | 0 | 0 | 0 | 0 | 0 | 0 | 0 | 0 | 324 | 335 | Oxidation (M) | M8:Oxidation (M):1000.00 | PEAKS DB |
| K.NQLTSNPENTVFDAK.R | Y | 50.60 | 1676.8005 | 15 | 0.1 | 839.4076 | 2 | 37.73 | 1 | F1:23391 | 13012022\_RID\_3262\_4a.raw | 6.3806E6 |  |  |  |  |  |  |  |  |  | 1 | 1 | 0 | 0 | 0 | 0 | 0 | 0 | 0 | 0 | 0 | 81 | 95 |  |  | PEAKS DB |
| K.VYEGERPLTK.D | Y | 50.32 | 1190.6295 | 10 | 0.6 | 596.3224 | 2 | 21.87 | 4 | F4:9030 | 17012022\_RID\_3262\_10a .raw |  |  |  | 2.9969E5 |  |  |  |  |  |  | 1 | 0 | 0 | 0 | 1 | 0 | 0 | 0 | 0 | 0 | 0 | 464 | 473 |  |  | PEAKS DB |
| R.ITPSYVAFTPEGER.L | Y | 49.55 | 1565.7725 | 14 | 0.6 | 783.8940 | 2 | 42.54 | 1 | F1:27752 | 13012022\_RID\_3262\_4a.raw | 8.2712E6 |  |  |  |  |  |  |  |  |  | 1 | 1 | 0 | 0 | 0 | 0 | 0 | 0 | 0 | 0 | 0 | 60 | 73 |  |  | PEAKS DB |
| R.VEIIANDQGNR.I | N | 48.68 | 1227.6207 | 11 | -0.1 | 614.8176 | 2 | 28.00 | 6 | F6:15050 | 17012022\_RID\_3262\_9a.raw | 9.9798E5 |  |  |  |  | 1.3198E5 |  |  | 7.0664E4 |  | 3 | 1 | 0 | 0 | 0 | 0 | 1 | 0 | 0 | 1 | 0 | 49 | 59 |  |  | PEAKS DB |
| K.TKPYIQVDIGGGQTK.T | Y | 48.32 | 1603.8569 | 15 | 0.9 | 535.6267 | 3 | 33.09 | 1 | F1:19277 | 13012022\_RID\_3262\_4a.raw | 3.2398E6 |  |  |  |  |  |  |  |  |  | 1 | 1 | 0 | 0 | 0 | 0 | 0 | 0 | 0 | 0 | 0 | 123 | 137 |  |  | PEAKS DB |
| K.MKETAEAYLGKK.V | Y | 47.88 | 1367.7118 | 12 | 0.4 | 456.9114 | 3 | 22.11 | 1 | F1:9440 | 13012022\_RID\_3262\_4a.raw | 1.7366E6 |  |  |  |  |  |  |  |  |  | 1 | 1 | 0 | 0 | 0 | 0 | 0 | 0 | 0 | 0 | 0 | 152 | 163 |  |  | PEAKS DB |
| K.DAGTIAGLNVMR.I | Y | 43.20 | 1216.6234 | 12 | 2.6 | 609.3206 | 2 | 44.39 | 1 | F1:30149 | 13012022\_RID\_3262\_4a.raw | 1.2625E7 |  |  |  |  |  |  |  |  |  | 2 | 2 | 0 | 0 | 0 | 0 | 0 | 0 | 0 | 0 | 0 | 185 | 196 |  |  | PEAKS DB |
| K.KTKPYIQVDIGGGQTK.T | Y | 40.93 | 1731.9519 | 16 | 1.5 | 578.3254 | 3 | 27.89 | 1 | F1:14640 | 13012022\_RID\_3262\_4a.raw | 6.5794E5 |  |  |  |  |  |  |  |  |  | 1 | 1 | 0 | 0 | 0 | 0 | 0 | 0 | 0 | 0 | 0 | 122 | 137 |  |  | PEAKS DB |
| total 18 peptides |  |  |  |  |  |  |  |  |  |  |  |  |  |  |  |  |  |  |  |  |  |  |  |  |  |  |  |  |  |  |  |  |  |  |  |  |  |
| --- | --- | --- | --- | --- | --- | --- | --- | --- | --- | --- | --- | --- | --- | --- | --- | --- | --- | --- | --- | --- | --- | --- | --- | --- | --- | --- | --- | --- | --- | --- | --- | --- | --- | --- | --- | --- | --- |

XP\_015681610.1

back to list

  

| Protein Coverage
| Supporting Peptides
|

Protein Coverage:

Supporting Peptides:

| Peptide | Uniq | -10lgP | Mass | Length | ppm | m/z | z | RT | Fraction | Scan | Source File | Area F4 | Area F5 | Area F6 | Area F10 | Area F8 | Area F9 | Area F7 | Area F1 | Area F2 | Area F3 | #Feature | #Feature F4 | #Feature F5 | #Feature F6 | #Feature F10 | #Feature F8 | #Feature F9 | #Feature F7 | #Feature F1 | #Feature F2 | #Feature F3 | Start | End | PTM | AScore | Found By |
| --- | --- | --- | --- | --- | --- | --- | --- | --- | --- | --- | --- | --- | --- | --- | --- | --- | --- | --- | --- | --- | --- | --- | --- | --- | --- | --- | --- | --- | --- | --- | --- | --- | --- | --- | --- | --- | --- |
| K.TFAPEEISAMVLTK.M | Y | 76.75 | 1535.7905 | 14 | 0.4 | 768.9028 | 2 | 54.76 | 1 | F1:38401 | 13012022\_RID\_3262\_4a.raw | 3.7208E6 |  |  |  |  |  |  |  |  |  | 1 | 1 | 0 | 0 | 0 | 0 | 0 | 0 | 0 | 0 | 0 | 138 | 151 |  |  | PEAKS DB |
| K.VLEDADLKKPDIDEIVLVGGSTR.I | Y | 72.81 | 2481.3325 | 23 | 0.8 | 828.1188 | 3 | 47.19 | 1 | F1:31786 | 13012022\_RID\_3262\_4a.raw | 4.5067E6 |  |  |  |  |  |  |  |  |  | 2 | 2 | 0 | 0 | 0 | 0 | 0 | 0 | 0 | 0 | 0 | 344 | 366 |  |  | PEAKS DB |
| R.IINEPTAAAIAYGLDKR.E | Y | 69.41 | 1814.9890 | 17 | 0.4 | 606.0038 | 3 | 43.28 | 1 | F1:28563 | 13012022\_RID\_3262\_4a.raw | 5.6277E6 |  |  |  |  |  |  |  |  |  | 2 | 2 | 0 | 0 | 0 | 0 | 0 | 0 | 0 | 0 | 0 | 197 | 213 |  |  | PEAKS DB |
| K.VTHAVVTVPAYFNDAQR.Q | Y | 69.06 | 1886.9639 | 17 | -0.2 | 629.9951 | 3 | 39.80 | 1 | F1:25353 | 13012022\_RID\_3262\_4a.raw | 4.8131E6 |  |  |  |  |  |  |  |  |  | 2 | 2 | 0 | 0 | 0 | 0 | 0 | 0 | 0 | 0 | 0 | 164 | 180 |  |  | PEAKS DB |
| R.IEIESFFEGEDFSETLTR.A | Y | 64.45 | 2147.9897 | 18 | 1.2 | 1075.0034 | 2 | 64.33 | 1 | F1:46230 | 13012022\_RID\_3262\_4a.raw | 6.9522E5 |  |  |  |  |  |  |  |  |  | 1 | 1 | 0 | 0 | 0 | 0 | 0 | 0 | 0 | 0 | 0 | 306 | 323 |  |  | PEAKS DB |
| K.NQLTSNPENTVFDAKR.L | Y | 59.96 | 1832.9016 | 16 | -0.7 | 611.9741 | 3 | 32.88 | 1 | F1:19052 | 13012022\_RID\_3262\_4a.raw | 1.4316E6 |  |  |  |  |  |  |  |  |  | 1 | 1 | 0 | 0 | 0 | 0 | 0 | 0 | 0 | 0 | 0 | 81 | 96 |  |  | PEAKS DB |
| R.TWNDPSVQQDIK.Y | Y | 58.15 | 1429.6837 | 12 | 0.3 | 715.8494 | 2 | 34.73 | 1 | F1:20927 | 13012022\_RID\_3262\_4a.raw | 4.7471E6 |  |  |  |  |  |  |  |  |  | 1 | 1 | 0 | 0 | 0 | 0 | 0 | 0 | 0 | 0 | 0 | 101 | 112 |  |  | PEAKS DB |
| K.KVTHAVVTVPAYFNDAQR.Q | Y | 57.12 | 2015.0588 | 18 | 0.0 | 504.7720 | 4 | 34.90 | 1 | F1:20930 | 13012022\_RID\_3262\_4a.raw | 2.9277E5 |  |  |  |  |  |  |  |  |  | 1 | 1 | 0 | 0 | 0 | 0 | 0 | 0 | 0 | 0 | 0 | 163 | 180 |  |  | PEAKS DB |
| R.IINEPTAAAIAYGLDK.R | N | 55.26 | 1658.8879 | 16 | 0.4 | 830.4515 | 2 | 48.16 | 1 | F1:32643 | 13012022\_RID\_3262\_4a.raw | 8.615E5 |  |  |  |  |  |  |  |  |  | 1 | 1 | 0 | 0 | 0 | 0 | 0 | 0 | 0 | 0 | 0 | 197 | 212 |  |  | PEAKS DB |
| R.AKFEELNM(+15.99)DLFR.S | Y | 54.16 | 1527.7391 | 12 | 0.5 | 764.8773 | 2 | 42.07 | 1 | F1:27411 | 13012022\_RID\_3262\_4a.raw | 1.0234E6 |  |  |  |  |  |  |  |  |  | 1 | 1 | 0 | 0 | 0 | 0 | 0 | 0 | 0 | 0 | 0 | 324 | 335 | Oxidation (M) | M8:Oxidation (M):1000.00 | PEAKS DB |
| K.NQLTSNPENTVFDAK.R | Y | 50.60 | 1676.8005 | 15 | 0.1 | 839.4076 | 2 | 37.73 | 1 | F1:23391 | 13012022\_RID\_3262\_4a.raw | 6.3806E6 |  |  |  |  |  |  |  |  |  | 1 | 1 | 0 | 0 | 0 | 0 | 0 | 0 | 0 | 0 | 0 | 81 | 95 |  |  | PEAKS DB |
| K.VYEGERPLTK.D | Y | 50.32 | 1190.6295 | 10 | 0.6 | 596.3224 | 2 | 21.87 | 4 | F4:9030 | 17012022\_RID\_3262\_10a .raw |  |  |  | 2.9969E5 |  |  |  |  |  |  | 1 | 0 | 0 | 0 | 1 | 0 | 0 | 0 | 0 | 0 | 0 | 464 | 473 |  |  | PEAKS DB |
| R.ITPSYVAFTPEGER.L | Y | 49.55 | 1565.7725 | 14 | 0.6 | 783.8940 | 2 | 42.54 | 1 | F1:27752 | 13012022\_RID\_3262\_4a.raw | 8.2712E6 |  |  |  |  |  |  |  |  |  | 1 | 1 | 0 | 0 | 0 | 0 | 0 | 0 | 0 | 0 | 0 | 60 | 73 |  |  | PEAKS DB |
| R.VEIIANDQGNR.I | N | 48.68 | 1227.6207 | 11 | -0.1 | 614.8176 | 2 | 28.00 | 6 | F6:15050 | 17012022\_RID\_3262\_9a.raw | 9.9798E5 |  |  |  |  | 1.3198E5 |  |  | 7.0664E4 |  | 3 | 1 | 0 | 0 | 0 | 0 | 1 | 0 | 0 | 1 | 0 | 49 | 59 |  |  | PEAKS DB |
| K.TKPYIQVDIGGGQTK.T | Y | 48.32 | 1603.8569 | 15 | 0.9 | 535.6267 | 3 | 33.09 | 1 | F1:19277 | 13012022\_RID\_3262\_4a.raw | 3.2398E6 |  |  |  |  |  |  |  |  |  | 1 | 1 | 0 | 0 | 0 | 0 | 0 | 0 | 0 | 0 | 0 | 123 | 137 |  |  | PEAKS DB |
| K.MKETAEAYLGKK.V | Y | 47.88 | 1367.7118 | 12 | 0.4 | 456.9114 | 3 | 22.11 | 1 | F1:9440 | 13012022\_RID\_3262\_4a.raw | 1.7366E6 |  |  |  |  |  |  |  |  |  | 1 | 1 | 0 | 0 | 0 | 0 | 0 | 0 | 0 | 0 | 0 | 152 | 163 |  |  | PEAKS DB |
| K.DAGTIAGLNVMR.I | Y | 43.20 | 1216.6234 | 12 | 2.6 | 609.3206 | 2 | 44.39 | 1 | F1:30149 | 13012022\_RID\_3262\_4a.raw | 1.2625E7 |  |  |  |  |  |  |  |  |  | 2 | 2 | 0 | 0 | 0 | 0 | 0 | 0 | 0 | 0 | 0 | 185 | 196 |  |  | PEAKS DB |
| K.KTKPYIQVDIGGGQTK.T | Y | 40.93 | 1731.9519 | 16 | 1.5 | 578.3254 | 3 | 27.89 | 1 | F1:14640 | 13012022\_RID\_3262\_4a.raw | 6.5794E5 |  |  |  |  |  |  |  |  |  | 1 | 1 | 0 | 0 | 0 | 0 | 0 | 0 | 0 | 0 | 0 | 122 | 137 |  |  | PEAKS DB |
| total 18 peptides |  |  |  |  |  |  |  |  |  |  |  |  |  |  |  |  |  |  |  |  |  |  |  |  |  |  |  |  |  |  |  |  |  |  |  |  |  |
| --- | --- | --- | --- | --- | --- | --- | --- | --- | --- | --- | --- | --- | --- | --- | --- | --- | --- | --- | --- | --- | --- | --- | --- | --- | --- | --- | --- | --- | --- | --- | --- | --- | --- | --- | --- | --- | --- |

AFJ50327.1

back to list

  

| Protein Coverage
| Supporting Peptides
|

Protein Coverage:

Supporting Peptides:

| Peptide | Uniq | -10lgP | Mass | Length | ppm | m/z | z | RT | Fraction | Scan | Source File | Area F4 | Area F5 | Area F6 | Area F10 | Area F8 | Area F9 | Area F7 | Area F1 | Area F2 | Area F3 | #Feature | #Feature F4 | #Feature F5 | #Feature F6 | #Feature F10 | #Feature F8 | #Feature F9 | #Feature F7 | #Feature F1 | #Feature F2 | #Feature F3 | Start | End | PTM | AScore | Found By |
| --- | --- | --- | --- | --- | --- | --- | --- | --- | --- | --- | --- | --- | --- | --- | --- | --- | --- | --- | --- | --- | --- | --- | --- | --- | --- | --- | --- | --- | --- | --- | --- | --- | --- | --- | --- | --- | --- |
| K.TFAPEEISAMVLTK.M | Y | 76.75 | 1535.7905 | 14 | 0.4 | 768.9028 | 2 | 54.76 | 1 | F1:38401 | 13012022\_RID\_3262\_4a.raw | 3.7208E6 |  |  |  |  |  |  |  |  |  | 1 | 1 | 0 | 0 | 0 | 0 | 0 | 0 | 0 | 0 | 0 | 138 | 151 |  |  | PEAKS DB |
| K.VLEDADLKKPDIDEIVLVGGSTR.I | Y | 72.81 | 2481.3325 | 23 | 0.8 | 828.1188 | 3 | 47.19 | 1 | F1:31786 | 13012022\_RID\_3262\_4a.raw | 4.5067E6 |  |  |  |  |  |  |  |  |  | 2 | 2 | 0 | 0 | 0 | 0 | 0 | 0 | 0 | 0 | 0 | 344 | 366 |  |  | PEAKS DB |
| R.IINEPTAAAIAYGLDKR.E | Y | 69.41 | 1814.9890 | 17 | 0.4 | 606.0038 | 3 | 43.28 | 1 | F1:28563 | 13012022\_RID\_3262\_4a.raw | 5.6277E6 |  |  |  |  |  |  |  |  |  | 2 | 2 | 0 | 0 | 0 | 0 | 0 | 0 | 0 | 0 | 0 | 197 | 213 |  |  | PEAKS DB |
| K.VTHAVVTVPAYFNDAQR.Q | Y | 69.06 | 1886.9639 | 17 | -0.2 | 629.9951 | 3 | 39.80 | 1 | F1:25353 | 13012022\_RID\_3262\_4a.raw | 4.8131E6 |  |  |  |  |  |  |  |  |  | 2 | 2 | 0 | 0 | 0 | 0 | 0 | 0 | 0 | 0 | 0 | 164 | 180 |  |  | PEAKS DB |
| R.IEIESFFEGEDFSETLTR.A | Y | 64.45 | 2147.9897 | 18 | 1.2 | 1075.0034 | 2 | 64.33 | 1 | F1:46230 | 13012022\_RID\_3262\_4a.raw | 6.9522E5 |  |  |  |  |  |  |  |  |  | 1 | 1 | 0 | 0 | 0 | 0 | 0 | 0 | 0 | 0 | 0 | 306 | 323 |  |  | PEAKS DB |
| K.NQLTSNPENTVFDAKR.L | Y | 59.96 | 1832.9016 | 16 | -0.7 | 611.9741 | 3 | 32.88 | 1 | F1:19052 | 13012022\_RID\_3262\_4a.raw | 1.4316E6 |  |  |  |  |  |  |  |  |  | 1 | 1 | 0 | 0 | 0 | 0 | 0 | 0 | 0 | 0 | 0 | 81 | 96 |  |  | PEAKS DB |
| R.TWNDPSVQQDIK.Y | Y | 58.15 | 1429.6837 | 12 | 0.3 | 715.8494 | 2 | 34.73 | 1 | F1:20927 | 13012022\_RID\_3262\_4a.raw | 4.7471E6 |  |  |  |  |  |  |  |  |  | 1 | 1 | 0 | 0 | 0 | 0 | 0 | 0 | 0 | 0 | 0 | 101 | 112 |  |  | PEAKS DB |
| K.KVTHAVVTVPAYFNDAQR.Q | Y | 57.12 | 2015.0588 | 18 | 0.0 | 504.7720 | 4 | 34.90 | 1 | F1:20930 | 13012022\_RID\_3262\_4a.raw | 2.9277E5 |  |  |  |  |  |  |  |  |  | 1 | 1 | 0 | 0 | 0 | 0 | 0 | 0 | 0 | 0 | 0 | 163 | 180 |  |  | PEAKS DB |
| R.IINEPTAAAIAYGLDK.R | N | 55.26 | 1658.8879 | 16 | 0.4 | 830.4515 | 2 | 48.16 | 1 | F1:32643 | 13012022\_RID\_3262\_4a.raw | 8.615E5 |  |  |  |  |  |  |  |  |  | 1 | 1 | 0 | 0 | 0 | 0 | 0 | 0 | 0 | 0 | 0 | 197 | 212 |  |  | PEAKS DB |
| R.AKFEELNM(+15.99)DLFR.S | Y | 54.16 | 1527.7391 | 12 | 0.5 | 764.8773 | 2 | 42.07 | 1 | F1:27411 | 13012022\_RID\_3262\_4a.raw | 1.0234E6 |  |  |  |  |  |  |  |  |  | 1 | 1 | 0 | 0 | 0 | 0 | 0 | 0 | 0 | 0 | 0 | 324 | 335 | Oxidation (M) | M8:Oxidation (M):1000.00 | PEAKS DB |
| K.NQLTSNPENTVFDAK.R | Y | 50.60 | 1676.8005 | 15 | 0.1 | 839.4076 | 2 | 37.73 | 1 | F1:23391 | 13012022\_RID\_3262\_4a.raw | 6.3806E6 |  |  |  |  |  |  |  |  |  | 1 | 1 | 0 | 0 | 0 | 0 | 0 | 0 | 0 | 0 | 0 | 81 | 95 |  |  | PEAKS DB |
| K.VYEGERPLTK.D | Y | 50.32 | 1190.6295 | 10 | 0.6 | 596.3224 | 2 | 21.87 | 4 | F4:9030 | 17012022\_RID\_3262\_10a .raw |  |  |  | 2.9969E5 |  |  |  |  |  |  | 1 | 0 | 0 | 0 | 1 | 0 | 0 | 0 | 0 | 0 | 0 | 464 | 473 |  |  | PEAKS DB |
| R.ITPSYVAFTPEGER.L | Y | 49.55 | 1565.7725 | 14 | 0.6 | 783.8940 | 2 | 42.54 | 1 | F1:27752 | 13012022\_RID\_3262\_4a.raw | 8.2712E6 |  |  |  |  |  |  |  |  |  | 1 | 1 | 0 | 0 | 0 | 0 | 0 | 0 | 0 | 0 | 0 | 60 | 73 |  |  | PEAKS DB |
| R.VEIIANDQGNR.I | N | 48.68 | 1227.6207 | 11 | -0.1 | 614.8176 | 2 | 28.00 | 6 | F6:15050 | 17012022\_RID\_3262\_9a.raw | 9.9798E5 |  |  |  |  | 1.3198E5 |  |  | 7.0664E4 |  | 3 | 1 | 0 | 0 | 0 | 0 | 1 | 0 | 0 | 1 | 0 | 49 | 59 |  |  | PEAKS DB |
| K.TKPYIQVDIGGGQTK.T | Y | 48.32 | 1603.8569 | 15 | 0.9 | 535.6267 | 3 | 33.09 | 1 | F1:19277 | 13012022\_RID\_3262\_4a.raw | 3.2398E6 |  |  |  |  |  |  |  |  |  | 1 | 1 | 0 | 0 | 0 | 0 | 0 | 0 | 0 | 0 | 0 | 123 | 137 |  |  | PEAKS DB |
| K.MKETAEAYLGKK.V | Y | 47.88 | 1367.7118 | 12 | 0.4 | 456.9114 | 3 | 22.11 | 1 | F1:9440 | 13012022\_RID\_3262\_4a.raw | 1.7366E6 |  |  |  |  |  |  |  |  |  | 1 | 1 | 0 | 0 | 0 | 0 | 0 | 0 | 0 | 0 | 0 | 152 | 163 |  |  | PEAKS DB |
| K.DAGTIAGLNVMR.I | Y | 43.20 | 1216.6234 | 12 | 2.6 | 609.3206 | 2 | 44.39 | 1 | F1:30149 | 13012022\_RID\_3262\_4a.raw | 1.2625E7 |  |  |  |  |  |  |  |  |  | 2 | 2 | 0 | 0 | 0 | 0 | 0 | 0 | 0 | 0 | 0 | 185 | 196 |  |  | PEAKS DB |
| K.KTKPYIQVDIGGGQTK.T | Y | 40.93 | 1731.9519 | 16 | 1.5 | 578.3254 | 3 | 27.89 | 1 | F1:14640 | 13012022\_RID\_3262\_4a.raw | 6.5794E5 |  |  |  |  |  |  |  |  |  | 1 | 1 | 0 | 0 | 0 | 0 | 0 | 0 | 0 | 0 | 0 | 122 | 137 |  |  | PEAKS DB |
| total 18 peptides |  |  |  |  |  |  |  |  |  |  |  |  |  |  |  |  |  |  |  |  |  |  |  |  |  |  |  |  |  |  |  |  |  |  |  |  |  |
| --- | --- | --- | --- | --- | --- | --- | --- | --- | --- | --- | --- | --- | --- | --- | --- | --- | --- | --- | --- | --- | --- | --- | --- | --- | --- | --- | --- | --- | --- | --- | --- | --- | --- | --- | --- | --- | --- |

AFJ50239.1

back to list

  

| Protein Coverage
| Supporting Peptides
|

Protein Coverage:

Supporting Peptides:

| Peptide | Uniq | -10lgP | Mass | Length | ppm | m/z | z | RT | Fraction | Scan | Source File | Area F4 | Area F5 | Area F6 | Area F10 | Area F8 | Area F9 | Area F7 | Area F1 | Area F2 | Area F3 | #Feature | #Feature F4 | #Feature F5 | #Feature F6 | #Feature F10 | #Feature F8 | #Feature F9 | #Feature F7 | #Feature F1 | #Feature F2 | #Feature F3 | Start | End | PTM | AScore | Found By |
| --- | --- | --- | --- | --- | --- | --- | --- | --- | --- | --- | --- | --- | --- | --- | --- | --- | --- | --- | --- | --- | --- | --- | --- | --- | --- | --- | --- | --- | --- | --- | --- | --- | --- | --- | --- | --- | --- |
| K.TFAPEEISAMVLTK.M | Y | 76.75 | 1535.7905 | 14 | 0.4 | 768.9028 | 2 | 54.76 | 1 | F1:38401 | 13012022\_RID\_3262\_4a.raw | 3.7208E6 |  |  |  |  |  |  |  |  |  | 1 | 1 | 0 | 0 | 0 | 0 | 0 | 0 | 0 | 0 | 0 | 138 | 151 |  |  | PEAKS DB |
| K.VLEDADLKKPDIDEIVLVGGSTR.I | Y | 72.81 | 2481.3325 | 23 | 0.8 | 828.1188 | 3 | 47.19 | 1 | F1:31786 | 13012022\_RID\_3262\_4a.raw | 4.5067E6 |  |  |  |  |  |  |  |  |  | 2 | 2 | 0 | 0 | 0 | 0 | 0 | 0 | 0 | 0 | 0 | 344 | 366 |  |  | PEAKS DB |
| R.IINEPTAAAIAYGLDKR.E | Y | 69.41 | 1814.9890 | 17 | 0.4 | 606.0038 | 3 | 43.28 | 1 | F1:28563 | 13012022\_RID\_3262\_4a.raw | 5.6277E6 |  |  |  |  |  |  |  |  |  | 2 | 2 | 0 | 0 | 0 | 0 | 0 | 0 | 0 | 0 | 0 | 197 | 213 |  |  | PEAKS DB |
| K.VTHAVVTVPAYFNDAQR.Q | Y | 69.06 | 1886.9639 | 17 | -0.2 | 629.9951 | 3 | 39.80 | 1 | F1:25353 | 13012022\_RID\_3262\_4a.raw | 4.8131E6 |  |  |  |  |  |  |  |  |  | 2 | 2 | 0 | 0 | 0 | 0 | 0 | 0 | 0 | 0 | 0 | 164 | 180 |  |  | PEAKS DB |
| R.IEIESFFEGEDFSETLTR.A | Y | 64.45 | 2147.9897 | 18 | 1.2 | 1075.0034 | 2 | 64.33 | 1 | F1:46230 | 13012022\_RID\_3262\_4a.raw | 6.9522E5 |  |  |  |  |  |  |  |  |  | 1 | 1 | 0 | 0 | 0 | 0 | 0 | 0 | 0 | 0 | 0 | 306 | 323 |  |  | PEAKS DB |
| K.NQLTSNPENTVFDAKR.L | Y | 59.96 | 1832.9016 | 16 | -0.7 | 611.9741 | 3 | 32.88 | 1 | F1:19052 | 13012022\_RID\_3262\_4a.raw | 1.4316E6 |  |  |  |  |  |  |  |  |  | 1 | 1 | 0 | 0 | 0 | 0 | 0 | 0 | 0 | 0 | 0 | 81 | 96 |  |  | PEAKS DB |
| R.TWNDPSVQQDIK.Y | Y | 58.15 | 1429.6837 | 12 | 0.3 | 715.8494 | 2 | 34.73 | 1 | F1:20927 | 13012022\_RID\_3262\_4a.raw | 4.7471E6 |  |  |  |  |  |  |  |  |  | 1 | 1 | 0 | 0 | 0 | 0 | 0 | 0 | 0 | 0 | 0 | 101 | 112 |  |  | PEAKS DB |
| K.KVTHAVVTVPAYFNDAQR.Q | Y | 57.12 | 2015.0588 | 18 | 0.0 | 504.7720 | 4 | 34.90 | 1 | F1:20930 | 13012022\_RID\_3262\_4a.raw | 2.9277E5 |  |  |  |  |  |  |  |  |  | 1 | 1 | 0 | 0 | 0 | 0 | 0 | 0 | 0 | 0 | 0 | 163 | 180 |  |  | PEAKS DB |
| R.IINEPTAAAIAYGLDK.R | N | 55.26 | 1658.8879 | 16 | 0.4 | 830.4515 | 2 | 48.16 | 1 | F1:32643 | 13012022\_RID\_3262\_4a.raw | 8.615E5 |  |  |  |  |  |  |  |  |  | 1 | 1 | 0 | 0 | 0 | 0 | 0 | 0 | 0 | 0 | 0 | 197 | 212 |  |  | PEAKS DB |
| R.AKFEELNM(+15.99)DLFR.S | Y | 54.16 | 1527.7391 | 12 | 0.5 | 764.8773 | 2 | 42.07 | 1 | F1:27411 | 13012022\_RID\_3262\_4a.raw | 1.0234E6 |  |  |  |  |  |  |  |  |  | 1 | 1 | 0 | 0 | 0 | 0 | 0 | 0 | 0 | 0 | 0 | 324 | 335 | Oxidation (M) | M8:Oxidation (M):1000.00 | PEAKS DB |
| K.NQLTSNPENTVFDAK.R | Y | 50.60 | 1676.8005 | 15 | 0.1 | 839.4076 | 2 | 37.73 | 1 | F1:23391 | 13012022\_RID\_3262\_4a.raw | 6.3806E6 |  |  |  |  |  |  |  |  |  | 1 | 1 | 0 | 0 | 0 | 0 | 0 | 0 | 0 | 0 | 0 | 81 | 95 |  |  | PEAKS DB |
| K.VYEGERPLTK.D | Y | 50.32 | 1190.6295 | 10 | 0.6 | 596.3224 | 2 | 21.87 | 4 | F4:9030 | 17012022\_RID\_3262\_10a .raw |  |  |  | 2.9969E5 |  |  |  |  |  |  | 1 | 0 | 0 | 0 | 1 | 0 | 0 | 0 | 0 | 0 | 0 | 464 | 473 |  |  | PEAKS DB |
| R.ITPSYVAFTPEGER.L | Y | 49.55 | 1565.7725 | 14 | 0.6 | 783.8940 | 2 | 42.54 | 1 | F1:27752 | 13012022\_RID\_3262\_4a.raw | 8.2712E6 |  |  |  |  |  |  |  |  |  | 1 | 1 | 0 | 0 | 0 | 0 | 0 | 0 | 0 | 0 | 0 | 60 | 73 |  |  | PEAKS DB |
| R.VEIIANDQGNR.I | N | 48.68 | 1227.6207 | 11 | -0.1 | 614.8176 | 2 | 28.00 | 6 | F6:15050 | 17012022\_RID\_3262\_9a.raw | 9.9798E5 |  |  |  |  | 1.3198E5 |  |  | 7.0664E4 |  | 3 | 1 | 0 | 0 | 0 | 0 | 1 | 0 | 0 | 1 | 0 | 49 | 59 |  |  | PEAKS DB |
| K.TKPYIQVDIGGGQTK.T | Y | 48.32 | 1603.8569 | 15 | 0.9 | 535.6267 | 3 | 33.09 | 1 | F1:19277 | 13012022\_RID\_3262\_4a.raw | 3.2398E6 |  |  |  |  |  |  |  |  |  | 1 | 1 | 0 | 0 | 0 | 0 | 0 | 0 | 0 | 0 | 0 | 123 | 137 |  |  | PEAKS DB |
| K.MKETAEAYLGKK.V | Y | 47.88 | 1367.7118 | 12 | 0.4 | 456.9114 | 3 | 22.11 | 1 | F1:9440 | 13012022\_RID\_3262\_4a.raw | 1.7366E6 |  |  |  |  |  |  |  |  |  | 1 | 1 | 0 | 0 | 0 | 0 | 0 | 0 | 0 | 0 | 0 | 152 | 163 |  |  | PEAKS DB |
| K.DAGTIAGLNVMR.I | Y | 43.20 | 1216.6234 | 12 | 2.6 | 609.3206 | 2 | 44.39 | 1 | F1:30149 | 13012022\_RID\_3262\_4a.raw | 1.2625E7 |  |  |  |  |  |  |  |  |  | 2 | 2 | 0 | 0 | 0 | 0 | 0 | 0 | 0 | 0 | 0 | 185 | 196 |  |  | PEAKS DB |
| K.KTKPYIQVDIGGGQTK.T | Y | 40.93 | 1731.9519 | 16 | 1.5 | 578.3254 | 3 | 27.89 | 1 | F1:14640 | 13012022\_RID\_3262\_4a.raw | 6.5794E5 |  |  |  |  |  |  |  |  |  | 1 | 1 | 0 | 0 | 0 | 0 | 0 | 0 | 0 | 0 | 0 | 122 | 137 |  |  | PEAKS DB |
| total 18 peptides |  |  |  |  |  |  |  |  |  |  |  |  |  |  |  |  |  |  |  |  |  |  |  |  |  |  |  |  |  |  |  |  |  |  |  |  |  |
| --- | --- | --- | --- | --- | --- | --- | --- | --- | --- | --- | --- | --- | --- | --- | --- | --- | --- | --- | --- | --- | --- | --- | --- | --- | --- | --- | --- | --- | --- | --- | --- | --- | --- | --- | --- | --- | --- |

JAI10406.1

back to list

  

| Protein Coverage
| Supporting Peptides
|

Protein Coverage:

Supporting Peptides:

| Peptide | Uniq | -10lgP | Mass | Length | ppm | m/z | z | RT | Fraction | Scan | Source File | Area F4 | Area F5 | Area F6 | Area F10 | Area F8 | Area F9 | Area F7 | Area F1 | Area F2 | Area F3 | #Feature | #Feature F4 | #Feature F5 | #Feature F6 | #Feature F10 | #Feature F8 | #Feature F9 | #Feature F7 | #Feature F1 | #Feature F2 | #Feature F3 | Start | End | PTM | AScore | Found By |
| --- | --- | --- | --- | --- | --- | --- | --- | --- | --- | --- | --- | --- | --- | --- | --- | --- | --- | --- | --- | --- | --- | --- | --- | --- | --- | --- | --- | --- | --- | --- | --- | --- | --- | --- | --- | --- | --- |
| K.HANFPILSANIRPK.G | N | 73.91 | 1576.8838 | 14 | 0.3 | 526.6354 | 3 | 33.47 | 9 | F9:16191 | 31122021\_RID\_3262\_2a.raw | 2.4791E6 |  |  |  |  |  |  | 7.6122E5 | 1.3586E7 |  | 5 | 1 | 0 | 0 | 0 | 0 | 0 | 0 | 2 | 2 | 0 | 148 | 161 |  |  | PEAKS DB |
| G.SFELTILHTNDVHAR.V | N | 71.46 | 1751.8955 | 15 | 0.2 | 584.9725 | 3 | 37.82 | 9 | F9:19802 | 31122021\_RID\_3262\_2a.raw |  |  |  |  |  |  |  |  | 9.8903E6 |  | 1 | 0 | 0 | 0 | 0 | 0 | 0 | 0 | 0 | 1 | 0 | 41 | 55 |  |  | PEAKS DB |
| K.IQLHNYSSQEIGK.T | N | 66.55 | 1515.7681 | 13 | 0.9 | 758.8920 | 2 | 25.89 | 9 | F9:9947 | 31122021\_RID\_3262\_2a.raw |  | 0 |  |  |  |  |  |  | 8.8417E5 |  | 2 | 0 | 0 | 0 | 0 | 0 | 0 | 0 | 0 | 2 | 0 | 343 | 355 |  |  | PEAKS DB |
| R.QVPVVQAYAFGK.Y | N | 64.12 | 1305.7081 | 12 | 0.7 | 653.8618 | 2 | 57.55 | 1 | F1:40709 | 13012022\_RID\_3262\_4a.raw | 4.6454E6 | 1.1825E6 |  |  |  |  |  | 3.0239E5 | 6.0962E6 |  | 5 | 2 | 1 | 0 | 0 | 0 | 0 | 0 | 1 | 1 | 0 | 288 | 299 |  |  | PEAKS DB |
| K.YLGYLNVIFDDKGNVIK.S | N | 63.64 | 1970.0513 | 17 | 0.7 | 986.0336 | 2 | 55.25 | 9 | F9:33414 | 31122021\_RID\_3262\_2a.raw |  |  |  |  |  |  |  |  | 5.214E6 |  | 2 | 0 | 0 | 0 | 0 | 0 | 0 | 0 | 0 | 2 | 0 | 300 | 316 |  |  | PEAKS DB |
| R.DEVEELQNHANK.L | N | 59.76 | 1424.6532 | 12 | 0.7 | 713.3344 | 2 | 23.15 | 9 | F9:7692 | 31122021\_RID\_3262\_2a.raw |  |  |  |  |  |  |  |  | 1.0268E6 |  | 1 | 0 | 0 | 0 | 0 | 0 | 0 | 0 | 0 | 1 | 0 | 209 | 220 |  |  | PEAKS DB |
| K.LLLPSFLAAGGDGYHML.K | N | 55.39 | 1773.9124 | 17 | 0.2 | 887.9636 | 2 | 68.35 | 9 | F9:42925 | 31122021\_RID\_3262\_2a.raw |  |  |  |  |  |  |  |  | 5.8724E5 |  | 1 | 0 | 0 | 0 | 0 | 0 | 0 | 0 | 0 | 1 | 0 | 510 | 526 |  |  | PEAKS DB |
| K.LLLPSFLAAGGDGYHM(+15.99)LK.G | N | 51.71 | 1918.0022 | 18 | -0.6 | 960.0078 | 2 | 57.15 | 9 | F9:34949 | 31122021\_RID\_3262\_2a.raw |  |  |  |  |  |  |  |  | 3.7896E5 |  | 1 | 0 | 0 | 0 | 0 | 0 | 0 | 0 | 0 | 1 | 0 | 510 | 527 | Oxidation (M) | M16:Oxidation (M):1000.00 | PEAKS DB |
| K.VGIIGYTTK.E | N | 51.25 | 950.5436 | 9 | 0.4 | 476.2793 | 2 | 33.16 | 9 | F9:16043 | 31122021\_RID\_3262\_2a.raw |  | 2.6943E6 | 7.1467E5 |  |  |  | 1.5898E5 |  | 8.0892E6 |  | 4 | 0 | 1 | 1 | 0 | 0 | 0 | 1 | 0 | 1 | 0 | 185 | 193 |  |  | PEAKS DB |
| K.LLLPSFLAAGGDGYH.M | N | 48.30 | 1529.7877 | 15 | 0.9 | 765.9019 | 2 | 61.99 | 9 | F9:38681 | 31122021\_RID\_3262\_2a.raw |  |  |  |  |  |  |  |  | 3.6135E5 |  | 1 | 0 | 0 | 0 | 0 | 0 | 0 | 0 | 0 | 1 | 0 | 510 | 524 |  |  | PEAKS DB |
| K.LLLPSFLAAGGDGYHM(+15.99)L.K | N | 46.54 | 1789.9072 | 17 | 1.0 | 895.9618 | 2 | 63.49 | 9 | F9:39581 | 31122021\_RID\_3262\_2a.raw |  |  |  |  |  |  |  |  | 1.84E5 |  | 1 | 0 | 0 | 0 | 0 | 0 | 0 | 0 | 0 | 1 | 0 | 510 | 526 | Oxidation (M) | M16:Oxidation (M):1000.00 | PEAKS DB |
| K.VVYDLSR.K | N | 46.31 | 850.4548 | 7 | 0.5 | 426.2349 | 2 | 30.36 | 1 | F1:16753 | 13012022\_RID\_3262\_4a.raw | 3.9579E6 | 3.0633E6 |  |  |  |  |  |  | 6.9401E6 |  | 3 | 1 | 1 | 0 | 0 | 0 | 0 | 0 | 0 | 1 | 0 | 472 | 478 |  |  | PEAKS DB |
| K.HANFPILSAN.I | N | 46.30 | 1082.5509 | 10 | 0.2 | 542.2828 | 2 | 40.87 | 9 | F9:22309 | 31122021\_RID\_3262\_2a.raw |  |  |  |  |  |  |  |  | 2.7202E6 |  | 1 | 0 | 0 | 0 | 0 | 0 | 0 | 0 | 0 | 1 | 0 | 148 | 157 |  |  | PEAKS DB |
| K.LLLPSFLAAGGDGYHM.L | N | 46.03 | 1660.8282 | 16 | 0.2 | 831.4216 | 2 | 65.01 | 9 | F9:40717 | 31122021\_RID\_3262\_2a.raw |  |  |  |  |  |  |  |  | 7.391E4 |  | 1 | 0 | 0 | 0 | 0 | 0 | 0 | 0 | 0 | 1 | 0 | 510 | 525 |  |  | PEAKS DB |
| K.LLLPSFLAAGGDGYHMLK.G | N | 45.84 | 1902.0073 | 18 | 0.1 | 635.0098 | 3 | 57.14 | 9 | F9:35003 | 31122021\_RID\_3262\_2a.raw | 6.1858E5 |  |  |  |  |  |  |  | 5.9658E6 |  | 2 | 1 | 0 | 0 | 0 | 0 | 0 | 0 | 0 | 1 | 0 | 510 | 527 |  |  | PEAKS DB |
| K.LLLPSFLAAGGD.G | N | 45.25 | 1172.6440 | 12 | -0.2 | 587.3292 | 2 | 72.39 | 9 | F9:44726 | 31122021\_RID\_3262\_2a.raw |  |  |  |  |  |  |  |  | 1.0233E5 |  | 1 | 0 | 0 | 0 | 0 | 0 | 0 | 0 | 0 | 1 | 0 | 510 | 521 |  |  | PEAKS DB |
| R.HGEGM(+15.99)GELLQVSGIK.V | Y | 44.08 | 1569.7821 | 15 | 1.0 | 524.2685 | 3 | 33.96 | 9 | F9:16600 | 31122021\_RID\_3262\_2a.raw |  |  |  |  |  |  |  |  | 1.2697E5 |  | 1 | 0 | 0 | 0 | 0 | 0 | 0 | 0 | 0 | 1 | 0 | 457 | 471 | Oxidation (M) | M5:Oxidation (M):1000.00 | PEAKS DB |
| N.LDISIVGDYIK.R | N | 44.01 | 1234.6809 | 11 | 0.5 | 618.3480 | 2 | 56.58 | 9 | F9:34546 | 31122021\_RID\_3262\_2a.raw |  |  |  |  |  |  |  |  | 2.949E4 |  | 1 | 0 | 0 | 0 | 0 | 0 | 0 | 0 | 0 | 1 | 0 | 538 | 548 |  |  | PEAKS DB |
| R.YDAMALGNHEFDNGLAGLLDPLLK.H | N | 43.69 | 2586.2788 | 24 | 0.6 | 863.1007 | 3 | 69.00 | 9 | F9:43247 | 31122021\_RID\_3262\_2a.raw |  |  |  |  |  |  |  |  | 9.895E5 |  | 1 | 0 | 0 | 0 | 0 | 0 | 0 | 0 | 0 | 1 | 0 | 124 | 147 |  |  | PEAKS DB |
| K.LTTLGVNK.I | N | 43.24 | 844.5018 | 8 | 0.8 | 423.2585 | 2 | 24.48 | 9 | F9:8941 | 31122021\_RID\_3262\_2a.raw |  |  |  |  |  |  |  |  | 1.1856E7 |  | 1 | 0 | 0 | 0 | 0 | 0 | 0 | 0 | 0 | 1 | 0 | 221 | 228 |  |  | PEAKS DB |
| F.GGTFDLLQIK.G | N | 43.16 | 1090.6022 | 10 | -0.1 | 546.3083 | 2 | 49.90 | 9 | F9:29347 | 31122021\_RID\_3262\_2a.raw |  |  |  |  |  |  |  |  | 6.6936E5 |  | 1 | 0 | 0 | 0 | 0 | 0 | 0 | 0 | 0 | 1 | 0 | 433 | 442 |  |  | PEAKS DB |
| K.YLGYLNVIFDDK.G | N | 41.85 | 1458.7394 | 12 | 1.3 | 730.3779 | 2 | 60.34 | 9 | F9:37272 | 31122021\_RID\_3262\_2a.raw |  |  |  |  |  |  |  |  | 2.5015E6 |  | 1 | 0 | 0 | 0 | 0 | 0 | 0 | 0 | 0 | 1 | 0 | 300 | 311 |  |  | PEAKS DB |
| total 22 peptides |  |  |  |  |  |  |  |  |  |  |  |  |  |  |  |  |  |  |  |  |  |  |  |  |  |  |  |  |  |  |  |  |  |  |  |  |  |
| --- | --- | --- | --- | --- | --- | --- | --- | --- | --- | --- | --- | --- | --- | --- | --- | --- | --- | --- | --- | --- | --- | --- | --- | --- | --- | --- | --- | --- | --- | --- | --- | --- | --- | --- | --- | --- | --- |

JAS04702.1

back to list

  

| Protein Coverage
| Supporting Peptides
|

Protein Coverage:

Supporting Peptides:

| Peptide | Uniq | -10lgP | Mass | Length | ppm | m/z | z | RT | Fraction | Scan | Source File | Area F4 | Area F5 | Area F6 | Area F10 | Area F8 | Area F9 | Area F7 | Area F1 | Area F2 | Area F3 | #Feature | #Feature F4 | #Feature F5 | #Feature F6 | #Feature F10 | #Feature F8 | #Feature F9 | #Feature F7 | #Feature F1 | #Feature F2 | #Feature F3 | Start | End | PTM | AScore | Found By |
| --- | --- | --- | --- | --- | --- | --- | --- | --- | --- | --- | --- | --- | --- | --- | --- | --- | --- | --- | --- | --- | --- | --- | --- | --- | --- | --- | --- | --- | --- | --- | --- | --- | --- | --- | --- | --- | --- |
| K.HANFPILSANIRPK.G | N | 73.91 | 1576.8838 | 14 | 0.3 | 526.6354 | 3 | 33.47 | 9 | F9:16191 | 31122021\_RID\_3262\_2a.raw | 2.4791E6 |  |  |  |  |  |  | 7.6122E5 | 1.3586E7 |  | 5 | 1 | 0 | 0 | 0 | 0 | 0 | 0 | 2 | 2 | 0 | 148 | 161 |  |  | PEAKS DB |
| G.SFELTILHTNDVHAR.V | N | 71.46 | 1751.8955 | 15 | 0.2 | 584.9725 | 3 | 37.82 | 9 | F9:19802 | 31122021\_RID\_3262\_2a.raw |  |  |  |  |  |  |  |  | 9.8903E6 |  | 1 | 0 | 0 | 0 | 0 | 0 | 0 | 0 | 0 | 1 | 0 | 41 | 55 |  |  | PEAKS DB |
| K.IQLHNYSSQEIGK.T | N | 66.55 | 1515.7681 | 13 | 0.9 | 758.8920 | 2 | 25.89 | 9 | F9:9947 | 31122021\_RID\_3262\_2a.raw |  | 0 |  |  |  |  |  |  | 8.8417E5 |  | 2 | 0 | 0 | 0 | 0 | 0 | 0 | 0 | 0 | 2 | 0 | 343 | 355 |  |  | PEAKS DB |
| R.QVPVVQAYAFGK.Y | N | 64.12 | 1305.7081 | 12 | 0.7 | 653.8618 | 2 | 57.55 | 1 | F1:40709 | 13012022\_RID\_3262\_4a.raw | 4.6454E6 | 1.1825E6 |  |  |  |  |  | 3.0239E5 | 6.0962E6 |  | 5 | 2 | 1 | 0 | 0 | 0 | 0 | 0 | 1 | 1 | 0 | 288 | 299 |  |  | PEAKS DB |
| K.YLGYLNVIFDDKGNVIK.S | N | 63.64 | 1970.0513 | 17 | 0.7 | 986.0336 | 2 | 55.25 | 9 | F9:33414 | 31122021\_RID\_3262\_2a.raw |  |  |  |  |  |  |  |  | 5.214E6 |  | 2 | 0 | 0 | 0 | 0 | 0 | 0 | 0 | 0 | 2 | 0 | 300 | 316 |  |  | PEAKS DB |
| R.DEVEELQNHANK.L | N | 59.76 | 1424.6532 | 12 | 0.7 | 713.3344 | 2 | 23.15 | 9 | F9:7692 | 31122021\_RID\_3262\_2a.raw |  |  |  |  |  |  |  |  | 1.0268E6 |  | 1 | 0 | 0 | 0 | 0 | 0 | 0 | 0 | 0 | 1 | 0 | 209 | 220 |  |  | PEAKS DB |
| K.LLLPSFLAAGGDGYHML.K | N | 55.39 | 1773.9124 | 17 | 0.2 | 887.9636 | 2 | 68.35 | 9 | F9:42925 | 31122021\_RID\_3262\_2a.raw |  |  |  |  |  |  |  |  | 5.8724E5 |  | 1 | 0 | 0 | 0 | 0 | 0 | 0 | 0 | 0 | 1 | 0 | 510 | 526 |  |  | PEAKS DB |
| K.LLLPSFLAAGGDGYHM(+15.99)LK.G | N | 51.71 | 1918.0022 | 18 | -0.6 | 960.0078 | 2 | 57.15 | 9 | F9:34949 | 31122021\_RID\_3262\_2a.raw |  |  |  |  |  |  |  |  | 3.7896E5 |  | 1 | 0 | 0 | 0 | 0 | 0 | 0 | 0 | 0 | 1 | 0 | 510 | 527 | Oxidation (M) | M16:Oxidation (M):1000.00 | PEAKS DB |
| K.VGIIGYTTK.E | N | 51.25 | 950.5436 | 9 | 0.4 | 476.2793 | 2 | 33.16 | 9 | F9:16043 | 31122021\_RID\_3262\_2a.raw |  | 2.6943E6 | 7.1467E5 |  |  |  | 1.5898E5 |  | 8.0892E6 |  | 4 | 0 | 1 | 1 | 0 | 0 | 0 | 1 | 0 | 1 | 0 | 185 | 193 |  |  | PEAKS DB |
| K.LLLPSFLAAGGDGYH.M | N | 48.30 | 1529.7877 | 15 | 0.9 | 765.9019 | 2 | 61.99 | 9 | F9:38681 | 31122021\_RID\_3262\_2a.raw |  |  |  |  |  |  |  |  | 3.6135E5 |  | 1 | 0 | 0 | 0 | 0 | 0 | 0 | 0 | 0 | 1 | 0 | 510 | 524 |  |  | PEAKS DB |
| K.LLLPSFLAAGGDGYHM(+15.99)L.K | N | 46.54 | 1789.9072 | 17 | 1.0 | 895.9618 | 2 | 63.49 | 9 | F9:39581 | 31122021\_RID\_3262\_2a.raw |  |  |  |  |  |  |  |  | 1.84E5 |  | 1 | 0 | 0 | 0 | 0 | 0 | 0 | 0 | 0 | 1 | 0 | 510 | 526 | Oxidation (M) | M16:Oxidation (M):1000.00 | PEAKS DB |
| K.VVYDLSR.K | N | 46.31 | 850.4548 | 7 | 0.5 | 426.2349 | 2 | 30.36 | 1 | F1:16753 | 13012022\_RID\_3262\_4a.raw | 3.9579E6 | 3.0633E6 |  |  |  |  |  |  | 6.9401E6 |  | 3 | 1 | 1 | 0 | 0 | 0 | 0 | 0 | 0 | 1 | 0 | 472 | 478 |  |  | PEAKS DB |
| K.HANFPILSAN.I | N | 46.30 | 1082.5509 | 10 | 0.2 | 542.2828 | 2 | 40.87 | 9 | F9:22309 | 31122021\_RID\_3262\_2a.raw |  |  |  |  |  |  |  |  | 2.7202E6 |  | 1 | 0 | 0 | 0 | 0 | 0 | 0 | 0 | 0 | 1 | 0 | 148 | 157 |  |  | PEAKS DB |
| K.LLLPSFLAAGGDGYHM.L | N | 46.03 | 1660.8282 | 16 | 0.2 | 831.4216 | 2 | 65.01 | 9 | F9:40717 | 31122021\_RID\_3262\_2a.raw |  |  |  |  |  |  |  |  | 7.391E4 |  | 1 | 0 | 0 | 0 | 0 | 0 | 0 | 0 | 0 | 1 | 0 | 510 | 525 |  |  | PEAKS DB |
| K.LLLPSFLAAGGDGYHMLK.G | N | 45.84 | 1902.0073 | 18 | 0.1 | 635.0098 | 3 | 57.14 | 9 | F9:35003 | 31122021\_RID\_3262\_2a.raw | 6.1858E5 |  |  |  |  |  |  |  | 5.9658E6 |  | 2 | 1 | 0 | 0 | 0 | 0 | 0 | 0 | 0 | 1 | 0 | 510 | 527 |  |  | PEAKS DB |
| K.LLLPSFLAAGGD.G | N | 45.25 | 1172.6440 | 12 | -0.2 | 587.3292 | 2 | 72.39 | 9 | F9:44726 | 31122021\_RID\_3262\_2a.raw |  |  |  |  |  |  |  |  | 1.0233E5 |  | 1 | 0 | 0 | 0 | 0 | 0 | 0 | 0 | 0 | 1 | 0 | 510 | 521 |  |  | PEAKS DB |
| R.HGEGM(+15.99)GELLQVSGIK.V | Y | 44.08 | 1569.7821 | 15 | 1.0 | 524.2685 | 3 | 33.96 | 9 | F9:16600 | 31122021\_RID\_3262\_2a.raw |  |  |  |  |  |  |  |  | 1.2697E5 |  | 1 | 0 | 0 | 0 | 0 | 0 | 0 | 0 | 0 | 1 | 0 | 457 | 471 | Oxidation (M) | M5:Oxidation (M):1000.00 | PEAKS DB |
| N.LDISIVGDYIK.R | N | 44.01 | 1234.6809 | 11 | 0.5 | 618.3480 | 2 | 56.58 | 9 | F9:34546 | 31122021\_RID\_3262\_2a.raw |  |  |  |  |  |  |  |  | 2.949E4 |  | 1 | 0 | 0 | 0 | 0 | 0 | 0 | 0 | 0 | 1 | 0 | 538 | 548 |  |  | PEAKS DB |
| R.YDAMALGNHEFDNGLAGLLDPLLK.H | N | 43.69 | 2586.2788 | 24 | 0.6 | 863.1007 | 3 | 69.00 | 9 | F9:43247 | 31122021\_RID\_3262\_2a.raw |  |  |  |  |  |  |  |  | 9.895E5 |  | 1 | 0 | 0 | 0 | 0 | 0 | 0 | 0 | 0 | 1 | 0 | 124 | 147 |  |  | PEAKS DB |
| K.LTTLGVNK.I | N | 43.24 | 844.5018 | 8 | 0.8 | 423.2585 | 2 | 24.48 | 9 | F9:8941 | 31122021\_RID\_3262\_2a.raw |  |  |  |  |  |  |  |  | 1.1856E7 |  | 1 | 0 | 0 | 0 | 0 | 0 | 0 | 0 | 0 | 1 | 0 | 221 | 228 |  |  | PEAKS DB |
| F.GGTFDLLQIK.G | N | 43.16 | 1090.6022 | 10 | -0.1 | 546.3083 | 2 | 49.90 | 9 | F9:29347 | 31122021\_RID\_3262\_2a.raw |  |  |  |  |  |  |  |  | 6.6936E5 |  | 1 | 0 | 0 | 0 | 0 | 0 | 0 | 0 | 0 | 1 | 0 | 433 | 442 |  |  | PEAKS DB |
| K.YLGYLNVIFDDK.G | N | 41.85 | 1458.7394 | 12 | 1.3 | 730.3779 | 2 | 60.34 | 9 | F9:37272 | 31122021\_RID\_3262\_2a.raw |  |  |  |  |  |  |  |  | 2.5015E6 |  | 1 | 0 | 0 | 0 | 0 | 0 | 0 | 0 | 0 | 1 | 0 | 300 | 311 |  |  | PEAKS DB |
| total 22 peptides |  |  |  |  |  |  |  |  |  |  |  |  |  |  |  |  |  |  |  |  |  |  |  |  |  |  |  |  |  |  |  |  |  |  |  |  |  |
| --- | --- | --- | --- | --- | --- | --- | --- | --- | --- | --- | --- | --- | --- | --- | --- | --- | --- | --- | --- | --- | --- | --- | --- | --- | --- | --- | --- | --- | --- | --- | --- | --- | --- | --- | --- | --- | --- |

AFJ49197.1

back to list

  

| Protein Coverage
| Supporting Peptides
|

Protein Coverage:

Supporting Peptides:

| Peptide | Uniq | -10lgP | Mass | Length | ppm | m/z | z | RT | Fraction | Scan | Source File | Area F4 | Area F5 | Area F6 | Area F10 | Area F8 | Area F9 | Area F7 | Area F1 | Area F2 | Area F3 | #Feature | #Feature F4 | #Feature F5 | #Feature F6 | #Feature F10 | #Feature F8 | #Feature F9 | #Feature F7 | #Feature F1 | #Feature F2 | #Feature F3 | Start | End | PTM | AScore | Found By |
| --- | --- | --- | --- | --- | --- | --- | --- | --- | --- | --- | --- | --- | --- | --- | --- | --- | --- | --- | --- | --- | --- | --- | --- | --- | --- | --- | --- | --- | --- | --- | --- | --- | --- | --- | --- | --- | --- |
| K.HANFPILSANIRPK.G | N | 73.91 | 1576.8838 | 14 | 0.3 | 526.6354 | 3 | 33.47 | 9 | F9:16191 | 31122021\_RID\_3262\_2a.raw | 2.4791E6 |  |  |  |  |  |  | 7.6122E5 | 1.3586E7 |  | 5 | 1 | 0 | 0 | 0 | 0 | 0 | 0 | 2 | 2 | 0 | 148 | 161 |  |  | PEAKS DB |
| G.SFELTILHTNDVHAR.V | N | 71.46 | 1751.8955 | 15 | 0.2 | 584.9725 | 3 | 37.82 | 9 | F9:19802 | 31122021\_RID\_3262\_2a.raw |  |  |  |  |  |  |  |  | 9.8903E6 |  | 1 | 0 | 0 | 0 | 0 | 0 | 0 | 0 | 0 | 1 | 0 | 41 | 55 |  |  | PEAKS DB |
| K.IQLHNYSSQEIGK.T | N | 66.55 | 1515.7681 | 13 | 0.9 | 758.8920 | 2 | 25.89 | 9 | F9:9947 | 31122021\_RID\_3262\_2a.raw |  | 0 |  |  |  |  |  |  | 8.8417E5 |  | 2 | 0 | 0 | 0 | 0 | 0 | 0 | 0 | 0 | 2 | 0 | 343 | 355 |  |  | PEAKS DB |
| R.QVPVVQAYAFGK.Y | N | 64.12 | 1305.7081 | 12 | 0.7 | 653.8618 | 2 | 57.55 | 1 | F1:40709 | 13012022\_RID\_3262\_4a.raw | 4.6454E6 | 1.1825E6 |  |  |  |  |  | 3.0239E5 | 6.0962E6 |  | 5 | 2 | 1 | 0 | 0 | 0 | 0 | 0 | 1 | 1 | 0 | 288 | 299 |  |  | PEAKS DB |
| K.YLGYLNVIFDDKGNVIK.S | N | 63.64 | 1970.0513 | 17 | 0.7 | 986.0336 | 2 | 55.25 | 9 | F9:33414 | 31122021\_RID\_3262\_2a.raw |  |  |  |  |  |  |  |  | 5.214E6 |  | 2 | 0 | 0 | 0 | 0 | 0 | 0 | 0 | 0 | 2 | 0 | 300 | 316 |  |  | PEAKS DB |
| R.DEVEELQNHANK.L | N | 59.76 | 1424.6532 | 12 | 0.7 | 713.3344 | 2 | 23.15 | 9 | F9:7692 | 31122021\_RID\_3262\_2a.raw |  |  |  |  |  |  |  |  | 1.0268E6 |  | 1 | 0 | 0 | 0 | 0 | 0 | 0 | 0 | 0 | 1 | 0 | 209 | 220 |  |  | PEAKS DB |
| K.LLLPSFLAAGGDGYHML.K | N | 55.39 | 1773.9124 | 17 | 0.2 | 887.9636 | 2 | 68.35 | 9 | F9:42925 | 31122021\_RID\_3262\_2a.raw |  |  |  |  |  |  |  |  | 5.8724E5 |  | 1 | 0 | 0 | 0 | 0 | 0 | 0 | 0 | 0 | 1 | 0 | 510 | 526 |  |  | PEAKS DB |
| K.LLLPSFLAAGGDGYHM(+15.99)LK.G | N | 51.71 | 1918.0022 | 18 | -0.6 | 960.0078 | 2 | 57.15 | 9 | F9:34949 | 31122021\_RID\_3262\_2a.raw |  |  |  |  |  |  |  |  | 3.7896E5 |  | 1 | 0 | 0 | 0 | 0 | 0 | 0 | 0 | 0 | 1 | 0 | 510 | 527 | Oxidation (M) | M16:Oxidation (M):1000.00 | PEAKS DB |
| K.VGIIGYTTK.E | N | 51.25 | 950.5436 | 9 | 0.4 | 476.2793 | 2 | 33.16 | 9 | F9:16043 | 31122021\_RID\_3262\_2a.raw |  | 2.6943E6 | 7.1467E5 |  |  |  | 1.5898E5 |  | 8.0892E6 |  | 4 | 0 | 1 | 1 | 0 | 0 | 0 | 1 | 0 | 1 | 0 | 185 | 193 |  |  | PEAKS DB |
| K.LLLPSFLAAGGDGYH.M | N | 48.30 | 1529.7877 | 15 | 0.9 | 765.9019 | 2 | 61.99 | 9 | F9:38681 | 31122021\_RID\_3262\_2a.raw |  |  |  |  |  |  |  |  | 3.6135E5 |  | 1 | 0 | 0 | 0 | 0 | 0 | 0 | 0 | 0 | 1 | 0 | 510 | 524 |  |  | PEAKS DB |
| K.LLLPSFLAAGGDGYHM(+15.99)L.K | N | 46.54 | 1789.9072 | 17 | 1.0 | 895.9618 | 2 | 63.49 | 9 | F9:39581 | 31122021\_RID\_3262\_2a.raw |  |  |  |  |  |  |  |  | 1.84E5 |  | 1 | 0 | 0 | 0 | 0 | 0 | 0 | 0 | 0 | 1 | 0 | 510 | 526 | Oxidation (M) | M16:Oxidation (M):1000.00 | PEAKS DB |
| K.VVYDLSR.K | N | 46.31 | 850.4548 | 7 | 0.5 | 426.2349 | 2 | 30.36 | 1 | F1:16753 | 13012022\_RID\_3262\_4a.raw | 3.9579E6 | 3.0633E6 |  |  |  |  |  |  | 6.9401E6 |  | 3 | 1 | 1 | 0 | 0 | 0 | 0 | 0 | 0 | 1 | 0 | 472 | 478 |  |  | PEAKS DB |
| K.HANFPILSAN.I | N | 46.30 | 1082.5509 | 10 | 0.2 | 542.2828 | 2 | 40.87 | 9 | F9:22309 | 31122021\_RID\_3262\_2a.raw |  |  |  |  |  |  |  |  | 2.7202E6 |  | 1 | 0 | 0 | 0 | 0 | 0 | 0 | 0 | 0 | 1 | 0 | 148 | 157 |  |  | PEAKS DB |
| K.LLLPSFLAAGGDGYHM.L | N | 46.03 | 1660.8282 | 16 | 0.2 | 831.4216 | 2 | 65.01 | 9 | F9:40717 | 31122021\_RID\_3262\_2a.raw |  |  |  |  |  |  |  |  | 7.391E4 |  | 1 | 0 | 0 | 0 | 0 | 0 | 0 | 0 | 0 | 1 | 0 | 510 | 525 |  |  | PEAKS DB |
| K.LLLPSFLAAGGDGYHMLK.G | N | 45.84 | 1902.0073 | 18 | 0.1 | 635.0098 | 3 | 57.14 | 9 | F9:35003 | 31122021\_RID\_3262\_2a.raw | 6.1858E5 |  |  |  |  |  |  |  | 5.9658E6 |  | 2 | 1 | 0 | 0 | 0 | 0 | 0 | 0 | 0 | 1 | 0 | 510 | 527 |  |  | PEAKS DB |
| K.LLLPSFLAAGGD.G | N | 45.25 | 1172.6440 | 12 | -0.2 | 587.3292 | 2 | 72.39 | 9 | F9:44726 | 31122021\_RID\_3262\_2a.raw |  |  |  |  |  |  |  |  | 1.0233E5 |  | 1 | 0 | 0 | 0 | 0 | 0 | 0 | 0 | 0 | 1 | 0 | 510 | 521 |  |  | PEAKS DB |
| R.HGEGM(+15.99)GELLQVSGIK.V | Y | 44.08 | 1569.7821 | 15 | 1.0 | 524.2685 | 3 | 33.96 | 9 | F9:16600 | 31122021\_RID\_3262\_2a.raw |  |  |  |  |  |  |  |  | 1.2697E5 |  | 1 | 0 | 0 | 0 | 0 | 0 | 0 | 0 | 0 | 1 | 0 | 457 | 471 | Oxidation (M) | M5:Oxidation (M):1000.00 | PEAKS DB |
| N.LDISIVGDYIK.R | N | 44.01 | 1234.6809 | 11 | 0.5 | 618.3480 | 2 | 56.58 | 9 | F9:34546 | 31122021\_RID\_3262\_2a.raw |  |  |  |  |  |  |  |  | 2.949E4 |  | 1 | 0 | 0 | 0 | 0 | 0 | 0 | 0 | 0 | 1 | 0 | 538 | 548 |  |  | PEAKS DB |
| R.YDAMALGNHEFDNGLAGLLDPLLK.H | N | 43.69 | 2586.2788 | 24 | 0.6 | 863.1007 | 3 | 69.00 | 9 | F9:43247 | 31122021\_RID\_3262\_2a.raw |  |  |  |  |  |  |  |  | 9.895E5 |  | 1 | 0 | 0 | 0 | 0 | 0 | 0 | 0 | 0 | 1 | 0 | 124 | 147 |  |  | PEAKS DB |
| K.LTTLGVNK.I | N | 43.24 | 844.5018 | 8 | 0.8 | 423.2585 | 2 | 24.48 | 9 | F9:8941 | 31122021\_RID\_3262\_2a.raw |  |  |  |  |  |  |  |  | 1.1856E7 |  | 1 | 0 | 0 | 0 | 0 | 0 | 0 | 0 | 0 | 1 | 0 | 221 | 228 |  |  | PEAKS DB |
| F.GGTFDLLQIK.G | N | 43.16 | 1090.6022 | 10 | -0.1 | 546.3083 | 2 | 49.90 | 9 | F9:29347 | 31122021\_RID\_3262\_2a.raw |  |  |  |  |  |  |  |  | 6.6936E5 |  | 1 | 0 | 0 | 0 | 0 | 0 | 0 | 0 | 0 | 1 | 0 | 433 | 442 |  |  | PEAKS DB |
| K.YLGYLNVIFDDK.G | N | 41.85 | 1458.7394 | 12 | 1.3 | 730.3779 | 2 | 60.34 | 9 | F9:37272 | 31122021\_RID\_3262\_2a.raw |  |  |  |  |  |  |  |  | 2.5015E6 |  | 1 | 0 | 0 | 0 | 0 | 0 | 0 | 0 | 0 | 1 | 0 | 300 | 311 |  |  | PEAKS DB |
| total 22 peptides |  |  |  |  |  |  |  |  |  |  |  |  |  |  |  |  |  |  |  |  |  |  |  |  |  |  |  |  |  |  |  |  |  |  |  |  |  |
| --- | --- | --- | --- | --- | --- | --- | --- | --- | --- | --- | --- | --- | --- | --- | --- | --- | --- | --- | --- | --- | --- | --- | --- | --- | --- | --- | --- | --- | --- | --- | --- | --- | --- | --- | --- | --- | --- |

F8S0Z7.2

back to list

  

| Protein Coverage
| Supporting Peptides
|

Protein Coverage:

Supporting Peptides:

| Peptide | Uniq | -10lgP | Mass | Length | ppm | m/z | z | RT | Fraction | Scan | Source File | Area F4 | Area F5 | Area F6 | Area F10 | Area F8 | Area F9 | Area F7 | Area F1 | Area F2 | Area F3 | #Feature | #Feature F4 | #Feature F5 | #Feature F6 | #Feature F10 | #Feature F8 | #Feature F9 | #Feature F7 | #Feature F1 | #Feature F2 | #Feature F3 | Start | End | PTM | AScore | Found By |
| --- | --- | --- | --- | --- | --- | --- | --- | --- | --- | --- | --- | --- | --- | --- | --- | --- | --- | --- | --- | --- | --- | --- | --- | --- | --- | --- | --- | --- | --- | --- | --- | --- | --- | --- | --- | --- | --- |
| K.HANFPILSANIRPK.G | N | 73.91 | 1576.8838 | 14 | 0.3 | 526.6354 | 3 | 33.47 | 9 | F9:16191 | 31122021\_RID\_3262\_2a.raw | 2.4791E6 |  |  |  |  |  |  | 7.6122E5 | 1.3586E7 |  | 5 | 1 | 0 | 0 | 0 | 0 | 0 | 0 | 2 | 2 | 0 | 148 | 161 |  |  | PEAKS DB |
| G.SFELTILHTNDVHAR.V | N | 71.46 | 1751.8955 | 15 | 0.2 | 584.9725 | 3 | 37.82 | 9 | F9:19802 | 31122021\_RID\_3262\_2a.raw |  |  |  |  |  |  |  |  | 9.8903E6 |  | 1 | 0 | 0 | 0 | 0 | 0 | 0 | 0 | 0 | 1 | 0 | 41 | 55 |  |  | PEAKS DB |
| K.IQLHNYSSQEIGK.T | N | 66.55 | 1515.7681 | 13 | 0.9 | 758.8920 | 2 | 25.89 | 9 | F9:9947 | 31122021\_RID\_3262\_2a.raw |  | 0 |  |  |  |  |  |  | 8.8417E5 |  | 2 | 0 | 0 | 0 | 0 | 0 | 0 | 0 | 0 | 2 | 0 | 343 | 355 |  |  | PEAKS DB |
| R.QVPVVQAYAFGK.Y | N | 64.12 | 1305.7081 | 12 | 0.7 | 653.8618 | 2 | 57.55 | 1 | F1:40709 | 13012022\_RID\_3262\_4a.raw | 4.6454E6 | 1.1825E6 |  |  |  |  |  | 3.0239E5 | 6.0962E6 |  | 5 | 2 | 1 | 0 | 0 | 0 | 0 | 0 | 1 | 1 | 0 | 288 | 299 |  |  | PEAKS DB |
| K.YLGYLNVIFDDKGNVIK.S | N | 63.64 | 1970.0513 | 17 | 0.7 | 986.0336 | 2 | 55.25 | 9 | F9:33414 | 31122021\_RID\_3262\_2a.raw |  |  |  |  |  |  |  |  | 5.214E6 |  | 2 | 0 | 0 | 0 | 0 | 0 | 0 | 0 | 0 | 2 | 0 | 300 | 316 |  |  | PEAKS DB |
| R.DEVEELQNHANK.L | N | 59.76 | 1424.6532 | 12 | 0.7 | 713.3344 | 2 | 23.15 | 9 | F9:7692 | 31122021\_RID\_3262\_2a.raw |  |  |  |  |  |  |  |  | 1.0268E6 |  | 1 | 0 | 0 | 0 | 0 | 0 | 0 | 0 | 0 | 1 | 0 | 209 | 220 |  |  | PEAKS DB |
| K.LLLPSFLAAGGDGYHML.K | N | 55.39 | 1773.9124 | 17 | 0.2 | 887.9636 | 2 | 68.35 | 9 | F9:42925 | 31122021\_RID\_3262\_2a.raw |  |  |  |  |  |  |  |  | 5.8724E5 |  | 1 | 0 | 0 | 0 | 0 | 0 | 0 | 0 | 0 | 1 | 0 | 510 | 526 |  |  | PEAKS DB |
| K.LLLPSFLAAGGDGYHM(+15.99)LK.G | N | 51.71 | 1918.0022 | 18 | -0.6 | 960.0078 | 2 | 57.15 | 9 | F9:34949 | 31122021\_RID\_3262\_2a.raw |  |  |  |  |  |  |  |  | 3.7896E5 |  | 1 | 0 | 0 | 0 | 0 | 0 | 0 | 0 | 0 | 1 | 0 | 510 | 527 | Oxidation (M) | M16:Oxidation (M):1000.00 | PEAKS DB |
| K.VGIIGYTTK.E | N | 51.25 | 950.5436 | 9 | 0.4 | 476.2793 | 2 | 33.16 | 9 | F9:16043 | 31122021\_RID\_3262\_2a.raw |  | 2.6943E6 | 7.1467E5 |  |  |  | 1.5898E5 |  | 8.0892E6 |  | 4 | 0 | 1 | 1 | 0 | 0 | 0 | 1 | 0 | 1 | 0 | 185 | 193 |  |  | PEAKS DB |
| K.LLLPSFLAAGGDGYH.M | N | 48.30 | 1529.7877 | 15 | 0.9 | 765.9019 | 2 | 61.99 | 9 | F9:38681 | 31122021\_RID\_3262\_2a.raw |  |  |  |  |  |  |  |  | 3.6135E5 |  | 1 | 0 | 0 | 0 | 0 | 0 | 0 | 0 | 0 | 1 | 0 | 510 | 524 |  |  | PEAKS DB |
| K.LLLPSFLAAGGDGYHM(+15.99)L.K | N | 46.54 | 1789.9072 | 17 | 1.0 | 895.9618 | 2 | 63.49 | 9 | F9:39581 | 31122021\_RID\_3262\_2a.raw |  |  |  |  |  |  |  |  | 1.84E5 |  | 1 | 0 | 0 | 0 | 0 | 0 | 0 | 0 | 0 | 1 | 0 | 510 | 526 | Oxidation (M) | M16:Oxidation (M):1000.00 | PEAKS DB |
| K.VVYDLSR.K | N | 46.31 | 850.4548 | 7 | 0.5 | 426.2349 | 2 | 30.36 | 1 | F1:16753 | 13012022\_RID\_3262\_4a.raw | 3.9579E6 | 3.0633E6 |  |  |  |  |  |  | 6.9401E6 |  | 3 | 1 | 1 | 0 | 0 | 0 | 0 | 0 | 0 | 1 | 0 | 472 | 478 |  |  | PEAKS DB |
| K.HANFPILSAN.I | N | 46.30 | 1082.5509 | 10 | 0.2 | 542.2828 | 2 | 40.87 | 9 | F9:22309 | 31122021\_RID\_3262\_2a.raw |  |  |  |  |  |  |  |  | 2.7202E6 |  | 1 | 0 | 0 | 0 | 0 | 0 | 0 | 0 | 0 | 1 | 0 | 148 | 157 |  |  | PEAKS DB |
| K.LLLPSFLAAGGDGYHM.L | N | 46.03 | 1660.8282 | 16 | 0.2 | 831.4216 | 2 | 65.01 | 9 | F9:40717 | 31122021\_RID\_3262\_2a.raw |  |  |  |  |  |  |  |  | 7.391E4 |  | 1 | 0 | 0 | 0 | 0 | 0 | 0 | 0 | 0 | 1 | 0 | 510 | 525 |  |  | PEAKS DB |
| K.LLLPSFLAAGGDGYHMLK.G | N | 45.84 | 1902.0073 | 18 | 0.1 | 635.0098 | 3 | 57.14 | 9 | F9:35003 | 31122021\_RID\_3262\_2a.raw | 6.1858E5 |  |  |  |  |  |  |  | 5.9658E6 |  | 2 | 1 | 0 | 0 | 0 | 0 | 0 | 0 | 0 | 1 | 0 | 510 | 527 |  |  | PEAKS DB |
| K.LLLPSFLAAGGD.G | N | 45.25 | 1172.6440 | 12 | -0.2 | 587.3292 | 2 | 72.39 | 9 | F9:44726 | 31122021\_RID\_3262\_2a.raw |  |  |  |  |  |  |  |  | 1.0233E5 |  | 1 | 0 | 0 | 0 | 0 | 0 | 0 | 0 | 0 | 1 | 0 | 510 | 521 |  |  | PEAKS DB |
| R.HGEGM(+15.99)GELLQVSGIK.V | Y | 44.08 | 1569.7821 | 15 | 1.0 | 524.2685 | 3 | 33.96 | 9 | F9:16600 | 31122021\_RID\_3262\_2a.raw |  |  |  |  |  |  |  |  | 1.2697E5 |  | 1 | 0 | 0 | 0 | 0 | 0 | 0 | 0 | 0 | 1 | 0 | 457 | 471 | Oxidation (M) | M5:Oxidation (M):1000.00 | PEAKS DB |
| N.LDISIVGDYIK.R | N | 44.01 | 1234.6809 | 11 | 0.5 | 618.3480 | 2 | 56.58 | 9 | F9:34546 | 31122021\_RID\_3262\_2a.raw |  |  |  |  |  |  |  |  | 2.949E4 |  | 1 | 0 | 0 | 0 | 0 | 0 | 0 | 0 | 0 | 1 | 0 | 538 | 548 |  |  | PEAKS DB |
| R.YDAMALGNHEFDNGLAGLLDPLLK.H | N | 43.69 | 2586.2788 | 24 | 0.6 | 863.1007 | 3 | 69.00 | 9 | F9:43247 | 31122021\_RID\_3262\_2a.raw |  |  |  |  |  |  |  |  | 9.895E5 |  | 1 | 0 | 0 | 0 | 0 | 0 | 0 | 0 | 0 | 1 | 0 | 124 | 147 |  |  | PEAKS DB |
| K.LTTLGVNK.I | N | 43.24 | 844.5018 | 8 | 0.8 | 423.2585 | 2 | 24.48 | 9 | F9:8941 | 31122021\_RID\_3262\_2a.raw |  |  |  |  |  |  |  |  | 1.1856E7 |  | 1 | 0 | 0 | 0 | 0 | 0 | 0 | 0 | 0 | 1 | 0 | 221 | 228 |  |  | PEAKS DB |
| F.GGTFDLLQIK.G | N | 43.16 | 1090.6022 | 10 | -0.1 | 546.3083 | 2 | 49.90 | 9 | F9:29347 | 31122021\_RID\_3262\_2a.raw |  |  |  |  |  |  |  |  | 6.6936E5 |  | 1 | 0 | 0 | 0 | 0 | 0 | 0 | 0 | 0 | 1 | 0 | 433 | 442 |  |  | PEAKS DB |
| K.YLGYLNVIFDDK.G | N | 41.85 | 1458.7394 | 12 | 1.3 | 730.3779 | 2 | 60.34 | 9 | F9:37272 | 31122021\_RID\_3262\_2a.raw |  |  |  |  |  |  |  |  | 2.5015E6 |  | 1 | 0 | 0 | 0 | 0 | 0 | 0 | 0 | 0 | 1 | 0 | 300 | 311 |  |  | PEAKS DB |
| total 22 peptides |  |  |  |  |  |  |  |  |  |  |  |  |  |  |  |  |  |  |  |  |  |  |  |  |  |  |  |  |  |  |  |  |  |  |  |  |  |
| --- | --- | --- | --- | --- | --- | --- | --- | --- | --- | --- | --- | --- | --- | --- | --- | --- | --- | --- | --- | --- | --- | --- | --- | --- | --- | --- | --- | --- | --- | --- | --- | --- | --- | --- | --- | --- | --- |

JAV48423.1

back to list

  

| Protein Coverage
| Supporting Peptides
|

Protein Coverage:

Supporting Peptides:

| Peptide | Uniq | -10lgP | Mass | Length | ppm | m/z | z | RT | Fraction | Scan | Source File | Area F4 | Area F5 | Area F6 | Area F10 | Area F8 | Area F9 | Area F7 | Area F1 | Area F2 | Area F3 | #Feature | #Feature F4 | #Feature F5 | #Feature F6 | #Feature F10 | #Feature F8 | #Feature F9 | #Feature F7 | #Feature F1 | #Feature F2 | #Feature F3 | Start | End | PTM | AScore | Found By |
| --- | --- | --- | --- | --- | --- | --- | --- | --- | --- | --- | --- | --- | --- | --- | --- | --- | --- | --- | --- | --- | --- | --- | --- | --- | --- | --- | --- | --- | --- | --- | --- | --- | --- | --- | --- | --- | --- |
| K.HANFPILSANIRPK.G | N | 73.91 | 1576.8838 | 14 | 0.3 | 526.6354 | 3 | 33.47 | 9 | F9:16191 | 31122021\_RID\_3262\_2a.raw | 2.4791E6 |  |  |  |  |  |  | 7.6122E5 | 1.3586E7 |  | 5 | 1 | 0 | 0 | 0 | 0 | 0 | 0 | 2 | 2 | 0 | 148 | 161 |  |  | PEAKS DB |
| G.SFELTILHTNDVHAR.V | N | 71.46 | 1751.8955 | 15 | 0.2 | 584.9725 | 3 | 37.82 | 9 | F9:19802 | 31122021\_RID\_3262\_2a.raw |  |  |  |  |  |  |  |  | 9.8903E6 |  | 1 | 0 | 0 | 0 | 0 | 0 | 0 | 0 | 0 | 1 | 0 | 41 | 55 |  |  | PEAKS DB |
| K.IQLHNYSSQEIGK.T | N | 66.55 | 1515.7681 | 13 | 0.9 | 758.8920 | 2 | 25.89 | 9 | F9:9947 | 31122021\_RID\_3262\_2a.raw |  | 0 |  |  |  |  |  |  | 8.8417E5 |  | 2 | 0 | 0 | 0 | 0 | 0 | 0 | 0 | 0 | 2 | 0 | 343 | 355 |  |  | PEAKS DB |
| R.QVPVVQAYAFGK.Y | N | 64.12 | 1305.7081 | 12 | 0.7 | 653.8618 | 2 | 57.55 | 1 | F1:40709 | 13012022\_RID\_3262\_4a.raw | 4.6454E6 | 1.1825E6 |  |  |  |  |  | 3.0239E5 | 6.0962E6 |  | 5 | 2 | 1 | 0 | 0 | 0 | 0 | 0 | 1 | 1 | 0 | 288 | 299 |  |  | PEAKS DB |
| K.YLGYLNVIFDDKGNVIK.S | N | 63.64 | 1970.0513 | 17 | 0.7 | 986.0336 | 2 | 55.25 | 9 | F9:33414 | 31122021\_RID\_3262\_2a.raw |  |  |  |  |  |  |  |  | 5.214E6 |  | 2 | 0 | 0 | 0 | 0 | 0 | 0 | 0 | 0 | 2 | 0 | 300 | 316 |  |  | PEAKS DB |
| R.DEVEELQNHANK.L | N | 59.76 | 1424.6532 | 12 | 0.7 | 713.3344 | 2 | 23.15 | 9 | F9:7692 | 31122021\_RID\_3262\_2a.raw |  |  |  |  |  |  |  |  | 1.0268E6 |  | 1 | 0 | 0 | 0 | 0 | 0 | 0 | 0 | 0 | 1 | 0 | 209 | 220 |  |  | PEAKS DB |
| K.LLLPSFLAAGGDGYHML.K | N | 55.39 | 1773.9124 | 17 | 0.2 | 887.9636 | 2 | 68.35 | 9 | F9:42925 | 31122021\_RID\_3262\_2a.raw |  |  |  |  |  |  |  |  | 5.8724E5 |  | 1 | 0 | 0 | 0 | 0 | 0 | 0 | 0 | 0 | 1 | 0 | 510 | 526 |  |  | PEAKS DB |
| K.LLLPSFLAAGGDGYHM(+15.99)LK.G | N | 51.71 | 1918.0022 | 18 | -0.6 | 960.0078 | 2 | 57.15 | 9 | F9:34949 | 31122021\_RID\_3262\_2a.raw |  |  |  |  |  |  |  |  | 3.7896E5 |  | 1 | 0 | 0 | 0 | 0 | 0 | 0 | 0 | 0 | 1 | 0 | 510 | 527 | Oxidation (M) | M16:Oxidation (M):1000.00 | PEAKS DB |
| K.VGIIGYTTK.E | N | 51.25 | 950.5436 | 9 | 0.4 | 476.2793 | 2 | 33.16 | 9 | F9:16043 | 31122021\_RID\_3262\_2a.raw |  | 2.6943E6 | 7.1467E5 |  |  |  | 1.5898E5 |  | 8.0892E6 |  | 4 | 0 | 1 | 1 | 0 | 0 | 0 | 1 | 0 | 1 | 0 | 185 | 193 |  |  | PEAKS DB |
| K.LLLPSFLAAGGDGYH.M | N | 48.30 | 1529.7877 | 15 | 0.9 | 765.9019 | 2 | 61.99 | 9 | F9:38681 | 31122021\_RID\_3262\_2a.raw |  |  |  |  |  |  |  |  | 3.6135E5 |  | 1 | 0 | 0 | 0 | 0 | 0 | 0 | 0 | 0 | 1 | 0 | 510 | 524 |  |  | PEAKS DB |
| K.LLLPSFLAAGGDGYHM(+15.99)L.K | N | 46.54 | 1789.9072 | 17 | 1.0 | 895.9618 | 2 | 63.49 | 9 | F9:39581 | 31122021\_RID\_3262\_2a.raw |  |  |  |  |  |  |  |  | 1.84E5 |  | 1 | 0 | 0 | 0 | 0 | 0 | 0 | 0 | 0 | 1 | 0 | 510 | 526 | Oxidation (M) | M16:Oxidation (M):1000.00 | PEAKS DB |
| K.VVYDLSR.K | N | 46.31 | 850.4548 | 7 | 0.5 | 426.2349 | 2 | 30.36 | 1 | F1:16753 | 13012022\_RID\_3262\_4a.raw | 3.9579E6 | 3.0633E6 |  |  |  |  |  |  | 6.9401E6 |  | 3 | 1 | 1 | 0 | 0 | 0 | 0 | 0 | 0 | 1 | 0 | 472 | 478 |  |  | PEAKS DB |
| K.HANFPILSAN.I | N | 46.30 | 1082.5509 | 10 | 0.2 | 542.2828 | 2 | 40.87 | 9 | F9:22309 | 31122021\_RID\_3262\_2a.raw |  |  |  |  |  |  |  |  | 2.7202E6 |  | 1 | 0 | 0 | 0 | 0 | 0 | 0 | 0 | 0 | 1 | 0 | 148 | 157 |  |  | PEAKS DB |
| K.LLLPSFLAAGGDGYHM.L | N | 46.03 | 1660.8282 | 16 | 0.2 | 831.4216 | 2 | 65.01 | 9 | F9:40717 | 31122021\_RID\_3262\_2a.raw |  |  |  |  |  |  |  |  | 7.391E4 |  | 1 | 0 | 0 | 0 | 0 | 0 | 0 | 0 | 0 | 1 | 0 | 510 | 525 |  |  | PEAKS DB |
| K.LLLPSFLAAGGDGYHMLK.G | N | 45.84 | 1902.0073 | 18 | 0.1 | 635.0098 | 3 | 57.14 | 9 | F9:35003 | 31122021\_RID\_3262\_2a.raw | 6.1858E5 |  |  |  |  |  |  |  | 5.9658E6 |  | 2 | 1 | 0 | 0 | 0 | 0 | 0 | 0 | 0 | 1 | 0 | 510 | 527 |  |  | PEAKS DB |
| K.LLLPSFLAAGGD.G | N | 45.25 | 1172.6440 | 12 | -0.2 | 587.3292 | 2 | 72.39 | 9 | F9:44726 | 31122021\_RID\_3262\_2a.raw |  |  |  |  |  |  |  |  | 1.0233E5 |  | 1 | 0 | 0 | 0 | 0 | 0 | 0 | 0 | 0 | 1 | 0 | 510 | 521 |  |  | PEAKS DB |
| R.HGEGM(+15.99)GELLQVSGIK.V | Y | 44.08 | 1569.7821 | 15 | 1.0 | 524.2685 | 3 | 33.96 | 9 | F9:16600 | 31122021\_RID\_3262\_2a.raw |  |  |  |  |  |  |  |  | 1.2697E5 |  | 1 | 0 | 0 | 0 | 0 | 0 | 0 | 0 | 0 | 1 | 0 | 457 | 471 | Oxidation (M) | M5:Oxidation (M):1000.00 | PEAKS DB |
| N.LDISIVGDYIK.R | N | 44.01 | 1234.6809 | 11 | 0.5 | 618.3480 | 2 | 56.58 | 9 | F9:34546 | 31122021\_RID\_3262\_2a.raw |  |  |  |  |  |  |  |  | 2.949E4 |  | 1 | 0 | 0 | 0 | 0 | 0 | 0 | 0 | 0 | 1 | 0 | 538 | 548 |  |  | PEAKS DB |
| R.YDAMALGNHEFDNGLAGLLDPLLK.H | N | 43.69 | 2586.2788 | 24 | 0.6 | 863.1007 | 3 | 69.00 | 9 | F9:43247 | 31122021\_RID\_3262\_2a.raw |  |  |  |  |  |  |  |  | 9.895E5 |  | 1 | 0 | 0 | 0 | 0 | 0 | 0 | 0 | 0 | 1 | 0 | 124 | 147 |  |  | PEAKS DB |
| K.LTTLGVNK.I | N | 43.24 | 844.5018 | 8 | 0.8 | 423.2585 | 2 | 24.48 | 9 | F9:8941 | 31122021\_RID\_3262\_2a.raw |  |  |  |  |  |  |  |  | 1.1856E7 |  | 1 | 0 | 0 | 0 | 0 | 0 | 0 | 0 | 0 | 1 | 0 | 221 | 228 |  |  | PEAKS DB |
| F.GGTFDLLQIK.G | N | 43.16 | 1090.6022 | 10 | -0.1 | 546.3083 | 2 | 49.90 | 9 | F9:29347 | 31122021\_RID\_3262\_2a.raw |  |  |  |  |  |  |  |  | 6.6936E5 |  | 1 | 0 | 0 | 0 | 0 | 0 | 0 | 0 | 0 | 1 | 0 | 433 | 442 |  |  | PEAKS DB |
| K.YLGYLNVIFDDK.G | N | 41.85 | 1458.7394 | 12 | 1.3 | 730.3779 | 2 | 60.34 | 9 | F9:37272 | 31122021\_RID\_3262\_2a.raw |  |  |  |  |  |  |  |  | 2.5015E6 |  | 1 | 0 | 0 | 0 | 0 | 0 | 0 | 0 | 0 | 1 | 0 | 300 | 311 |  |  | PEAKS DB |
| total 22 peptides |  |  |  |  |  |  |  |  |  |  |  |  |  |  |  |  |  |  |  |  |  |  |  |  |  |  |  |  |  |  |  |  |  |  |  |  |  |
| --- | --- | --- | --- | --- | --- | --- | --- | --- | --- | --- | --- | --- | --- | --- | --- | --- | --- | --- | --- | --- | --- | --- | --- | --- | --- | --- | --- | --- | --- | --- | --- | --- | --- | --- | --- | --- | --- |

SMD28261.1

back to list

  

| Protein Coverage
| Supporting Peptides
|

Protein Coverage:

Supporting Peptides:

| Peptide | Uniq | -10lgP | Mass | Length | ppm | m/z | z | RT | Fraction | Scan | Source File | Area F4 | Area F5 | Area F6 | Area F10 | Area F8 | Area F9 | Area F7 | Area F1 | Area F2 | Area F3 | #Feature | #Feature F4 | #Feature F5 | #Feature F6 | #Feature F10 | #Feature F8 | #Feature F9 | #Feature F7 | #Feature F1 | #Feature F2 | #Feature F3 | Start | End | PTM | AScore | Found By |
| --- | --- | --- | --- | --- | --- | --- | --- | --- | --- | --- | --- | --- | --- | --- | --- | --- | --- | --- | --- | --- | --- | --- | --- | --- | --- | --- | --- | --- | --- | --- | --- | --- | --- | --- | --- | --- | --- |
| K.HANFPILSANIRPK.G | N | 73.91 | 1576.8838 | 14 | 0.3 | 526.6354 | 3 | 33.47 | 9 | F9:16191 | 31122021\_RID\_3262\_2a.raw | 2.4791E6 |  |  |  |  |  |  | 7.6122E5 | 1.3586E7 |  | 5 | 1 | 0 | 0 | 0 | 0 | 0 | 0 | 2 | 2 | 0 | 148 | 161 |  |  | PEAKS DB |
| G.SFELTILHTNDVHAR.V | N | 71.46 | 1751.8955 | 15 | 0.2 | 584.9725 | 3 | 37.82 | 9 | F9:19802 | 31122021\_RID\_3262\_2a.raw |  |  |  |  |  |  |  |  | 9.8903E6 |  | 1 | 0 | 0 | 0 | 0 | 0 | 0 | 0 | 0 | 1 | 0 | 41 | 55 |  |  | PEAKS DB |
| K.IQLHNYSSQEIGK.T | N | 66.55 | 1515.7681 | 13 | 0.9 | 758.8920 | 2 | 25.89 | 9 | F9:9947 | 31122021\_RID\_3262\_2a.raw |  | 0 |  |  |  |  |  |  | 8.8417E5 |  | 2 | 0 | 0 | 0 | 0 | 0 | 0 | 0 | 0 | 2 | 0 | 343 | 355 |  |  | PEAKS DB |
| R.QVPVVQAYAFGK.Y | N | 64.12 | 1305.7081 | 12 | 0.7 | 653.8618 | 2 | 57.55 | 1 | F1:40709 | 13012022\_RID\_3262\_4a.raw | 4.6454E6 | 1.1825E6 |  |  |  |  |  | 3.0239E5 | 6.0962E6 |  | 5 | 2 | 1 | 0 | 0 | 0 | 0 | 0 | 1 | 1 | 0 | 288 | 299 |  |  | PEAKS DB |
| K.YLGYLNVIFDDKGNVIK.S | N | 63.64 | 1970.0513 | 17 | 0.7 | 986.0336 | 2 | 55.25 | 9 | F9:33414 | 31122021\_RID\_3262\_2a.raw |  |  |  |  |  |  |  |  | 5.214E6 |  | 2 | 0 | 0 | 0 | 0 | 0 | 0 | 0 | 0 | 2 | 0 | 300 | 316 |  |  | PEAKS DB |
| R.DEVEELQNHANK.L | N | 59.76 | 1424.6532 | 12 | 0.7 | 713.3344 | 2 | 23.15 | 9 | F9:7692 | 31122021\_RID\_3262\_2a.raw |  |  |  |  |  |  |  |  | 1.0268E6 |  | 1 | 0 | 0 | 0 | 0 | 0 | 0 | 0 | 0 | 1 | 0 | 209 | 220 |  |  | PEAKS DB |
| K.LLLPSFLAAGGDGYHML.K | N | 55.39 | 1773.9124 | 17 | 0.2 | 887.9636 | 2 | 68.35 | 9 | F9:42925 | 31122021\_RID\_3262\_2a.raw |  |  |  |  |  |  |  |  | 5.8724E5 |  | 1 | 0 | 0 | 0 | 0 | 0 | 0 | 0 | 0 | 1 | 0 | 510 | 526 |  |  | PEAKS DB |
| K.LLLPSFLAAGGDGYHM(+15.99)LK.G | N | 51.71 | 1918.0022 | 18 | -0.6 | 960.0078 | 2 | 57.15 | 9 | F9:34949 | 31122021\_RID\_3262\_2a.raw |  |  |  |  |  |  |  |  | 3.7896E5 |  | 1 | 0 | 0 | 0 | 0 | 0 | 0 | 0 | 0 | 1 | 0 | 510 | 527 | Oxidation (M) | M16:Oxidation (M):1000.00 | PEAKS DB |
| K.VGIIGYTTK.E | N | 51.25 | 950.5436 | 9 | 0.4 | 476.2793 | 2 | 33.16 | 9 | F9:16043 | 31122021\_RID\_3262\_2a.raw |  | 2.6943E6 | 7.1467E5 |  |  |  | 1.5898E5 |  | 8.0892E6 |  | 4 | 0 | 1 | 1 | 0 | 0 | 0 | 1 | 0 | 1 | 0 | 185 | 193 |  |  | PEAKS DB |
| K.LLLPSFLAAGGDGYH.M | N | 48.30 | 1529.7877 | 15 | 0.9 | 765.9019 | 2 | 61.99 | 9 | F9:38681 | 31122021\_RID\_3262\_2a.raw |  |  |  |  |  |  |  |  | 3.6135E5 |  | 1 | 0 | 0 | 0 | 0 | 0 | 0 | 0 | 0 | 1 | 0 | 510 | 524 |  |  | PEAKS DB |
| K.LLLPSFLAAGGDGYHM(+15.99)L.K | N | 46.54 | 1789.9072 | 17 | 1.0 | 895.9618 | 2 | 63.49 | 9 | F9:39581 | 31122021\_RID\_3262\_2a.raw |  |  |  |  |  |  |  |  | 1.84E5 |  | 1 | 0 | 0 | 0 | 0 | 0 | 0 | 0 | 0 | 1 | 0 | 510 | 526 | Oxidation (M) | M16:Oxidation (M):1000.00 | PEAKS DB |
| K.VVYDLSR.K | N | 46.31 | 850.4548 | 7 | 0.5 | 426.2349 | 2 | 30.36 | 1 | F1:16753 | 13012022\_RID\_3262\_4a.raw | 3.9579E6 | 3.0633E6 |  |  |  |  |  |  | 6.9401E6 |  | 3 | 1 | 1 | 0 | 0 | 0 | 0 | 0 | 0 | 1 | 0 | 472 | 478 |  |  | PEAKS DB |
| K.HANFPILSAN.I | N | 46.30 | 1082.5509 | 10 | 0.2 | 542.2828 | 2 | 40.87 | 9 | F9:22309 | 31122021\_RID\_3262\_2a.raw |  |  |  |  |  |  |  |  | 2.7202E6 |  | 1 | 0 | 0 | 0 | 0 | 0 | 0 | 0 | 0 | 1 | 0 | 148 | 157 |  |  | PEAKS DB |
| K.LLLPSFLAAGGDGYHM.L | N | 46.03 | 1660.8282 | 16 | 0.2 | 831.4216 | 2 | 65.01 | 9 | F9:40717 | 31122021\_RID\_3262\_2a.raw |  |  |  |  |  |  |  |  | 7.391E4 |  | 1 | 0 | 0 | 0 | 0 | 0 | 0 | 0 | 0 | 1 | 0 | 510 | 525 |  |  | PEAKS DB |
| K.LLLPSFLAAGGDGYHMLK.G | N | 45.84 | 1902.0073 | 18 | 0.1 | 635.0098 | 3 | 57.14 | 9 | F9:35003 | 31122021\_RID\_3262\_2a.raw | 6.1858E5 |  |  |  |  |  |  |  | 5.9658E6 |  | 2 | 1 | 0 | 0 | 0 | 0 | 0 | 0 | 0 | 1 | 0 | 510 | 527 |  |  | PEAKS DB |
| K.LLLPSFLAAGGD.G | N | 45.25 | 1172.6440 | 12 | -0.2 | 587.3292 | 2 | 72.39 | 9 | F9:44726 | 31122021\_RID\_3262\_2a.raw |  |  |  |  |  |  |  |  | 1.0233E5 |  | 1 | 0 | 0 | 0 | 0 | 0 | 0 | 0 | 0 | 1 | 0 | 510 | 521 |  |  | PEAKS DB |
| R.HGEGM(+15.99)GELLQVSGIK.V | Y | 44.08 | 1569.7821 | 15 | 1.0 | 524.2685 | 3 | 33.96 | 9 | F9:16600 | 31122021\_RID\_3262\_2a.raw |  |  |  |  |  |  |  |  | 1.2697E5 |  | 1 | 0 | 0 | 0 | 0 | 0 | 0 | 0 | 0 | 1 | 0 | 457 | 471 | Oxidation (M) | M5:Oxidation (M):1000.00 | PEAKS DB |
| N.LDISIVGDYIK.R | N | 44.01 | 1234.6809 | 11 | 0.5 | 618.3480 | 2 | 56.58 | 9 | F9:34546 | 31122021\_RID\_3262\_2a.raw |  |  |  |  |  |  |  |  | 2.949E4 |  | 1 | 0 | 0 | 0 | 0 | 0 | 0 | 0 | 0 | 1 | 0 | 538 | 548 |  |  | PEAKS DB |
| R.YDAMALGNHEFDNGLAGLLDPLLK.H | N | 43.69 | 2586.2788 | 24 | 0.6 | 863.1007 | 3 | 69.00 | 9 | F9:43247 | 31122021\_RID\_3262\_2a.raw |  |  |  |  |  |  |  |  | 9.895E5 |  | 1 | 0 | 0 | 0 | 0 | 0 | 0 | 0 | 0 | 1 | 0 | 124 | 147 |  |  | PEAKS DB |
| K.LTTLGVNK.I | N | 43.24 | 844.5018 | 8 | 0.8 | 423.2585 | 2 | 24.48 | 9 | F9:8941 | 31122021\_RID\_3262\_2a.raw |  |  |  |  |  |  |  |  | 1.1856E7 |  | 1 | 0 | 0 | 0 | 0 | 0 | 0 | 0 | 0 | 1 | 0 | 221 | 228 |  |  | PEAKS DB |
| F.GGTFDLLQIK.G | N | 43.16 | 1090.6022 | 10 | -0.1 | 546.3083 | 2 | 49.90 | 9 | F9:29347 | 31122021\_RID\_3262\_2a.raw |  |  |  |  |  |  |  |  | 6.6936E5 |  | 1 | 0 | 0 | 0 | 0 | 0 | 0 | 0 | 0 | 1 | 0 | 433 | 442 |  |  | PEAKS DB |
| K.YLGYLNVIFDDK.G | N | 41.85 | 1458.7394 | 12 | 1.3 | 730.3779 | 2 | 60.34 | 9 | F9:37272 | 31122021\_RID\_3262\_2a.raw |  |  |  |  |  |  |  |  | 2.5015E6 |  | 1 | 0 | 0 | 0 | 0 | 0 | 0 | 0 | 0 | 1 | 0 | 300 | 311 |  |  | PEAKS DB |
| total 22 peptides |  |  |  |  |  |  |  |  |  |  |  |  |  |  |  |  |  |  |  |  |  |  |  |  |  |  |  |  |  |  |  |  |  |  |  |  |  |
| --- | --- | --- | --- | --- | --- | --- | --- | --- | --- | --- | --- | --- | --- | --- | --- | --- | --- | --- | --- | --- | --- | --- | --- | --- | --- | --- | --- | --- | --- | --- | --- | --- | --- | --- | --- | --- | --- |

JAS05314.1

back to list

  

| Protein Coverage
| Supporting Peptides
|

Protein Coverage:

Supporting Peptides:

| Peptide | Uniq | -10lgP | Mass | Length | ppm | m/z | z | RT | Fraction | Scan | Source File | Area F4 | Area F5 | Area F6 | Area F10 | Area F8 | Area F9 | Area F7 | Area F1 | Area F2 | Area F3 | #Feature | #Feature F4 | #Feature F5 | #Feature F6 | #Feature F10 | #Feature F8 | #Feature F9 | #Feature F7 | #Feature F1 | #Feature F2 | #Feature F3 | Start | End | PTM | AScore | Found By |
| --- | --- | --- | --- | --- | --- | --- | --- | --- | --- | --- | --- | --- | --- | --- | --- | --- | --- | --- | --- | --- | --- | --- | --- | --- | --- | --- | --- | --- | --- | --- | --- | --- | --- | --- | --- | --- | --- |
| K.HANFPILSANIRPK.G | N | 73.91 | 1576.8838 | 14 | 0.3 | 526.6354 | 3 | 33.47 | 9 | F9:16191 | 31122021\_RID\_3262\_2a.raw | 2.4791E6 |  |  |  |  |  |  | 7.6122E5 | 1.3586E7 |  | 5 | 1 | 0 | 0 | 0 | 0 | 0 | 0 | 2 | 2 | 0 | 149 | 162 |  |  | PEAKS DB |
| G.SFELTILHTNDVHAR.V | N | 71.46 | 1751.8955 | 15 | 0.2 | 584.9725 | 3 | 37.82 | 9 | F9:19802 | 31122021\_RID\_3262\_2a.raw |  |  |  |  |  |  |  |  | 9.8903E6 |  | 1 | 0 | 0 | 0 | 0 | 0 | 0 | 0 | 0 | 1 | 0 | 42 | 56 |  |  | PEAKS DB |
| K.IQLHNYSSQEIGK.T | N | 66.55 | 1515.7681 | 13 | 0.9 | 758.8920 | 2 | 25.89 | 9 | F9:9947 | 31122021\_RID\_3262\_2a.raw |  | 0 |  |  |  |  |  |  | 8.8417E5 |  | 2 | 0 | 0 | 0 | 0 | 0 | 0 | 0 | 0 | 2 | 0 | 344 | 356 |  |  | PEAKS DB |
| R.QVPVVQAYAFGK.Y | N | 64.12 | 1305.7081 | 12 | 0.7 | 653.8618 | 2 | 57.55 | 1 | F1:40709 | 13012022\_RID\_3262\_4a.raw | 4.6454E6 | 1.1825E6 |  |  |  |  |  | 3.0239E5 | 6.0962E6 |  | 5 | 2 | 1 | 0 | 0 | 0 | 0 | 0 | 1 | 1 | 0 | 289 | 300 |  |  | PEAKS DB |
| K.YLGYLNVIFDDKGNVIK.S | N | 63.64 | 1970.0513 | 17 | 0.7 | 986.0336 | 2 | 55.25 | 9 | F9:33414 | 31122021\_RID\_3262\_2a.raw |  |  |  |  |  |  |  |  | 5.214E6 |  | 2 | 0 | 0 | 0 | 0 | 0 | 0 | 0 | 0 | 2 | 0 | 301 | 317 |  |  | PEAKS DB |
| R.DEVEELQNHANK.L | N | 59.76 | 1424.6532 | 12 | 0.7 | 713.3344 | 2 | 23.15 | 9 | F9:7692 | 31122021\_RID\_3262\_2a.raw |  |  |  |  |  |  |  |  | 1.0268E6 |  | 1 | 0 | 0 | 0 | 0 | 0 | 0 | 0 | 0 | 1 | 0 | 210 | 221 |  |  | PEAKS DB |
| K.LLLPSFLAAGGDGYHML.K | N | 55.39 | 1773.9124 | 17 | 0.2 | 887.9636 | 2 | 68.35 | 9 | F9:42925 | 31122021\_RID\_3262\_2a.raw |  |  |  |  |  |  |  |  | 5.8724E5 |  | 1 | 0 | 0 | 0 | 0 | 0 | 0 | 0 | 0 | 1 | 0 | 511 | 527 |  |  | PEAKS DB |
| K.LLLPSFLAAGGDGYHM(+15.99)LK.G | N | 51.71 | 1918.0022 | 18 | -0.6 | 960.0078 | 2 | 57.15 | 9 | F9:34949 | 31122021\_RID\_3262\_2a.raw |  |  |  |  |  |  |  |  | 3.7896E5 |  | 1 | 0 | 0 | 0 | 0 | 0 | 0 | 0 | 0 | 1 | 0 | 511 | 528 | Oxidation (M) | M16:Oxidation (M):1000.00 | PEAKS DB |
| K.VGIIGYTTK.E | N | 51.25 | 950.5436 | 9 | 0.4 | 476.2793 | 2 | 33.16 | 9 | F9:16043 | 31122021\_RID\_3262\_2a.raw |  | 2.6943E6 | 7.1467E5 |  |  |  | 1.5898E5 |  | 8.0892E6 |  | 4 | 0 | 1 | 1 | 0 | 0 | 0 | 1 | 0 | 1 | 0 | 186 | 194 |  |  | PEAKS DB |
| K.LLLPSFLAAGGDGYH.M | N | 48.30 | 1529.7877 | 15 | 0.9 | 765.9019 | 2 | 61.99 | 9 | F9:38681 | 31122021\_RID\_3262\_2a.raw |  |  |  |  |  |  |  |  | 3.6135E5 |  | 1 | 0 | 0 | 0 | 0 | 0 | 0 | 0 | 0 | 1 | 0 | 511 | 525 |  |  | PEAKS DB |
| K.LLLPSFLAAGGDGYHM(+15.99)L.K | N | 46.54 | 1789.9072 | 17 | 1.0 | 895.9618 | 2 | 63.49 | 9 | F9:39581 | 31122021\_RID\_3262\_2a.raw |  |  |  |  |  |  |  |  | 1.84E5 |  | 1 | 0 | 0 | 0 | 0 | 0 | 0 | 0 | 0 | 1 | 0 | 511 | 527 | Oxidation (M) | M16:Oxidation (M):1000.00 | PEAKS DB |
| K.VVYDLSR.K | N | 46.31 | 850.4548 | 7 | 0.5 | 426.2349 | 2 | 30.36 | 1 | F1:16753 | 13012022\_RID\_3262\_4a.raw | 3.9579E6 | 3.0633E6 |  |  |  |  |  |  | 6.9401E6 |  | 3 | 1 | 1 | 0 | 0 | 0 | 0 | 0 | 0 | 1 | 0 | 473 | 479 |  |  | PEAKS DB |
| K.HANFPILSAN.I | N | 46.30 | 1082.5509 | 10 | 0.2 | 542.2828 | 2 | 40.87 | 9 | F9:22309 | 31122021\_RID\_3262\_2a.raw |  |  |  |  |  |  |  |  | 2.7202E6 |  | 1 | 0 | 0 | 0 | 0 | 0 | 0 | 0 | 0 | 1 | 0 | 149 | 158 |  |  | PEAKS DB |
| K.LLLPSFLAAGGDGYHM.L | N | 46.03 | 1660.8282 | 16 | 0.2 | 831.4216 | 2 | 65.01 | 9 | F9:40717 | 31122021\_RID\_3262\_2a.raw |  |  |  |  |  |  |  |  | 7.391E4 |  | 1 | 0 | 0 | 0 | 0 | 0 | 0 | 0 | 0 | 1 | 0 | 511 | 526 |  |  | PEAKS DB |
| K.LLLPSFLAAGGDGYHMLK.G | N | 45.84 | 1902.0073 | 18 | 0.1 | 635.0098 | 3 | 57.14 | 9 | F9:35003 | 31122021\_RID\_3262\_2a.raw | 6.1858E5 |  |  |  |  |  |  |  | 5.9658E6 |  | 2 | 1 | 0 | 0 | 0 | 0 | 0 | 0 | 0 | 1 | 0 | 511 | 528 |  |  | PEAKS DB |
| K.LLLPSFLAAGGD.G | N | 45.25 | 1172.6440 | 12 | -0.2 | 587.3292 | 2 | 72.39 | 9 | F9:44726 | 31122021\_RID\_3262\_2a.raw |  |  |  |  |  |  |  |  | 1.0233E5 |  | 1 | 0 | 0 | 0 | 0 | 0 | 0 | 0 | 0 | 1 | 0 | 511 | 522 |  |  | PEAKS DB |
| R.HGEGM(+15.99)GELLQVSGIK.V | Y | 44.08 | 1569.7821 | 15 | 1.0 | 524.2685 | 3 | 33.96 | 9 | F9:16600 | 31122021\_RID\_3262\_2a.raw |  |  |  |  |  |  |  |  | 1.2697E5 |  | 1 | 0 | 0 | 0 | 0 | 0 | 0 | 0 | 0 | 1 | 0 | 458 | 472 | Oxidation (M) | M5:Oxidation (M):1000.00 | PEAKS DB |
| N.LDISIVGDYIK.R | N | 44.01 | 1234.6809 | 11 | 0.5 | 618.3480 | 2 | 56.58 | 9 | F9:34546 | 31122021\_RID\_3262\_2a.raw |  |  |  |  |  |  |  |  | 2.949E4 |  | 1 | 0 | 0 | 0 | 0 | 0 | 0 | 0 | 0 | 1 | 0 | 539 | 549 |  |  | PEAKS DB |
| R.YDAMALGNHEFDNGLAGLLDPLLK.H | N | 43.69 | 2586.2788 | 24 | 0.6 | 863.1007 | 3 | 69.00 | 9 | F9:43247 | 31122021\_RID\_3262\_2a.raw |  |  |  |  |  |  |  |  | 9.895E5 |  | 1 | 0 | 0 | 0 | 0 | 0 | 0 | 0 | 0 | 1 | 0 | 125 | 148 |  |  | PEAKS DB |
| K.LTTLGVNK.I | N | 43.24 | 844.5018 | 8 | 0.8 | 423.2585 | 2 | 24.48 | 9 | F9:8941 | 31122021\_RID\_3262\_2a.raw |  |  |  |  |  |  |  |  | 1.1856E7 |  | 1 | 0 | 0 | 0 | 0 | 0 | 0 | 0 | 0 | 1 | 0 | 222 | 229 |  |  | PEAKS DB |
| F.GGTFDLLQIK.G | N | 43.16 | 1090.6022 | 10 | -0.1 | 546.3083 | 2 | 49.90 | 9 | F9:29347 | 31122021\_RID\_3262\_2a.raw |  |  |  |  |  |  |  |  | 6.6936E5 |  | 1 | 0 | 0 | 0 | 0 | 0 | 0 | 0 | 0 | 1 | 0 | 434 | 443 |  |  | PEAKS DB |
| K.YLGYLNVIFDDK.G | N | 41.85 | 1458.7394 | 12 | 1.3 | 730.3779 | 2 | 60.34 | 9 | F9:37272 | 31122021\_RID\_3262\_2a.raw |  |  |  |  |  |  |  |  | 2.5015E6 |  | 1 | 0 | 0 | 0 | 0 | 0 | 0 | 0 | 0 | 1 | 0 | 301 | 312 |  |  | PEAKS DB |
| total 22 peptides |  |  |  |  |  |  |  |  |  |  |  |  |  |  |  |  |  |  |  |  |  |  |  |  |  |  |  |  |  |  |  |  |  |  |  |  |  |
| --- | --- | --- | --- | --- | --- | --- | --- | --- | --- | --- | --- | --- | --- | --- | --- | --- | --- | --- | --- | --- | --- | --- | --- | --- | --- | --- | --- | --- | --- | --- | --- | --- | --- | --- | --- | --- | --- |

JAS04863.1

back to list

  

| Protein Coverage
| Supporting Peptides
|

Protein Coverage:

Supporting Peptides:

| Peptide | Uniq | -10lgP | Mass | Length | ppm | m/z | z | RT | Fraction | Scan | Source File | Area F4 | Area F5 | Area F6 | Area F10 | Area F8 | Area F9 | Area F7 | Area F1 | Area F2 | Area F3 | #Feature | #Feature F4 | #Feature F5 | #Feature F6 | #Feature F10 | #Feature F8 | #Feature F9 | #Feature F7 | #Feature F1 | #Feature F2 | #Feature F3 | Start | End | PTM | AScore | Found By |
| --- | --- | --- | --- | --- | --- | --- | --- | --- | --- | --- | --- | --- | --- | --- | --- | --- | --- | --- | --- | --- | --- | --- | --- | --- | --- | --- | --- | --- | --- | --- | --- | --- | --- | --- | --- | --- | --- |
| K.HANFPILSANIRPK.G | N | 73.91 | 1576.8838 | 14 | 0.3 | 526.6354 | 3 | 33.47 | 9 | F9:16191 | 31122021\_RID\_3262\_2a.raw | 2.4791E6 |  |  |  |  |  |  | 7.6122E5 | 1.3586E7 |  | 5 | 1 | 0 | 0 | 0 | 0 | 0 | 0 | 2 | 2 | 0 | 150 | 163 |  |  | PEAKS DB |
| G.SFELTILHTNDVHAR.V | N | 71.46 | 1751.8955 | 15 | 0.2 | 584.9725 | 3 | 37.82 | 9 | F9:19802 | 31122021\_RID\_3262\_2a.raw |  |  |  |  |  |  |  |  | 9.8903E6 |  | 1 | 0 | 0 | 0 | 0 | 0 | 0 | 0 | 0 | 1 | 0 | 43 | 57 |  |  | PEAKS DB |
| K.IQLHNYSSQEIGK.T | N | 66.55 | 1515.7681 | 13 | 0.9 | 758.8920 | 2 | 25.89 | 9 | F9:9947 | 31122021\_RID\_3262\_2a.raw |  | 0 |  |  |  |  |  |  | 8.8417E5 |  | 2 | 0 | 0 | 0 | 0 | 0 | 0 | 0 | 0 | 2 | 0 | 345 | 357 |  |  | PEAKS DB |
| R.QVPVVQAYAFGK.Y | N | 64.12 | 1305.7081 | 12 | 0.7 | 653.8618 | 2 | 57.55 | 1 | F1:40709 | 13012022\_RID\_3262\_4a.raw | 4.6454E6 | 1.1825E6 |  |  |  |  |  | 3.0239E5 | 6.0962E6 |  | 5 | 2 | 1 | 0 | 0 | 0 | 0 | 0 | 1 | 1 | 0 | 290 | 301 |  |  | PEAKS DB |
| K.YLGYLNVIFDDKGNVIK.S | N | 63.64 | 1970.0513 | 17 | 0.7 | 986.0336 | 2 | 55.25 | 9 | F9:33414 | 31122021\_RID\_3262\_2a.raw |  |  |  |  |  |  |  |  | 5.214E6 |  | 2 | 0 | 0 | 0 | 0 | 0 | 0 | 0 | 0 | 2 | 0 | 302 | 318 |  |  | PEAKS DB |
| R.DEVEELQNHANK.L | N | 59.76 | 1424.6532 | 12 | 0.7 | 713.3344 | 2 | 23.15 | 9 | F9:7692 | 31122021\_RID\_3262\_2a.raw |  |  |  |  |  |  |  |  | 1.0268E6 |  | 1 | 0 | 0 | 0 | 0 | 0 | 0 | 0 | 0 | 1 | 0 | 211 | 222 |  |  | PEAKS DB |
| K.LLLPSFLAAGGDGYHML.K | N | 55.39 | 1773.9124 | 17 | 0.2 | 887.9636 | 2 | 68.35 | 9 | F9:42925 | 31122021\_RID\_3262\_2a.raw |  |  |  |  |  |  |  |  | 5.8724E5 |  | 1 | 0 | 0 | 0 | 0 | 0 | 0 | 0 | 0 | 1 | 0 | 512 | 528 |  |  | PEAKS DB |
| K.LLLPSFLAAGGDGYHM(+15.99)LK.G | N | 51.71 | 1918.0022 | 18 | -0.6 | 960.0078 | 2 | 57.15 | 9 | F9:34949 | 31122021\_RID\_3262\_2a.raw |  |  |  |  |  |  |  |  | 3.7896E5 |  | 1 | 0 | 0 | 0 | 0 | 0 | 0 | 0 | 0 | 1 | 0 | 512 | 529 | Oxidation (M) | M16:Oxidation (M):1000.00 | PEAKS DB |
| K.VGIIGYTTK.E | N | 51.25 | 950.5436 | 9 | 0.4 | 476.2793 | 2 | 33.16 | 9 | F9:16043 | 31122021\_RID\_3262\_2a.raw |  | 2.6943E6 | 7.1467E5 |  |  |  | 1.5898E5 |  | 8.0892E6 |  | 4 | 0 | 1 | 1 | 0 | 0 | 0 | 1 | 0 | 1 | 0 | 187 | 195 |  |  | PEAKS DB |
| K.LLLPSFLAAGGDGYH.M | N | 48.30 | 1529.7877 | 15 | 0.9 | 765.9019 | 2 | 61.99 | 9 | F9:38681 | 31122021\_RID\_3262\_2a.raw |  |  |  |  |  |  |  |  | 3.6135E5 |  | 1 | 0 | 0 | 0 | 0 | 0 | 0 | 0 | 0 | 1 | 0 | 512 | 526 |  |  | PEAKS DB |
| K.LLLPSFLAAGGDGYHM(+15.99)L.K | N | 46.54 | 1789.9072 | 17 | 1.0 | 895.9618 | 2 | 63.49 | 9 | F9:39581 | 31122021\_RID\_3262\_2a.raw |  |  |  |  |  |  |  |  | 1.84E5 |  | 1 | 0 | 0 | 0 | 0 | 0 | 0 | 0 | 0 | 1 | 0 | 512 | 528 | Oxidation (M) | M16:Oxidation (M):1000.00 | PEAKS DB |
| K.VVYDLSR.K | N | 46.31 | 850.4548 | 7 | 0.5 | 426.2349 | 2 | 30.36 | 1 | F1:16753 | 13012022\_RID\_3262\_4a.raw | 3.9579E6 | 3.0633E6 |  |  |  |  |  |  | 6.9401E6 |  | 3 | 1 | 1 | 0 | 0 | 0 | 0 | 0 | 0 | 1 | 0 | 474 | 480 |  |  | PEAKS DB |
| K.HANFPILSAN.I | N | 46.30 | 1082.5509 | 10 | 0.2 | 542.2828 | 2 | 40.87 | 9 | F9:22309 | 31122021\_RID\_3262\_2a.raw |  |  |  |  |  |  |  |  | 2.7202E6 |  | 1 | 0 | 0 | 0 | 0 | 0 | 0 | 0 | 0 | 1 | 0 | 150 | 159 |  |  | PEAKS DB |
| K.LLLPSFLAAGGDGYHM.L | N | 46.03 | 1660.8282 | 16 | 0.2 | 831.4216 | 2 | 65.01 | 9 | F9:40717 | 31122021\_RID\_3262\_2a.raw |  |  |  |  |  |  |  |  | 7.391E4 |  | 1 | 0 | 0 | 0 | 0 | 0 | 0 | 0 | 0 | 1 | 0 | 512 | 527 |  |  | PEAKS DB |
| K.LLLPSFLAAGGDGYHMLK.G | N | 45.84 | 1902.0073 | 18 | 0.1 | 635.0098 | 3 | 57.14 | 9 | F9:35003 | 31122021\_RID\_3262\_2a.raw | 6.1858E5 |  |  |  |  |  |  |  | 5.9658E6 |  | 2 | 1 | 0 | 0 | 0 | 0 | 0 | 0 | 0 | 1 | 0 | 512 | 529 |  |  | PEAKS DB |
| K.LLLPSFLAAGGD.G | N | 45.25 | 1172.6440 | 12 | -0.2 | 587.3292 | 2 | 72.39 | 9 | F9:44726 | 31122021\_RID\_3262\_2a.raw |  |  |  |  |  |  |  |  | 1.0233E5 |  | 1 | 0 | 0 | 0 | 0 | 0 | 0 | 0 | 0 | 1 | 0 | 512 | 523 |  |  | PEAKS DB |
| R.HGEGM(+15.99)GELLQVSGIK.V | Y | 44.08 | 1569.7821 | 15 | 1.0 | 524.2685 | 3 | 33.96 | 9 | F9:16600 | 31122021\_RID\_3262\_2a.raw |  |  |  |  |  |  |  |  | 1.2697E5 |  | 1 | 0 | 0 | 0 | 0 | 0 | 0 | 0 | 0 | 1 | 0 | 459 | 473 | Oxidation (M) | M5:Oxidation (M):1000.00 | PEAKS DB |
| N.LDISIVGDYIK.R | N | 44.01 | 1234.6809 | 11 | 0.5 | 618.3480 | 2 | 56.58 | 9 | F9:34546 | 31122021\_RID\_3262\_2a.raw |  |  |  |  |  |  |  |  | 2.949E4 |  | 1 | 0 | 0 | 0 | 0 | 0 | 0 | 0 | 0 | 1 | 0 | 540 | 550 |  |  | PEAKS DB |
| R.YDAMALGNHEFDNGLAGLLDPLLK.H | N | 43.69 | 2586.2788 | 24 | 0.6 | 863.1007 | 3 | 69.00 | 9 | F9:43247 | 31122021\_RID\_3262\_2a.raw |  |  |  |  |  |  |  |  | 9.895E5 |  | 1 | 0 | 0 | 0 | 0 | 0 | 0 | 0 | 0 | 1 | 0 | 126 | 149 |  |  | PEAKS DB |
| K.LTTLGVNK.I | N | 43.24 | 844.5018 | 8 | 0.8 | 423.2585 | 2 | 24.48 | 9 | F9:8941 | 31122021\_RID\_3262\_2a.raw |  |  |  |  |  |  |  |  | 1.1856E7 |  | 1 | 0 | 0 | 0 | 0 | 0 | 0 | 0 | 0 | 1 | 0 | 223 | 230 |  |  | PEAKS DB |
| F.GGTFDLLQIK.G | N | 43.16 | 1090.6022 | 10 | -0.1 | 546.3083 | 2 | 49.90 | 9 | F9:29347 | 31122021\_RID\_3262\_2a.raw |  |  |  |  |  |  |  |  | 6.6936E5 |  | 1 | 0 | 0 | 0 | 0 | 0 | 0 | 0 | 0 | 1 | 0 | 435 | 444 |  |  | PEAKS DB |
| K.YLGYLNVIFDDK.G | N | 41.85 | 1458.7394 | 12 | 1.3 | 730.3779 | 2 | 60.34 | 9 | F9:37272 | 31122021\_RID\_3262\_2a.raw |  |  |  |  |  |  |  |  | 2.5015E6 |  | 1 | 0 | 0 | 0 | 0 | 0 | 0 | 0 | 0 | 1 | 0 | 302 | 313 |  |  | PEAKS DB |
| total 22 peptides |  |  |  |  |  |  |  |  |  |  |  |  |  |  |  |  |  |  |  |  |  |  |  |  |  |  |  |  |  |  |  |  |  |  |  |  |  |
| --- | --- | --- | --- | --- | --- | --- | --- | --- | --- | --- | --- | --- | --- | --- | --- | --- | --- | --- | --- | --- | --- | --- | --- | --- | --- | --- | --- | --- | --- | --- | --- | --- | --- | --- | --- | --- | --- |

JAG46129.1

back to list

  

| Protein Coverage
| Supporting Peptides
|

Protein Coverage:

Supporting Peptides:

| Peptide | Uniq | -10lgP | Mass | Length | ppm | m/z | z | RT | Fraction | Scan | Source File | Area F4 | Area F5 | Area F6 | Area F10 | Area F8 | Area F9 | Area F7 | Area F1 | Area F2 | Area F3 | #Feature | #Feature F4 | #Feature F5 | #Feature F6 | #Feature F10 | #Feature F8 | #Feature F9 | #Feature F7 | #Feature F1 | #Feature F2 | #Feature F3 | Start | End | PTM | AScore | Found By |
| --- | --- | --- | --- | --- | --- | --- | --- | --- | --- | --- | --- | --- | --- | --- | --- | --- | --- | --- | --- | --- | --- | --- | --- | --- | --- | --- | --- | --- | --- | --- | --- | --- | --- | --- | --- | --- | --- |
| K.HANFPILSANIRPK.G | N | 73.91 | 1576.8838 | 14 | 0.3 | 526.6354 | 3 | 33.47 | 9 | F9:16191 | 31122021\_RID\_3262\_2a.raw | 2.4791E6 |  |  |  |  |  |  | 7.6122E5 | 1.3586E7 |  | 5 | 1 | 0 | 0 | 0 | 0 | 0 | 0 | 2 | 2 | 0 | 150 | 163 |  |  | PEAKS DB |
| G.SFELTILHTNDVHAR.V | N | 71.46 | 1751.8955 | 15 | 0.2 | 584.9725 | 3 | 37.82 | 9 | F9:19802 | 31122021\_RID\_3262\_2a.raw |  |  |  |  |  |  |  |  | 9.8903E6 |  | 1 | 0 | 0 | 0 | 0 | 0 | 0 | 0 | 0 | 1 | 0 | 43 | 57 |  |  | PEAKS DB |
| K.IQLHNYSSQEIGK.T | N | 66.55 | 1515.7681 | 13 | 0.9 | 758.8920 | 2 | 25.89 | 9 | F9:9947 | 31122021\_RID\_3262\_2a.raw |  | 0 |  |  |  |  |  |  | 8.8417E5 |  | 2 | 0 | 0 | 0 | 0 | 0 | 0 | 0 | 0 | 2 | 0 | 345 | 357 |  |  | PEAKS DB |
| R.QVPVVQAYAFGK.Y | N | 64.12 | 1305.7081 | 12 | 0.7 | 653.8618 | 2 | 57.55 | 1 | F1:40709 | 13012022\_RID\_3262\_4a.raw | 4.6454E6 | 1.1825E6 |  |  |  |  |  | 3.0239E5 | 6.0962E6 |  | 5 | 2 | 1 | 0 | 0 | 0 | 0 | 0 | 1 | 1 | 0 | 290 | 301 |  |  | PEAKS DB |
| K.YLGYLNVIFDDKGNVIK.S | N | 63.64 | 1970.0513 | 17 | 0.7 | 986.0336 | 2 | 55.25 | 9 | F9:33414 | 31122021\_RID\_3262\_2a.raw |  |  |  |  |  |  |  |  | 5.214E6 |  | 2 | 0 | 0 | 0 | 0 | 0 | 0 | 0 | 0 | 2 | 0 | 302 | 318 |  |  | PEAKS DB |
| R.DEVEELQNHANK.L | N | 59.76 | 1424.6532 | 12 | 0.7 | 713.3344 | 2 | 23.15 | 9 | F9:7692 | 31122021\_RID\_3262\_2a.raw |  |  |  |  |  |  |  |  | 1.0268E6 |  | 1 | 0 | 0 | 0 | 0 | 0 | 0 | 0 | 0 | 1 | 0 | 211 | 222 |  |  | PEAKS DB |
| K.LLLPSFLAAGGDGYHML.K | N | 55.39 | 1773.9124 | 17 | 0.2 | 887.9636 | 2 | 68.35 | 9 | F9:42925 | 31122021\_RID\_3262\_2a.raw |  |  |  |  |  |  |  |  | 5.8724E5 |  | 1 | 0 | 0 | 0 | 0 | 0 | 0 | 0 | 0 | 1 | 0 | 512 | 528 |  |  | PEAKS DB |
| K.LLLPSFLAAGGDGYHM(+15.99)LK.G | N | 51.71 | 1918.0022 | 18 | -0.6 | 960.0078 | 2 | 57.15 | 9 | F9:34949 | 31122021\_RID\_3262\_2a.raw |  |  |  |  |  |  |  |  | 3.7896E5 |  | 1 | 0 | 0 | 0 | 0 | 0 | 0 | 0 | 0 | 1 | 0 | 512 | 529 | Oxidation (M) | M16:Oxidation (M):1000.00 | PEAKS DB |
| K.VGIIGYTTK.E | N | 51.25 | 950.5436 | 9 | 0.4 | 476.2793 | 2 | 33.16 | 9 | F9:16043 | 31122021\_RID\_3262\_2a.raw |  | 2.6943E6 | 7.1467E5 |  |  |  | 1.5898E5 |  | 8.0892E6 |  | 4 | 0 | 1 | 1 | 0 | 0 | 0 | 1 | 0 | 1 | 0 | 187 | 195 |  |  | PEAKS DB |
| K.LLLPSFLAAGGDGYH.M | N | 48.30 | 1529.7877 | 15 | 0.9 | 765.9019 | 2 | 61.99 | 9 | F9:38681 | 31122021\_RID\_3262\_2a.raw |  |  |  |  |  |  |  |  | 3.6135E5 |  | 1 | 0 | 0 | 0 | 0 | 0 | 0 | 0 | 0 | 1 | 0 | 512 | 526 |  |  | PEAKS DB |
| K.LLLPSFLAAGGDGYHM(+15.99)L.K | N | 46.54 | 1789.9072 | 17 | 1.0 | 895.9618 | 2 | 63.49 | 9 | F9:39581 | 31122021\_RID\_3262\_2a.raw |  |  |  |  |  |  |  |  | 1.84E5 |  | 1 | 0 | 0 | 0 | 0 | 0 | 0 | 0 | 0 | 1 | 0 | 512 | 528 | Oxidation (M) | M16:Oxidation (M):1000.00 | PEAKS DB |
| K.VVYDLSR.K | N | 46.31 | 850.4548 | 7 | 0.5 | 426.2349 | 2 | 30.36 | 1 | F1:16753 | 13012022\_RID\_3262\_4a.raw | 3.9579E6 | 3.0633E6 |  |  |  |  |  |  | 6.9401E6 |  | 3 | 1 | 1 | 0 | 0 | 0 | 0 | 0 | 0 | 1 | 0 | 474 | 480 |  |  | PEAKS DB |
| K.HANFPILSAN.I | N | 46.30 | 1082.5509 | 10 | 0.2 | 542.2828 | 2 | 40.87 | 9 | F9:22309 | 31122021\_RID\_3262\_2a.raw |  |  |  |  |  |  |  |  | 2.7202E6 |  | 1 | 0 | 0 | 0 | 0 | 0 | 0 | 0 | 0 | 1 | 0 | 150 | 159 |  |  | PEAKS DB |
| K.LLLPSFLAAGGDGYHM.L | N | 46.03 | 1660.8282 | 16 | 0.2 | 831.4216 | 2 | 65.01 | 9 | F9:40717 | 31122021\_RID\_3262\_2a.raw |  |  |  |  |  |  |  |  | 7.391E4 |  | 1 | 0 | 0 | 0 | 0 | 0 | 0 | 0 | 0 | 1 | 0 | 512 | 527 |  |  | PEAKS DB |
| K.LLLPSFLAAGGDGYHMLK.G | N | 45.84 | 1902.0073 | 18 | 0.1 | 635.0098 | 3 | 57.14 | 9 | F9:35003 | 31122021\_RID\_3262\_2a.raw | 6.1858E5 |  |  |  |  |  |  |  | 5.9658E6 |  | 2 | 1 | 0 | 0 | 0 | 0 | 0 | 0 | 0 | 1 | 0 | 512 | 529 |  |  | PEAKS DB |
| K.LLLPSFLAAGGD.G | N | 45.25 | 1172.6440 | 12 | -0.2 | 587.3292 | 2 | 72.39 | 9 | F9:44726 | 31122021\_RID\_3262\_2a.raw |  |  |  |  |  |  |  |  | 1.0233E5 |  | 1 | 0 | 0 | 0 | 0 | 0 | 0 | 0 | 0 | 1 | 0 | 512 | 523 |  |  | PEAKS DB |
| R.HGEGM(+15.99)GELLQVSGIK.V | Y | 44.08 | 1569.7821 | 15 | 1.0 | 524.2685 | 3 | 33.96 | 9 | F9:16600 | 31122021\_RID\_3262\_2a.raw |  |  |  |  |  |  |  |  | 1.2697E5 |  | 1 | 0 | 0 | 0 | 0 | 0 | 0 | 0 | 0 | 1 | 0 | 459 | 473 | Oxidation (M) | M5:Oxidation (M):1000.00 | PEAKS DB |
| N.LDISIVGDYIK.R | N | 44.01 | 1234.6809 | 11 | 0.5 | 618.3480 | 2 | 56.58 | 9 | F9:34546 | 31122021\_RID\_3262\_2a.raw |  |  |  |  |  |  |  |  | 2.949E4 |  | 1 | 0 | 0 | 0 | 0 | 0 | 0 | 0 | 0 | 1 | 0 | 540 | 550 |  |  | PEAKS DB |
| R.YDAMALGNHEFDNGLAGLLDPLLK.H | N | 43.69 | 2586.2788 | 24 | 0.6 | 863.1007 | 3 | 69.00 | 9 | F9:43247 | 31122021\_RID\_3262\_2a.raw |  |  |  |  |  |  |  |  | 9.895E5 |  | 1 | 0 | 0 | 0 | 0 | 0 | 0 | 0 | 0 | 1 | 0 | 126 | 149 |  |  | PEAKS DB |
| K.LTTLGVNK.I | N | 43.24 | 844.5018 | 8 | 0.8 | 423.2585 | 2 | 24.48 | 9 | F9:8941 | 31122021\_RID\_3262\_2a.raw |  |  |  |  |  |  |  |  | 1.1856E7 |  | 1 | 0 | 0 | 0 | 0 | 0 | 0 | 0 | 0 | 1 | 0 | 223 | 230 |  |  | PEAKS DB |
| F.GGTFDLLQIK.G | N | 43.16 | 1090.6022 | 10 | -0.1 | 546.3083 | 2 | 49.90 | 9 | F9:29347 | 31122021\_RID\_3262\_2a.raw |  |  |  |  |  |  |  |  | 6.6936E5 |  | 1 | 0 | 0 | 0 | 0 | 0 | 0 | 0 | 0 | 1 | 0 | 435 | 444 |  |  | PEAKS DB |
| K.YLGYLNVIFDDK.G | N | 41.85 | 1458.7394 | 12 | 1.3 | 730.3779 | 2 | 60.34 | 9 | F9:37272 | 31122021\_RID\_3262\_2a.raw |  |  |  |  |  |  |  |  | 2.5015E6 |  | 1 | 0 | 0 | 0 | 0 | 0 | 0 | 0 | 0 | 1 | 0 | 302 | 313 |  |  | PEAKS DB |
| total 22 peptides |  |  |  |  |  |  |  |  |  |  |  |  |  |  |  |  |  |  |  |  |  |  |  |  |  |  |  |  |  |  |  |  |  |  |  |  |  |
| --- | --- | --- | --- | --- | --- | --- | --- | --- | --- | --- | --- | --- | --- | --- | --- | --- | --- | --- | --- | --- | --- | --- | --- | --- | --- | --- | --- | --- | --- | --- | --- | --- | --- | --- | --- | --- | --- |

JAG43650.1

back to list

  

| Protein Coverage
| Supporting Peptides
|

Protein Coverage:

Supporting Peptides:

| Peptide | Uniq | -10lgP | Mass | Length | ppm | m/z | z | RT | Fraction | Scan | Source File | Area F4 | Area F5 | Area F6 | Area F10 | Area F8 | Area F9 | Area F7 | Area F1 | Area F2 | Area F3 | #Feature | #Feature F4 | #Feature F5 | #Feature F6 | #Feature F10 | #Feature F8 | #Feature F9 | #Feature F7 | #Feature F1 | #Feature F2 | #Feature F3 | Start | End | PTM | AScore | Found By |
| --- | --- | --- | --- | --- | --- | --- | --- | --- | --- | --- | --- | --- | --- | --- | --- | --- | --- | --- | --- | --- | --- | --- | --- | --- | --- | --- | --- | --- | --- | --- | --- | --- | --- | --- | --- | --- | --- |
| K.HANFPILSANIRPK.G | N | 73.91 | 1576.8838 | 14 | 0.3 | 526.6354 | 3 | 33.47 | 9 | F9:16191 | 31122021\_RID\_3262\_2a.raw | 2.4791E6 |  |  |  |  |  |  | 7.6122E5 | 1.3586E7 |  | 5 | 1 | 0 | 0 | 0 | 0 | 0 | 0 | 2 | 2 | 0 | 150 | 163 |  |  | PEAKS DB |
| G.SFELTILHTNDVHAR.V | N | 71.46 | 1751.8955 | 15 | 0.2 | 584.9725 | 3 | 37.82 | 9 | F9:19802 | 31122021\_RID\_3262\_2a.raw |  |  |  |  |  |  |  |  | 9.8903E6 |  | 1 | 0 | 0 | 0 | 0 | 0 | 0 | 0 | 0 | 1 | 0 | 43 | 57 |  |  | PEAKS DB |
| K.IQLHNYSSQEIGK.T | N | 66.55 | 1515.7681 | 13 | 0.9 | 758.8920 | 2 | 25.89 | 9 | F9:9947 | 31122021\_RID\_3262\_2a.raw |  | 0 |  |  |  |  |  |  | 8.8417E5 |  | 2 | 0 | 0 | 0 | 0 | 0 | 0 | 0 | 0 | 2 | 0 | 345 | 357 |  |  | PEAKS DB |
| R.QVPVVQAYAFGK.Y | N | 64.12 | 1305.7081 | 12 | 0.7 | 653.8618 | 2 | 57.55 | 1 | F1:40709 | 13012022\_RID\_3262\_4a.raw | 4.6454E6 | 1.1825E6 |  |  |  |  |  | 3.0239E5 | 6.0962E6 |  | 5 | 2 | 1 | 0 | 0 | 0 | 0 | 0 | 1 | 1 | 0 | 290 | 301 |  |  | PEAKS DB |
| K.YLGYLNVIFDDKGNVIK.S | N | 63.64 | 1970.0513 | 17 | 0.7 | 986.0336 | 2 | 55.25 | 9 | F9:33414 | 31122021\_RID\_3262\_2a.raw |  |  |  |  |  |  |  |  | 5.214E6 |  | 2 | 0 | 0 | 0 | 0 | 0 | 0 | 0 | 0 | 2 | 0 | 302 | 318 |  |  | PEAKS DB |
| R.DEVEELQNHANK.L | N | 59.76 | 1424.6532 | 12 | 0.7 | 713.3344 | 2 | 23.15 | 9 | F9:7692 | 31122021\_RID\_3262\_2a.raw |  |  |  |  |  |  |  |  | 1.0268E6 |  | 1 | 0 | 0 | 0 | 0 | 0 | 0 | 0 | 0 | 1 | 0 | 211 | 222 |  |  | PEAKS DB |
| K.LLLPSFLAAGGDGYHML.K | N | 55.39 | 1773.9124 | 17 | 0.2 | 887.9636 | 2 | 68.35 | 9 | F9:42925 | 31122021\_RID\_3262\_2a.raw |  |  |  |  |  |  |  |  | 5.8724E5 |  | 1 | 0 | 0 | 0 | 0 | 0 | 0 | 0 | 0 | 1 | 0 | 512 | 528 |  |  | PEAKS DB |
| K.LLLPSFLAAGGDGYHM(+15.99)LK.G | N | 51.71 | 1918.0022 | 18 | -0.6 | 960.0078 | 2 | 57.15 | 9 | F9:34949 | 31122021\_RID\_3262\_2a.raw |  |  |  |  |  |  |  |  | 3.7896E5 |  | 1 | 0 | 0 | 0 | 0 | 0 | 0 | 0 | 0 | 1 | 0 | 512 | 529 | Oxidation (M) | M16:Oxidation (M):1000.00 | PEAKS DB |
| K.VGIIGYTTK.E | N | 51.25 | 950.5436 | 9 | 0.4 | 476.2793 | 2 | 33.16 | 9 | F9:16043 | 31122021\_RID\_3262\_2a.raw |  | 2.6943E6 | 7.1467E5 |  |  |  | 1.5898E5 |  | 8.0892E6 |  | 4 | 0 | 1 | 1 | 0 | 0 | 0 | 1 | 0 | 1 | 0 | 187 | 195 |  |  | PEAKS DB |
| K.LLLPSFLAAGGDGYH.M | N | 48.30 | 1529.7877 | 15 | 0.9 | 765.9019 | 2 | 61.99 | 9 | F9:38681 | 31122021\_RID\_3262\_2a.raw |  |  |  |  |  |  |  |  | 3.6135E5 |  | 1 | 0 | 0 | 0 | 0 | 0 | 0 | 0 | 0 | 1 | 0 | 512 | 526 |  |  | PEAKS DB |
| K.LLLPSFLAAGGDGYHM(+15.99)L.K | N | 46.54 | 1789.9072 | 17 | 1.0 | 895.9618 | 2 | 63.49 | 9 | F9:39581 | 31122021\_RID\_3262\_2a.raw |  |  |  |  |  |  |  |  | 1.84E5 |  | 1 | 0 | 0 | 0 | 0 | 0 | 0 | 0 | 0 | 1 | 0 | 512 | 528 | Oxidation (M) | M16:Oxidation (M):1000.00 | PEAKS DB |
| K.VVYDLSR.K | N | 46.31 | 850.4548 | 7 | 0.5 | 426.2349 | 2 | 30.36 | 1 | F1:16753 | 13012022\_RID\_3262\_4a.raw | 3.9579E6 | 3.0633E6 |  |  |  |  |  |  | 6.9401E6 |  | 3 | 1 | 1 | 0 | 0 | 0 | 0 | 0 | 0 | 1 | 0 | 474 | 480 |  |  | PEAKS DB |
| K.HANFPILSAN.I | N | 46.30 | 1082.5509 | 10 | 0.2 | 542.2828 | 2 | 40.87 | 9 | F9:22309 | 31122021\_RID\_3262\_2a.raw |  |  |  |  |  |  |  |  | 2.7202E6 |  | 1 | 0 | 0 | 0 | 0 | 0 | 0 | 0 | 0 | 1 | 0 | 150 | 159 |  |  | PEAKS DB |
| K.LLLPSFLAAGGDGYHM.L | N | 46.03 | 1660.8282 | 16 | 0.2 | 831.4216 | 2 | 65.01 | 9 | F9:40717 | 31122021\_RID\_3262\_2a.raw |  |  |  |  |  |  |  |  | 7.391E4 |  | 1 | 0 | 0 | 0 | 0 | 0 | 0 | 0 | 0 | 1 | 0 | 512 | 527 |  |  | PEAKS DB |
| K.LLLPSFLAAGGDGYHMLK.G | N | 45.84 | 1902.0073 | 18 | 0.1 | 635.0098 | 3 | 57.14 | 9 | F9:35003 | 31122021\_RID\_3262\_2a.raw | 6.1858E5 |  |  |  |  |  |  |  | 5.9658E6 |  | 2 | 1 | 0 | 0 | 0 | 0 | 0 | 0 | 0 | 1 | 0 | 512 | 529 |  |  | PEAKS DB |
| K.LLLPSFLAAGGD.G | N | 45.25 | 1172.6440 | 12 | -0.2 | 587.3292 | 2 | 72.39 | 9 | F9:44726 | 31122021\_RID\_3262\_2a.raw |  |  |  |  |  |  |  |  | 1.0233E5 |  | 1 | 0 | 0 | 0 | 0 | 0 | 0 | 0 | 0 | 1 | 0 | 512 | 523 |  |  | PEAKS DB |
| R.HGEGM(+15.99)GELLQVSGIK.V | Y | 44.08 | 1569.7821 | 15 | 1.0 | 524.2685 | 3 | 33.96 | 9 | F9:16600 | 31122021\_RID\_3262\_2a.raw |  |  |  |  |  |  |  |  | 1.2697E5 |  | 1 | 0 | 0 | 0 | 0 | 0 | 0 | 0 | 0 | 1 | 0 | 459 | 473 | Oxidation (M) | M5:Oxidation (M):1000.00 | PEAKS DB |
| N.LDISIVGDYIK.R | N | 44.01 | 1234.6809 | 11 | 0.5 | 618.3480 | 2 | 56.58 | 9 | F9:34546 | 31122021\_RID\_3262\_2a.raw |  |  |  |  |  |  |  |  | 2.949E4 |  | 1 | 0 | 0 | 0 | 0 | 0 | 0 | 0 | 0 | 1 | 0 | 540 | 550 |  |  | PEAKS DB |
| R.YDAMALGNHEFDNGLAGLLDPLLK.H | N | 43.69 | 2586.2788 | 24 | 0.6 | 863.1007 | 3 | 69.00 | 9 | F9:43247 | 31122021\_RID\_3262\_2a.raw |  |  |  |  |  |  |  |  | 9.895E5 |  | 1 | 0 | 0 | 0 | 0 | 0 | 0 | 0 | 0 | 1 | 0 | 126 | 149 |  |  | PEAKS DB |
| K.LTTLGVNK.I | N | 43.24 | 844.5018 | 8 | 0.8 | 423.2585 | 2 | 24.48 | 9 | F9:8941 | 31122021\_RID\_3262\_2a.raw |  |  |  |  |  |  |  |  | 1.1856E7 |  | 1 | 0 | 0 | 0 | 0 | 0 | 0 | 0 | 0 | 1 | 0 | 223 | 230 |  |  | PEAKS DB |
| F.GGTFDLLQIK.G | N | 43.16 | 1090.6022 | 10 | -0.1 | 546.3083 | 2 | 49.90 | 9 | F9:29347 | 31122021\_RID\_3262\_2a.raw |  |  |  |  |  |  |  |  | 6.6936E5 |  | 1 | 0 | 0 | 0 | 0 | 0 | 0 | 0 | 0 | 1 | 0 | 435 | 444 |  |  | PEAKS DB |
| K.YLGYLNVIFDDK.G | N | 41.85 | 1458.7394 | 12 | 1.3 | 730.3779 | 2 | 60.34 | 9 | F9:37272 | 31122021\_RID\_3262\_2a.raw |  |  |  |  |  |  |  |  | 2.5015E6 |  | 1 | 0 | 0 | 0 | 0 | 0 | 0 | 0 | 0 | 1 | 0 | 302 | 313 |  |  | PEAKS DB |
| total 22 peptides |  |  |  |  |  |  |  |  |  |  |  |  |  |  |  |  |  |  |  |  |  |  |  |  |  |  |  |  |  |  |  |  |  |  |  |  |  |
| --- | --- | --- | --- | --- | --- | --- | --- | --- | --- | --- | --- | --- | --- | --- | --- | --- | --- | --- | --- | --- | --- | --- | --- | --- | --- | --- | --- | --- | --- | --- | --- | --- | --- | --- | --- | --- | --- |

JAA94887.1

back to list

  

| Protein Coverage
| Supporting Peptides
|

Protein Coverage:

Supporting Peptides:

| Peptide | Uniq | -10lgP | Mass | Length | ppm | m/z | z | RT | Fraction | Scan | Source File | Area F4 | Area F5 | Area F6 | Area F10 | Area F8 | Area F9 | Area F7 | Area F1 | Area F2 | Area F3 | #Feature | #Feature F4 | #Feature F5 | #Feature F6 | #Feature F10 | #Feature F8 | #Feature F9 | #Feature F7 | #Feature F1 | #Feature F2 | #Feature F3 | Start | End | PTM | AScore | Found By |
| --- | --- | --- | --- | --- | --- | --- | --- | --- | --- | --- | --- | --- | --- | --- | --- | --- | --- | --- | --- | --- | --- | --- | --- | --- | --- | --- | --- | --- | --- | --- | --- | --- | --- | --- | --- | --- | --- |
| K.HANFPILSANIRPK.G | N | 73.91 | 1576.8838 | 14 | 0.3 | 526.6354 | 3 | 33.47 | 9 | F9:16191 | 31122021\_RID\_3262\_2a.raw | 2.4791E6 |  |  |  |  |  |  | 7.6122E5 | 1.3586E7 |  | 5 | 1 | 0 | 0 | 0 | 0 | 0 | 0 | 2 | 2 | 0 | 150 | 163 |  |  | PEAKS DB |
| G.SFELTILHTNDVHAR.V | N | 71.46 | 1751.8955 | 15 | 0.2 | 584.9725 | 3 | 37.82 | 9 | F9:19802 | 31122021\_RID\_3262\_2a.raw |  |  |  |  |  |  |  |  | 9.8903E6 |  | 1 | 0 | 0 | 0 | 0 | 0 | 0 | 0 | 0 | 1 | 0 | 43 | 57 |  |  | PEAKS DB |
| K.IQLHNYSSQEIGK.T | N | 66.55 | 1515.7681 | 13 | 0.9 | 758.8920 | 2 | 25.89 | 9 | F9:9947 | 31122021\_RID\_3262\_2a.raw |  | 0 |  |  |  |  |  |  | 8.8417E5 |  | 2 | 0 | 0 | 0 | 0 | 0 | 0 | 0 | 0 | 2 | 0 | 345 | 357 |  |  | PEAKS DB |
| R.QVPVVQAYAFGK.Y | N | 64.12 | 1305.7081 | 12 | 0.7 | 653.8618 | 2 | 57.55 | 1 | F1:40709 | 13012022\_RID\_3262\_4a.raw | 4.6454E6 | 1.1825E6 |  |  |  |  |  | 3.0239E5 | 6.0962E6 |  | 5 | 2 | 1 | 0 | 0 | 0 | 0 | 0 | 1 | 1 | 0 | 290 | 301 |  |  | PEAKS DB |
| K.YLGYLNVIFDDKGNVIK.S | N | 63.64 | 1970.0513 | 17 | 0.7 | 986.0336 | 2 | 55.25 | 9 | F9:33414 | 31122021\_RID\_3262\_2a.raw |  |  |  |  |  |  |  |  | 5.214E6 |  | 2 | 0 | 0 | 0 | 0 | 0 | 0 | 0 | 0 | 2 | 0 | 302 | 318 |  |  | PEAKS DB |
| R.DEVEELQNHANK.L | N | 59.76 | 1424.6532 | 12 | 0.7 | 713.3344 | 2 | 23.15 | 9 | F9:7692 | 31122021\_RID\_3262\_2a.raw |  |  |  |  |  |  |  |  | 1.0268E6 |  | 1 | 0 | 0 | 0 | 0 | 0 | 0 | 0 | 0 | 1 | 0 | 211 | 222 |  |  | PEAKS DB |
| K.LLLPSFLAAGGDGYHML.K | N | 55.39 | 1773.9124 | 17 | 0.2 | 887.9636 | 2 | 68.35 | 9 | F9:42925 | 31122021\_RID\_3262\_2a.raw |  |  |  |  |  |  |  |  | 5.8724E5 |  | 1 | 0 | 0 | 0 | 0 | 0 | 0 | 0 | 0 | 1 | 0 | 512 | 528 |  |  | PEAKS DB |
| K.LLLPSFLAAGGDGYHM(+15.99)LK.G | N | 51.71 | 1918.0022 | 18 | -0.6 | 960.0078 | 2 | 57.15 | 9 | F9:34949 | 31122021\_RID\_3262\_2a.raw |  |  |  |  |  |  |  |  | 3.7896E5 |  | 1 | 0 | 0 | 0 | 0 | 0 | 0 | 0 | 0 | 1 | 0 | 512 | 529 | Oxidation (M) | M16:Oxidation (M):1000.00 | PEAKS DB |
| K.VGIIGYTTK.E | N | 51.25 | 950.5436 | 9 | 0.4 | 476.2793 | 2 | 33.16 | 9 | F9:16043 | 31122021\_RID\_3262\_2a.raw |  | 2.6943E6 | 7.1467E5 |  |  |  | 1.5898E5 |  | 8.0892E6 |  | 4 | 0 | 1 | 1 | 0 | 0 | 0 | 1 | 0 | 1 | 0 | 187 | 195 |  |  | PEAKS DB |
| K.LLLPSFLAAGGDGYH.M | N | 48.30 | 1529.7877 | 15 | 0.9 | 765.9019 | 2 | 61.99 | 9 | F9:38681 | 31122021\_RID\_3262\_2a.raw |  |  |  |  |  |  |  |  | 3.6135E5 |  | 1 | 0 | 0 | 0 | 0 | 0 | 0 | 0 | 0 | 1 | 0 | 512 | 526 |  |  | PEAKS DB |
| K.LLLPSFLAAGGDGYHM(+15.99)L.K | N | 46.54 | 1789.9072 | 17 | 1.0 | 895.9618 | 2 | 63.49 | 9 | F9:39581 | 31122021\_RID\_3262\_2a.raw |  |  |  |  |  |  |  |  | 1.84E5 |  | 1 | 0 | 0 | 0 | 0 | 0 | 0 | 0 | 0 | 1 | 0 | 512 | 528 | Oxidation (M) | M16:Oxidation (M):1000.00 | PEAKS DB |
| K.VVYDLSR.K | N | 46.31 | 850.4548 | 7 | 0.5 | 426.2349 | 2 | 30.36 | 1 | F1:16753 | 13012022\_RID\_3262\_4a.raw | 3.9579E6 | 3.0633E6 |  |  |  |  |  |  | 6.9401E6 |  | 3 | 1 | 1 | 0 | 0 | 0 | 0 | 0 | 0 | 1 | 0 | 474 | 480 |  |  | PEAKS DB |
| K.HANFPILSAN.I | N | 46.30 | 1082.5509 | 10 | 0.2 | 542.2828 | 2 | 40.87 | 9 | F9:22309 | 31122021\_RID\_3262\_2a.raw |  |  |  |  |  |  |  |  | 2.7202E6 |  | 1 | 0 | 0 | 0 | 0 | 0 | 0 | 0 | 0 | 1 | 0 | 150 | 159 |  |  | PEAKS DB |
| K.LLLPSFLAAGGDGYHM.L | N | 46.03 | 1660.8282 | 16 | 0.2 | 831.4216 | 2 | 65.01 | 9 | F9:40717 | 31122021\_RID\_3262\_2a.raw |  |  |  |  |  |  |  |  | 7.391E4 |  | 1 | 0 | 0 | 0 | 0 | 0 | 0 | 0 | 0 | 1 | 0 | 512 | 527 |  |  | PEAKS DB |
| K.LLLPSFLAAGGDGYHMLK.G | N | 45.84 | 1902.0073 | 18 | 0.1 | 635.0098 | 3 | 57.14 | 9 | F9:35003 | 31122021\_RID\_3262\_2a.raw | 6.1858E5 |  |  |  |  |  |  |  | 5.9658E6 |  | 2 | 1 | 0 | 0 | 0 | 0 | 0 | 0 | 0 | 1 | 0 | 512 | 529 |  |  | PEAKS DB |
| K.LLLPSFLAAGGD.G | N | 45.25 | 1172.6440 | 12 | -0.2 | 587.3292 | 2 | 72.39 | 9 | F9:44726 | 31122021\_RID\_3262\_2a.raw |  |  |  |  |  |  |  |  | 1.0233E5 |  | 1 | 0 | 0 | 0 | 0 | 0 | 0 | 0 | 0 | 1 | 0 | 512 | 523 |  |  | PEAKS DB |
| R.HGEGM(+15.99)GELLQVSGIK.V | Y | 44.08 | 1569.7821 | 15 | 1.0 | 524.2685 | 3 | 33.96 | 9 | F9:16600 | 31122021\_RID\_3262\_2a.raw |  |  |  |  |  |  |  |  | 1.2697E5 |  | 1 | 0 | 0 | 0 | 0 | 0 | 0 | 0 | 0 | 1 | 0 | 459 | 473 | Oxidation (M) | M5:Oxidation (M):1000.00 | PEAKS DB |
| N.LDISIVGDYIK.R | N | 44.01 | 1234.6809 | 11 | 0.5 | 618.3480 | 2 | 56.58 | 9 | F9:34546 | 31122021\_RID\_3262\_2a.raw |  |  |  |  |  |  |  |  | 2.949E4 |  | 1 | 0 | 0 | 0 | 0 | 0 | 0 | 0 | 0 | 1 | 0 | 540 | 550 |  |  | PEAKS DB |
| R.YDAMALGNHEFDNGLAGLLDPLLK.H | N | 43.69 | 2586.2788 | 24 | 0.6 | 863.1007 | 3 | 69.00 | 9 | F9:43247 | 31122021\_RID\_3262\_2a.raw |  |  |  |  |  |  |  |  | 9.895E5 |  | 1 | 0 | 0 | 0 | 0 | 0 | 0 | 0 | 0 | 1 | 0 | 126 | 149 |  |  | PEAKS DB |
| K.LTTLGVNK.I | N | 43.24 | 844.5018 | 8 | 0.8 | 423.2585 | 2 | 24.48 | 9 | F9:8941 | 31122021\_RID\_3262\_2a.raw |  |  |  |  |  |  |  |  | 1.1856E7 |  | 1 | 0 | 0 | 0 | 0 | 0 | 0 | 0 | 0 | 1 | 0 | 223 | 230 |  |  | PEAKS DB |
| F.GGTFDLLQIK.G | N | 43.16 | 1090.6022 | 10 | -0.1 | 546.3083 | 2 | 49.90 | 9 | F9:29347 | 31122021\_RID\_3262\_2a.raw |  |  |  |  |  |  |  |  | 6.6936E5 |  | 1 | 0 | 0 | 0 | 0 | 0 | 0 | 0 | 0 | 1 | 0 | 435 | 444 |  |  | PEAKS DB |
| K.YLGYLNVIFDDK.G | N | 41.85 | 1458.7394 | 12 | 1.3 | 730.3779 | 2 | 60.34 | 9 | F9:37272 | 31122021\_RID\_3262\_2a.raw |  |  |  |  |  |  |  |  | 2.5015E6 |  | 1 | 0 | 0 | 0 | 0 | 0 | 0 | 0 | 0 | 1 | 0 | 302 | 313 |  |  | PEAKS DB |
| total 22 peptides |  |  |  |  |  |  |  |  |  |  |  |  |  |  |  |  |  |  |  |  |  |  |  |  |  |  |  |  |  |  |  |  |  |  |  |  |  |
| --- | --- | --- | --- | --- | --- | --- | --- | --- | --- | --- | --- | --- | --- | --- | --- | --- | --- | --- | --- | --- | --- | --- | --- | --- | --- | --- | --- | --- | --- | --- | --- | --- | --- | --- | --- | --- | --- |

JAS04780.1

back to list

  

| Protein Coverage
| Supporting Peptides
|

Protein Coverage:

Supporting Peptides:

| Peptide | Uniq | -10lgP | Mass | Length | ppm | m/z | z | RT | Fraction | Scan | Source File | Area F4 | Area F5 | Area F6 | Area F10 | Area F8 | Area F9 | Area F7 | Area F1 | Area F2 | Area F3 | #Feature | #Feature F4 | #Feature F5 | #Feature F6 | #Feature F10 | #Feature F8 | #Feature F9 | #Feature F7 | #Feature F1 | #Feature F2 | #Feature F3 | Start | End | PTM | AScore | Found By |
| --- | --- | --- | --- | --- | --- | --- | --- | --- | --- | --- | --- | --- | --- | --- | --- | --- | --- | --- | --- | --- | --- | --- | --- | --- | --- | --- | --- | --- | --- | --- | --- | --- | --- | --- | --- | --- | --- |
| K.HANFPILSANIRPK.G | N | 73.91 | 1576.8838 | 14 | 0.3 | 526.6354 | 3 | 33.47 | 9 | F9:16191 | 31122021\_RID\_3262\_2a.raw | 2.4791E6 |  |  |  |  |  |  | 7.6122E5 | 1.3586E7 |  | 5 | 1 | 0 | 0 | 0 | 0 | 0 | 0 | 2 | 2 | 0 | 150 | 163 |  |  | PEAKS DB |
| G.SFELTILHTNDVHAR.V | N | 71.46 | 1751.8955 | 15 | 0.2 | 584.9725 | 3 | 37.82 | 9 | F9:19802 | 31122021\_RID\_3262\_2a.raw |  |  |  |  |  |  |  |  | 9.8903E6 |  | 1 | 0 | 0 | 0 | 0 | 0 | 0 | 0 | 0 | 1 | 0 | 43 | 57 |  |  | PEAKS DB |
| K.IQLHNYSSQEIGK.T | N | 66.55 | 1515.7681 | 13 | 0.9 | 758.8920 | 2 | 25.89 | 9 | F9:9947 | 31122021\_RID\_3262\_2a.raw |  | 0 |  |  |  |  |  |  | 8.8417E5 |  | 2 | 0 | 0 | 0 | 0 | 0 | 0 | 0 | 0 | 2 | 0 | 345 | 357 |  |  | PEAKS DB |
| R.QVPVVQAYAFGK.Y | N | 64.12 | 1305.7081 | 12 | 0.7 | 653.8618 | 2 | 57.55 | 1 | F1:40709 | 13012022\_RID\_3262\_4a.raw | 4.6454E6 | 1.1825E6 |  |  |  |  |  | 3.0239E5 | 6.0962E6 |  | 5 | 2 | 1 | 0 | 0 | 0 | 0 | 0 | 1 | 1 | 0 | 290 | 301 |  |  | PEAKS DB |
| K.YLGYLNVIFDDKGNVIK.S | N | 63.64 | 1970.0513 | 17 | 0.7 | 986.0336 | 2 | 55.25 | 9 | F9:33414 | 31122021\_RID\_3262\_2a.raw |  |  |  |  |  |  |  |  | 5.214E6 |  | 2 | 0 | 0 | 0 | 0 | 0 | 0 | 0 | 0 | 2 | 0 | 302 | 318 |  |  | PEAKS DB |
| R.DEVEELQNHANK.L | N | 59.76 | 1424.6532 | 12 | 0.7 | 713.3344 | 2 | 23.15 | 9 | F9:7692 | 31122021\_RID\_3262\_2a.raw |  |  |  |  |  |  |  |  | 1.0268E6 |  | 1 | 0 | 0 | 0 | 0 | 0 | 0 | 0 | 0 | 1 | 0 | 211 | 222 |  |  | PEAKS DB |
| K.LLLPSFLAAGGDGYHML.K | N | 55.39 | 1773.9124 | 17 | 0.2 | 887.9636 | 2 | 68.35 | 9 | F9:42925 | 31122021\_RID\_3262\_2a.raw |  |  |  |  |  |  |  |  | 5.8724E5 |  | 1 | 0 | 0 | 0 | 0 | 0 | 0 | 0 | 0 | 1 | 0 | 512 | 528 |  |  | PEAKS DB |
| K.LLLPSFLAAGGDGYHM(+15.99)LK.G | N | 51.71 | 1918.0022 | 18 | -0.6 | 960.0078 | 2 | 57.15 | 9 | F9:34949 | 31122021\_RID\_3262\_2a.raw |  |  |  |  |  |  |  |  | 3.7896E5 |  | 1 | 0 | 0 | 0 | 0 | 0 | 0 | 0 | 0 | 1 | 0 | 512 | 529 | Oxidation (M) | M16:Oxidation (M):1000.00 | PEAKS DB |
| K.VGIIGYTTK.E | N | 51.25 | 950.5436 | 9 | 0.4 | 476.2793 | 2 | 33.16 | 9 | F9:16043 | 31122021\_RID\_3262\_2a.raw |  | 2.6943E6 | 7.1467E5 |  |  |  | 1.5898E5 |  | 8.0892E6 |  | 4 | 0 | 1 | 1 | 0 | 0 | 0 | 1 | 0 | 1 | 0 | 187 | 195 |  |  | PEAKS DB |
| K.LLLPSFLAAGGDGYH.M | N | 48.30 | 1529.7877 | 15 | 0.9 | 765.9019 | 2 | 61.99 | 9 | F9:38681 | 31122021\_RID\_3262\_2a.raw |  |  |  |  |  |  |  |  | 3.6135E5 |  | 1 | 0 | 0 | 0 | 0 | 0 | 0 | 0 | 0 | 1 | 0 | 512 | 526 |  |  | PEAKS DB |
| K.LLLPSFLAAGGDGYHM(+15.99)L.K | N | 46.54 | 1789.9072 | 17 | 1.0 | 895.9618 | 2 | 63.49 | 9 | F9:39581 | 31122021\_RID\_3262\_2a.raw |  |  |  |  |  |  |  |  | 1.84E5 |  | 1 | 0 | 0 | 0 | 0 | 0 | 0 | 0 | 0 | 1 | 0 | 512 | 528 | Oxidation (M) | M16:Oxidation (M):1000.00 | PEAKS DB |
| K.VVYDLSR.K | N | 46.31 | 850.4548 | 7 | 0.5 | 426.2349 | 2 | 30.36 | 1 | F1:16753 | 13012022\_RID\_3262\_4a.raw | 3.9579E6 | 3.0633E6 |  |  |  |  |  |  | 6.9401E6 |  | 3 | 1 | 1 | 0 | 0 | 0 | 0 | 0 | 0 | 1 | 0 | 474 | 480 |  |  | PEAKS DB |
| K.HANFPILSAN.I | N | 46.30 | 1082.5509 | 10 | 0.2 | 542.2828 | 2 | 40.87 | 9 | F9:22309 | 31122021\_RID\_3262\_2a.raw |  |  |  |  |  |  |  |  | 2.7202E6 |  | 1 | 0 | 0 | 0 | 0 | 0 | 0 | 0 | 0 | 1 | 0 | 150 | 159 |  |  | PEAKS DB |
| K.LLLPSFLAAGGDGYHM.L | N | 46.03 | 1660.8282 | 16 | 0.2 | 831.4216 | 2 | 65.01 | 9 | F9:40717 | 31122021\_RID\_3262\_2a.raw |  |  |  |  |  |  |  |  | 7.391E4 |  | 1 | 0 | 0 | 0 | 0 | 0 | 0 | 0 | 0 | 1 | 0 | 512 | 527 |  |  | PEAKS DB |
| K.LLLPSFLAAGGDGYHMLK.G | N | 45.84 | 1902.0073 | 18 | 0.1 | 635.0098 | 3 | 57.14 | 9 | F9:35003 | 31122021\_RID\_3262\_2a.raw | 6.1858E5 |  |  |  |  |  |  |  | 5.9658E6 |  | 2 | 1 | 0 | 0 | 0 | 0 | 0 | 0 | 0 | 1 | 0 | 512 | 529 |  |  | PEAKS DB |
| K.LLLPSFLAAGGD.G | N | 45.25 | 1172.6440 | 12 | -0.2 | 587.3292 | 2 | 72.39 | 9 | F9:44726 | 31122021\_RID\_3262\_2a.raw |  |  |  |  |  |  |  |  | 1.0233E5 |  | 1 | 0 | 0 | 0 | 0 | 0 | 0 | 0 | 0 | 1 | 0 | 512 | 523 |  |  | PEAKS DB |
| R.HGEGM(+15.99)GELLQVSGIK.V | Y | 44.08 | 1569.7821 | 15 | 1.0 | 524.2685 | 3 | 33.96 | 9 | F9:16600 | 31122021\_RID\_3262\_2a.raw |  |  |  |  |  |  |  |  | 1.2697E5 |  | 1 | 0 | 0 | 0 | 0 | 0 | 0 | 0 | 0 | 1 | 0 | 459 | 473 | Oxidation (M) | M5:Oxidation (M):1000.00 | PEAKS DB |
| N.LDISIVGDYIK.R | N | 44.01 | 1234.6809 | 11 | 0.5 | 618.3480 | 2 | 56.58 | 9 | F9:34546 | 31122021\_RID\_3262\_2a.raw |  |  |  |  |  |  |  |  | 2.949E4 |  | 1 | 0 | 0 | 0 | 0 | 0 | 0 | 0 | 0 | 1 | 0 | 540 | 550 |  |  | PEAKS DB |
| R.YDAMALGNHEFDNGLAGLLDPLLK.H | N | 43.69 | 2586.2788 | 24 | 0.6 | 863.1007 | 3 | 69.00 | 9 | F9:43247 | 31122021\_RID\_3262\_2a.raw |  |  |  |  |  |  |  |  | 9.895E5 |  | 1 | 0 | 0 | 0 | 0 | 0 | 0 | 0 | 0 | 1 | 0 | 126 | 149 |  |  | PEAKS DB |
| K.LTTLGVNK.I | N | 43.24 | 844.5018 | 8 | 0.8 | 423.2585 | 2 | 24.48 | 9 | F9:8941 | 31122021\_RID\_3262\_2a.raw |  |  |  |  |  |  |  |  | 1.1856E7 |  | 1 | 0 | 0 | 0 | 0 | 0 | 0 | 0 | 0 | 1 | 0 | 223 | 230 |  |  | PEAKS DB |
| F.GGTFDLLQIK.G | N | 43.16 | 1090.6022 | 10 | -0.1 | 546.3083 | 2 | 49.90 | 9 | F9:29347 | 31122021\_RID\_3262\_2a.raw |  |  |  |  |  |  |  |  | 6.6936E5 |  | 1 | 0 | 0 | 0 | 0 | 0 | 0 | 0 | 0 | 1 | 0 | 435 | 444 |  |  | PEAKS DB |
| K.YLGYLNVIFDDK.G | N | 41.85 | 1458.7394 | 12 | 1.3 | 730.3779 | 2 | 60.34 | 9 | F9:37272 | 31122021\_RID\_3262\_2a.raw |  |  |  |  |  |  |  |  | 2.5015E6 |  | 1 | 0 | 0 | 0 | 0 | 0 | 0 | 0 | 0 | 1 | 0 | 302 | 313 |  |  | PEAKS DB |
| total 22 peptides |  |  |  |  |  |  |  |  |  |  |  |  |  |  |  |  |  |  |  |  |  |  |  |  |  |  |  |  |  |  |  |  |  |  |  |  |  |
| --- | --- | --- | --- | --- | --- | --- | --- | --- | --- | --- | --- | --- | --- | --- | --- | --- | --- | --- | --- | --- | --- | --- | --- | --- | --- | --- | --- | --- | --- | --- | --- | --- | --- | --- | --- | --- | --- |

AMB36337.1

back to list

  

| Protein Coverage
| Supporting Peptides
|

Protein Coverage:

Supporting Peptides:

| Peptide | Uniq | -10lgP | Mass | Length | ppm | m/z | z | RT | Fraction | Scan | Source File | Area F4 | Area F5 | Area F6 | Area F10 | Area F8 | Area F9 | Area F7 | Area F1 | Area F2 | Area F3 | #Feature | #Feature F4 | #Feature F5 | #Feature F6 | #Feature F10 | #Feature F8 | #Feature F9 | #Feature F7 | #Feature F1 | #Feature F2 | #Feature F3 | Start | End | PTM | AScore | Found By |
| --- | --- | --- | --- | --- | --- | --- | --- | --- | --- | --- | --- | --- | --- | --- | --- | --- | --- | --- | --- | --- | --- | --- | --- | --- | --- | --- | --- | --- | --- | --- | --- | --- | --- | --- | --- | --- | --- |
| R.KPEIQNEIIDLHNSLR.R | Y | 89.03 | 1918.0272 | 16 | 0.3 | 640.3499 | 3 | 91.88 | 6 | F6:63195 | 17012022\_RID\_3262\_9a.raw | 8.3359E7 | 3.005E6 |  | 2.9998E7 | 8.7525E6 | 6.2366E7 | 9.1117E4 | 3.0252E7 | 2.5563E7 | 2.2549E7 | 23 | 5 | 1 | 0 | 2 | 1 | 8 | 1 | 1 | 3 | 1 | 30 | 45 |  |  | PEAKS DB |
| K.MEWYPEAAANAER.W | Y | 81.16 | 1536.6667 | 13 | 0.7 | 769.3412 | 2 | 40.90 | 8 | F8:21225 | 31122021\_RID\_3262\_1a.raw | 7.9122E7 | 1.6498E7 | 2.155E6 | 8.9092E6 |  | 1.6955E7 | 2.209E7 | 1.1392E7 | 4.6908E6 |  | 8 | 1 | 1 | 1 | 1 | 0 | 1 | 1 | 1 | 1 | 0 | 58 | 70 |  |  | PEAKS DB |
| K.M(+15.99)EWYPEAAANAER.W | Y | 69.85 | 1552.6616 | 13 | 0.3 | 777.3383 | 2 | 38.09 | 7 | F7:17968 | 21012022\_RID\_3262\_7a.raw | 2.0661E6 |  | 9.7814E5 | 3.1597E6 |  | 5.6555E6 | 1.1461E7 | 2.0431E6 | 5.669E5 |  | 11 | 1 | 0 | 1 | 1 | 0 | 1 | 3 | 3 | 1 | 0 | 58 | 70 | Oxidation (M) | M1:Oxidation (M):1000.00 | PEAKS DB |
| R.SVNPTASNM(+15.99)LK.M | Y | 68.64 | 1176.5808 | 11 | 0.0 | 589.2977 | 2 | 30.11 | 6 | F6:16925 | 17012022\_RID\_3262\_9a.raw |  | 4.2278E6 | 4.3197E6 | 8.5848E6 |  | 1.125E6 | 7.8837E7 | 3.6478E6 | 3.531E6 |  | 17 | 0 | 1 | 3 | 1 | 0 | 1 | 4 | 4 | 3 | 0 | 47 | 57 | Oxidation (M) | M9:Oxidation (M):1000.00 | PEAKS DB |
| G.SVDFDSESPR.K | Y | 65.80 | 1137.4938 | 10 | -0.2 | 569.7540 | 2 | 27.53 | 8 | F8:10617 | 31122021\_RID\_3262\_1a.raw | 8.5838E7 | 4.1373E7 | 6.2405E6 | 2.2716E7 |  | 3.301E7 | 1.2476E8 | 1.1032E7 | 1.0871E7 |  | 12 | 1 | 2 | 1 | 1 | 0 | 1 | 2 | 1 | 3 | 0 | 20 | 29 |  |  | PEAKS DB |
| R.RSVNPTASNMLK.M | Y | 64.72 | 1316.6870 | 12 | 0.8 | 439.9033 | 3 | 24.67 | 7 | F7:8627 | 21012022\_RID\_3262\_7a.raw | 1.3101E7 |  |  |  |  | 2.2245E6 | 4.7886E6 | 4.9394E5 |  |  | 7 | 2 | 0 | 0 | 0 | 0 | 2 | 2 | 1 | 0 | 0 | 46 | 57 |  |  | PEAKS DB |
| R.SVNPTASNMLK.M | Y | 62.70 | 1160.5859 | 11 | -0.3 | 581.3000 | 2 | 30.08 | 6 | F6:16781 | 17012022\_RID\_3262\_9a.raw | 9.3734E7 | 2.2937E7 | 6.7673E6 | 1.3614E7 |  | 2.4887E7 | 7.8663E7 | 1.1764E7 | 9.2651E6 |  | 9 | 2 | 1 | 1 | 1 | 0 | 1 | 1 | 1 | 1 | 0 | 47 | 57 |  |  | PEAKS DB |
| R.KPEIQNEIIDLHNSL.R | Y | 62.63 | 1761.9260 | 15 | 0.6 | 881.9708 | 2 | 45.07 | 1 | F1:29960 | 13012022\_RID\_3262\_4a.raw | 1.7257E6 |  |  |  |  |  |  | 4.7007E6 | 1.8566E6 |  | 4 | 1 | 0 | 0 | 0 | 0 | 0 | 0 | 2 | 1 | 0 | 30 | 44 |  |  | PEAKS DB |
| R.KPEIQNEIIDLHNSLRR.S | Y | 57.43 | 2074.1282 | 17 | -0.1 | 519.5392 | 4 | 36.09 | 1 | F1:21895 | 13012022\_RID\_3262\_4a.raw | 1.1176E7 |  |  |  |  |  |  |  |  |  | 2 | 2 | 0 | 0 | 0 | 0 | 0 | 0 | 0 | 0 | 0 | 30 | 46 |  |  | PEAKS DB |
| G.SVDFDSESPRKPEIQNEIIDLHNSLR.R | Y | 56.87 | 3037.5105 | 26 | 0.8 | 760.3855 | 4 | 43.45 | 1 | F1:28822 | 13012022\_RID\_3262\_4a.raw | 8.3116E6 |  |  |  |  |  |  |  |  |  | 1 | 1 | 0 | 0 | 0 | 0 | 0 | 0 | 0 | 0 | 0 | 20 | 45 |  |  | PEAKS DB |
| R.RSVNPTASNM(+15.99)LK.M | Y | 54.49 | 1332.6820 | 12 | 0.5 | 667.3486 | 2 | 19.59 | 7 | F7:5056 | 21012022\_RID\_3262\_7a.raw | 1.2907E6 |  |  |  |  | 3.596E5 | 2.3197E6 |  |  |  | 4 | 1 | 0 | 0 | 0 | 0 | 1 | 2 | 0 | 0 | 0 | 46 | 57 | Oxidation (M) | M10:Oxidation (M):1000.00 | PEAKS DB |
| S.VDFDSESPR.K | N | 54.45 | 1050.4618 | 9 | 0.7 | 526.2385 | 2 | 28.90 | 7 | F7:11521 | 21012022\_RID\_3262\_7a.raw |  |  | 3.3908E5 |  |  |  | 1.0581E7 | 6.71E5 | 5.9539E5 |  | 5 | 0 | 0 | 1 | 0 | 0 | 0 | 2 | 1 | 1 | 0 | 21 | 29 |  |  | PEAKS DB |
| R.KPEIQNEIIDLHN.S | Y | 52.17 | 1561.8099 | 13 | 0.0 | 781.9122 | 2 | 38.16 | 7 | F7:17950 | 21012022\_RID\_3262\_7a.raw |  |  |  |  |  |  | 6.4599E4 |  |  |  | 1 | 0 | 0 | 0 | 0 | 0 | 0 | 1 | 0 | 0 | 0 | 30 | 42 |  |  | PEAKS DB |
| P.EIQNEIIDLHNSLR.R | Y | 51.10 | 1692.8795 | 14 | 0.4 | 847.4474 | 2 | 42.68 | 8 | F8:22596 | 31122021\_RID\_3262\_1a.raw |  |  |  |  |  |  |  | 1.0267E5 |  |  | 1 | 0 | 0 | 0 | 0 | 0 | 0 | 0 | 1 | 0 | 0 | 32 | 45 |  |  | PEAKS DB |
| N.PTASNMLK.M | N | 44.10 | 860.4426 | 8 | -0.5 | 431.2283 | 2 | 23.21 | 7 | F7:7593 | 21012022\_RID\_3262\_7a.raw |  |  |  |  |  |  | 3.7964E5 |  |  |  | 1 | 0 | 0 | 0 | 0 | 0 | 0 | 1 | 0 | 0 | 0 | 50 | 57 |  |  | PEAKS DB |
| R.RSVNPTASNML.K | Y | 43.03 | 1188.5920 | 11 | 0.9 | 595.3038 | 2 | 32.28 | 1 | F1:18533 | 13012022\_RID\_3262\_4a.raw | 2.643E5 |  |  |  |  |  |  |  |  |  | 1 | 1 | 0 | 0 | 0 | 0 | 0 | 0 | 0 | 0 | 0 | 46 | 56 |  |  | PEAKS DB |
| total 16 peptides |  |  |  |  |  |  |  |  |  |  |  |  |  |  |  |  |  |  |  |  |  |  |  |  |  |  |  |  |  |  |  |  |  |  |  |  |  |
| --- | --- | --- | --- | --- | --- | --- | --- | --- | --- | --- | --- | --- | --- | --- | --- | --- | --- | --- | --- | --- | --- | --- | --- | --- | --- | --- | --- | --- | --- | --- | --- | --- | --- | --- | --- | --- | --- |

A0A024BTN9.1

back to list

  

| Protein Coverage
| Supporting Peptides
|

Protein Coverage:

Supporting Peptides:

| Peptide | Uniq | -10lgP | Mass | Length | ppm | m/z | z | RT | Fraction | Scan | Source File | Area F4 | Area F5 | Area F6 | Area F10 | Area F8 | Area F9 | Area F7 | Area F1 | Area F2 | Area F3 | #Feature | #Feature F4 | #Feature F5 | #Feature F6 | #Feature F10 | #Feature F8 | #Feature F9 | #Feature F7 | #Feature F1 | #Feature F2 | #Feature F3 | Start | End | PTM | AScore | Found By |
| --- | --- | --- | --- | --- | --- | --- | --- | --- | --- | --- | --- | --- | --- | --- | --- | --- | --- | --- | --- | --- | --- | --- | --- | --- | --- | --- | --- | --- | --- | --- | --- | --- | --- | --- | --- | --- | --- |
| K.KFWEDDGIHGGK.S | N | 70.84 | 1387.6520 | 12 | 1.0 | 694.8340 | 2 | 27.52 | 1 | F1:14353 | 13012022\_RID\_3262\_4a.raw | 2.2688E8 | 4.7295E4 |  |  |  |  |  | 1.1825E7 | 1.8101E7 | 8.0079E8 | 17 | 4 | 1 | 0 | 0 | 0 | 0 | 0 | 3 | 3 | 6 | 336 | 347 |  |  | PEAKS DB |
| Q.YPVKPSEEGK.S | N | 69.75 | 1132.5764 | 10 | 0.7 | 567.2959 | 2 | 17.88 | 6 | F6:5952 | 17012022\_RID\_3262\_9a.raw | 4.2299E8 | 5.2685E7 | 2.5077E5 | 3.2771E7 |  | 6.9462E7 | 2.576E7 |  |  |  | 12 | 2 | 2 | 1 | 3 | 0 | 2 | 2 | 0 | 0 | 0 | 144 | 153 |  |  | PEAKS DB |
| L.SAAYVLAGAGHQVTVLEASER.A | N | 67.91 | 2128.0913 | 21 | 0.0 | 1065.0530 | 2 | 41.69 | 10 | F10:10487 | 31122021\_RID\_3262\_3a.raw | 1.4728E6 |  |  |  |  |  |  | 6.4802E5 | 6.599E5 | 2.6305E7 | 6 | 1 | 0 | 0 | 0 | 0 | 0 | 0 | 2 | 1 | 2 | 49 | 69 |  |  | PEAKS DB |
| K.FWEDDGIHGGK.S | N | 65.97 | 1259.5570 | 11 | 0.4 | 630.7860 | 2 | 31.13 | 1 | F1:17598 | 13012022\_RID\_3262\_4a.raw | 7.5921E7 | 9.0996E6 |  |  |  | 1.9943E6 |  | 6.128E6 | 1.0893E7 | 5.5749E8 | 17 | 2 | 2 | 0 | 0 | 0 | 1 | 0 | 3 | 3 | 6 | 337 | 347 |  |  | PEAKS DB |
| A.YVLAGAGHQVTVLEASER.A | N | 64.94 | 1898.9850 | 18 | 0.8 | 950.5005 | 2 | 37.54 | 10 | F10:8957 | 31122021\_RID\_3262\_3a.raw | 9.515E5 |  |  |  |  |  |  |  |  | 6.9519E6 | 3 | 1 | 0 | 0 | 0 | 0 | 0 | 0 | 0 | 0 | 2 | 52 | 69 |  |  | PEAKS DB |
| K.SAGQLYEESLGK.V | N | 63.95 | 1280.6248 | 12 | 0.0 | 641.3196 | 2 | 33.66 | 2 | F2:17391 | 13012022\_RID\_3262\_5a.raw |  | 2.9891E7 |  |  |  |  |  | 1.4396E5 |  |  | 2 | 0 | 1 | 0 | 0 | 0 | 0 | 0 | 1 | 0 | 0 | 154 | 165 |  |  | PEAKS DB |
| Y.VLAGAGHQVTVLEASER.A | N | 61.46 | 1735.9216 | 17 | 0.9 | 579.6483 | 3 | 33.06 | 6 | F6:19337 | 17012022\_RID\_3262\_9a.raw | 2.8948E5 | 1.1934E6 |  | 6.6625E5 |  | 3.8459E6 |  | 1.4002E6 | 1.9495E6 | 3.1893E7 | 10 | 1 | 1 | 0 | 1 | 0 | 1 | 0 | 2 | 2 | 2 | 53 | 69 |  |  | PEAKS DB |
| D.YEEFLEIAR.N | N | 54.84 | 1168.5764 | 9 | 0.3 | 585.2957 | 2 | 46.57 | 4 | F4:30508 | 17012022\_RID\_3262\_10a .raw | 2.2869E6 |  |  | 1.3254E6 |  |  |  | 2.3858E5 | 1.5579E5 | 1.3646E7 | 5 | 1 | 0 | 0 | 1 | 0 | 0 | 0 | 1 | 1 | 1 | 18 | 26 |  |  | PEAKS DB |
| K.YDTYSTKEYLIK.E | N | 53.18 | 1522.7555 | 12 | 0.9 | 762.3857 | 2 | 34.13 | 1 | F1:20150 | 13012022\_RID\_3262\_4a.raw | 4.6273E6 |  |  |  |  |  |  |  |  |  | 2 | 2 | 0 | 0 | 0 | 0 | 0 | 0 | 0 | 0 | 0 | 182 | 193 |  |  | PEAKS DB |
| K.STTDLPSR.F | N | 52.16 | 875.4348 | 8 | 0.4 | 438.7249 | 2 | 21.40 | 6 | F6:8926 | 17012022\_RID\_3262\_9a.raw |  | 2.9854E7 |  | 4.8043E6 |  | 1.4473E7 | 1.9071E6 |  |  |  | 4 | 0 | 1 | 0 | 1 | 0 | 1 | 1 | 0 | 0 | 0 | 348 | 355 |  |  | PEAKS DB |
| S.AAYVLAGAGHQVTVLEASER.A | N | 50.51 | 2041.0592 | 20 | 1.3 | 681.3612 | 3 | 40.58 | 1 | F1:26109 | 13012022\_RID\_3262\_4a.raw | 5.4454E5 |  |  |  |  |  |  |  |  |  | 1 | 1 | 0 | 0 | 0 | 0 | 0 | 0 | 0 | 0 | 0 | 50 | 69 |  |  | PEAKS DB |
| A.GAGHQVTVLEASER.A | N | 49.80 | 1452.7321 | 14 | 0.7 | 727.3738 | 2 | 23.21 | 9 | F9:7765 | 31122021\_RID\_3262\_2a.raw | 4.3064E5 |  |  |  |  | 6.0642E5 |  | 6.5701E4 | 8.2054E4 |  | 4 | 1 | 0 | 0 | 0 | 0 | 1 | 0 | 1 | 1 | 0 | 56 | 69 |  |  | PEAKS DB |
| K.KDPGLLQYPVKPSEEGK.S | Y | 49.14 | 1883.9991 | 17 | -0.7 | 472.0067 | 4 | 24.47 | 1 | F1:11373 | 13012022\_RID\_3262\_4a.raw | 3.2487E6 |  |  |  |  |  |  |  |  |  | 1 | 1 | 0 | 0 | 0 | 0 | 0 | 0 | 0 | 0 | 0 | 137 | 153 |  |  | PEAKS DB |
| T.FEPPLPPK.K | N | 46.74 | 923.5116 | 8 | -0.6 | 462.7628 | 2 | 36.61 | 4 | F4:21855 | 17012022\_RID\_3262\_10a .raw | 8.6451E7 | 2.7491E7 |  | 4.4878E7 |  |  |  | 7.8215E6 | 8.5594E6 |  | 5 | 1 | 1 | 0 | 1 | 0 | 0 | 0 | 1 | 1 | 0 | 306 | 313 |  |  | PEAKS DB |
| T.FEPPLPPKK.A | N | 43.61 | 1051.6066 | 9 | 0.7 | 526.8109 | 2 | 29.21 | 4 | F4:15245 | 17012022\_RID\_3262\_10a .raw |  |  |  | 4.2511E6 |  |  |  |  |  |  | 1 | 0 | 0 | 0 | 1 | 0 | 0 | 0 | 0 | 0 | 0 | 306 | 314 |  |  | PEAKS DB |
| Q.VTVLEASER.A | N | 42.68 | 1002.5345 | 9 | -0.1 | 502.2745 | 2 | 28.85 | 7 | F7:11280 | 21012022\_RID\_3262\_7a.raw |  |  |  |  |  |  | 1.7683E5 |  |  |  | 1 | 0 | 0 | 0 | 0 | 0 | 0 | 1 | 0 | 0 | 0 | 61 | 69 |  |  | PEAKS DB |
| A.GHQVTVLEASER.A | N | 42.02 | 1324.6735 | 12 | 1.8 | 663.3452 | 2 | 22.45 | 8 | F8:6784 | 31122021\_RID\_3262\_1a.raw |  |  |  |  |  |  |  | 1.4851E4 |  |  | 1 | 0 | 0 | 0 | 0 | 0 | 0 | 0 | 1 | 0 | 0 | 58 | 69 |  |  | PEAKS DB |
| H.QVTVLEASER.A | N | 41.88 | 1130.5931 | 10 | -0.3 | 566.3036 | 2 | 28.32 | 9 | F9:11956 | 31122021\_RID\_3262\_2a.raw |  |  |  |  |  |  |  |  | 3.805E5 |  | 1 | 0 | 0 | 0 | 0 | 0 | 0 | 0 | 0 | 1 | 0 | 60 | 69 |  |  | PEAKS DB |
| A.AYVLAGAGHQVTVLEASER.A | N | 41.51 | 1970.0221 | 19 | -0.2 | 657.6812 | 3 | 38.48 | 10 | F10:9343 | 31122021\_RID\_3262\_3a.raw |  |  |  |  |  |  |  |  |  | 1.157E6 | 1 | 0 | 0 | 0 | 0 | 0 | 0 | 0 | 0 | 0 | 1 | 51 | 69 |  |  | PEAKS DB |
| total 19 peptides |  |  |  |  |  |  |  |  |  |  |  |  |  |  |  |  |  |  |  |  |  |  |  |  |  |  |  |  |  |  |  |  |  |  |  |  |  |
| --- | --- | --- | --- | --- | --- | --- | --- | --- | --- | --- | --- | --- | --- | --- | --- | --- | --- | --- | --- | --- | --- | --- | --- | --- | --- | --- | --- | --- | --- | --- | --- | --- | --- | --- | --- | --- | --- |

AHZ20792.1

back to list

  

| Protein Coverage
| Supporting Peptides
|

Protein Coverage:

Supporting Peptides:

| Peptide | Uniq | -10lgP | Mass | Length | ppm | m/z | z | RT | Fraction | Scan | Source File | Area F4 | Area F5 | Area F6 | Area F10 | Area F8 | Area F9 | Area F7 | Area F1 | Area F2 | Area F3 | #Feature | #Feature F4 | #Feature F5 | #Feature F6 | #Feature F10 | #Feature F8 | #Feature F9 | #Feature F7 | #Feature F1 | #Feature F2 | #Feature F3 | Start | End | PTM | AScore | Found By |
| --- | --- | --- | --- | --- | --- | --- | --- | --- | --- | --- | --- | --- | --- | --- | --- | --- | --- | --- | --- | --- | --- | --- | --- | --- | --- | --- | --- | --- | --- | --- | --- | --- | --- | --- | --- | --- | --- |
| K.KFWEDDGIHGGK.S | N | 70.84 | 1387.6520 | 12 | 1.0 | 694.8340 | 2 | 27.52 | 1 | F1:14353 | 13012022\_RID\_3262\_4a.raw | 2.2688E8 | 4.7295E4 |  |  |  |  |  | 1.1825E7 | 1.8101E7 | 8.0079E8 | 17 | 4 | 1 | 0 | 0 | 0 | 0 | 0 | 3 | 3 | 6 | 336 | 347 |  |  | PEAKS DB |
| Q.YPVKPSEEGK.S | N | 69.75 | 1132.5764 | 10 | 0.7 | 567.2959 | 2 | 17.88 | 6 | F6:5952 | 17012022\_RID\_3262\_9a.raw | 4.2299E8 | 5.2685E7 | 2.5077E5 | 3.2771E7 |  | 6.9462E7 | 2.576E7 |  |  |  | 12 | 2 | 2 | 1 | 3 | 0 | 2 | 2 | 0 | 0 | 0 | 144 | 153 |  |  | PEAKS DB |
| L.SAAYVLAGAGHQVTVLEASER.A | N | 67.91 | 2128.0913 | 21 | 0.0 | 1065.0530 | 2 | 41.69 | 10 | F10:10487 | 31122021\_RID\_3262\_3a.raw | 1.4728E6 |  |  |  |  |  |  | 6.4802E5 | 6.599E5 | 2.6305E7 | 6 | 1 | 0 | 0 | 0 | 0 | 0 | 0 | 2 | 1 | 2 | 49 | 69 |  |  | PEAKS DB |
| K.FWEDDGIHGGK.S | N | 65.97 | 1259.5570 | 11 | 0.4 | 630.7860 | 2 | 31.13 | 1 | F1:17598 | 13012022\_RID\_3262\_4a.raw | 7.5921E7 | 9.0996E6 |  |  |  | 1.9943E6 |  | 6.128E6 | 1.0893E7 | 5.5749E8 | 17 | 2 | 2 | 0 | 0 | 0 | 1 | 0 | 3 | 3 | 6 | 337 | 347 |  |  | PEAKS DB |
| A.YVLAGAGHQVTVLEASER.A | N | 64.94 | 1898.9850 | 18 | 0.8 | 950.5005 | 2 | 37.54 | 10 | F10:8957 | 31122021\_RID\_3262\_3a.raw | 9.515E5 |  |  |  |  |  |  |  |  | 6.9519E6 | 3 | 1 | 0 | 0 | 0 | 0 | 0 | 0 | 0 | 0 | 2 | 52 | 69 |  |  | PEAKS DB |
| K.SAGQLYEESLGK.V | N | 63.95 | 1280.6248 | 12 | 0.0 | 641.3196 | 2 | 33.66 | 2 | F2:17391 | 13012022\_RID\_3262\_5a.raw |  | 2.9891E7 |  |  |  |  |  | 1.4396E5 |  |  | 2 | 0 | 1 | 0 | 0 | 0 | 0 | 0 | 1 | 0 | 0 | 154 | 165 |  |  | PEAKS DB |
| Y.VLAGAGHQVTVLEASER.A | N | 61.46 | 1735.9216 | 17 | 0.9 | 579.6483 | 3 | 33.06 | 6 | F6:19337 | 17012022\_RID\_3262\_9a.raw | 2.8948E5 | 1.1934E6 |  | 6.6625E5 |  | 3.8459E6 |  | 1.4002E6 | 1.9495E6 | 3.1893E7 | 10 | 1 | 1 | 0 | 1 | 0 | 1 | 0 | 2 | 2 | 2 | 53 | 69 |  |  | PEAKS DB |
| D.YEEFLEIAR.N | N | 54.84 | 1168.5764 | 9 | 0.3 | 585.2957 | 2 | 46.57 | 4 | F4:30508 | 17012022\_RID\_3262\_10a .raw | 2.2869E6 |  |  | 1.3254E6 |  |  |  | 2.3858E5 | 1.5579E5 | 1.3646E7 | 5 | 1 | 0 | 0 | 1 | 0 | 0 | 0 | 1 | 1 | 1 | 18 | 26 |  |  | PEAKS DB |
| K.YDTYSTKEYLIK.E | N | 53.18 | 1522.7555 | 12 | 0.9 | 762.3857 | 2 | 34.13 | 1 | F1:20150 | 13012022\_RID\_3262\_4a.raw | 4.6273E6 |  |  |  |  |  |  |  |  |  | 2 | 2 | 0 | 0 | 0 | 0 | 0 | 0 | 0 | 0 | 0 | 182 | 193 |  |  | PEAKS DB |
| K.STTDLPSR.F | N | 52.16 | 875.4348 | 8 | 0.4 | 438.7249 | 2 | 21.40 | 6 | F6:8926 | 17012022\_RID\_3262\_9a.raw |  | 2.9854E7 |  | 4.8043E6 |  | 1.4473E7 | 1.9071E6 |  |  |  | 4 | 0 | 1 | 0 | 1 | 0 | 1 | 1 | 0 | 0 | 0 | 348 | 355 |  |  | PEAKS DB |
| S.AAYVLAGAGHQVTVLEASER.A | N | 50.51 | 2041.0592 | 20 | 1.3 | 681.3612 | 3 | 40.58 | 1 | F1:26109 | 13012022\_RID\_3262\_4a.raw | 5.4454E5 |  |  |  |  |  |  |  |  |  | 1 | 1 | 0 | 0 | 0 | 0 | 0 | 0 | 0 | 0 | 0 | 50 | 69 |  |  | PEAKS DB |
| A.GAGHQVTVLEASER.A | N | 49.80 | 1452.7321 | 14 | 0.7 | 727.3738 | 2 | 23.21 | 9 | F9:7765 | 31122021\_RID\_3262\_2a.raw | 4.3064E5 |  |  |  |  | 6.0642E5 |  | 6.5701E4 | 8.2054E4 |  | 4 | 1 | 0 | 0 | 0 | 0 | 1 | 0 | 1 | 1 | 0 | 56 | 69 |  |  | PEAKS DB |
| K.KDPGLLQYPVKPSEEGK.S | Y | 49.14 | 1883.9991 | 17 | -0.7 | 472.0067 | 4 | 24.47 | 1 | F1:11373 | 13012022\_RID\_3262\_4a.raw | 3.2487E6 |  |  |  |  |  |  |  |  |  | 1 | 1 | 0 | 0 | 0 | 0 | 0 | 0 | 0 | 0 | 0 | 137 | 153 |  |  | PEAKS DB |
| T.FEPPLPPK.K | N | 46.74 | 923.5116 | 8 | -0.6 | 462.7628 | 2 | 36.61 | 4 | F4:21855 | 17012022\_RID\_3262\_10a .raw | 8.6451E7 | 2.7491E7 |  | 4.4878E7 |  |  |  | 7.8215E6 | 8.5594E6 |  | 5 | 1 | 1 | 0 | 1 | 0 | 0 | 0 | 1 | 1 | 0 | 306 | 313 |  |  | PEAKS DB |
| T.FEPPLPPKK.A | N | 43.61 | 1051.6066 | 9 | 0.7 | 526.8109 | 2 | 29.21 | 4 | F4:15245 | 17012022\_RID\_3262\_10a .raw |  |  |  | 4.2511E6 |  |  |  |  |  |  | 1 | 0 | 0 | 0 | 1 | 0 | 0 | 0 | 0 | 0 | 0 | 306 | 314 |  |  | PEAKS DB |
| Q.VTVLEASER.A | N | 42.68 | 1002.5345 | 9 | -0.1 | 502.2745 | 2 | 28.85 | 7 | F7:11280 | 21012022\_RID\_3262\_7a.raw |  |  |  |  |  |  | 1.7683E5 |  |  |  | 1 | 0 | 0 | 0 | 0 | 0 | 0 | 1 | 0 | 0 | 0 | 61 | 69 |  |  | PEAKS DB |
| A.GHQVTVLEASER.A | N | 42.02 | 1324.6735 | 12 | 1.8 | 663.3452 | 2 | 22.45 | 8 | F8:6784 | 31122021\_RID\_3262\_1a.raw |  |  |  |  |  |  |  | 1.4851E4 |  |  | 1 | 0 | 0 | 0 | 0 | 0 | 0 | 0 | 1 | 0 | 0 | 58 | 69 |  |  | PEAKS DB |
| H.QVTVLEASER.A | N | 41.88 | 1130.5931 | 10 | -0.3 | 566.3036 | 2 | 28.32 | 9 | F9:11956 | 31122021\_RID\_3262\_2a.raw |  |  |  |  |  |  |  |  | 3.805E5 |  | 1 | 0 | 0 | 0 | 0 | 0 | 0 | 0 | 0 | 1 | 0 | 60 | 69 |  |  | PEAKS DB |
| A.AYVLAGAGHQVTVLEASER.A | N | 41.51 | 1970.0221 | 19 | -0.2 | 657.6812 | 3 | 38.48 | 10 | F10:9343 | 31122021\_RID\_3262\_3a.raw |  |  |  |  |  |  |  |  |  | 1.157E6 | 1 | 0 | 0 | 0 | 0 | 0 | 0 | 0 | 0 | 0 | 1 | 51 | 69 |  |  | PEAKS DB |
| total 19 peptides |  |  |  |  |  |  |  |  |  |  |  |  |  |  |  |  |  |  |  |  |  |  |  |  |  |  |  |  |  |  |  |  |  |  |  |  |  |
| --- | --- | --- | --- | --- | --- | --- | --- | --- | --- | --- | --- | --- | --- | --- | --- | --- | --- | --- | --- | --- | --- | --- | --- | --- | --- | --- | --- | --- | --- | --- | --- | --- | --- | --- | --- | --- | --- |

BAP39972.1

back to list

  

| Protein Coverage
| Supporting Peptides
|

Protein Coverage:

Supporting Peptides:

| Peptide | Uniq | -10lgP | Mass | Length | ppm | m/z | z | RT | Fraction | Scan | Source File | Area F4 | Area F5 | Area F6 | Area F10 | Area F8 | Area F9 | Area F7 | Area F1 | Area F2 | Area F3 | #Feature | #Feature F4 | #Feature F5 | #Feature F6 | #Feature F10 | #Feature F8 | #Feature F9 | #Feature F7 | #Feature F1 | #Feature F2 | #Feature F3 | Start | End | PTM | AScore | Found By |
| --- | --- | --- | --- | --- | --- | --- | --- | --- | --- | --- | --- | --- | --- | --- | --- | --- | --- | --- | --- | --- | --- | --- | --- | --- | --- | --- | --- | --- | --- | --- | --- | --- | --- | --- | --- | --- | --- |
| K.HANFPILSANIRPK.G | N | 73.91 | 1576.8838 | 14 | 0.3 | 526.6354 | 3 | 33.47 | 9 | F9:16191 | 31122021\_RID\_3262\_2a.raw | 2.4791E6 |  |  |  |  |  |  | 7.6122E5 | 1.3586E7 |  | 5 | 1 | 0 | 0 | 0 | 0 | 0 | 0 | 2 | 2 | 0 | 151 | 164 |  |  | PEAKS DB |
| G.SFELTILHTNDVHAR.V | N | 71.46 | 1751.8955 | 15 | 0.2 | 584.9725 | 3 | 37.82 | 9 | F9:19802 | 31122021\_RID\_3262\_2a.raw |  |  |  |  |  |  |  |  | 9.8903E6 |  | 1 | 0 | 0 | 0 | 0 | 0 | 0 | 0 | 0 | 1 | 0 | 44 | 58 |  |  | PEAKS DB |
| R.HGQGM(+15.99)GELLQVSGIK.V | N | 64.12 | 1568.7981 | 15 | 0.8 | 785.4069 | 2 | 32.16 | 9 | F9:15220 | 31122021\_RID\_3262\_2a.raw | 3.4878E6 |  |  |  |  |  |  |  | 5.8977E6 |  | 4 | 2 | 0 | 0 | 0 | 0 | 0 | 0 | 0 | 2 | 0 | 460 | 474 | Oxidation (M) | M5:Oxidation (M):1000.00 | PEAKS DB |
| R.QVPVVQAYAFGK.Y | N | 64.12 | 1305.7081 | 12 | 0.7 | 653.8618 | 2 | 57.55 | 1 | F1:40709 | 13012022\_RID\_3262\_4a.raw | 4.6454E6 | 1.1825E6 |  |  |  |  |  | 3.0239E5 | 6.0962E6 |  | 5 | 2 | 1 | 0 | 0 | 0 | 0 | 0 | 1 | 1 | 0 | 291 | 302 |  |  | PEAKS DB |
| K.YLGYLNVIFDDKGNVIK.A | N | 63.64 | 1970.0513 | 17 | 0.7 | 986.0336 | 2 | 55.25 | 9 | F9:33414 | 31122021\_RID\_3262\_2a.raw |  |  |  |  |  |  |  |  | 5.214E6 |  | 2 | 0 | 0 | 0 | 0 | 0 | 0 | 0 | 0 | 2 | 0 | 303 | 319 |  |  | PEAKS DB |
| R.DEVEELQNHANK.L | N | 59.76 | 1424.6532 | 12 | 0.7 | 713.3344 | 2 | 23.15 | 9 | F9:7692 | 31122021\_RID\_3262\_2a.raw |  |  |  |  |  |  |  |  | 1.0268E6 |  | 1 | 0 | 0 | 0 | 0 | 0 | 0 | 0 | 0 | 1 | 0 | 212 | 223 |  |  | PEAKS DB |
| K.LLLPSFLAAGGDGYHML.K | N | 55.39 | 1773.9124 | 17 | 0.2 | 887.9636 | 2 | 68.35 | 9 | F9:42925 | 31122021\_RID\_3262\_2a.raw |  |  |  |  |  |  |  |  | 5.8724E5 |  | 1 | 0 | 0 | 0 | 0 | 0 | 0 | 0 | 0 | 1 | 0 | 513 | 529 |  |  | PEAKS DB |
| K.VYPAVEGR.V | Y | 52.63 | 889.4657 | 8 | 0.2 | 445.7402 | 2 | 22.85 | 9 | F9:7430 | 31122021\_RID\_3262\_2a.raw | 1.2181E7 |  |  |  |  |  |  |  | 1.5359E7 |  | 2 | 1 | 0 | 0 | 0 | 0 | 0 | 0 | 0 | 1 | 0 | 556 | 563 |  |  | PEAKS DB |
| K.LLLPSFLAAGGDGYHM(+15.99)LK.G | N | 51.71 | 1918.0022 | 18 | -0.6 | 960.0078 | 2 | 57.15 | 9 | F9:34949 | 31122021\_RID\_3262\_2a.raw |  |  |  |  |  |  |  |  | 3.7896E5 |  | 1 | 0 | 0 | 0 | 0 | 0 | 0 | 0 | 0 | 1 | 0 | 513 | 530 | Oxidation (M) | M16:Oxidation (M):1000.00 | PEAKS DB |
| K.VGIIGYTTK.E | N | 51.25 | 950.5436 | 9 | 0.4 | 476.2793 | 2 | 33.16 | 9 | F9:16043 | 31122021\_RID\_3262\_2a.raw |  | 2.6943E6 | 7.1467E5 |  |  |  | 1.5898E5 |  | 8.0892E6 |  | 4 | 0 | 1 | 1 | 0 | 0 | 0 | 1 | 0 | 1 | 0 | 188 | 196 |  |  | PEAKS DB |
| K.LLLPSFLAAGGDGYH.M | N | 48.30 | 1529.7877 | 15 | 0.9 | 765.9019 | 2 | 61.99 | 9 | F9:38681 | 31122021\_RID\_3262\_2a.raw |  |  |  |  |  |  |  |  | 3.6135E5 |  | 1 | 0 | 0 | 0 | 0 | 0 | 0 | 0 | 0 | 1 | 0 | 513 | 527 |  |  | PEAKS DB |
| K.LLLPSFLAAGGDGYHM(+15.99)L.K | N | 46.54 | 1789.9072 | 17 | 1.0 | 895.9618 | 2 | 63.49 | 9 | F9:39581 | 31122021\_RID\_3262\_2a.raw |  |  |  |  |  |  |  |  | 1.84E5 |  | 1 | 0 | 0 | 0 | 0 | 0 | 0 | 0 | 0 | 1 | 0 | 513 | 529 | Oxidation (M) | M16:Oxidation (M):1000.00 | PEAKS DB |
| K.VVYDLSR.K | N | 46.31 | 850.4548 | 7 | 0.5 | 426.2349 | 2 | 30.36 | 1 | F1:16753 | 13012022\_RID\_3262\_4a.raw | 3.9579E6 | 3.0633E6 |  |  |  |  |  |  | 6.9401E6 |  | 3 | 1 | 1 | 0 | 0 | 0 | 0 | 0 | 0 | 1 | 0 | 475 | 481 |  |  | PEAKS DB |
| K.HANFPILSAN.I | N | 46.30 | 1082.5509 | 10 | 0.2 | 542.2828 | 2 | 40.87 | 9 | F9:22309 | 31122021\_RID\_3262\_2a.raw |  |  |  |  |  |  |  |  | 2.7202E6 |  | 1 | 0 | 0 | 0 | 0 | 0 | 0 | 0 | 0 | 1 | 0 | 151 | 160 |  |  | PEAKS DB |
| K.LLLPSFLAAGGDGYHM.L | N | 46.03 | 1660.8282 | 16 | 0.2 | 831.4216 | 2 | 65.01 | 9 | F9:40717 | 31122021\_RID\_3262\_2a.raw |  |  |  |  |  |  |  |  | 7.391E4 |  | 1 | 0 | 0 | 0 | 0 | 0 | 0 | 0 | 0 | 1 | 0 | 513 | 528 |  |  | PEAKS DB |
| K.LLLPSFLAAGGDGYHMLK.G | N | 45.84 | 1902.0073 | 18 | 0.1 | 635.0098 | 3 | 57.14 | 9 | F9:35003 | 31122021\_RID\_3262\_2a.raw | 6.1858E5 |  |  |  |  |  |  |  | 5.9658E6 |  | 2 | 1 | 0 | 0 | 0 | 0 | 0 | 0 | 0 | 1 | 0 | 513 | 530 |  |  | PEAKS DB |
| K.LLLPSFLAAGGD.G | N | 45.25 | 1172.6440 | 12 | -0.2 | 587.3292 | 2 | 72.39 | 9 | F9:44726 | 31122021\_RID\_3262\_2a.raw |  |  |  |  |  |  |  |  | 1.0233E5 |  | 1 | 0 | 0 | 0 | 0 | 0 | 0 | 0 | 0 | 1 | 0 | 513 | 524 |  |  | PEAKS DB |
| N.LDISIVGDYIK.R | N | 44.01 | 1234.6809 | 11 | 0.5 | 618.3480 | 2 | 56.58 | 9 | F9:34546 | 31122021\_RID\_3262\_2a.raw |  |  |  |  |  |  |  |  | 2.949E4 |  | 1 | 0 | 0 | 0 | 0 | 0 | 0 | 0 | 0 | 1 | 0 | 541 | 551 |  |  | PEAKS DB |
| R.YDAMALGNHEFDNGLAGLLDPLLK.H | N | 43.69 | 2586.2788 | 24 | 0.6 | 863.1007 | 3 | 69.00 | 9 | F9:43247 | 31122021\_RID\_3262\_2a.raw |  |  |  |  |  |  |  |  | 9.895E5 |  | 1 | 0 | 0 | 0 | 0 | 0 | 0 | 0 | 0 | 1 | 0 | 127 | 150 |  |  | PEAKS DB |
| K.LTTLGVNK.I | N | 43.24 | 844.5018 | 8 | 0.8 | 423.2585 | 2 | 24.48 | 9 | F9:8941 | 31122021\_RID\_3262\_2a.raw |  |  |  |  |  |  |  |  | 1.1856E7 |  | 1 | 0 | 0 | 0 | 0 | 0 | 0 | 0 | 0 | 1 | 0 | 224 | 231 |  |  | PEAKS DB |
| F.GGTFDLLQIK.G | N | 43.16 | 1090.6022 | 10 | -0.1 | 546.3083 | 2 | 49.90 | 9 | F9:29347 | 31122021\_RID\_3262\_2a.raw |  |  |  |  |  |  |  |  | 6.6936E5 |  | 1 | 0 | 0 | 0 | 0 | 0 | 0 | 0 | 0 | 1 | 0 | 436 | 445 |  |  | PEAKS DB |
| K.YLGYLNVIFDDK.G | N | 41.85 | 1458.7394 | 12 | 1.3 | 730.3779 | 2 | 60.34 | 9 | F9:37272 | 31122021\_RID\_3262\_2a.raw |  |  |  |  |  |  |  |  | 2.5015E6 |  | 1 | 0 | 0 | 0 | 0 | 0 | 0 | 0 | 0 | 1 | 0 | 303 | 314 |  |  | PEAKS DB |
| total 22 peptides |  |  |  |  |  |  |  |  |  |  |  |  |  |  |  |  |  |  |  |  |  |  |  |  |  |  |  |  |  |  |  |  |  |  |  |  |  |
| --- | --- | --- | --- | --- | --- | --- | --- | --- | --- | --- | --- | --- | --- | --- | --- | --- | --- | --- | --- | --- | --- | --- | --- | --- | --- | --- | --- | --- | --- | --- | --- | --- | --- | --- | --- | --- | --- |

XP\_015668350.1

back to list

  

| Protein Coverage
| Supporting Peptides
|

Protein Coverage:

Supporting Peptides:

| Peptide | Uniq | -10lgP | Mass | Length | ppm | m/z | z | RT | Fraction | Scan | Source File | Area F4 | Area F5 | Area F6 | Area F10 | Area F8 | Area F9 | Area F7 | Area F1 | Area F2 | Area F3 | #Feature | #Feature F4 | #Feature F5 | #Feature F6 | #Feature F10 | #Feature F8 | #Feature F9 | #Feature F7 | #Feature F1 | #Feature F2 | #Feature F3 | Start | End | PTM | AScore | Found By |
| --- | --- | --- | --- | --- | --- | --- | --- | --- | --- | --- | --- | --- | --- | --- | --- | --- | --- | --- | --- | --- | --- | --- | --- | --- | --- | --- | --- | --- | --- | --- | --- | --- | --- | --- | --- | --- | --- |
| K.HANFPILSANIRPK.G | N | 73.91 | 1576.8838 | 14 | 0.3 | 526.6354 | 3 | 33.47 | 9 | F9:16191 | 31122021\_RID\_3262\_2a.raw | 2.4791E6 |  |  |  |  |  |  | 7.6122E5 | 1.3586E7 |  | 5 | 1 | 0 | 0 | 0 | 0 | 0 | 0 | 2 | 2 | 0 | 148 | 161 |  |  | PEAKS DB |
| G.SFELTILHTNDVHAR.V | N | 71.46 | 1751.8955 | 15 | 0.2 | 584.9725 | 3 | 37.82 | 9 | F9:19802 | 31122021\_RID\_3262\_2a.raw |  |  |  |  |  |  |  |  | 9.8903E6 |  | 1 | 0 | 0 | 0 | 0 | 0 | 0 | 0 | 0 | 1 | 0 | 41 | 55 |  |  | PEAKS DB |
| R.HGQGM(+15.99)GELLQVSGIK.V | N | 64.12 | 1568.7981 | 15 | 0.8 | 785.4069 | 2 | 32.16 | 9 | F9:15220 | 31122021\_RID\_3262\_2a.raw | 3.4878E6 |  |  |  |  |  |  |  | 5.8977E6 |  | 4 | 2 | 0 | 0 | 0 | 0 | 0 | 0 | 0 | 2 | 0 | 457 | 471 | Oxidation (M) | M5:Oxidation (M):1000.00 | PEAKS DB |
| R.QVPVVQAYAFGK.Y | N | 64.12 | 1305.7081 | 12 | 0.7 | 653.8618 | 2 | 57.55 | 1 | F1:40709 | 13012022\_RID\_3262\_4a.raw | 4.6454E6 | 1.1825E6 |  |  |  |  |  | 3.0239E5 | 6.0962E6 |  | 5 | 2 | 1 | 0 | 0 | 0 | 0 | 0 | 1 | 1 | 0 | 288 | 299 |  |  | PEAKS DB |
| K.YLGYLNVIFDDKGNVIK.A | N | 63.64 | 1970.0513 | 17 | 0.7 | 986.0336 | 2 | 55.25 | 9 | F9:33414 | 31122021\_RID\_3262\_2a.raw |  |  |  |  |  |  |  |  | 5.214E6 |  | 2 | 0 | 0 | 0 | 0 | 0 | 0 | 0 | 0 | 2 | 0 | 300 | 316 |  |  | PEAKS DB |
| R.DEVEELQNHANK.L | N | 59.76 | 1424.6532 | 12 | 0.7 | 713.3344 | 2 | 23.15 | 9 | F9:7692 | 31122021\_RID\_3262\_2a.raw |  |  |  |  |  |  |  |  | 1.0268E6 |  | 1 | 0 | 0 | 0 | 0 | 0 | 0 | 0 | 0 | 1 | 0 | 209 | 220 |  |  | PEAKS DB |
| K.LLLPSFLAAGGDGYHML.K | N | 55.39 | 1773.9124 | 17 | 0.2 | 887.9636 | 2 | 68.35 | 9 | F9:42925 | 31122021\_RID\_3262\_2a.raw |  |  |  |  |  |  |  |  | 5.8724E5 |  | 1 | 0 | 0 | 0 | 0 | 0 | 0 | 0 | 0 | 1 | 0 | 510 | 526 |  |  | PEAKS DB |
| K.VYPAVEGR.V | Y | 52.63 | 889.4657 | 8 | 0.2 | 445.7402 | 2 | 22.85 | 9 | F9:7430 | 31122021\_RID\_3262\_2a.raw | 1.2181E7 |  |  |  |  |  |  |  | 1.5359E7 |  | 2 | 1 | 0 | 0 | 0 | 0 | 0 | 0 | 0 | 1 | 0 | 553 | 560 |  |  | PEAKS DB |
| K.LLLPSFLAAGGDGYHM(+15.99)LK.G | N | 51.71 | 1918.0022 | 18 | -0.6 | 960.0078 | 2 | 57.15 | 9 | F9:34949 | 31122021\_RID\_3262\_2a.raw |  |  |  |  |  |  |  |  | 3.7896E5 |  | 1 | 0 | 0 | 0 | 0 | 0 | 0 | 0 | 0 | 1 | 0 | 510 | 527 | Oxidation (M) | M16:Oxidation (M):1000.00 | PEAKS DB |
| K.VGIIGYTTK.E | N | 51.25 | 950.5436 | 9 | 0.4 | 476.2793 | 2 | 33.16 | 9 | F9:16043 | 31122021\_RID\_3262\_2a.raw |  | 2.6943E6 | 7.1467E5 |  |  |  | 1.5898E5 |  | 8.0892E6 |  | 4 | 0 | 1 | 1 | 0 | 0 | 0 | 1 | 0 | 1 | 0 | 185 | 193 |  |  | PEAKS DB |
| K.LLLPSFLAAGGDGYH.M | N | 48.30 | 1529.7877 | 15 | 0.9 | 765.9019 | 2 | 61.99 | 9 | F9:38681 | 31122021\_RID\_3262\_2a.raw |  |  |  |  |  |  |  |  | 3.6135E5 |  | 1 | 0 | 0 | 0 | 0 | 0 | 0 | 0 | 0 | 1 | 0 | 510 | 524 |  |  | PEAKS DB |
| K.LLLPSFLAAGGDGYHM(+15.99)L.K | N | 46.54 | 1789.9072 | 17 | 1.0 | 895.9618 | 2 | 63.49 | 9 | F9:39581 | 31122021\_RID\_3262\_2a.raw |  |  |  |  |  |  |  |  | 1.84E5 |  | 1 | 0 | 0 | 0 | 0 | 0 | 0 | 0 | 0 | 1 | 0 | 510 | 526 | Oxidation (M) | M16:Oxidation (M):1000.00 | PEAKS DB |
| K.VVYDLSR.K | N | 46.31 | 850.4548 | 7 | 0.5 | 426.2349 | 2 | 30.36 | 1 | F1:16753 | 13012022\_RID\_3262\_4a.raw | 3.9579E6 | 3.0633E6 |  |  |  |  |  |  | 6.9401E6 |  | 3 | 1 | 1 | 0 | 0 | 0 | 0 | 0 | 0 | 1 | 0 | 472 | 478 |  |  | PEAKS DB |
| K.HANFPILSAN.I | N | 46.30 | 1082.5509 | 10 | 0.2 | 542.2828 | 2 | 40.87 | 9 | F9:22309 | 31122021\_RID\_3262\_2a.raw |  |  |  |  |  |  |  |  | 2.7202E6 |  | 1 | 0 | 0 | 0 | 0 | 0 | 0 | 0 | 0 | 1 | 0 | 148 | 157 |  |  | PEAKS DB |
| K.LLLPSFLAAGGDGYHM.L | N | 46.03 | 1660.8282 | 16 | 0.2 | 831.4216 | 2 | 65.01 | 9 | F9:40717 | 31122021\_RID\_3262\_2a.raw |  |  |  |  |  |  |  |  | 7.391E4 |  | 1 | 0 | 0 | 0 | 0 | 0 | 0 | 0 | 0 | 1 | 0 | 510 | 525 |  |  | PEAKS DB |
| K.LLLPSFLAAGGDGYHMLK.G | N | 45.84 | 1902.0073 | 18 | 0.1 | 635.0098 | 3 | 57.14 | 9 | F9:35003 | 31122021\_RID\_3262\_2a.raw | 6.1858E5 |  |  |  |  |  |  |  | 5.9658E6 |  | 2 | 1 | 0 | 0 | 0 | 0 | 0 | 0 | 0 | 1 | 0 | 510 | 527 |  |  | PEAKS DB |
| K.LLLPSFLAAGGD.G | N | 45.25 | 1172.6440 | 12 | -0.2 | 587.3292 | 2 | 72.39 | 9 | F9:44726 | 31122021\_RID\_3262\_2a.raw |  |  |  |  |  |  |  |  | 1.0233E5 |  | 1 | 0 | 0 | 0 | 0 | 0 | 0 | 0 | 0 | 1 | 0 | 510 | 521 |  |  | PEAKS DB |
| N.LDISIVGDYIK.R | N | 44.01 | 1234.6809 | 11 | 0.5 | 618.3480 | 2 | 56.58 | 9 | F9:34546 | 31122021\_RID\_3262\_2a.raw |  |  |  |  |  |  |  |  | 2.949E4 |  | 1 | 0 | 0 | 0 | 0 | 0 | 0 | 0 | 0 | 1 | 0 | 538 | 548 |  |  | PEAKS DB |
| R.YDAMALGNHEFDNGLAGLLDPLLK.H | N | 43.69 | 2586.2788 | 24 | 0.6 | 863.1007 | 3 | 69.00 | 9 | F9:43247 | 31122021\_RID\_3262\_2a.raw |  |  |  |  |  |  |  |  | 9.895E5 |  | 1 | 0 | 0 | 0 | 0 | 0 | 0 | 0 | 0 | 1 | 0 | 124 | 147 |  |  | PEAKS DB |
| K.LTTLGVNK.I | N | 43.24 | 844.5018 | 8 | 0.8 | 423.2585 | 2 | 24.48 | 9 | F9:8941 | 31122021\_RID\_3262\_2a.raw |  |  |  |  |  |  |  |  | 1.1856E7 |  | 1 | 0 | 0 | 0 | 0 | 0 | 0 | 0 | 0 | 1 | 0 | 221 | 228 |  |  | PEAKS DB |
| F.GGTFDLLQIK.G | N | 43.16 | 1090.6022 | 10 | -0.1 | 546.3083 | 2 | 49.90 | 9 | F9:29347 | 31122021\_RID\_3262\_2a.raw |  |  |  |  |  |  |  |  | 6.6936E5 |  | 1 | 0 | 0 | 0 | 0 | 0 | 0 | 0 | 0 | 1 | 0 | 433 | 442 |  |  | PEAKS DB |
| K.YLGYLNVIFDDK.G | N | 41.85 | 1458.7394 | 12 | 1.3 | 730.3779 | 2 | 60.34 | 9 | F9:37272 | 31122021\_RID\_3262\_2a.raw |  |  |  |  |  |  |  |  | 2.5015E6 |  | 1 | 0 | 0 | 0 | 0 | 0 | 0 | 0 | 0 | 1 | 0 | 300 | 311 |  |  | PEAKS DB |
| total 22 peptides |  |  |  |  |  |  |  |  |  |  |  |  |  |  |  |  |  |  |  |  |  |  |  |  |  |  |  |  |  |  |  |  |  |  |  |  |  |
| --- | --- | --- | --- | --- | --- | --- | --- | --- | --- | --- | --- | --- | --- | --- | --- | --- | --- | --- | --- | --- | --- | --- | --- | --- | --- | --- | --- | --- | --- | --- | --- | --- | --- | --- | --- | --- | --- |

BAP39925.1

back to list

  

| Protein Coverage
| Supporting Peptides
|

Protein Coverage:

Supporting Peptides:

| Peptide | Uniq | -10lgP | Mass | Length | ppm | m/z | z | RT | Fraction | Scan | Source File | Area F4 | Area F5 | Area F6 | Area F10 | Area F8 | Area F9 | Area F7 | Area F1 | Area F2 | Area F3 | #Feature | #Feature F4 | #Feature F5 | #Feature F6 | #Feature F10 | #Feature F8 | #Feature F9 | #Feature F7 | #Feature F1 | #Feature F2 | #Feature F3 | Start | End | PTM | AScore | Found By |
| --- | --- | --- | --- | --- | --- | --- | --- | --- | --- | --- | --- | --- | --- | --- | --- | --- | --- | --- | --- | --- | --- | --- | --- | --- | --- | --- | --- | --- | --- | --- | --- | --- | --- | --- | --- | --- | --- |
| K.HANFPILSANIRPK.G | N | 73.91 | 1576.8838 | 14 | 0.3 | 526.6354 | 3 | 33.47 | 9 | F9:16191 | 31122021\_RID\_3262\_2a.raw | 2.4791E6 |  |  |  |  |  |  | 7.6122E5 | 1.3586E7 |  | 5 | 1 | 0 | 0 | 0 | 0 | 0 | 0 | 2 | 2 | 0 | 148 | 161 |  |  | PEAKS DB |
| G.SFELTILHTNDVHAR.V | N | 71.46 | 1751.8955 | 15 | 0.2 | 584.9725 | 3 | 37.82 | 9 | F9:19802 | 31122021\_RID\_3262\_2a.raw |  |  |  |  |  |  |  |  | 9.8903E6 |  | 1 | 0 | 0 | 0 | 0 | 0 | 0 | 0 | 0 | 1 | 0 | 41 | 55 |  |  | PEAKS DB |
| R.HGQGM(+15.99)GELLQVSGIK.V | N | 64.12 | 1568.7981 | 15 | 0.8 | 785.4069 | 2 | 32.16 | 9 | F9:15220 | 31122021\_RID\_3262\_2a.raw | 3.4878E6 |  |  |  |  |  |  |  | 5.8977E6 |  | 4 | 2 | 0 | 0 | 0 | 0 | 0 | 0 | 0 | 2 | 0 | 457 | 471 | Oxidation (M) | M5:Oxidation (M):1000.00 | PEAKS DB |
| R.QVPVVQAYAFGK.Y | N | 64.12 | 1305.7081 | 12 | 0.7 | 653.8618 | 2 | 57.55 | 1 | F1:40709 | 13012022\_RID\_3262\_4a.raw | 4.6454E6 | 1.1825E6 |  |  |  |  |  | 3.0239E5 | 6.0962E6 |  | 5 | 2 | 1 | 0 | 0 | 0 | 0 | 0 | 1 | 1 | 0 | 288 | 299 |  |  | PEAKS DB |
| K.YLGYLNVIFDDKGNVIK.A | N | 63.64 | 1970.0513 | 17 | 0.7 | 986.0336 | 2 | 55.25 | 9 | F9:33414 | 31122021\_RID\_3262\_2a.raw |  |  |  |  |  |  |  |  | 5.214E6 |  | 2 | 0 | 0 | 0 | 0 | 0 | 0 | 0 | 0 | 2 | 0 | 300 | 316 |  |  | PEAKS DB |
| R.DEVEELQNHANK.L | N | 59.76 | 1424.6532 | 12 | 0.7 | 713.3344 | 2 | 23.15 | 9 | F9:7692 | 31122021\_RID\_3262\_2a.raw |  |  |  |  |  |  |  |  | 1.0268E6 |  | 1 | 0 | 0 | 0 | 0 | 0 | 0 | 0 | 0 | 1 | 0 | 209 | 220 |  |  | PEAKS DB |
| K.LLLPSFLAAGGDGYHML.K | N | 55.39 | 1773.9124 | 17 | 0.2 | 887.9636 | 2 | 68.35 | 9 | F9:42925 | 31122021\_RID\_3262\_2a.raw |  |  |  |  |  |  |  |  | 5.8724E5 |  | 1 | 0 | 0 | 0 | 0 | 0 | 0 | 0 | 0 | 1 | 0 | 510 | 526 |  |  | PEAKS DB |
| K.VYPAVEGR.V | Y | 52.63 | 889.4657 | 8 | 0.2 | 445.7402 | 2 | 22.85 | 9 | F9:7430 | 31122021\_RID\_3262\_2a.raw | 1.2181E7 |  |  |  |  |  |  |  | 1.5359E7 |  | 2 | 1 | 0 | 0 | 0 | 0 | 0 | 0 | 0 | 1 | 0 | 553 | 560 |  |  | PEAKS DB |
| K.LLLPSFLAAGGDGYHM(+15.99)LK.G | N | 51.71 | 1918.0022 | 18 | -0.6 | 960.0078 | 2 | 57.15 | 9 | F9:34949 | 31122021\_RID\_3262\_2a.raw |  |  |  |  |  |  |  |  | 3.7896E5 |  | 1 | 0 | 0 | 0 | 0 | 0 | 0 | 0 | 0 | 1 | 0 | 510 | 527 | Oxidation (M) | M16:Oxidation (M):1000.00 | PEAKS DB |
| K.VGIIGYTTK.E | N | 51.25 | 950.5436 | 9 | 0.4 | 476.2793 | 2 | 33.16 | 9 | F9:16043 | 31122021\_RID\_3262\_2a.raw |  | 2.6943E6 | 7.1467E5 |  |  |  | 1.5898E5 |  | 8.0892E6 |  | 4 | 0 | 1 | 1 | 0 | 0 | 0 | 1 | 0 | 1 | 0 | 185 | 193 |  |  | PEAKS DB |
| K.LLLPSFLAAGGDGYH.M | N | 48.30 | 1529.7877 | 15 | 0.9 | 765.9019 | 2 | 61.99 | 9 | F9:38681 | 31122021\_RID\_3262\_2a.raw |  |  |  |  |  |  |  |  | 3.6135E5 |  | 1 | 0 | 0 | 0 | 0 | 0 | 0 | 0 | 0 | 1 | 0 | 510 | 524 |  |  | PEAKS DB |
| K.LLLPSFLAAGGDGYHM(+15.99)L.K | N | 46.54 | 1789.9072 | 17 | 1.0 | 895.9618 | 2 | 63.49 | 9 | F9:39581 | 31122021\_RID\_3262\_2a.raw |  |  |  |  |  |  |  |  | 1.84E5 |  | 1 | 0 | 0 | 0 | 0 | 0 | 0 | 0 | 0 | 1 | 0 | 510 | 526 | Oxidation (M) | M16:Oxidation (M):1000.00 | PEAKS DB |
| K.VVYDLSR.K | N | 46.31 | 850.4548 | 7 | 0.5 | 426.2349 | 2 | 30.36 | 1 | F1:16753 | 13012022\_RID\_3262\_4a.raw | 3.9579E6 | 3.0633E6 |  |  |  |  |  |  | 6.9401E6 |  | 3 | 1 | 1 | 0 | 0 | 0 | 0 | 0 | 0 | 1 | 0 | 472 | 478 |  |  | PEAKS DB |
| K.HANFPILSAN.I | N | 46.30 | 1082.5509 | 10 | 0.2 | 542.2828 | 2 | 40.87 | 9 | F9:22309 | 31122021\_RID\_3262\_2a.raw |  |  |  |  |  |  |  |  | 2.7202E6 |  | 1 | 0 | 0 | 0 | 0 | 0 | 0 | 0 | 0 | 1 | 0 | 148 | 157 |  |  | PEAKS DB |
| K.LLLPSFLAAGGDGYHM.L | N | 46.03 | 1660.8282 | 16 | 0.2 | 831.4216 | 2 | 65.01 | 9 | F9:40717 | 31122021\_RID\_3262\_2a.raw |  |  |  |  |  |  |  |  | 7.391E4 |  | 1 | 0 | 0 | 0 | 0 | 0 | 0 | 0 | 0 | 1 | 0 | 510 | 525 |  |  | PEAKS DB |
| K.LLLPSFLAAGGDGYHMLK.G | N | 45.84 | 1902.0073 | 18 | 0.1 | 635.0098 | 3 | 57.14 | 9 | F9:35003 | 31122021\_RID\_3262\_2a.raw | 6.1858E5 |  |  |  |  |  |  |  | 5.9658E6 |  | 2 | 1 | 0 | 0 | 0 | 0 | 0 | 0 | 0 | 1 | 0 | 510 | 527 |  |  | PEAKS DB |
| K.LLLPSFLAAGGD.G | N | 45.25 | 1172.6440 | 12 | -0.2 | 587.3292 | 2 | 72.39 | 9 | F9:44726 | 31122021\_RID\_3262\_2a.raw |  |  |  |  |  |  |  |  | 1.0233E5 |  | 1 | 0 | 0 | 0 | 0 | 0 | 0 | 0 | 0 | 1 | 0 | 510 | 521 |  |  | PEAKS DB |
| N.LDISIVGDYIK.R | N | 44.01 | 1234.6809 | 11 | 0.5 | 618.3480 | 2 | 56.58 | 9 | F9:34546 | 31122021\_RID\_3262\_2a.raw |  |  |  |  |  |  |  |  | 2.949E4 |  | 1 | 0 | 0 | 0 | 0 | 0 | 0 | 0 | 0 | 1 | 0 | 538 | 548 |  |  | PEAKS DB |
| R.YDAMALGNHEFDNGLAGLLDPLLK.H | N | 43.69 | 2586.2788 | 24 | 0.6 | 863.1007 | 3 | 69.00 | 9 | F9:43247 | 31122021\_RID\_3262\_2a.raw |  |  |  |  |  |  |  |  | 9.895E5 |  | 1 | 0 | 0 | 0 | 0 | 0 | 0 | 0 | 0 | 1 | 0 | 124 | 147 |  |  | PEAKS DB |
| K.LTTLGVNK.I | N | 43.24 | 844.5018 | 8 | 0.8 | 423.2585 | 2 | 24.48 | 9 | F9:8941 | 31122021\_RID\_3262\_2a.raw |  |  |  |  |  |  |  |  | 1.1856E7 |  | 1 | 0 | 0 | 0 | 0 | 0 | 0 | 0 | 0 | 1 | 0 | 221 | 228 |  |  | PEAKS DB |
| F.GGTFDLLQIK.G | N | 43.16 | 1090.6022 | 10 | -0.1 | 546.3083 | 2 | 49.90 | 9 | F9:29347 | 31122021\_RID\_3262\_2a.raw |  |  |  |  |  |  |  |  | 6.6936E5 |  | 1 | 0 | 0 | 0 | 0 | 0 | 0 | 0 | 0 | 1 | 0 | 433 | 442 |  |  | PEAKS DB |
| K.YLGYLNVIFDDK.G | N | 41.85 | 1458.7394 | 12 | 1.3 | 730.3779 | 2 | 60.34 | 9 | F9:37272 | 31122021\_RID\_3262\_2a.raw |  |  |  |  |  |  |  |  | 2.5015E6 |  | 1 | 0 | 0 | 0 | 0 | 0 | 0 | 0 | 0 | 1 | 0 | 300 | 311 |  |  | PEAKS DB |
| total 22 peptides |  |  |  |  |  |  |  |  |  |  |  |  |  |  |  |  |  |  |  |  |  |  |  |  |  |  |  |  |  |  |  |  |  |  |  |  |  |
| --- | --- | --- | --- | --- | --- | --- | --- | --- | --- | --- | --- | --- | --- | --- | --- | --- | --- | --- | --- | --- | --- | --- | --- | --- | --- | --- | --- | --- | --- | --- | --- | --- | --- | --- | --- | --- | --- |

JAV51280.1

back to list

  

| Protein Coverage
| Supporting Peptides
|

Protein Coverage:

Supporting Peptides:

| Peptide | Uniq | -10lgP | Mass | Length | ppm | m/z | z | RT | Fraction | Scan | Source File | Area F4 | Area F5 | Area F6 | Area F10 | Area F8 | Area F9 | Area F7 | Area F1 | Area F2 | Area F3 | #Feature | #Feature F4 | #Feature F5 | #Feature F6 | #Feature F10 | #Feature F8 | #Feature F9 | #Feature F7 | #Feature F1 | #Feature F2 | #Feature F3 | Start | End | PTM | AScore | Found By |
| --- | --- | --- | --- | --- | --- | --- | --- | --- | --- | --- | --- | --- | --- | --- | --- | --- | --- | --- | --- | --- | --- | --- | --- | --- | --- | --- | --- | --- | --- | --- | --- | --- | --- | --- | --- | --- | --- |
| R.AGFIDDAFALAR.A | N | 72.58 | 1265.6404 | 12 | 0.1 | 633.8275 | 2 | 56.96 | 8 | F8:33821 | 31122021\_RID\_3262\_1a.raw |  |  |  |  |  |  |  | 5.6661E6 | 2.6325E5 |  | 2 | 0 | 0 | 0 | 0 | 0 | 0 | 0 | 1 | 1 | 0 | 655 | 666 |  |  | PEAKS DB |
| K.SELAGITITRPSDLPPDSFLK.V | Y | 68.30 | 2256.2002 | 21 | 0.8 | 753.0746 | 3 | 52.71 | 8 | F8:30323 | 31122021\_RID\_3262\_1a.raw |  |  |  |  |  |  |  | 2.9127E6 |  |  | 1 | 0 | 0 | 0 | 0 | 0 | 0 | 0 | 1 | 0 | 0 | 597 | 617 |  |  | PEAKS DB |
| R.YPDAGAGEASR.K | N | 66.31 | 1092.4835 | 11 | 0.5 | 547.2493 | 2 | 19.80 | 8 | F8:4990 | 31122021\_RID\_3262\_1a.raw |  |  |  |  |  |  |  | 7.2199E5 |  |  | 1 | 0 | 0 | 0 | 0 | 0 | 0 | 0 | 1 | 0 | 0 | 914 | 924 |  |  | PEAKS DB |
| K.YIAATDHEPTDAR.K | N | 65.74 | 1458.6738 | 13 | 0.3 | 730.3444 | 2 | 20.97 | 8 | F8:5743 | 31122021\_RID\_3262\_1a.raw | 3.9385E5 | 5.7971E4 |  |  |  |  |  | 4.8623E5 | 2.3515E5 |  | 5 | 1 | 1 | 0 | 0 | 0 | 0 | 0 | 2 | 1 | 0 | 214 | 226 |  |  | PEAKS DB |
| K.IAIPDFGTGAMENWGLITYR.E | N | 59.87 | 2224.0986 | 20 | 1.1 | 1113.0579 | 2 | 69.19 | 8 | F8:43225 | 31122021\_RID\_3262\_1a.raw |  |  |  |  |  |  |  | 6.4859E5 |  |  | 1 | 0 | 0 | 0 | 0 | 0 | 0 | 0 | 1 | 0 | 0 | 347 | 366 |  |  | PEAKS DB |
| R.SQDVFTVLR.Y | N | 57.57 | 1063.5662 | 9 | 0.3 | 532.7905 | 2 | 43.09 | 8 | F8:22831 | 31122021\_RID\_3262\_1a.raw |  | 2.981E5 |  |  |  |  |  | 1.5248E6 | 8.2521E4 |  | 3 | 0 | 1 | 0 | 0 | 0 | 0 | 0 | 1 | 1 | 0 | 845 | 853 |  |  | PEAKS DB |
| R.YFGSLVKPIASE.L | N | 56.58 | 1309.6918 | 12 | 0.4 | 655.8534 | 2 | 44.52 | 8 | F8:23900 | 31122021\_RID\_3262\_1a.raw |  |  |  |  |  |  |  | 4.3145E5 |  |  | 1 | 0 | 0 | 0 | 0 | 0 | 0 | 0 | 1 | 0 | 0 | 718 | 729 |  |  | PEAKS DB |
| R.YFGSLVKPIASELK.W | N | 55.84 | 1550.8707 | 14 | 0.9 | 776.4434 | 2 | 43.59 | 8 | F8:23123 | 31122021\_RID\_3262\_1a.raw |  |  |  |  |  |  |  | 7.6327E5 |  |  | 1 | 0 | 0 | 0 | 0 | 0 | 0 | 0 | 1 | 0 | 0 | 718 | 731 |  |  | PEAKS DB |
| K.YLIDHYFK.N | N | 55.81 | 1097.5546 | 8 | -0.2 | 549.7844 | 2 | 37.86 | 8 | F8:18818 | 31122021\_RID\_3262\_1a.raw |  |  |  |  |  |  |  | 1.3914E6 |  |  | 1 | 0 | 0 | 0 | 0 | 0 | 0 | 0 | 1 | 0 | 0 | 503 | 510 |  |  | PEAKS DB |
| R.MLEDWISPDHFR.A | N | 55.23 | 1544.7081 | 12 | 0.2 | 515.9101 | 3 | 48.78 | 8 | F8:27275 | 31122021\_RID\_3262\_1a.raw |  |  |  |  |  |  |  | 2.1768E6 |  |  | 2 | 0 | 0 | 0 | 0 | 0 | 0 | 0 | 2 | 0 | 0 | 486 | 497 |  |  | PEAKS DB |
| R.ETNLLYDSR.E | N | 54.74 | 1109.5353 | 9 | 0.4 | 555.7751 | 2 | 32.33 | 8 | F8:14336 | 31122021\_RID\_3262\_1a.raw |  |  |  |  |  |  |  | 2.5631E6 | 2.035E5 |  | 2 | 0 | 0 | 0 | 0 | 0 | 0 | 0 | 1 | 1 | 0 | 367 | 375 |  |  | PEAKS DB |
| K.DHQNFNLADR.A | N | 52.66 | 1228.5585 | 10 | 0.3 | 615.2867 | 2 | 25.28 | 8 | F8:8849 | 31122021\_RID\_3262\_1a.raw |  |  |  |  |  |  |  | 4.9322E5 |  |  | 1 | 0 | 0 | 0 | 0 | 0 | 0 | 0 | 1 | 0 | 0 | 645 | 654 |  |  | PEAKS DB |
| R.LNWEYLVK.R | N | 52.08 | 1063.5702 | 8 | 0.7 | 532.7927 | 2 | 48.18 | 8 | F8:26890 | 31122021\_RID\_3262\_1a.raw |  |  |  |  |  |  |  | 1.1731E6 |  |  | 1 | 0 | 0 | 0 | 0 | 0 | 0 | 0 | 1 | 0 | 0 | 871 | 878 |  |  | PEAKS DB |
| R.MLEDWISPDHF.R | N | 51.09 | 1388.6071 | 11 | 0.5 | 695.3112 | 2 | 58.82 | 8 | F8:35162 | 31122021\_RID\_3262\_1a.raw |  |  |  |  |  |  |  | 2.1947E5 |  |  | 1 | 0 | 0 | 0 | 0 | 0 | 0 | 0 | 1 | 0 | 0 | 486 | 496 |  |  | PEAKS DB |
| K.VNKDHVGFYR.V | N | 50.28 | 1233.6254 | 10 | 0.1 | 412.2158 | 3 | 17.01 | 8 | F8:3494 | 31122021\_RID\_3262\_1a.raw |  |  |  |  |  |  |  | 1.4403E6 |  |  | 2 | 0 | 0 | 0 | 0 | 0 | 0 | 0 | 2 | 0 | 0 | 618 | 627 |  |  | PEAKS DB |
| K.AMEEVSGKPVR.E | N | 48.45 | 1201.6124 | 11 | 0.5 | 601.8138 | 2 | 17.87 | 9 | F9:3937 | 31122021\_RID\_3262\_2a.raw |  |  |  |  |  |  |  | 4.5191E5 | 8.126E4 |  | 2 | 0 | 0 | 0 | 0 | 0 | 0 | 0 | 1 | 1 | 0 | 520 | 530 |  |  | PEAKS DB |
| K.LLYGLASVK.N | N | 44.34 | 962.5800 | 9 | 0.8 | 482.2977 | 2 | 40.72 | 8 | F8:21128 | 31122021\_RID\_3262\_1a.raw |  |  |  |  |  |  |  | 1.6038E6 |  |  | 1 | 0 | 0 | 0 | 0 | 0 | 0 | 0 | 1 | 0 | 0 | 817 | 825 |  |  | PEAKS DB |
| total 17 peptides |  |  |  |  |  |  |  |  |  |  |  |  |  |  |  |  |  |  |  |  |  |  |  |  |  |  |  |  |  |  |  |  |  |  |  |  |  |
| --- | --- | --- | --- | --- | --- | --- | --- | --- | --- | --- | --- | --- | --- | --- | --- | --- | --- | --- | --- | --- | --- | --- | --- | --- | --- | --- | --- | --- | --- | --- | --- | --- | --- | --- | --- | --- | --- |

JAS04520.1

back to list

  

| Protein Coverage
| Supporting Peptides
|

Protein Coverage:

Supporting Peptides:

| Peptide | Uniq | -10lgP | Mass | Length | ppm | m/z | z | RT | Fraction | Scan | Source File | Area F4 | Area F5 | Area F6 | Area F10 | Area F8 | Area F9 | Area F7 | Area F1 | Area F2 | Area F3 | #Feature | #Feature F4 | #Feature F5 | #Feature F6 | #Feature F10 | #Feature F8 | #Feature F9 | #Feature F7 | #Feature F1 | #Feature F2 | #Feature F3 | Start | End | PTM | AScore | Found By |
| --- | --- | --- | --- | --- | --- | --- | --- | --- | --- | --- | --- | --- | --- | --- | --- | --- | --- | --- | --- | --- | --- | --- | --- | --- | --- | --- | --- | --- | --- | --- | --- | --- | --- | --- | --- | --- | --- |
| K.HANFPILSANIRPK.G | N | 73.91 | 1576.8838 | 14 | 0.3 | 526.6354 | 3 | 33.47 | 9 | F9:16191 | 31122021\_RID\_3262\_2a.raw | 2.4791E6 |  |  |  |  |  |  | 7.6122E5 | 1.3586E7 |  | 5 | 1 | 0 | 0 | 0 | 0 | 0 | 0 | 2 | 2 | 0 | 148 | 161 |  |  | PEAKS DB |
| G.SFELTILHTNDVHAR.V | N | 71.46 | 1751.8955 | 15 | 0.2 | 584.9725 | 3 | 37.82 | 9 | F9:19802 | 31122021\_RID\_3262\_2a.raw |  |  |  |  |  |  |  |  | 9.8903E6 |  | 1 | 0 | 0 | 0 | 0 | 0 | 0 | 0 | 0 | 1 | 0 | 41 | 55 |  |  | PEAKS DB |
| K.IQLHNYSSQEIGK.T | N | 66.55 | 1515.7681 | 13 | 0.9 | 758.8920 | 2 | 25.89 | 9 | F9:9947 | 31122021\_RID\_3262\_2a.raw |  | 0 |  |  |  |  |  |  | 8.8417E5 |  | 2 | 0 | 0 | 0 | 0 | 0 | 0 | 0 | 0 | 2 | 0 | 343 | 355 |  |  | PEAKS DB |
| R.HGQGM(+15.99)GELLQVSGIK.V | N | 64.12 | 1568.7981 | 15 | 0.8 | 785.4069 | 2 | 32.16 | 9 | F9:15220 | 31122021\_RID\_3262\_2a.raw | 3.4878E6 |  |  |  |  |  |  |  | 5.8977E6 |  | 4 | 2 | 0 | 0 | 0 | 0 | 0 | 0 | 0 | 2 | 0 | 457 | 471 | Oxidation (M) | M5:Oxidation (M):1000.00 | PEAKS DB |
| R.QVPVVQAYAFGK.Y | N | 64.12 | 1305.7081 | 12 | 0.7 | 653.8618 | 2 | 57.55 | 1 | F1:40709 | 13012022\_RID\_3262\_4a.raw | 4.6454E6 | 1.1825E6 |  |  |  |  |  | 3.0239E5 | 6.0962E6 |  | 5 | 2 | 1 | 0 | 0 | 0 | 0 | 0 | 1 | 1 | 0 | 288 | 299 |  |  | PEAKS DB |
| K.YLGYLNVIFDDKGNVIK.S | N | 63.64 | 1970.0513 | 17 | 0.7 | 986.0336 | 2 | 55.25 | 9 | F9:33414 | 31122021\_RID\_3262\_2a.raw |  |  |  |  |  |  |  |  | 5.214E6 |  | 2 | 0 | 0 | 0 | 0 | 0 | 0 | 0 | 0 | 2 | 0 | 300 | 316 |  |  | PEAKS DB |
| R.DEVEELQNHANK.L | N | 59.76 | 1424.6532 | 12 | 0.7 | 713.3344 | 2 | 23.15 | 9 | F9:7692 | 31122021\_RID\_3262\_2a.raw |  |  |  |  |  |  |  |  | 1.0268E6 |  | 1 | 0 | 0 | 0 | 0 | 0 | 0 | 0 | 0 | 1 | 0 | 209 | 220 |  |  | PEAKS DB |
| K.DISEDQDMKAEVNK.M | Y | 55.55 | 1620.7301 | 14 | 0.3 | 811.3726 | 2 | 26.96 | 1 | F1:13714 | 13012022\_RID\_3262\_4a.raw | 1.5655E6 |  |  |  |  |  |  |  |  |  | 2 | 2 | 0 | 0 | 0 | 0 | 0 | 0 | 0 | 0 | 0 | 327 | 340 |  |  | PEAKS DB |
| K.VGIIGYTTK.E | N | 51.25 | 950.5436 | 9 | 0.4 | 476.2793 | 2 | 33.16 | 9 | F9:16043 | 31122021\_RID\_3262\_2a.raw |  | 2.6943E6 | 7.1467E5 |  |  |  | 1.5898E5 |  | 8.0892E6 |  | 4 | 0 | 1 | 1 | 0 | 0 | 0 | 1 | 0 | 1 | 0 | 185 | 193 |  |  | PEAKS DB |
| K.DISEDQDM(+15.99)K.A | N | 50.30 | 1095.4390 | 9 | -0.1 | 548.7267 | 2 | 22.45 | 9 | F9:7124 | 31122021\_RID\_3262\_2a.raw |  |  |  |  |  |  |  |  | 4.7909E5 |  | 1 | 0 | 0 | 0 | 0 | 0 | 0 | 0 | 0 | 1 | 0 | 327 | 335 | Oxidation (M) | M8:Oxidation (M):1000.00 | PEAKS DB |
| K.DISEDQDMK.A | N | 48.46 | 1079.4441 | 9 | 0.3 | 540.7295 | 2 | 22.45 | 9 | F9:7062 | 31122021\_RID\_3262\_2a.raw | 4.4074E5 | 1.0473E5 |  |  |  |  |  |  | 2.3658E6 |  | 3 | 1 | 1 | 0 | 0 | 0 | 0 | 0 | 0 | 1 | 0 | 327 | 335 |  |  | PEAKS DB |
| K.VVYDLSR.K | N | 46.31 | 850.4548 | 7 | 0.5 | 426.2349 | 2 | 30.36 | 1 | F1:16753 | 13012022\_RID\_3262\_4a.raw | 3.9579E6 | 3.0633E6 |  |  |  |  |  |  | 6.9401E6 |  | 3 | 1 | 1 | 0 | 0 | 0 | 0 | 0 | 0 | 1 | 0 | 472 | 478 |  |  | PEAKS DB |
| K.HANFPILSAN.I | N | 46.30 | 1082.5509 | 10 | 0.2 | 542.2828 | 2 | 40.87 | 9 | F9:22309 | 31122021\_RID\_3262\_2a.raw |  |  |  |  |  |  |  |  | 2.7202E6 |  | 1 | 0 | 0 | 0 | 0 | 0 | 0 | 0 | 0 | 1 | 0 | 148 | 157 |  |  | PEAKS DB |
| N.LDISIVGDYIK.R | N | 44.01 | 1234.6809 | 11 | 0.5 | 618.3480 | 2 | 56.58 | 9 | F9:34546 | 31122021\_RID\_3262\_2a.raw |  |  |  |  |  |  |  |  | 2.949E4 |  | 1 | 0 | 0 | 0 | 0 | 0 | 0 | 0 | 0 | 1 | 0 | 538 | 548 |  |  | PEAKS DB |
| R.YDAMALGNHEFDNGLAGLLDPLLK.H | N | 43.69 | 2586.2788 | 24 | 0.6 | 863.1007 | 3 | 69.00 | 9 | F9:43247 | 31122021\_RID\_3262\_2a.raw |  |  |  |  |  |  |  |  | 9.895E5 |  | 1 | 0 | 0 | 0 | 0 | 0 | 0 | 0 | 0 | 1 | 0 | 124 | 147 |  |  | PEAKS DB |
| K.LTTLGVNK.I | N | 43.24 | 844.5018 | 8 | 0.8 | 423.2585 | 2 | 24.48 | 9 | F9:8941 | 31122021\_RID\_3262\_2a.raw |  |  |  |  |  |  |  |  | 1.1856E7 |  | 1 | 0 | 0 | 0 | 0 | 0 | 0 | 0 | 0 | 1 | 0 | 221 | 228 |  |  | PEAKS DB |
| F.GGTFDLLQIK.G | N | 43.16 | 1090.6022 | 10 | -0.1 | 546.3083 | 2 | 49.90 | 9 | F9:29347 | 31122021\_RID\_3262\_2a.raw |  |  |  |  |  |  |  |  | 6.6936E5 |  | 1 | 0 | 0 | 0 | 0 | 0 | 0 | 0 | 0 | 1 | 0 | 433 | 442 |  |  | PEAKS DB |
| K.YLGYLNVIFDDK.G | N | 41.85 | 1458.7394 | 12 | 1.3 | 730.3779 | 2 | 60.34 | 9 | F9:37272 | 31122021\_RID\_3262\_2a.raw |  |  |  |  |  |  |  |  | 2.5015E6 |  | 1 | 0 | 0 | 0 | 0 | 0 | 0 | 0 | 0 | 1 | 0 | 300 | 311 |  |  | PEAKS DB |
| total 18 peptides |  |  |  |  |  |  |  |  |  |  |  |  |  |  |  |  |  |  |  |  |  |  |  |  |  |  |  |  |  |  |  |  |  |  |  |  |  |
| --- | --- | --- | --- | --- | --- | --- | --- | --- | --- | --- | --- | --- | --- | --- | --- | --- | --- | --- | --- | --- | --- | --- | --- | --- | --- | --- | --- | --- | --- | --- | --- | --- | --- | --- | --- | --- | --- |

AAY89681.1

back to list

  

| Protein Coverage
| Supporting Peptides
|

Protein Coverage:

Supporting Peptides:

| Peptide | Uniq | -10lgP | Mass | Length | ppm | m/z | z | RT | Fraction | Scan | Source File | Area F4 | Area F5 | Area F6 | Area F10 | Area F8 | Area F9 | Area F7 | Area F1 | Area F2 | Area F3 | #Feature | #Feature F4 | #Feature F5 | #Feature F6 | #Feature F10 | #Feature F8 | #Feature F9 | #Feature F7 | #Feature F1 | #Feature F2 | #Feature F3 | Start | End | PTM | AScore | Found By |
| --- | --- | --- | --- | --- | --- | --- | --- | --- | --- | --- | --- | --- | --- | --- | --- | --- | --- | --- | --- | --- | --- | --- | --- | --- | --- | --- | --- | --- | --- | --- | --- | --- | --- | --- | --- | --- | --- |
| K.HVVVVGAGMAGLSAAYVLAGAGHNVTLLEASER.V | Y | 91.52 | 3218.6870 | 33 | 0.3 | 805.6793 | 4 | 65.12 | 1 | F1:47059 | 13012022\_RID\_3262\_4a.raw | 3.4843E7 |  |  |  |  |  |  | 0 |  | 4.1771E7 | 8 | 4 | 0 | 0 | 0 | 0 | 0 | 0 | 0 | 0 | 4 | 54 | 86 |  |  | PEAKS DB |
| K.HVVVVGAGM(+15.99)AGLSAAYVLAGAGHNVTLLEASER.V | Y | 87.40 | 3234.6819 | 33 | 0.6 | 809.6782 | 4 | 62.38 | 1 | F1:45514 | 13012022\_RID\_3262\_4a.raw | 3.7486E7 |  |  |  |  |  |  | 1.8957E6 |  | 1.7021E8 | 9 | 4 | 0 | 0 | 0 | 0 | 0 | 0 | 1 | 0 | 4 | 54 | 86 | Oxidation (M) | M9:Oxidation (M):1000.00 | PEAKS DB |
| Q.EADYEEFLEIAR.N | N | 83.87 | 1483.6830 | 12 | 0.7 | 742.8493 | 2 | 55.95 | 6 | F6:39873 | 17012022\_RID\_3262\_9a.raw | 2.7565E8 | 2.0308E7 | 1.2427E6 | 5.6557E7 |  | 5.9048E7 | 3.7778E5 | 4.8993E7 | 4.4393E7 | 2.2183E9 | 12 | 1 | 1 | 1 | 1 | 0 | 1 | 1 | 1 | 1 | 4 | 32 | 43 |  |  | PEAKS DB |
| D.YEEFLEIAR.N | N | 54.84 | 1168.5764 | 9 | 0.3 | 585.2957 | 2 | 46.57 | 4 | F4:30508 | 17012022\_RID\_3262\_10a .raw | 2.2869E6 |  |  | 1.3254E6 |  |  |  | 2.3858E5 | 1.5579E5 | 1.3646E7 | 5 | 1 | 0 | 0 | 1 | 0 | 0 | 0 | 1 | 1 | 1 | 35 | 43 |  |  | PEAKS DB |
| K.YDTYSTKEYLIK.E | N | 53.18 | 1522.7555 | 12 | 0.9 | 762.3857 | 2 | 34.13 | 1 | F1:20150 | 13012022\_RID\_3262\_4a.raw | 4.6273E6 |  |  |  |  |  |  |  |  |  | 2 | 2 | 0 | 0 | 0 | 0 | 0 | 0 | 0 | 0 | 0 | 199 | 210 |  |  | PEAKS DB |
| K.STTDLPSR.F | N | 52.16 | 875.4348 | 8 | 0.4 | 438.7249 | 2 | 21.40 | 6 | F6:8926 | 17012022\_RID\_3262\_9a.raw |  | 2.9854E7 |  | 4.8043E6 |  | 1.4473E7 | 1.9071E6 |  |  |  | 4 | 0 | 1 | 0 | 1 | 0 | 1 | 1 | 0 | 0 | 0 | 365 | 372 |  |  | PEAKS DB |
| N.DLSLIHQLPK.E | N | 51.65 | 1162.6710 | 10 | -0.7 | 582.3424 | 2 | 40.68 | 10 | F10:10098 | 31122021\_RID\_3262\_3a.raw |  |  |  |  |  |  |  | 1.197E6 | 2.7589E5 | 1.8174E7 | 3 | 0 | 0 | 0 | 0 | 0 | 0 | 0 | 1 | 1 | 1 | 415 | 424 |  |  | PEAKS DB |
| A.DIVINDLSLIHQLPK.E | N | 49.33 | 1716.9774 | 15 | 0.0 | 859.4960 | 2 | 55.52 | 8 | F8:32578 | 31122021\_RID\_3262\_1a.raw |  |  |  |  |  |  |  | 1.2793E5 | 1.1614E5 |  | 2 | 0 | 0 | 0 | 0 | 0 | 0 | 0 | 1 | 1 | 0 | 410 | 424 |  |  | PEAKS DB |
| K.HVVVVGAGMAGLSAAY.V | N | 48.78 | 1500.7759 | 16 | 0.1 | 751.3953 | 2 | 48.21 | 9 | F9:28042 | 31122021\_RID\_3262\_2a.raw |  |  |  |  |  |  |  | 3.8493E5 | 3.1875E5 | 7.767E6 | 3 | 0 | 0 | 0 | 0 | 0 | 0 | 0 | 1 | 1 | 1 | 54 | 69 |  |  | PEAKS DB |
| K.HVVVVGAGMAGL.S | N | 47.88 | 1108.6063 | 12 | 0.5 | 555.3107 | 2 | 43.58 | 9 | F9:24400 | 31122021\_RID\_3262\_2a.raw | 1.4776E6 |  |  |  |  |  |  |  | 3.0015E5 | 1.177E7 | 3 | 1 | 0 | 0 | 0 | 0 | 0 | 0 | 0 | 1 | 1 | 54 | 65 |  |  | PEAKS DB |
| Y.FEPPLPPK.K | N | 46.74 | 923.5116 | 8 | -0.6 | 462.7628 | 2 | 36.61 | 4 | F4:21855 | 17012022\_RID\_3262\_10a .raw | 8.6451E7 | 2.7491E7 |  | 4.4878E7 |  |  |  | 7.8215E6 | 8.5594E6 |  | 5 | 1 | 1 | 0 | 1 | 0 | 0 | 0 | 1 | 1 | 0 | 323 | 330 |  |  | PEAKS DB |
| Y.FEPPLPPKK.A | N | 43.61 | 1051.6066 | 9 | 0.7 | 526.8109 | 2 | 29.21 | 4 | F4:15245 | 17012022\_RID\_3262\_10a .raw |  |  |  | 4.2511E6 |  |  |  |  |  |  | 1 | 0 | 0 | 0 | 1 | 0 | 0 | 0 | 0 | 0 | 0 | 323 | 331 |  |  | PEAKS DB |
| total 12 peptides |  |  |  |  |  |  |  |  |  |  |  |  |  |  |  |  |  |  |  |  |  |  |  |  |  |  |  |  |  |  |  |  |  |  |  |  |  |
| --- | --- | --- | --- | --- | --- | --- | --- | --- | --- | --- | --- | --- | --- | --- | --- | --- | --- | --- | --- | --- | --- | --- | --- | --- | --- | --- | --- | --- | --- | --- | --- | --- | --- | --- | --- | --- | --- |

Q4JHE2.1

back to list

  

| Protein Coverage
| Supporting Peptides
|

Protein Coverage:

Supporting Peptides:

| Peptide | Uniq | -10lgP | Mass | Length | ppm | m/z | z | RT | Fraction | Scan | Source File | Area F4 | Area F5 | Area F6 | Area F10 | Area F8 | Area F9 | Area F7 | Area F1 | Area F2 | Area F3 | #Feature | #Feature F4 | #Feature F5 | #Feature F6 | #Feature F10 | #Feature F8 | #Feature F9 | #Feature F7 | #Feature F1 | #Feature F2 | #Feature F3 | Start | End | PTM | AScore | Found By |
| --- | --- | --- | --- | --- | --- | --- | --- | --- | --- | --- | --- | --- | --- | --- | --- | --- | --- | --- | --- | --- | --- | --- | --- | --- | --- | --- | --- | --- | --- | --- | --- | --- | --- | --- | --- | --- | --- |
| K.HVVVVGAGMAGLSAAYVLAGAGHNVTLLEASER.V | Y | 91.52 | 3218.6870 | 33 | 0.3 | 805.6793 | 4 | 65.12 | 1 | F1:47059 | 13012022\_RID\_3262\_4a.raw | 3.4843E7 |  |  |  |  |  |  | 0 |  | 4.1771E7 | 8 | 4 | 0 | 0 | 0 | 0 | 0 | 0 | 0 | 0 | 4 | 54 | 86 |  |  | PEAKS DB |
| K.HVVVVGAGM(+15.99)AGLSAAYVLAGAGHNVTLLEASER.V | Y | 87.40 | 3234.6819 | 33 | 0.6 | 809.6782 | 4 | 62.38 | 1 | F1:45514 | 13012022\_RID\_3262\_4a.raw | 3.7486E7 |  |  |  |  |  |  | 1.8957E6 |  | 1.7021E8 | 9 | 4 | 0 | 0 | 0 | 0 | 0 | 0 | 1 | 0 | 4 | 54 | 86 | Oxidation (M) | M9:Oxidation (M):1000.00 | PEAKS DB |
| Q.EADYEEFLEIAR.N | N | 83.87 | 1483.6830 | 12 | 0.7 | 742.8493 | 2 | 55.95 | 6 | F6:39873 | 17012022\_RID\_3262\_9a.raw | 2.7565E8 | 2.0308E7 | 1.2427E6 | 5.6557E7 |  | 5.9048E7 | 3.7778E5 | 4.8993E7 | 4.4393E7 | 2.2183E9 | 12 | 1 | 1 | 1 | 1 | 0 | 1 | 1 | 1 | 1 | 4 | 32 | 43 |  |  | PEAKS DB |
| D.YEEFLEIAR.N | N | 54.84 | 1168.5764 | 9 | 0.3 | 585.2957 | 2 | 46.57 | 4 | F4:30508 | 17012022\_RID\_3262\_10a .raw | 2.2869E6 |  |  | 1.3254E6 |  |  |  | 2.3858E5 | 1.5579E5 | 1.3646E7 | 5 | 1 | 0 | 0 | 1 | 0 | 0 | 0 | 1 | 1 | 1 | 35 | 43 |  |  | PEAKS DB |
| K.YDTYSTKEYLIK.E | N | 53.18 | 1522.7555 | 12 | 0.9 | 762.3857 | 2 | 34.13 | 1 | F1:20150 | 13012022\_RID\_3262\_4a.raw | 4.6273E6 |  |  |  |  |  |  |  |  |  | 2 | 2 | 0 | 0 | 0 | 0 | 0 | 0 | 0 | 0 | 0 | 199 | 210 |  |  | PEAKS DB |
| K.STTDLPSR.F | N | 52.16 | 875.4348 | 8 | 0.4 | 438.7249 | 2 | 21.40 | 6 | F6:8926 | 17012022\_RID\_3262\_9a.raw |  | 2.9854E7 |  | 4.8043E6 |  | 1.4473E7 | 1.9071E6 |  |  |  | 4 | 0 | 1 | 0 | 1 | 0 | 1 | 1 | 0 | 0 | 0 | 365 | 372 |  |  | PEAKS DB |
| N.DLSLIHQLPK.E | N | 51.65 | 1162.6710 | 10 | -0.7 | 582.3424 | 2 | 40.68 | 10 | F10:10098 | 31122021\_RID\_3262\_3a.raw |  |  |  |  |  |  |  | 1.197E6 | 2.7589E5 | 1.8174E7 | 3 | 0 | 0 | 0 | 0 | 0 | 0 | 0 | 1 | 1 | 1 | 415 | 424 |  |  | PEAKS DB |
| A.DIVINDLSLIHQLPK.E | N | 49.33 | 1716.9774 | 15 | 0.0 | 859.4960 | 2 | 55.52 | 8 | F8:32578 | 31122021\_RID\_3262\_1a.raw |  |  |  |  |  |  |  | 1.2793E5 | 1.1614E5 |  | 2 | 0 | 0 | 0 | 0 | 0 | 0 | 0 | 1 | 1 | 0 | 410 | 424 |  |  | PEAKS DB |
| K.HVVVVGAGMAGLSAAY.V | N | 48.78 | 1500.7759 | 16 | 0.1 | 751.3953 | 2 | 48.21 | 9 | F9:28042 | 31122021\_RID\_3262\_2a.raw |  |  |  |  |  |  |  | 3.8493E5 | 3.1875E5 | 7.767E6 | 3 | 0 | 0 | 0 | 0 | 0 | 0 | 0 | 1 | 1 | 1 | 54 | 69 |  |  | PEAKS DB |
| K.HVVVVGAGMAGL.S | N | 47.88 | 1108.6063 | 12 | 0.5 | 555.3107 | 2 | 43.58 | 9 | F9:24400 | 31122021\_RID\_3262\_2a.raw | 1.4776E6 |  |  |  |  |  |  |  | 3.0015E5 | 1.177E7 | 3 | 1 | 0 | 0 | 0 | 0 | 0 | 0 | 0 | 1 | 1 | 54 | 65 |  |  | PEAKS DB |
| Y.FEPPLPPK.K | N | 46.74 | 923.5116 | 8 | -0.6 | 462.7628 | 2 | 36.61 | 4 | F4:21855 | 17012022\_RID\_3262\_10a .raw | 8.6451E7 | 2.7491E7 |  | 4.4878E7 |  |  |  | 7.8215E6 | 8.5594E6 |  | 5 | 1 | 1 | 0 | 1 | 0 | 0 | 0 | 1 | 1 | 0 | 323 | 330 |  |  | PEAKS DB |
| Y.FEPPLPPKK.A | N | 43.61 | 1051.6066 | 9 | 0.7 | 526.8109 | 2 | 29.21 | 4 | F4:15245 | 17012022\_RID\_3262\_10a .raw |  |  |  | 4.2511E6 |  |  |  |  |  |  | 1 | 0 | 0 | 0 | 1 | 0 | 0 | 0 | 0 | 0 | 0 | 323 | 331 |  |  | PEAKS DB |
| total 12 peptides |  |  |  |  |  |  |  |  |  |  |  |  |  |  |  |  |  |  |  |  |  |  |  |  |  |  |  |  |  |  |  |  |  |  |  |  |  |
| --- | --- | --- | --- | --- | --- | --- | --- | --- | --- | --- | --- | --- | --- | --- | --- | --- | --- | --- | --- | --- | --- | --- | --- | --- | --- | --- | --- | --- | --- | --- | --- | --- | --- | --- | --- | --- | --- |

XP\_026538830.1

back to list

  

| Protein Coverage
| Supporting Peptides
|

Protein Coverage:

Supporting Peptides:

| Peptide | Uniq | -10lgP | Mass | Length | ppm | m/z | z | RT | Fraction | Scan | Source File | Area F4 | Area F5 | Area F6 | Area F10 | Area F8 | Area F9 | Area F7 | Area F1 | Area F2 | Area F3 | #Feature | #Feature F4 | #Feature F5 | #Feature F6 | #Feature F10 | #Feature F8 | #Feature F9 | #Feature F7 | #Feature F1 | #Feature F2 | #Feature F3 | Start | End | PTM | AScore | Found By |
| --- | --- | --- | --- | --- | --- | --- | --- | --- | --- | --- | --- | --- | --- | --- | --- | --- | --- | --- | --- | --- | --- | --- | --- | --- | --- | --- | --- | --- | --- | --- | --- | --- | --- | --- | --- | --- | --- |
| K.HVVVVGAGMAGLSAAYVLAGAGHNVTLLEASER.V | Y | 91.52 | 3218.6870 | 33 | 0.3 | 805.6793 | 4 | 65.12 | 1 | F1:47059 | 13012022\_RID\_3262\_4a.raw | 3.4843E7 |  |  |  |  |  |  | 0 |  | 4.1771E7 | 8 | 4 | 0 | 0 | 0 | 0 | 0 | 0 | 0 | 0 | 4 | 54 | 86 |  |  | PEAKS DB |
| K.HVVVVGAGM(+15.99)AGLSAAYVLAGAGHNVTLLEASER.V | Y | 87.40 | 3234.6819 | 33 | 0.6 | 809.6782 | 4 | 62.38 | 1 | F1:45514 | 13012022\_RID\_3262\_4a.raw | 3.7486E7 |  |  |  |  |  |  | 1.8957E6 |  | 1.7021E8 | 9 | 4 | 0 | 0 | 0 | 0 | 0 | 0 | 1 | 0 | 4 | 54 | 86 | Oxidation (M) | M9:Oxidation (M):1000.00 | PEAKS DB |
| Q.EADYEEFLEIAR.N | N | 83.87 | 1483.6830 | 12 | 0.7 | 742.8493 | 2 | 55.95 | 6 | F6:39873 | 17012022\_RID\_3262\_9a.raw | 2.7565E8 | 2.0308E7 | 1.2427E6 | 5.6557E7 |  | 5.9048E7 | 3.7778E5 | 4.8993E7 | 4.4393E7 | 2.2183E9 | 12 | 1 | 1 | 1 | 1 | 0 | 1 | 1 | 1 | 1 | 4 | 32 | 43 |  |  | PEAKS DB |
| D.YEEFLEIAR.N | N | 54.84 | 1168.5764 | 9 | 0.3 | 585.2957 | 2 | 46.57 | 4 | F4:30508 | 17012022\_RID\_3262\_10a .raw | 2.2869E6 |  |  | 1.3254E6 |  |  |  | 2.3858E5 | 1.5579E5 | 1.3646E7 | 5 | 1 | 0 | 0 | 1 | 0 | 0 | 0 | 1 | 1 | 1 | 35 | 43 |  |  | PEAKS DB |
| K.YDTYSTKEYLIK.E | N | 53.18 | 1522.7555 | 12 | 0.9 | 762.3857 | 2 | 34.13 | 1 | F1:20150 | 13012022\_RID\_3262\_4a.raw | 4.6273E6 |  |  |  |  |  |  |  |  |  | 2 | 2 | 0 | 0 | 0 | 0 | 0 | 0 | 0 | 0 | 0 | 199 | 210 |  |  | PEAKS DB |
| K.STTDLPSR.F | N | 52.16 | 875.4348 | 8 | 0.4 | 438.7249 | 2 | 21.40 | 6 | F6:8926 | 17012022\_RID\_3262\_9a.raw |  | 2.9854E7 |  | 4.8043E6 |  | 1.4473E7 | 1.9071E6 |  |  |  | 4 | 0 | 1 | 0 | 1 | 0 | 1 | 1 | 0 | 0 | 0 | 365 | 372 |  |  | PEAKS DB |
| N.DLSLIHQLPK.E | N | 51.65 | 1162.6710 | 10 | -0.7 | 582.3424 | 2 | 40.68 | 10 | F10:10098 | 31122021\_RID\_3262\_3a.raw |  |  |  |  |  |  |  | 1.197E6 | 2.7589E5 | 1.8174E7 | 3 | 0 | 0 | 0 | 0 | 0 | 0 | 0 | 1 | 1 | 1 | 415 | 424 |  |  | PEAKS DB |
| A.DIVINDLSLIHQLPK.E | N | 49.33 | 1716.9774 | 15 | 0.0 | 859.4960 | 2 | 55.52 | 8 | F8:32578 | 31122021\_RID\_3262\_1a.raw |  |  |  |  |  |  |  | 1.2793E5 | 1.1614E5 |  | 2 | 0 | 0 | 0 | 0 | 0 | 0 | 0 | 1 | 1 | 0 | 410 | 424 |  |  | PEAKS DB |
| K.HVVVVGAGMAGLSAAY.V | N | 48.78 | 1500.7759 | 16 | 0.1 | 751.3953 | 2 | 48.21 | 9 | F9:28042 | 31122021\_RID\_3262\_2a.raw |  |  |  |  |  |  |  | 3.8493E5 | 3.1875E5 | 7.767E6 | 3 | 0 | 0 | 0 | 0 | 0 | 0 | 0 | 1 | 1 | 1 | 54 | 69 |  |  | PEAKS DB |
| K.HVVVVGAGMAGL.S | N | 47.88 | 1108.6063 | 12 | 0.5 | 555.3107 | 2 | 43.58 | 9 | F9:24400 | 31122021\_RID\_3262\_2a.raw | 1.4776E6 |  |  |  |  |  |  |  | 3.0015E5 | 1.177E7 | 3 | 1 | 0 | 0 | 0 | 0 | 0 | 0 | 0 | 1 | 1 | 54 | 65 |  |  | PEAKS DB |
| Y.FEPPLPPK.K | N | 46.74 | 923.5116 | 8 | -0.6 | 462.7628 | 2 | 36.61 | 4 | F4:21855 | 17012022\_RID\_3262\_10a .raw | 8.6451E7 | 2.7491E7 |  | 4.4878E7 |  |  |  | 7.8215E6 | 8.5594E6 |  | 5 | 1 | 1 | 0 | 1 | 0 | 0 | 0 | 1 | 1 | 0 | 323 | 330 |  |  | PEAKS DB |
| Y.FEPPLPPKK.A | N | 43.61 | 1051.6066 | 9 | 0.7 | 526.8109 | 2 | 29.21 | 4 | F4:15245 | 17012022\_RID\_3262\_10a .raw |  |  |  | 4.2511E6 |  |  |  |  |  |  | 1 | 0 | 0 | 0 | 1 | 0 | 0 | 0 | 0 | 0 | 0 | 323 | 331 |  |  | PEAKS DB |
| total 12 peptides |  |  |  |  |  |  |  |  |  |  |  |  |  |  |  |  |  |  |  |  |  |  |  |  |  |  |  |  |  |  |  |  |  |  |  |  |  |
| --- | --- | --- | --- | --- | --- | --- | --- | --- | --- | --- | --- | --- | --- | --- | --- | --- | --- | --- | --- | --- | --- | --- | --- | --- | --- | --- | --- | --- | --- | --- | --- | --- | --- | --- | --- | --- | --- |

XP\_007427768.1

back to list

  

| Protein Coverage
| Supporting Peptides
|

Protein Coverage:

Supporting Peptides:

| Peptide | Uniq | -10lgP | Mass | Length | ppm | m/z | z | RT | Fraction | Scan | Source File | Area F4 | Area F5 | Area F6 | Area F10 | Area F8 | Area F9 | Area F7 | Area F1 | Area F2 | Area F3 | #Feature | #Feature F4 | #Feature F5 | #Feature F6 | #Feature F10 | #Feature F8 | #Feature F9 | #Feature F7 | #Feature F1 | #Feature F2 | #Feature F3 | Start | End | PTM | AScore | Found By |
| --- | --- | --- | --- | --- | --- | --- | --- | --- | --- | --- | --- | --- | --- | --- | --- | --- | --- | --- | --- | --- | --- | --- | --- | --- | --- | --- | --- | --- | --- | --- | --- | --- | --- | --- | --- | --- | --- |
| K.HQGLPESYNFDFVTMKPVL | N | 88.30 | 2221.0876 | 19 | 1.0 | 741.3705 | 3 | 60.75 | 1 | F1:42994 | 13012022\_RID\_3262\_4a.raw | 1.5758E8 |  |  |  |  |  |  | 9.1193E5 |  |  | 5 | 4 | 0 | 0 | 0 | 0 | 0 | 0 | 1 | 0 | 0 | 541 | 559 |  |  | PEAKS DB |
| K.HQGLPESYNFDFVTM(+15.99)KPVL | N | 82.55 | 2237.0828 | 19 | 0.9 | 746.7022 | 3 | 54.75 | 1 | F1:37685 | 13012022\_RID\_3262\_4a.raw | 8.3546E7 |  |  |  |  |  |  |  |  |  | 4 | 4 | 0 | 0 | 0 | 0 | 0 | 0 | 0 | 0 | 0 | 541 | 559 | Oxidation (M) | M15:Oxidation (M):1000.00 | PEAKS DB |
| K.QNSGTYNNQYMILDTK.K | N | 76.97 | 1888.8625 | 16 | -0.2 | 945.4384 | 2 | 40.58 | 1 | F1:26159 | 13012022\_RID\_3262\_4a.raw | 4.9824E7 |  |  |  |  |  |  |  |  |  | 3 | 3 | 0 | 0 | 0 | 0 | 0 | 0 | 0 | 0 | 0 | 357 | 372 |  |  | PEAKS DB |
| Q.VVPESLFAWER.V | N | 74.17 | 1331.6874 | 11 | 0.3 | 666.8512 | 2 | 53.43 | 1 | F1:37684 | 13012022\_RID\_3262\_4a.raw | 1.6375E8 | 3.8989E6 |  |  |  |  |  | 1.2567E6 |  |  | 3 | 1 | 1 | 0 | 0 | 0 | 0 | 0 | 1 | 0 | 0 | 326 | 336 |  |  | PEAKS DB |
| E.TKHQGLPESYNFDFVTMKPVL | N | 73.21 | 2450.2305 | 21 | 0.9 | 613.5654 | 4 | 61.28 | 1 | F1:43650 | 13012022\_RID\_3262\_4a.raw | 2.0337E7 |  |  |  |  |  |  |  |  |  | 3 | 3 | 0 | 0 | 0 | 0 | 0 | 0 | 0 | 0 | 0 | 539 | 559 |  |  | PEAKS DB |
| K.QNSGTYNNQYM(+15.99)ILDTK.K | N | 70.11 | 1904.8574 | 16 | 0.2 | 953.4362 | 2 | 36.64 | 1 | F1:22592 | 13012022\_RID\_3262\_4a.raw | 7.9472E6 |  |  |  |  |  |  |  |  |  | 2 | 2 | 0 | 0 | 0 | 0 | 0 | 0 | 0 | 0 | 0 | 357 | 372 | Oxidation (M) | M11:Oxidation (M):1000.00 | PEAKS DB |
| R.SLEDGTLYIVEQVPK.F | Y | 66.93 | 1689.8824 | 15 | -0.2 | 845.9484 | 2 | 54.43 | 2 | F2:33670 | 13012022\_RID\_3262\_5a.raw |  | 1.2067E6 |  |  |  |  |  |  |  |  | 1 | 0 | 1 | 0 | 0 | 0 | 0 | 0 | 0 | 0 | 0 | 379 | 393 |  |  | PEAKS DB |
| K.QNSGTYNNQYMILDTKK.I | N | 66.25 | 2016.9575 | 17 | 0.0 | 673.3265 | 3 | 33.86 | 1 | F1:19896 | 13012022\_RID\_3262\_4a.raw | 2.1321E7 |  |  |  |  |  |  |  |  |  | 2 | 2 | 0 | 0 | 0 | 0 | 0 | 0 | 0 | 0 | 0 | 357 | 373 |  |  | PEAKS DB |
| E.TKHQGLPESYNFDFVTM(+15.99)KPVL | N | 52.45 | 2466.2253 | 21 | 0.6 | 823.0829 | 3 | 46.88 | 1 | F1:31507 | 13012022\_RID\_3262\_4a.raw | 3.3945E6 |  |  |  |  |  |  |  |  |  | 2 | 2 | 0 | 0 | 0 | 0 | 0 | 0 | 0 | 0 | 0 | 539 | 559 | Oxidation (M) | M17:Oxidation (M):1000.00 | PEAKS DB |
| K.QNSGTYNNQYM(+15.99)ILDTKK.I | N | 51.05 | 2032.9524 | 17 | 0.1 | 678.6581 | 3 | 33.88 | 1 | F1:19963 | 13012022\_RID\_3262\_4a.raw | 1.3281E6 |  |  |  |  |  |  |  |  |  | 1 | 1 | 0 | 0 | 0 | 0 | 0 | 0 | 0 | 0 | 0 | 357 | 373 | Oxidation (M) | M11:Oxidation (M):1000.00 | PEAKS DB |
| K.HQGLPESYNFDFVTMKPV.L | N | 51.01 | 2108.0037 | 18 | 0.6 | 703.6756 | 3 | 51.54 | 1 | F1:35496 | 13012022\_RID\_3262\_4a.raw | 1.7469E6 |  |  |  |  |  |  |  |  |  | 1 | 1 | 0 | 0 | 0 | 0 | 0 | 0 | 0 | 0 | 0 | 541 | 558 |  |  | PEAKS DB |
| K.YGLDFSYEMAPR.A | N | 47.91 | 1447.6442 | 12 | 8.4 | 724.8354 | 2 | 41.27 | 1 | F1:26837 | 13012022\_RID\_3262\_4a.raw | 5.4775E6 |  |  |  |  |  |  |  |  |  | 1 | 1 | 0 | 0 | 0 | 0 | 0 | 0 | 0 | 0 | 0 | 433 | 444 |  |  | PEAKS DB |
| K.YGLDFSYEM(+15.99)APR.A | N | 46.81 | 1463.6390 | 12 | 8.5 | 732.8330 | 2 | 36.02 | 1 | F1:21843 | 13012022\_RID\_3262\_4a.raw | 3.0912E6 |  |  |  |  |  |  |  |  |  | 1 | 1 | 0 | 0 | 0 | 0 | 0 | 0 | 0 | 0 | 0 | 433 | 444 | Oxidation (M) | M9:Oxidation (M):1000.00 | PEAKS DB |
| K.VLPGYENIYFAH.S | N | 46.73 | 1421.6979 | 12 | 0.8 | 711.8568 | 2 | 49.22 | 1 | F1:33555 | 13012022\_RID\_3262\_4a.raw | 3.4702E6 |  |  |  |  |  |  |  |  |  | 1 | 1 | 0 | 0 | 0 | 0 | 0 | 0 | 0 | 0 | 0 | 245 | 256 |  |  | PEAKS DB |
| K.HQGLPESYNFDFVTM(+15.99)KPV.L | N | 44.49 | 2123.9985 | 18 | 0.3 | 709.0070 | 3 | 47.31 | 1 | F1:31932 | 13012022\_RID\_3262\_4a.raw | 9.0568E5 |  |  |  |  |  |  |  |  |  | 1 | 1 | 0 | 0 | 0 | 0 | 0 | 0 | 0 | 0 | 0 | 541 | 558 | Oxidation (M) | M15:Oxidation (M):1000.00 | PEAKS DB |
| N.FDFVTMKPVL | N | 43.97 | 1195.6311 | 10 | 0.3 | 598.8230 | 2 | 56.81 | 1 | F1:39982 | 13012022\_RID\_3262\_4a.raw | 8.4752E5 |  |  |  |  |  |  |  |  |  | 1 | 1 | 0 | 0 | 0 | 0 | 0 | 0 | 0 | 0 | 0 | 550 | 559 |  |  | PEAKS DB |
| total 16 peptides |  |  |  |  |  |  |  |  |  |  |  |  |  |  |  |  |  |  |  |  |  |  |  |  |  |  |  |  |  |  |  |  |  |  |  |  |  |
| --- | --- | --- | --- | --- | --- | --- | --- | --- | --- | --- | --- | --- | --- | --- | --- | --- | --- | --- | --- | --- | --- | --- | --- | --- | --- | --- | --- | --- | --- | --- | --- | --- | --- | --- | --- | --- | --- |

ETE70217.1

back to list

  

| Protein Coverage
| Supporting Peptides
|

Protein Coverage:

Supporting Peptides:

| Peptide | Uniq | -10lgP | Mass | Length | ppm | m/z | z | RT | Fraction | Scan | Source File | Area F4 | Area F5 | Area F6 | Area F10 | Area F8 | Area F9 | Area F7 | Area F1 | Area F2 | Area F3 | #Feature | #Feature F4 | #Feature F5 | #Feature F6 | #Feature F10 | #Feature F8 | #Feature F9 | #Feature F7 | #Feature F1 | #Feature F2 | #Feature F3 | Start | End | PTM | AScore | Found By |
| --- | --- | --- | --- | --- | --- | --- | --- | --- | --- | --- | --- | --- | --- | --- | --- | --- | --- | --- | --- | --- | --- | --- | --- | --- | --- | --- | --- | --- | --- | --- | --- | --- | --- | --- | --- | --- | --- |
| K.TRLEQEIATYR.N | N | 68.61 | 1378.7205 | 11 | 0.3 | 690.3677 | 2 | 32.51 | 1 | F1:18751 | 13012022\_RID\_3262\_4a.raw | 5.7848E6 |  |  | 5.9875E6 |  | 4.8518E6 |  | 6.6719E6 | 4.5332E6 | 1.9872E6 | 7 | 1 | 0 | 0 | 1 | 0 | 1 | 0 | 1 | 2 | 1 | 875 | 885 |  |  | PEAKS DB |
| R.ALEEANTELEVK.I | N | 64.18 | 1344.6772 | 12 | 0.6 | 673.3463 | 2 | 34.10 | 6 | F6:20277 | 17012022\_RID\_3262\_9a.raw |  |  |  | 4.8188E6 |  | 6.4935E6 |  | 3.4179E6 | 1.846E6 |  | 4 | 0 | 0 | 0 | 1 | 0 | 1 | 0 | 1 | 1 | 0 | 169 | 180 |  |  | PEAKS DB |
| R.LENEIETYR.H | N | 60.45 | 1165.5615 | 9 | 0.5 | 583.7883 | 2 | 30.98 | 6 | F6:17641 | 17012022\_RID\_3262\_9a.raw | 3.245E5 |  |  | 2.4101E6 |  | 2.2556E6 |  | 6.748E5 | 2.1401E6 |  | 6 | 1 | 0 | 0 | 1 | 0 | 1 | 0 | 1 | 2 | 0 | 2570 | 2578 |  |  | PEAKS DB |
| R.MSVEADINGLR.R | N | 59.23 | 1203.5918 | 11 | -1.1 | 602.8025 | 2 | 39.90 | 6 | F6:25459 | 17012022\_RID\_3262\_9a.raw |  |  |  |  |  | 2.8012E5 |  | 7.0924E5 |  |  | 2 | 0 | 0 | 0 | 0 | 0 | 1 | 0 | 1 | 0 | 0 | 246 | 256 |  |  | PEAKS DB |
| K.TKFETEQALR.M | N | 58.26 | 1221.6353 | 10 | 0.6 | 408.2193 | 3 | 24.06 | 4 | F4:11122 | 17012022\_RID\_3262\_10a .raw |  |  |  | 1.3535E6 |  |  | 3.5223E4 | 2.1967E5 | 1.5239E5 |  | 5 | 0 | 0 | 0 | 2 | 0 | 0 | 1 | 1 | 1 | 0 | 236 | 245 |  |  | PEAKS DB |
| R.TLQGLEIELQSQLSM(+15.99)K.T | N | 57.45 | 1832.9553 | 16 | 0.7 | 917.4855 | 2 | 55.09 | 8 | F8:32220 | 31122021\_RID\_3262\_1a.raw |  |  |  |  |  |  |  | 7.3664E4 |  |  | 1 | 0 | 0 | 0 | 0 | 0 | 0 | 0 | 1 | 0 | 0 | 371 | 386 | Oxidation (M) | M15:Oxidation (M):1000.00 | PEAKS DB |
| K.IRLENEIETYR.H | N | 56.27 | 1434.7467 | 11 | 0.5 | 718.3810 | 2 | 32.86 | 9 | F9:15718 | 31122021\_RID\_3262\_2a.raw |  |  |  |  |  |  |  | 1.4126E6 | 2.5524E5 |  | 2 | 0 | 0 | 0 | 0 | 0 | 0 | 0 | 1 | 1 | 0 | 2568 | 2578 |  |  | PEAKS DB |
| R.LEQEIATYR.N | N | 55.96 | 1121.5717 | 9 | 0.4 | 561.7933 | 2 | 28.83 | 8 | F8:11613 | 31122021\_RID\_3262\_1a.raw |  | 2.3732E6 | 1.2706E6 | 1.8026E7 |  | 1.4281E7 | 7.759E5 | 5.2291E6 | 5.2914E6 | 1.4632E7 | 9 | 0 | 1 | 1 | 1 | 0 | 1 | 1 | 1 | 1 | 2 | 877 | 885 |  |  | PEAKS DB |
| Q.LESLTEELAYLK.K | N | 52.86 | 1407.7496 | 12 | 0.8 | 704.8827 | 2 | 53.03 | 8 | F8:30615 | 31122021\_RID\_3262\_1a.raw |  |  |  |  |  |  |  | 2.8588E5 |  |  | 1 | 0 | 0 | 0 | 0 | 0 | 0 | 0 | 1 | 0 | 0 | 3368 | 3379 |  |  | PEAKS DB |
| R.M(+15.99)SVEADINGLR.R | N | 52.11 | 1219.5867 | 11 | -0.1 | 610.8005 | 2 | 34.73 | 8 | F8:16136 | 31122021\_RID\_3262\_1a.raw |  |  |  |  |  |  |  | 4.1628E5 |  |  | 1 | 0 | 0 | 0 | 0 | 0 | 0 | 0 | 1 | 0 | 0 | 246 | 256 | Oxidation (M) | M1:Oxidation (M):1000.00 | PEAKS DB |
| E.SLTEELAYLK.K | N | 50.77 | 1165.6230 | 10 | 0.7 | 583.8192 | 2 | 46.48 | 8 | F8:25304 | 31122021\_RID\_3262\_1a.raw |  |  |  |  |  | 1.764E6 |  | 2.1639E6 | 1.6718E5 |  | 3 | 0 | 0 | 0 | 0 | 0 | 1 | 0 | 1 | 1 | 0 | 3370 | 3379 |  |  | PEAKS DB |
| R.LAADDFR.L | N | 50.00 | 806.3922 | 7 | -0.2 | 404.2033 | 2 | 31.38 | 6 | F6:17916 | 17012022\_RID\_3262\_9a.raw | 1.3125E8 | 4.3624E7 | 1.735E7 | 3.5753E8 | 1.2868E6 | 3.2848E8 | 2.8818E7 | 8.1938E7 | 4.7597E7 | 9.2413E7 | 18 | 1 | 2 | 1 | 4 | 1 | 3 | 1 | 2 | 2 | 1 | 664 | 670 |  |  | PEAKS DB |
| K.ITMQNLNDR.L | N | 46.82 | 1103.5393 | 9 | 0.6 | 552.7773 | 2 | 29.51 | 6 | F6:16335 | 17012022\_RID\_3262\_9a.raw |  |  |  |  |  | 1.031E6 |  |  |  |  | 1 | 0 | 0 | 0 | 0 | 0 | 1 | 0 | 0 | 0 | 0 | 151 | 159 |  |  | PEAKS DB |
| R.ADLEM(+15.99)QIESLKEELAYLK.K | Y | 46.52 | 2138.0815 | 18 | 0.3 | 713.7014 | 3 | 63.24 | 8 | F8:38633 | 31122021\_RID\_3262\_1a.raw |  |  |  |  |  |  |  | 5.9702E5 |  |  | 1 | 0 | 0 | 0 | 0 | 0 | 0 | 0 | 1 | 0 | 0 | 267 | 284 | Oxidation (M) | M5:Oxidation (M):1000.00 | PEAKS DB |
| R.LASYLEK.V | N | 44.48 | 822.4487 | 7 | 0.3 | 412.2318 | 2 | 27.21 | 4 | F4:13951 | 17012022\_RID\_3262\_10a .raw |  |  |  | 3.0024E6 |  |  |  |  |  |  | 1 | 0 | 0 | 0 | 1 | 0 | 0 | 0 | 0 | 0 | 0 | 1070 | 1076 |  |  | PEAKS DB |
| K.SEVTELR.R | Y | 43.69 | 832.4290 | 7 | 0.8 | 417.2221 | 2 | 23.57 | 6 | F6:10963 | 17012022\_RID\_3262\_9a.raw |  |  |  | 4.869E6 |  | 3.3371E6 |  |  |  |  | 2 | 0 | 0 | 0 | 1 | 0 | 1 | 0 | 0 | 0 | 0 | 2991 | 2997 |  |  | PEAKS DB |
| R.ADLEMQIESLK.E | Y | 41.82 | 1275.6381 | 11 | 0.7 | 638.8268 | 2 | 45.14 | 9 | F9:25839 | 31122021\_RID\_3262\_2a.raw |  |  |  |  |  |  |  |  | 9.9682E5 |  | 2 | 0 | 0 | 0 | 0 | 0 | 0 | 0 | 0 | 2 | 0 | 267 | 277 |  |  | PEAKS DB |
| R.ADLEM(+15.99)QIESLK.E | Y | 41.44 | 1291.6329 | 11 | 1.0 | 646.8244 | 2 | 43.12 | 9 | F9:23932 | 31122021\_RID\_3262\_2a.raw |  |  |  |  |  |  |  |  | 1.6645E5 |  | 1 | 0 | 0 | 0 | 0 | 0 | 0 | 0 | 0 | 1 | 0 | 267 | 277 | Oxidation (M) | M5:Oxidation (M):1000.00 | PEAKS DB |
| total 18 peptides |  |  |  |  |  |  |  |  |  |  |  |  |  |  |  |  |  |  |  |  |  |  |  |  |  |  |  |  |  |  |  |  |  |  |  |  |  |
| --- | --- | --- | --- | --- | --- | --- | --- | --- | --- | --- | --- | --- | --- | --- | --- | --- | --- | --- | --- | --- | --- | --- | --- | --- | --- | --- | --- | --- | --- | --- | --- | --- | --- | --- | --- | --- | --- |

XP\_015676063.1

back to list

  

| Protein Coverage
| Supporting Peptides
|

Protein Coverage:

Supporting Peptides:

| Peptide | Uniq | -10lgP | Mass | Length | ppm | m/z | z | RT | Fraction | Scan | Source File | Area F4 | Area F5 | Area F6 | Area F10 | Area F8 | Area F9 | Area F7 | Area F1 | Area F2 | Area F3 | #Feature | #Feature F4 | #Feature F5 | #Feature F6 | #Feature F10 | #Feature F8 | #Feature F9 | #Feature F7 | #Feature F1 | #Feature F2 | #Feature F3 | Start | End | PTM | AScore | Found By |
| --- | --- | --- | --- | --- | --- | --- | --- | --- | --- | --- | --- | --- | --- | --- | --- | --- | --- | --- | --- | --- | --- | --- | --- | --- | --- | --- | --- | --- | --- | --- | --- | --- | --- | --- | --- | --- | --- |
| R.LADDFM(+15.99)GSTWQEK.V | Y | 69.37 | 1542.6660 | 13 | 0.4 | 772.3406 | 2 | 37.70 | 8 | F8:18715 | 31122021\_RID\_3262\_1a.raw |  |  |  |  |  |  |  | 1.6591E6 | 3.7408E5 |  | 2 | 0 | 0 | 0 | 0 | 0 | 0 | 0 | 1 | 1 | 0 | 208 | 220 | Oxidation (M) | M6:Oxidation (M):1000.00 | PEAKS DB |
| R.GDDIPYTPVFYAYTLLTK.T | N | 69.16 | 2076.0454 | 18 | 0.5 | 1039.0305 | 2 | 73.61 | 8 | F8:46146 | 31122021\_RID\_3262\_1a.raw |  |  |  |  |  |  |  | 3.5587E6 | 8.7984E5 |  | 3 | 0 | 0 | 0 | 0 | 0 | 0 | 0 | 2 | 1 | 0 | 254 | 271 |  |  | PEAKS DB |
| R.LSWMTGFSGSEGTGVITLQK.A | Y | 68.24 | 2098.0405 | 20 | 0.5 | 1050.0281 | 2 | 57.49 | 8 | F8:34287 | 31122021\_RID\_3262\_1a.raw |  |  |  |  |  |  |  | 1.4558E6 |  |  | 1 | 0 | 0 | 0 | 0 | 0 | 0 | 0 | 1 | 0 | 0 | 90 | 109 |  |  | PEAKS DB |
| R.LEDVALVVQAK.T | N | 65.14 | 1183.6812 | 11 | 0.4 | 592.8481 | 2 | 40.58 | 9 | F9:21971 | 31122021\_RID\_3262\_2a.raw |  |  |  |  |  |  |  | 6.1145E6 | 1.2348E6 |  | 2 | 0 | 0 | 0 | 0 | 0 | 0 | 0 | 1 | 1 | 0 | 570 | 580 |  |  | PEAKS DB |
| K.MMEAAHIR.D | N | 59.97 | 957.4525 | 8 | 0.4 | 479.7337 | 2 | 19.60 | 8 | F8:4812 | 31122021\_RID\_3262\_1a.raw |  |  |  |  |  |  |  | 3.7959E5 | 9.5521E4 |  | 2 | 0 | 0 | 0 | 0 | 0 | 0 | 0 | 1 | 1 | 0 | 366 | 373 |  |  | PEAKS DB |
| R.TIHWGEPTAFQK.E | N | 59.60 | 1413.7041 | 12 | 0.4 | 472.2422 | 3 | 34.27 | 8 | F8:15881 | 31122021\_RID\_3262\_1a.raw |  |  |  |  |  |  |  | 2.8249E6 | 0 |  | 3 | 0 | 0 | 0 | 0 | 0 | 0 | 0 | 3 | 0 | 0 | 467 | 478 |  |  | PEAKS DB |
| K.VVSLVPYAR.N | N | 58.37 | 1002.5862 | 9 | 0.3 | 502.3005 | 2 | 38.96 | 8 | F8:19771 | 31122021\_RID\_3262\_1a.raw | 1.0512E6 |  |  |  |  |  |  | 4.0529E6 | 7.1037E5 |  | 3 | 1 | 0 | 0 | 0 | 0 | 0 | 0 | 1 | 1 | 0 | 595 | 603 |  |  | PEAKS DB |
| R.VLM(+15.99)GNIDLSK.L | N | 56.40 | 1104.5848 | 10 | 1.7 | 553.3007 | 2 | 32.94 | 8 | F8:14847 | 31122021\_RID\_3262\_1a.raw |  |  |  |  |  |  |  | 2.0225E6 | 5.6608E5 |  | 3 | 0 | 0 | 0 | 0 | 0 | 0 | 0 | 1 | 2 | 0 | 484 | 493 | Oxidation (M) | M3:Oxidation (M):1000.00 | PEAKS DB |
| K.MM(+15.99)EAAHIR.D | N | 54.61 | 973.4473 | 8 | -0.3 | 487.7308 | 2 | 15.87 | 8 | F8:2900 | 31122021\_RID\_3262\_1a.raw |  |  |  |  |  |  |  | 1.3887E5 |  |  | 3 | 0 | 0 | 0 | 0 | 0 | 0 | 0 | 3 | 0 | 0 | 366 | 373 | Oxidation (M) | M2:Oxidation (M):14.02 | PEAKS DB |
| K.SVWINSIGQWILK.E | Y | 52.88 | 1542.8558 | 13 | 0.8 | 772.4358 | 2 | 64.25 | 8 | F8:39395 | 31122021\_RID\_3262\_1a.raw |  |  |  |  |  |  |  | 1.2817E6 | 2.4499E5 |  | 2 | 0 | 0 | 0 | 0 | 0 | 0 | 0 | 1 | 1 | 0 | 135 | 147 |  |  | PEAKS DB |
| R.VLMGNIDLSK.L | N | 50.63 | 1088.5900 | 10 | 0.9 | 545.3027 | 2 | 40.89 | 8 | F8:21051 | 31122021\_RID\_3262\_1a.raw |  |  |  |  |  |  |  | 3.1E6 | 6.693E5 |  | 2 | 0 | 0 | 0 | 0 | 0 | 0 | 0 | 1 | 1 | 0 | 484 | 493 |  |  | PEAKS DB |
| R.KLSLNEMYLLDSGGQYFDGTTDITR.T | Y | 50.09 | 2836.3589 | 25 | 1.0 | 946.4612 | 3 | 56.97 | 8 | F8:33674 | 31122021\_RID\_3262\_1a.raw |  |  |  |  |  |  |  | 1.7E6 |  |  | 1 | 0 | 0 | 0 | 0 | 0 | 0 | 0 | 1 | 0 | 0 | 442 | 466 |  |  | PEAKS DB |
| K.M(+15.99)MEAAHIR.D | N | 49.26 | 973.4473 | 8 | 0.0 | 487.7310 | 2 | 16.16 | 8 | F8:3110 | 31122021\_RID\_3262\_1a.raw |  |  |  |  |  |  |  | 1.1219E5 | 2.11E4 |  | 3 | 0 | 0 | 0 | 0 | 0 | 0 | 0 | 2 | 1 | 0 | 366 | 373 | Oxidation (M) | M1:Oxidation (M):12.28 | PEAKS DB |
| R.YWIQAER.Q | N | 47.48 | 964.4766 | 7 | -0.6 | 483.2453 | 2 | 31.76 | 9 | F9:14735 | 31122021\_RID\_3262\_2a.raw |  |  |  |  |  |  |  | 1.6204E6 | 4.2744E5 |  | 2 | 0 | 0 | 0 | 0 | 0 | 0 | 0 | 1 | 1 | 0 | 118 | 124 |  |  | PEAKS DB |
| K.NTEPFSHASLL.A | N | 46.54 | 1214.5931 | 11 | 0.1 | 608.3039 | 2 | 41.96 | 8 | F8:22010 | 31122021\_RID\_3262\_1a.raw |  |  |  |  |  |  |  | 3.7494E5 |  |  | 1 | 0 | 0 | 0 | 0 | 0 | 0 | 0 | 1 | 0 | 0 | 649 | 659 |  |  | PEAKS DB |
| R.LADDFMGSTWQEK.V | Y | 44.95 | 1526.6711 | 13 | 0.3 | 764.3431 | 2 | 45.42 | 8 | F8:24590 | 31122021\_RID\_3262\_1a.raw |  |  |  |  |  |  |  | 3.2704E5 |  |  | 1 | 0 | 0 | 0 | 0 | 0 | 0 | 0 | 1 | 0 | 0 | 208 | 220 |  |  | PEAKS DB |
| R.KLSLNEM(+15.99)YLLDSGGQYFDGTTDITR.T | Y | 44.28 | 2852.3538 | 25 | 0.8 | 951.7926 | 3 | 52.84 | 8 | F8:30629 | 31122021\_RID\_3262\_1a.raw |  |  |  |  |  |  |  | 3.6548E5 |  |  | 1 | 0 | 0 | 0 | 0 | 0 | 0 | 0 | 1 | 0 | 0 | 442 | 466 | Oxidation (M) | M7:Oxidation (M):1000.00 | PEAKS DB |
| K.GM(+15.99)FTSIEPGYYHDGEFGIR.L | N | 44.23 | 2190.9680 | 19 | 0.6 | 731.3304 | 3 | 45.51 | 8 | F8:24644 | 31122021\_RID\_3262\_1a.raw |  |  |  |  |  |  |  | 3.9301E5 |  |  | 1 | 0 | 0 | 0 | 0 | 0 | 0 | 0 | 1 | 0 | 0 | 551 | 569 | Oxidation (M) | M2:Oxidation (M):1000.00 | PEAKS DB |
| R.LPPPTNTIYR.L | N | 44.19 | 1170.6396 | 10 | 0.6 | 586.3275 | 2 | 32.12 | 8 | F8:14118 | 31122021\_RID\_3262\_1a.raw |  |  |  |  |  |  |  | 4.4687E6 |  |  | 1 | 0 | 0 | 0 | 0 | 0 | 0 | 0 | 1 | 0 | 0 | 198 | 207 |  |  | PEAKS DB |
| K.EVPAGETIGLDPFLF.S | Y | 42.25 | 1603.8134 | 15 | 1.3 | 802.9150 | 2 | 81.21 | 8 | F8:49296 | 31122021\_RID\_3262\_1a.raw |  |  |  |  |  |  |  | 6.4189E4 |  |  | 1 | 0 | 0 | 0 | 0 | 0 | 0 | 0 | 1 | 0 | 0 | 148 | 162 |  |  | PEAKS DB |
| R.YLVWLEK.N | N | 40.96 | 949.5273 | 7 | 0.1 | 475.7710 | 2 | 45.31 | 9 | F9:25829 | 31122021\_RID\_3262\_2a.raw |  |  |  |  |  |  |  |  | 7.199E5 |  | 1 | 0 | 0 | 0 | 0 | 0 | 0 | 0 | 0 | 1 | 0 | 381 | 387 |  |  | PEAKS DB |
| total 21 peptides |  |  |  |  |  |  |  |  |  |  |  |  |  |  |  |  |  |  |  |  |  |  |  |  |  |  |  |  |  |  |  |  |  |  |  |  |  |
| --- | --- | --- | --- | --- | --- | --- | --- | --- | --- | --- | --- | --- | --- | --- | --- | --- | --- | --- | --- | --- | --- | --- | --- | --- | --- | --- | --- | --- | --- | --- | --- | --- | --- | --- | --- | --- | --- |

JAG45871.1

back to list

  

| Protein Coverage
| Supporting Peptides
|

Protein Coverage:

Supporting Peptides:

| Peptide | Uniq | -10lgP | Mass | Length | ppm | m/z | z | RT | Fraction | Scan | Source File | Area F4 | Area F5 | Area F6 | Area F10 | Area F8 | Area F9 | Area F7 | Area F1 | Area F2 | Area F3 | #Feature | #Feature F4 | #Feature F5 | #Feature F6 | #Feature F10 | #Feature F8 | #Feature F9 | #Feature F7 | #Feature F1 | #Feature F2 | #Feature F3 | Start | End | PTM | AScore | Found By |
| --- | --- | --- | --- | --- | --- | --- | --- | --- | --- | --- | --- | --- | --- | --- | --- | --- | --- | --- | --- | --- | --- | --- | --- | --- | --- | --- | --- | --- | --- | --- | --- | --- | --- | --- | --- | --- | --- |
| R.AQIIDDAFNLAR.A | N | 75.56 | 1345.6990 | 12 | 0.3 | 673.8570 | 2 | 50.14 | 8 | F8:28478 | 31122021\_RID\_3262\_1a.raw |  |  |  |  |  |  |  | 3.2736E6 | 1.2252E5 |  | 2 | 0 | 0 | 0 | 0 | 0 | 0 | 0 | 1 | 1 | 0 | 673 | 684 |  |  | PEAKS DB |
| K.LLATTQMQAADAR.K | N | 74.49 | 1388.7081 | 13 | 0.0 | 695.3613 | 2 | 31.38 | 8 | F8:13582 | 31122021\_RID\_3262\_1a.raw |  |  |  |  |  |  |  | 7.5261E5 | 8.3505E4 |  | 2 | 0 | 0 | 0 | 0 | 0 | 0 | 0 | 1 | 1 | 0 | 219 | 231 |  |  | PEAKS DB |
| R.SSFSFSSLIQSVTQR.F | N | 71.95 | 1672.8420 | 15 | 0.9 | 837.4290 | 2 | 61.99 | 8 | F8:37658 | 31122021\_RID\_3262\_1a.raw |  |  |  |  |  |  |  | 1.0872E6 |  |  | 1 | 0 | 0 | 0 | 0 | 0 | 0 | 0 | 1 | 0 | 0 | 908 | 922 |  |  | PEAKS DB |
| K.LLATTQM(+15.99)QAADAR.K | N | 65.52 | 1404.7031 | 13 | -0.3 | 703.3586 | 2 | 24.51 | 8 | F8:8317 | 31122021\_RID\_3262\_1a.raw |  |  |  |  |  |  |  | 1.3418E5 |  |  | 2 | 0 | 0 | 0 | 0 | 0 | 0 | 0 | 2 | 0 | 0 | 219 | 231 | Oxidation (M) | M7:Oxidation (M):1000.00 | PEAKS DB |
| R.VNYDLENWQR.L | N | 60.46 | 1335.6207 | 10 | 1.2 | 668.8184 | 2 | 39.79 | 8 | F8:20296 | 31122021\_RID\_3262\_1a.raw |  |  |  |  |  |  |  | 5.408E5 |  |  | 1 | 0 | 0 | 0 | 0 | 0 | 0 | 0 | 1 | 0 | 0 | 646 | 655 |  |  | PEAKS DB |
| R.MLSEFLTEAVFR.E | N | 59.94 | 1441.7275 | 12 | 0.1 | 721.8711 | 2 | 61.39 | 8 | F8:37205 | 31122021\_RID\_3262\_1a.raw |  |  |  |  |  |  |  | 8.542E5 |  |  | 1 | 0 | 0 | 0 | 0 | 0 | 0 | 0 | 1 | 0 | 0 | 499 | 510 |  |  | PEAKS DB |
| R.EYLPWDTALDNLDYFR.L | N | 58.93 | 2029.9421 | 16 | 0.4 | 1015.9788 | 2 | 70.77 | 8 | F8:44367 | 31122021\_RID\_3262\_1a.raw |  |  |  |  |  |  |  | 8.8271E5 |  |  | 1 | 0 | 0 | 0 | 0 | 0 | 0 | 0 | 1 | 0 | 0 | 705 | 720 |  |  | PEAKS DB |
| R.DNADVGFGSATR.A | N | 58.75 | 1208.5421 | 12 | 0.0 | 605.2783 | 2 | 30.25 | 8 | F8:12548 | 31122021\_RID\_3262\_1a.raw |  |  |  |  |  |  |  | 3.9187E5 | 4.7892E4 |  | 2 | 0 | 0 | 0 | 0 | 0 | 0 | 0 | 1 | 1 | 0 | 938 | 949 |  |  | PEAKS DB |
| R.FSSPFELQQLEQFK.R | N | 56.17 | 1726.8566 | 14 | 0.2 | 864.4357 | 2 | 57.80 | 8 | F8:34343 | 31122021\_RID\_3262\_1a.raw |  |  |  |  |  |  |  | 6.2382E5 |  |  | 1 | 0 | 0 | 0 | 0 | 0 | 0 | 0 | 1 | 0 | 0 | 923 | 936 |  |  | PEAKS DB |
| R.AQIIDDAFNLA.R | N | 54.92 | 1189.5979 | 11 | 0.3 | 595.8064 | 2 | 60.82 | 8 | F8:36668 | 31122021\_RID\_3262\_1a.raw |  |  |  |  |  |  |  | 1.142E5 |  |  | 1 | 0 | 0 | 0 | 0 | 0 | 0 | 0 | 1 | 0 | 0 | 673 | 683 |  |  | PEAKS DB |
| R.M(+15.99)LSEFLTEAVFR.E | N | 53.04 | 1457.7224 | 12 | 0.4 | 729.8688 | 2 | 57.78 | 8 | F8:34334 | 31122021\_RID\_3262\_1a.raw |  |  |  |  |  |  |  | 1.8859E5 |  |  | 1 | 0 | 0 | 0 | 0 | 0 | 0 | 0 | 1 | 0 | 0 | 499 | 510 | Oxidation (M) | M1:Oxidation (M):1000.00 | PEAKS DB |
| K.TLFQQFGR.S | N | 52.24 | 995.5189 | 8 | 0.0 | 498.7667 | 2 | 39.45 | 8 | F8:19916 | 31122021\_RID\_3262\_1a.raw |  |  |  |  |  |  |  | 7.6108E5 |  |  | 1 | 0 | 0 | 0 | 0 | 0 | 0 | 0 | 1 | 0 | 0 | 900 | 907 |  |  | PEAKS DB |
| R.YLQYTLDPSK.I | Y | 50.36 | 1226.6183 | 10 | -0.3 | 614.3162 | 2 | 37.95 | 8 | F8:18716 | 31122021\_RID\_3262\_1a.raw |  |  |  |  |  |  |  | 7.1673E5 |  |  | 1 | 0 | 0 | 0 | 0 | 0 | 0 | 0 | 1 | 0 | 0 | 858 | 867 |  |  | PEAKS DB |
| R.SSFSFSSLIQSVTQ.R | N | 50.02 | 1516.7410 | 14 | 1.2 | 759.3787 | 2 | 71.85 | 8 | F8:45049 | 31122021\_RID\_3262\_1a.raw |  |  |  |  |  |  |  | 1.3092E5 |  |  | 1 | 0 | 0 | 0 | 0 | 0 | 0 | 0 | 1 | 0 | 0 | 908 | 921 |  |  | PEAKS DB |
| total 14 peptides |  |  |  |  |  |  |  |  |  |  |  |  |  |  |  |  |  |  |  |  |  |  |  |  |  |  |  |  |  |  |  |  |  |  |  |  |  |
| --- | --- | --- | --- | --- | --- | --- | --- | --- | --- | --- | --- | --- | --- | --- | --- | --- | --- | --- | --- | --- | --- | --- | --- | --- | --- | --- | --- | --- | --- | --- | --- | --- | --- | --- | --- | --- | --- |

JAA97801.1

back to list

  

| Protein Coverage
| Supporting Peptides
|

Protein Coverage:

Supporting Peptides:

| Peptide | Uniq | -10lgP | Mass | Length | ppm | m/z | z | RT | Fraction | Scan | Source File | Area F4 | Area F5 | Area F6 | Area F10 | Area F8 | Area F9 | Area F7 | Area F1 | Area F2 | Area F3 | #Feature | #Feature F4 | #Feature F5 | #Feature F6 | #Feature F10 | #Feature F8 | #Feature F9 | #Feature F7 | #Feature F1 | #Feature F2 | #Feature F3 | Start | End | PTM | AScore | Found By |
| --- | --- | --- | --- | --- | --- | --- | --- | --- | --- | --- | --- | --- | --- | --- | --- | --- | --- | --- | --- | --- | --- | --- | --- | --- | --- | --- | --- | --- | --- | --- | --- | --- | --- | --- | --- | --- | --- |
| R.AQIIDDAFNLAR.A | N | 75.56 | 1345.6990 | 12 | 0.3 | 673.8570 | 2 | 50.14 | 8 | F8:28478 | 31122021\_RID\_3262\_1a.raw |  |  |  |  |  |  |  | 3.2736E6 | 1.2252E5 |  | 2 | 0 | 0 | 0 | 0 | 0 | 0 | 0 | 1 | 1 | 0 | 673 | 684 |  |  | PEAKS DB |
| K.LLATTQMQAADAR.K | N | 74.49 | 1388.7081 | 13 | 0.0 | 695.3613 | 2 | 31.38 | 8 | F8:13582 | 31122021\_RID\_3262\_1a.raw |  |  |  |  |  |  |  | 7.5261E5 | 8.3505E4 |  | 2 | 0 | 0 | 0 | 0 | 0 | 0 | 0 | 1 | 1 | 0 | 219 | 231 |  |  | PEAKS DB |
| R.SSFSFSSLIQSVTQR.F | N | 71.95 | 1672.8420 | 15 | 0.9 | 837.4290 | 2 | 61.99 | 8 | F8:37658 | 31122021\_RID\_3262\_1a.raw |  |  |  |  |  |  |  | 1.0872E6 |  |  | 1 | 0 | 0 | 0 | 0 | 0 | 0 | 0 | 1 | 0 | 0 | 908 | 922 |  |  | PEAKS DB |
| K.LLATTQM(+15.99)QAADAR.K | N | 65.52 | 1404.7031 | 13 | -0.3 | 703.3586 | 2 | 24.51 | 8 | F8:8317 | 31122021\_RID\_3262\_1a.raw |  |  |  |  |  |  |  | 1.3418E5 |  |  | 2 | 0 | 0 | 0 | 0 | 0 | 0 | 0 | 2 | 0 | 0 | 219 | 231 | Oxidation (M) | M7:Oxidation (M):1000.00 | PEAKS DB |
| R.VNYDLENWQR.L | N | 60.46 | 1335.6207 | 10 | 1.2 | 668.8184 | 2 | 39.79 | 8 | F8:20296 | 31122021\_RID\_3262\_1a.raw |  |  |  |  |  |  |  | 5.408E5 |  |  | 1 | 0 | 0 | 0 | 0 | 0 | 0 | 0 | 1 | 0 | 0 | 646 | 655 |  |  | PEAKS DB |
| R.MLSEFLTEAVFR.E | N | 59.94 | 1441.7275 | 12 | 0.1 | 721.8711 | 2 | 61.39 | 8 | F8:37205 | 31122021\_RID\_3262\_1a.raw |  |  |  |  |  |  |  | 8.542E5 |  |  | 1 | 0 | 0 | 0 | 0 | 0 | 0 | 0 | 1 | 0 | 0 | 499 | 510 |  |  | PEAKS DB |
| R.EYLPWDTALDNLDYFR.L | N | 58.93 | 2029.9421 | 16 | 0.4 | 1015.9788 | 2 | 70.77 | 8 | F8:44367 | 31122021\_RID\_3262\_1a.raw |  |  |  |  |  |  |  | 8.8271E5 |  |  | 1 | 0 | 0 | 0 | 0 | 0 | 0 | 0 | 1 | 0 | 0 | 705 | 720 |  |  | PEAKS DB |
| R.DNADVGFGSATR.A | N | 58.75 | 1208.5421 | 12 | 0.0 | 605.2783 | 2 | 30.25 | 8 | F8:12548 | 31122021\_RID\_3262\_1a.raw |  |  |  |  |  |  |  | 3.9187E5 | 4.7892E4 |  | 2 | 0 | 0 | 0 | 0 | 0 | 0 | 0 | 1 | 1 | 0 | 938 | 949 |  |  | PEAKS DB |
| R.FSSPFELQQLEQFK.R | N | 56.17 | 1726.8566 | 14 | 0.2 | 864.4357 | 2 | 57.80 | 8 | F8:34343 | 31122021\_RID\_3262\_1a.raw |  |  |  |  |  |  |  | 6.2382E5 |  |  | 1 | 0 | 0 | 0 | 0 | 0 | 0 | 0 | 1 | 0 | 0 | 923 | 936 |  |  | PEAKS DB |
| R.AQIIDDAFNLA.R | N | 54.92 | 1189.5979 | 11 | 0.3 | 595.8064 | 2 | 60.82 | 8 | F8:36668 | 31122021\_RID\_3262\_1a.raw |  |  |  |  |  |  |  | 1.142E5 |  |  | 1 | 0 | 0 | 0 | 0 | 0 | 0 | 0 | 1 | 0 | 0 | 673 | 683 |  |  | PEAKS DB |
| R.M(+15.99)LSEFLTEAVFR.E | N | 53.04 | 1457.7224 | 12 | 0.4 | 729.8688 | 2 | 57.78 | 8 | F8:34334 | 31122021\_RID\_3262\_1a.raw |  |  |  |  |  |  |  | 1.8859E5 |  |  | 1 | 0 | 0 | 0 | 0 | 0 | 0 | 0 | 1 | 0 | 0 | 499 | 510 | Oxidation (M) | M1:Oxidation (M):1000.00 | PEAKS DB |
| K.TLFQQFGR.S | N | 52.24 | 995.5189 | 8 | 0.0 | 498.7667 | 2 | 39.45 | 8 | F8:19916 | 31122021\_RID\_3262\_1a.raw |  |  |  |  |  |  |  | 7.6108E5 |  |  | 1 | 0 | 0 | 0 | 0 | 0 | 0 | 0 | 1 | 0 | 0 | 900 | 907 |  |  | PEAKS DB |
| R.YLQYTLDPSK.I | Y | 50.36 | 1226.6183 | 10 | -0.3 | 614.3162 | 2 | 37.95 | 8 | F8:18716 | 31122021\_RID\_3262\_1a.raw |  |  |  |  |  |  |  | 7.1673E5 |  |  | 1 | 0 | 0 | 0 | 0 | 0 | 0 | 0 | 1 | 0 | 0 | 858 | 867 |  |  | PEAKS DB |
| R.SSFSFSSLIQSVTQ.R | N | 50.02 | 1516.7410 | 14 | 1.2 | 759.3787 | 2 | 71.85 | 8 | F8:45049 | 31122021\_RID\_3262\_1a.raw |  |  |  |  |  |  |  | 1.3092E5 |  |  | 1 | 0 | 0 | 0 | 0 | 0 | 0 | 0 | 1 | 0 | 0 | 908 | 921 |  |  | PEAKS DB |
| total 14 peptides |  |  |  |  |  |  |  |  |  |  |  |  |  |  |  |  |  |  |  |  |  |  |  |  |  |  |  |  |  |  |  |  |  |  |  |  |  |
| --- | --- | --- | --- | --- | --- | --- | --- | --- | --- | --- | --- | --- | --- | --- | --- | --- | --- | --- | --- | --- | --- | --- | --- | --- | --- | --- | --- | --- | --- | --- | --- | --- | --- | --- | --- | --- | --- |

JAG47501.1

back to list

  

| Protein Coverage
| Supporting Peptides
|

Protein Coverage:

Supporting Peptides:

| Peptide | Uniq | -10lgP | Mass | Length | ppm | m/z | z | RT | Fraction | Scan | Source File | Area F4 | Area F5 | Area F6 | Area F10 | Area F8 | Area F9 | Area F7 | Area F1 | Area F2 | Area F3 | #Feature | #Feature F4 | #Feature F5 | #Feature F6 | #Feature F10 | #Feature F8 | #Feature F9 | #Feature F7 | #Feature F1 | #Feature F2 | #Feature F3 | Start | End | PTM | AScore | Found By |
| --- | --- | --- | --- | --- | --- | --- | --- | --- | --- | --- | --- | --- | --- | --- | --- | --- | --- | --- | --- | --- | --- | --- | --- | --- | --- | --- | --- | --- | --- | --- | --- | --- | --- | --- | --- | --- | --- |
| R.AQIIDDAFNLAR.A | N | 75.56 | 1345.6990 | 12 | 0.3 | 673.8570 | 2 | 50.14 | 8 | F8:28478 | 31122021\_RID\_3262\_1a.raw |  |  |  |  |  |  |  | 3.2736E6 | 1.2252E5 |  | 2 | 0 | 0 | 0 | 0 | 0 | 0 | 0 | 1 | 1 | 0 | 673 | 684 |  |  | PEAKS DB |
| K.LLATTQMQAADAR.K | N | 74.49 | 1388.7081 | 13 | 0.0 | 695.3613 | 2 | 31.38 | 8 | F8:13582 | 31122021\_RID\_3262\_1a.raw |  |  |  |  |  |  |  | 7.5261E5 | 8.3505E4 |  | 2 | 0 | 0 | 0 | 0 | 0 | 0 | 0 | 1 | 1 | 0 | 219 | 231 |  |  | PEAKS DB |
| R.SSFSFSSLIQSVTQR.F | N | 71.95 | 1672.8420 | 15 | 0.9 | 837.4290 | 2 | 61.99 | 8 | F8:37658 | 31122021\_RID\_3262\_1a.raw |  |  |  |  |  |  |  | 1.0872E6 |  |  | 1 | 0 | 0 | 0 | 0 | 0 | 0 | 0 | 1 | 0 | 0 | 908 | 922 |  |  | PEAKS DB |
| K.LLATTQM(+15.99)QAADAR.K | N | 65.52 | 1404.7031 | 13 | -0.3 | 703.3586 | 2 | 24.51 | 8 | F8:8317 | 31122021\_RID\_3262\_1a.raw |  |  |  |  |  |  |  | 1.3418E5 |  |  | 2 | 0 | 0 | 0 | 0 | 0 | 0 | 0 | 2 | 0 | 0 | 219 | 231 | Oxidation (M) | M7:Oxidation (M):1000.00 | PEAKS DB |
| R.VNYDLENWQR.L | N | 60.46 | 1335.6207 | 10 | 1.2 | 668.8184 | 2 | 39.79 | 8 | F8:20296 | 31122021\_RID\_3262\_1a.raw |  |  |  |  |  |  |  | 5.408E5 |  |  | 1 | 0 | 0 | 0 | 0 | 0 | 0 | 0 | 1 | 0 | 0 | 646 | 655 |  |  | PEAKS DB |
| R.MLSEFLTEAVFR.E | N | 59.94 | 1441.7275 | 12 | 0.1 | 721.8711 | 2 | 61.39 | 8 | F8:37205 | 31122021\_RID\_3262\_1a.raw |  |  |  |  |  |  |  | 8.542E5 |  |  | 1 | 0 | 0 | 0 | 0 | 0 | 0 | 0 | 1 | 0 | 0 | 499 | 510 |  |  | PEAKS DB |
| R.EYLPWDTALDNLDYFR.L | N | 58.93 | 2029.9421 | 16 | 0.4 | 1015.9788 | 2 | 70.77 | 8 | F8:44367 | 31122021\_RID\_3262\_1a.raw |  |  |  |  |  |  |  | 8.8271E5 |  |  | 1 | 0 | 0 | 0 | 0 | 0 | 0 | 0 | 1 | 0 | 0 | 705 | 720 |  |  | PEAKS DB |
| R.DNADVGFGSATR.A | N | 58.75 | 1208.5421 | 12 | 0.0 | 605.2783 | 2 | 30.25 | 8 | F8:12548 | 31122021\_RID\_3262\_1a.raw |  |  |  |  |  |  |  | 3.9187E5 | 4.7892E4 |  | 2 | 0 | 0 | 0 | 0 | 0 | 0 | 0 | 1 | 1 | 0 | 938 | 949 |  |  | PEAKS DB |
| R.FSSPFELQQLEQFK.R | N | 56.17 | 1726.8566 | 14 | 0.2 | 864.4357 | 2 | 57.80 | 8 | F8:34343 | 31122021\_RID\_3262\_1a.raw |  |  |  |  |  |  |  | 6.2382E5 |  |  | 1 | 0 | 0 | 0 | 0 | 0 | 0 | 0 | 1 | 0 | 0 | 923 | 936 |  |  | PEAKS DB |
| R.AQIIDDAFNLA.R | N | 54.92 | 1189.5979 | 11 | 0.3 | 595.8064 | 2 | 60.82 | 8 | F8:36668 | 31122021\_RID\_3262\_1a.raw |  |  |  |  |  |  |  | 1.142E5 |  |  | 1 | 0 | 0 | 0 | 0 | 0 | 0 | 0 | 1 | 0 | 0 | 673 | 683 |  |  | PEAKS DB |
| R.M(+15.99)LSEFLTEAVFR.E | N | 53.04 | 1457.7224 | 12 | 0.4 | 729.8688 | 2 | 57.78 | 8 | F8:34334 | 31122021\_RID\_3262\_1a.raw |  |  |  |  |  |  |  | 1.8859E5 |  |  | 1 | 0 | 0 | 0 | 0 | 0 | 0 | 0 | 1 | 0 | 0 | 499 | 510 | Oxidation (M) | M1:Oxidation (M):1000.00 | PEAKS DB |
| K.TLFQQFGR.S | N | 52.24 | 995.5189 | 8 | 0.0 | 498.7667 | 2 | 39.45 | 8 | F8:19916 | 31122021\_RID\_3262\_1a.raw |  |  |  |  |  |  |  | 7.6108E5 |  |  | 1 | 0 | 0 | 0 | 0 | 0 | 0 | 0 | 1 | 0 | 0 | 900 | 907 |  |  | PEAKS DB |
| R.YLQYTLDPSK.I | Y | 50.36 | 1226.6183 | 10 | -0.3 | 614.3162 | 2 | 37.95 | 8 | F8:18716 | 31122021\_RID\_3262\_1a.raw |  |  |  |  |  |  |  | 7.1673E5 |  |  | 1 | 0 | 0 | 0 | 0 | 0 | 0 | 0 | 1 | 0 | 0 | 858 | 867 |  |  | PEAKS DB |
| R.SSFSFSSLIQSVTQ.R | N | 50.02 | 1516.7410 | 14 | 1.2 | 759.3787 | 2 | 71.85 | 8 | F8:45049 | 31122021\_RID\_3262\_1a.raw |  |  |  |  |  |  |  | 1.3092E5 |  |  | 1 | 0 | 0 | 0 | 0 | 0 | 0 | 0 | 1 | 0 | 0 | 908 | 921 |  |  | PEAKS DB |
| total 14 peptides |  |  |  |  |  |  |  |  |  |  |  |  |  |  |  |  |  |  |  |  |  |  |  |  |  |  |  |  |  |  |  |  |  |  |  |  |  |
| --- | --- | --- | --- | --- | --- | --- | --- | --- | --- | --- | --- | --- | --- | --- | --- | --- | --- | --- | --- | --- | --- | --- | --- | --- | --- | --- | --- | --- | --- | --- | --- | --- | --- | --- | --- | --- | --- |

XP\_032067281.1

back to list

  

| Protein Coverage
| Supporting Peptides
|

Protein Coverage:

Supporting Peptides:

| Peptide | Uniq | -10lgP | Mass | Length | ppm | m/z | z | RT | Fraction | Scan | Source File | Area F4 | Area F5 | Area F6 | Area F10 | Area F8 | Area F9 | Area F7 | Area F1 | Area F2 | Area F3 | #Feature | #Feature F4 | #Feature F5 | #Feature F6 | #Feature F10 | #Feature F8 | #Feature F9 | #Feature F7 | #Feature F1 | #Feature F2 | #Feature F3 | Start | End | PTM | AScore | Found By |
| --- | --- | --- | --- | --- | --- | --- | --- | --- | --- | --- | --- | --- | --- | --- | --- | --- | --- | --- | --- | --- | --- | --- | --- | --- | --- | --- | --- | --- | --- | --- | --- | --- | --- | --- | --- | --- | --- |
| K.LALDIEIATYR.K | N | 65.96 | 1276.7026 | 11 | 1.4 | 639.3595 | 2 | 52.91 | 8 | F8:30667 | 31122021\_RID\_3262\_1a.raw | 2.7385E7 | 1.0818E6 |  | 1.3337E7 |  |  |  | 4.2941E7 | 2.4275E7 | 6.0353E7 | 24 | 1 | 1 | 0 | 2 | 0 | 0 | 0 | 13 | 6 | 1 | 444 | 454 |  |  | PEAKS DB |
| R.LLKEYQELMNVK.L | N | 64.24 | 1506.8115 | 12 | 0.0 | 754.4130 | 2 | 39.68 | 8 | F8:20154 | 31122021\_RID\_3262\_1a.raw |  |  |  |  |  |  |  | 3.2141E6 | 1.0992E5 |  | 3 | 0 | 0 | 0 | 0 | 0 | 0 | 0 | 2 | 1 | 0 | 432 | 443 |  |  | PEAKS DB |
| K.EYQELMNVK.L | N | 62.87 | 1152.5485 | 9 | 0.7 | 577.2819 | 2 | 35.46 | 6 | F6:21360 | 17012022\_RID\_3262\_9a.raw |  |  |  | 3.3062E6 |  | 5.5812E6 |  | 1.6845E6 | 1.1435E6 |  | 4 | 0 | 0 | 0 | 1 | 0 | 1 | 0 | 1 | 1 | 0 | 435 | 443 |  |  | PEAKS DB |
| K.AQYEDIAQK.S | N | 62.68 | 1064.5138 | 9 | -0.5 | 533.2639 | 2 | 24.47 | 4 | F4:11436 | 17012022\_RID\_3262\_10a .raw | 2.7369E8 | 8.02E7 | 1.3508E7 | 8.8805E8 | 3.7376E6 | 7.4491E8 | 3.6578E7 | 1.9002E8 | 1.1631E8 | 2.661E8 | 15 | 1 | 1 | 1 | 4 | 1 | 2 | 1 | 2 | 1 | 1 | 327 | 335 |  |  | PEAKS DB |
| K.KDVDAAYMNK.V | N | 60.37 | 1153.5437 | 10 | -0.1 | 577.7791 | 2 | 20.89 | 6 | F6:8459 | 17012022\_RID\_3262\_9a.raw |  |  |  |  |  | 3.8686E5 |  |  |  |  | 1 | 0 | 0 | 0 | 0 | 0 | 1 | 0 | 0 | 0 | 0 | 260 | 269 |  |  | PEAKS DB |
| K.FASFIDKVR.F | N | 59.23 | 1081.5920 | 9 | 0.3 | 541.8035 | 2 | 35.45 | 6 | F6:21540 | 17012022\_RID\_3262\_9a.raw | 1.5335E7 |  |  | 9.5108E6 |  | 1.2654E7 |  | 1.0527E7 | 1.5026E6 |  | 9 | 1 | 0 | 0 | 3 | 0 | 1 | 0 | 3 | 1 | 0 | 161 | 169 |  |  | PEAKS DB |
| L.ALDIEIATYR.K | N | 57.72 | 1163.6187 | 10 | 0.7 | 582.8170 | 2 | 45.74 | 8 | F8:24835 | 31122021\_RID\_3262\_1a.raw |  |  |  |  |  |  |  | 2.3394E6 | 1.9465E5 |  | 2 | 0 | 0 | 0 | 0 | 0 | 0 | 0 | 1 | 1 | 0 | 445 | 454 |  |  | PEAKS DB |
| K.DVDAAYMNK.V | N | 56.55 | 1025.4487 | 9 | 0.6 | 513.7319 | 2 | 28.82 | 6 | F6:15725 | 17012022\_RID\_3262\_9a.raw |  |  |  |  |  | 4.6178E6 | 2.8794E5 |  | 6.4536E5 |  | 3 | 0 | 0 | 0 | 0 | 0 | 1 | 1 | 0 | 1 | 0 | 261 | 269 |  |  | PEAKS DB |
| R.LLKEYQELM(+15.99)NVK.L | N | 53.34 | 1522.8065 | 12 | -0.1 | 762.4105 | 2 | 33.16 | 8 | F8:14908 | 31122021\_RID\_3262\_1a.raw |  |  |  |  |  |  |  | 1.5111E5 |  |  | 1 | 0 | 0 | 0 | 0 | 0 | 0 | 0 | 1 | 0 | 0 | 432 | 443 | Oxidation (M) | M9:Oxidation (M):1000.00 | PEAKS DB |
| K.FASFIDK.V | N | 52.67 | 826.4225 | 7 | 0.7 | 414.2188 | 2 | 36.24 | 6 | F6:22289 | 17012022\_RID\_3262\_9a.raw | 2.5611E7 | 1.8348E7 | 6.6379E6 | 1.2204E8 |  | 1.3051E8 | 4.5017E6 | 4.1444E7 | 1.2872E7 |  | 15 | 2 | 2 | 1 | 4 | 0 | 3 | 1 | 1 | 1 | 0 | 161 | 167 |  |  | PEAKS DB |
| K.DVDAAYMNKVELQAK.L | N | 51.27 | 1693.8345 | 15 | 0.2 | 847.9247 | 2 | 37.76 | 6 | F6:23539 | 17012022\_RID\_3262\_9a.raw |  |  |  |  |  | 1.5702E5 |  |  | 2.0843E4 |  | 2 | 0 | 0 | 0 | 0 | 0 | 1 | 0 | 0 | 1 | 0 | 261 | 275 |  |  | PEAKS DB |
| R.GELALKDAR.E | N | 49.06 | 971.5399 | 9 | -0.6 | 486.7769 | 2 | 22.10 | 6 | F6:9646 | 17012022\_RID\_3262\_9a.raw |  |  |  | 1.6772E6 |  | 1.879E6 | 8.5932E4 |  |  |  | 3 | 0 | 0 | 0 | 1 | 0 | 1 | 1 | 0 | 0 | 0 | 404 | 412 |  |  | PEAKS DB |
| K.EYQELM(+15.99)NVK.L | N | 46.90 | 1168.5435 | 9 | -0.5 | 585.2787 | 2 | 27.89 | 4 | F4:14727 | 17012022\_RID\_3262\_10a .raw |  |  |  | 1.3738E6 |  |  |  | 6.2988E5 | 4.4468E5 |  | 4 | 0 | 0 | 0 | 1 | 0 | 0 | 0 | 2 | 1 | 0 | 435 | 443 | Oxidation (M) | M6:Oxidation (M):1000.00 | PEAKS DB |
| A.LDIEIATYR.K | N | 43.99 | 1092.5815 | 9 | 0.1 | 547.2981 | 2 | 45.00 | 8 | F8:24235 | 31122021\_RID\_3262\_1a.raw |  |  |  |  |  |  |  | 8.2061E5 |  |  | 1 | 0 | 0 | 0 | 0 | 0 | 0 | 0 | 1 | 0 | 0 | 446 | 454 |  |  | PEAKS DB |
| K.FASFIDKV.R | N | 43.37 | 925.4908 | 8 | 0.2 | 463.7528 | 2 | 44.26 | 4 | F4:28348 | 17012022\_RID\_3262\_10a .raw |  |  |  | 1.1458E7 |  |  |  | 5.4178E6 | 1.9415E6 |  | 3 | 0 | 0 | 0 | 1 | 0 | 0 | 0 | 1 | 1 | 0 | 161 | 168 |  |  | PEAKS DB |
| K.YEELQVSVGR.H | Y | 42.94 | 1178.5931 | 10 | -0.1 | 590.3038 | 2 | 36.67 | 3 | F3:16710 | 13012022\_RID\_3262\_6a.raw |  |  | 0 |  |  |  |  |  |  |  | 0 | 0 | 0 | 0 | 0 | 0 | 0 | 0 | 0 | 0 | 0 | 348 | 357 |  |  | PEAKS DB |
| V.KAQYEDIAQK.S | N | 41.09 | 1192.6088 | 10 | 0.6 | 597.3120 | 2 | 20.16 | 3 | F3:6968 | 13012022\_RID\_3262\_6a.raw |  |  | 3.7665E4 |  |  |  |  |  |  |  | 1 | 0 | 0 | 1 | 0 | 0 | 0 | 0 | 0 | 0 | 0 | 326 | 335 |  |  | PEAKS DB |
| total 17 peptides |  |  |  |  |  |  |  |  |  |  |  |  |  |  |  |  |  |  |  |  |  |  |  |  |  |  |  |  |  |  |  |  |  |  |  |  |  |
| --- | --- | --- | --- | --- | --- | --- | --- | --- | --- | --- | --- | --- | --- | --- | --- | --- | --- | --- | --- | --- | --- | --- | --- | --- | --- | --- | --- | --- | --- | --- | --- | --- | --- | --- | --- | --- | --- |

XP\_013915209.1

back to list

  

| Protein Coverage
| Supporting Peptides
|

Protein Coverage:

Supporting Peptides:

| Peptide | Uniq | -10lgP | Mass | Length | ppm | m/z | z | RT | Fraction | Scan | Source File | Area F4 | Area F5 | Area F6 | Area F10 | Area F8 | Area F9 | Area F7 | Area F1 | Area F2 | Area F3 | #Feature | #Feature F4 | #Feature F5 | #Feature F6 | #Feature F10 | #Feature F8 | #Feature F9 | #Feature F7 | #Feature F1 | #Feature F2 | #Feature F3 | Start | End | PTM | AScore | Found By |
| --- | --- | --- | --- | --- | --- | --- | --- | --- | --- | --- | --- | --- | --- | --- | --- | --- | --- | --- | --- | --- | --- | --- | --- | --- | --- | --- | --- | --- | --- | --- | --- | --- | --- | --- | --- | --- | --- |
| K.LALDIEIATYR.K | N | 65.96 | 1276.7026 | 11 | 1.4 | 639.3595 | 2 | 52.91 | 8 | F8:30667 | 31122021\_RID\_3262\_1a.raw | 2.7385E7 | 1.0818E6 |  | 1.3337E7 |  |  |  | 4.2941E7 | 2.4275E7 | 6.0353E7 | 24 | 1 | 1 | 0 | 2 | 0 | 0 | 0 | 13 | 6 | 1 | 444 | 454 |  |  | PEAKS DB |
| R.LLKEYQELMNVK.L | N | 64.24 | 1506.8115 | 12 | 0.0 | 754.4130 | 2 | 39.68 | 8 | F8:20154 | 31122021\_RID\_3262\_1a.raw |  |  |  |  |  |  |  | 3.2141E6 | 1.0992E5 |  | 3 | 0 | 0 | 0 | 0 | 0 | 0 | 0 | 2 | 1 | 0 | 432 | 443 |  |  | PEAKS DB |
| K.EYQELMNVK.L | N | 62.87 | 1152.5485 | 9 | 0.7 | 577.2819 | 2 | 35.46 | 6 | F6:21360 | 17012022\_RID\_3262\_9a.raw |  |  |  | 3.3062E6 |  | 5.5812E6 |  | 1.6845E6 | 1.1435E6 |  | 4 | 0 | 0 | 0 | 1 | 0 | 1 | 0 | 1 | 1 | 0 | 435 | 443 |  |  | PEAKS DB |
| K.AQYEDIAQK.S | N | 62.68 | 1064.5138 | 9 | -0.5 | 533.2639 | 2 | 24.47 | 4 | F4:11436 | 17012022\_RID\_3262\_10a .raw | 2.7369E8 | 8.02E7 | 1.3508E7 | 8.8805E8 | 3.7376E6 | 7.4491E8 | 3.6578E7 | 1.9002E8 | 1.1631E8 | 2.661E8 | 15 | 1 | 1 | 1 | 4 | 1 | 2 | 1 | 2 | 1 | 1 | 327 | 335 |  |  | PEAKS DB |
| K.KDVDAAYMNK.V | N | 60.37 | 1153.5437 | 10 | -0.1 | 577.7791 | 2 | 20.89 | 6 | F6:8459 | 17012022\_RID\_3262\_9a.raw |  |  |  |  |  | 3.8686E5 |  |  |  |  | 1 | 0 | 0 | 0 | 0 | 0 | 1 | 0 | 0 | 0 | 0 | 260 | 269 |  |  | PEAKS DB |
| K.FASFIDKVR.F | N | 59.23 | 1081.5920 | 9 | 0.3 | 541.8035 | 2 | 35.45 | 6 | F6:21540 | 17012022\_RID\_3262\_9a.raw | 1.5335E7 |  |  | 9.5108E6 |  | 1.2654E7 |  | 1.0527E7 | 1.5026E6 |  | 9 | 1 | 0 | 0 | 3 | 0 | 1 | 0 | 3 | 1 | 0 | 161 | 169 |  |  | PEAKS DB |
| L.ALDIEIATYR.K | N | 57.72 | 1163.6187 | 10 | 0.7 | 582.8170 | 2 | 45.74 | 8 | F8:24835 | 31122021\_RID\_3262\_1a.raw |  |  |  |  |  |  |  | 2.3394E6 | 1.9465E5 |  | 2 | 0 | 0 | 0 | 0 | 0 | 0 | 0 | 1 | 1 | 0 | 445 | 454 |  |  | PEAKS DB |
| K.DVDAAYMNK.V | N | 56.55 | 1025.4487 | 9 | 0.6 | 513.7319 | 2 | 28.82 | 6 | F6:15725 | 17012022\_RID\_3262\_9a.raw |  |  |  |  |  | 4.6178E6 | 2.8794E5 |  | 6.4536E5 |  | 3 | 0 | 0 | 0 | 0 | 0 | 1 | 1 | 0 | 1 | 0 | 261 | 269 |  |  | PEAKS DB |
| R.LLKEYQELM(+15.99)NVK.L | N | 53.34 | 1522.8065 | 12 | -0.1 | 762.4105 | 2 | 33.16 | 8 | F8:14908 | 31122021\_RID\_3262\_1a.raw |  |  |  |  |  |  |  | 1.5111E5 |  |  | 1 | 0 | 0 | 0 | 0 | 0 | 0 | 0 | 1 | 0 | 0 | 432 | 443 | Oxidation (M) | M9:Oxidation (M):1000.00 | PEAKS DB |
| K.FASFIDK.V | N | 52.67 | 826.4225 | 7 | 0.7 | 414.2188 | 2 | 36.24 | 6 | F6:22289 | 17012022\_RID\_3262\_9a.raw | 2.5611E7 | 1.8348E7 | 6.6379E6 | 1.2204E8 |  | 1.3051E8 | 4.5017E6 | 4.1444E7 | 1.2872E7 |  | 15 | 2 | 2 | 1 | 4 | 0 | 3 | 1 | 1 | 1 | 0 | 161 | 167 |  |  | PEAKS DB |
| K.DVDAAYMNKVELQAK.L | N | 51.27 | 1693.8345 | 15 | 0.2 | 847.9247 | 2 | 37.76 | 6 | F6:23539 | 17012022\_RID\_3262\_9a.raw |  |  |  |  |  | 1.5702E5 |  |  | 2.0843E4 |  | 2 | 0 | 0 | 0 | 0 | 0 | 1 | 0 | 0 | 1 | 0 | 261 | 275 |  |  | PEAKS DB |
| R.GELALKDAR.E | N | 49.06 | 971.5399 | 9 | -0.6 | 486.7769 | 2 | 22.10 | 6 | F6:9646 | 17012022\_RID\_3262\_9a.raw |  |  |  | 1.6772E6 |  | 1.879E6 | 8.5932E4 |  |  |  | 3 | 0 | 0 | 0 | 1 | 0 | 1 | 1 | 0 | 0 | 0 | 404 | 412 |  |  | PEAKS DB |
| K.EYQELM(+15.99)NVK.L | N | 46.90 | 1168.5435 | 9 | -0.5 | 585.2787 | 2 | 27.89 | 4 | F4:14727 | 17012022\_RID\_3262\_10a .raw |  |  |  | 1.3738E6 |  |  |  | 6.2988E5 | 4.4468E5 |  | 4 | 0 | 0 | 0 | 1 | 0 | 0 | 0 | 2 | 1 | 0 | 435 | 443 | Oxidation (M) | M6:Oxidation (M):1000.00 | PEAKS DB |
| A.LDIEIATYR.K | N | 43.99 | 1092.5815 | 9 | 0.1 | 547.2981 | 2 | 45.00 | 8 | F8:24235 | 31122021\_RID\_3262\_1a.raw |  |  |  |  |  |  |  | 8.2061E5 |  |  | 1 | 0 | 0 | 0 | 0 | 0 | 0 | 0 | 1 | 0 | 0 | 446 | 454 |  |  | PEAKS DB |
| K.FASFIDKV.R | N | 43.37 | 925.4908 | 8 | 0.2 | 463.7528 | 2 | 44.26 | 4 | F4:28348 | 17012022\_RID\_3262\_10a .raw |  |  |  | 1.1458E7 |  |  |  | 5.4178E6 | 1.9415E6 |  | 3 | 0 | 0 | 0 | 1 | 0 | 0 | 0 | 1 | 1 | 0 | 161 | 168 |  |  | PEAKS DB |
| K.YEELQVSVGR.H | Y | 42.94 | 1178.5931 | 10 | -0.1 | 590.3038 | 2 | 36.67 | 3 | F3:16710 | 13012022\_RID\_3262\_6a.raw |  |  | 0 |  |  |  |  |  |  |  | 0 | 0 | 0 | 0 | 0 | 0 | 0 | 0 | 0 | 0 | 0 | 348 | 357 |  |  | PEAKS DB |
| V.KAQYEDIAQK.S | N | 41.09 | 1192.6088 | 10 | 0.6 | 597.3120 | 2 | 20.16 | 3 | F3:6968 | 13012022\_RID\_3262\_6a.raw |  |  | 3.7665E4 |  |  |  |  |  |  |  | 1 | 0 | 0 | 1 | 0 | 0 | 0 | 0 | 0 | 0 | 0 | 326 | 335 |  |  | PEAKS DB |
| total 17 peptides |  |  |  |  |  |  |  |  |  |  |  |  |  |  |  |  |  |  |  |  |  |  |  |  |  |  |  |  |  |  |  |  |  |  |  |  |  |
| --- | --- | --- | --- | --- | --- | --- | --- | --- | --- | --- | --- | --- | --- | --- | --- | --- | --- | --- | --- | --- | --- | --- | --- | --- | --- | --- | --- | --- | --- | --- | --- | --- | --- | --- | --- | --- | --- |

O13063.1

back to list

  

| Protein Coverage
| Supporting Peptides
|

Protein Coverage:

Supporting Peptides:

| Peptide | Uniq | -10lgP | Mass | Length | ppm | m/z | z | RT | Fraction | Scan | Source File | Area F4 | Area F5 | Area F6 | Area F10 | Area F8 | Area F9 | Area F7 | Area F1 | Area F2 | Area F3 | #Feature | #Feature F4 | #Feature F5 | #Feature F6 | #Feature F10 | #Feature F8 | #Feature F9 | #Feature F7 | #Feature F1 | #Feature F2 | #Feature F3 | Start | End | PTM | AScore | Found By |
| --- | --- | --- | --- | --- | --- | --- | --- | --- | --- | --- | --- | --- | --- | --- | --- | --- | --- | --- | --- | --- | --- | --- | --- | --- | --- | --- | --- | --- | --- | --- | --- | --- | --- | --- | --- | --- | --- |
| L.GVHSASVLNDDEQARDPEEK.Y | N | 75.44 | 2195.0090 | 20 | 0.4 | 732.6772 | 3 | 24.08 | 1 | F1:11036 | 13012022\_RID\_3262\_4a.raw | 1.7822E7 |  |  |  |  |  |  |  |  |  | 2 | 2 | 0 | 0 | 0 | 0 | 0 | 0 | 0 | 0 | 0 | 76 | 95 |  |  | PEAKS DB |
| L.LGVHSASVLNDDEQARDPEEK.Y | N | 67.87 | 2308.0930 | 21 | 0.8 | 770.3722 | 3 | 28.73 | 1 | F1:15241 | 13012022\_RID\_3262\_4a.raw | 3.9488E7 |  |  |  |  |  |  |  |  |  | 2 | 2 | 0 | 0 | 0 | 0 | 0 | 0 | 0 | 0 | 0 | 75 | 95 |  |  | PEAKS DB |
| Q.ILLGVHSASVLNDDEQARDPEEK.Y | N | 63.86 | 2534.2612 | 23 | 0.3 | 634.5728 | 4 | 36.54 | 1 | F1:22421 | 13012022\_RID\_3262\_4a.raw | 4.254E6 |  |  |  |  |  |  |  |  |  | 2 | 2 | 0 | 0 | 0 | 0 | 0 | 0 | 0 | 0 | 0 | 73 | 95 |  |  | PEAKS DB |
| H.SASVLNDDEQARDPEEK.Y | N | 58.90 | 1901.8602 | 17 | -0.1 | 951.9373 | 2 | 25.24 | 1 | F1:12058 | 13012022\_RID\_3262\_4a.raw | 4.7582E7 |  |  |  |  |  |  |  |  |  | 2 | 2 | 0 | 0 | 0 | 0 | 0 | 0 | 0 | 0 | 0 | 79 | 95 |  |  | PEAKS DB |
| E.WDKDIMLIR.L | N | 57.88 | 1188.6324 | 9 | 0.5 | 595.3238 | 2 | 43.59 | 1 | F1:28744 | 13012022\_RID\_3262\_4a.raw | 6.6853E8 | 1.3316E6 |  |  |  |  | 2.7704E4 | 1.1405E7 | 1.608E6 |  | 12 | 5 | 3 | 0 | 0 | 0 | 0 | 1 | 2 | 1 | 0 | 107 | 115 |  |  | PEAKS DB |
| N.SVHIAPLSLPSSPPR.L | Y | 55.79 | 1556.8674 | 15 | 0.3 | 519.9633 | 3 | 40.22 | 1 | F1:25864 | 13012022\_RID\_3262\_4a.raw | 1.6372E8 |  |  |  |  |  |  |  |  |  | 2 | 2 | 0 | 0 | 0 | 0 | 0 | 0 | 0 | 0 | 0 | 123 | 137 |  |  | PEAKS DB |
| S.ASVLNDDEQARDPEEK.Y | N | 55.39 | 1814.8281 | 16 | 0.2 | 605.9501 | 3 | 24.88 | 1 | F1:11748 | 13012022\_RID\_3262\_4a.raw | 1.4418E6 |  |  |  |  |  |  |  |  |  | 1 | 1 | 0 | 0 | 0 | 0 | 0 | 0 | 0 | 0 | 0 | 80 | 95 |  |  | PEAKS DB |
| N.MQILLGVHSASVLNDDEQARDPEEK.Y | N | 54.04 | 2793.3604 | 25 | 0.4 | 699.3477 | 4 | 41.70 | 1 | F1:27094 | 13012022\_RID\_3262\_4a.raw | 3.1954E6 |  |  |  |  |  |  |  |  |  | 2 | 2 | 0 | 0 | 0 | 0 | 0 | 0 | 0 | 0 | 0 | 71 | 95 |  |  | PEAKS DB |
| G.VHSASVLNDDEQARDPEEK.Y | N | 51.20 | 2137.9875 | 19 | 0.7 | 713.6703 | 3 | 23.72 | 1 | F1:10792 | 13012022\_RID\_3262\_4a.raw | 8.2553E5 |  |  |  |  |  |  |  |  |  | 1 | 1 | 0 | 0 | 0 | 0 | 0 | 0 | 0 | 0 | 0 | 77 | 95 |  |  | PEAKS DB |
| V.HSASVLNDDEQARDPEEK.Y | N | 48.73 | 2038.9192 | 18 | 0.8 | 680.6476 | 3 | 22.30 | 1 | F1:9539 | 13012022\_RID\_3262\_4a.raw | 7.4669E5 |  |  |  |  |  |  |  |  |  | 1 | 1 | 0 | 0 | 0 | 0 | 0 | 0 | 0 | 0 | 0 | 78 | 95 |  |  | PEAKS DB |
| A.SVLNDDEQARDPEEK.Y | N | 45.09 | 1743.7910 | 15 | -0.3 | 582.2708 | 3 | 24.63 | 1 | F1:11456 | 13012022\_RID\_3262\_4a.raw | 1.8384E7 |  |  |  |  |  |  |  |  |  | 1 | 1 | 0 | 0 | 0 | 0 | 0 | 0 | 0 | 0 | 0 | 81 | 95 |  |  | PEAKS DB |
| E.WDKDIM(+15.99)LIR.L | N | 44.48 | 1204.6274 | 9 | 0.7 | 603.3214 | 2 | 37.71 | 2 | F2:20647 | 13012022\_RID\_3262\_5a.raw | 1.4316E8 | 4.5531E6 |  |  |  |  |  |  |  |  | 2 | 1 | 1 | 0 | 0 | 0 | 0 | 0 | 0 | 0 | 0 | 107 | 115 | Oxidation (M) | M6:Oxidation (M):1000.00 | PEAKS DB |
| N.NSVHIAPLSLPSSPPR.L | Y | 43.81 | 1670.9104 | 16 | -0.4 | 557.9772 | 3 | 39.97 | 1 | F1:25460 | 13012022\_RID\_3262\_4a.raw | 1.1729E6 |  |  |  |  |  |  |  |  |  | 1 | 1 | 0 | 0 | 0 | 0 | 0 | 0 | 0 | 0 | 0 | 122 | 137 |  |  | PEAKS DB |
| D.TEWDKDIMLIR.L | N | 42.16 | 1418.7228 | 11 | 0.0 | 473.9149 | 3 | 46.59 | 1 | F1:31460 | 13012022\_RID\_3262\_4a.raw | 4.7141E6 |  |  |  |  |  |  |  |  |  | 1 | 1 | 0 | 0 | 0 | 0 | 0 | 0 | 0 | 0 | 0 | 105 | 115 |  |  | PEAKS DB |
| W.DKDIMLIR.L | N | 41.99 | 1002.5532 | 8 | 0.7 | 502.2842 | 2 | 34.59 | 7 | F7:15514 | 21012022\_RID\_3262\_7a.raw |  |  |  |  |  |  | 2.6461E6 |  |  |  | 1 | 0 | 0 | 0 | 0 | 0 | 0 | 1 | 0 | 0 | 0 | 108 | 115 |  |  | PEAKS DB |
| I.LLGVHSASVLNDDEQARDPEEK.Y | N | 41.98 | 2421.1772 | 22 | 0.3 | 606.3018 | 4 | 33.30 | 1 | F1:19473 | 13012022\_RID\_3262\_4a.raw | 2.1612E6 |  |  |  |  |  |  |  |  |  | 1 | 1 | 0 | 0 | 0 | 0 | 0 | 0 | 0 | 0 | 0 | 74 | 95 |  |  | PEAKS DB |
| H.IAPLSLPSSPPR.L | N | 41.82 | 1233.7080 | 12 | -0.5 | 617.8610 | 2 | 41.68 | 1 | F1:27192 | 13012022\_RID\_3262\_4a.raw | 1.0328E7 |  |  |  |  |  |  |  |  |  | 1 | 1 | 0 | 0 | 0 | 0 | 0 | 0 | 0 | 0 | 0 | 126 | 137 |  |  | PEAKS DB |
| L.VIGGDEC(+57.02)NINEHR.S | N | 41.29 | 1511.6787 | 13 | 0.4 | 756.8469 | 2 | 21.60 | 2 | F2:8211 | 13012022\_RID\_3262\_5a.raw |  | 6.6457E6 |  |  |  |  |  |  |  |  | 1 | 0 | 1 | 0 | 0 | 0 | 0 | 0 | 0 | 0 | 0 | 25 | 37 | Carbamidomethylation | C7:Carbamidomethylation:1000.00 | PEAKS DB |
| total 18 peptides |  |  |  |  |  |  |  |  |  |  |  |  |  |  |  |  |  |  |  |  |  |  |  |  |  |  |  |  |  |  |  |  |  |  |  |  |  |
| --- | --- | --- | --- | --- | --- | --- | --- | --- | --- | --- | --- | --- | --- | --- | --- | --- | --- | --- | --- | --- | --- | --- | --- | --- | --- | --- | --- | --- | --- | --- | --- | --- | --- | --- | --- | --- | --- |

BAA19983.1

back to list

  

| Protein Coverage
| Supporting Peptides
|

Protein Coverage:

Supporting Peptides:

| Peptide | Uniq | -10lgP | Mass | Length | ppm | m/z | z | RT | Fraction | Scan | Source File | Area F4 | Area F5 | Area F6 | Area F10 | Area F8 | Area F9 | Area F7 | Area F1 | Area F2 | Area F3 | #Feature | #Feature F4 | #Feature F5 | #Feature F6 | #Feature F10 | #Feature F8 | #Feature F9 | #Feature F7 | #Feature F1 | #Feature F2 | #Feature F3 | Start | End | PTM | AScore | Found By |
| --- | --- | --- | --- | --- | --- | --- | --- | --- | --- | --- | --- | --- | --- | --- | --- | --- | --- | --- | --- | --- | --- | --- | --- | --- | --- | --- | --- | --- | --- | --- | --- | --- | --- | --- | --- | --- | --- |
| L.GVHSASVLNDDEQARDPEEK.Y | N | 75.44 | 2195.0090 | 20 | 0.4 | 732.6772 | 3 | 24.08 | 1 | F1:11036 | 13012022\_RID\_3262\_4a.raw | 1.7822E7 |  |  |  |  |  |  |  |  |  | 2 | 2 | 0 | 0 | 0 | 0 | 0 | 0 | 0 | 0 | 0 | 76 | 95 |  |  | PEAKS DB |
| L.LGVHSASVLNDDEQARDPEEK.Y | N | 67.87 | 2308.0930 | 21 | 0.8 | 770.3722 | 3 | 28.73 | 1 | F1:15241 | 13012022\_RID\_3262\_4a.raw | 3.9488E7 |  |  |  |  |  |  |  |  |  | 2 | 2 | 0 | 0 | 0 | 0 | 0 | 0 | 0 | 0 | 0 | 75 | 95 |  |  | PEAKS DB |
| Q.ILLGVHSASVLNDDEQARDPEEK.Y | N | 63.86 | 2534.2612 | 23 | 0.3 | 634.5728 | 4 | 36.54 | 1 | F1:22421 | 13012022\_RID\_3262\_4a.raw | 4.254E6 |  |  |  |  |  |  |  |  |  | 2 | 2 | 0 | 0 | 0 | 0 | 0 | 0 | 0 | 0 | 0 | 73 | 95 |  |  | PEAKS DB |
| H.SASVLNDDEQARDPEEK.Y | N | 58.90 | 1901.8602 | 17 | -0.1 | 951.9373 | 2 | 25.24 | 1 | F1:12058 | 13012022\_RID\_3262\_4a.raw | 4.7582E7 |  |  |  |  |  |  |  |  |  | 2 | 2 | 0 | 0 | 0 | 0 | 0 | 0 | 0 | 0 | 0 | 79 | 95 |  |  | PEAKS DB |
| E.WDKDIMLIR.L | N | 57.88 | 1188.6324 | 9 | 0.5 | 595.3238 | 2 | 43.59 | 1 | F1:28744 | 13012022\_RID\_3262\_4a.raw | 6.6853E8 | 1.3316E6 |  |  |  |  | 2.7704E4 | 1.1405E7 | 1.608E6 |  | 12 | 5 | 3 | 0 | 0 | 0 | 0 | 1 | 2 | 1 | 0 | 107 | 115 |  |  | PEAKS DB |
| N.SVHIAPLSLPSSPPR.L | Y | 55.79 | 1556.8674 | 15 | 0.3 | 519.9633 | 3 | 40.22 | 1 | F1:25864 | 13012022\_RID\_3262\_4a.raw | 1.6372E8 |  |  |  |  |  |  |  |  |  | 2 | 2 | 0 | 0 | 0 | 0 | 0 | 0 | 0 | 0 | 0 | 123 | 137 |  |  | PEAKS DB |
| S.ASVLNDDEQARDPEEK.Y | N | 55.39 | 1814.8281 | 16 | 0.2 | 605.9501 | 3 | 24.88 | 1 | F1:11748 | 13012022\_RID\_3262\_4a.raw | 1.4418E6 |  |  |  |  |  |  |  |  |  | 1 | 1 | 0 | 0 | 0 | 0 | 0 | 0 | 0 | 0 | 0 | 80 | 95 |  |  | PEAKS DB |
| N.MQILLGVHSASVLNDDEQARDPEEK.Y | N | 54.04 | 2793.3604 | 25 | 0.4 | 699.3477 | 4 | 41.70 | 1 | F1:27094 | 13012022\_RID\_3262\_4a.raw | 3.1954E6 |  |  |  |  |  |  |  |  |  | 2 | 2 | 0 | 0 | 0 | 0 | 0 | 0 | 0 | 0 | 0 | 71 | 95 |  |  | PEAKS DB |
| G.VHSASVLNDDEQARDPEEK.Y | N | 51.20 | 2137.9875 | 19 | 0.7 | 713.6703 | 3 | 23.72 | 1 | F1:10792 | 13012022\_RID\_3262\_4a.raw | 8.2553E5 |  |  |  |  |  |  |  |  |  | 1 | 1 | 0 | 0 | 0 | 0 | 0 | 0 | 0 | 0 | 0 | 77 | 95 |  |  | PEAKS DB |
| V.HSASVLNDDEQARDPEEK.Y | N | 48.73 | 2038.9192 | 18 | 0.8 | 680.6476 | 3 | 22.30 | 1 | F1:9539 | 13012022\_RID\_3262\_4a.raw | 7.4669E5 |  |  |  |  |  |  |  |  |  | 1 | 1 | 0 | 0 | 0 | 0 | 0 | 0 | 0 | 0 | 0 | 78 | 95 |  |  | PEAKS DB |
| A.SVLNDDEQARDPEEK.Y | N | 45.09 | 1743.7910 | 15 | -0.3 | 582.2708 | 3 | 24.63 | 1 | F1:11456 | 13012022\_RID\_3262\_4a.raw | 1.8384E7 |  |  |  |  |  |  |  |  |  | 1 | 1 | 0 | 0 | 0 | 0 | 0 | 0 | 0 | 0 | 0 | 81 | 95 |  |  | PEAKS DB |
| E.WDKDIM(+15.99)LIR.L | N | 44.48 | 1204.6274 | 9 | 0.7 | 603.3214 | 2 | 37.71 | 2 | F2:20647 | 13012022\_RID\_3262\_5a.raw | 1.4316E8 | 4.5531E6 |  |  |  |  |  |  |  |  | 2 | 1 | 1 | 0 | 0 | 0 | 0 | 0 | 0 | 0 | 0 | 107 | 115 | Oxidation (M) | M6:Oxidation (M):1000.00 | PEAKS DB |
| N.NSVHIAPLSLPSSPPR.L | Y | 43.81 | 1670.9104 | 16 | -0.4 | 557.9772 | 3 | 39.97 | 1 | F1:25460 | 13012022\_RID\_3262\_4a.raw | 1.1729E6 |  |  |  |  |  |  |  |  |  | 1 | 1 | 0 | 0 | 0 | 0 | 0 | 0 | 0 | 0 | 0 | 122 | 137 |  |  | PEAKS DB |
| D.TEWDKDIMLIR.L | N | 42.16 | 1418.7228 | 11 | 0.0 | 473.9149 | 3 | 46.59 | 1 | F1:31460 | 13012022\_RID\_3262\_4a.raw | 4.7141E6 |  |  |  |  |  |  |  |  |  | 1 | 1 | 0 | 0 | 0 | 0 | 0 | 0 | 0 | 0 | 0 | 105 | 115 |  |  | PEAKS DB |
| W.DKDIMLIR.L | N | 41.99 | 1002.5532 | 8 | 0.7 | 502.2842 | 2 | 34.59 | 7 | F7:15514 | 21012022\_RID\_3262\_7a.raw |  |  |  |  |  |  | 2.6461E6 |  |  |  | 1 | 0 | 0 | 0 | 0 | 0 | 0 | 1 | 0 | 0 | 0 | 108 | 115 |  |  | PEAKS DB |
| I.LLGVHSASVLNDDEQARDPEEK.Y | N | 41.98 | 2421.1772 | 22 | 0.3 | 606.3018 | 4 | 33.30 | 1 | F1:19473 | 13012022\_RID\_3262\_4a.raw | 2.1612E6 |  |  |  |  |  |  |  |  |  | 1 | 1 | 0 | 0 | 0 | 0 | 0 | 0 | 0 | 0 | 0 | 74 | 95 |  |  | PEAKS DB |
| H.IAPLSLPSSPPR.L | N | 41.82 | 1233.7080 | 12 | -0.5 | 617.8610 | 2 | 41.68 | 1 | F1:27192 | 13012022\_RID\_3262\_4a.raw | 1.0328E7 |  |  |  |  |  |  |  |  |  | 1 | 1 | 0 | 0 | 0 | 0 | 0 | 0 | 0 | 0 | 0 | 126 | 137 |  |  | PEAKS DB |
| L.VIGGDEC(+57.02)NINEHR.S | N | 41.29 | 1511.6787 | 13 | 0.4 | 756.8469 | 2 | 21.60 | 2 | F2:8211 | 13012022\_RID\_3262\_5a.raw |  | 6.6457E6 |  |  |  |  |  |  |  |  | 1 | 0 | 1 | 0 | 0 | 0 | 0 | 0 | 0 | 0 | 0 | 25 | 37 | Carbamidomethylation | C7:Carbamidomethylation:1000.00 | PEAKS DB |
| total 18 peptides |  |  |  |  |  |  |  |  |  |  |  |  |  |  |  |  |  |  |  |  |  |  |  |  |  |  |  |  |  |  |  |  |  |  |  |  |  |
| --- | --- | --- | --- | --- | --- | --- | --- | --- | --- | --- | --- | --- | --- | --- | --- | --- | --- | --- | --- | --- | --- | --- | --- | --- | --- | --- | --- | --- | --- | --- | --- | --- | --- | --- | --- | --- | --- |

AAG10789.1

back to list

  

| Protein Coverage
| Supporting Peptides
|

Protein Coverage:

Supporting Peptides:

| Peptide | Uniq | -10lgP | Mass | Length | ppm | m/z | z | RT | Fraction | Scan | Source File | Area F4 | Area F5 | Area F6 | Area F10 | Area F8 | Area F9 | Area F7 | Area F1 | Area F2 | Area F3 | #Feature | #Feature F4 | #Feature F5 | #Feature F6 | #Feature F10 | #Feature F8 | #Feature F9 | #Feature F7 | #Feature F1 | #Feature F2 | #Feature F3 | Start | End | PTM | AScore | Found By |
| --- | --- | --- | --- | --- | --- | --- | --- | --- | --- | --- | --- | --- | --- | --- | --- | --- | --- | --- | --- | --- | --- | --- | --- | --- | --- | --- | --- | --- | --- | --- | --- | --- | --- | --- | --- | --- | --- |
| K.NDDEVDKDIMLIK.L | Y | 77.76 | 1546.7549 | 13 | 1.0 | 774.3855 | 2 | 42.86 | 3 | F3:20363 | 13012022\_RID\_3262\_6a.raw | 1.9755E7 | 5.2448E7 | 1.1823E7 |  |  | 2.9314E6 | 1.0132E6 | 2.0487E6 | 5.7101E5 |  | 18 | 3 | 6 | 3 | 0 | 0 | 3 | 1 | 1 | 1 | 0 | 103 | 115 |  |  | PEAKS DB |
| R.KNDDEVDKDIMLIK.L | Y | 77.36 | 1674.8499 | 14 | 0.2 | 559.2906 | 3 | 35.89 | 2 | F2:19639 | 13012022\_RID\_3262\_5a.raw | 1.3944E7 | 1.4622E7 |  |  |  | 2.5045E6 | 8.1377E4 | 1.8474E6 |  |  | 12 | 3 | 4 | 0 | 0 | 0 | 1 | 1 | 3 | 0 | 0 | 102 | 115 |  |  | PEAKS DB |
| K.NDDEVDKDIM(+15.99)LIK.L | Y | 75.76 | 1562.7498 | 13 | 0.4 | 782.3824 | 2 | 38.57 | 2 | F2:21287 | 13012022\_RID\_3262\_5a.raw | 4.6227E5 | 1.1431E7 | 3.8674E6 |  |  | 4.5289E5 |  |  |  |  | 9 | 1 | 5 | 2 | 0 | 0 | 1 | 0 | 0 | 0 | 0 | 103 | 115 | Oxidation (M) | M10:Oxidation (M):1000.00 | PEAKS DB |
| R.KNDDEVDKDIM(+15.99)LIK.L | Y | 70.99 | 1690.8447 | 14 | -0.9 | 564.6217 | 3 | 33.61 | 2 | F2:17297 | 13012022\_RID\_3262\_5a.raw | 2.1955E6 | 7.4706E6 |  |  |  | 4.6287E5 |  |  |  |  | 7 | 2 | 4 | 0 | 0 | 0 | 1 | 0 | 0 | 0 | 0 | 102 | 115 | Oxidation (M) | M11:Oxidation (M):1000.00 | PEAKS DB |
| K.ILNEDEQTRDPK.E | N | 63.25 | 1456.7157 | 12 | 0.2 | 729.3652 | 2 | 20.40 | 2 | F2:7523 | 13012022\_RID\_3262\_5a.raw | 2.5611E7 | 5.5662E7 | 3.8171E6 | 7.1395E6 |  | 5.2046E6 | 7.5202E6 |  |  |  | 12 | 2 | 3 | 2 | 2 | 0 | 1 | 2 | 0 | 0 | 0 | 82 | 93 |  |  | PEAKS DB |
| K.ILNEDEQTR.D | N | 62.86 | 1116.5411 | 9 | -0.7 | 559.2775 | 2 | 22.53 | 4 | F4:9650 | 17012022\_RID\_3262\_10a .raw |  | 2.4557E7 | 3.3128E7 | 8.9808E6 |  | 5.8113E6 |  | 6.3735E5 | 9.79E5 | 0 | 7 | 0 | 1 | 2 | 1 | 0 | 1 | 0 | 1 | 1 | 0 | 82 | 90 |  |  | PEAKS DB |
| E.NFQLLFGVHSK.K | N | 62.43 | 1288.6927 | 11 | 0.4 | 645.3539 | 2 | 42.62 | 8 | F8:22782 | 31122021\_RID\_3262\_1a.raw | 1.2701E7 | 1.0353E6 | 2.5789E6 |  |  |  |  | 3.2275E6 | 1.1882E6 |  | 6 | 1 | 1 | 2 | 0 | 0 | 0 | 0 | 1 | 1 | 0 | 70 | 80 |  |  | PEAKS DB |
| K.KILNEDEQTR.D | N | 60.21 | 1244.6360 | 10 | -0.2 | 623.3251 | 2 | 18.75 | 2 | F2:6250 | 13012022\_RID\_3262\_5a.raw |  | 1.5363E7 |  | 8.4896E4 |  | 1.9432E6 | 2.5564E6 | 1.5673E4 |  |  | 8 | 0 | 3 | 0 | 1 | 0 | 1 | 2 | 1 | 0 | 0 | 81 | 90 |  |  | PEAKS DB |
| K.KILNEDEQTRDPK.E | N | 56.63 | 1584.8107 | 13 | 0.3 | 793.4128 | 2 | 19.17 | 7 | F7:4895 | 21012022\_RID\_3262\_7a.raw | 2.769E6 | 3.177E7 |  | 5.4009E6 |  | 7.2018E6 | 6.457E6 | 6.1213E4 |  |  | 8 | 1 | 2 | 0 | 1 | 0 | 1 | 2 | 1 | 0 | 0 | 81 | 93 |  |  | PEAKS DB |
| K.KILNEDEQTRD.P | N | 55.83 | 1359.6630 | 11 | 0.3 | 680.8390 | 2 | 20.13 | 7 | F7:5537 | 21012022\_RID\_3262\_7a.raw |  | 2.0037E6 |  | 4.1763E6 |  |  | 2.6434E6 |  |  |  | 4 | 0 | 1 | 0 | 1 | 0 | 0 | 2 | 0 | 0 | 0 | 81 | 91 |  |  | PEAKS DB |
| K.KILNEDEQTRDPKEK.F | N | 52.44 | 1841.9482 | 15 | 0.0 | 461.4943 | 4 | 16.95 | 1 | F1:5225 | 13012022\_RID\_3262\_4a.raw | 8.7274E6 |  |  |  |  |  |  |  |  |  | 2 | 2 | 0 | 0 | 0 | 0 | 0 | 0 | 0 | 0 | 0 | 81 | 95 |  |  | PEAKS DB |
| N.DDEVDKDIMLIK.L | N | 47.99 | 1432.7119 | 12 | 0.9 | 717.3639 | 2 | 44.20 | 2 | F2:25287 | 13012022\_RID\_3262\_5a.raw | 2.8885E5 | 3.1175E5 |  |  |  |  |  |  |  |  | 2 | 1 | 1 | 0 | 0 | 0 | 0 | 0 | 0 | 0 | 0 | 104 | 115 |  |  | PEAKS DB |
| K.ILNEDEQTRDPKEK.F | N | 47.76 | 1713.8533 | 14 | 0.4 | 572.2919 | 3 | 18.15 | 2 | F2:5610 | 13012022\_RID\_3262\_5a.raw |  | 1.6162E6 |  |  |  |  |  |  |  |  | 1 | 0 | 1 | 0 | 0 | 0 | 0 | 0 | 0 | 0 | 0 | 82 | 95 |  |  | PEAKS DB |
| total 13 peptides |  |  |  |  |  |  |  |  |  |  |  |  |  |  |  |  |  |  |  |  |  |  |  |  |  |  |  |  |  |  |  |  |  |  |  |  |  |
| --- | --- | --- | --- | --- | --- | --- | --- | --- | --- | --- | --- | --- | --- | --- | --- | --- | --- | --- | --- | --- | --- | --- | --- | --- | --- | --- | --- | --- | --- | --- | --- | --- | --- | --- | --- | --- | --- |

Q9DF67.1

back to list

  

| Protein Coverage
| Supporting Peptides
|

Protein Coverage:

Supporting Peptides:

| Peptide | Uniq | -10lgP | Mass | Length | ppm | m/z | z | RT | Fraction | Scan | Source File | Area F4 | Area F5 | Area F6 | Area F10 | Area F8 | Area F9 | Area F7 | Area F1 | Area F2 | Area F3 | #Feature | #Feature F4 | #Feature F5 | #Feature F6 | #Feature F10 | #Feature F8 | #Feature F9 | #Feature F7 | #Feature F1 | #Feature F2 | #Feature F3 | Start | End | PTM | AScore | Found By |
| --- | --- | --- | --- | --- | --- | --- | --- | --- | --- | --- | --- | --- | --- | --- | --- | --- | --- | --- | --- | --- | --- | --- | --- | --- | --- | --- | --- | --- | --- | --- | --- | --- | --- | --- | --- | --- | --- |
| K.NDDEVDKDIMLIK.L | Y | 77.76 | 1546.7549 | 13 | 1.0 | 774.3855 | 2 | 42.86 | 3 | F3:20363 | 13012022\_RID\_3262\_6a.raw | 1.9755E7 | 5.2448E7 | 1.1823E7 |  |  | 2.9314E6 | 1.0132E6 | 2.0487E6 | 5.7101E5 |  | 18 | 3 | 6 | 3 | 0 | 0 | 3 | 1 | 1 | 1 | 0 | 103 | 115 |  |  | PEAKS DB |
| R.KNDDEVDKDIMLIK.L | Y | 77.36 | 1674.8499 | 14 | 0.2 | 559.2906 | 3 | 35.89 | 2 | F2:19639 | 13012022\_RID\_3262\_5a.raw | 1.3944E7 | 1.4622E7 |  |  |  | 2.5045E6 | 8.1377E4 | 1.8474E6 |  |  | 12 | 3 | 4 | 0 | 0 | 0 | 1 | 1 | 3 | 0 | 0 | 102 | 115 |  |  | PEAKS DB |
| K.NDDEVDKDIM(+15.99)LIK.L | Y | 75.76 | 1562.7498 | 13 | 0.4 | 782.3824 | 2 | 38.57 | 2 | F2:21287 | 13012022\_RID\_3262\_5a.raw | 4.6227E5 | 1.1431E7 | 3.8674E6 |  |  | 4.5289E5 |  |  |  |  | 9 | 1 | 5 | 2 | 0 | 0 | 1 | 0 | 0 | 0 | 0 | 103 | 115 | Oxidation (M) | M10:Oxidation (M):1000.00 | PEAKS DB |
| R.KNDDEVDKDIM(+15.99)LIK.L | Y | 70.99 | 1690.8447 | 14 | -0.9 | 564.6217 | 3 | 33.61 | 2 | F2:17297 | 13012022\_RID\_3262\_5a.raw | 2.1955E6 | 7.4706E6 |  |  |  | 4.6287E5 |  |  |  |  | 7 | 2 | 4 | 0 | 0 | 0 | 1 | 0 | 0 | 0 | 0 | 102 | 115 | Oxidation (M) | M11:Oxidation (M):1000.00 | PEAKS DB |
| K.ILNEDEQTRDPK.E | N | 63.25 | 1456.7157 | 12 | 0.2 | 729.3652 | 2 | 20.40 | 2 | F2:7523 | 13012022\_RID\_3262\_5a.raw | 2.5611E7 | 5.5662E7 | 3.8171E6 | 7.1395E6 |  | 5.2046E6 | 7.5202E6 |  |  |  | 12 | 2 | 3 | 2 | 2 | 0 | 1 | 2 | 0 | 0 | 0 | 82 | 93 |  |  | PEAKS DB |
| K.ILNEDEQTR.D | N | 62.86 | 1116.5411 | 9 | -0.7 | 559.2775 | 2 | 22.53 | 4 | F4:9650 | 17012022\_RID\_3262\_10a .raw |  | 2.4557E7 | 3.3128E7 | 8.9808E6 |  | 5.8113E6 |  | 6.3735E5 | 9.79E5 | 0 | 7 | 0 | 1 | 2 | 1 | 0 | 1 | 0 | 1 | 1 | 0 | 82 | 90 |  |  | PEAKS DB |
| E.NFQLLFGVHSK.K | N | 62.43 | 1288.6927 | 11 | 0.4 | 645.3539 | 2 | 42.62 | 8 | F8:22782 | 31122021\_RID\_3262\_1a.raw | 1.2701E7 | 1.0353E6 | 2.5789E6 |  |  |  |  | 3.2275E6 | 1.1882E6 |  | 6 | 1 | 1 | 2 | 0 | 0 | 0 | 0 | 1 | 1 | 0 | 70 | 80 |  |  | PEAKS DB |
| K.KILNEDEQTR.D | N | 60.21 | 1244.6360 | 10 | -0.2 | 623.3251 | 2 | 18.75 | 2 | F2:6250 | 13012022\_RID\_3262\_5a.raw |  | 1.5363E7 |  | 8.4896E4 |  | 1.9432E6 | 2.5564E6 | 1.5673E4 |  |  | 8 | 0 | 3 | 0 | 1 | 0 | 1 | 2 | 1 | 0 | 0 | 81 | 90 |  |  | PEAKS DB |
| K.KILNEDEQTRDPK.E | N | 56.63 | 1584.8107 | 13 | 0.3 | 793.4128 | 2 | 19.17 | 7 | F7:4895 | 21012022\_RID\_3262\_7a.raw | 2.769E6 | 3.177E7 |  | 5.4009E6 |  | 7.2018E6 | 6.457E6 | 6.1213E4 |  |  | 8 | 1 | 2 | 0 | 1 | 0 | 1 | 2 | 1 | 0 | 0 | 81 | 93 |  |  | PEAKS DB |
| K.KILNEDEQTRD.P | N | 55.83 | 1359.6630 | 11 | 0.3 | 680.8390 | 2 | 20.13 | 7 | F7:5537 | 21012022\_RID\_3262\_7a.raw |  | 2.0037E6 |  | 4.1763E6 |  |  | 2.6434E6 |  |  |  | 4 | 0 | 1 | 0 | 1 | 0 | 0 | 2 | 0 | 0 | 0 | 81 | 91 |  |  | PEAKS DB |
| K.KILNEDEQTRDPKEK.F | N | 52.44 | 1841.9482 | 15 | 0.0 | 461.4943 | 4 | 16.95 | 1 | F1:5225 | 13012022\_RID\_3262\_4a.raw | 8.7274E6 |  |  |  |  |  |  |  |  |  | 2 | 2 | 0 | 0 | 0 | 0 | 0 | 0 | 0 | 0 | 0 | 81 | 95 |  |  | PEAKS DB |
| N.DDEVDKDIMLIK.L | N | 47.99 | 1432.7119 | 12 | 0.9 | 717.3639 | 2 | 44.20 | 2 | F2:25287 | 13012022\_RID\_3262\_5a.raw | 2.8885E5 | 3.1175E5 |  |  |  |  |  |  |  |  | 2 | 1 | 1 | 0 | 0 | 0 | 0 | 0 | 0 | 0 | 0 | 104 | 115 |  |  | PEAKS DB |
| K.ILNEDEQTRDPKEK.F | N | 47.76 | 1713.8533 | 14 | 0.4 | 572.2919 | 3 | 18.15 | 2 | F2:5610 | 13012022\_RID\_3262\_5a.raw |  | 1.6162E6 |  |  |  |  |  |  |  |  | 1 | 0 | 1 | 0 | 0 | 0 | 0 | 0 | 0 | 0 | 0 | 82 | 95 |  |  | PEAKS DB |
| total 13 peptides |  |  |  |  |  |  |  |  |  |  |  |  |  |  |  |  |  |  |  |  |  |  |  |  |  |  |  |  |  |  |  |  |  |  |  |  |  |
| --- | --- | --- | --- | --- | --- | --- | --- | --- | --- | --- | --- | --- | --- | --- | --- | --- | --- | --- | --- | --- | --- | --- | --- | --- | --- | --- | --- | --- | --- | --- | --- | --- | --- | --- | --- | --- | --- |

JAS05152.1

back to list

  

| Protein Coverage
| Supporting Peptides
|

Protein Coverage:

Supporting Peptides:

| Peptide | Uniq | -10lgP | Mass | Length | ppm | m/z | z | RT | Fraction | Scan | Source File | Area F4 | Area F5 | Area F6 | Area F10 | Area F8 | Area F9 | Area F7 | Area F1 | Area F2 | Area F3 | #Feature | #Feature F4 | #Feature F5 | #Feature F6 | #Feature F10 | #Feature F8 | #Feature F9 | #Feature F7 | #Feature F1 | #Feature F2 | #Feature F3 | Start | End | PTM | AScore | Found By |
| --- | --- | --- | --- | --- | --- | --- | --- | --- | --- | --- | --- | --- | --- | --- | --- | --- | --- | --- | --- | --- | --- | --- | --- | --- | --- | --- | --- | --- | --- | --- | --- | --- | --- | --- | --- | --- | --- |
| R.EADYEEFLEIAR.N | N | 83.87 | 1483.6830 | 12 | 0.7 | 742.8493 | 2 | 55.95 | 6 | F6:39873 | 17012022\_RID\_3262\_9a.raw | 2.7565E8 | 2.0308E7 | 1.2427E6 | 5.6557E7 |  | 5.9048E7 | 3.7778E5 | 4.8993E7 | 4.4393E7 | 2.2183E9 | 12 | 1 | 1 | 1 | 1 | 0 | 1 | 1 | 1 | 1 | 4 | 32 | 43 |  |  | PEAKS DB |
| K.YPVKPSEEGK.S | N | 69.75 | 1132.5764 | 10 | 0.7 | 567.2959 | 2 | 17.88 | 6 | F6:5952 | 17012022\_RID\_3262\_9a.raw | 4.2299E8 | 5.2685E7 | 2.5077E5 | 3.2771E7 |  | 6.9462E7 | 2.576E7 |  |  |  | 12 | 2 | 2 | 1 | 3 | 0 | 2 | 2 | 0 | 0 | 0 | 161 | 170 |  |  | PEAKS DB |
| D.YEEFLEIAR.N | N | 54.84 | 1168.5764 | 9 | 0.3 | 585.2957 | 2 | 46.57 | 4 | F4:30508 | 17012022\_RID\_3262\_10a .raw | 2.2869E6 |  |  | 1.3254E6 |  |  |  | 2.3858E5 | 1.5579E5 | 1.3646E7 | 5 | 1 | 0 | 0 | 1 | 0 | 0 | 0 | 1 | 1 | 1 | 35 | 43 |  |  | PEAKS DB |
| K.YDTYSTKEYLIK.E | N | 53.18 | 1522.7555 | 12 | 0.9 | 762.3857 | 2 | 34.13 | 1 | F1:20150 | 13012022\_RID\_3262\_4a.raw | 4.6273E6 |  |  |  |  |  |  |  |  |  | 2 | 2 | 0 | 0 | 0 | 0 | 0 | 0 | 0 | 0 | 0 | 199 | 210 |  |  | PEAKS DB |
| K.STTDLPSR.F | N | 52.16 | 875.4348 | 8 | 0.4 | 438.7249 | 2 | 21.40 | 6 | F6:8926 | 17012022\_RID\_3262\_9a.raw |  | 2.9854E7 |  | 4.8043E6 |  | 1.4473E7 | 1.9071E6 |  |  |  | 4 | 0 | 1 | 0 | 1 | 0 | 1 | 1 | 0 | 0 | 0 | 365 | 372 |  |  | PEAKS DB |
| N.DLSLIHQLPK.E | N | 51.65 | 1162.6710 | 10 | -0.7 | 582.3424 | 2 | 40.68 | 10 | F10:10098 | 31122021\_RID\_3262\_3a.raw |  |  |  |  |  |  |  | 1.197E6 | 2.7589E5 | 1.8174E7 | 3 | 0 | 0 | 0 | 0 | 0 | 0 | 0 | 1 | 1 | 1 | 415 | 424 |  |  | PEAKS DB |
| G.DIVINDLSLIHQLPK.E | N | 49.33 | 1716.9774 | 15 | 0.0 | 859.4960 | 2 | 55.52 | 8 | F8:32578 | 31122021\_RID\_3262\_1a.raw |  |  |  |  |  |  |  | 1.2793E5 | 1.1614E5 |  | 2 | 0 | 0 | 0 | 0 | 0 | 0 | 0 | 1 | 1 | 0 | 410 | 424 |  |  | PEAKS DB |
| K.HVVVVGAGMAGLSAAY.V | N | 48.78 | 1500.7759 | 16 | 0.1 | 751.3953 | 2 | 48.21 | 9 | F9:28042 | 31122021\_RID\_3262\_2a.raw |  |  |  |  |  |  |  | 3.8493E5 | 3.1875E5 | 7.767E6 | 3 | 0 | 0 | 0 | 0 | 0 | 0 | 0 | 1 | 1 | 1 | 54 | 69 |  |  | PEAKS DB |
| K.HVVVVGAGMAGL.S | N | 47.88 | 1108.6063 | 12 | 0.5 | 555.3107 | 2 | 43.58 | 9 | F9:24400 | 31122021\_RID\_3262\_2a.raw | 1.4776E6 |  |  |  |  |  |  |  | 3.0015E5 | 1.177E7 | 3 | 1 | 0 | 0 | 0 | 0 | 0 | 0 | 0 | 1 | 1 | 54 | 65 |  |  | PEAKS DB |
| K.TSGDIVINDLSLIHQLPK.E | Y | 42.83 | 1962.0786 | 18 | -8.8 | 655.0277 | 3 | 54.06 | 10 | F10:15420 | 31122021\_RID\_3262\_3a.raw |  |  |  |  |  |  |  |  |  | 9.2051E5 | 1 | 0 | 0 | 0 | 0 | 0 | 0 | 0 | 0 | 0 | 1 | 407 | 424 |  |  | PEAKS DB |
| total 10 peptides |  |  |  |  |  |  |  |  |  |  |  |  |  |  |  |  |  |  |  |  |  |  |  |  |  |  |  |  |  |  |  |  |  |  |  |  |  |
| --- | --- | --- | --- | --- | --- | --- | --- | --- | --- | --- | --- | --- | --- | --- | --- | --- | --- | --- | --- | --- | --- | --- | --- | --- | --- | --- | --- | --- | --- | --- | --- | --- | --- | --- | --- | --- | --- |

XP\_026539627.1

back to list

  

| Protein Coverage
| Supporting Peptides
|

Protein Coverage:

Supporting Peptides:

| Peptide | Uniq | -10lgP | Mass | Length | ppm | m/z | z | RT | Fraction | Scan | Source File | Area F4 | Area F5 | Area F6 | Area F10 | Area F8 | Area F9 | Area F7 | Area F1 | Area F2 | Area F3 | #Feature | #Feature F4 | #Feature F5 | #Feature F6 | #Feature F10 | #Feature F8 | #Feature F9 | #Feature F7 | #Feature F1 | #Feature F2 | #Feature F3 | Start | End | PTM | AScore | Found By |
| --- | --- | --- | --- | --- | --- | --- | --- | --- | --- | --- | --- | --- | --- | --- | --- | --- | --- | --- | --- | --- | --- | --- | --- | --- | --- | --- | --- | --- | --- | --- | --- | --- | --- | --- | --- | --- | --- |
| R.DYQELMNVK.L | N | 65.21 | 1138.5328 | 9 | 0.4 | 570.2739 | 2 | 41.02 | 4 | F4:25980 | 17012022\_RID\_3262\_10a .raw | 1.1187E7 | 8.8848E6 | 2.6711E6 | 4.9346E7 |  | 7.1078E7 | 1.1383E6 | 2.5131E7 | 5.6427E6 | 4.6395E7 | 9 | 1 | 1 | 1 | 1 | 0 | 1 | 1 | 1 | 1 | 1 | 445 | 453 |  |  | PEAKS DB |
| K.LALDVEIATYR.T | N | 63.88 | 1262.6870 | 11 | 0.8 | 632.3513 | 2 | 49.15 | 8 | F8:27481 | 31122021\_RID\_3262\_1a.raw | 1.1555E7 | 4.2265E5 |  | 6.3798E6 |  | 4.7079E5 |  | 1.6274E7 | 6.9194E6 | 1.6595E7 | 16 | 1 | 2 | 0 | 6 | 0 | 2 | 0 | 2 | 2 | 1 | 454 | 464 |  |  | PEAKS DB |
| K.NKYEDEINKR.T | N | 62.23 | 1307.6470 | 10 | -0.2 | 436.8895 | 3 | 19.57 | 6 | F6:7264 | 17012022\_RID\_3262\_9a.raw | 1.0196E8 | 9.8399E6 |  | 2.0043E8 |  | 2.2923E8 | 1.0038E7 | 4.397E7 | 3.6098E7 | 6.8711E7 | 17 | 3 | 1 | 0 | 3 | 0 | 2 | 2 | 2 | 2 | 2 | 249 | 258 |  |  | PEAKS DB |
| R.DYQELM(+15.99)NVK.L | N | 60.86 | 1154.5277 | 9 | -0.1 | 578.2711 | 2 | 31.71 | 4 | F4:18149 | 17012022\_RID\_3262\_10a .raw | 4.0442E6 | 2.0104E6 | 1.8647E6 | 1.8465E7 |  | 1.5647E7 | 9.5906E5 | 6.5695E6 | 4.2189E6 | 3.5004E6 | 15 | 1 | 1 | 2 | 1 | 0 | 2 | 1 | 4 | 2 | 1 | 445 | 453 | Oxidation (M) | M6:Oxidation (M):1000.00 | PEAKS DB |
| R.LLRDYQELMNVK.L | N | 60.01 | 1520.8021 | 12 | -0.1 | 507.9413 | 3 | 41.03 | 1 | F1:26617 | 13012022\_RID\_3262\_4a.raw | 7.7543E6 |  |  |  |  |  |  | 1.4523E7 |  |  | 3 | 1 | 0 | 0 | 0 | 0 | 0 | 0 | 2 | 0 | 0 | 442 | 453 |  |  | PEAKS DB |
| N.KYEDEINK.R | N | 59.49 | 1037.5029 | 8 | 0.1 | 519.7588 | 2 | 17.64 | 6 | F6:5589 | 17012022\_RID\_3262\_9a.raw |  |  |  | 7.1505E6 |  | 8.6198E6 |  |  |  |  | 2 | 0 | 0 | 0 | 1 | 0 | 1 | 0 | 0 | 0 | 0 | 250 | 257 |  |  | PEAKS DB |
| K.FASFIDKVR.F | N | 59.23 | 1081.5920 | 9 | 0.3 | 541.8035 | 2 | 35.45 | 6 | F6:21540 | 17012022\_RID\_3262\_9a.raw | 1.5335E7 |  |  | 9.5108E6 |  | 1.2654E7 |  | 1.0527E7 | 1.5026E6 |  | 9 | 1 | 0 | 0 | 3 | 0 | 1 | 0 | 3 | 1 | 0 | 172 | 180 |  |  | PEAKS DB |
| K.NKYEDEINK.R | N | 58.15 | 1151.5458 | 9 | -0.4 | 576.7799 | 2 | 19.99 | 6 | F6:7851 | 17012022\_RID\_3262\_9a.raw |  |  |  | 3.8328E7 |  | 2.7795E7 | 1.8307E6 | 3.6735E6 | 3.5275E6 | 3.3686E6 | 8 | 0 | 0 | 0 | 2 | 0 | 2 | 1 | 1 | 1 | 1 | 249 | 257 |  |  | PEAKS DB |
| N.KYEDEINKR.T | N | 54.63 | 1193.6040 | 9 | -0.3 | 597.8091 | 2 | 16.55 | 6 | F6:4717 | 17012022\_RID\_3262\_9a.raw | 2.4731E7 | 1.7851E6 |  | 5.25E7 |  | 7.1702E7 | 3.2062E6 |  |  |  | 9 | 1 | 1 | 0 | 2 | 0 | 3 | 2 | 0 | 0 | 0 | 250 | 258 |  |  | PEAKS DB |
| K.YEDEINKR.T | N | 54.27 | 1065.5090 | 8 | 0.2 | 533.7619 | 2 | 17.06 | 6 | F6:5136 | 17012022\_RID\_3262\_9a.raw |  |  |  | 3.7263E6 |  | 5.4143E6 | 1.8172E5 |  |  |  | 3 | 0 | 0 | 0 | 1 | 0 | 1 | 1 | 0 | 0 | 0 | 251 | 258 |  |  | PEAKS DB |
| R.DLNLDSIIAEVR.A | Y | 52.77 | 1356.7249 | 12 | 1.2 | 679.3705 | 2 | 61.24 | 9 | F9:37952 | 31122021\_RID\_3262\_2a.raw |  |  |  |  |  |  |  | 8.2015E5 | 2.3951E5 |  | 2 | 0 | 0 | 0 | 0 | 0 | 0 | 0 | 1 | 1 | 0 | 325 | 336 |  |  | PEAKS DB |
| K.FASFIDK.V | N | 52.67 | 826.4225 | 7 | 0.7 | 414.2188 | 2 | 36.24 | 6 | F6:22289 | 17012022\_RID\_3262\_9a.raw | 2.5611E7 | 1.8348E7 | 6.6379E6 | 1.2204E8 |  | 1.3051E8 | 4.5017E6 | 4.1444E7 | 1.2872E7 |  | 15 | 2 | 2 | 1 | 4 | 0 | 3 | 1 | 1 | 1 | 0 | 172 | 178 |  |  | PEAKS DB |
| A.LDVEIATYR.T | N | 51.71 | 1078.5658 | 9 | 5.1 | 540.2929 | 2 | 39.98 | 8 | F8:20433 | 31122021\_RID\_3262\_1a.raw |  |  |  |  |  |  |  | 2.3839E5 |  |  | 1 | 0 | 0 | 0 | 0 | 0 | 0 | 0 | 1 | 0 | 0 | 456 | 464 |  |  | PEAKS DB |
| L.ALDVEIATYR.T | N | 49.94 | 1149.6029 | 10 | 1.0 | 575.8093 | 2 | 40.83 | 9 | F9:22185 | 31122021\_RID\_3262\_2a.raw |  |  |  |  |  |  |  |  | 4.3303E4 |  | 1 | 0 | 0 | 0 | 0 | 0 | 0 | 0 | 0 | 1 | 0 | 455 | 464 |  |  | PEAKS DB |
| R.GELALKDAR.T | N | 49.06 | 971.5399 | 9 | -0.6 | 486.7769 | 2 | 22.10 | 6 | F6:9646 | 17012022\_RID\_3262\_9a.raw |  |  |  | 1.6772E6 |  | 1.879E6 | 8.5932E4 |  |  |  | 3 | 0 | 0 | 0 | 1 | 0 | 1 | 1 | 0 | 0 | 0 | 414 | 422 |  |  | PEAKS DB |
| R.LLRDYQELM(+15.99)NVK.L | N | 48.43 | 1536.7970 | 12 | 0.1 | 513.2730 | 3 | 34.30 | 6 | F6:20494 | 17012022\_RID\_3262\_9a.raw |  |  |  |  |  | 3.6586E6 |  | 2.0582E6 |  |  | 2 | 0 | 0 | 0 | 0 | 0 | 1 | 0 | 1 | 0 | 0 | 442 | 453 | Oxidation (M) | M9:Oxidation (M):1000.00 | PEAKS DB |
| K.FASFIDKV.R | N | 43.37 | 925.4908 | 8 | 0.2 | 463.7528 | 2 | 44.26 | 4 | F4:28348 | 17012022\_RID\_3262\_10a .raw |  |  |  | 1.1458E7 |  |  |  | 5.4178E6 | 1.9415E6 |  | 3 | 0 | 0 | 0 | 1 | 0 | 0 | 0 | 1 | 1 | 0 | 172 | 179 |  |  | PEAKS DB |
| total 17 peptides |  |  |  |  |  |  |  |  |  |  |  |  |  |  |  |  |  |  |  |  |  |  |  |  |  |  |  |  |  |  |  |  |  |  |  |  |  |
| --- | --- | --- | --- | --- | --- | --- | --- | --- | --- | --- | --- | --- | --- | --- | --- | --- | --- | --- | --- | --- | --- | --- | --- | --- | --- | --- | --- | --- | --- | --- | --- | --- | --- | --- | --- | --- | --- |

XP\_032080818.1

back to list

  

| Protein Coverage
| Supporting Peptides
|

Protein Coverage:

Supporting Peptides:

| Peptide | Uniq | -10lgP | Mass | Length | ppm | m/z | z | RT | Fraction | Scan | Source File | Area F4 | Area F5 | Area F6 | Area F10 | Area F8 | Area F9 | Area F7 | Area F1 | Area F2 | Area F3 | #Feature | #Feature F4 | #Feature F5 | #Feature F6 | #Feature F10 | #Feature F8 | #Feature F9 | #Feature F7 | #Feature F1 | #Feature F2 | #Feature F3 | Start | End | PTM | AScore | Found By |
| --- | --- | --- | --- | --- | --- | --- | --- | --- | --- | --- | --- | --- | --- | --- | --- | --- | --- | --- | --- | --- | --- | --- | --- | --- | --- | --- | --- | --- | --- | --- | --- | --- | --- | --- | --- | --- | --- |
| R.VQHASPAGNYAHVVNR.D | N | 80.89 | 1718.8601 | 16 | 0.0 | 573.9606 | 3 | 19.73 | 9 | F9:5169 | 31122021\_RID\_3262\_2a.raw | 5.9402E6 |  |  |  |  |  |  | 2.0488E5 | 2.9449E7 |  | 6 | 2 | 0 | 0 | 0 | 0 | 0 | 0 | 1 | 3 | 0 | 176 | 191 |  |  | PEAKS DB |
| H.ALTEAIEFDKAIER.A | N | 73.25 | 1604.8409 | 14 | 0.1 | 803.4279 | 2 | 41.60 | 9 | F9:22846 | 31122021\_RID\_3262\_2a.raw | 1.0763E6 |  |  |  |  |  |  |  | 3.2856E6 |  | 3 | 1 | 0 | 0 | 0 | 0 | 0 | 0 | 0 | 2 | 0 | 352 | 365 |  |  | PEAKS DB |
| H.ALTEAIEFDK.A | N | 66.55 | 1135.5760 | 10 | 0.3 | 568.7955 | 2 | 39.48 | 9 | F9:21119 | 31122021\_RID\_3262\_2a.raw | 4.5481E6 | 5.9828E5 |  |  |  |  |  |  | 3.4018E7 | 6.8465E6 | 5 | 1 | 1 | 0 | 0 | 0 | 0 | 0 | 0 | 2 | 1 | 352 | 361 |  |  | PEAKS DB |
| R.LMGLFEPSDMK.Y | N | 63.72 | 1266.5988 | 11 | 0.9 | 634.3073 | 2 | 49.66 | 9 | F9:29211 | 31122021\_RID\_3262\_2a.raw | 4.9848E6 |  |  |  |  |  |  |  | 1.3471E7 |  | 5 | 1 | 0 | 0 | 0 | 0 | 0 | 0 | 0 | 4 | 0 | 290 | 300 |  |  | PEAKS DB |
| R.LM(+15.99)GLFEPSDMK.Y | N | 63.14 | 1282.5938 | 11 | 0.7 | 642.3046 | 2 | 42.96 | 9 | F9:23996 | 31122021\_RID\_3262\_2a.raw |  |  |  |  |  |  |  |  | 4.4808E6 |  | 2 | 0 | 0 | 0 | 0 | 0 | 0 | 0 | 0 | 2 | 0 | 290 | 300 | Oxidation (M) | M2:Oxidation (M):192.66 | PEAKS DB |
| R.LMGLFEPSDM(+15.99)K.Y | N | 62.01 | 1282.5938 | 11 | 0.6 | 642.3046 | 2 | 45.18 | 9 | F9:25650 | 31122021\_RID\_3262\_2a.raw | 7.7041E5 |  |  |  |  |  |  |  | 1.3371E7 |  | 5 | 1 | 0 | 0 | 0 | 0 | 0 | 0 | 0 | 4 | 0 | 290 | 300 | Oxidation (M) | M10:Oxidation (M):139.69 | PEAKS DB |
| K.GFYLFVEGGR.I | N | 61.76 | 1143.5713 | 10 | 0.8 | 572.7934 | 2 | 50.09 | 9 | F9:29523 | 31122021\_RID\_3262\_2a.raw |  |  |  |  |  |  |  | 4.3408E5 | 1.1514E7 |  | 2 | 0 | 0 | 0 | 0 | 0 | 0 | 0 | 1 | 1 | 0 | 330 | 339 |  |  | PEAKS DB |
| R.LM(+15.99)GLFEPSDM(+15.99)K.Y | N | 57.65 | 1298.5886 | 11 | 0.4 | 650.3018 | 2 | 49.67 | 9 | F9:29202 | 31122021\_RID\_3262\_2a.raw |  |  |  |  |  |  |  |  | 6.5419E6 |  | 4 | 0 | 0 | 0 | 0 | 0 | 0 | 0 | 0 | 4 | 0 | 290 | 300 | Oxidation (M) | M2:Oxidation (M):1000.00;M10:Oxidation (M):1000.00 | PEAKS DB |
| K.SVGIVTTTR.V | N | 55.56 | 932.5291 | 9 | -0.6 | 467.2715 | 2 | 24.85 | 9 | F9:9035 | 31122021\_RID\_3262\_2a.raw | 4.7751E6 |  |  |  |  |  |  | 3.9398E5 | 2.2112E7 |  | 3 | 1 | 0 | 0 | 0 | 0 | 0 | 0 | 1 | 1 | 0 | 167 | 175 |  |  | PEAKS DB |
| R.GTSIFGLAPEK.A | Y | 55.23 | 1118.5972 | 11 | -0.1 | 560.3058 | 2 | 42.31 | 9 | F9:23392 | 31122021\_RID\_3262\_2a.raw |  |  | 4.641E5 |  |  |  |  | 1.1313E6 | 3.604E7 |  | 3 | 0 | 0 | 1 | 0 | 0 | 0 | 0 | 1 | 1 | 0 | 396 | 406 |  |  | PEAKS DB |
| R.RLMGLFEPSDMK.Y | N | 52.96 | 1422.7000 | 12 | -0.1 | 712.3572 | 2 | 41.16 | 9 | F9:22395 | 31122021\_RID\_3262\_2a.raw |  |  |  |  |  |  |  |  | 2.0343E6 |  | 1 | 0 | 0 | 0 | 0 | 0 | 0 | 0 | 0 | 1 | 0 | 289 | 300 |  |  | PEAKS DB |
| R.VQHASPAGNYAHVVN.R | N | 47.90 | 1562.7589 | 15 | 0.5 | 782.3871 | 2 | 22.55 | 9 | F9:7231 | 31122021\_RID\_3262\_2a.raw |  |  |  |  |  |  |  |  | 4.0973E6 |  | 2 | 0 | 0 | 0 | 0 | 0 | 0 | 0 | 0 | 2 | 0 | 176 | 190 |  |  | PEAKS DB |
| K.GPMAHLFH.G | N | 42.64 | 908.4327 | 8 | 0.6 | 455.2239 | 2 | 28.62 | 9 | F9:12184 | 31122021\_RID\_3262\_2a.raw |  |  |  |  |  |  |  |  | 2.3063E4 |  | 1 | 0 | 0 | 0 | 0 | 0 | 0 | 0 | 0 | 1 | 0 | 468 | 475 |  |  | PEAKS DB |
| total 13 peptides |  |  |  |  |  |  |  |  |  |  |  |  |  |  |  |  |  |  |  |  |  |  |  |  |  |  |  |  |  |  |  |  |  |  |  |  |  |
| --- | --- | --- | --- | --- | --- | --- | --- | --- | --- | --- | --- | --- | --- | --- | --- | --- | --- | --- | --- | --- | --- | --- | --- | --- | --- | --- | --- | --- | --- | --- | --- | --- | --- | --- | --- | --- | --- |

ETE57948.1

back to list

  

| Protein Coverage
| Supporting Peptides
|

Protein Coverage:

Supporting Peptides:

| Peptide | Uniq | -10lgP | Mass | Length | ppm | m/z | z | RT | Fraction | Scan | Source File | Area F4 | Area F5 | Area F6 | Area F10 | Area F8 | Area F9 | Area F7 | Area F1 | Area F2 | Area F3 | #Feature | #Feature F4 | #Feature F5 | #Feature F6 | #Feature F10 | #Feature F8 | #Feature F9 | #Feature F7 | #Feature F1 | #Feature F2 | #Feature F3 | Start | End | PTM | AScore | Found By |
| --- | --- | --- | --- | --- | --- | --- | --- | --- | --- | --- | --- | --- | --- | --- | --- | --- | --- | --- | --- | --- | --- | --- | --- | --- | --- | --- | --- | --- | --- | --- | --- | --- | --- | --- | --- | --- | --- |
| K.SYELPDGQVITIGNER.F | N | 82.78 | 1789.8846 | 16 | 0.4 | 895.9500 | 2 | 49.87 | 8 | F8:28149 | 31122021\_RID\_3262\_1a.raw | 4.7745E5 |  |  |  |  | 6.0954E5 |  | 1.0043E6 | 3.4371E5 |  | 4 | 1 | 0 | 0 | 0 | 0 | 1 | 0 | 1 | 1 | 0 | 224 | 239 |  |  | PEAKS DB |
| K.LC(+57.02)YVALDFEQEMATAASSSSLEK.S | Y | 65.71 | 2549.1665 | 23 | -0.5 | 1275.5898 | 2 | 62.33 | 7 | F7:28143 | 21012022\_RID\_3262\_7a.raw |  |  |  |  |  |  | 1.0541E6 |  |  |  | 2 | 0 | 0 | 0 | 0 | 0 | 0 | 2 | 0 | 0 | 0 | 201 | 223 | Carbamidomethylation | C2:Carbamidomethylation:1000.00 | PEAKS DB |
| K.DSYVGDEAQSK.R | N | 65.50 | 1197.5149 | 11 | 1.5 | 599.7656 | 2 | 23.16 | 4 | F4:10278 | 17012022\_RID\_3262\_10a .raw | 2.7572E5 |  |  | 3.783E5 |  | 4.8292E5 |  | 7.9712E4 | 7.8692E4 |  | 5 | 1 | 0 | 0 | 1 | 0 | 1 | 0 | 1 | 1 | 0 | 36 | 46 |  |  | PEAKS DB |
| R.GYSFTTTAER.E | Y | 60.44 | 1131.5197 | 10 | -0.1 | 566.7670 | 2 | 28.78 | 8 | F8:11438 | 31122021\_RID\_3262\_1a.raw |  |  |  | 1.3304E6 |  |  | 2.0729E5 | 2.9994E5 | 2.4553E5 |  | 4 | 0 | 0 | 0 | 1 | 0 | 0 | 1 | 1 | 1 | 0 | 182 | 191 |  |  | PEAKS DB |
| K.LC(+57.02)YVALDFEQEM(+15.99)ATAASSSSLEK.S | Y | 54.72 | 2565.1614 | 23 | 0.4 | 856.0614 | 3 | 62.38 | 7 | F7:28152 | 21012022\_RID\_3262\_7a.raw |  |  |  |  |  |  | 1.0591E5 |  |  |  | 1 | 0 | 0 | 0 | 0 | 0 | 0 | 1 | 0 | 0 | 0 | 201 | 223 | Carbamidomethylation; Oxidation (M) | C2:Carbamidomethylation:1000.00;M12:Oxidation (M):1000.00 | PEAKS DB |
| K.AGFAGDDAPR.A | N | 52.95 | 975.4410 | 10 | 3.9 | 488.7296 | 2 | 22.10 | 9 | F9:7134 | 31122021\_RID\_3262\_2a.raw |  | 3.3275E5 |  | 1.2847E6 |  |  |  |  | 2.7076E6 |  | 4 | 0 | 1 | 0 | 1 | 0 | 0 | 0 | 0 | 2 | 0 | 4 | 13 |  |  | PEAKS DB |
| R.VAPEEHPVLLTEAPLNPK.A | N | 51.47 | 1953.0570 | 18 | 0.5 | 652.0266 | 3 | 40.45 | 1 | F1:25900 | 13012022\_RID\_3262\_4a.raw | 8.5304E5 |  |  | 5.0748E5 |  |  |  | 7.2111E5 |  |  | 3 | 1 | 0 | 0 | 1 | 0 | 0 | 0 | 1 | 0 | 0 | 81 | 98 |  |  | PEAKS DB |
| K.EITALAPSTM(+15.99)K.I | N | 49.12 | 1176.6060 | 11 | 1.7 | 589.3113 | 2 | 30.85 | 6 | F6:17553 | 17012022\_RID\_3262\_9a.raw |  |  |  |  |  | 2.4825E5 |  |  |  |  | 1 | 0 | 0 | 0 | 0 | 0 | 1 | 0 | 0 | 0 | 0 | 301 | 311 | Oxidation (M) | M10:Oxidation (M):1000.00 | PEAKS DB |
| G.YALPHAILR.L | Y | 48.16 | 1052.6130 | 9 | 0.0 | 527.3138 | 2 | 35.63 | 1 | F1:21569 | 13012022\_RID\_3262\_4a.raw | 1.4154E6 |  |  |  |  |  |  |  |  |  | 1 | 1 | 0 | 0 | 0 | 0 | 0 | 0 | 0 | 0 | 0 | 154 | 162 |  |  | PEAKS DB |
| R.AVFPSIVGRPR.H | N | 45.70 | 1197.6981 | 11 | 1.5 | 400.2406 | 3 | 35.84 | 6 | F6:21794 | 17012022\_RID\_3262\_9a.raw | 1.4816E6 |  |  |  |  | 0 |  |  |  |  | 1 | 1 | 0 | 0 | 0 | 0 | 0 | 0 | 0 | 0 | 0 | 14 | 24 |  |  | PEAKS DB |
| R.DLTDYLMK.I | N | 43.13 | 997.4790 | 8 | 0.7 | 499.7471 | 2 | 51.80 | 6 | F6:36164 | 17012022\_RID\_3262\_9a.raw |  |  |  |  |  | 4.4466E5 |  |  |  |  | 1 | 0 | 0 | 0 | 0 | 0 | 1 | 0 | 0 | 0 | 0 | 169 | 176 |  |  | PEAKS DB |
| total 11 peptides |  |  |  |  |  |  |  |  |  |  |  |  |  |  |  |  |  |  |  |  |  |  |  |  |  |  |  |  |  |  |  |  |  |  |  |  |  |
| --- | --- | --- | --- | --- | --- | --- | --- | --- | --- | --- | --- | --- | --- | --- | --- | --- | --- | --- | --- | --- | --- | --- | --- | --- | --- | --- | --- | --- | --- | --- | --- | --- | --- | --- | --- | --- | --- |

LAA58652.1

back to list

  

| Protein Coverage
| Supporting Peptides
|

Protein Coverage:

Supporting Peptides:

| Peptide | Uniq | -10lgP | Mass | Length | ppm | m/z | z | RT | Fraction | Scan | Source File | Area F4 | Area F5 | Area F6 | Area F10 | Area F8 | Area F9 | Area F7 | Area F1 | Area F2 | Area F3 | #Feature | #Feature F4 | #Feature F5 | #Feature F6 | #Feature F10 | #Feature F8 | #Feature F9 | #Feature F7 | #Feature F1 | #Feature F2 | #Feature F3 | Start | End | PTM | AScore | Found By |
| --- | --- | --- | --- | --- | --- | --- | --- | --- | --- | --- | --- | --- | --- | --- | --- | --- | --- | --- | --- | --- | --- | --- | --- | --- | --- | --- | --- | --- | --- | --- | --- | --- | --- | --- | --- | --- | --- |
| K.SYELPDGQVITIGNER.F | N | 82.78 | 1789.8846 | 16 | 0.4 | 895.9500 | 2 | 49.87 | 8 | F8:28149 | 31122021\_RID\_3262\_1a.raw | 4.7745E5 |  |  |  |  | 6.0954E5 |  | 1.0043E6 | 3.4371E5 |  | 4 | 1 | 0 | 0 | 0 | 0 | 1 | 0 | 1 | 1 | 0 | 239 | 254 |  |  | PEAKS DB |
| K.LC(+57.02)YVALDFEQEMATAASSSSLEK.S | Y | 65.71 | 2549.1665 | 23 | -0.5 | 1275.5898 | 2 | 62.33 | 7 | F7:28143 | 21012022\_RID\_3262\_7a.raw |  |  |  |  |  |  | 1.0541E6 |  |  |  | 2 | 0 | 0 | 0 | 0 | 0 | 0 | 2 | 0 | 0 | 0 | 216 | 238 | Carbamidomethylation | C2:Carbamidomethylation:1000.00 | PEAKS DB |
| K.DSYVGDEAQSK.R | N | 65.50 | 1197.5149 | 11 | 1.5 | 599.7656 | 2 | 23.16 | 4 | F4:10278 | 17012022\_RID\_3262\_10a .raw | 2.7572E5 |  |  | 3.783E5 |  | 4.8292E5 |  | 7.9712E4 | 7.8692E4 |  | 5 | 1 | 0 | 0 | 1 | 0 | 1 | 0 | 1 | 1 | 0 | 51 | 61 |  |  | PEAKS DB |
| R.GYSFTTTAER.E | Y | 60.44 | 1131.5197 | 10 | -0.1 | 566.7670 | 2 | 28.78 | 8 | F8:11438 | 31122021\_RID\_3262\_1a.raw |  |  |  | 1.3304E6 |  |  | 2.0729E5 | 2.9994E5 | 2.4553E5 |  | 4 | 0 | 0 | 0 | 1 | 0 | 0 | 1 | 1 | 1 | 0 | 197 | 206 |  |  | PEAKS DB |
| K.LC(+57.02)YVALDFEQEM(+15.99)ATAASSSSLEK.S | Y | 54.72 | 2565.1614 | 23 | 0.4 | 856.0614 | 3 | 62.38 | 7 | F7:28152 | 21012022\_RID\_3262\_7a.raw |  |  |  |  |  |  | 1.0591E5 |  |  |  | 1 | 0 | 0 | 0 | 0 | 0 | 0 | 1 | 0 | 0 | 0 | 216 | 238 | Carbamidomethylation; Oxidation (M) | C2:Carbamidomethylation:1000.00;M12:Oxidation (M):1000.00 | PEAKS DB |
| K.AGFAGDDAPR.A | N | 52.95 | 975.4410 | 10 | 3.9 | 488.7296 | 2 | 22.10 | 9 | F9:7134 | 31122021\_RID\_3262\_2a.raw |  | 3.3275E5 |  | 1.2847E6 |  |  |  |  | 2.7076E6 |  | 4 | 0 | 1 | 0 | 1 | 0 | 0 | 0 | 0 | 2 | 0 | 19 | 28 |  |  | PEAKS DB |
| R.VAPEEHPVLLTEAPLNPK.A | N | 51.47 | 1953.0570 | 18 | 0.5 | 652.0266 | 3 | 40.45 | 1 | F1:25900 | 13012022\_RID\_3262\_4a.raw | 8.5304E5 |  |  | 5.0748E5 |  |  |  | 7.2111E5 |  |  | 3 | 1 | 0 | 0 | 1 | 0 | 0 | 0 | 1 | 0 | 0 | 96 | 113 |  |  | PEAKS DB |
| K.EITALAPSTM(+15.99)K.I | N | 49.12 | 1176.6060 | 11 | 1.7 | 589.3113 | 2 | 30.85 | 6 | F6:17553 | 17012022\_RID\_3262\_9a.raw |  |  |  |  |  | 2.4825E5 |  |  |  |  | 1 | 0 | 0 | 0 | 0 | 0 | 1 | 0 | 0 | 0 | 0 | 316 | 326 | Oxidation (M) | M10:Oxidation (M):1000.00 | PEAKS DB |
| G.YALPHAILR.L | Y | 48.16 | 1052.6130 | 9 | 0.0 | 527.3138 | 2 | 35.63 | 1 | F1:21569 | 13012022\_RID\_3262\_4a.raw | 1.4154E6 |  |  |  |  |  |  |  |  |  | 1 | 1 | 0 | 0 | 0 | 0 | 0 | 0 | 0 | 0 | 0 | 169 | 177 |  |  | PEAKS DB |
| R.AVFPSIVGRPR.H | N | 45.70 | 1197.6981 | 11 | 1.5 | 400.2406 | 3 | 35.84 | 6 | F6:21794 | 17012022\_RID\_3262\_9a.raw | 1.4816E6 |  |  |  |  | 0 |  |  |  |  | 1 | 1 | 0 | 0 | 0 | 0 | 0 | 0 | 0 | 0 | 0 | 29 | 39 |  |  | PEAKS DB |
| R.DLTDYLMK.I | N | 43.13 | 997.4790 | 8 | 0.7 | 499.7471 | 2 | 51.80 | 6 | F6:36164 | 17012022\_RID\_3262\_9a.raw |  |  |  |  |  | 4.4466E5 |  |  |  |  | 1 | 0 | 0 | 0 | 0 | 0 | 1 | 0 | 0 | 0 | 0 | 184 | 191 |  |  | PEAKS DB |
| total 11 peptides |  |  |  |  |  |  |  |  |  |  |  |  |  |  |  |  |  |  |  |  |  |  |  |  |  |  |  |  |  |  |  |  |  |  |  |  |  |
| --- | --- | --- | --- | --- | --- | --- | --- | --- | --- | --- | --- | --- | --- | --- | --- | --- | --- | --- | --- | --- | --- | --- | --- | --- | --- | --- | --- | --- | --- | --- | --- | --- | --- | --- | --- | --- | --- |

JAI10342.1

back to list

  

| Protein Coverage
| Supporting Peptides
|

Protein Coverage:

Supporting Peptides:

| Peptide | Uniq | -10lgP | Mass | Length | ppm | m/z | z | RT | Fraction | Scan | Source File | Area F4 | Area F5 | Area F6 | Area F10 | Area F8 | Area F9 | Area F7 | Area F1 | Area F2 | Area F3 | #Feature | #Feature F4 | #Feature F5 | #Feature F6 | #Feature F10 | #Feature F8 | #Feature F9 | #Feature F7 | #Feature F1 | #Feature F2 | #Feature F3 | Start | End | PTM | AScore | Found By |
| --- | --- | --- | --- | --- | --- | --- | --- | --- | --- | --- | --- | --- | --- | --- | --- | --- | --- | --- | --- | --- | --- | --- | --- | --- | --- | --- | --- | --- | --- | --- | --- | --- | --- | --- | --- | --- | --- |
| K.SYELPDGQVITIGNER.F | N | 82.78 | 1789.8846 | 16 | 0.4 | 895.9500 | 2 | 49.87 | 8 | F8:28149 | 31122021\_RID\_3262\_1a.raw | 4.7745E5 |  |  |  |  | 6.0954E5 |  | 1.0043E6 | 3.4371E5 |  | 4 | 1 | 0 | 0 | 0 | 0 | 1 | 0 | 1 | 1 | 0 | 239 | 254 |  |  | PEAKS DB |
| K.LC(+57.02)YVALDFEQEMATAASSSSLEK.S | Y | 65.71 | 2549.1665 | 23 | -0.5 | 1275.5898 | 2 | 62.33 | 7 | F7:28143 | 21012022\_RID\_3262\_7a.raw |  |  |  |  |  |  | 1.0541E6 |  |  |  | 2 | 0 | 0 | 0 | 0 | 0 | 0 | 2 | 0 | 0 | 0 | 216 | 238 | Carbamidomethylation | C2:Carbamidomethylation:1000.00 | PEAKS DB |
| K.DSYVGDEAQSK.R | N | 65.50 | 1197.5149 | 11 | 1.5 | 599.7656 | 2 | 23.16 | 4 | F4:10278 | 17012022\_RID\_3262\_10a .raw | 2.7572E5 |  |  | 3.783E5 |  | 4.8292E5 |  | 7.9712E4 | 7.8692E4 |  | 5 | 1 | 0 | 0 | 1 | 0 | 1 | 0 | 1 | 1 | 0 | 51 | 61 |  |  | PEAKS DB |
| R.GYSFTTTAER.E | Y | 60.44 | 1131.5197 | 10 | -0.1 | 566.7670 | 2 | 28.78 | 8 | F8:11438 | 31122021\_RID\_3262\_1a.raw |  |  |  | 1.3304E6 |  |  | 2.0729E5 | 2.9994E5 | 2.4553E5 |  | 4 | 0 | 0 | 0 | 1 | 0 | 0 | 1 | 1 | 1 | 0 | 197 | 206 |  |  | PEAKS DB |
| K.LC(+57.02)YVALDFEQEM(+15.99)ATAASSSSLEK.S | Y | 54.72 | 2565.1614 | 23 | 0.4 | 856.0614 | 3 | 62.38 | 7 | F7:28152 | 21012022\_RID\_3262\_7a.raw |  |  |  |  |  |  | 1.0591E5 |  |  |  | 1 | 0 | 0 | 0 | 0 | 0 | 0 | 1 | 0 | 0 | 0 | 216 | 238 | Carbamidomethylation; Oxidation (M) | C2:Carbamidomethylation:1000.00;M12:Oxidation (M):1000.00 | PEAKS DB |
| K.AGFAGDDAPR.A | N | 52.95 | 975.4410 | 10 | 3.9 | 488.7296 | 2 | 22.10 | 9 | F9:7134 | 31122021\_RID\_3262\_2a.raw |  | 3.3275E5 |  | 1.2847E6 |  |  |  |  | 2.7076E6 |  | 4 | 0 | 1 | 0 | 1 | 0 | 0 | 0 | 0 | 2 | 0 | 19 | 28 |  |  | PEAKS DB |
| R.VAPEEHPVLLTEAPLNPK.A | N | 51.47 | 1953.0570 | 18 | 0.5 | 652.0266 | 3 | 40.45 | 1 | F1:25900 | 13012022\_RID\_3262\_4a.raw | 8.5304E5 |  |  | 5.0748E5 |  |  |  | 7.2111E5 |  |  | 3 | 1 | 0 | 0 | 1 | 0 | 0 | 0 | 1 | 0 | 0 | 96 | 113 |  |  | PEAKS DB |
| K.EITALAPSTM(+15.99)K.I | N | 49.12 | 1176.6060 | 11 | 1.7 | 589.3113 | 2 | 30.85 | 6 | F6:17553 | 17012022\_RID\_3262\_9a.raw |  |  |  |  |  | 2.4825E5 |  |  |  |  | 1 | 0 | 0 | 0 | 0 | 0 | 1 | 0 | 0 | 0 | 0 | 316 | 326 | Oxidation (M) | M10:Oxidation (M):1000.00 | PEAKS DB |
| G.YALPHAILR.L | Y | 48.16 | 1052.6130 | 9 | 0.0 | 527.3138 | 2 | 35.63 | 1 | F1:21569 | 13012022\_RID\_3262\_4a.raw | 1.4154E6 |  |  |  |  |  |  |  |  |  | 1 | 1 | 0 | 0 | 0 | 0 | 0 | 0 | 0 | 0 | 0 | 169 | 177 |  |  | PEAKS DB |
| R.AVFPSIVGRPR.H | N | 45.70 | 1197.6981 | 11 | 1.5 | 400.2406 | 3 | 35.84 | 6 | F6:21794 | 17012022\_RID\_3262\_9a.raw | 1.4816E6 |  |  |  |  | 0 |  |  |  |  | 1 | 1 | 0 | 0 | 0 | 0 | 0 | 0 | 0 | 0 | 0 | 29 | 39 |  |  | PEAKS DB |
| R.DLTDYLMK.I | N | 43.13 | 997.4790 | 8 | 0.7 | 499.7471 | 2 | 51.80 | 6 | F6:36164 | 17012022\_RID\_3262\_9a.raw |  |  |  |  |  | 4.4466E5 |  |  |  |  | 1 | 0 | 0 | 0 | 0 | 0 | 1 | 0 | 0 | 0 | 0 | 184 | 191 |  |  | PEAKS DB |
| total 11 peptides |  |  |  |  |  |  |  |  |  |  |  |  |  |  |  |  |  |  |  |  |  |  |  |  |  |  |  |  |  |  |  |  |  |  |  |  |  |
| --- | --- | --- | --- | --- | --- | --- | --- | --- | --- | --- | --- | --- | --- | --- | --- | --- | --- | --- | --- | --- | --- | --- | --- | --- | --- | --- | --- | --- | --- | --- | --- | --- | --- | --- | --- | --- | --- |

JAB54771.1

back to list

  

| Protein Coverage
| Supporting Peptides
|

Protein Coverage:

Supporting Peptides:

| Peptide | Uniq | -10lgP | Mass | Length | ppm | m/z | z | RT | Fraction | Scan | Source File | Area F4 | Area F5 | Area F6 | Area F10 | Area F8 | Area F9 | Area F7 | Area F1 | Area F2 | Area F3 | #Feature | #Feature F4 | #Feature F5 | #Feature F6 | #Feature F10 | #Feature F8 | #Feature F9 | #Feature F7 | #Feature F1 | #Feature F2 | #Feature F3 | Start | End | PTM | AScore | Found By |
| --- | --- | --- | --- | --- | --- | --- | --- | --- | --- | --- | --- | --- | --- | --- | --- | --- | --- | --- | --- | --- | --- | --- | --- | --- | --- | --- | --- | --- | --- | --- | --- | --- | --- | --- | --- | --- | --- |
| K.SYELPDGQVITIGNER.F | N | 82.78 | 1789.8846 | 16 | 0.4 | 895.9500 | 2 | 49.87 | 8 | F8:28149 | 31122021\_RID\_3262\_1a.raw | 4.7745E5 |  |  |  |  | 6.0954E5 |  | 1.0043E6 | 3.4371E5 |  | 4 | 1 | 0 | 0 | 0 | 0 | 1 | 0 | 1 | 1 | 0 | 239 | 254 |  |  | PEAKS DB |
| K.LC(+57.02)YVALDFEQEMATAASSSSLEK.S | Y | 65.71 | 2549.1665 | 23 | -0.5 | 1275.5898 | 2 | 62.33 | 7 | F7:28143 | 21012022\_RID\_3262\_7a.raw |  |  |  |  |  |  | 1.0541E6 |  |  |  | 2 | 0 | 0 | 0 | 0 | 0 | 0 | 2 | 0 | 0 | 0 | 216 | 238 | Carbamidomethylation | C2:Carbamidomethylation:1000.00 | PEAKS DB |
| K.DSYVGDEAQSK.R | N | 65.50 | 1197.5149 | 11 | 1.5 | 599.7656 | 2 | 23.16 | 4 | F4:10278 | 17012022\_RID\_3262\_10a .raw | 2.7572E5 |  |  | 3.783E5 |  | 4.8292E5 |  | 7.9712E4 | 7.8692E4 |  | 5 | 1 | 0 | 0 | 1 | 0 | 1 | 0 | 1 | 1 | 0 | 51 | 61 |  |  | PEAKS DB |
| R.GYSFTTTAER.E | Y | 60.44 | 1131.5197 | 10 | -0.1 | 566.7670 | 2 | 28.78 | 8 | F8:11438 | 31122021\_RID\_3262\_1a.raw |  |  |  | 1.3304E6 |  |  | 2.0729E5 | 2.9994E5 | 2.4553E5 |  | 4 | 0 | 0 | 0 | 1 | 0 | 0 | 1 | 1 | 1 | 0 | 197 | 206 |  |  | PEAKS DB |
| K.LC(+57.02)YVALDFEQEM(+15.99)ATAASSSSLEK.S | Y | 54.72 | 2565.1614 | 23 | 0.4 | 856.0614 | 3 | 62.38 | 7 | F7:28152 | 21012022\_RID\_3262\_7a.raw |  |  |  |  |  |  | 1.0591E5 |  |  |  | 1 | 0 | 0 | 0 | 0 | 0 | 0 | 1 | 0 | 0 | 0 | 216 | 238 | Carbamidomethylation; Oxidation (M) | C2:Carbamidomethylation:1000.00;M12:Oxidation (M):1000.00 | PEAKS DB |
| K.AGFAGDDAPR.A | N | 52.95 | 975.4410 | 10 | 3.9 | 488.7296 | 2 | 22.10 | 9 | F9:7134 | 31122021\_RID\_3262\_2a.raw |  | 3.3275E5 |  | 1.2847E6 |  |  |  |  | 2.7076E6 |  | 4 | 0 | 1 | 0 | 1 | 0 | 0 | 0 | 0 | 2 | 0 | 19 | 28 |  |  | PEAKS DB |
| R.VAPEEHPVLLTEAPLNPK.A | N | 51.47 | 1953.0570 | 18 | 0.5 | 652.0266 | 3 | 40.45 | 1 | F1:25900 | 13012022\_RID\_3262\_4a.raw | 8.5304E5 |  |  | 5.0748E5 |  |  |  | 7.2111E5 |  |  | 3 | 1 | 0 | 0 | 1 | 0 | 0 | 0 | 1 | 0 | 0 | 96 | 113 |  |  | PEAKS DB |
| K.EITALAPSTM(+15.99)K.I | N | 49.12 | 1176.6060 | 11 | 1.7 | 589.3113 | 2 | 30.85 | 6 | F6:17553 | 17012022\_RID\_3262\_9a.raw |  |  |  |  |  | 2.4825E5 |  |  |  |  | 1 | 0 | 0 | 0 | 0 | 0 | 1 | 0 | 0 | 0 | 0 | 316 | 326 | Oxidation (M) | M10:Oxidation (M):1000.00 | PEAKS DB |
| G.YALPHAILR.L | Y | 48.16 | 1052.6130 | 9 | 0.0 | 527.3138 | 2 | 35.63 | 1 | F1:21569 | 13012022\_RID\_3262\_4a.raw | 1.4154E6 |  |  |  |  |  |  |  |  |  | 1 | 1 | 0 | 0 | 0 | 0 | 0 | 0 | 0 | 0 | 0 | 169 | 177 |  |  | PEAKS DB |
| R.AVFPSIVGRPR.H | N | 45.70 | 1197.6981 | 11 | 1.5 | 400.2406 | 3 | 35.84 | 6 | F6:21794 | 17012022\_RID\_3262\_9a.raw | 1.4816E6 |  |  |  |  | 0 |  |  |  |  | 1 | 1 | 0 | 0 | 0 | 0 | 0 | 0 | 0 | 0 | 0 | 29 | 39 |  |  | PEAKS DB |
| R.DLTDYLMK.I | N | 43.13 | 997.4790 | 8 | 0.7 | 499.7471 | 2 | 51.80 | 6 | F6:36164 | 17012022\_RID\_3262\_9a.raw |  |  |  |  |  | 4.4466E5 |  |  |  |  | 1 | 0 | 0 | 0 | 0 | 0 | 1 | 0 | 0 | 0 | 0 | 184 | 191 |  |  | PEAKS DB |
| total 11 peptides |  |  |  |  |  |  |  |  |  |  |  |  |  |  |  |  |  |  |  |  |  |  |  |  |  |  |  |  |  |  |  |  |  |  |  |  |  |
| --- | --- | --- | --- | --- | --- | --- | --- | --- | --- | --- | --- | --- | --- | --- | --- | --- | --- | --- | --- | --- | --- | --- | --- | --- | --- | --- | --- | --- | --- | --- | --- | --- | --- | --- | --- | --- | --- |

JAA97901.1

back to list

  

| Protein Coverage
| Supporting Peptides
|

Protein Coverage:

Supporting Peptides:

| Peptide | Uniq | -10lgP | Mass | Length | ppm | m/z | z | RT | Fraction | Scan | Source File | Area F4 | Area F5 | Area F6 | Area F10 | Area F8 | Area F9 | Area F7 | Area F1 | Area F2 | Area F3 | #Feature | #Feature F4 | #Feature F5 | #Feature F6 | #Feature F10 | #Feature F8 | #Feature F9 | #Feature F7 | #Feature F1 | #Feature F2 | #Feature F3 | Start | End | PTM | AScore | Found By |
| --- | --- | --- | --- | --- | --- | --- | --- | --- | --- | --- | --- | --- | --- | --- | --- | --- | --- | --- | --- | --- | --- | --- | --- | --- | --- | --- | --- | --- | --- | --- | --- | --- | --- | --- | --- | --- | --- |
| K.SYELPDGQVITIGNER.F | N | 82.78 | 1789.8846 | 16 | 0.4 | 895.9500 | 2 | 49.87 | 8 | F8:28149 | 31122021\_RID\_3262\_1a.raw | 4.7745E5 |  |  |  |  | 6.0954E5 |  | 1.0043E6 | 3.4371E5 |  | 4 | 1 | 0 | 0 | 0 | 0 | 1 | 0 | 1 | 1 | 0 | 239 | 254 |  |  | PEAKS DB |
| K.LC(+57.02)YVALDFEQEMATAASSSSLEK.S | Y | 65.71 | 2549.1665 | 23 | -0.5 | 1275.5898 | 2 | 62.33 | 7 | F7:28143 | 21012022\_RID\_3262\_7a.raw |  |  |  |  |  |  | 1.0541E6 |  |  |  | 2 | 0 | 0 | 0 | 0 | 0 | 0 | 2 | 0 | 0 | 0 | 216 | 238 | Carbamidomethylation | C2:Carbamidomethylation:1000.00 | PEAKS DB |
| K.DSYVGDEAQSK.R | N | 65.50 | 1197.5149 | 11 | 1.5 | 599.7656 | 2 | 23.16 | 4 | F4:10278 | 17012022\_RID\_3262\_10a .raw | 2.7572E5 |  |  | 3.783E5 |  | 4.8292E5 |  | 7.9712E4 | 7.8692E4 |  | 5 | 1 | 0 | 0 | 1 | 0 | 1 | 0 | 1 | 1 | 0 | 51 | 61 |  |  | PEAKS DB |
| R.GYSFTTTAER.E | Y | 60.44 | 1131.5197 | 10 | -0.1 | 566.7670 | 2 | 28.78 | 8 | F8:11438 | 31122021\_RID\_3262\_1a.raw |  |  |  | 1.3304E6 |  |  | 2.0729E5 | 2.9994E5 | 2.4553E5 |  | 4 | 0 | 0 | 0 | 1 | 0 | 0 | 1 | 1 | 1 | 0 | 197 | 206 |  |  | PEAKS DB |
| K.LC(+57.02)YVALDFEQEM(+15.99)ATAASSSSLEK.S | Y | 54.72 | 2565.1614 | 23 | 0.4 | 856.0614 | 3 | 62.38 | 7 | F7:28152 | 21012022\_RID\_3262\_7a.raw |  |  |  |  |  |  | 1.0591E5 |  |  |  | 1 | 0 | 0 | 0 | 0 | 0 | 0 | 1 | 0 | 0 | 0 | 216 | 238 | Carbamidomethylation; Oxidation (M) | C2:Carbamidomethylation:1000.00;M12:Oxidation (M):1000.00 | PEAKS DB |
| K.AGFAGDDAPR.A | N | 52.95 | 975.4410 | 10 | 3.9 | 488.7296 | 2 | 22.10 | 9 | F9:7134 | 31122021\_RID\_3262\_2a.raw |  | 3.3275E5 |  | 1.2847E6 |  |  |  |  | 2.7076E6 |  | 4 | 0 | 1 | 0 | 1 | 0 | 0 | 0 | 0 | 2 | 0 | 19 | 28 |  |  | PEAKS DB |
| R.VAPEEHPVLLTEAPLNPK.A | N | 51.47 | 1953.0570 | 18 | 0.5 | 652.0266 | 3 | 40.45 | 1 | F1:25900 | 13012022\_RID\_3262\_4a.raw | 8.5304E5 |  |  | 5.0748E5 |  |  |  | 7.2111E5 |  |  | 3 | 1 | 0 | 0 | 1 | 0 | 0 | 0 | 1 | 0 | 0 | 96 | 113 |  |  | PEAKS DB |
| K.EITALAPSTM(+15.99)K.I | N | 49.12 | 1176.6060 | 11 | 1.7 | 589.3113 | 2 | 30.85 | 6 | F6:17553 | 17012022\_RID\_3262\_9a.raw |  |  |  |  |  | 2.4825E5 |  |  |  |  | 1 | 0 | 0 | 0 | 0 | 0 | 1 | 0 | 0 | 0 | 0 | 316 | 326 | Oxidation (M) | M10:Oxidation (M):1000.00 | PEAKS DB |
| G.YALPHAILR.L | Y | 48.16 | 1052.6130 | 9 | 0.0 | 527.3138 | 2 | 35.63 | 1 | F1:21569 | 13012022\_RID\_3262\_4a.raw | 1.4154E6 |  |  |  |  |  |  |  |  |  | 1 | 1 | 0 | 0 | 0 | 0 | 0 | 0 | 0 | 0 | 0 | 169 | 177 |  |  | PEAKS DB |
| R.AVFPSIVGRPR.H | N | 45.70 | 1197.6981 | 11 | 1.5 | 400.2406 | 3 | 35.84 | 6 | F6:21794 | 17012022\_RID\_3262\_9a.raw | 1.4816E6 |  |  |  |  | 0 |  |  |  |  | 1 | 1 | 0 | 0 | 0 | 0 | 0 | 0 | 0 | 0 | 0 | 29 | 39 |  |  | PEAKS DB |
| R.DLTDYLMK.I | N | 43.13 | 997.4790 | 8 | 0.7 | 499.7471 | 2 | 51.80 | 6 | F6:36164 | 17012022\_RID\_3262\_9a.raw |  |  |  |  |  | 4.4466E5 |  |  |  |  | 1 | 0 | 0 | 0 | 0 | 0 | 1 | 0 | 0 | 0 | 0 | 184 | 191 |  |  | PEAKS DB |
| total 11 peptides |  |  |  |  |  |  |  |  |  |  |  |  |  |  |  |  |  |  |  |  |  |  |  |  |  |  |  |  |  |  |  |  |  |  |  |  |  |
| --- | --- | --- | --- | --- | --- | --- | --- | --- | --- | --- | --- | --- | --- | --- | --- | --- | --- | --- | --- | --- | --- | --- | --- | --- | --- | --- | --- | --- | --- | --- | --- | --- | --- | --- | --- | --- | --- |

JAG68844.1

back to list

  

| Protein Coverage
| Supporting Peptides
|

Protein Coverage:

Supporting Peptides:

| Peptide | Uniq | -10lgP | Mass | Length | ppm | m/z | z | RT | Fraction | Scan | Source File | Area F4 | Area F5 | Area F6 | Area F10 | Area F8 | Area F9 | Area F7 | Area F1 | Area F2 | Area F3 | #Feature | #Feature F4 | #Feature F5 | #Feature F6 | #Feature F10 | #Feature F8 | #Feature F9 | #Feature F7 | #Feature F1 | #Feature F2 | #Feature F3 | Start | End | PTM | AScore | Found By |
| --- | --- | --- | --- | --- | --- | --- | --- | --- | --- | --- | --- | --- | --- | --- | --- | --- | --- | --- | --- | --- | --- | --- | --- | --- | --- | --- | --- | --- | --- | --- | --- | --- | --- | --- | --- | --- | --- |
| K.SYELPDGQVITIGNER.F | N | 82.78 | 1789.8846 | 16 | 0.4 | 895.9500 | 2 | 49.87 | 8 | F8:28149 | 31122021\_RID\_3262\_1a.raw | 4.7745E5 |  |  |  |  | 6.0954E5 |  | 1.0043E6 | 3.4371E5 |  | 4 | 1 | 0 | 0 | 0 | 0 | 1 | 0 | 1 | 1 | 0 | 239 | 254 |  |  | PEAKS DB |
| K.LC(+57.02)YVALDFEQEMATAASSSSLEK.S | Y | 65.71 | 2549.1665 | 23 | -0.5 | 1275.5898 | 2 | 62.33 | 7 | F7:28143 | 21012022\_RID\_3262\_7a.raw |  |  |  |  |  |  | 1.0541E6 |  |  |  | 2 | 0 | 0 | 0 | 0 | 0 | 0 | 2 | 0 | 0 | 0 | 216 | 238 | Carbamidomethylation | C2:Carbamidomethylation:1000.00 | PEAKS DB |
| K.DSYVGDEAQSK.R | N | 65.50 | 1197.5149 | 11 | 1.5 | 599.7656 | 2 | 23.16 | 4 | F4:10278 | 17012022\_RID\_3262\_10a .raw | 2.7572E5 |  |  | 3.783E5 |  | 4.8292E5 |  | 7.9712E4 | 7.8692E4 |  | 5 | 1 | 0 | 0 | 1 | 0 | 1 | 0 | 1 | 1 | 0 | 51 | 61 |  |  | PEAKS DB |
| R.GYSFTTTAER.E | Y | 60.44 | 1131.5197 | 10 | -0.1 | 566.7670 | 2 | 28.78 | 8 | F8:11438 | 31122021\_RID\_3262\_1a.raw |  |  |  | 1.3304E6 |  |  | 2.0729E5 | 2.9994E5 | 2.4553E5 |  | 4 | 0 | 0 | 0 | 1 | 0 | 0 | 1 | 1 | 1 | 0 | 197 | 206 |  |  | PEAKS DB |
| K.LC(+57.02)YVALDFEQEM(+15.99)ATAASSSSLEK.S | Y | 54.72 | 2565.1614 | 23 | 0.4 | 856.0614 | 3 | 62.38 | 7 | F7:28152 | 21012022\_RID\_3262\_7a.raw |  |  |  |  |  |  | 1.0591E5 |  |  |  | 1 | 0 | 0 | 0 | 0 | 0 | 0 | 1 | 0 | 0 | 0 | 216 | 238 | Carbamidomethylation; Oxidation (M) | C2:Carbamidomethylation:1000.00;M12:Oxidation (M):1000.00 | PEAKS DB |
| K.AGFAGDDAPR.A | N | 52.95 | 975.4410 | 10 | 3.9 | 488.7296 | 2 | 22.10 | 9 | F9:7134 | 31122021\_RID\_3262\_2a.raw |  | 3.3275E5 |  | 1.2847E6 |  |  |  |  | 2.7076E6 |  | 4 | 0 | 1 | 0 | 1 | 0 | 0 | 0 | 0 | 2 | 0 | 19 | 28 |  |  | PEAKS DB |
| R.VAPEEHPVLLTEAPLNPK.A | N | 51.47 | 1953.0570 | 18 | 0.5 | 652.0266 | 3 | 40.45 | 1 | F1:25900 | 13012022\_RID\_3262\_4a.raw | 8.5304E5 |  |  | 5.0748E5 |  |  |  | 7.2111E5 |  |  | 3 | 1 | 0 | 0 | 1 | 0 | 0 | 0 | 1 | 0 | 0 | 96 | 113 |  |  | PEAKS DB |
| K.EITALAPSTM(+15.99)K.I | N | 49.12 | 1176.6060 | 11 | 1.7 | 589.3113 | 2 | 30.85 | 6 | F6:17553 | 17012022\_RID\_3262\_9a.raw |  |  |  |  |  | 2.4825E5 |  |  |  |  | 1 | 0 | 0 | 0 | 0 | 0 | 1 | 0 | 0 | 0 | 0 | 316 | 326 | Oxidation (M) | M10:Oxidation (M):1000.00 | PEAKS DB |
| G.YALPHAILR.L | Y | 48.16 | 1052.6130 | 9 | 0.0 | 527.3138 | 2 | 35.63 | 1 | F1:21569 | 13012022\_RID\_3262\_4a.raw | 1.4154E6 |  |  |  |  |  |  |  |  |  | 1 | 1 | 0 | 0 | 0 | 0 | 0 | 0 | 0 | 0 | 0 | 169 | 177 |  |  | PEAKS DB |
| R.AVFPSIVGRPR.H | N | 45.70 | 1197.6981 | 11 | 1.5 | 400.2406 | 3 | 35.84 | 6 | F6:21794 | 17012022\_RID\_3262\_9a.raw | 1.4816E6 |  |  |  |  | 0 |  |  |  |  | 1 | 1 | 0 | 0 | 0 | 0 | 0 | 0 | 0 | 0 | 0 | 29 | 39 |  |  | PEAKS DB |
| R.DLTDYLMK.I | N | 43.13 | 997.4790 | 8 | 0.7 | 499.7471 | 2 | 51.80 | 6 | F6:36164 | 17012022\_RID\_3262\_9a.raw |  |  |  |  |  | 4.4466E5 |  |  |  |  | 1 | 0 | 0 | 0 | 0 | 0 | 1 | 0 | 0 | 0 | 0 | 184 | 191 |  |  | PEAKS DB |
| total 11 peptides |  |  |  |  |  |  |  |  |  |  |  |  |  |  |  |  |  |  |  |  |  |  |  |  |  |  |  |  |  |  |  |  |  |  |  |  |  |
| --- | --- | --- | --- | --- | --- | --- | --- | --- | --- | --- | --- | --- | --- | --- | --- | --- | --- | --- | --- | --- | --- | --- | --- | --- | --- | --- | --- | --- | --- | --- | --- | --- | --- | --- | --- | --- | --- |

JAV51374.1

back to list

  

| Protein Coverage
| Supporting Peptides
|

Protein Coverage:

Supporting Peptides:

| Peptide | Uniq | -10lgP | Mass | Length | ppm | m/z | z | RT | Fraction | Scan | Source File | Area F4 | Area F5 | Area F6 | Area F10 | Area F8 | Area F9 | Area F7 | Area F1 | Area F2 | Area F3 | #Feature | #Feature F4 | #Feature F5 | #Feature F6 | #Feature F10 | #Feature F8 | #Feature F9 | #Feature F7 | #Feature F1 | #Feature F2 | #Feature F3 | Start | End | PTM | AScore | Found By |
| --- | --- | --- | --- | --- | --- | --- | --- | --- | --- | --- | --- | --- | --- | --- | --- | --- | --- | --- | --- | --- | --- | --- | --- | --- | --- | --- | --- | --- | --- | --- | --- | --- | --- | --- | --- | --- | --- |
| K.SYELPDGQVITIGNER.F | N | 82.78 | 1789.8846 | 16 | 0.4 | 895.9500 | 2 | 49.87 | 8 | F8:28149 | 31122021\_RID\_3262\_1a.raw | 4.7745E5 |  |  |  |  | 6.0954E5 |  | 1.0043E6 | 3.4371E5 |  | 4 | 1 | 0 | 0 | 0 | 0 | 1 | 0 | 1 | 1 | 0 | 239 | 254 |  |  | PEAKS DB |
| K.LC(+57.02)YVALDFEQEMATAASSSSLEK.S | Y | 65.71 | 2549.1665 | 23 | -0.5 | 1275.5898 | 2 | 62.33 | 7 | F7:28143 | 21012022\_RID\_3262\_7a.raw |  |  |  |  |  |  | 1.0541E6 |  |  |  | 2 | 0 | 0 | 0 | 0 | 0 | 0 | 2 | 0 | 0 | 0 | 216 | 238 | Carbamidomethylation | C2:Carbamidomethylation:1000.00 | PEAKS DB |
| K.DSYVGDEAQSK.R | N | 65.50 | 1197.5149 | 11 | 1.5 | 599.7656 | 2 | 23.16 | 4 | F4:10278 | 17012022\_RID\_3262\_10a .raw | 2.7572E5 |  |  | 3.783E5 |  | 4.8292E5 |  | 7.9712E4 | 7.8692E4 |  | 5 | 1 | 0 | 0 | 1 | 0 | 1 | 0 | 1 | 1 | 0 | 51 | 61 |  |  | PEAKS DB |
| R.GYSFTTTAER.E | Y | 60.44 | 1131.5197 | 10 | -0.1 | 566.7670 | 2 | 28.78 | 8 | F8:11438 | 31122021\_RID\_3262\_1a.raw |  |  |  | 1.3304E6 |  |  | 2.0729E5 | 2.9994E5 | 2.4553E5 |  | 4 | 0 | 0 | 0 | 1 | 0 | 0 | 1 | 1 | 1 | 0 | 197 | 206 |  |  | PEAKS DB |
| K.LC(+57.02)YVALDFEQEM(+15.99)ATAASSSSLEK.S | Y | 54.72 | 2565.1614 | 23 | 0.4 | 856.0614 | 3 | 62.38 | 7 | F7:28152 | 21012022\_RID\_3262\_7a.raw |  |  |  |  |  |  | 1.0591E5 |  |  |  | 1 | 0 | 0 | 0 | 0 | 0 | 0 | 1 | 0 | 0 | 0 | 216 | 238 | Carbamidomethylation; Oxidation (M) | C2:Carbamidomethylation:1000.00;M12:Oxidation (M):1000.00 | PEAKS DB |
| K.AGFAGDDAPR.A | N | 52.95 | 975.4410 | 10 | 3.9 | 488.7296 | 2 | 22.10 | 9 | F9:7134 | 31122021\_RID\_3262\_2a.raw |  | 3.3275E5 |  | 1.2847E6 |  |  |  |  | 2.7076E6 |  | 4 | 0 | 1 | 0 | 1 | 0 | 0 | 0 | 0 | 2 | 0 | 19 | 28 |  |  | PEAKS DB |
| R.VAPEEHPVLLTEAPLNPK.A | N | 51.47 | 1953.0570 | 18 | 0.5 | 652.0266 | 3 | 40.45 | 1 | F1:25900 | 13012022\_RID\_3262\_4a.raw | 8.5304E5 |  |  | 5.0748E5 |  |  |  | 7.2111E5 |  |  | 3 | 1 | 0 | 0 | 1 | 0 | 0 | 0 | 1 | 0 | 0 | 96 | 113 |  |  | PEAKS DB |
| K.EITALAPSTM(+15.99)K.I | N | 49.12 | 1176.6060 | 11 | 1.7 | 589.3113 | 2 | 30.85 | 6 | F6:17553 | 17012022\_RID\_3262\_9a.raw |  |  |  |  |  | 2.4825E5 |  |  |  |  | 1 | 0 | 0 | 0 | 0 | 0 | 1 | 0 | 0 | 0 | 0 | 316 | 326 | Oxidation (M) | M10:Oxidation (M):1000.00 | PEAKS DB |
| G.YALPHAILR.L | Y | 48.16 | 1052.6130 | 9 | 0.0 | 527.3138 | 2 | 35.63 | 1 | F1:21569 | 13012022\_RID\_3262\_4a.raw | 1.4154E6 |  |  |  |  |  |  |  |  |  | 1 | 1 | 0 | 0 | 0 | 0 | 0 | 0 | 0 | 0 | 0 | 169 | 177 |  |  | PEAKS DB |
| R.AVFPSIVGRPR.H | N | 45.70 | 1197.6981 | 11 | 1.5 | 400.2406 | 3 | 35.84 | 6 | F6:21794 | 17012022\_RID\_3262\_9a.raw | 1.4816E6 |  |  |  |  | 0 |  |  |  |  | 1 | 1 | 0 | 0 | 0 | 0 | 0 | 0 | 0 | 0 | 0 | 29 | 39 |  |  | PEAKS DB |
| R.DLTDYLMK.I | N | 43.13 | 997.4790 | 8 | 0.7 | 499.7471 | 2 | 51.80 | 6 | F6:36164 | 17012022\_RID\_3262\_9a.raw |  |  |  |  |  | 4.4466E5 |  |  |  |  | 1 | 0 | 0 | 0 | 0 | 0 | 1 | 0 | 0 | 0 | 0 | 184 | 191 |  |  | PEAKS DB |
| total 11 peptides |  |  |  |  |  |  |  |  |  |  |  |  |  |  |  |  |  |  |  |  |  |  |  |  |  |  |  |  |  |  |  |  |  |  |  |  |  |
| --- | --- | --- | --- | --- | --- | --- | --- | --- | --- | --- | --- | --- | --- | --- | --- | --- | --- | --- | --- | --- | --- | --- | --- | --- | --- | --- | --- | --- | --- | --- | --- | --- | --- | --- | --- | --- | --- |

JAG47580.1

back to list

  

| Protein Coverage
| Supporting Peptides
|

Protein Coverage:

Supporting Peptides:

| Peptide | Uniq | -10lgP | Mass | Length | ppm | m/z | z | RT | Fraction | Scan | Source File | Area F4 | Area F5 | Area F6 | Area F10 | Area F8 | Area F9 | Area F7 | Area F1 | Area F2 | Area F3 | #Feature | #Feature F4 | #Feature F5 | #Feature F6 | #Feature F10 | #Feature F8 | #Feature F9 | #Feature F7 | #Feature F1 | #Feature F2 | #Feature F3 | Start | End | PTM | AScore | Found By |
| --- | --- | --- | --- | --- | --- | --- | --- | --- | --- | --- | --- | --- | --- | --- | --- | --- | --- | --- | --- | --- | --- | --- | --- | --- | --- | --- | --- | --- | --- | --- | --- | --- | --- | --- | --- | --- | --- |
| K.SYELPDGQVITIGNER.F | N | 82.78 | 1789.8846 | 16 | 0.4 | 895.9500 | 2 | 49.87 | 8 | F8:28149 | 31122021\_RID\_3262\_1a.raw | 4.7745E5 |  |  |  |  | 6.0954E5 |  | 1.0043E6 | 3.4371E5 |  | 4 | 1 | 0 | 0 | 0 | 0 | 1 | 0 | 1 | 1 | 0 | 239 | 254 |  |  | PEAKS DB |
| K.LC(+57.02)YVALDFEQEMATAASSSSLEK.S | Y | 65.71 | 2549.1665 | 23 | -0.5 | 1275.5898 | 2 | 62.33 | 7 | F7:28143 | 21012022\_RID\_3262\_7a.raw |  |  |  |  |  |  | 1.0541E6 |  |  |  | 2 | 0 | 0 | 0 | 0 | 0 | 0 | 2 | 0 | 0 | 0 | 216 | 238 | Carbamidomethylation | C2:Carbamidomethylation:1000.00 | PEAKS DB |
| K.DSYVGDEAQSK.R | N | 65.50 | 1197.5149 | 11 | 1.5 | 599.7656 | 2 | 23.16 | 4 | F4:10278 | 17012022\_RID\_3262\_10a .raw | 2.7572E5 |  |  | 3.783E5 |  | 4.8292E5 |  | 7.9712E4 | 7.8692E4 |  | 5 | 1 | 0 | 0 | 1 | 0 | 1 | 0 | 1 | 1 | 0 | 51 | 61 |  |  | PEAKS DB |
| R.GYSFTTTAER.E | Y | 60.44 | 1131.5197 | 10 | -0.1 | 566.7670 | 2 | 28.78 | 8 | F8:11438 | 31122021\_RID\_3262\_1a.raw |  |  |  | 1.3304E6 |  |  | 2.0729E5 | 2.9994E5 | 2.4553E5 |  | 4 | 0 | 0 | 0 | 1 | 0 | 0 | 1 | 1 | 1 | 0 | 197 | 206 |  |  | PEAKS DB |
| K.LC(+57.02)YVALDFEQEM(+15.99)ATAASSSSLEK.S | Y | 54.72 | 2565.1614 | 23 | 0.4 | 856.0614 | 3 | 62.38 | 7 | F7:28152 | 21012022\_RID\_3262\_7a.raw |  |  |  |  |  |  | 1.0591E5 |  |  |  | 1 | 0 | 0 | 0 | 0 | 0 | 0 | 1 | 0 | 0 | 0 | 216 | 238 | Carbamidomethylation; Oxidation (M) | C2:Carbamidomethylation:1000.00;M12:Oxidation (M):1000.00 | PEAKS DB |
| K.AGFAGDDAPR.A | N | 52.95 | 975.4410 | 10 | 3.9 | 488.7296 | 2 | 22.10 | 9 | F9:7134 | 31122021\_RID\_3262\_2a.raw |  | 3.3275E5 |  | 1.2847E6 |  |  |  |  | 2.7076E6 |  | 4 | 0 | 1 | 0 | 1 | 0 | 0 | 0 | 0 | 2 | 0 | 19 | 28 |  |  | PEAKS DB |
| R.VAPEEHPVLLTEAPLNPK.A | N | 51.47 | 1953.0570 | 18 | 0.5 | 652.0266 | 3 | 40.45 | 1 | F1:25900 | 13012022\_RID\_3262\_4a.raw | 8.5304E5 |  |  | 5.0748E5 |  |  |  | 7.2111E5 |  |  | 3 | 1 | 0 | 0 | 1 | 0 | 0 | 0 | 1 | 0 | 0 | 96 | 113 |  |  | PEAKS DB |
| K.EITALAPSTM(+15.99)K.I | N | 49.12 | 1176.6060 | 11 | 1.7 | 589.3113 | 2 | 30.85 | 6 | F6:17553 | 17012022\_RID\_3262\_9a.raw |  |  |  |  |  | 2.4825E5 |  |  |  |  | 1 | 0 | 0 | 0 | 0 | 0 | 1 | 0 | 0 | 0 | 0 | 316 | 326 | Oxidation (M) | M10:Oxidation (M):1000.00 | PEAKS DB |
| G.YALPHAILR.L | Y | 48.16 | 1052.6130 | 9 | 0.0 | 527.3138 | 2 | 35.63 | 1 | F1:21569 | 13012022\_RID\_3262\_4a.raw | 1.4154E6 |  |  |  |  |  |  |  |  |  | 1 | 1 | 0 | 0 | 0 | 0 | 0 | 0 | 0 | 0 | 0 | 169 | 177 |  |  | PEAKS DB |
| R.AVFPSIVGRPR.H | N | 45.70 | 1197.6981 | 11 | 1.5 | 400.2406 | 3 | 35.84 | 6 | F6:21794 | 17012022\_RID\_3262\_9a.raw | 1.4816E6 |  |  |  |  | 0 |  |  |  |  | 1 | 1 | 0 | 0 | 0 | 0 | 0 | 0 | 0 | 0 | 0 | 29 | 39 |  |  | PEAKS DB |
| R.DLTDYLMK.I | N | 43.13 | 997.4790 | 8 | 0.7 | 499.7471 | 2 | 51.80 | 6 | F6:36164 | 17012022\_RID\_3262\_9a.raw |  |  |  |  |  | 4.4466E5 |  |  |  |  | 1 | 0 | 0 | 0 | 0 | 0 | 1 | 0 | 0 | 0 | 0 | 184 | 191 |  |  | PEAKS DB |
| total 11 peptides |  |  |  |  |  |  |  |  |  |  |  |  |  |  |  |  |  |  |  |  |  |  |  |  |  |  |  |  |  |  |  |  |  |  |  |  |  |
| --- | --- | --- | --- | --- | --- | --- | --- | --- | --- | --- | --- | --- | --- | --- | --- | --- | --- | --- | --- | --- | --- | --- | --- | --- | --- | --- | --- | --- | --- | --- | --- | --- | --- | --- | --- | --- | --- |

JAI14496.1

back to list

  

| Protein Coverage
| Supporting Peptides
|

Protein Coverage:

Supporting Peptides:

| Peptide | Uniq | -10lgP | Mass | Length | ppm | m/z | z | RT | Fraction | Scan | Source File | Area F4 | Area F5 | Area F6 | Area F10 | Area F8 | Area F9 | Area F7 | Area F1 | Area F2 | Area F3 | #Feature | #Feature F4 | #Feature F5 | #Feature F6 | #Feature F10 | #Feature F8 | #Feature F9 | #Feature F7 | #Feature F1 | #Feature F2 | #Feature F3 | Start | End | PTM | AScore | Found By |
| --- | --- | --- | --- | --- | --- | --- | --- | --- | --- | --- | --- | --- | --- | --- | --- | --- | --- | --- | --- | --- | --- | --- | --- | --- | --- | --- | --- | --- | --- | --- | --- | --- | --- | --- | --- | --- | --- |
| K.SYELPDGQVITIGNER.F | N | 82.78 | 1789.8846 | 16 | 0.4 | 895.9500 | 2 | 49.87 | 8 | F8:28149 | 31122021\_RID\_3262\_1a.raw | 4.7745E5 |  |  |  |  | 6.0954E5 |  | 1.0043E6 | 3.4371E5 |  | 4 | 1 | 0 | 0 | 0 | 0 | 1 | 0 | 1 | 1 | 0 | 239 | 254 |  |  | PEAKS DB |
| K.LC(+57.02)YVALDFEQEMATAASSSSLEK.S | Y | 65.71 | 2549.1665 | 23 | -0.5 | 1275.5898 | 2 | 62.33 | 7 | F7:28143 | 21012022\_RID\_3262\_7a.raw |  |  |  |  |  |  | 1.0541E6 |  |  |  | 2 | 0 | 0 | 0 | 0 | 0 | 0 | 2 | 0 | 0 | 0 | 216 | 238 | Carbamidomethylation | C2:Carbamidomethylation:1000.00 | PEAKS DB |
| K.DSYVGDEAQSK.R | N | 65.50 | 1197.5149 | 11 | 1.5 | 599.7656 | 2 | 23.16 | 4 | F4:10278 | 17012022\_RID\_3262\_10a .raw | 2.7572E5 |  |  | 3.783E5 |  | 4.8292E5 |  | 7.9712E4 | 7.8692E4 |  | 5 | 1 | 0 | 0 | 1 | 0 | 1 | 0 | 1 | 1 | 0 | 51 | 61 |  |  | PEAKS DB |
| R.GYSFTTTAER.E | Y | 60.44 | 1131.5197 | 10 | -0.1 | 566.7670 | 2 | 28.78 | 8 | F8:11438 | 31122021\_RID\_3262\_1a.raw |  |  |  | 1.3304E6 |  |  | 2.0729E5 | 2.9994E5 | 2.4553E5 |  | 4 | 0 | 0 | 0 | 1 | 0 | 0 | 1 | 1 | 1 | 0 | 197 | 206 |  |  | PEAKS DB |
| K.LC(+57.02)YVALDFEQEM(+15.99)ATAASSSSLEK.S | Y | 54.72 | 2565.1614 | 23 | 0.4 | 856.0614 | 3 | 62.38 | 7 | F7:28152 | 21012022\_RID\_3262\_7a.raw |  |  |  |  |  |  | 1.0591E5 |  |  |  | 1 | 0 | 0 | 0 | 0 | 0 | 0 | 1 | 0 | 0 | 0 | 216 | 238 | Carbamidomethylation; Oxidation (M) | C2:Carbamidomethylation:1000.00;M12:Oxidation (M):1000.00 | PEAKS DB |
| K.AGFAGDDAPR.A | N | 52.95 | 975.4410 | 10 | 3.9 | 488.7296 | 2 | 22.10 | 9 | F9:7134 | 31122021\_RID\_3262\_2a.raw |  | 3.3275E5 |  | 1.2847E6 |  |  |  |  | 2.7076E6 |  | 4 | 0 | 1 | 0 | 1 | 0 | 0 | 0 | 0 | 2 | 0 | 19 | 28 |  |  | PEAKS DB |
| R.VAPEEHPVLLTEAPLNPK.A | N | 51.47 | 1953.0570 | 18 | 0.5 | 652.0266 | 3 | 40.45 | 1 | F1:25900 | 13012022\_RID\_3262\_4a.raw | 8.5304E5 |  |  | 5.0748E5 |  |  |  | 7.2111E5 |  |  | 3 | 1 | 0 | 0 | 1 | 0 | 0 | 0 | 1 | 0 | 0 | 96 | 113 |  |  | PEAKS DB |
| K.EITALAPSTM(+15.99)K.I | N | 49.12 | 1176.6060 | 11 | 1.7 | 589.3113 | 2 | 30.85 | 6 | F6:17553 | 17012022\_RID\_3262\_9a.raw |  |  |  |  |  | 2.4825E5 |  |  |  |  | 1 | 0 | 0 | 0 | 0 | 0 | 1 | 0 | 0 | 0 | 0 | 316 | 326 | Oxidation (M) | M10:Oxidation (M):1000.00 | PEAKS DB |
| G.YALPHAILR.L | Y | 48.16 | 1052.6130 | 9 | 0.0 | 527.3138 | 2 | 35.63 | 1 | F1:21569 | 13012022\_RID\_3262\_4a.raw | 1.4154E6 |  |  |  |  |  |  |  |  |  | 1 | 1 | 0 | 0 | 0 | 0 | 0 | 0 | 0 | 0 | 0 | 169 | 177 |  |  | PEAKS DB |
| R.AVFPSIVGRPR.H | N | 45.70 | 1197.6981 | 11 | 1.5 | 400.2406 | 3 | 35.84 | 6 | F6:21794 | 17012022\_RID\_3262\_9a.raw | 1.4816E6 |  |  |  |  | 0 |  |  |  |  | 1 | 1 | 0 | 0 | 0 | 0 | 0 | 0 | 0 | 0 | 0 | 29 | 39 |  |  | PEAKS DB |
| R.DLTDYLMK.I | N | 43.13 | 997.4790 | 8 | 0.7 | 499.7471 | 2 | 51.80 | 6 | F6:36164 | 17012022\_RID\_3262\_9a.raw |  |  |  |  |  | 4.4466E5 |  |  |  |  | 1 | 0 | 0 | 0 | 0 | 0 | 1 | 0 | 0 | 0 | 0 | 184 | 191 |  |  | PEAKS DB |
| total 11 peptides |  |  |  |  |  |  |  |  |  |  |  |  |  |  |  |  |  |  |  |  |  |  |  |  |  |  |  |  |  |  |  |  |  |  |  |  |  |
| --- | --- | --- | --- | --- | --- | --- | --- | --- | --- | --- | --- | --- | --- | --- | --- | --- | --- | --- | --- | --- | --- | --- | --- | --- | --- | --- | --- | --- | --- | --- | --- | --- | --- | --- | --- | --- | --- |

XP\_007436312.1

back to list

  

| Protein Coverage
| Supporting Peptides
|

Protein Coverage:

Supporting Peptides:

| Peptide | Uniq | -10lgP | Mass | Length | ppm | m/z | z | RT | Fraction | Scan | Source File | Area F4 | Area F5 | Area F6 | Area F10 | Area F8 | Area F9 | Area F7 | Area F1 | Area F2 | Area F3 | #Feature | #Feature F4 | #Feature F5 | #Feature F6 | #Feature F10 | #Feature F8 | #Feature F9 | #Feature F7 | #Feature F1 | #Feature F2 | #Feature F3 | Start | End | PTM | AScore | Found By |
| --- | --- | --- | --- | --- | --- | --- | --- | --- | --- | --- | --- | --- | --- | --- | --- | --- | --- | --- | --- | --- | --- | --- | --- | --- | --- | --- | --- | --- | --- | --- | --- | --- | --- | --- | --- | --- | --- |
| K.SYELPDGQVITIGNER.F | N | 82.78 | 1789.8846 | 16 | 0.4 | 895.9500 | 2 | 49.87 | 8 | F8:28149 | 31122021\_RID\_3262\_1a.raw | 4.7745E5 |  |  |  |  | 6.0954E5 |  | 1.0043E6 | 3.4371E5 |  | 4 | 1 | 0 | 0 | 0 | 0 | 1 | 0 | 1 | 1 | 0 | 239 | 254 |  |  | PEAKS DB |
| K.LC(+57.02)YVALDFEQEMATAASSSSLEK.S | Y | 65.71 | 2549.1665 | 23 | -0.5 | 1275.5898 | 2 | 62.33 | 7 | F7:28143 | 21012022\_RID\_3262\_7a.raw |  |  |  |  |  |  | 1.0541E6 |  |  |  | 2 | 0 | 0 | 0 | 0 | 0 | 0 | 2 | 0 | 0 | 0 | 216 | 238 | Carbamidomethylation | C2:Carbamidomethylation:1000.00 | PEAKS DB |
| K.DSYVGDEAQSK.R | N | 65.50 | 1197.5149 | 11 | 1.5 | 599.7656 | 2 | 23.16 | 4 | F4:10278 | 17012022\_RID\_3262\_10a .raw | 2.7572E5 |  |  | 3.783E5 |  | 4.8292E5 |  | 7.9712E4 | 7.8692E4 |  | 5 | 1 | 0 | 0 | 1 | 0 | 1 | 0 | 1 | 1 | 0 | 51 | 61 |  |  | PEAKS DB |
| R.GYSFTTTAER.E | Y | 60.44 | 1131.5197 | 10 | -0.1 | 566.7670 | 2 | 28.78 | 8 | F8:11438 | 31122021\_RID\_3262\_1a.raw |  |  |  | 1.3304E6 |  |  | 2.0729E5 | 2.9994E5 | 2.4553E5 |  | 4 | 0 | 0 | 0 | 1 | 0 | 0 | 1 | 1 | 1 | 0 | 197 | 206 |  |  | PEAKS DB |
| K.LC(+57.02)YVALDFEQEM(+15.99)ATAASSSSLEK.S | Y | 54.72 | 2565.1614 | 23 | 0.4 | 856.0614 | 3 | 62.38 | 7 | F7:28152 | 21012022\_RID\_3262\_7a.raw |  |  |  |  |  |  | 1.0591E5 |  |  |  | 1 | 0 | 0 | 0 | 0 | 0 | 0 | 1 | 0 | 0 | 0 | 216 | 238 | Carbamidomethylation; Oxidation (M) | C2:Carbamidomethylation:1000.00;M12:Oxidation (M):1000.00 | PEAKS DB |
| K.AGFAGDDAPR.A | N | 52.95 | 975.4410 | 10 | 3.9 | 488.7296 | 2 | 22.10 | 9 | F9:7134 | 31122021\_RID\_3262\_2a.raw |  | 3.3275E5 |  | 1.2847E6 |  |  |  |  | 2.7076E6 |  | 4 | 0 | 1 | 0 | 1 | 0 | 0 | 0 | 0 | 2 | 0 | 19 | 28 |  |  | PEAKS DB |
| R.VAPEEHPVLLTEAPLNPK.A | N | 51.47 | 1953.0570 | 18 | 0.5 | 652.0266 | 3 | 40.45 | 1 | F1:25900 | 13012022\_RID\_3262\_4a.raw | 8.5304E5 |  |  | 5.0748E5 |  |  |  | 7.2111E5 |  |  | 3 | 1 | 0 | 0 | 1 | 0 | 0 | 0 | 1 | 0 | 0 | 96 | 113 |  |  | PEAKS DB |
| K.EITALAPSTM(+15.99)K.I | N | 49.12 | 1176.6060 | 11 | 1.7 | 589.3113 | 2 | 30.85 | 6 | F6:17553 | 17012022\_RID\_3262\_9a.raw |  |  |  |  |  | 2.4825E5 |  |  |  |  | 1 | 0 | 0 | 0 | 0 | 0 | 1 | 0 | 0 | 0 | 0 | 316 | 326 | Oxidation (M) | M10:Oxidation (M):1000.00 | PEAKS DB |
| G.YALPHAILR.L | Y | 48.16 | 1052.6130 | 9 | 0.0 | 527.3138 | 2 | 35.63 | 1 | F1:21569 | 13012022\_RID\_3262\_4a.raw | 1.4154E6 |  |  |  |  |  |  |  |  |  | 1 | 1 | 0 | 0 | 0 | 0 | 0 | 0 | 0 | 0 | 0 | 169 | 177 |  |  | PEAKS DB |
| R.AVFPSIVGRPR.H | N | 45.70 | 1197.6981 | 11 | 1.5 | 400.2406 | 3 | 35.84 | 6 | F6:21794 | 17012022\_RID\_3262\_9a.raw | 1.4816E6 |  |  |  |  | 0 |  |  |  |  | 1 | 1 | 0 | 0 | 0 | 0 | 0 | 0 | 0 | 0 | 0 | 29 | 39 |  |  | PEAKS DB |
| R.DLTDYLMK.I | N | 43.13 | 997.4790 | 8 | 0.7 | 499.7471 | 2 | 51.80 | 6 | F6:36164 | 17012022\_RID\_3262\_9a.raw |  |  |  |  |  | 4.4466E5 |  |  |  |  | 1 | 0 | 0 | 0 | 0 | 0 | 1 | 0 | 0 | 0 | 0 | 184 | 191 |  |  | PEAKS DB |
| total 11 peptides |  |  |  |  |  |  |  |  |  |  |  |  |  |  |  |  |  |  |  |  |  |  |  |  |  |  |  |  |  |  |  |  |  |  |  |  |  |
| --- | --- | --- | --- | --- | --- | --- | --- | --- | --- | --- | --- | --- | --- | --- | --- | --- | --- | --- | --- | --- | --- | --- | --- | --- | --- | --- | --- | --- | --- | --- | --- | --- | --- | --- | --- | --- | --- |

JAC96364.1

back to list

  

| Protein Coverage
| Supporting Peptides
|

Protein Coverage:

Supporting Peptides:

| Peptide | Uniq | -10lgP | Mass | Length | ppm | m/z | z | RT | Fraction | Scan | Source File | Area F4 | Area F5 | Area F6 | Area F10 | Area F8 | Area F9 | Area F7 | Area F1 | Area F2 | Area F3 | #Feature | #Feature F4 | #Feature F5 | #Feature F6 | #Feature F10 | #Feature F8 | #Feature F9 | #Feature F7 | #Feature F1 | #Feature F2 | #Feature F3 | Start | End | PTM | AScore | Found By |
| --- | --- | --- | --- | --- | --- | --- | --- | --- | --- | --- | --- | --- | --- | --- | --- | --- | --- | --- | --- | --- | --- | --- | --- | --- | --- | --- | --- | --- | --- | --- | --- | --- | --- | --- | --- | --- | --- |
| K.SYELPDGQVITIGNER.F | N | 82.78 | 1789.8846 | 16 | 0.4 | 895.9500 | 2 | 49.87 | 8 | F8:28149 | 31122021\_RID\_3262\_1a.raw | 4.7745E5 |  |  |  |  | 6.0954E5 |  | 1.0043E6 | 3.4371E5 |  | 4 | 1 | 0 | 0 | 0 | 0 | 1 | 0 | 1 | 1 | 0 | 239 | 254 |  |  | PEAKS DB |
| K.LC(+57.02)YVALDFEQEMATAASSSSLEK.S | Y | 65.71 | 2549.1665 | 23 | -0.5 | 1275.5898 | 2 | 62.33 | 7 | F7:28143 | 21012022\_RID\_3262\_7a.raw |  |  |  |  |  |  | 1.0541E6 |  |  |  | 2 | 0 | 0 | 0 | 0 | 0 | 0 | 2 | 0 | 0 | 0 | 216 | 238 | Carbamidomethylation | C2:Carbamidomethylation:1000.00 | PEAKS DB |
| K.DSYVGDEAQSK.R | N | 65.50 | 1197.5149 | 11 | 1.5 | 599.7656 | 2 | 23.16 | 4 | F4:10278 | 17012022\_RID\_3262\_10a .raw | 2.7572E5 |  |  | 3.783E5 |  | 4.8292E5 |  | 7.9712E4 | 7.8692E4 |  | 5 | 1 | 0 | 0 | 1 | 0 | 1 | 0 | 1 | 1 | 0 | 51 | 61 |  |  | PEAKS DB |
| R.GYSFTTTAER.E | Y | 60.44 | 1131.5197 | 10 | -0.1 | 566.7670 | 2 | 28.78 | 8 | F8:11438 | 31122021\_RID\_3262\_1a.raw |  |  |  | 1.3304E6 |  |  | 2.0729E5 | 2.9994E5 | 2.4553E5 |  | 4 | 0 | 0 | 0 | 1 | 0 | 0 | 1 | 1 | 1 | 0 | 197 | 206 |  |  | PEAKS DB |
| K.LC(+57.02)YVALDFEQEM(+15.99)ATAASSSSLEK.S | Y | 54.72 | 2565.1614 | 23 | 0.4 | 856.0614 | 3 | 62.38 | 7 | F7:28152 | 21012022\_RID\_3262\_7a.raw |  |  |  |  |  |  | 1.0591E5 |  |  |  | 1 | 0 | 0 | 0 | 0 | 0 | 0 | 1 | 0 | 0 | 0 | 216 | 238 | Carbamidomethylation; Oxidation (M) | C2:Carbamidomethylation:1000.00;M12:Oxidation (M):1000.00 | PEAKS DB |
| K.AGFAGDDAPR.A | N | 52.95 | 975.4410 | 10 | 3.9 | 488.7296 | 2 | 22.10 | 9 | F9:7134 | 31122021\_RID\_3262\_2a.raw |  | 3.3275E5 |  | 1.2847E6 |  |  |  |  | 2.7076E6 |  | 4 | 0 | 1 | 0 | 1 | 0 | 0 | 0 | 0 | 2 | 0 | 19 | 28 |  |  | PEAKS DB |
| R.VAPEEHPVLLTEAPLNPK.A | N | 51.47 | 1953.0570 | 18 | 0.5 | 652.0266 | 3 | 40.45 | 1 | F1:25900 | 13012022\_RID\_3262\_4a.raw | 8.5304E5 |  |  | 5.0748E5 |  |  |  | 7.2111E5 |  |  | 3 | 1 | 0 | 0 | 1 | 0 | 0 | 0 | 1 | 0 | 0 | 96 | 113 |  |  | PEAKS DB |
| K.EITALAPSTM(+15.99)K.I | N | 49.12 | 1176.6060 | 11 | 1.7 | 589.3113 | 2 | 30.85 | 6 | F6:17553 | 17012022\_RID\_3262\_9a.raw |  |  |  |  |  | 2.4825E5 |  |  |  |  | 1 | 0 | 0 | 0 | 0 | 0 | 1 | 0 | 0 | 0 | 0 | 316 | 326 | Oxidation (M) | M10:Oxidation (M):1000.00 | PEAKS DB |
| G.YALPHAILR.L | Y | 48.16 | 1052.6130 | 9 | 0.0 | 527.3138 | 2 | 35.63 | 1 | F1:21569 | 13012022\_RID\_3262\_4a.raw | 1.4154E6 |  |  |  |  |  |  |  |  |  | 1 | 1 | 0 | 0 | 0 | 0 | 0 | 0 | 0 | 0 | 0 | 169 | 177 |  |  | PEAKS DB |
| R.AVFPSIVGRPR.H | N | 45.70 | 1197.6981 | 11 | 1.5 | 400.2406 | 3 | 35.84 | 6 | F6:21794 | 17012022\_RID\_3262\_9a.raw | 1.4816E6 |  |  |  |  | 0 |  |  |  |  | 1 | 1 | 0 | 0 | 0 | 0 | 0 | 0 | 0 | 0 | 0 | 29 | 39 |  |  | PEAKS DB |
| R.DLTDYLMK.I | N | 43.13 | 997.4790 | 8 | 0.7 | 499.7471 | 2 | 51.80 | 6 | F6:36164 | 17012022\_RID\_3262\_9a.raw |  |  |  |  |  | 4.4466E5 |  |  |  |  | 1 | 0 | 0 | 0 | 0 | 0 | 1 | 0 | 0 | 0 | 0 | 184 | 191 |  |  | PEAKS DB |
| total 11 peptides |  |  |  |  |  |  |  |  |  |  |  |  |  |  |  |  |  |  |  |  |  |  |  |  |  |  |  |  |  |  |  |  |  |  |  |  |  |
| --- | --- | --- | --- | --- | --- | --- | --- | --- | --- | --- | --- | --- | --- | --- | --- | --- | --- | --- | --- | --- | --- | --- | --- | --- | --- | --- | --- | --- | --- | --- | --- | --- | --- | --- | --- | --- | --- |

AFJ49302.1

back to list

  

| Protein Coverage
| Supporting Peptides
|

Protein Coverage:

Supporting Peptides:

| Peptide | Uniq | -10lgP | Mass | Length | ppm | m/z | z | RT | Fraction | Scan | Source File | Area F4 | Area F5 | Area F6 | Area F10 | Area F8 | Area F9 | Area F7 | Area F1 | Area F2 | Area F3 | #Feature | #Feature F4 | #Feature F5 | #Feature F6 | #Feature F10 | #Feature F8 | #Feature F9 | #Feature F7 | #Feature F1 | #Feature F2 | #Feature F3 | Start | End | PTM | AScore | Found By |
| --- | --- | --- | --- | --- | --- | --- | --- | --- | --- | --- | --- | --- | --- | --- | --- | --- | --- | --- | --- | --- | --- | --- | --- | --- | --- | --- | --- | --- | --- | --- | --- | --- | --- | --- | --- | --- | --- |
| K.SYELPDGQVITIGNER.F | N | 82.78 | 1789.8846 | 16 | 0.4 | 895.9500 | 2 | 49.87 | 8 | F8:28149 | 31122021\_RID\_3262\_1a.raw | 4.7745E5 |  |  |  |  | 6.0954E5 |  | 1.0043E6 | 3.4371E5 |  | 4 | 1 | 0 | 0 | 0 | 0 | 1 | 0 | 1 | 1 | 0 | 239 | 254 |  |  | PEAKS DB |
| K.LC(+57.02)YVALDFEQEMATAASSSSLEK.S | Y | 65.71 | 2549.1665 | 23 | -0.5 | 1275.5898 | 2 | 62.33 | 7 | F7:28143 | 21012022\_RID\_3262\_7a.raw |  |  |  |  |  |  | 1.0541E6 |  |  |  | 2 | 0 | 0 | 0 | 0 | 0 | 0 | 2 | 0 | 0 | 0 | 216 | 238 | Carbamidomethylation | C2:Carbamidomethylation:1000.00 | PEAKS DB |
| K.DSYVGDEAQSK.R | N | 65.50 | 1197.5149 | 11 | 1.5 | 599.7656 | 2 | 23.16 | 4 | F4:10278 | 17012022\_RID\_3262\_10a .raw | 2.7572E5 |  |  | 3.783E5 |  | 4.8292E5 |  | 7.9712E4 | 7.8692E4 |  | 5 | 1 | 0 | 0 | 1 | 0 | 1 | 0 | 1 | 1 | 0 | 51 | 61 |  |  | PEAKS DB |
| R.GYSFTTTAER.E | Y | 60.44 | 1131.5197 | 10 | -0.1 | 566.7670 | 2 | 28.78 | 8 | F8:11438 | 31122021\_RID\_3262\_1a.raw |  |  |  | 1.3304E6 |  |  | 2.0729E5 | 2.9994E5 | 2.4553E5 |  | 4 | 0 | 0 | 0 | 1 | 0 | 0 | 1 | 1 | 1 | 0 | 197 | 206 |  |  | PEAKS DB |
| K.LC(+57.02)YVALDFEQEM(+15.99)ATAASSSSLEK.S | Y | 54.72 | 2565.1614 | 23 | 0.4 | 856.0614 | 3 | 62.38 | 7 | F7:28152 | 21012022\_RID\_3262\_7a.raw |  |  |  |  |  |  | 1.0591E5 |  |  |  | 1 | 0 | 0 | 0 | 0 | 0 | 0 | 1 | 0 | 0 | 0 | 216 | 238 | Carbamidomethylation; Oxidation (M) | C2:Carbamidomethylation:1000.00;M12:Oxidation (M):1000.00 | PEAKS DB |
| K.AGFAGDDAPR.A | N | 52.95 | 975.4410 | 10 | 3.9 | 488.7296 | 2 | 22.10 | 9 | F9:7134 | 31122021\_RID\_3262\_2a.raw |  | 3.3275E5 |  | 1.2847E6 |  |  |  |  | 2.7076E6 |  | 4 | 0 | 1 | 0 | 1 | 0 | 0 | 0 | 0 | 2 | 0 | 19 | 28 |  |  | PEAKS DB |
| R.VAPEEHPVLLTEAPLNPK.A | N | 51.47 | 1953.0570 | 18 | 0.5 | 652.0266 | 3 | 40.45 | 1 | F1:25900 | 13012022\_RID\_3262\_4a.raw | 8.5304E5 |  |  | 5.0748E5 |  |  |  | 7.2111E5 |  |  | 3 | 1 | 0 | 0 | 1 | 0 | 0 | 0 | 1 | 0 | 0 | 96 | 113 |  |  | PEAKS DB |
| K.EITALAPSTM(+15.99)K.I | N | 49.12 | 1176.6060 | 11 | 1.7 | 589.3113 | 2 | 30.85 | 6 | F6:17553 | 17012022\_RID\_3262\_9a.raw |  |  |  |  |  | 2.4825E5 |  |  |  |  | 1 | 0 | 0 | 0 | 0 | 0 | 1 | 0 | 0 | 0 | 0 | 316 | 326 | Oxidation (M) | M10:Oxidation (M):1000.00 | PEAKS DB |
| G.YALPHAILR.L | Y | 48.16 | 1052.6130 | 9 | 0.0 | 527.3138 | 2 | 35.63 | 1 | F1:21569 | 13012022\_RID\_3262\_4a.raw | 1.4154E6 |  |  |  |  |  |  |  |  |  | 1 | 1 | 0 | 0 | 0 | 0 | 0 | 0 | 0 | 0 | 0 | 169 | 177 |  |  | PEAKS DB |
| R.AVFPSIVGRPR.H | N | 45.70 | 1197.6981 | 11 | 1.5 | 400.2406 | 3 | 35.84 | 6 | F6:21794 | 17012022\_RID\_3262\_9a.raw | 1.4816E6 |  |  |  |  | 0 |  |  |  |  | 1 | 1 | 0 | 0 | 0 | 0 | 0 | 0 | 0 | 0 | 0 | 29 | 39 |  |  | PEAKS DB |
| R.DLTDYLMK.I | N | 43.13 | 997.4790 | 8 | 0.7 | 499.7471 | 2 | 51.80 | 6 | F6:36164 | 17012022\_RID\_3262\_9a.raw |  |  |  |  |  | 4.4466E5 |  |  |  |  | 1 | 0 | 0 | 0 | 0 | 0 | 1 | 0 | 0 | 0 | 0 | 184 | 191 |  |  | PEAKS DB |
| total 11 peptides |  |  |  |  |  |  |  |  |  |  |  |  |  |  |  |  |  |  |  |  |  |  |  |  |  |  |  |  |  |  |  |  |  |  |  |  |  |
| --- | --- | --- | --- | --- | --- | --- | --- | --- | --- | --- | --- | --- | --- | --- | --- | --- | --- | --- | --- | --- | --- | --- | --- | --- | --- | --- | --- | --- | --- | --- | --- | --- | --- | --- | --- | --- | --- |

BAQ22177.1

back to list

  

| Protein Coverage
| Supporting Peptides
|

Protein Coverage:

Supporting Peptides:

| Peptide | Uniq | -10lgP | Mass | Length | ppm | m/z | z | RT | Fraction | Scan | Source File | Area F4 | Area F5 | Area F6 | Area F10 | Area F8 | Area F9 | Area F7 | Area F1 | Area F2 | Area F3 | #Feature | #Feature F4 | #Feature F5 | #Feature F6 | #Feature F10 | #Feature F8 | #Feature F9 | #Feature F7 | #Feature F1 | #Feature F2 | #Feature F3 | Start | End | PTM | AScore | Found By |
| --- | --- | --- | --- | --- | --- | --- | --- | --- | --- | --- | --- | --- | --- | --- | --- | --- | --- | --- | --- | --- | --- | --- | --- | --- | --- | --- | --- | --- | --- | --- | --- | --- | --- | --- | --- | --- | --- |
| K.SYELPDGQVITIGNER.F | N | 82.78 | 1789.8846 | 16 | 0.4 | 895.9500 | 2 | 49.87 | 8 | F8:28149 | 31122021\_RID\_3262\_1a.raw | 4.7745E5 |  |  |  |  | 6.0954E5 |  | 1.0043E6 | 3.4371E5 |  | 4 | 1 | 0 | 0 | 0 | 0 | 1 | 0 | 1 | 1 | 0 | 239 | 254 |  |  | PEAKS DB |
| K.LC(+57.02)YVALDFEQEMATAASSSSLEK.S | Y | 65.71 | 2549.1665 | 23 | -0.5 | 1275.5898 | 2 | 62.33 | 7 | F7:28143 | 21012022\_RID\_3262\_7a.raw |  |  |  |  |  |  | 1.0541E6 |  |  |  | 2 | 0 | 0 | 0 | 0 | 0 | 0 | 2 | 0 | 0 | 0 | 216 | 238 | Carbamidomethylation | C2:Carbamidomethylation:1000.00 | PEAKS DB |
| K.DSYVGDEAQSK.R | N | 65.50 | 1197.5149 | 11 | 1.5 | 599.7656 | 2 | 23.16 | 4 | F4:10278 | 17012022\_RID\_3262\_10a .raw | 2.7572E5 |  |  | 3.783E5 |  | 4.8292E5 |  | 7.9712E4 | 7.8692E4 |  | 5 | 1 | 0 | 0 | 1 | 0 | 1 | 0 | 1 | 1 | 0 | 51 | 61 |  |  | PEAKS DB |
| R.GYSFTTTAER.E | Y | 60.44 | 1131.5197 | 10 | -0.1 | 566.7670 | 2 | 28.78 | 8 | F8:11438 | 31122021\_RID\_3262\_1a.raw |  |  |  | 1.3304E6 |  |  | 2.0729E5 | 2.9994E5 | 2.4553E5 |  | 4 | 0 | 0 | 0 | 1 | 0 | 0 | 1 | 1 | 1 | 0 | 197 | 206 |  |  | PEAKS DB |
| K.LC(+57.02)YVALDFEQEM(+15.99)ATAASSSSLEK.S | Y | 54.72 | 2565.1614 | 23 | 0.4 | 856.0614 | 3 | 62.38 | 7 | F7:28152 | 21012022\_RID\_3262\_7a.raw |  |  |  |  |  |  | 1.0591E5 |  |  |  | 1 | 0 | 0 | 0 | 0 | 0 | 0 | 1 | 0 | 0 | 0 | 216 | 238 | Carbamidomethylation; Oxidation (M) | C2:Carbamidomethylation:1000.00;M12:Oxidation (M):1000.00 | PEAKS DB |
| K.AGFAGDDAPR.A | N | 52.95 | 975.4410 | 10 | 3.9 | 488.7296 | 2 | 22.10 | 9 | F9:7134 | 31122021\_RID\_3262\_2a.raw |  | 3.3275E5 |  | 1.2847E6 |  |  |  |  | 2.7076E6 |  | 4 | 0 | 1 | 0 | 1 | 0 | 0 | 0 | 0 | 2 | 0 | 19 | 28 |  |  | PEAKS DB |
| R.VAPEEHPVLLTEAPLNPK.A | N | 51.47 | 1953.0570 | 18 | 0.5 | 652.0266 | 3 | 40.45 | 1 | F1:25900 | 13012022\_RID\_3262\_4a.raw | 8.5304E5 |  |  | 5.0748E5 |  |  |  | 7.2111E5 |  |  | 3 | 1 | 0 | 0 | 1 | 0 | 0 | 0 | 1 | 0 | 0 | 96 | 113 |  |  | PEAKS DB |
| K.EITALAPSTM(+15.99)K.I | N | 49.12 | 1176.6060 | 11 | 1.7 | 589.3113 | 2 | 30.85 | 6 | F6:17553 | 17012022\_RID\_3262\_9a.raw |  |  |  |  |  | 2.4825E5 |  |  |  |  | 1 | 0 | 0 | 0 | 0 | 0 | 1 | 0 | 0 | 0 | 0 | 316 | 326 | Oxidation (M) | M10:Oxidation (M):1000.00 | PEAKS DB |
| G.YALPHAILR.L | Y | 48.16 | 1052.6130 | 9 | 0.0 | 527.3138 | 2 | 35.63 | 1 | F1:21569 | 13012022\_RID\_3262\_4a.raw | 1.4154E6 |  |  |  |  |  |  |  |  |  | 1 | 1 | 0 | 0 | 0 | 0 | 0 | 0 | 0 | 0 | 0 | 169 | 177 |  |  | PEAKS DB |
| R.AVFPSIVGRPR.H | N | 45.70 | 1197.6981 | 11 | 1.5 | 400.2406 | 3 | 35.84 | 6 | F6:21794 | 17012022\_RID\_3262\_9a.raw | 1.4816E6 |  |  |  |  | 0 |  |  |  |  | 1 | 1 | 0 | 0 | 0 | 0 | 0 | 0 | 0 | 0 | 0 | 29 | 39 |  |  | PEAKS DB |
| R.DLTDYLMK.I | N | 43.13 | 997.4790 | 8 | 0.7 | 499.7471 | 2 | 51.80 | 6 | F6:36164 | 17012022\_RID\_3262\_9a.raw |  |  |  |  |  | 4.4466E5 |  |  |  |  | 1 | 0 | 0 | 0 | 0 | 0 | 1 | 0 | 0 | 0 | 0 | 184 | 191 |  |  | PEAKS DB |
| total 11 peptides |  |  |  |  |  |  |  |  |  |  |  |  |  |  |  |  |  |  |  |  |  |  |  |  |  |  |  |  |  |  |  |  |  |  |  |  |  |
| --- | --- | --- | --- | --- | --- | --- | --- | --- | --- | --- | --- | --- | --- | --- | --- | --- | --- | --- | --- | --- | --- | --- | --- | --- | --- | --- | --- | --- | --- | --- | --- | --- | --- | --- | --- | --- | --- |

JAG45999.1

back to list

  

| Protein Coverage
| Supporting Peptides
|

Protein Coverage:

Supporting Peptides:

| Peptide | Uniq | -10lgP | Mass | Length | ppm | m/z | z | RT | Fraction | Scan | Source File | Area F4 | Area F5 | Area F6 | Area F10 | Area F8 | Area F9 | Area F7 | Area F1 | Area F2 | Area F3 | #Feature | #Feature F4 | #Feature F5 | #Feature F6 | #Feature F10 | #Feature F8 | #Feature F9 | #Feature F7 | #Feature F1 | #Feature F2 | #Feature F3 | Start | End | PTM | AScore | Found By |
| --- | --- | --- | --- | --- | --- | --- | --- | --- | --- | --- | --- | --- | --- | --- | --- | --- | --- | --- | --- | --- | --- | --- | --- | --- | --- | --- | --- | --- | --- | --- | --- | --- | --- | --- | --- | --- | --- |
| K.SYELPDGQVITIGNER.F | N | 82.78 | 1789.8846 | 16 | 0.4 | 895.9500 | 2 | 49.87 | 8 | F8:28149 | 31122021\_RID\_3262\_1a.raw | 4.7745E5 |  |  |  |  | 6.0954E5 |  | 1.0043E6 | 3.4371E5 |  | 4 | 1 | 0 | 0 | 0 | 0 | 1 | 0 | 1 | 1 | 0 | 239 | 254 |  |  | PEAKS DB |
| K.LC(+57.02)YVALDFEQEMATAASSSSLEK.S | Y | 65.71 | 2549.1665 | 23 | -0.5 | 1275.5898 | 2 | 62.33 | 7 | F7:28143 | 21012022\_RID\_3262\_7a.raw |  |  |  |  |  |  | 1.0541E6 |  |  |  | 2 | 0 | 0 | 0 | 0 | 0 | 0 | 2 | 0 | 0 | 0 | 216 | 238 | Carbamidomethylation | C2:Carbamidomethylation:1000.00 | PEAKS DB |
| K.DSYVGDEAQSK.R | N | 65.50 | 1197.5149 | 11 | 1.5 | 599.7656 | 2 | 23.16 | 4 | F4:10278 | 17012022\_RID\_3262\_10a .raw | 2.7572E5 |  |  | 3.783E5 |  | 4.8292E5 |  | 7.9712E4 | 7.8692E4 |  | 5 | 1 | 0 | 0 | 1 | 0 | 1 | 0 | 1 | 1 | 0 | 51 | 61 |  |  | PEAKS DB |
| R.GYSFTTTAER.E | Y | 60.44 | 1131.5197 | 10 | -0.1 | 566.7670 | 2 | 28.78 | 8 | F8:11438 | 31122021\_RID\_3262\_1a.raw |  |  |  | 1.3304E6 |  |  | 2.0729E5 | 2.9994E5 | 2.4553E5 |  | 4 | 0 | 0 | 0 | 1 | 0 | 0 | 1 | 1 | 1 | 0 | 197 | 206 |  |  | PEAKS DB |
| K.LC(+57.02)YVALDFEQEM(+15.99)ATAASSSSLEK.S | Y | 54.72 | 2565.1614 | 23 | 0.4 | 856.0614 | 3 | 62.38 | 7 | F7:28152 | 21012022\_RID\_3262\_7a.raw |  |  |  |  |  |  | 1.0591E5 |  |  |  | 1 | 0 | 0 | 0 | 0 | 0 | 0 | 1 | 0 | 0 | 0 | 216 | 238 | Carbamidomethylation; Oxidation (M) | C2:Carbamidomethylation:1000.00;M12:Oxidation (M):1000.00 | PEAKS DB |
| K.AGFAGDDAPR.A | N | 52.95 | 975.4410 | 10 | 3.9 | 488.7296 | 2 | 22.10 | 9 | F9:7134 | 31122021\_RID\_3262\_2a.raw |  | 3.3275E5 |  | 1.2847E6 |  |  |  |  | 2.7076E6 |  | 4 | 0 | 1 | 0 | 1 | 0 | 0 | 0 | 0 | 2 | 0 | 19 | 28 |  |  | PEAKS DB |
| R.VAPEEHPVLLTEAPLNPK.A | N | 51.47 | 1953.0570 | 18 | 0.5 | 652.0266 | 3 | 40.45 | 1 | F1:25900 | 13012022\_RID\_3262\_4a.raw | 8.5304E5 |  |  | 5.0748E5 |  |  |  | 7.2111E5 |  |  | 3 | 1 | 0 | 0 | 1 | 0 | 0 | 0 | 1 | 0 | 0 | 96 | 113 |  |  | PEAKS DB |
| K.EITALAPSTM(+15.99)K.I | N | 49.12 | 1176.6060 | 11 | 1.7 | 589.3113 | 2 | 30.85 | 6 | F6:17553 | 17012022\_RID\_3262\_9a.raw |  |  |  |  |  | 2.4825E5 |  |  |  |  | 1 | 0 | 0 | 0 | 0 | 0 | 1 | 0 | 0 | 0 | 0 | 316 | 326 | Oxidation (M) | M10:Oxidation (M):1000.00 | PEAKS DB |
| G.YALPHAILR.L | Y | 48.16 | 1052.6130 | 9 | 0.0 | 527.3138 | 2 | 35.63 | 1 | F1:21569 | 13012022\_RID\_3262\_4a.raw | 1.4154E6 |  |  |  |  |  |  |  |  |  | 1 | 1 | 0 | 0 | 0 | 0 | 0 | 0 | 0 | 0 | 0 | 169 | 177 |  |  | PEAKS DB |
| R.AVFPSIVGRPR.H | N | 45.70 | 1197.6981 | 11 | 1.5 | 400.2406 | 3 | 35.84 | 6 | F6:21794 | 17012022\_RID\_3262\_9a.raw | 1.4816E6 |  |  |  |  | 0 |  |  |  |  | 1 | 1 | 0 | 0 | 0 | 0 | 0 | 0 | 0 | 0 | 0 | 29 | 39 |  |  | PEAKS DB |
| R.DLTDYLMK.I | N | 43.13 | 997.4790 | 8 | 0.7 | 499.7471 | 2 | 51.80 | 6 | F6:36164 | 17012022\_RID\_3262\_9a.raw |  |  |  |  |  | 4.4466E5 |  |  |  |  | 1 | 0 | 0 | 0 | 0 | 0 | 1 | 0 | 0 | 0 | 0 | 184 | 191 |  |  | PEAKS DB |
| total 11 peptides |  |  |  |  |  |  |  |  |  |  |  |  |  |  |  |  |  |  |  |  |  |  |  |  |  |  |  |  |  |  |  |  |  |  |  |  |  |
| --- | --- | --- | --- | --- | --- | --- | --- | --- | --- | --- | --- | --- | --- | --- | --- | --- | --- | --- | --- | --- | --- | --- | --- | --- | --- | --- | --- | --- | --- | --- | --- | --- | --- | --- | --- | --- | --- |

JAC96363.1

back to list

  

| Protein Coverage
| Supporting Peptides
|

Protein Coverage:

Supporting Peptides:

| Peptide | Uniq | -10lgP | Mass | Length | ppm | m/z | z | RT | Fraction | Scan | Source File | Area F4 | Area F5 | Area F6 | Area F10 | Area F8 | Area F9 | Area F7 | Area F1 | Area F2 | Area F3 | #Feature | #Feature F4 | #Feature F5 | #Feature F6 | #Feature F10 | #Feature F8 | #Feature F9 | #Feature F7 | #Feature F1 | #Feature F2 | #Feature F3 | Start | End | PTM | AScore | Found By |
| --- | --- | --- | --- | --- | --- | --- | --- | --- | --- | --- | --- | --- | --- | --- | --- | --- | --- | --- | --- | --- | --- | --- | --- | --- | --- | --- | --- | --- | --- | --- | --- | --- | --- | --- | --- | --- | --- |
| K.SYELPDGQVITIGNER.F | N | 82.78 | 1789.8846 | 16 | 0.4 | 895.9500 | 2 | 49.87 | 8 | F8:28149 | 31122021\_RID\_3262\_1a.raw | 4.7745E5 |  |  |  |  | 6.0954E5 |  | 1.0043E6 | 3.4371E5 |  | 4 | 1 | 0 | 0 | 0 | 0 | 1 | 0 | 1 | 1 | 0 | 239 | 254 |  |  | PEAKS DB |
| K.LC(+57.02)YVALDFEQEMATAASSSSLEK.S | Y | 65.71 | 2549.1665 | 23 | -0.5 | 1275.5898 | 2 | 62.33 | 7 | F7:28143 | 21012022\_RID\_3262\_7a.raw |  |  |  |  |  |  | 1.0541E6 |  |  |  | 2 | 0 | 0 | 0 | 0 | 0 | 0 | 2 | 0 | 0 | 0 | 216 | 238 | Carbamidomethylation | C2:Carbamidomethylation:1000.00 | PEAKS DB |
| K.DSYVGDEAQSK.R | N | 65.50 | 1197.5149 | 11 | 1.5 | 599.7656 | 2 | 23.16 | 4 | F4:10278 | 17012022\_RID\_3262\_10a .raw | 2.7572E5 |  |  | 3.783E5 |  | 4.8292E5 |  | 7.9712E4 | 7.8692E4 |  | 5 | 1 | 0 | 0 | 1 | 0 | 1 | 0 | 1 | 1 | 0 | 51 | 61 |  |  | PEAKS DB |
| R.GYSFTTTAER.E | Y | 60.44 | 1131.5197 | 10 | -0.1 | 566.7670 | 2 | 28.78 | 8 | F8:11438 | 31122021\_RID\_3262\_1a.raw |  |  |  | 1.3304E6 |  |  | 2.0729E5 | 2.9994E5 | 2.4553E5 |  | 4 | 0 | 0 | 0 | 1 | 0 | 0 | 1 | 1 | 1 | 0 | 197 | 206 |  |  | PEAKS DB |
| K.LC(+57.02)YVALDFEQEM(+15.99)ATAASSSSLEK.S | Y | 54.72 | 2565.1614 | 23 | 0.4 | 856.0614 | 3 | 62.38 | 7 | F7:28152 | 21012022\_RID\_3262\_7a.raw |  |  |  |  |  |  | 1.0591E5 |  |  |  | 1 | 0 | 0 | 0 | 0 | 0 | 0 | 1 | 0 | 0 | 0 | 216 | 238 | Carbamidomethylation; Oxidation (M) | C2:Carbamidomethylation:1000.00;M12:Oxidation (M):1000.00 | PEAKS DB |
| K.AGFAGDDAPR.A | N | 52.95 | 975.4410 | 10 | 3.9 | 488.7296 | 2 | 22.10 | 9 | F9:7134 | 31122021\_RID\_3262\_2a.raw |  | 3.3275E5 |  | 1.2847E6 |  |  |  |  | 2.7076E6 |  | 4 | 0 | 1 | 0 | 1 | 0 | 0 | 0 | 0 | 2 | 0 | 19 | 28 |  |  | PEAKS DB |
| R.VAPEEHPVLLTEAPLNPK.A | N | 51.47 | 1953.0570 | 18 | 0.5 | 652.0266 | 3 | 40.45 | 1 | F1:25900 | 13012022\_RID\_3262\_4a.raw | 8.5304E5 |  |  | 5.0748E5 |  |  |  | 7.2111E5 |  |  | 3 | 1 | 0 | 0 | 1 | 0 | 0 | 0 | 1 | 0 | 0 | 96 | 113 |  |  | PEAKS DB |
| K.EITALAPSTM(+15.99)K.I | N | 49.12 | 1176.6060 | 11 | 1.7 | 589.3113 | 2 | 30.85 | 6 | F6:17553 | 17012022\_RID\_3262\_9a.raw |  |  |  |  |  | 2.4825E5 |  |  |  |  | 1 | 0 | 0 | 0 | 0 | 0 | 1 | 0 | 0 | 0 | 0 | 316 | 326 | Oxidation (M) | M10:Oxidation (M):1000.00 | PEAKS DB |
| G.YALPHAILR.L | Y | 48.16 | 1052.6130 | 9 | 0.0 | 527.3138 | 2 | 35.63 | 1 | F1:21569 | 13012022\_RID\_3262\_4a.raw | 1.4154E6 |  |  |  |  |  |  |  |  |  | 1 | 1 | 0 | 0 | 0 | 0 | 0 | 0 | 0 | 0 | 0 | 169 | 177 |  |  | PEAKS DB |
| R.AVFPSIVGRPR.H | N | 45.70 | 1197.6981 | 11 | 1.5 | 400.2406 | 3 | 35.84 | 6 | F6:21794 | 17012022\_RID\_3262\_9a.raw | 1.4816E6 |  |  |  |  | 0 |  |  |  |  | 1 | 1 | 0 | 0 | 0 | 0 | 0 | 0 | 0 | 0 | 0 | 29 | 39 |  |  | PEAKS DB |
| R.DLTDYLMK.I | N | 43.13 | 997.4790 | 8 | 0.7 | 499.7471 | 2 | 51.80 | 6 | F6:36164 | 17012022\_RID\_3262\_9a.raw |  |  |  |  |  | 4.4466E5 |  |  |  |  | 1 | 0 | 0 | 0 | 0 | 0 | 1 | 0 | 0 | 0 | 0 | 184 | 191 |  |  | PEAKS DB |
| total 11 peptides |  |  |  |  |  |  |  |  |  |  |  |  |  |  |  |  |  |  |  |  |  |  |  |  |  |  |  |  |  |  |  |  |  |  |  |  |  |
| --- | --- | --- | --- | --- | --- | --- | --- | --- | --- | --- | --- | --- | --- | --- | --- | --- | --- | --- | --- | --- | --- | --- | --- | --- | --- | --- | --- | --- | --- | --- | --- | --- | --- | --- | --- | --- | --- |

JAA97900.1

back to list

  

| Protein Coverage
| Supporting Peptides
|

Protein Coverage:

Supporting Peptides:

| Peptide | Uniq | -10lgP | Mass | Length | ppm | m/z | z | RT | Fraction | Scan | Source File | Area F4 | Area F5 | Area F6 | Area F10 | Area F8 | Area F9 | Area F7 | Area F1 | Area F2 | Area F3 | #Feature | #Feature F4 | #Feature F5 | #Feature F6 | #Feature F10 | #Feature F8 | #Feature F9 | #Feature F7 | #Feature F1 | #Feature F2 | #Feature F3 | Start | End | PTM | AScore | Found By |
| --- | --- | --- | --- | --- | --- | --- | --- | --- | --- | --- | --- | --- | --- | --- | --- | --- | --- | --- | --- | --- | --- | --- | --- | --- | --- | --- | --- | --- | --- | --- | --- | --- | --- | --- | --- | --- | --- |
| K.SYELPDGQVITIGNER.F | N | 82.78 | 1789.8846 | 16 | 0.4 | 895.9500 | 2 | 49.87 | 8 | F8:28149 | 31122021\_RID\_3262\_1a.raw | 4.7745E5 |  |  |  |  | 6.0954E5 |  | 1.0043E6 | 3.4371E5 |  | 4 | 1 | 0 | 0 | 0 | 0 | 1 | 0 | 1 | 1 | 0 | 239 | 254 |  |  | PEAKS DB |
| K.LC(+57.02)YVALDFEQEMATAASSSSLEK.S | Y | 65.71 | 2549.1665 | 23 | -0.5 | 1275.5898 | 2 | 62.33 | 7 | F7:28143 | 21012022\_RID\_3262\_7a.raw |  |  |  |  |  |  | 1.0541E6 |  |  |  | 2 | 0 | 0 | 0 | 0 | 0 | 0 | 2 | 0 | 0 | 0 | 216 | 238 | Carbamidomethylation | C2:Carbamidomethylation:1000.00 | PEAKS DB |
| K.DSYVGDEAQSK.R | N | 65.50 | 1197.5149 | 11 | 1.5 | 599.7656 | 2 | 23.16 | 4 | F4:10278 | 17012022\_RID\_3262\_10a .raw | 2.7572E5 |  |  | 3.783E5 |  | 4.8292E5 |  | 7.9712E4 | 7.8692E4 |  | 5 | 1 | 0 | 0 | 1 | 0 | 1 | 0 | 1 | 1 | 0 | 51 | 61 |  |  | PEAKS DB |
| R.GYSFTTTAER.E | Y | 60.44 | 1131.5197 | 10 | -0.1 | 566.7670 | 2 | 28.78 | 8 | F8:11438 | 31122021\_RID\_3262\_1a.raw |  |  |  | 1.3304E6 |  |  | 2.0729E5 | 2.9994E5 | 2.4553E5 |  | 4 | 0 | 0 | 0 | 1 | 0 | 0 | 1 | 1 | 1 | 0 | 197 | 206 |  |  | PEAKS DB |
| K.LC(+57.02)YVALDFEQEM(+15.99)ATAASSSSLEK.S | Y | 54.72 | 2565.1614 | 23 | 0.4 | 856.0614 | 3 | 62.38 | 7 | F7:28152 | 21012022\_RID\_3262\_7a.raw |  |  |  |  |  |  | 1.0591E5 |  |  |  | 1 | 0 | 0 | 0 | 0 | 0 | 0 | 1 | 0 | 0 | 0 | 216 | 238 | Carbamidomethylation; Oxidation (M) | C2:Carbamidomethylation:1000.00;M12:Oxidation (M):1000.00 | PEAKS DB |
| K.AGFAGDDAPR.A | N | 52.95 | 975.4410 | 10 | 3.9 | 488.7296 | 2 | 22.10 | 9 | F9:7134 | 31122021\_RID\_3262\_2a.raw |  | 3.3275E5 |  | 1.2847E6 |  |  |  |  | 2.7076E6 |  | 4 | 0 | 1 | 0 | 1 | 0 | 0 | 0 | 0 | 2 | 0 | 19 | 28 |  |  | PEAKS DB |
| R.VAPEEHPVLLTEAPLNPK.A | N | 51.47 | 1953.0570 | 18 | 0.5 | 652.0266 | 3 | 40.45 | 1 | F1:25900 | 13012022\_RID\_3262\_4a.raw | 8.5304E5 |  |  | 5.0748E5 |  |  |  | 7.2111E5 |  |  | 3 | 1 | 0 | 0 | 1 | 0 | 0 | 0 | 1 | 0 | 0 | 96 | 113 |  |  | PEAKS DB |
| K.EITALAPSTM(+15.99)K.I | N | 49.12 | 1176.6060 | 11 | 1.7 | 589.3113 | 2 | 30.85 | 6 | F6:17553 | 17012022\_RID\_3262\_9a.raw |  |  |  |  |  | 2.4825E5 |  |  |  |  | 1 | 0 | 0 | 0 | 0 | 0 | 1 | 0 | 0 | 0 | 0 | 316 | 326 | Oxidation (M) | M10:Oxidation (M):1000.00 | PEAKS DB |
| G.YALPHAILR.L | Y | 48.16 | 1052.6130 | 9 | 0.0 | 527.3138 | 2 | 35.63 | 1 | F1:21569 | 13012022\_RID\_3262\_4a.raw | 1.4154E6 |  |  |  |  |  |  |  |  |  | 1 | 1 | 0 | 0 | 0 | 0 | 0 | 0 | 0 | 0 | 0 | 169 | 177 |  |  | PEAKS DB |
| R.AVFPSIVGRPR.H | N | 45.70 | 1197.6981 | 11 | 1.5 | 400.2406 | 3 | 35.84 | 6 | F6:21794 | 17012022\_RID\_3262\_9a.raw | 1.4816E6 |  |  |  |  | 0 |  |  |  |  | 1 | 1 | 0 | 0 | 0 | 0 | 0 | 0 | 0 | 0 | 0 | 29 | 39 |  |  | PEAKS DB |
| R.DLTDYLMK.I | N | 43.13 | 997.4790 | 8 | 0.7 | 499.7471 | 2 | 51.80 | 6 | F6:36164 | 17012022\_RID\_3262\_9a.raw |  |  |  |  |  | 4.4466E5 |  |  |  |  | 1 | 0 | 0 | 0 | 0 | 0 | 1 | 0 | 0 | 0 | 0 | 184 | 191 |  |  | PEAKS DB |
| total 11 peptides |  |  |  |  |  |  |  |  |  |  |  |  |  |  |  |  |  |  |  |  |  |  |  |  |  |  |  |  |  |  |  |  |  |  |  |  |  |
| --- | --- | --- | --- | --- | --- | --- | --- | --- | --- | --- | --- | --- | --- | --- | --- | --- | --- | --- | --- | --- | --- | --- | --- | --- | --- | --- | --- | --- | --- | --- | --- | --- | --- | --- | --- | --- | --- |

LAA21099.1

back to list

  

| Protein Coverage
| Supporting Peptides
|

Protein Coverage:

Supporting Peptides:

| Peptide | Uniq | -10lgP | Mass | Length | ppm | m/z | z | RT | Fraction | Scan | Source File | Area F4 | Area F5 | Area F6 | Area F10 | Area F8 | Area F9 | Area F7 | Area F1 | Area F2 | Area F3 | #Feature | #Feature F4 | #Feature F5 | #Feature F6 | #Feature F10 | #Feature F8 | #Feature F9 | #Feature F7 | #Feature F1 | #Feature F2 | #Feature F3 | Start | End | PTM | AScore | Found By |
| --- | --- | --- | --- | --- | --- | --- | --- | --- | --- | --- | --- | --- | --- | --- | --- | --- | --- | --- | --- | --- | --- | --- | --- | --- | --- | --- | --- | --- | --- | --- | --- | --- | --- | --- | --- | --- | --- |
| K.SYELPDGQVITIGNER.F | N | 82.78 | 1789.8846 | 16 | 0.4 | 895.9500 | 2 | 49.87 | 8 | F8:28149 | 31122021\_RID\_3262\_1a.raw | 4.7745E5 |  |  |  |  | 6.0954E5 |  | 1.0043E6 | 3.4371E5 |  | 4 | 1 | 0 | 0 | 0 | 0 | 1 | 0 | 1 | 1 | 0 | 239 | 254 |  |  | PEAKS DB |
| K.LC(+57.02)YVALDFEQEMATAASSSSLEK.S | Y | 65.71 | 2549.1665 | 23 | -0.5 | 1275.5898 | 2 | 62.33 | 7 | F7:28143 | 21012022\_RID\_3262\_7a.raw |  |  |  |  |  |  | 1.0541E6 |  |  |  | 2 | 0 | 0 | 0 | 0 | 0 | 0 | 2 | 0 | 0 | 0 | 216 | 238 | Carbamidomethylation | C2:Carbamidomethylation:1000.00 | PEAKS DB |
| K.DSYVGDEAQSK.R | N | 65.50 | 1197.5149 | 11 | 1.5 | 599.7656 | 2 | 23.16 | 4 | F4:10278 | 17012022\_RID\_3262\_10a .raw | 2.7572E5 |  |  | 3.783E5 |  | 4.8292E5 |  | 7.9712E4 | 7.8692E4 |  | 5 | 1 | 0 | 0 | 1 | 0 | 1 | 0 | 1 | 1 | 0 | 51 | 61 |  |  | PEAKS DB |
| R.GYSFTTTAER.E | Y | 60.44 | 1131.5197 | 10 | -0.1 | 566.7670 | 2 | 28.78 | 8 | F8:11438 | 31122021\_RID\_3262\_1a.raw |  |  |  | 1.3304E6 |  |  | 2.0729E5 | 2.9994E5 | 2.4553E5 |  | 4 | 0 | 0 | 0 | 1 | 0 | 0 | 1 | 1 | 1 | 0 | 197 | 206 |  |  | PEAKS DB |
| K.LC(+57.02)YVALDFEQEM(+15.99)ATAASSSSLEK.S | Y | 54.72 | 2565.1614 | 23 | 0.4 | 856.0614 | 3 | 62.38 | 7 | F7:28152 | 21012022\_RID\_3262\_7a.raw |  |  |  |  |  |  | 1.0591E5 |  |  |  | 1 | 0 | 0 | 0 | 0 | 0 | 0 | 1 | 0 | 0 | 0 | 216 | 238 | Carbamidomethylation; Oxidation (M) | C2:Carbamidomethylation:1000.00;M12:Oxidation (M):1000.00 | PEAKS DB |
| K.AGFAGDDAPR.A | N | 52.95 | 975.4410 | 10 | 3.9 | 488.7296 | 2 | 22.10 | 9 | F9:7134 | 31122021\_RID\_3262\_2a.raw |  | 3.3275E5 |  | 1.2847E6 |  |  |  |  | 2.7076E6 |  | 4 | 0 | 1 | 0 | 1 | 0 | 0 | 0 | 0 | 2 | 0 | 19 | 28 |  |  | PEAKS DB |
| R.VAPEEHPVLLTEAPLNPK.A | N | 51.47 | 1953.0570 | 18 | 0.5 | 652.0266 | 3 | 40.45 | 1 | F1:25900 | 13012022\_RID\_3262\_4a.raw | 8.5304E5 |  |  | 5.0748E5 |  |  |  | 7.2111E5 |  |  | 3 | 1 | 0 | 0 | 1 | 0 | 0 | 0 | 1 | 0 | 0 | 96 | 113 |  |  | PEAKS DB |
| K.EITALAPSTM(+15.99)K.I | N | 49.12 | 1176.6060 | 11 | 1.7 | 589.3113 | 2 | 30.85 | 6 | F6:17553 | 17012022\_RID\_3262\_9a.raw |  |  |  |  |  | 2.4825E5 |  |  |  |  | 1 | 0 | 0 | 0 | 0 | 0 | 1 | 0 | 0 | 0 | 0 | 316 | 326 | Oxidation (M) | M10:Oxidation (M):1000.00 | PEAKS DB |
| G.YALPHAILR.L | Y | 48.16 | 1052.6130 | 9 | 0.0 | 527.3138 | 2 | 35.63 | 1 | F1:21569 | 13012022\_RID\_3262\_4a.raw | 1.4154E6 |  |  |  |  |  |  |  |  |  | 1 | 1 | 0 | 0 | 0 | 0 | 0 | 0 | 0 | 0 | 0 | 169 | 177 |  |  | PEAKS DB |
| R.AVFPSIVGRPR.H | N | 45.70 | 1197.6981 | 11 | 1.5 | 400.2406 | 3 | 35.84 | 6 | F6:21794 | 17012022\_RID\_3262\_9a.raw | 1.4816E6 |  |  |  |  | 0 |  |  |  |  | 1 | 1 | 0 | 0 | 0 | 0 | 0 | 0 | 0 | 0 | 0 | 29 | 39 |  |  | PEAKS DB |
| R.DLTDYLMK.I | N | 43.13 | 997.4790 | 8 | 0.7 | 499.7471 | 2 | 51.80 | 6 | F6:36164 | 17012022\_RID\_3262\_9a.raw |  |  |  |  |  | 4.4466E5 |  |  |  |  | 1 | 0 | 0 | 0 | 0 | 0 | 1 | 0 | 0 | 0 | 0 | 184 | 191 |  |  | PEAKS DB |
| total 11 peptides |  |  |  |  |  |  |  |  |  |  |  |  |  |  |  |  |  |  |  |  |  |  |  |  |  |  |  |  |  |  |  |  |  |  |  |  |  |
| --- | --- | --- | --- | --- | --- | --- | --- | --- | --- | --- | --- | --- | --- | --- | --- | --- | --- | --- | --- | --- | --- | --- | --- | --- | --- | --- | --- | --- | --- | --- | --- | --- | --- | --- | --- | --- | --- |

JAG68843.1

back to list

  

| Protein Coverage
| Supporting Peptides
|

Protein Coverage:

Supporting Peptides:

| Peptide | Uniq | -10lgP | Mass | Length | ppm | m/z | z | RT | Fraction | Scan | Source File | Area F4 | Area F5 | Area F6 | Area F10 | Area F8 | Area F9 | Area F7 | Area F1 | Area F2 | Area F3 | #Feature | #Feature F4 | #Feature F5 | #Feature F6 | #Feature F10 | #Feature F8 | #Feature F9 | #Feature F7 | #Feature F1 | #Feature F2 | #Feature F3 | Start | End | PTM | AScore | Found By |
| --- | --- | --- | --- | --- | --- | --- | --- | --- | --- | --- | --- | --- | --- | --- | --- | --- | --- | --- | --- | --- | --- | --- | --- | --- | --- | --- | --- | --- | --- | --- | --- | --- | --- | --- | --- | --- | --- |
| K.SYELPDGQVITIGNER.F | N | 82.78 | 1789.8846 | 16 | 0.4 | 895.9500 | 2 | 49.87 | 8 | F8:28149 | 31122021\_RID\_3262\_1a.raw | 4.7745E5 |  |  |  |  | 6.0954E5 |  | 1.0043E6 | 3.4371E5 |  | 4 | 1 | 0 | 0 | 0 | 0 | 1 | 0 | 1 | 1 | 0 | 239 | 254 |  |  | PEAKS DB |
| K.LC(+57.02)YVALDFEQEMATAASSSSLEK.S | Y | 65.71 | 2549.1665 | 23 | -0.5 | 1275.5898 | 2 | 62.33 | 7 | F7:28143 | 21012022\_RID\_3262\_7a.raw |  |  |  |  |  |  | 1.0541E6 |  |  |  | 2 | 0 | 0 | 0 | 0 | 0 | 0 | 2 | 0 | 0 | 0 | 216 | 238 | Carbamidomethylation | C2:Carbamidomethylation:1000.00 | PEAKS DB |
| K.DSYVGDEAQSK.R | N | 65.50 | 1197.5149 | 11 | 1.5 | 599.7656 | 2 | 23.16 | 4 | F4:10278 | 17012022\_RID\_3262\_10a .raw | 2.7572E5 |  |  | 3.783E5 |  | 4.8292E5 |  | 7.9712E4 | 7.8692E4 |  | 5 | 1 | 0 | 0 | 1 | 0 | 1 | 0 | 1 | 1 | 0 | 51 | 61 |  |  | PEAKS DB |
| R.GYSFTTTAER.E | Y | 60.44 | 1131.5197 | 10 | -0.1 | 566.7670 | 2 | 28.78 | 8 | F8:11438 | 31122021\_RID\_3262\_1a.raw |  |  |  | 1.3304E6 |  |  | 2.0729E5 | 2.9994E5 | 2.4553E5 |  | 4 | 0 | 0 | 0 | 1 | 0 | 0 | 1 | 1 | 1 | 0 | 197 | 206 |  |  | PEAKS DB |
| K.LC(+57.02)YVALDFEQEM(+15.99)ATAASSSSLEK.S | Y | 54.72 | 2565.1614 | 23 | 0.4 | 856.0614 | 3 | 62.38 | 7 | F7:28152 | 21012022\_RID\_3262\_7a.raw |  |  |  |  |  |  | 1.0591E5 |  |  |  | 1 | 0 | 0 | 0 | 0 | 0 | 0 | 1 | 0 | 0 | 0 | 216 | 238 | Carbamidomethylation; Oxidation (M) | C2:Carbamidomethylation:1000.00;M12:Oxidation (M):1000.00 | PEAKS DB |
| K.AGFAGDDAPR.A | N | 52.95 | 975.4410 | 10 | 3.9 | 488.7296 | 2 | 22.10 | 9 | F9:7134 | 31122021\_RID\_3262\_2a.raw |  | 3.3275E5 |  | 1.2847E6 |  |  |  |  | 2.7076E6 |  | 4 | 0 | 1 | 0 | 1 | 0 | 0 | 0 | 0 | 2 | 0 | 19 | 28 |  |  | PEAKS DB |
| R.VAPEEHPVLLTEAPLNPK.A | N | 51.47 | 1953.0570 | 18 | 0.5 | 652.0266 | 3 | 40.45 | 1 | F1:25900 | 13012022\_RID\_3262\_4a.raw | 8.5304E5 |  |  | 5.0748E5 |  |  |  | 7.2111E5 |  |  | 3 | 1 | 0 | 0 | 1 | 0 | 0 | 0 | 1 | 0 | 0 | 96 | 113 |  |  | PEAKS DB |
| K.EITALAPSTM(+15.99)K.I | N | 49.12 | 1176.6060 | 11 | 1.7 | 589.3113 | 2 | 30.85 | 6 | F6:17553 | 17012022\_RID\_3262\_9a.raw |  |  |  |  |  | 2.4825E5 |  |  |  |  | 1 | 0 | 0 | 0 | 0 | 0 | 1 | 0 | 0 | 0 | 0 | 316 | 326 | Oxidation (M) | M10:Oxidation (M):1000.00 | PEAKS DB |
| G.YALPHAILR.L | Y | 48.16 | 1052.6130 | 9 | 0.0 | 527.3138 | 2 | 35.63 | 1 | F1:21569 | 13012022\_RID\_3262\_4a.raw | 1.4154E6 |  |  |  |  |  |  |  |  |  | 1 | 1 | 0 | 0 | 0 | 0 | 0 | 0 | 0 | 0 | 0 | 169 | 177 |  |  | PEAKS DB |
| R.AVFPSIVGRPR.H | N | 45.70 | 1197.6981 | 11 | 1.5 | 400.2406 | 3 | 35.84 | 6 | F6:21794 | 17012022\_RID\_3262\_9a.raw | 1.4816E6 |  |  |  |  | 0 |  |  |  |  | 1 | 1 | 0 | 0 | 0 | 0 | 0 | 0 | 0 | 0 | 0 | 29 | 39 |  |  | PEAKS DB |
| R.DLTDYLMK.I | N | 43.13 | 997.4790 | 8 | 0.7 | 499.7471 | 2 | 51.80 | 6 | F6:36164 | 17012022\_RID\_3262\_9a.raw |  |  |  |  |  | 4.4466E5 |  |  |  |  | 1 | 0 | 0 | 0 | 0 | 0 | 1 | 0 | 0 | 0 | 0 | 184 | 191 |  |  | PEAKS DB |
| total 11 peptides |  |  |  |  |  |  |  |  |  |  |  |  |  |  |  |  |  |  |  |  |  |  |  |  |  |  |  |  |  |  |  |  |  |  |  |  |  |
| --- | --- | --- | --- | --- | --- | --- | --- | --- | --- | --- | --- | --- | --- | --- | --- | --- | --- | --- | --- | --- | --- | --- | --- | --- | --- | --- | --- | --- | --- | --- | --- | --- | --- | --- | --- | --- | --- |

XP\_013921350.1

back to list

  

| Protein Coverage
| Supporting Peptides
|

Protein Coverage:

Supporting Peptides:

| Peptide | Uniq | -10lgP | Mass | Length | ppm | m/z | z | RT | Fraction | Scan | Source File | Area F4 | Area F5 | Area F6 | Area F10 | Area F8 | Area F9 | Area F7 | Area F1 | Area F2 | Area F3 | #Feature | #Feature F4 | #Feature F5 | #Feature F6 | #Feature F10 | #Feature F8 | #Feature F9 | #Feature F7 | #Feature F1 | #Feature F2 | #Feature F3 | Start | End | PTM | AScore | Found By |
| --- | --- | --- | --- | --- | --- | --- | --- | --- | --- | --- | --- | --- | --- | --- | --- | --- | --- | --- | --- | --- | --- | --- | --- | --- | --- | --- | --- | --- | --- | --- | --- | --- | --- | --- | --- | --- | --- |
| K.SYELPDGQVITIGNER.F | N | 82.78 | 1789.8846 | 16 | 0.4 | 895.9500 | 2 | 49.87 | 8 | F8:28149 | 31122021\_RID\_3262\_1a.raw | 4.7745E5 |  |  |  |  | 6.0954E5 |  | 1.0043E6 | 3.4371E5 |  | 4 | 1 | 0 | 0 | 0 | 0 | 1 | 0 | 1 | 1 | 0 | 239 | 254 |  |  | PEAKS DB |
| K.LC(+57.02)YVALDFEQEMATAASSSSLEK.S | Y | 65.71 | 2549.1665 | 23 | -0.5 | 1275.5898 | 2 | 62.33 | 7 | F7:28143 | 21012022\_RID\_3262\_7a.raw |  |  |  |  |  |  | 1.0541E6 |  |  |  | 2 | 0 | 0 | 0 | 0 | 0 | 0 | 2 | 0 | 0 | 0 | 216 | 238 | Carbamidomethylation | C2:Carbamidomethylation:1000.00 | PEAKS DB |
| K.DSYVGDEAQSK.R | N | 65.50 | 1197.5149 | 11 | 1.5 | 599.7656 | 2 | 23.16 | 4 | F4:10278 | 17012022\_RID\_3262\_10a .raw | 2.7572E5 |  |  | 3.783E5 |  | 4.8292E5 |  | 7.9712E4 | 7.8692E4 |  | 5 | 1 | 0 | 0 | 1 | 0 | 1 | 0 | 1 | 1 | 0 | 51 | 61 |  |  | PEAKS DB |
| R.GYSFTTTAER.E | Y | 60.44 | 1131.5197 | 10 | -0.1 | 566.7670 | 2 | 28.78 | 8 | F8:11438 | 31122021\_RID\_3262\_1a.raw |  |  |  | 1.3304E6 |  |  | 2.0729E5 | 2.9994E5 | 2.4553E5 |  | 4 | 0 | 0 | 0 | 1 | 0 | 0 | 1 | 1 | 1 | 0 | 197 | 206 |  |  | PEAKS DB |
| K.LC(+57.02)YVALDFEQEM(+15.99)ATAASSSSLEK.S | Y | 54.72 | 2565.1614 | 23 | 0.4 | 856.0614 | 3 | 62.38 | 7 | F7:28152 | 21012022\_RID\_3262\_7a.raw |  |  |  |  |  |  | 1.0591E5 |  |  |  | 1 | 0 | 0 | 0 | 0 | 0 | 0 | 1 | 0 | 0 | 0 | 216 | 238 | Carbamidomethylation; Oxidation (M) | C2:Carbamidomethylation:1000.00;M12:Oxidation (M):1000.00 | PEAKS DB |
| K.AGFAGDDAPR.A | N | 52.95 | 975.4410 | 10 | 3.9 | 488.7296 | 2 | 22.10 | 9 | F9:7134 | 31122021\_RID\_3262\_2a.raw |  | 3.3275E5 |  | 1.2847E6 |  |  |  |  | 2.7076E6 |  | 4 | 0 | 1 | 0 | 1 | 0 | 0 | 0 | 0 | 2 | 0 | 19 | 28 |  |  | PEAKS DB |
| R.VAPEEHPVLLTEAPLNPK.A | N | 51.47 | 1953.0570 | 18 | 0.5 | 652.0266 | 3 | 40.45 | 1 | F1:25900 | 13012022\_RID\_3262\_4a.raw | 8.5304E5 |  |  | 5.0748E5 |  |  |  | 7.2111E5 |  |  | 3 | 1 | 0 | 0 | 1 | 0 | 0 | 0 | 1 | 0 | 0 | 96 | 113 |  |  | PEAKS DB |
| K.EITALAPSTM(+15.99)K.I | N | 49.12 | 1176.6060 | 11 | 1.7 | 589.3113 | 2 | 30.85 | 6 | F6:17553 | 17012022\_RID\_3262\_9a.raw |  |  |  |  |  | 2.4825E5 |  |  |  |  | 1 | 0 | 0 | 0 | 0 | 0 | 1 | 0 | 0 | 0 | 0 | 316 | 326 | Oxidation (M) | M10:Oxidation (M):1000.00 | PEAKS DB |
| G.YALPHAILR.L | Y | 48.16 | 1052.6130 | 9 | 0.0 | 527.3138 | 2 | 35.63 | 1 | F1:21569 | 13012022\_RID\_3262\_4a.raw | 1.4154E6 |  |  |  |  |  |  |  |  |  | 1 | 1 | 0 | 0 | 0 | 0 | 0 | 0 | 0 | 0 | 0 | 169 | 177 |  |  | PEAKS DB |
| R.AVFPSIVGRPR.H | N | 45.70 | 1197.6981 | 11 | 1.5 | 400.2406 | 3 | 35.84 | 6 | F6:21794 | 17012022\_RID\_3262\_9a.raw | 1.4816E6 |  |  |  |  | 0 |  |  |  |  | 1 | 1 | 0 | 0 | 0 | 0 | 0 | 0 | 0 | 0 | 0 | 29 | 39 |  |  | PEAKS DB |
| R.DLTDYLMK.I | N | 43.13 | 997.4790 | 8 | 0.7 | 499.7471 | 2 | 51.80 | 6 | F6:36164 | 17012022\_RID\_3262\_9a.raw |  |  |  |  |  | 4.4466E5 |  |  |  |  | 1 | 0 | 0 | 0 | 0 | 0 | 1 | 0 | 0 | 0 | 0 | 184 | 191 |  |  | PEAKS DB |
| total 11 peptides |  |  |  |  |  |  |  |  |  |  |  |  |  |  |  |  |  |  |  |  |  |  |  |  |  |  |  |  |  |  |  |  |  |  |  |  |  |
| --- | --- | --- | --- | --- | --- | --- | --- | --- | --- | --- | --- | --- | --- | --- | --- | --- | --- | --- | --- | --- | --- | --- | --- | --- | --- | --- | --- | --- | --- | --- | --- | --- | --- | --- | --- | --- | --- |

XP\_026531551.1

back to list

  

| Protein Coverage
| Supporting Peptides
|

Protein Coverage:

Supporting Peptides:

| Peptide | Uniq | -10lgP | Mass | Length | ppm | m/z | z | RT | Fraction | Scan | Source File | Area F4 | Area F5 | Area F6 | Area F10 | Area F8 | Area F9 | Area F7 | Area F1 | Area F2 | Area F3 | #Feature | #Feature F4 | #Feature F5 | #Feature F6 | #Feature F10 | #Feature F8 | #Feature F9 | #Feature F7 | #Feature F1 | #Feature F2 | #Feature F3 | Start | End | PTM | AScore | Found By |
| --- | --- | --- | --- | --- | --- | --- | --- | --- | --- | --- | --- | --- | --- | --- | --- | --- | --- | --- | --- | --- | --- | --- | --- | --- | --- | --- | --- | --- | --- | --- | --- | --- | --- | --- | --- | --- | --- |
| K.SYELPDGQVITIGNER.F | N | 82.78 | 1789.8846 | 16 | 0.4 | 895.9500 | 2 | 49.87 | 8 | F8:28149 | 31122021\_RID\_3262\_1a.raw | 4.7745E5 |  |  |  |  | 6.0954E5 |  | 1.0043E6 | 3.4371E5 |  | 4 | 1 | 0 | 0 | 0 | 0 | 1 | 0 | 1 | 1 | 0 | 239 | 254 |  |  | PEAKS DB |
| K.LC(+57.02)YVALDFEQEMATAASSSSLEK.S | Y | 65.71 | 2549.1665 | 23 | -0.5 | 1275.5898 | 2 | 62.33 | 7 | F7:28143 | 21012022\_RID\_3262\_7a.raw |  |  |  |  |  |  | 1.0541E6 |  |  |  | 2 | 0 | 0 | 0 | 0 | 0 | 0 | 2 | 0 | 0 | 0 | 216 | 238 | Carbamidomethylation | C2:Carbamidomethylation:1000.00 | PEAKS DB |
| K.DSYVGDEAQSK.R | N | 65.50 | 1197.5149 | 11 | 1.5 | 599.7656 | 2 | 23.16 | 4 | F4:10278 | 17012022\_RID\_3262\_10a .raw | 2.7572E5 |  |  | 3.783E5 |  | 4.8292E5 |  | 7.9712E4 | 7.8692E4 |  | 5 | 1 | 0 | 0 | 1 | 0 | 1 | 0 | 1 | 1 | 0 | 51 | 61 |  |  | PEAKS DB |
| R.GYSFTTTAER.E | Y | 60.44 | 1131.5197 | 10 | -0.1 | 566.7670 | 2 | 28.78 | 8 | F8:11438 | 31122021\_RID\_3262\_1a.raw |  |  |  | 1.3304E6 |  |  | 2.0729E5 | 2.9994E5 | 2.4553E5 |  | 4 | 0 | 0 | 0 | 1 | 0 | 0 | 1 | 1 | 1 | 0 | 197 | 206 |  |  | PEAKS DB |
| K.LC(+57.02)YVALDFEQEM(+15.99)ATAASSSSLEK.S | Y | 54.72 | 2565.1614 | 23 | 0.4 | 856.0614 | 3 | 62.38 | 7 | F7:28152 | 21012022\_RID\_3262\_7a.raw |  |  |  |  |  |  | 1.0591E5 |  |  |  | 1 | 0 | 0 | 0 | 0 | 0 | 0 | 1 | 0 | 0 | 0 | 216 | 238 | Carbamidomethylation; Oxidation (M) | C2:Carbamidomethylation:1000.00;M12:Oxidation (M):1000.00 | PEAKS DB |
| K.AGFAGDDAPR.A | N | 52.95 | 975.4410 | 10 | 3.9 | 488.7296 | 2 | 22.10 | 9 | F9:7134 | 31122021\_RID\_3262\_2a.raw |  | 3.3275E5 |  | 1.2847E6 |  |  |  |  | 2.7076E6 |  | 4 | 0 | 1 | 0 | 1 | 0 | 0 | 0 | 0 | 2 | 0 | 19 | 28 |  |  | PEAKS DB |
| R.VAPEEHPVLLTEAPLNPK.A | N | 51.47 | 1953.0570 | 18 | 0.5 | 652.0266 | 3 | 40.45 | 1 | F1:25900 | 13012022\_RID\_3262\_4a.raw | 8.5304E5 |  |  | 5.0748E5 |  |  |  | 7.2111E5 |  |  | 3 | 1 | 0 | 0 | 1 | 0 | 0 | 0 | 1 | 0 | 0 | 96 | 113 |  |  | PEAKS DB |
| K.EITALAPSTM(+15.99)K.I | N | 49.12 | 1176.6060 | 11 | 1.7 | 589.3113 | 2 | 30.85 | 6 | F6:17553 | 17012022\_RID\_3262\_9a.raw |  |  |  |  |  | 2.4825E5 |  |  |  |  | 1 | 0 | 0 | 0 | 0 | 0 | 1 | 0 | 0 | 0 | 0 | 316 | 326 | Oxidation (M) | M10:Oxidation (M):1000.00 | PEAKS DB |
| G.YALPHAILR.L | Y | 48.16 | 1052.6130 | 9 | 0.0 | 527.3138 | 2 | 35.63 | 1 | F1:21569 | 13012022\_RID\_3262\_4a.raw | 1.4154E6 |  |  |  |  |  |  |  |  |  | 1 | 1 | 0 | 0 | 0 | 0 | 0 | 0 | 0 | 0 | 0 | 169 | 177 |  |  | PEAKS DB |
| R.AVFPSIVGRPR.H | N | 45.70 | 1197.6981 | 11 | 1.5 | 400.2406 | 3 | 35.84 | 6 | F6:21794 | 17012022\_RID\_3262\_9a.raw | 1.4816E6 |  |  |  |  | 0 |  |  |  |  | 1 | 1 | 0 | 0 | 0 | 0 | 0 | 0 | 0 | 0 | 0 | 29 | 39 |  |  | PEAKS DB |
| R.DLTDYLMK.I | N | 43.13 | 997.4790 | 8 | 0.7 | 499.7471 | 2 | 51.80 | 6 | F6:36164 | 17012022\_RID\_3262\_9a.raw |  |  |  |  |  | 4.4466E5 |  |  |  |  | 1 | 0 | 0 | 0 | 0 | 0 | 1 | 0 | 0 | 0 | 0 | 184 | 191 |  |  | PEAKS DB |
| total 11 peptides |  |  |  |  |  |  |  |  |  |  |  |  |  |  |  |  |  |  |  |  |  |  |  |  |  |  |  |  |  |  |  |  |  |  |  |  |  |
| --- | --- | --- | --- | --- | --- | --- | --- | --- | --- | --- | --- | --- | --- | --- | --- | --- | --- | --- | --- | --- | --- | --- | --- | --- | --- | --- | --- | --- | --- | --- | --- | --- | --- | --- | --- | --- | --- |

LAA21098.1

back to list

  

| Protein Coverage
| Supporting Peptides
|

Protein Coverage:

Supporting Peptides:

| Peptide | Uniq | -10lgP | Mass | Length | ppm | m/z | z | RT | Fraction | Scan | Source File | Area F4 | Area F5 | Area F6 | Area F10 | Area F8 | Area F9 | Area F7 | Area F1 | Area F2 | Area F3 | #Feature | #Feature F4 | #Feature F5 | #Feature F6 | #Feature F10 | #Feature F8 | #Feature F9 | #Feature F7 | #Feature F1 | #Feature F2 | #Feature F3 | Start | End | PTM | AScore | Found By |
| --- | --- | --- | --- | --- | --- | --- | --- | --- | --- | --- | --- | --- | --- | --- | --- | --- | --- | --- | --- | --- | --- | --- | --- | --- | --- | --- | --- | --- | --- | --- | --- | --- | --- | --- | --- | --- | --- |
| K.SYELPDGQVITIGNER.F | N | 82.78 | 1789.8846 | 16 | 0.4 | 895.9500 | 2 | 49.87 | 8 | F8:28149 | 31122021\_RID\_3262\_1a.raw | 4.7745E5 |  |  |  |  | 6.0954E5 |  | 1.0043E6 | 3.4371E5 |  | 4 | 1 | 0 | 0 | 0 | 0 | 1 | 0 | 1 | 1 | 0 | 239 | 254 |  |  | PEAKS DB |
| K.LC(+57.02)YVALDFEQEMATAASSSSLEK.S | Y | 65.71 | 2549.1665 | 23 | -0.5 | 1275.5898 | 2 | 62.33 | 7 | F7:28143 | 21012022\_RID\_3262\_7a.raw |  |  |  |  |  |  | 1.0541E6 |  |  |  | 2 | 0 | 0 | 0 | 0 | 0 | 0 | 2 | 0 | 0 | 0 | 216 | 238 | Carbamidomethylation | C2:Carbamidomethylation:1000.00 | PEAKS DB |
| K.DSYVGDEAQSK.R | N | 65.50 | 1197.5149 | 11 | 1.5 | 599.7656 | 2 | 23.16 | 4 | F4:10278 | 17012022\_RID\_3262\_10a .raw | 2.7572E5 |  |  | 3.783E5 |  | 4.8292E5 |  | 7.9712E4 | 7.8692E4 |  | 5 | 1 | 0 | 0 | 1 | 0 | 1 | 0 | 1 | 1 | 0 | 51 | 61 |  |  | PEAKS DB |
| R.GYSFTTTAER.E | Y | 60.44 | 1131.5197 | 10 | -0.1 | 566.7670 | 2 | 28.78 | 8 | F8:11438 | 31122021\_RID\_3262\_1a.raw |  |  |  | 1.3304E6 |  |  | 2.0729E5 | 2.9994E5 | 2.4553E5 |  | 4 | 0 | 0 | 0 | 1 | 0 | 0 | 1 | 1 | 1 | 0 | 197 | 206 |  |  | PEAKS DB |
| K.LC(+57.02)YVALDFEQEM(+15.99)ATAASSSSLEK.S | Y | 54.72 | 2565.1614 | 23 | 0.4 | 856.0614 | 3 | 62.38 | 7 | F7:28152 | 21012022\_RID\_3262\_7a.raw |  |  |  |  |  |  | 1.0591E5 |  |  |  | 1 | 0 | 0 | 0 | 0 | 0 | 0 | 1 | 0 | 0 | 0 | 216 | 238 | Carbamidomethylation; Oxidation (M) | C2:Carbamidomethylation:1000.00;M12:Oxidation (M):1000.00 | PEAKS DB |
| K.AGFAGDDAPR.A | N | 52.95 | 975.4410 | 10 | 3.9 | 488.7296 | 2 | 22.10 | 9 | F9:7134 | 31122021\_RID\_3262\_2a.raw |  | 3.3275E5 |  | 1.2847E6 |  |  |  |  | 2.7076E6 |  | 4 | 0 | 1 | 0 | 1 | 0 | 0 | 0 | 0 | 2 | 0 | 19 | 28 |  |  | PEAKS DB |
| R.VAPEEHPVLLTEAPLNPK.A | N | 51.47 | 1953.0570 | 18 | 0.5 | 652.0266 | 3 | 40.45 | 1 | F1:25900 | 13012022\_RID\_3262\_4a.raw | 8.5304E5 |  |  | 5.0748E5 |  |  |  | 7.2111E5 |  |  | 3 | 1 | 0 | 0 | 1 | 0 | 0 | 0 | 1 | 0 | 0 | 96 | 113 |  |  | PEAKS DB |
| K.EITALAPSTM(+15.99)K.I | N | 49.12 | 1176.6060 | 11 | 1.7 | 589.3113 | 2 | 30.85 | 6 | F6:17553 | 17012022\_RID\_3262\_9a.raw |  |  |  |  |  | 2.4825E5 |  |  |  |  | 1 | 0 | 0 | 0 | 0 | 0 | 1 | 0 | 0 | 0 | 0 | 316 | 326 | Oxidation (M) | M10:Oxidation (M):1000.00 | PEAKS DB |
| G.YALPHAILR.L | Y | 48.16 | 1052.6130 | 9 | 0.0 | 527.3138 | 2 | 35.63 | 1 | F1:21569 | 13012022\_RID\_3262\_4a.raw | 1.4154E6 |  |  |  |  |  |  |  |  |  | 1 | 1 | 0 | 0 | 0 | 0 | 0 | 0 | 0 | 0 | 0 | 169 | 177 |  |  | PEAKS DB |
| R.AVFPSIVGRPR.H | N | 45.70 | 1197.6981 | 11 | 1.5 | 400.2406 | 3 | 35.84 | 6 | F6:21794 | 17012022\_RID\_3262\_9a.raw | 1.4816E6 |  |  |  |  | 0 |  |  |  |  | 1 | 1 | 0 | 0 | 0 | 0 | 0 | 0 | 0 | 0 | 0 | 29 | 39 |  |  | PEAKS DB |
| R.DLTDYLMK.I | N | 43.13 | 997.4790 | 8 | 0.7 | 499.7471 | 2 | 51.80 | 6 | F6:36164 | 17012022\_RID\_3262\_9a.raw |  |  |  |  |  | 4.4466E5 |  |  |  |  | 1 | 0 | 0 | 0 | 0 | 0 | 1 | 0 | 0 | 0 | 0 | 184 | 191 |  |  | PEAKS DB |
| total 11 peptides |  |  |  |  |  |  |  |  |  |  |  |  |  |  |  |  |  |  |  |  |  |  |  |  |  |  |  |  |  |  |  |  |  |  |  |  |  |
| --- | --- | --- | --- | --- | --- | --- | --- | --- | --- | --- | --- | --- | --- | --- | --- | --- | --- | --- | --- | --- | --- | --- | --- | --- | --- | --- | --- | --- | --- | --- | --- | --- | --- | --- | --- | --- | --- |

AFJ49303.1

back to list

  

| Protein Coverage
| Supporting Peptides
|

Protein Coverage:

Supporting Peptides:

| Peptide | Uniq | -10lgP | Mass | Length | ppm | m/z | z | RT | Fraction | Scan | Source File | Area F4 | Area F5 | Area F6 | Area F10 | Area F8 | Area F9 | Area F7 | Area F1 | Area F2 | Area F3 | #Feature | #Feature F4 | #Feature F5 | #Feature F6 | #Feature F10 | #Feature F8 | #Feature F9 | #Feature F7 | #Feature F1 | #Feature F2 | #Feature F3 | Start | End | PTM | AScore | Found By |
| --- | --- | --- | --- | --- | --- | --- | --- | --- | --- | --- | --- | --- | --- | --- | --- | --- | --- | --- | --- | --- | --- | --- | --- | --- | --- | --- | --- | --- | --- | --- | --- | --- | --- | --- | --- | --- | --- |
| K.SYELPDGQVITIGNER.F | N | 82.78 | 1789.8846 | 16 | 0.4 | 895.9500 | 2 | 49.87 | 8 | F8:28149 | 31122021\_RID\_3262\_1a.raw | 4.7745E5 |  |  |  |  | 6.0954E5 |  | 1.0043E6 | 3.4371E5 |  | 4 | 1 | 0 | 0 | 0 | 0 | 1 | 0 | 1 | 1 | 0 | 239 | 254 |  |  | PEAKS DB |
| K.LC(+57.02)YVALDFEQEMATAASSSSLEK.S | Y | 65.71 | 2549.1665 | 23 | -0.5 | 1275.5898 | 2 | 62.33 | 7 | F7:28143 | 21012022\_RID\_3262\_7a.raw |  |  |  |  |  |  | 1.0541E6 |  |  |  | 2 | 0 | 0 | 0 | 0 | 0 | 0 | 2 | 0 | 0 | 0 | 216 | 238 | Carbamidomethylation | C2:Carbamidomethylation:1000.00 | PEAKS DB |
| K.DSYVGDEAQSK.R | N | 65.50 | 1197.5149 | 11 | 1.5 | 599.7656 | 2 | 23.16 | 4 | F4:10278 | 17012022\_RID\_3262\_10a .raw | 2.7572E5 |  |  | 3.783E5 |  | 4.8292E5 |  | 7.9712E4 | 7.8692E4 |  | 5 | 1 | 0 | 0 | 1 | 0 | 1 | 0 | 1 | 1 | 0 | 51 | 61 |  |  | PEAKS DB |
| R.GYSFTTTAER.E | Y | 60.44 | 1131.5197 | 10 | -0.1 | 566.7670 | 2 | 28.78 | 8 | F8:11438 | 31122021\_RID\_3262\_1a.raw |  |  |  | 1.3304E6 |  |  | 2.0729E5 | 2.9994E5 | 2.4553E5 |  | 4 | 0 | 0 | 0 | 1 | 0 | 0 | 1 | 1 | 1 | 0 | 197 | 206 |  |  | PEAKS DB |
| K.LC(+57.02)YVALDFEQEM(+15.99)ATAASSSSLEK.S | Y | 54.72 | 2565.1614 | 23 | 0.4 | 856.0614 | 3 | 62.38 | 7 | F7:28152 | 21012022\_RID\_3262\_7a.raw |  |  |  |  |  |  | 1.0591E5 |  |  |  | 1 | 0 | 0 | 0 | 0 | 0 | 0 | 1 | 0 | 0 | 0 | 216 | 238 | Carbamidomethylation; Oxidation (M) | C2:Carbamidomethylation:1000.00;M12:Oxidation (M):1000.00 | PEAKS DB |
| K.AGFAGDDAPR.A | N | 52.95 | 975.4410 | 10 | 3.9 | 488.7296 | 2 | 22.10 | 9 | F9:7134 | 31122021\_RID\_3262\_2a.raw |  | 3.3275E5 |  | 1.2847E6 |  |  |  |  | 2.7076E6 |  | 4 | 0 | 1 | 0 | 1 | 0 | 0 | 0 | 0 | 2 | 0 | 19 | 28 |  |  | PEAKS DB |
| R.VAPEEHPVLLTEAPLNPK.A | N | 51.47 | 1953.0570 | 18 | 0.5 | 652.0266 | 3 | 40.45 | 1 | F1:25900 | 13012022\_RID\_3262\_4a.raw | 8.5304E5 |  |  | 5.0748E5 |  |  |  | 7.2111E5 |  |  | 3 | 1 | 0 | 0 | 1 | 0 | 0 | 0 | 1 | 0 | 0 | 96 | 113 |  |  | PEAKS DB |
| K.EITALAPSTM(+15.99)K.I | N | 49.12 | 1176.6060 | 11 | 1.7 | 589.3113 | 2 | 30.85 | 6 | F6:17553 | 17012022\_RID\_3262\_9a.raw |  |  |  |  |  | 2.4825E5 |  |  |  |  | 1 | 0 | 0 | 0 | 0 | 0 | 1 | 0 | 0 | 0 | 0 | 316 | 326 | Oxidation (M) | M10:Oxidation (M):1000.00 | PEAKS DB |
| G.YALPHAILR.L | Y | 48.16 | 1052.6130 | 9 | 0.0 | 527.3138 | 2 | 35.63 | 1 | F1:21569 | 13012022\_RID\_3262\_4a.raw | 1.4154E6 |  |  |  |  |  |  |  |  |  | 1 | 1 | 0 | 0 | 0 | 0 | 0 | 0 | 0 | 0 | 0 | 169 | 177 |  |  | PEAKS DB |
| R.AVFPSIVGRPR.H | N | 45.70 | 1197.6981 | 11 | 1.5 | 400.2406 | 3 | 35.84 | 6 | F6:21794 | 17012022\_RID\_3262\_9a.raw | 1.4816E6 |  |  |  |  | 0 |  |  |  |  | 1 | 1 | 0 | 0 | 0 | 0 | 0 | 0 | 0 | 0 | 0 | 29 | 39 |  |  | PEAKS DB |
| R.DLTDYLMK.I | N | 43.13 | 997.4790 | 8 | 0.7 | 499.7471 | 2 | 51.80 | 6 | F6:36164 | 17012022\_RID\_3262\_9a.raw |  |  |  |  |  | 4.4466E5 |  |  |  |  | 1 | 0 | 0 | 0 | 0 | 0 | 1 | 0 | 0 | 0 | 0 | 184 | 191 |  |  | PEAKS DB |
| total 11 peptides |  |  |  |  |  |  |  |  |  |  |  |  |  |  |  |  |  |  |  |  |  |  |  |  |  |  |  |  |  |  |  |  |  |  |  |  |  |
| --- | --- | --- | --- | --- | --- | --- | --- | --- | --- | --- | --- | --- | --- | --- | --- | --- | --- | --- | --- | --- | --- | --- | --- | --- | --- | --- | --- | --- | --- | --- | --- | --- | --- | --- | --- | --- | --- |

JAI10341.1

back to list

  

| Protein Coverage
| Supporting Peptides
|

Protein Coverage:

Supporting Peptides:

| Peptide | Uniq | -10lgP | Mass | Length | ppm | m/z | z | RT | Fraction | Scan | Source File | Area F4 | Area F5 | Area F6 | Area F10 | Area F8 | Area F9 | Area F7 | Area F1 | Area F2 | Area F3 | #Feature | #Feature F4 | #Feature F5 | #Feature F6 | #Feature F10 | #Feature F8 | #Feature F9 | #Feature F7 | #Feature F1 | #Feature F2 | #Feature F3 | Start | End | PTM | AScore | Found By |
| --- | --- | --- | --- | --- | --- | --- | --- | --- | --- | --- | --- | --- | --- | --- | --- | --- | --- | --- | --- | --- | --- | --- | --- | --- | --- | --- | --- | --- | --- | --- | --- | --- | --- | --- | --- | --- | --- |
| K.SYELPDGQVITIGNER.F | N | 82.78 | 1789.8846 | 16 | 0.4 | 895.9500 | 2 | 49.87 | 8 | F8:28149 | 31122021\_RID\_3262\_1a.raw | 4.7745E5 |  |  |  |  | 6.0954E5 |  | 1.0043E6 | 3.4371E5 |  | 4 | 1 | 0 | 0 | 0 | 0 | 1 | 0 | 1 | 1 | 0 | 239 | 254 |  |  | PEAKS DB |
| K.LC(+57.02)YVALDFEQEMATAASSSSLEK.S | Y | 65.71 | 2549.1665 | 23 | -0.5 | 1275.5898 | 2 | 62.33 | 7 | F7:28143 | 21012022\_RID\_3262\_7a.raw |  |  |  |  |  |  | 1.0541E6 |  |  |  | 2 | 0 | 0 | 0 | 0 | 0 | 0 | 2 | 0 | 0 | 0 | 216 | 238 | Carbamidomethylation | C2:Carbamidomethylation:1000.00 | PEAKS DB |
| K.DSYVGDEAQSK.R | N | 65.50 | 1197.5149 | 11 | 1.5 | 599.7656 | 2 | 23.16 | 4 | F4:10278 | 17012022\_RID\_3262\_10a .raw | 2.7572E5 |  |  | 3.783E5 |  | 4.8292E5 |  | 7.9712E4 | 7.8692E4 |  | 5 | 1 | 0 | 0 | 1 | 0 | 1 | 0 | 1 | 1 | 0 | 51 | 61 |  |  | PEAKS DB |
| R.GYSFTTTAER.E | Y | 60.44 | 1131.5197 | 10 | -0.1 | 566.7670 | 2 | 28.78 | 8 | F8:11438 | 31122021\_RID\_3262\_1a.raw |  |  |  | 1.3304E6 |  |  | 2.0729E5 | 2.9994E5 | 2.4553E5 |  | 4 | 0 | 0 | 0 | 1 | 0 | 0 | 1 | 1 | 1 | 0 | 197 | 206 |  |  | PEAKS DB |
| K.LC(+57.02)YVALDFEQEM(+15.99)ATAASSSSLEK.S | Y | 54.72 | 2565.1614 | 23 | 0.4 | 856.0614 | 3 | 62.38 | 7 | F7:28152 | 21012022\_RID\_3262\_7a.raw |  |  |  |  |  |  | 1.0591E5 |  |  |  | 1 | 0 | 0 | 0 | 0 | 0 | 0 | 1 | 0 | 0 | 0 | 216 | 238 | Carbamidomethylation; Oxidation (M) | C2:Carbamidomethylation:1000.00;M12:Oxidation (M):1000.00 | PEAKS DB |
| K.AGFAGDDAPR.A | N | 52.95 | 975.4410 | 10 | 3.9 | 488.7296 | 2 | 22.10 | 9 | F9:7134 | 31122021\_RID\_3262\_2a.raw |  | 3.3275E5 |  | 1.2847E6 |  |  |  |  | 2.7076E6 |  | 4 | 0 | 1 | 0 | 1 | 0 | 0 | 0 | 0 | 2 | 0 | 19 | 28 |  |  | PEAKS DB |
| R.VAPEEHPVLLTEAPLNPK.A | N | 51.47 | 1953.0570 | 18 | 0.5 | 652.0266 | 3 | 40.45 | 1 | F1:25900 | 13012022\_RID\_3262\_4a.raw | 8.5304E5 |  |  | 5.0748E5 |  |  |  | 7.2111E5 |  |  | 3 | 1 | 0 | 0 | 1 | 0 | 0 | 0 | 1 | 0 | 0 | 96 | 113 |  |  | PEAKS DB |
| K.EITALAPSTM(+15.99)K.I | N | 49.12 | 1176.6060 | 11 | 1.7 | 589.3113 | 2 | 30.85 | 6 | F6:17553 | 17012022\_RID\_3262\_9a.raw |  |  |  |  |  | 2.4825E5 |  |  |  |  | 1 | 0 | 0 | 0 | 0 | 0 | 1 | 0 | 0 | 0 | 0 | 316 | 326 | Oxidation (M) | M10:Oxidation (M):1000.00 | PEAKS DB |
| G.YALPHAILR.L | Y | 48.16 | 1052.6130 | 9 | 0.0 | 527.3138 | 2 | 35.63 | 1 | F1:21569 | 13012022\_RID\_3262\_4a.raw | 1.4154E6 |  |  |  |  |  |  |  |  |  | 1 | 1 | 0 | 0 | 0 | 0 | 0 | 0 | 0 | 0 | 0 | 169 | 177 |  |  | PEAKS DB |
| R.AVFPSIVGRPR.H | N | 45.70 | 1197.6981 | 11 | 1.5 | 400.2406 | 3 | 35.84 | 6 | F6:21794 | 17012022\_RID\_3262\_9a.raw | 1.4816E6 |  |  |  |  | 0 |  |  |  |  | 1 | 1 | 0 | 0 | 0 | 0 | 0 | 0 | 0 | 0 | 0 | 29 | 39 |  |  | PEAKS DB |
| R.DLTDYLMK.I | N | 43.13 | 997.4790 | 8 | 0.7 | 499.7471 | 2 | 51.80 | 6 | F6:36164 | 17012022\_RID\_3262\_9a.raw |  |  |  |  |  | 4.4466E5 |  |  |  |  | 1 | 0 | 0 | 0 | 0 | 0 | 1 | 0 | 0 | 0 | 0 | 184 | 191 |  |  | PEAKS DB |
| total 11 peptides |  |  |  |  |  |  |  |  |  |  |  |  |  |  |  |  |  |  |  |  |  |  |  |  |  |  |  |  |  |  |  |  |  |  |  |  |  |
| --- | --- | --- | --- | --- | --- | --- | --- | --- | --- | --- | --- | --- | --- | --- | --- | --- | --- | --- | --- | --- | --- | --- | --- | --- | --- | --- | --- | --- | --- | --- | --- | --- | --- | --- | --- | --- | --- |

JAI14495.1

back to list

  

| Protein Coverage
| Supporting Peptides
|

Protein Coverage:

Supporting Peptides:

| Peptide | Uniq | -10lgP | Mass | Length | ppm | m/z | z | RT | Fraction | Scan | Source File | Area F4 | Area F5 | Area F6 | Area F10 | Area F8 | Area F9 | Area F7 | Area F1 | Area F2 | Area F3 | #Feature | #Feature F4 | #Feature F5 | #Feature F6 | #Feature F10 | #Feature F8 | #Feature F9 | #Feature F7 | #Feature F1 | #Feature F2 | #Feature F3 | Start | End | PTM | AScore | Found By |
| --- | --- | --- | --- | --- | --- | --- | --- | --- | --- | --- | --- | --- | --- | --- | --- | --- | --- | --- | --- | --- | --- | --- | --- | --- | --- | --- | --- | --- | --- | --- | --- | --- | --- | --- | --- | --- | --- |
| K.SYELPDGQVITIGNER.F | N | 82.78 | 1789.8846 | 16 | 0.4 | 895.9500 | 2 | 49.87 | 8 | F8:28149 | 31122021\_RID\_3262\_1a.raw | 4.7745E5 |  |  |  |  | 6.0954E5 |  | 1.0043E6 | 3.4371E5 |  | 4 | 1 | 0 | 0 | 0 | 0 | 1 | 0 | 1 | 1 | 0 | 239 | 254 |  |  | PEAKS DB |
| K.LC(+57.02)YVALDFEQEMATAASSSSLEK.S | Y | 65.71 | 2549.1665 | 23 | -0.5 | 1275.5898 | 2 | 62.33 | 7 | F7:28143 | 21012022\_RID\_3262\_7a.raw |  |  |  |  |  |  | 1.0541E6 |  |  |  | 2 | 0 | 0 | 0 | 0 | 0 | 0 | 2 | 0 | 0 | 0 | 216 | 238 | Carbamidomethylation | C2:Carbamidomethylation:1000.00 | PEAKS DB |
| K.DSYVGDEAQSK.R | N | 65.50 | 1197.5149 | 11 | 1.5 | 599.7656 | 2 | 23.16 | 4 | F4:10278 | 17012022\_RID\_3262\_10a .raw | 2.7572E5 |  |  | 3.783E5 |  | 4.8292E5 |  | 7.9712E4 | 7.8692E4 |  | 5 | 1 | 0 | 0 | 1 | 0 | 1 | 0 | 1 | 1 | 0 | 51 | 61 |  |  | PEAKS DB |
| R.GYSFTTTAER.E | Y | 60.44 | 1131.5197 | 10 | -0.1 | 566.7670 | 2 | 28.78 | 8 | F8:11438 | 31122021\_RID\_3262\_1a.raw |  |  |  | 1.3304E6 |  |  | 2.0729E5 | 2.9994E5 | 2.4553E5 |  | 4 | 0 | 0 | 0 | 1 | 0 | 0 | 1 | 1 | 1 | 0 | 197 | 206 |  |  | PEAKS DB |
| K.LC(+57.02)YVALDFEQEM(+15.99)ATAASSSSLEK.S | Y | 54.72 | 2565.1614 | 23 | 0.4 | 856.0614 | 3 | 62.38 | 7 | F7:28152 | 21012022\_RID\_3262\_7a.raw |  |  |  |  |  |  | 1.0591E5 |  |  |  | 1 | 0 | 0 | 0 | 0 | 0 | 0 | 1 | 0 | 0 | 0 | 216 | 238 | Carbamidomethylation; Oxidation (M) | C2:Carbamidomethylation:1000.00;M12:Oxidation (M):1000.00 | PEAKS DB |
| K.AGFAGDDAPR.A | N | 52.95 | 975.4410 | 10 | 3.9 | 488.7296 | 2 | 22.10 | 9 | F9:7134 | 31122021\_RID\_3262\_2a.raw |  | 3.3275E5 |  | 1.2847E6 |  |  |  |  | 2.7076E6 |  | 4 | 0 | 1 | 0 | 1 | 0 | 0 | 0 | 0 | 2 | 0 | 19 | 28 |  |  | PEAKS DB |
| R.VAPEEHPVLLTEAPLNPK.A | N | 51.47 | 1953.0570 | 18 | 0.5 | 652.0266 | 3 | 40.45 | 1 | F1:25900 | 13012022\_RID\_3262\_4a.raw | 8.5304E5 |  |  | 5.0748E5 |  |  |  | 7.2111E5 |  |  | 3 | 1 | 0 | 0 | 1 | 0 | 0 | 0 | 1 | 0 | 0 | 96 | 113 |  |  | PEAKS DB |
| K.EITALAPSTM(+15.99)K.I | N | 49.12 | 1176.6060 | 11 | 1.7 | 589.3113 | 2 | 30.85 | 6 | F6:17553 | 17012022\_RID\_3262\_9a.raw |  |  |  |  |  | 2.4825E5 |  |  |  |  | 1 | 0 | 0 | 0 | 0 | 0 | 1 | 0 | 0 | 0 | 0 | 316 | 326 | Oxidation (M) | M10:Oxidation (M):1000.00 | PEAKS DB |
| G.YALPHAILR.L | Y | 48.16 | 1052.6130 | 9 | 0.0 | 527.3138 | 2 | 35.63 | 1 | F1:21569 | 13012022\_RID\_3262\_4a.raw | 1.4154E6 |  |  |  |  |  |  |  |  |  | 1 | 1 | 0 | 0 | 0 | 0 | 0 | 0 | 0 | 0 | 0 | 169 | 177 |  |  | PEAKS DB |
| R.AVFPSIVGRPR.H | N | 45.70 | 1197.6981 | 11 | 1.5 | 400.2406 | 3 | 35.84 | 6 | F6:21794 | 17012022\_RID\_3262\_9a.raw | 1.4816E6 |  |  |  |  | 0 |  |  |  |  | 1 | 1 | 0 | 0 | 0 | 0 | 0 | 0 | 0 | 0 | 0 | 29 | 39 |  |  | PEAKS DB |
| R.DLTDYLMK.I | N | 43.13 | 997.4790 | 8 | 0.7 | 499.7471 | 2 | 51.80 | 6 | F6:36164 | 17012022\_RID\_3262\_9a.raw |  |  |  |  |  | 4.4466E5 |  |  |  |  | 1 | 0 | 0 | 0 | 0 | 0 | 1 | 0 | 0 | 0 | 0 | 184 | 191 |  |  | PEAKS DB |
| total 11 peptides |  |  |  |  |  |  |  |  |  |  |  |  |  |  |  |  |  |  |  |  |  |  |  |  |  |  |  |  |  |  |  |  |  |  |  |  |  |
| --- | --- | --- | --- | --- | --- | --- | --- | --- | --- | --- | --- | --- | --- | --- | --- | --- | --- | --- | --- | --- | --- | --- | --- | --- | --- | --- | --- | --- | --- | --- | --- | --- | --- | --- | --- | --- | --- |

XP\_007419915.2

back to list

  

| Protein Coverage
| Supporting Peptides
|

Protein Coverage:

Supporting Peptides:

| Peptide | Uniq | -10lgP | Mass | Length | ppm | m/z | z | RT | Fraction | Scan | Source File | Area F4 | Area F5 | Area F6 | Area F10 | Area F8 | Area F9 | Area F7 | Area F1 | Area F2 | Area F3 | #Feature | #Feature F4 | #Feature F5 | #Feature F6 | #Feature F10 | #Feature F8 | #Feature F9 | #Feature F7 | #Feature F1 | #Feature F2 | #Feature F3 | Start | End | PTM | AScore | Found By |
| --- | --- | --- | --- | --- | --- | --- | --- | --- | --- | --- | --- | --- | --- | --- | --- | --- | --- | --- | --- | --- | --- | --- | --- | --- | --- | --- | --- | --- | --- | --- | --- | --- | --- | --- | --- | --- | --- |
| K.SYELPDGQVITIGNER.F | N | 82.78 | 1789.8846 | 16 | 0.4 | 895.9500 | 2 | 49.87 | 8 | F8:28149 | 31122021\_RID\_3262\_1a.raw | 4.7745E5 |  |  |  |  | 6.0954E5 |  | 1.0043E6 | 3.4371E5 |  | 4 | 1 | 0 | 0 | 0 | 0 | 1 | 0 | 1 | 1 | 0 | 239 | 254 |  |  | PEAKS DB |
| K.LC(+57.02)YVALDFEQEMATAASSSSLEK.S | Y | 65.71 | 2549.1665 | 23 | -0.5 | 1275.5898 | 2 | 62.33 | 7 | F7:28143 | 21012022\_RID\_3262\_7a.raw |  |  |  |  |  |  | 1.0541E6 |  |  |  | 2 | 0 | 0 | 0 | 0 | 0 | 0 | 2 | 0 | 0 | 0 | 216 | 238 | Carbamidomethylation | C2:Carbamidomethylation:1000.00 | PEAKS DB |
| K.DSYVGDEAQSK.R | N | 65.50 | 1197.5149 | 11 | 1.5 | 599.7656 | 2 | 23.16 | 4 | F4:10278 | 17012022\_RID\_3262\_10a .raw | 2.7572E5 |  |  | 3.783E5 |  | 4.8292E5 |  | 7.9712E4 | 7.8692E4 |  | 5 | 1 | 0 | 0 | 1 | 0 | 1 | 0 | 1 | 1 | 0 | 51 | 61 |  |  | PEAKS DB |
| R.GYSFTTTAER.E | Y | 60.44 | 1131.5197 | 10 | -0.1 | 566.7670 | 2 | 28.78 | 8 | F8:11438 | 31122021\_RID\_3262\_1a.raw |  |  |  | 1.3304E6 |  |  | 2.0729E5 | 2.9994E5 | 2.4553E5 |  | 4 | 0 | 0 | 0 | 1 | 0 | 0 | 1 | 1 | 1 | 0 | 197 | 206 |  |  | PEAKS DB |
| K.LC(+57.02)YVALDFEQEM(+15.99)ATAASSSSLEK.S | Y | 54.72 | 2565.1614 | 23 | 0.4 | 856.0614 | 3 | 62.38 | 7 | F7:28152 | 21012022\_RID\_3262\_7a.raw |  |  |  |  |  |  | 1.0591E5 |  |  |  | 1 | 0 | 0 | 0 | 0 | 0 | 0 | 1 | 0 | 0 | 0 | 216 | 238 | Carbamidomethylation; Oxidation (M) | C2:Carbamidomethylation:1000.00;M12:Oxidation (M):1000.00 | PEAKS DB |
| K.AGFAGDDAPR.A | N | 52.95 | 975.4410 | 10 | 3.9 | 488.7296 | 2 | 22.10 | 9 | F9:7134 | 31122021\_RID\_3262\_2a.raw |  | 3.3275E5 |  | 1.2847E6 |  |  |  |  | 2.7076E6 |  | 4 | 0 | 1 | 0 | 1 | 0 | 0 | 0 | 0 | 2 | 0 | 19 | 28 |  |  | PEAKS DB |
| R.VAPEEHPVLLTEAPLNPK.A | N | 51.47 | 1953.0570 | 18 | 0.5 | 652.0266 | 3 | 40.45 | 1 | F1:25900 | 13012022\_RID\_3262\_4a.raw | 8.5304E5 |  |  | 5.0748E5 |  |  |  | 7.2111E5 |  |  | 3 | 1 | 0 | 0 | 1 | 0 | 0 | 0 | 1 | 0 | 0 | 96 | 113 |  |  | PEAKS DB |
| K.EITALAPSTM(+15.99)K.I | N | 49.12 | 1176.6060 | 11 | 1.7 | 589.3113 | 2 | 30.85 | 6 | F6:17553 | 17012022\_RID\_3262\_9a.raw |  |  |  |  |  | 2.4825E5 |  |  |  |  | 1 | 0 | 0 | 0 | 0 | 0 | 1 | 0 | 0 | 0 | 0 | 316 | 326 | Oxidation (M) | M10:Oxidation (M):1000.00 | PEAKS DB |
| G.YALPHAILR.L | Y | 48.16 | 1052.6130 | 9 | 0.0 | 527.3138 | 2 | 35.63 | 1 | F1:21569 | 13012022\_RID\_3262\_4a.raw | 1.4154E6 |  |  |  |  |  |  |  |  |  | 1 | 1 | 0 | 0 | 0 | 0 | 0 | 0 | 0 | 0 | 0 | 169 | 177 |  |  | PEAKS DB |
| R.AVFPSIVGRPR.H | N | 45.70 | 1197.6981 | 11 | 1.5 | 400.2406 | 3 | 35.84 | 6 | F6:21794 | 17012022\_RID\_3262\_9a.raw | 1.4816E6 |  |  |  |  | 0 |  |  |  |  | 1 | 1 | 0 | 0 | 0 | 0 | 0 | 0 | 0 | 0 | 0 | 29 | 39 |  |  | PEAKS DB |
| R.DLTDYLMK.I | N | 43.13 | 997.4790 | 8 | 0.7 | 499.7471 | 2 | 51.80 | 6 | F6:36164 | 17012022\_RID\_3262\_9a.raw |  |  |  |  |  | 4.4466E5 |  |  |  |  | 1 | 0 | 0 | 0 | 0 | 0 | 1 | 0 | 0 | 0 | 0 | 184 | 191 |  |  | PEAKS DB |
| total 11 peptides |  |  |  |  |  |  |  |  |  |  |  |  |  |  |  |  |  |  |  |  |  |  |  |  |  |  |  |  |  |  |  |  |  |  |  |  |  |
| --- | --- | --- | --- | --- | --- | --- | --- | --- | --- | --- | --- | --- | --- | --- | --- | --- | --- | --- | --- | --- | --- | --- | --- | --- | --- | --- | --- | --- | --- | --- | --- | --- | --- | --- | --- | --- | --- |

JAG47579.1

back to list

  

| Protein Coverage
| Supporting Peptides
|

Protein Coverage:

Supporting Peptides:

| Peptide | Uniq | -10lgP | Mass | Length | ppm | m/z | z | RT | Fraction | Scan | Source File | Area F4 | Area F5 | Area F6 | Area F10 | Area F8 | Area F9 | Area F7 | Area F1 | Area F2 | Area F3 | #Feature | #Feature F4 | #Feature F5 | #Feature F6 | #Feature F10 | #Feature F8 | #Feature F9 | #Feature F7 | #Feature F1 | #Feature F2 | #Feature F3 | Start | End | PTM | AScore | Found By |
| --- | --- | --- | --- | --- | --- | --- | --- | --- | --- | --- | --- | --- | --- | --- | --- | --- | --- | --- | --- | --- | --- | --- | --- | --- | --- | --- | --- | --- | --- | --- | --- | --- | --- | --- | --- | --- | --- |
| K.SYELPDGQVITIGNER.F | N | 82.78 | 1789.8846 | 16 | 0.4 | 895.9500 | 2 | 49.87 | 8 | F8:28149 | 31122021\_RID\_3262\_1a.raw | 4.7745E5 |  |  |  |  | 6.0954E5 |  | 1.0043E6 | 3.4371E5 |  | 4 | 1 | 0 | 0 | 0 | 0 | 1 | 0 | 1 | 1 | 0 | 239 | 254 |  |  | PEAKS DB |
| K.LC(+57.02)YVALDFEQEMATAASSSSLEK.S | Y | 65.71 | 2549.1665 | 23 | -0.5 | 1275.5898 | 2 | 62.33 | 7 | F7:28143 | 21012022\_RID\_3262\_7a.raw |  |  |  |  |  |  | 1.0541E6 |  |  |  | 2 | 0 | 0 | 0 | 0 | 0 | 0 | 2 | 0 | 0 | 0 | 216 | 238 | Carbamidomethylation | C2:Carbamidomethylation:1000.00 | PEAKS DB |
| K.DSYVGDEAQSK.R | N | 65.50 | 1197.5149 | 11 | 1.5 | 599.7656 | 2 | 23.16 | 4 | F4:10278 | 17012022\_RID\_3262\_10a .raw | 2.7572E5 |  |  | 3.783E5 |  | 4.8292E5 |  | 7.9712E4 | 7.8692E4 |  | 5 | 1 | 0 | 0 | 1 | 0 | 1 | 0 | 1 | 1 | 0 | 51 | 61 |  |  | PEAKS DB |
| R.GYSFTTTAER.E | Y | 60.44 | 1131.5197 | 10 | -0.1 | 566.7670 | 2 | 28.78 | 8 | F8:11438 | 31122021\_RID\_3262\_1a.raw |  |  |  | 1.3304E6 |  |  | 2.0729E5 | 2.9994E5 | 2.4553E5 |  | 4 | 0 | 0 | 0 | 1 | 0 | 0 | 1 | 1 | 1 | 0 | 197 | 206 |  |  | PEAKS DB |
| K.LC(+57.02)YVALDFEQEM(+15.99)ATAASSSSLEK.S | Y | 54.72 | 2565.1614 | 23 | 0.4 | 856.0614 | 3 | 62.38 | 7 | F7:28152 | 21012022\_RID\_3262\_7a.raw |  |  |  |  |  |  | 1.0591E5 |  |  |  | 1 | 0 | 0 | 0 | 0 | 0 | 0 | 1 | 0 | 0 | 0 | 216 | 238 | Carbamidomethylation; Oxidation (M) | C2:Carbamidomethylation:1000.00;M12:Oxidation (M):1000.00 | PEAKS DB |
| K.AGFAGDDAPR.A | N | 52.95 | 975.4410 | 10 | 3.9 | 488.7296 | 2 | 22.10 | 9 | F9:7134 | 31122021\_RID\_3262\_2a.raw |  | 3.3275E5 |  | 1.2847E6 |  |  |  |  | 2.7076E6 |  | 4 | 0 | 1 | 0 | 1 | 0 | 0 | 0 | 0 | 2 | 0 | 19 | 28 |  |  | PEAKS DB |
| R.VAPEEHPVLLTEAPLNPK.A | N | 51.47 | 1953.0570 | 18 | 0.5 | 652.0266 | 3 | 40.45 | 1 | F1:25900 | 13012022\_RID\_3262\_4a.raw | 8.5304E5 |  |  | 5.0748E5 |  |  |  | 7.2111E5 |  |  | 3 | 1 | 0 | 0 | 1 | 0 | 0 | 0 | 1 | 0 | 0 | 96 | 113 |  |  | PEAKS DB |
| K.EITALAPSTM(+15.99)K.I | N | 49.12 | 1176.6060 | 11 | 1.7 | 589.3113 | 2 | 30.85 | 6 | F6:17553 | 17012022\_RID\_3262\_9a.raw |  |  |  |  |  | 2.4825E5 |  |  |  |  | 1 | 0 | 0 | 0 | 0 | 0 | 1 | 0 | 0 | 0 | 0 | 316 | 326 | Oxidation (M) | M10:Oxidation (M):1000.00 | PEAKS DB |
| G.YALPHAILR.L | Y | 48.16 | 1052.6130 | 9 | 0.0 | 527.3138 | 2 | 35.63 | 1 | F1:21569 | 13012022\_RID\_3262\_4a.raw | 1.4154E6 |  |  |  |  |  |  |  |  |  | 1 | 1 | 0 | 0 | 0 | 0 | 0 | 0 | 0 | 0 | 0 | 169 | 177 |  |  | PEAKS DB |
| R.AVFPSIVGRPR.H | N | 45.70 | 1197.6981 | 11 | 1.5 | 400.2406 | 3 | 35.84 | 6 | F6:21794 | 17012022\_RID\_3262\_9a.raw | 1.4816E6 |  |  |  |  | 0 |  |  |  |  | 1 | 1 | 0 | 0 | 0 | 0 | 0 | 0 | 0 | 0 | 0 | 29 | 39 |  |  | PEAKS DB |
| R.DLTDYLMK.I | N | 43.13 | 997.4790 | 8 | 0.7 | 499.7471 | 2 | 51.80 | 6 | F6:36164 | 17012022\_RID\_3262\_9a.raw |  |  |  |  |  | 4.4466E5 |  |  |  |  | 1 | 0 | 0 | 0 | 0 | 0 | 1 | 0 | 0 | 0 | 0 | 184 | 191 |  |  | PEAKS DB |
| total 11 peptides |  |  |  |  |  |  |  |  |  |  |  |  |  |  |  |  |  |  |  |  |  |  |  |  |  |  |  |  |  |  |  |  |  |  |  |  |  |
| --- | --- | --- | --- | --- | --- | --- | --- | --- | --- | --- | --- | --- | --- | --- | --- | --- | --- | --- | --- | --- | --- | --- | --- | --- | --- | --- | --- | --- | --- | --- | --- | --- | --- | --- | --- | --- | --- |

JAV51372.1

back to list

  

| Protein Coverage
| Supporting Peptides
|

Protein Coverage:

Supporting Peptides:

| Peptide | Uniq | -10lgP | Mass | Length | ppm | m/z | z | RT | Fraction | Scan | Source File | Area F4 | Area F5 | Area F6 | Area F10 | Area F8 | Area F9 | Area F7 | Area F1 | Area F2 | Area F3 | #Feature | #Feature F4 | #Feature F5 | #Feature F6 | #Feature F10 | #Feature F8 | #Feature F9 | #Feature F7 | #Feature F1 | #Feature F2 | #Feature F3 | Start | End | PTM | AScore | Found By |
| --- | --- | --- | --- | --- | --- | --- | --- | --- | --- | --- | --- | --- | --- | --- | --- | --- | --- | --- | --- | --- | --- | --- | --- | --- | --- | --- | --- | --- | --- | --- | --- | --- | --- | --- | --- | --- | --- |
| K.SYELPDGQVITIGNER.F | N | 82.78 | 1789.8846 | 16 | 0.4 | 895.9500 | 2 | 49.87 | 8 | F8:28149 | 31122021\_RID\_3262\_1a.raw | 4.7745E5 |  |  |  |  | 6.0954E5 |  | 1.0043E6 | 3.4371E5 |  | 4 | 1 | 0 | 0 | 0 | 0 | 1 | 0 | 1 | 1 | 0 | 239 | 254 |  |  | PEAKS DB |
| K.LC(+57.02)YVALDFEQEMATAASSSSLEK.S | Y | 65.71 | 2549.1665 | 23 | -0.5 | 1275.5898 | 2 | 62.33 | 7 | F7:28143 | 21012022\_RID\_3262\_7a.raw |  |  |  |  |  |  | 1.0541E6 |  |  |  | 2 | 0 | 0 | 0 | 0 | 0 | 0 | 2 | 0 | 0 | 0 | 216 | 238 | Carbamidomethylation | C2:Carbamidomethylation:1000.00 | PEAKS DB |
| K.DSYVGDEAQSK.R | N | 65.50 | 1197.5149 | 11 | 1.5 | 599.7656 | 2 | 23.16 | 4 | F4:10278 | 17012022\_RID\_3262\_10a .raw | 2.7572E5 |  |  | 3.783E5 |  | 4.8292E5 |  | 7.9712E4 | 7.8692E4 |  | 5 | 1 | 0 | 0 | 1 | 0 | 1 | 0 | 1 | 1 | 0 | 51 | 61 |  |  | PEAKS DB |
| R.GYSFTTTAER.E | Y | 60.44 | 1131.5197 | 10 | -0.1 | 566.7670 | 2 | 28.78 | 8 | F8:11438 | 31122021\_RID\_3262\_1a.raw |  |  |  | 1.3304E6 |  |  | 2.0729E5 | 2.9994E5 | 2.4553E5 |  | 4 | 0 | 0 | 0 | 1 | 0 | 0 | 1 | 1 | 1 | 0 | 197 | 206 |  |  | PEAKS DB |
| K.LC(+57.02)YVALDFEQEM(+15.99)ATAASSSSLEK.S | Y | 54.72 | 2565.1614 | 23 | 0.4 | 856.0614 | 3 | 62.38 | 7 | F7:28152 | 21012022\_RID\_3262\_7a.raw |  |  |  |  |  |  | 1.0591E5 |  |  |  | 1 | 0 | 0 | 0 | 0 | 0 | 0 | 1 | 0 | 0 | 0 | 216 | 238 | Carbamidomethylation; Oxidation (M) | C2:Carbamidomethylation:1000.00;M12:Oxidation (M):1000.00 | PEAKS DB |
| K.AGFAGDDAPR.A | N | 52.95 | 975.4410 | 10 | 3.9 | 488.7296 | 2 | 22.10 | 9 | F9:7134 | 31122021\_RID\_3262\_2a.raw |  | 3.3275E5 |  | 1.2847E6 |  |  |  |  | 2.7076E6 |  | 4 | 0 | 1 | 0 | 1 | 0 | 0 | 0 | 0 | 2 | 0 | 19 | 28 |  |  | PEAKS DB |
| R.VAPEEHPVLLTEAPLNPK.A | N | 51.47 | 1953.0570 | 18 | 0.5 | 652.0266 | 3 | 40.45 | 1 | F1:25900 | 13012022\_RID\_3262\_4a.raw | 8.5304E5 |  |  | 5.0748E5 |  |  |  | 7.2111E5 |  |  | 3 | 1 | 0 | 0 | 1 | 0 | 0 | 0 | 1 | 0 | 0 | 96 | 113 |  |  | PEAKS DB |
| K.EITALAPSTM(+15.99)K.I | N | 49.12 | 1176.6060 | 11 | 1.7 | 589.3113 | 2 | 30.85 | 6 | F6:17553 | 17012022\_RID\_3262\_9a.raw |  |  |  |  |  | 2.4825E5 |  |  |  |  | 1 | 0 | 0 | 0 | 0 | 0 | 1 | 0 | 0 | 0 | 0 | 316 | 326 | Oxidation (M) | M10:Oxidation (M):1000.00 | PEAKS DB |
| G.YALPHAILR.L | Y | 48.16 | 1052.6130 | 9 | 0.0 | 527.3138 | 2 | 35.63 | 1 | F1:21569 | 13012022\_RID\_3262\_4a.raw | 1.4154E6 |  |  |  |  |  |  |  |  |  | 1 | 1 | 0 | 0 | 0 | 0 | 0 | 0 | 0 | 0 | 0 | 169 | 177 |  |  | PEAKS DB |
| R.AVFPSIVGRPR.H | N | 45.70 | 1197.6981 | 11 | 1.5 | 400.2406 | 3 | 35.84 | 6 | F6:21794 | 17012022\_RID\_3262\_9a.raw | 1.4816E6 |  |  |  |  | 0 |  |  |  |  | 1 | 1 | 0 | 0 | 0 | 0 | 0 | 0 | 0 | 0 | 0 | 29 | 39 |  |  | PEAKS DB |
| R.DLTDYLMK.I | N | 43.13 | 997.4790 | 8 | 0.7 | 499.7471 | 2 | 51.80 | 6 | F6:36164 | 17012022\_RID\_3262\_9a.raw |  |  |  |  |  | 4.4466E5 |  |  |  |  | 1 | 0 | 0 | 0 | 0 | 0 | 1 | 0 | 0 | 0 | 0 | 184 | 191 |  |  | PEAKS DB |
| total 11 peptides |  |  |  |  |  |  |  |  |  |  |  |  |  |  |  |  |  |  |  |  |  |  |  |  |  |  |  |  |  |  |  |  |  |  |  |  |  |
| --- | --- | --- | --- | --- | --- | --- | --- | --- | --- | --- | --- | --- | --- | --- | --- | --- | --- | --- | --- | --- | --- | --- | --- | --- | --- | --- | --- | --- | --- | --- | --- | --- | --- | --- | --- | --- | --- |

JAG45996.1

back to list

  

| Protein Coverage
| Supporting Peptides
|

Protein Coverage:

Supporting Peptides:

| Peptide | Uniq | -10lgP | Mass | Length | ppm | m/z | z | RT | Fraction | Scan | Source File | Area F4 | Area F5 | Area F6 | Area F10 | Area F8 | Area F9 | Area F7 | Area F1 | Area F2 | Area F3 | #Feature | #Feature F4 | #Feature F5 | #Feature F6 | #Feature F10 | #Feature F8 | #Feature F9 | #Feature F7 | #Feature F1 | #Feature F2 | #Feature F3 | Start | End | PTM | AScore | Found By |
| --- | --- | --- | --- | --- | --- | --- | --- | --- | --- | --- | --- | --- | --- | --- | --- | --- | --- | --- | --- | --- | --- | --- | --- | --- | --- | --- | --- | --- | --- | --- | --- | --- | --- | --- | --- | --- | --- |
| K.SYELPDGQVITIGNER.F | N | 82.78 | 1789.8846 | 16 | 0.4 | 895.9500 | 2 | 49.87 | 8 | F8:28149 | 31122021\_RID\_3262\_1a.raw | 4.7745E5 |  |  |  |  | 6.0954E5 |  | 1.0043E6 | 3.4371E5 |  | 4 | 1 | 0 | 0 | 0 | 0 | 1 | 0 | 1 | 1 | 0 | 239 | 254 |  |  | PEAKS DB |
| K.LC(+57.02)YVALDFEQEMATAASSSSLEK.S | Y | 65.71 | 2549.1665 | 23 | -0.5 | 1275.5898 | 2 | 62.33 | 7 | F7:28143 | 21012022\_RID\_3262\_7a.raw |  |  |  |  |  |  | 1.0541E6 |  |  |  | 2 | 0 | 0 | 0 | 0 | 0 | 0 | 2 | 0 | 0 | 0 | 216 | 238 | Carbamidomethylation | C2:Carbamidomethylation:1000.00 | PEAKS DB |
| K.DSYVGDEAQSK.R | N | 65.50 | 1197.5149 | 11 | 1.5 | 599.7656 | 2 | 23.16 | 4 | F4:10278 | 17012022\_RID\_3262\_10a .raw | 2.7572E5 |  |  | 3.783E5 |  | 4.8292E5 |  | 7.9712E4 | 7.8692E4 |  | 5 | 1 | 0 | 0 | 1 | 0 | 1 | 0 | 1 | 1 | 0 | 51 | 61 |  |  | PEAKS DB |
| R.GYSFTTTAER.E | Y | 60.44 | 1131.5197 | 10 | -0.1 | 566.7670 | 2 | 28.78 | 8 | F8:11438 | 31122021\_RID\_3262\_1a.raw |  |  |  | 1.3304E6 |  |  | 2.0729E5 | 2.9994E5 | 2.4553E5 |  | 4 | 0 | 0 | 0 | 1 | 0 | 0 | 1 | 1 | 1 | 0 | 197 | 206 |  |  | PEAKS DB |
| K.LC(+57.02)YVALDFEQEM(+15.99)ATAASSSSLEK.S | Y | 54.72 | 2565.1614 | 23 | 0.4 | 856.0614 | 3 | 62.38 | 7 | F7:28152 | 21012022\_RID\_3262\_7a.raw |  |  |  |  |  |  | 1.0591E5 |  |  |  | 1 | 0 | 0 | 0 | 0 | 0 | 0 | 1 | 0 | 0 | 0 | 216 | 238 | Carbamidomethylation; Oxidation (M) | C2:Carbamidomethylation:1000.00;M12:Oxidation (M):1000.00 | PEAKS DB |
| K.AGFAGDDAPR.A | N | 52.95 | 975.4410 | 10 | 3.9 | 488.7296 | 2 | 22.10 | 9 | F9:7134 | 31122021\_RID\_3262\_2a.raw |  | 3.3275E5 |  | 1.2847E6 |  |  |  |  | 2.7076E6 |  | 4 | 0 | 1 | 0 | 1 | 0 | 0 | 0 | 0 | 2 | 0 | 19 | 28 |  |  | PEAKS DB |
| R.VAPEEHPVLLTEAPLNPK.A | N | 51.47 | 1953.0570 | 18 | 0.5 | 652.0266 | 3 | 40.45 | 1 | F1:25900 | 13012022\_RID\_3262\_4a.raw | 8.5304E5 |  |  | 5.0748E5 |  |  |  | 7.2111E5 |  |  | 3 | 1 | 0 | 0 | 1 | 0 | 0 | 0 | 1 | 0 | 0 | 96 | 113 |  |  | PEAKS DB |
| K.EITALAPSTM(+15.99)K.I | N | 49.12 | 1176.6060 | 11 | 1.7 | 589.3113 | 2 | 30.85 | 6 | F6:17553 | 17012022\_RID\_3262\_9a.raw |  |  |  |  |  | 2.4825E5 |  |  |  |  | 1 | 0 | 0 | 0 | 0 | 0 | 1 | 0 | 0 | 0 | 0 | 316 | 326 | Oxidation (M) | M10:Oxidation (M):1000.00 | PEAKS DB |
| G.YALPHAILR.L | Y | 48.16 | 1052.6130 | 9 | 0.0 | 527.3138 | 2 | 35.63 | 1 | F1:21569 | 13012022\_RID\_3262\_4a.raw | 1.4154E6 |  |  |  |  |  |  |  |  |  | 1 | 1 | 0 | 0 | 0 | 0 | 0 | 0 | 0 | 0 | 0 | 169 | 177 |  |  | PEAKS DB |
| R.AVFPSIVGRPR.H | N | 45.70 | 1197.6981 | 11 | 1.5 | 400.2406 | 3 | 35.84 | 6 | F6:21794 | 17012022\_RID\_3262\_9a.raw | 1.4816E6 |  |  |  |  | 0 |  |  |  |  | 1 | 1 | 0 | 0 | 0 | 0 | 0 | 0 | 0 | 0 | 0 | 29 | 39 |  |  | PEAKS DB |
| R.DLTDYLMK.I | N | 43.13 | 997.4790 | 8 | 0.7 | 499.7471 | 2 | 51.80 | 6 | F6:36164 | 17012022\_RID\_3262\_9a.raw |  |  |  |  |  | 4.4466E5 |  |  |  |  | 1 | 0 | 0 | 0 | 0 | 0 | 1 | 0 | 0 | 0 | 0 | 184 | 191 |  |  | PEAKS DB |
| total 11 peptides |  |  |  |  |  |  |  |  |  |  |  |  |  |  |  |  |  |  |  |  |  |  |  |  |  |  |  |  |  |  |  |  |  |  |  |  |  |
| --- | --- | --- | --- | --- | --- | --- | --- | --- | --- | --- | --- | --- | --- | --- | --- | --- | --- | --- | --- | --- | --- | --- | --- | --- | --- | --- | --- | --- | --- | --- | --- | --- | --- | --- | --- | --- | --- |

JAB54395.1

back to list

  

| Protein Coverage
| Supporting Peptides
|

Protein Coverage:

Supporting Peptides:

| Peptide | Uniq | -10lgP | Mass | Length | ppm | m/z | z | RT | Fraction | Scan | Source File | Area F4 | Area F5 | Area F6 | Area F10 | Area F8 | Area F9 | Area F7 | Area F1 | Area F2 | Area F3 | #Feature | #Feature F4 | #Feature F5 | #Feature F6 | #Feature F10 | #Feature F8 | #Feature F9 | #Feature F7 | #Feature F1 | #Feature F2 | #Feature F3 | Start | End | PTM | AScore | Found By |
| --- | --- | --- | --- | --- | --- | --- | --- | --- | --- | --- | --- | --- | --- | --- | --- | --- | --- | --- | --- | --- | --- | --- | --- | --- | --- | --- | --- | --- | --- | --- | --- | --- | --- | --- | --- | --- | --- |
| K.SYELPDGQVITIGNER.F | N | 82.78 | 1789.8846 | 16 | 0.4 | 895.9500 | 2 | 49.87 | 8 | F8:28149 | 31122021\_RID\_3262\_1a.raw | 4.7745E5 |  |  |  |  | 6.0954E5 |  | 1.0043E6 | 3.4371E5 |  | 4 | 1 | 0 | 0 | 0 | 0 | 1 | 0 | 1 | 1 | 0 | 239 | 254 |  |  | PEAKS DB |
| K.LC(+57.02)YVALDFEQEMATAASSSSLEK.S | Y | 65.71 | 2549.1665 | 23 | -0.5 | 1275.5898 | 2 | 62.33 | 7 | F7:28143 | 21012022\_RID\_3262\_7a.raw |  |  |  |  |  |  | 1.0541E6 |  |  |  | 2 | 0 | 0 | 0 | 0 | 0 | 0 | 2 | 0 | 0 | 0 | 216 | 238 | Carbamidomethylation | C2:Carbamidomethylation:1000.00 | PEAKS DB |
| K.DSYVGDEAQSK.R | N | 65.50 | 1197.5149 | 11 | 1.5 | 599.7656 | 2 | 23.16 | 4 | F4:10278 | 17012022\_RID\_3262\_10a .raw | 2.7572E5 |  |  | 3.783E5 |  | 4.8292E5 |  | 7.9712E4 | 7.8692E4 |  | 5 | 1 | 0 | 0 | 1 | 0 | 1 | 0 | 1 | 1 | 0 | 51 | 61 |  |  | PEAKS DB |
| R.GYSFTTTAER.E | Y | 60.44 | 1131.5197 | 10 | -0.1 | 566.7670 | 2 | 28.78 | 8 | F8:11438 | 31122021\_RID\_3262\_1a.raw |  |  |  | 1.3304E6 |  |  | 2.0729E5 | 2.9994E5 | 2.4553E5 |  | 4 | 0 | 0 | 0 | 1 | 0 | 0 | 1 | 1 | 1 | 0 | 197 | 206 |  |  | PEAKS DB |
| K.LC(+57.02)YVALDFEQEM(+15.99)ATAASSSSLEK.S | Y | 54.72 | 2565.1614 | 23 | 0.4 | 856.0614 | 3 | 62.38 | 7 | F7:28152 | 21012022\_RID\_3262\_7a.raw |  |  |  |  |  |  | 1.0591E5 |  |  |  | 1 | 0 | 0 | 0 | 0 | 0 | 0 | 1 | 0 | 0 | 0 | 216 | 238 | Carbamidomethylation; Oxidation (M) | C2:Carbamidomethylation:1000.00;M12:Oxidation (M):1000.00 | PEAKS DB |
| K.AGFAGDDAPR.A | N | 52.95 | 975.4410 | 10 | 3.9 | 488.7296 | 2 | 22.10 | 9 | F9:7134 | 31122021\_RID\_3262\_2a.raw |  | 3.3275E5 |  | 1.2847E6 |  |  |  |  | 2.7076E6 |  | 4 | 0 | 1 | 0 | 1 | 0 | 0 | 0 | 0 | 2 | 0 | 19 | 28 |  |  | PEAKS DB |
| R.VAPEEHPVLLTEAPLNPK.A | N | 51.47 | 1953.0570 | 18 | 0.5 | 652.0266 | 3 | 40.45 | 1 | F1:25900 | 13012022\_RID\_3262\_4a.raw | 8.5304E5 |  |  | 5.0748E5 |  |  |  | 7.2111E5 |  |  | 3 | 1 | 0 | 0 | 1 | 0 | 0 | 0 | 1 | 0 | 0 | 96 | 113 |  |  | PEAKS DB |
| K.EITALAPSTM(+15.99)K.I | N | 49.12 | 1176.6060 | 11 | 1.7 | 589.3113 | 2 | 30.85 | 6 | F6:17553 | 17012022\_RID\_3262\_9a.raw |  |  |  |  |  | 2.4825E5 |  |  |  |  | 1 | 0 | 0 | 0 | 0 | 0 | 1 | 0 | 0 | 0 | 0 | 316 | 326 | Oxidation (M) | M10:Oxidation (M):1000.00 | PEAKS DB |
| G.YALPHAILR.L | Y | 48.16 | 1052.6130 | 9 | 0.0 | 527.3138 | 2 | 35.63 | 1 | F1:21569 | 13012022\_RID\_3262\_4a.raw | 1.4154E6 |  |  |  |  |  |  |  |  |  | 1 | 1 | 0 | 0 | 0 | 0 | 0 | 0 | 0 | 0 | 0 | 169 | 177 |  |  | PEAKS DB |
| R.AVFPSIVGRPR.H | N | 45.70 | 1197.6981 | 11 | 1.5 | 400.2406 | 3 | 35.84 | 6 | F6:21794 | 17012022\_RID\_3262\_9a.raw | 1.4816E6 |  |  |  |  | 0 |  |  |  |  | 1 | 1 | 0 | 0 | 0 | 0 | 0 | 0 | 0 | 0 | 0 | 29 | 39 |  |  | PEAKS DB |
| R.DLTDYLMK.I | N | 43.13 | 997.4790 | 8 | 0.7 | 499.7471 | 2 | 51.80 | 6 | F6:36164 | 17012022\_RID\_3262\_9a.raw |  |  |  |  |  | 4.4466E5 |  |  |  |  | 1 | 0 | 0 | 0 | 0 | 0 | 1 | 0 | 0 | 0 | 0 | 184 | 191 |  |  | PEAKS DB |
| total 11 peptides |  |  |  |  |  |  |  |  |  |  |  |  |  |  |  |  |  |  |  |  |  |  |  |  |  |  |  |  |  |  |  |  |  |  |  |  |  |
| --- | --- | --- | --- | --- | --- | --- | --- | --- | --- | --- | --- | --- | --- | --- | --- | --- | --- | --- | --- | --- | --- | --- | --- | --- | --- | --- | --- | --- | --- | --- | --- | --- | --- | --- | --- | --- | --- |

ETE74025.1

back to list

  

| Protein Coverage
| Supporting Peptides
|

Protein Coverage:

Supporting Peptides:

| Peptide | Uniq | -10lgP | Mass | Length | ppm | m/z | z | RT | Fraction | Scan | Source File | Area F4 | Area F5 | Area F6 | Area F10 | Area F8 | Area F9 | Area F7 | Area F1 | Area F2 | Area F3 | #Feature | #Feature F4 | #Feature F5 | #Feature F6 | #Feature F10 | #Feature F8 | #Feature F9 | #Feature F7 | #Feature F1 | #Feature F2 | #Feature F3 | Start | End | PTM | AScore | Found By |
| --- | --- | --- | --- | --- | --- | --- | --- | --- | --- | --- | --- | --- | --- | --- | --- | --- | --- | --- | --- | --- | --- | --- | --- | --- | --- | --- | --- | --- | --- | --- | --- | --- | --- | --- | --- | --- | --- |
| K.SYELPDGQVITIGNER.F | N | 82.78 | 1789.8846 | 16 | 0.4 | 895.9500 | 2 | 49.87 | 8 | F8:28149 | 31122021\_RID\_3262\_1a.raw | 4.7745E5 |  |  |  |  | 6.0954E5 |  | 1.0043E6 | 3.4371E5 |  | 4 | 1 | 0 | 0 | 0 | 0 | 1 | 0 | 1 | 1 | 0 | 239 | 254 |  |  | PEAKS DB |
| K.LC(+57.02)YVALDFEQEMATAASSSSLEK.S | Y | 65.71 | 2549.1665 | 23 | -0.5 | 1275.5898 | 2 | 62.33 | 7 | F7:28143 | 21012022\_RID\_3262\_7a.raw |  |  |  |  |  |  | 1.0541E6 |  |  |  | 2 | 0 | 0 | 0 | 0 | 0 | 0 | 2 | 0 | 0 | 0 | 216 | 238 | Carbamidomethylation | C2:Carbamidomethylation:1000.00 | PEAKS DB |
| K.DSYVGDEAQSK.R | N | 65.50 | 1197.5149 | 11 | 1.5 | 599.7656 | 2 | 23.16 | 4 | F4:10278 | 17012022\_RID\_3262\_10a .raw | 2.7572E5 |  |  | 3.783E5 |  | 4.8292E5 |  | 7.9712E4 | 7.8692E4 |  | 5 | 1 | 0 | 0 | 1 | 0 | 1 | 0 | 1 | 1 | 0 | 51 | 61 |  |  | PEAKS DB |
| R.GYSFTTTAER.E | Y | 60.44 | 1131.5197 | 10 | -0.1 | 566.7670 | 2 | 28.78 | 8 | F8:11438 | 31122021\_RID\_3262\_1a.raw |  |  |  | 1.3304E6 |  |  | 2.0729E5 | 2.9994E5 | 2.4553E5 |  | 4 | 0 | 0 | 0 | 1 | 0 | 0 | 1 | 1 | 1 | 0 | 197 | 206 |  |  | PEAKS DB |
| K.LC(+57.02)YVALDFEQEM(+15.99)ATAASSSSLEK.S | Y | 54.72 | 2565.1614 | 23 | 0.4 | 856.0614 | 3 | 62.38 | 7 | F7:28152 | 21012022\_RID\_3262\_7a.raw |  |  |  |  |  |  | 1.0591E5 |  |  |  | 1 | 0 | 0 | 0 | 0 | 0 | 0 | 1 | 0 | 0 | 0 | 216 | 238 | Carbamidomethylation; Oxidation (M) | C2:Carbamidomethylation:1000.00;M12:Oxidation (M):1000.00 | PEAKS DB |
| K.AGFAGDDAPR.A | N | 52.95 | 975.4410 | 10 | 3.9 | 488.7296 | 2 | 22.10 | 9 | F9:7134 | 31122021\_RID\_3262\_2a.raw |  | 3.3275E5 |  | 1.2847E6 |  |  |  |  | 2.7076E6 |  | 4 | 0 | 1 | 0 | 1 | 0 | 0 | 0 | 0 | 2 | 0 | 19 | 28 |  |  | PEAKS DB |
| R.VAPEEHPVLLTEAPLNPK.A | N | 51.47 | 1953.0570 | 18 | 0.5 | 652.0266 | 3 | 40.45 | 1 | F1:25900 | 13012022\_RID\_3262\_4a.raw | 8.5304E5 |  |  | 5.0748E5 |  |  |  | 7.2111E5 |  |  | 3 | 1 | 0 | 0 | 1 | 0 | 0 | 0 | 1 | 0 | 0 | 96 | 113 |  |  | PEAKS DB |
| K.EITALAPSTM(+15.99)K.I | N | 49.12 | 1176.6060 | 11 | 1.7 | 589.3113 | 2 | 30.85 | 6 | F6:17553 | 17012022\_RID\_3262\_9a.raw |  |  |  |  |  | 2.4825E5 |  |  |  |  | 1 | 0 | 0 | 0 | 0 | 0 | 1 | 0 | 0 | 0 | 0 | 316 | 326 | Oxidation (M) | M10:Oxidation (M):1000.00 | PEAKS DB |
| G.YALPHAILR.L | Y | 48.16 | 1052.6130 | 9 | 0.0 | 527.3138 | 2 | 35.63 | 1 | F1:21569 | 13012022\_RID\_3262\_4a.raw | 1.4154E6 |  |  |  |  |  |  |  |  |  | 1 | 1 | 0 | 0 | 0 | 0 | 0 | 0 | 0 | 0 | 0 | 169 | 177 |  |  | PEAKS DB |
| R.AVFPSIVGRPR.H | N | 45.70 | 1197.6981 | 11 | 1.5 | 400.2406 | 3 | 35.84 | 6 | F6:21794 | 17012022\_RID\_3262\_9a.raw | 1.4816E6 |  |  |  |  | 0 |  |  |  |  | 1 | 1 | 0 | 0 | 0 | 0 | 0 | 0 | 0 | 0 | 0 | 29 | 39 |  |  | PEAKS DB |
| R.DLTDYLMK.I | N | 43.13 | 997.4790 | 8 | 0.7 | 499.7471 | 2 | 51.80 | 6 | F6:36164 | 17012022\_RID\_3262\_9a.raw |  |  |  |  |  | 4.4466E5 |  |  |  |  | 1 | 0 | 0 | 0 | 0 | 0 | 1 | 0 | 0 | 0 | 0 | 184 | 191 |  |  | PEAKS DB |
| total 11 peptides |  |  |  |  |  |  |  |  |  |  |  |  |  |  |  |  |  |  |  |  |  |  |  |  |  |  |  |  |  |  |  |  |  |  |  |  |  |
| --- | --- | --- | --- | --- | --- | --- | --- | --- | --- | --- | --- | --- | --- | --- | --- | --- | --- | --- | --- | --- | --- | --- | --- | --- | --- | --- | --- | --- | --- | --- | --- | --- | --- | --- | --- | --- | --- |

XP\_026530513.1

back to list

  

| Protein Coverage
| Supporting Peptides
|

Protein Coverage:

Supporting Peptides:

| Peptide | Uniq | -10lgP | Mass | Length | ppm | m/z | z | RT | Fraction | Scan | Source File | Area F4 | Area F5 | Area F6 | Area F10 | Area F8 | Area F9 | Area F7 | Area F1 | Area F2 | Area F3 | #Feature | #Feature F4 | #Feature F5 | #Feature F6 | #Feature F10 | #Feature F8 | #Feature F9 | #Feature F7 | #Feature F1 | #Feature F2 | #Feature F3 | Start | End | PTM | AScore | Found By |
| --- | --- | --- | --- | --- | --- | --- | --- | --- | --- | --- | --- | --- | --- | --- | --- | --- | --- | --- | --- | --- | --- | --- | --- | --- | --- | --- | --- | --- | --- | --- | --- | --- | --- | --- | --- | --- | --- |
| R.GDDIPYTPVFYAYTLLTK.T | N | 69.16 | 2076.0454 | 18 | 0.5 | 1039.0305 | 2 | 73.61 | 8 | F8:46146 | 31122021\_RID\_3262\_1a.raw |  |  |  |  |  |  |  | 3.5587E6 | 8.7984E5 |  | 3 | 0 | 0 | 0 | 0 | 0 | 0 | 0 | 2 | 1 | 0 | 254 | 271 |  |  | PEAKS DB |
| R.IEDVALVVQAK.T | N | 65.14 | 1183.6812 | 11 | 0.4 | 592.8481 | 2 | 40.58 | 9 | F9:21971 | 31122021\_RID\_3262\_2a.raw |  |  |  |  |  |  |  | 6.1145E6 | 1.2348E6 |  | 2 | 0 | 0 | 0 | 0 | 0 | 0 | 0 | 1 | 1 | 0 | 570 | 580 |  |  | PEAKS DB |
| K.MMEAAHIR.D | N | 59.97 | 957.4525 | 8 | 0.4 | 479.7337 | 2 | 19.60 | 8 | F8:4812 | 31122021\_RID\_3262\_1a.raw |  |  |  |  |  |  |  | 3.7959E5 | 9.5521E4 |  | 2 | 0 | 0 | 0 | 0 | 0 | 0 | 0 | 1 | 1 | 0 | 366 | 373 |  |  | PEAKS DB |
| R.TIHWGEPTAFQK.E | N | 59.60 | 1413.7041 | 12 | 0.4 | 472.2422 | 3 | 34.27 | 8 | F8:15881 | 31122021\_RID\_3262\_1a.raw |  |  |  |  |  |  |  | 2.8249E6 | 0 |  | 3 | 0 | 0 | 0 | 0 | 0 | 0 | 0 | 3 | 0 | 0 | 467 | 478 |  |  | PEAKS DB |
| K.VVSLVPYAR.N | N | 58.37 | 1002.5862 | 9 | 0.3 | 502.3005 | 2 | 38.96 | 8 | F8:19771 | 31122021\_RID\_3262\_1a.raw | 1.0512E6 |  |  |  |  |  |  | 4.0529E6 | 7.1037E5 |  | 3 | 1 | 0 | 0 | 0 | 0 | 0 | 0 | 1 | 1 | 0 | 595 | 603 |  |  | PEAKS DB |
| R.VLM(+15.99)GNIDLSK.L | N | 56.40 | 1104.5848 | 10 | 1.7 | 553.3007 | 2 | 32.94 | 8 | F8:14847 | 31122021\_RID\_3262\_1a.raw |  |  |  |  |  |  |  | 2.0225E6 | 5.6608E5 |  | 3 | 0 | 0 | 0 | 0 | 0 | 0 | 0 | 1 | 2 | 0 | 484 | 493 | Oxidation (M) | M3:Oxidation (M):1000.00 | PEAKS DB |
| K.MM(+15.99)EAAHIR.D | N | 54.61 | 973.4473 | 8 | -0.3 | 487.7308 | 2 | 15.87 | 8 | F8:2900 | 31122021\_RID\_3262\_1a.raw |  |  |  |  |  |  |  | 1.3887E5 |  |  | 3 | 0 | 0 | 0 | 0 | 0 | 0 | 0 | 3 | 0 | 0 | 366 | 373 | Oxidation (M) | M2:Oxidation (M):14.02 | PEAKS DB |
| K.AVLFTDSR.Y | Y | 53.41 | 907.4763 | 8 | -0.4 | 454.7452 | 2 | 31.83 | 8 | F8:13814 | 31122021\_RID\_3262\_1a.raw |  |  |  |  |  |  |  | 2.1251E6 | 6.9519E5 |  | 2 | 0 | 0 | 0 | 0 | 0 | 0 | 0 | 1 | 1 | 0 | 110 | 117 |  |  | PEAKS DB |
| R.VLMGNIDLSK.L | N | 50.63 | 1088.5900 | 10 | 0.9 | 545.3027 | 2 | 40.89 | 8 | F8:21051 | 31122021\_RID\_3262\_1a.raw |  |  |  |  |  |  |  | 3.1E6 | 6.693E5 |  | 2 | 0 | 0 | 0 | 0 | 0 | 0 | 0 | 1 | 1 | 0 | 484 | 493 |  |  | PEAKS DB |
| K.M(+15.99)MEAAHIR.D | N | 49.26 | 973.4473 | 8 | 0.0 | 487.7310 | 2 | 16.16 | 8 | F8:3110 | 31122021\_RID\_3262\_1a.raw |  |  |  |  |  |  |  | 1.1219E5 | 2.11E4 |  | 3 | 0 | 0 | 0 | 0 | 0 | 0 | 0 | 2 | 1 | 0 | 366 | 373 | Oxidation (M) | M1:Oxidation (M):12.28 | PEAKS DB |
| R.YWIQAER.Q | N | 47.48 | 964.4766 | 7 | -0.6 | 483.2453 | 2 | 31.76 | 9 | F9:14735 | 31122021\_RID\_3262\_2a.raw |  |  |  |  |  |  |  | 1.6204E6 | 4.2744E5 |  | 2 | 0 | 0 | 0 | 0 | 0 | 0 | 0 | 1 | 1 | 0 | 118 | 124 |  |  | PEAKS DB |
| K.NTEPFSHASLL.A | N | 46.54 | 1214.5931 | 11 | 0.1 | 608.3039 | 2 | 41.96 | 8 | F8:22010 | 31122021\_RID\_3262\_1a.raw |  |  |  |  |  |  |  | 3.7494E5 |  |  | 1 | 0 | 0 | 0 | 0 | 0 | 0 | 0 | 1 | 0 | 0 | 649 | 659 |  |  | PEAKS DB |
| K.GM(+15.99)FTSIEPGYYHDGEFGIR.I | N | 44.23 | 2190.9680 | 19 | 0.6 | 731.3304 | 3 | 45.51 | 8 | F8:24644 | 31122021\_RID\_3262\_1a.raw |  |  |  |  |  |  |  | 3.9301E5 |  |  | 1 | 0 | 0 | 0 | 0 | 0 | 0 | 0 | 1 | 0 | 0 | 551 | 569 | Oxidation (M) | M2:Oxidation (M):1000.00 | PEAKS DB |
| R.LPPPTNTIYR.L | N | 44.19 | 1170.6396 | 10 | 0.6 | 586.3275 | 2 | 32.12 | 8 | F8:14118 | 31122021\_RID\_3262\_1a.raw |  |  |  |  |  |  |  | 4.4687E6 |  |  | 1 | 0 | 0 | 0 | 0 | 0 | 0 | 0 | 1 | 0 | 0 | 198 | 207 |  |  | PEAKS DB |
| Q.YLVWLEK.N | N | 40.96 | 949.5273 | 7 | 0.1 | 475.7710 | 2 | 45.31 | 9 | F9:25829 | 31122021\_RID\_3262\_2a.raw |  |  |  |  |  |  |  |  | 7.199E5 |  | 1 | 0 | 0 | 0 | 0 | 0 | 0 | 0 | 0 | 1 | 0 | 381 | 387 |  |  | PEAKS DB |
| total 15 peptides |  |  |  |  |  |  |  |  |  |  |  |  |  |  |  |  |  |  |  |  |  |  |  |  |  |  |  |  |  |  |  |  |  |  |  |  |  |
| --- | --- | --- | --- | --- | --- | --- | --- | --- | --- | --- | --- | --- | --- | --- | --- | --- | --- | --- | --- | --- | --- | --- | --- | --- | --- | --- | --- | --- | --- | --- | --- | --- | --- | --- | --- | --- | --- |

XP\_032067333.1

back to list

  

| Protein Coverage
| Supporting Peptides
|

Protein Coverage:

Supporting Peptides:

| Peptide | Uniq | -10lgP | Mass | Length | ppm | m/z | z | RT | Fraction | Scan | Source File | Area F4 | Area F5 | Area F6 | Area F10 | Area F8 | Area F9 | Area F7 | Area F1 | Area F2 | Area F3 | #Feature | #Feature F4 | #Feature F5 | #Feature F6 | #Feature F10 | #Feature F8 | #Feature F9 | #Feature F7 | #Feature F1 | #Feature F2 | #Feature F3 | Start | End | PTM | AScore | Found By |
| --- | --- | --- | --- | --- | --- | --- | --- | --- | --- | --- | --- | --- | --- | --- | --- | --- | --- | --- | --- | --- | --- | --- | --- | --- | --- | --- | --- | --- | --- | --- | --- | --- | --- | --- | --- | --- | --- |
| K.LSLDVEIATYR.K | Y | 63.62 | 1278.6819 | 11 | 0.1 | 640.3483 | 2 | 49.18 | 8 | F8:27724 | 31122021\_RID\_3262\_1a.raw |  |  |  |  |  |  |  | 7.118E5 |  |  | 1 | 0 | 0 | 0 | 0 | 0 | 0 | 0 | 1 | 0 | 0 | 448 | 458 |  |  | PEAKS DB |
| S.KYEDEINK.R | N | 59.49 | 1037.5029 | 8 | 0.1 | 519.7588 | 2 | 17.64 | 6 | F6:5589 | 17012022\_RID\_3262\_9a.raw |  |  |  | 7.1505E6 |  | 8.6198E6 |  |  |  |  | 2 | 0 | 0 | 0 | 1 | 0 | 1 | 0 | 0 | 0 | 0 | 244 | 251 |  |  | PEAKS DB |
| K.FASFIDKVR.F | N | 59.23 | 1081.5920 | 9 | 0.3 | 541.8035 | 2 | 35.45 | 6 | F6:21540 | 17012022\_RID\_3262\_9a.raw | 1.5335E7 |  |  | 9.5108E6 |  | 1.2654E7 |  | 1.0527E7 | 1.5026E6 |  | 9 | 1 | 0 | 0 | 3 | 0 | 1 | 0 | 3 | 1 | 0 | 166 | 174 |  |  | PEAKS DB |
| S.VILSMDNNR.Y | N | 54.65 | 1060.5336 | 9 | 0.7 | 531.2744 | 2 | 30.82 | 8 | F8:13227 | 31122021\_RID\_3262\_1a.raw |  |  |  |  |  | 2.3978E6 |  | 7.4496E5 | 2.5983E5 |  | 3 | 0 | 0 | 0 | 0 | 0 | 1 | 0 | 1 | 1 | 0 | 310 | 318 |  |  | PEAKS DB |
| S.KYEDEINKR.T | N | 54.63 | 1193.6040 | 9 | -0.3 | 597.8091 | 2 | 16.55 | 6 | F6:4717 | 17012022\_RID\_3262\_9a.raw | 2.4731E7 | 1.7851E6 |  | 5.25E7 |  | 7.1702E7 | 3.2062E6 |  |  |  | 9 | 1 | 1 | 0 | 2 | 0 | 3 | 2 | 0 | 0 | 0 | 244 | 252 |  |  | PEAKS DB |
| K.YEDEINKR.T | N | 54.27 | 1065.5090 | 8 | 0.2 | 533.7619 | 2 | 17.06 | 6 | F6:5136 | 17012022\_RID\_3262\_9a.raw |  |  |  | 3.7263E6 |  | 5.4143E6 | 1.8172E5 |  |  |  | 3 | 0 | 0 | 0 | 1 | 0 | 1 | 1 | 0 | 0 | 0 | 245 | 252 |  |  | PEAKS DB |
| K.FASFIDK.V | N | 52.67 | 826.4225 | 7 | 0.7 | 414.2188 | 2 | 36.24 | 6 | F6:22289 | 17012022\_RID\_3262\_9a.raw | 2.5611E7 | 1.8348E7 | 6.6379E6 | 1.2204E8 |  | 1.3051E8 | 4.5017E6 | 4.1444E7 | 1.2872E7 |  | 15 | 2 | 2 | 1 | 4 | 0 | 3 | 1 | 1 | 1 | 0 | 166 | 172 |  |  | PEAKS DB |
| S.LDVEIATYR.K | N | 51.71 | 1078.5658 | 9 | 5.1 | 540.2929 | 2 | 39.98 | 8 | F8:20433 | 31122021\_RID\_3262\_1a.raw |  |  |  |  |  |  |  | 2.3839E5 |  |  | 1 | 0 | 0 | 0 | 0 | 0 | 0 | 0 | 1 | 0 | 0 | 450 | 458 |  |  | PEAKS DB |
| R.SKYEDEINKR.T | N | 51.62 | 1280.6360 | 10 | 0.7 | 427.8862 | 3 | 18.28 | 4 | F4:6025 | 17012022\_RID\_3262\_10a .raw | 5.5028E5 |  |  | 9.8337E5 |  | 1.3366E6 |  | 3.7836E4 |  |  | 4 | 1 | 0 | 0 | 1 | 0 | 1 | 0 | 1 | 0 | 0 | 243 | 252 |  |  | PEAKS DB |
| R.SKYEDEINK.R | N | 49.31 | 1124.5349 | 9 | -0.2 | 563.2746 | 2 | 18.71 | 6 | F6:6604 | 17012022\_RID\_3262\_9a.raw |  |  |  | 1.1335E5 |  | 1.5841E5 |  |  |  |  | 2 | 0 | 0 | 0 | 1 | 0 | 1 | 0 | 0 | 0 | 0 | 243 | 251 |  |  | PEAKS DB |
| R.GELALKDAR.Q | N | 49.06 | 971.5399 | 9 | -0.6 | 486.7769 | 2 | 22.10 | 6 | F6:9646 | 17012022\_RID\_3262\_9a.raw |  |  |  | 1.6772E6 |  | 1.879E6 | 8.5932E4 |  |  |  | 3 | 0 | 0 | 0 | 1 | 0 | 1 | 1 | 0 | 0 | 0 | 408 | 416 |  |  | PEAKS DB |
| K.LAELEDALQK.A | N | 46.67 | 1128.6027 | 10 | 1.2 | 565.3093 | 2 | 38.43 | 6 | F6:24252 | 17012022\_RID\_3262\_9a.raw |  |  |  |  |  | 2.7711E5 |  |  |  |  | 1 | 0 | 0 | 0 | 0 | 0 | 1 | 0 | 0 | 0 | 0 | 419 | 428 |  |  | PEAKS DB |
| K.FASFIDKV.R | N | 43.37 | 925.4908 | 8 | 0.2 | 463.7528 | 2 | 44.26 | 4 | F4:28348 | 17012022\_RID\_3262\_10a .raw |  |  |  | 1.1458E7 |  |  |  | 5.4178E6 | 1.9415E6 |  | 3 | 0 | 0 | 0 | 1 | 0 | 0 | 0 | 1 | 1 | 0 | 166 | 173 |  |  | PEAKS DB |
| R.FLEQENKVLETK.W | N | 42.41 | 1476.7823 | 12 | 0.9 | 739.3991 | 2 | 35.03 | 4 | F4:20948 | 17012022\_RID\_3262\_10a .raw |  |  |  | 0 |  |  |  |  |  |  | 0 | 0 | 0 | 0 | 0 | 0 | 0 | 0 | 0 | 0 | 0 | 175 | 186 |  |  | PEAKS DB |
| R.KLLEGEEYR.L | N | 41.07 | 1135.5873 | 9 | 0.2 | 379.5364 | 3 | 24.42 | 4 | F4:11454 | 17012022\_RID\_3262\_10a .raw |  |  |  | 1.9768E5 |  |  |  |  |  |  | 1 | 0 | 0 | 0 | 1 | 0 | 0 | 0 | 0 | 0 | 0 | 459 | 467 |  |  | PEAKS DB |
| total 15 peptides |  |  |  |  |  |  |  |  |  |  |  |  |  |  |  |  |  |  |  |  |  |  |  |  |  |  |  |  |  |  |  |  |  |  |  |  |  |
| --- | --- | --- | --- | --- | --- | --- | --- | --- | --- | --- | --- | --- | --- | --- | --- | --- | --- | --- | --- | --- | --- | --- | --- | --- | --- | --- | --- | --- | --- | --- | --- | --- | --- | --- | --- | --- | --- |

XP\_026576301.1

back to list

  

| Protein Coverage
| Supporting Peptides
|

Protein Coverage:

Supporting Peptides:

| Peptide | Uniq | -10lgP | Mass | Length | ppm | m/z | z | RT | Fraction | Scan | Source File | Area F4 | Area F5 | Area F6 | Area F10 | Area F8 | Area F9 | Area F7 | Area F1 | Area F2 | Area F3 | #Feature | #Feature F4 | #Feature F5 | #Feature F6 | #Feature F10 | #Feature F8 | #Feature F9 | #Feature F7 | #Feature F1 | #Feature F2 | #Feature F3 | Start | End | PTM | AScore | Found By |
| --- | --- | --- | --- | --- | --- | --- | --- | --- | --- | --- | --- | --- | --- | --- | --- | --- | --- | --- | --- | --- | --- | --- | --- | --- | --- | --- | --- | --- | --- | --- | --- | --- | --- | --- | --- | --- | --- |
| R.NEGTATYAAAVLFR.I | Y | 74.08 | 1482.7467 | 14 | 0.2 | 742.3808 | 2 | 48.27 | 9 | F9:28127 | 31122021\_RID\_3262\_2a.raw | 4.317E5 |  |  |  |  | 1.024E6 |  | 1.4003E6 | 4.6202E5 |  | 5 | 1 | 0 | 0 | 0 | 0 | 1 | 0 | 2 | 1 | 0 | 638 | 651 |  |  | PEAKS DB |
| R.LNYGIPAIVK.L | Y | 65.66 | 1086.6437 | 10 | 0.0 | 544.3291 | 2 | 45.04 | 8 | F8:24415 | 31122021\_RID\_3262\_1a.raw |  |  |  | 2.5539E6 |  | 2.6128E6 |  | 1.8129E6 | 7.9277E5 |  | 4 | 0 | 0 | 0 | 1 | 0 | 1 | 0 | 1 | 1 | 0 | 478 | 487 |  |  | PEAKS DB |
| R.VSVELTNSLFK.H | Y | 60.86 | 1235.6761 | 11 | 0.7 | 618.8458 | 2 | 47.53 | 6 | F6:32450 | 17012022\_RID\_3262\_9a.raw |  |  |  | 1.1826E6 |  | 1.3838E6 |  | 2.5277E6 |  | 2.6941E6 | 4 | 0 | 0 | 0 | 1 | 0 | 1 | 0 | 1 | 0 | 1 | 664 | 674 |  |  | PEAKS DB |
| R.ISEDKNPDYR.K | Y | 59.00 | 1235.5781 | 10 | 0.9 | 618.7969 | 2 | 18.00 | 4 | F4:5768 | 17012022\_RID\_3262\_10a .raw | 2.2898E5 | 5.2363E4 |  | 5.724E5 |  | 6.4152E5 | 4.2912E4 |  |  |  | 5 | 1 | 1 | 0 | 1 | 0 | 1 | 1 | 0 | 0 | 0 | 652 | 661 |  |  | PEAKS DB |
| K.SAIVHLINYQDDAELATR.A | Y | 57.87 | 2028.0276 | 18 | 1.0 | 677.0172 | 3 | 43.76 | 1 | F1:28958 | 13012022\_RID\_3262\_4a.raw | 4.2034E5 |  |  |  |  |  |  | 1.3135E6 | 3.6387E5 |  | 4 | 1 | 0 | 0 | 0 | 0 | 0 | 0 | 2 | 1 | 0 | 125 | 142 |  |  | PEAKS DB |
| K.SGGIPALVR.M | Y | 56.02 | 868.5130 | 9 | 0.5 | 435.2640 | 2 | 35.68 | 6 | F6:21518 | 17012022\_RID\_3262\_9a.raw |  | 3.703E5 |  | 2.5461E6 |  | 2.1357E6 | 8.8669E4 | 0 | 6.1375E5 |  | 5 | 0 | 1 | 0 | 1 | 0 | 1 | 1 | 0 | 1 | 0 | 225 | 233 |  |  | PEAKS DB |
| R.TM(+15.99)QNTSDLDTAR.C | Y | 55.25 | 1367.5988 | 12 | -0.4 | 684.8064 | 2 | 21.71 | 4 | F4:8886 | 17012022\_RID\_3262\_10a .raw |  | 1.2132E5 |  | 1.0143E6 |  | 6.4155E5 |  | 2.1506E5 | 1.8157E5 |  | 5 | 0 | 1 | 0 | 1 | 0 | 1 | 0 | 1 | 1 | 0 | 192 | 203 | Oxidation (M) | M2:Oxidation (M):1000.00 | PEAKS DB |
| R.HPEAEM(+15.99)AQNSVR.L | Y | 53.70 | 1383.6201 | 12 | 1.0 | 692.8180 | 2 | 15.98 | 4 | F4:4154 | 17012022\_RID\_3262\_10a .raw |  |  |  | 4.0379E5 |  |  |  |  |  |  | 2 | 0 | 0 | 0 | 2 | 0 | 0 | 0 | 0 | 0 | 0 | 466 | 477 | Oxidation (M) | M6:Oxidation (M):1000.00 | PEAKS DB |
| K.AAM(+15.99)IVNQLSK.K | Y | 53.10 | 1089.5852 | 10 | 0.8 | 545.8003 | 2 | 28.59 | 4 | F4:15343 | 17012022\_RID\_3262\_10a .raw |  |  |  | 1.0996E6 |  |  |  |  | 1.8354E5 |  | 2 | 0 | 0 | 0 | 1 | 0 | 0 | 0 | 0 | 1 | 0 | 162 | 171 | Oxidation (M) | M3:Oxidation (M):1000.00 | PEAKS DB |
| R.TMQNTSDLDTAR.C | Y | 53.03 | 1351.6038 | 12 | 0.1 | 676.8092 | 2 | 22.98 | 9 | F9:7560 | 31122021\_RID\_3262\_2a.raw |  | 1.1062E5 |  |  |  | 9.3847E5 |  | 2.411E5 | 2.0476E5 |  | 4 | 0 | 1 | 0 | 0 | 0 | 1 | 0 | 1 | 1 | 0 | 192 | 203 |  |  | PEAKS DB |
| K.AAMIVNQLSK.K | Y | 52.58 | 1073.5903 | 10 | -0.4 | 537.8022 | 2 | 33.93 | 4 | F4:19962 | 17012022\_RID\_3262\_10a .raw |  |  |  | 1.2793E6 |  | 1.6681E6 |  |  |  |  | 2 | 0 | 0 | 0 | 1 | 0 | 1 | 0 | 0 | 0 | 0 | 162 | 171 |  |  | PEAKS DB |
| R.HPEAEMAQNSVR.L | Y | 51.71 | 1367.6251 | 12 | 0.0 | 684.8198 | 2 | 17.98 | 8 | F8:3971 | 31122021\_RID\_3262\_1a.raw |  |  |  | 3.542E5 |  |  |  | 1.8269E5 | 1.0075E5 |  | 3 | 0 | 0 | 0 | 1 | 0 | 0 | 0 | 1 | 1 | 0 | 466 | 477 |  |  | PEAKS DB |
| L.INYQDDAELATR.A | N | 45.89 | 1407.6630 | 12 | 0.5 | 704.8391 | 2 | 32.83 | 9 | F9:15651 | 31122021\_RID\_3262\_2a.raw |  |  |  |  |  |  |  | 6.9475E4 | 0 |  | 1 | 0 | 0 | 0 | 0 | 0 | 0 | 0 | 1 | 0 | 0 | 131 | 142 |  |  | PEAKS DB |
| total 13 peptides |  |  |  |  |  |  |  |  |  |  |  |  |  |  |  |  |  |  |  |  |  |  |  |  |  |  |  |  |  |  |  |  |  |  |  |  |  |
| --- | --- | --- | --- | --- | --- | --- | --- | --- | --- | --- | --- | --- | --- | --- | --- | --- | --- | --- | --- | --- | --- | --- | --- | --- | --- | --- | --- | --- | --- | --- | --- | --- | --- | --- | --- | --- | --- |

XP\_026525656.1

back to list

  

| Protein Coverage
| Supporting Peptides
|

Protein Coverage:

Supporting Peptides:

| Peptide | Uniq | -10lgP | Mass | Length | ppm | m/z | z | RT | Fraction | Scan | Source File | Area F4 | Area F5 | Area F6 | Area F10 | Area F8 | Area F9 | Area F7 | Area F1 | Area F2 | Area F3 | #Feature | #Feature F4 | #Feature F5 | #Feature F6 | #Feature F10 | #Feature F8 | #Feature F9 | #Feature F7 | #Feature F1 | #Feature F2 | #Feature F3 | Start | End | PTM | AScore | Found By |
| --- | --- | --- | --- | --- | --- | --- | --- | --- | --- | --- | --- | --- | --- | --- | --- | --- | --- | --- | --- | --- | --- | --- | --- | --- | --- | --- | --- | --- | --- | --- | --- | --- | --- | --- | --- | --- | --- |
| R.NEGTATYAAAVLFR.I | Y | 74.08 | 1482.7467 | 14 | 0.2 | 742.3808 | 2 | 48.27 | 9 | F9:28127 | 31122021\_RID\_3262\_2a.raw | 4.317E5 |  |  |  |  | 1.024E6 |  | 1.4003E6 | 4.6202E5 |  | 5 | 1 | 0 | 0 | 0 | 0 | 1 | 0 | 2 | 1 | 0 | 638 | 651 |  |  | PEAKS DB |
| R.LNYGIPAIVK.L | Y | 65.66 | 1086.6437 | 10 | 0.0 | 544.3291 | 2 | 45.04 | 8 | F8:24415 | 31122021\_RID\_3262\_1a.raw |  |  |  | 2.5539E6 |  | 2.6128E6 |  | 1.8129E6 | 7.9277E5 |  | 4 | 0 | 0 | 0 | 1 | 0 | 1 | 0 | 1 | 1 | 0 | 478 | 487 |  |  | PEAKS DB |
| R.VSVELTNSLFK.H | Y | 60.86 | 1235.6761 | 11 | 0.7 | 618.8458 | 2 | 47.53 | 6 | F6:32450 | 17012022\_RID\_3262\_9a.raw |  |  |  | 1.1826E6 |  | 1.3838E6 |  | 2.5277E6 |  | 2.6941E6 | 4 | 0 | 0 | 0 | 1 | 0 | 1 | 0 | 1 | 0 | 1 | 664 | 674 |  |  | PEAKS DB |
| R.ISEDKNPDYR.K | Y | 59.00 | 1235.5781 | 10 | 0.9 | 618.7969 | 2 | 18.00 | 4 | F4:5768 | 17012022\_RID\_3262\_10a .raw | 2.2898E5 | 5.2363E4 |  | 5.724E5 |  | 6.4152E5 | 4.2912E4 |  |  |  | 5 | 1 | 1 | 0 | 1 | 0 | 1 | 1 | 0 | 0 | 0 | 652 | 661 |  |  | PEAKS DB |
| K.SAIVHLINYQDDAELATR.A | Y | 57.87 | 2028.0276 | 18 | 1.0 | 677.0172 | 3 | 43.76 | 1 | F1:28958 | 13012022\_RID\_3262\_4a.raw | 4.2034E5 |  |  |  |  |  |  | 1.3135E6 | 3.6387E5 |  | 4 | 1 | 0 | 0 | 0 | 0 | 0 | 0 | 2 | 1 | 0 | 125 | 142 |  |  | PEAKS DB |
| K.SGGIPALVR.M | Y | 56.02 | 868.5130 | 9 | 0.5 | 435.2640 | 2 | 35.68 | 6 | F6:21518 | 17012022\_RID\_3262\_9a.raw |  | 3.703E5 |  | 2.5461E6 |  | 2.1357E6 | 8.8669E4 | 0 | 6.1375E5 |  | 5 | 0 | 1 | 0 | 1 | 0 | 1 | 1 | 0 | 1 | 0 | 225 | 233 |  |  | PEAKS DB |
| R.TM(+15.99)QNTSDLDTAR.C | Y | 55.25 | 1367.5988 | 12 | -0.4 | 684.8064 | 2 | 21.71 | 4 | F4:8886 | 17012022\_RID\_3262\_10a .raw |  | 1.2132E5 |  | 1.0143E6 |  | 6.4155E5 |  | 2.1506E5 | 1.8157E5 |  | 5 | 0 | 1 | 0 | 1 | 0 | 1 | 0 | 1 | 1 | 0 | 192 | 203 | Oxidation (M) | M2:Oxidation (M):1000.00 | PEAKS DB |
| R.HPEAEM(+15.99)AQNSVR.L | Y | 53.70 | 1383.6201 | 12 | 1.0 | 692.8180 | 2 | 15.98 | 4 | F4:4154 | 17012022\_RID\_3262\_10a .raw |  |  |  | 4.0379E5 |  |  |  |  |  |  | 2 | 0 | 0 | 0 | 2 | 0 | 0 | 0 | 0 | 0 | 0 | 466 | 477 | Oxidation (M) | M6:Oxidation (M):1000.00 | PEAKS DB |
| K.AAM(+15.99)IVNQLSK.K | Y | 53.10 | 1089.5852 | 10 | 0.8 | 545.8003 | 2 | 28.59 | 4 | F4:15343 | 17012022\_RID\_3262\_10a .raw |  |  |  | 1.0996E6 |  |  |  |  | 1.8354E5 |  | 2 | 0 | 0 | 0 | 1 | 0 | 0 | 0 | 0 | 1 | 0 | 162 | 171 | Oxidation (M) | M3:Oxidation (M):1000.00 | PEAKS DB |
| R.TMQNTSDLDTAR.C | Y | 53.03 | 1351.6038 | 12 | 0.1 | 676.8092 | 2 | 22.98 | 9 | F9:7560 | 31122021\_RID\_3262\_2a.raw |  | 1.1062E5 |  |  |  | 9.3847E5 |  | 2.411E5 | 2.0476E5 |  | 4 | 0 | 1 | 0 | 0 | 0 | 1 | 0 | 1 | 1 | 0 | 192 | 203 |  |  | PEAKS DB |
| K.AAMIVNQLSK.K | Y | 52.58 | 1073.5903 | 10 | -0.4 | 537.8022 | 2 | 33.93 | 4 | F4:19962 | 17012022\_RID\_3262\_10a .raw |  |  |  | 1.2793E6 |  | 1.6681E6 |  |  |  |  | 2 | 0 | 0 | 0 | 1 | 0 | 1 | 0 | 0 | 0 | 0 | 162 | 171 |  |  | PEAKS DB |
| R.HPEAEMAQNSVR.L | Y | 51.71 | 1367.6251 | 12 | 0.0 | 684.8198 | 2 | 17.98 | 8 | F8:3971 | 31122021\_RID\_3262\_1a.raw |  |  |  | 3.542E5 |  |  |  | 1.8269E5 | 1.0075E5 |  | 3 | 0 | 0 | 0 | 1 | 0 | 0 | 0 | 1 | 1 | 0 | 466 | 477 |  |  | PEAKS DB |
| L.INYQDDAELATR.A | N | 45.89 | 1407.6630 | 12 | 0.5 | 704.8391 | 2 | 32.83 | 9 | F9:15651 | 31122021\_RID\_3262\_2a.raw |  |  |  |  |  |  |  | 6.9475E4 | 0 |  | 1 | 0 | 0 | 0 | 0 | 0 | 0 | 0 | 1 | 0 | 0 | 131 | 142 |  |  | PEAKS DB |
| total 13 peptides |  |  |  |  |  |  |  |  |  |  |  |  |  |  |  |  |  |  |  |  |  |  |  |  |  |  |  |  |  |  |  |  |  |  |  |  |  |
| --- | --- | --- | --- | --- | --- | --- | --- | --- | --- | --- | --- | --- | --- | --- | --- | --- | --- | --- | --- | --- | --- | --- | --- | --- | --- | --- | --- | --- | --- | --- | --- | --- | --- | --- | --- | --- | --- |

XP\_026576299.1

back to list

  

| Protein Coverage
| Supporting Peptides
|

Protein Coverage:

Supporting Peptides:

| Peptide | Uniq | -10lgP | Mass | Length | ppm | m/z | z | RT | Fraction | Scan | Source File | Area F4 | Area F5 | Area F6 | Area F10 | Area F8 | Area F9 | Area F7 | Area F1 | Area F2 | Area F3 | #Feature | #Feature F4 | #Feature F5 | #Feature F6 | #Feature F10 | #Feature F8 | #Feature F9 | #Feature F7 | #Feature F1 | #Feature F2 | #Feature F3 | Start | End | PTM | AScore | Found By |
| --- | --- | --- | --- | --- | --- | --- | --- | --- | --- | --- | --- | --- | --- | --- | --- | --- | --- | --- | --- | --- | --- | --- | --- | --- | --- | --- | --- | --- | --- | --- | --- | --- | --- | --- | --- | --- | --- |
| R.NEGTATYAAAVLFR.I | Y | 74.08 | 1482.7467 | 14 | 0.2 | 742.3808 | 2 | 48.27 | 9 | F9:28127 | 31122021\_RID\_3262\_2a.raw | 4.317E5 |  |  |  |  | 1.024E6 |  | 1.4003E6 | 4.6202E5 |  | 5 | 1 | 0 | 0 | 0 | 0 | 1 | 0 | 2 | 1 | 0 | 639 | 652 |  |  | PEAKS DB |
| R.LNYGIPAIVK.L | Y | 65.66 | 1086.6437 | 10 | 0.0 | 544.3291 | 2 | 45.04 | 8 | F8:24415 | 31122021\_RID\_3262\_1a.raw |  |  |  | 2.5539E6 |  | 2.6128E6 |  | 1.8129E6 | 7.9277E5 |  | 4 | 0 | 0 | 0 | 1 | 0 | 1 | 0 | 1 | 1 | 0 | 479 | 488 |  |  | PEAKS DB |
| R.VSVELTNSLFK.H | Y | 60.86 | 1235.6761 | 11 | 0.7 | 618.8458 | 2 | 47.53 | 6 | F6:32450 | 17012022\_RID\_3262\_9a.raw |  |  |  | 1.1826E6 |  | 1.3838E6 |  | 2.5277E6 |  | 2.6941E6 | 4 | 0 | 0 | 0 | 1 | 0 | 1 | 0 | 1 | 0 | 1 | 665 | 675 |  |  | PEAKS DB |
| R.ISEDKNPDYR.K | Y | 59.00 | 1235.5781 | 10 | 0.9 | 618.7969 | 2 | 18.00 | 4 | F4:5768 | 17012022\_RID\_3262\_10a .raw | 2.2898E5 | 5.2363E4 |  | 5.724E5 |  | 6.4152E5 | 4.2912E4 |  |  |  | 5 | 1 | 1 | 0 | 1 | 0 | 1 | 1 | 0 | 0 | 0 | 653 | 662 |  |  | PEAKS DB |
| K.SAIVHLINYQDDAELATR.A | Y | 57.87 | 2028.0276 | 18 | 1.0 | 677.0172 | 3 | 43.76 | 1 | F1:28958 | 13012022\_RID\_3262\_4a.raw | 4.2034E5 |  |  |  |  |  |  | 1.3135E6 | 3.6387E5 |  | 4 | 1 | 0 | 0 | 0 | 0 | 0 | 0 | 2 | 1 | 0 | 126 | 143 |  |  | PEAKS DB |
| K.SGGIPALVR.M | Y | 56.02 | 868.5130 | 9 | 0.5 | 435.2640 | 2 | 35.68 | 6 | F6:21518 | 17012022\_RID\_3262\_9a.raw |  | 3.703E5 |  | 2.5461E6 |  | 2.1357E6 | 8.8669E4 | 0 | 6.1375E5 |  | 5 | 0 | 1 | 0 | 1 | 0 | 1 | 1 | 0 | 1 | 0 | 226 | 234 |  |  | PEAKS DB |
| R.TM(+15.99)QNTSDLDTAR.C | Y | 55.25 | 1367.5988 | 12 | -0.4 | 684.8064 | 2 | 21.71 | 4 | F4:8886 | 17012022\_RID\_3262\_10a .raw |  | 1.2132E5 |  | 1.0143E6 |  | 6.4155E5 |  | 2.1506E5 | 1.8157E5 |  | 5 | 0 | 1 | 0 | 1 | 0 | 1 | 0 | 1 | 1 | 0 | 193 | 204 | Oxidation (M) | M2:Oxidation (M):1000.00 | PEAKS DB |
| R.HPEAEM(+15.99)AQNSVR.L | Y | 53.70 | 1383.6201 | 12 | 1.0 | 692.8180 | 2 | 15.98 | 4 | F4:4154 | 17012022\_RID\_3262\_10a .raw |  |  |  | 4.0379E5 |  |  |  |  |  |  | 2 | 0 | 0 | 0 | 2 | 0 | 0 | 0 | 0 | 0 | 0 | 467 | 478 | Oxidation (M) | M6:Oxidation (M):1000.00 | PEAKS DB |
| K.AAM(+15.99)IVNQLSK.K | Y | 53.10 | 1089.5852 | 10 | 0.8 | 545.8003 | 2 | 28.59 | 4 | F4:15343 | 17012022\_RID\_3262\_10a .raw |  |  |  | 1.0996E6 |  |  |  |  | 1.8354E5 |  | 2 | 0 | 0 | 0 | 1 | 0 | 0 | 0 | 0 | 1 | 0 | 163 | 172 | Oxidation (M) | M3:Oxidation (M):1000.00 | PEAKS DB |
| R.TMQNTSDLDTAR.C | Y | 53.03 | 1351.6038 | 12 | 0.1 | 676.8092 | 2 | 22.98 | 9 | F9:7560 | 31122021\_RID\_3262\_2a.raw |  | 1.1062E5 |  |  |  | 9.3847E5 |  | 2.411E5 | 2.0476E5 |  | 4 | 0 | 1 | 0 | 0 | 0 | 1 | 0 | 1 | 1 | 0 | 193 | 204 |  |  | PEAKS DB |
| K.AAMIVNQLSK.K | Y | 52.58 | 1073.5903 | 10 | -0.4 | 537.8022 | 2 | 33.93 | 4 | F4:19962 | 17012022\_RID\_3262\_10a .raw |  |  |  | 1.2793E6 |  | 1.6681E6 |  |  |  |  | 2 | 0 | 0 | 0 | 1 | 0 | 1 | 0 | 0 | 0 | 0 | 163 | 172 |  |  | PEAKS DB |
| R.HPEAEMAQNSVR.L | Y | 51.71 | 1367.6251 | 12 | 0.0 | 684.8198 | 2 | 17.98 | 8 | F8:3971 | 31122021\_RID\_3262\_1a.raw |  |  |  | 3.542E5 |  |  |  | 1.8269E5 | 1.0075E5 |  | 3 | 0 | 0 | 0 | 1 | 0 | 0 | 0 | 1 | 1 | 0 | 467 | 478 |  |  | PEAKS DB |
| L.INYQDDAELATR.A | N | 45.89 | 1407.6630 | 12 | 0.5 | 704.8391 | 2 | 32.83 | 9 | F9:15651 | 31122021\_RID\_3262\_2a.raw |  |  |  |  |  |  |  | 6.9475E4 | 0 |  | 1 | 0 | 0 | 0 | 0 | 0 | 0 | 0 | 1 | 0 | 0 | 132 | 143 |  |  | PEAKS DB |
| total 13 peptides |  |  |  |  |  |  |  |  |  |  |  |  |  |  |  |  |  |  |  |  |  |  |  |  |  |  |  |  |  |  |  |  |  |  |  |  |  |
| --- | --- | --- | --- | --- | --- | --- | --- | --- | --- | --- | --- | --- | --- | --- | --- | --- | --- | --- | --- | --- | --- | --- | --- | --- | --- | --- | --- | --- | --- | --- | --- | --- | --- | --- | --- | --- | --- |

XP\_026525655.1

back to list

  

| Protein Coverage
| Supporting Peptides
|

Protein Coverage:

Supporting Peptides:

| Peptide | Uniq | -10lgP | Mass | Length | ppm | m/z | z | RT | Fraction | Scan | Source File | Area F4 | Area F5 | Area F6 | Area F10 | Area F8 | Area F9 | Area F7 | Area F1 | Area F2 | Area F3 | #Feature | #Feature F4 | #Feature F5 | #Feature F6 | #Feature F10 | #Feature F8 | #Feature F9 | #Feature F7 | #Feature F1 | #Feature F2 | #Feature F3 | Start | End | PTM | AScore | Found By |
| --- | --- | --- | --- | --- | --- | --- | --- | --- | --- | --- | --- | --- | --- | --- | --- | --- | --- | --- | --- | --- | --- | --- | --- | --- | --- | --- | --- | --- | --- | --- | --- | --- | --- | --- | --- | --- | --- |
| R.NEGTATYAAAVLFR.I | Y | 74.08 | 1482.7467 | 14 | 0.2 | 742.3808 | 2 | 48.27 | 9 | F9:28127 | 31122021\_RID\_3262\_2a.raw | 4.317E5 |  |  |  |  | 1.024E6 |  | 1.4003E6 | 4.6202E5 |  | 5 | 1 | 0 | 0 | 0 | 0 | 1 | 0 | 2 | 1 | 0 | 639 | 652 |  |  | PEAKS DB |
| R.LNYGIPAIVK.L | Y | 65.66 | 1086.6437 | 10 | 0.0 | 544.3291 | 2 | 45.04 | 8 | F8:24415 | 31122021\_RID\_3262\_1a.raw |  |  |  | 2.5539E6 |  | 2.6128E6 |  | 1.8129E6 | 7.9277E5 |  | 4 | 0 | 0 | 0 | 1 | 0 | 1 | 0 | 1 | 1 | 0 | 479 | 488 |  |  | PEAKS DB |
| R.VSVELTNSLFK.H | Y | 60.86 | 1235.6761 | 11 | 0.7 | 618.8458 | 2 | 47.53 | 6 | F6:32450 | 17012022\_RID\_3262\_9a.raw |  |  |  | 1.1826E6 |  | 1.3838E6 |  | 2.5277E6 |  | 2.6941E6 | 4 | 0 | 0 | 0 | 1 | 0 | 1 | 0 | 1 | 0 | 1 | 665 | 675 |  |  | PEAKS DB |
| R.ISEDKNPDYR.K | Y | 59.00 | 1235.5781 | 10 | 0.9 | 618.7969 | 2 | 18.00 | 4 | F4:5768 | 17012022\_RID\_3262\_10a .raw | 2.2898E5 | 5.2363E4 |  | 5.724E5 |  | 6.4152E5 | 4.2912E4 |  |  |  | 5 | 1 | 1 | 0 | 1 | 0 | 1 | 1 | 0 | 0 | 0 | 653 | 662 |  |  | PEAKS DB |
| K.SAIVHLINYQDDAELATR.A | Y | 57.87 | 2028.0276 | 18 | 1.0 | 677.0172 | 3 | 43.76 | 1 | F1:28958 | 13012022\_RID\_3262\_4a.raw | 4.2034E5 |  |  |  |  |  |  | 1.3135E6 | 3.6387E5 |  | 4 | 1 | 0 | 0 | 0 | 0 | 0 | 0 | 2 | 1 | 0 | 126 | 143 |  |  | PEAKS DB |
| K.SGGIPALVR.M | Y | 56.02 | 868.5130 | 9 | 0.5 | 435.2640 | 2 | 35.68 | 6 | F6:21518 | 17012022\_RID\_3262\_9a.raw |  | 3.703E5 |  | 2.5461E6 |  | 2.1357E6 | 8.8669E4 | 0 | 6.1375E5 |  | 5 | 0 | 1 | 0 | 1 | 0 | 1 | 1 | 0 | 1 | 0 | 226 | 234 |  |  | PEAKS DB |
| R.TM(+15.99)QNTSDLDTAR.C | Y | 55.25 | 1367.5988 | 12 | -0.4 | 684.8064 | 2 | 21.71 | 4 | F4:8886 | 17012022\_RID\_3262\_10a .raw |  | 1.2132E5 |  | 1.0143E6 |  | 6.4155E5 |  | 2.1506E5 | 1.8157E5 |  | 5 | 0 | 1 | 0 | 1 | 0 | 1 | 0 | 1 | 1 | 0 | 193 | 204 | Oxidation (M) | M2:Oxidation (M):1000.00 | PEAKS DB |
| R.HPEAEM(+15.99)AQNSVR.L | Y | 53.70 | 1383.6201 | 12 | 1.0 | 692.8180 | 2 | 15.98 | 4 | F4:4154 | 17012022\_RID\_3262\_10a .raw |  |  |  | 4.0379E5 |  |  |  |  |  |  | 2 | 0 | 0 | 0 | 2 | 0 | 0 | 0 | 0 | 0 | 0 | 467 | 478 | Oxidation (M) | M6:Oxidation (M):1000.00 | PEAKS DB |
| K.AAM(+15.99)IVNQLSK.K | Y | 53.10 | 1089.5852 | 10 | 0.8 | 545.8003 | 2 | 28.59 | 4 | F4:15343 | 17012022\_RID\_3262\_10a .raw |  |  |  | 1.0996E6 |  |  |  |  | 1.8354E5 |  | 2 | 0 | 0 | 0 | 1 | 0 | 0 | 0 | 0 | 1 | 0 | 163 | 172 | Oxidation (M) | M3:Oxidation (M):1000.00 | PEAKS DB |
| R.TMQNTSDLDTAR.C | Y | 53.03 | 1351.6038 | 12 | 0.1 | 676.8092 | 2 | 22.98 | 9 | F9:7560 | 31122021\_RID\_3262\_2a.raw |  | 1.1062E5 |  |  |  | 9.3847E5 |  | 2.411E5 | 2.0476E5 |  | 4 | 0 | 1 | 0 | 0 | 0 | 1 | 0 | 1 | 1 | 0 | 193 | 204 |  |  | PEAKS DB |
| K.AAMIVNQLSK.K | Y | 52.58 | 1073.5903 | 10 | -0.4 | 537.8022 | 2 | 33.93 | 4 | F4:19962 | 17012022\_RID\_3262\_10a .raw |  |  |  | 1.2793E6 |  | 1.6681E6 |  |  |  |  | 2 | 0 | 0 | 0 | 1 | 0 | 1 | 0 | 0 | 0 | 0 | 163 | 172 |  |  | PEAKS DB |
| R.HPEAEMAQNSVR.L | Y | 51.71 | 1367.6251 | 12 | 0.0 | 684.8198 | 2 | 17.98 | 8 | F8:3971 | 31122021\_RID\_3262\_1a.raw |  |  |  | 3.542E5 |  |  |  | 1.8269E5 | 1.0075E5 |  | 3 | 0 | 0 | 0 | 1 | 0 | 0 | 0 | 1 | 1 | 0 | 467 | 478 |  |  | PEAKS DB |
| L.INYQDDAELATR.A | N | 45.89 | 1407.6630 | 12 | 0.5 | 704.8391 | 2 | 32.83 | 9 | F9:15651 | 31122021\_RID\_3262\_2a.raw |  |  |  |  |  |  |  | 6.9475E4 | 0 |  | 1 | 0 | 0 | 0 | 0 | 0 | 0 | 0 | 1 | 0 | 0 | 132 | 143 |  |  | PEAKS DB |
| total 13 peptides |  |  |  |  |  |  |  |  |  |  |  |  |  |  |  |  |  |  |  |  |  |  |  |  |  |  |  |  |  |  |  |  |  |  |  |  |  |
| --- | --- | --- | --- | --- | --- | --- | --- | --- | --- | --- | --- | --- | --- | --- | --- | --- | --- | --- | --- | --- | --- | --- | --- | --- | --- | --- | --- | --- | --- | --- | --- | --- | --- | --- | --- | --- | --- |

XP\_026525654.1

back to list

  

| Protein Coverage
| Supporting Peptides
|

Protein Coverage:

Supporting Peptides:

| Peptide | Uniq | -10lgP | Mass | Length | ppm | m/z | z | RT | Fraction | Scan | Source File | Area F4 | Area F5 | Area F6 | Area F10 | Area F8 | Area F9 | Area F7 | Area F1 | Area F2 | Area F3 | #Feature | #Feature F4 | #Feature F5 | #Feature F6 | #Feature F10 | #Feature F8 | #Feature F9 | #Feature F7 | #Feature F1 | #Feature F2 | #Feature F3 | Start | End | PTM | AScore | Found By |
| --- | --- | --- | --- | --- | --- | --- | --- | --- | --- | --- | --- | --- | --- | --- | --- | --- | --- | --- | --- | --- | --- | --- | --- | --- | --- | --- | --- | --- | --- | --- | --- | --- | --- | --- | --- | --- | --- |
| R.NEGTATYAAAVLFR.I | Y | 74.08 | 1482.7467 | 14 | 0.2 | 742.3808 | 2 | 48.27 | 9 | F9:28127 | 31122021\_RID\_3262\_2a.raw | 4.317E5 |  |  |  |  | 1.024E6 |  | 1.4003E6 | 4.6202E5 |  | 5 | 1 | 0 | 0 | 0 | 0 | 1 | 0 | 2 | 1 | 0 | 639 | 652 |  |  | PEAKS DB |
| R.LNYGIPAIVK.L | Y | 65.66 | 1086.6437 | 10 | 0.0 | 544.3291 | 2 | 45.04 | 8 | F8:24415 | 31122021\_RID\_3262\_1a.raw |  |  |  | 2.5539E6 |  | 2.6128E6 |  | 1.8129E6 | 7.9277E5 |  | 4 | 0 | 0 | 0 | 1 | 0 | 1 | 0 | 1 | 1 | 0 | 479 | 488 |  |  | PEAKS DB |
| R.VSVELTNSLFK.H | Y | 60.86 | 1235.6761 | 11 | 0.7 | 618.8458 | 2 | 47.53 | 6 | F6:32450 | 17012022\_RID\_3262\_9a.raw |  |  |  | 1.1826E6 |  | 1.3838E6 |  | 2.5277E6 |  | 2.6941E6 | 4 | 0 | 0 | 0 | 1 | 0 | 1 | 0 | 1 | 0 | 1 | 665 | 675 |  |  | PEAKS DB |
| R.ISEDKNPDYR.K | Y | 59.00 | 1235.5781 | 10 | 0.9 | 618.7969 | 2 | 18.00 | 4 | F4:5768 | 17012022\_RID\_3262\_10a .raw | 2.2898E5 | 5.2363E4 |  | 5.724E5 |  | 6.4152E5 | 4.2912E4 |  |  |  | 5 | 1 | 1 | 0 | 1 | 0 | 1 | 1 | 0 | 0 | 0 | 653 | 662 |  |  | PEAKS DB |
| K.SAIVHLINYQDDAELATR.A | Y | 57.87 | 2028.0276 | 18 | 1.0 | 677.0172 | 3 | 43.76 | 1 | F1:28958 | 13012022\_RID\_3262\_4a.raw | 4.2034E5 |  |  |  |  |  |  | 1.3135E6 | 3.6387E5 |  | 4 | 1 | 0 | 0 | 0 | 0 | 0 | 0 | 2 | 1 | 0 | 126 | 143 |  |  | PEAKS DB |
| K.SGGIPALVR.M | Y | 56.02 | 868.5130 | 9 | 0.5 | 435.2640 | 2 | 35.68 | 6 | F6:21518 | 17012022\_RID\_3262\_9a.raw |  | 3.703E5 |  | 2.5461E6 |  | 2.1357E6 | 8.8669E4 | 0 | 6.1375E5 |  | 5 | 0 | 1 | 0 | 1 | 0 | 1 | 1 | 0 | 1 | 0 | 226 | 234 |  |  | PEAKS DB |
| R.TM(+15.99)QNTSDLDTAR.C | Y | 55.25 | 1367.5988 | 12 | -0.4 | 684.8064 | 2 | 21.71 | 4 | F4:8886 | 17012022\_RID\_3262\_10a .raw |  | 1.2132E5 |  | 1.0143E6 |  | 6.4155E5 |  | 2.1506E5 | 1.8157E5 |  | 5 | 0 | 1 | 0 | 1 | 0 | 1 | 0 | 1 | 1 | 0 | 193 | 204 | Oxidation (M) | M2:Oxidation (M):1000.00 | PEAKS DB |
| R.HPEAEM(+15.99)AQNSVR.L | Y | 53.70 | 1383.6201 | 12 | 1.0 | 692.8180 | 2 | 15.98 | 4 | F4:4154 | 17012022\_RID\_3262\_10a .raw |  |  |  | 4.0379E5 |  |  |  |  |  |  | 2 | 0 | 0 | 0 | 2 | 0 | 0 | 0 | 0 | 0 | 0 | 467 | 478 | Oxidation (M) | M6:Oxidation (M):1000.00 | PEAKS DB |
| K.AAM(+15.99)IVNQLSK.K | Y | 53.10 | 1089.5852 | 10 | 0.8 | 545.8003 | 2 | 28.59 | 4 | F4:15343 | 17012022\_RID\_3262\_10a .raw |  |  |  | 1.0996E6 |  |  |  |  | 1.8354E5 |  | 2 | 0 | 0 | 0 | 1 | 0 | 0 | 0 | 0 | 1 | 0 | 163 | 172 | Oxidation (M) | M3:Oxidation (M):1000.00 | PEAKS DB |
| R.TMQNTSDLDTAR.C | Y | 53.03 | 1351.6038 | 12 | 0.1 | 676.8092 | 2 | 22.98 | 9 | F9:7560 | 31122021\_RID\_3262\_2a.raw |  | 1.1062E5 |  |  |  | 9.3847E5 |  | 2.411E5 | 2.0476E5 |  | 4 | 0 | 1 | 0 | 0 | 0 | 1 | 0 | 1 | 1 | 0 | 193 | 204 |  |  | PEAKS DB |
| K.AAMIVNQLSK.K | Y | 52.58 | 1073.5903 | 10 | -0.4 | 537.8022 | 2 | 33.93 | 4 | F4:19962 | 17012022\_RID\_3262\_10a .raw |  |  |  | 1.2793E6 |  | 1.6681E6 |  |  |  |  | 2 | 0 | 0 | 0 | 1 | 0 | 1 | 0 | 0 | 0 | 0 | 163 | 172 |  |  | PEAKS DB |
| R.HPEAEMAQNSVR.L | Y | 51.71 | 1367.6251 | 12 | 0.0 | 684.8198 | 2 | 17.98 | 8 | F8:3971 | 31122021\_RID\_3262\_1a.raw |  |  |  | 3.542E5 |  |  |  | 1.8269E5 | 1.0075E5 |  | 3 | 0 | 0 | 0 | 1 | 0 | 0 | 0 | 1 | 1 | 0 | 467 | 478 |  |  | PEAKS DB |
| L.INYQDDAELATR.A | N | 45.89 | 1407.6630 | 12 | 0.5 | 704.8391 | 2 | 32.83 | 9 | F9:15651 | 31122021\_RID\_3262\_2a.raw |  |  |  |  |  |  |  | 6.9475E4 | 0 |  | 1 | 0 | 0 | 0 | 0 | 0 | 0 | 0 | 1 | 0 | 0 | 132 | 143 |  |  | PEAKS DB |
| total 13 peptides |  |  |  |  |  |  |  |  |  |  |  |  |  |  |  |  |  |  |  |  |  |  |  |  |  |  |  |  |  |  |  |  |  |  |  |  |  |
| --- | --- | --- | --- | --- | --- | --- | --- | --- | --- | --- | --- | --- | --- | --- | --- | --- | --- | --- | --- | --- | --- | --- | --- | --- | --- | --- | --- | --- | --- | --- | --- | --- | --- | --- | --- | --- | --- |

XP\_026576298.1

back to list

  

| Protein Coverage
| Supporting Peptides
|

Protein Coverage:

Supporting Peptides:

| Peptide | Uniq | -10lgP | Mass | Length | ppm | m/z | z | RT | Fraction | Scan | Source File | Area F4 | Area F5 | Area F6 | Area F10 | Area F8 | Area F9 | Area F7 | Area F1 | Area F2 | Area F3 | #Feature | #Feature F4 | #Feature F5 | #Feature F6 | #Feature F10 | #Feature F8 | #Feature F9 | #Feature F7 | #Feature F1 | #Feature F2 | #Feature F3 | Start | End | PTM | AScore | Found By |
| --- | --- | --- | --- | --- | --- | --- | --- | --- | --- | --- | --- | --- | --- | --- | --- | --- | --- | --- | --- | --- | --- | --- | --- | --- | --- | --- | --- | --- | --- | --- | --- | --- | --- | --- | --- | --- | --- |
| R.NEGTATYAAAVLFR.I | Y | 74.08 | 1482.7467 | 14 | 0.2 | 742.3808 | 2 | 48.27 | 9 | F9:28127 | 31122021\_RID\_3262\_2a.raw | 4.317E5 |  |  |  |  | 1.024E6 |  | 1.4003E6 | 4.6202E5 |  | 5 | 1 | 0 | 0 | 0 | 0 | 1 | 0 | 2 | 1 | 0 | 639 | 652 |  |  | PEAKS DB |
| R.LNYGIPAIVK.L | Y | 65.66 | 1086.6437 | 10 | 0.0 | 544.3291 | 2 | 45.04 | 8 | F8:24415 | 31122021\_RID\_3262\_1a.raw |  |  |  | 2.5539E6 |  | 2.6128E6 |  | 1.8129E6 | 7.9277E5 |  | 4 | 0 | 0 | 0 | 1 | 0 | 1 | 0 | 1 | 1 | 0 | 479 | 488 |  |  | PEAKS DB |
| R.VSVELTNSLFK.H | Y | 60.86 | 1235.6761 | 11 | 0.7 | 618.8458 | 2 | 47.53 | 6 | F6:32450 | 17012022\_RID\_3262\_9a.raw |  |  |  | 1.1826E6 |  | 1.3838E6 |  | 2.5277E6 |  | 2.6941E6 | 4 | 0 | 0 | 0 | 1 | 0 | 1 | 0 | 1 | 0 | 1 | 665 | 675 |  |  | PEAKS DB |
| R.ISEDKNPDYR.K | Y | 59.00 | 1235.5781 | 10 | 0.9 | 618.7969 | 2 | 18.00 | 4 | F4:5768 | 17012022\_RID\_3262\_10a .raw | 2.2898E5 | 5.2363E4 |  | 5.724E5 |  | 6.4152E5 | 4.2912E4 |  |  |  | 5 | 1 | 1 | 0 | 1 | 0 | 1 | 1 | 0 | 0 | 0 | 653 | 662 |  |  | PEAKS DB |
| K.SAIVHLINYQDDAELATR.A | Y | 57.87 | 2028.0276 | 18 | 1.0 | 677.0172 | 3 | 43.76 | 1 | F1:28958 | 13012022\_RID\_3262\_4a.raw | 4.2034E5 |  |  |  |  |  |  | 1.3135E6 | 3.6387E5 |  | 4 | 1 | 0 | 0 | 0 | 0 | 0 | 0 | 2 | 1 | 0 | 126 | 143 |  |  | PEAKS DB |
| K.SGGIPALVR.M | Y | 56.02 | 868.5130 | 9 | 0.5 | 435.2640 | 2 | 35.68 | 6 | F6:21518 | 17012022\_RID\_3262\_9a.raw |  | 3.703E5 |  | 2.5461E6 |  | 2.1357E6 | 8.8669E4 | 0 | 6.1375E5 |  | 5 | 0 | 1 | 0 | 1 | 0 | 1 | 1 | 0 | 1 | 0 | 226 | 234 |  |  | PEAKS DB |
| R.TM(+15.99)QNTSDLDTAR.C | Y | 55.25 | 1367.5988 | 12 | -0.4 | 684.8064 | 2 | 21.71 | 4 | F4:8886 | 17012022\_RID\_3262\_10a .raw |  | 1.2132E5 |  | 1.0143E6 |  | 6.4155E5 |  | 2.1506E5 | 1.8157E5 |  | 5 | 0 | 1 | 0 | 1 | 0 | 1 | 0 | 1 | 1 | 0 | 193 | 204 | Oxidation (M) | M2:Oxidation (M):1000.00 | PEAKS DB |
| R.HPEAEM(+15.99)AQNSVR.L | Y | 53.70 | 1383.6201 | 12 | 1.0 | 692.8180 | 2 | 15.98 | 4 | F4:4154 | 17012022\_RID\_3262\_10a .raw |  |  |  | 4.0379E5 |  |  |  |  |  |  | 2 | 0 | 0 | 0 | 2 | 0 | 0 | 0 | 0 | 0 | 0 | 467 | 478 | Oxidation (M) | M6:Oxidation (M):1000.00 | PEAKS DB |
| K.AAM(+15.99)IVNQLSK.K | Y | 53.10 | 1089.5852 | 10 | 0.8 | 545.8003 | 2 | 28.59 | 4 | F4:15343 | 17012022\_RID\_3262\_10a .raw |  |  |  | 1.0996E6 |  |  |  |  | 1.8354E5 |  | 2 | 0 | 0 | 0 | 1 | 0 | 0 | 0 | 0 | 1 | 0 | 163 | 172 | Oxidation (M) | M3:Oxidation (M):1000.00 | PEAKS DB |
| R.TMQNTSDLDTAR.C | Y | 53.03 | 1351.6038 | 12 | 0.1 | 676.8092 | 2 | 22.98 | 9 | F9:7560 | 31122021\_RID\_3262\_2a.raw |  | 1.1062E5 |  |  |  | 9.3847E5 |  | 2.411E5 | 2.0476E5 |  | 4 | 0 | 1 | 0 | 0 | 0 | 1 | 0 | 1 | 1 | 0 | 193 | 204 |  |  | PEAKS DB |
| K.AAMIVNQLSK.K | Y | 52.58 | 1073.5903 | 10 | -0.4 | 537.8022 | 2 | 33.93 | 4 | F4:19962 | 17012022\_RID\_3262\_10a .raw |  |  |  | 1.2793E6 |  | 1.6681E6 |  |  |  |  | 2 | 0 | 0 | 0 | 1 | 0 | 1 | 0 | 0 | 0 | 0 | 163 | 172 |  |  | PEAKS DB |
| R.HPEAEMAQNSVR.L | Y | 51.71 | 1367.6251 | 12 | 0.0 | 684.8198 | 2 | 17.98 | 8 | F8:3971 | 31122021\_RID\_3262\_1a.raw |  |  |  | 3.542E5 |  |  |  | 1.8269E5 | 1.0075E5 |  | 3 | 0 | 0 | 0 | 1 | 0 | 0 | 0 | 1 | 1 | 0 | 467 | 478 |  |  | PEAKS DB |
| L.INYQDDAELATR.A | N | 45.89 | 1407.6630 | 12 | 0.5 | 704.8391 | 2 | 32.83 | 9 | F9:15651 | 31122021\_RID\_3262\_2a.raw |  |  |  |  |  |  |  | 6.9475E4 | 0 |  | 1 | 0 | 0 | 0 | 0 | 0 | 0 | 0 | 1 | 0 | 0 | 132 | 143 |  |  | PEAKS DB |
| total 13 peptides |  |  |  |  |  |  |  |  |  |  |  |  |  |  |  |  |  |  |  |  |  |  |  |  |  |  |  |  |  |  |  |  |  |  |  |  |  |
| --- | --- | --- | --- | --- | --- | --- | --- | --- | --- | --- | --- | --- | --- | --- | --- | --- | --- | --- | --- | --- | --- | --- | --- | --- | --- | --- | --- | --- | --- | --- | --- | --- | --- | --- | --- | --- | --- |

JAG67632.1

back to list

  

| Protein Coverage
| Supporting Peptides
|

Protein Coverage:

Supporting Peptides:

| Peptide | Uniq | -10lgP | Mass | Length | ppm | m/z | z | RT | Fraction | Scan | Source File | Area F4 | Area F5 | Area F6 | Area F10 | Area F8 | Area F9 | Area F7 | Area F1 | Area F2 | Area F3 | #Feature | #Feature F4 | #Feature F5 | #Feature F6 | #Feature F10 | #Feature F8 | #Feature F9 | #Feature F7 | #Feature F1 | #Feature F2 | #Feature F3 | Start | End | PTM | AScore | Found By |
| --- | --- | --- | --- | --- | --- | --- | --- | --- | --- | --- | --- | --- | --- | --- | --- | --- | --- | --- | --- | --- | --- | --- | --- | --- | --- | --- | --- | --- | --- | --- | --- | --- | --- | --- | --- | --- | --- |
| R.NEGTATYAAAVLFR.I | Y | 74.08 | 1482.7467 | 14 | 0.2 | 742.3808 | 2 | 48.27 | 9 | F9:28127 | 31122021\_RID\_3262\_2a.raw | 4.317E5 |  |  |  |  | 1.024E6 |  | 1.4003E6 | 4.6202E5 |  | 5 | 1 | 0 | 0 | 0 | 0 | 1 | 0 | 2 | 1 | 0 | 639 | 652 |  |  | PEAKS DB |
| R.LNYGIPAIVK.L | Y | 65.66 | 1086.6437 | 10 | 0.0 | 544.3291 | 2 | 45.04 | 8 | F8:24415 | 31122021\_RID\_3262\_1a.raw |  |  |  | 2.5539E6 |  | 2.6128E6 |  | 1.8129E6 | 7.9277E5 |  | 4 | 0 | 0 | 0 | 1 | 0 | 1 | 0 | 1 | 1 | 0 | 479 | 488 |  |  | PEAKS DB |
| R.VSVELTNSLFK.H | Y | 60.86 | 1235.6761 | 11 | 0.7 | 618.8458 | 2 | 47.53 | 6 | F6:32450 | 17012022\_RID\_3262\_9a.raw |  |  |  | 1.1826E6 |  | 1.3838E6 |  | 2.5277E6 |  | 2.6941E6 | 4 | 0 | 0 | 0 | 1 | 0 | 1 | 0 | 1 | 0 | 1 | 665 | 675 |  |  | PEAKS DB |
| R.ISEDKNPDYR.K | Y | 59.00 | 1235.5781 | 10 | 0.9 | 618.7969 | 2 | 18.00 | 4 | F4:5768 | 17012022\_RID\_3262\_10a .raw | 2.2898E5 | 5.2363E4 |  | 5.724E5 |  | 6.4152E5 | 4.2912E4 |  |  |  | 5 | 1 | 1 | 0 | 1 | 0 | 1 | 1 | 0 | 0 | 0 | 653 | 662 |  |  | PEAKS DB |
| K.SAIVHLINYQDDAELATR.A | Y | 57.87 | 2028.0276 | 18 | 1.0 | 677.0172 | 3 | 43.76 | 1 | F1:28958 | 13012022\_RID\_3262\_4a.raw | 4.2034E5 |  |  |  |  |  |  | 1.3135E6 | 3.6387E5 |  | 4 | 1 | 0 | 0 | 0 | 0 | 0 | 0 | 2 | 1 | 0 | 126 | 143 |  |  | PEAKS DB |
| K.SGGIPALVR.M | Y | 56.02 | 868.5130 | 9 | 0.5 | 435.2640 | 2 | 35.68 | 6 | F6:21518 | 17012022\_RID\_3262\_9a.raw |  | 3.703E5 |  | 2.5461E6 |  | 2.1357E6 | 8.8669E4 | 0 | 6.1375E5 |  | 5 | 0 | 1 | 0 | 1 | 0 | 1 | 1 | 0 | 1 | 0 | 226 | 234 |  |  | PEAKS DB |
| R.TM(+15.99)QNTSDLDTAR.C | Y | 55.25 | 1367.5988 | 12 | -0.4 | 684.8064 | 2 | 21.71 | 4 | F4:8886 | 17012022\_RID\_3262\_10a .raw |  | 1.2132E5 |  | 1.0143E6 |  | 6.4155E5 |  | 2.1506E5 | 1.8157E5 |  | 5 | 0 | 1 | 0 | 1 | 0 | 1 | 0 | 1 | 1 | 0 | 193 | 204 | Oxidation (M) | M2:Oxidation (M):1000.00 | PEAKS DB |
| R.HPEAEM(+15.99)AQNSVR.L | Y | 53.70 | 1383.6201 | 12 | 1.0 | 692.8180 | 2 | 15.98 | 4 | F4:4154 | 17012022\_RID\_3262\_10a .raw |  |  |  | 4.0379E5 |  |  |  |  |  |  | 2 | 0 | 0 | 0 | 2 | 0 | 0 | 0 | 0 | 0 | 0 | 467 | 478 | Oxidation (M) | M6:Oxidation (M):1000.00 | PEAKS DB |
| K.AAM(+15.99)IVNQLSK.K | Y | 53.10 | 1089.5852 | 10 | 0.8 | 545.8003 | 2 | 28.59 | 4 | F4:15343 | 17012022\_RID\_3262\_10a .raw |  |  |  | 1.0996E6 |  |  |  |  | 1.8354E5 |  | 2 | 0 | 0 | 0 | 1 | 0 | 0 | 0 | 0 | 1 | 0 | 163 | 172 | Oxidation (M) | M3:Oxidation (M):1000.00 | PEAKS DB |
| R.TMQNTSDLDTAR.C | Y | 53.03 | 1351.6038 | 12 | 0.1 | 676.8092 | 2 | 22.98 | 9 | F9:7560 | 31122021\_RID\_3262\_2a.raw |  | 1.1062E5 |  |  |  | 9.3847E5 |  | 2.411E5 | 2.0476E5 |  | 4 | 0 | 1 | 0 | 0 | 0 | 1 | 0 | 1 | 1 | 0 | 193 | 204 |  |  | PEAKS DB |
| K.AAMIVNQLSK.K | Y | 52.58 | 1073.5903 | 10 | -0.4 | 537.8022 | 2 | 33.93 | 4 | F4:19962 | 17012022\_RID\_3262\_10a .raw |  |  |  | 1.2793E6 |  | 1.6681E6 |  |  |  |  | 2 | 0 | 0 | 0 | 1 | 0 | 1 | 0 | 0 | 0 | 0 | 163 | 172 |  |  | PEAKS DB |
| R.HPEAEMAQNSVR.L | Y | 51.71 | 1367.6251 | 12 | 0.0 | 684.8198 | 2 | 17.98 | 8 | F8:3971 | 31122021\_RID\_3262\_1a.raw |  |  |  | 3.542E5 |  |  |  | 1.8269E5 | 1.0075E5 |  | 3 | 0 | 0 | 0 | 1 | 0 | 0 | 0 | 1 | 1 | 0 | 467 | 478 |  |  | PEAKS DB |
| L.INYQDDAELATR.A | N | 45.89 | 1407.6630 | 12 | 0.5 | 704.8391 | 2 | 32.83 | 9 | F9:15651 | 31122021\_RID\_3262\_2a.raw |  |  |  |  |  |  |  | 6.9475E4 | 0 |  | 1 | 0 | 0 | 0 | 0 | 0 | 0 | 0 | 1 | 0 | 0 | 132 | 143 |  |  | PEAKS DB |
| total 13 peptides |  |  |  |  |  |  |  |  |  |  |  |  |  |  |  |  |  |  |  |  |  |  |  |  |  |  |  |  |  |  |  |  |  |  |  |  |  |
| --- | --- | --- | --- | --- | --- | --- | --- | --- | --- | --- | --- | --- | --- | --- | --- | --- | --- | --- | --- | --- | --- | --- | --- | --- | --- | --- | --- | --- | --- | --- | --- | --- | --- | --- | --- | --- | --- |

XP\_025022317.1

back to list

  

| Protein Coverage
| Supporting Peptides
|

Protein Coverage:

Supporting Peptides:

| Peptide | Uniq | -10lgP | Mass | Length | ppm | m/z | z | RT | Fraction | Scan | Source File | Area F4 | Area F5 | Area F6 | Area F10 | Area F8 | Area F9 | Area F7 | Area F1 | Area F2 | Area F3 | #Feature | #Feature F4 | #Feature F5 | #Feature F6 | #Feature F10 | #Feature F8 | #Feature F9 | #Feature F7 | #Feature F1 | #Feature F2 | #Feature F3 | Start | End | PTM | AScore | Found By |
| --- | --- | --- | --- | --- | --- | --- | --- | --- | --- | --- | --- | --- | --- | --- | --- | --- | --- | --- | --- | --- | --- | --- | --- | --- | --- | --- | --- | --- | --- | --- | --- | --- | --- | --- | --- | --- | --- |
| R.NEGTATYAAAVLFR.I | Y | 74.08 | 1482.7467 | 14 | 0.2 | 742.3808 | 2 | 48.27 | 9 | F9:28127 | 31122021\_RID\_3262\_2a.raw | 4.317E5 |  |  |  |  | 1.024E6 |  | 1.4003E6 | 4.6202E5 |  | 5 | 1 | 0 | 0 | 0 | 0 | 1 | 0 | 2 | 1 | 0 | 641 | 654 |  |  | PEAKS DB |
| R.LNYGIPAIVK.L | Y | 65.66 | 1086.6437 | 10 | 0.0 | 544.3291 | 2 | 45.04 | 8 | F8:24415 | 31122021\_RID\_3262\_1a.raw |  |  |  | 2.5539E6 |  | 2.6128E6 |  | 1.8129E6 | 7.9277E5 |  | 4 | 0 | 0 | 0 | 1 | 0 | 1 | 0 | 1 | 1 | 0 | 481 | 490 |  |  | PEAKS DB |
| R.VSVELTNSLFK.H | Y | 60.86 | 1235.6761 | 11 | 0.7 | 618.8458 | 2 | 47.53 | 6 | F6:32450 | 17012022\_RID\_3262\_9a.raw |  |  |  | 1.1826E6 |  | 1.3838E6 |  | 2.5277E6 |  | 2.6941E6 | 4 | 0 | 0 | 0 | 1 | 0 | 1 | 0 | 1 | 0 | 1 | 667 | 677 |  |  | PEAKS DB |
| R.ISEDKNPDYR.K | Y | 59.00 | 1235.5781 | 10 | 0.9 | 618.7969 | 2 | 18.00 | 4 | F4:5768 | 17012022\_RID\_3262\_10a .raw | 2.2898E5 | 5.2363E4 |  | 5.724E5 |  | 6.4152E5 | 4.2912E4 |  |  |  | 5 | 1 | 1 | 0 | 1 | 0 | 1 | 1 | 0 | 0 | 0 | 655 | 664 |  |  | PEAKS DB |
| K.SAIVHLINYQDDAELATR.A | Y | 57.87 | 2028.0276 | 18 | 1.0 | 677.0172 | 3 | 43.76 | 1 | F1:28958 | 13012022\_RID\_3262\_4a.raw | 4.2034E5 |  |  |  |  |  |  | 1.3135E6 | 3.6387E5 |  | 4 | 1 | 0 | 0 | 0 | 0 | 0 | 0 | 2 | 1 | 0 | 128 | 145 |  |  | PEAKS DB |
| K.SGGIPALVR.M | Y | 56.02 | 868.5130 | 9 | 0.5 | 435.2640 | 2 | 35.68 | 6 | F6:21518 | 17012022\_RID\_3262\_9a.raw |  | 3.703E5 |  | 2.5461E6 |  | 2.1357E6 | 8.8669E4 | 0 | 6.1375E5 |  | 5 | 0 | 1 | 0 | 1 | 0 | 1 | 1 | 0 | 1 | 0 | 228 | 236 |  |  | PEAKS DB |
| R.TM(+15.99)QNTSDLDTAR.C | Y | 55.25 | 1367.5988 | 12 | -0.4 | 684.8064 | 2 | 21.71 | 4 | F4:8886 | 17012022\_RID\_3262\_10a .raw |  | 1.2132E5 |  | 1.0143E6 |  | 6.4155E5 |  | 2.1506E5 | 1.8157E5 |  | 5 | 0 | 1 | 0 | 1 | 0 | 1 | 0 | 1 | 1 | 0 | 195 | 206 | Oxidation (M) | M2:Oxidation (M):1000.00 | PEAKS DB |
| R.HPEAEM(+15.99)AQNSVR.L | Y | 53.70 | 1383.6201 | 12 | 1.0 | 692.8180 | 2 | 15.98 | 4 | F4:4154 | 17012022\_RID\_3262\_10a .raw |  |  |  | 4.0379E5 |  |  |  |  |  |  | 2 | 0 | 0 | 0 | 2 | 0 | 0 | 0 | 0 | 0 | 0 | 469 | 480 | Oxidation (M) | M6:Oxidation (M):1000.00 | PEAKS DB |
| K.AAM(+15.99)IVNQLSK.K | Y | 53.10 | 1089.5852 | 10 | 0.8 | 545.8003 | 2 | 28.59 | 4 | F4:15343 | 17012022\_RID\_3262\_10a .raw |  |  |  | 1.0996E6 |  |  |  |  | 1.8354E5 |  | 2 | 0 | 0 | 0 | 1 | 0 | 0 | 0 | 0 | 1 | 0 | 165 | 174 | Oxidation (M) | M3:Oxidation (M):1000.00 | PEAKS DB |
| R.TMQNTSDLDTAR.C | Y | 53.03 | 1351.6038 | 12 | 0.1 | 676.8092 | 2 | 22.98 | 9 | F9:7560 | 31122021\_RID\_3262\_2a.raw |  | 1.1062E5 |  |  |  | 9.3847E5 |  | 2.411E5 | 2.0476E5 |  | 4 | 0 | 1 | 0 | 0 | 0 | 1 | 0 | 1 | 1 | 0 | 195 | 206 |  |  | PEAKS DB |
| K.AAMIVNQLSK.K | Y | 52.58 | 1073.5903 | 10 | -0.4 | 537.8022 | 2 | 33.93 | 4 | F4:19962 | 17012022\_RID\_3262\_10a .raw |  |  |  | 1.2793E6 |  | 1.6681E6 |  |  |  |  | 2 | 0 | 0 | 0 | 1 | 0 | 1 | 0 | 0 | 0 | 0 | 165 | 174 |  |  | PEAKS DB |
| R.HPEAEMAQNSVR.L | Y | 51.71 | 1367.6251 | 12 | 0.0 | 684.8198 | 2 | 17.98 | 8 | F8:3971 | 31122021\_RID\_3262\_1a.raw |  |  |  | 3.542E5 |  |  |  | 1.8269E5 | 1.0075E5 |  | 3 | 0 | 0 | 0 | 1 | 0 | 0 | 0 | 1 | 1 | 0 | 469 | 480 |  |  | PEAKS DB |
| L.INYQDDAELATR.A | N | 45.89 | 1407.6630 | 12 | 0.5 | 704.8391 | 2 | 32.83 | 9 | F9:15651 | 31122021\_RID\_3262\_2a.raw |  |  |  |  |  |  |  | 6.9475E4 | 0 |  | 1 | 0 | 0 | 0 | 0 | 0 | 0 | 0 | 1 | 0 | 0 | 134 | 145 |  |  | PEAKS DB |
| total 13 peptides |  |  |  |  |  |  |  |  |  |  |  |  |  |  |  |  |  |  |  |  |  |  |  |  |  |  |  |  |  |  |  |  |  |  |  |  |  |
| --- | --- | --- | --- | --- | --- | --- | --- | --- | --- | --- | --- | --- | --- | --- | --- | --- | --- | --- | --- | --- | --- | --- | --- | --- | --- | --- | --- | --- | --- | --- | --- | --- | --- | --- | --- | --- | --- |

XP\_007428111.1

back to list

  

| Protein Coverage
| Supporting Peptides
|

Protein Coverage:

Supporting Peptides:

| Peptide | Uniq | -10lgP | Mass | Length | ppm | m/z | z | RT | Fraction | Scan | Source File | Area F4 | Area F5 | Area F6 | Area F10 | Area F8 | Area F9 | Area F7 | Area F1 | Area F2 | Area F3 | #Feature | #Feature F4 | #Feature F5 | #Feature F6 | #Feature F10 | #Feature F8 | #Feature F9 | #Feature F7 | #Feature F1 | #Feature F2 | #Feature F3 | Start | End | PTM | AScore | Found By |
| --- | --- | --- | --- | --- | --- | --- | --- | --- | --- | --- | --- | --- | --- | --- | --- | --- | --- | --- | --- | --- | --- | --- | --- | --- | --- | --- | --- | --- | --- | --- | --- | --- | --- | --- | --- | --- | --- |
| R.NEGTATYAAAVLFR.I | Y | 74.08 | 1482.7467 | 14 | 0.2 | 742.3808 | 2 | 48.27 | 9 | F9:28127 | 31122021\_RID\_3262\_2a.raw | 4.317E5 |  |  |  |  | 1.024E6 |  | 1.4003E6 | 4.6202E5 |  | 5 | 1 | 0 | 0 | 0 | 0 | 1 | 0 | 2 | 1 | 0 | 641 | 654 |  |  | PEAKS DB |
| R.LNYGIPAIVK.L | Y | 65.66 | 1086.6437 | 10 | 0.0 | 544.3291 | 2 | 45.04 | 8 | F8:24415 | 31122021\_RID\_3262\_1a.raw |  |  |  | 2.5539E6 |  | 2.6128E6 |  | 1.8129E6 | 7.9277E5 |  | 4 | 0 | 0 | 0 | 1 | 0 | 1 | 0 | 1 | 1 | 0 | 481 | 490 |  |  | PEAKS DB |
| R.VSVELTNSLFK.H | Y | 60.86 | 1235.6761 | 11 | 0.7 | 618.8458 | 2 | 47.53 | 6 | F6:32450 | 17012022\_RID\_3262\_9a.raw |  |  |  | 1.1826E6 |  | 1.3838E6 |  | 2.5277E6 |  | 2.6941E6 | 4 | 0 | 0 | 0 | 1 | 0 | 1 | 0 | 1 | 0 | 1 | 667 | 677 |  |  | PEAKS DB |
| R.ISEDKNPDYR.K | Y | 59.00 | 1235.5781 | 10 | 0.9 | 618.7969 | 2 | 18.00 | 4 | F4:5768 | 17012022\_RID\_3262\_10a .raw | 2.2898E5 | 5.2363E4 |  | 5.724E5 |  | 6.4152E5 | 4.2912E4 |  |  |  | 5 | 1 | 1 | 0 | 1 | 0 | 1 | 1 | 0 | 0 | 0 | 655 | 664 |  |  | PEAKS DB |
| K.SAIVHLINYQDDAELATR.A | Y | 57.87 | 2028.0276 | 18 | 1.0 | 677.0172 | 3 | 43.76 | 1 | F1:28958 | 13012022\_RID\_3262\_4a.raw | 4.2034E5 |  |  |  |  |  |  | 1.3135E6 | 3.6387E5 |  | 4 | 1 | 0 | 0 | 0 | 0 | 0 | 0 | 2 | 1 | 0 | 128 | 145 |  |  | PEAKS DB |
| K.SGGIPALVR.M | Y | 56.02 | 868.5130 | 9 | 0.5 | 435.2640 | 2 | 35.68 | 6 | F6:21518 | 17012022\_RID\_3262\_9a.raw |  | 3.703E5 |  | 2.5461E6 |  | 2.1357E6 | 8.8669E4 | 0 | 6.1375E5 |  | 5 | 0 | 1 | 0 | 1 | 0 | 1 | 1 | 0 | 1 | 0 | 228 | 236 |  |  | PEAKS DB |
| R.TM(+15.99)QNTSDLDTAR.C | Y | 55.25 | 1367.5988 | 12 | -0.4 | 684.8064 | 2 | 21.71 | 4 | F4:8886 | 17012022\_RID\_3262\_10a .raw |  | 1.2132E5 |  | 1.0143E6 |  | 6.4155E5 |  | 2.1506E5 | 1.8157E5 |  | 5 | 0 | 1 | 0 | 1 | 0 | 1 | 0 | 1 | 1 | 0 | 195 | 206 | Oxidation (M) | M2:Oxidation (M):1000.00 | PEAKS DB |
| R.HPEAEM(+15.99)AQNSVR.L | Y | 53.70 | 1383.6201 | 12 | 1.0 | 692.8180 | 2 | 15.98 | 4 | F4:4154 | 17012022\_RID\_3262\_10a .raw |  |  |  | 4.0379E5 |  |  |  |  |  |  | 2 | 0 | 0 | 0 | 2 | 0 | 0 | 0 | 0 | 0 | 0 | 469 | 480 | Oxidation (M) | M6:Oxidation (M):1000.00 | PEAKS DB |
| K.AAM(+15.99)IVNQLSK.K | Y | 53.10 | 1089.5852 | 10 | 0.8 | 545.8003 | 2 | 28.59 | 4 | F4:15343 | 17012022\_RID\_3262\_10a .raw |  |  |  | 1.0996E6 |  |  |  |  | 1.8354E5 |  | 2 | 0 | 0 | 0 | 1 | 0 | 0 | 0 | 0 | 1 | 0 | 165 | 174 | Oxidation (M) | M3:Oxidation (M):1000.00 | PEAKS DB |
| R.TMQNTSDLDTAR.C | Y | 53.03 | 1351.6038 | 12 | 0.1 | 676.8092 | 2 | 22.98 | 9 | F9:7560 | 31122021\_RID\_3262\_2a.raw |  | 1.1062E5 |  |  |  | 9.3847E5 |  | 2.411E5 | 2.0476E5 |  | 4 | 0 | 1 | 0 | 0 | 0 | 1 | 0 | 1 | 1 | 0 | 195 | 206 |  |  | PEAKS DB |
| K.AAMIVNQLSK.K | Y | 52.58 | 1073.5903 | 10 | -0.4 | 537.8022 | 2 | 33.93 | 4 | F4:19962 | 17012022\_RID\_3262\_10a .raw |  |  |  | 1.2793E6 |  | 1.6681E6 |  |  |  |  | 2 | 0 | 0 | 0 | 1 | 0 | 1 | 0 | 0 | 0 | 0 | 165 | 174 |  |  | PEAKS DB |
| R.HPEAEMAQNSVR.L | Y | 51.71 | 1367.6251 | 12 | 0.0 | 684.8198 | 2 | 17.98 | 8 | F8:3971 | 31122021\_RID\_3262\_1a.raw |  |  |  | 3.542E5 |  |  |  | 1.8269E5 | 1.0075E5 |  | 3 | 0 | 0 | 0 | 1 | 0 | 0 | 0 | 1 | 1 | 0 | 469 | 480 |  |  | PEAKS DB |
| L.INYQDDAELATR.A | N | 45.89 | 1407.6630 | 12 | 0.5 | 704.8391 | 2 | 32.83 | 9 | F9:15651 | 31122021\_RID\_3262\_2a.raw |  |  |  |  |  |  |  | 6.9475E4 | 0 |  | 1 | 0 | 0 | 0 | 0 | 0 | 0 | 0 | 1 | 0 | 0 | 134 | 145 |  |  | PEAKS DB |
| total 13 peptides |  |  |  |  |  |  |  |  |  |  |  |  |  |  |  |  |  |  |  |  |  |  |  |  |  |  |  |  |  |  |  |  |  |  |  |  |  |
| --- | --- | --- | --- | --- | --- | --- | --- | --- | --- | --- | --- | --- | --- | --- | --- | --- | --- | --- | --- | --- | --- | --- | --- | --- | --- | --- | --- | --- | --- | --- | --- | --- | --- | --- | --- | --- | --- |

JAG44306.1

back to list

  

| Protein Coverage
| Supporting Peptides
|

Protein Coverage:

Supporting Peptides:

| Peptide | Uniq | -10lgP | Mass | Length | ppm | m/z | z | RT | Fraction | Scan | Source File | Area F4 | Area F5 | Area F6 | Area F10 | Area F8 | Area F9 | Area F7 | Area F1 | Area F2 | Area F3 | #Feature | #Feature F4 | #Feature F5 | #Feature F6 | #Feature F10 | #Feature F8 | #Feature F9 | #Feature F7 | #Feature F1 | #Feature F2 | #Feature F3 | Start | End | PTM | AScore | Found By |
| --- | --- | --- | --- | --- | --- | --- | --- | --- | --- | --- | --- | --- | --- | --- | --- | --- | --- | --- | --- | --- | --- | --- | --- | --- | --- | --- | --- | --- | --- | --- | --- | --- | --- | --- | --- | --- | --- |
| R.NEGTATYAAAVLFR.I | Y | 74.08 | 1482.7467 | 14 | 0.2 | 742.3808 | 2 | 48.27 | 9 | F9:28127 | 31122021\_RID\_3262\_2a.raw | 4.317E5 |  |  |  |  | 1.024E6 |  | 1.4003E6 | 4.6202E5 |  | 5 | 1 | 0 | 0 | 0 | 0 | 1 | 0 | 2 | 1 | 0 | 639 | 652 |  |  | PEAKS DB |
| R.LNYGIPAIVK.L | Y | 65.66 | 1086.6437 | 10 | 0.0 | 544.3291 | 2 | 45.04 | 8 | F8:24415 | 31122021\_RID\_3262\_1a.raw |  |  |  | 2.5539E6 |  | 2.6128E6 |  | 1.8129E6 | 7.9277E5 |  | 4 | 0 | 0 | 0 | 1 | 0 | 1 | 0 | 1 | 1 | 0 | 479 | 488 |  |  | PEAKS DB |
| R.VSVELTNSLFK.H | Y | 60.86 | 1235.6761 | 11 | 0.7 | 618.8458 | 2 | 47.53 | 6 | F6:32450 | 17012022\_RID\_3262\_9a.raw |  |  |  | 1.1826E6 |  | 1.3838E6 |  | 2.5277E6 |  | 2.6941E6 | 4 | 0 | 0 | 0 | 1 | 0 | 1 | 0 | 1 | 0 | 1 | 665 | 675 |  |  | PEAKS DB |
| R.ISEDKNPDYR.K | Y | 59.00 | 1235.5781 | 10 | 0.9 | 618.7969 | 2 | 18.00 | 4 | F4:5768 | 17012022\_RID\_3262\_10a .raw | 2.2898E5 | 5.2363E4 |  | 5.724E5 |  | 6.4152E5 | 4.2912E4 |  |  |  | 5 | 1 | 1 | 0 | 1 | 0 | 1 | 1 | 0 | 0 | 0 | 653 | 662 |  |  | PEAKS DB |
| K.SAIVHLINYQDDAELATR.A | Y | 57.87 | 2028.0276 | 18 | 1.0 | 677.0172 | 3 | 43.76 | 1 | F1:28958 | 13012022\_RID\_3262\_4a.raw | 4.2034E5 |  |  |  |  |  |  | 1.3135E6 | 3.6387E5 |  | 4 | 1 | 0 | 0 | 0 | 0 | 0 | 0 | 2 | 1 | 0 | 126 | 143 |  |  | PEAKS DB |
| K.SGGIPALVR.M | Y | 56.02 | 868.5130 | 9 | 0.5 | 435.2640 | 2 | 35.68 | 6 | F6:21518 | 17012022\_RID\_3262\_9a.raw |  | 3.703E5 |  | 2.5461E6 |  | 2.1357E6 | 8.8669E4 | 0 | 6.1375E5 |  | 5 | 0 | 1 | 0 | 1 | 0 | 1 | 1 | 0 | 1 | 0 | 226 | 234 |  |  | PEAKS DB |
| R.TM(+15.99)QNTSDLDTAR.C | Y | 55.25 | 1367.5988 | 12 | -0.4 | 684.8064 | 2 | 21.71 | 4 | F4:8886 | 17012022\_RID\_3262\_10a .raw |  | 1.2132E5 |  | 1.0143E6 |  | 6.4155E5 |  | 2.1506E5 | 1.8157E5 |  | 5 | 0 | 1 | 0 | 1 | 0 | 1 | 0 | 1 | 1 | 0 | 193 | 204 | Oxidation (M) | M2:Oxidation (M):1000.00 | PEAKS DB |
| R.HPEAEM(+15.99)AQNSVR.L | Y | 53.70 | 1383.6201 | 12 | 1.0 | 692.8180 | 2 | 15.98 | 4 | F4:4154 | 17012022\_RID\_3262\_10a .raw |  |  |  | 4.0379E5 |  |  |  |  |  |  | 2 | 0 | 0 | 0 | 2 | 0 | 0 | 0 | 0 | 0 | 0 | 467 | 478 | Oxidation (M) | M6:Oxidation (M):1000.00 | PEAKS DB |
| K.AAM(+15.99)IVNQLSK.K | Y | 53.10 | 1089.5852 | 10 | 0.8 | 545.8003 | 2 | 28.59 | 4 | F4:15343 | 17012022\_RID\_3262\_10a .raw |  |  |  | 1.0996E6 |  |  |  |  | 1.8354E5 |  | 2 | 0 | 0 | 0 | 1 | 0 | 0 | 0 | 0 | 1 | 0 | 163 | 172 | Oxidation (M) | M3:Oxidation (M):1000.00 | PEAKS DB |
| R.TMQNTSDLDTAR.C | Y | 53.03 | 1351.6038 | 12 | 0.1 | 676.8092 | 2 | 22.98 | 9 | F9:7560 | 31122021\_RID\_3262\_2a.raw |  | 1.1062E5 |  |  |  | 9.3847E5 |  | 2.411E5 | 2.0476E5 |  | 4 | 0 | 1 | 0 | 0 | 0 | 1 | 0 | 1 | 1 | 0 | 193 | 204 |  |  | PEAKS DB |
| K.AAMIVNQLSK.K | Y | 52.58 | 1073.5903 | 10 | -0.4 | 537.8022 | 2 | 33.93 | 4 | F4:19962 | 17012022\_RID\_3262\_10a .raw |  |  |  | 1.2793E6 |  | 1.6681E6 |  |  |  |  | 2 | 0 | 0 | 0 | 1 | 0 | 1 | 0 | 0 | 0 | 0 | 163 | 172 |  |  | PEAKS DB |
| R.HPEAEMAQNSVR.L | Y | 51.71 | 1367.6251 | 12 | 0.0 | 684.8198 | 2 | 17.98 | 8 | F8:3971 | 31122021\_RID\_3262\_1a.raw |  |  |  | 3.542E5 |  |  |  | 1.8269E5 | 1.0075E5 |  | 3 | 0 | 0 | 0 | 1 | 0 | 0 | 0 | 1 | 1 | 0 | 467 | 478 |  |  | PEAKS DB |
| L.INYQDDAELATR.A | N | 45.89 | 1407.6630 | 12 | 0.5 | 704.8391 | 2 | 32.83 | 9 | F9:15651 | 31122021\_RID\_3262\_2a.raw |  |  |  |  |  |  |  | 6.9475E4 | 0 |  | 1 | 0 | 0 | 0 | 0 | 0 | 0 | 0 | 1 | 0 | 0 | 132 | 143 |  |  | PEAKS DB |
| total 13 peptides |  |  |  |  |  |  |  |  |  |  |  |  |  |  |  |  |  |  |  |  |  |  |  |  |  |  |  |  |  |  |  |  |  |  |  |  |  |
| --- | --- | --- | --- | --- | --- | --- | --- | --- | --- | --- | --- | --- | --- | --- | --- | --- | --- | --- | --- | --- | --- | --- | --- | --- | --- | --- | --- | --- | --- | --- | --- | --- | --- | --- | --- | --- | --- |

JAA96758.1

back to list

  

| Protein Coverage
| Supporting Peptides
|

Protein Coverage:

Supporting Peptides:

| Peptide | Uniq | -10lgP | Mass | Length | ppm | m/z | z | RT | Fraction | Scan | Source File | Area F4 | Area F5 | Area F6 | Area F10 | Area F8 | Area F9 | Area F7 | Area F1 | Area F2 | Area F3 | #Feature | #Feature F4 | #Feature F5 | #Feature F6 | #Feature F10 | #Feature F8 | #Feature F9 | #Feature F7 | #Feature F1 | #Feature F2 | #Feature F3 | Start | End | PTM | AScore | Found By |
| --- | --- | --- | --- | --- | --- | --- | --- | --- | --- | --- | --- | --- | --- | --- | --- | --- | --- | --- | --- | --- | --- | --- | --- | --- | --- | --- | --- | --- | --- | --- | --- | --- | --- | --- | --- | --- | --- |
| R.NEGTATYAAAVLFR.I | Y | 74.08 | 1482.7467 | 14 | 0.2 | 742.3808 | 2 | 48.27 | 9 | F9:28127 | 31122021\_RID\_3262\_2a.raw | 4.317E5 |  |  |  |  | 1.024E6 |  | 1.4003E6 | 4.6202E5 |  | 5 | 1 | 0 | 0 | 0 | 0 | 1 | 0 | 2 | 1 | 0 | 639 | 652 |  |  | PEAKS DB |
| R.LNYGIPAIVK.L | Y | 65.66 | 1086.6437 | 10 | 0.0 | 544.3291 | 2 | 45.04 | 8 | F8:24415 | 31122021\_RID\_3262\_1a.raw |  |  |  | 2.5539E6 |  | 2.6128E6 |  | 1.8129E6 | 7.9277E5 |  | 4 | 0 | 0 | 0 | 1 | 0 | 1 | 0 | 1 | 1 | 0 | 479 | 488 |  |  | PEAKS DB |
| R.VSVELTNSLFK.H | Y | 60.86 | 1235.6761 | 11 | 0.7 | 618.8458 | 2 | 47.53 | 6 | F6:32450 | 17012022\_RID\_3262\_9a.raw |  |  |  | 1.1826E6 |  | 1.3838E6 |  | 2.5277E6 |  | 2.6941E6 | 4 | 0 | 0 | 0 | 1 | 0 | 1 | 0 | 1 | 0 | 1 | 665 | 675 |  |  | PEAKS DB |
| R.ISEDKNPDYR.K | Y | 59.00 | 1235.5781 | 10 | 0.9 | 618.7969 | 2 | 18.00 | 4 | F4:5768 | 17012022\_RID\_3262\_10a .raw | 2.2898E5 | 5.2363E4 |  | 5.724E5 |  | 6.4152E5 | 4.2912E4 |  |  |  | 5 | 1 | 1 | 0 | 1 | 0 | 1 | 1 | 0 | 0 | 0 | 653 | 662 |  |  | PEAKS DB |
| K.SAIVHLINYQDDAELATR.A | Y | 57.87 | 2028.0276 | 18 | 1.0 | 677.0172 | 3 | 43.76 | 1 | F1:28958 | 13012022\_RID\_3262\_4a.raw | 4.2034E5 |  |  |  |  |  |  | 1.3135E6 | 3.6387E5 |  | 4 | 1 | 0 | 0 | 0 | 0 | 0 | 0 | 2 | 1 | 0 | 126 | 143 |  |  | PEAKS DB |
| K.SGGIPALVR.M | Y | 56.02 | 868.5130 | 9 | 0.5 | 435.2640 | 2 | 35.68 | 6 | F6:21518 | 17012022\_RID\_3262\_9a.raw |  | 3.703E5 |  | 2.5461E6 |  | 2.1357E6 | 8.8669E4 | 0 | 6.1375E5 |  | 5 | 0 | 1 | 0 | 1 | 0 | 1 | 1 | 0 | 1 | 0 | 226 | 234 |  |  | PEAKS DB |
| R.TM(+15.99)QNTSDLDTAR.C | Y | 55.25 | 1367.5988 | 12 | -0.4 | 684.8064 | 2 | 21.71 | 4 | F4:8886 | 17012022\_RID\_3262\_10a .raw |  | 1.2132E5 |  | 1.0143E6 |  | 6.4155E5 |  | 2.1506E5 | 1.8157E5 |  | 5 | 0 | 1 | 0 | 1 | 0 | 1 | 0 | 1 | 1 | 0 | 193 | 204 | Oxidation (M) | M2:Oxidation (M):1000.00 | PEAKS DB |
| R.HPEAEM(+15.99)AQNSVR.L | Y | 53.70 | 1383.6201 | 12 | 1.0 | 692.8180 | 2 | 15.98 | 4 | F4:4154 | 17012022\_RID\_3262\_10a .raw |  |  |  | 4.0379E5 |  |  |  |  |  |  | 2 | 0 | 0 | 0 | 2 | 0 | 0 | 0 | 0 | 0 | 0 | 467 | 478 | Oxidation (M) | M6:Oxidation (M):1000.00 | PEAKS DB |
| K.AAM(+15.99)IVNQLSK.K | Y | 53.10 | 1089.5852 | 10 | 0.8 | 545.8003 | 2 | 28.59 | 4 | F4:15343 | 17012022\_RID\_3262\_10a .raw |  |  |  | 1.0996E6 |  |  |  |  | 1.8354E5 |  | 2 | 0 | 0 | 0 | 1 | 0 | 0 | 0 | 0 | 1 | 0 | 163 | 172 | Oxidation (M) | M3:Oxidation (M):1000.00 | PEAKS DB |
| R.TMQNTSDLDTAR.C | Y | 53.03 | 1351.6038 | 12 | 0.1 | 676.8092 | 2 | 22.98 | 9 | F9:7560 | 31122021\_RID\_3262\_2a.raw |  | 1.1062E5 |  |  |  | 9.3847E5 |  | 2.411E5 | 2.0476E5 |  | 4 | 0 | 1 | 0 | 0 | 0 | 1 | 0 | 1 | 1 | 0 | 193 | 204 |  |  | PEAKS DB |
| K.AAMIVNQLSK.K | Y | 52.58 | 1073.5903 | 10 | -0.4 | 537.8022 | 2 | 33.93 | 4 | F4:19962 | 17012022\_RID\_3262\_10a .raw |  |  |  | 1.2793E6 |  | 1.6681E6 |  |  |  |  | 2 | 0 | 0 | 0 | 1 | 0 | 1 | 0 | 0 | 0 | 0 | 163 | 172 |  |  | PEAKS DB |
| R.HPEAEMAQNSVR.L | Y | 51.71 | 1367.6251 | 12 | 0.0 | 684.8198 | 2 | 17.98 | 8 | F8:3971 | 31122021\_RID\_3262\_1a.raw |  |  |  | 3.542E5 |  |  |  | 1.8269E5 | 1.0075E5 |  | 3 | 0 | 0 | 0 | 1 | 0 | 0 | 0 | 1 | 1 | 0 | 467 | 478 |  |  | PEAKS DB |
| L.INYQDDAELATR.A | N | 45.89 | 1407.6630 | 12 | 0.5 | 704.8391 | 2 | 32.83 | 9 | F9:15651 | 31122021\_RID\_3262\_2a.raw |  |  |  |  |  |  |  | 6.9475E4 | 0 |  | 1 | 0 | 0 | 0 | 0 | 0 | 0 | 0 | 1 | 0 | 0 | 132 | 143 |  |  | PEAKS DB |
| total 13 peptides |  |  |  |  |  |  |  |  |  |  |  |  |  |  |  |  |  |  |  |  |  |  |  |  |  |  |  |  |  |  |  |  |  |  |  |  |  |
| --- | --- | --- | --- | --- | --- | --- | --- | --- | --- | --- | --- | --- | --- | --- | --- | --- | --- | --- | --- | --- | --- | --- | --- | --- | --- | --- | --- | --- | --- | --- | --- | --- | --- | --- | --- | --- | --- |

JAG46538.1

back to list

  

| Protein Coverage
| Supporting Peptides
|

Protein Coverage:

Supporting Peptides:

| Peptide | Uniq | -10lgP | Mass | Length | ppm | m/z | z | RT | Fraction | Scan | Source File | Area F4 | Area F5 | Area F6 | Area F10 | Area F8 | Area F9 | Area F7 | Area F1 | Area F2 | Area F3 | #Feature | #Feature F4 | #Feature F5 | #Feature F6 | #Feature F10 | #Feature F8 | #Feature F9 | #Feature F7 | #Feature F1 | #Feature F2 | #Feature F3 | Start | End | PTM | AScore | Found By |
| --- | --- | --- | --- | --- | --- | --- | --- | --- | --- | --- | --- | --- | --- | --- | --- | --- | --- | --- | --- | --- | --- | --- | --- | --- | --- | --- | --- | --- | --- | --- | --- | --- | --- | --- | --- | --- | --- |
| R.NEGTATYAAAVLFR.I | Y | 74.08 | 1482.7467 | 14 | 0.2 | 742.3808 | 2 | 48.27 | 9 | F9:28127 | 31122021\_RID\_3262\_2a.raw | 4.317E5 |  |  |  |  | 1.024E6 |  | 1.4003E6 | 4.6202E5 |  | 5 | 1 | 0 | 0 | 0 | 0 | 1 | 0 | 2 | 1 | 0 | 639 | 652 |  |  | PEAKS DB |
| R.LNYGIPAIVK.L | Y | 65.66 | 1086.6437 | 10 | 0.0 | 544.3291 | 2 | 45.04 | 8 | F8:24415 | 31122021\_RID\_3262\_1a.raw |  |  |  | 2.5539E6 |  | 2.6128E6 |  | 1.8129E6 | 7.9277E5 |  | 4 | 0 | 0 | 0 | 1 | 0 | 1 | 0 | 1 | 1 | 0 | 479 | 488 |  |  | PEAKS DB |
| R.VSVELTNSLFK.H | Y | 60.86 | 1235.6761 | 11 | 0.7 | 618.8458 | 2 | 47.53 | 6 | F6:32450 | 17012022\_RID\_3262\_9a.raw |  |  |  | 1.1826E6 |  | 1.3838E6 |  | 2.5277E6 |  | 2.6941E6 | 4 | 0 | 0 | 0 | 1 | 0 | 1 | 0 | 1 | 0 | 1 | 665 | 675 |  |  | PEAKS DB |
| R.ISEDKNPDYR.K | Y | 59.00 | 1235.5781 | 10 | 0.9 | 618.7969 | 2 | 18.00 | 4 | F4:5768 | 17012022\_RID\_3262\_10a .raw | 2.2898E5 | 5.2363E4 |  | 5.724E5 |  | 6.4152E5 | 4.2912E4 |  |  |  | 5 | 1 | 1 | 0 | 1 | 0 | 1 | 1 | 0 | 0 | 0 | 653 | 662 |  |  | PEAKS DB |
| K.SAIVHLINYQDDAELATR.A | Y | 57.87 | 2028.0276 | 18 | 1.0 | 677.0172 | 3 | 43.76 | 1 | F1:28958 | 13012022\_RID\_3262\_4a.raw | 4.2034E5 |  |  |  |  |  |  | 1.3135E6 | 3.6387E5 |  | 4 | 1 | 0 | 0 | 0 | 0 | 0 | 0 | 2 | 1 | 0 | 126 | 143 |  |  | PEAKS DB |
| K.SGGIPALVR.M | Y | 56.02 | 868.5130 | 9 | 0.5 | 435.2640 | 2 | 35.68 | 6 | F6:21518 | 17012022\_RID\_3262\_9a.raw |  | 3.703E5 |  | 2.5461E6 |  | 2.1357E6 | 8.8669E4 | 0 | 6.1375E5 |  | 5 | 0 | 1 | 0 | 1 | 0 | 1 | 1 | 0 | 1 | 0 | 226 | 234 |  |  | PEAKS DB |
| R.TM(+15.99)QNTSDLDTAR.C | Y | 55.25 | 1367.5988 | 12 | -0.4 | 684.8064 | 2 | 21.71 | 4 | F4:8886 | 17012022\_RID\_3262\_10a .raw |  | 1.2132E5 |  | 1.0143E6 |  | 6.4155E5 |  | 2.1506E5 | 1.8157E5 |  | 5 | 0 | 1 | 0 | 1 | 0 | 1 | 0 | 1 | 1 | 0 | 193 | 204 | Oxidation (M) | M2:Oxidation (M):1000.00 | PEAKS DB |
| R.HPEAEM(+15.99)AQNSVR.L | Y | 53.70 | 1383.6201 | 12 | 1.0 | 692.8180 | 2 | 15.98 | 4 | F4:4154 | 17012022\_RID\_3262\_10a .raw |  |  |  | 4.0379E5 |  |  |  |  |  |  | 2 | 0 | 0 | 0 | 2 | 0 | 0 | 0 | 0 | 0 | 0 | 467 | 478 | Oxidation (M) | M6:Oxidation (M):1000.00 | PEAKS DB |
| K.AAM(+15.99)IVNQLSK.K | Y | 53.10 | 1089.5852 | 10 | 0.8 | 545.8003 | 2 | 28.59 | 4 | F4:15343 | 17012022\_RID\_3262\_10a .raw |  |  |  | 1.0996E6 |  |  |  |  | 1.8354E5 |  | 2 | 0 | 0 | 0 | 1 | 0 | 0 | 0 | 0 | 1 | 0 | 163 | 172 | Oxidation (M) | M3:Oxidation (M):1000.00 | PEAKS DB |
| R.TMQNTSDLDTAR.C | Y | 53.03 | 1351.6038 | 12 | 0.1 | 676.8092 | 2 | 22.98 | 9 | F9:7560 | 31122021\_RID\_3262\_2a.raw |  | 1.1062E5 |  |  |  | 9.3847E5 |  | 2.411E5 | 2.0476E5 |  | 4 | 0 | 1 | 0 | 0 | 0 | 1 | 0 | 1 | 1 | 0 | 193 | 204 |  |  | PEAKS DB |
| K.AAMIVNQLSK.K | Y | 52.58 | 1073.5903 | 10 | -0.4 | 537.8022 | 2 | 33.93 | 4 | F4:19962 | 17012022\_RID\_3262\_10a .raw |  |  |  | 1.2793E6 |  | 1.6681E6 |  |  |  |  | 2 | 0 | 0 | 0 | 1 | 0 | 1 | 0 | 0 | 0 | 0 | 163 | 172 |  |  | PEAKS DB |
| R.HPEAEMAQNSVR.L | Y | 51.71 | 1367.6251 | 12 | 0.0 | 684.8198 | 2 | 17.98 | 8 | F8:3971 | 31122021\_RID\_3262\_1a.raw |  |  |  | 3.542E5 |  |  |  | 1.8269E5 | 1.0075E5 |  | 3 | 0 | 0 | 0 | 1 | 0 | 0 | 0 | 1 | 1 | 0 | 467 | 478 |  |  | PEAKS DB |
| L.INYQDDAELATR.A | N | 45.89 | 1407.6630 | 12 | 0.5 | 704.8391 | 2 | 32.83 | 9 | F9:15651 | 31122021\_RID\_3262\_2a.raw |  |  |  |  |  |  |  | 6.9475E4 | 0 |  | 1 | 0 | 0 | 0 | 0 | 0 | 0 | 0 | 1 | 0 | 0 | 132 | 143 |  |  | PEAKS DB |
| total 13 peptides |  |  |  |  |  |  |  |  |  |  |  |  |  |  |  |  |  |  |  |  |  |  |  |  |  |  |  |  |  |  |  |  |  |  |  |  |  |
| --- | --- | --- | --- | --- | --- | --- | --- | --- | --- | --- | --- | --- | --- | --- | --- | --- | --- | --- | --- | --- | --- | --- | --- | --- | --- | --- | --- | --- | --- | --- | --- | --- | --- | --- | --- | --- | --- |

JAV50234.1

back to list

  

| Protein Coverage
| Supporting Peptides
|

Protein Coverage:

Supporting Peptides:

| Peptide | Uniq | -10lgP | Mass | Length | ppm | m/z | z | RT | Fraction | Scan | Source File | Area F4 | Area F5 | Area F6 | Area F10 | Area F8 | Area F9 | Area F7 | Area F1 | Area F2 | Area F3 | #Feature | #Feature F4 | #Feature F5 | #Feature F6 | #Feature F10 | #Feature F8 | #Feature F9 | #Feature F7 | #Feature F1 | #Feature F2 | #Feature F3 | Start | End | PTM | AScore | Found By |
| --- | --- | --- | --- | --- | --- | --- | --- | --- | --- | --- | --- | --- | --- | --- | --- | --- | --- | --- | --- | --- | --- | --- | --- | --- | --- | --- | --- | --- | --- | --- | --- | --- | --- | --- | --- | --- | --- |
| R.NEGTATYAAAVLFR.I | Y | 74.08 | 1482.7467 | 14 | 0.2 | 742.3808 | 2 | 48.27 | 9 | F9:28127 | 31122021\_RID\_3262\_2a.raw | 4.317E5 |  |  |  |  | 1.024E6 |  | 1.4003E6 | 4.6202E5 |  | 5 | 1 | 0 | 0 | 0 | 0 | 1 | 0 | 2 | 1 | 0 | 639 | 652 |  |  | PEAKS DB |
| R.LNYGIPAIVK.L | Y | 65.66 | 1086.6437 | 10 | 0.0 | 544.3291 | 2 | 45.04 | 8 | F8:24415 | 31122021\_RID\_3262\_1a.raw |  |  |  | 2.5539E6 |  | 2.6128E6 |  | 1.8129E6 | 7.9277E5 |  | 4 | 0 | 0 | 0 | 1 | 0 | 1 | 0 | 1 | 1 | 0 | 479 | 488 |  |  | PEAKS DB |
| R.VSVELTNSLFK.H | Y | 60.86 | 1235.6761 | 11 | 0.7 | 618.8458 | 2 | 47.53 | 6 | F6:32450 | 17012022\_RID\_3262\_9a.raw |  |  |  | 1.1826E6 |  | 1.3838E6 |  | 2.5277E6 |  | 2.6941E6 | 4 | 0 | 0 | 0 | 1 | 0 | 1 | 0 | 1 | 0 | 1 | 665 | 675 |  |  | PEAKS DB |
| R.ISEDKNPDYR.K | Y | 59.00 | 1235.5781 | 10 | 0.9 | 618.7969 | 2 | 18.00 | 4 | F4:5768 | 17012022\_RID\_3262\_10a .raw | 2.2898E5 | 5.2363E4 |  | 5.724E5 |  | 6.4152E5 | 4.2912E4 |  |  |  | 5 | 1 | 1 | 0 | 1 | 0 | 1 | 1 | 0 | 0 | 0 | 653 | 662 |  |  | PEAKS DB |
| K.SAIVHLINYQDDAELATR.A | Y | 57.87 | 2028.0276 | 18 | 1.0 | 677.0172 | 3 | 43.76 | 1 | F1:28958 | 13012022\_RID\_3262\_4a.raw | 4.2034E5 |  |  |  |  |  |  | 1.3135E6 | 3.6387E5 |  | 4 | 1 | 0 | 0 | 0 | 0 | 0 | 0 | 2 | 1 | 0 | 126 | 143 |  |  | PEAKS DB |
| K.SGGIPALVR.M | Y | 56.02 | 868.5130 | 9 | 0.5 | 435.2640 | 2 | 35.68 | 6 | F6:21518 | 17012022\_RID\_3262\_9a.raw |  | 3.703E5 |  | 2.5461E6 |  | 2.1357E6 | 8.8669E4 | 0 | 6.1375E5 |  | 5 | 0 | 1 | 0 | 1 | 0 | 1 | 1 | 0 | 1 | 0 | 226 | 234 |  |  | PEAKS DB |
| R.TM(+15.99)QNTSDLDTAR.C | Y | 55.25 | 1367.5988 | 12 | -0.4 | 684.8064 | 2 | 21.71 | 4 | F4:8886 | 17012022\_RID\_3262\_10a .raw |  | 1.2132E5 |  | 1.0143E6 |  | 6.4155E5 |  | 2.1506E5 | 1.8157E5 |  | 5 | 0 | 1 | 0 | 1 | 0 | 1 | 0 | 1 | 1 | 0 | 193 | 204 | Oxidation (M) | M2:Oxidation (M):1000.00 | PEAKS DB |
| R.HPEAEM(+15.99)AQNSVR.L | Y | 53.70 | 1383.6201 | 12 | 1.0 | 692.8180 | 2 | 15.98 | 4 | F4:4154 | 17012022\_RID\_3262\_10a .raw |  |  |  | 4.0379E5 |  |  |  |  |  |  | 2 | 0 | 0 | 0 | 2 | 0 | 0 | 0 | 0 | 0 | 0 | 467 | 478 | Oxidation (M) | M6:Oxidation (M):1000.00 | PEAKS DB |
| K.AAM(+15.99)IVNQLSK.K | Y | 53.10 | 1089.5852 | 10 | 0.8 | 545.8003 | 2 | 28.59 | 4 | F4:15343 | 17012022\_RID\_3262\_10a .raw |  |  |  | 1.0996E6 |  |  |  |  | 1.8354E5 |  | 2 | 0 | 0 | 0 | 1 | 0 | 0 | 0 | 0 | 1 | 0 | 163 | 172 | Oxidation (M) | M3:Oxidation (M):1000.00 | PEAKS DB |
| R.TMQNTSDLDTAR.C | Y | 53.03 | 1351.6038 | 12 | 0.1 | 676.8092 | 2 | 22.98 | 9 | F9:7560 | 31122021\_RID\_3262\_2a.raw |  | 1.1062E5 |  |  |  | 9.3847E5 |  | 2.411E5 | 2.0476E5 |  | 4 | 0 | 1 | 0 | 0 | 0 | 1 | 0 | 1 | 1 | 0 | 193 | 204 |  |  | PEAKS DB |
| K.AAMIVNQLSK.K | Y | 52.58 | 1073.5903 | 10 | -0.4 | 537.8022 | 2 | 33.93 | 4 | F4:19962 | 17012022\_RID\_3262\_10a .raw |  |  |  | 1.2793E6 |  | 1.6681E6 |  |  |  |  | 2 | 0 | 0 | 0 | 1 | 0 | 1 | 0 | 0 | 0 | 0 | 163 | 172 |  |  | PEAKS DB |
| R.HPEAEMAQNSVR.L | Y | 51.71 | 1367.6251 | 12 | 0.0 | 684.8198 | 2 | 17.98 | 8 | F8:3971 | 31122021\_RID\_3262\_1a.raw |  |  |  | 3.542E5 |  |  |  | 1.8269E5 | 1.0075E5 |  | 3 | 0 | 0 | 0 | 1 | 0 | 0 | 0 | 1 | 1 | 0 | 467 | 478 |  |  | PEAKS DB |
| L.INYQDDAELATR.A | N | 45.89 | 1407.6630 | 12 | 0.5 | 704.8391 | 2 | 32.83 | 9 | F9:15651 | 31122021\_RID\_3262\_2a.raw |  |  |  |  |  |  |  | 6.9475E4 | 0 |  | 1 | 0 | 0 | 0 | 0 | 0 | 0 | 0 | 1 | 0 | 0 | 132 | 143 |  |  | PEAKS DB |
| total 13 peptides |  |  |  |  |  |  |  |  |  |  |  |  |  |  |  |  |  |  |  |  |  |  |  |  |  |  |  |  |  |  |  |  |  |  |  |  |  |
| --- | --- | --- | --- | --- | --- | --- | --- | --- | --- | --- | --- | --- | --- | --- | --- | --- | --- | --- | --- | --- | --- | --- | --- | --- | --- | --- | --- | --- | --- | --- | --- | --- | --- | --- | --- | --- | --- |

XP\_025022318.1

back to list

  

| Protein Coverage
| Supporting Peptides
|

Protein Coverage:

Supporting Peptides:

| Peptide | Uniq | -10lgP | Mass | Length | ppm | m/z | z | RT | Fraction | Scan | Source File | Area F4 | Area F5 | Area F6 | Area F10 | Area F8 | Area F9 | Area F7 | Area F1 | Area F2 | Area F3 | #Feature | #Feature F4 | #Feature F5 | #Feature F6 | #Feature F10 | #Feature F8 | #Feature F9 | #Feature F7 | #Feature F1 | #Feature F2 | #Feature F3 | Start | End | PTM | AScore | Found By |
| --- | --- | --- | --- | --- | --- | --- | --- | --- | --- | --- | --- | --- | --- | --- | --- | --- | --- | --- | --- | --- | --- | --- | --- | --- | --- | --- | --- | --- | --- | --- | --- | --- | --- | --- | --- | --- | --- |
| R.NEGTATYAAAVLFR.I | Y | 74.08 | 1482.7467 | 14 | 0.2 | 742.3808 | 2 | 48.27 | 9 | F9:28127 | 31122021\_RID\_3262\_2a.raw | 4.317E5 |  |  |  |  | 1.024E6 |  | 1.4003E6 | 4.6202E5 |  | 5 | 1 | 0 | 0 | 0 | 0 | 1 | 0 | 2 | 1 | 0 | 640 | 653 |  |  | PEAKS DB |
| R.LNYGIPAIVK.L | Y | 65.66 | 1086.6437 | 10 | 0.0 | 544.3291 | 2 | 45.04 | 8 | F8:24415 | 31122021\_RID\_3262\_1a.raw |  |  |  | 2.5539E6 |  | 2.6128E6 |  | 1.8129E6 | 7.9277E5 |  | 4 | 0 | 0 | 0 | 1 | 0 | 1 | 0 | 1 | 1 | 0 | 480 | 489 |  |  | PEAKS DB |
| R.VSVELTNSLFK.H | Y | 60.86 | 1235.6761 | 11 | 0.7 | 618.8458 | 2 | 47.53 | 6 | F6:32450 | 17012022\_RID\_3262\_9a.raw |  |  |  | 1.1826E6 |  | 1.3838E6 |  | 2.5277E6 |  | 2.6941E6 | 4 | 0 | 0 | 0 | 1 | 0 | 1 | 0 | 1 | 0 | 1 | 666 | 676 |  |  | PEAKS DB |
| R.ISEDKNPDYR.K | Y | 59.00 | 1235.5781 | 10 | 0.9 | 618.7969 | 2 | 18.00 | 4 | F4:5768 | 17012022\_RID\_3262\_10a .raw | 2.2898E5 | 5.2363E4 |  | 5.724E5 |  | 6.4152E5 | 4.2912E4 |  |  |  | 5 | 1 | 1 | 0 | 1 | 0 | 1 | 1 | 0 | 0 | 0 | 654 | 663 |  |  | PEAKS DB |
| K.SAIVHLINYQDDAELATR.A | Y | 57.87 | 2028.0276 | 18 | 1.0 | 677.0172 | 3 | 43.76 | 1 | F1:28958 | 13012022\_RID\_3262\_4a.raw | 4.2034E5 |  |  |  |  |  |  | 1.3135E6 | 3.6387E5 |  | 4 | 1 | 0 | 0 | 0 | 0 | 0 | 0 | 2 | 1 | 0 | 127 | 144 |  |  | PEAKS DB |
| K.SGGIPALVR.M | Y | 56.02 | 868.5130 | 9 | 0.5 | 435.2640 | 2 | 35.68 | 6 | F6:21518 | 17012022\_RID\_3262\_9a.raw |  | 3.703E5 |  | 2.5461E6 |  | 2.1357E6 | 8.8669E4 | 0 | 6.1375E5 |  | 5 | 0 | 1 | 0 | 1 | 0 | 1 | 1 | 0 | 1 | 0 | 227 | 235 |  |  | PEAKS DB |
| R.TM(+15.99)QNTSDLDTAR.C | Y | 55.25 | 1367.5988 | 12 | -0.4 | 684.8064 | 2 | 21.71 | 4 | F4:8886 | 17012022\_RID\_3262\_10a .raw |  | 1.2132E5 |  | 1.0143E6 |  | 6.4155E5 |  | 2.1506E5 | 1.8157E5 |  | 5 | 0 | 1 | 0 | 1 | 0 | 1 | 0 | 1 | 1 | 0 | 194 | 205 | Oxidation (M) | M2:Oxidation (M):1000.00 | PEAKS DB |
| R.HPEAEM(+15.99)AQNSVR.L | Y | 53.70 | 1383.6201 | 12 | 1.0 | 692.8180 | 2 | 15.98 | 4 | F4:4154 | 17012022\_RID\_3262\_10a .raw |  |  |  | 4.0379E5 |  |  |  |  |  |  | 2 | 0 | 0 | 0 | 2 | 0 | 0 | 0 | 0 | 0 | 0 | 468 | 479 | Oxidation (M) | M6:Oxidation (M):1000.00 | PEAKS DB |
| K.AAM(+15.99)IVNQLSK.K | Y | 53.10 | 1089.5852 | 10 | 0.8 | 545.8003 | 2 | 28.59 | 4 | F4:15343 | 17012022\_RID\_3262\_10a .raw |  |  |  | 1.0996E6 |  |  |  |  | 1.8354E5 |  | 2 | 0 | 0 | 0 | 1 | 0 | 0 | 0 | 0 | 1 | 0 | 164 | 173 | Oxidation (M) | M3:Oxidation (M):1000.00 | PEAKS DB |
| R.TMQNTSDLDTAR.C | Y | 53.03 | 1351.6038 | 12 | 0.1 | 676.8092 | 2 | 22.98 | 9 | F9:7560 | 31122021\_RID\_3262\_2a.raw |  | 1.1062E5 |  |  |  | 9.3847E5 |  | 2.411E5 | 2.0476E5 |  | 4 | 0 | 1 | 0 | 0 | 0 | 1 | 0 | 1 | 1 | 0 | 194 | 205 |  |  | PEAKS DB |
| K.AAMIVNQLSK.K | Y | 52.58 | 1073.5903 | 10 | -0.4 | 537.8022 | 2 | 33.93 | 4 | F4:19962 | 17012022\_RID\_3262\_10a .raw |  |  |  | 1.2793E6 |  | 1.6681E6 |  |  |  |  | 2 | 0 | 0 | 0 | 1 | 0 | 1 | 0 | 0 | 0 | 0 | 164 | 173 |  |  | PEAKS DB |
| R.HPEAEMAQNSVR.L | Y | 51.71 | 1367.6251 | 12 | 0.0 | 684.8198 | 2 | 17.98 | 8 | F8:3971 | 31122021\_RID\_3262\_1a.raw |  |  |  | 3.542E5 |  |  |  | 1.8269E5 | 1.0075E5 |  | 3 | 0 | 0 | 0 | 1 | 0 | 0 | 0 | 1 | 1 | 0 | 468 | 479 |  |  | PEAKS DB |
| L.INYQDDAELATR.A | N | 45.89 | 1407.6630 | 12 | 0.5 | 704.8391 | 2 | 32.83 | 9 | F9:15651 | 31122021\_RID\_3262\_2a.raw |  |  |  |  |  |  |  | 6.9475E4 | 0 |  | 1 | 0 | 0 | 0 | 0 | 0 | 0 | 0 | 1 | 0 | 0 | 133 | 144 |  |  | PEAKS DB |
| total 13 peptides |  |  |  |  |  |  |  |  |  |  |  |  |  |  |  |  |  |  |  |  |  |  |  |  |  |  |  |  |  |  |  |  |  |  |  |  |  |
| --- | --- | --- | --- | --- | --- | --- | --- | --- | --- | --- | --- | --- | --- | --- | --- | --- | --- | --- | --- | --- | --- | --- | --- | --- | --- | --- | --- | --- | --- | --- | --- | --- | --- | --- | --- | --- | --- |

XP\_034271617.1

back to list

  

| Protein Coverage
| Supporting Peptides
|

Protein Coverage:

Supporting Peptides:

| Peptide | Uniq | -10lgP | Mass | Length | ppm | m/z | z | RT | Fraction | Scan | Source File | Area F4 | Area F5 | Area F6 | Area F10 | Area F8 | Area F9 | Area F7 | Area F1 | Area F2 | Area F3 | #Feature | #Feature F4 | #Feature F5 | #Feature F6 | #Feature F10 | #Feature F8 | #Feature F9 | #Feature F7 | #Feature F1 | #Feature F2 | #Feature F3 | Start | End | PTM | AScore | Found By |
| --- | --- | --- | --- | --- | --- | --- | --- | --- | --- | --- | --- | --- | --- | --- | --- | --- | --- | --- | --- | --- | --- | --- | --- | --- | --- | --- | --- | --- | --- | --- | --- | --- | --- | --- | --- | --- | --- |
| R.NEGTATYAAAVLFR.I | Y | 74.08 | 1482.7467 | 14 | 0.2 | 742.3808 | 2 | 48.27 | 9 | F9:28127 | 31122021\_RID\_3262\_2a.raw | 4.317E5 |  |  |  |  | 1.024E6 |  | 1.4003E6 | 4.6202E5 |  | 5 | 1 | 0 | 0 | 0 | 0 | 1 | 0 | 2 | 1 | 0 | 638 | 651 |  |  | PEAKS DB |
| R.LNYGIPAIVK.L | Y | 65.66 | 1086.6437 | 10 | 0.0 | 544.3291 | 2 | 45.04 | 8 | F8:24415 | 31122021\_RID\_3262\_1a.raw |  |  |  | 2.5539E6 |  | 2.6128E6 |  | 1.8129E6 | 7.9277E5 |  | 4 | 0 | 0 | 0 | 1 | 0 | 1 | 0 | 1 | 1 | 0 | 478 | 487 |  |  | PEAKS DB |
| R.VSVELTNSLFK.H | Y | 60.86 | 1235.6761 | 11 | 0.7 | 618.8458 | 2 | 47.53 | 6 | F6:32450 | 17012022\_RID\_3262\_9a.raw |  |  |  | 1.1826E6 |  | 1.3838E6 |  | 2.5277E6 |  | 2.6941E6 | 4 | 0 | 0 | 0 | 1 | 0 | 1 | 0 | 1 | 0 | 1 | 664 | 674 |  |  | PEAKS DB |
| R.ISEDKNPDYR.K | Y | 59.00 | 1235.5781 | 10 | 0.9 | 618.7969 | 2 | 18.00 | 4 | F4:5768 | 17012022\_RID\_3262\_10a .raw | 2.2898E5 | 5.2363E4 |  | 5.724E5 |  | 6.4152E5 | 4.2912E4 |  |  |  | 5 | 1 | 1 | 0 | 1 | 0 | 1 | 1 | 0 | 0 | 0 | 652 | 661 |  |  | PEAKS DB |
| K.SAIVHLINYQDDAELATR.A | Y | 57.87 | 2028.0276 | 18 | 1.0 | 677.0172 | 3 | 43.76 | 1 | F1:28958 | 13012022\_RID\_3262\_4a.raw | 4.2034E5 |  |  |  |  |  |  | 1.3135E6 | 3.6387E5 |  | 4 | 1 | 0 | 0 | 0 | 0 | 0 | 0 | 2 | 1 | 0 | 125 | 142 |  |  | PEAKS DB |
| K.SGGIPALVR.M | Y | 56.02 | 868.5130 | 9 | 0.5 | 435.2640 | 2 | 35.68 | 6 | F6:21518 | 17012022\_RID\_3262\_9a.raw |  | 3.703E5 |  | 2.5461E6 |  | 2.1357E6 | 8.8669E4 | 0 | 6.1375E5 |  | 5 | 0 | 1 | 0 | 1 | 0 | 1 | 1 | 0 | 1 | 0 | 225 | 233 |  |  | PEAKS DB |
| R.TM(+15.99)QNTSDLDTAR.C | Y | 55.25 | 1367.5988 | 12 | -0.4 | 684.8064 | 2 | 21.71 | 4 | F4:8886 | 17012022\_RID\_3262\_10a .raw |  | 1.2132E5 |  | 1.0143E6 |  | 6.4155E5 |  | 2.1506E5 | 1.8157E5 |  | 5 | 0 | 1 | 0 | 1 | 0 | 1 | 0 | 1 | 1 | 0 | 192 | 203 | Oxidation (M) | M2:Oxidation (M):1000.00 | PEAKS DB |
| R.HPEAEM(+15.99)AQNSVR.L | Y | 53.70 | 1383.6201 | 12 | 1.0 | 692.8180 | 2 | 15.98 | 4 | F4:4154 | 17012022\_RID\_3262\_10a .raw |  |  |  | 4.0379E5 |  |  |  |  |  |  | 2 | 0 | 0 | 0 | 2 | 0 | 0 | 0 | 0 | 0 | 0 | 466 | 477 | Oxidation (M) | M6:Oxidation (M):1000.00 | PEAKS DB |
| K.AAM(+15.99)IVNQLSK.K | Y | 53.10 | 1089.5852 | 10 | 0.8 | 545.8003 | 2 | 28.59 | 4 | F4:15343 | 17012022\_RID\_3262\_10a .raw |  |  |  | 1.0996E6 |  |  |  |  | 1.8354E5 |  | 2 | 0 | 0 | 0 | 1 | 0 | 0 | 0 | 0 | 1 | 0 | 162 | 171 | Oxidation (M) | M3:Oxidation (M):1000.00 | PEAKS DB |
| R.TMQNTSDLDTAR.C | Y | 53.03 | 1351.6038 | 12 | 0.1 | 676.8092 | 2 | 22.98 | 9 | F9:7560 | 31122021\_RID\_3262\_2a.raw |  | 1.1062E5 |  |  |  | 9.3847E5 |  | 2.411E5 | 2.0476E5 |  | 4 | 0 | 1 | 0 | 0 | 0 | 1 | 0 | 1 | 1 | 0 | 192 | 203 |  |  | PEAKS DB |
| K.AAMIVNQLSK.K | Y | 52.58 | 1073.5903 | 10 | -0.4 | 537.8022 | 2 | 33.93 | 4 | F4:19962 | 17012022\_RID\_3262\_10a .raw |  |  |  | 1.2793E6 |  | 1.6681E6 |  |  |  |  | 2 | 0 | 0 | 0 | 1 | 0 | 1 | 0 | 0 | 0 | 0 | 162 | 171 |  |  | PEAKS DB |
| R.HPEAEMAQNSVR.L | Y | 51.71 | 1367.6251 | 12 | 0.0 | 684.8198 | 2 | 17.98 | 8 | F8:3971 | 31122021\_RID\_3262\_1a.raw |  |  |  | 3.542E5 |  |  |  | 1.8269E5 | 1.0075E5 |  | 3 | 0 | 0 | 0 | 1 | 0 | 0 | 0 | 1 | 1 | 0 | 466 | 477 |  |  | PEAKS DB |
| L.INYQDDAELATR.A | N | 45.89 | 1407.6630 | 12 | 0.5 | 704.8391 | 2 | 32.83 | 9 | F9:15651 | 31122021\_RID\_3262\_2a.raw |  |  |  |  |  |  |  | 6.9475E4 | 0 |  | 1 | 0 | 0 | 0 | 0 | 0 | 0 | 0 | 1 | 0 | 0 | 131 | 142 |  |  | PEAKS DB |
| total 13 peptides |  |  |  |  |  |  |  |  |  |  |  |  |  |  |  |  |  |  |  |  |  |  |  |  |  |  |  |  |  |  |  |  |  |  |  |  |  |
| --- | --- | --- | --- | --- | --- | --- | --- | --- | --- | --- | --- | --- | --- | --- | --- | --- | --- | --- | --- | --- | --- | --- | --- | --- | --- | --- | --- | --- | --- | --- | --- | --- | --- | --- | --- | --- | --- |

XP\_013919280.1

back to list

  

| Protein Coverage
| Supporting Peptides
|

Protein Coverage:

Supporting Peptides:

| Peptide | Uniq | -10lgP | Mass | Length | ppm | m/z | z | RT | Fraction | Scan | Source File | Area F4 | Area F5 | Area F6 | Area F10 | Area F8 | Area F9 | Area F7 | Area F1 | Area F2 | Area F3 | #Feature | #Feature F4 | #Feature F5 | #Feature F6 | #Feature F10 | #Feature F8 | #Feature F9 | #Feature F7 | #Feature F1 | #Feature F2 | #Feature F3 | Start | End | PTM | AScore | Found By |
| --- | --- | --- | --- | --- | --- | --- | --- | --- | --- | --- | --- | --- | --- | --- | --- | --- | --- | --- | --- | --- | --- | --- | --- | --- | --- | --- | --- | --- | --- | --- | --- | --- | --- | --- | --- | --- | --- |
| R.NEGTATYAAAVLFR.I | Y | 74.08 | 1482.7467 | 14 | 0.2 | 742.3808 | 2 | 48.27 | 9 | F9:28127 | 31122021\_RID\_3262\_2a.raw | 4.317E5 |  |  |  |  | 1.024E6 |  | 1.4003E6 | 4.6202E5 |  | 5 | 1 | 0 | 0 | 0 | 0 | 1 | 0 | 2 | 1 | 0 | 639 | 652 |  |  | PEAKS DB |
| R.LNYGIPAIVK.L | Y | 65.66 | 1086.6437 | 10 | 0.0 | 544.3291 | 2 | 45.04 | 8 | F8:24415 | 31122021\_RID\_3262\_1a.raw |  |  |  | 2.5539E6 |  | 2.6128E6 |  | 1.8129E6 | 7.9277E5 |  | 4 | 0 | 0 | 0 | 1 | 0 | 1 | 0 | 1 | 1 | 0 | 479 | 488 |  |  | PEAKS DB |
| R.VSVELTNSLFK.H | Y | 60.86 | 1235.6761 | 11 | 0.7 | 618.8458 | 2 | 47.53 | 6 | F6:32450 | 17012022\_RID\_3262\_9a.raw |  |  |  | 1.1826E6 |  | 1.3838E6 |  | 2.5277E6 |  | 2.6941E6 | 4 | 0 | 0 | 0 | 1 | 0 | 1 | 0 | 1 | 0 | 1 | 665 | 675 |  |  | PEAKS DB |
| R.ISEDKNPDYR.K | Y | 59.00 | 1235.5781 | 10 | 0.9 | 618.7969 | 2 | 18.00 | 4 | F4:5768 | 17012022\_RID\_3262\_10a .raw | 2.2898E5 | 5.2363E4 |  | 5.724E5 |  | 6.4152E5 | 4.2912E4 |  |  |  | 5 | 1 | 1 | 0 | 1 | 0 | 1 | 1 | 0 | 0 | 0 | 653 | 662 |  |  | PEAKS DB |
| K.SAIVHLINYQDDAELATR.A | Y | 57.87 | 2028.0276 | 18 | 1.0 | 677.0172 | 3 | 43.76 | 1 | F1:28958 | 13012022\_RID\_3262\_4a.raw | 4.2034E5 |  |  |  |  |  |  | 1.3135E6 | 3.6387E5 |  | 4 | 1 | 0 | 0 | 0 | 0 | 0 | 0 | 2 | 1 | 0 | 126 | 143 |  |  | PEAKS DB |
| K.SGGIPALVR.M | Y | 56.02 | 868.5130 | 9 | 0.5 | 435.2640 | 2 | 35.68 | 6 | F6:21518 | 17012022\_RID\_3262\_9a.raw |  | 3.703E5 |  | 2.5461E6 |  | 2.1357E6 | 8.8669E4 | 0 | 6.1375E5 |  | 5 | 0 | 1 | 0 | 1 | 0 | 1 | 1 | 0 | 1 | 0 | 226 | 234 |  |  | PEAKS DB |
| R.TM(+15.99)QNTSDLDTAR.C | Y | 55.25 | 1367.5988 | 12 | -0.4 | 684.8064 | 2 | 21.71 | 4 | F4:8886 | 17012022\_RID\_3262\_10a .raw |  | 1.2132E5 |  | 1.0143E6 |  | 6.4155E5 |  | 2.1506E5 | 1.8157E5 |  | 5 | 0 | 1 | 0 | 1 | 0 | 1 | 0 | 1 | 1 | 0 | 193 | 204 | Oxidation (M) | M2:Oxidation (M):1000.00 | PEAKS DB |
| R.HPEAEM(+15.99)AQNSVR.L | Y | 53.70 | 1383.6201 | 12 | 1.0 | 692.8180 | 2 | 15.98 | 4 | F4:4154 | 17012022\_RID\_3262\_10a .raw |  |  |  | 4.0379E5 |  |  |  |  |  |  | 2 | 0 | 0 | 0 | 2 | 0 | 0 | 0 | 0 | 0 | 0 | 467 | 478 | Oxidation (M) | M6:Oxidation (M):1000.00 | PEAKS DB |
| K.AAM(+15.99)IVNQLSK.K | Y | 53.10 | 1089.5852 | 10 | 0.8 | 545.8003 | 2 | 28.59 | 4 | F4:15343 | 17012022\_RID\_3262\_10a .raw |  |  |  | 1.0996E6 |  |  |  |  | 1.8354E5 |  | 2 | 0 | 0 | 0 | 1 | 0 | 0 | 0 | 0 | 1 | 0 | 163 | 172 | Oxidation (M) | M3:Oxidation (M):1000.00 | PEAKS DB |
| R.TMQNTSDLDTAR.C | Y | 53.03 | 1351.6038 | 12 | 0.1 | 676.8092 | 2 | 22.98 | 9 | F9:7560 | 31122021\_RID\_3262\_2a.raw |  | 1.1062E5 |  |  |  | 9.3847E5 |  | 2.411E5 | 2.0476E5 |  | 4 | 0 | 1 | 0 | 0 | 0 | 1 | 0 | 1 | 1 | 0 | 193 | 204 |  |  | PEAKS DB |
| K.AAMIVNQLSK.K | Y | 52.58 | 1073.5903 | 10 | -0.4 | 537.8022 | 2 | 33.93 | 4 | F4:19962 | 17012022\_RID\_3262\_10a .raw |  |  |  | 1.2793E6 |  | 1.6681E6 |  |  |  |  | 2 | 0 | 0 | 0 | 1 | 0 | 1 | 0 | 0 | 0 | 0 | 163 | 172 |  |  | PEAKS DB |
| R.HPEAEMAQNSVR.L | Y | 51.71 | 1367.6251 | 12 | 0.0 | 684.8198 | 2 | 17.98 | 8 | F8:3971 | 31122021\_RID\_3262\_1a.raw |  |  |  | 3.542E5 |  |  |  | 1.8269E5 | 1.0075E5 |  | 3 | 0 | 0 | 0 | 1 | 0 | 0 | 0 | 1 | 1 | 0 | 467 | 478 |  |  | PEAKS DB |
| L.INYQDDAELATR.A | N | 45.89 | 1407.6630 | 12 | 0.5 | 704.8391 | 2 | 32.83 | 9 | F9:15651 | 31122021\_RID\_3262\_2a.raw |  |  |  |  |  |  |  | 6.9475E4 | 0 |  | 1 | 0 | 0 | 0 | 0 | 0 | 0 | 0 | 1 | 0 | 0 | 132 | 143 |  |  | PEAKS DB |
| total 13 peptides |  |  |  |  |  |  |  |  |  |  |  |  |  |  |  |  |  |  |  |  |  |  |  |  |  |  |  |  |  |  |  |  |  |  |  |  |  |
| --- | --- | --- | --- | --- | --- | --- | --- | --- | --- | --- | --- | --- | --- | --- | --- | --- | --- | --- | --- | --- | --- | --- | --- | --- | --- | --- | --- | --- | --- | --- | --- | --- | --- | --- | --- | --- | --- |

XP\_034271615.1

back to list

  

| Protein Coverage
| Supporting Peptides
|

Protein Coverage:

Supporting Peptides:

| Peptide | Uniq | -10lgP | Mass | Length | ppm | m/z | z | RT | Fraction | Scan | Source File | Area F4 | Area F5 | Area F6 | Area F10 | Area F8 | Area F9 | Area F7 | Area F1 | Area F2 | Area F3 | #Feature | #Feature F4 | #Feature F5 | #Feature F6 | #Feature F10 | #Feature F8 | #Feature F9 | #Feature F7 | #Feature F1 | #Feature F2 | #Feature F3 | Start | End | PTM | AScore | Found By |
| --- | --- | --- | --- | --- | --- | --- | --- | --- | --- | --- | --- | --- | --- | --- | --- | --- | --- | --- | --- | --- | --- | --- | --- | --- | --- | --- | --- | --- | --- | --- | --- | --- | --- | --- | --- | --- | --- |
| R.NEGTATYAAAVLFR.I | Y | 74.08 | 1482.7467 | 14 | 0.2 | 742.3808 | 2 | 48.27 | 9 | F9:28127 | 31122021\_RID\_3262\_2a.raw | 4.317E5 |  |  |  |  | 1.024E6 |  | 1.4003E6 | 4.6202E5 |  | 5 | 1 | 0 | 0 | 0 | 0 | 1 | 0 | 2 | 1 | 0 | 639 | 652 |  |  | PEAKS DB |
| R.LNYGIPAIVK.L | Y | 65.66 | 1086.6437 | 10 | 0.0 | 544.3291 | 2 | 45.04 | 8 | F8:24415 | 31122021\_RID\_3262\_1a.raw |  |  |  | 2.5539E6 |  | 2.6128E6 |  | 1.8129E6 | 7.9277E5 |  | 4 | 0 | 0 | 0 | 1 | 0 | 1 | 0 | 1 | 1 | 0 | 479 | 488 |  |  | PEAKS DB |
| R.VSVELTNSLFK.H | Y | 60.86 | 1235.6761 | 11 | 0.7 | 618.8458 | 2 | 47.53 | 6 | F6:32450 | 17012022\_RID\_3262\_9a.raw |  |  |  | 1.1826E6 |  | 1.3838E6 |  | 2.5277E6 |  | 2.6941E6 | 4 | 0 | 0 | 0 | 1 | 0 | 1 | 0 | 1 | 0 | 1 | 665 | 675 |  |  | PEAKS DB |
| R.ISEDKNPDYR.K | Y | 59.00 | 1235.5781 | 10 | 0.9 | 618.7969 | 2 | 18.00 | 4 | F4:5768 | 17012022\_RID\_3262\_10a .raw | 2.2898E5 | 5.2363E4 |  | 5.724E5 |  | 6.4152E5 | 4.2912E4 |  |  |  | 5 | 1 | 1 | 0 | 1 | 0 | 1 | 1 | 0 | 0 | 0 | 653 | 662 |  |  | PEAKS DB |
| K.SAIVHLINYQDDAELATR.A | Y | 57.87 | 2028.0276 | 18 | 1.0 | 677.0172 | 3 | 43.76 | 1 | F1:28958 | 13012022\_RID\_3262\_4a.raw | 4.2034E5 |  |  |  |  |  |  | 1.3135E6 | 3.6387E5 |  | 4 | 1 | 0 | 0 | 0 | 0 | 0 | 0 | 2 | 1 | 0 | 126 | 143 |  |  | PEAKS DB |
| K.SGGIPALVR.M | Y | 56.02 | 868.5130 | 9 | 0.5 | 435.2640 | 2 | 35.68 | 6 | F6:21518 | 17012022\_RID\_3262\_9a.raw |  | 3.703E5 |  | 2.5461E6 |  | 2.1357E6 | 8.8669E4 | 0 | 6.1375E5 |  | 5 | 0 | 1 | 0 | 1 | 0 | 1 | 1 | 0 | 1 | 0 | 226 | 234 |  |  | PEAKS DB |
| R.TM(+15.99)QNTSDLDTAR.C | Y | 55.25 | 1367.5988 | 12 | -0.4 | 684.8064 | 2 | 21.71 | 4 | F4:8886 | 17012022\_RID\_3262\_10a .raw |  | 1.2132E5 |  | 1.0143E6 |  | 6.4155E5 |  | 2.1506E5 | 1.8157E5 |  | 5 | 0 | 1 | 0 | 1 | 0 | 1 | 0 | 1 | 1 | 0 | 193 | 204 | Oxidation (M) | M2:Oxidation (M):1000.00 | PEAKS DB |
| R.HPEAEM(+15.99)AQNSVR.L | Y | 53.70 | 1383.6201 | 12 | 1.0 | 692.8180 | 2 | 15.98 | 4 | F4:4154 | 17012022\_RID\_3262\_10a .raw |  |  |  | 4.0379E5 |  |  |  |  |  |  | 2 | 0 | 0 | 0 | 2 | 0 | 0 | 0 | 0 | 0 | 0 | 467 | 478 | Oxidation (M) | M6:Oxidation (M):1000.00 | PEAKS DB |
| K.AAM(+15.99)IVNQLSK.K | Y | 53.10 | 1089.5852 | 10 | 0.8 | 545.8003 | 2 | 28.59 | 4 | F4:15343 | 17012022\_RID\_3262\_10a .raw |  |  |  | 1.0996E6 |  |  |  |  | 1.8354E5 |  | 2 | 0 | 0 | 0 | 1 | 0 | 0 | 0 | 0 | 1 | 0 | 163 | 172 | Oxidation (M) | M3:Oxidation (M):1000.00 | PEAKS DB |
| R.TMQNTSDLDTAR.C | Y | 53.03 | 1351.6038 | 12 | 0.1 | 676.8092 | 2 | 22.98 | 9 | F9:7560 | 31122021\_RID\_3262\_2a.raw |  | 1.1062E5 |  |  |  | 9.3847E5 |  | 2.411E5 | 2.0476E5 |  | 4 | 0 | 1 | 0 | 0 | 0 | 1 | 0 | 1 | 1 | 0 | 193 | 204 |  |  | PEAKS DB |
| K.AAMIVNQLSK.K | Y | 52.58 | 1073.5903 | 10 | -0.4 | 537.8022 | 2 | 33.93 | 4 | F4:19962 | 17012022\_RID\_3262\_10a .raw |  |  |  | 1.2793E6 |  | 1.6681E6 |  |  |  |  | 2 | 0 | 0 | 0 | 1 | 0 | 1 | 0 | 0 | 0 | 0 | 163 | 172 |  |  | PEAKS DB |
| R.HPEAEMAQNSVR.L | Y | 51.71 | 1367.6251 | 12 | 0.0 | 684.8198 | 2 | 17.98 | 8 | F8:3971 | 31122021\_RID\_3262\_1a.raw |  |  |  | 3.542E5 |  |  |  | 1.8269E5 | 1.0075E5 |  | 3 | 0 | 0 | 0 | 1 | 0 | 0 | 0 | 1 | 1 | 0 | 467 | 478 |  |  | PEAKS DB |
| L.INYQDDAELATR.A | N | 45.89 | 1407.6630 | 12 | 0.5 | 704.8391 | 2 | 32.83 | 9 | F9:15651 | 31122021\_RID\_3262\_2a.raw |  |  |  |  |  |  |  | 6.9475E4 | 0 |  | 1 | 0 | 0 | 0 | 0 | 0 | 0 | 0 | 1 | 0 | 0 | 132 | 143 |  |  | PEAKS DB |
| total 13 peptides |  |  |  |  |  |  |  |  |  |  |  |  |  |  |  |  |  |  |  |  |  |  |  |  |  |  |  |  |  |  |  |  |  |  |  |  |  |
| --- | --- | --- | --- | --- | --- | --- | --- | --- | --- | --- | --- | --- | --- | --- | --- | --- | --- | --- | --- | --- | --- | --- | --- | --- | --- | --- | --- | --- | --- | --- | --- | --- | --- | --- | --- | --- | --- |

XP\_034271618.1

back to list

  

| Protein Coverage
| Supporting Peptides
|

Protein Coverage:

Supporting Peptides:

| Peptide | Uniq | -10lgP | Mass | Length | ppm | m/z | z | RT | Fraction | Scan | Source File | Area F4 | Area F5 | Area F6 | Area F10 | Area F8 | Area F9 | Area F7 | Area F1 | Area F2 | Area F3 | #Feature | #Feature F4 | #Feature F5 | #Feature F6 | #Feature F10 | #Feature F8 | #Feature F9 | #Feature F7 | #Feature F1 | #Feature F2 | #Feature F3 | Start | End | PTM | AScore | Found By |
| --- | --- | --- | --- | --- | --- | --- | --- | --- | --- | --- | --- | --- | --- | --- | --- | --- | --- | --- | --- | --- | --- | --- | --- | --- | --- | --- | --- | --- | --- | --- | --- | --- | --- | --- | --- | --- | --- |
| R.NEGTATYAAAVLFR.I | Y | 74.08 | 1482.7467 | 14 | 0.2 | 742.3808 | 2 | 48.27 | 9 | F9:28127 | 31122021\_RID\_3262\_2a.raw | 4.317E5 |  |  |  |  | 1.024E6 |  | 1.4003E6 | 4.6202E5 |  | 5 | 1 | 0 | 0 | 0 | 0 | 1 | 0 | 2 | 1 | 0 | 639 | 652 |  |  | PEAKS DB |
| R.LNYGIPAIVK.L | Y | 65.66 | 1086.6437 | 10 | 0.0 | 544.3291 | 2 | 45.04 | 8 | F8:24415 | 31122021\_RID\_3262\_1a.raw |  |  |  | 2.5539E6 |  | 2.6128E6 |  | 1.8129E6 | 7.9277E5 |  | 4 | 0 | 0 | 0 | 1 | 0 | 1 | 0 | 1 | 1 | 0 | 479 | 488 |  |  | PEAKS DB |
| R.VSVELTNSLFK.H | Y | 60.86 | 1235.6761 | 11 | 0.7 | 618.8458 | 2 | 47.53 | 6 | F6:32450 | 17012022\_RID\_3262\_9a.raw |  |  |  | 1.1826E6 |  | 1.3838E6 |  | 2.5277E6 |  | 2.6941E6 | 4 | 0 | 0 | 0 | 1 | 0 | 1 | 0 | 1 | 0 | 1 | 665 | 675 |  |  | PEAKS DB |
| R.ISEDKNPDYR.K | Y | 59.00 | 1235.5781 | 10 | 0.9 | 618.7969 | 2 | 18.00 | 4 | F4:5768 | 17012022\_RID\_3262\_10a .raw | 2.2898E5 | 5.2363E4 |  | 5.724E5 |  | 6.4152E5 | 4.2912E4 |  |  |  | 5 | 1 | 1 | 0 | 1 | 0 | 1 | 1 | 0 | 0 | 0 | 653 | 662 |  |  | PEAKS DB |
| K.SAIVHLINYQDDAELATR.A | Y | 57.87 | 2028.0276 | 18 | 1.0 | 677.0172 | 3 | 43.76 | 1 | F1:28958 | 13012022\_RID\_3262\_4a.raw | 4.2034E5 |  |  |  |  |  |  | 1.3135E6 | 3.6387E5 |  | 4 | 1 | 0 | 0 | 0 | 0 | 0 | 0 | 2 | 1 | 0 | 126 | 143 |  |  | PEAKS DB |
| K.SGGIPALVR.M | Y | 56.02 | 868.5130 | 9 | 0.5 | 435.2640 | 2 | 35.68 | 6 | F6:21518 | 17012022\_RID\_3262\_9a.raw |  | 3.703E5 |  | 2.5461E6 |  | 2.1357E6 | 8.8669E4 | 0 | 6.1375E5 |  | 5 | 0 | 1 | 0 | 1 | 0 | 1 | 1 | 0 | 1 | 0 | 226 | 234 |  |  | PEAKS DB |
| R.TM(+15.99)QNTSDLDTAR.C | Y | 55.25 | 1367.5988 | 12 | -0.4 | 684.8064 | 2 | 21.71 | 4 | F4:8886 | 17012022\_RID\_3262\_10a .raw |  | 1.2132E5 |  | 1.0143E6 |  | 6.4155E5 |  | 2.1506E5 | 1.8157E5 |  | 5 | 0 | 1 | 0 | 1 | 0 | 1 | 0 | 1 | 1 | 0 | 193 | 204 | Oxidation (M) | M2:Oxidation (M):1000.00 | PEAKS DB |
| R.HPEAEM(+15.99)AQNSVR.L | Y | 53.70 | 1383.6201 | 12 | 1.0 | 692.8180 | 2 | 15.98 | 4 | F4:4154 | 17012022\_RID\_3262\_10a .raw |  |  |  | 4.0379E5 |  |  |  |  |  |  | 2 | 0 | 0 | 0 | 2 | 0 | 0 | 0 | 0 | 0 | 0 | 467 | 478 | Oxidation (M) | M6:Oxidation (M):1000.00 | PEAKS DB |
| K.AAM(+15.99)IVNQLSK.K | Y | 53.10 | 1089.5852 | 10 | 0.8 | 545.8003 | 2 | 28.59 | 4 | F4:15343 | 17012022\_RID\_3262\_10a .raw |  |  |  | 1.0996E6 |  |  |  |  | 1.8354E5 |  | 2 | 0 | 0 | 0 | 1 | 0 | 0 | 0 | 0 | 1 | 0 | 163 | 172 | Oxidation (M) | M3:Oxidation (M):1000.00 | PEAKS DB |
| R.TMQNTSDLDTAR.C | Y | 53.03 | 1351.6038 | 12 | 0.1 | 676.8092 | 2 | 22.98 | 9 | F9:7560 | 31122021\_RID\_3262\_2a.raw |  | 1.1062E5 |  |  |  | 9.3847E5 |  | 2.411E5 | 2.0476E5 |  | 4 | 0 | 1 | 0 | 0 | 0 | 1 | 0 | 1 | 1 | 0 | 193 | 204 |  |  | PEAKS DB |
| K.AAMIVNQLSK.K | Y | 52.58 | 1073.5903 | 10 | -0.4 | 537.8022 | 2 | 33.93 | 4 | F4:19962 | 17012022\_RID\_3262\_10a .raw |  |  |  | 1.2793E6 |  | 1.6681E6 |  |  |  |  | 2 | 0 | 0 | 0 | 1 | 0 | 1 | 0 | 0 | 0 | 0 | 163 | 172 |  |  | PEAKS DB |
| R.HPEAEMAQNSVR.L | Y | 51.71 | 1367.6251 | 12 | 0.0 | 684.8198 | 2 | 17.98 | 8 | F8:3971 | 31122021\_RID\_3262\_1a.raw |  |  |  | 3.542E5 |  |  |  | 1.8269E5 | 1.0075E5 |  | 3 | 0 | 0 | 0 | 1 | 0 | 0 | 0 | 1 | 1 | 0 | 467 | 478 |  |  | PEAKS DB |
| L.INYQDDAELATR.A | N | 45.89 | 1407.6630 | 12 | 0.5 | 704.8391 | 2 | 32.83 | 9 | F9:15651 | 31122021\_RID\_3262\_2a.raw |  |  |  |  |  |  |  | 6.9475E4 | 0 |  | 1 | 0 | 0 | 0 | 0 | 0 | 0 | 0 | 1 | 0 | 0 | 132 | 143 |  |  | PEAKS DB |
| total 13 peptides |  |  |  |  |  |  |  |  |  |  |  |  |  |  |  |  |  |  |  |  |  |  |  |  |  |  |  |  |  |  |  |  |  |  |  |  |  |
| --- | --- | --- | --- | --- | --- | --- | --- | --- | --- | --- | --- | --- | --- | --- | --- | --- | --- | --- | --- | --- | --- | --- | --- | --- | --- | --- | --- | --- | --- | --- | --- | --- | --- | --- | --- | --- | --- |

XP\_034271616.1

back to list

  

| Protein Coverage
| Supporting Peptides
|

Protein Coverage:

Supporting Peptides:

| Peptide | Uniq | -10lgP | Mass | Length | ppm | m/z | z | RT | Fraction | Scan | Source File | Area F4 | Area F5 | Area F6 | Area F10 | Area F8 | Area F9 | Area F7 | Area F1 | Area F2 | Area F3 | #Feature | #Feature F4 | #Feature F5 | #Feature F6 | #Feature F10 | #Feature F8 | #Feature F9 | #Feature F7 | #Feature F1 | #Feature F2 | #Feature F3 | Start | End | PTM | AScore | Found By |
| --- | --- | --- | --- | --- | --- | --- | --- | --- | --- | --- | --- | --- | --- | --- | --- | --- | --- | --- | --- | --- | --- | --- | --- | --- | --- | --- | --- | --- | --- | --- | --- | --- | --- | --- | --- | --- | --- |
| R.NEGTATYAAAVLFR.I | Y | 74.08 | 1482.7467 | 14 | 0.2 | 742.3808 | 2 | 48.27 | 9 | F9:28127 | 31122021\_RID\_3262\_2a.raw | 4.317E5 |  |  |  |  | 1.024E6 |  | 1.4003E6 | 4.6202E5 |  | 5 | 1 | 0 | 0 | 0 | 0 | 1 | 0 | 2 | 1 | 0 | 639 | 652 |  |  | PEAKS DB |
| R.LNYGIPAIVK.L | Y | 65.66 | 1086.6437 | 10 | 0.0 | 544.3291 | 2 | 45.04 | 8 | F8:24415 | 31122021\_RID\_3262\_1a.raw |  |  |  | 2.5539E6 |  | 2.6128E6 |  | 1.8129E6 | 7.9277E5 |  | 4 | 0 | 0 | 0 | 1 | 0 | 1 | 0 | 1 | 1 | 0 | 479 | 488 |  |  | PEAKS DB |
| R.VSVELTNSLFK.H | Y | 60.86 | 1235.6761 | 11 | 0.7 | 618.8458 | 2 | 47.53 | 6 | F6:32450 | 17012022\_RID\_3262\_9a.raw |  |  |  | 1.1826E6 |  | 1.3838E6 |  | 2.5277E6 |  | 2.6941E6 | 4 | 0 | 0 | 0 | 1 | 0 | 1 | 0 | 1 | 0 | 1 | 665 | 675 |  |  | PEAKS DB |
| R.ISEDKNPDYR.K | Y | 59.00 | 1235.5781 | 10 | 0.9 | 618.7969 | 2 | 18.00 | 4 | F4:5768 | 17012022\_RID\_3262\_10a .raw | 2.2898E5 | 5.2363E4 |  | 5.724E5 |  | 6.4152E5 | 4.2912E4 |  |  |  | 5 | 1 | 1 | 0 | 1 | 0 | 1 | 1 | 0 | 0 | 0 | 653 | 662 |  |  | PEAKS DB |
| K.SAIVHLINYQDDAELATR.A | Y | 57.87 | 2028.0276 | 18 | 1.0 | 677.0172 | 3 | 43.76 | 1 | F1:28958 | 13012022\_RID\_3262\_4a.raw | 4.2034E5 |  |  |  |  |  |  | 1.3135E6 | 3.6387E5 |  | 4 | 1 | 0 | 0 | 0 | 0 | 0 | 0 | 2 | 1 | 0 | 126 | 143 |  |  | PEAKS DB |
| K.SGGIPALVR.M | Y | 56.02 | 868.5130 | 9 | 0.5 | 435.2640 | 2 | 35.68 | 6 | F6:21518 | 17012022\_RID\_3262\_9a.raw |  | 3.703E5 |  | 2.5461E6 |  | 2.1357E6 | 8.8669E4 | 0 | 6.1375E5 |  | 5 | 0 | 1 | 0 | 1 | 0 | 1 | 1 | 0 | 1 | 0 | 226 | 234 |  |  | PEAKS DB |
| R.TM(+15.99)QNTSDLDTAR.C | Y | 55.25 | 1367.5988 | 12 | -0.4 | 684.8064 | 2 | 21.71 | 4 | F4:8886 | 17012022\_RID\_3262\_10a .raw |  | 1.2132E5 |  | 1.0143E6 |  | 6.4155E5 |  | 2.1506E5 | 1.8157E5 |  | 5 | 0 | 1 | 0 | 1 | 0 | 1 | 0 | 1 | 1 | 0 | 193 | 204 | Oxidation (M) | M2:Oxidation (M):1000.00 | PEAKS DB |
| R.HPEAEM(+15.99)AQNSVR.L | Y | 53.70 | 1383.6201 | 12 | 1.0 | 692.8180 | 2 | 15.98 | 4 | F4:4154 | 17012022\_RID\_3262\_10a .raw |  |  |  | 4.0379E5 |  |  |  |  |  |  | 2 | 0 | 0 | 0 | 2 | 0 | 0 | 0 | 0 | 0 | 0 | 467 | 478 | Oxidation (M) | M6:Oxidation (M):1000.00 | PEAKS DB |
| K.AAM(+15.99)IVNQLSK.K | Y | 53.10 | 1089.5852 | 10 | 0.8 | 545.8003 | 2 | 28.59 | 4 | F4:15343 | 17012022\_RID\_3262\_10a .raw |  |  |  | 1.0996E6 |  |  |  |  | 1.8354E5 |  | 2 | 0 | 0 | 0 | 1 | 0 | 0 | 0 | 0 | 1 | 0 | 163 | 172 | Oxidation (M) | M3:Oxidation (M):1000.00 | PEAKS DB |
| R.TMQNTSDLDTAR.C | Y | 53.03 | 1351.6038 | 12 | 0.1 | 676.8092 | 2 | 22.98 | 9 | F9:7560 | 31122021\_RID\_3262\_2a.raw |  | 1.1062E5 |  |  |  | 9.3847E5 |  | 2.411E5 | 2.0476E5 |  | 4 | 0 | 1 | 0 | 0 | 0 | 1 | 0 | 1 | 1 | 0 | 193 | 204 |  |  | PEAKS DB |
| K.AAMIVNQLSK.K | Y | 52.58 | 1073.5903 | 10 | -0.4 | 537.8022 | 2 | 33.93 | 4 | F4:19962 | 17012022\_RID\_3262\_10a .raw |  |  |  | 1.2793E6 |  | 1.6681E6 |  |  |  |  | 2 | 0 | 0 | 0 | 1 | 0 | 1 | 0 | 0 | 0 | 0 | 163 | 172 |  |  | PEAKS DB |
| R.HPEAEMAQNSVR.L | Y | 51.71 | 1367.6251 | 12 | 0.0 | 684.8198 | 2 | 17.98 | 8 | F8:3971 | 31122021\_RID\_3262\_1a.raw |  |  |  | 3.542E5 |  |  |  | 1.8269E5 | 1.0075E5 |  | 3 | 0 | 0 | 0 | 1 | 0 | 0 | 0 | 1 | 1 | 0 | 467 | 478 |  |  | PEAKS DB |
| L.INYQDDAELATR.A | N | 45.89 | 1407.6630 | 12 | 0.5 | 704.8391 | 2 | 32.83 | 9 | F9:15651 | 31122021\_RID\_3262\_2a.raw |  |  |  |  |  |  |  | 6.9475E4 | 0 |  | 1 | 0 | 0 | 0 | 0 | 0 | 0 | 0 | 1 | 0 | 0 | 132 | 143 |  |  | PEAKS DB |
| total 13 peptides |  |  |  |  |  |  |  |  |  |  |  |  |  |  |  |  |  |  |  |  |  |  |  |  |  |  |  |  |  |  |  |  |  |  |  |  |  |
| --- | --- | --- | --- | --- | --- | --- | --- | --- | --- | --- | --- | --- | --- | --- | --- | --- | --- | --- | --- | --- | --- | --- | --- | --- | --- | --- | --- | --- | --- | --- | --- | --- | --- | --- | --- | --- | --- |

LAB43566.1

back to list

  

| Protein Coverage
| Supporting Peptides
|

Protein Coverage:

Supporting Peptides:

| Peptide | Uniq | -10lgP | Mass | Length | ppm | m/z | z | RT | Fraction | Scan | Source File | Area F4 | Area F5 | Area F6 | Area F10 | Area F8 | Area F9 | Area F7 | Area F1 | Area F2 | Area F3 | #Feature | #Feature F4 | #Feature F5 | #Feature F6 | #Feature F10 | #Feature F8 | #Feature F9 | #Feature F7 | #Feature F1 | #Feature F2 | #Feature F3 | Start | End | PTM | AScore | Found By |
| --- | --- | --- | --- | --- | --- | --- | --- | --- | --- | --- | --- | --- | --- | --- | --- | --- | --- | --- | --- | --- | --- | --- | --- | --- | --- | --- | --- | --- | --- | --- | --- | --- | --- | --- | --- | --- | --- |
| R.NEGTATYAAAVLFR.I | Y | 74.08 | 1482.7467 | 14 | 0.2 | 742.3808 | 2 | 48.27 | 9 | F9:28127 | 31122021\_RID\_3262\_2a.raw | 4.317E5 |  |  |  |  | 1.024E6 |  | 1.4003E6 | 4.6202E5 |  | 5 | 1 | 0 | 0 | 0 | 0 | 1 | 0 | 2 | 1 | 0 | 657 | 670 |  |  | PEAKS DB |
| R.LNYGIPAIVK.L | Y | 65.66 | 1086.6437 | 10 | 0.0 | 544.3291 | 2 | 45.04 | 8 | F8:24415 | 31122021\_RID\_3262\_1a.raw |  |  |  | 2.5539E6 |  | 2.6128E6 |  | 1.8129E6 | 7.9277E5 |  | 4 | 0 | 0 | 0 | 1 | 0 | 1 | 0 | 1 | 1 | 0 | 503 | 512 |  |  | PEAKS DB |
| R.VSVELTNSLFK.H | Y | 60.86 | 1235.6761 | 11 | 0.7 | 618.8458 | 2 | 47.53 | 6 | F6:32450 | 17012022\_RID\_3262\_9a.raw |  |  |  | 1.1826E6 |  | 1.3838E6 |  | 2.5277E6 |  | 2.6941E6 | 4 | 0 | 0 | 0 | 1 | 0 | 1 | 0 | 1 | 0 | 1 | 683 | 693 |  |  | PEAKS DB |
| R.ISEDKNPDYR.K | Y | 59.00 | 1235.5781 | 10 | 0.9 | 618.7969 | 2 | 18.00 | 4 | F4:5768 | 17012022\_RID\_3262\_10a .raw | 2.2898E5 | 5.2363E4 |  | 5.724E5 |  | 6.4152E5 | 4.2912E4 |  |  |  | 5 | 1 | 1 | 0 | 1 | 0 | 1 | 1 | 0 | 0 | 0 | 671 | 680 |  |  | PEAKS DB |
| K.SAIVHLINYQDDAELATR.A | Y | 57.87 | 2028.0276 | 18 | 1.0 | 677.0172 | 3 | 43.76 | 1 | F1:28958 | 13012022\_RID\_3262\_4a.raw | 4.2034E5 |  |  |  |  |  |  | 1.3135E6 | 3.6387E5 |  | 4 | 1 | 0 | 0 | 0 | 0 | 0 | 0 | 2 | 1 | 0 | 150 | 167 |  |  | PEAKS DB |
| K.SGGIPALVR.M | Y | 56.02 | 868.5130 | 9 | 0.5 | 435.2640 | 2 | 35.68 | 6 | F6:21518 | 17012022\_RID\_3262\_9a.raw |  | 3.703E5 |  | 2.5461E6 |  | 2.1357E6 | 8.8669E4 | 0 | 6.1375E5 |  | 5 | 0 | 1 | 0 | 1 | 0 | 1 | 1 | 0 | 1 | 0 | 250 | 258 |  |  | PEAKS DB |
| R.TM(+15.99)QNTSDLDTAR.C | Y | 55.25 | 1367.5988 | 12 | -0.4 | 684.8064 | 2 | 21.71 | 4 | F4:8886 | 17012022\_RID\_3262\_10a .raw |  | 1.2132E5 |  | 1.0143E6 |  | 6.4155E5 |  | 2.1506E5 | 1.8157E5 |  | 5 | 0 | 1 | 0 | 1 | 0 | 1 | 0 | 1 | 1 | 0 | 217 | 228 | Oxidation (M) | M2:Oxidation (M):1000.00 | PEAKS DB |
| R.HPEAEM(+15.99)AQNSVR.L | Y | 53.70 | 1383.6201 | 12 | 1.0 | 692.8180 | 2 | 15.98 | 4 | F4:4154 | 17012022\_RID\_3262\_10a .raw |  |  |  | 4.0379E5 |  |  |  |  |  |  | 2 | 0 | 0 | 0 | 2 | 0 | 0 | 0 | 0 | 0 | 0 | 491 | 502 | Oxidation (M) | M6:Oxidation (M):1000.00 | PEAKS DB |
| K.AAM(+15.99)IVNQLSK.K | Y | 53.10 | 1089.5852 | 10 | 0.8 | 545.8003 | 2 | 28.59 | 4 | F4:15343 | 17012022\_RID\_3262\_10a .raw |  |  |  | 1.0996E6 |  |  |  |  | 1.8354E5 |  | 2 | 0 | 0 | 0 | 1 | 0 | 0 | 0 | 0 | 1 | 0 | 187 | 196 | Oxidation (M) | M3:Oxidation (M):1000.00 | PEAKS DB |
| R.TMQNTSDLDTAR.C | Y | 53.03 | 1351.6038 | 12 | 0.1 | 676.8092 | 2 | 22.98 | 9 | F9:7560 | 31122021\_RID\_3262\_2a.raw |  | 1.1062E5 |  |  |  | 9.3847E5 |  | 2.411E5 | 2.0476E5 |  | 4 | 0 | 1 | 0 | 0 | 0 | 1 | 0 | 1 | 1 | 0 | 217 | 228 |  |  | PEAKS DB |
| K.AAMIVNQLSK.K | Y | 52.58 | 1073.5903 | 10 | -0.4 | 537.8022 | 2 | 33.93 | 4 | F4:19962 | 17012022\_RID\_3262\_10a .raw |  |  |  | 1.2793E6 |  | 1.6681E6 |  |  |  |  | 2 | 0 | 0 | 0 | 1 | 0 | 1 | 0 | 0 | 0 | 0 | 187 | 196 |  |  | PEAKS DB |
| R.HPEAEMAQNSVR.L | Y | 51.71 | 1367.6251 | 12 | 0.0 | 684.8198 | 2 | 17.98 | 8 | F8:3971 | 31122021\_RID\_3262\_1a.raw |  |  |  | 3.542E5 |  |  |  | 1.8269E5 | 1.0075E5 |  | 3 | 0 | 0 | 0 | 1 | 0 | 0 | 0 | 1 | 1 | 0 | 491 | 502 |  |  | PEAKS DB |
| L.INYQDDAELATR.A | N | 45.89 | 1407.6630 | 12 | 0.5 | 704.8391 | 2 | 32.83 | 9 | F9:15651 | 31122021\_RID\_3262\_2a.raw |  |  |  |  |  |  |  | 6.9475E4 | 0 |  | 1 | 0 | 0 | 0 | 0 | 0 | 0 | 0 | 1 | 0 | 0 | 156 | 167 |  |  | PEAKS DB |
| total 13 peptides |  |  |  |  |  |  |  |  |  |  |  |  |  |  |  |  |  |  |  |  |  |  |  |  |  |  |  |  |  |  |  |  |  |  |  |  |  |
| --- | --- | --- | --- | --- | --- | --- | --- | --- | --- | --- | --- | --- | --- | --- | --- | --- | --- | --- | --- | --- | --- | --- | --- | --- | --- | --- | --- | --- | --- | --- | --- | --- | --- | --- | --- | --- | --- |

XP\_032091066.1

back to list

  

| Protein Coverage
| Supporting Peptides
|

Protein Coverage:

Supporting Peptides:

| Peptide | Uniq | -10lgP | Mass | Length | ppm | m/z | z | RT | Fraction | Scan | Source File | Area F4 | Area F5 | Area F6 | Area F10 | Area F8 | Area F9 | Area F7 | Area F1 | Area F2 | Area F3 | #Feature | #Feature F4 | #Feature F5 | #Feature F6 | #Feature F10 | #Feature F8 | #Feature F9 | #Feature F7 | #Feature F1 | #Feature F2 | #Feature F3 | Start | End | PTM | AScore | Found By |
| --- | --- | --- | --- | --- | --- | --- | --- | --- | --- | --- | --- | --- | --- | --- | --- | --- | --- | --- | --- | --- | --- | --- | --- | --- | --- | --- | --- | --- | --- | --- | --- | --- | --- | --- | --- | --- | --- |
| R.NEGTATYAAAVLFR.I | Y | 74.08 | 1482.7467 | 14 | 0.2 | 742.3808 | 2 | 48.27 | 9 | F9:28127 | 31122021\_RID\_3262\_2a.raw | 4.317E5 |  |  |  |  | 1.024E6 |  | 1.4003E6 | 4.6202E5 |  | 5 | 1 | 0 | 0 | 0 | 0 | 1 | 0 | 2 | 1 | 0 | 682 | 695 |  |  | PEAKS DB |
| R.LNYGIPAIVK.L | Y | 65.66 | 1086.6437 | 10 | 0.0 | 544.3291 | 2 | 45.04 | 8 | F8:24415 | 31122021\_RID\_3262\_1a.raw |  |  |  | 2.5539E6 |  | 2.6128E6 |  | 1.8129E6 | 7.9277E5 |  | 4 | 0 | 0 | 0 | 1 | 0 | 1 | 0 | 1 | 1 | 0 | 522 | 531 |  |  | PEAKS DB |
| R.VSVELTNSLFK.H | Y | 60.86 | 1235.6761 | 11 | 0.7 | 618.8458 | 2 | 47.53 | 6 | F6:32450 | 17012022\_RID\_3262\_9a.raw |  |  |  | 1.1826E6 |  | 1.3838E6 |  | 2.5277E6 |  | 2.6941E6 | 4 | 0 | 0 | 0 | 1 | 0 | 1 | 0 | 1 | 0 | 1 | 708 | 718 |  |  | PEAKS DB |
| R.ISEDKNPDYR.K | Y | 59.00 | 1235.5781 | 10 | 0.9 | 618.7969 | 2 | 18.00 | 4 | F4:5768 | 17012022\_RID\_3262\_10a .raw | 2.2898E5 | 5.2363E4 |  | 5.724E5 |  | 6.4152E5 | 4.2912E4 |  |  |  | 5 | 1 | 1 | 0 | 1 | 0 | 1 | 1 | 0 | 0 | 0 | 696 | 705 |  |  | PEAKS DB |
| K.SAIVHLINYQDDAELATR.A | Y | 57.87 | 2028.0276 | 18 | 1.0 | 677.0172 | 3 | 43.76 | 1 | F1:28958 | 13012022\_RID\_3262\_4a.raw | 4.2034E5 |  |  |  |  |  |  | 1.3135E6 | 3.6387E5 |  | 4 | 1 | 0 | 0 | 0 | 0 | 0 | 0 | 2 | 1 | 0 | 169 | 186 |  |  | PEAKS DB |
| K.SGGIPALVR.M | Y | 56.02 | 868.5130 | 9 | 0.5 | 435.2640 | 2 | 35.68 | 6 | F6:21518 | 17012022\_RID\_3262\_9a.raw |  | 3.703E5 |  | 2.5461E6 |  | 2.1357E6 | 8.8669E4 | 0 | 6.1375E5 |  | 5 | 0 | 1 | 0 | 1 | 0 | 1 | 1 | 0 | 1 | 0 | 269 | 277 |  |  | PEAKS DB |
| R.TM(+15.99)QNTSDLDTAR.C | Y | 55.25 | 1367.5988 | 12 | -0.4 | 684.8064 | 2 | 21.71 | 4 | F4:8886 | 17012022\_RID\_3262\_10a .raw |  | 1.2132E5 |  | 1.0143E6 |  | 6.4155E5 |  | 2.1506E5 | 1.8157E5 |  | 5 | 0 | 1 | 0 | 1 | 0 | 1 | 0 | 1 | 1 | 0 | 236 | 247 | Oxidation (M) | M2:Oxidation (M):1000.00 | PEAKS DB |
| R.HPEAEM(+15.99)AQNSVR.L | Y | 53.70 | 1383.6201 | 12 | 1.0 | 692.8180 | 2 | 15.98 | 4 | F4:4154 | 17012022\_RID\_3262\_10a .raw |  |  |  | 4.0379E5 |  |  |  |  |  |  | 2 | 0 | 0 | 0 | 2 | 0 | 0 | 0 | 0 | 0 | 0 | 510 | 521 | Oxidation (M) | M6:Oxidation (M):1000.00 | PEAKS DB |
| K.AAM(+15.99)IVNQLSK.K | Y | 53.10 | 1089.5852 | 10 | 0.8 | 545.8003 | 2 | 28.59 | 4 | F4:15343 | 17012022\_RID\_3262\_10a .raw |  |  |  | 1.0996E6 |  |  |  |  | 1.8354E5 |  | 2 | 0 | 0 | 0 | 1 | 0 | 0 | 0 | 0 | 1 | 0 | 206 | 215 | Oxidation (M) | M3:Oxidation (M):1000.00 | PEAKS DB |
| R.TMQNTSDLDTAR.C | Y | 53.03 | 1351.6038 | 12 | 0.1 | 676.8092 | 2 | 22.98 | 9 | F9:7560 | 31122021\_RID\_3262\_2a.raw |  | 1.1062E5 |  |  |  | 9.3847E5 |  | 2.411E5 | 2.0476E5 |  | 4 | 0 | 1 | 0 | 0 | 0 | 1 | 0 | 1 | 1 | 0 | 236 | 247 |  |  | PEAKS DB |
| K.AAMIVNQLSK.K | Y | 52.58 | 1073.5903 | 10 | -0.4 | 537.8022 | 2 | 33.93 | 4 | F4:19962 | 17012022\_RID\_3262\_10a .raw |  |  |  | 1.2793E6 |  | 1.6681E6 |  |  |  |  | 2 | 0 | 0 | 0 | 1 | 0 | 1 | 0 | 0 | 0 | 0 | 206 | 215 |  |  | PEAKS DB |
| R.HPEAEMAQNSVR.L | Y | 51.71 | 1367.6251 | 12 | 0.0 | 684.8198 | 2 | 17.98 | 8 | F8:3971 | 31122021\_RID\_3262\_1a.raw |  |  |  | 3.542E5 |  |  |  | 1.8269E5 | 1.0075E5 |  | 3 | 0 | 0 | 0 | 1 | 0 | 0 | 0 | 1 | 1 | 0 | 510 | 521 |  |  | PEAKS DB |
| L.INYQDDAELATR.A | N | 45.89 | 1407.6630 | 12 | 0.5 | 704.8391 | 2 | 32.83 | 9 | F9:15651 | 31122021\_RID\_3262\_2a.raw |  |  |  |  |  |  |  | 6.9475E4 | 0 |  | 1 | 0 | 0 | 0 | 0 | 0 | 0 | 0 | 1 | 0 | 0 | 175 | 186 |  |  | PEAKS DB |
| total 13 peptides |  |  |  |  |  |  |  |  |  |  |  |  |  |  |  |  |  |  |  |  |  |  |  |  |  |  |  |  |  |  |  |  |  |  |  |  |  |
| --- | --- | --- | --- | --- | --- | --- | --- | --- | --- | --- | --- | --- | --- | --- | --- | --- | --- | --- | --- | --- | --- | --- | --- | --- | --- | --- | --- | --- | --- | --- | --- | --- | --- | --- | --- | --- | --- |

XP\_032091065.1

back to list

  

| Protein Coverage
| Supporting Peptides
|

Protein Coverage:

Supporting Peptides:

| Peptide | Uniq | -10lgP | Mass | Length | ppm | m/z | z | RT | Fraction | Scan | Source File | Area F4 | Area F5 | Area F6 | Area F10 | Area F8 | Area F9 | Area F7 | Area F1 | Area F2 | Area F3 | #Feature | #Feature F4 | #Feature F5 | #Feature F6 | #Feature F10 | #Feature F8 | #Feature F9 | #Feature F7 | #Feature F1 | #Feature F2 | #Feature F3 | Start | End | PTM | AScore | Found By |
| --- | --- | --- | --- | --- | --- | --- | --- | --- | --- | --- | --- | --- | --- | --- | --- | --- | --- | --- | --- | --- | --- | --- | --- | --- | --- | --- | --- | --- | --- | --- | --- | --- | --- | --- | --- | --- | --- |
| R.NEGTATYAAAVLFR.I | Y | 74.08 | 1482.7467 | 14 | 0.2 | 742.3808 | 2 | 48.27 | 9 | F9:28127 | 31122021\_RID\_3262\_2a.raw | 4.317E5 |  |  |  |  | 1.024E6 |  | 1.4003E6 | 4.6202E5 |  | 5 | 1 | 0 | 0 | 0 | 0 | 1 | 0 | 2 | 1 | 0 | 682 | 695 |  |  | PEAKS DB |
| R.LNYGIPAIVK.L | Y | 65.66 | 1086.6437 | 10 | 0.0 | 544.3291 | 2 | 45.04 | 8 | F8:24415 | 31122021\_RID\_3262\_1a.raw |  |  |  | 2.5539E6 |  | 2.6128E6 |  | 1.8129E6 | 7.9277E5 |  | 4 | 0 | 0 | 0 | 1 | 0 | 1 | 0 | 1 | 1 | 0 | 522 | 531 |  |  | PEAKS DB |
| R.VSVELTNSLFK.H | Y | 60.86 | 1235.6761 | 11 | 0.7 | 618.8458 | 2 | 47.53 | 6 | F6:32450 | 17012022\_RID\_3262\_9a.raw |  |  |  | 1.1826E6 |  | 1.3838E6 |  | 2.5277E6 |  | 2.6941E6 | 4 | 0 | 0 | 0 | 1 | 0 | 1 | 0 | 1 | 0 | 1 | 708 | 718 |  |  | PEAKS DB |
| R.ISEDKNPDYR.K | Y | 59.00 | 1235.5781 | 10 | 0.9 | 618.7969 | 2 | 18.00 | 4 | F4:5768 | 17012022\_RID\_3262\_10a .raw | 2.2898E5 | 5.2363E4 |  | 5.724E5 |  | 6.4152E5 | 4.2912E4 |  |  |  | 5 | 1 | 1 | 0 | 1 | 0 | 1 | 1 | 0 | 0 | 0 | 696 | 705 |  |  | PEAKS DB |
| K.SAIVHLINYQDDAELATR.A | Y | 57.87 | 2028.0276 | 18 | 1.0 | 677.0172 | 3 | 43.76 | 1 | F1:28958 | 13012022\_RID\_3262\_4a.raw | 4.2034E5 |  |  |  |  |  |  | 1.3135E6 | 3.6387E5 |  | 4 | 1 | 0 | 0 | 0 | 0 | 0 | 0 | 2 | 1 | 0 | 169 | 186 |  |  | PEAKS DB |
| K.SGGIPALVR.M | Y | 56.02 | 868.5130 | 9 | 0.5 | 435.2640 | 2 | 35.68 | 6 | F6:21518 | 17012022\_RID\_3262\_9a.raw |  | 3.703E5 |  | 2.5461E6 |  | 2.1357E6 | 8.8669E4 | 0 | 6.1375E5 |  | 5 | 0 | 1 | 0 | 1 | 0 | 1 | 1 | 0 | 1 | 0 | 269 | 277 |  |  | PEAKS DB |
| R.TM(+15.99)QNTSDLDTAR.C | Y | 55.25 | 1367.5988 | 12 | -0.4 | 684.8064 | 2 | 21.71 | 4 | F4:8886 | 17012022\_RID\_3262\_10a .raw |  | 1.2132E5 |  | 1.0143E6 |  | 6.4155E5 |  | 2.1506E5 | 1.8157E5 |  | 5 | 0 | 1 | 0 | 1 | 0 | 1 | 0 | 1 | 1 | 0 | 236 | 247 | Oxidation (M) | M2:Oxidation (M):1000.00 | PEAKS DB |
| R.HPEAEM(+15.99)AQNSVR.L | Y | 53.70 | 1383.6201 | 12 | 1.0 | 692.8180 | 2 | 15.98 | 4 | F4:4154 | 17012022\_RID\_3262\_10a .raw |  |  |  | 4.0379E5 |  |  |  |  |  |  | 2 | 0 | 0 | 0 | 2 | 0 | 0 | 0 | 0 | 0 | 0 | 510 | 521 | Oxidation (M) | M6:Oxidation (M):1000.00 | PEAKS DB |
| K.AAM(+15.99)IVNQLSK.K | Y | 53.10 | 1089.5852 | 10 | 0.8 | 545.8003 | 2 | 28.59 | 4 | F4:15343 | 17012022\_RID\_3262\_10a .raw |  |  |  | 1.0996E6 |  |  |  |  | 1.8354E5 |  | 2 | 0 | 0 | 0 | 1 | 0 | 0 | 0 | 0 | 1 | 0 | 206 | 215 | Oxidation (M) | M3:Oxidation (M):1000.00 | PEAKS DB |
| R.TMQNTSDLDTAR.C | Y | 53.03 | 1351.6038 | 12 | 0.1 | 676.8092 | 2 | 22.98 | 9 | F9:7560 | 31122021\_RID\_3262\_2a.raw |  | 1.1062E5 |  |  |  | 9.3847E5 |  | 2.411E5 | 2.0476E5 |  | 4 | 0 | 1 | 0 | 0 | 0 | 1 | 0 | 1 | 1 | 0 | 236 | 247 |  |  | PEAKS DB |
| K.AAMIVNQLSK.K | Y | 52.58 | 1073.5903 | 10 | -0.4 | 537.8022 | 2 | 33.93 | 4 | F4:19962 | 17012022\_RID\_3262\_10a .raw |  |  |  | 1.2793E6 |  | 1.6681E6 |  |  |  |  | 2 | 0 | 0 | 0 | 1 | 0 | 1 | 0 | 0 | 0 | 0 | 206 | 215 |  |  | PEAKS DB |
| R.HPEAEMAQNSVR.L | Y | 51.71 | 1367.6251 | 12 | 0.0 | 684.8198 | 2 | 17.98 | 8 | F8:3971 | 31122021\_RID\_3262\_1a.raw |  |  |  | 3.542E5 |  |  |  | 1.8269E5 | 1.0075E5 |  | 3 | 0 | 0 | 0 | 1 | 0 | 0 | 0 | 1 | 1 | 0 | 510 | 521 |  |  | PEAKS DB |
| L.INYQDDAELATR.A | N | 45.89 | 1407.6630 | 12 | 0.5 | 704.8391 | 2 | 32.83 | 9 | F9:15651 | 31122021\_RID\_3262\_2a.raw |  |  |  |  |  |  |  | 6.9475E4 | 0 |  | 1 | 0 | 0 | 0 | 0 | 0 | 0 | 0 | 1 | 0 | 0 | 175 | 186 |  |  | PEAKS DB |
| total 13 peptides |  |  |  |  |  |  |  |  |  |  |  |  |  |  |  |  |  |  |  |  |  |  |  |  |  |  |  |  |  |  |  |  |  |  |  |  |  |
| --- | --- | --- | --- | --- | --- | --- | --- | --- | --- | --- | --- | --- | --- | --- | --- | --- | --- | --- | --- | --- | --- | --- | --- | --- | --- | --- | --- | --- | --- | --- | --- | --- | --- | --- | --- | --- | --- |

ETE68218.1

back to list

  

| Protein Coverage
| Supporting Peptides
|

Protein Coverage:

Supporting Peptides:

| Peptide | Uniq | -10lgP | Mass | Length | ppm | m/z | z | RT | Fraction | Scan | Source File | Area F4 | Area F5 | Area F6 | Area F10 | Area F8 | Area F9 | Area F7 | Area F1 | Area F2 | Area F3 | #Feature | #Feature F4 | #Feature F5 | #Feature F6 | #Feature F10 | #Feature F8 | #Feature F9 | #Feature F7 | #Feature F1 | #Feature F2 | #Feature F3 | Start | End | PTM | AScore | Found By |
| --- | --- | --- | --- | --- | --- | --- | --- | --- | --- | --- | --- | --- | --- | --- | --- | --- | --- | --- | --- | --- | --- | --- | --- | --- | --- | --- | --- | --- | --- | --- | --- | --- | --- | --- | --- | --- | --- |
| R.NEGTATYAAAVLFR.I | Y | 74.08 | 1482.7467 | 14 | 0.2 | 742.3808 | 2 | 48.27 | 9 | F9:28127 | 31122021\_RID\_3262\_2a.raw | 4.317E5 |  |  |  |  | 1.024E6 |  | 1.4003E6 | 4.6202E5 |  | 5 | 1 | 0 | 0 | 0 | 0 | 1 | 0 | 2 | 1 | 0 | 647 | 660 |  |  | PEAKS DB |
| R.LNYGIPAIVK.L | Y | 65.66 | 1086.6437 | 10 | 0.0 | 544.3291 | 2 | 45.04 | 8 | F8:24415 | 31122021\_RID\_3262\_1a.raw |  |  |  | 2.5539E6 |  | 2.6128E6 |  | 1.8129E6 | 7.9277E5 |  | 4 | 0 | 0 | 0 | 1 | 0 | 1 | 0 | 1 | 1 | 0 | 478 | 487 |  |  | PEAKS DB |
| R.VSVELTNSLFK.H | Y | 60.86 | 1235.6761 | 11 | 0.7 | 618.8458 | 2 | 47.53 | 6 | F6:32450 | 17012022\_RID\_3262\_9a.raw |  |  |  | 1.1826E6 |  | 1.3838E6 |  | 2.5277E6 |  | 2.6941E6 | 4 | 0 | 0 | 0 | 1 | 0 | 1 | 0 | 1 | 0 | 1 | 673 | 683 |  |  | PEAKS DB |
| R.ISEDKNPDYR.K | Y | 59.00 | 1235.5781 | 10 | 0.9 | 618.7969 | 2 | 18.00 | 4 | F4:5768 | 17012022\_RID\_3262\_10a .raw | 2.2898E5 | 5.2363E4 |  | 5.724E5 |  | 6.4152E5 | 4.2912E4 |  |  |  | 5 | 1 | 1 | 0 | 1 | 0 | 1 | 1 | 0 | 0 | 0 | 661 | 670 |  |  | PEAKS DB |
| K.SAIVHLINYQDDAELATR.A | Y | 57.87 | 2028.0276 | 18 | 1.0 | 677.0172 | 3 | 43.76 | 1 | F1:28958 | 13012022\_RID\_3262\_4a.raw | 4.2034E5 |  |  |  |  |  |  | 1.3135E6 | 3.6387E5 |  | 4 | 1 | 0 | 0 | 0 | 0 | 0 | 0 | 2 | 1 | 0 | 125 | 142 |  |  | PEAKS DB |
| K.SGGIPALVR.M | Y | 56.02 | 868.5130 | 9 | 0.5 | 435.2640 | 2 | 35.68 | 6 | F6:21518 | 17012022\_RID\_3262\_9a.raw |  | 3.703E5 |  | 2.5461E6 |  | 2.1357E6 | 8.8669E4 | 0 | 6.1375E5 |  | 5 | 0 | 1 | 0 | 1 | 0 | 1 | 1 | 0 | 1 | 0 | 225 | 233 |  |  | PEAKS DB |
| R.TM(+15.99)QNTSDLDTAR.C | Y | 55.25 | 1367.5988 | 12 | -0.4 | 684.8064 | 2 | 21.71 | 4 | F4:8886 | 17012022\_RID\_3262\_10a .raw |  | 1.2132E5 |  | 1.0143E6 |  | 6.4155E5 |  | 2.1506E5 | 1.8157E5 |  | 5 | 0 | 1 | 0 | 1 | 0 | 1 | 0 | 1 | 1 | 0 | 192 | 203 | Oxidation (M) | M2:Oxidation (M):1000.00 | PEAKS DB |
| R.HPEAEM(+15.99)AQNSVR.L | Y | 53.70 | 1383.6201 | 12 | 1.0 | 692.8180 | 2 | 15.98 | 4 | F4:4154 | 17012022\_RID\_3262\_10a .raw |  |  |  | 4.0379E5 |  |  |  |  |  |  | 2 | 0 | 0 | 0 | 2 | 0 | 0 | 0 | 0 | 0 | 0 | 466 | 477 | Oxidation (M) | M6:Oxidation (M):1000.00 | PEAKS DB |
| K.AAM(+15.99)IVNQLSK.K | Y | 53.10 | 1089.5852 | 10 | 0.8 | 545.8003 | 2 | 28.59 | 4 | F4:15343 | 17012022\_RID\_3262\_10a .raw |  |  |  | 1.0996E6 |  |  |  |  | 1.8354E5 |  | 2 | 0 | 0 | 0 | 1 | 0 | 0 | 0 | 0 | 1 | 0 | 162 | 171 | Oxidation (M) | M3:Oxidation (M):1000.00 | PEAKS DB |
| R.TMQNTSDLDTAR.C | Y | 53.03 | 1351.6038 | 12 | 0.1 | 676.8092 | 2 | 22.98 | 9 | F9:7560 | 31122021\_RID\_3262\_2a.raw |  | 1.1062E5 |  |  |  | 9.3847E5 |  | 2.411E5 | 2.0476E5 |  | 4 | 0 | 1 | 0 | 0 | 0 | 1 | 0 | 1 | 1 | 0 | 192 | 203 |  |  | PEAKS DB |
| K.AAMIVNQLSK.K | Y | 52.58 | 1073.5903 | 10 | -0.4 | 537.8022 | 2 | 33.93 | 4 | F4:19962 | 17012022\_RID\_3262\_10a .raw |  |  |  | 1.2793E6 |  | 1.6681E6 |  |  |  |  | 2 | 0 | 0 | 0 | 1 | 0 | 1 | 0 | 0 | 0 | 0 | 162 | 171 |  |  | PEAKS DB |
| R.HPEAEMAQNSVR.L | Y | 51.71 | 1367.6251 | 12 | 0.0 | 684.8198 | 2 | 17.98 | 8 | F8:3971 | 31122021\_RID\_3262\_1a.raw |  |  |  | 3.542E5 |  |  |  | 1.8269E5 | 1.0075E5 |  | 3 | 0 | 0 | 0 | 1 | 0 | 0 | 0 | 1 | 1 | 0 | 466 | 477 |  |  | PEAKS DB |
| L.INYQDDAELATR.A | N | 45.89 | 1407.6630 | 12 | 0.5 | 704.8391 | 2 | 32.83 | 9 | F9:15651 | 31122021\_RID\_3262\_2a.raw |  |  |  |  |  |  |  | 6.9475E4 | 0 |  | 1 | 0 | 0 | 0 | 0 | 0 | 0 | 0 | 1 | 0 | 0 | 131 | 142 |  |  | PEAKS DB |
| total 13 peptides |  |  |  |  |  |  |  |  |  |  |  |  |  |  |  |  |  |  |  |  |  |  |  |  |  |  |  |  |  |  |  |  |  |  |  |  |  |
| --- | --- | --- | --- | --- | --- | --- | --- | --- | --- | --- | --- | --- | --- | --- | --- | --- | --- | --- | --- | --- | --- | --- | --- | --- | --- | --- | --- | --- | --- | --- | --- | --- | --- | --- | --- | --- | --- |

XP\_015687424.1

back to list

  

| Protein Coverage
| Supporting Peptides
|

Protein Coverage:

Supporting Peptides:

| Peptide | Uniq | -10lgP | Mass | Length | ppm | m/z | z | RT | Fraction | Scan | Source File | Area F4 | Area F5 | Area F6 | Area F10 | Area F8 | Area F9 | Area F7 | Area F1 | Area F2 | Area F3 | #Feature | #Feature F4 | #Feature F5 | #Feature F6 | #Feature F10 | #Feature F8 | #Feature F9 | #Feature F7 | #Feature F1 | #Feature F2 | #Feature F3 | Start | End | PTM | AScore | Found By |
| --- | --- | --- | --- | --- | --- | --- | --- | --- | --- | --- | --- | --- | --- | --- | --- | --- | --- | --- | --- | --- | --- | --- | --- | --- | --- | --- | --- | --- | --- | --- | --- | --- | --- | --- | --- | --- | --- |
| K.NVHLQQEHYSK.K | Y | 64.70 | 1381.6738 | 11 | 0.7 | 691.8447 | 2 | 17.10 | 1 | F1:5349 | 13012022\_RID\_3262\_4a.raw | 1.8116E5 |  |  |  |  |  |  |  |  |  | 1 | 1 | 0 | 0 | 0 | 0 | 0 | 0 | 0 | 0 | 0 | 528 | 538 |  |  | PEAKS DB |
| K.HESEITPVYQGLNELVPLYK.L | Y | 60.36 | 2328.2002 | 20 | 0.4 | 777.0743 | 3 | 54.95 | 1 | F1:38383 | 13012022\_RID\_3262\_4a.raw | 1.3421E6 |  |  |  |  |  |  |  |  |  | 2 | 2 | 0 | 0 | 0 | 0 | 0 | 0 | 0 | 0 | 0 | 653 | 672 |  |  | PEAKS DB |
| K.KFDFGSHSLAR.M | Y | 52.76 | 1263.6360 | 11 | -0.2 | 422.2192 | 3 | 29.19 | 1 | F1:15702 | 13012022\_RID\_3262\_4a.raw | 1.4305E6 |  |  |  |  |  |  |  |  |  | 1 | 1 | 0 | 0 | 0 | 0 | 0 | 0 | 0 | 0 | 0 | 850 | 860 |  |  | PEAKS DB |
| R.ASLINNAFQLVSAGK.L | Y | 51.57 | 1531.8358 | 15 | 0.9 | 766.9259 | 2 | 54.63 | 1 | F1:38148 | 13012022\_RID\_3262\_4a.raw | 4.2229E6 |  |  |  |  |  |  |  |  |  | 1 | 1 | 0 | 0 | 0 | 0 | 0 | 0 | 0 | 0 | 0 | 624 | 638 |  |  | PEAKS DB |
| R.MITGITNQYSTK.E | Y | 49.85 | 1355.6755 | 12 | 0.1 | 678.8451 | 2 | 33.68 | 1 | F1:19744 | 13012022\_RID\_3262\_4a.raw | 6.7377E6 |  |  |  |  |  |  |  |  |  | 1 | 1 | 0 | 0 | 0 | 0 | 0 | 0 | 0 | 0 | 0 | 861 | 872 |  |  | PEAKS DB |
| K.NMIDKQSWDDEGTVSEQMLR.T | Y | 49.07 | 2381.0627 | 20 | 0.4 | 794.6952 | 3 | 43.85 | 1 | F1:28975 | 13012022\_RID\_3262\_4a.raw | 3.8932E5 |  |  |  |  |  |  |  |  |  | 1 | 1 | 0 | 0 | 0 | 0 | 0 | 0 | 0 | 0 | 0 | 696 | 715 |  |  | PEAKS DB |
| R.AVQQAIETIEENIQWMDKNLER.I | Y | 47.91 | 2657.3118 | 22 | 0.9 | 886.7787 | 3 | 68.68 | 1 | F1:50027 | 13012022\_RID\_3262\_4a.raw | 1.1795E6 |  |  |  |  |  |  |  |  |  | 1 | 1 | 0 | 0 | 0 | 0 | 0 | 0 | 0 | 0 | 0 | 894 | 915 |  |  | PEAKS DB |
| K.TQELPSILFSVGR.N | Y | 47.70 | 1445.7878 | 13 | 0.8 | 723.9018 | 2 | 56.87 | 8 | F8:33718 | 31122021\_RID\_3262\_1a.raw | 2.3872E6 |  |  |  |  |  |  | 7.325E4 |  |  | 2 | 1 | 0 | 0 | 0 | 0 | 0 | 0 | 1 | 0 | 0 | 816 | 828 |  |  | PEAKS DB |
| R.LLKDNHETISSNDR.A | Y | 47.56 | 1640.8118 | 14 | 0.1 | 547.9446 | 3 | 19.66 | 1 | F1:7265 | 13012022\_RID\_3262\_4a.raw | 3.7244E6 |  |  |  |  |  |  |  |  |  | 1 | 1 | 0 | 0 | 0 | 0 | 0 | 0 | 0 | 0 | 0 | 610 | 623 |  |  | PEAKS DB |
| K.GFPLVTVTIK.G | Y | 44.29 | 1073.6484 | 10 | 0.8 | 537.8319 | 2 | 49.72 | 1 | F1:33970 | 13012022\_RID\_3262\_4a.raw | 3.002E6 |  |  |  |  |  |  |  |  |  | 1 | 1 | 0 | 0 | 0 | 0 | 0 | 0 | 0 | 0 | 0 | 516 | 525 |  |  | PEAKS DB |
| K.NM(+15.99)IDKQSWDDEGTVSEQMLR.T | Y | 41.37 | 2397.0576 | 20 | -1.0 | 800.0257 | 3 | 40.38 | 1 | F1:25906 | 13012022\_RID\_3262\_4a.raw | 1.4557E5 |  |  |  |  |  |  |  |  |  | 1 | 1 | 0 | 0 | 0 | 0 | 0 | 0 | 0 | 0 | 0 | 696 | 715 | Oxidation (M) | M2:Oxidation (M):139.37 | PEAKS DB |
| total 11 peptides |  |  |  |  |  |  |  |  |  |  |  |  |  |  |  |  |  |  |  |  |  |  |  |  |  |  |  |  |  |  |  |  |  |  |  |  |  |
| --- | --- | --- | --- | --- | --- | --- | --- | --- | --- | --- | --- | --- | --- | --- | --- | --- | --- | --- | --- | --- | --- | --- | --- | --- | --- | --- | --- | --- | --- | --- | --- | --- | --- | --- | --- | --- | --- |

JAG65874.1

back to list

  

| Protein Coverage
| Supporting Peptides
|

Protein Coverage:

Supporting Peptides:

| Peptide | Uniq | -10lgP | Mass | Length | ppm | m/z | z | RT | Fraction | Scan | Source File | Area F4 | Area F5 | Area F6 | Area F10 | Area F8 | Area F9 | Area F7 | Area F1 | Area F2 | Area F3 | #Feature | #Feature F4 | #Feature F5 | #Feature F6 | #Feature F10 | #Feature F8 | #Feature F9 | #Feature F7 | #Feature F1 | #Feature F2 | #Feature F3 | Start | End | PTM | AScore | Found By |
| --- | --- | --- | --- | --- | --- | --- | --- | --- | --- | --- | --- | --- | --- | --- | --- | --- | --- | --- | --- | --- | --- | --- | --- | --- | --- | --- | --- | --- | --- | --- | --- | --- | --- | --- | --- | --- | --- |
| R.GDDIPYTPVFYAYTLLTK.T | N | 69.16 | 2076.0454 | 18 | 0.5 | 1039.0305 | 2 | 73.61 | 8 | F8:46146 | 31122021\_RID\_3262\_1a.raw |  |  |  |  |  |  |  | 3.5587E6 | 8.7984E5 |  | 3 | 0 | 0 | 0 | 0 | 0 | 0 | 0 | 2 | 1 | 0 | 254 | 271 |  |  | PEAKS DB |
| R.TIHWGEPTAFQK.E | N | 59.60 | 1413.7041 | 12 | 0.4 | 472.2422 | 3 | 34.27 | 8 | F8:15881 | 31122021\_RID\_3262\_1a.raw |  |  |  |  |  |  |  | 2.8249E6 | 0 |  | 3 | 0 | 0 | 0 | 0 | 0 | 0 | 0 | 3 | 0 | 0 | 467 | 478 |  |  | PEAKS DB |
| K.VVSLVPYAR.N | N | 58.37 | 1002.5862 | 9 | 0.3 | 502.3005 | 2 | 38.96 | 8 | F8:19771 | 31122021\_RID\_3262\_1a.raw | 1.0512E6 |  |  |  |  |  |  | 4.0529E6 | 7.1037E5 |  | 3 | 1 | 0 | 0 | 0 | 0 | 0 | 0 | 1 | 1 | 0 | 595 | 603 |  |  | PEAKS DB |
| R.VLM(+15.99)GNIDLSK.L | N | 56.40 | 1104.5848 | 10 | 1.7 | 553.3007 | 2 | 32.94 | 8 | F8:14847 | 31122021\_RID\_3262\_1a.raw |  |  |  |  |  |  |  | 2.0225E6 | 5.6608E5 |  | 3 | 0 | 0 | 0 | 0 | 0 | 0 | 0 | 1 | 2 | 0 | 484 | 493 | Oxidation (M) | M3:Oxidation (M):1000.00 | PEAKS DB |
| R.VLMGNIDLSK.L | N | 50.63 | 1088.5900 | 10 | 0.9 | 545.3027 | 2 | 40.89 | 8 | F8:21051 | 31122021\_RID\_3262\_1a.raw |  |  |  |  |  |  |  | 3.1E6 | 6.693E5 |  | 2 | 0 | 0 | 0 | 0 | 0 | 0 | 0 | 1 | 1 | 0 | 484 | 493 |  |  | PEAKS DB |
| R.EIIGPELQR.R | Y | 49.09 | 1053.5818 | 9 | 0.8 | 527.7986 | 2 | 35.03 | 8 | F8:16343 | 31122021\_RID\_3262\_1a.raw |  |  |  |  |  |  |  | 2.0806E5 |  |  | 1 | 0 | 0 | 0 | 0 | 0 | 0 | 0 | 1 | 0 | 0 | 628 | 636 |  |  | PEAKS DB |
| R.YWIQAER.Q | N | 47.48 | 964.4766 | 7 | -0.6 | 483.2453 | 2 | 31.76 | 9 | F9:14735 | 31122021\_RID\_3262\_2a.raw |  |  |  |  |  |  |  | 1.6204E6 | 4.2744E5 |  | 2 | 0 | 0 | 0 | 0 | 0 | 0 | 0 | 1 | 1 | 0 | 118 | 124 |  |  | PEAKS DB |
| K.NTEPFSHASLL.A | N | 46.54 | 1214.5931 | 11 | 0.1 | 608.3039 | 2 | 41.96 | 8 | F8:22010 | 31122021\_RID\_3262\_1a.raw |  |  |  |  |  |  |  | 3.7494E5 |  |  | 1 | 0 | 0 | 0 | 0 | 0 | 0 | 0 | 1 | 0 | 0 | 649 | 659 |  |  | PEAKS DB |
| K.GM(+15.99)FTSIEPGYYHDGEFGIR.L | N | 44.23 | 2190.9680 | 19 | 0.6 | 731.3304 | 3 | 45.51 | 8 | F8:24644 | 31122021\_RID\_3262\_1a.raw |  |  |  |  |  |  |  | 3.9301E5 |  |  | 1 | 0 | 0 | 0 | 0 | 0 | 0 | 0 | 1 | 0 | 0 | 551 | 569 | Oxidation (M) | M2:Oxidation (M):1000.00 | PEAKS DB |
| R.YLVWLEK.K | N | 40.96 | 949.5273 | 7 | 0.1 | 475.7710 | 2 | 45.31 | 9 | F9:25829 | 31122021\_RID\_3262\_2a.raw |  |  |  |  |  |  |  |  | 7.199E5 |  | 1 | 0 | 0 | 0 | 0 | 0 | 0 | 0 | 0 | 1 | 0 | 381 | 387 |  |  | PEAKS DB |
| total 10 peptides |  |  |  |  |  |  |  |  |  |  |  |  |  |  |  |  |  |  |  |  |  |  |  |  |  |  |  |  |  |  |  |  |  |  |  |  |  |
| --- | --- | --- | --- | --- | --- | --- | --- | --- | --- | --- | --- | --- | --- | --- | --- | --- | --- | --- | --- | --- | --- | --- | --- | --- | --- | --- | --- | --- | --- | --- | --- | --- | --- | --- | --- | --- | --- |

XP\_015743663.1

back to list

  

| Protein Coverage
| Supporting Peptides
|

Protein Coverage:

Supporting Peptides:

| Peptide | Uniq | -10lgP | Mass | Length | ppm | m/z | z | RT | Fraction | Scan | Source File | Area F4 | Area F5 | Area F6 | Area F10 | Area F8 | Area F9 | Area F7 | Area F1 | Area F2 | Area F3 | #Feature | #Feature F4 | #Feature F5 | #Feature F6 | #Feature F10 | #Feature F8 | #Feature F9 | #Feature F7 | #Feature F1 | #Feature F2 | #Feature F3 | Start | End | PTM | AScore | Found By |
| --- | --- | --- | --- | --- | --- | --- | --- | --- | --- | --- | --- | --- | --- | --- | --- | --- | --- | --- | --- | --- | --- | --- | --- | --- | --- | --- | --- | --- | --- | --- | --- | --- | --- | --- | --- | --- | --- |
| R.VQHASPAGNYAHVVNR.N | N | 80.89 | 1718.8601 | 16 | 0.0 | 573.9606 | 3 | 19.73 | 9 | F9:5169 | 31122021\_RID\_3262\_2a.raw | 5.9402E6 |  |  |  |  |  |  | 2.0488E5 | 2.9449E7 |  | 6 | 2 | 0 | 0 | 0 | 0 | 0 | 0 | 1 | 3 | 0 | 169 | 184 |  |  | PEAKS DB |
| R.ALTEAIEFDK.A | N | 66.55 | 1135.5760 | 10 | 0.3 | 568.7955 | 2 | 39.48 | 9 | F9:21119 | 31122021\_RID\_3262\_2a.raw | 4.5481E6 | 5.9828E5 |  |  |  |  |  |  | 3.4018E7 | 6.8465E6 | 5 | 1 | 1 | 0 | 0 | 0 | 0 | 0 | 0 | 2 | 1 | 345 | 354 |  |  | PEAKS DB |
| K.SVGIVTTTR.V | N | 55.56 | 932.5291 | 9 | -0.6 | 467.2715 | 2 | 24.85 | 9 | F9:9035 | 31122021\_RID\_3262\_2a.raw | 4.7751E6 |  |  |  |  |  |  | 3.9398E5 | 2.2112E7 |  | 3 | 1 | 0 | 0 | 0 | 0 | 0 | 0 | 1 | 1 | 0 | 160 | 168 |  |  | PEAKS DB |
| K.RALTEAIEFDK.A | N | 53.46 | 1291.6771 | 11 | -0.5 | 431.5661 | 3 | 32.85 | 9 | F9:15657 | 31122021\_RID\_3262\_2a.raw |  |  |  |  |  |  |  |  | 2.1679E5 |  | 1 | 0 | 0 | 0 | 0 | 0 | 0 | 0 | 0 | 1 | 0 | 344 | 354 |  |  | PEAKS DB |
| R.GTSIYGLAPEK.A | Y | 50.53 | 1134.5920 | 11 | -1.7 | 568.3023 | 2 | 42.25 | 9 | F9:23320 | 31122021\_RID\_3262\_2a.raw |  |  |  |  |  |  |  |  | 0 |  | 0 | 0 | 0 | 0 | 0 | 0 | 0 | 0 | 0 | 0 | 0 | 389 | 399 |  |  | PEAKS DB |
| R.VQHASPAGNYAHVVN.R | N | 47.90 | 1562.7589 | 15 | 0.5 | 782.3871 | 2 | 22.55 | 9 | F9:7231 | 31122021\_RID\_3262\_2a.raw |  |  |  |  |  |  |  |  | 4.0973E6 |  | 2 | 0 | 0 | 0 | 0 | 0 | 0 | 0 | 0 | 2 | 0 | 169 | 183 |  |  | PEAKS DB |
| K.GPMAHLFH.G | N | 42.64 | 908.4327 | 8 | 0.6 | 455.2239 | 2 | 28.62 | 9 | F9:12184 | 31122021\_RID\_3262\_2a.raw |  |  |  |  |  |  |  |  | 2.3063E4 |  | 1 | 0 | 0 | 0 | 0 | 0 | 0 | 0 | 0 | 1 | 0 | 461 | 468 |  |  | PEAKS DB |
| total 7 peptides |  |  |  |  |  |  |  |  |  |  |  |  |  |  |  |  |  |  |  |  |  |  |  |  |  |  |  |  |  |  |  |  |  |  |  |  |  |
| --- | --- | --- | --- | --- | --- | --- | --- | --- | --- | --- | --- | --- | --- | --- | --- | --- | --- | --- | --- | --- | --- | --- | --- | --- | --- | --- | --- | --- | --- | --- | --- | --- | --- | --- | --- | --- | --- |

XP\_025022776.1

back to list

  

| Protein Coverage
| Supporting Peptides
|

Protein Coverage:

Supporting Peptides:

| Peptide | Uniq | -10lgP | Mass | Length | ppm | m/z | z | RT | Fraction | Scan | Source File | Area F4 | Area F5 | Area F6 | Area F10 | Area F8 | Area F9 | Area F7 | Area F1 | Area F2 | Area F3 | #Feature | #Feature F4 | #Feature F5 | #Feature F6 | #Feature F10 | #Feature F8 | #Feature F9 | #Feature F7 | #Feature F1 | #Feature F2 | #Feature F3 | Start | End | PTM | AScore | Found By |
| --- | --- | --- | --- | --- | --- | --- | --- | --- | --- | --- | --- | --- | --- | --- | --- | --- | --- | --- | --- | --- | --- | --- | --- | --- | --- | --- | --- | --- | --- | --- | --- | --- | --- | --- | --- | --- | --- |
| R.VQHASPAGNYAHVVNR.N | N | 80.89 | 1718.8601 | 16 | 0.0 | 573.9606 | 3 | 19.73 | 9 | F9:5169 | 31122021\_RID\_3262\_2a.raw | 5.9402E6 |  |  |  |  |  |  | 2.0488E5 | 2.9449E7 |  | 6 | 2 | 0 | 0 | 0 | 0 | 0 | 0 | 1 | 3 | 0 | 123 | 138 |  |  | PEAKS DB |
| R.ALTEAIEFDK.A | N | 66.55 | 1135.5760 | 10 | 0.3 | 568.7955 | 2 | 39.48 | 9 | F9:21119 | 31122021\_RID\_3262\_2a.raw | 4.5481E6 | 5.9828E5 |  |  |  |  |  |  | 3.4018E7 | 6.8465E6 | 5 | 1 | 1 | 0 | 0 | 0 | 0 | 0 | 0 | 2 | 1 | 299 | 308 |  |  | PEAKS DB |
| K.SVGIVTTTR.V | N | 55.56 | 932.5291 | 9 | -0.6 | 467.2715 | 2 | 24.85 | 9 | F9:9035 | 31122021\_RID\_3262\_2a.raw | 4.7751E6 |  |  |  |  |  |  | 3.9398E5 | 2.2112E7 |  | 3 | 1 | 0 | 0 | 0 | 0 | 0 | 0 | 1 | 1 | 0 | 114 | 122 |  |  | PEAKS DB |
| K.RALTEAIEFDK.A | N | 53.46 | 1291.6771 | 11 | -0.5 | 431.5661 | 3 | 32.85 | 9 | F9:15657 | 31122021\_RID\_3262\_2a.raw |  |  |  |  |  |  |  |  | 2.1679E5 |  | 1 | 0 | 0 | 0 | 0 | 0 | 0 | 0 | 0 | 1 | 0 | 298 | 308 |  |  | PEAKS DB |
| R.GTSIYGLAPEK.A | Y | 50.53 | 1134.5920 | 11 | -1.7 | 568.3023 | 2 | 42.25 | 9 | F9:23320 | 31122021\_RID\_3262\_2a.raw |  |  |  |  |  |  |  |  | 0 |  | 0 | 0 | 0 | 0 | 0 | 0 | 0 | 0 | 0 | 0 | 0 | 343 | 353 |  |  | PEAKS DB |
| R.VQHASPAGNYAHVVN.R | N | 47.90 | 1562.7589 | 15 | 0.5 | 782.3871 | 2 | 22.55 | 9 | F9:7231 | 31122021\_RID\_3262\_2a.raw |  |  |  |  |  |  |  |  | 4.0973E6 |  | 2 | 0 | 0 | 0 | 0 | 0 | 0 | 0 | 0 | 2 | 0 | 123 | 137 |  |  | PEAKS DB |
| K.GPMAHLFH.G | N | 42.64 | 908.4327 | 8 | 0.6 | 455.2239 | 2 | 28.62 | 9 | F9:12184 | 31122021\_RID\_3262\_2a.raw |  |  |  |  |  |  |  |  | 2.3063E4 |  | 1 | 0 | 0 | 0 | 0 | 0 | 0 | 0 | 0 | 1 | 0 | 415 | 422 |  |  | PEAKS DB |
| total 7 peptides |  |  |  |  |  |  |  |  |  |  |  |  |  |  |  |  |  |  |  |  |  |  |  |  |  |  |  |  |  |  |  |  |  |  |  |  |  |
| --- | --- | --- | --- | --- | --- | --- | --- | --- | --- | --- | --- | --- | --- | --- | --- | --- | --- | --- | --- | --- | --- | --- | --- | --- | --- | --- | --- | --- | --- | --- | --- | --- | --- | --- | --- | --- | --- |

XP\_025022775.1

back to list

  

| Protein Coverage
| Supporting Peptides
|

Protein Coverage:

Supporting Peptides:

| Peptide | Uniq | -10lgP | Mass | Length | ppm | m/z | z | RT | Fraction | Scan | Source File | Area F4 | Area F5 | Area F6 | Area F10 | Area F8 | Area F9 | Area F7 | Area F1 | Area F2 | Area F3 | #Feature | #Feature F4 | #Feature F5 | #Feature F6 | #Feature F10 | #Feature F8 | #Feature F9 | #Feature F7 | #Feature F1 | #Feature F2 | #Feature F3 | Start | End | PTM | AScore | Found By |
| --- | --- | --- | --- | --- | --- | --- | --- | --- | --- | --- | --- | --- | --- | --- | --- | --- | --- | --- | --- | --- | --- | --- | --- | --- | --- | --- | --- | --- | --- | --- | --- | --- | --- | --- | --- | --- | --- |
| R.VQHASPAGNYAHVVNR.N | N | 80.89 | 1718.8601 | 16 | 0.0 | 573.9606 | 3 | 19.73 | 9 | F9:5169 | 31122021\_RID\_3262\_2a.raw | 5.9402E6 |  |  |  |  |  |  | 2.0488E5 | 2.9449E7 |  | 6 | 2 | 0 | 0 | 0 | 0 | 0 | 0 | 1 | 3 | 0 | 209 | 224 |  |  | PEAKS DB |
| R.ALTEAIEFDK.A | N | 66.55 | 1135.5760 | 10 | 0.3 | 568.7955 | 2 | 39.48 | 9 | F9:21119 | 31122021\_RID\_3262\_2a.raw | 4.5481E6 | 5.9828E5 |  |  |  |  |  |  | 3.4018E7 | 6.8465E6 | 5 | 1 | 1 | 0 | 0 | 0 | 0 | 0 | 0 | 2 | 1 | 385 | 394 |  |  | PEAKS DB |
| K.SVGIVTTTR.V | N | 55.56 | 932.5291 | 9 | -0.6 | 467.2715 | 2 | 24.85 | 9 | F9:9035 | 31122021\_RID\_3262\_2a.raw | 4.7751E6 |  |  |  |  |  |  | 3.9398E5 | 2.2112E7 |  | 3 | 1 | 0 | 0 | 0 | 0 | 0 | 0 | 1 | 1 | 0 | 200 | 208 |  |  | PEAKS DB |
| K.RALTEAIEFDK.A | N | 53.46 | 1291.6771 | 11 | -0.5 | 431.5661 | 3 | 32.85 | 9 | F9:15657 | 31122021\_RID\_3262\_2a.raw |  |  |  |  |  |  |  |  | 2.1679E5 |  | 1 | 0 | 0 | 0 | 0 | 0 | 0 | 0 | 0 | 1 | 0 | 384 | 394 |  |  | PEAKS DB |
| R.GTSIYGLAPEK.A | Y | 50.53 | 1134.5920 | 11 | -1.7 | 568.3023 | 2 | 42.25 | 9 | F9:23320 | 31122021\_RID\_3262\_2a.raw |  |  |  |  |  |  |  |  | 0 |  | 0 | 0 | 0 | 0 | 0 | 0 | 0 | 0 | 0 | 0 | 0 | 429 | 439 |  |  | PEAKS DB |
| R.VQHASPAGNYAHVVN.R | N | 47.90 | 1562.7589 | 15 | 0.5 | 782.3871 | 2 | 22.55 | 9 | F9:7231 | 31122021\_RID\_3262\_2a.raw |  |  |  |  |  |  |  |  | 4.0973E6 |  | 2 | 0 | 0 | 0 | 0 | 0 | 0 | 0 | 0 | 2 | 0 | 209 | 223 |  |  | PEAKS DB |
| K.GPMAHLFH.G | N | 42.64 | 908.4327 | 8 | 0.6 | 455.2239 | 2 | 28.62 | 9 | F9:12184 | 31122021\_RID\_3262\_2a.raw |  |  |  |  |  |  |  |  | 2.3063E4 |  | 1 | 0 | 0 | 0 | 0 | 0 | 0 | 0 | 0 | 1 | 0 | 501 | 508 |  |  | PEAKS DB |
| total 7 peptides |  |  |  |  |  |  |  |  |  |  |  |  |  |  |  |  |  |  |  |  |  |  |  |  |  |  |  |  |  |  |  |  |  |  |  |  |  |
| --- | --- | --- | --- | --- | --- | --- | --- | --- | --- | --- | --- | --- | --- | --- | --- | --- | --- | --- | --- | --- | --- | --- | --- | --- | --- | --- | --- | --- | --- | --- | --- | --- | --- | --- | --- | --- | --- |

BAN89414.1

back to list

  

| Protein Coverage
| Supporting Peptides
|

Protein Coverage:

Supporting Peptides:

| Peptide | Uniq | -10lgP | Mass | Length | ppm | m/z | z | RT | Fraction | Scan | Source File | Area F4 | Area F5 | Area F6 | Area F10 | Area F8 | Area F9 | Area F7 | Area F1 | Area F2 | Area F3 | #Feature | #Feature F4 | #Feature F5 | #Feature F6 | #Feature F10 | #Feature F8 | #Feature F9 | #Feature F7 | #Feature F1 | #Feature F2 | #Feature F3 | Start | End | PTM | AScore | Found By |
| --- | --- | --- | --- | --- | --- | --- | --- | --- | --- | --- | --- | --- | --- | --- | --- | --- | --- | --- | --- | --- | --- | --- | --- | --- | --- | --- | --- | --- | --- | --- | --- | --- | --- | --- | --- | --- | --- |
| R.DSTALFPNIYLETILR.S | N | 76.87 | 1864.9934 | 16 | 0.4 | 933.5043 | 2 | 75.08 | 9 | F9:45769 | 31122021\_RID\_3262\_2a.raw | 1.5126E6 |  |  |  |  |  |  | 3.5567E5 | 2.0547E6 |  | 3 | 1 | 0 | 0 | 0 | 0 | 0 | 0 | 1 | 1 | 0 | 221 | 236 |  |  | PEAKS DB |
| R.NNQLLWLWR.D | Y | 54.91 | 1241.6669 | 9 | 0.2 | 621.8408 | 2 | 56.79 | 9 | F9:34769 | 31122021\_RID\_3262\_2a.raw |  |  |  |  |  |  |  |  | 1.1194E6 | 9.8456E6 | 2 | 0 | 0 | 0 | 0 | 0 | 0 | 0 | 0 | 1 | 1 | 212 | 220 |  |  | PEAKS DB |
| K.LELENLIFLR.N | Y | 53.64 | 1258.7285 | 10 | 0.8 | 630.3720 | 2 | 63.39 | 9 | F9:39486 | 31122021\_RID\_3262\_2a.raw |  |  |  |  |  |  |  |  | 3.4694E5 |  | 1 | 0 | 0 | 0 | 0 | 0 | 0 | 0 | 0 | 1 | 0 | 390 | 399 |  |  | PEAKS DB |
| K.GKLELENLIFLR.N | Y | 52.87 | 1443.8448 | 12 | 1.0 | 722.9304 | 2 | 53.82 | 9 | F9:32480 | 31122021\_RID\_3262\_2a.raw |  |  |  |  |  |  |  |  | 5.7774E5 |  | 1 | 0 | 0 | 0 | 0 | 0 | 0 | 0 | 0 | 1 | 0 | 388 | 399 |  |  | PEAKS DB |
| K.VKDYMDGPLGR.Y | Y | 52.30 | 1249.6124 | 11 | 0.3 | 417.5449 | 3 | 29.31 | 9 | F9:12742 | 31122021\_RID\_3262\_2a.raw |  |  |  |  |  |  |  |  | 7.4922E5 |  | 2 | 0 | 0 | 0 | 0 | 0 | 0 | 0 | 0 | 2 | 0 | 320 | 330 |  |  | PEAKS DB |
| K.DYMDGPLGR.Y | Y | 49.84 | 1022.4491 | 9 | 0.7 | 512.2322 | 2 | 38.45 | 9 | F9:20185 | 31122021\_RID\_3262\_2a.raw |  |  |  |  |  |  |  |  | 2.3887E5 |  | 1 | 0 | 0 | 0 | 0 | 0 | 0 | 0 | 0 | 1 | 0 | 322 | 330 |  |  | PEAKS DB |
| R.KDYALPVFVYAR.P | Y | 45.02 | 1440.7765 | 12 | 0.2 | 481.2662 | 3 | 45.92 | 9 | F9:26174 | 31122021\_RID\_3262\_2a.raw |  |  |  |  |  |  |  |  | 9.1169E5 |  | 1 | 0 | 0 | 0 | 0 | 0 | 0 | 0 | 0 | 1 | 0 | 261 | 272 |  |  | PEAKS DB |
| R.YKVDLDLK.T | N | 44.81 | 992.5542 | 8 | -0.6 | 497.2841 | 2 | 33.65 | 1 | F1:19752 | 13012022\_RID\_3262\_4a.raw | 1.0964E6 |  |  |  |  |  |  |  |  |  | 1 | 1 | 0 | 0 | 0 | 0 | 0 | 0 | 0 | 0 | 0 | 28 | 35 |  |  | PEAKS DB |
| K.VKDYM(+15.99)DGPLGR.Y | Y | 43.62 | 1265.6074 | 11 | 0.8 | 422.8767 | 3 | 29.33 | 9 | F9:12786 | 31122021\_RID\_3262\_2a.raw |  |  |  |  |  |  |  |  | 6.6806E4 |  | 1 | 0 | 0 | 0 | 0 | 0 | 0 | 0 | 0 | 1 | 0 | 320 | 330 | Oxidation (M) | M5:Oxidation (M):1000.00 | PEAKS DB |
| total 9 peptides |  |  |  |  |  |  |  |  |  |  |  |  |  |  |  |  |  |  |  |  |  |  |  |  |  |  |  |  |  |  |  |  |  |  |  |  |  |
| --- | --- | --- | --- | --- | --- | --- | --- | --- | --- | --- | --- | --- | --- | --- | --- | --- | --- | --- | --- | --- | --- | --- | --- | --- | --- | --- | --- | --- | --- | --- | --- | --- | --- | --- | --- | --- | --- |

BAN89413.1

back to list

  

| Protein Coverage
| Supporting Peptides
|

Protein Coverage:

Supporting Peptides:

| Peptide | Uniq | -10lgP | Mass | Length | ppm | m/z | z | RT | Fraction | Scan | Source File | Area F4 | Area F5 | Area F6 | Area F10 | Area F8 | Area F9 | Area F7 | Area F1 | Area F2 | Area F3 | #Feature | #Feature F4 | #Feature F5 | #Feature F6 | #Feature F10 | #Feature F8 | #Feature F9 | #Feature F7 | #Feature F1 | #Feature F2 | #Feature F3 | Start | End | PTM | AScore | Found By |
| --- | --- | --- | --- | --- | --- | --- | --- | --- | --- | --- | --- | --- | --- | --- | --- | --- | --- | --- | --- | --- | --- | --- | --- | --- | --- | --- | --- | --- | --- | --- | --- | --- | --- | --- | --- | --- | --- |
| R.DSTALFPNIYLETILR.S | N | 76.87 | 1864.9934 | 16 | 0.4 | 933.5043 | 2 | 75.08 | 9 | F9:45769 | 31122021\_RID\_3262\_2a.raw | 1.5126E6 |  |  |  |  |  |  | 3.5567E5 | 2.0547E6 |  | 3 | 1 | 0 | 0 | 0 | 0 | 0 | 0 | 1 | 1 | 0 | 244 | 259 |  |  | PEAKS DB |
| R.NNQLLWLWR.D | Y | 54.91 | 1241.6669 | 9 | 0.2 | 621.8408 | 2 | 56.79 | 9 | F9:34769 | 31122021\_RID\_3262\_2a.raw |  |  |  |  |  |  |  |  | 1.1194E6 | 9.8456E6 | 2 | 0 | 0 | 0 | 0 | 0 | 0 | 0 | 0 | 1 | 1 | 235 | 243 |  |  | PEAKS DB |
| K.LELENLIFLR.N | Y | 53.64 | 1258.7285 | 10 | 0.8 | 630.3720 | 2 | 63.39 | 9 | F9:39486 | 31122021\_RID\_3262\_2a.raw |  |  |  |  |  |  |  |  | 3.4694E5 |  | 1 | 0 | 0 | 0 | 0 | 0 | 0 | 0 | 0 | 1 | 0 | 413 | 422 |  |  | PEAKS DB |
| K.GKLELENLIFLR.N | Y | 52.87 | 1443.8448 | 12 | 1.0 | 722.9304 | 2 | 53.82 | 9 | F9:32480 | 31122021\_RID\_3262\_2a.raw |  |  |  |  |  |  |  |  | 5.7774E5 |  | 1 | 0 | 0 | 0 | 0 | 0 | 0 | 0 | 0 | 1 | 0 | 411 | 422 |  |  | PEAKS DB |
| K.VKDYMDGPLGR.Y | Y | 52.30 | 1249.6124 | 11 | 0.3 | 417.5449 | 3 | 29.31 | 9 | F9:12742 | 31122021\_RID\_3262\_2a.raw |  |  |  |  |  |  |  |  | 7.4922E5 |  | 2 | 0 | 0 | 0 | 0 | 0 | 0 | 0 | 0 | 2 | 0 | 343 | 353 |  |  | PEAKS DB |
| K.DYMDGPLGR.Y | Y | 49.84 | 1022.4491 | 9 | 0.7 | 512.2322 | 2 | 38.45 | 9 | F9:20185 | 31122021\_RID\_3262\_2a.raw |  |  |  |  |  |  |  |  | 2.3887E5 |  | 1 | 0 | 0 | 0 | 0 | 0 | 0 | 0 | 0 | 1 | 0 | 345 | 353 |  |  | PEAKS DB |
| R.KDYALPVFVYAR.P | Y | 45.02 | 1440.7765 | 12 | 0.2 | 481.2662 | 3 | 45.92 | 9 | F9:26174 | 31122021\_RID\_3262\_2a.raw |  |  |  |  |  |  |  |  | 9.1169E5 |  | 1 | 0 | 0 | 0 | 0 | 0 | 0 | 0 | 0 | 1 | 0 | 284 | 295 |  |  | PEAKS DB |
| R.YKVDLDLK.T | N | 44.81 | 992.5542 | 8 | -0.6 | 497.2841 | 2 | 33.65 | 1 | F1:19752 | 13012022\_RID\_3262\_4a.raw | 1.0964E6 |  |  |  |  |  |  |  |  |  | 1 | 1 | 0 | 0 | 0 | 0 | 0 | 0 | 0 | 0 | 0 | 51 | 58 |  |  | PEAKS DB |
| K.VKDYM(+15.99)DGPLGR.Y | Y | 43.62 | 1265.6074 | 11 | 0.8 | 422.8767 | 3 | 29.33 | 9 | F9:12786 | 31122021\_RID\_3262\_2a.raw |  |  |  |  |  |  |  |  | 6.6806E4 |  | 1 | 0 | 0 | 0 | 0 | 0 | 0 | 0 | 0 | 1 | 0 | 343 | 353 | Oxidation (M) | M5:Oxidation (M):1000.00 | PEAKS DB |
| total 9 peptides |  |  |  |  |  |  |  |  |  |  |  |  |  |  |  |  |  |  |  |  |  |  |  |  |  |  |  |  |  |  |  |  |  |  |  |  |  |
| --- | --- | --- | --- | --- | --- | --- | --- | --- | --- | --- | --- | --- | --- | --- | --- | --- | --- | --- | --- | --- | --- | --- | --- | --- | --- | --- | --- | --- | --- | --- | --- | --- | --- | --- | --- | --- | --- |

ASX97880.1

back to list

  

| Protein Coverage
| Supporting Peptides
|

Protein Coverage:

Supporting Peptides:

| Peptide | Uniq | -10lgP | Mass | Length | ppm | m/z | z | RT | Fraction | Scan | Source File | Area F4 | Area F5 | Area F6 | Area F10 | Area F8 | Area F9 | Area F7 | Area F1 | Area F2 | Area F3 | #Feature | #Feature F4 | #Feature F5 | #Feature F6 | #Feature F10 | #Feature F8 | #Feature F9 | #Feature F7 | #Feature F1 | #Feature F2 | #Feature F3 | Start | End | PTM | AScore | Found By |
| --- | --- | --- | --- | --- | --- | --- | --- | --- | --- | --- | --- | --- | --- | --- | --- | --- | --- | --- | --- | --- | --- | --- | --- | --- | --- | --- | --- | --- | --- | --- | --- | --- | --- | --- | --- | --- | --- |
| R.IM(+15.99)GWGTISSTK.E | N | 66.15 | 1195.5907 | 11 | -0.8 | 598.8021 | 2 | 35.74 | 7 | F7:16284 | 21012022\_RID\_3262\_7a.raw |  | 6.7622E5 | 1.701E6 |  |  | 4.1198E6 | 1.3032E7 |  |  |  | 6 | 0 | 1 | 1 | 0 | 0 | 1 | 3 | 0 | 0 | 0 | 144 | 154 | Oxidation (M) | M2:Oxidation (M):1000.00 | PEAKS DB |
| K.ETYPNVPR.C | Y | 65.08 | 974.4821 | 8 | 0.5 | 488.2486 | 2 | 28.46 | 7 | F7:11040 | 21012022\_RID\_3262\_7a.raw |  | 3.5572E6 | 4.5094E6 | 3.0855E6 |  | 2.2081E7 | 7.2467E7 |  |  |  | 5 | 0 | 1 | 1 | 1 | 0 | 1 | 1 | 0 | 0 | 0 | 155 | 162 |  |  | PEAKS DB |
| R.IMGWGTISSTK.E | N | 60.15 | 1179.5958 | 11 | 0.4 | 590.8054 | 2 | 42.00 | 3 | F3:19875 | 13012022\_RID\_3262\_6a.raw |  | 0 | 3.8883E6 |  |  | 1.7017E7 | 1.947E7 | 0 |  |  | 3 | 0 | 0 | 1 | 0 | 0 | 1 | 1 | 0 | 0 | 0 | 144 | 154 |  |  | PEAKS DB |
| A.HIAPLSLPSSPPSVGSLC(+57.02)R.I | N | 57.25 | 1974.0356 | 19 | -0.2 | 659.0190 | 3 | 45.79 | 3 | F3:22025 | 13012022\_RID\_3262\_6a.raw | 1.1698E7 | 2.4569E7 | 3.1622E7 |  |  |  |  |  |  |  | 5 | 2 | 1 | 2 | 0 | 0 | 0 | 0 | 0 | 0 | 0 | 125 | 143 | Carbamidomethylation | C18:Carbamidomethylation:1000.00 | PEAKS DB |
| R.AAYPWWPATTR.T | Y | 55.47 | 1318.6459 | 11 | 0.6 | 660.3306 | 2 | 50.49 | 7 | F7:24795 | 21012022\_RID\_3262\_7a.raw | 3.4114E6 |  |  |  |  | 2.0166E7 | 6.1853E6 | 1.16E6 | 3.9737E5 |  | 6 | 1 | 0 | 0 | 0 | 0 | 2 | 1 | 1 | 1 | 0 | 176 | 186 |  |  | PEAKS DB |
| L.SLPSSPPSVGSLC(+57.02)R.I | N | 55.06 | 1442.7188 | 14 | 0.4 | 722.3669 | 2 | 34.98 | 3 | F3:15674 | 13012022\_RID\_3262\_6a.raw |  | 1.1398E7 | 2.4327E6 |  |  |  | 2.4086E6 |  |  |  | 3 | 0 | 1 | 1 | 0 | 0 | 0 | 1 | 0 | 0 | 0 | 130 | 143 | Carbamidomethylation | C13:Carbamidomethylation:1000.00 | PEAKS DB |
| L.KPGFYTK.V | N | 48.32 | 839.4541 | 7 | -0.4 | 420.7342 | 2 | 17.97 | 7 | F7:4049 | 21012022\_RID\_3262\_7a.raw |  |  |  |  |  |  | 5.6489E5 |  |  |  | 1 | 0 | 0 | 0 | 0 | 0 | 0 | 1 | 0 | 0 | 0 | 230 | 236 |  |  | PEAKS DB |
| R.LKPGFYTK.V | Y | 43.23 | 952.5381 | 8 | 0.9 | 477.2768 | 2 | 23.89 | 7 | F7:8082 | 21012022\_RID\_3262\_7a.raw |  |  |  |  |  |  | 7.403E7 |  |  |  | 1 | 0 | 0 | 0 | 0 | 0 | 0 | 1 | 0 | 0 | 0 | 229 | 236 |  |  | PEAKS DB |
| total 8 peptides |  |  |  |  |  |  |  |  |  |  |  |  |  |  |  |  |  |  |  |  |  |  |  |  |  |  |  |  |  |  |  |  |  |  |  |  |  |
| --- | --- | --- | --- | --- | --- | --- | --- | --- | --- | --- | --- | --- | --- | --- | --- | --- | --- | --- | --- | --- | --- | --- | --- | --- | --- | --- | --- | --- | --- | --- | --- | --- | --- | --- | --- | --- | --- |

XP\_007435739.1

back to list

  

| Protein Coverage
| Supporting Peptides
|

Protein Coverage:

Supporting Peptides:

| Peptide | Uniq | -10lgP | Mass | Length | ppm | m/z | z | RT | Fraction | Scan | Source File | Area F4 | Area F5 | Area F6 | Area F10 | Area F8 | Area F9 | Area F7 | Area F1 | Area F2 | Area F3 | #Feature | #Feature F4 | #Feature F5 | #Feature F6 | #Feature F10 | #Feature F8 | #Feature F9 | #Feature F7 | #Feature F1 | #Feature F2 | #Feature F3 | Start | End | PTM | AScore | Found By |
| --- | --- | --- | --- | --- | --- | --- | --- | --- | --- | --- | --- | --- | --- | --- | --- | --- | --- | --- | --- | --- | --- | --- | --- | --- | --- | --- | --- | --- | --- | --- | --- | --- | --- | --- | --- | --- | --- |
| K.LALDIEIATYR.K | N | 65.96 | 1276.7026 | 11 | 1.4 | 639.3595 | 2 | 52.91 | 8 | F8:30667 | 31122021\_RID\_3262\_1a.raw | 2.7385E7 | 1.0818E6 |  | 1.3337E7 |  |  |  | 4.2941E7 | 2.4275E7 | 6.0353E7 | 24 | 1 | 1 | 0 | 2 | 0 | 0 | 0 | 13 | 6 | 1 | 380 | 390 |  |  | PEAKS DB |
| R.AQYEEIANR.S | Y | 63.32 | 1092.5199 | 9 | 0.6 | 547.2676 | 2 | 23.28 | 9 | F9:7964 | 31122021\_RID\_3262\_2a.raw |  | 5.7112E6 | 8.6592E5 | 5.2178E7 |  | 4.4375E7 | 2.6971E6 | 1.0894E7 | 5.7894E6 |  | 7 | 0 | 1 | 1 | 1 | 0 | 1 | 1 | 1 | 1 | 0 | 263 | 271 |  |  | PEAKS DB |
| R.EYQELMNVK.L | N | 62.87 | 1152.5485 | 9 | 0.7 | 577.2819 | 2 | 35.46 | 6 | F6:21360 | 17012022\_RID\_3262\_9a.raw |  |  |  | 3.3062E6 |  | 5.5812E6 |  | 1.6845E6 | 1.1435E6 |  | 4 | 0 | 0 | 0 | 1 | 0 | 1 | 0 | 1 | 1 | 0 | 371 | 379 |  |  | PEAKS DB |
| L.ALDIEIATYR.K | N | 57.72 | 1163.6187 | 10 | 0.7 | 582.8170 | 2 | 45.74 | 8 | F8:24835 | 31122021\_RID\_3262\_1a.raw |  |  |  |  |  |  |  | 2.3394E6 | 1.9465E5 |  | 2 | 0 | 0 | 0 | 0 | 0 | 0 | 0 | 1 | 1 | 0 | 381 | 390 |  |  | PEAKS DB |
| S.VILSMDNNR.S | N | 54.65 | 1060.5336 | 9 | 0.7 | 531.2744 | 2 | 30.82 | 8 | F8:13227 | 31122021\_RID\_3262\_1a.raw |  |  |  |  |  | 2.3978E6 |  | 7.4496E5 | 2.5983E5 |  | 3 | 0 | 0 | 0 | 0 | 0 | 1 | 0 | 1 | 1 | 0 | 242 | 250 |  |  | PEAKS DB |
| R.EYQELM(+15.99)NVK.L | N | 46.90 | 1168.5435 | 9 | -0.5 | 585.2787 | 2 | 27.89 | 4 | F4:14727 | 17012022\_RID\_3262\_10a .raw |  |  |  | 1.3738E6 |  |  |  | 6.2988E5 | 4.4468E5 |  | 4 | 0 | 0 | 0 | 1 | 0 | 0 | 0 | 2 | 1 | 0 | 371 | 379 | Oxidation (M) | M6:Oxidation (M):1000.00 | PEAKS DB |
| A.LDIEIATYR.K | N | 43.99 | 1092.5815 | 9 | 0.1 | 547.2981 | 2 | 45.00 | 8 | F8:24235 | 31122021\_RID\_3262\_1a.raw |  |  |  |  |  |  |  | 8.2061E5 |  |  | 1 | 0 | 0 | 0 | 0 | 0 | 0 | 0 | 1 | 0 | 0 | 382 | 390 |  |  | PEAKS DB |
| total 7 peptides |  |  |  |  |  |  |  |  |  |  |  |  |  |  |  |  |  |  |  |  |  |  |  |  |  |  |  |  |  |  |  |  |  |  |  |  |  |
| --- | --- | --- | --- | --- | --- | --- | --- | --- | --- | --- | --- | --- | --- | --- | --- | --- | --- | --- | --- | --- | --- | --- | --- | --- | --- | --- | --- | --- | --- | --- | --- | --- | --- | --- | --- | --- | --- |

AHJ09587.1

back to list

  

| Protein Coverage
| Supporting Peptides
|

Protein Coverage:

Supporting Peptides:

| Peptide | Uniq | -10lgP | Mass | Length | ppm | m/z | z | RT | Fraction | Scan | Source File | Area F4 | Area F5 | Area F6 | Area F10 | Area F8 | Area F9 | Area F7 | Area F1 | Area F2 | Area F3 | #Feature | #Feature F4 | #Feature F5 | #Feature F6 | #Feature F10 | #Feature F8 | #Feature F9 | #Feature F7 | #Feature F1 | #Feature F2 | #Feature F3 | Start | End | PTM | AScore | Found By |
| --- | --- | --- | --- | --- | --- | --- | --- | --- | --- | --- | --- | --- | --- | --- | --- | --- | --- | --- | --- | --- | --- | --- | --- | --- | --- | --- | --- | --- | --- | --- | --- | --- | --- | --- | --- | --- | --- |
| G.HLMQFETMIK.K | Y | 80.48 | 1276.6309 | 10 | 0.6 | 639.3231 | 2 | 45.09 | 6 | F6:31512 | 17012022\_RID\_3262\_9a.raw | 2.9209E7 |  |  | 6.4351E7 |  | 1.1303E9 |  | 1.0827E7 | 3.8511E6 |  | 7 | 1 | 0 | 0 | 1 | 0 | 2 | 0 | 2 | 1 | 0 | 17 | 26 |  |  | PEAKS DB |
| G.HLM(+15.99)QFETMIK.K | Y | 69.15 | 1292.6257 | 10 | 0.8 | 647.3207 | 2 | 39.27 | 6 | F6:24940 | 17012022\_RID\_3262\_9a.raw | 1.0138E8 | 1.1352E7 |  | 0 |  | 1.0351E10 | 1.6645E6 | 3.8365E7 | 2.524E7 |  | 12 | 1 | 1 | 0 | 0 | 0 | 4 | 1 | 3 | 2 | 0 | 17 | 26 | Oxidation (M) | M3:Oxidation (M):146.48 | PEAKS DB |
| G.HLMQFETMIKK.V | Y | 66.39 | 1404.7257 | 11 | -1.5 | 469.2485 | 3 | 43.00 | 6 | F6:29106 | 17012022\_RID\_3262\_9a.raw | 7.4025E6 |  |  |  |  | 4.1517E7 |  | 1.0052E6 | 3.1355E5 |  | 7 | 1 | 0 | 0 | 0 | 0 | 4 | 0 | 1 | 1 | 0 | 17 | 27 |  |  | PEAKS DB |
| G.HLM(+15.99)QFETM(+15.99)IK.K | Y | 62.82 | 1308.6206 | 10 | -0.6 | 655.3172 | 2 | 34.78 | 6 | F6:20893 | 17012022\_RID\_3262\_9a.raw | 2.4735E7 | 6.3752E6 |  | 1.9183E8 |  | 4.3869E9 | 1.8998E6 | 5.0145E6 | 8.2031E6 |  | 13 | 1 | 1 | 0 | 2 | 0 | 4 | 1 | 2 | 2 | 0 | 17 | 26 | Oxidation (M) | M3:Oxidation (M):1000.00;M8:Oxidation (M):1000.00 | PEAKS DB |
| G.HLMQFETM(+15.99)IK.K | Y | 62.10 | 1292.6257 | 10 | 0.1 | 647.3202 | 2 | 34.21 | 6 | F6:20479 | 17012022\_RID\_3262\_9a.raw |  |  |  |  |  | 3.3806E8 |  |  | 1.3103E6 |  | 2 | 0 | 0 | 0 | 0 | 0 | 1 | 0 | 0 | 1 | 0 | 17 | 26 | Oxidation (M) | M8:Oxidation (M):126.14 | PEAKS DB |
| G.HLM(+15.99)QFETMIKK.V | Y | 59.55 | 1420.7207 | 11 | 0.1 | 711.3677 | 2 | 28.91 | 6 | F6:15753 | 17012022\_RID\_3262\_9a.raw | 8.561E7 |  |  |  |  | 1.5412E9 |  |  | 1.8583E6 |  | 13 | 2 | 0 | 0 | 0 | 0 | 10 | 0 | 0 | 1 | 0 | 17 | 27 | Oxidation (M) | M3:Oxidation (M):139.79 | PEAKS DB |
| K.GGQGRPQDASDR.C | N | 55.16 | 1242.5701 | 12 | 0.2 | 415.1974 | 3 | 11.60 | 6 | F6:2151 | 17012022\_RID\_3262\_9a.raw |  |  |  |  |  | 9.079E4 |  |  |  |  | 1 | 0 | 0 | 0 | 0 | 0 | 1 | 0 | 0 | 0 | 0 | 47 | 58 |  |  | PEAKS DB |
| G.HLM(+15.99)QFETM(+15.99)IKK.V | Y | 51.89 | 1436.7156 | 11 | -0.4 | 479.9123 | 3 | 26.63 | 6 | F6:13867 | 17012022\_RID\_3262\_9a.raw |  |  |  | 6.9718E7 |  | 1.2834E9 |  |  |  |  | 4 | 0 | 0 | 0 | 1 | 0 | 3 | 0 | 0 | 0 | 0 | 17 | 27 | Oxidation (M) | M3:Oxidation (M):1000.00;M8:Oxidation (M):1000.00 | PEAKS DB |
| G.HLMQFETM(+15.99)IKK.V | Y | 51.69 | 1420.7207 | 11 | -0.1 | 711.3676 | 2 | 28.38 | 6 | F6:15371 | 17012022\_RID\_3262\_9a.raw |  |  |  |  |  | 1.5226E8 |  |  |  |  | 2 | 0 | 0 | 0 | 0 | 0 | 2 | 0 | 0 | 0 | 0 | 17 | 27 | Oxidation (M) | M8:Oxidation (M):100.21 | PEAKS DB |
| H.LMQFETMIK.K | Y | 49.39 | 1139.5719 | 9 | 0.4 | 570.7935 | 2 | 46.23 | 6 | F6:31332 | 17012022\_RID\_3262\_9a.raw |  |  |  |  |  | 8.4516E6 |  |  |  |  | 1 | 0 | 0 | 0 | 0 | 0 | 1 | 0 | 0 | 0 | 0 | 18 | 26 |  |  | PEAKS DB |
| H.LM(+15.99)QFETMIK.K | Y | 41.84 | 1155.5668 | 9 | -0.4 | 578.7904 | 2 | 39.46 | 6 | F6:24956 | 17012022\_RID\_3262\_9a.raw |  |  |  |  |  | 5.4763E7 |  |  |  |  | 1 | 0 | 0 | 0 | 0 | 0 | 1 | 0 | 0 | 0 | 0 | 18 | 26 | Oxidation (M) | M2:Oxidation (M):90.99 | PEAKS DB |
| total 11 peptides |  |  |  |  |  |  |  |  |  |  |  |  |  |  |  |  |  |  |  |  |  |  |  |  |  |  |  |  |  |  |  |  |  |  |  |  |  |
| --- | --- | --- | --- | --- | --- | --- | --- | --- | --- | --- | --- | --- | --- | --- | --- | --- | --- | --- | --- | --- | --- | --- | --- | --- | --- | --- | --- | --- | --- | --- | --- | --- | --- | --- | --- | --- | --- |

AHJ09591.1

back to list

  

| Protein Coverage
| Supporting Peptides
|

Protein Coverage:

Supporting Peptides:

| Peptide | Uniq | -10lgP | Mass | Length | ppm | m/z | z | RT | Fraction | Scan | Source File | Area F4 | Area F5 | Area F6 | Area F10 | Area F8 | Area F9 | Area F7 | Area F1 | Area F2 | Area F3 | #Feature | #Feature F4 | #Feature F5 | #Feature F6 | #Feature F10 | #Feature F8 | #Feature F9 | #Feature F7 | #Feature F1 | #Feature F2 | #Feature F3 | Start | End | PTM | AScore | Found By |
| --- | --- | --- | --- | --- | --- | --- | --- | --- | --- | --- | --- | --- | --- | --- | --- | --- | --- | --- | --- | --- | --- | --- | --- | --- | --- | --- | --- | --- | --- | --- | --- | --- | --- | --- | --- | --- | --- |
| G.HLMQFETMIK.K | Y | 80.48 | 1276.6309 | 10 | 0.6 | 639.3231 | 2 | 45.09 | 6 | F6:31512 | 17012022\_RID\_3262\_9a.raw | 2.9209E7 |  |  | 6.4351E7 |  | 1.1303E9 |  | 1.0827E7 | 3.8511E6 |  | 7 | 1 | 0 | 0 | 1 | 0 | 2 | 0 | 2 | 1 | 0 | 17 | 26 |  |  | PEAKS DB |
| G.HLM(+15.99)QFETMIK.K | Y | 69.15 | 1292.6257 | 10 | 0.8 | 647.3207 | 2 | 39.27 | 6 | F6:24940 | 17012022\_RID\_3262\_9a.raw | 1.0138E8 | 1.1352E7 |  | 0 |  | 1.0351E10 | 1.6645E6 | 3.8365E7 | 2.524E7 |  | 12 | 1 | 1 | 0 | 0 | 0 | 4 | 1 | 3 | 2 | 0 | 17 | 26 | Oxidation (M) | M3:Oxidation (M):146.48 | PEAKS DB |
| G.HLMQFETMIKK.V | Y | 66.39 | 1404.7257 | 11 | -1.5 | 469.2485 | 3 | 43.00 | 6 | F6:29106 | 17012022\_RID\_3262\_9a.raw | 7.4025E6 |  |  |  |  | 4.1517E7 |  | 1.0052E6 | 3.1355E5 |  | 7 | 1 | 0 | 0 | 0 | 0 | 4 | 0 | 1 | 1 | 0 | 17 | 27 |  |  | PEAKS DB |
| G.HLM(+15.99)QFETM(+15.99)IK.K | Y | 62.82 | 1308.6206 | 10 | -0.6 | 655.3172 | 2 | 34.78 | 6 | F6:20893 | 17012022\_RID\_3262\_9a.raw | 2.4735E7 | 6.3752E6 |  | 1.9183E8 |  | 4.3869E9 | 1.8998E6 | 5.0145E6 | 8.2031E6 |  | 13 | 1 | 1 | 0 | 2 | 0 | 4 | 1 | 2 | 2 | 0 | 17 | 26 | Oxidation (M) | M3:Oxidation (M):1000.00;M8:Oxidation (M):1000.00 | PEAKS DB |
| G.HLMQFETM(+15.99)IK.K | Y | 62.10 | 1292.6257 | 10 | 0.1 | 647.3202 | 2 | 34.21 | 6 | F6:20479 | 17012022\_RID\_3262\_9a.raw |  |  |  |  |  | 3.3806E8 |  |  | 1.3103E6 |  | 2 | 0 | 0 | 0 | 0 | 0 | 1 | 0 | 0 | 1 | 0 | 17 | 26 | Oxidation (M) | M8:Oxidation (M):126.14 | PEAKS DB |
| G.HLM(+15.99)QFETMIKK.V | Y | 59.55 | 1420.7207 | 11 | 0.1 | 711.3677 | 2 | 28.91 | 6 | F6:15753 | 17012022\_RID\_3262\_9a.raw | 8.561E7 |  |  |  |  | 1.5412E9 |  |  | 1.8583E6 |  | 13 | 2 | 0 | 0 | 0 | 0 | 10 | 0 | 0 | 1 | 0 | 17 | 27 | Oxidation (M) | M3:Oxidation (M):139.79 | PEAKS DB |
| K.GGQGRPQDASDR.C | N | 55.16 | 1242.5701 | 12 | 0.2 | 415.1974 | 3 | 11.60 | 6 | F6:2151 | 17012022\_RID\_3262\_9a.raw |  |  |  |  |  | 9.079E4 |  |  |  |  | 1 | 0 | 0 | 0 | 0 | 0 | 1 | 0 | 0 | 0 | 0 | 47 | 58 |  |  | PEAKS DB |
| G.HLM(+15.99)QFETM(+15.99)IKK.V | Y | 51.89 | 1436.7156 | 11 | -0.4 | 479.9123 | 3 | 26.63 | 6 | F6:13867 | 17012022\_RID\_3262\_9a.raw |  |  |  | 6.9718E7 |  | 1.2834E9 |  |  |  |  | 4 | 0 | 0 | 0 | 1 | 0 | 3 | 0 | 0 | 0 | 0 | 17 | 27 | Oxidation (M) | M3:Oxidation (M):1000.00;M8:Oxidation (M):1000.00 | PEAKS DB |
| G.HLMQFETM(+15.99)IKK.V | Y | 51.69 | 1420.7207 | 11 | -0.1 | 711.3676 | 2 | 28.38 | 6 | F6:15371 | 17012022\_RID\_3262\_9a.raw |  |  |  |  |  | 1.5226E8 |  |  |  |  | 2 | 0 | 0 | 0 | 0 | 0 | 2 | 0 | 0 | 0 | 0 | 17 | 27 | Oxidation (M) | M8:Oxidation (M):100.21 | PEAKS DB |
| H.LMQFETMIK.K | Y | 49.39 | 1139.5719 | 9 | 0.4 | 570.7935 | 2 | 46.23 | 6 | F6:31332 | 17012022\_RID\_3262\_9a.raw |  |  |  |  |  | 8.4516E6 |  |  |  |  | 1 | 0 | 0 | 0 | 0 | 0 | 1 | 0 | 0 | 0 | 0 | 18 | 26 |  |  | PEAKS DB |
| H.LM(+15.99)QFETMIK.K | Y | 41.84 | 1155.5668 | 9 | -0.4 | 578.7904 | 2 | 39.46 | 6 | F6:24956 | 17012022\_RID\_3262\_9a.raw |  |  |  |  |  | 5.4763E7 |  |  |  |  | 1 | 0 | 0 | 0 | 0 | 0 | 1 | 0 | 0 | 0 | 0 | 18 | 26 | Oxidation (M) | M2:Oxidation (M):90.99 | PEAKS DB |
| total 11 peptides |  |  |  |  |  |  |  |  |  |  |  |  |  |  |  |  |  |  |  |  |  |  |  |  |  |  |  |  |  |  |  |  |  |  |  |  |  |
| --- | --- | --- | --- | --- | --- | --- | --- | --- | --- | --- | --- | --- | --- | --- | --- | --- | --- | --- | --- | --- | --- | --- | --- | --- | --- | --- | --- | --- | --- | --- | --- | --- | --- | --- | --- | --- | --- |

AHJ09589.1

back to list

  

| Protein Coverage
| Supporting Peptides
|

Protein Coverage:

Supporting Peptides:

| Peptide | Uniq | -10lgP | Mass | Length | ppm | m/z | z | RT | Fraction | Scan | Source File | Area F4 | Area F5 | Area F6 | Area F10 | Area F8 | Area F9 | Area F7 | Area F1 | Area F2 | Area F3 | #Feature | #Feature F4 | #Feature F5 | #Feature F6 | #Feature F10 | #Feature F8 | #Feature F9 | #Feature F7 | #Feature F1 | #Feature F2 | #Feature F3 | Start | End | PTM | AScore | Found By |
| --- | --- | --- | --- | --- | --- | --- | --- | --- | --- | --- | --- | --- | --- | --- | --- | --- | --- | --- | --- | --- | --- | --- | --- | --- | --- | --- | --- | --- | --- | --- | --- | --- | --- | --- | --- | --- | --- |
| G.HLMQFETMIK.K | Y | 80.48 | 1276.6309 | 10 | 0.6 | 639.3231 | 2 | 45.09 | 6 | F6:31512 | 17012022\_RID\_3262\_9a.raw | 2.9209E7 |  |  | 6.4351E7 |  | 1.1303E9 |  | 1.0827E7 | 3.8511E6 |  | 7 | 1 | 0 | 0 | 1 | 0 | 2 | 0 | 2 | 1 | 0 | 17 | 26 |  |  | PEAKS DB |
| G.HLM(+15.99)QFETMIK.K | Y | 69.15 | 1292.6257 | 10 | 0.8 | 647.3207 | 2 | 39.27 | 6 | F6:24940 | 17012022\_RID\_3262\_9a.raw | 1.0138E8 | 1.1352E7 |  | 0 |  | 1.0351E10 | 1.6645E6 | 3.8365E7 | 2.524E7 |  | 12 | 1 | 1 | 0 | 0 | 0 | 4 | 1 | 3 | 2 | 0 | 17 | 26 | Oxidation (M) | M3:Oxidation (M):146.48 | PEAKS DB |
| G.HLMQFETMIKK.V | Y | 66.39 | 1404.7257 | 11 | -1.5 | 469.2485 | 3 | 43.00 | 6 | F6:29106 | 17012022\_RID\_3262\_9a.raw | 7.4025E6 |  |  |  |  | 4.1517E7 |  | 1.0052E6 | 3.1355E5 |  | 7 | 1 | 0 | 0 | 0 | 0 | 4 | 0 | 1 | 1 | 0 | 17 | 27 |  |  | PEAKS DB |
| G.HLM(+15.99)QFETM(+15.99)IK.K | Y | 62.82 | 1308.6206 | 10 | -0.6 | 655.3172 | 2 | 34.78 | 6 | F6:20893 | 17012022\_RID\_3262\_9a.raw | 2.4735E7 | 6.3752E6 |  | 1.9183E8 |  | 4.3869E9 | 1.8998E6 | 5.0145E6 | 8.2031E6 |  | 13 | 1 | 1 | 0 | 2 | 0 | 4 | 1 | 2 | 2 | 0 | 17 | 26 | Oxidation (M) | M3:Oxidation (M):1000.00;M8:Oxidation (M):1000.00 | PEAKS DB |
| G.HLMQFETM(+15.99)IK.K | Y | 62.10 | 1292.6257 | 10 | 0.1 | 647.3202 | 2 | 34.21 | 6 | F6:20479 | 17012022\_RID\_3262\_9a.raw |  |  |  |  |  | 3.3806E8 |  |  | 1.3103E6 |  | 2 | 0 | 0 | 0 | 0 | 0 | 1 | 0 | 0 | 1 | 0 | 17 | 26 | Oxidation (M) | M8:Oxidation (M):126.14 | PEAKS DB |
| G.HLM(+15.99)QFETMIKK.V | Y | 59.55 | 1420.7207 | 11 | 0.1 | 711.3677 | 2 | 28.91 | 6 | F6:15753 | 17012022\_RID\_3262\_9a.raw | 8.561E7 |  |  |  |  | 1.5412E9 |  |  | 1.8583E6 |  | 13 | 2 | 0 | 0 | 0 | 0 | 10 | 0 | 0 | 1 | 0 | 17 | 27 | Oxidation (M) | M3:Oxidation (M):139.79 | PEAKS DB |
| K.GGQGRPQDASDR.C | N | 55.16 | 1242.5701 | 12 | 0.2 | 415.1974 | 3 | 11.60 | 6 | F6:2151 | 17012022\_RID\_3262\_9a.raw |  |  |  |  |  | 9.079E4 |  |  |  |  | 1 | 0 | 0 | 0 | 0 | 0 | 1 | 0 | 0 | 0 | 0 | 47 | 58 |  |  | PEAKS DB |
| G.HLM(+15.99)QFETM(+15.99)IKK.V | Y | 51.89 | 1436.7156 | 11 | -0.4 | 479.9123 | 3 | 26.63 | 6 | F6:13867 | 17012022\_RID\_3262\_9a.raw |  |  |  | 6.9718E7 |  | 1.2834E9 |  |  |  |  | 4 | 0 | 0 | 0 | 1 | 0 | 3 | 0 | 0 | 0 | 0 | 17 | 27 | Oxidation (M) | M3:Oxidation (M):1000.00;M8:Oxidation (M):1000.00 | PEAKS DB |
| G.HLMQFETM(+15.99)IKK.V | Y | 51.69 | 1420.7207 | 11 | -0.1 | 711.3676 | 2 | 28.38 | 6 | F6:15371 | 17012022\_RID\_3262\_9a.raw |  |  |  |  |  | 1.5226E8 |  |  |  |  | 2 | 0 | 0 | 0 | 0 | 0 | 2 | 0 | 0 | 0 | 0 | 17 | 27 | Oxidation (M) | M8:Oxidation (M):100.21 | PEAKS DB |
| H.LMQFETMIK.K | Y | 49.39 | 1139.5719 | 9 | 0.4 | 570.7935 | 2 | 46.23 | 6 | F6:31332 | 17012022\_RID\_3262\_9a.raw |  |  |  |  |  | 8.4516E6 |  |  |  |  | 1 | 0 | 0 | 0 | 0 | 0 | 1 | 0 | 0 | 0 | 0 | 18 | 26 |  |  | PEAKS DB |
| H.LM(+15.99)QFETMIK.K | Y | 41.84 | 1155.5668 | 9 | -0.4 | 578.7904 | 2 | 39.46 | 6 | F6:24956 | 17012022\_RID\_3262\_9a.raw |  |  |  |  |  | 5.4763E7 |  |  |  |  | 1 | 0 | 0 | 0 | 0 | 0 | 1 | 0 | 0 | 0 | 0 | 18 | 26 | Oxidation (M) | M2:Oxidation (M):90.99 | PEAKS DB |
| total 11 peptides |  |  |  |  |  |  |  |  |  |  |  |  |  |  |  |  |  |  |  |  |  |  |  |  |  |  |  |  |  |  |  |  |  |  |  |  |  |
| --- | --- | --- | --- | --- | --- | --- | --- | --- | --- | --- | --- | --- | --- | --- | --- | --- | --- | --- | --- | --- | --- | --- | --- | --- | --- | --- | --- | --- | --- | --- | --- | --- | --- | --- | --- | --- | --- |

AHJ09585.1

back to list

  

| Protein Coverage
| Supporting Peptides
|

Protein Coverage:

Supporting Peptides:

| Peptide | Uniq | -10lgP | Mass | Length | ppm | m/z | z | RT | Fraction | Scan | Source File | Area F4 | Area F5 | Area F6 | Area F10 | Area F8 | Area F9 | Area F7 | Area F1 | Area F2 | Area F3 | #Feature | #Feature F4 | #Feature F5 | #Feature F6 | #Feature F10 | #Feature F8 | #Feature F9 | #Feature F7 | #Feature F1 | #Feature F2 | #Feature F3 | Start | End | PTM | AScore | Found By |
| --- | --- | --- | --- | --- | --- | --- | --- | --- | --- | --- | --- | --- | --- | --- | --- | --- | --- | --- | --- | --- | --- | --- | --- | --- | --- | --- | --- | --- | --- | --- | --- | --- | --- | --- | --- | --- | --- |
| G.HLMQFETMIK.K | Y | 80.48 | 1276.6309 | 10 | 0.6 | 639.3231 | 2 | 45.09 | 6 | F6:31512 | 17012022\_RID\_3262\_9a.raw | 2.9209E7 |  |  | 6.4351E7 |  | 1.1303E9 |  | 1.0827E7 | 3.8511E6 |  | 7 | 1 | 0 | 0 | 1 | 0 | 2 | 0 | 2 | 1 | 0 | 17 | 26 |  |  | PEAKS DB |
| G.HLM(+15.99)QFETMIK.K | Y | 69.15 | 1292.6257 | 10 | 0.8 | 647.3207 | 2 | 39.27 | 6 | F6:24940 | 17012022\_RID\_3262\_9a.raw | 1.0138E8 | 1.1352E7 |  | 0 |  | 1.0351E10 | 1.6645E6 | 3.8365E7 | 2.524E7 |  | 12 | 1 | 1 | 0 | 0 | 0 | 4 | 1 | 3 | 2 | 0 | 17 | 26 | Oxidation (M) | M3:Oxidation (M):146.48 | PEAKS DB |
| G.HLMQFETMIKK.V | Y | 66.39 | 1404.7257 | 11 | -1.5 | 469.2485 | 3 | 43.00 | 6 | F6:29106 | 17012022\_RID\_3262\_9a.raw | 7.4025E6 |  |  |  |  | 4.1517E7 |  | 1.0052E6 | 3.1355E5 |  | 7 | 1 | 0 | 0 | 0 | 0 | 4 | 0 | 1 | 1 | 0 | 17 | 27 |  |  | PEAKS DB |
| G.HLM(+15.99)QFETM(+15.99)IK.K | Y | 62.82 | 1308.6206 | 10 | -0.6 | 655.3172 | 2 | 34.78 | 6 | F6:20893 | 17012022\_RID\_3262\_9a.raw | 2.4735E7 | 6.3752E6 |  | 1.9183E8 |  | 4.3869E9 | 1.8998E6 | 5.0145E6 | 8.2031E6 |  | 13 | 1 | 1 | 0 | 2 | 0 | 4 | 1 | 2 | 2 | 0 | 17 | 26 | Oxidation (M) | M3:Oxidation (M):1000.00;M8:Oxidation (M):1000.00 | PEAKS DB |
| G.HLMQFETM(+15.99)IK.K | Y | 62.10 | 1292.6257 | 10 | 0.1 | 647.3202 | 2 | 34.21 | 6 | F6:20479 | 17012022\_RID\_3262\_9a.raw |  |  |  |  |  | 3.3806E8 |  |  | 1.3103E6 |  | 2 | 0 | 0 | 0 | 0 | 0 | 1 | 0 | 0 | 1 | 0 | 17 | 26 | Oxidation (M) | M8:Oxidation (M):126.14 | PEAKS DB |
| G.HLM(+15.99)QFETMIKK.V | Y | 59.55 | 1420.7207 | 11 | 0.1 | 711.3677 | 2 | 28.91 | 6 | F6:15753 | 17012022\_RID\_3262\_9a.raw | 8.561E7 |  |  |  |  | 1.5412E9 |  |  | 1.8583E6 |  | 13 | 2 | 0 | 0 | 0 | 0 | 10 | 0 | 0 | 1 | 0 | 17 | 27 | Oxidation (M) | M3:Oxidation (M):139.79 | PEAKS DB |
| K.GGQGRPQDASDR.C | N | 55.16 | 1242.5701 | 12 | 0.2 | 415.1974 | 3 | 11.60 | 6 | F6:2151 | 17012022\_RID\_3262\_9a.raw |  |  |  |  |  | 9.079E4 |  |  |  |  | 1 | 0 | 0 | 0 | 0 | 0 | 1 | 0 | 0 | 0 | 0 | 47 | 58 |  |  | PEAKS DB |
| G.HLM(+15.99)QFETM(+15.99)IKK.V | Y | 51.89 | 1436.7156 | 11 | -0.4 | 479.9123 | 3 | 26.63 | 6 | F6:13867 | 17012022\_RID\_3262\_9a.raw |  |  |  | 6.9718E7 |  | 1.2834E9 |  |  |  |  | 4 | 0 | 0 | 0 | 1 | 0 | 3 | 0 | 0 | 0 | 0 | 17 | 27 | Oxidation (M) | M3:Oxidation (M):1000.00;M8:Oxidation (M):1000.00 | PEAKS DB |
| G.HLMQFETM(+15.99)IKK.V | Y | 51.69 | 1420.7207 | 11 | -0.1 | 711.3676 | 2 | 28.38 | 6 | F6:15371 | 17012022\_RID\_3262\_9a.raw |  |  |  |  |  | 1.5226E8 |  |  |  |  | 2 | 0 | 0 | 0 | 0 | 0 | 2 | 0 | 0 | 0 | 0 | 17 | 27 | Oxidation (M) | M8:Oxidation (M):100.21 | PEAKS DB |
| H.LMQFETMIK.K | Y | 49.39 | 1139.5719 | 9 | 0.4 | 570.7935 | 2 | 46.23 | 6 | F6:31332 | 17012022\_RID\_3262\_9a.raw |  |  |  |  |  | 8.4516E6 |  |  |  |  | 1 | 0 | 0 | 0 | 0 | 0 | 1 | 0 | 0 | 0 | 0 | 18 | 26 |  |  | PEAKS DB |
| H.LM(+15.99)QFETMIK.K | Y | 41.84 | 1155.5668 | 9 | -0.4 | 578.7904 | 2 | 39.46 | 6 | F6:24956 | 17012022\_RID\_3262\_9a.raw |  |  |  |  |  | 5.4763E7 |  |  |  |  | 1 | 0 | 0 | 0 | 0 | 0 | 1 | 0 | 0 | 0 | 0 | 18 | 26 | Oxidation (M) | M2:Oxidation (M):90.99 | PEAKS DB |
| total 11 peptides |  |  |  |  |  |  |  |  |  |  |  |  |  |  |  |  |  |  |  |  |  |  |  |  |  |  |  |  |  |  |  |  |  |  |  |  |  |
| --- | --- | --- | --- | --- | --- | --- | --- | --- | --- | --- | --- | --- | --- | --- | --- | --- | --- | --- | --- | --- | --- | --- | --- | --- | --- | --- | --- | --- | --- | --- | --- | --- | --- | --- | --- | --- | --- |

AHJ09594.1

back to list

  

| Protein Coverage
| Supporting Peptides
|

Protein Coverage:

Supporting Peptides:

| Peptide | Uniq | -10lgP | Mass | Length | ppm | m/z | z | RT | Fraction | Scan | Source File | Area F4 | Area F5 | Area F6 | Area F10 | Area F8 | Area F9 | Area F7 | Area F1 | Area F2 | Area F3 | #Feature | #Feature F4 | #Feature F5 | #Feature F6 | #Feature F10 | #Feature F8 | #Feature F9 | #Feature F7 | #Feature F1 | #Feature F2 | #Feature F3 | Start | End | PTM | AScore | Found By |
| --- | --- | --- | --- | --- | --- | --- | --- | --- | --- | --- | --- | --- | --- | --- | --- | --- | --- | --- | --- | --- | --- | --- | --- | --- | --- | --- | --- | --- | --- | --- | --- | --- | --- | --- | --- | --- | --- |
| G.HLMQFETMIK.K | Y | 80.48 | 1276.6309 | 10 | 0.6 | 639.3231 | 2 | 45.09 | 6 | F6:31512 | 17012022\_RID\_3262\_9a.raw | 2.9209E7 |  |  | 6.4351E7 |  | 1.1303E9 |  | 1.0827E7 | 3.8511E6 |  | 7 | 1 | 0 | 0 | 1 | 0 | 2 | 0 | 2 | 1 | 0 | 17 | 26 |  |  | PEAKS DB |
| G.HLM(+15.99)QFETMIK.K | Y | 69.15 | 1292.6257 | 10 | 0.8 | 647.3207 | 2 | 39.27 | 6 | F6:24940 | 17012022\_RID\_3262\_9a.raw | 1.0138E8 | 1.1352E7 |  | 0 |  | 1.0351E10 | 1.6645E6 | 3.8365E7 | 2.524E7 |  | 12 | 1 | 1 | 0 | 0 | 0 | 4 | 1 | 3 | 2 | 0 | 17 | 26 | Oxidation (M) | M3:Oxidation (M):146.48 | PEAKS DB |
| G.HLMQFETMIKK.V | Y | 66.39 | 1404.7257 | 11 | -1.5 | 469.2485 | 3 | 43.00 | 6 | F6:29106 | 17012022\_RID\_3262\_9a.raw | 7.4025E6 |  |  |  |  | 4.1517E7 |  | 1.0052E6 | 3.1355E5 |  | 7 | 1 | 0 | 0 | 0 | 0 | 4 | 0 | 1 | 1 | 0 | 17 | 27 |  |  | PEAKS DB |
| G.HLM(+15.99)QFETM(+15.99)IK.K | Y | 62.82 | 1308.6206 | 10 | -0.6 | 655.3172 | 2 | 34.78 | 6 | F6:20893 | 17012022\_RID\_3262\_9a.raw | 2.4735E7 | 6.3752E6 |  | 1.9183E8 |  | 4.3869E9 | 1.8998E6 | 5.0145E6 | 8.2031E6 |  | 13 | 1 | 1 | 0 | 2 | 0 | 4 | 1 | 2 | 2 | 0 | 17 | 26 | Oxidation (M) | M3:Oxidation (M):1000.00;M8:Oxidation (M):1000.00 | PEAKS DB |
| G.HLMQFETM(+15.99)IK.K | Y | 62.10 | 1292.6257 | 10 | 0.1 | 647.3202 | 2 | 34.21 | 6 | F6:20479 | 17012022\_RID\_3262\_9a.raw |  |  |  |  |  | 3.3806E8 |  |  | 1.3103E6 |  | 2 | 0 | 0 | 0 | 0 | 0 | 1 | 0 | 0 | 1 | 0 | 17 | 26 | Oxidation (M) | M8:Oxidation (M):126.14 | PEAKS DB |
| G.HLM(+15.99)QFETMIKK.V | Y | 59.55 | 1420.7207 | 11 | 0.1 | 711.3677 | 2 | 28.91 | 6 | F6:15753 | 17012022\_RID\_3262\_9a.raw | 8.561E7 |  |  |  |  | 1.5412E9 |  |  | 1.8583E6 |  | 13 | 2 | 0 | 0 | 0 | 0 | 10 | 0 | 0 | 1 | 0 | 17 | 27 | Oxidation (M) | M3:Oxidation (M):139.79 | PEAKS DB |
| K.GGQGRPQDASDR.C | N | 55.16 | 1242.5701 | 12 | 0.2 | 415.1974 | 3 | 11.60 | 6 | F6:2151 | 17012022\_RID\_3262\_9a.raw |  |  |  |  |  | 9.079E4 |  |  |  |  | 1 | 0 | 0 | 0 | 0 | 0 | 1 | 0 | 0 | 0 | 0 | 47 | 58 |  |  | PEAKS DB |
| G.HLM(+15.99)QFETM(+15.99)IKK.V | Y | 51.89 | 1436.7156 | 11 | -0.4 | 479.9123 | 3 | 26.63 | 6 | F6:13867 | 17012022\_RID\_3262\_9a.raw |  |  |  | 6.9718E7 |  | 1.2834E9 |  |  |  |  | 4 | 0 | 0 | 0 | 1 | 0 | 3 | 0 | 0 | 0 | 0 | 17 | 27 | Oxidation (M) | M3:Oxidation (M):1000.00;M8:Oxidation (M):1000.00 | PEAKS DB |
| G.HLMQFETM(+15.99)IKK.V | Y | 51.69 | 1420.7207 | 11 | -0.1 | 711.3676 | 2 | 28.38 | 6 | F6:15371 | 17012022\_RID\_3262\_9a.raw |  |  |  |  |  | 1.5226E8 |  |  |  |  | 2 | 0 | 0 | 0 | 0 | 0 | 2 | 0 | 0 | 0 | 0 | 17 | 27 | Oxidation (M) | M8:Oxidation (M):100.21 | PEAKS DB |
| H.LMQFETMIK.K | Y | 49.39 | 1139.5719 | 9 | 0.4 | 570.7935 | 2 | 46.23 | 6 | F6:31332 | 17012022\_RID\_3262\_9a.raw |  |  |  |  |  | 8.4516E6 |  |  |  |  | 1 | 0 | 0 | 0 | 0 | 0 | 1 | 0 | 0 | 0 | 0 | 18 | 26 |  |  | PEAKS DB |
| H.LM(+15.99)QFETMIK.K | Y | 41.84 | 1155.5668 | 9 | -0.4 | 578.7904 | 2 | 39.46 | 6 | F6:24956 | 17012022\_RID\_3262\_9a.raw |  |  |  |  |  | 5.4763E7 |  |  |  |  | 1 | 0 | 0 | 0 | 0 | 0 | 1 | 0 | 0 | 0 | 0 | 18 | 26 | Oxidation (M) | M2:Oxidation (M):90.99 | PEAKS DB |
| total 11 peptides |  |  |  |  |  |  |  |  |  |  |  |  |  |  |  |  |  |  |  |  |  |  |  |  |  |  |  |  |  |  |  |  |  |  |  |  |  |
| --- | --- | --- | --- | --- | --- | --- | --- | --- | --- | --- | --- | --- | --- | --- | --- | --- | --- | --- | --- | --- | --- | --- | --- | --- | --- | --- | --- | --- | --- | --- | --- | --- | --- | --- | --- | --- | --- |

JAG67188.1

back to list

  

| Protein Coverage
| Supporting Peptides
|

Protein Coverage:

Supporting Peptides:

| Peptide | Uniq | -10lgP | Mass | Length | ppm | m/z | z | RT | Fraction | Scan | Source File | Area F4 | Area F5 | Area F6 | Area F10 | Area F8 | Area F9 | Area F7 | Area F1 | Area F2 | Area F3 | #Feature | #Feature F4 | #Feature F5 | #Feature F6 | #Feature F10 | #Feature F8 | #Feature F9 | #Feature F7 | #Feature F1 | #Feature F2 | #Feature F3 | Start | End | PTM | AScore | Found By |
| --- | --- | --- | --- | --- | --- | --- | --- | --- | --- | --- | --- | --- | --- | --- | --- | --- | --- | --- | --- | --- | --- | --- | --- | --- | --- | --- | --- | --- | --- | --- | --- | --- | --- | --- | --- | --- | --- |
| G.SFELTILHTNDVHAR.V | N | 71.46 | 1751.8955 | 15 | 0.2 | 584.9725 | 3 | 37.82 | 9 | F9:19802 | 31122021\_RID\_3262\_2a.raw |  |  |  |  |  |  |  |  | 9.8903E6 |  | 1 | 0 | 0 | 0 | 0 | 0 | 0 | 0 | 0 | 1 | 0 | 41 | 55 |  |  | PEAKS DB |
| R.QVPVVQAYAFGK.Y | N | 64.12 | 1305.7081 | 12 | 0.7 | 653.8618 | 2 | 57.55 | 1 | F1:40709 | 13012022\_RID\_3262\_4a.raw | 4.6454E6 | 1.1825E6 |  |  |  |  |  | 3.0239E5 | 6.0962E6 |  | 5 | 2 | 1 | 0 | 0 | 0 | 0 | 0 | 1 | 1 | 0 | 288 | 299 |  |  | PEAKS DB |
| K.TSGNPILLNK.D | Y | 62.94 | 1055.5975 | 10 | -0.1 | 528.8060 | 2 | 31.98 | 2 | F2:16058 | 13012022\_RID\_3262\_5a.raw | 7.609E6 | 1.8482E6 | 2.445E5 |  |  |  |  | 5.4762E5 | 1.8065E7 |  | 5 | 1 | 1 | 1 | 0 | 0 | 0 | 0 | 1 | 1 | 0 | 317 | 326 |  |  | PEAKS DB |
| K.VGIIGYTTK.E | N | 51.25 | 950.5436 | 9 | 0.4 | 476.2793 | 2 | 33.16 | 9 | F9:16043 | 31122021\_RID\_3262\_2a.raw |  | 2.6943E6 | 7.1467E5 |  |  |  | 1.5898E5 |  | 8.0892E6 |  | 4 | 0 | 1 | 1 | 0 | 0 | 0 | 1 | 0 | 1 | 0 | 185 | 193 |  |  | PEAKS DB |
| K.VVYDLSR.K | N | 46.31 | 850.4548 | 7 | 0.5 | 426.2349 | 2 | 30.36 | 1 | F1:16753 | 13012022\_RID\_3262\_4a.raw | 3.9579E6 | 3.0633E6 |  |  |  |  |  |  | 6.9401E6 |  | 3 | 1 | 1 | 0 | 0 | 0 | 0 | 0 | 0 | 1 | 0 | 472 | 478 |  |  | PEAKS DB |
| K.LTTLGVNK.I | N | 43.24 | 844.5018 | 8 | 0.8 | 423.2585 | 2 | 24.48 | 9 | F9:8941 | 31122021\_RID\_3262\_2a.raw |  |  |  |  |  |  |  |  | 1.1856E7 |  | 1 | 0 | 0 | 0 | 0 | 0 | 0 | 0 | 0 | 1 | 0 | 221 | 228 |  |  | PEAKS DB |
| total 6 peptides |  |  |  |  |  |  |  |  |  |  |  |  |  |  |  |  |  |  |  |  |  |  |  |  |  |  |  |  |  |  |  |  |  |  |  |  |  |
| --- | --- | --- | --- | --- | --- | --- | --- | --- | --- | --- | --- | --- | --- | --- | --- | --- | --- | --- | --- | --- | --- | --- | --- | --- | --- | --- | --- | --- | --- | --- | --- | --- | --- | --- | --- | --- | --- |

XP\_034265289.1

back to list

  

| Protein Coverage
| Supporting Peptides
|

Protein Coverage:

Supporting Peptides:

| Peptide | Uniq | -10lgP | Mass | Length | ppm | m/z | z | RT | Fraction | Scan | Source File | Area F4 | Area F5 | Area F6 | Area F10 | Area F8 | Area F9 | Area F7 | Area F1 | Area F2 | Area F3 | #Feature | #Feature F4 | #Feature F5 | #Feature F6 | #Feature F10 | #Feature F8 | #Feature F9 | #Feature F7 | #Feature F1 | #Feature F2 | #Feature F3 | Start | End | PTM | AScore | Found By |
| --- | --- | --- | --- | --- | --- | --- | --- | --- | --- | --- | --- | --- | --- | --- | --- | --- | --- | --- | --- | --- | --- | --- | --- | --- | --- | --- | --- | --- | --- | --- | --- | --- | --- | --- | --- | --- | --- |
| K.TPAQYDASELK.A | N | 78.63 | 1221.5876 | 11 | 0.6 | 611.8015 | 2 | 28.82 | 6 | F6:15708 | 17012022\_RID\_3262\_9a.raw |  |  |  | 2.2074E6 |  | 2.5005E6 | 1.4458E5 | 0 | 0 |  | 3 | 0 | 0 | 0 | 1 | 0 | 1 | 1 | 0 | 0 | 0 | 105 | 115 |  |  | PEAKS DB |
| K.GVDEVTIVNLLTNR.S | N | 64.57 | 1541.8413 | 14 | 0.5 | 771.9283 | 2 | 59.39 | 8 | F8:35757 | 31122021\_RID\_3262\_1a.raw | 3.1006E5 |  |  |  |  |  |  | 1.3513E6 | 4.6682E5 |  | 4 | 1 | 0 | 0 | 0 | 0 | 0 | 0 | 2 | 1 | 0 | 50 | 63 |  |  | PEAKS DB |
| K.DIISDTSGDFR.K | Y | 50.79 | 1224.5623 | 11 | 0.5 | 613.2887 | 2 | 43.82 | 4 | F4:28020 | 17012022\_RID\_3262\_10a .raw |  |  |  | 1.5803E5 |  |  |  |  |  |  | 1 | 0 | 0 | 0 | 1 | 0 | 0 | 0 | 0 | 0 | 0 | 158 | 168 |  |  | PEAKS DB |
| R.SEVDMLK.I | Y | 45.64 | 820.4000 | 7 | 0.2 | 411.2074 | 2 | 29.95 | 6 | F6:16782 | 17012022\_RID\_3262\_9a.raw |  |  |  |  |  | 5.945E5 |  |  |  |  | 1 | 0 | 0 | 0 | 0 | 0 | 1 | 0 | 0 | 0 | 0 | 296 | 302 |  |  | PEAKS DB |
| total 4 peptides |  |  |  |  |  |  |  |  |  |  |  |  |  |  |  |  |  |  |  |  |  |  |  |  |  |  |  |  |  |  |  |  |  |  |  |  |  |
| --- | --- | --- | --- | --- | --- | --- | --- | --- | --- | --- | --- | --- | --- | --- | --- | --- | --- | --- | --- | --- | --- | --- | --- | --- | --- | --- | --- | --- | --- | --- | --- | --- | --- | --- | --- | --- | --- |

XP\_026529665.1

back to list

  

| Protein Coverage
| Supporting Peptides
|

Protein Coverage:

Supporting Peptides:

| Peptide | Uniq | -10lgP | Mass | Length | ppm | m/z | z | RT | Fraction | Scan | Source File | Area F4 | Area F5 | Area F6 | Area F10 | Area F8 | Area F9 | Area F7 | Area F1 | Area F2 | Area F3 | #Feature | #Feature F4 | #Feature F5 | #Feature F6 | #Feature F10 | #Feature F8 | #Feature F9 | #Feature F7 | #Feature F1 | #Feature F2 | #Feature F3 | Start | End | PTM | AScore | Found By |
| --- | --- | --- | --- | --- | --- | --- | --- | --- | --- | --- | --- | --- | --- | --- | --- | --- | --- | --- | --- | --- | --- | --- | --- | --- | --- | --- | --- | --- | --- | --- | --- | --- | --- | --- | --- | --- | --- |
| K.LALDIEIATYR.K | N | 65.96 | 1276.7026 | 11 | 1.4 | 639.3595 | 2 | 52.91 | 8 | F8:30667 | 31122021\_RID\_3262\_1a.raw | 2.7385E7 | 1.0818E6 |  | 1.3337E7 |  |  |  | 4.2941E7 | 2.4275E7 | 6.0353E7 | 24 | 1 | 1 | 0 | 2 | 0 | 0 | 0 | 13 | 6 | 1 | 382 | 392 |  |  | PEAKS DB |
| R.EYQELMNVK.L | N | 62.87 | 1152.5485 | 9 | 0.7 | 577.2819 | 2 | 35.46 | 6 | F6:21360 | 17012022\_RID\_3262\_9a.raw |  |  |  | 3.3062E6 |  | 5.5812E6 |  | 1.6845E6 | 1.1435E6 |  | 4 | 0 | 0 | 0 | 1 | 0 | 1 | 0 | 1 | 1 | 0 | 373 | 381 |  |  | PEAKS DB |
| L.ALDIEIATYR.K | N | 57.72 | 1163.6187 | 10 | 0.7 | 582.8170 | 2 | 45.74 | 8 | F8:24835 | 31122021\_RID\_3262\_1a.raw |  |  |  |  |  |  |  | 2.3394E6 | 1.9465E5 |  | 2 | 0 | 0 | 0 | 0 | 0 | 0 | 0 | 1 | 1 | 0 | 383 | 392 |  |  | PEAKS DB |
| R.SLDLDGIIAEVK.A | Y | 56.02 | 1271.6973 | 12 | -1.0 | 636.8553 | 2 | 60.65 | 8 | F8:36661 | 31122021\_RID\_3262\_1a.raw |  |  |  |  |  |  |  | 3.1816E6 | 3.5252E6 |  | 4 | 0 | 0 | 0 | 0 | 0 | 0 | 0 | 3 | 1 | 0 | 253 | 264 |  |  | PEAKS DB |
| S.VILSMDNNR.S | N | 54.65 | 1060.5336 | 9 | 0.7 | 531.2744 | 2 | 30.82 | 8 | F8:13227 | 31122021\_RID\_3262\_1a.raw |  |  |  |  |  | 2.3978E6 |  | 7.4496E5 | 2.5983E5 |  | 3 | 0 | 0 | 0 | 0 | 0 | 1 | 0 | 1 | 1 | 0 | 244 | 252 |  |  | PEAKS DB |
| R.EYQELM(+15.99)NVK.L | N | 46.90 | 1168.5435 | 9 | -0.5 | 585.2787 | 2 | 27.89 | 4 | F4:14727 | 17012022\_RID\_3262\_10a .raw |  |  |  | 1.3738E6 |  |  |  | 6.2988E5 | 4.4468E5 |  | 4 | 0 | 0 | 0 | 1 | 0 | 0 | 0 | 2 | 1 | 0 | 373 | 381 | Oxidation (M) | M6:Oxidation (M):1000.00 | PEAKS DB |
| A.LDIEIATYR.K | N | 43.99 | 1092.5815 | 9 | 0.1 | 547.2981 | 2 | 45.00 | 8 | F8:24235 | 31122021\_RID\_3262\_1a.raw |  |  |  |  |  |  |  | 8.2061E5 |  |  | 1 | 0 | 0 | 0 | 0 | 0 | 0 | 0 | 1 | 0 | 0 | 384 | 392 |  |  | PEAKS DB |
| total 7 peptides |  |  |  |  |  |  |  |  |  |  |  |  |  |  |  |  |  |  |  |  |  |  |  |  |  |  |  |  |  |  |  |  |  |  |  |  |  |
| --- | --- | --- | --- | --- | --- | --- | --- | --- | --- | --- | --- | --- | --- | --- | --- | --- | --- | --- | --- | --- | --- | --- | --- | --- | --- | --- | --- | --- | --- | --- | --- | --- | --- | --- | --- | --- | --- |

XP\_029143063.1

back to list

  

| Protein Coverage
| Supporting Peptides
|

Protein Coverage:

Supporting Peptides:

| Peptide | Uniq | -10lgP | Mass | Length | ppm | m/z | z | RT | Fraction | Scan | Source File | Area F4 | Area F5 | Area F6 | Area F10 | Area F8 | Area F9 | Area F7 | Area F1 | Area F2 | Area F3 | #Feature | #Feature F4 | #Feature F5 | #Feature F6 | #Feature F10 | #Feature F8 | #Feature F9 | #Feature F7 | #Feature F1 | #Feature F2 | #Feature F3 | Start | End | PTM | AScore | Found By |
| --- | --- | --- | --- | --- | --- | --- | --- | --- | --- | --- | --- | --- | --- | --- | --- | --- | --- | --- | --- | --- | --- | --- | --- | --- | --- | --- | --- | --- | --- | --- | --- | --- | --- | --- | --- | --- | --- |
| K.EYNQLLSEMETIYSVAK.I | Y | 61.11 | 2016.9714 | 17 | 0.0 | 1009.4930 | 2 | 65.85 | 8 | F8:40499 | 31122021\_RID\_3262\_1a.raw |  |  |  |  |  |  |  | 2.9037E5 |  |  | 1 | 0 | 0 | 0 | 0 | 0 | 0 | 0 | 1 | 0 | 0 | 754 | 770 |  |  | PEAKS DB |
| R.DYDELLNTWK.G | Y | 56.97 | 1295.6034 | 10 | 0.9 | 648.8096 | 2 | 54.08 | 8 | F8:31492 | 31122021\_RID\_3262\_1a.raw |  |  |  |  |  |  |  | 8.2432E4 |  |  | 1 | 0 | 0 | 0 | 0 | 0 | 0 | 0 | 1 | 0 | 0 | 794 | 803 |  |  | PEAKS DB |
| K.AGSSQPWQDILQQLTGTNK.M | Y | 55.63 | 2071.0334 | 19 | 0.9 | 1036.5249 | 2 | 65.48 | 8 | F8:40203 | 31122021\_RID\_3262\_1a.raw |  |  |  |  |  |  |  | 8.9346E5 |  |  | 1 | 0 | 0 | 0 | 0 | 0 | 0 | 0 | 1 | 0 | 0 | 590 | 608 |  |  | PEAKS DB |
| K.ALDPDLTDTMAK.S | Y | 54.28 | 1289.6173 | 12 | 0.6 | 645.8163 | 2 | 39.38 | 8 | F8:19945 | 31122021\_RID\_3262\_1a.raw |  |  |  |  |  |  |  | 2.7304E5 |  |  | 1 | 0 | 0 | 0 | 0 | 0 | 0 | 0 | 1 | 0 | 0 | 780 | 791 |  |  | PEAKS DB |
| K.VDATPAMIQQGWTPK.K | Y | 53.94 | 1641.8185 | 15 | 0.2 | 821.9167 | 2 | 44.70 | 8 | F8:24017 | 31122021\_RID\_3262\_1a.raw |  |  |  |  |  |  |  | 1.3023E5 |  |  | 1 | 0 | 0 | 0 | 0 | 0 | 0 | 0 | 1 | 0 | 0 | 919 | 933 |  |  | PEAKS DB |
| Q.SLGLIPMPPEFWEK.S | Y | 48.38 | 1642.8429 | 14 | 1.0 | 822.4296 | 2 | 65.94 | 8 | F8:40682 | 31122021\_RID\_3262\_1a.raw |  |  |  |  |  |  |  | 4.7844E5 |  |  | 1 | 0 | 0 | 0 | 0 | 0 | 0 | 0 | 1 | 0 | 0 | 945 | 958 |  |  | PEAKS DB |
| K.SMLEKPSDGR.E | Y | 46.36 | 1118.5389 | 10 | -0.9 | 560.2762 | 2 | 18.02 | 8 | F8:3986 | 31122021\_RID\_3262\_1a.raw |  |  |  |  |  |  |  | 1.5281E4 |  |  | 1 | 0 | 0 | 0 | 0 | 0 | 0 | 0 | 1 | 0 | 0 | 959 | 968 |  |  | PEAKS DB |
| Q.SLGLIPM(+15.99)PPEFWEK.S | Y | 41.40 | 1658.8378 | 14 | 0.7 | 830.4268 | 2 | 60.16 | 8 | F8:36305 | 31122021\_RID\_3262\_1a.raw |  |  |  |  |  |  |  | 7.0056E4 |  |  | 1 | 0 | 0 | 0 | 0 | 0 | 0 | 0 | 1 | 0 | 0 | 945 | 958 | Oxidation (M) | M7:Oxidation (M):1000.00 | PEAKS DB |
| R.AALPEAELK.E | Y | 41.10 | 940.5229 | 9 | 0.4 | 471.2689 | 2 | 30.94 | 8 | F8:13102 | 31122021\_RID\_3262\_1a.raw |  |  |  |  |  |  |  | 2.0622E5 |  |  | 1 | 0 | 0 | 0 | 0 | 0 | 0 | 0 | 1 | 0 | 0 | 745 | 753 |  |  | PEAKS DB |
| total 9 peptides |  |  |  |  |  |  |  |  |  |  |  |  |  |  |  |  |  |  |  |  |  |  |  |  |  |  |  |  |  |  |  |  |  |  |  |  |  |
| --- | --- | --- | --- | --- | --- | --- | --- | --- | --- | --- | --- | --- | --- | --- | --- | --- | --- | --- | --- | --- | --- | --- | --- | --- | --- | --- | --- | --- | --- | --- | --- | --- | --- | --- | --- | --- | --- |

XP\_026537798.1

back to list

  

| Protein Coverage
| Supporting Peptides
|

Protein Coverage:

Supporting Peptides:

| Peptide | Uniq | -10lgP | Mass | Length | ppm | m/z | z | RT | Fraction | Scan | Source File | Area F4 | Area F5 | Area F6 | Area F10 | Area F8 | Area F9 | Area F7 | Area F1 | Area F2 | Area F3 | #Feature | #Feature F4 | #Feature F5 | #Feature F6 | #Feature F10 | #Feature F8 | #Feature F9 | #Feature F7 | #Feature F1 | #Feature F2 | #Feature F3 | Start | End | PTM | AScore | Found By |
| --- | --- | --- | --- | --- | --- | --- | --- | --- | --- | --- | --- | --- | --- | --- | --- | --- | --- | --- | --- | --- | --- | --- | --- | --- | --- | --- | --- | --- | --- | --- | --- | --- | --- | --- | --- | --- | --- |
| K.TPAQYDASELK.A | N | 78.63 | 1221.5876 | 11 | 0.6 | 611.8015 | 2 | 28.82 | 6 | F6:15708 | 17012022\_RID\_3262\_9a.raw |  |  |  | 2.2074E6 |  | 2.5005E6 | 1.4458E5 | 0 | 0 |  | 3 | 0 | 0 | 0 | 1 | 0 | 1 | 1 | 0 | 0 | 0 | 120 | 130 |  |  | PEAKS DB |
| K.GVDEVTIVNLLTNR.S | N | 64.57 | 1541.8413 | 14 | 0.5 | 771.9283 | 2 | 59.39 | 8 | F8:35757 | 31122021\_RID\_3262\_1a.raw | 3.1006E5 |  |  |  |  |  |  | 1.3513E6 | 4.6682E5 |  | 4 | 1 | 0 | 0 | 0 | 0 | 0 | 0 | 2 | 1 | 0 | 65 | 78 |  |  | PEAKS DB |
| K.DITSDTSGDFR.K | Y | 56.51 | 1212.5259 | 11 | 0.1 | 607.2703 | 2 | 33.53 | 8 | F8:15199 | 31122021\_RID\_3262\_1a.raw |  |  |  | 3.226E5 |  |  |  | 4.7166E4 | 2.2543E4 |  | 3 | 0 | 0 | 0 | 1 | 0 | 0 | 0 | 1 | 1 | 0 | 173 | 183 |  |  | PEAKS DB |
| total 3 peptides |  |  |  |  |  |  |  |  |  |  |  |  |  |  |  |  |  |  |  |  |  |  |  |  |  |  |  |  |  |  |  |  |  |  |  |  |  |
| --- | --- | --- | --- | --- | --- | --- | --- | --- | --- | --- | --- | --- | --- | --- | --- | --- | --- | --- | --- | --- | --- | --- | --- | --- | --- | --- | --- | --- | --- | --- | --- | --- | --- | --- | --- | --- | --- |

XP\_007424908.1

back to list

  

| Protein Coverage
| Supporting Peptides
|

Protein Coverage:

Supporting Peptides:

| Peptide | Uniq | -10lgP | Mass | Length | ppm | m/z | z | RT | Fraction | Scan | Source File | Area F4 | Area F5 | Area F6 | Area F10 | Area F8 | Area F9 | Area F7 | Area F1 | Area F2 | Area F3 | #Feature | #Feature F4 | #Feature F5 | #Feature F6 | #Feature F10 | #Feature F8 | #Feature F9 | #Feature F7 | #Feature F1 | #Feature F2 | #Feature F3 | Start | End | PTM | AScore | Found By |
| --- | --- | --- | --- | --- | --- | --- | --- | --- | --- | --- | --- | --- | --- | --- | --- | --- | --- | --- | --- | --- | --- | --- | --- | --- | --- | --- | --- | --- | --- | --- | --- | --- | --- | --- | --- | --- | --- |
| K.DGEAGAQGPPGPAGPAGER.G | Y | 79.22 | 1689.7706 | 19 | 0.2 | 845.8928 | 2 | 27.00 | 9 | F9:10809 | 31122021\_RID\_3262\_2a.raw |  |  |  |  |  |  |  |  | 6.2883E5 |  | 1 | 0 | 0 | 0 | 0 | 0 | 0 | 0 | 0 | 1 | 0 | 604 | 622 |  |  | PEAKS DB |
| R.GQAGVM(+15.99)GFPGPK.G | N | 61.60 | 1160.5648 | 12 | 1.2 | 581.2904 | 2 | 29.31 | 9 | F9:12753 | 31122021\_RID\_3262\_2a.raw |  |  |  |  |  |  |  |  | 4.9379E5 |  | 1 | 0 | 0 | 0 | 0 | 0 | 0 | 0 | 0 | 1 | 0 | 566 | 577 | Oxidation (M) | M6:Oxidation (M):1000.00 | PEAKS DB |
| K.GLTGSPGSPGPDGK.T | Y | 54.26 | 1225.5939 | 14 | 0.3 | 613.8044 | 2 | 22.24 | 9 | F9:7050 | 31122021\_RID\_3262\_2a.raw |  |  |  |  |  |  |  |  | 1.333E5 |  | 1 | 0 | 0 | 0 | 0 | 0 | 0 | 0 | 0 | 1 | 0 | 530 | 543 |  |  | PEAKS DB |
| R.GFSGLDGAK.G | N | 49.34 | 850.4185 | 9 | 0.7 | 426.2168 | 2 | 25.87 | 9 | F9:10014 | 31122021\_RID\_3262\_2a.raw |  |  |  |  |  |  |  |  | 2.44E6 |  | 1 | 0 | 0 | 0 | 0 | 0 | 0 | 0 | 0 | 1 | 0 | 260 | 268 |  |  | PEAKS DB |
| total 4 peptides |  |  |  |  |  |  |  |  |  |  |  |  |  |  |  |  |  |  |  |  |  |  |  |  |  |  |  |  |  |  |  |  |  |  |  |  |  |
| --- | --- | --- | --- | --- | --- | --- | --- | --- | --- | --- | --- | --- | --- | --- | --- | --- | --- | --- | --- | --- | --- | --- | --- | --- | --- | --- | --- | --- | --- | --- | --- | --- | --- | --- | --- | --- | --- |

AUS82539.1

back to list

  

| Protein Coverage
| Supporting Peptides
|

Protein Coverage:

Supporting Peptides:

| Peptide | Uniq | -10lgP | Mass | Length | ppm | m/z | z | RT | Fraction | Scan | Source File | Area F4 | Area F5 | Area F6 | Area F10 | Area F8 | Area F9 | Area F7 | Area F1 | Area F2 | Area F3 | #Feature | #Feature F4 | #Feature F5 | #Feature F6 | #Feature F10 | #Feature F8 | #Feature F9 | #Feature F7 | #Feature F1 | #Feature F2 | #Feature F3 | Start | End | PTM | AScore | Found By |
| --- | --- | --- | --- | --- | --- | --- | --- | --- | --- | --- | --- | --- | --- | --- | --- | --- | --- | --- | --- | --- | --- | --- | --- | --- | --- | --- | --- | --- | --- | --- | --- | --- | --- | --- | --- | --- | --- |
| R.IMGWGTISDTK.V | Y | 76.57 | 1207.5907 | 11 | 0.0 | 604.8026 | 2 | 43.11 | 8 | F8:22974 | 31122021\_RID\_3262\_1a.raw | 2.0348E9 | 3.8914E9 | 1.9345E7 | 9.4019E5 |  | 1.227E6 | 1.3061E7 | 1.2303E7 | 9.0652E6 | 2.5806E7 | 18 | 5 | 4 | 1 | 1 | 0 | 1 | 1 | 3 | 1 | 1 | 143 | 153 |  |  | PEAKS DB |
| R.IM(+15.99)GWGTISDTK.V | Y | 72.04 | 1223.5856 | 11 | -0.2 | 612.7999 | 2 | 38.28 | 7 | F7:18139 | 21012022\_RID\_3262\_7a.raw | 5.0821E8 | 1.2661E9 | 1.9724E7 |  |  |  | 1.4217E7 | 7.2759E6 | 2.8225E6 |  | 25 | 3 | 5 | 2 | 0 | 0 | 0 | 4 | 8 | 3 | 0 | 143 | 153 | Oxidation (M) | M2:Oxidation (M):1000.00 | PEAKS DB |
| F.WDKDIMLIR.L | N | 57.88 | 1188.6324 | 9 | 0.5 | 595.3238 | 2 | 43.59 | 1 | F1:28744 | 13012022\_RID\_3262\_4a.raw | 6.6853E8 | 1.3316E6 |  |  |  |  | 2.7704E4 | 1.1405E7 | 1.608E6 |  | 12 | 5 | 3 | 0 | 0 | 0 | 0 | 1 | 2 | 1 | 0 | 106 | 114 |  |  | PEAKS DB |
| R.EPALYTK.V | N | 56.50 | 820.4330 | 7 | 0.3 | 411.2239 | 2 | 24.64 | 1 | F1:11686 | 13012022\_RID\_3262\_4a.raw | 6.8741E6 |  |  |  |  | 1.0108E6 |  |  |  |  | 2 | 1 | 0 | 0 | 0 | 0 | 1 | 0 | 0 | 0 | 0 | 231 | 237 |  |  | PEAKS DB |
| F.WDKDIM(+15.99)LIR.L | N | 44.48 | 1204.6274 | 9 | 0.7 | 603.3214 | 2 | 37.71 | 2 | F2:20647 | 13012022\_RID\_3262\_5a.raw | 1.4316E8 | 4.5531E6 |  |  |  |  |  |  |  |  | 2 | 1 | 1 | 0 | 0 | 0 | 0 | 0 | 0 | 0 | 0 | 106 | 114 | Oxidation (M) | M6:Oxidation (M):1000.00 | PEAKS DB |
| W.DKDIMLIR.L | N | 41.99 | 1002.5532 | 8 | 0.7 | 502.2842 | 2 | 34.59 | 7 | F7:15514 | 21012022\_RID\_3262\_7a.raw |  |  |  |  |  |  | 2.6461E6 |  |  |  | 1 | 0 | 0 | 0 | 0 | 0 | 0 | 1 | 0 | 0 | 0 | 107 | 114 |  |  | PEAKS DB |
| total 6 peptides |  |  |  |  |  |  |  |  |  |  |  |  |  |  |  |  |  |  |  |  |  |  |  |  |  |  |  |  |  |  |  |  |  |  |  |  |  |
| --- | --- | --- | --- | --- | --- | --- | --- | --- | --- | --- | --- | --- | --- | --- | --- | --- | --- | --- | --- | --- | --- | --- | --- | --- | --- | --- | --- | --- | --- | --- | --- | --- | --- | --- | --- | --- | --- |

JAV01822.1

back to list

  

| Protein Coverage
| Supporting Peptides
|

Protein Coverage:

Supporting Peptides:

| Peptide | Uniq | -10lgP | Mass | Length | ppm | m/z | z | RT | Fraction | Scan | Source File | Area F4 | Area F5 | Area F6 | Area F10 | Area F8 | Area F9 | Area F7 | Area F1 | Area F2 | Area F3 | #Feature | #Feature F4 | #Feature F5 | #Feature F6 | #Feature F10 | #Feature F8 | #Feature F9 | #Feature F7 | #Feature F1 | #Feature F2 | #Feature F3 | Start | End | PTM | AScore | Found By |
| --- | --- | --- | --- | --- | --- | --- | --- | --- | --- | --- | --- | --- | --- | --- | --- | --- | --- | --- | --- | --- | --- | --- | --- | --- | --- | --- | --- | --- | --- | --- | --- | --- | --- | --- | --- | --- | --- |
| N.ILNEDEQTRDPK.E | N | 63.25 | 1456.7157 | 12 | 0.2 | 729.3652 | 2 | 20.40 | 2 | F2:7523 | 13012022\_RID\_3262\_5a.raw | 2.5611E7 | 5.5662E7 | 3.8171E6 | 7.1395E6 |  | 5.2046E6 | 7.5202E6 |  |  |  | 12 | 2 | 3 | 2 | 2 | 0 | 1 | 2 | 0 | 0 | 0 | 82 | 93 |  |  | PEAKS DB |
| N.ILNEDEQTR.D | N | 62.86 | 1116.5411 | 9 | -0.7 | 559.2775 | 2 | 22.53 | 4 | F4:9650 | 17012022\_RID\_3262\_10a .raw |  | 2.4557E7 | 3.3128E7 | 8.9808E6 |  | 5.8113E6 |  | 6.3735E5 | 9.79E5 | 0 | 7 | 0 | 1 | 2 | 1 | 0 | 1 | 0 | 1 | 1 | 0 | 82 | 90 |  |  | PEAKS DB |
| S.NSEHIAPLSLPSSPPSMGSLC(+57.02)R.I | Y | 54.17 | 2336.1252 | 22 | 0.2 | 779.7158 | 3 | 49.14 | 2 | F2:29112 | 13012022\_RID\_3262\_5a.raw | 1.3634E7 | 2.1926E7 |  |  |  |  |  |  |  |  | 2 | 1 | 1 | 0 | 0 | 0 | 0 | 0 | 0 | 0 | 0 | 122 | 143 | Carbamidomethylation | C21:Carbamidomethylation:1000.00 | PEAKS DB |
| L.SLPSSPPSM(+15.99)GSLC(+57.02)R.I | Y | 48.45 | 1490.6858 | 14 | 0.7 | 746.3507 | 2 | 28.70 | 1 | F1:15253 | 13012022\_RID\_3262\_4a.raw | 7.4432E6 |  |  |  |  |  |  |  |  |  | 1 | 1 | 0 | 0 | 0 | 0 | 0 | 0 | 0 | 0 | 0 | 130 | 143 | Oxidation (M); Carbamidomethylation | M9:Oxidation (M):1000.00;C13:Carbamidomethylation:1000.00 | PEAKS DB |
| N.ILNEDEQTRDPKEK.F | N | 47.76 | 1713.8533 | 14 | 0.4 | 572.2919 | 3 | 18.15 | 2 | F2:5610 | 13012022\_RID\_3262\_5a.raw |  | 1.6162E6 |  |  |  |  |  |  |  |  | 1 | 0 | 1 | 0 | 0 | 0 | 0 | 0 | 0 | 0 | 0 | 82 | 95 |  |  | PEAKS DB |
| L.DKDIMLIR.L | N | 41.99 | 1002.5532 | 8 | 0.7 | 502.2842 | 2 | 34.59 | 7 | F7:15514 | 21012022\_RID\_3262\_7a.raw |  |  |  |  |  |  | 2.6461E6 |  |  |  | 1 | 0 | 0 | 0 | 0 | 0 | 0 | 1 | 0 | 0 | 0 | 108 | 115 |  |  | PEAKS DB |
| total 6 peptides |  |  |  |  |  |  |  |  |  |  |  |  |  |  |  |  |  |  |  |  |  |  |  |  |  |  |  |  |  |  |  |  |  |  |  |  |  |
| --- | --- | --- | --- | --- | --- | --- | --- | --- | --- | --- | --- | --- | --- | --- | --- | --- | --- | --- | --- | --- | --- | --- | --- | --- | --- | --- | --- | --- | --- | --- | --- | --- | --- | --- | --- | --- | --- |

AFJ49950.1

back to list

  

| Protein Coverage
| Supporting Peptides
|

Protein Coverage:

Supporting Peptides:

| Peptide | Uniq | -10lgP | Mass | Length | ppm | m/z | z | RT | Fraction | Scan | Source File | Area F4 | Area F5 | Area F6 | Area F10 | Area F8 | Area F9 | Area F7 | Area F1 | Area F2 | Area F3 | #Feature | #Feature F4 | #Feature F5 | #Feature F6 | #Feature F10 | #Feature F8 | #Feature F9 | #Feature F7 | #Feature F1 | #Feature F2 | #Feature F3 | Start | End | PTM | AScore | Found By |
| --- | --- | --- | --- | --- | --- | --- | --- | --- | --- | --- | --- | --- | --- | --- | --- | --- | --- | --- | --- | --- | --- | --- | --- | --- | --- | --- | --- | --- | --- | --- | --- | --- | --- | --- | --- | --- | --- |
| K.IGGIGTVPVGR.V | Y | 63.63 | 1024.6029 | 11 | -0.7 | 513.3083 | 2 | 35.65 | 4 | F4:21489 | 17012022\_RID\_3262\_10a .raw |  |  |  | 1.4104E6 |  | 5.5207E5 |  |  |  |  | 2 | 0 | 0 | 0 | 1 | 0 | 1 | 0 | 0 | 0 | 0 | 256 | 266 |  |  | PEAKS DB |
| K.STTTGHLIYK.C | Y | 54.51 | 1119.5924 | 10 | 0.2 | 560.8036 | 2 | 22.46 | 4 | F4:9595 | 17012022\_RID\_3262\_10a .raw |  |  |  | 1.0764E6 |  |  |  | 2.1735E5 |  |  | 2 | 0 | 0 | 0 | 1 | 0 | 0 | 0 | 1 | 0 | 0 | 21 | 30 |  |  | PEAKS DB |
| R.EHALLAYTLGVK.Q | Y | 52.85 | 1313.7343 | 12 | 0.4 | 657.8747 | 2 | 39.97 | 8 | F8:20387 | 31122021\_RID\_3262\_1a.raw |  |  |  |  |  |  |  | 9.4565E4 | 1.3607E4 |  | 2 | 0 | 0 | 0 | 0 | 0 | 0 | 0 | 1 | 1 | 0 | 135 | 146 |  |  | PEAKS DB |
| R.LPLQDVYK.I | Y | 48.11 | 974.5436 | 8 | 0.0 | 488.2791 | 2 | 37.04 | 6 | F6:22905 | 17012022\_RID\_3262\_9a.raw |  |  |  | 4.7916E5 |  | 0 |  |  |  |  | 1 | 0 | 0 | 0 | 1 | 0 | 0 | 0 | 0 | 0 | 0 | 248 | 255 |  |  | PEAKS DB |
| K.YYVTIIDAPGHR.D | Y | 43.50 | 1403.7197 | 12 | 0.1 | 468.9139 | 3 | 37.42 | 6 | F6:23244 | 17012022\_RID\_3262\_9a.raw |  |  |  |  |  | 1.0411E5 |  | 4.0607E4 |  |  | 2 | 0 | 0 | 0 | 0 | 0 | 1 | 0 | 1 | 0 | 0 | 85 | 96 |  |  | PEAKS DB |
| total 5 peptides |  |  |  |  |  |  |  |  |  |  |  |  |  |  |  |  |  |  |  |  |  |  |  |  |  |  |  |  |  |  |  |  |  |  |  |  |  |
| --- | --- | --- | --- | --- | --- | --- | --- | --- | --- | --- | --- | --- | --- | --- | --- | --- | --- | --- | --- | --- | --- | --- | --- | --- | --- | --- | --- | --- | --- | --- | --- | --- | --- | --- | --- | --- | --- |

JAA97186.1

back to list

  

| Protein Coverage
| Supporting Peptides
|

Protein Coverage:

Supporting Peptides:

| Peptide | Uniq | -10lgP | Mass | Length | ppm | m/z | z | RT | Fraction | Scan | Source File | Area F4 | Area F5 | Area F6 | Area F10 | Area F8 | Area F9 | Area F7 | Area F1 | Area F2 | Area F3 | #Feature | #Feature F4 | #Feature F5 | #Feature F6 | #Feature F10 | #Feature F8 | #Feature F9 | #Feature F7 | #Feature F1 | #Feature F2 | #Feature F3 | Start | End | PTM | AScore | Found By |
| --- | --- | --- | --- | --- | --- | --- | --- | --- | --- | --- | --- | --- | --- | --- | --- | --- | --- | --- | --- | --- | --- | --- | --- | --- | --- | --- | --- | --- | --- | --- | --- | --- | --- | --- | --- | --- | --- |
| K.IGGIGTVPVGR.V | Y | 63.63 | 1024.6029 | 11 | -0.7 | 513.3083 | 2 | 35.65 | 4 | F4:21489 | 17012022\_RID\_3262\_10a .raw |  |  |  | 1.4104E6 |  | 5.5207E5 |  |  |  |  | 2 | 0 | 0 | 0 | 1 | 0 | 1 | 0 | 0 | 0 | 0 | 256 | 266 |  |  | PEAKS DB |
| K.STTTGHLIYK.C | Y | 54.51 | 1119.5924 | 10 | 0.2 | 560.8036 | 2 | 22.46 | 4 | F4:9595 | 17012022\_RID\_3262\_10a .raw |  |  |  | 1.0764E6 |  |  |  | 2.1735E5 |  |  | 2 | 0 | 0 | 0 | 1 | 0 | 0 | 0 | 1 | 0 | 0 | 21 | 30 |  |  | PEAKS DB |
| R.EHALLAYTLGVK.Q | Y | 52.85 | 1313.7343 | 12 | 0.4 | 657.8747 | 2 | 39.97 | 8 | F8:20387 | 31122021\_RID\_3262\_1a.raw |  |  |  |  |  |  |  | 9.4565E4 | 1.3607E4 |  | 2 | 0 | 0 | 0 | 0 | 0 | 0 | 0 | 1 | 1 | 0 | 135 | 146 |  |  | PEAKS DB |
| R.LPLQDVYK.I | Y | 48.11 | 974.5436 | 8 | 0.0 | 488.2791 | 2 | 37.04 | 6 | F6:22905 | 17012022\_RID\_3262\_9a.raw |  |  |  | 4.7916E5 |  | 0 |  |  |  |  | 1 | 0 | 0 | 0 | 1 | 0 | 0 | 0 | 0 | 0 | 0 | 248 | 255 |  |  | PEAKS DB |
| K.YYVTIIDAPGHR.D | Y | 43.50 | 1403.7197 | 12 | 0.1 | 468.9139 | 3 | 37.42 | 6 | F6:23244 | 17012022\_RID\_3262\_9a.raw |  |  |  |  |  | 1.0411E5 |  | 4.0607E4 |  |  | 2 | 0 | 0 | 0 | 0 | 0 | 1 | 0 | 1 | 0 | 0 | 85 | 96 |  |  | PEAKS DB |
| total 5 peptides |  |  |  |  |  |  |  |  |  |  |  |  |  |  |  |  |  |  |  |  |  |  |  |  |  |  |  |  |  |  |  |  |  |  |  |  |  |
| --- | --- | --- | --- | --- | --- | --- | --- | --- | --- | --- | --- | --- | --- | --- | --- | --- | --- | --- | --- | --- | --- | --- | --- | --- | --- | --- | --- | --- | --- | --- | --- | --- | --- | --- | --- | --- | --- |

JAI13488.1

back to list

  

| Protein Coverage
| Supporting Peptides
|

Protein Coverage:

Supporting Peptides:

| Peptide | Uniq | -10lgP | Mass | Length | ppm | m/z | z | RT | Fraction | Scan | Source File | Area F4 | Area F5 | Area F6 | Area F10 | Area F8 | Area F9 | Area F7 | Area F1 | Area F2 | Area F3 | #Feature | #Feature F4 | #Feature F5 | #Feature F6 | #Feature F10 | #Feature F8 | #Feature F9 | #Feature F7 | #Feature F1 | #Feature F2 | #Feature F3 | Start | End | PTM | AScore | Found By |
| --- | --- | --- | --- | --- | --- | --- | --- | --- | --- | --- | --- | --- | --- | --- | --- | --- | --- | --- | --- | --- | --- | --- | --- | --- | --- | --- | --- | --- | --- | --- | --- | --- | --- | --- | --- | --- | --- |
| K.IGGIGTVPVGR.V | Y | 63.63 | 1024.6029 | 11 | -0.7 | 513.3083 | 2 | 35.65 | 4 | F4:21489 | 17012022\_RID\_3262\_10a .raw |  |  |  | 1.4104E6 |  | 5.5207E5 |  |  |  |  | 2 | 0 | 0 | 0 | 1 | 0 | 1 | 0 | 0 | 0 | 0 | 256 | 266 |  |  | PEAKS DB |
| K.STTTGHLIYK.C | Y | 54.51 | 1119.5924 | 10 | 0.2 | 560.8036 | 2 | 22.46 | 4 | F4:9595 | 17012022\_RID\_3262\_10a .raw |  |  |  | 1.0764E6 |  |  |  | 2.1735E5 |  |  | 2 | 0 | 0 | 0 | 1 | 0 | 0 | 0 | 1 | 0 | 0 | 21 | 30 |  |  | PEAKS DB |
| R.EHALLAYTLGVK.Q | Y | 52.85 | 1313.7343 | 12 | 0.4 | 657.8747 | 2 | 39.97 | 8 | F8:20387 | 31122021\_RID\_3262\_1a.raw |  |  |  |  |  |  |  | 9.4565E4 | 1.3607E4 |  | 2 | 0 | 0 | 0 | 0 | 0 | 0 | 0 | 1 | 1 | 0 | 135 | 146 |  |  | PEAKS DB |
| R.LPLQDVYK.I | Y | 48.11 | 974.5436 | 8 | 0.0 | 488.2791 | 2 | 37.04 | 6 | F6:22905 | 17012022\_RID\_3262\_9a.raw |  |  |  | 4.7916E5 |  | 0 |  |  |  |  | 1 | 0 | 0 | 0 | 1 | 0 | 0 | 0 | 0 | 0 | 0 | 248 | 255 |  |  | PEAKS DB |
| K.YYVTIIDAPGHR.D | Y | 43.50 | 1403.7197 | 12 | 0.1 | 468.9139 | 3 | 37.42 | 6 | F6:23244 | 17012022\_RID\_3262\_9a.raw |  |  |  |  |  | 1.0411E5 |  | 4.0607E4 |  |  | 2 | 0 | 0 | 0 | 0 | 0 | 1 | 0 | 1 | 0 | 0 | 85 | 96 |  |  | PEAKS DB |
| total 5 peptides |  |  |  |  |  |  |  |  |  |  |  |  |  |  |  |  |  |  |  |  |  |  |  |  |  |  |  |  |  |  |  |  |  |  |  |  |  |
| --- | --- | --- | --- | --- | --- | --- | --- | --- | --- | --- | --- | --- | --- | --- | --- | --- | --- | --- | --- | --- | --- | --- | --- | --- | --- | --- | --- | --- | --- | --- | --- | --- | --- | --- | --- | --- | --- |

XP\_007431170.1

back to list

  

| Protein Coverage
| Supporting Peptides
|

Protein Coverage:

Supporting Peptides:

| Peptide | Uniq | -10lgP | Mass | Length | ppm | m/z | z | RT | Fraction | Scan | Source File | Area F4 | Area F5 | Area F6 | Area F10 | Area F8 | Area F9 | Area F7 | Area F1 | Area F2 | Area F3 | #Feature | #Feature F4 | #Feature F5 | #Feature F6 | #Feature F10 | #Feature F8 | #Feature F9 | #Feature F7 | #Feature F1 | #Feature F2 | #Feature F3 | Start | End | PTM | AScore | Found By |
| --- | --- | --- | --- | --- | --- | --- | --- | --- | --- | --- | --- | --- | --- | --- | --- | --- | --- | --- | --- | --- | --- | --- | --- | --- | --- | --- | --- | --- | --- | --- | --- | --- | --- | --- | --- | --- | --- |
| K.IGGIGTVPVGR.V | Y | 63.63 | 1024.6029 | 11 | -0.7 | 513.3083 | 2 | 35.65 | 4 | F4:21489 | 17012022\_RID\_3262\_10a .raw |  |  |  | 1.4104E6 |  | 5.5207E5 |  |  |  |  | 2 | 0 | 0 | 0 | 1 | 0 | 1 | 0 | 0 | 0 | 0 | 256 | 266 |  |  | PEAKS DB |
| K.STTTGHLIYK.C | Y | 54.51 | 1119.5924 | 10 | 0.2 | 560.8036 | 2 | 22.46 | 4 | F4:9595 | 17012022\_RID\_3262\_10a .raw |  |  |  | 1.0764E6 |  |  |  | 2.1735E5 |  |  | 2 | 0 | 0 | 0 | 1 | 0 | 0 | 0 | 1 | 0 | 0 | 21 | 30 |  |  | PEAKS DB |
| R.EHALLAYTLGVK.Q | Y | 52.85 | 1313.7343 | 12 | 0.4 | 657.8747 | 2 | 39.97 | 8 | F8:20387 | 31122021\_RID\_3262\_1a.raw |  |  |  |  |  |  |  | 9.4565E4 | 1.3607E4 |  | 2 | 0 | 0 | 0 | 0 | 0 | 0 | 0 | 1 | 1 | 0 | 135 | 146 |  |  | PEAKS DB |
| R.LPLQDVYK.I | Y | 48.11 | 974.5436 | 8 | 0.0 | 488.2791 | 2 | 37.04 | 6 | F6:22905 | 17012022\_RID\_3262\_9a.raw |  |  |  | 4.7916E5 |  | 0 |  |  |  |  | 1 | 0 | 0 | 0 | 1 | 0 | 0 | 0 | 0 | 0 | 0 | 248 | 255 |  |  | PEAKS DB |
| K.YYVTIIDAPGHR.D | Y | 43.50 | 1403.7197 | 12 | 0.1 | 468.9139 | 3 | 37.42 | 6 | F6:23244 | 17012022\_RID\_3262\_9a.raw |  |  |  |  |  | 1.0411E5 |  | 4.0607E4 |  |  | 2 | 0 | 0 | 0 | 0 | 0 | 1 | 0 | 1 | 0 | 0 | 85 | 96 |  |  | PEAKS DB |
| total 5 peptides |  |  |  |  |  |  |  |  |  |  |  |  |  |  |  |  |  |  |  |  |  |  |  |  |  |  |  |  |  |  |  |  |  |  |  |  |  |
| --- | --- | --- | --- | --- | --- | --- | --- | --- | --- | --- | --- | --- | --- | --- | --- | --- | --- | --- | --- | --- | --- | --- | --- | --- | --- | --- | --- | --- | --- | --- | --- | --- | --- | --- | --- | --- | --- |

JAG46923.1

back to list

  

| Protein Coverage
| Supporting Peptides
|

Protein Coverage:

Supporting Peptides:

| Peptide | Uniq | -10lgP | Mass | Length | ppm | m/z | z | RT | Fraction | Scan | Source File | Area F4 | Area F5 | Area F6 | Area F10 | Area F8 | Area F9 | Area F7 | Area F1 | Area F2 | Area F3 | #Feature | #Feature F4 | #Feature F5 | #Feature F6 | #Feature F10 | #Feature F8 | #Feature F9 | #Feature F7 | #Feature F1 | #Feature F2 | #Feature F3 | Start | End | PTM | AScore | Found By |
| --- | --- | --- | --- | --- | --- | --- | --- | --- | --- | --- | --- | --- | --- | --- | --- | --- | --- | --- | --- | --- | --- | --- | --- | --- | --- | --- | --- | --- | --- | --- | --- | --- | --- | --- | --- | --- | --- |
| K.IGGIGTVPVGR.V | Y | 63.63 | 1024.6029 | 11 | -0.7 | 513.3083 | 2 | 35.65 | 4 | F4:21489 | 17012022\_RID\_3262\_10a .raw |  |  |  | 1.4104E6 |  | 5.5207E5 |  |  |  |  | 2 | 0 | 0 | 0 | 1 | 0 | 1 | 0 | 0 | 0 | 0 | 256 | 266 |  |  | PEAKS DB |
| K.STTTGHLIYK.C | Y | 54.51 | 1119.5924 | 10 | 0.2 | 560.8036 | 2 | 22.46 | 4 | F4:9595 | 17012022\_RID\_3262\_10a .raw |  |  |  | 1.0764E6 |  |  |  | 2.1735E5 |  |  | 2 | 0 | 0 | 0 | 1 | 0 | 0 | 0 | 1 | 0 | 0 | 21 | 30 |  |  | PEAKS DB |
| R.EHALLAYTLGVK.Q | Y | 52.85 | 1313.7343 | 12 | 0.4 | 657.8747 | 2 | 39.97 | 8 | F8:20387 | 31122021\_RID\_3262\_1a.raw |  |  |  |  |  |  |  | 9.4565E4 | 1.3607E4 |  | 2 | 0 | 0 | 0 | 0 | 0 | 0 | 0 | 1 | 1 | 0 | 135 | 146 |  |  | PEAKS DB |
| R.LPLQDVYK.I | Y | 48.11 | 974.5436 | 8 | 0.0 | 488.2791 | 2 | 37.04 | 6 | F6:22905 | 17012022\_RID\_3262\_9a.raw |  |  |  | 4.7916E5 |  | 0 |  |  |  |  | 1 | 0 | 0 | 0 | 1 | 0 | 0 | 0 | 0 | 0 | 0 | 248 | 255 |  |  | PEAKS DB |
| K.YYVTIIDAPGHR.D | Y | 43.50 | 1403.7197 | 12 | 0.1 | 468.9139 | 3 | 37.42 | 6 | F6:23244 | 17012022\_RID\_3262\_9a.raw |  |  |  |  |  | 1.0411E5 |  | 4.0607E4 |  |  | 2 | 0 | 0 | 0 | 0 | 0 | 1 | 0 | 1 | 0 | 0 | 85 | 96 |  |  | PEAKS DB |
| total 5 peptides |  |  |  |  |  |  |  |  |  |  |  |  |  |  |  |  |  |  |  |  |  |  |  |  |  |  |  |  |  |  |  |  |  |  |  |  |  |
| --- | --- | --- | --- | --- | --- | --- | --- | --- | --- | --- | --- | --- | --- | --- | --- | --- | --- | --- | --- | --- | --- | --- | --- | --- | --- | --- | --- | --- | --- | --- | --- | --- | --- | --- | --- | --- | --- |

XP\_015672964.1

back to list

  

| Protein Coverage
| Supporting Peptides
|

Protein Coverage:

Supporting Peptides:

| Peptide | Uniq | -10lgP | Mass | Length | ppm | m/z | z | RT | Fraction | Scan | Source File | Area F4 | Area F5 | Area F6 | Area F10 | Area F8 | Area F9 | Area F7 | Area F1 | Area F2 | Area F3 | #Feature | #Feature F4 | #Feature F5 | #Feature F6 | #Feature F10 | #Feature F8 | #Feature F9 | #Feature F7 | #Feature F1 | #Feature F2 | #Feature F3 | Start | End | PTM | AScore | Found By |
| --- | --- | --- | --- | --- | --- | --- | --- | --- | --- | --- | --- | --- | --- | --- | --- | --- | --- | --- | --- | --- | --- | --- | --- | --- | --- | --- | --- | --- | --- | --- | --- | --- | --- | --- | --- | --- | --- |
| K.IGGIGTVPVGR.V | Y | 63.63 | 1024.6029 | 11 | -0.7 | 513.3083 | 2 | 35.65 | 4 | F4:21489 | 17012022\_RID\_3262\_10a .raw |  |  |  | 1.4104E6 |  | 5.5207E5 |  |  |  |  | 2 | 0 | 0 | 0 | 1 | 0 | 1 | 0 | 0 | 0 | 0 | 256 | 266 |  |  | PEAKS DB |
| K.STTTGHLIYK.C | Y | 54.51 | 1119.5924 | 10 | 0.2 | 560.8036 | 2 | 22.46 | 4 | F4:9595 | 17012022\_RID\_3262\_10a .raw |  |  |  | 1.0764E6 |  |  |  | 2.1735E5 |  |  | 2 | 0 | 0 | 0 | 1 | 0 | 0 | 0 | 1 | 0 | 0 | 21 | 30 |  |  | PEAKS DB |
| R.EHALLAYTLGVK.Q | Y | 52.85 | 1313.7343 | 12 | 0.4 | 657.8747 | 2 | 39.97 | 8 | F8:20387 | 31122021\_RID\_3262\_1a.raw |  |  |  |  |  |  |  | 9.4565E4 | 1.3607E4 |  | 2 | 0 | 0 | 0 | 0 | 0 | 0 | 0 | 1 | 1 | 0 | 135 | 146 |  |  | PEAKS DB |
| R.LPLQDVYK.I | Y | 48.11 | 974.5436 | 8 | 0.0 | 488.2791 | 2 | 37.04 | 6 | F6:22905 | 17012022\_RID\_3262\_9a.raw |  |  |  | 4.7916E5 |  | 0 |  |  |  |  | 1 | 0 | 0 | 0 | 1 | 0 | 0 | 0 | 0 | 0 | 0 | 248 | 255 |  |  | PEAKS DB |
| K.YYVTIIDAPGHR.D | Y | 43.50 | 1403.7197 | 12 | 0.1 | 468.9139 | 3 | 37.42 | 6 | F6:23244 | 17012022\_RID\_3262\_9a.raw |  |  |  |  |  | 1.0411E5 |  | 4.0607E4 |  |  | 2 | 0 | 0 | 0 | 0 | 0 | 1 | 0 | 1 | 0 | 0 | 85 | 96 |  |  | PEAKS DB |
| total 5 peptides |  |  |  |  |  |  |  |  |  |  |  |  |  |  |  |  |  |  |  |  |  |  |  |  |  |  |  |  |  |  |  |  |  |  |  |  |  |
| --- | --- | --- | --- | --- | --- | --- | --- | --- | --- | --- | --- | --- | --- | --- | --- | --- | --- | --- | --- | --- | --- | --- | --- | --- | --- | --- | --- | --- | --- | --- | --- | --- | --- | --- | --- | --- | --- |

JAG44960.1

back to list

  

| Protein Coverage
| Supporting Peptides
|

Protein Coverage:

Supporting Peptides:

| Peptide | Uniq | -10lgP | Mass | Length | ppm | m/z | z | RT | Fraction | Scan | Source File | Area F4 | Area F5 | Area F6 | Area F10 | Area F8 | Area F9 | Area F7 | Area F1 | Area F2 | Area F3 | #Feature | #Feature F4 | #Feature F5 | #Feature F6 | #Feature F10 | #Feature F8 | #Feature F9 | #Feature F7 | #Feature F1 | #Feature F2 | #Feature F3 | Start | End | PTM | AScore | Found By |
| --- | --- | --- | --- | --- | --- | --- | --- | --- | --- | --- | --- | --- | --- | --- | --- | --- | --- | --- | --- | --- | --- | --- | --- | --- | --- | --- | --- | --- | --- | --- | --- | --- | --- | --- | --- | --- | --- |
| K.IGGIGTVPVGR.V | Y | 63.63 | 1024.6029 | 11 | -0.7 | 513.3083 | 2 | 35.65 | 4 | F4:21489 | 17012022\_RID\_3262\_10a .raw |  |  |  | 1.4104E6 |  | 5.5207E5 |  |  |  |  | 2 | 0 | 0 | 0 | 1 | 0 | 1 | 0 | 0 | 0 | 0 | 256 | 266 |  |  | PEAKS DB |
| K.STTTGHLIYK.C | Y | 54.51 | 1119.5924 | 10 | 0.2 | 560.8036 | 2 | 22.46 | 4 | F4:9595 | 17012022\_RID\_3262\_10a .raw |  |  |  | 1.0764E6 |  |  |  | 2.1735E5 |  |  | 2 | 0 | 0 | 0 | 1 | 0 | 0 | 0 | 1 | 0 | 0 | 21 | 30 |  |  | PEAKS DB |
| R.EHALLAYTLGVK.Q | Y | 52.85 | 1313.7343 | 12 | 0.4 | 657.8747 | 2 | 39.97 | 8 | F8:20387 | 31122021\_RID\_3262\_1a.raw |  |  |  |  |  |  |  | 9.4565E4 | 1.3607E4 |  | 2 | 0 | 0 | 0 | 0 | 0 | 0 | 0 | 1 | 1 | 0 | 135 | 146 |  |  | PEAKS DB |
| R.LPLQDVYK.I | Y | 48.11 | 974.5436 | 8 | 0.0 | 488.2791 | 2 | 37.04 | 6 | F6:22905 | 17012022\_RID\_3262\_9a.raw |  |  |  | 4.7916E5 |  | 0 |  |  |  |  | 1 | 0 | 0 | 0 | 1 | 0 | 0 | 0 | 0 | 0 | 0 | 248 | 255 |  |  | PEAKS DB |
| K.YYVTIIDAPGHR.D | Y | 43.50 | 1403.7197 | 12 | 0.1 | 468.9139 | 3 | 37.42 | 6 | F6:23244 | 17012022\_RID\_3262\_9a.raw |  |  |  |  |  | 1.0411E5 |  | 4.0607E4 |  |  | 2 | 0 | 0 | 0 | 0 | 0 | 1 | 0 | 1 | 0 | 0 | 85 | 96 |  |  | PEAKS DB |
| total 5 peptides |  |  |  |  |  |  |  |  |  |  |  |  |  |  |  |  |  |  |  |  |  |  |  |  |  |  |  |  |  |  |  |  |  |  |  |  |  |
| --- | --- | --- | --- | --- | --- | --- | --- | --- | --- | --- | --- | --- | --- | --- | --- | --- | --- | --- | --- | --- | --- | --- | --- | --- | --- | --- | --- | --- | --- | --- | --- | --- | --- | --- | --- | --- | --- |

JAV50678.1

back to list

  

| Protein Coverage
| Supporting Peptides
|

Protein Coverage:

Supporting Peptides:

| Peptide | Uniq | -10lgP | Mass | Length | ppm | m/z | z | RT | Fraction | Scan | Source File | Area F4 | Area F5 | Area F6 | Area F10 | Area F8 | Area F9 | Area F7 | Area F1 | Area F2 | Area F3 | #Feature | #Feature F4 | #Feature F5 | #Feature F6 | #Feature F10 | #Feature F8 | #Feature F9 | #Feature F7 | #Feature F1 | #Feature F2 | #Feature F3 | Start | End | PTM | AScore | Found By |
| --- | --- | --- | --- | --- | --- | --- | --- | --- | --- | --- | --- | --- | --- | --- | --- | --- | --- | --- | --- | --- | --- | --- | --- | --- | --- | --- | --- | --- | --- | --- | --- | --- | --- | --- | --- | --- | --- |
| K.IGGIGTVPVGR.V | Y | 63.63 | 1024.6029 | 11 | -0.7 | 513.3083 | 2 | 35.65 | 4 | F4:21489 | 17012022\_RID\_3262\_10a .raw |  |  |  | 1.4104E6 |  | 5.5207E5 |  |  |  |  | 2 | 0 | 0 | 0 | 1 | 0 | 1 | 0 | 0 | 0 | 0 | 256 | 266 |  |  | PEAKS DB |
| K.STTTGHLIYK.C | Y | 54.51 | 1119.5924 | 10 | 0.2 | 560.8036 | 2 | 22.46 | 4 | F4:9595 | 17012022\_RID\_3262\_10a .raw |  |  |  | 1.0764E6 |  |  |  | 2.1735E5 |  |  | 2 | 0 | 0 | 0 | 1 | 0 | 0 | 0 | 1 | 0 | 0 | 21 | 30 |  |  | PEAKS DB |
| R.EHALLAYTLGVK.Q | Y | 52.85 | 1313.7343 | 12 | 0.4 | 657.8747 | 2 | 39.97 | 8 | F8:20387 | 31122021\_RID\_3262\_1a.raw |  |  |  |  |  |  |  | 9.4565E4 | 1.3607E4 |  | 2 | 0 | 0 | 0 | 0 | 0 | 0 | 0 | 1 | 1 | 0 | 135 | 146 |  |  | PEAKS DB |
| R.LPLQDVYK.I | Y | 48.11 | 974.5436 | 8 | 0.0 | 488.2791 | 2 | 37.04 | 6 | F6:22905 | 17012022\_RID\_3262\_9a.raw |  |  |  | 4.7916E5 |  | 0 |  |  |  |  | 1 | 0 | 0 | 0 | 1 | 0 | 0 | 0 | 0 | 0 | 0 | 248 | 255 |  |  | PEAKS DB |
| K.YYVTIIDAPGHR.D | Y | 43.50 | 1403.7197 | 12 | 0.1 | 468.9139 | 3 | 37.42 | 6 | F6:23244 | 17012022\_RID\_3262\_9a.raw |  |  |  |  |  | 1.0411E5 |  | 4.0607E4 |  |  | 2 | 0 | 0 | 0 | 0 | 0 | 1 | 0 | 1 | 0 | 0 | 85 | 96 |  |  | PEAKS DB |
| total 5 peptides |  |  |  |  |  |  |  |  |  |  |  |  |  |  |  |  |  |  |  |  |  |  |  |  |  |  |  |  |  |  |  |  |  |  |  |  |  |
| --- | --- | --- | --- | --- | --- | --- | --- | --- | --- | --- | --- | --- | --- | --- | --- | --- | --- | --- | --- | --- | --- | --- | --- | --- | --- | --- | --- | --- | --- | --- | --- | --- | --- | --- | --- | --- | --- |

XP\_026570509.1

back to list

  

| Protein Coverage
| Supporting Peptides
|

Protein Coverage:

Supporting Peptides:

| Peptide | Uniq | -10lgP | Mass | Length | ppm | m/z | z | RT | Fraction | Scan | Source File | Area F4 | Area F5 | Area F6 | Area F10 | Area F8 | Area F9 | Area F7 | Area F1 | Area F2 | Area F3 | #Feature | #Feature F4 | #Feature F5 | #Feature F6 | #Feature F10 | #Feature F8 | #Feature F9 | #Feature F7 | #Feature F1 | #Feature F2 | #Feature F3 | Start | End | PTM | AScore | Found By |
| --- | --- | --- | --- | --- | --- | --- | --- | --- | --- | --- | --- | --- | --- | --- | --- | --- | --- | --- | --- | --- | --- | --- | --- | --- | --- | --- | --- | --- | --- | --- | --- | --- | --- | --- | --- | --- | --- |
| E.FASFIDKVR.N | N | 59.23 | 1081.5920 | 9 | 0.3 | 541.8035 | 2 | 35.45 | 6 | F6:21540 | 17012022\_RID\_3262\_9a.raw | 1.5335E7 |  |  | 9.5108E6 |  | 1.2654E7 |  | 1.0527E7 | 1.5026E6 |  | 9 | 1 | 0 | 0 | 3 | 0 | 1 | 0 | 3 | 1 | 0 | 160 | 168 |  |  | PEAKS DB |
| K.TLLEGEESR | N | 57.54 | 1032.5087 | 9 | 0.1 | 517.2617 | 2 | 28.17 | 6 | F6:15234 | 17012022\_RID\_3262\_9a.raw | 2.503E8 | 1.2082E8 | 4.6408E7 | 1.0195E9 | 3.2941E6 | 8.0682E8 | 4.4201E7 | 2.2161E8 | 1.4877E8 | 3.5693E8 | 10 | 1 | 1 | 1 | 1 | 1 | 1 | 1 | 1 | 1 | 1 | 452 | 460 |  |  | PEAKS DB |
| E.FASFIDK.V | N | 52.67 | 826.4225 | 7 | 0.7 | 414.2188 | 2 | 36.24 | 6 | F6:22289 | 17012022\_RID\_3262\_9a.raw | 2.5611E7 | 1.8348E7 | 6.6379E6 | 1.2204E8 |  | 1.3051E8 | 4.5017E6 | 4.1444E7 | 1.2872E7 |  | 15 | 2 | 2 | 1 | 4 | 0 | 3 | 1 | 1 | 1 | 0 | 160 | 166 |  |  | PEAKS DB |
| K.NLNNEFASFIDK.V | Y | 44.75 | 1410.6779 | 12 | 0.7 | 706.3467 | 2 | 61.11 | 8 | F8:37016 | 31122021\_RID\_3262\_1a.raw |  |  |  |  |  |  |  | 5.4786E5 | 1.3457E5 |  | 2 | 0 | 0 | 0 | 0 | 0 | 0 | 0 | 1 | 1 | 0 | 155 | 166 |  |  | PEAKS DB |
| total 4 peptides |  |  |  |  |  |  |  |  |  |  |  |  |  |  |  |  |  |  |  |  |  |  |  |  |  |  |  |  |  |  |  |  |  |  |  |  |  |
| --- | --- | --- | --- | --- | --- | --- | --- | --- | --- | --- | --- | --- | --- | --- | --- | --- | --- | --- | --- | --- | --- | --- | --- | --- | --- | --- | --- | --- | --- | --- | --- | --- | --- | --- | --- | --- | --- |

XP\_026539658.1

back to list

  

| Protein Coverage
| Supporting Peptides
|

Protein Coverage:

Supporting Peptides:

| Peptide | Uniq | -10lgP | Mass | Length | ppm | m/z | z | RT | Fraction | Scan | Source File | Area F4 | Area F5 | Area F6 | Area F10 | Area F8 | Area F9 | Area F7 | Area F1 | Area F2 | Area F3 | #Feature | #Feature F4 | #Feature F5 | #Feature F6 | #Feature F10 | #Feature F8 | #Feature F9 | #Feature F7 | #Feature F1 | #Feature F2 | #Feature F3 | Start | End | PTM | AScore | Found By |
| --- | --- | --- | --- | --- | --- | --- | --- | --- | --- | --- | --- | --- | --- | --- | --- | --- | --- | --- | --- | --- | --- | --- | --- | --- | --- | --- | --- | --- | --- | --- | --- | --- | --- | --- | --- | --- | --- |
| E.FASFIDKVR.N | N | 59.23 | 1081.5920 | 9 | 0.3 | 541.8035 | 2 | 35.45 | 6 | F6:21540 | 17012022\_RID\_3262\_9a.raw | 1.5335E7 |  |  | 9.5108E6 |  | 1.2654E7 |  | 1.0527E7 | 1.5026E6 |  | 9 | 1 | 0 | 0 | 3 | 0 | 1 | 0 | 3 | 1 | 0 | 160 | 168 |  |  | PEAKS DB |
| K.TLLEGEESR | N | 57.54 | 1032.5087 | 9 | 0.1 | 517.2617 | 2 | 28.17 | 6 | F6:15234 | 17012022\_RID\_3262\_9a.raw | 2.503E8 | 1.2082E8 | 4.6408E7 | 1.0195E9 | 3.2941E6 | 8.0682E8 | 4.4201E7 | 2.2161E8 | 1.4877E8 | 3.5693E8 | 10 | 1 | 1 | 1 | 1 | 1 | 1 | 1 | 1 | 1 | 1 | 452 | 460 |  |  | PEAKS DB |
| E.FASFIDK.V | N | 52.67 | 826.4225 | 7 | 0.7 | 414.2188 | 2 | 36.24 | 6 | F6:22289 | 17012022\_RID\_3262\_9a.raw | 2.5611E7 | 1.8348E7 | 6.6379E6 | 1.2204E8 |  | 1.3051E8 | 4.5017E6 | 4.1444E7 | 1.2872E7 |  | 15 | 2 | 2 | 1 | 4 | 0 | 3 | 1 | 1 | 1 | 0 | 160 | 166 |  |  | PEAKS DB |
| K.NLNNEFASFIDK.V | Y | 44.75 | 1410.6779 | 12 | 0.7 | 706.3467 | 2 | 61.11 | 8 | F8:37016 | 31122021\_RID\_3262\_1a.raw |  |  |  |  |  |  |  | 5.4786E5 | 1.3457E5 |  | 2 | 0 | 0 | 0 | 0 | 0 | 0 | 0 | 1 | 1 | 0 | 155 | 166 |  |  | PEAKS DB |
| total 4 peptides |  |  |  |  |  |  |  |  |  |  |  |  |  |  |  |  |  |  |  |  |  |  |  |  |  |  |  |  |  |  |  |  |  |  |  |  |  |
| --- | --- | --- | --- | --- | --- | --- | --- | --- | --- | --- | --- | --- | --- | --- | --- | --- | --- | --- | --- | --- | --- | --- | --- | --- | --- | --- | --- | --- | --- | --- | --- | --- | --- | --- | --- | --- | --- |

P0DJJ5.1

back to list

  

| Protein Coverage
| Supporting Peptides
|

Protein Coverage:

Supporting Peptides:

| Peptide | Uniq | -10lgP | Mass | Length | ppm | m/z | z | RT | Fraction | Scan | Source File | Area F4 | Area F5 | Area F6 | Area F10 | Area F8 | Area F9 | Area F7 | Area F1 | Area F2 | Area F3 | #Feature | #Feature F4 | #Feature F5 | #Feature F6 | #Feature F10 | #Feature F8 | #Feature F9 | #Feature F7 | #Feature F1 | #Feature F2 | #Feature F3 | Start | End | PTM | AScore | Found By |
| --- | --- | --- | --- | --- | --- | --- | --- | --- | --- | --- | --- | --- | --- | --- | --- | --- | --- | --- | --- | --- | --- | --- | --- | --- | --- | --- | --- | --- | --- | --- | --- | --- | --- | --- | --- | --- | --- |
| SFELTILHTNDVHAR.V | N | 71.46 | 1751.8955 | 15 | 0.2 | 584.9725 | 3 | 37.82 | 9 | F9:19802 | 31122021\_RID\_3262\_2a.raw |  |  |  |  |  |  |  |  | 9.8903E6 |  | 1 | 0 | 0 | 0 | 0 | 0 | 0 | 0 | 0 | 1 | 0 | 1 | 15 |  |  | PEAKS DB |
| G.IQLHNYSSQEIGK | N | 66.55 | 1515.7681 | 13 | 0.9 | 758.8920 | 2 | 25.89 | 9 | F9:9947 | 31122021\_RID\_3262\_2a.raw |  | 0 |  |  |  |  |  |  | 8.8417E5 |  | 2 | 0 | 0 | 0 | 0 | 0 | 0 | 0 | 0 | 2 | 0 | 42 | 54 |  |  | PEAKS DB |
| SFELTILHTNDVHA.R | Y | 41.03 | 1595.7943 | 14 | -0.2 | 532.9386 | 3 | 45.26 | 9 | F9:25761 | 31122021\_RID\_3262\_2a.raw |  |  |  |  |  |  |  |  | 7.189E5 |  | 1 | 0 | 0 | 0 | 0 | 0 | 0 | 0 | 0 | 1 | 0 | 1 | 14 |  |  | PEAKS DB |
| total 3 peptides |  |  |  |  |  |  |  |  |  |  |  |  |  |  |  |  |  |  |  |  |  |  |  |  |  |  |  |  |  |  |  |  |  |  |  |  |  |
| --- | --- | --- | --- | --- | --- | --- | --- | --- | --- | --- | --- | --- | --- | --- | --- | --- | --- | --- | --- | --- | --- | --- | --- | --- | --- | --- | --- | --- | --- | --- | --- | --- | --- | --- | --- | --- | --- |

JAG47217.1

back to list

  

| Protein Coverage
| Supporting Peptides
|

Protein Coverage:

Supporting Peptides:

| Peptide | Uniq | -10lgP | Mass | Length | ppm | m/z | z | RT | Fraction | Scan | Source File | Area F4 | Area F5 | Area F6 | Area F10 | Area F8 | Area F9 | Area F7 | Area F1 | Area F2 | Area F3 | #Feature | #Feature F4 | #Feature F5 | #Feature F6 | #Feature F10 | #Feature F8 | #Feature F9 | #Feature F7 | #Feature F1 | #Feature F2 | #Feature F3 | Start | End | PTM | AScore | Found By |
| --- | --- | --- | --- | --- | --- | --- | --- | --- | --- | --- | --- | --- | --- | --- | --- | --- | --- | --- | --- | --- | --- | --- | --- | --- | --- | --- | --- | --- | --- | --- | --- | --- | --- | --- | --- | --- | --- |
| K.VAPFFDNVM(+15.99)EQGLLEK.N | N | 63.84 | 1851.9076 | 16 | 1.0 | 926.9620 | 2 | 52.44 | 1 | F1:36309 | 13012022\_RID\_3262\_4a.raw | 6.0231E5 |  |  |  |  |  |  |  |  |  | 1 | 1 | 0 | 0 | 0 | 0 | 0 | 0 | 0 | 0 | 0 | 198 | 213 | Oxidation (M) | M9:Oxidation (M):1000.00 | PEAKS DB |
| K.QPGITFIAAK.F | N | 51.81 | 1044.5967 | 10 | 0.6 | 523.3059 | 2 | 39.27 | 1 | F1:24810 | 13012022\_RID\_3262\_4a.raw | 7.5032E5 |  |  |  |  |  |  |  |  |  | 1 | 1 | 0 | 0 | 0 | 0 | 0 | 0 | 0 | 0 | 0 | 172 | 181 |  |  | PEAKS DB |
| K.NQLFGEATKQPGITFIAAK.F | N | 47.63 | 2033.0945 | 19 | 0.4 | 678.7057 | 3 | 45.22 | 1 | F1:30099 | 13012022\_RID\_3262\_4a.raw | 5.5813E5 |  |  |  |  |  |  |  |  |  | 1 | 1 | 0 | 0 | 0 | 0 | 0 | 0 | 0 | 0 | 0 | 163 | 181 |  |  | PEAKS DB |
| G.GPGAQIPTPEALK.N | Y | 46.72 | 1277.6979 | 13 | 0.4 | 639.8564 | 2 | 38.37 | 1 | F1:23997 | 13012022\_RID\_3262\_4a.raw | 9.9355E5 |  |  |  |  |  |  |  |  |  | 1 | 1 | 0 | 0 | 0 | 0 | 0 | 0 | 0 | 0 | 0 | 58 | 70 |  |  | PEAKS DB |
| K.NYM(+15.99)DAQYYGEIGIGTPQQK.F | Y | 45.84 | 2190.9893 | 19 | 0.5 | 1096.5024 | 2 | 42.95 | 1 | F1:28208 | 13012022\_RID\_3262\_4a.raw | 4.6138E4 |  |  |  |  |  |  |  |  |  | 1 | 1 | 0 | 0 | 0 | 0 | 0 | 0 | 0 | 0 | 0 | 71 | 89 | Oxidation (M) | M3:Oxidation (M):1000.00 | PEAKS DB |
| total 5 peptides |  |  |  |  |  |  |  |  |  |  |  |  |  |  |  |  |  |  |  |  |  |  |  |  |  |  |  |  |  |  |  |  |  |  |  |  |  |
| --- | --- | --- | --- | --- | --- | --- | --- | --- | --- | --- | --- | --- | --- | --- | --- | --- | --- | --- | --- | --- | --- | --- | --- | --- | --- | --- | --- | --- | --- | --- | --- | --- | --- | --- | --- | --- | --- |

P0CJ41.1

back to list

  

| Protein Coverage
| Supporting Peptides
|

Protein Coverage:

Supporting Peptides:

| Peptide | Uniq | -10lgP | Mass | Length | ppm | m/z | z | RT | Fraction | Scan | Source File | Area F4 | Area F5 | Area F6 | Area F10 | Area F8 | Area F9 | Area F7 | Area F1 | Area F2 | Area F3 | #Feature | #Feature F4 | #Feature F5 | #Feature F6 | #Feature F10 | #Feature F8 | #Feature F9 | #Feature F7 | #Feature F1 | #Feature F2 | #Feature F3 | Start | End | PTM | AScore | Found By |
| --- | --- | --- | --- | --- | --- | --- | --- | --- | --- | --- | --- | --- | --- | --- | --- | --- | --- | --- | --- | --- | --- | --- | --- | --- | --- | --- | --- | --- | --- | --- | --- | --- | --- | --- | --- | --- | --- |
| S.NEILDKDIMLIK.L | Y | 49.67 | 1443.8007 | 12 | 0.3 | 722.9078 | 2 | 44.70 | 8 | F8:24255 | 31122021\_RID\_3262\_1a.raw | 1.0071E7 |  |  |  |  | 1.611E6 | 1.8767E6 | 2.6266E6 |  |  | 4 | 1 | 0 | 0 | 0 | 0 | 1 | 1 | 1 | 0 | 0 | 104 | 115 |  |  | PEAKS DB |
| L.LNEDEQIRNPK.E | N | 48.30 | 1354.6841 | 11 | 0.2 | 678.3494 | 2 | 21.16 | 7 | F7:6218 | 21012022\_RID\_3262\_7a.raw |  |  |  |  |  |  | 4.369E4 |  |  |  | 1 | 0 | 0 | 0 | 0 | 0 | 0 | 1 | 0 | 0 | 0 | 83 | 93 |  |  | PEAKS DB |
| G.YPELLPEYR.T | N | 46.54 | 1178.5972 | 9 | 0.2 | 590.3060 | 2 | 45.44 | 7 | F7:22624 | 21012022\_RID\_3262\_7a.raw |  |  |  |  |  |  | 8.8577E5 |  |  |  | 1 | 0 | 0 | 0 | 0 | 0 | 0 | 1 | 0 | 0 | 0 | 178 | 186 |  |  | PEAKS DB |
| K.FGAHSKK.L | N | 43.53 | 773.4184 | 7 | -0.3 | 387.7163 | 2 | 14.54 | 7 | F7:2535 | 21012022\_RID\_3262\_7a.raw |  |  |  |  |  |  | 1.9648E7 |  |  |  | 1 | 0 | 0 | 0 | 0 | 0 | 0 | 1 | 0 | 0 | 0 | 75 | 81 |  |  | PEAKS DB |
| S.NEILDKDIM(+15.99)LIK.L | Y | 41.22 | 1459.7955 | 12 | -1.9 | 730.9037 | 2 | 44.70 | 8 | F8:24105 | 31122021\_RID\_3262\_1a.raw |  |  |  |  |  |  |  | 7.272E4 |  |  | 1 | 0 | 0 | 0 | 0 | 0 | 0 | 0 | 1 | 0 | 0 | 104 | 115 | Oxidation (M) | M9:Oxidation (M):1000.00 | PEAKS DB |
| total 5 peptides |  |  |  |  |  |  |  |  |  |  |  |  |  |  |  |  |  |  |  |  |  |  |  |  |  |  |  |  |  |  |  |  |  |  |  |  |  |
| --- | --- | --- | --- | --- | --- | --- | --- | --- | --- | --- | --- | --- | --- | --- | --- | --- | --- | --- | --- | --- | --- | --- | --- | --- | --- | --- | --- | --- | --- | --- | --- | --- | --- | --- | --- | --- | --- |

P0DJF5.1

back to list

  

| Protein Coverage
| Supporting Peptides
|

Protein Coverage:

Supporting Peptides:

| Peptide | Uniq | -10lgP | Mass | Length | ppm | m/z | z | RT | Fraction | Scan | Source File | Area F4 | Area F5 | Area F6 | Area F10 | Area F8 | Area F9 | Area F7 | Area F1 | Area F2 | Area F3 | #Feature | #Feature F4 | #Feature F5 | #Feature F6 | #Feature F10 | #Feature F8 | #Feature F9 | #Feature F7 | #Feature F1 | #Feature F2 | #Feature F3 | Start | End | PTM | AScore | Found By |
| --- | --- | --- | --- | --- | --- | --- | --- | --- | --- | --- | --- | --- | --- | --- | --- | --- | --- | --- | --- | --- | --- | --- | --- | --- | --- | --- | --- | --- | --- | --- | --- | --- | --- | --- | --- | --- | --- |
| N.NFQLLFGVHSK.K | N | 62.43 | 1288.6927 | 11 | 0.4 | 645.3539 | 2 | 42.62 | 8 | F8:22782 | 31122021\_RID\_3262\_1a.raw | 1.2701E7 | 1.0353E6 | 2.5789E6 |  |  |  |  | 3.2275E6 | 1.1882E6 |  | 6 | 1 | 1 | 2 | 0 | 0 | 0 | 0 | 1 | 1 | 0 | 70 | 80 |  |  | PEAKS DB |
| K.DDEVDKDIMLIK.L | N | 47.99 | 1432.7119 | 12 | 0.9 | 717.3639 | 2 | 44.20 | 2 | F2:25287 | 13012022\_RID\_3262\_5a.raw | 2.8885E5 | 3.1175E5 |  |  |  |  |  |  |  |  | 2 | 1 | 1 | 0 | 0 | 0 | 0 | 0 | 0 | 0 | 0 | 104 | 115 |  |  | PEAKS DB |
| K.TIPTKDIYPDVPH.C | Y | 42.71 | 1494.7719 | 13 | 0.3 | 499.2647 | 3 | 36.41 | 2 | F2:19646 | 13012022\_RID\_3262\_5a.raw |  | 1.0159E6 | 2.7071E6 |  |  |  |  |  |  |  | 2 | 0 | 1 | 1 | 0 | 0 | 0 | 0 | 0 | 0 | 0 | 150 | 162 |  |  | PEAKS DB |
| K.DIYPDVPH.C | Y | 42.03 | 954.4446 | 8 | 0.2 | 478.2297 | 2 | 39.84 | 3 | F3:18568 | 13012022\_RID\_3262\_6a.raw |  |  | 3.6063E6 |  |  |  |  |  |  |  | 1 | 0 | 0 | 1 | 0 | 0 | 0 | 0 | 0 | 0 | 0 | 155 | 162 |  |  | PEAKS DB |
| total 4 peptides |  |  |  |  |  |  |  |  |  |  |  |  |  |  |  |  |  |  |  |  |  |  |  |  |  |  |  |  |  |  |  |  |  |  |  |  |  |
| --- | --- | --- | --- | --- | --- | --- | --- | --- | --- | --- | --- | --- | --- | --- | --- | --- | --- | --- | --- | --- | --- | --- | --- | --- | --- | --- | --- | --- | --- | --- | --- | --- | --- | --- | --- | --- | --- |

AUS82501.1

back to list

  

| Protein Coverage
| Supporting Peptides
|

Protein Coverage:

Supporting Peptides:

| Peptide | Uniq | -10lgP | Mass | Length | ppm | m/z | z | RT | Fraction | Scan | Source File | Area F4 | Area F5 | Area F6 | Area F10 | Area F8 | Area F9 | Area F7 | Area F1 | Area F2 | Area F3 | #Feature | #Feature F4 | #Feature F5 | #Feature F6 | #Feature F10 | #Feature F8 | #Feature F9 | #Feature F7 | #Feature F1 | #Feature F2 | #Feature F3 | Start | End | PTM | AScore | Found By |
| --- | --- | --- | --- | --- | --- | --- | --- | --- | --- | --- | --- | --- | --- | --- | --- | --- | --- | --- | --- | --- | --- | --- | --- | --- | --- | --- | --- | --- | --- | --- | --- | --- | --- | --- | --- | --- | --- |
| H.VPALYTK.V | N | 58.85 | 790.4589 | 7 | -0.3 | 396.2366 | 2 | 27.94 | 2 | F2:12883 | 13012022\_RID\_3262\_5a.raw | 1.4799E8 | 5.0306E7 | 1.9273E6 |  |  |  |  | 5.4806E5 |  |  | 4 | 1 | 1 | 1 | 0 | 0 | 0 | 0 | 1 | 0 | 0 | 232 | 238 |  |  | PEAKS DB |
| K.WDKDIMLIR.L | N | 57.88 | 1188.6324 | 9 | 0.5 | 595.3238 | 2 | 43.59 | 1 | F1:28744 | 13012022\_RID\_3262\_4a.raw | 6.6853E8 | 1.3316E6 |  |  |  |  | 2.7704E4 | 1.1405E7 | 1.608E6 |  | 12 | 5 | 3 | 0 | 0 | 0 | 0 | 1 | 2 | 1 | 0 | 109 | 117 |  |  | PEAKS DB |
| C.IMGWGTISPTK.E | N | 47.73 | 1189.6166 | 11 | 1.3 | 595.8163 | 2 | 44.01 | 3 | F3:20987 | 13012022\_RID\_3262\_6a.raw |  | 2.3901E7 | 3.6021E5 |  |  |  |  |  |  |  | 2 | 0 | 1 | 1 | 0 | 0 | 0 | 0 | 0 | 0 | 0 | 146 | 156 |  |  | PEAKS DB |
| C.IM(+15.99)GWGTISPTK.E | N | 46.88 | 1205.6115 | 11 | 1.2 | 603.8137 | 2 | 37.85 | 2 | F2:20821 | 13012022\_RID\_3262\_5a.raw |  | 5.8537E6 |  |  |  |  |  |  |  |  | 1 | 0 | 1 | 0 | 0 | 0 | 0 | 0 | 0 | 0 | 0 | 146 | 156 | Oxidation (M) | M2:Oxidation (M):1000.00 | PEAKS DB |
| L.IIGGEEC(+57.02)NINEHR.F | Y | 46.59 | 1539.7100 | 13 | 0.1 | 770.8623 | 2 | 25.61 | 7 | F7:9178 | 21012022\_RID\_3262\_7a.raw |  |  |  |  |  |  | 9.9886E5 |  |  |  | 1 | 0 | 0 | 0 | 0 | 0 | 0 | 1 | 0 | 0 | 0 | 25 | 37 | Carbamidomethylation | C7:Carbamidomethylation:1000.00 | PEAKS DB |
| K.WDKDIM(+15.99)LIR.L | N | 44.48 | 1204.6274 | 9 | 0.7 | 603.3214 | 2 | 37.71 | 2 | F2:20647 | 13012022\_RID\_3262\_5a.raw | 1.4316E8 | 4.5531E6 |  |  |  |  |  |  |  |  | 2 | 1 | 1 | 0 | 0 | 0 | 0 | 0 | 0 | 0 | 0 | 109 | 117 | Oxidation (M) | M6:Oxidation (M):1000.00 | PEAKS DB |
| W.DKDIMLIR.L | N | 41.99 | 1002.5532 | 8 | 0.7 | 502.2842 | 2 | 34.59 | 7 | F7:15514 | 21012022\_RID\_3262\_7a.raw |  |  |  |  |  |  | 2.6461E6 |  |  |  | 1 | 0 | 0 | 0 | 0 | 0 | 0 | 1 | 0 | 0 | 0 | 110 | 117 |  |  | PEAKS DB |
| total 7 peptides |  |  |  |  |  |  |  |  |  |  |  |  |  |  |  |  |  |  |  |  |  |  |  |  |  |  |  |  |  |  |  |  |  |  |  |  |  |
| --- | --- | --- | --- | --- | --- | --- | --- | --- | --- | --- | --- | --- | --- | --- | --- | --- | --- | --- | --- | --- | --- | --- | --- | --- | --- | --- | --- | --- | --- | --- | --- | --- | --- | --- | --- | --- | --- |

AUS82565.1

back to list

  

| Protein Coverage
| Supporting Peptides
|

Protein Coverage:

Supporting Peptides:

| Peptide | Uniq | -10lgP | Mass | Length | ppm | m/z | z | RT | Fraction | Scan | Source File | Area F4 | Area F5 | Area F6 | Area F10 | Area F8 | Area F9 | Area F7 | Area F1 | Area F2 | Area F3 | #Feature | #Feature F4 | #Feature F5 | #Feature F6 | #Feature F10 | #Feature F8 | #Feature F9 | #Feature F7 | #Feature F1 | #Feature F2 | #Feature F3 | Start | End | PTM | AScore | Found By |
| --- | --- | --- | --- | --- | --- | --- | --- | --- | --- | --- | --- | --- | --- | --- | --- | --- | --- | --- | --- | --- | --- | --- | --- | --- | --- | --- | --- | --- | --- | --- | --- | --- | --- | --- | --- | --- | --- |
| K.NFQILFGVHSK.K | N | 62.43 | 1288.6927 | 11 | 0.4 | 645.3539 | 2 | 42.62 | 8 | F8:22782 | 31122021\_RID\_3262\_1a.raw | 1.2701E7 | 1.0353E6 | 2.5789E6 |  |  |  |  | 3.2275E6 | 1.1882E6 |  | 6 | 1 | 1 | 2 | 0 | 0 | 0 | 0 | 1 | 1 | 0 | 70 | 80 |  |  | PEAKS DB |
| R.IMGWGTISPTK.E | N | 47.73 | 1189.6166 | 11 | 1.3 | 595.8163 | 2 | 44.01 | 3 | F3:20987 | 13012022\_RID\_3262\_6a.raw |  | 2.3901E7 | 3.6021E5 |  |  |  |  |  |  |  | 2 | 0 | 1 | 1 | 0 | 0 | 0 | 0 | 0 | 0 | 0 | 144 | 154 |  |  | PEAKS DB |
| R.IM(+15.99)GWGTISPTK.E | N | 46.88 | 1205.6115 | 11 | 1.2 | 603.8137 | 2 | 37.85 | 2 | F2:20821 | 13012022\_RID\_3262\_5a.raw |  | 5.8537E6 |  |  |  |  |  |  |  |  | 1 | 0 | 1 | 0 | 0 | 0 | 0 | 0 | 0 | 0 | 0 | 144 | 154 | Oxidation (M) | M2:Oxidation (M):1000.00 | PEAKS DB |
| K.NFQILFGVHS.K | Y | 43.72 | 1160.5978 | 10 | -0.1 | 581.3061 | 2 | 55.26 | 8 | F8:32379 | 31122021\_RID\_3262\_1a.raw |  |  |  |  |  |  |  | 1.3612E5 |  |  | 1 | 0 | 0 | 0 | 0 | 0 | 0 | 0 | 1 | 0 | 0 | 70 | 79 |  |  | PEAKS DB |
| L.VVGGDEC(+57.02)NINEHR.S | N | 43.47 | 1497.6630 | 13 | 1.7 | 749.8400 | 2 | 21.08 | 7 | F7:6186 | 21012022\_RID\_3262\_7a.raw |  |  |  |  |  |  | 0 |  |  |  | 0 | 0 | 0 | 0 | 0 | 0 | 0 | 0 | 0 | 0 | 0 | 25 | 37 | Carbamidomethylation | C7:Carbamidomethylation:1000.00 | PEAKS DB |
| K.DKDIMLIR.L | N | 41.99 | 1002.5532 | 8 | 0.7 | 502.2842 | 2 | 34.59 | 7 | F7:15514 | 21012022\_RID\_3262\_7a.raw |  |  |  |  |  |  | 2.6461E6 |  |  |  | 1 | 0 | 0 | 0 | 0 | 0 | 0 | 1 | 0 | 0 | 0 | 108 | 115 |  |  | PEAKS DB |
| total 6 peptides |  |  |  |  |  |  |  |  |  |  |  |  |  |  |  |  |  |  |  |  |  |  |  |  |  |  |  |  |  |  |  |  |  |  |  |  |  |
| --- | --- | --- | --- | --- | --- | --- | --- | --- | --- | --- | --- | --- | --- | --- | --- | --- | --- | --- | --- | --- | --- | --- | --- | --- | --- | --- | --- | --- | --- | --- | --- | --- | --- | --- | --- | --- | --- |

XP\_015676332.1

back to list

  

| Protein Coverage
| Supporting Peptides
|

Protein Coverage:

Supporting Peptides:

| Peptide | Uniq | -10lgP | Mass | Length | ppm | m/z | z | RT | Fraction | Scan | Source File | Area F4 | Area F5 | Area F6 | Area F10 | Area F8 | Area F9 | Area F7 | Area F1 | Area F2 | Area F3 | #Feature | #Feature F4 | #Feature F5 | #Feature F6 | #Feature F10 | #Feature F8 | #Feature F9 | #Feature F7 | #Feature F1 | #Feature F2 | #Feature F3 | Start | End | PTM | AScore | Found By |
| --- | --- | --- | --- | --- | --- | --- | --- | --- | --- | --- | --- | --- | --- | --- | --- | --- | --- | --- | --- | --- | --- | --- | --- | --- | --- | --- | --- | --- | --- | --- | --- | --- | --- | --- | --- | --- | --- |
| K.IQEPYTFPALLK.S | Y | 61.98 | 1418.7809 | 12 | 0.5 | 710.3981 | 2 | 53.08 | 8 | F8:30647 | 31122021\_RID\_3262\_1a.raw |  |  |  |  |  |  |  | 1.3584E6 | 6.2212E4 |  | 2 | 0 | 0 | 0 | 0 | 0 | 0 | 0 | 1 | 1 | 0 | 118 | 129 |  |  | PEAKS DB |
| R.TLLSVDDLIEK.L | Y | 54.58 | 1244.6864 | 11 | 0.6 | 623.3508 | 2 | 52.37 | 8 | F8:30236 | 31122021\_RID\_3262\_1a.raw |  |  |  |  |  |  |  | 2.431E6 |  |  | 1 | 0 | 0 | 0 | 0 | 0 | 0 | 0 | 1 | 0 | 0 | 287 | 297 |  |  | PEAKS DB |
| K.IQEPYTFPALL.K | Y | 42.03 | 1290.6859 | 11 | 1.3 | 646.3511 | 2 | 69.27 | 8 | F8:43150 | 31122021\_RID\_3262\_1a.raw |  |  |  |  |  |  |  | 2.5791E4 |  |  | 1 | 0 | 0 | 0 | 0 | 0 | 0 | 0 | 1 | 0 | 0 | 118 | 128 |  |  | PEAKS DB |
| total 3 peptides |  |  |  |  |  |  |  |  |  |  |  |  |  |  |  |  |  |  |  |  |  |  |  |  |  |  |  |  |  |  |  |  |  |  |  |  |  |
| --- | --- | --- | --- | --- | --- | --- | --- | --- | --- | --- | --- | --- | --- | --- | --- | --- | --- | --- | --- | --- | --- | --- | --- | --- | --- | --- | --- | --- | --- | --- | --- | --- | --- | --- | --- | --- | --- |

LAA85609.1

back to list

  

| Protein Coverage
| Supporting Peptides
|

Protein Coverage:

Supporting Peptides:

| Peptide | Uniq | -10lgP | Mass | Length | ppm | m/z | z | RT | Fraction | Scan | Source File | Area F4 | Area F5 | Area F6 | Area F10 | Area F8 | Area F9 | Area F7 | Area F1 | Area F2 | Area F3 | #Feature | #Feature F4 | #Feature F5 | #Feature F6 | #Feature F10 | #Feature F8 | #Feature F9 | #Feature F7 | #Feature F1 | #Feature F2 | #Feature F3 | Start | End | PTM | AScore | Found By |
| --- | --- | --- | --- | --- | --- | --- | --- | --- | --- | --- | --- | --- | --- | --- | --- | --- | --- | --- | --- | --- | --- | --- | --- | --- | --- | --- | --- | --- | --- | --- | --- | --- | --- | --- | --- | --- | --- |
| K.IQEPYTFPALLK.S | Y | 61.98 | 1418.7809 | 12 | 0.5 | 710.3981 | 2 | 53.08 | 8 | F8:30647 | 31122021\_RID\_3262\_1a.raw |  |  |  |  |  |  |  | 1.3584E6 | 6.2212E4 |  | 2 | 0 | 0 | 0 | 0 | 0 | 0 | 0 | 1 | 1 | 0 | 118 | 129 |  |  | PEAKS DB |
| Q.TLLSVDDLIEK.L | Y | 54.58 | 1244.6864 | 11 | 0.6 | 623.3508 | 2 | 52.37 | 8 | F8:30236 | 31122021\_RID\_3262\_1a.raw |  |  |  |  |  |  |  | 2.431E6 |  |  | 1 | 0 | 0 | 0 | 0 | 0 | 0 | 0 | 1 | 0 | 0 | 287 | 297 |  |  | PEAKS DB |
| K.IQEPYTFPALL.K | Y | 42.03 | 1290.6859 | 11 | 1.3 | 646.3511 | 2 | 69.27 | 8 | F8:43150 | 31122021\_RID\_3262\_1a.raw |  |  |  |  |  |  |  | 2.5791E4 |  |  | 1 | 0 | 0 | 0 | 0 | 0 | 0 | 0 | 1 | 0 | 0 | 118 | 128 |  |  | PEAKS DB |
| total 3 peptides |  |  |  |  |  |  |  |  |  |  |  |  |  |  |  |  |  |  |  |  |  |  |  |  |  |  |  |  |  |  |  |  |  |  |  |  |  |
| --- | --- | --- | --- | --- | --- | --- | --- | --- | --- | --- | --- | --- | --- | --- | --- | --- | --- | --- | --- | --- | --- | --- | --- | --- | --- | --- | --- | --- | --- | --- | --- | --- | --- | --- | --- | --- | --- |

LAA85607.1

back to list

  

| Protein Coverage
| Supporting Peptides
|

Protein Coverage:

Supporting Peptides:

| Peptide | Uniq | -10lgP | Mass | Length | ppm | m/z | z | RT | Fraction | Scan | Source File | Area F4 | Area F5 | Area F6 | Area F10 | Area F8 | Area F9 | Area F7 | Area F1 | Area F2 | Area F3 | #Feature | #Feature F4 | #Feature F5 | #Feature F6 | #Feature F10 | #Feature F8 | #Feature F9 | #Feature F7 | #Feature F1 | #Feature F2 | #Feature F3 | Start | End | PTM | AScore | Found By |
| --- | --- | --- | --- | --- | --- | --- | --- | --- | --- | --- | --- | --- | --- | --- | --- | --- | --- | --- | --- | --- | --- | --- | --- | --- | --- | --- | --- | --- | --- | --- | --- | --- | --- | --- | --- | --- | --- |
| K.IQEPYTFPALLK.S | Y | 61.98 | 1418.7809 | 12 | 0.5 | 710.3981 | 2 | 53.08 | 8 | F8:30647 | 31122021\_RID\_3262\_1a.raw |  |  |  |  |  |  |  | 1.3584E6 | 6.2212E4 |  | 2 | 0 | 0 | 0 | 0 | 0 | 0 | 0 | 1 | 1 | 0 | 118 | 129 |  |  | PEAKS DB |
| Q.TLLSVDDLIEK.L | Y | 54.58 | 1244.6864 | 11 | 0.6 | 623.3508 | 2 | 52.37 | 8 | F8:30236 | 31122021\_RID\_3262\_1a.raw |  |  |  |  |  |  |  | 2.431E6 |  |  | 1 | 0 | 0 | 0 | 0 | 0 | 0 | 0 | 1 | 0 | 0 | 287 | 297 |  |  | PEAKS DB |
| K.IQEPYTFPALL.K | Y | 42.03 | 1290.6859 | 11 | 1.3 | 646.3511 | 2 | 69.27 | 8 | F8:43150 | 31122021\_RID\_3262\_1a.raw |  |  |  |  |  |  |  | 2.5791E4 |  |  | 1 | 0 | 0 | 0 | 0 | 0 | 0 | 0 | 1 | 0 | 0 | 118 | 128 |  |  | PEAKS DB |
| total 3 peptides |  |  |  |  |  |  |  |  |  |  |  |  |  |  |  |  |  |  |  |  |  |  |  |  |  |  |  |  |  |  |  |  |  |  |  |  |  |
| --- | --- | --- | --- | --- | --- | --- | --- | --- | --- | --- | --- | --- | --- | --- | --- | --- | --- | --- | --- | --- | --- | --- | --- | --- | --- | --- | --- | --- | --- | --- | --- | --- | --- | --- | --- | --- | --- |

XP\_007439537.1

back to list

  

| Protein Coverage
| Supporting Peptides
|

Protein Coverage:

Supporting Peptides:

| Peptide | Uniq | -10lgP | Mass | Length | ppm | m/z | z | RT | Fraction | Scan | Source File | Area F4 | Area F5 | Area F6 | Area F10 | Area F8 | Area F9 | Area F7 | Area F1 | Area F2 | Area F3 | #Feature | #Feature F4 | #Feature F5 | #Feature F6 | #Feature F10 | #Feature F8 | #Feature F9 | #Feature F7 | #Feature F1 | #Feature F2 | #Feature F3 | Start | End | PTM | AScore | Found By |
| --- | --- | --- | --- | --- | --- | --- | --- | --- | --- | --- | --- | --- | --- | --- | --- | --- | --- | --- | --- | --- | --- | --- | --- | --- | --- | --- | --- | --- | --- | --- | --- | --- | --- | --- | --- | --- | --- |
| Q.TVVYGITTGFGK.F | Y | 56.31 | 1241.6655 | 12 | 0.1 | 621.8401 | 2 | 42.93 | 8 | F8:22814 | 31122021\_RID\_3262\_1a.raw |  |  |  |  |  | 5.1027E5 |  | 2.5158E5 |  |  | 2 | 0 | 0 | 0 | 0 | 0 | 1 | 0 | 1 | 0 | 0 | 161 | 172 |  |  | PEAKS DB |
| R.FMAPDIEAAHR.L | Y | 52.02 | 1256.5972 | 11 | -0.3 | 419.8729 | 3 | 32.45 | 8 | F8:14310 | 31122021\_RID\_3262\_1a.raw |  |  |  |  |  |  |  | 7.5765E4 |  |  | 1 | 0 | 0 | 0 | 0 | 0 | 0 | 0 | 1 | 0 | 0 | 595 | 605 |  |  | PEAKS DB |
| K.LQELQVNLVR.S | Y | 51.42 | 1210.7034 | 10 | 0.8 | 606.3594 | 2 | 41.43 | 8 | F8:21563 | 31122021\_RID\_3262\_1a.raw |  |  |  |  |  |  |  | 5.5669E5 |  |  | 1 | 0 | 0 | 0 | 0 | 0 | 0 | 0 | 1 | 0 | 0 | 183 | 192 |  |  | PEAKS DB |
| S.LTTEDLVNLGK.G | Y | 44.45 | 1201.6554 | 11 | -0.1 | 601.8349 | 2 | 44.86 | 8 | F8:24204 | 31122021\_RID\_3262\_1a.raw |  |  |  |  |  |  |  | 3.2346E5 |  |  | 1 | 0 | 0 | 0 | 0 | 0 | 0 | 0 | 1 | 0 | 0 | 121 | 131 |  |  | PEAKS DB |
| R.FM(+15.99)APDIEAAHR.L | Y | 43.09 | 1272.5920 | 11 | -0.8 | 425.2043 | 3 | 31.69 | 4 | F4:17967 | 17012022\_RID\_3262\_10a .raw |  |  |  | 4.172E4 |  |  |  |  |  |  | 1 | 0 | 0 | 0 | 1 | 0 | 0 | 0 | 0 | 0 | 0 | 595 | 605 | Oxidation (M) | M2:Oxidation (M):1000.00 | PEAKS DB |
| total 5 peptides |  |  |  |  |  |  |  |  |  |  |  |  |  |  |  |  |  |  |  |  |  |  |  |  |  |  |  |  |  |  |  |  |  |  |  |  |  |
| --- | --- | --- | --- | --- | --- | --- | --- | --- | --- | --- | --- | --- | --- | --- | --- | --- | --- | --- | --- | --- | --- | --- | --- | --- | --- | --- | --- | --- | --- | --- | --- | --- | --- | --- | --- | --- | --- |

QBF53412.1

back to list

  

| Protein Coverage
| Supporting Peptides
|

Protein Coverage:

Supporting Peptides:

| Peptide | Uniq | -10lgP | Mass | Length | ppm | m/z | z | RT | Fraction | Scan | Source File | Area F4 | Area F5 | Area F6 | Area F10 | Area F8 | Area F9 | Area F7 | Area F1 | Area F2 | Area F3 | #Feature | #Feature F4 | #Feature F5 | #Feature F6 | #Feature F10 | #Feature F8 | #Feature F9 | #Feature F7 | #Feature F1 | #Feature F2 | #Feature F3 | Start | End | PTM | AScore | Found By |
| --- | --- | --- | --- | --- | --- | --- | --- | --- | --- | --- | --- | --- | --- | --- | --- | --- | --- | --- | --- | --- | --- | --- | --- | --- | --- | --- | --- | --- | --- | --- | --- | --- | --- | --- | --- | --- | --- |
| R.IM(+15.99)GWGTISSTK.V | N | 66.15 | 1195.5907 | 11 | -0.8 | 598.8021 | 2 | 35.74 | 7 | F7:16284 | 21012022\_RID\_3262\_7a.raw |  | 6.7622E5 | 1.701E6 |  |  | 4.1198E6 | 1.3032E7 |  |  |  | 6 | 0 | 1 | 1 | 0 | 0 | 1 | 3 | 0 | 0 | 0 | 143 | 153 | Oxidation (M) | M2:Oxidation (M):1000.00 | PEAKS DB |
| R.IMGWGTISSTK.V | N | 60.15 | 1179.5958 | 11 | 0.4 | 590.8054 | 2 | 42.00 | 3 | F3:19875 | 13012022\_RID\_3262\_6a.raw |  | 0 | 3.8883E6 |  |  | 1.7017E7 | 1.947E7 | 0 |  |  | 3 | 0 | 0 | 1 | 0 | 0 | 1 | 1 | 0 | 0 | 0 | 143 | 153 |  |  | PEAKS DB |
| M.WDKDIMLIR.L | N | 57.88 | 1188.6324 | 9 | 0.5 | 595.3238 | 2 | 43.59 | 1 | F1:28744 | 13012022\_RID\_3262\_4a.raw | 6.6853E8 | 1.3316E6 |  |  |  |  | 2.7704E4 | 1.1405E7 | 1.608E6 |  | 12 | 5 | 3 | 0 | 0 | 0 | 0 | 1 | 2 | 1 | 0 | 106 | 114 |  |  | PEAKS DB |
| R.C(+57.02)AGTLINQEWVLTAAH.C | Y | 53.51 | 1782.8723 | 16 | 1.1 | 892.4445 | 2 | 52.36 | 1 | F1:36235 | 13012022\_RID\_3262\_4a.raw | 1.0189E6 |  |  |  |  |  |  |  |  |  | 1 | 1 | 0 | 0 | 0 | 0 | 0 | 0 | 0 | 0 | 0 | 49 | 64 | Carbamidomethylation | C1:Carbamidomethylation:1000.00 | PEAKS DB |
| M.WDKDIM(+15.99)LIR.L | N | 44.48 | 1204.6274 | 9 | 0.7 | 603.3214 | 2 | 37.71 | 2 | F2:20647 | 13012022\_RID\_3262\_5a.raw | 1.4316E8 | 4.5531E6 |  |  |  |  |  |  |  |  | 2 | 1 | 1 | 0 | 0 | 0 | 0 | 0 | 0 | 0 | 0 | 106 | 114 | Oxidation (M) | M6:Oxidation (M):1000.00 | PEAKS DB |
| W.DKDIMLIR.L | N | 41.99 | 1002.5532 | 8 | 0.7 | 502.2842 | 2 | 34.59 | 7 | F7:15514 | 21012022\_RID\_3262\_7a.raw |  |  |  |  |  |  | 2.6461E6 |  |  |  | 1 | 0 | 0 | 0 | 0 | 0 | 0 | 1 | 0 | 0 | 0 | 107 | 114 |  |  | PEAKS DB |
| total 6 peptides |  |  |  |  |  |  |  |  |  |  |  |  |  |  |  |  |  |  |  |  |  |  |  |  |  |  |  |  |  |  |  |  |  |  |  |  |  |
| --- | --- | --- | --- | --- | --- | --- | --- | --- | --- | --- | --- | --- | --- | --- | --- | --- | --- | --- | --- | --- | --- | --- | --- | --- | --- | --- | --- | --- | --- | --- | --- | --- | --- | --- | --- | --- | --- |

BAA20283.1

back to list

  

| Protein Coverage
| Supporting Peptides
|

Protein Coverage:

Supporting Peptides:

| Peptide | Uniq | -10lgP | Mass | Length | ppm | m/z | z | RT | Fraction | Scan | Source File | Area F4 | Area F5 | Area F6 | Area F10 | Area F8 | Area F9 | Area F7 | Area F1 | Area F2 | Area F3 | #Feature | #Feature F4 | #Feature F5 | #Feature F6 | #Feature F10 | #Feature F8 | #Feature F9 | #Feature F7 | #Feature F1 | #Feature F2 | #Feature F3 | Start | End | PTM | AScore | Found By |
| --- | --- | --- | --- | --- | --- | --- | --- | --- | --- | --- | --- | --- | --- | --- | --- | --- | --- | --- | --- | --- | --- | --- | --- | --- | --- | --- | --- | --- | --- | --- | --- | --- | --- | --- | --- | --- | --- |
| K.WDKDIMLIR.L | N | 57.88 | 1188.6324 | 9 | 0.5 | 595.3238 | 2 | 43.59 | 1 | F1:28744 | 13012022\_RID\_3262\_4a.raw | 6.6853E8 | 1.3316E6 |  |  |  |  | 2.7704E4 | 1.1405E7 | 1.608E6 |  | 12 | 5 | 3 | 0 | 0 | 0 | 0 | 1 | 2 | 1 | 0 | 106 | 114 |  |  | PEAKS DB |
| L.EPALYTK.V | N | 56.50 | 820.4330 | 7 | 0.3 | 411.2239 | 2 | 24.64 | 1 | F1:11686 | 13012022\_RID\_3262\_4a.raw | 6.8741E6 |  |  |  |  | 1.0108E6 |  |  |  |  | 2 | 1 | 0 | 0 | 0 | 0 | 1 | 0 | 0 | 0 | 0 | 229 | 235 |  |  | PEAKS DB |
| R.IMGWGTISTSK.V | Y | 56.38 | 1179.5958 | 11 | 0.6 | 590.8055 | 2 | 42.13 | 7 | F7:20534 | 21012022\_RID\_3262\_7a.raw |  |  | 3.8883E6 |  |  |  | 1.947E7 |  | 2.6616E5 |  | 3 | 0 | 0 | 1 | 0 | 0 | 0 | 1 | 0 | 1 | 0 | 143 | 153 |  |  | PEAKS DB |
| R.IM(+15.99)GWGTISTSK.V | Y | 49.22 | 1195.5907 | 11 | -0.1 | 598.8026 | 2 | 33.85 | 8 | F8:15466 | 31122021\_RID\_3262\_1a.raw |  |  |  |  |  |  | 5.9332E6 | 0 |  |  | 1 | 0 | 0 | 0 | 0 | 0 | 0 | 1 | 0 | 0 | 0 | 143 | 153 | Oxidation (M) | M2:Oxidation (M):1000.00 | PEAKS DB |
| K.WDKDIM(+15.99)LIR.L | N | 44.48 | 1204.6274 | 9 | 0.7 | 603.3214 | 2 | 37.71 | 2 | F2:20647 | 13012022\_RID\_3262\_5a.raw | 1.4316E8 | 4.5531E6 |  |  |  |  |  |  |  |  | 2 | 1 | 1 | 0 | 0 | 0 | 0 | 0 | 0 | 0 | 0 | 106 | 114 | Oxidation (M) | M6:Oxidation (M):1000.00 | PEAKS DB |
| W.DKDIMLIR.L | N | 41.99 | 1002.5532 | 8 | 0.7 | 502.2842 | 2 | 34.59 | 7 | F7:15514 | 21012022\_RID\_3262\_7a.raw |  |  |  |  |  |  | 2.6461E6 |  |  |  | 1 | 0 | 0 | 0 | 0 | 0 | 0 | 1 | 0 | 0 | 0 | 107 | 114 |  |  | PEAKS DB |
| total 6 peptides |  |  |  |  |  |  |  |  |  |  |  |  |  |  |  |  |  |  |  |  |  |  |  |  |  |  |  |  |  |  |  |  |  |  |  |  |  |
| --- | --- | --- | --- | --- | --- | --- | --- | --- | --- | --- | --- | --- | --- | --- | --- | --- | --- | --- | --- | --- | --- | --- | --- | --- | --- | --- | --- | --- | --- | --- | --- | --- | --- | --- | --- | --- | --- |

O13069.1

back to list

  

| Protein Coverage
| Supporting Peptides
|

Protein Coverage:

Supporting Peptides:

| Peptide | Uniq | -10lgP | Mass | Length | ppm | m/z | z | RT | Fraction | Scan | Source File | Area F4 | Area F5 | Area F6 | Area F10 | Area F8 | Area F9 | Area F7 | Area F1 | Area F2 | Area F3 | #Feature | #Feature F4 | #Feature F5 | #Feature F6 | #Feature F10 | #Feature F8 | #Feature F9 | #Feature F7 | #Feature F1 | #Feature F2 | #Feature F3 | Start | End | PTM | AScore | Found By |
| --- | --- | --- | --- | --- | --- | --- | --- | --- | --- | --- | --- | --- | --- | --- | --- | --- | --- | --- | --- | --- | --- | --- | --- | --- | --- | --- | --- | --- | --- | --- | --- | --- | --- | --- | --- | --- | --- |
| K.WDKDIMLIR.L | N | 57.88 | 1188.6324 | 9 | 0.5 | 595.3238 | 2 | 43.59 | 1 | F1:28744 | 13012022\_RID\_3262\_4a.raw | 6.6853E8 | 1.3316E6 |  |  |  |  | 2.7704E4 | 1.1405E7 | 1.608E6 |  | 12 | 5 | 3 | 0 | 0 | 0 | 0 | 1 | 2 | 1 | 0 | 106 | 114 |  |  | PEAKS DB |
| L.EPALYTK.V | N | 56.50 | 820.4330 | 7 | 0.3 | 411.2239 | 2 | 24.64 | 1 | F1:11686 | 13012022\_RID\_3262\_4a.raw | 6.8741E6 |  |  |  |  | 1.0108E6 |  |  |  |  | 2 | 1 | 0 | 0 | 0 | 0 | 1 | 0 | 0 | 0 | 0 | 229 | 235 |  |  | PEAKS DB |
| R.IMGWGTISTSK.V | Y | 56.38 | 1179.5958 | 11 | 0.6 | 590.8055 | 2 | 42.13 | 7 | F7:20534 | 21012022\_RID\_3262\_7a.raw |  |  | 3.8883E6 |  |  |  | 1.947E7 |  | 2.6616E5 |  | 3 | 0 | 0 | 1 | 0 | 0 | 0 | 1 | 0 | 1 | 0 | 143 | 153 |  |  | PEAKS DB |
| R.IM(+15.99)GWGTISTSK.V | Y | 49.22 | 1195.5907 | 11 | -0.1 | 598.8026 | 2 | 33.85 | 8 | F8:15466 | 31122021\_RID\_3262\_1a.raw |  |  |  |  |  |  | 5.9332E6 | 0 |  |  | 1 | 0 | 0 | 0 | 0 | 0 | 0 | 1 | 0 | 0 | 0 | 143 | 153 | Oxidation (M) | M2:Oxidation (M):1000.00 | PEAKS DB |
| K.WDKDIM(+15.99)LIR.L | N | 44.48 | 1204.6274 | 9 | 0.7 | 603.3214 | 2 | 37.71 | 2 | F2:20647 | 13012022\_RID\_3262\_5a.raw | 1.4316E8 | 4.5531E6 |  |  |  |  |  |  |  |  | 2 | 1 | 1 | 0 | 0 | 0 | 0 | 0 | 0 | 0 | 0 | 106 | 114 | Oxidation (M) | M6:Oxidation (M):1000.00 | PEAKS DB |
| W.DKDIMLIR.L | N | 41.99 | 1002.5532 | 8 | 0.7 | 502.2842 | 2 | 34.59 | 7 | F7:15514 | 21012022\_RID\_3262\_7a.raw |  |  |  |  |  |  | 2.6461E6 |  |  |  | 1 | 0 | 0 | 0 | 0 | 0 | 0 | 1 | 0 | 0 | 0 | 107 | 114 |  |  | PEAKS DB |
| total 6 peptides |  |  |  |  |  |  |  |  |  |  |  |  |  |  |  |  |  |  |  |  |  |  |  |  |  |  |  |  |  |  |  |  |  |  |  |  |  |
| --- | --- | --- | --- | --- | --- | --- | --- | --- | --- | --- | --- | --- | --- | --- | --- | --- | --- | --- | --- | --- | --- | --- | --- | --- | --- | --- | --- | --- | --- | --- | --- | --- | --- | --- | --- | --- | --- |

ETE66900.1

back to list

  

| Protein Coverage
| Supporting Peptides
|

Protein Coverage:

Supporting Peptides:

| Peptide | Uniq | -10lgP | Mass | Length | ppm | m/z | z | RT | Fraction | Scan | Source File | Area F4 | Area F5 | Area F6 | Area F10 | Area F8 | Area F9 | Area F7 | Area F1 | Area F2 | Area F3 | #Feature | #Feature F4 | #Feature F5 | #Feature F6 | #Feature F10 | #Feature F8 | #Feature F9 | #Feature F7 | #Feature F1 | #Feature F2 | #Feature F3 | Start | End | PTM | AScore | Found By |
| --- | --- | --- | --- | --- | --- | --- | --- | --- | --- | --- | --- | --- | --- | --- | --- | --- | --- | --- | --- | --- | --- | --- | --- | --- | --- | --- | --- | --- | --- | --- | --- | --- | --- | --- | --- | --- | --- |
| K.VAPFFDNVM(+15.99)EQGLLEK.N | N | 63.84 | 1851.9076 | 16 | 1.0 | 926.9620 | 2 | 52.44 | 1 | F1:36309 | 13012022\_RID\_3262\_4a.raw | 6.0231E5 |  |  |  |  |  |  |  |  |  | 1 | 1 | 0 | 0 | 0 | 0 | 0 | 0 | 0 | 0 | 0 | 82 | 97 | Oxidation (M) | M9:Oxidation (M):1000.00 | PEAKS DB |
| K.QPGITFIAAK.F | N | 51.81 | 1044.5967 | 10 | 0.6 | 523.3059 | 2 | 39.27 | 1 | F1:24810 | 13012022\_RID\_3262\_4a.raw | 7.5032E5 |  |  |  |  |  |  |  |  |  | 1 | 1 | 0 | 0 | 0 | 0 | 0 | 0 | 0 | 0 | 0 | 56 | 65 |  |  | PEAKS DB |
| K.NQLFGEATKQPGITFIAAK.F | N | 47.63 | 2033.0945 | 19 | 0.4 | 678.7057 | 3 | 45.22 | 1 | F1:30099 | 13012022\_RID\_3262\_4a.raw | 5.5813E5 |  |  |  |  |  |  |  |  |  | 1 | 1 | 0 | 0 | 0 | 0 | 0 | 0 | 0 | 0 | 0 | 47 | 65 |  |  | PEAKS DB |
| K.FDGILGMAYPK.I | Y | 41.69 | 1210.6056 | 11 | 0.9 | 606.3106 | 2 | 51.28 | 1 | F1:35317 | 13012022\_RID\_3262\_4a.raw | 1.0944E6 |  |  |  |  |  |  |  |  |  | 1 | 1 | 0 | 0 | 0 | 0 | 0 | 0 | 0 | 0 | 0 | 66 | 76 |  |  | PEAKS DB |
| total 4 peptides |  |  |  |  |  |  |  |  |  |  |  |  |  |  |  |  |  |  |  |  |  |  |  |  |  |  |  |  |  |  |  |  |  |  |  |  |  |
| --- | --- | --- | --- | --- | --- | --- | --- | --- | --- | --- | --- | --- | --- | --- | --- | --- | --- | --- | --- | --- | --- | --- | --- | --- | --- | --- | --- | --- | --- | --- | --- | --- | --- | --- | --- | --- | --- |

ACZ34293.1

back to list

  

| Protein Coverage
| Supporting Peptides
|

Protein Coverage:

Supporting Peptides:

| Peptide | Uniq | -10lgP | Mass | Length | ppm | m/z | z | RT | Fraction | Scan | Source File | Area F4 | Area F5 | Area F6 | Area F10 | Area F8 | Area F9 | Area F7 | Area F1 | Area F2 | Area F3 | #Feature | #Feature F4 | #Feature F5 | #Feature F6 | #Feature F10 | #Feature F8 | #Feature F9 | #Feature F7 | #Feature F1 | #Feature F2 | #Feature F3 | Start | End | PTM | AScore | Found By |
| --- | --- | --- | --- | --- | --- | --- | --- | --- | --- | --- | --- | --- | --- | --- | --- | --- | --- | --- | --- | --- | --- | --- | --- | --- | --- | --- | --- | --- | --- | --- | --- | --- | --- | --- | --- | --- | --- |
| R.EAVFVAQLLSENIK.A | Y | 86.29 | 1559.8558 | 14 | 0.5 | 780.9355 | 2 | 58.76 | 6 | F6:42485 | 17012022\_RID\_3262\_9a.raw | 2.5025E6 | 4.3527E5 | 2.1003E5 | 7.7427E7 |  | 3.7591E7 |  | 4.8101E6 | 2.1619E6 |  | 13 | 1 | 1 | 1 | 6 | 0 | 2 | 0 | 1 | 1 | 0 | 70 | 83 |  |  | PEAKS DB |
| total 1 peptides |  |  |  |  |  |  |  |  |  |  |  |  |  |  |  |  |  |  |  |  |  |  |  |  |  |  |  |  |  |  |  |  |  |  |  |  |  |
| --- | --- | --- | --- | --- | --- | --- | --- | --- | --- | --- | --- | --- | --- | --- | --- | --- | --- | --- | --- | --- | --- | --- | --- | --- | --- | --- | --- | --- | --- | --- | --- | --- | --- | --- | --- | --- | --- |

D1MGU0.1

back to list

  

| Protein Coverage
| Supporting Peptides
|

Protein Coverage:

Supporting Peptides:

| Peptide | Uniq | -10lgP | Mass | Length | ppm | m/z | z | RT | Fraction | Scan | Source File | Area F4 | Area F5 | Area F6 | Area F10 | Area F8 | Area F9 | Area F7 | Area F1 | Area F2 | Area F3 | #Feature | #Feature F4 | #Feature F5 | #Feature F6 | #Feature F10 | #Feature F8 | #Feature F9 | #Feature F7 | #Feature F1 | #Feature F2 | #Feature F3 | Start | End | PTM | AScore | Found By |
| --- | --- | --- | --- | --- | --- | --- | --- | --- | --- | --- | --- | --- | --- | --- | --- | --- | --- | --- | --- | --- | --- | --- | --- | --- | --- | --- | --- | --- | --- | --- | --- | --- | --- | --- | --- | --- | --- |
| R.EAVFVAQLLSENIK.A | Y | 86.29 | 1559.8558 | 14 | 0.5 | 780.9355 | 2 | 58.76 | 6 | F6:42485 | 17012022\_RID\_3262\_9a.raw | 2.5025E6 | 4.3527E5 | 2.1003E5 | 7.7427E7 |  | 3.7591E7 |  | 4.8101E6 | 2.1619E6 |  | 13 | 1 | 1 | 1 | 6 | 0 | 2 | 0 | 1 | 1 | 0 | 70 | 83 |  |  | PEAKS DB |
| total 1 peptides |  |  |  |  |  |  |  |  |  |  |  |  |  |  |  |  |  |  |  |  |  |  |  |  |  |  |  |  |  |  |  |  |  |  |  |  |  |
| --- | --- | --- | --- | --- | --- | --- | --- | --- | --- | --- | --- | --- | --- | --- | --- | --- | --- | --- | --- | --- | --- | --- | --- | --- | --- | --- | --- | --- | --- | --- | --- | --- | --- | --- | --- | --- | --- |

BAP39913.1

back to list

  

| Protein Coverage
| Supporting Peptides
|

Protein Coverage:

Supporting Peptides:

| Peptide | Uniq | -10lgP | Mass | Length | ppm | m/z | z | RT | Fraction | Scan | Source File | Area F4 | Area F5 | Area F6 | Area F10 | Area F8 | Area F9 | Area F7 | Area F1 | Area F2 | Area F3 | #Feature | #Feature F4 | #Feature F5 | #Feature F6 | #Feature F10 | #Feature F8 | #Feature F9 | #Feature F7 | #Feature F1 | #Feature F2 | #Feature F3 | Start | End | PTM | AScore | Found By |
| --- | --- | --- | --- | --- | --- | --- | --- | --- | --- | --- | --- | --- | --- | --- | --- | --- | --- | --- | --- | --- | --- | --- | --- | --- | --- | --- | --- | --- | --- | --- | --- | --- | --- | --- | --- | --- | --- |
| V.YSPYDEDKGMVLPGTK.C | Y | 74.75 | 1798.8447 | 16 | 0.4 | 900.4300 | 2 | 37.68 | 1 | F1:23481 | 13012022\_RID\_3262\_4a.raw | 1.3379E9 | 9.1997E6 |  |  |  |  | 2.7801E6 | 8.2147E6 | 6.7646E5 | 7.0215E6 | 10 | 2 | 2 | 0 | 0 | 0 | 0 | 2 | 2 | 1 | 1 | 135 | 150 |  |  | PEAKS DB |
| V.YSPYDEDKGM(+15.99)VLPGTK.C | Y | 58.35 | 1814.8396 | 16 | -0.4 | 908.4268 | 2 | 32.59 | 1 | F1:18715 | 13012022\_RID\_3262\_4a.raw | 1.4258E9 | 1.1614E7 | 1.8361E6 | 2.5601E6 |  |  | 5.8087E6 | 2.8708E6 | 1.918E6 |  | 11 | 4 | 1 | 1 | 1 | 0 | 0 | 2 | 1 | 1 | 0 | 135 | 150 | Oxidation (M) | M10:Oxidation (M):1000.00 | PEAKS DB |
| K.GM(+15.99)VLPGTK.C | N | 47.53 | 817.4368 | 8 | -3.8 | 409.7241 | 2 | 30.58 | 2 | F2:15077 | 13012022\_RID\_3262\_5a.raw | 6.0504E7 | 3.227E6 | 7.6355E5 |  |  |  | 3.1499E5 |  |  |  | 7 | 3 | 2 | 1 | 0 | 0 | 0 | 1 | 0 | 0 | 0 | 143 | 150 | Oxidation (M) | M2:Oxidation (M):1000.00 | PEAKS DB |
| K.GMVLPGTK.C | N | 47.31 | 801.4418 | 8 | 0.3 | 401.7283 | 2 | 30.97 | 2 | F2:15254 | 13012022\_RID\_3262\_5a.raw | 1.0637E8 | 5.4594E6 | 1.626E6 |  |  |  | 1.5224E6 | 2.1234E6 |  |  | 5 | 1 | 1 | 1 | 0 | 0 | 0 | 1 | 1 | 0 | 0 | 143 | 150 |  |  | PEAKS DB |
| Y.SPYDEDKGM(+15.99)VLPGTK.C | Y | 44.87 | 1651.7762 | 15 | 0.1 | 826.8955 | 2 | 29.43 | 1 | F1:15924 | 13012022\_RID\_3262\_4a.raw | 2.9977E5 |  |  |  |  |  |  |  |  |  | 1 | 1 | 0 | 0 | 0 | 0 | 0 | 0 | 0 | 0 | 0 | 136 | 150 | Oxidation (M) | M9:Oxidation (M):1000.00 | PEAKS DB |
| Y.SPYDEDKGMVLPGTK.C | Y | 43.62 | 1635.7814 | 15 | 0.2 | 818.8981 | 2 | 34.72 | 1 | F1:20729 | 13012022\_RID\_3262\_4a.raw | 3.3033E5 |  |  |  |  |  |  |  |  |  | 1 | 1 | 0 | 0 | 0 | 0 | 0 | 0 | 0 | 0 | 0 | 136 | 150 |  |  | PEAKS DB |
| total 6 peptides |  |  |  |  |  |  |  |  |  |  |  |  |  |  |  |  |  |  |  |  |  |  |  |  |  |  |  |  |  |  |  |  |  |  |  |  |  |
| --- | --- | --- | --- | --- | --- | --- | --- | --- | --- | --- | --- | --- | --- | --- | --- | --- | --- | --- | --- | --- | --- | --- | --- | --- | --- | --- | --- | --- | --- | --- | --- | --- | --- | --- | --- | --- | --- |

P0DM87.1

back to list

  

| Protein Coverage
| Supporting Peptides
|

Protein Coverage:

Supporting Peptides:

| Peptide | Uniq | -10lgP | Mass | Length | ppm | m/z | z | RT | Fraction | Scan | Source File | Area F4 | Area F5 | Area F6 | Area F10 | Area F8 | Area F9 | Area F7 | Area F1 | Area F2 | Area F3 | #Feature | #Feature F4 | #Feature F5 | #Feature F6 | #Feature F10 | #Feature F8 | #Feature F9 | #Feature F7 | #Feature F1 | #Feature F2 | #Feature F3 | Start | End | PTM | AScore | Found By |
| --- | --- | --- | --- | --- | --- | --- | --- | --- | --- | --- | --- | --- | --- | --- | --- | --- | --- | --- | --- | --- | --- | --- | --- | --- | --- | --- | --- | --- | --- | --- | --- | --- | --- | --- | --- | --- | --- |
| R.MYTKYDGDETEISSK.I | Y | 81.40 | 1765.7716 | 15 | 0.1 | 883.8932 | 2 | 27.67 | 1 | F1:14517 | 13012022\_RID\_3262\_4a.raw | 8.6196E7 | 1.8962E6 |  |  |  |  | 6.0765E5 | 8.5103E4 |  |  | 9 | 4 | 2 | 0 | 0 | 0 | 0 | 2 | 1 | 0 | 0 | 206 | 220 |  |  | PEAKS DB |
| R.M(+15.99)YTKYDGDETEISSK.I | Y | 66.24 | 1781.7665 | 15 | 1.5 | 891.8918 | 2 | 27.68 | 1 | F1:14409 | 13012022\_RID\_3262\_4a.raw | 1.3792E7 | 3.4653E4 |  |  |  |  |  |  |  |  | 6 | 5 | 1 | 0 | 0 | 0 | 0 | 0 | 0 | 0 | 0 | 206 | 220 | Oxidation (M) | M1:Oxidation (M):1000.00 | PEAKS DB |
| K.YDGDETEISSK.I | Y | 59.69 | 1242.5251 | 11 | 0.2 | 622.2700 | 2 | 24.12 | 2 | F2:10012 | 13012022\_RID\_3262\_5a.raw | 3.1945E7 | 2.9516E6 | 9.5314E5 |  |  |  | 1.2557E6 | 3.5297E5 | 2.0418E4 |  | 8 | 3 | 1 | 1 | 0 | 0 | 0 | 1 | 1 | 1 | 0 | 210 | 220 |  |  | PEAKS DB |
| total 3 peptides |  |  |  |  |  |  |  |  |  |  |  |  |  |  |  |  |  |  |  |  |  |  |  |  |  |  |  |  |  |  |  |  |  |  |  |  |  |
| --- | --- | --- | --- | --- | --- | --- | --- | --- | --- | --- | --- | --- | --- | --- | --- | --- | --- | --- | --- | --- | --- | --- | --- | --- | --- | --- | --- | --- | --- | --- | --- | --- | --- | --- | --- | --- | --- |

AAF91498.1

back to list

  

| Protein Coverage
| Supporting Peptides
|

Protein Coverage:

Supporting Peptides:

| Peptide | Uniq | -10lgP | Mass | Length | ppm | m/z | z | RT | Fraction | Scan | Source File | Area F4 | Area F5 | Area F6 | Area F10 | Area F8 | Area F9 | Area F7 | Area F1 | Area F2 | Area F3 | #Feature | #Feature F4 | #Feature F5 | #Feature F6 | #Feature F10 | #Feature F8 | #Feature F9 | #Feature F7 | #Feature F1 | #Feature F2 | #Feature F3 | Start | End | PTM | AScore | Found By |
| --- | --- | --- | --- | --- | --- | --- | --- | --- | --- | --- | --- | --- | --- | --- | --- | --- | --- | --- | --- | --- | --- | --- | --- | --- | --- | --- | --- | --- | --- | --- | --- | --- | --- | --- | --- | --- | --- |
| S.GGQGRPQDASDR.C | N | 55.16 | 1242.5701 | 12 | 0.2 | 415.1974 | 3 | 11.60 | 6 | F6:2151 | 17012022\_RID\_3262\_9a.raw |  |  |  |  |  | 9.079E4 |  |  |  |  | 1 | 0 | 0 | 0 | 0 | 0 | 1 | 0 | 0 | 0 | 0 | 31 | 42 |  |  | PEAKS DB |
| R.DNKDTYDNK.Y | Y | 49.19 | 1111.4781 | 9 | 0.3 | 556.7465 | 2 | 11.63 | 6 | F6:2179 | 17012022\_RID\_3262\_9a.raw |  |  |  |  |  | 8.8117E4 |  |  |  |  | 1 | 0 | 0 | 0 | 0 | 0 | 1 | 0 | 0 | 0 | 0 | 98 | 106 |  |  | PEAKS DB |
| R.VAAVC(+57.02)FRDNKDTYDNK.Y | Y | 44.03 | 1914.8894 | 16 | -0.5 | 639.3034 | 3 | 26.66 | 6 | F6:13873 | 17012022\_RID\_3262\_9a.raw |  |  |  |  |  | 7.6234E6 |  |  |  |  | 1 | 0 | 0 | 0 | 0 | 0 | 1 | 0 | 0 | 0 | 0 | 91 | 106 | Carbamidomethylation | C5:Carbamidomethylation:1000.00 | PEAKS DB |
| R.DNKDTYDNKY.W | Y | 43.49 | 1274.5415 | 10 | 0.0 | 638.2780 | 2 | 20.02 | 6 | F6:7662 | 17012022\_RID\_3262\_9a.raw |  |  |  |  |  | 6.5547E6 |  |  |  |  | 1 | 0 | 0 | 0 | 0 | 0 | 1 | 0 | 0 | 0 | 0 | 98 | 107 |  |  | PEAKS DB |
| total 4 peptides |  |  |  |  |  |  |  |  |  |  |  |  |  |  |  |  |  |  |  |  |  |  |  |  |  |  |  |  |  |  |  |  |  |  |  |  |  |
| --- | --- | --- | --- | --- | --- | --- | --- | --- | --- | --- | --- | --- | --- | --- | --- | --- | --- | --- | --- | --- | --- | --- | --- | --- | --- | --- | --- | --- | --- | --- | --- | --- | --- | --- | --- | --- | --- |

Q9I8F8.1

back to list

  

| Protein Coverage
| Supporting Peptides
|

Protein Coverage:

Supporting Peptides:

| Peptide | Uniq | -10lgP | Mass | Length | ppm | m/z | z | RT | Fraction | Scan | Source File | Area F4 | Area F5 | Area F6 | Area F10 | Area F8 | Area F9 | Area F7 | Area F1 | Area F2 | Area F3 | #Feature | #Feature F4 | #Feature F5 | #Feature F6 | #Feature F10 | #Feature F8 | #Feature F9 | #Feature F7 | #Feature F1 | #Feature F2 | #Feature F3 | Start | End | PTM | AScore | Found By |
| --- | --- | --- | --- | --- | --- | --- | --- | --- | --- | --- | --- | --- | --- | --- | --- | --- | --- | --- | --- | --- | --- | --- | --- | --- | --- | --- | --- | --- | --- | --- | --- | --- | --- | --- | --- | --- | --- |
| S.GGQGRPQDASDR.C | N | 55.16 | 1242.5701 | 12 | 0.2 | 415.1974 | 3 | 11.60 | 6 | F6:2151 | 17012022\_RID\_3262\_9a.raw |  |  |  |  |  | 9.079E4 |  |  |  |  | 1 | 0 | 0 | 0 | 0 | 0 | 1 | 0 | 0 | 0 | 0 | 31 | 42 |  |  | PEAKS DB |
| R.DNKDTYDNK.Y | Y | 49.19 | 1111.4781 | 9 | 0.3 | 556.7465 | 2 | 11.63 | 6 | F6:2179 | 17012022\_RID\_3262\_9a.raw |  |  |  |  |  | 8.8117E4 |  |  |  |  | 1 | 0 | 0 | 0 | 0 | 0 | 1 | 0 | 0 | 0 | 0 | 98 | 106 |  |  | PEAKS DB |
| R.VAAVC(+57.02)FRDNKDTYDNK.Y | Y | 44.03 | 1914.8894 | 16 | -0.5 | 639.3034 | 3 | 26.66 | 6 | F6:13873 | 17012022\_RID\_3262\_9a.raw |  |  |  |  |  | 7.6234E6 |  |  |  |  | 1 | 0 | 0 | 0 | 0 | 0 | 1 | 0 | 0 | 0 | 0 | 91 | 106 | Carbamidomethylation | C5:Carbamidomethylation:1000.00 | PEAKS DB |
| R.DNKDTYDNKY.W | Y | 43.49 | 1274.5415 | 10 | 0.0 | 638.2780 | 2 | 20.02 | 6 | F6:7662 | 17012022\_RID\_3262\_9a.raw |  |  |  |  |  | 6.5547E6 |  |  |  |  | 1 | 0 | 0 | 0 | 0 | 0 | 1 | 0 | 0 | 0 | 0 | 98 | 107 |  |  | PEAKS DB |
| total 4 peptides |  |  |  |  |  |  |  |  |  |  |  |  |  |  |  |  |  |  |  |  |  |  |  |  |  |  |  |  |  |  |  |  |  |  |  |  |  |
| --- | --- | --- | --- | --- | --- | --- | --- | --- | --- | --- | --- | --- | --- | --- | --- | --- | --- | --- | --- | --- | --- | --- | --- | --- | --- | --- | --- | --- | --- | --- | --- | --- | --- | --- | --- | --- | --- |

XP\_015680726.1

back to list

  

| Protein Coverage
| Supporting Peptides
|

Protein Coverage:

Supporting Peptides:

| Peptide | Uniq | -10lgP | Mass | Length | ppm | m/z | z | RT | Fraction | Scan | Source File | Area F4 | Area F5 | Area F6 | Area F10 | Area F8 | Area F9 | Area F7 | Area F1 | Area F2 | Area F3 | #Feature | #Feature F4 | #Feature F5 | #Feature F6 | #Feature F10 | #Feature F8 | #Feature F9 | #Feature F7 | #Feature F1 | #Feature F2 | #Feature F3 | Start | End | PTM | AScore | Found By |
| --- | --- | --- | --- | --- | --- | --- | --- | --- | --- | --- | --- | --- | --- | --- | --- | --- | --- | --- | --- | --- | --- | --- | --- | --- | --- | --- | --- | --- | --- | --- | --- | --- | --- | --- | --- | --- | --- |
| K.AYTSIVYGNGPGYQITDK.G | Y | 72.40 | 1945.9421 | 18 | 0.7 | 973.9790 | 2 | 43.07 | 9 | F9:24061 | 31122021\_RID\_3262\_2a.raw |  |  |  |  |  |  |  |  | 1.9913E6 |  | 1 | 0 | 0 | 0 | 0 | 0 | 0 | 0 | 0 | 1 | 0 | 11 | 28 |  |  | PEAKS DB |
| N.GPGYQITDK.G | Y | 46.27 | 977.4818 | 9 | 0.1 | 489.7482 | 2 | 23.04 | 9 | F9:7580 | 31122021\_RID\_3262\_2a.raw |  |  |  |  |  |  |  |  | 5.2477E5 |  | 1 | 0 | 0 | 0 | 0 | 0 | 0 | 0 | 0 | 1 | 0 | 20 | 28 |  |  | PEAKS DB |
| K.GPMAHLFH.G | N | 42.64 | 908.4327 | 8 | 0.6 | 455.2239 | 2 | 28.62 | 9 | F9:12184 | 31122021\_RID\_3262\_2a.raw |  |  |  |  |  |  |  |  | 2.3063E4 |  | 1 | 0 | 0 | 0 | 0 | 0 | 0 | 0 | 0 | 1 | 0 | 67 | 74 |  |  | PEAKS DB |
| total 3 peptides |  |  |  |  |  |  |  |  |  |  |  |  |  |  |  |  |  |  |  |  |  |  |  |  |  |  |  |  |  |  |  |  |  |  |  |  |  |
| --- | --- | --- | --- | --- | --- | --- | --- | --- | --- | --- | --- | --- | --- | --- | --- | --- | --- | --- | --- | --- | --- | --- | --- | --- | --- | --- | --- | --- | --- | --- | --- | --- | --- | --- | --- | --- | --- |

XP\_032069201.1

back to list

  

| Protein Coverage
| Supporting Peptides
|

Protein Coverage:

Supporting Peptides:

| Peptide | Uniq | -10lgP | Mass | Length | ppm | m/z | z | RT | Fraction | Scan | Source File | Area F4 | Area F5 | Area F6 | Area F10 | Area F8 | Area F9 | Area F7 | Area F1 | Area F2 | Area F3 | #Feature | #Feature F4 | #Feature F5 | #Feature F6 | #Feature F10 | #Feature F8 | #Feature F9 | #Feature F7 | #Feature F1 | #Feature F2 | #Feature F3 | Start | End | PTM | AScore | Found By |
| --- | --- | --- | --- | --- | --- | --- | --- | --- | --- | --- | --- | --- | --- | --- | --- | --- | --- | --- | --- | --- | --- | --- | --- | --- | --- | --- | --- | --- | --- | --- | --- | --- | --- | --- | --- | --- | --- |
| K.LISWYDNEFGYSNR.V | Y | 69.58 | 1762.7950 | 14 | 1.2 | 882.4059 | 2 | 51.71 | 8 | F8:29501 | 31122021\_RID\_3262\_1a.raw | 3.7581E5 |  |  |  |  |  |  | 1.3561E6 | 2.7915E5 |  | 3 | 1 | 0 | 0 | 0 | 0 | 0 | 0 | 1 | 1 | 0 | 308 | 321 |  |  | PEAKS DB |
| K.LTGMAFR.V | N | 41.80 | 794.4109 | 7 | 0.1 | 398.2128 | 2 | 30.41 | 9 | F9:13699 | 31122021\_RID\_3262\_2a.raw |  |  | 5.1658E4 |  |  |  |  |  | 0 |  | 1 | 0 | 0 | 1 | 0 | 0 | 0 | 0 | 0 | 0 | 0 | 226 | 232 |  |  | PEAKS DB |
| total 2 peptides |  |  |  |  |  |  |  |  |  |  |  |  |  |  |  |  |  |  |  |  |  |  |  |  |  |  |  |  |  |  |  |  |  |  |  |  |  |
| --- | --- | --- | --- | --- | --- | --- | --- | --- | --- | --- | --- | --- | --- | --- | --- | --- | --- | --- | --- | --- | --- | --- | --- | --- | --- | --- | --- | --- | --- | --- | --- | --- | --- | --- | --- | --- | --- |

AUS82534.1

back to list

  

| Protein Coverage
| Supporting Peptides
|

Protein Coverage:

Supporting Peptides:

| Peptide | Uniq | -10lgP | Mass | Length | ppm | m/z | z | RT | Fraction | Scan | Source File | Area F4 | Area F5 | Area F6 | Area F10 | Area F8 | Area F9 | Area F7 | Area F1 | Area F2 | Area F3 | #Feature | #Feature F4 | #Feature F5 | #Feature F6 | #Feature F10 | #Feature F8 | #Feature F9 | #Feature F7 | #Feature F1 | #Feature F2 | #Feature F3 | Start | End | PTM | AScore | Found By |
| --- | --- | --- | --- | --- | --- | --- | --- | --- | --- | --- | --- | --- | --- | --- | --- | --- | --- | --- | --- | --- | --- | --- | --- | --- | --- | --- | --- | --- | --- | --- | --- | --- | --- | --- | --- | --- | --- |
| L.WDKDIMLIR.L | N | 57.88 | 1188.6324 | 9 | 0.5 | 595.3238 | 2 | 43.59 | 1 | F1:28744 | 13012022\_RID\_3262\_4a.raw | 6.6853E8 | 1.3316E6 |  |  |  |  | 2.7704E4 | 1.1405E7 | 1.608E6 |  | 12 | 5 | 3 | 0 | 0 | 0 | 0 | 1 | 2 | 1 | 0 | 107 | 115 |  |  | PEAKS DB |
| R.LPALYTK.V | Y | 55.00 | 804.4745 | 7 | -0.3 | 403.2444 | 2 | 31.50 | 6 | F6:17976 | 17012022\_RID\_3262\_9a.raw | 3.0178E9 | 8.3757E7 | 7.6285E6 | 7.8221E5 |  | 5.6121E6 |  | 1.0754E7 | 1.38E7 |  | 7 | 1 | 1 | 1 | 1 | 0 | 1 | 0 | 1 | 1 | 0 | 232 | 238 |  |  | PEAKS DB |
| L.WDKDIM(+15.99)LIR.L | N | 44.48 | 1204.6274 | 9 | 0.7 | 603.3214 | 2 | 37.71 | 2 | F2:20647 | 13012022\_RID\_3262\_5a.raw | 1.4316E8 | 4.5531E6 |  |  |  |  |  |  |  |  | 2 | 1 | 1 | 0 | 0 | 0 | 0 | 0 | 0 | 0 | 0 | 107 | 115 | Oxidation (M) | M6:Oxidation (M):1000.00 | PEAKS DB |
| W.DKDIMLIR.L | N | 41.99 | 1002.5532 | 8 | 0.7 | 502.2842 | 2 | 34.59 | 7 | F7:15514 | 21012022\_RID\_3262\_7a.raw |  |  |  |  |  |  | 2.6461E6 |  |  |  | 1 | 0 | 0 | 0 | 0 | 0 | 0 | 1 | 0 | 0 | 0 | 108 | 115 |  |  | PEAKS DB |
| L.VIGGDEC(+57.02)NINEHR.F | N | 41.29 | 1511.6787 | 13 | 0.4 | 756.8469 | 2 | 21.60 | 2 | F2:8211 | 13012022\_RID\_3262\_5a.raw |  | 6.6457E6 |  |  |  |  |  |  |  |  | 1 | 0 | 1 | 0 | 0 | 0 | 0 | 0 | 0 | 0 | 0 | 25 | 37 | Carbamidomethylation | C7:Carbamidomethylation:1000.00 | PEAKS DB |
| total 5 peptides |  |  |  |  |  |  |  |  |  |  |  |  |  |  |  |  |  |  |  |  |  |  |  |  |  |  |  |  |  |  |  |  |  |  |  |  |  |
| --- | --- | --- | --- | --- | --- | --- | --- | --- | --- | --- | --- | --- | --- | --- | --- | --- | --- | --- | --- | --- | --- | --- | --- | --- | --- | --- | --- | --- | --- | --- | --- | --- | --- | --- | --- | --- | --- |

XP\_026566978.1

back to list

  

| Protein Coverage
| Supporting Peptides
|

Protein Coverage:

Supporting Peptides:

| Peptide | Uniq | -10lgP | Mass | Length | ppm | m/z | z | RT | Fraction | Scan | Source File | Area F4 | Area F5 | Area F6 | Area F10 | Area F8 | Area F9 | Area F7 | Area F1 | Area F2 | Area F3 | #Feature | #Feature F4 | #Feature F5 | #Feature F6 | #Feature F10 | #Feature F8 | #Feature F9 | #Feature F7 | #Feature F1 | #Feature F2 | #Feature F3 | Start | End | PTM | AScore | Found By |
| --- | --- | --- | --- | --- | --- | --- | --- | --- | --- | --- | --- | --- | --- | --- | --- | --- | --- | --- | --- | --- | --- | --- | --- | --- | --- | --- | --- | --- | --- | --- | --- | --- | --- | --- | --- | --- | --- |
| R.LIGQIVSSITASLR.F | Y | 53.38 | 1456.8613 | 14 | 0.7 | 729.4384 | 2 | 60.41 | 7 | F7:27633 | 21012022\_RID\_3262\_7a.raw |  |  |  |  |  |  | 6.1041E4 |  |  |  | 1 | 0 | 0 | 0 | 0 | 0 | 0 | 1 | 0 | 0 | 0 | 195 | 208 |  |  | PEAKS DB |
| F.FSETGAGKHVPR.A | N | 46.34 | 1284.6575 | 12 | -0.3 | 429.2263 | 3 | 17.50 | 1 | F1:5633 | 13012022\_RID\_3262\_4a.raw | 5.8635E5 |  |  |  |  |  |  |  |  |  | 1 | 1 | 0 | 0 | 0 | 0 | 0 | 0 | 0 | 0 | 0 | 18 | 29 |  |  | PEAKS DB |
| K.EIIDLVLDR.I | N | 42.79 | 1084.6128 | 9 | 1.0 | 543.3142 | 2 | 54.32 | 8 | F8:31925 | 31122021\_RID\_3262\_1a.raw |  |  |  |  |  |  |  | 1.3935E5 |  |  | 1 | 0 | 0 | 0 | 0 | 0 | 0 | 0 | 1 | 0 | 0 | 78 | 86 |  |  | PEAKS DB |
| total 3 peptides |  |  |  |  |  |  |  |  |  |  |  |  |  |  |  |  |  |  |  |  |  |  |  |  |  |  |  |  |  |  |  |  |  |  |  |  |  |
| --- | --- | --- | --- | --- | --- | --- | --- | --- | --- | --- | --- | --- | --- | --- | --- | --- | --- | --- | --- | --- | --- | --- | --- | --- | --- | --- | --- | --- | --- | --- | --- | --- | --- | --- | --- | --- | --- |

XP\_026521383.1

back to list

  

| Protein Coverage
| Supporting Peptides
|

Protein Coverage:

Supporting Peptides:

| Peptide | Uniq | -10lgP | Mass | Length | ppm | m/z | z | RT | Fraction | Scan | Source File | Area F4 | Area F5 | Area F6 | Area F10 | Area F8 | Area F9 | Area F7 | Area F1 | Area F2 | Area F3 | #Feature | #Feature F4 | #Feature F5 | #Feature F6 | #Feature F10 | #Feature F8 | #Feature F9 | #Feature F7 | #Feature F1 | #Feature F2 | #Feature F3 | Start | End | PTM | AScore | Found By |
| --- | --- | --- | --- | --- | --- | --- | --- | --- | --- | --- | --- | --- | --- | --- | --- | --- | --- | --- | --- | --- | --- | --- | --- | --- | --- | --- | --- | --- | --- | --- | --- | --- | --- | --- | --- | --- | --- |
| R.LIGQIVSSITASLR.F | Y | 53.38 | 1456.8613 | 14 | 0.7 | 729.4384 | 2 | 60.41 | 7 | F7:27633 | 21012022\_RID\_3262\_7a.raw |  |  |  |  |  |  | 6.1041E4 |  |  |  | 1 | 0 | 0 | 0 | 0 | 0 | 0 | 1 | 0 | 0 | 0 | 195 | 208 |  |  | PEAKS DB |
| F.FSETGAGKHVPR.A | N | 46.34 | 1284.6575 | 12 | -0.3 | 429.2263 | 3 | 17.50 | 1 | F1:5633 | 13012022\_RID\_3262\_4a.raw | 5.8635E5 |  |  |  |  |  |  |  |  |  | 1 | 1 | 0 | 0 | 0 | 0 | 0 | 0 | 0 | 0 | 0 | 18 | 29 |  |  | PEAKS DB |
| K.EIIDLVLDR.I | N | 42.79 | 1084.6128 | 9 | 1.0 | 543.3142 | 2 | 54.32 | 8 | F8:31925 | 31122021\_RID\_3262\_1a.raw |  |  |  |  |  |  |  | 1.3935E5 |  |  | 1 | 0 | 0 | 0 | 0 | 0 | 0 | 0 | 1 | 0 | 0 | 78 | 86 |  |  | PEAKS DB |
| total 3 peptides |  |  |  |  |  |  |  |  |  |  |  |  |  |  |  |  |  |  |  |  |  |  |  |  |  |  |  |  |  |  |  |  |  |  |  |  |  |
| --- | --- | --- | --- | --- | --- | --- | --- | --- | --- | --- | --- | --- | --- | --- | --- | --- | --- | --- | --- | --- | --- | --- | --- | --- | --- | --- | --- | --- | --- | --- | --- | --- | --- | --- | --- | --- | --- |

XP\_025021189.1

back to list

  

| Protein Coverage
| Supporting Peptides
|

Protein Coverage:

Supporting Peptides:

| Peptide | Uniq | -10lgP | Mass | Length | ppm | m/z | z | RT | Fraction | Scan | Source File | Area F4 | Area F5 | Area F6 | Area F10 | Area F8 | Area F9 | Area F7 | Area F1 | Area F2 | Area F3 | #Feature | #Feature F4 | #Feature F5 | #Feature F6 | #Feature F10 | #Feature F8 | #Feature F9 | #Feature F7 | #Feature F1 | #Feature F2 | #Feature F3 | Start | End | PTM | AScore | Found By |
| --- | --- | --- | --- | --- | --- | --- | --- | --- | --- | --- | --- | --- | --- | --- | --- | --- | --- | --- | --- | --- | --- | --- | --- | --- | --- | --- | --- | --- | --- | --- | --- | --- | --- | --- | --- | --- | --- |
| R.LIGQIVSSITASLR.F | Y | 53.38 | 1456.8613 | 14 | 0.7 | 729.4384 | 2 | 60.41 | 7 | F7:27633 | 21012022\_RID\_3262\_7a.raw |  |  |  |  |  |  | 6.1041E4 |  |  |  | 1 | 0 | 0 | 0 | 0 | 0 | 0 | 1 | 0 | 0 | 0 | 230 | 243 |  |  | PEAKS DB |
| F.FSETGAGKHVPR.A | N | 46.34 | 1284.6575 | 12 | -0.3 | 429.2263 | 3 | 17.50 | 1 | F1:5633 | 13012022\_RID\_3262\_4a.raw | 5.8635E5 |  |  |  |  |  |  |  |  |  | 1 | 1 | 0 | 0 | 0 | 0 | 0 | 0 | 0 | 0 | 0 | 53 | 64 |  |  | PEAKS DB |
| K.EIIDLVLDR.I | N | 42.79 | 1084.6128 | 9 | 1.0 | 543.3142 | 2 | 54.32 | 8 | F8:31925 | 31122021\_RID\_3262\_1a.raw |  |  |  |  |  |  |  | 1.3935E5 |  |  | 1 | 0 | 0 | 0 | 0 | 0 | 0 | 0 | 1 | 0 | 0 | 113 | 121 |  |  | PEAKS DB |
| total 3 peptides |  |  |  |  |  |  |  |  |  |  |  |  |  |  |  |  |  |  |  |  |  |  |  |  |  |  |  |  |  |  |  |  |  |  |  |  |  |
| --- | --- | --- | --- | --- | --- | --- | --- | --- | --- | --- | --- | --- | --- | --- | --- | --- | --- | --- | --- | --- | --- | --- | --- | --- | --- | --- | --- | --- | --- | --- | --- | --- | --- | --- | --- | --- | --- |

XP\_007426281.1

back to list

  

| Protein Coverage
| Supporting Peptides
|

Protein Coverage:

Supporting Peptides:

| Peptide | Uniq | -10lgP | Mass | Length | ppm | m/z | z | RT | Fraction | Scan | Source File | Area F4 | Area F5 | Area F6 | Area F10 | Area F8 | Area F9 | Area F7 | Area F1 | Area F2 | Area F3 | #Feature | #Feature F4 | #Feature F5 | #Feature F6 | #Feature F10 | #Feature F8 | #Feature F9 | #Feature F7 | #Feature F1 | #Feature F2 | #Feature F3 | Start | End | PTM | AScore | Found By |
| --- | --- | --- | --- | --- | --- | --- | --- | --- | --- | --- | --- | --- | --- | --- | --- | --- | --- | --- | --- | --- | --- | --- | --- | --- | --- | --- | --- | --- | --- | --- | --- | --- | --- | --- | --- | --- | --- |
| R.LIGQIVSSITASLR.F | Y | 53.38 | 1456.8613 | 14 | 0.7 | 729.4384 | 2 | 60.41 | 7 | F7:27633 | 21012022\_RID\_3262\_7a.raw |  |  |  |  |  |  | 6.1041E4 |  |  |  | 1 | 0 | 0 | 0 | 0 | 0 | 0 | 1 | 0 | 0 | 0 | 230 | 243 |  |  | PEAKS DB |
| F.FSETGAGKHVPR.A | N | 46.34 | 1284.6575 | 12 | -0.3 | 429.2263 | 3 | 17.50 | 1 | F1:5633 | 13012022\_RID\_3262\_4a.raw | 5.8635E5 |  |  |  |  |  |  |  |  |  | 1 | 1 | 0 | 0 | 0 | 0 | 0 | 0 | 0 | 0 | 0 | 53 | 64 |  |  | PEAKS DB |
| K.EIIDLVLDR.I | N | 42.79 | 1084.6128 | 9 | 1.0 | 543.3142 | 2 | 54.32 | 8 | F8:31925 | 31122021\_RID\_3262\_1a.raw |  |  |  |  |  |  |  | 1.3935E5 |  |  | 1 | 0 | 0 | 0 | 0 | 0 | 0 | 0 | 1 | 0 | 0 | 113 | 121 |  |  | PEAKS DB |
| total 3 peptides |  |  |  |  |  |  |  |  |  |  |  |  |  |  |  |  |  |  |  |  |  |  |  |  |  |  |  |  |  |  |  |  |  |  |  |  |  |
| --- | --- | --- | --- | --- | --- | --- | --- | --- | --- | --- | --- | --- | --- | --- | --- | --- | --- | --- | --- | --- | --- | --- | --- | --- | --- | --- | --- | --- | --- | --- | --- | --- | --- | --- | --- | --- | --- |

XP\_026563732.1

back to list

  

| Protein Coverage
| Supporting Peptides
|

Protein Coverage:

Supporting Peptides:

| Peptide | Uniq | -10lgP | Mass | Length | ppm | m/z | z | RT | Fraction | Scan | Source File | Area F4 | Area F5 | Area F6 | Area F10 | Area F8 | Area F9 | Area F7 | Area F1 | Area F2 | Area F3 | #Feature | #Feature F4 | #Feature F5 | #Feature F6 | #Feature F10 | #Feature F8 | #Feature F9 | #Feature F7 | #Feature F1 | #Feature F2 | #Feature F3 | Start | End | PTM | AScore | Found By |
| --- | --- | --- | --- | --- | --- | --- | --- | --- | --- | --- | --- | --- | --- | --- | --- | --- | --- | --- | --- | --- | --- | --- | --- | --- | --- | --- | --- | --- | --- | --- | --- | --- | --- | --- | --- | --- | --- |
| R.LIGQIVSSITASLR.F | Y | 53.38 | 1456.8613 | 14 | 0.7 | 729.4384 | 2 | 60.41 | 7 | F7:27633 | 21012022\_RID\_3262\_7a.raw |  |  |  |  |  |  | 6.1041E4 |  |  |  | 1 | 0 | 0 | 0 | 0 | 0 | 0 | 1 | 0 | 0 | 0 | 239 | 252 |  |  | PEAKS DB |
| F.FSETGAGKHVPR.A | N | 46.34 | 1284.6575 | 12 | -0.3 | 429.2263 | 3 | 17.50 | 1 | F1:5633 | 13012022\_RID\_3262\_4a.raw | 5.8635E5 |  |  |  |  |  |  |  |  |  | 1 | 1 | 0 | 0 | 0 | 0 | 0 | 0 | 0 | 0 | 0 | 62 | 73 |  |  | PEAKS DB |
| K.EIIDLVLDR.I | N | 42.79 | 1084.6128 | 9 | 1.0 | 543.3142 | 2 | 54.32 | 8 | F8:31925 | 31122021\_RID\_3262\_1a.raw |  |  |  |  |  |  |  | 1.3935E5 |  |  | 1 | 0 | 0 | 0 | 0 | 0 | 0 | 0 | 1 | 0 | 0 | 122 | 130 |  |  | PEAKS DB |
| total 3 peptides |  |  |  |  |  |  |  |  |  |  |  |  |  |  |  |  |  |  |  |  |  |  |  |  |  |  |  |  |  |  |  |  |  |  |  |  |  |
| --- | --- | --- | --- | --- | --- | --- | --- | --- | --- | --- | --- | --- | --- | --- | --- | --- | --- | --- | --- | --- | --- | --- | --- | --- | --- | --- | --- | --- | --- | --- | --- | --- | --- | --- | --- | --- | --- |

XP\_007426279.2

back to list

  

| Protein Coverage
| Supporting Peptides
|

Protein Coverage:

Supporting Peptides:

| Peptide | Uniq | -10lgP | Mass | Length | ppm | m/z | z | RT | Fraction | Scan | Source File | Area F4 | Area F5 | Area F6 | Area F10 | Area F8 | Area F9 | Area F7 | Area F1 | Area F2 | Area F3 | #Feature | #Feature F4 | #Feature F5 | #Feature F6 | #Feature F10 | #Feature F8 | #Feature F9 | #Feature F7 | #Feature F1 | #Feature F2 | #Feature F3 | Start | End | PTM | AScore | Found By |
| --- | --- | --- | --- | --- | --- | --- | --- | --- | --- | --- | --- | --- | --- | --- | --- | --- | --- | --- | --- | --- | --- | --- | --- | --- | --- | --- | --- | --- | --- | --- | --- | --- | --- | --- | --- | --- | --- |
| R.LIGQIVSSITASLR.F | Y | 53.38 | 1456.8613 | 14 | 0.7 | 729.4384 | 2 | 60.41 | 7 | F7:27633 | 21012022\_RID\_3262\_7a.raw |  |  |  |  |  |  | 6.1041E4 |  |  |  | 1 | 0 | 0 | 0 | 0 | 0 | 0 | 1 | 0 | 0 | 0 | 230 | 243 |  |  | PEAKS DB |
| F.FSETGAGKHVPR.A | N | 46.34 | 1284.6575 | 12 | -0.3 | 429.2263 | 3 | 17.50 | 1 | F1:5633 | 13012022\_RID\_3262\_4a.raw | 5.8635E5 |  |  |  |  |  |  |  |  |  | 1 | 1 | 0 | 0 | 0 | 0 | 0 | 0 | 0 | 0 | 0 | 53 | 64 |  |  | PEAKS DB |
| K.EIIDLVLDR.I | N | 42.79 | 1084.6128 | 9 | 1.0 | 543.3142 | 2 | 54.32 | 8 | F8:31925 | 31122021\_RID\_3262\_1a.raw |  |  |  |  |  |  |  | 1.3935E5 |  |  | 1 | 0 | 0 | 0 | 0 | 0 | 0 | 0 | 1 | 0 | 0 | 113 | 121 |  |  | PEAKS DB |
| total 3 peptides |  |  |  |  |  |  |  |  |  |  |  |  |  |  |  |  |  |  |  |  |  |  |  |  |  |  |  |  |  |  |  |  |  |  |  |  |  |
| --- | --- | --- | --- | --- | --- | --- | --- | --- | --- | --- | --- | --- | --- | --- | --- | --- | --- | --- | --- | --- | --- | --- | --- | --- | --- | --- | --- | --- | --- | --- | --- | --- | --- | --- | --- | --- | --- |

Q8AY81.1

back to list

  

| Protein Coverage
| Supporting Peptides
|

Protein Coverage:

Supporting Peptides:

| Peptide | Uniq | -10lgP | Mass | Length | ppm | m/z | z | RT | Fraction | Scan | Source File | Area F4 | Area F5 | Area F6 | Area F10 | Area F8 | Area F9 | Area F7 | Area F1 | Area F2 | Area F3 | #Feature | #Feature F4 | #Feature F5 | #Feature F6 | #Feature F10 | #Feature F8 | #Feature F9 | #Feature F7 | #Feature F1 | #Feature F2 | #Feature F3 | Start | End | PTM | AScore | Found By |
| --- | --- | --- | --- | --- | --- | --- | --- | --- | --- | --- | --- | --- | --- | --- | --- | --- | --- | --- | --- | --- | --- | --- | --- | --- | --- | --- | --- | --- | --- | --- | --- | --- | --- | --- | --- | --- | --- |
| R.WDKDIMLIR.L | N | 57.88 | 1188.6324 | 9 | 0.5 | 595.3238 | 2 | 43.59 | 1 | F1:28744 | 13012022\_RID\_3262\_4a.raw | 6.6853E8 | 1.3316E6 |  |  |  |  | 2.7704E4 | 1.1405E7 | 1.608E6 |  | 12 | 5 | 3 | 0 | 0 | 0 | 0 | 1 | 2 | 1 | 0 | 109 | 117 |  |  | PEAKS DB |
| A.HIAPLSLPSSPPTVGSVC(+57.02)R.V | Y | 46.89 | 1974.0356 | 19 | 0.0 | 988.0251 | 2 | 45.82 | 3 | F3:22331 | 13012022\_RID\_3262\_6a.raw |  |  | 1.4947E6 |  |  |  |  |  |  |  | 1 | 0 | 0 | 1 | 0 | 0 | 0 | 0 | 0 | 0 | 0 | 127 | 145 | Carbamidomethylation | C18:Carbamidomethylation:1000.00 | PEAKS DB |
| R.WDKDIM(+15.99)LIR.L | N | 44.48 | 1204.6274 | 9 | 0.7 | 603.3214 | 2 | 37.71 | 2 | F2:20647 | 13012022\_RID\_3262\_5a.raw | 1.4316E8 | 4.5531E6 |  |  |  |  |  |  |  |  | 2 | 1 | 1 | 0 | 0 | 0 | 0 | 0 | 0 | 0 | 0 | 109 | 117 | Oxidation (M) | M6:Oxidation (M):1000.00 | PEAKS DB |
| W.DKDIMLIR.L | N | 41.99 | 1002.5532 | 8 | 0.7 | 502.2842 | 2 | 34.59 | 7 | F7:15514 | 21012022\_RID\_3262\_7a.raw |  |  |  |  |  |  | 2.6461E6 |  |  |  | 1 | 0 | 0 | 0 | 0 | 0 | 0 | 1 | 0 | 0 | 0 | 110 | 117 |  |  | PEAKS DB |
| L.VIGGDEC(+57.02)NINEHR.F | N | 41.29 | 1511.6787 | 13 | 0.4 | 756.8469 | 2 | 21.60 | 2 | F2:8211 | 13012022\_RID\_3262\_5a.raw |  | 6.6457E6 |  |  |  |  |  |  |  |  | 1 | 0 | 1 | 0 | 0 | 0 | 0 | 0 | 0 | 0 | 0 | 25 | 37 | Carbamidomethylation | C7:Carbamidomethylation:1000.00 | PEAKS DB |
| total 5 peptides |  |  |  |  |  |  |  |  |  |  |  |  |  |  |  |  |  |  |  |  |  |  |  |  |  |  |  |  |  |  |  |  |  |  |  |  |  |
| --- | --- | --- | --- | --- | --- | --- | --- | --- | --- | --- | --- | --- | --- | --- | --- | --- | --- | --- | --- | --- | --- | --- | --- | --- | --- | --- | --- | --- | --- | --- | --- | --- | --- | --- | --- | --- | --- |

AAN52347.1

back to list

  

| Protein Coverage
| Supporting Peptides
|

Protein Coverage:

Supporting Peptides:

| Peptide | Uniq | -10lgP | Mass | Length | ppm | m/z | z | RT | Fraction | Scan | Source File | Area F4 | Area F5 | Area F6 | Area F10 | Area F8 | Area F9 | Area F7 | Area F1 | Area F2 | Area F3 | #Feature | #Feature F4 | #Feature F5 | #Feature F6 | #Feature F10 | #Feature F8 | #Feature F9 | #Feature F7 | #Feature F1 | #Feature F2 | #Feature F3 | Start | End | PTM | AScore | Found By |
| --- | --- | --- | --- | --- | --- | --- | --- | --- | --- | --- | --- | --- | --- | --- | --- | --- | --- | --- | --- | --- | --- | --- | --- | --- | --- | --- | --- | --- | --- | --- | --- | --- | --- | --- | --- | --- | --- |
| R.WDKDIMLIR.L | N | 57.88 | 1188.6324 | 9 | 0.5 | 595.3238 | 2 | 43.59 | 1 | F1:28744 | 13012022\_RID\_3262\_4a.raw | 6.6853E8 | 1.3316E6 |  |  |  |  | 2.7704E4 | 1.1405E7 | 1.608E6 |  | 12 | 5 | 3 | 0 | 0 | 0 | 0 | 1 | 2 | 1 | 0 | 109 | 117 |  |  | PEAKS DB |
| A.HIAPLSLPSSPPTVGSVC(+57.02)R.V | Y | 46.89 | 1974.0356 | 19 | 0.0 | 988.0251 | 2 | 45.82 | 3 | F3:22331 | 13012022\_RID\_3262\_6a.raw |  |  | 1.4947E6 |  |  |  |  |  |  |  | 1 | 0 | 0 | 1 | 0 | 0 | 0 | 0 | 0 | 0 | 0 | 127 | 145 | Carbamidomethylation | C18:Carbamidomethylation:1000.00 | PEAKS DB |
| R.WDKDIM(+15.99)LIR.L | N | 44.48 | 1204.6274 | 9 | 0.7 | 603.3214 | 2 | 37.71 | 2 | F2:20647 | 13012022\_RID\_3262\_5a.raw | 1.4316E8 | 4.5531E6 |  |  |  |  |  |  |  |  | 2 | 1 | 1 | 0 | 0 | 0 | 0 | 0 | 0 | 0 | 0 | 109 | 117 | Oxidation (M) | M6:Oxidation (M):1000.00 | PEAKS DB |
| W.DKDIMLIR.L | N | 41.99 | 1002.5532 | 8 | 0.7 | 502.2842 | 2 | 34.59 | 7 | F7:15514 | 21012022\_RID\_3262\_7a.raw |  |  |  |  |  |  | 2.6461E6 |  |  |  | 1 | 0 | 0 | 0 | 0 | 0 | 0 | 1 | 0 | 0 | 0 | 110 | 117 |  |  | PEAKS DB |
| L.VIGGDEC(+57.02)NINEHR.F | N | 41.29 | 1511.6787 | 13 | 0.4 | 756.8469 | 2 | 21.60 | 2 | F2:8211 | 13012022\_RID\_3262\_5a.raw |  | 6.6457E6 |  |  |  |  |  |  |  |  | 1 | 0 | 1 | 0 | 0 | 0 | 0 | 0 | 0 | 0 | 0 | 25 | 37 | Carbamidomethylation | C7:Carbamidomethylation:1000.00 | PEAKS DB |
| total 5 peptides |  |  |  |  |  |  |  |  |  |  |  |  |  |  |  |  |  |  |  |  |  |  |  |  |  |  |  |  |  |  |  |  |  |  |  |  |  |
| --- | --- | --- | --- | --- | --- | --- | --- | --- | --- | --- | --- | --- | --- | --- | --- | --- | --- | --- | --- | --- | --- | --- | --- | --- | --- | --- | --- | --- | --- | --- | --- | --- | --- | --- | --- | --- | --- |

XP\_015746202.2

back to list

  

| Protein Coverage
| Supporting Peptides
|

Protein Coverage:

Supporting Peptides:

| Peptide | Uniq | -10lgP | Mass | Length | ppm | m/z | z | RT | Fraction | Scan | Source File | Area F4 | Area F5 | Area F6 | Area F10 | Area F8 | Area F9 | Area F7 | Area F1 | Area F2 | Area F3 | #Feature | #Feature F4 | #Feature F5 | #Feature F6 | #Feature F10 | #Feature F8 | #Feature F9 | #Feature F7 | #Feature F1 | #Feature F2 | #Feature F3 | Start | End | PTM | AScore | Found By |
| --- | --- | --- | --- | --- | --- | --- | --- | --- | --- | --- | --- | --- | --- | --- | --- | --- | --- | --- | --- | --- | --- | --- | --- | --- | --- | --- | --- | --- | --- | --- | --- | --- | --- | --- | --- | --- | --- |
| R.LPVDIAYK.R | Y | 46.99 | 917.5222 | 8 | 0.7 | 459.7687 | 2 | 38.07 | 6 | F6:23853 | 17012022\_RID\_3262\_9a.raw |  |  |  |  |  | 1.9801E6 |  |  |  |  | 1 | 0 | 0 | 0 | 0 | 0 | 1 | 0 | 0 | 0 | 0 | 2817 | 2824 |  |  | PEAKS DB |
| K.LTVEEAVR.M | N | 43.09 | 915.5025 | 8 | -0.9 | 458.7581 | 2 | 30.27 | 4 | F4:16752 | 17012022\_RID\_3262\_10a .raw |  |  |  | 2.3861E5 |  |  |  |  |  |  | 1 | 0 | 0 | 0 | 1 | 0 | 0 | 0 | 0 | 0 | 0 | 3977 | 3984 |  |  | PEAKS DB |
| total 2 peptides |  |  |  |  |  |  |  |  |  |  |  |  |  |  |  |  |  |  |  |  |  |  |  |  |  |  |  |  |  |  |  |  |  |  |  |  |  |
| --- | --- | --- | --- | --- | --- | --- | --- | --- | --- | --- | --- | --- | --- | --- | --- | --- | --- | --- | --- | --- | --- | --- | --- | --- | --- | --- | --- | --- | --- | --- | --- | --- | --- | --- | --- | --- | --- |

XP\_015746201.2

back to list

  

| Protein Coverage
| Supporting Peptides
|

Protein Coverage:

Supporting Peptides:

| Peptide | Uniq | -10lgP | Mass | Length | ppm | m/z | z | RT | Fraction | Scan | Source File | Area F4 | Area F5 | Area F6 | Area F10 | Area F8 | Area F9 | Area F7 | Area F1 | Area F2 | Area F3 | #Feature | #Feature F4 | #Feature F5 | #Feature F6 | #Feature F10 | #Feature F8 | #Feature F9 | #Feature F7 | #Feature F1 | #Feature F2 | #Feature F3 | Start | End | PTM | AScore | Found By |
| --- | --- | --- | --- | --- | --- | --- | --- | --- | --- | --- | --- | --- | --- | --- | --- | --- | --- | --- | --- | --- | --- | --- | --- | --- | --- | --- | --- | --- | --- | --- | --- | --- | --- | --- | --- | --- | --- |
| R.LPVDIAYK.R | Y | 46.99 | 917.5222 | 8 | 0.7 | 459.7687 | 2 | 38.07 | 6 | F6:23853 | 17012022\_RID\_3262\_9a.raw |  |  |  |  |  | 1.9801E6 |  |  |  |  | 1 | 0 | 0 | 0 | 0 | 0 | 1 | 0 | 0 | 0 | 0 | 2822 | 2829 |  |  | PEAKS DB |
| K.LTVEEAVR.M | N | 43.09 | 915.5025 | 8 | -0.9 | 458.7581 | 2 | 30.27 | 4 | F4:16752 | 17012022\_RID\_3262\_10a .raw |  |  |  | 2.3861E5 |  |  |  |  |  |  | 1 | 0 | 0 | 0 | 1 | 0 | 0 | 0 | 0 | 0 | 0 | 3982 | 3989 |  |  | PEAKS DB |
| total 2 peptides |  |  |  |  |  |  |  |  |  |  |  |  |  |  |  |  |  |  |  |  |  |  |  |  |  |  |  |  |  |  |  |  |  |  |  |  |  |
| --- | --- | --- | --- | --- | --- | --- | --- | --- | --- | --- | --- | --- | --- | --- | --- | --- | --- | --- | --- | --- | --- | --- | --- | --- | --- | --- | --- | --- | --- | --- | --- | --- | --- | --- | --- | --- | --- |

XP\_015746200.2

back to list

  

| Protein Coverage
| Supporting Peptides
|

Protein Coverage:

Supporting Peptides:

| Peptide | Uniq | -10lgP | Mass | Length | ppm | m/z | z | RT | Fraction | Scan | Source File | Area F4 | Area F5 | Area F6 | Area F10 | Area F8 | Area F9 | Area F7 | Area F1 | Area F2 | Area F3 | #Feature | #Feature F4 | #Feature F5 | #Feature F6 | #Feature F10 | #Feature F8 | #Feature F9 | #Feature F7 | #Feature F1 | #Feature F2 | #Feature F3 | Start | End | PTM | AScore | Found By |
| --- | --- | --- | --- | --- | --- | --- | --- | --- | --- | --- | --- | --- | --- | --- | --- | --- | --- | --- | --- | --- | --- | --- | --- | --- | --- | --- | --- | --- | --- | --- | --- | --- | --- | --- | --- | --- | --- |
| R.LPVDIAYK.R | Y | 46.99 | 917.5222 | 8 | 0.7 | 459.7687 | 2 | 38.07 | 6 | F6:23853 | 17012022\_RID\_3262\_9a.raw |  |  |  |  |  | 1.9801E6 |  |  |  |  | 1 | 0 | 0 | 0 | 0 | 0 | 1 | 0 | 0 | 0 | 0 | 2829 | 2836 |  |  | PEAKS DB |
| K.LTVEEAVR.M | N | 43.09 | 915.5025 | 8 | -0.9 | 458.7581 | 2 | 30.27 | 4 | F4:16752 | 17012022\_RID\_3262\_10a .raw |  |  |  | 2.3861E5 |  |  |  |  |  |  | 1 | 0 | 0 | 0 | 1 | 0 | 0 | 0 | 0 | 0 | 0 | 3989 | 3996 |  |  | PEAKS DB |
| total 2 peptides |  |  |  |  |  |  |  |  |  |  |  |  |  |  |  |  |  |  |  |  |  |  |  |  |  |  |  |  |  |  |  |  |  |  |  |  |  |
| --- | --- | --- | --- | --- | --- | --- | --- | --- | --- | --- | --- | --- | --- | --- | --- | --- | --- | --- | --- | --- | --- | --- | --- | --- | --- | --- | --- | --- | --- | --- | --- | --- | --- | --- | --- | --- | --- |

XP\_015746199.2

back to list

  

| Protein Coverage
| Supporting Peptides
|

Protein Coverage:

Supporting Peptides:

| Peptide | Uniq | -10lgP | Mass | Length | ppm | m/z | z | RT | Fraction | Scan | Source File | Area F4 | Area F5 | Area F6 | Area F10 | Area F8 | Area F9 | Area F7 | Area F1 | Area F2 | Area F3 | #Feature | #Feature F4 | #Feature F5 | #Feature F6 | #Feature F10 | #Feature F8 | #Feature F9 | #Feature F7 | #Feature F1 | #Feature F2 | #Feature F3 | Start | End | PTM | AScore | Found By |
| --- | --- | --- | --- | --- | --- | --- | --- | --- | --- | --- | --- | --- | --- | --- | --- | --- | --- | --- | --- | --- | --- | --- | --- | --- | --- | --- | --- | --- | --- | --- | --- | --- | --- | --- | --- | --- | --- |
| R.LPVDIAYK.R | Y | 46.99 | 917.5222 | 8 | 0.7 | 459.7687 | 2 | 38.07 | 6 | F6:23853 | 17012022\_RID\_3262\_9a.raw |  |  |  |  |  | 1.9801E6 |  |  |  |  | 1 | 0 | 0 | 0 | 0 | 0 | 1 | 0 | 0 | 0 | 0 | 2834 | 2841 |  |  | PEAKS DB |
| K.LTVEEAVR.M | N | 43.09 | 915.5025 | 8 | -0.9 | 458.7581 | 2 | 30.27 | 4 | F4:16752 | 17012022\_RID\_3262\_10a .raw |  |  |  | 2.3861E5 |  |  |  |  |  |  | 1 | 0 | 0 | 0 | 1 | 0 | 0 | 0 | 0 | 0 | 0 | 3994 | 4001 |  |  | PEAKS DB |
| total 2 peptides |  |  |  |  |  |  |  |  |  |  |  |  |  |  |  |  |  |  |  |  |  |  |  |  |  |  |  |  |  |  |  |  |  |  |  |  |  |
| --- | --- | --- | --- | --- | --- | --- | --- | --- | --- | --- | --- | --- | --- | --- | --- | --- | --- | --- | --- | --- | --- | --- | --- | --- | --- | --- | --- | --- | --- | --- | --- | --- | --- | --- | --- | --- | --- |

XP\_032091650.1

back to list

  

| Protein Coverage
| Supporting Peptides
|

Protein Coverage:

Supporting Peptides:

| Peptide | Uniq | -10lgP | Mass | Length | ppm | m/z | z | RT | Fraction | Scan | Source File | Area F4 | Area F5 | Area F6 | Area F10 | Area F8 | Area F9 | Area F7 | Area F1 | Area F2 | Area F3 | #Feature | #Feature F4 | #Feature F5 | #Feature F6 | #Feature F10 | #Feature F8 | #Feature F9 | #Feature F7 | #Feature F1 | #Feature F2 | #Feature F3 | Start | End | PTM | AScore | Found By |
| --- | --- | --- | --- | --- | --- | --- | --- | --- | --- | --- | --- | --- | --- | --- | --- | --- | --- | --- | --- | --- | --- | --- | --- | --- | --- | --- | --- | --- | --- | --- | --- | --- | --- | --- | --- | --- | --- |
| R.GQAGVM(+15.99)GFPGPK.G | N | 61.60 | 1160.5648 | 12 | 1.2 | 581.2904 | 2 | 29.31 | 9 | F9:12753 | 31122021\_RID\_3262\_2a.raw |  |  |  |  |  |  |  |  | 4.9379E5 |  | 1 | 0 | 0 | 0 | 0 | 0 | 0 | 0 | 0 | 1 | 0 | 501 | 512 | Oxidation (M) | M6:Oxidation (M):1000.00 | PEAKS DB |
| R.GFSGLDGAK.G | N | 49.34 | 850.4185 | 9 | 0.7 | 426.2168 | 2 | 25.87 | 9 | F9:10014 | 31122021\_RID\_3262\_2a.raw |  |  |  |  |  |  |  |  | 2.44E6 |  | 1 | 0 | 0 | 0 | 0 | 0 | 0 | 0 | 0 | 1 | 0 | 195 | 203 |  |  | PEAKS DB |
| R.GLTGPIGPPGPAGSPGDKGESGPSGPAGPTGAR.G | Y | 45.83 | 2852.4053 | 33 | 0.8 | 951.8098 | 3 | 33.81 | 9 | F9:16474 | 31122021\_RID\_3262\_2a.raw |  |  |  |  |  |  |  |  | 8.1161E6 |  | 1 | 0 | 0 | 0 | 0 | 0 | 0 | 0 | 0 | 1 | 0 | 690 | 722 |  |  | PEAKS DB |
| total 3 peptides |  |  |  |  |  |  |  |  |  |  |  |  |  |  |  |  |  |  |  |  |  |  |  |  |  |  |  |  |  |  |  |  |  |  |  |  |  |
| --- | --- | --- | --- | --- | --- | --- | --- | --- | --- | --- | --- | --- | --- | --- | --- | --- | --- | --- | --- | --- | --- | --- | --- | --- | --- | --- | --- | --- | --- | --- | --- | --- | --- | --- | --- | --- | --- |

XP\_032091649.1

back to list

  

| Protein Coverage
| Supporting Peptides
|

Protein Coverage:

Supporting Peptides:

| Peptide | Uniq | -10lgP | Mass | Length | ppm | m/z | z | RT | Fraction | Scan | Source File | Area F4 | Area F5 | Area F6 | Area F10 | Area F8 | Area F9 | Area F7 | Area F1 | Area F2 | Area F3 | #Feature | #Feature F4 | #Feature F5 | #Feature F6 | #Feature F10 | #Feature F8 | #Feature F9 | #Feature F7 | #Feature F1 | #Feature F2 | #Feature F3 | Start | End | PTM | AScore | Found By |
| --- | --- | --- | --- | --- | --- | --- | --- | --- | --- | --- | --- | --- | --- | --- | --- | --- | --- | --- | --- | --- | --- | --- | --- | --- | --- | --- | --- | --- | --- | --- | --- | --- | --- | --- | --- | --- | --- |
| R.GQAGVM(+15.99)GFPGPK.G | N | 61.60 | 1160.5648 | 12 | 1.2 | 581.2904 | 2 | 29.31 | 9 | F9:12753 | 31122021\_RID\_3262\_2a.raw |  |  |  |  |  |  |  |  | 4.9379E5 |  | 1 | 0 | 0 | 0 | 0 | 0 | 0 | 0 | 0 | 1 | 0 | 565 | 576 | Oxidation (M) | M6:Oxidation (M):1000.00 | PEAKS DB |
| R.GFSGLDGAK.G | N | 49.34 | 850.4185 | 9 | 0.7 | 426.2168 | 2 | 25.87 | 9 | F9:10014 | 31122021\_RID\_3262\_2a.raw |  |  |  |  |  |  |  |  | 2.44E6 |  | 1 | 0 | 0 | 0 | 0 | 0 | 0 | 0 | 0 | 1 | 0 | 259 | 267 |  |  | PEAKS DB |
| R.GLTGPIGPPGPAGSPGDKGESGPSGPAGPTGAR.G | Y | 45.83 | 2852.4053 | 33 | 0.8 | 951.8098 | 3 | 33.81 | 9 | F9:16474 | 31122021\_RID\_3262\_2a.raw |  |  |  |  |  |  |  |  | 8.1161E6 |  | 1 | 0 | 0 | 0 | 0 | 0 | 0 | 0 | 0 | 1 | 0 | 754 | 786 |  |  | PEAKS DB |
| total 3 peptides |  |  |  |  |  |  |  |  |  |  |  |  |  |  |  |  |  |  |  |  |  |  |  |  |  |  |  |  |  |  |  |  |  |  |  |  |  |
| --- | --- | --- | --- | --- | --- | --- | --- | --- | --- | --- | --- | --- | --- | --- | --- | --- | --- | --- | --- | --- | --- | --- | --- | --- | --- | --- | --- | --- | --- | --- | --- | --- | --- | --- | --- | --- | --- |

XP\_013916526.1

back to list

  

| Protein Coverage
| Supporting Peptides
|

Protein Coverage:

Supporting Peptides:

| Peptide | Uniq | -10lgP | Mass | Length | ppm | m/z | z | RT | Fraction | Scan | Source File | Area F4 | Area F5 | Area F6 | Area F10 | Area F8 | Area F9 | Area F7 | Area F1 | Area F2 | Area F3 | #Feature | #Feature F4 | #Feature F5 | #Feature F6 | #Feature F10 | #Feature F8 | #Feature F9 | #Feature F7 | #Feature F1 | #Feature F2 | #Feature F3 | Start | End | PTM | AScore | Found By |
| --- | --- | --- | --- | --- | --- | --- | --- | --- | --- | --- | --- | --- | --- | --- | --- | --- | --- | --- | --- | --- | --- | --- | --- | --- | --- | --- | --- | --- | --- | --- | --- | --- | --- | --- | --- | --- | --- |
| R.GQAGVM(+15.99)GFPGPK.G | N | 61.60 | 1160.5648 | 12 | 1.2 | 581.2904 | 2 | 29.31 | 9 | F9:12753 | 31122021\_RID\_3262\_2a.raw |  |  |  |  |  |  |  |  | 4.9379E5 |  | 1 | 0 | 0 | 0 | 0 | 0 | 0 | 0 | 0 | 1 | 0 | 565 | 576 | Oxidation (M) | M6:Oxidation (M):1000.00 | PEAKS DB |
| R.GFSGLDGAK.G | N | 49.34 | 850.4185 | 9 | 0.7 | 426.2168 | 2 | 25.87 | 9 | F9:10014 | 31122021\_RID\_3262\_2a.raw |  |  |  |  |  |  |  |  | 2.44E6 |  | 1 | 0 | 0 | 0 | 0 | 0 | 0 | 0 | 0 | 1 | 0 | 259 | 267 |  |  | PEAKS DB |
| R.GLTGPIGPPGPAGSPGDKGESGPSGPAGPTGAR.G | Y | 45.83 | 2852.4053 | 33 | 0.8 | 951.8098 | 3 | 33.81 | 9 | F9:16474 | 31122021\_RID\_3262\_2a.raw |  |  |  |  |  |  |  |  | 8.1161E6 |  | 1 | 0 | 0 | 0 | 0 | 0 | 0 | 0 | 0 | 1 | 0 | 754 | 786 |  |  | PEAKS DB |
| total 3 peptides |  |  |  |  |  |  |  |  |  |  |  |  |  |  |  |  |  |  |  |  |  |  |  |  |  |  |  |  |  |  |  |  |  |  |  |  |  |
| --- | --- | --- | --- | --- | --- | --- | --- | --- | --- | --- | --- | --- | --- | --- | --- | --- | --- | --- | --- | --- | --- | --- | --- | --- | --- | --- | --- | --- | --- | --- | --- | --- | --- | --- | --- | --- | --- |

XP\_015683679.2

back to list

  

| Protein Coverage
| Supporting Peptides
|

Protein Coverage:

Supporting Peptides:

| Peptide | Uniq | -10lgP | Mass | Length | ppm | m/z | z | RT | Fraction | Scan | Source File | Area F4 | Area F5 | Area F6 | Area F10 | Area F8 | Area F9 | Area F7 | Area F1 | Area F2 | Area F3 | #Feature | #Feature F4 | #Feature F5 | #Feature F6 | #Feature F10 | #Feature F8 | #Feature F9 | #Feature F7 | #Feature F1 | #Feature F2 | #Feature F3 | Start | End | PTM | AScore | Found By |
| --- | --- | --- | --- | --- | --- | --- | --- | --- | --- | --- | --- | --- | --- | --- | --- | --- | --- | --- | --- | --- | --- | --- | --- | --- | --- | --- | --- | --- | --- | --- | --- | --- | --- | --- | --- | --- | --- |
| R.DESIGM(+15.99)VDTGTK.C | Y | 74.77 | 1267.5602 | 12 | 0.2 | 634.7875 | 2 | 27.42 | 9 | F9:11334 | 31122021\_RID\_3262\_2a.raw |  | 2.4078E6 |  |  |  | 6.1248E5 | 1.0706E6 | 3.7863E6 | 6.861E7 | 6.4727E5 | 16 | 0 | 1 | 0 | 0 | 0 | 1 | 2 | 4 | 7 | 1 | 619 | 630 | Oxidation (M) | M6:Oxidation (M):1000.00 | PEAKS DB |
| R.DESIGMVDTGTK.C | Y | 69.09 | 1251.5653 | 12 | 0.4 | 626.7902 | 2 | 33.81 | 9 | F9:16582 | 31122021\_RID\_3262\_2a.raw |  | 2.4836E6 | 1.3339E6 |  |  | 8.7837E5 | 1.01E6 | 3.6874E6 | 5.1485E7 | 1.5243E7 | 9 | 0 | 1 | 1 | 0 | 0 | 1 | 1 | 1 | 3 | 1 | 619 | 630 |  |  | PEAKS DB |
| D.DTLDSFGEWR.K | N | 48.46 | 1224.5411 | 10 | 0.8 | 613.2783 | 2 | 49.42 | 9 | F9:28898 | 31122021\_RID\_3262\_2a.raw |  |  |  |  |  |  |  |  | 2.2842E5 |  | 1 | 0 | 0 | 0 | 0 | 0 | 0 | 0 | 0 | 1 | 0 | 304 | 313 |  |  | PEAKS DB |
| total 3 peptides |  |  |  |  |  |  |  |  |  |  |  |  |  |  |  |  |  |  |  |  |  |  |  |  |  |  |  |  |  |  |  |  |  |  |  |  |  |
| --- | --- | --- | --- | --- | --- | --- | --- | --- | --- | --- | --- | --- | --- | --- | --- | --- | --- | --- | --- | --- | --- | --- | --- | --- | --- | --- | --- | --- | --- | --- | --- | --- | --- | --- | --- | --- | --- |

XP\_026542921.1

back to list

  

| Protein Coverage
| Supporting Peptides
|

Protein Coverage:

Supporting Peptides:

| Peptide | Uniq | -10lgP | Mass | Length | ppm | m/z | z | RT | Fraction | Scan | Source File | Area F4 | Area F5 | Area F6 | Area F10 | Area F8 | Area F9 | Area F7 | Area F1 | Area F2 | Area F3 | #Feature | #Feature F4 | #Feature F5 | #Feature F6 | #Feature F10 | #Feature F8 | #Feature F9 | #Feature F7 | #Feature F1 | #Feature F2 | #Feature F3 | Start | End | PTM | AScore | Found By |
| --- | --- | --- | --- | --- | --- | --- | --- | --- | --- | --- | --- | --- | --- | --- | --- | --- | --- | --- | --- | --- | --- | --- | --- | --- | --- | --- | --- | --- | --- | --- | --- | --- | --- | --- | --- | --- | --- |
| K.LTVEEAVR.M | N | 43.09 | 915.5025 | 8 | -0.9 | 458.7581 | 2 | 30.27 | 4 | F4:16752 | 17012022\_RID\_3262\_10a .raw |  |  |  | 2.3861E5 |  |  |  |  |  |  | 1 | 0 | 0 | 0 | 1 | 0 | 0 | 0 | 0 | 0 | 0 | 4093 | 4100 |  |  | PEAKS DB |
| K.IISLFQAMK.K | Y | 42.69 | 1049.5944 | 9 | 0.4 | 525.8047 | 2 | 50.54 | 8 | F8:28666 | 31122021\_RID\_3262\_1a.raw |  |  |  |  |  |  |  | 7.3629E4 |  |  | 1 | 0 | 0 | 0 | 0 | 0 | 0 | 0 | 1 | 0 | 0 | 4130 | 4138 |  |  | PEAKS DB |
| total 2 peptides |  |  |  |  |  |  |  |  |  |  |  |  |  |  |  |  |  |  |  |  |  |  |  |  |  |  |  |  |  |  |  |  |  |  |  |  |  |
| --- | --- | --- | --- | --- | --- | --- | --- | --- | --- | --- | --- | --- | --- | --- | --- | --- | --- | --- | --- | --- | --- | --- | --- | --- | --- | --- | --- | --- | --- | --- | --- | --- | --- | --- | --- | --- | --- |

XP\_026577682.1

back to list

  

| Protein Coverage
| Supporting Peptides
|

Protein Coverage:

Supporting Peptides:

| Peptide | Uniq | -10lgP | Mass | Length | ppm | m/z | z | RT | Fraction | Scan | Source File | Area F4 | Area F5 | Area F6 | Area F10 | Area F8 | Area F9 | Area F7 | Area F1 | Area F2 | Area F3 | #Feature | #Feature F4 | #Feature F5 | #Feature F6 | #Feature F10 | #Feature F8 | #Feature F9 | #Feature F7 | #Feature F1 | #Feature F2 | #Feature F3 | Start | End | PTM | AScore | Found By |
| --- | --- | --- | --- | --- | --- | --- | --- | --- | --- | --- | --- | --- | --- | --- | --- | --- | --- | --- | --- | --- | --- | --- | --- | --- | --- | --- | --- | --- | --- | --- | --- | --- | --- | --- | --- | --- | --- |
| K.LTVEEAVR.M | N | 43.09 | 915.5025 | 8 | -0.9 | 458.7581 | 2 | 30.27 | 4 | F4:16752 | 17012022\_RID\_3262\_10a .raw |  |  |  | 2.3861E5 |  |  |  |  |  |  | 1 | 0 | 0 | 0 | 1 | 0 | 0 | 0 | 0 | 0 | 0 | 3893 | 3900 |  |  | PEAKS DB |
| K.IISLFQAMK.K | Y | 42.69 | 1049.5944 | 9 | 0.4 | 525.8047 | 2 | 50.54 | 8 | F8:28666 | 31122021\_RID\_3262\_1a.raw |  |  |  |  |  |  |  | 7.3629E4 |  |  | 1 | 0 | 0 | 0 | 0 | 0 | 0 | 0 | 1 | 0 | 0 | 3930 | 3938 |  |  | PEAKS DB |
| total 2 peptides |  |  |  |  |  |  |  |  |  |  |  |  |  |  |  |  |  |  |  |  |  |  |  |  |  |  |  |  |  |  |  |  |  |  |  |  |  |
| --- | --- | --- | --- | --- | --- | --- | --- | --- | --- | --- | --- | --- | --- | --- | --- | --- | --- | --- | --- | --- | --- | --- | --- | --- | --- | --- | --- | --- | --- | --- | --- | --- | --- | --- | --- | --- | --- |

XP\_026577681.1

back to list

  

| Protein Coverage
| Supporting Peptides
|

Protein Coverage:

Supporting Peptides:

| Peptide | Uniq | -10lgP | Mass | Length | ppm | m/z | z | RT | Fraction | Scan | Source File | Area F4 | Area F5 | Area F6 | Area F10 | Area F8 | Area F9 | Area F7 | Area F1 | Area F2 | Area F3 | #Feature | #Feature F4 | #Feature F5 | #Feature F6 | #Feature F10 | #Feature F8 | #Feature F9 | #Feature F7 | #Feature F1 | #Feature F2 | #Feature F3 | Start | End | PTM | AScore | Found By |
| --- | --- | --- | --- | --- | --- | --- | --- | --- | --- | --- | --- | --- | --- | --- | --- | --- | --- | --- | --- | --- | --- | --- | --- | --- | --- | --- | --- | --- | --- | --- | --- | --- | --- | --- | --- | --- | --- |
| K.LTVEEAVR.M | N | 43.09 | 915.5025 | 8 | -0.9 | 458.7581 | 2 | 30.27 | 4 | F4:16752 | 17012022\_RID\_3262\_10a .raw |  |  |  | 2.3861E5 |  |  |  |  |  |  | 1 | 0 | 0 | 0 | 1 | 0 | 0 | 0 | 0 | 0 | 0 | 3955 | 3962 |  |  | PEAKS DB |
| K.IISLFQAMK.K | Y | 42.69 | 1049.5944 | 9 | 0.4 | 525.8047 | 2 | 50.54 | 8 | F8:28666 | 31122021\_RID\_3262\_1a.raw |  |  |  |  |  |  |  | 7.3629E4 |  |  | 1 | 0 | 0 | 0 | 0 | 0 | 0 | 0 | 1 | 0 | 0 | 3992 | 4000 |  |  | PEAKS DB |
| total 2 peptides |  |  |  |  |  |  |  |  |  |  |  |  |  |  |  |  |  |  |  |  |  |  |  |  |  |  |  |  |  |  |  |  |  |  |  |  |  |
| --- | --- | --- | --- | --- | --- | --- | --- | --- | --- | --- | --- | --- | --- | --- | --- | --- | --- | --- | --- | --- | --- | --- | --- | --- | --- | --- | --- | --- | --- | --- | --- | --- | --- | --- | --- | --- | --- |

XP\_026577680.1

back to list

  

| Protein Coverage
| Supporting Peptides
|

Protein Coverage:

Supporting Peptides:

| Peptide | Uniq | -10lgP | Mass | Length | ppm | m/z | z | RT | Fraction | Scan | Source File | Area F4 | Area F5 | Area F6 | Area F10 | Area F8 | Area F9 | Area F7 | Area F1 | Area F2 | Area F3 | #Feature | #Feature F4 | #Feature F5 | #Feature F6 | #Feature F10 | #Feature F8 | #Feature F9 | #Feature F7 | #Feature F1 | #Feature F2 | #Feature F3 | Start | End | PTM | AScore | Found By |
| --- | --- | --- | --- | --- | --- | --- | --- | --- | --- | --- | --- | --- | --- | --- | --- | --- | --- | --- | --- | --- | --- | --- | --- | --- | --- | --- | --- | --- | --- | --- | --- | --- | --- | --- | --- | --- | --- |
| K.LTVEEAVR.M | N | 43.09 | 915.5025 | 8 | -0.9 | 458.7581 | 2 | 30.27 | 4 | F4:16752 | 17012022\_RID\_3262\_10a .raw |  |  |  | 2.3861E5 |  |  |  |  |  |  | 1 | 0 | 0 | 0 | 1 | 0 | 0 | 0 | 0 | 0 | 0 | 3960 | 3967 |  |  | PEAKS DB |
| K.IISLFQAMK.K | Y | 42.69 | 1049.5944 | 9 | 0.4 | 525.8047 | 2 | 50.54 | 8 | F8:28666 | 31122021\_RID\_3262\_1a.raw |  |  |  |  |  |  |  | 7.3629E4 |  |  | 1 | 0 | 0 | 0 | 0 | 0 | 0 | 0 | 1 | 0 | 0 | 3997 | 4005 |  |  | PEAKS DB |
| total 2 peptides |  |  |  |  |  |  |  |  |  |  |  |  |  |  |  |  |  |  |  |  |  |  |  |  |  |  |  |  |  |  |  |  |  |  |  |  |  |
| --- | --- | --- | --- | --- | --- | --- | --- | --- | --- | --- | --- | --- | --- | --- | --- | --- | --- | --- | --- | --- | --- | --- | --- | --- | --- | --- | --- | --- | --- | --- | --- | --- | --- | --- | --- | --- | --- |

XP\_029140206.1

back to list

  

| Protein Coverage
| Supporting Peptides
|

Protein Coverage:

Supporting Peptides:

| Peptide | Uniq | -10lgP | Mass | Length | ppm | m/z | z | RT | Fraction | Scan | Source File | Area F4 | Area F5 | Area F6 | Area F10 | Area F8 | Area F9 | Area F7 | Area F1 | Area F2 | Area F3 | #Feature | #Feature F4 | #Feature F5 | #Feature F6 | #Feature F10 | #Feature F8 | #Feature F9 | #Feature F7 | #Feature F1 | #Feature F2 | #Feature F3 | Start | End | PTM | AScore | Found By |
| --- | --- | --- | --- | --- | --- | --- | --- | --- | --- | --- | --- | --- | --- | --- | --- | --- | --- | --- | --- | --- | --- | --- | --- | --- | --- | --- | --- | --- | --- | --- | --- | --- | --- | --- | --- | --- | --- |
| K.LTVEEAVR.M | N | 43.09 | 915.5025 | 8 | -0.9 | 458.7581 | 2 | 30.27 | 4 | F4:16752 | 17012022\_RID\_3262\_10a .raw |  |  |  | 2.3861E5 |  |  |  |  |  |  | 1 | 0 | 0 | 0 | 1 | 0 | 0 | 0 | 0 | 0 | 0 | 3893 | 3900 |  |  | PEAKS DB |
| K.IISLFQAMK.K | Y | 42.69 | 1049.5944 | 9 | 0.4 | 525.8047 | 2 | 50.54 | 8 | F8:28666 | 31122021\_RID\_3262\_1a.raw |  |  |  |  |  |  |  | 7.3629E4 |  |  | 1 | 0 | 0 | 0 | 0 | 0 | 0 | 0 | 1 | 0 | 0 | 3930 | 3938 |  |  | PEAKS DB |
| total 2 peptides |  |  |  |  |  |  |  |  |  |  |  |  |  |  |  |  |  |  |  |  |  |  |  |  |  |  |  |  |  |  |  |  |  |  |  |  |  |
| --- | --- | --- | --- | --- | --- | --- | --- | --- | --- | --- | --- | --- | --- | --- | --- | --- | --- | --- | --- | --- | --- | --- | --- | --- | --- | --- | --- | --- | --- | --- | --- | --- | --- | --- | --- | --- | --- |

XP\_026577679.1

back to list

  

| Protein Coverage
| Supporting Peptides
|

Protein Coverage:

Supporting Peptides:

| Peptide | Uniq | -10lgP | Mass | Length | ppm | m/z | z | RT | Fraction | Scan | Source File | Area F4 | Area F5 | Area F6 | Area F10 | Area F8 | Area F9 | Area F7 | Area F1 | Area F2 | Area F3 | #Feature | #Feature F4 | #Feature F5 | #Feature F6 | #Feature F10 | #Feature F8 | #Feature F9 | #Feature F7 | #Feature F1 | #Feature F2 | #Feature F3 | Start | End | PTM | AScore | Found By |
| --- | --- | --- | --- | --- | --- | --- | --- | --- | --- | --- | --- | --- | --- | --- | --- | --- | --- | --- | --- | --- | --- | --- | --- | --- | --- | --- | --- | --- | --- | --- | --- | --- | --- | --- | --- | --- | --- |
| K.LTVEEAVR.M | N | 43.09 | 915.5025 | 8 | -0.9 | 458.7581 | 2 | 30.27 | 4 | F4:16752 | 17012022\_RID\_3262\_10a .raw |  |  |  | 2.3861E5 |  |  |  |  |  |  | 1 | 0 | 0 | 0 | 1 | 0 | 0 | 0 | 0 | 0 | 0 | 3980 | 3987 |  |  | PEAKS DB |
| K.IISLFQAMK.K | Y | 42.69 | 1049.5944 | 9 | 0.4 | 525.8047 | 2 | 50.54 | 8 | F8:28666 | 31122021\_RID\_3262\_1a.raw |  |  |  |  |  |  |  | 7.3629E4 |  |  | 1 | 0 | 0 | 0 | 0 | 0 | 0 | 0 | 1 | 0 | 0 | 4017 | 4025 |  |  | PEAKS DB |
| total 2 peptides |  |  |  |  |  |  |  |  |  |  |  |  |  |  |  |  |  |  |  |  |  |  |  |  |  |  |  |  |  |  |  |  |  |  |  |  |  |
| --- | --- | --- | --- | --- | --- | --- | --- | --- | --- | --- | --- | --- | --- | --- | --- | --- | --- | --- | --- | --- | --- | --- | --- | --- | --- | --- | --- | --- | --- | --- | --- | --- | --- | --- | --- | --- | --- |

XP\_026577678.1

back to list

  

| Protein Coverage
| Supporting Peptides
|

Protein Coverage:

Supporting Peptides:

| Peptide | Uniq | -10lgP | Mass | Length | ppm | m/z | z | RT | Fraction | Scan | Source File | Area F4 | Area F5 | Area F6 | Area F10 | Area F8 | Area F9 | Area F7 | Area F1 | Area F2 | Area F3 | #Feature | #Feature F4 | #Feature F5 | #Feature F6 | #Feature F10 | #Feature F8 | #Feature F9 | #Feature F7 | #Feature F1 | #Feature F2 | #Feature F3 | Start | End | PTM | AScore | Found By |
| --- | --- | --- | --- | --- | --- | --- | --- | --- | --- | --- | --- | --- | --- | --- | --- | --- | --- | --- | --- | --- | --- | --- | --- | --- | --- | --- | --- | --- | --- | --- | --- | --- | --- | --- | --- | --- | --- |
| K.LTVEEAVR.M | N | 43.09 | 915.5025 | 8 | -0.9 | 458.7581 | 2 | 30.27 | 4 | F4:16752 | 17012022\_RID\_3262\_10a .raw |  |  |  | 2.3861E5 |  |  |  |  |  |  | 1 | 0 | 0 | 0 | 1 | 0 | 0 | 0 | 0 | 0 | 0 | 3990 | 3997 |  |  | PEAKS DB |
| K.IISLFQAMK.K | Y | 42.69 | 1049.5944 | 9 | 0.4 | 525.8047 | 2 | 50.54 | 8 | F8:28666 | 31122021\_RID\_3262\_1a.raw |  |  |  |  |  |  |  | 7.3629E4 |  |  | 1 | 0 | 0 | 0 | 0 | 0 | 0 | 0 | 1 | 0 | 0 | 4027 | 4035 |  |  | PEAKS DB |
| total 2 peptides |  |  |  |  |  |  |  |  |  |  |  |  |  |  |  |  |  |  |  |  |  |  |  |  |  |  |  |  |  |  |  |  |  |  |  |  |  |
| --- | --- | --- | --- | --- | --- | --- | --- | --- | --- | --- | --- | --- | --- | --- | --- | --- | --- | --- | --- | --- | --- | --- | --- | --- | --- | --- | --- | --- | --- | --- | --- | --- | --- | --- | --- | --- | --- |

XP\_026577677.1

back to list

  

| Protein Coverage
| Supporting Peptides
|

Protein Coverage:

Supporting Peptides:

| Peptide | Uniq | -10lgP | Mass | Length | ppm | m/z | z | RT | Fraction | Scan | Source File | Area F4 | Area F5 | Area F6 | Area F10 | Area F8 | Area F9 | Area F7 | Area F1 | Area F2 | Area F3 | #Feature | #Feature F4 | #Feature F5 | #Feature F6 | #Feature F10 | #Feature F8 | #Feature F9 | #Feature F7 | #Feature F1 | #Feature F2 | #Feature F3 | Start | End | PTM | AScore | Found By |
| --- | --- | --- | --- | --- | --- | --- | --- | --- | --- | --- | --- | --- | --- | --- | --- | --- | --- | --- | --- | --- | --- | --- | --- | --- | --- | --- | --- | --- | --- | --- | --- | --- | --- | --- | --- | --- | --- |
| K.LTVEEAVR.M | N | 43.09 | 915.5025 | 8 | -0.9 | 458.7581 | 2 | 30.27 | 4 | F4:16752 | 17012022\_RID\_3262\_10a .raw |  |  |  | 2.3861E5 |  |  |  |  |  |  | 1 | 0 | 0 | 0 | 1 | 0 | 0 | 0 | 0 | 0 | 0 | 3992 | 3999 |  |  | PEAKS DB |
| K.IISLFQAMK.K | Y | 42.69 | 1049.5944 | 9 | 0.4 | 525.8047 | 2 | 50.54 | 8 | F8:28666 | 31122021\_RID\_3262\_1a.raw |  |  |  |  |  |  |  | 7.3629E4 |  |  | 1 | 0 | 0 | 0 | 0 | 0 | 0 | 0 | 1 | 0 | 0 | 4029 | 4037 |  |  | PEAKS DB |
| total 2 peptides |  |  |  |  |  |  |  |  |  |  |  |  |  |  |  |  |  |  |  |  |  |  |  |  |  |  |  |  |  |  |  |  |  |  |  |  |  |
| --- | --- | --- | --- | --- | --- | --- | --- | --- | --- | --- | --- | --- | --- | --- | --- | --- | --- | --- | --- | --- | --- | --- | --- | --- | --- | --- | --- | --- | --- | --- | --- | --- | --- | --- | --- | --- | --- |

XP\_026577675.1

back to list

  

| Protein Coverage
| Supporting Peptides
|

Protein Coverage:

Supporting Peptides:

| Peptide | Uniq | -10lgP | Mass | Length | ppm | m/z | z | RT | Fraction | Scan | Source File | Area F4 | Area F5 | Area F6 | Area F10 | Area F8 | Area F9 | Area F7 | Area F1 | Area F2 | Area F3 | #Feature | #Feature F4 | #Feature F5 | #Feature F6 | #Feature F10 | #Feature F8 | #Feature F9 | #Feature F7 | #Feature F1 | #Feature F2 | #Feature F3 | Start | End | PTM | AScore | Found By |
| --- | --- | --- | --- | --- | --- | --- | --- | --- | --- | --- | --- | --- | --- | --- | --- | --- | --- | --- | --- | --- | --- | --- | --- | --- | --- | --- | --- | --- | --- | --- | --- | --- | --- | --- | --- | --- | --- |
| K.LTVEEAVR.M | N | 43.09 | 915.5025 | 8 | -0.9 | 458.7581 | 2 | 30.27 | 4 | F4:16752 | 17012022\_RID\_3262\_10a .raw |  |  |  | 2.3861E5 |  |  |  |  |  |  | 1 | 0 | 0 | 0 | 1 | 0 | 0 | 0 | 0 | 0 | 0 | 3993 | 4000 |  |  | PEAKS DB |
| K.IISLFQAMK.K | Y | 42.69 | 1049.5944 | 9 | 0.4 | 525.8047 | 2 | 50.54 | 8 | F8:28666 | 31122021\_RID\_3262\_1a.raw |  |  |  |  |  |  |  | 7.3629E4 |  |  | 1 | 0 | 0 | 0 | 0 | 0 | 0 | 0 | 1 | 0 | 0 | 4030 | 4038 |  |  | PEAKS DB |
| total 2 peptides |  |  |  |  |  |  |  |  |  |  |  |  |  |  |  |  |  |  |  |  |  |  |  |  |  |  |  |  |  |  |  |  |  |  |  |  |  |
| --- | --- | --- | --- | --- | --- | --- | --- | --- | --- | --- | --- | --- | --- | --- | --- | --- | --- | --- | --- | --- | --- | --- | --- | --- | --- | --- | --- | --- | --- | --- | --- | --- | --- | --- | --- | --- | --- |

XP\_026577674.1

back to list

  

| Protein Coverage
| Supporting Peptides
|

Protein Coverage:

Supporting Peptides:

| Peptide | Uniq | -10lgP | Mass | Length | ppm | m/z | z | RT | Fraction | Scan | Source File | Area F4 | Area F5 | Area F6 | Area F10 | Area F8 | Area F9 | Area F7 | Area F1 | Area F2 | Area F3 | #Feature | #Feature F4 | #Feature F5 | #Feature F6 | #Feature F10 | #Feature F8 | #Feature F9 | #Feature F7 | #Feature F1 | #Feature F2 | #Feature F3 | Start | End | PTM | AScore | Found By |
| --- | --- | --- | --- | --- | --- | --- | --- | --- | --- | --- | --- | --- | --- | --- | --- | --- | --- | --- | --- | --- | --- | --- | --- | --- | --- | --- | --- | --- | --- | --- | --- | --- | --- | --- | --- | --- | --- |
| K.LTVEEAVR.M | N | 43.09 | 915.5025 | 8 | -0.9 | 458.7581 | 2 | 30.27 | 4 | F4:16752 | 17012022\_RID\_3262\_10a .raw |  |  |  | 2.3861E5 |  |  |  |  |  |  | 1 | 0 | 0 | 0 | 1 | 0 | 0 | 0 | 0 | 0 | 0 | 3995 | 4002 |  |  | PEAKS DB |
| K.IISLFQAMK.K | Y | 42.69 | 1049.5944 | 9 | 0.4 | 525.8047 | 2 | 50.54 | 8 | F8:28666 | 31122021\_RID\_3262\_1a.raw |  |  |  |  |  |  |  | 7.3629E4 |  |  | 1 | 0 | 0 | 0 | 0 | 0 | 0 | 0 | 1 | 0 | 0 | 4032 | 4040 |  |  | PEAKS DB |
| total 2 peptides |  |  |  |  |  |  |  |  |  |  |  |  |  |  |  |  |  |  |  |  |  |  |  |  |  |  |  |  |  |  |  |  |  |  |  |  |  |
| --- | --- | --- | --- | --- | --- | --- | --- | --- | --- | --- | --- | --- | --- | --- | --- | --- | --- | --- | --- | --- | --- | --- | --- | --- | --- | --- | --- | --- | --- | --- | --- | --- | --- | --- | --- | --- | --- |

XP\_026577673.1

back to list

  

| Protein Coverage
| Supporting Peptides
|

Protein Coverage:

Supporting Peptides:

| Peptide | Uniq | -10lgP | Mass | Length | ppm | m/z | z | RT | Fraction | Scan | Source File | Area F4 | Area F5 | Area F6 | Area F10 | Area F8 | Area F9 | Area F7 | Area F1 | Area F2 | Area F3 | #Feature | #Feature F4 | #Feature F5 | #Feature F6 | #Feature F10 | #Feature F8 | #Feature F9 | #Feature F7 | #Feature F1 | #Feature F2 | #Feature F3 | Start | End | PTM | AScore | Found By |
| --- | --- | --- | --- | --- | --- | --- | --- | --- | --- | --- | --- | --- | --- | --- | --- | --- | --- | --- | --- | --- | --- | --- | --- | --- | --- | --- | --- | --- | --- | --- | --- | --- | --- | --- | --- | --- | --- |
| K.LTVEEAVR.M | N | 43.09 | 915.5025 | 8 | -0.9 | 458.7581 | 2 | 30.27 | 4 | F4:16752 | 17012022\_RID\_3262\_10a .raw |  |  |  | 2.3861E5 |  |  |  |  |  |  | 1 | 0 | 0 | 0 | 1 | 0 | 0 | 0 | 0 | 0 | 0 | 3997 | 4004 |  |  | PEAKS DB |
| K.IISLFQAMK.K | Y | 42.69 | 1049.5944 | 9 | 0.4 | 525.8047 | 2 | 50.54 | 8 | F8:28666 | 31122021\_RID\_3262\_1a.raw |  |  |  |  |  |  |  | 7.3629E4 |  |  | 1 | 0 | 0 | 0 | 0 | 0 | 0 | 0 | 1 | 0 | 0 | 4034 | 4042 |  |  | PEAKS DB |
| total 2 peptides |  |  |  |  |  |  |  |  |  |  |  |  |  |  |  |  |  |  |  |  |  |  |  |  |  |  |  |  |  |  |  |  |  |  |  |  |  |
| --- | --- | --- | --- | --- | --- | --- | --- | --- | --- | --- | --- | --- | --- | --- | --- | --- | --- | --- | --- | --- | --- | --- | --- | --- | --- | --- | --- | --- | --- | --- | --- | --- | --- | --- | --- | --- | --- |

XP\_026577672.1

back to list

  

| Protein Coverage
| Supporting Peptides
|

Protein Coverage:

Supporting Peptides:

| Peptide | Uniq | -10lgP | Mass | Length | ppm | m/z | z | RT | Fraction | Scan | Source File | Area F4 | Area F5 | Area F6 | Area F10 | Area F8 | Area F9 | Area F7 | Area F1 | Area F2 | Area F3 | #Feature | #Feature F4 | #Feature F5 | #Feature F6 | #Feature F10 | #Feature F8 | #Feature F9 | #Feature F7 | #Feature F1 | #Feature F2 | #Feature F3 | Start | End | PTM | AScore | Found By |
| --- | --- | --- | --- | --- | --- | --- | --- | --- | --- | --- | --- | --- | --- | --- | --- | --- | --- | --- | --- | --- | --- | --- | --- | --- | --- | --- | --- | --- | --- | --- | --- | --- | --- | --- | --- | --- | --- |
| K.LTVEEAVR.M | N | 43.09 | 915.5025 | 8 | -0.9 | 458.7581 | 2 | 30.27 | 4 | F4:16752 | 17012022\_RID\_3262\_10a .raw |  |  |  | 2.3861E5 |  |  |  |  |  |  | 1 | 0 | 0 | 0 | 1 | 0 | 0 | 0 | 0 | 0 | 0 | 3998 | 4005 |  |  | PEAKS DB |
| K.IISLFQAMK.K | Y | 42.69 | 1049.5944 | 9 | 0.4 | 525.8047 | 2 | 50.54 | 8 | F8:28666 | 31122021\_RID\_3262\_1a.raw |  |  |  |  |  |  |  | 7.3629E4 |  |  | 1 | 0 | 0 | 0 | 0 | 0 | 0 | 0 | 1 | 0 | 0 | 4035 | 4043 |  |  | PEAKS DB |
| total 2 peptides |  |  |  |  |  |  |  |  |  |  |  |  |  |  |  |  |  |  |  |  |  |  |  |  |  |  |  |  |  |  |  |  |  |  |  |  |  |
| --- | --- | --- | --- | --- | --- | --- | --- | --- | --- | --- | --- | --- | --- | --- | --- | --- | --- | --- | --- | --- | --- | --- | --- | --- | --- | --- | --- | --- | --- | --- | --- | --- | --- | --- | --- | --- | --- |

XP\_026577671.1

back to list

  

| Protein Coverage
| Supporting Peptides
|

Protein Coverage:

Supporting Peptides:

| Peptide | Uniq | -10lgP | Mass | Length | ppm | m/z | z | RT | Fraction | Scan | Source File | Area F4 | Area F5 | Area F6 | Area F10 | Area F8 | Area F9 | Area F7 | Area F1 | Area F2 | Area F3 | #Feature | #Feature F4 | #Feature F5 | #Feature F6 | #Feature F10 | #Feature F8 | #Feature F9 | #Feature F7 | #Feature F1 | #Feature F2 | #Feature F3 | Start | End | PTM | AScore | Found By |
| --- | --- | --- | --- | --- | --- | --- | --- | --- | --- | --- | --- | --- | --- | --- | --- | --- | --- | --- | --- | --- | --- | --- | --- | --- | --- | --- | --- | --- | --- | --- | --- | --- | --- | --- | --- | --- | --- |
| K.LTVEEAVR.M | N | 43.09 | 915.5025 | 8 | -0.9 | 458.7581 | 2 | 30.27 | 4 | F4:16752 | 17012022\_RID\_3262\_10a .raw |  |  |  | 2.3861E5 |  |  |  |  |  |  | 1 | 0 | 0 | 0 | 1 | 0 | 0 | 0 | 0 | 0 | 0 | 4014 | 4021 |  |  | PEAKS DB |
| K.IISLFQAMK.K | Y | 42.69 | 1049.5944 | 9 | 0.4 | 525.8047 | 2 | 50.54 | 8 | F8:28666 | 31122021\_RID\_3262\_1a.raw |  |  |  |  |  |  |  | 7.3629E4 |  |  | 1 | 0 | 0 | 0 | 0 | 0 | 0 | 0 | 1 | 0 | 0 | 4051 | 4059 |  |  | PEAKS DB |
| total 2 peptides |  |  |  |  |  |  |  |  |  |  |  |  |  |  |  |  |  |  |  |  |  |  |  |  |  |  |  |  |  |  |  |  |  |  |  |  |  |
| --- | --- | --- | --- | --- | --- | --- | --- | --- | --- | --- | --- | --- | --- | --- | --- | --- | --- | --- | --- | --- | --- | --- | --- | --- | --- | --- | --- | --- | --- | --- | --- | --- | --- | --- | --- | --- | --- |

XP\_026577670.1

back to list

  

| Protein Coverage
| Supporting Peptides
|

Protein Coverage:

Supporting Peptides:

| Peptide | Uniq | -10lgP | Mass | Length | ppm | m/z | z | RT | Fraction | Scan | Source File | Area F4 | Area F5 | Area F6 | Area F10 | Area F8 | Area F9 | Area F7 | Area F1 | Area F2 | Area F3 | #Feature | #Feature F4 | #Feature F5 | #Feature F6 | #Feature F10 | #Feature F8 | #Feature F9 | #Feature F7 | #Feature F1 | #Feature F2 | #Feature F3 | Start | End | PTM | AScore | Found By |
| --- | --- | --- | --- | --- | --- | --- | --- | --- | --- | --- | --- | --- | --- | --- | --- | --- | --- | --- | --- | --- | --- | --- | --- | --- | --- | --- | --- | --- | --- | --- | --- | --- | --- | --- | --- | --- | --- |
| K.LTVEEAVR.M | N | 43.09 | 915.5025 | 8 | -0.9 | 458.7581 | 2 | 30.27 | 4 | F4:16752 | 17012022\_RID\_3262\_10a .raw |  |  |  | 2.3861E5 |  |  |  |  |  |  | 1 | 0 | 0 | 0 | 1 | 0 | 0 | 0 | 0 | 0 | 0 | 4019 | 4026 |  |  | PEAKS DB |
| K.IISLFQAMK.K | Y | 42.69 | 1049.5944 | 9 | 0.4 | 525.8047 | 2 | 50.54 | 8 | F8:28666 | 31122021\_RID\_3262\_1a.raw |  |  |  |  |  |  |  | 7.3629E4 |  |  | 1 | 0 | 0 | 0 | 0 | 0 | 0 | 0 | 1 | 0 | 0 | 4056 | 4064 |  |  | PEAKS DB |
| total 2 peptides |  |  |  |  |  |  |  |  |  |  |  |  |  |  |  |  |  |  |  |  |  |  |  |  |  |  |  |  |  |  |  |  |  |  |  |  |  |
| --- | --- | --- | --- | --- | --- | --- | --- | --- | --- | --- | --- | --- | --- | --- | --- | --- | --- | --- | --- | --- | --- | --- | --- | --- | --- | --- | --- | --- | --- | --- | --- | --- | --- | --- | --- | --- | --- |

XP\_034275868.1

back to list

  

| Protein Coverage
| Supporting Peptides
|

Protein Coverage:

Supporting Peptides:

| Peptide | Uniq | -10lgP | Mass | Length | ppm | m/z | z | RT | Fraction | Scan | Source File | Area F4 | Area F5 | Area F6 | Area F10 | Area F8 | Area F9 | Area F7 | Area F1 | Area F2 | Area F3 | #Feature | #Feature F4 | #Feature F5 | #Feature F6 | #Feature F10 | #Feature F8 | #Feature F9 | #Feature F7 | #Feature F1 | #Feature F2 | #Feature F3 | Start | End | PTM | AScore | Found By |
| --- | --- | --- | --- | --- | --- | --- | --- | --- | --- | --- | --- | --- | --- | --- | --- | --- | --- | --- | --- | --- | --- | --- | --- | --- | --- | --- | --- | --- | --- | --- | --- | --- | --- | --- | --- | --- | --- |
| K.LTVEEAVR.M | N | 43.09 | 915.5025 | 8 | -0.9 | 458.7581 | 2 | 30.27 | 4 | F4:16752 | 17012022\_RID\_3262\_10a .raw |  |  |  | 2.3861E5 |  |  |  |  |  |  | 1 | 0 | 0 | 0 | 1 | 0 | 0 | 0 | 0 | 0 | 0 | 3967 | 3974 |  |  | PEAKS DB |
| K.IISLFQAMK.K | Y | 42.69 | 1049.5944 | 9 | 0.4 | 525.8047 | 2 | 50.54 | 8 | F8:28666 | 31122021\_RID\_3262\_1a.raw |  |  |  |  |  |  |  | 7.3629E4 |  |  | 1 | 0 | 0 | 0 | 0 | 0 | 0 | 0 | 1 | 0 | 0 | 4004 | 4012 |  |  | PEAKS DB |
| total 2 peptides |  |  |  |  |  |  |  |  |  |  |  |  |  |  |  |  |  |  |  |  |  |  |  |  |  |  |  |  |  |  |  |  |  |  |  |  |  |
| --- | --- | --- | --- | --- | --- | --- | --- | --- | --- | --- | --- | --- | --- | --- | --- | --- | --- | --- | --- | --- | --- | --- | --- | --- | --- | --- | --- | --- | --- | --- | --- | --- | --- | --- | --- | --- | --- |

XP\_034275867.1

back to list

  

| Protein Coverage
| Supporting Peptides
|

Protein Coverage:

Supporting Peptides:

| Peptide | Uniq | -10lgP | Mass | Length | ppm | m/z | z | RT | Fraction | Scan | Source File | Area F4 | Area F5 | Area F6 | Area F10 | Area F8 | Area F9 | Area F7 | Area F1 | Area F2 | Area F3 | #Feature | #Feature F4 | #Feature F5 | #Feature F6 | #Feature F10 | #Feature F8 | #Feature F9 | #Feature F7 | #Feature F1 | #Feature F2 | #Feature F3 | Start | End | PTM | AScore | Found By |
| --- | --- | --- | --- | --- | --- | --- | --- | --- | --- | --- | --- | --- | --- | --- | --- | --- | --- | --- | --- | --- | --- | --- | --- | --- | --- | --- | --- | --- | --- | --- | --- | --- | --- | --- | --- | --- | --- |
| K.LTVEEAVR.M | N | 43.09 | 915.5025 | 8 | -0.9 | 458.7581 | 2 | 30.27 | 4 | F4:16752 | 17012022\_RID\_3262\_10a .raw |  |  |  | 2.3861E5 |  |  |  |  |  |  | 1 | 0 | 0 | 0 | 1 | 0 | 0 | 0 | 0 | 0 | 0 | 3972 | 3979 |  |  | PEAKS DB |
| K.IISLFQAMK.K | Y | 42.69 | 1049.5944 | 9 | 0.4 | 525.8047 | 2 | 50.54 | 8 | F8:28666 | 31122021\_RID\_3262\_1a.raw |  |  |  |  |  |  |  | 7.3629E4 |  |  | 1 | 0 | 0 | 0 | 0 | 0 | 0 | 0 | 1 | 0 | 0 | 4009 | 4017 |  |  | PEAKS DB |
| total 2 peptides |  |  |  |  |  |  |  |  |  |  |  |  |  |  |  |  |  |  |  |  |  |  |  |  |  |  |  |  |  |  |  |  |  |  |  |  |  |
| --- | --- | --- | --- | --- | --- | --- | --- | --- | --- | --- | --- | --- | --- | --- | --- | --- | --- | --- | --- | --- | --- | --- | --- | --- | --- | --- | --- | --- | --- | --- | --- | --- | --- | --- | --- | --- | --- |

XP\_034275879.1

back to list

  

| Protein Coverage
| Supporting Peptides
|

Protein Coverage:

Supporting Peptides:

| Peptide | Uniq | -10lgP | Mass | Length | ppm | m/z | z | RT | Fraction | Scan | Source File | Area F4 | Area F5 | Area F6 | Area F10 | Area F8 | Area F9 | Area F7 | Area F1 | Area F2 | Area F3 | #Feature | #Feature F4 | #Feature F5 | #Feature F6 | #Feature F10 | #Feature F8 | #Feature F9 | #Feature F7 | #Feature F1 | #Feature F2 | #Feature F3 | Start | End | PTM | AScore | Found By |
| --- | --- | --- | --- | --- | --- | --- | --- | --- | --- | --- | --- | --- | --- | --- | --- | --- | --- | --- | --- | --- | --- | --- | --- | --- | --- | --- | --- | --- | --- | --- | --- | --- | --- | --- | --- | --- | --- |
| K.LTVEEAVR.M | N | 43.09 | 915.5025 | 8 | -0.9 | 458.7581 | 2 | 30.27 | 4 | F4:16752 | 17012022\_RID\_3262\_10a .raw |  |  |  | 2.3861E5 |  |  |  |  |  |  | 1 | 0 | 0 | 0 | 1 | 0 | 0 | 0 | 0 | 0 | 0 | 3992 | 3999 |  |  | PEAKS DB |
| K.IISLFQAMK.K | Y | 42.69 | 1049.5944 | 9 | 0.4 | 525.8047 | 2 | 50.54 | 8 | F8:28666 | 31122021\_RID\_3262\_1a.raw |  |  |  |  |  |  |  | 7.3629E4 |  |  | 1 | 0 | 0 | 0 | 0 | 0 | 0 | 0 | 1 | 0 | 0 | 4029 | 4037 |  |  | PEAKS DB |
| total 2 peptides |  |  |  |  |  |  |  |  |  |  |  |  |  |  |  |  |  |  |  |  |  |  |  |  |  |  |  |  |  |  |  |  |  |  |  |  |  |
| --- | --- | --- | --- | --- | --- | --- | --- | --- | --- | --- | --- | --- | --- | --- | --- | --- | --- | --- | --- | --- | --- | --- | --- | --- | --- | --- | --- | --- | --- | --- | --- | --- | --- | --- | --- | --- | --- |

XP\_026577669.1

back to list

  

| Protein Coverage
| Supporting Peptides
|

Protein Coverage:

Supporting Peptides:

| Peptide | Uniq | -10lgP | Mass | Length | ppm | m/z | z | RT | Fraction | Scan | Source File | Area F4 | Area F5 | Area F6 | Area F10 | Area F8 | Area F9 | Area F7 | Area F1 | Area F2 | Area F3 | #Feature | #Feature F4 | #Feature F5 | #Feature F6 | #Feature F10 | #Feature F8 | #Feature F9 | #Feature F7 | #Feature F1 | #Feature F2 | #Feature F3 | Start | End | PTM | AScore | Found By |
| --- | --- | --- | --- | --- | --- | --- | --- | --- | --- | --- | --- | --- | --- | --- | --- | --- | --- | --- | --- | --- | --- | --- | --- | --- | --- | --- | --- | --- | --- | --- | --- | --- | --- | --- | --- | --- | --- |
| K.LTVEEAVR.M | N | 43.09 | 915.5025 | 8 | -0.9 | 458.7581 | 2 | 30.27 | 4 | F4:16752 | 17012022\_RID\_3262\_10a .raw |  |  |  | 2.3861E5 |  |  |  |  |  |  | 1 | 0 | 0 | 0 | 1 | 0 | 0 | 0 | 0 | 0 | 0 | 4113 | 4120 |  |  | PEAKS DB |
| K.IISLFQAMK.K | Y | 42.69 | 1049.5944 | 9 | 0.4 | 525.8047 | 2 | 50.54 | 8 | F8:28666 | 31122021\_RID\_3262\_1a.raw |  |  |  |  |  |  |  | 7.3629E4 |  |  | 1 | 0 | 0 | 0 | 0 | 0 | 0 | 0 | 1 | 0 | 0 | 4150 | 4158 |  |  | PEAKS DB |
| total 2 peptides |  |  |  |  |  |  |  |  |  |  |  |  |  |  |  |  |  |  |  |  |  |  |  |  |  |  |  |  |  |  |  |  |  |  |  |  |  |
| --- | --- | --- | --- | --- | --- | --- | --- | --- | --- | --- | --- | --- | --- | --- | --- | --- | --- | --- | --- | --- | --- | --- | --- | --- | --- | --- | --- | --- | --- | --- | --- | --- | --- | --- | --- | --- | --- |

XP\_026577668.1

back to list

  

| Protein Coverage
| Supporting Peptides
|

Protein Coverage:

Supporting Peptides:

| Peptide | Uniq | -10lgP | Mass | Length | ppm | m/z | z | RT | Fraction | Scan | Source File | Area F4 | Area F5 | Area F6 | Area F10 | Area F8 | Area F9 | Area F7 | Area F1 | Area F2 | Area F3 | #Feature | #Feature F4 | #Feature F5 | #Feature F6 | #Feature F10 | #Feature F8 | #Feature F9 | #Feature F7 | #Feature F1 | #Feature F2 | #Feature F3 | Start | End | PTM | AScore | Found By |
| --- | --- | --- | --- | --- | --- | --- | --- | --- | --- | --- | --- | --- | --- | --- | --- | --- | --- | --- | --- | --- | --- | --- | --- | --- | --- | --- | --- | --- | --- | --- | --- | --- | --- | --- | --- | --- | --- |
| K.LTVEEAVR.M | N | 43.09 | 915.5025 | 8 | -0.9 | 458.7581 | 2 | 30.27 | 4 | F4:16752 | 17012022\_RID\_3262\_10a .raw |  |  |  | 2.3861E5 |  |  |  |  |  |  | 1 | 0 | 0 | 0 | 1 | 0 | 0 | 0 | 0 | 0 | 0 | 4118 | 4125 |  |  | PEAKS DB |
| K.IISLFQAMK.K | Y | 42.69 | 1049.5944 | 9 | 0.4 | 525.8047 | 2 | 50.54 | 8 | F8:28666 | 31122021\_RID\_3262\_1a.raw |  |  |  |  |  |  |  | 7.3629E4 |  |  | 1 | 0 | 0 | 0 | 0 | 0 | 0 | 0 | 1 | 0 | 0 | 4155 | 4163 |  |  | PEAKS DB |
| total 2 peptides |  |  |  |  |  |  |  |  |  |  |  |  |  |  |  |  |  |  |  |  |  |  |  |  |  |  |  |  |  |  |  |  |  |  |  |  |  |
| --- | --- | --- | --- | --- | --- | --- | --- | --- | --- | --- | --- | --- | --- | --- | --- | --- | --- | --- | --- | --- | --- | --- | --- | --- | --- | --- | --- | --- | --- | --- | --- | --- | --- | --- | --- | --- | --- |

XP\_034275875.1

back to list

  

| Protein Coverage
| Supporting Peptides
|

Protein Coverage:

Supporting Peptides:

| Peptide | Uniq | -10lgP | Mass | Length | ppm | m/z | z | RT | Fraction | Scan | Source File | Area F4 | Area F5 | Area F6 | Area F10 | Area F8 | Area F9 | Area F7 | Area F1 | Area F2 | Area F3 | #Feature | #Feature F4 | #Feature F5 | #Feature F6 | #Feature F10 | #Feature F8 | #Feature F9 | #Feature F7 | #Feature F1 | #Feature F2 | #Feature F3 | Start | End | PTM | AScore | Found By |
| --- | --- | --- | --- | --- | --- | --- | --- | --- | --- | --- | --- | --- | --- | --- | --- | --- | --- | --- | --- | --- | --- | --- | --- | --- | --- | --- | --- | --- | --- | --- | --- | --- | --- | --- | --- | --- | --- |
| K.LTVEEAVR.M | N | 43.09 | 915.5025 | 8 | -0.9 | 458.7581 | 2 | 30.27 | 4 | F4:16752 | 17012022\_RID\_3262\_10a .raw |  |  |  | 2.3861E5 |  |  |  |  |  |  | 1 | 0 | 0 | 0 | 1 | 0 | 0 | 0 | 0 | 0 | 0 | 4004 | 4011 |  |  | PEAKS DB |
| K.IISLFQAMK.K | Y | 42.69 | 1049.5944 | 9 | 0.4 | 525.8047 | 2 | 50.54 | 8 | F8:28666 | 31122021\_RID\_3262\_1a.raw |  |  |  |  |  |  |  | 7.3629E4 |  |  | 1 | 0 | 0 | 0 | 0 | 0 | 0 | 0 | 1 | 0 | 0 | 4041 | 4049 |  |  | PEAKS DB |
| total 2 peptides |  |  |  |  |  |  |  |  |  |  |  |  |  |  |  |  |  |  |  |  |  |  |  |  |  |  |  |  |  |  |  |  |  |  |  |  |  |
| --- | --- | --- | --- | --- | --- | --- | --- | --- | --- | --- | --- | --- | --- | --- | --- | --- | --- | --- | --- | --- | --- | --- | --- | --- | --- | --- | --- | --- | --- | --- | --- | --- | --- | --- | --- | --- | --- |

XP\_034275865.1

back to list

  

| Protein Coverage
| Supporting Peptides
|

Protein Coverage:

Supporting Peptides:

| Peptide | Uniq | -10lgP | Mass | Length | ppm | m/z | z | RT | Fraction | Scan | Source File | Area F4 | Area F5 | Area F6 | Area F10 | Area F8 | Area F9 | Area F7 | Area F1 | Area F2 | Area F3 | #Feature | #Feature F4 | #Feature F5 | #Feature F6 | #Feature F10 | #Feature F8 | #Feature F9 | #Feature F7 | #Feature F1 | #Feature F2 | #Feature F3 | Start | End | PTM | AScore | Found By |
| --- | --- | --- | --- | --- | --- | --- | --- | --- | --- | --- | --- | --- | --- | --- | --- | --- | --- | --- | --- | --- | --- | --- | --- | --- | --- | --- | --- | --- | --- | --- | --- | --- | --- | --- | --- | --- | --- |
| K.LTVEEAVR.M | N | 43.09 | 915.5025 | 8 | -0.9 | 458.7581 | 2 | 30.27 | 4 | F4:16752 | 17012022\_RID\_3262\_10a .raw |  |  |  | 2.3861E5 |  |  |  |  |  |  | 1 | 0 | 0 | 0 | 1 | 0 | 0 | 0 | 0 | 0 | 0 | 4113 | 4120 |  |  | PEAKS DB |
| K.IISLFQAMK.K | Y | 42.69 | 1049.5944 | 9 | 0.4 | 525.8047 | 2 | 50.54 | 8 | F8:28666 | 31122021\_RID\_3262\_1a.raw |  |  |  |  |  |  |  | 7.3629E4 |  |  | 1 | 0 | 0 | 0 | 0 | 0 | 0 | 0 | 1 | 0 | 0 | 4150 | 4158 |  |  | PEAKS DB |
| total 2 peptides |  |  |  |  |  |  |  |  |  |  |  |  |  |  |  |  |  |  |  |  |  |  |  |  |  |  |  |  |  |  |  |  |  |  |  |  |  |
| --- | --- | --- | --- | --- | --- | --- | --- | --- | --- | --- | --- | --- | --- | --- | --- | --- | --- | --- | --- | --- | --- | --- | --- | --- | --- | --- | --- | --- | --- | --- | --- | --- | --- | --- | --- | --- | --- |

XP\_034275864.1

back to list

  

| Protein Coverage
| Supporting Peptides
|

Protein Coverage:

Supporting Peptides:

| Peptide | Uniq | -10lgP | Mass | Length | ppm | m/z | z | RT | Fraction | Scan | Source File | Area F4 | Area F5 | Area F6 | Area F10 | Area F8 | Area F9 | Area F7 | Area F1 | Area F2 | Area F3 | #Feature | #Feature F4 | #Feature F5 | #Feature F6 | #Feature F10 | #Feature F8 | #Feature F9 | #Feature F7 | #Feature F1 | #Feature F2 | #Feature F3 | Start | End | PTM | AScore | Found By |
| --- | --- | --- | --- | --- | --- | --- | --- | --- | --- | --- | --- | --- | --- | --- | --- | --- | --- | --- | --- | --- | --- | --- | --- | --- | --- | --- | --- | --- | --- | --- | --- | --- | --- | --- | --- | --- | --- |
| K.LTVEEAVR.M | N | 43.09 | 915.5025 | 8 | -0.9 | 458.7581 | 2 | 30.27 | 4 | F4:16752 | 17012022\_RID\_3262\_10a .raw |  |  |  | 2.3861E5 |  |  |  |  |  |  | 1 | 0 | 0 | 0 | 1 | 0 | 0 | 0 | 0 | 0 | 0 | 4118 | 4125 |  |  | PEAKS DB |
| K.IISLFQAMK.K | Y | 42.69 | 1049.5944 | 9 | 0.4 | 525.8047 | 2 | 50.54 | 8 | F8:28666 | 31122021\_RID\_3262\_1a.raw |  |  |  |  |  |  |  | 7.3629E4 |  |  | 1 | 0 | 0 | 0 | 0 | 0 | 0 | 0 | 1 | 0 | 0 | 4155 | 4163 |  |  | PEAKS DB |
| total 2 peptides |  |  |  |  |  |  |  |  |  |  |  |  |  |  |  |  |  |  |  |  |  |  |  |  |  |  |  |  |  |  |  |  |  |  |  |  |  |
| --- | --- | --- | --- | --- | --- | --- | --- | --- | --- | --- | --- | --- | --- | --- | --- | --- | --- | --- | --- | --- | --- | --- | --- | --- | --- | --- | --- | --- | --- | --- | --- | --- | --- | --- | --- | --- | --- |

XP\_034275862.1

back to list

  

| Protein Coverage
| Supporting Peptides
|

Protein Coverage:

Supporting Peptides:

| Peptide | Uniq | -10lgP | Mass | Length | ppm | m/z | z | RT | Fraction | Scan | Source File | Area F4 | Area F5 | Area F6 | Area F10 | Area F8 | Area F9 | Area F7 | Area F1 | Area F2 | Area F3 | #Feature | #Feature F4 | #Feature F5 | #Feature F6 | #Feature F10 | #Feature F8 | #Feature F9 | #Feature F7 | #Feature F1 | #Feature F2 | #Feature F3 | Start | End | PTM | AScore | Found By |
| --- | --- | --- | --- | --- | --- | --- | --- | --- | --- | --- | --- | --- | --- | --- | --- | --- | --- | --- | --- | --- | --- | --- | --- | --- | --- | --- | --- | --- | --- | --- | --- | --- | --- | --- | --- | --- | --- |
| K.LTVEEAVR.M | N | 43.09 | 915.5025 | 8 | -0.9 | 458.7581 | 2 | 30.27 | 4 | F4:16752 | 17012022\_RID\_3262\_10a .raw |  |  |  | 2.3861E5 |  |  |  |  |  |  | 1 | 0 | 0 | 0 | 1 | 0 | 0 | 0 | 0 | 0 | 0 | 4125 | 4132 |  |  | PEAKS DB |
| K.IISLFQAMK.K | Y | 42.69 | 1049.5944 | 9 | 0.4 | 525.8047 | 2 | 50.54 | 8 | F8:28666 | 31122021\_RID\_3262\_1a.raw |  |  |  |  |  |  |  | 7.3629E4 |  |  | 1 | 0 | 0 | 0 | 0 | 0 | 0 | 0 | 1 | 0 | 0 | 4162 | 4170 |  |  | PEAKS DB |
| total 2 peptides |  |  |  |  |  |  |  |  |  |  |  |  |  |  |  |  |  |  |  |  |  |  |  |  |  |  |  |  |  |  |  |  |  |  |  |  |  |
| --- | --- | --- | --- | --- | --- | --- | --- | --- | --- | --- | --- | --- | --- | --- | --- | --- | --- | --- | --- | --- | --- | --- | --- | --- | --- | --- | --- | --- | --- | --- | --- | --- | --- | --- | --- | --- | --- |

XP\_034275860.1

back to list

  

| Protein Coverage
| Supporting Peptides
|

Protein Coverage:

Supporting Peptides:

| Peptide | Uniq | -10lgP | Mass | Length | ppm | m/z | z | RT | Fraction | Scan | Source File | Area F4 | Area F5 | Area F6 | Area F10 | Area F8 | Area F9 | Area F7 | Area F1 | Area F2 | Area F3 | #Feature | #Feature F4 | #Feature F5 | #Feature F6 | #Feature F10 | #Feature F8 | #Feature F9 | #Feature F7 | #Feature F1 | #Feature F2 | #Feature F3 | Start | End | PTM | AScore | Found By |
| --- | --- | --- | --- | --- | --- | --- | --- | --- | --- | --- | --- | --- | --- | --- | --- | --- | --- | --- | --- | --- | --- | --- | --- | --- | --- | --- | --- | --- | --- | --- | --- | --- | --- | --- | --- | --- | --- |
| K.LTVEEAVR.M | N | 43.09 | 915.5025 | 8 | -0.9 | 458.7581 | 2 | 30.27 | 4 | F4:16752 | 17012022\_RID\_3262\_10a .raw |  |  |  | 2.3861E5 |  |  |  |  |  |  | 1 | 0 | 0 | 0 | 1 | 0 | 0 | 0 | 0 | 0 | 0 | 4130 | 4137 |  |  | PEAKS DB |
| K.IISLFQAMK.K | Y | 42.69 | 1049.5944 | 9 | 0.4 | 525.8047 | 2 | 50.54 | 8 | F8:28666 | 31122021\_RID\_3262\_1a.raw |  |  |  |  |  |  |  | 7.3629E4 |  |  | 1 | 0 | 0 | 0 | 0 | 0 | 0 | 0 | 1 | 0 | 0 | 4167 | 4175 |  |  | PEAKS DB |
| total 2 peptides |  |  |  |  |  |  |  |  |  |  |  |  |  |  |  |  |  |  |  |  |  |  |  |  |  |  |  |  |  |  |  |  |  |  |  |  |  |
| --- | --- | --- | --- | --- | --- | --- | --- | --- | --- | --- | --- | --- | --- | --- | --- | --- | --- | --- | --- | --- | --- | --- | --- | --- | --- | --- | --- | --- | --- | --- | --- | --- | --- | --- | --- | --- | --- |

XP\_034275866.1

back to list

  

| Protein Coverage
| Supporting Peptides
|

Protein Coverage:

Supporting Peptides:

| Peptide | Uniq | -10lgP | Mass | Length | ppm | m/z | z | RT | Fraction | Scan | Source File | Area F4 | Area F5 | Area F6 | Area F10 | Area F8 | Area F9 | Area F7 | Area F1 | Area F2 | Area F3 | #Feature | #Feature F4 | #Feature F5 | #Feature F6 | #Feature F10 | #Feature F8 | #Feature F9 | #Feature F7 | #Feature F1 | #Feature F2 | #Feature F3 | Start | End | PTM | AScore | Found By |
| --- | --- | --- | --- | --- | --- | --- | --- | --- | --- | --- | --- | --- | --- | --- | --- | --- | --- | --- | --- | --- | --- | --- | --- | --- | --- | --- | --- | --- | --- | --- | --- | --- | --- | --- | --- | --- | --- |
| K.LTVEEAVR.M | N | 43.09 | 915.5025 | 8 | -0.9 | 458.7581 | 2 | 30.27 | 4 | F4:16752 | 17012022\_RID\_3262\_10a .raw |  |  |  | 2.3861E5 |  |  |  |  |  |  | 1 | 0 | 0 | 0 | 1 | 0 | 0 | 0 | 0 | 0 | 0 | 4111 | 4118 |  |  | PEAKS DB |
| K.IISLFQAMK.K | Y | 42.69 | 1049.5944 | 9 | 0.4 | 525.8047 | 2 | 50.54 | 8 | F8:28666 | 31122021\_RID\_3262\_1a.raw |  |  |  |  |  |  |  | 7.3629E4 |  |  | 1 | 0 | 0 | 0 | 0 | 0 | 0 | 0 | 1 | 0 | 0 | 4148 | 4156 |  |  | PEAKS DB |
| total 2 peptides |  |  |  |  |  |  |  |  |  |  |  |  |  |  |  |  |  |  |  |  |  |  |  |  |  |  |  |  |  |  |  |  |  |  |  |  |  |
| --- | --- | --- | --- | --- | --- | --- | --- | --- | --- | --- | --- | --- | --- | --- | --- | --- | --- | --- | --- | --- | --- | --- | --- | --- | --- | --- | --- | --- | --- | --- | --- | --- | --- | --- | --- | --- | --- |

XP\_034275863.1

back to list

  

| Protein Coverage
| Supporting Peptides
|

Protein Coverage:

Supporting Peptides:

| Peptide | Uniq | -10lgP | Mass | Length | ppm | m/z | z | RT | Fraction | Scan | Source File | Area F4 | Area F5 | Area F6 | Area F10 | Area F8 | Area F9 | Area F7 | Area F1 | Area F2 | Area F3 | #Feature | #Feature F4 | #Feature F5 | #Feature F6 | #Feature F10 | #Feature F8 | #Feature F9 | #Feature F7 | #Feature F1 | #Feature F2 | #Feature F3 | Start | End | PTM | AScore | Found By |
| --- | --- | --- | --- | --- | --- | --- | --- | --- | --- | --- | --- | --- | --- | --- | --- | --- | --- | --- | --- | --- | --- | --- | --- | --- | --- | --- | --- | --- | --- | --- | --- | --- | --- | --- | --- | --- | --- |
| K.LTVEEAVR.M | N | 43.09 | 915.5025 | 8 | -0.9 | 458.7581 | 2 | 30.27 | 4 | F4:16752 | 17012022\_RID\_3262\_10a .raw |  |  |  | 2.3861E5 |  |  |  |  |  |  | 1 | 0 | 0 | 0 | 1 | 0 | 0 | 0 | 0 | 0 | 0 | 4123 | 4130 |  |  | PEAKS DB |
| K.IISLFQAMK.K | Y | 42.69 | 1049.5944 | 9 | 0.4 | 525.8047 | 2 | 50.54 | 8 | F8:28666 | 31122021\_RID\_3262\_1a.raw |  |  |  |  |  |  |  | 7.3629E4 |  |  | 1 | 0 | 0 | 0 | 0 | 0 | 0 | 0 | 1 | 0 | 0 | 4160 | 4168 |  |  | PEAKS DB |
| total 2 peptides |  |  |  |  |  |  |  |  |  |  |  |  |  |  |  |  |  |  |  |  |  |  |  |  |  |  |  |  |  |  |  |  |  |  |  |  |  |
| --- | --- | --- | --- | --- | --- | --- | --- | --- | --- | --- | --- | --- | --- | --- | --- | --- | --- | --- | --- | --- | --- | --- | --- | --- | --- | --- | --- | --- | --- | --- | --- | --- | --- | --- | --- | --- | --- |

XP\_034275861.1

back to list

  

| Protein Coverage
| Supporting Peptides
|

Protein Coverage:

Supporting Peptides:

| Peptide | Uniq | -10lgP | Mass | Length | ppm | m/z | z | RT | Fraction | Scan | Source File | Area F4 | Area F5 | Area F6 | Area F10 | Area F8 | Area F9 | Area F7 | Area F1 | Area F2 | Area F3 | #Feature | #Feature F4 | #Feature F5 | #Feature F6 | #Feature F10 | #Feature F8 | #Feature F9 | #Feature F7 | #Feature F1 | #Feature F2 | #Feature F3 | Start | End | PTM | AScore | Found By |
| --- | --- | --- | --- | --- | --- | --- | --- | --- | --- | --- | --- | --- | --- | --- | --- | --- | --- | --- | --- | --- | --- | --- | --- | --- | --- | --- | --- | --- | --- | --- | --- | --- | --- | --- | --- | --- | --- |
| K.LTVEEAVR.M | N | 43.09 | 915.5025 | 8 | -0.9 | 458.7581 | 2 | 30.27 | 4 | F4:16752 | 17012022\_RID\_3262\_10a .raw |  |  |  | 2.3861E5 |  |  |  |  |  |  | 1 | 0 | 0 | 0 | 1 | 0 | 0 | 0 | 0 | 0 | 0 | 4128 | 4135 |  |  | PEAKS DB |
| K.IISLFQAMK.K | Y | 42.69 | 1049.5944 | 9 | 0.4 | 525.8047 | 2 | 50.54 | 8 | F8:28666 | 31122021\_RID\_3262\_1a.raw |  |  |  |  |  |  |  | 7.3629E4 |  |  | 1 | 0 | 0 | 0 | 0 | 0 | 0 | 0 | 1 | 0 | 0 | 4165 | 4173 |  |  | PEAKS DB |
| total 2 peptides |  |  |  |  |  |  |  |  |  |  |  |  |  |  |  |  |  |  |  |  |  |  |  |  |  |  |  |  |  |  |  |  |  |  |  |  |  |
| --- | --- | --- | --- | --- | --- | --- | --- | --- | --- | --- | --- | --- | --- | --- | --- | --- | --- | --- | --- | --- | --- | --- | --- | --- | --- | --- | --- | --- | --- | --- | --- | --- | --- | --- | --- | --- | --- |

XP\_015682690.1

back to list

  

| Protein Coverage
| Supporting Peptides
|

Protein Coverage:

Supporting Peptides:

| Peptide | Uniq | -10lgP | Mass | Length | ppm | m/z | z | RT | Fraction | Scan | Source File | Area F4 | Area F5 | Area F6 | Area F10 | Area F8 | Area F9 | Area F7 | Area F1 | Area F2 | Area F3 | #Feature | #Feature F4 | #Feature F5 | #Feature F6 | #Feature F10 | #Feature F8 | #Feature F9 | #Feature F7 | #Feature F1 | #Feature F2 | #Feature F3 | Start | End | PTM | AScore | Found By |
| --- | --- | --- | --- | --- | --- | --- | --- | --- | --- | --- | --- | --- | --- | --- | --- | --- | --- | --- | --- | --- | --- | --- | --- | --- | --- | --- | --- | --- | --- | --- | --- | --- | --- | --- | --- | --- | --- |
| K.LMNQNMDILEDAIQK.A | Y | 72.72 | 1774.8594 | 15 | 0.7 | 888.4376 | 2 | 52.23 | 8 | F8:29977 | 31122021\_RID\_3262\_1a.raw |  |  |  |  |  |  |  | 2.4656E5 | 8.5492E5 |  | 2 | 0 | 0 | 0 | 0 | 0 | 0 | 0 | 1 | 1 | 0 | 54 | 68 |  |  | PEAKS DB |
| K.LM(+15.99)NQNM(+15.99)DILEDAIQK.A | Y | 51.44 | 1806.8491 | 15 | 0.8 | 904.4326 | 2 | 51.91 | 9 | F9:30819 | 31122021\_RID\_3262\_2a.raw |  |  |  |  |  |  |  |  | 5.8872E5 |  | 1 | 0 | 0 | 0 | 0 | 0 | 0 | 0 | 0 | 1 | 0 | 54 | 68 | Oxidation (M) | M2:Oxidation (M):1000.00;M6:Oxidation (M):1000.00 | PEAKS DB |
| K.LMNQNM(+15.99)DILEDAIQK.A | Y | 47.63 | 1790.8542 | 15 | 0.5 | 896.4348 | 2 | 45.12 | 9 | F9:25608 | 31122021\_RID\_3262\_2a.raw |  |  |  |  |  |  |  |  | 9.4582E4 |  | 1 | 0 | 0 | 0 | 0 | 0 | 0 | 0 | 0 | 1 | 0 | 54 | 68 | Oxidation (M) | M6:Oxidation (M):38.03 | PEAKS DB |
| R.FGPAPVQK.R | Y | 44.69 | 842.4650 | 8 | 0.6 | 422.2400 | 2 | 25.32 | 1 | F1:12209 | 13012022\_RID\_3262\_4a.raw | 1.1731E5 |  |  |  |  |  |  |  |  |  | 1 | 1 | 0 | 0 | 0 | 0 | 0 | 0 | 0 | 0 | 0 | 117 | 124 |  |  | PEAKS DB |
| total 4 peptides |  |  |  |  |  |  |  |  |  |  |  |  |  |  |  |  |  |  |  |  |  |  |  |  |  |  |  |  |  |  |  |  |  |  |  |  |  |
| --- | --- | --- | --- | --- | --- | --- | --- | --- | --- | --- | --- | --- | --- | --- | --- | --- | --- | --- | --- | --- | --- | --- | --- | --- | --- | --- | --- | --- | --- | --- | --- | --- | --- | --- | --- | --- | --- |

JAG66642.1

back to list

  

| Protein Coverage
| Supporting Peptides
|

Protein Coverage:

Supporting Peptides:

| Peptide | Uniq | -10lgP | Mass | Length | ppm | m/z | z | RT | Fraction | Scan | Source File | Area F4 | Area F5 | Area F6 | Area F10 | Area F8 | Area F9 | Area F7 | Area F1 | Area F2 | Area F3 | #Feature | #Feature F4 | #Feature F5 | #Feature F6 | #Feature F10 | #Feature F8 | #Feature F9 | #Feature F7 | #Feature F1 | #Feature F2 | #Feature F3 | Start | End | PTM | AScore | Found By |
| --- | --- | --- | --- | --- | --- | --- | --- | --- | --- | --- | --- | --- | --- | --- | --- | --- | --- | --- | --- | --- | --- | --- | --- | --- | --- | --- | --- | --- | --- | --- | --- | --- | --- | --- | --- | --- | --- |
| R.TLSDYNIQK.E | Y | 60.06 | 1080.5452 | 9 | 1.2 | 541.2805 | 2 | 29.38 | 6 | F6:16185 | 17012022\_RID\_3262\_9a.raw |  |  |  |  |  | 1.2579E6 |  | 4.0613E5 |  |  | 2 | 0 | 0 | 0 | 0 | 0 | 1 | 0 | 1 | 0 | 0 | 55 | 63 |  |  | PEAKS DB |
| K.ESTLHLVLR.L | Y | 56.05 | 1066.6135 | 9 | -0.1 | 534.3140 | 2 | 34.82 | 6 | F6:20918 | 17012022\_RID\_3262\_9a.raw |  |  |  |  |  | 2.5278E5 |  |  |  |  | 1 | 0 | 0 | 0 | 0 | 0 | 1 | 0 | 0 | 0 | 0 | 64 | 72 |  |  | PEAKS DB |
| K.TITLEVEPSDTIENVK.A | Y | 50.54 | 1786.9200 | 16 | 0.5 | 894.4678 | 2 | 46.47 | 8 | F8:25378 | 31122021\_RID\_3262\_1a.raw |  |  |  |  |  |  |  | 1.3488E6 |  |  | 1 | 0 | 0 | 0 | 0 | 0 | 0 | 0 | 1 | 0 | 0 | 12 | 27 |  |  | PEAKS DB |
| total 3 peptides |  |  |  |  |  |  |  |  |  |  |  |  |  |  |  |  |  |  |  |  |  |  |  |  |  |  |  |  |  |  |  |  |  |  |  |  |  |
| --- | --- | --- | --- | --- | --- | --- | --- | --- | --- | --- | --- | --- | --- | --- | --- | --- | --- | --- | --- | --- | --- | --- | --- | --- | --- | --- | --- | --- | --- | --- | --- | --- | --- | --- | --- | --- | --- |

JAA95751.1

back to list

  

| Protein Coverage
| Supporting Peptides
|

Protein Coverage:

Supporting Peptides:

| Peptide | Uniq | -10lgP | Mass | Length | ppm | m/z | z | RT | Fraction | Scan | Source File | Area F4 | Area F5 | Area F6 | Area F10 | Area F8 | Area F9 | Area F7 | Area F1 | Area F2 | Area F3 | #Feature | #Feature F4 | #Feature F5 | #Feature F6 | #Feature F10 | #Feature F8 | #Feature F9 | #Feature F7 | #Feature F1 | #Feature F2 | #Feature F3 | Start | End | PTM | AScore | Found By |
| --- | --- | --- | --- | --- | --- | --- | --- | --- | --- | --- | --- | --- | --- | --- | --- | --- | --- | --- | --- | --- | --- | --- | --- | --- | --- | --- | --- | --- | --- | --- | --- | --- | --- | --- | --- | --- | --- |
| R.TLSDYNIQK.E | Y | 60.06 | 1080.5452 | 9 | 1.2 | 541.2805 | 2 | 29.38 | 6 | F6:16185 | 17012022\_RID\_3262\_9a.raw |  |  |  |  |  | 1.2579E6 |  | 4.0613E5 |  |  | 2 | 0 | 0 | 0 | 0 | 0 | 1 | 0 | 1 | 0 | 0 | 55 | 63 |  |  | PEAKS DB |
| K.ESTLHLVLR.L | Y | 56.05 | 1066.6135 | 9 | -0.1 | 534.3140 | 2 | 34.82 | 6 | F6:20918 | 17012022\_RID\_3262\_9a.raw |  |  |  |  |  | 2.5278E5 |  |  |  |  | 1 | 0 | 0 | 0 | 0 | 0 | 1 | 0 | 0 | 0 | 0 | 64 | 72 |  |  | PEAKS DB |
| K.TITLEVEPSDTIENVK.A | Y | 50.54 | 1786.9200 | 16 | 0.5 | 894.4678 | 2 | 46.47 | 8 | F8:25378 | 31122021\_RID\_3262\_1a.raw |  |  |  |  |  |  |  | 1.3488E6 |  |  | 1 | 0 | 0 | 0 | 0 | 0 | 0 | 0 | 1 | 0 | 0 | 12 | 27 |  |  | PEAKS DB |
| total 3 peptides |  |  |  |  |  |  |  |  |  |  |  |  |  |  |  |  |  |  |  |  |  |  |  |  |  |  |  |  |  |  |  |  |  |  |  |  |  |
| --- | --- | --- | --- | --- | --- | --- | --- | --- | --- | --- | --- | --- | --- | --- | --- | --- | --- | --- | --- | --- | --- | --- | --- | --- | --- | --- | --- | --- | --- | --- | --- | --- | --- | --- | --- | --- | --- |

JAB53407.1

back to list

  

| Protein Coverage
| Supporting Peptides
|

Protein Coverage:

Supporting Peptides:

| Peptide | Uniq | -10lgP | Mass | Length | ppm | m/z | z | RT | Fraction | Scan | Source File | Area F4 | Area F5 | Area F6 | Area F10 | Area F8 | Area F9 | Area F7 | Area F1 | Area F2 | Area F3 | #Feature | #Feature F4 | #Feature F5 | #Feature F6 | #Feature F10 | #Feature F8 | #Feature F9 | #Feature F7 | #Feature F1 | #Feature F2 | #Feature F3 | Start | End | PTM | AScore | Found By |
| --- | --- | --- | --- | --- | --- | --- | --- | --- | --- | --- | --- | --- | --- | --- | --- | --- | --- | --- | --- | --- | --- | --- | --- | --- | --- | --- | --- | --- | --- | --- | --- | --- | --- | --- | --- | --- | --- |
| R.TLSDYNIQK.E | Y | 60.06 | 1080.5452 | 9 | 1.2 | 541.2805 | 2 | 29.38 | 6 | F6:16185 | 17012022\_RID\_3262\_9a.raw |  |  |  |  |  | 1.2579E6 |  | 4.0613E5 |  |  | 2 | 0 | 0 | 0 | 0 | 0 | 1 | 0 | 1 | 0 | 0 | 55 | 63 |  |  | PEAKS DB |
| K.ESTLHLVLR.L | Y | 56.05 | 1066.6135 | 9 | -0.1 | 534.3140 | 2 | 34.82 | 6 | F6:20918 | 17012022\_RID\_3262\_9a.raw |  |  |  |  |  | 2.5278E5 |  |  |  |  | 1 | 0 | 0 | 0 | 0 | 0 | 1 | 0 | 0 | 0 | 0 | 64 | 72 |  |  | PEAKS DB |
| K.TITLEVEPSDTIENVK.A | Y | 50.54 | 1786.9200 | 16 | 0.5 | 894.4678 | 2 | 46.47 | 8 | F8:25378 | 31122021\_RID\_3262\_1a.raw |  |  |  |  |  |  |  | 1.3488E6 |  |  | 1 | 0 | 0 | 0 | 0 | 0 | 0 | 0 | 1 | 0 | 0 | 12 | 27 |  |  | PEAKS DB |
| total 3 peptides |  |  |  |  |  |  |  |  |  |  |  |  |  |  |  |  |  |  |  |  |  |  |  |  |  |  |  |  |  |  |  |  |  |  |  |  |  |
| --- | --- | --- | --- | --- | --- | --- | --- | --- | --- | --- | --- | --- | --- | --- | --- | --- | --- | --- | --- | --- | --- | --- | --- | --- | --- | --- | --- | --- | --- | --- | --- | --- | --- | --- | --- | --- | --- |

JAI11580.1

back to list

  

| Protein Coverage
| Supporting Peptides
|

Protein Coverage:

Supporting Peptides:

| Peptide | Uniq | -10lgP | Mass | Length | ppm | m/z | z | RT | Fraction | Scan | Source File | Area F4 | Area F5 | Area F6 | Area F10 | Area F8 | Area F9 | Area F7 | Area F1 | Area F2 | Area F3 | #Feature | #Feature F4 | #Feature F5 | #Feature F6 | #Feature F10 | #Feature F8 | #Feature F9 | #Feature F7 | #Feature F1 | #Feature F2 | #Feature F3 | Start | End | PTM | AScore | Found By |
| --- | --- | --- | --- | --- | --- | --- | --- | --- | --- | --- | --- | --- | --- | --- | --- | --- | --- | --- | --- | --- | --- | --- | --- | --- | --- | --- | --- | --- | --- | --- | --- | --- | --- | --- | --- | --- | --- |
| R.TLSDYNIQK.E | Y | 60.06 | 1080.5452 | 9 | 1.2 | 541.2805 | 2 | 29.38 | 6 | F6:16185 | 17012022\_RID\_3262\_9a.raw |  |  |  |  |  | 1.2579E6 |  | 4.0613E5 |  |  | 2 | 0 | 0 | 0 | 0 | 0 | 1 | 0 | 1 | 0 | 0 | 55 | 63 |  |  | PEAKS DB |
| K.ESTLHLVLR.L | Y | 56.05 | 1066.6135 | 9 | -0.1 | 534.3140 | 2 | 34.82 | 6 | F6:20918 | 17012022\_RID\_3262\_9a.raw |  |  |  |  |  | 2.5278E5 |  |  |  |  | 1 | 0 | 0 | 0 | 0 | 0 | 1 | 0 | 0 | 0 | 0 | 64 | 72 |  |  | PEAKS DB |
| K.TITLEVEPSDTIENVK.A | Y | 50.54 | 1786.9200 | 16 | 0.5 | 894.4678 | 2 | 46.47 | 8 | F8:25378 | 31122021\_RID\_3262\_1a.raw |  |  |  |  |  |  |  | 1.3488E6 |  |  | 1 | 0 | 0 | 0 | 0 | 0 | 0 | 0 | 1 | 0 | 0 | 12 | 27 |  |  | PEAKS DB |
| total 3 peptides |  |  |  |  |  |  |  |  |  |  |  |  |  |  |  |  |  |  |  |  |  |  |  |  |  |  |  |  |  |  |  |  |  |  |  |  |  |
| --- | --- | --- | --- | --- | --- | --- | --- | --- | --- | --- | --- | --- | --- | --- | --- | --- | --- | --- | --- | --- | --- | --- | --- | --- | --- | --- | --- | --- | --- | --- | --- | --- | --- | --- | --- | --- | --- |

JAI09470.1

back to list

  

| Protein Coverage
| Supporting Peptides
|

Protein Coverage:

Supporting Peptides:

| Peptide | Uniq | -10lgP | Mass | Length | ppm | m/z | z | RT | Fraction | Scan | Source File | Area F4 | Area F5 | Area F6 | Area F10 | Area F8 | Area F9 | Area F7 | Area F1 | Area F2 | Area F3 | #Feature | #Feature F4 | #Feature F5 | #Feature F6 | #Feature F10 | #Feature F8 | #Feature F9 | #Feature F7 | #Feature F1 | #Feature F2 | #Feature F3 | Start | End | PTM | AScore | Found By |
| --- | --- | --- | --- | --- | --- | --- | --- | --- | --- | --- | --- | --- | --- | --- | --- | --- | --- | --- | --- | --- | --- | --- | --- | --- | --- | --- | --- | --- | --- | --- | --- | --- | --- | --- | --- | --- | --- |
| R.TLSDYNIQK.E | Y | 60.06 | 1080.5452 | 9 | 1.2 | 541.2805 | 2 | 29.38 | 6 | F6:16185 | 17012022\_RID\_3262\_9a.raw |  |  |  |  |  | 1.2579E6 |  | 4.0613E5 |  |  | 2 | 0 | 0 | 0 | 0 | 0 | 1 | 0 | 1 | 0 | 0 | 55 | 63 |  |  | PEAKS DB |
| K.ESTLHLVLR.L | Y | 56.05 | 1066.6135 | 9 | -0.1 | 534.3140 | 2 | 34.82 | 6 | F6:20918 | 17012022\_RID\_3262\_9a.raw |  |  |  |  |  | 2.5278E5 |  |  |  |  | 1 | 0 | 0 | 0 | 0 | 0 | 1 | 0 | 0 | 0 | 0 | 64 | 72 |  |  | PEAKS DB |
| K.TITLEVEPSDTIENVK.A | Y | 50.54 | 1786.9200 | 16 | 0.5 | 894.4678 | 2 | 46.47 | 8 | F8:25378 | 31122021\_RID\_3262\_1a.raw |  |  |  |  |  |  |  | 1.3488E6 |  |  | 1 | 0 | 0 | 0 | 0 | 0 | 0 | 0 | 1 | 0 | 0 | 12 | 27 |  |  | PEAKS DB |
| total 3 peptides |  |  |  |  |  |  |  |  |  |  |  |  |  |  |  |  |  |  |  |  |  |  |  |  |  |  |  |  |  |  |  |  |  |  |  |  |  |
| --- | --- | --- | --- | --- | --- | --- | --- | --- | --- | --- | --- | --- | --- | --- | --- | --- | --- | --- | --- | --- | --- | --- | --- | --- | --- | --- | --- | --- | --- | --- | --- | --- | --- | --- | --- | --- | --- |

AFJ51304.1

back to list

  

| Protein Coverage
| Supporting Peptides
|

Protein Coverage:

Supporting Peptides:

| Peptide | Uniq | -10lgP | Mass | Length | ppm | m/z | z | RT | Fraction | Scan | Source File | Area F4 | Area F5 | Area F6 | Area F10 | Area F8 | Area F9 | Area F7 | Area F1 | Area F2 | Area F3 | #Feature | #Feature F4 | #Feature F5 | #Feature F6 | #Feature F10 | #Feature F8 | #Feature F9 | #Feature F7 | #Feature F1 | #Feature F2 | #Feature F3 | Start | End | PTM | AScore | Found By |
| --- | --- | --- | --- | --- | --- | --- | --- | --- | --- | --- | --- | --- | --- | --- | --- | --- | --- | --- | --- | --- | --- | --- | --- | --- | --- | --- | --- | --- | --- | --- | --- | --- | --- | --- | --- | --- | --- |
| R.TLSDYNIQK.E | Y | 60.06 | 1080.5452 | 9 | 1.2 | 541.2805 | 2 | 29.38 | 6 | F6:16185 | 17012022\_RID\_3262\_9a.raw |  |  |  |  |  | 1.2579E6 |  | 4.0613E5 |  |  | 2 | 0 | 0 | 0 | 0 | 0 | 1 | 0 | 1 | 0 | 0 | 55 | 63 |  |  | PEAKS DB |
| K.ESTLHLVLR.L | Y | 56.05 | 1066.6135 | 9 | -0.1 | 534.3140 | 2 | 34.82 | 6 | F6:20918 | 17012022\_RID\_3262\_9a.raw |  |  |  |  |  | 2.5278E5 |  |  |  |  | 1 | 0 | 0 | 0 | 0 | 0 | 1 | 0 | 0 | 0 | 0 | 64 | 72 |  |  | PEAKS DB |
| K.TITLEVEPSDTIENVK.A | Y | 50.54 | 1786.9200 | 16 | 0.5 | 894.4678 | 2 | 46.47 | 8 | F8:25378 | 31122021\_RID\_3262\_1a.raw |  |  |  |  |  |  |  | 1.3488E6 |  |  | 1 | 0 | 0 | 0 | 0 | 0 | 0 | 0 | 1 | 0 | 0 | 12 | 27 |  |  | PEAKS DB |
| total 3 peptides |  |  |  |  |  |  |  |  |  |  |  |  |  |  |  |  |  |  |  |  |  |  |  |  |  |  |  |  |  |  |  |  |  |  |  |  |  |
| --- | --- | --- | --- | --- | --- | --- | --- | --- | --- | --- | --- | --- | --- | --- | --- | --- | --- | --- | --- | --- | --- | --- | --- | --- | --- | --- | --- | --- | --- | --- | --- | --- | --- | --- | --- | --- | --- |

JAI10738.1

back to list

  

| Protein Coverage
| Supporting Peptides
|

Protein Coverage:

Supporting Peptides:

| Peptide | Uniq | -10lgP | Mass | Length | ppm | m/z | z | RT | Fraction | Scan | Source File | Area F4 | Area F5 | Area F6 | Area F10 | Area F8 | Area F9 | Area F7 | Area F1 | Area F2 | Area F3 | #Feature | #Feature F4 | #Feature F5 | #Feature F6 | #Feature F10 | #Feature F8 | #Feature F9 | #Feature F7 | #Feature F1 | #Feature F2 | #Feature F3 | Start | End | PTM | AScore | Found By |
| --- | --- | --- | --- | --- | --- | --- | --- | --- | --- | --- | --- | --- | --- | --- | --- | --- | --- | --- | --- | --- | --- | --- | --- | --- | --- | --- | --- | --- | --- | --- | --- | --- | --- | --- | --- | --- | --- |
| R.TLSDYNIQK.E | Y | 60.06 | 1080.5452 | 9 | 1.2 | 541.2805 | 2 | 29.38 | 6 | F6:16185 | 17012022\_RID\_3262\_9a.raw |  |  |  |  |  | 1.2579E6 |  | 4.0613E5 |  |  | 2 | 0 | 0 | 0 | 0 | 0 | 1 | 0 | 1 | 0 | 0 | 55 | 63 |  |  | PEAKS DB |
| K.ESTLHLVLR.L | Y | 56.05 | 1066.6135 | 9 | -0.1 | 534.3140 | 2 | 34.82 | 6 | F6:20918 | 17012022\_RID\_3262\_9a.raw |  |  |  |  |  | 2.5278E5 |  |  |  |  | 1 | 0 | 0 | 0 | 0 | 0 | 1 | 0 | 0 | 0 | 0 | 64 | 72 |  |  | PEAKS DB |
| K.TITLEVEPSDTIENVK.A | Y | 50.54 | 1786.9200 | 16 | 0.5 | 894.4678 | 2 | 46.47 | 8 | F8:25378 | 31122021\_RID\_3262\_1a.raw |  |  |  |  |  |  |  | 1.3488E6 |  |  | 1 | 0 | 0 | 0 | 0 | 0 | 0 | 0 | 1 | 0 | 0 | 12 | 27 |  |  | PEAKS DB |
| total 3 peptides |  |  |  |  |  |  |  |  |  |  |  |  |  |  |  |  |  |  |  |  |  |  |  |  |  |  |  |  |  |  |  |  |  |  |  |  |  |
| --- | --- | --- | --- | --- | --- | --- | --- | --- | --- | --- | --- | --- | --- | --- | --- | --- | --- | --- | --- | --- | --- | --- | --- | --- | --- | --- | --- | --- | --- | --- | --- | --- | --- | --- | --- | --- | --- |

JAA95781.1

back to list

  

| Protein Coverage
| Supporting Peptides
|

Protein Coverage:

Supporting Peptides:

| Peptide | Uniq | -10lgP | Mass | Length | ppm | m/z | z | RT | Fraction | Scan | Source File | Area F4 | Area F5 | Area F6 | Area F10 | Area F8 | Area F9 | Area F7 | Area F1 | Area F2 | Area F3 | #Feature | #Feature F4 | #Feature F5 | #Feature F6 | #Feature F10 | #Feature F8 | #Feature F9 | #Feature F7 | #Feature F1 | #Feature F2 | #Feature F3 | Start | End | PTM | AScore | Found By |
| --- | --- | --- | --- | --- | --- | --- | --- | --- | --- | --- | --- | --- | --- | --- | --- | --- | --- | --- | --- | --- | --- | --- | --- | --- | --- | --- | --- | --- | --- | --- | --- | --- | --- | --- | --- | --- | --- |
| R.TLSDYNIQK.E | Y | 60.06 | 1080.5452 | 9 | 1.2 | 541.2805 | 2 | 29.38 | 6 | F6:16185 | 17012022\_RID\_3262\_9a.raw |  |  |  |  |  | 1.2579E6 |  | 4.0613E5 |  |  | 2 | 0 | 0 | 0 | 0 | 0 | 1 | 0 | 1 | 0 | 0 | 55 | 63 |  |  | PEAKS DB |
| K.ESTLHLVLR.L | Y | 56.05 | 1066.6135 | 9 | -0.1 | 534.3140 | 2 | 34.82 | 6 | F6:20918 | 17012022\_RID\_3262\_9a.raw |  |  |  |  |  | 2.5278E5 |  |  |  |  | 1 | 0 | 0 | 0 | 0 | 0 | 1 | 0 | 0 | 0 | 0 | 64 | 72 |  |  | PEAKS DB |
| K.TITLEVEPSDTIENVK.A | Y | 50.54 | 1786.9200 | 16 | 0.5 | 894.4678 | 2 | 46.47 | 8 | F8:25378 | 31122021\_RID\_3262\_1a.raw |  |  |  |  |  |  |  | 1.3488E6 |  |  | 1 | 0 | 0 | 0 | 0 | 0 | 0 | 0 | 1 | 0 | 0 | 12 | 27 |  |  | PEAKS DB |
| total 3 peptides |  |  |  |  |  |  |  |  |  |  |  |  |  |  |  |  |  |  |  |  |  |  |  |  |  |  |  |  |  |  |  |  |  |  |  |  |  |
| --- | --- | --- | --- | --- | --- | --- | --- | --- | --- | --- | --- | --- | --- | --- | --- | --- | --- | --- | --- | --- | --- | --- | --- | --- | --- | --- | --- | --- | --- | --- | --- | --- | --- | --- | --- | --- | --- |

XP\_013929631.1

back to list

  

| Protein Coverage
| Supporting Peptides
|

Protein Coverage:

Supporting Peptides:

| Peptide | Uniq | -10lgP | Mass | Length | ppm | m/z | z | RT | Fraction | Scan | Source File | Area F4 | Area F5 | Area F6 | Area F10 | Area F8 | Area F9 | Area F7 | Area F1 | Area F2 | Area F3 | #Feature | #Feature F4 | #Feature F5 | #Feature F6 | #Feature F10 | #Feature F8 | #Feature F9 | #Feature F7 | #Feature F1 | #Feature F2 | #Feature F3 | Start | End | PTM | AScore | Found By |
| --- | --- | --- | --- | --- | --- | --- | --- | --- | --- | --- | --- | --- | --- | --- | --- | --- | --- | --- | --- | --- | --- | --- | --- | --- | --- | --- | --- | --- | --- | --- | --- | --- | --- | --- | --- | --- | --- |
| R.TLSDYNIQK.E | Y | 60.06 | 1080.5452 | 9 | 1.2 | 541.2805 | 2 | 29.38 | 6 | F6:16185 | 17012022\_RID\_3262\_9a.raw |  |  |  |  |  | 1.2579E6 |  | 4.0613E5 |  |  | 2 | 0 | 0 | 0 | 0 | 0 | 1 | 0 | 1 | 0 | 0 | 55 | 63 |  |  | PEAKS DB |
| K.ESTLHLVLR.L | Y | 56.05 | 1066.6135 | 9 | -0.1 | 534.3140 | 2 | 34.82 | 6 | F6:20918 | 17012022\_RID\_3262\_9a.raw |  |  |  |  |  | 2.5278E5 |  |  |  |  | 1 | 0 | 0 | 0 | 0 | 0 | 1 | 0 | 0 | 0 | 0 | 64 | 72 |  |  | PEAKS DB |
| K.TITLEVEPSDTIENVK.A | Y | 50.54 | 1786.9200 | 16 | 0.5 | 894.4678 | 2 | 46.47 | 8 | F8:25378 | 31122021\_RID\_3262\_1a.raw |  |  |  |  |  |  |  | 1.3488E6 |  |  | 1 | 0 | 0 | 0 | 0 | 0 | 0 | 0 | 1 | 0 | 0 | 12 | 27 |  |  | PEAKS DB |
| total 3 peptides |  |  |  |  |  |  |  |  |  |  |  |  |  |  |  |  |  |  |  |  |  |  |  |  |  |  |  |  |  |  |  |  |  |  |  |  |  |
| --- | --- | --- | --- | --- | --- | --- | --- | --- | --- | --- | --- | --- | --- | --- | --- | --- | --- | --- | --- | --- | --- | --- | --- | --- | --- | --- | --- | --- | --- | --- | --- | --- | --- | --- | --- | --- | --- |

JAB53440.1

back to list

  

| Protein Coverage
| Supporting Peptides
|

Protein Coverage:

Supporting Peptides:

| Peptide | Uniq | -10lgP | Mass | Length | ppm | m/z | z | RT | Fraction | Scan | Source File | Area F4 | Area F5 | Area F6 | Area F10 | Area F8 | Area F9 | Area F7 | Area F1 | Area F2 | Area F3 | #Feature | #Feature F4 | #Feature F5 | #Feature F6 | #Feature F10 | #Feature F8 | #Feature F9 | #Feature F7 | #Feature F1 | #Feature F2 | #Feature F3 | Start | End | PTM | AScore | Found By |
| --- | --- | --- | --- | --- | --- | --- | --- | --- | --- | --- | --- | --- | --- | --- | --- | --- | --- | --- | --- | --- | --- | --- | --- | --- | --- | --- | --- | --- | --- | --- | --- | --- | --- | --- | --- | --- | --- |
| R.TLSDYNIQK.E | Y | 60.06 | 1080.5452 | 9 | 1.2 | 541.2805 | 2 | 29.38 | 6 | F6:16185 | 17012022\_RID\_3262\_9a.raw |  |  |  |  |  | 1.2579E6 |  | 4.0613E5 |  |  | 2 | 0 | 0 | 0 | 0 | 0 | 1 | 0 | 1 | 0 | 0 | 55 | 63 |  |  | PEAKS DB |
| K.ESTLHLVLR.L | Y | 56.05 | 1066.6135 | 9 | -0.1 | 534.3140 | 2 | 34.82 | 6 | F6:20918 | 17012022\_RID\_3262\_9a.raw |  |  |  |  |  | 2.5278E5 |  |  |  |  | 1 | 0 | 0 | 0 | 0 | 0 | 1 | 0 | 0 | 0 | 0 | 64 | 72 |  |  | PEAKS DB |
| K.TITLEVEPSDTIENVK.A | Y | 50.54 | 1786.9200 | 16 | 0.5 | 894.4678 | 2 | 46.47 | 8 | F8:25378 | 31122021\_RID\_3262\_1a.raw |  |  |  |  |  |  |  | 1.3488E6 |  |  | 1 | 0 | 0 | 0 | 0 | 0 | 0 | 0 | 1 | 0 | 0 | 12 | 27 |  |  | PEAKS DB |
| total 3 peptides |  |  |  |  |  |  |  |  |  |  |  |  |  |  |  |  |  |  |  |  |  |  |  |  |  |  |  |  |  |  |  |  |  |  |  |  |  |
| --- | --- | --- | --- | --- | --- | --- | --- | --- | --- | --- | --- | --- | --- | --- | --- | --- | --- | --- | --- | --- | --- | --- | --- | --- | --- | --- | --- | --- | --- | --- | --- | --- | --- | --- | --- | --- | --- |

JAI09497.1

back to list

  

| Protein Coverage
| Supporting Peptides
|

Protein Coverage:

Supporting Peptides:

| Peptide | Uniq | -10lgP | Mass | Length | ppm | m/z | z | RT | Fraction | Scan | Source File | Area F4 | Area F5 | Area F6 | Area F10 | Area F8 | Area F9 | Area F7 | Area F1 | Area F2 | Area F3 | #Feature | #Feature F4 | #Feature F5 | #Feature F6 | #Feature F10 | #Feature F8 | #Feature F9 | #Feature F7 | #Feature F1 | #Feature F2 | #Feature F3 | Start | End | PTM | AScore | Found By |
| --- | --- | --- | --- | --- | --- | --- | --- | --- | --- | --- | --- | --- | --- | --- | --- | --- | --- | --- | --- | --- | --- | --- | --- | --- | --- | --- | --- | --- | --- | --- | --- | --- | --- | --- | --- | --- | --- |
| R.TLSDYNIQK.E | Y | 60.06 | 1080.5452 | 9 | 1.2 | 541.2805 | 2 | 29.38 | 6 | F6:16185 | 17012022\_RID\_3262\_9a.raw |  |  |  |  |  | 1.2579E6 |  | 4.0613E5 |  |  | 2 | 0 | 0 | 0 | 0 | 0 | 1 | 0 | 1 | 0 | 0 | 55 | 63 |  |  | PEAKS DB |
| K.ESTLHLVLR.L | Y | 56.05 | 1066.6135 | 9 | -0.1 | 534.3140 | 2 | 34.82 | 6 | F6:20918 | 17012022\_RID\_3262\_9a.raw |  |  |  |  |  | 2.5278E5 |  |  |  |  | 1 | 0 | 0 | 0 | 0 | 0 | 1 | 0 | 0 | 0 | 0 | 64 | 72 |  |  | PEAKS DB |
| K.TITLEVEPSDTIENVK.A | Y | 50.54 | 1786.9200 | 16 | 0.5 | 894.4678 | 2 | 46.47 | 8 | F8:25378 | 31122021\_RID\_3262\_1a.raw |  |  |  |  |  |  |  | 1.3488E6 |  |  | 1 | 0 | 0 | 0 | 0 | 0 | 0 | 0 | 1 | 0 | 0 | 12 | 27 |  |  | PEAKS DB |
| total 3 peptides |  |  |  |  |  |  |  |  |  |  |  |  |  |  |  |  |  |  |  |  |  |  |  |  |  |  |  |  |  |  |  |  |  |  |  |  |  |
| --- | --- | --- | --- | --- | --- | --- | --- | --- | --- | --- | --- | --- | --- | --- | --- | --- | --- | --- | --- | --- | --- | --- | --- | --- | --- | --- | --- | --- | --- | --- | --- | --- | --- | --- | --- | --- | --- |

JAB53439.1

back to list

  

| Protein Coverage
| Supporting Peptides
|

Protein Coverage:

Supporting Peptides:

| Peptide | Uniq | -10lgP | Mass | Length | ppm | m/z | z | RT | Fraction | Scan | Source File | Area F4 | Area F5 | Area F6 | Area F10 | Area F8 | Area F9 | Area F7 | Area F1 | Area F2 | Area F3 | #Feature | #Feature F4 | #Feature F5 | #Feature F6 | #Feature F10 | #Feature F8 | #Feature F9 | #Feature F7 | #Feature F1 | #Feature F2 | #Feature F3 | Start | End | PTM | AScore | Found By |
| --- | --- | --- | --- | --- | --- | --- | --- | --- | --- | --- | --- | --- | --- | --- | --- | --- | --- | --- | --- | --- | --- | --- | --- | --- | --- | --- | --- | --- | --- | --- | --- | --- | --- | --- | --- | --- | --- |
| R.TLSDYNIQK.E | Y | 60.06 | 1080.5452 | 9 | 1.2 | 541.2805 | 2 | 29.38 | 6 | F6:16185 | 17012022\_RID\_3262\_9a.raw |  |  |  |  |  | 1.2579E6 |  | 4.0613E5 |  |  | 2 | 0 | 0 | 0 | 0 | 0 | 1 | 0 | 1 | 0 | 0 | 55 | 63 |  |  | PEAKS DB |
| K.ESTLHLVLR.L | Y | 56.05 | 1066.6135 | 9 | -0.1 | 534.3140 | 2 | 34.82 | 6 | F6:20918 | 17012022\_RID\_3262\_9a.raw |  |  |  |  |  | 2.5278E5 |  |  |  |  | 1 | 0 | 0 | 0 | 0 | 0 | 1 | 0 | 0 | 0 | 0 | 64 | 72 |  |  | PEAKS DB |
| K.TITLEVEPSDTIENVK.A | Y | 50.54 | 1786.9200 | 16 | 0.5 | 894.4678 | 2 | 46.47 | 8 | F8:25378 | 31122021\_RID\_3262\_1a.raw |  |  |  |  |  |  |  | 1.3488E6 |  |  | 1 | 0 | 0 | 0 | 0 | 0 | 0 | 0 | 1 | 0 | 0 | 12 | 27 |  |  | PEAKS DB |
| total 3 peptides |  |  |  |  |  |  |  |  |  |  |  |  |  |  |  |  |  |  |  |  |  |  |  |  |  |  |  |  |  |  |  |  |  |  |  |  |  |
| --- | --- | --- | --- | --- | --- | --- | --- | --- | --- | --- | --- | --- | --- | --- | --- | --- | --- | --- | --- | --- | --- | --- | --- | --- | --- | --- | --- | --- | --- | --- | --- | --- | --- | --- | --- | --- | --- |

JAI11613.1

back to list

  

| Protein Coverage
| Supporting Peptides
|

Protein Coverage:

Supporting Peptides:

| Peptide | Uniq | -10lgP | Mass | Length | ppm | m/z | z | RT | Fraction | Scan | Source File | Area F4 | Area F5 | Area F6 | Area F10 | Area F8 | Area F9 | Area F7 | Area F1 | Area F2 | Area F3 | #Feature | #Feature F4 | #Feature F5 | #Feature F6 | #Feature F10 | #Feature F8 | #Feature F9 | #Feature F7 | #Feature F1 | #Feature F2 | #Feature F3 | Start | End | PTM | AScore | Found By |
| --- | --- | --- | --- | --- | --- | --- | --- | --- | --- | --- | --- | --- | --- | --- | --- | --- | --- | --- | --- | --- | --- | --- | --- | --- | --- | --- | --- | --- | --- | --- | --- | --- | --- | --- | --- | --- | --- |
| R.TLSDYNIQK.E | Y | 60.06 | 1080.5452 | 9 | 1.2 | 541.2805 | 2 | 29.38 | 6 | F6:16185 | 17012022\_RID\_3262\_9a.raw |  |  |  |  |  | 1.2579E6 |  | 4.0613E5 |  |  | 2 | 0 | 0 | 0 | 0 | 0 | 1 | 0 | 1 | 0 | 0 | 55 | 63 |  |  | PEAKS DB |
| K.ESTLHLVLR.L | Y | 56.05 | 1066.6135 | 9 | -0.1 | 534.3140 | 2 | 34.82 | 6 | F6:20918 | 17012022\_RID\_3262\_9a.raw |  |  |  |  |  | 2.5278E5 |  |  |  |  | 1 | 0 | 0 | 0 | 0 | 0 | 1 | 0 | 0 | 0 | 0 | 64 | 72 |  |  | PEAKS DB |
| K.TITLEVEPSDTIENVK.A | Y | 50.54 | 1786.9200 | 16 | 0.5 | 894.4678 | 2 | 46.47 | 8 | F8:25378 | 31122021\_RID\_3262\_1a.raw |  |  |  |  |  |  |  | 1.3488E6 |  |  | 1 | 0 | 0 | 0 | 0 | 0 | 0 | 0 | 1 | 0 | 0 | 12 | 27 |  |  | PEAKS DB |
| total 3 peptides |  |  |  |  |  |  |  |  |  |  |  |  |  |  |  |  |  |  |  |  |  |  |  |  |  |  |  |  |  |  |  |  |  |  |  |  |  |
| --- | --- | --- | --- | --- | --- | --- | --- | --- | --- | --- | --- | --- | --- | --- | --- | --- | --- | --- | --- | --- | --- | --- | --- | --- | --- | --- | --- | --- | --- | --- | --- | --- | --- | --- | --- | --- | --- |

XP\_013929630.1

back to list

  

| Protein Coverage
| Supporting Peptides
|

Protein Coverage:

Supporting Peptides:

| Peptide | Uniq | -10lgP | Mass | Length | ppm | m/z | z | RT | Fraction | Scan | Source File | Area F4 | Area F5 | Area F6 | Area F10 | Area F8 | Area F9 | Area F7 | Area F1 | Area F2 | Area F3 | #Feature | #Feature F4 | #Feature F5 | #Feature F6 | #Feature F10 | #Feature F8 | #Feature F9 | #Feature F7 | #Feature F1 | #Feature F2 | #Feature F3 | Start | End | PTM | AScore | Found By |
| --- | --- | --- | --- | --- | --- | --- | --- | --- | --- | --- | --- | --- | --- | --- | --- | --- | --- | --- | --- | --- | --- | --- | --- | --- | --- | --- | --- | --- | --- | --- | --- | --- | --- | --- | --- | --- | --- |
| R.TLSDYNIQK.E | Y | 60.06 | 1080.5452 | 9 | 1.2 | 541.2805 | 2 | 29.38 | 6 | F6:16185 | 17012022\_RID\_3262\_9a.raw |  |  |  |  |  | 1.2579E6 |  | 4.0613E5 |  |  | 2 | 0 | 0 | 0 | 0 | 0 | 1 | 0 | 1 | 0 | 0 | 55 | 63 |  |  | PEAKS DB |
| K.ESTLHLVLR.L | Y | 56.05 | 1066.6135 | 9 | -0.1 | 534.3140 | 2 | 34.82 | 6 | F6:20918 | 17012022\_RID\_3262\_9a.raw |  |  |  |  |  | 2.5278E5 |  |  |  |  | 1 | 0 | 0 | 0 | 0 | 0 | 1 | 0 | 0 | 0 | 0 | 64 | 72 |  |  | PEAKS DB |
| K.TITLEVEPSDTIENVK.A | Y | 50.54 | 1786.9200 | 16 | 0.5 | 894.4678 | 2 | 46.47 | 8 | F8:25378 | 31122021\_RID\_3262\_1a.raw |  |  |  |  |  |  |  | 1.3488E6 |  |  | 1 | 0 | 0 | 0 | 0 | 0 | 0 | 0 | 1 | 0 | 0 | 12 | 27 |  |  | PEAKS DB |
| total 3 peptides |  |  |  |  |  |  |  |  |  |  |  |  |  |  |  |  |  |  |  |  |  |  |  |  |  |  |  |  |  |  |  |  |  |  |  |  |  |
| --- | --- | --- | --- | --- | --- | --- | --- | --- | --- | --- | --- | --- | --- | --- | --- | --- | --- | --- | --- | --- | --- | --- | --- | --- | --- | --- | --- | --- | --- | --- | --- | --- | --- | --- | --- | --- | --- |

AFJ51273.1

back to list

  

| Protein Coverage
| Supporting Peptides
|

Protein Coverage:

Supporting Peptides:

| Peptide | Uniq | -10lgP | Mass | Length | ppm | m/z | z | RT | Fraction | Scan | Source File | Area F4 | Area F5 | Area F6 | Area F10 | Area F8 | Area F9 | Area F7 | Area F1 | Area F2 | Area F3 | #Feature | #Feature F4 | #Feature F5 | #Feature F6 | #Feature F10 | #Feature F8 | #Feature F9 | #Feature F7 | #Feature F1 | #Feature F2 | #Feature F3 | Start | End | PTM | AScore | Found By |
| --- | --- | --- | --- | --- | --- | --- | --- | --- | --- | --- | --- | --- | --- | --- | --- | --- | --- | --- | --- | --- | --- | --- | --- | --- | --- | --- | --- | --- | --- | --- | --- | --- | --- | --- | --- | --- | --- |
| R.TLSDYNIQK.E | Y | 60.06 | 1080.5452 | 9 | 1.2 | 541.2805 | 2 | 29.38 | 6 | F6:16185 | 17012022\_RID\_3262\_9a.raw |  |  |  |  |  | 1.2579E6 |  | 4.0613E5 |  |  | 2 | 0 | 0 | 0 | 0 | 0 | 1 | 0 | 1 | 0 | 0 | 55 | 63 |  |  | PEAKS DB |
| K.ESTLHLVLR.L | Y | 56.05 | 1066.6135 | 9 | -0.1 | 534.3140 | 2 | 34.82 | 6 | F6:20918 | 17012022\_RID\_3262\_9a.raw |  |  |  |  |  | 2.5278E5 |  |  |  |  | 1 | 0 | 0 | 0 | 0 | 0 | 1 | 0 | 0 | 0 | 0 | 64 | 72 |  |  | PEAKS DB |
| K.TITLEVEPSDTIENVK.A | Y | 50.54 | 1786.9200 | 16 | 0.5 | 894.4678 | 2 | 46.47 | 8 | F8:25378 | 31122021\_RID\_3262\_1a.raw |  |  |  |  |  |  |  | 1.3488E6 |  |  | 1 | 0 | 0 | 0 | 0 | 0 | 0 | 0 | 1 | 0 | 0 | 12 | 27 |  |  | PEAKS DB |
| total 3 peptides |  |  |  |  |  |  |  |  |  |  |  |  |  |  |  |  |  |  |  |  |  |  |  |  |  |  |  |  |  |  |  |  |  |  |  |  |  |
| --- | --- | --- | --- | --- | --- | --- | --- | --- | --- | --- | --- | --- | --- | --- | --- | --- | --- | --- | --- | --- | --- | --- | --- | --- | --- | --- | --- | --- | --- | --- | --- | --- | --- | --- | --- | --- | --- |

JAG66673.1

back to list

  

| Protein Coverage
| Supporting Peptides
|

Protein Coverage:

Supporting Peptides:

| Peptide | Uniq | -10lgP | Mass | Length | ppm | m/z | z | RT | Fraction | Scan | Source File | Area F4 | Area F5 | Area F6 | Area F10 | Area F8 | Area F9 | Area F7 | Area F1 | Area F2 | Area F3 | #Feature | #Feature F4 | #Feature F5 | #Feature F6 | #Feature F10 | #Feature F8 | #Feature F9 | #Feature F7 | #Feature F1 | #Feature F2 | #Feature F3 | Start | End | PTM | AScore | Found By |
| --- | --- | --- | --- | --- | --- | --- | --- | --- | --- | --- | --- | --- | --- | --- | --- | --- | --- | --- | --- | --- | --- | --- | --- | --- | --- | --- | --- | --- | --- | --- | --- | --- | --- | --- | --- | --- | --- |
| R.TLSDYNIQK.E | Y | 60.06 | 1080.5452 | 9 | 1.2 | 541.2805 | 2 | 29.38 | 6 | F6:16185 | 17012022\_RID\_3262\_9a.raw |  |  |  |  |  | 1.2579E6 |  | 4.0613E5 |  |  | 2 | 0 | 0 | 0 | 0 | 0 | 1 | 0 | 1 | 0 | 0 | 55 | 63 |  |  | PEAKS DB |
| K.ESTLHLVLR.L | Y | 56.05 | 1066.6135 | 9 | -0.1 | 534.3140 | 2 | 34.82 | 6 | F6:20918 | 17012022\_RID\_3262\_9a.raw |  |  |  |  |  | 2.5278E5 |  |  |  |  | 1 | 0 | 0 | 0 | 0 | 0 | 1 | 0 | 0 | 0 | 0 | 64 | 72 |  |  | PEAKS DB |
| K.TITLEVEPSDTIENVK.A | Y | 50.54 | 1786.9200 | 16 | 0.5 | 894.4678 | 2 | 46.47 | 8 | F8:25378 | 31122021\_RID\_3262\_1a.raw |  |  |  |  |  |  |  | 1.3488E6 |  |  | 1 | 0 | 0 | 0 | 0 | 0 | 0 | 0 | 1 | 0 | 0 | 12 | 27 |  |  | PEAKS DB |
| total 3 peptides |  |  |  |  |  |  |  |  |  |  |  |  |  |  |  |  |  |  |  |  |  |  |  |  |  |  |  |  |  |  |  |  |  |  |  |  |  |
| --- | --- | --- | --- | --- | --- | --- | --- | --- | --- | --- | --- | --- | --- | --- | --- | --- | --- | --- | --- | --- | --- | --- | --- | --- | --- | --- | --- | --- | --- | --- | --- | --- | --- | --- | --- | --- | --- |

AAG17445.1

back to list

  

| Protein Coverage
| Supporting Peptides
|

Protein Coverage:

Supporting Peptides:

| Peptide | Uniq | -10lgP | Mass | Length | ppm | m/z | z | RT | Fraction | Scan | Source File | Area F4 | Area F5 | Area F6 | Area F10 | Area F8 | Area F9 | Area F7 | Area F1 | Area F2 | Area F3 | #Feature | #Feature F4 | #Feature F5 | #Feature F6 | #Feature F10 | #Feature F8 | #Feature F9 | #Feature F7 | #Feature F1 | #Feature F2 | #Feature F3 | Start | End | PTM | AScore | Found By |
| --- | --- | --- | --- | --- | --- | --- | --- | --- | --- | --- | --- | --- | --- | --- | --- | --- | --- | --- | --- | --- | --- | --- | --- | --- | --- | --- | --- | --- | --- | --- | --- | --- | --- | --- | --- | --- | --- |
| R.TLSDYNIQK.E | Y | 60.06 | 1080.5452 | 9 | 1.2 | 541.2805 | 2 | 29.38 | 6 | F6:16185 | 17012022\_RID\_3262\_9a.raw |  |  |  |  |  | 1.2579E6 |  | 4.0613E5 |  |  | 2 | 0 | 0 | 0 | 0 | 0 | 1 | 0 | 1 | 0 | 0 | 55 | 63 |  |  | PEAKS DB |
| K.ESTLHLVLR.L | Y | 56.05 | 1066.6135 | 9 | -0.1 | 534.3140 | 2 | 34.82 | 6 | F6:20918 | 17012022\_RID\_3262\_9a.raw |  |  |  |  |  | 2.5278E5 |  |  |  |  | 1 | 0 | 0 | 0 | 0 | 0 | 1 | 0 | 0 | 0 | 0 | 64 | 72 |  |  | PEAKS DB |
| K.TITLEVEPSDTIENVK.A | Y | 50.54 | 1786.9200 | 16 | 0.5 | 894.4678 | 2 | 46.47 | 8 | F8:25378 | 31122021\_RID\_3262\_1a.raw |  |  |  |  |  |  |  | 1.3488E6 |  |  | 1 | 0 | 0 | 0 | 0 | 0 | 0 | 0 | 1 | 0 | 0 | 12 | 27 |  |  | PEAKS DB |
| total 3 peptides |  |  |  |  |  |  |  |  |  |  |  |  |  |  |  |  |  |  |  |  |  |  |  |  |  |  |  |  |  |  |  |  |  |  |  |  |  |
| --- | --- | --- | --- | --- | --- | --- | --- | --- | --- | --- | --- | --- | --- | --- | --- | --- | --- | --- | --- | --- | --- | --- | --- | --- | --- | --- | --- | --- | --- | --- | --- | --- | --- | --- | --- | --- | --- |

AFJ51941.1

back to list

  

| Protein Coverage
| Supporting Peptides
|

Protein Coverage:

Supporting Peptides:

| Peptide | Uniq | -10lgP | Mass | Length | ppm | m/z | z | RT | Fraction | Scan | Source File | Area F4 | Area F5 | Area F6 | Area F10 | Area F8 | Area F9 | Area F7 | Area F1 | Area F2 | Area F3 | #Feature | #Feature F4 | #Feature F5 | #Feature F6 | #Feature F10 | #Feature F8 | #Feature F9 | #Feature F7 | #Feature F1 | #Feature F2 | #Feature F3 | Start | End | PTM | AScore | Found By |
| --- | --- | --- | --- | --- | --- | --- | --- | --- | --- | --- | --- | --- | --- | --- | --- | --- | --- | --- | --- | --- | --- | --- | --- | --- | --- | --- | --- | --- | --- | --- | --- | --- | --- | --- | --- | --- | --- |
| R.TLSDYNIQK.E | Y | 60.06 | 1080.5452 | 9 | 1.2 | 541.2805 | 2 | 29.38 | 6 | F6:16185 | 17012022\_RID\_3262\_9a.raw |  |  |  |  |  | 1.2579E6 |  | 4.0613E5 |  |  | 2 | 0 | 0 | 0 | 0 | 0 | 1 | 0 | 1 | 0 | 0 | 55 | 63 |  |  | PEAKS DB |
| K.ESTLHLVLR.L | Y | 56.05 | 1066.6135 | 9 | -0.1 | 534.3140 | 2 | 34.82 | 6 | F6:20918 | 17012022\_RID\_3262\_9a.raw |  |  |  |  |  | 2.5278E5 |  |  |  |  | 1 | 0 | 0 | 0 | 0 | 0 | 1 | 0 | 0 | 0 | 0 | 64 | 72 |  |  | PEAKS DB |
| K.TITLEVEPSDTIENVK.A | Y | 50.54 | 1786.9200 | 16 | 0.5 | 894.4678 | 2 | 46.47 | 8 | F8:25378 | 31122021\_RID\_3262\_1a.raw |  |  |  |  |  |  |  | 1.3488E6 |  |  | 1 | 0 | 0 | 0 | 0 | 0 | 0 | 0 | 1 | 0 | 0 | 12 | 27 |  |  | PEAKS DB |
| total 3 peptides |  |  |  |  |  |  |  |  |  |  |  |  |  |  |  |  |  |  |  |  |  |  |  |  |  |  |  |  |  |  |  |  |  |  |  |  |  |
| --- | --- | --- | --- | --- | --- | --- | --- | --- | --- | --- | --- | --- | --- | --- | --- | --- | --- | --- | --- | --- | --- | --- | --- | --- | --- | --- | --- | --- | --- | --- | --- | --- | --- | --- | --- | --- | --- |

XP\_013928637.1

back to list

  

| Protein Coverage
| Supporting Peptides
|

Protein Coverage:

Supporting Peptides:

| Peptide | Uniq | -10lgP | Mass | Length | ppm | m/z | z | RT | Fraction | Scan | Source File | Area F4 | Area F5 | Area F6 | Area F10 | Area F8 | Area F9 | Area F7 | Area F1 | Area F2 | Area F3 | #Feature | #Feature F4 | #Feature F5 | #Feature F6 | #Feature F10 | #Feature F8 | #Feature F9 | #Feature F7 | #Feature F1 | #Feature F2 | #Feature F3 | Start | End | PTM | AScore | Found By |
| --- | --- | --- | --- | --- | --- | --- | --- | --- | --- | --- | --- | --- | --- | --- | --- | --- | --- | --- | --- | --- | --- | --- | --- | --- | --- | --- | --- | --- | --- | --- | --- | --- | --- | --- | --- | --- | --- |
| R.TLSDYNIQK.E | Y | 60.06 | 1080.5452 | 9 | 1.2 | 541.2805 | 2 | 29.38 | 6 | F6:16185 | 17012022\_RID\_3262\_9a.raw |  |  |  |  |  | 1.2579E6 |  | 4.0613E5 |  |  | 2 | 0 | 0 | 0 | 0 | 0 | 1 | 0 | 1 | 0 | 0 | 55 | 63 |  |  | PEAKS DB |
| K.ESTLHLVLR.L | Y | 56.05 | 1066.6135 | 9 | -0.1 | 534.3140 | 2 | 34.82 | 6 | F6:20918 | 17012022\_RID\_3262\_9a.raw |  |  |  |  |  | 2.5278E5 |  |  |  |  | 1 | 0 | 0 | 0 | 0 | 0 | 1 | 0 | 0 | 0 | 0 | 64 | 72 |  |  | PEAKS DB |
| K.TITLEVEPSDTIENVK.A | Y | 50.54 | 1786.9200 | 16 | 0.5 | 894.4678 | 2 | 46.47 | 8 | F8:25378 | 31122021\_RID\_3262\_1a.raw |  |  |  |  |  |  |  | 1.3488E6 |  |  | 1 | 0 | 0 | 0 | 0 | 0 | 0 | 0 | 1 | 0 | 0 | 12 | 27 |  |  | PEAKS DB |
| total 3 peptides |  |  |  |  |  |  |  |  |  |  |  |  |  |  |  |  |  |  |  |  |  |  |  |  |  |  |  |  |  |  |  |  |  |  |  |  |  |
| --- | --- | --- | --- | --- | --- | --- | --- | --- | --- | --- | --- | --- | --- | --- | --- | --- | --- | --- | --- | --- | --- | --- | --- | --- | --- | --- | --- | --- | --- | --- | --- | --- | --- | --- | --- | --- | --- |

JAB53023.1

back to list

  

| Protein Coverage
| Supporting Peptides
|

Protein Coverage:

Supporting Peptides:

| Peptide | Uniq | -10lgP | Mass | Length | ppm | m/z | z | RT | Fraction | Scan | Source File | Area F4 | Area F5 | Area F6 | Area F10 | Area F8 | Area F9 | Area F7 | Area F1 | Area F2 | Area F3 | #Feature | #Feature F4 | #Feature F5 | #Feature F6 | #Feature F10 | #Feature F8 | #Feature F9 | #Feature F7 | #Feature F1 | #Feature F2 | #Feature F3 | Start | End | PTM | AScore | Found By |
| --- | --- | --- | --- | --- | --- | --- | --- | --- | --- | --- | --- | --- | --- | --- | --- | --- | --- | --- | --- | --- | --- | --- | --- | --- | --- | --- | --- | --- | --- | --- | --- | --- | --- | --- | --- | --- | --- |
| R.TLSDYNIQK.E | Y | 60.06 | 1080.5452 | 9 | 1.2 | 541.2805 | 2 | 29.38 | 6 | F6:16185 | 17012022\_RID\_3262\_9a.raw |  |  |  |  |  | 1.2579E6 |  | 4.0613E5 |  |  | 2 | 0 | 0 | 0 | 0 | 0 | 1 | 0 | 1 | 0 | 0 | 55 | 63 |  |  | PEAKS DB |
| K.ESTLHLVLR.L | Y | 56.05 | 1066.6135 | 9 | -0.1 | 534.3140 | 2 | 34.82 | 6 | F6:20918 | 17012022\_RID\_3262\_9a.raw |  |  |  |  |  | 2.5278E5 |  |  |  |  | 1 | 0 | 0 | 0 | 0 | 0 | 1 | 0 | 0 | 0 | 0 | 64 | 72 |  |  | PEAKS DB |
| K.TITLEVEPSDTIENVK.A | Y | 50.54 | 1786.9200 | 16 | 0.5 | 894.4678 | 2 | 46.47 | 8 | F8:25378 | 31122021\_RID\_3262\_1a.raw |  |  |  |  |  |  |  | 1.3488E6 |  |  | 1 | 0 | 0 | 0 | 0 | 0 | 0 | 0 | 1 | 0 | 0 | 12 | 27 |  |  | PEAKS DB |
| total 3 peptides |  |  |  |  |  |  |  |  |  |  |  |  |  |  |  |  |  |  |  |  |  |  |  |  |  |  |  |  |  |  |  |  |  |  |  |  |  |
| --- | --- | --- | --- | --- | --- | --- | --- | --- | --- | --- | --- | --- | --- | --- | --- | --- | --- | --- | --- | --- | --- | --- | --- | --- | --- | --- | --- | --- | --- | --- | --- | --- | --- | --- | --- | --- | --- |

XP\_032091816.1

back to list

  

| Protein Coverage
| Supporting Peptides
|

Protein Coverage:

Supporting Peptides:

| Peptide | Uniq | -10lgP | Mass | Length | ppm | m/z | z | RT | Fraction | Scan | Source File | Area F4 | Area F5 | Area F6 | Area F10 | Area F8 | Area F9 | Area F7 | Area F1 | Area F2 | Area F3 | #Feature | #Feature F4 | #Feature F5 | #Feature F6 | #Feature F10 | #Feature F8 | #Feature F9 | #Feature F7 | #Feature F1 | #Feature F2 | #Feature F3 | Start | End | PTM | AScore | Found By |
| --- | --- | --- | --- | --- | --- | --- | --- | --- | --- | --- | --- | --- | --- | --- | --- | --- | --- | --- | --- | --- | --- | --- | --- | --- | --- | --- | --- | --- | --- | --- | --- | --- | --- | --- | --- | --- | --- |
| R.LENEIETYR.C | N | 60.45 | 1165.5615 | 9 | 0.5 | 583.7883 | 2 | 30.98 | 6 | F6:17641 | 17012022\_RID\_3262\_9a.raw | 3.245E5 |  |  | 2.4101E6 |  | 2.2556E6 |  | 6.748E5 | 2.1401E6 |  | 6 | 1 | 0 | 0 | 1 | 0 | 1 | 0 | 1 | 2 | 0 | 382 | 390 |  |  | PEAKS DB |
| K.IRLENEIETYR.C | N | 56.27 | 1434.7467 | 11 | 0.5 | 718.3810 | 2 | 32.86 | 9 | F9:15718 | 31122021\_RID\_3262\_2a.raw |  |  |  |  |  |  |  | 1.4126E6 | 2.5524E5 |  | 2 | 0 | 0 | 0 | 0 | 0 | 0 | 0 | 1 | 1 | 0 | 380 | 390 |  |  | PEAKS DB |
| R.LAVDDFR.M | Y | 43.97 | 834.4235 | 7 | -0.8 | 418.2187 | 2 | 34.47 | 8 | F8:15844 | 31122021\_RID\_3262\_1a.raw |  |  |  |  |  |  |  | 2.8262E5 |  |  | 1 | 0 | 0 | 0 | 0 | 0 | 0 | 0 | 1 | 0 | 0 | 169 | 175 |  |  | PEAKS DB |
| total 3 peptides |  |  |  |  |  |  |  |  |  |  |  |  |  |  |  |  |  |  |  |  |  |  |  |  |  |  |  |  |  |  |  |  |  |  |  |  |  |
| --- | --- | --- | --- | --- | --- | --- | --- | --- | --- | --- | --- | --- | --- | --- | --- | --- | --- | --- | --- | --- | --- | --- | --- | --- | --- | --- | --- | --- | --- | --- | --- | --- | --- | --- | --- | --- | --- |

XP\_013917303.1

back to list

  

| Protein Coverage
| Supporting Peptides
|

Protein Coverage:

Supporting Peptides:

| Peptide | Uniq | -10lgP | Mass | Length | ppm | m/z | z | RT | Fraction | Scan | Source File | Area F4 | Area F5 | Area F6 | Area F10 | Area F8 | Area F9 | Area F7 | Area F1 | Area F2 | Area F3 | #Feature | #Feature F4 | #Feature F5 | #Feature F6 | #Feature F10 | #Feature F8 | #Feature F9 | #Feature F7 | #Feature F1 | #Feature F2 | #Feature F3 | Start | End | PTM | AScore | Found By |
| --- | --- | --- | --- | --- | --- | --- | --- | --- | --- | --- | --- | --- | --- | --- | --- | --- | --- | --- | --- | --- | --- | --- | --- | --- | --- | --- | --- | --- | --- | --- | --- | --- | --- | --- | --- | --- | --- |
| R.LENEIETYR.R | N | 60.45 | 1165.5615 | 9 | 0.5 | 583.7883 | 2 | 30.98 | 6 | F6:17641 | 17012022\_RID\_3262\_9a.raw | 3.245E5 |  |  | 2.4101E6 |  | 2.2556E6 |  | 6.748E5 | 2.1401E6 |  | 6 | 1 | 0 | 0 | 1 | 0 | 1 | 0 | 1 | 2 | 0 | 293 | 301 |  |  | PEAKS DB |
| K.IRLENEIETYR.R | N | 56.27 | 1434.7467 | 11 | 0.5 | 718.3810 | 2 | 32.86 | 9 | F9:15718 | 31122021\_RID\_3262\_2a.raw |  |  |  |  |  |  |  | 1.4126E6 | 2.5524E5 |  | 2 | 0 | 0 | 0 | 0 | 0 | 0 | 0 | 1 | 1 | 0 | 291 | 301 |  |  | PEAKS DB |
| R.LAVDDFR.M | Y | 43.97 | 834.4235 | 7 | -0.8 | 418.2187 | 2 | 34.47 | 8 | F8:15844 | 31122021\_RID\_3262\_1a.raw |  |  |  |  |  |  |  | 2.8262E5 |  |  | 1 | 0 | 0 | 0 | 0 | 0 | 0 | 0 | 1 | 0 | 0 | 80 | 86 |  |  | PEAKS DB |
| total 3 peptides |  |  |  |  |  |  |  |  |  |  |  |  |  |  |  |  |  |  |  |  |  |  |  |  |  |  |  |  |  |  |  |  |  |  |  |  |  |
| --- | --- | --- | --- | --- | --- | --- | --- | --- | --- | --- | --- | --- | --- | --- | --- | --- | --- | --- | --- | --- | --- | --- | --- | --- | --- | --- | --- | --- | --- | --- | --- | --- | --- | --- | --- | --- | --- |

XP\_034271639.1

back to list

  

| Protein Coverage
| Supporting Peptides
|

Protein Coverage:

Supporting Peptides:

| Peptide | Uniq | -10lgP | Mass | Length | ppm | m/z | z | RT | Fraction | Scan | Source File | Area F4 | Area F5 | Area F6 | Area F10 | Area F8 | Area F9 | Area F7 | Area F1 | Area F2 | Area F3 | #Feature | #Feature F4 | #Feature F5 | #Feature F6 | #Feature F10 | #Feature F8 | #Feature F9 | #Feature F7 | #Feature F1 | #Feature F2 | #Feature F3 | Start | End | PTM | AScore | Found By |
| --- | --- | --- | --- | --- | --- | --- | --- | --- | --- | --- | --- | --- | --- | --- | --- | --- | --- | --- | --- | --- | --- | --- | --- | --- | --- | --- | --- | --- | --- | --- | --- | --- | --- | --- | --- | --- | --- |
| R.LENEIETYR.R | N | 60.45 | 1165.5615 | 9 | 0.5 | 583.7883 | 2 | 30.98 | 6 | F6:17641 | 17012022\_RID\_3262\_9a.raw | 3.245E5 |  |  | 2.4101E6 |  | 2.2556E6 |  | 6.748E5 | 2.1401E6 |  | 6 | 1 | 0 | 0 | 1 | 0 | 1 | 0 | 1 | 2 | 0 | 290 | 298 |  |  | PEAKS DB |
| K.IRLENEIETYR.R | N | 56.27 | 1434.7467 | 11 | 0.5 | 718.3810 | 2 | 32.86 | 9 | F9:15718 | 31122021\_RID\_3262\_2a.raw |  |  |  |  |  |  |  | 1.4126E6 | 2.5524E5 |  | 2 | 0 | 0 | 0 | 0 | 0 | 0 | 0 | 1 | 1 | 0 | 288 | 298 |  |  | PEAKS DB |
| R.LAVDDFR.M | Y | 43.97 | 834.4235 | 7 | -0.8 | 418.2187 | 2 | 34.47 | 8 | F8:15844 | 31122021\_RID\_3262\_1a.raw |  |  |  |  |  |  |  | 2.8262E5 |  |  | 1 | 0 | 0 | 0 | 0 | 0 | 0 | 0 | 1 | 0 | 0 | 80 | 86 |  |  | PEAKS DB |
| total 3 peptides |  |  |  |  |  |  |  |  |  |  |  |  |  |  |  |  |  |  |  |  |  |  |  |  |  |  |  |  |  |  |  |  |  |  |  |  |  |
| --- | --- | --- | --- | --- | --- | --- | --- | --- | --- | --- | --- | --- | --- | --- | --- | --- | --- | --- | --- | --- | --- | --- | --- | --- | --- | --- | --- | --- | --- | --- | --- | --- | --- | --- | --- | --- | --- |

P0DJL3.1

back to list

  

| Protein Coverage
| Supporting Peptides
|

Protein Coverage:

Supporting Peptides:

| Peptide | Uniq | -10lgP | Mass | Length | ppm | m/z | z | RT | Fraction | Scan | Source File | Area F4 | Area F5 | Area F6 | Area F10 | Area F8 | Area F9 | Area F7 | Area F1 | Area F2 | Area F3 | #Feature | #Feature F4 | #Feature F5 | #Feature F6 | #Feature F10 | #Feature F8 | #Feature F9 | #Feature F7 | #Feature F1 | #Feature F2 | #Feature F3 | Start | End | PTM | AScore | Found By |
| --- | --- | --- | --- | --- | --- | --- | --- | --- | --- | --- | --- | --- | --- | --- | --- | --- | --- | --- | --- | --- | --- | --- | --- | --- | --- | --- | --- | --- | --- | --- | --- | --- | --- | --- | --- | --- | --- |
| R.LQWSDGTELK.Y | Y | 61.93 | 1175.5823 | 10 | 0.5 | 588.7987 | 2 | 38.12 | 4 | F4:23634 | 17012022\_RID\_3262\_10a .raw |  | 0 |  | 1.9965E7 |  |  |  |  |  |  | 2 | 0 | 0 | 0 | 2 | 0 | 0 | 0 | 0 | 0 | 0 | 77 | 86 |  |  | PEAKS DB |
| K.TTDNQWWTR.S | Y | 57.71 | 1206.5417 | 9 | 0.1 | 604.2782 | 2 | 38.01 | 4 | F4:23288 | 17012022\_RID\_3262\_10a .raw |  |  |  | 3.414E8 |  | 6.4193E6 |  |  |  |  | 3 | 0 | 0 | 0 | 2 | 0 | 1 | 0 | 0 | 0 | 0 | 101 | 109 |  |  | PEAKS DB |
| total 2 peptides |  |  |  |  |  |  |  |  |  |  |  |  |  |  |  |  |  |  |  |  |  |  |  |  |  |  |  |  |  |  |  |  |  |  |  |  |  |
| --- | --- | --- | --- | --- | --- | --- | --- | --- | --- | --- | --- | --- | --- | --- | --- | --- | --- | --- | --- | --- | --- | --- | --- | --- | --- | --- | --- | --- | --- | --- | --- | --- | --- | --- | --- | --- | --- |

P81116.2

back to list

  

| Protein Coverage
| Supporting Peptides
|

Protein Coverage:

Supporting Peptides:

| Peptide | Uniq | -10lgP | Mass | Length | ppm | m/z | z | RT | Fraction | Scan | Source File | Area F4 | Area F5 | Area F6 | Area F10 | Area F8 | Area F9 | Area F7 | Area F1 | Area F2 | Area F3 | #Feature | #Feature F4 | #Feature F5 | #Feature F6 | #Feature F10 | #Feature F8 | #Feature F9 | #Feature F7 | #Feature F1 | #Feature F2 | #Feature F3 | Start | End | PTM | AScore | Found By |
| --- | --- | --- | --- | --- | --- | --- | --- | --- | --- | --- | --- | --- | --- | --- | --- | --- | --- | --- | --- | --- | --- | --- | --- | --- | --- | --- | --- | --- | --- | --- | --- | --- | --- | --- | --- | --- | --- |
| R.LQWSDGTELK.Y | Y | 61.93 | 1175.5823 | 10 | 0.5 | 588.7987 | 2 | 38.12 | 4 | F4:23634 | 17012022\_RID\_3262\_10a .raw |  | 0 |  | 1.9965E7 |  |  |  |  |  |  | 2 | 0 | 0 | 0 | 2 | 0 | 0 | 0 | 0 | 0 | 0 | 100 | 109 |  |  | PEAKS DB |
| K.TTDNQWWTR.S | Y | 57.71 | 1206.5417 | 9 | 0.1 | 604.2782 | 2 | 38.01 | 4 | F4:23288 | 17012022\_RID\_3262\_10a .raw |  |  |  | 3.414E8 |  | 6.4193E6 |  |  |  |  | 3 | 0 | 0 | 0 | 2 | 0 | 1 | 0 | 0 | 0 | 0 | 124 | 132 |  |  | PEAKS DB |
| total 2 peptides |  |  |  |  |  |  |  |  |  |  |  |  |  |  |  |  |  |  |  |  |  |  |  |  |  |  |  |  |  |  |  |  |  |  |  |  |  |
| --- | --- | --- | --- | --- | --- | --- | --- | --- | --- | --- | --- | --- | --- | --- | --- | --- | --- | --- | --- | --- | --- | --- | --- | --- | --- | --- | --- | --- | --- | --- | --- | --- | --- | --- | --- | --- | --- |

ABS12077.1

back to list

  

| Protein Coverage
| Supporting Peptides
|

Protein Coverage:

Supporting Peptides:

| Peptide | Uniq | -10lgP | Mass | Length | ppm | m/z | z | RT | Fraction | Scan | Source File | Area F4 | Area F5 | Area F6 | Area F10 | Area F8 | Area F9 | Area F7 | Area F1 | Area F2 | Area F3 | #Feature | #Feature F4 | #Feature F5 | #Feature F6 | #Feature F10 | #Feature F8 | #Feature F9 | #Feature F7 | #Feature F1 | #Feature F2 | #Feature F3 | Start | End | PTM | AScore | Found By |
| --- | --- | --- | --- | --- | --- | --- | --- | --- | --- | --- | --- | --- | --- | --- | --- | --- | --- | --- | --- | --- | --- | --- | --- | --- | --- | --- | --- | --- | --- | --- | --- | --- | --- | --- | --- | --- | --- |
| R.LQWSDGTELK.Y | Y | 61.93 | 1175.5823 | 10 | 0.5 | 588.7987 | 2 | 38.12 | 4 | F4:23634 | 17012022\_RID\_3262\_10a .raw |  | 0 |  | 1.9965E7 |  |  |  |  |  |  | 2 | 0 | 0 | 0 | 2 | 0 | 0 | 0 | 0 | 0 | 0 | 100 | 109 |  |  | PEAKS DB |
| K.TTDNQWWTR.S | Y | 57.71 | 1206.5417 | 9 | 0.1 | 604.2782 | 2 | 38.01 | 4 | F4:23288 | 17012022\_RID\_3262\_10a .raw |  |  |  | 3.414E8 |  | 6.4193E6 |  |  |  |  | 3 | 0 | 0 | 0 | 2 | 0 | 1 | 0 | 0 | 0 | 0 | 124 | 132 |  |  | PEAKS DB |
| total 2 peptides |  |  |  |  |  |  |  |  |  |  |  |  |  |  |  |  |  |  |  |  |  |  |  |  |  |  |  |  |  |  |  |  |  |  |  |  |  |
| --- | --- | --- | --- | --- | --- | --- | --- | --- | --- | --- | --- | --- | --- | --- | --- | --- | --- | --- | --- | --- | --- | --- | --- | --- | --- | --- | --- | --- | --- | --- | --- | --- | --- | --- | --- | --- | --- |

BAN89403.1

back to list

  

| Protein Coverage
| Supporting Peptides
|

Protein Coverage:

Supporting Peptides:

| Peptide | Uniq | -10lgP | Mass | Length | ppm | m/z | z | RT | Fraction | Scan | Source File | Area F4 | Area F5 | Area F6 | Area F10 | Area F8 | Area F9 | Area F7 | Area F1 | Area F2 | Area F3 | #Feature | #Feature F4 | #Feature F5 | #Feature F6 | #Feature F10 | #Feature F8 | #Feature F9 | #Feature F7 | #Feature F1 | #Feature F2 | #Feature F3 | Start | End | PTM | AScore | Found By |
| --- | --- | --- | --- | --- | --- | --- | --- | --- | --- | --- | --- | --- | --- | --- | --- | --- | --- | --- | --- | --- | --- | --- | --- | --- | --- | --- | --- | --- | --- | --- | --- | --- | --- | --- | --- | --- | --- |
| K.SHDNAHLLTGINFN.G | Y | 64.47 | 1551.7430 | 14 | 0.2 | 518.2551 | 3 | 40.64 | 1 | F1:26366 | 13012022\_RID\_3262\_4a.raw | 6.8904E6 |  |  |  |  |  |  |  |  |  | 3 | 3 | 0 | 0 | 0 | 0 | 0 | 0 | 0 | 0 | 0 | 285 | 298 |  |  | PEAKS DB |
| K.GM(+15.99)VLPGTK.C | N | 47.53 | 817.4368 | 8 | -3.8 | 409.7241 | 2 | 30.58 | 2 | F2:15077 | 13012022\_RID\_3262\_5a.raw | 6.0504E7 | 3.227E6 | 7.6355E5 |  |  |  | 3.1499E5 |  |  |  | 7 | 3 | 2 | 1 | 0 | 0 | 0 | 1 | 0 | 0 | 0 | 585 | 592 | Oxidation (M) | M2:Oxidation (M):1000.00 | PEAKS DB |
| K.GMVLPGTK.C | N | 47.31 | 801.4418 | 8 | 0.3 | 401.7283 | 2 | 30.97 | 2 | F2:15254 | 13012022\_RID\_3262\_5a.raw | 1.0637E8 | 5.4594E6 | 1.626E6 |  |  |  | 1.5224E6 | 2.1234E6 |  |  | 5 | 1 | 1 | 1 | 0 | 0 | 0 | 1 | 1 | 0 | 0 | 585 | 592 |  |  | PEAKS DB |
| total 3 peptides |  |  |  |  |  |  |  |  |  |  |  |  |  |  |  |  |  |  |  |  |  |  |  |  |  |  |  |  |  |  |  |  |  |  |  |  |  |
| --- | --- | --- | --- | --- | --- | --- | --- | --- | --- | --- | --- | --- | --- | --- | --- | --- | --- | --- | --- | --- | --- | --- | --- | --- | --- | --- | --- | --- | --- | --- | --- | --- | --- | --- | --- | --- | --- |

LAA93895.1

back to list

  

| Protein Coverage
| Supporting Peptides
|

Protein Coverage:

Supporting Peptides:

| Peptide | Uniq | -10lgP | Mass | Length | ppm | m/z | z | RT | Fraction | Scan | Source File | Area F4 | Area F5 | Area F6 | Area F10 | Area F8 | Area F9 | Area F7 | Area F1 | Area F2 | Area F3 | #Feature | #Feature F4 | #Feature F5 | #Feature F6 | #Feature F10 | #Feature F8 | #Feature F9 | #Feature F7 | #Feature F1 | #Feature F2 | #Feature F3 | Start | End | PTM | AScore | Found By |
| --- | --- | --- | --- | --- | --- | --- | --- | --- | --- | --- | --- | --- | --- | --- | --- | --- | --- | --- | --- | --- | --- | --- | --- | --- | --- | --- | --- | --- | --- | --- | --- | --- | --- | --- | --- | --- | --- |
| K.LAELEEALQK.A | Y | 55.88 | 1142.6183 | 10 | -0.4 | 572.3162 | 2 | 38.89 | 4 | F4:24277 | 17012022\_RID\_3262\_10a .raw | 7.2289E6 | 5.7848E6 | 2.9885E6 | 2.9771E7 |  | 3.548E7 | 6.434E5 | 2.3478E7 | 7.6904E6 |  | 8 | 1 | 1 | 1 | 1 | 0 | 1 | 1 | 1 | 1 | 0 | 68 | 77 |  |  | PEAKS DB |
| R.GELALKDAR.Q | N | 49.06 | 971.5399 | 9 | -0.6 | 486.7769 | 2 | 22.10 | 6 | F6:9646 | 17012022\_RID\_3262\_9a.raw |  |  |  | 1.6772E6 |  | 1.879E6 | 8.5932E4 |  |  |  | 3 | 0 | 0 | 0 | 1 | 0 | 1 | 1 | 0 | 0 | 0 | 57 | 65 |  |  | PEAKS DB |
| K.KLLEGEEYR.L | N | 41.07 | 1135.5873 | 9 | 0.2 | 379.5364 | 3 | 24.42 | 4 | F4:11454 | 17012022\_RID\_3262\_10a .raw |  |  |  | 1.9768E5 |  |  |  |  |  |  | 1 | 0 | 0 | 0 | 1 | 0 | 0 | 0 | 0 | 0 | 0 | 108 | 116 |  |  | PEAKS DB |
| total 3 peptides |  |  |  |  |  |  |  |  |  |  |  |  |  |  |  |  |  |  |  |  |  |  |  |  |  |  |  |  |  |  |  |  |  |  |  |  |  |
| --- | --- | --- | --- | --- | --- | --- | --- | --- | --- | --- | --- | --- | --- | --- | --- | --- | --- | --- | --- | --- | --- | --- | --- | --- | --- | --- | --- | --- | --- | --- | --- | --- | --- | --- | --- | --- | --- |

Q2YHJ2.1

back to list

  

| Protein Coverage
| Supporting Peptides
|

Protein Coverage:

Supporting Peptides:

| Peptide | Uniq | -10lgP | Mass | Length | ppm | m/z | z | RT | Fraction | Scan | Source File | Area F4 | Area F5 | Area F6 | Area F10 | Area F8 | Area F9 | Area F7 | Area F1 | Area F2 | Area F3 | #Feature | #Feature F4 | #Feature F5 | #Feature F6 | #Feature F10 | #Feature F8 | #Feature F9 | #Feature F7 | #Feature F1 | #Feature F2 | #Feature F3 | Start | End | PTM | AScore | Found By |
| --- | --- | --- | --- | --- | --- | --- | --- | --- | --- | --- | --- | --- | --- | --- | --- | --- | --- | --- | --- | --- | --- | --- | --- | --- | --- | --- | --- | --- | --- | --- | --- | --- | --- | --- | --- | --- | --- |
| K.TDIYFYYR.K | Y | 57.84 | 1139.5287 | 8 | -0.1 | 570.7715 | 2 | 43.78 | 9 | F9:24555 | 31122021\_RID\_3262\_2a.raw | 1.7238E7 | 1.1289E6 | 2.5258E5 | 2.8026E8 |  | 7.7444E7 |  | 9.5005E6 | 4.0119E6 | 7.3435E6 | 10 | 1 | 1 | 1 | 2 | 0 | 1 | 0 | 2 | 1 | 1 | 77 | 84 |  |  | PEAKS DB |
| G.SLLEFGR.M | N | 44.54 | 820.4443 | 7 | 1.1 | 411.2299 | 2 | 41.75 | 4 | F4:26641 | 17012022\_RID\_3262\_10a .raw |  |  |  | 1.1809E10 |  | 6.3702E8 |  |  | 1.5471E7 |  | 4 | 0 | 0 | 0 | 1 | 0 | 2 | 0 | 0 | 1 | 0 | 17 | 23 |  |  | PEAKS DB |
| K.TDIYFYYRK.N | Y | 41.99 | 1267.6237 | 9 | 0.8 | 634.8196 | 2 | 36.35 | 6 | F6:22253 | 17012022\_RID\_3262\_9a.raw |  |  |  |  |  | 1.5536E6 |  |  |  |  | 1 | 0 | 0 | 0 | 0 | 0 | 1 | 0 | 0 | 0 | 0 | 77 | 85 |  |  | PEAKS DB |
| total 3 peptides |  |  |  |  |  |  |  |  |  |  |  |  |  |  |  |  |  |  |  |  |  |  |  |  |  |  |  |  |  |  |  |  |  |  |  |  |  |
| --- | --- | --- | --- | --- | --- | --- | --- | --- | --- | --- | --- | --- | --- | --- | --- | --- | --- | --- | --- | --- | --- | --- | --- | --- | --- | --- | --- | --- | --- | --- | --- | --- | --- | --- | --- | --- | --- |

AAR14173.1

back to list

  

| Protein Coverage
| Supporting Peptides
|

Protein Coverage:

Supporting Peptides:

| Peptide | Uniq | -10lgP | Mass | Length | ppm | m/z | z | RT | Fraction | Scan | Source File | Area F4 | Area F5 | Area F6 | Area F10 | Area F8 | Area F9 | Area F7 | Area F1 | Area F2 | Area F3 | #Feature | #Feature F4 | #Feature F5 | #Feature F6 | #Feature F10 | #Feature F8 | #Feature F9 | #Feature F7 | #Feature F1 | #Feature F2 | #Feature F3 | Start | End | PTM | AScore | Found By |
| --- | --- | --- | --- | --- | --- | --- | --- | --- | --- | --- | --- | --- | --- | --- | --- | --- | --- | --- | --- | --- | --- | --- | --- | --- | --- | --- | --- | --- | --- | --- | --- | --- | --- | --- | --- | --- | --- |
| K.TDIYFYYR.K | Y | 57.84 | 1139.5287 | 8 | -0.1 | 570.7715 | 2 | 43.78 | 9 | F9:24555 | 31122021\_RID\_3262\_2a.raw | 1.7238E7 | 1.1289E6 | 2.5258E5 | 2.8026E8 |  | 7.7444E7 |  | 9.5005E6 | 4.0119E6 | 7.3435E6 | 10 | 1 | 1 | 1 | 2 | 0 | 1 | 0 | 2 | 1 | 1 | 77 | 84 |  |  | PEAKS DB |
| G.SLLEFGR.M | N | 44.54 | 820.4443 | 7 | 1.1 | 411.2299 | 2 | 41.75 | 4 | F4:26641 | 17012022\_RID\_3262\_10a .raw |  |  |  | 1.1809E10 |  | 6.3702E8 |  |  | 1.5471E7 |  | 4 | 0 | 0 | 0 | 1 | 0 | 2 | 0 | 0 | 1 | 0 | 17 | 23 |  |  | PEAKS DB |
| K.TDIYFYYRK.N | Y | 41.99 | 1267.6237 | 9 | 0.8 | 634.8196 | 2 | 36.35 | 6 | F6:22253 | 17012022\_RID\_3262\_9a.raw |  |  |  |  |  | 1.5536E6 |  |  |  |  | 1 | 0 | 0 | 0 | 0 | 0 | 1 | 0 | 0 | 0 | 0 | 77 | 85 |  |  | PEAKS DB |
| total 3 peptides |  |  |  |  |  |  |  |  |  |  |  |  |  |  |  |  |  |  |  |  |  |  |  |  |  |  |  |  |  |  |  |  |  |  |  |  |  |
| --- | --- | --- | --- | --- | --- | --- | --- | --- | --- | --- | --- | --- | --- | --- | --- | --- | --- | --- | --- | --- | --- | --- | --- | --- | --- | --- | --- | --- | --- | --- | --- | --- | --- | --- | --- | --- | --- |

Q2YHJ7.1

back to list

  

| Protein Coverage
| Supporting Peptides
|

Protein Coverage:

Supporting Peptides:

| Peptide | Uniq | -10lgP | Mass | Length | ppm | m/z | z | RT | Fraction | Scan | Source File | Area F4 | Area F5 | Area F6 | Area F10 | Area F8 | Area F9 | Area F7 | Area F1 | Area F2 | Area F3 | #Feature | #Feature F4 | #Feature F5 | #Feature F6 | #Feature F10 | #Feature F8 | #Feature F9 | #Feature F7 | #Feature F1 | #Feature F2 | #Feature F3 | Start | End | PTM | AScore | Found By |
| --- | --- | --- | --- | --- | --- | --- | --- | --- | --- | --- | --- | --- | --- | --- | --- | --- | --- | --- | --- | --- | --- | --- | --- | --- | --- | --- | --- | --- | --- | --- | --- | --- | --- | --- | --- | --- | --- |
| K.TDIYFYYR.K | Y | 57.84 | 1139.5287 | 8 | -0.1 | 570.7715 | 2 | 43.78 | 9 | F9:24555 | 31122021\_RID\_3262\_2a.raw | 1.7238E7 | 1.1289E6 | 2.5258E5 | 2.8026E8 |  | 7.7444E7 |  | 9.5005E6 | 4.0119E6 | 7.3435E6 | 10 | 1 | 1 | 1 | 2 | 0 | 1 | 0 | 2 | 1 | 1 | 77 | 84 |  |  | PEAKS DB |
| G.SLLEFGR.M | N | 44.54 | 820.4443 | 7 | 1.1 | 411.2299 | 2 | 41.75 | 4 | F4:26641 | 17012022\_RID\_3262\_10a .raw |  |  |  | 1.1809E10 |  | 6.3702E8 |  |  | 1.5471E7 |  | 4 | 0 | 0 | 0 | 1 | 0 | 2 | 0 | 0 | 1 | 0 | 17 | 23 |  |  | PEAKS DB |
| K.TDIYFYYRK.N | Y | 41.99 | 1267.6237 | 9 | 0.8 | 634.8196 | 2 | 36.35 | 6 | F6:22253 | 17012022\_RID\_3262\_9a.raw |  |  |  |  |  | 1.5536E6 |  |  |  |  | 1 | 0 | 0 | 0 | 0 | 0 | 1 | 0 | 0 | 0 | 0 | 77 | 85 |  |  | PEAKS DB |
| total 3 peptides |  |  |  |  |  |  |  |  |  |  |  |  |  |  |  |  |  |  |  |  |  |  |  |  |  |  |  |  |  |  |  |  |  |  |  |  |  |
| --- | --- | --- | --- | --- | --- | --- | --- | --- | --- | --- | --- | --- | --- | --- | --- | --- | --- | --- | --- | --- | --- | --- | --- | --- | --- | --- | --- | --- | --- | --- | --- | --- | --- | --- | --- | --- | --- |

AAR14167.1

back to list

  

| Protein Coverage
| Supporting Peptides
|

Protein Coverage:

Supporting Peptides:

| Peptide | Uniq | -10lgP | Mass | Length | ppm | m/z | z | RT | Fraction | Scan | Source File | Area F4 | Area F5 | Area F6 | Area F10 | Area F8 | Area F9 | Area F7 | Area F1 | Area F2 | Area F3 | #Feature | #Feature F4 | #Feature F5 | #Feature F6 | #Feature F10 | #Feature F8 | #Feature F9 | #Feature F7 | #Feature F1 | #Feature F2 | #Feature F3 | Start | End | PTM | AScore | Found By |
| --- | --- | --- | --- | --- | --- | --- | --- | --- | --- | --- | --- | --- | --- | --- | --- | --- | --- | --- | --- | --- | --- | --- | --- | --- | --- | --- | --- | --- | --- | --- | --- | --- | --- | --- | --- | --- | --- |
| K.TDIYFYYR.K | Y | 57.84 | 1139.5287 | 8 | -0.1 | 570.7715 | 2 | 43.78 | 9 | F9:24555 | 31122021\_RID\_3262\_2a.raw | 1.7238E7 | 1.1289E6 | 2.5258E5 | 2.8026E8 |  | 7.7444E7 |  | 9.5005E6 | 4.0119E6 | 7.3435E6 | 10 | 1 | 1 | 1 | 2 | 0 | 1 | 0 | 2 | 1 | 1 | 77 | 84 |  |  | PEAKS DB |
| G.SLLEFGR.M | N | 44.54 | 820.4443 | 7 | 1.1 | 411.2299 | 2 | 41.75 | 4 | F4:26641 | 17012022\_RID\_3262\_10a .raw |  |  |  | 1.1809E10 |  | 6.3702E8 |  |  | 1.5471E7 |  | 4 | 0 | 0 | 0 | 1 | 0 | 2 | 0 | 0 | 1 | 0 | 17 | 23 |  |  | PEAKS DB |
| K.TDIYFYYRK.N | Y | 41.99 | 1267.6237 | 9 | 0.8 | 634.8196 | 2 | 36.35 | 6 | F6:22253 | 17012022\_RID\_3262\_9a.raw |  |  |  |  |  | 1.5536E6 |  |  |  |  | 1 | 0 | 0 | 0 | 0 | 0 | 1 | 0 | 0 | 0 | 0 | 77 | 85 |  |  | PEAKS DB |
| total 3 peptides |  |  |  |  |  |  |  |  |  |  |  |  |  |  |  |  |  |  |  |  |  |  |  |  |  |  |  |  |  |  |  |  |  |  |  |  |  |
| --- | --- | --- | --- | --- | --- | --- | --- | --- | --- | --- | --- | --- | --- | --- | --- | --- | --- | --- | --- | --- | --- | --- | --- | --- | --- | --- | --- | --- | --- | --- | --- | --- | --- | --- | --- | --- | --- |

JAB53718.1

back to list

  

| Protein Coverage
| Supporting Peptides
|

Protein Coverage:

Supporting Peptides:

| Peptide | Uniq | -10lgP | Mass | Length | ppm | m/z | z | RT | Fraction | Scan | Source File | Area F4 | Area F5 | Area F6 | Area F10 | Area F8 | Area F9 | Area F7 | Area F1 | Area F2 | Area F3 | #Feature | #Feature F4 | #Feature F5 | #Feature F6 | #Feature F10 | #Feature F8 | #Feature F9 | #Feature F7 | #Feature F1 | #Feature F2 | #Feature F3 | Start | End | PTM | AScore | Found By |
| --- | --- | --- | --- | --- | --- | --- | --- | --- | --- | --- | --- | --- | --- | --- | --- | --- | --- | --- | --- | --- | --- | --- | --- | --- | --- | --- | --- | --- | --- | --- | --- | --- | --- | --- | --- | --- | --- |
| R.LVQAFQFTDK.N | N | 58.02 | 1195.6237 | 10 | 0.9 | 598.8196 | 2 | 40.96 | 8 | F8:21197 | 31122021\_RID\_3262\_1a.raw |  |  |  |  |  |  |  | 2.1849E5 |  |  | 1 | 0 | 0 | 0 | 0 | 0 | 0 | 0 | 1 | 0 | 0 | 159 | 168 |  |  | PEAKS DB |
| K.ATAVMPDGQFK.D | N | 41.76 | 1163.5645 | 11 | 0.1 | 582.7896 | 2 | 33.47 | 6 | F6:19724 | 17012022\_RID\_3262\_9a.raw |  |  |  |  |  | 5.1945E5 |  |  |  |  | 1 | 0 | 0 | 0 | 0 | 0 | 1 | 0 | 0 | 0 | 0 | 17 | 27 |  |  | PEAKS DB |
| F.IGKPAPDFK.A | Y | 40.93 | 971.5439 | 9 | -0.9 | 486.7788 | 2 | 22.53 | 6 | F6:10011 | 17012022\_RID\_3262\_9a.raw |  |  |  |  |  | 5.7206E5 |  |  |  |  | 1 | 0 | 0 | 0 | 0 | 0 | 1 | 0 | 0 | 0 | 0 | 8 | 16 |  |  | PEAKS DB |
| total 3 peptides |  |  |  |  |  |  |  |  |  |  |  |  |  |  |  |  |  |  |  |  |  |  |  |  |  |  |  |  |  |  |  |  |  |  |  |  |  |
| --- | --- | --- | --- | --- | --- | --- | --- | --- | --- | --- | --- | --- | --- | --- | --- | --- | --- | --- | --- | --- | --- | --- | --- | --- | --- | --- | --- | --- | --- | --- | --- | --- | --- | --- | --- | --- | --- |

XP\_026521545.1

back to list

  

| Protein Coverage
| Supporting Peptides
|

Protein Coverage:

Supporting Peptides:

| Peptide | Uniq | -10lgP | Mass | Length | ppm | m/z | z | RT | Fraction | Scan | Source File | Area F4 | Area F5 | Area F6 | Area F10 | Area F8 | Area F9 | Area F7 | Area F1 | Area F2 | Area F3 | #Feature | #Feature F4 | #Feature F5 | #Feature F6 | #Feature F10 | #Feature F8 | #Feature F9 | #Feature F7 | #Feature F1 | #Feature F2 | #Feature F3 | Start | End | PTM | AScore | Found By |
| --- | --- | --- | --- | --- | --- | --- | --- | --- | --- | --- | --- | --- | --- | --- | --- | --- | --- | --- | --- | --- | --- | --- | --- | --- | --- | --- | --- | --- | --- | --- | --- | --- | --- | --- | --- | --- | --- |
| R.LVQAFQFTDK.N | N | 58.02 | 1195.6237 | 10 | 0.9 | 598.8196 | 2 | 40.96 | 8 | F8:21197 | 31122021\_RID\_3262\_1a.raw |  |  |  |  |  |  |  | 2.1849E5 |  |  | 1 | 0 | 0 | 0 | 0 | 0 | 0 | 0 | 1 | 0 | 0 | 159 | 168 |  |  | PEAKS DB |
| K.ATAVMPDGQFK.D | N | 41.76 | 1163.5645 | 11 | 0.1 | 582.7896 | 2 | 33.47 | 6 | F6:19724 | 17012022\_RID\_3262\_9a.raw |  |  |  |  |  | 5.1945E5 |  |  |  |  | 1 | 0 | 0 | 0 | 0 | 0 | 1 | 0 | 0 | 0 | 0 | 17 | 27 |  |  | PEAKS DB |
| F.IGKPAPDFK.A | Y | 40.93 | 971.5439 | 9 | -0.9 | 486.7788 | 2 | 22.53 | 6 | F6:10011 | 17012022\_RID\_3262\_9a.raw |  |  |  |  |  | 5.7206E5 |  |  |  |  | 1 | 0 | 0 | 0 | 0 | 0 | 1 | 0 | 0 | 0 | 0 | 8 | 16 |  |  | PEAKS DB |
| total 3 peptides |  |  |  |  |  |  |  |  |  |  |  |  |  |  |  |  |  |  |  |  |  |  |  |  |  |  |  |  |  |  |  |  |  |  |  |  |  |
| --- | --- | --- | --- | --- | --- | --- | --- | --- | --- | --- | --- | --- | --- | --- | --- | --- | --- | --- | --- | --- | --- | --- | --- | --- | --- | --- | --- | --- | --- | --- | --- | --- | --- | --- | --- | --- | --- |

JAI09680.1

back to list

  

| Protein Coverage
| Supporting Peptides
|

Protein Coverage:

Supporting Peptides:

| Peptide | Uniq | -10lgP | Mass | Length | ppm | m/z | z | RT | Fraction | Scan | Source File | Area F4 | Area F5 | Area F6 | Area F10 | Area F8 | Area F9 | Area F7 | Area F1 | Area F2 | Area F3 | #Feature | #Feature F4 | #Feature F5 | #Feature F6 | #Feature F10 | #Feature F8 | #Feature F9 | #Feature F7 | #Feature F1 | #Feature F2 | #Feature F3 | Start | End | PTM | AScore | Found By |
| --- | --- | --- | --- | --- | --- | --- | --- | --- | --- | --- | --- | --- | --- | --- | --- | --- | --- | --- | --- | --- | --- | --- | --- | --- | --- | --- | --- | --- | --- | --- | --- | --- | --- | --- | --- | --- | --- |
| R.LVQAFQFTDK.N | N | 58.02 | 1195.6237 | 10 | 0.9 | 598.8196 | 2 | 40.96 | 8 | F8:21197 | 31122021\_RID\_3262\_1a.raw |  |  |  |  |  |  |  | 2.1849E5 |  |  | 1 | 0 | 0 | 0 | 0 | 0 | 0 | 0 | 1 | 0 | 0 | 159 | 168 |  |  | PEAKS DB |
| K.ATAVMPDGQFK.D | N | 41.76 | 1163.5645 | 11 | 0.1 | 582.7896 | 2 | 33.47 | 6 | F6:19724 | 17012022\_RID\_3262\_9a.raw |  |  |  |  |  | 5.1945E5 |  |  |  |  | 1 | 0 | 0 | 0 | 0 | 0 | 1 | 0 | 0 | 0 | 0 | 17 | 27 |  |  | PEAKS DB |
| F.IGKPAPDFK.A | Y | 40.93 | 971.5439 | 9 | -0.9 | 486.7788 | 2 | 22.53 | 6 | F6:10011 | 17012022\_RID\_3262\_9a.raw |  |  |  |  |  | 5.7206E5 |  |  |  |  | 1 | 0 | 0 | 0 | 0 | 0 | 1 | 0 | 0 | 0 | 0 | 8 | 16 |  |  | PEAKS DB |
| total 3 peptides |  |  |  |  |  |  |  |  |  |  |  |  |  |  |  |  |  |  |  |  |  |  |  |  |  |  |  |  |  |  |  |  |  |  |  |  |  |
| --- | --- | --- | --- | --- | --- | --- | --- | --- | --- | --- | --- | --- | --- | --- | --- | --- | --- | --- | --- | --- | --- | --- | --- | --- | --- | --- | --- | --- | --- | --- | --- | --- | --- | --- | --- | --- | --- |

XP\_026562803.1

back to list

  

| Protein Coverage
| Supporting Peptides
|

Protein Coverage:

Supporting Peptides:

| Peptide | Uniq | -10lgP | Mass | Length | ppm | m/z | z | RT | Fraction | Scan | Source File | Area F4 | Area F5 | Area F6 | Area F10 | Area F8 | Area F9 | Area F7 | Area F1 | Area F2 | Area F3 | #Feature | #Feature F4 | #Feature F5 | #Feature F6 | #Feature F10 | #Feature F8 | #Feature F9 | #Feature F7 | #Feature F1 | #Feature F2 | #Feature F3 | Start | End | PTM | AScore | Found By |
| --- | --- | --- | --- | --- | --- | --- | --- | --- | --- | --- | --- | --- | --- | --- | --- | --- | --- | --- | --- | --- | --- | --- | --- | --- | --- | --- | --- | --- | --- | --- | --- | --- | --- | --- | --- | --- | --- |
| R.LVQAFQFTDK.H | N | 58.02 | 1195.6237 | 10 | 0.9 | 598.8196 | 2 | 40.96 | 8 | F8:21197 | 31122021\_RID\_3262\_1a.raw |  |  |  |  |  |  |  | 2.1849E5 |  |  | 1 | 0 | 0 | 0 | 0 | 0 | 0 | 0 | 1 | 0 | 0 | 159 | 168 |  |  | PEAKS DB |
| K.ATAVMPDGQFK.D | N | 41.76 | 1163.5645 | 11 | 0.1 | 582.7896 | 2 | 33.47 | 6 | F6:19724 | 17012022\_RID\_3262\_9a.raw |  |  |  |  |  | 5.1945E5 |  |  |  |  | 1 | 0 | 0 | 0 | 0 | 0 | 1 | 0 | 0 | 0 | 0 | 17 | 27 |  |  | PEAKS DB |
| F.IGKPAPDFK.A | Y | 40.93 | 971.5439 | 9 | -0.9 | 486.7788 | 2 | 22.53 | 6 | F6:10011 | 17012022\_RID\_3262\_9a.raw |  |  |  |  |  | 5.7206E5 |  |  |  |  | 1 | 0 | 0 | 0 | 0 | 0 | 1 | 0 | 0 | 0 | 0 | 8 | 16 |  |  | PEAKS DB |
| total 3 peptides |  |  |  |  |  |  |  |  |  |  |  |  |  |  |  |  |  |  |  |  |  |  |  |  |  |  |  |  |  |  |  |  |  |  |  |  |  |
| --- | --- | --- | --- | --- | --- | --- | --- | --- | --- | --- | --- | --- | --- | --- | --- | --- | --- | --- | --- | --- | --- | --- | --- | --- | --- | --- | --- | --- | --- | --- | --- | --- | --- | --- | --- | --- | --- |

XP\_026521546.1

back to list

  

| Protein Coverage
| Supporting Peptides
|

Protein Coverage:

Supporting Peptides:

| Peptide | Uniq | -10lgP | Mass | Length | ppm | m/z | z | RT | Fraction | Scan | Source File | Area F4 | Area F5 | Area F6 | Area F10 | Area F8 | Area F9 | Area F7 | Area F1 | Area F2 | Area F3 | #Feature | #Feature F4 | #Feature F5 | #Feature F6 | #Feature F10 | #Feature F8 | #Feature F9 | #Feature F7 | #Feature F1 | #Feature F2 | #Feature F3 | Start | End | PTM | AScore | Found By |
| --- | --- | --- | --- | --- | --- | --- | --- | --- | --- | --- | --- | --- | --- | --- | --- | --- | --- | --- | --- | --- | --- | --- | --- | --- | --- | --- | --- | --- | --- | --- | --- | --- | --- | --- | --- | --- | --- |
| R.LVQAFQFTDK.N | N | 58.02 | 1195.6237 | 10 | 0.9 | 598.8196 | 2 | 40.96 | 8 | F8:21197 | 31122021\_RID\_3262\_1a.raw |  |  |  |  |  |  |  | 2.1849E5 |  |  | 1 | 0 | 0 | 0 | 0 | 0 | 0 | 0 | 1 | 0 | 0 | 159 | 168 |  |  | PEAKS DB |
| K.ATAVMPDGQFK.D | N | 41.76 | 1163.5645 | 11 | 0.1 | 582.7896 | 2 | 33.47 | 6 | F6:19724 | 17012022\_RID\_3262\_9a.raw |  |  |  |  |  | 5.1945E5 |  |  |  |  | 1 | 0 | 0 | 0 | 0 | 0 | 1 | 0 | 0 | 0 | 0 | 17 | 27 |  |  | PEAKS DB |
| F.IGKPAPDFK.A | Y | 40.93 | 971.5439 | 9 | -0.9 | 486.7788 | 2 | 22.53 | 6 | F6:10011 | 17012022\_RID\_3262\_9a.raw |  |  |  |  |  | 5.7206E5 |  |  |  |  | 1 | 0 | 0 | 0 | 0 | 0 | 1 | 0 | 0 | 0 | 0 | 8 | 16 |  |  | PEAKS DB |
| total 3 peptides |  |  |  |  |  |  |  |  |  |  |  |  |  |  |  |  |  |  |  |  |  |  |  |  |  |  |  |  |  |  |  |  |  |  |  |  |  |
| --- | --- | --- | --- | --- | --- | --- | --- | --- | --- | --- | --- | --- | --- | --- | --- | --- | --- | --- | --- | --- | --- | --- | --- | --- | --- | --- | --- | --- | --- | --- | --- | --- | --- | --- | --- | --- | --- |

XP\_007420267.1

back to list

  

| Protein Coverage
| Supporting Peptides
|

Protein Coverage:

Supporting Peptides:

| Peptide | Uniq | -10lgP | Mass | Length | ppm | m/z | z | RT | Fraction | Scan | Source File | Area F4 | Area F5 | Area F6 | Area F10 | Area F8 | Area F9 | Area F7 | Area F1 | Area F2 | Area F3 | #Feature | #Feature F4 | #Feature F5 | #Feature F6 | #Feature F10 | #Feature F8 | #Feature F9 | #Feature F7 | #Feature F1 | #Feature F2 | #Feature F3 | Start | End | PTM | AScore | Found By |
| --- | --- | --- | --- | --- | --- | --- | --- | --- | --- | --- | --- | --- | --- | --- | --- | --- | --- | --- | --- | --- | --- | --- | --- | --- | --- | --- | --- | --- | --- | --- | --- | --- | --- | --- | --- | --- | --- |
| R.LVQAFQFTDK.H | N | 58.02 | 1195.6237 | 10 | 0.9 | 598.8196 | 2 | 40.96 | 8 | F8:21197 | 31122021\_RID\_3262\_1a.raw |  |  |  |  |  |  |  | 2.1849E5 |  |  | 1 | 0 | 0 | 0 | 0 | 0 | 0 | 0 | 1 | 0 | 0 | 159 | 168 |  |  | PEAKS DB |
| K.ATAVMPDGQFK.D | N | 41.76 | 1163.5645 | 11 | 0.1 | 582.7896 | 2 | 33.47 | 6 | F6:19724 | 17012022\_RID\_3262\_9a.raw |  |  |  |  |  | 5.1945E5 |  |  |  |  | 1 | 0 | 0 | 0 | 0 | 0 | 1 | 0 | 0 | 0 | 0 | 17 | 27 |  |  | PEAKS DB |
| F.IGKPAPDFK.A | Y | 40.93 | 971.5439 | 9 | -0.9 | 486.7788 | 2 | 22.53 | 6 | F6:10011 | 17012022\_RID\_3262\_9a.raw |  |  |  |  |  | 5.7206E5 |  |  |  |  | 1 | 0 | 0 | 0 | 0 | 0 | 1 | 0 | 0 | 0 | 0 | 8 | 16 |  |  | PEAKS DB |
| total 3 peptides |  |  |  |  |  |  |  |  |  |  |  |  |  |  |  |  |  |  |  |  |  |  |  |  |  |  |  |  |  |  |  |  |  |  |  |  |  |
| --- | --- | --- | --- | --- | --- | --- | --- | --- | --- | --- | --- | --- | --- | --- | --- | --- | --- | --- | --- | --- | --- | --- | --- | --- | --- | --- | --- | --- | --- | --- | --- | --- | --- | --- | --- | --- | --- |

JAV49689.1

back to list

  

| Protein Coverage
| Supporting Peptides
|

Protein Coverage:

Supporting Peptides:

| Peptide | Uniq | -10lgP | Mass | Length | ppm | m/z | z | RT | Fraction | Scan | Source File | Area F4 | Area F5 | Area F6 | Area F10 | Area F8 | Area F9 | Area F7 | Area F1 | Area F2 | Area F3 | #Feature | #Feature F4 | #Feature F5 | #Feature F6 | #Feature F10 | #Feature F8 | #Feature F9 | #Feature F7 | #Feature F1 | #Feature F2 | #Feature F3 | Start | End | PTM | AScore | Found By |
| --- | --- | --- | --- | --- | --- | --- | --- | --- | --- | --- | --- | --- | --- | --- | --- | --- | --- | --- | --- | --- | --- | --- | --- | --- | --- | --- | --- | --- | --- | --- | --- | --- | --- | --- | --- | --- | --- |
| R.LVQAFQFTDK.H | N | 58.02 | 1195.6237 | 10 | 0.9 | 598.8196 | 2 | 40.96 | 8 | F8:21197 | 31122021\_RID\_3262\_1a.raw |  |  |  |  |  |  |  | 2.1849E5 |  |  | 1 | 0 | 0 | 0 | 0 | 0 | 0 | 0 | 1 | 0 | 0 | 159 | 168 |  |  | PEAKS DB |
| K.ATAVMPDGQFK.D | N | 41.76 | 1163.5645 | 11 | 0.1 | 582.7896 | 2 | 33.47 | 6 | F6:19724 | 17012022\_RID\_3262\_9a.raw |  |  |  |  |  | 5.1945E5 |  |  |  |  | 1 | 0 | 0 | 0 | 0 | 0 | 1 | 0 | 0 | 0 | 0 | 17 | 27 |  |  | PEAKS DB |
| F.IGKPAPDFK.A | Y | 40.93 | 971.5439 | 9 | -0.9 | 486.7788 | 2 | 22.53 | 6 | F6:10011 | 17012022\_RID\_3262\_9a.raw |  |  |  |  |  | 5.7206E5 |  |  |  |  | 1 | 0 | 0 | 0 | 0 | 0 | 1 | 0 | 0 | 0 | 0 | 8 | 16 |  |  | PEAKS DB |
| total 3 peptides |  |  |  |  |  |  |  |  |  |  |  |  |  |  |  |  |  |  |  |  |  |  |  |  |  |  |  |  |  |  |  |  |  |  |  |  |  |
| --- | --- | --- | --- | --- | --- | --- | --- | --- | --- | --- | --- | --- | --- | --- | --- | --- | --- | --- | --- | --- | --- | --- | --- | --- | --- | --- | --- | --- | --- | --- | --- | --- | --- | --- | --- | --- | --- |

XP\_015666821.1

back to list

  

| Protein Coverage
| Supporting Peptides
|

Protein Coverage:

Supporting Peptides:

| Peptide | Uniq | -10lgP | Mass | Length | ppm | m/z | z | RT | Fraction | Scan | Source File | Area F4 | Area F5 | Area F6 | Area F10 | Area F8 | Area F9 | Area F7 | Area F1 | Area F2 | Area F3 | #Feature | #Feature F4 | #Feature F5 | #Feature F6 | #Feature F10 | #Feature F8 | #Feature F9 | #Feature F7 | #Feature F1 | #Feature F2 | #Feature F3 | Start | End | PTM | AScore | Found By |
| --- | --- | --- | --- | --- | --- | --- | --- | --- | --- | --- | --- | --- | --- | --- | --- | --- | --- | --- | --- | --- | --- | --- | --- | --- | --- | --- | --- | --- | --- | --- | --- | --- | --- | --- | --- | --- | --- |
| R.LVQAFQFTDK.H | N | 58.02 | 1195.6237 | 10 | 0.9 | 598.8196 | 2 | 40.96 | 8 | F8:21197 | 31122021\_RID\_3262\_1a.raw |  |  |  |  |  |  |  | 2.1849E5 |  |  | 1 | 0 | 0 | 0 | 0 | 0 | 0 | 0 | 1 | 0 | 0 | 159 | 168 |  |  | PEAKS DB |
| K.ATAVMPDGQFK.D | N | 41.76 | 1163.5645 | 11 | 0.1 | 582.7896 | 2 | 33.47 | 6 | F6:19724 | 17012022\_RID\_3262\_9a.raw |  |  |  |  |  | 5.1945E5 |  |  |  |  | 1 | 0 | 0 | 0 | 0 | 0 | 1 | 0 | 0 | 0 | 0 | 17 | 27 |  |  | PEAKS DB |
| F.IGKPAPDFK.A | Y | 40.93 | 971.5439 | 9 | -0.9 | 486.7788 | 2 | 22.53 | 6 | F6:10011 | 17012022\_RID\_3262\_9a.raw |  |  |  |  |  | 5.7206E5 |  |  |  |  | 1 | 0 | 0 | 0 | 0 | 0 | 1 | 0 | 0 | 0 | 0 | 8 | 16 |  |  | PEAKS DB |
| total 3 peptides |  |  |  |  |  |  |  |  |  |  |  |  |  |  |  |  |  |  |  |  |  |  |  |  |  |  |  |  |  |  |  |  |  |  |  |  |  |
| --- | --- | --- | --- | --- | --- | --- | --- | --- | --- | --- | --- | --- | --- | --- | --- | --- | --- | --- | --- | --- | --- | --- | --- | --- | --- | --- | --- | --- | --- | --- | --- | --- | --- | --- | --- | --- | --- |

JAI12123.1

back to list

  

| Protein Coverage
| Supporting Peptides
|

Protein Coverage:

Supporting Peptides:

| Peptide | Uniq | -10lgP | Mass | Length | ppm | m/z | z | RT | Fraction | Scan | Source File | Area F4 | Area F5 | Area F6 | Area F10 | Area F8 | Area F9 | Area F7 | Area F1 | Area F2 | Area F3 | #Feature | #Feature F4 | #Feature F5 | #Feature F6 | #Feature F10 | #Feature F8 | #Feature F9 | #Feature F7 | #Feature F1 | #Feature F2 | #Feature F3 | Start | End | PTM | AScore | Found By |
| --- | --- | --- | --- | --- | --- | --- | --- | --- | --- | --- | --- | --- | --- | --- | --- | --- | --- | --- | --- | --- | --- | --- | --- | --- | --- | --- | --- | --- | --- | --- | --- | --- | --- | --- | --- | --- | --- |
| R.LVQAFQFTDK.H | N | 58.02 | 1195.6237 | 10 | 0.9 | 598.8196 | 2 | 40.96 | 8 | F8:21197 | 31122021\_RID\_3262\_1a.raw |  |  |  |  |  |  |  | 2.1849E5 |  |  | 1 | 0 | 0 | 0 | 0 | 0 | 0 | 0 | 1 | 0 | 0 | 159 | 168 |  |  | PEAKS DB |
| K.ATAVMPDGQFK.D | N | 41.76 | 1163.5645 | 11 | 0.1 | 582.7896 | 2 | 33.47 | 6 | F6:19724 | 17012022\_RID\_3262\_9a.raw |  |  |  |  |  | 5.1945E5 |  |  |  |  | 1 | 0 | 0 | 0 | 0 | 0 | 1 | 0 | 0 | 0 | 0 | 17 | 27 |  |  | PEAKS DB |
| F.IGKPAPDFK.A | Y | 40.93 | 971.5439 | 9 | -0.9 | 486.7788 | 2 | 22.53 | 6 | F6:10011 | 17012022\_RID\_3262\_9a.raw |  |  |  |  |  | 5.7206E5 |  |  |  |  | 1 | 0 | 0 | 0 | 0 | 0 | 1 | 0 | 0 | 0 | 0 | 8 | 16 |  |  | PEAKS DB |
| total 3 peptides |  |  |  |  |  |  |  |  |  |  |  |  |  |  |  |  |  |  |  |  |  |  |  |  |  |  |  |  |  |  |  |  |  |  |  |  |  |
| --- | --- | --- | --- | --- | --- | --- | --- | --- | --- | --- | --- | --- | --- | --- | --- | --- | --- | --- | --- | --- | --- | --- | --- | --- | --- | --- | --- | --- | --- | --- | --- | --- | --- | --- | --- | --- | --- |

AFJ50897.1

back to list

  

| Protein Coverage
| Supporting Peptides
|

Protein Coverage:

Supporting Peptides:

| Peptide | Uniq | -10lgP | Mass | Length | ppm | m/z | z | RT | Fraction | Scan | Source File | Area F4 | Area F5 | Area F6 | Area F10 | Area F8 | Area F9 | Area F7 | Area F1 | Area F2 | Area F3 | #Feature | #Feature F4 | #Feature F5 | #Feature F6 | #Feature F10 | #Feature F8 | #Feature F9 | #Feature F7 | #Feature F1 | #Feature F2 | #Feature F3 | Start | End | PTM | AScore | Found By |
| --- | --- | --- | --- | --- | --- | --- | --- | --- | --- | --- | --- | --- | --- | --- | --- | --- | --- | --- | --- | --- | --- | --- | --- | --- | --- | --- | --- | --- | --- | --- | --- | --- | --- | --- | --- | --- | --- |
| R.LVQAFQFTDK.H | N | 58.02 | 1195.6237 | 10 | 0.9 | 598.8196 | 2 | 40.96 | 8 | F8:21197 | 31122021\_RID\_3262\_1a.raw |  |  |  |  |  |  |  | 2.1849E5 |  |  | 1 | 0 | 0 | 0 | 0 | 0 | 0 | 0 | 1 | 0 | 0 | 159 | 168 |  |  | PEAKS DB |
| K.ATAVMPDGQFK.D | N | 41.76 | 1163.5645 | 11 | 0.1 | 582.7896 | 2 | 33.47 | 6 | F6:19724 | 17012022\_RID\_3262\_9a.raw |  |  |  |  |  | 5.1945E5 |  |  |  |  | 1 | 0 | 0 | 0 | 0 | 0 | 1 | 0 | 0 | 0 | 0 | 17 | 27 |  |  | PEAKS DB |
| F.IGKPAPDFK.A | Y | 40.93 | 971.5439 | 9 | -0.9 | 486.7788 | 2 | 22.53 | 6 | F6:10011 | 17012022\_RID\_3262\_9a.raw |  |  |  |  |  | 5.7206E5 |  |  |  |  | 1 | 0 | 0 | 0 | 0 | 0 | 1 | 0 | 0 | 0 | 0 | 8 | 16 |  |  | PEAKS DB |
| total 3 peptides |  |  |  |  |  |  |  |  |  |  |  |  |  |  |  |  |  |  |  |  |  |  |  |  |  |  |  |  |  |  |  |  |  |  |  |  |  |
| --- | --- | --- | --- | --- | --- | --- | --- | --- | --- | --- | --- | --- | --- | --- | --- | --- | --- | --- | --- | --- | --- | --- | --- | --- | --- | --- | --- | --- | --- | --- | --- | --- | --- | --- | --- | --- | --- |

JAA96190.1

back to list

  

| Protein Coverage
| Supporting Peptides
|

Protein Coverage:

Supporting Peptides:

| Peptide | Uniq | -10lgP | Mass | Length | ppm | m/z | z | RT | Fraction | Scan | Source File | Area F4 | Area F5 | Area F6 | Area F10 | Area F8 | Area F9 | Area F7 | Area F1 | Area F2 | Area F3 | #Feature | #Feature F4 | #Feature F5 | #Feature F6 | #Feature F10 | #Feature F8 | #Feature F9 | #Feature F7 | #Feature F1 | #Feature F2 | #Feature F3 | Start | End | PTM | AScore | Found By |
| --- | --- | --- | --- | --- | --- | --- | --- | --- | --- | --- | --- | --- | --- | --- | --- | --- | --- | --- | --- | --- | --- | --- | --- | --- | --- | --- | --- | --- | --- | --- | --- | --- | --- | --- | --- | --- | --- |
| R.LVQAFQFTDK.H | N | 58.02 | 1195.6237 | 10 | 0.9 | 598.8196 | 2 | 40.96 | 8 | F8:21197 | 31122021\_RID\_3262\_1a.raw |  |  |  |  |  |  |  | 2.1849E5 |  |  | 1 | 0 | 0 | 0 | 0 | 0 | 0 | 0 | 1 | 0 | 0 | 159 | 168 |  |  | PEAKS DB |
| K.ATAVMPDGQFK.D | N | 41.76 | 1163.5645 | 11 | 0.1 | 582.7896 | 2 | 33.47 | 6 | F6:19724 | 17012022\_RID\_3262\_9a.raw |  |  |  |  |  | 5.1945E5 |  |  |  |  | 1 | 0 | 0 | 0 | 0 | 0 | 1 | 0 | 0 | 0 | 0 | 17 | 27 |  |  | PEAKS DB |
| F.IGKPAPDFK.A | Y | 40.93 | 971.5439 | 9 | -0.9 | 486.7788 | 2 | 22.53 | 6 | F6:10011 | 17012022\_RID\_3262\_9a.raw |  |  |  |  |  | 5.7206E5 |  |  |  |  | 1 | 0 | 0 | 0 | 0 | 0 | 1 | 0 | 0 | 0 | 0 | 8 | 16 |  |  | PEAKS DB |
| total 3 peptides |  |  |  |  |  |  |  |  |  |  |  |  |  |  |  |  |  |  |  |  |  |  |  |  |  |  |  |  |  |  |  |  |  |  |  |  |  |
| --- | --- | --- | --- | --- | --- | --- | --- | --- | --- | --- | --- | --- | --- | --- | --- | --- | --- | --- | --- | --- | --- | --- | --- | --- | --- | --- | --- | --- | --- | --- | --- | --- | --- | --- | --- | --- | --- |

XP\_007420268.1

back to list

  

| Protein Coverage
| Supporting Peptides
|

Protein Coverage:

Supporting Peptides:

| Peptide | Uniq | -10lgP | Mass | Length | ppm | m/z | z | RT | Fraction | Scan | Source File | Area F4 | Area F5 | Area F6 | Area F10 | Area F8 | Area F9 | Area F7 | Area F1 | Area F2 | Area F3 | #Feature | #Feature F4 | #Feature F5 | #Feature F6 | #Feature F10 | #Feature F8 | #Feature F9 | #Feature F7 | #Feature F1 | #Feature F2 | #Feature F3 | Start | End | PTM | AScore | Found By |
| --- | --- | --- | --- | --- | --- | --- | --- | --- | --- | --- | --- | --- | --- | --- | --- | --- | --- | --- | --- | --- | --- | --- | --- | --- | --- | --- | --- | --- | --- | --- | --- | --- | --- | --- | --- | --- | --- |
| R.LVQAFQFTDK.H | N | 58.02 | 1195.6237 | 10 | 0.9 | 598.8196 | 2 | 40.96 | 8 | F8:21197 | 31122021\_RID\_3262\_1a.raw |  |  |  |  |  |  |  | 2.1849E5 |  |  | 1 | 0 | 0 | 0 | 0 | 0 | 0 | 0 | 1 | 0 | 0 | 159 | 168 |  |  | PEAKS DB |
| K.ATAVMPDGQFK.D | N | 41.76 | 1163.5645 | 11 | 0.1 | 582.7896 | 2 | 33.47 | 6 | F6:19724 | 17012022\_RID\_3262\_9a.raw |  |  |  |  |  | 5.1945E5 |  |  |  |  | 1 | 0 | 0 | 0 | 0 | 0 | 1 | 0 | 0 | 0 | 0 | 17 | 27 |  |  | PEAKS DB |
| F.IGKPAPDFK.A | Y | 40.93 | 971.5439 | 9 | -0.9 | 486.7788 | 2 | 22.53 | 6 | F6:10011 | 17012022\_RID\_3262\_9a.raw |  |  |  |  |  | 5.7206E5 |  |  |  |  | 1 | 0 | 0 | 0 | 0 | 0 | 1 | 0 | 0 | 0 | 0 | 8 | 16 |  |  | PEAKS DB |
| total 3 peptides |  |  |  |  |  |  |  |  |  |  |  |  |  |  |  |  |  |  |  |  |  |  |  |  |  |  |  |  |  |  |  |  |  |  |  |  |  |
| --- | --- | --- | --- | --- | --- | --- | --- | --- | --- | --- | --- | --- | --- | --- | --- | --- | --- | --- | --- | --- | --- | --- | --- | --- | --- | --- | --- | --- | --- | --- | --- | --- | --- | --- | --- | --- | --- |

XP\_015666820.1

back to list

  

| Protein Coverage
| Supporting Peptides
|

Protein Coverage:

Supporting Peptides:

| Peptide | Uniq | -10lgP | Mass | Length | ppm | m/z | z | RT | Fraction | Scan | Source File | Area F4 | Area F5 | Area F6 | Area F10 | Area F8 | Area F9 | Area F7 | Area F1 | Area F2 | Area F3 | #Feature | #Feature F4 | #Feature F5 | #Feature F6 | #Feature F10 | #Feature F8 | #Feature F9 | #Feature F7 | #Feature F1 | #Feature F2 | #Feature F3 | Start | End | PTM | AScore | Found By |
| --- | --- | --- | --- | --- | --- | --- | --- | --- | --- | --- | --- | --- | --- | --- | --- | --- | --- | --- | --- | --- | --- | --- | --- | --- | --- | --- | --- | --- | --- | --- | --- | --- | --- | --- | --- | --- | --- |
| R.LVQAFQFTDK.H | N | 58.02 | 1195.6237 | 10 | 0.9 | 598.8196 | 2 | 40.96 | 8 | F8:21197 | 31122021\_RID\_3262\_1a.raw |  |  |  |  |  |  |  | 2.1849E5 |  |  | 1 | 0 | 0 | 0 | 0 | 0 | 0 | 0 | 1 | 0 | 0 | 159 | 168 |  |  | PEAKS DB |
| K.ATAVMPDGQFK.D | N | 41.76 | 1163.5645 | 11 | 0.1 | 582.7896 | 2 | 33.47 | 6 | F6:19724 | 17012022\_RID\_3262\_9a.raw |  |  |  |  |  | 5.1945E5 |  |  |  |  | 1 | 0 | 0 | 0 | 0 | 0 | 1 | 0 | 0 | 0 | 0 | 17 | 27 |  |  | PEAKS DB |
| F.IGKPAPDFK.A | Y | 40.93 | 971.5439 | 9 | -0.9 | 486.7788 | 2 | 22.53 | 6 | F6:10011 | 17012022\_RID\_3262\_9a.raw |  |  |  |  |  | 5.7206E5 |  |  |  |  | 1 | 0 | 0 | 0 | 0 | 0 | 1 | 0 | 0 | 0 | 0 | 8 | 16 |  |  | PEAKS DB |
| total 3 peptides |  |  |  |  |  |  |  |  |  |  |  |  |  |  |  |  |  |  |  |  |  |  |  |  |  |  |  |  |  |  |  |  |  |  |  |  |  |
| --- | --- | --- | --- | --- | --- | --- | --- | --- | --- | --- | --- | --- | --- | --- | --- | --- | --- | --- | --- | --- | --- | --- | --- | --- | --- | --- | --- | --- | --- | --- | --- | --- | --- | --- | --- | --- | --- |

AAZ75600.1

back to list

  

| Protein Coverage
| Supporting Peptides
|

Protein Coverage:

Supporting Peptides:

| Peptide | Uniq | -10lgP | Mass | Length | ppm | m/z | z | RT | Fraction | Scan | Source File | Area F4 | Area F5 | Area F6 | Area F10 | Area F8 | Area F9 | Area F7 | Area F1 | Area F2 | Area F3 | #Feature | #Feature F4 | #Feature F5 | #Feature F6 | #Feature F10 | #Feature F8 | #Feature F9 | #Feature F7 | #Feature F1 | #Feature F2 | #Feature F3 | Start | End | PTM | AScore | Found By |
| --- | --- | --- | --- | --- | --- | --- | --- | --- | --- | --- | --- | --- | --- | --- | --- | --- | --- | --- | --- | --- | --- | --- | --- | --- | --- | --- | --- | --- | --- | --- | --- | --- | --- | --- | --- | --- | --- |
| T.VDFDSESPR.R | N | 54.45 | 1050.4618 | 9 | 0.7 | 526.2385 | 2 | 28.90 | 7 | F7:11521 | 21012022\_RID\_3262\_7a.raw |  |  | 3.3908E5 |  |  |  | 1.0581E7 | 6.71E5 | 5.9539E5 |  | 5 | 0 | 0 | 1 | 0 | 0 | 0 | 2 | 1 | 1 | 0 | 20 | 28 |  |  | PEAKS DB |
| G.TVDFDSESPR.R | Y | 46.23 | 1151.5094 | 10 | 0.6 | 576.7623 | 2 | 30.61 | 2 | F2:14842 | 13012022\_RID\_3262\_5a.raw |  | 2.0776E5 |  |  |  |  | 1.1494E6 |  |  |  | 2 | 0 | 1 | 0 | 0 | 0 | 0 | 1 | 0 | 0 | 0 | 19 | 28 |  |  | PEAKS DB |
| S.PTASNMLK.M | N | 44.10 | 860.4426 | 8 | -0.5 | 431.2283 | 2 | 23.21 | 7 | F7:7593 | 21012022\_RID\_3262\_7a.raw |  |  |  |  |  |  | 3.7964E5 |  |  |  | 1 | 0 | 0 | 0 | 0 | 0 | 0 | 1 | 0 | 0 | 0 | 49 | 56 |  |  | PEAKS DB |
| total 3 peptides |  |  |  |  |  |  |  |  |  |  |  |  |  |  |  |  |  |  |  |  |  |  |  |  |  |  |  |  |  |  |  |  |  |  |  |  |  |
| --- | --- | --- | --- | --- | --- | --- | --- | --- | --- | --- | --- | --- | --- | --- | --- | --- | --- | --- | --- | --- | --- | --- | --- | --- | --- | --- | --- | --- | --- | --- | --- | --- | --- | --- | --- | --- | --- |

Q2XXQ1.1

back to list

  

| Protein Coverage
| Supporting Peptides
|

Protein Coverage:

Supporting Peptides:

| Peptide | Uniq | -10lgP | Mass | Length | ppm | m/z | z | RT | Fraction | Scan | Source File | Area F4 | Area F5 | Area F6 | Area F10 | Area F8 | Area F9 | Area F7 | Area F1 | Area F2 | Area F3 | #Feature | #Feature F4 | #Feature F5 | #Feature F6 | #Feature F10 | #Feature F8 | #Feature F9 | #Feature F7 | #Feature F1 | #Feature F2 | #Feature F3 | Start | End | PTM | AScore | Found By |
| --- | --- | --- | --- | --- | --- | --- | --- | --- | --- | --- | --- | --- | --- | --- | --- | --- | --- | --- | --- | --- | --- | --- | --- | --- | --- | --- | --- | --- | --- | --- | --- | --- | --- | --- | --- | --- | --- |
| T.VDFDSESPR.R | N | 54.45 | 1050.4618 | 9 | 0.7 | 526.2385 | 2 | 28.90 | 7 | F7:11521 | 21012022\_RID\_3262\_7a.raw |  |  | 3.3908E5 |  |  |  | 1.0581E7 | 6.71E5 | 5.9539E5 |  | 5 | 0 | 0 | 1 | 0 | 0 | 0 | 2 | 1 | 1 | 0 | 20 | 28 |  |  | PEAKS DB |
| G.TVDFDSESPR.R | Y | 46.23 | 1151.5094 | 10 | 0.6 | 576.7623 | 2 | 30.61 | 2 | F2:14842 | 13012022\_RID\_3262\_5a.raw |  | 2.0776E5 |  |  |  |  | 1.1494E6 |  |  |  | 2 | 0 | 1 | 0 | 0 | 0 | 0 | 1 | 0 | 0 | 0 | 19 | 28 |  |  | PEAKS DB |
| S.PTASNMLK.M | N | 44.10 | 860.4426 | 8 | -0.5 | 431.2283 | 2 | 23.21 | 7 | F7:7593 | 21012022\_RID\_3262\_7a.raw |  |  |  |  |  |  | 3.7964E5 |  |  |  | 1 | 0 | 0 | 0 | 0 | 0 | 0 | 1 | 0 | 0 | 0 | 49 | 56 |  |  | PEAKS DB |
| total 3 peptides |  |  |  |  |  |  |  |  |  |  |  |  |  |  |  |  |  |  |  |  |  |  |  |  |  |  |  |  |  |  |  |  |  |  |  |  |  |
| --- | --- | --- | --- | --- | --- | --- | --- | --- | --- | --- | --- | --- | --- | --- | --- | --- | --- | --- | --- | --- | --- | --- | --- | --- | --- | --- | --- | --- | --- | --- | --- | --- | --- | --- | --- | --- | --- |

JAV01828.1

back to list

  

| Protein Coverage
| Supporting Peptides
|

Protein Coverage:

Supporting Peptides:

| Peptide | Uniq | -10lgP | Mass | Length | ppm | m/z | z | RT | Fraction | Scan | Source File | Area F4 | Area F5 | Area F6 | Area F10 | Area F8 | Area F9 | Area F7 | Area F1 | Area F2 | Area F3 | #Feature | #Feature F4 | #Feature F5 | #Feature F6 | #Feature F10 | #Feature F8 | #Feature F9 | #Feature F7 | #Feature F1 | #Feature F2 | #Feature F3 | Start | End | PTM | AScore | Found By |
| --- | --- | --- | --- | --- | --- | --- | --- | --- | --- | --- | --- | --- | --- | --- | --- | --- | --- | --- | --- | --- | --- | --- | --- | --- | --- | --- | --- | --- | --- | --- | --- | --- | --- | --- | --- | --- | --- |
| R.KPGFYTK.V | N | 48.32 | 839.4541 | 7 | -0.4 | 420.7342 | 2 | 17.97 | 7 | F7:4049 | 21012022\_RID\_3262\_7a.raw |  |  |  |  |  |  | 5.6489E5 |  |  |  | 1 | 0 | 0 | 0 | 0 | 0 | 0 | 1 | 0 | 0 | 0 | 229 | 235 |  |  | PEAKS DB |
| R.FLALVYSDR.F | Y | 45.68 | 1082.5760 | 9 | 1.0 | 542.2958 | 2 | 47.03 | 1 | F1:31692 | 13012022\_RID\_3262\_4a.raw | 1.7494E5 |  |  |  |  |  |  |  |  |  | 1 | 1 | 0 | 0 | 0 | 0 | 0 | 0 | 0 | 0 | 0 | 38 | 46 |  |  | PEAKS DB |
| L.DKDIMLIR.L | N | 41.99 | 1002.5532 | 8 | 0.7 | 502.2842 | 2 | 34.59 | 7 | F7:15514 | 21012022\_RID\_3262\_7a.raw |  |  |  |  |  |  | 2.6461E6 |  |  |  | 1 | 0 | 0 | 0 | 0 | 0 | 0 | 1 | 0 | 0 | 0 | 107 | 114 |  |  | PEAKS DB |
| total 3 peptides |  |  |  |  |  |  |  |  |  |  |  |  |  |  |  |  |  |  |  |  |  |  |  |  |  |  |  |  |  |  |  |  |  |  |  |  |  |
| --- | --- | --- | --- | --- | --- | --- | --- | --- | --- | --- | --- | --- | --- | --- | --- | --- | --- | --- | --- | --- | --- | --- | --- | --- | --- | --- | --- | --- | --- | --- | --- | --- | --- | --- | --- | --- | --- |

JAB53603.1

back to list

  

| Protein Coverage
| Supporting Peptides
|

Protein Coverage:

Supporting Peptides:

| Peptide | Uniq | -10lgP | Mass | Length | ppm | m/z | z | RT | Fraction | Scan | Source File | Area F4 | Area F5 | Area F6 | Area F10 | Area F8 | Area F9 | Area F7 | Area F1 | Area F2 | Area F3 | #Feature | #Feature F4 | #Feature F5 | #Feature F6 | #Feature F10 | #Feature F8 | #Feature F9 | #Feature F7 | #Feature F1 | #Feature F2 | #Feature F3 | Start | End | PTM | AScore | Found By |
| --- | --- | --- | --- | --- | --- | --- | --- | --- | --- | --- | --- | --- | --- | --- | --- | --- | --- | --- | --- | --- | --- | --- | --- | --- | --- | --- | --- | --- | --- | --- | --- | --- | --- | --- | --- | --- | --- |
| K.ALLEVVQSGGK.N | Y | 52.87 | 1099.6237 | 11 | 0.6 | 550.8195 | 2 | 37.23 | 6 | F6:23073 | 17012022\_RID\_3262\_9a.raw |  |  |  |  |  | 3.0807E5 |  | 1.5924E5 |  |  | 2 | 0 | 0 | 0 | 0 | 0 | 1 | 0 | 1 | 0 | 0 | 194 | 204 |  |  | PEAKS DB |
| R.LTVEDPVTVEYITR.Y | Y | 47.38 | 1633.8563 | 14 | 0.7 | 817.9360 | 2 | 49.47 | 8 | F8:27799 | 31122021\_RID\_3262\_1a.raw |  |  |  |  |  |  |  | 1.803E5 |  |  | 1 | 0 | 0 | 0 | 0 | 0 | 0 | 0 | 1 | 0 | 0 | 96 | 109 |  |  | PEAKS DB |
| total 2 peptides |  |  |  |  |  |  |  |  |  |  |  |  |  |  |  |  |  |  |  |  |  |  |  |  |  |  |  |  |  |  |  |  |  |  |  |  |  |
| --- | --- | --- | --- | --- | --- | --- | --- | --- | --- | --- | --- | --- | --- | --- | --- | --- | --- | --- | --- | --- | --- | --- | --- | --- | --- | --- | --- | --- | --- | --- | --- | --- | --- | --- | --- | --- | --- |

JAA96031.1

back to list

  

| Protein Coverage
| Supporting Peptides
|

Protein Coverage:

Supporting Peptides:

| Peptide | Uniq | -10lgP | Mass | Length | ppm | m/z | z | RT | Fraction | Scan | Source File | Area F4 | Area F5 | Area F6 | Area F10 | Area F8 | Area F9 | Area F7 | Area F1 | Area F2 | Area F3 | #Feature | #Feature F4 | #Feature F5 | #Feature F6 | #Feature F10 | #Feature F8 | #Feature F9 | #Feature F7 | #Feature F1 | #Feature F2 | #Feature F3 | Start | End | PTM | AScore | Found By |
| --- | --- | --- | --- | --- | --- | --- | --- | --- | --- | --- | --- | --- | --- | --- | --- | --- | --- | --- | --- | --- | --- | --- | --- | --- | --- | --- | --- | --- | --- | --- | --- | --- | --- | --- | --- | --- | --- |
| K.ALLEVVQSGGK.N | Y | 52.87 | 1099.6237 | 11 | 0.6 | 550.8195 | 2 | 37.23 | 6 | F6:23073 | 17012022\_RID\_3262\_9a.raw |  |  |  |  |  | 3.0807E5 |  | 1.5924E5 |  |  | 2 | 0 | 0 | 0 | 0 | 0 | 1 | 0 | 1 | 0 | 0 | 194 | 204 |  |  | PEAKS DB |
| R.LTVEDPVTVEYITR.Y | Y | 47.38 | 1633.8563 | 14 | 0.7 | 817.9360 | 2 | 49.47 | 8 | F8:27799 | 31122021\_RID\_3262\_1a.raw |  |  |  |  |  |  |  | 1.803E5 |  |  | 1 | 0 | 0 | 0 | 0 | 0 | 0 | 0 | 1 | 0 | 0 | 96 | 109 |  |  | PEAKS DB |
| total 2 peptides |  |  |  |  |  |  |  |  |  |  |  |  |  |  |  |  |  |  |  |  |  |  |  |  |  |  |  |  |  |  |  |  |  |  |  |  |  |
| --- | --- | --- | --- | --- | --- | --- | --- | --- | --- | --- | --- | --- | --- | --- | --- | --- | --- | --- | --- | --- | --- | --- | --- | --- | --- | --- | --- | --- | --- | --- | --- | --- | --- | --- | --- | --- | --- |

AFJ51040.1

back to list

  

| Protein Coverage
| Supporting Peptides
|

Protein Coverage:

Supporting Peptides:

| Peptide | Uniq | -10lgP | Mass | Length | ppm | m/z | z | RT | Fraction | Scan | Source File | Area F4 | Area F5 | Area F6 | Area F10 | Area F8 | Area F9 | Area F7 | Area F1 | Area F2 | Area F3 | #Feature | #Feature F4 | #Feature F5 | #Feature F6 | #Feature F10 | #Feature F8 | #Feature F9 | #Feature F7 | #Feature F1 | #Feature F2 | #Feature F3 | Start | End | PTM | AScore | Found By |
| --- | --- | --- | --- | --- | --- | --- | --- | --- | --- | --- | --- | --- | --- | --- | --- | --- | --- | --- | --- | --- | --- | --- | --- | --- | --- | --- | --- | --- | --- | --- | --- | --- | --- | --- | --- | --- | --- |
| K.ALLEVVQSGGK.N | Y | 52.87 | 1099.6237 | 11 | 0.6 | 550.8195 | 2 | 37.23 | 6 | F6:23073 | 17012022\_RID\_3262\_9a.raw |  |  |  |  |  | 3.0807E5 |  | 1.5924E5 |  |  | 2 | 0 | 0 | 0 | 0 | 0 | 1 | 0 | 1 | 0 | 0 | 194 | 204 |  |  | PEAKS DB |
| R.LTVEDPVTVEYITR.Y | Y | 47.38 | 1633.8563 | 14 | 0.7 | 817.9360 | 2 | 49.47 | 8 | F8:27799 | 31122021\_RID\_3262\_1a.raw |  |  |  |  |  |  |  | 1.803E5 |  |  | 1 | 0 | 0 | 0 | 0 | 0 | 0 | 0 | 1 | 0 | 0 | 96 | 109 |  |  | PEAKS DB |
| total 2 peptides |  |  |  |  |  |  |  |  |  |  |  |  |  |  |  |  |  |  |  |  |  |  |  |  |  |  |  |  |  |  |  |  |  |  |  |  |  |
| --- | --- | --- | --- | --- | --- | --- | --- | --- | --- | --- | --- | --- | --- | --- | --- | --- | --- | --- | --- | --- | --- | --- | --- | --- | --- | --- | --- | --- | --- | --- | --- | --- | --- | --- | --- | --- | --- |

ETE69560.1

back to list

  

| Protein Coverage
| Supporting Peptides
|

Protein Coverage:

Supporting Peptides:

| Peptide | Uniq | -10lgP | Mass | Length | ppm | m/z | z | RT | Fraction | Scan | Source File | Area F4 | Area F5 | Area F6 | Area F10 | Area F8 | Area F9 | Area F7 | Area F1 | Area F2 | Area F3 | #Feature | #Feature F4 | #Feature F5 | #Feature F6 | #Feature F10 | #Feature F8 | #Feature F9 | #Feature F7 | #Feature F1 | #Feature F2 | #Feature F3 | Start | End | PTM | AScore | Found By |
| --- | --- | --- | --- | --- | --- | --- | --- | --- | --- | --- | --- | --- | --- | --- | --- | --- | --- | --- | --- | --- | --- | --- | --- | --- | --- | --- | --- | --- | --- | --- | --- | --- | --- | --- | --- | --- | --- |
| K.ALLEVVQSGGK.N | Y | 52.87 | 1099.6237 | 11 | 0.6 | 550.8195 | 2 | 37.23 | 6 | F6:23073 | 17012022\_RID\_3262\_9a.raw |  |  |  |  |  | 3.0807E5 |  | 1.5924E5 |  |  | 2 | 0 | 0 | 0 | 0 | 0 | 1 | 0 | 1 | 0 | 0 | 194 | 204 |  |  | PEAKS DB |
| R.LTVEDPVTVEYITR.Y | Y | 47.38 | 1633.8563 | 14 | 0.7 | 817.9360 | 2 | 49.47 | 8 | F8:27799 | 31122021\_RID\_3262\_1a.raw |  |  |  |  |  |  |  | 1.803E5 |  |  | 1 | 0 | 0 | 0 | 0 | 0 | 0 | 0 | 1 | 0 | 0 | 96 | 109 |  |  | PEAKS DB |
| total 2 peptides |  |  |  |  |  |  |  |  |  |  |  |  |  |  |  |  |  |  |  |  |  |  |  |  |  |  |  |  |  |  |  |  |  |  |  |  |  |
| --- | --- | --- | --- | --- | --- | --- | --- | --- | --- | --- | --- | --- | --- | --- | --- | --- | --- | --- | --- | --- | --- | --- | --- | --- | --- | --- | --- | --- | --- | --- | --- | --- | --- | --- | --- | --- | --- |

XP\_015673624.1

back to list

  

| Protein Coverage
| Supporting Peptides
|

Protein Coverage:

Supporting Peptides:

| Peptide | Uniq | -10lgP | Mass | Length | ppm | m/z | z | RT | Fraction | Scan | Source File | Area F4 | Area F5 | Area F6 | Area F10 | Area F8 | Area F9 | Area F7 | Area F1 | Area F2 | Area F3 | #Feature | #Feature F4 | #Feature F5 | #Feature F6 | #Feature F10 | #Feature F8 | #Feature F9 | #Feature F7 | #Feature F1 | #Feature F2 | #Feature F3 | Start | End | PTM | AScore | Found By |
| --- | --- | --- | --- | --- | --- | --- | --- | --- | --- | --- | --- | --- | --- | --- | --- | --- | --- | --- | --- | --- | --- | --- | --- | --- | --- | --- | --- | --- | --- | --- | --- | --- | --- | --- | --- | --- | --- |
| K.ALLEVVQSGGK.N | Y | 52.87 | 1099.6237 | 11 | 0.6 | 550.8195 | 2 | 37.23 | 6 | F6:23073 | 17012022\_RID\_3262\_9a.raw |  |  |  |  |  | 3.0807E5 |  | 1.5924E5 |  |  | 2 | 0 | 0 | 0 | 0 | 0 | 1 | 0 | 1 | 0 | 0 | 194 | 204 |  |  | PEAKS DB |
| R.LTVEDPVTVEYITR.Y | Y | 47.38 | 1633.8563 | 14 | 0.7 | 817.9360 | 2 | 49.47 | 8 | F8:27799 | 31122021\_RID\_3262\_1a.raw |  |  |  |  |  |  |  | 1.803E5 |  |  | 1 | 0 | 0 | 0 | 0 | 0 | 0 | 0 | 1 | 0 | 0 | 96 | 109 |  |  | PEAKS DB |
| total 2 peptides |  |  |  |  |  |  |  |  |  |  |  |  |  |  |  |  |  |  |  |  |  |  |  |  |  |  |  |  |  |  |  |  |  |  |  |  |  |
| --- | --- | --- | --- | --- | --- | --- | --- | --- | --- | --- | --- | --- | --- | --- | --- | --- | --- | --- | --- | --- | --- | --- | --- | --- | --- | --- | --- | --- | --- | --- | --- | --- | --- | --- | --- | --- | --- |

JAI09615.1

back to list

  

| Protein Coverage
| Supporting Peptides
|

Protein Coverage:

Supporting Peptides:

| Peptide | Uniq | -10lgP | Mass | Length | ppm | m/z | z | RT | Fraction | Scan | Source File | Area F4 | Area F5 | Area F6 | Area F10 | Area F8 | Area F9 | Area F7 | Area F1 | Area F2 | Area F3 | #Feature | #Feature F4 | #Feature F5 | #Feature F6 | #Feature F10 | #Feature F8 | #Feature F9 | #Feature F7 | #Feature F1 | #Feature F2 | #Feature F3 | Start | End | PTM | AScore | Found By |
| --- | --- | --- | --- | --- | --- | --- | --- | --- | --- | --- | --- | --- | --- | --- | --- | --- | --- | --- | --- | --- | --- | --- | --- | --- | --- | --- | --- | --- | --- | --- | --- | --- | --- | --- | --- | --- | --- |
| K.ALLEVVQSGGK.N | Y | 52.87 | 1099.6237 | 11 | 0.6 | 550.8195 | 2 | 37.23 | 6 | F6:23073 | 17012022\_RID\_3262\_9a.raw |  |  |  |  |  | 3.0807E5 |  | 1.5924E5 |  |  | 2 | 0 | 0 | 0 | 0 | 0 | 1 | 0 | 1 | 0 | 0 | 194 | 204 |  |  | PEAKS DB |
| R.LTVEDPVTVEYITR.Y | Y | 47.38 | 1633.8563 | 14 | 0.7 | 817.9360 | 2 | 49.47 | 8 | F8:27799 | 31122021\_RID\_3262\_1a.raw |  |  |  |  |  |  |  | 1.803E5 |  |  | 1 | 0 | 0 | 0 | 0 | 0 | 0 | 0 | 1 | 0 | 0 | 96 | 109 |  |  | PEAKS DB |
| total 2 peptides |  |  |  |  |  |  |  |  |  |  |  |  |  |  |  |  |  |  |  |  |  |  |  |  |  |  |  |  |  |  |  |  |  |  |  |  |  |
| --- | --- | --- | --- | --- | --- | --- | --- | --- | --- | --- | --- | --- | --- | --- | --- | --- | --- | --- | --- | --- | --- | --- | --- | --- | --- | --- | --- | --- | --- | --- | --- | --- | --- | --- | --- | --- | --- |

JAI11926.1

back to list

  

| Protein Coverage
| Supporting Peptides
|

Protein Coverage:

Supporting Peptides:

| Peptide | Uniq | -10lgP | Mass | Length | ppm | m/z | z | RT | Fraction | Scan | Source File | Area F4 | Area F5 | Area F6 | Area F10 | Area F8 | Area F9 | Area F7 | Area F1 | Area F2 | Area F3 | #Feature | #Feature F4 | #Feature F5 | #Feature F6 | #Feature F10 | #Feature F8 | #Feature F9 | #Feature F7 | #Feature F1 | #Feature F2 | #Feature F3 | Start | End | PTM | AScore | Found By |
| --- | --- | --- | --- | --- | --- | --- | --- | --- | --- | --- | --- | --- | --- | --- | --- | --- | --- | --- | --- | --- | --- | --- | --- | --- | --- | --- | --- | --- | --- | --- | --- | --- | --- | --- | --- | --- | --- |
| K.ALLEVVQSGGK.N | Y | 52.87 | 1099.6237 | 11 | 0.6 | 550.8195 | 2 | 37.23 | 6 | F6:23073 | 17012022\_RID\_3262\_9a.raw |  |  |  |  |  | 3.0807E5 |  | 1.5924E5 |  |  | 2 | 0 | 0 | 0 | 0 | 0 | 1 | 0 | 1 | 0 | 0 | 194 | 204 |  |  | PEAKS DB |
| R.LTVEDPVTVEYITR.Y | Y | 47.38 | 1633.8563 | 14 | 0.7 | 817.9360 | 2 | 49.47 | 8 | F8:27799 | 31122021\_RID\_3262\_1a.raw |  |  |  |  |  |  |  | 1.803E5 |  |  | 1 | 0 | 0 | 0 | 0 | 0 | 0 | 0 | 1 | 0 | 0 | 96 | 109 |  |  | PEAKS DB |
| total 2 peptides |  |  |  |  |  |  |  |  |  |  |  |  |  |  |  |  |  |  |  |  |  |  |  |  |  |  |  |  |  |  |  |  |  |  |  |  |  |
| --- | --- | --- | --- | --- | --- | --- | --- | --- | --- | --- | --- | --- | --- | --- | --- | --- | --- | --- | --- | --- | --- | --- | --- | --- | --- | --- | --- | --- | --- | --- | --- | --- | --- | --- | --- | --- | --- |

JAG66915.1

back to list

  

| Protein Coverage
| Supporting Peptides
|

Protein Coverage:

Supporting Peptides:

| Peptide | Uniq | -10lgP | Mass | Length | ppm | m/z | z | RT | Fraction | Scan | Source File | Area F4 | Area F5 | Area F6 | Area F10 | Area F8 | Area F9 | Area F7 | Area F1 | Area F2 | Area F3 | #Feature | #Feature F4 | #Feature F5 | #Feature F6 | #Feature F10 | #Feature F8 | #Feature F9 | #Feature F7 | #Feature F1 | #Feature F2 | #Feature F3 | Start | End | PTM | AScore | Found By |
| --- | --- | --- | --- | --- | --- | --- | --- | --- | --- | --- | --- | --- | --- | --- | --- | --- | --- | --- | --- | --- | --- | --- | --- | --- | --- | --- | --- | --- | --- | --- | --- | --- | --- | --- | --- | --- | --- |
| K.ALLEVVQSGGK.N | Y | 52.87 | 1099.6237 | 11 | 0.6 | 550.8195 | 2 | 37.23 | 6 | F6:23073 | 17012022\_RID\_3262\_9a.raw |  |  |  |  |  | 3.0807E5 |  | 1.5924E5 |  |  | 2 | 0 | 0 | 0 | 0 | 0 | 1 | 0 | 1 | 0 | 0 | 194 | 204 |  |  | PEAKS DB |
| R.LTVEDPVTVEYITR.Y | Y | 47.38 | 1633.8563 | 14 | 0.7 | 817.9360 | 2 | 49.47 | 8 | F8:27799 | 31122021\_RID\_3262\_1a.raw |  |  |  |  |  |  |  | 1.803E5 |  |  | 1 | 0 | 0 | 0 | 0 | 0 | 0 | 0 | 1 | 0 | 0 | 96 | 109 |  |  | PEAKS DB |
| total 2 peptides |  |  |  |  |  |  |  |  |  |  |  |  |  |  |  |  |  |  |  |  |  |  |  |  |  |  |  |  |  |  |  |  |  |  |  |  |  |
| --- | --- | --- | --- | --- | --- | --- | --- | --- | --- | --- | --- | --- | --- | --- | --- | --- | --- | --- | --- | --- | --- | --- | --- | --- | --- | --- | --- | --- | --- | --- | --- | --- | --- | --- | --- | --- | --- |

JAV49551.1

back to list

  

| Protein Coverage
| Supporting Peptides
|

Protein Coverage:

Supporting Peptides:

| Peptide | Uniq | -10lgP | Mass | Length | ppm | m/z | z | RT | Fraction | Scan | Source File | Area F4 | Area F5 | Area F6 | Area F10 | Area F8 | Area F9 | Area F7 | Area F1 | Area F2 | Area F3 | #Feature | #Feature F4 | #Feature F5 | #Feature F6 | #Feature F10 | #Feature F8 | #Feature F9 | #Feature F7 | #Feature F1 | #Feature F2 | #Feature F3 | Start | End | PTM | AScore | Found By |
| --- | --- | --- | --- | --- | --- | --- | --- | --- | --- | --- | --- | --- | --- | --- | --- | --- | --- | --- | --- | --- | --- | --- | --- | --- | --- | --- | --- | --- | --- | --- | --- | --- | --- | --- | --- | --- | --- |
| K.ALLEVVQSGGK.N | Y | 52.87 | 1099.6237 | 11 | 0.6 | 550.8195 | 2 | 37.23 | 6 | F6:23073 | 17012022\_RID\_3262\_9a.raw |  |  |  |  |  | 3.0807E5 |  | 1.5924E5 |  |  | 2 | 0 | 0 | 0 | 0 | 0 | 1 | 0 | 1 | 0 | 0 | 194 | 204 |  |  | PEAKS DB |
| R.LTVEDPVTVEYITR.Y | Y | 47.38 | 1633.8563 | 14 | 0.7 | 817.9360 | 2 | 49.47 | 8 | F8:27799 | 31122021\_RID\_3262\_1a.raw |  |  |  |  |  |  |  | 1.803E5 |  |  | 1 | 0 | 0 | 0 | 0 | 0 | 0 | 0 | 1 | 0 | 0 | 96 | 109 |  |  | PEAKS DB |
| total 2 peptides |  |  |  |  |  |  |  |  |  |  |  |  |  |  |  |  |  |  |  |  |  |  |  |  |  |  |  |  |  |  |  |  |  |  |  |  |  |
| --- | --- | --- | --- | --- | --- | --- | --- | --- | --- | --- | --- | --- | --- | --- | --- | --- | --- | --- | --- | --- | --- | --- | --- | --- | --- | --- | --- | --- | --- | --- | --- | --- | --- | --- | --- | --- | --- |

XP\_026553199.1

back to list

  

| Protein Coverage
| Supporting Peptides
|

Protein Coverage:

Supporting Peptides:

| Peptide | Uniq | -10lgP | Mass | Length | ppm | m/z | z | RT | Fraction | Scan | Source File | Area F4 | Area F5 | Area F6 | Area F10 | Area F8 | Area F9 | Area F7 | Area F1 | Area F2 | Area F3 | #Feature | #Feature F4 | #Feature F5 | #Feature F6 | #Feature F10 | #Feature F8 | #Feature F9 | #Feature F7 | #Feature F1 | #Feature F2 | #Feature F3 | Start | End | PTM | AScore | Found By |
| --- | --- | --- | --- | --- | --- | --- | --- | --- | --- | --- | --- | --- | --- | --- | --- | --- | --- | --- | --- | --- | --- | --- | --- | --- | --- | --- | --- | --- | --- | --- | --- | --- | --- | --- | --- | --- | --- |
| K.ALLEVVQSGGK.N | Y | 52.87 | 1099.6237 | 11 | 0.6 | 550.8195 | 2 | 37.23 | 6 | F6:23073 | 17012022\_RID\_3262\_9a.raw |  |  |  |  |  | 3.0807E5 |  | 1.5924E5 |  |  | 2 | 0 | 0 | 0 | 0 | 0 | 1 | 0 | 1 | 0 | 0 | 194 | 204 |  |  | PEAKS DB |
| R.LTVEDPVTVEYITR.Y | Y | 47.38 | 1633.8563 | 14 | 0.7 | 817.9360 | 2 | 49.47 | 8 | F8:27799 | 31122021\_RID\_3262\_1a.raw |  |  |  |  |  |  |  | 1.803E5 |  |  | 1 | 0 | 0 | 0 | 0 | 0 | 0 | 0 | 1 | 0 | 0 | 96 | 109 |  |  | PEAKS DB |
| total 2 peptides |  |  |  |  |  |  |  |  |  |  |  |  |  |  |  |  |  |  |  |  |  |  |  |  |  |  |  |  |  |  |  |  |  |  |  |  |  |
| --- | --- | --- | --- | --- | --- | --- | --- | --- | --- | --- | --- | --- | --- | --- | --- | --- | --- | --- | --- | --- | --- | --- | --- | --- | --- | --- | --- | --- | --- | --- | --- | --- | --- | --- | --- | --- | --- |

XP\_034289033.1

back to list

  

| Protein Coverage
| Supporting Peptides
|

Protein Coverage:

Supporting Peptides:

| Peptide | Uniq | -10lgP | Mass | Length | ppm | m/z | z | RT | Fraction | Scan | Source File | Area F4 | Area F5 | Area F6 | Area F10 | Area F8 | Area F9 | Area F7 | Area F1 | Area F2 | Area F3 | #Feature | #Feature F4 | #Feature F5 | #Feature F6 | #Feature F10 | #Feature F8 | #Feature F9 | #Feature F7 | #Feature F1 | #Feature F2 | #Feature F3 | Start | End | PTM | AScore | Found By |
| --- | --- | --- | --- | --- | --- | --- | --- | --- | --- | --- | --- | --- | --- | --- | --- | --- | --- | --- | --- | --- | --- | --- | --- | --- | --- | --- | --- | --- | --- | --- | --- | --- | --- | --- | --- | --- | --- |
| K.ALLEVVQSGGK.N | Y | 52.87 | 1099.6237 | 11 | 0.6 | 550.8195 | 2 | 37.23 | 6 | F6:23073 | 17012022\_RID\_3262\_9a.raw |  |  |  |  |  | 3.0807E5 |  | 1.5924E5 |  |  | 2 | 0 | 0 | 0 | 0 | 0 | 1 | 0 | 1 | 0 | 0 | 194 | 204 |  |  | PEAKS DB |
| R.LTVEDPVTVEYITR.Y | Y | 47.38 | 1633.8563 | 14 | 0.7 | 817.9360 | 2 | 49.47 | 8 | F8:27799 | 31122021\_RID\_3262\_1a.raw |  |  |  |  |  |  |  | 1.803E5 |  |  | 1 | 0 | 0 | 0 | 0 | 0 | 0 | 0 | 1 | 0 | 0 | 96 | 109 |  |  | PEAKS DB |
| total 2 peptides |  |  |  |  |  |  |  |  |  |  |  |  |  |  |  |  |  |  |  |  |  |  |  |  |  |  |  |  |  |  |  |  |  |  |  |  |  |
| --- | --- | --- | --- | --- | --- | --- | --- | --- | --- | --- | --- | --- | --- | --- | --- | --- | --- | --- | --- | --- | --- | --- | --- | --- | --- | --- | --- | --- | --- | --- | --- | --- | --- | --- | --- | --- | --- |

XP\_032074995.1

back to list

  

| Protein Coverage
| Supporting Peptides
|

Protein Coverage:

Supporting Peptides:

| Peptide | Uniq | -10lgP | Mass | Length | ppm | m/z | z | RT | Fraction | Scan | Source File | Area F4 | Area F5 | Area F6 | Area F10 | Area F8 | Area F9 | Area F7 | Area F1 | Area F2 | Area F3 | #Feature | #Feature F4 | #Feature F5 | #Feature F6 | #Feature F10 | #Feature F8 | #Feature F9 | #Feature F7 | #Feature F1 | #Feature F2 | #Feature F3 | Start | End | PTM | AScore | Found By |
| --- | --- | --- | --- | --- | --- | --- | --- | --- | --- | --- | --- | --- | --- | --- | --- | --- | --- | --- | --- | --- | --- | --- | --- | --- | --- | --- | --- | --- | --- | --- | --- | --- | --- | --- | --- | --- | --- |
| K.ALLEVVQSGGK.N | Y | 52.87 | 1099.6237 | 11 | 0.6 | 550.8195 | 2 | 37.23 | 6 | F6:23073 | 17012022\_RID\_3262\_9a.raw |  |  |  |  |  | 3.0807E5 |  | 1.5924E5 |  |  | 2 | 0 | 0 | 0 | 0 | 0 | 1 | 0 | 1 | 0 | 0 | 194 | 204 |  |  | PEAKS DB |
| R.LTVEDPVTVEYITR.Y | Y | 47.38 | 1633.8563 | 14 | 0.7 | 817.9360 | 2 | 49.47 | 8 | F8:27799 | 31122021\_RID\_3262\_1a.raw |  |  |  |  |  |  |  | 1.803E5 |  |  | 1 | 0 | 0 | 0 | 0 | 0 | 0 | 0 | 1 | 0 | 0 | 96 | 109 |  |  | PEAKS DB |
| total 2 peptides |  |  |  |  |  |  |  |  |  |  |  |  |  |  |  |  |  |  |  |  |  |  |  |  |  |  |  |  |  |  |  |  |  |  |  |  |  |
| --- | --- | --- | --- | --- | --- | --- | --- | --- | --- | --- | --- | --- | --- | --- | --- | --- | --- | --- | --- | --- | --- | --- | --- | --- | --- | --- | --- | --- | --- | --- | --- | --- | --- | --- | --- | --- | --- |

BAN82080.1

back to list

  

| Protein Coverage
| Supporting Peptides
|

Protein Coverage:

Supporting Peptides:

| Peptide | Uniq | -10lgP | Mass | Length | ppm | m/z | z | RT | Fraction | Scan | Source File | Area F4 | Area F5 | Area F6 | Area F10 | Area F8 | Area F9 | Area F7 | Area F1 | Area F2 | Area F3 | #Feature | #Feature F4 | #Feature F5 | #Feature F6 | #Feature F10 | #Feature F8 | #Feature F9 | #Feature F7 | #Feature F1 | #Feature F2 | #Feature F3 | Start | End | PTM | AScore | Found By |
| --- | --- | --- | --- | --- | --- | --- | --- | --- | --- | --- | --- | --- | --- | --- | --- | --- | --- | --- | --- | --- | --- | --- | --- | --- | --- | --- | --- | --- | --- | --- | --- | --- | --- | --- | --- | --- | --- |
| K.ALLEVVQSGGK.N | Y | 52.87 | 1099.6237 | 11 | 0.6 | 550.8195 | 2 | 37.23 | 6 | F6:23073 | 17012022\_RID\_3262\_9a.raw |  |  |  |  |  | 3.0807E5 |  | 1.5924E5 |  |  | 2 | 0 | 0 | 0 | 0 | 0 | 1 | 0 | 1 | 0 | 0 | 194 | 204 |  |  | PEAKS DB |
| R.LTVEDPVTVEYITR.Y | Y | 47.38 | 1633.8563 | 14 | 0.7 | 817.9360 | 2 | 49.47 | 8 | F8:27799 | 31122021\_RID\_3262\_1a.raw |  |  |  |  |  |  |  | 1.803E5 |  |  | 1 | 0 | 0 | 0 | 0 | 0 | 0 | 0 | 1 | 0 | 0 | 96 | 109 |  |  | PEAKS DB |
| total 2 peptides |  |  |  |  |  |  |  |  |  |  |  |  |  |  |  |  |  |  |  |  |  |  |  |  |  |  |  |  |  |  |  |  |  |  |  |  |  |
| --- | --- | --- | --- | --- | --- | --- | --- | --- | --- | --- | --- | --- | --- | --- | --- | --- | --- | --- | --- | --- | --- | --- | --- | --- | --- | --- | --- | --- | --- | --- | --- | --- | --- | --- | --- | --- | --- |

XP\_013917261.1

back to list

  

| Protein Coverage
| Supporting Peptides
|

Protein Coverage:

Supporting Peptides:

| Peptide | Uniq | -10lgP | Mass | Length | ppm | m/z | z | RT | Fraction | Scan | Source File | Area F4 | Area F5 | Area F6 | Area F10 | Area F8 | Area F9 | Area F7 | Area F1 | Area F2 | Area F3 | #Feature | #Feature F4 | #Feature F5 | #Feature F6 | #Feature F10 | #Feature F8 | #Feature F9 | #Feature F7 | #Feature F1 | #Feature F2 | #Feature F3 | Start | End | PTM | AScore | Found By |
| --- | --- | --- | --- | --- | --- | --- | --- | --- | --- | --- | --- | --- | --- | --- | --- | --- | --- | --- | --- | --- | --- | --- | --- | --- | --- | --- | --- | --- | --- | --- | --- | --- | --- | --- | --- | --- | --- |
| K.ALLEVVQSGGK.N | Y | 52.87 | 1099.6237 | 11 | 0.6 | 550.8195 | 2 | 37.23 | 6 | F6:23073 | 17012022\_RID\_3262\_9a.raw |  |  |  |  |  | 3.0807E5 |  | 1.5924E5 |  |  | 2 | 0 | 0 | 0 | 0 | 0 | 1 | 0 | 1 | 0 | 0 | 194 | 204 |  |  | PEAKS DB |
| R.LTVEDPVTVEYITR.Y | Y | 47.38 | 1633.8563 | 14 | 0.7 | 817.9360 | 2 | 49.47 | 8 | F8:27799 | 31122021\_RID\_3262\_1a.raw |  |  |  |  |  |  |  | 1.803E5 |  |  | 1 | 0 | 0 | 0 | 0 | 0 | 0 | 0 | 1 | 0 | 0 | 96 | 109 |  |  | PEAKS DB |
| total 2 peptides |  |  |  |  |  |  |  |  |  |  |  |  |  |  |  |  |  |  |  |  |  |  |  |  |  |  |  |  |  |  |  |  |  |  |  |  |  |
| --- | --- | --- | --- | --- | --- | --- | --- | --- | --- | --- | --- | --- | --- | --- | --- | --- | --- | --- | --- | --- | --- | --- | --- | --- | --- | --- | --- | --- | --- | --- | --- | --- | --- | --- | --- | --- | --- |

Q6H3C5.2

back to list

  

| Protein Coverage
| Supporting Peptides
|

Protein Coverage:

Supporting Peptides:

| Peptide | Uniq | -10lgP | Mass | Length | ppm | m/z | z | RT | Fraction | Scan | Source File | Area F4 | Area F5 | Area F6 | Area F10 | Area F8 | Area F9 | Area F7 | Area F1 | Area F2 | Area F3 | #Feature | #Feature F4 | #Feature F5 | #Feature F6 | #Feature F10 | #Feature F8 | #Feature F9 | #Feature F7 | #Feature F1 | #Feature F2 | #Feature F3 | Start | End | PTM | AScore | Found By |
| --- | --- | --- | --- | --- | --- | --- | --- | --- | --- | --- | --- | --- | --- | --- | --- | --- | --- | --- | --- | --- | --- | --- | --- | --- | --- | --- | --- | --- | --- | --- | --- | --- | --- | --- | --- | --- | --- |
| K.EEYHSYGK.S | Y | 48.08 | 1011.4297 | 8 | 0.4 | 506.7224 | 2 | 20.15 | 4 | F4:7715 | 17012022\_RID\_3262\_10a .raw |  |  |  | 8.5341E5 |  |  |  |  |  |  | 1 | 0 | 0 | 0 | 1 | 0 | 0 | 0 | 0 | 0 | 0 | 105 | 112 |  |  | PEAKS DB |
| SLLEFGR.M | N | 44.54 | 820.4443 | 7 | 1.1 | 411.2299 | 2 | 41.75 | 4 | F4:26641 | 17012022\_RID\_3262\_10a .raw |  |  |  | 1.1809E10 |  | 6.3702E8 |  |  | 1.5471E7 |  | 4 | 0 | 0 | 0 | 1 | 0 | 2 | 0 | 0 | 1 | 0 | 1 | 7 |  |  | PEAKS DB |
| total 2 peptides |  |  |  |  |  |  |  |  |  |  |  |  |  |  |  |  |  |  |  |  |  |  |  |  |  |  |  |  |  |  |  |  |  |  |  |  |  |
| --- | --- | --- | --- | --- | --- | --- | --- | --- | --- | --- | --- | --- | --- | --- | --- | --- | --- | --- | --- | --- | --- | --- | --- | --- | --- | --- | --- | --- | --- | --- | --- | --- | --- | --- | --- | --- | --- |

AFJ51714.1

back to list

  

| Protein Coverage
| Supporting Peptides
|

Protein Coverage:

Supporting Peptides:

| Peptide | Uniq | -10lgP | Mass | Length | ppm | m/z | z | RT | Fraction | Scan | Source File | Area F4 | Area F5 | Area F6 | Area F10 | Area F8 | Area F9 | Area F7 | Area F1 | Area F2 | Area F3 | #Feature | #Feature F4 | #Feature F5 | #Feature F6 | #Feature F10 | #Feature F8 | #Feature F9 | #Feature F7 | #Feature F1 | #Feature F2 | #Feature F3 | Start | End | PTM | AScore | Found By |
| --- | --- | --- | --- | --- | --- | --- | --- | --- | --- | --- | --- | --- | --- | --- | --- | --- | --- | --- | --- | --- | --- | --- | --- | --- | --- | --- | --- | --- | --- | --- | --- | --- | --- | --- | --- | --- | --- |
| R.HGLLVPNNTTDQELQHIR.N | Y | 73.44 | 2084.0762 | 18 | 0.0 | 695.6993 | 3 | 33.67 | 8 | F8:15319 | 31122021\_RID\_3262\_1a.raw |  |  |  |  |  |  |  | 1.1516E5 |  |  | 1 | 0 | 0 | 0 | 0 | 0 | 0 | 0 | 1 | 0 | 0 | 68 | 85 |  |  | PEAKS DB |
| total 1 peptides |  |  |  |  |  |  |  |  |  |  |  |  |  |  |  |  |  |  |  |  |  |  |  |  |  |  |  |  |  |  |  |  |  |  |  |  |  |
| --- | --- | --- | --- | --- | --- | --- | --- | --- | --- | --- | --- | --- | --- | --- | --- | --- | --- | --- | --- | --- | --- | --- | --- | --- | --- | --- | --- | --- | --- | --- | --- | --- | --- | --- | --- | --- | --- |

JAI11019.1

back to list

  

| Protein Coverage
| Supporting Peptides
|

Protein Coverage:

Supporting Peptides:

| Peptide | Uniq | -10lgP | Mass | Length | ppm | m/z | z | RT | Fraction | Scan | Source File | Area F4 | Area F5 | Area F6 | Area F10 | Area F8 | Area F9 | Area F7 | Area F1 | Area F2 | Area F3 | #Feature | #Feature F4 | #Feature F5 | #Feature F6 | #Feature F10 | #Feature F8 | #Feature F9 | #Feature F7 | #Feature F1 | #Feature F2 | #Feature F3 | Start | End | PTM | AScore | Found By |
| --- | --- | --- | --- | --- | --- | --- | --- | --- | --- | --- | --- | --- | --- | --- | --- | --- | --- | --- | --- | --- | --- | --- | --- | --- | --- | --- | --- | --- | --- | --- | --- | --- | --- | --- | --- | --- | --- |
| R.HGLLVPNNTTDQELQHIR.N | Y | 73.44 | 2084.0762 | 18 | 0.0 | 695.6993 | 3 | 33.67 | 8 | F8:15319 | 31122021\_RID\_3262\_1a.raw |  |  |  |  |  |  |  | 1.1516E5 |  |  | 1 | 0 | 0 | 0 | 0 | 0 | 0 | 0 | 1 | 0 | 0 | 68 | 85 |  |  | PEAKS DB |
| total 1 peptides |  |  |  |  |  |  |  |  |  |  |  |  |  |  |  |  |  |  |  |  |  |  |  |  |  |  |  |  |  |  |  |  |  |  |  |  |  |
| --- | --- | --- | --- | --- | --- | --- | --- | --- | --- | --- | --- | --- | --- | --- | --- | --- | --- | --- | --- | --- | --- | --- | --- | --- | --- | --- | --- | --- | --- | --- | --- | --- | --- | --- | --- | --- | --- |

JAA95343.1

back to list

  

| Protein Coverage
| Supporting Peptides
|

Protein Coverage:

Supporting Peptides:

| Peptide | Uniq | -10lgP | Mass | Length | ppm | m/z | z | RT | Fraction | Scan | Source File | Area F4 | Area F5 | Area F6 | Area F10 | Area F8 | Area F9 | Area F7 | Area F1 | Area F2 | Area F3 | #Feature | #Feature F4 | #Feature F5 | #Feature F6 | #Feature F10 | #Feature F8 | #Feature F9 | #Feature F7 | #Feature F1 | #Feature F2 | #Feature F3 | Start | End | PTM | AScore | Found By |
| --- | --- | --- | --- | --- | --- | --- | --- | --- | --- | --- | --- | --- | --- | --- | --- | --- | --- | --- | --- | --- | --- | --- | --- | --- | --- | --- | --- | --- | --- | --- | --- | --- | --- | --- | --- | --- | --- |
| R.HGLLVPNNTTDQELQHIR.N | Y | 73.44 | 2084.0762 | 18 | 0.0 | 695.6993 | 3 | 33.67 | 8 | F8:15319 | 31122021\_RID\_3262\_1a.raw |  |  |  |  |  |  |  | 1.1516E5 |  |  | 1 | 0 | 0 | 0 | 0 | 0 | 0 | 0 | 1 | 0 | 0 | 68 | 85 |  |  | PEAKS DB |
| total 1 peptides |  |  |  |  |  |  |  |  |  |  |  |  |  |  |  |  |  |  |  |  |  |  |  |  |  |  |  |  |  |  |  |  |  |  |  |  |  |
| --- | --- | --- | --- | --- | --- | --- | --- | --- | --- | --- | --- | --- | --- | --- | --- | --- | --- | --- | --- | --- | --- | --- | --- | --- | --- | --- | --- | --- | --- | --- | --- | --- | --- | --- | --- | --- | --- |

LAB21681.1

back to list

  

| Protein Coverage
| Supporting Peptides
|

Protein Coverage:

Supporting Peptides:

| Peptide | Uniq | -10lgP | Mass | Length | ppm | m/z | z | RT | Fraction | Scan | Source File | Area F4 | Area F5 | Area F6 | Area F10 | Area F8 | Area F9 | Area F7 | Area F1 | Area F2 | Area F3 | #Feature | #Feature F4 | #Feature F5 | #Feature F6 | #Feature F10 | #Feature F8 | #Feature F9 | #Feature F7 | #Feature F1 | #Feature F2 | #Feature F3 | Start | End | PTM | AScore | Found By |
| --- | --- | --- | --- | --- | --- | --- | --- | --- | --- | --- | --- | --- | --- | --- | --- | --- | --- | --- | --- | --- | --- | --- | --- | --- | --- | --- | --- | --- | --- | --- | --- | --- | --- | --- | --- | --- | --- |
| R.HGLLVPNNTTDQELQHIR.N | Y | 73.44 | 2084.0762 | 18 | 0.0 | 695.6993 | 3 | 33.67 | 8 | F8:15319 | 31122021\_RID\_3262\_1a.raw |  |  |  |  |  |  |  | 1.1516E5 |  |  | 1 | 0 | 0 | 0 | 0 | 0 | 0 | 0 | 1 | 0 | 0 | 53 | 70 |  |  | PEAKS DB |
| total 1 peptides |  |  |  |  |  |  |  |  |  |  |  |  |  |  |  |  |  |  |  |  |  |  |  |  |  |  |  |  |  |  |  |  |  |  |  |  |  |
| --- | --- | --- | --- | --- | --- | --- | --- | --- | --- | --- | --- | --- | --- | --- | --- | --- | --- | --- | --- | --- | --- | --- | --- | --- | --- | --- | --- | --- | --- | --- | --- | --- | --- | --- | --- | --- | --- |

JAV48857.1

back to list

  

| Protein Coverage
| Supporting Peptides
|

Protein Coverage:

Supporting Peptides:

| Peptide | Uniq | -10lgP | Mass | Length | ppm | m/z | z | RT | Fraction | Scan | Source File | Area F4 | Area F5 | Area F6 | Area F10 | Area F8 | Area F9 | Area F7 | Area F1 | Area F2 | Area F3 | #Feature | #Feature F4 | #Feature F5 | #Feature F6 | #Feature F10 | #Feature F8 | #Feature F9 | #Feature F7 | #Feature F1 | #Feature F2 | #Feature F3 | Start | End | PTM | AScore | Found By |
| --- | --- | --- | --- | --- | --- | --- | --- | --- | --- | --- | --- | --- | --- | --- | --- | --- | --- | --- | --- | --- | --- | --- | --- | --- | --- | --- | --- | --- | --- | --- | --- | --- | --- | --- | --- | --- | --- |
| R.HGLLVPNNTTDQELQHIR.N | Y | 73.44 | 2084.0762 | 18 | 0.0 | 695.6993 | 3 | 33.67 | 8 | F8:15319 | 31122021\_RID\_3262\_1a.raw |  |  |  |  |  |  |  | 1.1516E5 |  |  | 1 | 0 | 0 | 0 | 0 | 0 | 0 | 0 | 1 | 0 | 0 | 68 | 85 |  |  | PEAKS DB |
| total 1 peptides |  |  |  |  |  |  |  |  |  |  |  |  |  |  |  |  |  |  |  |  |  |  |  |  |  |  |  |  |  |  |  |  |  |  |  |  |  |
| --- | --- | --- | --- | --- | --- | --- | --- | --- | --- | --- | --- | --- | --- | --- | --- | --- | --- | --- | --- | --- | --- | --- | --- | --- | --- | --- | --- | --- | --- | --- | --- | --- | --- | --- | --- | --- | --- |

XP\_026553507.1

back to list

  

| Protein Coverage
| Supporting Peptides
|

Protein Coverage:

Supporting Peptides:

| Peptide | Uniq | -10lgP | Mass | Length | ppm | m/z | z | RT | Fraction | Scan | Source File | Area F4 | Area F5 | Area F6 | Area F10 | Area F8 | Area F9 | Area F7 | Area F1 | Area F2 | Area F3 | #Feature | #Feature F4 | #Feature F5 | #Feature F6 | #Feature F10 | #Feature F8 | #Feature F9 | #Feature F7 | #Feature F1 | #Feature F2 | #Feature F3 | Start | End | PTM | AScore | Found By |
| --- | --- | --- | --- | --- | --- | --- | --- | --- | --- | --- | --- | --- | --- | --- | --- | --- | --- | --- | --- | --- | --- | --- | --- | --- | --- | --- | --- | --- | --- | --- | --- | --- | --- | --- | --- | --- | --- |
| R.HGLLVPNNTTDQELQHIR.N | Y | 73.44 | 2084.0762 | 18 | 0.0 | 695.6993 | 3 | 33.67 | 8 | F8:15319 | 31122021\_RID\_3262\_1a.raw |  |  |  |  |  |  |  | 1.1516E5 |  |  | 1 | 0 | 0 | 0 | 0 | 0 | 0 | 0 | 1 | 0 | 0 | 68 | 85 |  |  | PEAKS DB |
| total 1 peptides |  |  |  |  |  |  |  |  |  |  |  |  |  |  |  |  |  |  |  |  |  |  |  |  |  |  |  |  |  |  |  |  |  |  |  |  |  |
| --- | --- | --- | --- | --- | --- | --- | --- | --- | --- | --- | --- | --- | --- | --- | --- | --- | --- | --- | --- | --- | --- | --- | --- | --- | --- | --- | --- | --- | --- | --- | --- | --- | --- | --- | --- | --- | --- |

XP\_026540970.1

back to list

  

| Protein Coverage
| Supporting Peptides
|

Protein Coverage:

Supporting Peptides:

| Peptide | Uniq | -10lgP | Mass | Length | ppm | m/z | z | RT | Fraction | Scan | Source File | Area F4 | Area F5 | Area F6 | Area F10 | Area F8 | Area F9 | Area F7 | Area F1 | Area F2 | Area F3 | #Feature | #Feature F4 | #Feature F5 | #Feature F6 | #Feature F10 | #Feature F8 | #Feature F9 | #Feature F7 | #Feature F1 | #Feature F2 | #Feature F3 | Start | End | PTM | AScore | Found By |
| --- | --- | --- | --- | --- | --- | --- | --- | --- | --- | --- | --- | --- | --- | --- | --- | --- | --- | --- | --- | --- | --- | --- | --- | --- | --- | --- | --- | --- | --- | --- | --- | --- | --- | --- | --- | --- | --- |
| R.HGLLVPNNTTDQELQHIR.N | Y | 73.44 | 2084.0762 | 18 | 0.0 | 695.6993 | 3 | 33.67 | 8 | F8:15319 | 31122021\_RID\_3262\_1a.raw |  |  |  |  |  |  |  | 1.1516E5 |  |  | 1 | 0 | 0 | 0 | 0 | 0 | 0 | 0 | 1 | 0 | 0 | 68 | 85 |  |  | PEAKS DB |
| total 1 peptides |  |  |  |  |  |  |  |  |  |  |  |  |  |  |  |  |  |  |  |  |  |  |  |  |  |  |  |  |  |  |  |  |  |  |  |  |  |
| --- | --- | --- | --- | --- | --- | --- | --- | --- | --- | --- | --- | --- | --- | --- | --- | --- | --- | --- | --- | --- | --- | --- | --- | --- | --- | --- | --- | --- | --- | --- | --- | --- | --- | --- | --- | --- | --- |

XP\_015671062.1

back to list

  

| Protein Coverage
| Supporting Peptides
|

Protein Coverage:

Supporting Peptides:

| Peptide | Uniq | -10lgP | Mass | Length | ppm | m/z | z | RT | Fraction | Scan | Source File | Area F4 | Area F5 | Area F6 | Area F10 | Area F8 | Area F9 | Area F7 | Area F1 | Area F2 | Area F3 | #Feature | #Feature F4 | #Feature F5 | #Feature F6 | #Feature F10 | #Feature F8 | #Feature F9 | #Feature F7 | #Feature F1 | #Feature F2 | #Feature F3 | Start | End | PTM | AScore | Found By |
| --- | --- | --- | --- | --- | --- | --- | --- | --- | --- | --- | --- | --- | --- | --- | --- | --- | --- | --- | --- | --- | --- | --- | --- | --- | --- | --- | --- | --- | --- | --- | --- | --- | --- | --- | --- | --- | --- |
| R.HGLLVPNNTTDQELQHIR.N | Y | 73.44 | 2084.0762 | 18 | 0.0 | 695.6993 | 3 | 33.67 | 8 | F8:15319 | 31122021\_RID\_3262\_1a.raw |  |  |  |  |  |  |  | 1.1516E5 |  |  | 1 | 0 | 0 | 0 | 0 | 0 | 0 | 0 | 1 | 0 | 0 | 68 | 85 |  |  | PEAKS DB |
| total 1 peptides |  |  |  |  |  |  |  |  |  |  |  |  |  |  |  |  |  |  |  |  |  |  |  |  |  |  |  |  |  |  |  |  |  |  |  |  |  |
| --- | --- | --- | --- | --- | --- | --- | --- | --- | --- | --- | --- | --- | --- | --- | --- | --- | --- | --- | --- | --- | --- | --- | --- | --- | --- | --- | --- | --- | --- | --- | --- | --- | --- | --- | --- | --- | --- |

XP\_007439387.1

back to list

  

| Protein Coverage
| Supporting Peptides
|

Protein Coverage:

Supporting Peptides:

| Peptide | Uniq | -10lgP | Mass | Length | ppm | m/z | z | RT | Fraction | Scan | Source File | Area F4 | Area F5 | Area F6 | Area F10 | Area F8 | Area F9 | Area F7 | Area F1 | Area F2 | Area F3 | #Feature | #Feature F4 | #Feature F5 | #Feature F6 | #Feature F10 | #Feature F8 | #Feature F9 | #Feature F7 | #Feature F1 | #Feature F2 | #Feature F3 | Start | End | PTM | AScore | Found By |
| --- | --- | --- | --- | --- | --- | --- | --- | --- | --- | --- | --- | --- | --- | --- | --- | --- | --- | --- | --- | --- | --- | --- | --- | --- | --- | --- | --- | --- | --- | --- | --- | --- | --- | --- | --- | --- | --- |
| R.HGLLVPNNTTDQELQHIR.N | Y | 73.44 | 2084.0762 | 18 | 0.0 | 695.6993 | 3 | 33.67 | 8 | F8:15319 | 31122021\_RID\_3262\_1a.raw |  |  |  |  |  |  |  | 1.1516E5 |  |  | 1 | 0 | 0 | 0 | 0 | 0 | 0 | 0 | 1 | 0 | 0 | 70 | 87 |  |  | PEAKS DB |
| total 1 peptides |  |  |  |  |  |  |  |  |  |  |  |  |  |  |  |  |  |  |  |  |  |  |  |  |  |  |  |  |  |  |  |  |  |  |  |  |  |
| --- | --- | --- | --- | --- | --- | --- | --- | --- | --- | --- | --- | --- | --- | --- | --- | --- | --- | --- | --- | --- | --- | --- | --- | --- | --- | --- | --- | --- | --- | --- | --- | --- | --- | --- | --- | --- | --- |

LAA26046.1

back to list

  

| Protein Coverage
| Supporting Peptides
|

Protein Coverage:

Supporting Peptides:

| Peptide | Uniq | -10lgP | Mass | Length | ppm | m/z | z | RT | Fraction | Scan | Source File | Area F4 | Area F5 | Area F6 | Area F10 | Area F8 | Area F9 | Area F7 | Area F1 | Area F2 | Area F3 | #Feature | #Feature F4 | #Feature F5 | #Feature F6 | #Feature F10 | #Feature F8 | #Feature F9 | #Feature F7 | #Feature F1 | #Feature F2 | #Feature F3 | Start | End | PTM | AScore | Found By |
| --- | --- | --- | --- | --- | --- | --- | --- | --- | --- | --- | --- | --- | --- | --- | --- | --- | --- | --- | --- | --- | --- | --- | --- | --- | --- | --- | --- | --- | --- | --- | --- | --- | --- | --- | --- | --- | --- |
| R.HGLLVPNNTTDQELQHIR.N | Y | 73.44 | 2084.0762 | 18 | 0.0 | 695.6993 | 3 | 33.67 | 8 | F8:15319 | 31122021\_RID\_3262\_1a.raw |  |  |  |  |  |  |  | 1.1516E5 |  |  | 1 | 0 | 0 | 0 | 0 | 0 | 0 | 0 | 1 | 0 | 0 | 101 | 118 |  |  | PEAKS DB |
| total 1 peptides |  |  |  |  |  |  |  |  |  |  |  |  |  |  |  |  |  |  |  |  |  |  |  |  |  |  |  |  |  |  |  |  |  |  |  |  |  |
| --- | --- | --- | --- | --- | --- | --- | --- | --- | --- | --- | --- | --- | --- | --- | --- | --- | --- | --- | --- | --- | --- | --- | --- | --- | --- | --- | --- | --- | --- | --- | --- | --- | --- | --- | --- | --- | --- |

AFJ51037.1

back to list

  

| Protein Coverage
| Supporting Peptides
|

Protein Coverage:

Supporting Peptides:

| Peptide | Uniq | -10lgP | Mass | Length | ppm | m/z | z | RT | Fraction | Scan | Source File | Area F4 | Area F5 | Area F6 | Area F10 | Area F8 | Area F9 | Area F7 | Area F1 | Area F2 | Area F3 | #Feature | #Feature F4 | #Feature F5 | #Feature F6 | #Feature F10 | #Feature F8 | #Feature F9 | #Feature F7 | #Feature F1 | #Feature F2 | #Feature F3 | Start | End | PTM | AScore | Found By |
| --- | --- | --- | --- | --- | --- | --- | --- | --- | --- | --- | --- | --- | --- | --- | --- | --- | --- | --- | --- | --- | --- | --- | --- | --- | --- | --- | --- | --- | --- | --- | --- | --- | --- | --- | --- | --- | --- |
| K.LLDEVFFSEK.I | Y | 54.87 | 1225.6230 | 10 | 0.5 | 613.8191 | 2 | 51.90 | 8 | F8:29772 | 31122021\_RID\_3262\_1a.raw |  |  |  |  |  |  |  | 1.9782E5 |  |  | 1 | 0 | 0 | 0 | 0 | 0 | 0 | 0 | 1 | 0 | 0 | 55 | 64 |  |  | PEAKS DB |
| K.VEIATLTR.E | Y | 41.83 | 901.5233 | 8 | -0.1 | 451.7689 | 2 | 33.77 | 6 | F6:20061 | 17012022\_RID\_3262\_9a.raw |  |  |  |  |  | 1.4105E5 |  |  |  |  | 1 | 0 | 0 | 0 | 0 | 0 | 1 | 0 | 0 | 0 | 0 | 211 | 218 |  |  | PEAKS DB |
| total 2 peptides |  |  |  |  |  |  |  |  |  |  |  |  |  |  |  |  |  |  |  |  |  |  |  |  |  |  |  |  |  |  |  |  |  |  |  |  |  |
| --- | --- | --- | --- | --- | --- | --- | --- | --- | --- | --- | --- | --- | --- | --- | --- | --- | --- | --- | --- | --- | --- | --- | --- | --- | --- | --- | --- | --- | --- | --- | --- | --- | --- | --- | --- | --- | --- |

JAA96034.1

back to list

  

| Protein Coverage
| Supporting Peptides
|

Protein Coverage:

Supporting Peptides:

| Peptide | Uniq | -10lgP | Mass | Length | ppm | m/z | z | RT | Fraction | Scan | Source File | Area F4 | Area F5 | Area F6 | Area F10 | Area F8 | Area F9 | Area F7 | Area F1 | Area F2 | Area F3 | #Feature | #Feature F4 | #Feature F5 | #Feature F6 | #Feature F10 | #Feature F8 | #Feature F9 | #Feature F7 | #Feature F1 | #Feature F2 | #Feature F3 | Start | End | PTM | AScore | Found By |
| --- | --- | --- | --- | --- | --- | --- | --- | --- | --- | --- | --- | --- | --- | --- | --- | --- | --- | --- | --- | --- | --- | --- | --- | --- | --- | --- | --- | --- | --- | --- | --- | --- | --- | --- | --- | --- | --- |
| K.LLDEVFFSEK.I | Y | 54.87 | 1225.6230 | 10 | 0.5 | 613.8191 | 2 | 51.90 | 8 | F8:29772 | 31122021\_RID\_3262\_1a.raw |  |  |  |  |  |  |  | 1.9782E5 |  |  | 1 | 0 | 0 | 0 | 0 | 0 | 0 | 0 | 1 | 0 | 0 | 55 | 64 |  |  | PEAKS DB |
| K.VEIATLTR.E | Y | 41.83 | 901.5233 | 8 | -0.1 | 451.7689 | 2 | 33.77 | 6 | F6:20061 | 17012022\_RID\_3262\_9a.raw |  |  |  |  |  | 1.4105E5 |  |  |  |  | 1 | 0 | 0 | 0 | 0 | 0 | 1 | 0 | 0 | 0 | 0 | 211 | 218 |  |  | PEAKS DB |
| total 2 peptides |  |  |  |  |  |  |  |  |  |  |  |  |  |  |  |  |  |  |  |  |  |  |  |  |  |  |  |  |  |  |  |  |  |  |  |  |  |
| --- | --- | --- | --- | --- | --- | --- | --- | --- | --- | --- | --- | --- | --- | --- | --- | --- | --- | --- | --- | --- | --- | --- | --- | --- | --- | --- | --- | --- | --- | --- | --- | --- | --- | --- | --- | --- | --- |

JAV49554.1

back to list

  

| Protein Coverage
| Supporting Peptides
|

Protein Coverage:

Supporting Peptides:

| Peptide | Uniq | -10lgP | Mass | Length | ppm | m/z | z | RT | Fraction | Scan | Source File | Area F4 | Area F5 | Area F6 | Area F10 | Area F8 | Area F9 | Area F7 | Area F1 | Area F2 | Area F3 | #Feature | #Feature F4 | #Feature F5 | #Feature F6 | #Feature F10 | #Feature F8 | #Feature F9 | #Feature F7 | #Feature F1 | #Feature F2 | #Feature F3 | Start | End | PTM | AScore | Found By |
| --- | --- | --- | --- | --- | --- | --- | --- | --- | --- | --- | --- | --- | --- | --- | --- | --- | --- | --- | --- | --- | --- | --- | --- | --- | --- | --- | --- | --- | --- | --- | --- | --- | --- | --- | --- | --- | --- |
| K.LLDEVFFSEK.I | Y | 54.87 | 1225.6230 | 10 | 0.5 | 613.8191 | 2 | 51.90 | 8 | F8:29772 | 31122021\_RID\_3262\_1a.raw |  |  |  |  |  |  |  | 1.9782E5 |  |  | 1 | 0 | 0 | 0 | 0 | 0 | 0 | 0 | 1 | 0 | 0 | 55 | 64 |  |  | PEAKS DB |
| K.VEIATLTR.E | Y | 41.83 | 901.5233 | 8 | -0.1 | 451.7689 | 2 | 33.77 | 6 | F6:20061 | 17012022\_RID\_3262\_9a.raw |  |  |  |  |  | 1.4105E5 |  |  |  |  | 1 | 0 | 0 | 0 | 0 | 0 | 1 | 0 | 0 | 0 | 0 | 211 | 218 |  |  | PEAKS DB |
| total 2 peptides |  |  |  |  |  |  |  |  |  |  |  |  |  |  |  |  |  |  |  |  |  |  |  |  |  |  |  |  |  |  |  |  |  |  |  |  |  |
| --- | --- | --- | --- | --- | --- | --- | --- | --- | --- | --- | --- | --- | --- | --- | --- | --- | --- | --- | --- | --- | --- | --- | --- | --- | --- | --- | --- | --- | --- | --- | --- | --- | --- | --- | --- | --- | --- |

JAI11929.1

back to list

  

| Protein Coverage
| Supporting Peptides
|

Protein Coverage:

Supporting Peptides:

| Peptide | Uniq | -10lgP | Mass | Length | ppm | m/z | z | RT | Fraction | Scan | Source File | Area F4 | Area F5 | Area F6 | Area F10 | Area F8 | Area F9 | Area F7 | Area F1 | Area F2 | Area F3 | #Feature | #Feature F4 | #Feature F5 | #Feature F6 | #Feature F10 | #Feature F8 | #Feature F9 | #Feature F7 | #Feature F1 | #Feature F2 | #Feature F3 | Start | End | PTM | AScore | Found By |
| --- | --- | --- | --- | --- | --- | --- | --- | --- | --- | --- | --- | --- | --- | --- | --- | --- | --- | --- | --- | --- | --- | --- | --- | --- | --- | --- | --- | --- | --- | --- | --- | --- | --- | --- | --- | --- | --- |
| K.LLDEVFFSEK.I | Y | 54.87 | 1225.6230 | 10 | 0.5 | 613.8191 | 2 | 51.90 | 8 | F8:29772 | 31122021\_RID\_3262\_1a.raw |  |  |  |  |  |  |  | 1.9782E5 |  |  | 1 | 0 | 0 | 0 | 0 | 0 | 0 | 0 | 1 | 0 | 0 | 55 | 64 |  |  | PEAKS DB |
| K.VEIATLTR.E | Y | 41.83 | 901.5233 | 8 | -0.1 | 451.7689 | 2 | 33.77 | 6 | F6:20061 | 17012022\_RID\_3262\_9a.raw |  |  |  |  |  | 1.4105E5 |  |  |  |  | 1 | 0 | 0 | 0 | 0 | 0 | 1 | 0 | 0 | 0 | 0 | 211 | 218 |  |  | PEAKS DB |
| total 2 peptides |  |  |  |  |  |  |  |  |  |  |  |  |  |  |  |  |  |  |  |  |  |  |  |  |  |  |  |  |  |  |  |  |  |  |  |  |  |
| --- | --- | --- | --- | --- | --- | --- | --- | --- | --- | --- | --- | --- | --- | --- | --- | --- | --- | --- | --- | --- | --- | --- | --- | --- | --- | --- | --- | --- | --- | --- | --- | --- | --- | --- | --- | --- | --- |

AEJ31991.1

back to list

  

| Protein Coverage
| Supporting Peptides
|

Protein Coverage:

Supporting Peptides:

| Peptide | Uniq | -10lgP | Mass | Length | ppm | m/z | z | RT | Fraction | Scan | Source File | Area F4 | Area F5 | Area F6 | Area F10 | Area F8 | Area F9 | Area F7 | Area F1 | Area F2 | Area F3 | #Feature | #Feature F4 | #Feature F5 | #Feature F6 | #Feature F10 | #Feature F8 | #Feature F9 | #Feature F7 | #Feature F1 | #Feature F2 | #Feature F3 | Start | End | PTM | AScore | Found By |
| --- | --- | --- | --- | --- | --- | --- | --- | --- | --- | --- | --- | --- | --- | --- | --- | --- | --- | --- | --- | --- | --- | --- | --- | --- | --- | --- | --- | --- | --- | --- | --- | --- | --- | --- | --- | --- | --- |
| R.TDIVSPPIC(+57.02)GNELLEVG.E | Y | 61.10 | 1811.8975 | 17 | 6.5 | 906.9619 | 2 | 47.81 | 9 | F9:27707 | 31122021\_RID\_3262\_2a.raw | 6.6124E6 |  |  |  |  |  |  | 1.2944E7 | 1.0548E8 | 7.1088E7 | 5 | 1 | 0 | 0 | 0 | 0 | 0 | 0 | 1 | 2 | 1 | 275 | 291 | Carbamidomethylation | C9:Carbamidomethylation:1000.00 | PEAKS DB |
| D.PDYGMVDHGTK.C | Y | 49.91 | 1218.5339 | 11 | -0.4 | 610.2740 | 2 | 23.44 | 4 | F4:10536 | 17012022\_RID\_3262\_10a .raw |  |  |  | 2.2861E5 |  |  |  | 2.43E5 | 2.2473E5 |  | 3 | 0 | 0 | 0 | 1 | 0 | 0 | 0 | 1 | 1 | 0 | 454 | 464 |  |  | PEAKS DB |
| D.PDYGM(+15.99)VDHGTK.C | Y | 42.90 | 1234.5288 | 11 | 0.5 | 412.5171 | 3 | 18.61 | 7 | F7:4766 | 21012022\_RID\_3262\_7a.raw |  |  |  |  |  |  | 4.066E5 |  |  |  | 1 | 0 | 0 | 0 | 0 | 0 | 0 | 1 | 0 | 0 | 0 | 454 | 464 | Oxidation (M) | M5:Oxidation (M):1000.00 | PEAKS DB |
| total 3 peptides |  |  |  |  |  |  |  |  |  |  |  |  |  |  |  |  |  |  |  |  |  |  |  |  |  |  |  |  |  |  |  |  |  |  |  |  |  |
| --- | --- | --- | --- | --- | --- | --- | --- | --- | --- | --- | --- | --- | --- | --- | --- | --- | --- | --- | --- | --- | --- | --- | --- | --- | --- | --- | --- | --- | --- | --- | --- | --- | --- | --- | --- | --- | --- |

AFJ49243.1

back to list

  

| Protein Coverage
| Supporting Peptides
|

Protein Coverage:

Supporting Peptides:

| Peptide | Uniq | -10lgP | Mass | Length | ppm | m/z | z | RT | Fraction | Scan | Source File | Area F4 | Area F5 | Area F6 | Area F10 | Area F8 | Area F9 | Area F7 | Area F1 | Area F2 | Area F3 | #Feature | #Feature F4 | #Feature F5 | #Feature F6 | #Feature F10 | #Feature F8 | #Feature F9 | #Feature F7 | #Feature F1 | #Feature F2 | #Feature F3 | Start | End | PTM | AScore | Found By |
| --- | --- | --- | --- | --- | --- | --- | --- | --- | --- | --- | --- | --- | --- | --- | --- | --- | --- | --- | --- | --- | --- | --- | --- | --- | --- | --- | --- | --- | --- | --- | --- | --- | --- | --- | --- | --- | --- |
| R.TDIVSPPIC(+57.02)GNELLEVG.E | Y | 61.10 | 1811.8975 | 17 | 6.5 | 906.9619 | 2 | 47.81 | 9 | F9:27707 | 31122021\_RID\_3262\_2a.raw | 6.6124E6 |  |  |  |  |  |  | 1.2944E7 | 1.0548E8 | 7.1088E7 | 5 | 1 | 0 | 0 | 0 | 0 | 0 | 0 | 1 | 2 | 1 | 399 | 415 | Carbamidomethylation | C9:Carbamidomethylation:1000.00 | PEAKS DB |
| D.PDYGMVDHGTK.C | Y | 49.91 | 1218.5339 | 11 | -0.4 | 610.2740 | 2 | 23.44 | 4 | F4:10536 | 17012022\_RID\_3262\_10a .raw |  |  |  | 2.2861E5 |  |  |  | 2.43E5 | 2.2473E5 |  | 3 | 0 | 0 | 0 | 1 | 0 | 0 | 0 | 1 | 1 | 0 | 578 | 588 |  |  | PEAKS DB |
| D.PDYGM(+15.99)VDHGTK.C | Y | 42.90 | 1234.5288 | 11 | 0.5 | 412.5171 | 3 | 18.61 | 7 | F7:4766 | 21012022\_RID\_3262\_7a.raw |  |  |  |  |  |  | 4.066E5 |  |  |  | 1 | 0 | 0 | 0 | 0 | 0 | 0 | 1 | 0 | 0 | 0 | 578 | 588 | Oxidation (M) | M5:Oxidation (M):1000.00 | PEAKS DB |
| total 3 peptides |  |  |  |  |  |  |  |  |  |  |  |  |  |  |  |  |  |  |  |  |  |  |  |  |  |  |  |  |  |  |  |  |  |  |  |  |  |
| --- | --- | --- | --- | --- | --- | --- | --- | --- | --- | --- | --- | --- | --- | --- | --- | --- | --- | --- | --- | --- | --- | --- | --- | --- | --- | --- | --- | --- | --- | --- | --- | --- | --- | --- | --- | --- | --- |

JAV48412.1

back to list

  

| Protein Coverage
| Supporting Peptides
|

Protein Coverage:

Supporting Peptides:

| Peptide | Uniq | -10lgP | Mass | Length | ppm | m/z | z | RT | Fraction | Scan | Source File | Area F4 | Area F5 | Area F6 | Area F10 | Area F8 | Area F9 | Area F7 | Area F1 | Area F2 | Area F3 | #Feature | #Feature F4 | #Feature F5 | #Feature F6 | #Feature F10 | #Feature F8 | #Feature F9 | #Feature F7 | #Feature F1 | #Feature F2 | #Feature F3 | Start | End | PTM | AScore | Found By |
| --- | --- | --- | --- | --- | --- | --- | --- | --- | --- | --- | --- | --- | --- | --- | --- | --- | --- | --- | --- | --- | --- | --- | --- | --- | --- | --- | --- | --- | --- | --- | --- | --- | --- | --- | --- | --- | --- |
| R.TDIVSPPIC(+57.02)GNELLEVG.E | Y | 61.10 | 1811.8975 | 17 | 6.5 | 906.9619 | 2 | 47.81 | 9 | F9:27707 | 31122021\_RID\_3262\_2a.raw | 6.6124E6 |  |  |  |  |  |  | 1.2944E7 | 1.0548E8 | 7.1088E7 | 5 | 1 | 0 | 0 | 0 | 0 | 0 | 0 | 1 | 2 | 1 | 399 | 415 | Carbamidomethylation | C9:Carbamidomethylation:1000.00 | PEAKS DB |
| D.PDYGMVDHGTK.C | Y | 49.91 | 1218.5339 | 11 | -0.4 | 610.2740 | 2 | 23.44 | 4 | F4:10536 | 17012022\_RID\_3262\_10a .raw |  |  |  | 2.2861E5 |  |  |  | 2.43E5 | 2.2473E5 |  | 3 | 0 | 0 | 0 | 1 | 0 | 0 | 0 | 1 | 1 | 0 | 578 | 588 |  |  | PEAKS DB |
| D.PDYGM(+15.99)VDHGTK.C | Y | 42.90 | 1234.5288 | 11 | 0.5 | 412.5171 | 3 | 18.61 | 7 | F7:4766 | 21012022\_RID\_3262\_7a.raw |  |  |  |  |  |  | 4.066E5 |  |  |  | 1 | 0 | 0 | 0 | 0 | 0 | 0 | 1 | 0 | 0 | 0 | 578 | 588 | Oxidation (M) | M5:Oxidation (M):1000.00 | PEAKS DB |
| total 3 peptides |  |  |  |  |  |  |  |  |  |  |  |  |  |  |  |  |  |  |  |  |  |  |  |  |  |  |  |  |  |  |  |  |  |  |  |  |  |
| --- | --- | --- | --- | --- | --- | --- | --- | --- | --- | --- | --- | --- | --- | --- | --- | --- | --- | --- | --- | --- | --- | --- | --- | --- | --- | --- | --- | --- | --- | --- | --- | --- | --- | --- | --- | --- | --- |

AUS82484.1

back to list

  

| Protein Coverage
| Supporting Peptides
|

Protein Coverage:

Supporting Peptides:

| Peptide | Uniq | -10lgP | Mass | Length | ppm | m/z | z | RT | Fraction | Scan | Source File | Area F4 | Area F5 | Area F6 | Area F10 | Area F8 | Area F9 | Area F7 | Area F1 | Area F2 | Area F3 | #Feature | #Feature F4 | #Feature F5 | #Feature F6 | #Feature F10 | #Feature F8 | #Feature F9 | #Feature F7 | #Feature F1 | #Feature F2 | #Feature F3 | Start | End | PTM | AScore | Found By |
| --- | --- | --- | --- | --- | --- | --- | --- | --- | --- | --- | --- | --- | --- | --- | --- | --- | --- | --- | --- | --- | --- | --- | --- | --- | --- | --- | --- | --- | --- | --- | --- | --- | --- | --- | --- | --- | --- |
| K.KDDVLDQDIMLIK.L | Y | 50.08 | 1544.8120 | 13 | 0.6 | 515.9449 | 3 | 48.82 | 2 | F2:28329 | 13012022\_RID\_3262\_5a.raw |  | 1.1075E7 |  |  |  |  |  |  |  |  | 3 | 0 | 3 | 0 | 0 | 0 | 0 | 0 | 0 | 0 | 0 | 103 | 115 |  |  | PEAKS DB |
| L.VVGGDEC(+57.02)NINEHR.S | N | 43.47 | 1497.6630 | 13 | 1.7 | 749.8400 | 2 | 21.08 | 7 | F7:6186 | 21012022\_RID\_3262\_7a.raw |  |  |  |  |  |  | 0 |  |  |  | 0 | 0 | 0 | 0 | 0 | 0 | 0 | 0 | 0 | 0 | 0 | 25 | 37 | Carbamidomethylation | C7:Carbamidomethylation:1000.00 | PEAKS DB |
| total 2 peptides |  |  |  |  |  |  |  |  |  |  |  |  |  |  |  |  |  |  |  |  |  |  |  |  |  |  |  |  |  |  |  |  |  |  |  |  |  |
| --- | --- | --- | --- | --- | --- | --- | --- | --- | --- | --- | --- | --- | --- | --- | --- | --- | --- | --- | --- | --- | --- | --- | --- | --- | --- | --- | --- | --- | --- | --- | --- | --- | --- | --- | --- | --- | --- |

Q9PVK7.1

back to list

  

| Protein Coverage
| Supporting Peptides
|

Protein Coverage:

Supporting Peptides:

| Peptide | Uniq | -10lgP | Mass | Length | ppm | m/z | z | RT | Fraction | Scan | Source File | Area F4 | Area F5 | Area F6 | Area F10 | Area F8 | Area F9 | Area F7 | Area F1 | Area F2 | Area F3 | #Feature | #Feature F4 | #Feature F5 | #Feature F6 | #Feature F10 | #Feature F8 | #Feature F9 | #Feature F7 | #Feature F1 | #Feature F2 | #Feature F3 | Start | End | PTM | AScore | Found By |
| --- | --- | --- | --- | --- | --- | --- | --- | --- | --- | --- | --- | --- | --- | --- | --- | --- | --- | --- | --- | --- | --- | --- | --- | --- | --- | --- | --- | --- | --- | --- | --- | --- | --- | --- | --- | --- | --- |
| K.ATLDLFGEWR.E | Y | 58.92 | 1206.6033 | 10 | 0.1 | 604.3090 | 2 | 56.94 | 8 | F8:33726 | 31122021\_RID\_3262\_1a.raw |  |  |  |  |  |  |  | 1.2214E5 | 7.0812E4 |  | 2 | 0 | 0 | 0 | 0 | 0 | 0 | 0 | 1 | 1 | 0 | 260 | 269 |  |  | PEAKS DB |
| K.TSAAVVQDYSK.S | Y | 43.77 | 1167.5771 | 11 | 0.8 | 584.7963 | 2 | 24.53 | 8 | F8:8318 | 31122021\_RID\_3262\_1a.raw |  |  |  |  |  |  |  | 2.4891E4 |  |  | 1 | 0 | 0 | 0 | 0 | 0 | 0 | 0 | 1 | 0 | 0 | 308 | 318 |  |  | PEAKS DB |
| total 2 peptides |  |  |  |  |  |  |  |  |  |  |  |  |  |  |  |  |  |  |  |  |  |  |  |  |  |  |  |  |  |  |  |  |  |  |  |  |  |
| --- | --- | --- | --- | --- | --- | --- | --- | --- | --- | --- | --- | --- | --- | --- | --- | --- | --- | --- | --- | --- | --- | --- | --- | --- | --- | --- | --- | --- | --- | --- | --- | --- | --- | --- | --- | --- | --- |

AAF00693.1

back to list

  

| Protein Coverage
| Supporting Peptides
|

Protein Coverage:

Supporting Peptides:

| Peptide | Uniq | -10lgP | Mass | Length | ppm | m/z | z | RT | Fraction | Scan | Source File | Area F4 | Area F5 | Area F6 | Area F10 | Area F8 | Area F9 | Area F7 | Area F1 | Area F2 | Area F3 | #Feature | #Feature F4 | #Feature F5 | #Feature F6 | #Feature F10 | #Feature F8 | #Feature F9 | #Feature F7 | #Feature F1 | #Feature F2 | #Feature F3 | Start | End | PTM | AScore | Found By |
| --- | --- | --- | --- | --- | --- | --- | --- | --- | --- | --- | --- | --- | --- | --- | --- | --- | --- | --- | --- | --- | --- | --- | --- | --- | --- | --- | --- | --- | --- | --- | --- | --- | --- | --- | --- | --- | --- |
| K.ATLDLFGEWR.E | Y | 58.92 | 1206.6033 | 10 | 0.1 | 604.3090 | 2 | 56.94 | 8 | F8:33726 | 31122021\_RID\_3262\_1a.raw |  |  |  |  |  |  |  | 1.2214E5 | 7.0812E4 |  | 2 | 0 | 0 | 0 | 0 | 0 | 0 | 0 | 1 | 1 | 0 | 260 | 269 |  |  | PEAKS DB |
| K.TSAAVVQDYSK.S | Y | 43.77 | 1167.5771 | 11 | 0.8 | 584.7963 | 2 | 24.53 | 8 | F8:8318 | 31122021\_RID\_3262\_1a.raw |  |  |  |  |  |  |  | 2.4891E4 |  |  | 1 | 0 | 0 | 0 | 0 | 0 | 0 | 0 | 1 | 0 | 0 | 308 | 318 |  |  | PEAKS DB |
| total 2 peptides |  |  |  |  |  |  |  |  |  |  |  |  |  |  |  |  |  |  |  |  |  |  |  |  |  |  |  |  |  |  |  |  |  |  |  |  |  |
| --- | --- | --- | --- | --- | --- | --- | --- | --- | --- | --- | --- | --- | --- | --- | --- | --- | --- | --- | --- | --- | --- | --- | --- | --- | --- | --- | --- | --- | --- | --- | --- | --- | --- | --- | --- | --- | --- |

XP\_026544503.1

back to list

  

| Protein Coverage
| Supporting Peptides
|

Protein Coverage:

Supporting Peptides:

| Peptide | Uniq | -10lgP | Mass | Length | ppm | m/z | z | RT | Fraction | Scan | Source File | Area F4 | Area F5 | Area F6 | Area F10 | Area F8 | Area F9 | Area F7 | Area F1 | Area F2 | Area F3 | #Feature | #Feature F4 | #Feature F5 | #Feature F6 | #Feature F10 | #Feature F8 | #Feature F9 | #Feature F7 | #Feature F1 | #Feature F2 | #Feature F3 | Start | End | PTM | AScore | Found By |
| --- | --- | --- | --- | --- | --- | --- | --- | --- | --- | --- | --- | --- | --- | --- | --- | --- | --- | --- | --- | --- | --- | --- | --- | --- | --- | --- | --- | --- | --- | --- | --- | --- | --- | --- | --- | --- | --- |
| R.AAVPSGASTGIYEALELR.D | Y | 45.59 | 1803.9366 | 18 | 0.3 | 902.9759 | 2 | 51.67 | 9 | F9:30712 | 31122021\_RID\_3262\_2a.raw |  |  |  |  |  |  |  |  | 9.5933E4 |  | 1 | 0 | 0 | 0 | 0 | 0 | 0 | 0 | 0 | 1 | 0 | 33 | 50 |  |  | PEAKS DB |
| R.IGAEVYHNLK.N | Y | 43.95 | 1142.6084 | 10 | 1.2 | 572.3121 | 2 | 24.75 | 8 | F8:8475 | 31122021\_RID\_3262\_1a.raw |  |  |  |  |  |  |  | 2.1124E4 |  |  | 1 | 0 | 0 | 0 | 0 | 0 | 0 | 0 | 1 | 0 | 0 | 184 | 193 |  |  | PEAKS DB |
| total 2 peptides |  |  |  |  |  |  |  |  |  |  |  |  |  |  |  |  |  |  |  |  |  |  |  |  |  |  |  |  |  |  |  |  |  |  |  |  |  |
| --- | --- | --- | --- | --- | --- | --- | --- | --- | --- | --- | --- | --- | --- | --- | --- | --- | --- | --- | --- | --- | --- | --- | --- | --- | --- | --- | --- | --- | --- | --- | --- | --- | --- | --- | --- | --- | --- |

JAG68757.1

back to list

  

| Protein Coverage
| Supporting Peptides
|

Protein Coverage:

Supporting Peptides:

| Peptide | Uniq | -10lgP | Mass | Length | ppm | m/z | z | RT | Fraction | Scan | Source File | Area F4 | Area F5 | Area F6 | Area F10 | Area F8 | Area F9 | Area F7 | Area F1 | Area F2 | Area F3 | #Feature | #Feature F4 | #Feature F5 | #Feature F6 | #Feature F10 | #Feature F8 | #Feature F9 | #Feature F7 | #Feature F1 | #Feature F2 | #Feature F3 | Start | End | PTM | AScore | Found By |
| --- | --- | --- | --- | --- | --- | --- | --- | --- | --- | --- | --- | --- | --- | --- | --- | --- | --- | --- | --- | --- | --- | --- | --- | --- | --- | --- | --- | --- | --- | --- | --- | --- | --- | --- | --- | --- | --- |
| R.AAVPSGASTGIYEALELR.D | Y | 45.59 | 1803.9366 | 18 | 0.3 | 902.9759 | 2 | 51.67 | 9 | F9:30712 | 31122021\_RID\_3262\_2a.raw |  |  |  |  |  |  |  |  | 9.5933E4 |  | 1 | 0 | 0 | 0 | 0 | 0 | 0 | 0 | 0 | 1 | 0 | 33 | 50 |  |  | PEAKS DB |
| R.IGAEVYHNLK.N | Y | 43.95 | 1142.6084 | 10 | 1.2 | 572.3121 | 2 | 24.75 | 8 | F8:8475 | 31122021\_RID\_3262\_1a.raw |  |  |  |  |  |  |  | 2.1124E4 |  |  | 1 | 0 | 0 | 0 | 0 | 0 | 0 | 0 | 1 | 0 | 0 | 184 | 193 |  |  | PEAKS DB |
| total 2 peptides |  |  |  |  |  |  |  |  |  |  |  |  |  |  |  |  |  |  |  |  |  |  |  |  |  |  |  |  |  |  |  |  |  |  |  |  |  |
| --- | --- | --- | --- | --- | --- | --- | --- | --- | --- | --- | --- | --- | --- | --- | --- | --- | --- | --- | --- | --- | --- | --- | --- | --- | --- | --- | --- | --- | --- | --- | --- | --- | --- | --- | --- | --- | --- |

JAC96284.1

back to list

  

| Protein Coverage
| Supporting Peptides
|

Protein Coverage:

Supporting Peptides:

| Peptide | Uniq | -10lgP | Mass | Length | ppm | m/z | z | RT | Fraction | Scan | Source File | Area F4 | Area F5 | Area F6 | Area F10 | Area F8 | Area F9 | Area F7 | Area F1 | Area F2 | Area F3 | #Feature | #Feature F4 | #Feature F5 | #Feature F6 | #Feature F10 | #Feature F8 | #Feature F9 | #Feature F7 | #Feature F1 | #Feature F2 | #Feature F3 | Start | End | PTM | AScore | Found By |
| --- | --- | --- | --- | --- | --- | --- | --- | --- | --- | --- | --- | --- | --- | --- | --- | --- | --- | --- | --- | --- | --- | --- | --- | --- | --- | --- | --- | --- | --- | --- | --- | --- | --- | --- | --- | --- | --- |
| R.AAVPSGASTGIYEALELR.D | Y | 45.59 | 1803.9366 | 18 | 0.3 | 902.9759 | 2 | 51.67 | 9 | F9:30712 | 31122021\_RID\_3262\_2a.raw |  |  |  |  |  |  |  |  | 9.5933E4 |  | 1 | 0 | 0 | 0 | 0 | 0 | 0 | 0 | 0 | 1 | 0 | 33 | 50 |  |  | PEAKS DB |
| R.IGAEVYHNLK.N | Y | 43.95 | 1142.6084 | 10 | 1.2 | 572.3121 | 2 | 24.75 | 8 | F8:8475 | 31122021\_RID\_3262\_1a.raw |  |  |  |  |  |  |  | 2.1124E4 |  |  | 1 | 0 | 0 | 0 | 0 | 0 | 0 | 0 | 1 | 0 | 0 | 184 | 193 |  |  | PEAKS DB |
| total 2 peptides |  |  |  |  |  |  |  |  |  |  |  |  |  |  |  |  |  |  |  |  |  |  |  |  |  |  |  |  |  |  |  |  |  |  |  |  |  |
| --- | --- | --- | --- | --- | --- | --- | --- | --- | --- | --- | --- | --- | --- | --- | --- | --- | --- | --- | --- | --- | --- | --- | --- | --- | --- | --- | --- | --- | --- | --- | --- | --- | --- | --- | --- | --- | --- |

XP\_015685587.1

back to list

  

| Protein Coverage
| Supporting Peptides
|

Protein Coverage:

Supporting Peptides:

| Peptide | Uniq | -10lgP | Mass | Length | ppm | m/z | z | RT | Fraction | Scan | Source File | Area F4 | Area F5 | Area F6 | Area F10 | Area F8 | Area F9 | Area F7 | Area F1 | Area F2 | Area F3 | #Feature | #Feature F4 | #Feature F5 | #Feature F6 | #Feature F10 | #Feature F8 | #Feature F9 | #Feature F7 | #Feature F1 | #Feature F2 | #Feature F3 | Start | End | PTM | AScore | Found By |
| --- | --- | --- | --- | --- | --- | --- | --- | --- | --- | --- | --- | --- | --- | --- | --- | --- | --- | --- | --- | --- | --- | --- | --- | --- | --- | --- | --- | --- | --- | --- | --- | --- | --- | --- | --- | --- | --- |
| K.FNSNLTEVK.T | Y | 53.94 | 1050.5345 | 9 | -0.7 | 526.2742 | 2 | 28.15 | 9 | F9:11934 | 31122021\_RID\_3262\_2a.raw |  |  |  |  |  |  |  |  | 6.0805E6 |  | 1 | 0 | 0 | 0 | 0 | 0 | 0 | 0 | 0 | 1 | 0 | 253 | 261 |  |  | PEAKS DB |
| Y.DTLDSFGEWR.K | N | 48.46 | 1224.5411 | 10 | 0.8 | 613.2783 | 2 | 49.42 | 9 | F9:28898 | 31122021\_RID\_3262\_2a.raw |  |  |  |  |  |  |  |  | 2.2842E5 |  | 1 | 0 | 0 | 0 | 0 | 0 | 0 | 0 | 0 | 1 | 0 | 304 | 313 |  |  | PEAKS DB |
| total 2 peptides |  |  |  |  |  |  |  |  |  |  |  |  |  |  |  |  |  |  |  |  |  |  |  |  |  |  |  |  |  |  |  |  |  |  |  |  |  |
| --- | --- | --- | --- | --- | --- | --- | --- | --- | --- | --- | --- | --- | --- | --- | --- | --- | --- | --- | --- | --- | --- | --- | --- | --- | --- | --- | --- | --- | --- | --- | --- | --- | --- | --- | --- | --- | --- |

BAD82814.1

back to list

  

| Protein Coverage
| Supporting Peptides
|

Protein Coverage:

Supporting Peptides:

| Peptide | Uniq | -10lgP | Mass | Length | ppm | m/z | z | RT | Fraction | Scan | Source File | Area F4 | Area F5 | Area F6 | Area F10 | Area F8 | Area F9 | Area F7 | Area F1 | Area F2 | Area F3 | #Feature | #Feature F4 | #Feature F5 | #Feature F6 | #Feature F10 | #Feature F8 | #Feature F9 | #Feature F7 | #Feature F1 | #Feature F2 | #Feature F3 | Start | End | PTM | AScore | Found By |
| --- | --- | --- | --- | --- | --- | --- | --- | --- | --- | --- | --- | --- | --- | --- | --- | --- | --- | --- | --- | --- | --- | --- | --- | --- | --- | --- | --- | --- | --- | --- | --- | --- | --- | --- | --- | --- | --- |
| K.LSADIIALHQK.C | Y | 44.38 | 1207.6924 | 11 | 1.4 | 604.8543 | 2 | 33.58 | 9 | F9:16282 | 31122021\_RID\_3262\_2a.raw |  |  |  |  |  |  |  |  | 6.9991E4 |  | 1 | 0 | 0 | 0 | 0 | 0 | 0 | 0 | 0 | 1 | 0 | 69 | 79 |  |  | PEAKS DB |
| total 1 peptides |  |  |  |  |  |  |  |  |  |  |  |  |  |  |  |  |  |  |  |  |  |  |  |  |  |  |  |  |  |  |  |  |  |  |  |  |  |
| --- | --- | --- | --- | --- | --- | --- | --- | --- | --- | --- | --- | --- | --- | --- | --- | --- | --- | --- | --- | --- | --- | --- | --- | --- | --- | --- | --- | --- | --- | --- | --- | --- | --- | --- | --- | --- | --- |

XP\_026551425.1

back to list

  

| Protein Coverage
| Supporting Peptides
|

Protein Coverage:

Supporting Peptides:

| Peptide | Uniq | -10lgP | Mass | Length | ppm | m/z | z | RT | Fraction | Scan | Source File | Area F4 | Area F5 | Area F6 | Area F10 | Area F8 | Area F9 | Area F7 | Area F1 | Area F2 | Area F3 | #Feature | #Feature F4 | #Feature F5 | #Feature F6 | #Feature F10 | #Feature F8 | #Feature F9 | #Feature F7 | #Feature F1 | #Feature F2 | #Feature F3 | Start | End | PTM | AScore | Found By |
| --- | --- | --- | --- | --- | --- | --- | --- | --- | --- | --- | --- | --- | --- | --- | --- | --- | --- | --- | --- | --- | --- | --- | --- | --- | --- | --- | --- | --- | --- | --- | --- | --- | --- | --- | --- | --- | --- |
| K.TQLEELEDELQATEDAK.L | Y | 55.38 | 1960.9113 | 17 | 2.1 | 981.4650 | 2 | 53.67 | 8 | F8:31140 | 31122021\_RID\_3262\_1a.raw |  |  |  |  |  |  |  | 1.3593E4 |  |  | 1 | 0 | 0 | 0 | 0 | 0 | 0 | 0 | 1 | 0 | 0 | 1539 | 1555 |  |  | PEAKS DB |
| R.IAEFTTNLTEEEEK.S | Y | 41.18 | 1652.7781 | 14 | 0.9 | 827.3970 | 2 | 39.35 | 8 | F8:19948 | 31122021\_RID\_3262\_1a.raw |  |  |  |  |  |  |  | 4.4528E4 |  |  | 1 | 0 | 0 | 0 | 0 | 0 | 0 | 0 | 1 | 0 | 0 | 1001 | 1014 |  |  | PEAKS DB |
| total 2 peptides |  |  |  |  |  |  |  |  |  |  |  |  |  |  |  |  |  |  |  |  |  |  |  |  |  |  |  |  |  |  |  |  |  |  |  |  |  |
| --- | --- | --- | --- | --- | --- | --- | --- | --- | --- | --- | --- | --- | --- | --- | --- | --- | --- | --- | --- | --- | --- | --- | --- | --- | --- | --- | --- | --- | --- | --- | --- | --- | --- | --- | --- | --- | --- |

XP\_026551417.1

back to list

  

| Protein Coverage
| Supporting Peptides
|

Protein Coverage:

Supporting Peptides:

| Peptide | Uniq | -10lgP | Mass | Length | ppm | m/z | z | RT | Fraction | Scan | Source File | Area F4 | Area F5 | Area F6 | Area F10 | Area F8 | Area F9 | Area F7 | Area F1 | Area F2 | Area F3 | #Feature | #Feature F4 | #Feature F5 | #Feature F6 | #Feature F10 | #Feature F8 | #Feature F9 | #Feature F7 | #Feature F1 | #Feature F2 | #Feature F3 | Start | End | PTM | AScore | Found By |
| --- | --- | --- | --- | --- | --- | --- | --- | --- | --- | --- | --- | --- | --- | --- | --- | --- | --- | --- | --- | --- | --- | --- | --- | --- | --- | --- | --- | --- | --- | --- | --- | --- | --- | --- | --- | --- | --- |
| K.TQLEELEDELQATEDAK.L | Y | 55.38 | 1960.9113 | 17 | 2.1 | 981.4650 | 2 | 53.67 | 8 | F8:31140 | 31122021\_RID\_3262\_1a.raw |  |  |  |  |  |  |  | 1.3593E4 |  |  | 1 | 0 | 0 | 0 | 0 | 0 | 0 | 0 | 1 | 0 | 0 | 1560 | 1576 |  |  | PEAKS DB |
| R.IAEFTTNLTEEEEK.S | Y | 41.18 | 1652.7781 | 14 | 0.9 | 827.3970 | 2 | 39.35 | 8 | F8:19948 | 31122021\_RID\_3262\_1a.raw |  |  |  |  |  |  |  | 4.4528E4 |  |  | 1 | 0 | 0 | 0 | 0 | 0 | 0 | 0 | 1 | 0 | 0 | 1022 | 1035 |  |  | PEAKS DB |
| total 2 peptides |  |  |  |  |  |  |  |  |  |  |  |  |  |  |  |  |  |  |  |  |  |  |  |  |  |  |  |  |  |  |  |  |  |  |  |  |  |
| --- | --- | --- | --- | --- | --- | --- | --- | --- | --- | --- | --- | --- | --- | --- | --- | --- | --- | --- | --- | --- | --- | --- | --- | --- | --- | --- | --- | --- | --- | --- | --- | --- | --- | --- | --- | --- | --- |

BAN82211.1

back to list

  

| Protein Coverage
| Supporting Peptides
|

Protein Coverage:

Supporting Peptides:

| Peptide | Uniq | -10lgP | Mass | Length | ppm | m/z | z | RT | Fraction | Scan | Source File | Area F4 | Area F5 | Area F6 | Area F10 | Area F8 | Area F9 | Area F7 | Area F1 | Area F2 | Area F3 | #Feature | #Feature F4 | #Feature F5 | #Feature F6 | #Feature F10 | #Feature F8 | #Feature F9 | #Feature F7 | #Feature F1 | #Feature F2 | #Feature F3 | Start | End | PTM | AScore | Found By |
| --- | --- | --- | --- | --- | --- | --- | --- | --- | --- | --- | --- | --- | --- | --- | --- | --- | --- | --- | --- | --- | --- | --- | --- | --- | --- | --- | --- | --- | --- | --- | --- | --- | --- | --- | --- | --- | --- |
| K.TQLEELEDELQATEDAK.L | Y | 55.38 | 1960.9113 | 17 | 2.1 | 981.4650 | 2 | 53.67 | 8 | F8:31140 | 31122021\_RID\_3262\_1a.raw |  |  |  |  |  |  |  | 1.3593E4 |  |  | 1 | 0 | 0 | 0 | 0 | 0 | 0 | 0 | 1 | 0 | 0 | 1539 | 1555 |  |  | PEAKS DB |
| R.IAEFTTNLTEEEEK.S | Y | 41.18 | 1652.7781 | 14 | 0.9 | 827.3970 | 2 | 39.35 | 8 | F8:19948 | 31122021\_RID\_3262\_1a.raw |  |  |  |  |  |  |  | 4.4528E4 |  |  | 1 | 0 | 0 | 0 | 0 | 0 | 0 | 0 | 1 | 0 | 0 | 1001 | 1014 |  |  | PEAKS DB |
| total 2 peptides |  |  |  |  |  |  |  |  |  |  |  |  |  |  |  |  |  |  |  |  |  |  |  |  |  |  |  |  |  |  |  |  |  |  |  |  |  |
| --- | --- | --- | --- | --- | --- | --- | --- | --- | --- | --- | --- | --- | --- | --- | --- | --- | --- | --- | --- | --- | --- | --- | --- | --- | --- | --- | --- | --- | --- | --- | --- | --- | --- | --- | --- | --- | --- |

JAI12437.1

back to list

  

| Protein Coverage
| Supporting Peptides
|

Protein Coverage:

Supporting Peptides:

| Peptide | Uniq | -10lgP | Mass | Length | ppm | m/z | z | RT | Fraction | Scan | Source File | Area F4 | Area F5 | Area F6 | Area F10 | Area F8 | Area F9 | Area F7 | Area F1 | Area F2 | Area F3 | #Feature | #Feature F4 | #Feature F5 | #Feature F6 | #Feature F10 | #Feature F8 | #Feature F9 | #Feature F7 | #Feature F1 | #Feature F2 | #Feature F3 | Start | End | PTM | AScore | Found By |
| --- | --- | --- | --- | --- | --- | --- | --- | --- | --- | --- | --- | --- | --- | --- | --- | --- | --- | --- | --- | --- | --- | --- | --- | --- | --- | --- | --- | --- | --- | --- | --- | --- | --- | --- | --- | --- | --- |
| K.TQLEELEDELQATEDAK.L | Y | 55.38 | 1960.9113 | 17 | 2.1 | 981.4650 | 2 | 53.67 | 8 | F8:31140 | 31122021\_RID\_3262\_1a.raw |  |  |  |  |  |  |  | 1.3593E4 |  |  | 1 | 0 | 0 | 0 | 0 | 0 | 0 | 0 | 1 | 0 | 0 | 1539 | 1555 |  |  | PEAKS DB |
| R.IAEFTTNLTEEEEK.S | Y | 41.18 | 1652.7781 | 14 | 0.9 | 827.3970 | 2 | 39.35 | 8 | F8:19948 | 31122021\_RID\_3262\_1a.raw |  |  |  |  |  |  |  | 4.4528E4 |  |  | 1 | 0 | 0 | 0 | 0 | 0 | 0 | 0 | 1 | 0 | 0 | 1001 | 1014 |  |  | PEAKS DB |
| total 2 peptides |  |  |  |  |  |  |  |  |  |  |  |  |  |  |  |  |  |  |  |  |  |  |  |  |  |  |  |  |  |  |  |  |  |  |  |  |  |
| --- | --- | --- | --- | --- | --- | --- | --- | --- | --- | --- | --- | --- | --- | --- | --- | --- | --- | --- | --- | --- | --- | --- | --- | --- | --- | --- | --- | --- | --- | --- | --- | --- | --- | --- | --- | --- | --- |

XP\_015687322.1

back to list

  

| Protein Coverage
| Supporting Peptides
|

Protein Coverage:

Supporting Peptides:

| Peptide | Uniq | -10lgP | Mass | Length | ppm | m/z | z | RT | Fraction | Scan | Source File | Area F4 | Area F5 | Area F6 | Area F10 | Area F8 | Area F9 | Area F7 | Area F1 | Area F2 | Area F3 | #Feature | #Feature F4 | #Feature F5 | #Feature F6 | #Feature F10 | #Feature F8 | #Feature F9 | #Feature F7 | #Feature F1 | #Feature F2 | #Feature F3 | Start | End | PTM | AScore | Found By |
| --- | --- | --- | --- | --- | --- | --- | --- | --- | --- | --- | --- | --- | --- | --- | --- | --- | --- | --- | --- | --- | --- | --- | --- | --- | --- | --- | --- | --- | --- | --- | --- | --- | --- | --- | --- | --- | --- |
| K.TQLEELEDELQATEDAK.L | Y | 55.38 | 1960.9113 | 17 | 2.1 | 981.4650 | 2 | 53.67 | 8 | F8:31140 | 31122021\_RID\_3262\_1a.raw |  |  |  |  |  |  |  | 1.3593E4 |  |  | 1 | 0 | 0 | 0 | 0 | 0 | 0 | 0 | 1 | 0 | 0 | 1539 | 1555 |  |  | PEAKS DB |
| R.IAEFTTNLTEEEEK.S | Y | 41.18 | 1652.7781 | 14 | 0.9 | 827.3970 | 2 | 39.35 | 8 | F8:19948 | 31122021\_RID\_3262\_1a.raw |  |  |  |  |  |  |  | 4.4528E4 |  |  | 1 | 0 | 0 | 0 | 0 | 0 | 0 | 0 | 1 | 0 | 0 | 1001 | 1014 |  |  | PEAKS DB |
| total 2 peptides |  |  |  |  |  |  |  |  |  |  |  |  |  |  |  |  |  |  |  |  |  |  |  |  |  |  |  |  |  |  |  |  |  |  |  |  |  |
| --- | --- | --- | --- | --- | --- | --- | --- | --- | --- | --- | --- | --- | --- | --- | --- | --- | --- | --- | --- | --- | --- | --- | --- | --- | --- | --- | --- | --- | --- | --- | --- | --- | --- | --- | --- | --- | --- |

JAV49917.1

back to list

  

| Protein Coverage
| Supporting Peptides
|

Protein Coverage:

Supporting Peptides:

| Peptide | Uniq | -10lgP | Mass | Length | ppm | m/z | z | RT | Fraction | Scan | Source File | Area F4 | Area F5 | Area F6 | Area F10 | Area F8 | Area F9 | Area F7 | Area F1 | Area F2 | Area F3 | #Feature | #Feature F4 | #Feature F5 | #Feature F6 | #Feature F10 | #Feature F8 | #Feature F9 | #Feature F7 | #Feature F1 | #Feature F2 | #Feature F3 | Start | End | PTM | AScore | Found By |
| --- | --- | --- | --- | --- | --- | --- | --- | --- | --- | --- | --- | --- | --- | --- | --- | --- | --- | --- | --- | --- | --- | --- | --- | --- | --- | --- | --- | --- | --- | --- | --- | --- | --- | --- | --- | --- | --- |
| K.TQLEELEDELQATEDAK.L | Y | 55.38 | 1960.9113 | 17 | 2.1 | 981.4650 | 2 | 53.67 | 8 | F8:31140 | 31122021\_RID\_3262\_1a.raw |  |  |  |  |  |  |  | 1.3593E4 |  |  | 1 | 0 | 0 | 0 | 0 | 0 | 0 | 0 | 1 | 0 | 0 | 1539 | 1555 |  |  | PEAKS DB |
| R.IAEFTTNLTEEEEK.S | Y | 41.18 | 1652.7781 | 14 | 0.9 | 827.3970 | 2 | 39.35 | 8 | F8:19948 | 31122021\_RID\_3262\_1a.raw |  |  |  |  |  |  |  | 4.4528E4 |  |  | 1 | 0 | 0 | 0 | 0 | 0 | 0 | 0 | 1 | 0 | 0 | 1001 | 1014 |  |  | PEAKS DB |
| total 2 peptides |  |  |  |  |  |  |  |  |  |  |  |  |  |  |  |  |  |  |  |  |  |  |  |  |  |  |  |  |  |  |  |  |  |  |  |  |  |
| --- | --- | --- | --- | --- | --- | --- | --- | --- | --- | --- | --- | --- | --- | --- | --- | --- | --- | --- | --- | --- | --- | --- | --- | --- | --- | --- | --- | --- | --- | --- | --- | --- | --- | --- | --- | --- | --- |

JAG46253.1

back to list

  

| Protein Coverage
| Supporting Peptides
|

Protein Coverage:

Supporting Peptides:

| Peptide | Uniq | -10lgP | Mass | Length | ppm | m/z | z | RT | Fraction | Scan | Source File | Area F4 | Area F5 | Area F6 | Area F10 | Area F8 | Area F9 | Area F7 | Area F1 | Area F2 | Area F3 | #Feature | #Feature F4 | #Feature F5 | #Feature F6 | #Feature F10 | #Feature F8 | #Feature F9 | #Feature F7 | #Feature F1 | #Feature F2 | #Feature F3 | Start | End | PTM | AScore | Found By |
| --- | --- | --- | --- | --- | --- | --- | --- | --- | --- | --- | --- | --- | --- | --- | --- | --- | --- | --- | --- | --- | --- | --- | --- | --- | --- | --- | --- | --- | --- | --- | --- | --- | --- | --- | --- | --- | --- |
| K.TQLEELEDELQATEDAK.L | Y | 55.38 | 1960.9113 | 17 | 2.1 | 981.4650 | 2 | 53.67 | 8 | F8:31140 | 31122021\_RID\_3262\_1a.raw |  |  |  |  |  |  |  | 1.3593E4 |  |  | 1 | 0 | 0 | 0 | 0 | 0 | 0 | 0 | 1 | 0 | 0 | 1539 | 1555 |  |  | PEAKS DB |
| R.IAEFTTNLTEEEEK.S | Y | 41.18 | 1652.7781 | 14 | 0.9 | 827.3970 | 2 | 39.35 | 8 | F8:19948 | 31122021\_RID\_3262\_1a.raw |  |  |  |  |  |  |  | 4.4528E4 |  |  | 1 | 0 | 0 | 0 | 0 | 0 | 0 | 0 | 1 | 0 | 0 | 1001 | 1014 |  |  | PEAKS DB |
| total 2 peptides |  |  |  |  |  |  |  |  |  |  |  |  |  |  |  |  |  |  |  |  |  |  |  |  |  |  |  |  |  |  |  |  |  |  |  |  |  |
| --- | --- | --- | --- | --- | --- | --- | --- | --- | --- | --- | --- | --- | --- | --- | --- | --- | --- | --- | --- | --- | --- | --- | --- | --- | --- | --- | --- | --- | --- | --- | --- | --- | --- | --- | --- | --- | --- |

XP\_015687313.1

back to list

  

| Protein Coverage
| Supporting Peptides
|

Protein Coverage:

Supporting Peptides:

| Peptide | Uniq | -10lgP | Mass | Length | ppm | m/z | z | RT | Fraction | Scan | Source File | Area F4 | Area F5 | Area F6 | Area F10 | Area F8 | Area F9 | Area F7 | Area F1 | Area F2 | Area F3 | #Feature | #Feature F4 | #Feature F5 | #Feature F6 | #Feature F10 | #Feature F8 | #Feature F9 | #Feature F7 | #Feature F1 | #Feature F2 | #Feature F3 | Start | End | PTM | AScore | Found By |
| --- | --- | --- | --- | --- | --- | --- | --- | --- | --- | --- | --- | --- | --- | --- | --- | --- | --- | --- | --- | --- | --- | --- | --- | --- | --- | --- | --- | --- | --- | --- | --- | --- | --- | --- | --- | --- | --- |
| K.TQLEELEDELQATEDAK.L | Y | 55.38 | 1960.9113 | 17 | 2.1 | 981.4650 | 2 | 53.67 | 8 | F8:31140 | 31122021\_RID\_3262\_1a.raw |  |  |  |  |  |  |  | 1.3593E4 |  |  | 1 | 0 | 0 | 0 | 0 | 0 | 0 | 0 | 1 | 0 | 0 | 1560 | 1576 |  |  | PEAKS DB |
| R.IAEFTTNLTEEEEK.S | Y | 41.18 | 1652.7781 | 14 | 0.9 | 827.3970 | 2 | 39.35 | 8 | F8:19948 | 31122021\_RID\_3262\_1a.raw |  |  |  |  |  |  |  | 4.4528E4 |  |  | 1 | 0 | 0 | 0 | 0 | 0 | 0 | 0 | 1 | 0 | 0 | 1022 | 1035 |  |  | PEAKS DB |
| total 2 peptides |  |  |  |  |  |  |  |  |  |  |  |  |  |  |  |  |  |  |  |  |  |  |  |  |  |  |  |  |  |  |  |  |  |  |  |  |  |
| --- | --- | --- | --- | --- | --- | --- | --- | --- | --- | --- | --- | --- | --- | --- | --- | --- | --- | --- | --- | --- | --- | --- | --- | --- | --- | --- | --- | --- | --- | --- | --- | --- | --- | --- | --- | --- | --- |

JAB53856.1

back to list

  

| Protein Coverage
| Supporting Peptides
|

Protein Coverage:

Supporting Peptides:

| Peptide | Uniq | -10lgP | Mass | Length | ppm | m/z | z | RT | Fraction | Scan | Source File | Area F4 | Area F5 | Area F6 | Area F10 | Area F8 | Area F9 | Area F7 | Area F1 | Area F2 | Area F3 | #Feature | #Feature F4 | #Feature F5 | #Feature F6 | #Feature F10 | #Feature F8 | #Feature F9 | #Feature F7 | #Feature F1 | #Feature F2 | #Feature F3 | Start | End | PTM | AScore | Found By |
| --- | --- | --- | --- | --- | --- | --- | --- | --- | --- | --- | --- | --- | --- | --- | --- | --- | --- | --- | --- | --- | --- | --- | --- | --- | --- | --- | --- | --- | --- | --- | --- | --- | --- | --- | --- | --- | --- |
| K.TQLEELEDELQATEDAK.L | Y | 55.38 | 1960.9113 | 17 | 2.1 | 981.4650 | 2 | 53.67 | 8 | F8:31140 | 31122021\_RID\_3262\_1a.raw |  |  |  |  |  |  |  | 1.3593E4 |  |  | 1 | 0 | 0 | 0 | 0 | 0 | 0 | 0 | 1 | 0 | 0 | 1539 | 1555 |  |  | PEAKS DB |
| R.IAEFTTNLTEEEEK.S | Y | 41.18 | 1652.7781 | 14 | 0.9 | 827.3970 | 2 | 39.35 | 8 | F8:19948 | 31122021\_RID\_3262\_1a.raw |  |  |  |  |  |  |  | 4.4528E4 |  |  | 1 | 0 | 0 | 0 | 0 | 0 | 0 | 0 | 1 | 0 | 0 | 1001 | 1014 |  |  | PEAKS DB |
| total 2 peptides |  |  |  |  |  |  |  |  |  |  |  |  |  |  |  |  |  |  |  |  |  |  |  |  |  |  |  |  |  |  |  |  |  |  |  |  |  |
| --- | --- | --- | --- | --- | --- | --- | --- | --- | --- | --- | --- | --- | --- | --- | --- | --- | --- | --- | --- | --- | --- | --- | --- | --- | --- | --- | --- | --- | --- | --- | --- | --- | --- | --- | --- | --- | --- |

LAB26995.1

back to list

  

| Protein Coverage
| Supporting Peptides
|

Protein Coverage:

Supporting Peptides:

| Peptide | Uniq | -10lgP | Mass | Length | ppm | m/z | z | RT | Fraction | Scan | Source File | Area F4 | Area F5 | Area F6 | Area F10 | Area F8 | Area F9 | Area F7 | Area F1 | Area F2 | Area F3 | #Feature | #Feature F4 | #Feature F5 | #Feature F6 | #Feature F10 | #Feature F8 | #Feature F9 | #Feature F7 | #Feature F1 | #Feature F2 | #Feature F3 | Start | End | PTM | AScore | Found By |
| --- | --- | --- | --- | --- | --- | --- | --- | --- | --- | --- | --- | --- | --- | --- | --- | --- | --- | --- | --- | --- | --- | --- | --- | --- | --- | --- | --- | --- | --- | --- | --- | --- | --- | --- | --- | --- | --- |
| K.TQLEELEDELQATEDAK.L | Y | 55.38 | 1960.9113 | 17 | 2.1 | 981.4650 | 2 | 53.67 | 8 | F8:31140 | 31122021\_RID\_3262\_1a.raw |  |  |  |  |  |  |  | 1.3593E4 |  |  | 1 | 0 | 0 | 0 | 0 | 0 | 0 | 0 | 1 | 0 | 0 | 1539 | 1555 |  |  | PEAKS DB |
| R.IAEFTTNLTEEEEK.S | Y | 41.18 | 1652.7781 | 14 | 0.9 | 827.3970 | 2 | 39.35 | 8 | F8:19948 | 31122021\_RID\_3262\_1a.raw |  |  |  |  |  |  |  | 4.4528E4 |  |  | 1 | 0 | 0 | 0 | 0 | 0 | 0 | 0 | 1 | 0 | 0 | 1001 | 1014 |  |  | PEAKS DB |
| total 2 peptides |  |  |  |  |  |  |  |  |  |  |  |  |  |  |  |  |  |  |  |  |  |  |  |  |  |  |  |  |  |  |  |  |  |  |  |  |  |
| --- | --- | --- | --- | --- | --- | --- | --- | --- | --- | --- | --- | --- | --- | --- | --- | --- | --- | --- | --- | --- | --- | --- | --- | --- | --- | --- | --- | --- | --- | --- | --- | --- | --- | --- | --- | --- | --- |

LAB26993.1

back to list

  

| Protein Coverage
| Supporting Peptides
|

Protein Coverage:

Supporting Peptides:

| Peptide | Uniq | -10lgP | Mass | Length | ppm | m/z | z | RT | Fraction | Scan | Source File | Area F4 | Area F5 | Area F6 | Area F10 | Area F8 | Area F9 | Area F7 | Area F1 | Area F2 | Area F3 | #Feature | #Feature F4 | #Feature F5 | #Feature F6 | #Feature F10 | #Feature F8 | #Feature F9 | #Feature F7 | #Feature F1 | #Feature F2 | #Feature F3 | Start | End | PTM | AScore | Found By |
| --- | --- | --- | --- | --- | --- | --- | --- | --- | --- | --- | --- | --- | --- | --- | --- | --- | --- | --- | --- | --- | --- | --- | --- | --- | --- | --- | --- | --- | --- | --- | --- | --- | --- | --- | --- | --- | --- |
| K.TQLEELEDELQATEDAK.L | Y | 55.38 | 1960.9113 | 17 | 2.1 | 981.4650 | 2 | 53.67 | 8 | F8:31140 | 31122021\_RID\_3262\_1a.raw |  |  |  |  |  |  |  | 1.3593E4 |  |  | 1 | 0 | 0 | 0 | 0 | 0 | 0 | 0 | 1 | 0 | 0 | 1539 | 1555 |  |  | PEAKS DB |
| R.IAEFTTNLTEEEEK.S | Y | 41.18 | 1652.7781 | 14 | 0.9 | 827.3970 | 2 | 39.35 | 8 | F8:19948 | 31122021\_RID\_3262\_1a.raw |  |  |  |  |  |  |  | 4.4528E4 |  |  | 1 | 0 | 0 | 0 | 0 | 0 | 0 | 0 | 1 | 0 | 0 | 1001 | 1014 |  |  | PEAKS DB |
| total 2 peptides |  |  |  |  |  |  |  |  |  |  |  |  |  |  |  |  |  |  |  |  |  |  |  |  |  |  |  |  |  |  |  |  |  |  |  |  |  |
| --- | --- | --- | --- | --- | --- | --- | --- | --- | --- | --- | --- | --- | --- | --- | --- | --- | --- | --- | --- | --- | --- | --- | --- | --- | --- | --- | --- | --- | --- | --- | --- | --- | --- | --- | --- | --- | --- |

LAA85344.1

back to list

  

| Protein Coverage
| Supporting Peptides
|

Protein Coverage:

Supporting Peptides:

| Peptide | Uniq | -10lgP | Mass | Length | ppm | m/z | z | RT | Fraction | Scan | Source File | Area F4 | Area F5 | Area F6 | Area F10 | Area F8 | Area F9 | Area F7 | Area F1 | Area F2 | Area F3 | #Feature | #Feature F4 | #Feature F5 | #Feature F6 | #Feature F10 | #Feature F8 | #Feature F9 | #Feature F7 | #Feature F1 | #Feature F2 | #Feature F3 | Start | End | PTM | AScore | Found By |
| --- | --- | --- | --- | --- | --- | --- | --- | --- | --- | --- | --- | --- | --- | --- | --- | --- | --- | --- | --- | --- | --- | --- | --- | --- | --- | --- | --- | --- | --- | --- | --- | --- | --- | --- | --- | --- | --- |
| K.TQLEELEDELQATEDAK.L | Y | 55.38 | 1960.9113 | 17 | 2.1 | 981.4650 | 2 | 53.67 | 8 | F8:31140 | 31122021\_RID\_3262\_1a.raw |  |  |  |  |  |  |  | 1.3593E4 |  |  | 1 | 0 | 0 | 0 | 0 | 0 | 0 | 0 | 1 | 0 | 0 | 1539 | 1555 |  |  | PEAKS DB |
| R.IAEFTTNLTEEEEK.S | Y | 41.18 | 1652.7781 | 14 | 0.9 | 827.3970 | 2 | 39.35 | 8 | F8:19948 | 31122021\_RID\_3262\_1a.raw |  |  |  |  |  |  |  | 4.4528E4 |  |  | 1 | 0 | 0 | 0 | 0 | 0 | 0 | 0 | 1 | 0 | 0 | 1001 | 1014 |  |  | PEAKS DB |
| total 2 peptides |  |  |  |  |  |  |  |  |  |  |  |  |  |  |  |  |  |  |  |  |  |  |  |  |  |  |  |  |  |  |  |  |  |  |  |  |  |
| --- | --- | --- | --- | --- | --- | --- | --- | --- | --- | --- | --- | --- | --- | --- | --- | --- | --- | --- | --- | --- | --- | --- | --- | --- | --- | --- | --- | --- | --- | --- | --- | --- | --- | --- | --- | --- | --- |

XP\_026550725.1

back to list

  

| Protein Coverage
| Supporting Peptides
|

Protein Coverage:

Supporting Peptides:

| Peptide | Uniq | -10lgP | Mass | Length | ppm | m/z | z | RT | Fraction | Scan | Source File | Area F4 | Area F5 | Area F6 | Area F10 | Area F8 | Area F9 | Area F7 | Area F1 | Area F2 | Area F3 | #Feature | #Feature F4 | #Feature F5 | #Feature F6 | #Feature F10 | #Feature F8 | #Feature F9 | #Feature F7 | #Feature F1 | #Feature F2 | #Feature F3 | Start | End | PTM | AScore | Found By |
| --- | --- | --- | --- | --- | --- | --- | --- | --- | --- | --- | --- | --- | --- | --- | --- | --- | --- | --- | --- | --- | --- | --- | --- | --- | --- | --- | --- | --- | --- | --- | --- | --- | --- | --- | --- | --- | --- |
| K.TQLEELEDELQATEDAK.L | Y | 55.38 | 1960.9113 | 17 | 2.1 | 981.4650 | 2 | 53.67 | 8 | F8:31140 | 31122021\_RID\_3262\_1a.raw |  |  |  |  |  |  |  | 1.3593E4 |  |  | 1 | 0 | 0 | 0 | 0 | 0 | 0 | 0 | 1 | 0 | 0 | 1539 | 1555 |  |  | PEAKS DB |
| R.IAEFTTNLTEEEEK.S | Y | 41.18 | 1652.7781 | 14 | 0.9 | 827.3970 | 2 | 39.35 | 8 | F8:19948 | 31122021\_RID\_3262\_1a.raw |  |  |  |  |  |  |  | 4.4528E4 |  |  | 1 | 0 | 0 | 0 | 0 | 0 | 0 | 0 | 1 | 0 | 0 | 1001 | 1014 |  |  | PEAKS DB |
| total 2 peptides |  |  |  |  |  |  |  |  |  |  |  |  |  |  |  |  |  |  |  |  |  |  |  |  |  |  |  |  |  |  |  |  |  |  |  |  |  |
| --- | --- | --- | --- | --- | --- | --- | --- | --- | --- | --- | --- | --- | --- | --- | --- | --- | --- | --- | --- | --- | --- | --- | --- | --- | --- | --- | --- | --- | --- | --- | --- | --- | --- | --- | --- | --- | --- |

XP\_026550724.1

back to list

  

| Protein Coverage
| Supporting Peptides
|

Protein Coverage:

Supporting Peptides:

| Peptide | Uniq | -10lgP | Mass | Length | ppm | m/z | z | RT | Fraction | Scan | Source File | Area F4 | Area F5 | Area F6 | Area F10 | Area F8 | Area F9 | Area F7 | Area F1 | Area F2 | Area F3 | #Feature | #Feature F4 | #Feature F5 | #Feature F6 | #Feature F10 | #Feature F8 | #Feature F9 | #Feature F7 | #Feature F1 | #Feature F2 | #Feature F3 | Start | End | PTM | AScore | Found By |
| --- | --- | --- | --- | --- | --- | --- | --- | --- | --- | --- | --- | --- | --- | --- | --- | --- | --- | --- | --- | --- | --- | --- | --- | --- | --- | --- | --- | --- | --- | --- | --- | --- | --- | --- | --- | --- | --- |
| K.TQLEELEDELQATEDAK.L | Y | 55.38 | 1960.9113 | 17 | 2.1 | 981.4650 | 2 | 53.67 | 8 | F8:31140 | 31122021\_RID\_3262\_1a.raw |  |  |  |  |  |  |  | 1.3593E4 |  |  | 1 | 0 | 0 | 0 | 0 | 0 | 0 | 0 | 1 | 0 | 0 | 1560 | 1576 |  |  | PEAKS DB |
| R.IAEFTTNLTEEEEK.S | Y | 41.18 | 1652.7781 | 14 | 0.9 | 827.3970 | 2 | 39.35 | 8 | F8:19948 | 31122021\_RID\_3262\_1a.raw |  |  |  |  |  |  |  | 4.4528E4 |  |  | 1 | 0 | 0 | 0 | 0 | 0 | 0 | 0 | 1 | 0 | 0 | 1022 | 1035 |  |  | PEAKS DB |
| total 2 peptides |  |  |  |  |  |  |  |  |  |  |  |  |  |  |  |  |  |  |  |  |  |  |  |  |  |  |  |  |  |  |  |  |  |  |  |  |  |
| --- | --- | --- | --- | --- | --- | --- | --- | --- | --- | --- | --- | --- | --- | --- | --- | --- | --- | --- | --- | --- | --- | --- | --- | --- | --- | --- | --- | --- | --- | --- | --- | --- | --- | --- | --- | --- | --- |

LAB55089.1

back to list

  

| Protein Coverage
| Supporting Peptides
|

Protein Coverage:

Supporting Peptides:

| Peptide | Uniq | -10lgP | Mass | Length | ppm | m/z | z | RT | Fraction | Scan | Source File | Area F4 | Area F5 | Area F6 | Area F10 | Area F8 | Area F9 | Area F7 | Area F1 | Area F2 | Area F3 | #Feature | #Feature F4 | #Feature F5 | #Feature F6 | #Feature F10 | #Feature F8 | #Feature F9 | #Feature F7 | #Feature F1 | #Feature F2 | #Feature F3 | Start | End | PTM | AScore | Found By |
| --- | --- | --- | --- | --- | --- | --- | --- | --- | --- | --- | --- | --- | --- | --- | --- | --- | --- | --- | --- | --- | --- | --- | --- | --- | --- | --- | --- | --- | --- | --- | --- | --- | --- | --- | --- | --- | --- |
| K.TQLEELEDELQATEDAK.L | Y | 55.38 | 1960.9113 | 17 | 2.1 | 981.4650 | 2 | 53.67 | 8 | F8:31140 | 31122021\_RID\_3262\_1a.raw |  |  |  |  |  |  |  | 1.3593E4 |  |  | 1 | 0 | 0 | 0 | 0 | 0 | 0 | 0 | 1 | 0 | 0 | 896 | 912 |  |  | PEAKS DB |
| R.IAEFTTNLTEEEEK.S | Y | 41.18 | 1652.7781 | 14 | 0.9 | 827.3970 | 2 | 39.35 | 8 | F8:19948 | 31122021\_RID\_3262\_1a.raw |  |  |  |  |  |  |  | 4.4528E4 |  |  | 1 | 0 | 0 | 0 | 0 | 0 | 0 | 0 | 1 | 0 | 0 | 358 | 371 |  |  | PEAKS DB |
| total 2 peptides |  |  |  |  |  |  |  |  |  |  |  |  |  |  |  |  |  |  |  |  |  |  |  |  |  |  |  |  |  |  |  |  |  |  |  |  |  |
| --- | --- | --- | --- | --- | --- | --- | --- | --- | --- | --- | --- | --- | --- | --- | --- | --- | --- | --- | --- | --- | --- | --- | --- | --- | --- | --- | --- | --- | --- | --- | --- | --- | --- | --- | --- | --- | --- |

XP\_007430787.1

back to list

  

| Protein Coverage
| Supporting Peptides
|

Protein Coverage:

Supporting Peptides:

| Peptide | Uniq | -10lgP | Mass | Length | ppm | m/z | z | RT | Fraction | Scan | Source File | Area F4 | Area F5 | Area F6 | Area F10 | Area F8 | Area F9 | Area F7 | Area F1 | Area F2 | Area F3 | #Feature | #Feature F4 | #Feature F5 | #Feature F6 | #Feature F10 | #Feature F8 | #Feature F9 | #Feature F7 | #Feature F1 | #Feature F2 | #Feature F3 | Start | End | PTM | AScore | Found By |
| --- | --- | --- | --- | --- | --- | --- | --- | --- | --- | --- | --- | --- | --- | --- | --- | --- | --- | --- | --- | --- | --- | --- | --- | --- | --- | --- | --- | --- | --- | --- | --- | --- | --- | --- | --- | --- | --- |
| K.TQLEELEDELQATEDAK.L | Y | 55.38 | 1960.9113 | 17 | 2.1 | 981.4650 | 2 | 53.67 | 8 | F8:31140 | 31122021\_RID\_3262\_1a.raw |  |  |  |  |  |  |  | 1.3593E4 |  |  | 1 | 0 | 0 | 0 | 0 | 0 | 0 | 0 | 1 | 0 | 0 | 1539 | 1555 |  |  | PEAKS DB |
| R.IAEFTTNLTEEEEK.S | Y | 41.18 | 1652.7781 | 14 | 0.9 | 827.3970 | 2 | 39.35 | 8 | F8:19948 | 31122021\_RID\_3262\_1a.raw |  |  |  |  |  |  |  | 4.4528E4 |  |  | 1 | 0 | 0 | 0 | 0 | 0 | 0 | 0 | 1 | 0 | 0 | 1001 | 1014 |  |  | PEAKS DB |
| total 2 peptides |  |  |  |  |  |  |  |  |  |  |  |  |  |  |  |  |  |  |  |  |  |  |  |  |  |  |  |  |  |  |  |  |  |  |  |  |  |
| --- | --- | --- | --- | --- | --- | --- | --- | --- | --- | --- | --- | --- | --- | --- | --- | --- | --- | --- | --- | --- | --- | --- | --- | --- | --- | --- | --- | --- | --- | --- | --- | --- | --- | --- | --- | --- | --- |

XP\_015744014.1

back to list

  

| Protein Coverage
| Supporting Peptides
|

Protein Coverage:

Supporting Peptides:

| Peptide | Uniq | -10lgP | Mass | Length | ppm | m/z | z | RT | Fraction | Scan | Source File | Area F4 | Area F5 | Area F6 | Area F10 | Area F8 | Area F9 | Area F7 | Area F1 | Area F2 | Area F3 | #Feature | #Feature F4 | #Feature F5 | #Feature F6 | #Feature F10 | #Feature F8 | #Feature F9 | #Feature F7 | #Feature F1 | #Feature F2 | #Feature F3 | Start | End | PTM | AScore | Found By |
| --- | --- | --- | --- | --- | --- | --- | --- | --- | --- | --- | --- | --- | --- | --- | --- | --- | --- | --- | --- | --- | --- | --- | --- | --- | --- | --- | --- | --- | --- | --- | --- | --- | --- | --- | --- | --- | --- |
| K.TQLEELEDELQATEDAK.L | Y | 55.38 | 1960.9113 | 17 | 2.1 | 981.4650 | 2 | 53.67 | 8 | F8:31140 | 31122021\_RID\_3262\_1a.raw |  |  |  |  |  |  |  | 1.3593E4 |  |  | 1 | 0 | 0 | 0 | 0 | 0 | 0 | 0 | 1 | 0 | 0 | 1539 | 1555 |  |  | PEAKS DB |
| R.IAEFTTNLTEEEEK.S | Y | 41.18 | 1652.7781 | 14 | 0.9 | 827.3970 | 2 | 39.35 | 8 | F8:19948 | 31122021\_RID\_3262\_1a.raw |  |  |  |  |  |  |  | 4.4528E4 |  |  | 1 | 0 | 0 | 0 | 0 | 0 | 0 | 0 | 1 | 0 | 0 | 1001 | 1014 |  |  | PEAKS DB |
| total 2 peptides |  |  |  |  |  |  |  |  |  |  |  |  |  |  |  |  |  |  |  |  |  |  |  |  |  |  |  |  |  |  |  |  |  |  |  |  |  |
| --- | --- | --- | --- | --- | --- | --- | --- | --- | --- | --- | --- | --- | --- | --- | --- | --- | --- | --- | --- | --- | --- | --- | --- | --- | --- | --- | --- | --- | --- | --- | --- | --- | --- | --- | --- | --- | --- |

XP\_015744015.1

back to list

  

| Protein Coverage
| Supporting Peptides
|

Protein Coverage:

Supporting Peptides:

| Peptide | Uniq | -10lgP | Mass | Length | ppm | m/z | z | RT | Fraction | Scan | Source File | Area F4 | Area F5 | Area F6 | Area F10 | Area F8 | Area F9 | Area F7 | Area F1 | Area F2 | Area F3 | #Feature | #Feature F4 | #Feature F5 | #Feature F6 | #Feature F10 | #Feature F8 | #Feature F9 | #Feature F7 | #Feature F1 | #Feature F2 | #Feature F3 | Start | End | PTM | AScore | Found By |
| --- | --- | --- | --- | --- | --- | --- | --- | --- | --- | --- | --- | --- | --- | --- | --- | --- | --- | --- | --- | --- | --- | --- | --- | --- | --- | --- | --- | --- | --- | --- | --- | --- | --- | --- | --- | --- | --- |
| K.TQLEELEDELQATEDAK.L | Y | 55.38 | 1960.9113 | 17 | 2.1 | 981.4650 | 2 | 53.67 | 8 | F8:31140 | 31122021\_RID\_3262\_1a.raw |  |  |  |  |  |  |  | 1.3593E4 |  |  | 1 | 0 | 0 | 0 | 0 | 0 | 0 | 0 | 1 | 0 | 0 | 1539 | 1555 |  |  | PEAKS DB |
| R.IAEFTTNLTEEEEK.S | Y | 41.18 | 1652.7781 | 14 | 0.9 | 827.3970 | 2 | 39.35 | 8 | F8:19948 | 31122021\_RID\_3262\_1a.raw |  |  |  |  |  |  |  | 4.4528E4 |  |  | 1 | 0 | 0 | 0 | 0 | 0 | 0 | 0 | 1 | 0 | 0 | 1001 | 1014 |  |  | PEAKS DB |
| total 2 peptides |  |  |  |  |  |  |  |  |  |  |  |  |  |  |  |  |  |  |  |  |  |  |  |  |  |  |  |  |  |  |  |  |  |  |  |  |  |
| --- | --- | --- | --- | --- | --- | --- | --- | --- | --- | --- | --- | --- | --- | --- | --- | --- | --- | --- | --- | --- | --- | --- | --- | --- | --- | --- | --- | --- | --- | --- | --- | --- | --- | --- | --- | --- | --- |

ETE73299.1

back to list

  

| Protein Coverage
| Supporting Peptides
|

Protein Coverage:

Supporting Peptides:

| Peptide | Uniq | -10lgP | Mass | Length | ppm | m/z | z | RT | Fraction | Scan | Source File | Area F4 | Area F5 | Area F6 | Area F10 | Area F8 | Area F9 | Area F7 | Area F1 | Area F2 | Area F3 | #Feature | #Feature F4 | #Feature F5 | #Feature F6 | #Feature F10 | #Feature F8 | #Feature F9 | #Feature F7 | #Feature F1 | #Feature F2 | #Feature F3 | Start | End | PTM | AScore | Found By |
| --- | --- | --- | --- | --- | --- | --- | --- | --- | --- | --- | --- | --- | --- | --- | --- | --- | --- | --- | --- | --- | --- | --- | --- | --- | --- | --- | --- | --- | --- | --- | --- | --- | --- | --- | --- | --- | --- |
| K.TQLEELEDELQATEDAK.L | Y | 55.38 | 1960.9113 | 17 | 2.1 | 981.4650 | 2 | 53.67 | 8 | F8:31140 | 31122021\_RID\_3262\_1a.raw |  |  |  |  |  |  |  | 1.3593E4 |  |  | 1 | 0 | 0 | 0 | 0 | 0 | 0 | 0 | 1 | 0 | 0 | 1487 | 1503 |  |  | PEAKS DB |
| R.IAEFTTNLTEEEEK.S | Y | 41.18 | 1652.7781 | 14 | 0.9 | 827.3970 | 2 | 39.35 | 8 | F8:19948 | 31122021\_RID\_3262\_1a.raw |  |  |  |  |  |  |  | 4.4528E4 |  |  | 1 | 0 | 0 | 0 | 0 | 0 | 0 | 0 | 1 | 0 | 0 | 949 | 962 |  |  | PEAKS DB |
| total 2 peptides |  |  |  |  |  |  |  |  |  |  |  |  |  |  |  |  |  |  |  |  |  |  |  |  |  |  |  |  |  |  |  |  |  |  |  |  |  |
| --- | --- | --- | --- | --- | --- | --- | --- | --- | --- | --- | --- | --- | --- | --- | --- | --- | --- | --- | --- | --- | --- | --- | --- | --- | --- | --- | --- | --- | --- | --- | --- | --- | --- | --- | --- | --- | --- |

XP\_034268135.1

back to list

  

| Protein Coverage
| Supporting Peptides
|

Protein Coverage:

Supporting Peptides:

| Peptide | Uniq | -10lgP | Mass | Length | ppm | m/z | z | RT | Fraction | Scan | Source File | Area F4 | Area F5 | Area F6 | Area F10 | Area F8 | Area F9 | Area F7 | Area F1 | Area F2 | Area F3 | #Feature | #Feature F4 | #Feature F5 | #Feature F6 | #Feature F10 | #Feature F8 | #Feature F9 | #Feature F7 | #Feature F1 | #Feature F2 | #Feature F3 | Start | End | PTM | AScore | Found By |
| --- | --- | --- | --- | --- | --- | --- | --- | --- | --- | --- | --- | --- | --- | --- | --- | --- | --- | --- | --- | --- | --- | --- | --- | --- | --- | --- | --- | --- | --- | --- | --- | --- | --- | --- | --- | --- | --- |
| K.EYQELMNTK.L | Y | 50.56 | 1154.5277 | 9 | -0.1 | 578.2711 | 2 | 28.79 | 8 | F8:11590 | 31122021\_RID\_3262\_1a.raw |  |  | 5.9763E5 |  |  |  |  | 5.3046E6 | 3.1299E6 |  | 3 | 0 | 0 | 1 | 0 | 0 | 0 | 0 | 1 | 1 | 0 | 380 | 388 |  |  | PEAKS DB |
| K.EYQELM(+15.99)NTK.L | Y | 45.96 | 1170.5227 | 9 | -0.4 | 586.2684 | 2 | 23.20 | 4 | F4:10461 | 17012022\_RID\_3262\_10a .raw |  |  |  | 4.2669E6 |  |  |  | 1.2002E6 |  |  | 2 | 0 | 0 | 0 | 1 | 0 | 0 | 0 | 1 | 0 | 0 | 380 | 388 | Oxidation (M) | M6:Oxidation (M):1000.00 | PEAKS DB |
| total 2 peptides |  |  |  |  |  |  |  |  |  |  |  |  |  |  |  |  |  |  |  |  |  |  |  |  |  |  |  |  |  |  |  |  |  |  |  |  |  |
| --- | --- | --- | --- | --- | --- | --- | --- | --- | --- | --- | --- | --- | --- | --- | --- | --- | --- | --- | --- | --- | --- | --- | --- | --- | --- | --- | --- | --- | --- | --- | --- | --- | --- | --- | --- | --- | --- |

XP\_013924292.1

back to list

  

| Protein Coverage
| Supporting Peptides
|

Protein Coverage:

Supporting Peptides:

| Peptide | Uniq | -10lgP | Mass | Length | ppm | m/z | z | RT | Fraction | Scan | Source File | Area F4 | Area F5 | Area F6 | Area F10 | Area F8 | Area F9 | Area F7 | Area F1 | Area F2 | Area F3 | #Feature | #Feature F4 | #Feature F5 | #Feature F6 | #Feature F10 | #Feature F8 | #Feature F9 | #Feature F7 | #Feature F1 | #Feature F2 | #Feature F3 | Start | End | PTM | AScore | Found By |
| --- | --- | --- | --- | --- | --- | --- | --- | --- | --- | --- | --- | --- | --- | --- | --- | --- | --- | --- | --- | --- | --- | --- | --- | --- | --- | --- | --- | --- | --- | --- | --- | --- | --- | --- | --- | --- | --- |
| K.EYQELMNTK.L | Y | 50.56 | 1154.5277 | 9 | -0.1 | 578.2711 | 2 | 28.79 | 8 | F8:11590 | 31122021\_RID\_3262\_1a.raw |  |  | 5.9763E5 |  |  |  |  | 5.3046E6 | 3.1299E6 |  | 3 | 0 | 0 | 1 | 0 | 0 | 0 | 0 | 1 | 1 | 0 | 380 | 388 |  |  | PEAKS DB |
| K.EYQELM(+15.99)NTK.L | Y | 45.96 | 1170.5227 | 9 | -0.4 | 586.2684 | 2 | 23.20 | 4 | F4:10461 | 17012022\_RID\_3262\_10a .raw |  |  |  | 4.2669E6 |  |  |  | 1.2002E6 |  |  | 2 | 0 | 0 | 0 | 1 | 0 | 0 | 0 | 1 | 0 | 0 | 380 | 388 | Oxidation (M) | M6:Oxidation (M):1000.00 | PEAKS DB |
| total 2 peptides |  |  |  |  |  |  |  |  |  |  |  |  |  |  |  |  |  |  |  |  |  |  |  |  |  |  |  |  |  |  |  |  |  |  |  |  |  |
| --- | --- | --- | --- | --- | --- | --- | --- | --- | --- | --- | --- | --- | --- | --- | --- | --- | --- | --- | --- | --- | --- | --- | --- | --- | --- | --- | --- | --- | --- | --- | --- | --- | --- | --- | --- | --- | --- |

XP\_032067334.1

back to list

  

| Protein Coverage
| Supporting Peptides
|

Protein Coverage:

Supporting Peptides:

| Peptide | Uniq | -10lgP | Mass | Length | ppm | m/z | z | RT | Fraction | Scan | Source File | Area F4 | Area F5 | Area F6 | Area F10 | Area F8 | Area F9 | Area F7 | Area F1 | Area F2 | Area F3 | #Feature | #Feature F4 | #Feature F5 | #Feature F6 | #Feature F10 | #Feature F8 | #Feature F9 | #Feature F7 | #Feature F1 | #Feature F2 | #Feature F3 | Start | End | PTM | AScore | Found By |
| --- | --- | --- | --- | --- | --- | --- | --- | --- | --- | --- | --- | --- | --- | --- | --- | --- | --- | --- | --- | --- | --- | --- | --- | --- | --- | --- | --- | --- | --- | --- | --- | --- | --- | --- | --- | --- | --- |
| K.EYQELMNTK.L | Y | 50.56 | 1154.5277 | 9 | -0.1 | 578.2711 | 2 | 28.79 | 8 | F8:11590 | 31122021\_RID\_3262\_1a.raw |  |  | 5.9763E5 |  |  |  |  | 5.3046E6 | 3.1299E6 |  | 3 | 0 | 0 | 1 | 0 | 0 | 0 | 0 | 1 | 1 | 0 | 336 | 344 |  |  | PEAKS DB |
| K.EYQELM(+15.99)NTK.L | Y | 45.96 | 1170.5227 | 9 | -0.4 | 586.2684 | 2 | 23.20 | 4 | F4:10461 | 17012022\_RID\_3262\_10a .raw |  |  |  | 4.2669E6 |  |  |  | 1.2002E6 |  |  | 2 | 0 | 0 | 0 | 1 | 0 | 0 | 0 | 1 | 0 | 0 | 336 | 344 | Oxidation (M) | M6:Oxidation (M):1000.00 | PEAKS DB |
| total 2 peptides |  |  |  |  |  |  |  |  |  |  |  |  |  |  |  |  |  |  |  |  |  |  |  |  |  |  |  |  |  |  |  |  |  |  |  |  |  |
| --- | --- | --- | --- | --- | --- | --- | --- | --- | --- | --- | --- | --- | --- | --- | --- | --- | --- | --- | --- | --- | --- | --- | --- | --- | --- | --- | --- | --- | --- | --- | --- | --- | --- | --- | --- | --- | --- |

XP\_026573192.1

back to list

  

| Protein Coverage
| Supporting Peptides
|

Protein Coverage:

Supporting Peptides:

| Peptide | Uniq | -10lgP | Mass | Length | ppm | m/z | z | RT | Fraction | Scan | Source File | Area F4 | Area F5 | Area F6 | Area F10 | Area F8 | Area F9 | Area F7 | Area F1 | Area F2 | Area F3 | #Feature | #Feature F4 | #Feature F5 | #Feature F6 | #Feature F10 | #Feature F8 | #Feature F9 | #Feature F7 | #Feature F1 | #Feature F2 | #Feature F3 | Start | End | PTM | AScore | Found By |
| --- | --- | --- | --- | --- | --- | --- | --- | --- | --- | --- | --- | --- | --- | --- | --- | --- | --- | --- | --- | --- | --- | --- | --- | --- | --- | --- | --- | --- | --- | --- | --- | --- | --- | --- | --- | --- | --- |
| K.EYQELMNTK.L | Y | 50.56 | 1154.5277 | 9 | -0.1 | 578.2711 | 2 | 28.79 | 8 | F8:11590 | 31122021\_RID\_3262\_1a.raw |  |  | 5.9763E5 |  |  |  |  | 5.3046E6 | 3.1299E6 |  | 3 | 0 | 0 | 1 | 0 | 0 | 0 | 0 | 1 | 1 | 0 | 379 | 387 |  |  | PEAKS DB |
| K.EYQELM(+15.99)NTK.L | Y | 45.96 | 1170.5227 | 9 | -0.4 | 586.2684 | 2 | 23.20 | 4 | F4:10461 | 17012022\_RID\_3262\_10a .raw |  |  |  | 4.2669E6 |  |  |  | 1.2002E6 |  |  | 2 | 0 | 0 | 0 | 1 | 0 | 0 | 0 | 1 | 0 | 0 | 379 | 387 | Oxidation (M) | M6:Oxidation (M):1000.00 | PEAKS DB |
| total 2 peptides |  |  |  |  |  |  |  |  |  |  |  |  |  |  |  |  |  |  |  |  |  |  |  |  |  |  |  |  |  |  |  |  |  |  |  |  |  |
| --- | --- | --- | --- | --- | --- | --- | --- | --- | --- | --- | --- | --- | --- | --- | --- | --- | --- | --- | --- | --- | --- | --- | --- | --- | --- | --- | --- | --- | --- | --- | --- | --- | --- | --- | --- | --- | --- |

XP\_026535749.1

back to list

  

| Protein Coverage
| Supporting Peptides
|

Protein Coverage:

Supporting Peptides:

| Peptide | Uniq | -10lgP | Mass | Length | ppm | m/z | z | RT | Fraction | Scan | Source File | Area F4 | Area F5 | Area F6 | Area F10 | Area F8 | Area F9 | Area F7 | Area F1 | Area F2 | Area F3 | #Feature | #Feature F4 | #Feature F5 | #Feature F6 | #Feature F10 | #Feature F8 | #Feature F9 | #Feature F7 | #Feature F1 | #Feature F2 | #Feature F3 | Start | End | PTM | AScore | Found By |
| --- | --- | --- | --- | --- | --- | --- | --- | --- | --- | --- | --- | --- | --- | --- | --- | --- | --- | --- | --- | --- | --- | --- | --- | --- | --- | --- | --- | --- | --- | --- | --- | --- | --- | --- | --- | --- | --- |
| K.EYQELMNTK.L | Y | 50.56 | 1154.5277 | 9 | -0.1 | 578.2711 | 2 | 28.79 | 8 | F8:11590 | 31122021\_RID\_3262\_1a.raw |  |  | 5.9763E5 |  |  |  |  | 5.3046E6 | 3.1299E6 |  | 3 | 0 | 0 | 1 | 0 | 0 | 0 | 0 | 1 | 1 | 0 | 379 | 387 |  |  | PEAKS DB |
| K.EYQELM(+15.99)NTK.L | Y | 45.96 | 1170.5227 | 9 | -0.4 | 586.2684 | 2 | 23.20 | 4 | F4:10461 | 17012022\_RID\_3262\_10a .raw |  |  |  | 4.2669E6 |  |  |  | 1.2002E6 |  |  | 2 | 0 | 0 | 0 | 1 | 0 | 0 | 0 | 1 | 0 | 0 | 379 | 387 | Oxidation (M) | M6:Oxidation (M):1000.00 | PEAKS DB |
| total 2 peptides |  |  |  |  |  |  |  |  |  |  |  |  |  |  |  |  |  |  |  |  |  |  |  |  |  |  |  |  |  |  |  |  |  |  |  |  |  |
| --- | --- | --- | --- | --- | --- | --- | --- | --- | --- | --- | --- | --- | --- | --- | --- | --- | --- | --- | --- | --- | --- | --- | --- | --- | --- | --- | --- | --- | --- | --- | --- | --- | --- | --- | --- | --- | --- |

ETE71916.1

back to list

  

| Protein Coverage
| Supporting Peptides
|

Protein Coverage:

Supporting Peptides:

| Peptide | Uniq | -10lgP | Mass | Length | ppm | m/z | z | RT | Fraction | Scan | Source File | Area F4 | Area F5 | Area F6 | Area F10 | Area F8 | Area F9 | Area F7 | Area F1 | Area F2 | Area F3 | #Feature | #Feature F4 | #Feature F5 | #Feature F6 | #Feature F10 | #Feature F8 | #Feature F9 | #Feature F7 | #Feature F1 | #Feature F2 | #Feature F3 | Start | End | PTM | AScore | Found By |
| --- | --- | --- | --- | --- | --- | --- | --- | --- | --- | --- | --- | --- | --- | --- | --- | --- | --- | --- | --- | --- | --- | --- | --- | --- | --- | --- | --- | --- | --- | --- | --- | --- | --- | --- | --- | --- | --- |
| K.EYQELMNTK.L | Y | 50.56 | 1154.5277 | 9 | -0.1 | 578.2711 | 2 | 28.79 | 8 | F8:11590 | 31122021\_RID\_3262\_1a.raw |  |  | 5.9763E5 |  |  |  |  | 5.3046E6 | 3.1299E6 |  | 3 | 0 | 0 | 1 | 0 | 0 | 0 | 0 | 1 | 1 | 0 | 68 | 76 |  |  | PEAKS DB |
| K.EYQELM(+15.99)NTK.L | Y | 45.96 | 1170.5227 | 9 | -0.4 | 586.2684 | 2 | 23.20 | 4 | F4:10461 | 17012022\_RID\_3262\_10a .raw |  |  |  | 4.2669E6 |  |  |  | 1.2002E6 |  |  | 2 | 0 | 0 | 0 | 1 | 0 | 0 | 0 | 1 | 0 | 0 | 68 | 76 | Oxidation (M) | M6:Oxidation (M):1000.00 | PEAKS DB |
| total 2 peptides |  |  |  |  |  |  |  |  |  |  |  |  |  |  |  |  |  |  |  |  |  |  |  |  |  |  |  |  |  |  |  |  |  |  |  |  |  |
| --- | --- | --- | --- | --- | --- | --- | --- | --- | --- | --- | --- | --- | --- | --- | --- | --- | --- | --- | --- | --- | --- | --- | --- | --- | --- | --- | --- | --- | --- | --- | --- | --- | --- | --- | --- | --- | --- |

XP\_029142905.1

back to list

  

| Protein Coverage
| Supporting Peptides
|

Protein Coverage:

Supporting Peptides:

| Peptide | Uniq | -10lgP | Mass | Length | ppm | m/z | z | RT | Fraction | Scan | Source File | Area F4 | Area F5 | Area F6 | Area F10 | Area F8 | Area F9 | Area F7 | Area F1 | Area F2 | Area F3 | #Feature | #Feature F4 | #Feature F5 | #Feature F6 | #Feature F10 | #Feature F8 | #Feature F9 | #Feature F7 | #Feature F1 | #Feature F2 | #Feature F3 | Start | End | PTM | AScore | Found By |
| --- | --- | --- | --- | --- | --- | --- | --- | --- | --- | --- | --- | --- | --- | --- | --- | --- | --- | --- | --- | --- | --- | --- | --- | --- | --- | --- | --- | --- | --- | --- | --- | --- | --- | --- | --- | --- | --- |
| K.EYQELMNTK.L | Y | 50.56 | 1154.5277 | 9 | -0.1 | 578.2711 | 2 | 28.79 | 8 | F8:11590 | 31122021\_RID\_3262\_1a.raw |  |  | 5.9763E5 |  |  |  |  | 5.3046E6 | 3.1299E6 |  | 3 | 0 | 0 | 1 | 0 | 0 | 0 | 0 | 1 | 1 | 0 | 176 | 184 |  |  | PEAKS DB |
| K.EYQELM(+15.99)NTK.L | Y | 45.96 | 1170.5227 | 9 | -0.4 | 586.2684 | 2 | 23.20 | 4 | F4:10461 | 17012022\_RID\_3262\_10a .raw |  |  |  | 4.2669E6 |  |  |  | 1.2002E6 |  |  | 2 | 0 | 0 | 0 | 1 | 0 | 0 | 0 | 1 | 0 | 0 | 176 | 184 | Oxidation (M) | M6:Oxidation (M):1000.00 | PEAKS DB |
| total 2 peptides |  |  |  |  |  |  |  |  |  |  |  |  |  |  |  |  |  |  |  |  |  |  |  |  |  |  |  |  |  |  |  |  |  |  |  |  |  |
| --- | --- | --- | --- | --- | --- | --- | --- | --- | --- | --- | --- | --- | --- | --- | --- | --- | --- | --- | --- | --- | --- | --- | --- | --- | --- | --- | --- | --- | --- | --- | --- | --- | --- | --- | --- | --- | --- |

JAS03130.1

back to list

  

| Protein Coverage
| Supporting Peptides
|

Protein Coverage:

Supporting Peptides:

| Peptide | Uniq | -10lgP | Mass | Length | ppm | m/z | z | RT | Fraction | Scan | Source File | Area F4 | Area F5 | Area F6 | Area F10 | Area F8 | Area F9 | Area F7 | Area F1 | Area F2 | Area F3 | #Feature | #Feature F4 | #Feature F5 | #Feature F6 | #Feature F10 | #Feature F8 | #Feature F9 | #Feature F7 | #Feature F1 | #Feature F2 | #Feature F3 | Start | End | PTM | AScore | Found By |
| --- | --- | --- | --- | --- | --- | --- | --- | --- | --- | --- | --- | --- | --- | --- | --- | --- | --- | --- | --- | --- | --- | --- | --- | --- | --- | --- | --- | --- | --- | --- | --- | --- | --- | --- | --- | --- | --- |
| Q.QGEVIPFMK.V | Y | 49.78 | 1047.5422 | 9 | -0.5 | 524.7781 | 2 | 44.15 | 4 | F4:28365 | 17012022\_RID\_3262\_10a .raw |  |  |  | 1.1042E6 |  |  |  |  |  |  | 1 | 0 | 0 | 0 | 1 | 0 | 0 | 0 | 0 | 0 | 0 | 37 | 45 |  |  | PEAKS DB |
| total 1 peptides |  |  |  |  |  |  |  |  |  |  |  |  |  |  |  |  |  |  |  |  |  |  |  |  |  |  |  |  |  |  |  |  |  |  |  |  |  |
| --- | --- | --- | --- | --- | --- | --- | --- | --- | --- | --- | --- | --- | --- | --- | --- | --- | --- | --- | --- | --- | --- | --- | --- | --- | --- | --- | --- | --- | --- | --- | --- | --- | --- | --- | --- | --- | --- |

JAV51434.1

back to list

  

| Protein Coverage
| Supporting Peptides
|

Protein Coverage:

Supporting Peptides:

| Peptide | Uniq | -10lgP | Mass | Length | ppm | m/z | z | RT | Fraction | Scan | Source File | Area F4 | Area F5 | Area F6 | Area F10 | Area F8 | Area F9 | Area F7 | Area F1 | Area F2 | Area F3 | #Feature | #Feature F4 | #Feature F5 | #Feature F6 | #Feature F10 | #Feature F8 | #Feature F9 | #Feature F7 | #Feature F1 | #Feature F2 | #Feature F3 | Start | End | PTM | AScore | Found By |
| --- | --- | --- | --- | --- | --- | --- | --- | --- | --- | --- | --- | --- | --- | --- | --- | --- | --- | --- | --- | --- | --- | --- | --- | --- | --- | --- | --- | --- | --- | --- | --- | --- | --- | --- | --- | --- | --- |
| A.SQLVVTPEQQR.Y | Y | 48.83 | 1283.6833 | 11 | 0.7 | 642.8494 | 2 | 28.01 | 9 | F9:11693 | 31122021\_RID\_3262\_2a.raw |  |  |  |  |  |  |  |  | 2.1629E5 |  | 1 | 0 | 0 | 0 | 0 | 0 | 0 | 0 | 0 | 1 | 0 | 184 | 194 |  |  | PEAKS DB |
| K.HSVGIVQDHGK.T | Y | 42.10 | 1175.6047 | 11 | 0.5 | 392.8757 | 3 | 15.41 | 4 | F4:3693 | 17012022\_RID\_3262\_10a .raw |  |  |  | 1.8544E5 |  |  |  |  |  |  | 1 | 0 | 0 | 0 | 1 | 0 | 0 | 0 | 0 | 0 | 0 | 315 | 325 |  |  | PEAKS DB |
| total 2 peptides |  |  |  |  |  |  |  |  |  |  |  |  |  |  |  |  |  |  |  |  |  |  |  |  |  |  |  |  |  |  |  |  |  |  |  |  |  |
| --- | --- | --- | --- | --- | --- | --- | --- | --- | --- | --- | --- | --- | --- | --- | --- | --- | --- | --- | --- | --- | --- | --- | --- | --- | --- | --- | --- | --- | --- | --- | --- | --- | --- | --- | --- | --- | --- |

JAS04446.1

back to list

  

| Protein Coverage
| Supporting Peptides
|

Protein Coverage:

Supporting Peptides:

| Peptide | Uniq | -10lgP | Mass | Length | ppm | m/z | z | RT | Fraction | Scan | Source File | Area F4 | Area F5 | Area F6 | Area F10 | Area F8 | Area F9 | Area F7 | Area F1 | Area F2 | Area F3 | #Feature | #Feature F4 | #Feature F5 | #Feature F6 | #Feature F10 | #Feature F8 | #Feature F9 | #Feature F7 | #Feature F1 | #Feature F2 | #Feature F3 | Start | End | PTM | AScore | Found By |
| --- | --- | --- | --- | --- | --- | --- | --- | --- | --- | --- | --- | --- | --- | --- | --- | --- | --- | --- | --- | --- | --- | --- | --- | --- | --- | --- | --- | --- | --- | --- | --- | --- | --- | --- | --- | --- | --- |
| A.SQLVVTPEQQR.Y | Y | 48.83 | 1283.6833 | 11 | 0.7 | 642.8494 | 2 | 28.01 | 9 | F9:11693 | 31122021\_RID\_3262\_2a.raw |  |  |  |  |  |  |  |  | 2.1629E5 |  | 1 | 0 | 0 | 0 | 0 | 0 | 0 | 0 | 0 | 1 | 0 | 184 | 194 |  |  | PEAKS DB |
| K.HSVGIVQDHGK.T | Y | 42.10 | 1175.6047 | 11 | 0.5 | 392.8757 | 3 | 15.41 | 4 | F4:3693 | 17012022\_RID\_3262\_10a .raw |  |  |  | 1.8544E5 |  |  |  |  |  |  | 1 | 0 | 0 | 0 | 1 | 0 | 0 | 0 | 0 | 0 | 0 | 315 | 325 |  |  | PEAKS DB |
| total 2 peptides |  |  |  |  |  |  |  |  |  |  |  |  |  |  |  |  |  |  |  |  |  |  |  |  |  |  |  |  |  |  |  |  |  |  |  |  |  |
| --- | --- | --- | --- | --- | --- | --- | --- | --- | --- | --- | --- | --- | --- | --- | --- | --- | --- | --- | --- | --- | --- | --- | --- | --- | --- | --- | --- | --- | --- | --- | --- | --- | --- | --- | --- | --- | --- |

AEJ31985.1

back to list

  

| Protein Coverage
| Supporting Peptides
|

Protein Coverage:

Supporting Peptides:

| Peptide | Uniq | -10lgP | Mass | Length | ppm | m/z | z | RT | Fraction | Scan | Source File | Area F4 | Area F5 | Area F6 | Area F10 | Area F8 | Area F9 | Area F7 | Area F1 | Area F2 | Area F3 | #Feature | #Feature F4 | #Feature F5 | #Feature F6 | #Feature F10 | #Feature F8 | #Feature F9 | #Feature F7 | #Feature F1 | #Feature F2 | #Feature F3 | Start | End | PTM | AScore | Found By |
| --- | --- | --- | --- | --- | --- | --- | --- | --- | --- | --- | --- | --- | --- | --- | --- | --- | --- | --- | --- | --- | --- | --- | --- | --- | --- | --- | --- | --- | --- | --- | --- | --- | --- | --- | --- | --- | --- |
| K.HSVGIVR.D | Y | 49.98 | 766.4449 | 7 | 0.7 | 384.2300 | 2 | 17.97 | 7 | F7:4065 | 21012022\_RID\_3262\_7a.raw |  |  |  |  |  |  | 3.442E6 |  |  |  | 1 | 0 | 0 | 0 | 0 | 0 | 0 | 1 | 0 | 0 | 0 | 313 | 319 |  |  | PEAKS DB |
| total 1 peptides |  |  |  |  |  |  |  |  |  |  |  |  |  |  |  |  |  |  |  |  |  |  |  |  |  |  |  |  |  |  |  |  |  |  |  |  |  |
| --- | --- | --- | --- | --- | --- | --- | --- | --- | --- | --- | --- | --- | --- | --- | --- | --- | --- | --- | --- | --- | --- | --- | --- | --- | --- | --- | --- | --- | --- | --- | --- | --- | --- | --- | --- | --- | --- |

AFJ49219.1

back to list

  

| Protein Coverage
| Supporting Peptides
|

Protein Coverage:

Supporting Peptides:

| Peptide | Uniq | -10lgP | Mass | Length | ppm | m/z | z | RT | Fraction | Scan | Source File | Area F4 | Area F5 | Area F6 | Area F10 | Area F8 | Area F9 | Area F7 | Area F1 | Area F2 | Area F3 | #Feature | #Feature F4 | #Feature F5 | #Feature F6 | #Feature F10 | #Feature F8 | #Feature F9 | #Feature F7 | #Feature F1 | #Feature F2 | #Feature F3 | Start | End | PTM | AScore | Found By |
| --- | --- | --- | --- | --- | --- | --- | --- | --- | --- | --- | --- | --- | --- | --- | --- | --- | --- | --- | --- | --- | --- | --- | --- | --- | --- | --- | --- | --- | --- | --- | --- | --- | --- | --- | --- | --- | --- |
| K.HSVGIVR.D | Y | 49.98 | 766.4449 | 7 | 0.7 | 384.2300 | 2 | 17.97 | 7 | F7:4065 | 21012022\_RID\_3262\_7a.raw |  |  |  |  |  |  | 3.442E6 |  |  |  | 1 | 0 | 0 | 0 | 0 | 0 | 0 | 1 | 0 | 0 | 0 | 313 | 319 |  |  | PEAKS DB |
| total 1 peptides |  |  |  |  |  |  |  |  |  |  |  |  |  |  |  |  |  |  |  |  |  |  |  |  |  |  |  |  |  |  |  |  |  |  |  |  |  |
| --- | --- | --- | --- | --- | --- | --- | --- | --- | --- | --- | --- | --- | --- | --- | --- | --- | --- | --- | --- | --- | --- | --- | --- | --- | --- | --- | --- | --- | --- | --- | --- | --- | --- | --- | --- | --- | --- |

JAS05303.1

back to list

  

| Protein Coverage
| Supporting Peptides
|

Protein Coverage:

Supporting Peptides:

| Peptide | Uniq | -10lgP | Mass | Length | ppm | m/z | z | RT | Fraction | Scan | Source File | Area F4 | Area F5 | Area F6 | Area F10 | Area F8 | Area F9 | Area F7 | Area F1 | Area F2 | Area F3 | #Feature | #Feature F4 | #Feature F5 | #Feature F6 | #Feature F10 | #Feature F8 | #Feature F9 | #Feature F7 | #Feature F1 | #Feature F2 | #Feature F3 | Start | End | PTM | AScore | Found By |
| --- | --- | --- | --- | --- | --- | --- | --- | --- | --- | --- | --- | --- | --- | --- | --- | --- | --- | --- | --- | --- | --- | --- | --- | --- | --- | --- | --- | --- | --- | --- | --- | --- | --- | --- | --- | --- | --- |
| K.HSVGIVR.D | Y | 49.98 | 766.4449 | 7 | 0.7 | 384.2300 | 2 | 17.97 | 7 | F7:4065 | 21012022\_RID\_3262\_7a.raw |  |  |  |  |  |  | 3.442E6 |  |  |  | 1 | 0 | 0 | 0 | 0 | 0 | 0 | 1 | 0 | 0 | 0 | 343 | 349 |  |  | PEAKS DB |
| total 1 peptides |  |  |  |  |  |  |  |  |  |  |  |  |  |  |  |  |  |  |  |  |  |  |  |  |  |  |  |  |  |  |  |  |  |  |  |  |  |
| --- | --- | --- | --- | --- | --- | --- | --- | --- | --- | --- | --- | --- | --- | --- | --- | --- | --- | --- | --- | --- | --- | --- | --- | --- | --- | --- | --- | --- | --- | --- | --- | --- | --- | --- | --- | --- | --- |

AFS49715.1

back to list

  

| Protein Coverage
| Supporting Peptides
|

Protein Coverage:

Supporting Peptides:

| Peptide | Uniq | -10lgP | Mass | Length | ppm | m/z | z | RT | Fraction | Scan | Source File | Area F4 | Area F5 | Area F6 | Area F10 | Area F8 | Area F9 | Area F7 | Area F1 | Area F2 | Area F3 | #Feature | #Feature F4 | #Feature F5 | #Feature F6 | #Feature F10 | #Feature F8 | #Feature F9 | #Feature F7 | #Feature F1 | #Feature F2 | #Feature F3 | Start | End | PTM | AScore | Found By |
| --- | --- | --- | --- | --- | --- | --- | --- | --- | --- | --- | --- | --- | --- | --- | --- | --- | --- | --- | --- | --- | --- | --- | --- | --- | --- | --- | --- | --- | --- | --- | --- | --- | --- | --- | --- | --- | --- |
| K.HSVGIVR.D | Y | 49.98 | 766.4449 | 7 | 0.7 | 384.2300 | 2 | 17.97 | 7 | F7:4065 | 21012022\_RID\_3262\_7a.raw |  |  |  |  |  |  | 3.442E6 |  |  |  | 1 | 0 | 0 | 0 | 0 | 0 | 0 | 1 | 0 | 0 | 0 | 313 | 319 |  |  | PEAKS DB |
| total 1 peptides |  |  |  |  |  |  |  |  |  |  |  |  |  |  |  |  |  |  |  |  |  |  |  |  |  |  |  |  |  |  |  |  |  |  |  |  |  |
| --- | --- | --- | --- | --- | --- | --- | --- | --- | --- | --- | --- | --- | --- | --- | --- | --- | --- | --- | --- | --- | --- | --- | --- | --- | --- | --- | --- | --- | --- | --- | --- | --- | --- | --- | --- | --- | --- |

AFJ49230.1

back to list

  

| Protein Coverage
| Supporting Peptides
|

Protein Coverage:

Supporting Peptides:

| Peptide | Uniq | -10lgP | Mass | Length | ppm | m/z | z | RT | Fraction | Scan | Source File | Area F4 | Area F5 | Area F6 | Area F10 | Area F8 | Area F9 | Area F7 | Area F1 | Area F2 | Area F3 | #Feature | #Feature F4 | #Feature F5 | #Feature F6 | #Feature F10 | #Feature F8 | #Feature F9 | #Feature F7 | #Feature F1 | #Feature F2 | #Feature F3 | Start | End | PTM | AScore | Found By |
| --- | --- | --- | --- | --- | --- | --- | --- | --- | --- | --- | --- | --- | --- | --- | --- | --- | --- | --- | --- | --- | --- | --- | --- | --- | --- | --- | --- | --- | --- | --- | --- | --- | --- | --- | --- | --- | --- |
| K.HSVGIVR.D | Y | 49.98 | 766.4449 | 7 | 0.7 | 384.2300 | 2 | 17.97 | 7 | F7:4065 | 21012022\_RID\_3262\_7a.raw |  |  |  |  |  |  | 3.442E6 |  |  |  | 1 | 0 | 0 | 0 | 0 | 0 | 0 | 1 | 0 | 0 | 0 | 313 | 319 |  |  | PEAKS DB |
| total 1 peptides |  |  |  |  |  |  |  |  |  |  |  |  |  |  |  |  |  |  |  |  |  |  |  |  |  |  |  |  |  |  |  |  |  |  |  |  |  |
| --- | --- | --- | --- | --- | --- | --- | --- | --- | --- | --- | --- | --- | --- | --- | --- | --- | --- | --- | --- | --- | --- | --- | --- | --- | --- | --- | --- | --- | --- | --- | --- | --- | --- | --- | --- | --- | --- |

JAI10398.1

back to list

  

| Protein Coverage
| Supporting Peptides
|

Protein Coverage:

Supporting Peptides:

| Peptide | Uniq | -10lgP | Mass | Length | ppm | m/z | z | RT | Fraction | Scan | Source File | Area F4 | Area F5 | Area F6 | Area F10 | Area F8 | Area F9 | Area F7 | Area F1 | Area F2 | Area F3 | #Feature | #Feature F4 | #Feature F5 | #Feature F6 | #Feature F10 | #Feature F8 | #Feature F9 | #Feature F7 | #Feature F1 | #Feature F2 | #Feature F3 | Start | End | PTM | AScore | Found By |
| --- | --- | --- | --- | --- | --- | --- | --- | --- | --- | --- | --- | --- | --- | --- | --- | --- | --- | --- | --- | --- | --- | --- | --- | --- | --- | --- | --- | --- | --- | --- | --- | --- | --- | --- | --- | --- | --- |
| K.HSVGIVR.D | Y | 49.98 | 766.4449 | 7 | 0.7 | 384.2300 | 2 | 17.97 | 7 | F7:4065 | 21012022\_RID\_3262\_7a.raw |  |  |  |  |  |  | 3.442E6 |  |  |  | 1 | 0 | 0 | 0 | 0 | 0 | 0 | 1 | 0 | 0 | 0 | 313 | 319 |  |  | PEAKS DB |
| total 1 peptides |  |  |  |  |  |  |  |  |  |  |  |  |  |  |  |  |  |  |  |  |  |  |  |  |  |  |  |  |  |  |  |  |  |  |  |  |  |
| --- | --- | --- | --- | --- | --- | --- | --- | --- | --- | --- | --- | --- | --- | --- | --- | --- | --- | --- | --- | --- | --- | --- | --- | --- | --- | --- | --- | --- | --- | --- | --- | --- | --- | --- | --- | --- | --- |

JAI10397.1

back to list

  

| Protein Coverage
| Supporting Peptides
|

Protein Coverage:

Supporting Peptides:

| Peptide | Uniq | -10lgP | Mass | Length | ppm | m/z | z | RT | Fraction | Scan | Source File | Area F4 | Area F5 | Area F6 | Area F10 | Area F8 | Area F9 | Area F7 | Area F1 | Area F2 | Area F3 | #Feature | #Feature F4 | #Feature F5 | #Feature F6 | #Feature F10 | #Feature F8 | #Feature F9 | #Feature F7 | #Feature F1 | #Feature F2 | #Feature F3 | Start | End | PTM | AScore | Found By |
| --- | --- | --- | --- | --- | --- | --- | --- | --- | --- | --- | --- | --- | --- | --- | --- | --- | --- | --- | --- | --- | --- | --- | --- | --- | --- | --- | --- | --- | --- | --- | --- | --- | --- | --- | --- | --- | --- |
| K.HSVGIVR.D | Y | 49.98 | 766.4449 | 7 | 0.7 | 384.2300 | 2 | 17.97 | 7 | F7:4065 | 21012022\_RID\_3262\_7a.raw |  |  |  |  |  |  | 3.442E6 |  |  |  | 1 | 0 | 0 | 0 | 0 | 0 | 0 | 1 | 0 | 0 | 0 | 313 | 319 |  |  | PEAKS DB |
| total 1 peptides |  |  |  |  |  |  |  |  |  |  |  |  |  |  |  |  |  |  |  |  |  |  |  |  |  |  |  |  |  |  |  |  |  |  |  |  |  |
| --- | --- | --- | --- | --- | --- | --- | --- | --- | --- | --- | --- | --- | --- | --- | --- | --- | --- | --- | --- | --- | --- | --- | --- | --- | --- | --- | --- | --- | --- | --- | --- | --- | --- | --- | --- | --- | --- |

JAI10396.1

back to list

  

| Protein Coverage
| Supporting Peptides
|

Protein Coverage:

Supporting Peptides:

| Peptide | Uniq | -10lgP | Mass | Length | ppm | m/z | z | RT | Fraction | Scan | Source File | Area F4 | Area F5 | Area F6 | Area F10 | Area F8 | Area F9 | Area F7 | Area F1 | Area F2 | Area F3 | #Feature | #Feature F4 | #Feature F5 | #Feature F6 | #Feature F10 | #Feature F8 | #Feature F9 | #Feature F7 | #Feature F1 | #Feature F2 | #Feature F3 | Start | End | PTM | AScore | Found By |
| --- | --- | --- | --- | --- | --- | --- | --- | --- | --- | --- | --- | --- | --- | --- | --- | --- | --- | --- | --- | --- | --- | --- | --- | --- | --- | --- | --- | --- | --- | --- | --- | --- | --- | --- | --- | --- | --- |
| K.HSVGIVR.D | Y | 49.98 | 766.4449 | 7 | 0.7 | 384.2300 | 2 | 17.97 | 7 | F7:4065 | 21012022\_RID\_3262\_7a.raw |  |  |  |  |  |  | 3.442E6 |  |  |  | 1 | 0 | 0 | 0 | 0 | 0 | 0 | 1 | 0 | 0 | 0 | 313 | 319 |  |  | PEAKS DB |
| total 1 peptides |  |  |  |  |  |  |  |  |  |  |  |  |  |  |  |  |  |  |  |  |  |  |  |  |  |  |  |  |  |  |  |  |  |  |  |  |  |
| --- | --- | --- | --- | --- | --- | --- | --- | --- | --- | --- | --- | --- | --- | --- | --- | --- | --- | --- | --- | --- | --- | --- | --- | --- | --- | --- | --- | --- | --- | --- | --- | --- | --- | --- | --- | --- | --- |

JAS04694.1

back to list

  

| Protein Coverage
| Supporting Peptides
|

Protein Coverage:

Supporting Peptides:

| Peptide | Uniq | -10lgP | Mass | Length | ppm | m/z | z | RT | Fraction | Scan | Source File | Area F4 | Area F5 | Area F6 | Area F10 | Area F8 | Area F9 | Area F7 | Area F1 | Area F2 | Area F3 | #Feature | #Feature F4 | #Feature F5 | #Feature F6 | #Feature F10 | #Feature F8 | #Feature F9 | #Feature F7 | #Feature F1 | #Feature F2 | #Feature F3 | Start | End | PTM | AScore | Found By |
| --- | --- | --- | --- | --- | --- | --- | --- | --- | --- | --- | --- | --- | --- | --- | --- | --- | --- | --- | --- | --- | --- | --- | --- | --- | --- | --- | --- | --- | --- | --- | --- | --- | --- | --- | --- | --- | --- |
| K.HSVGIVR.D | Y | 49.98 | 766.4449 | 7 | 0.7 | 384.2300 | 2 | 17.97 | 7 | F7:4065 | 21012022\_RID\_3262\_7a.raw |  |  |  |  |  |  | 3.442E6 |  |  |  | 1 | 0 | 0 | 0 | 0 | 0 | 0 | 1 | 0 | 0 | 0 | 313 | 319 |  |  | PEAKS DB |
| total 1 peptides |  |  |  |  |  |  |  |  |  |  |  |  |  |  |  |  |  |  |  |  |  |  |  |  |  |  |  |  |  |  |  |  |  |  |  |  |  |
| --- | --- | --- | --- | --- | --- | --- | --- | --- | --- | --- | --- | --- | --- | --- | --- | --- | --- | --- | --- | --- | --- | --- | --- | --- | --- | --- | --- | --- | --- | --- | --- | --- | --- | --- | --- | --- | --- |

JAI10395.1

back to list

  

| Protein Coverage
| Supporting Peptides
|

Protein Coverage:

Supporting Peptides:

| Peptide | Uniq | -10lgP | Mass | Length | ppm | m/z | z | RT | Fraction | Scan | Source File | Area F4 | Area F5 | Area F6 | Area F10 | Area F8 | Area F9 | Area F7 | Area F1 | Area F2 | Area F3 | #Feature | #Feature F4 | #Feature F5 | #Feature F6 | #Feature F10 | #Feature F8 | #Feature F9 | #Feature F7 | #Feature F1 | #Feature F2 | #Feature F3 | Start | End | PTM | AScore | Found By |
| --- | --- | --- | --- | --- | --- | --- | --- | --- | --- | --- | --- | --- | --- | --- | --- | --- | --- | --- | --- | --- | --- | --- | --- | --- | --- | --- | --- | --- | --- | --- | --- | --- | --- | --- | --- | --- | --- |
| K.HSVGIVR.D | Y | 49.98 | 766.4449 | 7 | 0.7 | 384.2300 | 2 | 17.97 | 7 | F7:4065 | 21012022\_RID\_3262\_7a.raw |  |  |  |  |  |  | 3.442E6 |  |  |  | 1 | 0 | 0 | 0 | 0 | 0 | 0 | 1 | 0 | 0 | 0 | 313 | 319 |  |  | PEAKS DB |
| total 1 peptides |  |  |  |  |  |  |  |  |  |  |  |  |  |  |  |  |  |  |  |  |  |  |  |  |  |  |  |  |  |  |  |  |  |  |  |  |  |
| --- | --- | --- | --- | --- | --- | --- | --- | --- | --- | --- | --- | --- | --- | --- | --- | --- | --- | --- | --- | --- | --- | --- | --- | --- | --- | --- | --- | --- | --- | --- | --- | --- | --- | --- | --- | --- | --- |

AFJ49217.1

back to list

  

| Protein Coverage
| Supporting Peptides
|

Protein Coverage:

Supporting Peptides:

| Peptide | Uniq | -10lgP | Mass | Length | ppm | m/z | z | RT | Fraction | Scan | Source File | Area F4 | Area F5 | Area F6 | Area F10 | Area F8 | Area F9 | Area F7 | Area F1 | Area F2 | Area F3 | #Feature | #Feature F4 | #Feature F5 | #Feature F6 | #Feature F10 | #Feature F8 | #Feature F9 | #Feature F7 | #Feature F1 | #Feature F2 | #Feature F3 | Start | End | PTM | AScore | Found By |
| --- | --- | --- | --- | --- | --- | --- | --- | --- | --- | --- | --- | --- | --- | --- | --- | --- | --- | --- | --- | --- | --- | --- | --- | --- | --- | --- | --- | --- | --- | --- | --- | --- | --- | --- | --- | --- | --- |
| K.HSVGIVR.D | Y | 49.98 | 766.4449 | 7 | 0.7 | 384.2300 | 2 | 17.97 | 7 | F7:4065 | 21012022\_RID\_3262\_7a.raw |  |  |  |  |  |  | 3.442E6 |  |  |  | 1 | 0 | 0 | 0 | 0 | 0 | 0 | 1 | 0 | 0 | 0 | 313 | 319 |  |  | PEAKS DB |
| total 1 peptides |  |  |  |  |  |  |  |  |  |  |  |  |  |  |  |  |  |  |  |  |  |  |  |  |  |  |  |  |  |  |  |  |  |  |  |  |  |
| --- | --- | --- | --- | --- | --- | --- | --- | --- | --- | --- | --- | --- | --- | --- | --- | --- | --- | --- | --- | --- | --- | --- | --- | --- | --- | --- | --- | --- | --- | --- | --- | --- | --- | --- | --- | --- | --- |

JAS04693.1

back to list

  

| Protein Coverage
| Supporting Peptides
|

Protein Coverage:

Supporting Peptides:

| Peptide | Uniq | -10lgP | Mass | Length | ppm | m/z | z | RT | Fraction | Scan | Source File | Area F4 | Area F5 | Area F6 | Area F10 | Area F8 | Area F9 | Area F7 | Area F1 | Area F2 | Area F3 | #Feature | #Feature F4 | #Feature F5 | #Feature F6 | #Feature F10 | #Feature F8 | #Feature F9 | #Feature F7 | #Feature F1 | #Feature F2 | #Feature F3 | Start | End | PTM | AScore | Found By |
| --- | --- | --- | --- | --- | --- | --- | --- | --- | --- | --- | --- | --- | --- | --- | --- | --- | --- | --- | --- | --- | --- | --- | --- | --- | --- | --- | --- | --- | --- | --- | --- | --- | --- | --- | --- | --- | --- |
| K.HSVGIVR.D | Y | 49.98 | 766.4449 | 7 | 0.7 | 384.2300 | 2 | 17.97 | 7 | F7:4065 | 21012022\_RID\_3262\_7a.raw |  |  |  |  |  |  | 3.442E6 |  |  |  | 1 | 0 | 0 | 0 | 0 | 0 | 0 | 1 | 0 | 0 | 0 | 313 | 319 |  |  | PEAKS DB |
| total 1 peptides |  |  |  |  |  |  |  |  |  |  |  |  |  |  |  |  |  |  |  |  |  |  |  |  |  |  |  |  |  |  |  |  |  |  |  |  |  |
| --- | --- | --- | --- | --- | --- | --- | --- | --- | --- | --- | --- | --- | --- | --- | --- | --- | --- | --- | --- | --- | --- | --- | --- | --- | --- | --- | --- | --- | --- | --- | --- | --- | --- | --- | --- | --- | --- |

AEJ31984.1

back to list

  

| Protein Coverage
| Supporting Peptides
|

Protein Coverage:

Supporting Peptides:

| Peptide | Uniq | -10lgP | Mass | Length | ppm | m/z | z | RT | Fraction | Scan | Source File | Area F4 | Area F5 | Area F6 | Area F10 | Area F8 | Area F9 | Area F7 | Area F1 | Area F2 | Area F3 | #Feature | #Feature F4 | #Feature F5 | #Feature F6 | #Feature F10 | #Feature F8 | #Feature F9 | #Feature F7 | #Feature F1 | #Feature F2 | #Feature F3 | Start | End | PTM | AScore | Found By |
| --- | --- | --- | --- | --- | --- | --- | --- | --- | --- | --- | --- | --- | --- | --- | --- | --- | --- | --- | --- | --- | --- | --- | --- | --- | --- | --- | --- | --- | --- | --- | --- | --- | --- | --- | --- | --- | --- |
| K.HSVGIVR.D | Y | 49.98 | 766.4449 | 7 | 0.7 | 384.2300 | 2 | 17.97 | 7 | F7:4065 | 21012022\_RID\_3262\_7a.raw |  |  |  |  |  |  | 3.442E6 |  |  |  | 1 | 0 | 0 | 0 | 0 | 0 | 0 | 1 | 0 | 0 | 0 | 313 | 319 |  |  | PEAKS DB |
| total 1 peptides |  |  |  |  |  |  |  |  |  |  |  |  |  |  |  |  |  |  |  |  |  |  |  |  |  |  |  |  |  |  |  |  |  |  |  |  |  |
| --- | --- | --- | --- | --- | --- | --- | --- | --- | --- | --- | --- | --- | --- | --- | --- | --- | --- | --- | --- | --- | --- | --- | --- | --- | --- | --- | --- | --- | --- | --- | --- | --- | --- | --- | --- | --- | --- |

JAS04691.1

back to list

  

| Protein Coverage
| Supporting Peptides
|

Protein Coverage:

Supporting Peptides:

| Peptide | Uniq | -10lgP | Mass | Length | ppm | m/z | z | RT | Fraction | Scan | Source File | Area F4 | Area F5 | Area F6 | Area F10 | Area F8 | Area F9 | Area F7 | Area F1 | Area F2 | Area F3 | #Feature | #Feature F4 | #Feature F5 | #Feature F6 | #Feature F10 | #Feature F8 | #Feature F9 | #Feature F7 | #Feature F1 | #Feature F2 | #Feature F3 | Start | End | PTM | AScore | Found By |
| --- | --- | --- | --- | --- | --- | --- | --- | --- | --- | --- | --- | --- | --- | --- | --- | --- | --- | --- | --- | --- | --- | --- | --- | --- | --- | --- | --- | --- | --- | --- | --- | --- | --- | --- | --- | --- | --- |
| K.HSVGIVR.D | Y | 49.98 | 766.4449 | 7 | 0.7 | 384.2300 | 2 | 17.97 | 7 | F7:4065 | 21012022\_RID\_3262\_7a.raw |  |  |  |  |  |  | 3.442E6 |  |  |  | 1 | 0 | 0 | 0 | 0 | 0 | 0 | 1 | 0 | 0 | 0 | 313 | 319 |  |  | PEAKS DB |
| total 1 peptides |  |  |  |  |  |  |  |  |  |  |  |  |  |  |  |  |  |  |  |  |  |  |  |  |  |  |  |  |  |  |  |  |  |  |  |  |  |
| --- | --- | --- | --- | --- | --- | --- | --- | --- | --- | --- | --- | --- | --- | --- | --- | --- | --- | --- | --- | --- | --- | --- | --- | --- | --- | --- | --- | --- | --- | --- | --- | --- | --- | --- | --- | --- | --- |

AFJ49216.1

back to list

  

| Protein Coverage
| Supporting Peptides
|

Protein Coverage:

Supporting Peptides:

| Peptide | Uniq | -10lgP | Mass | Length | ppm | m/z | z | RT | Fraction | Scan | Source File | Area F4 | Area F5 | Area F6 | Area F10 | Area F8 | Area F9 | Area F7 | Area F1 | Area F2 | Area F3 | #Feature | #Feature F4 | #Feature F5 | #Feature F6 | #Feature F10 | #Feature F8 | #Feature F9 | #Feature F7 | #Feature F1 | #Feature F2 | #Feature F3 | Start | End | PTM | AScore | Found By |
| --- | --- | --- | --- | --- | --- | --- | --- | --- | --- | --- | --- | --- | --- | --- | --- | --- | --- | --- | --- | --- | --- | --- | --- | --- | --- | --- | --- | --- | --- | --- | --- | --- | --- | --- | --- | --- | --- |
| K.HSVGIVR.D | Y | 49.98 | 766.4449 | 7 | 0.7 | 384.2300 | 2 | 17.97 | 7 | F7:4065 | 21012022\_RID\_3262\_7a.raw |  |  |  |  |  |  | 3.442E6 |  |  |  | 1 | 0 | 0 | 0 | 0 | 0 | 0 | 1 | 0 | 0 | 0 | 313 | 319 |  |  | PEAKS DB |
| total 1 peptides |  |  |  |  |  |  |  |  |  |  |  |  |  |  |  |  |  |  |  |  |  |  |  |  |  |  |  |  |  |  |  |  |  |  |  |  |  |
| --- | --- | --- | --- | --- | --- | --- | --- | --- | --- | --- | --- | --- | --- | --- | --- | --- | --- | --- | --- | --- | --- | --- | --- | --- | --- | --- | --- | --- | --- | --- | --- | --- | --- | --- | --- | --- | --- |

AFJ49218.1

back to list

  

| Protein Coverage
| Supporting Peptides
|

Protein Coverage:

Supporting Peptides:

| Peptide | Uniq | -10lgP | Mass | Length | ppm | m/z | z | RT | Fraction | Scan | Source File | Area F4 | Area F5 | Area F6 | Area F10 | Area F8 | Area F9 | Area F7 | Area F1 | Area F2 | Area F3 | #Feature | #Feature F4 | #Feature F5 | #Feature F6 | #Feature F10 | #Feature F8 | #Feature F9 | #Feature F7 | #Feature F1 | #Feature F2 | #Feature F3 | Start | End | PTM | AScore | Found By |
| --- | --- | --- | --- | --- | --- | --- | --- | --- | --- | --- | --- | --- | --- | --- | --- | --- | --- | --- | --- | --- | --- | --- | --- | --- | --- | --- | --- | --- | --- | --- | --- | --- | --- | --- | --- | --- | --- |
| K.HSVGIVR.D | Y | 49.98 | 766.4449 | 7 | 0.7 | 384.2300 | 2 | 17.97 | 7 | F7:4065 | 21012022\_RID\_3262\_7a.raw |  |  |  |  |  |  | 3.442E6 |  |  |  | 1 | 0 | 0 | 0 | 0 | 0 | 0 | 1 | 0 | 0 | 0 | 313 | 319 |  |  | PEAKS DB |
| total 1 peptides |  |  |  |  |  |  |  |  |  |  |  |  |  |  |  |  |  |  |  |  |  |  |  |  |  |  |  |  |  |  |  |  |  |  |  |  |  |
| --- | --- | --- | --- | --- | --- | --- | --- | --- | --- | --- | --- | --- | --- | --- | --- | --- | --- | --- | --- | --- | --- | --- | --- | --- | --- | --- | --- | --- | --- | --- | --- | --- | --- | --- | --- | --- | --- |

AFJ49215.1

back to list

  

| Protein Coverage
| Supporting Peptides
|

Protein Coverage:

Supporting Peptides:

| Peptide | Uniq | -10lgP | Mass | Length | ppm | m/z | z | RT | Fraction | Scan | Source File | Area F4 | Area F5 | Area F6 | Area F10 | Area F8 | Area F9 | Area F7 | Area F1 | Area F2 | Area F3 | #Feature | #Feature F4 | #Feature F5 | #Feature F6 | #Feature F10 | #Feature F8 | #Feature F9 | #Feature F7 | #Feature F1 | #Feature F2 | #Feature F3 | Start | End | PTM | AScore | Found By |
| --- | --- | --- | --- | --- | --- | --- | --- | --- | --- | --- | --- | --- | --- | --- | --- | --- | --- | --- | --- | --- | --- | --- | --- | --- | --- | --- | --- | --- | --- | --- | --- | --- | --- | --- | --- | --- | --- |
| K.HSVGIVR.D | Y | 49.98 | 766.4449 | 7 | 0.7 | 384.2300 | 2 | 17.97 | 7 | F7:4065 | 21012022\_RID\_3262\_7a.raw |  |  |  |  |  |  | 3.442E6 |  |  |  | 1 | 0 | 0 | 0 | 0 | 0 | 0 | 1 | 0 | 0 | 0 | 313 | 319 |  |  | PEAKS DB |
| total 1 peptides |  |  |  |  |  |  |  |  |  |  |  |  |  |  |  |  |  |  |  |  |  |  |  |  |  |  |  |  |  |  |  |  |  |  |  |  |  |
| --- | --- | --- | --- | --- | --- | --- | --- | --- | --- | --- | --- | --- | --- | --- | --- | --- | --- | --- | --- | --- | --- | --- | --- | --- | --- | --- | --- | --- | --- | --- | --- | --- | --- | --- | --- | --- | --- |

JAS04692.1

back to list

  

| Protein Coverage
| Supporting Peptides
|

Protein Coverage:

Supporting Peptides:

| Peptide | Uniq | -10lgP | Mass | Length | ppm | m/z | z | RT | Fraction | Scan | Source File | Area F4 | Area F5 | Area F6 | Area F10 | Area F8 | Area F9 | Area F7 | Area F1 | Area F2 | Area F3 | #Feature | #Feature F4 | #Feature F5 | #Feature F6 | #Feature F10 | #Feature F8 | #Feature F9 | #Feature F7 | #Feature F1 | #Feature F2 | #Feature F3 | Start | End | PTM | AScore | Found By |
| --- | --- | --- | --- | --- | --- | --- | --- | --- | --- | --- | --- | --- | --- | --- | --- | --- | --- | --- | --- | --- | --- | --- | --- | --- | --- | --- | --- | --- | --- | --- | --- | --- | --- | --- | --- | --- | --- |
| K.HSVGIVR.D | Y | 49.98 | 766.4449 | 7 | 0.7 | 384.2300 | 2 | 17.97 | 7 | F7:4065 | 21012022\_RID\_3262\_7a.raw |  |  |  |  |  |  | 3.442E6 |  |  |  | 1 | 0 | 0 | 0 | 0 | 0 | 0 | 1 | 0 | 0 | 0 | 313 | 319 |  |  | PEAKS DB |
| total 1 peptides |  |  |  |  |  |  |  |  |  |  |  |  |  |  |  |  |  |  |  |  |  |  |  |  |  |  |  |  |  |  |  |  |  |  |  |  |  |
| --- | --- | --- | --- | --- | --- | --- | --- | --- | --- | --- | --- | --- | --- | --- | --- | --- | --- | --- | --- | --- | --- | --- | --- | --- | --- | --- | --- | --- | --- | --- | --- | --- | --- | --- | --- | --- | --- |

AFJ49211.1

back to list

  

| Protein Coverage
| Supporting Peptides
|

Protein Coverage:

Supporting Peptides:

| Peptide | Uniq | -10lgP | Mass | Length | ppm | m/z | z | RT | Fraction | Scan | Source File | Area F4 | Area F5 | Area F6 | Area F10 | Area F8 | Area F9 | Area F7 | Area F1 | Area F2 | Area F3 | #Feature | #Feature F4 | #Feature F5 | #Feature F6 | #Feature F10 | #Feature F8 | #Feature F9 | #Feature F7 | #Feature F1 | #Feature F2 | #Feature F3 | Start | End | PTM | AScore | Found By |
| --- | --- | --- | --- | --- | --- | --- | --- | --- | --- | --- | --- | --- | --- | --- | --- | --- | --- | --- | --- | --- | --- | --- | --- | --- | --- | --- | --- | --- | --- | --- | --- | --- | --- | --- | --- | --- | --- |
| K.HSVGIVR.D | Y | 49.98 | 766.4449 | 7 | 0.7 | 384.2300 | 2 | 17.97 | 7 | F7:4065 | 21012022\_RID\_3262\_7a.raw |  |  |  |  |  |  | 3.442E6 |  |  |  | 1 | 0 | 0 | 0 | 0 | 0 | 0 | 1 | 0 | 0 | 0 | 312 | 318 |  |  | PEAKS DB |
| total 1 peptides |  |  |  |  |  |  |  |  |  |  |  |  |  |  |  |  |  |  |  |  |  |  |  |  |  |  |  |  |  |  |  |  |  |  |  |  |  |
| --- | --- | --- | --- | --- | --- | --- | --- | --- | --- | --- | --- | --- | --- | --- | --- | --- | --- | --- | --- | --- | --- | --- | --- | --- | --- | --- | --- | --- | --- | --- | --- | --- | --- | --- | --- | --- | --- |

AFJ49208.1

back to list

  

| Protein Coverage
| Supporting Peptides
|

Protein Coverage:

Supporting Peptides:

| Peptide | Uniq | -10lgP | Mass | Length | ppm | m/z | z | RT | Fraction | Scan | Source File | Area F4 | Area F5 | Area F6 | Area F10 | Area F8 | Area F9 | Area F7 | Area F1 | Area F2 | Area F3 | #Feature | #Feature F4 | #Feature F5 | #Feature F6 | #Feature F10 | #Feature F8 | #Feature F9 | #Feature F7 | #Feature F1 | #Feature F2 | #Feature F3 | Start | End | PTM | AScore | Found By |
| --- | --- | --- | --- | --- | --- | --- | --- | --- | --- | --- | --- | --- | --- | --- | --- | --- | --- | --- | --- | --- | --- | --- | --- | --- | --- | --- | --- | --- | --- | --- | --- | --- | --- | --- | --- | --- | --- |
| K.HSVGIVR.D | Y | 49.98 | 766.4449 | 7 | 0.7 | 384.2300 | 2 | 17.97 | 7 | F7:4065 | 21012022\_RID\_3262\_7a.raw |  |  |  |  |  |  | 3.442E6 |  |  |  | 1 | 0 | 0 | 0 | 0 | 0 | 0 | 1 | 0 | 0 | 0 | 313 | 319 |  |  | PEAKS DB |
| total 1 peptides |  |  |  |  |  |  |  |  |  |  |  |  |  |  |  |  |  |  |  |  |  |  |  |  |  |  |  |  |  |  |  |  |  |  |  |  |  |
| --- | --- | --- | --- | --- | --- | --- | --- | --- | --- | --- | --- | --- | --- | --- | --- | --- | --- | --- | --- | --- | --- | --- | --- | --- | --- | --- | --- | --- | --- | --- | --- | --- | --- | --- | --- | --- | --- |

AFJ49209.1

back to list

  

| Protein Coverage
| Supporting Peptides
|

Protein Coverage:

Supporting Peptides:

| Peptide | Uniq | -10lgP | Mass | Length | ppm | m/z | z | RT | Fraction | Scan | Source File | Area F4 | Area F5 | Area F6 | Area F10 | Area F8 | Area F9 | Area F7 | Area F1 | Area F2 | Area F3 | #Feature | #Feature F4 | #Feature F5 | #Feature F6 | #Feature F10 | #Feature F8 | #Feature F9 | #Feature F7 | #Feature F1 | #Feature F2 | #Feature F3 | Start | End | PTM | AScore | Found By |
| --- | --- | --- | --- | --- | --- | --- | --- | --- | --- | --- | --- | --- | --- | --- | --- | --- | --- | --- | --- | --- | --- | --- | --- | --- | --- | --- | --- | --- | --- | --- | --- | --- | --- | --- | --- | --- | --- |
| K.HSVGIVR.D | Y | 49.98 | 766.4449 | 7 | 0.7 | 384.2300 | 2 | 17.97 | 7 | F7:4065 | 21012022\_RID\_3262\_7a.raw |  |  |  |  |  |  | 3.442E6 |  |  |  | 1 | 0 | 0 | 0 | 0 | 0 | 0 | 1 | 0 | 0 | 0 | 313 | 319 |  |  | PEAKS DB |
| total 1 peptides |  |  |  |  |  |  |  |  |  |  |  |  |  |  |  |  |  |  |  |  |  |  |  |  |  |  |  |  |  |  |  |  |  |  |  |  |  |
| --- | --- | --- | --- | --- | --- | --- | --- | --- | --- | --- | --- | --- | --- | --- | --- | --- | --- | --- | --- | --- | --- | --- | --- | --- | --- | --- | --- | --- | --- | --- | --- | --- | --- | --- | --- | --- | --- |

AFJ49210.1

back to list

  

| Protein Coverage
| Supporting Peptides
|

Protein Coverage:

Supporting Peptides:

| Peptide | Uniq | -10lgP | Mass | Length | ppm | m/z | z | RT | Fraction | Scan | Source File | Area F4 | Area F5 | Area F6 | Area F10 | Area F8 | Area F9 | Area F7 | Area F1 | Area F2 | Area F3 | #Feature | #Feature F4 | #Feature F5 | #Feature F6 | #Feature F10 | #Feature F8 | #Feature F9 | #Feature F7 | #Feature F1 | #Feature F2 | #Feature F3 | Start | End | PTM | AScore | Found By |
| --- | --- | --- | --- | --- | --- | --- | --- | --- | --- | --- | --- | --- | --- | --- | --- | --- | --- | --- | --- | --- | --- | --- | --- | --- | --- | --- | --- | --- | --- | --- | --- | --- | --- | --- | --- | --- | --- |
| K.HSVGIVR.D | Y | 49.98 | 766.4449 | 7 | 0.7 | 384.2300 | 2 | 17.97 | 7 | F7:4065 | 21012022\_RID\_3262\_7a.raw |  |  |  |  |  |  | 3.442E6 |  |  |  | 1 | 0 | 0 | 0 | 0 | 0 | 0 | 1 | 0 | 0 | 0 | 313 | 319 |  |  | PEAKS DB |
| total 1 peptides |  |  |  |  |  |  |  |  |  |  |  |  |  |  |  |  |  |  |  |  |  |  |  |  |  |  |  |  |  |  |  |  |  |  |  |  |  |
| --- | --- | --- | --- | --- | --- | --- | --- | --- | --- | --- | --- | --- | --- | --- | --- | --- | --- | --- | --- | --- | --- | --- | --- | --- | --- | --- | --- | --- | --- | --- | --- | --- | --- | --- | --- | --- | --- |

AFJ49212.1

back to list

  

| Protein Coverage
| Supporting Peptides
|

Protein Coverage:

Supporting Peptides:

| Peptide | Uniq | -10lgP | Mass | Length | ppm | m/z | z | RT | Fraction | Scan | Source File | Area F4 | Area F5 | Area F6 | Area F10 | Area F8 | Area F9 | Area F7 | Area F1 | Area F2 | Area F3 | #Feature | #Feature F4 | #Feature F5 | #Feature F6 | #Feature F10 | #Feature F8 | #Feature F9 | #Feature F7 | #Feature F1 | #Feature F2 | #Feature F3 | Start | End | PTM | AScore | Found By |
| --- | --- | --- | --- | --- | --- | --- | --- | --- | --- | --- | --- | --- | --- | --- | --- | --- | --- | --- | --- | --- | --- | --- | --- | --- | --- | --- | --- | --- | --- | --- | --- | --- | --- | --- | --- | --- | --- |
| K.HSVGIVR.D | Y | 49.98 | 766.4449 | 7 | 0.7 | 384.2300 | 2 | 17.97 | 7 | F7:4065 | 21012022\_RID\_3262\_7a.raw |  |  |  |  |  |  | 3.442E6 |  |  |  | 1 | 0 | 0 | 0 | 0 | 0 | 0 | 1 | 0 | 0 | 0 | 313 | 319 |  |  | PEAKS DB |
| total 1 peptides |  |  |  |  |  |  |  |  |  |  |  |  |  |  |  |  |  |  |  |  |  |  |  |  |  |  |  |  |  |  |  |  |  |  |  |  |  |
| --- | --- | --- | --- | --- | --- | --- | --- | --- | --- | --- | --- | --- | --- | --- | --- | --- | --- | --- | --- | --- | --- | --- | --- | --- | --- | --- | --- | --- | --- | --- | --- | --- | --- | --- | --- | --- | --- |

JAS05427.1

back to list

  

| Protein Coverage
| Supporting Peptides
|

Protein Coverage:

Supporting Peptides:

| Peptide | Uniq | -10lgP | Mass | Length | ppm | m/z | z | RT | Fraction | Scan | Source File | Area F4 | Area F5 | Area F6 | Area F10 | Area F8 | Area F9 | Area F7 | Area F1 | Area F2 | Area F3 | #Feature | #Feature F4 | #Feature F5 | #Feature F6 | #Feature F10 | #Feature F8 | #Feature F9 | #Feature F7 | #Feature F1 | #Feature F2 | #Feature F3 | Start | End | PTM | AScore | Found By |
| --- | --- | --- | --- | --- | --- | --- | --- | --- | --- | --- | --- | --- | --- | --- | --- | --- | --- | --- | --- | --- | --- | --- | --- | --- | --- | --- | --- | --- | --- | --- | --- | --- | --- | --- | --- | --- | --- |
| K.HSVGIVR.D | Y | 49.98 | 766.4449 | 7 | 0.7 | 384.2300 | 2 | 17.97 | 7 | F7:4065 | 21012022\_RID\_3262\_7a.raw |  |  |  |  |  |  | 3.442E6 |  |  |  | 1 | 0 | 0 | 0 | 0 | 0 | 0 | 1 | 0 | 0 | 0 | 343 | 349 |  |  | PEAKS DB |
| total 1 peptides |  |  |  |  |  |  |  |  |  |  |  |  |  |  |  |  |  |  |  |  |  |  |  |  |  |  |  |  |  |  |  |  |  |  |  |  |  |
| --- | --- | --- | --- | --- | --- | --- | --- | --- | --- | --- | --- | --- | --- | --- | --- | --- | --- | --- | --- | --- | --- | --- | --- | --- | --- | --- | --- | --- | --- | --- | --- | --- | --- | --- | --- | --- | --- |

JAS05426.1

back to list

  

| Protein Coverage
| Supporting Peptides
|

Protein Coverage:

Supporting Peptides:

| Peptide | Uniq | -10lgP | Mass | Length | ppm | m/z | z | RT | Fraction | Scan | Source File | Area F4 | Area F5 | Area F6 | Area F10 | Area F8 | Area F9 | Area F7 | Area F1 | Area F2 | Area F3 | #Feature | #Feature F4 | #Feature F5 | #Feature F6 | #Feature F10 | #Feature F8 | #Feature F9 | #Feature F7 | #Feature F1 | #Feature F2 | #Feature F3 | Start | End | PTM | AScore | Found By |
| --- | --- | --- | --- | --- | --- | --- | --- | --- | --- | --- | --- | --- | --- | --- | --- | --- | --- | --- | --- | --- | --- | --- | --- | --- | --- | --- | --- | --- | --- | --- | --- | --- | --- | --- | --- | --- | --- |
| K.HSVGIVR.D | Y | 49.98 | 766.4449 | 7 | 0.7 | 384.2300 | 2 | 17.97 | 7 | F7:4065 | 21012022\_RID\_3262\_7a.raw |  |  |  |  |  |  | 3.442E6 |  |  |  | 1 | 0 | 0 | 0 | 0 | 0 | 0 | 1 | 0 | 0 | 0 | 343 | 349 |  |  | PEAKS DB |
| total 1 peptides |  |  |  |  |  |  |  |  |  |  |  |  |  |  |  |  |  |  |  |  |  |  |  |  |  |  |  |  |  |  |  |  |  |  |  |  |  |
| --- | --- | --- | --- | --- | --- | --- | --- | --- | --- | --- | --- | --- | --- | --- | --- | --- | --- | --- | --- | --- | --- | --- | --- | --- | --- | --- | --- | --- | --- | --- | --- | --- | --- | --- | --- | --- | --- |

JAS05428.1

back to list

  

| Protein Coverage
| Supporting Peptides
|

Protein Coverage:

Supporting Peptides:

| Peptide | Uniq | -10lgP | Mass | Length | ppm | m/z | z | RT | Fraction | Scan | Source File | Area F4 | Area F5 | Area F6 | Area F10 | Area F8 | Area F9 | Area F7 | Area F1 | Area F2 | Area F3 | #Feature | #Feature F4 | #Feature F5 | #Feature F6 | #Feature F10 | #Feature F8 | #Feature F9 | #Feature F7 | #Feature F1 | #Feature F2 | #Feature F3 | Start | End | PTM | AScore | Found By |
| --- | --- | --- | --- | --- | --- | --- | --- | --- | --- | --- | --- | --- | --- | --- | --- | --- | --- | --- | --- | --- | --- | --- | --- | --- | --- | --- | --- | --- | --- | --- | --- | --- | --- | --- | --- | --- | --- |
| K.HSVGIVR.D | Y | 49.98 | 766.4449 | 7 | 0.7 | 384.2300 | 2 | 17.97 | 7 | F7:4065 | 21012022\_RID\_3262\_7a.raw |  |  |  |  |  |  | 3.442E6 |  |  |  | 1 | 0 | 0 | 0 | 0 | 0 | 0 | 1 | 0 | 0 | 0 | 343 | 349 |  |  | PEAKS DB |
| total 1 peptides |  |  |  |  |  |  |  |  |  |  |  |  |  |  |  |  |  |  |  |  |  |  |  |  |  |  |  |  |  |  |  |  |  |  |  |  |  |
| --- | --- | --- | --- | --- | --- | --- | --- | --- | --- | --- | --- | --- | --- | --- | --- | --- | --- | --- | --- | --- | --- | --- | --- | --- | --- | --- | --- | --- | --- | --- | --- | --- | --- | --- | --- | --- | --- |

XP\_026539659.1

back to list

  

| Protein Coverage
| Supporting Peptides
|

Protein Coverage:

Supporting Peptides:

| Peptide | Uniq | -10lgP | Mass | Length | ppm | m/z | z | RT | Fraction | Scan | Source File | Area F4 | Area F5 | Area F6 | Area F10 | Area F8 | Area F9 | Area F7 | Area F1 | Area F2 | Area F3 | #Feature | #Feature F4 | #Feature F5 | #Feature F6 | #Feature F10 | #Feature F8 | #Feature F9 | #Feature F7 | #Feature F1 | #Feature F2 | #Feature F3 | Start | End | PTM | AScore | Found By |
| --- | --- | --- | --- | --- | --- | --- | --- | --- | --- | --- | --- | --- | --- | --- | --- | --- | --- | --- | --- | --- | --- | --- | --- | --- | --- | --- | --- | --- | --- | --- | --- | --- | --- | --- | --- | --- | --- |
| R.SQYEEIAQR.S | Y | 50.41 | 1122.5305 | 9 | 0.3 | 562.2727 | 2 | 23.76 | 9 | F9:8231 | 31122021\_RID\_3262\_2a.raw |  |  |  |  |  |  |  |  | 2.213E5 |  | 1 | 0 | 0 | 0 | 0 | 0 | 0 | 0 | 0 | 1 | 0 | 353 | 361 |  |  | PEAKS DB |
| total 1 peptides |  |  |  |  |  |  |  |  |  |  |  |  |  |  |  |  |  |  |  |  |  |  |  |  |  |  |  |  |  |  |  |  |  |  |  |  |  |
| --- | --- | --- | --- | --- | --- | --- | --- | --- | --- | --- | --- | --- | --- | --- | --- | --- | --- | --- | --- | --- | --- | --- | --- | --- | --- | --- | --- | --- | --- | --- | --- | --- | --- | --- | --- | --- | --- |

XP\_026570510.1

back to list

  

| Protein Coverage
| Supporting Peptides
|

Protein Coverage:

Supporting Peptides:

| Peptide | Uniq | -10lgP | Mass | Length | ppm | m/z | z | RT | Fraction | Scan | Source File | Area F4 | Area F5 | Area F6 | Area F10 | Area F8 | Area F9 | Area F7 | Area F1 | Area F2 | Area F3 | #Feature | #Feature F4 | #Feature F5 | #Feature F6 | #Feature F10 | #Feature F8 | #Feature F9 | #Feature F7 | #Feature F1 | #Feature F2 | #Feature F3 | Start | End | PTM | AScore | Found By |
| --- | --- | --- | --- | --- | --- | --- | --- | --- | --- | --- | --- | --- | --- | --- | --- | --- | --- | --- | --- | --- | --- | --- | --- | --- | --- | --- | --- | --- | --- | --- | --- | --- | --- | --- | --- | --- | --- |
| R.SQYEEIAQR.S | Y | 50.41 | 1122.5305 | 9 | 0.3 | 562.2727 | 2 | 23.76 | 9 | F9:8231 | 31122021\_RID\_3262\_2a.raw |  |  |  |  |  |  |  |  | 2.213E5 |  | 1 | 0 | 0 | 0 | 0 | 0 | 0 | 0 | 0 | 1 | 0 | 353 | 361 |  |  | PEAKS DB |
| total 1 peptides |  |  |  |  |  |  |  |  |  |  |  |  |  |  |  |  |  |  |  |  |  |  |  |  |  |  |  |  |  |  |  |  |  |  |  |  |  |
| --- | --- | --- | --- | --- | --- | --- | --- | --- | --- | --- | --- | --- | --- | --- | --- | --- | --- | --- | --- | --- | --- | --- | --- | --- | --- | --- | --- | --- | --- | --- | --- | --- | --- | --- | --- | --- | --- |

P26324.1

back to list

  

| Protein Coverage
| Supporting Peptides
|

Protein Coverage:

Supporting Peptides:

| Peptide | Uniq | -10lgP | Mass | Length | ppm | m/z | z | RT | Fraction | Scan | Source File | Area F4 | Area F5 | Area F6 | Area F10 | Area F8 | Area F9 | Area F7 | Area F1 | Area F2 | Area F3 | #Feature | #Feature F4 | #Feature F5 | #Feature F6 | #Feature F10 | #Feature F8 | #Feature F9 | #Feature F7 | #Feature F1 | #Feature F2 | #Feature F3 | Start | End | PTM | AScore | Found By |
| --- | --- | --- | --- | --- | --- | --- | --- | --- | --- | --- | --- | --- | --- | --- | --- | --- | --- | --- | --- | --- | --- | --- | --- | --- | --- | --- | --- | --- | --- | --- | --- | --- | --- | --- | --- | --- | --- |
| R.VMGWGSINR.R | Y | 45.67 | 1018.5018 | 9 | -0.6 | 510.2579 | 2 | 37.99 | 2 | F2:20882 | 13012022\_RID\_3262\_5a.raw |  | 7.0834E6 |  |  |  |  |  |  |  |  | 1 | 0 | 1 | 0 | 0 | 0 | 0 | 0 | 0 | 0 | 0 | 122 | 130 |  |  | PEAKS DB |
| VIGGDEC(+57.02)NINEHR.F | N | 41.29 | 1511.6787 | 13 | 0.4 | 756.8469 | 2 | 21.60 | 2 | F2:8211 | 13012022\_RID\_3262\_5a.raw |  | 6.6457E6 |  |  |  |  |  |  |  |  | 1 | 0 | 1 | 0 | 0 | 0 | 0 | 0 | 0 | 0 | 0 | 1 | 13 | Carbamidomethylation | C7:Carbamidomethylation:1000.00 | PEAKS DB |
| total 2 peptides |  |  |  |  |  |  |  |  |  |  |  |  |  |  |  |  |  |  |  |  |  |  |  |  |  |  |  |  |  |  |  |  |  |  |  |  |  |
| --- | --- | --- | --- | --- | --- | --- | --- | --- | --- | --- | --- | --- | --- | --- | --- | --- | --- | --- | --- | --- | --- | --- | --- | --- | --- | --- | --- | --- | --- | --- | --- | --- | --- | --- | --- | --- | --- |

AAA03254.1

back to list

  

| Protein Coverage
| Supporting Peptides
|

Protein Coverage:

Supporting Peptides:

| Peptide | Uniq | -10lgP | Mass | Length | ppm | m/z | z | RT | Fraction | Scan | Source File | Area F4 | Area F5 | Area F6 | Area F10 | Area F8 | Area F9 | Area F7 | Area F1 | Area F2 | Area F3 | #Feature | #Feature F4 | #Feature F5 | #Feature F6 | #Feature F10 | #Feature F8 | #Feature F9 | #Feature F7 | #Feature F1 | #Feature F2 | #Feature F3 | Start | End | PTM | AScore | Found By |
| --- | --- | --- | --- | --- | --- | --- | --- | --- | --- | --- | --- | --- | --- | --- | --- | --- | --- | --- | --- | --- | --- | --- | --- | --- | --- | --- | --- | --- | --- | --- | --- | --- | --- | --- | --- | --- | --- |
| R.VMGWGSINR.R | Y | 45.67 | 1018.5018 | 9 | -0.6 | 510.2579 | 2 | 37.99 | 2 | F2:20882 | 13012022\_RID\_3262\_5a.raw |  | 7.0834E6 |  |  |  |  |  |  |  |  | 1 | 0 | 1 | 0 | 0 | 0 | 0 | 0 | 0 | 0 | 0 | 122 | 130 |  |  | PEAKS DB |
| VIGGDEC(+57.02)NINEHR.F | N | 41.29 | 1511.6787 | 13 | 0.4 | 756.8469 | 2 | 21.60 | 2 | F2:8211 | 13012022\_RID\_3262\_5a.raw |  | 6.6457E6 |  |  |  |  |  |  |  |  | 1 | 0 | 1 | 0 | 0 | 0 | 0 | 0 | 0 | 0 | 0 | 1 | 13 | Carbamidomethylation | C7:Carbamidomethylation:1000.00 | PEAKS DB |
| total 2 peptides |  |  |  |  |  |  |  |  |  |  |  |  |  |  |  |  |  |  |  |  |  |  |  |  |  |  |  |  |  |  |  |  |  |  |  |  |  |
| --- | --- | --- | --- | --- | --- | --- | --- | --- | --- | --- | --- | --- | --- | --- | --- | --- | --- | --- | --- | --- | --- | --- | --- | --- | --- | --- | --- | --- | --- | --- | --- | --- | --- | --- | --- | --- | --- |

XP\_013919189.1

back to list

  

| Protein Coverage
| Supporting Peptides
|

Protein Coverage:

Supporting Peptides:

| Peptide | Uniq | -10lgP | Mass | Length | ppm | m/z | z | RT | Fraction | Scan | Source File | Area F4 | Area F5 | Area F6 | Area F10 | Area F8 | Area F9 | Area F7 | Area F1 | Area F2 | Area F3 | #Feature | #Feature F4 | #Feature F5 | #Feature F6 | #Feature F10 | #Feature F8 | #Feature F9 | #Feature F7 | #Feature F1 | #Feature F2 | #Feature F3 | Start | End | PTM | AScore | Found By |
| --- | --- | --- | --- | --- | --- | --- | --- | --- | --- | --- | --- | --- | --- | --- | --- | --- | --- | --- | --- | --- | --- | --- | --- | --- | --- | --- | --- | --- | --- | --- | --- | --- | --- | --- | --- | --- | --- |
| R.AGLQFPVGR.V | Y | 48.32 | 943.5239 | 9 | 0.1 | 472.7693 | 2 | 38.48 | 9 | F9:20306 | 31122021\_RID\_3262\_2a.raw |  |  |  |  |  |  |  |  | 2.2355E5 |  | 1 | 0 | 0 | 0 | 0 | 0 | 0 | 0 | 0 | 1 | 0 | 22 | 30 |  |  | PEAKS DB |
| total 1 peptides |  |  |  |  |  |  |  |  |  |  |  |  |  |  |  |  |  |  |  |  |  |  |  |  |  |  |  |  |  |  |  |  |  |  |  |  |  |
| --- | --- | --- | --- | --- | --- | --- | --- | --- | --- | --- | --- | --- | --- | --- | --- | --- | --- | --- | --- | --- | --- | --- | --- | --- | --- | --- | --- | --- | --- | --- | --- | --- | --- | --- | --- | --- | --- |

XP\_013919188.1

back to list

  

| Protein Coverage
| Supporting Peptides
|

Protein Coverage:

Supporting Peptides:

| Peptide | Uniq | -10lgP | Mass | Length | ppm | m/z | z | RT | Fraction | Scan | Source File | Area F4 | Area F5 | Area F6 | Area F10 | Area F8 | Area F9 | Area F7 | Area F1 | Area F2 | Area F3 | #Feature | #Feature F4 | #Feature F5 | #Feature F6 | #Feature F10 | #Feature F8 | #Feature F9 | #Feature F7 | #Feature F1 | #Feature F2 | #Feature F3 | Start | End | PTM | AScore | Found By |
| --- | --- | --- | --- | --- | --- | --- | --- | --- | --- | --- | --- | --- | --- | --- | --- | --- | --- | --- | --- | --- | --- | --- | --- | --- | --- | --- | --- | --- | --- | --- | --- | --- | --- | --- | --- | --- | --- |
| R.AGLQFPVGR.V | Y | 48.32 | 943.5239 | 9 | 0.1 | 472.7693 | 2 | 38.48 | 9 | F9:20306 | 31122021\_RID\_3262\_2a.raw |  |  |  |  |  |  |  |  | 2.2355E5 |  | 1 | 0 | 0 | 0 | 0 | 0 | 0 | 0 | 0 | 1 | 0 | 22 | 30 |  |  | PEAKS DB |
| total 1 peptides |  |  |  |  |  |  |  |  |  |  |  |  |  |  |  |  |  |  |  |  |  |  |  |  |  |  |  |  |  |  |  |  |  |  |  |  |  |
| --- | --- | --- | --- | --- | --- | --- | --- | --- | --- | --- | --- | --- | --- | --- | --- | --- | --- | --- | --- | --- | --- | --- | --- | --- | --- | --- | --- | --- | --- | --- | --- | --- | --- | --- | --- | --- | --- |

ETE65301.1

back to list

  

| Protein Coverage
| Supporting Peptides
|

Protein Coverage:

Supporting Peptides:

| Peptide | Uniq | -10lgP | Mass | Length | ppm | m/z | z | RT | Fraction | Scan | Source File | Area F4 | Area F5 | Area F6 | Area F10 | Area F8 | Area F9 | Area F7 | Area F1 | Area F2 | Area F3 | #Feature | #Feature F4 | #Feature F5 | #Feature F6 | #Feature F10 | #Feature F8 | #Feature F9 | #Feature F7 | #Feature F1 | #Feature F2 | #Feature F3 | Start | End | PTM | AScore | Found By |
| --- | --- | --- | --- | --- | --- | --- | --- | --- | --- | --- | --- | --- | --- | --- | --- | --- | --- | --- | --- | --- | --- | --- | --- | --- | --- | --- | --- | --- | --- | --- | --- | --- | --- | --- | --- | --- | --- |
| R.AGLQFPVGR.V | Y | 48.32 | 943.5239 | 9 | 0.1 | 472.7693 | 2 | 38.48 | 9 | F9:20306 | 31122021\_RID\_3262\_2a.raw |  |  |  |  |  |  |  |  | 2.2355E5 |  | 1 | 0 | 0 | 0 | 0 | 0 | 0 | 0 | 0 | 1 | 0 | 22 | 30 |  |  | PEAKS DB |
| total 1 peptides |  |  |  |  |  |  |  |  |  |  |  |  |  |  |  |  |  |  |  |  |  |  |  |  |  |  |  |  |  |  |  |  |  |  |  |  |  |
| --- | --- | --- | --- | --- | --- | --- | --- | --- | --- | --- | --- | --- | --- | --- | --- | --- | --- | --- | --- | --- | --- | --- | --- | --- | --- | --- | --- | --- | --- | --- | --- | --- | --- | --- | --- | --- | --- |

XP\_013931116.1

back to list

  

| Protein Coverage
| Supporting Peptides
|

Protein Coverage:

Supporting Peptides:

| Peptide | Uniq | -10lgP | Mass | Length | ppm | m/z | z | RT | Fraction | Scan | Source File | Area F4 | Area F5 | Area F6 | Area F10 | Area F8 | Area F9 | Area F7 | Area F1 | Area F2 | Area F3 | #Feature | #Feature F4 | #Feature F5 | #Feature F6 | #Feature F10 | #Feature F8 | #Feature F9 | #Feature F7 | #Feature F1 | #Feature F2 | #Feature F3 | Start | End | PTM | AScore | Found By |
| --- | --- | --- | --- | --- | --- | --- | --- | --- | --- | --- | --- | --- | --- | --- | --- | --- | --- | --- | --- | --- | --- | --- | --- | --- | --- | --- | --- | --- | --- | --- | --- | --- | --- | --- | --- | --- | --- |
| R.AGLQFPVGR.V | Y | 48.32 | 943.5239 | 9 | 0.1 | 472.7693 | 2 | 38.48 | 9 | F9:20306 | 31122021\_RID\_3262\_2a.raw |  |  |  |  |  |  |  |  | 2.2355E5 |  | 1 | 0 | 0 | 0 | 0 | 0 | 0 | 0 | 0 | 1 | 0 | 22 | 30 |  |  | PEAKS DB |
| total 1 peptides |  |  |  |  |  |  |  |  |  |  |  |  |  |  |  |  |  |  |  |  |  |  |  |  |  |  |  |  |  |  |  |  |  |  |  |  |  |
| --- | --- | --- | --- | --- | --- | --- | --- | --- | --- | --- | --- | --- | --- | --- | --- | --- | --- | --- | --- | --- | --- | --- | --- | --- | --- | --- | --- | --- | --- | --- | --- | --- | --- | --- | --- | --- | --- |

XP\_013908942.1

back to list

  

| Protein Coverage
| Supporting Peptides
|

Protein Coverage:

Supporting Peptides:

| Peptide | Uniq | -10lgP | Mass | Length | ppm | m/z | z | RT | Fraction | Scan | Source File | Area F4 | Area F5 | Area F6 | Area F10 | Area F8 | Area F9 | Area F7 | Area F1 | Area F2 | Area F3 | #Feature | #Feature F4 | #Feature F5 | #Feature F6 | #Feature F10 | #Feature F8 | #Feature F9 | #Feature F7 | #Feature F1 | #Feature F2 | #Feature F3 | Start | End | PTM | AScore | Found By |
| --- | --- | --- | --- | --- | --- | --- | --- | --- | --- | --- | --- | --- | --- | --- | --- | --- | --- | --- | --- | --- | --- | --- | --- | --- | --- | --- | --- | --- | --- | --- | --- | --- | --- | --- | --- | --- | --- |
| R.AGLQFPVGR.V | Y | 48.32 | 943.5239 | 9 | 0.1 | 472.7693 | 2 | 38.48 | 9 | F9:20306 | 31122021\_RID\_3262\_2a.raw |  |  |  |  |  |  |  |  | 2.2355E5 |  | 1 | 0 | 0 | 0 | 0 | 0 | 0 | 0 | 0 | 1 | 0 | 22 | 30 |  |  | PEAKS DB |
| total 1 peptides |  |  |  |  |  |  |  |  |  |  |  |  |  |  |  |  |  |  |  |  |  |  |  |  |  |  |  |  |  |  |  |  |  |  |  |  |  |
| --- | --- | --- | --- | --- | --- | --- | --- | --- | --- | --- | --- | --- | --- | --- | --- | --- | --- | --- | --- | --- | --- | --- | --- | --- | --- | --- | --- | --- | --- | --- | --- | --- | --- | --- | --- | --- | --- |

ETE65060.1

back to list

  

| Protein Coverage
| Supporting Peptides
|

Protein Coverage:

Supporting Peptides:

| Peptide | Uniq | -10lgP | Mass | Length | ppm | m/z | z | RT | Fraction | Scan | Source File | Area F4 | Area F5 | Area F6 | Area F10 | Area F8 | Area F9 | Area F7 | Area F1 | Area F2 | Area F3 | #Feature | #Feature F4 | #Feature F5 | #Feature F6 | #Feature F10 | #Feature F8 | #Feature F9 | #Feature F7 | #Feature F1 | #Feature F2 | #Feature F3 | Start | End | PTM | AScore | Found By |
| --- | --- | --- | --- | --- | --- | --- | --- | --- | --- | --- | --- | --- | --- | --- | --- | --- | --- | --- | --- | --- | --- | --- | --- | --- | --- | --- | --- | --- | --- | --- | --- | --- | --- | --- | --- | --- | --- |
| R.AGLQFPVGR.V | Y | 48.32 | 943.5239 | 9 | 0.1 | 472.7693 | 2 | 38.48 | 9 | F9:20306 | 31122021\_RID\_3262\_2a.raw |  |  |  |  |  |  |  |  | 2.2355E5 |  | 1 | 0 | 0 | 0 | 0 | 0 | 0 | 0 | 0 | 1 | 0 | 22 | 30 |  |  | PEAKS DB |
| total 1 peptides |  |  |  |  |  |  |  |  |  |  |  |  |  |  |  |  |  |  |  |  |  |  |  |  |  |  |  |  |  |  |  |  |  |  |  |  |  |
| --- | --- | --- | --- | --- | --- | --- | --- | --- | --- | --- | --- | --- | --- | --- | --- | --- | --- | --- | --- | --- | --- | --- | --- | --- | --- | --- | --- | --- | --- | --- | --- | --- | --- | --- | --- | --- | --- |

XP\_013915314.1

back to list

  

| Protein Coverage
| Supporting Peptides
|

Protein Coverage:

Supporting Peptides:

| Peptide | Uniq | -10lgP | Mass | Length | ppm | m/z | z | RT | Fraction | Scan | Source File | Area F4 | Area F5 | Area F6 | Area F10 | Area F8 | Area F9 | Area F7 | Area F1 | Area F2 | Area F3 | #Feature | #Feature F4 | #Feature F5 | #Feature F6 | #Feature F10 | #Feature F8 | #Feature F9 | #Feature F7 | #Feature F1 | #Feature F2 | #Feature F3 | Start | End | PTM | AScore | Found By |
| --- | --- | --- | --- | --- | --- | --- | --- | --- | --- | --- | --- | --- | --- | --- | --- | --- | --- | --- | --- | --- | --- | --- | --- | --- | --- | --- | --- | --- | --- | --- | --- | --- | --- | --- | --- | --- | --- |
| R.AGLQFPVGR.V | Y | 48.32 | 943.5239 | 9 | 0.1 | 472.7693 | 2 | 38.48 | 9 | F9:20306 | 31122021\_RID\_3262\_2a.raw |  |  |  |  |  |  |  |  | 2.2355E5 |  | 1 | 0 | 0 | 0 | 0 | 0 | 0 | 0 | 0 | 1 | 0 | 22 | 30 |  |  | PEAKS DB |
| total 1 peptides |  |  |  |  |  |  |  |  |  |  |  |  |  |  |  |  |  |  |  |  |  |  |  |  |  |  |  |  |  |  |  |  |  |  |  |  |  |
| --- | --- | --- | --- | --- | --- | --- | --- | --- | --- | --- | --- | --- | --- | --- | --- | --- | --- | --- | --- | --- | --- | --- | --- | --- | --- | --- | --- | --- | --- | --- | --- | --- | --- | --- | --- | --- | --- |

JAS04797.1

back to list

  

| Protein Coverage
| Supporting Peptides
|

Protein Coverage:

Supporting Peptides:

| Peptide | Uniq | -10lgP | Mass | Length | ppm | m/z | z | RT | Fraction | Scan | Source File | Area F4 | Area F5 | Area F6 | Area F10 | Area F8 | Area F9 | Area F7 | Area F1 | Area F2 | Area F3 | #Feature | #Feature F4 | #Feature F5 | #Feature F6 | #Feature F10 | #Feature F8 | #Feature F9 | #Feature F7 | #Feature F1 | #Feature F2 | #Feature F3 | Start | End | PTM | AScore | Found By |
| --- | --- | --- | --- | --- | --- | --- | --- | --- | --- | --- | --- | --- | --- | --- | --- | --- | --- | --- | --- | --- | --- | --- | --- | --- | --- | --- | --- | --- | --- | --- | --- | --- | --- | --- | --- | --- | --- |
| K.GGHLVSIK.S | Y | 42.92 | 809.4759 | 8 | -0.3 | 405.7451 | 2 | 19.86 | 6 | F6:7480 | 17012022\_RID\_3262\_9a.raw |  |  |  | 1.7937E7 |  | 7.5976E7 |  |  |  |  | 2 | 0 | 0 | 0 | 1 | 0 | 1 | 0 | 0 | 0 | 0 | 61 | 68 |  |  | PEAKS DB |
| total 1 peptides |  |  |  |  |  |  |  |  |  |  |  |  |  |  |  |  |  |  |  |  |  |  |  |  |  |  |  |  |  |  |  |  |  |  |  |  |  |
| --- | --- | --- | --- | --- | --- | --- | --- | --- | --- | --- | --- | --- | --- | --- | --- | --- | --- | --- | --- | --- | --- | --- | --- | --- | --- | --- | --- | --- | --- | --- | --- | --- | --- | --- | --- | --- | --- |

JAA94903.1

back to list

  

| Protein Coverage
| Supporting Peptides
|

Protein Coverage:

Supporting Peptides:

| Peptide | Uniq | -10lgP | Mass | Length | ppm | m/z | z | RT | Fraction | Scan | Source File | Area F4 | Area F5 | Area F6 | Area F10 | Area F8 | Area F9 | Area F7 | Area F1 | Area F2 | Area F3 | #Feature | #Feature F4 | #Feature F5 | #Feature F6 | #Feature F10 | #Feature F8 | #Feature F9 | #Feature F7 | #Feature F1 | #Feature F2 | #Feature F3 | Start | End | PTM | AScore | Found By |
| --- | --- | --- | --- | --- | --- | --- | --- | --- | --- | --- | --- | --- | --- | --- | --- | --- | --- | --- | --- | --- | --- | --- | --- | --- | --- | --- | --- | --- | --- | --- | --- | --- | --- | --- | --- | --- | --- |
| K.GGHLVSIK.S | Y | 42.92 | 809.4759 | 8 | -0.3 | 405.7451 | 2 | 19.86 | 6 | F6:7480 | 17012022\_RID\_3262\_9a.raw |  |  |  | 1.7937E7 |  | 7.5976E7 |  |  |  |  | 2 | 0 | 0 | 0 | 1 | 0 | 1 | 0 | 0 | 0 | 0 | 61 | 68 |  |  | PEAKS DB |
| total 1 peptides |  |  |  |  |  |  |  |  |  |  |  |  |  |  |  |  |  |  |  |  |  |  |  |  |  |  |  |  |  |  |  |  |  |  |  |  |  |
| --- | --- | --- | --- | --- | --- | --- | --- | --- | --- | --- | --- | --- | --- | --- | --- | --- | --- | --- | --- | --- | --- | --- | --- | --- | --- | --- | --- | --- | --- | --- | --- | --- | --- | --- | --- | --- | --- |

JAG43667.1

back to list

  

| Protein Coverage
| Supporting Peptides
|

Protein Coverage:

Supporting Peptides:

| Peptide | Uniq | -10lgP | Mass | Length | ppm | m/z | z | RT | Fraction | Scan | Source File | Area F4 | Area F5 | Area F6 | Area F10 | Area F8 | Area F9 | Area F7 | Area F1 | Area F2 | Area F3 | #Feature | #Feature F4 | #Feature F5 | #Feature F6 | #Feature F10 | #Feature F8 | #Feature F9 | #Feature F7 | #Feature F1 | #Feature F2 | #Feature F3 | Start | End | PTM | AScore | Found By |
| --- | --- | --- | --- | --- | --- | --- | --- | --- | --- | --- | --- | --- | --- | --- | --- | --- | --- | --- | --- | --- | --- | --- | --- | --- | --- | --- | --- | --- | --- | --- | --- | --- | --- | --- | --- | --- | --- |
| K.GGHLVSIK.S | Y | 42.92 | 809.4759 | 8 | -0.3 | 405.7451 | 2 | 19.86 | 6 | F6:7480 | 17012022\_RID\_3262\_9a.raw |  |  |  | 1.7937E7 |  | 7.5976E7 |  |  |  |  | 2 | 0 | 0 | 0 | 1 | 0 | 1 | 0 | 0 | 0 | 0 | 61 | 68 |  |  | PEAKS DB |
| total 1 peptides |  |  |  |  |  |  |  |  |  |  |  |  |  |  |  |  |  |  |  |  |  |  |  |  |  |  |  |  |  |  |  |  |  |  |  |  |  |
| --- | --- | --- | --- | --- | --- | --- | --- | --- | --- | --- | --- | --- | --- | --- | --- | --- | --- | --- | --- | --- | --- | --- | --- | --- | --- | --- | --- | --- | --- | --- | --- | --- | --- | --- | --- | --- | --- |

JAG46149.1

back to list

  

| Protein Coverage
| Supporting Peptides
|

Protein Coverage:

Supporting Peptides:

| Peptide | Uniq | -10lgP | Mass | Length | ppm | m/z | z | RT | Fraction | Scan | Source File | Area F4 | Area F5 | Area F6 | Area F10 | Area F8 | Area F9 | Area F7 | Area F1 | Area F2 | Area F3 | #Feature | #Feature F4 | #Feature F5 | #Feature F6 | #Feature F10 | #Feature F8 | #Feature F9 | #Feature F7 | #Feature F1 | #Feature F2 | #Feature F3 | Start | End | PTM | AScore | Found By |
| --- | --- | --- | --- | --- | --- | --- | --- | --- | --- | --- | --- | --- | --- | --- | --- | --- | --- | --- | --- | --- | --- | --- | --- | --- | --- | --- | --- | --- | --- | --- | --- | --- | --- | --- | --- | --- | --- |
| K.GGHLVSIK.S | Y | 42.92 | 809.4759 | 8 | -0.3 | 405.7451 | 2 | 19.86 | 6 | F6:7480 | 17012022\_RID\_3262\_9a.raw |  |  |  | 1.7937E7 |  | 7.5976E7 |  |  |  |  | 2 | 0 | 0 | 0 | 1 | 0 | 1 | 0 | 0 | 0 | 0 | 61 | 68 |  |  | PEAKS DB |
| total 1 peptides |  |  |  |  |  |  |  |  |  |  |  |  |  |  |  |  |  |  |  |  |  |  |  |  |  |  |  |  |  |  |  |  |  |  |  |  |  |
| --- | --- | --- | --- | --- | --- | --- | --- | --- | --- | --- | --- | --- | --- | --- | --- | --- | --- | --- | --- | --- | --- | --- | --- | --- | --- | --- | --- | --- | --- | --- | --- | --- | --- | --- | --- | --- | --- |

JAS04883.1

back to list

  

| Protein Coverage
| Supporting Peptides
|

Protein Coverage:

Supporting Peptides:

| Peptide | Uniq | -10lgP | Mass | Length | ppm | m/z | z | RT | Fraction | Scan | Source File | Area F4 | Area F5 | Area F6 | Area F10 | Area F8 | Area F9 | Area F7 | Area F1 | Area F2 | Area F3 | #Feature | #Feature F4 | #Feature F5 | #Feature F6 | #Feature F10 | #Feature F8 | #Feature F9 | #Feature F7 | #Feature F1 | #Feature F2 | #Feature F3 | Start | End | PTM | AScore | Found By |
| --- | --- | --- | --- | --- | --- | --- | --- | --- | --- | --- | --- | --- | --- | --- | --- | --- | --- | --- | --- | --- | --- | --- | --- | --- | --- | --- | --- | --- | --- | --- | --- | --- | --- | --- | --- | --- | --- |
| K.GGHLVSIK.S | Y | 42.92 | 809.4759 | 8 | -0.3 | 405.7451 | 2 | 19.86 | 6 | F6:7480 | 17012022\_RID\_3262\_9a.raw |  |  |  | 1.7937E7 |  | 7.5976E7 |  |  |  |  | 2 | 0 | 0 | 0 | 1 | 0 | 1 | 0 | 0 | 0 | 0 | 61 | 68 |  |  | PEAKS DB |
| total 1 peptides |  |  |  |  |  |  |  |  |  |  |  |  |  |  |  |  |  |  |  |  |  |  |  |  |  |  |  |  |  |  |  |  |  |  |  |  |  |
| --- | --- | --- | --- | --- | --- | --- | --- | --- | --- | --- | --- | --- | --- | --- | --- | --- | --- | --- | --- | --- | --- | --- | --- | --- | --- | --- | --- | --- | --- | --- | --- | --- | --- | --- | --- | --- | --- |

JAA94904.1

back to list

  

| Protein Coverage
| Supporting Peptides
|

Protein Coverage:

Supporting Peptides:

| Peptide | Uniq | -10lgP | Mass | Length | ppm | m/z | z | RT | Fraction | Scan | Source File | Area F4 | Area F5 | Area F6 | Area F10 | Area F8 | Area F9 | Area F7 | Area F1 | Area F2 | Area F3 | #Feature | #Feature F4 | #Feature F5 | #Feature F6 | #Feature F10 | #Feature F8 | #Feature F9 | #Feature F7 | #Feature F1 | #Feature F2 | #Feature F3 | Start | End | PTM | AScore | Found By |
| --- | --- | --- | --- | --- | --- | --- | --- | --- | --- | --- | --- | --- | --- | --- | --- | --- | --- | --- | --- | --- | --- | --- | --- | --- | --- | --- | --- | --- | --- | --- | --- | --- | --- | --- | --- | --- | --- |
| K.GGHLVSIK.S | Y | 42.92 | 809.4759 | 8 | -0.3 | 405.7451 | 2 | 19.86 | 6 | F6:7480 | 17012022\_RID\_3262\_9a.raw |  |  |  | 1.7937E7 |  | 7.5976E7 |  |  |  |  | 2 | 0 | 0 | 0 | 1 | 0 | 1 | 0 | 0 | 0 | 0 | 61 | 68 |  |  | PEAKS DB |
| total 1 peptides |  |  |  |  |  |  |  |  |  |  |  |  |  |  |  |  |  |  |  |  |  |  |  |  |  |  |  |  |  |  |  |  |  |  |  |  |  |
| --- | --- | --- | --- | --- | --- | --- | --- | --- | --- | --- | --- | --- | --- | --- | --- | --- | --- | --- | --- | --- | --- | --- | --- | --- | --- | --- | --- | --- | --- | --- | --- | --- | --- | --- | --- | --- | --- |

XP\_032091818.1

back to list

  

| Protein Coverage
| Supporting Peptides
|

Protein Coverage:

Supporting Peptides:

| Peptide | Uniq | -10lgP | Mass | Length | ppm | m/z | z | RT | Fraction | Scan | Source File | Area F4 | Area F5 | Area F6 | Area F10 | Area F8 | Area F9 | Area F7 | Area F1 | Area F2 | Area F3 | #Feature | #Feature F4 | #Feature F5 | #Feature F6 | #Feature F10 | #Feature F8 | #Feature F9 | #Feature F7 | #Feature F1 | #Feature F2 | #Feature F3 | Start | End | PTM | AScore | Found By |
| --- | --- | --- | --- | --- | --- | --- | --- | --- | --- | --- | --- | --- | --- | --- | --- | --- | --- | --- | --- | --- | --- | --- | --- | --- | --- | --- | --- | --- | --- | --- | --- | --- | --- | --- | --- | --- | --- |
| K.ATMQNLNDR.L | Y | 62.51 | 1061.4924 | 9 | -0.4 | 531.7533 | 2 | 22.42 | 4 | F4:9541 | 17012022\_RID\_3262\_10a .raw |  |  |  | 2.1836E6 |  | 2.8352E6 |  |  |  |  | 2 | 0 | 0 | 0 | 1 | 0 | 1 | 0 | 0 | 0 | 0 | 141 | 149 |  |  | PEAKS DB |
| total 1 peptides |  |  |  |  |  |  |  |  |  |  |  |  |  |  |  |  |  |  |  |  |  |  |  |  |  |  |  |  |  |  |  |  |  |  |  |  |  |
| --- | --- | --- | --- | --- | --- | --- | --- | --- | --- | --- | --- | --- | --- | --- | --- | --- | --- | --- | --- | --- | --- | --- | --- | --- | --- | --- | --- | --- | --- | --- | --- | --- | --- | --- | --- | --- | --- |

XP\_026541829.1

back to list

  

| Protein Coverage
| Supporting Peptides
|

Protein Coverage:

Supporting Peptides:

| Peptide | Uniq | -10lgP | Mass | Length | ppm | m/z | z | RT | Fraction | Scan | Source File | Area F4 | Area F5 | Area F6 | Area F10 | Area F8 | Area F9 | Area F7 | Area F1 | Area F2 | Area F3 | #Feature | #Feature F4 | #Feature F5 | #Feature F6 | #Feature F10 | #Feature F8 | #Feature F9 | #Feature F7 | #Feature F1 | #Feature F2 | #Feature F3 | Start | End | PTM | AScore | Found By |
| --- | --- | --- | --- | --- | --- | --- | --- | --- | --- | --- | --- | --- | --- | --- | --- | --- | --- | --- | --- | --- | --- | --- | --- | --- | --- | --- | --- | --- | --- | --- | --- | --- | --- | --- | --- | --- | --- |
| K.ATMQNLNDR.L | Y | 62.51 | 1061.4924 | 9 | -0.4 | 531.7533 | 2 | 22.42 | 4 | F4:9541 | 17012022\_RID\_3262\_10a .raw |  |  |  | 2.1836E6 |  | 2.8352E6 |  |  |  |  | 2 | 0 | 0 | 0 | 1 | 0 | 1 | 0 | 0 | 0 | 0 | 133 | 141 |  |  | PEAKS DB |
| total 1 peptides |  |  |  |  |  |  |  |  |  |  |  |  |  |  |  |  |  |  |  |  |  |  |  |  |  |  |  |  |  |  |  |  |  |  |  |  |  |
| --- | --- | --- | --- | --- | --- | --- | --- | --- | --- | --- | --- | --- | --- | --- | --- | --- | --- | --- | --- | --- | --- | --- | --- | --- | --- | --- | --- | --- | --- | --- | --- | --- | --- | --- | --- | --- | --- |

XP\_032091819.1

back to list

  

| Protein Coverage
| Supporting Peptides
|

Protein Coverage:

Supporting Peptides:

| Peptide | Uniq | -10lgP | Mass | Length | ppm | m/z | z | RT | Fraction | Scan | Source File | Area F4 | Area F5 | Area F6 | Area F10 | Area F8 | Area F9 | Area F7 | Area F1 | Area F2 | Area F3 | #Feature | #Feature F4 | #Feature F5 | #Feature F6 | #Feature F10 | #Feature F8 | #Feature F9 | #Feature F7 | #Feature F1 | #Feature F2 | #Feature F3 | Start | End | PTM | AScore | Found By |
| --- | --- | --- | --- | --- | --- | --- | --- | --- | --- | --- | --- | --- | --- | --- | --- | --- | --- | --- | --- | --- | --- | --- | --- | --- | --- | --- | --- | --- | --- | --- | --- | --- | --- | --- | --- | --- | --- |
| K.ATMQNLNDR.L | Y | 62.51 | 1061.4924 | 9 | -0.4 | 531.7533 | 2 | 22.42 | 4 | F4:9541 | 17012022\_RID\_3262\_10a .raw |  |  |  | 2.1836E6 |  | 2.8352E6 |  |  |  |  | 2 | 0 | 0 | 0 | 1 | 0 | 1 | 0 | 0 | 0 | 0 | 141 | 149 |  |  | PEAKS DB |
| total 1 peptides |  |  |  |  |  |  |  |  |  |  |  |  |  |  |  |  |  |  |  |  |  |  |  |  |  |  |  |  |  |  |  |  |  |  |  |  |  |
| --- | --- | --- | --- | --- | --- | --- | --- | --- | --- | --- | --- | --- | --- | --- | --- | --- | --- | --- | --- | --- | --- | --- | --- | --- | --- | --- | --- | --- | --- | --- | --- | --- | --- | --- | --- | --- | --- |

XP\_013921793.1

back to list

  

| Protein Coverage
| Supporting Peptides
|

Protein Coverage:

Supporting Peptides:

| Peptide | Uniq | -10lgP | Mass | Length | ppm | m/z | z | RT | Fraction | Scan | Source File | Area F4 | Area F5 | Area F6 | Area F10 | Area F8 | Area F9 | Area F7 | Area F1 | Area F2 | Area F3 | #Feature | #Feature F4 | #Feature F5 | #Feature F6 | #Feature F10 | #Feature F8 | #Feature F9 | #Feature F7 | #Feature F1 | #Feature F2 | #Feature F3 | Start | End | PTM | AScore | Found By |
| --- | --- | --- | --- | --- | --- | --- | --- | --- | --- | --- | --- | --- | --- | --- | --- | --- | --- | --- | --- | --- | --- | --- | --- | --- | --- | --- | --- | --- | --- | --- | --- | --- | --- | --- | --- | --- | --- |
| K.ATMQNLNDR.L | Y | 62.51 | 1061.4924 | 9 | -0.4 | 531.7533 | 2 | 22.42 | 4 | F4:9541 | 17012022\_RID\_3262\_10a .raw |  |  |  | 2.1836E6 |  | 2.8352E6 |  |  |  |  | 2 | 0 | 0 | 0 | 1 | 0 | 1 | 0 | 0 | 0 | 0 | 141 | 149 |  |  | PEAKS DB |
| total 1 peptides |  |  |  |  |  |  |  |  |  |  |  |  |  |  |  |  |  |  |  |  |  |  |  |  |  |  |  |  |  |  |  |  |  |  |  |  |  |
| --- | --- | --- | --- | --- | --- | --- | --- | --- | --- | --- | --- | --- | --- | --- | --- | --- | --- | --- | --- | --- | --- | --- | --- | --- | --- | --- | --- | --- | --- | --- | --- | --- | --- | --- | --- | --- | --- |

XP\_034263292.1

back to list

  

| Protein Coverage
| Supporting Peptides
|

Protein Coverage:

Supporting Peptides:

| Peptide | Uniq | -10lgP | Mass | Length | ppm | m/z | z | RT | Fraction | Scan | Source File | Area F4 | Area F5 | Area F6 | Area F10 | Area F8 | Area F9 | Area F7 | Area F1 | Area F2 | Area F3 | #Feature | #Feature F4 | #Feature F5 | #Feature F6 | #Feature F10 | #Feature F8 | #Feature F9 | #Feature F7 | #Feature F1 | #Feature F2 | #Feature F3 | Start | End | PTM | AScore | Found By |
| --- | --- | --- | --- | --- | --- | --- | --- | --- | --- | --- | --- | --- | --- | --- | --- | --- | --- | --- | --- | --- | --- | --- | --- | --- | --- | --- | --- | --- | --- | --- | --- | --- | --- | --- | --- | --- | --- |
| K.ATMQNLNDR.L | Y | 62.51 | 1061.4924 | 9 | -0.4 | 531.7533 | 2 | 22.42 | 4 | F4:9541 | 17012022\_RID\_3262\_10a .raw |  |  |  | 2.1836E6 |  | 2.8352E6 |  |  |  |  | 2 | 0 | 0 | 0 | 1 | 0 | 1 | 0 | 0 | 0 | 0 | 126 | 134 |  |  | PEAKS DB |
| total 1 peptides |  |  |  |  |  |  |  |  |  |  |  |  |  |  |  |  |  |  |  |  |  |  |  |  |  |  |  |  |  |  |  |  |  |  |  |  |  |
| --- | --- | --- | --- | --- | --- | --- | --- | --- | --- | --- | --- | --- | --- | --- | --- | --- | --- | --- | --- | --- | --- | --- | --- | --- | --- | --- | --- | --- | --- | --- | --- | --- | --- | --- | --- | --- | --- |

LAB46984.1

back to list

  

| Protein Coverage
| Supporting Peptides
|

Protein Coverage:

Supporting Peptides:

| Peptide | Uniq | -10lgP | Mass | Length | ppm | m/z | z | RT | Fraction | Scan | Source File | Area F4 | Area F5 | Area F6 | Area F10 | Area F8 | Area F9 | Area F7 | Area F1 | Area F2 | Area F3 | #Feature | #Feature F4 | #Feature F5 | #Feature F6 | #Feature F10 | #Feature F8 | #Feature F9 | #Feature F7 | #Feature F1 | #Feature F2 | #Feature F3 | Start | End | PTM | AScore | Found By |
| --- | --- | --- | --- | --- | --- | --- | --- | --- | --- | --- | --- | --- | --- | --- | --- | --- | --- | --- | --- | --- | --- | --- | --- | --- | --- | --- | --- | --- | --- | --- | --- | --- | --- | --- | --- | --- | --- |
| K.ATMQNLNDR.L | Y | 62.51 | 1061.4924 | 9 | -0.4 | 531.7533 | 2 | 22.42 | 4 | F4:9541 | 17012022\_RID\_3262\_10a .raw |  |  |  | 2.1836E6 |  | 2.8352E6 |  |  |  |  | 2 | 0 | 0 | 0 | 1 | 0 | 1 | 0 | 0 | 0 | 0 | 133 | 141 |  |  | PEAKS DB |
| total 1 peptides |  |  |  |  |  |  |  |  |  |  |  |  |  |  |  |  |  |  |  |  |  |  |  |  |  |  |  |  |  |  |  |  |  |  |  |  |  |
| --- | --- | --- | --- | --- | --- | --- | --- | --- | --- | --- | --- | --- | --- | --- | --- | --- | --- | --- | --- | --- | --- | --- | --- | --- | --- | --- | --- | --- | --- | --- | --- | --- | --- | --- | --- | --- | --- |

XP\_025025771.1

back to list

  

| Protein Coverage
| Supporting Peptides
|

Protein Coverage:

Supporting Peptides:

| Peptide | Uniq | -10lgP | Mass | Length | ppm | m/z | z | RT | Fraction | Scan | Source File | Area F4 | Area F5 | Area F6 | Area F10 | Area F8 | Area F9 | Area F7 | Area F1 | Area F2 | Area F3 | #Feature | #Feature F4 | #Feature F5 | #Feature F6 | #Feature F10 | #Feature F8 | #Feature F9 | #Feature F7 | #Feature F1 | #Feature F2 | #Feature F3 | Start | End | PTM | AScore | Found By |
| --- | --- | --- | --- | --- | --- | --- | --- | --- | --- | --- | --- | --- | --- | --- | --- | --- | --- | --- | --- | --- | --- | --- | --- | --- | --- | --- | --- | --- | --- | --- | --- | --- | --- | --- | --- | --- | --- |
| K.ATMQNLNDR.L | Y | 62.51 | 1061.4924 | 9 | -0.4 | 531.7533 | 2 | 22.42 | 4 | F4:9541 | 17012022\_RID\_3262\_10a .raw |  |  |  | 2.1836E6 |  | 2.8352E6 |  |  |  |  | 2 | 0 | 0 | 0 | 1 | 0 | 1 | 0 | 0 | 0 | 0 | 119 | 127 |  |  | PEAKS DB |
| total 1 peptides |  |  |  |  |  |  |  |  |  |  |  |  |  |  |  |  |  |  |  |  |  |  |  |  |  |  |  |  |  |  |  |  |  |  |  |  |  |
| --- | --- | --- | --- | --- | --- | --- | --- | --- | --- | --- | --- | --- | --- | --- | --- | --- | --- | --- | --- | --- | --- | --- | --- | --- | --- | --- | --- | --- | --- | --- | --- | --- | --- | --- | --- | --- | --- |

LAA25601.1

back to list

  

| Protein Coverage
| Supporting Peptides
|

Protein Coverage:

Supporting Peptides:

| Peptide | Uniq | -10lgP | Mass | Length | ppm | m/z | z | RT | Fraction | Scan | Source File | Area F4 | Area F5 | Area F6 | Area F10 | Area F8 | Area F9 | Area F7 | Area F1 | Area F2 | Area F3 | #Feature | #Feature F4 | #Feature F5 | #Feature F6 | #Feature F10 | #Feature F8 | #Feature F9 | #Feature F7 | #Feature F1 | #Feature F2 | #Feature F3 | Start | End | PTM | AScore | Found By |
| --- | --- | --- | --- | --- | --- | --- | --- | --- | --- | --- | --- | --- | --- | --- | --- | --- | --- | --- | --- | --- | --- | --- | --- | --- | --- | --- | --- | --- | --- | --- | --- | --- | --- | --- | --- | --- | --- |
| K.ATMQNLNDR.L | Y | 62.51 | 1061.4924 | 9 | -0.4 | 531.7533 | 2 | 22.42 | 4 | F4:9541 | 17012022\_RID\_3262\_10a .raw |  |  |  | 2.1836E6 |  | 2.8352E6 |  |  |  |  | 2 | 0 | 0 | 0 | 1 | 0 | 1 | 0 | 0 | 0 | 0 | 133 | 141 |  |  | PEAKS DB |
| total 1 peptides |  |  |  |  |  |  |  |  |  |  |  |  |  |  |  |  |  |  |  |  |  |  |  |  |  |  |  |  |  |  |  |  |  |  |  |  |  |
| --- | --- | --- | --- | --- | --- | --- | --- | --- | --- | --- | --- | --- | --- | --- | --- | --- | --- | --- | --- | --- | --- | --- | --- | --- | --- | --- | --- | --- | --- | --- | --- | --- | --- | --- | --- | --- | --- |

JAV50411.1

back to list

  

| Protein Coverage
| Supporting Peptides
|

Protein Coverage:

Supporting Peptides:

| Peptide | Uniq | -10lgP | Mass | Length | ppm | m/z | z | RT | Fraction | Scan | Source File | Area F4 | Area F5 | Area F6 | Area F10 | Area F8 | Area F9 | Area F7 | Area F1 | Area F2 | Area F3 | #Feature | #Feature F4 | #Feature F5 | #Feature F6 | #Feature F10 | #Feature F8 | #Feature F9 | #Feature F7 | #Feature F1 | #Feature F2 | #Feature F3 | Start | End | PTM | AScore | Found By |
| --- | --- | --- | --- | --- | --- | --- | --- | --- | --- | --- | --- | --- | --- | --- | --- | --- | --- | --- | --- | --- | --- | --- | --- | --- | --- | --- | --- | --- | --- | --- | --- | --- | --- | --- | --- | --- | --- |
| K.SAYGTVQAPR.D | Y | 59.65 | 1048.5302 | 10 | 0.0 | 525.2723 | 2 | 23.15 | 9 | F9:7734 | 31122021\_RID\_3262\_2a.raw |  |  |  |  |  |  |  |  | 1.1072E5 |  | 1 | 0 | 0 | 0 | 0 | 0 | 0 | 0 | 0 | 1 | 0 | 240 | 249 |  |  | PEAKS DB |
| total 1 peptides |  |  |  |  |  |  |  |  |  |  |  |  |  |  |  |  |  |  |  |  |  |  |  |  |  |  |  |  |  |  |  |  |  |  |  |  |  |
| --- | --- | --- | --- | --- | --- | --- | --- | --- | --- | --- | --- | --- | --- | --- | --- | --- | --- | --- | --- | --- | --- | --- | --- | --- | --- | --- | --- | --- | --- | --- | --- | --- | --- | --- | --- | --- | --- |

JAS03131.1

back to list

  

| Protein Coverage
| Supporting Peptides
|

Protein Coverage:

Supporting Peptides:

| Peptide | Uniq | -10lgP | Mass | Length | ppm | m/z | z | RT | Fraction | Scan | Source File | Area F4 | Area F5 | Area F6 | Area F10 | Area F8 | Area F9 | Area F7 | Area F1 | Area F2 | Area F3 | #Feature | #Feature F4 | #Feature F5 | #Feature F6 | #Feature F10 | #Feature F8 | #Feature F9 | #Feature F7 | #Feature F1 | #Feature F2 | #Feature F3 | Start | End | PTM | AScore | Found By |
| --- | --- | --- | --- | --- | --- | --- | --- | --- | --- | --- | --- | --- | --- | --- | --- | --- | --- | --- | --- | --- | --- | --- | --- | --- | --- | --- | --- | --- | --- | --- | --- | --- | --- | --- | --- | --- | --- |
| R.VMGWGTITSPK.R | Y | 59.06 | 1175.6008 | 11 | -0.1 | 588.8076 | 2 | 41.70 | 2 | F2:23769 | 13012022\_RID\_3262\_5a.raw |  | 3.6855E8 |  |  |  |  |  |  |  |  | 1 | 0 | 1 | 0 | 0 | 0 | 0 | 0 | 0 | 0 | 0 | 139 | 149 |  |  | PEAKS DB |
| R.VM(+15.99)GWGTITSPK.R | Y | 55.96 | 1191.5958 | 11 | 1.4 | 596.8060 | 2 | 37.86 | 2 | F2:22908 | 13012022\_RID\_3262\_5a.raw |  | 8.3127E7 |  |  |  |  |  |  |  |  | 3 | 0 | 3 | 0 | 0 | 0 | 0 | 0 | 0 | 0 | 0 | 139 | 149 | Oxidation (M) | M2:Oxidation (M):1000.00 | PEAKS DB |
| total 2 peptides |  |  |  |  |  |  |  |  |  |  |  |  |  |  |  |  |  |  |  |  |  |  |  |  |  |  |  |  |  |  |  |  |  |  |  |  |  |
| --- | --- | --- | --- | --- | --- | --- | --- | --- | --- | --- | --- | --- | --- | --- | --- | --- | --- | --- | --- | --- | --- | --- | --- | --- | --- | --- | --- | --- | --- | --- | --- | --- | --- | --- | --- | --- | --- |

ABN72545.1

back to list

  

| Protein Coverage
| Supporting Peptides
|

Protein Coverage:

Supporting Peptides:

| Peptide | Uniq | -10lgP | Mass | Length | ppm | m/z | z | RT | Fraction | Scan | Source File | Area F4 | Area F5 | Area F6 | Area F10 | Area F8 | Area F9 | Area F7 | Area F1 | Area F2 | Area F3 | #Feature | #Feature F4 | #Feature F5 | #Feature F6 | #Feature F10 | #Feature F8 | #Feature F9 | #Feature F7 | #Feature F1 | #Feature F2 | #Feature F3 | Start | End | PTM | AScore | Found By |
| --- | --- | --- | --- | --- | --- | --- | --- | --- | --- | --- | --- | --- | --- | --- | --- | --- | --- | --- | --- | --- | --- | --- | --- | --- | --- | --- | --- | --- | --- | --- | --- | --- | --- | --- | --- | --- | --- |
| T.VMGWGTITSPK.V | Y | 59.06 | 1175.6008 | 11 | -0.1 | 588.8076 | 2 | 41.70 | 2 | F2:23769 | 13012022\_RID\_3262\_5a.raw |  | 3.6855E8 |  |  |  |  |  |  |  |  | 1 | 0 | 1 | 0 | 0 | 0 | 0 | 0 | 0 | 0 | 0 | 176 | 186 |  |  | PEAKS DB |
| T.VM(+15.99)GWGTITSPK.V | Y | 55.96 | 1191.5958 | 11 | 1.4 | 596.8060 | 2 | 37.86 | 2 | F2:22908 | 13012022\_RID\_3262\_5a.raw |  | 8.3127E7 |  |  |  |  |  |  |  |  | 3 | 0 | 3 | 0 | 0 | 0 | 0 | 0 | 0 | 0 | 0 | 176 | 186 | Oxidation (M) | M2:Oxidation (M):1000.00 | PEAKS DB |
| total 2 peptides |  |  |  |  |  |  |  |  |  |  |  |  |  |  |  |  |  |  |  |  |  |  |  |  |  |  |  |  |  |  |  |  |  |  |  |  |  |
| --- | --- | --- | --- | --- | --- | --- | --- | --- | --- | --- | --- | --- | --- | --- | --- | --- | --- | --- | --- | --- | --- | --- | --- | --- | --- | --- | --- | --- | --- | --- | --- | --- | --- | --- | --- | --- | --- |

A8QL57.1

back to list

  

| Protein Coverage
| Supporting Peptides
|

Protein Coverage:

Supporting Peptides:

| Peptide | Uniq | -10lgP | Mass | Length | ppm | m/z | z | RT | Fraction | Scan | Source File | Area F4 | Area F5 | Area F6 | Area F10 | Area F8 | Area F9 | Area F7 | Area F1 | Area F2 | Area F3 | #Feature | #Feature F4 | #Feature F5 | #Feature F6 | #Feature F10 | #Feature F8 | #Feature F9 | #Feature F7 | #Feature F1 | #Feature F2 | #Feature F3 | Start | End | PTM | AScore | Found By |
| --- | --- | --- | --- | --- | --- | --- | --- | --- | --- | --- | --- | --- | --- | --- | --- | --- | --- | --- | --- | --- | --- | --- | --- | --- | --- | --- | --- | --- | --- | --- | --- | --- | --- | --- | --- | --- | --- |
| T.VMGWGTITSPK.V | Y | 59.06 | 1175.6008 | 11 | -0.1 | 588.8076 | 2 | 41.70 | 2 | F2:23769 | 13012022\_RID\_3262\_5a.raw |  | 3.6855E8 |  |  |  |  |  |  |  |  | 1 | 0 | 1 | 0 | 0 | 0 | 0 | 0 | 0 | 0 | 0 | 176 | 186 |  |  | PEAKS DB |
| T.VM(+15.99)GWGTITSPK.V | Y | 55.96 | 1191.5958 | 11 | 1.4 | 596.8060 | 2 | 37.86 | 2 | F2:22908 | 13012022\_RID\_3262\_5a.raw |  | 8.3127E7 |  |  |  |  |  |  |  |  | 3 | 0 | 3 | 0 | 0 | 0 | 0 | 0 | 0 | 0 | 0 | 176 | 186 | Oxidation (M) | M2:Oxidation (M):1000.00 | PEAKS DB |
| total 2 peptides |  |  |  |  |  |  |  |  |  |  |  |  |  |  |  |  |  |  |  |  |  |  |  |  |  |  |  |  |  |  |  |  |  |  |  |  |  |
| --- | --- | --- | --- | --- | --- | --- | --- | --- | --- | --- | --- | --- | --- | --- | --- | --- | --- | --- | --- | --- | --- | --- | --- | --- | --- | --- | --- | --- | --- | --- | --- | --- | --- | --- | --- | --- | --- |

ABN72541.1

back to list

  

| Protein Coverage
| Supporting Peptides
|

Protein Coverage:

Supporting Peptides:

| Peptide | Uniq | -10lgP | Mass | Length | ppm | m/z | z | RT | Fraction | Scan | Source File | Area F4 | Area F5 | Area F6 | Area F10 | Area F8 | Area F9 | Area F7 | Area F1 | Area F2 | Area F3 | #Feature | #Feature F4 | #Feature F5 | #Feature F6 | #Feature F10 | #Feature F8 | #Feature F9 | #Feature F7 | #Feature F1 | #Feature F2 | #Feature F3 | Start | End | PTM | AScore | Found By |
| --- | --- | --- | --- | --- | --- | --- | --- | --- | --- | --- | --- | --- | --- | --- | --- | --- | --- | --- | --- | --- | --- | --- | --- | --- | --- | --- | --- | --- | --- | --- | --- | --- | --- | --- | --- | --- | --- |
| T.VMGWGTITSPK.V | Y | 59.06 | 1175.6008 | 11 | -0.1 | 588.8076 | 2 | 41.70 | 2 | F2:23769 | 13012022\_RID\_3262\_5a.raw |  | 3.6855E8 |  |  |  |  |  |  |  |  | 1 | 0 | 1 | 0 | 0 | 0 | 0 | 0 | 0 | 0 | 0 | 176 | 186 |  |  | PEAKS DB |
| T.VM(+15.99)GWGTITSPK.V | Y | 55.96 | 1191.5958 | 11 | 1.4 | 596.8060 | 2 | 37.86 | 2 | F2:22908 | 13012022\_RID\_3262\_5a.raw |  | 8.3127E7 |  |  |  |  |  |  |  |  | 3 | 0 | 3 | 0 | 0 | 0 | 0 | 0 | 0 | 0 | 0 | 176 | 186 | Oxidation (M) | M2:Oxidation (M):1000.00 | PEAKS DB |
| total 2 peptides |  |  |  |  |  |  |  |  |  |  |  |  |  |  |  |  |  |  |  |  |  |  |  |  |  |  |  |  |  |  |  |  |  |  |  |  |  |
| --- | --- | --- | --- | --- | --- | --- | --- | --- | --- | --- | --- | --- | --- | --- | --- | --- | --- | --- | --- | --- | --- | --- | --- | --- | --- | --- | --- | --- | --- | --- | --- | --- | --- | --- | --- | --- | --- |

A8QL53.1

back to list

  

| Protein Coverage
| Supporting Peptides
|

Protein Coverage:

Supporting Peptides:

| Peptide | Uniq | -10lgP | Mass | Length | ppm | m/z | z | RT | Fraction | Scan | Source File | Area F4 | Area F5 | Area F6 | Area F10 | Area F8 | Area F9 | Area F7 | Area F1 | Area F2 | Area F3 | #Feature | #Feature F4 | #Feature F5 | #Feature F6 | #Feature F10 | #Feature F8 | #Feature F9 | #Feature F7 | #Feature F1 | #Feature F2 | #Feature F3 | Start | End | PTM | AScore | Found By |
| --- | --- | --- | --- | --- | --- | --- | --- | --- | --- | --- | --- | --- | --- | --- | --- | --- | --- | --- | --- | --- | --- | --- | --- | --- | --- | --- | --- | --- | --- | --- | --- | --- | --- | --- | --- | --- | --- |
| T.VMGWGTITSPK.V | Y | 59.06 | 1175.6008 | 11 | -0.1 | 588.8076 | 2 | 41.70 | 2 | F2:23769 | 13012022\_RID\_3262\_5a.raw |  | 3.6855E8 |  |  |  |  |  |  |  |  | 1 | 0 | 1 | 0 | 0 | 0 | 0 | 0 | 0 | 0 | 0 | 176 | 186 |  |  | PEAKS DB |
| T.VM(+15.99)GWGTITSPK.V | Y | 55.96 | 1191.5958 | 11 | 1.4 | 596.8060 | 2 | 37.86 | 2 | F2:22908 | 13012022\_RID\_3262\_5a.raw |  | 8.3127E7 |  |  |  |  |  |  |  |  | 3 | 0 | 3 | 0 | 0 | 0 | 0 | 0 | 0 | 0 | 0 | 176 | 186 | Oxidation (M) | M2:Oxidation (M):1000.00 | PEAKS DB |
| total 2 peptides |  |  |  |  |  |  |  |  |  |  |  |  |  |  |  |  |  |  |  |  |  |  |  |  |  |  |  |  |  |  |  |  |  |  |  |  |  |
| --- | --- | --- | --- | --- | --- | --- | --- | --- | --- | --- | --- | --- | --- | --- | --- | --- | --- | --- | --- | --- | --- | --- | --- | --- | --- | --- | --- | --- | --- | --- | --- | --- | --- | --- | --- | --- | --- |

E3P6N7.1

back to list

  

| Protein Coverage
| Supporting Peptides
|

Protein Coverage:

Supporting Peptides:

| Peptide | Uniq | -10lgP | Mass | Length | ppm | m/z | z | RT | Fraction | Scan | Source File | Area F4 | Area F5 | Area F6 | Area F10 | Area F8 | Area F9 | Area F7 | Area F1 | Area F2 | Area F3 | #Feature | #Feature F4 | #Feature F5 | #Feature F6 | #Feature F10 | #Feature F8 | #Feature F9 | #Feature F7 | #Feature F1 | #Feature F2 | #Feature F3 | Start | End | PTM | AScore | Found By |
| --- | --- | --- | --- | --- | --- | --- | --- | --- | --- | --- | --- | --- | --- | --- | --- | --- | --- | --- | --- | --- | --- | --- | --- | --- | --- | --- | --- | --- | --- | --- | --- | --- | --- | --- | --- | --- | --- |
| R.IVEAQSQVVSGAK.Y | Y | 58.84 | 1314.7144 | 13 | -0.4 | 658.3642 | 2 | 25.54 | 4 | F4:12503 | 17012022\_RID\_3262\_10a .raw |  |  |  | 6.295E5 |  |  |  |  |  |  | 1 | 0 | 0 | 0 | 1 | 0 | 0 | 0 | 0 | 0 | 0 | 67 | 79 |  |  | PEAKS DB |
| total 1 peptides |  |  |  |  |  |  |  |  |  |  |  |  |  |  |  |  |  |  |  |  |  |  |  |  |  |  |  |  |  |  |  |  |  |  |  |  |  |
| --- | --- | --- | --- | --- | --- | --- | --- | --- | --- | --- | --- | --- | --- | --- | --- | --- | --- | --- | --- | --- | --- | --- | --- | --- | --- | --- | --- | --- | --- | --- | --- | --- | --- | --- | --- | --- | --- |

ACR83843.1

back to list

  

| Protein Coverage
| Supporting Peptides
|

Protein Coverage:

Supporting Peptides:

| Peptide | Uniq | -10lgP | Mass | Length | ppm | m/z | z | RT | Fraction | Scan | Source File | Area F4 | Area F5 | Area F6 | Area F10 | Area F8 | Area F9 | Area F7 | Area F1 | Area F2 | Area F3 | #Feature | #Feature F4 | #Feature F5 | #Feature F6 | #Feature F10 | #Feature F8 | #Feature F9 | #Feature F7 | #Feature F1 | #Feature F2 | #Feature F3 | Start | End | PTM | AScore | Found By |
| --- | --- | --- | --- | --- | --- | --- | --- | --- | --- | --- | --- | --- | --- | --- | --- | --- | --- | --- | --- | --- | --- | --- | --- | --- | --- | --- | --- | --- | --- | --- | --- | --- | --- | --- | --- | --- | --- |
| R.IVEAQSQVVSGAK.Y | Y | 58.84 | 1314.7144 | 13 | -0.4 | 658.3642 | 2 | 25.54 | 4 | F4:12503 | 17012022\_RID\_3262\_10a .raw |  |  |  | 6.295E5 |  |  |  |  |  |  | 1 | 0 | 0 | 0 | 1 | 0 | 0 | 0 | 0 | 0 | 0 | 67 | 79 |  |  | PEAKS DB |
| total 1 peptides |  |  |  |  |  |  |  |  |  |  |  |  |  |  |  |  |  |  |  |  |  |  |  |  |  |  |  |  |  |  |  |  |  |  |  |  |  |
| --- | --- | --- | --- | --- | --- | --- | --- | --- | --- | --- | --- | --- | --- | --- | --- | --- | --- | --- | --- | --- | --- | --- | --- | --- | --- | --- | --- | --- | --- | --- | --- | --- | --- | --- | --- | --- | --- |

E3P6P3.1

back to list

  

| Protein Coverage
| Supporting Peptides
|

Protein Coverage:

Supporting Peptides:

| Peptide | Uniq | -10lgP | Mass | Length | ppm | m/z | z | RT | Fraction | Scan | Source File | Area F4 | Area F5 | Area F6 | Area F10 | Area F8 | Area F9 | Area F7 | Area F1 | Area F2 | Area F3 | #Feature | #Feature F4 | #Feature F5 | #Feature F6 | #Feature F10 | #Feature F8 | #Feature F9 | #Feature F7 | #Feature F1 | #Feature F2 | #Feature F3 | Start | End | PTM | AScore | Found By |
| --- | --- | --- | --- | --- | --- | --- | --- | --- | --- | --- | --- | --- | --- | --- | --- | --- | --- | --- | --- | --- | --- | --- | --- | --- | --- | --- | --- | --- | --- | --- | --- | --- | --- | --- | --- | --- | --- |
| R.IVEAQSQVVSGAK.Y | Y | 58.84 | 1314.7144 | 13 | -0.4 | 658.3642 | 2 | 25.54 | 4 | F4:12503 | 17012022\_RID\_3262\_10a .raw |  |  |  | 6.295E5 |  |  |  |  |  |  | 1 | 0 | 0 | 0 | 1 | 0 | 0 | 0 | 0 | 0 | 0 | 67 | 79 |  |  | PEAKS DB |
| total 1 peptides |  |  |  |  |  |  |  |  |  |  |  |  |  |  |  |  |  |  |  |  |  |  |  |  |  |  |  |  |  |  |  |  |  |  |  |  |  |
| --- | --- | --- | --- | --- | --- | --- | --- | --- | --- | --- | --- | --- | --- | --- | --- | --- | --- | --- | --- | --- | --- | --- | --- | --- | --- | --- | --- | --- | --- | --- | --- | --- | --- | --- | --- | --- | --- |

ACR83849.1

back to list

  

| Protein Coverage
| Supporting Peptides
|

Protein Coverage:

Supporting Peptides:

| Peptide | Uniq | -10lgP | Mass | Length | ppm | m/z | z | RT | Fraction | Scan | Source File | Area F4 | Area F5 | Area F6 | Area F10 | Area F8 | Area F9 | Area F7 | Area F1 | Area F2 | Area F3 | #Feature | #Feature F4 | #Feature F5 | #Feature F6 | #Feature F10 | #Feature F8 | #Feature F9 | #Feature F7 | #Feature F1 | #Feature F2 | #Feature F3 | Start | End | PTM | AScore | Found By |
| --- | --- | --- | --- | --- | --- | --- | --- | --- | --- | --- | --- | --- | --- | --- | --- | --- | --- | --- | --- | --- | --- | --- | --- | --- | --- | --- | --- | --- | --- | --- | --- | --- | --- | --- | --- | --- | --- |
| R.IVEAQSQVVSGAK.Y | Y | 58.84 | 1314.7144 | 13 | -0.4 | 658.3642 | 2 | 25.54 | 4 | F4:12503 | 17012022\_RID\_3262\_10a .raw |  |  |  | 6.295E5 |  |  |  |  |  |  | 1 | 0 | 0 | 0 | 1 | 0 | 0 | 0 | 0 | 0 | 0 | 67 | 79 |  |  | PEAKS DB |
| total 1 peptides |  |  |  |  |  |  |  |  |  |  |  |  |  |  |  |  |  |  |  |  |  |  |  |  |  |  |  |  |  |  |  |  |  |  |  |  |  |
| --- | --- | --- | --- | --- | --- | --- | --- | --- | --- | --- | --- | --- | --- | --- | --- | --- | --- | --- | --- | --- | --- | --- | --- | --- | --- | --- | --- | --- | --- | --- | --- | --- | --- | --- | --- | --- | --- |

ACR83844.1

back to list

  

| Protein Coverage
| Supporting Peptides
|

Protein Coverage:

Supporting Peptides:

| Peptide | Uniq | -10lgP | Mass | Length | ppm | m/z | z | RT | Fraction | Scan | Source File | Area F4 | Area F5 | Area F6 | Area F10 | Area F8 | Area F9 | Area F7 | Area F1 | Area F2 | Area F3 | #Feature | #Feature F4 | #Feature F5 | #Feature F6 | #Feature F10 | #Feature F8 | #Feature F9 | #Feature F7 | #Feature F1 | #Feature F2 | #Feature F3 | Start | End | PTM | AScore | Found By |
| --- | --- | --- | --- | --- | --- | --- | --- | --- | --- | --- | --- | --- | --- | --- | --- | --- | --- | --- | --- | --- | --- | --- | --- | --- | --- | --- | --- | --- | --- | --- | --- | --- | --- | --- | --- | --- | --- |
| R.IVEAQSQVVSGAK.Y | Y | 58.84 | 1314.7144 | 13 | -0.4 | 658.3642 | 2 | 25.54 | 4 | F4:12503 | 17012022\_RID\_3262\_10a .raw |  |  |  | 6.295E5 |  |  |  |  |  |  | 1 | 0 | 0 | 0 | 1 | 0 | 0 | 0 | 0 | 0 | 0 | 67 | 79 |  |  | PEAKS DB |
| total 1 peptides |  |  |  |  |  |  |  |  |  |  |  |  |  |  |  |  |  |  |  |  |  |  |  |  |  |  |  |  |  |  |  |  |  |  |  |  |  |
| --- | --- | --- | --- | --- | --- | --- | --- | --- | --- | --- | --- | --- | --- | --- | --- | --- | --- | --- | --- | --- | --- | --- | --- | --- | --- | --- | --- | --- | --- | --- | --- | --- | --- | --- | --- | --- | --- |

E3P6N8.1

back to list

  

| Protein Coverage
| Supporting Peptides
|

Protein Coverage:

Supporting Peptides:

| Peptide | Uniq | -10lgP | Mass | Length | ppm | m/z | z | RT | Fraction | Scan | Source File | Area F4 | Area F5 | Area F6 | Area F10 | Area F8 | Area F9 | Area F7 | Area F1 | Area F2 | Area F3 | #Feature | #Feature F4 | #Feature F5 | #Feature F6 | #Feature F10 | #Feature F8 | #Feature F9 | #Feature F7 | #Feature F1 | #Feature F2 | #Feature F3 | Start | End | PTM | AScore | Found By |
| --- | --- | --- | --- | --- | --- | --- | --- | --- | --- | --- | --- | --- | --- | --- | --- | --- | --- | --- | --- | --- | --- | --- | --- | --- | --- | --- | --- | --- | --- | --- | --- | --- | --- | --- | --- | --- | --- |
| R.IVEAQSQVVSGAK.Y | Y | 58.84 | 1314.7144 | 13 | -0.4 | 658.3642 | 2 | 25.54 | 4 | F4:12503 | 17012022\_RID\_3262\_10a .raw |  |  |  | 6.295E5 |  |  |  |  |  |  | 1 | 0 | 0 | 0 | 1 | 0 | 0 | 0 | 0 | 0 | 0 | 67 | 79 |  |  | PEAKS DB |
| total 1 peptides |  |  |  |  |  |  |  |  |  |  |  |  |  |  |  |  |  |  |  |  |  |  |  |  |  |  |  |  |  |  |  |  |  |  |  |  |  |
| --- | --- | --- | --- | --- | --- | --- | --- | --- | --- | --- | --- | --- | --- | --- | --- | --- | --- | --- | --- | --- | --- | --- | --- | --- | --- | --- | --- | --- | --- | --- | --- | --- | --- | --- | --- | --- | --- |

JAA97708.1

back to list

  

| Protein Coverage
| Supporting Peptides
|

Protein Coverage:

Supporting Peptides:

| Peptide | Uniq | -10lgP | Mass | Length | ppm | m/z | z | RT | Fraction | Scan | Source File | Area F4 | Area F5 | Area F6 | Area F10 | Area F8 | Area F9 | Area F7 | Area F1 | Area F2 | Area F3 | #Feature | #Feature F4 | #Feature F5 | #Feature F6 | #Feature F10 | #Feature F8 | #Feature F9 | #Feature F7 | #Feature F1 | #Feature F2 | #Feature F3 | Start | End | PTM | AScore | Found By |
| --- | --- | --- | --- | --- | --- | --- | --- | --- | --- | --- | --- | --- | --- | --- | --- | --- | --- | --- | --- | --- | --- | --- | --- | --- | --- | --- | --- | --- | --- | --- | --- | --- | --- | --- | --- | --- | --- |
| K.AVDSLVPIGR.G | Y | 42.56 | 1025.5869 | 10 | 0.2 | 513.8008 | 2 | 41.00 | 4 | F4:25892 | 17012022\_RID\_3262\_10a .raw |  |  |  | 5.6349E4 |  |  |  |  |  |  | 1 | 0 | 0 | 0 | 1 | 0 | 0 | 0 | 0 | 0 | 0 | 195 | 204 |  |  | PEAKS DB |
| total 1 peptides |  |  |  |  |  |  |  |  |  |  |  |  |  |  |  |  |  |  |  |  |  |  |  |  |  |  |  |  |  |  |  |  |  |  |  |  |  |
| --- | --- | --- | --- | --- | --- | --- | --- | --- | --- | --- | --- | --- | --- | --- | --- | --- | --- | --- | --- | --- | --- | --- | --- | --- | --- | --- | --- | --- | --- | --- | --- | --- | --- | --- | --- | --- | --- |

XP\_013918352.1

back to list

  

| Protein Coverage
| Supporting Peptides
|

Protein Coverage:

Supporting Peptides:

| Peptide | Uniq | -10lgP | Mass | Length | ppm | m/z | z | RT | Fraction | Scan | Source File | Area F4 | Area F5 | Area F6 | Area F10 | Area F8 | Area F9 | Area F7 | Area F1 | Area F2 | Area F3 | #Feature | #Feature F4 | #Feature F5 | #Feature F6 | #Feature F10 | #Feature F8 | #Feature F9 | #Feature F7 | #Feature F1 | #Feature F2 | #Feature F3 | Start | End | PTM | AScore | Found By |
| --- | --- | --- | --- | --- | --- | --- | --- | --- | --- | --- | --- | --- | --- | --- | --- | --- | --- | --- | --- | --- | --- | --- | --- | --- | --- | --- | --- | --- | --- | --- | --- | --- | --- | --- | --- | --- | --- |
| K.TPAQFDADELR.Y | Y | 56.49 | 1261.5939 | 11 | 0.2 | 631.8043 | 2 | 36.76 | 7 | F7:16919 | 21012022\_RID\_3262\_7a.raw |  |  |  |  |  |  | 9.1107E4 |  |  |  | 1 | 0 | 0 | 0 | 0 | 0 | 0 | 1 | 0 | 0 | 0 | 86 | 96 |  |  | PEAKS DB |
| total 1 peptides |  |  |  |  |  |  |  |  |  |  |  |  |  |  |  |  |  |  |  |  |  |  |  |  |  |  |  |  |  |  |  |  |  |  |  |  |  |
| --- | --- | --- | --- | --- | --- | --- | --- | --- | --- | --- | --- | --- | --- | --- | --- | --- | --- | --- | --- | --- | --- | --- | --- | --- | --- | --- | --- | --- | --- | --- | --- | --- | --- | --- | --- | --- | --- |

XP\_032070448.1

back to list

  

| Protein Coverage
| Supporting Peptides
|

Protein Coverage:

Supporting Peptides:

| Peptide | Uniq | -10lgP | Mass | Length | ppm | m/z | z | RT | Fraction | Scan | Source File | Area F4 | Area F5 | Area F6 | Area F10 | Area F8 | Area F9 | Area F7 | Area F1 | Area F2 | Area F3 | #Feature | #Feature F4 | #Feature F5 | #Feature F6 | #Feature F10 | #Feature F8 | #Feature F9 | #Feature F7 | #Feature F1 | #Feature F2 | #Feature F3 | Start | End | PTM | AScore | Found By |
| --- | --- | --- | --- | --- | --- | --- | --- | --- | --- | --- | --- | --- | --- | --- | --- | --- | --- | --- | --- | --- | --- | --- | --- | --- | --- | --- | --- | --- | --- | --- | --- | --- | --- | --- | --- | --- | --- |
| K.TPAQFDADELR.Y | Y | 56.49 | 1261.5939 | 11 | 0.2 | 631.8043 | 2 | 36.76 | 7 | F7:16919 | 21012022\_RID\_3262\_7a.raw |  |  |  |  |  |  | 9.1107E4 |  |  |  | 1 | 0 | 0 | 0 | 0 | 0 | 0 | 1 | 0 | 0 | 0 | 109 | 119 |  |  | PEAKS DB |
| total 1 peptides |  |  |  |  |  |  |  |  |  |  |  |  |  |  |  |  |  |  |  |  |  |  |  |  |  |  |  |  |  |  |  |  |  |  |  |  |  |
| --- | --- | --- | --- | --- | --- | --- | --- | --- | --- | --- | --- | --- | --- | --- | --- | --- | --- | --- | --- | --- | --- | --- | --- | --- | --- | --- | --- | --- | --- | --- | --- | --- | --- | --- | --- | --- | --- |

XP\_007430670.1

back to list

  

| Protein Coverage
| Supporting Peptides
|

Protein Coverage:

Supporting Peptides:

| Peptide | Uniq | -10lgP | Mass | Length | ppm | m/z | z | RT | Fraction | Scan | Source File | Area F4 | Area F5 | Area F6 | Area F10 | Area F8 | Area F9 | Area F7 | Area F1 | Area F2 | Area F3 | #Feature | #Feature F4 | #Feature F5 | #Feature F6 | #Feature F10 | #Feature F8 | #Feature F9 | #Feature F7 | #Feature F1 | #Feature F2 | #Feature F3 | Start | End | PTM | AScore | Found By |
| --- | --- | --- | --- | --- | --- | --- | --- | --- | --- | --- | --- | --- | --- | --- | --- | --- | --- | --- | --- | --- | --- | --- | --- | --- | --- | --- | --- | --- | --- | --- | --- | --- | --- | --- | --- | --- | --- |
| K.LTLSALLDGK.N | Y | 54.85 | 1029.6069 | 10 | 0.6 | 515.8110 | 2 | 51.10 | 8 | F8:29079 | 31122021\_RID\_3262\_1a.raw |  |  |  |  |  |  |  | 7.3889E4 |  |  | 1 | 0 | 0 | 0 | 0 | 0 | 0 | 0 | 1 | 0 | 0 | 299 | 308 |  |  | PEAKS DB |
| total 1 peptides |  |  |  |  |  |  |  |  |  |  |  |  |  |  |  |  |  |  |  |  |  |  |  |  |  |  |  |  |  |  |  |  |  |  |  |  |  |
| --- | --- | --- | --- | --- | --- | --- | --- | --- | --- | --- | --- | --- | --- | --- | --- | --- | --- | --- | --- | --- | --- | --- | --- | --- | --- | --- | --- | --- | --- | --- | --- | --- | --- | --- | --- | --- | --- |

XP\_026543118.1

back to list

  

| Protein Coverage
| Supporting Peptides
|

Protein Coverage:

Supporting Peptides:

| Peptide | Uniq | -10lgP | Mass | Length | ppm | m/z | z | RT | Fraction | Scan | Source File | Area F4 | Area F5 | Area F6 | Area F10 | Area F8 | Area F9 | Area F7 | Area F1 | Area F2 | Area F3 | #Feature | #Feature F4 | #Feature F5 | #Feature F6 | #Feature F10 | #Feature F8 | #Feature F9 | #Feature F7 | #Feature F1 | #Feature F2 | #Feature F3 | Start | End | PTM | AScore | Found By |
| --- | --- | --- | --- | --- | --- | --- | --- | --- | --- | --- | --- | --- | --- | --- | --- | --- | --- | --- | --- | --- | --- | --- | --- | --- | --- | --- | --- | --- | --- | --- | --- | --- | --- | --- | --- | --- | --- |
| K.LTLSALLDGK.N | Y | 54.85 | 1029.6069 | 10 | 0.6 | 515.8110 | 2 | 51.10 | 8 | F8:29079 | 31122021\_RID\_3262\_1a.raw |  |  |  |  |  |  |  | 7.3889E4 |  |  | 1 | 0 | 0 | 0 | 0 | 0 | 0 | 0 | 1 | 0 | 0 | 257 | 266 |  |  | PEAKS DB |
| total 1 peptides |  |  |  |  |  |  |  |  |  |  |  |  |  |  |  |  |  |  |  |  |  |  |  |  |  |  |  |  |  |  |  |  |  |  |  |  |  |
| --- | --- | --- | --- | --- | --- | --- | --- | --- | --- | --- | --- | --- | --- | --- | --- | --- | --- | --- | --- | --- | --- | --- | --- | --- | --- | --- | --- | --- | --- | --- | --- | --- | --- | --- | --- | --- | --- |

JAI09157.1

back to list

  

| Protein Coverage
| Supporting Peptides
|

Protein Coverage:

Supporting Peptides:

| Peptide | Uniq | -10lgP | Mass | Length | ppm | m/z | z | RT | Fraction | Scan | Source File | Area F4 | Area F5 | Area F6 | Area F10 | Area F8 | Area F9 | Area F7 | Area F1 | Area F2 | Area F3 | #Feature | #Feature F4 | #Feature F5 | #Feature F6 | #Feature F10 | #Feature F8 | #Feature F9 | #Feature F7 | #Feature F1 | #Feature F2 | #Feature F3 | Start | End | PTM | AScore | Found By |
| --- | --- | --- | --- | --- | --- | --- | --- | --- | --- | --- | --- | --- | --- | --- | --- | --- | --- | --- | --- | --- | --- | --- | --- | --- | --- | --- | --- | --- | --- | --- | --- | --- | --- | --- | --- | --- | --- |
| K.LTLSALLDGK.N | Y | 54.85 | 1029.6069 | 10 | 0.6 | 515.8110 | 2 | 51.10 | 8 | F8:29079 | 31122021\_RID\_3262\_1a.raw |  |  |  |  |  |  |  | 7.3889E4 |  |  | 1 | 0 | 0 | 0 | 0 | 0 | 0 | 0 | 1 | 0 | 0 | 257 | 266 |  |  | PEAKS DB |
| total 1 peptides |  |  |  |  |  |  |  |  |  |  |  |  |  |  |  |  |  |  |  |  |  |  |  |  |  |  |  |  |  |  |  |  |  |  |  |  |  |
| --- | --- | --- | --- | --- | --- | --- | --- | --- | --- | --- | --- | --- | --- | --- | --- | --- | --- | --- | --- | --- | --- | --- | --- | --- | --- | --- | --- | --- | --- | --- | --- | --- | --- | --- | --- | --- | --- |

JAB52928.1

back to list

  

| Protein Coverage
| Supporting Peptides
|

Protein Coverage:

Supporting Peptides:

| Peptide | Uniq | -10lgP | Mass | Length | ppm | m/z | z | RT | Fraction | Scan | Source File | Area F4 | Area F5 | Area F6 | Area F10 | Area F8 | Area F9 | Area F7 | Area F1 | Area F2 | Area F3 | #Feature | #Feature F4 | #Feature F5 | #Feature F6 | #Feature F10 | #Feature F8 | #Feature F9 | #Feature F7 | #Feature F1 | #Feature F2 | #Feature F3 | Start | End | PTM | AScore | Found By |
| --- | --- | --- | --- | --- | --- | --- | --- | --- | --- | --- | --- | --- | --- | --- | --- | --- | --- | --- | --- | --- | --- | --- | --- | --- | --- | --- | --- | --- | --- | --- | --- | --- | --- | --- | --- | --- | --- |
| K.LTLSALLDGK.N | Y | 54.85 | 1029.6069 | 10 | 0.6 | 515.8110 | 2 | 51.10 | 8 | F8:29079 | 31122021\_RID\_3262\_1a.raw |  |  |  |  |  |  |  | 7.3889E4 |  |  | 1 | 0 | 0 | 0 | 0 | 0 | 0 | 0 | 1 | 0 | 0 | 257 | 266 |  |  | PEAKS DB |
| total 1 peptides |  |  |  |  |  |  |  |  |  |  |  |  |  |  |  |  |  |  |  |  |  |  |  |  |  |  |  |  |  |  |  |  |  |  |  |  |  |
| --- | --- | --- | --- | --- | --- | --- | --- | --- | --- | --- | --- | --- | --- | --- | --- | --- | --- | --- | --- | --- | --- | --- | --- | --- | --- | --- | --- | --- | --- | --- | --- | --- | --- | --- | --- | --- | --- |

XP\_026552260.1

back to list

  

| Protein Coverage
| Supporting Peptides
|

Protein Coverage:

Supporting Peptides:

| Peptide | Uniq | -10lgP | Mass | Length | ppm | m/z | z | RT | Fraction | Scan | Source File | Area F4 | Area F5 | Area F6 | Area F10 | Area F8 | Area F9 | Area F7 | Area F1 | Area F2 | Area F3 | #Feature | #Feature F4 | #Feature F5 | #Feature F6 | #Feature F10 | #Feature F8 | #Feature F9 | #Feature F7 | #Feature F1 | #Feature F2 | #Feature F3 | Start | End | PTM | AScore | Found By |
| --- | --- | --- | --- | --- | --- | --- | --- | --- | --- | --- | --- | --- | --- | --- | --- | --- | --- | --- | --- | --- | --- | --- | --- | --- | --- | --- | --- | --- | --- | --- | --- | --- | --- | --- | --- | --- | --- |
| K.LTLSALLDGK.N | Y | 54.85 | 1029.6069 | 10 | 0.6 | 515.8110 | 2 | 51.10 | 8 | F8:29079 | 31122021\_RID\_3262\_1a.raw |  |  |  |  |  |  |  | 7.3889E4 |  |  | 1 | 0 | 0 | 0 | 0 | 0 | 0 | 0 | 1 | 0 | 0 | 299 | 308 |  |  | PEAKS DB |
| total 1 peptides |  |  |  |  |  |  |  |  |  |  |  |  |  |  |  |  |  |  |  |  |  |  |  |  |  |  |  |  |  |  |  |  |  |  |  |  |  |
| --- | --- | --- | --- | --- | --- | --- | --- | --- | --- | --- | --- | --- | --- | --- | --- | --- | --- | --- | --- | --- | --- | --- | --- | --- | --- | --- | --- | --- | --- | --- | --- | --- | --- | --- | --- | --- | --- |

LAA28449.1

back to list

  

| Protein Coverage
| Supporting Peptides
|

Protein Coverage:

Supporting Peptides:

| Peptide | Uniq | -10lgP | Mass | Length | ppm | m/z | z | RT | Fraction | Scan | Source File | Area F4 | Area F5 | Area F6 | Area F10 | Area F8 | Area F9 | Area F7 | Area F1 | Area F2 | Area F3 | #Feature | #Feature F4 | #Feature F5 | #Feature F6 | #Feature F10 | #Feature F8 | #Feature F9 | #Feature F7 | #Feature F1 | #Feature F2 | #Feature F3 | Start | End | PTM | AScore | Found By |
| --- | --- | --- | --- | --- | --- | --- | --- | --- | --- | --- | --- | --- | --- | --- | --- | --- | --- | --- | --- | --- | --- | --- | --- | --- | --- | --- | --- | --- | --- | --- | --- | --- | --- | --- | --- | --- | --- |
| K.LTLSALLDGK.N | Y | 54.85 | 1029.6069 | 10 | 0.6 | 515.8110 | 2 | 51.10 | 8 | F8:29079 | 31122021\_RID\_3262\_1a.raw |  |  |  |  |  |  |  | 7.3889E4 |  |  | 1 | 0 | 0 | 0 | 0 | 0 | 0 | 0 | 1 | 0 | 0 | 341 | 350 |  |  | PEAKS DB |
| total 1 peptides |  |  |  |  |  |  |  |  |  |  |  |  |  |  |  |  |  |  |  |  |  |  |  |  |  |  |  |  |  |  |  |  |  |  |  |  |  |
| --- | --- | --- | --- | --- | --- | --- | --- | --- | --- | --- | --- | --- | --- | --- | --- | --- | --- | --- | --- | --- | --- | --- | --- | --- | --- | --- | --- | --- | --- | --- | --- | --- | --- | --- | --- | --- | --- |

JAI10615.1

back to list

  

| Protein Coverage
| Supporting Peptides
|

Protein Coverage:

Supporting Peptides:

| Peptide | Uniq | -10lgP | Mass | Length | ppm | m/z | z | RT | Fraction | Scan | Source File | Area F4 | Area F5 | Area F6 | Area F10 | Area F8 | Area F9 | Area F7 | Area F1 | Area F2 | Area F3 | #Feature | #Feature F4 | #Feature F5 | #Feature F6 | #Feature F10 | #Feature F8 | #Feature F9 | #Feature F7 | #Feature F1 | #Feature F2 | #Feature F3 | Start | End | PTM | AScore | Found By |
| --- | --- | --- | --- | --- | --- | --- | --- | --- | --- | --- | --- | --- | --- | --- | --- | --- | --- | --- | --- | --- | --- | --- | --- | --- | --- | --- | --- | --- | --- | --- | --- | --- | --- | --- | --- | --- | --- |
| K.LTLSALLDGK.N | Y | 54.85 | 1029.6069 | 10 | 0.6 | 515.8110 | 2 | 51.10 | 8 | F8:29079 | 31122021\_RID\_3262\_1a.raw |  |  |  |  |  |  |  | 7.3889E4 |  |  | 1 | 0 | 0 | 0 | 0 | 0 | 0 | 0 | 1 | 0 | 0 | 257 | 266 |  |  | PEAKS DB |
| total 1 peptides |  |  |  |  |  |  |  |  |  |  |  |  |  |  |  |  |  |  |  |  |  |  |  |  |  |  |  |  |  |  |  |  |  |  |  |  |  |
| --- | --- | --- | --- | --- | --- | --- | --- | --- | --- | --- | --- | --- | --- | --- | --- | --- | --- | --- | --- | --- | --- | --- | --- | --- | --- | --- | --- | --- | --- | --- | --- | --- | --- | --- | --- | --- | --- |

JAG65932.1

back to list

  

| Protein Coverage
| Supporting Peptides
|

Protein Coverage:

Supporting Peptides:

| Peptide | Uniq | -10lgP | Mass | Length | ppm | m/z | z | RT | Fraction | Scan | Source File | Area F4 | Area F5 | Area F6 | Area F10 | Area F8 | Area F9 | Area F7 | Area F1 | Area F2 | Area F3 | #Feature | #Feature F4 | #Feature F5 | #Feature F6 | #Feature F10 | #Feature F8 | #Feature F9 | #Feature F7 | #Feature F1 | #Feature F2 | #Feature F3 | Start | End | PTM | AScore | Found By |
| --- | --- | --- | --- | --- | --- | --- | --- | --- | --- | --- | --- | --- | --- | --- | --- | --- | --- | --- | --- | --- | --- | --- | --- | --- | --- | --- | --- | --- | --- | --- | --- | --- | --- | --- | --- | --- | --- |
| K.LTLSALLDGK.N | Y | 54.85 | 1029.6069 | 10 | 0.6 | 515.8110 | 2 | 51.10 | 8 | F8:29079 | 31122021\_RID\_3262\_1a.raw |  |  |  |  |  |  |  | 7.3889E4 |  |  | 1 | 0 | 0 | 0 | 0 | 0 | 0 | 0 | 1 | 0 | 0 | 257 | 266 |  |  | PEAKS DB |
| total 1 peptides |  |  |  |  |  |  |  |  |  |  |  |  |  |  |  |  |  |  |  |  |  |  |  |  |  |  |  |  |  |  |  |  |  |  |  |  |  |
| --- | --- | --- | --- | --- | --- | --- | --- | --- | --- | --- | --- | --- | --- | --- | --- | --- | --- | --- | --- | --- | --- | --- | --- | --- | --- | --- | --- | --- | --- | --- | --- | --- | --- | --- | --- | --- | --- |

JAV48559.1

back to list

  

| Protein Coverage
| Supporting Peptides
|

Protein Coverage:

Supporting Peptides:

| Peptide | Uniq | -10lgP | Mass | Length | ppm | m/z | z | RT | Fraction | Scan | Source File | Area F4 | Area F5 | Area F6 | Area F10 | Area F8 | Area F9 | Area F7 | Area F1 | Area F2 | Area F3 | #Feature | #Feature F4 | #Feature F5 | #Feature F6 | #Feature F10 | #Feature F8 | #Feature F9 | #Feature F7 | #Feature F1 | #Feature F2 | #Feature F3 | Start | End | PTM | AScore | Found By |
| --- | --- | --- | --- | --- | --- | --- | --- | --- | --- | --- | --- | --- | --- | --- | --- | --- | --- | --- | --- | --- | --- | --- | --- | --- | --- | --- | --- | --- | --- | --- | --- | --- | --- | --- | --- | --- | --- |
| K.LTLSALLDGK.N | Y | 54.85 | 1029.6069 | 10 | 0.6 | 515.8110 | 2 | 51.10 | 8 | F8:29079 | 31122021\_RID\_3262\_1a.raw |  |  |  |  |  |  |  | 7.3889E4 |  |  | 1 | 0 | 0 | 0 | 0 | 0 | 0 | 0 | 1 | 0 | 0 | 257 | 266 |  |  | PEAKS DB |
| total 1 peptides |  |  |  |  |  |  |  |  |  |  |  |  |  |  |  |  |  |  |  |  |  |  |  |  |  |  |  |  |  |  |  |  |  |  |  |  |  |
| --- | --- | --- | --- | --- | --- | --- | --- | --- | --- | --- | --- | --- | --- | --- | --- | --- | --- | --- | --- | --- | --- | --- | --- | --- | --- | --- | --- | --- | --- | --- | --- | --- | --- | --- | --- | --- | --- |

XP\_034260052.1

back to list

  

| Protein Coverage
| Supporting Peptides
|

Protein Coverage:

Supporting Peptides:

| Peptide | Uniq | -10lgP | Mass | Length | ppm | m/z | z | RT | Fraction | Scan | Source File | Area F4 | Area F5 | Area F6 | Area F10 | Area F8 | Area F9 | Area F7 | Area F1 | Area F2 | Area F3 | #Feature | #Feature F4 | #Feature F5 | #Feature F6 | #Feature F10 | #Feature F8 | #Feature F9 | #Feature F7 | #Feature F1 | #Feature F2 | #Feature F3 | Start | End | PTM | AScore | Found By |
| --- | --- | --- | --- | --- | --- | --- | --- | --- | --- | --- | --- | --- | --- | --- | --- | --- | --- | --- | --- | --- | --- | --- | --- | --- | --- | --- | --- | --- | --- | --- | --- | --- | --- | --- | --- | --- | --- |
| K.LTLSALLDGK.N | Y | 54.85 | 1029.6069 | 10 | 0.6 | 515.8110 | 2 | 51.10 | 8 | F8:29079 | 31122021\_RID\_3262\_1a.raw |  |  |  |  |  |  |  | 7.3889E4 |  |  | 1 | 0 | 0 | 0 | 0 | 0 | 0 | 0 | 1 | 0 | 0 | 257 | 266 |  |  | PEAKS DB |
| total 1 peptides |  |  |  |  |  |  |  |  |  |  |  |  |  |  |  |  |  |  |  |  |  |  |  |  |  |  |  |  |  |  |  |  |  |  |  |  |  |
| --- | --- | --- | --- | --- | --- | --- | --- | --- | --- | --- | --- | --- | --- | --- | --- | --- | --- | --- | --- | --- | --- | --- | --- | --- | --- | --- | --- | --- | --- | --- | --- | --- | --- | --- | --- | --- | --- |

XP\_034260044.1

back to list

  

| Protein Coverage
| Supporting Peptides
|

Protein Coverage:

Supporting Peptides:

| Peptide | Uniq | -10lgP | Mass | Length | ppm | m/z | z | RT | Fraction | Scan | Source File | Area F4 | Area F5 | Area F6 | Area F10 | Area F8 | Area F9 | Area F7 | Area F1 | Area F2 | Area F3 | #Feature | #Feature F4 | #Feature F5 | #Feature F6 | #Feature F10 | #Feature F8 | #Feature F9 | #Feature F7 | #Feature F1 | #Feature F2 | #Feature F3 | Start | End | PTM | AScore | Found By |
| --- | --- | --- | --- | --- | --- | --- | --- | --- | --- | --- | --- | --- | --- | --- | --- | --- | --- | --- | --- | --- | --- | --- | --- | --- | --- | --- | --- | --- | --- | --- | --- | --- | --- | --- | --- | --- | --- |
| K.LTLSALLDGK.N | Y | 54.85 | 1029.6069 | 10 | 0.6 | 515.8110 | 2 | 51.10 | 8 | F8:29079 | 31122021\_RID\_3262\_1a.raw |  |  |  |  |  |  |  | 7.3889E4 |  |  | 1 | 0 | 0 | 0 | 0 | 0 | 0 | 0 | 1 | 0 | 0 | 257 | 266 |  |  | PEAKS DB |
| total 1 peptides |  |  |  |  |  |  |  |  |  |  |  |  |  |  |  |  |  |  |  |  |  |  |  |  |  |  |  |  |  |  |  |  |  |  |  |  |  |
| --- | --- | --- | --- | --- | --- | --- | --- | --- | --- | --- | --- | --- | --- | --- | --- | --- | --- | --- | --- | --- | --- | --- | --- | --- | --- | --- | --- | --- | --- | --- | --- | --- | --- | --- | --- | --- | --- |

JAA95034.1

back to list

  

| Protein Coverage
| Supporting Peptides
|

Protein Coverage:

Supporting Peptides:

| Peptide | Uniq | -10lgP | Mass | Length | ppm | m/z | z | RT | Fraction | Scan | Source File | Area F4 | Area F5 | Area F6 | Area F10 | Area F8 | Area F9 | Area F7 | Area F1 | Area F2 | Area F3 | #Feature | #Feature F4 | #Feature F5 | #Feature F6 | #Feature F10 | #Feature F8 | #Feature F9 | #Feature F7 | #Feature F1 | #Feature F2 | #Feature F3 | Start | End | PTM | AScore | Found By |
| --- | --- | --- | --- | --- | --- | --- | --- | --- | --- | --- | --- | --- | --- | --- | --- | --- | --- | --- | --- | --- | --- | --- | --- | --- | --- | --- | --- | --- | --- | --- | --- | --- | --- | --- | --- | --- | --- |
| K.LTLSALLDGK.N | Y | 54.85 | 1029.6069 | 10 | 0.6 | 515.8110 | 2 | 51.10 | 8 | F8:29079 | 31122021\_RID\_3262\_1a.raw |  |  |  |  |  |  |  | 7.3889E4 |  |  | 1 | 0 | 0 | 0 | 0 | 0 | 0 | 0 | 1 | 0 | 0 | 257 | 266 |  |  | PEAKS DB |
| total 1 peptides |  |  |  |  |  |  |  |  |  |  |  |  |  |  |  |  |  |  |  |  |  |  |  |  |  |  |  |  |  |  |  |  |  |  |  |  |  |
| --- | --- | --- | --- | --- | --- | --- | --- | --- | --- | --- | --- | --- | --- | --- | --- | --- | --- | --- | --- | --- | --- | --- | --- | --- | --- | --- | --- | --- | --- | --- | --- | --- | --- | --- | --- | --- | --- |

BAN82166.1

back to list

  

| Protein Coverage
| Supporting Peptides
|

Protein Coverage:

Supporting Peptides:

| Peptide | Uniq | -10lgP | Mass | Length | ppm | m/z | z | RT | Fraction | Scan | Source File | Area F4 | Area F5 | Area F6 | Area F10 | Area F8 | Area F9 | Area F7 | Area F1 | Area F2 | Area F3 | #Feature | #Feature F4 | #Feature F5 | #Feature F6 | #Feature F10 | #Feature F8 | #Feature F9 | #Feature F7 | #Feature F1 | #Feature F2 | #Feature F3 | Start | End | PTM | AScore | Found By |
| --- | --- | --- | --- | --- | --- | --- | --- | --- | --- | --- | --- | --- | --- | --- | --- | --- | --- | --- | --- | --- | --- | --- | --- | --- | --- | --- | --- | --- | --- | --- | --- | --- | --- | --- | --- | --- | --- |
| K.LTLSALLDGK.N | Y | 54.85 | 1029.6069 | 10 | 0.6 | 515.8110 | 2 | 51.10 | 8 | F8:29079 | 31122021\_RID\_3262\_1a.raw |  |  |  |  |  |  |  | 7.3889E4 |  |  | 1 | 0 | 0 | 0 | 0 | 0 | 0 | 0 | 1 | 0 | 0 | 299 | 308 |  |  | PEAKS DB |
| total 1 peptides |  |  |  |  |  |  |  |  |  |  |  |  |  |  |  |  |  |  |  |  |  |  |  |  |  |  |  |  |  |  |  |  |  |  |  |  |  |
| --- | --- | --- | --- | --- | --- | --- | --- | --- | --- | --- | --- | --- | --- | --- | --- | --- | --- | --- | --- | --- | --- | --- | --- | --- | --- | --- | --- | --- | --- | --- | --- | --- | --- | --- | --- | --- | --- |

XP\_032066440.1

back to list

  

| Protein Coverage
| Supporting Peptides
|

Protein Coverage:

Supporting Peptides:

| Peptide | Uniq | -10lgP | Mass | Length | ppm | m/z | z | RT | Fraction | Scan | Source File | Area F4 | Area F5 | Area F6 | Area F10 | Area F8 | Area F9 | Area F7 | Area F1 | Area F2 | Area F3 | #Feature | #Feature F4 | #Feature F5 | #Feature F6 | #Feature F10 | #Feature F8 | #Feature F9 | #Feature F7 | #Feature F1 | #Feature F2 | #Feature F3 | Start | End | PTM | AScore | Found By |
| --- | --- | --- | --- | --- | --- | --- | --- | --- | --- | --- | --- | --- | --- | --- | --- | --- | --- | --- | --- | --- | --- | --- | --- | --- | --- | --- | --- | --- | --- | --- | --- | --- | --- | --- | --- | --- | --- |
| K.LTLSALLDGK.N | Y | 54.85 | 1029.6069 | 10 | 0.6 | 515.8110 | 2 | 51.10 | 8 | F8:29079 | 31122021\_RID\_3262\_1a.raw |  |  |  |  |  |  |  | 7.3889E4 |  |  | 1 | 0 | 0 | 0 | 0 | 0 | 0 | 0 | 1 | 0 | 0 | 299 | 308 |  |  | PEAKS DB |
| total 1 peptides |  |  |  |  |  |  |  |  |  |  |  |  |  |  |  |  |  |  |  |  |  |  |  |  |  |  |  |  |  |  |  |  |  |  |  |  |  |
| --- | --- | --- | --- | --- | --- | --- | --- | --- | --- | --- | --- | --- | --- | --- | --- | --- | --- | --- | --- | --- | --- | --- | --- | --- | --- | --- | --- | --- | --- | --- | --- | --- | --- | --- | --- | --- | --- |

XP\_015679656.1

back to list

  

| Protein Coverage
| Supporting Peptides
|

Protein Coverage:

Supporting Peptides:

| Peptide | Uniq | -10lgP | Mass | Length | ppm | m/z | z | RT | Fraction | Scan | Source File | Area F4 | Area F5 | Area F6 | Area F10 | Area F8 | Area F9 | Area F7 | Area F1 | Area F2 | Area F3 | #Feature | #Feature F4 | #Feature F5 | #Feature F6 | #Feature F10 | #Feature F8 | #Feature F9 | #Feature F7 | #Feature F1 | #Feature F2 | #Feature F3 | Start | End | PTM | AScore | Found By |
| --- | --- | --- | --- | --- | --- | --- | --- | --- | --- | --- | --- | --- | --- | --- | --- | --- | --- | --- | --- | --- | --- | --- | --- | --- | --- | --- | --- | --- | --- | --- | --- | --- | --- | --- | --- | --- | --- |
| K.LTLSALLDGK.N | Y | 54.85 | 1029.6069 | 10 | 0.6 | 515.8110 | 2 | 51.10 | 8 | F8:29079 | 31122021\_RID\_3262\_1a.raw |  |  |  |  |  |  |  | 7.3889E4 |  |  | 1 | 0 | 0 | 0 | 0 | 0 | 0 | 0 | 1 | 0 | 0 | 299 | 308 |  |  | PEAKS DB |
| total 1 peptides |  |  |  |  |  |  |  |  |  |  |  |  |  |  |  |  |  |  |  |  |  |  |  |  |  |  |  |  |  |  |  |  |  |  |  |  |  |
| --- | --- | --- | --- | --- | --- | --- | --- | --- | --- | --- | --- | --- | --- | --- | --- | --- | --- | --- | --- | --- | --- | --- | --- | --- | --- | --- | --- | --- | --- | --- | --- | --- | --- | --- | --- | --- | --- |

XP\_034260037.1

back to list

  

| Protein Coverage
| Supporting Peptides
|

Protein Coverage:

Supporting Peptides:

| Peptide | Uniq | -10lgP | Mass | Length | ppm | m/z | z | RT | Fraction | Scan | Source File | Area F4 | Area F5 | Area F6 | Area F10 | Area F8 | Area F9 | Area F7 | Area F1 | Area F2 | Area F3 | #Feature | #Feature F4 | #Feature F5 | #Feature F6 | #Feature F10 | #Feature F8 | #Feature F9 | #Feature F7 | #Feature F1 | #Feature F2 | #Feature F3 | Start | End | PTM | AScore | Found By |
| --- | --- | --- | --- | --- | --- | --- | --- | --- | --- | --- | --- | --- | --- | --- | --- | --- | --- | --- | --- | --- | --- | --- | --- | --- | --- | --- | --- | --- | --- | --- | --- | --- | --- | --- | --- | --- | --- |
| K.LTLSALLDGK.N | Y | 54.85 | 1029.6069 | 10 | 0.6 | 515.8110 | 2 | 51.10 | 8 | F8:29079 | 31122021\_RID\_3262\_1a.raw |  |  |  |  |  |  |  | 7.3889E4 |  |  | 1 | 0 | 0 | 0 | 0 | 0 | 0 | 0 | 1 | 0 | 0 | 299 | 308 |  |  | PEAKS DB |
| total 1 peptides |  |  |  |  |  |  |  |  |  |  |  |  |  |  |  |  |  |  |  |  |  |  |  |  |  |  |  |  |  |  |  |  |  |  |  |  |  |
| --- | --- | --- | --- | --- | --- | --- | --- | --- | --- | --- | --- | --- | --- | --- | --- | --- | --- | --- | --- | --- | --- | --- | --- | --- | --- | --- | --- | --- | --- | --- | --- | --- | --- | --- | --- | --- | --- |

XP\_013913912.1

back to list

  

| Protein Coverage
| Supporting Peptides
|

Protein Coverage:

Supporting Peptides:

| Peptide | Uniq | -10lgP | Mass | Length | ppm | m/z | z | RT | Fraction | Scan | Source File | Area F4 | Area F5 | Area F6 | Area F10 | Area F8 | Area F9 | Area F7 | Area F1 | Area F2 | Area F3 | #Feature | #Feature F4 | #Feature F5 | #Feature F6 | #Feature F10 | #Feature F8 | #Feature F9 | #Feature F7 | #Feature F1 | #Feature F2 | #Feature F3 | Start | End | PTM | AScore | Found By |
| --- | --- | --- | --- | --- | --- | --- | --- | --- | --- | --- | --- | --- | --- | --- | --- | --- | --- | --- | --- | --- | --- | --- | --- | --- | --- | --- | --- | --- | --- | --- | --- | --- | --- | --- | --- | --- | --- |
| K.LTLSALLDGK.N | Y | 54.85 | 1029.6069 | 10 | 0.6 | 515.8110 | 2 | 51.10 | 8 | F8:29079 | 31122021\_RID\_3262\_1a.raw |  |  |  |  |  |  |  | 7.3889E4 |  |  | 1 | 0 | 0 | 0 | 0 | 0 | 0 | 0 | 1 | 0 | 0 | 299 | 308 |  |  | PEAKS DB |
| total 1 peptides |  |  |  |  |  |  |  |  |  |  |  |  |  |  |  |  |  |  |  |  |  |  |  |  |  |  |  |  |  |  |  |  |  |  |  |  |  |
| --- | --- | --- | --- | --- | --- | --- | --- | --- | --- | --- | --- | --- | --- | --- | --- | --- | --- | --- | --- | --- | --- | --- | --- | --- | --- | --- | --- | --- | --- | --- | --- | --- | --- | --- | --- | --- | --- |

XP\_026577138.1

back to list

  

| Protein Coverage
| Supporting Peptides
|

Protein Coverage:

Supporting Peptides:

| Peptide | Uniq | -10lgP | Mass | Length | ppm | m/z | z | RT | Fraction | Scan | Source File | Area F4 | Area F5 | Area F6 | Area F10 | Area F8 | Area F9 | Area F7 | Area F1 | Area F2 | Area F3 | #Feature | #Feature F4 | #Feature F5 | #Feature F6 | #Feature F10 | #Feature F8 | #Feature F9 | #Feature F7 | #Feature F1 | #Feature F2 | #Feature F3 | Start | End | PTM | AScore | Found By |
| --- | --- | --- | --- | --- | --- | --- | --- | --- | --- | --- | --- | --- | --- | --- | --- | --- | --- | --- | --- | --- | --- | --- | --- | --- | --- | --- | --- | --- | --- | --- | --- | --- | --- | --- | --- | --- | --- |
| K.HVNLVQVLEEK.T | Y | 53.19 | 1306.7245 | 11 | -0.4 | 654.3693 | 2 | 40.16 | 1 | F1:25610 | 13012022\_RID\_3262\_4a.raw | 3.9013E5 |  |  |  |  |  |  |  |  |  | 1 | 1 | 0 | 0 | 0 | 0 | 0 | 0 | 0 | 0 | 0 | 114 | 124 |  |  | PEAKS DB |
| total 1 peptides |  |  |  |  |  |  |  |  |  |  |  |  |  |  |  |  |  |  |  |  |  |  |  |  |  |  |  |  |  |  |  |  |  |  |  |  |  |
| --- | --- | --- | --- | --- | --- | --- | --- | --- | --- | --- | --- | --- | --- | --- | --- | --- | --- | --- | --- | --- | --- | --- | --- | --- | --- | --- | --- | --- | --- | --- | --- | --- | --- | --- | --- | --- | --- |

XP\_029140647.1

back to list

  

| Protein Coverage
| Supporting Peptides
|

Protein Coverage:

Supporting Peptides:

| Peptide | Uniq | -10lgP | Mass | Length | ppm | m/z | z | RT | Fraction | Scan | Source File | Area F4 | Area F5 | Area F6 | Area F10 | Area F8 | Area F9 | Area F7 | Area F1 | Area F2 | Area F3 | #Feature | #Feature F4 | #Feature F5 | #Feature F6 | #Feature F10 | #Feature F8 | #Feature F9 | #Feature F7 | #Feature F1 | #Feature F2 | #Feature F3 | Start | End | PTM | AScore | Found By |
| --- | --- | --- | --- | --- | --- | --- | --- | --- | --- | --- | --- | --- | --- | --- | --- | --- | --- | --- | --- | --- | --- | --- | --- | --- | --- | --- | --- | --- | --- | --- | --- | --- | --- | --- | --- | --- | --- |
| K.HVNLVQVLEEK.T | Y | 53.19 | 1306.7245 | 11 | -0.4 | 654.3693 | 2 | 40.16 | 1 | F1:25610 | 13012022\_RID\_3262\_4a.raw | 3.9013E5 |  |  |  |  |  |  |  |  |  | 1 | 1 | 0 | 0 | 0 | 0 | 0 | 0 | 0 | 0 | 0 | 114 | 124 |  |  | PEAKS DB |
| total 1 peptides |  |  |  |  |  |  |  |  |  |  |  |  |  |  |  |  |  |  |  |  |  |  |  |  |  |  |  |  |  |  |  |  |  |  |  |  |  |
| --- | --- | --- | --- | --- | --- | --- | --- | --- | --- | --- | --- | --- | --- | --- | --- | --- | --- | --- | --- | --- | --- | --- | --- | --- | --- | --- | --- | --- | --- | --- | --- | --- | --- | --- | --- | --- | --- |

XP\_026534386.1

back to list

  

| Protein Coverage
| Supporting Peptides
|

Protein Coverage:

Supporting Peptides:

| Peptide | Uniq | -10lgP | Mass | Length | ppm | m/z | z | RT | Fraction | Scan | Source File | Area F4 | Area F5 | Area F6 | Area F10 | Area F8 | Area F9 | Area F7 | Area F1 | Area F2 | Area F3 | #Feature | #Feature F4 | #Feature F5 | #Feature F6 | #Feature F10 | #Feature F8 | #Feature F9 | #Feature F7 | #Feature F1 | #Feature F2 | #Feature F3 | Start | End | PTM | AScore | Found By |
| --- | --- | --- | --- | --- | --- | --- | --- | --- | --- | --- | --- | --- | --- | --- | --- | --- | --- | --- | --- | --- | --- | --- | --- | --- | --- | --- | --- | --- | --- | --- | --- | --- | --- | --- | --- | --- | --- |
| T.LVVADESMVK.F | Y | 48.58 | 1089.5740 | 10 | 0.3 | 545.7944 | 2 | 33.37 | 2 | F2:17086 | 13012022\_RID\_3262\_5a.raw |  | 5.3478E6 |  |  |  |  |  |  |  |  | 1 | 0 | 1 | 0 | 0 | 0 | 0 | 0 | 0 | 0 | 0 | 237 | 246 |  |  | PEAKS DB |
| total 1 peptides |  |  |  |  |  |  |  |  |  |  |  |  |  |  |  |  |  |  |  |  |  |  |  |  |  |  |  |  |  |  |  |  |  |  |  |  |  |
| --- | --- | --- | --- | --- | --- | --- | --- | --- | --- | --- | --- | --- | --- | --- | --- | --- | --- | --- | --- | --- | --- | --- | --- | --- | --- | --- | --- | --- | --- | --- | --- | --- | --- | --- | --- | --- | --- |

XP\_026534385.1

back to list

  

| Protein Coverage
| Supporting Peptides
|

Protein Coverage:

Supporting Peptides:

| Peptide | Uniq | -10lgP | Mass | Length | ppm | m/z | z | RT | Fraction | Scan | Source File | Area F4 | Area F5 | Area F6 | Area F10 | Area F8 | Area F9 | Area F7 | Area F1 | Area F2 | Area F3 | #Feature | #Feature F4 | #Feature F5 | #Feature F6 | #Feature F10 | #Feature F8 | #Feature F9 | #Feature F7 | #Feature F1 | #Feature F2 | #Feature F3 | Start | End | PTM | AScore | Found By |
| --- | --- | --- | --- | --- | --- | --- | --- | --- | --- | --- | --- | --- | --- | --- | --- | --- | --- | --- | --- | --- | --- | --- | --- | --- | --- | --- | --- | --- | --- | --- | --- | --- | --- | --- | --- | --- | --- |
| T.LVVADESMVK.F | Y | 48.58 | 1089.5740 | 10 | 0.3 | 545.7944 | 2 | 33.37 | 2 | F2:17086 | 13012022\_RID\_3262\_5a.raw |  | 5.3478E6 |  |  |  |  |  |  |  |  | 1 | 0 | 1 | 0 | 0 | 0 | 0 | 0 | 0 | 0 | 0 | 237 | 246 |  |  | PEAKS DB |
| total 1 peptides |  |  |  |  |  |  |  |  |  |  |  |  |  |  |  |  |  |  |  |  |  |  |  |  |  |  |  |  |  |  |  |  |  |  |  |  |  |
| --- | --- | --- | --- | --- | --- | --- | --- | --- | --- | --- | --- | --- | --- | --- | --- | --- | --- | --- | --- | --- | --- | --- | --- | --- | --- | --- | --- | --- | --- | --- | --- | --- | --- | --- | --- | --- | --- |

XP\_015673523.1

back to list

  

| Protein Coverage
| Supporting Peptides
|

Protein Coverage:

Supporting Peptides:

| Peptide | Uniq | -10lgP | Mass | Length | ppm | m/z | z | RT | Fraction | Scan | Source File | Area F4 | Area F5 | Area F6 | Area F10 | Area F8 | Area F9 | Area F7 | Area F1 | Area F2 | Area F3 | #Feature | #Feature F4 | #Feature F5 | #Feature F6 | #Feature F10 | #Feature F8 | #Feature F9 | #Feature F7 | #Feature F1 | #Feature F2 | #Feature F3 | Start | End | PTM | AScore | Found By |
| --- | --- | --- | --- | --- | --- | --- | --- | --- | --- | --- | --- | --- | --- | --- | --- | --- | --- | --- | --- | --- | --- | --- | --- | --- | --- | --- | --- | --- | --- | --- | --- | --- | --- | --- | --- | --- | --- |
| T.LVVADESMVK.F | Y | 48.58 | 1089.5740 | 10 | 0.3 | 545.7944 | 2 | 33.37 | 2 | F2:17086 | 13012022\_RID\_3262\_5a.raw |  | 5.3478E6 |  |  |  |  |  |  |  |  | 1 | 0 | 1 | 0 | 0 | 0 | 0 | 0 | 0 | 0 | 0 | 237 | 246 |  |  | PEAKS DB |
| total 1 peptides |  |  |  |  |  |  |  |  |  |  |  |  |  |  |  |  |  |  |  |  |  |  |  |  |  |  |  |  |  |  |  |  |  |  |  |  |  |
| --- | --- | --- | --- | --- | --- | --- | --- | --- | --- | --- | --- | --- | --- | --- | --- | --- | --- | --- | --- | --- | --- | --- | --- | --- | --- | --- | --- | --- | --- | --- | --- | --- | --- | --- | --- | --- | --- |

XP\_026564825.1

back to list

  

| Protein Coverage
| Supporting Peptides
|

Protein Coverage:

Supporting Peptides:

| Peptide | Uniq | -10lgP | Mass | Length | ppm | m/z | z | RT | Fraction | Scan | Source File | Area F4 | Area F5 | Area F6 | Area F10 | Area F8 | Area F9 | Area F7 | Area F1 | Area F2 | Area F3 | #Feature | #Feature F4 | #Feature F5 | #Feature F6 | #Feature F10 | #Feature F8 | #Feature F9 | #Feature F7 | #Feature F1 | #Feature F2 | #Feature F3 | Start | End | PTM | AScore | Found By |
| --- | --- | --- | --- | --- | --- | --- | --- | --- | --- | --- | --- | --- | --- | --- | --- | --- | --- | --- | --- | --- | --- | --- | --- | --- | --- | --- | --- | --- | --- | --- | --- | --- | --- | --- | --- | --- | --- |
| T.LVVADESMVK.F | Y | 48.58 | 1089.5740 | 10 | 0.3 | 545.7944 | 2 | 33.37 | 2 | F2:17086 | 13012022\_RID\_3262\_5a.raw |  | 5.3478E6 |  |  |  |  |  |  |  |  | 1 | 0 | 1 | 0 | 0 | 0 | 0 | 0 | 0 | 0 | 0 | 240 | 249 |  |  | PEAKS DB |
| total 1 peptides |  |  |  |  |  |  |  |  |  |  |  |  |  |  |  |  |  |  |  |  |  |  |  |  |  |  |  |  |  |  |  |  |  |  |  |  |  |
| --- | --- | --- | --- | --- | --- | --- | --- | --- | --- | --- | --- | --- | --- | --- | --- | --- | --- | --- | --- | --- | --- | --- | --- | --- | --- | --- | --- | --- | --- | --- | --- | --- | --- | --- | --- | --- | --- |

XP\_026564824.1

back to list

  

| Protein Coverage
| Supporting Peptides
|

Protein Coverage:

Supporting Peptides:

| Peptide | Uniq | -10lgP | Mass | Length | ppm | m/z | z | RT | Fraction | Scan | Source File | Area F4 | Area F5 | Area F6 | Area F10 | Area F8 | Area F9 | Area F7 | Area F1 | Area F2 | Area F3 | #Feature | #Feature F4 | #Feature F5 | #Feature F6 | #Feature F10 | #Feature F8 | #Feature F9 | #Feature F7 | #Feature F1 | #Feature F2 | #Feature F3 | Start | End | PTM | AScore | Found By |
| --- | --- | --- | --- | --- | --- | --- | --- | --- | --- | --- | --- | --- | --- | --- | --- | --- | --- | --- | --- | --- | --- | --- | --- | --- | --- | --- | --- | --- | --- | --- | --- | --- | --- | --- | --- | --- | --- |
| T.LVVADESMVK.F | Y | 48.58 | 1089.5740 | 10 | 0.3 | 545.7944 | 2 | 33.37 | 2 | F2:17086 | 13012022\_RID\_3262\_5a.raw |  | 5.3478E6 |  |  |  |  |  |  |  |  | 1 | 0 | 1 | 0 | 0 | 0 | 0 | 0 | 0 | 0 | 0 | 240 | 249 |  |  | PEAKS DB |
| total 1 peptides |  |  |  |  |  |  |  |  |  |  |  |  |  |  |  |  |  |  |  |  |  |  |  |  |  |  |  |  |  |  |  |  |  |  |  |  |  |
| --- | --- | --- | --- | --- | --- | --- | --- | --- | --- | --- | --- | --- | --- | --- | --- | --- | --- | --- | --- | --- | --- | --- | --- | --- | --- | --- | --- | --- | --- | --- | --- | --- | --- | --- | --- | --- | --- |

XP\_034277870.1

back to list

  

| Protein Coverage
| Supporting Peptides
|

Protein Coverage:

Supporting Peptides:

| Peptide | Uniq | -10lgP | Mass | Length | ppm | m/z | z | RT | Fraction | Scan | Source File | Area F4 | Area F5 | Area F6 | Area F10 | Area F8 | Area F9 | Area F7 | Area F1 | Area F2 | Area F3 | #Feature | #Feature F4 | #Feature F5 | #Feature F6 | #Feature F10 | #Feature F8 | #Feature F9 | #Feature F7 | #Feature F1 | #Feature F2 | #Feature F3 | Start | End | PTM | AScore | Found By |
| --- | --- | --- | --- | --- | --- | --- | --- | --- | --- | --- | --- | --- | --- | --- | --- | --- | --- | --- | --- | --- | --- | --- | --- | --- | --- | --- | --- | --- | --- | --- | --- | --- | --- | --- | --- | --- | --- |
| T.LVVADESMVK.F | Y | 48.58 | 1089.5740 | 10 | 0.3 | 545.7944 | 2 | 33.37 | 2 | F2:17086 | 13012022\_RID\_3262\_5a.raw |  | 5.3478E6 |  |  |  |  |  |  |  |  | 1 | 0 | 1 | 0 | 0 | 0 | 0 | 0 | 0 | 0 | 0 | 234 | 243 |  |  | PEAKS DB |
| total 1 peptides |  |  |  |  |  |  |  |  |  |  |  |  |  |  |  |  |  |  |  |  |  |  |  |  |  |  |  |  |  |  |  |  |  |  |  |  |  |
| --- | --- | --- | --- | --- | --- | --- | --- | --- | --- | --- | --- | --- | --- | --- | --- | --- | --- | --- | --- | --- | --- | --- | --- | --- | --- | --- | --- | --- | --- | --- | --- | --- | --- | --- | --- | --- | --- |

XP\_007438168.1

back to list

  

| Protein Coverage
| Supporting Peptides
|

Protein Coverage:

Supporting Peptides:

| Peptide | Uniq | -10lgP | Mass | Length | ppm | m/z | z | RT | Fraction | Scan | Source File | Area F4 | Area F5 | Area F6 | Area F10 | Area F8 | Area F9 | Area F7 | Area F1 | Area F2 | Area F3 | #Feature | #Feature F4 | #Feature F5 | #Feature F6 | #Feature F10 | #Feature F8 | #Feature F9 | #Feature F7 | #Feature F1 | #Feature F2 | #Feature F3 | Start | End | PTM | AScore | Found By |
| --- | --- | --- | --- | --- | --- | --- | --- | --- | --- | --- | --- | --- | --- | --- | --- | --- | --- | --- | --- | --- | --- | --- | --- | --- | --- | --- | --- | --- | --- | --- | --- | --- | --- | --- | --- | --- | --- |
| T.LVVADESMVK.F | Y | 48.58 | 1089.5740 | 10 | 0.3 | 545.7944 | 2 | 33.37 | 2 | F2:17086 | 13012022\_RID\_3262\_5a.raw |  | 5.3478E6 |  |  |  |  |  |  |  |  | 1 | 0 | 1 | 0 | 0 | 0 | 0 | 0 | 0 | 0 | 0 | 157 | 166 |  |  | PEAKS DB |
| total 1 peptides |  |  |  |  |  |  |  |  |  |  |  |  |  |  |  |  |  |  |  |  |  |  |  |  |  |  |  |  |  |  |  |  |  |  |  |  |  |
| --- | --- | --- | --- | --- | --- | --- | --- | --- | --- | --- | --- | --- | --- | --- | --- | --- | --- | --- | --- | --- | --- | --- | --- | --- | --- | --- | --- | --- | --- | --- | --- | --- | --- | --- | --- | --- | --- |

LAA53077.1

back to list

  

| Protein Coverage
| Supporting Peptides
|

Protein Coverage:

Supporting Peptides:

| Peptide | Uniq | -10lgP | Mass | Length | ppm | m/z | z | RT | Fraction | Scan | Source File | Area F4 | Area F5 | Area F6 | Area F10 | Area F8 | Area F9 | Area F7 | Area F1 | Area F2 | Area F3 | #Feature | #Feature F4 | #Feature F5 | #Feature F6 | #Feature F10 | #Feature F8 | #Feature F9 | #Feature F7 | #Feature F1 | #Feature F2 | #Feature F3 | Start | End | PTM | AScore | Found By |
| --- | --- | --- | --- | --- | --- | --- | --- | --- | --- | --- | --- | --- | --- | --- | --- | --- | --- | --- | --- | --- | --- | --- | --- | --- | --- | --- | --- | --- | --- | --- | --- | --- | --- | --- | --- | --- | --- |
| T.LVVADESMVK.F | Y | 48.58 | 1089.5740 | 10 | 0.3 | 545.7944 | 2 | 33.37 | 2 | F2:17086 | 13012022\_RID\_3262\_5a.raw |  | 5.3478E6 |  |  |  |  |  |  |  |  | 1 | 0 | 1 | 0 | 0 | 0 | 0 | 0 | 0 | 0 | 0 | 8 | 17 |  |  | PEAKS DB |
| total 1 peptides |  |  |  |  |  |  |  |  |  |  |  |  |  |  |  |  |  |  |  |  |  |  |  |  |  |  |  |  |  |  |  |  |  |  |  |  |  |
| --- | --- | --- | --- | --- | --- | --- | --- | --- | --- | --- | --- | --- | --- | --- | --- | --- | --- | --- | --- | --- | --- | --- | --- | --- | --- | --- | --- | --- | --- | --- | --- | --- | --- | --- | --- | --- | --- |

XP\_032084777.1

back to list

  

| Protein Coverage
| Supporting Peptides
|

Protein Coverage:

Supporting Peptides:

| Peptide | Uniq | -10lgP | Mass | Length | ppm | m/z | z | RT | Fraction | Scan | Source File | Area F4 | Area F5 | Area F6 | Area F10 | Area F8 | Area F9 | Area F7 | Area F1 | Area F2 | Area F3 | #Feature | #Feature F4 | #Feature F5 | #Feature F6 | #Feature F10 | #Feature F8 | #Feature F9 | #Feature F7 | #Feature F1 | #Feature F2 | #Feature F3 | Start | End | PTM | AScore | Found By |
| --- | --- | --- | --- | --- | --- | --- | --- | --- | --- | --- | --- | --- | --- | --- | --- | --- | --- | --- | --- | --- | --- | --- | --- | --- | --- | --- | --- | --- | --- | --- | --- | --- | --- | --- | --- | --- | --- |
| T.LVVADESMVK.F | Y | 48.58 | 1089.5740 | 10 | 0.3 | 545.7944 | 2 | 33.37 | 2 | F2:17086 | 13012022\_RID\_3262\_5a.raw |  | 5.3478E6 |  |  |  |  |  |  |  |  | 1 | 0 | 1 | 0 | 0 | 0 | 0 | 0 | 0 | 0 | 0 | 228 | 237 |  |  | PEAKS DB |
| total 1 peptides |  |  |  |  |  |  |  |  |  |  |  |  |  |  |  |  |  |  |  |  |  |  |  |  |  |  |  |  |  |  |  |  |  |  |  |  |  |
| --- | --- | --- | --- | --- | --- | --- | --- | --- | --- | --- | --- | --- | --- | --- | --- | --- | --- | --- | --- | --- | --- | --- | --- | --- | --- | --- | --- | --- | --- | --- | --- | --- | --- | --- | --- | --- | --- |

XP\_034292227.1

back to list

  

| Protein Coverage
| Supporting Peptides
|

Protein Coverage:

Supporting Peptides:

| Peptide | Uniq | -10lgP | Mass | Length | ppm | m/z | z | RT | Fraction | Scan | Source File | Area F4 | Area F5 | Area F6 | Area F10 | Area F8 | Area F9 | Area F7 | Area F1 | Area F2 | Area F3 | #Feature | #Feature F4 | #Feature F5 | #Feature F6 | #Feature F10 | #Feature F8 | #Feature F9 | #Feature F7 | #Feature F1 | #Feature F2 | #Feature F3 | Start | End | PTM | AScore | Found By |
| --- | --- | --- | --- | --- | --- | --- | --- | --- | --- | --- | --- | --- | --- | --- | --- | --- | --- | --- | --- | --- | --- | --- | --- | --- | --- | --- | --- | --- | --- | --- | --- | --- | --- | --- | --- | --- | --- |
| R.LAEVALAYAK.A | Y | 46.75 | 1047.5964 | 10 | 0.8 | 524.8059 | 2 | 37.71 | 8 | F8:18594 | 31122021\_RID\_3262\_1a.raw |  |  |  |  |  |  |  | 6.0544E4 | 1.1426E4 |  | 2 | 0 | 0 | 0 | 0 | 0 | 0 | 0 | 1 | 1 | 0 | 150 | 159 |  |  | PEAKS DB |
| total 1 peptides |  |  |  |  |  |  |  |  |  |  |  |  |  |  |  |  |  |  |  |  |  |  |  |  |  |  |  |  |  |  |  |  |  |  |  |  |  |
| --- | --- | --- | --- | --- | --- | --- | --- | --- | --- | --- | --- | --- | --- | --- | --- | --- | --- | --- | --- | --- | --- | --- | --- | --- | --- | --- | --- | --- | --- | --- | --- | --- | --- | --- | --- | --- | --- |

XP\_015687137.1

back to list

  

| Protein Coverage
| Supporting Peptides
|

Protein Coverage:

Supporting Peptides:

| Peptide | Uniq | -10lgP | Mass | Length | ppm | m/z | z | RT | Fraction | Scan | Source File | Area F4 | Area F5 | Area F6 | Area F10 | Area F8 | Area F9 | Area F7 | Area F1 | Area F2 | Area F3 | #Feature | #Feature F4 | #Feature F5 | #Feature F6 | #Feature F10 | #Feature F8 | #Feature F9 | #Feature F7 | #Feature F1 | #Feature F2 | #Feature F3 | Start | End | PTM | AScore | Found By |
| --- | --- | --- | --- | --- | --- | --- | --- | --- | --- | --- | --- | --- | --- | --- | --- | --- | --- | --- | --- | --- | --- | --- | --- | --- | --- | --- | --- | --- | --- | --- | --- | --- | --- | --- | --- | --- | --- |
| R.LAEVALAYAK.A | Y | 46.75 | 1047.5964 | 10 | 0.8 | 524.8059 | 2 | 37.71 | 8 | F8:18594 | 31122021\_RID\_3262\_1a.raw |  |  |  |  |  |  |  | 6.0544E4 | 1.1426E4 |  | 2 | 0 | 0 | 0 | 0 | 0 | 0 | 0 | 1 | 1 | 0 | 150 | 159 |  |  | PEAKS DB |
| total 1 peptides |  |  |  |  |  |  |  |  |  |  |  |  |  |  |  |  |  |  |  |  |  |  |  |  |  |  |  |  |  |  |  |  |  |  |  |  |  |
| --- | --- | --- | --- | --- | --- | --- | --- | --- | --- | --- | --- | --- | --- | --- | --- | --- | --- | --- | --- | --- | --- | --- | --- | --- | --- | --- | --- | --- | --- | --- | --- | --- | --- | --- | --- | --- | --- |

JAG68750.1

back to list

  

| Protein Coverage
| Supporting Peptides
|

Protein Coverage:

Supporting Peptides:

| Peptide | Uniq | -10lgP | Mass | Length | ppm | m/z | z | RT | Fraction | Scan | Source File | Area F4 | Area F5 | Area F6 | Area F10 | Area F8 | Area F9 | Area F7 | Area F1 | Area F2 | Area F3 | #Feature | #Feature F4 | #Feature F5 | #Feature F6 | #Feature F10 | #Feature F8 | #Feature F9 | #Feature F7 | #Feature F1 | #Feature F2 | #Feature F3 | Start | End | PTM | AScore | Found By |
| --- | --- | --- | --- | --- | --- | --- | --- | --- | --- | --- | --- | --- | --- | --- | --- | --- | --- | --- | --- | --- | --- | --- | --- | --- | --- | --- | --- | --- | --- | --- | --- | --- | --- | --- | --- | --- | --- |
| R.LAEVALAYAK.A | Y | 46.75 | 1047.5964 | 10 | 0.8 | 524.8059 | 2 | 37.71 | 8 | F8:18594 | 31122021\_RID\_3262\_1a.raw |  |  |  |  |  |  |  | 6.0544E4 | 1.1426E4 |  | 2 | 0 | 0 | 0 | 0 | 0 | 0 | 0 | 1 | 1 | 0 | 150 | 159 |  |  | PEAKS DB |
| total 1 peptides |  |  |  |  |  |  |  |  |  |  |  |  |  |  |  |  |  |  |  |  |  |  |  |  |  |  |  |  |  |  |  |  |  |  |  |  |  |
| --- | --- | --- | --- | --- | --- | --- | --- | --- | --- | --- | --- | --- | --- | --- | --- | --- | --- | --- | --- | --- | --- | --- | --- | --- | --- | --- | --- | --- | --- | --- | --- | --- | --- | --- | --- | --- | --- |

LAA91247.1

back to list

  

| Protein Coverage
| Supporting Peptides
|

Protein Coverage:

Supporting Peptides:

| Peptide | Uniq | -10lgP | Mass | Length | ppm | m/z | z | RT | Fraction | Scan | Source File | Area F4 | Area F5 | Area F6 | Area F10 | Area F8 | Area F9 | Area F7 | Area F1 | Area F2 | Area F3 | #Feature | #Feature F4 | #Feature F5 | #Feature F6 | #Feature F10 | #Feature F8 | #Feature F9 | #Feature F7 | #Feature F1 | #Feature F2 | #Feature F3 | Start | End | PTM | AScore | Found By |
| --- | --- | --- | --- | --- | --- | --- | --- | --- | --- | --- | --- | --- | --- | --- | --- | --- | --- | --- | --- | --- | --- | --- | --- | --- | --- | --- | --- | --- | --- | --- | --- | --- | --- | --- | --- | --- | --- |
| R.LAEVALAYAK.A | Y | 46.75 | 1047.5964 | 10 | 0.8 | 524.8059 | 2 | 37.71 | 8 | F8:18594 | 31122021\_RID\_3262\_1a.raw |  |  |  |  |  |  |  | 6.0544E4 | 1.1426E4 |  | 2 | 0 | 0 | 0 | 0 | 0 | 0 | 0 | 1 | 1 | 0 | 119 | 128 |  |  | PEAKS DB |
| total 1 peptides |  |  |  |  |  |  |  |  |  |  |  |  |  |  |  |  |  |  |  |  |  |  |  |  |  |  |  |  |  |  |  |  |  |  |  |  |  |
| --- | --- | --- | --- | --- | --- | --- | --- | --- | --- | --- | --- | --- | --- | --- | --- | --- | --- | --- | --- | --- | --- | --- | --- | --- | --- | --- | --- | --- | --- | --- | --- | --- | --- | --- | --- | --- | --- |

XP\_007443339.1

back to list

  

| Protein Coverage
| Supporting Peptides
|

Protein Coverage:

Supporting Peptides:

| Peptide | Uniq | -10lgP | Mass | Length | ppm | m/z | z | RT | Fraction | Scan | Source File | Area F4 | Area F5 | Area F6 | Area F10 | Area F8 | Area F9 | Area F7 | Area F1 | Area F2 | Area F3 | #Feature | #Feature F4 | #Feature F5 | #Feature F6 | #Feature F10 | #Feature F8 | #Feature F9 | #Feature F7 | #Feature F1 | #Feature F2 | #Feature F3 | Start | End | PTM | AScore | Found By |
| --- | --- | --- | --- | --- | --- | --- | --- | --- | --- | --- | --- | --- | --- | --- | --- | --- | --- | --- | --- | --- | --- | --- | --- | --- | --- | --- | --- | --- | --- | --- | --- | --- | --- | --- | --- | --- | --- |
| R.LAEVALAYAK.A | Y | 46.75 | 1047.5964 | 10 | 0.8 | 524.8059 | 2 | 37.71 | 8 | F8:18594 | 31122021\_RID\_3262\_1a.raw |  |  |  |  |  |  |  | 6.0544E4 | 1.1426E4 |  | 2 | 0 | 0 | 0 | 0 | 0 | 0 | 0 | 1 | 1 | 0 | 256 | 265 |  |  | PEAKS DB |
| total 1 peptides |  |  |  |  |  |  |  |  |  |  |  |  |  |  |  |  |  |  |  |  |  |  |  |  |  |  |  |  |  |  |  |  |  |  |  |  |  |
| --- | --- | --- | --- | --- | --- | --- | --- | --- | --- | --- | --- | --- | --- | --- | --- | --- | --- | --- | --- | --- | --- | --- | --- | --- | --- | --- | --- | --- | --- | --- | --- | --- | --- | --- | --- | --- | --- |

JAV51283.1

back to list

  

| Protein Coverage
| Supporting Peptides
|

Protein Coverage:

Supporting Peptides:

| Peptide | Uniq | -10lgP | Mass | Length | ppm | m/z | z | RT | Fraction | Scan | Source File | Area F4 | Area F5 | Area F6 | Area F10 | Area F8 | Area F9 | Area F7 | Area F1 | Area F2 | Area F3 | #Feature | #Feature F4 | #Feature F5 | #Feature F6 | #Feature F10 | #Feature F8 | #Feature F9 | #Feature F7 | #Feature F1 | #Feature F2 | #Feature F3 | Start | End | PTM | AScore | Found By |
| --- | --- | --- | --- | --- | --- | --- | --- | --- | --- | --- | --- | --- | --- | --- | --- | --- | --- | --- | --- | --- | --- | --- | --- | --- | --- | --- | --- | --- | --- | --- | --- | --- | --- | --- | --- | --- | --- |
| R.LAEVALAYAK.A | Y | 46.75 | 1047.5964 | 10 | 0.8 | 524.8059 | 2 | 37.71 | 8 | F8:18594 | 31122021\_RID\_3262\_1a.raw |  |  |  |  |  |  |  | 6.0544E4 | 1.1426E4 |  | 2 | 0 | 0 | 0 | 0 | 0 | 0 | 0 | 1 | 1 | 0 | 150 | 159 |  |  | PEAKS DB |
| total 1 peptides |  |  |  |  |  |  |  |  |  |  |  |  |  |  |  |  |  |  |  |  |  |  |  |  |  |  |  |  |  |  |  |  |  |  |  |  |  |
| --- | --- | --- | --- | --- | --- | --- | --- | --- | --- | --- | --- | --- | --- | --- | --- | --- | --- | --- | --- | --- | --- | --- | --- | --- | --- | --- | --- | --- | --- | --- | --- | --- | --- | --- | --- | --- | --- |

JAC96276.1

back to list

  

| Protein Coverage
| Supporting Peptides
|

Protein Coverage:

Supporting Peptides:

| Peptide | Uniq | -10lgP | Mass | Length | ppm | m/z | z | RT | Fraction | Scan | Source File | Area F4 | Area F5 | Area F6 | Area F10 | Area F8 | Area F9 | Area F7 | Area F1 | Area F2 | Area F3 | #Feature | #Feature F4 | #Feature F5 | #Feature F6 | #Feature F10 | #Feature F8 | #Feature F9 | #Feature F7 | #Feature F1 | #Feature F2 | #Feature F3 | Start | End | PTM | AScore | Found By |
| --- | --- | --- | --- | --- | --- | --- | --- | --- | --- | --- | --- | --- | --- | --- | --- | --- | --- | --- | --- | --- | --- | --- | --- | --- | --- | --- | --- | --- | --- | --- | --- | --- | --- | --- | --- | --- | --- |
| R.LAEVALAYAK.A | Y | 46.75 | 1047.5964 | 10 | 0.8 | 524.8059 | 2 | 37.71 | 8 | F8:18594 | 31122021\_RID\_3262\_1a.raw |  |  |  |  |  |  |  | 6.0544E4 | 1.1426E4 |  | 2 | 0 | 0 | 0 | 0 | 0 | 0 | 0 | 1 | 1 | 0 | 150 | 159 |  |  | PEAKS DB |
| total 1 peptides |  |  |  |  |  |  |  |  |  |  |  |  |  |  |  |  |  |  |  |  |  |  |  |  |  |  |  |  |  |  |  |  |  |  |  |  |  |
| --- | --- | --- | --- | --- | --- | --- | --- | --- | --- | --- | --- | --- | --- | --- | --- | --- | --- | --- | --- | --- | --- | --- | --- | --- | --- | --- | --- | --- | --- | --- | --- | --- | --- | --- | --- | --- | --- |

XP\_032088448.1

back to list

  

| Protein Coverage
| Supporting Peptides
|

Protein Coverage:

Supporting Peptides:

| Peptide | Uniq | -10lgP | Mass | Length | ppm | m/z | z | RT | Fraction | Scan | Source File | Area F4 | Area F5 | Area F6 | Area F10 | Area F8 | Area F9 | Area F7 | Area F1 | Area F2 | Area F3 | #Feature | #Feature F4 | #Feature F5 | #Feature F6 | #Feature F10 | #Feature F8 | #Feature F9 | #Feature F7 | #Feature F1 | #Feature F2 | #Feature F3 | Start | End | PTM | AScore | Found By |
| --- | --- | --- | --- | --- | --- | --- | --- | --- | --- | --- | --- | --- | --- | --- | --- | --- | --- | --- | --- | --- | --- | --- | --- | --- | --- | --- | --- | --- | --- | --- | --- | --- | --- | --- | --- | --- | --- |
| R.LAEVALAYAK.A | Y | 46.75 | 1047.5964 | 10 | 0.8 | 524.8059 | 2 | 37.71 | 8 | F8:18594 | 31122021\_RID\_3262\_1a.raw |  |  |  |  |  |  |  | 6.0544E4 | 1.1426E4 |  | 2 | 0 | 0 | 0 | 0 | 0 | 0 | 0 | 1 | 1 | 0 | 150 | 159 |  |  | PEAKS DB |
| total 1 peptides |  |  |  |  |  |  |  |  |  |  |  |  |  |  |  |  |  |  |  |  |  |  |  |  |  |  |  |  |  |  |  |  |  |  |  |  |  |
| --- | --- | --- | --- | --- | --- | --- | --- | --- | --- | --- | --- | --- | --- | --- | --- | --- | --- | --- | --- | --- | --- | --- | --- | --- | --- | --- | --- | --- | --- | --- | --- | --- | --- | --- | --- | --- | --- |

AFJ49864.1

back to list

  

| Protein Coverage
| Supporting Peptides
|

Protein Coverage:

Supporting Peptides:

| Peptide | Uniq | -10lgP | Mass | Length | ppm | m/z | z | RT | Fraction | Scan | Source File | Area F4 | Area F5 | Area F6 | Area F10 | Area F8 | Area F9 | Area F7 | Area F1 | Area F2 | Area F3 | #Feature | #Feature F4 | #Feature F5 | #Feature F6 | #Feature F10 | #Feature F8 | #Feature F9 | #Feature F7 | #Feature F1 | #Feature F2 | #Feature F3 | Start | End | PTM | AScore | Found By |
| --- | --- | --- | --- | --- | --- | --- | --- | --- | --- | --- | --- | --- | --- | --- | --- | --- | --- | --- | --- | --- | --- | --- | --- | --- | --- | --- | --- | --- | --- | --- | --- | --- | --- | --- | --- | --- | --- |
| R.LAEVALAYAK.A | Y | 46.75 | 1047.5964 | 10 | 0.8 | 524.8059 | 2 | 37.71 | 8 | F8:18594 | 31122021\_RID\_3262\_1a.raw |  |  |  |  |  |  |  | 6.0544E4 | 1.1426E4 |  | 2 | 0 | 0 | 0 | 0 | 0 | 0 | 0 | 1 | 1 | 0 | 150 | 159 |  |  | PEAKS DB |
| total 1 peptides |  |  |  |  |  |  |  |  |  |  |  |  |  |  |  |  |  |  |  |  |  |  |  |  |  |  |  |  |  |  |  |  |  |  |  |  |  |
| --- | --- | --- | --- | --- | --- | --- | --- | --- | --- | --- | --- | --- | --- | --- | --- | --- | --- | --- | --- | --- | --- | --- | --- | --- | --- | --- | --- | --- | --- | --- | --- | --- | --- | --- | --- | --- | --- |

XP\_026568189.1

back to list

  

| Protein Coverage
| Supporting Peptides
|

Protein Coverage:

Supporting Peptides:

| Peptide | Uniq | -10lgP | Mass | Length | ppm | m/z | z | RT | Fraction | Scan | Source File | Area F4 | Area F5 | Area F6 | Area F10 | Area F8 | Area F9 | Area F7 | Area F1 | Area F2 | Area F3 | #Feature | #Feature F4 | #Feature F5 | #Feature F6 | #Feature F10 | #Feature F8 | #Feature F9 | #Feature F7 | #Feature F1 | #Feature F2 | #Feature F3 | Start | End | PTM | AScore | Found By |
| --- | --- | --- | --- | --- | --- | --- | --- | --- | --- | --- | --- | --- | --- | --- | --- | --- | --- | --- | --- | --- | --- | --- | --- | --- | --- | --- | --- | --- | --- | --- | --- | --- | --- | --- | --- | --- | --- |
| R.LAEVALAYAK.A | Y | 46.75 | 1047.5964 | 10 | 0.8 | 524.8059 | 2 | 37.71 | 8 | F8:18594 | 31122021\_RID\_3262\_1a.raw |  |  |  |  |  |  |  | 6.0544E4 | 1.1426E4 |  | 2 | 0 | 0 | 0 | 0 | 0 | 0 | 0 | 1 | 1 | 0 | 150 | 159 |  |  | PEAKS DB |
| total 1 peptides |  |  |  |  |  |  |  |  |  |  |  |  |  |  |  |  |  |  |  |  |  |  |  |  |  |  |  |  |  |  |  |  |  |  |  |  |  |
| --- | --- | --- | --- | --- | --- | --- | --- | --- | --- | --- | --- | --- | --- | --- | --- | --- | --- | --- | --- | --- | --- | --- | --- | --- | --- | --- | --- | --- | --- | --- | --- | --- | --- | --- | --- | --- | --- |

JAI12578.1

back to list

  

| Protein Coverage
| Supporting Peptides
|

Protein Coverage:

Supporting Peptides:

| Peptide | Uniq | -10lgP | Mass | Length | ppm | m/z | z | RT | Fraction | Scan | Source File | Area F4 | Area F5 | Area F6 | Area F10 | Area F8 | Area F9 | Area F7 | Area F1 | Area F2 | Area F3 | #Feature | #Feature F4 | #Feature F5 | #Feature F6 | #Feature F10 | #Feature F8 | #Feature F9 | #Feature F7 | #Feature F1 | #Feature F2 | #Feature F3 | Start | End | PTM | AScore | Found By |
| --- | --- | --- | --- | --- | --- | --- | --- | --- | --- | --- | --- | --- | --- | --- | --- | --- | --- | --- | --- | --- | --- | --- | --- | --- | --- | --- | --- | --- | --- | --- | --- | --- | --- | --- | --- | --- | --- |
| R.LAEVALAYAK.A | Y | 46.75 | 1047.5964 | 10 | 0.8 | 524.8059 | 2 | 37.71 | 8 | F8:18594 | 31122021\_RID\_3262\_1a.raw |  |  |  |  |  |  |  | 6.0544E4 | 1.1426E4 |  | 2 | 0 | 0 | 0 | 0 | 0 | 0 | 0 | 1 | 1 | 0 | 150 | 159 |  |  | PEAKS DB |
| total 1 peptides |  |  |  |  |  |  |  |  |  |  |  |  |  |  |  |  |  |  |  |  |  |  |  |  |  |  |  |  |  |  |  |  |  |  |  |  |  |
| --- | --- | --- | --- | --- | --- | --- | --- | --- | --- | --- | --- | --- | --- | --- | --- | --- | --- | --- | --- | --- | --- | --- | --- | --- | --- | --- | --- | --- | --- | --- | --- | --- | --- | --- | --- | --- | --- |

XP\_026542345.1

back to list

  

| Protein Coverage
| Supporting Peptides
|

Protein Coverage:

Supporting Peptides:

| Peptide | Uniq | -10lgP | Mass | Length | ppm | m/z | z | RT | Fraction | Scan | Source File | Area F4 | Area F5 | Area F6 | Area F10 | Area F8 | Area F9 | Area F7 | Area F1 | Area F2 | Area F3 | #Feature | #Feature F4 | #Feature F5 | #Feature F6 | #Feature F10 | #Feature F8 | #Feature F9 | #Feature F7 | #Feature F1 | #Feature F2 | #Feature F3 | Start | End | PTM | AScore | Found By |
| --- | --- | --- | --- | --- | --- | --- | --- | --- | --- | --- | --- | --- | --- | --- | --- | --- | --- | --- | --- | --- | --- | --- | --- | --- | --- | --- | --- | --- | --- | --- | --- | --- | --- | --- | --- | --- | --- |
| R.LAEVALAYAK.A | Y | 46.75 | 1047.5964 | 10 | 0.8 | 524.8059 | 2 | 37.71 | 8 | F8:18594 | 31122021\_RID\_3262\_1a.raw |  |  |  |  |  |  |  | 6.0544E4 | 1.1426E4 |  | 2 | 0 | 0 | 0 | 0 | 0 | 0 | 0 | 1 | 1 | 0 | 150 | 159 |  |  | PEAKS DB |
| total 1 peptides |  |  |  |  |  |  |  |  |  |  |  |  |  |  |  |  |  |  |  |  |  |  |  |  |  |  |  |  |  |  |  |  |  |  |  |  |  |
| --- | --- | --- | --- | --- | --- | --- | --- | --- | --- | --- | --- | --- | --- | --- | --- | --- | --- | --- | --- | --- | --- | --- | --- | --- | --- | --- | --- | --- | --- | --- | --- | --- | --- | --- | --- | --- | --- |

XP\_034287864.1

back to list

  

| Protein Coverage
| Supporting Peptides
|

Protein Coverage:

Supporting Peptides:

| Peptide | Uniq | -10lgP | Mass | Length | ppm | m/z | z | RT | Fraction | Scan | Source File | Area F4 | Area F5 | Area F6 | Area F10 | Area F8 | Area F9 | Area F7 | Area F1 | Area F2 | Area F3 | #Feature | #Feature F4 | #Feature F5 | #Feature F6 | #Feature F10 | #Feature F8 | #Feature F9 | #Feature F7 | #Feature F1 | #Feature F2 | #Feature F3 | Start | End | PTM | AScore | Found By |
| --- | --- | --- | --- | --- | --- | --- | --- | --- | --- | --- | --- | --- | --- | --- | --- | --- | --- | --- | --- | --- | --- | --- | --- | --- | --- | --- | --- | --- | --- | --- | --- | --- | --- | --- | --- | --- | --- |
| P.GPVGPPGSPGK.T | Y | 46.22 | 948.5028 | 11 | -0.2 | 475.2586 | 2 | 19.59 | 9 | F9:5010 | 31122021\_RID\_3262\_2a.raw |  |  |  |  |  |  |  |  | 5.6673E5 |  | 1 | 0 | 0 | 0 | 0 | 0 | 0 | 0 | 0 | 1 | 0 | 631 | 641 |  |  | PEAKS DB |
| total 1 peptides |  |  |  |  |  |  |  |  |  |  |  |  |  |  |  |  |  |  |  |  |  |  |  |  |  |  |  |  |  |  |  |  |  |  |  |  |  |
| --- | --- | --- | --- | --- | --- | --- | --- | --- | --- | --- | --- | --- | --- | --- | --- | --- | --- | --- | --- | --- | --- | --- | --- | --- | --- | --- | --- | --- | --- | --- | --- | --- | --- | --- | --- | --- | --- |

P0C2D7.1

back to list

  

| Protein Coverage
| Supporting Peptides
|

Protein Coverage:

Supporting Peptides:

| Peptide | Uniq | -10lgP | Mass | Length | ppm | m/z | z | RT | Fraction | Scan | Source File | Area F4 | Area F5 | Area F6 | Area F10 | Area F8 | Area F9 | Area F7 | Area F1 | Area F2 | Area F3 | #Feature | #Feature F4 | #Feature F5 | #Feature F6 | #Feature F10 | #Feature F8 | #Feature F9 | #Feature F7 | #Feature F1 | #Feature F2 | #Feature F3 | Start | End | PTM | AScore | Found By |
| --- | --- | --- | --- | --- | --- | --- | --- | --- | --- | --- | --- | --- | --- | --- | --- | --- | --- | --- | --- | --- | --- | --- | --- | --- | --- | --- | --- | --- | --- | --- | --- | --- | --- | --- | --- | --- | --- |
| P.SEQLYEESLR.D | Y | 45.16 | 1252.5935 | 10 | -0.3 | 627.3038 | 2 | 33.57 | 10 | F10:7512 | 31122021\_RID\_3262\_3a.raw |  |  |  |  |  |  |  |  |  | 8.4843E7 | 1 | 0 | 0 | 0 | 0 | 0 | 0 | 0 | 0 | 0 | 1 | 43 | 52 |  |  | PEAKS DB |
| total 1 peptides |  |  |  |  |  |  |  |  |  |  |  |  |  |  |  |  |  |  |  |  |  |  |  |  |  |  |  |  |  |  |  |  |  |  |  |  |  |
| --- | --- | --- | --- | --- | --- | --- | --- | --- | --- | --- | --- | --- | --- | --- | --- | --- | --- | --- | --- | --- | --- | --- | --- | --- | --- | --- | --- | --- | --- | --- | --- | --- | --- | --- | --- | --- | --- |

ACC85814.1

back to list

  

| Protein Coverage
| Supporting Peptides
|

Protein Coverage:

Supporting Peptides:

| Peptide | Uniq | -10lgP | Mass | Length | ppm | m/z | z | RT | Fraction | Scan | Source File | Area F4 | Area F5 | Area F6 | Area F10 | Area F8 | Area F9 | Area F7 | Area F1 | Area F2 | Area F3 | #Feature | #Feature F4 | #Feature F5 | #Feature F6 | #Feature F10 | #Feature F8 | #Feature F9 | #Feature F7 | #Feature F1 | #Feature F2 | #Feature F3 | Start | End | PTM | AScore | Found By |
| --- | --- | --- | --- | --- | --- | --- | --- | --- | --- | --- | --- | --- | --- | --- | --- | --- | --- | --- | --- | --- | --- | --- | --- | --- | --- | --- | --- | --- | --- | --- | --- | --- | --- | --- | --- | --- | --- |
| K.QYFFETK.C | Y | 44.88 | 961.4545 | 7 | 0.0 | 481.7345 | 2 | 37.69 | 6 | F6:23416 | 17012022\_RID\_3262\_9a.raw |  |  |  |  |  | 9.0689E6 |  |  |  |  | 1 | 0 | 0 | 0 | 0 | 0 | 1 | 0 | 0 | 0 | 0 | 164 | 170 |  |  | PEAKS DB |
| total 1 peptides |  |  |  |  |  |  |  |  |  |  |  |  |  |  |  |  |  |  |  |  |  |  |  |  |  |  |  |  |  |  |  |  |  |  |  |  |  |
| --- | --- | --- | --- | --- | --- | --- | --- | --- | --- | --- | --- | --- | --- | --- | --- | --- | --- | --- | --- | --- | --- | --- | --- | --- | --- | --- | --- | --- | --- | --- | --- | --- | --- | --- | --- | --- | --- |

JAG46130.1

back to list

  

| Protein Coverage
| Supporting Peptides
|

Protein Coverage:

Supporting Peptides:

| Peptide | Uniq | -10lgP | Mass | Length | ppm | m/z | z | RT | Fraction | Scan | Source File | Area F4 | Area F5 | Area F6 | Area F10 | Area F8 | Area F9 | Area F7 | Area F1 | Area F2 | Area F3 | #Feature | #Feature F4 | #Feature F5 | #Feature F6 | #Feature F10 | #Feature F8 | #Feature F9 | #Feature F7 | #Feature F1 | #Feature F2 | #Feature F3 | Start | End | PTM | AScore | Found By |
| --- | --- | --- | --- | --- | --- | --- | --- | --- | --- | --- | --- | --- | --- | --- | --- | --- | --- | --- | --- | --- | --- | --- | --- | --- | --- | --- | --- | --- | --- | --- | --- | --- | --- | --- | --- | --- | --- |
| K.QYFFETK.C | Y | 44.88 | 961.4545 | 7 | 0.0 | 481.7345 | 2 | 37.69 | 6 | F6:23416 | 17012022\_RID\_3262\_9a.raw |  |  |  |  |  | 9.0689E6 |  |  |  |  | 1 | 0 | 0 | 0 | 0 | 0 | 1 | 0 | 0 | 0 | 0 | 172 | 178 |  |  | PEAKS DB |
| total 1 peptides |  |  |  |  |  |  |  |  |  |  |  |  |  |  |  |  |  |  |  |  |  |  |  |  |  |  |  |  |  |  |  |  |  |  |  |  |  |
| --- | --- | --- | --- | --- | --- | --- | --- | --- | --- | --- | --- | --- | --- | --- | --- | --- | --- | --- | --- | --- | --- | --- | --- | --- | --- | --- | --- | --- | --- | --- | --- | --- | --- | --- | --- | --- | --- |

XP\_015686279.2

back to list

  

| Protein Coverage
| Supporting Peptides
|

Protein Coverage:

Supporting Peptides:

| Peptide | Uniq | -10lgP | Mass | Length | ppm | m/z | z | RT | Fraction | Scan | Source File | Area F4 | Area F5 | Area F6 | Area F10 | Area F8 | Area F9 | Area F7 | Area F1 | Area F2 | Area F3 | #Feature | #Feature F4 | #Feature F5 | #Feature F6 | #Feature F10 | #Feature F8 | #Feature F9 | #Feature F7 | #Feature F1 | #Feature F2 | #Feature F3 | Start | End | PTM | AScore | Found By |
| --- | --- | --- | --- | --- | --- | --- | --- | --- | --- | --- | --- | --- | --- | --- | --- | --- | --- | --- | --- | --- | --- | --- | --- | --- | --- | --- | --- | --- | --- | --- | --- | --- | --- | --- | --- | --- | --- |
| K.IPGIYVLSLK.I | Y | 43.78 | 1101.6797 | 10 | 1.0 | 551.8477 | 2 | 53.17 | 3 | F3:26237 | 13012022\_RID\_3262\_6a.raw |  |  | 1.2301E5 |  |  |  |  |  |  |  | 1 | 0 | 0 | 1 | 0 | 0 | 0 | 0 | 0 | 0 | 0 | 22 | 31 |  |  | PEAKS DB |
| total 1 peptides |  |  |  |  |  |  |  |  |  |  |  |  |  |  |  |  |  |  |  |  |  |  |  |  |  |  |  |  |  |  |  |  |  |  |  |  |  |
| --- | --- | --- | --- | --- | --- | --- | --- | --- | --- | --- | --- | --- | --- | --- | --- | --- | --- | --- | --- | --- | --- | --- | --- | --- | --- | --- | --- | --- | --- | --- | --- | --- | --- | --- | --- | --- | --- |

XP\_026536429.1

back to list

  

| Protein Coverage
| Supporting Peptides
|

Protein Coverage:

Supporting Peptides:

| Peptide | Uniq | -10lgP | Mass | Length | ppm | m/z | z | RT | Fraction | Scan | Source File | Area F4 | Area F5 | Area F6 | Area F10 | Area F8 | Area F9 | Area F7 | Area F1 | Area F2 | Area F3 | #Feature | #Feature F4 | #Feature F5 | #Feature F6 | #Feature F10 | #Feature F8 | #Feature F9 | #Feature F7 | #Feature F1 | #Feature F2 | #Feature F3 | Start | End | PTM | AScore | Found By |
| --- | --- | --- | --- | --- | --- | --- | --- | --- | --- | --- | --- | --- | --- | --- | --- | --- | --- | --- | --- | --- | --- | --- | --- | --- | --- | --- | --- | --- | --- | --- | --- | --- | --- | --- | --- | --- | --- |
| K.IPGIYVLSLK.I | Y | 43.78 | 1101.6797 | 10 | 1.0 | 551.8477 | 2 | 53.17 | 3 | F3:26237 | 13012022\_RID\_3262\_6a.raw |  |  | 1.2301E5 |  |  |  |  |  |  |  | 1 | 0 | 0 | 1 | 0 | 0 | 0 | 0 | 0 | 0 | 0 | 60 | 69 |  |  | PEAKS DB |
| total 1 peptides |  |  |  |  |  |  |  |  |  |  |  |  |  |  |  |  |  |  |  |  |  |  |  |  |  |  |  |  |  |  |  |  |  |  |  |  |  |
| --- | --- | --- | --- | --- | --- | --- | --- | --- | --- | --- | --- | --- | --- | --- | --- | --- | --- | --- | --- | --- | --- | --- | --- | --- | --- | --- | --- | --- | --- | --- | --- | --- | --- | --- | --- | --- | --- |

XP\_026558426.1

back to list

  

| Protein Coverage
| Supporting Peptides
|

Protein Coverage:

Supporting Peptides:

| Peptide | Uniq | -10lgP | Mass | Length | ppm | m/z | z | RT | Fraction | Scan | Source File | Area F4 | Area F5 | Area F6 | Area F10 | Area F8 | Area F9 | Area F7 | Area F1 | Area F2 | Area F3 | #Feature | #Feature F4 | #Feature F5 | #Feature F6 | #Feature F10 | #Feature F8 | #Feature F9 | #Feature F7 | #Feature F1 | #Feature F2 | #Feature F3 | Start | End | PTM | AScore | Found By |
| --- | --- | --- | --- | --- | --- | --- | --- | --- | --- | --- | --- | --- | --- | --- | --- | --- | --- | --- | --- | --- | --- | --- | --- | --- | --- | --- | --- | --- | --- | --- | --- | --- | --- | --- | --- | --- | --- |
| K.IPGIYVLSLK.I | Y | 43.78 | 1101.6797 | 10 | 1.0 | 551.8477 | 2 | 53.17 | 3 | F3:26237 | 13012022\_RID\_3262\_6a.raw |  |  | 1.2301E5 |  |  |  |  |  |  |  | 1 | 0 | 0 | 1 | 0 | 0 | 0 | 0 | 0 | 0 | 0 | 60 | 69 |  |  | PEAKS DB |
| total 1 peptides |  |  |  |  |  |  |  |  |  |  |  |  |  |  |  |  |  |  |  |  |  |  |  |  |  |  |  |  |  |  |  |  |  |  |  |  |  |
| --- | --- | --- | --- | --- | --- | --- | --- | --- | --- | --- | --- | --- | --- | --- | --- | --- | --- | --- | --- | --- | --- | --- | --- | --- | --- | --- | --- | --- | --- | --- | --- | --- | --- | --- | --- | --- | --- |

XP\_034269326.1

back to list

  

| Protein Coverage
| Supporting Peptides
|

Protein Coverage:

Supporting Peptides:

| Peptide | Uniq | -10lgP | Mass | Length | ppm | m/z | z | RT | Fraction | Scan | Source File | Area F4 | Area F5 | Area F6 | Area F10 | Area F8 | Area F9 | Area F7 | Area F1 | Area F2 | Area F3 | #Feature | #Feature F4 | #Feature F5 | #Feature F6 | #Feature F10 | #Feature F8 | #Feature F9 | #Feature F7 | #Feature F1 | #Feature F2 | #Feature F3 | Start | End | PTM | AScore | Found By |
| --- | --- | --- | --- | --- | --- | --- | --- | --- | --- | --- | --- | --- | --- | --- | --- | --- | --- | --- | --- | --- | --- | --- | --- | --- | --- | --- | --- | --- | --- | --- | --- | --- | --- | --- | --- | --- | --- |
| K.IPGIYVLSLK.I | Y | 43.78 | 1101.6797 | 10 | 1.0 | 551.8477 | 2 | 53.17 | 3 | F3:26237 | 13012022\_RID\_3262\_6a.raw |  |  | 1.2301E5 |  |  |  |  |  |  |  | 1 | 0 | 0 | 1 | 0 | 0 | 0 | 0 | 0 | 0 | 0 | 60 | 69 |  |  | PEAKS DB |
| total 1 peptides |  |  |  |  |  |  |  |  |  |  |  |  |  |  |  |  |  |  |  |  |  |  |  |  |  |  |  |  |  |  |  |  |  |  |  |  |  |
| --- | --- | --- | --- | --- | --- | --- | --- | --- | --- | --- | --- | --- | --- | --- | --- | --- | --- | --- | --- | --- | --- | --- | --- | --- | --- | --- | --- | --- | --- | --- | --- | --- | --- | --- | --- | --- | --- |

XP\_034269327.1

back to list

  

| Protein Coverage
| Supporting Peptides
|

Protein Coverage:

Supporting Peptides:

| Peptide | Uniq | -10lgP | Mass | Length | ppm | m/z | z | RT | Fraction | Scan | Source File | Area F4 | Area F5 | Area F6 | Area F10 | Area F8 | Area F9 | Area F7 | Area F1 | Area F2 | Area F3 | #Feature | #Feature F4 | #Feature F5 | #Feature F6 | #Feature F10 | #Feature F8 | #Feature F9 | #Feature F7 | #Feature F1 | #Feature F2 | #Feature F3 | Start | End | PTM | AScore | Found By |
| --- | --- | --- | --- | --- | --- | --- | --- | --- | --- | --- | --- | --- | --- | --- | --- | --- | --- | --- | --- | --- | --- | --- | --- | --- | --- | --- | --- | --- | --- | --- | --- | --- | --- | --- | --- | --- | --- |
| K.IPGIYVLSLK.I | Y | 43.78 | 1101.6797 | 10 | 1.0 | 551.8477 | 2 | 53.17 | 3 | F3:26237 | 13012022\_RID\_3262\_6a.raw |  |  | 1.2301E5 |  |  |  |  |  |  |  | 1 | 0 | 0 | 1 | 0 | 0 | 0 | 0 | 0 | 0 | 0 | 12 | 21 |  |  | PEAKS DB |
| total 1 peptides |  |  |  |  |  |  |  |  |  |  |  |  |  |  |  |  |  |  |  |  |  |  |  |  |  |  |  |  |  |  |  |  |  |  |  |  |  |
| --- | --- | --- | --- | --- | --- | --- | --- | --- | --- | --- | --- | --- | --- | --- | --- | --- | --- | --- | --- | --- | --- | --- | --- | --- | --- | --- | --- | --- | --- | --- | --- | --- | --- | --- | --- | --- | --- |

XP\_032084406.1

back to list

  

| Protein Coverage
| Supporting Peptides
|

Protein Coverage:

Supporting Peptides:

| Peptide | Uniq | -10lgP | Mass | Length | ppm | m/z | z | RT | Fraction | Scan | Source File | Area F4 | Area F5 | Area F6 | Area F10 | Area F8 | Area F9 | Area F7 | Area F1 | Area F2 | Area F3 | #Feature | #Feature F4 | #Feature F5 | #Feature F6 | #Feature F10 | #Feature F8 | #Feature F9 | #Feature F7 | #Feature F1 | #Feature F2 | #Feature F3 | Start | End | PTM | AScore | Found By |
| --- | --- | --- | --- | --- | --- | --- | --- | --- | --- | --- | --- | --- | --- | --- | --- | --- | --- | --- | --- | --- | --- | --- | --- | --- | --- | --- | --- | --- | --- | --- | --- | --- | --- | --- | --- | --- | --- |
| K.IPGIYVLSLK.I | Y | 43.78 | 1101.6797 | 10 | 1.0 | 551.8477 | 2 | 53.17 | 3 | F3:26237 | 13012022\_RID\_3262\_6a.raw |  |  | 1.2301E5 |  |  |  |  |  |  |  | 1 | 0 | 0 | 1 | 0 | 0 | 0 | 0 | 0 | 0 | 0 | 59 | 68 |  |  | PEAKS DB |
| total 1 peptides |  |  |  |  |  |  |  |  |  |  |  |  |  |  |  |  |  |  |  |  |  |  |  |  |  |  |  |  |  |  |  |  |  |  |  |  |  |
| --- | --- | --- | --- | --- | --- | --- | --- | --- | --- | --- | --- | --- | --- | --- | --- | --- | --- | --- | --- | --- | --- | --- | --- | --- | --- | --- | --- | --- | --- | --- | --- | --- | --- | --- | --- | --- | --- |

XP\_013915921.1

back to list

  

| Protein Coverage
| Supporting Peptides
|

Protein Coverage:

Supporting Peptides:

| Peptide | Uniq | -10lgP | Mass | Length | ppm | m/z | z | RT | Fraction | Scan | Source File | Area F4 | Area F5 | Area F6 | Area F10 | Area F8 | Area F9 | Area F7 | Area F1 | Area F2 | Area F3 | #Feature | #Feature F4 | #Feature F5 | #Feature F6 | #Feature F10 | #Feature F8 | #Feature F9 | #Feature F7 | #Feature F1 | #Feature F2 | #Feature F3 | Start | End | PTM | AScore | Found By |
| --- | --- | --- | --- | --- | --- | --- | --- | --- | --- | --- | --- | --- | --- | --- | --- | --- | --- | --- | --- | --- | --- | --- | --- | --- | --- | --- | --- | --- | --- | --- | --- | --- | --- | --- | --- | --- | --- |
| K.IPGIYVLSLK.I | Y | 43.78 | 1101.6797 | 10 | 1.0 | 551.8477 | 2 | 53.17 | 3 | F3:26237 | 13012022\_RID\_3262\_6a.raw |  |  | 1.2301E5 |  |  |  |  |  |  |  | 1 | 0 | 0 | 1 | 0 | 0 | 0 | 0 | 0 | 0 | 0 | 22 | 31 |  |  | PEAKS DB |
| total 1 peptides |  |  |  |  |  |  |  |  |  |  |  |  |  |  |  |  |  |  |  |  |  |  |  |  |  |  |  |  |  |  |  |  |  |  |  |  |  |
| --- | --- | --- | --- | --- | --- | --- | --- | --- | --- | --- | --- | --- | --- | --- | --- | --- | --- | --- | --- | --- | --- | --- | --- | --- | --- | --- | --- | --- | --- | --- | --- | --- | --- | --- | --- | --- | --- |

XP\_007430564.1

back to list

  

| Protein Coverage
| Supporting Peptides
|

Protein Coverage:

Supporting Peptides:

| Peptide | Uniq | -10lgP | Mass | Length | ppm | m/z | z | RT | Fraction | Scan | Source File | Area F4 | Area F5 | Area F6 | Area F10 | Area F8 | Area F9 | Area F7 | Area F1 | Area F2 | Area F3 | #Feature | #Feature F4 | #Feature F5 | #Feature F6 | #Feature F10 | #Feature F8 | #Feature F9 | #Feature F7 | #Feature F1 | #Feature F2 | #Feature F3 | Start | End | PTM | AScore | Found By |
| --- | --- | --- | --- | --- | --- | --- | --- | --- | --- | --- | --- | --- | --- | --- | --- | --- | --- | --- | --- | --- | --- | --- | --- | --- | --- | --- | --- | --- | --- | --- | --- | --- | --- | --- | --- | --- | --- |
| K.IPGIYVLSLK.I | Y | 43.78 | 1101.6797 | 10 | 1.0 | 551.8477 | 2 | 53.17 | 3 | F3:26237 | 13012022\_RID\_3262\_6a.raw |  |  | 1.2301E5 |  |  |  |  |  |  |  | 1 | 0 | 0 | 1 | 0 | 0 | 0 | 0 | 0 | 0 | 0 | 63 | 72 |  |  | PEAKS DB |
| total 1 peptides |  |  |  |  |  |  |  |  |  |  |  |  |  |  |  |  |  |  |  |  |  |  |  |  |  |  |  |  |  |  |  |  |  |  |  |  |  |
| --- | --- | --- | --- | --- | --- | --- | --- | --- | --- | --- | --- | --- | --- | --- | --- | --- | --- | --- | --- | --- | --- | --- | --- | --- | --- | --- | --- | --- | --- | --- | --- | --- | --- | --- | --- | --- | --- |

XP\_026556752.1

back to list

  

| Protein Coverage
| Supporting Peptides
|

Protein Coverage:

Supporting Peptides:

| Peptide | Uniq | -10lgP | Mass | Length | ppm | m/z | z | RT | Fraction | Scan | Source File | Area F4 | Area F5 | Area F6 | Area F10 | Area F8 | Area F9 | Area F7 | Area F1 | Area F2 | Area F3 | #Feature | #Feature F4 | #Feature F5 | #Feature F6 | #Feature F10 | #Feature F8 | #Feature F9 | #Feature F7 | #Feature F1 | #Feature F2 | #Feature F3 | Start | End | PTM | AScore | Found By |
| --- | --- | --- | --- | --- | --- | --- | --- | --- | --- | --- | --- | --- | --- | --- | --- | --- | --- | --- | --- | --- | --- | --- | --- | --- | --- | --- | --- | --- | --- | --- | --- | --- | --- | --- | --- | --- | --- |
| R.LPSYLDK.I | Y | 42.85 | 834.4487 | 7 | -0.2 | 418.2315 | 2 | 38.50 | 6 | F6:24206 | 17012022\_RID\_3262\_9a.raw |  |  |  |  |  | 1.3731E6 |  |  |  |  | 1 | 0 | 0 | 0 | 0 | 0 | 1 | 0 | 0 | 0 | 0 | 393 | 399 |  |  | PEAKS DB |
| total 1 peptides |  |  |  |  |  |  |  |  |  |  |  |  |  |  |  |  |  |  |  |  |  |  |  |  |  |  |  |  |  |  |  |  |  |  |  |  |  |
| --- | --- | --- | --- | --- | --- | --- | --- | --- | --- | --- | --- | --- | --- | --- | --- | --- | --- | --- | --- | --- | --- | --- | --- | --- | --- | --- | --- | --- | --- | --- | --- | --- | --- | --- | --- | --- | --- |

XP\_034291152.1

back to list

  

| Protein Coverage
| Supporting Peptides
|

Protein Coverage:

Supporting Peptides:

| Peptide | Uniq | -10lgP | Mass | Length | ppm | m/z | z | RT | Fraction | Scan | Source File | Area F4 | Area F5 | Area F6 | Area F10 | Area F8 | Area F9 | Area F7 | Area F1 | Area F2 | Area F3 | #Feature | #Feature F4 | #Feature F5 | #Feature F6 | #Feature F10 | #Feature F8 | #Feature F9 | #Feature F7 | #Feature F1 | #Feature F2 | #Feature F3 | Start | End | PTM | AScore | Found By |
| --- | --- | --- | --- | --- | --- | --- | --- | --- | --- | --- | --- | --- | --- | --- | --- | --- | --- | --- | --- | --- | --- | --- | --- | --- | --- | --- | --- | --- | --- | --- | --- | --- | --- | --- | --- | --- | --- |
| R.SEIDLVQIK.Q | Y | 42.76 | 1043.5862 | 9 | -0.4 | 522.8002 | 2 | 41.30 | 8 | F8:21525 | 31122021\_RID\_3262\_1a.raw |  |  |  |  |  |  |  | 0 |  |  | 0 | 0 | 0 | 0 | 0 | 0 | 0 | 0 | 0 | 0 | 0 | 332 | 340 |  |  | PEAKS DB |
| total 1 peptides |  |  |  |  |  |  |  |  |  |  |  |  |  |  |  |  |  |  |  |  |  |  |  |  |  |  |  |  |  |  |  |  |  |  |  |  |  |
| --- | --- | --- | --- | --- | --- | --- | --- | --- | --- | --- | --- | --- | --- | --- | --- | --- | --- | --- | --- | --- | --- | --- | --- | --- | --- | --- | --- | --- | --- | --- | --- | --- | --- | --- | --- | --- | --- |

XP\_034291151.1

back to list

  

| Protein Coverage
| Supporting Peptides
|

Protein Coverage:

Supporting Peptides:

| Peptide | Uniq | -10lgP | Mass | Length | ppm | m/z | z | RT | Fraction | Scan | Source File | Area F4 | Area F5 | Area F6 | Area F10 | Area F8 | Area F9 | Area F7 | Area F1 | Area F2 | Area F3 | #Feature | #Feature F4 | #Feature F5 | #Feature F6 | #Feature F10 | #Feature F8 | #Feature F9 | #Feature F7 | #Feature F1 | #Feature F2 | #Feature F3 | Start | End | PTM | AScore | Found By |
| --- | --- | --- | --- | --- | --- | --- | --- | --- | --- | --- | --- | --- | --- | --- | --- | --- | --- | --- | --- | --- | --- | --- | --- | --- | --- | --- | --- | --- | --- | --- | --- | --- | --- | --- | --- | --- | --- |
| R.SEIDLVQIK.Q | Y | 42.76 | 1043.5862 | 9 | -0.4 | 522.8002 | 2 | 41.30 | 8 | F8:21525 | 31122021\_RID\_3262\_1a.raw |  |  |  |  |  |  |  | 0 |  |  | 0 | 0 | 0 | 0 | 0 | 0 | 0 | 0 | 0 | 0 | 0 | 418 | 426 |  |  | PEAKS DB |
| total 1 peptides |  |  |  |  |  |  |  |  |  |  |  |  |  |  |  |  |  |  |  |  |  |  |  |  |  |  |  |  |  |  |  |  |  |  |  |  |  |
| --- | --- | --- | --- | --- | --- | --- | --- | --- | --- | --- | --- | --- | --- | --- | --- | --- | --- | --- | --- | --- | --- | --- | --- | --- | --- | --- | --- | --- | --- | --- | --- | --- | --- | --- | --- | --- | --- |

XP\_034291150.1

back to list

  

| Protein Coverage
| Supporting Peptides
|

Protein Coverage:

Supporting Peptides:

| Peptide | Uniq | -10lgP | Mass | Length | ppm | m/z | z | RT | Fraction | Scan | Source File | Area F4 | Area F5 | Area F6 | Area F10 | Area F8 | Area F9 | Area F7 | Area F1 | Area F2 | Area F3 | #Feature | #Feature F4 | #Feature F5 | #Feature F6 | #Feature F10 | #Feature F8 | #Feature F9 | #Feature F7 | #Feature F1 | #Feature F2 | #Feature F3 | Start | End | PTM | AScore | Found By |
| --- | --- | --- | --- | --- | --- | --- | --- | --- | --- | --- | --- | --- | --- | --- | --- | --- | --- | --- | --- | --- | --- | --- | --- | --- | --- | --- | --- | --- | --- | --- | --- | --- | --- | --- | --- | --- | --- |
| R.SEIDLVQIK.Q | Y | 42.76 | 1043.5862 | 9 | -0.4 | 522.8002 | 2 | 41.30 | 8 | F8:21525 | 31122021\_RID\_3262\_1a.raw |  |  |  |  |  |  |  | 0 |  |  | 0 | 0 | 0 | 0 | 0 | 0 | 0 | 0 | 0 | 0 | 0 | 418 | 426 |  |  | PEAKS DB |
| total 1 peptides |  |  |  |  |  |  |  |  |  |  |  |  |  |  |  |  |  |  |  |  |  |  |  |  |  |  |  |  |  |  |  |  |  |  |  |  |  |
| --- | --- | --- | --- | --- | --- | --- | --- | --- | --- | --- | --- | --- | --- | --- | --- | --- | --- | --- | --- | --- | --- | --- | --- | --- | --- | --- | --- | --- | --- | --- | --- | --- | --- | --- | --- | --- | --- |

XP\_034291149.1

back to list

  

| Protein Coverage
| Supporting Peptides
|

Protein Coverage:

Supporting Peptides:

| Peptide | Uniq | -10lgP | Mass | Length | ppm | m/z | z | RT | Fraction | Scan | Source File | Area F4 | Area F5 | Area F6 | Area F10 | Area F8 | Area F9 | Area F7 | Area F1 | Area F2 | Area F3 | #Feature | #Feature F4 | #Feature F5 | #Feature F6 | #Feature F10 | #Feature F8 | #Feature F9 | #Feature F7 | #Feature F1 | #Feature F2 | #Feature F3 | Start | End | PTM | AScore | Found By |
| --- | --- | --- | --- | --- | --- | --- | --- | --- | --- | --- | --- | --- | --- | --- | --- | --- | --- | --- | --- | --- | --- | --- | --- | --- | --- | --- | --- | --- | --- | --- | --- | --- | --- | --- | --- | --- | --- |
| R.SEIDLVQIK.Q | Y | 42.76 | 1043.5862 | 9 | -0.4 | 522.8002 | 2 | 41.30 | 8 | F8:21525 | 31122021\_RID\_3262\_1a.raw |  |  |  |  |  |  |  | 0 |  |  | 0 | 0 | 0 | 0 | 0 | 0 | 0 | 0 | 0 | 0 | 0 | 433 | 441 |  |  | PEAKS DB |
| total 1 peptides |  |  |  |  |  |  |  |  |  |  |  |  |  |  |  |  |  |  |  |  |  |  |  |  |  |  |  |  |  |  |  |  |  |  |  |  |  |
| --- | --- | --- | --- | --- | --- | --- | --- | --- | --- | --- | --- | --- | --- | --- | --- | --- | --- | --- | --- | --- | --- | --- | --- | --- | --- | --- | --- | --- | --- | --- | --- | --- | --- | --- | --- | --- | --- |

XP\_034291148.1

back to list

  

| Protein Coverage
| Supporting Peptides
|

Protein Coverage:

Supporting Peptides:

| Peptide | Uniq | -10lgP | Mass | Length | ppm | m/z | z | RT | Fraction | Scan | Source File | Area F4 | Area F5 | Area F6 | Area F10 | Area F8 | Area F9 | Area F7 | Area F1 | Area F2 | Area F3 | #Feature | #Feature F4 | #Feature F5 | #Feature F6 | #Feature F10 | #Feature F8 | #Feature F9 | #Feature F7 | #Feature F1 | #Feature F2 | #Feature F3 | Start | End | PTM | AScore | Found By |
| --- | --- | --- | --- | --- | --- | --- | --- | --- | --- | --- | --- | --- | --- | --- | --- | --- | --- | --- | --- | --- | --- | --- | --- | --- | --- | --- | --- | --- | --- | --- | --- | --- | --- | --- | --- | --- | --- |
| R.SEIDLVQIK.Q | Y | 42.76 | 1043.5862 | 9 | -0.4 | 522.8002 | 2 | 41.30 | 8 | F8:21525 | 31122021\_RID\_3262\_1a.raw |  |  |  |  |  |  |  | 0 |  |  | 0 | 0 | 0 | 0 | 0 | 0 | 0 | 0 | 0 | 0 | 0 | 437 | 445 |  |  | PEAKS DB |
| total 1 peptides |  |  |  |  |  |  |  |  |  |  |  |  |  |  |  |  |  |  |  |  |  |  |  |  |  |  |  |  |  |  |  |  |  |  |  |  |  |
| --- | --- | --- | --- | --- | --- | --- | --- | --- | --- | --- | --- | --- | --- | --- | --- | --- | --- | --- | --- | --- | --- | --- | --- | --- | --- | --- | --- | --- | --- | --- | --- | --- | --- | --- | --- | --- | --- |

XP\_013914127.1

back to list

  

| Protein Coverage
| Supporting Peptides
|

Protein Coverage:

Supporting Peptides:

| Peptide | Uniq | -10lgP | Mass | Length | ppm | m/z | z | RT | Fraction | Scan | Source File | Area F4 | Area F5 | Area F6 | Area F10 | Area F8 | Area F9 | Area F7 | Area F1 | Area F2 | Area F3 | #Feature | #Feature F4 | #Feature F5 | #Feature F6 | #Feature F10 | #Feature F8 | #Feature F9 | #Feature F7 | #Feature F1 | #Feature F2 | #Feature F3 | Start | End | PTM | AScore | Found By |
| --- | --- | --- | --- | --- | --- | --- | --- | --- | --- | --- | --- | --- | --- | --- | --- | --- | --- | --- | --- | --- | --- | --- | --- | --- | --- | --- | --- | --- | --- | --- | --- | --- | --- | --- | --- | --- | --- |
| K.TKDGVVEITGK.H | Y | 42.34 | 1145.6292 | 11 | 1.6 | 573.8228 | 2 | 20.96 | 8 | F8:5748 | 31122021\_RID\_3262\_1a.raw |  |  |  |  |  | 1.3494E5 |  | 2.5834E4 |  |  | 2 | 0 | 0 | 0 | 0 | 0 | 1 | 0 | 1 | 0 | 0 | 118 | 128 |  |  | PEAKS DB |
| total 1 peptides |  |  |  |  |  |  |  |  |  |  |  |  |  |  |  |  |  |  |  |  |  |  |  |  |  |  |  |  |  |  |  |  |  |  |  |  |  |
| --- | --- | --- | --- | --- | --- | --- | --- | --- | --- | --- | --- | --- | --- | --- | --- | --- | --- | --- | --- | --- | --- | --- | --- | --- | --- | --- | --- | --- | --- | --- | --- | --- | --- | --- | --- | --- | --- |

XP\_032072257.1

back to list

  

| Protein Coverage
| Supporting Peptides
|

Protein Coverage:

Supporting Peptides:

| Peptide | Uniq | -10lgP | Mass | Length | ppm | m/z | z | RT | Fraction | Scan | Source File | Area F4 | Area F5 | Area F6 | Area F10 | Area F8 | Area F9 | Area F7 | Area F1 | Area F2 | Area F3 | #Feature | #Feature F4 | #Feature F5 | #Feature F6 | #Feature F10 | #Feature F8 | #Feature F9 | #Feature F7 | #Feature F1 | #Feature F2 | #Feature F3 | Start | End | PTM | AScore | Found By |
| --- | --- | --- | --- | --- | --- | --- | --- | --- | --- | --- | --- | --- | --- | --- | --- | --- | --- | --- | --- | --- | --- | --- | --- | --- | --- | --- | --- | --- | --- | --- | --- | --- | --- | --- | --- | --- | --- |
| K.TKDGVVEITGK.H | Y | 42.34 | 1145.6292 | 11 | 1.6 | 573.8228 | 2 | 20.96 | 8 | F8:5748 | 31122021\_RID\_3262\_1a.raw |  |  |  |  |  | 1.3494E5 |  | 2.5834E4 |  |  | 2 | 0 | 0 | 0 | 0 | 0 | 1 | 0 | 1 | 0 | 0 | 118 | 128 |  |  | PEAKS DB |
| total 1 peptides |  |  |  |  |  |  |  |  |  |  |  |  |  |  |  |  |  |  |  |  |  |  |  |  |  |  |  |  |  |  |  |  |  |  |  |  |  |
| --- | --- | --- | --- | --- | --- | --- | --- | --- | --- | --- | --- | --- | --- | --- | --- | --- | --- | --- | --- | --- | --- | --- | --- | --- | --- | --- | --- | --- | --- | --- | --- | --- | --- | --- | --- | --- | --- |

XP\_015679331.1

back to list

  

| Protein Coverage
| Supporting Peptides
|

Protein Coverage:

Supporting Peptides:

| Peptide | Uniq | -10lgP | Mass | Length | ppm | m/z | z | RT | Fraction | Scan | Source File | Area F4 | Area F5 | Area F6 | Area F10 | Area F8 | Area F9 | Area F7 | Area F1 | Area F2 | Area F3 | #Feature | #Feature F4 | #Feature F5 | #Feature F6 | #Feature F10 | #Feature F8 | #Feature F9 | #Feature F7 | #Feature F1 | #Feature F2 | #Feature F3 | Start | End | PTM | AScore | Found By |
| --- | --- | --- | --- | --- | --- | --- | --- | --- | --- | --- | --- | --- | --- | --- | --- | --- | --- | --- | --- | --- | --- | --- | --- | --- | --- | --- | --- | --- | --- | --- | --- | --- | --- | --- | --- | --- | --- |
| K.TKDGVVEITGK.H | Y | 42.34 | 1145.6292 | 11 | 1.6 | 573.8228 | 2 | 20.96 | 8 | F8:5748 | 31122021\_RID\_3262\_1a.raw |  |  |  |  |  | 1.3494E5 |  | 2.5834E4 |  |  | 2 | 0 | 0 | 0 | 0 | 0 | 1 | 0 | 1 | 0 | 0 | 120 | 130 |  |  | PEAKS DB |
| total 1 peptides |  |  |  |  |  |  |  |  |  |  |  |  |  |  |  |  |  |  |  |  |  |  |  |  |  |  |  |  |  |  |  |  |  |  |  |  |  |
| --- | --- | --- | --- | --- | --- | --- | --- | --- | --- | --- | --- | --- | --- | --- | --- | --- | --- | --- | --- | --- | --- | --- | --- | --- | --- | --- | --- | --- | --- | --- | --- | --- | --- | --- | --- | --- | --- |

XP\_015685588.1

back to list

  

| Protein Coverage
| Supporting Peptides
|

Protein Coverage:

Supporting Peptides:

| Peptide | Uniq | -10lgP | Mass | Length | ppm | m/z | z | RT | Fraction | Scan | Source File | Area F4 | Area F5 | Area F6 | Area F10 | Area F8 | Area F9 | Area F7 | Area F1 | Area F2 | Area F3 | #Feature | #Feature F4 | #Feature F5 | #Feature F6 | #Feature F10 | #Feature F8 | #Feature F9 | #Feature F7 | #Feature F1 | #Feature F2 | #Feature F3 | Start | End | PTM | AScore | Found By |
| --- | --- | --- | --- | --- | --- | --- | --- | --- | --- | --- | --- | --- | --- | --- | --- | --- | --- | --- | --- | --- | --- | --- | --- | --- | --- | --- | --- | --- | --- | --- | --- | --- | --- | --- | --- | --- | --- |
| R.EAVFVAQLLSEDIK.T | Y | 42.21 | 1560.8398 | 14 | 0.8 | 781.4279 | 2 | 60.51 | 8 | F8:36485 | 31122021\_RID\_3262\_1a.raw |  |  |  |  |  |  |  | 1.3789E4 |  |  | 1 | 0 | 0 | 0 | 0 | 0 | 0 | 0 | 1 | 0 | 0 | 70 | 83 |  |  | PEAKS DB |
| total 1 peptides |  |  |  |  |  |  |  |  |  |  |  |  |  |  |  |  |  |  |  |  |  |  |  |  |  |  |  |  |  |  |  |  |  |  |  |  |  |
| --- | --- | --- | --- | --- | --- | --- | --- | --- | --- | --- | --- | --- | --- | --- | --- | --- | --- | --- | --- | --- | --- | --- | --- | --- | --- | --- | --- | --- | --- | --- | --- | --- | --- | --- | --- | --- | --- |

BAP39929.1

back to list

  

| Protein Coverage
| Supporting Peptides
|

Protein Coverage:

Supporting Peptides:

| Peptide | Uniq | -10lgP | Mass | Length | ppm | m/z | z | RT | Fraction | Scan | Source File | Area F4 | Area F5 | Area F6 | Area F10 | Area F8 | Area F9 | Area F7 | Area F1 | Area F2 | Area F3 | #Feature | #Feature F4 | #Feature F5 | #Feature F6 | #Feature F10 | #Feature F8 | #Feature F9 | #Feature F7 | #Feature F1 | #Feature F2 | #Feature F3 | Start | End | PTM | AScore | Found By |
| --- | --- | --- | --- | --- | --- | --- | --- | --- | --- | --- | --- | --- | --- | --- | --- | --- | --- | --- | --- | --- | --- | --- | --- | --- | --- | --- | --- | --- | --- | --- | --- | --- | --- | --- | --- | --- | --- |
| R.EAVFVAQLLSEDIK.T | Y | 42.21 | 1560.8398 | 14 | 0.8 | 781.4279 | 2 | 60.51 | 8 | F8:36485 | 31122021\_RID\_3262\_1a.raw |  |  |  |  |  |  |  | 1.3789E4 |  |  | 1 | 0 | 0 | 0 | 0 | 0 | 0 | 0 | 1 | 0 | 0 | 98 | 111 |  |  | PEAKS DB |
| total 1 peptides |  |  |  |  |  |  |  |  |  |  |  |  |  |  |  |  |  |  |  |  |  |  |  |  |  |  |  |  |  |  |  |  |  |  |  |  |  |
| --- | --- | --- | --- | --- | --- | --- | --- | --- | --- | --- | --- | --- | --- | --- | --- | --- | --- | --- | --- | --- | --- | --- | --- | --- | --- | --- | --- | --- | --- | --- | --- | --- | --- | --- | --- | --- | --- |

ETE73522.1

back to list

  

| Protein Coverage
| Supporting Peptides
|

Protein Coverage:

Supporting Peptides:

| Peptide | Uniq | -10lgP | Mass | Length | ppm | m/z | z | RT | Fraction | Scan | Source File | Area F4 | Area F5 | Area F6 | Area F10 | Area F8 | Area F9 | Area F7 | Area F1 | Area F2 | Area F3 | #Feature | #Feature F4 | #Feature F5 | #Feature F6 | #Feature F10 | #Feature F8 | #Feature F9 | #Feature F7 | #Feature F1 | #Feature F2 | #Feature F3 | Start | End | PTM | AScore | Found By |
| --- | --- | --- | --- | --- | --- | --- | --- | --- | --- | --- | --- | --- | --- | --- | --- | --- | --- | --- | --- | --- | --- | --- | --- | --- | --- | --- | --- | --- | --- | --- | --- | --- | --- | --- | --- | --- | --- |
| K.AVENSSTAIGIR.C | Y | 41.53 | 1216.6411 | 12 | -0.3 | 609.3276 | 2 | 29.75 | 4 | F4:16295 | 17012022\_RID\_3262\_10a .raw |  |  |  | 2.1242E5 |  |  |  |  |  |  | 1 | 0 | 0 | 0 | 1 | 0 | 0 | 0 | 0 | 0 | 0 | 30 | 41 |  |  | PEAKS DB |
| total 1 peptides |  |  |  |  |  |  |  |  |  |  |  |  |  |  |  |  |  |  |  |  |  |  |  |  |  |  |  |  |  |  |  |  |  |  |  |  |  |
| --- | --- | --- | --- | --- | --- | --- | --- | --- | --- | --- | --- | --- | --- | --- | --- | --- | --- | --- | --- | --- | --- | --- | --- | --- | --- | --- | --- | --- | --- | --- | --- | --- | --- | --- | --- | --- | --- |

XP\_013912175.1

back to list

  

| Protein Coverage
| Supporting Peptides
|

Protein Coverage:

Supporting Peptides:

| Peptide | Uniq | -10lgP | Mass | Length | ppm | m/z | z | RT | Fraction | Scan | Source File | Area F4 | Area F5 | Area F6 | Area F10 | Area F8 | Area F9 | Area F7 | Area F1 | Area F2 | Area F3 | #Feature | #Feature F4 | #Feature F5 | #Feature F6 | #Feature F10 | #Feature F8 | #Feature F9 | #Feature F7 | #Feature F1 | #Feature F2 | #Feature F3 | Start | End | PTM | AScore | Found By |
| --- | --- | --- | --- | --- | --- | --- | --- | --- | --- | --- | --- | --- | --- | --- | --- | --- | --- | --- | --- | --- | --- | --- | --- | --- | --- | --- | --- | --- | --- | --- | --- | --- | --- | --- | --- | --- | --- |
| K.AVENSSTAIGIR.C | Y | 41.53 | 1216.6411 | 12 | -0.3 | 609.3276 | 2 | 29.75 | 4 | F4:16295 | 17012022\_RID\_3262\_10a .raw |  |  |  | 2.1242E5 |  |  |  |  |  |  | 1 | 0 | 0 | 0 | 1 | 0 | 0 | 0 | 0 | 0 | 0 | 30 | 41 |  |  | PEAKS DB |
| total 1 peptides |  |  |  |  |  |  |  |  |  |  |  |  |  |  |  |  |  |  |  |  |  |  |  |  |  |  |  |  |  |  |  |  |  |  |  |  |  |
| --- | --- | --- | --- | --- | --- | --- | --- | --- | --- | --- | --- | --- | --- | --- | --- | --- | --- | --- | --- | --- | --- | --- | --- | --- | --- | --- | --- | --- | --- | --- | --- | --- | --- | --- | --- | --- | --- |

JAG66919.1

back to list

  

| Protein Coverage
| Supporting Peptides
|

Protein Coverage:

Supporting Peptides:

| Peptide | Uniq | -10lgP | Mass | Length | ppm | m/z | z | RT | Fraction | Scan | Source File | Area F4 | Area F5 | Area F6 | Area F10 | Area F8 | Area F9 | Area F7 | Area F1 | Area F2 | Area F3 | #Feature | #Feature F4 | #Feature F5 | #Feature F6 | #Feature F10 | #Feature F8 | #Feature F9 | #Feature F7 | #Feature F1 | #Feature F2 | #Feature F3 | Start | End | PTM | AScore | Found By |
| --- | --- | --- | --- | --- | --- | --- | --- | --- | --- | --- | --- | --- | --- | --- | --- | --- | --- | --- | --- | --- | --- | --- | --- | --- | --- | --- | --- | --- | --- | --- | --- | --- | --- | --- | --- | --- | --- |
| K.AVENSSTAIGIR.C | Y | 41.53 | 1216.6411 | 12 | -0.3 | 609.3276 | 2 | 29.75 | 4 | F4:16295 | 17012022\_RID\_3262\_10a .raw |  |  |  | 2.1242E5 |  |  |  |  |  |  | 1 | 0 | 0 | 0 | 1 | 0 | 0 | 0 | 0 | 0 | 0 | 30 | 41 |  |  | PEAKS DB |
| total 1 peptides |  |  |  |  |  |  |  |  |  |  |  |  |  |  |  |  |  |  |  |  |  |  |  |  |  |  |  |  |  |  |  |  |  |  |  |  |  |
| --- | --- | --- | --- | --- | --- | --- | --- | --- | --- | --- | --- | --- | --- | --- | --- | --- | --- | --- | --- | --- | --- | --- | --- | --- | --- | --- | --- | --- | --- | --- | --- | --- | --- | --- | --- | --- | --- |

XP\_032092012.1

back to list

  

| Protein Coverage
| Supporting Peptides
|

Protein Coverage:

Supporting Peptides:

| Peptide | Uniq | -10lgP | Mass | Length | ppm | m/z | z | RT | Fraction | Scan | Source File | Area F4 | Area F5 | Area F6 | Area F10 | Area F8 | Area F9 | Area F7 | Area F1 | Area F2 | Area F3 | #Feature | #Feature F4 | #Feature F5 | #Feature F6 | #Feature F10 | #Feature F8 | #Feature F9 | #Feature F7 | #Feature F1 | #Feature F2 | #Feature F3 | Start | End | PTM | AScore | Found By |
| --- | --- | --- | --- | --- | --- | --- | --- | --- | --- | --- | --- | --- | --- | --- | --- | --- | --- | --- | --- | --- | --- | --- | --- | --- | --- | --- | --- | --- | --- | --- | --- | --- | --- | --- | --- | --- | --- |
| K.AVENSSTAIGIR.C | Y | 41.53 | 1216.6411 | 12 | -0.3 | 609.3276 | 2 | 29.75 | 4 | F4:16295 | 17012022\_RID\_3262\_10a .raw |  |  |  | 2.1242E5 |  |  |  |  |  |  | 1 | 0 | 0 | 0 | 1 | 0 | 0 | 0 | 0 | 0 | 0 | 30 | 41 |  |  | PEAKS DB |
| total 1 peptides |  |  |  |  |  |  |  |  |  |  |  |  |  |  |  |  |  |  |  |  |  |  |  |  |  |  |  |  |  |  |  |  |  |  |  |  |  |
| --- | --- | --- | --- | --- | --- | --- | --- | --- | --- | --- | --- | --- | --- | --- | --- | --- | --- | --- | --- | --- | --- | --- | --- | --- | --- | --- | --- | --- | --- | --- | --- | --- | --- | --- | --- | --- | --- |

XP\_034289368.1

back to list

  

| Protein Coverage
| Supporting Peptides
|

Protein Coverage:

Supporting Peptides:

| Peptide | Uniq | -10lgP | Mass | Length | ppm | m/z | z | RT | Fraction | Scan | Source File | Area F4 | Area F5 | Area F6 | Area F10 | Area F8 | Area F9 | Area F7 | Area F1 | Area F2 | Area F3 | #Feature | #Feature F4 | #Feature F5 | #Feature F6 | #Feature F10 | #Feature F8 | #Feature F9 | #Feature F7 | #Feature F1 | #Feature F2 | #Feature F3 | Start | End | PTM | AScore | Found By |
| --- | --- | --- | --- | --- | --- | --- | --- | --- | --- | --- | --- | --- | --- | --- | --- | --- | --- | --- | --- | --- | --- | --- | --- | --- | --- | --- | --- | --- | --- | --- | --- | --- | --- | --- | --- | --- | --- |
| K.AVENSSTAIGIR.C | Y | 41.53 | 1216.6411 | 12 | -0.3 | 609.3276 | 2 | 29.75 | 4 | F4:16295 | 17012022\_RID\_3262\_10a .raw |  |  |  | 2.1242E5 |  |  |  |  |  |  | 1 | 0 | 0 | 0 | 1 | 0 | 0 | 0 | 0 | 0 | 0 | 30 | 41 |  |  | PEAKS DB |
| total 1 peptides |  |  |  |  |  |  |  |  |  |  |  |  |  |  |  |  |  |  |  |  |  |  |  |  |  |  |  |  |  |  |  |  |  |  |  |  |  |
| --- | --- | --- | --- | --- | --- | --- | --- | --- | --- | --- | --- | --- | --- | --- | --- | --- | --- | --- | --- | --- | --- | --- | --- | --- | --- | --- | --- | --- | --- | --- | --- | --- | --- | --- | --- | --- | --- |

XP\_026554461.1

back to list

  

| Protein Coverage
| Supporting Peptides
|

Protein Coverage:

Supporting Peptides:

| Peptide | Uniq | -10lgP | Mass | Length | ppm | m/z | z | RT | Fraction | Scan | Source File | Area F4 | Area F5 | Area F6 | Area F10 | Area F8 | Area F9 | Area F7 | Area F1 | Area F2 | Area F3 | #Feature | #Feature F4 | #Feature F5 | #Feature F6 | #Feature F10 | #Feature F8 | #Feature F9 | #Feature F7 | #Feature F1 | #Feature F2 | #Feature F3 | Start | End | PTM | AScore | Found By |
| --- | --- | --- | --- | --- | --- | --- | --- | --- | --- | --- | --- | --- | --- | --- | --- | --- | --- | --- | --- | --- | --- | --- | --- | --- | --- | --- | --- | --- | --- | --- | --- | --- | --- | --- | --- | --- | --- |
| K.AVENSSTAIGIR.C | Y | 41.53 | 1216.6411 | 12 | -0.3 | 609.3276 | 2 | 29.75 | 4 | F4:16295 | 17012022\_RID\_3262\_10a .raw |  |  |  | 2.1242E5 |  |  |  |  |  |  | 1 | 0 | 0 | 0 | 1 | 0 | 0 | 0 | 0 | 0 | 0 | 30 | 41 |  |  | PEAKS DB |
| total 1 peptides |  |  |  |  |  |  |  |  |  |  |  |  |  |  |  |  |  |  |  |  |  |  |  |  |  |  |  |  |  |  |  |  |  |  |  |  |  |
| --- | --- | --- | --- | --- | --- | --- | --- | --- | --- | --- | --- | --- | --- | --- | --- | --- | --- | --- | --- | --- | --- | --- | --- | --- | --- | --- | --- | --- | --- | --- | --- | --- | --- | --- | --- | --- | --- |

JAI09619.1

back to list

  

| Protein Coverage
| Supporting Peptides
|

Protein Coverage:

Supporting Peptides:

| Peptide | Uniq | -10lgP | Mass | Length | ppm | m/z | z | RT | Fraction | Scan | Source File | Area F4 | Area F5 | Area F6 | Area F10 | Area F8 | Area F9 | Area F7 | Area F1 | Area F2 | Area F3 | #Feature | #Feature F4 | #Feature F5 | #Feature F6 | #Feature F10 | #Feature F8 | #Feature F9 | #Feature F7 | #Feature F1 | #Feature F2 | #Feature F3 | Start | End | PTM | AScore | Found By |
| --- | --- | --- | --- | --- | --- | --- | --- | --- | --- | --- | --- | --- | --- | --- | --- | --- | --- | --- | --- | --- | --- | --- | --- | --- | --- | --- | --- | --- | --- | --- | --- | --- | --- | --- | --- | --- | --- |
| K.AVENSSTAIGIR.C | Y | 41.53 | 1216.6411 | 12 | -0.3 | 609.3276 | 2 | 29.75 | 4 | F4:16295 | 17012022\_RID\_3262\_10a .raw |  |  |  | 2.1242E5 |  |  |  |  |  |  | 1 | 0 | 0 | 0 | 1 | 0 | 0 | 0 | 0 | 0 | 0 | 30 | 41 |  |  | PEAKS DB |
| total 1 peptides |  |  |  |  |  |  |  |  |  |  |  |  |  |  |  |  |  |  |  |  |  |  |  |  |  |  |  |  |  |  |  |  |  |  |  |  |  |
| --- | --- | --- | --- | --- | --- | --- | --- | --- | --- | --- | --- | --- | --- | --- | --- | --- | --- | --- | --- | --- | --- | --- | --- | --- | --- | --- | --- | --- | --- | --- | --- | --- | --- | --- | --- | --- | --- |

XP\_007422519.1

back to list

  

| Protein Coverage
| Supporting Peptides
|

Protein Coverage:

Supporting Peptides:

| Peptide | Uniq | -10lgP | Mass | Length | ppm | m/z | z | RT | Fraction | Scan | Source File | Area F4 | Area F5 | Area F6 | Area F10 | Area F8 | Area F9 | Area F7 | Area F1 | Area F2 | Area F3 | #Feature | #Feature F4 | #Feature F5 | #Feature F6 | #Feature F10 | #Feature F8 | #Feature F9 | #Feature F7 | #Feature F1 | #Feature F2 | #Feature F3 | Start | End | PTM | AScore | Found By |
| --- | --- | --- | --- | --- | --- | --- | --- | --- | --- | --- | --- | --- | --- | --- | --- | --- | --- | --- | --- | --- | --- | --- | --- | --- | --- | --- | --- | --- | --- | --- | --- | --- | --- | --- | --- | --- | --- |
| K.AVENSSTAIGIR.C | Y | 41.53 | 1216.6411 | 12 | -0.3 | 609.3276 | 2 | 29.75 | 4 | F4:16295 | 17012022\_RID\_3262\_10a .raw |  |  |  | 2.1242E5 |  |  |  |  |  |  | 1 | 0 | 0 | 0 | 1 | 0 | 0 | 0 | 0 | 0 | 0 | 30 | 41 |  |  | PEAKS DB |
| total 1 peptides |  |  |  |  |  |  |  |  |  |  |  |  |  |  |  |  |  |  |  |  |  |  |  |  |  |  |  |  |  |  |  |  |  |  |  |  |  |
| --- | --- | --- | --- | --- | --- | --- | --- | --- | --- | --- | --- | --- | --- | --- | --- | --- | --- | --- | --- | --- | --- | --- | --- | --- | --- | --- | --- | --- | --- | --- | --- | --- | --- | --- | --- | --- | --- |

XP\_026525981.1

back to list

  

| Protein Coverage
| Supporting Peptides
|

Protein Coverage:

Supporting Peptides:

| Peptide | Uniq | -10lgP | Mass | Length | ppm | m/z | z | RT | Fraction | Scan | Source File | Area F4 | Area F5 | Area F6 | Area F10 | Area F8 | Area F9 | Area F7 | Area F1 | Area F2 | Area F3 | #Feature | #Feature F4 | #Feature F5 | #Feature F6 | #Feature F10 | #Feature F8 | #Feature F9 | #Feature F7 | #Feature F1 | #Feature F2 | #Feature F3 | Start | End | PTM | AScore | Found By |
| --- | --- | --- | --- | --- | --- | --- | --- | --- | --- | --- | --- | --- | --- | --- | --- | --- | --- | --- | --- | --- | --- | --- | --- | --- | --- | --- | --- | --- | --- | --- | --- | --- | --- | --- | --- | --- | --- |
| K.AVENSSTAIGIR.C | Y | 41.53 | 1216.6411 | 12 | -0.3 | 609.3276 | 2 | 29.75 | 4 | F4:16295 | 17012022\_RID\_3262\_10a .raw |  |  |  | 2.1242E5 |  |  |  |  |  |  | 1 | 0 | 0 | 0 | 1 | 0 | 0 | 0 | 0 | 0 | 0 | 30 | 41 |  |  | PEAKS DB |
| total 1 peptides |  |  |  |  |  |  |  |  |  |  |  |  |  |  |  |  |  |  |  |  |  |  |  |  |  |  |  |  |  |  |  |  |  |  |  |  |  |
| --- | --- | --- | --- | --- | --- | --- | --- | --- | --- | --- | --- | --- | --- | --- | --- | --- | --- | --- | --- | --- | --- | --- | --- | --- | --- | --- | --- | --- | --- | --- | --- | --- | --- | --- | --- | --- | --- |

JAG66904.1

back to list

  

| Protein Coverage
| Supporting Peptides
|

Protein Coverage:

Supporting Peptides:

| Peptide | Uniq | -10lgP | Mass | Length | ppm | m/z | z | RT | Fraction | Scan | Source File | Area F4 | Area F5 | Area F6 | Area F10 | Area F8 | Area F9 | Area F7 | Area F1 | Area F2 | Area F3 | #Feature | #Feature F4 | #Feature F5 | #Feature F6 | #Feature F10 | #Feature F8 | #Feature F9 | #Feature F7 | #Feature F1 | #Feature F2 | #Feature F3 | Start | End | PTM | AScore | Found By |
| --- | --- | --- | --- | --- | --- | --- | --- | --- | --- | --- | --- | --- | --- | --- | --- | --- | --- | --- | --- | --- | --- | --- | --- | --- | --- | --- | --- | --- | --- | --- | --- | --- | --- | --- | --- | --- | --- |
| K.AVENSSTAIGIR.C | Y | 41.53 | 1216.6411 | 12 | -0.3 | 609.3276 | 2 | 29.75 | 4 | F4:16295 | 17012022\_RID\_3262\_10a .raw |  |  |  | 2.1242E5 |  |  |  |  |  |  | 1 | 0 | 0 | 0 | 1 | 0 | 0 | 0 | 0 | 0 | 0 | 30 | 41 |  |  | PEAKS DB |
| total 1 peptides |  |  |  |  |  |  |  |  |  |  |  |  |  |  |  |  |  |  |  |  |  |  |  |  |  |  |  |  |  |  |  |  |  |  |  |  |  |
| --- | --- | --- | --- | --- | --- | --- | --- | --- | --- | --- | --- | --- | --- | --- | --- | --- | --- | --- | --- | --- | --- | --- | --- | --- | --- | --- | --- | --- | --- | --- | --- | --- | --- | --- | --- | --- | --- |

JAB53606.1

back to list

  

| Protein Coverage
| Supporting Peptides
|

Protein Coverage:

Supporting Peptides:

| Peptide | Uniq | -10lgP | Mass | Length | ppm | m/z | z | RT | Fraction | Scan | Source File | Area F4 | Area F5 | Area F6 | Area F10 | Area F8 | Area F9 | Area F7 | Area F1 | Area F2 | Area F3 | #Feature | #Feature F4 | #Feature F5 | #Feature F6 | #Feature F10 | #Feature F8 | #Feature F9 | #Feature F7 | #Feature F1 | #Feature F2 | #Feature F3 | Start | End | PTM | AScore | Found By |
| --- | --- | --- | --- | --- | --- | --- | --- | --- | --- | --- | --- | --- | --- | --- | --- | --- | --- | --- | --- | --- | --- | --- | --- | --- | --- | --- | --- | --- | --- | --- | --- | --- | --- | --- | --- | --- | --- |
| K.AVENSSTAIGIR.C | Y | 41.53 | 1216.6411 | 12 | -0.3 | 609.3276 | 2 | 29.75 | 4 | F4:16295 | 17012022\_RID\_3262\_10a .raw |  |  |  | 2.1242E5 |  |  |  |  |  |  | 1 | 0 | 0 | 0 | 1 | 0 | 0 | 0 | 0 | 0 | 0 | 30 | 41 |  |  | PEAKS DB |
| total 1 peptides |  |  |  |  |  |  |  |  |  |  |  |  |  |  |  |  |  |  |  |  |  |  |  |  |  |  |  |  |  |  |  |  |  |  |  |  |  |
| --- | --- | --- | --- | --- | --- | --- | --- | --- | --- | --- | --- | --- | --- | --- | --- | --- | --- | --- | --- | --- | --- | --- | --- | --- | --- | --- | --- | --- | --- | --- | --- | --- | --- | --- | --- | --- | --- |

JAA96035.1

back to list

  

| Protein Coverage
| Supporting Peptides
|

Protein Coverage:

Supporting Peptides:

| Peptide | Uniq | -10lgP | Mass | Length | ppm | m/z | z | RT | Fraction | Scan | Source File | Area F4 | Area F5 | Area F6 | Area F10 | Area F8 | Area F9 | Area F7 | Area F1 | Area F2 | Area F3 | #Feature | #Feature F4 | #Feature F5 | #Feature F6 | #Feature F10 | #Feature F8 | #Feature F9 | #Feature F7 | #Feature F1 | #Feature F2 | #Feature F3 | Start | End | PTM | AScore | Found By |
| --- | --- | --- | --- | --- | --- | --- | --- | --- | --- | --- | --- | --- | --- | --- | --- | --- | --- | --- | --- | --- | --- | --- | --- | --- | --- | --- | --- | --- | --- | --- | --- | --- | --- | --- | --- | --- | --- |
| K.AVENSSTAIGIR.C | Y | 41.53 | 1216.6411 | 12 | -0.3 | 609.3276 | 2 | 29.75 | 4 | F4:16295 | 17012022\_RID\_3262\_10a .raw |  |  |  | 2.1242E5 |  |  |  |  |  |  | 1 | 0 | 0 | 0 | 1 | 0 | 0 | 0 | 0 | 0 | 0 | 30 | 41 |  |  | PEAKS DB |
| total 1 peptides |  |  |  |  |  |  |  |  |  |  |  |  |  |  |  |  |  |  |  |  |  |  |  |  |  |  |  |  |  |  |  |  |  |  |  |  |  |
| --- | --- | --- | --- | --- | --- | --- | --- | --- | --- | --- | --- | --- | --- | --- | --- | --- | --- | --- | --- | --- | --- | --- | --- | --- | --- | --- | --- | --- | --- | --- | --- | --- | --- | --- | --- | --- | --- |

XP\_015676739.1

back to list

  

| Protein Coverage
| Supporting Peptides
|

Protein Coverage:

Supporting Peptides:

| Peptide | Uniq | -10lgP | Mass | Length | ppm | m/z | z | RT | Fraction | Scan | Source File | Area F4 | Area F5 | Area F6 | Area F10 | Area F8 | Area F9 | Area F7 | Area F1 | Area F2 | Area F3 | #Feature | #Feature F4 | #Feature F5 | #Feature F6 | #Feature F10 | #Feature F8 | #Feature F9 | #Feature F7 | #Feature F1 | #Feature F2 | #Feature F3 | Start | End | PTM | AScore | Found By |
| --- | --- | --- | --- | --- | --- | --- | --- | --- | --- | --- | --- | --- | --- | --- | --- | --- | --- | --- | --- | --- | --- | --- | --- | --- | --- | --- | --- | --- | --- | --- | --- | --- | --- | --- | --- | --- | --- |
| K.AVENSSTAIGIR.C | Y | 41.53 | 1216.6411 | 12 | -0.3 | 609.3276 | 2 | 29.75 | 4 | F4:16295 | 17012022\_RID\_3262\_10a .raw |  |  |  | 2.1242E5 |  |  |  |  |  |  | 1 | 0 | 0 | 0 | 1 | 0 | 0 | 0 | 0 | 0 | 0 | 30 | 41 |  |  | PEAKS DB |
| total 1 peptides |  |  |  |  |  |  |  |  |  |  |  |  |  |  |  |  |  |  |  |  |  |  |  |  |  |  |  |  |  |  |  |  |  |  |  |  |  |
| --- | --- | --- | --- | --- | --- | --- | --- | --- | --- | --- | --- | --- | --- | --- | --- | --- | --- | --- | --- | --- | --- | --- | --- | --- | --- | --- | --- | --- | --- | --- | --- | --- | --- | --- | --- | --- | --- |

JAV49555.1

back to list

  

| Protein Coverage
| Supporting Peptides
|

Protein Coverage:

Supporting Peptides:

| Peptide | Uniq | -10lgP | Mass | Length | ppm | m/z | z | RT | Fraction | Scan | Source File | Area F4 | Area F5 | Area F6 | Area F10 | Area F8 | Area F9 | Area F7 | Area F1 | Area F2 | Area F3 | #Feature | #Feature F4 | #Feature F5 | #Feature F6 | #Feature F10 | #Feature F8 | #Feature F9 | #Feature F7 | #Feature F1 | #Feature F2 | #Feature F3 | Start | End | PTM | AScore | Found By |
| --- | --- | --- | --- | --- | --- | --- | --- | --- | --- | --- | --- | --- | --- | --- | --- | --- | --- | --- | --- | --- | --- | --- | --- | --- | --- | --- | --- | --- | --- | --- | --- | --- | --- | --- | --- | --- | --- |
| K.AVENSSTAIGIR.C | Y | 41.53 | 1216.6411 | 12 | -0.3 | 609.3276 | 2 | 29.75 | 4 | F4:16295 | 17012022\_RID\_3262\_10a .raw |  |  |  | 2.1242E5 |  |  |  |  |  |  | 1 | 0 | 0 | 0 | 1 | 0 | 0 | 0 | 0 | 0 | 0 | 30 | 41 |  |  | PEAKS DB |
| total 1 peptides |  |  |  |  |  |  |  |  |  |  |  |  |  |  |  |  |  |  |  |  |  |  |  |  |  |  |  |  |  |  |  |  |  |  |  |  |  |
| --- | --- | --- | --- | --- | --- | --- | --- | --- | --- | --- | --- | --- | --- | --- | --- | --- | --- | --- | --- | --- | --- | --- | --- | --- | --- | --- | --- | --- | --- | --- | --- | --- | --- | --- | --- | --- | --- |

JAI11930.1

back to list

  

| Protein Coverage
| Supporting Peptides
|

Protein Coverage:

Supporting Peptides:

| Peptide | Uniq | -10lgP | Mass | Length | ppm | m/z | z | RT | Fraction | Scan | Source File | Area F4 | Area F5 | Area F6 | Area F10 | Area F8 | Area F9 | Area F7 | Area F1 | Area F2 | Area F3 | #Feature | #Feature F4 | #Feature F5 | #Feature F6 | #Feature F10 | #Feature F8 | #Feature F9 | #Feature F7 | #Feature F1 | #Feature F2 | #Feature F3 | Start | End | PTM | AScore | Found By |
| --- | --- | --- | --- | --- | --- | --- | --- | --- | --- | --- | --- | --- | --- | --- | --- | --- | --- | --- | --- | --- | --- | --- | --- | --- | --- | --- | --- | --- | --- | --- | --- | --- | --- | --- | --- | --- | --- |
| K.AVENSSTAIGIR.C | Y | 41.53 | 1216.6411 | 12 | -0.3 | 609.3276 | 2 | 29.75 | 4 | F4:16295 | 17012022\_RID\_3262\_10a .raw |  |  |  | 2.1242E5 |  |  |  |  |  |  | 1 | 0 | 0 | 0 | 1 | 0 | 0 | 0 | 0 | 0 | 0 | 30 | 41 |  |  | PEAKS DB |
| total 1 peptides |  |  |  |  |  |  |  |  |  |  |  |  |  |  |  |  |  |  |  |  |  |  |  |  |  |  |  |  |  |  |  |  |  |  |  |  |  |
| --- | --- | --- | --- | --- | --- | --- | --- | --- | --- | --- | --- | --- | --- | --- | --- | --- | --- | --- | --- | --- | --- | --- | --- | --- | --- | --- | --- | --- | --- | --- | --- | --- | --- | --- | --- | --- | --- |

AFJ51036.1

back to list

  

| Protein Coverage
| Supporting Peptides
|

Protein Coverage:

Supporting Peptides:

| Peptide | Uniq | -10lgP | Mass | Length | ppm | m/z | z | RT | Fraction | Scan | Source File | Area F4 | Area F5 | Area F6 | Area F10 | Area F8 | Area F9 | Area F7 | Area F1 | Area F2 | Area F3 | #Feature | #Feature F4 | #Feature F5 | #Feature F6 | #Feature F10 | #Feature F8 | #Feature F9 | #Feature F7 | #Feature F1 | #Feature F2 | #Feature F3 | Start | End | PTM | AScore | Found By |
| --- | --- | --- | --- | --- | --- | --- | --- | --- | --- | --- | --- | --- | --- | --- | --- | --- | --- | --- | --- | --- | --- | --- | --- | --- | --- | --- | --- | --- | --- | --- | --- | --- | --- | --- | --- | --- | --- |
| K.AVENSSTAIGIR.C | Y | 41.53 | 1216.6411 | 12 | -0.3 | 609.3276 | 2 | 29.75 | 4 | F4:16295 | 17012022\_RID\_3262\_10a .raw |  |  |  | 2.1242E5 |  |  |  |  |  |  | 1 | 0 | 0 | 0 | 1 | 0 | 0 | 0 | 0 | 0 | 0 | 30 | 41 |  |  | PEAKS DB |
| total 1 peptides |  |  |  |  |  |  |  |  |  |  |  |  |  |  |  |  |  |  |  |  |  |  |  |  |  |  |  |  |  |  |  |  |  |  |  |  |  |
| --- | --- | --- | --- | --- | --- | --- | --- | --- | --- | --- | --- | --- | --- | --- | --- | --- | --- | --- | --- | --- | --- | --- | --- | --- | --- | --- | --- | --- | --- | --- | --- | --- | --- | --- | --- | --- | --- |

Peptide List

  
  

---

Prepared with PEAKS ™ (bioinfor.com)
