# Supplementary material for: Fangs in the Ghats: Preclinical Insights into the Medical Importance of Pit Vipers from the Western Ghats
Source: Int J Mol Sci. 2023 May 30;24(11):9516. doi: 10.3390/ijms24119516 (PMC10253985; doi:10.3390/ijms24119516)
Supplement: Supplementary file 1 [file ijms-24-09516-s001.zip › Supplementary File S1.zip!/Supplementary File S1/HYHY/HYHY.html]

proteins


Summary

  

# 1. Notes

# 2. Result Statistics

**Figure 1.**
False discovery rate (FDR) curve. X axis is the number of peptide-spectrum matches (PSM) being kept. Y axis is the corresponding FDR.

  


  

**Figure 2.**
PSM score distribution.  **(a)**
Distribution of PEAKS peptide score;  **(b)**
Scatterplot of PEAKS peptide score versus precursor mass error.

**(a)**

**(b)**

**Table 1.**
Statistics of data.


|  |  |  |  |  |  |  |  |  |  |  |  |
| --- | --- | --- | --- | --- | --- | --- | --- | --- | --- | --- | --- |
|  | #Scans | | #Features | Identified | | | #Peptides | #Sequences | #Proteins^\*^ | | |
|  | MS1 | MS2 |  | #PSMs | #Scans | #Features |  |  | Groups | All | Top |
| Total | 104768 | 142072 | 196064 | 2708 | 2648 | 15837 | 536 | 481 | 91 | 833 | 444 |
| F2 | 15803 | 36786 | 48410 | 976 | 959 | 4334 | 363 | 328 | 68 | 651 | 327 |
| F1 | 17629 | 13931 | 29045 | 453 | 447 | 1183 | 171 | 157 | 30 | 225 | 109 |
| F3 | 19583 | 13929 | 8440 | 320 | 306 | 1211 | 159 | 149 | 27 | 145 | 96 |
| F4 | 19346 | 10028 | 8767 | 200 | 191 | 872 | 116 | 109 | 21 | 119 | 32 |
| F5 | 16211 | 34703 | 52483 | 398 | 392 | 4170 | 225 | 206 | 55 | 449 | 212 |
| F6 | 16196 | 32695 | 48919 | 361 | 353 | 4067 | 196 | 182 | 56 | 531 | 244 |

^\* proteins with significant peptides are used in counts.^

**Figure 3.**
Sample overlap for Proteins and Peptides (up to 8 samples).  **(a)**
All Proteins;  **(b)**
Top Proteins;  **(c)**
Peptides;

**(a)**

|  |  |  |  |  |  |  |  |  |  |  |  |  |  |  |  |  |  |  |  |  |  |  |  |  |  |  |  |  |  |  |  |  |  |  |  |  |  |  |  |  |  |  |  |  |  |  |  |  |  |  |  |  |  |  |  |  |
| --- | --- | --- | --- | --- | --- | --- | --- | --- | --- | --- | --- | --- | --- | --- | --- | --- | --- | --- | --- | --- | --- | --- | --- | --- | --- | --- | --- | --- | --- | --- | --- | --- | --- | --- | --- | --- | --- | --- | --- | --- | --- | --- | --- | --- | --- | --- | --- | --- | --- | --- | --- | --- | --- | --- | --- | --- |
|  | 1 | 2 | 3 | 4 | 5 | 6 | Unique | \|  \|  \|  \|  \|  \|  \|  \|  \| \| --- \| --- \| --- \| --- \| --- \| --- \| --- \| --- \| \| 1 \| 651 \| 225 \| 133 \| 119 \| 345 \| 377 \| 174 \| \| 2 \| 225 \| 225 \| 105 \| 99 \| 189 \| 167 \| 0 \| \| 3 \| 133 \| 105 \| 145 \| 56 \| 131 \| 107 \| 0 \| \| 4 \| 119 \| 99 \| 56 \| 119 \| 118 \| 117 \| 0 \| \| 5 \| 345 \| 189 \| 131 \| 118 \| 449 \| 363 \| 19 \| \| 6 \| 377 \| 167 \| 107 \| 117 \| 363 \| 531 \| 75 \| |

**(b)**

|  |  |  |  |  |  |  |  |  |  |  |  |  |  |  |  |  |  |  |  |  |  |  |  |  |  |  |  |  |  |  |  |  |  |  |  |  |  |  |  |  |  |  |  |  |  |  |  |  |  |  |  |  |  |  |  |  |
| --- | --- | --- | --- | --- | --- | --- | --- | --- | --- | --- | --- | --- | --- | --- | --- | --- | --- | --- | --- | --- | --- | --- | --- | --- | --- | --- | --- | --- | --- | --- | --- | --- | --- | --- | --- | --- | --- | --- | --- | --- | --- | --- | --- | --- | --- | --- | --- | --- | --- | --- | --- | --- | --- | --- | --- | --- |
|  | 1 | 2 | 3 | 4 | 5 | 6 | Unique | \|  \|  \|  \|  \|  \|  \|  \|  \| \| --- \| --- \| --- \| --- \| --- \| --- \| --- \| --- \| \| 1 \| 327 \| 109 \| 84 \| 32 \| 152 \| 150 \| 115 \| \| 2 \| 109 \| 109 \| 69 \| 30 \| 82 \| 72 \| 0 \| \| 3 \| 84 \| 69 \| 96 \| 27 \| 87 \| 69 \| 0 \| \| 4 \| 32 \| 30 \| 27 \| 32 \| 31 \| 30 \| 0 \| \| 5 \| 152 \| 82 \| 87 \| 31 \| 212 \| 158 \| 14 \| \| 6 \| 150 \| 72 \| 69 \| 30 \| 158 \| 244 \| 54 \| |

**(c)**

|  |  |  |  |  |  |  |  |  |  |  |  |  |  |  |  |  |  |  |  |  |  |  |  |  |  |  |  |  |  |  |  |  |  |  |  |  |  |  |  |  |  |  |  |  |  |  |  |  |  |  |  |  |  |  |  |  |
| --- | --- | --- | --- | --- | --- | --- | --- | --- | --- | --- | --- | --- | --- | --- | --- | --- | --- | --- | --- | --- | --- | --- | --- | --- | --- | --- | --- | --- | --- | --- | --- | --- | --- | --- | --- | --- | --- | --- | --- | --- | --- | --- | --- | --- | --- | --- | --- | --- | --- | --- | --- | --- | --- | --- | --- | --- |
|  | 1 | 2 | 3 | 4 | 5 | 6 | Unique | \|  \|  \|  \|  \|  \|  \|  \|  \| \| --- \| --- \| --- \| --- \| --- \| --- \| --- \| --- \| \| 1 \| 363 \| 141 \| 124 \| 95 \| 148 \| 131 \| 136 \| \| 2 \| 141 \| 171 \| 86 \| 68 \| 97 \| 89 \| 27 \| \| 3 \| 124 \| 86 \| 159 \| 94 \| 99 \| 87 \| 19 \| \| 4 \| 95 \| 68 \| 94 \| 116 \| 89 \| 83 \| 6 \| \| 5 \| 148 \| 97 \| 99 \| 89 \| 225 \| 121 \| 43 \| \| 6 \| 131 \| 89 \| 87 \| 83 \| 121 \| 196 \| 35 \| |

**Figure 4.**
Distribution of peptide feature detection.  **(a)**
Feature m/z distribution;  **(b)**
Feature RT distribution.

**(a)**

**(b)**

**Figure 5.**
Distribution of identified peptide features.  **(a)**
Feature abundance distribution;  **(b)**
 *De novo*
sequencing validation.

**(a)**

**(b)**

**Table 2.**
Result filtration parameters.

|  |  |
| --- | --- |
| Peptide -10lgP | ≥42.3 |
| PTM Ascore | ≥0 |
| Protein -10lgP | ≥20 |
| Proteins unique peptides | ≥1 |
| De novo score(%) | ≥50% |


**Table 3.**
Statistics of filtered result.

|  |  |
| --- | --- |
| FDR (Peptide-Spectrum Matches) | 0.1% |
| FDR (Peptide Sequences) | 0.4% |
| FDR (Protein Group) | 2.7% |
| De Novo Only Spectra | 25621 |

**Table 4.**
PTM profile.

| Name | ∆Mass | Position | #PSM | -10lgP | Abundance | AScore || Oxidation | 15.99 | M | 403 | 80.93 | 1.93E6 | 1000.00 |
| Carbamidomethyl | 57.02 | C | 26 | 61.83 | 1.71E5 | 1000.00 |

# 3. Experiment Control

**Figure 6.**
Precursor mass error of peptide-spectrum matches (PSM) in filtered result.  **(a)**
Distribution of precursor mass error in ppm;  **(b)**
Scatterplot of precursor m/z versus precursor mass error in ppm.

**(a)**

**(b)**

**Table 5.**
Number of identified peptides in each sample by the number of missed cleavages.

|  |  |  |  |  |  |
| --- | --- | --- | --- | --- | --- |
| Missed Cleavages | 0 | 1 | 2 | 3 | 4+ |
| F2 | 285 | 72 | 6 | 0 | 0 |
| F1 | 148 | 20 | 3 | 0 | 0 |
| F3 | 114 | 38 | 7 | 0 | 0 |
| F4 | 85 | 27 | 4 | 0 | 0 |
| F5 | 168 | 51 | 6 | 0 | 0 |
| F6 | 157 | 35 | 4 | 0 | 0 |

# 4. Other Information

**Table 6.**
Search parameters.

Search Engine Name: PEAKS  
Parent Mass Error Tolerance: 10.0 ppm  
Fragment Mass Error Tolerance: 0.6 Da  
Precursor Mass Search Type: monoisotopic  
Enzyme: Trypsin  
Max Missed Cleavages: 3  
Digest Mode: Semispecific  
Fixed Modifications:   
  Carbamidomethylation: 57.02  
Variable Modifications:   
  Oxidation (M): 15.99  
Max Variable PTM Per Peptide: 3  
Database: NCBI\_Serpentes\_NR  
Taxon: All  
Contaminant Database: cRAP\_contaminants  
Searched Entry: 410049  
FDR Estimation: Enabled  
Merge Options: no merge  
Precursor Options: corrected  
Charge Options: no correction  
Filter Charge: 2 - 8  
Process: true  
Associate chimera: yes

**Table 7.**
Instrument parameters.

Fractions: 28122021\_RID\_2349\_2C.raw, 30122021\_RID\_2349\_1C.raw,   
31122021\_RID\_2349\_3C.raw, 31122021\_RID\_2349\_4C.raw, 31122021\_  
RID\_2349\_5C.raw, 31122021\_RID\_2349\_6C.raw  
Ion Source: ESI(nano-spray)  
Fragmentation Mode: CID, CAD(y and b ions)  
MS Scan Mode: FT-ICR/Orbitrap  
MS/MS Scan Mode: FT-ICR/Orbitrap

  

Protein List

  

Protein Accession Contains:

Protein Description Contains:

Protein Sample Area >=

Protein PTM Contains:

| Protein Group | Protein ID | Accession | -10lgP | Coverage (%) | Coverage (%) F2 | Coverage (%) F1 | Coverage (%) F3 | Coverage (%) F4 | Coverage (%) F5 | Coverage (%) F6 | Area F2 | Area F1 | Area F3 | Area F4 | Area F5 | Area F6 | #Peptides | #Unique | #Spec F2 | #Spec F1 | #Spec F3 | #Spec F4 | #Spec F5 | #Spec F6 | PTM | Avg. Mass | Description |
| --- | --- | --- | --- | --- | --- | --- | --- | --- | --- | --- | --- | --- | --- | --- | --- | --- | --- | --- | --- | --- | --- | --- | --- | --- | --- | --- | --- |
| 6 | 28 | Q90W54.1 | 311.92 | 30 | 19 | 21 | 17 | 12 | 16 | 12 |  |  | 9.9657E6 | 1.1078E6 | 2.5437E6 | 3.5235E5 | 28 | 1 | 72 | 74 | 13 | 4 | 22 | 12 | N | 57092 | RecName: Full=L-amino-acid oxidase; Short=LAAO; Short=M-LAO; Flags: Precursor |
| 6 | 29 | BAB69450.1 | 311.92 | 30 | 19 | 21 | 17 | 12 | 16 | 12 |  |  | 9.9657E6 | 1.1078E6 | 2.5437E6 | 3.5235E5 | 28 | 1 | 72 | 74 | 13 | 4 | 22 | 12 | N | 57092 | M-LAO [Gloydius blomhoffii] |
| 6 | 30 | AID53087.1 | 311.92 | 30 | 19 | 21 | 17 | 12 | 16 | 12 |  |  | 9.9657E6 | 1.1078E6 | 2.5437E6 | 3.5235E5 | 28 | 1 | 72 | 74 | 13 | 4 | 22 | 12 | N | 57096 | L-amino acid oxidase [Gloydius intermedius] |
| 23 | 6 | AFJ50957.1 | 300.91 | 40 | 29 | 0 | 0 | 0 | 13 | 11 | 2.1874E6 |  |  |  |  |  | 23 | 1 | 80 | 0 | 0 | 0 | 11 | 7 | Y | 64091 | Phospholipase B [Crotalus adamanteus] |
| 23 | 7 | AEJ31983.1 | 300.91 | 40 | 29 | 0 | 0 | 0 | 13 | 11 | 2.1874E6 |  |  |  |  |  | 23 | 1 | 80 | 0 | 0 | 0 | 11 | 7 | Y | 64049 | phospholipase B [Crotalus adamanteus] |
| 23 | 8 | JAI12031.1 | 300.91 | 40 | 29 | 0 | 0 | 0 | 13 | 11 | 2.1874E6 |  |  |  |  |  | 23 | 1 | 80 | 0 | 0 | 0 | 11 | 7 | Y | 64091 | Phospholipase B [Crotalus adamanteus] |
| 23 | 9 | SMD28240.1 | 300.91 | 40 | 29 | 0 | 0 | 0 | 13 | 11 | 2.1874E6 |  |  |  |  |  | 23 | 1 | 80 | 0 | 0 | 0 | 11 | 7 | Y | 64104 | phospholipase B CdtPLB1 [Crotalus durissus terrificus] |
| 23 | 10 | F8S101.1 | 300.91 | 40 | 29 | 0 | 0 | 0 | 13 | 11 | 2.1874E6 |  |  |  |  |  | 23 | 1 | 80 | 0 | 0 | 0 | 11 | 7 | Y | 64049 | RecName: Full=Phospholipase B; Short=PLB; Flags: Precursor |
| 23 | 11 | JAV49636.1 | 300.91 | 40 | 29 | 0 | 0 | 0 | 13 | 11 | 2.1874E6 |  |  |  |  |  | 23 | 1 | 80 | 0 | 0 | 0 | 11 | 7 | Y | 64129 | Phospholipase B [Agkistrodon contortrix contortrix] |
| 23 | 12 | JAV48419.1 | 300.91 | 40 | 29 | 0 | 0 | 0 | 13 | 11 | 2.1874E6 |  |  |  |  |  | 23 | 1 | 80 | 0 | 0 | 0 | 11 | 7 | Y | 64110 | Cadam10\_PLB-1 [Crotalus adamanteus] |
| 5 | 22 | 2IID | 282.58 | 32 | 23 | 20 | 16 | 2 | 9 | 9 | 4.62E6 | 1.7029E7 |  |  | 1.8334E6 | 1.8954E6 | 24 | 2 | 68 | 126 | 9 | 1 | 19 | 6 | Y | 56228 | Chain D, L-amino-acid Oxidase |
| 5 | 23 | 1F8S | 282.58 | 32 | 23 | 20 | 16 | 2 | 9 | 9 | 4.62E6 | 1.7029E7 |  |  | 1.8334E6 | 1.8954E6 | 24 | 2 | 68 | 126 | 9 | 1 | 19 | 6 | Y | 56228 | Chain H, L-amino Acid Oxidase |
| 5 | 24 | 1F8R | 282.58 | 32 | 23 | 20 | 16 | 2 | 9 | 9 | 4.62E6 | 1.7029E7 |  |  | 1.8334E6 | 1.8954E6 | 24 | 2 | 68 | 126 | 9 | 1 | 19 | 6 | Y | 56228 | Chain D, L-amino Acid Oxidase |
| 5 | 25 | P81382.2 | 282.58 | 30 | 22 | 19 | 15 | 2 | 9 | 9 | 4.62E6 | 1.7029E7 |  |  | 1.8334E6 | 1.8954E6 | 24 | 2 | 68 | 126 | 9 | 1 | 19 | 6 | Y | 58221 | RecName: Full=L-amino-acid oxidase; Short=LAO; Short=MPV-LAAO; Flags: Precursor |
| 5 | 26 | CAB71136.1 | 282.58 | 30 | 22 | 19 | 15 | 2 | 9 | 9 | 4.62E6 | 1.7029E7 |  |  | 1.8334E6 | 1.8954E6 | 24 | 2 | 68 | 126 | 9 | 1 | 19 | 6 | Y | 58221 | L-amino acid oxidase [Calloselasma rhodostoma] |
| 40 | 218 | XP\_007427768.1 | 245.44 | 15 | 13 | 0 | 3 | 0 | 7 | 5 | 4.7342E7 |  | 4.5566E6 |  | 3.3485E5 | 1.4268E5 | 12 | 3 | 38 | 0 | 1 | 0 | 5 | 2 | Y | 64842 | phospholipase B-like 1 [Python bivittatus] |
| 44 | 503 | AMB36337.1 | 235.19 | 21 | 14 | 4 | 0 | 15 | 21 | 21 | 6.2934E5 |  |  | 7.3698E7 | 1.412E8 | 1.0786E7 | 11 | 7 | 3 | 1 | 0 | 6 | 30 | 6 | Y | 26517 | cysteine-rich secretory protein CRISP-1 [Vipera ammodytes ammodytes] |
| 55 | 98 | Q90YA8.1 | 221.66 | 32 | 32 | 0 | 0 | 0 | 8 | 10 | 1.0019E7 |  |  |  | 0 |  | 8 | 2 | 24 | 0 | 0 | 0 | 2 | 3 | Y | 42267 | RecName: Full=Glutaminyl-peptide cyclotransferase; AltName: Full=Glutaminyl cyclase; Short=QC; AltName: Full=Glutaminyl-tRNA cyclotransferase; Flags: Precursor |
| 55 | 99 | BAB69586.1 | 221.66 | 32 | 32 | 0 | 0 | 0 | 8 | 10 | 1.0019E7 |  |  |  | 0 |  | 8 | 2 | 24 | 0 | 0 | 0 | 2 | 3 | Y | 42267 | glutaminyl cyclase [Gloydius blomhoffii] |
| 55 | 100 | BAN89376.1 | 221.66 | 39 | 39 | 0 | 0 | 0 | 10 | 12 | 1.0019E7 |  |  |  | 0 |  | 8 | 2 | 24 | 0 | 0 | 0 | 2 | 3 | Y | 34563 | glutaminyl cyclase [Protobothrops flavoviridis] |
| 55 | 101 | BAN82005.1 | 221.66 | 36 | 36 | 0 | 0 | 0 | 9 | 11 | 1.0019E7 |  |  |  | 0 |  | 8 | 2 | 24 | 0 | 0 | 0 | 2 | 3 | Y | 38114 | glutaminyl cyclase, partial [Protobothrops flavoviridis] |
| 55 | 102 | JAG46763.1 | 221.66 | 32 | 32 | 0 | 0 | 0 | 8 | 10 | 1.0019E7 |  |  |  | 0 |  | 8 | 2 | 24 | 0 | 0 | 0 | 2 | 3 | Y | 42370 | glutaminyl-peptide cyclotransferases [Crotalus horridus] |
| 55 | 103 | BAP39934.1 | 221.66 | 32 | 32 | 0 | 0 | 0 | 8 | 10 | 1.0019E7 |  |  |  | 0 |  | 8 | 2 | 24 | 0 | 0 | 0 | 2 | 3 | Y | 42305 | glutaminyl-peptide cyclotransferase [Protobothrops elegans] |
| 55 | 104 | AFE84763.1 | 221.66 | 32 | 32 | 0 | 0 | 0 | 8 | 10 | 1.0019E7 |  |  |  | 0 |  | 8 | 2 | 24 | 0 | 0 | 0 | 2 | 3 | Y | 42295 | glutaminyl-peptide cyclotransferases [Trimeresurus gracilis] |
| 55 | 105 | BAP39982.1 | 221.66 | 32 | 32 | 0 | 0 | 0 | 8 | 10 | 1.0019E7 |  |  |  | 0 |  | 8 | 2 | 24 | 0 | 0 | 0 | 2 | 3 | Y | 42219 | glutaminyl-peptide cyclotransferase [Protobothrops flavoviridis] |
| 55 | 106 | AFE84765.1 | 221.66 | 32 | 32 | 0 | 0 | 0 | 8 | 10 | 1.0019E7 |  |  |  | 0 |  | 8 | 2 | 24 | 0 | 0 | 0 | 2 | 3 | Y | 42297 | glutaminyl-peptide cyclotransferases [Sistrurus tergeminus] |
| 55 | 107 | AFJ49187.1 | 221.66 | 32 | 32 | 0 | 0 | 0 | 8 | 10 | 1.0019E7 |  |  |  | 0 |  | 8 | 2 | 24 | 0 | 0 | 0 | 2 | 3 | Y | 42354 | Glutaminyl-peptide cyclotransferase [Crotalus adamanteus] |
| 55 | 108 | JAA94895.1 | 221.66 | 32 | 32 | 0 | 0 | 0 | 8 | 10 | 1.0019E7 |  |  |  | 0 |  | 8 | 2 | 24 | 0 | 0 | 0 | 2 | 3 | Y | 42369 | Glutaminyl-peptide cyclotransferase [Crotalus horridus] |
| 55 | 109 | JAI13216.1 | 221.66 | 32 | 32 | 0 | 0 | 0 | 8 | 10 | 1.0019E7 |  |  |  | 0 |  | 8 | 2 | 24 | 0 | 0 | 0 | 2 | 3 | Y | 42354 | glutaminyl-peptide cyclotransferases [Crotalus adamanteus] |
| 55 | 110 | BAN82004.1 | 221.66 | 32 | 32 | 0 | 0 | 0 | 8 | 10 | 1.0019E7 |  |  |  | 0 |  | 8 | 2 | 24 | 0 | 0 | 0 | 2 | 3 | Y | 42219 | glutaminyl cyclase [Protobothrops flavoviridis] |
| 55 | 111 | JAG44660.1 | 221.66 | 32 | 32 | 0 | 0 | 0 | 8 | 10 | 1.0019E7 |  |  |  | 0 |  | 8 | 2 | 24 | 0 | 0 | 0 | 2 | 3 | Y | 42369 | Glutaminyl-peptide cyclotransferase [Crotalus horridus] |
| 55 | 112 | BAN89375.1 | 221.66 | 32 | 32 | 0 | 0 | 0 | 8 | 10 | 1.0019E7 |  |  |  | 0 |  | 8 | 2 | 24 | 0 | 0 | 0 | 2 | 3 | Y | 42219 | glutaminyl cyclase [Protobothrops flavoviridis] |
| 36 | 177 | ETE67131.1 | 217.68 | 4 | 3 | 2 | 4 | 2 | 3 | 3 |  |  | 0 | 2.8331E5 |  |  | 14 | 1 | 17 | 8 | 15 | 6 | 14 | 7 | N | 240642 | Keratin, type II cytoskeletal 1, partial [Ophiophagus hannah] |
| 65 | 175 | XP\_026551250.1 | 204.64 | 26 | 26 | 0 | 0 | 0 | 7 | 10 | 3.417E6 |  |  |  | 1.318E5 |  | 7 | 1 | 19 | 0 | 0 | 0 | 2 | 3 | N | 42075 | glutaminyl-peptide cyclotransferase [Pseudonaja textilis] |
| 69 | 113 | ETE57948.1 | 199.83 | 32 | 30 | 3 | 4 | 0 | 7 | 4 | 2.9886E6 | 1.8624E4 |  |  |  |  | 10 | 3 | 12 | 1 | 1 | 0 | 2 | 2 | Y | 40263 | hypothetical protein L345\_16333, partial [Ophiophagus hannah] |
| 69 | 114 | LAA58652.1 | 199.83 | 31 | 29 | 3 | 4 | 0 | 6 | 4 | 2.9886E6 | 1.8624E4 |  |  |  |  | 10 | 3 | 12 | 1 | 1 | 0 | 2 | 2 | Y | 41737 | hypothetical protein [Micrurus corallinus] |
| 69 | 115 | JAC96363.1 | 199.83 | 31 | 29 | 3 | 4 | 0 | 6 | 4 | 2.9886E6 | 1.8624E4 |  |  |  |  | 10 | 3 | 12 | 1 | 1 | 0 | 2 | 2 | Y | 41793 | Actin, cytoplasmic 2 [Hypsiglena sp. JMG-2014] |
| 69 | 116 | JAA97900.1 | 199.83 | 31 | 29 | 3 | 4 | 0 | 6 | 4 | 2.9886E6 | 1.8624E4 |  |  |  |  | 10 | 3 | 12 | 1 | 1 | 0 | 2 | 2 | Y | 41793 | Actin, cytoplasmic 2 [Crotalus horridus] |
| 69 | 117 | LAA21099.1 | 199.83 | 31 | 29 | 3 | 4 | 0 | 6 | 4 | 2.9886E6 | 1.8624E4 |  |  |  |  | 10 | 3 | 12 | 1 | 1 | 0 | 2 | 2 | Y | 41793 | hypothetical protein [Micrurus lemniscatus carvalhoi] |
| 69 | 118 | JAI10342.1 | 199.83 | 31 | 29 | 3 | 4 | 0 | 6 | 4 | 2.9886E6 | 1.8624E4 |  |  |  |  | 10 | 3 | 12 | 1 | 1 | 0 | 2 | 2 | Y | 41737 | Actin, cytoplasmic 1 [Micrurus fulvius] |
| 69 | 119 | JAG68843.1 | 199.83 | 31 | 29 | 3 | 4 | 0 | 6 | 4 | 2.9886E6 | 1.8624E4 |  |  |  |  | 10 | 3 | 12 | 1 | 1 | 0 | 2 | 2 | Y | 41793 | Actin, cytoplasmic 2 [Boiga irregularis] |
| 69 | 120 | XP\_013921350.1 | 199.83 | 31 | 29 | 3 | 4 | 0 | 6 | 4 | 2.9886E6 | 1.8624E4 |  |  |  |  | 10 | 3 | 12 | 1 | 1 | 0 | 2 | 2 | Y | 41793 | PREDICTED: actin, cytoplasmic 2 [Thamnophis sirtalis] |
| 69 | 121 | JAB54771.1 | 199.83 | 31 | 29 | 3 | 4 | 0 | 6 | 4 | 2.9886E6 | 1.8624E4 |  |  |  |  | 10 | 3 | 12 | 1 | 1 | 0 | 2 | 2 | Y | 41737 | Actin [Micrurus fulvius] |
| 69 | 122 | JAA97901.1 | 199.83 | 31 | 29 | 3 | 4 | 0 | 6 | 4 | 2.9886E6 | 1.8624E4 |  |  |  |  | 10 | 3 | 12 | 1 | 1 | 0 | 2 | 2 | Y | 41737 | Actin, cytoplasmic 1 [Crotalus horridus] |
| 69 | 123 | JAG68844.1 | 199.83 | 31 | 29 | 3 | 4 | 0 | 6 | 4 | 2.9886E6 | 1.8624E4 |  |  |  |  | 10 | 3 | 12 | 1 | 1 | 0 | 2 | 2 | Y | 41737 | Actin, cytoplasmic 1 [Boiga irregularis] |
| 69 | 124 | XP\_026531551.1 | 199.83 | 31 | 29 | 3 | 4 | 0 | 6 | 4 | 2.9886E6 | 1.8624E4 |  |  |  |  | 10 | 3 | 12 | 1 | 1 | 0 | 2 | 2 | Y | 41793 | actin, cytoplasmic 2 [Notechis scutatus] |
| 69 | 125 | LAA21098.1 | 199.83 | 31 | 29 | 3 | 4 | 0 | 6 | 4 | 2.9886E6 | 1.8624E4 |  |  |  |  | 10 | 3 | 12 | 1 | 1 | 0 | 2 | 2 | Y | 41793 | hypothetical protein [Micrurus lemniscatus carvalhoi] |
| 69 | 126 | JAV51374.1 | 199.83 | 31 | 29 | 3 | 4 | 0 | 6 | 4 | 2.9886E6 | 1.8624E4 |  |  |  |  | 10 | 3 | 12 | 1 | 1 | 0 | 2 | 2 | Y | 41737 | Actin, cytoplasmic 1 [Agkistrodon contortrix contortrix] |
| 69 | 127 | JAG47580.1 | 199.83 | 31 | 29 | 3 | 4 | 0 | 6 | 4 | 2.9886E6 | 1.8624E4 |  |  |  |  | 10 | 3 | 12 | 1 | 1 | 0 | 2 | 2 | Y | 41737 | Actin, cytoplasmic 1 [Crotalus horridus] |
| 69 | 128 | AFJ49303.1 | 199.83 | 31 | 29 | 3 | 4 | 0 | 6 | 4 | 2.9886E6 | 1.8624E4 |  |  |  |  | 10 | 3 | 12 | 1 | 1 | 0 | 2 | 2 | Y | 41793 | Actin, cytoplasmic 2 [Crotalus adamanteus] |
| 69 | 129 | JAI14496.1 | 199.83 | 31 | 29 | 3 | 4 | 0 | 6 | 4 | 2.9886E6 | 1.8624E4 |  |  |  |  | 10 | 3 | 12 | 1 | 1 | 0 | 2 | 2 | Y | 41737 | Actin, cytoplasmic 1 [Crotalus adamanteus] |
| 69 | 130 | JAI10341.1 | 199.83 | 31 | 29 | 3 | 4 | 0 | 6 | 4 | 2.9886E6 | 1.8624E4 |  |  |  |  | 10 | 3 | 12 | 1 | 1 | 0 | 2 | 2 | Y | 41793 | Actin, cytoplasmic 2 [Micrurus fulvius] |
| 69 | 131 | JAI14495.1 | 199.83 | 31 | 29 | 3 | 4 | 0 | 6 | 4 | 2.9886E6 | 1.8624E4 |  |  |  |  | 10 | 3 | 12 | 1 | 1 | 0 | 2 | 2 | Y | 41793 | Actin, cytoplasmic 2 [Crotalus adamanteus] |
| 69 | 132 | XP\_007436312.1 | 199.83 | 31 | 29 | 3 | 4 | 0 | 6 | 4 | 2.9886E6 | 1.8624E4 |  |  |  |  | 10 | 3 | 12 | 1 | 1 | 0 | 2 | 2 | Y | 41737 | actin-like protein 8 [Python bivittatus] |
| 69 | 133 | XP\_007419915.2 | 199.83 | 31 | 29 | 3 | 4 | 0 | 6 | 4 | 2.9886E6 | 1.8624E4 |  |  |  |  | 10 | 3 | 12 | 1 | 1 | 0 | 2 | 2 | Y | 41793 | actin, cytoplasmic 2 [Python bivittatus] |
| 69 | 134 | JAC96364.1 | 199.83 | 31 | 29 | 3 | 4 | 0 | 6 | 4 | 2.9886E6 | 1.8624E4 |  |  |  |  | 10 | 3 | 12 | 1 | 1 | 0 | 2 | 2 | Y | 41737 | Actin, cytoplasmic 1 [Hypsiglena sp. JMG-2014] |
| 69 | 135 | JAG47579.1 | 199.83 | 31 | 29 | 3 | 4 | 0 | 6 | 4 | 2.9886E6 | 1.8624E4 |  |  |  |  | 10 | 3 | 12 | 1 | 1 | 0 | 2 | 2 | Y | 41793 | Actin, cytoplasmic 2 [Crotalus horridus] |
| 69 | 136 | JAV51372.1 | 199.83 | 31 | 29 | 3 | 4 | 0 | 6 | 4 | 2.9886E6 | 1.8624E4 |  |  |  |  | 10 | 3 | 12 | 1 | 1 | 0 | 2 | 2 | Y | 41793 | Actin, cytoplasmic 2 [Agkistrodon contortrix contortrix] |
| 69 | 137 | AFJ49302.1 | 199.83 | 31 | 29 | 3 | 4 | 0 | 6 | 4 | 2.9886E6 | 1.8624E4 |  |  |  |  | 10 | 3 | 12 | 1 | 1 | 0 | 2 | 2 | Y | 41737 | Actin, cytoplasmic 1 [Crotalus adamanteus] |
| 69 | 138 | BAQ22177.1 | 199.83 | 31 | 29 | 3 | 4 | 0 | 6 | 4 | 2.9886E6 | 1.8624E4 |  |  |  |  | 10 | 3 | 12 | 1 | 1 | 0 | 2 | 2 | Y | 41737 | beta-actin [Protobothrops flavoviridis] |
| 69 | 139 | JAG45996.1 | 199.83 | 31 | 29 | 3 | 4 | 0 | 6 | 4 | 2.9886E6 | 1.8624E4 |  |  |  |  | 10 | 3 | 12 | 1 | 1 | 0 | 2 | 2 | Y | 41793 | Actin, cytoplasmic 2 [Crotalus horridus] |
| 69 | 140 | JAG45999.1 | 199.83 | 31 | 29 | 3 | 4 | 0 | 6 | 4 | 2.9886E6 | 1.8624E4 |  |  |  |  | 10 | 3 | 12 | 1 | 1 | 0 | 2 | 2 | Y | 41737 | actin, cytoplasmic 1 [Crotalus horridus] |
| 69 | 141 | JAB54395.1 | 199.83 | 31 | 29 | 3 | 4 | 0 | 6 | 4 | 2.9886E6 | 1.8624E4 |  |  |  |  | 10 | 3 | 12 | 1 | 1 | 0 | 2 | 2 | Y | 41793 | cytoplasmic 2 [Micrurus fulvius] |
| 69 | 142 | ETE74025.1 | 199.83 | 31 | 29 | 3 | 4 | 0 | 6 | 4 | 2.9886E6 | 1.8624E4 |  |  |  |  | 10 | 3 | 12 | 1 | 1 | 0 | 2 | 2 | Y | 41793 | Actin, cytoplasmic 2 [Ophiophagus hannah] |
| 41 | 272 | XP\_007442357.1 | 192.83 | 12 | 10 | 7 | 12 | 5 | 7 | 8 |  |  | 0 |  |  |  | 10 | 1 | 15 | 7 | 11 | 3 | 8 | 6 | N | 60285 | keratin, type II cytoskeletal 6A-like [Python bivittatus] |
| 58 | 284 | JAV51432.1 | 187.50 | 10 | 6 | 2 | 0 | 0 | 2 | 8 | 7.95E5 |  |  |  |  | 2.2921E6 | 8 | 4 | 6 | 1 | 0 | 0 | 1 | 7 | Y | 68106 | Metalloproteinase (type III) 6a [Agkistrodon contortrix contortrix] |
| 66 | 489 | P82981.1 | 179.40 | 12 | 5 | 0 | 6 | 0 | 12 | 5 | 8.9947E4 |  |  |  | 1.9247E6 | 4.8116E4 | 6 | 4 | 1 | 0 | 11 | 0 | 9 | 1 | Y | 25413 | RecName: Full=Thrombin-like enzyme contortrixobin; Short=SVTLE; AltName: Full=Fibrinogen-clotting enzyme; AltName: Full=Snake venom serine protease; Short=SVSP; AltName: Full=Venombin B |
| 66 | 490 | JAV51419.1 | 179.40 | 10 | 5 | 0 | 5 | 0 | 10 | 5 | 8.9947E4 |  |  |  | 1.9247E6 | 4.8116E4 | 6 | 4 | 1 | 0 | 11 | 0 | 9 | 1 | Y | 28037 | Serine proteinase 3a [Agkistrodon contortrix contortrix] |
| 66 | 724 | BAA19976.1 | 179.40 | 10 | 5 | 0 | 5 | 0 | 10 | 5 | 8.9947E4 |  |  |  | 1.9247E6 | 4.8116E4 | 6 | 4 | 1 | 0 | 11 | 0 | 9 | 1 | Y | 28398 | serine protease [Protobothrops flavoviridis] |
| 66 | 725 | BAN82033.1 | 179.40 | 10 | 5 | 0 | 5 | 0 | 10 | 5 | 8.9947E4 |  |  |  | 1.9247E6 | 4.8116E4 | 6 | 4 | 1 | 0 | 11 | 0 | 9 | 1 | Y | 28398 | serine protease [Protobothrops flavoviridis] |
| 66 | 726 | BAP39955.1 | 179.40 | 10 | 5 | 0 | 5 | 0 | 10 | 5 | 8.9947E4 |  |  |  | 1.9247E6 | 4.8116E4 | 6 | 4 | 1 | 0 | 11 | 0 | 9 | 1 | Y | 28308 | serine protease [Protobothrops flavoviridis] |
| 66 | 727 | P05620.2 | 179.40 | 10 | 5 | 0 | 5 | 0 | 10 | 5 | 8.9947E4 |  |  |  | 1.9247E6 | 4.8116E4 | 6 | 4 | 1 | 0 | 11 | 0 | 9 | 1 | Y | 28398 | RecName: Full=Thrombin-like enzyme flavoxobin; Short=SVTLE; AltName: Full=Fibrinogen-clotting enzyme; AltName: Full=Habutobin; AltName: Full=Snake venom serine protease 1; Short=SVSP; Flags: Precursor |
| 54 | 242 | AXL95287.1 | 174.25 | 9 | 3 | 4 | 0 | 0 | 4 | 4 |  | 3.3005E5 |  |  |  |  | 7 | 1 | 5 | 6 | 0 | 0 | 3 | 2 | N | 58594 | L-amino acid oxidase 1, partial [Spilotes sulphureus] |
| 54 | 243 | AXL95288.1 | 174.25 | 9 | 3 | 4 | 0 | 0 | 4 | 4 |  | 3.3005E5 |  |  |  |  | 7 | 1 | 5 | 6 | 0 | 0 | 3 | 2 | N | 58594 | L-amino acid oxidase 2, partial [Spilotes sulphureus] |
| 116 | 403 | ETE68810.1 | 171.17 | 19 | 0 | 0 | 0 | 0 | 19 | 0 |  |  |  |  | 1.448E7 |  | 5 | 2 | 0 | 0 | 0 | 0 | 9 | 0 | N | 29588 | Glutathione peroxidase 3, partial [Ophiophagus hannah] |
| 114 | 536 | XP\_034258146.1 | 170.33 | 16 | 0 | 0 | 0 | 0 | 16 | 7 |  |  |  |  | 2.6733E7 | 1.8888E6 | 4 | 1 | 0 | 0 | 0 | 0 | 7 | 2 | N | 28406 | glutathione peroxidase 3 [Pantherophis guttatus] |
| 97 | 165 | XP\_015675298.1 | 167.87 | 22 | 17 | 0 | 0 | 0 | 10 | 0 | 6.4884E6 |  |  |  | 9.4163E5 |  | 8 | 8 | 10 | 0 | 0 | 0 | 4 | 0 | N | 53899 | carboxypeptidase E [Protobothrops mucrosquamatus] |
| 97 | 166 | AFJ49609.1 | 167.87 | 22 | 17 | 0 | 0 | 0 | 10 | 0 | 6.4884E6 |  |  |  | 9.4163E5 |  | 8 | 8 | 10 | 0 | 0 | 0 | 4 | 0 | N | 53839 | Carboxypeptidase E-like [Crotalus adamanteus] |
| 57 | 673 | JAS04545.1 | 156.01 | 13 | 0 | 0 | 0 | 0 | 7 | 13 |  |  |  |  | 2.5212E5 | 8.5611E7 | 5 | 5 | 0 | 0 | 0 | 0 | 2 | 25 | Y | 18656 | C-type lectin 2 [Agkistrodon piscivorus conanti] |
| 86 | 666 | ACE73567.1 | 153.68 | 16 | 10 | 4 | 0 | 4 | 16 | 10 |  |  |  |  | 0 |  | 5 | 1 | 2 | 1 | 0 | 1 | 11 | 2 | Y | 26688 | cysteine-rich seceretory protein Dr-CRPK [Daboia russelii] |
| 92 | 678 | P0DM87.1 | 153.23 | 10 | 6 | 2 | 3 | 0 | 5 | 4 | 5.7959E6 | 8.3464E3 |  |  | 1.6666E5 |  | 4 | 2 | 11 | 1 | 1 | 0 | 2 | 1 | Y | 54436 | RecName: Full=Zinc metalloproteinase-disintegrin stejnitin; AltName: Full=Snake venom metalloproteinase; Short=SVMP; Flags: Precursor |
| 63 | 337 | ETE70217.1 | 150.12 | 1 | 1 | 1 | 0 | 0 | 0 | 0 |  | 9.7875E3 |  |  |  |  | 5 | 1 | 10 | 8 | 5 | 1 | 1 | 2 | N | 436388 | Keratin, type I cytoskeletal 19, partial [Ophiophagus hannah] |
| 87 | 574 | XP\_026573193.1 | 146.36 | 8 | 6 | 0 | 4 | 0 | 4 | 4 |  |  |  |  |  | 2.8074E4 | 6 | 1 | 5 | 0 | 2 | 0 | 3 | 2 | N | 50631 | keratin, type II cytoskeletal 1-like [Pseudonaja textilis] |
| 110 | 472 | P0CB14.1 | 145.67 | 16 | 5 | 2 | 0 | 0 | 9 | 9 | 3.3086E5 |  |  |  | 2.2296E7 | 5.6819E4 | 4 | 2 | 3 | 1 | 0 | 0 | 3 | 4 | Y | 47446 | RecName: Full=Snake venom metalloproteinase kistomin; Short=SVMP; Flags: Precursor |
| 100 | 250 | XP\_034285001.1 | 138.87 | 2 | 2 | 1 | 0 | 0 | 1 | 0 | 6.6895E5 | 1.1289E5 | 1.0871E6 |  | 0 |  | 4 | 4 | 5 | 2 | 1 | 0 | 1 | 0 | N | 276847 | desmoplakin isoform X2 [Pantherophis guttatus] |
| 100 | 252 | XP\_007422201.1 | 138.87 | 2 | 2 | 1 | 0 | 0 | 1 | 0 | 6.6895E5 | 1.1289E5 | 1.0871E6 |  | 0 |  | 4 | 4 | 5 | 2 | 1 | 0 | 1 | 0 | N | 329911 | desmoplakin isoform X1 [Python bivittatus] |
| 100 | 254 | XP\_007422202.1 | 138.87 | 2 | 2 | 1 | 0 | 0 | 1 | 0 | 6.6895E5 | 1.1289E5 | 1.0871E6 |  | 0 |  | 4 | 4 | 5 | 2 | 1 | 0 | 1 | 0 | N | 277098 | desmoplakin isoform X2 [Python bivittatus] |
| 100 | 255 | XP\_034285000.1 | 138.87 | 2 | 2 | 1 | 0 | 0 | 1 | 0 | 6.6895E5 | 1.1289E5 | 1.0871E6 |  | 0 |  | 4 | 4 | 5 | 2 | 1 | 0 | 1 | 0 | N | 329611 | desmoplakin isoform X1 [Pantherophis guttatus] |
| 100 | 256 | XP\_015666325.1 | 138.87 | 2 | 2 | 1 | 0 | 0 | 1 | 0 | 6.6895E5 | 1.1289E5 | 1.0871E6 |  | 0 |  | 4 | 4 | 5 | 2 | 1 | 0 | 1 | 0 | N | 329546 | desmoplakin [Protobothrops mucrosquamatus] |
| 100 | 258 | XP\_032079162.1 | 138.87 | 2 | 2 | 1 | 0 | 0 | 1 | 0 | 6.6895E5 | 1.1289E5 | 1.0871E6 |  | 0 |  | 4 | 4 | 5 | 2 | 1 | 0 | 1 | 0 | N | 277026 | desmoplakin isoform X2 [Thamnophis elegans] |
| 100 | 259 | XP\_032079161.1 | 138.87 | 2 | 2 | 1 | 0 | 0 | 1 | 0 | 6.6895E5 | 1.1289E5 | 1.0871E6 |  | 0 |  | 4 | 4 | 5 | 2 | 1 | 0 | 1 | 0 | N | 329798 | desmoplakin isoform X1 [Thamnophis elegans] |
| 100 | 260 | XP\_034285002.1 | 138.87 | 2 | 2 | 1 | 0 | 0 | 1 | 0 | 6.6895E5 | 1.1289E5 | 1.0871E6 |  | 0 |  | 4 | 4 | 5 | 2 | 1 | 0 | 1 | 0 | N | 258226 | desmoplakin isoform X3 [Pantherophis guttatus] |
| 100 | 261 | XP\_007422203.1 | 138.87 | 2 | 2 | 1 | 0 | 0 | 1 | 0 | 6.6895E5 | 1.1289E5 | 1.0871E6 |  | 0 |  | 4 | 4 | 5 | 2 | 1 | 0 | 1 | 0 | N | 258600 | desmoplakin isoform X3 [Python bivittatus] |
| 100 | 268 | JAC95787.1 | 138.87 | 2 | 2 | 1 | 0 | 0 | 1 | 0 | 6.6895E5 | 1.1289E5 | 1.0871E6 |  | 0 |  | 4 | 4 | 5 | 2 | 1 | 0 | 1 | 0 | N | 257947 | desmoplakin isoform II [Hypsiglena sp. JMG-2014] |
| 100 | 269 | XP\_032079163.1 | 138.87 | 2 | 2 | 1 | 0 | 0 | 1 | 0 | 6.6895E5 | 1.1289E5 | 1.0871E6 |  | 0 |  | 4 | 4 | 5 | 2 | 1 | 0 | 1 | 0 | N | 258448 | desmoplakin isoform X3 [Thamnophis elegans] |
| 145 | 567 | JAG47218.1 | 135.24 | 11 | 6 | 0 | 5 | 0 | 0 | 0 | 3.1425E5 |  | 9.3929E5 |  |  |  | 3 | 3 | 2 | 0 | 1 | 0 | 0 | 0 | N | 37477 | Cathepsin B-like protein [Crotalus horridus] |
| 145 | 568 | JAA97493.1 | 135.24 | 11 | 6 | 0 | 5 | 0 | 0 | 0 | 3.1425E5 |  | 9.3929E5 |  |  |  | 3 | 3 | 2 | 0 | 1 | 0 | 0 | 0 | N | 37477 | Cathepsin B-like protein [Crotalus horridus] |
| 145 | 569 | JAI13964.1 | 135.24 | 11 | 6 | 0 | 5 | 0 | 0 | 0 | 3.1425E5 |  | 9.3929E5 |  |  |  | 3 | 3 | 2 | 0 | 1 | 0 | 0 | 0 | N | 37477 | Cathepsin B-like protein [Crotalus adamanteus] |
| 145 | 570 | JAV51001.1 | 135.24 | 11 | 6 | 0 | 5 | 0 | 0 | 0 | 3.1425E5 |  | 9.3929E5 |  |  |  | 3 | 3 | 2 | 0 | 1 | 0 | 0 | 0 | N | 37351 | Cathepsin B-like protein [Agkistrodon contortrix contortrix] |
| 145 | 571 | JAG45448.1 | 135.24 | 11 | 6 | 0 | 5 | 0 | 0 | 0 | 3.1425E5 |  | 9.3929E5 |  |  |  | 3 | 3 | 2 | 0 | 1 | 0 | 0 | 0 | N | 37477 | Cathepsin B-like protein [Crotalus horridus] |
| 148 | 1147 | JAG46154.1 | 133.59 | 16 | 0 | 0 | 0 | 0 | 0 | 16 |  |  |  |  |  | 1.081E7 | 3 | 3 | 0 | 0 | 0 | 0 | 0 | 4 | N | 17459 | C-type lectin 2 [Crotalus horridus] |
| 148 | 1148 | JAS04715.1 | 133.59 | 16 | 0 | 0 | 0 | 0 | 0 | 16 |  |  |  |  |  | 1.081E7 | 3 | 3 | 0 | 0 | 0 | 0 | 0 | 4 | N | 17461 | C-type lectin 4b [Crotalus adamanteus] |
| 148 | 1149 | JAS05341.1 | 133.59 | 16 | 0 | 0 | 0 | 0 | 0 | 16 |  |  |  |  |  | 1.081E7 | 3 | 3 | 0 | 0 | 0 | 0 | 0 | 4 | N | 17459 | C-type lectin 19b [Sistrurus tergeminus] |
| 148 | 1150 | JAI10419.1 | 133.59 | 16 | 0 | 0 | 0 | 0 | 0 | 16 |  |  |  |  |  | 1.081E7 | 3 | 3 | 0 | 0 | 0 | 0 | 0 | 4 | N | 17461 | C-type lectin 4b [Crotalus adamanteus] |
| 148 | 1151 | AEJ31972.1 | 133.59 | 16 | 0 | 0 | 0 | 0 | 0 | 16 |  |  |  |  |  | 1.081E7 | 3 | 3 | 0 | 0 | 0 | 0 | 0 | 4 | N | 17461 | C-type lectin 6 [Crotalus adamanteus] |
| 148 | 1152 | JAS04888.1 | 133.59 | 16 | 0 | 0 | 0 | 0 | 0 | 16 |  |  |  |  |  | 1.081E7 | 3 | 3 | 0 | 0 | 0 | 0 | 0 | 4 | N | 17459 | C-type lectin 2 [Crotalus horridus] |
| 148 | 1153 | JAS05342.1 | 133.59 | 16 | 0 | 0 | 0 | 0 | 0 | 16 |  |  |  |  |  | 1.081E7 | 3 | 3 | 0 | 0 | 0 | 0 | 0 | 4 | N | 17489 | C-type lectin 19a [Sistrurus tergeminus] |
| 148 | 1154 | JAS04716.1 | 133.59 | 16 | 0 | 0 | 0 | 0 | 0 | 16 |  |  |  |  |  | 1.081E7 | 3 | 3 | 0 | 0 | 0 | 0 | 0 | 4 | N | 17429 | C-type lectin 4a [Crotalus adamanteus] |
| 148 | 1155 | AFJ49153.1 | 133.59 | 16 | 0 | 0 | 0 | 0 | 0 | 16 |  |  |  |  |  | 1.081E7 | 3 | 3 | 0 | 0 | 0 | 0 | 0 | 4 | N | 17461 | C-type lectin 12b [Crotalus adamanteus] |
| 148 | 1156 | JAV48435.1 | 133.59 | 16 | 0 | 0 | 0 | 0 | 0 | 16 |  |  |  |  |  | 1.081E7 | 3 | 3 | 0 | 0 | 0 | 0 | 0 | 4 | N | 17461 | Cadam10\_CTL-9 [Crotalus adamanteus] |
| 148 | 1157 | JAI10420.1 | 133.59 | 16 | 0 | 0 | 0 | 0 | 0 | 16 |  |  |  |  |  | 1.081E7 | 3 | 3 | 0 | 0 | 0 | 0 | 0 | 4 | N | 17429 | C-type lectin 4a [Crotalus adamanteus] |
| 148 | 1158 | JAA97993.1 | 133.59 | 16 | 0 | 0 | 0 | 0 | 0 | 16 |  |  |  |  |  | 1.081E7 | 3 | 3 | 0 | 0 | 0 | 0 | 0 | 4 | N | 17415 | Lectin\_beta-CohPH-6 [Crotalus oreganus helleri] |
| 148 | 1159 | AFJ49152.1 | 133.59 | 16 | 0 | 0 | 0 | 0 | 0 | 16 |  |  |  |  |  | 1.081E7 | 3 | 3 | 0 | 0 | 0 | 0 | 0 | 4 | N | 17429 | C-type lectin 12a [Crotalus adamanteus] |
| 148 | 1160 | CAD35498.1 | 133.59 | 16 | 0 | 0 | 0 | 0 | 0 | 16 |  |  |  |  |  | 1.081E7 | 3 | 3 | 0 | 0 | 0 | 0 | 0 | 4 | N | 17548 | unnamed protein product, partial [Sistrurus miliarius] |
| 148 | 1161 | JAS05482.1 | 133.59 | 15 | 0 | 0 | 0 | 0 | 0 | 15 |  |  |  |  |  | 1.081E7 | 3 | 3 | 0 | 0 | 0 | 0 | 0 | 4 | N | 18072 | C-type lectin 10b [Sistrurus miliarius barbouri] |
| 148 | 1442 | JAS05481.1 | 133.59 | 15 | 0 | 0 | 0 | 0 | 0 | 15 |  |  |  |  |  | 1.081E7 | 3 | 3 | 0 | 0 | 0 | 0 | 0 | 4 | N | 18044 | C-type lectin 10c [Sistrurus miliarius barbouri] |
| 148 | 1443 | JAS05483.1 | 133.59 | 15 | 0 | 0 | 0 | 0 | 0 | 15 |  |  |  |  |  | 1.081E7 | 3 | 3 | 0 | 0 | 0 | 0 | 0 | 4 | N | 18058 | C-type lectin 10a [Sistrurus miliarius barbouri] |
| 108 | 979 | XP\_007439730.1 | 124.09 | 5 | 2 | 0 | 0 | 0 | 5 | 2 | 5.1752E5 |  |  |  | 1.298E8 | 4.3086E6 | 4 | 4 | 2 | 0 | 0 | 0 | 9 | 2 | N | 57056 | cysteine-rich secretory protein LCCL domain-containing 2 [Python bivittatus] |
| 108 | 984 | ETE66074.1 | 124.09 | 5 | 2 | 0 | 0 | 0 | 5 | 2 | 5.1752E5 |  |  |  | 1.298E8 | 4.3086E6 | 4 | 4 | 2 | 0 | 0 | 0 | 9 | 2 | N | 53285 | Cysteine-rich secretory protein LCCL domain-containing 2 [Ophiophagus hannah] |
| 108 | 985 | JAI13759.1 | 124.09 | 5 | 2 | 0 | 0 | 0 | 5 | 2 | 5.1752E5 |  |  |  | 1.298E8 | 4.3086E6 | 4 | 4 | 2 | 0 | 0 | 0 | 9 | 2 | N | 55896 | cysteine-rich secretory protein LCCL domain-containing 2 [Crotalus adamanteus] |
| 108 | 986 | XP\_026569732.1 | 124.09 | 5 | 2 | 0 | 0 | 0 | 5 | 2 | 5.1752E5 |  |  |  | 1.298E8 | 4.3086E6 | 4 | 4 | 2 | 0 | 0 | 0 | 9 | 2 | N | 55739 | cysteine-rich secretory protein LCCL domain-containing 2 [Pseudonaja textilis] |
| 108 | 987 | XP\_015666034.1 | 124.09 | 5 | 2 | 0 | 0 | 0 | 5 | 2 | 5.1752E5 |  |  |  | 1.298E8 | 4.3086E6 | 4 | 4 | 2 | 0 | 0 | 0 | 9 | 2 | N | 55757 | cysteine-rich secretory protein LCCL domain-containing 2 [Protobothrops mucrosquamatus] |
| 108 | 988 | XP\_026539804.1 | 124.09 | 5 | 2 | 0 | 0 | 0 | 5 | 2 | 5.1752E5 |  |  |  | 1.298E8 | 4.3086E6 | 4 | 4 | 2 | 0 | 0 | 0 | 9 | 2 | N | 55954 | cysteine-rich secretory protein LCCL domain-containing 2 [Notechis scutatus] |
| 108 | 989 | XP\_034282442.1 | 124.09 | 5 | 2 | 0 | 0 | 0 | 5 | 2 | 5.1752E5 |  |  |  | 1.298E8 | 4.3086E6 | 4 | 4 | 2 | 0 | 0 | 0 | 9 | 2 | N | 55902 | cysteine-rich secretory protein LCCL domain-containing 2 [Pantherophis guttatus] |
| 108 | 999 | QHR82746.1 | 124.09 | 10 | 4 | 0 | 0 | 0 | 10 | 4 | 5.1752E5 |  |  |  | 1.298E8 | 4.3086E6 | 4 | 4 | 2 | 0 | 0 | 0 | 9 | 2 | N | 26590 | cysteine-rich secretory protein 2, partial [Vipera anatolica senliki] |
| 108 | 1000 | XP\_032086736.1 | 124.09 | 7 | 3 | 0 | 0 | 0 | 7 | 3 | 5.1752E5 |  |  |  | 1.298E8 | 4.3086E6 | 4 | 4 | 2 | 0 | 0 | 0 | 9 | 2 | N | 41744 | LOW QUALITY PROTEIN: cysteine-rich secretory protein LCCL domain-containing 2-like [Thamnophis elegans] |
| 108 | 1003 | XP\_032087342.1 | 124.09 | 5 | 2 | 0 | 0 | 0 | 5 | 2 | 5.1752E5 |  |  |  | 1.298E8 | 4.3086E6 | 4 | 4 | 2 | 0 | 0 | 0 | 9 | 2 | N | 55746 | LOW QUALITY PROTEIN: cysteine-rich secretory protein LCCL domain-containing 2-like [Thamnophis elegans] |
| 83 | 572 | XP\_007435739.1 | 121.70 | 6 | 6 | 4 | 6 | 2 | 2 | 4 | 2.9111E6 | 8.3407E5 | 3.1651E7 |  |  | 2.1515E5 | 3 | 1 | 4 | 3 | 5 | 1 | 3 | 3 | N | 53751 | keratin, type II cytoskeletal 8 [Python bivittatus] |
| 119 | 798 | P0DJL3.1 | 121.63 | 15 | 0 | 0 | 0 | 0 | 7 | 15 |  |  |  |  | 1.0259E6 | 1.2753E7 | 2 | 1 | 0 | 0 | 0 | 0 | 1 | 6 | N | 14498 | RecName: Full=Snaclec purpureotin subunit beta |
| 119 | 807 | P81116.2 | 121.63 | 13 | 0 | 0 | 0 | 0 | 6 | 13 |  |  |  |  | 1.0259E6 | 1.2753E7 | 2 | 1 | 0 | 0 | 0 | 0 | 1 | 6 | N | 16871 | RecName: Full=Snaclec alboaggregin-B subunit beta; Short=AL-B subunit beta; AltName: Full=25-kDa alboaggregin; Flags: Precursor |
| 119 | 808 | ABS12077.1 | 121.63 | 13 | 0 | 0 | 0 | 0 | 6 | 13 |  |  |  |  | 1.0259E6 | 1.2753E7 | 2 | 1 | 0 | 0 | 0 | 0 | 1 | 6 | N | 16871 | alboaggregin B beta subunit [Trimeresurus albolabris] |
| 89 | 575 | JAV48393.1 | 116.63 | 12 | 8 | 0 | 5 | 0 | 3 | 0 | 1.6435E7 |  | 1.6322E8 |  |  |  | 4 | 1 | 8 | 0 | 1 | 0 | 1 | 0 | Y | 28642 | Cadam10\_SVSP-11 [Crotalus adamanteus] |
| 16 | 768 | P26324.1 | 116.46 | 13 | 13 | 13 | 4 | 8 | 8 | 6 | 1.4609E9 | 1.0046E7 | 4.0038E7 | 2.55E7 | 1.1301E7 |  | 3 | 2 | 108 | 6 | 1 | 3 | 7 | 1 | Y | 26570 | RecName: Full=Thrombin-like enzyme ancrod; Short=SVTLE; AltName: Full=Fibrinogen-clotting enzyme; AltName: Full=Snake venom serine protease; Short=SVSP; AltName: Full=Venombin A |
| 16 | 769 | AAA03254.1 | 116.46 | 13 | 13 | 13 | 4 | 8 | 8 | 6 | 1.4609E9 | 1.0046E7 | 4.0038E7 | 2.55E7 | 1.1301E7 |  | 3 | 2 | 108 | 6 | 1 | 3 | 7 | 1 | Y | 26570 | Ancrod=thrombin-like alpha-fibrinogenase [Akistrodon rhodostoma, venom, Peptide, 234 aa] |
| 126 | 286 | XP\_034262421.1 | 115.85 | 7 | 4 | 0 | 0 | 0 | 0 | 3 | 2.0275E5 |  |  |  |  | 3.0923E4 | 3 | 3 | 2 | 0 | 0 | 0 | 0 | 1 | N | 68299 | endoplasmic reticulum chaperone BiP, partial [Pantherophis guttatus] |
| 126 | 288 | JAV50419.1 | 115.85 | 7 | 4 | 0 | 0 | 0 | 0 | 3 | 2.0275E5 |  |  |  |  | 3.0923E4 | 3 | 3 | 2 | 0 | 0 | 0 | 0 | 1 | N | 72067 | 78 kDa glucose-regulated protein [Agkistrodon contortrix contortrix] |
| 126 | 289 | JAG44540.1 | 115.85 | 7 | 4 | 0 | 0 | 0 | 0 | 3 | 2.0275E5 |  |  |  |  | 3.0923E4 | 3 | 3 | 2 | 0 | 0 | 0 | 0 | 1 | N | 72105 | 78 kDa glucose-regulated protein precursor [Crotalus horridus] |
| 126 | 290 | JAI13107.1 | 115.85 | 7 | 4 | 0 | 0 | 0 | 0 | 3 | 2.0275E5 |  |  |  |  | 3.0923E4 | 3 | 3 | 2 | 0 | 0 | 0 | 0 | 1 | N | 72105 | 78 kDa glucose-regulated protein [Crotalus adamanteus] |
| 126 | 291 | JAG46692.1 | 115.85 | 7 | 4 | 0 | 0 | 0 | 0 | 3 | 2.0275E5 |  |  |  |  | 3.0923E4 | 3 | 3 | 2 | 0 | 0 | 0 | 0 | 1 | N | 72105 | 78 kDa glucose-regulated protein [Crotalus horridus] |
| 126 | 292 | JAA96909.1 | 115.85 | 7 | 4 | 0 | 0 | 0 | 0 | 3 | 2.0275E5 |  |  |  |  | 3.0923E4 | 3 | 3 | 2 | 0 | 0 | 0 | 0 | 1 | N | 72105 | 78 kDa glucose-regulated protein [Crotalus horridus] |
| 126 | 293 | XP\_015681610.1 | 115.85 | 7 | 4 | 0 | 0 | 0 | 0 | 3 | 2.0275E5 |  |  |  |  | 3.0923E4 | 3 | 3 | 2 | 0 | 0 | 0 | 0 | 1 | N | 72155 | endoplasmic reticulum chaperone BiP [Protobothrops mucrosquamatus] |
| 126 | 294 | AFJ50327.1 | 115.85 | 7 | 4 | 0 | 0 | 0 | 0 | 3 | 2.0275E5 |  |  |  |  | 3.0923E4 | 3 | 3 | 2 | 0 | 0 | 0 | 0 | 1 | N | 72105 | Heat shock protein 5 [Crotalus adamanteus] |
| 126 | 295 | JAB54174.1 | 115.85 | 7 | 4 | 0 | 0 | 0 | 0 | 3 | 2.0275E5 |  |  |  |  | 3.0923E4 | 3 | 3 | 2 | 0 | 0 | 0 | 0 | 1 | N | 72106 | glucose-regulated protein [Micrurus fulvius] |
| 126 | 296 | XP\_026542482.1 | 115.85 | 7 | 4 | 0 | 0 | 0 | 0 | 3 | 2.0275E5 |  |  |  |  | 3.0923E4 | 3 | 3 | 2 | 0 | 0 | 0 | 0 | 1 | N | 72286 | endoplasmic reticulum chaperone BiP [Notechis scutatus] |
| 126 | 297 | JAC95528.1 | 115.85 | 7 | 4 | 0 | 0 | 0 | 0 | 3 | 2.0275E5 |  |  |  |  | 3.0923E4 | 3 | 3 | 2 | 0 | 0 | 0 | 0 | 1 | N | 72299 | 78 kDa glucose-regulated protein-like [Hypsiglena sp. JMG-2014] |
| 126 | 298 | AFJ50239.1 | 115.85 | 6 | 4 | 0 | 0 | 0 | 0 | 2 | 2.0275E5 |  |  |  |  | 3.0923E4 | 3 | 3 | 2 | 0 | 0 | 0 | 0 | 1 | N | 75480 | 78 kDa glucose-regulated protein precursor [Crotalus adamanteus] |
| 126 | 299 | XP\_026568326.1 | 115.85 | 7 | 4 | 0 | 0 | 0 | 0 | 3 | 2.0275E5 |  |  |  |  | 3.0923E4 | 3 | 3 | 2 | 0 | 0 | 0 | 0 | 1 | N | 72187 | endoplasmic reticulum chaperone BiP [Pseudonaja textilis] |
| 126 | 301 | XP\_025022112.1 | 115.85 | 7 | 4 | 0 | 0 | 0 | 0 | 3 | 2.0275E5 |  |  |  |  | 3.0923E4 | 3 | 3 | 2 | 0 | 0 | 0 | 0 | 1 | N | 71864 | endoplasmic reticulum chaperone BiP [Python bivittatus] |
| 126 | 302 | JAG67806.1 | 115.85 | 7 | 4 | 0 | 0 | 0 | 0 | 3 | 2.0275E5 |  |  |  |  | 3.0923E4 | 3 | 3 | 2 | 0 | 0 | 0 | 0 | 1 | N | 71941 | 78 kDa glucose-regulated protein [Boiga irregularis] |
| 126 | 303 | XP\_032088936.1 | 115.85 | 7 | 4 | 0 | 0 | 0 | 0 | 3 | 2.0275E5 |  |  |  |  | 3.0923E4 | 3 | 3 | 2 | 0 | 0 | 0 | 0 | 1 | N | 72006 | endoplasmic reticulum chaperone BiP [Thamnophis elegans] |
| 126 | 304 | XP\_013910585.1 | 115.85 | 7 | 4 | 0 | 0 | 0 | 0 | 3 | 2.0275E5 |  |  |  |  | 3.0923E4 | 3 | 3 | 2 | 0 | 0 | 0 | 0 | 1 | N | 72006 | PREDICTED: 78 kDa glucose-regulated protein [Thamnophis sirtalis] |
| 113 | 1277 | ACZ34293.1 | 115.29 | 9 | 9 | 0 | 0 | 0 | 0 | 9 | 6.7822E4 |  |  |  |  | 7.2926E6 | 1 | 1 | 1 | 0 | 0 | 0 | 0 | 5 | N | 18146 | TJ-GPIb-bp alpha chain [Protobothrops jerdonii] |
| 113 | 1278 | D1MGU0.1 | 115.29 | 9 | 9 | 0 | 0 | 0 | 0 | 9 | 6.7822E4 |  |  |  |  | 7.2926E6 | 1 | 1 | 1 | 0 | 0 | 0 | 0 | 5 | N | 18146 | RecName: Full=Snaclec jerdonibitin subunit alpha; AltName: Full=TJ-GPIb-bp subunit alpha; Flags: Precursor |
| 163 | 1076 | Q8JJ51.1 | 107.02 | 8 | 0 | 0 | 4 | 0 | 0 | 4 |  |  | 8.1989E6 |  |  |  | 2 | 1 | 0 | 0 | 1 | 0 | 0 | 1 | N | 46908 | RecName: Full=Snake venom metalloproteinase; Short=SVMP; Flags: Precursor |
| 163 | 1077 | AAM27041.1 | 107.02 | 8 | 0 | 0 | 4 | 0 | 0 | 4 |  |  | 8.1989E6 |  |  |  | 2 | 1 | 0 | 0 | 1 | 0 | 0 | 1 | N | 46908 | metalloproteinase precursor [Crotalus molossus molossus] |
| 163 | 1162 | ABD75822.1 | 107.02 | 8 | 0 | 0 | 4 | 0 | 0 | 4 |  |  | 8.1989E6 |  |  |  | 2 | 1 | 0 | 0 | 1 | 0 | 0 | 1 | N | 46856 | metalloproteinase P-I [Crotalus durissus durissus] |
| 143 | 1240 | P0DJL2.1 | 105.88 | 9 | 0 | 0 | 0 | 0 | 0 | 9 |  |  |  |  |  | 3.386E5 | 1 | 1 | 0 | 0 | 0 | 0 | 0 | 2 | N | 15613 | RecName: Full=Snaclec purpureotin subunit alpha |
| 94 | 594 | AAQ02908.1 | 105.29 | 12 | 12 | 5 | 0 | 0 | 3 | 5 | 8.1648E6 |  |  |  | 0 |  | 3 | 1 | 10 | 1 | 0 | 0 | 1 | 1 | Y | 28512 | serine protease KN14 precursor [Trimeresurus stejnegeri] |
| 94 | 595 | Q71QH9.1 | 105.29 | 12 | 12 | 5 | 0 | 0 | 3 | 5 | 8.1648E6 |  |  |  | 0 |  | 3 | 1 | 10 | 1 | 0 | 0 | 1 | 1 | Y | 28512 | RecName: Full=Snake venom serine protease KN14; Short=SVSP; Flags: Precursor |
| 94 | 606 | Q71QJ2.1 | 105.29 | 12 | 12 | 5 | 0 | 0 | 3 | 5 | 8.1648E6 |  |  |  | 0 |  | 3 | 1 | 10 | 1 | 0 | 0 | 1 | 1 | Y | 28541 | RecName: Full=Snake venom serine protease KN6; Short=SVSP; Flags: Precursor |
| 94 | 607 | AAQ02903.1 | 105.29 | 12 | 12 | 5 | 0 | 0 | 3 | 5 | 8.1648E6 |  |  |  | 0 |  | 3 | 1 | 10 | 1 | 0 | 0 | 1 | 1 | Y | 28510 | serine protease KN5 precursor [Trimeresurus stejnegeri] |
| 94 | 608 | Q71QI4.1 | 105.29 | 12 | 12 | 5 | 0 | 0 | 3 | 5 | 8.1648E6 |  |  |  | 0 |  | 3 | 1 | 10 | 1 | 0 | 0 | 1 | 1 | Y | 28510 | RecName: Full=Snake venom serine protease KN5; Short=SVSP; Flags: Precursor |
| 94 | 609 | AAQ02895.1 | 105.29 | 12 | 12 | 5 | 0 | 0 | 3 | 5 | 8.1648E6 |  |  |  | 0 |  | 3 | 1 | 10 | 1 | 0 | 0 | 1 | 1 | Y | 28541 | serine protease KN6 precursor [Trimeresurus stejnegeri] |
| 111 | 1324 | J3RYA3.1 | 100.31 | 8 | 8 | 0 | 0 | 0 | 0 | 0 | 2.2168E6 |  |  |  |  |  | 3 | 1 | 6 | 0 | 0 | 0 | 0 | 0 | Y | 29530 | RecName: Full=Snake venom serine proteinase 9; Short=SVSP; Flags: Precursor |
| 111 | 1325 | AEJ31999.1 | 100.31 | 8 | 8 | 0 | 0 | 0 | 0 | 0 | 2.2168E6 |  |  |  |  |  | 3 | 1 | 6 | 0 | 0 | 0 | 0 | 0 | Y | 29514 | serine proteinase 5 [Crotalus adamanteus] |
| 111 | 1326 | AFJ49264.1 | 100.31 | 8 | 8 | 0 | 0 | 0 | 0 | 0 | 2.2168E6 |  |  |  |  |  | 3 | 1 | 6 | 0 | 0 | 0 | 0 | 0 | Y | 29530 | Snake venom serine proteinase 9 [Crotalus adamanteus] |
| 115 | 1854 | BAC77707.1 | 100.18 | 8 | 0 | 0 | 0 | 0 | 0 | 8 |  |  |  |  |  | 7.1552E6 | 1 | 1 | 0 | 0 | 0 | 0 | 0 | 10 | Y | 17761 | EMS16 B chain [Echis multisquamatus] |
| 115 | 1855 | Q7T2Q0.1 | 100.18 | 8 | 0 | 0 | 0 | 0 | 0 | 8 |  |  |  |  |  | 7.1552E6 | 1 | 1 | 0 | 0 | 0 | 0 | 0 | 10 | Y | 17761 | RecName: Full=Snaclec EMS16 subunit beta; Short=EMS16B; Flags: Precursor |
| 115 | 2143 | 1UKM | 100.18 | 10 | 0 | 0 | 0 | 0 | 0 | 10 |  |  |  |  |  | 7.1552E6 | 1 | 1 | 0 | 0 | 0 | 0 | 0 | 10 | Y | 15099 | Chain B, EMS16 B chain |
| 115 | 2144 | 1V7P | 100.18 | 10 | 0 | 0 | 0 | 0 | 0 | 10 |  |  |  |  |  | 7.1552E6 | 1 | 1 | 0 | 0 | 0 | 0 | 0 | 10 | Y | 15099 | Chain B, Ems16 B Chain |
| 109 | 866 | AUS82539.1 | 98.08 | 8 | 8 | 0 | 0 | 0 | 4 | 0 | 0 |  |  |  | 0 |  | 3 | 1 | 6 | 0 | 0 | 0 | 1 | 0 | Y | 28390 | serine endopeptidase [Crotalus scutulatus] |
| 135 | 990 | ABA60128.1 | 97.89 | 8 | 0 | 0 | 0 | 0 | 8 | 8 |  |  |  |  | 5.3342E5 | 2.701E6 | 2 | 2 | 0 | 0 | 0 | 0 | 2 | 4 | Y | 27776 | venom nerve growth factor precursor [Hoplocephalus stephensii] |
| 135 | 1226 | JAA74661.1 | 97.89 | 8 | 0 | 0 | 0 | 0 | 8 | 8 |  |  |  |  | 5.3342E5 | 2.701E6 | 2 | 2 | 0 | 0 | 0 | 0 | 2 | 4 | Y | 27752 | NGF-Bra-7 [Brachyurophis roperi] |
| 135 | 1231 | ABA60119.1 | 97.89 | 8 | 0 | 0 | 0 | 0 | 8 | 8 |  |  |  |  | 5.3342E5 | 2.701E6 | 2 | 2 | 0 | 0 | 0 | 0 | 2 | 4 | Y | 27472 | venom nerve growth factor precursor [Pseudonaja textilis] |
| 135 | 1238 | AAG12169.1 | 97.89 | 8 | 0 | 0 | 0 | 0 | 8 | 8 |  |  |  |  | 5.3342E5 | 2.701E6 | 2 | 2 | 0 | 0 | 0 | 0 | 2 | 4 | Y | 27162 | putative neurotrophic growth factor precursor [Bothrops jararacussu] |
| 135 | 1271 | ABA60132.1 | 97.89 | 8 | 0 | 0 | 0 | 0 | 8 | 8 |  |  |  |  | 5.3342E5 | 2.701E6 | 2 | 2 | 0 | 0 | 0 | 0 | 2 | 4 | Y | 27629 | venom nerve growth factor precursor [Tropidechis carinatus] |
| 135 | 1272 | ABA60120.1 | 97.89 | 8 | 0 | 0 | 0 | 0 | 8 | 8 |  |  |  |  | 5.3342E5 | 2.701E6 | 2 | 2 | 0 | 0 | 0 | 0 | 2 | 4 | Y | 27770 | venom nerve growth factor precursor [Notechis scutatus] |
| 135 | 1273 | ABA60122.1 | 97.89 | 8 | 0 | 0 | 0 | 0 | 8 | 8 |  |  |  |  | 5.3342E5 | 2.701E6 | 2 | 2 | 0 | 0 | 0 | 0 | 2 | 4 | Y | 27789 | venom nerve growth factor precursor [Notechis scutatus] |
| 135 | 1291 | AAZ39009.1 | 97.89 | 8 | 0 | 0 | 0 | 0 | 8 | 8 |  |  |  |  | 5.3342E5 | 2.701E6 | 2 | 2 | 0 | 0 | 0 | 0 | 2 | 4 | Y | 27382 | venom nerve growth factor precursor [Oxyuranus scutellatus] |
| 135 | 1292 | ABA60117.1 | 97.89 | 8 | 0 | 0 | 0 | 0 | 8 | 8 |  |  |  |  | 5.3342E5 | 2.701E6 | 2 | 2 | 0 | 0 | 0 | 0 | 2 | 4 | Y | 27334 | venom nerve growth factor precursor [Oxyuranus microlepidotus] |
| 135 | 1293 | ABA60116.1 | 97.89 | 8 | 0 | 0 | 0 | 0 | 8 | 8 |  |  |  |  | 5.3342E5 | 2.701E6 | 2 | 2 | 0 | 0 | 0 | 0 | 2 | 4 | Y | 27353 | venom nerve growth factor precursor [Oxyuranus microlepidotus] |
| 135 | 1298 | ABA60133.1 | 97.89 | 8 | 0 | 0 | 0 | 0 | 8 | 8 |  |  |  |  | 5.3342E5 | 2.701E6 | 2 | 2 | 0 | 0 | 0 | 0 | 2 | 4 | Y | 27593 | venom nerve growth factor precursor [Tropidechis carinatus] |
| 135 | 1302 | ABA60121.1 | 97.89 | 8 | 0 | 0 | 0 | 0 | 8 | 8 |  |  |  |  | 5.3342E5 | 2.701E6 | 2 | 2 | 0 | 0 | 0 | 0 | 2 | 4 | Y | 27795 | venom nerve growth factor precursor [Notechis scutatus] |
| 135 | 1303 | ABA60130.1 | 97.89 | 8 | 0 | 0 | 0 | 0 | 8 | 8 |  |  |  |  | 5.3342E5 | 2.701E6 | 2 | 2 | 0 | 0 | 0 | 0 | 2 | 4 | Y | 27676 | venom nerve growth factor precursor [Tropidechis carinatus] |
| 135 | 1370 | ABA60123.1 | 97.89 | 8 | 0 | 0 | 0 | 0 | 8 | 8 |  |  |  |  | 5.3342E5 | 2.701E6 | 2 | 2 | 0 | 0 | 0 | 0 | 2 | 4 | Y | 26753 | venom nerve growth factor precursor [Pseudechis porphyriacus] |
| 135 | 1371 | ABA60126.1 | 97.89 | 8 | 0 | 0 | 0 | 0 | 8 | 8 |  |  |  |  | 5.3342E5 | 2.701E6 | 2 | 2 | 0 | 0 | 0 | 0 | 2 | 4 | Y | 27138 | venom nerve growth factor precursor [Pseudechis australis] |
| 135 | 1372 | ABA60125.1 | 97.89 | 8 | 0 | 0 | 0 | 0 | 8 | 8 |  |  |  |  | 5.3342E5 | 2.701E6 | 2 | 2 | 0 | 0 | 0 | 0 | 2 | 4 | Y | 27207 | venom nerve growth factor precursor [Pseudechis australis] |
| 135 | 1373 | ABA60124.1 | 97.89 | 8 | 0 | 0 | 0 | 0 | 8 | 8 |  |  |  |  | 5.3342E5 | 2.701E6 | 2 | 2 | 0 | 0 | 0 | 0 | 2 | 4 | Y | 27156 | venom nerve growth factor precursor [Pseudechis australis] |
| 135 | 1374 | JAB52817.1 | 97.89 | 8 | 0 | 0 | 0 | 0 | 8 | 8 |  |  |  |  | 5.3342E5 | 2.701E6 | 2 | 2 | 0 | 0 | 0 | 0 | 2 | 4 | Y | 27188 | Venom nerve growth factor 1b [Micrurus fulvius] |
| 135 | 1470 | AAS94269.1 | 97.89 | 8 | 0 | 0 | 0 | 0 | 8 | 8 |  |  |  |  | 5.3342E5 | 2.701E6 | 2 | 2 | 0 | 0 | 0 | 0 | 2 | 4 | Y | 27030 | nerve growth factor II [Naja sputatrix] |
| 135 | 1471 | JAA98018.1 | 97.89 | 8 | 0 | 0 | 0 | 0 | 8 | 8 |  |  |  |  | 5.3342E5 | 2.701E6 | 2 | 2 | 0 | 0 | 0 | 0 | 2 | 4 | Y | 27168 | NGF-CohLL-2 [Crotalus oreganus helleri] |
| 135 | 1472 | AAG30924.1 | 97.89 | 8 | 0 | 0 | 0 | 0 | 8 | 8 |  |  |  |  | 5.3342E5 | 2.701E6 | 2 | 2 | 0 | 0 | 0 | 0 | 2 | 4 | Y | 27119 | nerve growth factor [Crotalus durissus terrificus] |
| 135 | 1473 | JAA98019.1 | 97.89 | 8 | 0 | 0 | 0 | 0 | 8 | 8 |  |  |  |  | 5.3342E5 | 2.701E6 | 2 | 2 | 0 | 0 | 0 | 0 | 2 | 4 | Y | 27167 | NGF-CohLL-1 [Crotalus oreganus helleri] |
| 135 | 1474 | JAA94888.1 | 97.89 | 8 | 0 | 0 | 0 | 0 | 8 | 8 |  |  |  |  | 5.3342E5 | 2.701E6 | 2 | 2 | 0 | 0 | 0 | 0 | 2 | 4 | Y | 27141 | Venom nerve growth factor [Crotalus horridus] |
| 135 | 1475 | AFJ49196.1 | 97.89 | 8 | 0 | 0 | 0 | 0 | 8 | 8 |  |  |  |  | 5.3342E5 | 2.701E6 | 2 | 2 | 0 | 0 | 0 | 0 | 2 | 4 | Y | 27107 | venom nerve growth factor [Crotalus adamanteus] |
| 135 | 1476 | JAA97946.1 | 97.89 | 8 | 0 | 0 | 0 | 0 | 8 | 8 |  |  |  |  | 5.3342E5 | 2.701E6 | 2 | 2 | 0 | 0 | 0 | 0 | 2 | 4 | Y | 27167 | NGF-CohCI-3 [Crotalus oreganus helleri] |
| 135 | 1477 | AAV64846.1 | 97.89 | 8 | 0 | 0 | 0 | 0 | 8 | 8 |  |  |  |  | 5.3342E5 | 2.701E6 | 2 | 2 | 0 | 0 | 0 | 0 | 2 | 4 | Y | 27318 | nerve growth factor [Macrovipera lebetina] |
| 135 | 1478 | A59218 | 97.89 | 8 | 0 | 0 | 0 | 0 | 8 | 8 |  |  |  |  | 5.3342E5 | 2.701E6 | 2 | 2 | 0 | 0 | 0 | 0 | 2 | 4 | Y | 27648 | nerve growth factor beta chain precursor - monocled cobra |
| 136 | 1449 | AAQ15159.1 | 97.81 | 16 | 0 | 0 | 0 | 0 | 0 | 16 |  |  |  |  |  | 1.0397E6 | 2 | 1 | 0 | 0 | 0 | 0 | 0 | 4 | Y | 16820 | stejaggregin-B beta chain-2 [Trimeresurus stejnegeri] |
| 136 | 1450 | AAQ15158.1 | 97.81 | 16 | 0 | 0 | 0 | 0 | 0 | 16 |  |  |  |  |  | 1.0397E6 | 2 | 1 | 0 | 0 | 0 | 0 | 0 | 4 | Y | 16806 | stejaggregin-B beta chain-1 [Trimeresurus stejnegeri] |
| 136 | 1451 | Q71RQ8.1 | 97.81 | 16 | 0 | 0 | 0 | 0 | 0 | 16 |  |  |  |  |  | 1.0397E6 | 2 | 1 | 0 | 0 | 0 | 0 | 0 | 4 | Y | 16820 | RecName: Full=Snaclec stejaggregin-B subunit beta-2; Flags: Precursor |
| 136 | 1452 | Q71RQ9.1 | 97.81 | 16 | 0 | 0 | 0 | 0 | 0 | 16 |  |  |  |  |  | 1.0397E6 | 2 | 1 | 0 | 0 | 0 | 0 | 0 | 4 | Y | 16806 | RecName: Full=Snaclec stejaggregin-B subunit beta-1; Flags: Precursor |
| 132 | 729 | XP\_015675625.1 | 96.55 | 3 | 3 | 3 | 0 | 0 | 0 | 0 | 3.0245E4 | 1.4342E5 |  |  |  |  | 1 | 1 | 1 | 2 | 0 | 0 | 0 | 0 | Y | 50021 | beta-hexosaminidase subunit beta [Protobothrops mucrosquamatus] |
| 132 | 730 | XP\_032068596.1 | 96.55 | 2 | 2 | 2 | 0 | 0 | 0 | 0 | 3.0245E4 | 1.4342E5 |  |  |  |  | 1 | 1 | 1 | 2 | 0 | 0 | 0 | 0 | Y | 62017 | beta-hexosaminidase subunit beta [Thamnophis elegans] |
| 132 | 784 | XP\_025021294.1 | 96.55 | 2 | 2 | 2 | 0 | 0 | 0 | 0 | 3.0245E4 | 1.4342E5 |  |  |  |  | 1 | 1 | 1 | 2 | 0 | 0 | 0 | 0 | Y | 62228 | beta-hexosaminidase subunit beta [Python bivittatus] |
| 153 | 638 | XP\_015687424.1 | 96.43 | 4 | 4 | 0 | 0 | 0 | 0 | 0 | 1.8899E5 |  |  |  |  |  | 3 | 3 | 3 | 0 | 0 | 0 | 0 | 0 | N | 106458 | endoplasmic reticulum aminopeptidase 1 [Protobothrops mucrosquamatus] |
| 153 | 639 | JAI13418.1 | 96.43 | 4 | 4 | 0 | 0 | 0 | 0 | 0 | 1.8899E5 |  |  |  |  |  | 3 | 3 | 3 | 0 | 0 | 0 | 0 | 0 | N | 107364 | endoplasmic reticulum aminopeptidase 1-like protein [Crotalus adamanteus] |
| 153 | 640 | JAG44871.1 | 96.43 | 4 | 4 | 0 | 0 | 0 | 0 | 0 | 1.8899E5 |  |  |  |  |  | 3 | 3 | 3 | 0 | 0 | 0 | 0 | 0 | N | 107397 | endoplasmic reticulum aminopeptidase 1-like protein [Crotalus horridus] |
| 153 | 641 | JAG46882.1 | 96.43 | 4 | 4 | 0 | 0 | 0 | 0 | 0 | 1.8899E5 |  |  |  |  |  | 3 | 3 | 3 | 0 | 0 | 0 | 0 | 0 | N | 107397 | endoplasmic reticulum aminopeptidase 1-like protein [Crotalus horridus] |
| 153 | 642 | JAA97127.1 | 96.43 | 4 | 4 | 0 | 0 | 0 | 0 | 0 | 1.8899E5 |  |  |  |  |  | 3 | 3 | 3 | 0 | 0 | 0 | 0 | 0 | N | 107397 | endoplasmic reticulum aminopeptidase 1-like protein [Crotalus horridus] |
| 104 | 700 | CAA01524.1 | 95.36 | 8 | 8 | 0 | 0 | 0 | 0 | 0 | 3.8579E7 |  |  |  |  |  | 2 | 1 | 10 | 0 | 0 | 0 | 0 | 0 | Y | 25261 | ancrod-like protein [Calloselasma rhodostoma] |
| 141 | 452 | BAN89426.1 | 95.06 | 4 | 1 | 3 | 0 | 0 | 0 | 0 | 2.6454E5 | 1.1555E5 |  |  |  |  | 3 | 3 | 1 | 2 | 0 | 0 | 0 | 0 | N | 96239 | phosphodiesterase [Ovophis okinavensis] |
| 141 | 453 | BAN89425.1 | 95.06 | 4 | 1 | 2 | 0 | 0 | 0 | 0 | 2.6454E5 | 1.1555E5 |  |  |  |  | 3 | 3 | 1 | 2 | 0 | 0 | 0 | 0 | N | 100879 | phosphodiesterase [Ovophis okinavensis] |
| 141 | 465 | BAN82023.1 | 95.06 | 4 | 1 | 3 | 0 | 0 | 0 | 0 | 2.6454E5 | 1.1555E5 |  |  |  |  | 3 | 3 | 1 | 2 | 0 | 0 | 0 | 0 | N | 91910 | phosphodiesterase [Protobothrops flavoviridis] |
| 141 | 466 | BAN82024.1 | 95.06 | 4 | 1 | 3 | 0 | 0 | 0 | 0 | 2.6454E5 | 1.1555E5 |  |  |  |  | 3 | 3 | 1 | 2 | 0 | 0 | 0 | 0 | N | 92129 | phosphodiesterase [Protobothrops flavoviridis] |
| 141 | 467 | BAP39977.1 | 95.06 | 4 | 1 | 3 | 0 | 0 | 0 | 0 | 2.6454E5 | 1.1555E5 |  |  |  |  | 3 | 3 | 1 | 2 | 0 | 0 | 0 | 0 | N | 96588 | phosphodiesterase [Protobothrops flavoviridis] |
| 141 | 468 | BAN82021.1 | 95.06 | 4 | 1 | 3 | 0 | 0 | 0 | 0 | 2.6454E5 | 1.1555E5 |  |  |  |  | 3 | 3 | 1 | 2 | 0 | 0 | 0 | 0 | N | 96588 | phosphodiesterase [Protobothrops flavoviridis] |
| 141 | 469 | BAN82022.1 | 95.06 | 4 | 1 | 3 | 0 | 0 | 0 | 0 | 2.6454E5 | 1.1555E5 |  |  |  |  | 3 | 3 | 1 | 2 | 0 | 0 | 0 | 0 | N | 96808 | phosphodiesterase [Protobothrops flavoviridis] |
| 141 | 474 | JAV48422.1 | 95.06 | 4 | 1 | 3 | 0 | 0 | 0 | 0 | 2.6454E5 | 1.1555E5 |  |  |  |  | 3 | 3 | 1 | 2 | 0 | 0 | 0 | 0 | N | 91752 | Cadam10\_PDE-1 [Crotalus adamanteus] |
| 141 | 475 | AFJ49200.1 | 95.06 | 4 | 1 | 3 | 0 | 0 | 0 | 0 | 2.6454E5 | 1.1555E5 |  |  |  |  | 3 | 3 | 1 | 2 | 0 | 0 | 0 | 0 | N | 91752 | Phosphodiesterase [Crotalus adamanteus] |
| 141 | 476 | J3SBP3.1 | 95.06 | 4 | 1 | 3 | 0 | 0 | 0 | 0 | 2.6454E5 | 1.1555E5 |  |  |  |  | 3 | 3 | 1 | 2 | 0 | 0 | 0 | 0 | N | 91752 | RecName: Full=Venom phosphodiesterase 2; Short=PDE; Flags: Precursor |
| 141 | 477 | JAS05450.1 | 95.06 | 4 | 1 | 3 | 0 | 0 | 0 | 0 | 2.6454E5 | 1.1555E5 |  |  |  |  | 3 | 3 | 1 | 2 | 0 | 0 | 0 | 0 | N | 91767 | Phosphodiesterase [Sistrurus miliarius barbouri] |
| 141 | 478 | JAA96215.1 | 95.06 | 4 | 1 | 3 | 0 | 0 | 0 | 0 | 2.6454E5 | 1.1555E5 |  |  |  |  | 3 | 3 | 1 | 2 | 0 | 0 | 0 | 0 | N | 93754 | Ectonucleotide pyrophosphatase/phosphodiesterase family member 3-like protein [Crotalus horridus] |
| 141 | 479 | JAS04779.1 | 95.06 | 4 | 1 | 3 | 0 | 0 | 0 | 0 | 2.6454E5 | 1.1555E5 |  |  |  |  | 3 | 3 | 1 | 2 | 0 | 0 | 0 | 0 | N | 96387 | Phosphodiesterase [Crotalus horridus] |
| 141 | 480 | JAA94884.1 | 95.06 | 4 | 1 | 3 | 0 | 0 | 0 | 0 | 2.6454E5 | 1.1555E5 |  |  |  |  | 3 | 3 | 1 | 2 | 0 | 0 | 0 | 0 | N | 96387 | Phosphodiesterase [Crotalus horridus] |
| 141 | 481 | AEJ31980.1 | 95.06 | 4 | 1 | 3 | 0 | 0 | 0 | 0 | 2.6454E5 | 1.1555E5 |  |  |  |  | 3 | 3 | 1 | 2 | 0 | 0 | 0 | 0 | N | 96373 | phosphodiesterase 1 [Crotalus adamanteus] |
| 141 | 482 | JAI10403.1 | 95.06 | 4 | 1 | 3 | 0 | 0 | 0 | 0 | 2.6454E5 | 1.1555E5 |  |  |  |  | 3 | 3 | 1 | 2 | 0 | 0 | 0 | 0 | N | 96373 | phosphodiesterase [Crotalus adamanteus] |
| 141 | 483 | J3SEZ3.2 | 95.06 | 4 | 1 | 3 | 0 | 0 | 0 | 0 | 2.6454E5 | 1.1555E5 |  |  |  |  | 3 | 3 | 1 | 2 | 0 | 0 | 0 | 0 | N | 96373 | RecName: Full=Venom phosphodiesterase 1; Short=PDE; Flags: Precursor |
| 141 | 484 | JAS04861.1 | 95.06 | 4 | 1 | 3 | 0 | 0 | 0 | 0 | 2.6454E5 | 1.1555E5 |  |  |  |  | 3 | 3 | 1 | 2 | 0 | 0 | 0 | 0 | N | 96387 | Phosphodiesterase [Crotalus horridus] |
| 141 | 485 | JAG46127.1 | 95.06 | 4 | 1 | 3 | 0 | 0 | 0 | 0 | 2.6454E5 | 1.1555E5 |  |  |  |  | 3 | 3 | 1 | 2 | 0 | 0 | 0 | 0 | N | 96387 | Phosphodiesterase [Crotalus horridus] |
| 141 | 486 | JAG43649.1 | 95.06 | 4 | 1 | 3 | 0 | 0 | 0 | 0 | 2.6454E5 | 1.1555E5 |  |  |  |  | 3 | 3 | 1 | 2 | 0 | 0 | 0 | 0 | N | 96387 | Phosphodiesterase [Crotalus horridus] |
| 141 | 487 | JAS04699.1 | 95.06 | 4 | 1 | 3 | 0 | 0 | 0 | 0 | 2.6454E5 | 1.1555E5 |  |  |  |  | 3 | 3 | 1 | 2 | 0 | 0 | 0 | 0 | N | 96373 | phosphodiesterase [Crotalus adamanteus] |
| 141 | 488 | AFJ50876.1 | 95.06 | 4 | 1 | 3 | 0 | 0 | 0 | 0 | 2.6454E5 | 1.1555E5 |  |  |  |  | 3 | 3 | 1 | 2 | 0 | 0 | 0 | 0 | N | 99618 | Ectonucleotide pyrophosphatase/phosphodiesterase family member 3 [Crotalus adamanteus] |
| 168 | 776 | XP\_015672096.1 | 93.04 | 16 | 0 | 0 | 0 | 0 | 0 | 16 |  |  |  |  |  | 2.1973E5 | 2 | 2 | 0 | 0 | 0 | 0 | 0 | 2 | N | 15686 | cystatin-1 [Protobothrops mucrosquamatus] |
| 152 | 1227 | XP\_026561295.1 | 90.66 | 5 | 2 | 0 | 0 | 0 | 0 | 3 | 4.7087E5 |  |  |  |  | 3.3639E4 | 2 | 2 | 1 | 0 | 0 | 0 | 0 | 1 | N | 53982 | pantetheinase isoform X2 [Pseudonaja textilis] |
| 152 | 1228 | XP\_026561296.1 | 90.66 | 5 | 2 | 0 | 0 | 0 | 0 | 3 | 4.7087E5 |  |  |  |  | 3.3639E4 | 2 | 2 | 1 | 0 | 0 | 0 | 0 | 1 | N | 53982 | pantetheinase isoform X2 [Pseudonaja textilis] |
| 152 | 1229 | XP\_026561293.1 | 90.66 | 5 | 2 | 0 | 0 | 0 | 0 | 3 | 4.7087E5 |  |  |  |  | 3.3639E4 | 2 | 2 | 1 | 0 | 0 | 0 | 0 | 1 | N | 55550 | pantetheinase isoform X1 [Pseudonaja textilis] |
| 98 | 1279 | AHJ09589.1 | 89.75 | 8 | 7 | 0 | 0 | 0 | 8 | 8 | 1.1323E6 |  |  |  | 8.6449E6 | 7.9876E6 | 2 | 2 | 1 | 0 | 0 | 0 | 5 | 7 | Y | 15864 | phospholipase A2 [Trimeresurus cardamomensis] |
| 98 | 1280 | AHJ09585.1 | 89.75 | 8 | 7 | 0 | 0 | 0 | 8 | 8 | 1.1323E6 |  |  |  | 8.6449E6 | 7.9876E6 | 2 | 2 | 1 | 0 | 0 | 0 | 5 | 7 | Y | 15864 | phospholipase A2 [Trimeresurus cardamomensis] |
| 98 | 1281 | AHJ09594.1 | 89.75 | 8 | 7 | 0 | 0 | 0 | 8 | 8 | 1.1323E6 |  |  |  | 8.6449E6 | 7.9876E6 | 2 | 2 | 1 | 0 | 0 | 0 | 5 | 7 | Y | 15864 | phospholipase A2 [Trimeresurus cardamomensis] |
| 98 | 1282 | AHJ09590.1 | 89.75 | 8 | 7 | 0 | 0 | 0 | 8 | 8 | 1.1323E6 |  |  |  | 8.6449E6 | 7.9876E6 | 2 | 2 | 1 | 0 | 0 | 0 | 5 | 7 | Y | 15922 | phospholipase A2 [Trimeresurus cardamomensis] |
| 98 | 1283 | AHJ09520.1 | 89.75 | 8 | 7 | 0 | 0 | 0 | 8 | 8 | 1.1323E6 |  |  |  | 8.6449E6 | 7.9876E6 | 2 | 2 | 1 | 0 | 0 | 0 | 5 | 7 | Y | 15813 | phospholipase A2 [Trimeresurus venustus] |
| 98 | 1602 | AHJ09591.1 | 89.75 | 8 | 7 | 0 | 0 | 0 | 8 | 8 | 1.1323E6 |  |  |  | 8.6449E6 | 7.9876E6 | 2 | 2 | 1 | 0 | 0 | 0 | 5 | 7 | Y | 15804 | phospholipase A2 [Trimeresurus cardamomensis] |
| 98 | 1712 | AHJ09587.1 | 89.75 | 8 | 7 | 0 | 0 | 0 | 8 | 8 | 1.1323E6 |  |  |  | 8.6449E6 | 7.9876E6 | 2 | 2 | 1 | 0 | 0 | 0 | 5 | 7 | Y | 15070 | phospholipase A2 [Trimeresurus cardamomensis] |
| 98 | 1759 | AHJ09549.1 | 89.75 | 8 | 7 | 0 | 0 | 0 | 8 | 8 | 1.1323E6 |  |  |  | 8.6449E6 | 7.9876E6 | 2 | 2 | 1 | 0 | 0 | 0 | 5 | 7 | Y | 15336 | phospholipase A2 [Protobothrops mangshanensis] |
| 139 | 413 | XP\_026576301.1 | 88.93 | 2 | 2 | 0 | 0 | 0 | 0 | 0 | 1.4398E5 |  |  |  |  |  | 2 | 2 | 2 | 0 | 0 | 0 | 0 | 0 | N | 81304 | junction plakoglobin isoform X2 [Pseudonaja textilis] |
| 139 | 414 | XP\_026525656.1 | 88.93 | 2 | 2 | 0 | 0 | 0 | 0 | 0 | 1.4398E5 |  |  |  |  |  | 2 | 2 | 2 | 0 | 0 | 0 | 0 | 0 | N | 81304 | junction plakoglobin isoform X2 [Notechis scutatus] |
| 139 | 415 | XP\_026576299.1 | 88.93 | 2 | 2 | 0 | 0 | 0 | 0 | 0 | 1.4398E5 |  |  |  |  |  | 2 | 2 | 2 | 0 | 0 | 0 | 0 | 0 | N | 81375 | junction plakoglobin isoform X1 [Pseudonaja textilis] |
| 139 | 416 | XP\_026525655.1 | 88.93 | 2 | 2 | 0 | 0 | 0 | 0 | 0 | 1.4398E5 |  |  |  |  |  | 2 | 2 | 2 | 0 | 0 | 0 | 0 | 0 | N | 81375 | junction plakoglobin isoform X1 [Notechis scutatus] |
| 139 | 417 | XP\_026525654.1 | 88.93 | 2 | 2 | 0 | 0 | 0 | 0 | 0 | 1.4398E5 |  |  |  |  |  | 2 | 2 | 2 | 0 | 0 | 0 | 0 | 0 | N | 81375 | junction plakoglobin isoform X1 [Notechis scutatus] |
| 139 | 418 | XP\_026576298.1 | 88.93 | 2 | 2 | 0 | 0 | 0 | 0 | 0 | 1.4398E5 |  |  |  |  |  | 2 | 2 | 2 | 0 | 0 | 0 | 0 | 0 | N | 81375 | junction plakoglobin isoform X1 [Pseudonaja textilis] |
| 139 | 419 | JAG67632.1 | 88.93 | 2 | 2 | 0 | 0 | 0 | 0 | 0 | 1.4398E5 |  |  |  |  |  | 2 | 2 | 2 | 0 | 0 | 0 | 0 | 0 | N | 81861 | junction plakoglobin [Boiga irregularis] |
| 139 | 420 | JAV50234.1 | 88.93 | 2 | 2 | 0 | 0 | 0 | 0 | 0 | 1.4398E5 |  |  |  |  |  | 2 | 2 | 2 | 0 | 0 | 0 | 0 | 0 | N | 81358 | JUP protein [Agkistrodon contortrix contortrix] |
| 139 | 421 | XP\_034271617.1 | 88.93 | 2 | 2 | 0 | 0 | 0 | 0 | 0 | 1.4398E5 |  |  |  |  |  | 2 | 2 | 2 | 0 | 0 | 0 | 0 | 0 | N | 81870 | junction plakoglobin isoform X2 [Pantherophis guttatus] |
| 139 | 422 | JAG44306.1 | 88.93 | 2 | 2 | 0 | 0 | 0 | 0 | 0 | 1.4398E5 |  |  |  |  |  | 2 | 2 | 2 | 0 | 0 | 0 | 0 | 0 | N | 81903 | junction plakoglobin [Crotalus horridus] |
| 139 | 423 | XP\_034271615.1 | 88.93 | 2 | 2 | 0 | 0 | 0 | 0 | 0 | 1.4398E5 |  |  |  |  |  | 2 | 2 | 2 | 0 | 0 | 0 | 0 | 0 | N | 81941 | junction plakoglobin isoform X1 [Pantherophis guttatus] |
| 139 | 424 | XP\_013919280.1 | 88.93 | 2 | 2 | 0 | 0 | 0 | 0 | 0 | 1.4398E5 |  |  |  |  |  | 2 | 2 | 2 | 0 | 0 | 0 | 0 | 0 | N | 81965 | PREDICTED: junction plakoglobin [Thamnophis sirtalis] |
| 139 | 425 | XP\_034271618.1 | 88.93 | 2 | 2 | 0 | 0 | 0 | 0 | 0 | 1.4398E5 |  |  |  |  |  | 2 | 2 | 2 | 0 | 0 | 0 | 0 | 0 | N | 81941 | junction plakoglobin isoform X1 [Pantherophis guttatus] |
| 139 | 426 | JAA96758.1 | 88.93 | 2 | 2 | 0 | 0 | 0 | 0 | 0 | 1.4398E5 |  |  |  |  |  | 2 | 2 | 2 | 0 | 0 | 0 | 0 | 0 | N | 81903 | junction plakoglobin [Crotalus horridus] |
| 139 | 427 | JAG46538.1 | 88.93 | 2 | 2 | 0 | 0 | 0 | 0 | 0 | 1.4398E5 |  |  |  |  |  | 2 | 2 | 2 | 0 | 0 | 0 | 0 | 0 | N | 81903 | junction plakoglobin [Crotalus horridus] |
| 139 | 428 | XP\_034271616.1 | 88.93 | 2 | 2 | 0 | 0 | 0 | 0 | 0 | 1.4398E5 |  |  |  |  |  | 2 | 2 | 2 | 0 | 0 | 0 | 0 | 0 | N | 81941 | junction plakoglobin isoform X1 [Pantherophis guttatus] |
| 139 | 429 | XP\_025022318.1 | 88.93 | 2 | 2 | 0 | 0 | 0 | 0 | 0 | 1.4398E5 |  |  |  |  |  | 2 | 2 | 2 | 0 | 0 | 0 | 0 | 0 | N | 81985 | junction plakoglobin isoform X2 [Python bivittatus] |
| 139 | 430 | XP\_025022317.1 | 88.93 | 2 | 2 | 0 | 0 | 0 | 0 | 0 | 1.4398E5 |  |  |  |  |  | 2 | 2 | 2 | 0 | 0 | 0 | 0 | 0 | N | 82056 | junction plakoglobin isoform X1 [Python bivittatus] |
| 139 | 431 | XP\_007428111.1 | 88.93 | 2 | 2 | 0 | 0 | 0 | 0 | 0 | 1.4398E5 |  |  |  |  |  | 2 | 2 | 2 | 0 | 0 | 0 | 0 | 0 | N | 82056 | junction plakoglobin isoform X1 [Python bivittatus] |
| 139 | 432 | ETE68218.1 | 88.93 | 2 | 2 | 0 | 0 | 0 | 0 | 0 | 1.4398E5 |  |  |  |  |  | 2 | 2 | 2 | 0 | 0 | 0 | 0 | 0 | N | 83009 | Junction plakoglobin [Ophiophagus hannah] |
| 139 | 433 | XP\_032091066.1 | 88.93 | 2 | 2 | 0 | 0 | 0 | 0 | 0 | 1.4398E5 |  |  |  |  |  | 2 | 2 | 2 | 0 | 0 | 0 | 0 | 0 | N | 86365 | junction plakoglobin [Thamnophis elegans] |
| 139 | 434 | XP\_032091065.1 | 88.93 | 2 | 2 | 0 | 0 | 0 | 0 | 0 | 1.4398E5 |  |  |  |  |  | 2 | 2 | 2 | 0 | 0 | 0 | 0 | 0 | N | 86365 | junction plakoglobin [Thamnophis elegans] |
| 139 | 435 | LAB43566.1 | 88.93 | 2 | 2 | 0 | 0 | 0 | 0 | 0 | 1.4398E5 |  |  |  |  |  | 2 | 2 | 2 | 0 | 0 | 0 | 0 | 0 | N | 83463 | hypothetical protein, partial [Micrurus spixii] |
| 139 | 444 | XP\_015687775.1 | 88.93 | 2 | 2 | 0 | 0 | 0 | 0 | 0 | 1.4398E5 |  |  |  |  |  | 2 | 2 | 2 | 0 | 0 | 0 | 0 | 0 | N | 81935 | junction plakoglobin [Protobothrops mucrosquamatus] |
| 139 | 445 | XP\_015687778.1 | 88.93 | 2 | 2 | 0 | 0 | 0 | 0 | 0 | 1.4398E5 |  |  |  |  |  | 2 | 2 | 2 | 0 | 0 | 0 | 0 | 0 | N | 81935 | junction plakoglobin [Protobothrops mucrosquamatus] |
| 139 | 446 | XP\_015687777.1 | 88.93 | 2 | 2 | 0 | 0 | 0 | 0 | 0 | 1.4398E5 |  |  |  |  |  | 2 | 2 | 2 | 0 | 0 | 0 | 0 | 0 | N | 81935 | junction plakoglobin [Protobothrops mucrosquamatus] |
| 139 | 447 | XP\_015687776.1 | 88.93 | 2 | 2 | 0 | 0 | 0 | 0 | 0 | 1.4398E5 |  |  |  |  |  | 2 | 2 | 2 | 0 | 0 | 0 | 0 | 0 | N | 81935 | junction plakoglobin [Protobothrops mucrosquamatus] |
| 139 | 448 | JAI12884.1 | 88.93 | 2 | 2 | 0 | 0 | 0 | 0 | 0 | 1.4398E5 |  |  |  |  |  | 2 | 2 | 2 | 0 | 0 | 0 | 0 | 0 | N | 81917 | junction plakoglobin [Crotalus adamanteus] |
| 156 | 1202 | BAP39929.1 | 86.88 | 8 | 0 | 0 | 0 | 0 | 0 | 8 |  |  |  |  |  | 2.5305E4 | 1 | 1 | 0 | 0 | 0 | 0 | 0 | 1 | N | 21332 | C-type lectin B subunit, partial [Protobothrops elegans] |
| 156 | 1495 | XP\_015685588.1 | 86.88 | 9 | 0 | 0 | 0 | 0 | 0 | 9 |  |  |  |  |  | 2.5305E4 | 1 | 1 | 0 | 0 | 0 | 0 | 0 | 1 | N | 18348 | snaclec jerdonibitin subunit alpha [Protobothrops mucrosquamatus] |
| 154 | 898 | JAC94969.1 | 85.05 | 5 | 5 | 0 | 0 | 0 | 0 | 0 | 2.3383E5 |  |  |  |  |  | 2 | 2 | 3 | 0 | 0 | 0 | 0 | 0 | N | 43342 | Cathepsin D [Opheodrys aestivus] |
| 154 | 906 | ETE66900.1 | 85.05 | 7 | 7 | 0 | 0 | 0 | 0 | 0 | 2.3383E5 |  |  |  |  |  | 2 | 2 | 3 | 0 | 0 | 0 | 0 | 0 | N | 30492 | Cathepsin D, partial [Ophiophagus hannah] |
| 154 | 963 | JAB54523.1 | 85.05 | 5 | 5 | 0 | 0 | 0 | 0 | 0 | 2.3383E5 |  |  |  |  |  | 2 | 2 | 3 | 0 | 0 | 0 | 0 | 0 | N | 43244 | Cathepsin D [Micrurus fulvius] |
| 154 | 964 | JAI10168.1 | 85.05 | 5 | 5 | 0 | 0 | 0 | 0 | 0 | 2.3383E5 |  |  |  |  |  | 2 | 2 | 3 | 0 | 0 | 0 | 0 | 0 | N | 43375 | Cathepsin D [Micrurus fulvius] |
| 154 | 978 | JAC96615.1 | 85.05 | 5 | 5 | 0 | 0 | 0 | 0 | 0 | 2.3383E5 |  |  |  |  |  | 2 | 2 | 3 | 0 | 0 | 0 | 0 | 0 | N | 43777 | Cathepsin D [Echis coloratus] |
| 154 | 1146 | LAA50656.1 | 85.05 | 10 | 10 | 0 | 0 | 0 | 0 | 0 | 2.3383E5 |  |  |  |  |  | 2 | 2 | 3 | 0 | 0 | 0 | 0 | 0 | N | 22136 | hypothetical protein, partial [Micrurus corallinus] |
| 164 | 848 | JAG46182.1 | 78.69 | 6 | 6 | 0 | 0 | 0 | 0 | 0 | 1.0312E5 |  |  |  |  |  | 2 | 2 | 2 | 0 | 0 | 0 | 0 | 0 | N | 44172 | Glia-derived nexin-like protein [Crotalus horridus] |
| 164 | 849 | JAG43702.1 | 78.69 | 6 | 6 | 0 | 0 | 0 | 0 | 0 | 1.0312E5 |  |  |  |  |  | 2 | 2 | 2 | 0 | 0 | 0 | 0 | 0 | N | 44172 | Glia-derived nexin-like protein [Crotalus horridus] |
| 164 | 850 | JAA96331.1 | 78.69 | 6 | 6 | 0 | 0 | 0 | 0 | 0 | 1.0312E5 |  |  |  |  |  | 2 | 2 | 2 | 0 | 0 | 0 | 0 | 0 | N | 44172 | Glia-derived nexin-like protein [Crotalus horridus] |
| 164 | 854 | XP\_015674504.1 | 78.69 | 6 | 6 | 0 | 0 | 0 | 0 | 0 | 1.0312E5 |  |  |  |  |  | 2 | 2 | 2 | 0 | 0 | 0 | 0 | 0 | N | 44068 | glia-derived nexin [Protobothrops mucrosquamatus] |
| 164 | 855 | XP\_015674505.1 | 78.69 | 6 | 6 | 0 | 0 | 0 | 0 | 0 | 1.0312E5 |  |  |  |  |  | 2 | 2 | 2 | 0 | 0 | 0 | 0 | 0 | N | 44068 | glia-derived nexin [Protobothrops mucrosquamatus] |
| 164 | 856 | JAV49839.1 | 78.69 | 6 | 6 | 0 | 0 | 0 | 0 | 0 | 1.0312E5 |  |  |  |  |  | 2 | 2 | 2 | 0 | 0 | 0 | 0 | 0 | N | 44202 | Glia-derived nexin-like protein [Agkistrodon contortrix contortrix] |
| 133 | 750 | BAP39986.1 | 77.71 | 4 | 4 | 0 | 0 | 0 | 0 | 4 | 4.5894E5 |  |  |  |  | 1.8231E5 | 2 | 2 | 2 | 0 | 0 | 0 | 0 | 3 | N | 60400 | hyaluronidase, partial [Protobothrops flavoviridis] |
| 133 | 751 | BAN89379.1 | 77.71 | 4 | 4 | 0 | 0 | 0 | 0 | 4 | 4.5894E5 |  |  |  |  | 1.8231E5 | 2 | 2 | 2 | 0 | 0 | 0 | 0 | 3 | N | 61065 | hyaluronidase, partial [Protobothrops flavoviridis] |
| 133 | 752 | JAV01892.1 | 77.71 | 5 | 5 | 0 | 0 | 0 | 0 | 5 | 4.5894E5 |  |  |  |  | 1.8231E5 | 2 | 2 | 2 | 0 | 0 | 0 | 0 | 3 | N | 52550 | BATXHYAL1 [Bothrops atrox] |
| 133 | 753 | ATU85542.1 | 77.71 | 5 | 5 | 0 | 0 | 0 | 0 | 5 | 4.5894E5 |  |  |  |  | 1.8231E5 | 2 | 2 | 2 | 0 | 0 | 0 | 0 | 3 | N | 52550 | Hyaluronidase BmooHyal-1, partial [Bothrops moojeni] |
| 133 | 842 | BAN89414.1 | 77.71 | 5 | 5 | 0 | 0 | 0 | 0 | 5 | 4.5894E5 |  |  |  |  | 1.8231E5 | 2 | 2 | 2 | 0 | 0 | 0 | 0 | 3 | N | 49636 | hyaluronidase [Ovophis okinavensis] |
| 133 | 843 | BAN89413.1 | 77.71 | 5 | 5 | 0 | 0 | 0 | 0 | 5 | 4.5894E5 |  |  |  |  | 1.8231E5 | 2 | 2 | 2 | 0 | 0 | 0 | 0 | 3 | N | 52464 | hyaluronidase [Ovophis okinavensis] |
| 38 | 1977 | JAS03131.1 | 77.07 | 4 | 4 | 4 | 4 | 4 | 4 | 4 | 2.0919E7 | 4.8904E5 | 3.2665E9 | 9.9907E7 | 9.4069E7 | 1.5347E6 | 1 | 1 | 10 | 1 | 24 | 4 | 17 | 3 | Y | 28382 | Snake Venom Serine Proteinase [Phalotris mertensi] |
| 38 | 1978 | ABN72541.1 | 77.07 | 4 | 4 | 4 | 4 | 4 | 4 | 4 | 2.0919E7 | 4.8904E5 | 3.2665E9 | 9.9907E7 | 9.4069E7 | 1.5347E6 | 1 | 1 | 10 | 1 | 24 | 4 | 17 | 3 | Y | 31137 | putative serine protease, partial [Naja atra] |
| 38 | 1979 | A8QL53.1 | 77.07 | 4 | 4 | 4 | 4 | 4 | 4 | 4 | 2.0919E7 | 4.8904E5 | 3.2665E9 | 9.9907E7 | 9.4069E7 | 1.5347E6 | 1 | 1 | 10 | 1 | 24 | 4 | 17 | 3 | Y | 31137 | RecName: Full=Snake venom serine protease NaSP; Short=SVSP; Flags: Precursor |
| 38 | 1980 | ABN72545.1 | 77.07 | 4 | 4 | 4 | 4 | 4 | 4 | 4 | 2.0919E7 | 4.8904E5 | 3.2665E9 | 9.9907E7 | 9.4069E7 | 1.5347E6 | 1 | 1 | 10 | 1 | 24 | 4 | 17 | 3 | Y | 31010 | putative serine protease, partial [Bungarus multicinctus] |
| 38 | 1981 | A8QL57.1 | 77.07 | 4 | 4 | 4 | 4 | 4 | 4 | 4 | 2.0919E7 | 4.8904E5 | 3.2665E9 | 9.9907E7 | 9.4069E7 | 1.5347E6 | 1 | 1 | 10 | 1 | 24 | 4 | 17 | 3 | Y | 31010 | RecName: Full=Snake venom serine protease BmSP; Short=SVSP; Flags: Precursor |
| 167 | 1336 | AFJ50447.1 | 76.77 | 3 | 3 | 0 | 0 | 0 | 0 | 0 | 1.7651E5 |  |  |  |  |  | 2 | 2 | 2 | 0 | 0 | 0 | 0 | 0 | N | 44744 | Lysosome-associated membrane glycoprotein 1-like [Crotalus adamanteus] |
| 167 | 1964 | XP\_007423023.1 | 76.77 | 3 | 3 | 0 | 0 | 0 | 0 | 0 | 1.7651E5 |  |  |  |  |  | 2 | 2 | 2 | 0 | 0 | 0 | 0 | 0 | N | 44199 | lysosome-associated membrane glycoprotein 1 [Python bivittatus] |
| 138 | 1926 | D2YW39.2 | 74.99 | 8 | 0 | 0 | 0 | 0 | 0 | 8 |  |  |  |  |  | 4.8598E6 | 1 | 1 | 0 | 0 | 0 | 0 | 0 | 4 | N | 15725 | RecName: Full=Snaclec rhodocetin subunit gamma |
| 138 | 2115 | XP\_015686565.1 | 74.99 | 14 | 0 | 0 | 0 | 0 | 0 | 14 |  |  |  |  |  | 4.8598E6 | 1 | 1 | 0 | 0 | 0 | 0 | 0 | 4 | N | 9652 | snaclec stejaggregin-A subunit alpha [Protobothrops mucrosquamatus] |
| 138 | 2149 | BAN81991.1 | 74.99 | 7 | 0 | 0 | 0 | 0 | 0 | 7 |  |  |  |  |  | 4.8598E6 | 1 | 1 | 0 | 0 | 0 | 0 | 0 | 4 | N | 17364 | C-type lectin alpha subunit, partial [Protobothrops flavoviridis] |
| 138 | 2150 | AAQ15166.1 | 74.99 | 7 | 0 | 0 | 0 | 0 | 0 | 7 |  |  |  |  |  | 4.8598E6 | 1 | 1 | 0 | 0 | 0 | 0 | 0 | 4 | N | 18055 | stejaggregin-A alpha chain [Trimeresurus stejnegeri] |
| 138 | 2151 | Q71RQ1.1 | 74.99 | 7 | 0 | 0 | 0 | 0 | 0 | 7 |  |  |  |  |  | 4.8598E6 | 1 | 1 | 0 | 0 | 0 | 0 | 0 | 4 | N | 18055 | RecName: Full=Snaclec stejaggregin-A subunit alpha; Flags: Precursor |
| 188 | 1433 | XP\_032069561.1 | 74.78 | 3 | 0 | 0 | 0 | 0 | 3 | 0 |  |  |  |  | 5.6006E4 |  | 1 | 1 | 0 | 0 | 0 | 0 | 1 | 0 | N | 62259 | cochlin [Thamnophis elegans] |
| 188 | 1480 | XP\_013923615.1 | 74.78 | 3 | 0 | 0 | 0 | 0 | 3 | 0 |  |  |  |  | 5.6006E4 |  | 1 | 1 | 0 | 0 | 0 | 0 | 1 | 0 | N | 59675 | PREDICTED: cochlin [Thamnophis sirtalis] |
| 188 | 1481 | XP\_026520312.1 | 74.78 | 3 | 0 | 0 | 0 | 0 | 3 | 0 |  |  |  |  | 5.6006E4 |  | 1 | 1 | 0 | 0 | 0 | 0 | 1 | 0 | N | 59883 | cochlin [Notechis scutatus] |
| 188 | 1738 | XP\_007429085.1 | 74.78 | 3 | 0 | 0 | 0 | 0 | 3 | 0 |  |  |  |  | 5.6006E4 |  | 1 | 1 | 0 | 0 | 0 | 0 | 1 | 0 | N | 59718 | cochlin [Python bivittatus] |
| 188 | 1814 | LAA86986.1 | 74.78 | 7 | 0 | 0 | 0 | 0 | 7 | 0 |  |  |  |  | 5.6006E4 |  | 1 | 1 | 0 | 0 | 0 | 0 | 1 | 0 | N | 28508 | hypothetical protein, partial [Micrurus lemniscatus lemniscatus] |
| 188 | 1815 | LAB57337.1 | 74.78 | 6 | 0 | 0 | 0 | 0 | 6 | 0 |  |  |  |  | 5.6006E4 |  | 1 | 1 | 0 | 0 | 0 | 0 | 1 | 0 | N | 30451 | hypothetical protein, partial [Micrurus surinamensis] |
| 188 | 1816 | XP\_026566518.1 | 74.78 | 3 | 0 | 0 | 0 | 0 | 3 | 0 |  |  |  |  | 5.6006E4 |  | 1 | 1 | 0 | 0 | 0 | 0 | 1 | 0 | N | 59709 | cochlin [Pseudonaja textilis] |
| 62 | 1185 | BAO23490.1 | 73.75 | 3 | 3 | 0 | 2 | 2 | 2 | 0 | 1.3298E6 |  | 3.4695E7 | 2.0569E5 | 2.7273E6 |  | 2 | 1 | 5 | 0 | 6 | 1 | 3 | 0 | Y | 67765 | metalloproteinase [Deinagkistrodon acutus] |
| 169 | 1514 | ACN93671.1 | 67.61 | 7 | 0 | 0 | 0 | 0 | 7 | 0 |  |  |  |  | 1.2008E5 |  | 1 | 1 | 0 | 0 | 0 | 0 | 1 | 0 | N | 26305 | opharin precursor [Ophiophagus hannah] |
| 169 | 1515 | ACH73168.1 | 67.61 | 7 | 0 | 0 | 0 | 0 | 7 | 0 |  |  |  |  | 1.2008E5 |  | 1 | 1 | 0 | 0 | 0 | 0 | 1 | 0 | N | 26216 | kaouthin-2 precursor [Naja kaouthia] |
| 169 | 1516 | AAP20603.1 | 67.61 | 7 | 0 | 0 | 0 | 0 | 7 | 0 |  |  |  |  | 1.2008E5 |  | 1 | 1 | 0 | 0 | 0 | 0 | 1 | 0 | N | 26246 | cysteine-rich venom protein [Naja atra] |
| 160 | 580 | BAN82159.1 | 64.57 | 1 | 1 | 0 | 0 | 0 | 0 | 0 | 0 |  |  |  |  |  | 1 | 1 | 1 | 0 | 0 | 0 | 0 | 0 | N | 109541 | aminopeptidase A [Ovophis okinavensis] |
| 144 | 1552 | Q2YHJ2.1 | 59.65 | 6 | 0 | 0 | 0 | 0 | 6 | 6 |  |  |  |  | 3.5993E5 | 7.9455E5 | 1 | 1 | 0 | 0 | 0 | 0 | 1 | 2 | N | 15742 | RecName: Full=Basic phospholipase A2 Tbo-G6D49; Short=svPLA2; AltName: Full=Phosphatidylcholine 2-acylhydrolase; Flags: Precursor |
| 144 | 1553 | Q2YHJ7.1 | 59.65 | 6 | 0 | 0 | 0 | 0 | 6 | 6 |  |  |  |  | 3.5993E5 | 7.9455E5 | 1 | 1 | 0 | 0 | 0 | 0 | 1 | 2 | N | 15699 | RecName: Full=Basic phospholipase A2 Tpu-G6D49; Short=svPLA2; AltName: Full=Phosphatidylcholine 2-acylhydrolase; Flags: Precursor |
| 144 | 1554 | AAR14173.1 | 59.65 | 6 | 0 | 0 | 0 | 0 | 6 | 6 |  |  |  |  | 3.5993E5 | 7.9455E5 | 1 | 1 | 0 | 0 | 0 | 0 | 1 | 2 | N | 15742 | G6D49 phospholipase A2 [Trimeresurus borneensis] |
| 144 | 1555 | AAR14167.1 | 59.65 | 6 | 0 | 0 | 0 | 0 | 6 | 6 |  |  |  |  | 3.5993E5 | 7.9455E5 | 1 | 1 | 0 | 0 | 0 | 0 | 1 | 2 | N | 15699 | G6D49 phospholipase A2 [Trimeresurus puniceus] |
| 120 | 1315 | LAA93895.1 | 58.73 | 5 | 5 | 5 | 5 | 5 | 0 | 5 | 1.2127E6 | 6.8999E5 | 1.4775E7 | 4.3508E6 |  | 3.242E5 | 1 | 1 | 2 | 1 | 2 | 1 | 0 | 2 | N | 19716 | hypothetical protein, partial [Micrurus lemniscatus lemniscatus] |
| 121 | 1411 | XP\_026564825.1 | 58.54 | 1 | 0 | 0 | 1 | 0 | 1 | 0 |  |  | 9.3953E5 |  | 3.0398E7 |  | 1 | 1 | 0 | 0 | 1 | 0 | 6 | 0 | Y | 106234 | A disintegrin and metalloproteinase with thrombospondin motifs 15 isoform X2 [Pseudonaja textilis] |
| 121 | 1423 | XP\_026534386.1 | 58.54 | 1 | 0 | 0 | 1 | 0 | 1 | 0 |  |  | 9.3953E5 |  | 3.0398E7 |  | 1 | 1 | 0 | 0 | 1 | 0 | 6 | 0 | Y | 106048 | A disintegrin and metalloproteinase with thrombospondin motifs 15 isoform X2 [Notechis scutatus] |
| 121 | 1424 | XP\_026564824.1 | 58.54 | 1 | 0 | 0 | 1 | 0 | 1 | 0 |  |  | 9.3953E5 |  | 3.0398E7 |  | 1 | 1 | 0 | 0 | 1 | 0 | 6 | 0 | Y | 106430 | A disintegrin and metalloproteinase with thrombospondin motifs 15 isoform X1 [Pseudonaja textilis] |
| 121 | 1425 | XP\_007438168.1 | 58.54 | 1 | 0 | 0 | 1 | 0 | 1 | 0 |  |  | 9.3953E5 |  | 3.0398E7 |  | 1 | 1 | 0 | 0 | 1 | 0 | 6 | 0 | Y | 98239 | A disintegrin and metalloproteinase with thrombospondin motifs 15 [Python bivittatus] |
| 121 | 1432 | XP\_026534385.1 | 58.54 | 1 | 0 | 0 | 1 | 0 | 1 | 0 |  |  | 9.3953E5 |  | 3.0398E7 |  | 1 | 1 | 0 | 0 | 1 | 0 | 6 | 0 | Y | 106245 | A disintegrin and metalloproteinase with thrombospondin motifs 15 isoform X1 [Notechis scutatus] |
| 121 | 1518 | XP\_034277870.1 | 58.54 | 1 | 0 | 0 | 1 | 0 | 1 | 0 |  |  | 9.3953E5 |  | 3.0398E7 |  | 1 | 1 | 0 | 0 | 1 | 0 | 6 | 0 | Y | 105888 | A disintegrin and metalloproteinase with thrombospondin motifs 15 [Pantherophis guttatus] |
| 121 | 1537 | XP\_015673523.1 | 58.54 | 1 | 0 | 0 | 1 | 0 | 1 | 0 |  |  | 9.3953E5 |  | 3.0398E7 |  | 1 | 1 | 0 | 0 | 1 | 0 | 6 | 0 | Y | 105960 | A disintegrin and metalloproteinase with thrombospondin motifs 15 [Protobothrops mucrosquamatus] |
| 121 | 1908 | XP\_032084777.1 | 58.54 | 4 | 0 | 0 | 4 | 0 | 4 | 0 |  |  | 9.3953E5 |  | 3.0398E7 |  | 1 | 1 | 0 | 0 | 1 | 0 | 6 | 0 | Y | 31212 | A disintegrin and metalloproteinase with thrombospondin motifs 15-like [Thamnophis elegans] |
| 121 | 2177 | LAA53077.1 | 58.54 | 9 | 0 | 0 | 9 | 0 | 9 | 0 |  |  | 9.3953E5 |  | 3.0398E7 |  | 1 | 1 | 0 | 0 | 1 | 0 | 6 | 0 | Y | 12463 | hypothetical protein, partial [Micrurus corallinus] |
| 176 | 1397 | QBF53415.1 | 55.16 | 2 | 2 | 0 | 0 | 0 | 0 | 0 | 1.382E5 |  |  |  |  |  | 1 | 1 | 1 | 0 | 0 | 0 | 0 | 0 | N | 69218 | metalloproteinase of class P-III MPIII-2 [Vipera ammodytes ammodytes] |
| 176 | 1768 | QHR82833.1 | 55.16 | 4 | 4 | 0 | 0 | 0 | 0 | 0 | 1.382E5 |  |  |  |  |  | 1 | 1 | 1 | 0 | 0 | 0 | 0 | 0 | N | 28232 | snake venom metalloprotease 2, partial [Vipera anatolica senliki] |
| 187 | 1130 | BAG82600.1 | 53.64 | 1 | 1 | 0 | 0 | 0 | 0 | 0 | 4.7764E4 |  |  |  |  |  | 1 | 1 | 1 | 0 | 0 | 0 | 0 | 0 | N | 106105 | aminopeptidase N, partial [Gloydius brevicaudus] |
| 187 | 1967 | XP\_015745309.1 | 53.64 | 1 | 1 | 0 | 0 | 0 | 0 | 0 | 4.7764E4 |  |  |  |  |  | 1 | 1 | 1 | 0 | 0 | 0 | 0 | 0 | N | 112663 | aminopeptidase N [Python bivittatus] |
| 170 | 2202 | AAK09384.1 | 53.25 | 9 | 0 | 0 | 0 | 0 | 0 | 9 |  |  |  |  |  | 2.3395E5 | 1 | 1 | 0 | 0 | 0 | 0 | 0 | 2 | N | 12003 | thioredoxin protein [Ophiophagus hannah] |
| 170 | 2203 | Q98TX1.3 | 53.25 | 9 | 0 | 0 | 0 | 0 | 0 | 9 |  |  |  |  |  | 2.3395E5 | 1 | 1 | 0 | 0 | 0 | 0 | 0 | 2 | N | 12003 | RecName: Full=Thioredoxin; Short=Trx |
| 170 | 2206 | XP\_007424609.1 | 53.25 | 5 | 0 | 0 | 0 | 0 | 0 | 5 |  |  |  |  |  | 2.3395E5 | 1 | 1 | 0 | 0 | 0 | 0 | 0 | 2 | N | 17514 | thioredoxin [Python bivittatus] |
| 170 | 2207 | XP\_034266461.1 | 53.25 | 5 | 0 | 0 | 0 | 0 | 0 | 5 |  |  |  |  |  | 2.3395E5 | 1 | 1 | 0 | 0 | 0 | 0 | 0 | 2 | N | 21200 | thioredoxin isoform X1 [Pantherophis guttatus] |
| 170 | 2208 | XP\_015674703.1 | 53.25 | 4 | 0 | 0 | 0 | 0 | 0 | 4 |  |  |  |  |  | 2.3395E5 | 1 | 1 | 0 | 0 | 0 | 0 | 0 | 2 | N | 21592 | thioredoxin [Protobothrops mucrosquamatus] |
| 170 | 2209 | XP\_026569170.1 | 53.25 | 4 | 0 | 0 | 0 | 0 | 0 | 4 |  |  |  |  |  | 2.3395E5 | 1 | 1 | 0 | 0 | 0 | 0 | 0 | 2 | N | 23415 | thioredoxin domain-containing protein 2-like [Pseudonaja textilis] |
| 170 | 2210 | XP\_026528084.1 | 53.25 | 4 | 0 | 0 | 0 | 0 | 0 | 4 |  |  |  |  |  | 2.3395E5 | 1 | 1 | 0 | 0 | 0 | 0 | 0 | 2 | N | 23631 | thioredoxin domain-containing protein 2-like [Notechis scutatus] |
| 194 | 1995 | 3K7L | 51.48 | 3 | 3 | 0 | 0 | 0 | 0 | 0 | 0 |  |  |  |  |  | 1 | 1 | 1 | 0 | 0 | 0 | 0 | 0 | N | 47685 | Chain A, Structures Of Two Elapid Snake Venom Metalloproteases With Distinct Activities Highlight The Disulfide Patterns In The D Domain Of Adamalysin Family Proteins |
| 194 | 1996 | ACN50006.1 | 51.48 | 2 | 2 | 0 | 0 | 0 | 0 | 0 | 0 |  |  |  |  |  | 1 | 1 | 1 | 0 | 0 | 0 | 0 | 0 | N | 69181 | atragin precursor, partial [Naja atra] |
| 194 | 1997 | D3TTC2.1 | 51.48 | 2 | 2 | 0 | 0 | 0 | 0 | 0 | 0 |  |  |  |  |  | 1 | 1 | 1 | 0 | 0 | 0 | 0 | 0 | N | 69181 | RecName: Full=Zinc metalloproteinase-disintegrin-like atragin; AltName: Full=Snake venom metalloproteinase; Short=SVMP; Flags: Precursor |
| 189 | 1630 | XP\_013927550.1 | 50.36 | 6 | 6 | 0 | 0 | 0 | 0 | 0 | 2.2215E5 |  |  |  |  |  | 1 | 1 | 1 | 0 | 0 | 0 | 0 | 0 | N | 30267 | PREDICTED: prostasin-like [Thamnophis sirtalis] |
| 189 | 1903 | XP\_032093652.1 | 50.36 | 5 | 5 | 0 | 0 | 0 | 0 | 0 | 2.2215E5 |  |  |  |  |  | 1 | 1 | 1 | 0 | 0 | 0 | 0 | 0 | N | 38272 | prostasin [Thamnophis elegans] |
| 189 | 2197 | XP\_013908018.1 | 50.36 | 11 | 11 | 0 | 0 | 0 | 0 | 0 | 2.2215E5 |  |  |  |  |  | 1 | 1 | 1 | 0 | 0 | 0 | 0 | 0 | N | 17847 | PREDICTED: prostasin-like [Thamnophis sirtalis] |
| 189 | 2198 | BAN82108.1 | 50.36 | 6 | 6 | 0 | 0 | 0 | 0 | 0 | 2.2215E5 |  |  |  |  |  | 1 | 1 | 1 | 0 | 0 | 0 | 0 | 0 | N | 33471 | hypothetical protein [Protobothrops flavoviridis] |
| 189 | 2199 | XP\_034275696.1 | 50.36 | 5 | 5 | 0 | 0 | 0 | 0 | 0 | 2.2215E5 |  |  |  |  |  | 1 | 1 | 1 | 0 | 0 | 0 | 0 | 0 | N | 38122 | prostasin [Pantherophis guttatus] |
| 196 | 2260 | AAZ53176.1 | 49.56 | 9 | 0 | 0 | 0 | 0 | 0 | 9 |  |  |  |  |  | 6.5019E4 | 1 | 1 | 0 | 0 | 0 | 0 | 0 | 1 | N | 15586 | acidic phospholipase A2 [Daboia siamensis] |
| 196 | 2261 | A8CG78.1 | 49.56 | 9 | 0 | 0 | 0 | 0 | 0 | 9 |  |  |  |  |  | 6.5019E4 | 1 | 1 | 0 | 0 | 0 | 0 | 0 | 1 | N | 15586 | RecName: Full=Acidic phospholipase A2 DsM-a2/DsM-a2'; Short=svPLA2; AltName: Full=Phosphatidylcholine 2-acylhydrolase; Flags: Precursor |
| 196 | 2262 | AAZ53181.1 | 49.56 | 9 | 0 | 0 | 0 | 0 | 0 | 9 |  |  |  |  |  | 6.5019E4 | 1 | 1 | 0 | 0 | 0 | 0 | 0 | 1 | N | 15586 | acidic phospholipase A2 [Daboia russelii] |
| 196 | 2263 | AAP13805.1 | 49.56 | 9 | 0 | 0 | 0 | 0 | 0 | 9 |  |  |  |  |  | 6.5019E4 | 1 | 1 | 0 | 0 | 0 | 0 | 0 | 1 | N | 15401 | phospholipase A2-I [Daboia siamensis] |
| 196 | 2264 | A8CG87.1 | 49.56 | 9 | 0 | 0 | 0 | 0 | 0 | 9 |  |  |  |  |  | 6.5019E4 | 1 | 1 | 0 | 0 | 0 | 0 | 0 | 1 | N | 15586 | RecName: Full=Acidic phospholipase A2 Drk-a2; Short=svPLA2; AltName: Full=Phosphatidylcholine 2-acylhydrolase; Flags: Precursor |
| 130 | 1796 | P86541.2 | 49.23 | 13 | 13 | 0 | 0 | 0 | 13 | 0 | 6.7482E4 |  |  |  | 3.4891E5 |  | 1 | 1 | 1 | 0 | 0 | 0 | 3 | 0 | Y | 6764 | RecName: Full=Cytotoxin 10; Short=CTX10 |
| 130 | 1797 | P01440.1 | 49.23 | 13 | 13 | 0 | 0 | 0 | 13 | 0 | 6.7482E4 |  |  |  | 3.4891E5 |  | 1 | 1 | 1 | 0 | 0 | 0 | 3 | 0 | Y | 6763 | RecName: Full=Cytotoxin 2; AltName: Full=Cobramine-B; AltName: Full=Cytotoxin II |
| 130 | 1798 | AAC61315.1 | 49.23 | 13 | 13 | 0 | 0 | 0 | 13 | 0 | 6.7482E4 |  |  |  | 3.4891E5 |  | 1 | 1 | 1 | 0 | 0 | 0 | 3 | 0 | Y | 7028 | cardiotoxin 2A precursor, partial [Naja sputatrix] |
| 130 | 1799 | BAU24665.1 | 49.23 | 11 | 11 | 0 | 0 | 0 | 11 | 0 | 6.7482E4 |  |  |  | 3.4891E5 |  | 1 | 1 | 1 | 0 | 0 | 0 | 3 | 0 | Y | 8041 | cytotoxin 9, partial [Naja naja] |
| 130 | 1800 | A0A0U5AUY6.1 | 49.23 | 11 | 11 | 0 | 0 | 0 | 11 | 0 | 6.7482E4 |  |  |  | 3.4891E5 |  | 1 | 1 | 1 | 0 | 0 | 0 | 3 | 0 | Y | 8041 | RecName: Full=Cytotoxin 9; Short=CTX9; Flags: Precursor |
| 130 | 1801 | O93472.1 | 49.23 | 10 | 10 | 0 | 0 | 0 | 10 | 0 | 6.7482E4 |  |  |  | 3.4891E5 |  | 1 | 1 | 1 | 0 | 0 | 0 | 3 | 0 | Y | 9128 | RecName: Full=Cytotoxin 2c; AltName: Full=Cardiotoxin-2c; Short=CTX-2c; Short=Ctx2c; Flags: Precursor |
| 130 | 1802 | Q9PST3.1 | 49.23 | 10 | 10 | 0 | 0 | 0 | 10 | 0 | 6.7482E4 |  |  |  | 3.4891E5 |  | 1 | 1 | 1 | 0 | 0 | 0 | 3 | 0 | Y | 9042 | RecName: Full=Cytotoxin 2b; AltName: Full=Cardiotoxin-2b; Short=CTX-2b; Short=Ctx2b; Flags: Precursor |
| 130 | 1803 | AAK49439.1 | 49.23 | 10 | 10 | 0 | 0 | 0 | 10 | 0 | 6.7482E4 |  |  |  | 3.4891E5 |  | 1 | 1 | 1 | 0 | 0 | 0 | 3 | 0 | Y | 9054 | cardiotoxin [Naja sputatrix] |
| 130 | 1804 | AAC27686.1 | 49.23 | 10 | 10 | 0 | 0 | 0 | 10 | 0 | 6.7482E4 |  |  |  | 3.4891E5 |  | 1 | 1 | 1 | 0 | 0 | 0 | 3 | 0 | Y | 9042 | cardiotoxin-2b [Naja sputatrix] |
| 130 | 1805 | Q9PST4.1 | 49.23 | 10 | 10 | 0 | 0 | 0 | 10 | 0 | 6.7482E4 |  |  |  | 3.4891E5 |  | 1 | 1 | 1 | 0 | 0 | 0 | 3 | 0 | Y | 9054 | RecName: Full=Cytotoxin 2a; AltName: Full=Cardiotoxin 2a; Short=CTX-2a; Short=Ctx2a; Flags: Precursor |
| 130 | 1806 | AAC27687.1 | 49.23 | 10 | 10 | 0 | 0 | 0 | 10 | 0 | 6.7482E4 |  |  |  | 3.4891E5 |  | 1 | 1 | 1 | 0 | 0 | 0 | 3 | 0 | Y | 9128 | cardiotoxin-2c [Naja sputatrix] |
| 130 | 1807 | AAC27685.1 | 49.23 | 10 | 10 | 0 | 0 | 0 | 10 | 0 | 6.7482E4 |  |  |  | 3.4891E5 |  | 1 | 1 | 1 | 0 | 0 | 0 | 3 | 0 | Y | 9054 | cardiotoxin-2a [Naja sputatrix] |
| 166 | 865 | XP\_032080818.1 | 48.83 | 2 | 2 | 0 | 0 | 0 | 0 | 0 | 6.3404E4 |  |  |  |  |  | 1 | 1 | 2 | 0 | 0 | 0 | 0 | 0 | N | 57576 | intestinal-type alkaline phosphatase 1-like [Thamnophis elegans] |
| 166 | 1164 | XP\_015674366.1 | 48.83 | 3 | 3 | 0 | 0 | 0 | 0 | 0 | 6.3404E4 |  |  |  |  |  | 1 | 1 | 2 | 0 | 0 | 0 | 0 | 0 | N | 37677 | intestinal-type alkaline phosphatase-like [Protobothrops mucrosquamatus] |
| 166 | 1583 | XP\_026545273.1 | 48.83 | 2 | 2 | 0 | 0 | 0 | 0 | 0 | 6.3404E4 |  |  |  |  |  | 1 | 1 | 2 | 0 | 0 | 0 | 0 | 0 | N | 56767 | intestinal-type alkaline phosphatase [Notechis scutatus] |
| 166 | 1584 | XP\_026576786.1 | 48.83 | 2 | 2 | 0 | 0 | 0 | 0 | 0 | 6.3404E4 |  |  |  |  |  | 1 | 1 | 2 | 0 | 0 | 0 | 0 | 0 | N | 56737 | intestinal-type alkaline phosphatase [Pseudonaja textilis] |
| 166 | 1866 | XP\_025022776.1 | 48.83 | 2 | 2 | 0 | 0 | 0 | 0 | 0 | 6.3404E4 |  |  |  |  |  | 1 | 1 | 2 | 0 | 0 | 0 | 0 | 0 | N | 50994 | intestinal-type alkaline phosphatase isoform X3 [Python bivittatus] |
| 166 | 1867 | XP\_015743663.1 | 48.83 | 2 | 2 | 0 | 0 | 0 | 0 | 0 | 6.3404E4 |  |  |  |  |  | 1 | 1 | 2 | 0 | 0 | 0 | 0 | 0 | N | 56333 | intestinal-type alkaline phosphatase isoform X2 [Python bivittatus] |
| 166 | 1868 | XP\_025022775.1 | 48.83 | 2 | 2 | 0 | 0 | 0 | 0 | 0 | 6.3404E4 |  |  |  |  |  | 1 | 1 | 2 | 0 | 0 | 0 | 0 | 0 | N | 60659 | intestinal-type alkaline phosphatase isoform X1 [Python bivittatus] |
| 166 | 2213 | XP\_034261216.1 | 48.83 | 7 | 7 | 0 | 0 | 0 | 0 | 0 | 6.3404E4 |  |  |  |  |  | 1 | 1 | 2 | 0 | 0 | 0 | 0 | 0 | N | 15574 | intestinal-type alkaline phosphatase-like, partial [Pantherophis guttatus] |
| 166 | 2214 | LAA85037.1 | 48.83 | 5 | 5 | 0 | 0 | 0 | 0 | 0 | 6.3404E4 |  |  |  |  |  | 1 | 1 | 2 | 0 | 0 | 0 | 0 | 0 | N | 22625 | hypothetical protein, partial [Micrurus lemniscatus lemniscatus] |
| 158 | 2081 | AAR14169.1 | 48.20 | 6 | 0 | 0 | 0 | 0 | 6 | 0 |  |  |  |  | 1.6735E6 |  | 1 | 1 | 0 | 0 | 0 | 0 | 1 | 0 | N | 13943 | E6b acidic phospholipase A2, partial [Trimeresurus puniceus] |
| 158 | 2082 | Q2YHJ5.1 | 48.20 | 6 | 0 | 0 | 0 | 0 | 6 | 0 |  |  |  |  | 1.6735E6 |  | 1 | 1 | 0 | 0 | 0 | 0 | 1 | 0 | N | 13943 | RecName: Full=Acidic phospholipase A2 Tpu-E6b; Short=svPLA2; AltName: Full=Phosphatidylcholine 2-acylhydrolase |
| 183 | 1695 | AFJ50263.1 | 48.15 | 3 | 3 | 0 | 0 | 0 | 0 | 0 | 5.6366E4 |  |  |  |  |  | 1 | 1 | 1 | 0 | 0 | 0 | 0 | 0 | N | 43886 | Guanine nucleotide-binding protein subunit alpha-13-like [Crotalus adamanteus] |
| 183 | 1700 | JAA96948.1 | 48.15 | 3 | 3 | 0 | 0 | 0 | 0 | 0 | 5.6366E4 |  |  |  |  |  | 1 | 1 | 1 | 0 | 0 | 0 | 0 | 0 | N | 43946 | Guanine nucleotide-binding protein subunit alpha-13-like protein [Crotalus horridus] |
| 183 | 1701 | JAG44584.1 | 48.15 | 3 | 3 | 0 | 0 | 0 | 0 | 0 | 5.6366E4 |  |  |  |  |  | 1 | 1 | 1 | 0 | 0 | 0 | 0 | 0 | N | 43946 | Guanine nucleotide-binding protein subunit alpha-13 -like protein [Crotalus horridus] |
| 183 | 1702 | ETE71869.1 | 48.15 | 3 | 3 | 0 | 0 | 0 | 0 | 0 | 5.6366E4 |  |  |  |  |  | 1 | 1 | 1 | 0 | 0 | 0 | 0 | 0 | N | 43990 | Guanine nucleotide-binding protein subunit alpha-13, partial [Ophiophagus hannah] |
| 183 | 1834 | JAC95458.1 | 48.15 | 3 | 3 | 0 | 0 | 0 | 0 | 0 | 5.6366E4 |  |  |  |  |  | 1 | 1 | 1 | 0 | 0 | 0 | 0 | 0 | N | 44356 | guanine nucleotide-binding protein G(s) subunit alpha isoforms short-like protein [Hypsiglena sp. JMG-2014] |
| 183 | 1835 | JAG67788.1 | 48.15 | 3 | 3 | 0 | 0 | 0 | 0 | 0 | 5.6366E4 |  |  |  |  |  | 1 | 1 | 1 | 0 | 0 | 0 | 0 | 0 | N | 44384 | guanine nucleotide-binding protein G(s) subunit alpha isoforms short-like protein [Boiga irregularis] |
| 183 | 1895 | ETE73579.1 | 48.15 | 3 | 3 | 0 | 0 | 0 | 0 | 0 | 5.6366E4 |  |  |  |  |  | 1 | 1 | 1 | 0 | 0 | 0 | 0 | 0 | N | 41603 | Guanine nucleotide-binding protein G(t) subunit alpha-2, partial [Ophiophagus hannah] |
| 183 | 1942 | XP\_034285323.1 | 48.15 | 2 | 2 | 0 | 0 | 0 | 0 | 0 | 5.6366E4 |  |  |  |  |  | 1 | 1 | 1 | 0 | 0 | 0 | 0 | 0 | N | 54712 | guanine nucleotide-binding protein G(olf) subunit alpha isoform X2 [Pantherophis guttatus] |
| 183 | 1965 | ETE68023.1 | 48.15 | 4 | 4 | 0 | 0 | 0 | 0 | 0 | 5.6366E4 |  |  |  |  |  | 1 | 1 | 1 | 0 | 0 | 0 | 0 | 0 | N | 35663 | Guanine nucleotide-binding protein G(t) subunit alpha-1, partial [Ophiophagus hannah] |
| 183 | 2118 | JAG46680.1 | 48.15 | 3 | 3 | 0 | 0 | 0 | 0 | 0 | 5.6366E4 |  |  |  |  |  | 1 | 1 | 1 | 0 | 0 | 0 | 0 | 0 | N | 44427 | guanine nucleotide-binding protein G(s) subunit alpha isoforms short-like protein [Crotalus horridus] |
| 183 | 2119 | JAI13087.1 | 48.15 | 3 | 3 | 0 | 0 | 0 | 0 | 0 | 5.6366E4 |  |  |  |  |  | 1 | 1 | 1 | 0 | 0 | 0 | 0 | 0 | N | 44427 | guanine nucleotide-binding protein G(s) subunit alpha isoforms short-like protein [Crotalus adamanteus] |
| 183 | 2120 | JAA96898.1 | 48.15 | 3 | 3 | 0 | 0 | 0 | 0 | 0 | 5.6366E4 |  |  |  |  |  | 1 | 1 | 1 | 0 | 0 | 0 | 0 | 0 | N | 44427 | guanine nucleotide-binding protein G(s) subunit alpha isoforms short-like protein [Crotalus horridus] |
| 183 | 2121 | JAG44520.1 | 48.15 | 3 | 3 | 0 | 0 | 0 | 0 | 0 | 5.6366E4 |  |  |  |  |  | 1 | 1 | 1 | 0 | 0 | 0 | 0 | 0 | N | 44427 | guanine nucleotide-binding protein G(s) subunit alpha isoforms short-like protein [Crotalus horridus] |
| 183 | 2122 | AFJ50208.1 | 48.15 | 3 | 3 | 0 | 0 | 0 | 0 | 0 | 5.6366E4 |  |  |  |  |  | 1 | 1 | 1 | 0 | 0 | 0 | 0 | 0 | N | 44427 | GNAS complex locus [Crotalus adamanteus] |
| 183 | 2216 | BAM28742.1 | 48.15 | 3 | 3 | 0 | 0 | 0 | 0 | 0 | 5.6366E4 |  |  |  |  |  | 1 | 1 | 1 | 0 | 0 | 0 | 0 | 0 | N | 36966 | guanine nucleotide-binding protein G(i2) subunit alpha, partial [Elaphe quadrivirgata] |
| 183 | 2217 | JAA96947.1 | 48.15 | 3 | 3 | 0 | 0 | 0 | 0 | 0 | 5.6366E4 |  |  |  |  |  | 1 | 1 | 1 | 0 | 0 | 0 | 0 | 0 | N | 40671 | Guanine nucleotide-binding protein G(k) subunit alpha-like protein [Crotalus horridus] |
| 183 | 2218 | JAI09966.1 | 48.15 | 3 | 3 | 0 | 0 | 0 | 0 | 0 | 5.6366E4 |  |  |  |  |  | 1 | 1 | 1 | 0 | 0 | 0 | 0 | 0 | N | 40638 | Guanine nucleotide-binding protein G(k) subunit alpha-like protein [Micrurus fulvius] |
| 183 | 2219 | AFJ50262.1 | 48.15 | 3 | 3 | 0 | 0 | 0 | 0 | 0 | 5.6366E4 |  |  |  |  |  | 1 | 1 | 1 | 0 | 0 | 0 | 0 | 0 | N | 40671 | Guanine nucleotide-binding protein G(k) subunit alpha-like [Crotalus adamanteus] |
| 183 | 2220 | JAG46724.1 | 48.15 | 3 | 3 | 0 | 0 | 0 | 0 | 0 | 5.6366E4 |  |  |  |  |  | 1 | 1 | 1 | 0 | 0 | 0 | 0 | 0 | N | 40671 | Guanine nucleotide-binding protein G(k) subunit alpha-like protein [Crotalus horridus] |
| 183 | 2221 | JAC95504.1 | 48.15 | 3 | 3 | 0 | 0 | 0 | 0 | 0 | 5.6366E4 |  |  |  |  |  | 1 | 1 | 1 | 0 | 0 | 0 | 0 | 0 | N | 40668 | Guanine nucleotide-binding protein G(k) subunit alpha-like protein [Hypsiglena sp. JMG-2014] |
| 183 | 2222 | JAB54125.1 | 48.15 | 3 | 3 | 0 | 0 | 0 | 0 | 0 | 5.6366E4 |  |  |  |  |  | 1 | 1 | 1 | 0 | 0 | 0 | 0 | 0 | N | 40638 | Guanine nucleotide-binding protein subunit alpha [Micrurus fulvius] |
| 183 | 2223 | JAG67837.1 | 48.15 | 3 | 3 | 0 | 0 | 0 | 0 | 0 | 5.6366E4 |  |  |  |  |  | 1 | 1 | 1 | 0 | 0 | 0 | 0 | 0 | N | 40638 | Guanine nucleotide-binding protein G(k) subunit alpha-like protein [Boiga irregularis] |
| 183 | 2224 | JAG44583.1 | 48.15 | 3 | 3 | 0 | 0 | 0 | 0 | 0 | 5.6366E4 |  |  |  |  |  | 1 | 1 | 1 | 0 | 0 | 0 | 0 | 0 | N | 40671 | Guanine nucleotide-binding protein G(k) subunit alpha-like protein [Crotalus horridus] |
| 183 | 2225 | JAG44582.1 | 48.15 | 3 | 3 | 0 | 0 | 0 | 0 | 0 | 5.6366E4 |  |  |  |  |  | 1 | 1 | 1 | 0 | 0 | 0 | 0 | 0 | N | 40629 | Guanine nucleotide-binding protein G(i) subunit alpha-2 [Crotalus horridus] |
| 183 | 2226 | JAI09965.1 | 48.15 | 3 | 3 | 0 | 0 | 0 | 0 | 0 | 5.6366E4 |  |  |  |  |  | 1 | 1 | 1 | 0 | 0 | 0 | 0 | 0 | N | 40597 | Guanine nucleotide-binding protein G(i) subunit alpha-2 [Micrurus fulvius] |
| 183 | 2227 | JAA96946.1 | 48.15 | 3 | 3 | 0 | 0 | 0 | 0 | 0 | 5.6366E4 |  |  |  |  |  | 1 | 1 | 1 | 0 | 0 | 0 | 0 | 0 | N | 40629 | Guanine nucleotide-binding protein G(i) subunit alpha-2 [Crotalus horridus] |
| 183 | 2228 | JAG46723.1 | 48.15 | 3 | 3 | 0 | 0 | 0 | 0 | 0 | 5.6366E4 |  |  |  |  |  | 1 | 1 | 1 | 0 | 0 | 0 | 0 | 0 | N | 40629 | Guanine nucleotide-binding protein G(i) subunit alpha-2 [Crotalus horridus] |
| 183 | 2229 | JAG44556.1 | 48.15 | 3 | 3 | 0 | 0 | 0 | 0 | 0 | 5.6366E4 |  |  |  |  |  | 1 | 1 | 1 | 0 | 0 | 0 | 0 | 0 | N | 44109 | guanine nucleotide binding protein (G protein) alpha 12 [Crotalus horridus] |
| 183 | 2230 | JAI13125.1 | 48.15 | 3 | 3 | 0 | 0 | 0 | 0 | 0 | 5.6366E4 |  |  |  |  |  | 1 | 1 | 1 | 0 | 0 | 0 | 0 | 0 | N | 44109 | guanine nucleotide binding protein (G protein) alpha 12 [Crotalus adamanteus] |
| 183 | 2231 | JAA96922.1 | 48.15 | 3 | 3 | 0 | 0 | 0 | 0 | 0 | 5.6366E4 |  |  |  |  |  | 1 | 1 | 1 | 0 | 0 | 0 | 0 | 0 | N | 44109 | guanine nucleotide binding protein (G protein) alpha 12 [Crotalus horridus] |
| 183 | 2232 | JAG67838.1 | 48.15 | 3 | 3 | 0 | 0 | 0 | 0 | 0 | 5.6366E4 |  |  |  |  |  | 1 | 1 | 1 | 0 | 0 | 0 | 0 | 0 | N | 46749 | guanine nucleotide-binding protein subunit alpha-12 [Boiga irregularis] |
| 172 | 1297 | XP\_026564997.1 | 43.98 | 1 | 1 | 0 | 0 | 0 | 0 | 0 | 2.6135E4 |  |  |  |  |  | 1 | 1 | 1 | 0 | 0 | 0 | 0 | 0 | N | 236499 | sortilin-related receptor [Pseudonaja textilis] |
| 172 | 1333 | XP\_025024673.1 | 43.98 | 1 | 1 | 0 | 0 | 0 | 0 | 0 | 2.6135E4 |  |  |  |  |  | 1 | 1 | 1 | 0 | 0 | 0 | 0 | 0 | N | 236002 | sortilin-related receptor isoform X2 [Python bivittatus] |
| 172 | 1334 | XP\_007431813.2 | 43.98 | 1 | 1 | 0 | 0 | 0 | 0 | 0 | 2.6135E4 |  |  |  |  |  | 1 | 1 | 1 | 0 | 0 | 0 | 0 | 0 | N | 236514 | sortilin-related receptor isoform X1 [Python bivittatus] |
| 172 | 1340 | XP\_026537699.1 | 43.98 | 1 | 1 | 0 | 0 | 0 | 0 | 0 | 2.6135E4 |  |  |  |  |  | 1 | 1 | 1 | 0 | 0 | 0 | 0 | 0 | N | 236512 | sortilin-related receptor [Notechis scutatus] |
| 172 | 1350 | XP\_032085080.1 | 43.98 | 1 | 1 | 0 | 0 | 0 | 0 | 0 | 2.6135E4 |  |  |  |  |  | 1 | 1 | 1 | 0 | 0 | 0 | 0 | 0 | N | 192086 | sortilin-related receptor isoform X2 [Thamnophis elegans] |
| 172 | 1351 | XP\_032085079.1 | 43.98 | 0 | 0 | 0 | 0 | 0 | 0 | 0 | 2.6135E4 |  |  |  |  |  | 1 | 1 | 1 | 0 | 0 | 0 | 0 | 0 | N | 247880 | sortilin-related receptor isoform X1 [Thamnophis elegans] |
| 172 | 1356 | XP\_034286370.1 | 43.98 | 1 | 1 | 0 | 0 | 0 | 0 | 0 | 2.6135E4 |  |  |  |  |  | 1 | 1 | 1 | 0 | 0 | 0 | 0 | 0 | N | 235167 | sortilin-related receptor [Pantherophis guttatus] |
| 172 | 1489 | XP\_015676891.1 | 43.98 | 1 | 1 | 0 | 0 | 0 | 0 | 0 | 2.6135E4 |  |  |  |  |  | 1 | 1 | 1 | 0 | 0 | 0 | 0 | 0 | N | 152809 | sortilin-related receptor, partial [Protobothrops mucrosquamatus] |
| 172 | 1572 | ETE61149.1 | 43.98 | 1 | 1 | 0 | 0 | 0 | 0 | 0 | 2.6135E4 |  |  |  |  |  | 1 | 1 | 1 | 0 | 0 | 0 | 0 | 0 | N | 129350 | Sortilin-related receptor, partial [Ophiophagus hannah] |
| 172 | 1621 | XP\_013929765.1 | 43.98 | 1 | 1 | 0 | 0 | 0 | 0 | 0 | 2.6135E4 |  |  |  |  |  | 1 | 1 | 1 | 0 | 0 | 0 | 0 | 0 | N | 151597 | PREDICTED: sortilin-related receptor, partial [Thamnophis sirtalis] |
| 195 | 2078 | XP\_032078096.1 | 43.57 | 7 | 0 | 0 | 0 | 0 | 0 | 7 |  |  |  |  |  | 5.8274E4 | 1 | 1 | 0 | 0 | 0 | 0 | 0 | 1 | N | 14835 | fatty acid-binding protein, adipocyte-like isoform X2 [Thamnophis elegans] |
| 195 | 2079 | XP\_013920450.1 | 43.57 | 7 | 0 | 0 | 0 | 0 | 0 | 7 |  |  |  |  |  | 5.8274E4 | 1 | 1 | 0 | 0 | 0 | 0 | 0 | 1 | N | 14833 | PREDICTED: fatty acid-binding protein, adipocyte-like isoform X2 [Thamnophis sirtalis] |
| 195 | 2314 | XP\_026547098.1 | 43.57 | 7 | 0 | 0 | 0 | 0 | 0 | 7 |  |  |  |  |  | 5.8274E4 | 1 | 1 | 0 | 0 | 0 | 0 | 0 | 1 | N | 14782 | fatty acid-binding protein, adipocyte-like [Notechis scutatus] |
| 195 | 2315 | XP\_026561817.1 | 43.57 | 7 | 0 | 0 | 0 | 0 | 0 | 7 |  |  |  |  |  | 5.8274E4 | 1 | 1 | 0 | 0 | 0 | 0 | 0 | 1 | N | 14794 | fatty acid-binding protein, adipocyte-like [Pseudonaja textilis] |
| 195 | 2316 | XP\_007432588.1 | 43.57 | 7 | 0 | 0 | 0 | 0 | 0 | 7 |  |  |  |  |  | 5.8274E4 | 1 | 1 | 0 | 0 | 0 | 0 | 0 | 1 | N | 14966 | fatty acid-binding protein, adipocyte [Python bivittatus] |
| 195 | 2317 | XP\_034278224.1 | 43.57 | 7 | 0 | 0 | 0 | 0 | 0 | 7 |  |  |  |  |  | 5.8274E4 | 1 | 1 | 0 | 0 | 0 | 0 | 0 | 1 | N | 14859 | fatty acid-binding protein, adipocyte-like [Pantherophis guttatus] |
| 195 | 2318 | XP\_015687553.1 | 43.57 | 7 | 0 | 0 | 0 | 0 | 0 | 7 |  |  |  |  |  | 5.8274E4 | 1 | 1 | 0 | 0 | 0 | 0 | 0 | 1 | N | 14823 | fatty acid-binding protein, adipocyte [Protobothrops mucrosquamatus] |
| 195 | 2319 | ETE71857.1 | 43.57 | 3 | 0 | 0 | 0 | 0 | 0 | 3 |  |  |  |  |  | 5.8274E4 | 1 | 1 | 0 | 0 | 0 | 0 | 0 | 1 | N | 29827 | Myelin P2 protein [Ophiophagus hannah] |
| 174 | 1683 | P86802.1 | 43.00 | 8 | 0 | 0 | 0 | 0 | 8 | 0 |  |  |  |  | 2.5748E5 |  | 1 | 1 | 0 | 0 | 0 | 0 | 1 | 0 | N | 27266 | RecName: Full=Snake venom metalloproteinase HF-1; Short=BmHF-1; Short=SVMP |
| total 428 proteins |  |  |  |  |  |  |  |  |  |  |  |  |  |  |  |  |  |  |  |  |  |  |  |  |  |  |  |
| --- | --- | --- | --- | --- | --- | --- | --- | --- | --- | --- | --- | --- | --- | --- | --- | --- | --- | --- | --- | --- | --- | --- | --- | --- | --- | --- | --- |

  

Q90W54.1

back to list

  

| Protein Coverage
| Supporting Peptides
|

Protein Coverage:

Supporting Peptides:

| Peptide | Uniq | -10lgP | Mass | Length | ppm | m/z | z | RT | Fraction | Scan | Source File | Area F2 | Area F1 | Area F3 | Area F4 | Area F5 | Area F6 | #Feature | #Feature F2 | #Feature F1 | #Feature F3 | #Feature F4 | #Feature F5 | #Feature F6 | Start | End | PTM | AScore | Found By |
| --- | --- | --- | --- | --- | --- | --- | --- | --- | --- | --- | --- | --- | --- | --- | --- | --- | --- | --- | --- | --- | --- | --- | --- | --- | --- | --- | --- | --- | --- |
| R.KFGLQLNEFSQENDNAWYFIK.N | Y | 106.65 | 2590.2493 | 21 | 0.4 | 864.4240 | 3 | 58.46 | 5 | F5:33481 | 31122021\_RID\_2349\_5C.raw |  |  | 9.9657E6 | 1.1078E6 | 2.5437E6 | 3.5235E5 | 4 | 0 | 0 | 1 | 1 | 1 | 1 | 122 | 142 |  |  | PEAKS DB |
| K.SAGQLYEESLGK.V | N | 80.33 | 1280.6248 | 12 | -0.1 | 641.3196 | 2 | 32.32 | 5 | F5:14229 | 31122021\_RID\_2349\_5C.raw | 3.9268E8 | 5.6746E8 | 8.8153E7 | 1.109E8 | 1.2738E8 | 1.0719E5 | 12 | 3 | 3 | 1 | 1 | 3 | 1 | 170 | 181 |  |  | PEAKS DB |
| Y.GIGDDANFFQALDFK.D | N | 75.31 | 1656.7783 | 15 | 0.2 | 829.3966 | 2 | 64.60 | 3 | F3:23470 | 31122021\_RID\_2349\_3C.raw |  |  | 1.1679E5 |  |  |  | 1 | 0 | 0 | 1 | 0 | 0 | 0 | 391 | 405 |  |  | PEAKS DB |
| R.HDDIFAYEK.R | N | 72.69 | 1136.5138 | 9 | -0.1 | 569.2641 | 2 | 30.15 | 2 | F2:8868 | 30122021\_RID\_2349\_1C.raw | 5.9259E7 | 1.5095E8 | 7.8164E7 |  |  | 1.3576E6 | 7 | 3 | 2 | 1 | 0 | 0 | 1 | 241 | 249 |  |  | PEAKS DB |
| K.KDPGVLKYPVKPSEEGK.S | N | 69.54 | 1870.0199 | 17 | 0.6 | 624.3476 | 3 | 22.58 | 1 | F1:7284 | 28122021\_RID\_2349\_2C.raw | 4.0206E7 | 6.6249E6 | 4.6122E6 | 7.8889E6 | 5.5106E6 |  | 8 | 3 | 1 | 1 | 1 | 2 | 0 | 153 | 169 |  |  | PEAKS DB |
| D.PGVLKYPVKPSEEGK.S | N | 68.71 | 1626.8981 | 15 | 0.0 | 407.7318 | 4 | 23.75 | 1 | F1:8065 | 28122021\_RID\_2349\_2C.raw | 1.9885E7 |  |  |  |  |  | 3 | 3 | 0 | 0 | 0 | 0 | 0 | 155 | 169 |  |  | PEAKS DB |
| K.DPGVLKYPVKPSEEGK.S | N | 68.31 | 1741.9249 | 16 | 0.4 | 581.6492 | 3 | 28.35 | 1 | F1:11687 | 28122021\_RID\_2349\_2C.raw | 3.0191E6 | 1.5465E6 | 5.0737E5 |  | 1.0906E6 |  | 5 | 2 | 1 | 1 | 0 | 1 | 0 | 154 | 169 |  |  | PEAKS DB |
| R.KFGLQLNEFSQEND.N | N | 67.11 | 1667.7791 | 14 | 0.6 | 834.8973 | 2 | 47.96 | 6 | F6:25528 | 31122021\_RID\_2349\_6C.raw |  |  |  |  |  | 3.2405E6 | 1 | 0 | 0 | 0 | 0 | 0 | 1 | 122 | 135 |  |  | PEAKS DB |
| R.HDDIFAYEKR.F | N | 60.61 | 1292.6149 | 10 | 0.6 | 431.8792 | 3 | 25.32 | 1 | F1:9449 | 28122021\_RID\_2349\_2C.raw | 9.7777E5 | 4.5859E5 |  |  |  |  | 2 | 1 | 1 | 0 | 0 | 0 | 0 | 241 | 250 |  |  | PEAKS DB |
| R.IKFEPPLPPK.K | N | 58.77 | 1164.6907 | 10 | 0.3 | 583.3528 | 2 | 36.36 | 1 | F1:18064 | 28122021\_RID\_2349\_2C.raw | 6.5252E7 | 6.2344E7 | 1.1354E8 | 5.5586E6 | 1.9599E6 | 2.0974E6 | 11 | 3 | 2 | 3 | 1 | 1 | 1 | 320 | 329 |  |  | PEAKS DB |
| D.YEEFLEIAR.N | N | 56.31 | 1168.5764 | 9 | 0.7 | 585.2959 | 2 | 45.58 | 2 | F2:15608 | 30122021\_RID\_2349\_1C.raw |  | 2.3921E5 |  |  |  |  | 1 | 0 | 1 | 0 | 0 | 0 | 0 | 34 | 42 |  |  | PEAKS DB |
| T.DYEEFLEIAR.N | N | 56.01 | 1283.6033 | 10 | 0.8 | 642.8094 | 2 | 56.80 | 5 | F5:32140 | 31122021\_RID\_2349\_5C.raw | 1.3399E5 | 2.7826E5 |  |  | 9.5716E4 | 7.7266E4 | 4 | 1 | 1 | 0 | 0 | 1 | 1 | 33 | 42 |  |  | PEAKS DB |
| A.GQLYEESLGK.V | N | 54.58 | 1122.5557 | 10 | 1.2 | 562.2858 | 2 | 29.95 | 1 | F1:13018 | 28122021\_RID\_2349\_2C.raw | 4.0796E5 | 6.449E5 |  |  |  |  | 2 | 1 | 1 | 0 | 0 | 0 | 0 | 172 | 181 |  |  | PEAKS DB |
| N.EFSQENDNAWYFIK.N | N | 53.57 | 1789.7947 | 14 | 0.5 | 895.9051 | 2 | 53.07 | 1 | F1:31357 | 28122021\_RID\_2349\_2C.raw | 2.5591E5 | 2.9753E5 |  |  |  |  | 2 | 1 | 1 | 0 | 0 | 0 | 0 | 129 | 142 |  |  | PEAKS DB |
| F.SQENDNAWYFIK.N | N | 53.25 | 1513.6837 | 12 | 1.0 | 757.8499 | 2 | 47.51 | 2 | F2:16263 | 30122021\_RID\_2349\_1C.raw |  | 7.0312E5 |  |  |  |  | 1 | 0 | 1 | 0 | 0 | 0 | 0 | 131 | 142 |  |  | PEAKS DB |
| K.KFWEDEGIHGGK.S | N | 51.61 | 1401.6677 | 12 | -0.6 | 468.2296 | 3 | 24.58 | 1 | F1:8904 | 28122021\_RID\_2349\_2C.raw | 4.5254E5 |  |  |  |  |  | 1 | 1 | 0 | 0 | 0 | 0 | 0 | 352 | 363 |  |  | PEAKS DB |
| R.IKFEPPLPPKK.A | N | 50.54 | 1292.7855 | 11 | 0.1 | 431.9358 | 3 | 29.13 | 1 | F1:12372 | 28122021\_RID\_2349\_2C.raw | 9.1587E6 | 3.2038E6 | 8.8306E5 |  |  |  | 4 | 2 | 1 | 1 | 0 | 0 | 0 | 320 | 330 |  |  | PEAKS DB |
| K.YDTYSTKEY.L | N | 49.76 | 1168.4924 | 9 | 1.0 | 585.2541 | 2 | 25.91 | 5 | F5:9933 | 31122021\_RID\_2349\_5C.raw |  |  |  |  | 2.4008E6 |  | 1 | 0 | 0 | 0 | 0 | 1 | 0 | 198 | 206 |  |  | PEAKS DB |
| K.FGLQLNEFSQEND.N | N | 47.16 | 1539.6841 | 13 | 0.5 | 770.8497 | 2 | 59.71 | 6 | F6:34055 | 31122021\_RID\_2349\_6C.raw |  |  |  |  |  | 4.3601E5 | 1 | 0 | 0 | 0 | 0 | 0 | 1 | 123 | 135 |  |  | PEAKS DB |
| E.AHGWIDSTIK.S | N | 45.39 | 1126.5771 | 10 | 1.2 | 564.2965 | 2 | 28.81 | 2 | F2:8087 | 30122021\_RID\_2349\_1C.raw |  | 3.6217E5 |  |  |  |  | 1 | 0 | 1 | 0 | 0 | 0 | 0 | 480 | 489 |  |  | PEAKS DB |
| H.QVTVLEASER.A | N | 45.38 | 1130.5931 | 10 | -0.2 | 566.3037 | 2 | 28.45 | 2 | F2:7775 | 30122021\_RID\_2349\_1C.raw |  | 6.9878E5 |  |  |  |  | 1 | 0 | 1 | 0 | 0 | 0 | 0 | 76 | 85 |  |  | PEAKS DB |
| N.FFQALDFK.D | N | 45.08 | 1014.5174 | 8 | 0.8 | 508.2664 | 2 | 48.21 | 2 | F2:16516 | 30122021\_RID\_2349\_1C.raw |  | 1.1251E4 |  |  |  |  | 1 | 0 | 1 | 0 | 0 | 0 | 0 | 398 | 405 |  |  | PEAKS DB |
| K.FWEDEGIHGGK.S | N | 44.74 | 1273.5728 | 11 | 0.4 | 637.7939 | 2 | 28.56 | 1 | F1:11862 | 28122021\_RID\_2349\_2C.raw | 1.1259E5 |  |  |  |  |  | 1 | 1 | 0 | 0 | 0 | 0 | 0 | 353 | 363 |  |  | PEAKS DB |
| G.AGHQVTVLEASER.A | N | 44.71 | 1395.7106 | 13 | 0.9 | 698.8632 | 2 | 22.59 | 2 | F2:5163 | 30122021\_RID\_2349\_1C.raw |  | 3.5423E4 |  |  |  |  | 1 | 0 | 1 | 0 | 0 | 0 | 0 | 73 | 85 |  |  | PEAKS DB |
| K.FEPPLPPK.K | N | 43.76 | 923.5116 | 8 | -0.4 | 462.7629 | 2 | 36.36 | 1 | F1:17621 | 28122021\_RID\_2349\_2C.raw | 2.2198E7 |  |  |  |  |  | 1 | 1 | 0 | 0 | 0 | 0 | 0 | 322 | 329 |  |  | PEAKS DB |
| K.FGLQLNEFSQEN.D | N | 43.25 | 1424.6572 | 12 | 1.1 | 713.3367 | 2 | 58.34 | 6 | F6:33069 | 31122021\_RID\_2349\_6C.raw |  |  |  |  |  | 9.3396E4 | 1 | 0 | 0 | 0 | 0 | 0 | 1 | 123 | 134 |  |  | PEAKS DB |
| E.GWYANLGPMR.L | N | 42.97 | 1163.5546 | 10 | 0.2 | 582.7847 | 2 | 43.50 | 1 | F1:23833 | 28122021\_RID\_2349\_2C.raw | 3.4804E4 |  |  |  |  |  | 1 | 1 | 0 | 0 | 0 | 0 | 0 | 99 | 108 |  |  | PEAKS DB |
| S.AGQLYEESLGK.V | N | 42.75 | 1193.5928 | 11 | -0.1 | 597.8036 | 2 | 30.49 | 2 | F2:8743 | 30122021\_RID\_2349\_1C.raw |  | 1.8392E5 |  |  |  |  | 1 | 0 | 1 | 0 | 0 | 0 | 0 | 171 | 181 |  |  | PEAKS DB |
| total 28 peptides |  |  |  |  |  |  |  |  |  |  |  |  |  |  |  |  |  |  |  |  |  |  |  |  |  |  |  |  |  |
| --- | --- | --- | --- | --- | --- | --- | --- | --- | --- | --- | --- | --- | --- | --- | --- | --- | --- | --- | --- | --- | --- | --- | --- | --- | --- | --- | --- | --- | --- |

BAB69450.1

back to list

  

| Protein Coverage
| Supporting Peptides
|

Protein Coverage:

Supporting Peptides:

| Peptide | Uniq | -10lgP | Mass | Length | ppm | m/z | z | RT | Fraction | Scan | Source File | Area F2 | Area F1 | Area F3 | Area F4 | Area F5 | Area F6 | #Feature | #Feature F2 | #Feature F1 | #Feature F3 | #Feature F4 | #Feature F5 | #Feature F6 | Start | End | PTM | AScore | Found By |
| --- | --- | --- | --- | --- | --- | --- | --- | --- | --- | --- | --- | --- | --- | --- | --- | --- | --- | --- | --- | --- | --- | --- | --- | --- | --- | --- | --- | --- | --- |
| R.KFGLQLNEFSQENDNAWYFIK.N | Y | 106.65 | 2590.2493 | 21 | 0.4 | 864.4240 | 3 | 58.46 | 5 | F5:33481 | 31122021\_RID\_2349\_5C.raw |  |  | 9.9657E6 | 1.1078E6 | 2.5437E6 | 3.5235E5 | 4 | 0 | 0 | 1 | 1 | 1 | 1 | 122 | 142 |  |  | PEAKS DB |
| K.SAGQLYEESLGK.V | N | 80.33 | 1280.6248 | 12 | -0.1 | 641.3196 | 2 | 32.32 | 5 | F5:14229 | 31122021\_RID\_2349\_5C.raw | 3.9268E8 | 5.6746E8 | 8.8153E7 | 1.109E8 | 1.2738E8 | 1.0719E5 | 12 | 3 | 3 | 1 | 1 | 3 | 1 | 170 | 181 |  |  | PEAKS DB |
| Y.GIGDDANFFQALDFK.D | N | 75.31 | 1656.7783 | 15 | 0.2 | 829.3966 | 2 | 64.60 | 3 | F3:23470 | 31122021\_RID\_2349\_3C.raw |  |  | 1.1679E5 |  |  |  | 1 | 0 | 0 | 1 | 0 | 0 | 0 | 391 | 405 |  |  | PEAKS DB |
| R.HDDIFAYEK.R | N | 72.69 | 1136.5138 | 9 | -0.1 | 569.2641 | 2 | 30.15 | 2 | F2:8868 | 30122021\_RID\_2349\_1C.raw | 5.9259E7 | 1.5095E8 | 7.8164E7 |  |  | 1.3576E6 | 7 | 3 | 2 | 1 | 0 | 0 | 1 | 241 | 249 |  |  | PEAKS DB |
| K.KDPGVLKYPVKPSEEGK.S | N | 69.54 | 1870.0199 | 17 | 0.6 | 624.3476 | 3 | 22.58 | 1 | F1:7284 | 28122021\_RID\_2349\_2C.raw | 4.0206E7 | 6.6249E6 | 4.6122E6 | 7.8889E6 | 5.5106E6 |  | 8 | 3 | 1 | 1 | 1 | 2 | 0 | 153 | 169 |  |  | PEAKS DB |
| D.PGVLKYPVKPSEEGK.S | N | 68.71 | 1626.8981 | 15 | 0.0 | 407.7318 | 4 | 23.75 | 1 | F1:8065 | 28122021\_RID\_2349\_2C.raw | 1.9885E7 |  |  |  |  |  | 3 | 3 | 0 | 0 | 0 | 0 | 0 | 155 | 169 |  |  | PEAKS DB |
| K.DPGVLKYPVKPSEEGK.S | N | 68.31 | 1741.9249 | 16 | 0.4 | 581.6492 | 3 | 28.35 | 1 | F1:11687 | 28122021\_RID\_2349\_2C.raw | 3.0191E6 | 1.5465E6 | 5.0737E5 |  | 1.0906E6 |  | 5 | 2 | 1 | 1 | 0 | 1 | 0 | 154 | 169 |  |  | PEAKS DB |
| R.KFGLQLNEFSQEND.N | N | 67.11 | 1667.7791 | 14 | 0.6 | 834.8973 | 2 | 47.96 | 6 | F6:25528 | 31122021\_RID\_2349\_6C.raw |  |  |  |  |  | 3.2405E6 | 1 | 0 | 0 | 0 | 0 | 0 | 1 | 122 | 135 |  |  | PEAKS DB |
| R.HDDIFAYEKR.F | N | 60.61 | 1292.6149 | 10 | 0.6 | 431.8792 | 3 | 25.32 | 1 | F1:9449 | 28122021\_RID\_2349\_2C.raw | 9.7777E5 | 4.5859E5 |  |  |  |  | 2 | 1 | 1 | 0 | 0 | 0 | 0 | 241 | 250 |  |  | PEAKS DB |
| R.IKFEPPLPPK.K | N | 58.77 | 1164.6907 | 10 | 0.3 | 583.3528 | 2 | 36.36 | 1 | F1:18064 | 28122021\_RID\_2349\_2C.raw | 6.5252E7 | 6.2344E7 | 1.1354E8 | 5.5586E6 | 1.9599E6 | 2.0974E6 | 11 | 3 | 2 | 3 | 1 | 1 | 1 | 320 | 329 |  |  | PEAKS DB |
| D.YEEFLEIAR.N | N | 56.31 | 1168.5764 | 9 | 0.7 | 585.2959 | 2 | 45.58 | 2 | F2:15608 | 30122021\_RID\_2349\_1C.raw |  | 2.3921E5 |  |  |  |  | 1 | 0 | 1 | 0 | 0 | 0 | 0 | 34 | 42 |  |  | PEAKS DB |
| T.DYEEFLEIAR.N | N | 56.01 | 1283.6033 | 10 | 0.8 | 642.8094 | 2 | 56.80 | 5 | F5:32140 | 31122021\_RID\_2349\_5C.raw | 1.3399E5 | 2.7826E5 |  |  | 9.5716E4 | 7.7266E4 | 4 | 1 | 1 | 0 | 0 | 1 | 1 | 33 | 42 |  |  | PEAKS DB |
| A.GQLYEESLGK.V | N | 54.58 | 1122.5557 | 10 | 1.2 | 562.2858 | 2 | 29.95 | 1 | F1:13018 | 28122021\_RID\_2349\_2C.raw | 4.0796E5 | 6.449E5 |  |  |  |  | 2 | 1 | 1 | 0 | 0 | 0 | 0 | 172 | 181 |  |  | PEAKS DB |
| N.EFSQENDNAWYFIK.N | N | 53.57 | 1789.7947 | 14 | 0.5 | 895.9051 | 2 | 53.07 | 1 | F1:31357 | 28122021\_RID\_2349\_2C.raw | 2.5591E5 | 2.9753E5 |  |  |  |  | 2 | 1 | 1 | 0 | 0 | 0 | 0 | 129 | 142 |  |  | PEAKS DB |
| F.SQENDNAWYFIK.N | N | 53.25 | 1513.6837 | 12 | 1.0 | 757.8499 | 2 | 47.51 | 2 | F2:16263 | 30122021\_RID\_2349\_1C.raw |  | 7.0312E5 |  |  |  |  | 1 | 0 | 1 | 0 | 0 | 0 | 0 | 131 | 142 |  |  | PEAKS DB |
| K.KFWEDEGIHGGK.S | N | 51.61 | 1401.6677 | 12 | -0.6 | 468.2296 | 3 | 24.58 | 1 | F1:8904 | 28122021\_RID\_2349\_2C.raw | 4.5254E5 |  |  |  |  |  | 1 | 1 | 0 | 0 | 0 | 0 | 0 | 352 | 363 |  |  | PEAKS DB |
| R.IKFEPPLPPKK.A | N | 50.54 | 1292.7855 | 11 | 0.1 | 431.9358 | 3 | 29.13 | 1 | F1:12372 | 28122021\_RID\_2349\_2C.raw | 9.1587E6 | 3.2038E6 | 8.8306E5 |  |  |  | 4 | 2 | 1 | 1 | 0 | 0 | 0 | 320 | 330 |  |  | PEAKS DB |
| K.YDTYSTKEY.L | N | 49.76 | 1168.4924 | 9 | 1.0 | 585.2541 | 2 | 25.91 | 5 | F5:9933 | 31122021\_RID\_2349\_5C.raw |  |  |  |  | 2.4008E6 |  | 1 | 0 | 0 | 0 | 0 | 1 | 0 | 198 | 206 |  |  | PEAKS DB |
| K.FGLQLNEFSQEND.N | N | 47.16 | 1539.6841 | 13 | 0.5 | 770.8497 | 2 | 59.71 | 6 | F6:34055 | 31122021\_RID\_2349\_6C.raw |  |  |  |  |  | 4.3601E5 | 1 | 0 | 0 | 0 | 0 | 0 | 1 | 123 | 135 |  |  | PEAKS DB |
| E.AHGWIDSTIK.S | N | 45.39 | 1126.5771 | 10 | 1.2 | 564.2965 | 2 | 28.81 | 2 | F2:8087 | 30122021\_RID\_2349\_1C.raw |  | 3.6217E5 |  |  |  |  | 1 | 0 | 1 | 0 | 0 | 0 | 0 | 480 | 489 |  |  | PEAKS DB |
| H.QVTVLEASER.A | N | 45.38 | 1130.5931 | 10 | -0.2 | 566.3037 | 2 | 28.45 | 2 | F2:7775 | 30122021\_RID\_2349\_1C.raw |  | 6.9878E5 |  |  |  |  | 1 | 0 | 1 | 0 | 0 | 0 | 0 | 76 | 85 |  |  | PEAKS DB |
| N.FFQALDFK.D | N | 45.08 | 1014.5174 | 8 | 0.8 | 508.2664 | 2 | 48.21 | 2 | F2:16516 | 30122021\_RID\_2349\_1C.raw |  | 1.1251E4 |  |  |  |  | 1 | 0 | 1 | 0 | 0 | 0 | 0 | 398 | 405 |  |  | PEAKS DB |
| K.FWEDEGIHGGK.S | N | 44.74 | 1273.5728 | 11 | 0.4 | 637.7939 | 2 | 28.56 | 1 | F1:11862 | 28122021\_RID\_2349\_2C.raw | 1.1259E5 |  |  |  |  |  | 1 | 1 | 0 | 0 | 0 | 0 | 0 | 353 | 363 |  |  | PEAKS DB |
| G.AGHQVTVLEASER.A | N | 44.71 | 1395.7106 | 13 | 0.9 | 698.8632 | 2 | 22.59 | 2 | F2:5163 | 30122021\_RID\_2349\_1C.raw |  | 3.5423E4 |  |  |  |  | 1 | 0 | 1 | 0 | 0 | 0 | 0 | 73 | 85 |  |  | PEAKS DB |
| K.FEPPLPPK.K | N | 43.76 | 923.5116 | 8 | -0.4 | 462.7629 | 2 | 36.36 | 1 | F1:17621 | 28122021\_RID\_2349\_2C.raw | 2.2198E7 |  |  |  |  |  | 1 | 1 | 0 | 0 | 0 | 0 | 0 | 322 | 329 |  |  | PEAKS DB |
| K.FGLQLNEFSQEN.D | N | 43.25 | 1424.6572 | 12 | 1.1 | 713.3367 | 2 | 58.34 | 6 | F6:33069 | 31122021\_RID\_2349\_6C.raw |  |  |  |  |  | 9.3396E4 | 1 | 0 | 0 | 0 | 0 | 0 | 1 | 123 | 134 |  |  | PEAKS DB |
| E.GWYANLGPMR.L | N | 42.97 | 1163.5546 | 10 | 0.2 | 582.7847 | 2 | 43.50 | 1 | F1:23833 | 28122021\_RID\_2349\_2C.raw | 3.4804E4 |  |  |  |  |  | 1 | 1 | 0 | 0 | 0 | 0 | 0 | 99 | 108 |  |  | PEAKS DB |
| S.AGQLYEESLGK.V | N | 42.75 | 1193.5928 | 11 | -0.1 | 597.8036 | 2 | 30.49 | 2 | F2:8743 | 30122021\_RID\_2349\_1C.raw |  | 1.8392E5 |  |  |  |  | 1 | 0 | 1 | 0 | 0 | 0 | 0 | 171 | 181 |  |  | PEAKS DB |
| total 28 peptides |  |  |  |  |  |  |  |  |  |  |  |  |  |  |  |  |  |  |  |  |  |  |  |  |  |  |  |  |  |
| --- | --- | --- | --- | --- | --- | --- | --- | --- | --- | --- | --- | --- | --- | --- | --- | --- | --- | --- | --- | --- | --- | --- | --- | --- | --- | --- | --- | --- | --- |

AID53087.1

back to list

  

| Protein Coverage
| Supporting Peptides
|

Protein Coverage:

Supporting Peptides:

| Peptide | Uniq | -10lgP | Mass | Length | ppm | m/z | z | RT | Fraction | Scan | Source File | Area F2 | Area F1 | Area F3 | Area F4 | Area F5 | Area F6 | #Feature | #Feature F2 | #Feature F1 | #Feature F3 | #Feature F4 | #Feature F5 | #Feature F6 | Start | End | PTM | AScore | Found By |
| --- | --- | --- | --- | --- | --- | --- | --- | --- | --- | --- | --- | --- | --- | --- | --- | --- | --- | --- | --- | --- | --- | --- | --- | --- | --- | --- | --- | --- | --- |
| R.KFGLQLNEFSQENDNAWYFIK.N | Y | 106.65 | 2590.2493 | 21 | 0.4 | 864.4240 | 3 | 58.46 | 5 | F5:33481 | 31122021\_RID\_2349\_5C.raw |  |  | 9.9657E6 | 1.1078E6 | 2.5437E6 | 3.5235E5 | 4 | 0 | 0 | 1 | 1 | 1 | 1 | 122 | 142 |  |  | PEAKS DB |
| K.SAGQLYEESLGK.V | N | 80.33 | 1280.6248 | 12 | -0.1 | 641.3196 | 2 | 32.32 | 5 | F5:14229 | 31122021\_RID\_2349\_5C.raw | 3.9268E8 | 5.6746E8 | 8.8153E7 | 1.109E8 | 1.2738E8 | 1.0719E5 | 12 | 3 | 3 | 1 | 1 | 3 | 1 | 170 | 181 |  |  | PEAKS DB |
| Y.GIGDDANFFQALDFK.D | N | 75.31 | 1656.7783 | 15 | 0.2 | 829.3966 | 2 | 64.60 | 3 | F3:23470 | 31122021\_RID\_2349\_3C.raw |  |  | 1.1679E5 |  |  |  | 1 | 0 | 0 | 1 | 0 | 0 | 0 | 391 | 405 |  |  | PEAKS DB |
| R.HDDIFAYEK.R | N | 72.69 | 1136.5138 | 9 | -0.1 | 569.2641 | 2 | 30.15 | 2 | F2:8868 | 30122021\_RID\_2349\_1C.raw | 5.9259E7 | 1.5095E8 | 7.8164E7 |  |  | 1.3576E6 | 7 | 3 | 2 | 1 | 0 | 0 | 1 | 241 | 249 |  |  | PEAKS DB |
| K.KDPGVLKYPVKPSEEGK.S | N | 69.54 | 1870.0199 | 17 | 0.6 | 624.3476 | 3 | 22.58 | 1 | F1:7284 | 28122021\_RID\_2349\_2C.raw | 4.0206E7 | 6.6249E6 | 4.6122E6 | 7.8889E6 | 5.5106E6 |  | 8 | 3 | 1 | 1 | 1 | 2 | 0 | 153 | 169 |  |  | PEAKS DB |
| D.PGVLKYPVKPSEEGK.S | N | 68.71 | 1626.8981 | 15 | 0.0 | 407.7318 | 4 | 23.75 | 1 | F1:8065 | 28122021\_RID\_2349\_2C.raw | 1.9885E7 |  |  |  |  |  | 3 | 3 | 0 | 0 | 0 | 0 | 0 | 155 | 169 |  |  | PEAKS DB |
| K.DPGVLKYPVKPSEEGK.S | N | 68.31 | 1741.9249 | 16 | 0.4 | 581.6492 | 3 | 28.35 | 1 | F1:11687 | 28122021\_RID\_2349\_2C.raw | 3.0191E6 | 1.5465E6 | 5.0737E5 |  | 1.0906E6 |  | 5 | 2 | 1 | 1 | 0 | 1 | 0 | 154 | 169 |  |  | PEAKS DB |
| R.KFGLQLNEFSQEND.N | N | 67.11 | 1667.7791 | 14 | 0.6 | 834.8973 | 2 | 47.96 | 6 | F6:25528 | 31122021\_RID\_2349\_6C.raw |  |  |  |  |  | 3.2405E6 | 1 | 0 | 0 | 0 | 0 | 0 | 1 | 122 | 135 |  |  | PEAKS DB |
| R.HDDIFAYEKR.F | N | 60.61 | 1292.6149 | 10 | 0.6 | 431.8792 | 3 | 25.32 | 1 | F1:9449 | 28122021\_RID\_2349\_2C.raw | 9.7777E5 | 4.5859E5 |  |  |  |  | 2 | 1 | 1 | 0 | 0 | 0 | 0 | 241 | 250 |  |  | PEAKS DB |
| R.IKFEPPLPPK.K | N | 58.77 | 1164.6907 | 10 | 0.3 | 583.3528 | 2 | 36.36 | 1 | F1:18064 | 28122021\_RID\_2349\_2C.raw | 6.5252E7 | 6.2344E7 | 1.1354E8 | 5.5586E6 | 1.9599E6 | 2.0974E6 | 11 | 3 | 2 | 3 | 1 | 1 | 1 | 320 | 329 |  |  | PEAKS DB |
| D.YEEFLEIAR.N | N | 56.31 | 1168.5764 | 9 | 0.7 | 585.2959 | 2 | 45.58 | 2 | F2:15608 | 30122021\_RID\_2349\_1C.raw |  | 2.3921E5 |  |  |  |  | 1 | 0 | 1 | 0 | 0 | 0 | 0 | 34 | 42 |  |  | PEAKS DB |
| T.DYEEFLEIAR.N | N | 56.01 | 1283.6033 | 10 | 0.8 | 642.8094 | 2 | 56.80 | 5 | F5:32140 | 31122021\_RID\_2349\_5C.raw | 1.3399E5 | 2.7826E5 |  |  | 9.5716E4 | 7.7266E4 | 4 | 1 | 1 | 0 | 0 | 1 | 1 | 33 | 42 |  |  | PEAKS DB |
| A.GQLYEESLGK.V | N | 54.58 | 1122.5557 | 10 | 1.2 | 562.2858 | 2 | 29.95 | 1 | F1:13018 | 28122021\_RID\_2349\_2C.raw | 4.0796E5 | 6.449E5 |  |  |  |  | 2 | 1 | 1 | 0 | 0 | 0 | 0 | 172 | 181 |  |  | PEAKS DB |
| N.EFSQENDNAWYFIK.N | N | 53.57 | 1789.7947 | 14 | 0.5 | 895.9051 | 2 | 53.07 | 1 | F1:31357 | 28122021\_RID\_2349\_2C.raw | 2.5591E5 | 2.9753E5 |  |  |  |  | 2 | 1 | 1 | 0 | 0 | 0 | 0 | 129 | 142 |  |  | PEAKS DB |
| F.SQENDNAWYFIK.N | N | 53.25 | 1513.6837 | 12 | 1.0 | 757.8499 | 2 | 47.51 | 2 | F2:16263 | 30122021\_RID\_2349\_1C.raw |  | 7.0312E5 |  |  |  |  | 1 | 0 | 1 | 0 | 0 | 0 | 0 | 131 | 142 |  |  | PEAKS DB |
| K.KFWEDEGIHGGK.S | N | 51.61 | 1401.6677 | 12 | -0.6 | 468.2296 | 3 | 24.58 | 1 | F1:8904 | 28122021\_RID\_2349\_2C.raw | 4.5254E5 |  |  |  |  |  | 1 | 1 | 0 | 0 | 0 | 0 | 0 | 352 | 363 |  |  | PEAKS DB |
| R.IKFEPPLPPKK.A | N | 50.54 | 1292.7855 | 11 | 0.1 | 431.9358 | 3 | 29.13 | 1 | F1:12372 | 28122021\_RID\_2349\_2C.raw | 9.1587E6 | 3.2038E6 | 8.8306E5 |  |  |  | 4 | 2 | 1 | 1 | 0 | 0 | 0 | 320 | 330 |  |  | PEAKS DB |
| K.YDTYSTKEY.L | N | 49.76 | 1168.4924 | 9 | 1.0 | 585.2541 | 2 | 25.91 | 5 | F5:9933 | 31122021\_RID\_2349\_5C.raw |  |  |  |  | 2.4008E6 |  | 1 | 0 | 0 | 0 | 0 | 1 | 0 | 198 | 206 |  |  | PEAKS DB |
| K.FGLQLNEFSQEND.N | N | 47.16 | 1539.6841 | 13 | 0.5 | 770.8497 | 2 | 59.71 | 6 | F6:34055 | 31122021\_RID\_2349\_6C.raw |  |  |  |  |  | 4.3601E5 | 1 | 0 | 0 | 0 | 0 | 0 | 1 | 123 | 135 |  |  | PEAKS DB |
| E.AHGWIDSTIK.S | N | 45.39 | 1126.5771 | 10 | 1.2 | 564.2965 | 2 | 28.81 | 2 | F2:8087 | 30122021\_RID\_2349\_1C.raw |  | 3.6217E5 |  |  |  |  | 1 | 0 | 1 | 0 | 0 | 0 | 0 | 480 | 489 |  |  | PEAKS DB |
| H.QVTVLEASER.A | N | 45.38 | 1130.5931 | 10 | -0.2 | 566.3037 | 2 | 28.45 | 2 | F2:7775 | 30122021\_RID\_2349\_1C.raw |  | 6.9878E5 |  |  |  |  | 1 | 0 | 1 | 0 | 0 | 0 | 0 | 76 | 85 |  |  | PEAKS DB |
| N.FFQALDFK.D | N | 45.08 | 1014.5174 | 8 | 0.8 | 508.2664 | 2 | 48.21 | 2 | F2:16516 | 30122021\_RID\_2349\_1C.raw |  | 1.1251E4 |  |  |  |  | 1 | 0 | 1 | 0 | 0 | 0 | 0 | 398 | 405 |  |  | PEAKS DB |
| K.FWEDEGIHGGK.S | N | 44.74 | 1273.5728 | 11 | 0.4 | 637.7939 | 2 | 28.56 | 1 | F1:11862 | 28122021\_RID\_2349\_2C.raw | 1.1259E5 |  |  |  |  |  | 1 | 1 | 0 | 0 | 0 | 0 | 0 | 353 | 363 |  |  | PEAKS DB |
| G.AGHQVTVLEASER.A | N | 44.71 | 1395.7106 | 13 | 0.9 | 698.8632 | 2 | 22.59 | 2 | F2:5163 | 30122021\_RID\_2349\_1C.raw |  | 3.5423E4 |  |  |  |  | 1 | 0 | 1 | 0 | 0 | 0 | 0 | 73 | 85 |  |  | PEAKS DB |
| K.FEPPLPPK.K | N | 43.76 | 923.5116 | 8 | -0.4 | 462.7629 | 2 | 36.36 | 1 | F1:17621 | 28122021\_RID\_2349\_2C.raw | 2.2198E7 |  |  |  |  |  | 1 | 1 | 0 | 0 | 0 | 0 | 0 | 322 | 329 |  |  | PEAKS DB |
| K.FGLQLNEFSQEN.D | N | 43.25 | 1424.6572 | 12 | 1.1 | 713.3367 | 2 | 58.34 | 6 | F6:33069 | 31122021\_RID\_2349\_6C.raw |  |  |  |  |  | 9.3396E4 | 1 | 0 | 0 | 0 | 0 | 0 | 1 | 123 | 134 |  |  | PEAKS DB |
| E.GWYANLGPMR.L | N | 42.97 | 1163.5546 | 10 | 0.2 | 582.7847 | 2 | 43.50 | 1 | F1:23833 | 28122021\_RID\_2349\_2C.raw | 3.4804E4 |  |  |  |  |  | 1 | 1 | 0 | 0 | 0 | 0 | 0 | 99 | 108 |  |  | PEAKS DB |
| S.AGQLYEESLGK.V | N | 42.75 | 1193.5928 | 11 | -0.1 | 597.8036 | 2 | 30.49 | 2 | F2:8743 | 30122021\_RID\_2349\_1C.raw |  | 1.8392E5 |  |  |  |  | 1 | 0 | 1 | 0 | 0 | 0 | 0 | 171 | 181 |  |  | PEAKS DB |
| total 28 peptides |  |  |  |  |  |  |  |  |  |  |  |  |  |  |  |  |  |  |  |  |  |  |  |  |  |  |  |  |  |
| --- | --- | --- | --- | --- | --- | --- | --- | --- | --- | --- | --- | --- | --- | --- | --- | --- | --- | --- | --- | --- | --- | --- | --- | --- | --- | --- | --- | --- | --- |

AFJ50957.1

back to list

  

| Protein Coverage
| Supporting Peptides
|

Protein Coverage:

Supporting Peptides:

| Peptide | Uniq | -10lgP | Mass | Length | ppm | m/z | z | RT | Fraction | Scan | Source File | Area F2 | Area F1 | Area F3 | Area F4 | Area F5 | Area F6 | #Feature | #Feature F2 | #Feature F1 | #Feature F3 | #Feature F4 | #Feature F5 | #Feature F6 | Start | End | PTM | AScore | Found By |
| --- | --- | --- | --- | --- | --- | --- | --- | --- | --- | --- | --- | --- | --- | --- | --- | --- | --- | --- | --- | --- | --- | --- | --- | --- | --- | --- | --- | --- | --- |
| K.QNSGTYNNQYMILDTK.K | N | 89.37 | 1888.8625 | 16 | 0.2 | 945.4387 | 2 | 40.12 | 1 | F1:21225 | 28122021\_RID\_2349\_2C.raw | 1.0877E7 |  |  |  | 1.1423E5 |  | 3 | 2 | 0 | 0 | 0 | 1 | 0 | 351 | 366 |  |  | PEAKS DB |
| A.DIHYATVYWLEAEK.S | N | 86.70 | 1736.8409 | 14 | 0.4 | 869.4281 | 2 | 50.82 | 6 | F6:27720 | 31122021\_RID\_2349\_6C.raw |  |  |  |  | 2.5122E6 | 1.489E6 | 4 | 0 | 0 | 0 | 0 | 2 | 2 | 37 | 50 |  |  | PEAKS DB |
| K.VKDFIQKQDEWTR.Q | N | 83.49 | 1691.8630 | 13 | 0.5 | 564.9619 | 3 | 27.81 | 5 | F5:11246 | 31122021\_RID\_2349\_5C.raw |  |  |  |  | 1.6688E6 | 1.7564E5 | 3 | 0 | 0 | 0 | 0 | 2 | 1 | 133 | 145 |  |  | PEAKS DB |
| K.FTAYAINGPPVEK.G | N | 79.89 | 1405.7241 | 13 | 0.6 | 703.8698 | 2 | 39.33 | 1 | F1:20625 | 28122021\_RID\_2349\_2C.raw | 1.0693E7 |  |  |  |  | 7.4802E4 | 2 | 1 | 0 | 0 | 0 | 0 | 1 | 508 | 520 |  |  | PEAKS DB |
| K.VVPESLFAWER.V | N | 78.21 | 1331.6874 | 11 | 0.6 | 666.8514 | 2 | 53.73 | 1 | F1:31675 | 28122021\_RID\_2349\_2C.raw | 2.3421E7 |  |  |  |  | 1.2729E6 | 2 | 1 | 0 | 0 | 0 | 0 | 1 | 320 | 330 |  |  | PEAKS DB |
| R.SLEDGTLYIIEQVPK.L | Y | 76.84 | 1703.8981 | 15 | 0.4 | 852.9567 | 2 | 59.24 | 1 | F1:35518 | 28122021\_RID\_2349\_2C.raw | 2.1874E6 |  |  |  |  |  | 2 | 2 | 0 | 0 | 0 | 0 | 0 | 373 | 387 |  |  | PEAKS DB |
| R.IANMMADSGK.T | N | 75.60 | 1036.4681 | 10 | -1.0 | 519.2408 | 2 | 24.14 | 1 | F1:8353 | 28122021\_RID\_2349\_2C.raw | 1.8547E7 |  |  |  |  |  | 1 | 1 | 0 | 0 | 0 | 0 | 0 | 333 | 342 |  |  | PEAKS DB |
| K.QNSGTYNNQYM(+15.99)ILDTK.K | N | 75.10 | 1904.8574 | 16 | 0.7 | 953.4366 | 2 | 36.00 | 1 | F1:17991 | 28122021\_RID\_2349\_2C.raw | 4.466E6 |  |  |  |  |  | 4 | 4 | 0 | 0 | 0 | 0 | 0 | 351 | 366 | Oxidation (M) | M11:Oxidation (M):1000.00 | PEAKS DB |
| K.HQGLPESYNFDFVTMKPVL | N | 73.59 | 2221.0876 | 19 | 1.1 | 741.3707 | 3 | 57.46 | 1 | F1:34351 | 28122021\_RID\_2349\_2C.raw | 1.288E7 |  |  |  |  |  | 2 | 2 | 0 | 0 | 0 | 0 | 0 | 535 | 553 |  |  | PEAKS DB |
| R.NGYWPSYNIPFDK.E | N | 72.13 | 1599.7357 | 13 | -0.3 | 800.8749 | 2 | 53.44 | 1 | F1:31595 | 28122021\_RID\_2349\_2C.raw | 6.1512E6 |  |  |  |  |  | 1 | 1 | 0 | 0 | 0 | 0 | 0 | 400 | 412 |  |  | PEAKS DB |
| K.HQGLPESYNFDFVTM(+15.99)KPVL | N | 71.00 | 2237.0828 | 19 | 0.5 | 746.7019 | 3 | 52.61 | 1 | F1:30998 | 28122021\_RID\_2349\_2C.raw | 4.8497E6 |  |  |  |  |  | 2 | 2 | 0 | 0 | 0 | 0 | 0 | 535 | 553 | Oxidation (M) | M15:Oxidation (M):1000.00 | PEAKS DB |
| R.IANM(+15.99)MADSGK.T | N | 69.25 | 1052.4630 | 10 | 0.1 | 527.2388 | 2 | 19.75 | 1 | F1:4522 | 28122021\_RID\_2349\_2C.raw | 2.4471E7 |  |  |  |  |  | 6 | 6 | 0 | 0 | 0 | 0 | 0 | 333 | 342 | Oxidation (M) | M4:Oxidation (M):30.46 | PEAKS DB |
| K.VADISMAAK.F | N | 69.21 | 904.4688 | 9 | 0.2 | 453.2418 | 2 | 26.21 | 1 | F1:10174 | 28122021\_RID\_2349\_2C.raw | 2.4604E7 |  |  |  |  | 8.0654E4 | 2 | 1 | 0 | 0 | 0 | 0 | 1 | 499 | 507 |  |  | PEAKS DB |
| R.HGLEFSYEMAPR.A | N | 68.50 | 1435.6554 | 12 | 0.7 | 718.8354 | 2 | 37.79 | 1 | F1:19391 | 28122021\_RID\_2349\_2C.raw | 1.1944E6 |  |  |  |  |  | 2 | 2 | 0 | 0 | 0 | 0 | 0 | 427 | 438 |  |  | PEAKS DB |
| K.KVVPESLFAWER.V | N | 67.95 | 1459.7823 | 12 | 0.3 | 730.8987 | 2 | 44.90 | 1 | F1:25054 | 28122021\_RID\_2349\_2C.raw | 1.3139E7 |  |  |  |  | 1.8426E5 | 3 | 2 | 0 | 0 | 0 | 0 | 1 | 319 | 330 |  |  | PEAKS DB |
| R.IANMM(+15.99)ADSGK.T | N | 67.88 | 1052.4630 | 10 | 0.1 | 527.2388 | 2 | 19.75 | 1 | F1:4789 | 28122021\_RID\_2349\_2C.raw | 1.7957E7 |  |  |  |  |  | 1 | 1 | 0 | 0 | 0 | 0 | 0 | 333 | 342 | Oxidation (M) | M5:Oxidation (M):18.17 | PEAKS DB |
| K.NNKDDPFWR.N | N | 67.25 | 1190.5469 | 9 | 0.5 | 596.2810 | 2 | 28.10 | 5 | F5:11429 | 31122021\_RID\_2349\_5C.raw |  |  |  |  | 5.6786E6 |  | 2 | 0 | 0 | 0 | 0 | 2 | 0 | 150 | 158 |  |  | PEAKS DB |
| N.SGTYNNQYMILDTK.K | N | 65.95 | 1646.7610 | 14 | 0.9 | 824.3885 | 2 | 40.39 | 1 | F1:21338 | 28122021\_RID\_2349\_2C.raw | 2.6394E5 |  |  |  |  |  | 1 | 1 | 0 | 0 | 0 | 0 | 0 | 353 | 366 |  |  | PEAKS DB |
| R.DQGKVTDMESMK.F | N | 64.71 | 1367.6061 | 12 | 0.9 | 684.8109 | 2 | 24.00 | 1 | F1:8415 | 28122021\_RID\_2349\_2C.raw | 2.2724E6 |  |  |  |  |  | 1 | 1 | 0 | 0 | 0 | 0 | 0 | 445 | 456 |  |  | PEAKS DB |
| R.DQGKVTDMESM(+15.99)K.F | N | 62.42 | 1383.6010 | 12 | 0.0 | 692.8077 | 2 | 18.14 | 1 | F1:4116 | 28122021\_RID\_2349\_2C.raw | 7.9689E5 |  |  |  |  |  | 2 | 2 | 0 | 0 | 0 | 0 | 0 | 445 | 456 | Oxidation (M) | M11:Oxidation (M):43.31 | PEAKS DB |
| R.NAGYVIAQLDGLYMGNVEWAK.R | N | 60.60 | 2311.1306 | 21 | 0.8 | 1156.5735 | 2 | 65.79 | 5 | F5:38140 | 31122021\_RID\_2349\_5C.raw |  |  |  |  | 9.6071E4 |  | 1 | 0 | 0 | 0 | 0 | 1 | 0 | 159 | 179 |  |  | PEAKS DB |
| K.VADISM(+15.99)AAK.F | N | 58.55 | 920.4637 | 9 | -0.8 | 461.2387 | 2 | 26.23 | 1 | F1:10049 | 28122021\_RID\_2349\_2C.raw | 1.8947E6 |  |  |  |  |  | 1 | 1 | 0 | 0 | 0 | 0 | 0 | 499 | 507 | Oxidation (M) | M6:Oxidation (M):1000.00 | PEAKS DB |
| R.HGLEFSYEM(+15.99)APR.A | N | 57.54 | 1451.6503 | 12 | -0.2 | 726.8323 | 2 | 32.44 | 1 | F1:15087 | 28122021\_RID\_2349\_2C.raw | 1.3899E6 |  |  |  |  |  | 3 | 3 | 0 | 0 | 0 | 0 | 0 | 427 | 438 | Oxidation (M) | M9:Oxidation (M):1000.00 | PEAKS DB |
| K.TWAETFEK.Q | N | 55.89 | 1010.4709 | 8 | -0.7 | 506.2424 | 2 | 34.11 | 1 | F1:16345 | 28122021\_RID\_2349\_2C.raw | 3.866E7 |  |  |  |  |  | 1 | 1 | 0 | 0 | 0 | 0 | 0 | 343 | 350 |  |  | PEAKS DB |
| R.IANMMADSGKTWAETFEK.Q | N | 55.68 | 2028.9285 | 18 | 0.4 | 677.3170 | 3 | 44.54 | 1 | F1:24655 | 28122021\_RID\_2349\_2C.raw | 4.0453E5 |  |  |  |  |  | 1 | 1 | 0 | 0 | 0 | 0 | 0 | 333 | 350 |  |  | PEAKS DB |
| R.YNNYKEDPYAK.H | N | 55.54 | 1403.6357 | 11 | 0.2 | 702.8253 | 2 | 19.40 | 1 | F1:4792 | 28122021\_RID\_2349\_2C.raw | 1.2416E6 |  |  |  |  |  | 2 | 2 | 0 | 0 | 0 | 0 | 0 | 461 | 471 |  |  | PEAKS DB |
| N.NQYMILDTK.K | N | 51.50 | 1124.5536 | 9 | -0.3 | 563.2839 | 2 | 37.24 | 1 | F1:18793 | 28122021\_RID\_2349\_2C.raw | 1.1739E5 |  |  |  |  |  | 1 | 1 | 0 | 0 | 0 | 0 | 0 | 358 | 366 |  |  | PEAKS DB |
| H.SSWFTYAATLR.I | N | 48.67 | 1301.6404 | 11 | 0.3 | 651.8276 | 2 | 51.40 | 1 | F1:29950 | 28122021\_RID\_2349\_2C.raw | 2.188E5 |  |  |  |  |  | 1 | 1 | 0 | 0 | 0 | 0 | 0 | 251 | 261 |  |  | PEAKS DB |
| Y.NFDFVTMKPVL | N | 47.54 | 1309.6741 | 11 | 0.3 | 655.8445 | 2 | 58.32 | 1 | F1:34760 | 28122021\_RID\_2349\_2C.raw | 6.2109E4 |  |  |  |  |  | 1 | 1 | 0 | 0 | 0 | 0 | 0 | 543 | 553 |  |  | PEAKS DB |
| K.QNSGTYNNQYMILDTKK.I | N | 45.69 | 2016.9575 | 17 | 0.6 | 673.3268 | 3 | 32.64 | 1 | F1:15031 | 28122021\_RID\_2349\_2C.raw | 2.6845E6 |  |  |  |  |  | 1 | 1 | 0 | 0 | 0 | 0 | 0 | 351 | 367 |  |  | PEAKS DB |
| total 30 peptides |  |  |  |  |  |  |  |  |  |  |  |  |  |  |  |  |  |  |  |  |  |  |  |  |  |  |  |  |  |
| --- | --- | --- | --- | --- | --- | --- | --- | --- | --- | --- | --- | --- | --- | --- | --- | --- | --- | --- | --- | --- | --- | --- | --- | --- | --- | --- | --- | --- | --- |

AEJ31983.1

back to list

  

| Protein Coverage
| Supporting Peptides
|

Protein Coverage:

Supporting Peptides:

| Peptide | Uniq | -10lgP | Mass | Length | ppm | m/z | z | RT | Fraction | Scan | Source File | Area F2 | Area F1 | Area F3 | Area F4 | Area F5 | Area F6 | #Feature | #Feature F2 | #Feature F1 | #Feature F3 | #Feature F4 | #Feature F5 | #Feature F6 | Start | End | PTM | AScore | Found By |
| --- | --- | --- | --- | --- | --- | --- | --- | --- | --- | --- | --- | --- | --- | --- | --- | --- | --- | --- | --- | --- | --- | --- | --- | --- | --- | --- | --- | --- | --- |
| K.QNSGTYNNQYMILDTK.K | N | 89.37 | 1888.8625 | 16 | 0.2 | 945.4387 | 2 | 40.12 | 1 | F1:21225 | 28122021\_RID\_2349\_2C.raw | 1.0877E7 |  |  |  | 1.1423E5 |  | 3 | 2 | 0 | 0 | 0 | 1 | 0 | 351 | 366 |  |  | PEAKS DB |
| A.DIHYATVYWLEAEK.S | N | 86.70 | 1736.8409 | 14 | 0.4 | 869.4281 | 2 | 50.82 | 6 | F6:27720 | 31122021\_RID\_2349\_6C.raw |  |  |  |  | 2.5122E6 | 1.489E6 | 4 | 0 | 0 | 0 | 0 | 2 | 2 | 37 | 50 |  |  | PEAKS DB |
| K.VKDFIQKQDEWTR.Q | N | 83.49 | 1691.8630 | 13 | 0.5 | 564.9619 | 3 | 27.81 | 5 | F5:11246 | 31122021\_RID\_2349\_5C.raw |  |  |  |  | 1.6688E6 | 1.7564E5 | 3 | 0 | 0 | 0 | 0 | 2 | 1 | 133 | 145 |  |  | PEAKS DB |
| K.FTAYAINGPPVEK.G | N | 79.89 | 1405.7241 | 13 | 0.6 | 703.8698 | 2 | 39.33 | 1 | F1:20625 | 28122021\_RID\_2349\_2C.raw | 1.0693E7 |  |  |  |  | 7.4802E4 | 2 | 1 | 0 | 0 | 0 | 0 | 1 | 508 | 520 |  |  | PEAKS DB |
| K.VVPESLFAWER.V | N | 78.21 | 1331.6874 | 11 | 0.6 | 666.8514 | 2 | 53.73 | 1 | F1:31675 | 28122021\_RID\_2349\_2C.raw | 2.3421E7 |  |  |  |  | 1.2729E6 | 2 | 1 | 0 | 0 | 0 | 0 | 1 | 320 | 330 |  |  | PEAKS DB |
| R.SLEDGTLYIIEQVPK.L | Y | 76.84 | 1703.8981 | 15 | 0.4 | 852.9567 | 2 | 59.24 | 1 | F1:35518 | 28122021\_RID\_2349\_2C.raw | 2.1874E6 |  |  |  |  |  | 2 | 2 | 0 | 0 | 0 | 0 | 0 | 373 | 387 |  |  | PEAKS DB |
| R.IANMMADSGK.T | N | 75.60 | 1036.4681 | 10 | -1.0 | 519.2408 | 2 | 24.14 | 1 | F1:8353 | 28122021\_RID\_2349\_2C.raw | 1.8547E7 |  |  |  |  |  | 1 | 1 | 0 | 0 | 0 | 0 | 0 | 333 | 342 |  |  | PEAKS DB |
| K.QNSGTYNNQYM(+15.99)ILDTK.K | N | 75.10 | 1904.8574 | 16 | 0.7 | 953.4366 | 2 | 36.00 | 1 | F1:17991 | 28122021\_RID\_2349\_2C.raw | 4.466E6 |  |  |  |  |  | 4 | 4 | 0 | 0 | 0 | 0 | 0 | 351 | 366 | Oxidation (M) | M11:Oxidation (M):1000.00 | PEAKS DB |
| K.HQGLPESYNFDFVTMKPVL | N | 73.59 | 2221.0876 | 19 | 1.1 | 741.3707 | 3 | 57.46 | 1 | F1:34351 | 28122021\_RID\_2349\_2C.raw | 1.288E7 |  |  |  |  |  | 2 | 2 | 0 | 0 | 0 | 0 | 0 | 535 | 553 |  |  | PEAKS DB |
| R.NGYWPSYNIPFDK.E | N | 72.13 | 1599.7357 | 13 | -0.3 | 800.8749 | 2 | 53.44 | 1 | F1:31595 | 28122021\_RID\_2349\_2C.raw | 6.1512E6 |  |  |  |  |  | 1 | 1 | 0 | 0 | 0 | 0 | 0 | 400 | 412 |  |  | PEAKS DB |
| K.HQGLPESYNFDFVTM(+15.99)KPVL | N | 71.00 | 2237.0828 | 19 | 0.5 | 746.7019 | 3 | 52.61 | 1 | F1:30998 | 28122021\_RID\_2349\_2C.raw | 4.8497E6 |  |  |  |  |  | 2 | 2 | 0 | 0 | 0 | 0 | 0 | 535 | 553 | Oxidation (M) | M15:Oxidation (M):1000.00 | PEAKS DB |
| R.IANM(+15.99)MADSGK.T | N | 69.25 | 1052.4630 | 10 | 0.1 | 527.2388 | 2 | 19.75 | 1 | F1:4522 | 28122021\_RID\_2349\_2C.raw | 2.4471E7 |  |  |  |  |  | 6 | 6 | 0 | 0 | 0 | 0 | 0 | 333 | 342 | Oxidation (M) | M4:Oxidation (M):30.46 | PEAKS DB |
| K.VADISMAAK.F | N | 69.21 | 904.4688 | 9 | 0.2 | 453.2418 | 2 | 26.21 | 1 | F1:10174 | 28122021\_RID\_2349\_2C.raw | 2.4604E7 |  |  |  |  | 8.0654E4 | 2 | 1 | 0 | 0 | 0 | 0 | 1 | 499 | 507 |  |  | PEAKS DB |
| R.HGLEFSYEMAPR.A | N | 68.50 | 1435.6554 | 12 | 0.7 | 718.8354 | 2 | 37.79 | 1 | F1:19391 | 28122021\_RID\_2349\_2C.raw | 1.1944E6 |  |  |  |  |  | 2 | 2 | 0 | 0 | 0 | 0 | 0 | 427 | 438 |  |  | PEAKS DB |
| K.KVVPESLFAWER.V | N | 67.95 | 1459.7823 | 12 | 0.3 | 730.8987 | 2 | 44.90 | 1 | F1:25054 | 28122021\_RID\_2349\_2C.raw | 1.3139E7 |  |  |  |  | 1.8426E5 | 3 | 2 | 0 | 0 | 0 | 0 | 1 | 319 | 330 |  |  | PEAKS DB |
| R.IANMM(+15.99)ADSGK.T | N | 67.88 | 1052.4630 | 10 | 0.1 | 527.2388 | 2 | 19.75 | 1 | F1:4789 | 28122021\_RID\_2349\_2C.raw | 1.7957E7 |  |  |  |  |  | 1 | 1 | 0 | 0 | 0 | 0 | 0 | 333 | 342 | Oxidation (M) | M5:Oxidation (M):18.17 | PEAKS DB |
| K.NNKDDPFWR.N | N | 67.25 | 1190.5469 | 9 | 0.5 | 596.2810 | 2 | 28.10 | 5 | F5:11429 | 31122021\_RID\_2349\_5C.raw |  |  |  |  | 5.6786E6 |  | 2 | 0 | 0 | 0 | 0 | 2 | 0 | 150 | 158 |  |  | PEAKS DB |
| N.SGTYNNQYMILDTK.K | N | 65.95 | 1646.7610 | 14 | 0.9 | 824.3885 | 2 | 40.39 | 1 | F1:21338 | 28122021\_RID\_2349\_2C.raw | 2.6394E5 |  |  |  |  |  | 1 | 1 | 0 | 0 | 0 | 0 | 0 | 353 | 366 |  |  | PEAKS DB |
| R.DQGKVTDMESMK.F | N | 64.71 | 1367.6061 | 12 | 0.9 | 684.8109 | 2 | 24.00 | 1 | F1:8415 | 28122021\_RID\_2349\_2C.raw | 2.2724E6 |  |  |  |  |  | 1 | 1 | 0 | 0 | 0 | 0 | 0 | 445 | 456 |  |  | PEAKS DB |
| R.DQGKVTDMESM(+15.99)K.F | N | 62.42 | 1383.6010 | 12 | 0.0 | 692.8077 | 2 | 18.14 | 1 | F1:4116 | 28122021\_RID\_2349\_2C.raw | 7.9689E5 |  |  |  |  |  | 2 | 2 | 0 | 0 | 0 | 0 | 0 | 445 | 456 | Oxidation (M) | M11:Oxidation (M):43.31 | PEAKS DB |
| R.NAGYVIAQLDGLYMGNVEWAK.R | N | 60.60 | 2311.1306 | 21 | 0.8 | 1156.5735 | 2 | 65.79 | 5 | F5:38140 | 31122021\_RID\_2349\_5C.raw |  |  |  |  | 9.6071E4 |  | 1 | 0 | 0 | 0 | 0 | 1 | 0 | 159 | 179 |  |  | PEAKS DB |
| K.VADISM(+15.99)AAK.F | N | 58.55 | 920.4637 | 9 | -0.8 | 461.2387 | 2 | 26.23 | 1 | F1:10049 | 28122021\_RID\_2349\_2C.raw | 1.8947E6 |  |  |  |  |  | 1 | 1 | 0 | 0 | 0 | 0 | 0 | 499 | 507 | Oxidation (M) | M6:Oxidation (M):1000.00 | PEAKS DB |
| R.HGLEFSYEM(+15.99)APR.A | N | 57.54 | 1451.6503 | 12 | -0.2 | 726.8323 | 2 | 32.44 | 1 | F1:15087 | 28122021\_RID\_2349\_2C.raw | 1.3899E6 |  |  |  |  |  | 3 | 3 | 0 | 0 | 0 | 0 | 0 | 427 | 438 | Oxidation (M) | M9:Oxidation (M):1000.00 | PEAKS DB |
| K.TWAETFEK.Q | N | 55.89 | 1010.4709 | 8 | -0.7 | 506.2424 | 2 | 34.11 | 1 | F1:16345 | 28122021\_RID\_2349\_2C.raw | 3.866E7 |  |  |  |  |  | 1 | 1 | 0 | 0 | 0 | 0 | 0 | 343 | 350 |  |  | PEAKS DB |
| R.IANMMADSGKTWAETFEK.Q | N | 55.68 | 2028.9285 | 18 | 0.4 | 677.3170 | 3 | 44.54 | 1 | F1:24655 | 28122021\_RID\_2349\_2C.raw | 4.0453E5 |  |  |  |  |  | 1 | 1 | 0 | 0 | 0 | 0 | 0 | 333 | 350 |  |  | PEAKS DB |
| R.YNNYKEDPYAK.H | N | 55.54 | 1403.6357 | 11 | 0.2 | 702.8253 | 2 | 19.40 | 1 | F1:4792 | 28122021\_RID\_2349\_2C.raw | 1.2416E6 |  |  |  |  |  | 2 | 2 | 0 | 0 | 0 | 0 | 0 | 461 | 471 |  |  | PEAKS DB |
| N.NQYMILDTK.K | N | 51.50 | 1124.5536 | 9 | -0.3 | 563.2839 | 2 | 37.24 | 1 | F1:18793 | 28122021\_RID\_2349\_2C.raw | 1.1739E5 |  |  |  |  |  | 1 | 1 | 0 | 0 | 0 | 0 | 0 | 358 | 366 |  |  | PEAKS DB |
| H.SSWFTYAATLR.I | N | 48.67 | 1301.6404 | 11 | 0.3 | 651.8276 | 2 | 51.40 | 1 | F1:29950 | 28122021\_RID\_2349\_2C.raw | 2.188E5 |  |  |  |  |  | 1 | 1 | 0 | 0 | 0 | 0 | 0 | 251 | 261 |  |  | PEAKS DB |
| Y.NFDFVTMKPVL | N | 47.54 | 1309.6741 | 11 | 0.3 | 655.8445 | 2 | 58.32 | 1 | F1:34760 | 28122021\_RID\_2349\_2C.raw | 6.2109E4 |  |  |  |  |  | 1 | 1 | 0 | 0 | 0 | 0 | 0 | 543 | 553 |  |  | PEAKS DB |
| K.QNSGTYNNQYMILDTKK.I | N | 45.69 | 2016.9575 | 17 | 0.6 | 673.3268 | 3 | 32.64 | 1 | F1:15031 | 28122021\_RID\_2349\_2C.raw | 2.6845E6 |  |  |  |  |  | 1 | 1 | 0 | 0 | 0 | 0 | 0 | 351 | 367 |  |  | PEAKS DB |
| total 30 peptides |  |  |  |  |  |  |  |  |  |  |  |  |  |  |  |  |  |  |  |  |  |  |  |  |  |  |  |  |  |
| --- | --- | --- | --- | --- | --- | --- | --- | --- | --- | --- | --- | --- | --- | --- | --- | --- | --- | --- | --- | --- | --- | --- | --- | --- | --- | --- | --- | --- | --- |

JAI12031.1

back to list

  

| Protein Coverage
| Supporting Peptides
|

Protein Coverage:

Supporting Peptides:

| Peptide | Uniq | -10lgP | Mass | Length | ppm | m/z | z | RT | Fraction | Scan | Source File | Area F2 | Area F1 | Area F3 | Area F4 | Area F5 | Area F6 | #Feature | #Feature F2 | #Feature F1 | #Feature F3 | #Feature F4 | #Feature F5 | #Feature F6 | Start | End | PTM | AScore | Found By |
| --- | --- | --- | --- | --- | --- | --- | --- | --- | --- | --- | --- | --- | --- | --- | --- | --- | --- | --- | --- | --- | --- | --- | --- | --- | --- | --- | --- | --- | --- |
| K.QNSGTYNNQYMILDTK.K | N | 89.37 | 1888.8625 | 16 | 0.2 | 945.4387 | 2 | 40.12 | 1 | F1:21225 | 28122021\_RID\_2349\_2C.raw | 1.0877E7 |  |  |  | 1.1423E5 |  | 3 | 2 | 0 | 0 | 0 | 1 | 0 | 351 | 366 |  |  | PEAKS DB |
| A.DIHYATVYWLEAEK.S | N | 86.70 | 1736.8409 | 14 | 0.4 | 869.4281 | 2 | 50.82 | 6 | F6:27720 | 31122021\_RID\_2349\_6C.raw |  |  |  |  | 2.5122E6 | 1.489E6 | 4 | 0 | 0 | 0 | 0 | 2 | 2 | 37 | 50 |  |  | PEAKS DB |
| K.VKDFIQKQDEWTR.Q | N | 83.49 | 1691.8630 | 13 | 0.5 | 564.9619 | 3 | 27.81 | 5 | F5:11246 | 31122021\_RID\_2349\_5C.raw |  |  |  |  | 1.6688E6 | 1.7564E5 | 3 | 0 | 0 | 0 | 0 | 2 | 1 | 133 | 145 |  |  | PEAKS DB |
| K.FTAYAINGPPVEK.G | N | 79.89 | 1405.7241 | 13 | 0.6 | 703.8698 | 2 | 39.33 | 1 | F1:20625 | 28122021\_RID\_2349\_2C.raw | 1.0693E7 |  |  |  |  | 7.4802E4 | 2 | 1 | 0 | 0 | 0 | 0 | 1 | 508 | 520 |  |  | PEAKS DB |
| K.VVPESLFAWER.V | N | 78.21 | 1331.6874 | 11 | 0.6 | 666.8514 | 2 | 53.73 | 1 | F1:31675 | 28122021\_RID\_2349\_2C.raw | 2.3421E7 |  |  |  |  | 1.2729E6 | 2 | 1 | 0 | 0 | 0 | 0 | 1 | 320 | 330 |  |  | PEAKS DB |
| R.SLEDGTLYIIEQVPK.L | Y | 76.84 | 1703.8981 | 15 | 0.4 | 852.9567 | 2 | 59.24 | 1 | F1:35518 | 28122021\_RID\_2349\_2C.raw | 2.1874E6 |  |  |  |  |  | 2 | 2 | 0 | 0 | 0 | 0 | 0 | 373 | 387 |  |  | PEAKS DB |
| R.IANMMADSGK.T | N | 75.60 | 1036.4681 | 10 | -1.0 | 519.2408 | 2 | 24.14 | 1 | F1:8353 | 28122021\_RID\_2349\_2C.raw | 1.8547E7 |  |  |  |  |  | 1 | 1 | 0 | 0 | 0 | 0 | 0 | 333 | 342 |  |  | PEAKS DB |
| K.QNSGTYNNQYM(+15.99)ILDTK.K | N | 75.10 | 1904.8574 | 16 | 0.7 | 953.4366 | 2 | 36.00 | 1 | F1:17991 | 28122021\_RID\_2349\_2C.raw | 4.466E6 |  |  |  |  |  | 4 | 4 | 0 | 0 | 0 | 0 | 0 | 351 | 366 | Oxidation (M) | M11:Oxidation (M):1000.00 | PEAKS DB |
| K.HQGLPESYNFDFVTMKPVL | N | 73.59 | 2221.0876 | 19 | 1.1 | 741.3707 | 3 | 57.46 | 1 | F1:34351 | 28122021\_RID\_2349\_2C.raw | 1.288E7 |  |  |  |  |  | 2 | 2 | 0 | 0 | 0 | 0 | 0 | 535 | 553 |  |  | PEAKS DB |
| R.NGYWPSYNIPFDK.E | N | 72.13 | 1599.7357 | 13 | -0.3 | 800.8749 | 2 | 53.44 | 1 | F1:31595 | 28122021\_RID\_2349\_2C.raw | 6.1512E6 |  |  |  |  |  | 1 | 1 | 0 | 0 | 0 | 0 | 0 | 400 | 412 |  |  | PEAKS DB |
| K.HQGLPESYNFDFVTM(+15.99)KPVL | N | 71.00 | 2237.0828 | 19 | 0.5 | 746.7019 | 3 | 52.61 | 1 | F1:30998 | 28122021\_RID\_2349\_2C.raw | 4.8497E6 |  |  |  |  |  | 2 | 2 | 0 | 0 | 0 | 0 | 0 | 535 | 553 | Oxidation (M) | M15:Oxidation (M):1000.00 | PEAKS DB |
| R.IANM(+15.99)MADSGK.T | N | 69.25 | 1052.4630 | 10 | 0.1 | 527.2388 | 2 | 19.75 | 1 | F1:4522 | 28122021\_RID\_2349\_2C.raw | 2.4471E7 |  |  |  |  |  | 6 | 6 | 0 | 0 | 0 | 0 | 0 | 333 | 342 | Oxidation (M) | M4:Oxidation (M):30.46 | PEAKS DB |
| K.VADISMAAK.F | N | 69.21 | 904.4688 | 9 | 0.2 | 453.2418 | 2 | 26.21 | 1 | F1:10174 | 28122021\_RID\_2349\_2C.raw | 2.4604E7 |  |  |  |  | 8.0654E4 | 2 | 1 | 0 | 0 | 0 | 0 | 1 | 499 | 507 |  |  | PEAKS DB |
| R.HGLEFSYEMAPR.A | N | 68.50 | 1435.6554 | 12 | 0.7 | 718.8354 | 2 | 37.79 | 1 | F1:19391 | 28122021\_RID\_2349\_2C.raw | 1.1944E6 |  |  |  |  |  | 2 | 2 | 0 | 0 | 0 | 0 | 0 | 427 | 438 |  |  | PEAKS DB |
| K.KVVPESLFAWER.V | N | 67.95 | 1459.7823 | 12 | 0.3 | 730.8987 | 2 | 44.90 | 1 | F1:25054 | 28122021\_RID\_2349\_2C.raw | 1.3139E7 |  |  |  |  | 1.8426E5 | 3 | 2 | 0 | 0 | 0 | 0 | 1 | 319 | 330 |  |  | PEAKS DB |
| R.IANMM(+15.99)ADSGK.T | N | 67.88 | 1052.4630 | 10 | 0.1 | 527.2388 | 2 | 19.75 | 1 | F1:4789 | 28122021\_RID\_2349\_2C.raw | 1.7957E7 |  |  |  |  |  | 1 | 1 | 0 | 0 | 0 | 0 | 0 | 333 | 342 | Oxidation (M) | M5:Oxidation (M):18.17 | PEAKS DB |
| K.NNKDDPFWR.N | N | 67.25 | 1190.5469 | 9 | 0.5 | 596.2810 | 2 | 28.10 | 5 | F5:11429 | 31122021\_RID\_2349\_5C.raw |  |  |  |  | 5.6786E6 |  | 2 | 0 | 0 | 0 | 0 | 2 | 0 | 150 | 158 |  |  | PEAKS DB |
| N.SGTYNNQYMILDTK.K | N | 65.95 | 1646.7610 | 14 | 0.9 | 824.3885 | 2 | 40.39 | 1 | F1:21338 | 28122021\_RID\_2349\_2C.raw | 2.6394E5 |  |  |  |  |  | 1 | 1 | 0 | 0 | 0 | 0 | 0 | 353 | 366 |  |  | PEAKS DB |
| R.DQGKVTDMESMK.F | N | 64.71 | 1367.6061 | 12 | 0.9 | 684.8109 | 2 | 24.00 | 1 | F1:8415 | 28122021\_RID\_2349\_2C.raw | 2.2724E6 |  |  |  |  |  | 1 | 1 | 0 | 0 | 0 | 0 | 0 | 445 | 456 |  |  | PEAKS DB |
| R.DQGKVTDMESM(+15.99)K.F | N | 62.42 | 1383.6010 | 12 | 0.0 | 692.8077 | 2 | 18.14 | 1 | F1:4116 | 28122021\_RID\_2349\_2C.raw | 7.9689E5 |  |  |  |  |  | 2 | 2 | 0 | 0 | 0 | 0 | 0 | 445 | 456 | Oxidation (M) | M11:Oxidation (M):43.31 | PEAKS DB |
| R.NAGYVIAQLDGLYMGNVEWAK.R | N | 60.60 | 2311.1306 | 21 | 0.8 | 1156.5735 | 2 | 65.79 | 5 | F5:38140 | 31122021\_RID\_2349\_5C.raw |  |  |  |  | 9.6071E4 |  | 1 | 0 | 0 | 0 | 0 | 1 | 0 | 159 | 179 |  |  | PEAKS DB |
| K.VADISM(+15.99)AAK.F | N | 58.55 | 920.4637 | 9 | -0.8 | 461.2387 | 2 | 26.23 | 1 | F1:10049 | 28122021\_RID\_2349\_2C.raw | 1.8947E6 |  |  |  |  |  | 1 | 1 | 0 | 0 | 0 | 0 | 0 | 499 | 507 | Oxidation (M) | M6:Oxidation (M):1000.00 | PEAKS DB |
| R.HGLEFSYEM(+15.99)APR.A | N | 57.54 | 1451.6503 | 12 | -0.2 | 726.8323 | 2 | 32.44 | 1 | F1:15087 | 28122021\_RID\_2349\_2C.raw | 1.3899E6 |  |  |  |  |  | 3 | 3 | 0 | 0 | 0 | 0 | 0 | 427 | 438 | Oxidation (M) | M9:Oxidation (M):1000.00 | PEAKS DB |
| K.TWAETFEK.Q | N | 55.89 | 1010.4709 | 8 | -0.7 | 506.2424 | 2 | 34.11 | 1 | F1:16345 | 28122021\_RID\_2349\_2C.raw | 3.866E7 |  |  |  |  |  | 1 | 1 | 0 | 0 | 0 | 0 | 0 | 343 | 350 |  |  | PEAKS DB |
| R.IANMMADSGKTWAETFEK.Q | N | 55.68 | 2028.9285 | 18 | 0.4 | 677.3170 | 3 | 44.54 | 1 | F1:24655 | 28122021\_RID\_2349\_2C.raw | 4.0453E5 |  |  |  |  |  | 1 | 1 | 0 | 0 | 0 | 0 | 0 | 333 | 350 |  |  | PEAKS DB |
| R.YNNYKEDPYAK.H | N | 55.54 | 1403.6357 | 11 | 0.2 | 702.8253 | 2 | 19.40 | 1 | F1:4792 | 28122021\_RID\_2349\_2C.raw | 1.2416E6 |  |  |  |  |  | 2 | 2 | 0 | 0 | 0 | 0 | 0 | 461 | 471 |  |  | PEAKS DB |
| N.NQYMILDTK.K | N | 51.50 | 1124.5536 | 9 | -0.3 | 563.2839 | 2 | 37.24 | 1 | F1:18793 | 28122021\_RID\_2349\_2C.raw | 1.1739E5 |  |  |  |  |  | 1 | 1 | 0 | 0 | 0 | 0 | 0 | 358 | 366 |  |  | PEAKS DB |
| H.SSWFTYAATLR.I | N | 48.67 | 1301.6404 | 11 | 0.3 | 651.8276 | 2 | 51.40 | 1 | F1:29950 | 28122021\_RID\_2349\_2C.raw | 2.188E5 |  |  |  |  |  | 1 | 1 | 0 | 0 | 0 | 0 | 0 | 251 | 261 |  |  | PEAKS DB |
| Y.NFDFVTMKPVL | N | 47.54 | 1309.6741 | 11 | 0.3 | 655.8445 | 2 | 58.32 | 1 | F1:34760 | 28122021\_RID\_2349\_2C.raw | 6.2109E4 |  |  |  |  |  | 1 | 1 | 0 | 0 | 0 | 0 | 0 | 543 | 553 |  |  | PEAKS DB |
| K.QNSGTYNNQYMILDTKK.I | N | 45.69 | 2016.9575 | 17 | 0.6 | 673.3268 | 3 | 32.64 | 1 | F1:15031 | 28122021\_RID\_2349\_2C.raw | 2.6845E6 |  |  |  |  |  | 1 | 1 | 0 | 0 | 0 | 0 | 0 | 351 | 367 |  |  | PEAKS DB |
| total 30 peptides |  |  |  |  |  |  |  |  |  |  |  |  |  |  |  |  |  |  |  |  |  |  |  |  |  |  |  |  |  |
| --- | --- | --- | --- | --- | --- | --- | --- | --- | --- | --- | --- | --- | --- | --- | --- | --- | --- | --- | --- | --- | --- | --- | --- | --- | --- | --- | --- | --- | --- |

SMD28240.1

back to list

  

| Protein Coverage
| Supporting Peptides
|

Protein Coverage:

Supporting Peptides:

| Peptide | Uniq | -10lgP | Mass | Length | ppm | m/z | z | RT | Fraction | Scan | Source File | Area F2 | Area F1 | Area F3 | Area F4 | Area F5 | Area F6 | #Feature | #Feature F2 | #Feature F1 | #Feature F3 | #Feature F4 | #Feature F5 | #Feature F6 | Start | End | PTM | AScore | Found By |
| --- | --- | --- | --- | --- | --- | --- | --- | --- | --- | --- | --- | --- | --- | --- | --- | --- | --- | --- | --- | --- | --- | --- | --- | --- | --- | --- | --- | --- | --- |
| K.QNSGTYNNQYMILDTK.K | N | 89.37 | 1888.8625 | 16 | 0.2 | 945.4387 | 2 | 40.12 | 1 | F1:21225 | 28122021\_RID\_2349\_2C.raw | 1.0877E7 |  |  |  | 1.1423E5 |  | 3 | 2 | 0 | 0 | 0 | 1 | 0 | 351 | 366 |  |  | PEAKS DB |
| A.DIHYATVYWLEAEK.S | N | 86.70 | 1736.8409 | 14 | 0.4 | 869.4281 | 2 | 50.82 | 6 | F6:27720 | 31122021\_RID\_2349\_6C.raw |  |  |  |  | 2.5122E6 | 1.489E6 | 4 | 0 | 0 | 0 | 0 | 2 | 2 | 37 | 50 |  |  | PEAKS DB |
| K.VKDFIQKQDEWTR.Q | N | 83.49 | 1691.8630 | 13 | 0.5 | 564.9619 | 3 | 27.81 | 5 | F5:11246 | 31122021\_RID\_2349\_5C.raw |  |  |  |  | 1.6688E6 | 1.7564E5 | 3 | 0 | 0 | 0 | 0 | 2 | 1 | 133 | 145 |  |  | PEAKS DB |
| K.FTAYAINGPPVEK.G | N | 79.89 | 1405.7241 | 13 | 0.6 | 703.8698 | 2 | 39.33 | 1 | F1:20625 | 28122021\_RID\_2349\_2C.raw | 1.0693E7 |  |  |  |  | 7.4802E4 | 2 | 1 | 0 | 0 | 0 | 0 | 1 | 508 | 520 |  |  | PEAKS DB |
| K.VVPESLFAWER.V | N | 78.21 | 1331.6874 | 11 | 0.6 | 666.8514 | 2 | 53.73 | 1 | F1:31675 | 28122021\_RID\_2349\_2C.raw | 2.3421E7 |  |  |  |  | 1.2729E6 | 2 | 1 | 0 | 0 | 0 | 0 | 1 | 320 | 330 |  |  | PEAKS DB |
| R.SLEDGTLYIIEQVPK.L | Y | 76.84 | 1703.8981 | 15 | 0.4 | 852.9567 | 2 | 59.24 | 1 | F1:35518 | 28122021\_RID\_2349\_2C.raw | 2.1874E6 |  |  |  |  |  | 2 | 2 | 0 | 0 | 0 | 0 | 0 | 373 | 387 |  |  | PEAKS DB |
| R.IANMMADSGK.T | N | 75.60 | 1036.4681 | 10 | -1.0 | 519.2408 | 2 | 24.14 | 1 | F1:8353 | 28122021\_RID\_2349\_2C.raw | 1.8547E7 |  |  |  |  |  | 1 | 1 | 0 | 0 | 0 | 0 | 0 | 333 | 342 |  |  | PEAKS DB |
| K.QNSGTYNNQYM(+15.99)ILDTK.K | N | 75.10 | 1904.8574 | 16 | 0.7 | 953.4366 | 2 | 36.00 | 1 | F1:17991 | 28122021\_RID\_2349\_2C.raw | 4.466E6 |  |  |  |  |  | 4 | 4 | 0 | 0 | 0 | 0 | 0 | 351 | 366 | Oxidation (M) | M11:Oxidation (M):1000.00 | PEAKS DB |
| K.HQGLPESYNFDFVTMKPVL | N | 73.59 | 2221.0876 | 19 | 1.1 | 741.3707 | 3 | 57.46 | 1 | F1:34351 | 28122021\_RID\_2349\_2C.raw | 1.288E7 |  |  |  |  |  | 2 | 2 | 0 | 0 | 0 | 0 | 0 | 535 | 553 |  |  | PEAKS DB |
| R.NGYWPSYNIPFDK.E | N | 72.13 | 1599.7357 | 13 | -0.3 | 800.8749 | 2 | 53.44 | 1 | F1:31595 | 28122021\_RID\_2349\_2C.raw | 6.1512E6 |  |  |  |  |  | 1 | 1 | 0 | 0 | 0 | 0 | 0 | 400 | 412 |  |  | PEAKS DB |
| K.HQGLPESYNFDFVTM(+15.99)KPVL | N | 71.00 | 2237.0828 | 19 | 0.5 | 746.7019 | 3 | 52.61 | 1 | F1:30998 | 28122021\_RID\_2349\_2C.raw | 4.8497E6 |  |  |  |  |  | 2 | 2 | 0 | 0 | 0 | 0 | 0 | 535 | 553 | Oxidation (M) | M15:Oxidation (M):1000.00 | PEAKS DB |
| R.IANM(+15.99)MADSGK.T | N | 69.25 | 1052.4630 | 10 | 0.1 | 527.2388 | 2 | 19.75 | 1 | F1:4522 | 28122021\_RID\_2349\_2C.raw | 2.4471E7 |  |  |  |  |  | 6 | 6 | 0 | 0 | 0 | 0 | 0 | 333 | 342 | Oxidation (M) | M4:Oxidation (M):30.46 | PEAKS DB |
| K.VADISMAAK.F | N | 69.21 | 904.4688 | 9 | 0.2 | 453.2418 | 2 | 26.21 | 1 | F1:10174 | 28122021\_RID\_2349\_2C.raw | 2.4604E7 |  |  |  |  | 8.0654E4 | 2 | 1 | 0 | 0 | 0 | 0 | 1 | 499 | 507 |  |  | PEAKS DB |
| R.HGLEFSYEMAPR.A | N | 68.50 | 1435.6554 | 12 | 0.7 | 718.8354 | 2 | 37.79 | 1 | F1:19391 | 28122021\_RID\_2349\_2C.raw | 1.1944E6 |  |  |  |  |  | 2 | 2 | 0 | 0 | 0 | 0 | 0 | 427 | 438 |  |  | PEAKS DB |
| K.KVVPESLFAWER.V | N | 67.95 | 1459.7823 | 12 | 0.3 | 730.8987 | 2 | 44.90 | 1 | F1:25054 | 28122021\_RID\_2349\_2C.raw | 1.3139E7 |  |  |  |  | 1.8426E5 | 3 | 2 | 0 | 0 | 0 | 0 | 1 | 319 | 330 |  |  | PEAKS DB |
| R.IANMM(+15.99)ADSGK.T | N | 67.88 | 1052.4630 | 10 | 0.1 | 527.2388 | 2 | 19.75 | 1 | F1:4789 | 28122021\_RID\_2349\_2C.raw | 1.7957E7 |  |  |  |  |  | 1 | 1 | 0 | 0 | 0 | 0 | 0 | 333 | 342 | Oxidation (M) | M5:Oxidation (M):18.17 | PEAKS DB |
| K.NNKDDPFWR.N | N | 67.25 | 1190.5469 | 9 | 0.5 | 596.2810 | 2 | 28.10 | 5 | F5:11429 | 31122021\_RID\_2349\_5C.raw |  |  |  |  | 5.6786E6 |  | 2 | 0 | 0 | 0 | 0 | 2 | 0 | 150 | 158 |  |  | PEAKS DB |
| N.SGTYNNQYMILDTK.K | N | 65.95 | 1646.7610 | 14 | 0.9 | 824.3885 | 2 | 40.39 | 1 | F1:21338 | 28122021\_RID\_2349\_2C.raw | 2.6394E5 |  |  |  |  |  | 1 | 1 | 0 | 0 | 0 | 0 | 0 | 353 | 366 |  |  | PEAKS DB |
| R.DQGKVTDMESMK.F | N | 64.71 | 1367.6061 | 12 | 0.9 | 684.8109 | 2 | 24.00 | 1 | F1:8415 | 28122021\_RID\_2349\_2C.raw | 2.2724E6 |  |  |  |  |  | 1 | 1 | 0 | 0 | 0 | 0 | 0 | 445 | 456 |  |  | PEAKS DB |
| R.DQGKVTDMESM(+15.99)K.F | N | 62.42 | 1383.6010 | 12 | 0.0 | 692.8077 | 2 | 18.14 | 1 | F1:4116 | 28122021\_RID\_2349\_2C.raw | 7.9689E5 |  |  |  |  |  | 2 | 2 | 0 | 0 | 0 | 0 | 0 | 445 | 456 | Oxidation (M) | M11:Oxidation (M):43.31 | PEAKS DB |
| R.NAGYVIAQLDGLYMGNVEWAK.R | N | 60.60 | 2311.1306 | 21 | 0.8 | 1156.5735 | 2 | 65.79 | 5 | F5:38140 | 31122021\_RID\_2349\_5C.raw |  |  |  |  | 9.6071E4 |  | 1 | 0 | 0 | 0 | 0 | 1 | 0 | 159 | 179 |  |  | PEAKS DB |
| K.VADISM(+15.99)AAK.F | N | 58.55 | 920.4637 | 9 | -0.8 | 461.2387 | 2 | 26.23 | 1 | F1:10049 | 28122021\_RID\_2349\_2C.raw | 1.8947E6 |  |  |  |  |  | 1 | 1 | 0 | 0 | 0 | 0 | 0 | 499 | 507 | Oxidation (M) | M6:Oxidation (M):1000.00 | PEAKS DB |
| R.HGLEFSYEM(+15.99)APR.A | N | 57.54 | 1451.6503 | 12 | -0.2 | 726.8323 | 2 | 32.44 | 1 | F1:15087 | 28122021\_RID\_2349\_2C.raw | 1.3899E6 |  |  |  |  |  | 3 | 3 | 0 | 0 | 0 | 0 | 0 | 427 | 438 | Oxidation (M) | M9:Oxidation (M):1000.00 | PEAKS DB |
| K.TWAETFEK.Q | N | 55.89 | 1010.4709 | 8 | -0.7 | 506.2424 | 2 | 34.11 | 1 | F1:16345 | 28122021\_RID\_2349\_2C.raw | 3.866E7 |  |  |  |  |  | 1 | 1 | 0 | 0 | 0 | 0 | 0 | 343 | 350 |  |  | PEAKS DB |
| R.IANMMADSGKTWAETFEK.Q | N | 55.68 | 2028.9285 | 18 | 0.4 | 677.3170 | 3 | 44.54 | 1 | F1:24655 | 28122021\_RID\_2349\_2C.raw | 4.0453E5 |  |  |  |  |  | 1 | 1 | 0 | 0 | 0 | 0 | 0 | 333 | 350 |  |  | PEAKS DB |
| R.YNNYKEDPYAK.R | N | 55.54 | 1403.6357 | 11 | 0.2 | 702.8253 | 2 | 19.40 | 1 | F1:4792 | 28122021\_RID\_2349\_2C.raw | 1.2416E6 |  |  |  |  |  | 2 | 2 | 0 | 0 | 0 | 0 | 0 | 461 | 471 |  |  | PEAKS DB |
| N.NQYMILDTK.K | N | 51.50 | 1124.5536 | 9 | -0.3 | 563.2839 | 2 | 37.24 | 1 | F1:18793 | 28122021\_RID\_2349\_2C.raw | 1.1739E5 |  |  |  |  |  | 1 | 1 | 0 | 0 | 0 | 0 | 0 | 358 | 366 |  |  | PEAKS DB |
| H.SSWFTYAATLR.I | N | 48.67 | 1301.6404 | 11 | 0.3 | 651.8276 | 2 | 51.40 | 1 | F1:29950 | 28122021\_RID\_2349\_2C.raw | 2.188E5 |  |  |  |  |  | 1 | 1 | 0 | 0 | 0 | 0 | 0 | 251 | 261 |  |  | PEAKS DB |
| Y.NFDFVTMKPVL | N | 47.54 | 1309.6741 | 11 | 0.3 | 655.8445 | 2 | 58.32 | 1 | F1:34760 | 28122021\_RID\_2349\_2C.raw | 6.2109E4 |  |  |  |  |  | 1 | 1 | 0 | 0 | 0 | 0 | 0 | 543 | 553 |  |  | PEAKS DB |
| K.QNSGTYNNQYMILDTKK.I | N | 45.69 | 2016.9575 | 17 | 0.6 | 673.3268 | 3 | 32.64 | 1 | F1:15031 | 28122021\_RID\_2349\_2C.raw | 2.6845E6 |  |  |  |  |  | 1 | 1 | 0 | 0 | 0 | 0 | 0 | 351 | 367 |  |  | PEAKS DB |
| total 30 peptides |  |  |  |  |  |  |  |  |  |  |  |  |  |  |  |  |  |  |  |  |  |  |  |  |  |  |  |  |  |
| --- | --- | --- | --- | --- | --- | --- | --- | --- | --- | --- | --- | --- | --- | --- | --- | --- | --- | --- | --- | --- | --- | --- | --- | --- | --- | --- | --- | --- | --- |

F8S101.1

back to list

  

| Protein Coverage
| Supporting Peptides
|

Protein Coverage:

Supporting Peptides:

| Peptide | Uniq | -10lgP | Mass | Length | ppm | m/z | z | RT | Fraction | Scan | Source File | Area F2 | Area F1 | Area F3 | Area F4 | Area F5 | Area F6 | #Feature | #Feature F2 | #Feature F1 | #Feature F3 | #Feature F4 | #Feature F5 | #Feature F6 | Start | End | PTM | AScore | Found By |
| --- | --- | --- | --- | --- | --- | --- | --- | --- | --- | --- | --- | --- | --- | --- | --- | --- | --- | --- | --- | --- | --- | --- | --- | --- | --- | --- | --- | --- | --- |
| K.QNSGTYNNQYMILDTK.K | N | 89.37 | 1888.8625 | 16 | 0.2 | 945.4387 | 2 | 40.12 | 1 | F1:21225 | 28122021\_RID\_2349\_2C.raw | 1.0877E7 |  |  |  | 1.1423E5 |  | 3 | 2 | 0 | 0 | 0 | 1 | 0 | 351 | 366 |  |  | PEAKS DB |
| A.DIHYATVYWLEAEK.S | N | 86.70 | 1736.8409 | 14 | 0.4 | 869.4281 | 2 | 50.82 | 6 | F6:27720 | 31122021\_RID\_2349\_6C.raw |  |  |  |  | 2.5122E6 | 1.489E6 | 4 | 0 | 0 | 0 | 0 | 2 | 2 | 37 | 50 |  |  | PEAKS DB |
| K.VKDFIQKQDEWTR.Q | N | 83.49 | 1691.8630 | 13 | 0.5 | 564.9619 | 3 | 27.81 | 5 | F5:11246 | 31122021\_RID\_2349\_5C.raw |  |  |  |  | 1.6688E6 | 1.7564E5 | 3 | 0 | 0 | 0 | 0 | 2 | 1 | 133 | 145 |  |  | PEAKS DB |
| K.FTAYAINGPPVEK.G | N | 79.89 | 1405.7241 | 13 | 0.6 | 703.8698 | 2 | 39.33 | 1 | F1:20625 | 28122021\_RID\_2349\_2C.raw | 1.0693E7 |  |  |  |  | 7.4802E4 | 2 | 1 | 0 | 0 | 0 | 0 | 1 | 508 | 520 |  |  | PEAKS DB |
| K.VVPESLFAWER.V | N | 78.21 | 1331.6874 | 11 | 0.6 | 666.8514 | 2 | 53.73 | 1 | F1:31675 | 28122021\_RID\_2349\_2C.raw | 2.3421E7 |  |  |  |  | 1.2729E6 | 2 | 1 | 0 | 0 | 0 | 0 | 1 | 320 | 330 |  |  | PEAKS DB |
| R.SLEDGTLYIIEQVPK.L | Y | 76.84 | 1703.8981 | 15 | 0.4 | 852.9567 | 2 | 59.24 | 1 | F1:35518 | 28122021\_RID\_2349\_2C.raw | 2.1874E6 |  |  |  |  |  | 2 | 2 | 0 | 0 | 0 | 0 | 0 | 373 | 387 |  |  | PEAKS DB |
| R.IANMMADSGK.T | N | 75.60 | 1036.4681 | 10 | -1.0 | 519.2408 | 2 | 24.14 | 1 | F1:8353 | 28122021\_RID\_2349\_2C.raw | 1.8547E7 |  |  |  |  |  | 1 | 1 | 0 | 0 | 0 | 0 | 0 | 333 | 342 |  |  | PEAKS DB |
| K.QNSGTYNNQYM(+15.99)ILDTK.K | N | 75.10 | 1904.8574 | 16 | 0.7 | 953.4366 | 2 | 36.00 | 1 | F1:17991 | 28122021\_RID\_2349\_2C.raw | 4.466E6 |  |  |  |  |  | 4 | 4 | 0 | 0 | 0 | 0 | 0 | 351 | 366 | Oxidation (M) | M11:Oxidation (M):1000.00 | PEAKS DB |
| K.HQGLPESYNFDFVTMKPVL | N | 73.59 | 2221.0876 | 19 | 1.1 | 741.3707 | 3 | 57.46 | 1 | F1:34351 | 28122021\_RID\_2349\_2C.raw | 1.288E7 |  |  |  |  |  | 2 | 2 | 0 | 0 | 0 | 0 | 0 | 535 | 553 |  |  | PEAKS DB |
| R.NGYWPSYNIPFDK.E | N | 72.13 | 1599.7357 | 13 | -0.3 | 800.8749 | 2 | 53.44 | 1 | F1:31595 | 28122021\_RID\_2349\_2C.raw | 6.1512E6 |  |  |  |  |  | 1 | 1 | 0 | 0 | 0 | 0 | 0 | 400 | 412 |  |  | PEAKS DB |
| K.HQGLPESYNFDFVTM(+15.99)KPVL | N | 71.00 | 2237.0828 | 19 | 0.5 | 746.7019 | 3 | 52.61 | 1 | F1:30998 | 28122021\_RID\_2349\_2C.raw | 4.8497E6 |  |  |  |  |  | 2 | 2 | 0 | 0 | 0 | 0 | 0 | 535 | 553 | Oxidation (M) | M15:Oxidation (M):1000.00 | PEAKS DB |
| R.IANM(+15.99)MADSGK.T | N | 69.25 | 1052.4630 | 10 | 0.1 | 527.2388 | 2 | 19.75 | 1 | F1:4522 | 28122021\_RID\_2349\_2C.raw | 2.4471E7 |  |  |  |  |  | 6 | 6 | 0 | 0 | 0 | 0 | 0 | 333 | 342 | Oxidation (M) | M4:Oxidation (M):30.46 | PEAKS DB |
| K.VADISMAAK.F | N | 69.21 | 904.4688 | 9 | 0.2 | 453.2418 | 2 | 26.21 | 1 | F1:10174 | 28122021\_RID\_2349\_2C.raw | 2.4604E7 |  |  |  |  | 8.0654E4 | 2 | 1 | 0 | 0 | 0 | 0 | 1 | 499 | 507 |  |  | PEAKS DB |
| R.HGLEFSYEMAPR.A | N | 68.50 | 1435.6554 | 12 | 0.7 | 718.8354 | 2 | 37.79 | 1 | F1:19391 | 28122021\_RID\_2349\_2C.raw | 1.1944E6 |  |  |  |  |  | 2 | 2 | 0 | 0 | 0 | 0 | 0 | 427 | 438 |  |  | PEAKS DB |
| K.KVVPESLFAWER.V | N | 67.95 | 1459.7823 | 12 | 0.3 | 730.8987 | 2 | 44.90 | 1 | F1:25054 | 28122021\_RID\_2349\_2C.raw | 1.3139E7 |  |  |  |  | 1.8426E5 | 3 | 2 | 0 | 0 | 0 | 0 | 1 | 319 | 330 |  |  | PEAKS DB |
| R.IANMM(+15.99)ADSGK.T | N | 67.88 | 1052.4630 | 10 | 0.1 | 527.2388 | 2 | 19.75 | 1 | F1:4789 | 28122021\_RID\_2349\_2C.raw | 1.7957E7 |  |  |  |  |  | 1 | 1 | 0 | 0 | 0 | 0 | 0 | 333 | 342 | Oxidation (M) | M5:Oxidation (M):18.17 | PEAKS DB |
| K.NNKDDPFWR.N | N | 67.25 | 1190.5469 | 9 | 0.5 | 596.2810 | 2 | 28.10 | 5 | F5:11429 | 31122021\_RID\_2349\_5C.raw |  |  |  |  | 5.6786E6 |  | 2 | 0 | 0 | 0 | 0 | 2 | 0 | 150 | 158 |  |  | PEAKS DB |
| N.SGTYNNQYMILDTK.K | N | 65.95 | 1646.7610 | 14 | 0.9 | 824.3885 | 2 | 40.39 | 1 | F1:21338 | 28122021\_RID\_2349\_2C.raw | 2.6394E5 |  |  |  |  |  | 1 | 1 | 0 | 0 | 0 | 0 | 0 | 353 | 366 |  |  | PEAKS DB |
| R.DQGKVTDMESMK.F | N | 64.71 | 1367.6061 | 12 | 0.9 | 684.8109 | 2 | 24.00 | 1 | F1:8415 | 28122021\_RID\_2349\_2C.raw | 2.2724E6 |  |  |  |  |  | 1 | 1 | 0 | 0 | 0 | 0 | 0 | 445 | 456 |  |  | PEAKS DB |
| R.DQGKVTDMESM(+15.99)K.F | N | 62.42 | 1383.6010 | 12 | 0.0 | 692.8077 | 2 | 18.14 | 1 | F1:4116 | 28122021\_RID\_2349\_2C.raw | 7.9689E5 |  |  |  |  |  | 2 | 2 | 0 | 0 | 0 | 0 | 0 | 445 | 456 | Oxidation (M) | M11:Oxidation (M):43.31 | PEAKS DB |
| R.NAGYVIAQLDGLYMGNVEWAK.R | N | 60.60 | 2311.1306 | 21 | 0.8 | 1156.5735 | 2 | 65.79 | 5 | F5:38140 | 31122021\_RID\_2349\_5C.raw |  |  |  |  | 9.6071E4 |  | 1 | 0 | 0 | 0 | 0 | 1 | 0 | 159 | 179 |  |  | PEAKS DB |
| K.VADISM(+15.99)AAK.F | N | 58.55 | 920.4637 | 9 | -0.8 | 461.2387 | 2 | 26.23 | 1 | F1:10049 | 28122021\_RID\_2349\_2C.raw | 1.8947E6 |  |  |  |  |  | 1 | 1 | 0 | 0 | 0 | 0 | 0 | 499 | 507 | Oxidation (M) | M6:Oxidation (M):1000.00 | PEAKS DB |
| R.HGLEFSYEM(+15.99)APR.A | N | 57.54 | 1451.6503 | 12 | -0.2 | 726.8323 | 2 | 32.44 | 1 | F1:15087 | 28122021\_RID\_2349\_2C.raw | 1.3899E6 |  |  |  |  |  | 3 | 3 | 0 | 0 | 0 | 0 | 0 | 427 | 438 | Oxidation (M) | M9:Oxidation (M):1000.00 | PEAKS DB |
| K.TWAETFEK.Q | N | 55.89 | 1010.4709 | 8 | -0.7 | 506.2424 | 2 | 34.11 | 1 | F1:16345 | 28122021\_RID\_2349\_2C.raw | 3.866E7 |  |  |  |  |  | 1 | 1 | 0 | 0 | 0 | 0 | 0 | 343 | 350 |  |  | PEAKS DB |
| R.IANMMADSGKTWAETFEK.Q | N | 55.68 | 2028.9285 | 18 | 0.4 | 677.3170 | 3 | 44.54 | 1 | F1:24655 | 28122021\_RID\_2349\_2C.raw | 4.0453E5 |  |  |  |  |  | 1 | 1 | 0 | 0 | 0 | 0 | 0 | 333 | 350 |  |  | PEAKS DB |
| R.YNNYKEDPYAK.H | N | 55.54 | 1403.6357 | 11 | 0.2 | 702.8253 | 2 | 19.40 | 1 | F1:4792 | 28122021\_RID\_2349\_2C.raw | 1.2416E6 |  |  |  |  |  | 2 | 2 | 0 | 0 | 0 | 0 | 0 | 461 | 471 |  |  | PEAKS DB |
| N.NQYMILDTK.K | N | 51.50 | 1124.5536 | 9 | -0.3 | 563.2839 | 2 | 37.24 | 1 | F1:18793 | 28122021\_RID\_2349\_2C.raw | 1.1739E5 |  |  |  |  |  | 1 | 1 | 0 | 0 | 0 | 0 | 0 | 358 | 366 |  |  | PEAKS DB |
| H.SSWFTYAATLR.I | N | 48.67 | 1301.6404 | 11 | 0.3 | 651.8276 | 2 | 51.40 | 1 | F1:29950 | 28122021\_RID\_2349\_2C.raw | 2.188E5 |  |  |  |  |  | 1 | 1 | 0 | 0 | 0 | 0 | 0 | 251 | 261 |  |  | PEAKS DB |
| Y.NFDFVTMKPVL | N | 47.54 | 1309.6741 | 11 | 0.3 | 655.8445 | 2 | 58.32 | 1 | F1:34760 | 28122021\_RID\_2349\_2C.raw | 6.2109E4 |  |  |  |  |  | 1 | 1 | 0 | 0 | 0 | 0 | 0 | 543 | 553 |  |  | PEAKS DB |
| K.QNSGTYNNQYMILDTKK.I | N | 45.69 | 2016.9575 | 17 | 0.6 | 673.3268 | 3 | 32.64 | 1 | F1:15031 | 28122021\_RID\_2349\_2C.raw | 2.6845E6 |  |  |  |  |  | 1 | 1 | 0 | 0 | 0 | 0 | 0 | 351 | 367 |  |  | PEAKS DB |
| total 30 peptides |  |  |  |  |  |  |  |  |  |  |  |  |  |  |  |  |  |  |  |  |  |  |  |  |  |  |  |  |  |
| --- | --- | --- | --- | --- | --- | --- | --- | --- | --- | --- | --- | --- | --- | --- | --- | --- | --- | --- | --- | --- | --- | --- | --- | --- | --- | --- | --- | --- | --- |

JAV49636.1

back to list

  

| Protein Coverage
| Supporting Peptides
|

Protein Coverage:

Supporting Peptides:

| Peptide | Uniq | -10lgP | Mass | Length | ppm | m/z | z | RT | Fraction | Scan | Source File | Area F2 | Area F1 | Area F3 | Area F4 | Area F5 | Area F6 | #Feature | #Feature F2 | #Feature F1 | #Feature F3 | #Feature F4 | #Feature F5 | #Feature F6 | Start | End | PTM | AScore | Found By |
| --- | --- | --- | --- | --- | --- | --- | --- | --- | --- | --- | --- | --- | --- | --- | --- | --- | --- | --- | --- | --- | --- | --- | --- | --- | --- | --- | --- | --- | --- |
| K.QNSGTYNNQYMILDTK.K | N | 89.37 | 1888.8625 | 16 | 0.2 | 945.4387 | 2 | 40.12 | 1 | F1:21225 | 28122021\_RID\_2349\_2C.raw | 1.0877E7 |  |  |  | 1.1423E5 |  | 3 | 2 | 0 | 0 | 0 | 1 | 0 | 351 | 366 |  |  | PEAKS DB |
| A.DIHYATVYWLEAEK.S | N | 86.70 | 1736.8409 | 14 | 0.4 | 869.4281 | 2 | 50.82 | 6 | F6:27720 | 31122021\_RID\_2349\_6C.raw |  |  |  |  | 2.5122E6 | 1.489E6 | 4 | 0 | 0 | 0 | 0 | 2 | 2 | 37 | 50 |  |  | PEAKS DB |
| K.VKDFIQKQDEWTR.Q | N | 83.49 | 1691.8630 | 13 | 0.5 | 564.9619 | 3 | 27.81 | 5 | F5:11246 | 31122021\_RID\_2349\_5C.raw |  |  |  |  | 1.6688E6 | 1.7564E5 | 3 | 0 | 0 | 0 | 0 | 2 | 1 | 133 | 145 |  |  | PEAKS DB |
| K.FTAYAINGPPVEK.G | N | 79.89 | 1405.7241 | 13 | 0.6 | 703.8698 | 2 | 39.33 | 1 | F1:20625 | 28122021\_RID\_2349\_2C.raw | 1.0693E7 |  |  |  |  | 7.4802E4 | 2 | 1 | 0 | 0 | 0 | 0 | 1 | 508 | 520 |  |  | PEAKS DB |
| K.VVPESLFAWER.V | N | 78.21 | 1331.6874 | 11 | 0.6 | 666.8514 | 2 | 53.73 | 1 | F1:31675 | 28122021\_RID\_2349\_2C.raw | 2.3421E7 |  |  |  |  | 1.2729E6 | 2 | 1 | 0 | 0 | 0 | 0 | 1 | 320 | 330 |  |  | PEAKS DB |
| R.SLEDGTLYIIEQVPK.L | Y | 76.84 | 1703.8981 | 15 | 0.4 | 852.9567 | 2 | 59.24 | 1 | F1:35518 | 28122021\_RID\_2349\_2C.raw | 2.1874E6 |  |  |  |  |  | 2 | 2 | 0 | 0 | 0 | 0 | 0 | 373 | 387 |  |  | PEAKS DB |
| R.IANMMADSGK.T | N | 75.60 | 1036.4681 | 10 | -1.0 | 519.2408 | 2 | 24.14 | 1 | F1:8353 | 28122021\_RID\_2349\_2C.raw | 1.8547E7 |  |  |  |  |  | 1 | 1 | 0 | 0 | 0 | 0 | 0 | 333 | 342 |  |  | PEAKS DB |
| K.QNSGTYNNQYM(+15.99)ILDTK.K | N | 75.10 | 1904.8574 | 16 | 0.7 | 953.4366 | 2 | 36.00 | 1 | F1:17991 | 28122021\_RID\_2349\_2C.raw | 4.466E6 |  |  |  |  |  | 4 | 4 | 0 | 0 | 0 | 0 | 0 | 351 | 366 | Oxidation (M) | M11:Oxidation (M):1000.00 | PEAKS DB |
| K.HQGLPESYNFDFVTMKPVL | N | 73.59 | 2221.0876 | 19 | 1.1 | 741.3707 | 3 | 57.46 | 1 | F1:34351 | 28122021\_RID\_2349\_2C.raw | 1.288E7 |  |  |  |  |  | 2 | 2 | 0 | 0 | 0 | 0 | 0 | 535 | 553 |  |  | PEAKS DB |
| R.NGYWPSYNIPFDK.E | N | 72.13 | 1599.7357 | 13 | -0.3 | 800.8749 | 2 | 53.44 | 1 | F1:31595 | 28122021\_RID\_2349\_2C.raw | 6.1512E6 |  |  |  |  |  | 1 | 1 | 0 | 0 | 0 | 0 | 0 | 400 | 412 |  |  | PEAKS DB |
| K.HQGLPESYNFDFVTM(+15.99)KPVL | N | 71.00 | 2237.0828 | 19 | 0.5 | 746.7019 | 3 | 52.61 | 1 | F1:30998 | 28122021\_RID\_2349\_2C.raw | 4.8497E6 |  |  |  |  |  | 2 | 2 | 0 | 0 | 0 | 0 | 0 | 535 | 553 | Oxidation (M) | M15:Oxidation (M):1000.00 | PEAKS DB |
| R.IANM(+15.99)MADSGK.T | N | 69.25 | 1052.4630 | 10 | 0.1 | 527.2388 | 2 | 19.75 | 1 | F1:4522 | 28122021\_RID\_2349\_2C.raw | 2.4471E7 |  |  |  |  |  | 6 | 6 | 0 | 0 | 0 | 0 | 0 | 333 | 342 | Oxidation (M) | M4:Oxidation (M):30.46 | PEAKS DB |
| K.VADISMAAK.F | N | 69.21 | 904.4688 | 9 | 0.2 | 453.2418 | 2 | 26.21 | 1 | F1:10174 | 28122021\_RID\_2349\_2C.raw | 2.4604E7 |  |  |  |  | 8.0654E4 | 2 | 1 | 0 | 0 | 0 | 0 | 1 | 499 | 507 |  |  | PEAKS DB |
| R.HGLEFSYEMAPR.A | N | 68.50 | 1435.6554 | 12 | 0.7 | 718.8354 | 2 | 37.79 | 1 | F1:19391 | 28122021\_RID\_2349\_2C.raw | 1.1944E6 |  |  |  |  |  | 2 | 2 | 0 | 0 | 0 | 0 | 0 | 427 | 438 |  |  | PEAKS DB |
| K.KVVPESLFAWER.V | N | 67.95 | 1459.7823 | 12 | 0.3 | 730.8987 | 2 | 44.90 | 1 | F1:25054 | 28122021\_RID\_2349\_2C.raw | 1.3139E7 |  |  |  |  | 1.8426E5 | 3 | 2 | 0 | 0 | 0 | 0 | 1 | 319 | 330 |  |  | PEAKS DB |
| R.IANMM(+15.99)ADSGK.T | N | 67.88 | 1052.4630 | 10 | 0.1 | 527.2388 | 2 | 19.75 | 1 | F1:4789 | 28122021\_RID\_2349\_2C.raw | 1.7957E7 |  |  |  |  |  | 1 | 1 | 0 | 0 | 0 | 0 | 0 | 333 | 342 | Oxidation (M) | M5:Oxidation (M):18.17 | PEAKS DB |
| K.NNKDDPFWR.N | N | 67.25 | 1190.5469 | 9 | 0.5 | 596.2810 | 2 | 28.10 | 5 | F5:11429 | 31122021\_RID\_2349\_5C.raw |  |  |  |  | 5.6786E6 |  | 2 | 0 | 0 | 0 | 0 | 2 | 0 | 150 | 158 |  |  | PEAKS DB |
| N.SGTYNNQYMILDTK.K | N | 65.95 | 1646.7610 | 14 | 0.9 | 824.3885 | 2 | 40.39 | 1 | F1:21338 | 28122021\_RID\_2349\_2C.raw | 2.6394E5 |  |  |  |  |  | 1 | 1 | 0 | 0 | 0 | 0 | 0 | 353 | 366 |  |  | PEAKS DB |
| R.DQGKVTDMESMK.F | N | 64.71 | 1367.6061 | 12 | 0.9 | 684.8109 | 2 | 24.00 | 1 | F1:8415 | 28122021\_RID\_2349\_2C.raw | 2.2724E6 |  |  |  |  |  | 1 | 1 | 0 | 0 | 0 | 0 | 0 | 445 | 456 |  |  | PEAKS DB |
| R.DQGKVTDMESM(+15.99)K.F | N | 62.42 | 1383.6010 | 12 | 0.0 | 692.8077 | 2 | 18.14 | 1 | F1:4116 | 28122021\_RID\_2349\_2C.raw | 7.9689E5 |  |  |  |  |  | 2 | 2 | 0 | 0 | 0 | 0 | 0 | 445 | 456 | Oxidation (M) | M11:Oxidation (M):43.31 | PEAKS DB |
| R.NAGYVIAQLDGLYMGNVEWAK.R | N | 60.60 | 2311.1306 | 21 | 0.8 | 1156.5735 | 2 | 65.79 | 5 | F5:38140 | 31122021\_RID\_2349\_5C.raw |  |  |  |  | 9.6071E4 |  | 1 | 0 | 0 | 0 | 0 | 1 | 0 | 159 | 179 |  |  | PEAKS DB |
| K.VADISM(+15.99)AAK.F | N | 58.55 | 920.4637 | 9 | -0.8 | 461.2387 | 2 | 26.23 | 1 | F1:10049 | 28122021\_RID\_2349\_2C.raw | 1.8947E6 |  |  |  |  |  | 1 | 1 | 0 | 0 | 0 | 0 | 0 | 499 | 507 | Oxidation (M) | M6:Oxidation (M):1000.00 | PEAKS DB |
| R.HGLEFSYEM(+15.99)APR.A | N | 57.54 | 1451.6503 | 12 | -0.2 | 726.8323 | 2 | 32.44 | 1 | F1:15087 | 28122021\_RID\_2349\_2C.raw | 1.3899E6 |  |  |  |  |  | 3 | 3 | 0 | 0 | 0 | 0 | 0 | 427 | 438 | Oxidation (M) | M9:Oxidation (M):1000.00 | PEAKS DB |
| K.TWAETFEK.Q | N | 55.89 | 1010.4709 | 8 | -0.7 | 506.2424 | 2 | 34.11 | 1 | F1:16345 | 28122021\_RID\_2349\_2C.raw | 3.866E7 |  |  |  |  |  | 1 | 1 | 0 | 0 | 0 | 0 | 0 | 343 | 350 |  |  | PEAKS DB |
| R.IANMMADSGKTWAETFEK.Q | N | 55.68 | 2028.9285 | 18 | 0.4 | 677.3170 | 3 | 44.54 | 1 | F1:24655 | 28122021\_RID\_2349\_2C.raw | 4.0453E5 |  |  |  |  |  | 1 | 1 | 0 | 0 | 0 | 0 | 0 | 333 | 350 |  |  | PEAKS DB |
| R.YNNYKEDPYAK.R | N | 55.54 | 1403.6357 | 11 | 0.2 | 702.8253 | 2 | 19.40 | 1 | F1:4792 | 28122021\_RID\_2349\_2C.raw | 1.2416E6 |  |  |  |  |  | 2 | 2 | 0 | 0 | 0 | 0 | 0 | 461 | 471 |  |  | PEAKS DB |
| N.NQYMILDTK.K | N | 51.50 | 1124.5536 | 9 | -0.3 | 563.2839 | 2 | 37.24 | 1 | F1:18793 | 28122021\_RID\_2349\_2C.raw | 1.1739E5 |  |  |  |  |  | 1 | 1 | 0 | 0 | 0 | 0 | 0 | 358 | 366 |  |  | PEAKS DB |
| H.SSWFTYAATLR.I | N | 48.67 | 1301.6404 | 11 | 0.3 | 651.8276 | 2 | 51.40 | 1 | F1:29950 | 28122021\_RID\_2349\_2C.raw | 2.188E5 |  |  |  |  |  | 1 | 1 | 0 | 0 | 0 | 0 | 0 | 251 | 261 |  |  | PEAKS DB |
| Y.NFDFVTMKPVL | N | 47.54 | 1309.6741 | 11 | 0.3 | 655.8445 | 2 | 58.32 | 1 | F1:34760 | 28122021\_RID\_2349\_2C.raw | 6.2109E4 |  |  |  |  |  | 1 | 1 | 0 | 0 | 0 | 0 | 0 | 543 | 553 |  |  | PEAKS DB |
| K.QNSGTYNNQYMILDTKK.I | N | 45.69 | 2016.9575 | 17 | 0.6 | 673.3268 | 3 | 32.64 | 1 | F1:15031 | 28122021\_RID\_2349\_2C.raw | 2.6845E6 |  |  |  |  |  | 1 | 1 | 0 | 0 | 0 | 0 | 0 | 351 | 367 |  |  | PEAKS DB |
| total 30 peptides |  |  |  |  |  |  |  |  |  |  |  |  |  |  |  |  |  |  |  |  |  |  |  |  |  |  |  |  |  |
| --- | --- | --- | --- | --- | --- | --- | --- | --- | --- | --- | --- | --- | --- | --- | --- | --- | --- | --- | --- | --- | --- | --- | --- | --- | --- | --- | --- | --- | --- |

JAV48419.1

back to list

  

| Protein Coverage
| Supporting Peptides
|

Protein Coverage:

Supporting Peptides:

| Peptide | Uniq | -10lgP | Mass | Length | ppm | m/z | z | RT | Fraction | Scan | Source File | Area F2 | Area F1 | Area F3 | Area F4 | Area F5 | Area F6 | #Feature | #Feature F2 | #Feature F1 | #Feature F3 | #Feature F4 | #Feature F5 | #Feature F6 | Start | End | PTM | AScore | Found By |
| --- | --- | --- | --- | --- | --- | --- | --- | --- | --- | --- | --- | --- | --- | --- | --- | --- | --- | --- | --- | --- | --- | --- | --- | --- | --- | --- | --- | --- | --- |
| K.QNSGTYNNQYMILDTK.K | N | 89.37 | 1888.8625 | 16 | 0.2 | 945.4387 | 2 | 40.12 | 1 | F1:21225 | 28122021\_RID\_2349\_2C.raw | 1.0877E7 |  |  |  | 1.1423E5 |  | 3 | 2 | 0 | 0 | 0 | 1 | 0 | 351 | 366 |  |  | PEAKS DB |
| A.DIHYATVYWLEAEK.S | N | 86.70 | 1736.8409 | 14 | 0.4 | 869.4281 | 2 | 50.82 | 6 | F6:27720 | 31122021\_RID\_2349\_6C.raw |  |  |  |  | 2.5122E6 | 1.489E6 | 4 | 0 | 0 | 0 | 0 | 2 | 2 | 37 | 50 |  |  | PEAKS DB |
| K.VKDFIQKQDEWTR.Q | N | 83.49 | 1691.8630 | 13 | 0.5 | 564.9619 | 3 | 27.81 | 5 | F5:11246 | 31122021\_RID\_2349\_5C.raw |  |  |  |  | 1.6688E6 | 1.7564E5 | 3 | 0 | 0 | 0 | 0 | 2 | 1 | 133 | 145 |  |  | PEAKS DB |
| K.FTAYAINGPPVEK.G | N | 79.89 | 1405.7241 | 13 | 0.6 | 703.8698 | 2 | 39.33 | 1 | F1:20625 | 28122021\_RID\_2349\_2C.raw | 1.0693E7 |  |  |  |  | 7.4802E4 | 2 | 1 | 0 | 0 | 0 | 0 | 1 | 508 | 520 |  |  | PEAKS DB |
| K.VVPESLFAWER.V | N | 78.21 | 1331.6874 | 11 | 0.6 | 666.8514 | 2 | 53.73 | 1 | F1:31675 | 28122021\_RID\_2349\_2C.raw | 2.3421E7 |  |  |  |  | 1.2729E6 | 2 | 1 | 0 | 0 | 0 | 0 | 1 | 320 | 330 |  |  | PEAKS DB |
| R.SLEDGTLYIIEQVPK.L | Y | 76.84 | 1703.8981 | 15 | 0.4 | 852.9567 | 2 | 59.24 | 1 | F1:35518 | 28122021\_RID\_2349\_2C.raw | 2.1874E6 |  |  |  |  |  | 2 | 2 | 0 | 0 | 0 | 0 | 0 | 373 | 387 |  |  | PEAKS DB |
| R.IANMMADSGK.T | N | 75.60 | 1036.4681 | 10 | -1.0 | 519.2408 | 2 | 24.14 | 1 | F1:8353 | 28122021\_RID\_2349\_2C.raw | 1.8547E7 |  |  |  |  |  | 1 | 1 | 0 | 0 | 0 | 0 | 0 | 333 | 342 |  |  | PEAKS DB |
| K.QNSGTYNNQYM(+15.99)ILDTK.K | N | 75.10 | 1904.8574 | 16 | 0.7 | 953.4366 | 2 | 36.00 | 1 | F1:17991 | 28122021\_RID\_2349\_2C.raw | 4.466E6 |  |  |  |  |  | 4 | 4 | 0 | 0 | 0 | 0 | 0 | 351 | 366 | Oxidation (M) | M11:Oxidation (M):1000.00 | PEAKS DB |
| K.HQGLPESYNFDFVTMKPVL | N | 73.59 | 2221.0876 | 19 | 1.1 | 741.3707 | 3 | 57.46 | 1 | F1:34351 | 28122021\_RID\_2349\_2C.raw | 1.288E7 |  |  |  |  |  | 2 | 2 | 0 | 0 | 0 | 0 | 0 | 535 | 553 |  |  | PEAKS DB |
| R.NGYWPSYNIPFDK.E | N | 72.13 | 1599.7357 | 13 | -0.3 | 800.8749 | 2 | 53.44 | 1 | F1:31595 | 28122021\_RID\_2349\_2C.raw | 6.1512E6 |  |  |  |  |  | 1 | 1 | 0 | 0 | 0 | 0 | 0 | 400 | 412 |  |  | PEAKS DB |
| K.HQGLPESYNFDFVTM(+15.99)KPVL | N | 71.00 | 2237.0828 | 19 | 0.5 | 746.7019 | 3 | 52.61 | 1 | F1:30998 | 28122021\_RID\_2349\_2C.raw | 4.8497E6 |  |  |  |  |  | 2 | 2 | 0 | 0 | 0 | 0 | 0 | 535 | 553 | Oxidation (M) | M15:Oxidation (M):1000.00 | PEAKS DB |
| R.IANM(+15.99)MADSGK.T | N | 69.25 | 1052.4630 | 10 | 0.1 | 527.2388 | 2 | 19.75 | 1 | F1:4522 | 28122021\_RID\_2349\_2C.raw | 2.4471E7 |  |  |  |  |  | 6 | 6 | 0 | 0 | 0 | 0 | 0 | 333 | 342 | Oxidation (M) | M4:Oxidation (M):30.46 | PEAKS DB |
| K.VADISMAAK.F | N | 69.21 | 904.4688 | 9 | 0.2 | 453.2418 | 2 | 26.21 | 1 | F1:10174 | 28122021\_RID\_2349\_2C.raw | 2.4604E7 |  |  |  |  | 8.0654E4 | 2 | 1 | 0 | 0 | 0 | 0 | 1 | 499 | 507 |  |  | PEAKS DB |
| R.HGLEFSYEMAPR.A | N | 68.50 | 1435.6554 | 12 | 0.7 | 718.8354 | 2 | 37.79 | 1 | F1:19391 | 28122021\_RID\_2349\_2C.raw | 1.1944E6 |  |  |  |  |  | 2 | 2 | 0 | 0 | 0 | 0 | 0 | 427 | 438 |  |  | PEAKS DB |
| K.KVVPESLFAWER.V | N | 67.95 | 1459.7823 | 12 | 0.3 | 730.8987 | 2 | 44.90 | 1 | F1:25054 | 28122021\_RID\_2349\_2C.raw | 1.3139E7 |  |  |  |  | 1.8426E5 | 3 | 2 | 0 | 0 | 0 | 0 | 1 | 319 | 330 |  |  | PEAKS DB |
| R.IANMM(+15.99)ADSGK.T | N | 67.88 | 1052.4630 | 10 | 0.1 | 527.2388 | 2 | 19.75 | 1 | F1:4789 | 28122021\_RID\_2349\_2C.raw | 1.7957E7 |  |  |  |  |  | 1 | 1 | 0 | 0 | 0 | 0 | 0 | 333 | 342 | Oxidation (M) | M5:Oxidation (M):18.17 | PEAKS DB |
| K.NNKDDPFWR.N | N | 67.25 | 1190.5469 | 9 | 0.5 | 596.2810 | 2 | 28.10 | 5 | F5:11429 | 31122021\_RID\_2349\_5C.raw |  |  |  |  | 5.6786E6 |  | 2 | 0 | 0 | 0 | 0 | 2 | 0 | 150 | 158 |  |  | PEAKS DB |
| N.SGTYNNQYMILDTK.K | N | 65.95 | 1646.7610 | 14 | 0.9 | 824.3885 | 2 | 40.39 | 1 | F1:21338 | 28122021\_RID\_2349\_2C.raw | 2.6394E5 |  |  |  |  |  | 1 | 1 | 0 | 0 | 0 | 0 | 0 | 353 | 366 |  |  | PEAKS DB |
| R.DQGKVTDMESMK.F | N | 64.71 | 1367.6061 | 12 | 0.9 | 684.8109 | 2 | 24.00 | 1 | F1:8415 | 28122021\_RID\_2349\_2C.raw | 2.2724E6 |  |  |  |  |  | 1 | 1 | 0 | 0 | 0 | 0 | 0 | 445 | 456 |  |  | PEAKS DB |
| R.DQGKVTDMESM(+15.99)K.F | N | 62.42 | 1383.6010 | 12 | 0.0 | 692.8077 | 2 | 18.14 | 1 | F1:4116 | 28122021\_RID\_2349\_2C.raw | 7.9689E5 |  |  |  |  |  | 2 | 2 | 0 | 0 | 0 | 0 | 0 | 445 | 456 | Oxidation (M) | M11:Oxidation (M):43.31 | PEAKS DB |
| R.NAGYVIAQLDGLYMGNVEWAK.R | N | 60.60 | 2311.1306 | 21 | 0.8 | 1156.5735 | 2 | 65.79 | 5 | F5:38140 | 31122021\_RID\_2349\_5C.raw |  |  |  |  | 9.6071E4 |  | 1 | 0 | 0 | 0 | 0 | 1 | 0 | 159 | 179 |  |  | PEAKS DB |
| K.VADISM(+15.99)AAK.F | N | 58.55 | 920.4637 | 9 | -0.8 | 461.2387 | 2 | 26.23 | 1 | F1:10049 | 28122021\_RID\_2349\_2C.raw | 1.8947E6 |  |  |  |  |  | 1 | 1 | 0 | 0 | 0 | 0 | 0 | 499 | 507 | Oxidation (M) | M6:Oxidation (M):1000.00 | PEAKS DB |
| R.HGLEFSYEM(+15.99)APR.A | N | 57.54 | 1451.6503 | 12 | -0.2 | 726.8323 | 2 | 32.44 | 1 | F1:15087 | 28122021\_RID\_2349\_2C.raw | 1.3899E6 |  |  |  |  |  | 3 | 3 | 0 | 0 | 0 | 0 | 0 | 427 | 438 | Oxidation (M) | M9:Oxidation (M):1000.00 | PEAKS DB |
| K.TWAETFEK.Q | N | 55.89 | 1010.4709 | 8 | -0.7 | 506.2424 | 2 | 34.11 | 1 | F1:16345 | 28122021\_RID\_2349\_2C.raw | 3.866E7 |  |  |  |  |  | 1 | 1 | 0 | 0 | 0 | 0 | 0 | 343 | 350 |  |  | PEAKS DB |
| R.IANMMADSGKTWAETFEK.Q | N | 55.68 | 2028.9285 | 18 | 0.4 | 677.3170 | 3 | 44.54 | 1 | F1:24655 | 28122021\_RID\_2349\_2C.raw | 4.0453E5 |  |  |  |  |  | 1 | 1 | 0 | 0 | 0 | 0 | 0 | 333 | 350 |  |  | PEAKS DB |
| R.YNNYKEDPYAK.R | N | 55.54 | 1403.6357 | 11 | 0.2 | 702.8253 | 2 | 19.40 | 1 | F1:4792 | 28122021\_RID\_2349\_2C.raw | 1.2416E6 |  |  |  |  |  | 2 | 2 | 0 | 0 | 0 | 0 | 0 | 461 | 471 |  |  | PEAKS DB |
| N.NQYMILDTK.K | N | 51.50 | 1124.5536 | 9 | -0.3 | 563.2839 | 2 | 37.24 | 1 | F1:18793 | 28122021\_RID\_2349\_2C.raw | 1.1739E5 |  |  |  |  |  | 1 | 1 | 0 | 0 | 0 | 0 | 0 | 358 | 366 |  |  | PEAKS DB |
| H.SSWFTYAATLR.I | N | 48.67 | 1301.6404 | 11 | 0.3 | 651.8276 | 2 | 51.40 | 1 | F1:29950 | 28122021\_RID\_2349\_2C.raw | 2.188E5 |  |  |  |  |  | 1 | 1 | 0 | 0 | 0 | 0 | 0 | 251 | 261 |  |  | PEAKS DB |
| Y.NFDFVTMKPVL | N | 47.54 | 1309.6741 | 11 | 0.3 | 655.8445 | 2 | 58.32 | 1 | F1:34760 | 28122021\_RID\_2349\_2C.raw | 6.2109E4 |  |  |  |  |  | 1 | 1 | 0 | 0 | 0 | 0 | 0 | 543 | 553 |  |  | PEAKS DB |
| K.QNSGTYNNQYMILDTKK.I | N | 45.69 | 2016.9575 | 17 | 0.6 | 673.3268 | 3 | 32.64 | 1 | F1:15031 | 28122021\_RID\_2349\_2C.raw | 2.6845E6 |  |  |  |  |  | 1 | 1 | 0 | 0 | 0 | 0 | 0 | 351 | 367 |  |  | PEAKS DB |
| total 30 peptides |  |  |  |  |  |  |  |  |  |  |  |  |  |  |  |  |  |  |  |  |  |  |  |  |  |  |  |  |  |
| --- | --- | --- | --- | --- | --- | --- | --- | --- | --- | --- | --- | --- | --- | --- | --- | --- | --- | --- | --- | --- | --- | --- | --- | --- | --- | --- | --- | --- | --- |

2IID

back to list

  

| Protein Coverage
| Supporting Peptides
|

Protein Coverage:

Supporting Peptides:

| Peptide | Uniq | -10lgP | Mass | Length | ppm | m/z | z | RT | Fraction | Scan | Source File | Area F2 | Area F1 | Area F3 | Area F4 | Area F5 | Area F6 | #Feature | #Feature F2 | #Feature F1 | #Feature F3 | #Feature F4 | #Feature F5 | #Feature F6 | Start | End | PTM | AScore | Found By |
| --- | --- | --- | --- | --- | --- | --- | --- | --- | --- | --- | --- | --- | --- | --- | --- | --- | --- | --- | --- | --- | --- | --- | --- | --- | --- | --- | --- | --- | --- |
| T.KFWEDDGIHGGK.S | N | 83.09 | 1387.6520 | 12 | 0.2 | 463.5580 | 3 | 24.69 | 5 | F5:8977 | 31122021\_RID\_2349\_5C.raw | 3.2966E7 | 1.6951E7 | 4.7218E7 |  | 1.4906E6 |  | 10 | 4 | 3 | 1 | 0 | 2 | 0 | 334 | 345 |  |  | PEAKS DB |
| K.SAGQLYEESLGK.V | N | 80.33 | 1280.6248 | 12 | -0.1 | 641.3196 | 2 | 32.32 | 5 | F5:14229 | 31122021\_RID\_2349\_5C.raw | 3.9268E8 | 5.6746E8 | 8.8153E7 | 1.109E8 | 1.2738E8 | 1.0719E5 | 12 | 3 | 3 | 1 | 1 | 3 | 1 | 152 | 163 |  |  | PEAKS DB |
| Y.GIGDDANFFQALDFK.D | N | 75.31 | 1656.7783 | 15 | 0.2 | 829.3966 | 2 | 64.60 | 3 | F3:23470 | 31122021\_RID\_2349\_3C.raw |  |  | 1.1679E5 |  |  |  | 1 | 0 | 0 | 1 | 0 | 0 | 0 | 373 | 387 |  |  | PEAKS DB |
| K.RFDEIVDGMDK.L | N | 74.01 | 1323.6129 | 11 | 0.3 | 662.8139 | 2 | 35.47 | 1 | F1:17511 | 28122021\_RID\_2349\_2C.raw | 9.5788E6 | 2.9071E7 | 1.7532E7 |  |  | 4.2754E5 | 7 | 2 | 3 | 1 | 0 | 0 | 1 | 232 | 242 |  |  | PEAKS DB |
| K.RFDEIVDGM(+15.99)DK.L | N | 73.28 | 1339.6078 | 11 | 0.3 | 670.8114 | 2 | 34.96 | 1 | F1:17438 | 28122021\_RID\_2349\_2C.raw | 4.3666E6 | 1.0728E7 |  |  |  |  | 9 | 4 | 5 | 0 | 0 | 0 | 0 | 232 | 242 | Oxidation (M) | M9:Oxidation (M):1000.00 | PEAKS DB |
| K.HDDIFAYEK.R | N | 72.69 | 1136.5138 | 9 | -0.1 | 569.2641 | 2 | 30.15 | 2 | F2:8868 | 30122021\_RID\_2349\_1C.raw | 5.9259E7 | 1.5095E8 | 7.8164E7 |  |  | 1.3576E6 | 7 | 3 | 2 | 1 | 0 | 0 | 1 | 223 | 231 |  |  | PEAKS DB |
| K.FWEDDGIHGGK.S | N | 71.93 | 1259.5570 | 11 | 0.4 | 630.7860 | 2 | 28.94 | 1 | F1:12222 | 28122021\_RID\_2349\_2C.raw | 1.3683E7 | 2.9767E7 | 9.0355E6 |  |  |  | 10 | 4 | 5 | 1 | 0 | 0 | 0 | 335 | 345 |  |  | PEAKS DB |
| R.IYFAGEYTAQ.A | N | 68.04 | 1161.5342 | 10 | 0.2 | 581.7745 | 2 | 47.04 | 2 | F2:16101 | 30122021\_RID\_2349\_1C.raw |  | 4.684E5 |  |  |  |  | 1 | 0 | 1 | 0 | 0 | 0 | 0 | 452 | 461 |  |  | PEAKS DB |
| R.IYFAGEYTAQAH.G | N | 65.33 | 1369.6302 | 12 | 0.8 | 685.8229 | 2 | 38.89 | 2 | F2:12898 | 30122021\_RID\_2349\_1C.raw |  | 1.6051E6 |  |  |  |  | 1 | 0 | 1 | 0 | 0 | 0 | 0 | 452 | 463 |  |  | PEAKS DB |
| R.FDEIVDGMDK.L | N | 64.83 | 1167.5117 | 10 | 0.9 | 584.7637 | 2 | 38.63 | 1 | F1:19868 | 28122021\_RID\_2349\_2C.raw | 3.5946E6 | 1.093E7 | 3.5028E6 |  |  | 1.3745E5 | 6 | 2 | 2 | 1 | 0 | 0 | 1 | 233 | 242 |  |  | PEAKS DB |
| K.HDDIFAYEKR.F | N | 60.61 | 1292.6149 | 10 | 0.6 | 431.8792 | 3 | 25.32 | 1 | F1:9449 | 28122021\_RID\_2349\_2C.raw | 9.7777E5 | 4.5859E5 |  |  |  |  | 2 | 1 | 1 | 0 | 0 | 0 | 0 | 223 | 232 |  |  | PEAKS DB |
| Q.ENDYEEFLEIAR.N | Y | 59.88 | 1526.6888 | 12 | 0.3 | 764.3519 | 2 | 55.90 | 1 | F1:33325 | 28122021\_RID\_2349\_2C.raw | 3.2858E6 | 1.7029E7 |  |  | 1.8334E6 | 1.8954E6 | 4 | 1 | 1 | 0 | 0 | 1 | 1 | 13 | 24 |  |  | PEAKS DB |
| R.FDEIVDGM(+15.99)DK.L | N | 57.69 | 1183.5067 | 10 | 0.0 | 592.7606 | 2 | 31.41 | 1 | F1:14146 | 28122021\_RID\_2349\_2C.raw | 1.0488E6 | 2.2712E6 |  |  |  |  | 2 | 1 | 1 | 0 | 0 | 0 | 0 | 233 | 242 | Oxidation (M) | M8:Oxidation (M):1000.00 | PEAKS DB |
| D.YEEFLEIAR.N | N | 56.31 | 1168.5764 | 9 | 0.7 | 585.2959 | 2 | 45.58 | 2 | F2:15608 | 30122021\_RID\_2349\_1C.raw |  | 2.3921E5 |  |  |  |  | 1 | 0 | 1 | 0 | 0 | 0 | 0 | 16 | 24 |  |  | PEAKS DB |
| N.DYEEFLEIAR.N | N | 56.01 | 1283.6033 | 10 | 0.8 | 642.8094 | 2 | 56.80 | 5 | F5:32140 | 31122021\_RID\_2349\_5C.raw | 1.3399E5 | 2.7826E5 |  |  | 9.5716E4 | 7.7266E4 | 4 | 1 | 1 | 0 | 0 | 1 | 1 | 15 | 24 |  |  | PEAKS DB |
| A.GQLYEESLGK.V | N | 54.58 | 1122.5557 | 10 | 1.2 | 562.2858 | 2 | 29.95 | 1 | F1:13018 | 28122021\_RID\_2349\_2C.raw | 4.0796E5 | 6.449E5 |  |  |  |  | 2 | 1 | 1 | 0 | 0 | 0 | 0 | 154 | 163 |  |  | PEAKS DB |
| N.EFSQENDNAWYFIK.N | N | 53.57 | 1789.7947 | 14 | 0.5 | 895.9051 | 2 | 53.07 | 1 | F1:31357 | 28122021\_RID\_2349\_2C.raw | 2.5591E5 | 2.9753E5 |  |  |  |  | 2 | 1 | 1 | 0 | 0 | 0 | 0 | 111 | 124 |  |  | PEAKS DB |
| F.SQENDNAWYFIK.N | N | 53.25 | 1513.6837 | 12 | 1.0 | 757.8499 | 2 | 47.51 | 2 | F2:16263 | 30122021\_RID\_2349\_1C.raw |  | 7.0312E5 |  |  |  |  | 1 | 0 | 1 | 0 | 0 | 0 | 0 | 113 | 124 |  |  | PEAKS DB |
| N.EDSGYYVSFIESLK.H | N | 53.12 | 1635.7667 | 14 | 0.7 | 818.8912 | 2 | 61.10 | 1 | F1:36406 | 28122021\_RID\_2349\_2C.raw | 7.7617E4 |  |  |  |  |  | 1 | 1 | 0 | 0 | 0 | 0 | 0 | 209 | 222 |  |  | PEAKS DB |
| R.IYFAGEYTAQAHGWIDSTIK.S | N | 51.08 | 2270.1008 | 20 | 0.0 | 757.7076 | 3 | 50.32 | 3 | F3:17332 | 31122021\_RID\_2349\_3C.raw |  |  | 1.0543E7 |  |  |  | 1 | 0 | 0 | 1 | 0 | 0 | 0 | 452 | 471 |  |  | PEAKS DB |
| K.YDTYSTKEY.L | N | 49.76 | 1168.4924 | 9 | 1.0 | 585.2541 | 2 | 25.91 | 5 | F5:9933 | 31122021\_RID\_2349\_5C.raw |  |  |  |  | 2.4008E6 |  | 1 | 0 | 0 | 0 | 0 | 1 | 0 | 180 | 188 |  |  | PEAKS DB |
| K.YAMGGITTFTPYQFQHFSD.P | Y | 48.98 | 2209.9778 | 19 | 0.6 | 1105.9968 | 2 | 60.23 | 1 | F1:36094 | 28122021\_RID\_2349\_2C.raw | 1.3342E6 |  |  |  |  |  | 1 | 1 | 0 | 0 | 0 | 0 | 0 | 425 | 443 |  |  | PEAKS DB |
| Q.AHGWIDSTIK.S | N | 45.39 | 1126.5771 | 10 | 1.2 | 564.2965 | 2 | 28.81 | 2 | F2:8087 | 30122021\_RID\_2349\_1C.raw |  | 3.6217E5 |  |  |  |  | 1 | 0 | 1 | 0 | 0 | 0 | 0 | 462 | 471 |  |  | PEAKS DB |
| N.FFQALDFK.D | N | 45.08 | 1014.5174 | 8 | 0.8 | 508.2664 | 2 | 48.21 | 2 | F2:16516 | 30122021\_RID\_2349\_1C.raw |  | 1.1251E4 |  |  |  |  | 1 | 0 | 1 | 0 | 0 | 0 | 0 | 380 | 387 |  |  | PEAKS DB |
| A.GWYANLGPMR.L | N | 42.97 | 1163.5546 | 10 | 0.2 | 582.7847 | 2 | 43.50 | 1 | F1:23833 | 28122021\_RID\_2349\_2C.raw | 3.4804E4 |  |  |  |  |  | 1 | 1 | 0 | 0 | 0 | 0 | 0 | 81 | 90 |  |  | PEAKS DB |
| S.AGQLYEESLGK.V | N | 42.75 | 1193.5928 | 11 | -0.1 | 597.8036 | 2 | 30.49 | 2 | F2:8743 | 30122021\_RID\_2349\_1C.raw |  | 1.8392E5 |  |  |  |  | 1 | 0 | 1 | 0 | 0 | 0 | 0 | 153 | 163 |  |  | PEAKS DB |
| total 26 peptides |  |  |  |  |  |  |  |  |  |  |  |  |  |  |  |  |  |  |  |  |  |  |  |  |  |  |  |  |  |
| --- | --- | --- | --- | --- | --- | --- | --- | --- | --- | --- | --- | --- | --- | --- | --- | --- | --- | --- | --- | --- | --- | --- | --- | --- | --- | --- | --- | --- | --- |

1F8S

back to list

  

| Protein Coverage
| Supporting Peptides
|

Protein Coverage:

Supporting Peptides:

| Peptide | Uniq | -10lgP | Mass | Length | ppm | m/z | z | RT | Fraction | Scan | Source File | Area F2 | Area F1 | Area F3 | Area F4 | Area F5 | Area F6 | #Feature | #Feature F2 | #Feature F1 | #Feature F3 | #Feature F4 | #Feature F5 | #Feature F6 | Start | End | PTM | AScore | Found By |
| --- | --- | --- | --- | --- | --- | --- | --- | --- | --- | --- | --- | --- | --- | --- | --- | --- | --- | --- | --- | --- | --- | --- | --- | --- | --- | --- | --- | --- | --- |
| T.KFWEDDGIHGGK.S | N | 83.09 | 1387.6520 | 12 | 0.2 | 463.5580 | 3 | 24.69 | 5 | F5:8977 | 31122021\_RID\_2349\_5C.raw | 3.2966E7 | 1.6951E7 | 4.7218E7 |  | 1.4906E6 |  | 10 | 4 | 3 | 1 | 0 | 2 | 0 | 334 | 345 |  |  | PEAKS DB |
| K.SAGQLYEESLGK.V | N | 80.33 | 1280.6248 | 12 | -0.1 | 641.3196 | 2 | 32.32 | 5 | F5:14229 | 31122021\_RID\_2349\_5C.raw | 3.9268E8 | 5.6746E8 | 8.8153E7 | 1.109E8 | 1.2738E8 | 1.0719E5 | 12 | 3 | 3 | 1 | 1 | 3 | 1 | 152 | 163 |  |  | PEAKS DB |
| Y.GIGDDANFFQALDFK.D | N | 75.31 | 1656.7783 | 15 | 0.2 | 829.3966 | 2 | 64.60 | 3 | F3:23470 | 31122021\_RID\_2349\_3C.raw |  |  | 1.1679E5 |  |  |  | 1 | 0 | 0 | 1 | 0 | 0 | 0 | 373 | 387 |  |  | PEAKS DB |
| K.RFDEIVDGMDK.L | N | 74.01 | 1323.6129 | 11 | 0.3 | 662.8139 | 2 | 35.47 | 1 | F1:17511 | 28122021\_RID\_2349\_2C.raw | 9.5788E6 | 2.9071E7 | 1.7532E7 |  |  | 4.2754E5 | 7 | 2 | 3 | 1 | 0 | 0 | 1 | 232 | 242 |  |  | PEAKS DB |
| K.RFDEIVDGM(+15.99)DK.L | N | 73.28 | 1339.6078 | 11 | 0.3 | 670.8114 | 2 | 34.96 | 1 | F1:17438 | 28122021\_RID\_2349\_2C.raw | 4.3666E6 | 1.0728E7 |  |  |  |  | 9 | 4 | 5 | 0 | 0 | 0 | 0 | 232 | 242 | Oxidation (M) | M9:Oxidation (M):1000.00 | PEAKS DB |
| K.HDDIFAYEK.R | N | 72.69 | 1136.5138 | 9 | -0.1 | 569.2641 | 2 | 30.15 | 2 | F2:8868 | 30122021\_RID\_2349\_1C.raw | 5.9259E7 | 1.5095E8 | 7.8164E7 |  |  | 1.3576E6 | 7 | 3 | 2 | 1 | 0 | 0 | 1 | 223 | 231 |  |  | PEAKS DB |
| K.FWEDDGIHGGK.S | N | 71.93 | 1259.5570 | 11 | 0.4 | 630.7860 | 2 | 28.94 | 1 | F1:12222 | 28122021\_RID\_2349\_2C.raw | 1.3683E7 | 2.9767E7 | 9.0355E6 |  |  |  | 10 | 4 | 5 | 1 | 0 | 0 | 0 | 335 | 345 |  |  | PEAKS DB |
| R.IYFAGEYTAQ.A | N | 68.04 | 1161.5342 | 10 | 0.2 | 581.7745 | 2 | 47.04 | 2 | F2:16101 | 30122021\_RID\_2349\_1C.raw |  | 4.684E5 |  |  |  |  | 1 | 0 | 1 | 0 | 0 | 0 | 0 | 452 | 461 |  |  | PEAKS DB |
| R.IYFAGEYTAQAH.G | N | 65.33 | 1369.6302 | 12 | 0.8 | 685.8229 | 2 | 38.89 | 2 | F2:12898 | 30122021\_RID\_2349\_1C.raw |  | 1.6051E6 |  |  |  |  | 1 | 0 | 1 | 0 | 0 | 0 | 0 | 452 | 463 |  |  | PEAKS DB |
| R.FDEIVDGMDK.L | N | 64.83 | 1167.5117 | 10 | 0.9 | 584.7637 | 2 | 38.63 | 1 | F1:19868 | 28122021\_RID\_2349\_2C.raw | 3.5946E6 | 1.093E7 | 3.5028E6 |  |  | 1.3745E5 | 6 | 2 | 2 | 1 | 0 | 0 | 1 | 233 | 242 |  |  | PEAKS DB |
| K.HDDIFAYEKR.F | N | 60.61 | 1292.6149 | 10 | 0.6 | 431.8792 | 3 | 25.32 | 1 | F1:9449 | 28122021\_RID\_2349\_2C.raw | 9.7777E5 | 4.5859E5 |  |  |  |  | 2 | 1 | 1 | 0 | 0 | 0 | 0 | 223 | 232 |  |  | PEAKS DB |
| Q.ENDYEEFLEIAR.N | Y | 59.88 | 1526.6888 | 12 | 0.3 | 764.3519 | 2 | 55.90 | 1 | F1:33325 | 28122021\_RID\_2349\_2C.raw | 3.2858E6 | 1.7029E7 |  |  | 1.8334E6 | 1.8954E6 | 4 | 1 | 1 | 0 | 0 | 1 | 1 | 13 | 24 |  |  | PEAKS DB |
| R.FDEIVDGM(+15.99)DK.L | N | 57.69 | 1183.5067 | 10 | 0.0 | 592.7606 | 2 | 31.41 | 1 | F1:14146 | 28122021\_RID\_2349\_2C.raw | 1.0488E6 | 2.2712E6 |  |  |  |  | 2 | 1 | 1 | 0 | 0 | 0 | 0 | 233 | 242 | Oxidation (M) | M8:Oxidation (M):1000.00 | PEAKS DB |
| D.YEEFLEIAR.N | N | 56.31 | 1168.5764 | 9 | 0.7 | 585.2959 | 2 | 45.58 | 2 | F2:15608 | 30122021\_RID\_2349\_1C.raw |  | 2.3921E5 |  |  |  |  | 1 | 0 | 1 | 0 | 0 | 0 | 0 | 16 | 24 |  |  | PEAKS DB |
| N.DYEEFLEIAR.N | N | 56.01 | 1283.6033 | 10 | 0.8 | 642.8094 | 2 | 56.80 | 5 | F5:32140 | 31122021\_RID\_2349\_5C.raw | 1.3399E5 | 2.7826E5 |  |  | 9.5716E4 | 7.7266E4 | 4 | 1 | 1 | 0 | 0 | 1 | 1 | 15 | 24 |  |  | PEAKS DB |
| A.GQLYEESLGK.V | N | 54.58 | 1122.5557 | 10 | 1.2 | 562.2858 | 2 | 29.95 | 1 | F1:13018 | 28122021\_RID\_2349\_2C.raw | 4.0796E5 | 6.449E5 |  |  |  |  | 2 | 1 | 1 | 0 | 0 | 0 | 0 | 154 | 163 |  |  | PEAKS DB |
| N.EFSQENDNAWYFIK.N | N | 53.57 | 1789.7947 | 14 | 0.5 | 895.9051 | 2 | 53.07 | 1 | F1:31357 | 28122021\_RID\_2349\_2C.raw | 2.5591E5 | 2.9753E5 |  |  |  |  | 2 | 1 | 1 | 0 | 0 | 0 | 0 | 111 | 124 |  |  | PEAKS DB |
| F.SQENDNAWYFIK.N | N | 53.25 | 1513.6837 | 12 | 1.0 | 757.8499 | 2 | 47.51 | 2 | F2:16263 | 30122021\_RID\_2349\_1C.raw |  | 7.0312E5 |  |  |  |  | 1 | 0 | 1 | 0 | 0 | 0 | 0 | 113 | 124 |  |  | PEAKS DB |
| N.EDSGYYVSFIESLK.H | N | 53.12 | 1635.7667 | 14 | 0.7 | 818.8912 | 2 | 61.10 | 1 | F1:36406 | 28122021\_RID\_2349\_2C.raw | 7.7617E4 |  |  |  |  |  | 1 | 1 | 0 | 0 | 0 | 0 | 0 | 209 | 222 |  |  | PEAKS DB |
| R.IYFAGEYTAQAHGWIDSTIK.S | N | 51.08 | 2270.1008 | 20 | 0.0 | 757.7076 | 3 | 50.32 | 3 | F3:17332 | 31122021\_RID\_2349\_3C.raw |  |  | 1.0543E7 |  |  |  | 1 | 0 | 0 | 1 | 0 | 0 | 0 | 452 | 471 |  |  | PEAKS DB |
| K.YDTYSTKEY.L | N | 49.76 | 1168.4924 | 9 | 1.0 | 585.2541 | 2 | 25.91 | 5 | F5:9933 | 31122021\_RID\_2349\_5C.raw |  |  |  |  | 2.4008E6 |  | 1 | 0 | 0 | 0 | 0 | 1 | 0 | 180 | 188 |  |  | PEAKS DB |
| K.YAMGGITTFTPYQFQHFSD.P | Y | 48.98 | 2209.9778 | 19 | 0.6 | 1105.9968 | 2 | 60.23 | 1 | F1:36094 | 28122021\_RID\_2349\_2C.raw | 1.3342E6 |  |  |  |  |  | 1 | 1 | 0 | 0 | 0 | 0 | 0 | 425 | 443 |  |  | PEAKS DB |
| Q.AHGWIDSTIK.S | N | 45.39 | 1126.5771 | 10 | 1.2 | 564.2965 | 2 | 28.81 | 2 | F2:8087 | 30122021\_RID\_2349\_1C.raw |  | 3.6217E5 |  |  |  |  | 1 | 0 | 1 | 0 | 0 | 0 | 0 | 462 | 471 |  |  | PEAKS DB |
| N.FFQALDFK.D | N | 45.08 | 1014.5174 | 8 | 0.8 | 508.2664 | 2 | 48.21 | 2 | F2:16516 | 30122021\_RID\_2349\_1C.raw |  | 1.1251E4 |  |  |  |  | 1 | 0 | 1 | 0 | 0 | 0 | 0 | 380 | 387 |  |  | PEAKS DB |
| A.GWYANLGPMR.L | N | 42.97 | 1163.5546 | 10 | 0.2 | 582.7847 | 2 | 43.50 | 1 | F1:23833 | 28122021\_RID\_2349\_2C.raw | 3.4804E4 |  |  |  |  |  | 1 | 1 | 0 | 0 | 0 | 0 | 0 | 81 | 90 |  |  | PEAKS DB |
| S.AGQLYEESLGK.V | N | 42.75 | 1193.5928 | 11 | -0.1 | 597.8036 | 2 | 30.49 | 2 | F2:8743 | 30122021\_RID\_2349\_1C.raw |  | 1.8392E5 |  |  |  |  | 1 | 0 | 1 | 0 | 0 | 0 | 0 | 153 | 163 |  |  | PEAKS DB |
| total 26 peptides |  |  |  |  |  |  |  |  |  |  |  |  |  |  |  |  |  |  |  |  |  |  |  |  |  |  |  |  |  |
| --- | --- | --- | --- | --- | --- | --- | --- | --- | --- | --- | --- | --- | --- | --- | --- | --- | --- | --- | --- | --- | --- | --- | --- | --- | --- | --- | --- | --- | --- |

1F8R

back to list

  

| Protein Coverage
| Supporting Peptides
|

Protein Coverage:

Supporting Peptides:

| Peptide | Uniq | -10lgP | Mass | Length | ppm | m/z | z | RT | Fraction | Scan | Source File | Area F2 | Area F1 | Area F3 | Area F4 | Area F5 | Area F6 | #Feature | #Feature F2 | #Feature F1 | #Feature F3 | #Feature F4 | #Feature F5 | #Feature F6 | Start | End | PTM | AScore | Found By |
| --- | --- | --- | --- | --- | --- | --- | --- | --- | --- | --- | --- | --- | --- | --- | --- | --- | --- | --- | --- | --- | --- | --- | --- | --- | --- | --- | --- | --- | --- |
| T.KFWEDDGIHGGK.S | N | 83.09 | 1387.6520 | 12 | 0.2 | 463.5580 | 3 | 24.69 | 5 | F5:8977 | 31122021\_RID\_2349\_5C.raw | 3.2966E7 | 1.6951E7 | 4.7218E7 |  | 1.4906E6 |  | 10 | 4 | 3 | 1 | 0 | 2 | 0 | 334 | 345 |  |  | PEAKS DB |
| K.SAGQLYEESLGK.V | N | 80.33 | 1280.6248 | 12 | -0.1 | 641.3196 | 2 | 32.32 | 5 | F5:14229 | 31122021\_RID\_2349\_5C.raw | 3.9268E8 | 5.6746E8 | 8.8153E7 | 1.109E8 | 1.2738E8 | 1.0719E5 | 12 | 3 | 3 | 1 | 1 | 3 | 1 | 152 | 163 |  |  | PEAKS DB |
| Y.GIGDDANFFQALDFK.D | N | 75.31 | 1656.7783 | 15 | 0.2 | 829.3966 | 2 | 64.60 | 3 | F3:23470 | 31122021\_RID\_2349\_3C.raw |  |  | 1.1679E5 |  |  |  | 1 | 0 | 0 | 1 | 0 | 0 | 0 | 373 | 387 |  |  | PEAKS DB |
| K.RFDEIVDGMDK.L | N | 74.01 | 1323.6129 | 11 | 0.3 | 662.8139 | 2 | 35.47 | 1 | F1:17511 | 28122021\_RID\_2349\_2C.raw | 9.5788E6 | 2.9071E7 | 1.7532E7 |  |  | 4.2754E5 | 7 | 2 | 3 | 1 | 0 | 0 | 1 | 232 | 242 |  |  | PEAKS DB |
| K.RFDEIVDGM(+15.99)DK.L | N | 73.28 | 1339.6078 | 11 | 0.3 | 670.8114 | 2 | 34.96 | 1 | F1:17438 | 28122021\_RID\_2349\_2C.raw | 4.3666E6 | 1.0728E7 |  |  |  |  | 9 | 4 | 5 | 0 | 0 | 0 | 0 | 232 | 242 | Oxidation (M) | M9:Oxidation (M):1000.00 | PEAKS DB |
| K.HDDIFAYEK.R | N | 72.69 | 1136.5138 | 9 | -0.1 | 569.2641 | 2 | 30.15 | 2 | F2:8868 | 30122021\_RID\_2349\_1C.raw | 5.9259E7 | 1.5095E8 | 7.8164E7 |  |  | 1.3576E6 | 7 | 3 | 2 | 1 | 0 | 0 | 1 | 223 | 231 |  |  | PEAKS DB |
| K.FWEDDGIHGGK.S | N | 71.93 | 1259.5570 | 11 | 0.4 | 630.7860 | 2 | 28.94 | 1 | F1:12222 | 28122021\_RID\_2349\_2C.raw | 1.3683E7 | 2.9767E7 | 9.0355E6 |  |  |  | 10 | 4 | 5 | 1 | 0 | 0 | 0 | 335 | 345 |  |  | PEAKS DB |
| R.IYFAGEYTAQ.A | N | 68.04 | 1161.5342 | 10 | 0.2 | 581.7745 | 2 | 47.04 | 2 | F2:16101 | 30122021\_RID\_2349\_1C.raw |  | 4.684E5 |  |  |  |  | 1 | 0 | 1 | 0 | 0 | 0 | 0 | 452 | 461 |  |  | PEAKS DB |
| R.IYFAGEYTAQAH.G | N | 65.33 | 1369.6302 | 12 | 0.8 | 685.8229 | 2 | 38.89 | 2 | F2:12898 | 30122021\_RID\_2349\_1C.raw |  | 1.6051E6 |  |  |  |  | 1 | 0 | 1 | 0 | 0 | 0 | 0 | 452 | 463 |  |  | PEAKS DB |
| R.FDEIVDGMDK.L | N | 64.83 | 1167.5117 | 10 | 0.9 | 584.7637 | 2 | 38.63 | 1 | F1:19868 | 28122021\_RID\_2349\_2C.raw | 3.5946E6 | 1.093E7 | 3.5028E6 |  |  | 1.3745E5 | 6 | 2 | 2 | 1 | 0 | 0 | 1 | 233 | 242 |  |  | PEAKS DB |
| K.HDDIFAYEKR.F | N | 60.61 | 1292.6149 | 10 | 0.6 | 431.8792 | 3 | 25.32 | 1 | F1:9449 | 28122021\_RID\_2349\_2C.raw | 9.7777E5 | 4.5859E5 |  |  |  |  | 2 | 1 | 1 | 0 | 0 | 0 | 0 | 223 | 232 |  |  | PEAKS DB |
| Q.ENDYEEFLEIAR.N | Y | 59.88 | 1526.6888 | 12 | 0.3 | 764.3519 | 2 | 55.90 | 1 | F1:33325 | 28122021\_RID\_2349\_2C.raw | 3.2858E6 | 1.7029E7 |  |  | 1.8334E6 | 1.8954E6 | 4 | 1 | 1 | 0 | 0 | 1 | 1 | 13 | 24 |  |  | PEAKS DB |
| R.FDEIVDGM(+15.99)DK.L | N | 57.69 | 1183.5067 | 10 | 0.0 | 592.7606 | 2 | 31.41 | 1 | F1:14146 | 28122021\_RID\_2349\_2C.raw | 1.0488E6 | 2.2712E6 |  |  |  |  | 2 | 1 | 1 | 0 | 0 | 0 | 0 | 233 | 242 | Oxidation (M) | M8:Oxidation (M):1000.00 | PEAKS DB |
| D.YEEFLEIAR.N | N | 56.31 | 1168.5764 | 9 | 0.7 | 585.2959 | 2 | 45.58 | 2 | F2:15608 | 30122021\_RID\_2349\_1C.raw |  | 2.3921E5 |  |  |  |  | 1 | 0 | 1 | 0 | 0 | 0 | 0 | 16 | 24 |  |  | PEAKS DB |
| N.DYEEFLEIAR.N | N | 56.01 | 1283.6033 | 10 | 0.8 | 642.8094 | 2 | 56.80 | 5 | F5:32140 | 31122021\_RID\_2349\_5C.raw | 1.3399E5 | 2.7826E5 |  |  | 9.5716E4 | 7.7266E4 | 4 | 1 | 1 | 0 | 0 | 1 | 1 | 15 | 24 |  |  | PEAKS DB |
| A.GQLYEESLGK.V | N | 54.58 | 1122.5557 | 10 | 1.2 | 562.2858 | 2 | 29.95 | 1 | F1:13018 | 28122021\_RID\_2349\_2C.raw | 4.0796E5 | 6.449E5 |  |  |  |  | 2 | 1 | 1 | 0 | 0 | 0 | 0 | 154 | 163 |  |  | PEAKS DB |
| N.EFSQENDNAWYFIK.N | N | 53.57 | 1789.7947 | 14 | 0.5 | 895.9051 | 2 | 53.07 | 1 | F1:31357 | 28122021\_RID\_2349\_2C.raw | 2.5591E5 | 2.9753E5 |  |  |  |  | 2 | 1 | 1 | 0 | 0 | 0 | 0 | 111 | 124 |  |  | PEAKS DB |
| F.SQENDNAWYFIK.N | N | 53.25 | 1513.6837 | 12 | 1.0 | 757.8499 | 2 | 47.51 | 2 | F2:16263 | 30122021\_RID\_2349\_1C.raw |  | 7.0312E5 |  |  |  |  | 1 | 0 | 1 | 0 | 0 | 0 | 0 | 113 | 124 |  |  | PEAKS DB |
| N.EDSGYYVSFIESLK.H | N | 53.12 | 1635.7667 | 14 | 0.7 | 818.8912 | 2 | 61.10 | 1 | F1:36406 | 28122021\_RID\_2349\_2C.raw | 7.7617E4 |  |  |  |  |  | 1 | 1 | 0 | 0 | 0 | 0 | 0 | 209 | 222 |  |  | PEAKS DB |
| R.IYFAGEYTAQAHGWIDSTIK.S | N | 51.08 | 2270.1008 | 20 | 0.0 | 757.7076 | 3 | 50.32 | 3 | F3:17332 | 31122021\_RID\_2349\_3C.raw |  |  | 1.0543E7 |  |  |  | 1 | 0 | 0 | 1 | 0 | 0 | 0 | 452 | 471 |  |  | PEAKS DB |
| K.YDTYSTKEY.L | N | 49.76 | 1168.4924 | 9 | 1.0 | 585.2541 | 2 | 25.91 | 5 | F5:9933 | 31122021\_RID\_2349\_5C.raw |  |  |  |  | 2.4008E6 |  | 1 | 0 | 0 | 0 | 0 | 1 | 0 | 180 | 188 |  |  | PEAKS DB |
| K.YAMGGITTFTPYQFQHFSD.P | Y | 48.98 | 2209.9778 | 19 | 0.6 | 1105.9968 | 2 | 60.23 | 1 | F1:36094 | 28122021\_RID\_2349\_2C.raw | 1.3342E6 |  |  |  |  |  | 1 | 1 | 0 | 0 | 0 | 0 | 0 | 425 | 443 |  |  | PEAKS DB |
| Q.AHGWIDSTIK.S | N | 45.39 | 1126.5771 | 10 | 1.2 | 564.2965 | 2 | 28.81 | 2 | F2:8087 | 30122021\_RID\_2349\_1C.raw |  | 3.6217E5 |  |  |  |  | 1 | 0 | 1 | 0 | 0 | 0 | 0 | 462 | 471 |  |  | PEAKS DB |
| N.FFQALDFK.D | N | 45.08 | 1014.5174 | 8 | 0.8 | 508.2664 | 2 | 48.21 | 2 | F2:16516 | 30122021\_RID\_2349\_1C.raw |  | 1.1251E4 |  |  |  |  | 1 | 0 | 1 | 0 | 0 | 0 | 0 | 380 | 387 |  |  | PEAKS DB |
| A.GWYANLGPMR.L | N | 42.97 | 1163.5546 | 10 | 0.2 | 582.7847 | 2 | 43.50 | 1 | F1:23833 | 28122021\_RID\_2349\_2C.raw | 3.4804E4 |  |  |  |  |  | 1 | 1 | 0 | 0 | 0 | 0 | 0 | 81 | 90 |  |  | PEAKS DB |
| S.AGQLYEESLGK.V | N | 42.75 | 1193.5928 | 11 | -0.1 | 597.8036 | 2 | 30.49 | 2 | F2:8743 | 30122021\_RID\_2349\_1C.raw |  | 1.8392E5 |  |  |  |  | 1 | 0 | 1 | 0 | 0 | 0 | 0 | 153 | 163 |  |  | PEAKS DB |
| total 26 peptides |  |  |  |  |  |  |  |  |  |  |  |  |  |  |  |  |  |  |  |  |  |  |  |  |  |  |  |  |  |
| --- | --- | --- | --- | --- | --- | --- | --- | --- | --- | --- | --- | --- | --- | --- | --- | --- | --- | --- | --- | --- | --- | --- | --- | --- | --- | --- | --- | --- | --- |

P81382.2

back to list

  

| Protein Coverage
| Supporting Peptides
|

Protein Coverage:

Supporting Peptides:

| Peptide | Uniq | -10lgP | Mass | Length | ppm | m/z | z | RT | Fraction | Scan | Source File | Area F2 | Area F1 | Area F3 | Area F4 | Area F5 | Area F6 | #Feature | #Feature F2 | #Feature F1 | #Feature F3 | #Feature F4 | #Feature F5 | #Feature F6 | Start | End | PTM | AScore | Found By |
| --- | --- | --- | --- | --- | --- | --- | --- | --- | --- | --- | --- | --- | --- | --- | --- | --- | --- | --- | --- | --- | --- | --- | --- | --- | --- | --- | --- | --- | --- |
| T.KFWEDDGIHGGK.S | N | 83.09 | 1387.6520 | 12 | 0.2 | 463.5580 | 3 | 24.69 | 5 | F5:8977 | 31122021\_RID\_2349\_5C.raw | 3.2966E7 | 1.6951E7 | 4.7218E7 |  | 1.4906E6 |  | 10 | 4 | 3 | 1 | 0 | 2 | 0 | 352 | 363 |  |  | PEAKS DB |
| K.SAGQLYEESLGK.V | N | 80.33 | 1280.6248 | 12 | -0.1 | 641.3196 | 2 | 32.32 | 5 | F5:14229 | 31122021\_RID\_2349\_5C.raw | 3.9268E8 | 5.6746E8 | 8.8153E7 | 1.109E8 | 1.2738E8 | 1.0719E5 | 12 | 3 | 3 | 1 | 1 | 3 | 1 | 170 | 181 |  |  | PEAKS DB |
| Y.GIGDDANFFQALDFK.D | N | 75.31 | 1656.7783 | 15 | 0.2 | 829.3966 | 2 | 64.60 | 3 | F3:23470 | 31122021\_RID\_2349\_3C.raw |  |  | 1.1679E5 |  |  |  | 1 | 0 | 0 | 1 | 0 | 0 | 0 | 391 | 405 |  |  | PEAKS DB |
| K.RFDEIVDGMDK.L | N | 74.01 | 1323.6129 | 11 | 0.3 | 662.8139 | 2 | 35.47 | 1 | F1:17511 | 28122021\_RID\_2349\_2C.raw | 9.5788E6 | 2.9071E7 | 1.7532E7 |  |  | 4.2754E5 | 7 | 2 | 3 | 1 | 0 | 0 | 1 | 250 | 260 |  |  | PEAKS DB |
| K.RFDEIVDGM(+15.99)DK.L | N | 73.28 | 1339.6078 | 11 | 0.3 | 670.8114 | 2 | 34.96 | 1 | F1:17438 | 28122021\_RID\_2349\_2C.raw | 4.3666E6 | 1.0728E7 |  |  |  |  | 9 | 4 | 5 | 0 | 0 | 0 | 0 | 250 | 260 | Oxidation (M) | M9:Oxidation (M):1000.00 | PEAKS DB |
| K.HDDIFAYEK.R | N | 72.69 | 1136.5138 | 9 | -0.1 | 569.2641 | 2 | 30.15 | 2 | F2:8868 | 30122021\_RID\_2349\_1C.raw | 5.9259E7 | 1.5095E8 | 7.8164E7 |  |  | 1.3576E6 | 7 | 3 | 2 | 1 | 0 | 0 | 1 | 241 | 249 |  |  | PEAKS DB |
| K.FWEDDGIHGGK.S | N | 71.93 | 1259.5570 | 11 | 0.4 | 630.7860 | 2 | 28.94 | 1 | F1:12222 | 28122021\_RID\_2349\_2C.raw | 1.3683E7 | 2.9767E7 | 9.0355E6 |  |  |  | 10 | 4 | 5 | 1 | 0 | 0 | 0 | 353 | 363 |  |  | PEAKS DB |
| R.IYFAGEYTAQ.A | N | 68.04 | 1161.5342 | 10 | 0.2 | 581.7745 | 2 | 47.04 | 2 | F2:16101 | 30122021\_RID\_2349\_1C.raw |  | 4.684E5 |  |  |  |  | 1 | 0 | 1 | 0 | 0 | 0 | 0 | 470 | 479 |  |  | PEAKS DB |
| R.IYFAGEYTAQAH.G | N | 65.33 | 1369.6302 | 12 | 0.8 | 685.8229 | 2 | 38.89 | 2 | F2:12898 | 30122021\_RID\_2349\_1C.raw |  | 1.6051E6 |  |  |  |  | 1 | 0 | 1 | 0 | 0 | 0 | 0 | 470 | 481 |  |  | PEAKS DB |
| R.FDEIVDGMDK.L | N | 64.83 | 1167.5117 | 10 | 0.9 | 584.7637 | 2 | 38.63 | 1 | F1:19868 | 28122021\_RID\_2349\_2C.raw | 3.5946E6 | 1.093E7 | 3.5028E6 |  |  | 1.3745E5 | 6 | 2 | 2 | 1 | 0 | 0 | 1 | 251 | 260 |  |  | PEAKS DB |
| K.HDDIFAYEKR.F | N | 60.61 | 1292.6149 | 10 | 0.6 | 431.8792 | 3 | 25.32 | 1 | F1:9449 | 28122021\_RID\_2349\_2C.raw | 9.7777E5 | 4.5859E5 |  |  |  |  | 2 | 1 | 1 | 0 | 0 | 0 | 0 | 241 | 250 |  |  | PEAKS DB |
| Q.ENDYEEFLEIAR.N | Y | 59.88 | 1526.6888 | 12 | 0.3 | 764.3519 | 2 | 55.90 | 1 | F1:33325 | 28122021\_RID\_2349\_2C.raw | 3.2858E6 | 1.7029E7 |  |  | 1.8334E6 | 1.8954E6 | 4 | 1 | 1 | 0 | 0 | 1 | 1 | 31 | 42 |  |  | PEAKS DB |
| R.FDEIVDGM(+15.99)DK.L | N | 57.69 | 1183.5067 | 10 | 0.0 | 592.7606 | 2 | 31.41 | 1 | F1:14146 | 28122021\_RID\_2349\_2C.raw | 1.0488E6 | 2.2712E6 |  |  |  |  | 2 | 1 | 1 | 0 | 0 | 0 | 0 | 251 | 260 | Oxidation (M) | M8:Oxidation (M):1000.00 | PEAKS DB |
| D.YEEFLEIAR.N | N | 56.31 | 1168.5764 | 9 | 0.7 | 585.2959 | 2 | 45.58 | 2 | F2:15608 | 30122021\_RID\_2349\_1C.raw |  | 2.3921E5 |  |  |  |  | 1 | 0 | 1 | 0 | 0 | 0 | 0 | 34 | 42 |  |  | PEAKS DB |
| N.DYEEFLEIAR.N | N | 56.01 | 1283.6033 | 10 | 0.8 | 642.8094 | 2 | 56.80 | 5 | F5:32140 | 31122021\_RID\_2349\_5C.raw | 1.3399E5 | 2.7826E5 |  |  | 9.5716E4 | 7.7266E4 | 4 | 1 | 1 | 0 | 0 | 1 | 1 | 33 | 42 |  |  | PEAKS DB |
| A.GQLYEESLGK.V | N | 54.58 | 1122.5557 | 10 | 1.2 | 562.2858 | 2 | 29.95 | 1 | F1:13018 | 28122021\_RID\_2349\_2C.raw | 4.0796E5 | 6.449E5 |  |  |  |  | 2 | 1 | 1 | 0 | 0 | 0 | 0 | 172 | 181 |  |  | PEAKS DB |
| N.EFSQENDNAWYFIK.N | N | 53.57 | 1789.7947 | 14 | 0.5 | 895.9051 | 2 | 53.07 | 1 | F1:31357 | 28122021\_RID\_2349\_2C.raw | 2.5591E5 | 2.9753E5 |  |  |  |  | 2 | 1 | 1 | 0 | 0 | 0 | 0 | 129 | 142 |  |  | PEAKS DB |
| F.SQENDNAWYFIK.N | N | 53.25 | 1513.6837 | 12 | 1.0 | 757.8499 | 2 | 47.51 | 2 | F2:16263 | 30122021\_RID\_2349\_1C.raw |  | 7.0312E5 |  |  |  |  | 1 | 0 | 1 | 0 | 0 | 0 | 0 | 131 | 142 |  |  | PEAKS DB |
| N.EDSGYYVSFIESLK.H | N | 53.12 | 1635.7667 | 14 | 0.7 | 818.8912 | 2 | 61.10 | 1 | F1:36406 | 28122021\_RID\_2349\_2C.raw | 7.7617E4 |  |  |  |  |  | 1 | 1 | 0 | 0 | 0 | 0 | 0 | 227 | 240 |  |  | PEAKS DB |
| R.IYFAGEYTAQAHGWIDSTIK.S | N | 51.08 | 2270.1008 | 20 | 0.0 | 757.7076 | 3 | 50.32 | 3 | F3:17332 | 31122021\_RID\_2349\_3C.raw |  |  | 1.0543E7 |  |  |  | 1 | 0 | 0 | 1 | 0 | 0 | 0 | 470 | 489 |  |  | PEAKS DB |
| K.YDTYSTKEY.L | N | 49.76 | 1168.4924 | 9 | 1.0 | 585.2541 | 2 | 25.91 | 5 | F5:9933 | 31122021\_RID\_2349\_5C.raw |  |  |  |  | 2.4008E6 |  | 1 | 0 | 0 | 0 | 0 | 1 | 0 | 198 | 206 |  |  | PEAKS DB |
| K.YAMGGITTFTPYQFQHFSD.P | Y | 48.98 | 2209.9778 | 19 | 0.6 | 1105.9968 | 2 | 60.23 | 1 | F1:36094 | 28122021\_RID\_2349\_2C.raw | 1.3342E6 |  |  |  |  |  | 1 | 1 | 0 | 0 | 0 | 0 | 0 | 443 | 461 |  |  | PEAKS DB |
| Q.AHGWIDSTIK.S | N | 45.39 | 1126.5771 | 10 | 1.2 | 564.2965 | 2 | 28.81 | 2 | F2:8087 | 30122021\_RID\_2349\_1C.raw |  | 3.6217E5 |  |  |  |  | 1 | 0 | 1 | 0 | 0 | 0 | 0 | 480 | 489 |  |  | PEAKS DB |
| N.FFQALDFK.D | N | 45.08 | 1014.5174 | 8 | 0.8 | 508.2664 | 2 | 48.21 | 2 | F2:16516 | 30122021\_RID\_2349\_1C.raw |  | 1.1251E4 |  |  |  |  | 1 | 0 | 1 | 0 | 0 | 0 | 0 | 398 | 405 |  |  | PEAKS DB |
| A.GWYANLGPMR.L | N | 42.97 | 1163.5546 | 10 | 0.2 | 582.7847 | 2 | 43.50 | 1 | F1:23833 | 28122021\_RID\_2349\_2C.raw | 3.4804E4 |  |  |  |  |  | 1 | 1 | 0 | 0 | 0 | 0 | 0 | 99 | 108 |  |  | PEAKS DB |
| S.AGQLYEESLGK.V | N | 42.75 | 1193.5928 | 11 | -0.1 | 597.8036 | 2 | 30.49 | 2 | F2:8743 | 30122021\_RID\_2349\_1C.raw |  | 1.8392E5 |  |  |  |  | 1 | 0 | 1 | 0 | 0 | 0 | 0 | 171 | 181 |  |  | PEAKS DB |
| total 26 peptides |  |  |  |  |  |  |  |  |  |  |  |  |  |  |  |  |  |  |  |  |  |  |  |  |  |  |  |  |  |
| --- | --- | --- | --- | --- | --- | --- | --- | --- | --- | --- | --- | --- | --- | --- | --- | --- | --- | --- | --- | --- | --- | --- | --- | --- | --- | --- | --- | --- | --- |

CAB71136.1

back to list

  

| Protein Coverage
| Supporting Peptides
|

Protein Coverage:

Supporting Peptides:

| Peptide | Uniq | -10lgP | Mass | Length | ppm | m/z | z | RT | Fraction | Scan | Source File | Area F2 | Area F1 | Area F3 | Area F4 | Area F5 | Area F6 | #Feature | #Feature F2 | #Feature F1 | #Feature F3 | #Feature F4 | #Feature F5 | #Feature F6 | Start | End | PTM | AScore | Found By |
| --- | --- | --- | --- | --- | --- | --- | --- | --- | --- | --- | --- | --- | --- | --- | --- | --- | --- | --- | --- | --- | --- | --- | --- | --- | --- | --- | --- | --- | --- |
| T.KFWEDDGIHGGK.S | N | 83.09 | 1387.6520 | 12 | 0.2 | 463.5580 | 3 | 24.69 | 5 | F5:8977 | 31122021\_RID\_2349\_5C.raw | 3.2966E7 | 1.6951E7 | 4.7218E7 |  | 1.4906E6 |  | 10 | 4 | 3 | 1 | 0 | 2 | 0 | 352 | 363 |  |  | PEAKS DB |
| K.SAGQLYEESLGK.V | N | 80.33 | 1280.6248 | 12 | -0.1 | 641.3196 | 2 | 32.32 | 5 | F5:14229 | 31122021\_RID\_2349\_5C.raw | 3.9268E8 | 5.6746E8 | 8.8153E7 | 1.109E8 | 1.2738E8 | 1.0719E5 | 12 | 3 | 3 | 1 | 1 | 3 | 1 | 170 | 181 |  |  | PEAKS DB |
| Y.GIGDDANFFQALDFK.D | N | 75.31 | 1656.7783 | 15 | 0.2 | 829.3966 | 2 | 64.60 | 3 | F3:23470 | 31122021\_RID\_2349\_3C.raw |  |  | 1.1679E5 |  |  |  | 1 | 0 | 0 | 1 | 0 | 0 | 0 | 391 | 405 |  |  | PEAKS DB |
| K.RFDEIVDGMDK.L | N | 74.01 | 1323.6129 | 11 | 0.3 | 662.8139 | 2 | 35.47 | 1 | F1:17511 | 28122021\_RID\_2349\_2C.raw | 9.5788E6 | 2.9071E7 | 1.7532E7 |  |  | 4.2754E5 | 7 | 2 | 3 | 1 | 0 | 0 | 1 | 250 | 260 |  |  | PEAKS DB |
| K.RFDEIVDGM(+15.99)DK.L | N | 73.28 | 1339.6078 | 11 | 0.3 | 670.8114 | 2 | 34.96 | 1 | F1:17438 | 28122021\_RID\_2349\_2C.raw | 4.3666E6 | 1.0728E7 |  |  |  |  | 9 | 4 | 5 | 0 | 0 | 0 | 0 | 250 | 260 | Oxidation (M) | M9:Oxidation (M):1000.00 | PEAKS DB |
| K.HDDIFAYEK.R | N | 72.69 | 1136.5138 | 9 | -0.1 | 569.2641 | 2 | 30.15 | 2 | F2:8868 | 30122021\_RID\_2349\_1C.raw | 5.9259E7 | 1.5095E8 | 7.8164E7 |  |  | 1.3576E6 | 7 | 3 | 2 | 1 | 0 | 0 | 1 | 241 | 249 |  |  | PEAKS DB |
| K.FWEDDGIHGGK.S | N | 71.93 | 1259.5570 | 11 | 0.4 | 630.7860 | 2 | 28.94 | 1 | F1:12222 | 28122021\_RID\_2349\_2C.raw | 1.3683E7 | 2.9767E7 | 9.0355E6 |  |  |  | 10 | 4 | 5 | 1 | 0 | 0 | 0 | 353 | 363 |  |  | PEAKS DB |
| R.IYFAGEYTAQ.A | N | 68.04 | 1161.5342 | 10 | 0.2 | 581.7745 | 2 | 47.04 | 2 | F2:16101 | 30122021\_RID\_2349\_1C.raw |  | 4.684E5 |  |  |  |  | 1 | 0 | 1 | 0 | 0 | 0 | 0 | 470 | 479 |  |  | PEAKS DB |
| R.IYFAGEYTAQAH.G | N | 65.33 | 1369.6302 | 12 | 0.8 | 685.8229 | 2 | 38.89 | 2 | F2:12898 | 30122021\_RID\_2349\_1C.raw |  | 1.6051E6 |  |  |  |  | 1 | 0 | 1 | 0 | 0 | 0 | 0 | 470 | 481 |  |  | PEAKS DB |
| R.FDEIVDGMDK.L | N | 64.83 | 1167.5117 | 10 | 0.9 | 584.7637 | 2 | 38.63 | 1 | F1:19868 | 28122021\_RID\_2349\_2C.raw | 3.5946E6 | 1.093E7 | 3.5028E6 |  |  | 1.3745E5 | 6 | 2 | 2 | 1 | 0 | 0 | 1 | 251 | 260 |  |  | PEAKS DB |
| K.HDDIFAYEKR.F | N | 60.61 | 1292.6149 | 10 | 0.6 | 431.8792 | 3 | 25.32 | 1 | F1:9449 | 28122021\_RID\_2349\_2C.raw | 9.7777E5 | 4.5859E5 |  |  |  |  | 2 | 1 | 1 | 0 | 0 | 0 | 0 | 241 | 250 |  |  | PEAKS DB |
| Q.ENDYEEFLEIAR.N | Y | 59.88 | 1526.6888 | 12 | 0.3 | 764.3519 | 2 | 55.90 | 1 | F1:33325 | 28122021\_RID\_2349\_2C.raw | 3.2858E6 | 1.7029E7 |  |  | 1.8334E6 | 1.8954E6 | 4 | 1 | 1 | 0 | 0 | 1 | 1 | 31 | 42 |  |  | PEAKS DB |
| R.FDEIVDGM(+15.99)DK.L | N | 57.69 | 1183.5067 | 10 | 0.0 | 592.7606 | 2 | 31.41 | 1 | F1:14146 | 28122021\_RID\_2349\_2C.raw | 1.0488E6 | 2.2712E6 |  |  |  |  | 2 | 1 | 1 | 0 | 0 | 0 | 0 | 251 | 260 | Oxidation (M) | M8:Oxidation (M):1000.00 | PEAKS DB |
| D.YEEFLEIAR.N | N | 56.31 | 1168.5764 | 9 | 0.7 | 585.2959 | 2 | 45.58 | 2 | F2:15608 | 30122021\_RID\_2349\_1C.raw |  | 2.3921E5 |  |  |  |  | 1 | 0 | 1 | 0 | 0 | 0 | 0 | 34 | 42 |  |  | PEAKS DB |
| N.DYEEFLEIAR.N | N | 56.01 | 1283.6033 | 10 | 0.8 | 642.8094 | 2 | 56.80 | 5 | F5:32140 | 31122021\_RID\_2349\_5C.raw | 1.3399E5 | 2.7826E5 |  |  | 9.5716E4 | 7.7266E4 | 4 | 1 | 1 | 0 | 0 | 1 | 1 | 33 | 42 |  |  | PEAKS DB |
| A.GQLYEESLGK.V | N | 54.58 | 1122.5557 | 10 | 1.2 | 562.2858 | 2 | 29.95 | 1 | F1:13018 | 28122021\_RID\_2349\_2C.raw | 4.0796E5 | 6.449E5 |  |  |  |  | 2 | 1 | 1 | 0 | 0 | 0 | 0 | 172 | 181 |  |  | PEAKS DB |
| N.EFSQENDNAWYFIK.N | N | 53.57 | 1789.7947 | 14 | 0.5 | 895.9051 | 2 | 53.07 | 1 | F1:31357 | 28122021\_RID\_2349\_2C.raw | 2.5591E5 | 2.9753E5 |  |  |  |  | 2 | 1 | 1 | 0 | 0 | 0 | 0 | 129 | 142 |  |  | PEAKS DB |
| F.SQENDNAWYFIK.N | N | 53.25 | 1513.6837 | 12 | 1.0 | 757.8499 | 2 | 47.51 | 2 | F2:16263 | 30122021\_RID\_2349\_1C.raw |  | 7.0312E5 |  |  |  |  | 1 | 0 | 1 | 0 | 0 | 0 | 0 | 131 | 142 |  |  | PEAKS DB |
| N.EDSGYYVSFIESLK.H | N | 53.12 | 1635.7667 | 14 | 0.7 | 818.8912 | 2 | 61.10 | 1 | F1:36406 | 28122021\_RID\_2349\_2C.raw | 7.7617E4 |  |  |  |  |  | 1 | 1 | 0 | 0 | 0 | 0 | 0 | 227 | 240 |  |  | PEAKS DB |
| R.IYFAGEYTAQAHGWIDSTIK.S | N | 51.08 | 2270.1008 | 20 | 0.0 | 757.7076 | 3 | 50.32 | 3 | F3:17332 | 31122021\_RID\_2349\_3C.raw |  |  | 1.0543E7 |  |  |  | 1 | 0 | 0 | 1 | 0 | 0 | 0 | 470 | 489 |  |  | PEAKS DB |
| K.YDTYSTKEY.L | N | 49.76 | 1168.4924 | 9 | 1.0 | 585.2541 | 2 | 25.91 | 5 | F5:9933 | 31122021\_RID\_2349\_5C.raw |  |  |  |  | 2.4008E6 |  | 1 | 0 | 0 | 0 | 0 | 1 | 0 | 198 | 206 |  |  | PEAKS DB |
| K.YAMGGITTFTPYQFQHFSD.P | Y | 48.98 | 2209.9778 | 19 | 0.6 | 1105.9968 | 2 | 60.23 | 1 | F1:36094 | 28122021\_RID\_2349\_2C.raw | 1.3342E6 |  |  |  |  |  | 1 | 1 | 0 | 0 | 0 | 0 | 0 | 443 | 461 |  |  | PEAKS DB |
| Q.AHGWIDSTIK.S | N | 45.39 | 1126.5771 | 10 | 1.2 | 564.2965 | 2 | 28.81 | 2 | F2:8087 | 30122021\_RID\_2349\_1C.raw |  | 3.6217E5 |  |  |  |  | 1 | 0 | 1 | 0 | 0 | 0 | 0 | 480 | 489 |  |  | PEAKS DB |
| N.FFQALDFK.D | N | 45.08 | 1014.5174 | 8 | 0.8 | 508.2664 | 2 | 48.21 | 2 | F2:16516 | 30122021\_RID\_2349\_1C.raw |  | 1.1251E4 |  |  |  |  | 1 | 0 | 1 | 0 | 0 | 0 | 0 | 398 | 405 |  |  | PEAKS DB |
| A.GWYANLGPMR.L | N | 42.97 | 1163.5546 | 10 | 0.2 | 582.7847 | 2 | 43.50 | 1 | F1:23833 | 28122021\_RID\_2349\_2C.raw | 3.4804E4 |  |  |  |  |  | 1 | 1 | 0 | 0 | 0 | 0 | 0 | 99 | 108 |  |  | PEAKS DB |
| S.AGQLYEESLGK.V | N | 42.75 | 1193.5928 | 11 | -0.1 | 597.8036 | 2 | 30.49 | 2 | F2:8743 | 30122021\_RID\_2349\_1C.raw |  | 1.8392E5 |  |  |  |  | 1 | 0 | 1 | 0 | 0 | 0 | 0 | 171 | 181 |  |  | PEAKS DB |
| total 26 peptides |  |  |  |  |  |  |  |  |  |  |  |  |  |  |  |  |  |  |  |  |  |  |  |  |  |  |  |  |  |
| --- | --- | --- | --- | --- | --- | --- | --- | --- | --- | --- | --- | --- | --- | --- | --- | --- | --- | --- | --- | --- | --- | --- | --- | --- | --- | --- | --- | --- | --- |

XP\_007427768.1

back to list

  

| Protein Coverage
| Supporting Peptides
|

Protein Coverage:

Supporting Peptides:

| Peptide | Uniq | -10lgP | Mass | Length | ppm | m/z | z | RT | Fraction | Scan | Source File | Area F2 | Area F1 | Area F3 | Area F4 | Area F5 | Area F6 | #Feature | #Feature F2 | #Feature F1 | #Feature F3 | #Feature F4 | #Feature F5 | #Feature F6 | Start | End | PTM | AScore | Found By |
| --- | --- | --- | --- | --- | --- | --- | --- | --- | --- | --- | --- | --- | --- | --- | --- | --- | --- | --- | --- | --- | --- | --- | --- | --- | --- | --- | --- | --- | --- |
| K.QNSGTYNNQYMILDTK.K | N | 89.37 | 1888.8625 | 16 | 0.2 | 945.4387 | 2 | 40.12 | 1 | F1:21225 | 28122021\_RID\_2349\_2C.raw | 1.0877E7 |  |  |  | 1.1423E5 |  | 3 | 2 | 0 | 0 | 0 | 1 | 0 | 357 | 372 |  |  | PEAKS DB |
| R.SLEDGTLYIVEQVPK.F | Y | 86.64 | 1689.8824 | 15 | 0.3 | 845.9488 | 2 | 55.25 | 1 | F1:32836 | 28122021\_RID\_2349\_2C.raw | 4.019E7 |  | 4.5566E6 |  | 3.3485E5 | 1.4268E5 | 7 | 4 | 0 | 1 | 0 | 1 | 1 | 379 | 393 |  |  | PEAKS DB |
| Q.VVPESLFAWER.V | N | 78.21 | 1331.6874 | 11 | 0.6 | 666.8514 | 2 | 53.73 | 1 | F1:31675 | 28122021\_RID\_2349\_2C.raw | 2.3421E7 |  |  |  |  | 1.2729E6 | 2 | 1 | 0 | 0 | 0 | 0 | 1 | 326 | 336 |  |  | PEAKS DB |
| K.QNSGTYNNQYM(+15.99)ILDTK.K | N | 75.10 | 1904.8574 | 16 | 0.7 | 953.4366 | 2 | 36.00 | 1 | F1:17991 | 28122021\_RID\_2349\_2C.raw | 4.466E6 |  |  |  |  |  | 4 | 4 | 0 | 0 | 0 | 0 | 0 | 357 | 372 | Oxidation (M) | M11:Oxidation (M):1000.00 | PEAKS DB |
| K.HQGLPESYNFDFVTMKPVL | N | 73.59 | 2221.0876 | 19 | 1.1 | 741.3707 | 3 | 57.46 | 1 | F1:34351 | 28122021\_RID\_2349\_2C.raw | 1.288E7 |  |  |  |  |  | 2 | 2 | 0 | 0 | 0 | 0 | 0 | 541 | 559 |  |  | PEAKS DB |
| K.HQGLPESYNFDFVTM(+15.99)KPVL | N | 71.00 | 2237.0828 | 19 | 0.5 | 746.7019 | 3 | 52.61 | 1 | F1:30998 | 28122021\_RID\_2349\_2C.raw | 4.8497E6 |  |  |  |  |  | 2 | 2 | 0 | 0 | 0 | 0 | 0 | 541 | 559 | Oxidation (M) | M15:Oxidation (M):1000.00 | PEAKS DB |
| K.NNKDDPFWR.N | N | 67.25 | 1190.5469 | 9 | 0.5 | 596.2810 | 2 | 28.10 | 5 | F5:11429 | 31122021\_RID\_2349\_5C.raw |  |  |  |  | 5.6786E6 |  | 2 | 0 | 0 | 0 | 0 | 2 | 0 | 156 | 164 |  |  | PEAKS DB |
| Q.RSLEDGTLYIVEQVPK.F | Y | 66.19 | 1845.9835 | 16 | 0.5 | 616.3354 | 3 | 46.94 | 1 | F1:26659 | 28122021\_RID\_2349\_2C.raw | 6.9251E6 |  |  |  |  |  | 1 | 1 | 0 | 0 | 0 | 0 | 0 | 378 | 393 |  |  | PEAKS DB |
| N.SGTYNNQYMILDTK.K | N | 65.95 | 1646.7610 | 14 | 0.9 | 824.3885 | 2 | 40.39 | 1 | F1:21338 | 28122021\_RID\_2349\_2C.raw | 2.6394E5 |  |  |  |  |  | 1 | 1 | 0 | 0 | 0 | 0 | 0 | 359 | 372 |  |  | PEAKS DB |
| D.GTLYIVEQVPK.F | Y | 57.86 | 1245.6969 | 11 | 0.4 | 623.8560 | 2 | 42.10 | 1 | F1:22722 | 28122021\_RID\_2349\_2C.raw | 2.2694E5 |  |  |  |  |  | 1 | 1 | 0 | 0 | 0 | 0 | 0 | 383 | 393 |  |  | PEAKS DB |
| N.NQYMILDTK.K | N | 51.50 | 1124.5536 | 9 | -0.3 | 563.2839 | 2 | 37.24 | 1 | F1:18793 | 28122021\_RID\_2349\_2C.raw | 1.1739E5 |  |  |  |  |  | 1 | 1 | 0 | 0 | 0 | 0 | 0 | 364 | 372 |  |  | PEAKS DB |
| H.SSWFTYAATLR.I | N | 48.67 | 1301.6404 | 11 | 0.3 | 651.8276 | 2 | 51.40 | 1 | F1:29950 | 28122021\_RID\_2349\_2C.raw | 2.188E5 |  |  |  |  |  | 1 | 1 | 0 | 0 | 0 | 0 | 0 | 257 | 267 |  |  | PEAKS DB |
| Y.NFDFVTMKPVL | N | 47.54 | 1309.6741 | 11 | 0.3 | 655.8445 | 2 | 58.32 | 1 | F1:34760 | 28122021\_RID\_2349\_2C.raw | 6.2109E4 |  |  |  |  |  | 1 | 1 | 0 | 0 | 0 | 0 | 0 | 549 | 559 |  |  | PEAKS DB |
| K.QNSGTYNNQYMILDTKK.I | N | 45.69 | 2016.9575 | 17 | 0.6 | 673.3268 | 3 | 32.64 | 1 | F1:15031 | 28122021\_RID\_2349\_2C.raw | 2.6845E6 |  |  |  |  |  | 1 | 1 | 0 | 0 | 0 | 0 | 0 | 357 | 373 |  |  | PEAKS DB |
| total 14 peptides |  |  |  |  |  |  |  |  |  |  |  |  |  |  |  |  |  |  |  |  |  |  |  |  |  |  |  |  |  |
| --- | --- | --- | --- | --- | --- | --- | --- | --- | --- | --- | --- | --- | --- | --- | --- | --- | --- | --- | --- | --- | --- | --- | --- | --- | --- | --- | --- | --- | --- |

AMB36337.1

back to list

  

| Protein Coverage
| Supporting Peptides
|

Protein Coverage:

Supporting Peptides:

| Peptide | Uniq | -10lgP | Mass | Length | ppm | m/z | z | RT | Fraction | Scan | Source File | Area F2 | Area F1 | Area F3 | Area F4 | Area F5 | Area F6 | #Feature | #Feature F2 | #Feature F1 | #Feature F3 | #Feature F4 | #Feature F5 | #Feature F6 | Start | End | PTM | AScore | Found By |
| --- | --- | --- | --- | --- | --- | --- | --- | --- | --- | --- | --- | --- | --- | --- | --- | --- | --- | --- | --- | --- | --- | --- | --- | --- | --- | --- | --- | --- | --- |
| R.KPEIQNEIIDLHNSLR.R | Y | 101.21 | 1918.0272 | 16 | 0.1 | 640.3497 | 3 | 38.51 | 5 | F5:18814 | 31122021\_RID\_2349\_5C.raw |  |  |  | 3.5279E7 | 1.1708E8 | 1.0245E7 | 8 | 0 | 0 | 0 | 1 | 6 | 1 | 30 | 45 |  |  | PEAKS DB |
| R.SVNPTASNMLK.M | Y | 74.92 | 1160.5859 | 11 | -0.3 | 581.3000 | 2 | 28.86 | 5 | F5:12057 | 31122021\_RID\_2349\_5C.raw | 6.2934E5 |  |  | 3.8419E7 | 1.8211E7 | 5.4123E5 | 4 | 1 | 0 | 0 | 1 | 1 | 1 | 47 | 57 |  |  | PEAKS DB |
| K.MEWYPEAAANAER.W | N | 74.89 | 1536.6667 | 13 | 0.1 | 769.3408 | 2 | 41.29 | 5 | F5:20631 | 31122021\_RID\_2349\_5C.raw | 8.9957E4 |  |  |  | 3.8512E6 | 3.9844E5 | 3 | 1 | 0 | 0 | 0 | 1 | 1 | 58 | 70 |  |  | PEAKS DB |
| G.SVDFDSESPR.K | N | 71.72 | 1137.4938 | 10 | 0.2 | 569.7543 | 2 | 27.74 | 4 | F4:5760 | 31122021\_RID\_2349\_4C.raw | 9.0181E5 | 1.111E5 |  | 3.8035E7 | 1.1757E7 | 3.4766E5 | 7 | 1 | 1 | 0 | 1 | 3 | 1 | 20 | 29 |  |  | PEAKS DB |
| R.RSVNPTASNMLK.M | Y | 66.73 | 1316.6870 | 12 | 0.6 | 439.9032 | 3 | 22.38 | 5 | F5:7210 | 31122021\_RID\_2349\_5C.raw |  |  |  |  | 1.7924E6 |  | 2 | 0 | 0 | 0 | 0 | 2 | 0 | 46 | 57 |  |  | PEAKS DB |
| R.SVNPTASNM(+15.99)LK.M | Y | 66.46 | 1176.5808 | 11 | 0.9 | 589.2982 | 2 | 21.61 | 5 | F5:6785 | 31122021\_RID\_2349\_5C.raw |  |  |  |  | 2.3283E6 |  | 1 | 0 | 0 | 0 | 0 | 1 | 0 | 47 | 57 | Oxidation (M) | M9:Oxidation (M):1000.00 | PEAKS DB |
| K.M(+15.99)EWYPEAAANAER.W | N | 63.14 | 1552.6616 | 13 | 0.6 | 777.3386 | 2 | 36.95 | 5 | F5:17377 | 31122021\_RID\_2349\_5C.raw |  |  |  |  | 2.2518E5 |  | 1 | 0 | 0 | 0 | 0 | 1 | 0 | 58 | 70 | Oxidation (M) | M1:Oxidation (M):1000.00 | PEAKS DB |
| E.WYPEAAANAER.W | N | 62.44 | 1276.5836 | 11 | 0.4 | 639.2993 | 2 | 31.93 | 5 | F5:14009 | 31122021\_RID\_2349\_5C.raw |  |  |  |  | 1.3008E6 |  | 1 | 0 | 0 | 0 | 0 | 1 | 0 | 60 | 70 |  |  | PEAKS DB |
| P.EIQNEIIDLHNSLR.R | Y | 55.40 | 1692.8795 | 14 | 0.7 | 847.4476 | 2 | 42.96 | 5 | F5:21786 | 31122021\_RID\_2349\_5C.raw |  |  |  |  | 5.7366E4 |  | 1 | 0 | 0 | 0 | 0 | 1 | 0 | 32 | 45 |  |  | PEAKS DB |
| G.SVDFDSESPRKPEIQNEIIDLHNSLR.R | Y | 53.71 | 3037.5105 | 26 | 0.1 | 760.3850 | 4 | 43.18 | 5 | F5:22029 | 31122021\_RID\_2349\_5C.raw |  |  |  |  | 1.4823E6 |  | 1 | 0 | 0 | 0 | 0 | 1 | 0 | 20 | 45 |  |  | PEAKS DB |
| S.VDFDSESPR.K | N | 52.15 | 1050.4618 | 9 | 0.0 | 526.2382 | 2 | 27.13 | 5 | F5:10640 | 31122021\_RID\_2349\_5C.raw |  |  |  |  | 6.9151E5 |  | 1 | 0 | 0 | 0 | 0 | 1 | 0 | 21 | 29 |  |  | PEAKS DB |
| R.KPEIQNEIIDLHNSLRR.S | Y | 51.64 | 2074.1282 | 17 | 0.5 | 692.3837 | 3 | 34.63 | 5 | F5:15899 | 31122021\_RID\_2349\_5C.raw |  |  |  |  | 0 |  | 0 | 0 | 0 | 0 | 0 | 0 | 0 | 30 | 46 |  |  | PEAKS DB |
| R.KPEIQNEIIDLHNSL.R | Y | 46.99 | 1761.9260 | 15 | 0.9 | 881.9711 | 2 | 44.80 | 5 | F5:23188 | 31122021\_RID\_2349\_5C.raw |  |  |  |  | 2.5133E5 |  | 1 | 0 | 0 | 0 | 0 | 1 | 0 | 30 | 44 |  |  | PEAKS DB |
| total 13 peptides |  |  |  |  |  |  |  |  |  |  |  |  |  |  |  |  |  |  |  |  |  |  |  |  |  |  |  |  |  |
| --- | --- | --- | --- | --- | --- | --- | --- | --- | --- | --- | --- | --- | --- | --- | --- | --- | --- | --- | --- | --- | --- | --- | --- | --- | --- | --- | --- | --- | --- |

Q90YA8.1

back to list

  

| Protein Coverage
| Supporting Peptides
|

Protein Coverage:

Supporting Peptides:

| Peptide | Uniq | -10lgP | Mass | Length | ppm | m/z | z | RT | Fraction | Scan | Source File | Area F2 | Area F1 | Area F3 | Area F4 | Area F5 | Area F6 | #Feature | #Feature F2 | #Feature F1 | #Feature F3 | #Feature F4 | #Feature F5 | #Feature F6 | Start | End | PTM | AScore | Found By |
| --- | --- | --- | --- | --- | --- | --- | --- | --- | --- | --- | --- | --- | --- | --- | --- | --- | --- | --- | --- | --- | --- | --- | --- | --- | --- | --- | --- | --- | --- |
| Q.NLYDLGLLNNYSSER.Q | Y | 87.50 | 1769.8584 | 15 | 0.7 | 885.9371 | 2 | 55.06 | 1 | F1:32757 | 28122021\_RID\_2349\_2C.raw | 2.221E6 |  |  |  | 0 |  | 1 | 1 | 0 | 0 | 0 | 0 | 0 | 283 | 297 |  |  | PEAKS DB |
| K.LIFFDGEEAFVR.W | N | 85.67 | 1441.7241 | 12 | 0.6 | 721.8698 | 2 | 59.72 | 1 | F1:35753 | 28122021\_RID\_2349\_2C.raw | 3.5159E6 |  |  |  |  | 2.168E4 | 5 | 4 | 0 | 0 | 0 | 0 | 1 | 201 | 212 |  |  | PEAKS DB |
| R.VWHTMEDNEENLDKPTIDNLSK.I | Y | 84.37 | 2627.2173 | 22 | 1.1 | 876.7473 | 3 | 37.39 | 1 | F1:19045 | 28122021\_RID\_2349\_2C.raw | 6.149E6 |  |  |  |  |  | 2 | 2 | 0 | 0 | 0 | 0 | 0 | 334 | 355 |  |  | PEAKS DB |
| R.YPGSPGSYAVR.Q | N | 69.00 | 1152.5563 | 11 | 0.1 | 577.2855 | 2 | 28.48 | 1 | F1:11793 | 28122021\_RID\_2349\_2C.raw | 7.1634E6 |  |  |  |  | 3.2738E5 | 2 | 1 | 0 | 0 | 0 | 0 | 1 | 82 | 92 |  |  | PEAKS DB |
| R.TFSNIISTLNPLAK.R | N | 68.92 | 1517.8453 | 14 | 1.1 | 759.9308 | 2 | 58.63 | 1 | F1:35258 | 28122021\_RID\_2349\_2C.raw | 2.8095E6 |  |  |  | 1.6857E5 | 8.0094E4 | 3 | 1 | 0 | 0 | 0 | 1 | 1 | 123 | 136 |  |  | PEAKS DB |
| R.WSPSDSLYGSR.S | N | 68.08 | 1253.5676 | 11 | 0.3 | 627.7913 | 2 | 38.76 | 1 | F1:20122 | 28122021\_RID\_2349\_2C.raw | 4.7528E6 |  |  |  |  |  | 1 | 1 | 0 | 0 | 0 | 0 | 0 | 213 | 223 |  |  | PEAKS DB |
| R.VWHTM(+15.99)EDNEENLDKPTIDNLSK.I | Y | 54.31 | 2643.2122 | 22 | 0.8 | 882.0787 | 3 | 33.87 | 1 | F1:16176 | 28122021\_RID\_2349\_2C.raw | 1.6494E6 |  |  |  |  |  | 2 | 2 | 0 | 0 | 0 | 0 | 0 | 334 | 355 | Oxidation (M) | M5:Oxidation (M):1000.00 | PEAKS DB |
| K.YFPPQLDGK.V | N | 50.09 | 1063.5338 | 9 | 0.9 | 532.7747 | 2 | 36.56 | 1 | F1:18056 | 28122021\_RID\_2349\_2C.raw | 5.8019E6 |  |  |  |  |  | 1 | 1 | 0 | 0 | 0 | 0 | 0 | 149 | 157 |  |  | PEAKS DB |
| R.LQGLQAGWLVEEDTFQSHTPYGYR.T | N | 42.74 | 2794.3350 | 24 | 1.1 | 932.4532 | 3 | 54.10 | 1 | F1:31900 | 28122021\_RID\_2349\_2C.raw | 8.091E5 |  |  |  |  |  | 1 | 1 | 0 | 0 | 0 | 0 | 0 | 99 | 122 |  |  | PEAKS DB |
| total 9 peptides |  |  |  |  |  |  |  |  |  |  |  |  |  |  |  |  |  |  |  |  |  |  |  |  |  |  |  |  |  |
| --- | --- | --- | --- | --- | --- | --- | --- | --- | --- | --- | --- | --- | --- | --- | --- | --- | --- | --- | --- | --- | --- | --- | --- | --- | --- | --- | --- | --- | --- |

BAB69586.1

back to list

  

| Protein Coverage
| Supporting Peptides
|

Protein Coverage:

Supporting Peptides:

| Peptide | Uniq | -10lgP | Mass | Length | ppm | m/z | z | RT | Fraction | Scan | Source File | Area F2 | Area F1 | Area F3 | Area F4 | Area F5 | Area F6 | #Feature | #Feature F2 | #Feature F1 | #Feature F3 | #Feature F4 | #Feature F5 | #Feature F6 | Start | End | PTM | AScore | Found By |
| --- | --- | --- | --- | --- | --- | --- | --- | --- | --- | --- | --- | --- | --- | --- | --- | --- | --- | --- | --- | --- | --- | --- | --- | --- | --- | --- | --- | --- | --- |
| Q.NLYDLGLLNNYSSER.Q | Y | 87.50 | 1769.8584 | 15 | 0.7 | 885.9371 | 2 | 55.06 | 1 | F1:32757 | 28122021\_RID\_2349\_2C.raw | 2.221E6 |  |  |  | 0 |  | 1 | 1 | 0 | 0 | 0 | 0 | 0 | 283 | 297 |  |  | PEAKS DB |
| K.LIFFDGEEAFVR.W | N | 85.67 | 1441.7241 | 12 | 0.6 | 721.8698 | 2 | 59.72 | 1 | F1:35753 | 28122021\_RID\_2349\_2C.raw | 3.5159E6 |  |  |  |  | 2.168E4 | 5 | 4 | 0 | 0 | 0 | 0 | 1 | 201 | 212 |  |  | PEAKS DB |
| R.VWHTMEDNEENLDKPTIDNLSK.I | Y | 84.37 | 2627.2173 | 22 | 1.1 | 876.7473 | 3 | 37.39 | 1 | F1:19045 | 28122021\_RID\_2349\_2C.raw | 6.149E6 |  |  |  |  |  | 2 | 2 | 0 | 0 | 0 | 0 | 0 | 334 | 355 |  |  | PEAKS DB |
| R.YPGSPGSYAVR.Q | N | 69.00 | 1152.5563 | 11 | 0.1 | 577.2855 | 2 | 28.48 | 1 | F1:11793 | 28122021\_RID\_2349\_2C.raw | 7.1634E6 |  |  |  |  | 3.2738E5 | 2 | 1 | 0 | 0 | 0 | 0 | 1 | 82 | 92 |  |  | PEAKS DB |
| R.TFSNIISTLNPLAK.R | N | 68.92 | 1517.8453 | 14 | 1.1 | 759.9308 | 2 | 58.63 | 1 | F1:35258 | 28122021\_RID\_2349\_2C.raw | 2.8095E6 |  |  |  | 1.6857E5 | 8.0094E4 | 3 | 1 | 0 | 0 | 0 | 1 | 1 | 123 | 136 |  |  | PEAKS DB |
| R.WSPSDSLYGSR.S | N | 68.08 | 1253.5676 | 11 | 0.3 | 627.7913 | 2 | 38.76 | 1 | F1:20122 | 28122021\_RID\_2349\_2C.raw | 4.7528E6 |  |  |  |  |  | 1 | 1 | 0 | 0 | 0 | 0 | 0 | 213 | 223 |  |  | PEAKS DB |
| R.VWHTM(+15.99)EDNEENLDKPTIDNLSK.I | Y | 54.31 | 2643.2122 | 22 | 0.8 | 882.0787 | 3 | 33.87 | 1 | F1:16176 | 28122021\_RID\_2349\_2C.raw | 1.6494E6 |  |  |  |  |  | 2 | 2 | 0 | 0 | 0 | 0 | 0 | 334 | 355 | Oxidation (M) | M5:Oxidation (M):1000.00 | PEAKS DB |
| K.YFPPQLDGK.V | N | 50.09 | 1063.5338 | 9 | 0.9 | 532.7747 | 2 | 36.56 | 1 | F1:18056 | 28122021\_RID\_2349\_2C.raw | 5.8019E6 |  |  |  |  |  | 1 | 1 | 0 | 0 | 0 | 0 | 0 | 149 | 157 |  |  | PEAKS DB |
| R.LQGLQAGWLVEEDTFQSHTPYGYR.T | N | 42.74 | 2794.3350 | 24 | 1.1 | 932.4532 | 3 | 54.10 | 1 | F1:31900 | 28122021\_RID\_2349\_2C.raw | 8.091E5 |  |  |  |  |  | 1 | 1 | 0 | 0 | 0 | 0 | 0 | 99 | 122 |  |  | PEAKS DB |
| total 9 peptides |  |  |  |  |  |  |  |  |  |  |  |  |  |  |  |  |  |  |  |  |  |  |  |  |  |  |  |  |  |
| --- | --- | --- | --- | --- | --- | --- | --- | --- | --- | --- | --- | --- | --- | --- | --- | --- | --- | --- | --- | --- | --- | --- | --- | --- | --- | --- | --- | --- | --- |

BAN89376.1

back to list

  

| Protein Coverage
| Supporting Peptides
|

Protein Coverage:

Supporting Peptides:

| Peptide | Uniq | -10lgP | Mass | Length | ppm | m/z | z | RT | Fraction | Scan | Source File | Area F2 | Area F1 | Area F3 | Area F4 | Area F5 | Area F6 | #Feature | #Feature F2 | #Feature F1 | #Feature F3 | #Feature F4 | #Feature F5 | #Feature F6 | Start | End | PTM | AScore | Found By |
| --- | --- | --- | --- | --- | --- | --- | --- | --- | --- | --- | --- | --- | --- | --- | --- | --- | --- | --- | --- | --- | --- | --- | --- | --- | --- | --- | --- | --- | --- |
| R.NLYDLGLLNNYSSER.Q | Y | 87.50 | 1769.8584 | 15 | 0.7 | 885.9371 | 2 | 55.06 | 1 | F1:32757 | 28122021\_RID\_2349\_2C.raw | 2.221E6 |  |  |  | 0 |  | 1 | 1 | 0 | 0 | 0 | 0 | 0 | 215 | 229 |  |  | PEAKS DB |
| K.LIFFDGEEAFVR.W | N | 85.67 | 1441.7241 | 12 | 0.6 | 721.8698 | 2 | 59.72 | 1 | F1:35753 | 28122021\_RID\_2349\_2C.raw | 3.5159E6 |  |  |  |  | 2.168E4 | 5 | 4 | 0 | 0 | 0 | 0 | 1 | 133 | 144 |  |  | PEAKS DB |
| R.VWHTMEDNEENLDKPTIDNLSK.I | Y | 84.37 | 2627.2173 | 22 | 1.1 | 876.7473 | 3 | 37.39 | 1 | F1:19045 | 28122021\_RID\_2349\_2C.raw | 6.149E6 |  |  |  |  |  | 2 | 2 | 0 | 0 | 0 | 0 | 0 | 266 | 287 |  |  | PEAKS DB |
| R.YPGSPGSYAVR.Q | N | 69.00 | 1152.5563 | 11 | 0.1 | 577.2855 | 2 | 28.48 | 1 | F1:11793 | 28122021\_RID\_2349\_2C.raw | 7.1634E6 |  |  |  |  | 3.2738E5 | 2 | 1 | 0 | 0 | 0 | 0 | 1 | 14 | 24 |  |  | PEAKS DB |
| R.TFSNIISTLNPLAK.R | N | 68.92 | 1517.8453 | 14 | 1.1 | 759.9308 | 2 | 58.63 | 1 | F1:35258 | 28122021\_RID\_2349\_2C.raw | 2.8095E6 |  |  |  | 1.6857E5 | 8.0094E4 | 3 | 1 | 0 | 0 | 0 | 1 | 1 | 55 | 68 |  |  | PEAKS DB |
| R.WSPSDSLYGSR.S | N | 68.08 | 1253.5676 | 11 | 0.3 | 627.7913 | 2 | 38.76 | 1 | F1:20122 | 28122021\_RID\_2349\_2C.raw | 4.7528E6 |  |  |  |  |  | 1 | 1 | 0 | 0 | 0 | 0 | 0 | 145 | 155 |  |  | PEAKS DB |
| R.VWHTM(+15.99)EDNEENLDKPTIDNLSK.I | Y | 54.31 | 2643.2122 | 22 | 0.8 | 882.0787 | 3 | 33.87 | 1 | F1:16176 | 28122021\_RID\_2349\_2C.raw | 1.6494E6 |  |  |  |  |  | 2 | 2 | 0 | 0 | 0 | 0 | 0 | 266 | 287 | Oxidation (M) | M5:Oxidation (M):1000.00 | PEAKS DB |
| K.YFPPQLDGK.V | N | 50.09 | 1063.5338 | 9 | 0.9 | 532.7747 | 2 | 36.56 | 1 | F1:18056 | 28122021\_RID\_2349\_2C.raw | 5.8019E6 |  |  |  |  |  | 1 | 1 | 0 | 0 | 0 | 0 | 0 | 81 | 89 |  |  | PEAKS DB |
| R.LQGLQAGWLVEEDTFQSHTPYGYR.T | N | 42.74 | 2794.3350 | 24 | 1.1 | 932.4532 | 3 | 54.10 | 1 | F1:31900 | 28122021\_RID\_2349\_2C.raw | 8.091E5 |  |  |  |  |  | 1 | 1 | 0 | 0 | 0 | 0 | 0 | 31 | 54 |  |  | PEAKS DB |
| total 9 peptides |  |  |  |  |  |  |  |  |  |  |  |  |  |  |  |  |  |  |  |  |  |  |  |  |  |  |  |  |  |
| --- | --- | --- | --- | --- | --- | --- | --- | --- | --- | --- | --- | --- | --- | --- | --- | --- | --- | --- | --- | --- | --- | --- | --- | --- | --- | --- | --- | --- | --- |

BAN82005.1

back to list

  

| Protein Coverage
| Supporting Peptides
|

Protein Coverage:

Supporting Peptides:

| Peptide | Uniq | -10lgP | Mass | Length | ppm | m/z | z | RT | Fraction | Scan | Source File | Area F2 | Area F1 | Area F3 | Area F4 | Area F5 | Area F6 | #Feature | #Feature F2 | #Feature F1 | #Feature F3 | #Feature F4 | #Feature F5 | #Feature F6 | Start | End | PTM | AScore | Found By |
| --- | --- | --- | --- | --- | --- | --- | --- | --- | --- | --- | --- | --- | --- | --- | --- | --- | --- | --- | --- | --- | --- | --- | --- | --- | --- | --- | --- | --- | --- |
| R.NLYDLGLLNNYSSER.Q | Y | 87.50 | 1769.8584 | 15 | 0.7 | 885.9371 | 2 | 55.06 | 1 | F1:32757 | 28122021\_RID\_2349\_2C.raw | 2.221E6 |  |  |  | 0 |  | 1 | 1 | 0 | 0 | 0 | 0 | 0 | 247 | 261 |  |  | PEAKS DB |
| K.LIFFDGEEAFVR.W | N | 85.67 | 1441.7241 | 12 | 0.6 | 721.8698 | 2 | 59.72 | 1 | F1:35753 | 28122021\_RID\_2349\_2C.raw | 3.5159E6 |  |  |  |  | 2.168E4 | 5 | 4 | 0 | 0 | 0 | 0 | 1 | 165 | 176 |  |  | PEAKS DB |
| R.VWHTMEDNEENLDKPTIDNLSK.I | Y | 84.37 | 2627.2173 | 22 | 1.1 | 876.7473 | 3 | 37.39 | 1 | F1:19045 | 28122021\_RID\_2349\_2C.raw | 6.149E6 |  |  |  |  |  | 2 | 2 | 0 | 0 | 0 | 0 | 0 | 298 | 319 |  |  | PEAKS DB |
| R.YPGSPGSYAVR.Q | N | 69.00 | 1152.5563 | 11 | 0.1 | 577.2855 | 2 | 28.48 | 1 | F1:11793 | 28122021\_RID\_2349\_2C.raw | 7.1634E6 |  |  |  |  | 3.2738E5 | 2 | 1 | 0 | 0 | 0 | 0 | 1 | 46 | 56 |  |  | PEAKS DB |
| R.TFSNIISTLNPLAK.R | N | 68.92 | 1517.8453 | 14 | 1.1 | 759.9308 | 2 | 58.63 | 1 | F1:35258 | 28122021\_RID\_2349\_2C.raw | 2.8095E6 |  |  |  | 1.6857E5 | 8.0094E4 | 3 | 1 | 0 | 0 | 0 | 1 | 1 | 87 | 100 |  |  | PEAKS DB |
| R.WSPSDSLYGSR.S | N | 68.08 | 1253.5676 | 11 | 0.3 | 627.7913 | 2 | 38.76 | 1 | F1:20122 | 28122021\_RID\_2349\_2C.raw | 4.7528E6 |  |  |  |  |  | 1 | 1 | 0 | 0 | 0 | 0 | 0 | 177 | 187 |  |  | PEAKS DB |
| R.VWHTM(+15.99)EDNEENLDKPTIDNLSK.I | Y | 54.31 | 2643.2122 | 22 | 0.8 | 882.0787 | 3 | 33.87 | 1 | F1:16176 | 28122021\_RID\_2349\_2C.raw | 1.6494E6 |  |  |  |  |  | 2 | 2 | 0 | 0 | 0 | 0 | 0 | 298 | 319 | Oxidation (M) | M5:Oxidation (M):1000.00 | PEAKS DB |
| K.YFPPQLDGK.V | N | 50.09 | 1063.5338 | 9 | 0.9 | 532.7747 | 2 | 36.56 | 1 | F1:18056 | 28122021\_RID\_2349\_2C.raw | 5.8019E6 |  |  |  |  |  | 1 | 1 | 0 | 0 | 0 | 0 | 0 | 113 | 121 |  |  | PEAKS DB |
| R.LQGLQAGWLVEEDTFQSHTPYGYR.T | N | 42.74 | 2794.3350 | 24 | 1.1 | 932.4532 | 3 | 54.10 | 1 | F1:31900 | 28122021\_RID\_2349\_2C.raw | 8.091E5 |  |  |  |  |  | 1 | 1 | 0 | 0 | 0 | 0 | 0 | 63 | 86 |  |  | PEAKS DB |
| total 9 peptides |  |  |  |  |  |  |  |  |  |  |  |  |  |  |  |  |  |  |  |  |  |  |  |  |  |  |  |  |  |
| --- | --- | --- | --- | --- | --- | --- | --- | --- | --- | --- | --- | --- | --- | --- | --- | --- | --- | --- | --- | --- | --- | --- | --- | --- | --- | --- | --- | --- | --- |

JAG46763.1

back to list

  

| Protein Coverage
| Supporting Peptides
|

Protein Coverage:

Supporting Peptides:

| Peptide | Uniq | -10lgP | Mass | Length | ppm | m/z | z | RT | Fraction | Scan | Source File | Area F2 | Area F1 | Area F3 | Area F4 | Area F5 | Area F6 | #Feature | #Feature F2 | #Feature F1 | #Feature F3 | #Feature F4 | #Feature F5 | #Feature F6 | Start | End | PTM | AScore | Found By |
| --- | --- | --- | --- | --- | --- | --- | --- | --- | --- | --- | --- | --- | --- | --- | --- | --- | --- | --- | --- | --- | --- | --- | --- | --- | --- | --- | --- | --- | --- |
| R.NLYDLGLLNNYSSER.Q | Y | 87.50 | 1769.8584 | 15 | 0.7 | 885.9371 | 2 | 55.06 | 1 | F1:32757 | 28122021\_RID\_2349\_2C.raw | 2.221E6 |  |  |  | 0 |  | 1 | 1 | 0 | 0 | 0 | 0 | 0 | 283 | 297 |  |  | PEAKS DB |
| K.LIFFDGEEAFVR.W | N | 85.67 | 1441.7241 | 12 | 0.6 | 721.8698 | 2 | 59.72 | 1 | F1:35753 | 28122021\_RID\_2349\_2C.raw | 3.5159E6 |  |  |  |  | 2.168E4 | 5 | 4 | 0 | 0 | 0 | 0 | 1 | 201 | 212 |  |  | PEAKS DB |
| R.VWHTMEDNEENLDKPTIDNLSK.I | Y | 84.37 | 2627.2173 | 22 | 1.1 | 876.7473 | 3 | 37.39 | 1 | F1:19045 | 28122021\_RID\_2349\_2C.raw | 6.149E6 |  |  |  |  |  | 2 | 2 | 0 | 0 | 0 | 0 | 0 | 334 | 355 |  |  | PEAKS DB |
| R.YPGSPGSYAVR.Q | N | 69.00 | 1152.5563 | 11 | 0.1 | 577.2855 | 2 | 28.48 | 1 | F1:11793 | 28122021\_RID\_2349\_2C.raw | 7.1634E6 |  |  |  |  | 3.2738E5 | 2 | 1 | 0 | 0 | 0 | 0 | 1 | 82 | 92 |  |  | PEAKS DB |
| R.TFSNIISTLNPLAK.R | N | 68.92 | 1517.8453 | 14 | 1.1 | 759.9308 | 2 | 58.63 | 1 | F1:35258 | 28122021\_RID\_2349\_2C.raw | 2.8095E6 |  |  |  | 1.6857E5 | 8.0094E4 | 3 | 1 | 0 | 0 | 0 | 1 | 1 | 123 | 136 |  |  | PEAKS DB |
| R.WSPSDSLYGSR.S | N | 68.08 | 1253.5676 | 11 | 0.3 | 627.7913 | 2 | 38.76 | 1 | F1:20122 | 28122021\_RID\_2349\_2C.raw | 4.7528E6 |  |  |  |  |  | 1 | 1 | 0 | 0 | 0 | 0 | 0 | 213 | 223 |  |  | PEAKS DB |
| R.VWHTM(+15.99)EDNEENLDKPTIDNLSK.I | Y | 54.31 | 2643.2122 | 22 | 0.8 | 882.0787 | 3 | 33.87 | 1 | F1:16176 | 28122021\_RID\_2349\_2C.raw | 1.6494E6 |  |  |  |  |  | 2 | 2 | 0 | 0 | 0 | 0 | 0 | 334 | 355 | Oxidation (M) | M5:Oxidation (M):1000.00 | PEAKS DB |
| K.YFPPQLDGK.V | N | 50.09 | 1063.5338 | 9 | 0.9 | 532.7747 | 2 | 36.56 | 1 | F1:18056 | 28122021\_RID\_2349\_2C.raw | 5.8019E6 |  |  |  |  |  | 1 | 1 | 0 | 0 | 0 | 0 | 0 | 149 | 157 |  |  | PEAKS DB |
| R.LQGLQAGWLVEEDTFQSHTPYGYR.T | N | 42.74 | 2794.3350 | 24 | 1.1 | 932.4532 | 3 | 54.10 | 1 | F1:31900 | 28122021\_RID\_2349\_2C.raw | 8.091E5 |  |  |  |  |  | 1 | 1 | 0 | 0 | 0 | 0 | 0 | 99 | 122 |  |  | PEAKS DB |
| total 9 peptides |  |  |  |  |  |  |  |  |  |  |  |  |  |  |  |  |  |  |  |  |  |  |  |  |  |  |  |  |  |
| --- | --- | --- | --- | --- | --- | --- | --- | --- | --- | --- | --- | --- | --- | --- | --- | --- | --- | --- | --- | --- | --- | --- | --- | --- | --- | --- | --- | --- | --- |

BAP39934.1

back to list

  

| Protein Coverage
| Supporting Peptides
|

Protein Coverage:

Supporting Peptides:

| Peptide | Uniq | -10lgP | Mass | Length | ppm | m/z | z | RT | Fraction | Scan | Source File | Area F2 | Area F1 | Area F3 | Area F4 | Area F5 | Area F6 | #Feature | #Feature F2 | #Feature F1 | #Feature F3 | #Feature F4 | #Feature F5 | #Feature F6 | Start | End | PTM | AScore | Found By |
| --- | --- | --- | --- | --- | --- | --- | --- | --- | --- | --- | --- | --- | --- | --- | --- | --- | --- | --- | --- | --- | --- | --- | --- | --- | --- | --- | --- | --- | --- |
| R.NLYDLGLLNNYSSER.Q | Y | 87.50 | 1769.8584 | 15 | 0.7 | 885.9371 | 2 | 55.06 | 1 | F1:32757 | 28122021\_RID\_2349\_2C.raw | 2.221E6 |  |  |  | 0 |  | 1 | 1 | 0 | 0 | 0 | 0 | 0 | 283 | 297 |  |  | PEAKS DB |
| K.LIFFDGEEAFVR.W | N | 85.67 | 1441.7241 | 12 | 0.6 | 721.8698 | 2 | 59.72 | 1 | F1:35753 | 28122021\_RID\_2349\_2C.raw | 3.5159E6 |  |  |  |  | 2.168E4 | 5 | 4 | 0 | 0 | 0 | 0 | 1 | 201 | 212 |  |  | PEAKS DB |
| R.VWHTMEDNEENLDKPTIDNLSK.I | Y | 84.37 | 2627.2173 | 22 | 1.1 | 876.7473 | 3 | 37.39 | 1 | F1:19045 | 28122021\_RID\_2349\_2C.raw | 6.149E6 |  |  |  |  |  | 2 | 2 | 0 | 0 | 0 | 0 | 0 | 334 | 355 |  |  | PEAKS DB |
| R.YPGSPGSYAVR.Q | N | 69.00 | 1152.5563 | 11 | 0.1 | 577.2855 | 2 | 28.48 | 1 | F1:11793 | 28122021\_RID\_2349\_2C.raw | 7.1634E6 |  |  |  |  | 3.2738E5 | 2 | 1 | 0 | 0 | 0 | 0 | 1 | 82 | 92 |  |  | PEAKS DB |
| R.TFSNIISTLNPLAK.R | N | 68.92 | 1517.8453 | 14 | 1.1 | 759.9308 | 2 | 58.63 | 1 | F1:35258 | 28122021\_RID\_2349\_2C.raw | 2.8095E6 |  |  |  | 1.6857E5 | 8.0094E4 | 3 | 1 | 0 | 0 | 0 | 1 | 1 | 123 | 136 |  |  | PEAKS DB |
| R.WSPSDSLYGSR.S | N | 68.08 | 1253.5676 | 11 | 0.3 | 627.7913 | 2 | 38.76 | 1 | F1:20122 | 28122021\_RID\_2349\_2C.raw | 4.7528E6 |  |  |  |  |  | 1 | 1 | 0 | 0 | 0 | 0 | 0 | 213 | 223 |  |  | PEAKS DB |
| R.VWHTM(+15.99)EDNEENLDKPTIDNLSK.I | Y | 54.31 | 2643.2122 | 22 | 0.8 | 882.0787 | 3 | 33.87 | 1 | F1:16176 | 28122021\_RID\_2349\_2C.raw | 1.6494E6 |  |  |  |  |  | 2 | 2 | 0 | 0 | 0 | 0 | 0 | 334 | 355 | Oxidation (M) | M5:Oxidation (M):1000.00 | PEAKS DB |
| K.YFPPQLDGK.V | N | 50.09 | 1063.5338 | 9 | 0.9 | 532.7747 | 2 | 36.56 | 1 | F1:18056 | 28122021\_RID\_2349\_2C.raw | 5.8019E6 |  |  |  |  |  | 1 | 1 | 0 | 0 | 0 | 0 | 0 | 149 | 157 |  |  | PEAKS DB |
| R.LQGLQAGWLVEEDTFQSHTPYGYR.T | N | 42.74 | 2794.3350 | 24 | 1.1 | 932.4532 | 3 | 54.10 | 1 | F1:31900 | 28122021\_RID\_2349\_2C.raw | 8.091E5 |  |  |  |  |  | 1 | 1 | 0 | 0 | 0 | 0 | 0 | 99 | 122 |  |  | PEAKS DB |
| total 9 peptides |  |  |  |  |  |  |  |  |  |  |  |  |  |  |  |  |  |  |  |  |  |  |  |  |  |  |  |  |  |
| --- | --- | --- | --- | --- | --- | --- | --- | --- | --- | --- | --- | --- | --- | --- | --- | --- | --- | --- | --- | --- | --- | --- | --- | --- | --- | --- | --- | --- | --- |

AFE84763.1

back to list

  

| Protein Coverage
| Supporting Peptides
|

Protein Coverage:

Supporting Peptides:

| Peptide | Uniq | -10lgP | Mass | Length | ppm | m/z | z | RT | Fraction | Scan | Source File | Area F2 | Area F1 | Area F3 | Area F4 | Area F5 | Area F6 | #Feature | #Feature F2 | #Feature F1 | #Feature F3 | #Feature F4 | #Feature F5 | #Feature F6 | Start | End | PTM | AScore | Found By |
| --- | --- | --- | --- | --- | --- | --- | --- | --- | --- | --- | --- | --- | --- | --- | --- | --- | --- | --- | --- | --- | --- | --- | --- | --- | --- | --- | --- | --- | --- |
| R.NLYDLGLLNNYSSER.Q | Y | 87.50 | 1769.8584 | 15 | 0.7 | 885.9371 | 2 | 55.06 | 1 | F1:32757 | 28122021\_RID\_2349\_2C.raw | 2.221E6 |  |  |  | 0 |  | 1 | 1 | 0 | 0 | 0 | 0 | 0 | 283 | 297 |  |  | PEAKS DB |
| K.LIFFDGEEAFVR.W | N | 85.67 | 1441.7241 | 12 | 0.6 | 721.8698 | 2 | 59.72 | 1 | F1:35753 | 28122021\_RID\_2349\_2C.raw | 3.5159E6 |  |  |  |  | 2.168E4 | 5 | 4 | 0 | 0 | 0 | 0 | 1 | 201 | 212 |  |  | PEAKS DB |
| R.VWHTMEDNEENLDKPTIDNLSK.I | Y | 84.37 | 2627.2173 | 22 | 1.1 | 876.7473 | 3 | 37.39 | 1 | F1:19045 | 28122021\_RID\_2349\_2C.raw | 6.149E6 |  |  |  |  |  | 2 | 2 | 0 | 0 | 0 | 0 | 0 | 334 | 355 |  |  | PEAKS DB |
| R.YPGSPGSYAVR.Q | N | 69.00 | 1152.5563 | 11 | 0.1 | 577.2855 | 2 | 28.48 | 1 | F1:11793 | 28122021\_RID\_2349\_2C.raw | 7.1634E6 |  |  |  |  | 3.2738E5 | 2 | 1 | 0 | 0 | 0 | 0 | 1 | 82 | 92 |  |  | PEAKS DB |
| R.TFSNIISTLNPLAK.R | N | 68.92 | 1517.8453 | 14 | 1.1 | 759.9308 | 2 | 58.63 | 1 | F1:35258 | 28122021\_RID\_2349\_2C.raw | 2.8095E6 |  |  |  | 1.6857E5 | 8.0094E4 | 3 | 1 | 0 | 0 | 0 | 1 | 1 | 123 | 136 |  |  | PEAKS DB |
| R.WSPSDSLYGSR.S | N | 68.08 | 1253.5676 | 11 | 0.3 | 627.7913 | 2 | 38.76 | 1 | F1:20122 | 28122021\_RID\_2349\_2C.raw | 4.7528E6 |  |  |  |  |  | 1 | 1 | 0 | 0 | 0 | 0 | 0 | 213 | 223 |  |  | PEAKS DB |
| R.VWHTM(+15.99)EDNEENLDKPTIDNLSK.I | Y | 54.31 | 2643.2122 | 22 | 0.8 | 882.0787 | 3 | 33.87 | 1 | F1:16176 | 28122021\_RID\_2349\_2C.raw | 1.6494E6 |  |  |  |  |  | 2 | 2 | 0 | 0 | 0 | 0 | 0 | 334 | 355 | Oxidation (M) | M5:Oxidation (M):1000.00 | PEAKS DB |
| K.YFPPQLDGK.V | N | 50.09 | 1063.5338 | 9 | 0.9 | 532.7747 | 2 | 36.56 | 1 | F1:18056 | 28122021\_RID\_2349\_2C.raw | 5.8019E6 |  |  |  |  |  | 1 | 1 | 0 | 0 | 0 | 0 | 0 | 149 | 157 |  |  | PEAKS DB |
| R.LQGLQAGWLVEEDTFQSHTPYGYR.T | N | 42.74 | 2794.3350 | 24 | 1.1 | 932.4532 | 3 | 54.10 | 1 | F1:31900 | 28122021\_RID\_2349\_2C.raw | 8.091E5 |  |  |  |  |  | 1 | 1 | 0 | 0 | 0 | 0 | 0 | 99 | 122 |  |  | PEAKS DB |
| total 9 peptides |  |  |  |  |  |  |  |  |  |  |  |  |  |  |  |  |  |  |  |  |  |  |  |  |  |  |  |  |  |
| --- | --- | --- | --- | --- | --- | --- | --- | --- | --- | --- | --- | --- | --- | --- | --- | --- | --- | --- | --- | --- | --- | --- | --- | --- | --- | --- | --- | --- | --- |

BAP39982.1

back to list

  

| Protein Coverage
| Supporting Peptides
|

Protein Coverage:

Supporting Peptides:

| Peptide | Uniq | -10lgP | Mass | Length | ppm | m/z | z | RT | Fraction | Scan | Source File | Area F2 | Area F1 | Area F3 | Area F4 | Area F5 | Area F6 | #Feature | #Feature F2 | #Feature F1 | #Feature F3 | #Feature F4 | #Feature F5 | #Feature F6 | Start | End | PTM | AScore | Found By |
| --- | --- | --- | --- | --- | --- | --- | --- | --- | --- | --- | --- | --- | --- | --- | --- | --- | --- | --- | --- | --- | --- | --- | --- | --- | --- | --- | --- | --- | --- |
| R.NLYDLGLLNNYSSER.Q | Y | 87.50 | 1769.8584 | 15 | 0.7 | 885.9371 | 2 | 55.06 | 1 | F1:32757 | 28122021\_RID\_2349\_2C.raw | 2.221E6 |  |  |  | 0 |  | 1 | 1 | 0 | 0 | 0 | 0 | 0 | 283 | 297 |  |  | PEAKS DB |
| K.LIFFDGEEAFVR.W | N | 85.67 | 1441.7241 | 12 | 0.6 | 721.8698 | 2 | 59.72 | 1 | F1:35753 | 28122021\_RID\_2349\_2C.raw | 3.5159E6 |  |  |  |  | 2.168E4 | 5 | 4 | 0 | 0 | 0 | 0 | 1 | 201 | 212 |  |  | PEAKS DB |
| R.VWHTMEDNEENLDKPTIDNLSK.I | Y | 84.37 | 2627.2173 | 22 | 1.1 | 876.7473 | 3 | 37.39 | 1 | F1:19045 | 28122021\_RID\_2349\_2C.raw | 6.149E6 |  |  |  |  |  | 2 | 2 | 0 | 0 | 0 | 0 | 0 | 334 | 355 |  |  | PEAKS DB |
| R.YPGSPGSYAVR.Q | N | 69.00 | 1152.5563 | 11 | 0.1 | 577.2855 | 2 | 28.48 | 1 | F1:11793 | 28122021\_RID\_2349\_2C.raw | 7.1634E6 |  |  |  |  | 3.2738E5 | 2 | 1 | 0 | 0 | 0 | 0 | 1 | 82 | 92 |  |  | PEAKS DB |
| R.TFSNIISTLNPLAK.R | N | 68.92 | 1517.8453 | 14 | 1.1 | 759.9308 | 2 | 58.63 | 1 | F1:35258 | 28122021\_RID\_2349\_2C.raw | 2.8095E6 |  |  |  | 1.6857E5 | 8.0094E4 | 3 | 1 | 0 | 0 | 0 | 1 | 1 | 123 | 136 |  |  | PEAKS DB |
| R.WSPSDSLYGSR.S | N | 68.08 | 1253.5676 | 11 | 0.3 | 627.7913 | 2 | 38.76 | 1 | F1:20122 | 28122021\_RID\_2349\_2C.raw | 4.7528E6 |  |  |  |  |  | 1 | 1 | 0 | 0 | 0 | 0 | 0 | 213 | 223 |  |  | PEAKS DB |
| R.VWHTM(+15.99)EDNEENLDKPTIDNLSK.I | Y | 54.31 | 2643.2122 | 22 | 0.8 | 882.0787 | 3 | 33.87 | 1 | F1:16176 | 28122021\_RID\_2349\_2C.raw | 1.6494E6 |  |  |  |  |  | 2 | 2 | 0 | 0 | 0 | 0 | 0 | 334 | 355 | Oxidation (M) | M5:Oxidation (M):1000.00 | PEAKS DB |
| K.YFPPQLDGK.V | N | 50.09 | 1063.5338 | 9 | 0.9 | 532.7747 | 2 | 36.56 | 1 | F1:18056 | 28122021\_RID\_2349\_2C.raw | 5.8019E6 |  |  |  |  |  | 1 | 1 | 0 | 0 | 0 | 0 | 0 | 149 | 157 |  |  | PEAKS DB |
| R.LQGLQAGWLVEEDTFQSHTPYGYR.T | N | 42.74 | 2794.3350 | 24 | 1.1 | 932.4532 | 3 | 54.10 | 1 | F1:31900 | 28122021\_RID\_2349\_2C.raw | 8.091E5 |  |  |  |  |  | 1 | 1 | 0 | 0 | 0 | 0 | 0 | 99 | 122 |  |  | PEAKS DB |
| total 9 peptides |  |  |  |  |  |  |  |  |  |  |  |  |  |  |  |  |  |  |  |  |  |  |  |  |  |  |  |  |  |
| --- | --- | --- | --- | --- | --- | --- | --- | --- | --- | --- | --- | --- | --- | --- | --- | --- | --- | --- | --- | --- | --- | --- | --- | --- | --- | --- | --- | --- | --- |

AFE84765.1

back to list

  

| Protein Coverage
| Supporting Peptides
|

Protein Coverage:

Supporting Peptides:

| Peptide | Uniq | -10lgP | Mass | Length | ppm | m/z | z | RT | Fraction | Scan | Source File | Area F2 | Area F1 | Area F3 | Area F4 | Area F5 | Area F6 | #Feature | #Feature F2 | #Feature F1 | #Feature F3 | #Feature F4 | #Feature F5 | #Feature F6 | Start | End | PTM | AScore | Found By |
| --- | --- | --- | --- | --- | --- | --- | --- | --- | --- | --- | --- | --- | --- | --- | --- | --- | --- | --- | --- | --- | --- | --- | --- | --- | --- | --- | --- | --- | --- |
| R.NLYDLGLLNNYSSER.Q | Y | 87.50 | 1769.8584 | 15 | 0.7 | 885.9371 | 2 | 55.06 | 1 | F1:32757 | 28122021\_RID\_2349\_2C.raw | 2.221E6 |  |  |  | 0 |  | 1 | 1 | 0 | 0 | 0 | 0 | 0 | 283 | 297 |  |  | PEAKS DB |
| K.LIFFDGEEAFVR.W | N | 85.67 | 1441.7241 | 12 | 0.6 | 721.8698 | 2 | 59.72 | 1 | F1:35753 | 28122021\_RID\_2349\_2C.raw | 3.5159E6 |  |  |  |  | 2.168E4 | 5 | 4 | 0 | 0 | 0 | 0 | 1 | 201 | 212 |  |  | PEAKS DB |
| R.VWHTMEDNEENLDKPTIDNLSK.I | Y | 84.37 | 2627.2173 | 22 | 1.1 | 876.7473 | 3 | 37.39 | 1 | F1:19045 | 28122021\_RID\_2349\_2C.raw | 6.149E6 |  |  |  |  |  | 2 | 2 | 0 | 0 | 0 | 0 | 0 | 334 | 355 |  |  | PEAKS DB |
| R.YPGSPGSYAVR.Q | N | 69.00 | 1152.5563 | 11 | 0.1 | 577.2855 | 2 | 28.48 | 1 | F1:11793 | 28122021\_RID\_2349\_2C.raw | 7.1634E6 |  |  |  |  | 3.2738E5 | 2 | 1 | 0 | 0 | 0 | 0 | 1 | 82 | 92 |  |  | PEAKS DB |
| R.TFSNIISTLNPLAK.R | N | 68.92 | 1517.8453 | 14 | 1.1 | 759.9308 | 2 | 58.63 | 1 | F1:35258 | 28122021\_RID\_2349\_2C.raw | 2.8095E6 |  |  |  | 1.6857E5 | 8.0094E4 | 3 | 1 | 0 | 0 | 0 | 1 | 1 | 123 | 136 |  |  | PEAKS DB |
| R.WSPSDSLYGSR.S | N | 68.08 | 1253.5676 | 11 | 0.3 | 627.7913 | 2 | 38.76 | 1 | F1:20122 | 28122021\_RID\_2349\_2C.raw | 4.7528E6 |  |  |  |  |  | 1 | 1 | 0 | 0 | 0 | 0 | 0 | 213 | 223 |  |  | PEAKS DB |
| R.VWHTM(+15.99)EDNEENLDKPTIDNLSK.I | Y | 54.31 | 2643.2122 | 22 | 0.8 | 882.0787 | 3 | 33.87 | 1 | F1:16176 | 28122021\_RID\_2349\_2C.raw | 1.6494E6 |  |  |  |  |  | 2 | 2 | 0 | 0 | 0 | 0 | 0 | 334 | 355 | Oxidation (M) | M5:Oxidation (M):1000.00 | PEAKS DB |
| K.YFPPQLDGK.V | N | 50.09 | 1063.5338 | 9 | 0.9 | 532.7747 | 2 | 36.56 | 1 | F1:18056 | 28122021\_RID\_2349\_2C.raw | 5.8019E6 |  |  |  |  |  | 1 | 1 | 0 | 0 | 0 | 0 | 0 | 149 | 157 |  |  | PEAKS DB |
| R.LQGLQAGWLVEEDTFQSHTPYGYR.T | N | 42.74 | 2794.3350 | 24 | 1.1 | 932.4532 | 3 | 54.10 | 1 | F1:31900 | 28122021\_RID\_2349\_2C.raw | 8.091E5 |  |  |  |  |  | 1 | 1 | 0 | 0 | 0 | 0 | 0 | 99 | 122 |  |  | PEAKS DB |
| total 9 peptides |  |  |  |  |  |  |  |  |  |  |  |  |  |  |  |  |  |  |  |  |  |  |  |  |  |  |  |  |  |
| --- | --- | --- | --- | --- | --- | --- | --- | --- | --- | --- | --- | --- | --- | --- | --- | --- | --- | --- | --- | --- | --- | --- | --- | --- | --- | --- | --- | --- | --- |

AFJ49187.1

back to list

  

| Protein Coverage
| Supporting Peptides
|

Protein Coverage:

Supporting Peptides:

| Peptide | Uniq | -10lgP | Mass | Length | ppm | m/z | z | RT | Fraction | Scan | Source File | Area F2 | Area F1 | Area F3 | Area F4 | Area F5 | Area F6 | #Feature | #Feature F2 | #Feature F1 | #Feature F3 | #Feature F4 | #Feature F5 | #Feature F6 | Start | End | PTM | AScore | Found By |
| --- | --- | --- | --- | --- | --- | --- | --- | --- | --- | --- | --- | --- | --- | --- | --- | --- | --- | --- | --- | --- | --- | --- | --- | --- | --- | --- | --- | --- | --- |
| R.NLYDLGLLNNYSSER.Q | Y | 87.50 | 1769.8584 | 15 | 0.7 | 885.9371 | 2 | 55.06 | 1 | F1:32757 | 28122021\_RID\_2349\_2C.raw | 2.221E6 |  |  |  | 0 |  | 1 | 1 | 0 | 0 | 0 | 0 | 0 | 283 | 297 |  |  | PEAKS DB |
| K.LIFFDGEEAFVR.W | N | 85.67 | 1441.7241 | 12 | 0.6 | 721.8698 | 2 | 59.72 | 1 | F1:35753 | 28122021\_RID\_2349\_2C.raw | 3.5159E6 |  |  |  |  | 2.168E4 | 5 | 4 | 0 | 0 | 0 | 0 | 1 | 201 | 212 |  |  | PEAKS DB |
| R.VWHTMEDNEENLDKPTIDNLSK.I | Y | 84.37 | 2627.2173 | 22 | 1.1 | 876.7473 | 3 | 37.39 | 1 | F1:19045 | 28122021\_RID\_2349\_2C.raw | 6.149E6 |  |  |  |  |  | 2 | 2 | 0 | 0 | 0 | 0 | 0 | 334 | 355 |  |  | PEAKS DB |
| R.YPGSPGSYAVR.Q | N | 69.00 | 1152.5563 | 11 | 0.1 | 577.2855 | 2 | 28.48 | 1 | F1:11793 | 28122021\_RID\_2349\_2C.raw | 7.1634E6 |  |  |  |  | 3.2738E5 | 2 | 1 | 0 | 0 | 0 | 0 | 1 | 82 | 92 |  |  | PEAKS DB |
| R.TFSNIISTLNPLAK.R | N | 68.92 | 1517.8453 | 14 | 1.1 | 759.9308 | 2 | 58.63 | 1 | F1:35258 | 28122021\_RID\_2349\_2C.raw | 2.8095E6 |  |  |  | 1.6857E5 | 8.0094E4 | 3 | 1 | 0 | 0 | 0 | 1 | 1 | 123 | 136 |  |  | PEAKS DB |
| R.WSPSDSLYGSR.S | N | 68.08 | 1253.5676 | 11 | 0.3 | 627.7913 | 2 | 38.76 | 1 | F1:20122 | 28122021\_RID\_2349\_2C.raw | 4.7528E6 |  |  |  |  |  | 1 | 1 | 0 | 0 | 0 | 0 | 0 | 213 | 223 |  |  | PEAKS DB |
| R.VWHTM(+15.99)EDNEENLDKPTIDNLSK.I | Y | 54.31 | 2643.2122 | 22 | 0.8 | 882.0787 | 3 | 33.87 | 1 | F1:16176 | 28122021\_RID\_2349\_2C.raw | 1.6494E6 |  |  |  |  |  | 2 | 2 | 0 | 0 | 0 | 0 | 0 | 334 | 355 | Oxidation (M) | M5:Oxidation (M):1000.00 | PEAKS DB |
| K.YFPPQLDGK.V | N | 50.09 | 1063.5338 | 9 | 0.9 | 532.7747 | 2 | 36.56 | 1 | F1:18056 | 28122021\_RID\_2349\_2C.raw | 5.8019E6 |  |  |  |  |  | 1 | 1 | 0 | 0 | 0 | 0 | 0 | 149 | 157 |  |  | PEAKS DB |
| R.LQGLQAGWLVEEDTFQSHTPYGYR.T | N | 42.74 | 2794.3350 | 24 | 1.1 | 932.4532 | 3 | 54.10 | 1 | F1:31900 | 28122021\_RID\_2349\_2C.raw | 8.091E5 |  |  |  |  |  | 1 | 1 | 0 | 0 | 0 | 0 | 0 | 99 | 122 |  |  | PEAKS DB |
| total 9 peptides |  |  |  |  |  |  |  |  |  |  |  |  |  |  |  |  |  |  |  |  |  |  |  |  |  |  |  |  |  |
| --- | --- | --- | --- | --- | --- | --- | --- | --- | --- | --- | --- | --- | --- | --- | --- | --- | --- | --- | --- | --- | --- | --- | --- | --- | --- | --- | --- | --- | --- |

JAA94895.1

back to list

  

| Protein Coverage
| Supporting Peptides
|

Protein Coverage:

Supporting Peptides:

| Peptide | Uniq | -10lgP | Mass | Length | ppm | m/z | z | RT | Fraction | Scan | Source File | Area F2 | Area F1 | Area F3 | Area F4 | Area F5 | Area F6 | #Feature | #Feature F2 | #Feature F1 | #Feature F3 | #Feature F4 | #Feature F5 | #Feature F6 | Start | End | PTM | AScore | Found By |
| --- | --- | --- | --- | --- | --- | --- | --- | --- | --- | --- | --- | --- | --- | --- | --- | --- | --- | --- | --- | --- | --- | --- | --- | --- | --- | --- | --- | --- | --- |
| R.NLYDLGLLNNYSSER.Q | Y | 87.50 | 1769.8584 | 15 | 0.7 | 885.9371 | 2 | 55.06 | 1 | F1:32757 | 28122021\_RID\_2349\_2C.raw | 2.221E6 |  |  |  | 0 |  | 1 | 1 | 0 | 0 | 0 | 0 | 0 | 283 | 297 |  |  | PEAKS DB |
| K.LIFFDGEEAFVR.W | N | 85.67 | 1441.7241 | 12 | 0.6 | 721.8698 | 2 | 59.72 | 1 | F1:35753 | 28122021\_RID\_2349\_2C.raw | 3.5159E6 |  |  |  |  | 2.168E4 | 5 | 4 | 0 | 0 | 0 | 0 | 1 | 201 | 212 |  |  | PEAKS DB |
| R.VWHTMEDNEENLDKPTIDNLSK.I | Y | 84.37 | 2627.2173 | 22 | 1.1 | 876.7473 | 3 | 37.39 | 1 | F1:19045 | 28122021\_RID\_2349\_2C.raw | 6.149E6 |  |  |  |  |  | 2 | 2 | 0 | 0 | 0 | 0 | 0 | 334 | 355 |  |  | PEAKS DB |
| R.YPGSPGSYAVR.Q | N | 69.00 | 1152.5563 | 11 | 0.1 | 577.2855 | 2 | 28.48 | 1 | F1:11793 | 28122021\_RID\_2349\_2C.raw | 7.1634E6 |  |  |  |  | 3.2738E5 | 2 | 1 | 0 | 0 | 0 | 0 | 1 | 82 | 92 |  |  | PEAKS DB |
| R.TFSNIISTLNPLAK.R | N | 68.92 | 1517.8453 | 14 | 1.1 | 759.9308 | 2 | 58.63 | 1 | F1:35258 | 28122021\_RID\_2349\_2C.raw | 2.8095E6 |  |  |  | 1.6857E5 | 8.0094E4 | 3 | 1 | 0 | 0 | 0 | 1 | 1 | 123 | 136 |  |  | PEAKS DB |
| R.WSPSDSLYGSR.S | N | 68.08 | 1253.5676 | 11 | 0.3 | 627.7913 | 2 | 38.76 | 1 | F1:20122 | 28122021\_RID\_2349\_2C.raw | 4.7528E6 |  |  |  |  |  | 1 | 1 | 0 | 0 | 0 | 0 | 0 | 213 | 223 |  |  | PEAKS DB |
| R.VWHTM(+15.99)EDNEENLDKPTIDNLSK.I | Y | 54.31 | 2643.2122 | 22 | 0.8 | 882.0787 | 3 | 33.87 | 1 | F1:16176 | 28122021\_RID\_2349\_2C.raw | 1.6494E6 |  |  |  |  |  | 2 | 2 | 0 | 0 | 0 | 0 | 0 | 334 | 355 | Oxidation (M) | M5:Oxidation (M):1000.00 | PEAKS DB |
| K.YFPPQLDGK.V | N | 50.09 | 1063.5338 | 9 | 0.9 | 532.7747 | 2 | 36.56 | 1 | F1:18056 | 28122021\_RID\_2349\_2C.raw | 5.8019E6 |  |  |  |  |  | 1 | 1 | 0 | 0 | 0 | 0 | 0 | 149 | 157 |  |  | PEAKS DB |
| R.LQGLQAGWLVEEDTFQSHTPYGYR.T | N | 42.74 | 2794.3350 | 24 | 1.1 | 932.4532 | 3 | 54.10 | 1 | F1:31900 | 28122021\_RID\_2349\_2C.raw | 8.091E5 |  |  |  |  |  | 1 | 1 | 0 | 0 | 0 | 0 | 0 | 99 | 122 |  |  | PEAKS DB |
| total 9 peptides |  |  |  |  |  |  |  |  |  |  |  |  |  |  |  |  |  |  |  |  |  |  |  |  |  |  |  |  |  |
| --- | --- | --- | --- | --- | --- | --- | --- | --- | --- | --- | --- | --- | --- | --- | --- | --- | --- | --- | --- | --- | --- | --- | --- | --- | --- | --- | --- | --- | --- |

JAI13216.1

back to list

  

| Protein Coverage
| Supporting Peptides
|

Protein Coverage:

Supporting Peptides:

| Peptide | Uniq | -10lgP | Mass | Length | ppm | m/z | z | RT | Fraction | Scan | Source File | Area F2 | Area F1 | Area F3 | Area F4 | Area F5 | Area F6 | #Feature | #Feature F2 | #Feature F1 | #Feature F3 | #Feature F4 | #Feature F5 | #Feature F6 | Start | End | PTM | AScore | Found By |
| --- | --- | --- | --- | --- | --- | --- | --- | --- | --- | --- | --- | --- | --- | --- | --- | --- | --- | --- | --- | --- | --- | --- | --- | --- | --- | --- | --- | --- | --- |
| R.NLYDLGLLNNYSSER.Q | Y | 87.50 | 1769.8584 | 15 | 0.7 | 885.9371 | 2 | 55.06 | 1 | F1:32757 | 28122021\_RID\_2349\_2C.raw | 2.221E6 |  |  |  | 0 |  | 1 | 1 | 0 | 0 | 0 | 0 | 0 | 283 | 297 |  |  | PEAKS DB |
| K.LIFFDGEEAFVR.W | N | 85.67 | 1441.7241 | 12 | 0.6 | 721.8698 | 2 | 59.72 | 1 | F1:35753 | 28122021\_RID\_2349\_2C.raw | 3.5159E6 |  |  |  |  | 2.168E4 | 5 | 4 | 0 | 0 | 0 | 0 | 1 | 201 | 212 |  |  | PEAKS DB |
| R.VWHTMEDNEENLDKPTIDNLSK.I | Y | 84.37 | 2627.2173 | 22 | 1.1 | 876.7473 | 3 | 37.39 | 1 | F1:19045 | 28122021\_RID\_2349\_2C.raw | 6.149E6 |  |  |  |  |  | 2 | 2 | 0 | 0 | 0 | 0 | 0 | 334 | 355 |  |  | PEAKS DB |
| R.YPGSPGSYAVR.Q | N | 69.00 | 1152.5563 | 11 | 0.1 | 577.2855 | 2 | 28.48 | 1 | F1:11793 | 28122021\_RID\_2349\_2C.raw | 7.1634E6 |  |  |  |  | 3.2738E5 | 2 | 1 | 0 | 0 | 0 | 0 | 1 | 82 | 92 |  |  | PEAKS DB |
| R.TFSNIISTLNPLAK.R | N | 68.92 | 1517.8453 | 14 | 1.1 | 759.9308 | 2 | 58.63 | 1 | F1:35258 | 28122021\_RID\_2349\_2C.raw | 2.8095E6 |  |  |  | 1.6857E5 | 8.0094E4 | 3 | 1 | 0 | 0 | 0 | 1 | 1 | 123 | 136 |  |  | PEAKS DB |
| R.WSPSDSLYGSR.S | N | 68.08 | 1253.5676 | 11 | 0.3 | 627.7913 | 2 | 38.76 | 1 | F1:20122 | 28122021\_RID\_2349\_2C.raw | 4.7528E6 |  |  |  |  |  | 1 | 1 | 0 | 0 | 0 | 0 | 0 | 213 | 223 |  |  | PEAKS DB |
| R.VWHTM(+15.99)EDNEENLDKPTIDNLSK.I | Y | 54.31 | 2643.2122 | 22 | 0.8 | 882.0787 | 3 | 33.87 | 1 | F1:16176 | 28122021\_RID\_2349\_2C.raw | 1.6494E6 |  |  |  |  |  | 2 | 2 | 0 | 0 | 0 | 0 | 0 | 334 | 355 | Oxidation (M) | M5:Oxidation (M):1000.00 | PEAKS DB |
| K.YFPPQLDGK.V | N | 50.09 | 1063.5338 | 9 | 0.9 | 532.7747 | 2 | 36.56 | 1 | F1:18056 | 28122021\_RID\_2349\_2C.raw | 5.8019E6 |  |  |  |  |  | 1 | 1 | 0 | 0 | 0 | 0 | 0 | 149 | 157 |  |  | PEAKS DB |
| R.LQGLQAGWLVEEDTFQSHTPYGYR.T | N | 42.74 | 2794.3350 | 24 | 1.1 | 932.4532 | 3 | 54.10 | 1 | F1:31900 | 28122021\_RID\_2349\_2C.raw | 8.091E5 |  |  |  |  |  | 1 | 1 | 0 | 0 | 0 | 0 | 0 | 99 | 122 |  |  | PEAKS DB |
| total 9 peptides |  |  |  |  |  |  |  |  |  |  |  |  |  |  |  |  |  |  |  |  |  |  |  |  |  |  |  |  |  |
| --- | --- | --- | --- | --- | --- | --- | --- | --- | --- | --- | --- | --- | --- | --- | --- | --- | --- | --- | --- | --- | --- | --- | --- | --- | --- | --- | --- | --- | --- |

BAN82004.1

back to list

  

| Protein Coverage
| Supporting Peptides
|

Protein Coverage:

Supporting Peptides:

| Peptide | Uniq | -10lgP | Mass | Length | ppm | m/z | z | RT | Fraction | Scan | Source File | Area F2 | Area F1 | Area F3 | Area F4 | Area F5 | Area F6 | #Feature | #Feature F2 | #Feature F1 | #Feature F3 | #Feature F4 | #Feature F5 | #Feature F6 | Start | End | PTM | AScore | Found By |
| --- | --- | --- | --- | --- | --- | --- | --- | --- | --- | --- | --- | --- | --- | --- | --- | --- | --- | --- | --- | --- | --- | --- | --- | --- | --- | --- | --- | --- | --- |
| R.NLYDLGLLNNYSSER.Q | Y | 87.50 | 1769.8584 | 15 | 0.7 | 885.9371 | 2 | 55.06 | 1 | F1:32757 | 28122021\_RID\_2349\_2C.raw | 2.221E6 |  |  |  | 0 |  | 1 | 1 | 0 | 0 | 0 | 0 | 0 | 283 | 297 |  |  | PEAKS DB |
| K.LIFFDGEEAFVR.W | N | 85.67 | 1441.7241 | 12 | 0.6 | 721.8698 | 2 | 59.72 | 1 | F1:35753 | 28122021\_RID\_2349\_2C.raw | 3.5159E6 |  |  |  |  | 2.168E4 | 5 | 4 | 0 | 0 | 0 | 0 | 1 | 201 | 212 |  |  | PEAKS DB |
| R.VWHTMEDNEENLDKPTIDNLSK.I | Y | 84.37 | 2627.2173 | 22 | 1.1 | 876.7473 | 3 | 37.39 | 1 | F1:19045 | 28122021\_RID\_2349\_2C.raw | 6.149E6 |  |  |  |  |  | 2 | 2 | 0 | 0 | 0 | 0 | 0 | 334 | 355 |  |  | PEAKS DB |
| R.YPGSPGSYAVR.Q | N | 69.00 | 1152.5563 | 11 | 0.1 | 577.2855 | 2 | 28.48 | 1 | F1:11793 | 28122021\_RID\_2349\_2C.raw | 7.1634E6 |  |  |  |  | 3.2738E5 | 2 | 1 | 0 | 0 | 0 | 0 | 1 | 82 | 92 |  |  | PEAKS DB |
| R.TFSNIISTLNPLAK.R | N | 68.92 | 1517.8453 | 14 | 1.1 | 759.9308 | 2 | 58.63 | 1 | F1:35258 | 28122021\_RID\_2349\_2C.raw | 2.8095E6 |  |  |  | 1.6857E5 | 8.0094E4 | 3 | 1 | 0 | 0 | 0 | 1 | 1 | 123 | 136 |  |  | PEAKS DB |
| R.WSPSDSLYGSR.S | N | 68.08 | 1253.5676 | 11 | 0.3 | 627.7913 | 2 | 38.76 | 1 | F1:20122 | 28122021\_RID\_2349\_2C.raw | 4.7528E6 |  |  |  |  |  | 1 | 1 | 0 | 0 | 0 | 0 | 0 | 213 | 223 |  |  | PEAKS DB |
| R.VWHTM(+15.99)EDNEENLDKPTIDNLSK.I | Y | 54.31 | 2643.2122 | 22 | 0.8 | 882.0787 | 3 | 33.87 | 1 | F1:16176 | 28122021\_RID\_2349\_2C.raw | 1.6494E6 |  |  |  |  |  | 2 | 2 | 0 | 0 | 0 | 0 | 0 | 334 | 355 | Oxidation (M) | M5:Oxidation (M):1000.00 | PEAKS DB |
| K.YFPPQLDGK.V | N | 50.09 | 1063.5338 | 9 | 0.9 | 532.7747 | 2 | 36.56 | 1 | F1:18056 | 28122021\_RID\_2349\_2C.raw | 5.8019E6 |  |  |  |  |  | 1 | 1 | 0 | 0 | 0 | 0 | 0 | 149 | 157 |  |  | PEAKS DB |
| R.LQGLQAGWLVEEDTFQSHTPYGYR.T | N | 42.74 | 2794.3350 | 24 | 1.1 | 932.4532 | 3 | 54.10 | 1 | F1:31900 | 28122021\_RID\_2349\_2C.raw | 8.091E5 |  |  |  |  |  | 1 | 1 | 0 | 0 | 0 | 0 | 0 | 99 | 122 |  |  | PEAKS DB |
| total 9 peptides |  |  |  |  |  |  |  |  |  |  |  |  |  |  |  |  |  |  |  |  |  |  |  |  |  |  |  |  |  |
| --- | --- | --- | --- | --- | --- | --- | --- | --- | --- | --- | --- | --- | --- | --- | --- | --- | --- | --- | --- | --- | --- | --- | --- | --- | --- | --- | --- | --- | --- |

JAG44660.1

back to list

  

| Protein Coverage
| Supporting Peptides
|

Protein Coverage:

Supporting Peptides:

| Peptide | Uniq | -10lgP | Mass | Length | ppm | m/z | z | RT | Fraction | Scan | Source File | Area F2 | Area F1 | Area F3 | Area F4 | Area F5 | Area F6 | #Feature | #Feature F2 | #Feature F1 | #Feature F3 | #Feature F4 | #Feature F5 | #Feature F6 | Start | End | PTM | AScore | Found By |
| --- | --- | --- | --- | --- | --- | --- | --- | --- | --- | --- | --- | --- | --- | --- | --- | --- | --- | --- | --- | --- | --- | --- | --- | --- | --- | --- | --- | --- | --- |
| R.NLYDLGLLNNYSSER.Q | Y | 87.50 | 1769.8584 | 15 | 0.7 | 885.9371 | 2 | 55.06 | 1 | F1:32757 | 28122021\_RID\_2349\_2C.raw | 2.221E6 |  |  |  | 0 |  | 1 | 1 | 0 | 0 | 0 | 0 | 0 | 283 | 297 |  |  | PEAKS DB |
| K.LIFFDGEEAFVR.W | N | 85.67 | 1441.7241 | 12 | 0.6 | 721.8698 | 2 | 59.72 | 1 | F1:35753 | 28122021\_RID\_2349\_2C.raw | 3.5159E6 |  |  |  |  | 2.168E4 | 5 | 4 | 0 | 0 | 0 | 0 | 1 | 201 | 212 |  |  | PEAKS DB |
| R.VWHTMEDNEENLDKPTIDNLSK.I | Y | 84.37 | 2627.2173 | 22 | 1.1 | 876.7473 | 3 | 37.39 | 1 | F1:19045 | 28122021\_RID\_2349\_2C.raw | 6.149E6 |  |  |  |  |  | 2 | 2 | 0 | 0 | 0 | 0 | 0 | 334 | 355 |  |  | PEAKS DB |
| R.YPGSPGSYAVR.Q | N | 69.00 | 1152.5563 | 11 | 0.1 | 577.2855 | 2 | 28.48 | 1 | F1:11793 | 28122021\_RID\_2349\_2C.raw | 7.1634E6 |  |  |  |  | 3.2738E5 | 2 | 1 | 0 | 0 | 0 | 0 | 1 | 82 | 92 |  |  | PEAKS DB |
| R.TFSNIISTLNPLAK.R | N | 68.92 | 1517.8453 | 14 | 1.1 | 759.9308 | 2 | 58.63 | 1 | F1:35258 | 28122021\_RID\_2349\_2C.raw | 2.8095E6 |  |  |  | 1.6857E5 | 8.0094E4 | 3 | 1 | 0 | 0 | 0 | 1 | 1 | 123 | 136 |  |  | PEAKS DB |
| R.WSPSDSLYGSR.S | N | 68.08 | 1253.5676 | 11 | 0.3 | 627.7913 | 2 | 38.76 | 1 | F1:20122 | 28122021\_RID\_2349\_2C.raw | 4.7528E6 |  |  |  |  |  | 1 | 1 | 0 | 0 | 0 | 0 | 0 | 213 | 223 |  |  | PEAKS DB |
| R.VWHTM(+15.99)EDNEENLDKPTIDNLSK.I | Y | 54.31 | 2643.2122 | 22 | 0.8 | 882.0787 | 3 | 33.87 | 1 | F1:16176 | 28122021\_RID\_2349\_2C.raw | 1.6494E6 |  |  |  |  |  | 2 | 2 | 0 | 0 | 0 | 0 | 0 | 334 | 355 | Oxidation (M) | M5:Oxidation (M):1000.00 | PEAKS DB |
| K.YFPPQLDGK.V | N | 50.09 | 1063.5338 | 9 | 0.9 | 532.7747 | 2 | 36.56 | 1 | F1:18056 | 28122021\_RID\_2349\_2C.raw | 5.8019E6 |  |  |  |  |  | 1 | 1 | 0 | 0 | 0 | 0 | 0 | 149 | 157 |  |  | PEAKS DB |
| R.LQGLQAGWLVEEDTFQSHTPYGYR.T | N | 42.74 | 2794.3350 | 24 | 1.1 | 932.4532 | 3 | 54.10 | 1 | F1:31900 | 28122021\_RID\_2349\_2C.raw | 8.091E5 |  |  |  |  |  | 1 | 1 | 0 | 0 | 0 | 0 | 0 | 99 | 122 |  |  | PEAKS DB |
| total 9 peptides |  |  |  |  |  |  |  |  |  |  |  |  |  |  |  |  |  |  |  |  |  |  |  |  |  |  |  |  |  |
| --- | --- | --- | --- | --- | --- | --- | --- | --- | --- | --- | --- | --- | --- | --- | --- | --- | --- | --- | --- | --- | --- | --- | --- | --- | --- | --- | --- | --- | --- |

BAN89375.1

back to list

  

| Protein Coverage
| Supporting Peptides
|

Protein Coverage:

Supporting Peptides:

| Peptide | Uniq | -10lgP | Mass | Length | ppm | m/z | z | RT | Fraction | Scan | Source File | Area F2 | Area F1 | Area F3 | Area F4 | Area F5 | Area F6 | #Feature | #Feature F2 | #Feature F1 | #Feature F3 | #Feature F4 | #Feature F5 | #Feature F6 | Start | End | PTM | AScore | Found By |
| --- | --- | --- | --- | --- | --- | --- | --- | --- | --- | --- | --- | --- | --- | --- | --- | --- | --- | --- | --- | --- | --- | --- | --- | --- | --- | --- | --- | --- | --- |
| R.NLYDLGLLNNYSSER.Q | Y | 87.50 | 1769.8584 | 15 | 0.7 | 885.9371 | 2 | 55.06 | 1 | F1:32757 | 28122021\_RID\_2349\_2C.raw | 2.221E6 |  |  |  | 0 |  | 1 | 1 | 0 | 0 | 0 | 0 | 0 | 283 | 297 |  |  | PEAKS DB |
| K.LIFFDGEEAFVR.W | N | 85.67 | 1441.7241 | 12 | 0.6 | 721.8698 | 2 | 59.72 | 1 | F1:35753 | 28122021\_RID\_2349\_2C.raw | 3.5159E6 |  |  |  |  | 2.168E4 | 5 | 4 | 0 | 0 | 0 | 0 | 1 | 201 | 212 |  |  | PEAKS DB |
| R.VWHTMEDNEENLDKPTIDNLSK.I | Y | 84.37 | 2627.2173 | 22 | 1.1 | 876.7473 | 3 | 37.39 | 1 | F1:19045 | 28122021\_RID\_2349\_2C.raw | 6.149E6 |  |  |  |  |  | 2 | 2 | 0 | 0 | 0 | 0 | 0 | 334 | 355 |  |  | PEAKS DB |
| R.YPGSPGSYAVR.Q | N | 69.00 | 1152.5563 | 11 | 0.1 | 577.2855 | 2 | 28.48 | 1 | F1:11793 | 28122021\_RID\_2349\_2C.raw | 7.1634E6 |  |  |  |  | 3.2738E5 | 2 | 1 | 0 | 0 | 0 | 0 | 1 | 82 | 92 |  |  | PEAKS DB |
| R.TFSNIISTLNPLAK.R | N | 68.92 | 1517.8453 | 14 | 1.1 | 759.9308 | 2 | 58.63 | 1 | F1:35258 | 28122021\_RID\_2349\_2C.raw | 2.8095E6 |  |  |  | 1.6857E5 | 8.0094E4 | 3 | 1 | 0 | 0 | 0 | 1 | 1 | 123 | 136 |  |  | PEAKS DB |
| R.WSPSDSLYGSR.S | N | 68.08 | 1253.5676 | 11 | 0.3 | 627.7913 | 2 | 38.76 | 1 | F1:20122 | 28122021\_RID\_2349\_2C.raw | 4.7528E6 |  |  |  |  |  | 1 | 1 | 0 | 0 | 0 | 0 | 0 | 213 | 223 |  |  | PEAKS DB |
| R.VWHTM(+15.99)EDNEENLDKPTIDNLSK.I | Y | 54.31 | 2643.2122 | 22 | 0.8 | 882.0787 | 3 | 33.87 | 1 | F1:16176 | 28122021\_RID\_2349\_2C.raw | 1.6494E6 |  |  |  |  |  | 2 | 2 | 0 | 0 | 0 | 0 | 0 | 334 | 355 | Oxidation (M) | M5:Oxidation (M):1000.00 | PEAKS DB |
| K.YFPPQLDGK.V | N | 50.09 | 1063.5338 | 9 | 0.9 | 532.7747 | 2 | 36.56 | 1 | F1:18056 | 28122021\_RID\_2349\_2C.raw | 5.8019E6 |  |  |  |  |  | 1 | 1 | 0 | 0 | 0 | 0 | 0 | 149 | 157 |  |  | PEAKS DB |
| R.LQGLQAGWLVEEDTFQSHTPYGYR.T | N | 42.74 | 2794.3350 | 24 | 1.1 | 932.4532 | 3 | 54.10 | 1 | F1:31900 | 28122021\_RID\_2349\_2C.raw | 8.091E5 |  |  |  |  |  | 1 | 1 | 0 | 0 | 0 | 0 | 0 | 99 | 122 |  |  | PEAKS DB |
| total 9 peptides |  |  |  |  |  |  |  |  |  |  |  |  |  |  |  |  |  |  |  |  |  |  |  |  |  |  |  |  |  |
| --- | --- | --- | --- | --- | --- | --- | --- | --- | --- | --- | --- | --- | --- | --- | --- | --- | --- | --- | --- | --- | --- | --- | --- | --- | --- | --- | --- | --- | --- |

ETE67131.1

back to list

  

| Protein Coverage
| Supporting Peptides
|

Protein Coverage:

Supporting Peptides:

| Peptide | Uniq | -10lgP | Mass | Length | ppm | m/z | z | RT | Fraction | Scan | Source File | Area F2 | Area F1 | Area F3 | Area F4 | Area F5 | Area F6 | #Feature | #Feature F2 | #Feature F1 | #Feature F3 | #Feature F4 | #Feature F5 | #Feature F6 | Start | End | PTM | AScore | Found By |
| --- | --- | --- | --- | --- | --- | --- | --- | --- | --- | --- | --- | --- | --- | --- | --- | --- | --- | --- | --- | --- | --- | --- | --- | --- | --- | --- | --- | --- | --- |
| K.AQYEDIAQK.S | N | 72.21 | 1064.5138 | 9 | 0.4 | 533.2644 | 2 | 22.89 | 6 | F6:6606 | 31122021\_RID\_2349\_6C.raw | 3.9872E7 | 1.3826E7 | 1.946E8 | 1.0751E8 | 7.0662E6 | 3.7146E6 | 6 | 1 | 1 | 1 | 1 | 1 | 1 | 334 | 342 |  |  | PEAKS DB |
| K.LALDIEIATYR.K | N | 71.69 | 1276.7026 | 11 | 0.5 | 639.3589 | 2 | 53.27 | 1 | F1:31324 | 28122021\_RID\_2349\_2C.raw | 9.0531E5 | 2.8506E4 | 2.692E7 | 1.4957E7 | 5.3148E5 | 1.8027E5 | 6 | 1 | 1 | 1 | 1 | 1 | 1 | 446 | 456 |  |  | PEAKS DB |
| R.DYQELMNVK.L | N | 66.55 | 1138.5328 | 9 | 0.4 | 570.2739 | 2 | 40.16 | 2 | F2:13387 | 30122021\_RID\_2349\_1C.raw | 1.3419E6 | 1.4984E6 | 1.645E7 |  |  | 1.7412E5 | 4 | 1 | 1 | 1 | 0 | 0 | 1 | 2043 | 2051 |  |  | PEAKS DB |
| K.LALDVEIATYR.T | N | 65.93 | 1262.6870 | 11 | 0.9 | 632.3514 | 2 | 49.29 | 1 | F1:28385 | 28122021\_RID\_2349\_2C.raw | 2.3891E5 |  | 1.2459E7 | 3.5375E6 | 8.7538E5 | 1.1533E5 | 6 | 1 | 0 | 1 | 1 | 2 | 1 | 2052 | 2062 |  |  | PEAKS DB |
| R.LLRDYQELMNVK.L | N | 62.26 | 1520.8021 | 12 | 0.9 | 507.9418 | 3 | 39.66 | 5 | F5:19467 | 31122021\_RID\_2349\_5C.raw |  |  |  |  | 6.0538E5 |  | 2 | 0 | 0 | 0 | 0 | 2 | 0 | 2040 | 2051 |  |  | PEAKS DB |
| R.LLKEYQELMNVK.L | N | 61.54 | 1506.8115 | 12 | 0.1 | 754.4131 | 2 | 40.19 | 3 | F3:11620 | 31122021\_RID\_2349\_3C.raw |  |  | 2.9093E6 |  |  |  | 1 | 0 | 0 | 1 | 0 | 0 | 0 | 434 | 445 |  |  | PEAKS DB |
| D.LDLDSIIAEVK.A | N | 61.19 | 1214.6758 | 11 | 0.8 | 608.3456 | 2 | 61.57 | 5 | F5:35620 | 31122021\_RID\_2349\_5C.raw |  |  |  |  | 2.2258E5 |  | 1 | 0 | 0 | 0 | 0 | 1 | 0 | 1924 | 1934 |  |  | PEAKS DB |
| R.DLDLDSIIAEVK.A | Y | 60.08 | 1329.7028 | 12 | 0.3 | 665.8589 | 2 | 75.31 | 4 | F4:18951 | 31122021\_RID\_2349\_4C.raw |  |  | 0 | 2.8331E5 |  |  | 1 | 0 | 0 | 0 | 1 | 0 | 0 | 1923 | 1934 |  |  | PEAKS DB |
| K.FASFIDKVR.F | N | 59.84 | 1081.5920 | 9 | 1.2 | 541.8040 | 2 | 33.97 | 6 | F6:14555 | 31122021\_RID\_2349\_6C.raw | 6.7057E5 |  | 1.5615E7 |  | 6.0374E5 | 2.7185E5 | 4 | 1 | 0 | 1 | 0 | 1 | 1 | 200 | 208 |  |  | PEAKS DB |
| K.NKYEDEINKR.T | N | 59.38 | 1307.6470 | 10 | 0.8 | 654.8313 | 2 | 16.32 | 1 | F1:3281 | 28122021\_RID\_2349\_2C.raw | 1.8679E7 | 2.4367E6 | 8.2281E7 | 3.4977E7 | 1.6788E6 | 9.6273E5 | 8 | 2 | 1 | 1 | 1 | 2 | 1 | 1847 | 1856 |  |  | PEAKS DB |
| L.DLDSIIAEVK.A | N | 59.18 | 1101.5917 | 10 | -0.1 | 551.8030 | 2 | 55.07 | 1 | F1:32794 | 28122021\_RID\_2349\_2C.raw | 3.6044E5 |  |  |  |  |  | 1 | 1 | 0 | 0 | 0 | 0 | 0 | 1925 | 1934 |  |  | PEAKS DB |
| K.NKYEDEINK.R | N | 53.36 | 1151.5458 | 9 | 0.4 | 576.7804 | 2 | 16.89 | 1 | F1:3601 | 28122021\_RID\_2349\_2C.raw | 1.1716E6 |  | 1.3715E7 |  |  |  | 4 | 2 | 0 | 2 | 0 | 0 | 0 | 1847 | 1855 |  |  | PEAKS DB |
| K.EYQELMNVK.L | N | 49.62 | 1152.5485 | 9 | 0.7 | 577.2819 | 2 | 34.25 | 1 | F1:16408 | 28122021\_RID\_2349\_2C.raw | 1.4449E5 |  | 7.4495E6 |  |  |  | 2 | 1 | 0 | 1 | 0 | 0 | 0 | 437 | 445 |  |  | PEAKS DB |
| K.FASFIDK.V | N | 45.14 | 826.4225 | 7 | -0.5 | 414.2183 | 2 | 34.78 | 1 | F1:16890 | 28122021\_RID\_2349\_2C.raw | 6.9087E6 |  |  |  | 9.8962E5 |  | 2 | 1 | 0 | 0 | 0 | 1 | 0 | 200 | 206 |  |  | PEAKS DB |
| total 14 peptides |  |  |  |  |  |  |  |  |  |  |  |  |  |  |  |  |  |  |  |  |  |  |  |  |  |  |  |  |  |
| --- | --- | --- | --- | --- | --- | --- | --- | --- | --- | --- | --- | --- | --- | --- | --- | --- | --- | --- | --- | --- | --- | --- | --- | --- | --- | --- | --- | --- | --- |

XP\_026551250.1

back to list

  

| Protein Coverage
| Supporting Peptides
|

Protein Coverage:

Supporting Peptides:

| Peptide | Uniq | -10lgP | Mass | Length | ppm | m/z | z | RT | Fraction | Scan | Source File | Area F2 | Area F1 | Area F3 | Area F4 | Area F5 | Area F6 | #Feature | #Feature F2 | #Feature F1 | #Feature F3 | #Feature F4 | #Feature F5 | #Feature F6 | Start | End | PTM | AScore | Found By |
| --- | --- | --- | --- | --- | --- | --- | --- | --- | --- | --- | --- | --- | --- | --- | --- | --- | --- | --- | --- | --- | --- | --- | --- | --- | --- | --- | --- | --- | --- |
| K.LIFFDGEEAFVR.W | N | 85.67 | 1441.7241 | 12 | 0.6 | 721.8698 | 2 | 59.72 | 1 | F1:35753 | 28122021\_RID\_2349\_2C.raw | 3.5159E6 |  |  |  |  | 2.168E4 | 5 | 4 | 0 | 0 | 0 | 0 | 1 | 201 | 212 |  |  | PEAKS DB |
| R.NPIFPVYFLNTAR.W | Y | 81.81 | 1550.8245 | 13 | 0.8 | 776.4202 | 2 | 61.30 | 1 | F1:36625 | 28122021\_RID\_2349\_2C.raw | 3.417E6 |  |  |  | 1.318E5 |  | 2 | 1 | 0 | 0 | 0 | 1 | 0 | 260 | 272 |  |  | PEAKS DB |
| R.YPGSPGSYAVR.Q | N | 69.00 | 1152.5563 | 11 | 0.1 | 577.2855 | 2 | 28.48 | 1 | F1:11793 | 28122021\_RID\_2349\_2C.raw | 7.1634E6 |  |  |  |  | 3.2738E5 | 2 | 1 | 0 | 0 | 0 | 0 | 1 | 82 | 92 |  |  | PEAKS DB |
| R.TFSNIISTLNPLAK.R | N | 68.92 | 1517.8453 | 14 | 1.1 | 759.9308 | 2 | 58.63 | 1 | F1:35258 | 28122021\_RID\_2349\_2C.raw | 2.8095E6 |  |  |  | 1.6857E5 | 8.0094E4 | 3 | 1 | 0 | 0 | 0 | 1 | 1 | 123 | 136 |  |  | PEAKS DB |
| R.WSPSDSLYGSR.S | N | 68.08 | 1253.5676 | 11 | 0.3 | 627.7913 | 2 | 38.76 | 1 | F1:20122 | 28122021\_RID\_2349\_2C.raw | 4.7528E6 |  |  |  |  |  | 1 | 1 | 0 | 0 | 0 | 0 | 0 | 213 | 223 |  |  | PEAKS DB |
| K.YFPPQLDGK.V | N | 50.09 | 1063.5338 | 9 | 0.9 | 532.7747 | 2 | 36.56 | 1 | F1:18056 | 28122021\_RID\_2349\_2C.raw | 5.8019E6 |  |  |  |  |  | 1 | 1 | 0 | 0 | 0 | 0 | 0 | 149 | 157 |  |  | PEAKS DB |
| R.LQGLQAGWLVEEDTFQSHTPYGYR.T | N | 42.74 | 2794.3350 | 24 | 1.1 | 932.4532 | 3 | 54.10 | 1 | F1:31900 | 28122021\_RID\_2349\_2C.raw | 8.091E5 |  |  |  |  |  | 1 | 1 | 0 | 0 | 0 | 0 | 0 | 99 | 122 |  |  | PEAKS DB |
| total 7 peptides |  |  |  |  |  |  |  |  |  |  |  |  |  |  |  |  |  |  |  |  |  |  |  |  |  |  |  |  |  |
| --- | --- | --- | --- | --- | --- | --- | --- | --- | --- | --- | --- | --- | --- | --- | --- | --- | --- | --- | --- | --- | --- | --- | --- | --- | --- | --- | --- | --- | --- |

ETE57948.1

back to list

  

| Protein Coverage
| Supporting Peptides
|

Protein Coverage:

Supporting Peptides:

| Peptide | Uniq | -10lgP | Mass | Length | ppm | m/z | z | RT | Fraction | Scan | Source File | Area F2 | Area F1 | Area F3 | Area F4 | Area F5 | Area F6 | #Feature | #Feature F2 | #Feature F1 | #Feature F3 | #Feature F4 | #Feature F5 | #Feature F6 | Start | End | PTM | AScore | Found By |
| --- | --- | --- | --- | --- | --- | --- | --- | --- | --- | --- | --- | --- | --- | --- | --- | --- | --- | --- | --- | --- | --- | --- | --- | --- | --- | --- | --- | --- | --- |
| R.GYSFTTTAER.E | Y | 73.07 | 1131.5197 | 10 | 0.2 | 566.7672 | 2 | 28.89 | 1 | F1:12159 | 28122021\_RID\_2349\_2C.raw | 9.9209E5 | 1.8624E4 |  |  |  |  | 2 | 1 | 1 | 0 | 0 | 0 | 0 | 182 | 191 |  |  | PEAKS DB |
| K.SYELPDGQVITIGNER.F | N | 71.59 | 1789.8846 | 16 | 0.9 | 895.9504 | 2 | 50.05 | 1 | F1:29089 | 28122021\_RID\_2349\_2C.raw | 6.9813E5 |  | 4.7278E5 |  | 1.5455E5 | 3.4204E5 | 4 | 1 | 0 | 1 | 0 | 1 | 1 | 224 | 239 |  |  | PEAKS DB |
| K.DSYVGDEAQSK.R | N | 71.53 | 1197.5149 | 11 | 0.8 | 599.7652 | 2 | 21.07 | 1 | F1:6133 | 28122021\_RID\_2349\_2C.raw | 9.3415E5 |  |  |  |  |  | 1 | 1 | 0 | 0 | 0 | 0 | 0 | 36 | 46 |  |  | PEAKS DB |
| R.HQGVMVGMGQK.D | N | 65.34 | 1170.5638 | 11 | 0.1 | 586.2892 | 2 | 20.03 | 1 | F1:5302 | 28122021\_RID\_2349\_2C.raw | 1.0514E5 |  |  |  |  |  | 1 | 1 | 0 | 0 | 0 | 0 | 0 | 25 | 35 |  |  | PEAKS DB |
| R.AVFPSIVGRPR.H | N | 62.54 | 1197.6981 | 11 | 0.6 | 599.8567 | 2 | 34.45 | 1 | F1:16654 | 28122021\_RID\_2349\_2C.raw | 5.9717E5 |  |  |  |  |  | 1 | 1 | 0 | 0 | 0 | 0 | 0 | 14 | 24 |  |  | PEAKS DB |
| K.AGFAGDDAPR.A | N | 60.47 | 975.4410 | 10 | 0.3 | 488.7279 | 2 | 22.55 | 1 | F1:7091 | 28122021\_RID\_2349\_2C.raw | 1.4103E6 |  |  |  |  |  | 1 | 1 | 0 | 0 | 0 | 0 | 0 | 4 | 13 |  |  | PEAKS DB |
| R.HQGVM(+15.99)VGMGQK.D | N | 55.56 | 1186.5587 | 11 | -0.4 | 396.5267 | 3 | 16.87 | 1 | F1:3534 | 28122021\_RID\_2349\_2C.raw | 0 |  |  |  |  |  | 0 | 0 | 0 | 0 | 0 | 0 | 0 | 25 | 35 | Oxidation (M) | M5:Oxidation (M):34.94 | PEAKS DB |
| K.DLYANTVLSGGTTM(+15.99)YPGIADR.M | Y | 54.04 | 2230.0576 | 21 | 0.2 | 1116.0363 | 2 | 53.98 | 1 | F1:31887 | 28122021\_RID\_2349\_2C.raw | 4.0971E4 |  |  |  |  |  | 1 | 1 | 0 | 0 | 0 | 0 | 0 | 277 | 297 | Oxidation (M) | M14:Oxidation (M):1000.00 | PEAKS DB |
| R.VAPEEHPVLLTEAPLNPK.A | Y | 53.40 | 1953.0570 | 18 | 0.5 | 652.0266 | 3 | 40.65 | 1 | F1:21676 | 28122021\_RID\_2349\_2C.raw | 1.9556E6 |  |  |  |  |  | 1 | 1 | 0 | 0 | 0 | 0 | 0 | 81 | 98 |  |  | PEAKS DB |
| K.DSYVGDEAQSKR.G | N | 52.16 | 1353.6160 | 12 | 0.7 | 677.8157 | 2 | 17.11 | 1 | F1:3748 | 28122021\_RID\_2349\_2C.raw | 1.2575E5 |  |  |  |  |  | 1 | 1 | 0 | 0 | 0 | 0 | 0 | 36 | 47 |  |  | PEAKS DB |
| R.DLTDYLMK.I | N | 43.76 | 997.4790 | 8 | -0.1 | 499.7467 | 2 | 52.05 | 5 | F5:28689 | 31122021\_RID\_2349\_5C.raw |  |  |  |  | 9.6944E4 |  | 1 | 0 | 0 | 0 | 0 | 1 | 0 | 169 | 176 |  |  | PEAKS DB |
| total 11 peptides |  |  |  |  |  |  |  |  |  |  |  |  |  |  |  |  |  |  |  |  |  |  |  |  |  |  |  |  |  |
| --- | --- | --- | --- | --- | --- | --- | --- | --- | --- | --- | --- | --- | --- | --- | --- | --- | --- | --- | --- | --- | --- | --- | --- | --- | --- | --- | --- | --- | --- |

LAA58652.1

back to list

  

| Protein Coverage
| Supporting Peptides
|

Protein Coverage:

Supporting Peptides:

| Peptide | Uniq | -10lgP | Mass | Length | ppm | m/z | z | RT | Fraction | Scan | Source File | Area F2 | Area F1 | Area F3 | Area F4 | Area F5 | Area F6 | #Feature | #Feature F2 | #Feature F1 | #Feature F3 | #Feature F4 | #Feature F5 | #Feature F6 | Start | End | PTM | AScore | Found By |
| --- | --- | --- | --- | --- | --- | --- | --- | --- | --- | --- | --- | --- | --- | --- | --- | --- | --- | --- | --- | --- | --- | --- | --- | --- | --- | --- | --- | --- | --- |
| R.GYSFTTTAER.E | Y | 73.07 | 1131.5197 | 10 | 0.2 | 566.7672 | 2 | 28.89 | 1 | F1:12159 | 28122021\_RID\_2349\_2C.raw | 9.9209E5 | 1.8624E4 |  |  |  |  | 2 | 1 | 1 | 0 | 0 | 0 | 0 | 197 | 206 |  |  | PEAKS DB |
| K.SYELPDGQVITIGNER.F | N | 71.59 | 1789.8846 | 16 | 0.9 | 895.9504 | 2 | 50.05 | 1 | F1:29089 | 28122021\_RID\_2349\_2C.raw | 6.9813E5 |  | 4.7278E5 |  | 1.5455E5 | 3.4204E5 | 4 | 1 | 0 | 1 | 0 | 1 | 1 | 239 | 254 |  |  | PEAKS DB |
| K.DSYVGDEAQSK.R | N | 71.53 | 1197.5149 | 11 | 0.8 | 599.7652 | 2 | 21.07 | 1 | F1:6133 | 28122021\_RID\_2349\_2C.raw | 9.3415E5 |  |  |  |  |  | 1 | 1 | 0 | 0 | 0 | 0 | 0 | 51 | 61 |  |  | PEAKS DB |
| R.HQGVMVGMGQK.D | N | 65.34 | 1170.5638 | 11 | 0.1 | 586.2892 | 2 | 20.03 | 1 | F1:5302 | 28122021\_RID\_2349\_2C.raw | 1.0514E5 |  |  |  |  |  | 1 | 1 | 0 | 0 | 0 | 0 | 0 | 40 | 50 |  |  | PEAKS DB |
| R.AVFPSIVGRPR.H | N | 62.54 | 1197.6981 | 11 | 0.6 | 599.8567 | 2 | 34.45 | 1 | F1:16654 | 28122021\_RID\_2349\_2C.raw | 5.9717E5 |  |  |  |  |  | 1 | 1 | 0 | 0 | 0 | 0 | 0 | 29 | 39 |  |  | PEAKS DB |
| K.AGFAGDDAPR.A | N | 60.47 | 975.4410 | 10 | 0.3 | 488.7279 | 2 | 22.55 | 1 | F1:7091 | 28122021\_RID\_2349\_2C.raw | 1.4103E6 |  |  |  |  |  | 1 | 1 | 0 | 0 | 0 | 0 | 0 | 19 | 28 |  |  | PEAKS DB |
| R.HQGVM(+15.99)VGMGQK.D | N | 55.56 | 1186.5587 | 11 | -0.4 | 396.5267 | 3 | 16.87 | 1 | F1:3534 | 28122021\_RID\_2349\_2C.raw | 0 |  |  |  |  |  | 0 | 0 | 0 | 0 | 0 | 0 | 0 | 40 | 50 | Oxidation (M) | M5:Oxidation (M):34.94 | PEAKS DB |
| K.DLYANTVLSGGTTM(+15.99)YPGIADR.M | Y | 54.04 | 2230.0576 | 21 | 0.2 | 1116.0363 | 2 | 53.98 | 1 | F1:31887 | 28122021\_RID\_2349\_2C.raw | 4.0971E4 |  |  |  |  |  | 1 | 1 | 0 | 0 | 0 | 0 | 0 | 292 | 312 | Oxidation (M) | M14:Oxidation (M):1000.00 | PEAKS DB |
| R.VAPEEHPVLLTEAPLNPK.A | Y | 53.40 | 1953.0570 | 18 | 0.5 | 652.0266 | 3 | 40.65 | 1 | F1:21676 | 28122021\_RID\_2349\_2C.raw | 1.9556E6 |  |  |  |  |  | 1 | 1 | 0 | 0 | 0 | 0 | 0 | 96 | 113 |  |  | PEAKS DB |
| K.DSYVGDEAQSKR.G | N | 52.16 | 1353.6160 | 12 | 0.7 | 677.8157 | 2 | 17.11 | 1 | F1:3748 | 28122021\_RID\_2349\_2C.raw | 1.2575E5 |  |  |  |  |  | 1 | 1 | 0 | 0 | 0 | 0 | 0 | 51 | 62 |  |  | PEAKS DB |
| R.DLTDYLMK.I | N | 43.76 | 997.4790 | 8 | -0.1 | 499.7467 | 2 | 52.05 | 5 | F5:28689 | 31122021\_RID\_2349\_5C.raw |  |  |  |  | 9.6944E4 |  | 1 | 0 | 0 | 0 | 0 | 1 | 0 | 184 | 191 |  |  | PEAKS DB |
| total 11 peptides |  |  |  |  |  |  |  |  |  |  |  |  |  |  |  |  |  |  |  |  |  |  |  |  |  |  |  |  |  |
| --- | --- | --- | --- | --- | --- | --- | --- | --- | --- | --- | --- | --- | --- | --- | --- | --- | --- | --- | --- | --- | --- | --- | --- | --- | --- | --- | --- | --- | --- |

JAC96363.1

back to list

  

| Protein Coverage
| Supporting Peptides
|

Protein Coverage:

Supporting Peptides:

| Peptide | Uniq | -10lgP | Mass | Length | ppm | m/z | z | RT | Fraction | Scan | Source File | Area F2 | Area F1 | Area F3 | Area F4 | Area F5 | Area F6 | #Feature | #Feature F2 | #Feature F1 | #Feature F3 | #Feature F4 | #Feature F5 | #Feature F6 | Start | End | PTM | AScore | Found By |
| --- | --- | --- | --- | --- | --- | --- | --- | --- | --- | --- | --- | --- | --- | --- | --- | --- | --- | --- | --- | --- | --- | --- | --- | --- | --- | --- | --- | --- | --- |
| R.GYSFTTTAER.E | Y | 73.07 | 1131.5197 | 10 | 0.2 | 566.7672 | 2 | 28.89 | 1 | F1:12159 | 28122021\_RID\_2349\_2C.raw | 9.9209E5 | 1.8624E4 |  |  |  |  | 2 | 1 | 1 | 0 | 0 | 0 | 0 | 197 | 206 |  |  | PEAKS DB |
| K.SYELPDGQVITIGNER.F | N | 71.59 | 1789.8846 | 16 | 0.9 | 895.9504 | 2 | 50.05 | 1 | F1:29089 | 28122021\_RID\_2349\_2C.raw | 6.9813E5 |  | 4.7278E5 |  | 1.5455E5 | 3.4204E5 | 4 | 1 | 0 | 1 | 0 | 1 | 1 | 239 | 254 |  |  | PEAKS DB |
| K.DSYVGDEAQSK.R | N | 71.53 | 1197.5149 | 11 | 0.8 | 599.7652 | 2 | 21.07 | 1 | F1:6133 | 28122021\_RID\_2349\_2C.raw | 9.3415E5 |  |  |  |  |  | 1 | 1 | 0 | 0 | 0 | 0 | 0 | 51 | 61 |  |  | PEAKS DB |
| R.HQGVMVGMGQK.D | N | 65.34 | 1170.5638 | 11 | 0.1 | 586.2892 | 2 | 20.03 | 1 | F1:5302 | 28122021\_RID\_2349\_2C.raw | 1.0514E5 |  |  |  |  |  | 1 | 1 | 0 | 0 | 0 | 0 | 0 | 40 | 50 |  |  | PEAKS DB |
| R.AVFPSIVGRPR.H | N | 62.54 | 1197.6981 | 11 | 0.6 | 599.8567 | 2 | 34.45 | 1 | F1:16654 | 28122021\_RID\_2349\_2C.raw | 5.9717E5 |  |  |  |  |  | 1 | 1 | 0 | 0 | 0 | 0 | 0 | 29 | 39 |  |  | PEAKS DB |
| K.AGFAGDDAPR.A | N | 60.47 | 975.4410 | 10 | 0.3 | 488.7279 | 2 | 22.55 | 1 | F1:7091 | 28122021\_RID\_2349\_2C.raw | 1.4103E6 |  |  |  |  |  | 1 | 1 | 0 | 0 | 0 | 0 | 0 | 19 | 28 |  |  | PEAKS DB |
| R.HQGVM(+15.99)VGMGQK.D | N | 55.56 | 1186.5587 | 11 | -0.4 | 396.5267 | 3 | 16.87 | 1 | F1:3534 | 28122021\_RID\_2349\_2C.raw | 0 |  |  |  |  |  | 0 | 0 | 0 | 0 | 0 | 0 | 0 | 40 | 50 | Oxidation (M) | M5:Oxidation (M):34.94 | PEAKS DB |
| K.DLYANTVLSGGTTM(+15.99)YPGIADR.M | Y | 54.04 | 2230.0576 | 21 | 0.2 | 1116.0363 | 2 | 53.98 | 1 | F1:31887 | 28122021\_RID\_2349\_2C.raw | 4.0971E4 |  |  |  |  |  | 1 | 1 | 0 | 0 | 0 | 0 | 0 | 292 | 312 | Oxidation (M) | M14:Oxidation (M):1000.00 | PEAKS DB |
| R.VAPEEHPVLLTEAPLNPK.A | Y | 53.40 | 1953.0570 | 18 | 0.5 | 652.0266 | 3 | 40.65 | 1 | F1:21676 | 28122021\_RID\_2349\_2C.raw | 1.9556E6 |  |  |  |  |  | 1 | 1 | 0 | 0 | 0 | 0 | 0 | 96 | 113 |  |  | PEAKS DB |
| K.DSYVGDEAQSKR.G | N | 52.16 | 1353.6160 | 12 | 0.7 | 677.8157 | 2 | 17.11 | 1 | F1:3748 | 28122021\_RID\_2349\_2C.raw | 1.2575E5 |  |  |  |  |  | 1 | 1 | 0 | 0 | 0 | 0 | 0 | 51 | 62 |  |  | PEAKS DB |
| R.DLTDYLMK.I | N | 43.76 | 997.4790 | 8 | -0.1 | 499.7467 | 2 | 52.05 | 5 | F5:28689 | 31122021\_RID\_2349\_5C.raw |  |  |  |  | 9.6944E4 |  | 1 | 0 | 0 | 0 | 0 | 1 | 0 | 184 | 191 |  |  | PEAKS DB |
| total 11 peptides |  |  |  |  |  |  |  |  |  |  |  |  |  |  |  |  |  |  |  |  |  |  |  |  |  |  |  |  |  |
| --- | --- | --- | --- | --- | --- | --- | --- | --- | --- | --- | --- | --- | --- | --- | --- | --- | --- | --- | --- | --- | --- | --- | --- | --- | --- | --- | --- | --- | --- |

JAA97900.1

back to list

  

| Protein Coverage
| Supporting Peptides
|

Protein Coverage:

Supporting Peptides:

| Peptide | Uniq | -10lgP | Mass | Length | ppm | m/z | z | RT | Fraction | Scan | Source File | Area F2 | Area F1 | Area F3 | Area F4 | Area F5 | Area F6 | #Feature | #Feature F2 | #Feature F1 | #Feature F3 | #Feature F4 | #Feature F5 | #Feature F6 | Start | End | PTM | AScore | Found By |
| --- | --- | --- | --- | --- | --- | --- | --- | --- | --- | --- | --- | --- | --- | --- | --- | --- | --- | --- | --- | --- | --- | --- | --- | --- | --- | --- | --- | --- | --- |
| R.GYSFTTTAER.E | Y | 73.07 | 1131.5197 | 10 | 0.2 | 566.7672 | 2 | 28.89 | 1 | F1:12159 | 28122021\_RID\_2349\_2C.raw | 9.9209E5 | 1.8624E4 |  |  |  |  | 2 | 1 | 1 | 0 | 0 | 0 | 0 | 197 | 206 |  |  | PEAKS DB |
| K.SYELPDGQVITIGNER.F | N | 71.59 | 1789.8846 | 16 | 0.9 | 895.9504 | 2 | 50.05 | 1 | F1:29089 | 28122021\_RID\_2349\_2C.raw | 6.9813E5 |  | 4.7278E5 |  | 1.5455E5 | 3.4204E5 | 4 | 1 | 0 | 1 | 0 | 1 | 1 | 239 | 254 |  |  | PEAKS DB |
| K.DSYVGDEAQSK.R | N | 71.53 | 1197.5149 | 11 | 0.8 | 599.7652 | 2 | 21.07 | 1 | F1:6133 | 28122021\_RID\_2349\_2C.raw | 9.3415E5 |  |  |  |  |  | 1 | 1 | 0 | 0 | 0 | 0 | 0 | 51 | 61 |  |  | PEAKS DB |
| R.HQGVMVGMGQK.D | N | 65.34 | 1170.5638 | 11 | 0.1 | 586.2892 | 2 | 20.03 | 1 | F1:5302 | 28122021\_RID\_2349\_2C.raw | 1.0514E5 |  |  |  |  |  | 1 | 1 | 0 | 0 | 0 | 0 | 0 | 40 | 50 |  |  | PEAKS DB |
| R.AVFPSIVGRPR.H | N | 62.54 | 1197.6981 | 11 | 0.6 | 599.8567 | 2 | 34.45 | 1 | F1:16654 | 28122021\_RID\_2349\_2C.raw | 5.9717E5 |  |  |  |  |  | 1 | 1 | 0 | 0 | 0 | 0 | 0 | 29 | 39 |  |  | PEAKS DB |
| K.AGFAGDDAPR.A | N | 60.47 | 975.4410 | 10 | 0.3 | 488.7279 | 2 | 22.55 | 1 | F1:7091 | 28122021\_RID\_2349\_2C.raw | 1.4103E6 |  |  |  |  |  | 1 | 1 | 0 | 0 | 0 | 0 | 0 | 19 | 28 |  |  | PEAKS DB |
| R.HQGVM(+15.99)VGMGQK.D | N | 55.56 | 1186.5587 | 11 | -0.4 | 396.5267 | 3 | 16.87 | 1 | F1:3534 | 28122021\_RID\_2349\_2C.raw | 0 |  |  |  |  |  | 0 | 0 | 0 | 0 | 0 | 0 | 0 | 40 | 50 | Oxidation (M) | M5:Oxidation (M):34.94 | PEAKS DB |
| K.DLYANTVLSGGTTM(+15.99)YPGIADR.M | Y | 54.04 | 2230.0576 | 21 | 0.2 | 1116.0363 | 2 | 53.98 | 1 | F1:31887 | 28122021\_RID\_2349\_2C.raw | 4.0971E4 |  |  |  |  |  | 1 | 1 | 0 | 0 | 0 | 0 | 0 | 292 | 312 | Oxidation (M) | M14:Oxidation (M):1000.00 | PEAKS DB |
| R.VAPEEHPVLLTEAPLNPK.A | Y | 53.40 | 1953.0570 | 18 | 0.5 | 652.0266 | 3 | 40.65 | 1 | F1:21676 | 28122021\_RID\_2349\_2C.raw | 1.9556E6 |  |  |  |  |  | 1 | 1 | 0 | 0 | 0 | 0 | 0 | 96 | 113 |  |  | PEAKS DB |
| K.DSYVGDEAQSKR.G | N | 52.16 | 1353.6160 | 12 | 0.7 | 677.8157 | 2 | 17.11 | 1 | F1:3748 | 28122021\_RID\_2349\_2C.raw | 1.2575E5 |  |  |  |  |  | 1 | 1 | 0 | 0 | 0 | 0 | 0 | 51 | 62 |  |  | PEAKS DB |
| R.DLTDYLMK.I | N | 43.76 | 997.4790 | 8 | -0.1 | 499.7467 | 2 | 52.05 | 5 | F5:28689 | 31122021\_RID\_2349\_5C.raw |  |  |  |  | 9.6944E4 |  | 1 | 0 | 0 | 0 | 0 | 1 | 0 | 184 | 191 |  |  | PEAKS DB |
| total 11 peptides |  |  |  |  |  |  |  |  |  |  |  |  |  |  |  |  |  |  |  |  |  |  |  |  |  |  |  |  |  |
| --- | --- | --- | --- | --- | --- | --- | --- | --- | --- | --- | --- | --- | --- | --- | --- | --- | --- | --- | --- | --- | --- | --- | --- | --- | --- | --- | --- | --- | --- |

LAA21099.1

back to list

  

| Protein Coverage
| Supporting Peptides
|

Protein Coverage:

Supporting Peptides:

| Peptide | Uniq | -10lgP | Mass | Length | ppm | m/z | z | RT | Fraction | Scan | Source File | Area F2 | Area F1 | Area F3 | Area F4 | Area F5 | Area F6 | #Feature | #Feature F2 | #Feature F1 | #Feature F3 | #Feature F4 | #Feature F5 | #Feature F6 | Start | End | PTM | AScore | Found By |
| --- | --- | --- | --- | --- | --- | --- | --- | --- | --- | --- | --- | --- | --- | --- | --- | --- | --- | --- | --- | --- | --- | --- | --- | --- | --- | --- | --- | --- | --- |
| R.GYSFTTTAER.E | Y | 73.07 | 1131.5197 | 10 | 0.2 | 566.7672 | 2 | 28.89 | 1 | F1:12159 | 28122021\_RID\_2349\_2C.raw | 9.9209E5 | 1.8624E4 |  |  |  |  | 2 | 1 | 1 | 0 | 0 | 0 | 0 | 197 | 206 |  |  | PEAKS DB |
| K.SYELPDGQVITIGNER.F | N | 71.59 | 1789.8846 | 16 | 0.9 | 895.9504 | 2 | 50.05 | 1 | F1:29089 | 28122021\_RID\_2349\_2C.raw | 6.9813E5 |  | 4.7278E5 |  | 1.5455E5 | 3.4204E5 | 4 | 1 | 0 | 1 | 0 | 1 | 1 | 239 | 254 |  |  | PEAKS DB |
| K.DSYVGDEAQSK.R | N | 71.53 | 1197.5149 | 11 | 0.8 | 599.7652 | 2 | 21.07 | 1 | F1:6133 | 28122021\_RID\_2349\_2C.raw | 9.3415E5 |  |  |  |  |  | 1 | 1 | 0 | 0 | 0 | 0 | 0 | 51 | 61 |  |  | PEAKS DB |
| R.HQGVMVGMGQK.D | N | 65.34 | 1170.5638 | 11 | 0.1 | 586.2892 | 2 | 20.03 | 1 | F1:5302 | 28122021\_RID\_2349\_2C.raw | 1.0514E5 |  |  |  |  |  | 1 | 1 | 0 | 0 | 0 | 0 | 0 | 40 | 50 |  |  | PEAKS DB |
| R.AVFPSIVGRPR.H | N | 62.54 | 1197.6981 | 11 | 0.6 | 599.8567 | 2 | 34.45 | 1 | F1:16654 | 28122021\_RID\_2349\_2C.raw | 5.9717E5 |  |  |  |  |  | 1 | 1 | 0 | 0 | 0 | 0 | 0 | 29 | 39 |  |  | PEAKS DB |
| K.AGFAGDDAPR.A | N | 60.47 | 975.4410 | 10 | 0.3 | 488.7279 | 2 | 22.55 | 1 | F1:7091 | 28122021\_RID\_2349\_2C.raw | 1.4103E6 |  |  |  |  |  | 1 | 1 | 0 | 0 | 0 | 0 | 0 | 19 | 28 |  |  | PEAKS DB |
| R.HQGVM(+15.99)VGMGQK.D | N | 55.56 | 1186.5587 | 11 | -0.4 | 396.5267 | 3 | 16.87 | 1 | F1:3534 | 28122021\_RID\_2349\_2C.raw | 0 |  |  |  |  |  | 0 | 0 | 0 | 0 | 0 | 0 | 0 | 40 | 50 | Oxidation (M) | M5:Oxidation (M):34.94 | PEAKS DB |
| K.DLYANTVLSGGTTM(+15.99)YPGIADR.M | Y | 54.04 | 2230.0576 | 21 | 0.2 | 1116.0363 | 2 | 53.98 | 1 | F1:31887 | 28122021\_RID\_2349\_2C.raw | 4.0971E4 |  |  |  |  |  | 1 | 1 | 0 | 0 | 0 | 0 | 0 | 292 | 312 | Oxidation (M) | M14:Oxidation (M):1000.00 | PEAKS DB |
| R.VAPEEHPVLLTEAPLNPK.A | Y | 53.40 | 1953.0570 | 18 | 0.5 | 652.0266 | 3 | 40.65 | 1 | F1:21676 | 28122021\_RID\_2349\_2C.raw | 1.9556E6 |  |  |  |  |  | 1 | 1 | 0 | 0 | 0 | 0 | 0 | 96 | 113 |  |  | PEAKS DB |
| K.DSYVGDEAQSKR.G | N | 52.16 | 1353.6160 | 12 | 0.7 | 677.8157 | 2 | 17.11 | 1 | F1:3748 | 28122021\_RID\_2349\_2C.raw | 1.2575E5 |  |  |  |  |  | 1 | 1 | 0 | 0 | 0 | 0 | 0 | 51 | 62 |  |  | PEAKS DB |
| R.DLTDYLMK.I | N | 43.76 | 997.4790 | 8 | -0.1 | 499.7467 | 2 | 52.05 | 5 | F5:28689 | 31122021\_RID\_2349\_5C.raw |  |  |  |  | 9.6944E4 |  | 1 | 0 | 0 | 0 | 0 | 1 | 0 | 184 | 191 |  |  | PEAKS DB |
| total 11 peptides |  |  |  |  |  |  |  |  |  |  |  |  |  |  |  |  |  |  |  |  |  |  |  |  |  |  |  |  |  |
| --- | --- | --- | --- | --- | --- | --- | --- | --- | --- | --- | --- | --- | --- | --- | --- | --- | --- | --- | --- | --- | --- | --- | --- | --- | --- | --- | --- | --- | --- |

JAI10342.1

back to list

  

| Protein Coverage
| Supporting Peptides
|

Protein Coverage:

Supporting Peptides:

| Peptide | Uniq | -10lgP | Mass | Length | ppm | m/z | z | RT | Fraction | Scan | Source File | Area F2 | Area F1 | Area F3 | Area F4 | Area F5 | Area F6 | #Feature | #Feature F2 | #Feature F1 | #Feature F3 | #Feature F4 | #Feature F5 | #Feature F6 | Start | End | PTM | AScore | Found By |
| --- | --- | --- | --- | --- | --- | --- | --- | --- | --- | --- | --- | --- | --- | --- | --- | --- | --- | --- | --- | --- | --- | --- | --- | --- | --- | --- | --- | --- | --- |
| R.GYSFTTTAER.E | Y | 73.07 | 1131.5197 | 10 | 0.2 | 566.7672 | 2 | 28.89 | 1 | F1:12159 | 28122021\_RID\_2349\_2C.raw | 9.9209E5 | 1.8624E4 |  |  |  |  | 2 | 1 | 1 | 0 | 0 | 0 | 0 | 197 | 206 |  |  | PEAKS DB |
| K.SYELPDGQVITIGNER.F | N | 71.59 | 1789.8846 | 16 | 0.9 | 895.9504 | 2 | 50.05 | 1 | F1:29089 | 28122021\_RID\_2349\_2C.raw | 6.9813E5 |  | 4.7278E5 |  | 1.5455E5 | 3.4204E5 | 4 | 1 | 0 | 1 | 0 | 1 | 1 | 239 | 254 |  |  | PEAKS DB |
| K.DSYVGDEAQSK.R | N | 71.53 | 1197.5149 | 11 | 0.8 | 599.7652 | 2 | 21.07 | 1 | F1:6133 | 28122021\_RID\_2349\_2C.raw | 9.3415E5 |  |  |  |  |  | 1 | 1 | 0 | 0 | 0 | 0 | 0 | 51 | 61 |  |  | PEAKS DB |
| R.HQGVMVGMGQK.D | N | 65.34 | 1170.5638 | 11 | 0.1 | 586.2892 | 2 | 20.03 | 1 | F1:5302 | 28122021\_RID\_2349\_2C.raw | 1.0514E5 |  |  |  |  |  | 1 | 1 | 0 | 0 | 0 | 0 | 0 | 40 | 50 |  |  | PEAKS DB |
| R.AVFPSIVGRPR.H | N | 62.54 | 1197.6981 | 11 | 0.6 | 599.8567 | 2 | 34.45 | 1 | F1:16654 | 28122021\_RID\_2349\_2C.raw | 5.9717E5 |  |  |  |  |  | 1 | 1 | 0 | 0 | 0 | 0 | 0 | 29 | 39 |  |  | PEAKS DB |
| K.AGFAGDDAPR.A | N | 60.47 | 975.4410 | 10 | 0.3 | 488.7279 | 2 | 22.55 | 1 | F1:7091 | 28122021\_RID\_2349\_2C.raw | 1.4103E6 |  |  |  |  |  | 1 | 1 | 0 | 0 | 0 | 0 | 0 | 19 | 28 |  |  | PEAKS DB |
| R.HQGVM(+15.99)VGMGQK.D | N | 55.56 | 1186.5587 | 11 | -0.4 | 396.5267 | 3 | 16.87 | 1 | F1:3534 | 28122021\_RID\_2349\_2C.raw | 0 |  |  |  |  |  | 0 | 0 | 0 | 0 | 0 | 0 | 0 | 40 | 50 | Oxidation (M) | M5:Oxidation (M):34.94 | PEAKS DB |
| K.DLYANTVLSGGTTM(+15.99)YPGIADR.M | Y | 54.04 | 2230.0576 | 21 | 0.2 | 1116.0363 | 2 | 53.98 | 1 | F1:31887 | 28122021\_RID\_2349\_2C.raw | 4.0971E4 |  |  |  |  |  | 1 | 1 | 0 | 0 | 0 | 0 | 0 | 292 | 312 | Oxidation (M) | M14:Oxidation (M):1000.00 | PEAKS DB |
| R.VAPEEHPVLLTEAPLNPK.A | Y | 53.40 | 1953.0570 | 18 | 0.5 | 652.0266 | 3 | 40.65 | 1 | F1:21676 | 28122021\_RID\_2349\_2C.raw | 1.9556E6 |  |  |  |  |  | 1 | 1 | 0 | 0 | 0 | 0 | 0 | 96 | 113 |  |  | PEAKS DB |
| K.DSYVGDEAQSKR.G | N | 52.16 | 1353.6160 | 12 | 0.7 | 677.8157 | 2 | 17.11 | 1 | F1:3748 | 28122021\_RID\_2349\_2C.raw | 1.2575E5 |  |  |  |  |  | 1 | 1 | 0 | 0 | 0 | 0 | 0 | 51 | 62 |  |  | PEAKS DB |
| R.DLTDYLMK.I | N | 43.76 | 997.4790 | 8 | -0.1 | 499.7467 | 2 | 52.05 | 5 | F5:28689 | 31122021\_RID\_2349\_5C.raw |  |  |  |  | 9.6944E4 |  | 1 | 0 | 0 | 0 | 0 | 1 | 0 | 184 | 191 |  |  | PEAKS DB |
| total 11 peptides |  |  |  |  |  |  |  |  |  |  |  |  |  |  |  |  |  |  |  |  |  |  |  |  |  |  |  |  |  |
| --- | --- | --- | --- | --- | --- | --- | --- | --- | --- | --- | --- | --- | --- | --- | --- | --- | --- | --- | --- | --- | --- | --- | --- | --- | --- | --- | --- | --- | --- |

JAG68843.1

back to list

  

| Protein Coverage
| Supporting Peptides
|

Protein Coverage:

Supporting Peptides:

| Peptide | Uniq | -10lgP | Mass | Length | ppm | m/z | z | RT | Fraction | Scan | Source File | Area F2 | Area F1 | Area F3 | Area F4 | Area F5 | Area F6 | #Feature | #Feature F2 | #Feature F1 | #Feature F3 | #Feature F4 | #Feature F5 | #Feature F6 | Start | End | PTM | AScore | Found By |
| --- | --- | --- | --- | --- | --- | --- | --- | --- | --- | --- | --- | --- | --- | --- | --- | --- | --- | --- | --- | --- | --- | --- | --- | --- | --- | --- | --- | --- | --- |
| R.GYSFTTTAER.E | Y | 73.07 | 1131.5197 | 10 | 0.2 | 566.7672 | 2 | 28.89 | 1 | F1:12159 | 28122021\_RID\_2349\_2C.raw | 9.9209E5 | 1.8624E4 |  |  |  |  | 2 | 1 | 1 | 0 | 0 | 0 | 0 | 197 | 206 |  |  | PEAKS DB |
| K.SYELPDGQVITIGNER.F | N | 71.59 | 1789.8846 | 16 | 0.9 | 895.9504 | 2 | 50.05 | 1 | F1:29089 | 28122021\_RID\_2349\_2C.raw | 6.9813E5 |  | 4.7278E5 |  | 1.5455E5 | 3.4204E5 | 4 | 1 | 0 | 1 | 0 | 1 | 1 | 239 | 254 |  |  | PEAKS DB |
| K.DSYVGDEAQSK.R | N | 71.53 | 1197.5149 | 11 | 0.8 | 599.7652 | 2 | 21.07 | 1 | F1:6133 | 28122021\_RID\_2349\_2C.raw | 9.3415E5 |  |  |  |  |  | 1 | 1 | 0 | 0 | 0 | 0 | 0 | 51 | 61 |  |  | PEAKS DB |
| R.HQGVMVGMGQK.D | N | 65.34 | 1170.5638 | 11 | 0.1 | 586.2892 | 2 | 20.03 | 1 | F1:5302 | 28122021\_RID\_2349\_2C.raw | 1.0514E5 |  |  |  |  |  | 1 | 1 | 0 | 0 | 0 | 0 | 0 | 40 | 50 |  |  | PEAKS DB |
| R.AVFPSIVGRPR.H | N | 62.54 | 1197.6981 | 11 | 0.6 | 599.8567 | 2 | 34.45 | 1 | F1:16654 | 28122021\_RID\_2349\_2C.raw | 5.9717E5 |  |  |  |  |  | 1 | 1 | 0 | 0 | 0 | 0 | 0 | 29 | 39 |  |  | PEAKS DB |
| K.AGFAGDDAPR.A | N | 60.47 | 975.4410 | 10 | 0.3 | 488.7279 | 2 | 22.55 | 1 | F1:7091 | 28122021\_RID\_2349\_2C.raw | 1.4103E6 |  |  |  |  |  | 1 | 1 | 0 | 0 | 0 | 0 | 0 | 19 | 28 |  |  | PEAKS DB |
| R.HQGVM(+15.99)VGMGQK.D | N | 55.56 | 1186.5587 | 11 | -0.4 | 396.5267 | 3 | 16.87 | 1 | F1:3534 | 28122021\_RID\_2349\_2C.raw | 0 |  |  |  |  |  | 0 | 0 | 0 | 0 | 0 | 0 | 0 | 40 | 50 | Oxidation (M) | M5:Oxidation (M):34.94 | PEAKS DB |
| K.DLYANTVLSGGTTM(+15.99)YPGIADR.M | Y | 54.04 | 2230.0576 | 21 | 0.2 | 1116.0363 | 2 | 53.98 | 1 | F1:31887 | 28122021\_RID\_2349\_2C.raw | 4.0971E4 |  |  |  |  |  | 1 | 1 | 0 | 0 | 0 | 0 | 0 | 292 | 312 | Oxidation (M) | M14:Oxidation (M):1000.00 | PEAKS DB |
| R.VAPEEHPVLLTEAPLNPK.A | Y | 53.40 | 1953.0570 | 18 | 0.5 | 652.0266 | 3 | 40.65 | 1 | F1:21676 | 28122021\_RID\_2349\_2C.raw | 1.9556E6 |  |  |  |  |  | 1 | 1 | 0 | 0 | 0 | 0 | 0 | 96 | 113 |  |  | PEAKS DB |
| K.DSYVGDEAQSKR.G | N | 52.16 | 1353.6160 | 12 | 0.7 | 677.8157 | 2 | 17.11 | 1 | F1:3748 | 28122021\_RID\_2349\_2C.raw | 1.2575E5 |  |  |  |  |  | 1 | 1 | 0 | 0 | 0 | 0 | 0 | 51 | 62 |  |  | PEAKS DB |
| R.DLTDYLMK.I | N | 43.76 | 997.4790 | 8 | -0.1 | 499.7467 | 2 | 52.05 | 5 | F5:28689 | 31122021\_RID\_2349\_5C.raw |  |  |  |  | 9.6944E4 |  | 1 | 0 | 0 | 0 | 0 | 1 | 0 | 184 | 191 |  |  | PEAKS DB |
| total 11 peptides |  |  |  |  |  |  |  |  |  |  |  |  |  |  |  |  |  |  |  |  |  |  |  |  |  |  |  |  |  |
| --- | --- | --- | --- | --- | --- | --- | --- | --- | --- | --- | --- | --- | --- | --- | --- | --- | --- | --- | --- | --- | --- | --- | --- | --- | --- | --- | --- | --- | --- |

XP\_013921350.1

back to list

  

| Protein Coverage
| Supporting Peptides
|

Protein Coverage:

Supporting Peptides:

| Peptide | Uniq | -10lgP | Mass | Length | ppm | m/z | z | RT | Fraction | Scan | Source File | Area F2 | Area F1 | Area F3 | Area F4 | Area F5 | Area F6 | #Feature | #Feature F2 | #Feature F1 | #Feature F3 | #Feature F4 | #Feature F5 | #Feature F6 | Start | End | PTM | AScore | Found By |
| --- | --- | --- | --- | --- | --- | --- | --- | --- | --- | --- | --- | --- | --- | --- | --- | --- | --- | --- | --- | --- | --- | --- | --- | --- | --- | --- | --- | --- | --- |
| R.GYSFTTTAER.E | Y | 73.07 | 1131.5197 | 10 | 0.2 | 566.7672 | 2 | 28.89 | 1 | F1:12159 | 28122021\_RID\_2349\_2C.raw | 9.9209E5 | 1.8624E4 |  |  |  |  | 2 | 1 | 1 | 0 | 0 | 0 | 0 | 197 | 206 |  |  | PEAKS DB |
| K.SYELPDGQVITIGNER.F | N | 71.59 | 1789.8846 | 16 | 0.9 | 895.9504 | 2 | 50.05 | 1 | F1:29089 | 28122021\_RID\_2349\_2C.raw | 6.9813E5 |  | 4.7278E5 |  | 1.5455E5 | 3.4204E5 | 4 | 1 | 0 | 1 | 0 | 1 | 1 | 239 | 254 |  |  | PEAKS DB |
| K.DSYVGDEAQSK.R | N | 71.53 | 1197.5149 | 11 | 0.8 | 599.7652 | 2 | 21.07 | 1 | F1:6133 | 28122021\_RID\_2349\_2C.raw | 9.3415E5 |  |  |  |  |  | 1 | 1 | 0 | 0 | 0 | 0 | 0 | 51 | 61 |  |  | PEAKS DB |
| R.HQGVMVGMGQK.D | N | 65.34 | 1170.5638 | 11 | 0.1 | 586.2892 | 2 | 20.03 | 1 | F1:5302 | 28122021\_RID\_2349\_2C.raw | 1.0514E5 |  |  |  |  |  | 1 | 1 | 0 | 0 | 0 | 0 | 0 | 40 | 50 |  |  | PEAKS DB |
| R.AVFPSIVGRPR.H | N | 62.54 | 1197.6981 | 11 | 0.6 | 599.8567 | 2 | 34.45 | 1 | F1:16654 | 28122021\_RID\_2349\_2C.raw | 5.9717E5 |  |  |  |  |  | 1 | 1 | 0 | 0 | 0 | 0 | 0 | 29 | 39 |  |  | PEAKS DB |
| K.AGFAGDDAPR.A | N | 60.47 | 975.4410 | 10 | 0.3 | 488.7279 | 2 | 22.55 | 1 | F1:7091 | 28122021\_RID\_2349\_2C.raw | 1.4103E6 |  |  |  |  |  | 1 | 1 | 0 | 0 | 0 | 0 | 0 | 19 | 28 |  |  | PEAKS DB |
| R.HQGVM(+15.99)VGMGQK.D | N | 55.56 | 1186.5587 | 11 | -0.4 | 396.5267 | 3 | 16.87 | 1 | F1:3534 | 28122021\_RID\_2349\_2C.raw | 0 |  |  |  |  |  | 0 | 0 | 0 | 0 | 0 | 0 | 0 | 40 | 50 | Oxidation (M) | M5:Oxidation (M):34.94 | PEAKS DB |
| K.DLYANTVLSGGTTM(+15.99)YPGIADR.M | Y | 54.04 | 2230.0576 | 21 | 0.2 | 1116.0363 | 2 | 53.98 | 1 | F1:31887 | 28122021\_RID\_2349\_2C.raw | 4.0971E4 |  |  |  |  |  | 1 | 1 | 0 | 0 | 0 | 0 | 0 | 292 | 312 | Oxidation (M) | M14:Oxidation (M):1000.00 | PEAKS DB |
| R.VAPEEHPVLLTEAPLNPK.A | Y | 53.40 | 1953.0570 | 18 | 0.5 | 652.0266 | 3 | 40.65 | 1 | F1:21676 | 28122021\_RID\_2349\_2C.raw | 1.9556E6 |  |  |  |  |  | 1 | 1 | 0 | 0 | 0 | 0 | 0 | 96 | 113 |  |  | PEAKS DB |
| K.DSYVGDEAQSKR.G | N | 52.16 | 1353.6160 | 12 | 0.7 | 677.8157 | 2 | 17.11 | 1 | F1:3748 | 28122021\_RID\_2349\_2C.raw | 1.2575E5 |  |  |  |  |  | 1 | 1 | 0 | 0 | 0 | 0 | 0 | 51 | 62 |  |  | PEAKS DB |
| R.DLTDYLMK.I | N | 43.76 | 997.4790 | 8 | -0.1 | 499.7467 | 2 | 52.05 | 5 | F5:28689 | 31122021\_RID\_2349\_5C.raw |  |  |  |  | 9.6944E4 |  | 1 | 0 | 0 | 0 | 0 | 1 | 0 | 184 | 191 |  |  | PEAKS DB |
| total 11 peptides |  |  |  |  |  |  |  |  |  |  |  |  |  |  |  |  |  |  |  |  |  |  |  |  |  |  |  |  |  |
| --- | --- | --- | --- | --- | --- | --- | --- | --- | --- | --- | --- | --- | --- | --- | --- | --- | --- | --- | --- | --- | --- | --- | --- | --- | --- | --- | --- | --- | --- |

JAB54771.1

back to list

  

| Protein Coverage
| Supporting Peptides
|

Protein Coverage:

Supporting Peptides:

| Peptide | Uniq | -10lgP | Mass | Length | ppm | m/z | z | RT | Fraction | Scan | Source File | Area F2 | Area F1 | Area F3 | Area F4 | Area F5 | Area F6 | #Feature | #Feature F2 | #Feature F1 | #Feature F3 | #Feature F4 | #Feature F5 | #Feature F6 | Start | End | PTM | AScore | Found By |
| --- | --- | --- | --- | --- | --- | --- | --- | --- | --- | --- | --- | --- | --- | --- | --- | --- | --- | --- | --- | --- | --- | --- | --- | --- | --- | --- | --- | --- | --- |
| R.GYSFTTTAER.E | Y | 73.07 | 1131.5197 | 10 | 0.2 | 566.7672 | 2 | 28.89 | 1 | F1:12159 | 28122021\_RID\_2349\_2C.raw | 9.9209E5 | 1.8624E4 |  |  |  |  | 2 | 1 | 1 | 0 | 0 | 0 | 0 | 197 | 206 |  |  | PEAKS DB |
| K.SYELPDGQVITIGNER.F | N | 71.59 | 1789.8846 | 16 | 0.9 | 895.9504 | 2 | 50.05 | 1 | F1:29089 | 28122021\_RID\_2349\_2C.raw | 6.9813E5 |  | 4.7278E5 |  | 1.5455E5 | 3.4204E5 | 4 | 1 | 0 | 1 | 0 | 1 | 1 | 239 | 254 |  |  | PEAKS DB |
| K.DSYVGDEAQSK.R | N | 71.53 | 1197.5149 | 11 | 0.8 | 599.7652 | 2 | 21.07 | 1 | F1:6133 | 28122021\_RID\_2349\_2C.raw | 9.3415E5 |  |  |  |  |  | 1 | 1 | 0 | 0 | 0 | 0 | 0 | 51 | 61 |  |  | PEAKS DB |
| R.HQGVMVGMGQK.D | N | 65.34 | 1170.5638 | 11 | 0.1 | 586.2892 | 2 | 20.03 | 1 | F1:5302 | 28122021\_RID\_2349\_2C.raw | 1.0514E5 |  |  |  |  |  | 1 | 1 | 0 | 0 | 0 | 0 | 0 | 40 | 50 |  |  | PEAKS DB |
| R.AVFPSIVGRPR.H | N | 62.54 | 1197.6981 | 11 | 0.6 | 599.8567 | 2 | 34.45 | 1 | F1:16654 | 28122021\_RID\_2349\_2C.raw | 5.9717E5 |  |  |  |  |  | 1 | 1 | 0 | 0 | 0 | 0 | 0 | 29 | 39 |  |  | PEAKS DB |
| K.AGFAGDDAPR.A | N | 60.47 | 975.4410 | 10 | 0.3 | 488.7279 | 2 | 22.55 | 1 | F1:7091 | 28122021\_RID\_2349\_2C.raw | 1.4103E6 |  |  |  |  |  | 1 | 1 | 0 | 0 | 0 | 0 | 0 | 19 | 28 |  |  | PEAKS DB |
| R.HQGVM(+15.99)VGMGQK.D | N | 55.56 | 1186.5587 | 11 | -0.4 | 396.5267 | 3 | 16.87 | 1 | F1:3534 | 28122021\_RID\_2349\_2C.raw | 0 |  |  |  |  |  | 0 | 0 | 0 | 0 | 0 | 0 | 0 | 40 | 50 | Oxidation (M) | M5:Oxidation (M):34.94 | PEAKS DB |
| K.DLYANTVLSGGTTM(+15.99)YPGIADR.M | Y | 54.04 | 2230.0576 | 21 | 0.2 | 1116.0363 | 2 | 53.98 | 1 | F1:31887 | 28122021\_RID\_2349\_2C.raw | 4.0971E4 |  |  |  |  |  | 1 | 1 | 0 | 0 | 0 | 0 | 0 | 292 | 312 | Oxidation (M) | M14:Oxidation (M):1000.00 | PEAKS DB |
| R.VAPEEHPVLLTEAPLNPK.A | Y | 53.40 | 1953.0570 | 18 | 0.5 | 652.0266 | 3 | 40.65 | 1 | F1:21676 | 28122021\_RID\_2349\_2C.raw | 1.9556E6 |  |  |  |  |  | 1 | 1 | 0 | 0 | 0 | 0 | 0 | 96 | 113 |  |  | PEAKS DB |
| K.DSYVGDEAQSKR.G | N | 52.16 | 1353.6160 | 12 | 0.7 | 677.8157 | 2 | 17.11 | 1 | F1:3748 | 28122021\_RID\_2349\_2C.raw | 1.2575E5 |  |  |  |  |  | 1 | 1 | 0 | 0 | 0 | 0 | 0 | 51 | 62 |  |  | PEAKS DB |
| R.DLTDYLMK.I | N | 43.76 | 997.4790 | 8 | -0.1 | 499.7467 | 2 | 52.05 | 5 | F5:28689 | 31122021\_RID\_2349\_5C.raw |  |  |  |  | 9.6944E4 |  | 1 | 0 | 0 | 0 | 0 | 1 | 0 | 184 | 191 |  |  | PEAKS DB |
| total 11 peptides |  |  |  |  |  |  |  |  |  |  |  |  |  |  |  |  |  |  |  |  |  |  |  |  |  |  |  |  |  |
| --- | --- | --- | --- | --- | --- | --- | --- | --- | --- | --- | --- | --- | --- | --- | --- | --- | --- | --- | --- | --- | --- | --- | --- | --- | --- | --- | --- | --- | --- |

JAA97901.1

back to list

  

| Protein Coverage
| Supporting Peptides
|

Protein Coverage:

Supporting Peptides:

| Peptide | Uniq | -10lgP | Mass | Length | ppm | m/z | z | RT | Fraction | Scan | Source File | Area F2 | Area F1 | Area F3 | Area F4 | Area F5 | Area F6 | #Feature | #Feature F2 | #Feature F1 | #Feature F3 | #Feature F4 | #Feature F5 | #Feature F6 | Start | End | PTM | AScore | Found By |
| --- | --- | --- | --- | --- | --- | --- | --- | --- | --- | --- | --- | --- | --- | --- | --- | --- | --- | --- | --- | --- | --- | --- | --- | --- | --- | --- | --- | --- | --- |
| R.GYSFTTTAER.E | Y | 73.07 | 1131.5197 | 10 | 0.2 | 566.7672 | 2 | 28.89 | 1 | F1:12159 | 28122021\_RID\_2349\_2C.raw | 9.9209E5 | 1.8624E4 |  |  |  |  | 2 | 1 | 1 | 0 | 0 | 0 | 0 | 197 | 206 |  |  | PEAKS DB |
| K.SYELPDGQVITIGNER.F | N | 71.59 | 1789.8846 | 16 | 0.9 | 895.9504 | 2 | 50.05 | 1 | F1:29089 | 28122021\_RID\_2349\_2C.raw | 6.9813E5 |  | 4.7278E5 |  | 1.5455E5 | 3.4204E5 | 4 | 1 | 0 | 1 | 0 | 1 | 1 | 239 | 254 |  |  | PEAKS DB |
| K.DSYVGDEAQSK.R | N | 71.53 | 1197.5149 | 11 | 0.8 | 599.7652 | 2 | 21.07 | 1 | F1:6133 | 28122021\_RID\_2349\_2C.raw | 9.3415E5 |  |  |  |  |  | 1 | 1 | 0 | 0 | 0 | 0 | 0 | 51 | 61 |  |  | PEAKS DB |
| R.HQGVMVGMGQK.D | N | 65.34 | 1170.5638 | 11 | 0.1 | 586.2892 | 2 | 20.03 | 1 | F1:5302 | 28122021\_RID\_2349\_2C.raw | 1.0514E5 |  |  |  |  |  | 1 | 1 | 0 | 0 | 0 | 0 | 0 | 40 | 50 |  |  | PEAKS DB |
| R.AVFPSIVGRPR.H | N | 62.54 | 1197.6981 | 11 | 0.6 | 599.8567 | 2 | 34.45 | 1 | F1:16654 | 28122021\_RID\_2349\_2C.raw | 5.9717E5 |  |  |  |  |  | 1 | 1 | 0 | 0 | 0 | 0 | 0 | 29 | 39 |  |  | PEAKS DB |
| K.AGFAGDDAPR.A | N | 60.47 | 975.4410 | 10 | 0.3 | 488.7279 | 2 | 22.55 | 1 | F1:7091 | 28122021\_RID\_2349\_2C.raw | 1.4103E6 |  |  |  |  |  | 1 | 1 | 0 | 0 | 0 | 0 | 0 | 19 | 28 |  |  | PEAKS DB |
| R.HQGVM(+15.99)VGMGQK.D | N | 55.56 | 1186.5587 | 11 | -0.4 | 396.5267 | 3 | 16.87 | 1 | F1:3534 | 28122021\_RID\_2349\_2C.raw | 0 |  |  |  |  |  | 0 | 0 | 0 | 0 | 0 | 0 | 0 | 40 | 50 | Oxidation (M) | M5:Oxidation (M):34.94 | PEAKS DB |
| K.DLYANTVLSGGTTM(+15.99)YPGIADR.M | Y | 54.04 | 2230.0576 | 21 | 0.2 | 1116.0363 | 2 | 53.98 | 1 | F1:31887 | 28122021\_RID\_2349\_2C.raw | 4.0971E4 |  |  |  |  |  | 1 | 1 | 0 | 0 | 0 | 0 | 0 | 292 | 312 | Oxidation (M) | M14:Oxidation (M):1000.00 | PEAKS DB |
| R.VAPEEHPVLLTEAPLNPK.A | Y | 53.40 | 1953.0570 | 18 | 0.5 | 652.0266 | 3 | 40.65 | 1 | F1:21676 | 28122021\_RID\_2349\_2C.raw | 1.9556E6 |  |  |  |  |  | 1 | 1 | 0 | 0 | 0 | 0 | 0 | 96 | 113 |  |  | PEAKS DB |
| K.DSYVGDEAQSKR.G | N | 52.16 | 1353.6160 | 12 | 0.7 | 677.8157 | 2 | 17.11 | 1 | F1:3748 | 28122021\_RID\_2349\_2C.raw | 1.2575E5 |  |  |  |  |  | 1 | 1 | 0 | 0 | 0 | 0 | 0 | 51 | 62 |  |  | PEAKS DB |
| R.DLTDYLMK.I | N | 43.76 | 997.4790 | 8 | -0.1 | 499.7467 | 2 | 52.05 | 5 | F5:28689 | 31122021\_RID\_2349\_5C.raw |  |  |  |  | 9.6944E4 |  | 1 | 0 | 0 | 0 | 0 | 1 | 0 | 184 | 191 |  |  | PEAKS DB |
| total 11 peptides |  |  |  |  |  |  |  |  |  |  |  |  |  |  |  |  |  |  |  |  |  |  |  |  |  |  |  |  |  |
| --- | --- | --- | --- | --- | --- | --- | --- | --- | --- | --- | --- | --- | --- | --- | --- | --- | --- | --- | --- | --- | --- | --- | --- | --- | --- | --- | --- | --- | --- |

JAG68844.1

back to list

  

| Protein Coverage
| Supporting Peptides
|

Protein Coverage:

Supporting Peptides:

| Peptide | Uniq | -10lgP | Mass | Length | ppm | m/z | z | RT | Fraction | Scan | Source File | Area F2 | Area F1 | Area F3 | Area F4 | Area F5 | Area F6 | #Feature | #Feature F2 | #Feature F1 | #Feature F3 | #Feature F4 | #Feature F5 | #Feature F6 | Start | End | PTM | AScore | Found By |
| --- | --- | --- | --- | --- | --- | --- | --- | --- | --- | --- | --- | --- | --- | --- | --- | --- | --- | --- | --- | --- | --- | --- | --- | --- | --- | --- | --- | --- | --- |
| R.GYSFTTTAER.E | Y | 73.07 | 1131.5197 | 10 | 0.2 | 566.7672 | 2 | 28.89 | 1 | F1:12159 | 28122021\_RID\_2349\_2C.raw | 9.9209E5 | 1.8624E4 |  |  |  |  | 2 | 1 | 1 | 0 | 0 | 0 | 0 | 197 | 206 |  |  | PEAKS DB |
| K.SYELPDGQVITIGNER.F | N | 71.59 | 1789.8846 | 16 | 0.9 | 895.9504 | 2 | 50.05 | 1 | F1:29089 | 28122021\_RID\_2349\_2C.raw | 6.9813E5 |  | 4.7278E5 |  | 1.5455E5 | 3.4204E5 | 4 | 1 | 0 | 1 | 0 | 1 | 1 | 239 | 254 |  |  | PEAKS DB |
| K.DSYVGDEAQSK.R | N | 71.53 | 1197.5149 | 11 | 0.8 | 599.7652 | 2 | 21.07 | 1 | F1:6133 | 28122021\_RID\_2349\_2C.raw | 9.3415E5 |  |  |  |  |  | 1 | 1 | 0 | 0 | 0 | 0 | 0 | 51 | 61 |  |  | PEAKS DB |
| R.HQGVMVGMGQK.D | N | 65.34 | 1170.5638 | 11 | 0.1 | 586.2892 | 2 | 20.03 | 1 | F1:5302 | 28122021\_RID\_2349\_2C.raw | 1.0514E5 |  |  |  |  |  | 1 | 1 | 0 | 0 | 0 | 0 | 0 | 40 | 50 |  |  | PEAKS DB |
| R.AVFPSIVGRPR.H | N | 62.54 | 1197.6981 | 11 | 0.6 | 599.8567 | 2 | 34.45 | 1 | F1:16654 | 28122021\_RID\_2349\_2C.raw | 5.9717E5 |  |  |  |  |  | 1 | 1 | 0 | 0 | 0 | 0 | 0 | 29 | 39 |  |  | PEAKS DB |
| K.AGFAGDDAPR.A | N | 60.47 | 975.4410 | 10 | 0.3 | 488.7279 | 2 | 22.55 | 1 | F1:7091 | 28122021\_RID\_2349\_2C.raw | 1.4103E6 |  |  |  |  |  | 1 | 1 | 0 | 0 | 0 | 0 | 0 | 19 | 28 |  |  | PEAKS DB |
| R.HQGVM(+15.99)VGMGQK.D | N | 55.56 | 1186.5587 | 11 | -0.4 | 396.5267 | 3 | 16.87 | 1 | F1:3534 | 28122021\_RID\_2349\_2C.raw | 0 |  |  |  |  |  | 0 | 0 | 0 | 0 | 0 | 0 | 0 | 40 | 50 | Oxidation (M) | M5:Oxidation (M):34.94 | PEAKS DB |
| K.DLYANTVLSGGTTM(+15.99)YPGIADR.M | Y | 54.04 | 2230.0576 | 21 | 0.2 | 1116.0363 | 2 | 53.98 | 1 | F1:31887 | 28122021\_RID\_2349\_2C.raw | 4.0971E4 |  |  |  |  |  | 1 | 1 | 0 | 0 | 0 | 0 | 0 | 292 | 312 | Oxidation (M) | M14:Oxidation (M):1000.00 | PEAKS DB |
| R.VAPEEHPVLLTEAPLNPK.A | Y | 53.40 | 1953.0570 | 18 | 0.5 | 652.0266 | 3 | 40.65 | 1 | F1:21676 | 28122021\_RID\_2349\_2C.raw | 1.9556E6 |  |  |  |  |  | 1 | 1 | 0 | 0 | 0 | 0 | 0 | 96 | 113 |  |  | PEAKS DB |
| K.DSYVGDEAQSKR.G | N | 52.16 | 1353.6160 | 12 | 0.7 | 677.8157 | 2 | 17.11 | 1 | F1:3748 | 28122021\_RID\_2349\_2C.raw | 1.2575E5 |  |  |  |  |  | 1 | 1 | 0 | 0 | 0 | 0 | 0 | 51 | 62 |  |  | PEAKS DB |
| R.DLTDYLMK.I | N | 43.76 | 997.4790 | 8 | -0.1 | 499.7467 | 2 | 52.05 | 5 | F5:28689 | 31122021\_RID\_2349\_5C.raw |  |  |  |  | 9.6944E4 |  | 1 | 0 | 0 | 0 | 0 | 1 | 0 | 184 | 191 |  |  | PEAKS DB |
| total 11 peptides |  |  |  |  |  |  |  |  |  |  |  |  |  |  |  |  |  |  |  |  |  |  |  |  |  |  |  |  |  |
| --- | --- | --- | --- | --- | --- | --- | --- | --- | --- | --- | --- | --- | --- | --- | --- | --- | --- | --- | --- | --- | --- | --- | --- | --- | --- | --- | --- | --- | --- |

XP\_026531551.1

back to list

  

| Protein Coverage
| Supporting Peptides
|

Protein Coverage:

Supporting Peptides:

| Peptide | Uniq | -10lgP | Mass | Length | ppm | m/z | z | RT | Fraction | Scan | Source File | Area F2 | Area F1 | Area F3 | Area F4 | Area F5 | Area F6 | #Feature | #Feature F2 | #Feature F1 | #Feature F3 | #Feature F4 | #Feature F5 | #Feature F6 | Start | End | PTM | AScore | Found By |
| --- | --- | --- | --- | --- | --- | --- | --- | --- | --- | --- | --- | --- | --- | --- | --- | --- | --- | --- | --- | --- | --- | --- | --- | --- | --- | --- | --- | --- | --- |
| R.GYSFTTTAER.E | Y | 73.07 | 1131.5197 | 10 | 0.2 | 566.7672 | 2 | 28.89 | 1 | F1:12159 | 28122021\_RID\_2349\_2C.raw | 9.9209E5 | 1.8624E4 |  |  |  |  | 2 | 1 | 1 | 0 | 0 | 0 | 0 | 197 | 206 |  |  | PEAKS DB |
| K.SYELPDGQVITIGNER.F | N | 71.59 | 1789.8846 | 16 | 0.9 | 895.9504 | 2 | 50.05 | 1 | F1:29089 | 28122021\_RID\_2349\_2C.raw | 6.9813E5 |  | 4.7278E5 |  | 1.5455E5 | 3.4204E5 | 4 | 1 | 0 | 1 | 0 | 1 | 1 | 239 | 254 |  |  | PEAKS DB |
| K.DSYVGDEAQSK.R | N | 71.53 | 1197.5149 | 11 | 0.8 | 599.7652 | 2 | 21.07 | 1 | F1:6133 | 28122021\_RID\_2349\_2C.raw | 9.3415E5 |  |  |  |  |  | 1 | 1 | 0 | 0 | 0 | 0 | 0 | 51 | 61 |  |  | PEAKS DB |
| R.HQGVMVGMGQK.D | N | 65.34 | 1170.5638 | 11 | 0.1 | 586.2892 | 2 | 20.03 | 1 | F1:5302 | 28122021\_RID\_2349\_2C.raw | 1.0514E5 |  |  |  |  |  | 1 | 1 | 0 | 0 | 0 | 0 | 0 | 40 | 50 |  |  | PEAKS DB |
| R.AVFPSIVGRPR.H | N | 62.54 | 1197.6981 | 11 | 0.6 | 599.8567 | 2 | 34.45 | 1 | F1:16654 | 28122021\_RID\_2349\_2C.raw | 5.9717E5 |  |  |  |  |  | 1 | 1 | 0 | 0 | 0 | 0 | 0 | 29 | 39 |  |  | PEAKS DB |
| K.AGFAGDDAPR.A | N | 60.47 | 975.4410 | 10 | 0.3 | 488.7279 | 2 | 22.55 | 1 | F1:7091 | 28122021\_RID\_2349\_2C.raw | 1.4103E6 |  |  |  |  |  | 1 | 1 | 0 | 0 | 0 | 0 | 0 | 19 | 28 |  |  | PEAKS DB |
| R.HQGVM(+15.99)VGMGQK.D | N | 55.56 | 1186.5587 | 11 | -0.4 | 396.5267 | 3 | 16.87 | 1 | F1:3534 | 28122021\_RID\_2349\_2C.raw | 0 |  |  |  |  |  | 0 | 0 | 0 | 0 | 0 | 0 | 0 | 40 | 50 | Oxidation (M) | M5:Oxidation (M):34.94 | PEAKS DB |
| K.DLYANTVLSGGTTM(+15.99)YPGIADR.M | Y | 54.04 | 2230.0576 | 21 | 0.2 | 1116.0363 | 2 | 53.98 | 1 | F1:31887 | 28122021\_RID\_2349\_2C.raw | 4.0971E4 |  |  |  |  |  | 1 | 1 | 0 | 0 | 0 | 0 | 0 | 292 | 312 | Oxidation (M) | M14:Oxidation (M):1000.00 | PEAKS DB |
| R.VAPEEHPVLLTEAPLNPK.A | Y | 53.40 | 1953.0570 | 18 | 0.5 | 652.0266 | 3 | 40.65 | 1 | F1:21676 | 28122021\_RID\_2349\_2C.raw | 1.9556E6 |  |  |  |  |  | 1 | 1 | 0 | 0 | 0 | 0 | 0 | 96 | 113 |  |  | PEAKS DB |
| K.DSYVGDEAQSKR.G | N | 52.16 | 1353.6160 | 12 | 0.7 | 677.8157 | 2 | 17.11 | 1 | F1:3748 | 28122021\_RID\_2349\_2C.raw | 1.2575E5 |  |  |  |  |  | 1 | 1 | 0 | 0 | 0 | 0 | 0 | 51 | 62 |  |  | PEAKS DB |
| R.DLTDYLMK.I | N | 43.76 | 997.4790 | 8 | -0.1 | 499.7467 | 2 | 52.05 | 5 | F5:28689 | 31122021\_RID\_2349\_5C.raw |  |  |  |  | 9.6944E4 |  | 1 | 0 | 0 | 0 | 0 | 1 | 0 | 184 | 191 |  |  | PEAKS DB |
| total 11 peptides |  |  |  |  |  |  |  |  |  |  |  |  |  |  |  |  |  |  |  |  |  |  |  |  |  |  |  |  |  |
| --- | --- | --- | --- | --- | --- | --- | --- | --- | --- | --- | --- | --- | --- | --- | --- | --- | --- | --- | --- | --- | --- | --- | --- | --- | --- | --- | --- | --- | --- |

LAA21098.1

back to list

  

| Protein Coverage
| Supporting Peptides
|

Protein Coverage:

Supporting Peptides:

| Peptide | Uniq | -10lgP | Mass | Length | ppm | m/z | z | RT | Fraction | Scan | Source File | Area F2 | Area F1 | Area F3 | Area F4 | Area F5 | Area F6 | #Feature | #Feature F2 | #Feature F1 | #Feature F3 | #Feature F4 | #Feature F5 | #Feature F6 | Start | End | PTM | AScore | Found By |
| --- | --- | --- | --- | --- | --- | --- | --- | --- | --- | --- | --- | --- | --- | --- | --- | --- | --- | --- | --- | --- | --- | --- | --- | --- | --- | --- | --- | --- | --- |
| R.GYSFTTTAER.E | Y | 73.07 | 1131.5197 | 10 | 0.2 | 566.7672 | 2 | 28.89 | 1 | F1:12159 | 28122021\_RID\_2349\_2C.raw | 9.9209E5 | 1.8624E4 |  |  |  |  | 2 | 1 | 1 | 0 | 0 | 0 | 0 | 197 | 206 |  |  | PEAKS DB |
| K.SYELPDGQVITIGNER.F | N | 71.59 | 1789.8846 | 16 | 0.9 | 895.9504 | 2 | 50.05 | 1 | F1:29089 | 28122021\_RID\_2349\_2C.raw | 6.9813E5 |  | 4.7278E5 |  | 1.5455E5 | 3.4204E5 | 4 | 1 | 0 | 1 | 0 | 1 | 1 | 239 | 254 |  |  | PEAKS DB |
| K.DSYVGDEAQSK.R | N | 71.53 | 1197.5149 | 11 | 0.8 | 599.7652 | 2 | 21.07 | 1 | F1:6133 | 28122021\_RID\_2349\_2C.raw | 9.3415E5 |  |  |  |  |  | 1 | 1 | 0 | 0 | 0 | 0 | 0 | 51 | 61 |  |  | PEAKS DB |
| R.HQGVMVGMGQK.D | N | 65.34 | 1170.5638 | 11 | 0.1 | 586.2892 | 2 | 20.03 | 1 | F1:5302 | 28122021\_RID\_2349\_2C.raw | 1.0514E5 |  |  |  |  |  | 1 | 1 | 0 | 0 | 0 | 0 | 0 | 40 | 50 |  |  | PEAKS DB |
| R.AVFPSIVGRPR.H | N | 62.54 | 1197.6981 | 11 | 0.6 | 599.8567 | 2 | 34.45 | 1 | F1:16654 | 28122021\_RID\_2349\_2C.raw | 5.9717E5 |  |  |  |  |  | 1 | 1 | 0 | 0 | 0 | 0 | 0 | 29 | 39 |  |  | PEAKS DB |
| K.AGFAGDDAPR.A | N | 60.47 | 975.4410 | 10 | 0.3 | 488.7279 | 2 | 22.55 | 1 | F1:7091 | 28122021\_RID\_2349\_2C.raw | 1.4103E6 |  |  |  |  |  | 1 | 1 | 0 | 0 | 0 | 0 | 0 | 19 | 28 |  |  | PEAKS DB |
| R.HQGVM(+15.99)VGMGQK.D | N | 55.56 | 1186.5587 | 11 | -0.4 | 396.5267 | 3 | 16.87 | 1 | F1:3534 | 28122021\_RID\_2349\_2C.raw | 0 |  |  |  |  |  | 0 | 0 | 0 | 0 | 0 | 0 | 0 | 40 | 50 | Oxidation (M) | M5:Oxidation (M):34.94 | PEAKS DB |
| K.DLYANTVLSGGTTM(+15.99)YPGIADR.M | Y | 54.04 | 2230.0576 | 21 | 0.2 | 1116.0363 | 2 | 53.98 | 1 | F1:31887 | 28122021\_RID\_2349\_2C.raw | 4.0971E4 |  |  |  |  |  | 1 | 1 | 0 | 0 | 0 | 0 | 0 | 292 | 312 | Oxidation (M) | M14:Oxidation (M):1000.00 | PEAKS DB |
| R.VAPEEHPVLLTEAPLNPK.A | Y | 53.40 | 1953.0570 | 18 | 0.5 | 652.0266 | 3 | 40.65 | 1 | F1:21676 | 28122021\_RID\_2349\_2C.raw | 1.9556E6 |  |  |  |  |  | 1 | 1 | 0 | 0 | 0 | 0 | 0 | 96 | 113 |  |  | PEAKS DB |
| K.DSYVGDEAQSKR.G | N | 52.16 | 1353.6160 | 12 | 0.7 | 677.8157 | 2 | 17.11 | 1 | F1:3748 | 28122021\_RID\_2349\_2C.raw | 1.2575E5 |  |  |  |  |  | 1 | 1 | 0 | 0 | 0 | 0 | 0 | 51 | 62 |  |  | PEAKS DB |
| R.DLTDYLMK.I | N | 43.76 | 997.4790 | 8 | -0.1 | 499.7467 | 2 | 52.05 | 5 | F5:28689 | 31122021\_RID\_2349\_5C.raw |  |  |  |  | 9.6944E4 |  | 1 | 0 | 0 | 0 | 0 | 1 | 0 | 184 | 191 |  |  | PEAKS DB |
| total 11 peptides |  |  |  |  |  |  |  |  |  |  |  |  |  |  |  |  |  |  |  |  |  |  |  |  |  |  |  |  |  |
| --- | --- | --- | --- | --- | --- | --- | --- | --- | --- | --- | --- | --- | --- | --- | --- | --- | --- | --- | --- | --- | --- | --- | --- | --- | --- | --- | --- | --- | --- |

JAV51374.1

back to list

  

| Protein Coverage
| Supporting Peptides
|

Protein Coverage:

Supporting Peptides:

| Peptide | Uniq | -10lgP | Mass | Length | ppm | m/z | z | RT | Fraction | Scan | Source File | Area F2 | Area F1 | Area F3 | Area F4 | Area F5 | Area F6 | #Feature | #Feature F2 | #Feature F1 | #Feature F3 | #Feature F4 | #Feature F5 | #Feature F6 | Start | End | PTM | AScore | Found By |
| --- | --- | --- | --- | --- | --- | --- | --- | --- | --- | --- | --- | --- | --- | --- | --- | --- | --- | --- | --- | --- | --- | --- | --- | --- | --- | --- | --- | --- | --- |
| R.GYSFTTTAER.E | Y | 73.07 | 1131.5197 | 10 | 0.2 | 566.7672 | 2 | 28.89 | 1 | F1:12159 | 28122021\_RID\_2349\_2C.raw | 9.9209E5 | 1.8624E4 |  |  |  |  | 2 | 1 | 1 | 0 | 0 | 0 | 0 | 197 | 206 |  |  | PEAKS DB |
| K.SYELPDGQVITIGNER.F | N | 71.59 | 1789.8846 | 16 | 0.9 | 895.9504 | 2 | 50.05 | 1 | F1:29089 | 28122021\_RID\_2349\_2C.raw | 6.9813E5 |  | 4.7278E5 |  | 1.5455E5 | 3.4204E5 | 4 | 1 | 0 | 1 | 0 | 1 | 1 | 239 | 254 |  |  | PEAKS DB |
| K.DSYVGDEAQSK.R | N | 71.53 | 1197.5149 | 11 | 0.8 | 599.7652 | 2 | 21.07 | 1 | F1:6133 | 28122021\_RID\_2349\_2C.raw | 9.3415E5 |  |  |  |  |  | 1 | 1 | 0 | 0 | 0 | 0 | 0 | 51 | 61 |  |  | PEAKS DB |
| R.HQGVMVGMGQK.D | N | 65.34 | 1170.5638 | 11 | 0.1 | 586.2892 | 2 | 20.03 | 1 | F1:5302 | 28122021\_RID\_2349\_2C.raw | 1.0514E5 |  |  |  |  |  | 1 | 1 | 0 | 0 | 0 | 0 | 0 | 40 | 50 |  |  | PEAKS DB |
| R.AVFPSIVGRPR.H | N | 62.54 | 1197.6981 | 11 | 0.6 | 599.8567 | 2 | 34.45 | 1 | F1:16654 | 28122021\_RID\_2349\_2C.raw | 5.9717E5 |  |  |  |  |  | 1 | 1 | 0 | 0 | 0 | 0 | 0 | 29 | 39 |  |  | PEAKS DB |
| K.AGFAGDDAPR.A | N | 60.47 | 975.4410 | 10 | 0.3 | 488.7279 | 2 | 22.55 | 1 | F1:7091 | 28122021\_RID\_2349\_2C.raw | 1.4103E6 |  |  |  |  |  | 1 | 1 | 0 | 0 | 0 | 0 | 0 | 19 | 28 |  |  | PEAKS DB |
| R.HQGVM(+15.99)VGMGQK.D | N | 55.56 | 1186.5587 | 11 | -0.4 | 396.5267 | 3 | 16.87 | 1 | F1:3534 | 28122021\_RID\_2349\_2C.raw | 0 |  |  |  |  |  | 0 | 0 | 0 | 0 | 0 | 0 | 0 | 40 | 50 | Oxidation (M) | M5:Oxidation (M):34.94 | PEAKS DB |
| K.DLYANTVLSGGTTM(+15.99)YPGIADR.M | Y | 54.04 | 2230.0576 | 21 | 0.2 | 1116.0363 | 2 | 53.98 | 1 | F1:31887 | 28122021\_RID\_2349\_2C.raw | 4.0971E4 |  |  |  |  |  | 1 | 1 | 0 | 0 | 0 | 0 | 0 | 292 | 312 | Oxidation (M) | M14:Oxidation (M):1000.00 | PEAKS DB |
| R.VAPEEHPVLLTEAPLNPK.A | Y | 53.40 | 1953.0570 | 18 | 0.5 | 652.0266 | 3 | 40.65 | 1 | F1:21676 | 28122021\_RID\_2349\_2C.raw | 1.9556E6 |  |  |  |  |  | 1 | 1 | 0 | 0 | 0 | 0 | 0 | 96 | 113 |  |  | PEAKS DB |
| K.DSYVGDEAQSKR.G | N | 52.16 | 1353.6160 | 12 | 0.7 | 677.8157 | 2 | 17.11 | 1 | F1:3748 | 28122021\_RID\_2349\_2C.raw | 1.2575E5 |  |  |  |  |  | 1 | 1 | 0 | 0 | 0 | 0 | 0 | 51 | 62 |  |  | PEAKS DB |
| R.DLTDYLMK.I | N | 43.76 | 997.4790 | 8 | -0.1 | 499.7467 | 2 | 52.05 | 5 | F5:28689 | 31122021\_RID\_2349\_5C.raw |  |  |  |  | 9.6944E4 |  | 1 | 0 | 0 | 0 | 0 | 1 | 0 | 184 | 191 |  |  | PEAKS DB |
| total 11 peptides |  |  |  |  |  |  |  |  |  |  |  |  |  |  |  |  |  |  |  |  |  |  |  |  |  |  |  |  |  |
| --- | --- | --- | --- | --- | --- | --- | --- | --- | --- | --- | --- | --- | --- | --- | --- | --- | --- | --- | --- | --- | --- | --- | --- | --- | --- | --- | --- | --- | --- |

JAG47580.1

back to list

  

| Protein Coverage
| Supporting Peptides
|

Protein Coverage:

Supporting Peptides:

| Peptide | Uniq | -10lgP | Mass | Length | ppm | m/z | z | RT | Fraction | Scan | Source File | Area F2 | Area F1 | Area F3 | Area F4 | Area F5 | Area F6 | #Feature | #Feature F2 | #Feature F1 | #Feature F3 | #Feature F4 | #Feature F5 | #Feature F6 | Start | End | PTM | AScore | Found By |
| --- | --- | --- | --- | --- | --- | --- | --- | --- | --- | --- | --- | --- | --- | --- | --- | --- | --- | --- | --- | --- | --- | --- | --- | --- | --- | --- | --- | --- | --- |
| R.GYSFTTTAER.E | Y | 73.07 | 1131.5197 | 10 | 0.2 | 566.7672 | 2 | 28.89 | 1 | F1:12159 | 28122021\_RID\_2349\_2C.raw | 9.9209E5 | 1.8624E4 |  |  |  |  | 2 | 1 | 1 | 0 | 0 | 0 | 0 | 197 | 206 |  |  | PEAKS DB |
| K.SYELPDGQVITIGNER.F | N | 71.59 | 1789.8846 | 16 | 0.9 | 895.9504 | 2 | 50.05 | 1 | F1:29089 | 28122021\_RID\_2349\_2C.raw | 6.9813E5 |  | 4.7278E5 |  | 1.5455E5 | 3.4204E5 | 4 | 1 | 0 | 1 | 0 | 1 | 1 | 239 | 254 |  |  | PEAKS DB |
| K.DSYVGDEAQSK.R | N | 71.53 | 1197.5149 | 11 | 0.8 | 599.7652 | 2 | 21.07 | 1 | F1:6133 | 28122021\_RID\_2349\_2C.raw | 9.3415E5 |  |  |  |  |  | 1 | 1 | 0 | 0 | 0 | 0 | 0 | 51 | 61 |  |  | PEAKS DB |
| R.HQGVMVGMGQK.D | N | 65.34 | 1170.5638 | 11 | 0.1 | 586.2892 | 2 | 20.03 | 1 | F1:5302 | 28122021\_RID\_2349\_2C.raw | 1.0514E5 |  |  |  |  |  | 1 | 1 | 0 | 0 | 0 | 0 | 0 | 40 | 50 |  |  | PEAKS DB |
| R.AVFPSIVGRPR.H | N | 62.54 | 1197.6981 | 11 | 0.6 | 599.8567 | 2 | 34.45 | 1 | F1:16654 | 28122021\_RID\_2349\_2C.raw | 5.9717E5 |  |  |  |  |  | 1 | 1 | 0 | 0 | 0 | 0 | 0 | 29 | 39 |  |  | PEAKS DB |
| K.AGFAGDDAPR.A | N | 60.47 | 975.4410 | 10 | 0.3 | 488.7279 | 2 | 22.55 | 1 | F1:7091 | 28122021\_RID\_2349\_2C.raw | 1.4103E6 |  |  |  |  |  | 1 | 1 | 0 | 0 | 0 | 0 | 0 | 19 | 28 |  |  | PEAKS DB |
| R.HQGVM(+15.99)VGMGQK.D | N | 55.56 | 1186.5587 | 11 | -0.4 | 396.5267 | 3 | 16.87 | 1 | F1:3534 | 28122021\_RID\_2349\_2C.raw | 0 |  |  |  |  |  | 0 | 0 | 0 | 0 | 0 | 0 | 0 | 40 | 50 | Oxidation (M) | M5:Oxidation (M):34.94 | PEAKS DB |
| K.DLYANTVLSGGTTM(+15.99)YPGIADR.M | Y | 54.04 | 2230.0576 | 21 | 0.2 | 1116.0363 | 2 | 53.98 | 1 | F1:31887 | 28122021\_RID\_2349\_2C.raw | 4.0971E4 |  |  |  |  |  | 1 | 1 | 0 | 0 | 0 | 0 | 0 | 292 | 312 | Oxidation (M) | M14:Oxidation (M):1000.00 | PEAKS DB |
| R.VAPEEHPVLLTEAPLNPK.A | Y | 53.40 | 1953.0570 | 18 | 0.5 | 652.0266 | 3 | 40.65 | 1 | F1:21676 | 28122021\_RID\_2349\_2C.raw | 1.9556E6 |  |  |  |  |  | 1 | 1 | 0 | 0 | 0 | 0 | 0 | 96 | 113 |  |  | PEAKS DB |
| K.DSYVGDEAQSKR.G | N | 52.16 | 1353.6160 | 12 | 0.7 | 677.8157 | 2 | 17.11 | 1 | F1:3748 | 28122021\_RID\_2349\_2C.raw | 1.2575E5 |  |  |  |  |  | 1 | 1 | 0 | 0 | 0 | 0 | 0 | 51 | 62 |  |  | PEAKS DB |
| R.DLTDYLMK.I | N | 43.76 | 997.4790 | 8 | -0.1 | 499.7467 | 2 | 52.05 | 5 | F5:28689 | 31122021\_RID\_2349\_5C.raw |  |  |  |  | 9.6944E4 |  | 1 | 0 | 0 | 0 | 0 | 1 | 0 | 184 | 191 |  |  | PEAKS DB |
| total 11 peptides |  |  |  |  |  |  |  |  |  |  |  |  |  |  |  |  |  |  |  |  |  |  |  |  |  |  |  |  |  |
| --- | --- | --- | --- | --- | --- | --- | --- | --- | --- | --- | --- | --- | --- | --- | --- | --- | --- | --- | --- | --- | --- | --- | --- | --- | --- | --- | --- | --- | --- |

AFJ49303.1

back to list

  

| Protein Coverage
| Supporting Peptides
|

Protein Coverage:

Supporting Peptides:

| Peptide | Uniq | -10lgP | Mass | Length | ppm | m/z | z | RT | Fraction | Scan | Source File | Area F2 | Area F1 | Area F3 | Area F4 | Area F5 | Area F6 | #Feature | #Feature F2 | #Feature F1 | #Feature F3 | #Feature F4 | #Feature F5 | #Feature F6 | Start | End | PTM | AScore | Found By |
| --- | --- | --- | --- | --- | --- | --- | --- | --- | --- | --- | --- | --- | --- | --- | --- | --- | --- | --- | --- | --- | --- | --- | --- | --- | --- | --- | --- | --- | --- |
| R.GYSFTTTAER.E | Y | 73.07 | 1131.5197 | 10 | 0.2 | 566.7672 | 2 | 28.89 | 1 | F1:12159 | 28122021\_RID\_2349\_2C.raw | 9.9209E5 | 1.8624E4 |  |  |  |  | 2 | 1 | 1 | 0 | 0 | 0 | 0 | 197 | 206 |  |  | PEAKS DB |
| K.SYELPDGQVITIGNER.F | N | 71.59 | 1789.8846 | 16 | 0.9 | 895.9504 | 2 | 50.05 | 1 | F1:29089 | 28122021\_RID\_2349\_2C.raw | 6.9813E5 |  | 4.7278E5 |  | 1.5455E5 | 3.4204E5 | 4 | 1 | 0 | 1 | 0 | 1 | 1 | 239 | 254 |  |  | PEAKS DB |
| K.DSYVGDEAQSK.R | N | 71.53 | 1197.5149 | 11 | 0.8 | 599.7652 | 2 | 21.07 | 1 | F1:6133 | 28122021\_RID\_2349\_2C.raw | 9.3415E5 |  |  |  |  |  | 1 | 1 | 0 | 0 | 0 | 0 | 0 | 51 | 61 |  |  | PEAKS DB |
| R.HQGVMVGMGQK.D | N | 65.34 | 1170.5638 | 11 | 0.1 | 586.2892 | 2 | 20.03 | 1 | F1:5302 | 28122021\_RID\_2349\_2C.raw | 1.0514E5 |  |  |  |  |  | 1 | 1 | 0 | 0 | 0 | 0 | 0 | 40 | 50 |  |  | PEAKS DB |
| R.AVFPSIVGRPR.H | N | 62.54 | 1197.6981 | 11 | 0.6 | 599.8567 | 2 | 34.45 | 1 | F1:16654 | 28122021\_RID\_2349\_2C.raw | 5.9717E5 |  |  |  |  |  | 1 | 1 | 0 | 0 | 0 | 0 | 0 | 29 | 39 |  |  | PEAKS DB |
| K.AGFAGDDAPR.A | N | 60.47 | 975.4410 | 10 | 0.3 | 488.7279 | 2 | 22.55 | 1 | F1:7091 | 28122021\_RID\_2349\_2C.raw | 1.4103E6 |  |  |  |  |  | 1 | 1 | 0 | 0 | 0 | 0 | 0 | 19 | 28 |  |  | PEAKS DB |
| R.HQGVM(+15.99)VGMGQK.D | N | 55.56 | 1186.5587 | 11 | -0.4 | 396.5267 | 3 | 16.87 | 1 | F1:3534 | 28122021\_RID\_2349\_2C.raw | 0 |  |  |  |  |  | 0 | 0 | 0 | 0 | 0 | 0 | 0 | 40 | 50 | Oxidation (M) | M5:Oxidation (M):34.94 | PEAKS DB |
| K.DLYANTVLSGGTTM(+15.99)YPGIADR.M | Y | 54.04 | 2230.0576 | 21 | 0.2 | 1116.0363 | 2 | 53.98 | 1 | F1:31887 | 28122021\_RID\_2349\_2C.raw | 4.0971E4 |  |  |  |  |  | 1 | 1 | 0 | 0 | 0 | 0 | 0 | 292 | 312 | Oxidation (M) | M14:Oxidation (M):1000.00 | PEAKS DB |
| R.VAPEEHPVLLTEAPLNPK.A | Y | 53.40 | 1953.0570 | 18 | 0.5 | 652.0266 | 3 | 40.65 | 1 | F1:21676 | 28122021\_RID\_2349\_2C.raw | 1.9556E6 |  |  |  |  |  | 1 | 1 | 0 | 0 | 0 | 0 | 0 | 96 | 113 |  |  | PEAKS DB |
| K.DSYVGDEAQSKR.G | N | 52.16 | 1353.6160 | 12 | 0.7 | 677.8157 | 2 | 17.11 | 1 | F1:3748 | 28122021\_RID\_2349\_2C.raw | 1.2575E5 |  |  |  |  |  | 1 | 1 | 0 | 0 | 0 | 0 | 0 | 51 | 62 |  |  | PEAKS DB |
| R.DLTDYLMK.I | N | 43.76 | 997.4790 | 8 | -0.1 | 499.7467 | 2 | 52.05 | 5 | F5:28689 | 31122021\_RID\_2349\_5C.raw |  |  |  |  | 9.6944E4 |  | 1 | 0 | 0 | 0 | 0 | 1 | 0 | 184 | 191 |  |  | PEAKS DB |
| total 11 peptides |  |  |  |  |  |  |  |  |  |  |  |  |  |  |  |  |  |  |  |  |  |  |  |  |  |  |  |  |  |
| --- | --- | --- | --- | --- | --- | --- | --- | --- | --- | --- | --- | --- | --- | --- | --- | --- | --- | --- | --- | --- | --- | --- | --- | --- | --- | --- | --- | --- | --- |

JAI14496.1

back to list

  

| Protein Coverage
| Supporting Peptides
|

Protein Coverage:

Supporting Peptides:

| Peptide | Uniq | -10lgP | Mass | Length | ppm | m/z | z | RT | Fraction | Scan | Source File | Area F2 | Area F1 | Area F3 | Area F4 | Area F5 | Area F6 | #Feature | #Feature F2 | #Feature F1 | #Feature F3 | #Feature F4 | #Feature F5 | #Feature F6 | Start | End | PTM | AScore | Found By |
| --- | --- | --- | --- | --- | --- | --- | --- | --- | --- | --- | --- | --- | --- | --- | --- | --- | --- | --- | --- | --- | --- | --- | --- | --- | --- | --- | --- | --- | --- |
| R.GYSFTTTAER.E | Y | 73.07 | 1131.5197 | 10 | 0.2 | 566.7672 | 2 | 28.89 | 1 | F1:12159 | 28122021\_RID\_2349\_2C.raw | 9.9209E5 | 1.8624E4 |  |  |  |  | 2 | 1 | 1 | 0 | 0 | 0 | 0 | 197 | 206 |  |  | PEAKS DB |
| K.SYELPDGQVITIGNER.F | N | 71.59 | 1789.8846 | 16 | 0.9 | 895.9504 | 2 | 50.05 | 1 | F1:29089 | 28122021\_RID\_2349\_2C.raw | 6.9813E5 |  | 4.7278E5 |  | 1.5455E5 | 3.4204E5 | 4 | 1 | 0 | 1 | 0 | 1 | 1 | 239 | 254 |  |  | PEAKS DB |
| K.DSYVGDEAQSK.R | N | 71.53 | 1197.5149 | 11 | 0.8 | 599.7652 | 2 | 21.07 | 1 | F1:6133 | 28122021\_RID\_2349\_2C.raw | 9.3415E5 |  |  |  |  |  | 1 | 1 | 0 | 0 | 0 | 0 | 0 | 51 | 61 |  |  | PEAKS DB |
| R.HQGVMVGMGQK.D | N | 65.34 | 1170.5638 | 11 | 0.1 | 586.2892 | 2 | 20.03 | 1 | F1:5302 | 28122021\_RID\_2349\_2C.raw | 1.0514E5 |  |  |  |  |  | 1 | 1 | 0 | 0 | 0 | 0 | 0 | 40 | 50 |  |  | PEAKS DB |
| R.AVFPSIVGRPR.H | N | 62.54 | 1197.6981 | 11 | 0.6 | 599.8567 | 2 | 34.45 | 1 | F1:16654 | 28122021\_RID\_2349\_2C.raw | 5.9717E5 |  |  |  |  |  | 1 | 1 | 0 | 0 | 0 | 0 | 0 | 29 | 39 |  |  | PEAKS DB |
| K.AGFAGDDAPR.A | N | 60.47 | 975.4410 | 10 | 0.3 | 488.7279 | 2 | 22.55 | 1 | F1:7091 | 28122021\_RID\_2349\_2C.raw | 1.4103E6 |  |  |  |  |  | 1 | 1 | 0 | 0 | 0 | 0 | 0 | 19 | 28 |  |  | PEAKS DB |
| R.HQGVM(+15.99)VGMGQK.D | N | 55.56 | 1186.5587 | 11 | -0.4 | 396.5267 | 3 | 16.87 | 1 | F1:3534 | 28122021\_RID\_2349\_2C.raw | 0 |  |  |  |  |  | 0 | 0 | 0 | 0 | 0 | 0 | 0 | 40 | 50 | Oxidation (M) | M5:Oxidation (M):34.94 | PEAKS DB |
| K.DLYANTVLSGGTTM(+15.99)YPGIADR.M | Y | 54.04 | 2230.0576 | 21 | 0.2 | 1116.0363 | 2 | 53.98 | 1 | F1:31887 | 28122021\_RID\_2349\_2C.raw | 4.0971E4 |  |  |  |  |  | 1 | 1 | 0 | 0 | 0 | 0 | 0 | 292 | 312 | Oxidation (M) | M14:Oxidation (M):1000.00 | PEAKS DB |
| R.VAPEEHPVLLTEAPLNPK.A | Y | 53.40 | 1953.0570 | 18 | 0.5 | 652.0266 | 3 | 40.65 | 1 | F1:21676 | 28122021\_RID\_2349\_2C.raw | 1.9556E6 |  |  |  |  |  | 1 | 1 | 0 | 0 | 0 | 0 | 0 | 96 | 113 |  |  | PEAKS DB |
| K.DSYVGDEAQSKR.G | N | 52.16 | 1353.6160 | 12 | 0.7 | 677.8157 | 2 | 17.11 | 1 | F1:3748 | 28122021\_RID\_2349\_2C.raw | 1.2575E5 |  |  |  |  |  | 1 | 1 | 0 | 0 | 0 | 0 | 0 | 51 | 62 |  |  | PEAKS DB |
| R.DLTDYLMK.I | N | 43.76 | 997.4790 | 8 | -0.1 | 499.7467 | 2 | 52.05 | 5 | F5:28689 | 31122021\_RID\_2349\_5C.raw |  |  |  |  | 9.6944E4 |  | 1 | 0 | 0 | 0 | 0 | 1 | 0 | 184 | 191 |  |  | PEAKS DB |
| total 11 peptides |  |  |  |  |  |  |  |  |  |  |  |  |  |  |  |  |  |  |  |  |  |  |  |  |  |  |  |  |  |
| --- | --- | --- | --- | --- | --- | --- | --- | --- | --- | --- | --- | --- | --- | --- | --- | --- | --- | --- | --- | --- | --- | --- | --- | --- | --- | --- | --- | --- | --- |

JAI10341.1

back to list

  

| Protein Coverage
| Supporting Peptides
|

Protein Coverage:

Supporting Peptides:

| Peptide | Uniq | -10lgP | Mass | Length | ppm | m/z | z | RT | Fraction | Scan | Source File | Area F2 | Area F1 | Area F3 | Area F4 | Area F5 | Area F6 | #Feature | #Feature F2 | #Feature F1 | #Feature F3 | #Feature F4 | #Feature F5 | #Feature F6 | Start | End | PTM | AScore | Found By |
| --- | --- | --- | --- | --- | --- | --- | --- | --- | --- | --- | --- | --- | --- | --- | --- | --- | --- | --- | --- | --- | --- | --- | --- | --- | --- | --- | --- | --- | --- |
| R.GYSFTTTAER.E | Y | 73.07 | 1131.5197 | 10 | 0.2 | 566.7672 | 2 | 28.89 | 1 | F1:12159 | 28122021\_RID\_2349\_2C.raw | 9.9209E5 | 1.8624E4 |  |  |  |  | 2 | 1 | 1 | 0 | 0 | 0 | 0 | 197 | 206 |  |  | PEAKS DB |
| K.SYELPDGQVITIGNER.F | N | 71.59 | 1789.8846 | 16 | 0.9 | 895.9504 | 2 | 50.05 | 1 | F1:29089 | 28122021\_RID\_2349\_2C.raw | 6.9813E5 |  | 4.7278E5 |  | 1.5455E5 | 3.4204E5 | 4 | 1 | 0 | 1 | 0 | 1 | 1 | 239 | 254 |  |  | PEAKS DB |
| K.DSYVGDEAQSK.R | N | 71.53 | 1197.5149 | 11 | 0.8 | 599.7652 | 2 | 21.07 | 1 | F1:6133 | 28122021\_RID\_2349\_2C.raw | 9.3415E5 |  |  |  |  |  | 1 | 1 | 0 | 0 | 0 | 0 | 0 | 51 | 61 |  |  | PEAKS DB |
| R.HQGVMVGMGQK.D | N | 65.34 | 1170.5638 | 11 | 0.1 | 586.2892 | 2 | 20.03 | 1 | F1:5302 | 28122021\_RID\_2349\_2C.raw | 1.0514E5 |  |  |  |  |  | 1 | 1 | 0 | 0 | 0 | 0 | 0 | 40 | 50 |  |  | PEAKS DB |
| R.AVFPSIVGRPR.H | N | 62.54 | 1197.6981 | 11 | 0.6 | 599.8567 | 2 | 34.45 | 1 | F1:16654 | 28122021\_RID\_2349\_2C.raw | 5.9717E5 |  |  |  |  |  | 1 | 1 | 0 | 0 | 0 | 0 | 0 | 29 | 39 |  |  | PEAKS DB |
| K.AGFAGDDAPR.A | N | 60.47 | 975.4410 | 10 | 0.3 | 488.7279 | 2 | 22.55 | 1 | F1:7091 | 28122021\_RID\_2349\_2C.raw | 1.4103E6 |  |  |  |  |  | 1 | 1 | 0 | 0 | 0 | 0 | 0 | 19 | 28 |  |  | PEAKS DB |
| R.HQGVM(+15.99)VGMGQK.D | N | 55.56 | 1186.5587 | 11 | -0.4 | 396.5267 | 3 | 16.87 | 1 | F1:3534 | 28122021\_RID\_2349\_2C.raw | 0 |  |  |  |  |  | 0 | 0 | 0 | 0 | 0 | 0 | 0 | 40 | 50 | Oxidation (M) | M5:Oxidation (M):34.94 | PEAKS DB |
| K.DLYANTVLSGGTTM(+15.99)YPGIADR.M | Y | 54.04 | 2230.0576 | 21 | 0.2 | 1116.0363 | 2 | 53.98 | 1 | F1:31887 | 28122021\_RID\_2349\_2C.raw | 4.0971E4 |  |  |  |  |  | 1 | 1 | 0 | 0 | 0 | 0 | 0 | 292 | 312 | Oxidation (M) | M14:Oxidation (M):1000.00 | PEAKS DB |
| R.VAPEEHPVLLTEAPLNPK.A | Y | 53.40 | 1953.0570 | 18 | 0.5 | 652.0266 | 3 | 40.65 | 1 | F1:21676 | 28122021\_RID\_2349\_2C.raw | 1.9556E6 |  |  |  |  |  | 1 | 1 | 0 | 0 | 0 | 0 | 0 | 96 | 113 |  |  | PEAKS DB |
| K.DSYVGDEAQSKR.G | N | 52.16 | 1353.6160 | 12 | 0.7 | 677.8157 | 2 | 17.11 | 1 | F1:3748 | 28122021\_RID\_2349\_2C.raw | 1.2575E5 |  |  |  |  |  | 1 | 1 | 0 | 0 | 0 | 0 | 0 | 51 | 62 |  |  | PEAKS DB |
| R.DLTDYLMK.I | N | 43.76 | 997.4790 | 8 | -0.1 | 499.7467 | 2 | 52.05 | 5 | F5:28689 | 31122021\_RID\_2349\_5C.raw |  |  |  |  | 9.6944E4 |  | 1 | 0 | 0 | 0 | 0 | 1 | 0 | 184 | 191 |  |  | PEAKS DB |
| total 11 peptides |  |  |  |  |  |  |  |  |  |  |  |  |  |  |  |  |  |  |  |  |  |  |  |  |  |  |  |  |  |
| --- | --- | --- | --- | --- | --- | --- | --- | --- | --- | --- | --- | --- | --- | --- | --- | --- | --- | --- | --- | --- | --- | --- | --- | --- | --- | --- | --- | --- | --- |

JAI14495.1

back to list

  

| Protein Coverage
| Supporting Peptides
|

Protein Coverage:

Supporting Peptides:

| Peptide | Uniq | -10lgP | Mass | Length | ppm | m/z | z | RT | Fraction | Scan | Source File | Area F2 | Area F1 | Area F3 | Area F4 | Area F5 | Area F6 | #Feature | #Feature F2 | #Feature F1 | #Feature F3 | #Feature F4 | #Feature F5 | #Feature F6 | Start | End | PTM | AScore | Found By |
| --- | --- | --- | --- | --- | --- | --- | --- | --- | --- | --- | --- | --- | --- | --- | --- | --- | --- | --- | --- | --- | --- | --- | --- | --- | --- | --- | --- | --- | --- |
| R.GYSFTTTAER.E | Y | 73.07 | 1131.5197 | 10 | 0.2 | 566.7672 | 2 | 28.89 | 1 | F1:12159 | 28122021\_RID\_2349\_2C.raw | 9.9209E5 | 1.8624E4 |  |  |  |  | 2 | 1 | 1 | 0 | 0 | 0 | 0 | 197 | 206 |  |  | PEAKS DB |
| K.SYELPDGQVITIGNER.F | N | 71.59 | 1789.8846 | 16 | 0.9 | 895.9504 | 2 | 50.05 | 1 | F1:29089 | 28122021\_RID\_2349\_2C.raw | 6.9813E5 |  | 4.7278E5 |  | 1.5455E5 | 3.4204E5 | 4 | 1 | 0 | 1 | 0 | 1 | 1 | 239 | 254 |  |  | PEAKS DB |
| K.DSYVGDEAQSK.R | N | 71.53 | 1197.5149 | 11 | 0.8 | 599.7652 | 2 | 21.07 | 1 | F1:6133 | 28122021\_RID\_2349\_2C.raw | 9.3415E5 |  |  |  |  |  | 1 | 1 | 0 | 0 | 0 | 0 | 0 | 51 | 61 |  |  | PEAKS DB |
| R.HQGVMVGMGQK.D | N | 65.34 | 1170.5638 | 11 | 0.1 | 586.2892 | 2 | 20.03 | 1 | F1:5302 | 28122021\_RID\_2349\_2C.raw | 1.0514E5 |  |  |  |  |  | 1 | 1 | 0 | 0 | 0 | 0 | 0 | 40 | 50 |  |  | PEAKS DB |
| R.AVFPSIVGRPR.H | N | 62.54 | 1197.6981 | 11 | 0.6 | 599.8567 | 2 | 34.45 | 1 | F1:16654 | 28122021\_RID\_2349\_2C.raw | 5.9717E5 |  |  |  |  |  | 1 | 1 | 0 | 0 | 0 | 0 | 0 | 29 | 39 |  |  | PEAKS DB |
| K.AGFAGDDAPR.A | N | 60.47 | 975.4410 | 10 | 0.3 | 488.7279 | 2 | 22.55 | 1 | F1:7091 | 28122021\_RID\_2349\_2C.raw | 1.4103E6 |  |  |  |  |  | 1 | 1 | 0 | 0 | 0 | 0 | 0 | 19 | 28 |  |  | PEAKS DB |
| R.HQGVM(+15.99)VGMGQK.D | N | 55.56 | 1186.5587 | 11 | -0.4 | 396.5267 | 3 | 16.87 | 1 | F1:3534 | 28122021\_RID\_2349\_2C.raw | 0 |  |  |  |  |  | 0 | 0 | 0 | 0 | 0 | 0 | 0 | 40 | 50 | Oxidation (M) | M5:Oxidation (M):34.94 | PEAKS DB |
| K.DLYANTVLSGGTTM(+15.99)YPGIADR.M | Y | 54.04 | 2230.0576 | 21 | 0.2 | 1116.0363 | 2 | 53.98 | 1 | F1:31887 | 28122021\_RID\_2349\_2C.raw | 4.0971E4 |  |  |  |  |  | 1 | 1 | 0 | 0 | 0 | 0 | 0 | 292 | 312 | Oxidation (M) | M14:Oxidation (M):1000.00 | PEAKS DB |
| R.VAPEEHPVLLTEAPLNPK.A | Y | 53.40 | 1953.0570 | 18 | 0.5 | 652.0266 | 3 | 40.65 | 1 | F1:21676 | 28122021\_RID\_2349\_2C.raw | 1.9556E6 |  |  |  |  |  | 1 | 1 | 0 | 0 | 0 | 0 | 0 | 96 | 113 |  |  | PEAKS DB |
| K.DSYVGDEAQSKR.G | N | 52.16 | 1353.6160 | 12 | 0.7 | 677.8157 | 2 | 17.11 | 1 | F1:3748 | 28122021\_RID\_2349\_2C.raw | 1.2575E5 |  |  |  |  |  | 1 | 1 | 0 | 0 | 0 | 0 | 0 | 51 | 62 |  |  | PEAKS DB |
| R.DLTDYLMK.I | N | 43.76 | 997.4790 | 8 | -0.1 | 499.7467 | 2 | 52.05 | 5 | F5:28689 | 31122021\_RID\_2349\_5C.raw |  |  |  |  | 9.6944E4 |  | 1 | 0 | 0 | 0 | 0 | 1 | 0 | 184 | 191 |  |  | PEAKS DB |
| total 11 peptides |  |  |  |  |  |  |  |  |  |  |  |  |  |  |  |  |  |  |  |  |  |  |  |  |  |  |  |  |  |
| --- | --- | --- | --- | --- | --- | --- | --- | --- | --- | --- | --- | --- | --- | --- | --- | --- | --- | --- | --- | --- | --- | --- | --- | --- | --- | --- | --- | --- | --- |

XP\_007436312.1

back to list

  

| Protein Coverage
| Supporting Peptides
|

Protein Coverage:

Supporting Peptides:

| Peptide | Uniq | -10lgP | Mass | Length | ppm | m/z | z | RT | Fraction | Scan | Source File | Area F2 | Area F1 | Area F3 | Area F4 | Area F5 | Area F6 | #Feature | #Feature F2 | #Feature F1 | #Feature F3 | #Feature F4 | #Feature F5 | #Feature F6 | Start | End | PTM | AScore | Found By |
| --- | --- | --- | --- | --- | --- | --- | --- | --- | --- | --- | --- | --- | --- | --- | --- | --- | --- | --- | --- | --- | --- | --- | --- | --- | --- | --- | --- | --- | --- |
| R.GYSFTTTAER.E | Y | 73.07 | 1131.5197 | 10 | 0.2 | 566.7672 | 2 | 28.89 | 1 | F1:12159 | 28122021\_RID\_2349\_2C.raw | 9.9209E5 | 1.8624E4 |  |  |  |  | 2 | 1 | 1 | 0 | 0 | 0 | 0 | 197 | 206 |  |  | PEAKS DB |
| K.SYELPDGQVITIGNER.F | N | 71.59 | 1789.8846 | 16 | 0.9 | 895.9504 | 2 | 50.05 | 1 | F1:29089 | 28122021\_RID\_2349\_2C.raw | 6.9813E5 |  | 4.7278E5 |  | 1.5455E5 | 3.4204E5 | 4 | 1 | 0 | 1 | 0 | 1 | 1 | 239 | 254 |  |  | PEAKS DB |
| K.DSYVGDEAQSK.R | N | 71.53 | 1197.5149 | 11 | 0.8 | 599.7652 | 2 | 21.07 | 1 | F1:6133 | 28122021\_RID\_2349\_2C.raw | 9.3415E5 |  |  |  |  |  | 1 | 1 | 0 | 0 | 0 | 0 | 0 | 51 | 61 |  |  | PEAKS DB |
| R.HQGVMVGMGQK.D | N | 65.34 | 1170.5638 | 11 | 0.1 | 586.2892 | 2 | 20.03 | 1 | F1:5302 | 28122021\_RID\_2349\_2C.raw | 1.0514E5 |  |  |  |  |  | 1 | 1 | 0 | 0 | 0 | 0 | 0 | 40 | 50 |  |  | PEAKS DB |
| R.AVFPSIVGRPR.H | N | 62.54 | 1197.6981 | 11 | 0.6 | 599.8567 | 2 | 34.45 | 1 | F1:16654 | 28122021\_RID\_2349\_2C.raw | 5.9717E5 |  |  |  |  |  | 1 | 1 | 0 | 0 | 0 | 0 | 0 | 29 | 39 |  |  | PEAKS DB |
| K.AGFAGDDAPR.A | N | 60.47 | 975.4410 | 10 | 0.3 | 488.7279 | 2 | 22.55 | 1 | F1:7091 | 28122021\_RID\_2349\_2C.raw | 1.4103E6 |  |  |  |  |  | 1 | 1 | 0 | 0 | 0 | 0 | 0 | 19 | 28 |  |  | PEAKS DB |
| R.HQGVM(+15.99)VGMGQK.D | N | 55.56 | 1186.5587 | 11 | -0.4 | 396.5267 | 3 | 16.87 | 1 | F1:3534 | 28122021\_RID\_2349\_2C.raw | 0 |  |  |  |  |  | 0 | 0 | 0 | 0 | 0 | 0 | 0 | 40 | 50 | Oxidation (M) | M5:Oxidation (M):34.94 | PEAKS DB |
| K.DLYANTVLSGGTTM(+15.99)YPGIADR.M | Y | 54.04 | 2230.0576 | 21 | 0.2 | 1116.0363 | 2 | 53.98 | 1 | F1:31887 | 28122021\_RID\_2349\_2C.raw | 4.0971E4 |  |  |  |  |  | 1 | 1 | 0 | 0 | 0 | 0 | 0 | 292 | 312 | Oxidation (M) | M14:Oxidation (M):1000.00 | PEAKS DB |
| R.VAPEEHPVLLTEAPLNPK.A | Y | 53.40 | 1953.0570 | 18 | 0.5 | 652.0266 | 3 | 40.65 | 1 | F1:21676 | 28122021\_RID\_2349\_2C.raw | 1.9556E6 |  |  |  |  |  | 1 | 1 | 0 | 0 | 0 | 0 | 0 | 96 | 113 |  |  | PEAKS DB |
| K.DSYVGDEAQSKR.G | N | 52.16 | 1353.6160 | 12 | 0.7 | 677.8157 | 2 | 17.11 | 1 | F1:3748 | 28122021\_RID\_2349\_2C.raw | 1.2575E5 |  |  |  |  |  | 1 | 1 | 0 | 0 | 0 | 0 | 0 | 51 | 62 |  |  | PEAKS DB |
| R.DLTDYLMK.I | N | 43.76 | 997.4790 | 8 | -0.1 | 499.7467 | 2 | 52.05 | 5 | F5:28689 | 31122021\_RID\_2349\_5C.raw |  |  |  |  | 9.6944E4 |  | 1 | 0 | 0 | 0 | 0 | 1 | 0 | 184 | 191 |  |  | PEAKS DB |
| total 11 peptides |  |  |  |  |  |  |  |  |  |  |  |  |  |  |  |  |  |  |  |  |  |  |  |  |  |  |  |  |  |
| --- | --- | --- | --- | --- | --- | --- | --- | --- | --- | --- | --- | --- | --- | --- | --- | --- | --- | --- | --- | --- | --- | --- | --- | --- | --- | --- | --- | --- | --- |

XP\_007419915.2

back to list

  

| Protein Coverage
| Supporting Peptides
|

Protein Coverage:

Supporting Peptides:

| Peptide | Uniq | -10lgP | Mass | Length | ppm | m/z | z | RT | Fraction | Scan | Source File | Area F2 | Area F1 | Area F3 | Area F4 | Area F5 | Area F6 | #Feature | #Feature F2 | #Feature F1 | #Feature F3 | #Feature F4 | #Feature F5 | #Feature F6 | Start | End | PTM | AScore | Found By |
| --- | --- | --- | --- | --- | --- | --- | --- | --- | --- | --- | --- | --- | --- | --- | --- | --- | --- | --- | --- | --- | --- | --- | --- | --- | --- | --- | --- | --- | --- |
| R.GYSFTTTAER.E | Y | 73.07 | 1131.5197 | 10 | 0.2 | 566.7672 | 2 | 28.89 | 1 | F1:12159 | 28122021\_RID\_2349\_2C.raw | 9.9209E5 | 1.8624E4 |  |  |  |  | 2 | 1 | 1 | 0 | 0 | 0 | 0 | 197 | 206 |  |  | PEAKS DB |
| K.SYELPDGQVITIGNER.F | N | 71.59 | 1789.8846 | 16 | 0.9 | 895.9504 | 2 | 50.05 | 1 | F1:29089 | 28122021\_RID\_2349\_2C.raw | 6.9813E5 |  | 4.7278E5 |  | 1.5455E5 | 3.4204E5 | 4 | 1 | 0 | 1 | 0 | 1 | 1 | 239 | 254 |  |  | PEAKS DB |
| K.DSYVGDEAQSK.R | N | 71.53 | 1197.5149 | 11 | 0.8 | 599.7652 | 2 | 21.07 | 1 | F1:6133 | 28122021\_RID\_2349\_2C.raw | 9.3415E5 |  |  |  |  |  | 1 | 1 | 0 | 0 | 0 | 0 | 0 | 51 | 61 |  |  | PEAKS DB |
| R.HQGVMVGMGQK.D | N | 65.34 | 1170.5638 | 11 | 0.1 | 586.2892 | 2 | 20.03 | 1 | F1:5302 | 28122021\_RID\_2349\_2C.raw | 1.0514E5 |  |  |  |  |  | 1 | 1 | 0 | 0 | 0 | 0 | 0 | 40 | 50 |  |  | PEAKS DB |
| R.AVFPSIVGRPR.H | N | 62.54 | 1197.6981 | 11 | 0.6 | 599.8567 | 2 | 34.45 | 1 | F1:16654 | 28122021\_RID\_2349\_2C.raw | 5.9717E5 |  |  |  |  |  | 1 | 1 | 0 | 0 | 0 | 0 | 0 | 29 | 39 |  |  | PEAKS DB |
| K.AGFAGDDAPR.A | N | 60.47 | 975.4410 | 10 | 0.3 | 488.7279 | 2 | 22.55 | 1 | F1:7091 | 28122021\_RID\_2349\_2C.raw | 1.4103E6 |  |  |  |  |  | 1 | 1 | 0 | 0 | 0 | 0 | 0 | 19 | 28 |  |  | PEAKS DB |
| R.HQGVM(+15.99)VGMGQK.D | N | 55.56 | 1186.5587 | 11 | -0.4 | 396.5267 | 3 | 16.87 | 1 | F1:3534 | 28122021\_RID\_2349\_2C.raw | 0 |  |  |  |  |  | 0 | 0 | 0 | 0 | 0 | 0 | 0 | 40 | 50 | Oxidation (M) | M5:Oxidation (M):34.94 | PEAKS DB |
| K.DLYANTVLSGGTTM(+15.99)YPGIADR.M | Y | 54.04 | 2230.0576 | 21 | 0.2 | 1116.0363 | 2 | 53.98 | 1 | F1:31887 | 28122021\_RID\_2349\_2C.raw | 4.0971E4 |  |  |  |  |  | 1 | 1 | 0 | 0 | 0 | 0 | 0 | 292 | 312 | Oxidation (M) | M14:Oxidation (M):1000.00 | PEAKS DB |
| R.VAPEEHPVLLTEAPLNPK.A | Y | 53.40 | 1953.0570 | 18 | 0.5 | 652.0266 | 3 | 40.65 | 1 | F1:21676 | 28122021\_RID\_2349\_2C.raw | 1.9556E6 |  |  |  |  |  | 1 | 1 | 0 | 0 | 0 | 0 | 0 | 96 | 113 |  |  | PEAKS DB |
| K.DSYVGDEAQSKR.G | N | 52.16 | 1353.6160 | 12 | 0.7 | 677.8157 | 2 | 17.11 | 1 | F1:3748 | 28122021\_RID\_2349\_2C.raw | 1.2575E5 |  |  |  |  |  | 1 | 1 | 0 | 0 | 0 | 0 | 0 | 51 | 62 |  |  | PEAKS DB |
| R.DLTDYLMK.I | N | 43.76 | 997.4790 | 8 | -0.1 | 499.7467 | 2 | 52.05 | 5 | F5:28689 | 31122021\_RID\_2349\_5C.raw |  |  |  |  | 9.6944E4 |  | 1 | 0 | 0 | 0 | 0 | 1 | 0 | 184 | 191 |  |  | PEAKS DB |
| total 11 peptides |  |  |  |  |  |  |  |  |  |  |  |  |  |  |  |  |  |  |  |  |  |  |  |  |  |  |  |  |  |
| --- | --- | --- | --- | --- | --- | --- | --- | --- | --- | --- | --- | --- | --- | --- | --- | --- | --- | --- | --- | --- | --- | --- | --- | --- | --- | --- | --- | --- | --- |

JAC96364.1

back to list

  

| Protein Coverage
| Supporting Peptides
|

Protein Coverage:

Supporting Peptides:

| Peptide | Uniq | -10lgP | Mass | Length | ppm | m/z | z | RT | Fraction | Scan | Source File | Area F2 | Area F1 | Area F3 | Area F4 | Area F5 | Area F6 | #Feature | #Feature F2 | #Feature F1 | #Feature F3 | #Feature F4 | #Feature F5 | #Feature F6 | Start | End | PTM | AScore | Found By |
| --- | --- | --- | --- | --- | --- | --- | --- | --- | --- | --- | --- | --- | --- | --- | --- | --- | --- | --- | --- | --- | --- | --- | --- | --- | --- | --- | --- | --- | --- |
| R.GYSFTTTAER.E | Y | 73.07 | 1131.5197 | 10 | 0.2 | 566.7672 | 2 | 28.89 | 1 | F1:12159 | 28122021\_RID\_2349\_2C.raw | 9.9209E5 | 1.8624E4 |  |  |  |  | 2 | 1 | 1 | 0 | 0 | 0 | 0 | 197 | 206 |  |  | PEAKS DB |
| K.SYELPDGQVITIGNER.F | N | 71.59 | 1789.8846 | 16 | 0.9 | 895.9504 | 2 | 50.05 | 1 | F1:29089 | 28122021\_RID\_2349\_2C.raw | 6.9813E5 |  | 4.7278E5 |  | 1.5455E5 | 3.4204E5 | 4 | 1 | 0 | 1 | 0 | 1 | 1 | 239 | 254 |  |  | PEAKS DB |
| K.DSYVGDEAQSK.R | N | 71.53 | 1197.5149 | 11 | 0.8 | 599.7652 | 2 | 21.07 | 1 | F1:6133 | 28122021\_RID\_2349\_2C.raw | 9.3415E5 |  |  |  |  |  | 1 | 1 | 0 | 0 | 0 | 0 | 0 | 51 | 61 |  |  | PEAKS DB |
| R.HQGVMVGMGQK.D | N | 65.34 | 1170.5638 | 11 | 0.1 | 586.2892 | 2 | 20.03 | 1 | F1:5302 | 28122021\_RID\_2349\_2C.raw | 1.0514E5 |  |  |  |  |  | 1 | 1 | 0 | 0 | 0 | 0 | 0 | 40 | 50 |  |  | PEAKS DB |
| R.AVFPSIVGRPR.H | N | 62.54 | 1197.6981 | 11 | 0.6 | 599.8567 | 2 | 34.45 | 1 | F1:16654 | 28122021\_RID\_2349\_2C.raw | 5.9717E5 |  |  |  |  |  | 1 | 1 | 0 | 0 | 0 | 0 | 0 | 29 | 39 |  |  | PEAKS DB |
| K.AGFAGDDAPR.A | N | 60.47 | 975.4410 | 10 | 0.3 | 488.7279 | 2 | 22.55 | 1 | F1:7091 | 28122021\_RID\_2349\_2C.raw | 1.4103E6 |  |  |  |  |  | 1 | 1 | 0 | 0 | 0 | 0 | 0 | 19 | 28 |  |  | PEAKS DB |
| R.HQGVM(+15.99)VGMGQK.D | N | 55.56 | 1186.5587 | 11 | -0.4 | 396.5267 | 3 | 16.87 | 1 | F1:3534 | 28122021\_RID\_2349\_2C.raw | 0 |  |  |  |  |  | 0 | 0 | 0 | 0 | 0 | 0 | 0 | 40 | 50 | Oxidation (M) | M5:Oxidation (M):34.94 | PEAKS DB |
| K.DLYANTVLSGGTTM(+15.99)YPGIADR.M | Y | 54.04 | 2230.0576 | 21 | 0.2 | 1116.0363 | 2 | 53.98 | 1 | F1:31887 | 28122021\_RID\_2349\_2C.raw | 4.0971E4 |  |  |  |  |  | 1 | 1 | 0 | 0 | 0 | 0 | 0 | 292 | 312 | Oxidation (M) | M14:Oxidation (M):1000.00 | PEAKS DB |
| R.VAPEEHPVLLTEAPLNPK.A | Y | 53.40 | 1953.0570 | 18 | 0.5 | 652.0266 | 3 | 40.65 | 1 | F1:21676 | 28122021\_RID\_2349\_2C.raw | 1.9556E6 |  |  |  |  |  | 1 | 1 | 0 | 0 | 0 | 0 | 0 | 96 | 113 |  |  | PEAKS DB |
| K.DSYVGDEAQSKR.G | N | 52.16 | 1353.6160 | 12 | 0.7 | 677.8157 | 2 | 17.11 | 1 | F1:3748 | 28122021\_RID\_2349\_2C.raw | 1.2575E5 |  |  |  |  |  | 1 | 1 | 0 | 0 | 0 | 0 | 0 | 51 | 62 |  |  | PEAKS DB |
| R.DLTDYLMK.I | N | 43.76 | 997.4790 | 8 | -0.1 | 499.7467 | 2 | 52.05 | 5 | F5:28689 | 31122021\_RID\_2349\_5C.raw |  |  |  |  | 9.6944E4 |  | 1 | 0 | 0 | 0 | 0 | 1 | 0 | 184 | 191 |  |  | PEAKS DB |
| total 11 peptides |  |  |  |  |  |  |  |  |  |  |  |  |  |  |  |  |  |  |  |  |  |  |  |  |  |  |  |  |  |
| --- | --- | --- | --- | --- | --- | --- | --- | --- | --- | --- | --- | --- | --- | --- | --- | --- | --- | --- | --- | --- | --- | --- | --- | --- | --- | --- | --- | --- | --- |

JAG47579.1

back to list

  

| Protein Coverage
| Supporting Peptides
|

Protein Coverage:

Supporting Peptides:

| Peptide | Uniq | -10lgP | Mass | Length | ppm | m/z | z | RT | Fraction | Scan | Source File | Area F2 | Area F1 | Area F3 | Area F4 | Area F5 | Area F6 | #Feature | #Feature F2 | #Feature F1 | #Feature F3 | #Feature F4 | #Feature F5 | #Feature F6 | Start | End | PTM | AScore | Found By |
| --- | --- | --- | --- | --- | --- | --- | --- | --- | --- | --- | --- | --- | --- | --- | --- | --- | --- | --- | --- | --- | --- | --- | --- | --- | --- | --- | --- | --- | --- |
| R.GYSFTTTAER.E | Y | 73.07 | 1131.5197 | 10 | 0.2 | 566.7672 | 2 | 28.89 | 1 | F1:12159 | 28122021\_RID\_2349\_2C.raw | 9.9209E5 | 1.8624E4 |  |  |  |  | 2 | 1 | 1 | 0 | 0 | 0 | 0 | 197 | 206 |  |  | PEAKS DB |
| K.SYELPDGQVITIGNER.F | N | 71.59 | 1789.8846 | 16 | 0.9 | 895.9504 | 2 | 50.05 | 1 | F1:29089 | 28122021\_RID\_2349\_2C.raw | 6.9813E5 |  | 4.7278E5 |  | 1.5455E5 | 3.4204E5 | 4 | 1 | 0 | 1 | 0 | 1 | 1 | 239 | 254 |  |  | PEAKS DB |
| K.DSYVGDEAQSK.R | N | 71.53 | 1197.5149 | 11 | 0.8 | 599.7652 | 2 | 21.07 | 1 | F1:6133 | 28122021\_RID\_2349\_2C.raw | 9.3415E5 |  |  |  |  |  | 1 | 1 | 0 | 0 | 0 | 0 | 0 | 51 | 61 |  |  | PEAKS DB |
| R.HQGVMVGMGQK.D | N | 65.34 | 1170.5638 | 11 | 0.1 | 586.2892 | 2 | 20.03 | 1 | F1:5302 | 28122021\_RID\_2349\_2C.raw | 1.0514E5 |  |  |  |  |  | 1 | 1 | 0 | 0 | 0 | 0 | 0 | 40 | 50 |  |  | PEAKS DB |
| R.AVFPSIVGRPR.H | N | 62.54 | 1197.6981 | 11 | 0.6 | 599.8567 | 2 | 34.45 | 1 | F1:16654 | 28122021\_RID\_2349\_2C.raw | 5.9717E5 |  |  |  |  |  | 1 | 1 | 0 | 0 | 0 | 0 | 0 | 29 | 39 |  |  | PEAKS DB |
| K.AGFAGDDAPR.A | N | 60.47 | 975.4410 | 10 | 0.3 | 488.7279 | 2 | 22.55 | 1 | F1:7091 | 28122021\_RID\_2349\_2C.raw | 1.4103E6 |  |  |  |  |  | 1 | 1 | 0 | 0 | 0 | 0 | 0 | 19 | 28 |  |  | PEAKS DB |
| R.HQGVM(+15.99)VGMGQK.D | N | 55.56 | 1186.5587 | 11 | -0.4 | 396.5267 | 3 | 16.87 | 1 | F1:3534 | 28122021\_RID\_2349\_2C.raw | 0 |  |  |  |  |  | 0 | 0 | 0 | 0 | 0 | 0 | 0 | 40 | 50 | Oxidation (M) | M5:Oxidation (M):34.94 | PEAKS DB |
| K.DLYANTVLSGGTTM(+15.99)YPGIADR.M | Y | 54.04 | 2230.0576 | 21 | 0.2 | 1116.0363 | 2 | 53.98 | 1 | F1:31887 | 28122021\_RID\_2349\_2C.raw | 4.0971E4 |  |  |  |  |  | 1 | 1 | 0 | 0 | 0 | 0 | 0 | 292 | 312 | Oxidation (M) | M14:Oxidation (M):1000.00 | PEAKS DB |
| R.VAPEEHPVLLTEAPLNPK.A | Y | 53.40 | 1953.0570 | 18 | 0.5 | 652.0266 | 3 | 40.65 | 1 | F1:21676 | 28122021\_RID\_2349\_2C.raw | 1.9556E6 |  |  |  |  |  | 1 | 1 | 0 | 0 | 0 | 0 | 0 | 96 | 113 |  |  | PEAKS DB |
| K.DSYVGDEAQSKR.G | N | 52.16 | 1353.6160 | 12 | 0.7 | 677.8157 | 2 | 17.11 | 1 | F1:3748 | 28122021\_RID\_2349\_2C.raw | 1.2575E5 |  |  |  |  |  | 1 | 1 | 0 | 0 | 0 | 0 | 0 | 51 | 62 |  |  | PEAKS DB |
| R.DLTDYLMK.I | N | 43.76 | 997.4790 | 8 | -0.1 | 499.7467 | 2 | 52.05 | 5 | F5:28689 | 31122021\_RID\_2349\_5C.raw |  |  |  |  | 9.6944E4 |  | 1 | 0 | 0 | 0 | 0 | 1 | 0 | 184 | 191 |  |  | PEAKS DB |
| total 11 peptides |  |  |  |  |  |  |  |  |  |  |  |  |  |  |  |  |  |  |  |  |  |  |  |  |  |  |  |  |  |
| --- | --- | --- | --- | --- | --- | --- | --- | --- | --- | --- | --- | --- | --- | --- | --- | --- | --- | --- | --- | --- | --- | --- | --- | --- | --- | --- | --- | --- | --- |

JAV51372.1

back to list

  

| Protein Coverage
| Supporting Peptides
|

Protein Coverage:

Supporting Peptides:

| Peptide | Uniq | -10lgP | Mass | Length | ppm | m/z | z | RT | Fraction | Scan | Source File | Area F2 | Area F1 | Area F3 | Area F4 | Area F5 | Area F6 | #Feature | #Feature F2 | #Feature F1 | #Feature F3 | #Feature F4 | #Feature F5 | #Feature F6 | Start | End | PTM | AScore | Found By |
| --- | --- | --- | --- | --- | --- | --- | --- | --- | --- | --- | --- | --- | --- | --- | --- | --- | --- | --- | --- | --- | --- | --- | --- | --- | --- | --- | --- | --- | --- |
| R.GYSFTTTAER.E | Y | 73.07 | 1131.5197 | 10 | 0.2 | 566.7672 | 2 | 28.89 | 1 | F1:12159 | 28122021\_RID\_2349\_2C.raw | 9.9209E5 | 1.8624E4 |  |  |  |  | 2 | 1 | 1 | 0 | 0 | 0 | 0 | 197 | 206 |  |  | PEAKS DB |
| K.SYELPDGQVITIGNER.F | N | 71.59 | 1789.8846 | 16 | 0.9 | 895.9504 | 2 | 50.05 | 1 | F1:29089 | 28122021\_RID\_2349\_2C.raw | 6.9813E5 |  | 4.7278E5 |  | 1.5455E5 | 3.4204E5 | 4 | 1 | 0 | 1 | 0 | 1 | 1 | 239 | 254 |  |  | PEAKS DB |
| K.DSYVGDEAQSK.R | N | 71.53 | 1197.5149 | 11 | 0.8 | 599.7652 | 2 | 21.07 | 1 | F1:6133 | 28122021\_RID\_2349\_2C.raw | 9.3415E5 |  |  |  |  |  | 1 | 1 | 0 | 0 | 0 | 0 | 0 | 51 | 61 |  |  | PEAKS DB |
| R.HQGVMVGMGQK.D | N | 65.34 | 1170.5638 | 11 | 0.1 | 586.2892 | 2 | 20.03 | 1 | F1:5302 | 28122021\_RID\_2349\_2C.raw | 1.0514E5 |  |  |  |  |  | 1 | 1 | 0 | 0 | 0 | 0 | 0 | 40 | 50 |  |  | PEAKS DB |
| R.AVFPSIVGRPR.H | N | 62.54 | 1197.6981 | 11 | 0.6 | 599.8567 | 2 | 34.45 | 1 | F1:16654 | 28122021\_RID\_2349\_2C.raw | 5.9717E5 |  |  |  |  |  | 1 | 1 | 0 | 0 | 0 | 0 | 0 | 29 | 39 |  |  | PEAKS DB |
| K.AGFAGDDAPR.A | N | 60.47 | 975.4410 | 10 | 0.3 | 488.7279 | 2 | 22.55 | 1 | F1:7091 | 28122021\_RID\_2349\_2C.raw | 1.4103E6 |  |  |  |  |  | 1 | 1 | 0 | 0 | 0 | 0 | 0 | 19 | 28 |  |  | PEAKS DB |
| R.HQGVM(+15.99)VGMGQK.D | N | 55.56 | 1186.5587 | 11 | -0.4 | 396.5267 | 3 | 16.87 | 1 | F1:3534 | 28122021\_RID\_2349\_2C.raw | 0 |  |  |  |  |  | 0 | 0 | 0 | 0 | 0 | 0 | 0 | 40 | 50 | Oxidation (M) | M5:Oxidation (M):34.94 | PEAKS DB |
| K.DLYANTVLSGGTTM(+15.99)YPGIADR.M | Y | 54.04 | 2230.0576 | 21 | 0.2 | 1116.0363 | 2 | 53.98 | 1 | F1:31887 | 28122021\_RID\_2349\_2C.raw | 4.0971E4 |  |  |  |  |  | 1 | 1 | 0 | 0 | 0 | 0 | 0 | 292 | 312 | Oxidation (M) | M14:Oxidation (M):1000.00 | PEAKS DB |
| R.VAPEEHPVLLTEAPLNPK.A | Y | 53.40 | 1953.0570 | 18 | 0.5 | 652.0266 | 3 | 40.65 | 1 | F1:21676 | 28122021\_RID\_2349\_2C.raw | 1.9556E6 |  |  |  |  |  | 1 | 1 | 0 | 0 | 0 | 0 | 0 | 96 | 113 |  |  | PEAKS DB |
| K.DSYVGDEAQSKR.G | N | 52.16 | 1353.6160 | 12 | 0.7 | 677.8157 | 2 | 17.11 | 1 | F1:3748 | 28122021\_RID\_2349\_2C.raw | 1.2575E5 |  |  |  |  |  | 1 | 1 | 0 | 0 | 0 | 0 | 0 | 51 | 62 |  |  | PEAKS DB |
| R.DLTDYLMK.I | N | 43.76 | 997.4790 | 8 | -0.1 | 499.7467 | 2 | 52.05 | 5 | F5:28689 | 31122021\_RID\_2349\_5C.raw |  |  |  |  | 9.6944E4 |  | 1 | 0 | 0 | 0 | 0 | 1 | 0 | 184 | 191 |  |  | PEAKS DB |
| total 11 peptides |  |  |  |  |  |  |  |  |  |  |  |  |  |  |  |  |  |  |  |  |  |  |  |  |  |  |  |  |  |
| --- | --- | --- | --- | --- | --- | --- | --- | --- | --- | --- | --- | --- | --- | --- | --- | --- | --- | --- | --- | --- | --- | --- | --- | --- | --- | --- | --- | --- | --- |

AFJ49302.1

back to list

  

| Protein Coverage
| Supporting Peptides
|

Protein Coverage:

Supporting Peptides:

| Peptide | Uniq | -10lgP | Mass | Length | ppm | m/z | z | RT | Fraction | Scan | Source File | Area F2 | Area F1 | Area F3 | Area F4 | Area F5 | Area F6 | #Feature | #Feature F2 | #Feature F1 | #Feature F3 | #Feature F4 | #Feature F5 | #Feature F6 | Start | End | PTM | AScore | Found By |
| --- | --- | --- | --- | --- | --- | --- | --- | --- | --- | --- | --- | --- | --- | --- | --- | --- | --- | --- | --- | --- | --- | --- | --- | --- | --- | --- | --- | --- | --- |
| R.GYSFTTTAER.E | Y | 73.07 | 1131.5197 | 10 | 0.2 | 566.7672 | 2 | 28.89 | 1 | F1:12159 | 28122021\_RID\_2349\_2C.raw | 9.9209E5 | 1.8624E4 |  |  |  |  | 2 | 1 | 1 | 0 | 0 | 0 | 0 | 197 | 206 |  |  | PEAKS DB |
| K.SYELPDGQVITIGNER.F | N | 71.59 | 1789.8846 | 16 | 0.9 | 895.9504 | 2 | 50.05 | 1 | F1:29089 | 28122021\_RID\_2349\_2C.raw | 6.9813E5 |  | 4.7278E5 |  | 1.5455E5 | 3.4204E5 | 4 | 1 | 0 | 1 | 0 | 1 | 1 | 239 | 254 |  |  | PEAKS DB |
| K.DSYVGDEAQSK.R | N | 71.53 | 1197.5149 | 11 | 0.8 | 599.7652 | 2 | 21.07 | 1 | F1:6133 | 28122021\_RID\_2349\_2C.raw | 9.3415E5 |  |  |  |  |  | 1 | 1 | 0 | 0 | 0 | 0 | 0 | 51 | 61 |  |  | PEAKS DB |
| R.HQGVMVGMGQK.D | N | 65.34 | 1170.5638 | 11 | 0.1 | 586.2892 | 2 | 20.03 | 1 | F1:5302 | 28122021\_RID\_2349\_2C.raw | 1.0514E5 |  |  |  |  |  | 1 | 1 | 0 | 0 | 0 | 0 | 0 | 40 | 50 |  |  | PEAKS DB |
| R.AVFPSIVGRPR.H | N | 62.54 | 1197.6981 | 11 | 0.6 | 599.8567 | 2 | 34.45 | 1 | F1:16654 | 28122021\_RID\_2349\_2C.raw | 5.9717E5 |  |  |  |  |  | 1 | 1 | 0 | 0 | 0 | 0 | 0 | 29 | 39 |  |  | PEAKS DB |
| K.AGFAGDDAPR.A | N | 60.47 | 975.4410 | 10 | 0.3 | 488.7279 | 2 | 22.55 | 1 | F1:7091 | 28122021\_RID\_2349\_2C.raw | 1.4103E6 |  |  |  |  |  | 1 | 1 | 0 | 0 | 0 | 0 | 0 | 19 | 28 |  |  | PEAKS DB |
| R.HQGVM(+15.99)VGMGQK.D | N | 55.56 | 1186.5587 | 11 | -0.4 | 396.5267 | 3 | 16.87 | 1 | F1:3534 | 28122021\_RID\_2349\_2C.raw | 0 |  |  |  |  |  | 0 | 0 | 0 | 0 | 0 | 0 | 0 | 40 | 50 | Oxidation (M) | M5:Oxidation (M):34.94 | PEAKS DB |
| K.DLYANTVLSGGTTM(+15.99)YPGIADR.M | Y | 54.04 | 2230.0576 | 21 | 0.2 | 1116.0363 | 2 | 53.98 | 1 | F1:31887 | 28122021\_RID\_2349\_2C.raw | 4.0971E4 |  |  |  |  |  | 1 | 1 | 0 | 0 | 0 | 0 | 0 | 292 | 312 | Oxidation (M) | M14:Oxidation (M):1000.00 | PEAKS DB |
| R.VAPEEHPVLLTEAPLNPK.A | Y | 53.40 | 1953.0570 | 18 | 0.5 | 652.0266 | 3 | 40.65 | 1 | F1:21676 | 28122021\_RID\_2349\_2C.raw | 1.9556E6 |  |  |  |  |  | 1 | 1 | 0 | 0 | 0 | 0 | 0 | 96 | 113 |  |  | PEAKS DB |
| K.DSYVGDEAQSKR.G | N | 52.16 | 1353.6160 | 12 | 0.7 | 677.8157 | 2 | 17.11 | 1 | F1:3748 | 28122021\_RID\_2349\_2C.raw | 1.2575E5 |  |  |  |  |  | 1 | 1 | 0 | 0 | 0 | 0 | 0 | 51 | 62 |  |  | PEAKS DB |
| R.DLTDYLMK.I | N | 43.76 | 997.4790 | 8 | -0.1 | 499.7467 | 2 | 52.05 | 5 | F5:28689 | 31122021\_RID\_2349\_5C.raw |  |  |  |  | 9.6944E4 |  | 1 | 0 | 0 | 0 | 0 | 1 | 0 | 184 | 191 |  |  | PEAKS DB |
| total 11 peptides |  |  |  |  |  |  |  |  |  |  |  |  |  |  |  |  |  |  |  |  |  |  |  |  |  |  |  |  |  |
| --- | --- | --- | --- | --- | --- | --- | --- | --- | --- | --- | --- | --- | --- | --- | --- | --- | --- | --- | --- | --- | --- | --- | --- | --- | --- | --- | --- | --- | --- |

BAQ22177.1

back to list

  

| Protein Coverage
| Supporting Peptides
|

Protein Coverage:

Supporting Peptides:

| Peptide | Uniq | -10lgP | Mass | Length | ppm | m/z | z | RT | Fraction | Scan | Source File | Area F2 | Area F1 | Area F3 | Area F4 | Area F5 | Area F6 | #Feature | #Feature F2 | #Feature F1 | #Feature F3 | #Feature F4 | #Feature F5 | #Feature F6 | Start | End | PTM | AScore | Found By |
| --- | --- | --- | --- | --- | --- | --- | --- | --- | --- | --- | --- | --- | --- | --- | --- | --- | --- | --- | --- | --- | --- | --- | --- | --- | --- | --- | --- | --- | --- |
| R.GYSFTTTAER.E | Y | 73.07 | 1131.5197 | 10 | 0.2 | 566.7672 | 2 | 28.89 | 1 | F1:12159 | 28122021\_RID\_2349\_2C.raw | 9.9209E5 | 1.8624E4 |  |  |  |  | 2 | 1 | 1 | 0 | 0 | 0 | 0 | 197 | 206 |  |  | PEAKS DB |
| K.SYELPDGQVITIGNER.F | N | 71.59 | 1789.8846 | 16 | 0.9 | 895.9504 | 2 | 50.05 | 1 | F1:29089 | 28122021\_RID\_2349\_2C.raw | 6.9813E5 |  | 4.7278E5 |  | 1.5455E5 | 3.4204E5 | 4 | 1 | 0 | 1 | 0 | 1 | 1 | 239 | 254 |  |  | PEAKS DB |
| K.DSYVGDEAQSK.R | N | 71.53 | 1197.5149 | 11 | 0.8 | 599.7652 | 2 | 21.07 | 1 | F1:6133 | 28122021\_RID\_2349\_2C.raw | 9.3415E5 |  |  |  |  |  | 1 | 1 | 0 | 0 | 0 | 0 | 0 | 51 | 61 |  |  | PEAKS DB |
| R.HQGVMVGMGQK.D | N | 65.34 | 1170.5638 | 11 | 0.1 | 586.2892 | 2 | 20.03 | 1 | F1:5302 | 28122021\_RID\_2349\_2C.raw | 1.0514E5 |  |  |  |  |  | 1 | 1 | 0 | 0 | 0 | 0 | 0 | 40 | 50 |  |  | PEAKS DB |
| R.AVFPSIVGRPR.H | N | 62.54 | 1197.6981 | 11 | 0.6 | 599.8567 | 2 | 34.45 | 1 | F1:16654 | 28122021\_RID\_2349\_2C.raw | 5.9717E5 |  |  |  |  |  | 1 | 1 | 0 | 0 | 0 | 0 | 0 | 29 | 39 |  |  | PEAKS DB |
| K.AGFAGDDAPR.A | N | 60.47 | 975.4410 | 10 | 0.3 | 488.7279 | 2 | 22.55 | 1 | F1:7091 | 28122021\_RID\_2349\_2C.raw | 1.4103E6 |  |  |  |  |  | 1 | 1 | 0 | 0 | 0 | 0 | 0 | 19 | 28 |  |  | PEAKS DB |
| R.HQGVM(+15.99)VGMGQK.D | N | 55.56 | 1186.5587 | 11 | -0.4 | 396.5267 | 3 | 16.87 | 1 | F1:3534 | 28122021\_RID\_2349\_2C.raw | 0 |  |  |  |  |  | 0 | 0 | 0 | 0 | 0 | 0 | 0 | 40 | 50 | Oxidation (M) | M5:Oxidation (M):34.94 | PEAKS DB |
| K.DLYANTVLSGGTTM(+15.99)YPGIADR.M | Y | 54.04 | 2230.0576 | 21 | 0.2 | 1116.0363 | 2 | 53.98 | 1 | F1:31887 | 28122021\_RID\_2349\_2C.raw | 4.0971E4 |  |  |  |  |  | 1 | 1 | 0 | 0 | 0 | 0 | 0 | 292 | 312 | Oxidation (M) | M14:Oxidation (M):1000.00 | PEAKS DB |
| R.VAPEEHPVLLTEAPLNPK.A | Y | 53.40 | 1953.0570 | 18 | 0.5 | 652.0266 | 3 | 40.65 | 1 | F1:21676 | 28122021\_RID\_2349\_2C.raw | 1.9556E6 |  |  |  |  |  | 1 | 1 | 0 | 0 | 0 | 0 | 0 | 96 | 113 |  |  | PEAKS DB |
| K.DSYVGDEAQSKR.G | N | 52.16 | 1353.6160 | 12 | 0.7 | 677.8157 | 2 | 17.11 | 1 | F1:3748 | 28122021\_RID\_2349\_2C.raw | 1.2575E5 |  |  |  |  |  | 1 | 1 | 0 | 0 | 0 | 0 | 0 | 51 | 62 |  |  | PEAKS DB |
| R.DLTDYLMK.I | N | 43.76 | 997.4790 | 8 | -0.1 | 499.7467 | 2 | 52.05 | 5 | F5:28689 | 31122021\_RID\_2349\_5C.raw |  |  |  |  | 9.6944E4 |  | 1 | 0 | 0 | 0 | 0 | 1 | 0 | 184 | 191 |  |  | PEAKS DB |
| total 11 peptides |  |  |  |  |  |  |  |  |  |  |  |  |  |  |  |  |  |  |  |  |  |  |  |  |  |  |  |  |  |
| --- | --- | --- | --- | --- | --- | --- | --- | --- | --- | --- | --- | --- | --- | --- | --- | --- | --- | --- | --- | --- | --- | --- | --- | --- | --- | --- | --- | --- | --- |

JAG45996.1

back to list

  

| Protein Coverage
| Supporting Peptides
|

Protein Coverage:

Supporting Peptides:

| Peptide | Uniq | -10lgP | Mass | Length | ppm | m/z | z | RT | Fraction | Scan | Source File | Area F2 | Area F1 | Area F3 | Area F4 | Area F5 | Area F6 | #Feature | #Feature F2 | #Feature F1 | #Feature F3 | #Feature F4 | #Feature F5 | #Feature F6 | Start | End | PTM | AScore | Found By |
| --- | --- | --- | --- | --- | --- | --- | --- | --- | --- | --- | --- | --- | --- | --- | --- | --- | --- | --- | --- | --- | --- | --- | --- | --- | --- | --- | --- | --- | --- |
| R.GYSFTTTAER.E | Y | 73.07 | 1131.5197 | 10 | 0.2 | 566.7672 | 2 | 28.89 | 1 | F1:12159 | 28122021\_RID\_2349\_2C.raw | 9.9209E5 | 1.8624E4 |  |  |  |  | 2 | 1 | 1 | 0 | 0 | 0 | 0 | 197 | 206 |  |  | PEAKS DB |
| K.SYELPDGQVITIGNER.F | N | 71.59 | 1789.8846 | 16 | 0.9 | 895.9504 | 2 | 50.05 | 1 | F1:29089 | 28122021\_RID\_2349\_2C.raw | 6.9813E5 |  | 4.7278E5 |  | 1.5455E5 | 3.4204E5 | 4 | 1 | 0 | 1 | 0 | 1 | 1 | 239 | 254 |  |  | PEAKS DB |
| K.DSYVGDEAQSK.R | N | 71.53 | 1197.5149 | 11 | 0.8 | 599.7652 | 2 | 21.07 | 1 | F1:6133 | 28122021\_RID\_2349\_2C.raw | 9.3415E5 |  |  |  |  |  | 1 | 1 | 0 | 0 | 0 | 0 | 0 | 51 | 61 |  |  | PEAKS DB |
| R.HQGVMVGMGQK.D | N | 65.34 | 1170.5638 | 11 | 0.1 | 586.2892 | 2 | 20.03 | 1 | F1:5302 | 28122021\_RID\_2349\_2C.raw | 1.0514E5 |  |  |  |  |  | 1 | 1 | 0 | 0 | 0 | 0 | 0 | 40 | 50 |  |  | PEAKS DB |
| R.AVFPSIVGRPR.H | N | 62.54 | 1197.6981 | 11 | 0.6 | 599.8567 | 2 | 34.45 | 1 | F1:16654 | 28122021\_RID\_2349\_2C.raw | 5.9717E5 |  |  |  |  |  | 1 | 1 | 0 | 0 | 0 | 0 | 0 | 29 | 39 |  |  | PEAKS DB |
| K.AGFAGDDAPR.A | N | 60.47 | 975.4410 | 10 | 0.3 | 488.7279 | 2 | 22.55 | 1 | F1:7091 | 28122021\_RID\_2349\_2C.raw | 1.4103E6 |  |  |  |  |  | 1 | 1 | 0 | 0 | 0 | 0 | 0 | 19 | 28 |  |  | PEAKS DB |
| R.HQGVM(+15.99)VGMGQK.D | N | 55.56 | 1186.5587 | 11 | -0.4 | 396.5267 | 3 | 16.87 | 1 | F1:3534 | 28122021\_RID\_2349\_2C.raw | 0 |  |  |  |  |  | 0 | 0 | 0 | 0 | 0 | 0 | 0 | 40 | 50 | Oxidation (M) | M5:Oxidation (M):34.94 | PEAKS DB |
| K.DLYANTVLSGGTTM(+15.99)YPGIADR.M | Y | 54.04 | 2230.0576 | 21 | 0.2 | 1116.0363 | 2 | 53.98 | 1 | F1:31887 | 28122021\_RID\_2349\_2C.raw | 4.0971E4 |  |  |  |  |  | 1 | 1 | 0 | 0 | 0 | 0 | 0 | 292 | 312 | Oxidation (M) | M14:Oxidation (M):1000.00 | PEAKS DB |
| R.VAPEEHPVLLTEAPLNPK.A | Y | 53.40 | 1953.0570 | 18 | 0.5 | 652.0266 | 3 | 40.65 | 1 | F1:21676 | 28122021\_RID\_2349\_2C.raw | 1.9556E6 |  |  |  |  |  | 1 | 1 | 0 | 0 | 0 | 0 | 0 | 96 | 113 |  |  | PEAKS DB |
| K.DSYVGDEAQSKR.G | N | 52.16 | 1353.6160 | 12 | 0.7 | 677.8157 | 2 | 17.11 | 1 | F1:3748 | 28122021\_RID\_2349\_2C.raw | 1.2575E5 |  |  |  |  |  | 1 | 1 | 0 | 0 | 0 | 0 | 0 | 51 | 62 |  |  | PEAKS DB |
| R.DLTDYLMK.I | N | 43.76 | 997.4790 | 8 | -0.1 | 499.7467 | 2 | 52.05 | 5 | F5:28689 | 31122021\_RID\_2349\_5C.raw |  |  |  |  | 9.6944E4 |  | 1 | 0 | 0 | 0 | 0 | 1 | 0 | 184 | 191 |  |  | PEAKS DB |
| total 11 peptides |  |  |  |  |  |  |  |  |  |  |  |  |  |  |  |  |  |  |  |  |  |  |  |  |  |  |  |  |  |
| --- | --- | --- | --- | --- | --- | --- | --- | --- | --- | --- | --- | --- | --- | --- | --- | --- | --- | --- | --- | --- | --- | --- | --- | --- | --- | --- | --- | --- | --- |

JAG45999.1

back to list

  

| Protein Coverage
| Supporting Peptides
|

Protein Coverage:

Supporting Peptides:

| Peptide | Uniq | -10lgP | Mass | Length | ppm | m/z | z | RT | Fraction | Scan | Source File | Area F2 | Area F1 | Area F3 | Area F4 | Area F5 | Area F6 | #Feature | #Feature F2 | #Feature F1 | #Feature F3 | #Feature F4 | #Feature F5 | #Feature F6 | Start | End | PTM | AScore | Found By |
| --- | --- | --- | --- | --- | --- | --- | --- | --- | --- | --- | --- | --- | --- | --- | --- | --- | --- | --- | --- | --- | --- | --- | --- | --- | --- | --- | --- | --- | --- |
| R.GYSFTTTAER.E | Y | 73.07 | 1131.5197 | 10 | 0.2 | 566.7672 | 2 | 28.89 | 1 | F1:12159 | 28122021\_RID\_2349\_2C.raw | 9.9209E5 | 1.8624E4 |  |  |  |  | 2 | 1 | 1 | 0 | 0 | 0 | 0 | 197 | 206 |  |  | PEAKS DB |
| K.SYELPDGQVITIGNER.F | N | 71.59 | 1789.8846 | 16 | 0.9 | 895.9504 | 2 | 50.05 | 1 | F1:29089 | 28122021\_RID\_2349\_2C.raw | 6.9813E5 |  | 4.7278E5 |  | 1.5455E5 | 3.4204E5 | 4 | 1 | 0 | 1 | 0 | 1 | 1 | 239 | 254 |  |  | PEAKS DB |
| K.DSYVGDEAQSK.R | N | 71.53 | 1197.5149 | 11 | 0.8 | 599.7652 | 2 | 21.07 | 1 | F1:6133 | 28122021\_RID\_2349\_2C.raw | 9.3415E5 |  |  |  |  |  | 1 | 1 | 0 | 0 | 0 | 0 | 0 | 51 | 61 |  |  | PEAKS DB |
| R.HQGVMVGMGQK.D | N | 65.34 | 1170.5638 | 11 | 0.1 | 586.2892 | 2 | 20.03 | 1 | F1:5302 | 28122021\_RID\_2349\_2C.raw | 1.0514E5 |  |  |  |  |  | 1 | 1 | 0 | 0 | 0 | 0 | 0 | 40 | 50 |  |  | PEAKS DB |
| R.AVFPSIVGRPR.H | N | 62.54 | 1197.6981 | 11 | 0.6 | 599.8567 | 2 | 34.45 | 1 | F1:16654 | 28122021\_RID\_2349\_2C.raw | 5.9717E5 |  |  |  |  |  | 1 | 1 | 0 | 0 | 0 | 0 | 0 | 29 | 39 |  |  | PEAKS DB |
| K.AGFAGDDAPR.A | N | 60.47 | 975.4410 | 10 | 0.3 | 488.7279 | 2 | 22.55 | 1 | F1:7091 | 28122021\_RID\_2349\_2C.raw | 1.4103E6 |  |  |  |  |  | 1 | 1 | 0 | 0 | 0 | 0 | 0 | 19 | 28 |  |  | PEAKS DB |
| R.HQGVM(+15.99)VGMGQK.D | N | 55.56 | 1186.5587 | 11 | -0.4 | 396.5267 | 3 | 16.87 | 1 | F1:3534 | 28122021\_RID\_2349\_2C.raw | 0 |  |  |  |  |  | 0 | 0 | 0 | 0 | 0 | 0 | 0 | 40 | 50 | Oxidation (M) | M5:Oxidation (M):34.94 | PEAKS DB |
| K.DLYANTVLSGGTTM(+15.99)YPGIADR.M | Y | 54.04 | 2230.0576 | 21 | 0.2 | 1116.0363 | 2 | 53.98 | 1 | F1:31887 | 28122021\_RID\_2349\_2C.raw | 4.0971E4 |  |  |  |  |  | 1 | 1 | 0 | 0 | 0 | 0 | 0 | 292 | 312 | Oxidation (M) | M14:Oxidation (M):1000.00 | PEAKS DB |
| R.VAPEEHPVLLTEAPLNPK.A | Y | 53.40 | 1953.0570 | 18 | 0.5 | 652.0266 | 3 | 40.65 | 1 | F1:21676 | 28122021\_RID\_2349\_2C.raw | 1.9556E6 |  |  |  |  |  | 1 | 1 | 0 | 0 | 0 | 0 | 0 | 96 | 113 |  |  | PEAKS DB |
| K.DSYVGDEAQSKR.G | N | 52.16 | 1353.6160 | 12 | 0.7 | 677.8157 | 2 | 17.11 | 1 | F1:3748 | 28122021\_RID\_2349\_2C.raw | 1.2575E5 |  |  |  |  |  | 1 | 1 | 0 | 0 | 0 | 0 | 0 | 51 | 62 |  |  | PEAKS DB |
| R.DLTDYLMK.I | N | 43.76 | 997.4790 | 8 | -0.1 | 499.7467 | 2 | 52.05 | 5 | F5:28689 | 31122021\_RID\_2349\_5C.raw |  |  |  |  | 9.6944E4 |  | 1 | 0 | 0 | 0 | 0 | 1 | 0 | 184 | 191 |  |  | PEAKS DB |
| total 11 peptides |  |  |  |  |  |  |  |  |  |  |  |  |  |  |  |  |  |  |  |  |  |  |  |  |  |  |  |  |  |
| --- | --- | --- | --- | --- | --- | --- | --- | --- | --- | --- | --- | --- | --- | --- | --- | --- | --- | --- | --- | --- | --- | --- | --- | --- | --- | --- | --- | --- | --- |

JAB54395.1

back to list

  

| Protein Coverage
| Supporting Peptides
|

Protein Coverage:

Supporting Peptides:

| Peptide | Uniq | -10lgP | Mass | Length | ppm | m/z | z | RT | Fraction | Scan | Source File | Area F2 | Area F1 | Area F3 | Area F4 | Area F5 | Area F6 | #Feature | #Feature F2 | #Feature F1 | #Feature F3 | #Feature F4 | #Feature F5 | #Feature F6 | Start | End | PTM | AScore | Found By |
| --- | --- | --- | --- | --- | --- | --- | --- | --- | --- | --- | --- | --- | --- | --- | --- | --- | --- | --- | --- | --- | --- | --- | --- | --- | --- | --- | --- | --- | --- |
| R.GYSFTTTAER.E | Y | 73.07 | 1131.5197 | 10 | 0.2 | 566.7672 | 2 | 28.89 | 1 | F1:12159 | 28122021\_RID\_2349\_2C.raw | 9.9209E5 | 1.8624E4 |  |  |  |  | 2 | 1 | 1 | 0 | 0 | 0 | 0 | 197 | 206 |  |  | PEAKS DB |
| K.SYELPDGQVITIGNER.F | N | 71.59 | 1789.8846 | 16 | 0.9 | 895.9504 | 2 | 50.05 | 1 | F1:29089 | 28122021\_RID\_2349\_2C.raw | 6.9813E5 |  | 4.7278E5 |  | 1.5455E5 | 3.4204E5 | 4 | 1 | 0 | 1 | 0 | 1 | 1 | 239 | 254 |  |  | PEAKS DB |
| K.DSYVGDEAQSK.R | N | 71.53 | 1197.5149 | 11 | 0.8 | 599.7652 | 2 | 21.07 | 1 | F1:6133 | 28122021\_RID\_2349\_2C.raw | 9.3415E5 |  |  |  |  |  | 1 | 1 | 0 | 0 | 0 | 0 | 0 | 51 | 61 |  |  | PEAKS DB |
| R.HQGVMVGMGQK.D | N | 65.34 | 1170.5638 | 11 | 0.1 | 586.2892 | 2 | 20.03 | 1 | F1:5302 | 28122021\_RID\_2349\_2C.raw | 1.0514E5 |  |  |  |  |  | 1 | 1 | 0 | 0 | 0 | 0 | 0 | 40 | 50 |  |  | PEAKS DB |
| R.AVFPSIVGRPR.H | N | 62.54 | 1197.6981 | 11 | 0.6 | 599.8567 | 2 | 34.45 | 1 | F1:16654 | 28122021\_RID\_2349\_2C.raw | 5.9717E5 |  |  |  |  |  | 1 | 1 | 0 | 0 | 0 | 0 | 0 | 29 | 39 |  |  | PEAKS DB |
| K.AGFAGDDAPR.A | N | 60.47 | 975.4410 | 10 | 0.3 | 488.7279 | 2 | 22.55 | 1 | F1:7091 | 28122021\_RID\_2349\_2C.raw | 1.4103E6 |  |  |  |  |  | 1 | 1 | 0 | 0 | 0 | 0 | 0 | 19 | 28 |  |  | PEAKS DB |
| R.HQGVM(+15.99)VGMGQK.D | N | 55.56 | 1186.5587 | 11 | -0.4 | 396.5267 | 3 | 16.87 | 1 | F1:3534 | 28122021\_RID\_2349\_2C.raw | 0 |  |  |  |  |  | 0 | 0 | 0 | 0 | 0 | 0 | 0 | 40 | 50 | Oxidation (M) | M5:Oxidation (M):34.94 | PEAKS DB |
| K.DLYANTVLSGGTTM(+15.99)YPGIADR.M | Y | 54.04 | 2230.0576 | 21 | 0.2 | 1116.0363 | 2 | 53.98 | 1 | F1:31887 | 28122021\_RID\_2349\_2C.raw | 4.0971E4 |  |  |  |  |  | 1 | 1 | 0 | 0 | 0 | 0 | 0 | 292 | 312 | Oxidation (M) | M14:Oxidation (M):1000.00 | PEAKS DB |
| R.VAPEEHPVLLTEAPLNPK.A | Y | 53.40 | 1953.0570 | 18 | 0.5 | 652.0266 | 3 | 40.65 | 1 | F1:21676 | 28122021\_RID\_2349\_2C.raw | 1.9556E6 |  |  |  |  |  | 1 | 1 | 0 | 0 | 0 | 0 | 0 | 96 | 113 |  |  | PEAKS DB |
| K.DSYVGDEAQSKR.G | N | 52.16 | 1353.6160 | 12 | 0.7 | 677.8157 | 2 | 17.11 | 1 | F1:3748 | 28122021\_RID\_2349\_2C.raw | 1.2575E5 |  |  |  |  |  | 1 | 1 | 0 | 0 | 0 | 0 | 0 | 51 | 62 |  |  | PEAKS DB |
| R.DLTDYLMK.I | N | 43.76 | 997.4790 | 8 | -0.1 | 499.7467 | 2 | 52.05 | 5 | F5:28689 | 31122021\_RID\_2349\_5C.raw |  |  |  |  | 9.6944E4 |  | 1 | 0 | 0 | 0 | 0 | 1 | 0 | 184 | 191 |  |  | PEAKS DB |
| total 11 peptides |  |  |  |  |  |  |  |  |  |  |  |  |  |  |  |  |  |  |  |  |  |  |  |  |  |  |  |  |  |
| --- | --- | --- | --- | --- | --- | --- | --- | --- | --- | --- | --- | --- | --- | --- | --- | --- | --- | --- | --- | --- | --- | --- | --- | --- | --- | --- | --- | --- | --- |

ETE74025.1

back to list

  

| Protein Coverage
| Supporting Peptides
|

Protein Coverage:

Supporting Peptides:

| Peptide | Uniq | -10lgP | Mass | Length | ppm | m/z | z | RT | Fraction | Scan | Source File | Area F2 | Area F1 | Area F3 | Area F4 | Area F5 | Area F6 | #Feature | #Feature F2 | #Feature F1 | #Feature F3 | #Feature F4 | #Feature F5 | #Feature F6 | Start | End | PTM | AScore | Found By |
| --- | --- | --- | --- | --- | --- | --- | --- | --- | --- | --- | --- | --- | --- | --- | --- | --- | --- | --- | --- | --- | --- | --- | --- | --- | --- | --- | --- | --- | --- |
| R.GYSFTTTAER.E | Y | 73.07 | 1131.5197 | 10 | 0.2 | 566.7672 | 2 | 28.89 | 1 | F1:12159 | 28122021\_RID\_2349\_2C.raw | 9.9209E5 | 1.8624E4 |  |  |  |  | 2 | 1 | 1 | 0 | 0 | 0 | 0 | 197 | 206 |  |  | PEAKS DB |
| K.SYELPDGQVITIGNER.F | N | 71.59 | 1789.8846 | 16 | 0.9 | 895.9504 | 2 | 50.05 | 1 | F1:29089 | 28122021\_RID\_2349\_2C.raw | 6.9813E5 |  | 4.7278E5 |  | 1.5455E5 | 3.4204E5 | 4 | 1 | 0 | 1 | 0 | 1 | 1 | 239 | 254 |  |  | PEAKS DB |
| K.DSYVGDEAQSK.R | N | 71.53 | 1197.5149 | 11 | 0.8 | 599.7652 | 2 | 21.07 | 1 | F1:6133 | 28122021\_RID\_2349\_2C.raw | 9.3415E5 |  |  |  |  |  | 1 | 1 | 0 | 0 | 0 | 0 | 0 | 51 | 61 |  |  | PEAKS DB |
| R.HQGVMVGMGQK.D | N | 65.34 | 1170.5638 | 11 | 0.1 | 586.2892 | 2 | 20.03 | 1 | F1:5302 | 28122021\_RID\_2349\_2C.raw | 1.0514E5 |  |  |  |  |  | 1 | 1 | 0 | 0 | 0 | 0 | 0 | 40 | 50 |  |  | PEAKS DB |
| R.AVFPSIVGRPR.H | N | 62.54 | 1197.6981 | 11 | 0.6 | 599.8567 | 2 | 34.45 | 1 | F1:16654 | 28122021\_RID\_2349\_2C.raw | 5.9717E5 |  |  |  |  |  | 1 | 1 | 0 | 0 | 0 | 0 | 0 | 29 | 39 |  |  | PEAKS DB |
| K.AGFAGDDAPR.A | N | 60.47 | 975.4410 | 10 | 0.3 | 488.7279 | 2 | 22.55 | 1 | F1:7091 | 28122021\_RID\_2349\_2C.raw | 1.4103E6 |  |  |  |  |  | 1 | 1 | 0 | 0 | 0 | 0 | 0 | 19 | 28 |  |  | PEAKS DB |
| R.HQGVM(+15.99)VGMGQK.D | N | 55.56 | 1186.5587 | 11 | -0.4 | 396.5267 | 3 | 16.87 | 1 | F1:3534 | 28122021\_RID\_2349\_2C.raw | 0 |  |  |  |  |  | 0 | 0 | 0 | 0 | 0 | 0 | 0 | 40 | 50 | Oxidation (M) | M5:Oxidation (M):34.94 | PEAKS DB |
| K.DLYANTVLSGGTTM(+15.99)YPGIADR.M | Y | 54.04 | 2230.0576 | 21 | 0.2 | 1116.0363 | 2 | 53.98 | 1 | F1:31887 | 28122021\_RID\_2349\_2C.raw | 4.0971E4 |  |  |  |  |  | 1 | 1 | 0 | 0 | 0 | 0 | 0 | 292 | 312 | Oxidation (M) | M14:Oxidation (M):1000.00 | PEAKS DB |
| R.VAPEEHPVLLTEAPLNPK.A | Y | 53.40 | 1953.0570 | 18 | 0.5 | 652.0266 | 3 | 40.65 | 1 | F1:21676 | 28122021\_RID\_2349\_2C.raw | 1.9556E6 |  |  |  |  |  | 1 | 1 | 0 | 0 | 0 | 0 | 0 | 96 | 113 |  |  | PEAKS DB |
| K.DSYVGDEAQSKR.G | N | 52.16 | 1353.6160 | 12 | 0.7 | 677.8157 | 2 | 17.11 | 1 | F1:3748 | 28122021\_RID\_2349\_2C.raw | 1.2575E5 |  |  |  |  |  | 1 | 1 | 0 | 0 | 0 | 0 | 0 | 51 | 62 |  |  | PEAKS DB |
| R.DLTDYLMK.I | N | 43.76 | 997.4790 | 8 | -0.1 | 499.7467 | 2 | 52.05 | 5 | F5:28689 | 31122021\_RID\_2349\_5C.raw |  |  |  |  | 9.6944E4 |  | 1 | 0 | 0 | 0 | 0 | 1 | 0 | 184 | 191 |  |  | PEAKS DB |
| total 11 peptides |  |  |  |  |  |  |  |  |  |  |  |  |  |  |  |  |  |  |  |  |  |  |  |  |  |  |  |  |  |
| --- | --- | --- | --- | --- | --- | --- | --- | --- | --- | --- | --- | --- | --- | --- | --- | --- | --- | --- | --- | --- | --- | --- | --- | --- | --- | --- | --- | --- | --- |

XP\_007442357.1

back to list

  

| Protein Coverage
| Supporting Peptides
|

Protein Coverage:

Supporting Peptides:

| Peptide | Uniq | -10lgP | Mass | Length | ppm | m/z | z | RT | Fraction | Scan | Source File | Area F2 | Area F1 | Area F3 | Area F4 | Area F5 | Area F6 | #Feature | #Feature F2 | #Feature F1 | #Feature F3 | #Feature F4 | #Feature F5 | #Feature F6 | Start | End | PTM | AScore | Found By |
| --- | --- | --- | --- | --- | --- | --- | --- | --- | --- | --- | --- | --- | --- | --- | --- | --- | --- | --- | --- | --- | --- | --- | --- | --- | --- | --- | --- | --- | --- |
| K.AQYEDIAQK.S | N | 72.21 | 1064.5138 | 9 | 0.4 | 533.2644 | 2 | 22.89 | 6 | F6:6606 | 31122021\_RID\_2349\_6C.raw | 3.9872E7 | 1.3826E7 | 1.946E8 | 1.0751E8 | 7.0662E6 | 3.7146E6 | 6 | 1 | 1 | 1 | 1 | 1 | 1 | 331 | 339 |  |  | PEAKS DB |
| K.LALDIEIATYR.K | N | 71.69 | 1276.7026 | 11 | 0.5 | 639.3589 | 2 | 53.27 | 1 | F1:31324 | 28122021\_RID\_2349\_2C.raw | 9.0531E5 | 2.8506E4 | 2.692E7 | 1.4957E7 | 5.3148E5 | 1.8027E5 | 6 | 1 | 1 | 1 | 1 | 1 | 1 | 448 | 458 |  |  | PEAKS DB |
| R.LLKEYQELMNVK.L | N | 61.54 | 1506.8115 | 12 | 0.1 | 754.4131 | 2 | 40.19 | 3 | F3:11620 | 31122021\_RID\_2349\_3C.raw |  |  | 2.9093E6 |  |  |  | 1 | 0 | 0 | 1 | 0 | 0 | 0 | 436 | 447 |  |  | PEAKS DB |
| K.DVDAAYMNK.V | N | 61.08 | 1025.4487 | 9 | 1.0 | 513.7322 | 2 | 27.22 | 1 | F1:10874 | 28122021\_RID\_2349\_2C.raw | 2.0775E5 | 5.2491E4 |  |  |  | 3.9562E4 | 3 | 1 | 1 | 0 | 0 | 0 | 1 | 265 | 273 |  |  | PEAKS DB |
| K.FASFIDKVR.F | N | 59.84 | 1081.5920 | 9 | 1.2 | 541.8040 | 2 | 33.97 | 6 | F6:14555 | 31122021\_RID\_2349\_6C.raw | 6.7057E5 |  | 1.5615E7 |  | 6.0374E5 | 2.7185E5 | 4 | 1 | 0 | 1 | 0 | 1 | 1 | 164 | 172 |  |  | PEAKS DB |
| K.NKYEDEINKR.T | N | 59.38 | 1307.6470 | 10 | 0.8 | 654.8313 | 2 | 16.32 | 1 | F1:3281 | 28122021\_RID\_2349\_2C.raw | 1.8679E7 | 2.4367E6 | 8.2281E7 | 3.4977E7 | 1.6788E6 | 9.6273E5 | 8 | 2 | 1 | 1 | 1 | 2 | 1 | 243 | 252 |  |  | PEAKS DB |
| K.NKYEDEINK.R | N | 53.36 | 1151.5458 | 9 | 0.4 | 576.7804 | 2 | 16.89 | 1 | F1:3601 | 28122021\_RID\_2349\_2C.raw | 1.1716E6 |  | 1.3715E7 |  |  |  | 4 | 2 | 0 | 2 | 0 | 0 | 0 | 243 | 251 |  |  | PEAKS DB |
| K.EYQELMNVK.L | N | 49.62 | 1152.5485 | 9 | 0.7 | 577.2819 | 2 | 34.25 | 1 | F1:16408 | 28122021\_RID\_2349\_2C.raw | 1.4449E5 |  | 7.4495E6 |  |  |  | 2 | 1 | 0 | 1 | 0 | 0 | 0 | 439 | 447 |  |  | PEAKS DB |
| K.DVDAAYMNKVELQAK.L | Y | 48.03 | 1693.8345 | 15 | 1.3 | 847.9256 | 2 | 37.54 | 3 | F3:10585 | 31122021\_RID\_2349\_3C.raw |  |  | 0 |  |  |  | 0 | 0 | 0 | 0 | 0 | 0 | 0 | 265 | 279 |  |  | PEAKS DB |
| K.FASFIDK.V | N | 45.14 | 826.4225 | 7 | -0.5 | 414.2183 | 2 | 34.78 | 1 | F1:16890 | 28122021\_RID\_2349\_2C.raw | 6.9087E6 |  |  |  | 9.8962E5 |  | 2 | 1 | 0 | 0 | 0 | 1 | 0 | 164 | 170 |  |  | PEAKS DB |
| total 10 peptides |  |  |  |  |  |  |  |  |  |  |  |  |  |  |  |  |  |  |  |  |  |  |  |  |  |  |  |  |  |
| --- | --- | --- | --- | --- | --- | --- | --- | --- | --- | --- | --- | --- | --- | --- | --- | --- | --- | --- | --- | --- | --- | --- | --- | --- | --- | --- | --- | --- | --- |

JAV51432.1

back to list

  

| Protein Coverage
| Supporting Peptides
|

Protein Coverage:

Supporting Peptides:

| Peptide | Uniq | -10lgP | Mass | Length | ppm | m/z | z | RT | Fraction | Scan | Source File | Area F2 | Area F1 | Area F3 | Area F4 | Area F5 | Area F6 | #Feature | #Feature F2 | #Feature F1 | #Feature F3 | #Feature F4 | #Feature F5 | #Feature F6 | Start | End | PTM | AScore | Found By |
| --- | --- | --- | --- | --- | --- | --- | --- | --- | --- | --- | --- | --- | --- | --- | --- | --- | --- | --- | --- | --- | --- | --- | --- | --- | --- | --- | --- | --- | --- |
| S.IILESGNVNDYEVVYPR.K | N | 79.46 | 1979.0000 | 17 | 0.8 | 990.5081 | 2 | 50.36 | 6 | F6:27387 | 31122021\_RID\_2349\_6C.raw |  |  |  |  |  | 7.374E5 | 1 | 0 | 0 | 0 | 0 | 0 | 1 | 21 | 37 |  |  | PEAKS DB |
| K.YEDTMQYEFK.V | N | 70.38 | 1352.5594 | 10 | 0.5 | 677.2874 | 2 | 37.13 | 1 | F1:18721 | 28122021\_RID\_2349\_2C.raw | 3.5439E5 | 7.7616E3 |  |  | 1.6811E5 | 5.9028E5 | 4 | 1 | 1 | 0 | 0 | 1 | 1 | 51 | 60 |  |  | PEAKS DB |
| N.DEDKGMVLPGTK.C | N | 58.32 | 1288.6333 | 12 | 0.5 | 645.3242 | 2 | 29.12 | 1 | F1:12339 | 28122021\_RID\_2349\_2C.raw | 6.3922E5 |  |  |  |  |  | 1 | 1 | 0 | 0 | 0 | 0 | 0 | 580 | 591 |  |  | PEAKS DB |
| K.SHDNAQLLTGINFN.G | Y | 51.02 | 1542.7427 | 14 | 0.2 | 772.3788 | 2 | 48.95 | 1 | F1:28129 | 28122021\_RID\_2349\_2C.raw | 7.3415E5 |  |  |  |  | 2.2009E5 | 2 | 1 | 0 | 0 | 0 | 0 | 1 | 284 | 297 |  |  | PEAKS DB |
| K.SHDNAQLLTGINFNGP.T | Y | 46.99 | 1696.8169 | 16 | 0.2 | 849.4159 | 2 | 50.48 | 1 | F1:29297 | 28122021\_RID\_2349\_2C.raw | 6.0855E4 |  |  |  |  | 2.6907E5 | 2 | 1 | 0 | 0 | 0 | 0 | 1 | 284 | 299 |  |  | PEAKS DB |
| K.SHDNAQLLTGINFNGPTAG.L | Y | 46.68 | 1925.9231 | 19 | 0.6 | 963.9694 | 2 | 50.59 | 6 | F6:27641 | 31122021\_RID\_2349\_6C.raw |  |  |  |  |  | 1.1555E6 | 1 | 0 | 0 | 0 | 0 | 0 | 1 | 284 | 302 |  |  | PEAKS DB |
| K.SHDNAQLLTGINFNGPTAGLA.Y | Y | 43.59 | 2110.0442 | 21 | 0.9 | 1056.0303 | 2 | 56.96 | 6 | F6:32263 | 31122021\_RID\_2349\_6C.raw |  |  |  |  |  | 6.4737E5 | 1 | 0 | 0 | 0 | 0 | 0 | 1 | 284 | 304 |  |  | PEAKS DB |
| K.GM(+15.99)VLPGTK.C | N | 43.14 | 817.4368 | 8 | -0.6 | 409.7254 | 2 | 23.59 | 1 | F1:8201 | 28122021\_RID\_2349\_2C.raw | 2.8841E7 |  |  |  |  |  | 1 | 1 | 0 | 0 | 0 | 0 | 0 | 584 | 591 | Oxidation (M) | M2:Oxidation (M):1000.00 | PEAKS DB |
| total 8 peptides |  |  |  |  |  |  |  |  |  |  |  |  |  |  |  |  |  |  |  |  |  |  |  |  |  |  |  |  |  |
| --- | --- | --- | --- | --- | --- | --- | --- | --- | --- | --- | --- | --- | --- | --- | --- | --- | --- | --- | --- | --- | --- | --- | --- | --- | --- | --- | --- | --- | --- |

P82981.1

back to list

  

| Protein Coverage
| Supporting Peptides
|

Protein Coverage:

Supporting Peptides:

| Peptide | Uniq | -10lgP | Mass | Length | ppm | m/z | z | RT | Fraction | Scan | Source File | Area F2 | Area F1 | Area F3 | Area F4 | Area F5 | Area F6 | #Feature | #Feature F2 | #Feature F1 | #Feature F3 | #Feature F4 | #Feature F5 | #Feature F6 | Start | End | PTM | AScore | Found By |
| --- | --- | --- | --- | --- | --- | --- | --- | --- | --- | --- | --- | --- | --- | --- | --- | --- | --- | --- | --- | --- | --- | --- | --- | --- | --- | --- | --- | --- | --- |
| K.NDEVLDKDIMLIK.L | N | 90.84 | 1544.8120 | 13 | 0.1 | 773.4133 | 2 | 45.16 | 3 | F3:14095 | 31122021\_RID\_2349\_3C.raw |  |  | 1.9011E8 |  | 1.3152E7 |  | 7 | 0 | 0 | 4 | 0 | 3 | 0 | 79 | 91 |  |  | PEAKS DB |
| K.NDEVLDKDIM(+15.99)LIK.L | N | 70.24 | 1560.8069 | 13 | 0.3 | 781.4109 | 2 | 42.53 | 3 | F3:12773 | 31122021\_RID\_2349\_3C.raw |  |  | 6.8271E6 |  | 2.693E5 |  | 3 | 0 | 0 | 2 | 0 | 1 | 0 | 79 | 91 | Oxidation (M) | M10:Oxidation (M):1000.00 | PEAKS DB |
| K.VLNEDEQIRNPK.E | Y | 67.72 | 1453.7524 | 12 | 0.5 | 727.8839 | 2 | 21.52 | 1 | F1:6415 | 28122021\_RID\_2349\_2C.raw | 8.9947E4 |  |  |  | 3.2956E5 | 4.8116E4 | 3 | 1 | 0 | 0 | 0 | 1 | 1 | 58 | 69 |  |  | PEAKS DB |
| K.KNDEVLDKDIMLIK.L | N | 67.48 | 1672.9069 | 14 | 0.1 | 558.6429 | 3 | 38.36 | 5 | F5:18445 | 31122021\_RID\_2349\_5C.raw |  |  | 2.1269E7 |  | 1.0581E6 |  | 2 | 0 | 0 | 1 | 0 | 1 | 0 | 78 | 91 |  |  | PEAKS DB |
| K.VLNEDEQIR.N | Y | 59.81 | 1114.5618 | 9 | 0.4 | 558.2884 | 2 | 24.74 | 5 | F5:8986 | 31122021\_RID\_2349\_5C.raw |  |  |  |  | 0 |  | 0 | 0 | 0 | 0 | 0 | 0 | 0 | 58 | 66 |  |  | PEAKS DB |
| K.KVLNEDEQIRNPK.E | Y | 48.15 | 1581.8474 | 13 | 0.1 | 528.2898 | 3 | 18.38 | 5 | F5:4790 | 31122021\_RID\_2349\_5C.raw |  |  |  |  | 1.4253E6 |  | 1 | 0 | 0 | 0 | 0 | 1 | 0 | 57 | 69 |  |  | PEAKS DB |
| K.KVLNEDEQIR.N | Y | 45.67 | 1242.6567 | 10 | 0.3 | 415.2263 | 3 | 20.00 | 5 | F5:5661 | 31122021\_RID\_2349\_5C.raw |  |  |  |  | 1.6986E5 |  | 1 | 0 | 0 | 0 | 0 | 1 | 0 | 57 | 66 |  |  | PEAKS DB |
| total 7 peptides |  |  |  |  |  |  |  |  |  |  |  |  |  |  |  |  |  |  |  |  |  |  |  |  |  |  |  |  |  |
| --- | --- | --- | --- | --- | --- | --- | --- | --- | --- | --- | --- | --- | --- | --- | --- | --- | --- | --- | --- | --- | --- | --- | --- | --- | --- | --- | --- | --- | --- |

JAV51419.1

back to list

  

| Protein Coverage
| Supporting Peptides
|

Protein Coverage:

Supporting Peptides:

| Peptide | Uniq | -10lgP | Mass | Length | ppm | m/z | z | RT | Fraction | Scan | Source File | Area F2 | Area F1 | Area F3 | Area F4 | Area F5 | Area F6 | #Feature | #Feature F2 | #Feature F1 | #Feature F3 | #Feature F4 | #Feature F5 | #Feature F6 | Start | End | PTM | AScore | Found By |
| --- | --- | --- | --- | --- | --- | --- | --- | --- | --- | --- | --- | --- | --- | --- | --- | --- | --- | --- | --- | --- | --- | --- | --- | --- | --- | --- | --- | --- | --- |
| K.NDEVLDKDIMLIK.L | N | 90.84 | 1544.8120 | 13 | 0.1 | 773.4133 | 2 | 45.16 | 3 | F3:14095 | 31122021\_RID\_2349\_3C.raw |  |  | 1.9011E8 |  | 1.3152E7 |  | 7 | 0 | 0 | 4 | 0 | 3 | 0 | 103 | 115 |  |  | PEAKS DB |
| K.NDEVLDKDIM(+15.99)LIK.L | N | 70.24 | 1560.8069 | 13 | 0.3 | 781.4109 | 2 | 42.53 | 3 | F3:12773 | 31122021\_RID\_2349\_3C.raw |  |  | 6.8271E6 |  | 2.693E5 |  | 3 | 0 | 0 | 2 | 0 | 1 | 0 | 103 | 115 | Oxidation (M) | M10:Oxidation (M):1000.00 | PEAKS DB |
| K.VLNEDEQIRNPK.E | Y | 67.72 | 1453.7524 | 12 | 0.5 | 727.8839 | 2 | 21.52 | 1 | F1:6415 | 28122021\_RID\_2349\_2C.raw | 8.9947E4 |  |  |  | 3.2956E5 | 4.8116E4 | 3 | 1 | 0 | 0 | 0 | 1 | 1 | 82 | 93 |  |  | PEAKS DB |
| K.KNDEVLDKDIMLIK.L | N | 67.48 | 1672.9069 | 14 | 0.1 | 558.6429 | 3 | 38.36 | 5 | F5:18445 | 31122021\_RID\_2349\_5C.raw |  |  | 2.1269E7 |  | 1.0581E6 |  | 2 | 0 | 0 | 1 | 0 | 1 | 0 | 102 | 115 |  |  | PEAKS DB |
| K.VLNEDEQIR.N | Y | 59.81 | 1114.5618 | 9 | 0.4 | 558.2884 | 2 | 24.74 | 5 | F5:8986 | 31122021\_RID\_2349\_5C.raw |  |  |  |  | 0 |  | 0 | 0 | 0 | 0 | 0 | 0 | 0 | 82 | 90 |  |  | PEAKS DB |
| K.KVLNEDEQIRNPK.E | Y | 48.15 | 1581.8474 | 13 | 0.1 | 528.2898 | 3 | 18.38 | 5 | F5:4790 | 31122021\_RID\_2349\_5C.raw |  |  |  |  | 1.4253E6 |  | 1 | 0 | 0 | 0 | 0 | 1 | 0 | 81 | 93 |  |  | PEAKS DB |
| K.KVLNEDEQIR.N | Y | 45.67 | 1242.6567 | 10 | 0.3 | 415.2263 | 3 | 20.00 | 5 | F5:5661 | 31122021\_RID\_2349\_5C.raw |  |  |  |  | 1.6986E5 |  | 1 | 0 | 0 | 0 | 0 | 1 | 0 | 81 | 90 |  |  | PEAKS DB |
| total 7 peptides |  |  |  |  |  |  |  |  |  |  |  |  |  |  |  |  |  |  |  |  |  |  |  |  |  |  |  |  |  |
| --- | --- | --- | --- | --- | --- | --- | --- | --- | --- | --- | --- | --- | --- | --- | --- | --- | --- | --- | --- | --- | --- | --- | --- | --- | --- | --- | --- | --- | --- |

BAA19976.1

back to list

  

| Protein Coverage
| Supporting Peptides
|

Protein Coverage:

Supporting Peptides:

| Peptide | Uniq | -10lgP | Mass | Length | ppm | m/z | z | RT | Fraction | Scan | Source File | Area F2 | Area F1 | Area F3 | Area F4 | Area F5 | Area F6 | #Feature | #Feature F2 | #Feature F1 | #Feature F3 | #Feature F4 | #Feature F5 | #Feature F6 | Start | End | PTM | AScore | Found By |
| --- | --- | --- | --- | --- | --- | --- | --- | --- | --- | --- | --- | --- | --- | --- | --- | --- | --- | --- | --- | --- | --- | --- | --- | --- | --- | --- | --- | --- | --- |
| K.NDEVLDKDIMLIK.L | N | 90.84 | 1544.8120 | 13 | 0.1 | 773.4133 | 2 | 45.16 | 3 | F3:14095 | 31122021\_RID\_2349\_3C.raw |  |  | 1.9011E8 |  | 1.3152E7 |  | 7 | 0 | 0 | 4 | 0 | 3 | 0 | 105 | 117 |  |  | PEAKS DB |
| K.NDEVLDKDIM(+15.99)LIK.L | N | 70.24 | 1560.8069 | 13 | 0.3 | 781.4109 | 2 | 42.53 | 3 | F3:12773 | 31122021\_RID\_2349\_3C.raw |  |  | 6.8271E6 |  | 2.693E5 |  | 3 | 0 | 0 | 2 | 0 | 1 | 0 | 105 | 117 | Oxidation (M) | M10:Oxidation (M):1000.00 | PEAKS DB |
| K.VLNEDEQIRNPK.E | Y | 67.72 | 1453.7524 | 12 | 0.5 | 727.8839 | 2 | 21.52 | 1 | F1:6415 | 28122021\_RID\_2349\_2C.raw | 8.9947E4 |  |  |  | 3.2956E5 | 4.8116E4 | 3 | 1 | 0 | 0 | 0 | 1 | 1 | 84 | 95 |  |  | PEAKS DB |
| K.KNDEVLDKDIMLIK.L | N | 67.48 | 1672.9069 | 14 | 0.1 | 558.6429 | 3 | 38.36 | 5 | F5:18445 | 31122021\_RID\_2349\_5C.raw |  |  | 2.1269E7 |  | 1.0581E6 |  | 2 | 0 | 0 | 1 | 0 | 1 | 0 | 104 | 117 |  |  | PEAKS DB |
| K.VLNEDEQIR.N | Y | 59.81 | 1114.5618 | 9 | 0.4 | 558.2884 | 2 | 24.74 | 5 | F5:8986 | 31122021\_RID\_2349\_5C.raw |  |  |  |  | 0 |  | 0 | 0 | 0 | 0 | 0 | 0 | 0 | 84 | 92 |  |  | PEAKS DB |
| K.KVLNEDEQIRNPK.E | Y | 48.15 | 1581.8474 | 13 | 0.1 | 528.2898 | 3 | 18.38 | 5 | F5:4790 | 31122021\_RID\_2349\_5C.raw |  |  |  |  | 1.4253E6 |  | 1 | 0 | 0 | 0 | 0 | 1 | 0 | 83 | 95 |  |  | PEAKS DB |
| K.KVLNEDEQIR.N | Y | 45.67 | 1242.6567 | 10 | 0.3 | 415.2263 | 3 | 20.00 | 5 | F5:5661 | 31122021\_RID\_2349\_5C.raw |  |  |  |  | 1.6986E5 |  | 1 | 0 | 0 | 0 | 0 | 1 | 0 | 83 | 92 |  |  | PEAKS DB |
| total 7 peptides |  |  |  |  |  |  |  |  |  |  |  |  |  |  |  |  |  |  |  |  |  |  |  |  |  |  |  |  |  |
| --- | --- | --- | --- | --- | --- | --- | --- | --- | --- | --- | --- | --- | --- | --- | --- | --- | --- | --- | --- | --- | --- | --- | --- | --- | --- | --- | --- | --- | --- |

BAN82033.1

back to list

  

| Protein Coverage
| Supporting Peptides
|

Protein Coverage:

Supporting Peptides:

| Peptide | Uniq | -10lgP | Mass | Length | ppm | m/z | z | RT | Fraction | Scan | Source File | Area F2 | Area F1 | Area F3 | Area F4 | Area F5 | Area F6 | #Feature | #Feature F2 | #Feature F1 | #Feature F3 | #Feature F4 | #Feature F5 | #Feature F6 | Start | End | PTM | AScore | Found By |
| --- | --- | --- | --- | --- | --- | --- | --- | --- | --- | --- | --- | --- | --- | --- | --- | --- | --- | --- | --- | --- | --- | --- | --- | --- | --- | --- | --- | --- | --- |
| K.NDEVLDKDIMLIK.L | N | 90.84 | 1544.8120 | 13 | 0.1 | 773.4133 | 2 | 45.16 | 3 | F3:14095 | 31122021\_RID\_2349\_3C.raw |  |  | 1.9011E8 |  | 1.3152E7 |  | 7 | 0 | 0 | 4 | 0 | 3 | 0 | 105 | 117 |  |  | PEAKS DB |
| K.NDEVLDKDIM(+15.99)LIK.L | N | 70.24 | 1560.8069 | 13 | 0.3 | 781.4109 | 2 | 42.53 | 3 | F3:12773 | 31122021\_RID\_2349\_3C.raw |  |  | 6.8271E6 |  | 2.693E5 |  | 3 | 0 | 0 | 2 | 0 | 1 | 0 | 105 | 117 | Oxidation (M) | M10:Oxidation (M):1000.00 | PEAKS DB |
| K.VLNEDEQIRNPK.E | Y | 67.72 | 1453.7524 | 12 | 0.5 | 727.8839 | 2 | 21.52 | 1 | F1:6415 | 28122021\_RID\_2349\_2C.raw | 8.9947E4 |  |  |  | 3.2956E5 | 4.8116E4 | 3 | 1 | 0 | 0 | 0 | 1 | 1 | 84 | 95 |  |  | PEAKS DB |
| K.KNDEVLDKDIMLIK.L | N | 67.48 | 1672.9069 | 14 | 0.1 | 558.6429 | 3 | 38.36 | 5 | F5:18445 | 31122021\_RID\_2349\_5C.raw |  |  | 2.1269E7 |  | 1.0581E6 |  | 2 | 0 | 0 | 1 | 0 | 1 | 0 | 104 | 117 |  |  | PEAKS DB |
| K.VLNEDEQIR.N | Y | 59.81 | 1114.5618 | 9 | 0.4 | 558.2884 | 2 | 24.74 | 5 | F5:8986 | 31122021\_RID\_2349\_5C.raw |  |  |  |  | 0 |  | 0 | 0 | 0 | 0 | 0 | 0 | 0 | 84 | 92 |  |  | PEAKS DB |
| K.KVLNEDEQIRNPK.E | Y | 48.15 | 1581.8474 | 13 | 0.1 | 528.2898 | 3 | 18.38 | 5 | F5:4790 | 31122021\_RID\_2349\_5C.raw |  |  |  |  | 1.4253E6 |  | 1 | 0 | 0 | 0 | 0 | 1 | 0 | 83 | 95 |  |  | PEAKS DB |
| K.KVLNEDEQIR.N | Y | 45.67 | 1242.6567 | 10 | 0.3 | 415.2263 | 3 | 20.00 | 5 | F5:5661 | 31122021\_RID\_2349\_5C.raw |  |  |  |  | 1.6986E5 |  | 1 | 0 | 0 | 0 | 0 | 1 | 0 | 83 | 92 |  |  | PEAKS DB |
| total 7 peptides |  |  |  |  |  |  |  |  |  |  |  |  |  |  |  |  |  |  |  |  |  |  |  |  |  |  |  |  |  |
| --- | --- | --- | --- | --- | --- | --- | --- | --- | --- | --- | --- | --- | --- | --- | --- | --- | --- | --- | --- | --- | --- | --- | --- | --- | --- | --- | --- | --- | --- |

BAP39955.1

back to list

  

| Protein Coverage
| Supporting Peptides
|

Protein Coverage:

Supporting Peptides:

| Peptide | Uniq | -10lgP | Mass | Length | ppm | m/z | z | RT | Fraction | Scan | Source File | Area F2 | Area F1 | Area F3 | Area F4 | Area F5 | Area F6 | #Feature | #Feature F2 | #Feature F1 | #Feature F3 | #Feature F4 | #Feature F5 | #Feature F6 | Start | End | PTM | AScore | Found By |
| --- | --- | --- | --- | --- | --- | --- | --- | --- | --- | --- | --- | --- | --- | --- | --- | --- | --- | --- | --- | --- | --- | --- | --- | --- | --- | --- | --- | --- | --- |
| K.NDEVLDKDIMLIK.L | N | 90.84 | 1544.8120 | 13 | 0.1 | 773.4133 | 2 | 45.16 | 3 | F3:14095 | 31122021\_RID\_2349\_3C.raw |  |  | 1.9011E8 |  | 1.3152E7 |  | 7 | 0 | 0 | 4 | 0 | 3 | 0 | 105 | 117 |  |  | PEAKS DB |
| K.NDEVLDKDIM(+15.99)LIK.L | N | 70.24 | 1560.8069 | 13 | 0.3 | 781.4109 | 2 | 42.53 | 3 | F3:12773 | 31122021\_RID\_2349\_3C.raw |  |  | 6.8271E6 |  | 2.693E5 |  | 3 | 0 | 0 | 2 | 0 | 1 | 0 | 105 | 117 | Oxidation (M) | M10:Oxidation (M):1000.00 | PEAKS DB |
| K.VLNEDEQIRNPK.E | Y | 67.72 | 1453.7524 | 12 | 0.5 | 727.8839 | 2 | 21.52 | 1 | F1:6415 | 28122021\_RID\_2349\_2C.raw | 8.9947E4 |  |  |  | 3.2956E5 | 4.8116E4 | 3 | 1 | 0 | 0 | 0 | 1 | 1 | 84 | 95 |  |  | PEAKS DB |
| K.KNDEVLDKDIMLIK.L | N | 67.48 | 1672.9069 | 14 | 0.1 | 558.6429 | 3 | 38.36 | 5 | F5:18445 | 31122021\_RID\_2349\_5C.raw |  |  | 2.1269E7 |  | 1.0581E6 |  | 2 | 0 | 0 | 1 | 0 | 1 | 0 | 104 | 117 |  |  | PEAKS DB |
| K.VLNEDEQIR.N | Y | 59.81 | 1114.5618 | 9 | 0.4 | 558.2884 | 2 | 24.74 | 5 | F5:8986 | 31122021\_RID\_2349\_5C.raw |  |  |  |  | 0 |  | 0 | 0 | 0 | 0 | 0 | 0 | 0 | 84 | 92 |  |  | PEAKS DB |
| K.KVLNEDEQIRNPK.E | Y | 48.15 | 1581.8474 | 13 | 0.1 | 528.2898 | 3 | 18.38 | 5 | F5:4790 | 31122021\_RID\_2349\_5C.raw |  |  |  |  | 1.4253E6 |  | 1 | 0 | 0 | 0 | 0 | 1 | 0 | 83 | 95 |  |  | PEAKS DB |
| K.KVLNEDEQIR.N | Y | 45.67 | 1242.6567 | 10 | 0.3 | 415.2263 | 3 | 20.00 | 5 | F5:5661 | 31122021\_RID\_2349\_5C.raw |  |  |  |  | 1.6986E5 |  | 1 | 0 | 0 | 0 | 0 | 1 | 0 | 83 | 92 |  |  | PEAKS DB |
| total 7 peptides |  |  |  |  |  |  |  |  |  |  |  |  |  |  |  |  |  |  |  |  |  |  |  |  |  |  |  |  |  |
| --- | --- | --- | --- | --- | --- | --- | --- | --- | --- | --- | --- | --- | --- | --- | --- | --- | --- | --- | --- | --- | --- | --- | --- | --- | --- | --- | --- | --- | --- |

P05620.2

back to list

  

| Protein Coverage
| Supporting Peptides
|

Protein Coverage:

Supporting Peptides:

| Peptide | Uniq | -10lgP | Mass | Length | ppm | m/z | z | RT | Fraction | Scan | Source File | Area F2 | Area F1 | Area F3 | Area F4 | Area F5 | Area F6 | #Feature | #Feature F2 | #Feature F1 | #Feature F3 | #Feature F4 | #Feature F5 | #Feature F6 | Start | End | PTM | AScore | Found By |
| --- | --- | --- | --- | --- | --- | --- | --- | --- | --- | --- | --- | --- | --- | --- | --- | --- | --- | --- | --- | --- | --- | --- | --- | --- | --- | --- | --- | --- | --- |
| K.NDEVLDKDIMLIK.L | N | 90.84 | 1544.8120 | 13 | 0.1 | 773.4133 | 2 | 45.16 | 3 | F3:14095 | 31122021\_RID\_2349\_3C.raw |  |  | 1.9011E8 |  | 1.3152E7 |  | 7 | 0 | 0 | 4 | 0 | 3 | 0 | 105 | 117 |  |  | PEAKS DB |
| K.NDEVLDKDIM(+15.99)LIK.L | N | 70.24 | 1560.8069 | 13 | 0.3 | 781.4109 | 2 | 42.53 | 3 | F3:12773 | 31122021\_RID\_2349\_3C.raw |  |  | 6.8271E6 |  | 2.693E5 |  | 3 | 0 | 0 | 2 | 0 | 1 | 0 | 105 | 117 | Oxidation (M) | M10:Oxidation (M):1000.00 | PEAKS DB |
| K.VLNEDEQIRNPK.E | Y | 67.72 | 1453.7524 | 12 | 0.5 | 727.8839 | 2 | 21.52 | 1 | F1:6415 | 28122021\_RID\_2349\_2C.raw | 8.9947E4 |  |  |  | 3.2956E5 | 4.8116E4 | 3 | 1 | 0 | 0 | 0 | 1 | 1 | 84 | 95 |  |  | PEAKS DB |
| K.KNDEVLDKDIMLIK.L | N | 67.48 | 1672.9069 | 14 | 0.1 | 558.6429 | 3 | 38.36 | 5 | F5:18445 | 31122021\_RID\_2349\_5C.raw |  |  | 2.1269E7 |  | 1.0581E6 |  | 2 | 0 | 0 | 1 | 0 | 1 | 0 | 104 | 117 |  |  | PEAKS DB |
| K.VLNEDEQIR.N | Y | 59.81 | 1114.5618 | 9 | 0.4 | 558.2884 | 2 | 24.74 | 5 | F5:8986 | 31122021\_RID\_2349\_5C.raw |  |  |  |  | 0 |  | 0 | 0 | 0 | 0 | 0 | 0 | 0 | 84 | 92 |  |  | PEAKS DB |
| K.KVLNEDEQIRNPK.E | Y | 48.15 | 1581.8474 | 13 | 0.1 | 528.2898 | 3 | 18.38 | 5 | F5:4790 | 31122021\_RID\_2349\_5C.raw |  |  |  |  | 1.4253E6 |  | 1 | 0 | 0 | 0 | 0 | 1 | 0 | 83 | 95 |  |  | PEAKS DB |
| K.KVLNEDEQIR.N | Y | 45.67 | 1242.6567 | 10 | 0.3 | 415.2263 | 3 | 20.00 | 5 | F5:5661 | 31122021\_RID\_2349\_5C.raw |  |  |  |  | 1.6986E5 |  | 1 | 0 | 0 | 0 | 0 | 1 | 0 | 83 | 92 |  |  | PEAKS DB |
| total 7 peptides |  |  |  |  |  |  |  |  |  |  |  |  |  |  |  |  |  |  |  |  |  |  |  |  |  |  |  |  |  |
| --- | --- | --- | --- | --- | --- | --- | --- | --- | --- | --- | --- | --- | --- | --- | --- | --- | --- | --- | --- | --- | --- | --- | --- | --- | --- | --- | --- | --- | --- |

AXL95287.1

back to list

  

| Protein Coverage
| Supporting Peptides
|

Protein Coverage:

Supporting Peptides:

| Peptide | Uniq | -10lgP | Mass | Length | ppm | m/z | z | RT | Fraction | Scan | Source File | Area F2 | Area F1 | Area F3 | Area F4 | Area F5 | Area F6 | #Feature | #Feature F2 | #Feature F1 | #Feature F3 | #Feature F4 | #Feature F5 | #Feature F6 | Start | End | PTM | AScore | Found By |
| --- | --- | --- | --- | --- | --- | --- | --- | --- | --- | --- | --- | --- | --- | --- | --- | --- | --- | --- | --- | --- | --- | --- | --- | --- | --- | --- | --- | --- | --- |
| Q.EDDYEEFLEIAR.N | Y | 61.68 | 1527.6729 | 12 | 1.3 | 764.8447 | 2 | 60.09 | 2 | F2:19611 | 30122021\_RID\_2349\_1C.raw |  | 3.3005E5 |  |  |  |  | 1 | 0 | 1 | 0 | 0 | 0 | 0 | 31 | 42 |  |  | PEAKS DB |
| R.EGWYVNLGPMR.L | N | 59.29 | 1320.6284 | 11 | 0.6 | 661.3219 | 2 | 51.43 | 6 | F6:28069 | 31122021\_RID\_2349\_6C.raw |  |  |  |  |  | 4.6915E4 | 1 | 0 | 0 | 0 | 0 | 0 | 1 | 98 | 108 |  |  | PEAKS DB |
| D.YEEFLEIAR.N | N | 56.31 | 1168.5764 | 9 | 0.7 | 585.2959 | 2 | 45.58 | 2 | F2:15608 | 30122021\_RID\_2349\_1C.raw |  | 2.3921E5 |  |  |  |  | 1 | 0 | 1 | 0 | 0 | 0 | 0 | 34 | 42 |  |  | PEAKS DB |
| D.DYEEFLEIAR.N | N | 56.01 | 1283.6033 | 10 | 0.8 | 642.8094 | 2 | 56.80 | 5 | F5:32140 | 31122021\_RID\_2349\_5C.raw | 1.3399E5 | 2.7826E5 |  |  | 9.5716E4 | 7.7266E4 | 4 | 1 | 1 | 0 | 0 | 1 | 1 | 33 | 42 |  |  | PEAKS DB |
| K.YDTYSTKEY.L | N | 49.76 | 1168.4924 | 9 | 1.0 | 585.2541 | 2 | 25.91 | 5 | F5:9933 | 31122021\_RID\_2349\_5C.raw |  |  |  |  | 2.4008E6 |  | 1 | 0 | 0 | 0 | 0 | 1 | 0 | 198 | 206 |  |  | PEAKS DB |
| D.FFQALDFK.S | N | 45.08 | 1014.5174 | 8 | 0.8 | 508.2664 | 2 | 48.21 | 2 | F2:16516 | 30122021\_RID\_2349\_1C.raw |  | 1.1251E4 |  |  |  |  | 1 | 0 | 1 | 0 | 0 | 0 | 0 | 398 | 405 |  |  | PEAKS DB |
| N.FEPPLPPK.K | N | 43.76 | 923.5116 | 8 | -0.4 | 462.7629 | 2 | 36.36 | 1 | F1:17621 | 28122021\_RID\_2349\_2C.raw | 2.2198E7 |  |  |  |  |  | 1 | 1 | 0 | 0 | 0 | 0 | 0 | 322 | 329 |  |  | PEAKS DB |
| total 7 peptides |  |  |  |  |  |  |  |  |  |  |  |  |  |  |  |  |  |  |  |  |  |  |  |  |  |  |  |  |  |
| --- | --- | --- | --- | --- | --- | --- | --- | --- | --- | --- | --- | --- | --- | --- | --- | --- | --- | --- | --- | --- | --- | --- | --- | --- | --- | --- | --- | --- | --- |

AXL95288.1

back to list

  

| Protein Coverage
| Supporting Peptides
|

Protein Coverage:

Supporting Peptides:

| Peptide | Uniq | -10lgP | Mass | Length | ppm | m/z | z | RT | Fraction | Scan | Source File | Area F2 | Area F1 | Area F3 | Area F4 | Area F5 | Area F6 | #Feature | #Feature F2 | #Feature F1 | #Feature F3 | #Feature F4 | #Feature F5 | #Feature F6 | Start | End | PTM | AScore | Found By |
| --- | --- | --- | --- | --- | --- | --- | --- | --- | --- | --- | --- | --- | --- | --- | --- | --- | --- | --- | --- | --- | --- | --- | --- | --- | --- | --- | --- | --- | --- |
| Q.EDDYEEFLEIAR.N | Y | 61.68 | 1527.6729 | 12 | 1.3 | 764.8447 | 2 | 60.09 | 2 | F2:19611 | 30122021\_RID\_2349\_1C.raw |  | 3.3005E5 |  |  |  |  | 1 | 0 | 1 | 0 | 0 | 0 | 0 | 31 | 42 |  |  | PEAKS DB |
| R.EGWYVNLGPMR.L | N | 59.29 | 1320.6284 | 11 | 0.6 | 661.3219 | 2 | 51.43 | 6 | F6:28069 | 31122021\_RID\_2349\_6C.raw |  |  |  |  |  | 4.6915E4 | 1 | 0 | 0 | 0 | 0 | 0 | 1 | 98 | 108 |  |  | PEAKS DB |
| D.YEEFLEIAR.N | N | 56.31 | 1168.5764 | 9 | 0.7 | 585.2959 | 2 | 45.58 | 2 | F2:15608 | 30122021\_RID\_2349\_1C.raw |  | 2.3921E5 |  |  |  |  | 1 | 0 | 1 | 0 | 0 | 0 | 0 | 34 | 42 |  |  | PEAKS DB |
| D.DYEEFLEIAR.N | N | 56.01 | 1283.6033 | 10 | 0.8 | 642.8094 | 2 | 56.80 | 5 | F5:32140 | 31122021\_RID\_2349\_5C.raw | 1.3399E5 | 2.7826E5 |  |  | 9.5716E4 | 7.7266E4 | 4 | 1 | 1 | 0 | 0 | 1 | 1 | 33 | 42 |  |  | PEAKS DB |
| K.YDTYSTKEY.L | N | 49.76 | 1168.4924 | 9 | 1.0 | 585.2541 | 2 | 25.91 | 5 | F5:9933 | 31122021\_RID\_2349\_5C.raw |  |  |  |  | 2.4008E6 |  | 1 | 0 | 0 | 0 | 0 | 1 | 0 | 198 | 206 |  |  | PEAKS DB |
| D.FFQALDFK.S | N | 45.08 | 1014.5174 | 8 | 0.8 | 508.2664 | 2 | 48.21 | 2 | F2:16516 | 30122021\_RID\_2349\_1C.raw |  | 1.1251E4 |  |  |  |  | 1 | 0 | 1 | 0 | 0 | 0 | 0 | 398 | 405 |  |  | PEAKS DB |
| N.FEPPLPPK.K | N | 43.76 | 923.5116 | 8 | -0.4 | 462.7629 | 2 | 36.36 | 1 | F1:17621 | 28122021\_RID\_2349\_2C.raw | 2.2198E7 |  |  |  |  |  | 1 | 1 | 0 | 0 | 0 | 0 | 0 | 322 | 329 |  |  | PEAKS DB |
| total 7 peptides |  |  |  |  |  |  |  |  |  |  |  |  |  |  |  |  |  |  |  |  |  |  |  |  |  |  |  |  |  |
| --- | --- | --- | --- | --- | --- | --- | --- | --- | --- | --- | --- | --- | --- | --- | --- | --- | --- | --- | --- | --- | --- | --- | --- | --- | --- | --- | --- | --- | --- |

ETE68810.1

back to list

  

| Protein Coverage
| Supporting Peptides
|

Protein Coverage:

Supporting Peptides:

| Peptide | Uniq | -10lgP | Mass | Length | ppm | m/z | z | RT | Fraction | Scan | Source File | Area F2 | Area F1 | Area F3 | Area F4 | Area F5 | Area F6 | #Feature | #Feature F2 | #Feature F1 | #Feature F3 | #Feature F4 | #Feature F5 | #Feature F6 | Start | End | PTM | AScore | Found By |
| --- | --- | --- | --- | --- | --- | --- | --- | --- | --- | --- | --- | --- | --- | --- | --- | --- | --- | --- | --- | --- | --- | --- | --- | --- | --- | --- | --- | --- | --- |
| K.QEPGQNSEILQGIK.H | N | 89.67 | 1539.7893 | 14 | 1.1 | 770.9028 | 2 | 37.67 | 5 | F5:17986 | 31122021\_RID\_2349\_5C.raw |  |  |  |  | 1.5715E7 |  | 1 | 0 | 0 | 0 | 0 | 1 | 0 | 150 | 163 |  |  | PEAKS DB |
| R.PGGGFVPNFQLFQK.G | N | 71.59 | 1534.7932 | 14 | 0.4 | 768.4042 | 2 | 56.25 | 5 | F5:31879 | 31122021\_RID\_2349\_5C.raw |  |  |  |  | 1.1749E6 |  | 1 | 0 | 0 | 0 | 0 | 1 | 0 | 167 | 180 |  |  | PEAKS DB |
| K.IHDIKWNFEK.F | N | 63.66 | 1328.6877 | 10 | 0.1 | 665.3512 | 2 | 30.71 | 5 | F5:13153 | 31122021\_RID\_2349\_5C.raw |  |  |  |  | 0 |  | 0 | 0 | 0 | 0 | 0 | 0 | 0 | 219 | 228 |  |  | PEAKS DB |
| K.FLVNPQGKPVMR.W | Y | 61.92 | 1384.7649 | 12 | 0.6 | 693.3901 | 2 | 31.63 | 5 | F5:13833 | 31122021\_RID\_2349\_5C.raw |  |  |  |  | 1.2481E7 |  | 2 | 0 | 0 | 0 | 0 | 2 | 0 | 229 | 240 |  |  | PEAKS DB |
| K.FLVNPQGKPVM.R | Y | 45.00 | 1228.6638 | 11 | -0.3 | 615.3390 | 2 | 41.06 | 5 | F5:20387 | 31122021\_RID\_2349\_5C.raw |  |  |  |  | 1.9986E6 |  | 1 | 0 | 0 | 0 | 0 | 1 | 0 | 229 | 239 |  |  | PEAKS DB |
| total 5 peptides |  |  |  |  |  |  |  |  |  |  |  |  |  |  |  |  |  |  |  |  |  |  |  |  |  |  |  |  |  |
| --- | --- | --- | --- | --- | --- | --- | --- | --- | --- | --- | --- | --- | --- | --- | --- | --- | --- | --- | --- | --- | --- | --- | --- | --- | --- | --- | --- | --- | --- |

XP\_034258146.1

back to list

  

| Protein Coverage
| Supporting Peptides
|

Protein Coverage:

Supporting Peptides:

| Peptide | Uniq | -10lgP | Mass | Length | ppm | m/z | z | RT | Fraction | Scan | Source File | Area F2 | Area F1 | Area F3 | Area F4 | Area F5 | Area F6 | #Feature | #Feature F2 | #Feature F1 | #Feature F3 | #Feature F4 | #Feature F5 | #Feature F6 | Start | End | PTM | AScore | Found By |
| --- | --- | --- | --- | --- | --- | --- | --- | --- | --- | --- | --- | --- | --- | --- | --- | --- | --- | --- | --- | --- | --- | --- | --- | --- | --- | --- | --- | --- | --- |
| K.QEPGQNSEILQGIK.Y | N | 89.67 | 1539.7893 | 14 | 1.1 | 770.9028 | 2 | 37.67 | 5 | F5:17986 | 31122021\_RID\_2349\_5C.raw |  |  |  |  | 1.5715E7 |  | 1 | 0 | 0 | 0 | 0 | 1 | 0 | 137 | 150 |  |  | PEAKS DB |
| K.YVRPGGGFVPNFQLFQK.I | Y | 81.75 | 1953.0260 | 17 | 3.5 | 652.0182 | 3 | 50.31 | 5 | F5:27408 | 31122021\_RID\_2349\_5C.raw |  |  |  |  | 2.6733E7 | 1.8888E6 | 3 | 0 | 0 | 0 | 0 | 2 | 1 | 151 | 167 |  |  | PEAKS DB |
| R.PGGGFVPNFQLFQK.I | N | 71.59 | 1534.7932 | 14 | 0.4 | 768.4042 | 2 | 56.25 | 5 | F5:31879 | 31122021\_RID\_2349\_5C.raw |  |  |  |  | 1.1749E6 |  | 1 | 0 | 0 | 0 | 0 | 1 | 0 | 154 | 167 |  |  | PEAKS DB |
| K.IHDIKWNFEK.F | N | 63.66 | 1328.6877 | 10 | 0.1 | 665.3512 | 2 | 30.71 | 5 | F5:13153 | 31122021\_RID\_2349\_5C.raw |  |  |  |  | 0 |  | 0 | 0 | 0 | 0 | 0 | 0 | 0 | 206 | 215 |  |  | PEAKS DB |
| total 4 peptides |  |  |  |  |  |  |  |  |  |  |  |  |  |  |  |  |  |  |  |  |  |  |  |  |  |  |  |  |  |
| --- | --- | --- | --- | --- | --- | --- | --- | --- | --- | --- | --- | --- | --- | --- | --- | --- | --- | --- | --- | --- | --- | --- | --- | --- | --- | --- | --- | --- | --- |

XP\_015675298.1

back to list

  

| Protein Coverage
| Supporting Peptides
|

Protein Coverage:

Supporting Peptides:

| Peptide | Uniq | -10lgP | Mass | Length | ppm | m/z | z | RT | Fraction | Scan | Source File | Area F2 | Area F1 | Area F3 | Area F4 | Area F5 | Area F6 | #Feature | #Feature F2 | #Feature F1 | #Feature F3 | #Feature F4 | #Feature F5 | #Feature F6 | Start | End | PTM | AScore | Found By |
| --- | --- | --- | --- | --- | --- | --- | --- | --- | --- | --- | --- | --- | --- | --- | --- | --- | --- | --- | --- | --- | --- | --- | --- | --- | --- | --- | --- | --- | --- |
| R.VDFDLESLSER.K | Y | 68.52 | 1308.6198 | 11 | 0.3 | 655.3173 | 2 | 50.36 | 1 | F1:29143 | 28122021\_RID\_2349\_2C.raw | 7.5467E5 |  |  |  | 7.9237E4 |  | 2 | 1 | 0 | 0 | 0 | 1 | 0 | 449 | 459 |  |  | PEAKS DB |
| K.YVGNMHGNEAVGR.E | Y | 66.69 | 1402.6411 | 13 | 1.1 | 702.3286 | 2 | 19.22 | 1 | F1:4678 | 28122021\_RID\_2349\_2C.raw | 8.6129E4 |  |  |  |  |  | 1 | 1 | 0 | 0 | 0 | 0 | 0 | 113 | 125 |  |  | PEAKS DB |
| K.AASQPGELKDWFVGR.S | Y | 64.21 | 1659.8368 | 15 | 0.5 | 554.2865 | 3 | 42.51 | 1 | F1:23240 | 28122021\_RID\_2349\_2C.raw | 9.7399E5 |  |  |  | 6.3604E5 |  | 3 | 1 | 0 | 0 | 0 | 2 | 0 | 169 | 183 |  |  | PEAKS DB |
| R.ELLVIEVSDNPGEHEPGEPEFK.Y | Y | 59.10 | 2463.1804 | 22 | 0.8 | 822.0681 | 3 | 49.17 | 5 | F5:26473 | 31122021\_RID\_2349\_5C.raw |  |  |  |  | 2.2636E5 |  | 1 | 0 | 0 | 0 | 0 | 1 | 0 | 91 | 112 |  |  | PEAKS DB |
| L.SQEDGISFEYHR.Y | Y | 51.30 | 1466.6426 | 12 | 0.4 | 734.3289 | 2 | 30.89 | 1 | F1:13886 | 28122021\_RID\_2349\_2C.raw | 6.2852E5 |  |  |  |  |  | 1 | 1 | 0 | 0 | 0 | 0 | 0 | 48 | 59 |  |  | PEAKS DB |
| R.IVYVNER.E | Y | 46.30 | 891.4814 | 7 | -0.3 | 446.7478 | 2 | 23.75 | 1 | F1:8099 | 28122021\_RID\_2349\_2C.raw | 2.7553E6 |  |  |  |  |  | 1 | 1 | 0 | 0 | 0 | 0 | 0 | 201 | 207 |  |  | PEAKS DB |
| R.IHIMPSLNPDGFEK.A | Y | 45.85 | 1596.7970 | 14 | 0.6 | 799.4062 | 2 | 43.34 | 1 | F1:23673 | 28122021\_RID\_2349\_2C.raw | 1.7055E5 |  |  |  |  |  | 1 | 1 | 0 | 0 | 0 | 0 | 0 | 155 | 168 |  |  | PEAKS DB |
| K.VAVPFSPAIR.V | Y | 42.31 | 1055.6127 | 10 | 0.2 | 528.8137 | 2 | 43.23 | 1 | F1:23759 | 28122021\_RID\_2349\_2C.raw | 1.1193E6 |  |  |  |  |  | 1 | 1 | 0 | 0 | 0 | 0 | 0 | 439 | 448 |  |  | PEAKS DB |
| total 8 peptides |  |  |  |  |  |  |  |  |  |  |  |  |  |  |  |  |  |  |  |  |  |  |  |  |  |  |  |  |  |
| --- | --- | --- | --- | --- | --- | --- | --- | --- | --- | --- | --- | --- | --- | --- | --- | --- | --- | --- | --- | --- | --- | --- | --- | --- | --- | --- | --- | --- | --- |

AFJ49609.1

back to list

  

| Protein Coverage
| Supporting Peptides
|

Protein Coverage:

Supporting Peptides:

| Peptide | Uniq | -10lgP | Mass | Length | ppm | m/z | z | RT | Fraction | Scan | Source File | Area F2 | Area F1 | Area F3 | Area F4 | Area F5 | Area F6 | #Feature | #Feature F2 | #Feature F1 | #Feature F3 | #Feature F4 | #Feature F5 | #Feature F6 | Start | End | PTM | AScore | Found By |
| --- | --- | --- | --- | --- | --- | --- | --- | --- | --- | --- | --- | --- | --- | --- | --- | --- | --- | --- | --- | --- | --- | --- | --- | --- | --- | --- | --- | --- | --- |
| R.VDFDLESLSER.K | Y | 68.52 | 1308.6198 | 11 | 0.3 | 655.3173 | 2 | 50.36 | 1 | F1:29143 | 28122021\_RID\_2349\_2C.raw | 7.5467E5 |  |  |  | 7.9237E4 |  | 2 | 1 | 0 | 0 | 0 | 1 | 0 | 449 | 459 |  |  | PEAKS DB |
| K.YVGNMHGNEAVGR.E | Y | 66.69 | 1402.6411 | 13 | 1.1 | 702.3286 | 2 | 19.22 | 1 | F1:4678 | 28122021\_RID\_2349\_2C.raw | 8.6129E4 |  |  |  |  |  | 1 | 1 | 0 | 0 | 0 | 0 | 0 | 113 | 125 |  |  | PEAKS DB |
| K.AASQPGELKDWFVGR.S | Y | 64.21 | 1659.8368 | 15 | 0.5 | 554.2865 | 3 | 42.51 | 1 | F1:23240 | 28122021\_RID\_2349\_2C.raw | 9.7399E5 |  |  |  | 6.3604E5 |  | 3 | 1 | 0 | 0 | 0 | 2 | 0 | 169 | 183 |  |  | PEAKS DB |
| R.ELLVIEVSDNPGEHEPGEPEFK.Y | Y | 59.10 | 2463.1804 | 22 | 0.8 | 822.0681 | 3 | 49.17 | 5 | F5:26473 | 31122021\_RID\_2349\_5C.raw |  |  |  |  | 2.2636E5 |  | 1 | 0 | 0 | 0 | 0 | 1 | 0 | 91 | 112 |  |  | PEAKS DB |
| L.SQEDGISFEYHR.Y | Y | 51.30 | 1466.6426 | 12 | 0.4 | 734.3289 | 2 | 30.89 | 1 | F1:13886 | 28122021\_RID\_2349\_2C.raw | 6.2852E5 |  |  |  |  |  | 1 | 1 | 0 | 0 | 0 | 0 | 0 | 48 | 59 |  |  | PEAKS DB |
| R.IVYVNER.E | Y | 46.30 | 891.4814 | 7 | -0.3 | 446.7478 | 2 | 23.75 | 1 | F1:8099 | 28122021\_RID\_2349\_2C.raw | 2.7553E6 |  |  |  |  |  | 1 | 1 | 0 | 0 | 0 | 0 | 0 | 201 | 207 |  |  | PEAKS DB |
| R.IHIMPSLNPDGFEK.A | Y | 45.85 | 1596.7970 | 14 | 0.6 | 799.4062 | 2 | 43.34 | 1 | F1:23673 | 28122021\_RID\_2349\_2C.raw | 1.7055E5 |  |  |  |  |  | 1 | 1 | 0 | 0 | 0 | 0 | 0 | 155 | 168 |  |  | PEAKS DB |
| K.VAVPFSPAIR.V | Y | 42.31 | 1055.6127 | 10 | 0.2 | 528.8137 | 2 | 43.23 | 1 | F1:23759 | 28122021\_RID\_2349\_2C.raw | 1.1193E6 |  |  |  |  |  | 1 | 1 | 0 | 0 | 0 | 0 | 0 | 439 | 448 |  |  | PEAKS DB |
| total 8 peptides |  |  |  |  |  |  |  |  |  |  |  |  |  |  |  |  |  |  |  |  |  |  |  |  |  |  |  |  |  |
| --- | --- | --- | --- | --- | --- | --- | --- | --- | --- | --- | --- | --- | --- | --- | --- | --- | --- | --- | --- | --- | --- | --- | --- | --- | --- | --- | --- | --- | --- |

JAS04545.1

back to list

  

| Protein Coverage
| Supporting Peptides
|

Protein Coverage:

Supporting Peptides:

| Peptide | Uniq | -10lgP | Mass | Length | ppm | m/z | z | RT | Fraction | Scan | Source File | Area F2 | Area F1 | Area F3 | Area F4 | Area F5 | Area F6 | #Feature | #Feature F2 | #Feature F1 | #Feature F3 | #Feature F4 | #Feature F5 | #Feature F6 | Start | End | PTM | AScore | Found By |
| --- | --- | --- | --- | --- | --- | --- | --- | --- | --- | --- | --- | --- | --- | --- | --- | --- | --- | --- | --- | --- | --- | --- | --- | --- | --- | --- | --- | --- | --- |
| K.KKDFSWEWTDR.S | Y | 75.90 | 1496.7048 | 11 | 1.0 | 749.3605 | 2 | 33.31 | 6 | F6:14165 | 31122021\_RID\_2349\_6C.raw |  |  |  |  | 2.2255E4 | 2.713E7 | 4 | 0 | 0 | 0 | 0 | 1 | 3 | 97 | 107 |  |  | PEAKS DB |
| K.DFSWEWTDR.S | Y | 57.89 | 1240.5149 | 9 | 0.2 | 621.2648 | 2 | 54.27 | 6 | F6:30059 | 31122021\_RID\_2349\_6C.raw |  |  |  |  | 2.2986E5 | 3.8252E7 | 3 | 0 | 0 | 0 | 0 | 1 | 2 | 99 | 107 |  |  | PEAKS DB |
| K.KDFSWEWTDR.S | Y | 55.84 | 1368.6099 | 10 | 0.4 | 685.3125 | 2 | 40.48 | 6 | F6:19731 | 31122021\_RID\_2349\_6C.raw |  |  |  |  |  | 1.9221E7 | 2 | 0 | 0 | 0 | 0 | 0 | 2 | 98 | 107 |  |  | PEAKS DB |
| D.RSC(+57.02)TDYLTWDK.N | Y | 46.27 | 1443.6453 | 11 | -6.5 | 722.8252 | 2 | 39.49 | 6 | F6:18826 | 31122021\_RID\_2349\_6C.raw |  |  |  |  |  | 7.383E5 | 1 | 0 | 0 | 0 | 0 | 0 | 1 | 107 | 117 | Carbamidomethylation | C3:Carbamidomethylation:1000.00 | PEAKS DB |
| D.FSWEWTDR.S | Y | 45.18 | 1125.4879 | 8 | 1.1 | 563.7518 | 2 | 48.02 | 6 | F6:25547 | 31122021\_RID\_2349\_6C.raw |  |  |  |  |  | 2.6976E5 | 1 | 0 | 0 | 0 | 0 | 0 | 1 | 100 | 107 |  |  | PEAKS DB |
| total 5 peptides |  |  |  |  |  |  |  |  |  |  |  |  |  |  |  |  |  |  |  |  |  |  |  |  |  |  |  |  |  |
| --- | --- | --- | --- | --- | --- | --- | --- | --- | --- | --- | --- | --- | --- | --- | --- | --- | --- | --- | --- | --- | --- | --- | --- | --- | --- | --- | --- | --- | --- |

ACE73567.1

back to list

  

| Protein Coverage
| Supporting Peptides
|

Protein Coverage:

Supporting Peptides:

| Peptide | Uniq | -10lgP | Mass | Length | ppm | m/z | z | RT | Fraction | Scan | Source File | Area F2 | Area F1 | Area F3 | Area F4 | Area F5 | Area F6 | #Feature | #Feature F2 | #Feature F1 | #Feature F3 | #Feature F4 | #Feature F5 | #Feature F6 | Start | End | PTM | AScore | Found By |
| --- | --- | --- | --- | --- | --- | --- | --- | --- | --- | --- | --- | --- | --- | --- | --- | --- | --- | --- | --- | --- | --- | --- | --- | --- | --- | --- | --- | --- | --- |
| K.MEWYPEAAANAER.W | N | 74.89 | 1536.6667 | 13 | 0.1 | 769.3408 | 2 | 41.29 | 5 | F5:20631 | 31122021\_RID\_2349\_5C.raw | 8.9957E4 |  |  |  | 3.8512E6 | 3.9844E5 | 3 | 1 | 0 | 0 | 0 | 1 | 1 | 58 | 70 |  |  | PEAKS DB |
| G.SVDFDSESPR.R | N | 71.72 | 1137.4938 | 10 | 0.2 | 569.7543 | 2 | 27.74 | 4 | F4:5760 | 31122021\_RID\_2349\_4C.raw | 9.0181E5 | 1.111E5 |  | 3.8035E7 | 1.1757E7 | 3.4766E5 | 7 | 1 | 1 | 0 | 1 | 3 | 1 | 20 | 29 |  |  | PEAKS DB |
| K.M(+15.99)EWYPEAAANAER.W | N | 63.14 | 1552.6616 | 13 | 0.6 | 777.3386 | 2 | 36.95 | 5 | F5:17377 | 31122021\_RID\_2349\_5C.raw |  |  |  |  | 2.2518E5 |  | 1 | 0 | 0 | 0 | 0 | 1 | 0 | 58 | 70 | Oxidation (M) | M1:Oxidation (M):1000.00 | PEAKS DB |
| E.WYPEAAANAER.W | N | 62.44 | 1276.5836 | 11 | 0.4 | 639.2993 | 2 | 31.93 | 5 | F5:14009 | 31122021\_RID\_2349\_5C.raw |  |  |  |  | 1.3008E6 |  | 1 | 0 | 0 | 0 | 0 | 1 | 0 | 60 | 70 |  |  | PEAKS DB |
| S.VDFDSESPR.R | N | 52.15 | 1050.4618 | 9 | 0.0 | 526.2382 | 2 | 27.13 | 5 | F5:10640 | 31122021\_RID\_2349\_5C.raw |  |  |  |  | 6.9151E5 |  | 1 | 0 | 0 | 0 | 0 | 1 | 0 | 21 | 29 |  |  | PEAKS DB |
| R.RPEIQNEIVDLHNSLR.R | Y | 45.37 | 1932.0177 | 16 | 0.8 | 645.0137 | 3 | 37.33 | 5 | F5:17754 | 31122021\_RID\_2349\_5C.raw |  |  |  |  | 0 |  | 0 | 0 | 0 | 0 | 0 | 0 | 0 | 30 | 45 |  |  | PEAKS DB |
| total 6 peptides |  |  |  |  |  |  |  |  |  |  |  |  |  |  |  |  |  |  |  |  |  |  |  |  |  |  |  |  |  |
| --- | --- | --- | --- | --- | --- | --- | --- | --- | --- | --- | --- | --- | --- | --- | --- | --- | --- | --- | --- | --- | --- | --- | --- | --- | --- | --- | --- | --- | --- |

P0DM87.1

back to list

  

| Protein Coverage
| Supporting Peptides
|

Protein Coverage:

Supporting Peptides:

| Peptide | Uniq | -10lgP | Mass | Length | ppm | m/z | z | RT | Fraction | Scan | Source File | Area F2 | Area F1 | Area F3 | Area F4 | Area F5 | Area F6 | #Feature | #Feature F2 | #Feature F1 | #Feature F3 | #Feature F4 | #Feature F5 | #Feature F6 | Start | End | PTM | AScore | Found By |
| --- | --- | --- | --- | --- | --- | --- | --- | --- | --- | --- | --- | --- | --- | --- | --- | --- | --- | --- | --- | --- | --- | --- | --- | --- | --- | --- | --- | --- | --- |
| S.IILESGNVNDYEVVYPR.K | N | 79.46 | 1979.0000 | 17 | 0.8 | 990.5081 | 2 | 50.36 | 6 | F6:27387 | 31122021\_RID\_2349\_6C.raw |  |  |  |  |  | 7.374E5 | 1 | 0 | 0 | 0 | 0 | 0 | 1 | 21 | 37 |  |  | PEAKS DB |
| K.YDGDETEISSK.I | Y | 72.80 | 1242.5251 | 11 | 0.0 | 622.2698 | 2 | 22.32 | 1 | F1:7079 | 28122021\_RID\_2349\_2C.raw | 2.8421E6 | 8.3464E3 |  |  | 1.6666E5 |  | 3 | 1 | 1 | 0 | 0 | 1 | 0 | 210 | 220 |  |  | PEAKS DB |
| R.HDNAQLLTGM(+15.99)IFNEK.I | N | 65.26 | 1745.8407 | 15 | -0.3 | 582.9540 | 3 | 41.33 | 1 | F1:22069 | 28122021\_RID\_2349\_2C.raw | 2.6908E6 |  | 0 |  | 0 |  | 2 | 2 | 0 | 0 | 0 | 0 | 0 | 280 | 294 | Oxidation (M) | M10:Oxidation (M):1000.00 | PEAKS DB |
| R.MYTKYDGDETEISSK.I | Y | 62.43 | 1765.7716 | 15 | -0.2 | 883.8929 | 2 | 26.45 | 1 | F1:10166 | 28122021\_RID\_2349\_2C.raw | 2.8954E6 |  |  |  |  |  | 2 | 2 | 0 | 0 | 0 | 0 | 0 | 206 | 220 |  |  | PEAKS DB |
| R.M(+15.99)YTKYDGDETEISSK.I | Y | 50.20 | 1781.7665 | 15 | 0.7 | 891.8911 | 2 | 24.18 | 1 | F1:8372 | 28122021\_RID\_2349\_2C.raw | 5.8349E4 |  |  |  |  |  | 1 | 1 | 0 | 0 | 0 | 0 | 0 | 206 | 220 | Oxidation (M) | M1:Oxidation (M):1000.00 | PEAKS DB |
| R.HDNAQLLTGMIFNEK.I | N | 45.45 | 1729.8457 | 15 | 0.9 | 577.6230 | 3 | 47.96 | 1 | F1:27379 | 28122021\_RID\_2349\_2C.raw | 1.4232E5 |  |  |  |  |  | 1 | 1 | 0 | 0 | 0 | 0 | 0 | 280 | 294 |  |  | PEAKS DB |
| total 6 peptides |  |  |  |  |  |  |  |  |  |  |  |  |  |  |  |  |  |  |  |  |  |  |  |  |  |  |  |  |  |
| --- | --- | --- | --- | --- | --- | --- | --- | --- | --- | --- | --- | --- | --- | --- | --- | --- | --- | --- | --- | --- | --- | --- | --- | --- | --- | --- | --- | --- | --- |

ETE70217.1

back to list

  

| Protein Coverage
| Supporting Peptides
|

Protein Coverage:

Supporting Peptides:

| Peptide | Uniq | -10lgP | Mass | Length | ppm | m/z | z | RT | Fraction | Scan | Source File | Area F2 | Area F1 | Area F3 | Area F4 | Area F5 | Area F6 | #Feature | #Feature F2 | #Feature F1 | #Feature F3 | #Feature F4 | #Feature F5 | #Feature F6 | Start | End | PTM | AScore | Found By |
| --- | --- | --- | --- | --- | --- | --- | --- | --- | --- | --- | --- | --- | --- | --- | --- | --- | --- | --- | --- | --- | --- | --- | --- | --- | --- | --- | --- | --- | --- |
| K.TRLEQEIATYR.N | N | 77.74 | 1378.7205 | 11 | 0.3 | 690.3677 | 2 | 30.32 | 3 | F3:7816 | 31122021\_RID\_2349\_3C.raw | 8.1799E5 | 1.1063E5 | 2.8494E7 |  |  |  | 4 | 1 | 1 | 2 | 0 | 0 | 0 | 875 | 885 |  |  | PEAKS DB |
| R.ALEEANTELEVK.I | N | 62.47 | 1344.6772 | 12 | 0.8 | 673.3464 | 2 | 32.99 | 1 | F1:15408 | 28122021\_RID\_2349\_2C.raw | 2.9938E5 |  |  |  |  |  | 1 | 1 | 0 | 0 | 0 | 0 | 0 | 169 | 180 |  |  | PEAKS DB |
| R.LAADDFR.L | N | 56.81 | 806.3922 | 7 | 0.6 | 404.2036 | 2 | 27.88 | 6 | F6:9922 | 31122021\_RID\_2349\_6C.raw | 1.4717E7 | 5.6851E6 | 6.5111E7 | 4.2079E7 | 2.9775E6 | 1.738E6 | 7 | 2 | 1 | 1 | 1 | 1 | 1 | 664 | 670 |  |  | PEAKS DB |
| R.LEQEIATYR.N | N | 54.19 | 1121.5717 | 9 | 0.4 | 561.7933 | 2 | 28.94 | 1 | F1:12152 | 28122021\_RID\_2349\_2C.raw | 6.9312E5 | 4.7439E5 | 1.0975E7 |  |  |  | 3 | 1 | 1 | 1 | 0 | 0 | 0 | 877 | 885 |  |  | PEAKS DB |
| K.LEVEIATYR.R | Y | 43.31 | 1092.5815 | 9 | 0.5 | 547.2983 | 2 | 45.21 | 2 | F2:15440 | 30122021\_RID\_2349\_1C.raw |  | 9.7875E3 |  |  |  |  | 1 | 0 | 1 | 0 | 0 | 0 | 0 | 1789 | 1797 |  |  | PEAKS DB |
| total 5 peptides |  |  |  |  |  |  |  |  |  |  |  |  |  |  |  |  |  |  |  |  |  |  |  |  |  |  |  |  |  |
| --- | --- | --- | --- | --- | --- | --- | --- | --- | --- | --- | --- | --- | --- | --- | --- | --- | --- | --- | --- | --- | --- | --- | --- | --- | --- | --- | --- | --- | --- |

XP\_026573193.1

back to list

  

| Protein Coverage
| Supporting Peptides
|

Protein Coverage:

Supporting Peptides:

| Peptide | Uniq | -10lgP | Mass | Length | ppm | m/z | z | RT | Fraction | Scan | Source File | Area F2 | Area F1 | Area F3 | Area F4 | Area F5 | Area F6 | #Feature | #Feature F2 | #Feature F1 | #Feature F3 | #Feature F4 | #Feature F5 | #Feature F6 | Start | End | PTM | AScore | Found By |
| --- | --- | --- | --- | --- | --- | --- | --- | --- | --- | --- | --- | --- | --- | --- | --- | --- | --- | --- | --- | --- | --- | --- | --- | --- | --- | --- | --- | --- | --- |
| Y.LDLDSIIAEVK.A | N | 61.19 | 1214.6758 | 11 | 0.8 | 608.3456 | 2 | 61.57 | 5 | F5:35620 | 31122021\_RID\_2349\_5C.raw |  |  |  |  | 2.2258E5 |  | 1 | 0 | 0 | 0 | 0 | 1 | 0 | 218 | 228 |  |  | PEAKS DB |
| K.FASFIDKVR.F | N | 59.84 | 1081.5920 | 9 | 1.2 | 541.8040 | 2 | 33.97 | 6 | F6:14555 | 31122021\_RID\_2349\_6C.raw | 6.7057E5 |  | 1.5615E7 |  | 6.0374E5 | 2.7185E5 | 4 | 1 | 0 | 1 | 0 | 1 | 1 | 166 | 174 |  |  | PEAKS DB |
| L.DLDSIIAEVK.A | N | 59.18 | 1101.5917 | 10 | -0.1 | 551.8030 | 2 | 55.07 | 1 | F1:32794 | 28122021\_RID\_2349\_2C.raw | 3.6044E5 |  |  |  |  |  | 1 | 1 | 0 | 0 | 0 | 0 | 0 | 219 | 228 |  |  | PEAKS DB |
| K.LAELEDALQK.T | Y | 50.62 | 1128.6027 | 10 | -0.8 | 565.3082 | 2 | 38.15 | 6 | F6:17725 | 31122021\_RID\_2349\_6C.raw |  |  |  |  |  | 2.8074E4 | 1 | 0 | 0 | 0 | 0 | 0 | 1 | 317 | 326 |  |  | PEAKS DB |
| K.EYQELMNVK.L | N | 49.62 | 1152.5485 | 9 | 0.7 | 577.2819 | 2 | 34.25 | 1 | F1:16408 | 28122021\_RID\_2349\_2C.raw | 1.4449E5 |  | 7.4495E6 |  |  |  | 2 | 1 | 0 | 1 | 0 | 0 | 0 | 337 | 345 |  |  | PEAKS DB |
| K.FASFIDK.V | N | 45.14 | 826.4225 | 7 | -0.5 | 414.2183 | 2 | 34.78 | 1 | F1:16890 | 28122021\_RID\_2349\_2C.raw | 6.9087E6 |  |  |  | 9.8962E5 |  | 2 | 1 | 0 | 0 | 0 | 1 | 0 | 166 | 172 |  |  | PEAKS DB |
| total 6 peptides |  |  |  |  |  |  |  |  |  |  |  |  |  |  |  |  |  |  |  |  |  |  |  |  |  |  |  |  |  |
| --- | --- | --- | --- | --- | --- | --- | --- | --- | --- | --- | --- | --- | --- | --- | --- | --- | --- | --- | --- | --- | --- | --- | --- | --- | --- | --- | --- | --- | --- |

P0CB14.1

back to list

  

| Protein Coverage
| Supporting Peptides
|

Protein Coverage:

Supporting Peptides:

| Peptide | Uniq | -10lgP | Mass | Length | ppm | m/z | z | RT | Fraction | Scan | Source File | Area F2 | Area F1 | Area F3 | Area F4 | Area F5 | Area F6 | #Feature | #Feature F2 | #Feature F1 | #Feature F3 | #Feature F4 | #Feature F5 | #Feature F6 | Start | End | PTM | AScore | Found By |
| --- | --- | --- | --- | --- | --- | --- | --- | --- | --- | --- | --- | --- | --- | --- | --- | --- | --- | --- | --- | --- | --- | --- | --- | --- | --- | --- | --- | --- | --- |
| S.IILESGNVNDYEVVYPR.K | N | 79.46 | 1979.0000 | 17 | 0.8 | 990.5081 | 2 | 50.36 | 6 | F6:27387 | 31122021\_RID\_2349\_6C.raw |  |  |  |  |  | 7.374E5 | 1 | 0 | 0 | 0 | 0 | 0 | 1 | 21 | 37 |  |  | PEAKS DB |
| K.YEDTMQYEFK.V | N | 70.38 | 1352.5594 | 10 | 0.5 | 677.2874 | 2 | 37.13 | 1 | F1:18721 | 28122021\_RID\_2349\_2C.raw | 3.5439E5 | 7.7616E3 |  |  | 1.6811E5 | 5.9028E5 | 4 | 1 | 1 | 0 | 0 | 1 | 1 | 51 | 60 |  |  | PEAKS DB |
| K.YENIEKEDETPK.M | Y | 57.78 | 1493.6885 | 12 | 1.1 | 747.8524 | 2 | 20.15 | 1 | F1:5349 | 28122021\_RID\_2349\_2C.raw | 3.3086E5 |  |  |  |  | 5.6819E4 | 3 | 2 | 0 | 0 | 0 | 0 | 1 | 154 | 165 |  |  | PEAKS DB |
| K.VPLLMAITMGHEIGHNLGM(+15.99)EHDEANC(+57.02)K.C | Y | 47.02 | 3032.3977 | 27 | 1.7 | 759.1080 | 4 | 49.55 | 5 | F5:26827 | 31122021\_RID\_2349\_5C.raw |  |  |  |  | 2.2296E7 |  | 2 | 0 | 0 | 0 | 0 | 2 | 0 | 323 | 349 | Oxidation (M); Carbamidomethylation | M19:Oxidation (M):7.40;C26:Carbamidomethylation:1000.00 | PEAKS DB |
| total 4 peptides |  |  |  |  |  |  |  |  |  |  |  |  |  |  |  |  |  |  |  |  |  |  |  |  |  |  |  |  |  |
| --- | --- | --- | --- | --- | --- | --- | --- | --- | --- | --- | --- | --- | --- | --- | --- | --- | --- | --- | --- | --- | --- | --- | --- | --- | --- | --- | --- | --- | --- |

XP\_034285001.1

back to list

  

| Protein Coverage
| Supporting Peptides
|

Protein Coverage:

Supporting Peptides:

| Peptide | Uniq | -10lgP | Mass | Length | ppm | m/z | z | RT | Fraction | Scan | Source File | Area F2 | Area F1 | Area F3 | Area F4 | Area F5 | Area F6 | #Feature | #Feature F2 | #Feature F1 | #Feature F3 | #Feature F4 | #Feature F5 | #Feature F6 | Start | End | PTM | AScore | Found By |
| --- | --- | --- | --- | --- | --- | --- | --- | --- | --- | --- | --- | --- | --- | --- | --- | --- | --- | --- | --- | --- | --- | --- | --- | --- | --- | --- | --- | --- | --- |
| R.LLEAQIATGGIIDPK.E | Y | 62.27 | 1537.8715 | 15 | 0.8 | 769.9436 | 2 | 48.96 | 5 | F5:26330 | 31122021\_RID\_2349\_5C.raw | 1.7815E5 | 3.2439E4 |  |  | 0 |  | 2 | 1 | 1 | 0 | 0 | 0 | 0 | 1917 | 1931 |  |  | PEAKS DB |
| R.QLQNIIQATSR.E | Y | 58.31 | 1270.6993 | 11 | -0.4 | 636.3567 | 2 | 34.80 | 1 | F1:16901 | 28122021\_RID\_2349\_2C.raw | 1.9015E5 |  | 1.0871E6 |  |  |  | 2 | 1 | 0 | 1 | 0 | 0 | 0 | 283 | 293 |  |  | PEAKS DB |
| R.AELIVQPELK.Y | Y | 46.23 | 1138.6597 | 10 | 1.2 | 570.3378 | 2 | 40.56 | 2 | F2:13481 | 30122021\_RID\_2349\_1C.raw | 1.5689E5 | 8.0452E4 |  |  |  |  | 2 | 1 | 1 | 0 | 0 | 0 | 0 | 98 | 107 |  |  | PEAKS DB |
| R.GLVGIEFK.E | Y | 45.71 | 861.4960 | 8 | 0.2 | 431.7553 | 2 | 42.22 | 1 | F1:22806 | 28122021\_RID\_2349\_2C.raw | 1.4375E5 |  |  |  |  |  | 1 | 1 | 0 | 0 | 0 | 0 | 0 | 1869 | 1876 |  |  | PEAKS DB |
| total 4 peptides |  |  |  |  |  |  |  |  |  |  |  |  |  |  |  |  |  |  |  |  |  |  |  |  |  |  |  |  |  |
| --- | --- | --- | --- | --- | --- | --- | --- | --- | --- | --- | --- | --- | --- | --- | --- | --- | --- | --- | --- | --- | --- | --- | --- | --- | --- | --- | --- | --- | --- |

XP\_007422201.1

back to list

  

| Protein Coverage
| Supporting Peptides
|

Protein Coverage:

Supporting Peptides:

| Peptide | Uniq | -10lgP | Mass | Length | ppm | m/z | z | RT | Fraction | Scan | Source File | Area F2 | Area F1 | Area F3 | Area F4 | Area F5 | Area F6 | #Feature | #Feature F2 | #Feature F1 | #Feature F3 | #Feature F4 | #Feature F5 | #Feature F6 | Start | End | PTM | AScore | Found By |
| --- | --- | --- | --- | --- | --- | --- | --- | --- | --- | --- | --- | --- | --- | --- | --- | --- | --- | --- | --- | --- | --- | --- | --- | --- | --- | --- | --- | --- | --- |
| R.LLEAQIATGGIIDPK.E | Y | 62.27 | 1537.8715 | 15 | 0.8 | 769.9436 | 2 | 48.96 | 5 | F5:26330 | 31122021\_RID\_2349\_5C.raw | 1.7815E5 | 3.2439E4 |  |  | 0 |  | 2 | 1 | 1 | 0 | 0 | 0 | 0 | 2360 | 2374 |  |  | PEAKS DB |
| R.QLQNIIQATSR.E | Y | 58.31 | 1270.6993 | 11 | -0.4 | 636.3567 | 2 | 34.80 | 1 | F1:16901 | 28122021\_RID\_2349\_2C.raw | 1.9015E5 |  | 1.0871E6 |  |  |  | 2 | 1 | 0 | 1 | 0 | 0 | 0 | 279 | 289 |  |  | PEAKS DB |
| R.AELIVQPELK.Y | Y | 46.23 | 1138.6597 | 10 | 1.2 | 570.3378 | 2 | 40.56 | 2 | F2:13481 | 30122021\_RID\_2349\_1C.raw | 1.5689E5 | 8.0452E4 |  |  |  |  | 2 | 1 | 1 | 0 | 0 | 0 | 0 | 94 | 103 |  |  | PEAKS DB |
| R.GLVGIEFK.E | Y | 45.71 | 861.4960 | 8 | 0.2 | 431.7553 | 2 | 42.22 | 1 | F1:22806 | 28122021\_RID\_2349\_2C.raw | 1.4375E5 |  |  |  |  |  | 1 | 1 | 0 | 0 | 0 | 0 | 0 | 2312 | 2319 |  |  | PEAKS DB |
| total 4 peptides |  |  |  |  |  |  |  |  |  |  |  |  |  |  |  |  |  |  |  |  |  |  |  |  |  |  |  |  |  |
| --- | --- | --- | --- | --- | --- | --- | --- | --- | --- | --- | --- | --- | --- | --- | --- | --- | --- | --- | --- | --- | --- | --- | --- | --- | --- | --- | --- | --- | --- |

XP\_007422202.1

back to list

  

| Protein Coverage
| Supporting Peptides
|

Protein Coverage:

Supporting Peptides:

| Peptide | Uniq | -10lgP | Mass | Length | ppm | m/z | z | RT | Fraction | Scan | Source File | Area F2 | Area F1 | Area F3 | Area F4 | Area F5 | Area F6 | #Feature | #Feature F2 | #Feature F1 | #Feature F3 | #Feature F4 | #Feature F5 | #Feature F6 | Start | End | PTM | AScore | Found By |
| --- | --- | --- | --- | --- | --- | --- | --- | --- | --- | --- | --- | --- | --- | --- | --- | --- | --- | --- | --- | --- | --- | --- | --- | --- | --- | --- | --- | --- | --- |
| R.LLEAQIATGGIIDPK.E | Y | 62.27 | 1537.8715 | 15 | 0.8 | 769.9436 | 2 | 48.96 | 5 | F5:26330 | 31122021\_RID\_2349\_5C.raw | 1.7815E5 | 3.2439E4 |  |  | 0 |  | 2 | 1 | 1 | 0 | 0 | 0 | 0 | 1917 | 1931 |  |  | PEAKS DB |
| R.QLQNIIQATSR.E | Y | 58.31 | 1270.6993 | 11 | -0.4 | 636.3567 | 2 | 34.80 | 1 | F1:16901 | 28122021\_RID\_2349\_2C.raw | 1.9015E5 |  | 1.0871E6 |  |  |  | 2 | 1 | 0 | 1 | 0 | 0 | 0 | 279 | 289 |  |  | PEAKS DB |
| R.AELIVQPELK.Y | Y | 46.23 | 1138.6597 | 10 | 1.2 | 570.3378 | 2 | 40.56 | 2 | F2:13481 | 30122021\_RID\_2349\_1C.raw | 1.5689E5 | 8.0452E4 |  |  |  |  | 2 | 1 | 1 | 0 | 0 | 0 | 0 | 94 | 103 |  |  | PEAKS DB |
| R.GLVGIEFK.E | Y | 45.71 | 861.4960 | 8 | 0.2 | 431.7553 | 2 | 42.22 | 1 | F1:22806 | 28122021\_RID\_2349\_2C.raw | 1.4375E5 |  |  |  |  |  | 1 | 1 | 0 | 0 | 0 | 0 | 0 | 1869 | 1876 |  |  | PEAKS DB |
| total 4 peptides |  |  |  |  |  |  |  |  |  |  |  |  |  |  |  |  |  |  |  |  |  |  |  |  |  |  |  |  |  |
| --- | --- | --- | --- | --- | --- | --- | --- | --- | --- | --- | --- | --- | --- | --- | --- | --- | --- | --- | --- | --- | --- | --- | --- | --- | --- | --- | --- | --- | --- |

XP\_034285000.1

back to list

  

| Protein Coverage
| Supporting Peptides
|

Protein Coverage:

Supporting Peptides:

| Peptide | Uniq | -10lgP | Mass | Length | ppm | m/z | z | RT | Fraction | Scan | Source File | Area F2 | Area F1 | Area F3 | Area F4 | Area F5 | Area F6 | #Feature | #Feature F2 | #Feature F1 | #Feature F3 | #Feature F4 | #Feature F5 | #Feature F6 | Start | End | PTM | AScore | Found By |
| --- | --- | --- | --- | --- | --- | --- | --- | --- | --- | --- | --- | --- | --- | --- | --- | --- | --- | --- | --- | --- | --- | --- | --- | --- | --- | --- | --- | --- | --- |
| R.LLEAQIATGGIIDPK.E | Y | 62.27 | 1537.8715 | 15 | 0.8 | 769.9436 | 2 | 48.96 | 5 | F5:26330 | 31122021\_RID\_2349\_5C.raw | 1.7815E5 | 3.2439E4 |  |  | 0 |  | 2 | 1 | 1 | 0 | 0 | 0 | 0 | 2360 | 2374 |  |  | PEAKS DB |
| R.QLQNIIQATSR.E | Y | 58.31 | 1270.6993 | 11 | -0.4 | 636.3567 | 2 | 34.80 | 1 | F1:16901 | 28122021\_RID\_2349\_2C.raw | 1.9015E5 |  | 1.0871E6 |  |  |  | 2 | 1 | 0 | 1 | 0 | 0 | 0 | 283 | 293 |  |  | PEAKS DB |
| R.AELIVQPELK.Y | Y | 46.23 | 1138.6597 | 10 | 1.2 | 570.3378 | 2 | 40.56 | 2 | F2:13481 | 30122021\_RID\_2349\_1C.raw | 1.5689E5 | 8.0452E4 |  |  |  |  | 2 | 1 | 1 | 0 | 0 | 0 | 0 | 98 | 107 |  |  | PEAKS DB |
| R.GLVGIEFK.E | Y | 45.71 | 861.4960 | 8 | 0.2 | 431.7553 | 2 | 42.22 | 1 | F1:22806 | 28122021\_RID\_2349\_2C.raw | 1.4375E5 |  |  |  |  |  | 1 | 1 | 0 | 0 | 0 | 0 | 0 | 2312 | 2319 |  |  | PEAKS DB |
| total 4 peptides |  |  |  |  |  |  |  |  |  |  |  |  |  |  |  |  |  |  |  |  |  |  |  |  |  |  |  |  |  |
| --- | --- | --- | --- | --- | --- | --- | --- | --- | --- | --- | --- | --- | --- | --- | --- | --- | --- | --- | --- | --- | --- | --- | --- | --- | --- | --- | --- | --- | --- |

XP\_015666325.1

back to list

  

| Protein Coverage
| Supporting Peptides
|

Protein Coverage:

Supporting Peptides:

| Peptide | Uniq | -10lgP | Mass | Length | ppm | m/z | z | RT | Fraction | Scan | Source File | Area F2 | Area F1 | Area F3 | Area F4 | Area F5 | Area F6 | #Feature | #Feature F2 | #Feature F1 | #Feature F3 | #Feature F4 | #Feature F5 | #Feature F6 | Start | End | PTM | AScore | Found By |
| --- | --- | --- | --- | --- | --- | --- | --- | --- | --- | --- | --- | --- | --- | --- | --- | --- | --- | --- | --- | --- | --- | --- | --- | --- | --- | --- | --- | --- | --- |
| R.LLEAQIATGGIIDPK.E | Y | 62.27 | 1537.8715 | 15 | 0.8 | 769.9436 | 2 | 48.96 | 5 | F5:26330 | 31122021\_RID\_2349\_5C.raw | 1.7815E5 | 3.2439E4 |  |  | 0 |  | 2 | 1 | 1 | 0 | 0 | 0 | 0 | 2359 | 2373 |  |  | PEAKS DB |
| R.QLQNIIQATSR.E | Y | 58.31 | 1270.6993 | 11 | -0.4 | 636.3567 | 2 | 34.80 | 1 | F1:16901 | 28122021\_RID\_2349\_2C.raw | 1.9015E5 |  | 1.0871E6 |  |  |  | 2 | 1 | 0 | 1 | 0 | 0 | 0 | 281 | 291 |  |  | PEAKS DB |
| R.AELIVQPELK.Y | Y | 46.23 | 1138.6597 | 10 | 1.2 | 570.3378 | 2 | 40.56 | 2 | F2:13481 | 30122021\_RID\_2349\_1C.raw | 1.5689E5 | 8.0452E4 |  |  |  |  | 2 | 1 | 1 | 0 | 0 | 0 | 0 | 96 | 105 |  |  | PEAKS DB |
| R.GLVGIEFK.E | Y | 45.71 | 861.4960 | 8 | 0.2 | 431.7553 | 2 | 42.22 | 1 | F1:22806 | 28122021\_RID\_2349\_2C.raw | 1.4375E5 |  |  |  |  |  | 1 | 1 | 0 | 0 | 0 | 0 | 0 | 2311 | 2318 |  |  | PEAKS DB |
| total 4 peptides |  |  |  |  |  |  |  |  |  |  |  |  |  |  |  |  |  |  |  |  |  |  |  |  |  |  |  |  |  |
| --- | --- | --- | --- | --- | --- | --- | --- | --- | --- | --- | --- | --- | --- | --- | --- | --- | --- | --- | --- | --- | --- | --- | --- | --- | --- | --- | --- | --- | --- |

XP\_032079162.1

back to list

  

| Protein Coverage
| Supporting Peptides
|

Protein Coverage:

Supporting Peptides:

| Peptide | Uniq | -10lgP | Mass | Length | ppm | m/z | z | RT | Fraction | Scan | Source File | Area F2 | Area F1 | Area F3 | Area F4 | Area F5 | Area F6 | #Feature | #Feature F2 | #Feature F1 | #Feature F3 | #Feature F4 | #Feature F5 | #Feature F6 | Start | End | PTM | AScore | Found By |
| --- | --- | --- | --- | --- | --- | --- | --- | --- | --- | --- | --- | --- | --- | --- | --- | --- | --- | --- | --- | --- | --- | --- | --- | --- | --- | --- | --- | --- | --- |
| R.LLEAQIATGGIIDPK.E | Y | 62.27 | 1537.8715 | 15 | 0.8 | 769.9436 | 2 | 48.96 | 5 | F5:26330 | 31122021\_RID\_2349\_5C.raw | 1.7815E5 | 3.2439E4 |  |  | 0 |  | 2 | 1 | 1 | 0 | 0 | 0 | 0 | 1915 | 1929 |  |  | PEAKS DB |
| R.QLQNIIQATSR.E | Y | 58.31 | 1270.6993 | 11 | -0.4 | 636.3567 | 2 | 34.80 | 1 | F1:16901 | 28122021\_RID\_2349\_2C.raw | 1.9015E5 |  | 1.0871E6 |  |  |  | 2 | 1 | 0 | 1 | 0 | 0 | 0 | 282 | 292 |  |  | PEAKS DB |
| R.AELIVQPELK.Y | Y | 46.23 | 1138.6597 | 10 | 1.2 | 570.3378 | 2 | 40.56 | 2 | F2:13481 | 30122021\_RID\_2349\_1C.raw | 1.5689E5 | 8.0452E4 |  |  |  |  | 2 | 1 | 1 | 0 | 0 | 0 | 0 | 97 | 106 |  |  | PEAKS DB |
| R.GLVGIEFK.E | Y | 45.71 | 861.4960 | 8 | 0.2 | 431.7553 | 2 | 42.22 | 1 | F1:22806 | 28122021\_RID\_2349\_2C.raw | 1.4375E5 |  |  |  |  |  | 1 | 1 | 0 | 0 | 0 | 0 | 0 | 1867 | 1874 |  |  | PEAKS DB |
| total 4 peptides |  |  |  |  |  |  |  |  |  |  |  |  |  |  |  |  |  |  |  |  |  |  |  |  |  |  |  |  |  |
| --- | --- | --- | --- | --- | --- | --- | --- | --- | --- | --- | --- | --- | --- | --- | --- | --- | --- | --- | --- | --- | --- | --- | --- | --- | --- | --- | --- | --- | --- |

XP\_032079161.1

back to list

  

| Protein Coverage
| Supporting Peptides
|

Protein Coverage:

Supporting Peptides:

| Peptide | Uniq | -10lgP | Mass | Length | ppm | m/z | z | RT | Fraction | Scan | Source File | Area F2 | Area F1 | Area F3 | Area F4 | Area F5 | Area F6 | #Feature | #Feature F2 | #Feature F1 | #Feature F3 | #Feature F4 | #Feature F5 | #Feature F6 | Start | End | PTM | AScore | Found By |
| --- | --- | --- | --- | --- | --- | --- | --- | --- | --- | --- | --- | --- | --- | --- | --- | --- | --- | --- | --- | --- | --- | --- | --- | --- | --- | --- | --- | --- | --- |
| R.LLEAQIATGGIIDPK.E | Y | 62.27 | 1537.8715 | 15 | 0.8 | 769.9436 | 2 | 48.96 | 5 | F5:26330 | 31122021\_RID\_2349\_5C.raw | 1.7815E5 | 3.2439E4 |  |  | 0 |  | 2 | 1 | 1 | 0 | 0 | 0 | 0 | 2358 | 2372 |  |  | PEAKS DB |
| R.QLQNIIQATSR.E | Y | 58.31 | 1270.6993 | 11 | -0.4 | 636.3567 | 2 | 34.80 | 1 | F1:16901 | 28122021\_RID\_2349\_2C.raw | 1.9015E5 |  | 1.0871E6 |  |  |  | 2 | 1 | 0 | 1 | 0 | 0 | 0 | 282 | 292 |  |  | PEAKS DB |
| R.AELIVQPELK.Y | Y | 46.23 | 1138.6597 | 10 | 1.2 | 570.3378 | 2 | 40.56 | 2 | F2:13481 | 30122021\_RID\_2349\_1C.raw | 1.5689E5 | 8.0452E4 |  |  |  |  | 2 | 1 | 1 | 0 | 0 | 0 | 0 | 97 | 106 |  |  | PEAKS DB |
| R.GLVGIEFK.E | Y | 45.71 | 861.4960 | 8 | 0.2 | 431.7553 | 2 | 42.22 | 1 | F1:22806 | 28122021\_RID\_2349\_2C.raw | 1.4375E5 |  |  |  |  |  | 1 | 1 | 0 | 0 | 0 | 0 | 0 | 2310 | 2317 |  |  | PEAKS DB |
| total 4 peptides |  |  |  |  |  |  |  |  |  |  |  |  |  |  |  |  |  |  |  |  |  |  |  |  |  |  |  |  |  |
| --- | --- | --- | --- | --- | --- | --- | --- | --- | --- | --- | --- | --- | --- | --- | --- | --- | --- | --- | --- | --- | --- | --- | --- | --- | --- | --- | --- | --- | --- |

XP\_034285002.1

back to list

  

| Protein Coverage
| Supporting Peptides
|

Protein Coverage:

Supporting Peptides:

| Peptide | Uniq | -10lgP | Mass | Length | ppm | m/z | z | RT | Fraction | Scan | Source File | Area F2 | Area F1 | Area F3 | Area F4 | Area F5 | Area F6 | #Feature | #Feature F2 | #Feature F1 | #Feature F3 | #Feature F4 | #Feature F5 | #Feature F6 | Start | End | PTM | AScore | Found By |
| --- | --- | --- | --- | --- | --- | --- | --- | --- | --- | --- | --- | --- | --- | --- | --- | --- | --- | --- | --- | --- | --- | --- | --- | --- | --- | --- | --- | --- | --- |
| R.LLEAQIATGGIIDPK.E | Y | 62.27 | 1537.8715 | 15 | 0.8 | 769.9436 | 2 | 48.96 | 5 | F5:26330 | 31122021\_RID\_2349\_5C.raw | 1.7815E5 | 3.2439E4 |  |  | 0 |  | 2 | 1 | 1 | 0 | 0 | 0 | 0 | 1761 | 1775 |  |  | PEAKS DB |
| R.QLQNIIQATSR.E | Y | 58.31 | 1270.6993 | 11 | -0.4 | 636.3567 | 2 | 34.80 | 1 | F1:16901 | 28122021\_RID\_2349\_2C.raw | 1.9015E5 |  | 1.0871E6 |  |  |  | 2 | 1 | 0 | 1 | 0 | 0 | 0 | 283 | 293 |  |  | PEAKS DB |
| R.AELIVQPELK.Y | Y | 46.23 | 1138.6597 | 10 | 1.2 | 570.3378 | 2 | 40.56 | 2 | F2:13481 | 30122021\_RID\_2349\_1C.raw | 1.5689E5 | 8.0452E4 |  |  |  |  | 2 | 1 | 1 | 0 | 0 | 0 | 0 | 98 | 107 |  |  | PEAKS DB |
| R.GLVGIEFK.E | Y | 45.71 | 861.4960 | 8 | 0.2 | 431.7553 | 2 | 42.22 | 1 | F1:22806 | 28122021\_RID\_2349\_2C.raw | 1.4375E5 |  |  |  |  |  | 1 | 1 | 0 | 0 | 0 | 0 | 0 | 1713 | 1720 |  |  | PEAKS DB |
| total 4 peptides |  |  |  |  |  |  |  |  |  |  |  |  |  |  |  |  |  |  |  |  |  |  |  |  |  |  |  |  |  |
| --- | --- | --- | --- | --- | --- | --- | --- | --- | --- | --- | --- | --- | --- | --- | --- | --- | --- | --- | --- | --- | --- | --- | --- | --- | --- | --- | --- | --- | --- |

XP\_007422203.1

back to list

  

| Protein Coverage
| Supporting Peptides
|

Protein Coverage:

Supporting Peptides:

| Peptide | Uniq | -10lgP | Mass | Length | ppm | m/z | z | RT | Fraction | Scan | Source File | Area F2 | Area F1 | Area F3 | Area F4 | Area F5 | Area F6 | #Feature | #Feature F2 | #Feature F1 | #Feature F3 | #Feature F4 | #Feature F5 | #Feature F6 | Start | End | PTM | AScore | Found By |
| --- | --- | --- | --- | --- | --- | --- | --- | --- | --- | --- | --- | --- | --- | --- | --- | --- | --- | --- | --- | --- | --- | --- | --- | --- | --- | --- | --- | --- | --- |
| R.LLEAQIATGGIIDPK.E | Y | 62.27 | 1537.8715 | 15 | 0.8 | 769.9436 | 2 | 48.96 | 5 | F5:26330 | 31122021\_RID\_2349\_5C.raw | 1.7815E5 | 3.2439E4 |  |  | 0 |  | 2 | 1 | 1 | 0 | 0 | 0 | 0 | 1761 | 1775 |  |  | PEAKS DB |
| R.QLQNIIQATSR.E | Y | 58.31 | 1270.6993 | 11 | -0.4 | 636.3567 | 2 | 34.80 | 1 | F1:16901 | 28122021\_RID\_2349\_2C.raw | 1.9015E5 |  | 1.0871E6 |  |  |  | 2 | 1 | 0 | 1 | 0 | 0 | 0 | 279 | 289 |  |  | PEAKS DB |
| R.AELIVQPELK.Y | Y | 46.23 | 1138.6597 | 10 | 1.2 | 570.3378 | 2 | 40.56 | 2 | F2:13481 | 30122021\_RID\_2349\_1C.raw | 1.5689E5 | 8.0452E4 |  |  |  |  | 2 | 1 | 1 | 0 | 0 | 0 | 0 | 94 | 103 |  |  | PEAKS DB |
| R.GLVGIEFK.E | Y | 45.71 | 861.4960 | 8 | 0.2 | 431.7553 | 2 | 42.22 | 1 | F1:22806 | 28122021\_RID\_2349\_2C.raw | 1.4375E5 |  |  |  |  |  | 1 | 1 | 0 | 0 | 0 | 0 | 0 | 1713 | 1720 |  |  | PEAKS DB |
| total 4 peptides |  |  |  |  |  |  |  |  |  |  |  |  |  |  |  |  |  |  |  |  |  |  |  |  |  |  |  |  |  |
| --- | --- | --- | --- | --- | --- | --- | --- | --- | --- | --- | --- | --- | --- | --- | --- | --- | --- | --- | --- | --- | --- | --- | --- | --- | --- | --- | --- | --- | --- |

JAC95787.1

back to list

  

| Protein Coverage
| Supporting Peptides
|

Protein Coverage:

Supporting Peptides:

| Peptide | Uniq | -10lgP | Mass | Length | ppm | m/z | z | RT | Fraction | Scan | Source File | Area F2 | Area F1 | Area F3 | Area F4 | Area F5 | Area F6 | #Feature | #Feature F2 | #Feature F1 | #Feature F3 | #Feature F4 | #Feature F5 | #Feature F6 | Start | End | PTM | AScore | Found By |
| --- | --- | --- | --- | --- | --- | --- | --- | --- | --- | --- | --- | --- | --- | --- | --- | --- | --- | --- | --- | --- | --- | --- | --- | --- | --- | --- | --- | --- | --- |
| R.LLEAQIATGGIIDPK.E | Y | 62.27 | 1537.8715 | 15 | 0.8 | 769.9436 | 2 | 48.96 | 5 | F5:26330 | 31122021\_RID\_2349\_5C.raw | 1.7815E5 | 3.2439E4 |  |  | 0 |  | 2 | 1 | 1 | 0 | 0 | 0 | 0 | 1759 | 1773 |  |  | PEAKS DB |
| R.QLQNIIQATSR.E | Y | 58.31 | 1270.6993 | 11 | -0.4 | 636.3567 | 2 | 34.80 | 1 | F1:16901 | 28122021\_RID\_2349\_2C.raw | 1.9015E5 |  | 1.0871E6 |  |  |  | 2 | 1 | 0 | 1 | 0 | 0 | 0 | 281 | 291 |  |  | PEAKS DB |
| R.AELIVQPELK.Y | Y | 46.23 | 1138.6597 | 10 | 1.2 | 570.3378 | 2 | 40.56 | 2 | F2:13481 | 30122021\_RID\_2349\_1C.raw | 1.5689E5 | 8.0452E4 |  |  |  |  | 2 | 1 | 1 | 0 | 0 | 0 | 0 | 96 | 105 |  |  | PEAKS DB |
| R.GLVGIEFK.E | Y | 45.71 | 861.4960 | 8 | 0.2 | 431.7553 | 2 | 42.22 | 1 | F1:22806 | 28122021\_RID\_2349\_2C.raw | 1.4375E5 |  |  |  |  |  | 1 | 1 | 0 | 0 | 0 | 0 | 0 | 1711 | 1718 |  |  | PEAKS DB |
| total 4 peptides |  |  |  |  |  |  |  |  |  |  |  |  |  |  |  |  |  |  |  |  |  |  |  |  |  |  |  |  |  |
| --- | --- | --- | --- | --- | --- | --- | --- | --- | --- | --- | --- | --- | --- | --- | --- | --- | --- | --- | --- | --- | --- | --- | --- | --- | --- | --- | --- | --- | --- |

XP\_032079163.1

back to list

  

| Protein Coverage
| Supporting Peptides
|

Protein Coverage:

Supporting Peptides:

| Peptide | Uniq | -10lgP | Mass | Length | ppm | m/z | z | RT | Fraction | Scan | Source File | Area F2 | Area F1 | Area F3 | Area F4 | Area F5 | Area F6 | #Feature | #Feature F2 | #Feature F1 | #Feature F3 | #Feature F4 | #Feature F5 | #Feature F6 | Start | End | PTM | AScore | Found By |
| --- | --- | --- | --- | --- | --- | --- | --- | --- | --- | --- | --- | --- | --- | --- | --- | --- | --- | --- | --- | --- | --- | --- | --- | --- | --- | --- | --- | --- | --- |
| R.LLEAQIATGGIIDPK.E | Y | 62.27 | 1537.8715 | 15 | 0.8 | 769.9436 | 2 | 48.96 | 5 | F5:26330 | 31122021\_RID\_2349\_5C.raw | 1.7815E5 | 3.2439E4 |  |  | 0 |  | 2 | 1 | 1 | 0 | 0 | 0 | 0 | 1759 | 1773 |  |  | PEAKS DB |
| R.QLQNIIQATSR.E | Y | 58.31 | 1270.6993 | 11 | -0.4 | 636.3567 | 2 | 34.80 | 1 | F1:16901 | 28122021\_RID\_2349\_2C.raw | 1.9015E5 |  | 1.0871E6 |  |  |  | 2 | 1 | 0 | 1 | 0 | 0 | 0 | 282 | 292 |  |  | PEAKS DB |
| R.AELIVQPELK.Y | Y | 46.23 | 1138.6597 | 10 | 1.2 | 570.3378 | 2 | 40.56 | 2 | F2:13481 | 30122021\_RID\_2349\_1C.raw | 1.5689E5 | 8.0452E4 |  |  |  |  | 2 | 1 | 1 | 0 | 0 | 0 | 0 | 97 | 106 |  |  | PEAKS DB |
| R.GLVGIEFK.E | Y | 45.71 | 861.4960 | 8 | 0.2 | 431.7553 | 2 | 42.22 | 1 | F1:22806 | 28122021\_RID\_2349\_2C.raw | 1.4375E5 |  |  |  |  |  | 1 | 1 | 0 | 0 | 0 | 0 | 0 | 1711 | 1718 |  |  | PEAKS DB |
| total 4 peptides |  |  |  |  |  |  |  |  |  |  |  |  |  |  |  |  |  |  |  |  |  |  |  |  |  |  |  |  |  |
| --- | --- | --- | --- | --- | --- | --- | --- | --- | --- | --- | --- | --- | --- | --- | --- | --- | --- | --- | --- | --- | --- | --- | --- | --- | --- | --- | --- | --- | --- |

JAG47218.1

back to list

  

| Protein Coverage
| Supporting Peptides
|

Protein Coverage:

Supporting Peptides:

| Peptide | Uniq | -10lgP | Mass | Length | ppm | m/z | z | RT | Fraction | Scan | Source File | Area F2 | Area F1 | Area F3 | Area F4 | Area F5 | Area F6 | #Feature | #Feature F2 | #Feature F1 | #Feature F3 | #Feature F4 | #Feature F5 | #Feature F6 | Start | End | PTM | AScore | Found By |
| --- | --- | --- | --- | --- | --- | --- | --- | --- | --- | --- | --- | --- | --- | --- | --- | --- | --- | --- | --- | --- | --- | --- | --- | --- | --- | --- | --- | --- | --- |
| K.SGVYQHVSGESVGGHAIR.I | Y | 76.21 | 1838.9023 | 18 | 0.8 | 613.9752 | 3 | 23.25 | 3 | F3:4929 | 31122021\_RID\_2349\_3C.raw |  |  | 9.3929E5 |  |  |  | 1 | 0 | 0 | 1 | 0 | 0 | 0 | 265 | 282 |  |  | PEAKS DB |
| R.FEFAGDLVLPK.S | Y | 69.09 | 1234.6597 | 11 | 0.7 | 618.3375 | 2 | 54.89 | 1 | F1:32582 | 28122021\_RID\_2349\_2C.raw | 1.4066E5 |  |  |  |  |  | 1 | 1 | 0 | 0 | 0 | 0 | 0 | 72 | 82 |  |  | PEAKS DB |
| K.EIMAEIYK.N | Y | 43.48 | 995.4998 | 8 | 0.8 | 498.7575 | 2 | 36.81 | 1 | F1:18465 | 28122021\_RID\_2349\_2C.raw | 1.7359E5 |  |  |  |  |  | 1 | 1 | 0 | 0 | 0 | 0 | 0 | 239 | 246 |  |  | PEAKS DB |
| total 3 peptides |  |  |  |  |  |  |  |  |  |  |  |  |  |  |  |  |  |  |  |  |  |  |  |  |  |  |  |  |  |
| --- | --- | --- | --- | --- | --- | --- | --- | --- | --- | --- | --- | --- | --- | --- | --- | --- | --- | --- | --- | --- | --- | --- | --- | --- | --- | --- | --- | --- | --- |

JAA97493.1

back to list

  

| Protein Coverage
| Supporting Peptides
|

Protein Coverage:

Supporting Peptides:

| Peptide | Uniq | -10lgP | Mass | Length | ppm | m/z | z | RT | Fraction | Scan | Source File | Area F2 | Area F1 | Area F3 | Area F4 | Area F5 | Area F6 | #Feature | #Feature F2 | #Feature F1 | #Feature F3 | #Feature F4 | #Feature F5 | #Feature F6 | Start | End | PTM | AScore | Found By |
| --- | --- | --- | --- | --- | --- | --- | --- | --- | --- | --- | --- | --- | --- | --- | --- | --- | --- | --- | --- | --- | --- | --- | --- | --- | --- | --- | --- | --- | --- |
| K.SGVYQHVSGESVGGHAIR.I | Y | 76.21 | 1838.9023 | 18 | 0.8 | 613.9752 | 3 | 23.25 | 3 | F3:4929 | 31122021\_RID\_2349\_3C.raw |  |  | 9.3929E5 |  |  |  | 1 | 0 | 0 | 1 | 0 | 0 | 0 | 265 | 282 |  |  | PEAKS DB |
| R.FEFAGDLVLPK.S | Y | 69.09 | 1234.6597 | 11 | 0.7 | 618.3375 | 2 | 54.89 | 1 | F1:32582 | 28122021\_RID\_2349\_2C.raw | 1.4066E5 |  |  |  |  |  | 1 | 1 | 0 | 0 | 0 | 0 | 0 | 72 | 82 |  |  | PEAKS DB |
| K.EIMAEIYK.N | Y | 43.48 | 995.4998 | 8 | 0.8 | 498.7575 | 2 | 36.81 | 1 | F1:18465 | 28122021\_RID\_2349\_2C.raw | 1.7359E5 |  |  |  |  |  | 1 | 1 | 0 | 0 | 0 | 0 | 0 | 239 | 246 |  |  | PEAKS DB |
| total 3 peptides |  |  |  |  |  |  |  |  |  |  |  |  |  |  |  |  |  |  |  |  |  |  |  |  |  |  |  |  |  |
| --- | --- | --- | --- | --- | --- | --- | --- | --- | --- | --- | --- | --- | --- | --- | --- | --- | --- | --- | --- | --- | --- | --- | --- | --- | --- | --- | --- | --- | --- |

JAI13964.1

back to list

  

| Protein Coverage
| Supporting Peptides
|

Protein Coverage:

Supporting Peptides:

| Peptide | Uniq | -10lgP | Mass | Length | ppm | m/z | z | RT | Fraction | Scan | Source File | Area F2 | Area F1 | Area F3 | Area F4 | Area F5 | Area F6 | #Feature | #Feature F2 | #Feature F1 | #Feature F3 | #Feature F4 | #Feature F5 | #Feature F6 | Start | End | PTM | AScore | Found By |
| --- | --- | --- | --- | --- | --- | --- | --- | --- | --- | --- | --- | --- | --- | --- | --- | --- | --- | --- | --- | --- | --- | --- | --- | --- | --- | --- | --- | --- | --- |
| K.SGVYQHVSGESVGGHAIR.I | Y | 76.21 | 1838.9023 | 18 | 0.8 | 613.9752 | 3 | 23.25 | 3 | F3:4929 | 31122021\_RID\_2349\_3C.raw |  |  | 9.3929E5 |  |  |  | 1 | 0 | 0 | 1 | 0 | 0 | 0 | 265 | 282 |  |  | PEAKS DB |
| R.FEFAGDLVLPK.S | Y | 69.09 | 1234.6597 | 11 | 0.7 | 618.3375 | 2 | 54.89 | 1 | F1:32582 | 28122021\_RID\_2349\_2C.raw | 1.4066E5 |  |  |  |  |  | 1 | 1 | 0 | 0 | 0 | 0 | 0 | 72 | 82 |  |  | PEAKS DB |
| K.EIMAEIYK.N | Y | 43.48 | 995.4998 | 8 | 0.8 | 498.7575 | 2 | 36.81 | 1 | F1:18465 | 28122021\_RID\_2349\_2C.raw | 1.7359E5 |  |  |  |  |  | 1 | 1 | 0 | 0 | 0 | 0 | 0 | 239 | 246 |  |  | PEAKS DB |
| total 3 peptides |  |  |  |  |  |  |  |  |  |  |  |  |  |  |  |  |  |  |  |  |  |  |  |  |  |  |  |  |  |
| --- | --- | --- | --- | --- | --- | --- | --- | --- | --- | --- | --- | --- | --- | --- | --- | --- | --- | --- | --- | --- | --- | --- | --- | --- | --- | --- | --- | --- | --- |

JAV51001.1

back to list

  

| Protein Coverage
| Supporting Peptides
|

Protein Coverage:

Supporting Peptides:

| Peptide | Uniq | -10lgP | Mass | Length | ppm | m/z | z | RT | Fraction | Scan | Source File | Area F2 | Area F1 | Area F3 | Area F4 | Area F5 | Area F6 | #Feature | #Feature F2 | #Feature F1 | #Feature F3 | #Feature F4 | #Feature F5 | #Feature F6 | Start | End | PTM | AScore | Found By |
| --- | --- | --- | --- | --- | --- | --- | --- | --- | --- | --- | --- | --- | --- | --- | --- | --- | --- | --- | --- | --- | --- | --- | --- | --- | --- | --- | --- | --- | --- |
| K.SGVYQHVSGESVGGHAIR.I | Y | 76.21 | 1838.9023 | 18 | 0.8 | 613.9752 | 3 | 23.25 | 3 | F3:4929 | 31122021\_RID\_2349\_3C.raw |  |  | 9.3929E5 |  |  |  | 1 | 0 | 0 | 1 | 0 | 0 | 0 | 265 | 282 |  |  | PEAKS DB |
| R.FEFAGDLVLPK.S | Y | 69.09 | 1234.6597 | 11 | 0.7 | 618.3375 | 2 | 54.89 | 1 | F1:32582 | 28122021\_RID\_2349\_2C.raw | 1.4066E5 |  |  |  |  |  | 1 | 1 | 0 | 0 | 0 | 0 | 0 | 72 | 82 |  |  | PEAKS DB |
| K.EIMAEIYK.N | Y | 43.48 | 995.4998 | 8 | 0.8 | 498.7575 | 2 | 36.81 | 1 | F1:18465 | 28122021\_RID\_2349\_2C.raw | 1.7359E5 |  |  |  |  |  | 1 | 1 | 0 | 0 | 0 | 0 | 0 | 239 | 246 |  |  | PEAKS DB |
| total 3 peptides |  |  |  |  |  |  |  |  |  |  |  |  |  |  |  |  |  |  |  |  |  |  |  |  |  |  |  |  |  |
| --- | --- | --- | --- | --- | --- | --- | --- | --- | --- | --- | --- | --- | --- | --- | --- | --- | --- | --- | --- | --- | --- | --- | --- | --- | --- | --- | --- | --- | --- |

JAG45448.1

back to list

  

| Protein Coverage
| Supporting Peptides
|

Protein Coverage:

Supporting Peptides:

| Peptide | Uniq | -10lgP | Mass | Length | ppm | m/z | z | RT | Fraction | Scan | Source File | Area F2 | Area F1 | Area F3 | Area F4 | Area F5 | Area F6 | #Feature | #Feature F2 | #Feature F1 | #Feature F3 | #Feature F4 | #Feature F5 | #Feature F6 | Start | End | PTM | AScore | Found By |
| --- | --- | --- | --- | --- | --- | --- | --- | --- | --- | --- | --- | --- | --- | --- | --- | --- | --- | --- | --- | --- | --- | --- | --- | --- | --- | --- | --- | --- | --- |
| K.SGVYQHVSGESVGGHAIR.I | Y | 76.21 | 1838.9023 | 18 | 0.8 | 613.9752 | 3 | 23.25 | 3 | F3:4929 | 31122021\_RID\_2349\_3C.raw |  |  | 9.3929E5 |  |  |  | 1 | 0 | 0 | 1 | 0 | 0 | 0 | 265 | 282 |  |  | PEAKS DB |
| R.FEFAGDLVLPK.S | Y | 69.09 | 1234.6597 | 11 | 0.7 | 618.3375 | 2 | 54.89 | 1 | F1:32582 | 28122021\_RID\_2349\_2C.raw | 1.4066E5 |  |  |  |  |  | 1 | 1 | 0 | 0 | 0 | 0 | 0 | 72 | 82 |  |  | PEAKS DB |
| K.EIMAEIYK.N | Y | 43.48 | 995.4998 | 8 | 0.8 | 498.7575 | 2 | 36.81 | 1 | F1:18465 | 28122021\_RID\_2349\_2C.raw | 1.7359E5 |  |  |  |  |  | 1 | 1 | 0 | 0 | 0 | 0 | 0 | 239 | 246 |  |  | PEAKS DB |
| total 3 peptides |  |  |  |  |  |  |  |  |  |  |  |  |  |  |  |  |  |  |  |  |  |  |  |  |  |  |  |  |  |
| --- | --- | --- | --- | --- | --- | --- | --- | --- | --- | --- | --- | --- | --- | --- | --- | --- | --- | --- | --- | --- | --- | --- | --- | --- | --- | --- | --- | --- | --- |

JAG46154.1

back to list

  

| Protein Coverage
| Supporting Peptides
|

Protein Coverage:

Supporting Peptides:

| Peptide | Uniq | -10lgP | Mass | Length | ppm | m/z | z | RT | Fraction | Scan | Source File | Area F2 | Area F1 | Area F3 | Area F4 | Area F5 | Area F6 | #Feature | #Feature F2 | #Feature F1 | #Feature F3 | #Feature F4 | #Feature F5 | #Feature F6 | Start | End | PTM | AScore | Found By |
| --- | --- | --- | --- | --- | --- | --- | --- | --- | --- | --- | --- | --- | --- | --- | --- | --- | --- | --- | --- | --- | --- | --- | --- | --- | --- | --- | --- | --- | --- |
| R.LASIHSSEEEAFVGK.L | Y | 90.38 | 1602.7889 | 15 | 0.4 | 802.4021 | 2 | 30.99 | 6 | F6:12299 | 31122021\_RID\_2349\_6C.raw |  |  |  |  |  | 1.0596E7 | 2 | 0 | 0 | 0 | 0 | 0 | 2 | 64 | 78 |  |  | PEAKS DB |
| K.FTSMWIGLK.D | Y | 55.64 | 1081.5630 | 9 | 0.4 | 541.7890 | 2 | 51.46 | 6 | F6:28029 | 31122021\_RID\_2349\_6C.raw |  |  |  |  |  | 2.1382E5 | 1 | 0 | 0 | 0 | 0 | 0 | 1 | 86 | 94 |  |  | PEAKS DB |
| H.SSEEEAFVGK.L | Y | 46.15 | 1081.4927 | 10 | -0.2 | 541.7535 | 2 | 25.83 | 6 | F6:8519 | 31122021\_RID\_2349\_6C.raw |  |  |  |  |  | 0 | 0 | 0 | 0 | 0 | 0 | 0 | 0 | 69 | 78 |  |  | PEAKS DB |
| total 3 peptides |  |  |  |  |  |  |  |  |  |  |  |  |  |  |  |  |  |  |  |  |  |  |  |  |  |  |  |  |  |
| --- | --- | --- | --- | --- | --- | --- | --- | --- | --- | --- | --- | --- | --- | --- | --- | --- | --- | --- | --- | --- | --- | --- | --- | --- | --- | --- | --- | --- | --- |

JAS04715.1

back to list

  

| Protein Coverage
| Supporting Peptides
|

Protein Coverage:

Supporting Peptides:

| Peptide | Uniq | -10lgP | Mass | Length | ppm | m/z | z | RT | Fraction | Scan | Source File | Area F2 | Area F1 | Area F3 | Area F4 | Area F5 | Area F6 | #Feature | #Feature F2 | #Feature F1 | #Feature F3 | #Feature F4 | #Feature F5 | #Feature F6 | Start | End | PTM | AScore | Found By |
| --- | --- | --- | --- | --- | --- | --- | --- | --- | --- | --- | --- | --- | --- | --- | --- | --- | --- | --- | --- | --- | --- | --- | --- | --- | --- | --- | --- | --- | --- |
| R.LASIHSSEEEAFVGK.L | Y | 90.38 | 1602.7889 | 15 | 0.4 | 802.4021 | 2 | 30.99 | 6 | F6:12299 | 31122021\_RID\_2349\_6C.raw |  |  |  |  |  | 1.0596E7 | 2 | 0 | 0 | 0 | 0 | 0 | 2 | 64 | 78 |  |  | PEAKS DB |
| K.FTSMWIGLK.D | Y | 55.64 | 1081.5630 | 9 | 0.4 | 541.7890 | 2 | 51.46 | 6 | F6:28029 | 31122021\_RID\_2349\_6C.raw |  |  |  |  |  | 2.1382E5 | 1 | 0 | 0 | 0 | 0 | 0 | 1 | 86 | 94 |  |  | PEAKS DB |
| H.SSEEEAFVGK.L | Y | 46.15 | 1081.4927 | 10 | -0.2 | 541.7535 | 2 | 25.83 | 6 | F6:8519 | 31122021\_RID\_2349\_6C.raw |  |  |  |  |  | 0 | 0 | 0 | 0 | 0 | 0 | 0 | 0 | 69 | 78 |  |  | PEAKS DB |
| total 3 peptides |  |  |  |  |  |  |  |  |  |  |  |  |  |  |  |  |  |  |  |  |  |  |  |  |  |  |  |  |  |
| --- | --- | --- | --- | --- | --- | --- | --- | --- | --- | --- | --- | --- | --- | --- | --- | --- | --- | --- | --- | --- | --- | --- | --- | --- | --- | --- | --- | --- | --- |

JAS05341.1

back to list

  

| Protein Coverage
| Supporting Peptides
|

Protein Coverage:

Supporting Peptides:

| Peptide | Uniq | -10lgP | Mass | Length | ppm | m/z | z | RT | Fraction | Scan | Source File | Area F2 | Area F1 | Area F3 | Area F4 | Area F5 | Area F6 | #Feature | #Feature F2 | #Feature F1 | #Feature F3 | #Feature F4 | #Feature F5 | #Feature F6 | Start | End | PTM | AScore | Found By |
| --- | --- | --- | --- | --- | --- | --- | --- | --- | --- | --- | --- | --- | --- | --- | --- | --- | --- | --- | --- | --- | --- | --- | --- | --- | --- | --- | --- | --- | --- |
| R.LASIHSSEEEAFVGK.L | Y | 90.38 | 1602.7889 | 15 | 0.4 | 802.4021 | 2 | 30.99 | 6 | F6:12299 | 31122021\_RID\_2349\_6C.raw |  |  |  |  |  | 1.0596E7 | 2 | 0 | 0 | 0 | 0 | 0 | 2 | 64 | 78 |  |  | PEAKS DB |
| K.FTSMWIGLK.D | Y | 55.64 | 1081.5630 | 9 | 0.4 | 541.7890 | 2 | 51.46 | 6 | F6:28029 | 31122021\_RID\_2349\_6C.raw |  |  |  |  |  | 2.1382E5 | 1 | 0 | 0 | 0 | 0 | 0 | 1 | 86 | 94 |  |  | PEAKS DB |
| H.SSEEEAFVGK.L | Y | 46.15 | 1081.4927 | 10 | -0.2 | 541.7535 | 2 | 25.83 | 6 | F6:8519 | 31122021\_RID\_2349\_6C.raw |  |  |  |  |  | 0 | 0 | 0 | 0 | 0 | 0 | 0 | 0 | 69 | 78 |  |  | PEAKS DB |
| total 3 peptides |  |  |  |  |  |  |  |  |  |  |  |  |  |  |  |  |  |  |  |  |  |  |  |  |  |  |  |  |  |
| --- | --- | --- | --- | --- | --- | --- | --- | --- | --- | --- | --- | --- | --- | --- | --- | --- | --- | --- | --- | --- | --- | --- | --- | --- | --- | --- | --- | --- | --- |

JAI10419.1

back to list

  

| Protein Coverage
| Supporting Peptides
|

Protein Coverage:

Supporting Peptides:

| Peptide | Uniq | -10lgP | Mass | Length | ppm | m/z | z | RT | Fraction | Scan | Source File | Area F2 | Area F1 | Area F3 | Area F4 | Area F5 | Area F6 | #Feature | #Feature F2 | #Feature F1 | #Feature F3 | #Feature F4 | #Feature F5 | #Feature F6 | Start | End | PTM | AScore | Found By |
| --- | --- | --- | --- | --- | --- | --- | --- | --- | --- | --- | --- | --- | --- | --- | --- | --- | --- | --- | --- | --- | --- | --- | --- | --- | --- | --- | --- | --- | --- |
| R.LASIHSSEEEAFVGK.L | Y | 90.38 | 1602.7889 | 15 | 0.4 | 802.4021 | 2 | 30.99 | 6 | F6:12299 | 31122021\_RID\_2349\_6C.raw |  |  |  |  |  | 1.0596E7 | 2 | 0 | 0 | 0 | 0 | 0 | 2 | 64 | 78 |  |  | PEAKS DB |
| K.FTSMWIGLK.D | Y | 55.64 | 1081.5630 | 9 | 0.4 | 541.7890 | 2 | 51.46 | 6 | F6:28029 | 31122021\_RID\_2349\_6C.raw |  |  |  |  |  | 2.1382E5 | 1 | 0 | 0 | 0 | 0 | 0 | 1 | 86 | 94 |  |  | PEAKS DB |
| H.SSEEEAFVGK.L | Y | 46.15 | 1081.4927 | 10 | -0.2 | 541.7535 | 2 | 25.83 | 6 | F6:8519 | 31122021\_RID\_2349\_6C.raw |  |  |  |  |  | 0 | 0 | 0 | 0 | 0 | 0 | 0 | 0 | 69 | 78 |  |  | PEAKS DB |
| total 3 peptides |  |  |  |  |  |  |  |  |  |  |  |  |  |  |  |  |  |  |  |  |  |  |  |  |  |  |  |  |  |
| --- | --- | --- | --- | --- | --- | --- | --- | --- | --- | --- | --- | --- | --- | --- | --- | --- | --- | --- | --- | --- | --- | --- | --- | --- | --- | --- | --- | --- | --- |

AEJ31972.1

back to list

  

| Protein Coverage
| Supporting Peptides
|

Protein Coverage:

Supporting Peptides:

| Peptide | Uniq | -10lgP | Mass | Length | ppm | m/z | z | RT | Fraction | Scan | Source File | Area F2 | Area F1 | Area F3 | Area F4 | Area F5 | Area F6 | #Feature | #Feature F2 | #Feature F1 | #Feature F3 | #Feature F4 | #Feature F5 | #Feature F6 | Start | End | PTM | AScore | Found By |
| --- | --- | --- | --- | --- | --- | --- | --- | --- | --- | --- | --- | --- | --- | --- | --- | --- | --- | --- | --- | --- | --- | --- | --- | --- | --- | --- | --- | --- | --- |
| R.LASIHSSEEEAFVGK.L | Y | 90.38 | 1602.7889 | 15 | 0.4 | 802.4021 | 2 | 30.99 | 6 | F6:12299 | 31122021\_RID\_2349\_6C.raw |  |  |  |  |  | 1.0596E7 | 2 | 0 | 0 | 0 | 0 | 0 | 2 | 64 | 78 |  |  | PEAKS DB |
| K.FTSMWIGLK.D | Y | 55.64 | 1081.5630 | 9 | 0.4 | 541.7890 | 2 | 51.46 | 6 | F6:28029 | 31122021\_RID\_2349\_6C.raw |  |  |  |  |  | 2.1382E5 | 1 | 0 | 0 | 0 | 0 | 0 | 1 | 86 | 94 |  |  | PEAKS DB |
| H.SSEEEAFVGK.L | Y | 46.15 | 1081.4927 | 10 | -0.2 | 541.7535 | 2 | 25.83 | 6 | F6:8519 | 31122021\_RID\_2349\_6C.raw |  |  |  |  |  | 0 | 0 | 0 | 0 | 0 | 0 | 0 | 0 | 69 | 78 |  |  | PEAKS DB |
| total 3 peptides |  |  |  |  |  |  |  |  |  |  |  |  |  |  |  |  |  |  |  |  |  |  |  |  |  |  |  |  |  |
| --- | --- | --- | --- | --- | --- | --- | --- | --- | --- | --- | --- | --- | --- | --- | --- | --- | --- | --- | --- | --- | --- | --- | --- | --- | --- | --- | --- | --- | --- |

JAS04888.1

back to list

  

| Protein Coverage
| Supporting Peptides
|

Protein Coverage:

Supporting Peptides:

| Peptide | Uniq | -10lgP | Mass | Length | ppm | m/z | z | RT | Fraction | Scan | Source File | Area F2 | Area F1 | Area F3 | Area F4 | Area F5 | Area F6 | #Feature | #Feature F2 | #Feature F1 | #Feature F3 | #Feature F4 | #Feature F5 | #Feature F6 | Start | End | PTM | AScore | Found By |
| --- | --- | --- | --- | --- | --- | --- | --- | --- | --- | --- | --- | --- | --- | --- | --- | --- | --- | --- | --- | --- | --- | --- | --- | --- | --- | --- | --- | --- | --- |
| R.LASIHSSEEEAFVGK.L | Y | 90.38 | 1602.7889 | 15 | 0.4 | 802.4021 | 2 | 30.99 | 6 | F6:12299 | 31122021\_RID\_2349\_6C.raw |  |  |  |  |  | 1.0596E7 | 2 | 0 | 0 | 0 | 0 | 0 | 2 | 64 | 78 |  |  | PEAKS DB |
| K.FTSMWIGLK.D | Y | 55.64 | 1081.5630 | 9 | 0.4 | 541.7890 | 2 | 51.46 | 6 | F6:28029 | 31122021\_RID\_2349\_6C.raw |  |  |  |  |  | 2.1382E5 | 1 | 0 | 0 | 0 | 0 | 0 | 1 | 86 | 94 |  |  | PEAKS DB |
| H.SSEEEAFVGK.L | Y | 46.15 | 1081.4927 | 10 | -0.2 | 541.7535 | 2 | 25.83 | 6 | F6:8519 | 31122021\_RID\_2349\_6C.raw |  |  |  |  |  | 0 | 0 | 0 | 0 | 0 | 0 | 0 | 0 | 69 | 78 |  |  | PEAKS DB |
| total 3 peptides |  |  |  |  |  |  |  |  |  |  |  |  |  |  |  |  |  |  |  |  |  |  |  |  |  |  |  |  |  |
| --- | --- | --- | --- | --- | --- | --- | --- | --- | --- | --- | --- | --- | --- | --- | --- | --- | --- | --- | --- | --- | --- | --- | --- | --- | --- | --- | --- | --- | --- |

JAS05342.1

back to list

  

| Protein Coverage
| Supporting Peptides
|

Protein Coverage:

Supporting Peptides:

| Peptide | Uniq | -10lgP | Mass | Length | ppm | m/z | z | RT | Fraction | Scan | Source File | Area F2 | Area F1 | Area F3 | Area F4 | Area F5 | Area F6 | #Feature | #Feature F2 | #Feature F1 | #Feature F3 | #Feature F4 | #Feature F5 | #Feature F6 | Start | End | PTM | AScore | Found By |
| --- | --- | --- | --- | --- | --- | --- | --- | --- | --- | --- | --- | --- | --- | --- | --- | --- | --- | --- | --- | --- | --- | --- | --- | --- | --- | --- | --- | --- | --- |
| R.LASIHSSEEEAFVGK.L | Y | 90.38 | 1602.7889 | 15 | 0.4 | 802.4021 | 2 | 30.99 | 6 | F6:12299 | 31122021\_RID\_2349\_6C.raw |  |  |  |  |  | 1.0596E7 | 2 | 0 | 0 | 0 | 0 | 0 | 2 | 64 | 78 |  |  | PEAKS DB |
| K.FTSMWIGLK.D | Y | 55.64 | 1081.5630 | 9 | 0.4 | 541.7890 | 2 | 51.46 | 6 | F6:28029 | 31122021\_RID\_2349\_6C.raw |  |  |  |  |  | 2.1382E5 | 1 | 0 | 0 | 0 | 0 | 0 | 1 | 86 | 94 |  |  | PEAKS DB |
| H.SSEEEAFVGK.L | Y | 46.15 | 1081.4927 | 10 | -0.2 | 541.7535 | 2 | 25.83 | 6 | F6:8519 | 31122021\_RID\_2349\_6C.raw |  |  |  |  |  | 0 | 0 | 0 | 0 | 0 | 0 | 0 | 0 | 69 | 78 |  |  | PEAKS DB |
| total 3 peptides |  |  |  |  |  |  |  |  |  |  |  |  |  |  |  |  |  |  |  |  |  |  |  |  |  |  |  |  |  |
| --- | --- | --- | --- | --- | --- | --- | --- | --- | --- | --- | --- | --- | --- | --- | --- | --- | --- | --- | --- | --- | --- | --- | --- | --- | --- | --- | --- | --- | --- |

JAS04716.1

back to list

  

| Protein Coverage
| Supporting Peptides
|

Protein Coverage:

Supporting Peptides:

| Peptide | Uniq | -10lgP | Mass | Length | ppm | m/z | z | RT | Fraction | Scan | Source File | Area F2 | Area F1 | Area F3 | Area F4 | Area F5 | Area F6 | #Feature | #Feature F2 | #Feature F1 | #Feature F3 | #Feature F4 | #Feature F5 | #Feature F6 | Start | End | PTM | AScore | Found By |
| --- | --- | --- | --- | --- | --- | --- | --- | --- | --- | --- | --- | --- | --- | --- | --- | --- | --- | --- | --- | --- | --- | --- | --- | --- | --- | --- | --- | --- | --- |
| R.LASIHSSEEEAFVGK.L | Y | 90.38 | 1602.7889 | 15 | 0.4 | 802.4021 | 2 | 30.99 | 6 | F6:12299 | 31122021\_RID\_2349\_6C.raw |  |  |  |  |  | 1.0596E7 | 2 | 0 | 0 | 0 | 0 | 0 | 2 | 64 | 78 |  |  | PEAKS DB |
| K.FTSMWIGLK.D | Y | 55.64 | 1081.5630 | 9 | 0.4 | 541.7890 | 2 | 51.46 | 6 | F6:28029 | 31122021\_RID\_2349\_6C.raw |  |  |  |  |  | 2.1382E5 | 1 | 0 | 0 | 0 | 0 | 0 | 1 | 86 | 94 |  |  | PEAKS DB |
| H.SSEEEAFVGK.L | Y | 46.15 | 1081.4927 | 10 | -0.2 | 541.7535 | 2 | 25.83 | 6 | F6:8519 | 31122021\_RID\_2349\_6C.raw |  |  |  |  |  | 0 | 0 | 0 | 0 | 0 | 0 | 0 | 0 | 69 | 78 |  |  | PEAKS DB |
| total 3 peptides |  |  |  |  |  |  |  |  |  |  |  |  |  |  |  |  |  |  |  |  |  |  |  |  |  |  |  |  |  |
| --- | --- | --- | --- | --- | --- | --- | --- | --- | --- | --- | --- | --- | --- | --- | --- | --- | --- | --- | --- | --- | --- | --- | --- | --- | --- | --- | --- | --- | --- |

AFJ49153.1

back to list

  

| Protein Coverage
| Supporting Peptides
|

Protein Coverage:

Supporting Peptides:

| Peptide | Uniq | -10lgP | Mass | Length | ppm | m/z | z | RT | Fraction | Scan | Source File | Area F2 | Area F1 | Area F3 | Area F4 | Area F5 | Area F6 | #Feature | #Feature F2 | #Feature F1 | #Feature F3 | #Feature F4 | #Feature F5 | #Feature F6 | Start | End | PTM | AScore | Found By |
| --- | --- | --- | --- | --- | --- | --- | --- | --- | --- | --- | --- | --- | --- | --- | --- | --- | --- | --- | --- | --- | --- | --- | --- | --- | --- | --- | --- | --- | --- |
| R.LASIHSSEEEAFVGK.L | Y | 90.38 | 1602.7889 | 15 | 0.4 | 802.4021 | 2 | 30.99 | 6 | F6:12299 | 31122021\_RID\_2349\_6C.raw |  |  |  |  |  | 1.0596E7 | 2 | 0 | 0 | 0 | 0 | 0 | 2 | 64 | 78 |  |  | PEAKS DB |
| K.FTSMWIGLK.D | Y | 55.64 | 1081.5630 | 9 | 0.4 | 541.7890 | 2 | 51.46 | 6 | F6:28029 | 31122021\_RID\_2349\_6C.raw |  |  |  |  |  | 2.1382E5 | 1 | 0 | 0 | 0 | 0 | 0 | 1 | 86 | 94 |  |  | PEAKS DB |
| H.SSEEEAFVGK.L | Y | 46.15 | 1081.4927 | 10 | -0.2 | 541.7535 | 2 | 25.83 | 6 | F6:8519 | 31122021\_RID\_2349\_6C.raw |  |  |  |  |  | 0 | 0 | 0 | 0 | 0 | 0 | 0 | 0 | 69 | 78 |  |  | PEAKS DB |
| total 3 peptides |  |  |  |  |  |  |  |  |  |  |  |  |  |  |  |  |  |  |  |  |  |  |  |  |  |  |  |  |  |
| --- | --- | --- | --- | --- | --- | --- | --- | --- | --- | --- | --- | --- | --- | --- | --- | --- | --- | --- | --- | --- | --- | --- | --- | --- | --- | --- | --- | --- | --- |

JAV48435.1

back to list

  

| Protein Coverage
| Supporting Peptides
|

Protein Coverage:

Supporting Peptides:

| Peptide | Uniq | -10lgP | Mass | Length | ppm | m/z | z | RT | Fraction | Scan | Source File | Area F2 | Area F1 | Area F3 | Area F4 | Area F5 | Area F6 | #Feature | #Feature F2 | #Feature F1 | #Feature F3 | #Feature F4 | #Feature F5 | #Feature F6 | Start | End | PTM | AScore | Found By |
| --- | --- | --- | --- | --- | --- | --- | --- | --- | --- | --- | --- | --- | --- | --- | --- | --- | --- | --- | --- | --- | --- | --- | --- | --- | --- | --- | --- | --- | --- |
| R.LASIHSSEEEAFVGK.L | Y | 90.38 | 1602.7889 | 15 | 0.4 | 802.4021 | 2 | 30.99 | 6 | F6:12299 | 31122021\_RID\_2349\_6C.raw |  |  |  |  |  | 1.0596E7 | 2 | 0 | 0 | 0 | 0 | 0 | 2 | 64 | 78 |  |  | PEAKS DB |
| K.FTSMWIGLK.D | Y | 55.64 | 1081.5630 | 9 | 0.4 | 541.7890 | 2 | 51.46 | 6 | F6:28029 | 31122021\_RID\_2349\_6C.raw |  |  |  |  |  | 2.1382E5 | 1 | 0 | 0 | 0 | 0 | 0 | 1 | 86 | 94 |  |  | PEAKS DB |
| H.SSEEEAFVGK.L | Y | 46.15 | 1081.4927 | 10 | -0.2 | 541.7535 | 2 | 25.83 | 6 | F6:8519 | 31122021\_RID\_2349\_6C.raw |  |  |  |  |  | 0 | 0 | 0 | 0 | 0 | 0 | 0 | 0 | 69 | 78 |  |  | PEAKS DB |
| total 3 peptides |  |  |  |  |  |  |  |  |  |  |  |  |  |  |  |  |  |  |  |  |  |  |  |  |  |  |  |  |  |
| --- | --- | --- | --- | --- | --- | --- | --- | --- | --- | --- | --- | --- | --- | --- | --- | --- | --- | --- | --- | --- | --- | --- | --- | --- | --- | --- | --- | --- | --- |

JAI10420.1

back to list

  

| Protein Coverage
| Supporting Peptides
|

Protein Coverage:

Supporting Peptides:

| Peptide | Uniq | -10lgP | Mass | Length | ppm | m/z | z | RT | Fraction | Scan | Source File | Area F2 | Area F1 | Area F3 | Area F4 | Area F5 | Area F6 | #Feature | #Feature F2 | #Feature F1 | #Feature F3 | #Feature F4 | #Feature F5 | #Feature F6 | Start | End | PTM | AScore | Found By |
| --- | --- | --- | --- | --- | --- | --- | --- | --- | --- | --- | --- | --- | --- | --- | --- | --- | --- | --- | --- | --- | --- | --- | --- | --- | --- | --- | --- | --- | --- |
| R.LASIHSSEEEAFVGK.L | Y | 90.38 | 1602.7889 | 15 | 0.4 | 802.4021 | 2 | 30.99 | 6 | F6:12299 | 31122021\_RID\_2349\_6C.raw |  |  |  |  |  | 1.0596E7 | 2 | 0 | 0 | 0 | 0 | 0 | 2 | 64 | 78 |  |  | PEAKS DB |
| K.FTSMWIGLK.D | Y | 55.64 | 1081.5630 | 9 | 0.4 | 541.7890 | 2 | 51.46 | 6 | F6:28029 | 31122021\_RID\_2349\_6C.raw |  |  |  |  |  | 2.1382E5 | 1 | 0 | 0 | 0 | 0 | 0 | 1 | 86 | 94 |  |  | PEAKS DB |
| H.SSEEEAFVGK.L | Y | 46.15 | 1081.4927 | 10 | -0.2 | 541.7535 | 2 | 25.83 | 6 | F6:8519 | 31122021\_RID\_2349\_6C.raw |  |  |  |  |  | 0 | 0 | 0 | 0 | 0 | 0 | 0 | 0 | 69 | 78 |  |  | PEAKS DB |
| total 3 peptides |  |  |  |  |  |  |  |  |  |  |  |  |  |  |  |  |  |  |  |  |  |  |  |  |  |  |  |  |  |
| --- | --- | --- | --- | --- | --- | --- | --- | --- | --- | --- | --- | --- | --- | --- | --- | --- | --- | --- | --- | --- | --- | --- | --- | --- | --- | --- | --- | --- | --- |

JAA97993.1

back to list

  

| Protein Coverage
| Supporting Peptides
|

Protein Coverage:

Supporting Peptides:

| Peptide | Uniq | -10lgP | Mass | Length | ppm | m/z | z | RT | Fraction | Scan | Source File | Area F2 | Area F1 | Area F3 | Area F4 | Area F5 | Area F6 | #Feature | #Feature F2 | #Feature F1 | #Feature F3 | #Feature F4 | #Feature F5 | #Feature F6 | Start | End | PTM | AScore | Found By |
| --- | --- | --- | --- | --- | --- | --- | --- | --- | --- | --- | --- | --- | --- | --- | --- | --- | --- | --- | --- | --- | --- | --- | --- | --- | --- | --- | --- | --- | --- |
| R.LASIHSSEEEAFVGK.L | Y | 90.38 | 1602.7889 | 15 | 0.4 | 802.4021 | 2 | 30.99 | 6 | F6:12299 | 31122021\_RID\_2349\_6C.raw |  |  |  |  |  | 1.0596E7 | 2 | 0 | 0 | 0 | 0 | 0 | 2 | 64 | 78 |  |  | PEAKS DB |
| K.FTSMWIGLK.D | Y | 55.64 | 1081.5630 | 9 | 0.4 | 541.7890 | 2 | 51.46 | 6 | F6:28029 | 31122021\_RID\_2349\_6C.raw |  |  |  |  |  | 2.1382E5 | 1 | 0 | 0 | 0 | 0 | 0 | 1 | 86 | 94 |  |  | PEAKS DB |
| H.SSEEEAFVGK.L | Y | 46.15 | 1081.4927 | 10 | -0.2 | 541.7535 | 2 | 25.83 | 6 | F6:8519 | 31122021\_RID\_2349\_6C.raw |  |  |  |  |  | 0 | 0 | 0 | 0 | 0 | 0 | 0 | 0 | 69 | 78 |  |  | PEAKS DB |
| total 3 peptides |  |  |  |  |  |  |  |  |  |  |  |  |  |  |  |  |  |  |  |  |  |  |  |  |  |  |  |  |  |
| --- | --- | --- | --- | --- | --- | --- | --- | --- | --- | --- | --- | --- | --- | --- | --- | --- | --- | --- | --- | --- | --- | --- | --- | --- | --- | --- | --- | --- | --- |

AFJ49152.1

back to list

  

| Protein Coverage
| Supporting Peptides
|

Protein Coverage:

Supporting Peptides:

| Peptide | Uniq | -10lgP | Mass | Length | ppm | m/z | z | RT | Fraction | Scan | Source File | Area F2 | Area F1 | Area F3 | Area F4 | Area F5 | Area F6 | #Feature | #Feature F2 | #Feature F1 | #Feature F3 | #Feature F4 | #Feature F5 | #Feature F6 | Start | End | PTM | AScore | Found By |
| --- | --- | --- | --- | --- | --- | --- | --- | --- | --- | --- | --- | --- | --- | --- | --- | --- | --- | --- | --- | --- | --- | --- | --- | --- | --- | --- | --- | --- | --- |
| R.LASIHSSEEEAFVGK.L | Y | 90.38 | 1602.7889 | 15 | 0.4 | 802.4021 | 2 | 30.99 | 6 | F6:12299 | 31122021\_RID\_2349\_6C.raw |  |  |  |  |  | 1.0596E7 | 2 | 0 | 0 | 0 | 0 | 0 | 2 | 64 | 78 |  |  | PEAKS DB |
| K.FTSMWIGLK.D | Y | 55.64 | 1081.5630 | 9 | 0.4 | 541.7890 | 2 | 51.46 | 6 | F6:28029 | 31122021\_RID\_2349\_6C.raw |  |  |  |  |  | 2.1382E5 | 1 | 0 | 0 | 0 | 0 | 0 | 1 | 86 | 94 |  |  | PEAKS DB |
| H.SSEEEAFVGK.L | Y | 46.15 | 1081.4927 | 10 | -0.2 | 541.7535 | 2 | 25.83 | 6 | F6:8519 | 31122021\_RID\_2349\_6C.raw |  |  |  |  |  | 0 | 0 | 0 | 0 | 0 | 0 | 0 | 0 | 69 | 78 |  |  | PEAKS DB |
| total 3 peptides |  |  |  |  |  |  |  |  |  |  |  |  |  |  |  |  |  |  |  |  |  |  |  |  |  |  |  |  |  |
| --- | --- | --- | --- | --- | --- | --- | --- | --- | --- | --- | --- | --- | --- | --- | --- | --- | --- | --- | --- | --- | --- | --- | --- | --- | --- | --- | --- | --- | --- |

CAD35498.1

back to list

  

| Protein Coverage
| Supporting Peptides
|

Protein Coverage:

Supporting Peptides:

| Peptide | Uniq | -10lgP | Mass | Length | ppm | m/z | z | RT | Fraction | Scan | Source File | Area F2 | Area F1 | Area F3 | Area F4 | Area F5 | Area F6 | #Feature | #Feature F2 | #Feature F1 | #Feature F3 | #Feature F4 | #Feature F5 | #Feature F6 | Start | End | PTM | AScore | Found By |
| --- | --- | --- | --- | --- | --- | --- | --- | --- | --- | --- | --- | --- | --- | --- | --- | --- | --- | --- | --- | --- | --- | --- | --- | --- | --- | --- | --- | --- | --- |
| R.LASIHSSEEEAFVGK.L | Y | 90.38 | 1602.7889 | 15 | 0.4 | 802.4021 | 2 | 30.99 | 6 | F6:12299 | 31122021\_RID\_2349\_6C.raw |  |  |  |  |  | 1.0596E7 | 2 | 0 | 0 | 0 | 0 | 0 | 2 | 58 | 72 |  |  | PEAKS DB |
| K.FTSMWIGLK.D | Y | 55.64 | 1081.5630 | 9 | 0.4 | 541.7890 | 2 | 51.46 | 6 | F6:28029 | 31122021\_RID\_2349\_6C.raw |  |  |  |  |  | 2.1382E5 | 1 | 0 | 0 | 0 | 0 | 0 | 1 | 80 | 88 |  |  | PEAKS DB |
| H.SSEEEAFVGK.L | Y | 46.15 | 1081.4927 | 10 | -0.2 | 541.7535 | 2 | 25.83 | 6 | F6:8519 | 31122021\_RID\_2349\_6C.raw |  |  |  |  |  | 0 | 0 | 0 | 0 | 0 | 0 | 0 | 0 | 63 | 72 |  |  | PEAKS DB |
| total 3 peptides |  |  |  |  |  |  |  |  |  |  |  |  |  |  |  |  |  |  |  |  |  |  |  |  |  |  |  |  |  |
| --- | --- | --- | --- | --- | --- | --- | --- | --- | --- | --- | --- | --- | --- | --- | --- | --- | --- | --- | --- | --- | --- | --- | --- | --- | --- | --- | --- | --- | --- |

JAS05482.1

back to list

  

| Protein Coverage
| Supporting Peptides
|

Protein Coverage:

Supporting Peptides:

| Peptide | Uniq | -10lgP | Mass | Length | ppm | m/z | z | RT | Fraction | Scan | Source File | Area F2 | Area F1 | Area F3 | Area F4 | Area F5 | Area F6 | #Feature | #Feature F2 | #Feature F1 | #Feature F3 | #Feature F4 | #Feature F5 | #Feature F6 | Start | End | PTM | AScore | Found By |
| --- | --- | --- | --- | --- | --- | --- | --- | --- | --- | --- | --- | --- | --- | --- | --- | --- | --- | --- | --- | --- | --- | --- | --- | --- | --- | --- | --- | --- | --- |
| R.LASIHSSEEEAFVGK.L | Y | 90.38 | 1602.7889 | 15 | 0.4 | 802.4021 | 2 | 30.99 | 6 | F6:12299 | 31122021\_RID\_2349\_6C.raw |  |  |  |  |  | 1.0596E7 | 2 | 0 | 0 | 0 | 0 | 0 | 2 | 62 | 76 |  |  | PEAKS DB |
| K.FTSMWIGLK.D | Y | 55.64 | 1081.5630 | 9 | 0.4 | 541.7890 | 2 | 51.46 | 6 | F6:28029 | 31122021\_RID\_2349\_6C.raw |  |  |  |  |  | 2.1382E5 | 1 | 0 | 0 | 0 | 0 | 0 | 1 | 84 | 92 |  |  | PEAKS DB |
| H.SSEEEAFVGK.L | Y | 46.15 | 1081.4927 | 10 | -0.2 | 541.7535 | 2 | 25.83 | 6 | F6:8519 | 31122021\_RID\_2349\_6C.raw |  |  |  |  |  | 0 | 0 | 0 | 0 | 0 | 0 | 0 | 0 | 67 | 76 |  |  | PEAKS DB |
| total 3 peptides |  |  |  |  |  |  |  |  |  |  |  |  |  |  |  |  |  |  |  |  |  |  |  |  |  |  |  |  |  |
| --- | --- | --- | --- | --- | --- | --- | --- | --- | --- | --- | --- | --- | --- | --- | --- | --- | --- | --- | --- | --- | --- | --- | --- | --- | --- | --- | --- | --- | --- |

JAS05481.1

back to list

  

| Protein Coverage
| Supporting Peptides
|

Protein Coverage:

Supporting Peptides:

| Peptide | Uniq | -10lgP | Mass | Length | ppm | m/z | z | RT | Fraction | Scan | Source File | Area F2 | Area F1 | Area F3 | Area F4 | Area F5 | Area F6 | #Feature | #Feature F2 | #Feature F1 | #Feature F3 | #Feature F4 | #Feature F5 | #Feature F6 | Start | End | PTM | AScore | Found By |
| --- | --- | --- | --- | --- | --- | --- | --- | --- | --- | --- | --- | --- | --- | --- | --- | --- | --- | --- | --- | --- | --- | --- | --- | --- | --- | --- | --- | --- | --- |
| R.LASIHSSEEEAFVGK.L | Y | 90.38 | 1602.7889 | 15 | 0.4 | 802.4021 | 2 | 30.99 | 6 | F6:12299 | 31122021\_RID\_2349\_6C.raw |  |  |  |  |  | 1.0596E7 | 2 | 0 | 0 | 0 | 0 | 0 | 2 | 62 | 76 |  |  | PEAKS DB |
| K.FTSMWIGLK.D | Y | 55.64 | 1081.5630 | 9 | 0.4 | 541.7890 | 2 | 51.46 | 6 | F6:28029 | 31122021\_RID\_2349\_6C.raw |  |  |  |  |  | 2.1382E5 | 1 | 0 | 0 | 0 | 0 | 0 | 1 | 84 | 92 |  |  | PEAKS DB |
| H.SSEEEAFVGK.L | Y | 46.15 | 1081.4927 | 10 | -0.2 | 541.7535 | 2 | 25.83 | 6 | F6:8519 | 31122021\_RID\_2349\_6C.raw |  |  |  |  |  | 0 | 0 | 0 | 0 | 0 | 0 | 0 | 0 | 67 | 76 |  |  | PEAKS DB |
| total 3 peptides |  |  |  |  |  |  |  |  |  |  |  |  |  |  |  |  |  |  |  |  |  |  |  |  |  |  |  |  |  |
| --- | --- | --- | --- | --- | --- | --- | --- | --- | --- | --- | --- | --- | --- | --- | --- | --- | --- | --- | --- | --- | --- | --- | --- | --- | --- | --- | --- | --- | --- |

JAS05483.1

back to list

  

| Protein Coverage
| Supporting Peptides
|

Protein Coverage:

Supporting Peptides:

| Peptide | Uniq | -10lgP | Mass | Length | ppm | m/z | z | RT | Fraction | Scan | Source File | Area F2 | Area F1 | Area F3 | Area F4 | Area F5 | Area F6 | #Feature | #Feature F2 | #Feature F1 | #Feature F3 | #Feature F4 | #Feature F5 | #Feature F6 | Start | End | PTM | AScore | Found By |
| --- | --- | --- | --- | --- | --- | --- | --- | --- | --- | --- | --- | --- | --- | --- | --- | --- | --- | --- | --- | --- | --- | --- | --- | --- | --- | --- | --- | --- | --- |
| R.LASIHSSEEEAFVGK.L | Y | 90.38 | 1602.7889 | 15 | 0.4 | 802.4021 | 2 | 30.99 | 6 | F6:12299 | 31122021\_RID\_2349\_6C.raw |  |  |  |  |  | 1.0596E7 | 2 | 0 | 0 | 0 | 0 | 0 | 2 | 62 | 76 |  |  | PEAKS DB |
| K.FTSMWIGLK.D | Y | 55.64 | 1081.5630 | 9 | 0.4 | 541.7890 | 2 | 51.46 | 6 | F6:28029 | 31122021\_RID\_2349\_6C.raw |  |  |  |  |  | 2.1382E5 | 1 | 0 | 0 | 0 | 0 | 0 | 1 | 84 | 92 |  |  | PEAKS DB |
| H.SSEEEAFVGK.L | Y | 46.15 | 1081.4927 | 10 | -0.2 | 541.7535 | 2 | 25.83 | 6 | F6:8519 | 31122021\_RID\_2349\_6C.raw |  |  |  |  |  | 0 | 0 | 0 | 0 | 0 | 0 | 0 | 0 | 67 | 76 |  |  | PEAKS DB |
| total 3 peptides |  |  |  |  |  |  |  |  |  |  |  |  |  |  |  |  |  |  |  |  |  |  |  |  |  |  |  |  |  |
| --- | --- | --- | --- | --- | --- | --- | --- | --- | --- | --- | --- | --- | --- | --- | --- | --- | --- | --- | --- | --- | --- | --- | --- | --- | --- | --- | --- | --- | --- |

XP\_007439730.1

back to list

  

| Protein Coverage
| Supporting Peptides
|

Protein Coverage:

Supporting Peptides:

| Peptide | Uniq | -10lgP | Mass | Length | ppm | m/z | z | RT | Fraction | Scan | Source File | Area F2 | Area F1 | Area F3 | Area F4 | Area F5 | Area F6 | #Feature | #Feature F2 | #Feature F1 | #Feature F3 | #Feature F4 | #Feature F5 | #Feature F6 | Start | End | PTM | AScore | Found By |
| --- | --- | --- | --- | --- | --- | --- | --- | --- | --- | --- | --- | --- | --- | --- | --- | --- | --- | --- | --- | --- | --- | --- | --- | --- | --- | --- | --- | --- | --- |
| K.GNWIGEAPYK.N | Y | 71.22 | 1133.5505 | 10 | 0.4 | 567.7828 | 2 | 35.95 | 5 | F5:16763 | 31122021\_RID\_2349\_5C.raw | 5.1752E5 |  |  |  | 1.2794E8 | 4.3086E6 | 3 | 1 | 0 | 0 | 0 | 1 | 1 | 207 | 216 |  |  | PEAKS DB |
| K.LRGEVYPSASN.M | Y | 53.71 | 1191.5884 | 11 | -0.1 | 596.8014 | 2 | 24.95 | 5 | F5:9103 | 31122021\_RID\_2349\_5C.raw |  |  |  |  | 6.976E5 |  | 1 | 0 | 0 | 0 | 0 | 1 | 0 | 69 | 79 |  |  | PEAKS DB |
| K.GNWIGEAPYKNGR.S | Y | 45.10 | 1460.7161 | 13 | 0.7 | 731.3658 | 2 | 28.96 | 5 | F5:11987 | 31122021\_RID\_2349\_5C.raw |  |  |  |  | 7.7949E4 |  | 1 | 0 | 0 | 0 | 0 | 1 | 0 | 207 | 219 |  |  | PEAKS DB |
| K.GNWIGEAPYKN.G | Y | 43.88 | 1247.5935 | 11 | 0.6 | 624.8044 | 2 | 35.40 | 5 | F5:16343 | 31122021\_RID\_2349\_5C.raw |  |  |  |  | 1.0897E6 |  | 1 | 0 | 0 | 0 | 0 | 1 | 0 | 207 | 217 |  |  | PEAKS DB |
| total 4 peptides |  |  |  |  |  |  |  |  |  |  |  |  |  |  |  |  |  |  |  |  |  |  |  |  |  |  |  |  |  |
| --- | --- | --- | --- | --- | --- | --- | --- | --- | --- | --- | --- | --- | --- | --- | --- | --- | --- | --- | --- | --- | --- | --- | --- | --- | --- | --- | --- | --- | --- |

ETE66074.1

back to list

  

| Protein Coverage
| Supporting Peptides
|

Protein Coverage:

Supporting Peptides:

| Peptide | Uniq | -10lgP | Mass | Length | ppm | m/z | z | RT | Fraction | Scan | Source File | Area F2 | Area F1 | Area F3 | Area F4 | Area F5 | Area F6 | #Feature | #Feature F2 | #Feature F1 | #Feature F3 | #Feature F4 | #Feature F5 | #Feature F6 | Start | End | PTM | AScore | Found By |
| --- | --- | --- | --- | --- | --- | --- | --- | --- | --- | --- | --- | --- | --- | --- | --- | --- | --- | --- | --- | --- | --- | --- | --- | --- | --- | --- | --- | --- | --- |
| K.GNWIGEAPYK.N | Y | 71.22 | 1133.5505 | 10 | 0.4 | 567.7828 | 2 | 35.95 | 5 | F5:16763 | 31122021\_RID\_2349\_5C.raw | 5.1752E5 |  |  |  | 1.2794E8 | 4.3086E6 | 3 | 1 | 0 | 0 | 0 | 1 | 1 | 183 | 192 |  |  | PEAKS DB |
| K.LRGEVYPSASN.M | Y | 53.71 | 1191.5884 | 11 | -0.1 | 596.8014 | 2 | 24.95 | 5 | F5:9103 | 31122021\_RID\_2349\_5C.raw |  |  |  |  | 6.976E5 |  | 1 | 0 | 0 | 0 | 0 | 1 | 0 | 69 | 79 |  |  | PEAKS DB |
| K.GNWIGEAPYKNGR.S | Y | 45.10 | 1460.7161 | 13 | 0.7 | 731.3658 | 2 | 28.96 | 5 | F5:11987 | 31122021\_RID\_2349\_5C.raw |  |  |  |  | 7.7949E4 |  | 1 | 0 | 0 | 0 | 0 | 1 | 0 | 183 | 195 |  |  | PEAKS DB |
| K.GNWIGEAPYKN.G | Y | 43.88 | 1247.5935 | 11 | 0.6 | 624.8044 | 2 | 35.40 | 5 | F5:16343 | 31122021\_RID\_2349\_5C.raw |  |  |  |  | 1.0897E6 |  | 1 | 0 | 0 | 0 | 0 | 1 | 0 | 183 | 193 |  |  | PEAKS DB |
| total 4 peptides |  |  |  |  |  |  |  |  |  |  |  |  |  |  |  |  |  |  |  |  |  |  |  |  |  |  |  |  |  |
| --- | --- | --- | --- | --- | --- | --- | --- | --- | --- | --- | --- | --- | --- | --- | --- | --- | --- | --- | --- | --- | --- | --- | --- | --- | --- | --- | --- | --- | --- |

JAI13759.1

back to list

  

| Protein Coverage
| Supporting Peptides
|

Protein Coverage:

Supporting Peptides:

| Peptide | Uniq | -10lgP | Mass | Length | ppm | m/z | z | RT | Fraction | Scan | Source File | Area F2 | Area F1 | Area F3 | Area F4 | Area F5 | Area F6 | #Feature | #Feature F2 | #Feature F1 | #Feature F3 | #Feature F4 | #Feature F5 | #Feature F6 | Start | End | PTM | AScore | Found By |
| --- | --- | --- | --- | --- | --- | --- | --- | --- | --- | --- | --- | --- | --- | --- | --- | --- | --- | --- | --- | --- | --- | --- | --- | --- | --- | --- | --- | --- | --- |
| K.GNWIGEAPYK.N | Y | 71.22 | 1133.5505 | 10 | 0.4 | 567.7828 | 2 | 35.95 | 5 | F5:16763 | 31122021\_RID\_2349\_5C.raw | 5.1752E5 |  |  |  | 1.2794E8 | 4.3086E6 | 3 | 1 | 0 | 0 | 0 | 1 | 1 | 207 | 216 |  |  | PEAKS DB |
| K.LRGEVYPSASN.M | Y | 53.71 | 1191.5884 | 11 | -0.1 | 596.8014 | 2 | 24.95 | 5 | F5:9103 | 31122021\_RID\_2349\_5C.raw |  |  |  |  | 6.976E5 |  | 1 | 0 | 0 | 0 | 0 | 1 | 0 | 69 | 79 |  |  | PEAKS DB |
| K.GNWIGEAPYKNGR.S | Y | 45.10 | 1460.7161 | 13 | 0.7 | 731.3658 | 2 | 28.96 | 5 | F5:11987 | 31122021\_RID\_2349\_5C.raw |  |  |  |  | 7.7949E4 |  | 1 | 0 | 0 | 0 | 0 | 1 | 0 | 207 | 219 |  |  | PEAKS DB |
| K.GNWIGEAPYKN.G | Y | 43.88 | 1247.5935 | 11 | 0.6 | 624.8044 | 2 | 35.40 | 5 | F5:16343 | 31122021\_RID\_2349\_5C.raw |  |  |  |  | 1.0897E6 |  | 1 | 0 | 0 | 0 | 0 | 1 | 0 | 207 | 217 |  |  | PEAKS DB |
| total 4 peptides |  |  |  |  |  |  |  |  |  |  |  |  |  |  |  |  |  |  |  |  |  |  |  |  |  |  |  |  |  |
| --- | --- | --- | --- | --- | --- | --- | --- | --- | --- | --- | --- | --- | --- | --- | --- | --- | --- | --- | --- | --- | --- | --- | --- | --- | --- | --- | --- | --- | --- |

XP\_026569732.1

back to list

  

| Protein Coverage
| Supporting Peptides
|

Protein Coverage:

Supporting Peptides:

| Peptide | Uniq | -10lgP | Mass | Length | ppm | m/z | z | RT | Fraction | Scan | Source File | Area F2 | Area F1 | Area F3 | Area F4 | Area F5 | Area F6 | #Feature | #Feature F2 | #Feature F1 | #Feature F3 | #Feature F4 | #Feature F5 | #Feature F6 | Start | End | PTM | AScore | Found By |
| --- | --- | --- | --- | --- | --- | --- | --- | --- | --- | --- | --- | --- | --- | --- | --- | --- | --- | --- | --- | --- | --- | --- | --- | --- | --- | --- | --- | --- | --- |
| K.GNWIGEAPYK.N | Y | 71.22 | 1133.5505 | 10 | 0.4 | 567.7828 | 2 | 35.95 | 5 | F5:16763 | 31122021\_RID\_2349\_5C.raw | 5.1752E5 |  |  |  | 1.2794E8 | 4.3086E6 | 3 | 1 | 0 | 0 | 0 | 1 | 1 | 207 | 216 |  |  | PEAKS DB |
| K.LRGEVYPSASN.M | Y | 53.71 | 1191.5884 | 11 | -0.1 | 596.8014 | 2 | 24.95 | 5 | F5:9103 | 31122021\_RID\_2349\_5C.raw |  |  |  |  | 6.976E5 |  | 1 | 0 | 0 | 0 | 0 | 1 | 0 | 69 | 79 |  |  | PEAKS DB |
| K.GNWIGEAPYKNGR.S | Y | 45.10 | 1460.7161 | 13 | 0.7 | 731.3658 | 2 | 28.96 | 5 | F5:11987 | 31122021\_RID\_2349\_5C.raw |  |  |  |  | 7.7949E4 |  | 1 | 0 | 0 | 0 | 0 | 1 | 0 | 207 | 219 |  |  | PEAKS DB |
| K.GNWIGEAPYKN.G | Y | 43.88 | 1247.5935 | 11 | 0.6 | 624.8044 | 2 | 35.40 | 5 | F5:16343 | 31122021\_RID\_2349\_5C.raw |  |  |  |  | 1.0897E6 |  | 1 | 0 | 0 | 0 | 0 | 1 | 0 | 207 | 217 |  |  | PEAKS DB |
| total 4 peptides |  |  |  |  |  |  |  |  |  |  |  |  |  |  |  |  |  |  |  |  |  |  |  |  |  |  |  |  |  |
| --- | --- | --- | --- | --- | --- | --- | --- | --- | --- | --- | --- | --- | --- | --- | --- | --- | --- | --- | --- | --- | --- | --- | --- | --- | --- | --- | --- | --- | --- |

XP\_015666034.1

back to list

  

| Protein Coverage
| Supporting Peptides
|

Protein Coverage:

Supporting Peptides:

| Peptide | Uniq | -10lgP | Mass | Length | ppm | m/z | z | RT | Fraction | Scan | Source File | Area F2 | Area F1 | Area F3 | Area F4 | Area F5 | Area F6 | #Feature | #Feature F2 | #Feature F1 | #Feature F3 | #Feature F4 | #Feature F5 | #Feature F6 | Start | End | PTM | AScore | Found By |
| --- | --- | --- | --- | --- | --- | --- | --- | --- | --- | --- | --- | --- | --- | --- | --- | --- | --- | --- | --- | --- | --- | --- | --- | --- | --- | --- | --- | --- | --- |
| K.GNWIGEAPYK.N | Y | 71.22 | 1133.5505 | 10 | 0.4 | 567.7828 | 2 | 35.95 | 5 | F5:16763 | 31122021\_RID\_2349\_5C.raw | 5.1752E5 |  |  |  | 1.2794E8 | 4.3086E6 | 3 | 1 | 0 | 0 | 0 | 1 | 1 | 207 | 216 |  |  | PEAKS DB |
| K.LRGEVYPSASN.M | Y | 53.71 | 1191.5884 | 11 | -0.1 | 596.8014 | 2 | 24.95 | 5 | F5:9103 | 31122021\_RID\_2349\_5C.raw |  |  |  |  | 6.976E5 |  | 1 | 0 | 0 | 0 | 0 | 1 | 0 | 69 | 79 |  |  | PEAKS DB |
| K.GNWIGEAPYKNGR.S | Y | 45.10 | 1460.7161 | 13 | 0.7 | 731.3658 | 2 | 28.96 | 5 | F5:11987 | 31122021\_RID\_2349\_5C.raw |  |  |  |  | 7.7949E4 |  | 1 | 0 | 0 | 0 | 0 | 1 | 0 | 207 | 219 |  |  | PEAKS DB |
| K.GNWIGEAPYKN.G | Y | 43.88 | 1247.5935 | 11 | 0.6 | 624.8044 | 2 | 35.40 | 5 | F5:16343 | 31122021\_RID\_2349\_5C.raw |  |  |  |  | 1.0897E6 |  | 1 | 0 | 0 | 0 | 0 | 1 | 0 | 207 | 217 |  |  | PEAKS DB |
| total 4 peptides |  |  |  |  |  |  |  |  |  |  |  |  |  |  |  |  |  |  |  |  |  |  |  |  |  |  |  |  |  |
| --- | --- | --- | --- | --- | --- | --- | --- | --- | --- | --- | --- | --- | --- | --- | --- | --- | --- | --- | --- | --- | --- | --- | --- | --- | --- | --- | --- | --- | --- |

XP\_026539804.1

back to list

  

| Protein Coverage
| Supporting Peptides
|

Protein Coverage:

Supporting Peptides:

| Peptide | Uniq | -10lgP | Mass | Length | ppm | m/z | z | RT | Fraction | Scan | Source File | Area F2 | Area F1 | Area F3 | Area F4 | Area F5 | Area F6 | #Feature | #Feature F2 | #Feature F1 | #Feature F3 | #Feature F4 | #Feature F5 | #Feature F6 | Start | End | PTM | AScore | Found By |
| --- | --- | --- | --- | --- | --- | --- | --- | --- | --- | --- | --- | --- | --- | --- | --- | --- | --- | --- | --- | --- | --- | --- | --- | --- | --- | --- | --- | --- | --- |
| K.GNWIGEAPYK.N | Y | 71.22 | 1133.5505 | 10 | 0.4 | 567.7828 | 2 | 35.95 | 5 | F5:16763 | 31122021\_RID\_2349\_5C.raw | 5.1752E5 |  |  |  | 1.2794E8 | 4.3086E6 | 3 | 1 | 0 | 0 | 0 | 1 | 1 | 207 | 216 |  |  | PEAKS DB |
| K.LRGEVYPSASN.M | Y | 53.71 | 1191.5884 | 11 | -0.1 | 596.8014 | 2 | 24.95 | 5 | F5:9103 | 31122021\_RID\_2349\_5C.raw |  |  |  |  | 6.976E5 |  | 1 | 0 | 0 | 0 | 0 | 1 | 0 | 69 | 79 |  |  | PEAKS DB |
| K.GNWIGEAPYKNGR.S | Y | 45.10 | 1460.7161 | 13 | 0.7 | 731.3658 | 2 | 28.96 | 5 | F5:11987 | 31122021\_RID\_2349\_5C.raw |  |  |  |  | 7.7949E4 |  | 1 | 0 | 0 | 0 | 0 | 1 | 0 | 207 | 219 |  |  | PEAKS DB |
| K.GNWIGEAPYKN.G | Y | 43.88 | 1247.5935 | 11 | 0.6 | 624.8044 | 2 | 35.40 | 5 | F5:16343 | 31122021\_RID\_2349\_5C.raw |  |  |  |  | 1.0897E6 |  | 1 | 0 | 0 | 0 | 0 | 1 | 0 | 207 | 217 |  |  | PEAKS DB |
| total 4 peptides |  |  |  |  |  |  |  |  |  |  |  |  |  |  |  |  |  |  |  |  |  |  |  |  |  |  |  |  |  |
| --- | --- | --- | --- | --- | --- | --- | --- | --- | --- | --- | --- | --- | --- | --- | --- | --- | --- | --- | --- | --- | --- | --- | --- | --- | --- | --- | --- | --- | --- |

XP\_034282442.1

back to list

  

| Protein Coverage
| Supporting Peptides
|

Protein Coverage:

Supporting Peptides:

| Peptide | Uniq | -10lgP | Mass | Length | ppm | m/z | z | RT | Fraction | Scan | Source File | Area F2 | Area F1 | Area F3 | Area F4 | Area F5 | Area F6 | #Feature | #Feature F2 | #Feature F1 | #Feature F3 | #Feature F4 | #Feature F5 | #Feature F6 | Start | End | PTM | AScore | Found By |
| --- | --- | --- | --- | --- | --- | --- | --- | --- | --- | --- | --- | --- | --- | --- | --- | --- | --- | --- | --- | --- | --- | --- | --- | --- | --- | --- | --- | --- | --- |
| K.GNWIGEAPYK.N | Y | 71.22 | 1133.5505 | 10 | 0.4 | 567.7828 | 2 | 35.95 | 5 | F5:16763 | 31122021\_RID\_2349\_5C.raw | 5.1752E5 |  |  |  | 1.2794E8 | 4.3086E6 | 3 | 1 | 0 | 0 | 0 | 1 | 1 | 207 | 216 |  |  | PEAKS DB |
| K.LRGEVYPSASN.M | Y | 53.71 | 1191.5884 | 11 | -0.1 | 596.8014 | 2 | 24.95 | 5 | F5:9103 | 31122021\_RID\_2349\_5C.raw |  |  |  |  | 6.976E5 |  | 1 | 0 | 0 | 0 | 0 | 1 | 0 | 69 | 79 |  |  | PEAKS DB |
| K.GNWIGEAPYKNGR.S | Y | 45.10 | 1460.7161 | 13 | 0.7 | 731.3658 | 2 | 28.96 | 5 | F5:11987 | 31122021\_RID\_2349\_5C.raw |  |  |  |  | 7.7949E4 |  | 1 | 0 | 0 | 0 | 0 | 1 | 0 | 207 | 219 |  |  | PEAKS DB |
| K.GNWIGEAPYKN.G | Y | 43.88 | 1247.5935 | 11 | 0.6 | 624.8044 | 2 | 35.40 | 5 | F5:16343 | 31122021\_RID\_2349\_5C.raw |  |  |  |  | 1.0897E6 |  | 1 | 0 | 0 | 0 | 0 | 1 | 0 | 207 | 217 |  |  | PEAKS DB |
| total 4 peptides |  |  |  |  |  |  |  |  |  |  |  |  |  |  |  |  |  |  |  |  |  |  |  |  |  |  |  |  |  |
| --- | --- | --- | --- | --- | --- | --- | --- | --- | --- | --- | --- | --- | --- | --- | --- | --- | --- | --- | --- | --- | --- | --- | --- | --- | --- | --- | --- | --- | --- |

QHR82746.1

back to list

  

| Protein Coverage
| Supporting Peptides
|

Protein Coverage:

Supporting Peptides:

| Peptide | Uniq | -10lgP | Mass | Length | ppm | m/z | z | RT | Fraction | Scan | Source File | Area F2 | Area F1 | Area F3 | Area F4 | Area F5 | Area F6 | #Feature | #Feature F2 | #Feature F1 | #Feature F3 | #Feature F4 | #Feature F5 | #Feature F6 | Start | End | PTM | AScore | Found By |
| --- | --- | --- | --- | --- | --- | --- | --- | --- | --- | --- | --- | --- | --- | --- | --- | --- | --- | --- | --- | --- | --- | --- | --- | --- | --- | --- | --- | --- | --- |
| K.GNWIGEAPYK.N | Y | 71.22 | 1133.5505 | 10 | 0.4 | 567.7828 | 2 | 35.95 | 5 | F5:16763 | 31122021\_RID\_2349\_5C.raw | 5.1752E5 |  |  |  | 1.2794E8 | 4.3086E6 | 3 | 1 | 0 | 0 | 0 | 1 | 1 | 207 | 216 |  |  | PEAKS DB |
| K.LRGEVYPSASN.M | Y | 53.71 | 1191.5884 | 11 | -0.1 | 596.8014 | 2 | 24.95 | 5 | F5:9103 | 31122021\_RID\_2349\_5C.raw |  |  |  |  | 6.976E5 |  | 1 | 0 | 0 | 0 | 0 | 1 | 0 | 69 | 79 |  |  | PEAKS DB |
| K.GNWIGEAPYKNGR.S | Y | 45.10 | 1460.7161 | 13 | 0.7 | 731.3658 | 2 | 28.96 | 5 | F5:11987 | 31122021\_RID\_2349\_5C.raw |  |  |  |  | 7.7949E4 |  | 1 | 0 | 0 | 0 | 0 | 1 | 0 | 207 | 219 |  |  | PEAKS DB |
| K.GNWIGEAPYKN.G | Y | 43.88 | 1247.5935 | 11 | 0.6 | 624.8044 | 2 | 35.40 | 5 | F5:16343 | 31122021\_RID\_2349\_5C.raw |  |  |  |  | 1.0897E6 |  | 1 | 0 | 0 | 0 | 0 | 1 | 0 | 207 | 217 |  |  | PEAKS DB |
| total 4 peptides |  |  |  |  |  |  |  |  |  |  |  |  |  |  |  |  |  |  |  |  |  |  |  |  |  |  |  |  |  |
| --- | --- | --- | --- | --- | --- | --- | --- | --- | --- | --- | --- | --- | --- | --- | --- | --- | --- | --- | --- | --- | --- | --- | --- | --- | --- | --- | --- | --- | --- |

XP\_032086736.1

back to list

  

| Protein Coverage
| Supporting Peptides
|

Protein Coverage:

Supporting Peptides:

| Peptide | Uniq | -10lgP | Mass | Length | ppm | m/z | z | RT | Fraction | Scan | Source File | Area F2 | Area F1 | Area F3 | Area F4 | Area F5 | Area F6 | #Feature | #Feature F2 | #Feature F1 | #Feature F3 | #Feature F4 | #Feature F5 | #Feature F6 | Start | End | PTM | AScore | Found By |
| --- | --- | --- | --- | --- | --- | --- | --- | --- | --- | --- | --- | --- | --- | --- | --- | --- | --- | --- | --- | --- | --- | --- | --- | --- | --- | --- | --- | --- | --- |
| K.GNWIGEAPYK.N | Y | 71.22 | 1133.5505 | 10 | 0.4 | 567.7828 | 2 | 35.95 | 5 | F5:16763 | 31122021\_RID\_2349\_5C.raw | 5.1752E5 |  |  |  | 1.2794E8 | 4.3086E6 | 3 | 1 | 0 | 0 | 0 | 1 | 1 | 207 | 216 |  |  | PEAKS DB |
| K.LRGEVYPSASN.M | Y | 53.71 | 1191.5884 | 11 | -0.1 | 596.8014 | 2 | 24.95 | 5 | F5:9103 | 31122021\_RID\_2349\_5C.raw |  |  |  |  | 6.976E5 |  | 1 | 0 | 0 | 0 | 0 | 1 | 0 | 69 | 79 |  |  | PEAKS DB |
| K.GNWIGEAPYKNGR.S | Y | 45.10 | 1460.7161 | 13 | 0.7 | 731.3658 | 2 | 28.96 | 5 | F5:11987 | 31122021\_RID\_2349\_5C.raw |  |  |  |  | 7.7949E4 |  | 1 | 0 | 0 | 0 | 0 | 1 | 0 | 207 | 219 |  |  | PEAKS DB |
| K.GNWIGEAPYKN.G | Y | 43.88 | 1247.5935 | 11 | 0.6 | 624.8044 | 2 | 35.40 | 5 | F5:16343 | 31122021\_RID\_2349\_5C.raw |  |  |  |  | 1.0897E6 |  | 1 | 0 | 0 | 0 | 0 | 1 | 0 | 207 | 217 |  |  | PEAKS DB |
| total 4 peptides |  |  |  |  |  |  |  |  |  |  |  |  |  |  |  |  |  |  |  |  |  |  |  |  |  |  |  |  |  |
| --- | --- | --- | --- | --- | --- | --- | --- | --- | --- | --- | --- | --- | --- | --- | --- | --- | --- | --- | --- | --- | --- | --- | --- | --- | --- | --- | --- | --- | --- |

XP\_032087342.1

back to list

  

| Protein Coverage
| Supporting Peptides
|

Protein Coverage:

Supporting Peptides:

| Peptide | Uniq | -10lgP | Mass | Length | ppm | m/z | z | RT | Fraction | Scan | Source File | Area F2 | Area F1 | Area F3 | Area F4 | Area F5 | Area F6 | #Feature | #Feature F2 | #Feature F1 | #Feature F3 | #Feature F4 | #Feature F5 | #Feature F6 | Start | End | PTM | AScore | Found By |
| --- | --- | --- | --- | --- | --- | --- | --- | --- | --- | --- | --- | --- | --- | --- | --- | --- | --- | --- | --- | --- | --- | --- | --- | --- | --- | --- | --- | --- | --- |
| K.GNWIGEAPYK.N | Y | 71.22 | 1133.5505 | 10 | 0.4 | 567.7828 | 2 | 35.95 | 5 | F5:16763 | 31122021\_RID\_2349\_5C.raw | 5.1752E5 |  |  |  | 1.2794E8 | 4.3086E6 | 3 | 1 | 0 | 0 | 0 | 1 | 1 | 206 | 215 |  |  | PEAKS DB |
| K.LRGEVYPSASN.M | Y | 53.71 | 1191.5884 | 11 | -0.1 | 596.8014 | 2 | 24.95 | 5 | F5:9103 | 31122021\_RID\_2349\_5C.raw |  |  |  |  | 6.976E5 |  | 1 | 0 | 0 | 0 | 0 | 1 | 0 | 68 | 78 |  |  | PEAKS DB |
| K.GNWIGEAPYKNGR.S | Y | 45.10 | 1460.7161 | 13 | 0.7 | 731.3658 | 2 | 28.96 | 5 | F5:11987 | 31122021\_RID\_2349\_5C.raw |  |  |  |  | 7.7949E4 |  | 1 | 0 | 0 | 0 | 0 | 1 | 0 | 206 | 218 |  |  | PEAKS DB |
| K.GNWIGEAPYKN.G | Y | 43.88 | 1247.5935 | 11 | 0.6 | 624.8044 | 2 | 35.40 | 5 | F5:16343 | 31122021\_RID\_2349\_5C.raw |  |  |  |  | 1.0897E6 |  | 1 | 0 | 0 | 0 | 0 | 1 | 0 | 206 | 216 |  |  | PEAKS DB |
| total 4 peptides |  |  |  |  |  |  |  |  |  |  |  |  |  |  |  |  |  |  |  |  |  |  |  |  |  |  |  |  |  |
| --- | --- | --- | --- | --- | --- | --- | --- | --- | --- | --- | --- | --- | --- | --- | --- | --- | --- | --- | --- | --- | --- | --- | --- | --- | --- | --- | --- | --- | --- |

XP\_007435739.1

back to list

  

| Protein Coverage
| Supporting Peptides
|

Protein Coverage:

Supporting Peptides:

| Peptide | Uniq | -10lgP | Mass | Length | ppm | m/z | z | RT | Fraction | Scan | Source File | Area F2 | Area F1 | Area F3 | Area F4 | Area F5 | Area F6 | #Feature | #Feature F2 | #Feature F1 | #Feature F3 | #Feature F4 | #Feature F5 | #Feature F6 | Start | End | PTM | AScore | Found By |
| --- | --- | --- | --- | --- | --- | --- | --- | --- | --- | --- | --- | --- | --- | --- | --- | --- | --- | --- | --- | --- | --- | --- | --- | --- | --- | --- | --- | --- | --- |
| K.LALDIEIATYR.K | N | 71.69 | 1276.7026 | 11 | 0.5 | 639.3589 | 2 | 53.27 | 1 | F1:31324 | 28122021\_RID\_2349\_2C.raw | 9.0531E5 | 2.8506E4 | 2.692E7 | 1.4957E7 | 5.3148E5 | 1.8027E5 | 6 | 1 | 1 | 1 | 1 | 1 | 1 | 380 | 390 |  |  | PEAKS DB |
| R.AQYEEIANR.S | Y | 66.93 | 1092.5199 | 9 | -0.2 | 547.2671 | 2 | 23.69 | 6 | F6:7113 | 31122021\_RID\_2349\_6C.raw | 2.9111E6 | 8.3407E5 | 3.1651E7 |  |  | 2.1515E5 | 4 | 1 | 1 | 1 | 0 | 0 | 1 | 263 | 271 |  |  | PEAKS DB |
| R.EYQELMNVK.L | N | 49.62 | 1152.5485 | 9 | 0.7 | 577.2819 | 2 | 34.25 | 1 | F1:16408 | 28122021\_RID\_2349\_2C.raw | 1.4449E5 |  | 7.4495E6 |  |  |  | 2 | 1 | 0 | 1 | 0 | 0 | 0 | 371 | 379 |  |  | PEAKS DB |
| total 3 peptides |  |  |  |  |  |  |  |  |  |  |  |  |  |  |  |  |  |  |  |  |  |  |  |  |  |  |  |  |  |
| --- | --- | --- | --- | --- | --- | --- | --- | --- | --- | --- | --- | --- | --- | --- | --- | --- | --- | --- | --- | --- | --- | --- | --- | --- | --- | --- | --- | --- | --- |

P0DJL3.1

back to list

  

| Protein Coverage
| Supporting Peptides
|

Protein Coverage:

Supporting Peptides:

| Peptide | Uniq | -10lgP | Mass | Length | ppm | m/z | z | RT | Fraction | Scan | Source File | Area F2 | Area F1 | Area F3 | Area F4 | Area F5 | Area F6 | #Feature | #Feature F2 | #Feature F1 | #Feature F3 | #Feature F4 | #Feature F5 | #Feature F6 | Start | End | PTM | AScore | Found By |
| --- | --- | --- | --- | --- | --- | --- | --- | --- | --- | --- | --- | --- | --- | --- | --- | --- | --- | --- | --- | --- | --- | --- | --- | --- | --- | --- | --- | --- | --- |
| K.TTDNQWWTR.S | Y | 72.92 | 1206.5417 | 9 | 0.1 | 604.2782 | 2 | 36.13 | 6 | F6:16258 | 31122021\_RID\_2349\_6C.raw |  |  |  |  | 1.0259E6 | 1.2753E7 | 2 | 0 | 0 | 0 | 0 | 1 | 1 | 101 | 109 |  |  | PEAKS DB |
| R.LQWSDGTELK.Y | N | 71.99 | 1175.5823 | 10 | 0.5 | 588.7987 | 2 | 37.04 | 6 | F6:16834 | 31122021\_RID\_2349\_6C.raw |  |  |  |  |  | 1.8205E7 | 2 | 0 | 0 | 0 | 0 | 0 | 2 | 77 | 86 |  |  | PEAKS DB |
| total 2 peptides |  |  |  |  |  |  |  |  |  |  |  |  |  |  |  |  |  |  |  |  |  |  |  |  |  |  |  |  |  |
| --- | --- | --- | --- | --- | --- | --- | --- | --- | --- | --- | --- | --- | --- | --- | --- | --- | --- | --- | --- | --- | --- | --- | --- | --- | --- | --- | --- | --- | --- |

P81116.2

back to list

  

| Protein Coverage
| Supporting Peptides
|

Protein Coverage:

Supporting Peptides:

| Peptide | Uniq | -10lgP | Mass | Length | ppm | m/z | z | RT | Fraction | Scan | Source File | Area F2 | Area F1 | Area F3 | Area F4 | Area F5 | Area F6 | #Feature | #Feature F2 | #Feature F1 | #Feature F3 | #Feature F4 | #Feature F5 | #Feature F6 | Start | End | PTM | AScore | Found By |
| --- | --- | --- | --- | --- | --- | --- | --- | --- | --- | --- | --- | --- | --- | --- | --- | --- | --- | --- | --- | --- | --- | --- | --- | --- | --- | --- | --- | --- | --- |
| K.TTDNQWWTR.S | Y | 72.92 | 1206.5417 | 9 | 0.1 | 604.2782 | 2 | 36.13 | 6 | F6:16258 | 31122021\_RID\_2349\_6C.raw |  |  |  |  | 1.0259E6 | 1.2753E7 | 2 | 0 | 0 | 0 | 0 | 1 | 1 | 124 | 132 |  |  | PEAKS DB |
| R.LQWSDGTELK.Y | N | 71.99 | 1175.5823 | 10 | 0.5 | 588.7987 | 2 | 37.04 | 6 | F6:16834 | 31122021\_RID\_2349\_6C.raw |  |  |  |  |  | 1.8205E7 | 2 | 0 | 0 | 0 | 0 | 0 | 2 | 100 | 109 |  |  | PEAKS DB |
| total 2 peptides |  |  |  |  |  |  |  |  |  |  |  |  |  |  |  |  |  |  |  |  |  |  |  |  |  |  |  |  |  |
| --- | --- | --- | --- | --- | --- | --- | --- | --- | --- | --- | --- | --- | --- | --- | --- | --- | --- | --- | --- | --- | --- | --- | --- | --- | --- | --- | --- | --- | --- |

ABS12077.1

back to list

  

| Protein Coverage
| Supporting Peptides
|

Protein Coverage:

Supporting Peptides:

| Peptide | Uniq | -10lgP | Mass | Length | ppm | m/z | z | RT | Fraction | Scan | Source File | Area F2 | Area F1 | Area F3 | Area F4 | Area F5 | Area F6 | #Feature | #Feature F2 | #Feature F1 | #Feature F3 | #Feature F4 | #Feature F5 | #Feature F6 | Start | End | PTM | AScore | Found By |
| --- | --- | --- | --- | --- | --- | --- | --- | --- | --- | --- | --- | --- | --- | --- | --- | --- | --- | --- | --- | --- | --- | --- | --- | --- | --- | --- | --- | --- | --- |
| K.TTDNQWWTR.S | Y | 72.92 | 1206.5417 | 9 | 0.1 | 604.2782 | 2 | 36.13 | 6 | F6:16258 | 31122021\_RID\_2349\_6C.raw |  |  |  |  | 1.0259E6 | 1.2753E7 | 2 | 0 | 0 | 0 | 0 | 1 | 1 | 124 | 132 |  |  | PEAKS DB |
| R.LQWSDGTELK.Y | N | 71.99 | 1175.5823 | 10 | 0.5 | 588.7987 | 2 | 37.04 | 6 | F6:16834 | 31122021\_RID\_2349\_6C.raw |  |  |  |  |  | 1.8205E7 | 2 | 0 | 0 | 0 | 0 | 0 | 2 | 100 | 109 |  |  | PEAKS DB |
| total 2 peptides |  |  |  |  |  |  |  |  |  |  |  |  |  |  |  |  |  |  |  |  |  |  |  |  |  |  |  |  |  |
| --- | --- | --- | --- | --- | --- | --- | --- | --- | --- | --- | --- | --- | --- | --- | --- | --- | --- | --- | --- | --- | --- | --- | --- | --- | --- | --- | --- | --- | --- |

JAV48393.1

back to list

  

| Protein Coverage
| Supporting Peptides
|

Protein Coverage:

Supporting Peptides:

| Peptide | Uniq | -10lgP | Mass | Length | ppm | m/z | z | RT | Fraction | Scan | Source File | Area F2 | Area F1 | Area F3 | Area F4 | Area F5 | Area F6 | #Feature | #Feature F2 | #Feature F1 | #Feature F3 | #Feature F4 | #Feature F5 | #Feature F6 | Start | End | PTM | AScore | Found By |
| --- | --- | --- | --- | --- | --- | --- | --- | --- | --- | --- | --- | --- | --- | --- | --- | --- | --- | --- | --- | --- | --- | --- | --- | --- | --- | --- | --- | --- | --- |
| K.WDKDIMLIR.L | N | 56.29 | 1188.6324 | 9 | 0.8 | 595.3240 | 2 | 41.38 | 1 | F1:22123 | 28122021\_RID\_2349\_2C.raw | 6.4894E6 |  |  |  |  |  | 2 | 2 | 0 | 0 | 0 | 0 | 0 | 109 | 117 |  |  | PEAKS DB |
| L.VIGGEEC(+57.02)NINEHR.F | Y | 49.60 | 1525.6943 | 13 | 0.7 | 763.8550 | 2 | 22.00 | 1 | F1:6778 | 28122021\_RID\_2349\_2C.raw | 1.6435E7 |  | 1.6322E8 |  |  |  | 2 | 1 | 0 | 1 | 0 | 0 | 0 | 25 | 37 | Carbamidomethylation | C7:Carbamidomethylation:1000.00 | PEAKS DB |
| K.ETYPDVPH.C | N | 47.14 | 956.4240 | 8 | 0.5 | 479.2195 | 2 | 30.30 | 5 | F5:12945 | 31122021\_RID\_2349\_5C.raw |  |  |  |  | 4.0519E6 |  | 1 | 0 | 0 | 0 | 0 | 1 | 0 | 157 | 164 |  |  | PEAKS DB |
| K.WDKDIM(+15.99)LIR.L | N | 46.72 | 1204.6274 | 9 | 0.3 | 402.5499 | 3 | 36.54 | 1 | F1:18303 | 28122021\_RID\_2349\_2C.raw | 1.1053E6 |  |  |  |  |  | 2 | 2 | 0 | 0 | 0 | 0 | 0 | 109 | 117 | Oxidation (M) | M6:Oxidation (M):1000.00 | PEAKS DB |
| W.DKDIMLIR.L | N | 46.50 | 1002.5532 | 8 | 1.1 | 502.2844 | 2 | 33.08 | 1 | F1:15497 | 28122021\_RID\_2349\_2C.raw | 1.4232E5 |  |  |  |  |  | 1 | 1 | 0 | 0 | 0 | 0 | 0 | 110 | 117 |  |  | PEAKS DB |
| total 5 peptides |  |  |  |  |  |  |  |  |  |  |  |  |  |  |  |  |  |  |  |  |  |  |  |  |  |  |  |  |  |
| --- | --- | --- | --- | --- | --- | --- | --- | --- | --- | --- | --- | --- | --- | --- | --- | --- | --- | --- | --- | --- | --- | --- | --- | --- | --- | --- | --- | --- | --- |

P26324.1

back to list

  

| Protein Coverage
| Supporting Peptides
|

Protein Coverage:

Supporting Peptides:

| Peptide | Uniq | -10lgP | Mass | Length | ppm | m/z | z | RT | Fraction | Scan | Source File | Area F2 | Area F1 | Area F3 | Area F4 | Area F5 | Area F6 | #Feature | #Feature F2 | #Feature F1 | #Feature F3 | #Feature F4 | #Feature F5 | #Feature F6 | Start | End | PTM | AScore | Found By |
| --- | --- | --- | --- | --- | --- | --- | --- | --- | --- | --- | --- | --- | --- | --- | --- | --- | --- | --- | --- | --- | --- | --- | --- | --- | --- | --- | --- | --- | --- |
| R.VMGWGSINR.R | Y | 66.14 | 1018.5018 | 9 | 0.5 | 510.2584 | 2 | 36.41 | 5 | F5:17166 | 31122021\_RID\_2349\_5C.raw | 6.7919E8 | 6.0579E6 | 4.0038E7 | 1.4897E7 | 8.289E6 |  | 7 | 3 | 1 | 1 | 1 | 1 | 0 | 122 | 130 |  |  | PEAKS DB |
| R.VM(+15.99)GWGSINR.R | Y | 63.73 | 1034.4967 | 9 | -0.5 | 518.2554 | 2 | 32.78 | 1 | F1:15786 | 28122021\_RID\_2349\_2C.raw | 2.351E8 |  |  |  |  |  | 4 | 4 | 0 | 0 | 0 | 0 | 0 | 122 | 130 | Oxidation (M) | M2:Oxidation (M):1000.00 | PEAKS DB |
| VIGGDEC(+57.02)NINEHR.F | N | 61.83 | 1511.6787 | 13 | 0.7 | 756.8472 | 2 | 19.75 | 6 | F6:4533 | 31122021\_RID\_2349\_6C.raw | 2.5261E8 | 1.2178E6 |  |  |  | 1.7091E5 | 5 | 3 | 1 | 0 | 0 | 0 | 1 | 1 | 13 | Carbamidomethylation | C7:Carbamidomethylation:1000.00 | PEAKS DB |
| S.WDEDIM(+15.99)LIR.L | Y | 58.18 | 1205.5751 | 9 | 0.5 | 603.7951 | 2 | 47.57 | 1 | F1:27110 | 28122021\_RID\_2349\_2C.raw | 1.3656E8 | 1.3631E6 |  |  | 4.2623E5 |  | 4 | 2 | 1 | 0 | 0 | 1 | 0 | 85 | 93 | Oxidation (M) | M6:Oxidation (M):1000.00 | PEAKS DB |
| S.WDEDIMLIR.L | Y | 57.76 | 1189.5801 | 9 | 0.0 | 595.7973 | 2 | 53.48 | 4 | F4:13737 | 31122021\_RID\_2349\_4C.raw | 4.1007E8 | 2.6251E6 |  | 1.0603E7 | 2.5862E6 |  | 4 | 1 | 1 | 0 | 1 | 1 | 0 | 85 | 93 |  |  | PEAKS DB |
| total 5 peptides |  |  |  |  |  |  |  |  |  |  |  |  |  |  |  |  |  |  |  |  |  |  |  |  |  |  |  |  |  |
| --- | --- | --- | --- | --- | --- | --- | --- | --- | --- | --- | --- | --- | --- | --- | --- | --- | --- | --- | --- | --- | --- | --- | --- | --- | --- | --- | --- | --- | --- |

AAA03254.1

back to list

  

| Protein Coverage
| Supporting Peptides
|

Protein Coverage:

Supporting Peptides:

| Peptide | Uniq | -10lgP | Mass | Length | ppm | m/z | z | RT | Fraction | Scan | Source File | Area F2 | Area F1 | Area F3 | Area F4 | Area F5 | Area F6 | #Feature | #Feature F2 | #Feature F1 | #Feature F3 | #Feature F4 | #Feature F5 | #Feature F6 | Start | End | PTM | AScore | Found By |
| --- | --- | --- | --- | --- | --- | --- | --- | --- | --- | --- | --- | --- | --- | --- | --- | --- | --- | --- | --- | --- | --- | --- | --- | --- | --- | --- | --- | --- | --- |
| R.VMGWGSINR.R | Y | 66.14 | 1018.5018 | 9 | 0.5 | 510.2584 | 2 | 36.41 | 5 | F5:17166 | 31122021\_RID\_2349\_5C.raw | 6.7919E8 | 6.0579E6 | 4.0038E7 | 1.4897E7 | 8.289E6 |  | 7 | 3 | 1 | 1 | 1 | 1 | 0 | 122 | 130 |  |  | PEAKS DB |
| R.VM(+15.99)GWGSINR.R | Y | 63.73 | 1034.4967 | 9 | -0.5 | 518.2554 | 2 | 32.78 | 1 | F1:15786 | 28122021\_RID\_2349\_2C.raw | 2.351E8 |  |  |  |  |  | 4 | 4 | 0 | 0 | 0 | 0 | 0 | 122 | 130 | Oxidation (M) | M2:Oxidation (M):1000.00 | PEAKS DB |
| VIGGDEC(+57.02)NINEHR.F | N | 61.83 | 1511.6787 | 13 | 0.7 | 756.8472 | 2 | 19.75 | 6 | F6:4533 | 31122021\_RID\_2349\_6C.raw | 2.5261E8 | 1.2178E6 |  |  |  | 1.7091E5 | 5 | 3 | 1 | 0 | 0 | 0 | 1 | 1 | 13 | Carbamidomethylation | C7:Carbamidomethylation:1000.00 | PEAKS DB |
| S.WDEDIM(+15.99)LIR.L | Y | 58.18 | 1205.5751 | 9 | 0.5 | 603.7951 | 2 | 47.57 | 1 | F1:27110 | 28122021\_RID\_2349\_2C.raw | 1.3656E8 | 1.3631E6 |  |  | 4.2623E5 |  | 4 | 2 | 1 | 0 | 0 | 1 | 0 | 85 | 93 | Oxidation (M) | M6:Oxidation (M):1000.00 | PEAKS DB |
| S.WDEDIMLIR.L | Y | 57.76 | 1189.5801 | 9 | 0.0 | 595.7973 | 2 | 53.48 | 4 | F4:13737 | 31122021\_RID\_2349\_4C.raw | 4.1007E8 | 2.6251E6 |  | 1.0603E7 | 2.5862E6 |  | 4 | 1 | 1 | 0 | 1 | 1 | 0 | 85 | 93 |  |  | PEAKS DB |
| total 5 peptides |  |  |  |  |  |  |  |  |  |  |  |  |  |  |  |  |  |  |  |  |  |  |  |  |  |  |  |  |  |
| --- | --- | --- | --- | --- | --- | --- | --- | --- | --- | --- | --- | --- | --- | --- | --- | --- | --- | --- | --- | --- | --- | --- | --- | --- | --- | --- | --- | --- | --- |

XP\_034262421.1

back to list

  

| Protein Coverage
| Supporting Peptides
|

Protein Coverage:

Supporting Peptides:

| Peptide | Uniq | -10lgP | Mass | Length | ppm | m/z | z | RT | Fraction | Scan | Source File | Area F2 | Area F1 | Area F3 | Area F4 | Area F5 | Area F6 | #Feature | #Feature F2 | #Feature F1 | #Feature F3 | #Feature F4 | #Feature F5 | #Feature F6 | Start | End | PTM | AScore | Found By |
| --- | --- | --- | --- | --- | --- | --- | --- | --- | --- | --- | --- | --- | --- | --- | --- | --- | --- | --- | --- | --- | --- | --- | --- | --- | --- | --- | --- | --- | --- |
| K.SQIFSTASDNQPTVTIK.V | Y | 52.26 | 1835.9265 | 17 | -0.7 | 918.9698 | 2 | 40.68 | 6 | F6:19789 | 31122021\_RID\_2349\_6C.raw |  |  |  |  |  | 3.0923E4 | 1 | 0 | 0 | 0 | 0 | 0 | 1 | 413 | 429 |  |  | PEAKS DB |
| K.NQLTSNPENTVFDAK.R | Y | 48.13 | 1676.8005 | 15 | 2.0 | 839.4092 | 2 | 37.18 | 1 | F1:18792 | 28122021\_RID\_2349\_2C.raw | 1.2492E5 |  |  |  |  |  | 1 | 1 | 0 | 0 | 0 | 0 | 0 | 47 | 61 |  |  | PEAKS DB |
| R.TWNDPSVQQDIK.Y | Y | 46.24 | 1429.6837 | 12 | 0.2 | 715.8492 | 2 | 34.09 | 1 | F1:16305 | 28122021\_RID\_2349\_2C.raw | 7.7839E4 |  |  |  |  |  | 1 | 1 | 0 | 0 | 0 | 0 | 0 | 67 | 78 |  |  | PEAKS DB |
| total 3 peptides |  |  |  |  |  |  |  |  |  |  |  |  |  |  |  |  |  |  |  |  |  |  |  |  |  |  |  |  |  |
| --- | --- | --- | --- | --- | --- | --- | --- | --- | --- | --- | --- | --- | --- | --- | --- | --- | --- | --- | --- | --- | --- | --- | --- | --- | --- | --- | --- | --- | --- |

JAV50419.1

back to list

  

| Protein Coverage
| Supporting Peptides
|

Protein Coverage:

Supporting Peptides:

| Peptide | Uniq | -10lgP | Mass | Length | ppm | m/z | z | RT | Fraction | Scan | Source File | Area F2 | Area F1 | Area F3 | Area F4 | Area F5 | Area F6 | #Feature | #Feature F2 | #Feature F1 | #Feature F3 | #Feature F4 | #Feature F5 | #Feature F6 | Start | End | PTM | AScore | Found By |
| --- | --- | --- | --- | --- | --- | --- | --- | --- | --- | --- | --- | --- | --- | --- | --- | --- | --- | --- | --- | --- | --- | --- | --- | --- | --- | --- | --- | --- | --- |
| K.SQIFSTASDNQPTVTIK.V | Y | 52.26 | 1835.9265 | 17 | -0.7 | 918.9698 | 2 | 40.68 | 6 | F6:19789 | 31122021\_RID\_2349\_6C.raw |  |  |  |  |  | 3.0923E4 | 1 | 0 | 0 | 0 | 0 | 0 | 1 | 447 | 463 |  |  | PEAKS DB |
| K.NQLTSNPENTVFDAK.R | Y | 48.13 | 1676.8005 | 15 | 2.0 | 839.4092 | 2 | 37.18 | 1 | F1:18792 | 28122021\_RID\_2349\_2C.raw | 1.2492E5 |  |  |  |  |  | 1 | 1 | 0 | 0 | 0 | 0 | 0 | 81 | 95 |  |  | PEAKS DB |
| R.TWNDPSVQQDIK.Y | Y | 46.24 | 1429.6837 | 12 | 0.2 | 715.8492 | 2 | 34.09 | 1 | F1:16305 | 28122021\_RID\_2349\_2C.raw | 7.7839E4 |  |  |  |  |  | 1 | 1 | 0 | 0 | 0 | 0 | 0 | 101 | 112 |  |  | PEAKS DB |
| total 3 peptides |  |  |  |  |  |  |  |  |  |  |  |  |  |  |  |  |  |  |  |  |  |  |  |  |  |  |  |  |  |
| --- | --- | --- | --- | --- | --- | --- | --- | --- | --- | --- | --- | --- | --- | --- | --- | --- | --- | --- | --- | --- | --- | --- | --- | --- | --- | --- | --- | --- | --- |

JAG44540.1

back to list

  

| Protein Coverage
| Supporting Peptides
|

Protein Coverage:

Supporting Peptides:

| Peptide | Uniq | -10lgP | Mass | Length | ppm | m/z | z | RT | Fraction | Scan | Source File | Area F2 | Area F1 | Area F3 | Area F4 | Area F5 | Area F6 | #Feature | #Feature F2 | #Feature F1 | #Feature F3 | #Feature F4 | #Feature F5 | #Feature F6 | Start | End | PTM | AScore | Found By |
| --- | --- | --- | --- | --- | --- | --- | --- | --- | --- | --- | --- | --- | --- | --- | --- | --- | --- | --- | --- | --- | --- | --- | --- | --- | --- | --- | --- | --- | --- |
| K.SQIFSTASDNQPTVTIK.V | Y | 52.26 | 1835.9265 | 17 | -0.7 | 918.9698 | 2 | 40.68 | 6 | F6:19789 | 31122021\_RID\_2349\_6C.raw |  |  |  |  |  | 3.0923E4 | 1 | 0 | 0 | 0 | 0 | 0 | 1 | 447 | 463 |  |  | PEAKS DB |
| K.NQLTSNPENTVFDAK.R | Y | 48.13 | 1676.8005 | 15 | 2.0 | 839.4092 | 2 | 37.18 | 1 | F1:18792 | 28122021\_RID\_2349\_2C.raw | 1.2492E5 |  |  |  |  |  | 1 | 1 | 0 | 0 | 0 | 0 | 0 | 81 | 95 |  |  | PEAKS DB |
| R.TWNDPSVQQDIK.Y | Y | 46.24 | 1429.6837 | 12 | 0.2 | 715.8492 | 2 | 34.09 | 1 | F1:16305 | 28122021\_RID\_2349\_2C.raw | 7.7839E4 |  |  |  |  |  | 1 | 1 | 0 | 0 | 0 | 0 | 0 | 101 | 112 |  |  | PEAKS DB |
| total 3 peptides |  |  |  |  |  |  |  |  |  |  |  |  |  |  |  |  |  |  |  |  |  |  |  |  |  |  |  |  |  |
| --- | --- | --- | --- | --- | --- | --- | --- | --- | --- | --- | --- | --- | --- | --- | --- | --- | --- | --- | --- | --- | --- | --- | --- | --- | --- | --- | --- | --- | --- |

JAI13107.1

back to list

  

| Protein Coverage
| Supporting Peptides
|

Protein Coverage:

Supporting Peptides:

| Peptide | Uniq | -10lgP | Mass | Length | ppm | m/z | z | RT | Fraction | Scan | Source File | Area F2 | Area F1 | Area F3 | Area F4 | Area F5 | Area F6 | #Feature | #Feature F2 | #Feature F1 | #Feature F3 | #Feature F4 | #Feature F5 | #Feature F6 | Start | End | PTM | AScore | Found By |
| --- | --- | --- | --- | --- | --- | --- | --- | --- | --- | --- | --- | --- | --- | --- | --- | --- | --- | --- | --- | --- | --- | --- | --- | --- | --- | --- | --- | --- | --- |
| K.SQIFSTASDNQPTVTIK.V | Y | 52.26 | 1835.9265 | 17 | -0.7 | 918.9698 | 2 | 40.68 | 6 | F6:19789 | 31122021\_RID\_2349\_6C.raw |  |  |  |  |  | 3.0923E4 | 1 | 0 | 0 | 0 | 0 | 0 | 1 | 447 | 463 |  |  | PEAKS DB |
| K.NQLTSNPENTVFDAK.R | Y | 48.13 | 1676.8005 | 15 | 2.0 | 839.4092 | 2 | 37.18 | 1 | F1:18792 | 28122021\_RID\_2349\_2C.raw | 1.2492E5 |  |  |  |  |  | 1 | 1 | 0 | 0 | 0 | 0 | 0 | 81 | 95 |  |  | PEAKS DB |
| R.TWNDPSVQQDIK.Y | Y | 46.24 | 1429.6837 | 12 | 0.2 | 715.8492 | 2 | 34.09 | 1 | F1:16305 | 28122021\_RID\_2349\_2C.raw | 7.7839E4 |  |  |  |  |  | 1 | 1 | 0 | 0 | 0 | 0 | 0 | 101 | 112 |  |  | PEAKS DB |
| total 3 peptides |  |  |  |  |  |  |  |  |  |  |  |  |  |  |  |  |  |  |  |  |  |  |  |  |  |  |  |  |  |
| --- | --- | --- | --- | --- | --- | --- | --- | --- | --- | --- | --- | --- | --- | --- | --- | --- | --- | --- | --- | --- | --- | --- | --- | --- | --- | --- | --- | --- | --- |

JAG46692.1

back to list

  

| Protein Coverage
| Supporting Peptides
|

Protein Coverage:

Supporting Peptides:

| Peptide | Uniq | -10lgP | Mass | Length | ppm | m/z | z | RT | Fraction | Scan | Source File | Area F2 | Area F1 | Area F3 | Area F4 | Area F5 | Area F6 | #Feature | #Feature F2 | #Feature F1 | #Feature F3 | #Feature F4 | #Feature F5 | #Feature F6 | Start | End | PTM | AScore | Found By |
| --- | --- | --- | --- | --- | --- | --- | --- | --- | --- | --- | --- | --- | --- | --- | --- | --- | --- | --- | --- | --- | --- | --- | --- | --- | --- | --- | --- | --- | --- |
| K.SQIFSTASDNQPTVTIK.V | Y | 52.26 | 1835.9265 | 17 | -0.7 | 918.9698 | 2 | 40.68 | 6 | F6:19789 | 31122021\_RID\_2349\_6C.raw |  |  |  |  |  | 3.0923E4 | 1 | 0 | 0 | 0 | 0 | 0 | 1 | 447 | 463 |  |  | PEAKS DB |
| K.NQLTSNPENTVFDAK.R | Y | 48.13 | 1676.8005 | 15 | 2.0 | 839.4092 | 2 | 37.18 | 1 | F1:18792 | 28122021\_RID\_2349\_2C.raw | 1.2492E5 |  |  |  |  |  | 1 | 1 | 0 | 0 | 0 | 0 | 0 | 81 | 95 |  |  | PEAKS DB |
| R.TWNDPSVQQDIK.Y | Y | 46.24 | 1429.6837 | 12 | 0.2 | 715.8492 | 2 | 34.09 | 1 | F1:16305 | 28122021\_RID\_2349\_2C.raw | 7.7839E4 |  |  |  |  |  | 1 | 1 | 0 | 0 | 0 | 0 | 0 | 101 | 112 |  |  | PEAKS DB |
| total 3 peptides |  |  |  |  |  |  |  |  |  |  |  |  |  |  |  |  |  |  |  |  |  |  |  |  |  |  |  |  |  |
| --- | --- | --- | --- | --- | --- | --- | --- | --- | --- | --- | --- | --- | --- | --- | --- | --- | --- | --- | --- | --- | --- | --- | --- | --- | --- | --- | --- | --- | --- |

JAA96909.1

back to list

  

| Protein Coverage
| Supporting Peptides
|

Protein Coverage:

Supporting Peptides:

| Peptide | Uniq | -10lgP | Mass | Length | ppm | m/z | z | RT | Fraction | Scan | Source File | Area F2 | Area F1 | Area F3 | Area F4 | Area F5 | Area F6 | #Feature | #Feature F2 | #Feature F1 | #Feature F3 | #Feature F4 | #Feature F5 | #Feature F6 | Start | End | PTM | AScore | Found By |
| --- | --- | --- | --- | --- | --- | --- | --- | --- | --- | --- | --- | --- | --- | --- | --- | --- | --- | --- | --- | --- | --- | --- | --- | --- | --- | --- | --- | --- | --- |
| K.SQIFSTASDNQPTVTIK.V | Y | 52.26 | 1835.9265 | 17 | -0.7 | 918.9698 | 2 | 40.68 | 6 | F6:19789 | 31122021\_RID\_2349\_6C.raw |  |  |  |  |  | 3.0923E4 | 1 | 0 | 0 | 0 | 0 | 0 | 1 | 447 | 463 |  |  | PEAKS DB |
| K.NQLTSNPENTVFDAK.R | Y | 48.13 | 1676.8005 | 15 | 2.0 | 839.4092 | 2 | 37.18 | 1 | F1:18792 | 28122021\_RID\_2349\_2C.raw | 1.2492E5 |  |  |  |  |  | 1 | 1 | 0 | 0 | 0 | 0 | 0 | 81 | 95 |  |  | PEAKS DB |
| R.TWNDPSVQQDIK.Y | Y | 46.24 | 1429.6837 | 12 | 0.2 | 715.8492 | 2 | 34.09 | 1 | F1:16305 | 28122021\_RID\_2349\_2C.raw | 7.7839E4 |  |  |  |  |  | 1 | 1 | 0 | 0 | 0 | 0 | 0 | 101 | 112 |  |  | PEAKS DB |
| total 3 peptides |  |  |  |  |  |  |  |  |  |  |  |  |  |  |  |  |  |  |  |  |  |  |  |  |  |  |  |  |  |
| --- | --- | --- | --- | --- | --- | --- | --- | --- | --- | --- | --- | --- | --- | --- | --- | --- | --- | --- | --- | --- | --- | --- | --- | --- | --- | --- | --- | --- | --- |

XP\_015681610.1

back to list

  

| Protein Coverage
| Supporting Peptides
|

Protein Coverage:

Supporting Peptides:

| Peptide | Uniq | -10lgP | Mass | Length | ppm | m/z | z | RT | Fraction | Scan | Source File | Area F2 | Area F1 | Area F3 | Area F4 | Area F5 | Area F6 | #Feature | #Feature F2 | #Feature F1 | #Feature F3 | #Feature F4 | #Feature F5 | #Feature F6 | Start | End | PTM | AScore | Found By |
| --- | --- | --- | --- | --- | --- | --- | --- | --- | --- | --- | --- | --- | --- | --- | --- | --- | --- | --- | --- | --- | --- | --- | --- | --- | --- | --- | --- | --- | --- |
| K.SQIFSTASDNQPTVTIK.V | Y | 52.26 | 1835.9265 | 17 | -0.7 | 918.9698 | 2 | 40.68 | 6 | F6:19789 | 31122021\_RID\_2349\_6C.raw |  |  |  |  |  | 3.0923E4 | 1 | 0 | 0 | 0 | 0 | 0 | 1 | 447 | 463 |  |  | PEAKS DB |
| K.NQLTSNPENTVFDAK.R | Y | 48.13 | 1676.8005 | 15 | 2.0 | 839.4092 | 2 | 37.18 | 1 | F1:18792 | 28122021\_RID\_2349\_2C.raw | 1.2492E5 |  |  |  |  |  | 1 | 1 | 0 | 0 | 0 | 0 | 0 | 81 | 95 |  |  | PEAKS DB |
| R.TWNDPSVQQDIK.Y | Y | 46.24 | 1429.6837 | 12 | 0.2 | 715.8492 | 2 | 34.09 | 1 | F1:16305 | 28122021\_RID\_2349\_2C.raw | 7.7839E4 |  |  |  |  |  | 1 | 1 | 0 | 0 | 0 | 0 | 0 | 101 | 112 |  |  | PEAKS DB |
| total 3 peptides |  |  |  |  |  |  |  |  |  |  |  |  |  |  |  |  |  |  |  |  |  |  |  |  |  |  |  |  |  |
| --- | --- | --- | --- | --- | --- | --- | --- | --- | --- | --- | --- | --- | --- | --- | --- | --- | --- | --- | --- | --- | --- | --- | --- | --- | --- | --- | --- | --- | --- |

AFJ50327.1

back to list

  

| Protein Coverage
| Supporting Peptides
|

Protein Coverage:

Supporting Peptides:

| Peptide | Uniq | -10lgP | Mass | Length | ppm | m/z | z | RT | Fraction | Scan | Source File | Area F2 | Area F1 | Area F3 | Area F4 | Area F5 | Area F6 | #Feature | #Feature F2 | #Feature F1 | #Feature F3 | #Feature F4 | #Feature F5 | #Feature F6 | Start | End | PTM | AScore | Found By |
| --- | --- | --- | --- | --- | --- | --- | --- | --- | --- | --- | --- | --- | --- | --- | --- | --- | --- | --- | --- | --- | --- | --- | --- | --- | --- | --- | --- | --- | --- |
| K.SQIFSTASDNQPTVTIK.V | Y | 52.26 | 1835.9265 | 17 | -0.7 | 918.9698 | 2 | 40.68 | 6 | F6:19789 | 31122021\_RID\_2349\_6C.raw |  |  |  |  |  | 3.0923E4 | 1 | 0 | 0 | 0 | 0 | 0 | 1 | 447 | 463 |  |  | PEAKS DB |
| K.NQLTSNPENTVFDAK.R | Y | 48.13 | 1676.8005 | 15 | 2.0 | 839.4092 | 2 | 37.18 | 1 | F1:18792 | 28122021\_RID\_2349\_2C.raw | 1.2492E5 |  |  |  |  |  | 1 | 1 | 0 | 0 | 0 | 0 | 0 | 81 | 95 |  |  | PEAKS DB |
| R.TWNDPSVQQDIK.Y | Y | 46.24 | 1429.6837 | 12 | 0.2 | 715.8492 | 2 | 34.09 | 1 | F1:16305 | 28122021\_RID\_2349\_2C.raw | 7.7839E4 |  |  |  |  |  | 1 | 1 | 0 | 0 | 0 | 0 | 0 | 101 | 112 |  |  | PEAKS DB |
| total 3 peptides |  |  |  |  |  |  |  |  |  |  |  |  |  |  |  |  |  |  |  |  |  |  |  |  |  |  |  |  |  |
| --- | --- | --- | --- | --- | --- | --- | --- | --- | --- | --- | --- | --- | --- | --- | --- | --- | --- | --- | --- | --- | --- | --- | --- | --- | --- | --- | --- | --- | --- |

JAB54174.1

back to list

  

| Protein Coverage
| Supporting Peptides
|

Protein Coverage:

Supporting Peptides:

| Peptide | Uniq | -10lgP | Mass | Length | ppm | m/z | z | RT | Fraction | Scan | Source File | Area F2 | Area F1 | Area F3 | Area F4 | Area F5 | Area F6 | #Feature | #Feature F2 | #Feature F1 | #Feature F3 | #Feature F4 | #Feature F5 | #Feature F6 | Start | End | PTM | AScore | Found By |
| --- | --- | --- | --- | --- | --- | --- | --- | --- | --- | --- | --- | --- | --- | --- | --- | --- | --- | --- | --- | --- | --- | --- | --- | --- | --- | --- | --- | --- | --- |
| K.SQIFSTASDNQPTVTIK.V | Y | 52.26 | 1835.9265 | 17 | -0.7 | 918.9698 | 2 | 40.68 | 6 | F6:19789 | 31122021\_RID\_2349\_6C.raw |  |  |  |  |  | 3.0923E4 | 1 | 0 | 0 | 0 | 0 | 0 | 1 | 448 | 464 |  |  | PEAKS DB |
| K.NQLTSNPENTVFDAK.R | Y | 48.13 | 1676.8005 | 15 | 2.0 | 839.4092 | 2 | 37.18 | 1 | F1:18792 | 28122021\_RID\_2349\_2C.raw | 1.2492E5 |  |  |  |  |  | 1 | 1 | 0 | 0 | 0 | 0 | 0 | 82 | 96 |  |  | PEAKS DB |
| R.TWNDPSVQQDIK.Y | Y | 46.24 | 1429.6837 | 12 | 0.2 | 715.8492 | 2 | 34.09 | 1 | F1:16305 | 28122021\_RID\_2349\_2C.raw | 7.7839E4 |  |  |  |  |  | 1 | 1 | 0 | 0 | 0 | 0 | 0 | 102 | 113 |  |  | PEAKS DB |
| total 3 peptides |  |  |  |  |  |  |  |  |  |  |  |  |  |  |  |  |  |  |  |  |  |  |  |  |  |  |  |  |  |
| --- | --- | --- | --- | --- | --- | --- | --- | --- | --- | --- | --- | --- | --- | --- | --- | --- | --- | --- | --- | --- | --- | --- | --- | --- | --- | --- | --- | --- | --- |

XP\_026542482.1

back to list

  

| Protein Coverage
| Supporting Peptides
|

Protein Coverage:

Supporting Peptides:

| Peptide | Uniq | -10lgP | Mass | Length | ppm | m/z | z | RT | Fraction | Scan | Source File | Area F2 | Area F1 | Area F3 | Area F4 | Area F5 | Area F6 | #Feature | #Feature F2 | #Feature F1 | #Feature F3 | #Feature F4 | #Feature F5 | #Feature F6 | Start | End | PTM | AScore | Found By |
| --- | --- | --- | --- | --- | --- | --- | --- | --- | --- | --- | --- | --- | --- | --- | --- | --- | --- | --- | --- | --- | --- | --- | --- | --- | --- | --- | --- | --- | --- |
| K.SQIFSTASDNQPTVTIK.V | Y | 52.26 | 1835.9265 | 17 | -0.7 | 918.9698 | 2 | 40.68 | 6 | F6:19789 | 31122021\_RID\_2349\_6C.raw |  |  |  |  |  | 3.0923E4 | 1 | 0 | 0 | 0 | 0 | 0 | 1 | 448 | 464 |  |  | PEAKS DB |
| K.NQLTSNPENTVFDAK.R | Y | 48.13 | 1676.8005 | 15 | 2.0 | 839.4092 | 2 | 37.18 | 1 | F1:18792 | 28122021\_RID\_2349\_2C.raw | 1.2492E5 |  |  |  |  |  | 1 | 1 | 0 | 0 | 0 | 0 | 0 | 82 | 96 |  |  | PEAKS DB |
| R.TWNDPSVQQDIK.Y | Y | 46.24 | 1429.6837 | 12 | 0.2 | 715.8492 | 2 | 34.09 | 1 | F1:16305 | 28122021\_RID\_2349\_2C.raw | 7.7839E4 |  |  |  |  |  | 1 | 1 | 0 | 0 | 0 | 0 | 0 | 102 | 113 |  |  | PEAKS DB |
| total 3 peptides |  |  |  |  |  |  |  |  |  |  |  |  |  |  |  |  |  |  |  |  |  |  |  |  |  |  |  |  |  |
| --- | --- | --- | --- | --- | --- | --- | --- | --- | --- | --- | --- | --- | --- | --- | --- | --- | --- | --- | --- | --- | --- | --- | --- | --- | --- | --- | --- | --- | --- |

JAC95528.1

back to list

  

| Protein Coverage
| Supporting Peptides
|

Protein Coverage:

Supporting Peptides:

| Peptide | Uniq | -10lgP | Mass | Length | ppm | m/z | z | RT | Fraction | Scan | Source File | Area F2 | Area F1 | Area F3 | Area F4 | Area F5 | Area F6 | #Feature | #Feature F2 | #Feature F1 | #Feature F3 | #Feature F4 | #Feature F5 | #Feature F6 | Start | End | PTM | AScore | Found By |
| --- | --- | --- | --- | --- | --- | --- | --- | --- | --- | --- | --- | --- | --- | --- | --- | --- | --- | --- | --- | --- | --- | --- | --- | --- | --- | --- | --- | --- | --- |
| K.SQIFSTASDNQPTVTIK.V | Y | 52.26 | 1835.9265 | 17 | -0.7 | 918.9698 | 2 | 40.68 | 6 | F6:19789 | 31122021\_RID\_2349\_6C.raw |  |  |  |  |  | 3.0923E4 | 1 | 0 | 0 | 0 | 0 | 0 | 1 | 448 | 464 |  |  | PEAKS DB |
| K.NQLTSNPENTVFDAK.R | Y | 48.13 | 1676.8005 | 15 | 2.0 | 839.4092 | 2 | 37.18 | 1 | F1:18792 | 28122021\_RID\_2349\_2C.raw | 1.2492E5 |  |  |  |  |  | 1 | 1 | 0 | 0 | 0 | 0 | 0 | 82 | 96 |  |  | PEAKS DB |
| R.TWNDPSVQQDIK.Y | Y | 46.24 | 1429.6837 | 12 | 0.2 | 715.8492 | 2 | 34.09 | 1 | F1:16305 | 28122021\_RID\_2349\_2C.raw | 7.7839E4 |  |  |  |  |  | 1 | 1 | 0 | 0 | 0 | 0 | 0 | 102 | 113 |  |  | PEAKS DB |
| total 3 peptides |  |  |  |  |  |  |  |  |  |  |  |  |  |  |  |  |  |  |  |  |  |  |  |  |  |  |  |  |  |
| --- | --- | --- | --- | --- | --- | --- | --- | --- | --- | --- | --- | --- | --- | --- | --- | --- | --- | --- | --- | --- | --- | --- | --- | --- | --- | --- | --- | --- | --- |

AFJ50239.1

back to list

  

| Protein Coverage
| Supporting Peptides
|

Protein Coverage:

Supporting Peptides:

| Peptide | Uniq | -10lgP | Mass | Length | ppm | m/z | z | RT | Fraction | Scan | Source File | Area F2 | Area F1 | Area F3 | Area F4 | Area F5 | Area F6 | #Feature | #Feature F2 | #Feature F1 | #Feature F3 | #Feature F4 | #Feature F5 | #Feature F6 | Start | End | PTM | AScore | Found By |
| --- | --- | --- | --- | --- | --- | --- | --- | --- | --- | --- | --- | --- | --- | --- | --- | --- | --- | --- | --- | --- | --- | --- | --- | --- | --- | --- | --- | --- | --- |
| K.SQIFSTASDNQPTVTIK.V | Y | 52.26 | 1835.9265 | 17 | -0.7 | 918.9698 | 2 | 40.68 | 6 | F6:19789 | 31122021\_RID\_2349\_6C.raw |  |  |  |  |  | 3.0923E4 | 1 | 0 | 0 | 0 | 0 | 0 | 1 | 447 | 463 |  |  | PEAKS DB |
| K.NQLTSNPENTVFDAK.R | Y | 48.13 | 1676.8005 | 15 | 2.0 | 839.4092 | 2 | 37.18 | 1 | F1:18792 | 28122021\_RID\_2349\_2C.raw | 1.2492E5 |  |  |  |  |  | 1 | 1 | 0 | 0 | 0 | 0 | 0 | 81 | 95 |  |  | PEAKS DB |
| R.TWNDPSVQQDIK.Y | Y | 46.24 | 1429.6837 | 12 | 0.2 | 715.8492 | 2 | 34.09 | 1 | F1:16305 | 28122021\_RID\_2349\_2C.raw | 7.7839E4 |  |  |  |  |  | 1 | 1 | 0 | 0 | 0 | 0 | 0 | 101 | 112 |  |  | PEAKS DB |
| total 3 peptides |  |  |  |  |  |  |  |  |  |  |  |  |  |  |  |  |  |  |  |  |  |  |  |  |  |  |  |  |  |
| --- | --- | --- | --- | --- | --- | --- | --- | --- | --- | --- | --- | --- | --- | --- | --- | --- | --- | --- | --- | --- | --- | --- | --- | --- | --- | --- | --- | --- | --- |

XP\_026568326.1

back to list

  

| Protein Coverage
| Supporting Peptides
|

Protein Coverage:

Supporting Peptides:

| Peptide | Uniq | -10lgP | Mass | Length | ppm | m/z | z | RT | Fraction | Scan | Source File | Area F2 | Area F1 | Area F3 | Area F4 | Area F5 | Area F6 | #Feature | #Feature F2 | #Feature F1 | #Feature F3 | #Feature F4 | #Feature F5 | #Feature F6 | Start | End | PTM | AScore | Found By |
| --- | --- | --- | --- | --- | --- | --- | --- | --- | --- | --- | --- | --- | --- | --- | --- | --- | --- | --- | --- | --- | --- | --- | --- | --- | --- | --- | --- | --- | --- |
| K.SQIFSTASDNQPTVTIK.V | Y | 52.26 | 1835.9265 | 17 | -0.7 | 918.9698 | 2 | 40.68 | 6 | F6:19789 | 31122021\_RID\_2349\_6C.raw |  |  |  |  |  | 3.0923E4 | 1 | 0 | 0 | 0 | 0 | 0 | 1 | 448 | 464 |  |  | PEAKS DB |
| K.NQLTSNPENTVFDAK.R | Y | 48.13 | 1676.8005 | 15 | 2.0 | 839.4092 | 2 | 37.18 | 1 | F1:18792 | 28122021\_RID\_2349\_2C.raw | 1.2492E5 |  |  |  |  |  | 1 | 1 | 0 | 0 | 0 | 0 | 0 | 82 | 96 |  |  | PEAKS DB |
| R.TWNDPSVQQDIK.Y | Y | 46.24 | 1429.6837 | 12 | 0.2 | 715.8492 | 2 | 34.09 | 1 | F1:16305 | 28122021\_RID\_2349\_2C.raw | 7.7839E4 |  |  |  |  |  | 1 | 1 | 0 | 0 | 0 | 0 | 0 | 102 | 113 |  |  | PEAKS DB |
| total 3 peptides |  |  |  |  |  |  |  |  |  |  |  |  |  |  |  |  |  |  |  |  |  |  |  |  |  |  |  |  |  |
| --- | --- | --- | --- | --- | --- | --- | --- | --- | --- | --- | --- | --- | --- | --- | --- | --- | --- | --- | --- | --- | --- | --- | --- | --- | --- | --- | --- | --- | --- |

XP\_025022112.1

back to list

  

| Protein Coverage
| Supporting Peptides
|

Protein Coverage:

Supporting Peptides:

| Peptide | Uniq | -10lgP | Mass | Length | ppm | m/z | z | RT | Fraction | Scan | Source File | Area F2 | Area F1 | Area F3 | Area F4 | Area F5 | Area F6 | #Feature | #Feature F2 | #Feature F1 | #Feature F3 | #Feature F4 | #Feature F5 | #Feature F6 | Start | End | PTM | AScore | Found By |
| --- | --- | --- | --- | --- | --- | --- | --- | --- | --- | --- | --- | --- | --- | --- | --- | --- | --- | --- | --- | --- | --- | --- | --- | --- | --- | --- | --- | --- | --- |
| K.SQIFSTASDNQPTVTIK.V | Y | 52.26 | 1835.9265 | 17 | -0.7 | 918.9698 | 2 | 40.68 | 6 | F6:19789 | 31122021\_RID\_2349\_6C.raw |  |  |  |  |  | 3.0923E4 | 1 | 0 | 0 | 0 | 0 | 0 | 1 | 446 | 462 |  |  | PEAKS DB |
| K.NQLTSNPENTVFDAK.R | Y | 48.13 | 1676.8005 | 15 | 2.0 | 839.4092 | 2 | 37.18 | 1 | F1:18792 | 28122021\_RID\_2349\_2C.raw | 1.2492E5 |  |  |  |  |  | 1 | 1 | 0 | 0 | 0 | 0 | 0 | 80 | 94 |  |  | PEAKS DB |
| R.TWNDPSVQQDIK.Y | Y | 46.24 | 1429.6837 | 12 | 0.2 | 715.8492 | 2 | 34.09 | 1 | F1:16305 | 28122021\_RID\_2349\_2C.raw | 7.7839E4 |  |  |  |  |  | 1 | 1 | 0 | 0 | 0 | 0 | 0 | 100 | 111 |  |  | PEAKS DB |
| total 3 peptides |  |  |  |  |  |  |  |  |  |  |  |  |  |  |  |  |  |  |  |  |  |  |  |  |  |  |  |  |  |
| --- | --- | --- | --- | --- | --- | --- | --- | --- | --- | --- | --- | --- | --- | --- | --- | --- | --- | --- | --- | --- | --- | --- | --- | --- | --- | --- | --- | --- | --- |

JAG67806.1

back to list

  

| Protein Coverage
| Supporting Peptides
|

Protein Coverage:

Supporting Peptides:

| Peptide | Uniq | -10lgP | Mass | Length | ppm | m/z | z | RT | Fraction | Scan | Source File | Area F2 | Area F1 | Area F3 | Area F4 | Area F5 | Area F6 | #Feature | #Feature F2 | #Feature F1 | #Feature F3 | #Feature F4 | #Feature F5 | #Feature F6 | Start | End | PTM | AScore | Found By |
| --- | --- | --- | --- | --- | --- | --- | --- | --- | --- | --- | --- | --- | --- | --- | --- | --- | --- | --- | --- | --- | --- | --- | --- | --- | --- | --- | --- | --- | --- |
| K.SQIFSTASDNQPTVTIK.V | Y | 52.26 | 1835.9265 | 17 | -0.7 | 918.9698 | 2 | 40.68 | 6 | F6:19789 | 31122021\_RID\_2349\_6C.raw |  |  |  |  |  | 3.0923E4 | 1 | 0 | 0 | 0 | 0 | 0 | 1 | 447 | 463 |  |  | PEAKS DB |
| K.NQLTSNPENTVFDAK.R | Y | 48.13 | 1676.8005 | 15 | 2.0 | 839.4092 | 2 | 37.18 | 1 | F1:18792 | 28122021\_RID\_2349\_2C.raw | 1.2492E5 |  |  |  |  |  | 1 | 1 | 0 | 0 | 0 | 0 | 0 | 81 | 95 |  |  | PEAKS DB |
| R.TWNDPSVQQDIK.Y | Y | 46.24 | 1429.6837 | 12 | 0.2 | 715.8492 | 2 | 34.09 | 1 | F1:16305 | 28122021\_RID\_2349\_2C.raw | 7.7839E4 |  |  |  |  |  | 1 | 1 | 0 | 0 | 0 | 0 | 0 | 101 | 112 |  |  | PEAKS DB |
| total 3 peptides |  |  |  |  |  |  |  |  |  |  |  |  |  |  |  |  |  |  |  |  |  |  |  |  |  |  |  |  |  |
| --- | --- | --- | --- | --- | --- | --- | --- | --- | --- | --- | --- | --- | --- | --- | --- | --- | --- | --- | --- | --- | --- | --- | --- | --- | --- | --- | --- | --- | --- |

XP\_032088936.1

back to list

  

| Protein Coverage
| Supporting Peptides
|

Protein Coverage:

Supporting Peptides:

| Peptide | Uniq | -10lgP | Mass | Length | ppm | m/z | z | RT | Fraction | Scan | Source File | Area F2 | Area F1 | Area F3 | Area F4 | Area F5 | Area F6 | #Feature | #Feature F2 | #Feature F1 | #Feature F3 | #Feature F4 | #Feature F5 | #Feature F6 | Start | End | PTM | AScore | Found By |
| --- | --- | --- | --- | --- | --- | --- | --- | --- | --- | --- | --- | --- | --- | --- | --- | --- | --- | --- | --- | --- | --- | --- | --- | --- | --- | --- | --- | --- | --- |
| K.SQIFSTASDNQPTVTIK.V | Y | 52.26 | 1835.9265 | 17 | -0.7 | 918.9698 | 2 | 40.68 | 6 | F6:19789 | 31122021\_RID\_2349\_6C.raw |  |  |  |  |  | 3.0923E4 | 1 | 0 | 0 | 0 | 0 | 0 | 1 | 447 | 463 |  |  | PEAKS DB |
| K.NQLTSNPENTVFDAK.R | Y | 48.13 | 1676.8005 | 15 | 2.0 | 839.4092 | 2 | 37.18 | 1 | F1:18792 | 28122021\_RID\_2349\_2C.raw | 1.2492E5 |  |  |  |  |  | 1 | 1 | 0 | 0 | 0 | 0 | 0 | 81 | 95 |  |  | PEAKS DB |
| R.TWNDPSVQQDIK.Y | Y | 46.24 | 1429.6837 | 12 | 0.2 | 715.8492 | 2 | 34.09 | 1 | F1:16305 | 28122021\_RID\_2349\_2C.raw | 7.7839E4 |  |  |  |  |  | 1 | 1 | 0 | 0 | 0 | 0 | 0 | 101 | 112 |  |  | PEAKS DB |
| total 3 peptides |  |  |  |  |  |  |  |  |  |  |  |  |  |  |  |  |  |  |  |  |  |  |  |  |  |  |  |  |  |
| --- | --- | --- | --- | --- | --- | --- | --- | --- | --- | --- | --- | --- | --- | --- | --- | --- | --- | --- | --- | --- | --- | --- | --- | --- | --- | --- | --- | --- | --- |

XP\_013910585.1

back to list

  

| Protein Coverage
| Supporting Peptides
|

Protein Coverage:

Supporting Peptides:

| Peptide | Uniq | -10lgP | Mass | Length | ppm | m/z | z | RT | Fraction | Scan | Source File | Area F2 | Area F1 | Area F3 | Area F4 | Area F5 | Area F6 | #Feature | #Feature F2 | #Feature F1 | #Feature F3 | #Feature F4 | #Feature F5 | #Feature F6 | Start | End | PTM | AScore | Found By |
| --- | --- | --- | --- | --- | --- | --- | --- | --- | --- | --- | --- | --- | --- | --- | --- | --- | --- | --- | --- | --- | --- | --- | --- | --- | --- | --- | --- | --- | --- |
| K.SQIFSTASDNQPTVTIK.V | Y | 52.26 | 1835.9265 | 17 | -0.7 | 918.9698 | 2 | 40.68 | 6 | F6:19789 | 31122021\_RID\_2349\_6C.raw |  |  |  |  |  | 3.0923E4 | 1 | 0 | 0 | 0 | 0 | 0 | 1 | 447 | 463 |  |  | PEAKS DB |
| K.NQLTSNPENTVFDAK.R | Y | 48.13 | 1676.8005 | 15 | 2.0 | 839.4092 | 2 | 37.18 | 1 | F1:18792 | 28122021\_RID\_2349\_2C.raw | 1.2492E5 |  |  |  |  |  | 1 | 1 | 0 | 0 | 0 | 0 | 0 | 81 | 95 |  |  | PEAKS DB |
| R.TWNDPSVQQDIK.Y | Y | 46.24 | 1429.6837 | 12 | 0.2 | 715.8492 | 2 | 34.09 | 1 | F1:16305 | 28122021\_RID\_2349\_2C.raw | 7.7839E4 |  |  |  |  |  | 1 | 1 | 0 | 0 | 0 | 0 | 0 | 101 | 112 |  |  | PEAKS DB |
| total 3 peptides |  |  |  |  |  |  |  |  |  |  |  |  |  |  |  |  |  |  |  |  |  |  |  |  |  |  |  |  |  |
| --- | --- | --- | --- | --- | --- | --- | --- | --- | --- | --- | --- | --- | --- | --- | --- | --- | --- | --- | --- | --- | --- | --- | --- | --- | --- | --- | --- | --- | --- |

ACZ34293.1

back to list

  

| Protein Coverage
| Supporting Peptides
|

Protein Coverage:

Supporting Peptides:

| Peptide | Uniq | -10lgP | Mass | Length | ppm | m/z | z | RT | Fraction | Scan | Source File | Area F2 | Area F1 | Area F3 | Area F4 | Area F5 | Area F6 | #Feature | #Feature F2 | #Feature F1 | #Feature F3 | #Feature F4 | #Feature F5 | #Feature F6 | Start | End | PTM | AScore | Found By |
| --- | --- | --- | --- | --- | --- | --- | --- | --- | --- | --- | --- | --- | --- | --- | --- | --- | --- | --- | --- | --- | --- | --- | --- | --- | --- | --- | --- | --- | --- |
| R.EAVFVAQLLSENIK.A | Y | 83.00 | 1559.8558 | 14 | 0.6 | 780.9357 | 2 | 59.72 | 6 | F6:34028 | 31122021\_RID\_2349\_6C.raw | 6.7822E4 |  |  |  |  | 7.2926E6 | 2 | 1 | 0 | 0 | 0 | 0 | 1 | 70 | 83 |  |  | PEAKS DB |
| total 1 peptides |  |  |  |  |  |  |  |  |  |  |  |  |  |  |  |  |  |  |  |  |  |  |  |  |  |  |  |  |  |
| --- | --- | --- | --- | --- | --- | --- | --- | --- | --- | --- | --- | --- | --- | --- | --- | --- | --- | --- | --- | --- | --- | --- | --- | --- | --- | --- | --- | --- | --- |

D1MGU0.1

back to list

  

| Protein Coverage
| Supporting Peptides
|

Protein Coverage:

Supporting Peptides:

| Peptide | Uniq | -10lgP | Mass | Length | ppm | m/z | z | RT | Fraction | Scan | Source File | Area F2 | Area F1 | Area F3 | Area F4 | Area F5 | Area F6 | #Feature | #Feature F2 | #Feature F1 | #Feature F3 | #Feature F4 | #Feature F5 | #Feature F6 | Start | End | PTM | AScore | Found By |
| --- | --- | --- | --- | --- | --- | --- | --- | --- | --- | --- | --- | --- | --- | --- | --- | --- | --- | --- | --- | --- | --- | --- | --- | --- | --- | --- | --- | --- | --- |
| R.EAVFVAQLLSENIK.A | Y | 83.00 | 1559.8558 | 14 | 0.6 | 780.9357 | 2 | 59.72 | 6 | F6:34028 | 31122021\_RID\_2349\_6C.raw | 6.7822E4 |  |  |  |  | 7.2926E6 | 2 | 1 | 0 | 0 | 0 | 0 | 1 | 70 | 83 |  |  | PEAKS DB |
| total 1 peptides |  |  |  |  |  |  |  |  |  |  |  |  |  |  |  |  |  |  |  |  |  |  |  |  |  |  |  |  |  |
| --- | --- | --- | --- | --- | --- | --- | --- | --- | --- | --- | --- | --- | --- | --- | --- | --- | --- | --- | --- | --- | --- | --- | --- | --- | --- | --- | --- | --- | --- |

Q8JJ51.1

back to list

  

| Protein Coverage
| Supporting Peptides
|

Protein Coverage:

Supporting Peptides:

| Peptide | Uniq | -10lgP | Mass | Length | ppm | m/z | z | RT | Fraction | Scan | Source File | Area F2 | Area F1 | Area F3 | Area F4 | Area F5 | Area F6 | #Feature | #Feature F2 | #Feature F1 | #Feature F3 | #Feature F4 | #Feature F5 | #Feature F6 | Start | End | PTM | AScore | Found By |
| --- | --- | --- | --- | --- | --- | --- | --- | --- | --- | --- | --- | --- | --- | --- | --- | --- | --- | --- | --- | --- | --- | --- | --- | --- | --- | --- | --- | --- | --- |
| S.IILESGNVNDYEVVYPR.K | N | 79.46 | 1979.0000 | 17 | 0.8 | 990.5081 | 2 | 50.36 | 6 | F6:27387 | 31122021\_RID\_2349\_6C.raw |  |  |  |  |  | 7.374E5 | 1 | 0 | 0 | 0 | 0 | 0 | 1 | 21 | 37 |  |  | PEAKS DB |
| M.GVTMAHELGHNLGMK.H | Y | 55.12 | 1593.7755 | 15 | 1.0 | 797.8958 | 2 | 26.66 | 3 | F3:6395 | 31122021\_RID\_2349\_3C.raw |  |  | 8.1989E6 |  |  |  | 1 | 0 | 0 | 1 | 0 | 0 | 0 | 328 | 342 |  |  | PEAKS DB |
| total 2 peptides |  |  |  |  |  |  |  |  |  |  |  |  |  |  |  |  |  |  |  |  |  |  |  |  |  |  |  |  |  |
| --- | --- | --- | --- | --- | --- | --- | --- | --- | --- | --- | --- | --- | --- | --- | --- | --- | --- | --- | --- | --- | --- | --- | --- | --- | --- | --- | --- | --- | --- |

AAM27041.1

back to list

  

| Protein Coverage
| Supporting Peptides
|

Protein Coverage:

Supporting Peptides:

| Peptide | Uniq | -10lgP | Mass | Length | ppm | m/z | z | RT | Fraction | Scan | Source File | Area F2 | Area F1 | Area F3 | Area F4 | Area F5 | Area F6 | #Feature | #Feature F2 | #Feature F1 | #Feature F3 | #Feature F4 | #Feature F5 | #Feature F6 | Start | End | PTM | AScore | Found By |
| --- | --- | --- | --- | --- | --- | --- | --- | --- | --- | --- | --- | --- | --- | --- | --- | --- | --- | --- | --- | --- | --- | --- | --- | --- | --- | --- | --- | --- | --- |
| S.IILESGNVNDYEVVYPR.K | N | 79.46 | 1979.0000 | 17 | 0.8 | 990.5081 | 2 | 50.36 | 6 | F6:27387 | 31122021\_RID\_2349\_6C.raw |  |  |  |  |  | 7.374E5 | 1 | 0 | 0 | 0 | 0 | 0 | 1 | 21 | 37 |  |  | PEAKS DB |
| M.GVTMAHELGHNLGMK.H | Y | 55.12 | 1593.7755 | 15 | 1.0 | 797.8958 | 2 | 26.66 | 3 | F3:6395 | 31122021\_RID\_2349\_3C.raw |  |  | 8.1989E6 |  |  |  | 1 | 0 | 0 | 1 | 0 | 0 | 0 | 328 | 342 |  |  | PEAKS DB |
| total 2 peptides |  |  |  |  |  |  |  |  |  |  |  |  |  |  |  |  |  |  |  |  |  |  |  |  |  |  |  |  |  |
| --- | --- | --- | --- | --- | --- | --- | --- | --- | --- | --- | --- | --- | --- | --- | --- | --- | --- | --- | --- | --- | --- | --- | --- | --- | --- | --- | --- | --- | --- |

ABD75822.1

back to list

  

| Protein Coverage
| Supporting Peptides
|

Protein Coverage:

Supporting Peptides:

| Peptide | Uniq | -10lgP | Mass | Length | ppm | m/z | z | RT | Fraction | Scan | Source File | Area F2 | Area F1 | Area F3 | Area F4 | Area F5 | Area F6 | #Feature | #Feature F2 | #Feature F1 | #Feature F3 | #Feature F4 | #Feature F5 | #Feature F6 | Start | End | PTM | AScore | Found By |
| --- | --- | --- | --- | --- | --- | --- | --- | --- | --- | --- | --- | --- | --- | --- | --- | --- | --- | --- | --- | --- | --- | --- | --- | --- | --- | --- | --- | --- | --- |
| S.IILESGNVNDYEVVYPR.K | N | 79.46 | 1979.0000 | 17 | 0.8 | 990.5081 | 2 | 50.36 | 6 | F6:27387 | 31122021\_RID\_2349\_6C.raw |  |  |  |  |  | 7.374E5 | 1 | 0 | 0 | 0 | 0 | 0 | 1 | 21 | 37 |  |  | PEAKS DB |
| M.GVTMAHELGHNLGMK.H | Y | 55.12 | 1593.7755 | 15 | 1.0 | 797.8958 | 2 | 26.66 | 3 | F3:6395 | 31122021\_RID\_2349\_3C.raw |  |  | 8.1989E6 |  |  |  | 1 | 0 | 0 | 1 | 0 | 0 | 0 | 328 | 342 |  |  | PEAKS DB |
| total 2 peptides |  |  |  |  |  |  |  |  |  |  |  |  |  |  |  |  |  |  |  |  |  |  |  |  |  |  |  |  |  |
| --- | --- | --- | --- | --- | --- | --- | --- | --- | --- | --- | --- | --- | --- | --- | --- | --- | --- | --- | --- | --- | --- | --- | --- | --- | --- | --- | --- | --- | --- |

P0DJL2.1

back to list

  

| Protein Coverage
| Supporting Peptides
|

Protein Coverage:

Supporting Peptides:

| Peptide | Uniq | -10lgP | Mass | Length | ppm | m/z | z | RT | Fraction | Scan | Source File | Area F2 | Area F1 | Area F3 | Area F4 | Area F5 | Area F6 | #Feature | #Feature F2 | #Feature F1 | #Feature F3 | #Feature F4 | #Feature F5 | #Feature F6 | Start | End | PTM | AScore | Found By |
| --- | --- | --- | --- | --- | --- | --- | --- | --- | --- | --- | --- | --- | --- | --- | --- | --- | --- | --- | --- | --- | --- | --- | --- | --- | --- | --- | --- | --- | --- |
| K.YHVWIGLSVQNK.G | Y | 73.58 | 1442.7670 | 12 | 0.7 | 722.3912 | 2 | 39.80 | 6 | F6:19286 | 31122021\_RID\_2349\_6C.raw |  |  |  |  |  | 3.386E5 | 1 | 0 | 0 | 0 | 0 | 0 | 1 | 64 | 75 |  |  | PEAKS DB |
| total 1 peptides |  |  |  |  |  |  |  |  |  |  |  |  |  |  |  |  |  |  |  |  |  |  |  |  |  |  |  |  |  |
| --- | --- | --- | --- | --- | --- | --- | --- | --- | --- | --- | --- | --- | --- | --- | --- | --- | --- | --- | --- | --- | --- | --- | --- | --- | --- | --- | --- | --- | --- |

AAQ02908.1

back to list

  

| Protein Coverage
| Supporting Peptides
|

Protein Coverage:

Supporting Peptides:

| Peptide | Uniq | -10lgP | Mass | Length | ppm | m/z | z | RT | Fraction | Scan | Source File | Area F2 | Area F1 | Area F3 | Area F4 | Area F5 | Area F6 | #Feature | #Feature F2 | #Feature F1 | #Feature F3 | #Feature F4 | #Feature F5 | #Feature F6 | Start | End | PTM | AScore | Found By |
| --- | --- | --- | --- | --- | --- | --- | --- | --- | --- | --- | --- | --- | --- | --- | --- | --- | --- | --- | --- | --- | --- | --- | --- | --- | --- | --- | --- | --- | --- |
| L.VIGGDEC(+57.02)NINEHR.F | N | 61.83 | 1511.6787 | 13 | 0.7 | 756.8472 | 2 | 19.75 | 6 | F6:4533 | 31122021\_RID\_2349\_6C.raw | 2.5261E8 | 1.2178E6 |  |  |  | 1.7091E5 | 5 | 3 | 1 | 0 | 0 | 0 | 1 | 25 | 37 | Carbamidomethylation | C7:Carbamidomethylation:1000.00 | PEAKS DB |
| Q.AAYPELPAK.S | Y | 56.72 | 958.5123 | 9 | -0.2 | 480.2634 | 2 | 30.72 | 1 | F1:13684 | 28122021\_RID\_2349\_2C.raw | 8.1648E6 |  |  |  | 0 |  | 1 | 1 | 0 | 0 | 0 | 0 | 0 | 178 | 186 |  |  | PEAKS DB |
| K.LQFGLHSK.R | N | 45.29 | 928.5130 | 8 | 0.3 | 465.2639 | 2 | 24.70 | 1 | F1:8792 | 28122021\_RID\_2349\_2C.raw | 2.0047E6 |  |  |  |  |  | 1 | 1 | 0 | 0 | 0 | 0 | 0 | 75 | 82 |  |  | PEAKS DB |
| total 3 peptides |  |  |  |  |  |  |  |  |  |  |  |  |  |  |  |  |  |  |  |  |  |  |  |  |  |  |  |  |  |
| --- | --- | --- | --- | --- | --- | --- | --- | --- | --- | --- | --- | --- | --- | --- | --- | --- | --- | --- | --- | --- | --- | --- | --- | --- | --- | --- | --- | --- | --- |

Q71QH9.1

back to list

  

| Protein Coverage
| Supporting Peptides
|

Protein Coverage:

Supporting Peptides:

| Peptide | Uniq | -10lgP | Mass | Length | ppm | m/z | z | RT | Fraction | Scan | Source File | Area F2 | Area F1 | Area F3 | Area F4 | Area F5 | Area F6 | #Feature | #Feature F2 | #Feature F1 | #Feature F3 | #Feature F4 | #Feature F5 | #Feature F6 | Start | End | PTM | AScore | Found By |
| --- | --- | --- | --- | --- | --- | --- | --- | --- | --- | --- | --- | --- | --- | --- | --- | --- | --- | --- | --- | --- | --- | --- | --- | --- | --- | --- | --- | --- | --- |
| L.VIGGDEC(+57.02)NINEHR.F | N | 61.83 | 1511.6787 | 13 | 0.7 | 756.8472 | 2 | 19.75 | 6 | F6:4533 | 31122021\_RID\_2349\_6C.raw | 2.5261E8 | 1.2178E6 |  |  |  | 1.7091E5 | 5 | 3 | 1 | 0 | 0 | 0 | 1 | 25 | 37 | Carbamidomethylation | C7:Carbamidomethylation:1000.00 | PEAKS DB |
| Q.AAYPELPAK.S | Y | 56.72 | 958.5123 | 9 | -0.2 | 480.2634 | 2 | 30.72 | 1 | F1:13684 | 28122021\_RID\_2349\_2C.raw | 8.1648E6 |  |  |  | 0 |  | 1 | 1 | 0 | 0 | 0 | 0 | 0 | 178 | 186 |  |  | PEAKS DB |
| K.LQFGLHSK.R | N | 45.29 | 928.5130 | 8 | 0.3 | 465.2639 | 2 | 24.70 | 1 | F1:8792 | 28122021\_RID\_2349\_2C.raw | 2.0047E6 |  |  |  |  |  | 1 | 1 | 0 | 0 | 0 | 0 | 0 | 75 | 82 |  |  | PEAKS DB |
| total 3 peptides |  |  |  |  |  |  |  |  |  |  |  |  |  |  |  |  |  |  |  |  |  |  |  |  |  |  |  |  |  |
| --- | --- | --- | --- | --- | --- | --- | --- | --- | --- | --- | --- | --- | --- | --- | --- | --- | --- | --- | --- | --- | --- | --- | --- | --- | --- | --- | --- | --- | --- |

Q71QJ2.1

back to list

  

| Protein Coverage
| Supporting Peptides
|

Protein Coverage:

Supporting Peptides:

| Peptide | Uniq | -10lgP | Mass | Length | ppm | m/z | z | RT | Fraction | Scan | Source File | Area F2 | Area F1 | Area F3 | Area F4 | Area F5 | Area F6 | #Feature | #Feature F2 | #Feature F1 | #Feature F3 | #Feature F4 | #Feature F5 | #Feature F6 | Start | End | PTM | AScore | Found By |
| --- | --- | --- | --- | --- | --- | --- | --- | --- | --- | --- | --- | --- | --- | --- | --- | --- | --- | --- | --- | --- | --- | --- | --- | --- | --- | --- | --- | --- | --- |
| L.VIGGDEC(+57.02)NINEHR.F | N | 61.83 | 1511.6787 | 13 | 0.7 | 756.8472 | 2 | 19.75 | 6 | F6:4533 | 31122021\_RID\_2349\_6C.raw | 2.5261E8 | 1.2178E6 |  |  |  | 1.7091E5 | 5 | 3 | 1 | 0 | 0 | 0 | 1 | 25 | 37 | Carbamidomethylation | C7:Carbamidomethylation:1000.00 | PEAKS DB |
| Q.AAYPELPAK.S | Y | 56.72 | 958.5123 | 9 | -0.2 | 480.2634 | 2 | 30.72 | 1 | F1:13684 | 28122021\_RID\_2349\_2C.raw | 8.1648E6 |  |  |  | 0 |  | 1 | 1 | 0 | 0 | 0 | 0 | 0 | 178 | 186 |  |  | PEAKS DB |
| K.LQFGLHSK.R | N | 45.29 | 928.5130 | 8 | 0.3 | 465.2639 | 2 | 24.70 | 1 | F1:8792 | 28122021\_RID\_2349\_2C.raw | 2.0047E6 |  |  |  |  |  | 1 | 1 | 0 | 0 | 0 | 0 | 0 | 75 | 82 |  |  | PEAKS DB |
| total 3 peptides |  |  |  |  |  |  |  |  |  |  |  |  |  |  |  |  |  |  |  |  |  |  |  |  |  |  |  |  |  |
| --- | --- | --- | --- | --- | --- | --- | --- | --- | --- | --- | --- | --- | --- | --- | --- | --- | --- | --- | --- | --- | --- | --- | --- | --- | --- | --- | --- | --- | --- |

AAQ02903.1

back to list

  

| Protein Coverage
| Supporting Peptides
|

Protein Coverage:

Supporting Peptides:

| Peptide | Uniq | -10lgP | Mass | Length | ppm | m/z | z | RT | Fraction | Scan | Source File | Area F2 | Area F1 | Area F3 | Area F4 | Area F5 | Area F6 | #Feature | #Feature F2 | #Feature F1 | #Feature F3 | #Feature F4 | #Feature F5 | #Feature F6 | Start | End | PTM | AScore | Found By |
| --- | --- | --- | --- | --- | --- | --- | --- | --- | --- | --- | --- | --- | --- | --- | --- | --- | --- | --- | --- | --- | --- | --- | --- | --- | --- | --- | --- | --- | --- |
| L.VIGGDEC(+57.02)NINEHR.F | N | 61.83 | 1511.6787 | 13 | 0.7 | 756.8472 | 2 | 19.75 | 6 | F6:4533 | 31122021\_RID\_2349\_6C.raw | 2.5261E8 | 1.2178E6 |  |  |  | 1.7091E5 | 5 | 3 | 1 | 0 | 0 | 0 | 1 | 25 | 37 | Carbamidomethylation | C7:Carbamidomethylation:1000.00 | PEAKS DB |
| Q.AAYPELPAK.S | Y | 56.72 | 958.5123 | 9 | -0.2 | 480.2634 | 2 | 30.72 | 1 | F1:13684 | 28122021\_RID\_2349\_2C.raw | 8.1648E6 |  |  |  | 0 |  | 1 | 1 | 0 | 0 | 0 | 0 | 0 | 178 | 186 |  |  | PEAKS DB |
| K.LQFGLHSK.R | N | 45.29 | 928.5130 | 8 | 0.3 | 465.2639 | 2 | 24.70 | 1 | F1:8792 | 28122021\_RID\_2349\_2C.raw | 2.0047E6 |  |  |  |  |  | 1 | 1 | 0 | 0 | 0 | 0 | 0 | 75 | 82 |  |  | PEAKS DB |
| total 3 peptides |  |  |  |  |  |  |  |  |  |  |  |  |  |  |  |  |  |  |  |  |  |  |  |  |  |  |  |  |  |
| --- | --- | --- | --- | --- | --- | --- | --- | --- | --- | --- | --- | --- | --- | --- | --- | --- | --- | --- | --- | --- | --- | --- | --- | --- | --- | --- | --- | --- | --- |

Q71QI4.1

back to list

  

| Protein Coverage
| Supporting Peptides
|

Protein Coverage:

Supporting Peptides:

| Peptide | Uniq | -10lgP | Mass | Length | ppm | m/z | z | RT | Fraction | Scan | Source File | Area F2 | Area F1 | Area F3 | Area F4 | Area F5 | Area F6 | #Feature | #Feature F2 | #Feature F1 | #Feature F3 | #Feature F4 | #Feature F5 | #Feature F6 | Start | End | PTM | AScore | Found By |
| --- | --- | --- | --- | --- | --- | --- | --- | --- | --- | --- | --- | --- | --- | --- | --- | --- | --- | --- | --- | --- | --- | --- | --- | --- | --- | --- | --- | --- | --- |
| L.VIGGDEC(+57.02)NINEHR.F | N | 61.83 | 1511.6787 | 13 | 0.7 | 756.8472 | 2 | 19.75 | 6 | F6:4533 | 31122021\_RID\_2349\_6C.raw | 2.5261E8 | 1.2178E6 |  |  |  | 1.7091E5 | 5 | 3 | 1 | 0 | 0 | 0 | 1 | 25 | 37 | Carbamidomethylation | C7:Carbamidomethylation:1000.00 | PEAKS DB |
| Q.AAYPELPAK.S | Y | 56.72 | 958.5123 | 9 | -0.2 | 480.2634 | 2 | 30.72 | 1 | F1:13684 | 28122021\_RID\_2349\_2C.raw | 8.1648E6 |  |  |  | 0 |  | 1 | 1 | 0 | 0 | 0 | 0 | 0 | 178 | 186 |  |  | PEAKS DB |
| K.LQFGLHSK.R | N | 45.29 | 928.5130 | 8 | 0.3 | 465.2639 | 2 | 24.70 | 1 | F1:8792 | 28122021\_RID\_2349\_2C.raw | 2.0047E6 |  |  |  |  |  | 1 | 1 | 0 | 0 | 0 | 0 | 0 | 75 | 82 |  |  | PEAKS DB |
| total 3 peptides |  |  |  |  |  |  |  |  |  |  |  |  |  |  |  |  |  |  |  |  |  |  |  |  |  |  |  |  |  |
| --- | --- | --- | --- | --- | --- | --- | --- | --- | --- | --- | --- | --- | --- | --- | --- | --- | --- | --- | --- | --- | --- | --- | --- | --- | --- | --- | --- | --- | --- |

AAQ02895.1

back to list

  

| Protein Coverage
| Supporting Peptides
|

Protein Coverage:

Supporting Peptides:

| Peptide | Uniq | -10lgP | Mass | Length | ppm | m/z | z | RT | Fraction | Scan | Source File | Area F2 | Area F1 | Area F3 | Area F4 | Area F5 | Area F6 | #Feature | #Feature F2 | #Feature F1 | #Feature F3 | #Feature F4 | #Feature F5 | #Feature F6 | Start | End | PTM | AScore | Found By |
| --- | --- | --- | --- | --- | --- | --- | --- | --- | --- | --- | --- | --- | --- | --- | --- | --- | --- | --- | --- | --- | --- | --- | --- | --- | --- | --- | --- | --- | --- |
| L.VIGGDEC(+57.02)NINEHR.F | N | 61.83 | 1511.6787 | 13 | 0.7 | 756.8472 | 2 | 19.75 | 6 | F6:4533 | 31122021\_RID\_2349\_6C.raw | 2.5261E8 | 1.2178E6 |  |  |  | 1.7091E5 | 5 | 3 | 1 | 0 | 0 | 0 | 1 | 25 | 37 | Carbamidomethylation | C7:Carbamidomethylation:1000.00 | PEAKS DB |
| Q.AAYPELPAK.S | Y | 56.72 | 958.5123 | 9 | -0.2 | 480.2634 | 2 | 30.72 | 1 | F1:13684 | 28122021\_RID\_2349\_2C.raw | 8.1648E6 |  |  |  | 0 |  | 1 | 1 | 0 | 0 | 0 | 0 | 0 | 178 | 186 |  |  | PEAKS DB |
| K.LQFGLHSK.R | N | 45.29 | 928.5130 | 8 | 0.3 | 465.2639 | 2 | 24.70 | 1 | F1:8792 | 28122021\_RID\_2349\_2C.raw | 2.0047E6 |  |  |  |  |  | 1 | 1 | 0 | 0 | 0 | 0 | 0 | 75 | 82 |  |  | PEAKS DB |
| total 3 peptides |  |  |  |  |  |  |  |  |  |  |  |  |  |  |  |  |  |  |  |  |  |  |  |  |  |  |  |  |  |
| --- | --- | --- | --- | --- | --- | --- | --- | --- | --- | --- | --- | --- | --- | --- | --- | --- | --- | --- | --- | --- | --- | --- | --- | --- | --- | --- | --- | --- | --- |

J3RYA3.1

back to list

  

| Protein Coverage
| Supporting Peptides
|

Protein Coverage:

Supporting Peptides:

| Peptide | Uniq | -10lgP | Mass | Length | ppm | m/z | z | RT | Fraction | Scan | Source File | Area F2 | Area F1 | Area F3 | Area F4 | Area F5 | Area F6 | #Feature | #Feature F2 | #Feature F1 | #Feature F3 | #Feature F4 | #Feature F5 | #Feature F6 | Start | End | PTM | AScore | Found By |
| --- | --- | --- | --- | --- | --- | --- | --- | --- | --- | --- | --- | --- | --- | --- | --- | --- | --- | --- | --- | --- | --- | --- | --- | --- | --- | --- | --- | --- | --- |
| L.VIGGDEC(+57.02)NIDEHR.F | Y | 56.66 | 1512.6627 | 13 | 0.2 | 757.3388 | 2 | 21.58 | 1 | F1:6456 | 28122021\_RID\_2349\_2C.raw | 2.2168E6 |  |  |  |  |  | 1 | 1 | 0 | 0 | 0 | 0 | 0 | 25 | 37 | Carbamidomethylation | C7:Carbamidomethylation:1000.00 | PEAKS DB |
| R.WDKDIMLIR.L | N | 56.29 | 1188.6324 | 9 | 0.8 | 595.3240 | 2 | 41.38 | 1 | F1:22123 | 28122021\_RID\_2349\_2C.raw | 6.4894E6 |  |  |  |  |  | 2 | 2 | 0 | 0 | 0 | 0 | 0 | 109 | 117 |  |  | PEAKS DB |
| R.WDKDIM(+15.99)LIR.L | N | 46.72 | 1204.6274 | 9 | 0.3 | 402.5499 | 3 | 36.54 | 1 | F1:18303 | 28122021\_RID\_2349\_2C.raw | 1.1053E6 |  |  |  |  |  | 2 | 2 | 0 | 0 | 0 | 0 | 0 | 109 | 117 | Oxidation (M) | M6:Oxidation (M):1000.00 | PEAKS DB |
| W.DKDIMLIR.L | N | 46.50 | 1002.5532 | 8 | 1.1 | 502.2844 | 2 | 33.08 | 1 | F1:15497 | 28122021\_RID\_2349\_2C.raw | 1.4232E5 |  |  |  |  |  | 1 | 1 | 0 | 0 | 0 | 0 | 0 | 110 | 117 |  |  | PEAKS DB |
| total 4 peptides |  |  |  |  |  |  |  |  |  |  |  |  |  |  |  |  |  |  |  |  |  |  |  |  |  |  |  |  |  |
| --- | --- | --- | --- | --- | --- | --- | --- | --- | --- | --- | --- | --- | --- | --- | --- | --- | --- | --- | --- | --- | --- | --- | --- | --- | --- | --- | --- | --- | --- |

AEJ31999.1

back to list

  

| Protein Coverage
| Supporting Peptides
|

Protein Coverage:

Supporting Peptides:

| Peptide | Uniq | -10lgP | Mass | Length | ppm | m/z | z | RT | Fraction | Scan | Source File | Area F2 | Area F1 | Area F3 | Area F4 | Area F5 | Area F6 | #Feature | #Feature F2 | #Feature F1 | #Feature F3 | #Feature F4 | #Feature F5 | #Feature F6 | Start | End | PTM | AScore | Found By |
| --- | --- | --- | --- | --- | --- | --- | --- | --- | --- | --- | --- | --- | --- | --- | --- | --- | --- | --- | --- | --- | --- | --- | --- | --- | --- | --- | --- | --- | --- |
| L.VIGGDEC(+57.02)NIDEHR.F | Y | 56.66 | 1512.6627 | 13 | 0.2 | 757.3388 | 2 | 21.58 | 1 | F1:6456 | 28122021\_RID\_2349\_2C.raw | 2.2168E6 |  |  |  |  |  | 1 | 1 | 0 | 0 | 0 | 0 | 0 | 25 | 37 | Carbamidomethylation | C7:Carbamidomethylation:1000.00 | PEAKS DB |
| R.WDKDIMLIR.L | N | 56.29 | 1188.6324 | 9 | 0.8 | 595.3240 | 2 | 41.38 | 1 | F1:22123 | 28122021\_RID\_2349\_2C.raw | 6.4894E6 |  |  |  |  |  | 2 | 2 | 0 | 0 | 0 | 0 | 0 | 109 | 117 |  |  | PEAKS DB |
| R.WDKDIM(+15.99)LIR.L | N | 46.72 | 1204.6274 | 9 | 0.3 | 402.5499 | 3 | 36.54 | 1 | F1:18303 | 28122021\_RID\_2349\_2C.raw | 1.1053E6 |  |  |  |  |  | 2 | 2 | 0 | 0 | 0 | 0 | 0 | 109 | 117 | Oxidation (M) | M6:Oxidation (M):1000.00 | PEAKS DB |
| W.DKDIMLIR.L | N | 46.50 | 1002.5532 | 8 | 1.1 | 502.2844 | 2 | 33.08 | 1 | F1:15497 | 28122021\_RID\_2349\_2C.raw | 1.4232E5 |  |  |  |  |  | 1 | 1 | 0 | 0 | 0 | 0 | 0 | 110 | 117 |  |  | PEAKS DB |
| total 4 peptides |  |  |  |  |  |  |  |  |  |  |  |  |  |  |  |  |  |  |  |  |  |  |  |  |  |  |  |  |  |
| --- | --- | --- | --- | --- | --- | --- | --- | --- | --- | --- | --- | --- | --- | --- | --- | --- | --- | --- | --- | --- | --- | --- | --- | --- | --- | --- | --- | --- | --- |

AFJ49264.1

back to list

  

| Protein Coverage
| Supporting Peptides
|

Protein Coverage:

Supporting Peptides:

| Peptide | Uniq | -10lgP | Mass | Length | ppm | m/z | z | RT | Fraction | Scan | Source File | Area F2 | Area F1 | Area F3 | Area F4 | Area F5 | Area F6 | #Feature | #Feature F2 | #Feature F1 | #Feature F3 | #Feature F4 | #Feature F5 | #Feature F6 | Start | End | PTM | AScore | Found By |
| --- | --- | --- | --- | --- | --- | --- | --- | --- | --- | --- | --- | --- | --- | --- | --- | --- | --- | --- | --- | --- | --- | --- | --- | --- | --- | --- | --- | --- | --- |
| L.VIGGDEC(+57.02)NIDEHR.F | Y | 56.66 | 1512.6627 | 13 | 0.2 | 757.3388 | 2 | 21.58 | 1 | F1:6456 | 28122021\_RID\_2349\_2C.raw | 2.2168E6 |  |  |  |  |  | 1 | 1 | 0 | 0 | 0 | 0 | 0 | 25 | 37 | Carbamidomethylation | C7:Carbamidomethylation:1000.00 | PEAKS DB |
| R.WDKDIMLIR.L | N | 56.29 | 1188.6324 | 9 | 0.8 | 595.3240 | 2 | 41.38 | 1 | F1:22123 | 28122021\_RID\_2349\_2C.raw | 6.4894E6 |  |  |  |  |  | 2 | 2 | 0 | 0 | 0 | 0 | 0 | 109 | 117 |  |  | PEAKS DB |
| R.WDKDIM(+15.99)LIR.L | N | 46.72 | 1204.6274 | 9 | 0.3 | 402.5499 | 3 | 36.54 | 1 | F1:18303 | 28122021\_RID\_2349\_2C.raw | 1.1053E6 |  |  |  |  |  | 2 | 2 | 0 | 0 | 0 | 0 | 0 | 109 | 117 | Oxidation (M) | M6:Oxidation (M):1000.00 | PEAKS DB |
| W.DKDIMLIR.L | N | 46.50 | 1002.5532 | 8 | 1.1 | 502.2844 | 2 | 33.08 | 1 | F1:15497 | 28122021\_RID\_2349\_2C.raw | 1.4232E5 |  |  |  |  |  | 1 | 1 | 0 | 0 | 0 | 0 | 0 | 110 | 117 |  |  | PEAKS DB |
| total 4 peptides |  |  |  |  |  |  |  |  |  |  |  |  |  |  |  |  |  |  |  |  |  |  |  |  |  |  |  |  |  |
| --- | --- | --- | --- | --- | --- | --- | --- | --- | --- | --- | --- | --- | --- | --- | --- | --- | --- | --- | --- | --- | --- | --- | --- | --- | --- | --- | --- | --- | --- |

BAC77707.1

back to list

  

| Protein Coverage
| Supporting Peptides
|

Protein Coverage:

Supporting Peptides:

| Peptide | Uniq | -10lgP | Mass | Length | ppm | m/z | z | RT | Fraction | Scan | Source File | Area F2 | Area F1 | Area F3 | Area F4 | Area F5 | Area F6 | #Feature | #Feature F2 | #Feature F1 | #Feature F3 | #Feature F4 | #Feature F5 | #Feature F6 | Start | End | PTM | AScore | Found By |
| --- | --- | --- | --- | --- | --- | --- | --- | --- | --- | --- | --- | --- | --- | --- | --- | --- | --- | --- | --- | --- | --- | --- | --- | --- | --- | --- | --- | --- | --- |
| K.FTSMWIGLNNPWK.D | Y | 100.18 | 1592.7810 | 13 | 0.4 | 797.3981 | 2 | 60.50 | 6 | F6:34527 | 31122021\_RID\_2349\_6C.raw |  |  |  |  |  | 4.7033E6 | 1 | 0 | 0 | 0 | 0 | 0 | 1 | 86 | 98 |  |  | PEAKS DB |
| K.FTSM(+15.99)WIGLNNPWK.D | Y | 77.71 | 1608.7759 | 13 | 1.4 | 805.3964 | 2 | 55.67 | 6 | F6:31266 | 31122021\_RID\_2349\_6C.raw |  |  |  |  |  | 2.4519E6 | 3 | 0 | 0 | 0 | 0 | 0 | 3 | 86 | 98 | Oxidation (M) | M4:Oxidation (M):1000.00 | PEAKS DB |
| total 2 peptides |  |  |  |  |  |  |  |  |  |  |  |  |  |  |  |  |  |  |  |  |  |  |  |  |  |  |  |  |  |
| --- | --- | --- | --- | --- | --- | --- | --- | --- | --- | --- | --- | --- | --- | --- | --- | --- | --- | --- | --- | --- | --- | --- | --- | --- | --- | --- | --- | --- | --- |

Q7T2Q0.1

back to list

  

| Protein Coverage
| Supporting Peptides
|

Protein Coverage:

Supporting Peptides:

| Peptide | Uniq | -10lgP | Mass | Length | ppm | m/z | z | RT | Fraction | Scan | Source File | Area F2 | Area F1 | Area F3 | Area F4 | Area F5 | Area F6 | #Feature | #Feature F2 | #Feature F1 | #Feature F3 | #Feature F4 | #Feature F5 | #Feature F6 | Start | End | PTM | AScore | Found By |
| --- | --- | --- | --- | --- | --- | --- | --- | --- | --- | --- | --- | --- | --- | --- | --- | --- | --- | --- | --- | --- | --- | --- | --- | --- | --- | --- | --- | --- | --- |
| K.FTSMWIGLNNPWK.D | Y | 100.18 | 1592.7810 | 13 | 0.4 | 797.3981 | 2 | 60.50 | 6 | F6:34527 | 31122021\_RID\_2349\_6C.raw |  |  |  |  |  | 4.7033E6 | 1 | 0 | 0 | 0 | 0 | 0 | 1 | 86 | 98 |  |  | PEAKS DB |
| K.FTSM(+15.99)WIGLNNPWK.D | Y | 77.71 | 1608.7759 | 13 | 1.4 | 805.3964 | 2 | 55.67 | 6 | F6:31266 | 31122021\_RID\_2349\_6C.raw |  |  |  |  |  | 2.4519E6 | 3 | 0 | 0 | 0 | 0 | 0 | 3 | 86 | 98 | Oxidation (M) | M4:Oxidation (M):1000.00 | PEAKS DB |
| total 2 peptides |  |  |  |  |  |  |  |  |  |  |  |  |  |  |  |  |  |  |  |  |  |  |  |  |  |  |  |  |  |
| --- | --- | --- | --- | --- | --- | --- | --- | --- | --- | --- | --- | --- | --- | --- | --- | --- | --- | --- | --- | --- | --- | --- | --- | --- | --- | --- | --- | --- | --- |

1UKM

back to list

  

| Protein Coverage
| Supporting Peptides
|

Protein Coverage:

Supporting Peptides:

| Peptide | Uniq | -10lgP | Mass | Length | ppm | m/z | z | RT | Fraction | Scan | Source File | Area F2 | Area F1 | Area F3 | Area F4 | Area F5 | Area F6 | #Feature | #Feature F2 | #Feature F1 | #Feature F3 | #Feature F4 | #Feature F5 | #Feature F6 | Start | End | PTM | AScore | Found By |
| --- | --- | --- | --- | --- | --- | --- | --- | --- | --- | --- | --- | --- | --- | --- | --- | --- | --- | --- | --- | --- | --- | --- | --- | --- | --- | --- | --- | --- | --- |
| K.FTSMWIGLNNPWK.D | Y | 100.18 | 1592.7810 | 13 | 0.4 | 797.3981 | 2 | 60.50 | 6 | F6:34527 | 31122021\_RID\_2349\_6C.raw |  |  |  |  |  | 4.7033E6 | 1 | 0 | 0 | 0 | 0 | 0 | 1 | 60 | 72 |  |  | PEAKS DB |
| K.FTSM(+15.99)WIGLNNPWK.D | Y | 77.71 | 1608.7759 | 13 | 1.4 | 805.3964 | 2 | 55.67 | 6 | F6:31266 | 31122021\_RID\_2349\_6C.raw |  |  |  |  |  | 2.4519E6 | 3 | 0 | 0 | 0 | 0 | 0 | 3 | 60 | 72 | Oxidation (M) | M4:Oxidation (M):1000.00 | PEAKS DB |
| total 2 peptides |  |  |  |  |  |  |  |  |  |  |  |  |  |  |  |  |  |  |  |  |  |  |  |  |  |  |  |  |  |
| --- | --- | --- | --- | --- | --- | --- | --- | --- | --- | --- | --- | --- | --- | --- | --- | --- | --- | --- | --- | --- | --- | --- | --- | --- | --- | --- | --- | --- | --- |

1V7P

back to list

  

| Protein Coverage
| Supporting Peptides
|

Protein Coverage:

Supporting Peptides:

| Peptide | Uniq | -10lgP | Mass | Length | ppm | m/z | z | RT | Fraction | Scan | Source File | Area F2 | Area F1 | Area F3 | Area F4 | Area F5 | Area F6 | #Feature | #Feature F2 | #Feature F1 | #Feature F3 | #Feature F4 | #Feature F5 | #Feature F6 | Start | End | PTM | AScore | Found By |
| --- | --- | --- | --- | --- | --- | --- | --- | --- | --- | --- | --- | --- | --- | --- | --- | --- | --- | --- | --- | --- | --- | --- | --- | --- | --- | --- | --- | --- | --- |
| K.FTSMWIGLNNPWK.D | Y | 100.18 | 1592.7810 | 13 | 0.4 | 797.3981 | 2 | 60.50 | 6 | F6:34527 | 31122021\_RID\_2349\_6C.raw |  |  |  |  |  | 4.7033E6 | 1 | 0 | 0 | 0 | 0 | 0 | 1 | 60 | 72 |  |  | PEAKS DB |
| K.FTSM(+15.99)WIGLNNPWK.D | Y | 77.71 | 1608.7759 | 13 | 1.4 | 805.3964 | 2 | 55.67 | 6 | F6:31266 | 31122021\_RID\_2349\_6C.raw |  |  |  |  |  | 2.4519E6 | 3 | 0 | 0 | 0 | 0 | 0 | 3 | 60 | 72 | Oxidation (M) | M4:Oxidation (M):1000.00 | PEAKS DB |
| total 2 peptides |  |  |  |  |  |  |  |  |  |  |  |  |  |  |  |  |  |  |  |  |  |  |  |  |  |  |  |  |  |
| --- | --- | --- | --- | --- | --- | --- | --- | --- | --- | --- | --- | --- | --- | --- | --- | --- | --- | --- | --- | --- | --- | --- | --- | --- | --- | --- | --- | --- | --- |

AUS82539.1

back to list

  

| Protein Coverage
| Supporting Peptides
|

Protein Coverage:

Supporting Peptides:

| Peptide | Uniq | -10lgP | Mass | Length | ppm | m/z | z | RT | Fraction | Scan | Source File | Area F2 | Area F1 | Area F3 | Area F4 | Area F5 | Area F6 | #Feature | #Feature F2 | #Feature F1 | #Feature F3 | #Feature F4 | #Feature F5 | #Feature F6 | Start | End | PTM | AScore | Found By |
| --- | --- | --- | --- | --- | --- | --- | --- | --- | --- | --- | --- | --- | --- | --- | --- | --- | --- | --- | --- | --- | --- | --- | --- | --- | --- | --- | --- | --- | --- |
| F.WDKDIMLIR.L | N | 56.29 | 1188.6324 | 9 | 0.8 | 595.3240 | 2 | 41.38 | 1 | F1:22123 | 28122021\_RID\_2349\_2C.raw | 6.4894E6 |  |  |  |  |  | 2 | 2 | 0 | 0 | 0 | 0 | 0 | 106 | 114 |  |  | PEAKS DB |
| R.IMGWGTISDTK.V | Y | 52.56 | 1207.5907 | 11 | 0.9 | 604.8032 | 2 | 43.28 | 5 | F5:22048 | 31122021\_RID\_2349\_5C.raw | 0 |  |  |  | 0 |  | 0 | 0 | 0 | 0 | 0 | 0 | 0 | 143 | 153 |  |  | PEAKS DB |
| F.WDKDIM(+15.99)LIR.L | N | 46.72 | 1204.6274 | 9 | 0.3 | 402.5499 | 3 | 36.54 | 1 | F1:18303 | 28122021\_RID\_2349\_2C.raw | 1.1053E6 |  |  |  |  |  | 2 | 2 | 0 | 0 | 0 | 0 | 0 | 106 | 114 | Oxidation (M) | M6:Oxidation (M):1000.00 | PEAKS DB |
| W.DKDIMLIR.L | N | 46.50 | 1002.5532 | 8 | 1.1 | 502.2844 | 2 | 33.08 | 1 | F1:15497 | 28122021\_RID\_2349\_2C.raw | 1.4232E5 |  |  |  |  |  | 1 | 1 | 0 | 0 | 0 | 0 | 0 | 107 | 114 |  |  | PEAKS DB |
| total 4 peptides |  |  |  |  |  |  |  |  |  |  |  |  |  |  |  |  |  |  |  |  |  |  |  |  |  |  |  |  |  |
| --- | --- | --- | --- | --- | --- | --- | --- | --- | --- | --- | --- | --- | --- | --- | --- | --- | --- | --- | --- | --- | --- | --- | --- | --- | --- | --- | --- | --- | --- |

ABA60128.1

back to list

  

| Protein Coverage
| Supporting Peptides
|

Protein Coverage:

Supporting Peptides:

| Peptide | Uniq | -10lgP | Mass | Length | ppm | m/z | z | RT | Fraction | Scan | Source File | Area F2 | Area F1 | Area F3 | Area F4 | Area F5 | Area F6 | #Feature | #Feature F2 | #Feature F1 | #Feature F3 | #Feature F4 | #Feature F5 | #Feature F6 | Start | End | PTM | AScore | Found By |
| --- | --- | --- | --- | --- | --- | --- | --- | --- | --- | --- | --- | --- | --- | --- | --- | --- | --- | --- | --- | --- | --- | --- | --- | --- | --- | --- | --- | --- | --- |
| K.ALTMEGNQASWR.F | Y | 74.63 | 1362.6350 | 12 | 0.5 | 682.3251 | 2 | 34.48 | 5 | F5:15784 | 31122021\_RID\_2349\_5C.raw |  |  |  |  | 1.7409E5 | 1.0442E6 | 2 | 0 | 0 | 0 | 0 | 1 | 1 | 215 | 226 |  |  | PEAKS DB |
| K.ALTM(+15.99)EGNQASWR.F | Y | 47.13 | 1378.6299 | 12 | 0.6 | 690.3226 | 2 | 27.92 | 6 | F6:9991 | 31122021\_RID\_2349\_6C.raw |  |  |  |  |  | 8.9439E4 | 1 | 0 | 0 | 0 | 0 | 0 | 1 | 215 | 226 | Oxidation (M) | M4:Oxidation (M):1000.00 | PEAKS DB |
| K.QYFFETK.C | Y | 46.50 | 961.4545 | 7 | -0.2 | 481.7344 | 2 | 36.16 | 6 | F6:16404 | 31122021\_RID\_2349\_6C.raw |  |  |  |  | 3.5933E5 | 1.5674E6 | 2 | 0 | 0 | 0 | 0 | 1 | 1 | 175 | 181 |  |  | PEAKS DB |
| total 3 peptides |  |  |  |  |  |  |  |  |  |  |  |  |  |  |  |  |  |  |  |  |  |  |  |  |  |  |  |  |  |
| --- | --- | --- | --- | --- | --- | --- | --- | --- | --- | --- | --- | --- | --- | --- | --- | --- | --- | --- | --- | --- | --- | --- | --- | --- | --- | --- | --- | --- | --- |

JAA74661.1

back to list

  

| Protein Coverage
| Supporting Peptides
|

Protein Coverage:

Supporting Peptides:

| Peptide | Uniq | -10lgP | Mass | Length | ppm | m/z | z | RT | Fraction | Scan | Source File | Area F2 | Area F1 | Area F3 | Area F4 | Area F5 | Area F6 | #Feature | #Feature F2 | #Feature F1 | #Feature F3 | #Feature F4 | #Feature F5 | #Feature F6 | Start | End | PTM | AScore | Found By |
| --- | --- | --- | --- | --- | --- | --- | --- | --- | --- | --- | --- | --- | --- | --- | --- | --- | --- | --- | --- | --- | --- | --- | --- | --- | --- | --- | --- | --- | --- |
| K.ALTMEGNQASWR.F | Y | 74.63 | 1362.6350 | 12 | 0.5 | 682.3251 | 2 | 34.48 | 5 | F5:15784 | 31122021\_RID\_2349\_5C.raw |  |  |  |  | 1.7409E5 | 1.0442E6 | 2 | 0 | 0 | 0 | 0 | 1 | 1 | 216 | 227 |  |  | PEAKS DB |
| K.ALTM(+15.99)EGNQASWR.F | Y | 47.13 | 1378.6299 | 12 | 0.6 | 690.3226 | 2 | 27.92 | 6 | F6:9991 | 31122021\_RID\_2349\_6C.raw |  |  |  |  |  | 8.9439E4 | 1 | 0 | 0 | 0 | 0 | 0 | 1 | 216 | 227 | Oxidation (M) | M4:Oxidation (M):1000.00 | PEAKS DB |
| K.QYFFETK.C | Y | 46.50 | 961.4545 | 7 | -0.2 | 481.7344 | 2 | 36.16 | 6 | F6:16404 | 31122021\_RID\_2349\_6C.raw |  |  |  |  | 3.5933E5 | 1.5674E6 | 2 | 0 | 0 | 0 | 0 | 1 | 1 | 178 | 184 |  |  | PEAKS DB |
| total 3 peptides |  |  |  |  |  |  |  |  |  |  |  |  |  |  |  |  |  |  |  |  |  |  |  |  |  |  |  |  |  |
| --- | --- | --- | --- | --- | --- | --- | --- | --- | --- | --- | --- | --- | --- | --- | --- | --- | --- | --- | --- | --- | --- | --- | --- | --- | --- | --- | --- | --- | --- |

ABA60119.1

back to list

  

| Protein Coverage
| Supporting Peptides
|

Protein Coverage:

Supporting Peptides:

| Peptide | Uniq | -10lgP | Mass | Length | ppm | m/z | z | RT | Fraction | Scan | Source File | Area F2 | Area F1 | Area F3 | Area F4 | Area F5 | Area F6 | #Feature | #Feature F2 | #Feature F1 | #Feature F3 | #Feature F4 | #Feature F5 | #Feature F6 | Start | End | PTM | AScore | Found By |
| --- | --- | --- | --- | --- | --- | --- | --- | --- | --- | --- | --- | --- | --- | --- | --- | --- | --- | --- | --- | --- | --- | --- | --- | --- | --- | --- | --- | --- | --- |
| R.ALTMEGNQASWR.F | Y | 74.63 | 1362.6350 | 12 | 0.5 | 682.3251 | 2 | 34.48 | 5 | F5:15784 | 31122021\_RID\_2349\_5C.raw |  |  |  |  | 1.7409E5 | 1.0442E6 | 2 | 0 | 0 | 0 | 0 | 1 | 1 | 213 | 224 |  |  | PEAKS DB |
| R.ALTM(+15.99)EGNQASWR.F | Y | 47.13 | 1378.6299 | 12 | 0.6 | 690.3226 | 2 | 27.92 | 6 | F6:9991 | 31122021\_RID\_2349\_6C.raw |  |  |  |  |  | 8.9439E4 | 1 | 0 | 0 | 0 | 0 | 0 | 1 | 213 | 224 | Oxidation (M) | M4:Oxidation (M):1000.00 | PEAKS DB |
| K.QYFFETK.Y | Y | 46.50 | 961.4545 | 7 | -0.2 | 481.7344 | 2 | 36.16 | 6 | F6:16404 | 31122021\_RID\_2349\_6C.raw |  |  |  |  | 3.5933E5 | 1.5674E6 | 2 | 0 | 0 | 0 | 0 | 1 | 1 | 175 | 181 |  |  | PEAKS DB |
| total 3 peptides |  |  |  |  |  |  |  |  |  |  |  |  |  |  |  |  |  |  |  |  |  |  |  |  |  |  |  |  |  |
| --- | --- | --- | --- | --- | --- | --- | --- | --- | --- | --- | --- | --- | --- | --- | --- | --- | --- | --- | --- | --- | --- | --- | --- | --- | --- | --- | --- | --- | --- |

AAG12169.1

back to list

  

| Protein Coverage
| Supporting Peptides
|

Protein Coverage:

Supporting Peptides:

| Peptide | Uniq | -10lgP | Mass | Length | ppm | m/z | z | RT | Fraction | Scan | Source File | Area F2 | Area F1 | Area F3 | Area F4 | Area F5 | Area F6 | #Feature | #Feature F2 | #Feature F1 | #Feature F3 | #Feature F4 | #Feature F5 | #Feature F6 | Start | End | PTM | AScore | Found By |
| --- | --- | --- | --- | --- | --- | --- | --- | --- | --- | --- | --- | --- | --- | --- | --- | --- | --- | --- | --- | --- | --- | --- | --- | --- | --- | --- | --- | --- | --- |
| K.ALTMEGNQASWR.F | Y | 74.63 | 1362.6350 | 12 | 0.5 | 682.3251 | 2 | 34.48 | 5 | F5:15784 | 31122021\_RID\_2349\_5C.raw |  |  |  |  | 1.7409E5 | 1.0442E6 | 2 | 0 | 0 | 0 | 0 | 1 | 1 | 210 | 221 |  |  | PEAKS DB |
| K.ALTM(+15.99)EGNQASWR.F | Y | 47.13 | 1378.6299 | 12 | 0.6 | 690.3226 | 2 | 27.92 | 6 | F6:9991 | 31122021\_RID\_2349\_6C.raw |  |  |  |  |  | 8.9439E4 | 1 | 0 | 0 | 0 | 0 | 0 | 1 | 210 | 221 | Oxidation (M) | M4:Oxidation (M):1000.00 | PEAKS DB |
| K.QYFFETK.C | Y | 46.50 | 961.4545 | 7 | -0.2 | 481.7344 | 2 | 36.16 | 6 | F6:16404 | 31122021\_RID\_2349\_6C.raw |  |  |  |  | 3.5933E5 | 1.5674E6 | 2 | 0 | 0 | 0 | 0 | 1 | 1 | 172 | 178 |  |  | PEAKS DB |
| total 3 peptides |  |  |  |  |  |  |  |  |  |  |  |  |  |  |  |  |  |  |  |  |  |  |  |  |  |  |  |  |  |
| --- | --- | --- | --- | --- | --- | --- | --- | --- | --- | --- | --- | --- | --- | --- | --- | --- | --- | --- | --- | --- | --- | --- | --- | --- | --- | --- | --- | --- | --- |

ABA60132.1

back to list

  

| Protein Coverage
| Supporting Peptides
|

Protein Coverage:

Supporting Peptides:

| Peptide | Uniq | -10lgP | Mass | Length | ppm | m/z | z | RT | Fraction | Scan | Source File | Area F2 | Area F1 | Area F3 | Area F4 | Area F5 | Area F6 | #Feature | #Feature F2 | #Feature F1 | #Feature F3 | #Feature F4 | #Feature F5 | #Feature F6 | Start | End | PTM | AScore | Found By |
| --- | --- | --- | --- | --- | --- | --- | --- | --- | --- | --- | --- | --- | --- | --- | --- | --- | --- | --- | --- | --- | --- | --- | --- | --- | --- | --- | --- | --- | --- |
| R.ALTMEGNQASWR.F | Y | 74.63 | 1362.6350 | 12 | 0.5 | 682.3251 | 2 | 34.48 | 5 | F5:15784 | 31122021\_RID\_2349\_5C.raw |  |  |  |  | 1.7409E5 | 1.0442E6 | 2 | 0 | 0 | 0 | 0 | 1 | 1 | 214 | 225 |  |  | PEAKS DB |
| R.ALTM(+15.99)EGNQASWR.F | Y | 47.13 | 1378.6299 | 12 | 0.6 | 690.3226 | 2 | 27.92 | 6 | F6:9991 | 31122021\_RID\_2349\_6C.raw |  |  |  |  |  | 8.9439E4 | 1 | 0 | 0 | 0 | 0 | 0 | 1 | 214 | 225 | Oxidation (M) | M4:Oxidation (M):1000.00 | PEAKS DB |
| K.QYFFETK.C | Y | 46.50 | 961.4545 | 7 | -0.2 | 481.7344 | 2 | 36.16 | 6 | F6:16404 | 31122021\_RID\_2349\_6C.raw |  |  |  |  | 3.5933E5 | 1.5674E6 | 2 | 0 | 0 | 0 | 0 | 1 | 1 | 174 | 180 |  |  | PEAKS DB |
| total 3 peptides |  |  |  |  |  |  |  |  |  |  |  |  |  |  |  |  |  |  |  |  |  |  |  |  |  |  |  |  |  |
| --- | --- | --- | --- | --- | --- | --- | --- | --- | --- | --- | --- | --- | --- | --- | --- | --- | --- | --- | --- | --- | --- | --- | --- | --- | --- | --- | --- | --- | --- |

ABA60120.1

back to list

  

| Protein Coverage
| Supporting Peptides
|

Protein Coverage:

Supporting Peptides:

| Peptide | Uniq | -10lgP | Mass | Length | ppm | m/z | z | RT | Fraction | Scan | Source File | Area F2 | Area F1 | Area F3 | Area F4 | Area F5 | Area F6 | #Feature | #Feature F2 | #Feature F1 | #Feature F3 | #Feature F4 | #Feature F5 | #Feature F6 | Start | End | PTM | AScore | Found By |
| --- | --- | --- | --- | --- | --- | --- | --- | --- | --- | --- | --- | --- | --- | --- | --- | --- | --- | --- | --- | --- | --- | --- | --- | --- | --- | --- | --- | --- | --- |
| R.ALTMEGNQASWR.F | Y | 74.63 | 1362.6350 | 12 | 0.5 | 682.3251 | 2 | 34.48 | 5 | F5:15784 | 31122021\_RID\_2349\_5C.raw |  |  |  |  | 1.7409E5 | 1.0442E6 | 2 | 0 | 0 | 0 | 0 | 1 | 1 | 214 | 225 |  |  | PEAKS DB |
| R.ALTM(+15.99)EGNQASWR.F | Y | 47.13 | 1378.6299 | 12 | 0.6 | 690.3226 | 2 | 27.92 | 6 | F6:9991 | 31122021\_RID\_2349\_6C.raw |  |  |  |  |  | 8.9439E4 | 1 | 0 | 0 | 0 | 0 | 0 | 1 | 214 | 225 | Oxidation (M) | M4:Oxidation (M):1000.00 | PEAKS DB |
| K.QYFFETK.C | Y | 46.50 | 961.4545 | 7 | -0.2 | 481.7344 | 2 | 36.16 | 6 | F6:16404 | 31122021\_RID\_2349\_6C.raw |  |  |  |  | 3.5933E5 | 1.5674E6 | 2 | 0 | 0 | 0 | 0 | 1 | 1 | 174 | 180 |  |  | PEAKS DB |
| total 3 peptides |  |  |  |  |  |  |  |  |  |  |  |  |  |  |  |  |  |  |  |  |  |  |  |  |  |  |  |  |  |
| --- | --- | --- | --- | --- | --- | --- | --- | --- | --- | --- | --- | --- | --- | --- | --- | --- | --- | --- | --- | --- | --- | --- | --- | --- | --- | --- | --- | --- | --- |

ABA60122.1

back to list

  

| Protein Coverage
| Supporting Peptides
|

Protein Coverage:

Supporting Peptides:

| Peptide | Uniq | -10lgP | Mass | Length | ppm | m/z | z | RT | Fraction | Scan | Source File | Area F2 | Area F1 | Area F3 | Area F4 | Area F5 | Area F6 | #Feature | #Feature F2 | #Feature F1 | #Feature F3 | #Feature F4 | #Feature F5 | #Feature F6 | Start | End | PTM | AScore | Found By |
| --- | --- | --- | --- | --- | --- | --- | --- | --- | --- | --- | --- | --- | --- | --- | --- | --- | --- | --- | --- | --- | --- | --- | --- | --- | --- | --- | --- | --- | --- |
| R.ALTMEGNQASWR.F | Y | 74.63 | 1362.6350 | 12 | 0.5 | 682.3251 | 2 | 34.48 | 5 | F5:15784 | 31122021\_RID\_2349\_5C.raw |  |  |  |  | 1.7409E5 | 1.0442E6 | 2 | 0 | 0 | 0 | 0 | 1 | 1 | 214 | 225 |  |  | PEAKS DB |
| R.ALTM(+15.99)EGNQASWR.F | Y | 47.13 | 1378.6299 | 12 | 0.6 | 690.3226 | 2 | 27.92 | 6 | F6:9991 | 31122021\_RID\_2349\_6C.raw |  |  |  |  |  | 8.9439E4 | 1 | 0 | 0 | 0 | 0 | 0 | 1 | 214 | 225 | Oxidation (M) | M4:Oxidation (M):1000.00 | PEAKS DB |
| K.QYFFETK.C | Y | 46.50 | 961.4545 | 7 | -0.2 | 481.7344 | 2 | 36.16 | 6 | F6:16404 | 31122021\_RID\_2349\_6C.raw |  |  |  |  | 3.5933E5 | 1.5674E6 | 2 | 0 | 0 | 0 | 0 | 1 | 1 | 174 | 180 |  |  | PEAKS DB |
| total 3 peptides |  |  |  |  |  |  |  |  |  |  |  |  |  |  |  |  |  |  |  |  |  |  |  |  |  |  |  |  |  |
| --- | --- | --- | --- | --- | --- | --- | --- | --- | --- | --- | --- | --- | --- | --- | --- | --- | --- | --- | --- | --- | --- | --- | --- | --- | --- | --- | --- | --- | --- |

AAZ39009.1

back to list

  

| Protein Coverage
| Supporting Peptides
|

Protein Coverage:

Supporting Peptides:

| Peptide | Uniq | -10lgP | Mass | Length | ppm | m/z | z | RT | Fraction | Scan | Source File | Area F2 | Area F1 | Area F3 | Area F4 | Area F5 | Area F6 | #Feature | #Feature F2 | #Feature F1 | #Feature F3 | #Feature F4 | #Feature F5 | #Feature F6 | Start | End | PTM | AScore | Found By |
| --- | --- | --- | --- | --- | --- | --- | --- | --- | --- | --- | --- | --- | --- | --- | --- | --- | --- | --- | --- | --- | --- | --- | --- | --- | --- | --- | --- | --- | --- |
| R.ALTMEGNQASWR.F | Y | 74.63 | 1362.6350 | 12 | 0.5 | 682.3251 | 2 | 34.48 | 5 | F5:15784 | 31122021\_RID\_2349\_5C.raw |  |  |  |  | 1.7409E5 | 1.0442E6 | 2 | 0 | 0 | 0 | 0 | 1 | 1 | 213 | 224 |  |  | PEAKS DB |
| R.ALTM(+15.99)EGNQASWR.F | Y | 47.13 | 1378.6299 | 12 | 0.6 | 690.3226 | 2 | 27.92 | 6 | F6:9991 | 31122021\_RID\_2349\_6C.raw |  |  |  |  |  | 8.9439E4 | 1 | 0 | 0 | 0 | 0 | 0 | 1 | 213 | 224 | Oxidation (M) | M4:Oxidation (M):1000.00 | PEAKS DB |
| K.QYFFETK.C | Y | 46.50 | 961.4545 | 7 | -0.2 | 481.7344 | 2 | 36.16 | 6 | F6:16404 | 31122021\_RID\_2349\_6C.raw |  |  |  |  | 3.5933E5 | 1.5674E6 | 2 | 0 | 0 | 0 | 0 | 1 | 1 | 175 | 181 |  |  | PEAKS DB |
| total 3 peptides |  |  |  |  |  |  |  |  |  |  |  |  |  |  |  |  |  |  |  |  |  |  |  |  |  |  |  |  |  |
| --- | --- | --- | --- | --- | --- | --- | --- | --- | --- | --- | --- | --- | --- | --- | --- | --- | --- | --- | --- | --- | --- | --- | --- | --- | --- | --- | --- | --- | --- |

ABA60117.1

back to list

  

| Protein Coverage
| Supporting Peptides
|

Protein Coverage:

Supporting Peptides:

| Peptide | Uniq | -10lgP | Mass | Length | ppm | m/z | z | RT | Fraction | Scan | Source File | Area F2 | Area F1 | Area F3 | Area F4 | Area F5 | Area F6 | #Feature | #Feature F2 | #Feature F1 | #Feature F3 | #Feature F4 | #Feature F5 | #Feature F6 | Start | End | PTM | AScore | Found By |
| --- | --- | --- | --- | --- | --- | --- | --- | --- | --- | --- | --- | --- | --- | --- | --- | --- | --- | --- | --- | --- | --- | --- | --- | --- | --- | --- | --- | --- | --- |
| R.ALTMEGNQASWR.F | Y | 74.63 | 1362.6350 | 12 | 0.5 | 682.3251 | 2 | 34.48 | 5 | F5:15784 | 31122021\_RID\_2349\_5C.raw |  |  |  |  | 1.7409E5 | 1.0442E6 | 2 | 0 | 0 | 0 | 0 | 1 | 1 | 213 | 224 |  |  | PEAKS DB |
| R.ALTM(+15.99)EGNQASWR.F | Y | 47.13 | 1378.6299 | 12 | 0.6 | 690.3226 | 2 | 27.92 | 6 | F6:9991 | 31122021\_RID\_2349\_6C.raw |  |  |  |  |  | 8.9439E4 | 1 | 0 | 0 | 0 | 0 | 0 | 1 | 213 | 224 | Oxidation (M) | M4:Oxidation (M):1000.00 | PEAKS DB |
| K.QYFFETK.C | Y | 46.50 | 961.4545 | 7 | -0.2 | 481.7344 | 2 | 36.16 | 6 | F6:16404 | 31122021\_RID\_2349\_6C.raw |  |  |  |  | 3.5933E5 | 1.5674E6 | 2 | 0 | 0 | 0 | 0 | 1 | 1 | 175 | 181 |  |  | PEAKS DB |
| total 3 peptides |  |  |  |  |  |  |  |  |  |  |  |  |  |  |  |  |  |  |  |  |  |  |  |  |  |  |  |  |  |
| --- | --- | --- | --- | --- | --- | --- | --- | --- | --- | --- | --- | --- | --- | --- | --- | --- | --- | --- | --- | --- | --- | --- | --- | --- | --- | --- | --- | --- | --- |

ABA60116.1

back to list

  

| Protein Coverage
| Supporting Peptides
|

Protein Coverage:

Supporting Peptides:

| Peptide | Uniq | -10lgP | Mass | Length | ppm | m/z | z | RT | Fraction | Scan | Source File | Area F2 | Area F1 | Area F3 | Area F4 | Area F5 | Area F6 | #Feature | #Feature F2 | #Feature F1 | #Feature F3 | #Feature F4 | #Feature F5 | #Feature F6 | Start | End | PTM | AScore | Found By |
| --- | --- | --- | --- | --- | --- | --- | --- | --- | --- | --- | --- | --- | --- | --- | --- | --- | --- | --- | --- | --- | --- | --- | --- | --- | --- | --- | --- | --- | --- |
| R.ALTMEGNQASWR.F | Y | 74.63 | 1362.6350 | 12 | 0.5 | 682.3251 | 2 | 34.48 | 5 | F5:15784 | 31122021\_RID\_2349\_5C.raw |  |  |  |  | 1.7409E5 | 1.0442E6 | 2 | 0 | 0 | 0 | 0 | 1 | 1 | 213 | 224 |  |  | PEAKS DB |
| R.ALTM(+15.99)EGNQASWR.F | Y | 47.13 | 1378.6299 | 12 | 0.6 | 690.3226 | 2 | 27.92 | 6 | F6:9991 | 31122021\_RID\_2349\_6C.raw |  |  |  |  |  | 8.9439E4 | 1 | 0 | 0 | 0 | 0 | 0 | 1 | 213 | 224 | Oxidation (M) | M4:Oxidation (M):1000.00 | PEAKS DB |
| K.QYFFETK.C | Y | 46.50 | 961.4545 | 7 | -0.2 | 481.7344 | 2 | 36.16 | 6 | F6:16404 | 31122021\_RID\_2349\_6C.raw |  |  |  |  | 3.5933E5 | 1.5674E6 | 2 | 0 | 0 | 0 | 0 | 1 | 1 | 175 | 181 |  |  | PEAKS DB |
| total 3 peptides |  |  |  |  |  |  |  |  |  |  |  |  |  |  |  |  |  |  |  |  |  |  |  |  |  |  |  |  |  |
| --- | --- | --- | --- | --- | --- | --- | --- | --- | --- | --- | --- | --- | --- | --- | --- | --- | --- | --- | --- | --- | --- | --- | --- | --- | --- | --- | --- | --- | --- |

ABA60133.1

back to list

  

| Protein Coverage
| Supporting Peptides
|

Protein Coverage:

Supporting Peptides:

| Peptide | Uniq | -10lgP | Mass | Length | ppm | m/z | z | RT | Fraction | Scan | Source File | Area F2 | Area F1 | Area F3 | Area F4 | Area F5 | Area F6 | #Feature | #Feature F2 | #Feature F1 | #Feature F3 | #Feature F4 | #Feature F5 | #Feature F6 | Start | End | PTM | AScore | Found By |
| --- | --- | --- | --- | --- | --- | --- | --- | --- | --- | --- | --- | --- | --- | --- | --- | --- | --- | --- | --- | --- | --- | --- | --- | --- | --- | --- | --- | --- | --- |
| R.ALTMEGNQASWR.F | Y | 74.63 | 1362.6350 | 12 | 0.5 | 682.3251 | 2 | 34.48 | 5 | F5:15784 | 31122021\_RID\_2349\_5C.raw |  |  |  |  | 1.7409E5 | 1.0442E6 | 2 | 0 | 0 | 0 | 0 | 1 | 1 | 213 | 224 |  |  | PEAKS DB |
| R.ALTM(+15.99)EGNQASWR.F | Y | 47.13 | 1378.6299 | 12 | 0.6 | 690.3226 | 2 | 27.92 | 6 | F6:9991 | 31122021\_RID\_2349\_6C.raw |  |  |  |  |  | 8.9439E4 | 1 | 0 | 0 | 0 | 0 | 0 | 1 | 213 | 224 | Oxidation (M) | M4:Oxidation (M):1000.00 | PEAKS DB |
| K.QYFFETK.C | Y | 46.50 | 961.4545 | 7 | -0.2 | 481.7344 | 2 | 36.16 | 6 | F6:16404 | 31122021\_RID\_2349\_6C.raw |  |  |  |  | 3.5933E5 | 1.5674E6 | 2 | 0 | 0 | 0 | 0 | 1 | 1 | 175 | 181 |  |  | PEAKS DB |
| total 3 peptides |  |  |  |  |  |  |  |  |  |  |  |  |  |  |  |  |  |  |  |  |  |  |  |  |  |  |  |  |  |
| --- | --- | --- | --- | --- | --- | --- | --- | --- | --- | --- | --- | --- | --- | --- | --- | --- | --- | --- | --- | --- | --- | --- | --- | --- | --- | --- | --- | --- | --- |

ABA60121.1

back to list

  

| Protein Coverage
| Supporting Peptides
|

Protein Coverage:

Supporting Peptides:

| Peptide | Uniq | -10lgP | Mass | Length | ppm | m/z | z | RT | Fraction | Scan | Source File | Area F2 | Area F1 | Area F3 | Area F4 | Area F5 | Area F6 | #Feature | #Feature F2 | #Feature F1 | #Feature F3 | #Feature F4 | #Feature F5 | #Feature F6 | Start | End | PTM | AScore | Found By |
| --- | --- | --- | --- | --- | --- | --- | --- | --- | --- | --- | --- | --- | --- | --- | --- | --- | --- | --- | --- | --- | --- | --- | --- | --- | --- | --- | --- | --- | --- |
| R.ALTMEGNQASWR.F | Y | 74.63 | 1362.6350 | 12 | 0.5 | 682.3251 | 2 | 34.48 | 5 | F5:15784 | 31122021\_RID\_2349\_5C.raw |  |  |  |  | 1.7409E5 | 1.0442E6 | 2 | 0 | 0 | 0 | 0 | 1 | 1 | 214 | 225 |  |  | PEAKS DB |
| R.ALTM(+15.99)EGNQASWR.F | Y | 47.13 | 1378.6299 | 12 | 0.6 | 690.3226 | 2 | 27.92 | 6 | F6:9991 | 31122021\_RID\_2349\_6C.raw |  |  |  |  |  | 8.9439E4 | 1 | 0 | 0 | 0 | 0 | 0 | 1 | 214 | 225 | Oxidation (M) | M4:Oxidation (M):1000.00 | PEAKS DB |
| K.QYFFETK.C | Y | 46.50 | 961.4545 | 7 | -0.2 | 481.7344 | 2 | 36.16 | 6 | F6:16404 | 31122021\_RID\_2349\_6C.raw |  |  |  |  | 3.5933E5 | 1.5674E6 | 2 | 0 | 0 | 0 | 0 | 1 | 1 | 174 | 180 |  |  | PEAKS DB |
| total 3 peptides |  |  |  |  |  |  |  |  |  |  |  |  |  |  |  |  |  |  |  |  |  |  |  |  |  |  |  |  |  |
| --- | --- | --- | --- | --- | --- | --- | --- | --- | --- | --- | --- | --- | --- | --- | --- | --- | --- | --- | --- | --- | --- | --- | --- | --- | --- | --- | --- | --- | --- |

ABA60130.1

back to list

  

| Protein Coverage
| Supporting Peptides
|

Protein Coverage:

Supporting Peptides:

| Peptide | Uniq | -10lgP | Mass | Length | ppm | m/z | z | RT | Fraction | Scan | Source File | Area F2 | Area F1 | Area F3 | Area F4 | Area F5 | Area F6 | #Feature | #Feature F2 | #Feature F1 | #Feature F3 | #Feature F4 | #Feature F5 | #Feature F6 | Start | End | PTM | AScore | Found By |
| --- | --- | --- | --- | --- | --- | --- | --- | --- | --- | --- | --- | --- | --- | --- | --- | --- | --- | --- | --- | --- | --- | --- | --- | --- | --- | --- | --- | --- | --- |
| R.ALTMEGNQASWR.F | Y | 74.63 | 1362.6350 | 12 | 0.5 | 682.3251 | 2 | 34.48 | 5 | F5:15784 | 31122021\_RID\_2349\_5C.raw |  |  |  |  | 1.7409E5 | 1.0442E6 | 2 | 0 | 0 | 0 | 0 | 1 | 1 | 214 | 225 |  |  | PEAKS DB |
| R.ALTM(+15.99)EGNQASWR.F | Y | 47.13 | 1378.6299 | 12 | 0.6 | 690.3226 | 2 | 27.92 | 6 | F6:9991 | 31122021\_RID\_2349\_6C.raw |  |  |  |  |  | 8.9439E4 | 1 | 0 | 0 | 0 | 0 | 0 | 1 | 214 | 225 | Oxidation (M) | M4:Oxidation (M):1000.00 | PEAKS DB |
| K.QYFFETK.C | Y | 46.50 | 961.4545 | 7 | -0.2 | 481.7344 | 2 | 36.16 | 6 | F6:16404 | 31122021\_RID\_2349\_6C.raw |  |  |  |  | 3.5933E5 | 1.5674E6 | 2 | 0 | 0 | 0 | 0 | 1 | 1 | 174 | 180 |  |  | PEAKS DB |
| total 3 peptides |  |  |  |  |  |  |  |  |  |  |  |  |  |  |  |  |  |  |  |  |  |  |  |  |  |  |  |  |  |
| --- | --- | --- | --- | --- | --- | --- | --- | --- | --- | --- | --- | --- | --- | --- | --- | --- | --- | --- | --- | --- | --- | --- | --- | --- | --- | --- | --- | --- | --- |

ABA60123.1

back to list

  

| Protein Coverage
| Supporting Peptides
|

Protein Coverage:

Supporting Peptides:

| Peptide | Uniq | -10lgP | Mass | Length | ppm | m/z | z | RT | Fraction | Scan | Source File | Area F2 | Area F1 | Area F3 | Area F4 | Area F5 | Area F6 | #Feature | #Feature F2 | #Feature F1 | #Feature F3 | #Feature F4 | #Feature F5 | #Feature F6 | Start | End | PTM | AScore | Found By |
| --- | --- | --- | --- | --- | --- | --- | --- | --- | --- | --- | --- | --- | --- | --- | --- | --- | --- | --- | --- | --- | --- | --- | --- | --- | --- | --- | --- | --- | --- |
| R.ALTMEGNQASWR.F | Y | 74.63 | 1362.6350 | 12 | 0.5 | 682.3251 | 2 | 34.48 | 5 | F5:15784 | 31122021\_RID\_2349\_5C.raw |  |  |  |  | 1.7409E5 | 1.0442E6 | 2 | 0 | 0 | 0 | 0 | 1 | 1 | 212 | 223 |  |  | PEAKS DB |
| R.ALTM(+15.99)EGNQASWR.F | Y | 47.13 | 1378.6299 | 12 | 0.6 | 690.3226 | 2 | 27.92 | 6 | F6:9991 | 31122021\_RID\_2349\_6C.raw |  |  |  |  |  | 8.9439E4 | 1 | 0 | 0 | 0 | 0 | 0 | 1 | 212 | 223 | Oxidation (M) | M4:Oxidation (M):1000.00 | PEAKS DB |
| K.QYFFETK.C | Y | 46.50 | 961.4545 | 7 | -0.2 | 481.7344 | 2 | 36.16 | 6 | F6:16404 | 31122021\_RID\_2349\_6C.raw |  |  |  |  | 3.5933E5 | 1.5674E6 | 2 | 0 | 0 | 0 | 0 | 1 | 1 | 174 | 180 |  |  | PEAKS DB |
| total 3 peptides |  |  |  |  |  |  |  |  |  |  |  |  |  |  |  |  |  |  |  |  |  |  |  |  |  |  |  |  |  |
| --- | --- | --- | --- | --- | --- | --- | --- | --- | --- | --- | --- | --- | --- | --- | --- | --- | --- | --- | --- | --- | --- | --- | --- | --- | --- | --- | --- | --- | --- |

ABA60126.1

back to list

  

| Protein Coverage
| Supporting Peptides
|

Protein Coverage:

Supporting Peptides:

| Peptide | Uniq | -10lgP | Mass | Length | ppm | m/z | z | RT | Fraction | Scan | Source File | Area F2 | Area F1 | Area F3 | Area F4 | Area F5 | Area F6 | #Feature | #Feature F2 | #Feature F1 | #Feature F3 | #Feature F4 | #Feature F5 | #Feature F6 | Start | End | PTM | AScore | Found By |
| --- | --- | --- | --- | --- | --- | --- | --- | --- | --- | --- | --- | --- | --- | --- | --- | --- | --- | --- | --- | --- | --- | --- | --- | --- | --- | --- | --- | --- | --- |
| K.ALTMEGNQASWR.F | Y | 74.63 | 1362.6350 | 12 | 0.5 | 682.3251 | 2 | 34.48 | 5 | F5:15784 | 31122021\_RID\_2349\_5C.raw |  |  |  |  | 1.7409E5 | 1.0442E6 | 2 | 0 | 0 | 0 | 0 | 1 | 1 | 212 | 223 |  |  | PEAKS DB |
| K.ALTM(+15.99)EGNQASWR.F | Y | 47.13 | 1378.6299 | 12 | 0.6 | 690.3226 | 2 | 27.92 | 6 | F6:9991 | 31122021\_RID\_2349\_6C.raw |  |  |  |  |  | 8.9439E4 | 1 | 0 | 0 | 0 | 0 | 0 | 1 | 212 | 223 | Oxidation (M) | M4:Oxidation (M):1000.00 | PEAKS DB |
| K.QYFFETK.C | Y | 46.50 | 961.4545 | 7 | -0.2 | 481.7344 | 2 | 36.16 | 6 | F6:16404 | 31122021\_RID\_2349\_6C.raw |  |  |  |  | 3.5933E5 | 1.5674E6 | 2 | 0 | 0 | 0 | 0 | 1 | 1 | 174 | 180 |  |  | PEAKS DB |
| total 3 peptides |  |  |  |  |  |  |  |  |  |  |  |  |  |  |  |  |  |  |  |  |  |  |  |  |  |  |  |  |  |
| --- | --- | --- | --- | --- | --- | --- | --- | --- | --- | --- | --- | --- | --- | --- | --- | --- | --- | --- | --- | --- | --- | --- | --- | --- | --- | --- | --- | --- | --- |

ABA60125.1

back to list

  

| Protein Coverage
| Supporting Peptides
|

Protein Coverage:

Supporting Peptides:

| Peptide | Uniq | -10lgP | Mass | Length | ppm | m/z | z | RT | Fraction | Scan | Source File | Area F2 | Area F1 | Area F3 | Area F4 | Area F5 | Area F6 | #Feature | #Feature F2 | #Feature F1 | #Feature F3 | #Feature F4 | #Feature F5 | #Feature F6 | Start | End | PTM | AScore | Found By |
| --- | --- | --- | --- | --- | --- | --- | --- | --- | --- | --- | --- | --- | --- | --- | --- | --- | --- | --- | --- | --- | --- | --- | --- | --- | --- | --- | --- | --- | --- |
| R.ALTMEGNQASWR.F | Y | 74.63 | 1362.6350 | 12 | 0.5 | 682.3251 | 2 | 34.48 | 5 | F5:15784 | 31122021\_RID\_2349\_5C.raw |  |  |  |  | 1.7409E5 | 1.0442E6 | 2 | 0 | 0 | 0 | 0 | 1 | 1 | 212 | 223 |  |  | PEAKS DB |
| R.ALTM(+15.99)EGNQASWR.F | Y | 47.13 | 1378.6299 | 12 | 0.6 | 690.3226 | 2 | 27.92 | 6 | F6:9991 | 31122021\_RID\_2349\_6C.raw |  |  |  |  |  | 8.9439E4 | 1 | 0 | 0 | 0 | 0 | 0 | 1 | 212 | 223 | Oxidation (M) | M4:Oxidation (M):1000.00 | PEAKS DB |
| K.QYFFETK.C | Y | 46.50 | 961.4545 | 7 | -0.2 | 481.7344 | 2 | 36.16 | 6 | F6:16404 | 31122021\_RID\_2349\_6C.raw |  |  |  |  | 3.5933E5 | 1.5674E6 | 2 | 0 | 0 | 0 | 0 | 1 | 1 | 174 | 180 |  |  | PEAKS DB |
| total 3 peptides |  |  |  |  |  |  |  |  |  |  |  |  |  |  |  |  |  |  |  |  |  |  |  |  |  |  |  |  |  |
| --- | --- | --- | --- | --- | --- | --- | --- | --- | --- | --- | --- | --- | --- | --- | --- | --- | --- | --- | --- | --- | --- | --- | --- | --- | --- | --- | --- | --- | --- |

ABA60124.1

back to list

  

| Protein Coverage
| Supporting Peptides
|

Protein Coverage:

Supporting Peptides:

| Peptide | Uniq | -10lgP | Mass | Length | ppm | m/z | z | RT | Fraction | Scan | Source File | Area F2 | Area F1 | Area F3 | Area F4 | Area F5 | Area F6 | #Feature | #Feature F2 | #Feature F1 | #Feature F3 | #Feature F4 | #Feature F5 | #Feature F6 | Start | End | PTM | AScore | Found By |
| --- | --- | --- | --- | --- | --- | --- | --- | --- | --- | --- | --- | --- | --- | --- | --- | --- | --- | --- | --- | --- | --- | --- | --- | --- | --- | --- | --- | --- | --- |
| K.ALTMEGNQASWR.F | Y | 74.63 | 1362.6350 | 12 | 0.5 | 682.3251 | 2 | 34.48 | 5 | F5:15784 | 31122021\_RID\_2349\_5C.raw |  |  |  |  | 1.7409E5 | 1.0442E6 | 2 | 0 | 0 | 0 | 0 | 1 | 1 | 212 | 223 |  |  | PEAKS DB |
| K.ALTM(+15.99)EGNQASWR.F | Y | 47.13 | 1378.6299 | 12 | 0.6 | 690.3226 | 2 | 27.92 | 6 | F6:9991 | 31122021\_RID\_2349\_6C.raw |  |  |  |  |  | 8.9439E4 | 1 | 0 | 0 | 0 | 0 | 0 | 1 | 212 | 223 | Oxidation (M) | M4:Oxidation (M):1000.00 | PEAKS DB |
| K.QYFFETK.C | Y | 46.50 | 961.4545 | 7 | -0.2 | 481.7344 | 2 | 36.16 | 6 | F6:16404 | 31122021\_RID\_2349\_6C.raw |  |  |  |  | 3.5933E5 | 1.5674E6 | 2 | 0 | 0 | 0 | 0 | 1 | 1 | 174 | 180 |  |  | PEAKS DB |
| total 3 peptides |  |  |  |  |  |  |  |  |  |  |  |  |  |  |  |  |  |  |  |  |  |  |  |  |  |  |  |  |  |
| --- | --- | --- | --- | --- | --- | --- | --- | --- | --- | --- | --- | --- | --- | --- | --- | --- | --- | --- | --- | --- | --- | --- | --- | --- | --- | --- | --- | --- | --- |

JAB52817.1

back to list

  

| Protein Coverage
| Supporting Peptides
|

Protein Coverage:

Supporting Peptides:

| Peptide | Uniq | -10lgP | Mass | Length | ppm | m/z | z | RT | Fraction | Scan | Source File | Area F2 | Area F1 | Area F3 | Area F4 | Area F5 | Area F6 | #Feature | #Feature F2 | #Feature F1 | #Feature F3 | #Feature F4 | #Feature F5 | #Feature F6 | Start | End | PTM | AScore | Found By |
| --- | --- | --- | --- | --- | --- | --- | --- | --- | --- | --- | --- | --- | --- | --- | --- | --- | --- | --- | --- | --- | --- | --- | --- | --- | --- | --- | --- | --- | --- |
| K.ALTMEGNQASWR.F | Y | 74.63 | 1362.6350 | 12 | 0.5 | 682.3251 | 2 | 34.48 | 5 | F5:15784 | 31122021\_RID\_2349\_5C.raw |  |  |  |  | 1.7409E5 | 1.0442E6 | 2 | 0 | 0 | 0 | 0 | 1 | 1 | 213 | 224 |  |  | PEAKS DB |
| K.ALTM(+15.99)EGNQASWR.F | Y | 47.13 | 1378.6299 | 12 | 0.6 | 690.3226 | 2 | 27.92 | 6 | F6:9991 | 31122021\_RID\_2349\_6C.raw |  |  |  |  |  | 8.9439E4 | 1 | 0 | 0 | 0 | 0 | 0 | 1 | 213 | 224 | Oxidation (M) | M4:Oxidation (M):1000.00 | PEAKS DB |
| K.QYFFETK.C | Y | 46.50 | 961.4545 | 7 | -0.2 | 481.7344 | 2 | 36.16 | 6 | F6:16404 | 31122021\_RID\_2349\_6C.raw |  |  |  |  | 3.5933E5 | 1.5674E6 | 2 | 0 | 0 | 0 | 0 | 1 | 1 | 175 | 181 |  |  | PEAKS DB |
| total 3 peptides |  |  |  |  |  |  |  |  |  |  |  |  |  |  |  |  |  |  |  |  |  |  |  |  |  |  |  |  |  |
| --- | --- | --- | --- | --- | --- | --- | --- | --- | --- | --- | --- | --- | --- | --- | --- | --- | --- | --- | --- | --- | --- | --- | --- | --- | --- | --- | --- | --- | --- |

AAS94269.1

back to list

  

| Protein Coverage
| Supporting Peptides
|

Protein Coverage:

Supporting Peptides:

| Peptide | Uniq | -10lgP | Mass | Length | ppm | m/z | z | RT | Fraction | Scan | Source File | Area F2 | Area F1 | Area F3 | Area F4 | Area F5 | Area F6 | #Feature | #Feature F2 | #Feature F1 | #Feature F3 | #Feature F4 | #Feature F5 | #Feature F6 | Start | End | PTM | AScore | Found By |
| --- | --- | --- | --- | --- | --- | --- | --- | --- | --- | --- | --- | --- | --- | --- | --- | --- | --- | --- | --- | --- | --- | --- | --- | --- | --- | --- | --- | --- | --- |
| K.ALTMEGNQASWR.F | Y | 74.63 | 1362.6350 | 12 | 0.5 | 682.3251 | 2 | 34.48 | 5 | F5:15784 | 31122021\_RID\_2349\_5C.raw |  |  |  |  | 1.7409E5 | 1.0442E6 | 2 | 0 | 0 | 0 | 0 | 1 | 1 | 212 | 223 |  |  | PEAKS DB |
| K.ALTM(+15.99)EGNQASWR.F | Y | 47.13 | 1378.6299 | 12 | 0.6 | 690.3226 | 2 | 27.92 | 6 | F6:9991 | 31122021\_RID\_2349\_6C.raw |  |  |  |  |  | 8.9439E4 | 1 | 0 | 0 | 0 | 0 | 0 | 1 | 212 | 223 | Oxidation (M) | M4:Oxidation (M):1000.00 | PEAKS DB |
| K.QYFFETK.C | Y | 46.50 | 961.4545 | 7 | -0.2 | 481.7344 | 2 | 36.16 | 6 | F6:16404 | 31122021\_RID\_2349\_6C.raw |  |  |  |  | 3.5933E5 | 1.5674E6 | 2 | 0 | 0 | 0 | 0 | 1 | 1 | 174 | 180 |  |  | PEAKS DB |
| total 3 peptides |  |  |  |  |  |  |  |  |  |  |  |  |  |  |  |  |  |  |  |  |  |  |  |  |  |  |  |  |  |
| --- | --- | --- | --- | --- | --- | --- | --- | --- | --- | --- | --- | --- | --- | --- | --- | --- | --- | --- | --- | --- | --- | --- | --- | --- | --- | --- | --- | --- | --- |

JAA98018.1

back to list

  

| Protein Coverage
| Supporting Peptides
|

Protein Coverage:

Supporting Peptides:

| Peptide | Uniq | -10lgP | Mass | Length | ppm | m/z | z | RT | Fraction | Scan | Source File | Area F2 | Area F1 | Area F3 | Area F4 | Area F5 | Area F6 | #Feature | #Feature F2 | #Feature F1 | #Feature F3 | #Feature F4 | #Feature F5 | #Feature F6 | Start | End | PTM | AScore | Found By |
| --- | --- | --- | --- | --- | --- | --- | --- | --- | --- | --- | --- | --- | --- | --- | --- | --- | --- | --- | --- | --- | --- | --- | --- | --- | --- | --- | --- | --- | --- |
| K.ALTMEGNQASWR.F | Y | 74.63 | 1362.6350 | 12 | 0.5 | 682.3251 | 2 | 34.48 | 5 | F5:15784 | 31122021\_RID\_2349\_5C.raw |  |  |  |  | 1.7409E5 | 1.0442E6 | 2 | 0 | 0 | 0 | 0 | 1 | 1 | 210 | 221 |  |  | PEAKS DB |
| K.ALTM(+15.99)EGNQASWR.F | Y | 47.13 | 1378.6299 | 12 | 0.6 | 690.3226 | 2 | 27.92 | 6 | F6:9991 | 31122021\_RID\_2349\_6C.raw |  |  |  |  |  | 8.9439E4 | 1 | 0 | 0 | 0 | 0 | 0 | 1 | 210 | 221 | Oxidation (M) | M4:Oxidation (M):1000.00 | PEAKS DB |
| K.QYFFETK.C | Y | 46.50 | 961.4545 | 7 | -0.2 | 481.7344 | 2 | 36.16 | 6 | F6:16404 | 31122021\_RID\_2349\_6C.raw |  |  |  |  | 3.5933E5 | 1.5674E6 | 2 | 0 | 0 | 0 | 0 | 1 | 1 | 172 | 178 |  |  | PEAKS DB |
| total 3 peptides |  |  |  |  |  |  |  |  |  |  |  |  |  |  |  |  |  |  |  |  |  |  |  |  |  |  |  |  |  |
| --- | --- | --- | --- | --- | --- | --- | --- | --- | --- | --- | --- | --- | --- | --- | --- | --- | --- | --- | --- | --- | --- | --- | --- | --- | --- | --- | --- | --- | --- |

AAG30924.1

back to list

  

| Protein Coverage
| Supporting Peptides
|

Protein Coverage:

Supporting Peptides:

| Peptide | Uniq | -10lgP | Mass | Length | ppm | m/z | z | RT | Fraction | Scan | Source File | Area F2 | Area F1 | Area F3 | Area F4 | Area F5 | Area F6 | #Feature | #Feature F2 | #Feature F1 | #Feature F3 | #Feature F4 | #Feature F5 | #Feature F6 | Start | End | PTM | AScore | Found By |
| --- | --- | --- | --- | --- | --- | --- | --- | --- | --- | --- | --- | --- | --- | --- | --- | --- | --- | --- | --- | --- | --- | --- | --- | --- | --- | --- | --- | --- | --- |
| K.ALTMEGNQASWR.F | Y | 74.63 | 1362.6350 | 12 | 0.5 | 682.3251 | 2 | 34.48 | 5 | F5:15784 | 31122021\_RID\_2349\_5C.raw |  |  |  |  | 1.7409E5 | 1.0442E6 | 2 | 0 | 0 | 0 | 0 | 1 | 1 | 210 | 221 |  |  | PEAKS DB |
| K.ALTM(+15.99)EGNQASWR.F | Y | 47.13 | 1378.6299 | 12 | 0.6 | 690.3226 | 2 | 27.92 | 6 | F6:9991 | 31122021\_RID\_2349\_6C.raw |  |  |  |  |  | 8.9439E4 | 1 | 0 | 0 | 0 | 0 | 0 | 1 | 210 | 221 | Oxidation (M) | M4:Oxidation (M):1000.00 | PEAKS DB |
| K.QYFFETK.C | Y | 46.50 | 961.4545 | 7 | -0.2 | 481.7344 | 2 | 36.16 | 6 | F6:16404 | 31122021\_RID\_2349\_6C.raw |  |  |  |  | 3.5933E5 | 1.5674E6 | 2 | 0 | 0 | 0 | 0 | 1 | 1 | 172 | 178 |  |  | PEAKS DB |
| total 3 peptides |  |  |  |  |  |  |  |  |  |  |  |  |  |  |  |  |  |  |  |  |  |  |  |  |  |  |  |  |  |
| --- | --- | --- | --- | --- | --- | --- | --- | --- | --- | --- | --- | --- | --- | --- | --- | --- | --- | --- | --- | --- | --- | --- | --- | --- | --- | --- | --- | --- | --- |

JAA98019.1

back to list

  

| Protein Coverage
| Supporting Peptides
|

Protein Coverage:

Supporting Peptides:

| Peptide | Uniq | -10lgP | Mass | Length | ppm | m/z | z | RT | Fraction | Scan | Source File | Area F2 | Area F1 | Area F3 | Area F4 | Area F5 | Area F6 | #Feature | #Feature F2 | #Feature F1 | #Feature F3 | #Feature F4 | #Feature F5 | #Feature F6 | Start | End | PTM | AScore | Found By |
| --- | --- | --- | --- | --- | --- | --- | --- | --- | --- | --- | --- | --- | --- | --- | --- | --- | --- | --- | --- | --- | --- | --- | --- | --- | --- | --- | --- | --- | --- |
| K.ALTMEGNQASWR.F | Y | 74.63 | 1362.6350 | 12 | 0.5 | 682.3251 | 2 | 34.48 | 5 | F5:15784 | 31122021\_RID\_2349\_5C.raw |  |  |  |  | 1.7409E5 | 1.0442E6 | 2 | 0 | 0 | 0 | 0 | 1 | 1 | 210 | 221 |  |  | PEAKS DB |
| K.ALTM(+15.99)EGNQASWR.F | Y | 47.13 | 1378.6299 | 12 | 0.6 | 690.3226 | 2 | 27.92 | 6 | F6:9991 | 31122021\_RID\_2349\_6C.raw |  |  |  |  |  | 8.9439E4 | 1 | 0 | 0 | 0 | 0 | 0 | 1 | 210 | 221 | Oxidation (M) | M4:Oxidation (M):1000.00 | PEAKS DB |
| K.QYFFETK.C | Y | 46.50 | 961.4545 | 7 | -0.2 | 481.7344 | 2 | 36.16 | 6 | F6:16404 | 31122021\_RID\_2349\_6C.raw |  |  |  |  | 3.5933E5 | 1.5674E6 | 2 | 0 | 0 | 0 | 0 | 1 | 1 | 172 | 178 |  |  | PEAKS DB |
| total 3 peptides |  |  |  |  |  |  |  |  |  |  |  |  |  |  |  |  |  |  |  |  |  |  |  |  |  |  |  |  |  |
| --- | --- | --- | --- | --- | --- | --- | --- | --- | --- | --- | --- | --- | --- | --- | --- | --- | --- | --- | --- | --- | --- | --- | --- | --- | --- | --- | --- | --- | --- |

JAA94888.1

back to list

  

| Protein Coverage
| Supporting Peptides
|

Protein Coverage:

Supporting Peptides:

| Peptide | Uniq | -10lgP | Mass | Length | ppm | m/z | z | RT | Fraction | Scan | Source File | Area F2 | Area F1 | Area F3 | Area F4 | Area F5 | Area F6 | #Feature | #Feature F2 | #Feature F1 | #Feature F3 | #Feature F4 | #Feature F5 | #Feature F6 | Start | End | PTM | AScore | Found By |
| --- | --- | --- | --- | --- | --- | --- | --- | --- | --- | --- | --- | --- | --- | --- | --- | --- | --- | --- | --- | --- | --- | --- | --- | --- | --- | --- | --- | --- | --- |
| K.ALTMEGNQASWR.F | Y | 74.63 | 1362.6350 | 12 | 0.5 | 682.3251 | 2 | 34.48 | 5 | F5:15784 | 31122021\_RID\_2349\_5C.raw |  |  |  |  | 1.7409E5 | 1.0442E6 | 2 | 0 | 0 | 0 | 0 | 1 | 1 | 210 | 221 |  |  | PEAKS DB |
| K.ALTM(+15.99)EGNQASWR.F | Y | 47.13 | 1378.6299 | 12 | 0.6 | 690.3226 | 2 | 27.92 | 6 | F6:9991 | 31122021\_RID\_2349\_6C.raw |  |  |  |  |  | 8.9439E4 | 1 | 0 | 0 | 0 | 0 | 0 | 1 | 210 | 221 | Oxidation (M) | M4:Oxidation (M):1000.00 | PEAKS DB |
| K.QYFFETK.C | Y | 46.50 | 961.4545 | 7 | -0.2 | 481.7344 | 2 | 36.16 | 6 | F6:16404 | 31122021\_RID\_2349\_6C.raw |  |  |  |  | 3.5933E5 | 1.5674E6 | 2 | 0 | 0 | 0 | 0 | 1 | 1 | 172 | 178 |  |  | PEAKS DB |
| total 3 peptides |  |  |  |  |  |  |  |  |  |  |  |  |  |  |  |  |  |  |  |  |  |  |  |  |  |  |  |  |  |
| --- | --- | --- | --- | --- | --- | --- | --- | --- | --- | --- | --- | --- | --- | --- | --- | --- | --- | --- | --- | --- | --- | --- | --- | --- | --- | --- | --- | --- | --- |

AFJ49196.1

back to list

  

| Protein Coverage
| Supporting Peptides
|

Protein Coverage:

Supporting Peptides:

| Peptide | Uniq | -10lgP | Mass | Length | ppm | m/z | z | RT | Fraction | Scan | Source File | Area F2 | Area F1 | Area F3 | Area F4 | Area F5 | Area F6 | #Feature | #Feature F2 | #Feature F1 | #Feature F3 | #Feature F4 | #Feature F5 | #Feature F6 | Start | End | PTM | AScore | Found By |
| --- | --- | --- | --- | --- | --- | --- | --- | --- | --- | --- | --- | --- | --- | --- | --- | --- | --- | --- | --- | --- | --- | --- | --- | --- | --- | --- | --- | --- | --- |
| K.ALTMEGNQASWR.F | Y | 74.63 | 1362.6350 | 12 | 0.5 | 682.3251 | 2 | 34.48 | 5 | F5:15784 | 31122021\_RID\_2349\_5C.raw |  |  |  |  | 1.7409E5 | 1.0442E6 | 2 | 0 | 0 | 0 | 0 | 1 | 1 | 210 | 221 |  |  | PEAKS DB |
| K.ALTM(+15.99)EGNQASWR.F | Y | 47.13 | 1378.6299 | 12 | 0.6 | 690.3226 | 2 | 27.92 | 6 | F6:9991 | 31122021\_RID\_2349\_6C.raw |  |  |  |  |  | 8.9439E4 | 1 | 0 | 0 | 0 | 0 | 0 | 1 | 210 | 221 | Oxidation (M) | M4:Oxidation (M):1000.00 | PEAKS DB |
| K.QYFFETK.C | Y | 46.50 | 961.4545 | 7 | -0.2 | 481.7344 | 2 | 36.16 | 6 | F6:16404 | 31122021\_RID\_2349\_6C.raw |  |  |  |  | 3.5933E5 | 1.5674E6 | 2 | 0 | 0 | 0 | 0 | 1 | 1 | 172 | 178 |  |  | PEAKS DB |
| total 3 peptides |  |  |  |  |  |  |  |  |  |  |  |  |  |  |  |  |  |  |  |  |  |  |  |  |  |  |  |  |  |
| --- | --- | --- | --- | --- | --- | --- | --- | --- | --- | --- | --- | --- | --- | --- | --- | --- | --- | --- | --- | --- | --- | --- | --- | --- | --- | --- | --- | --- | --- |

JAA97946.1

back to list

  

| Protein Coverage
| Supporting Peptides
|

Protein Coverage:

Supporting Peptides:

| Peptide | Uniq | -10lgP | Mass | Length | ppm | m/z | z | RT | Fraction | Scan | Source File | Area F2 | Area F1 | Area F3 | Area F4 | Area F5 | Area F6 | #Feature | #Feature F2 | #Feature F1 | #Feature F3 | #Feature F4 | #Feature F5 | #Feature F6 | Start | End | PTM | AScore | Found By |
| --- | --- | --- | --- | --- | --- | --- | --- | --- | --- | --- | --- | --- | --- | --- | --- | --- | --- | --- | --- | --- | --- | --- | --- | --- | --- | --- | --- | --- | --- |
| K.ALTMEGNQASWR.F | Y | 74.63 | 1362.6350 | 12 | 0.5 | 682.3251 | 2 | 34.48 | 5 | F5:15784 | 31122021\_RID\_2349\_5C.raw |  |  |  |  | 1.7409E5 | 1.0442E6 | 2 | 0 | 0 | 0 | 0 | 1 | 1 | 210 | 221 |  |  | PEAKS DB |
| K.ALTM(+15.99)EGNQASWR.F | Y | 47.13 | 1378.6299 | 12 | 0.6 | 690.3226 | 2 | 27.92 | 6 | F6:9991 | 31122021\_RID\_2349\_6C.raw |  |  |  |  |  | 8.9439E4 | 1 | 0 | 0 | 0 | 0 | 0 | 1 | 210 | 221 | Oxidation (M) | M4:Oxidation (M):1000.00 | PEAKS DB |
| K.QYFFETK.C | Y | 46.50 | 961.4545 | 7 | -0.2 | 481.7344 | 2 | 36.16 | 6 | F6:16404 | 31122021\_RID\_2349\_6C.raw |  |  |  |  | 3.5933E5 | 1.5674E6 | 2 | 0 | 0 | 0 | 0 | 1 | 1 | 172 | 178 |  |  | PEAKS DB |
| total 3 peptides |  |  |  |  |  |  |  |  |  |  |  |  |  |  |  |  |  |  |  |  |  |  |  |  |  |  |  |  |  |
| --- | --- | --- | --- | --- | --- | --- | --- | --- | --- | --- | --- | --- | --- | --- | --- | --- | --- | --- | --- | --- | --- | --- | --- | --- | --- | --- | --- | --- | --- |

AAV64846.1

back to list

  

| Protein Coverage
| Supporting Peptides
|

Protein Coverage:

Supporting Peptides:

| Peptide | Uniq | -10lgP | Mass | Length | ppm | m/z | z | RT | Fraction | Scan | Source File | Area F2 | Area F1 | Area F3 | Area F4 | Area F5 | Area F6 | #Feature | #Feature F2 | #Feature F1 | #Feature F3 | #Feature F4 | #Feature F5 | #Feature F6 | Start | End | PTM | AScore | Found By |
| --- | --- | --- | --- | --- | --- | --- | --- | --- | --- | --- | --- | --- | --- | --- | --- | --- | --- | --- | --- | --- | --- | --- | --- | --- | --- | --- | --- | --- | --- |
| R.ALTMEGNQASWR.F | Y | 74.63 | 1362.6350 | 12 | 0.5 | 682.3251 | 2 | 34.48 | 5 | F5:15784 | 31122021\_RID\_2349\_5C.raw |  |  |  |  | 1.7409E5 | 1.0442E6 | 2 | 0 | 0 | 0 | 0 | 1 | 1 | 213 | 224 |  |  | PEAKS DB |
| R.ALTM(+15.99)EGNQASWR.F | Y | 47.13 | 1378.6299 | 12 | 0.6 | 690.3226 | 2 | 27.92 | 6 | F6:9991 | 31122021\_RID\_2349\_6C.raw |  |  |  |  |  | 8.9439E4 | 1 | 0 | 0 | 0 | 0 | 0 | 1 | 213 | 224 | Oxidation (M) | M4:Oxidation (M):1000.00 | PEAKS DB |
| R.QYFFETK.C | Y | 46.50 | 961.4545 | 7 | -0.2 | 481.7344 | 2 | 36.16 | 6 | F6:16404 | 31122021\_RID\_2349\_6C.raw |  |  |  |  | 3.5933E5 | 1.5674E6 | 2 | 0 | 0 | 0 | 0 | 1 | 1 | 175 | 181 |  |  | PEAKS DB |
| total 3 peptides |  |  |  |  |  |  |  |  |  |  |  |  |  |  |  |  |  |  |  |  |  |  |  |  |  |  |  |  |  |
| --- | --- | --- | --- | --- | --- | --- | --- | --- | --- | --- | --- | --- | --- | --- | --- | --- | --- | --- | --- | --- | --- | --- | --- | --- | --- | --- | --- | --- | --- |

A59218

back to list

  

| Protein Coverage
| Supporting Peptides
|

Protein Coverage:

Supporting Peptides:

| Peptide | Uniq | -10lgP | Mass | Length | ppm | m/z | z | RT | Fraction | Scan | Source File | Area F2 | Area F1 | Area F3 | Area F4 | Area F5 | Area F6 | #Feature | #Feature F2 | #Feature F1 | #Feature F3 | #Feature F4 | #Feature F5 | #Feature F6 | Start | End | PTM | AScore | Found By |
| --- | --- | --- | --- | --- | --- | --- | --- | --- | --- | --- | --- | --- | --- | --- | --- | --- | --- | --- | --- | --- | --- | --- | --- | --- | --- | --- | --- | --- | --- |
| K.ALTMEGNQASWR.F | Y | 74.63 | 1362.6350 | 12 | 0.5 | 682.3251 | 2 | 34.48 | 5 | F5:15784 | 31122021\_RID\_2349\_5C.raw |  |  |  |  | 1.7409E5 | 1.0442E6 | 2 | 0 | 0 | 0 | 0 | 1 | 1 | 217 | 228 |  |  | PEAKS DB |
| K.ALTM(+15.99)EGNQASWR.F | Y | 47.13 | 1378.6299 | 12 | 0.6 | 690.3226 | 2 | 27.92 | 6 | F6:9991 | 31122021\_RID\_2349\_6C.raw |  |  |  |  |  | 8.9439E4 | 1 | 0 | 0 | 0 | 0 | 0 | 1 | 217 | 228 | Oxidation (M) | M4:Oxidation (M):1000.00 | PEAKS DB |
| K.QYFFETK.C | Y | 46.50 | 961.4545 | 7 | -0.2 | 481.7344 | 2 | 36.16 | 6 | F6:16404 | 31122021\_RID\_2349\_6C.raw |  |  |  |  | 3.5933E5 | 1.5674E6 | 2 | 0 | 0 | 0 | 0 | 1 | 1 | 179 | 185 |  |  | PEAKS DB |
| total 3 peptides |  |  |  |  |  |  |  |  |  |  |  |  |  |  |  |  |  |  |  |  |  |  |  |  |  |  |  |  |  |
| --- | --- | --- | --- | --- | --- | --- | --- | --- | --- | --- | --- | --- | --- | --- | --- | --- | --- | --- | --- | --- | --- | --- | --- | --- | --- | --- | --- | --- | --- |

AAQ15159.1

back to list

  

| Protein Coverage
| Supporting Peptides
|

Protein Coverage:

Supporting Peptides:

| Peptide | Uniq | -10lgP | Mass | Length | ppm | m/z | z | RT | Fraction | Scan | Source File | Area F2 | Area F1 | Area F3 | Area F4 | Area F5 | Area F6 | #Feature | #Feature F2 | #Feature F1 | #Feature F3 | #Feature F4 | #Feature F5 | #Feature F6 | Start | End | PTM | AScore | Found By |
| --- | --- | --- | --- | --- | --- | --- | --- | --- | --- | --- | --- | --- | --- | --- | --- | --- | --- | --- | --- | --- | --- | --- | --- | --- | --- | --- | --- | --- | --- |
| K.LQWSDGTELK.Y | N | 71.99 | 1175.5823 | 10 | 0.5 | 588.7987 | 2 | 37.04 | 6 | F6:16834 | 31122021\_RID\_2349\_6C.raw |  |  |  |  |  | 1.8205E7 | 2 | 0 | 0 | 0 | 0 | 0 | 2 | 100 | 109 |  |  | PEAKS DB |
| K.YNAWSAESEC(+57.02)ITSK.S | Y | 51.63 | 1644.7090 | 14 | 0.7 | 823.3623 | 2 | 35.57 | 6 | F6:15720 | 31122021\_RID\_2349\_6C.raw |  |  |  |  |  | 1.0397E6 | 1 | 0 | 0 | 0 | 0 | 0 | 1 | 110 | 123 | Carbamidomethylation | C10:Carbamidomethylation:1000.00 | PEAKS DB |
| total 2 peptides |  |  |  |  |  |  |  |  |  |  |  |  |  |  |  |  |  |  |  |  |  |  |  |  |  |  |  |  |  |
| --- | --- | --- | --- | --- | --- | --- | --- | --- | --- | --- | --- | --- | --- | --- | --- | --- | --- | --- | --- | --- | --- | --- | --- | --- | --- | --- | --- | --- | --- |

AAQ15158.1

back to list

  

| Protein Coverage
| Supporting Peptides
|

Protein Coverage:

Supporting Peptides:

| Peptide | Uniq | -10lgP | Mass | Length | ppm | m/z | z | RT | Fraction | Scan | Source File | Area F2 | Area F1 | Area F3 | Area F4 | Area F5 | Area F6 | #Feature | #Feature F2 | #Feature F1 | #Feature F3 | #Feature F4 | #Feature F5 | #Feature F6 | Start | End | PTM | AScore | Found By |
| --- | --- | --- | --- | --- | --- | --- | --- | --- | --- | --- | --- | --- | --- | --- | --- | --- | --- | --- | --- | --- | --- | --- | --- | --- | --- | --- | --- | --- | --- |
| K.LQWSDGTELK.Y | N | 71.99 | 1175.5823 | 10 | 0.5 | 588.7987 | 2 | 37.04 | 6 | F6:16834 | 31122021\_RID\_2349\_6C.raw |  |  |  |  |  | 1.8205E7 | 2 | 0 | 0 | 0 | 0 | 0 | 2 | 100 | 109 |  |  | PEAKS DB |
| K.YNAWSAESEC(+57.02)ITSK.S | Y | 51.63 | 1644.7090 | 14 | 0.7 | 823.3623 | 2 | 35.57 | 6 | F6:15720 | 31122021\_RID\_2349\_6C.raw |  |  |  |  |  | 1.0397E6 | 1 | 0 | 0 | 0 | 0 | 0 | 1 | 110 | 123 | Carbamidomethylation | C10:Carbamidomethylation:1000.00 | PEAKS DB |
| total 2 peptides |  |  |  |  |  |  |  |  |  |  |  |  |  |  |  |  |  |  |  |  |  |  |  |  |  |  |  |  |  |
| --- | --- | --- | --- | --- | --- | --- | --- | --- | --- | --- | --- | --- | --- | --- | --- | --- | --- | --- | --- | --- | --- | --- | --- | --- | --- | --- | --- | --- | --- |

Q71RQ8.1

back to list

  

| Protein Coverage
| Supporting Peptides
|

Protein Coverage:

Supporting Peptides:

| Peptide | Uniq | -10lgP | Mass | Length | ppm | m/z | z | RT | Fraction | Scan | Source File | Area F2 | Area F1 | Area F3 | Area F4 | Area F5 | Area F6 | #Feature | #Feature F2 | #Feature F1 | #Feature F3 | #Feature F4 | #Feature F5 | #Feature F6 | Start | End | PTM | AScore | Found By |
| --- | --- | --- | --- | --- | --- | --- | --- | --- | --- | --- | --- | --- | --- | --- | --- | --- | --- | --- | --- | --- | --- | --- | --- | --- | --- | --- | --- | --- | --- |
| K.LQWSDGTELK.Y | N | 71.99 | 1175.5823 | 10 | 0.5 | 588.7987 | 2 | 37.04 | 6 | F6:16834 | 31122021\_RID\_2349\_6C.raw |  |  |  |  |  | 1.8205E7 | 2 | 0 | 0 | 0 | 0 | 0 | 2 | 100 | 109 |  |  | PEAKS DB |
| K.YNAWSAESEC(+57.02)ITSK.S | Y | 51.63 | 1644.7090 | 14 | 0.7 | 823.3623 | 2 | 35.57 | 6 | F6:15720 | 31122021\_RID\_2349\_6C.raw |  |  |  |  |  | 1.0397E6 | 1 | 0 | 0 | 0 | 0 | 0 | 1 | 110 | 123 | Carbamidomethylation | C10:Carbamidomethylation:1000.00 | PEAKS DB |
| total 2 peptides |  |  |  |  |  |  |  |  |  |  |  |  |  |  |  |  |  |  |  |  |  |  |  |  |  |  |  |  |  |
| --- | --- | --- | --- | --- | --- | --- | --- | --- | --- | --- | --- | --- | --- | --- | --- | --- | --- | --- | --- | --- | --- | --- | --- | --- | --- | --- | --- | --- | --- |

Q71RQ9.1

back to list

  

| Protein Coverage
| Supporting Peptides
|

Protein Coverage:

Supporting Peptides:

| Peptide | Uniq | -10lgP | Mass | Length | ppm | m/z | z | RT | Fraction | Scan | Source File | Area F2 | Area F1 | Area F3 | Area F4 | Area F5 | Area F6 | #Feature | #Feature F2 | #Feature F1 | #Feature F3 | #Feature F4 | #Feature F5 | #Feature F6 | Start | End | PTM | AScore | Found By |
| --- | --- | --- | --- | --- | --- | --- | --- | --- | --- | --- | --- | --- | --- | --- | --- | --- | --- | --- | --- | --- | --- | --- | --- | --- | --- | --- | --- | --- | --- |
| K.LQWSDGTELK.Y | N | 71.99 | 1175.5823 | 10 | 0.5 | 588.7987 | 2 | 37.04 | 6 | F6:16834 | 31122021\_RID\_2349\_6C.raw |  |  |  |  |  | 1.8205E7 | 2 | 0 | 0 | 0 | 0 | 0 | 2 | 100 | 109 |  |  | PEAKS DB |
| K.YNAWSAESEC(+57.02)ITSK.S | Y | 51.63 | 1644.7090 | 14 | 0.7 | 823.3623 | 2 | 35.57 | 6 | F6:15720 | 31122021\_RID\_2349\_6C.raw |  |  |  |  |  | 1.0397E6 | 1 | 0 | 0 | 0 | 0 | 0 | 1 | 110 | 123 | Carbamidomethylation | C10:Carbamidomethylation:1000.00 | PEAKS DB |
| total 2 peptides |  |  |  |  |  |  |  |  |  |  |  |  |  |  |  |  |  |  |  |  |  |  |  |  |  |  |  |  |  |
| --- | --- | --- | --- | --- | --- | --- | --- | --- | --- | --- | --- | --- | --- | --- | --- | --- | --- | --- | --- | --- | --- | --- | --- | --- | --- | --- | --- | --- | --- |

XP\_015675625.1

back to list

  

| Protein Coverage
| Supporting Peptides
|

Protein Coverage:

Supporting Peptides:

| Peptide | Uniq | -10lgP | Mass | Length | ppm | m/z | z | RT | Fraction | Scan | Source File | Area F2 | Area F1 | Area F3 | Area F4 | Area F5 | Area F6 | #Feature | #Feature F2 | #Feature F1 | #Feature F3 | #Feature F4 | #Feature F5 | #Feature F6 | Start | End | PTM | AScore | Found By |
| --- | --- | --- | --- | --- | --- | --- | --- | --- | --- | --- | --- | --- | --- | --- | --- | --- | --- | --- | --- | --- | --- | --- | --- | --- | --- | --- | --- | --- | --- |
| K.VILTNLDAMAFNK.F | Y | 61.50 | 1448.7698 | 13 | 0.7 | 725.3927 | 2 | 52.05 | 2 | F2:17625 | 30122021\_RID\_2349\_1C.raw | 3.0245E4 | 8.053E4 |  |  |  |  | 2 | 1 | 1 | 0 | 0 | 0 | 0 | 116 | 128 |  |  | PEAKS DB |
| K.VILTNLDAM(+15.99)AFNK.F | Y | 46.54 | 1464.7646 | 13 | 1.1 | 733.3904 | 2 | 45.59 | 2 | F2:15561 | 30122021\_RID\_2349\_1C.raw |  | 6.2887E4 |  |  |  |  | 1 | 0 | 1 | 0 | 0 | 0 | 0 | 116 | 128 | Oxidation (M) | M9:Oxidation (M):1000.00 | PEAKS DB |
| total 2 peptides |  |  |  |  |  |  |  |  |  |  |  |  |  |  |  |  |  |  |  |  |  |  |  |  |  |  |  |  |  |
| --- | --- | --- | --- | --- | --- | --- | --- | --- | --- | --- | --- | --- | --- | --- | --- | --- | --- | --- | --- | --- | --- | --- | --- | --- | --- | --- | --- | --- | --- |

XP\_032068596.1

back to list

  

| Protein Coverage
| Supporting Peptides
|

Protein Coverage:

Supporting Peptides:

| Peptide | Uniq | -10lgP | Mass | Length | ppm | m/z | z | RT | Fraction | Scan | Source File | Area F2 | Area F1 | Area F3 | Area F4 | Area F5 | Area F6 | #Feature | #Feature F2 | #Feature F1 | #Feature F3 | #Feature F4 | #Feature F5 | #Feature F6 | Start | End | PTM | AScore | Found By |
| --- | --- | --- | --- | --- | --- | --- | --- | --- | --- | --- | --- | --- | --- | --- | --- | --- | --- | --- | --- | --- | --- | --- | --- | --- | --- | --- | --- | --- | --- |
| K.VILTNLDAMAFNK.F | Y | 61.50 | 1448.7698 | 13 | 0.7 | 725.3927 | 2 | 52.05 | 2 | F2:17625 | 30122021\_RID\_2349\_1C.raw | 3.0245E4 | 8.053E4 |  |  |  |  | 2 | 1 | 1 | 0 | 0 | 0 | 0 | 210 | 222 |  |  | PEAKS DB |
| K.VILTNLDAM(+15.99)AFNK.F | Y | 46.54 | 1464.7646 | 13 | 1.1 | 733.3904 | 2 | 45.59 | 2 | F2:15561 | 30122021\_RID\_2349\_1C.raw |  | 6.2887E4 |  |  |  |  | 1 | 0 | 1 | 0 | 0 | 0 | 0 | 210 | 222 | Oxidation (M) | M9:Oxidation (M):1000.00 | PEAKS DB |
| total 2 peptides |  |  |  |  |  |  |  |  |  |  |  |  |  |  |  |  |  |  |  |  |  |  |  |  |  |  |  |  |  |
| --- | --- | --- | --- | --- | --- | --- | --- | --- | --- | --- | --- | --- | --- | --- | --- | --- | --- | --- | --- | --- | --- | --- | --- | --- | --- | --- | --- | --- | --- |

XP\_025021294.1

back to list

  

| Protein Coverage
| Supporting Peptides
|

Protein Coverage:

Supporting Peptides:

| Peptide | Uniq | -10lgP | Mass | Length | ppm | m/z | z | RT | Fraction | Scan | Source File | Area F2 | Area F1 | Area F3 | Area F4 | Area F5 | Area F6 | #Feature | #Feature F2 | #Feature F1 | #Feature F3 | #Feature F4 | #Feature F5 | #Feature F6 | Start | End | PTM | AScore | Found By |
| --- | --- | --- | --- | --- | --- | --- | --- | --- | --- | --- | --- | --- | --- | --- | --- | --- | --- | --- | --- | --- | --- | --- | --- | --- | --- | --- | --- | --- | --- |
| K.VILTNLDAMAFNK.F | Y | 61.50 | 1448.7698 | 13 | 0.7 | 725.3927 | 2 | 52.05 | 2 | F2:17625 | 30122021\_RID\_2349\_1C.raw | 3.0245E4 | 8.053E4 |  |  |  |  | 2 | 1 | 1 | 0 | 0 | 0 | 0 | 210 | 222 |  |  | PEAKS DB |
| K.VILTNLDAM(+15.99)AFNK.F | Y | 46.54 | 1464.7646 | 13 | 1.1 | 733.3904 | 2 | 45.59 | 2 | F2:15561 | 30122021\_RID\_2349\_1C.raw |  | 6.2887E4 |  |  |  |  | 1 | 0 | 1 | 0 | 0 | 0 | 0 | 210 | 222 | Oxidation (M) | M9:Oxidation (M):1000.00 | PEAKS DB |
| total 2 peptides |  |  |  |  |  |  |  |  |  |  |  |  |  |  |  |  |  |  |  |  |  |  |  |  |  |  |  |  |  |
| --- | --- | --- | --- | --- | --- | --- | --- | --- | --- | --- | --- | --- | --- | --- | --- | --- | --- | --- | --- | --- | --- | --- | --- | --- | --- | --- | --- | --- | --- |

XP\_015687424.1

back to list

  

| Protein Coverage
| Supporting Peptides
|

Protein Coverage:

Supporting Peptides:

| Peptide | Uniq | -10lgP | Mass | Length | ppm | m/z | z | RT | Fraction | Scan | Source File | Area F2 | Area F1 | Area F3 | Area F4 | Area F5 | Area F6 | #Feature | #Feature F2 | #Feature F1 | #Feature F3 | #Feature F4 | #Feature F5 | #Feature F6 | Start | End | PTM | AScore | Found By |
| --- | --- | --- | --- | --- | --- | --- | --- | --- | --- | --- | --- | --- | --- | --- | --- | --- | --- | --- | --- | --- | --- | --- | --- | --- | --- | --- | --- | --- | --- |
| K.AYIVNLFK.N | Y | 57.70 | 966.5538 | 8 | -0.3 | 484.2841 | 2 | 47.92 | 1 | F1:27265 | 28122021\_RID\_2349\_2C.raw | 8.1966E4 |  |  |  |  |  | 1 | 1 | 0 | 0 | 0 | 0 | 0 | 688 | 695 |  |  | PEAKS DB |
| K.QAEYALDAAVK.L | Y | 47.96 | 1177.5979 | 11 | 0.0 | 589.8062 | 2 | 33.00 | 1 | F1:15450 | 28122021\_RID\_2349\_2C.raw | 2.801E4 |  |  |  |  |  | 1 | 1 | 0 | 0 | 0 | 0 | 0 | 263 | 273 |  |  | PEAKS DB |
| R.ASLINNAFQLVSAGK.L | Y | 44.22 | 1531.8358 | 15 | 1.1 | 766.9260 | 2 | 55.07 | 1 | F1:32635 | 28122021\_RID\_2349\_2C.raw | 7.9015E4 |  |  |  |  |  | 1 | 1 | 0 | 0 | 0 | 0 | 0 | 624 | 638 |  |  | PEAKS DB |
| total 3 peptides |  |  |  |  |  |  |  |  |  |  |  |  |  |  |  |  |  |  |  |  |  |  |  |  |  |  |  |  |  |
| --- | --- | --- | --- | --- | --- | --- | --- | --- | --- | --- | --- | --- | --- | --- | --- | --- | --- | --- | --- | --- | --- | --- | --- | --- | --- | --- | --- | --- | --- |

JAI13418.1

back to list

  

| Protein Coverage
| Supporting Peptides
|

Protein Coverage:

Supporting Peptides:

| Peptide | Uniq | -10lgP | Mass | Length | ppm | m/z | z | RT | Fraction | Scan | Source File | Area F2 | Area F1 | Area F3 | Area F4 | Area F5 | Area F6 | #Feature | #Feature F2 | #Feature F1 | #Feature F3 | #Feature F4 | #Feature F5 | #Feature F6 | Start | End | PTM | AScore | Found By |
| --- | --- | --- | --- | --- | --- | --- | --- | --- | --- | --- | --- | --- | --- | --- | --- | --- | --- | --- | --- | --- | --- | --- | --- | --- | --- | --- | --- | --- | --- |
| K.AYIVNLFK.N | Y | 57.70 | 966.5538 | 8 | -0.3 | 484.2841 | 2 | 47.92 | 1 | F1:27265 | 28122021\_RID\_2349\_2C.raw | 8.1966E4 |  |  |  |  |  | 1 | 1 | 0 | 0 | 0 | 0 | 0 | 696 | 703 |  |  | PEAKS DB |
| K.QAEYALDAAVK.L | Y | 47.96 | 1177.5979 | 11 | 0.0 | 589.8062 | 2 | 33.00 | 1 | F1:15450 | 28122021\_RID\_2349\_2C.raw | 2.801E4 |  |  |  |  |  | 1 | 1 | 0 | 0 | 0 | 0 | 0 | 271 | 281 |  |  | PEAKS DB |
| R.ASLINNAFQLVSAGK.L | Y | 44.22 | 1531.8358 | 15 | 1.1 | 766.9260 | 2 | 55.07 | 1 | F1:32635 | 28122021\_RID\_2349\_2C.raw | 7.9015E4 |  |  |  |  |  | 1 | 1 | 0 | 0 | 0 | 0 | 0 | 632 | 646 |  |  | PEAKS DB |
| total 3 peptides |  |  |  |  |  |  |  |  |  |  |  |  |  |  |  |  |  |  |  |  |  |  |  |  |  |  |  |  |  |
| --- | --- | --- | --- | --- | --- | --- | --- | --- | --- | --- | --- | --- | --- | --- | --- | --- | --- | --- | --- | --- | --- | --- | --- | --- | --- | --- | --- | --- | --- |

JAG44871.1

back to list

  

| Protein Coverage
| Supporting Peptides
|

Protein Coverage:

Supporting Peptides:

| Peptide | Uniq | -10lgP | Mass | Length | ppm | m/z | z | RT | Fraction | Scan | Source File | Area F2 | Area F1 | Area F3 | Area F4 | Area F5 | Area F6 | #Feature | #Feature F2 | #Feature F1 | #Feature F3 | #Feature F4 | #Feature F5 | #Feature F6 | Start | End | PTM | AScore | Found By |
| --- | --- | --- | --- | --- | --- | --- | --- | --- | --- | --- | --- | --- | --- | --- | --- | --- | --- | --- | --- | --- | --- | --- | --- | --- | --- | --- | --- | --- | --- |
| K.AYIVNLFK.N | Y | 57.70 | 966.5538 | 8 | -0.3 | 484.2841 | 2 | 47.92 | 1 | F1:27265 | 28122021\_RID\_2349\_2C.raw | 8.1966E4 |  |  |  |  |  | 1 | 1 | 0 | 0 | 0 | 0 | 0 | 696 | 703 |  |  | PEAKS DB |
| K.QAEYALDAAVK.L | Y | 47.96 | 1177.5979 | 11 | 0.0 | 589.8062 | 2 | 33.00 | 1 | F1:15450 | 28122021\_RID\_2349\_2C.raw | 2.801E4 |  |  |  |  |  | 1 | 1 | 0 | 0 | 0 | 0 | 0 | 271 | 281 |  |  | PEAKS DB |
| R.ASLINNAFQLVSAGK.L | Y | 44.22 | 1531.8358 | 15 | 1.1 | 766.9260 | 2 | 55.07 | 1 | F1:32635 | 28122021\_RID\_2349\_2C.raw | 7.9015E4 |  |  |  |  |  | 1 | 1 | 0 | 0 | 0 | 0 | 0 | 632 | 646 |  |  | PEAKS DB |
| total 3 peptides |  |  |  |  |  |  |  |  |  |  |  |  |  |  |  |  |  |  |  |  |  |  |  |  |  |  |  |  |  |
| --- | --- | --- | --- | --- | --- | --- | --- | --- | --- | --- | --- | --- | --- | --- | --- | --- | --- | --- | --- | --- | --- | --- | --- | --- | --- | --- | --- | --- | --- |

JAG46882.1

back to list

  

| Protein Coverage
| Supporting Peptides
|

Protein Coverage:

Supporting Peptides:

| Peptide | Uniq | -10lgP | Mass | Length | ppm | m/z | z | RT | Fraction | Scan | Source File | Area F2 | Area F1 | Area F3 | Area F4 | Area F5 | Area F6 | #Feature | #Feature F2 | #Feature F1 | #Feature F3 | #Feature F4 | #Feature F5 | #Feature F6 | Start | End | PTM | AScore | Found By |
| --- | --- | --- | --- | --- | --- | --- | --- | --- | --- | --- | --- | --- | --- | --- | --- | --- | --- | --- | --- | --- | --- | --- | --- | --- | --- | --- | --- | --- | --- |
| K.AYIVNLFK.N | Y | 57.70 | 966.5538 | 8 | -0.3 | 484.2841 | 2 | 47.92 | 1 | F1:27265 | 28122021\_RID\_2349\_2C.raw | 8.1966E4 |  |  |  |  |  | 1 | 1 | 0 | 0 | 0 | 0 | 0 | 696 | 703 |  |  | PEAKS DB |
| K.QAEYALDAAVK.L | Y | 47.96 | 1177.5979 | 11 | 0.0 | 589.8062 | 2 | 33.00 | 1 | F1:15450 | 28122021\_RID\_2349\_2C.raw | 2.801E4 |  |  |  |  |  | 1 | 1 | 0 | 0 | 0 | 0 | 0 | 271 | 281 |  |  | PEAKS DB |
| R.ASLINNAFQLVSAGK.L | Y | 44.22 | 1531.8358 | 15 | 1.1 | 766.9260 | 2 | 55.07 | 1 | F1:32635 | 28122021\_RID\_2349\_2C.raw | 7.9015E4 |  |  |  |  |  | 1 | 1 | 0 | 0 | 0 | 0 | 0 | 632 | 646 |  |  | PEAKS DB |
| total 3 peptides |  |  |  |  |  |  |  |  |  |  |  |  |  |  |  |  |  |  |  |  |  |  |  |  |  |  |  |  |  |
| --- | --- | --- | --- | --- | --- | --- | --- | --- | --- | --- | --- | --- | --- | --- | --- | --- | --- | --- | --- | --- | --- | --- | --- | --- | --- | --- | --- | --- | --- |

JAA97127.1

back to list

  

| Protein Coverage
| Supporting Peptides
|

Protein Coverage:

Supporting Peptides:

| Peptide | Uniq | -10lgP | Mass | Length | ppm | m/z | z | RT | Fraction | Scan | Source File | Area F2 | Area F1 | Area F3 | Area F4 | Area F5 | Area F6 | #Feature | #Feature F2 | #Feature F1 | #Feature F3 | #Feature F4 | #Feature F5 | #Feature F6 | Start | End | PTM | AScore | Found By |
| --- | --- | --- | --- | --- | --- | --- | --- | --- | --- | --- | --- | --- | --- | --- | --- | --- | --- | --- | --- | --- | --- | --- | --- | --- | --- | --- | --- | --- | --- |
| K.AYIVNLFK.N | Y | 57.70 | 966.5538 | 8 | -0.3 | 484.2841 | 2 | 47.92 | 1 | F1:27265 | 28122021\_RID\_2349\_2C.raw | 8.1966E4 |  |  |  |  |  | 1 | 1 | 0 | 0 | 0 | 0 | 0 | 696 | 703 |  |  | PEAKS DB |
| K.QAEYALDAAVK.L | Y | 47.96 | 1177.5979 | 11 | 0.0 | 589.8062 | 2 | 33.00 | 1 | F1:15450 | 28122021\_RID\_2349\_2C.raw | 2.801E4 |  |  |  |  |  | 1 | 1 | 0 | 0 | 0 | 0 | 0 | 271 | 281 |  |  | PEAKS DB |
| R.ASLINNAFQLVSAGK.L | Y | 44.22 | 1531.8358 | 15 | 1.1 | 766.9260 | 2 | 55.07 | 1 | F1:32635 | 28122021\_RID\_2349\_2C.raw | 7.9015E4 |  |  |  |  |  | 1 | 1 | 0 | 0 | 0 | 0 | 0 | 632 | 646 |  |  | PEAKS DB |
| total 3 peptides |  |  |  |  |  |  |  |  |  |  |  |  |  |  |  |  |  |  |  |  |  |  |  |  |  |  |  |  |  |
| --- | --- | --- | --- | --- | --- | --- | --- | --- | --- | --- | --- | --- | --- | --- | --- | --- | --- | --- | --- | --- | --- | --- | --- | --- | --- | --- | --- | --- | --- |

CAA01524.1

back to list

  

| Protein Coverage
| Supporting Peptides
|

Protein Coverage:

Supporting Peptides:

| Peptide | Uniq | -10lgP | Mass | Length | ppm | m/z | z | RT | Fraction | Scan | Source File | Area F2 | Area F1 | Area F3 | Area F4 | Area F5 | Area F6 | #Feature | #Feature F2 | #Feature F1 | #Feature F3 | #Feature F4 | #Feature F5 | #Feature F6 | Start | End | PTM | AScore | Found By |
| --- | --- | --- | --- | --- | --- | --- | --- | --- | --- | --- | --- | --- | --- | --- | --- | --- | --- | --- | --- | --- | --- | --- | --- | --- | --- | --- | --- | --- | --- |
| K.VADYTDWIK.S | Y | 67.35 | 1109.5393 | 9 | 1.2 | 555.7776 | 2 | 44.97 | 1 | F1:25084 | 28122021\_RID\_2349\_2C.raw | 3.8579E7 |  |  |  |  |  | 1 | 1 | 0 | 0 | 0 | 0 | 0 | 214 | 222 |  |  | PEAKS DB |
| K.WDKDIMLIK.L | N | 56.01 | 1160.6263 | 9 | 0.9 | 581.3210 | 2 | 40.39 | 1 | F1:21507 | 28122021\_RID\_2349\_2C.raw | 5.1473E6 |  |  |  |  |  | 2 | 2 | 0 | 0 | 0 | 0 | 0 | 84 | 92 |  |  | PEAKS DB |
| K.WDKDIM(+15.99)LIK.L | N | 47.61 | 1176.6212 | 9 | 0.0 | 589.3179 | 2 | 35.74 | 1 | F1:17512 | 28122021\_RID\_2349\_2C.raw | 7.9357E5 |  |  |  |  |  | 1 | 1 | 0 | 0 | 0 | 0 | 0 | 84 | 92 | Oxidation (M) | M6:Oxidation (M):1000.00 | PEAKS DB |
| total 3 peptides |  |  |  |  |  |  |  |  |  |  |  |  |  |  |  |  |  |  |  |  |  |  |  |  |  |  |  |  |  |
| --- | --- | --- | --- | --- | --- | --- | --- | --- | --- | --- | --- | --- | --- | --- | --- | --- | --- | --- | --- | --- | --- | --- | --- | --- | --- | --- | --- | --- | --- |

BAN89426.1

back to list

  

| Protein Coverage
| Supporting Peptides
|

Protein Coverage:

Supporting Peptides:

| Peptide | Uniq | -10lgP | Mass | Length | ppm | m/z | z | RT | Fraction | Scan | Source File | Area F2 | Area F1 | Area F3 | Area F4 | Area F5 | Area F6 | #Feature | #Feature F2 | #Feature F1 | #Feature F3 | #Feature F4 | #Feature F5 | #Feature F6 | Start | End | PTM | AScore | Found By |
| --- | --- | --- | --- | --- | --- | --- | --- | --- | --- | --- | --- | --- | --- | --- | --- | --- | --- | --- | --- | --- | --- | --- | --- | --- | --- | --- | --- | --- | --- |
| K.YGPVSGEIIK.A | Y | 54.54 | 1061.5757 | 10 | -0.3 | 531.7950 | 2 | 34.19 | 2 | F2:10532 | 30122021\_RID\_2349\_1C.raw |  | 9.1292E4 |  |  |  |  | 1 | 0 | 1 | 0 | 0 | 0 | 0 | 308 | 317 |  |  | PEAKS DB |
| K.DFYTFDSEGIVK.N | Y | 49.58 | 1419.6558 | 12 | 0.4 | 710.8354 | 2 | 52.85 | 2 | F2:17870 | 30122021\_RID\_2349\_1C.raw |  | 2.4259E4 |  |  |  |  | 1 | 0 | 1 | 0 | 0 | 0 | 0 | 390 | 401 |  |  | PEAKS DB |
| K.AATYFWPGSEVK.I | Y | 47.15 | 1354.6558 | 12 | 1.0 | 678.3358 | 2 | 45.04 | 1 | F1:25040 | 28122021\_RID\_2349\_2C.raw | 2.6454E5 |  |  |  |  |  | 1 | 1 | 0 | 0 | 0 | 0 | 0 | 243 | 254 |  |  | PEAKS DB |
| total 3 peptides |  |  |  |  |  |  |  |  |  |  |  |  |  |  |  |  |  |  |  |  |  |  |  |  |  |  |  |  |  |
| --- | --- | --- | --- | --- | --- | --- | --- | --- | --- | --- | --- | --- | --- | --- | --- | --- | --- | --- | --- | --- | --- | --- | --- | --- | --- | --- | --- | --- | --- |

BAN89425.1

back to list

  

| Protein Coverage
| Supporting Peptides
|

Protein Coverage:

Supporting Peptides:

| Peptide | Uniq | -10lgP | Mass | Length | ppm | m/z | z | RT | Fraction | Scan | Source File | Area F2 | Area F1 | Area F3 | Area F4 | Area F5 | Area F6 | #Feature | #Feature F2 | #Feature F1 | #Feature F3 | #Feature F4 | #Feature F5 | #Feature F6 | Start | End | PTM | AScore | Found By |
| --- | --- | --- | --- | --- | --- | --- | --- | --- | --- | --- | --- | --- | --- | --- | --- | --- | --- | --- | --- | --- | --- | --- | --- | --- | --- | --- | --- | --- | --- |
| K.YGPVSGEIIK.A | Y | 54.54 | 1061.5757 | 10 | -0.3 | 531.7950 | 2 | 34.19 | 2 | F2:10532 | 30122021\_RID\_2349\_1C.raw |  | 9.1292E4 |  |  |  |  | 1 | 0 | 1 | 0 | 0 | 0 | 0 | 349 | 358 |  |  | PEAKS DB |
| K.DFYTFDSEGIVK.N | Y | 49.58 | 1419.6558 | 12 | 0.4 | 710.8354 | 2 | 52.85 | 2 | F2:17870 | 30122021\_RID\_2349\_1C.raw |  | 2.4259E4 |  |  |  |  | 1 | 0 | 1 | 0 | 0 | 0 | 0 | 431 | 442 |  |  | PEAKS DB |
| K.AATYFWPGSEVK.I | Y | 47.15 | 1354.6558 | 12 | 1.0 | 678.3358 | 2 | 45.04 | 1 | F1:25040 | 28122021\_RID\_2349\_2C.raw | 2.6454E5 |  |  |  |  |  | 1 | 1 | 0 | 0 | 0 | 0 | 0 | 284 | 295 |  |  | PEAKS DB |
| total 3 peptides |  |  |  |  |  |  |  |  |  |  |  |  |  |  |  |  |  |  |  |  |  |  |  |  |  |  |  |  |  |
| --- | --- | --- | --- | --- | --- | --- | --- | --- | --- | --- | --- | --- | --- | --- | --- | --- | --- | --- | --- | --- | --- | --- | --- | --- | --- | --- | --- | --- | --- |

BAN82023.1

back to list

  

| Protein Coverage
| Supporting Peptides
|

Protein Coverage:

Supporting Peptides:

| Peptide | Uniq | -10lgP | Mass | Length | ppm | m/z | z | RT | Fraction | Scan | Source File | Area F2 | Area F1 | Area F3 | Area F4 | Area F5 | Area F6 | #Feature | #Feature F2 | #Feature F1 | #Feature F3 | #Feature F4 | #Feature F5 | #Feature F6 | Start | End | PTM | AScore | Found By |
| --- | --- | --- | --- | --- | --- | --- | --- | --- | --- | --- | --- | --- | --- | --- | --- | --- | --- | --- | --- | --- | --- | --- | --- | --- | --- | --- | --- | --- | --- |
| K.YGPVSGEIIK.A | Y | 54.54 | 1061.5757 | 10 | -0.3 | 531.7950 | 2 | 34.19 | 2 | F2:10532 | 30122021\_RID\_2349\_1C.raw |  | 9.1292E4 |  |  |  |  | 1 | 0 | 1 | 0 | 0 | 0 | 0 | 270 | 279 |  |  | PEAKS DB |
| K.DFYTFDSEGIVK.N | Y | 49.58 | 1419.6558 | 12 | 0.4 | 710.8354 | 2 | 52.85 | 2 | F2:17870 | 30122021\_RID\_2349\_1C.raw |  | 2.4259E4 |  |  |  |  | 1 | 0 | 1 | 0 | 0 | 0 | 0 | 352 | 363 |  |  | PEAKS DB |
| K.AATYFWPGSEVK.I | Y | 47.15 | 1354.6558 | 12 | 1.0 | 678.3358 | 2 | 45.04 | 1 | F1:25040 | 28122021\_RID\_2349\_2C.raw | 2.6454E5 |  |  |  |  |  | 1 | 1 | 0 | 0 | 0 | 0 | 0 | 205 | 216 |  |  | PEAKS DB |
| total 3 peptides |  |  |  |  |  |  |  |  |  |  |  |  |  |  |  |  |  |  |  |  |  |  |  |  |  |  |  |  |  |
| --- | --- | --- | --- | --- | --- | --- | --- | --- | --- | --- | --- | --- | --- | --- | --- | --- | --- | --- | --- | --- | --- | --- | --- | --- | --- | --- | --- | --- | --- |

BAN82024.1

back to list

  

| Protein Coverage
| Supporting Peptides
|

Protein Coverage:

Supporting Peptides:

| Peptide | Uniq | -10lgP | Mass | Length | ppm | m/z | z | RT | Fraction | Scan | Source File | Area F2 | Area F1 | Area F3 | Area F4 | Area F5 | Area F6 | #Feature | #Feature F2 | #Feature F1 | #Feature F3 | #Feature F4 | #Feature F5 | #Feature F6 | Start | End | PTM | AScore | Found By |
| --- | --- | --- | --- | --- | --- | --- | --- | --- | --- | --- | --- | --- | --- | --- | --- | --- | --- | --- | --- | --- | --- | --- | --- | --- | --- | --- | --- | --- | --- |
| K.YGPVSGEIIK.A | Y | 54.54 | 1061.5757 | 10 | -0.3 | 531.7950 | 2 | 34.19 | 2 | F2:10532 | 30122021\_RID\_2349\_1C.raw |  | 9.1292E4 |  |  |  |  | 1 | 0 | 1 | 0 | 0 | 0 | 0 | 271 | 280 |  |  | PEAKS DB |
| K.DFYTFDSEGIVK.N | Y | 49.58 | 1419.6558 | 12 | 0.4 | 710.8354 | 2 | 52.85 | 2 | F2:17870 | 30122021\_RID\_2349\_1C.raw |  | 2.4259E4 |  |  |  |  | 1 | 0 | 1 | 0 | 0 | 0 | 0 | 353 | 364 |  |  | PEAKS DB |
| K.AATYFWPGSEVK.I | Y | 47.15 | 1354.6558 | 12 | 1.0 | 678.3358 | 2 | 45.04 | 1 | F1:25040 | 28122021\_RID\_2349\_2C.raw | 2.6454E5 |  |  |  |  |  | 1 | 1 | 0 | 0 | 0 | 0 | 0 | 206 | 217 |  |  | PEAKS DB |
| total 3 peptides |  |  |  |  |  |  |  |  |  |  |  |  |  |  |  |  |  |  |  |  |  |  |  |  |  |  |  |  |  |
| --- | --- | --- | --- | --- | --- | --- | --- | --- | --- | --- | --- | --- | --- | --- | --- | --- | --- | --- | --- | --- | --- | --- | --- | --- | --- | --- | --- | --- | --- |

BAP39977.1

back to list

  

| Protein Coverage
| Supporting Peptides
|

Protein Coverage:

Supporting Peptides:

| Peptide | Uniq | -10lgP | Mass | Length | ppm | m/z | z | RT | Fraction | Scan | Source File | Area F2 | Area F1 | Area F3 | Area F4 | Area F5 | Area F6 | #Feature | #Feature F2 | #Feature F1 | #Feature F3 | #Feature F4 | #Feature F5 | #Feature F6 | Start | End | PTM | AScore | Found By |
| --- | --- | --- | --- | --- | --- | --- | --- | --- | --- | --- | --- | --- | --- | --- | --- | --- | --- | --- | --- | --- | --- | --- | --- | --- | --- | --- | --- | --- | --- |
| K.YGPVSGEIIK.A | Y | 54.54 | 1061.5757 | 10 | -0.3 | 531.7950 | 2 | 34.19 | 2 | F2:10532 | 30122021\_RID\_2349\_1C.raw |  | 9.1292E4 |  |  |  |  | 1 | 0 | 1 | 0 | 0 | 0 | 0 | 311 | 320 |  |  | PEAKS DB |
| K.DFYTFDSEGIVK.N | Y | 49.58 | 1419.6558 | 12 | 0.4 | 710.8354 | 2 | 52.85 | 2 | F2:17870 | 30122021\_RID\_2349\_1C.raw |  | 2.4259E4 |  |  |  |  | 1 | 0 | 1 | 0 | 0 | 0 | 0 | 393 | 404 |  |  | PEAKS DB |
| K.AATYFWPGSEVK.I | Y | 47.15 | 1354.6558 | 12 | 1.0 | 678.3358 | 2 | 45.04 | 1 | F1:25040 | 28122021\_RID\_2349\_2C.raw | 2.6454E5 |  |  |  |  |  | 1 | 1 | 0 | 0 | 0 | 0 | 0 | 246 | 257 |  |  | PEAKS DB |
| total 3 peptides |  |  |  |  |  |  |  |  |  |  |  |  |  |  |  |  |  |  |  |  |  |  |  |  |  |  |  |  |  |
| --- | --- | --- | --- | --- | --- | --- | --- | --- | --- | --- | --- | --- | --- | --- | --- | --- | --- | --- | --- | --- | --- | --- | --- | --- | --- | --- | --- | --- | --- |

BAN82021.1

back to list

  

| Protein Coverage
| Supporting Peptides
|

Protein Coverage:

Supporting Peptides:

| Peptide | Uniq | -10lgP | Mass | Length | ppm | m/z | z | RT | Fraction | Scan | Source File | Area F2 | Area F1 | Area F3 | Area F4 | Area F5 | Area F6 | #Feature | #Feature F2 | #Feature F1 | #Feature F3 | #Feature F4 | #Feature F5 | #Feature F6 | Start | End | PTM | AScore | Found By |
| --- | --- | --- | --- | --- | --- | --- | --- | --- | --- | --- | --- | --- | --- | --- | --- | --- | --- | --- | --- | --- | --- | --- | --- | --- | --- | --- | --- | --- | --- |
| K.YGPVSGEIIK.A | Y | 54.54 | 1061.5757 | 10 | -0.3 | 531.7950 | 2 | 34.19 | 2 | F2:10532 | 30122021\_RID\_2349\_1C.raw |  | 9.1292E4 |  |  |  |  | 1 | 0 | 1 | 0 | 0 | 0 | 0 | 311 | 320 |  |  | PEAKS DB |
| K.DFYTFDSEGIVK.N | Y | 49.58 | 1419.6558 | 12 | 0.4 | 710.8354 | 2 | 52.85 | 2 | F2:17870 | 30122021\_RID\_2349\_1C.raw |  | 2.4259E4 |  |  |  |  | 1 | 0 | 1 | 0 | 0 | 0 | 0 | 393 | 404 |  |  | PEAKS DB |
| K.AATYFWPGSEVK.I | Y | 47.15 | 1354.6558 | 12 | 1.0 | 678.3358 | 2 | 45.04 | 1 | F1:25040 | 28122021\_RID\_2349\_2C.raw | 2.6454E5 |  |  |  |  |  | 1 | 1 | 0 | 0 | 0 | 0 | 0 | 246 | 257 |  |  | PEAKS DB |
| total 3 peptides |  |  |  |  |  |  |  |  |  |  |  |  |  |  |  |  |  |  |  |  |  |  |  |  |  |  |  |  |  |
| --- | --- | --- | --- | --- | --- | --- | --- | --- | --- | --- | --- | --- | --- | --- | --- | --- | --- | --- | --- | --- | --- | --- | --- | --- | --- | --- | --- | --- | --- |

BAN82022.1

back to list

  

| Protein Coverage
| Supporting Peptides
|

Protein Coverage:

Supporting Peptides:

| Peptide | Uniq | -10lgP | Mass | Length | ppm | m/z | z | RT | Fraction | Scan | Source File | Area F2 | Area F1 | Area F3 | Area F4 | Area F5 | Area F6 | #Feature | #Feature F2 | #Feature F1 | #Feature F3 | #Feature F4 | #Feature F5 | #Feature F6 | Start | End | PTM | AScore | Found By |
| --- | --- | --- | --- | --- | --- | --- | --- | --- | --- | --- | --- | --- | --- | --- | --- | --- | --- | --- | --- | --- | --- | --- | --- | --- | --- | --- | --- | --- | --- |
| K.YGPVSGEIIK.A | Y | 54.54 | 1061.5757 | 10 | -0.3 | 531.7950 | 2 | 34.19 | 2 | F2:10532 | 30122021\_RID\_2349\_1C.raw |  | 9.1292E4 |  |  |  |  | 1 | 0 | 1 | 0 | 0 | 0 | 0 | 312 | 321 |  |  | PEAKS DB |
| K.DFYTFDSEGIVK.N | Y | 49.58 | 1419.6558 | 12 | 0.4 | 710.8354 | 2 | 52.85 | 2 | F2:17870 | 30122021\_RID\_2349\_1C.raw |  | 2.4259E4 |  |  |  |  | 1 | 0 | 1 | 0 | 0 | 0 | 0 | 394 | 405 |  |  | PEAKS DB |
| K.AATYFWPGSEVK.I | Y | 47.15 | 1354.6558 | 12 | 1.0 | 678.3358 | 2 | 45.04 | 1 | F1:25040 | 28122021\_RID\_2349\_2C.raw | 2.6454E5 |  |  |  |  |  | 1 | 1 | 0 | 0 | 0 | 0 | 0 | 247 | 258 |  |  | PEAKS DB |
| total 3 peptides |  |  |  |  |  |  |  |  |  |  |  |  |  |  |  |  |  |  |  |  |  |  |  |  |  |  |  |  |  |
| --- | --- | --- | --- | --- | --- | --- | --- | --- | --- | --- | --- | --- | --- | --- | --- | --- | --- | --- | --- | --- | --- | --- | --- | --- | --- | --- | --- | --- | --- |

JAV48422.1

back to list

  

| Protein Coverage
| Supporting Peptides
|

Protein Coverage:

Supporting Peptides:

| Peptide | Uniq | -10lgP | Mass | Length | ppm | m/z | z | RT | Fraction | Scan | Source File | Area F2 | Area F1 | Area F3 | Area F4 | Area F5 | Area F6 | #Feature | #Feature F2 | #Feature F1 | #Feature F3 | #Feature F4 | #Feature F5 | #Feature F6 | Start | End | PTM | AScore | Found By |
| --- | --- | --- | --- | --- | --- | --- | --- | --- | --- | --- | --- | --- | --- | --- | --- | --- | --- | --- | --- | --- | --- | --- | --- | --- | --- | --- | --- | --- | --- |
| K.YGPVSGEIIK.A | Y | 54.54 | 1061.5757 | 10 | -0.3 | 531.7950 | 2 | 34.19 | 2 | F2:10532 | 30122021\_RID\_2349\_1C.raw |  | 9.1292E4 |  |  |  |  | 1 | 0 | 1 | 0 | 0 | 0 | 0 | 270 | 279 |  |  | PEAKS DB |
| K.DFYTFDSEGIVK.N | Y | 49.58 | 1419.6558 | 12 | 0.4 | 710.8354 | 2 | 52.85 | 2 | F2:17870 | 30122021\_RID\_2349\_1C.raw |  | 2.4259E4 |  |  |  |  | 1 | 0 | 1 | 0 | 0 | 0 | 0 | 352 | 363 |  |  | PEAKS DB |
| K.AATYFWPGSEVK.I | Y | 47.15 | 1354.6558 | 12 | 1.0 | 678.3358 | 2 | 45.04 | 1 | F1:25040 | 28122021\_RID\_2349\_2C.raw | 2.6454E5 |  |  |  |  |  | 1 | 1 | 0 | 0 | 0 | 0 | 0 | 205 | 216 |  |  | PEAKS DB |
| total 3 peptides |  |  |  |  |  |  |  |  |  |  |  |  |  |  |  |  |  |  |  |  |  |  |  |  |  |  |  |  |  |
| --- | --- | --- | --- | --- | --- | --- | --- | --- | --- | --- | --- | --- | --- | --- | --- | --- | --- | --- | --- | --- | --- | --- | --- | --- | --- | --- | --- | --- | --- |

AFJ49200.1

back to list

  

| Protein Coverage
| Supporting Peptides
|

Protein Coverage:

Supporting Peptides:

| Peptide | Uniq | -10lgP | Mass | Length | ppm | m/z | z | RT | Fraction | Scan | Source File | Area F2 | Area F1 | Area F3 | Area F4 | Area F5 | Area F6 | #Feature | #Feature F2 | #Feature F1 | #Feature F3 | #Feature F4 | #Feature F5 | #Feature F6 | Start | End | PTM | AScore | Found By |
| --- | --- | --- | --- | --- | --- | --- | --- | --- | --- | --- | --- | --- | --- | --- | --- | --- | --- | --- | --- | --- | --- | --- | --- | --- | --- | --- | --- | --- | --- |
| K.YGPVSGEIIK.A | Y | 54.54 | 1061.5757 | 10 | -0.3 | 531.7950 | 2 | 34.19 | 2 | F2:10532 | 30122021\_RID\_2349\_1C.raw |  | 9.1292E4 |  |  |  |  | 1 | 0 | 1 | 0 | 0 | 0 | 0 | 270 | 279 |  |  | PEAKS DB |
| K.DFYTFDSEGIVK.N | Y | 49.58 | 1419.6558 | 12 | 0.4 | 710.8354 | 2 | 52.85 | 2 | F2:17870 | 30122021\_RID\_2349\_1C.raw |  | 2.4259E4 |  |  |  |  | 1 | 0 | 1 | 0 | 0 | 0 | 0 | 352 | 363 |  |  | PEAKS DB |
| K.AATYFWPGSEVK.I | Y | 47.15 | 1354.6558 | 12 | 1.0 | 678.3358 | 2 | 45.04 | 1 | F1:25040 | 28122021\_RID\_2349\_2C.raw | 2.6454E5 |  |  |  |  |  | 1 | 1 | 0 | 0 | 0 | 0 | 0 | 205 | 216 |  |  | PEAKS DB |
| total 3 peptides |  |  |  |  |  |  |  |  |  |  |  |  |  |  |  |  |  |  |  |  |  |  |  |  |  |  |  |  |  |
| --- | --- | --- | --- | --- | --- | --- | --- | --- | --- | --- | --- | --- | --- | --- | --- | --- | --- | --- | --- | --- | --- | --- | --- | --- | --- | --- | --- | --- | --- |

J3SBP3.1

back to list

  

| Protein Coverage
| Supporting Peptides
|

Protein Coverage:

Supporting Peptides:

| Peptide | Uniq | -10lgP | Mass | Length | ppm | m/z | z | RT | Fraction | Scan | Source File | Area F2 | Area F1 | Area F3 | Area F4 | Area F5 | Area F6 | #Feature | #Feature F2 | #Feature F1 | #Feature F3 | #Feature F4 | #Feature F5 | #Feature F6 | Start | End | PTM | AScore | Found By |
| --- | --- | --- | --- | --- | --- | --- | --- | --- | --- | --- | --- | --- | --- | --- | --- | --- | --- | --- | --- | --- | --- | --- | --- | --- | --- | --- | --- | --- | --- |
| K.YGPVSGEIIK.A | Y | 54.54 | 1061.5757 | 10 | -0.3 | 531.7950 | 2 | 34.19 | 2 | F2:10532 | 30122021\_RID\_2349\_1C.raw |  | 9.1292E4 |  |  |  |  | 1 | 0 | 1 | 0 | 0 | 0 | 0 | 270 | 279 |  |  | PEAKS DB |
| K.DFYTFDSEGIVK.N | Y | 49.58 | 1419.6558 | 12 | 0.4 | 710.8354 | 2 | 52.85 | 2 | F2:17870 | 30122021\_RID\_2349\_1C.raw |  | 2.4259E4 |  |  |  |  | 1 | 0 | 1 | 0 | 0 | 0 | 0 | 352 | 363 |  |  | PEAKS DB |
| K.AATYFWPGSEVK.I | Y | 47.15 | 1354.6558 | 12 | 1.0 | 678.3358 | 2 | 45.04 | 1 | F1:25040 | 28122021\_RID\_2349\_2C.raw | 2.6454E5 |  |  |  |  |  | 1 | 1 | 0 | 0 | 0 | 0 | 0 | 205 | 216 |  |  | PEAKS DB |
| total 3 peptides |  |  |  |  |  |  |  |  |  |  |  |  |  |  |  |  |  |  |  |  |  |  |  |  |  |  |  |  |  |
| --- | --- | --- | --- | --- | --- | --- | --- | --- | --- | --- | --- | --- | --- | --- | --- | --- | --- | --- | --- | --- | --- | --- | --- | --- | --- | --- | --- | --- | --- |

JAS05450.1

back to list

  

| Protein Coverage
| Supporting Peptides
|

Protein Coverage:

Supporting Peptides:

| Peptide | Uniq | -10lgP | Mass | Length | ppm | m/z | z | RT | Fraction | Scan | Source File | Area F2 | Area F1 | Area F3 | Area F4 | Area F5 | Area F6 | #Feature | #Feature F2 | #Feature F1 | #Feature F3 | #Feature F4 | #Feature F5 | #Feature F6 | Start | End | PTM | AScore | Found By |
| --- | --- | --- | --- | --- | --- | --- | --- | --- | --- | --- | --- | --- | --- | --- | --- | --- | --- | --- | --- | --- | --- | --- | --- | --- | --- | --- | --- | --- | --- |
| K.YGPVSGEIIK.A | Y | 54.54 | 1061.5757 | 10 | -0.3 | 531.7950 | 2 | 34.19 | 2 | F2:10532 | 30122021\_RID\_2349\_1C.raw |  | 9.1292E4 |  |  |  |  | 1 | 0 | 1 | 0 | 0 | 0 | 0 | 270 | 279 |  |  | PEAKS DB |
| K.DFYTFDSEGIVK.N | Y | 49.58 | 1419.6558 | 12 | 0.4 | 710.8354 | 2 | 52.85 | 2 | F2:17870 | 30122021\_RID\_2349\_1C.raw |  | 2.4259E4 |  |  |  |  | 1 | 0 | 1 | 0 | 0 | 0 | 0 | 352 | 363 |  |  | PEAKS DB |
| K.AATYFWPGSEVK.I | Y | 47.15 | 1354.6558 | 12 | 1.0 | 678.3358 | 2 | 45.04 | 1 | F1:25040 | 28122021\_RID\_2349\_2C.raw | 2.6454E5 |  |  |  |  |  | 1 | 1 | 0 | 0 | 0 | 0 | 0 | 205 | 216 |  |  | PEAKS DB |
| total 3 peptides |  |  |  |  |  |  |  |  |  |  |  |  |  |  |  |  |  |  |  |  |  |  |  |  |  |  |  |  |  |
| --- | --- | --- | --- | --- | --- | --- | --- | --- | --- | --- | --- | --- | --- | --- | --- | --- | --- | --- | --- | --- | --- | --- | --- | --- | --- | --- | --- | --- | --- |

JAA96215.1

back to list

  

| Protein Coverage
| Supporting Peptides
|

Protein Coverage:

Supporting Peptides:

| Peptide | Uniq | -10lgP | Mass | Length | ppm | m/z | z | RT | Fraction | Scan | Source File | Area F2 | Area F1 | Area F3 | Area F4 | Area F5 | Area F6 | #Feature | #Feature F2 | #Feature F1 | #Feature F3 | #Feature F4 | #Feature F5 | #Feature F6 | Start | End | PTM | AScore | Found By |
| --- | --- | --- | --- | --- | --- | --- | --- | --- | --- | --- | --- | --- | --- | --- | --- | --- | --- | --- | --- | --- | --- | --- | --- | --- | --- | --- | --- | --- | --- |
| K.YGPVSGEIIK.A | Y | 54.54 | 1061.5757 | 10 | -0.3 | 531.7950 | 2 | 34.19 | 2 | F2:10532 | 30122021\_RID\_2349\_1C.raw |  | 9.1292E4 |  |  |  |  | 1 | 0 | 1 | 0 | 0 | 0 | 0 | 287 | 296 |  |  | PEAKS DB |
| K.DFYTFDSEGIVK.N | Y | 49.58 | 1419.6558 | 12 | 0.4 | 710.8354 | 2 | 52.85 | 2 | F2:17870 | 30122021\_RID\_2349\_1C.raw |  | 2.4259E4 |  |  |  |  | 1 | 0 | 1 | 0 | 0 | 0 | 0 | 369 | 380 |  |  | PEAKS DB |
| K.AATYFWPGSEVK.I | Y | 47.15 | 1354.6558 | 12 | 1.0 | 678.3358 | 2 | 45.04 | 1 | F1:25040 | 28122021\_RID\_2349\_2C.raw | 2.6454E5 |  |  |  |  |  | 1 | 1 | 0 | 0 | 0 | 0 | 0 | 222 | 233 |  |  | PEAKS DB |
| total 3 peptides |  |  |  |  |  |  |  |  |  |  |  |  |  |  |  |  |  |  |  |  |  |  |  |  |  |  |  |  |  |
| --- | --- | --- | --- | --- | --- | --- | --- | --- | --- | --- | --- | --- | --- | --- | --- | --- | --- | --- | --- | --- | --- | --- | --- | --- | --- | --- | --- | --- | --- |

JAS04779.1

back to list

  

| Protein Coverage
| Supporting Peptides
|

Protein Coverage:

Supporting Peptides:

| Peptide | Uniq | -10lgP | Mass | Length | ppm | m/z | z | RT | Fraction | Scan | Source File | Area F2 | Area F1 | Area F3 | Area F4 | Area F5 | Area F6 | #Feature | #Feature F2 | #Feature F1 | #Feature F3 | #Feature F4 | #Feature F5 | #Feature F6 | Start | End | PTM | AScore | Found By |
| --- | --- | --- | --- | --- | --- | --- | --- | --- | --- | --- | --- | --- | --- | --- | --- | --- | --- | --- | --- | --- | --- | --- | --- | --- | --- | --- | --- | --- | --- |
| K.YGPVSGEIIK.A | Y | 54.54 | 1061.5757 | 10 | -0.3 | 531.7950 | 2 | 34.19 | 2 | F2:10532 | 30122021\_RID\_2349\_1C.raw |  | 9.1292E4 |  |  |  |  | 1 | 0 | 1 | 0 | 0 | 0 | 0 | 311 | 320 |  |  | PEAKS DB |
| K.DFYTFDSEGIVK.N | Y | 49.58 | 1419.6558 | 12 | 0.4 | 710.8354 | 2 | 52.85 | 2 | F2:17870 | 30122021\_RID\_2349\_1C.raw |  | 2.4259E4 |  |  |  |  | 1 | 0 | 1 | 0 | 0 | 0 | 0 | 393 | 404 |  |  | PEAKS DB |
| K.AATYFWPGSEVK.I | Y | 47.15 | 1354.6558 | 12 | 1.0 | 678.3358 | 2 | 45.04 | 1 | F1:25040 | 28122021\_RID\_2349\_2C.raw | 2.6454E5 |  |  |  |  |  | 1 | 1 | 0 | 0 | 0 | 0 | 0 | 246 | 257 |  |  | PEAKS DB |
| total 3 peptides |  |  |  |  |  |  |  |  |  |  |  |  |  |  |  |  |  |  |  |  |  |  |  |  |  |  |  |  |  |
| --- | --- | --- | --- | --- | --- | --- | --- | --- | --- | --- | --- | --- | --- | --- | --- | --- | --- | --- | --- | --- | --- | --- | --- | --- | --- | --- | --- | --- | --- |

JAA94884.1

back to list

  

| Protein Coverage
| Supporting Peptides
|

Protein Coverage:

Supporting Peptides:

| Peptide | Uniq | -10lgP | Mass | Length | ppm | m/z | z | RT | Fraction | Scan | Source File | Area F2 | Area F1 | Area F3 | Area F4 | Area F5 | Area F6 | #Feature | #Feature F2 | #Feature F1 | #Feature F3 | #Feature F4 | #Feature F5 | #Feature F6 | Start | End | PTM | AScore | Found By |
| --- | --- | --- | --- | --- | --- | --- | --- | --- | --- | --- | --- | --- | --- | --- | --- | --- | --- | --- | --- | --- | --- | --- | --- | --- | --- | --- | --- | --- | --- |
| K.YGPVSGEIIK.A | Y | 54.54 | 1061.5757 | 10 | -0.3 | 531.7950 | 2 | 34.19 | 2 | F2:10532 | 30122021\_RID\_2349\_1C.raw |  | 9.1292E4 |  |  |  |  | 1 | 0 | 1 | 0 | 0 | 0 | 0 | 311 | 320 |  |  | PEAKS DB |
| K.DFYTFDSEGIVK.N | Y | 49.58 | 1419.6558 | 12 | 0.4 | 710.8354 | 2 | 52.85 | 2 | F2:17870 | 30122021\_RID\_2349\_1C.raw |  | 2.4259E4 |  |  |  |  | 1 | 0 | 1 | 0 | 0 | 0 | 0 | 393 | 404 |  |  | PEAKS DB |
| K.AATYFWPGSEVK.I | Y | 47.15 | 1354.6558 | 12 | 1.0 | 678.3358 | 2 | 45.04 | 1 | F1:25040 | 28122021\_RID\_2349\_2C.raw | 2.6454E5 |  |  |  |  |  | 1 | 1 | 0 | 0 | 0 | 0 | 0 | 246 | 257 |  |  | PEAKS DB |
| total 3 peptides |  |  |  |  |  |  |  |  |  |  |  |  |  |  |  |  |  |  |  |  |  |  |  |  |  |  |  |  |  |
| --- | --- | --- | --- | --- | --- | --- | --- | --- | --- | --- | --- | --- | --- | --- | --- | --- | --- | --- | --- | --- | --- | --- | --- | --- | --- | --- | --- | --- | --- |

AEJ31980.1

back to list

  

| Protein Coverage
| Supporting Peptides
|

Protein Coverage:

Supporting Peptides:

| Peptide | Uniq | -10lgP | Mass | Length | ppm | m/z | z | RT | Fraction | Scan | Source File | Area F2 | Area F1 | Area F3 | Area F4 | Area F5 | Area F6 | #Feature | #Feature F2 | #Feature F1 | #Feature F3 | #Feature F4 | #Feature F5 | #Feature F6 | Start | End | PTM | AScore | Found By |
| --- | --- | --- | --- | --- | --- | --- | --- | --- | --- | --- | --- | --- | --- | --- | --- | --- | --- | --- | --- | --- | --- | --- | --- | --- | --- | --- | --- | --- | --- |
| K.YGPVSGEIIK.A | Y | 54.54 | 1061.5757 | 10 | -0.3 | 531.7950 | 2 | 34.19 | 2 | F2:10532 | 30122021\_RID\_2349\_1C.raw |  | 9.1292E4 |  |  |  |  | 1 | 0 | 1 | 0 | 0 | 0 | 0 | 311 | 320 |  |  | PEAKS DB |
| K.DFYTFDSEGIVK.N | Y | 49.58 | 1419.6558 | 12 | 0.4 | 710.8354 | 2 | 52.85 | 2 | F2:17870 | 30122021\_RID\_2349\_1C.raw |  | 2.4259E4 |  |  |  |  | 1 | 0 | 1 | 0 | 0 | 0 | 0 | 393 | 404 |  |  | PEAKS DB |
| K.AATYFWPGSEVK.I | Y | 47.15 | 1354.6558 | 12 | 1.0 | 678.3358 | 2 | 45.04 | 1 | F1:25040 | 28122021\_RID\_2349\_2C.raw | 2.6454E5 |  |  |  |  |  | 1 | 1 | 0 | 0 | 0 | 0 | 0 | 246 | 257 |  |  | PEAKS DB |
| total 3 peptides |  |  |  |  |  |  |  |  |  |  |  |  |  |  |  |  |  |  |  |  |  |  |  |  |  |  |  |  |  |
| --- | --- | --- | --- | --- | --- | --- | --- | --- | --- | --- | --- | --- | --- | --- | --- | --- | --- | --- | --- | --- | --- | --- | --- | --- | --- | --- | --- | --- | --- |

JAI10403.1

back to list

  

| Protein Coverage
| Supporting Peptides
|

Protein Coverage:

Supporting Peptides:

| Peptide | Uniq | -10lgP | Mass | Length | ppm | m/z | z | RT | Fraction | Scan | Source File | Area F2 | Area F1 | Area F3 | Area F4 | Area F5 | Area F6 | #Feature | #Feature F2 | #Feature F1 | #Feature F3 | #Feature F4 | #Feature F5 | #Feature F6 | Start | End | PTM | AScore | Found By |
| --- | --- | --- | --- | --- | --- | --- | --- | --- | --- | --- | --- | --- | --- | --- | --- | --- | --- | --- | --- | --- | --- | --- | --- | --- | --- | --- | --- | --- | --- |
| K.YGPVSGEIIK.A | Y | 54.54 | 1061.5757 | 10 | -0.3 | 531.7950 | 2 | 34.19 | 2 | F2:10532 | 30122021\_RID\_2349\_1C.raw |  | 9.1292E4 |  |  |  |  | 1 | 0 | 1 | 0 | 0 | 0 | 0 | 311 | 320 |  |  | PEAKS DB |
| K.DFYTFDSEGIVK.N | Y | 49.58 | 1419.6558 | 12 | 0.4 | 710.8354 | 2 | 52.85 | 2 | F2:17870 | 30122021\_RID\_2349\_1C.raw |  | 2.4259E4 |  |  |  |  | 1 | 0 | 1 | 0 | 0 | 0 | 0 | 393 | 404 |  |  | PEAKS DB |
| K.AATYFWPGSEVK.I | Y | 47.15 | 1354.6558 | 12 | 1.0 | 678.3358 | 2 | 45.04 | 1 | F1:25040 | 28122021\_RID\_2349\_2C.raw | 2.6454E5 |  |  |  |  |  | 1 | 1 | 0 | 0 | 0 | 0 | 0 | 246 | 257 |  |  | PEAKS DB |
| total 3 peptides |  |  |  |  |  |  |  |  |  |  |  |  |  |  |  |  |  |  |  |  |  |  |  |  |  |  |  |  |  |
| --- | --- | --- | --- | --- | --- | --- | --- | --- | --- | --- | --- | --- | --- | --- | --- | --- | --- | --- | --- | --- | --- | --- | --- | --- | --- | --- | --- | --- | --- |

J3SEZ3.2

back to list

  

| Protein Coverage
| Supporting Peptides
|

Protein Coverage:

Supporting Peptides:

| Peptide | Uniq | -10lgP | Mass | Length | ppm | m/z | z | RT | Fraction | Scan | Source File | Area F2 | Area F1 | Area F3 | Area F4 | Area F5 | Area F6 | #Feature | #Feature F2 | #Feature F1 | #Feature F3 | #Feature F4 | #Feature F5 | #Feature F6 | Start | End | PTM | AScore | Found By |
| --- | --- | --- | --- | --- | --- | --- | --- | --- | --- | --- | --- | --- | --- | --- | --- | --- | --- | --- | --- | --- | --- | --- | --- | --- | --- | --- | --- | --- | --- |
| K.YGPVSGEIIK.A | Y | 54.54 | 1061.5757 | 10 | -0.3 | 531.7950 | 2 | 34.19 | 2 | F2:10532 | 30122021\_RID\_2349\_1C.raw |  | 9.1292E4 |  |  |  |  | 1 | 0 | 1 | 0 | 0 | 0 | 0 | 311 | 320 |  |  | PEAKS DB |
| K.DFYTFDSEGIVK.N | Y | 49.58 | 1419.6558 | 12 | 0.4 | 710.8354 | 2 | 52.85 | 2 | F2:17870 | 30122021\_RID\_2349\_1C.raw |  | 2.4259E4 |  |  |  |  | 1 | 0 | 1 | 0 | 0 | 0 | 0 | 393 | 404 |  |  | PEAKS DB |
| K.AATYFWPGSEVK.I | Y | 47.15 | 1354.6558 | 12 | 1.0 | 678.3358 | 2 | 45.04 | 1 | F1:25040 | 28122021\_RID\_2349\_2C.raw | 2.6454E5 |  |  |  |  |  | 1 | 1 | 0 | 0 | 0 | 0 | 0 | 246 | 257 |  |  | PEAKS DB |
| total 3 peptides |  |  |  |  |  |  |  |  |  |  |  |  |  |  |  |  |  |  |  |  |  |  |  |  |  |  |  |  |  |
| --- | --- | --- | --- | --- | --- | --- | --- | --- | --- | --- | --- | --- | --- | --- | --- | --- | --- | --- | --- | --- | --- | --- | --- | --- | --- | --- | --- | --- | --- |

JAS04861.1

back to list

  

| Protein Coverage
| Supporting Peptides
|

Protein Coverage:

Supporting Peptides:

| Peptide | Uniq | -10lgP | Mass | Length | ppm | m/z | z | RT | Fraction | Scan | Source File | Area F2 | Area F1 | Area F3 | Area F4 | Area F5 | Area F6 | #Feature | #Feature F2 | #Feature F1 | #Feature F3 | #Feature F4 | #Feature F5 | #Feature F6 | Start | End | PTM | AScore | Found By |
| --- | --- | --- | --- | --- | --- | --- | --- | --- | --- | --- | --- | --- | --- | --- | --- | --- | --- | --- | --- | --- | --- | --- | --- | --- | --- | --- | --- | --- | --- |
| K.YGPVSGEIIK.A | Y | 54.54 | 1061.5757 | 10 | -0.3 | 531.7950 | 2 | 34.19 | 2 | F2:10532 | 30122021\_RID\_2349\_1C.raw |  | 9.1292E4 |  |  |  |  | 1 | 0 | 1 | 0 | 0 | 0 | 0 | 311 | 320 |  |  | PEAKS DB |
| K.DFYTFDSEGIVK.N | Y | 49.58 | 1419.6558 | 12 | 0.4 | 710.8354 | 2 | 52.85 | 2 | F2:17870 | 30122021\_RID\_2349\_1C.raw |  | 2.4259E4 |  |  |  |  | 1 | 0 | 1 | 0 | 0 | 0 | 0 | 393 | 404 |  |  | PEAKS DB |
| K.AATYFWPGSEVK.I | Y | 47.15 | 1354.6558 | 12 | 1.0 | 678.3358 | 2 | 45.04 | 1 | F1:25040 | 28122021\_RID\_2349\_2C.raw | 2.6454E5 |  |  |  |  |  | 1 | 1 | 0 | 0 | 0 | 0 | 0 | 246 | 257 |  |  | PEAKS DB |
| total 3 peptides |  |  |  |  |  |  |  |  |  |  |  |  |  |  |  |  |  |  |  |  |  |  |  |  |  |  |  |  |  |
| --- | --- | --- | --- | --- | --- | --- | --- | --- | --- | --- | --- | --- | --- | --- | --- | --- | --- | --- | --- | --- | --- | --- | --- | --- | --- | --- | --- | --- | --- |

JAG46127.1

back to list

  

| Protein Coverage
| Supporting Peptides
|

Protein Coverage:

Supporting Peptides:

| Peptide | Uniq | -10lgP | Mass | Length | ppm | m/z | z | RT | Fraction | Scan | Source File | Area F2 | Area F1 | Area F3 | Area F4 | Area F5 | Area F6 | #Feature | #Feature F2 | #Feature F1 | #Feature F3 | #Feature F4 | #Feature F5 | #Feature F6 | Start | End | PTM | AScore | Found By |
| --- | --- | --- | --- | --- | --- | --- | --- | --- | --- | --- | --- | --- | --- | --- | --- | --- | --- | --- | --- | --- | --- | --- | --- | --- | --- | --- | --- | --- | --- |
| K.YGPVSGEIIK.A | Y | 54.54 | 1061.5757 | 10 | -0.3 | 531.7950 | 2 | 34.19 | 2 | F2:10532 | 30122021\_RID\_2349\_1C.raw |  | 9.1292E4 |  |  |  |  | 1 | 0 | 1 | 0 | 0 | 0 | 0 | 311 | 320 |  |  | PEAKS DB |
| K.DFYTFDSEGIVK.N | Y | 49.58 | 1419.6558 | 12 | 0.4 | 710.8354 | 2 | 52.85 | 2 | F2:17870 | 30122021\_RID\_2349\_1C.raw |  | 2.4259E4 |  |  |  |  | 1 | 0 | 1 | 0 | 0 | 0 | 0 | 393 | 404 |  |  | PEAKS DB |
| K.AATYFWPGSEVK.I | Y | 47.15 | 1354.6558 | 12 | 1.0 | 678.3358 | 2 | 45.04 | 1 | F1:25040 | 28122021\_RID\_2349\_2C.raw | 2.6454E5 |  |  |  |  |  | 1 | 1 | 0 | 0 | 0 | 0 | 0 | 246 | 257 |  |  | PEAKS DB |
| total 3 peptides |  |  |  |  |  |  |  |  |  |  |  |  |  |  |  |  |  |  |  |  |  |  |  |  |  |  |  |  |  |
| --- | --- | --- | --- | --- | --- | --- | --- | --- | --- | --- | --- | --- | --- | --- | --- | --- | --- | --- | --- | --- | --- | --- | --- | --- | --- | --- | --- | --- | --- |

JAG43649.1

back to list

  

| Protein Coverage
| Supporting Peptides
|

Protein Coverage:

Supporting Peptides:

| Peptide | Uniq | -10lgP | Mass | Length | ppm | m/z | z | RT | Fraction | Scan | Source File | Area F2 | Area F1 | Area F3 | Area F4 | Area F5 | Area F6 | #Feature | #Feature F2 | #Feature F1 | #Feature F3 | #Feature F4 | #Feature F5 | #Feature F6 | Start | End | PTM | AScore | Found By |
| --- | --- | --- | --- | --- | --- | --- | --- | --- | --- | --- | --- | --- | --- | --- | --- | --- | --- | --- | --- | --- | --- | --- | --- | --- | --- | --- | --- | --- | --- |
| K.YGPVSGEIIK.A | Y | 54.54 | 1061.5757 | 10 | -0.3 | 531.7950 | 2 | 34.19 | 2 | F2:10532 | 30122021\_RID\_2349\_1C.raw |  | 9.1292E4 |  |  |  |  | 1 | 0 | 1 | 0 | 0 | 0 | 0 | 311 | 320 |  |  | PEAKS DB |
| K.DFYTFDSEGIVK.N | Y | 49.58 | 1419.6558 | 12 | 0.4 | 710.8354 | 2 | 52.85 | 2 | F2:17870 | 30122021\_RID\_2349\_1C.raw |  | 2.4259E4 |  |  |  |  | 1 | 0 | 1 | 0 | 0 | 0 | 0 | 393 | 404 |  |  | PEAKS DB |
| K.AATYFWPGSEVK.I | Y | 47.15 | 1354.6558 | 12 | 1.0 | 678.3358 | 2 | 45.04 | 1 | F1:25040 | 28122021\_RID\_2349\_2C.raw | 2.6454E5 |  |  |  |  |  | 1 | 1 | 0 | 0 | 0 | 0 | 0 | 246 | 257 |  |  | PEAKS DB |
| total 3 peptides |  |  |  |  |  |  |  |  |  |  |  |  |  |  |  |  |  |  |  |  |  |  |  |  |  |  |  |  |  |
| --- | --- | --- | --- | --- | --- | --- | --- | --- | --- | --- | --- | --- | --- | --- | --- | --- | --- | --- | --- | --- | --- | --- | --- | --- | --- | --- | --- | --- | --- |

JAS04699.1

back to list

  

| Protein Coverage
| Supporting Peptides
|

Protein Coverage:

Supporting Peptides:

| Peptide | Uniq | -10lgP | Mass | Length | ppm | m/z | z | RT | Fraction | Scan | Source File | Area F2 | Area F1 | Area F3 | Area F4 | Area F5 | Area F6 | #Feature | #Feature F2 | #Feature F1 | #Feature F3 | #Feature F4 | #Feature F5 | #Feature F6 | Start | End | PTM | AScore | Found By |
| --- | --- | --- | --- | --- | --- | --- | --- | --- | --- | --- | --- | --- | --- | --- | --- | --- | --- | --- | --- | --- | --- | --- | --- | --- | --- | --- | --- | --- | --- |
| K.YGPVSGEIIK.A | Y | 54.54 | 1061.5757 | 10 | -0.3 | 531.7950 | 2 | 34.19 | 2 | F2:10532 | 30122021\_RID\_2349\_1C.raw |  | 9.1292E4 |  |  |  |  | 1 | 0 | 1 | 0 | 0 | 0 | 0 | 311 | 320 |  |  | PEAKS DB |
| K.DFYTFDSEGIVK.N | Y | 49.58 | 1419.6558 | 12 | 0.4 | 710.8354 | 2 | 52.85 | 2 | F2:17870 | 30122021\_RID\_2349\_1C.raw |  | 2.4259E4 |  |  |  |  | 1 | 0 | 1 | 0 | 0 | 0 | 0 | 393 | 404 |  |  | PEAKS DB |
| K.AATYFWPGSEVK.I | Y | 47.15 | 1354.6558 | 12 | 1.0 | 678.3358 | 2 | 45.04 | 1 | F1:25040 | 28122021\_RID\_2349\_2C.raw | 2.6454E5 |  |  |  |  |  | 1 | 1 | 0 | 0 | 0 | 0 | 0 | 246 | 257 |  |  | PEAKS DB |
| total 3 peptides |  |  |  |  |  |  |  |  |  |  |  |  |  |  |  |  |  |  |  |  |  |  |  |  |  |  |  |  |  |
| --- | --- | --- | --- | --- | --- | --- | --- | --- | --- | --- | --- | --- | --- | --- | --- | --- | --- | --- | --- | --- | --- | --- | --- | --- | --- | --- | --- | --- | --- |

AFJ50876.1

back to list

  

| Protein Coverage
| Supporting Peptides
|

Protein Coverage:

Supporting Peptides:

| Peptide | Uniq | -10lgP | Mass | Length | ppm | m/z | z | RT | Fraction | Scan | Source File | Area F2 | Area F1 | Area F3 | Area F4 | Area F5 | Area F6 | #Feature | #Feature F2 | #Feature F1 | #Feature F3 | #Feature F4 | #Feature F5 | #Feature F6 | Start | End | PTM | AScore | Found By |
| --- | --- | --- | --- | --- | --- | --- | --- | --- | --- | --- | --- | --- | --- | --- | --- | --- | --- | --- | --- | --- | --- | --- | --- | --- | --- | --- | --- | --- | --- |
| K.YGPVSGEIIK.A | Y | 54.54 | 1061.5757 | 10 | -0.3 | 531.7950 | 2 | 34.19 | 2 | F2:10532 | 30122021\_RID\_2349\_1C.raw |  | 9.1292E4 |  |  |  |  | 1 | 0 | 1 | 0 | 0 | 0 | 0 | 339 | 348 |  |  | PEAKS DB |
| K.DFYTFDSEGIVK.N | Y | 49.58 | 1419.6558 | 12 | 0.4 | 710.8354 | 2 | 52.85 | 2 | F2:17870 | 30122021\_RID\_2349\_1C.raw |  | 2.4259E4 |  |  |  |  | 1 | 0 | 1 | 0 | 0 | 0 | 0 | 421 | 432 |  |  | PEAKS DB |
| K.AATYFWPGSEVK.I | Y | 47.15 | 1354.6558 | 12 | 1.0 | 678.3358 | 2 | 45.04 | 1 | F1:25040 | 28122021\_RID\_2349\_2C.raw | 2.6454E5 |  |  |  |  |  | 1 | 1 | 0 | 0 | 0 | 0 | 0 | 274 | 285 |  |  | PEAKS DB |
| total 3 peptides |  |  |  |  |  |  |  |  |  |  |  |  |  |  |  |  |  |  |  |  |  |  |  |  |  |  |  |  |  |
| --- | --- | --- | --- | --- | --- | --- | --- | --- | --- | --- | --- | --- | --- | --- | --- | --- | --- | --- | --- | --- | --- | --- | --- | --- | --- | --- | --- | --- | --- |

XP\_015672096.1

back to list

  

| Protein Coverage
| Supporting Peptides
|

Protein Coverage:

Supporting Peptides:

| Peptide | Uniq | -10lgP | Mass | Length | ppm | m/z | z | RT | Fraction | Scan | Source File | Area F2 | Area F1 | Area F3 | Area F4 | Area F5 | Area F6 | #Feature | #Feature F2 | #Feature F1 | #Feature F3 | #Feature F4 | #Feature F5 | #Feature F6 | Start | End | PTM | AScore | Found By |
| --- | --- | --- | --- | --- | --- | --- | --- | --- | --- | --- | --- | --- | --- | --- | --- | --- | --- | --- | --- | --- | --- | --- | --- | --- | --- | --- | --- | --- | --- |
| R.VVEAQSQVVSGAK.Y | Y | 67.99 | 1300.6986 | 13 | 1.0 | 651.3572 | 2 | 23.04 | 6 | F6:6584 | 31122021\_RID\_2349\_6C.raw |  |  |  |  |  | 1.3292E5 | 1 | 0 | 0 | 0 | 0 | 0 | 1 | 65 | 77 |  |  | PEAKS DB |
| K.YYLTMELVK.T | Y | 50.09 | 1158.5995 | 9 | 1.0 | 580.3076 | 2 | 47.98 | 6 | F6:25390 | 31122021\_RID\_2349\_6C.raw |  |  |  |  |  | 8.6814E4 | 1 | 0 | 0 | 0 | 0 | 0 | 1 | 78 | 86 |  |  | PEAKS DB |
| total 2 peptides |  |  |  |  |  |  |  |  |  |  |  |  |  |  |  |  |  |  |  |  |  |  |  |  |  |  |  |  |  |
| --- | --- | --- | --- | --- | --- | --- | --- | --- | --- | --- | --- | --- | --- | --- | --- | --- | --- | --- | --- | --- | --- | --- | --- | --- | --- | --- | --- | --- | --- |

XP\_026561295.1

back to list

  

| Protein Coverage
| Supporting Peptides
|

Protein Coverage:

Supporting Peptides:

| Peptide | Uniq | -10lgP | Mass | Length | ppm | m/z | z | RT | Fraction | Scan | Source File | Area F2 | Area F1 | Area F3 | Area F4 | Area F5 | Area F6 | #Feature | #Feature F2 | #Feature F1 | #Feature F3 | #Feature F4 | #Feature F5 | #Feature F6 | Start | End | PTM | AScore | Found By |
| --- | --- | --- | --- | --- | --- | --- | --- | --- | --- | --- | --- | --- | --- | --- | --- | --- | --- | --- | --- | --- | --- | --- | --- | --- | --- | --- | --- | --- | --- |
| K.LMNQNIDILEDAIQK.A | Y | 67.56 | 1756.9030 | 15 | 1.4 | 879.4600 | 2 | 54.14 | 6 | F6:30141 | 31122021\_RID\_2349\_6C.raw |  |  |  |  |  | 3.3639E4 | 1 | 0 | 0 | 0 | 0 | 0 | 1 | 55 | 69 |  |  | PEAKS DB |
| R.FGPAPVQK.R | Y | 46.19 | 842.4650 | 8 | -0.3 | 422.2396 | 2 | 23.51 | 1 | F1:7852 | 28122021\_RID\_2349\_2C.raw | 4.7087E5 |  |  |  |  |  | 1 | 1 | 0 | 0 | 0 | 0 | 0 | 118 | 125 |  |  | PEAKS DB |
| total 2 peptides |  |  |  |  |  |  |  |  |  |  |  |  |  |  |  |  |  |  |  |  |  |  |  |  |  |  |  |  |  |
| --- | --- | --- | --- | --- | --- | --- | --- | --- | --- | --- | --- | --- | --- | --- | --- | --- | --- | --- | --- | --- | --- | --- | --- | --- | --- | --- | --- | --- | --- |

XP\_026561296.1

back to list

  

| Protein Coverage
| Supporting Peptides
|

Protein Coverage:

Supporting Peptides:

| Peptide | Uniq | -10lgP | Mass | Length | ppm | m/z | z | RT | Fraction | Scan | Source File | Area F2 | Area F1 | Area F3 | Area F4 | Area F5 | Area F6 | #Feature | #Feature F2 | #Feature F1 | #Feature F3 | #Feature F4 | #Feature F5 | #Feature F6 | Start | End | PTM | AScore | Found By |
| --- | --- | --- | --- | --- | --- | --- | --- | --- | --- | --- | --- | --- | --- | --- | --- | --- | --- | --- | --- | --- | --- | --- | --- | --- | --- | --- | --- | --- | --- |
| K.LMNQNIDILEDAIQK.A | Y | 67.56 | 1756.9030 | 15 | 1.4 | 879.4600 | 2 | 54.14 | 6 | F6:30141 | 31122021\_RID\_2349\_6C.raw |  |  |  |  |  | 3.3639E4 | 1 | 0 | 0 | 0 | 0 | 0 | 1 | 55 | 69 |  |  | PEAKS DB |
| R.FGPAPVQK.R | Y | 46.19 | 842.4650 | 8 | -0.3 | 422.2396 | 2 | 23.51 | 1 | F1:7852 | 28122021\_RID\_2349\_2C.raw | 4.7087E5 |  |  |  |  |  | 1 | 1 | 0 | 0 | 0 | 0 | 0 | 118 | 125 |  |  | PEAKS DB |
| total 2 peptides |  |  |  |  |  |  |  |  |  |  |  |  |  |  |  |  |  |  |  |  |  |  |  |  |  |  |  |  |  |
| --- | --- | --- | --- | --- | --- | --- | --- | --- | --- | --- | --- | --- | --- | --- | --- | --- | --- | --- | --- | --- | --- | --- | --- | --- | --- | --- | --- | --- | --- |

XP\_026561293.1

back to list

  

| Protein Coverage
| Supporting Peptides
|

Protein Coverage:

Supporting Peptides:

| Peptide | Uniq | -10lgP | Mass | Length | ppm | m/z | z | RT | Fraction | Scan | Source File | Area F2 | Area F1 | Area F3 | Area F4 | Area F5 | Area F6 | #Feature | #Feature F2 | #Feature F1 | #Feature F3 | #Feature F4 | #Feature F5 | #Feature F6 | Start | End | PTM | AScore | Found By |
| --- | --- | --- | --- | --- | --- | --- | --- | --- | --- | --- | --- | --- | --- | --- | --- | --- | --- | --- | --- | --- | --- | --- | --- | --- | --- | --- | --- | --- | --- |
| K.LMNQNIDILEDAIQK.A | Y | 67.56 | 1756.9030 | 15 | 1.4 | 879.4600 | 2 | 54.14 | 6 | F6:30141 | 31122021\_RID\_2349\_6C.raw |  |  |  |  |  | 3.3639E4 | 1 | 0 | 0 | 0 | 0 | 0 | 1 | 55 | 69 |  |  | PEAKS DB |
| R.FGPAPVQK.R | Y | 46.19 | 842.4650 | 8 | -0.3 | 422.2396 | 2 | 23.51 | 1 | F1:7852 | 28122021\_RID\_2349\_2C.raw | 4.7087E5 |  |  |  |  |  | 1 | 1 | 0 | 0 | 0 | 0 | 0 | 118 | 125 |  |  | PEAKS DB |
| total 2 peptides |  |  |  |  |  |  |  |  |  |  |  |  |  |  |  |  |  |  |  |  |  |  |  |  |  |  |  |  |  |
| --- | --- | --- | --- | --- | --- | --- | --- | --- | --- | --- | --- | --- | --- | --- | --- | --- | --- | --- | --- | --- | --- | --- | --- | --- | --- | --- | --- | --- | --- |

AHJ09589.1

back to list

  

| Protein Coverage
| Supporting Peptides
|

Protein Coverage:

Supporting Peptides:

| Peptide | Uniq | -10lgP | Mass | Length | ppm | m/z | z | RT | Fraction | Scan | Source File | Area F2 | Area F1 | Area F3 | Area F4 | Area F5 | Area F6 | #Feature | #Feature F2 | #Feature F1 | #Feature F3 | #Feature F4 | #Feature F5 | #Feature F6 | Start | End | PTM | AScore | Found By |
| --- | --- | --- | --- | --- | --- | --- | --- | --- | --- | --- | --- | --- | --- | --- | --- | --- | --- | --- | --- | --- | --- | --- | --- | --- | --- | --- | --- | --- | --- |
| G.HLM(+15.99)QFETMIK.K | Y | 64.50 | 1292.6257 | 10 | -0.1 | 647.3201 | 2 | 33.27 | 5 | F5:14943 | 31122021\_RID\_2349\_5C.raw | 1.1323E6 |  |  |  | 5.3875E6 | 5.0321E6 | 3 | 1 | 0 | 0 | 0 | 1 | 1 | 17 | 26 | Oxidation (M) | M3:Oxidation (M):127.44 | PEAKS DB |
| G.HLMQFETMIK.K | Y | 56.72 | 1276.6309 | 10 | 0.7 | 639.3232 | 2 | 39.39 | 6 | F6:18928 | 31122021\_RID\_2349\_6C.raw |  |  |  |  | 1.8542E6 | 1.7142E6 | 2 | 0 | 0 | 0 | 0 | 1 | 1 | 17 | 26 |  |  | PEAKS DB |
| G.HLM(+15.99)QFETMIKK.V | Y | 50.50 | 1420.7207 | 11 | 0.3 | 474.5810 | 3 | 27.28 | 6 | F6:9566 | 31122021\_RID\_2349\_6C.raw |  |  |  |  | 9.215E5 | 6.357E5 | 2 | 0 | 0 | 0 | 0 | 1 | 1 | 17 | 27 | Oxidation (M) | M3:Oxidation (M):79.02 | PEAKS DB |
| G.HLM(+15.99)QFETM(+15.99)IK.K | Y | 50.24 | 1308.6206 | 10 | 0.2 | 655.3177 | 2 | 26.84 | 6 | F6:9287 | 31122021\_RID\_2349\_6C.raw |  |  |  |  | 4.8168E5 | 6.0555E5 | 2 | 0 | 0 | 0 | 0 | 1 | 1 | 17 | 26 | Oxidation (M) | M3:Oxidation (M):1000.00;M8:Oxidation (M):1000.00 | PEAKS DB |
| total 4 peptides |  |  |  |  |  |  |  |  |  |  |  |  |  |  |  |  |  |  |  |  |  |  |  |  |  |  |  |  |  |
| --- | --- | --- | --- | --- | --- | --- | --- | --- | --- | --- | --- | --- | --- | --- | --- | --- | --- | --- | --- | --- | --- | --- | --- | --- | --- | --- | --- | --- | --- |

AHJ09585.1

back to list

  

| Protein Coverage
| Supporting Peptides
|

Protein Coverage:

Supporting Peptides:

| Peptide | Uniq | -10lgP | Mass | Length | ppm | m/z | z | RT | Fraction | Scan | Source File | Area F2 | Area F1 | Area F3 | Area F4 | Area F5 | Area F6 | #Feature | #Feature F2 | #Feature F1 | #Feature F3 | #Feature F4 | #Feature F5 | #Feature F6 | Start | End | PTM | AScore | Found By |
| --- | --- | --- | --- | --- | --- | --- | --- | --- | --- | --- | --- | --- | --- | --- | --- | --- | --- | --- | --- | --- | --- | --- | --- | --- | --- | --- | --- | --- | --- |
| G.HLM(+15.99)QFETMIK.K | Y | 64.50 | 1292.6257 | 10 | -0.1 | 647.3201 | 2 | 33.27 | 5 | F5:14943 | 31122021\_RID\_2349\_5C.raw | 1.1323E6 |  |  |  | 5.3875E6 | 5.0321E6 | 3 | 1 | 0 | 0 | 0 | 1 | 1 | 17 | 26 | Oxidation (M) | M3:Oxidation (M):127.44 | PEAKS DB |
| G.HLMQFETMIK.K | Y | 56.72 | 1276.6309 | 10 | 0.7 | 639.3232 | 2 | 39.39 | 6 | F6:18928 | 31122021\_RID\_2349\_6C.raw |  |  |  |  | 1.8542E6 | 1.7142E6 | 2 | 0 | 0 | 0 | 0 | 1 | 1 | 17 | 26 |  |  | PEAKS DB |
| G.HLM(+15.99)QFETMIKK.V | Y | 50.50 | 1420.7207 | 11 | 0.3 | 474.5810 | 3 | 27.28 | 6 | F6:9566 | 31122021\_RID\_2349\_6C.raw |  |  |  |  | 9.215E5 | 6.357E5 | 2 | 0 | 0 | 0 | 0 | 1 | 1 | 17 | 27 | Oxidation (M) | M3:Oxidation (M):79.02 | PEAKS DB |
| G.HLM(+15.99)QFETM(+15.99)IK.K | Y | 50.24 | 1308.6206 | 10 | 0.2 | 655.3177 | 2 | 26.84 | 6 | F6:9287 | 31122021\_RID\_2349\_6C.raw |  |  |  |  | 4.8168E5 | 6.0555E5 | 2 | 0 | 0 | 0 | 0 | 1 | 1 | 17 | 26 | Oxidation (M) | M3:Oxidation (M):1000.00;M8:Oxidation (M):1000.00 | PEAKS DB |
| total 4 peptides |  |  |  |  |  |  |  |  |  |  |  |  |  |  |  |  |  |  |  |  |  |  |  |  |  |  |  |  |  |
| --- | --- | --- | --- | --- | --- | --- | --- | --- | --- | --- | --- | --- | --- | --- | --- | --- | --- | --- | --- | --- | --- | --- | --- | --- | --- | --- | --- | --- | --- |

AHJ09594.1

back to list

  

| Protein Coverage
| Supporting Peptides
|

Protein Coverage:

Supporting Peptides:

| Peptide | Uniq | -10lgP | Mass | Length | ppm | m/z | z | RT | Fraction | Scan | Source File | Area F2 | Area F1 | Area F3 | Area F4 | Area F5 | Area F6 | #Feature | #Feature F2 | #Feature F1 | #Feature F3 | #Feature F4 | #Feature F5 | #Feature F6 | Start | End | PTM | AScore | Found By |
| --- | --- | --- | --- | --- | --- | --- | --- | --- | --- | --- | --- | --- | --- | --- | --- | --- | --- | --- | --- | --- | --- | --- | --- | --- | --- | --- | --- | --- | --- |
| G.HLM(+15.99)QFETMIK.K | Y | 64.50 | 1292.6257 | 10 | -0.1 | 647.3201 | 2 | 33.27 | 5 | F5:14943 | 31122021\_RID\_2349\_5C.raw | 1.1323E6 |  |  |  | 5.3875E6 | 5.0321E6 | 3 | 1 | 0 | 0 | 0 | 1 | 1 | 17 | 26 | Oxidation (M) | M3:Oxidation (M):127.44 | PEAKS DB |
| G.HLMQFETMIK.K | Y | 56.72 | 1276.6309 | 10 | 0.7 | 639.3232 | 2 | 39.39 | 6 | F6:18928 | 31122021\_RID\_2349\_6C.raw |  |  |  |  | 1.8542E6 | 1.7142E6 | 2 | 0 | 0 | 0 | 0 | 1 | 1 | 17 | 26 |  |  | PEAKS DB |
| G.HLM(+15.99)QFETMIKK.V | Y | 50.50 | 1420.7207 | 11 | 0.3 | 474.5810 | 3 | 27.28 | 6 | F6:9566 | 31122021\_RID\_2349\_6C.raw |  |  |  |  | 9.215E5 | 6.357E5 | 2 | 0 | 0 | 0 | 0 | 1 | 1 | 17 | 27 | Oxidation (M) | M3:Oxidation (M):79.02 | PEAKS DB |
| G.HLM(+15.99)QFETM(+15.99)IK.K | Y | 50.24 | 1308.6206 | 10 | 0.2 | 655.3177 | 2 | 26.84 | 6 | F6:9287 | 31122021\_RID\_2349\_6C.raw |  |  |  |  | 4.8168E5 | 6.0555E5 | 2 | 0 | 0 | 0 | 0 | 1 | 1 | 17 | 26 | Oxidation (M) | M3:Oxidation (M):1000.00;M8:Oxidation (M):1000.00 | PEAKS DB |
| total 4 peptides |  |  |  |  |  |  |  |  |  |  |  |  |  |  |  |  |  |  |  |  |  |  |  |  |  |  |  |  |  |
| --- | --- | --- | --- | --- | --- | --- | --- | --- | --- | --- | --- | --- | --- | --- | --- | --- | --- | --- | --- | --- | --- | --- | --- | --- | --- | --- | --- | --- | --- |

AHJ09590.1

back to list

  

| Protein Coverage
| Supporting Peptides
|

Protein Coverage:

Supporting Peptides:

| Peptide | Uniq | -10lgP | Mass | Length | ppm | m/z | z | RT | Fraction | Scan | Source File | Area F2 | Area F1 | Area F3 | Area F4 | Area F5 | Area F6 | #Feature | #Feature F2 | #Feature F1 | #Feature F3 | #Feature F4 | #Feature F5 | #Feature F6 | Start | End | PTM | AScore | Found By |
| --- | --- | --- | --- | --- | --- | --- | --- | --- | --- | --- | --- | --- | --- | --- | --- | --- | --- | --- | --- | --- | --- | --- | --- | --- | --- | --- | --- | --- | --- |
| G.HLM(+15.99)QFETMIK.K | Y | 64.50 | 1292.6257 | 10 | -0.1 | 647.3201 | 2 | 33.27 | 5 | F5:14943 | 31122021\_RID\_2349\_5C.raw | 1.1323E6 |  |  |  | 5.3875E6 | 5.0321E6 | 3 | 1 | 0 | 0 | 0 | 1 | 1 | 17 | 26 | Oxidation (M) | M3:Oxidation (M):127.44 | PEAKS DB |
| G.HLMQFETMIK.K | Y | 56.72 | 1276.6309 | 10 | 0.7 | 639.3232 | 2 | 39.39 | 6 | F6:18928 | 31122021\_RID\_2349\_6C.raw |  |  |  |  | 1.8542E6 | 1.7142E6 | 2 | 0 | 0 | 0 | 0 | 1 | 1 | 17 | 26 |  |  | PEAKS DB |
| G.HLM(+15.99)QFETMIKK.V | Y | 50.50 | 1420.7207 | 11 | 0.3 | 474.5810 | 3 | 27.28 | 6 | F6:9566 | 31122021\_RID\_2349\_6C.raw |  |  |  |  | 9.215E5 | 6.357E5 | 2 | 0 | 0 | 0 | 0 | 1 | 1 | 17 | 27 | Oxidation (M) | M3:Oxidation (M):79.02 | PEAKS DB |
| G.HLM(+15.99)QFETM(+15.99)IK.K | Y | 50.24 | 1308.6206 | 10 | 0.2 | 655.3177 | 2 | 26.84 | 6 | F6:9287 | 31122021\_RID\_2349\_6C.raw |  |  |  |  | 4.8168E5 | 6.0555E5 | 2 | 0 | 0 | 0 | 0 | 1 | 1 | 17 | 26 | Oxidation (M) | M3:Oxidation (M):1000.00;M8:Oxidation (M):1000.00 | PEAKS DB |
| total 4 peptides |  |  |  |  |  |  |  |  |  |  |  |  |  |  |  |  |  |  |  |  |  |  |  |  |  |  |  |  |  |
| --- | --- | --- | --- | --- | --- | --- | --- | --- | --- | --- | --- | --- | --- | --- | --- | --- | --- | --- | --- | --- | --- | --- | --- | --- | --- | --- | --- | --- | --- |

AHJ09520.1

back to list

  

| Protein Coverage
| Supporting Peptides
|

Protein Coverage:

Supporting Peptides:

| Peptide | Uniq | -10lgP | Mass | Length | ppm | m/z | z | RT | Fraction | Scan | Source File | Area F2 | Area F1 | Area F3 | Area F4 | Area F5 | Area F6 | #Feature | #Feature F2 | #Feature F1 | #Feature F3 | #Feature F4 | #Feature F5 | #Feature F6 | Start | End | PTM | AScore | Found By |
| --- | --- | --- | --- | --- | --- | --- | --- | --- | --- | --- | --- | --- | --- | --- | --- | --- | --- | --- | --- | --- | --- | --- | --- | --- | --- | --- | --- | --- | --- |
| G.HLM(+15.99)QFETMIK.K | Y | 64.50 | 1292.6257 | 10 | -0.1 | 647.3201 | 2 | 33.27 | 5 | F5:14943 | 31122021\_RID\_2349\_5C.raw | 1.1323E6 |  |  |  | 5.3875E6 | 5.0321E6 | 3 | 1 | 0 | 0 | 0 | 1 | 1 | 17 | 26 | Oxidation (M) | M3:Oxidation (M):127.44 | PEAKS DB |
| G.HLMQFETMIK.K | Y | 56.72 | 1276.6309 | 10 | 0.7 | 639.3232 | 2 | 39.39 | 6 | F6:18928 | 31122021\_RID\_2349\_6C.raw |  |  |  |  | 1.8542E6 | 1.7142E6 | 2 | 0 | 0 | 0 | 0 | 1 | 1 | 17 | 26 |  |  | PEAKS DB |
| G.HLM(+15.99)QFETMIKK.V | Y | 50.50 | 1420.7207 | 11 | 0.3 | 474.5810 | 3 | 27.28 | 6 | F6:9566 | 31122021\_RID\_2349\_6C.raw |  |  |  |  | 9.215E5 | 6.357E5 | 2 | 0 | 0 | 0 | 0 | 1 | 1 | 17 | 27 | Oxidation (M) | M3:Oxidation (M):79.02 | PEAKS DB |
| G.HLM(+15.99)QFETM(+15.99)IK.K | Y | 50.24 | 1308.6206 | 10 | 0.2 | 655.3177 | 2 | 26.84 | 6 | F6:9287 | 31122021\_RID\_2349\_6C.raw |  |  |  |  | 4.8168E5 | 6.0555E5 | 2 | 0 | 0 | 0 | 0 | 1 | 1 | 17 | 26 | Oxidation (M) | M3:Oxidation (M):1000.00;M8:Oxidation (M):1000.00 | PEAKS DB |
| total 4 peptides |  |  |  |  |  |  |  |  |  |  |  |  |  |  |  |  |  |  |  |  |  |  |  |  |  |  |  |  |  |
| --- | --- | --- | --- | --- | --- | --- | --- | --- | --- | --- | --- | --- | --- | --- | --- | --- | --- | --- | --- | --- | --- | --- | --- | --- | --- | --- | --- | --- | --- |

AHJ09591.1

back to list

  

| Protein Coverage
| Supporting Peptides
|

Protein Coverage:

Supporting Peptides:

| Peptide | Uniq | -10lgP | Mass | Length | ppm | m/z | z | RT | Fraction | Scan | Source File | Area F2 | Area F1 | Area F3 | Area F4 | Area F5 | Area F6 | #Feature | #Feature F2 | #Feature F1 | #Feature F3 | #Feature F4 | #Feature F5 | #Feature F6 | Start | End | PTM | AScore | Found By |
| --- | --- | --- | --- | --- | --- | --- | --- | --- | --- | --- | --- | --- | --- | --- | --- | --- | --- | --- | --- | --- | --- | --- | --- | --- | --- | --- | --- | --- | --- |
| G.HLM(+15.99)QFETMIK.K | Y | 64.50 | 1292.6257 | 10 | -0.1 | 647.3201 | 2 | 33.27 | 5 | F5:14943 | 31122021\_RID\_2349\_5C.raw | 1.1323E6 |  |  |  | 5.3875E6 | 5.0321E6 | 3 | 1 | 0 | 0 | 0 | 1 | 1 | 17 | 26 | Oxidation (M) | M3:Oxidation (M):127.44 | PEAKS DB |
| G.HLMQFETMIK.K | Y | 56.72 | 1276.6309 | 10 | 0.7 | 639.3232 | 2 | 39.39 | 6 | F6:18928 | 31122021\_RID\_2349\_6C.raw |  |  |  |  | 1.8542E6 | 1.7142E6 | 2 | 0 | 0 | 0 | 0 | 1 | 1 | 17 | 26 |  |  | PEAKS DB |
| G.HLM(+15.99)QFETMIKK.V | Y | 50.50 | 1420.7207 | 11 | 0.3 | 474.5810 | 3 | 27.28 | 6 | F6:9566 | 31122021\_RID\_2349\_6C.raw |  |  |  |  | 9.215E5 | 6.357E5 | 2 | 0 | 0 | 0 | 0 | 1 | 1 | 17 | 27 | Oxidation (M) | M3:Oxidation (M):79.02 | PEAKS DB |
| G.HLM(+15.99)QFETM(+15.99)IK.K | Y | 50.24 | 1308.6206 | 10 | 0.2 | 655.3177 | 2 | 26.84 | 6 | F6:9287 | 31122021\_RID\_2349\_6C.raw |  |  |  |  | 4.8168E5 | 6.0555E5 | 2 | 0 | 0 | 0 | 0 | 1 | 1 | 17 | 26 | Oxidation (M) | M3:Oxidation (M):1000.00;M8:Oxidation (M):1000.00 | PEAKS DB |
| total 4 peptides |  |  |  |  |  |  |  |  |  |  |  |  |  |  |  |  |  |  |  |  |  |  |  |  |  |  |  |  |  |
| --- | --- | --- | --- | --- | --- | --- | --- | --- | --- | --- | --- | --- | --- | --- | --- | --- | --- | --- | --- | --- | --- | --- | --- | --- | --- | --- | --- | --- | --- |

AHJ09587.1

back to list

  

| Protein Coverage
| Supporting Peptides
|

Protein Coverage:

Supporting Peptides:

| Peptide | Uniq | -10lgP | Mass | Length | ppm | m/z | z | RT | Fraction | Scan | Source File | Area F2 | Area F1 | Area F3 | Area F4 | Area F5 | Area F6 | #Feature | #Feature F2 | #Feature F1 | #Feature F3 | #Feature F4 | #Feature F5 | #Feature F6 | Start | End | PTM | AScore | Found By |
| --- | --- | --- | --- | --- | --- | --- | --- | --- | --- | --- | --- | --- | --- | --- | --- | --- | --- | --- | --- | --- | --- | --- | --- | --- | --- | --- | --- | --- | --- |
| G.HLM(+15.99)QFETMIK.K | Y | 64.50 | 1292.6257 | 10 | -0.1 | 647.3201 | 2 | 33.27 | 5 | F5:14943 | 31122021\_RID\_2349\_5C.raw | 1.1323E6 |  |  |  | 5.3875E6 | 5.0321E6 | 3 | 1 | 0 | 0 | 0 | 1 | 1 | 17 | 26 | Oxidation (M) | M3:Oxidation (M):127.44 | PEAKS DB |
| G.HLMQFETMIK.K | Y | 56.72 | 1276.6309 | 10 | 0.7 | 639.3232 | 2 | 39.39 | 6 | F6:18928 | 31122021\_RID\_2349\_6C.raw |  |  |  |  | 1.8542E6 | 1.7142E6 | 2 | 0 | 0 | 0 | 0 | 1 | 1 | 17 | 26 |  |  | PEAKS DB |
| G.HLM(+15.99)QFETMIKK.V | Y | 50.50 | 1420.7207 | 11 | 0.3 | 474.5810 | 3 | 27.28 | 6 | F6:9566 | 31122021\_RID\_2349\_6C.raw |  |  |  |  | 9.215E5 | 6.357E5 | 2 | 0 | 0 | 0 | 0 | 1 | 1 | 17 | 27 | Oxidation (M) | M3:Oxidation (M):79.02 | PEAKS DB |
| G.HLM(+15.99)QFETM(+15.99)IK.K | Y | 50.24 | 1308.6206 | 10 | 0.2 | 655.3177 | 2 | 26.84 | 6 | F6:9287 | 31122021\_RID\_2349\_6C.raw |  |  |  |  | 4.8168E5 | 6.0555E5 | 2 | 0 | 0 | 0 | 0 | 1 | 1 | 17 | 26 | Oxidation (M) | M3:Oxidation (M):1000.00;M8:Oxidation (M):1000.00 | PEAKS DB |
| total 4 peptides |  |  |  |  |  |  |  |  |  |  |  |  |  |  |  |  |  |  |  |  |  |  |  |  |  |  |  |  |  |
| --- | --- | --- | --- | --- | --- | --- | --- | --- | --- | --- | --- | --- | --- | --- | --- | --- | --- | --- | --- | --- | --- | --- | --- | --- | --- | --- | --- | --- | --- |

AHJ09549.1

back to list

  

| Protein Coverage
| Supporting Peptides
|

Protein Coverage:

Supporting Peptides:

| Peptide | Uniq | -10lgP | Mass | Length | ppm | m/z | z | RT | Fraction | Scan | Source File | Area F2 | Area F1 | Area F3 | Area F4 | Area F5 | Area F6 | #Feature | #Feature F2 | #Feature F1 | #Feature F3 | #Feature F4 | #Feature F5 | #Feature F6 | Start | End | PTM | AScore | Found By |
| --- | --- | --- | --- | --- | --- | --- | --- | --- | --- | --- | --- | --- | --- | --- | --- | --- | --- | --- | --- | --- | --- | --- | --- | --- | --- | --- | --- | --- | --- |
| G.HLM(+15.99)QFETMIK.K | Y | 64.50 | 1292.6257 | 10 | -0.1 | 647.3201 | 2 | 33.27 | 5 | F5:14943 | 31122021\_RID\_2349\_5C.raw | 1.1323E6 |  |  |  | 5.3875E6 | 5.0321E6 | 3 | 1 | 0 | 0 | 0 | 1 | 1 | 17 | 26 | Oxidation (M) | M3:Oxidation (M):127.44 | PEAKS DB |
| G.HLMQFETMIK.K | Y | 56.72 | 1276.6309 | 10 | 0.7 | 639.3232 | 2 | 39.39 | 6 | F6:18928 | 31122021\_RID\_2349\_6C.raw |  |  |  |  | 1.8542E6 | 1.7142E6 | 2 | 0 | 0 | 0 | 0 | 1 | 1 | 17 | 26 |  |  | PEAKS DB |
| G.HLM(+15.99)QFETMIKK.A | Y | 50.50 | 1420.7207 | 11 | 0.3 | 474.5810 | 3 | 27.28 | 6 | F6:9566 | 31122021\_RID\_2349\_6C.raw |  |  |  |  | 9.215E5 | 6.357E5 | 2 | 0 | 0 | 0 | 0 | 1 | 1 | 17 | 27 | Oxidation (M) | M3:Oxidation (M):79.02 | PEAKS DB |
| G.HLM(+15.99)QFETM(+15.99)IK.K | Y | 50.24 | 1308.6206 | 10 | 0.2 | 655.3177 | 2 | 26.84 | 6 | F6:9287 | 31122021\_RID\_2349\_6C.raw |  |  |  |  | 4.8168E5 | 6.0555E5 | 2 | 0 | 0 | 0 | 0 | 1 | 1 | 17 | 26 | Oxidation (M) | M3:Oxidation (M):1000.00;M8:Oxidation (M):1000.00 | PEAKS DB |
| total 4 peptides |  |  |  |  |  |  |  |  |  |  |  |  |  |  |  |  |  |  |  |  |  |  |  |  |  |  |  |  |  |
| --- | --- | --- | --- | --- | --- | --- | --- | --- | --- | --- | --- | --- | --- | --- | --- | --- | --- | --- | --- | --- | --- | --- | --- | --- | --- | --- | --- | --- | --- |

XP\_026576301.1

back to list

  

| Protein Coverage
| Supporting Peptides
|

Protein Coverage:

Supporting Peptides:

| Peptide | Uniq | -10lgP | Mass | Length | ppm | m/z | z | RT | Fraction | Scan | Source File | Area F2 | Area F1 | Area F3 | Area F4 | Area F5 | Area F6 | #Feature | #Feature F2 | #Feature F1 | #Feature F3 | #Feature F4 | #Feature F5 | #Feature F6 | Start | End | PTM | AScore | Found By |
| --- | --- | --- | --- | --- | --- | --- | --- | --- | --- | --- | --- | --- | --- | --- | --- | --- | --- | --- | --- | --- | --- | --- | --- | --- | --- | --- | --- | --- | --- |
| R.LNYGIPAIVK.L | Y | 55.09 | 1086.6437 | 10 | 0.7 | 544.3295 | 2 | 45.40 | 1 | F1:25328 | 28122021\_RID\_2349\_2C.raw | 1.1506E5 |  |  |  |  |  | 1 | 1 | 0 | 0 | 0 | 0 | 0 | 478 | 487 |  |  | PEAKS DB |
| K.LLWTTSR.V | Y | 42.34 | 875.4865 | 7 | 0.5 | 438.7508 | 2 | 35.52 | 1 | F1:17478 | 28122021\_RID\_2349\_2C.raw | 2.8921E4 |  |  |  |  |  | 1 | 1 | 0 | 0 | 0 | 0 | 0 | 327 | 333 |  |  | PEAKS DB |
| total 2 peptides |  |  |  |  |  |  |  |  |  |  |  |  |  |  |  |  |  |  |  |  |  |  |  |  |  |  |  |  |  |
| --- | --- | --- | --- | --- | --- | --- | --- | --- | --- | --- | --- | --- | --- | --- | --- | --- | --- | --- | --- | --- | --- | --- | --- | --- | --- | --- | --- | --- | --- |

XP\_026525656.1

back to list

  

| Protein Coverage
| Supporting Peptides
|

Protein Coverage:

Supporting Peptides:

| Peptide | Uniq | -10lgP | Mass | Length | ppm | m/z | z | RT | Fraction | Scan | Source File | Area F2 | Area F1 | Area F3 | Area F4 | Area F5 | Area F6 | #Feature | #Feature F2 | #Feature F1 | #Feature F3 | #Feature F4 | #Feature F5 | #Feature F6 | Start | End | PTM | AScore | Found By |
| --- | --- | --- | --- | --- | --- | --- | --- | --- | --- | --- | --- | --- | --- | --- | --- | --- | --- | --- | --- | --- | --- | --- | --- | --- | --- | --- | --- | --- | --- |
| R.LNYGIPAIVK.L | Y | 55.09 | 1086.6437 | 10 | 0.7 | 544.3295 | 2 | 45.40 | 1 | F1:25328 | 28122021\_RID\_2349\_2C.raw | 1.1506E5 |  |  |  |  |  | 1 | 1 | 0 | 0 | 0 | 0 | 0 | 478 | 487 |  |  | PEAKS DB |
| K.LLWTTSR.V | Y | 42.34 | 875.4865 | 7 | 0.5 | 438.7508 | 2 | 35.52 | 1 | F1:17478 | 28122021\_RID\_2349\_2C.raw | 2.8921E4 |  |  |  |  |  | 1 | 1 | 0 | 0 | 0 | 0 | 0 | 327 | 333 |  |  | PEAKS DB |
| total 2 peptides |  |  |  |  |  |  |  |  |  |  |  |  |  |  |  |  |  |  |  |  |  |  |  |  |  |  |  |  |  |
| --- | --- | --- | --- | --- | --- | --- | --- | --- | --- | --- | --- | --- | --- | --- | --- | --- | --- | --- | --- | --- | --- | --- | --- | --- | --- | --- | --- | --- | --- |

XP\_026576299.1

back to list

  

| Protein Coverage
| Supporting Peptides
|

Protein Coverage:

Supporting Peptides:

| Peptide | Uniq | -10lgP | Mass | Length | ppm | m/z | z | RT | Fraction | Scan | Source File | Area F2 | Area F1 | Area F3 | Area F4 | Area F5 | Area F6 | #Feature | #Feature F2 | #Feature F1 | #Feature F3 | #Feature F4 | #Feature F5 | #Feature F6 | Start | End | PTM | AScore | Found By |
| --- | --- | --- | --- | --- | --- | --- | --- | --- | --- | --- | --- | --- | --- | --- | --- | --- | --- | --- | --- | --- | --- | --- | --- | --- | --- | --- | --- | --- | --- |
| R.LNYGIPAIVK.L | Y | 55.09 | 1086.6437 | 10 | 0.7 | 544.3295 | 2 | 45.40 | 1 | F1:25328 | 28122021\_RID\_2349\_2C.raw | 1.1506E5 |  |  |  |  |  | 1 | 1 | 0 | 0 | 0 | 0 | 0 | 479 | 488 |  |  | PEAKS DB |
| K.LLWTTSR.V | Y | 42.34 | 875.4865 | 7 | 0.5 | 438.7508 | 2 | 35.52 | 1 | F1:17478 | 28122021\_RID\_2349\_2C.raw | 2.8921E4 |  |  |  |  |  | 1 | 1 | 0 | 0 | 0 | 0 | 0 | 328 | 334 |  |  | PEAKS DB |
| total 2 peptides |  |  |  |  |  |  |  |  |  |  |  |  |  |  |  |  |  |  |  |  |  |  |  |  |  |  |  |  |  |
| --- | --- | --- | --- | --- | --- | --- | --- | --- | --- | --- | --- | --- | --- | --- | --- | --- | --- | --- | --- | --- | --- | --- | --- | --- | --- | --- | --- | --- | --- |

XP\_026525655.1

back to list

  

| Protein Coverage
| Supporting Peptides
|

Protein Coverage:

Supporting Peptides:

| Peptide | Uniq | -10lgP | Mass | Length | ppm | m/z | z | RT | Fraction | Scan | Source File | Area F2 | Area F1 | Area F3 | Area F4 | Area F5 | Area F6 | #Feature | #Feature F2 | #Feature F1 | #Feature F3 | #Feature F4 | #Feature F5 | #Feature F6 | Start | End | PTM | AScore | Found By |
| --- | --- | --- | --- | --- | --- | --- | --- | --- | --- | --- | --- | --- | --- | --- | --- | --- | --- | --- | --- | --- | --- | --- | --- | --- | --- | --- | --- | --- | --- |
| R.LNYGIPAIVK.L | Y | 55.09 | 1086.6437 | 10 | 0.7 | 544.3295 | 2 | 45.40 | 1 | F1:25328 | 28122021\_RID\_2349\_2C.raw | 1.1506E5 |  |  |  |  |  | 1 | 1 | 0 | 0 | 0 | 0 | 0 | 479 | 488 |  |  | PEAKS DB |
| K.LLWTTSR.V | Y | 42.34 | 875.4865 | 7 | 0.5 | 438.7508 | 2 | 35.52 | 1 | F1:17478 | 28122021\_RID\_2349\_2C.raw | 2.8921E4 |  |  |  |  |  | 1 | 1 | 0 | 0 | 0 | 0 | 0 | 328 | 334 |  |  | PEAKS DB |
| total 2 peptides |  |  |  |  |  |  |  |  |  |  |  |  |  |  |  |  |  |  |  |  |  |  |  |  |  |  |  |  |  |
| --- | --- | --- | --- | --- | --- | --- | --- | --- | --- | --- | --- | --- | --- | --- | --- | --- | --- | --- | --- | --- | --- | --- | --- | --- | --- | --- | --- | --- | --- |

XP\_026525654.1

back to list

  

| Protein Coverage
| Supporting Peptides
|

Protein Coverage:

Supporting Peptides:

| Peptide | Uniq | -10lgP | Mass | Length | ppm | m/z | z | RT | Fraction | Scan | Source File | Area F2 | Area F1 | Area F3 | Area F4 | Area F5 | Area F6 | #Feature | #Feature F2 | #Feature F1 | #Feature F3 | #Feature F4 | #Feature F5 | #Feature F6 | Start | End | PTM | AScore | Found By |
| --- | --- | --- | --- | --- | --- | --- | --- | --- | --- | --- | --- | --- | --- | --- | --- | --- | --- | --- | --- | --- | --- | --- | --- | --- | --- | --- | --- | --- | --- |
| R.LNYGIPAIVK.L | Y | 55.09 | 1086.6437 | 10 | 0.7 | 544.3295 | 2 | 45.40 | 1 | F1:25328 | 28122021\_RID\_2349\_2C.raw | 1.1506E5 |  |  |  |  |  | 1 | 1 | 0 | 0 | 0 | 0 | 0 | 479 | 488 |  |  | PEAKS DB |
| K.LLWTTSR.V | Y | 42.34 | 875.4865 | 7 | 0.5 | 438.7508 | 2 | 35.52 | 1 | F1:17478 | 28122021\_RID\_2349\_2C.raw | 2.8921E4 |  |  |  |  |  | 1 | 1 | 0 | 0 | 0 | 0 | 0 | 328 | 334 |  |  | PEAKS DB |
| total 2 peptides |  |  |  |  |  |  |  |  |  |  |  |  |  |  |  |  |  |  |  |  |  |  |  |  |  |  |  |  |  |
| --- | --- | --- | --- | --- | --- | --- | --- | --- | --- | --- | --- | --- | --- | --- | --- | --- | --- | --- | --- | --- | --- | --- | --- | --- | --- | --- | --- | --- | --- |

XP\_026576298.1

back to list

  

| Protein Coverage
| Supporting Peptides
|

Protein Coverage:

Supporting Peptides:

| Peptide | Uniq | -10lgP | Mass | Length | ppm | m/z | z | RT | Fraction | Scan | Source File | Area F2 | Area F1 | Area F3 | Area F4 | Area F5 | Area F6 | #Feature | #Feature F2 | #Feature F1 | #Feature F3 | #Feature F4 | #Feature F5 | #Feature F6 | Start | End | PTM | AScore | Found By |
| --- | --- | --- | --- | --- | --- | --- | --- | --- | --- | --- | --- | --- | --- | --- | --- | --- | --- | --- | --- | --- | --- | --- | --- | --- | --- | --- | --- | --- | --- |
| R.LNYGIPAIVK.L | Y | 55.09 | 1086.6437 | 10 | 0.7 | 544.3295 | 2 | 45.40 | 1 | F1:25328 | 28122021\_RID\_2349\_2C.raw | 1.1506E5 |  |  |  |  |  | 1 | 1 | 0 | 0 | 0 | 0 | 0 | 479 | 488 |  |  | PEAKS DB |
| K.LLWTTSR.V | Y | 42.34 | 875.4865 | 7 | 0.5 | 438.7508 | 2 | 35.52 | 1 | F1:17478 | 28122021\_RID\_2349\_2C.raw | 2.8921E4 |  |  |  |  |  | 1 | 1 | 0 | 0 | 0 | 0 | 0 | 328 | 334 |  |  | PEAKS DB |
| total 2 peptides |  |  |  |  |  |  |  |  |  |  |  |  |  |  |  |  |  |  |  |  |  |  |  |  |  |  |  |  |  |
| --- | --- | --- | --- | --- | --- | --- | --- | --- | --- | --- | --- | --- | --- | --- | --- | --- | --- | --- | --- | --- | --- | --- | --- | --- | --- | --- | --- | --- | --- |

JAG67632.1

back to list

  

| Protein Coverage
| Supporting Peptides
|

Protein Coverage:

Supporting Peptides:

| Peptide | Uniq | -10lgP | Mass | Length | ppm | m/z | z | RT | Fraction | Scan | Source File | Area F2 | Area F1 | Area F3 | Area F4 | Area F5 | Area F6 | #Feature | #Feature F2 | #Feature F1 | #Feature F3 | #Feature F4 | #Feature F5 | #Feature F6 | Start | End | PTM | AScore | Found By |
| --- | --- | --- | --- | --- | --- | --- | --- | --- | --- | --- | --- | --- | --- | --- | --- | --- | --- | --- | --- | --- | --- | --- | --- | --- | --- | --- | --- | --- | --- |
| R.LNYGIPAIVK.L | Y | 55.09 | 1086.6437 | 10 | 0.7 | 544.3295 | 2 | 45.40 | 1 | F1:25328 | 28122021\_RID\_2349\_2C.raw | 1.1506E5 |  |  |  |  |  | 1 | 1 | 0 | 0 | 0 | 0 | 0 | 479 | 488 |  |  | PEAKS DB |
| K.LLWTTSR.V | Y | 42.34 | 875.4865 | 7 | 0.5 | 438.7508 | 2 | 35.52 | 1 | F1:17478 | 28122021\_RID\_2349\_2C.raw | 2.8921E4 |  |  |  |  |  | 1 | 1 | 0 | 0 | 0 | 0 | 0 | 328 | 334 |  |  | PEAKS DB |
| total 2 peptides |  |  |  |  |  |  |  |  |  |  |  |  |  |  |  |  |  |  |  |  |  |  |  |  |  |  |  |  |  |
| --- | --- | --- | --- | --- | --- | --- | --- | --- | --- | --- | --- | --- | --- | --- | --- | --- | --- | --- | --- | --- | --- | --- | --- | --- | --- | --- | --- | --- | --- |

JAV50234.1

back to list

  

| Protein Coverage
| Supporting Peptides
|

Protein Coverage:

Supporting Peptides:

| Peptide | Uniq | -10lgP | Mass | Length | ppm | m/z | z | RT | Fraction | Scan | Source File | Area F2 | Area F1 | Area F3 | Area F4 | Area F5 | Area F6 | #Feature | #Feature F2 | #Feature F1 | #Feature F3 | #Feature F4 | #Feature F5 | #Feature F6 | Start | End | PTM | AScore | Found By |
| --- | --- | --- | --- | --- | --- | --- | --- | --- | --- | --- | --- | --- | --- | --- | --- | --- | --- | --- | --- | --- | --- | --- | --- | --- | --- | --- | --- | --- | --- |
| R.LNYGIPAIVK.L | Y | 55.09 | 1086.6437 | 10 | 0.7 | 544.3295 | 2 | 45.40 | 1 | F1:25328 | 28122021\_RID\_2349\_2C.raw | 1.1506E5 |  |  |  |  |  | 1 | 1 | 0 | 0 | 0 | 0 | 0 | 479 | 488 |  |  | PEAKS DB |
| K.LLWTTSR.V | Y | 42.34 | 875.4865 | 7 | 0.5 | 438.7508 | 2 | 35.52 | 1 | F1:17478 | 28122021\_RID\_2349\_2C.raw | 2.8921E4 |  |  |  |  |  | 1 | 1 | 0 | 0 | 0 | 0 | 0 | 328 | 334 |  |  | PEAKS DB |
| total 2 peptides |  |  |  |  |  |  |  |  |  |  |  |  |  |  |  |  |  |  |  |  |  |  |  |  |  |  |  |  |  |
| --- | --- | --- | --- | --- | --- | --- | --- | --- | --- | --- | --- | --- | --- | --- | --- | --- | --- | --- | --- | --- | --- | --- | --- | --- | --- | --- | --- | --- | --- |

XP\_034271617.1

back to list

  

| Protein Coverage
| Supporting Peptides
|

Protein Coverage:

Supporting Peptides:

| Peptide | Uniq | -10lgP | Mass | Length | ppm | m/z | z | RT | Fraction | Scan | Source File | Area F2 | Area F1 | Area F3 | Area F4 | Area F5 | Area F6 | #Feature | #Feature F2 | #Feature F1 | #Feature F3 | #Feature F4 | #Feature F5 | #Feature F6 | Start | End | PTM | AScore | Found By |
| --- | --- | --- | --- | --- | --- | --- | --- | --- | --- | --- | --- | --- | --- | --- | --- | --- | --- | --- | --- | --- | --- | --- | --- | --- | --- | --- | --- | --- | --- |
| R.LNYGIPAIVK.L | Y | 55.09 | 1086.6437 | 10 | 0.7 | 544.3295 | 2 | 45.40 | 1 | F1:25328 | 28122021\_RID\_2349\_2C.raw | 1.1506E5 |  |  |  |  |  | 1 | 1 | 0 | 0 | 0 | 0 | 0 | 478 | 487 |  |  | PEAKS DB |
| K.LLWTTSR.V | Y | 42.34 | 875.4865 | 7 | 0.5 | 438.7508 | 2 | 35.52 | 1 | F1:17478 | 28122021\_RID\_2349\_2C.raw | 2.8921E4 |  |  |  |  |  | 1 | 1 | 0 | 0 | 0 | 0 | 0 | 327 | 333 |  |  | PEAKS DB |
| total 2 peptides |  |  |  |  |  |  |  |  |  |  |  |  |  |  |  |  |  |  |  |  |  |  |  |  |  |  |  |  |  |
| --- | --- | --- | --- | --- | --- | --- | --- | --- | --- | --- | --- | --- | --- | --- | --- | --- | --- | --- | --- | --- | --- | --- | --- | --- | --- | --- | --- | --- | --- |

JAG44306.1

back to list

  

| Protein Coverage
| Supporting Peptides
|

Protein Coverage:

Supporting Peptides:

| Peptide | Uniq | -10lgP | Mass | Length | ppm | m/z | z | RT | Fraction | Scan | Source File | Area F2 | Area F1 | Area F3 | Area F4 | Area F5 | Area F6 | #Feature | #Feature F2 | #Feature F1 | #Feature F3 | #Feature F4 | #Feature F5 | #Feature F6 | Start | End | PTM | AScore | Found By |
| --- | --- | --- | --- | --- | --- | --- | --- | --- | --- | --- | --- | --- | --- | --- | --- | --- | --- | --- | --- | --- | --- | --- | --- | --- | --- | --- | --- | --- | --- |
| R.LNYGIPAIVK.L | Y | 55.09 | 1086.6437 | 10 | 0.7 | 544.3295 | 2 | 45.40 | 1 | F1:25328 | 28122021\_RID\_2349\_2C.raw | 1.1506E5 |  |  |  |  |  | 1 | 1 | 0 | 0 | 0 | 0 | 0 | 479 | 488 |  |  | PEAKS DB |
| K.LLWTTSR.V | Y | 42.34 | 875.4865 | 7 | 0.5 | 438.7508 | 2 | 35.52 | 1 | F1:17478 | 28122021\_RID\_2349\_2C.raw | 2.8921E4 |  |  |  |  |  | 1 | 1 | 0 | 0 | 0 | 0 | 0 | 328 | 334 |  |  | PEAKS DB |
| total 2 peptides |  |  |  |  |  |  |  |  |  |  |  |  |  |  |  |  |  |  |  |  |  |  |  |  |  |  |  |  |  |
| --- | --- | --- | --- | --- | --- | --- | --- | --- | --- | --- | --- | --- | --- | --- | --- | --- | --- | --- | --- | --- | --- | --- | --- | --- | --- | --- | --- | --- | --- |

XP\_034271615.1

back to list

  

| Protein Coverage
| Supporting Peptides
|

Protein Coverage:

Supporting Peptides:

| Peptide | Uniq | -10lgP | Mass | Length | ppm | m/z | z | RT | Fraction | Scan | Source File | Area F2 | Area F1 | Area F3 | Area F4 | Area F5 | Area F6 | #Feature | #Feature F2 | #Feature F1 | #Feature F3 | #Feature F4 | #Feature F5 | #Feature F6 | Start | End | PTM | AScore | Found By |
| --- | --- | --- | --- | --- | --- | --- | --- | --- | --- | --- | --- | --- | --- | --- | --- | --- | --- | --- | --- | --- | --- | --- | --- | --- | --- | --- | --- | --- | --- |
| R.LNYGIPAIVK.L | Y | 55.09 | 1086.6437 | 10 | 0.7 | 544.3295 | 2 | 45.40 | 1 | F1:25328 | 28122021\_RID\_2349\_2C.raw | 1.1506E5 |  |  |  |  |  | 1 | 1 | 0 | 0 | 0 | 0 | 0 | 479 | 488 |  |  | PEAKS DB |
| K.LLWTTSR.V | Y | 42.34 | 875.4865 | 7 | 0.5 | 438.7508 | 2 | 35.52 | 1 | F1:17478 | 28122021\_RID\_2349\_2C.raw | 2.8921E4 |  |  |  |  |  | 1 | 1 | 0 | 0 | 0 | 0 | 0 | 328 | 334 |  |  | PEAKS DB |
| total 2 peptides |  |  |  |  |  |  |  |  |  |  |  |  |  |  |  |  |  |  |  |  |  |  |  |  |  |  |  |  |  |
| --- | --- | --- | --- | --- | --- | --- | --- | --- | --- | --- | --- | --- | --- | --- | --- | --- | --- | --- | --- | --- | --- | --- | --- | --- | --- | --- | --- | --- | --- |

XP\_013919280.1

back to list

  

| Protein Coverage
| Supporting Peptides
|

Protein Coverage:

Supporting Peptides:

| Peptide | Uniq | -10lgP | Mass | Length | ppm | m/z | z | RT | Fraction | Scan | Source File | Area F2 | Area F1 | Area F3 | Area F4 | Area F5 | Area F6 | #Feature | #Feature F2 | #Feature F1 | #Feature F3 | #Feature F4 | #Feature F5 | #Feature F6 | Start | End | PTM | AScore | Found By |
| --- | --- | --- | --- | --- | --- | --- | --- | --- | --- | --- | --- | --- | --- | --- | --- | --- | --- | --- | --- | --- | --- | --- | --- | --- | --- | --- | --- | --- | --- |
| R.LNYGIPAIVK.L | Y | 55.09 | 1086.6437 | 10 | 0.7 | 544.3295 | 2 | 45.40 | 1 | F1:25328 | 28122021\_RID\_2349\_2C.raw | 1.1506E5 |  |  |  |  |  | 1 | 1 | 0 | 0 | 0 | 0 | 0 | 479 | 488 |  |  | PEAKS DB |
| K.LLWTTSR.V | Y | 42.34 | 875.4865 | 7 | 0.5 | 438.7508 | 2 | 35.52 | 1 | F1:17478 | 28122021\_RID\_2349\_2C.raw | 2.8921E4 |  |  |  |  |  | 1 | 1 | 0 | 0 | 0 | 0 | 0 | 328 | 334 |  |  | PEAKS DB |
| total 2 peptides |  |  |  |  |  |  |  |  |  |  |  |  |  |  |  |  |  |  |  |  |  |  |  |  |  |  |  |  |  |
| --- | --- | --- | --- | --- | --- | --- | --- | --- | --- | --- | --- | --- | --- | --- | --- | --- | --- | --- | --- | --- | --- | --- | --- | --- | --- | --- | --- | --- | --- |

XP\_034271618.1

back to list

  

| Protein Coverage
| Supporting Peptides
|

Protein Coverage:

Supporting Peptides:

| Peptide | Uniq | -10lgP | Mass | Length | ppm | m/z | z | RT | Fraction | Scan | Source File | Area F2 | Area F1 | Area F3 | Area F4 | Area F5 | Area F6 | #Feature | #Feature F2 | #Feature F1 | #Feature F3 | #Feature F4 | #Feature F5 | #Feature F6 | Start | End | PTM | AScore | Found By |
| --- | --- | --- | --- | --- | --- | --- | --- | --- | --- | --- | --- | --- | --- | --- | --- | --- | --- | --- | --- | --- | --- | --- | --- | --- | --- | --- | --- | --- | --- |
| R.LNYGIPAIVK.L | Y | 55.09 | 1086.6437 | 10 | 0.7 | 544.3295 | 2 | 45.40 | 1 | F1:25328 | 28122021\_RID\_2349\_2C.raw | 1.1506E5 |  |  |  |  |  | 1 | 1 | 0 | 0 | 0 | 0 | 0 | 479 | 488 |  |  | PEAKS DB |
| K.LLWTTSR.V | Y | 42.34 | 875.4865 | 7 | 0.5 | 438.7508 | 2 | 35.52 | 1 | F1:17478 | 28122021\_RID\_2349\_2C.raw | 2.8921E4 |  |  |  |  |  | 1 | 1 | 0 | 0 | 0 | 0 | 0 | 328 | 334 |  |  | PEAKS DB |
| total 2 peptides |  |  |  |  |  |  |  |  |  |  |  |  |  |  |  |  |  |  |  |  |  |  |  |  |  |  |  |  |  |
| --- | --- | --- | --- | --- | --- | --- | --- | --- | --- | --- | --- | --- | --- | --- | --- | --- | --- | --- | --- | --- | --- | --- | --- | --- | --- | --- | --- | --- | --- |

JAA96758.1

back to list

  

| Protein Coverage
| Supporting Peptides
|

Protein Coverage:

Supporting Peptides:

| Peptide | Uniq | -10lgP | Mass | Length | ppm | m/z | z | RT | Fraction | Scan | Source File | Area F2 | Area F1 | Area F3 | Area F4 | Area F5 | Area F6 | #Feature | #Feature F2 | #Feature F1 | #Feature F3 | #Feature F4 | #Feature F5 | #Feature F6 | Start | End | PTM | AScore | Found By |
| --- | --- | --- | --- | --- | --- | --- | --- | --- | --- | --- | --- | --- | --- | --- | --- | --- | --- | --- | --- | --- | --- | --- | --- | --- | --- | --- | --- | --- | --- |
| R.LNYGIPAIVK.L | Y | 55.09 | 1086.6437 | 10 | 0.7 | 544.3295 | 2 | 45.40 | 1 | F1:25328 | 28122021\_RID\_2349\_2C.raw | 1.1506E5 |  |  |  |  |  | 1 | 1 | 0 | 0 | 0 | 0 | 0 | 479 | 488 |  |  | PEAKS DB |
| K.LLWTTSR.V | Y | 42.34 | 875.4865 | 7 | 0.5 | 438.7508 | 2 | 35.52 | 1 | F1:17478 | 28122021\_RID\_2349\_2C.raw | 2.8921E4 |  |  |  |  |  | 1 | 1 | 0 | 0 | 0 | 0 | 0 | 328 | 334 |  |  | PEAKS DB |
| total 2 peptides |  |  |  |  |  |  |  |  |  |  |  |  |  |  |  |  |  |  |  |  |  |  |  |  |  |  |  |  |  |
| --- | --- | --- | --- | --- | --- | --- | --- | --- | --- | --- | --- | --- | --- | --- | --- | --- | --- | --- | --- | --- | --- | --- | --- | --- | --- | --- | --- | --- | --- |

JAG46538.1

back to list

  

| Protein Coverage
| Supporting Peptides
|

Protein Coverage:

Supporting Peptides:

| Peptide | Uniq | -10lgP | Mass | Length | ppm | m/z | z | RT | Fraction | Scan | Source File | Area F2 | Area F1 | Area F3 | Area F4 | Area F5 | Area F6 | #Feature | #Feature F2 | #Feature F1 | #Feature F3 | #Feature F4 | #Feature F5 | #Feature F6 | Start | End | PTM | AScore | Found By |
| --- | --- | --- | --- | --- | --- | --- | --- | --- | --- | --- | --- | --- | --- | --- | --- | --- | --- | --- | --- | --- | --- | --- | --- | --- | --- | --- | --- | --- | --- |
| R.LNYGIPAIVK.L | Y | 55.09 | 1086.6437 | 10 | 0.7 | 544.3295 | 2 | 45.40 | 1 | F1:25328 | 28122021\_RID\_2349\_2C.raw | 1.1506E5 |  |  |  |  |  | 1 | 1 | 0 | 0 | 0 | 0 | 0 | 479 | 488 |  |  | PEAKS DB |
| K.LLWTTSR.V | Y | 42.34 | 875.4865 | 7 | 0.5 | 438.7508 | 2 | 35.52 | 1 | F1:17478 | 28122021\_RID\_2349\_2C.raw | 2.8921E4 |  |  |  |  |  | 1 | 1 | 0 | 0 | 0 | 0 | 0 | 328 | 334 |  |  | PEAKS DB |
| total 2 peptides |  |  |  |  |  |  |  |  |  |  |  |  |  |  |  |  |  |  |  |  |  |  |  |  |  |  |  |  |  |
| --- | --- | --- | --- | --- | --- | --- | --- | --- | --- | --- | --- | --- | --- | --- | --- | --- | --- | --- | --- | --- | --- | --- | --- | --- | --- | --- | --- | --- | --- |

XP\_034271616.1

back to list

  

| Protein Coverage
| Supporting Peptides
|

Protein Coverage:

Supporting Peptides:

| Peptide | Uniq | -10lgP | Mass | Length | ppm | m/z | z | RT | Fraction | Scan | Source File | Area F2 | Area F1 | Area F3 | Area F4 | Area F5 | Area F6 | #Feature | #Feature F2 | #Feature F1 | #Feature F3 | #Feature F4 | #Feature F5 | #Feature F6 | Start | End | PTM | AScore | Found By |
| --- | --- | --- | --- | --- | --- | --- | --- | --- | --- | --- | --- | --- | --- | --- | --- | --- | --- | --- | --- | --- | --- | --- | --- | --- | --- | --- | --- | --- | --- |
| R.LNYGIPAIVK.L | Y | 55.09 | 1086.6437 | 10 | 0.7 | 544.3295 | 2 | 45.40 | 1 | F1:25328 | 28122021\_RID\_2349\_2C.raw | 1.1506E5 |  |  |  |  |  | 1 | 1 | 0 | 0 | 0 | 0 | 0 | 479 | 488 |  |  | PEAKS DB |
| K.LLWTTSR.V | Y | 42.34 | 875.4865 | 7 | 0.5 | 438.7508 | 2 | 35.52 | 1 | F1:17478 | 28122021\_RID\_2349\_2C.raw | 2.8921E4 |  |  |  |  |  | 1 | 1 | 0 | 0 | 0 | 0 | 0 | 328 | 334 |  |  | PEAKS DB |
| total 2 peptides |  |  |  |  |  |  |  |  |  |  |  |  |  |  |  |  |  |  |  |  |  |  |  |  |  |  |  |  |  |
| --- | --- | --- | --- | --- | --- | --- | --- | --- | --- | --- | --- | --- | --- | --- | --- | --- | --- | --- | --- | --- | --- | --- | --- | --- | --- | --- | --- | --- | --- |

XP\_025022318.1

back to list

  

| Protein Coverage
| Supporting Peptides
|

Protein Coverage:

Supporting Peptides:

| Peptide | Uniq | -10lgP | Mass | Length | ppm | m/z | z | RT | Fraction | Scan | Source File | Area F2 | Area F1 | Area F3 | Area F4 | Area F5 | Area F6 | #Feature | #Feature F2 | #Feature F1 | #Feature F3 | #Feature F4 | #Feature F5 | #Feature F6 | Start | End | PTM | AScore | Found By |
| --- | --- | --- | --- | --- | --- | --- | --- | --- | --- | --- | --- | --- | --- | --- | --- | --- | --- | --- | --- | --- | --- | --- | --- | --- | --- | --- | --- | --- | --- |
| R.LNYGIPAIVK.L | Y | 55.09 | 1086.6437 | 10 | 0.7 | 544.3295 | 2 | 45.40 | 1 | F1:25328 | 28122021\_RID\_2349\_2C.raw | 1.1506E5 |  |  |  |  |  | 1 | 1 | 0 | 0 | 0 | 0 | 0 | 480 | 489 |  |  | PEAKS DB |
| K.LLWTTSR.V | Y | 42.34 | 875.4865 | 7 | 0.5 | 438.7508 | 2 | 35.52 | 1 | F1:17478 | 28122021\_RID\_2349\_2C.raw | 2.8921E4 |  |  |  |  |  | 1 | 1 | 0 | 0 | 0 | 0 | 0 | 329 | 335 |  |  | PEAKS DB |
| total 2 peptides |  |  |  |  |  |  |  |  |  |  |  |  |  |  |  |  |  |  |  |  |  |  |  |  |  |  |  |  |  |
| --- | --- | --- | --- | --- | --- | --- | --- | --- | --- | --- | --- | --- | --- | --- | --- | --- | --- | --- | --- | --- | --- | --- | --- | --- | --- | --- | --- | --- | --- |

XP\_025022317.1

back to list

  

| Protein Coverage
| Supporting Peptides
|

Protein Coverage:

Supporting Peptides:

| Peptide | Uniq | -10lgP | Mass | Length | ppm | m/z | z | RT | Fraction | Scan | Source File | Area F2 | Area F1 | Area F3 | Area F4 | Area F5 | Area F6 | #Feature | #Feature F2 | #Feature F1 | #Feature F3 | #Feature F4 | #Feature F5 | #Feature F6 | Start | End | PTM | AScore | Found By |
| --- | --- | --- | --- | --- | --- | --- | --- | --- | --- | --- | --- | --- | --- | --- | --- | --- | --- | --- | --- | --- | --- | --- | --- | --- | --- | --- | --- | --- | --- |
| R.LNYGIPAIVK.L | Y | 55.09 | 1086.6437 | 10 | 0.7 | 544.3295 | 2 | 45.40 | 1 | F1:25328 | 28122021\_RID\_2349\_2C.raw | 1.1506E5 |  |  |  |  |  | 1 | 1 | 0 | 0 | 0 | 0 | 0 | 481 | 490 |  |  | PEAKS DB |
| K.LLWTTSR.V | Y | 42.34 | 875.4865 | 7 | 0.5 | 438.7508 | 2 | 35.52 | 1 | F1:17478 | 28122021\_RID\_2349\_2C.raw | 2.8921E4 |  |  |  |  |  | 1 | 1 | 0 | 0 | 0 | 0 | 0 | 330 | 336 |  |  | PEAKS DB |
| total 2 peptides |  |  |  |  |  |  |  |  |  |  |  |  |  |  |  |  |  |  |  |  |  |  |  |  |  |  |  |  |  |
| --- | --- | --- | --- | --- | --- | --- | --- | --- | --- | --- | --- | --- | --- | --- | --- | --- | --- | --- | --- | --- | --- | --- | --- | --- | --- | --- | --- | --- | --- |

XP\_007428111.1

back to list

  

| Protein Coverage
| Supporting Peptides
|

Protein Coverage:

Supporting Peptides:

| Peptide | Uniq | -10lgP | Mass | Length | ppm | m/z | z | RT | Fraction | Scan | Source File | Area F2 | Area F1 | Area F3 | Area F4 | Area F5 | Area F6 | #Feature | #Feature F2 | #Feature F1 | #Feature F3 | #Feature F4 | #Feature F5 | #Feature F6 | Start | End | PTM | AScore | Found By |
| --- | --- | --- | --- | --- | --- | --- | --- | --- | --- | --- | --- | --- | --- | --- | --- | --- | --- | --- | --- | --- | --- | --- | --- | --- | --- | --- | --- | --- | --- |
| R.LNYGIPAIVK.L | Y | 55.09 | 1086.6437 | 10 | 0.7 | 544.3295 | 2 | 45.40 | 1 | F1:25328 | 28122021\_RID\_2349\_2C.raw | 1.1506E5 |  |  |  |  |  | 1 | 1 | 0 | 0 | 0 | 0 | 0 | 481 | 490 |  |  | PEAKS DB |
| K.LLWTTSR.V | Y | 42.34 | 875.4865 | 7 | 0.5 | 438.7508 | 2 | 35.52 | 1 | F1:17478 | 28122021\_RID\_2349\_2C.raw | 2.8921E4 |  |  |  |  |  | 1 | 1 | 0 | 0 | 0 | 0 | 0 | 330 | 336 |  |  | PEAKS DB |
| total 2 peptides |  |  |  |  |  |  |  |  |  |  |  |  |  |  |  |  |  |  |  |  |  |  |  |  |  |  |  |  |  |
| --- | --- | --- | --- | --- | --- | --- | --- | --- | --- | --- | --- | --- | --- | --- | --- | --- | --- | --- | --- | --- | --- | --- | --- | --- | --- | --- | --- | --- | --- |

ETE68218.1

back to list

  

| Protein Coverage
| Supporting Peptides
|

Protein Coverage:

Supporting Peptides:

| Peptide | Uniq | -10lgP | Mass | Length | ppm | m/z | z | RT | Fraction | Scan | Source File | Area F2 | Area F1 | Area F3 | Area F4 | Area F5 | Area F6 | #Feature | #Feature F2 | #Feature F1 | #Feature F3 | #Feature F4 | #Feature F5 | #Feature F6 | Start | End | PTM | AScore | Found By |
| --- | --- | --- | --- | --- | --- | --- | --- | --- | --- | --- | --- | --- | --- | --- | --- | --- | --- | --- | --- | --- | --- | --- | --- | --- | --- | --- | --- | --- | --- |
| R.LNYGIPAIVK.L | Y | 55.09 | 1086.6437 | 10 | 0.7 | 544.3295 | 2 | 45.40 | 1 | F1:25328 | 28122021\_RID\_2349\_2C.raw | 1.1506E5 |  |  |  |  |  | 1 | 1 | 0 | 0 | 0 | 0 | 0 | 478 | 487 |  |  | PEAKS DB |
| K.LLWTTSR.V | Y | 42.34 | 875.4865 | 7 | 0.5 | 438.7508 | 2 | 35.52 | 1 | F1:17478 | 28122021\_RID\_2349\_2C.raw | 2.8921E4 |  |  |  |  |  | 1 | 1 | 0 | 0 | 0 | 0 | 0 | 327 | 333 |  |  | PEAKS DB |
| total 2 peptides |  |  |  |  |  |  |  |  |  |  |  |  |  |  |  |  |  |  |  |  |  |  |  |  |  |  |  |  |  |
| --- | --- | --- | --- | --- | --- | --- | --- | --- | --- | --- | --- | --- | --- | --- | --- | --- | --- | --- | --- | --- | --- | --- | --- | --- | --- | --- | --- | --- | --- |

XP\_032091066.1

back to list

  

| Protein Coverage
| Supporting Peptides
|

Protein Coverage:

Supporting Peptides:

| Peptide | Uniq | -10lgP | Mass | Length | ppm | m/z | z | RT | Fraction | Scan | Source File | Area F2 | Area F1 | Area F3 | Area F4 | Area F5 | Area F6 | #Feature | #Feature F2 | #Feature F1 | #Feature F3 | #Feature F4 | #Feature F5 | #Feature F6 | Start | End | PTM | AScore | Found By |
| --- | --- | --- | --- | --- | --- | --- | --- | --- | --- | --- | --- | --- | --- | --- | --- | --- | --- | --- | --- | --- | --- | --- | --- | --- | --- | --- | --- | --- | --- |
| R.LNYGIPAIVK.L | Y | 55.09 | 1086.6437 | 10 | 0.7 | 544.3295 | 2 | 45.40 | 1 | F1:25328 | 28122021\_RID\_2349\_2C.raw | 1.1506E5 |  |  |  |  |  | 1 | 1 | 0 | 0 | 0 | 0 | 0 | 522 | 531 |  |  | PEAKS DB |
| K.LLWTTSR.V | Y | 42.34 | 875.4865 | 7 | 0.5 | 438.7508 | 2 | 35.52 | 1 | F1:17478 | 28122021\_RID\_2349\_2C.raw | 2.8921E4 |  |  |  |  |  | 1 | 1 | 0 | 0 | 0 | 0 | 0 | 371 | 377 |  |  | PEAKS DB |
| total 2 peptides |  |  |  |  |  |  |  |  |  |  |  |  |  |  |  |  |  |  |  |  |  |  |  |  |  |  |  |  |  |
| --- | --- | --- | --- | --- | --- | --- | --- | --- | --- | --- | --- | --- | --- | --- | --- | --- | --- | --- | --- | --- | --- | --- | --- | --- | --- | --- | --- | --- | --- |

XP\_032091065.1

back to list

  

| Protein Coverage
| Supporting Peptides
|

Protein Coverage:

Supporting Peptides:

| Peptide | Uniq | -10lgP | Mass | Length | ppm | m/z | z | RT | Fraction | Scan | Source File | Area F2 | Area F1 | Area F3 | Area F4 | Area F5 | Area F6 | #Feature | #Feature F2 | #Feature F1 | #Feature F3 | #Feature F4 | #Feature F5 | #Feature F6 | Start | End | PTM | AScore | Found By |
| --- | --- | --- | --- | --- | --- | --- | --- | --- | --- | --- | --- | --- | --- | --- | --- | --- | --- | --- | --- | --- | --- | --- | --- | --- | --- | --- | --- | --- | --- |
| R.LNYGIPAIVK.L | Y | 55.09 | 1086.6437 | 10 | 0.7 | 544.3295 | 2 | 45.40 | 1 | F1:25328 | 28122021\_RID\_2349\_2C.raw | 1.1506E5 |  |  |  |  |  | 1 | 1 | 0 | 0 | 0 | 0 | 0 | 522 | 531 |  |  | PEAKS DB |
| K.LLWTTSR.V | Y | 42.34 | 875.4865 | 7 | 0.5 | 438.7508 | 2 | 35.52 | 1 | F1:17478 | 28122021\_RID\_2349\_2C.raw | 2.8921E4 |  |  |  |  |  | 1 | 1 | 0 | 0 | 0 | 0 | 0 | 371 | 377 |  |  | PEAKS DB |
| total 2 peptides |  |  |  |  |  |  |  |  |  |  |  |  |  |  |  |  |  |  |  |  |  |  |  |  |  |  |  |  |  |
| --- | --- | --- | --- | --- | --- | --- | --- | --- | --- | --- | --- | --- | --- | --- | --- | --- | --- | --- | --- | --- | --- | --- | --- | --- | --- | --- | --- | --- | --- |

LAB43566.1

back to list

  

| Protein Coverage
| Supporting Peptides
|

Protein Coverage:

Supporting Peptides:

| Peptide | Uniq | -10lgP | Mass | Length | ppm | m/z | z | RT | Fraction | Scan | Source File | Area F2 | Area F1 | Area F3 | Area F4 | Area F5 | Area F6 | #Feature | #Feature F2 | #Feature F1 | #Feature F3 | #Feature F4 | #Feature F5 | #Feature F6 | Start | End | PTM | AScore | Found By |
| --- | --- | --- | --- | --- | --- | --- | --- | --- | --- | --- | --- | --- | --- | --- | --- | --- | --- | --- | --- | --- | --- | --- | --- | --- | --- | --- | --- | --- | --- |
| R.LNYGIPAIVK.L | Y | 55.09 | 1086.6437 | 10 | 0.7 | 544.3295 | 2 | 45.40 | 1 | F1:25328 | 28122021\_RID\_2349\_2C.raw | 1.1506E5 |  |  |  |  |  | 1 | 1 | 0 | 0 | 0 | 0 | 0 | 503 | 512 |  |  | PEAKS DB |
| K.LLWTTSR.V | Y | 42.34 | 875.4865 | 7 | 0.5 | 438.7508 | 2 | 35.52 | 1 | F1:17478 | 28122021\_RID\_2349\_2C.raw | 2.8921E4 |  |  |  |  |  | 1 | 1 | 0 | 0 | 0 | 0 | 0 | 352 | 358 |  |  | PEAKS DB |
| total 2 peptides |  |  |  |  |  |  |  |  |  |  |  |  |  |  |  |  |  |  |  |  |  |  |  |  |  |  |  |  |  |
| --- | --- | --- | --- | --- | --- | --- | --- | --- | --- | --- | --- | --- | --- | --- | --- | --- | --- | --- | --- | --- | --- | --- | --- | --- | --- | --- | --- | --- | --- |

XP\_015687775.1

back to list

  

| Protein Coverage
| Supporting Peptides
|

Protein Coverage:

Supporting Peptides:

| Peptide | Uniq | -10lgP | Mass | Length | ppm | m/z | z | RT | Fraction | Scan | Source File | Area F2 | Area F1 | Area F3 | Area F4 | Area F5 | Area F6 | #Feature | #Feature F2 | #Feature F1 | #Feature F3 | #Feature F4 | #Feature F5 | #Feature F6 | Start | End | PTM | AScore | Found By |
| --- | --- | --- | --- | --- | --- | --- | --- | --- | --- | --- | --- | --- | --- | --- | --- | --- | --- | --- | --- | --- | --- | --- | --- | --- | --- | --- | --- | --- | --- |
| R.LNYGIPAIVK.L | Y | 55.09 | 1086.6437 | 10 | 0.7 | 544.3295 | 2 | 45.40 | 1 | F1:25328 | 28122021\_RID\_2349\_2C.raw | 1.1506E5 |  |  |  |  |  | 1 | 1 | 0 | 0 | 0 | 0 | 0 | 479 | 488 |  |  | PEAKS DB |
| K.LLWTTSR.V | Y | 42.34 | 875.4865 | 7 | 0.5 | 438.7508 | 2 | 35.52 | 1 | F1:17478 | 28122021\_RID\_2349\_2C.raw | 2.8921E4 |  |  |  |  |  | 1 | 1 | 0 | 0 | 0 | 0 | 0 | 328 | 334 |  |  | PEAKS DB |
| total 2 peptides |  |  |  |  |  |  |  |  |  |  |  |  |  |  |  |  |  |  |  |  |  |  |  |  |  |  |  |  |  |
| --- | --- | --- | --- | --- | --- | --- | --- | --- | --- | --- | --- | --- | --- | --- | --- | --- | --- | --- | --- | --- | --- | --- | --- | --- | --- | --- | --- | --- | --- |

XP\_015687778.1

back to list

  

| Protein Coverage
| Supporting Peptides
|

Protein Coverage:

Supporting Peptides:

| Peptide | Uniq | -10lgP | Mass | Length | ppm | m/z | z | RT | Fraction | Scan | Source File | Area F2 | Area F1 | Area F3 | Area F4 | Area F5 | Area F6 | #Feature | #Feature F2 | #Feature F1 | #Feature F3 | #Feature F4 | #Feature F5 | #Feature F6 | Start | End | PTM | AScore | Found By |
| --- | --- | --- | --- | --- | --- | --- | --- | --- | --- | --- | --- | --- | --- | --- | --- | --- | --- | --- | --- | --- | --- | --- | --- | --- | --- | --- | --- | --- | --- |
| R.LNYGIPAIVK.L | Y | 55.09 | 1086.6437 | 10 | 0.7 | 544.3295 | 2 | 45.40 | 1 | F1:25328 | 28122021\_RID\_2349\_2C.raw | 1.1506E5 |  |  |  |  |  | 1 | 1 | 0 | 0 | 0 | 0 | 0 | 479 | 488 |  |  | PEAKS DB |
| K.LLWTTSR.V | Y | 42.34 | 875.4865 | 7 | 0.5 | 438.7508 | 2 | 35.52 | 1 | F1:17478 | 28122021\_RID\_2349\_2C.raw | 2.8921E4 |  |  |  |  |  | 1 | 1 | 0 | 0 | 0 | 0 | 0 | 328 | 334 |  |  | PEAKS DB |
| total 2 peptides |  |  |  |  |  |  |  |  |  |  |  |  |  |  |  |  |  |  |  |  |  |  |  |  |  |  |  |  |  |
| --- | --- | --- | --- | --- | --- | --- | --- | --- | --- | --- | --- | --- | --- | --- | --- | --- | --- | --- | --- | --- | --- | --- | --- | --- | --- | --- | --- | --- | --- |

XP\_015687777.1

back to list

  

| Protein Coverage
| Supporting Peptides
|

Protein Coverage:

Supporting Peptides:

| Peptide | Uniq | -10lgP | Mass | Length | ppm | m/z | z | RT | Fraction | Scan | Source File | Area F2 | Area F1 | Area F3 | Area F4 | Area F5 | Area F6 | #Feature | #Feature F2 | #Feature F1 | #Feature F3 | #Feature F4 | #Feature F5 | #Feature F6 | Start | End | PTM | AScore | Found By |
| --- | --- | --- | --- | --- | --- | --- | --- | --- | --- | --- | --- | --- | --- | --- | --- | --- | --- | --- | --- | --- | --- | --- | --- | --- | --- | --- | --- | --- | --- |
| R.LNYGIPAIVK.L | Y | 55.09 | 1086.6437 | 10 | 0.7 | 544.3295 | 2 | 45.40 | 1 | F1:25328 | 28122021\_RID\_2349\_2C.raw | 1.1506E5 |  |  |  |  |  | 1 | 1 | 0 | 0 | 0 | 0 | 0 | 479 | 488 |  |  | PEAKS DB |
| K.LLWTTSR.V | Y | 42.34 | 875.4865 | 7 | 0.5 | 438.7508 | 2 | 35.52 | 1 | F1:17478 | 28122021\_RID\_2349\_2C.raw | 2.8921E4 |  |  |  |  |  | 1 | 1 | 0 | 0 | 0 | 0 | 0 | 328 | 334 |  |  | PEAKS DB |
| total 2 peptides |  |  |  |  |  |  |  |  |  |  |  |  |  |  |  |  |  |  |  |  |  |  |  |  |  |  |  |  |  |
| --- | --- | --- | --- | --- | --- | --- | --- | --- | --- | --- | --- | --- | --- | --- | --- | --- | --- | --- | --- | --- | --- | --- | --- | --- | --- | --- | --- | --- | --- |

XP\_015687776.1

back to list

  

| Protein Coverage
| Supporting Peptides
|

Protein Coverage:

Supporting Peptides:

| Peptide | Uniq | -10lgP | Mass | Length | ppm | m/z | z | RT | Fraction | Scan | Source File | Area F2 | Area F1 | Area F3 | Area F4 | Area F5 | Area F6 | #Feature | #Feature F2 | #Feature F1 | #Feature F3 | #Feature F4 | #Feature F5 | #Feature F6 | Start | End | PTM | AScore | Found By |
| --- | --- | --- | --- | --- | --- | --- | --- | --- | --- | --- | --- | --- | --- | --- | --- | --- | --- | --- | --- | --- | --- | --- | --- | --- | --- | --- | --- | --- | --- |
| R.LNYGIPAIVK.L | Y | 55.09 | 1086.6437 | 10 | 0.7 | 544.3295 | 2 | 45.40 | 1 | F1:25328 | 28122021\_RID\_2349\_2C.raw | 1.1506E5 |  |  |  |  |  | 1 | 1 | 0 | 0 | 0 | 0 | 0 | 479 | 488 |  |  | PEAKS DB |
| K.LLWTTSR.V | Y | 42.34 | 875.4865 | 7 | 0.5 | 438.7508 | 2 | 35.52 | 1 | F1:17478 | 28122021\_RID\_2349\_2C.raw | 2.8921E4 |  |  |  |  |  | 1 | 1 | 0 | 0 | 0 | 0 | 0 | 328 | 334 |  |  | PEAKS DB |
| total 2 peptides |  |  |  |  |  |  |  |  |  |  |  |  |  |  |  |  |  |  |  |  |  |  |  |  |  |  |  |  |  |
| --- | --- | --- | --- | --- | --- | --- | --- | --- | --- | --- | --- | --- | --- | --- | --- | --- | --- | --- | --- | --- | --- | --- | --- | --- | --- | --- | --- | --- | --- |

JAI12884.1

back to list

  

| Protein Coverage
| Supporting Peptides
|

Protein Coverage:

Supporting Peptides:

| Peptide | Uniq | -10lgP | Mass | Length | ppm | m/z | z | RT | Fraction | Scan | Source File | Area F2 | Area F1 | Area F3 | Area F4 | Area F5 | Area F6 | #Feature | #Feature F2 | #Feature F1 | #Feature F3 | #Feature F4 | #Feature F5 | #Feature F6 | Start | End | PTM | AScore | Found By |
| --- | --- | --- | --- | --- | --- | --- | --- | --- | --- | --- | --- | --- | --- | --- | --- | --- | --- | --- | --- | --- | --- | --- | --- | --- | --- | --- | --- | --- | --- |
| R.LNYGIPAIVK.L | Y | 55.09 | 1086.6437 | 10 | 0.7 | 544.3295 | 2 | 45.40 | 1 | F1:25328 | 28122021\_RID\_2349\_2C.raw | 1.1506E5 |  |  |  |  |  | 1 | 1 | 0 | 0 | 0 | 0 | 0 | 479 | 488 |  |  | PEAKS DB |
| K.LLWTTSR.V | Y | 42.34 | 875.4865 | 7 | 0.5 | 438.7508 | 2 | 35.52 | 1 | F1:17478 | 28122021\_RID\_2349\_2C.raw | 2.8921E4 |  |  |  |  |  | 1 | 1 | 0 | 0 | 0 | 0 | 0 | 328 | 334 |  |  | PEAKS DB |
| total 2 peptides |  |  |  |  |  |  |  |  |  |  |  |  |  |  |  |  |  |  |  |  |  |  |  |  |  |  |  |  |  |
| --- | --- | --- | --- | --- | --- | --- | --- | --- | --- | --- | --- | --- | --- | --- | --- | --- | --- | --- | --- | --- | --- | --- | --- | --- | --- | --- | --- | --- | --- |

BAP39929.1

back to list

  

| Protein Coverage
| Supporting Peptides
|

Protein Coverage:

Supporting Peptides:

| Peptide | Uniq | -10lgP | Mass | Length | ppm | m/z | z | RT | Fraction | Scan | Source File | Area F2 | Area F1 | Area F3 | Area F4 | Area F5 | Area F6 | #Feature | #Feature F2 | #Feature F1 | #Feature F3 | #Feature F4 | #Feature F5 | #Feature F6 | Start | End | PTM | AScore | Found By |
| --- | --- | --- | --- | --- | --- | --- | --- | --- | --- | --- | --- | --- | --- | --- | --- | --- | --- | --- | --- | --- | --- | --- | --- | --- | --- | --- | --- | --- | --- |
| R.EAVFVAQLLSEDIK.T | Y | 54.58 | 1560.8398 | 14 | 1.6 | 781.4285 | 2 | 61.08 | 6 | F6:34838 | 31122021\_RID\_2349\_6C.raw |  |  |  |  |  | 2.5305E4 | 1 | 0 | 0 | 0 | 0 | 0 | 1 | 98 | 111 |  |  | PEAKS DB |
| total 1 peptides |  |  |  |  |  |  |  |  |  |  |  |  |  |  |  |  |  |  |  |  |  |  |  |  |  |  |  |  |  |
| --- | --- | --- | --- | --- | --- | --- | --- | --- | --- | --- | --- | --- | --- | --- | --- | --- | --- | --- | --- | --- | --- | --- | --- | --- | --- | --- | --- | --- | --- |

XP\_015685588.1

back to list

  

| Protein Coverage
| Supporting Peptides
|

Protein Coverage:

Supporting Peptides:

| Peptide | Uniq | -10lgP | Mass | Length | ppm | m/z | z | RT | Fraction | Scan | Source File | Area F2 | Area F1 | Area F3 | Area F4 | Area F5 | Area F6 | #Feature | #Feature F2 | #Feature F1 | #Feature F3 | #Feature F4 | #Feature F5 | #Feature F6 | Start | End | PTM | AScore | Found By |
| --- | --- | --- | --- | --- | --- | --- | --- | --- | --- | --- | --- | --- | --- | --- | --- | --- | --- | --- | --- | --- | --- | --- | --- | --- | --- | --- | --- | --- | --- |
| R.EAVFVAQLLSEDIK.T | Y | 54.58 | 1560.8398 | 14 | 1.6 | 781.4285 | 2 | 61.08 | 6 | F6:34838 | 31122021\_RID\_2349\_6C.raw |  |  |  |  |  | 2.5305E4 | 1 | 0 | 0 | 0 | 0 | 0 | 1 | 70 | 83 |  |  | PEAKS DB |
| total 1 peptides |  |  |  |  |  |  |  |  |  |  |  |  |  |  |  |  |  |  |  |  |  |  |  |  |  |  |  |  |  |
| --- | --- | --- | --- | --- | --- | --- | --- | --- | --- | --- | --- | --- | --- | --- | --- | --- | --- | --- | --- | --- | --- | --- | --- | --- | --- | --- | --- | --- | --- |

JAC94969.1

back to list

  

| Protein Coverage
| Supporting Peptides
|

Protein Coverage:

Supporting Peptides:

| Peptide | Uniq | -10lgP | Mass | Length | ppm | m/z | z | RT | Fraction | Scan | Source File | Area F2 | Area F1 | Area F3 | Area F4 | Area F5 | Area F6 | #Feature | #Feature F2 | #Feature F1 | #Feature F3 | #Feature F4 | #Feature F5 | #Feature F6 | Start | End | PTM | AScore | Found By |
| --- | --- | --- | --- | --- | --- | --- | --- | --- | --- | --- | --- | --- | --- | --- | --- | --- | --- | --- | --- | --- | --- | --- | --- | --- | --- | --- | --- | --- | --- |
| K.QPGITFIAAK.F | Y | 59.75 | 1044.5967 | 10 | -0.1 | 523.3055 | 2 | 38.53 | 1 | F1:19890 | 28122021\_RID\_2349\_2C.raw | 1.4738E5 |  |  |  |  |  | 1 | 1 | 0 | 0 | 0 | 0 | 0 | 172 | 181 |  |  | PEAKS DB |
| K.FDGILGMAYPK.I | Y | 50.59 | 1210.6056 | 11 | 0.2 | 606.3102 | 2 | 51.76 | 1 | F1:30350 | 28122021\_RID\_2349\_2C.raw | 8.6444E4 |  |  |  |  |  | 1 | 1 | 0 | 0 | 0 | 0 | 0 | 182 | 192 |  |  | PEAKS DB |
| total 2 peptides |  |  |  |  |  |  |  |  |  |  |  |  |  |  |  |  |  |  |  |  |  |  |  |  |  |  |  |  |  |
| --- | --- | --- | --- | --- | --- | --- | --- | --- | --- | --- | --- | --- | --- | --- | --- | --- | --- | --- | --- | --- | --- | --- | --- | --- | --- | --- | --- | --- | --- |

ETE66900.1

back to list

  

| Protein Coverage
| Supporting Peptides
|

Protein Coverage:

Supporting Peptides:

| Peptide | Uniq | -10lgP | Mass | Length | ppm | m/z | z | RT | Fraction | Scan | Source File | Area F2 | Area F1 | Area F3 | Area F4 | Area F5 | Area F6 | #Feature | #Feature F2 | #Feature F1 | #Feature F3 | #Feature F4 | #Feature F5 | #Feature F6 | Start | End | PTM | AScore | Found By |
| --- | --- | --- | --- | --- | --- | --- | --- | --- | --- | --- | --- | --- | --- | --- | --- | --- | --- | --- | --- | --- | --- | --- | --- | --- | --- | --- | --- | --- | --- |
| K.QPGITFIAAK.F | Y | 59.75 | 1044.5967 | 10 | -0.1 | 523.3055 | 2 | 38.53 | 1 | F1:19890 | 28122021\_RID\_2349\_2C.raw | 1.4738E5 |  |  |  |  |  | 1 | 1 | 0 | 0 | 0 | 0 | 0 | 56 | 65 |  |  | PEAKS DB |
| K.FDGILGMAYPK.I | Y | 50.59 | 1210.6056 | 11 | 0.2 | 606.3102 | 2 | 51.76 | 1 | F1:30350 | 28122021\_RID\_2349\_2C.raw | 8.6444E4 |  |  |  |  |  | 1 | 1 | 0 | 0 | 0 | 0 | 0 | 66 | 76 |  |  | PEAKS DB |
| total 2 peptides |  |  |  |  |  |  |  |  |  |  |  |  |  |  |  |  |  |  |  |  |  |  |  |  |  |  |  |  |  |
| --- | --- | --- | --- | --- | --- | --- | --- | --- | --- | --- | --- | --- | --- | --- | --- | --- | --- | --- | --- | --- | --- | --- | --- | --- | --- | --- | --- | --- | --- |

JAB54523.1

back to list

  

| Protein Coverage
| Supporting Peptides
|

Protein Coverage:

Supporting Peptides:

| Peptide | Uniq | -10lgP | Mass | Length | ppm | m/z | z | RT | Fraction | Scan | Source File | Area F2 | Area F1 | Area F3 | Area F4 | Area F5 | Area F6 | #Feature | #Feature F2 | #Feature F1 | #Feature F3 | #Feature F4 | #Feature F5 | #Feature F6 | Start | End | PTM | AScore | Found By |
| --- | --- | --- | --- | --- | --- | --- | --- | --- | --- | --- | --- | --- | --- | --- | --- | --- | --- | --- | --- | --- | --- | --- | --- | --- | --- | --- | --- | --- | --- |
| K.QPGITFIAAK.F | Y | 59.75 | 1044.5967 | 10 | -0.1 | 523.3055 | 2 | 38.53 | 1 | F1:19890 | 28122021\_RID\_2349\_2C.raw | 1.4738E5 |  |  |  |  |  | 1 | 1 | 0 | 0 | 0 | 0 | 0 | 171 | 180 |  |  | PEAKS DB |
| K.FDGILGMAYPK.I | Y | 50.59 | 1210.6056 | 11 | 0.2 | 606.3102 | 2 | 51.76 | 1 | F1:30350 | 28122021\_RID\_2349\_2C.raw | 8.6444E4 |  |  |  |  |  | 1 | 1 | 0 | 0 | 0 | 0 | 0 | 181 | 191 |  |  | PEAKS DB |
| total 2 peptides |  |  |  |  |  |  |  |  |  |  |  |  |  |  |  |  |  |  |  |  |  |  |  |  |  |  |  |  |  |
| --- | --- | --- | --- | --- | --- | --- | --- | --- | --- | --- | --- | --- | --- | --- | --- | --- | --- | --- | --- | --- | --- | --- | --- | --- | --- | --- | --- | --- | --- |

JAI10168.1

back to list

  

| Protein Coverage
| Supporting Peptides
|

Protein Coverage:

Supporting Peptides:

| Peptide | Uniq | -10lgP | Mass | Length | ppm | m/z | z | RT | Fraction | Scan | Source File | Area F2 | Area F1 | Area F3 | Area F4 | Area F5 | Area F6 | #Feature | #Feature F2 | #Feature F1 | #Feature F3 | #Feature F4 | #Feature F5 | #Feature F6 | Start | End | PTM | AScore | Found By |
| --- | --- | --- | --- | --- | --- | --- | --- | --- | --- | --- | --- | --- | --- | --- | --- | --- | --- | --- | --- | --- | --- | --- | --- | --- | --- | --- | --- | --- | --- |
| K.QPGITFIAAK.F | Y | 59.75 | 1044.5967 | 10 | -0.1 | 523.3055 | 2 | 38.53 | 1 | F1:19890 | 28122021\_RID\_2349\_2C.raw | 1.4738E5 |  |  |  |  |  | 1 | 1 | 0 | 0 | 0 | 0 | 0 | 172 | 181 |  |  | PEAKS DB |
| K.FDGILGMAYPK.I | Y | 50.59 | 1210.6056 | 11 | 0.2 | 606.3102 | 2 | 51.76 | 1 | F1:30350 | 28122021\_RID\_2349\_2C.raw | 8.6444E4 |  |  |  |  |  | 1 | 1 | 0 | 0 | 0 | 0 | 0 | 182 | 192 |  |  | PEAKS DB |
| total 2 peptides |  |  |  |  |  |  |  |  |  |  |  |  |  |  |  |  |  |  |  |  |  |  |  |  |  |  |  |  |  |
| --- | --- | --- | --- | --- | --- | --- | --- | --- | --- | --- | --- | --- | --- | --- | --- | --- | --- | --- | --- | --- | --- | --- | --- | --- | --- | --- | --- | --- | --- |

JAC96615.1

back to list

  

| Protein Coverage
| Supporting Peptides
|

Protein Coverage:

Supporting Peptides:

| Peptide | Uniq | -10lgP | Mass | Length | ppm | m/z | z | RT | Fraction | Scan | Source File | Area F2 | Area F1 | Area F3 | Area F4 | Area F5 | Area F6 | #Feature | #Feature F2 | #Feature F1 | #Feature F3 | #Feature F4 | #Feature F5 | #Feature F6 | Start | End | PTM | AScore | Found By |
| --- | --- | --- | --- | --- | --- | --- | --- | --- | --- | --- | --- | --- | --- | --- | --- | --- | --- | --- | --- | --- | --- | --- | --- | --- | --- | --- | --- | --- | --- |
| K.QPGITFIAAK.F | Y | 59.75 | 1044.5967 | 10 | -0.1 | 523.3055 | 2 | 38.53 | 1 | F1:19890 | 28122021\_RID\_2349\_2C.raw | 1.4738E5 |  |  |  |  |  | 1 | 1 | 0 | 0 | 0 | 0 | 0 | 172 | 181 |  |  | PEAKS DB |
| K.FDGILGMAYPK.I | Y | 50.59 | 1210.6056 | 11 | 0.2 | 606.3102 | 2 | 51.76 | 1 | F1:30350 | 28122021\_RID\_2349\_2C.raw | 8.6444E4 |  |  |  |  |  | 1 | 1 | 0 | 0 | 0 | 0 | 0 | 182 | 192 |  |  | PEAKS DB |
| total 2 peptides |  |  |  |  |  |  |  |  |  |  |  |  |  |  |  |  |  |  |  |  |  |  |  |  |  |  |  |  |  |
| --- | --- | --- | --- | --- | --- | --- | --- | --- | --- | --- | --- | --- | --- | --- | --- | --- | --- | --- | --- | --- | --- | --- | --- | --- | --- | --- | --- | --- | --- |

LAA50656.1

back to list

  

| Protein Coverage
| Supporting Peptides
|

Protein Coverage:

Supporting Peptides:

| Peptide | Uniq | -10lgP | Mass | Length | ppm | m/z | z | RT | Fraction | Scan | Source File | Area F2 | Area F1 | Area F3 | Area F4 | Area F5 | Area F6 | #Feature | #Feature F2 | #Feature F1 | #Feature F3 | #Feature F4 | #Feature F5 | #Feature F6 | Start | End | PTM | AScore | Found By |
| --- | --- | --- | --- | --- | --- | --- | --- | --- | --- | --- | --- | --- | --- | --- | --- | --- | --- | --- | --- | --- | --- | --- | --- | --- | --- | --- | --- | --- | --- |
| K.QPGITFIAAK.F | Y | 59.75 | 1044.5967 | 10 | -0.1 | 523.3055 | 2 | 38.53 | 1 | F1:19890 | 28122021\_RID\_2349\_2C.raw | 1.4738E5 |  |  |  |  |  | 1 | 1 | 0 | 0 | 0 | 0 | 0 | 46 | 55 |  |  | PEAKS DB |
| K.FDGILGMAYPK.I | Y | 50.59 | 1210.6056 | 11 | 0.2 | 606.3102 | 2 | 51.76 | 1 | F1:30350 | 28122021\_RID\_2349\_2C.raw | 8.6444E4 |  |  |  |  |  | 1 | 1 | 0 | 0 | 0 | 0 | 0 | 56 | 66 |  |  | PEAKS DB |
| total 2 peptides |  |  |  |  |  |  |  |  |  |  |  |  |  |  |  |  |  |  |  |  |  |  |  |  |  |  |  |  |  |
| --- | --- | --- | --- | --- | --- | --- | --- | --- | --- | --- | --- | --- | --- | --- | --- | --- | --- | --- | --- | --- | --- | --- | --- | --- | --- | --- | --- | --- | --- |

JAG46182.1

back to list

  

| Protein Coverage
| Supporting Peptides
|

Protein Coverage:

Supporting Peptides:

| Peptide | Uniq | -10lgP | Mass | Length | ppm | m/z | z | RT | Fraction | Scan | Source File | Area F2 | Area F1 | Area F3 | Area F4 | Area F5 | Area F6 | #Feature | #Feature F2 | #Feature F1 | #Feature F3 | #Feature F4 | #Feature F5 | #Feature F6 | Start | End | PTM | AScore | Found By |
| --- | --- | --- | --- | --- | --- | --- | --- | --- | --- | --- | --- | --- | --- | --- | --- | --- | --- | --- | --- | --- | --- | --- | --- | --- | --- | --- | --- | --- | --- |
| K.ASAATTAIVTAR.S | Y | 53.07 | 1131.6248 | 12 | 0.8 | 566.8201 | 2 | 26.78 | 1 | F1:10507 | 28122021\_RID\_2349\_2C.raw | 5.2581E4 |  |  |  |  |  | 1 | 1 | 0 | 0 | 0 | 0 | 0 | 352 | 363 |  |  | PEAKS DB |
| K.IANVVFANSGLK.M | Y | 51.22 | 1231.6924 | 12 | 0.8 | 616.8539 | 2 | 40.14 | 1 | F1:21188 | 28122021\_RID\_2349\_2C.raw | 5.0539E4 |  |  |  |  |  | 1 | 1 | 0 | 0 | 0 | 0 | 0 | 109 | 120 |  |  | PEAKS DB |
| total 2 peptides |  |  |  |  |  |  |  |  |  |  |  |  |  |  |  |  |  |  |  |  |  |  |  |  |  |  |  |  |  |
| --- | --- | --- | --- | --- | --- | --- | --- | --- | --- | --- | --- | --- | --- | --- | --- | --- | --- | --- | --- | --- | --- | --- | --- | --- | --- | --- | --- | --- | --- |

JAG43702.1

back to list

  

| Protein Coverage
| Supporting Peptides
|

Protein Coverage:

Supporting Peptides:

| Peptide | Uniq | -10lgP | Mass | Length | ppm | m/z | z | RT | Fraction | Scan | Source File | Area F2 | Area F1 | Area F3 | Area F4 | Area F5 | Area F6 | #Feature | #Feature F2 | #Feature F1 | #Feature F3 | #Feature F4 | #Feature F5 | #Feature F6 | Start | End | PTM | AScore | Found By |
| --- | --- | --- | --- | --- | --- | --- | --- | --- | --- | --- | --- | --- | --- | --- | --- | --- | --- | --- | --- | --- | --- | --- | --- | --- | --- | --- | --- | --- | --- |
| K.ASAATTAIVTAR.S | Y | 53.07 | 1131.6248 | 12 | 0.8 | 566.8201 | 2 | 26.78 | 1 | F1:10507 | 28122021\_RID\_2349\_2C.raw | 5.2581E4 |  |  |  |  |  | 1 | 1 | 0 | 0 | 0 | 0 | 0 | 352 | 363 |  |  | PEAKS DB |
| K.IANVVFANSGLK.M | Y | 51.22 | 1231.6924 | 12 | 0.8 | 616.8539 | 2 | 40.14 | 1 | F1:21188 | 28122021\_RID\_2349\_2C.raw | 5.0539E4 |  |  |  |  |  | 1 | 1 | 0 | 0 | 0 | 0 | 0 | 109 | 120 |  |  | PEAKS DB |
| total 2 peptides |  |  |  |  |  |  |  |  |  |  |  |  |  |  |  |  |  |  |  |  |  |  |  |  |  |  |  |  |  |
| --- | --- | --- | --- | --- | --- | --- | --- | --- | --- | --- | --- | --- | --- | --- | --- | --- | --- | --- | --- | --- | --- | --- | --- | --- | --- | --- | --- | --- | --- |

JAA96331.1

back to list

  

| Protein Coverage
| Supporting Peptides
|

Protein Coverage:

Supporting Peptides:

| Peptide | Uniq | -10lgP | Mass | Length | ppm | m/z | z | RT | Fraction | Scan | Source File | Area F2 | Area F1 | Area F3 | Area F4 | Area F5 | Area F6 | #Feature | #Feature F2 | #Feature F1 | #Feature F3 | #Feature F4 | #Feature F5 | #Feature F6 | Start | End | PTM | AScore | Found By |
| --- | --- | --- | --- | --- | --- | --- | --- | --- | --- | --- | --- | --- | --- | --- | --- | --- | --- | --- | --- | --- | --- | --- | --- | --- | --- | --- | --- | --- | --- |
| K.ASAATTAIVTAR.S | Y | 53.07 | 1131.6248 | 12 | 0.8 | 566.8201 | 2 | 26.78 | 1 | F1:10507 | 28122021\_RID\_2349\_2C.raw | 5.2581E4 |  |  |  |  |  | 1 | 1 | 0 | 0 | 0 | 0 | 0 | 352 | 363 |  |  | PEAKS DB |
| K.IANVVFANSGLK.M | Y | 51.22 | 1231.6924 | 12 | 0.8 | 616.8539 | 2 | 40.14 | 1 | F1:21188 | 28122021\_RID\_2349\_2C.raw | 5.0539E4 |  |  |  |  |  | 1 | 1 | 0 | 0 | 0 | 0 | 0 | 109 | 120 |  |  | PEAKS DB |
| total 2 peptides |  |  |  |  |  |  |  |  |  |  |  |  |  |  |  |  |  |  |  |  |  |  |  |  |  |  |  |  |  |
| --- | --- | --- | --- | --- | --- | --- | --- | --- | --- | --- | --- | --- | --- | --- | --- | --- | --- | --- | --- | --- | --- | --- | --- | --- | --- | --- | --- | --- | --- |

XP\_015674504.1

back to list

  

| Protein Coverage
| Supporting Peptides
|

Protein Coverage:

Supporting Peptides:

| Peptide | Uniq | -10lgP | Mass | Length | ppm | m/z | z | RT | Fraction | Scan | Source File | Area F2 | Area F1 | Area F3 | Area F4 | Area F5 | Area F6 | #Feature | #Feature F2 | #Feature F1 | #Feature F3 | #Feature F4 | #Feature F5 | #Feature F6 | Start | End | PTM | AScore | Found By |
| --- | --- | --- | --- | --- | --- | --- | --- | --- | --- | --- | --- | --- | --- | --- | --- | --- | --- | --- | --- | --- | --- | --- | --- | --- | --- | --- | --- | --- | --- |
| K.ASAATTAIVTAR.S | Y | 53.07 | 1131.6248 | 12 | 0.8 | 566.8201 | 2 | 26.78 | 1 | F1:10507 | 28122021\_RID\_2349\_2C.raw | 5.2581E4 |  |  |  |  |  | 1 | 1 | 0 | 0 | 0 | 0 | 0 | 352 | 363 |  |  | PEAKS DB |
| K.IANVVFANSGLK.M | Y | 51.22 | 1231.6924 | 12 | 0.8 | 616.8539 | 2 | 40.14 | 1 | F1:21188 | 28122021\_RID\_2349\_2C.raw | 5.0539E4 |  |  |  |  |  | 1 | 1 | 0 | 0 | 0 | 0 | 0 | 109 | 120 |  |  | PEAKS DB |
| total 2 peptides |  |  |  |  |  |  |  |  |  |  |  |  |  |  |  |  |  |  |  |  |  |  |  |  |  |  |  |  |  |
| --- | --- | --- | --- | --- | --- | --- | --- | --- | --- | --- | --- | --- | --- | --- | --- | --- | --- | --- | --- | --- | --- | --- | --- | --- | --- | --- | --- | --- | --- |

XP\_015674505.1

back to list

  

| Protein Coverage
| Supporting Peptides
|

Protein Coverage:

Supporting Peptides:

| Peptide | Uniq | -10lgP | Mass | Length | ppm | m/z | z | RT | Fraction | Scan | Source File | Area F2 | Area F1 | Area F3 | Area F4 | Area F5 | Area F6 | #Feature | #Feature F2 | #Feature F1 | #Feature F3 | #Feature F4 | #Feature F5 | #Feature F6 | Start | End | PTM | AScore | Found By |
| --- | --- | --- | --- | --- | --- | --- | --- | --- | --- | --- | --- | --- | --- | --- | --- | --- | --- | --- | --- | --- | --- | --- | --- | --- | --- | --- | --- | --- | --- |
| K.ASAATTAIVTAR.S | Y | 53.07 | 1131.6248 | 12 | 0.8 | 566.8201 | 2 | 26.78 | 1 | F1:10507 | 28122021\_RID\_2349\_2C.raw | 5.2581E4 |  |  |  |  |  | 1 | 1 | 0 | 0 | 0 | 0 | 0 | 352 | 363 |  |  | PEAKS DB |
| K.IANVVFANSGLK.M | Y | 51.22 | 1231.6924 | 12 | 0.8 | 616.8539 | 2 | 40.14 | 1 | F1:21188 | 28122021\_RID\_2349\_2C.raw | 5.0539E4 |  |  |  |  |  | 1 | 1 | 0 | 0 | 0 | 0 | 0 | 109 | 120 |  |  | PEAKS DB |
| total 2 peptides |  |  |  |  |  |  |  |  |  |  |  |  |  |  |  |  |  |  |  |  |  |  |  |  |  |  |  |  |  |
| --- | --- | --- | --- | --- | --- | --- | --- | --- | --- | --- | --- | --- | --- | --- | --- | --- | --- | --- | --- | --- | --- | --- | --- | --- | --- | --- | --- | --- | --- |

JAV49839.1

back to list

  

| Protein Coverage
| Supporting Peptides
|

Protein Coverage:

Supporting Peptides:

| Peptide | Uniq | -10lgP | Mass | Length | ppm | m/z | z | RT | Fraction | Scan | Source File | Area F2 | Area F1 | Area F3 | Area F4 | Area F5 | Area F6 | #Feature | #Feature F2 | #Feature F1 | #Feature F3 | #Feature F4 | #Feature F5 | #Feature F6 | Start | End | PTM | AScore | Found By |
| --- | --- | --- | --- | --- | --- | --- | --- | --- | --- | --- | --- | --- | --- | --- | --- | --- | --- | --- | --- | --- | --- | --- | --- | --- | --- | --- | --- | --- | --- |
| K.ASAATTAIVTAR.S | Y | 53.07 | 1131.6248 | 12 | 0.8 | 566.8201 | 2 | 26.78 | 1 | F1:10507 | 28122021\_RID\_2349\_2C.raw | 5.2581E4 |  |  |  |  |  | 1 | 1 | 0 | 0 | 0 | 0 | 0 | 352 | 363 |  |  | PEAKS DB |
| K.IANVVFANSGLK.M | Y | 51.22 | 1231.6924 | 12 | 0.8 | 616.8539 | 2 | 40.14 | 1 | F1:21188 | 28122021\_RID\_2349\_2C.raw | 5.0539E4 |  |  |  |  |  | 1 | 1 | 0 | 0 | 0 | 0 | 0 | 109 | 120 |  |  | PEAKS DB |
| total 2 peptides |  |  |  |  |  |  |  |  |  |  |  |  |  |  |  |  |  |  |  |  |  |  |  |  |  |  |  |  |  |
| --- | --- | --- | --- | --- | --- | --- | --- | --- | --- | --- | --- | --- | --- | --- | --- | --- | --- | --- | --- | --- | --- | --- | --- | --- | --- | --- | --- | --- | --- |

BAP39986.1

back to list

  

| Protein Coverage
| Supporting Peptides
|

Protein Coverage:

Supporting Peptides:

| Peptide | Uniq | -10lgP | Mass | Length | ppm | m/z | z | RT | Fraction | Scan | Source File | Area F2 | Area F1 | Area F3 | Area F4 | Area F5 | Area F6 | #Feature | #Feature F2 | #Feature F1 | #Feature F3 | #Feature F4 | #Feature F5 | #Feature F6 | Start | End | PTM | AScore | Found By |
| --- | --- | --- | --- | --- | --- | --- | --- | --- | --- | --- | --- | --- | --- | --- | --- | --- | --- | --- | --- | --- | --- | --- | --- | --- | --- | --- | --- | --- | --- |
| R.NNQLLWLWR.E | Y | 55.53 | 1241.6669 | 9 | 0.0 | 621.8407 | 2 | 57.68 | 6 | F6:32658 | 31122021\_RID\_2349\_6C.raw | 1.013E5 |  |  |  |  | 9.5587E4 | 2 | 1 | 0 | 0 | 0 | 0 | 1 | 305 | 313 |  |  | PEAKS DB |
| R.KDYALPVFVYAR.P | Y | 44.35 | 1440.7765 | 12 | 0.1 | 481.2661 | 3 | 46.64 | 6 | F6:24365 | 31122021\_RID\_2349\_6C.raw | 3.5764E5 |  |  |  |  | 8.6724E4 | 2 | 1 | 0 | 0 | 0 | 0 | 1 | 354 | 365 |  |  | PEAKS DB |
| total 2 peptides |  |  |  |  |  |  |  |  |  |  |  |  |  |  |  |  |  |  |  |  |  |  |  |  |  |  |  |  |  |
| --- | --- | --- | --- | --- | --- | --- | --- | --- | --- | --- | --- | --- | --- | --- | --- | --- | --- | --- | --- | --- | --- | --- | --- | --- | --- | --- | --- | --- | --- |

BAN89379.1

back to list

  

| Protein Coverage
| Supporting Peptides
|

Protein Coverage:

Supporting Peptides:

| Peptide | Uniq | -10lgP | Mass | Length | ppm | m/z | z | RT | Fraction | Scan | Source File | Area F2 | Area F1 | Area F3 | Area F4 | Area F5 | Area F6 | #Feature | #Feature F2 | #Feature F1 | #Feature F3 | #Feature F4 | #Feature F5 | #Feature F6 | Start | End | PTM | AScore | Found By |
| --- | --- | --- | --- | --- | --- | --- | --- | --- | --- | --- | --- | --- | --- | --- | --- | --- | --- | --- | --- | --- | --- | --- | --- | --- | --- | --- | --- | --- | --- |
| R.NNQLLWLWR.E | Y | 55.53 | 1241.6669 | 9 | 0.0 | 621.8407 | 2 | 57.68 | 6 | F6:32658 | 31122021\_RID\_2349\_6C.raw | 1.013E5 |  |  |  |  | 9.5587E4 | 2 | 1 | 0 | 0 | 0 | 0 | 1 | 311 | 319 |  |  | PEAKS DB |
| R.KDYALPVFVYAR.P | Y | 44.35 | 1440.7765 | 12 | 0.1 | 481.2661 | 3 | 46.64 | 6 | F6:24365 | 31122021\_RID\_2349\_6C.raw | 3.5764E5 |  |  |  |  | 8.6724E4 | 2 | 1 | 0 | 0 | 0 | 0 | 1 | 360 | 371 |  |  | PEAKS DB |
| total 2 peptides |  |  |  |  |  |  |  |  |  |  |  |  |  |  |  |  |  |  |  |  |  |  |  |  |  |  |  |  |  |
| --- | --- | --- | --- | --- | --- | --- | --- | --- | --- | --- | --- | --- | --- | --- | --- | --- | --- | --- | --- | --- | --- | --- | --- | --- | --- | --- | --- | --- | --- |

JAV01892.1

back to list

  

| Protein Coverage
| Supporting Peptides
|

Protein Coverage:

Supporting Peptides:

| Peptide | Uniq | -10lgP | Mass | Length | ppm | m/z | z | RT | Fraction | Scan | Source File | Area F2 | Area F1 | Area F3 | Area F4 | Area F5 | Area F6 | #Feature | #Feature F2 | #Feature F1 | #Feature F3 | #Feature F4 | #Feature F5 | #Feature F6 | Start | End | PTM | AScore | Found By |
| --- | --- | --- | --- | --- | --- | --- | --- | --- | --- | --- | --- | --- | --- | --- | --- | --- | --- | --- | --- | --- | --- | --- | --- | --- | --- | --- | --- | --- | --- |
| R.NNQLLWLWR.D | Y | 55.53 | 1241.6669 | 9 | 0.0 | 621.8407 | 2 | 57.68 | 6 | F6:32658 | 31122021\_RID\_2349\_6C.raw | 1.013E5 |  |  |  |  | 9.5587E4 | 2 | 1 | 0 | 0 | 0 | 0 | 1 | 235 | 243 |  |  | PEAKS DB |
| R.KDYALPVFVYAR.P | Y | 44.35 | 1440.7765 | 12 | 0.1 | 481.2661 | 3 | 46.64 | 6 | F6:24365 | 31122021\_RID\_2349\_6C.raw | 3.5764E5 |  |  |  |  | 8.6724E4 | 2 | 1 | 0 | 0 | 0 | 0 | 1 | 284 | 295 |  |  | PEAKS DB |
| total 2 peptides |  |  |  |  |  |  |  |  |  |  |  |  |  |  |  |  |  |  |  |  |  |  |  |  |  |  |  |  |  |
| --- | --- | --- | --- | --- | --- | --- | --- | --- | --- | --- | --- | --- | --- | --- | --- | --- | --- | --- | --- | --- | --- | --- | --- | --- | --- | --- | --- | --- | --- |

ATU85542.1

back to list

  

| Protein Coverage
| Supporting Peptides
|

Protein Coverage:

Supporting Peptides:

| Peptide | Uniq | -10lgP | Mass | Length | ppm | m/z | z | RT | Fraction | Scan | Source File | Area F2 | Area F1 | Area F3 | Area F4 | Area F5 | Area F6 | #Feature | #Feature F2 | #Feature F1 | #Feature F3 | #Feature F4 | #Feature F5 | #Feature F6 | Start | End | PTM | AScore | Found By |
| --- | --- | --- | --- | --- | --- | --- | --- | --- | --- | --- | --- | --- | --- | --- | --- | --- | --- | --- | --- | --- | --- | --- | --- | --- | --- | --- | --- | --- | --- |
| R.NNQLLWLWR.D | Y | 55.53 | 1241.6669 | 9 | 0.0 | 621.8407 | 2 | 57.68 | 6 | F6:32658 | 31122021\_RID\_2349\_6C.raw | 1.013E5 |  |  |  |  | 9.5587E4 | 2 | 1 | 0 | 0 | 0 | 0 | 1 | 235 | 243 |  |  | PEAKS DB |
| R.KDYALPVFVYAR.P | Y | 44.35 | 1440.7765 | 12 | 0.1 | 481.2661 | 3 | 46.64 | 6 | F6:24365 | 31122021\_RID\_2349\_6C.raw | 3.5764E5 |  |  |  |  | 8.6724E4 | 2 | 1 | 0 | 0 | 0 | 0 | 1 | 284 | 295 |  |  | PEAKS DB |
| total 2 peptides |  |  |  |  |  |  |  |  |  |  |  |  |  |  |  |  |  |  |  |  |  |  |  |  |  |  |  |  |  |
| --- | --- | --- | --- | --- | --- | --- | --- | --- | --- | --- | --- | --- | --- | --- | --- | --- | --- | --- | --- | --- | --- | --- | --- | --- | --- | --- | --- | --- | --- |

BAN89414.1

back to list

  

| Protein Coverage
| Supporting Peptides
|

Protein Coverage:

Supporting Peptides:

| Peptide | Uniq | -10lgP | Mass | Length | ppm | m/z | z | RT | Fraction | Scan | Source File | Area F2 | Area F1 | Area F3 | Area F4 | Area F5 | Area F6 | #Feature | #Feature F2 | #Feature F1 | #Feature F3 | #Feature F4 | #Feature F5 | #Feature F6 | Start | End | PTM | AScore | Found By |
| --- | --- | --- | --- | --- | --- | --- | --- | --- | --- | --- | --- | --- | --- | --- | --- | --- | --- | --- | --- | --- | --- | --- | --- | --- | --- | --- | --- | --- | --- |
| R.NNQLLWLWR.D | Y | 55.53 | 1241.6669 | 9 | 0.0 | 621.8407 | 2 | 57.68 | 6 | F6:32658 | 31122021\_RID\_2349\_6C.raw | 1.013E5 |  |  |  |  | 9.5587E4 | 2 | 1 | 0 | 0 | 0 | 0 | 1 | 212 | 220 |  |  | PEAKS DB |
| R.KDYALPVFVYAR.P | Y | 44.35 | 1440.7765 | 12 | 0.1 | 481.2661 | 3 | 46.64 | 6 | F6:24365 | 31122021\_RID\_2349\_6C.raw | 3.5764E5 |  |  |  |  | 8.6724E4 | 2 | 1 | 0 | 0 | 0 | 0 | 1 | 261 | 272 |  |  | PEAKS DB |
| total 2 peptides |  |  |  |  |  |  |  |  |  |  |  |  |  |  |  |  |  |  |  |  |  |  |  |  |  |  |  |  |  |
| --- | --- | --- | --- | --- | --- | --- | --- | --- | --- | --- | --- | --- | --- | --- | --- | --- | --- | --- | --- | --- | --- | --- | --- | --- | --- | --- | --- | --- | --- |

BAN89413.1

back to list

  

| Protein Coverage
| Supporting Peptides
|

Protein Coverage:

Supporting Peptides:

| Peptide | Uniq | -10lgP | Mass | Length | ppm | m/z | z | RT | Fraction | Scan | Source File | Area F2 | Area F1 | Area F3 | Area F4 | Area F5 | Area F6 | #Feature | #Feature F2 | #Feature F1 | #Feature F3 | #Feature F4 | #Feature F5 | #Feature F6 | Start | End | PTM | AScore | Found By |
| --- | --- | --- | --- | --- | --- | --- | --- | --- | --- | --- | --- | --- | --- | --- | --- | --- | --- | --- | --- | --- | --- | --- | --- | --- | --- | --- | --- | --- | --- |
| R.NNQLLWLWR.D | Y | 55.53 | 1241.6669 | 9 | 0.0 | 621.8407 | 2 | 57.68 | 6 | F6:32658 | 31122021\_RID\_2349\_6C.raw | 1.013E5 |  |  |  |  | 9.5587E4 | 2 | 1 | 0 | 0 | 0 | 0 | 1 | 235 | 243 |  |  | PEAKS DB |
| R.KDYALPVFVYAR.P | Y | 44.35 | 1440.7765 | 12 | 0.1 | 481.2661 | 3 | 46.64 | 6 | F6:24365 | 31122021\_RID\_2349\_6C.raw | 3.5764E5 |  |  |  |  | 8.6724E4 | 2 | 1 | 0 | 0 | 0 | 0 | 1 | 284 | 295 |  |  | PEAKS DB |
| total 2 peptides |  |  |  |  |  |  |  |  |  |  |  |  |  |  |  |  |  |  |  |  |  |  |  |  |  |  |  |  |  |
| --- | --- | --- | --- | --- | --- | --- | --- | --- | --- | --- | --- | --- | --- | --- | --- | --- | --- | --- | --- | --- | --- | --- | --- | --- | --- | --- | --- | --- | --- |

JAS03131.1

back to list

  

| Protein Coverage
| Supporting Peptides
|

Protein Coverage:

Supporting Peptides:

| Peptide | Uniq | -10lgP | Mass | Length | ppm | m/z | z | RT | Fraction | Scan | Source File | Area F2 | Area F1 | Area F3 | Area F4 | Area F5 | Area F6 | #Feature | #Feature F2 | #Feature F1 | #Feature F3 | #Feature F4 | #Feature F5 | #Feature F6 | Start | End | PTM | AScore | Found By |
| --- | --- | --- | --- | --- | --- | --- | --- | --- | --- | --- | --- | --- | --- | --- | --- | --- | --- | --- | --- | --- | --- | --- | --- | --- | --- | --- | --- | --- | --- |
| R.VMGWGTITSPK.R | Y | 77.07 | 1175.6008 | 11 | -0.3 | 588.8075 | 2 | 41.06 | 4 | F4:9792 | 31122021\_RID\_2349\_4C.raw | 1.7051E7 | 4.8904E5 | 2.6803E9 | 8.9339E7 | 8.3713E7 | 1.4262E6 | 6 | 1 | 1 | 1 | 1 | 1 | 1 | 139 | 149 |  |  | PEAKS DB |
| R.VM(+15.99)GWGTITSPK.R | Y | 65.04 | 1191.5958 | 11 | 0.8 | 596.8057 | 2 | 35.11 | 3 | F3:9576 | 31122021\_RID\_2349\_3C.raw | 3.8682E6 |  | 5.862E8 | 1.0568E7 | 1.0355E7 | 1.0852E5 | 19 | 5 | 0 | 6 | 2 | 5 | 1 | 139 | 149 | Oxidation (M) | M2:Oxidation (M):1000.00 | PEAKS DB |
| total 2 peptides |  |  |  |  |  |  |  |  |  |  |  |  |  |  |  |  |  |  |  |  |  |  |  |  |  |  |  |  |  |
| --- | --- | --- | --- | --- | --- | --- | --- | --- | --- | --- | --- | --- | --- | --- | --- | --- | --- | --- | --- | --- | --- | --- | --- | --- | --- | --- | --- | --- | --- |

ABN72541.1

back to list

  

| Protein Coverage
| Supporting Peptides
|

Protein Coverage:

Supporting Peptides:

| Peptide | Uniq | -10lgP | Mass | Length | ppm | m/z | z | RT | Fraction | Scan | Source File | Area F2 | Area F1 | Area F3 | Area F4 | Area F5 | Area F6 | #Feature | #Feature F2 | #Feature F1 | #Feature F3 | #Feature F4 | #Feature F5 | #Feature F6 | Start | End | PTM | AScore | Found By |
| --- | --- | --- | --- | --- | --- | --- | --- | --- | --- | --- | --- | --- | --- | --- | --- | --- | --- | --- | --- | --- | --- | --- | --- | --- | --- | --- | --- | --- | --- |
| T.VMGWGTITSPK.V | Y | 77.07 | 1175.6008 | 11 | -0.3 | 588.8075 | 2 | 41.06 | 4 | F4:9792 | 31122021\_RID\_2349\_4C.raw | 1.7051E7 | 4.8904E5 | 2.6803E9 | 8.9339E7 | 8.3713E7 | 1.4262E6 | 6 | 1 | 1 | 1 | 1 | 1 | 1 | 176 | 186 |  |  | PEAKS DB |
| T.VM(+15.99)GWGTITSPK.V | Y | 65.04 | 1191.5958 | 11 | 0.8 | 596.8057 | 2 | 35.11 | 3 | F3:9576 | 31122021\_RID\_2349\_3C.raw | 3.8682E6 |  | 5.862E8 | 1.0568E7 | 1.0355E7 | 1.0852E5 | 19 | 5 | 0 | 6 | 2 | 5 | 1 | 176 | 186 | Oxidation (M) | M2:Oxidation (M):1000.00 | PEAKS DB |
| total 2 peptides |  |  |  |  |  |  |  |  |  |  |  |  |  |  |  |  |  |  |  |  |  |  |  |  |  |  |  |  |  |
| --- | --- | --- | --- | --- | --- | --- | --- | --- | --- | --- | --- | --- | --- | --- | --- | --- | --- | --- | --- | --- | --- | --- | --- | --- | --- | --- | --- | --- | --- |

A8QL53.1

back to list

  

| Protein Coverage
| Supporting Peptides
|

Protein Coverage:

Supporting Peptides:

| Peptide | Uniq | -10lgP | Mass | Length | ppm | m/z | z | RT | Fraction | Scan | Source File | Area F2 | Area F1 | Area F3 | Area F4 | Area F5 | Area F6 | #Feature | #Feature F2 | #Feature F1 | #Feature F3 | #Feature F4 | #Feature F5 | #Feature F6 | Start | End | PTM | AScore | Found By |
| --- | --- | --- | --- | --- | --- | --- | --- | --- | --- | --- | --- | --- | --- | --- | --- | --- | --- | --- | --- | --- | --- | --- | --- | --- | --- | --- | --- | --- | --- |
| T.VMGWGTITSPK.V | Y | 77.07 | 1175.6008 | 11 | -0.3 | 588.8075 | 2 | 41.06 | 4 | F4:9792 | 31122021\_RID\_2349\_4C.raw | 1.7051E7 | 4.8904E5 | 2.6803E9 | 8.9339E7 | 8.3713E7 | 1.4262E6 | 6 | 1 | 1 | 1 | 1 | 1 | 1 | 176 | 186 |  |  | PEAKS DB |
| T.VM(+15.99)GWGTITSPK.V | Y | 65.04 | 1191.5958 | 11 | 0.8 | 596.8057 | 2 | 35.11 | 3 | F3:9576 | 31122021\_RID\_2349\_3C.raw | 3.8682E6 |  | 5.862E8 | 1.0568E7 | 1.0355E7 | 1.0852E5 | 19 | 5 | 0 | 6 | 2 | 5 | 1 | 176 | 186 | Oxidation (M) | M2:Oxidation (M):1000.00 | PEAKS DB |
| total 2 peptides |  |  |  |  |  |  |  |  |  |  |  |  |  |  |  |  |  |  |  |  |  |  |  |  |  |  |  |  |  |
| --- | --- | --- | --- | --- | --- | --- | --- | --- | --- | --- | --- | --- | --- | --- | --- | --- | --- | --- | --- | --- | --- | --- | --- | --- | --- | --- | --- | --- | --- |

ABN72545.1

back to list

  

| Protein Coverage
| Supporting Peptides
|

Protein Coverage:

Supporting Peptides:

| Peptide | Uniq | -10lgP | Mass | Length | ppm | m/z | z | RT | Fraction | Scan | Source File | Area F2 | Area F1 | Area F3 | Area F4 | Area F5 | Area F6 | #Feature | #Feature F2 | #Feature F1 | #Feature F3 | #Feature F4 | #Feature F5 | #Feature F6 | Start | End | PTM | AScore | Found By |
| --- | --- | --- | --- | --- | --- | --- | --- | --- | --- | --- | --- | --- | --- | --- | --- | --- | --- | --- | --- | --- | --- | --- | --- | --- | --- | --- | --- | --- | --- |
| T.VMGWGTITSPK.V | Y | 77.07 | 1175.6008 | 11 | -0.3 | 588.8075 | 2 | 41.06 | 4 | F4:9792 | 31122021\_RID\_2349\_4C.raw | 1.7051E7 | 4.8904E5 | 2.6803E9 | 8.9339E7 | 8.3713E7 | 1.4262E6 | 6 | 1 | 1 | 1 | 1 | 1 | 1 | 176 | 186 |  |  | PEAKS DB |
| T.VM(+15.99)GWGTITSPK.V | Y | 65.04 | 1191.5958 | 11 | 0.8 | 596.8057 | 2 | 35.11 | 3 | F3:9576 | 31122021\_RID\_2349\_3C.raw | 3.8682E6 |  | 5.862E8 | 1.0568E7 | 1.0355E7 | 1.0852E5 | 19 | 5 | 0 | 6 | 2 | 5 | 1 | 176 | 186 | Oxidation (M) | M2:Oxidation (M):1000.00 | PEAKS DB |
| total 2 peptides |  |  |  |  |  |  |  |  |  |  |  |  |  |  |  |  |  |  |  |  |  |  |  |  |  |  |  |  |  |
| --- | --- | --- | --- | --- | --- | --- | --- | --- | --- | --- | --- | --- | --- | --- | --- | --- | --- | --- | --- | --- | --- | --- | --- | --- | --- | --- | --- | --- | --- |

A8QL57.1

back to list

  

| Protein Coverage
| Supporting Peptides
|

Protein Coverage:

Supporting Peptides:

| Peptide | Uniq | -10lgP | Mass | Length | ppm | m/z | z | RT | Fraction | Scan | Source File | Area F2 | Area F1 | Area F3 | Area F4 | Area F5 | Area F6 | #Feature | #Feature F2 | #Feature F1 | #Feature F3 | #Feature F4 | #Feature F5 | #Feature F6 | Start | End | PTM | AScore | Found By |
| --- | --- | --- | --- | --- | --- | --- | --- | --- | --- | --- | --- | --- | --- | --- | --- | --- | --- | --- | --- | --- | --- | --- | --- | --- | --- | --- | --- | --- | --- |
| T.VMGWGTITSPK.V | Y | 77.07 | 1175.6008 | 11 | -0.3 | 588.8075 | 2 | 41.06 | 4 | F4:9792 | 31122021\_RID\_2349\_4C.raw | 1.7051E7 | 4.8904E5 | 2.6803E9 | 8.9339E7 | 8.3713E7 | 1.4262E6 | 6 | 1 | 1 | 1 | 1 | 1 | 1 | 176 | 186 |  |  | PEAKS DB |
| T.VM(+15.99)GWGTITSPK.V | Y | 65.04 | 1191.5958 | 11 | 0.8 | 596.8057 | 2 | 35.11 | 3 | F3:9576 | 31122021\_RID\_2349\_3C.raw | 3.8682E6 |  | 5.862E8 | 1.0568E7 | 1.0355E7 | 1.0852E5 | 19 | 5 | 0 | 6 | 2 | 5 | 1 | 176 | 186 | Oxidation (M) | M2:Oxidation (M):1000.00 | PEAKS DB |
| total 2 peptides |  |  |  |  |  |  |  |  |  |  |  |  |  |  |  |  |  |  |  |  |  |  |  |  |  |  |  |  |  |
| --- | --- | --- | --- | --- | --- | --- | --- | --- | --- | --- | --- | --- | --- | --- | --- | --- | --- | --- | --- | --- | --- | --- | --- | --- | --- | --- | --- | --- | --- |

AFJ50447.1

back to list

  

| Protein Coverage
| Supporting Peptides
|

Protein Coverage:

Supporting Peptides:

| Peptide | Uniq | -10lgP | Mass | Length | ppm | m/z | z | RT | Fraction | Scan | Source File | Area F2 | Area F1 | Area F3 | Area F4 | Area F5 | Area F6 | #Feature | #Feature F2 | #Feature F1 | #Feature F3 | #Feature F4 | #Feature F5 | #Feature F6 | Start | End | PTM | AScore | Found By |
| --- | --- | --- | --- | --- | --- | --- | --- | --- | --- | --- | --- | --- | --- | --- | --- | --- | --- | --- | --- | --- | --- | --- | --- | --- | --- | --- | --- | --- | --- |
| K.KGSDIQADLNTK.Y | Y | 54.62 | 1288.6622 | 12 | 1.1 | 645.3391 | 2 | 21.04 | 1 | F1:6049 | 28122021\_RID\_2349\_2C.raw | 8.2634E4 |  |  |  |  |  | 1 | 1 | 0 | 0 | 0 | 0 | 0 | 134 | 145 |  |  | PEAKS DB |
| K.GSDIQADLNTK.Y | Y | 44.30 | 1160.5673 | 11 | 0.1 | 581.2910 | 2 | 27.03 | 1 | F1:10682 | 28122021\_RID\_2349\_2C.raw | 9.3876E4 |  |  |  |  |  | 1 | 1 | 0 | 0 | 0 | 0 | 0 | 135 | 145 |  |  | PEAKS DB |
| total 2 peptides |  |  |  |  |  |  |  |  |  |  |  |  |  |  |  |  |  |  |  |  |  |  |  |  |  |  |  |  |  |
| --- | --- | --- | --- | --- | --- | --- | --- | --- | --- | --- | --- | --- | --- | --- | --- | --- | --- | --- | --- | --- | --- | --- | --- | --- | --- | --- | --- | --- | --- |

XP\_007423023.1

back to list

  

| Protein Coverage
| Supporting Peptides
|

Protein Coverage:

Supporting Peptides:

| Peptide | Uniq | -10lgP | Mass | Length | ppm | m/z | z | RT | Fraction | Scan | Source File | Area F2 | Area F1 | Area F3 | Area F4 | Area F5 | Area F6 | #Feature | #Feature F2 | #Feature F1 | #Feature F3 | #Feature F4 | #Feature F5 | #Feature F6 | Start | End | PTM | AScore | Found By |
| --- | --- | --- | --- | --- | --- | --- | --- | --- | --- | --- | --- | --- | --- | --- | --- | --- | --- | --- | --- | --- | --- | --- | --- | --- | --- | --- | --- | --- | --- |
| K.KGSDIQADLNTK.Y | Y | 54.62 | 1288.6622 | 12 | 1.1 | 645.3391 | 2 | 21.04 | 1 | F1:6049 | 28122021\_RID\_2349\_2C.raw | 8.2634E4 |  |  |  |  |  | 1 | 1 | 0 | 0 | 0 | 0 | 0 | 135 | 146 |  |  | PEAKS DB |
| K.GSDIQADLNTK.Y | Y | 44.30 | 1160.5673 | 11 | 0.1 | 581.2910 | 2 | 27.03 | 1 | F1:10682 | 28122021\_RID\_2349\_2C.raw | 9.3876E4 |  |  |  |  |  | 1 | 1 | 0 | 0 | 0 | 0 | 0 | 136 | 146 |  |  | PEAKS DB |
| total 2 peptides |  |  |  |  |  |  |  |  |  |  |  |  |  |  |  |  |  |  |  |  |  |  |  |  |  |  |  |  |  |
| --- | --- | --- | --- | --- | --- | --- | --- | --- | --- | --- | --- | --- | --- | --- | --- | --- | --- | --- | --- | --- | --- | --- | --- | --- | --- | --- | --- | --- | --- |

D2YW39.2

back to list

  

| Protein Coverage
| Supporting Peptides
|

Protein Coverage:

Supporting Peptides:

| Peptide | Uniq | -10lgP | Mass | Length | ppm | m/z | z | RT | Fraction | Scan | Source File | Area F2 | Area F1 | Area F3 | Area F4 | Area F5 | Area F6 | #Feature | #Feature F2 | #Feature F1 | #Feature F3 | #Feature F4 | #Feature F5 | #Feature F6 | Start | End | PTM | AScore | Found By |
| --- | --- | --- | --- | --- | --- | --- | --- | --- | --- | --- | --- | --- | --- | --- | --- | --- | --- | --- | --- | --- | --- | --- | --- | --- | --- | --- | --- | --- | --- |
| K.RPELYVWIGLR.D | Y | 74.99 | 1400.7928 | 11 | 0.6 | 701.4041 | 2 | 50.06 | 6 | F6:27001 | 31122021\_RID\_2349\_6C.raw |  |  |  |  |  | 4.8598E6 | 2 | 0 | 0 | 0 | 0 | 0 | 2 | 63 | 73 |  |  | PEAKS DB |
| total 1 peptides |  |  |  |  |  |  |  |  |  |  |  |  |  |  |  |  |  |  |  |  |  |  |  |  |  |  |  |  |  |
| --- | --- | --- | --- | --- | --- | --- | --- | --- | --- | --- | --- | --- | --- | --- | --- | --- | --- | --- | --- | --- | --- | --- | --- | --- | --- | --- | --- | --- | --- |

XP\_015686565.1

back to list

  

| Protein Coverage
| Supporting Peptides
|

Protein Coverage:

Supporting Peptides:

| Peptide | Uniq | -10lgP | Mass | Length | ppm | m/z | z | RT | Fraction | Scan | Source File | Area F2 | Area F1 | Area F3 | Area F4 | Area F5 | Area F6 | #Feature | #Feature F2 | #Feature F1 | #Feature F3 | #Feature F4 | #Feature F5 | #Feature F6 | Start | End | PTM | AScore | Found By |
| --- | --- | --- | --- | --- | --- | --- | --- | --- | --- | --- | --- | --- | --- | --- | --- | --- | --- | --- | --- | --- | --- | --- | --- | --- | --- | --- | --- | --- | --- |
| H.RPEIYVWIGLR.D | Y | 74.99 | 1400.7928 | 11 | 0.6 | 701.4041 | 2 | 50.06 | 6 | F6:27001 | 31122021\_RID\_2349\_6C.raw |  |  |  |  |  | 4.8598E6 | 2 | 0 | 0 | 0 | 0 | 0 | 2 | 8 | 18 |  |  | PEAKS DB |
| total 1 peptides |  |  |  |  |  |  |  |  |  |  |  |  |  |  |  |  |  |  |  |  |  |  |  |  |  |  |  |  |  |
| --- | --- | --- | --- | --- | --- | --- | --- | --- | --- | --- | --- | --- | --- | --- | --- | --- | --- | --- | --- | --- | --- | --- | --- | --- | --- | --- | --- | --- | --- |

BAN81991.1

back to list

  

| Protein Coverage
| Supporting Peptides
|

Protein Coverage:

Supporting Peptides:

| Peptide | Uniq | -10lgP | Mass | Length | ppm | m/z | z | RT | Fraction | Scan | Source File | Area F2 | Area F1 | Area F3 | Area F4 | Area F5 | Area F6 | #Feature | #Feature F2 | #Feature F1 | #Feature F3 | #Feature F4 | #Feature F5 | #Feature F6 | Start | End | PTM | AScore | Found By |
| --- | --- | --- | --- | --- | --- | --- | --- | --- | --- | --- | --- | --- | --- | --- | --- | --- | --- | --- | --- | --- | --- | --- | --- | --- | --- | --- | --- | --- | --- |
| H.RPEIYVWIGLR.D | Y | 74.99 | 1400.7928 | 11 | 0.6 | 701.4041 | 2 | 50.06 | 6 | F6:27001 | 31122021\_RID\_2349\_6C.raw |  |  |  |  |  | 4.8598E6 | 2 | 0 | 0 | 0 | 0 | 0 | 2 | 77 | 87 |  |  | PEAKS DB |
| total 1 peptides |  |  |  |  |  |  |  |  |  |  |  |  |  |  |  |  |  |  |  |  |  |  |  |  |  |  |  |  |  |
| --- | --- | --- | --- | --- | --- | --- | --- | --- | --- | --- | --- | --- | --- | --- | --- | --- | --- | --- | --- | --- | --- | --- | --- | --- | --- | --- | --- | --- | --- |

AAQ15166.1

back to list

  

| Protein Coverage
| Supporting Peptides
|

Protein Coverage:

Supporting Peptides:

| Peptide | Uniq | -10lgP | Mass | Length | ppm | m/z | z | RT | Fraction | Scan | Source File | Area F2 | Area F1 | Area F3 | Area F4 | Area F5 | Area F6 | #Feature | #Feature F2 | #Feature F1 | #Feature F3 | #Feature F4 | #Feature F5 | #Feature F6 | Start | End | PTM | AScore | Found By |
| --- | --- | --- | --- | --- | --- | --- | --- | --- | --- | --- | --- | --- | --- | --- | --- | --- | --- | --- | --- | --- | --- | --- | --- | --- | --- | --- | --- | --- | --- |
| Q.RPEIYVWIGLR.D | Y | 74.99 | 1400.7928 | 11 | 0.6 | 701.4041 | 2 | 50.06 | 6 | F6:27001 | 31122021\_RID\_2349\_6C.raw |  |  |  |  |  | 4.8598E6 | 2 | 0 | 0 | 0 | 0 | 0 | 2 | 86 | 96 |  |  | PEAKS DB |
| total 1 peptides |  |  |  |  |  |  |  |  |  |  |  |  |  |  |  |  |  |  |  |  |  |  |  |  |  |  |  |  |  |
| --- | --- | --- | --- | --- | --- | --- | --- | --- | --- | --- | --- | --- | --- | --- | --- | --- | --- | --- | --- | --- | --- | --- | --- | --- | --- | --- | --- | --- | --- |

Q71RQ1.1

back to list

  

| Protein Coverage
| Supporting Peptides
|

Protein Coverage:

Supporting Peptides:

| Peptide | Uniq | -10lgP | Mass | Length | ppm | m/z | z | RT | Fraction | Scan | Source File | Area F2 | Area F1 | Area F3 | Area F4 | Area F5 | Area F6 | #Feature | #Feature F2 | #Feature F1 | #Feature F3 | #Feature F4 | #Feature F5 | #Feature F6 | Start | End | PTM | AScore | Found By |
| --- | --- | --- | --- | --- | --- | --- | --- | --- | --- | --- | --- | --- | --- | --- | --- | --- | --- | --- | --- | --- | --- | --- | --- | --- | --- | --- | --- | --- | --- |
| Q.RPEIYVWIGLR.D | Y | 74.99 | 1400.7928 | 11 | 0.6 | 701.4041 | 2 | 50.06 | 6 | F6:27001 | 31122021\_RID\_2349\_6C.raw |  |  |  |  |  | 4.8598E6 | 2 | 0 | 0 | 0 | 0 | 0 | 2 | 86 | 96 |  |  | PEAKS DB |
| total 1 peptides |  |  |  |  |  |  |  |  |  |  |  |  |  |  |  |  |  |  |  |  |  |  |  |  |  |  |  |  |  |
| --- | --- | --- | --- | --- | --- | --- | --- | --- | --- | --- | --- | --- | --- | --- | --- | --- | --- | --- | --- | --- | --- | --- | --- | --- | --- | --- | --- | --- | --- |

XP\_032069561.1

back to list

  

| Protein Coverage
| Supporting Peptides
|

Protein Coverage:

Supporting Peptides:

| Peptide | Uniq | -10lgP | Mass | Length | ppm | m/z | z | RT | Fraction | Scan | Source File | Area F2 | Area F1 | Area F3 | Area F4 | Area F5 | Area F6 | #Feature | #Feature F2 | #Feature F1 | #Feature F3 | #Feature F4 | #Feature F5 | #Feature F6 | Start | End | PTM | AScore | Found By |
| --- | --- | --- | --- | --- | --- | --- | --- | --- | --- | --- | --- | --- | --- | --- | --- | --- | --- | --- | --- | --- | --- | --- | --- | --- | --- | --- | --- | --- | --- |
| R.YMSGGTATGEAITYVTK.N | Y | 74.78 | 1748.8291 | 17 | 0.5 | 875.4222 | 2 | 39.50 | 5 | F5:19347 | 31122021\_RID\_2349\_5C.raw |  |  |  |  | 5.6006E4 |  | 1 | 0 | 0 | 0 | 0 | 1 | 0 | 454 | 470 |  |  | PEAKS DB |
| total 1 peptides |  |  |  |  |  |  |  |  |  |  |  |  |  |  |  |  |  |  |  |  |  |  |  |  |  |  |  |  |  |
| --- | --- | --- | --- | --- | --- | --- | --- | --- | --- | --- | --- | --- | --- | --- | --- | --- | --- | --- | --- | --- | --- | --- | --- | --- | --- | --- | --- | --- | --- |

XP\_013923615.1

back to list

  

| Protein Coverage
| Supporting Peptides
|

Protein Coverage:

Supporting Peptides:

| Peptide | Uniq | -10lgP | Mass | Length | ppm | m/z | z | RT | Fraction | Scan | Source File | Area F2 | Area F1 | Area F3 | Area F4 | Area F5 | Area F6 | #Feature | #Feature F2 | #Feature F1 | #Feature F3 | #Feature F4 | #Feature F5 | #Feature F6 | Start | End | PTM | AScore | Found By |
| --- | --- | --- | --- | --- | --- | --- | --- | --- | --- | --- | --- | --- | --- | --- | --- | --- | --- | --- | --- | --- | --- | --- | --- | --- | --- | --- | --- | --- | --- |
| R.YMSGGTATGEAITYVTK.N | Y | 74.78 | 1748.8291 | 17 | 0.5 | 875.4222 | 2 | 39.50 | 5 | F5:19347 | 31122021\_RID\_2349\_5C.raw |  |  |  |  | 5.6006E4 |  | 1 | 0 | 0 | 0 | 0 | 1 | 0 | 435 | 451 |  |  | PEAKS DB |
| total 1 peptides |  |  |  |  |  |  |  |  |  |  |  |  |  |  |  |  |  |  |  |  |  |  |  |  |  |  |  |  |  |
| --- | --- | --- | --- | --- | --- | --- | --- | --- | --- | --- | --- | --- | --- | --- | --- | --- | --- | --- | --- | --- | --- | --- | --- | --- | --- | --- | --- | --- | --- |

XP\_026520312.1

back to list

  

| Protein Coverage
| Supporting Peptides
|

Protein Coverage:

Supporting Peptides:

| Peptide | Uniq | -10lgP | Mass | Length | ppm | m/z | z | RT | Fraction | Scan | Source File | Area F2 | Area F1 | Area F3 | Area F4 | Area F5 | Area F6 | #Feature | #Feature F2 | #Feature F1 | #Feature F3 | #Feature F4 | #Feature F5 | #Feature F6 | Start | End | PTM | AScore | Found By |
| --- | --- | --- | --- | --- | --- | --- | --- | --- | --- | --- | --- | --- | --- | --- | --- | --- | --- | --- | --- | --- | --- | --- | --- | --- | --- | --- | --- | --- | --- |
| R.YMSGGTATGEAITYVTK.N | Y | 74.78 | 1748.8291 | 17 | 0.5 | 875.4222 | 2 | 39.50 | 5 | F5:19347 | 31122021\_RID\_2349\_5C.raw |  |  |  |  | 5.6006E4 |  | 1 | 0 | 0 | 0 | 0 | 1 | 0 | 435 | 451 |  |  | PEAKS DB |
| total 1 peptides |  |  |  |  |  |  |  |  |  |  |  |  |  |  |  |  |  |  |  |  |  |  |  |  |  |  |  |  |  |
| --- | --- | --- | --- | --- | --- | --- | --- | --- | --- | --- | --- | --- | --- | --- | --- | --- | --- | --- | --- | --- | --- | --- | --- | --- | --- | --- | --- | --- | --- |

XP\_007429085.1

back to list

  

| Protein Coverage
| Supporting Peptides
|

Protein Coverage:

Supporting Peptides:

| Peptide | Uniq | -10lgP | Mass | Length | ppm | m/z | z | RT | Fraction | Scan | Source File | Area F2 | Area F1 | Area F3 | Area F4 | Area F5 | Area F6 | #Feature | #Feature F2 | #Feature F1 | #Feature F3 | #Feature F4 | #Feature F5 | #Feature F6 | Start | End | PTM | AScore | Found By |
| --- | --- | --- | --- | --- | --- | --- | --- | --- | --- | --- | --- | --- | --- | --- | --- | --- | --- | --- | --- | --- | --- | --- | --- | --- | --- | --- | --- | --- | --- |
| R.YMSGGTATGEAITYVTK.N | Y | 74.78 | 1748.8291 | 17 | 0.5 | 875.4222 | 2 | 39.50 | 5 | F5:19347 | 31122021\_RID\_2349\_5C.raw |  |  |  |  | 5.6006E4 |  | 1 | 0 | 0 | 0 | 0 | 1 | 0 | 435 | 451 |  |  | PEAKS DB |
| total 1 peptides |  |  |  |  |  |  |  |  |  |  |  |  |  |  |  |  |  |  |  |  |  |  |  |  |  |  |  |  |  |
| --- | --- | --- | --- | --- | --- | --- | --- | --- | --- | --- | --- | --- | --- | --- | --- | --- | --- | --- | --- | --- | --- | --- | --- | --- | --- | --- | --- | --- | --- |

LAA86986.1

back to list

  

| Protein Coverage
| Supporting Peptides
|

Protein Coverage:

Supporting Peptides:

| Peptide | Uniq | -10lgP | Mass | Length | ppm | m/z | z | RT | Fraction | Scan | Source File | Area F2 | Area F1 | Area F3 | Area F4 | Area F5 | Area F6 | #Feature | #Feature F2 | #Feature F1 | #Feature F3 | #Feature F4 | #Feature F5 | #Feature F6 | Start | End | PTM | AScore | Found By |
| --- | --- | --- | --- | --- | --- | --- | --- | --- | --- | --- | --- | --- | --- | --- | --- | --- | --- | --- | --- | --- | --- | --- | --- | --- | --- | --- | --- | --- | --- |
| R.YMSGGTATGEAITYVTK.N | Y | 74.78 | 1748.8291 | 17 | 0.5 | 875.4222 | 2 | 39.50 | 5 | F5:19347 | 31122021\_RID\_2349\_5C.raw |  |  |  |  | 5.6006E4 |  | 1 | 0 | 0 | 0 | 0 | 1 | 0 | 146 | 162 |  |  | PEAKS DB |
| total 1 peptides |  |  |  |  |  |  |  |  |  |  |  |  |  |  |  |  |  |  |  |  |  |  |  |  |  |  |  |  |  |
| --- | --- | --- | --- | --- | --- | --- | --- | --- | --- | --- | --- | --- | --- | --- | --- | --- | --- | --- | --- | --- | --- | --- | --- | --- | --- | --- | --- | --- | --- |

LAB57337.1

back to list

  

| Protein Coverage
| Supporting Peptides
|

Protein Coverage:

Supporting Peptides:

| Peptide | Uniq | -10lgP | Mass | Length | ppm | m/z | z | RT | Fraction | Scan | Source File | Area F2 | Area F1 | Area F3 | Area F4 | Area F5 | Area F6 | #Feature | #Feature F2 | #Feature F1 | #Feature F3 | #Feature F4 | #Feature F5 | #Feature F6 | Start | End | PTM | AScore | Found By |
| --- | --- | --- | --- | --- | --- | --- | --- | --- | --- | --- | --- | --- | --- | --- | --- | --- | --- | --- | --- | --- | --- | --- | --- | --- | --- | --- | --- | --- | --- |
| R.YMSGGTATGEAITYVTK.N | Y | 74.78 | 1748.8291 | 17 | 0.5 | 875.4222 | 2 | 39.50 | 5 | F5:19347 | 31122021\_RID\_2349\_5C.raw |  |  |  |  | 5.6006E4 |  | 1 | 0 | 0 | 0 | 0 | 1 | 0 | 164 | 180 |  |  | PEAKS DB |
| total 1 peptides |  |  |  |  |  |  |  |  |  |  |  |  |  |  |  |  |  |  |  |  |  |  |  |  |  |  |  |  |  |
| --- | --- | --- | --- | --- | --- | --- | --- | --- | --- | --- | --- | --- | --- | --- | --- | --- | --- | --- | --- | --- | --- | --- | --- | --- | --- | --- | --- | --- | --- |

XP\_026566518.1

back to list

  

| Protein Coverage
| Supporting Peptides
|

Protein Coverage:

Supporting Peptides:

| Peptide | Uniq | -10lgP | Mass | Length | ppm | m/z | z | RT | Fraction | Scan | Source File | Area F2 | Area F1 | Area F3 | Area F4 | Area F5 | Area F6 | #Feature | #Feature F2 | #Feature F1 | #Feature F3 | #Feature F4 | #Feature F5 | #Feature F6 | Start | End | PTM | AScore | Found By |
| --- | --- | --- | --- | --- | --- | --- | --- | --- | --- | --- | --- | --- | --- | --- | --- | --- | --- | --- | --- | --- | --- | --- | --- | --- | --- | --- | --- | --- | --- |
| R.YMSGGTATGEAITYVTK.N | Y | 74.78 | 1748.8291 | 17 | 0.5 | 875.4222 | 2 | 39.50 | 5 | F5:19347 | 31122021\_RID\_2349\_5C.raw |  |  |  |  | 5.6006E4 |  | 1 | 0 | 0 | 0 | 0 | 1 | 0 | 435 | 451 |  |  | PEAKS DB |
| total 1 peptides |  |  |  |  |  |  |  |  |  |  |  |  |  |  |  |  |  |  |  |  |  |  |  |  |  |  |  |  |  |
| --- | --- | --- | --- | --- | --- | --- | --- | --- | --- | --- | --- | --- | --- | --- | --- | --- | --- | --- | --- | --- | --- | --- | --- | --- | --- | --- | --- | --- | --- |

BAO23490.1

back to list

  

| Protein Coverage
| Supporting Peptides
|

Protein Coverage:

Supporting Peptides:

| Peptide | Uniq | -10lgP | Mass | Length | ppm | m/z | z | RT | Fraction | Scan | Source File | Area F2 | Area F1 | Area F3 | Area F4 | Area F5 | Area F6 | #Feature | #Feature F2 | #Feature F1 | #Feature F3 | #Feature F4 | #Feature F5 | #Feature F6 | Start | End | PTM | AScore | Found By |
| --- | --- | --- | --- | --- | --- | --- | --- | --- | --- | --- | --- | --- | --- | --- | --- | --- | --- | --- | --- | --- | --- | --- | --- | --- | --- | --- | --- | --- | --- |
| R.YIELIIVADHR.M | Y | 52.17 | 1340.7452 | 11 | 0.3 | 671.3801 | 2 | 45.78 | 3 | F3:14554 | 31122021\_RID\_2349\_3C.raw | 1.3298E6 |  | 3.4695E7 | 2.0569E5 | 2.7273E6 |  | 8 | 2 | 0 | 3 | 1 | 2 | 0 | 199 | 209 |  |  | PEAKS DB |
| K.GM(+15.99)VLPGTK.C | N | 43.14 | 817.4368 | 8 | -0.6 | 409.7254 | 2 | 23.59 | 1 | F1:8201 | 28122021\_RID\_2349\_2C.raw | 2.8841E7 |  |  |  |  |  | 1 | 1 | 0 | 0 | 0 | 0 | 0 | 581 | 588 | Oxidation (M) | M2:Oxidation (M):1000.00 | PEAKS DB |
| total 2 peptides |  |  |  |  |  |  |  |  |  |  |  |  |  |  |  |  |  |  |  |  |  |  |  |  |  |  |  |  |  |
| --- | --- | --- | --- | --- | --- | --- | --- | --- | --- | --- | --- | --- | --- | --- | --- | --- | --- | --- | --- | --- | --- | --- | --- | --- | --- | --- | --- | --- | --- |

ACN93671.1

back to list

  

| Protein Coverage
| Supporting Peptides
|

Protein Coverage:

Supporting Peptides:

| Peptide | Uniq | -10lgP | Mass | Length | ppm | m/z | z | RT | Fraction | Scan | Source File | Area F2 | Area F1 | Area F3 | Area F4 | Area F5 | Area F6 | #Feature | #Feature F2 | #Feature F1 | #Feature F3 | #Feature F4 | #Feature F5 | #Feature F6 | Start | End | PTM | AScore | Found By |
| --- | --- | --- | --- | --- | --- | --- | --- | --- | --- | --- | --- | --- | --- | --- | --- | --- | --- | --- | --- | --- | --- | --- | --- | --- | --- | --- | --- | --- | --- |
| R.NMLQMEWNSNAAQNAK.R | Y | 46.68 | 1848.8247 | 16 | 0.2 | 925.4198 | 2 | 42.20 | 5 | F5:21185 | 31122021\_RID\_2349\_5C.raw |  |  |  |  | 1.2008E5 |  | 1 | 0 | 0 | 0 | 0 | 1 | 0 | 53 | 68 |  |  | PEAKS DB |
| total 1 peptides |  |  |  |  |  |  |  |  |  |  |  |  |  |  |  |  |  |  |  |  |  |  |  |  |  |  |  |  |  |
| --- | --- | --- | --- | --- | --- | --- | --- | --- | --- | --- | --- | --- | --- | --- | --- | --- | --- | --- | --- | --- | --- | --- | --- | --- | --- | --- | --- | --- | --- |

ACH73168.1

back to list

  

| Protein Coverage
| Supporting Peptides
|

Protein Coverage:

Supporting Peptides:

| Peptide | Uniq | -10lgP | Mass | Length | ppm | m/z | z | RT | Fraction | Scan | Source File | Area F2 | Area F1 | Area F3 | Area F4 | Area F5 | Area F6 | #Feature | #Feature F2 | #Feature F1 | #Feature F3 | #Feature F4 | #Feature F5 | #Feature F6 | Start | End | PTM | AScore | Found By |
| --- | --- | --- | --- | --- | --- | --- | --- | --- | --- | --- | --- | --- | --- | --- | --- | --- | --- | --- | --- | --- | --- | --- | --- | --- | --- | --- | --- | --- | --- |
| R.NMLQMEWNSNAAQNAK.R | Y | 46.68 | 1848.8247 | 16 | 0.2 | 925.4198 | 2 | 42.20 | 5 | F5:21185 | 31122021\_RID\_2349\_5C.raw |  |  |  |  | 1.2008E5 |  | 1 | 0 | 0 | 0 | 0 | 1 | 0 | 54 | 69 |  |  | PEAKS DB |
| total 1 peptides |  |  |  |  |  |  |  |  |  |  |  |  |  |  |  |  |  |  |  |  |  |  |  |  |  |  |  |  |  |
| --- | --- | --- | --- | --- | --- | --- | --- | --- | --- | --- | --- | --- | --- | --- | --- | --- | --- | --- | --- | --- | --- | --- | --- | --- | --- | --- | --- | --- | --- |

AAP20603.1

back to list

  

| Protein Coverage
| Supporting Peptides
|

Protein Coverage:

Supporting Peptides:

| Peptide | Uniq | -10lgP | Mass | Length | ppm | m/z | z | RT | Fraction | Scan | Source File | Area F2 | Area F1 | Area F3 | Area F4 | Area F5 | Area F6 | #Feature | #Feature F2 | #Feature F1 | #Feature F3 | #Feature F4 | #Feature F5 | #Feature F6 | Start | End | PTM | AScore | Found By |
| --- | --- | --- | --- | --- | --- | --- | --- | --- | --- | --- | --- | --- | --- | --- | --- | --- | --- | --- | --- | --- | --- | --- | --- | --- | --- | --- | --- | --- | --- |
| R.NMLQMEWNSNAAQNAK.R | Y | 46.68 | 1848.8247 | 16 | 0.2 | 925.4198 | 2 | 42.20 | 5 | F5:21185 | 31122021\_RID\_2349\_5C.raw |  |  |  |  | 1.2008E5 |  | 1 | 0 | 0 | 0 | 0 | 1 | 0 | 54 | 69 |  |  | PEAKS DB |
| total 1 peptides |  |  |  |  |  |  |  |  |  |  |  |  |  |  |  |  |  |  |  |  |  |  |  |  |  |  |  |  |  |
| --- | --- | --- | --- | --- | --- | --- | --- | --- | --- | --- | --- | --- | --- | --- | --- | --- | --- | --- | --- | --- | --- | --- | --- | --- | --- | --- | --- | --- | --- |

BAN82159.1

back to list

  

| Protein Coverage
| Supporting Peptides
|

Protein Coverage:

Supporting Peptides:

| Peptide | Uniq | -10lgP | Mass | Length | ppm | m/z | z | RT | Fraction | Scan | Source File | Area F2 | Area F1 | Area F3 | Area F4 | Area F5 | Area F6 | #Feature | #Feature F2 | #Feature F1 | #Feature F3 | #Feature F4 | #Feature F5 | #Feature F6 | Start | End | PTM | AScore | Found By |
| --- | --- | --- | --- | --- | --- | --- | --- | --- | --- | --- | --- | --- | --- | --- | --- | --- | --- | --- | --- | --- | --- | --- | --- | --- | --- | --- | --- | --- | --- |
| R.ETNLLYDSR.E | Y | 43.74 | 1109.5353 | 9 | -0.6 | 555.7746 | 2 | 32.25 | 1 | F1:14874 | 28122021\_RID\_2349\_2C.raw | 0 |  |  |  |  |  | 0 | 0 | 0 | 0 | 0 | 0 | 0 | 367 | 375 |  |  | PEAKS DB |
| total 1 peptides |  |  |  |  |  |  |  |  |  |  |  |  |  |  |  |  |  |  |  |  |  |  |  |  |  |  |  |  |  |
| --- | --- | --- | --- | --- | --- | --- | --- | --- | --- | --- | --- | --- | --- | --- | --- | --- | --- | --- | --- | --- | --- | --- | --- | --- | --- | --- | --- | --- | --- |

Q2YHJ2.1

back to list

  

| Protein Coverage
| Supporting Peptides
|

Protein Coverage:

Supporting Peptides:

| Peptide | Uniq | -10lgP | Mass | Length | ppm | m/z | z | RT | Fraction | Scan | Source File | Area F2 | Area F1 | Area F3 | Area F4 | Area F5 | Area F6 | #Feature | #Feature F2 | #Feature F1 | #Feature F3 | #Feature F4 | #Feature F5 | #Feature F6 | Start | End | PTM | AScore | Found By |
| --- | --- | --- | --- | --- | --- | --- | --- | --- | --- | --- | --- | --- | --- | --- | --- | --- | --- | --- | --- | --- | --- | --- | --- | --- | --- | --- | --- | --- | --- |
| K.TDIYFYYR.K | Y | 59.65 | 1139.5287 | 8 | 0.0 | 570.7716 | 2 | 44.42 | 6 | F6:22726 | 31122021\_RID\_2349\_6C.raw |  |  |  |  | 3.5993E5 | 7.9455E5 | 2 | 0 | 0 | 0 | 0 | 1 | 1 | 77 | 84 |  |  | PEAKS DB |
| total 1 peptides |  |  |  |  |  |  |  |  |  |  |  |  |  |  |  |  |  |  |  |  |  |  |  |  |  |  |  |  |  |
| --- | --- | --- | --- | --- | --- | --- | --- | --- | --- | --- | --- | --- | --- | --- | --- | --- | --- | --- | --- | --- | --- | --- | --- | --- | --- | --- | --- | --- | --- |

Q2YHJ7.1

back to list

  

| Protein Coverage
| Supporting Peptides
|

Protein Coverage:

Supporting Peptides:

| Peptide | Uniq | -10lgP | Mass | Length | ppm | m/z | z | RT | Fraction | Scan | Source File | Area F2 | Area F1 | Area F3 | Area F4 | Area F5 | Area F6 | #Feature | #Feature F2 | #Feature F1 | #Feature F3 | #Feature F4 | #Feature F5 | #Feature F6 | Start | End | PTM | AScore | Found By |
| --- | --- | --- | --- | --- | --- | --- | --- | --- | --- | --- | --- | --- | --- | --- | --- | --- | --- | --- | --- | --- | --- | --- | --- | --- | --- | --- | --- | --- | --- |
| K.TDIYFYYR.K | Y | 59.65 | 1139.5287 | 8 | 0.0 | 570.7716 | 2 | 44.42 | 6 | F6:22726 | 31122021\_RID\_2349\_6C.raw |  |  |  |  | 3.5993E5 | 7.9455E5 | 2 | 0 | 0 | 0 | 0 | 1 | 1 | 77 | 84 |  |  | PEAKS DB |
| total 1 peptides |  |  |  |  |  |  |  |  |  |  |  |  |  |  |  |  |  |  |  |  |  |  |  |  |  |  |  |  |  |
| --- | --- | --- | --- | --- | --- | --- | --- | --- | --- | --- | --- | --- | --- | --- | --- | --- | --- | --- | --- | --- | --- | --- | --- | --- | --- | --- | --- | --- | --- |

AAR14173.1

back to list

  

| Protein Coverage
| Supporting Peptides
|

Protein Coverage:

Supporting Peptides:

| Peptide | Uniq | -10lgP | Mass | Length | ppm | m/z | z | RT | Fraction | Scan | Source File | Area F2 | Area F1 | Area F3 | Area F4 | Area F5 | Area F6 | #Feature | #Feature F2 | #Feature F1 | #Feature F3 | #Feature F4 | #Feature F5 | #Feature F6 | Start | End | PTM | AScore | Found By |
| --- | --- | --- | --- | --- | --- | --- | --- | --- | --- | --- | --- | --- | --- | --- | --- | --- | --- | --- | --- | --- | --- | --- | --- | --- | --- | --- | --- | --- | --- |
| K.TDIYFYYR.K | Y | 59.65 | 1139.5287 | 8 | 0.0 | 570.7716 | 2 | 44.42 | 6 | F6:22726 | 31122021\_RID\_2349\_6C.raw |  |  |  |  | 3.5993E5 | 7.9455E5 | 2 | 0 | 0 | 0 | 0 | 1 | 1 | 77 | 84 |  |  | PEAKS DB |
| total 1 peptides |  |  |  |  |  |  |  |  |  |  |  |  |  |  |  |  |  |  |  |  |  |  |  |  |  |  |  |  |  |
| --- | --- | --- | --- | --- | --- | --- | --- | --- | --- | --- | --- | --- | --- | --- | --- | --- | --- | --- | --- | --- | --- | --- | --- | --- | --- | --- | --- | --- | --- |

AAR14167.1

back to list

  

| Protein Coverage
| Supporting Peptides
|

Protein Coverage:

Supporting Peptides:

| Peptide | Uniq | -10lgP | Mass | Length | ppm | m/z | z | RT | Fraction | Scan | Source File | Area F2 | Area F1 | Area F3 | Area F4 | Area F5 | Area F6 | #Feature | #Feature F2 | #Feature F1 | #Feature F3 | #Feature F4 | #Feature F5 | #Feature F6 | Start | End | PTM | AScore | Found By |
| --- | --- | --- | --- | --- | --- | --- | --- | --- | --- | --- | --- | --- | --- | --- | --- | --- | --- | --- | --- | --- | --- | --- | --- | --- | --- | --- | --- | --- | --- |
| K.TDIYFYYR.K | Y | 59.65 | 1139.5287 | 8 | 0.0 | 570.7716 | 2 | 44.42 | 6 | F6:22726 | 31122021\_RID\_2349\_6C.raw |  |  |  |  | 3.5993E5 | 7.9455E5 | 2 | 0 | 0 | 0 | 0 | 1 | 1 | 77 | 84 |  |  | PEAKS DB |
| total 1 peptides |  |  |  |  |  |  |  |  |  |  |  |  |  |  |  |  |  |  |  |  |  |  |  |  |  |  |  |  |  |
| --- | --- | --- | --- | --- | --- | --- | --- | --- | --- | --- | --- | --- | --- | --- | --- | --- | --- | --- | --- | --- | --- | --- | --- | --- | --- | --- | --- | --- | --- |

LAA93895.1

back to list

  

| Protein Coverage
| Supporting Peptides
|

Protein Coverage:

Supporting Peptides:

| Peptide | Uniq | -10lgP | Mass | Length | ppm | m/z | z | RT | Fraction | Scan | Source File | Area F2 | Area F1 | Area F3 | Area F4 | Area F5 | Area F6 | #Feature | #Feature F2 | #Feature F1 | #Feature F3 | #Feature F4 | #Feature F5 | #Feature F6 | Start | End | PTM | AScore | Found By |
| --- | --- | --- | --- | --- | --- | --- | --- | --- | --- | --- | --- | --- | --- | --- | --- | --- | --- | --- | --- | --- | --- | --- | --- | --- | --- | --- | --- | --- | --- |
| K.LAELEEALQK.A | Y | 58.73 | 1142.6183 | 10 | 0.3 | 572.3166 | 2 | 38.05 | 3 | F3:10817 | 31122021\_RID\_2349\_3C.raw | 1.2127E6 | 6.8999E5 | 1.4775E7 | 4.3508E6 |  | 3.242E5 | 5 | 1 | 1 | 1 | 1 | 0 | 1 | 68 | 77 |  |  | PEAKS DB |
| total 1 peptides |  |  |  |  |  |  |  |  |  |  |  |  |  |  |  |  |  |  |  |  |  |  |  |  |  |  |  |  |  |
| --- | --- | --- | --- | --- | --- | --- | --- | --- | --- | --- | --- | --- | --- | --- | --- | --- | --- | --- | --- | --- | --- | --- | --- | --- | --- | --- | --- | --- | --- |

XP\_026564825.1

back to list

  

| Protein Coverage
| Supporting Peptides
|

Protein Coverage:

Supporting Peptides:

| Peptide | Uniq | -10lgP | Mass | Length | ppm | m/z | z | RT | Fraction | Scan | Source File | Area F2 | Area F1 | Area F3 | Area F4 | Area F5 | Area F6 | #Feature | #Feature F2 | #Feature F1 | #Feature F3 | #Feature F4 | #Feature F5 | #Feature F6 | Start | End | PTM | AScore | Found By |
| --- | --- | --- | --- | --- | --- | --- | --- | --- | --- | --- | --- | --- | --- | --- | --- | --- | --- | --- | --- | --- | --- | --- | --- | --- | --- | --- | --- | --- | --- |
| T.LVVADESM(+15.99)VK.F | Y | 58.54 | 1105.5688 | 10 | -0.7 | 553.7913 | 2 | 32.72 | 5 | F5:14460 | 31122021\_RID\_2349\_5C.raw |  |  | 9.3953E5 |  | 3.0708E6 |  | 4 | 0 | 0 | 1 | 0 | 3 | 0 | 240 | 249 | Oxidation (M) | M8:Oxidation (M):1000.00 | PEAKS DB |
| T.LVVADESMVK.F | Y | 56.49 | 1089.5740 | 10 | 1.0 | 545.7948 | 2 | 32.72 | 5 | F5:14403 | 31122021\_RID\_2349\_5C.raw |  |  |  |  | 2.7327E7 |  | 1 | 0 | 0 | 0 | 0 | 1 | 0 | 240 | 249 |  |  | PEAKS DB |
| total 2 peptides |  |  |  |  |  |  |  |  |  |  |  |  |  |  |  |  |  |  |  |  |  |  |  |  |  |  |  |  |  |
| --- | --- | --- | --- | --- | --- | --- | --- | --- | --- | --- | --- | --- | --- | --- | --- | --- | --- | --- | --- | --- | --- | --- | --- | --- | --- | --- | --- | --- | --- |

XP\_026534386.1

back to list

  

| Protein Coverage
| Supporting Peptides
|

Protein Coverage:

Supporting Peptides:

| Peptide | Uniq | -10lgP | Mass | Length | ppm | m/z | z | RT | Fraction | Scan | Source File | Area F2 | Area F1 | Area F3 | Area F4 | Area F5 | Area F6 | #Feature | #Feature F2 | #Feature F1 | #Feature F3 | #Feature F4 | #Feature F5 | #Feature F6 | Start | End | PTM | AScore | Found By |
| --- | --- | --- | --- | --- | --- | --- | --- | --- | --- | --- | --- | --- | --- | --- | --- | --- | --- | --- | --- | --- | --- | --- | --- | --- | --- | --- | --- | --- | --- |
| T.LVVADESM(+15.99)VK.F | Y | 58.54 | 1105.5688 | 10 | -0.7 | 553.7913 | 2 | 32.72 | 5 | F5:14460 | 31122021\_RID\_2349\_5C.raw |  |  | 9.3953E5 |  | 3.0708E6 |  | 4 | 0 | 0 | 1 | 0 | 3 | 0 | 237 | 246 | Oxidation (M) | M8:Oxidation (M):1000.00 | PEAKS DB |
| T.LVVADESMVK.F | Y | 56.49 | 1089.5740 | 10 | 1.0 | 545.7948 | 2 | 32.72 | 5 | F5:14403 | 31122021\_RID\_2349\_5C.raw |  |  |  |  | 2.7327E7 |  | 1 | 0 | 0 | 0 | 0 | 1 | 0 | 237 | 246 |  |  | PEAKS DB |
| total 2 peptides |  |  |  |  |  |  |  |  |  |  |  |  |  |  |  |  |  |  |  |  |  |  |  |  |  |  |  |  |  |
| --- | --- | --- | --- | --- | --- | --- | --- | --- | --- | --- | --- | --- | --- | --- | --- | --- | --- | --- | --- | --- | --- | --- | --- | --- | --- | --- | --- | --- | --- |

XP\_026564824.1

back to list

  

| Protein Coverage
| Supporting Peptides
|

Protein Coverage:

Supporting Peptides:

| Peptide | Uniq | -10lgP | Mass | Length | ppm | m/z | z | RT | Fraction | Scan | Source File | Area F2 | Area F1 | Area F3 | Area F4 | Area F5 | Area F6 | #Feature | #Feature F2 | #Feature F1 | #Feature F3 | #Feature F4 | #Feature F5 | #Feature F6 | Start | End | PTM | AScore | Found By |
| --- | --- | --- | --- | --- | --- | --- | --- | --- | --- | --- | --- | --- | --- | --- | --- | --- | --- | --- | --- | --- | --- | --- | --- | --- | --- | --- | --- | --- | --- |
| T.LVVADESM(+15.99)VK.F | Y | 58.54 | 1105.5688 | 10 | -0.7 | 553.7913 | 2 | 32.72 | 5 | F5:14460 | 31122021\_RID\_2349\_5C.raw |  |  | 9.3953E5 |  | 3.0708E6 |  | 4 | 0 | 0 | 1 | 0 | 3 | 0 | 240 | 249 | Oxidation (M) | M8:Oxidation (M):1000.00 | PEAKS DB |
| T.LVVADESMVK.F | Y | 56.49 | 1089.5740 | 10 | 1.0 | 545.7948 | 2 | 32.72 | 5 | F5:14403 | 31122021\_RID\_2349\_5C.raw |  |  |  |  | 2.7327E7 |  | 1 | 0 | 0 | 0 | 0 | 1 | 0 | 240 | 249 |  |  | PEAKS DB |
| total 2 peptides |  |  |  |  |  |  |  |  |  |  |  |  |  |  |  |  |  |  |  |  |  |  |  |  |  |  |  |  |  |
| --- | --- | --- | --- | --- | --- | --- | --- | --- | --- | --- | --- | --- | --- | --- | --- | --- | --- | --- | --- | --- | --- | --- | --- | --- | --- | --- | --- | --- | --- |

XP\_007438168.1

back to list

  

| Protein Coverage
| Supporting Peptides
|

Protein Coverage:

Supporting Peptides:

| Peptide | Uniq | -10lgP | Mass | Length | ppm | m/z | z | RT | Fraction | Scan | Source File | Area F2 | Area F1 | Area F3 | Area F4 | Area F5 | Area F6 | #Feature | #Feature F2 | #Feature F1 | #Feature F3 | #Feature F4 | #Feature F5 | #Feature F6 | Start | End | PTM | AScore | Found By |
| --- | --- | --- | --- | --- | --- | --- | --- | --- | --- | --- | --- | --- | --- | --- | --- | --- | --- | --- | --- | --- | --- | --- | --- | --- | --- | --- | --- | --- | --- |
| T.LVVADESM(+15.99)VK.F | Y | 58.54 | 1105.5688 | 10 | -0.7 | 553.7913 | 2 | 32.72 | 5 | F5:14460 | 31122021\_RID\_2349\_5C.raw |  |  | 9.3953E5 |  | 3.0708E6 |  | 4 | 0 | 0 | 1 | 0 | 3 | 0 | 157 | 166 | Oxidation (M) | M8:Oxidation (M):1000.00 | PEAKS DB |
| T.LVVADESMVK.F | Y | 56.49 | 1089.5740 | 10 | 1.0 | 545.7948 | 2 | 32.72 | 5 | F5:14403 | 31122021\_RID\_2349\_5C.raw |  |  |  |  | 2.7327E7 |  | 1 | 0 | 0 | 0 | 0 | 1 | 0 | 157 | 166 |  |  | PEAKS DB |
| total 2 peptides |  |  |  |  |  |  |  |  |  |  |  |  |  |  |  |  |  |  |  |  |  |  |  |  |  |  |  |  |  |
| --- | --- | --- | --- | --- | --- | --- | --- | --- | --- | --- | --- | --- | --- | --- | --- | --- | --- | --- | --- | --- | --- | --- | --- | --- | --- | --- | --- | --- | --- |

XP\_026534385.1

back to list

  

| Protein Coverage
| Supporting Peptides
|

Protein Coverage:

Supporting Peptides:

| Peptide | Uniq | -10lgP | Mass | Length | ppm | m/z | z | RT | Fraction | Scan | Source File | Area F2 | Area F1 | Area F3 | Area F4 | Area F5 | Area F6 | #Feature | #Feature F2 | #Feature F1 | #Feature F3 | #Feature F4 | #Feature F5 | #Feature F6 | Start | End | PTM | AScore | Found By |
| --- | --- | --- | --- | --- | --- | --- | --- | --- | --- | --- | --- | --- | --- | --- | --- | --- | --- | --- | --- | --- | --- | --- | --- | --- | --- | --- | --- | --- | --- |
| T.LVVADESM(+15.99)VK.F | Y | 58.54 | 1105.5688 | 10 | -0.7 | 553.7913 | 2 | 32.72 | 5 | F5:14460 | 31122021\_RID\_2349\_5C.raw |  |  | 9.3953E5 |  | 3.0708E6 |  | 4 | 0 | 0 | 1 | 0 | 3 | 0 | 237 | 246 | Oxidation (M) | M8:Oxidation (M):1000.00 | PEAKS DB |
| T.LVVADESMVK.F | Y | 56.49 | 1089.5740 | 10 | 1.0 | 545.7948 | 2 | 32.72 | 5 | F5:14403 | 31122021\_RID\_2349\_5C.raw |  |  |  |  | 2.7327E7 |  | 1 | 0 | 0 | 0 | 0 | 1 | 0 | 237 | 246 |  |  | PEAKS DB |
| total 2 peptides |  |  |  |  |  |  |  |  |  |  |  |  |  |  |  |  |  |  |  |  |  |  |  |  |  |  |  |  |  |
| --- | --- | --- | --- | --- | --- | --- | --- | --- | --- | --- | --- | --- | --- | --- | --- | --- | --- | --- | --- | --- | --- | --- | --- | --- | --- | --- | --- | --- | --- |

XP\_034277870.1

back to list

  

| Protein Coverage
| Supporting Peptides
|

Protein Coverage:

Supporting Peptides:

| Peptide | Uniq | -10lgP | Mass | Length | ppm | m/z | z | RT | Fraction | Scan | Source File | Area F2 | Area F1 | Area F3 | Area F4 | Area F5 | Area F6 | #Feature | #Feature F2 | #Feature F1 | #Feature F3 | #Feature F4 | #Feature F5 | #Feature F6 | Start | End | PTM | AScore | Found By |
| --- | --- | --- | --- | --- | --- | --- | --- | --- | --- | --- | --- | --- | --- | --- | --- | --- | --- | --- | --- | --- | --- | --- | --- | --- | --- | --- | --- | --- | --- |
| T.LVVADESM(+15.99)VK.F | Y | 58.54 | 1105.5688 | 10 | -0.7 | 553.7913 | 2 | 32.72 | 5 | F5:14460 | 31122021\_RID\_2349\_5C.raw |  |  | 9.3953E5 |  | 3.0708E6 |  | 4 | 0 | 0 | 1 | 0 | 3 | 0 | 234 | 243 | Oxidation (M) | M8:Oxidation (M):1000.00 | PEAKS DB |
| T.LVVADESMVK.F | Y | 56.49 | 1089.5740 | 10 | 1.0 | 545.7948 | 2 | 32.72 | 5 | F5:14403 | 31122021\_RID\_2349\_5C.raw |  |  |  |  | 2.7327E7 |  | 1 | 0 | 0 | 0 | 0 | 1 | 0 | 234 | 243 |  |  | PEAKS DB |
| total 2 peptides |  |  |  |  |  |  |  |  |  |  |  |  |  |  |  |  |  |  |  |  |  |  |  |  |  |  |  |  |  |
| --- | --- | --- | --- | --- | --- | --- | --- | --- | --- | --- | --- | --- | --- | --- | --- | --- | --- | --- | --- | --- | --- | --- | --- | --- | --- | --- | --- | --- | --- |

XP\_015673523.1

back to list

  

| Protein Coverage
| Supporting Peptides
|

Protein Coverage:

Supporting Peptides:

| Peptide | Uniq | -10lgP | Mass | Length | ppm | m/z | z | RT | Fraction | Scan | Source File | Area F2 | Area F1 | Area F3 | Area F4 | Area F5 | Area F6 | #Feature | #Feature F2 | #Feature F1 | #Feature F3 | #Feature F4 | #Feature F5 | #Feature F6 | Start | End | PTM | AScore | Found By |
| --- | --- | --- | --- | --- | --- | --- | --- | --- | --- | --- | --- | --- | --- | --- | --- | --- | --- | --- | --- | --- | --- | --- | --- | --- | --- | --- | --- | --- | --- |
| T.LVVADESM(+15.99)VK.F | Y | 58.54 | 1105.5688 | 10 | -0.7 | 553.7913 | 2 | 32.72 | 5 | F5:14460 | 31122021\_RID\_2349\_5C.raw |  |  | 9.3953E5 |  | 3.0708E6 |  | 4 | 0 | 0 | 1 | 0 | 3 | 0 | 237 | 246 | Oxidation (M) | M8:Oxidation (M):1000.00 | PEAKS DB |
| T.LVVADESMVK.F | Y | 56.49 | 1089.5740 | 10 | 1.0 | 545.7948 | 2 | 32.72 | 5 | F5:14403 | 31122021\_RID\_2349\_5C.raw |  |  |  |  | 2.7327E7 |  | 1 | 0 | 0 | 0 | 0 | 1 | 0 | 237 | 246 |  |  | PEAKS DB |
| total 2 peptides |  |  |  |  |  |  |  |  |  |  |  |  |  |  |  |  |  |  |  |  |  |  |  |  |  |  |  |  |  |
| --- | --- | --- | --- | --- | --- | --- | --- | --- | --- | --- | --- | --- | --- | --- | --- | --- | --- | --- | --- | --- | --- | --- | --- | --- | --- | --- | --- | --- | --- |

XP\_032084777.1

back to list

  

| Protein Coverage
| Supporting Peptides
|

Protein Coverage:

Supporting Peptides:

| Peptide | Uniq | -10lgP | Mass | Length | ppm | m/z | z | RT | Fraction | Scan | Source File | Area F2 | Area F1 | Area F3 | Area F4 | Area F5 | Area F6 | #Feature | #Feature F2 | #Feature F1 | #Feature F3 | #Feature F4 | #Feature F5 | #Feature F6 | Start | End | PTM | AScore | Found By |
| --- | --- | --- | --- | --- | --- | --- | --- | --- | --- | --- | --- | --- | --- | --- | --- | --- | --- | --- | --- | --- | --- | --- | --- | --- | --- | --- | --- | --- | --- |
| T.LVVADESM(+15.99)VK.F | Y | 58.54 | 1105.5688 | 10 | -0.7 | 553.7913 | 2 | 32.72 | 5 | F5:14460 | 31122021\_RID\_2349\_5C.raw |  |  | 9.3953E5 |  | 3.0708E6 |  | 4 | 0 | 0 | 1 | 0 | 3 | 0 | 228 | 237 | Oxidation (M) | M8:Oxidation (M):1000.00 | PEAKS DB |
| T.LVVADESMVK.F | Y | 56.49 | 1089.5740 | 10 | 1.0 | 545.7948 | 2 | 32.72 | 5 | F5:14403 | 31122021\_RID\_2349\_5C.raw |  |  |  |  | 2.7327E7 |  | 1 | 0 | 0 | 0 | 0 | 1 | 0 | 228 | 237 |  |  | PEAKS DB |
| total 2 peptides |  |  |  |  |  |  |  |  |  |  |  |  |  |  |  |  |  |  |  |  |  |  |  |  |  |  |  |  |  |
| --- | --- | --- | --- | --- | --- | --- | --- | --- | --- | --- | --- | --- | --- | --- | --- | --- | --- | --- | --- | --- | --- | --- | --- | --- | --- | --- | --- | --- | --- |

LAA53077.1

back to list

  

| Protein Coverage
| Supporting Peptides
|

Protein Coverage:

Supporting Peptides:

| Peptide | Uniq | -10lgP | Mass | Length | ppm | m/z | z | RT | Fraction | Scan | Source File | Area F2 | Area F1 | Area F3 | Area F4 | Area F5 | Area F6 | #Feature | #Feature F2 | #Feature F1 | #Feature F3 | #Feature F4 | #Feature F5 | #Feature F6 | Start | End | PTM | AScore | Found By |
| --- | --- | --- | --- | --- | --- | --- | --- | --- | --- | --- | --- | --- | --- | --- | --- | --- | --- | --- | --- | --- | --- | --- | --- | --- | --- | --- | --- | --- | --- |
| T.LVVADESM(+15.99)VK.F | Y | 58.54 | 1105.5688 | 10 | -0.7 | 553.7913 | 2 | 32.72 | 5 | F5:14460 | 31122021\_RID\_2349\_5C.raw |  |  | 9.3953E5 |  | 3.0708E6 |  | 4 | 0 | 0 | 1 | 0 | 3 | 0 | 8 | 17 | Oxidation (M) | M8:Oxidation (M):1000.00 | PEAKS DB |
| T.LVVADESMVK.F | Y | 56.49 | 1089.5740 | 10 | 1.0 | 545.7948 | 2 | 32.72 | 5 | F5:14403 | 31122021\_RID\_2349\_5C.raw |  |  |  |  | 2.7327E7 |  | 1 | 0 | 0 | 0 | 0 | 1 | 0 | 8 | 17 |  |  | PEAKS DB |
| total 2 peptides |  |  |  |  |  |  |  |  |  |  |  |  |  |  |  |  |  |  |  |  |  |  |  |  |  |  |  |  |  |
| --- | --- | --- | --- | --- | --- | --- | --- | --- | --- | --- | --- | --- | --- | --- | --- | --- | --- | --- | --- | --- | --- | --- | --- | --- | --- | --- | --- | --- | --- |

QBF53415.1

back to list

  

| Protein Coverage
| Supporting Peptides
|

Protein Coverage:

Supporting Peptides:

| Peptide | Uniq | -10lgP | Mass | Length | ppm | m/z | z | RT | Fraction | Scan | Source File | Area F2 | Area F1 | Area F3 | Area F4 | Area F5 | Area F6 | #Feature | #Feature F2 | #Feature F1 | #Feature F3 | #Feature F4 | #Feature F5 | #Feature F6 | Start | End | PTM | AScore | Found By |
| --- | --- | --- | --- | --- | --- | --- | --- | --- | --- | --- | --- | --- | --- | --- | --- | --- | --- | --- | --- | --- | --- | --- | --- | --- | --- | --- | --- | --- | --- |
| D.DTLDSFAEWR.G | Y | 55.16 | 1238.5568 | 10 | 1.6 | 620.2866 | 2 | 52.28 | 1 | F1:30648 | 28122021\_RID\_2349\_2C.raw | 1.382E5 |  |  |  |  |  | 1 | 1 | 0 | 0 | 0 | 0 | 0 | 268 | 277 |  |  | PEAKS DB |
| total 1 peptides |  |  |  |  |  |  |  |  |  |  |  |  |  |  |  |  |  |  |  |  |  |  |  |  |  |  |  |  |  |
| --- | --- | --- | --- | --- | --- | --- | --- | --- | --- | --- | --- | --- | --- | --- | --- | --- | --- | --- | --- | --- | --- | --- | --- | --- | --- | --- | --- | --- | --- |

QHR82833.1

back to list

  

| Protein Coverage
| Supporting Peptides
|

Protein Coverage:

Supporting Peptides:

| Peptide | Uniq | -10lgP | Mass | Length | ppm | m/z | z | RT | Fraction | Scan | Source File | Area F2 | Area F1 | Area F3 | Area F4 | Area F5 | Area F6 | #Feature | #Feature F2 | #Feature F1 | #Feature F3 | #Feature F4 | #Feature F5 | #Feature F6 | Start | End | PTM | AScore | Found By |
| --- | --- | --- | --- | --- | --- | --- | --- | --- | --- | --- | --- | --- | --- | --- | --- | --- | --- | --- | --- | --- | --- | --- | --- | --- | --- | --- | --- | --- | --- |
| D.DTLDSFAEWR.G | Y | 55.16 | 1238.5568 | 10 | 1.6 | 620.2866 | 2 | 52.28 | 1 | F1:30648 | 28122021\_RID\_2349\_2C.raw | 1.382E5 |  |  |  |  |  | 1 | 1 | 0 | 0 | 0 | 0 | 0 | 100 | 109 |  |  | PEAKS DB |
| total 1 peptides |  |  |  |  |  |  |  |  |  |  |  |  |  |  |  |  |  |  |  |  |  |  |  |  |  |  |  |  |  |
| --- | --- | --- | --- | --- | --- | --- | --- | --- | --- | --- | --- | --- | --- | --- | --- | --- | --- | --- | --- | --- | --- | --- | --- | --- | --- | --- | --- | --- | --- |

BAG82600.1

back to list

  

| Protein Coverage
| Supporting Peptides
|

Protein Coverage:

Supporting Peptides:

| Peptide | Uniq | -10lgP | Mass | Length | ppm | m/z | z | RT | Fraction | Scan | Source File | Area F2 | Area F1 | Area F3 | Area F4 | Area F5 | Area F6 | #Feature | #Feature F2 | #Feature F1 | #Feature F3 | #Feature F4 | #Feature F5 | #Feature F6 | Start | End | PTM | AScore | Found By |
| --- | --- | --- | --- | --- | --- | --- | --- | --- | --- | --- | --- | --- | --- | --- | --- | --- | --- | --- | --- | --- | --- | --- | --- | --- | --- | --- | --- | --- | --- |
| R.AQIIDDAFNLAR.A | Y | 53.64 | 1345.6990 | 12 | 0.3 | 673.8569 | 2 | 50.36 | 1 | F1:29185 | 28122021\_RID\_2349\_2C.raw | 4.7764E4 |  |  |  |  |  | 1 | 1 | 0 | 0 | 0 | 0 | 0 | 621 | 632 |  |  | PEAKS DB |
| total 1 peptides |  |  |  |  |  |  |  |  |  |  |  |  |  |  |  |  |  |  |  |  |  |  |  |  |  |  |  |  |  |
| --- | --- | --- | --- | --- | --- | --- | --- | --- | --- | --- | --- | --- | --- | --- | --- | --- | --- | --- | --- | --- | --- | --- | --- | --- | --- | --- | --- | --- | --- |

XP\_015745309.1

back to list

  

| Protein Coverage
| Supporting Peptides
|

Protein Coverage:

Supporting Peptides:

| Peptide | Uniq | -10lgP | Mass | Length | ppm | m/z | z | RT | Fraction | Scan | Source File | Area F2 | Area F1 | Area F3 | Area F4 | Area F5 | Area F6 | #Feature | #Feature F2 | #Feature F1 | #Feature F3 | #Feature F4 | #Feature F5 | #Feature F6 | Start | End | PTM | AScore | Found By |
| --- | --- | --- | --- | --- | --- | --- | --- | --- | --- | --- | --- | --- | --- | --- | --- | --- | --- | --- | --- | --- | --- | --- | --- | --- | --- | --- | --- | --- | --- |
| R.AQIIDDAFNLAR.A | Y | 53.64 | 1345.6990 | 12 | 0.3 | 673.8569 | 2 | 50.36 | 1 | F1:29185 | 28122021\_RID\_2349\_2C.raw | 4.7764E4 |  |  |  |  |  | 1 | 1 | 0 | 0 | 0 | 0 | 0 | 696 | 707 |  |  | PEAKS DB |
| total 1 peptides |  |  |  |  |  |  |  |  |  |  |  |  |  |  |  |  |  |  |  |  |  |  |  |  |  |  |  |  |  |
| --- | --- | --- | --- | --- | --- | --- | --- | --- | --- | --- | --- | --- | --- | --- | --- | --- | --- | --- | --- | --- | --- | --- | --- | --- | --- | --- | --- | --- | --- |

AAK09384.1

back to list

  

| Protein Coverage
| Supporting Peptides
|

Protein Coverage:

Supporting Peptides:

| Peptide | Uniq | -10lgP | Mass | Length | ppm | m/z | z | RT | Fraction | Scan | Source File | Area F2 | Area F1 | Area F3 | Area F4 | Area F5 | Area F6 | #Feature | #Feature F2 | #Feature F1 | #Feature F3 | #Feature F4 | #Feature F5 | #Feature F6 | Start | End | PTM | AScore | Found By |
| --- | --- | --- | --- | --- | --- | --- | --- | --- | --- | --- | --- | --- | --- | --- | --- | --- | --- | --- | --- | --- | --- | --- | --- | --- | --- | --- | --- | --- | --- |
| K.IVGDLTEFR.A | Y | 53.25 | 1048.5553 | 9 | 0.4 | 525.2852 | 2 | 41.25 | 6 | F6:20172 | 31122021\_RID\_2349\_6C.raw |  |  |  |  |  | 2.3395E5 | 1 | 0 | 0 | 0 | 0 | 0 | 1 | 4 | 12 |  |  | PEAKS DB |
| total 1 peptides |  |  |  |  |  |  |  |  |  |  |  |  |  |  |  |  |  |  |  |  |  |  |  |  |  |  |  |  |  |
| --- | --- | --- | --- | --- | --- | --- | --- | --- | --- | --- | --- | --- | --- | --- | --- | --- | --- | --- | --- | --- | --- | --- | --- | --- | --- | --- | --- | --- | --- |

Q98TX1.3

back to list

  

| Protein Coverage
| Supporting Peptides
|

Protein Coverage:

Supporting Peptides:

| Peptide | Uniq | -10lgP | Mass | Length | ppm | m/z | z | RT | Fraction | Scan | Source File | Area F2 | Area F1 | Area F3 | Area F4 | Area F5 | Area F6 | #Feature | #Feature F2 | #Feature F1 | #Feature F3 | #Feature F4 | #Feature F5 | #Feature F6 | Start | End | PTM | AScore | Found By |
| --- | --- | --- | --- | --- | --- | --- | --- | --- | --- | --- | --- | --- | --- | --- | --- | --- | --- | --- | --- | --- | --- | --- | --- | --- | --- | --- | --- | --- | --- |
| K.IVGDLTEFR.A | Y | 53.25 | 1048.5553 | 9 | 0.4 | 525.2852 | 2 | 41.25 | 6 | F6:20172 | 31122021\_RID\_2349\_6C.raw |  |  |  |  |  | 2.3395E5 | 1 | 0 | 0 | 0 | 0 | 0 | 1 | 4 | 12 |  |  | PEAKS DB |
| total 1 peptides |  |  |  |  |  |  |  |  |  |  |  |  |  |  |  |  |  |  |  |  |  |  |  |  |  |  |  |  |  |
| --- | --- | --- | --- | --- | --- | --- | --- | --- | --- | --- | --- | --- | --- | --- | --- | --- | --- | --- | --- | --- | --- | --- | --- | --- | --- | --- | --- | --- | --- |

XP\_007424609.1

back to list

  

| Protein Coverage
| Supporting Peptides
|

Protein Coverage:

Supporting Peptides:

| Peptide | Uniq | -10lgP | Mass | Length | ppm | m/z | z | RT | Fraction | Scan | Source File | Area F2 | Area F1 | Area F3 | Area F4 | Area F5 | Area F6 | #Feature | #Feature F2 | #Feature F1 | #Feature F3 | #Feature F4 | #Feature F5 | #Feature F6 | Start | End | PTM | AScore | Found By |
| --- | --- | --- | --- | --- | --- | --- | --- | --- | --- | --- | --- | --- | --- | --- | --- | --- | --- | --- | --- | --- | --- | --- | --- | --- | --- | --- | --- | --- | --- |
| K.IVGDLTEFR.T | Y | 53.25 | 1048.5553 | 9 | 0.4 | 525.2852 | 2 | 41.25 | 6 | F6:20172 | 31122021\_RID\_2349\_6C.raw |  |  |  |  |  | 2.3395E5 | 1 | 0 | 0 | 0 | 0 | 0 | 1 | 63 | 71 |  |  | PEAKS DB |
| total 1 peptides |  |  |  |  |  |  |  |  |  |  |  |  |  |  |  |  |  |  |  |  |  |  |  |  |  |  |  |  |  |
| --- | --- | --- | --- | --- | --- | --- | --- | --- | --- | --- | --- | --- | --- | --- | --- | --- | --- | --- | --- | --- | --- | --- | --- | --- | --- | --- | --- | --- | --- |

XP\_034266461.1

back to list

  

| Protein Coverage
| Supporting Peptides
|

Protein Coverage:

Supporting Peptides:

| Peptide | Uniq | -10lgP | Mass | Length | ppm | m/z | z | RT | Fraction | Scan | Source File | Area F2 | Area F1 | Area F3 | Area F4 | Area F5 | Area F6 | #Feature | #Feature F2 | #Feature F1 | #Feature F3 | #Feature F4 | #Feature F5 | #Feature F6 | Start | End | PTM | AScore | Found By |
| --- | --- | --- | --- | --- | --- | --- | --- | --- | --- | --- | --- | --- | --- | --- | --- | --- | --- | --- | --- | --- | --- | --- | --- | --- | --- | --- | --- | --- | --- |
| K.IVGDLTEFR.T | Y | 53.25 | 1048.5553 | 9 | 0.4 | 525.2852 | 2 | 41.25 | 6 | F6:20172 | 31122021\_RID\_2349\_6C.raw |  |  |  |  |  | 2.3395E5 | 1 | 0 | 0 | 0 | 0 | 0 | 1 | 98 | 106 |  |  | PEAKS DB |
| total 1 peptides |  |  |  |  |  |  |  |  |  |  |  |  |  |  |  |  |  |  |  |  |  |  |  |  |  |  |  |  |  |
| --- | --- | --- | --- | --- | --- | --- | --- | --- | --- | --- | --- | --- | --- | --- | --- | --- | --- | --- | --- | --- | --- | --- | --- | --- | --- | --- | --- | --- | --- |

XP\_015674703.1

back to list

  

| Protein Coverage
| Supporting Peptides
|

Protein Coverage:

Supporting Peptides:

| Peptide | Uniq | -10lgP | Mass | Length | ppm | m/z | z | RT | Fraction | Scan | Source File | Area F2 | Area F1 | Area F3 | Area F4 | Area F5 | Area F6 | #Feature | #Feature F2 | #Feature F1 | #Feature F3 | #Feature F4 | #Feature F5 | #Feature F6 | Start | End | PTM | AScore | Found By |
| --- | --- | --- | --- | --- | --- | --- | --- | --- | --- | --- | --- | --- | --- | --- | --- | --- | --- | --- | --- | --- | --- | --- | --- | --- | --- | --- | --- | --- | --- |
| K.IVGDLTEFR.S | Y | 53.25 | 1048.5553 | 9 | 0.4 | 525.2852 | 2 | 41.25 | 6 | F6:20172 | 31122021\_RID\_2349\_6C.raw |  |  |  |  |  | 2.3395E5 | 1 | 0 | 0 | 0 | 0 | 0 | 1 | 103 | 111 |  |  | PEAKS DB |
| total 1 peptides |  |  |  |  |  |  |  |  |  |  |  |  |  |  |  |  |  |  |  |  |  |  |  |  |  |  |  |  |  |
| --- | --- | --- | --- | --- | --- | --- | --- | --- | --- | --- | --- | --- | --- | --- | --- | --- | --- | --- | --- | --- | --- | --- | --- | --- | --- | --- | --- | --- | --- |

XP\_026569170.1

back to list

  

| Protein Coverage
| Supporting Peptides
|

Protein Coverage:

Supporting Peptides:

| Peptide | Uniq | -10lgP | Mass | Length | ppm | m/z | z | RT | Fraction | Scan | Source File | Area F2 | Area F1 | Area F3 | Area F4 | Area F5 | Area F6 | #Feature | #Feature F2 | #Feature F1 | #Feature F3 | #Feature F4 | #Feature F5 | #Feature F6 | Start | End | PTM | AScore | Found By |
| --- | --- | --- | --- | --- | --- | --- | --- | --- | --- | --- | --- | --- | --- | --- | --- | --- | --- | --- | --- | --- | --- | --- | --- | --- | --- | --- | --- | --- | --- |
| K.IVGDLTEFR.A | Y | 53.25 | 1048.5553 | 9 | 0.4 | 525.2852 | 2 | 41.25 | 6 | F6:20172 | 31122021\_RID\_2349\_6C.raw |  |  |  |  |  | 2.3395E5 | 1 | 0 | 0 | 0 | 0 | 0 | 1 | 120 | 128 |  |  | PEAKS DB |
| total 1 peptides |  |  |  |  |  |  |  |  |  |  |  |  |  |  |  |  |  |  |  |  |  |  |  |  |  |  |  |  |  |
| --- | --- | --- | --- | --- | --- | --- | --- | --- | --- | --- | --- | --- | --- | --- | --- | --- | --- | --- | --- | --- | --- | --- | --- | --- | --- | --- | --- | --- | --- |

XP\_026528084.1

back to list

  

| Protein Coverage
| Supporting Peptides
|

Protein Coverage:

Supporting Peptides:

| Peptide | Uniq | -10lgP | Mass | Length | ppm | m/z | z | RT | Fraction | Scan | Source File | Area F2 | Area F1 | Area F3 | Area F4 | Area F5 | Area F6 | #Feature | #Feature F2 | #Feature F1 | #Feature F3 | #Feature F4 | #Feature F5 | #Feature F6 | Start | End | PTM | AScore | Found By |
| --- | --- | --- | --- | --- | --- | --- | --- | --- | --- | --- | --- | --- | --- | --- | --- | --- | --- | --- | --- | --- | --- | --- | --- | --- | --- | --- | --- | --- | --- |
| K.IVGDLTEFR.A | Y | 53.25 | 1048.5553 | 9 | 0.4 | 525.2852 | 2 | 41.25 | 6 | F6:20172 | 31122021\_RID\_2349\_6C.raw |  |  |  |  |  | 2.3395E5 | 1 | 0 | 0 | 0 | 0 | 0 | 1 | 122 | 130 |  |  | PEAKS DB |
| total 1 peptides |  |  |  |  |  |  |  |  |  |  |  |  |  |  |  |  |  |  |  |  |  |  |  |  |  |  |  |  |  |
| --- | --- | --- | --- | --- | --- | --- | --- | --- | --- | --- | --- | --- | --- | --- | --- | --- | --- | --- | --- | --- | --- | --- | --- | --- | --- | --- | --- | --- | --- |

3K7L

back to list

  

| Protein Coverage
| Supporting Peptides
|

Protein Coverage:

Supporting Peptides:

| Peptide | Uniq | -10lgP | Mass | Length | ppm | m/z | z | RT | Fraction | Scan | Source File | Area F2 | Area F1 | Area F3 | Area F4 | Area F5 | Area F6 | #Feature | #Feature F2 | #Feature F1 | #Feature F3 | #Feature F4 | #Feature F5 | #Feature F6 | Start | End | PTM | AScore | Found By |
| --- | --- | --- | --- | --- | --- | --- | --- | --- | --- | --- | --- | --- | --- | --- | --- | --- | --- | --- | --- | --- | --- | --- | --- | --- | --- | --- | --- | --- | --- |
| R.DPNYGMVEPGTK.C | Y | 51.48 | 1306.5863 | 12 | -0.1 | 654.3004 | 2 | 33.70 | 1 | F1:16026 | 28122021\_RID\_2349\_2C.raw | 0 |  |  |  |  |  | 0 | 0 | 0 | 0 | 0 | 0 | 0 | 392 | 403 |  |  | PEAKS DB |
| total 1 peptides |  |  |  |  |  |  |  |  |  |  |  |  |  |  |  |  |  |  |  |  |  |  |  |  |  |  |  |  |  |
| --- | --- | --- | --- | --- | --- | --- | --- | --- | --- | --- | --- | --- | --- | --- | --- | --- | --- | --- | --- | --- | --- | --- | --- | --- | --- | --- | --- | --- | --- |

ACN50006.1

back to list

  

| Protein Coverage
| Supporting Peptides
|

Protein Coverage:

Supporting Peptides:

| Peptide | Uniq | -10lgP | Mass | Length | ppm | m/z | z | RT | Fraction | Scan | Source File | Area F2 | Area F1 | Area F3 | Area F4 | Area F5 | Area F6 | #Feature | #Feature F2 | #Feature F1 | #Feature F3 | #Feature F4 | #Feature F5 | #Feature F6 | Start | End | PTM | AScore | Found By |
| --- | --- | --- | --- | --- | --- | --- | --- | --- | --- | --- | --- | --- | --- | --- | --- | --- | --- | --- | --- | --- | --- | --- | --- | --- | --- | --- | --- | --- | --- |
| R.DPNYGMVEPGTK.C | Y | 51.48 | 1306.5863 | 12 | -0.1 | 654.3004 | 2 | 33.70 | 1 | F1:16026 | 28122021\_RID\_2349\_2C.raw | 0 |  |  |  |  |  | 0 | 0 | 0 | 0 | 0 | 0 | 0 | 583 | 594 |  |  | PEAKS DB |
| total 1 peptides |  |  |  |  |  |  |  |  |  |  |  |  |  |  |  |  |  |  |  |  |  |  |  |  |  |  |  |  |  |
| --- | --- | --- | --- | --- | --- | --- | --- | --- | --- | --- | --- | --- | --- | --- | --- | --- | --- | --- | --- | --- | --- | --- | --- | --- | --- | --- | --- | --- | --- |

D3TTC2.1

back to list

  

| Protein Coverage
| Supporting Peptides
|

Protein Coverage:

Supporting Peptides:

| Peptide | Uniq | -10lgP | Mass | Length | ppm | m/z | z | RT | Fraction | Scan | Source File | Area F2 | Area F1 | Area F3 | Area F4 | Area F5 | Area F6 | #Feature | #Feature F2 | #Feature F1 | #Feature F3 | #Feature F4 | #Feature F5 | #Feature F6 | Start | End | PTM | AScore | Found By |
| --- | --- | --- | --- | --- | --- | --- | --- | --- | --- | --- | --- | --- | --- | --- | --- | --- | --- | --- | --- | --- | --- | --- | --- | --- | --- | --- | --- | --- | --- |
| R.DPNYGMVEPGTK.C | Y | 51.48 | 1306.5863 | 12 | -0.1 | 654.3004 | 2 | 33.70 | 1 | F1:16026 | 28122021\_RID\_2349\_2C.raw | 0 |  |  |  |  |  | 0 | 0 | 0 | 0 | 0 | 0 | 0 | 583 | 594 |  |  | PEAKS DB |
| total 1 peptides |  |  |  |  |  |  |  |  |  |  |  |  |  |  |  |  |  |  |  |  |  |  |  |  |  |  |  |  |  |
| --- | --- | --- | --- | --- | --- | --- | --- | --- | --- | --- | --- | --- | --- | --- | --- | --- | --- | --- | --- | --- | --- | --- | --- | --- | --- | --- | --- | --- | --- |

XP\_013927550.1

back to list

  

| Protein Coverage
| Supporting Peptides
|

Protein Coverage:

Supporting Peptides:

| Peptide | Uniq | -10lgP | Mass | Length | ppm | m/z | z | RT | Fraction | Scan | Source File | Area F2 | Area F1 | Area F3 | Area F4 | Area F5 | Area F6 | #Feature | #Feature F2 | #Feature F1 | #Feature F3 | #Feature F4 | #Feature F5 | #Feature F6 | Start | End | PTM | AScore | Found By |
| --- | --- | --- | --- | --- | --- | --- | --- | --- | --- | --- | --- | --- | --- | --- | --- | --- | --- | --- | --- | --- | --- | --- | --- | --- | --- | --- | --- | --- | --- |
| K.HPDFNEDEGSQGDIALVK.L | Y | 50.36 | 1969.9017 | 18 | 0.6 | 657.6416 | 3 | 36.17 | 1 | F1:18046 | 28122021\_RID\_2349\_2C.raw | 2.2215E5 |  |  |  |  |  | 1 | 1 | 0 | 0 | 0 | 0 | 0 | 44 | 61 |  |  | PEAKS DB |
| total 1 peptides |  |  |  |  |  |  |  |  |  |  |  |  |  |  |  |  |  |  |  |  |  |  |  |  |  |  |  |  |  |
| --- | --- | --- | --- | --- | --- | --- | --- | --- | --- | --- | --- | --- | --- | --- | --- | --- | --- | --- | --- | --- | --- | --- | --- | --- | --- | --- | --- | --- | --- |

XP\_032093652.1

back to list

  

| Protein Coverage
| Supporting Peptides
|

Protein Coverage:

Supporting Peptides:

| Peptide | Uniq | -10lgP | Mass | Length | ppm | m/z | z | RT | Fraction | Scan | Source File | Area F2 | Area F1 | Area F3 | Area F4 | Area F5 | Area F6 | #Feature | #Feature F2 | #Feature F1 | #Feature F3 | #Feature F4 | #Feature F5 | #Feature F6 | Start | End | PTM | AScore | Found By |
| --- | --- | --- | --- | --- | --- | --- | --- | --- | --- | --- | --- | --- | --- | --- | --- | --- | --- | --- | --- | --- | --- | --- | --- | --- | --- | --- | --- | --- | --- |
| K.HPDFNEDEGSQGDIALVK.L | Y | 50.36 | 1969.9017 | 18 | 0.6 | 657.6416 | 3 | 36.17 | 1 | F1:18046 | 28122021\_RID\_2349\_2C.raw | 2.2215E5 |  |  |  |  |  | 1 | 1 | 0 | 0 | 0 | 0 | 0 | 119 | 136 |  |  | PEAKS DB |
| total 1 peptides |  |  |  |  |  |  |  |  |  |  |  |  |  |  |  |  |  |  |  |  |  |  |  |  |  |  |  |  |  |
| --- | --- | --- | --- | --- | --- | --- | --- | --- | --- | --- | --- | --- | --- | --- | --- | --- | --- | --- | --- | --- | --- | --- | --- | --- | --- | --- | --- | --- | --- |

XP\_013908018.1

back to list

  

| Protein Coverage
| Supporting Peptides
|

Protein Coverage:

Supporting Peptides:

| Peptide | Uniq | -10lgP | Mass | Length | ppm | m/z | z | RT | Fraction | Scan | Source File | Area F2 | Area F1 | Area F3 | Area F4 | Area F5 | Area F6 | #Feature | #Feature F2 | #Feature F1 | #Feature F3 | #Feature F4 | #Feature F5 | #Feature F6 | Start | End | PTM | AScore | Found By |
| --- | --- | --- | --- | --- | --- | --- | --- | --- | --- | --- | --- | --- | --- | --- | --- | --- | --- | --- | --- | --- | --- | --- | --- | --- | --- | --- | --- | --- | --- |
| K.HPDFNEDEGSQGDIALVK.L | Y | 50.36 | 1969.9017 | 18 | 0.6 | 657.6416 | 3 | 36.17 | 1 | F1:18046 | 28122021\_RID\_2349\_2C.raw | 2.2215E5 |  |  |  |  |  | 1 | 1 | 0 | 0 | 0 | 0 | 0 | 98 | 115 |  |  | PEAKS DB |
| total 1 peptides |  |  |  |  |  |  |  |  |  |  |  |  |  |  |  |  |  |  |  |  |  |  |  |  |  |  |  |  |  |
| --- | --- | --- | --- | --- | --- | --- | --- | --- | --- | --- | --- | --- | --- | --- | --- | --- | --- | --- | --- | --- | --- | --- | --- | --- | --- | --- | --- | --- | --- |

BAN82108.1

back to list

  

| Protein Coverage
| Supporting Peptides
|

Protein Coverage:

Supporting Peptides:

| Peptide | Uniq | -10lgP | Mass | Length | ppm | m/z | z | RT | Fraction | Scan | Source File | Area F2 | Area F1 | Area F3 | Area F4 | Area F5 | Area F6 | #Feature | #Feature F2 | #Feature F1 | #Feature F3 | #Feature F4 | #Feature F5 | #Feature F6 | Start | End | PTM | AScore | Found By |
| --- | --- | --- | --- | --- | --- | --- | --- | --- | --- | --- | --- | --- | --- | --- | --- | --- | --- | --- | --- | --- | --- | --- | --- | --- | --- | --- | --- | --- | --- |
| K.HPDFNEDEGSQGDIALVK.L | Y | 50.36 | 1969.9017 | 18 | 0.6 | 657.6416 | 3 | 36.17 | 1 | F1:18046 | 28122021\_RID\_2349\_2C.raw | 2.2215E5 |  |  |  |  |  | 1 | 1 | 0 | 0 | 0 | 0 | 0 | 70 | 87 |  |  | PEAKS DB |
| total 1 peptides |  |  |  |  |  |  |  |  |  |  |  |  |  |  |  |  |  |  |  |  |  |  |  |  |  |  |  |  |  |
| --- | --- | --- | --- | --- | --- | --- | --- | --- | --- | --- | --- | --- | --- | --- | --- | --- | --- | --- | --- | --- | --- | --- | --- | --- | --- | --- | --- | --- | --- |

XP\_034275696.1

back to list

  

| Protein Coverage
| Supporting Peptides
|

Protein Coverage:

Supporting Peptides:

| Peptide | Uniq | -10lgP | Mass | Length | ppm | m/z | z | RT | Fraction | Scan | Source File | Area F2 | Area F1 | Area F3 | Area F4 | Area F5 | Area F6 | #Feature | #Feature F2 | #Feature F1 | #Feature F3 | #Feature F4 | #Feature F5 | #Feature F6 | Start | End | PTM | AScore | Found By |
| --- | --- | --- | --- | --- | --- | --- | --- | --- | --- | --- | --- | --- | --- | --- | --- | --- | --- | --- | --- | --- | --- | --- | --- | --- | --- | --- | --- | --- | --- |
| K.HPDFNEDEGSQGDIALVK.L | Y | 50.36 | 1969.9017 | 18 | 0.6 | 657.6416 | 3 | 36.17 | 1 | F1:18046 | 28122021\_RID\_2349\_2C.raw | 2.2215E5 |  |  |  |  |  | 1 | 1 | 0 | 0 | 0 | 0 | 0 | 118 | 135 |  |  | PEAKS DB |
| total 1 peptides |  |  |  |  |  |  |  |  |  |  |  |  |  |  |  |  |  |  |  |  |  |  |  |  |  |  |  |  |  |
| --- | --- | --- | --- | --- | --- | --- | --- | --- | --- | --- | --- | --- | --- | --- | --- | --- | --- | --- | --- | --- | --- | --- | --- | --- | --- | --- | --- | --- | --- |

AAZ53176.1

back to list

  

| Protein Coverage
| Supporting Peptides
|

Protein Coverage:

Supporting Peptides:

| Peptide | Uniq | -10lgP | Mass | Length | ppm | m/z | z | RT | Fraction | Scan | Source File | Area F2 | Area F1 | Area F3 | Area F4 | Area F5 | Area F6 | #Feature | #Feature F2 | #Feature F1 | #Feature F3 | #Feature F4 | #Feature F5 | #Feature F6 | Start | End | PTM | AScore | Found By |
| --- | --- | --- | --- | --- | --- | --- | --- | --- | --- | --- | --- | --- | --- | --- | --- | --- | --- | --- | --- | --- | --- | --- | --- | --- | --- | --- | --- | --- | --- |
| G.NLYQFGEMINQK.T | Y | 49.56 | 1483.7129 | 12 | 0.9 | 742.8644 | 2 | 46.57 | 6 | F6:24269 | 31122021\_RID\_2349\_6C.raw |  |  |  |  |  | 6.5019E4 | 1 | 0 | 0 | 0 | 0 | 0 | 1 | 17 | 28 |  |  | PEAKS DB |
| total 1 peptides |  |  |  |  |  |  |  |  |  |  |  |  |  |  |  |  |  |  |  |  |  |  |  |  |  |  |  |  |  |
| --- | --- | --- | --- | --- | --- | --- | --- | --- | --- | --- | --- | --- | --- | --- | --- | --- | --- | --- | --- | --- | --- | --- | --- | --- | --- | --- | --- | --- | --- |

A8CG78.1

back to list

  

| Protein Coverage
| Supporting Peptides
|

Protein Coverage:

Supporting Peptides:

| Peptide | Uniq | -10lgP | Mass | Length | ppm | m/z | z | RT | Fraction | Scan | Source File | Area F2 | Area F1 | Area F3 | Area F4 | Area F5 | Area F6 | #Feature | #Feature F2 | #Feature F1 | #Feature F3 | #Feature F4 | #Feature F5 | #Feature F6 | Start | End | PTM | AScore | Found By |
| --- | --- | --- | --- | --- | --- | --- | --- | --- | --- | --- | --- | --- | --- | --- | --- | --- | --- | --- | --- | --- | --- | --- | --- | --- | --- | --- | --- | --- | --- |
| G.NLYQFGEMINQK.T | Y | 49.56 | 1483.7129 | 12 | 0.9 | 742.8644 | 2 | 46.57 | 6 | F6:24269 | 31122021\_RID\_2349\_6C.raw |  |  |  |  |  | 6.5019E4 | 1 | 0 | 0 | 0 | 0 | 0 | 1 | 17 | 28 |  |  | PEAKS DB |
| total 1 peptides |  |  |  |  |  |  |  |  |  |  |  |  |  |  |  |  |  |  |  |  |  |  |  |  |  |  |  |  |  |
| --- | --- | --- | --- | --- | --- | --- | --- | --- | --- | --- | --- | --- | --- | --- | --- | --- | --- | --- | --- | --- | --- | --- | --- | --- | --- | --- | --- | --- | --- |

AAZ53181.1

back to list

  

| Protein Coverage
| Supporting Peptides
|

Protein Coverage:

Supporting Peptides:

| Peptide | Uniq | -10lgP | Mass | Length | ppm | m/z | z | RT | Fraction | Scan | Source File | Area F2 | Area F1 | Area F3 | Area F4 | Area F5 | Area F6 | #Feature | #Feature F2 | #Feature F1 | #Feature F3 | #Feature F4 | #Feature F5 | #Feature F6 | Start | End | PTM | AScore | Found By |
| --- | --- | --- | --- | --- | --- | --- | --- | --- | --- | --- | --- | --- | --- | --- | --- | --- | --- | --- | --- | --- | --- | --- | --- | --- | --- | --- | --- | --- | --- |
| G.NLYQFGEMINQK.T | Y | 49.56 | 1483.7129 | 12 | 0.9 | 742.8644 | 2 | 46.57 | 6 | F6:24269 | 31122021\_RID\_2349\_6C.raw |  |  |  |  |  | 6.5019E4 | 1 | 0 | 0 | 0 | 0 | 0 | 1 | 17 | 28 |  |  | PEAKS DB |
| total 1 peptides |  |  |  |  |  |  |  |  |  |  |  |  |  |  |  |  |  |  |  |  |  |  |  |  |  |  |  |  |  |
| --- | --- | --- | --- | --- | --- | --- | --- | --- | --- | --- | --- | --- | --- | --- | --- | --- | --- | --- | --- | --- | --- | --- | --- | --- | --- | --- | --- | --- | --- |

AAP13805.1

back to list

  

| Protein Coverage
| Supporting Peptides
|

Protein Coverage:

Supporting Peptides:

| Peptide | Uniq | -10lgP | Mass | Length | ppm | m/z | z | RT | Fraction | Scan | Source File | Area F2 | Area F1 | Area F3 | Area F4 | Area F5 | Area F6 | #Feature | #Feature F2 | #Feature F1 | #Feature F3 | #Feature F4 | #Feature F5 | #Feature F6 | Start | End | PTM | AScore | Found By |
| --- | --- | --- | --- | --- | --- | --- | --- | --- | --- | --- | --- | --- | --- | --- | --- | --- | --- | --- | --- | --- | --- | --- | --- | --- | --- | --- | --- | --- | --- |
| G.NLYQFGEMINQK.T | Y | 49.56 | 1483.7129 | 12 | 0.9 | 742.8644 | 2 | 46.57 | 6 | F6:24269 | 31122021\_RID\_2349\_6C.raw |  |  |  |  |  | 6.5019E4 | 1 | 0 | 0 | 0 | 0 | 0 | 1 | 17 | 28 |  |  | PEAKS DB |
| total 1 peptides |  |  |  |  |  |  |  |  |  |  |  |  |  |  |  |  |  |  |  |  |  |  |  |  |  |  |  |  |  |
| --- | --- | --- | --- | --- | --- | --- | --- | --- | --- | --- | --- | --- | --- | --- | --- | --- | --- | --- | --- | --- | --- | --- | --- | --- | --- | --- | --- | --- | --- |

A8CG87.1

back to list

  

| Protein Coverage
| Supporting Peptides
|

Protein Coverage:

Supporting Peptides:

| Peptide | Uniq | -10lgP | Mass | Length | ppm | m/z | z | RT | Fraction | Scan | Source File | Area F2 | Area F1 | Area F3 | Area F4 | Area F5 | Area F6 | #Feature | #Feature F2 | #Feature F1 | #Feature F3 | #Feature F4 | #Feature F5 | #Feature F6 | Start | End | PTM | AScore | Found By |
| --- | --- | --- | --- | --- | --- | --- | --- | --- | --- | --- | --- | --- | --- | --- | --- | --- | --- | --- | --- | --- | --- | --- | --- | --- | --- | --- | --- | --- | --- |
| G.NLYQFGEMINQK.T | Y | 49.56 | 1483.7129 | 12 | 0.9 | 742.8644 | 2 | 46.57 | 6 | F6:24269 | 31122021\_RID\_2349\_6C.raw |  |  |  |  |  | 6.5019E4 | 1 | 0 | 0 | 0 | 0 | 0 | 1 | 17 | 28 |  |  | PEAKS DB |
| total 1 peptides |  |  |  |  |  |  |  |  |  |  |  |  |  |  |  |  |  |  |  |  |  |  |  |  |  |  |  |  |  |
| --- | --- | --- | --- | --- | --- | --- | --- | --- | --- | --- | --- | --- | --- | --- | --- | --- | --- | --- | --- | --- | --- | --- | --- | --- | --- | --- | --- | --- | --- |

P86541.2

back to list

  

| Protein Coverage
| Supporting Peptides
|

Protein Coverage:

Supporting Peptides:

| Peptide | Uniq | -10lgP | Mass | Length | ppm | m/z | z | RT | Fraction | Scan | Source File | Area F2 | Area F1 | Area F3 | Area F4 | Area F5 | Area F6 | #Feature | #Feature F2 | #Feature F1 | #Feature F3 | #Feature F4 | #Feature F5 | #Feature F6 | Start | End | PTM | AScore | Found By |
| --- | --- | --- | --- | --- | --- | --- | --- | --- | --- | --- | --- | --- | --- | --- | --- | --- | --- | --- | --- | --- | --- | --- | --- | --- | --- | --- | --- | --- | --- |
| K.MYMVATPK.V | Y | 49.23 | 939.4558 | 8 | 0.5 | 470.7354 | 2 | 31.41 | 5 | F5:13504 | 31122021\_RID\_2349\_5C.raw | 6.7482E4 |  |  |  | 2.1332E5 |  | 2 | 1 | 0 | 0 | 0 | 1 | 0 | 24 | 31 |  |  | PEAKS DB |
| K.MYM(+15.99)VATPK.V | Y | 47.21 | 955.4507 | 8 | 0.1 | 478.7327 | 2 | 26.37 | 5 | F5:10025 | 31122021\_RID\_2349\_5C.raw |  |  |  |  | 1.356E5 |  | 1 | 0 | 0 | 0 | 0 | 1 | 0 | 24 | 31 | Oxidation (M) | M3:Oxidation (M):28.86 | PEAKS DB |
| total 2 peptides |  |  |  |  |  |  |  |  |  |  |  |  |  |  |  |  |  |  |  |  |  |  |  |  |  |  |  |  |  |
| --- | --- | --- | --- | --- | --- | --- | --- | --- | --- | --- | --- | --- | --- | --- | --- | --- | --- | --- | --- | --- | --- | --- | --- | --- | --- | --- | --- | --- | --- |

P01440.1

back to list

  

| Protein Coverage
| Supporting Peptides
|

Protein Coverage:

Supporting Peptides:

| Peptide | Uniq | -10lgP | Mass | Length | ppm | m/z | z | RT | Fraction | Scan | Source File | Area F2 | Area F1 | Area F3 | Area F4 | Area F5 | Area F6 | #Feature | #Feature F2 | #Feature F1 | #Feature F3 | #Feature F4 | #Feature F5 | #Feature F6 | Start | End | PTM | AScore | Found By |
| --- | --- | --- | --- | --- | --- | --- | --- | --- | --- | --- | --- | --- | --- | --- | --- | --- | --- | --- | --- | --- | --- | --- | --- | --- | --- | --- | --- | --- | --- |
| K.MYMVATPK.V | Y | 49.23 | 939.4558 | 8 | 0.5 | 470.7354 | 2 | 31.41 | 5 | F5:13504 | 31122021\_RID\_2349\_5C.raw | 6.7482E4 |  |  |  | 2.1332E5 |  | 2 | 1 | 0 | 0 | 0 | 1 | 0 | 24 | 31 |  |  | PEAKS DB |
| K.MYM(+15.99)VATPK.V | Y | 47.21 | 955.4507 | 8 | 0.1 | 478.7327 | 2 | 26.37 | 5 | F5:10025 | 31122021\_RID\_2349\_5C.raw |  |  |  |  | 1.356E5 |  | 1 | 0 | 0 | 0 | 0 | 1 | 0 | 24 | 31 | Oxidation (M) | M3:Oxidation (M):28.86 | PEAKS DB |
| total 2 peptides |  |  |  |  |  |  |  |  |  |  |  |  |  |  |  |  |  |  |  |  |  |  |  |  |  |  |  |  |  |
| --- | --- | --- | --- | --- | --- | --- | --- | --- | --- | --- | --- | --- | --- | --- | --- | --- | --- | --- | --- | --- | --- | --- | --- | --- | --- | --- | --- | --- | --- |

AAC61315.1

back to list

  

| Protein Coverage
| Supporting Peptides
|

Protein Coverage:

Supporting Peptides:

| Peptide | Uniq | -10lgP | Mass | Length | ppm | m/z | z | RT | Fraction | Scan | Source File | Area F2 | Area F1 | Area F3 | Area F4 | Area F5 | Area F6 | #Feature | #Feature F2 | #Feature F1 | #Feature F3 | #Feature F4 | #Feature F5 | #Feature F6 | Start | End | PTM | AScore | Found By |
| --- | --- | --- | --- | --- | --- | --- | --- | --- | --- | --- | --- | --- | --- | --- | --- | --- | --- | --- | --- | --- | --- | --- | --- | --- | --- | --- | --- | --- | --- |
| K.MYMVATPK.V | Y | 49.23 | 939.4558 | 8 | 0.5 | 470.7354 | 2 | 31.41 | 5 | F5:13504 | 31122021\_RID\_2349\_5C.raw | 6.7482E4 |  |  |  | 2.1332E5 |  | 2 | 1 | 0 | 0 | 0 | 1 | 0 | 26 | 33 |  |  | PEAKS DB |
| K.MYM(+15.99)VATPK.V | Y | 47.21 | 955.4507 | 8 | 0.1 | 478.7327 | 2 | 26.37 | 5 | F5:10025 | 31122021\_RID\_2349\_5C.raw |  |  |  |  | 1.356E5 |  | 1 | 0 | 0 | 0 | 0 | 1 | 0 | 26 | 33 | Oxidation (M) | M3:Oxidation (M):28.86 | PEAKS DB |
| total 2 peptides |  |  |  |  |  |  |  |  |  |  |  |  |  |  |  |  |  |  |  |  |  |  |  |  |  |  |  |  |  |
| --- | --- | --- | --- | --- | --- | --- | --- | --- | --- | --- | --- | --- | --- | --- | --- | --- | --- | --- | --- | --- | --- | --- | --- | --- | --- | --- | --- | --- | --- |

BAU24665.1

back to list

  

| Protein Coverage
| Supporting Peptides
|

Protein Coverage:

Supporting Peptides:

| Peptide | Uniq | -10lgP | Mass | Length | ppm | m/z | z | RT | Fraction | Scan | Source File | Area F2 | Area F1 | Area F3 | Area F4 | Area F5 | Area F6 | #Feature | #Feature F2 | #Feature F1 | #Feature F3 | #Feature F4 | #Feature F5 | #Feature F6 | Start | End | PTM | AScore | Found By |
| --- | --- | --- | --- | --- | --- | --- | --- | --- | --- | --- | --- | --- | --- | --- | --- | --- | --- | --- | --- | --- | --- | --- | --- | --- | --- | --- | --- | --- | --- |
| K.MYMVATPK.V | Y | 49.23 | 939.4558 | 8 | 0.5 | 470.7354 | 2 | 31.41 | 5 | F5:13504 | 31122021\_RID\_2349\_5C.raw | 6.7482E4 |  |  |  | 2.1332E5 |  | 2 | 1 | 0 | 0 | 0 | 1 | 0 | 36 | 43 |  |  | PEAKS DB |
| K.MYM(+15.99)VATPK.V | Y | 47.21 | 955.4507 | 8 | 0.1 | 478.7327 | 2 | 26.37 | 5 | F5:10025 | 31122021\_RID\_2349\_5C.raw |  |  |  |  | 1.356E5 |  | 1 | 0 | 0 | 0 | 0 | 1 | 0 | 36 | 43 | Oxidation (M) | M3:Oxidation (M):28.86 | PEAKS DB |
| total 2 peptides |  |  |  |  |  |  |  |  |  |  |  |  |  |  |  |  |  |  |  |  |  |  |  |  |  |  |  |  |  |
| --- | --- | --- | --- | --- | --- | --- | --- | --- | --- | --- | --- | --- | --- | --- | --- | --- | --- | --- | --- | --- | --- | --- | --- | --- | --- | --- | --- | --- | --- |

A0A0U5AUY6.1

back to list

  

| Protein Coverage
| Supporting Peptides
|

Protein Coverage:

Supporting Peptides:

| Peptide | Uniq | -10lgP | Mass | Length | ppm | m/z | z | RT | Fraction | Scan | Source File | Area F2 | Area F1 | Area F3 | Area F4 | Area F5 | Area F6 | #Feature | #Feature F2 | #Feature F1 | #Feature F3 | #Feature F4 | #Feature F5 | #Feature F6 | Start | End | PTM | AScore | Found By |
| --- | --- | --- | --- | --- | --- | --- | --- | --- | --- | --- | --- | --- | --- | --- | --- | --- | --- | --- | --- | --- | --- | --- | --- | --- | --- | --- | --- | --- | --- |
| K.MYMVATPK.V | Y | 49.23 | 939.4558 | 8 | 0.5 | 470.7354 | 2 | 31.41 | 5 | F5:13504 | 31122021\_RID\_2349\_5C.raw | 6.7482E4 |  |  |  | 2.1332E5 |  | 2 | 1 | 0 | 0 | 0 | 1 | 0 | 36 | 43 |  |  | PEAKS DB |
| K.MYM(+15.99)VATPK.V | Y | 47.21 | 955.4507 | 8 | 0.1 | 478.7327 | 2 | 26.37 | 5 | F5:10025 | 31122021\_RID\_2349\_5C.raw |  |  |  |  | 1.356E5 |  | 1 | 0 | 0 | 0 | 0 | 1 | 0 | 36 | 43 | Oxidation (M) | M3:Oxidation (M):28.86 | PEAKS DB |
| total 2 peptides |  |  |  |  |  |  |  |  |  |  |  |  |  |  |  |  |  |  |  |  |  |  |  |  |  |  |  |  |  |
| --- | --- | --- | --- | --- | --- | --- | --- | --- | --- | --- | --- | --- | --- | --- | --- | --- | --- | --- | --- | --- | --- | --- | --- | --- | --- | --- | --- | --- | --- |

O93472.1

back to list

  

| Protein Coverage
| Supporting Peptides
|

Protein Coverage:

Supporting Peptides:

| Peptide | Uniq | -10lgP | Mass | Length | ppm | m/z | z | RT | Fraction | Scan | Source File | Area F2 | Area F1 | Area F3 | Area F4 | Area F5 | Area F6 | #Feature | #Feature F2 | #Feature F1 | #Feature F3 | #Feature F4 | #Feature F5 | #Feature F6 | Start | End | PTM | AScore | Found By |
| --- | --- | --- | --- | --- | --- | --- | --- | --- | --- | --- | --- | --- | --- | --- | --- | --- | --- | --- | --- | --- | --- | --- | --- | --- | --- | --- | --- | --- | --- |
| K.MYMVATPK.V | Y | 49.23 | 939.4558 | 8 | 0.5 | 470.7354 | 2 | 31.41 | 5 | F5:13504 | 31122021\_RID\_2349\_5C.raw | 6.7482E4 |  |  |  | 2.1332E5 |  | 2 | 1 | 0 | 0 | 0 | 1 | 0 | 45 | 52 |  |  | PEAKS DB |
| K.MYM(+15.99)VATPK.V | Y | 47.21 | 955.4507 | 8 | 0.1 | 478.7327 | 2 | 26.37 | 5 | F5:10025 | 31122021\_RID\_2349\_5C.raw |  |  |  |  | 1.356E5 |  | 1 | 0 | 0 | 0 | 0 | 1 | 0 | 45 | 52 | Oxidation (M) | M3:Oxidation (M):28.86 | PEAKS DB |
| total 2 peptides |  |  |  |  |  |  |  |  |  |  |  |  |  |  |  |  |  |  |  |  |  |  |  |  |  |  |  |  |  |
| --- | --- | --- | --- | --- | --- | --- | --- | --- | --- | --- | --- | --- | --- | --- | --- | --- | --- | --- | --- | --- | --- | --- | --- | --- | --- | --- | --- | --- | --- |

Q9PST3.1

back to list

  

| Protein Coverage
| Supporting Peptides
|

Protein Coverage:

Supporting Peptides:

| Peptide | Uniq | -10lgP | Mass | Length | ppm | m/z | z | RT | Fraction | Scan | Source File | Area F2 | Area F1 | Area F3 | Area F4 | Area F5 | Area F6 | #Feature | #Feature F2 | #Feature F1 | #Feature F3 | #Feature F4 | #Feature F5 | #Feature F6 | Start | End | PTM | AScore | Found By |
| --- | --- | --- | --- | --- | --- | --- | --- | --- | --- | --- | --- | --- | --- | --- | --- | --- | --- | --- | --- | --- | --- | --- | --- | --- | --- | --- | --- | --- | --- |
| K.MYMVATPK.V | Y | 49.23 | 939.4558 | 8 | 0.5 | 470.7354 | 2 | 31.41 | 5 | F5:13504 | 31122021\_RID\_2349\_5C.raw | 6.7482E4 |  |  |  | 2.1332E5 |  | 2 | 1 | 0 | 0 | 0 | 1 | 0 | 45 | 52 |  |  | PEAKS DB |
| K.MYM(+15.99)VATPK.V | Y | 47.21 | 955.4507 | 8 | 0.1 | 478.7327 | 2 | 26.37 | 5 | F5:10025 | 31122021\_RID\_2349\_5C.raw |  |  |  |  | 1.356E5 |  | 1 | 0 | 0 | 0 | 0 | 1 | 0 | 45 | 52 | Oxidation (M) | M3:Oxidation (M):28.86 | PEAKS DB |
| total 2 peptides |  |  |  |  |  |  |  |  |  |  |  |  |  |  |  |  |  |  |  |  |  |  |  |  |  |  |  |  |  |
| --- | --- | --- | --- | --- | --- | --- | --- | --- | --- | --- | --- | --- | --- | --- | --- | --- | --- | --- | --- | --- | --- | --- | --- | --- | --- | --- | --- | --- | --- |

AAK49439.1

back to list

  

| Protein Coverage
| Supporting Peptides
|

Protein Coverage:

Supporting Peptides:

| Peptide | Uniq | -10lgP | Mass | Length | ppm | m/z | z | RT | Fraction | Scan | Source File | Area F2 | Area F1 | Area F3 | Area F4 | Area F5 | Area F6 | #Feature | #Feature F2 | #Feature F1 | #Feature F3 | #Feature F4 | #Feature F5 | #Feature F6 | Start | End | PTM | AScore | Found By |
| --- | --- | --- | --- | --- | --- | --- | --- | --- | --- | --- | --- | --- | --- | --- | --- | --- | --- | --- | --- | --- | --- | --- | --- | --- | --- | --- | --- | --- | --- |
| K.MYMVATPK.V | Y | 49.23 | 939.4558 | 8 | 0.5 | 470.7354 | 2 | 31.41 | 5 | F5:13504 | 31122021\_RID\_2349\_5C.raw | 6.7482E4 |  |  |  | 2.1332E5 |  | 2 | 1 | 0 | 0 | 0 | 1 | 0 | 45 | 52 |  |  | PEAKS DB |
| K.MYM(+15.99)VATPK.V | Y | 47.21 | 955.4507 | 8 | 0.1 | 478.7327 | 2 | 26.37 | 5 | F5:10025 | 31122021\_RID\_2349\_5C.raw |  |  |  |  | 1.356E5 |  | 1 | 0 | 0 | 0 | 0 | 1 | 0 | 45 | 52 | Oxidation (M) | M3:Oxidation (M):28.86 | PEAKS DB |
| total 2 peptides |  |  |  |  |  |  |  |  |  |  |  |  |  |  |  |  |  |  |  |  |  |  |  |  |  |  |  |  |  |
| --- | --- | --- | --- | --- | --- | --- | --- | --- | --- | --- | --- | --- | --- | --- | --- | --- | --- | --- | --- | --- | --- | --- | --- | --- | --- | --- | --- | --- | --- |

AAC27686.1

back to list

  

| Protein Coverage
| Supporting Peptides
|

Protein Coverage:

Supporting Peptides:

| Peptide | Uniq | -10lgP | Mass | Length | ppm | m/z | z | RT | Fraction | Scan | Source File | Area F2 | Area F1 | Area F3 | Area F4 | Area F5 | Area F6 | #Feature | #Feature F2 | #Feature F1 | #Feature F3 | #Feature F4 | #Feature F5 | #Feature F6 | Start | End | PTM | AScore | Found By |
| --- | --- | --- | --- | --- | --- | --- | --- | --- | --- | --- | --- | --- | --- | --- | --- | --- | --- | --- | --- | --- | --- | --- | --- | --- | --- | --- | --- | --- | --- |
| K.MYMVATPK.V | Y | 49.23 | 939.4558 | 8 | 0.5 | 470.7354 | 2 | 31.41 | 5 | F5:13504 | 31122021\_RID\_2349\_5C.raw | 6.7482E4 |  |  |  | 2.1332E5 |  | 2 | 1 | 0 | 0 | 0 | 1 | 0 | 45 | 52 |  |  | PEAKS DB |
| K.MYM(+15.99)VATPK.V | Y | 47.21 | 955.4507 | 8 | 0.1 | 478.7327 | 2 | 26.37 | 5 | F5:10025 | 31122021\_RID\_2349\_5C.raw |  |  |  |  | 1.356E5 |  | 1 | 0 | 0 | 0 | 0 | 1 | 0 | 45 | 52 | Oxidation (M) | M3:Oxidation (M):28.86 | PEAKS DB |
| total 2 peptides |  |  |  |  |  |  |  |  |  |  |  |  |  |  |  |  |  |  |  |  |  |  |  |  |  |  |  |  |  |
| --- | --- | --- | --- | --- | --- | --- | --- | --- | --- | --- | --- | --- | --- | --- | --- | --- | --- | --- | --- | --- | --- | --- | --- | --- | --- | --- | --- | --- | --- |

Q9PST4.1

back to list

  

| Protein Coverage
| Supporting Peptides
|

Protein Coverage:

Supporting Peptides:

| Peptide | Uniq | -10lgP | Mass | Length | ppm | m/z | z | RT | Fraction | Scan | Source File | Area F2 | Area F1 | Area F3 | Area F4 | Area F5 | Area F6 | #Feature | #Feature F2 | #Feature F1 | #Feature F3 | #Feature F4 | #Feature F5 | #Feature F6 | Start | End | PTM | AScore | Found By |
| --- | --- | --- | --- | --- | --- | --- | --- | --- | --- | --- | --- | --- | --- | --- | --- | --- | --- | --- | --- | --- | --- | --- | --- | --- | --- | --- | --- | --- | --- |
| K.MYMVATPK.V | Y | 49.23 | 939.4558 | 8 | 0.5 | 470.7354 | 2 | 31.41 | 5 | F5:13504 | 31122021\_RID\_2349\_5C.raw | 6.7482E4 |  |  |  | 2.1332E5 |  | 2 | 1 | 0 | 0 | 0 | 1 | 0 | 45 | 52 |  |  | PEAKS DB |
| K.MYM(+15.99)VATPK.V | Y | 47.21 | 955.4507 | 8 | 0.1 | 478.7327 | 2 | 26.37 | 5 | F5:10025 | 31122021\_RID\_2349\_5C.raw |  |  |  |  | 1.356E5 |  | 1 | 0 | 0 | 0 | 0 | 1 | 0 | 45 | 52 | Oxidation (M) | M3:Oxidation (M):28.86 | PEAKS DB |
| total 2 peptides |  |  |  |  |  |  |  |  |  |  |  |  |  |  |  |  |  |  |  |  |  |  |  |  |  |  |  |  |  |
| --- | --- | --- | --- | --- | --- | --- | --- | --- | --- | --- | --- | --- | --- | --- | --- | --- | --- | --- | --- | --- | --- | --- | --- | --- | --- | --- | --- | --- | --- |

AAC27687.1

back to list

  

| Protein Coverage
| Supporting Peptides
|

Protein Coverage:

Supporting Peptides:

| Peptide | Uniq | -10lgP | Mass | Length | ppm | m/z | z | RT | Fraction | Scan | Source File | Area F2 | Area F1 | Area F3 | Area F4 | Area F5 | Area F6 | #Feature | #Feature F2 | #Feature F1 | #Feature F3 | #Feature F4 | #Feature F5 | #Feature F6 | Start | End | PTM | AScore | Found By |
| --- | --- | --- | --- | --- | --- | --- | --- | --- | --- | --- | --- | --- | --- | --- | --- | --- | --- | --- | --- | --- | --- | --- | --- | --- | --- | --- | --- | --- | --- |
| K.MYMVATPK.V | Y | 49.23 | 939.4558 | 8 | 0.5 | 470.7354 | 2 | 31.41 | 5 | F5:13504 | 31122021\_RID\_2349\_5C.raw | 6.7482E4 |  |  |  | 2.1332E5 |  | 2 | 1 | 0 | 0 | 0 | 1 | 0 | 45 | 52 |  |  | PEAKS DB |
| K.MYM(+15.99)VATPK.V | Y | 47.21 | 955.4507 | 8 | 0.1 | 478.7327 | 2 | 26.37 | 5 | F5:10025 | 31122021\_RID\_2349\_5C.raw |  |  |  |  | 1.356E5 |  | 1 | 0 | 0 | 0 | 0 | 1 | 0 | 45 | 52 | Oxidation (M) | M3:Oxidation (M):28.86 | PEAKS DB |
| total 2 peptides |  |  |  |  |  |  |  |  |  |  |  |  |  |  |  |  |  |  |  |  |  |  |  |  |  |  |  |  |  |
| --- | --- | --- | --- | --- | --- | --- | --- | --- | --- | --- | --- | --- | --- | --- | --- | --- | --- | --- | --- | --- | --- | --- | --- | --- | --- | --- | --- | --- | --- |

AAC27685.1

back to list

  

| Protein Coverage
| Supporting Peptides
|

Protein Coverage:

Supporting Peptides:

| Peptide | Uniq | -10lgP | Mass | Length | ppm | m/z | z | RT | Fraction | Scan | Source File | Area F2 | Area F1 | Area F3 | Area F4 | Area F5 | Area F6 | #Feature | #Feature F2 | #Feature F1 | #Feature F3 | #Feature F4 | #Feature F5 | #Feature F6 | Start | End | PTM | AScore | Found By |
| --- | --- | --- | --- | --- | --- | --- | --- | --- | --- | --- | --- | --- | --- | --- | --- | --- | --- | --- | --- | --- | --- | --- | --- | --- | --- | --- | --- | --- | --- |
| K.MYMVATPK.V | Y | 49.23 | 939.4558 | 8 | 0.5 | 470.7354 | 2 | 31.41 | 5 | F5:13504 | 31122021\_RID\_2349\_5C.raw | 6.7482E4 |  |  |  | 2.1332E5 |  | 2 | 1 | 0 | 0 | 0 | 1 | 0 | 45 | 52 |  |  | PEAKS DB |
| K.MYM(+15.99)VATPK.V | Y | 47.21 | 955.4507 | 8 | 0.1 | 478.7327 | 2 | 26.37 | 5 | F5:10025 | 31122021\_RID\_2349\_5C.raw |  |  |  |  | 1.356E5 |  | 1 | 0 | 0 | 0 | 0 | 1 | 0 | 45 | 52 | Oxidation (M) | M3:Oxidation (M):28.86 | PEAKS DB |
| total 2 peptides |  |  |  |  |  |  |  |  |  |  |  |  |  |  |  |  |  |  |  |  |  |  |  |  |  |  |  |  |  |
| --- | --- | --- | --- | --- | --- | --- | --- | --- | --- | --- | --- | --- | --- | --- | --- | --- | --- | --- | --- | --- | --- | --- | --- | --- | --- | --- | --- | --- | --- |

XP\_032080818.1

back to list

  

| Protein Coverage
| Supporting Peptides
|

Protein Coverage:

Supporting Peptides:

| Peptide | Uniq | -10lgP | Mass | Length | ppm | m/z | z | RT | Fraction | Scan | Source File | Area F2 | Area F1 | Area F3 | Area F4 | Area F5 | Area F6 | #Feature | #Feature F2 | #Feature F1 | #Feature F3 | #Feature F4 | #Feature F5 | #Feature F6 | Start | End | PTM | AScore | Found By |
| --- | --- | --- | --- | --- | --- | --- | --- | --- | --- | --- | --- | --- | --- | --- | --- | --- | --- | --- | --- | --- | --- | --- | --- | --- | --- | --- | --- | --- | --- |
| H.ALTEAIEFDK.A | Y | 48.83 | 1135.5760 | 10 | 0.7 | 568.7957 | 2 | 39.90 | 1 | F1:21054 | 28122021\_RID\_2349\_2C.raw | 6.3404E4 |  |  |  |  |  | 1 | 1 | 0 | 0 | 0 | 0 | 0 | 352 | 361 |  |  | PEAKS DB |
| total 1 peptides |  |  |  |  |  |  |  |  |  |  |  |  |  |  |  |  |  |  |  |  |  |  |  |  |  |  |  |  |  |
| --- | --- | --- | --- | --- | --- | --- | --- | --- | --- | --- | --- | --- | --- | --- | --- | --- | --- | --- | --- | --- | --- | --- | --- | --- | --- | --- | --- | --- | --- |

XP\_015674366.1

back to list

  

| Protein Coverage
| Supporting Peptides
|

Protein Coverage:

Supporting Peptides:

| Peptide | Uniq | -10lgP | Mass | Length | ppm | m/z | z | RT | Fraction | Scan | Source File | Area F2 | Area F1 | Area F3 | Area F4 | Area F5 | Area F6 | #Feature | #Feature F2 | #Feature F1 | #Feature F3 | #Feature F4 | #Feature F5 | #Feature F6 | Start | End | PTM | AScore | Found By |
| --- | --- | --- | --- | --- | --- | --- | --- | --- | --- | --- | --- | --- | --- | --- | --- | --- | --- | --- | --- | --- | --- | --- | --- | --- | --- | --- | --- | --- | --- |
| R.ALTEAIEFDK.A | Y | 48.83 | 1135.5760 | 10 | 0.7 | 568.7957 | 2 | 39.90 | 1 | F1:21054 | 28122021\_RID\_2349\_2C.raw | 6.3404E4 |  |  |  |  |  | 1 | 1 | 0 | 0 | 0 | 0 | 0 | 290 | 299 |  |  | PEAKS DB |
| total 1 peptides |  |  |  |  |  |  |  |  |  |  |  |  |  |  |  |  |  |  |  |  |  |  |  |  |  |  |  |  |  |
| --- | --- | --- | --- | --- | --- | --- | --- | --- | --- | --- | --- | --- | --- | --- | --- | --- | --- | --- | --- | --- | --- | --- | --- | --- | --- | --- | --- | --- | --- |

XP\_026545273.1

back to list

  

| Protein Coverage
| Supporting Peptides
|

Protein Coverage:

Supporting Peptides:

| Peptide | Uniq | -10lgP | Mass | Length | ppm | m/z | z | RT | Fraction | Scan | Source File | Area F2 | Area F1 | Area F3 | Area F4 | Area F5 | Area F6 | #Feature | #Feature F2 | #Feature F1 | #Feature F3 | #Feature F4 | #Feature F5 | #Feature F6 | Start | End | PTM | AScore | Found By |
| --- | --- | --- | --- | --- | --- | --- | --- | --- | --- | --- | --- | --- | --- | --- | --- | --- | --- | --- | --- | --- | --- | --- | --- | --- | --- | --- | --- | --- | --- |
| R.ALTEAIEFDK.A | Y | 48.83 | 1135.5760 | 10 | 0.7 | 568.7957 | 2 | 39.90 | 1 | F1:21054 | 28122021\_RID\_2349\_2C.raw | 6.3404E4 |  |  |  |  |  | 1 | 1 | 0 | 0 | 0 | 0 | 0 | 343 | 352 |  |  | PEAKS DB |
| total 1 peptides |  |  |  |  |  |  |  |  |  |  |  |  |  |  |  |  |  |  |  |  |  |  |  |  |  |  |  |  |  |
| --- | --- | --- | --- | --- | --- | --- | --- | --- | --- | --- | --- | --- | --- | --- | --- | --- | --- | --- | --- | --- | --- | --- | --- | --- | --- | --- | --- | --- | --- |

XP\_026576786.1

back to list

  

| Protein Coverage
| Supporting Peptides
|

Protein Coverage:

Supporting Peptides:

| Peptide | Uniq | -10lgP | Mass | Length | ppm | m/z | z | RT | Fraction | Scan | Source File | Area F2 | Area F1 | Area F3 | Area F4 | Area F5 | Area F6 | #Feature | #Feature F2 | #Feature F1 | #Feature F3 | #Feature F4 | #Feature F5 | #Feature F6 | Start | End | PTM | AScore | Found By |
| --- | --- | --- | --- | --- | --- | --- | --- | --- | --- | --- | --- | --- | --- | --- | --- | --- | --- | --- | --- | --- | --- | --- | --- | --- | --- | --- | --- | --- | --- |
| R.ALTEAIEFDK.A | Y | 48.83 | 1135.5760 | 10 | 0.7 | 568.7957 | 2 | 39.90 | 1 | F1:21054 | 28122021\_RID\_2349\_2C.raw | 6.3404E4 |  |  |  |  |  | 1 | 1 | 0 | 0 | 0 | 0 | 0 | 343 | 352 |  |  | PEAKS DB |
| total 1 peptides |  |  |  |  |  |  |  |  |  |  |  |  |  |  |  |  |  |  |  |  |  |  |  |  |  |  |  |  |  |
| --- | --- | --- | --- | --- | --- | --- | --- | --- | --- | --- | --- | --- | --- | --- | --- | --- | --- | --- | --- | --- | --- | --- | --- | --- | --- | --- | --- | --- | --- |

XP\_025022776.1

back to list

  

| Protein Coverage
| Supporting Peptides
|

Protein Coverage:

Supporting Peptides:

| Peptide | Uniq | -10lgP | Mass | Length | ppm | m/z | z | RT | Fraction | Scan | Source File | Area F2 | Area F1 | Area F3 | Area F4 | Area F5 | Area F6 | #Feature | #Feature F2 | #Feature F1 | #Feature F3 | #Feature F4 | #Feature F5 | #Feature F6 | Start | End | PTM | AScore | Found By |
| --- | --- | --- | --- | --- | --- | --- | --- | --- | --- | --- | --- | --- | --- | --- | --- | --- | --- | --- | --- | --- | --- | --- | --- | --- | --- | --- | --- | --- | --- |
| R.ALTEAIEFDK.A | Y | 48.83 | 1135.5760 | 10 | 0.7 | 568.7957 | 2 | 39.90 | 1 | F1:21054 | 28122021\_RID\_2349\_2C.raw | 6.3404E4 |  |  |  |  |  | 1 | 1 | 0 | 0 | 0 | 0 | 0 | 299 | 308 |  |  | PEAKS DB |
| total 1 peptides |  |  |  |  |  |  |  |  |  |  |  |  |  |  |  |  |  |  |  |  |  |  |  |  |  |  |  |  |  |
| --- | --- | --- | --- | --- | --- | --- | --- | --- | --- | --- | --- | --- | --- | --- | --- | --- | --- | --- | --- | --- | --- | --- | --- | --- | --- | --- | --- | --- | --- |

XP\_015743663.1

back to list

  

| Protein Coverage
| Supporting Peptides
|

Protein Coverage:

Supporting Peptides:

| Peptide | Uniq | -10lgP | Mass | Length | ppm | m/z | z | RT | Fraction | Scan | Source File | Area F2 | Area F1 | Area F3 | Area F4 | Area F5 | Area F6 | #Feature | #Feature F2 | #Feature F1 | #Feature F3 | #Feature F4 | #Feature F5 | #Feature F6 | Start | End | PTM | AScore | Found By |
| --- | --- | --- | --- | --- | --- | --- | --- | --- | --- | --- | --- | --- | --- | --- | --- | --- | --- | --- | --- | --- | --- | --- | --- | --- | --- | --- | --- | --- | --- |
| R.ALTEAIEFDK.A | Y | 48.83 | 1135.5760 | 10 | 0.7 | 568.7957 | 2 | 39.90 | 1 | F1:21054 | 28122021\_RID\_2349\_2C.raw | 6.3404E4 |  |  |  |  |  | 1 | 1 | 0 | 0 | 0 | 0 | 0 | 345 | 354 |  |  | PEAKS DB |
| total 1 peptides |  |  |  |  |  |  |  |  |  |  |  |  |  |  |  |  |  |  |  |  |  |  |  |  |  |  |  |  |  |
| --- | --- | --- | --- | --- | --- | --- | --- | --- | --- | --- | --- | --- | --- | --- | --- | --- | --- | --- | --- | --- | --- | --- | --- | --- | --- | --- | --- | --- | --- |

XP\_025022775.1

back to list

  

| Protein Coverage
| Supporting Peptides
|

Protein Coverage:

Supporting Peptides:

| Peptide | Uniq | -10lgP | Mass | Length | ppm | m/z | z | RT | Fraction | Scan | Source File | Area F2 | Area F1 | Area F3 | Area F4 | Area F5 | Area F6 | #Feature | #Feature F2 | #Feature F1 | #Feature F3 | #Feature F4 | #Feature F5 | #Feature F6 | Start | End | PTM | AScore | Found By |
| --- | --- | --- | --- | --- | --- | --- | --- | --- | --- | --- | --- | --- | --- | --- | --- | --- | --- | --- | --- | --- | --- | --- | --- | --- | --- | --- | --- | --- | --- |
| R.ALTEAIEFDK.A | Y | 48.83 | 1135.5760 | 10 | 0.7 | 568.7957 | 2 | 39.90 | 1 | F1:21054 | 28122021\_RID\_2349\_2C.raw | 6.3404E4 |  |  |  |  |  | 1 | 1 | 0 | 0 | 0 | 0 | 0 | 385 | 394 |  |  | PEAKS DB |
| total 1 peptides |  |  |  |  |  |  |  |  |  |  |  |  |  |  |  |  |  |  |  |  |  |  |  |  |  |  |  |  |  |
| --- | --- | --- | --- | --- | --- | --- | --- | --- | --- | --- | --- | --- | --- | --- | --- | --- | --- | --- | --- | --- | --- | --- | --- | --- | --- | --- | --- | --- | --- |

XP\_034261216.1

back to list

  

| Protein Coverage
| Supporting Peptides
|

Protein Coverage:

Supporting Peptides:

| Peptide | Uniq | -10lgP | Mass | Length | ppm | m/z | z | RT | Fraction | Scan | Source File | Area F2 | Area F1 | Area F3 | Area F4 | Area F5 | Area F6 | #Feature | #Feature F2 | #Feature F1 | #Feature F3 | #Feature F4 | #Feature F5 | #Feature F6 | Start | End | PTM | AScore | Found By |
| --- | --- | --- | --- | --- | --- | --- | --- | --- | --- | --- | --- | --- | --- | --- | --- | --- | --- | --- | --- | --- | --- | --- | --- | --- | --- | --- | --- | --- | --- |
| R.ALTEAIEFDK.A | Y | 48.83 | 1135.5760 | 10 | 0.7 | 568.7957 | 2 | 39.90 | 1 | F1:21054 | 28122021\_RID\_2349\_2C.raw | 6.3404E4 |  |  |  |  |  | 1 | 1 | 0 | 0 | 0 | 0 | 0 | 60 | 69 |  |  | PEAKS DB |
| total 1 peptides |  |  |  |  |  |  |  |  |  |  |  |  |  |  |  |  |  |  |  |  |  |  |  |  |  |  |  |  |  |
| --- | --- | --- | --- | --- | --- | --- | --- | --- | --- | --- | --- | --- | --- | --- | --- | --- | --- | --- | --- | --- | --- | --- | --- | --- | --- | --- | --- | --- | --- |

LAA85037.1

back to list

  

| Protein Coverage
| Supporting Peptides
|

Protein Coverage:

Supporting Peptides:

| Peptide | Uniq | -10lgP | Mass | Length | ppm | m/z | z | RT | Fraction | Scan | Source File | Area F2 | Area F1 | Area F3 | Area F4 | Area F5 | Area F6 | #Feature | #Feature F2 | #Feature F1 | #Feature F3 | #Feature F4 | #Feature F5 | #Feature F6 | Start | End | PTM | AScore | Found By |
| --- | --- | --- | --- | --- | --- | --- | --- | --- | --- | --- | --- | --- | --- | --- | --- | --- | --- | --- | --- | --- | --- | --- | --- | --- | --- | --- | --- | --- | --- |
| R.ALTEAIEFDK.A | Y | 48.83 | 1135.5760 | 10 | 0.7 | 568.7957 | 2 | 39.90 | 1 | F1:21054 | 28122021\_RID\_2349\_2C.raw | 6.3404E4 |  |  |  |  |  | 1 | 1 | 0 | 0 | 0 | 0 | 0 | 30 | 39 |  |  | PEAKS DB |
| total 1 peptides |  |  |  |  |  |  |  |  |  |  |  |  |  |  |  |  |  |  |  |  |  |  |  |  |  |  |  |  |  |
| --- | --- | --- | --- | --- | --- | --- | --- | --- | --- | --- | --- | --- | --- | --- | --- | --- | --- | --- | --- | --- | --- | --- | --- | --- | --- | --- | --- | --- | --- |

AAR14169.1

back to list

  

| Protein Coverage
| Supporting Peptides
|

Protein Coverage:

Supporting Peptides:

| Peptide | Uniq | -10lgP | Mass | Length | ppm | m/z | z | RT | Fraction | Scan | Source File | Area F2 | Area F1 | Area F3 | Area F4 | Area F5 | Area F6 | #Feature | #Feature F2 | #Feature F1 | #Feature F3 | #Feature F4 | #Feature F5 | #Feature F6 | Start | End | PTM | AScore | Found By |
| --- | --- | --- | --- | --- | --- | --- | --- | --- | --- | --- | --- | --- | --- | --- | --- | --- | --- | --- | --- | --- | --- | --- | --- | --- | --- | --- | --- | --- | --- |
| K.YWFYPTK.Y | Y | 48.20 | 1003.4803 | 7 | -0.1 | 502.7474 | 2 | 44.91 | 5 | F5:23339 | 31122021\_RID\_2349\_5C.raw |  |  |  |  | 1.6735E6 |  | 1 | 0 | 0 | 0 | 0 | 1 | 0 | 107 | 113 |  |  | PEAKS DB |
| total 1 peptides |  |  |  |  |  |  |  |  |  |  |  |  |  |  |  |  |  |  |  |  |  |  |  |  |  |  |  |  |  |
| --- | --- | --- | --- | --- | --- | --- | --- | --- | --- | --- | --- | --- | --- | --- | --- | --- | --- | --- | --- | --- | --- | --- | --- | --- | --- | --- | --- | --- | --- |

Q2YHJ5.1

back to list

  

| Protein Coverage
| Supporting Peptides
|

Protein Coverage:

Supporting Peptides:

| Peptide | Uniq | -10lgP | Mass | Length | ppm | m/z | z | RT | Fraction | Scan | Source File | Area F2 | Area F1 | Area F3 | Area F4 | Area F5 | Area F6 | #Feature | #Feature F2 | #Feature F1 | #Feature F3 | #Feature F4 | #Feature F5 | #Feature F6 | Start | End | PTM | AScore | Found By |
| --- | --- | --- | --- | --- | --- | --- | --- | --- | --- | --- | --- | --- | --- | --- | --- | --- | --- | --- | --- | --- | --- | --- | --- | --- | --- | --- | --- | --- | --- |
| K.YWFYPTK.Y | Y | 48.20 | 1003.4803 | 7 | -0.1 | 502.7474 | 2 | 44.91 | 5 | F5:23339 | 31122021\_RID\_2349\_5C.raw |  |  |  |  | 1.6735E6 |  | 1 | 0 | 0 | 0 | 0 | 1 | 0 | 107 | 113 |  |  | PEAKS DB |
| total 1 peptides |  |  |  |  |  |  |  |  |  |  |  |  |  |  |  |  |  |  |  |  |  |  |  |  |  |  |  |  |  |
| --- | --- | --- | --- | --- | --- | --- | --- | --- | --- | --- | --- | --- | --- | --- | --- | --- | --- | --- | --- | --- | --- | --- | --- | --- | --- | --- | --- | --- | --- |

AFJ50263.1

back to list

  

| Protein Coverage
| Supporting Peptides
|

Protein Coverage:

Supporting Peptides:

| Peptide | Uniq | -10lgP | Mass | Length | ppm | m/z | z | RT | Fraction | Scan | Source File | Area F2 | Area F1 | Area F3 | Area F4 | Area F5 | Area F6 | #Feature | #Feature F2 | #Feature F1 | #Feature F3 | #Feature F4 | #Feature F5 | #Feature F6 | Start | End | PTM | AScore | Found By |
| --- | --- | --- | --- | --- | --- | --- | --- | --- | --- | --- | --- | --- | --- | --- | --- | --- | --- | --- | --- | --- | --- | --- | --- | --- | --- | --- | --- | --- | --- |
| K.ILLLGAGESGK.S | Y | 48.15 | 1056.6179 | 11 | 0.2 | 529.3163 | 2 | 40.45 | 1 | F1:21467 | 28122021\_RID\_2349\_2C.raw | 5.6366E4 |  |  |  |  |  | 1 | 1 | 0 | 0 | 0 | 0 | 0 | 51 | 61 |  |  | PEAKS DB |
| total 1 peptides |  |  |  |  |  |  |  |  |  |  |  |  |  |  |  |  |  |  |  |  |  |  |  |  |  |  |  |  |  |
| --- | --- | --- | --- | --- | --- | --- | --- | --- | --- | --- | --- | --- | --- | --- | --- | --- | --- | --- | --- | --- | --- | --- | --- | --- | --- | --- | --- | --- | --- |

JAA96948.1

back to list

  

| Protein Coverage
| Supporting Peptides
|

Protein Coverage:

Supporting Peptides:

| Peptide | Uniq | -10lgP | Mass | Length | ppm | m/z | z | RT | Fraction | Scan | Source File | Area F2 | Area F1 | Area F3 | Area F4 | Area F5 | Area F6 | #Feature | #Feature F2 | #Feature F1 | #Feature F3 | #Feature F4 | #Feature F5 | #Feature F6 | Start | End | PTM | AScore | Found By |
| --- | --- | --- | --- | --- | --- | --- | --- | --- | --- | --- | --- | --- | --- | --- | --- | --- | --- | --- | --- | --- | --- | --- | --- | --- | --- | --- | --- | --- | --- |
| K.ILLLGAGESGK.S | Y | 48.15 | 1056.6179 | 11 | 0.2 | 529.3163 | 2 | 40.45 | 1 | F1:21467 | 28122021\_RID\_2349\_2C.raw | 5.6366E4 |  |  |  |  |  | 1 | 1 | 0 | 0 | 0 | 0 | 0 | 51 | 61 |  |  | PEAKS DB |
| total 1 peptides |  |  |  |  |  |  |  |  |  |  |  |  |  |  |  |  |  |  |  |  |  |  |  |  |  |  |  |  |  |
| --- | --- | --- | --- | --- | --- | --- | --- | --- | --- | --- | --- | --- | --- | --- | --- | --- | --- | --- | --- | --- | --- | --- | --- | --- | --- | --- | --- | --- | --- |

JAG44584.1

back to list

  

| Protein Coverage
| Supporting Peptides
|

Protein Coverage:

Supporting Peptides:

| Peptide | Uniq | -10lgP | Mass | Length | ppm | m/z | z | RT | Fraction | Scan | Source File | Area F2 | Area F1 | Area F3 | Area F4 | Area F5 | Area F6 | #Feature | #Feature F2 | #Feature F1 | #Feature F3 | #Feature F4 | #Feature F5 | #Feature F6 | Start | End | PTM | AScore | Found By |
| --- | --- | --- | --- | --- | --- | --- | --- | --- | --- | --- | --- | --- | --- | --- | --- | --- | --- | --- | --- | --- | --- | --- | --- | --- | --- | --- | --- | --- | --- |
| K.ILLLGAGESGK.S | Y | 48.15 | 1056.6179 | 11 | 0.2 | 529.3163 | 2 | 40.45 | 1 | F1:21467 | 28122021\_RID\_2349\_2C.raw | 5.6366E4 |  |  |  |  |  | 1 | 1 | 0 | 0 | 0 | 0 | 0 | 51 | 61 |  |  | PEAKS DB |
| total 1 peptides |  |  |  |  |  |  |  |  |  |  |  |  |  |  |  |  |  |  |  |  |  |  |  |  |  |  |  |  |  |
| --- | --- | --- | --- | --- | --- | --- | --- | --- | --- | --- | --- | --- | --- | --- | --- | --- | --- | --- | --- | --- | --- | --- | --- | --- | --- | --- | --- | --- | --- |

ETE71869.1

back to list

  

| Protein Coverage
| Supporting Peptides
|

Protein Coverage:

Supporting Peptides:

| Peptide | Uniq | -10lgP | Mass | Length | ppm | m/z | z | RT | Fraction | Scan | Source File | Area F2 | Area F1 | Area F3 | Area F4 | Area F5 | Area F6 | #Feature | #Feature F2 | #Feature F1 | #Feature F3 | #Feature F4 | #Feature F5 | #Feature F6 | Start | End | PTM | AScore | Found By |
| --- | --- | --- | --- | --- | --- | --- | --- | --- | --- | --- | --- | --- | --- | --- | --- | --- | --- | --- | --- | --- | --- | --- | --- | --- | --- | --- | --- | --- | --- |
| K.ILLLGAGESGK.S | Y | 48.15 | 1056.6179 | 11 | 0.2 | 529.3163 | 2 | 40.45 | 1 | F1:21467 | 28122021\_RID\_2349\_2C.raw | 5.6366E4 |  |  |  |  |  | 1 | 1 | 0 | 0 | 0 | 0 | 0 | 51 | 61 |  |  | PEAKS DB |
| total 1 peptides |  |  |  |  |  |  |  |  |  |  |  |  |  |  |  |  |  |  |  |  |  |  |  |  |  |  |  |  |  |
| --- | --- | --- | --- | --- | --- | --- | --- | --- | --- | --- | --- | --- | --- | --- | --- | --- | --- | --- | --- | --- | --- | --- | --- | --- | --- | --- | --- | --- | --- |

JAC95458.1

back to list

  

| Protein Coverage
| Supporting Peptides
|

Protein Coverage:

Supporting Peptides:

| Peptide | Uniq | -10lgP | Mass | Length | ppm | m/z | z | RT | Fraction | Scan | Source File | Area F2 | Area F1 | Area F3 | Area F4 | Area F5 | Area F6 | #Feature | #Feature F2 | #Feature F1 | #Feature F3 | #Feature F4 | #Feature F5 | #Feature F6 | Start | End | PTM | AScore | Found By |
| --- | --- | --- | --- | --- | --- | --- | --- | --- | --- | --- | --- | --- | --- | --- | --- | --- | --- | --- | --- | --- | --- | --- | --- | --- | --- | --- | --- | --- | --- |
| R.LLLLGAGESGK.S | Y | 48.15 | 1056.6179 | 11 | 0.2 | 529.3163 | 2 | 40.45 | 1 | F1:21467 | 28122021\_RID\_2349\_2C.raw | 5.6366E4 |  |  |  |  |  | 1 | 1 | 0 | 0 | 0 | 0 | 0 | 43 | 53 |  |  | PEAKS DB |
| total 1 peptides |  |  |  |  |  |  |  |  |  |  |  |  |  |  |  |  |  |  |  |  |  |  |  |  |  |  |  |  |  |
| --- | --- | --- | --- | --- | --- | --- | --- | --- | --- | --- | --- | --- | --- | --- | --- | --- | --- | --- | --- | --- | --- | --- | --- | --- | --- | --- | --- | --- | --- |

JAG67788.1

back to list

  

| Protein Coverage
| Supporting Peptides
|

Protein Coverage:

Supporting Peptides:

| Peptide | Uniq | -10lgP | Mass | Length | ppm | m/z | z | RT | Fraction | Scan | Source File | Area F2 | Area F1 | Area F3 | Area F4 | Area F5 | Area F6 | #Feature | #Feature F2 | #Feature F1 | #Feature F3 | #Feature F4 | #Feature F5 | #Feature F6 | Start | End | PTM | AScore | Found By |
| --- | --- | --- | --- | --- | --- | --- | --- | --- | --- | --- | --- | --- | --- | --- | --- | --- | --- | --- | --- | --- | --- | --- | --- | --- | --- | --- | --- | --- | --- |
| R.LLLLGAGESGK.S | Y | 48.15 | 1056.6179 | 11 | 0.2 | 529.3163 | 2 | 40.45 | 1 | F1:21467 | 28122021\_RID\_2349\_2C.raw | 5.6366E4 |  |  |  |  |  | 1 | 1 | 0 | 0 | 0 | 0 | 0 | 43 | 53 |  |  | PEAKS DB |
| total 1 peptides |  |  |  |  |  |  |  |  |  |  |  |  |  |  |  |  |  |  |  |  |  |  |  |  |  |  |  |  |  |
| --- | --- | --- | --- | --- | --- | --- | --- | --- | --- | --- | --- | --- | --- | --- | --- | --- | --- | --- | --- | --- | --- | --- | --- | --- | --- | --- | --- | --- | --- |

ETE73579.1

back to list

  

| Protein Coverage
| Supporting Peptides
|

Protein Coverage:

Supporting Peptides:

| Peptide | Uniq | -10lgP | Mass | Length | ppm | m/z | z | RT | Fraction | Scan | Source File | Area F2 | Area F1 | Area F3 | Area F4 | Area F5 | Area F6 | #Feature | #Feature F2 | #Feature F1 | #Feature F3 | #Feature F4 | #Feature F5 | #Feature F6 | Start | End | PTM | AScore | Found By |
| --- | --- | --- | --- | --- | --- | --- | --- | --- | --- | --- | --- | --- | --- | --- | --- | --- | --- | --- | --- | --- | --- | --- | --- | --- | --- | --- | --- | --- | --- |
| K.LLLLGAGESGK.S | Y | 48.15 | 1056.6179 | 11 | 0.2 | 529.3163 | 2 | 40.45 | 1 | F1:21467 | 28122021\_RID\_2349\_2C.raw | 5.6366E4 |  |  |  |  |  | 1 | 1 | 0 | 0 | 0 | 0 | 0 | 81 | 91 |  |  | PEAKS DB |
| total 1 peptides |  |  |  |  |  |  |  |  |  |  |  |  |  |  |  |  |  |  |  |  |  |  |  |  |  |  |  |  |  |
| --- | --- | --- | --- | --- | --- | --- | --- | --- | --- | --- | --- | --- | --- | --- | --- | --- | --- | --- | --- | --- | --- | --- | --- | --- | --- | --- | --- | --- | --- |

XP\_034285323.1

back to list

  

| Protein Coverage
| Supporting Peptides
|

Protein Coverage:

Supporting Peptides:

| Peptide | Uniq | -10lgP | Mass | Length | ppm | m/z | z | RT | Fraction | Scan | Source File | Area F2 | Area F1 | Area F3 | Area F4 | Area F5 | Area F6 | #Feature | #Feature F2 | #Feature F1 | #Feature F3 | #Feature F4 | #Feature F5 | #Feature F6 | Start | End | PTM | AScore | Found By |
| --- | --- | --- | --- | --- | --- | --- | --- | --- | --- | --- | --- | --- | --- | --- | --- | --- | --- | --- | --- | --- | --- | --- | --- | --- | --- | --- | --- | --- | --- |
| R.LLLLGAGESGK.S | Y | 48.15 | 1056.6179 | 11 | 0.2 | 529.3163 | 2 | 40.45 | 1 | F1:21467 | 28122021\_RID\_2349\_2C.raw | 5.6366E4 |  |  |  |  |  | 1 | 1 | 0 | 0 | 0 | 0 | 0 | 182 | 192 |  |  | PEAKS DB |
| total 1 peptides |  |  |  |  |  |  |  |  |  |  |  |  |  |  |  |  |  |  |  |  |  |  |  |  |  |  |  |  |  |
| --- | --- | --- | --- | --- | --- | --- | --- | --- | --- | --- | --- | --- | --- | --- | --- | --- | --- | --- | --- | --- | --- | --- | --- | --- | --- | --- | --- | --- | --- |

ETE68023.1

back to list

  

| Protein Coverage
| Supporting Peptides
|

Protein Coverage:

Supporting Peptides:

| Peptide | Uniq | -10lgP | Mass | Length | ppm | m/z | z | RT | Fraction | Scan | Source File | Area F2 | Area F1 | Area F3 | Area F4 | Area F5 | Area F6 | #Feature | #Feature F2 | #Feature F1 | #Feature F3 | #Feature F4 | #Feature F5 | #Feature F6 | Start | End | PTM | AScore | Found By |
| --- | --- | --- | --- | --- | --- | --- | --- | --- | --- | --- | --- | --- | --- | --- | --- | --- | --- | --- | --- | --- | --- | --- | --- | --- | --- | --- | --- | --- | --- |
| K.LLLLGAGESGK.S | Y | 48.15 | 1056.6179 | 11 | 0.2 | 529.3163 | 2 | 40.45 | 1 | F1:21467 | 28122021\_RID\_2349\_2C.raw | 5.6366E4 |  |  |  |  |  | 1 | 1 | 0 | 0 | 0 | 0 | 0 | 32 | 42 |  |  | PEAKS DB |
| total 1 peptides |  |  |  |  |  |  |  |  |  |  |  |  |  |  |  |  |  |  |  |  |  |  |  |  |  |  |  |  |  |
| --- | --- | --- | --- | --- | --- | --- | --- | --- | --- | --- | --- | --- | --- | --- | --- | --- | --- | --- | --- | --- | --- | --- | --- | --- | --- | --- | --- | --- | --- |

JAG46680.1

back to list

  

| Protein Coverage
| Supporting Peptides
|

Protein Coverage:

Supporting Peptides:

| Peptide | Uniq | -10lgP | Mass | Length | ppm | m/z | z | RT | Fraction | Scan | Source File | Area F2 | Area F1 | Area F3 | Area F4 | Area F5 | Area F6 | #Feature | #Feature F2 | #Feature F1 | #Feature F3 | #Feature F4 | #Feature F5 | #Feature F6 | Start | End | PTM | AScore | Found By |
| --- | --- | --- | --- | --- | --- | --- | --- | --- | --- | --- | --- | --- | --- | --- | --- | --- | --- | --- | --- | --- | --- | --- | --- | --- | --- | --- | --- | --- | --- |
| R.LLLLGAGESGK.S | Y | 48.15 | 1056.6179 | 11 | 0.2 | 529.3163 | 2 | 40.45 | 1 | F1:21467 | 28122021\_RID\_2349\_2C.raw | 5.6366E4 |  |  |  |  |  | 1 | 1 | 0 | 0 | 0 | 0 | 0 | 43 | 53 |  |  | PEAKS DB |
| total 1 peptides |  |  |  |  |  |  |  |  |  |  |  |  |  |  |  |  |  |  |  |  |  |  |  |  |  |  |  |  |  |
| --- | --- | --- | --- | --- | --- | --- | --- | --- | --- | --- | --- | --- | --- | --- | --- | --- | --- | --- | --- | --- | --- | --- | --- | --- | --- | --- | --- | --- | --- |

JAI13087.1

back to list

  

| Protein Coverage
| Supporting Peptides
|

Protein Coverage:

Supporting Peptides:

| Peptide | Uniq | -10lgP | Mass | Length | ppm | m/z | z | RT | Fraction | Scan | Source File | Area F2 | Area F1 | Area F3 | Area F4 | Area F5 | Area F6 | #Feature | #Feature F2 | #Feature F1 | #Feature F3 | #Feature F4 | #Feature F5 | #Feature F6 | Start | End | PTM | AScore | Found By |
| --- | --- | --- | --- | --- | --- | --- | --- | --- | --- | --- | --- | --- | --- | --- | --- | --- | --- | --- | --- | --- | --- | --- | --- | --- | --- | --- | --- | --- | --- |
| R.LLLLGAGESGK.S | Y | 48.15 | 1056.6179 | 11 | 0.2 | 529.3163 | 2 | 40.45 | 1 | F1:21467 | 28122021\_RID\_2349\_2C.raw | 5.6366E4 |  |  |  |  |  | 1 | 1 | 0 | 0 | 0 | 0 | 0 | 43 | 53 |  |  | PEAKS DB |
| total 1 peptides |  |  |  |  |  |  |  |  |  |  |  |  |  |  |  |  |  |  |  |  |  |  |  |  |  |  |  |  |  |
| --- | --- | --- | --- | --- | --- | --- | --- | --- | --- | --- | --- | --- | --- | --- | --- | --- | --- | --- | --- | --- | --- | --- | --- | --- | --- | --- | --- | --- | --- |

JAA96898.1

back to list

  

| Protein Coverage
| Supporting Peptides
|

Protein Coverage:

Supporting Peptides:

| Peptide | Uniq | -10lgP | Mass | Length | ppm | m/z | z | RT | Fraction | Scan | Source File | Area F2 | Area F1 | Area F3 | Area F4 | Area F5 | Area F6 | #Feature | #Feature F2 | #Feature F1 | #Feature F3 | #Feature F4 | #Feature F5 | #Feature F6 | Start | End | PTM | AScore | Found By |
| --- | --- | --- | --- | --- | --- | --- | --- | --- | --- | --- | --- | --- | --- | --- | --- | --- | --- | --- | --- | --- | --- | --- | --- | --- | --- | --- | --- | --- | --- |
| R.LLLLGAGESGK.S | Y | 48.15 | 1056.6179 | 11 | 0.2 | 529.3163 | 2 | 40.45 | 1 | F1:21467 | 28122021\_RID\_2349\_2C.raw | 5.6366E4 |  |  |  |  |  | 1 | 1 | 0 | 0 | 0 | 0 | 0 | 43 | 53 |  |  | PEAKS DB |
| total 1 peptides |  |  |  |  |  |  |  |  |  |  |  |  |  |  |  |  |  |  |  |  |  |  |  |  |  |  |  |  |  |
| --- | --- | --- | --- | --- | --- | --- | --- | --- | --- | --- | --- | --- | --- | --- | --- | --- | --- | --- | --- | --- | --- | --- | --- | --- | --- | --- | --- | --- | --- |

JAG44520.1

back to list

  

| Protein Coverage
| Supporting Peptides
|

Protein Coverage:

Supporting Peptides:

| Peptide | Uniq | -10lgP | Mass | Length | ppm | m/z | z | RT | Fraction | Scan | Source File | Area F2 | Area F1 | Area F3 | Area F4 | Area F5 | Area F6 | #Feature | #Feature F2 | #Feature F1 | #Feature F3 | #Feature F4 | #Feature F5 | #Feature F6 | Start | End | PTM | AScore | Found By |
| --- | --- | --- | --- | --- | --- | --- | --- | --- | --- | --- | --- | --- | --- | --- | --- | --- | --- | --- | --- | --- | --- | --- | --- | --- | --- | --- | --- | --- | --- |
| R.LLLLGAGESGK.S | Y | 48.15 | 1056.6179 | 11 | 0.2 | 529.3163 | 2 | 40.45 | 1 | F1:21467 | 28122021\_RID\_2349\_2C.raw | 5.6366E4 |  |  |  |  |  | 1 | 1 | 0 | 0 | 0 | 0 | 0 | 43 | 53 |  |  | PEAKS DB |
| total 1 peptides |  |  |  |  |  |  |  |  |  |  |  |  |  |  |  |  |  |  |  |  |  |  |  |  |  |  |  |  |  |
| --- | --- | --- | --- | --- | --- | --- | --- | --- | --- | --- | --- | --- | --- | --- | --- | --- | --- | --- | --- | --- | --- | --- | --- | --- | --- | --- | --- | --- | --- |

AFJ50208.1

back to list

  

| Protein Coverage
| Supporting Peptides
|

Protein Coverage:

Supporting Peptides:

| Peptide | Uniq | -10lgP | Mass | Length | ppm | m/z | z | RT | Fraction | Scan | Source File | Area F2 | Area F1 | Area F3 | Area F4 | Area F5 | Area F6 | #Feature | #Feature F2 | #Feature F1 | #Feature F3 | #Feature F4 | #Feature F5 | #Feature F6 | Start | End | PTM | AScore | Found By |
| --- | --- | --- | --- | --- | --- | --- | --- | --- | --- | --- | --- | --- | --- | --- | --- | --- | --- | --- | --- | --- | --- | --- | --- | --- | --- | --- | --- | --- | --- |
| R.LLLLGAGESGK.S | Y | 48.15 | 1056.6179 | 11 | 0.2 | 529.3163 | 2 | 40.45 | 1 | F1:21467 | 28122021\_RID\_2349\_2C.raw | 5.6366E4 |  |  |  |  |  | 1 | 1 | 0 | 0 | 0 | 0 | 0 | 43 | 53 |  |  | PEAKS DB |
| total 1 peptides |  |  |  |  |  |  |  |  |  |  |  |  |  |  |  |  |  |  |  |  |  |  |  |  |  |  |  |  |  |
| --- | --- | --- | --- | --- | --- | --- | --- | --- | --- | --- | --- | --- | --- | --- | --- | --- | --- | --- | --- | --- | --- | --- | --- | --- | --- | --- | --- | --- | --- |

BAM28742.1

back to list

  

| Protein Coverage
| Supporting Peptides
|

Protein Coverage:

Supporting Peptides:

| Peptide | Uniq | -10lgP | Mass | Length | ppm | m/z | z | RT | Fraction | Scan | Source File | Area F2 | Area F1 | Area F3 | Area F4 | Area F5 | Area F6 | #Feature | #Feature F2 | #Feature F1 | #Feature F3 | #Feature F4 | #Feature F5 | #Feature F6 | Start | End | PTM | AScore | Found By |
| --- | --- | --- | --- | --- | --- | --- | --- | --- | --- | --- | --- | --- | --- | --- | --- | --- | --- | --- | --- | --- | --- | --- | --- | --- | --- | --- | --- | --- | --- |
| K.LLLLGAGESGK.S | Y | 48.15 | 1056.6179 | 11 | 0.2 | 529.3163 | 2 | 40.45 | 1 | F1:21467 | 28122021\_RID\_2349\_2C.raw | 5.6366E4 |  |  |  |  |  | 1 | 1 | 0 | 0 | 0 | 0 | 0 | 36 | 46 |  |  | PEAKS DB |
| total 1 peptides |  |  |  |  |  |  |  |  |  |  |  |  |  |  |  |  |  |  |  |  |  |  |  |  |  |  |  |  |  |
| --- | --- | --- | --- | --- | --- | --- | --- | --- | --- | --- | --- | --- | --- | --- | --- | --- | --- | --- | --- | --- | --- | --- | --- | --- | --- | --- | --- | --- | --- |

JAA96947.1

back to list

  

| Protein Coverage
| Supporting Peptides
|

Protein Coverage:

Supporting Peptides:

| Peptide | Uniq | -10lgP | Mass | Length | ppm | m/z | z | RT | Fraction | Scan | Source File | Area F2 | Area F1 | Area F3 | Area F4 | Area F5 | Area F6 | #Feature | #Feature F2 | #Feature F1 | #Feature F3 | #Feature F4 | #Feature F5 | #Feature F6 | Start | End | PTM | AScore | Found By |
| --- | --- | --- | --- | --- | --- | --- | --- | --- | --- | --- | --- | --- | --- | --- | --- | --- | --- | --- | --- | --- | --- | --- | --- | --- | --- | --- | --- | --- | --- |
| K.LLLLGAGESGK.S | Y | 48.15 | 1056.6179 | 11 | 0.2 | 529.3163 | 2 | 40.45 | 1 | F1:21467 | 28122021\_RID\_2349\_2C.raw | 5.6366E4 |  |  |  |  |  | 1 | 1 | 0 | 0 | 0 | 0 | 0 | 36 | 46 |  |  | PEAKS DB |
| total 1 peptides |  |  |  |  |  |  |  |  |  |  |  |  |  |  |  |  |  |  |  |  |  |  |  |  |  |  |  |  |  |
| --- | --- | --- | --- | --- | --- | --- | --- | --- | --- | --- | --- | --- | --- | --- | --- | --- | --- | --- | --- | --- | --- | --- | --- | --- | --- | --- | --- | --- | --- |

JAI09966.1

back to list

  

| Protein Coverage
| Supporting Peptides
|

Protein Coverage:

Supporting Peptides:

| Peptide | Uniq | -10lgP | Mass | Length | ppm | m/z | z | RT | Fraction | Scan | Source File | Area F2 | Area F1 | Area F3 | Area F4 | Area F5 | Area F6 | #Feature | #Feature F2 | #Feature F1 | #Feature F3 | #Feature F4 | #Feature F5 | #Feature F6 | Start | End | PTM | AScore | Found By |
| --- | --- | --- | --- | --- | --- | --- | --- | --- | --- | --- | --- | --- | --- | --- | --- | --- | --- | --- | --- | --- | --- | --- | --- | --- | --- | --- | --- | --- | --- |
| K.LLLLGAGESGK.S | Y | 48.15 | 1056.6179 | 11 | 0.2 | 529.3163 | 2 | 40.45 | 1 | F1:21467 | 28122021\_RID\_2349\_2C.raw | 5.6366E4 |  |  |  |  |  | 1 | 1 | 0 | 0 | 0 | 0 | 0 | 36 | 46 |  |  | PEAKS DB |
| total 1 peptides |  |  |  |  |  |  |  |  |  |  |  |  |  |  |  |  |  |  |  |  |  |  |  |  |  |  |  |  |  |
| --- | --- | --- | --- | --- | --- | --- | --- | --- | --- | --- | --- | --- | --- | --- | --- | --- | --- | --- | --- | --- | --- | --- | --- | --- | --- | --- | --- | --- | --- |

AFJ50262.1

back to list

  

| Protein Coverage
| Supporting Peptides
|

Protein Coverage:

Supporting Peptides:

| Peptide | Uniq | -10lgP | Mass | Length | ppm | m/z | z | RT | Fraction | Scan | Source File | Area F2 | Area F1 | Area F3 | Area F4 | Area F5 | Area F6 | #Feature | #Feature F2 | #Feature F1 | #Feature F3 | #Feature F4 | #Feature F5 | #Feature F6 | Start | End | PTM | AScore | Found By |
| --- | --- | --- | --- | --- | --- | --- | --- | --- | --- | --- | --- | --- | --- | --- | --- | --- | --- | --- | --- | --- | --- | --- | --- | --- | --- | --- | --- | --- | --- |
| K.LLLLGAGESGK.S | Y | 48.15 | 1056.6179 | 11 | 0.2 | 529.3163 | 2 | 40.45 | 1 | F1:21467 | 28122021\_RID\_2349\_2C.raw | 5.6366E4 |  |  |  |  |  | 1 | 1 | 0 | 0 | 0 | 0 | 0 | 36 | 46 |  |  | PEAKS DB |
| total 1 peptides |  |  |  |  |  |  |  |  |  |  |  |  |  |  |  |  |  |  |  |  |  |  |  |  |  |  |  |  |  |
| --- | --- | --- | --- | --- | --- | --- | --- | --- | --- | --- | --- | --- | --- | --- | --- | --- | --- | --- | --- | --- | --- | --- | --- | --- | --- | --- | --- | --- | --- |

JAG46724.1

back to list

  

| Protein Coverage
| Supporting Peptides
|

Protein Coverage:

Supporting Peptides:

| Peptide | Uniq | -10lgP | Mass | Length | ppm | m/z | z | RT | Fraction | Scan | Source File | Area F2 | Area F1 | Area F3 | Area F4 | Area F5 | Area F6 | #Feature | #Feature F2 | #Feature F1 | #Feature F3 | #Feature F4 | #Feature F5 | #Feature F6 | Start | End | PTM | AScore | Found By |
| --- | --- | --- | --- | --- | --- | --- | --- | --- | --- | --- | --- | --- | --- | --- | --- | --- | --- | --- | --- | --- | --- | --- | --- | --- | --- | --- | --- | --- | --- |
| K.LLLLGAGESGK.S | Y | 48.15 | 1056.6179 | 11 | 0.2 | 529.3163 | 2 | 40.45 | 1 | F1:21467 | 28122021\_RID\_2349\_2C.raw | 5.6366E4 |  |  |  |  |  | 1 | 1 | 0 | 0 | 0 | 0 | 0 | 36 | 46 |  |  | PEAKS DB |
| total 1 peptides |  |  |  |  |  |  |  |  |  |  |  |  |  |  |  |  |  |  |  |  |  |  |  |  |  |  |  |  |  |
| --- | --- | --- | --- | --- | --- | --- | --- | --- | --- | --- | --- | --- | --- | --- | --- | --- | --- | --- | --- | --- | --- | --- | --- | --- | --- | --- | --- | --- | --- |

JAC95504.1

back to list

  

| Protein Coverage
| Supporting Peptides
|

Protein Coverage:

Supporting Peptides:

| Peptide | Uniq | -10lgP | Mass | Length | ppm | m/z | z | RT | Fraction | Scan | Source File | Area F2 | Area F1 | Area F3 | Area F4 | Area F5 | Area F6 | #Feature | #Feature F2 | #Feature F1 | #Feature F3 | #Feature F4 | #Feature F5 | #Feature F6 | Start | End | PTM | AScore | Found By |
| --- | --- | --- | --- | --- | --- | --- | --- | --- | --- | --- | --- | --- | --- | --- | --- | --- | --- | --- | --- | --- | --- | --- | --- | --- | --- | --- | --- | --- | --- |
| K.LLLLGAGESGK.S | Y | 48.15 | 1056.6179 | 11 | 0.2 | 529.3163 | 2 | 40.45 | 1 | F1:21467 | 28122021\_RID\_2349\_2C.raw | 5.6366E4 |  |  |  |  |  | 1 | 1 | 0 | 0 | 0 | 0 | 0 | 36 | 46 |  |  | PEAKS DB |
| total 1 peptides |  |  |  |  |  |  |  |  |  |  |  |  |  |  |  |  |  |  |  |  |  |  |  |  |  |  |  |  |  |
| --- | --- | --- | --- | --- | --- | --- | --- | --- | --- | --- | --- | --- | --- | --- | --- | --- | --- | --- | --- | --- | --- | --- | --- | --- | --- | --- | --- | --- | --- |

JAB54125.1

back to list

  

| Protein Coverage
| Supporting Peptides
|

Protein Coverage:

Supporting Peptides:

| Peptide | Uniq | -10lgP | Mass | Length | ppm | m/z | z | RT | Fraction | Scan | Source File | Area F2 | Area F1 | Area F3 | Area F4 | Area F5 | Area F6 | #Feature | #Feature F2 | #Feature F1 | #Feature F3 | #Feature F4 | #Feature F5 | #Feature F6 | Start | End | PTM | AScore | Found By |
| --- | --- | --- | --- | --- | --- | --- | --- | --- | --- | --- | --- | --- | --- | --- | --- | --- | --- | --- | --- | --- | --- | --- | --- | --- | --- | --- | --- | --- | --- |
| K.LLLLGAGESGK.S | Y | 48.15 | 1056.6179 | 11 | 0.2 | 529.3163 | 2 | 40.45 | 1 | F1:21467 | 28122021\_RID\_2349\_2C.raw | 5.6366E4 |  |  |  |  |  | 1 | 1 | 0 | 0 | 0 | 0 | 0 | 36 | 46 |  |  | PEAKS DB |
| total 1 peptides |  |  |  |  |  |  |  |  |  |  |  |  |  |  |  |  |  |  |  |  |  |  |  |  |  |  |  |  |  |
| --- | --- | --- | --- | --- | --- | --- | --- | --- | --- | --- | --- | --- | --- | --- | --- | --- | --- | --- | --- | --- | --- | --- | --- | --- | --- | --- | --- | --- | --- |

JAG67837.1

back to list

  

| Protein Coverage
| Supporting Peptides
|

Protein Coverage:

Supporting Peptides:

| Peptide | Uniq | -10lgP | Mass | Length | ppm | m/z | z | RT | Fraction | Scan | Source File | Area F2 | Area F1 | Area F3 | Area F4 | Area F5 | Area F6 | #Feature | #Feature F2 | #Feature F1 | #Feature F3 | #Feature F4 | #Feature F5 | #Feature F6 | Start | End | PTM | AScore | Found By |
| --- | --- | --- | --- | --- | --- | --- | --- | --- | --- | --- | --- | --- | --- | --- | --- | --- | --- | --- | --- | --- | --- | --- | --- | --- | --- | --- | --- | --- | --- |
| K.LLLLGAGESGK.S | Y | 48.15 | 1056.6179 | 11 | 0.2 | 529.3163 | 2 | 40.45 | 1 | F1:21467 | 28122021\_RID\_2349\_2C.raw | 5.6366E4 |  |  |  |  |  | 1 | 1 | 0 | 0 | 0 | 0 | 0 | 36 | 46 |  |  | PEAKS DB |
| total 1 peptides |  |  |  |  |  |  |  |  |  |  |  |  |  |  |  |  |  |  |  |  |  |  |  |  |  |  |  |  |  |
| --- | --- | --- | --- | --- | --- | --- | --- | --- | --- | --- | --- | --- | --- | --- | --- | --- | --- | --- | --- | --- | --- | --- | --- | --- | --- | --- | --- | --- | --- |

JAG44583.1

back to list

  

| Protein Coverage
| Supporting Peptides
|

Protein Coverage:

Supporting Peptides:

| Peptide | Uniq | -10lgP | Mass | Length | ppm | m/z | z | RT | Fraction | Scan | Source File | Area F2 | Area F1 | Area F3 | Area F4 | Area F5 | Area F6 | #Feature | #Feature F2 | #Feature F1 | #Feature F3 | #Feature F4 | #Feature F5 | #Feature F6 | Start | End | PTM | AScore | Found By |
| --- | --- | --- | --- | --- | --- | --- | --- | --- | --- | --- | --- | --- | --- | --- | --- | --- | --- | --- | --- | --- | --- | --- | --- | --- | --- | --- | --- | --- | --- |
| K.LLLLGAGESGK.S | Y | 48.15 | 1056.6179 | 11 | 0.2 | 529.3163 | 2 | 40.45 | 1 | F1:21467 | 28122021\_RID\_2349\_2C.raw | 5.6366E4 |  |  |  |  |  | 1 | 1 | 0 | 0 | 0 | 0 | 0 | 36 | 46 |  |  | PEAKS DB |
| total 1 peptides |  |  |  |  |  |  |  |  |  |  |  |  |  |  |  |  |  |  |  |  |  |  |  |  |  |  |  |  |  |
| --- | --- | --- | --- | --- | --- | --- | --- | --- | --- | --- | --- | --- | --- | --- | --- | --- | --- | --- | --- | --- | --- | --- | --- | --- | --- | --- | --- | --- | --- |

JAG44582.1

back to list

  

| Protein Coverage
| Supporting Peptides
|

Protein Coverage:

Supporting Peptides:

| Peptide | Uniq | -10lgP | Mass | Length | ppm | m/z | z | RT | Fraction | Scan | Source File | Area F2 | Area F1 | Area F3 | Area F4 | Area F5 | Area F6 | #Feature | #Feature F2 | #Feature F1 | #Feature F3 | #Feature F4 | #Feature F5 | #Feature F6 | Start | End | PTM | AScore | Found By |
| --- | --- | --- | --- | --- | --- | --- | --- | --- | --- | --- | --- | --- | --- | --- | --- | --- | --- | --- | --- | --- | --- | --- | --- | --- | --- | --- | --- | --- | --- |
| K.LLLLGAGESGK.S | Y | 48.15 | 1056.6179 | 11 | 0.2 | 529.3163 | 2 | 40.45 | 1 | F1:21467 | 28122021\_RID\_2349\_2C.raw | 5.6366E4 |  |  |  |  |  | 1 | 1 | 0 | 0 | 0 | 0 | 0 | 36 | 46 |  |  | PEAKS DB |
| total 1 peptides |  |  |  |  |  |  |  |  |  |  |  |  |  |  |  |  |  |  |  |  |  |  |  |  |  |  |  |  |  |
| --- | --- | --- | --- | --- | --- | --- | --- | --- | --- | --- | --- | --- | --- | --- | --- | --- | --- | --- | --- | --- | --- | --- | --- | --- | --- | --- | --- | --- | --- |

JAI09965.1

back to list

  

| Protein Coverage
| Supporting Peptides
|

Protein Coverage:

Supporting Peptides:

| Peptide | Uniq | -10lgP | Mass | Length | ppm | m/z | z | RT | Fraction | Scan | Source File | Area F2 | Area F1 | Area F3 | Area F4 | Area F5 | Area F6 | #Feature | #Feature F2 | #Feature F1 | #Feature F3 | #Feature F4 | #Feature F5 | #Feature F6 | Start | End | PTM | AScore | Found By |
| --- | --- | --- | --- | --- | --- | --- | --- | --- | --- | --- | --- | --- | --- | --- | --- | --- | --- | --- | --- | --- | --- | --- | --- | --- | --- | --- | --- | --- | --- |
| K.LLLLGAGESGK.S | Y | 48.15 | 1056.6179 | 11 | 0.2 | 529.3163 | 2 | 40.45 | 1 | F1:21467 | 28122021\_RID\_2349\_2C.raw | 5.6366E4 |  |  |  |  |  | 1 | 1 | 0 | 0 | 0 | 0 | 0 | 36 | 46 |  |  | PEAKS DB |
| total 1 peptides |  |  |  |  |  |  |  |  |  |  |  |  |  |  |  |  |  |  |  |  |  |  |  |  |  |  |  |  |  |
| --- | --- | --- | --- | --- | --- | --- | --- | --- | --- | --- | --- | --- | --- | --- | --- | --- | --- | --- | --- | --- | --- | --- | --- | --- | --- | --- | --- | --- | --- |

JAA96946.1

back to list

  

| Protein Coverage
| Supporting Peptides
|

Protein Coverage:

Supporting Peptides:

| Peptide | Uniq | -10lgP | Mass | Length | ppm | m/z | z | RT | Fraction | Scan | Source File | Area F2 | Area F1 | Area F3 | Area F4 | Area F5 | Area F6 | #Feature | #Feature F2 | #Feature F1 | #Feature F3 | #Feature F4 | #Feature F5 | #Feature F6 | Start | End | PTM | AScore | Found By |
| --- | --- | --- | --- | --- | --- | --- | --- | --- | --- | --- | --- | --- | --- | --- | --- | --- | --- | --- | --- | --- | --- | --- | --- | --- | --- | --- | --- | --- | --- |
| K.LLLLGAGESGK.S | Y | 48.15 | 1056.6179 | 11 | 0.2 | 529.3163 | 2 | 40.45 | 1 | F1:21467 | 28122021\_RID\_2349\_2C.raw | 5.6366E4 |  |  |  |  |  | 1 | 1 | 0 | 0 | 0 | 0 | 0 | 36 | 46 |  |  | PEAKS DB |
| total 1 peptides |  |  |  |  |  |  |  |  |  |  |  |  |  |  |  |  |  |  |  |  |  |  |  |  |  |  |  |  |  |
| --- | --- | --- | --- | --- | --- | --- | --- | --- | --- | --- | --- | --- | --- | --- | --- | --- | --- | --- | --- | --- | --- | --- | --- | --- | --- | --- | --- | --- | --- |

JAG46723.1

back to list

  

| Protein Coverage
| Supporting Peptides
|

Protein Coverage:

Supporting Peptides:

| Peptide | Uniq | -10lgP | Mass | Length | ppm | m/z | z | RT | Fraction | Scan | Source File | Area F2 | Area F1 | Area F3 | Area F4 | Area F5 | Area F6 | #Feature | #Feature F2 | #Feature F1 | #Feature F3 | #Feature F4 | #Feature F5 | #Feature F6 | Start | End | PTM | AScore | Found By |
| --- | --- | --- | --- | --- | --- | --- | --- | --- | --- | --- | --- | --- | --- | --- | --- | --- | --- | --- | --- | --- | --- | --- | --- | --- | --- | --- | --- | --- | --- |
| K.LLLLGAGESGK.S | Y | 48.15 | 1056.6179 | 11 | 0.2 | 529.3163 | 2 | 40.45 | 1 | F1:21467 | 28122021\_RID\_2349\_2C.raw | 5.6366E4 |  |  |  |  |  | 1 | 1 | 0 | 0 | 0 | 0 | 0 | 36 | 46 |  |  | PEAKS DB |
| total 1 peptides |  |  |  |  |  |  |  |  |  |  |  |  |  |  |  |  |  |  |  |  |  |  |  |  |  |  |  |  |  |
| --- | --- | --- | --- | --- | --- | --- | --- | --- | --- | --- | --- | --- | --- | --- | --- | --- | --- | --- | --- | --- | --- | --- | --- | --- | --- | --- | --- | --- | --- |

JAG44556.1

back to list

  

| Protein Coverage
| Supporting Peptides
|

Protein Coverage:

Supporting Peptides:

| Peptide | Uniq | -10lgP | Mass | Length | ppm | m/z | z | RT | Fraction | Scan | Source File | Area F2 | Area F1 | Area F3 | Area F4 | Area F5 | Area F6 | #Feature | #Feature F2 | #Feature F1 | #Feature F3 | #Feature F4 | #Feature F5 | #Feature F6 | Start | End | PTM | AScore | Found By |
| --- | --- | --- | --- | --- | --- | --- | --- | --- | --- | --- | --- | --- | --- | --- | --- | --- | --- | --- | --- | --- | --- | --- | --- | --- | --- | --- | --- | --- | --- |
| R.ILLLGAGESGK.S | Y | 48.15 | 1056.6179 | 11 | 0.2 | 529.3163 | 2 | 40.45 | 1 | F1:21467 | 28122021\_RID\_2349\_2C.raw | 5.6366E4 |  |  |  |  |  | 1 | 1 | 0 | 0 | 0 | 0 | 0 | 56 | 66 |  |  | PEAKS DB |
| total 1 peptides |  |  |  |  |  |  |  |  |  |  |  |  |  |  |  |  |  |  |  |  |  |  |  |  |  |  |  |  |  |
| --- | --- | --- | --- | --- | --- | --- | --- | --- | --- | --- | --- | --- | --- | --- | --- | --- | --- | --- | --- | --- | --- | --- | --- | --- | --- | --- | --- | --- | --- |

JAI13125.1

back to list

  

| Protein Coverage
| Supporting Peptides
|

Protein Coverage:

Supporting Peptides:

| Peptide | Uniq | -10lgP | Mass | Length | ppm | m/z | z | RT | Fraction | Scan | Source File | Area F2 | Area F1 | Area F3 | Area F4 | Area F5 | Area F6 | #Feature | #Feature F2 | #Feature F1 | #Feature F3 | #Feature F4 | #Feature F5 | #Feature F6 | Start | End | PTM | AScore | Found By |
| --- | --- | --- | --- | --- | --- | --- | --- | --- | --- | --- | --- | --- | --- | --- | --- | --- | --- | --- | --- | --- | --- | --- | --- | --- | --- | --- | --- | --- | --- |
| R.ILLLGAGESGK.S | Y | 48.15 | 1056.6179 | 11 | 0.2 | 529.3163 | 2 | 40.45 | 1 | F1:21467 | 28122021\_RID\_2349\_2C.raw | 5.6366E4 |  |  |  |  |  | 1 | 1 | 0 | 0 | 0 | 0 | 0 | 56 | 66 |  |  | PEAKS DB |
| total 1 peptides |  |  |  |  |  |  |  |  |  |  |  |  |  |  |  |  |  |  |  |  |  |  |  |  |  |  |  |  |  |
| --- | --- | --- | --- | --- | --- | --- | --- | --- | --- | --- | --- | --- | --- | --- | --- | --- | --- | --- | --- | --- | --- | --- | --- | --- | --- | --- | --- | --- | --- |

JAA96922.1

back to list

  

| Protein Coverage
| Supporting Peptides
|

Protein Coverage:

Supporting Peptides:

| Peptide | Uniq | -10lgP | Mass | Length | ppm | m/z | z | RT | Fraction | Scan | Source File | Area F2 | Area F1 | Area F3 | Area F4 | Area F5 | Area F6 | #Feature | #Feature F2 | #Feature F1 | #Feature F3 | #Feature F4 | #Feature F5 | #Feature F6 | Start | End | PTM | AScore | Found By |
| --- | --- | --- | --- | --- | --- | --- | --- | --- | --- | --- | --- | --- | --- | --- | --- | --- | --- | --- | --- | --- | --- | --- | --- | --- | --- | --- | --- | --- | --- |
| R.ILLLGAGESGK.S | Y | 48.15 | 1056.6179 | 11 | 0.2 | 529.3163 | 2 | 40.45 | 1 | F1:21467 | 28122021\_RID\_2349\_2C.raw | 5.6366E4 |  |  |  |  |  | 1 | 1 | 0 | 0 | 0 | 0 | 0 | 56 | 66 |  |  | PEAKS DB |
| total 1 peptides |  |  |  |  |  |  |  |  |  |  |  |  |  |  |  |  |  |  |  |  |  |  |  |  |  |  |  |  |  |
| --- | --- | --- | --- | --- | --- | --- | --- | --- | --- | --- | --- | --- | --- | --- | --- | --- | --- | --- | --- | --- | --- | --- | --- | --- | --- | --- | --- | --- | --- |

JAG67838.1

back to list

  

| Protein Coverage
| Supporting Peptides
|

Protein Coverage:

Supporting Peptides:

| Peptide | Uniq | -10lgP | Mass | Length | ppm | m/z | z | RT | Fraction | Scan | Source File | Area F2 | Area F1 | Area F3 | Area F4 | Area F5 | Area F6 | #Feature | #Feature F2 | #Feature F1 | #Feature F3 | #Feature F4 | #Feature F5 | #Feature F6 | Start | End | PTM | AScore | Found By |
| --- | --- | --- | --- | --- | --- | --- | --- | --- | --- | --- | --- | --- | --- | --- | --- | --- | --- | --- | --- | --- | --- | --- | --- | --- | --- | --- | --- | --- | --- |
| R.ILLLGAGESGK.S | Y | 48.15 | 1056.6179 | 11 | 0.2 | 529.3163 | 2 | 40.45 | 1 | F1:21467 | 28122021\_RID\_2349\_2C.raw | 5.6366E4 |  |  |  |  |  | 1 | 1 | 0 | 0 | 0 | 0 | 0 | 83 | 93 |  |  | PEAKS DB |
| total 1 peptides |  |  |  |  |  |  |  |  |  |  |  |  |  |  |  |  |  |  |  |  |  |  |  |  |  |  |  |  |  |
| --- | --- | --- | --- | --- | --- | --- | --- | --- | --- | --- | --- | --- | --- | --- | --- | --- | --- | --- | --- | --- | --- | --- | --- | --- | --- | --- | --- | --- | --- |

XP\_026564997.1

back to list

  

| Protein Coverage
| Supporting Peptides
|

Protein Coverage:

Supporting Peptides:

| Peptide | Uniq | -10lgP | Mass | Length | ppm | m/z | z | RT | Fraction | Scan | Source File | Area F2 | Area F1 | Area F3 | Area F4 | Area F5 | Area F6 | #Feature | #Feature F2 | #Feature F1 | #Feature F3 | #Feature F4 | #Feature F5 | #Feature F6 | Start | End | PTM | AScore | Found By |
| --- | --- | --- | --- | --- | --- | --- | --- | --- | --- | --- | --- | --- | --- | --- | --- | --- | --- | --- | --- | --- | --- | --- | --- | --- | --- | --- | --- | --- | --- |
| K.IEVANPDGDLR.L | Y | 43.98 | 1197.5989 | 11 | 0.7 | 599.8071 | 2 | 32.61 | 1 | F1:15125 | 28122021\_RID\_2349\_2C.raw | 2.6135E4 |  |  |  |  |  | 1 | 1 | 0 | 0 | 0 | 0 | 0 | 752 | 762 |  |  | PEAKS DB |
| total 1 peptides |  |  |  |  |  |  |  |  |  |  |  |  |  |  |  |  |  |  |  |  |  |  |  |  |  |  |  |  |  |
| --- | --- | --- | --- | --- | --- | --- | --- | --- | --- | --- | --- | --- | --- | --- | --- | --- | --- | --- | --- | --- | --- | --- | --- | --- | --- | --- | --- | --- | --- |

XP\_025024673.1

back to list

  

| Protein Coverage
| Supporting Peptides
|

Protein Coverage:

Supporting Peptides:

| Peptide | Uniq | -10lgP | Mass | Length | ppm | m/z | z | RT | Fraction | Scan | Source File | Area F2 | Area F1 | Area F3 | Area F4 | Area F5 | Area F6 | #Feature | #Feature F2 | #Feature F1 | #Feature F3 | #Feature F4 | #Feature F5 | #Feature F6 | Start | End | PTM | AScore | Found By |
| --- | --- | --- | --- | --- | --- | --- | --- | --- | --- | --- | --- | --- | --- | --- | --- | --- | --- | --- | --- | --- | --- | --- | --- | --- | --- | --- | --- | --- | --- |
| K.IEVANPDGDLR.L | Y | 43.98 | 1197.5989 | 11 | 0.7 | 599.8071 | 2 | 32.61 | 1 | F1:15125 | 28122021\_RID\_2349\_2C.raw | 2.6135E4 |  |  |  |  |  | 1 | 1 | 0 | 0 | 0 | 0 | 0 | 752 | 762 |  |  | PEAKS DB |
| total 1 peptides |  |  |  |  |  |  |  |  |  |  |  |  |  |  |  |  |  |  |  |  |  |  |  |  |  |  |  |  |  |
| --- | --- | --- | --- | --- | --- | --- | --- | --- | --- | --- | --- | --- | --- | --- | --- | --- | --- | --- | --- | --- | --- | --- | --- | --- | --- | --- | --- | --- | --- |

XP\_007431813.2

back to list

  

| Protein Coverage
| Supporting Peptides
|

Protein Coverage:

Supporting Peptides:

| Peptide | Uniq | -10lgP | Mass | Length | ppm | m/z | z | RT | Fraction | Scan | Source File | Area F2 | Area F1 | Area F3 | Area F4 | Area F5 | Area F6 | #Feature | #Feature F2 | #Feature F1 | #Feature F3 | #Feature F4 | #Feature F5 | #Feature F6 | Start | End | PTM | AScore | Found By |
| --- | --- | --- | --- | --- | --- | --- | --- | --- | --- | --- | --- | --- | --- | --- | --- | --- | --- | --- | --- | --- | --- | --- | --- | --- | --- | --- | --- | --- | --- |
| K.IEVANPDGDLR.L | Y | 43.98 | 1197.5989 | 11 | 0.7 | 599.8071 | 2 | 32.61 | 1 | F1:15125 | 28122021\_RID\_2349\_2C.raw | 2.6135E4 |  |  |  |  |  | 1 | 1 | 0 | 0 | 0 | 0 | 0 | 752 | 762 |  |  | PEAKS DB |
| total 1 peptides |  |  |  |  |  |  |  |  |  |  |  |  |  |  |  |  |  |  |  |  |  |  |  |  |  |  |  |  |  |
| --- | --- | --- | --- | --- | --- | --- | --- | --- | --- | --- | --- | --- | --- | --- | --- | --- | --- | --- | --- | --- | --- | --- | --- | --- | --- | --- | --- | --- | --- |

XP\_026537699.1

back to list

  

| Protein Coverage
| Supporting Peptides
|

Protein Coverage:

Supporting Peptides:

| Peptide | Uniq | -10lgP | Mass | Length | ppm | m/z | z | RT | Fraction | Scan | Source File | Area F2 | Area F1 | Area F3 | Area F4 | Area F5 | Area F6 | #Feature | #Feature F2 | #Feature F1 | #Feature F3 | #Feature F4 | #Feature F5 | #Feature F6 | Start | End | PTM | AScore | Found By |
| --- | --- | --- | --- | --- | --- | --- | --- | --- | --- | --- | --- | --- | --- | --- | --- | --- | --- | --- | --- | --- | --- | --- | --- | --- | --- | --- | --- | --- | --- |
| K.IEVANPDGDLR.L | Y | 43.98 | 1197.5989 | 11 | 0.7 | 599.8071 | 2 | 32.61 | 1 | F1:15125 | 28122021\_RID\_2349\_2C.raw | 2.6135E4 |  |  |  |  |  | 1 | 1 | 0 | 0 | 0 | 0 | 0 | 752 | 762 |  |  | PEAKS DB |
| total 1 peptides |  |  |  |  |  |  |  |  |  |  |  |  |  |  |  |  |  |  |  |  |  |  |  |  |  |  |  |  |  |
| --- | --- | --- | --- | --- | --- | --- | --- | --- | --- | --- | --- | --- | --- | --- | --- | --- | --- | --- | --- | --- | --- | --- | --- | --- | --- | --- | --- | --- | --- |

XP\_032085080.1

back to list

  

| Protein Coverage
| Supporting Peptides
|

Protein Coverage:

Supporting Peptides:

| Peptide | Uniq | -10lgP | Mass | Length | ppm | m/z | z | RT | Fraction | Scan | Source File | Area F2 | Area F1 | Area F3 | Area F4 | Area F5 | Area F6 | #Feature | #Feature F2 | #Feature F1 | #Feature F3 | #Feature F4 | #Feature F5 | #Feature F6 | Start | End | PTM | AScore | Found By |
| --- | --- | --- | --- | --- | --- | --- | --- | --- | --- | --- | --- | --- | --- | --- | --- | --- | --- | --- | --- | --- | --- | --- | --- | --- | --- | --- | --- | --- | --- |
| K.IEVANPDGDLR.L | Y | 43.98 | 1197.5989 | 11 | 0.7 | 599.8071 | 2 | 32.61 | 1 | F1:15125 | 28122021\_RID\_2349\_2C.raw | 2.6135E4 |  |  |  |  |  | 1 | 1 | 0 | 0 | 0 | 0 | 0 | 365 | 375 |  |  | PEAKS DB |
| total 1 peptides |  |  |  |  |  |  |  |  |  |  |  |  |  |  |  |  |  |  |  |  |  |  |  |  |  |  |  |  |  |
| --- | --- | --- | --- | --- | --- | --- | --- | --- | --- | --- | --- | --- | --- | --- | --- | --- | --- | --- | --- | --- | --- | --- | --- | --- | --- | --- | --- | --- | --- |

XP\_032085079.1

back to list

  

| Protein Coverage
| Supporting Peptides
|

Protein Coverage:

Supporting Peptides:

| Peptide | Uniq | -10lgP | Mass | Length | ppm | m/z | z | RT | Fraction | Scan | Source File | Area F2 | Area F1 | Area F3 | Area F4 | Area F5 | Area F6 | #Feature | #Feature F2 | #Feature F1 | #Feature F3 | #Feature F4 | #Feature F5 | #Feature F6 | Start | End | PTM | AScore | Found By |
| --- | --- | --- | --- | --- | --- | --- | --- | --- | --- | --- | --- | --- | --- | --- | --- | --- | --- | --- | --- | --- | --- | --- | --- | --- | --- | --- | --- | --- | --- |
| K.IEVANPDGDLR.L | Y | 43.98 | 1197.5989 | 11 | 0.7 | 599.8071 | 2 | 32.61 | 1 | F1:15125 | 28122021\_RID\_2349\_2C.raw | 2.6135E4 |  |  |  |  |  | 1 | 1 | 0 | 0 | 0 | 0 | 0 | 863 | 873 |  |  | PEAKS DB |
| total 1 peptides |  |  |  |  |  |  |  |  |  |  |  |  |  |  |  |  |  |  |  |  |  |  |  |  |  |  |  |  |  |
| --- | --- | --- | --- | --- | --- | --- | --- | --- | --- | --- | --- | --- | --- | --- | --- | --- | --- | --- | --- | --- | --- | --- | --- | --- | --- | --- | --- | --- | --- |

XP\_034286370.1

back to list

  

| Protein Coverage
| Supporting Peptides
|

Protein Coverage:

Supporting Peptides:

| Peptide | Uniq | -10lgP | Mass | Length | ppm | m/z | z | RT | Fraction | Scan | Source File | Area F2 | Area F1 | Area F3 | Area F4 | Area F5 | Area F6 | #Feature | #Feature F2 | #Feature F1 | #Feature F3 | #Feature F4 | #Feature F5 | #Feature F6 | Start | End | PTM | AScore | Found By |
| --- | --- | --- | --- | --- | --- | --- | --- | --- | --- | --- | --- | --- | --- | --- | --- | --- | --- | --- | --- | --- | --- | --- | --- | --- | --- | --- | --- | --- | --- |
| K.IEVANPDGDLR.L | Y | 43.98 | 1197.5989 | 11 | 0.7 | 599.8071 | 2 | 32.61 | 1 | F1:15125 | 28122021\_RID\_2349\_2C.raw | 2.6135E4 |  |  |  |  |  | 1 | 1 | 0 | 0 | 0 | 0 | 0 | 752 | 762 |  |  | PEAKS DB |
| total 1 peptides |  |  |  |  |  |  |  |  |  |  |  |  |  |  |  |  |  |  |  |  |  |  |  |  |  |  |  |  |  |
| --- | --- | --- | --- | --- | --- | --- | --- | --- | --- | --- | --- | --- | --- | --- | --- | --- | --- | --- | --- | --- | --- | --- | --- | --- | --- | --- | --- | --- | --- |

XP\_015676891.1

back to list

  

| Protein Coverage
| Supporting Peptides
|

Protein Coverage:

Supporting Peptides:

| Peptide | Uniq | -10lgP | Mass | Length | ppm | m/z | z | RT | Fraction | Scan | Source File | Area F2 | Area F1 | Area F3 | Area F4 | Area F5 | Area F6 | #Feature | #Feature F2 | #Feature F1 | #Feature F3 | #Feature F4 | #Feature F5 | #Feature F6 | Start | End | PTM | AScore | Found By |
| --- | --- | --- | --- | --- | --- | --- | --- | --- | --- | --- | --- | --- | --- | --- | --- | --- | --- | --- | --- | --- | --- | --- | --- | --- | --- | --- | --- | --- | --- |
| K.IEVANPDGDLR.L | Y | 43.98 | 1197.5989 | 11 | 0.7 | 599.8071 | 2 | 32.61 | 1 | F1:15125 | 28122021\_RID\_2349\_2C.raw | 2.6135E4 |  |  |  |  |  | 1 | 1 | 0 | 0 | 0 | 0 | 0 | 78 | 88 |  |  | PEAKS DB |
| total 1 peptides |  |  |  |  |  |  |  |  |  |  |  |  |  |  |  |  |  |  |  |  |  |  |  |  |  |  |  |  |  |
| --- | --- | --- | --- | --- | --- | --- | --- | --- | --- | --- | --- | --- | --- | --- | --- | --- | --- | --- | --- | --- | --- | --- | --- | --- | --- | --- | --- | --- | --- |

ETE61149.1

back to list

  

| Protein Coverage
| Supporting Peptides
|

Protein Coverage:

Supporting Peptides:

| Peptide | Uniq | -10lgP | Mass | Length | ppm | m/z | z | RT | Fraction | Scan | Source File | Area F2 | Area F1 | Area F3 | Area F4 | Area F5 | Area F6 | #Feature | #Feature F2 | #Feature F1 | #Feature F3 | #Feature F4 | #Feature F5 | #Feature F6 | Start | End | PTM | AScore | Found By |
| --- | --- | --- | --- | --- | --- | --- | --- | --- | --- | --- | --- | --- | --- | --- | --- | --- | --- | --- | --- | --- | --- | --- | --- | --- | --- | --- | --- | --- | --- |
| K.IEVANPDGDLR.L | Y | 43.98 | 1197.5989 | 11 | 0.7 | 599.8071 | 2 | 32.61 | 1 | F1:15125 | 28122021\_RID\_2349\_2C.raw | 2.6135E4 |  |  |  |  |  | 1 | 1 | 0 | 0 | 0 | 0 | 0 | 324 | 334 |  |  | PEAKS DB |
| total 1 peptides |  |  |  |  |  |  |  |  |  |  |  |  |  |  |  |  |  |  |  |  |  |  |  |  |  |  |  |  |  |
| --- | --- | --- | --- | --- | --- | --- | --- | --- | --- | --- | --- | --- | --- | --- | --- | --- | --- | --- | --- | --- | --- | --- | --- | --- | --- | --- | --- | --- | --- |

XP\_013929765.1

back to list

  

| Protein Coverage
| Supporting Peptides
|

Protein Coverage:

Supporting Peptides:

| Peptide | Uniq | -10lgP | Mass | Length | ppm | m/z | z | RT | Fraction | Scan | Source File | Area F2 | Area F1 | Area F3 | Area F4 | Area F5 | Area F6 | #Feature | #Feature F2 | #Feature F1 | #Feature F3 | #Feature F4 | #Feature F5 | #Feature F6 | Start | End | PTM | AScore | Found By |
| --- | --- | --- | --- | --- | --- | --- | --- | --- | --- | --- | --- | --- | --- | --- | --- | --- | --- | --- | --- | --- | --- | --- | --- | --- | --- | --- | --- | --- | --- |
| K.IEVANPDGDLR.L | Y | 43.98 | 1197.5989 | 11 | 0.7 | 599.8071 | 2 | 32.61 | 1 | F1:15125 | 28122021\_RID\_2349\_2C.raw | 2.6135E4 |  |  |  |  |  | 1 | 1 | 0 | 0 | 0 | 0 | 0 | 307 | 317 |  |  | PEAKS DB |
| total 1 peptides |  |  |  |  |  |  |  |  |  |  |  |  |  |  |  |  |  |  |  |  |  |  |  |  |  |  |  |  |  |
| --- | --- | --- | --- | --- | --- | --- | --- | --- | --- | --- | --- | --- | --- | --- | --- | --- | --- | --- | --- | --- | --- | --- | --- | --- | --- | --- | --- | --- | --- |

XP\_032078096.1

back to list

  

| Protein Coverage
| Supporting Peptides
|

Protein Coverage:

Supporting Peptides:

| Peptide | Uniq | -10lgP | Mass | Length | ppm | m/z | z | RT | Fraction | Scan | Source File | Area F2 | Area F1 | Area F3 | Area F4 | Area F5 | Area F6 | #Feature | #Feature F2 | #Feature F1 | #Feature F3 | #Feature F4 | #Feature F5 | #Feature F6 | Start | End | PTM | AScore | Found By |
| --- | --- | --- | --- | --- | --- | --- | --- | --- | --- | --- | --- | --- | --- | --- | --- | --- | --- | --- | --- | --- | --- | --- | --- | --- | --- | --- | --- | --- | --- |
| K.ELGVGFATR.K | Y | 43.57 | 948.5029 | 9 | -0.4 | 475.2585 | 2 | 36.46 | 6 | F6:16444 | 31122021\_RID\_2349\_6C.raw |  |  |  |  |  | 5.8274E4 | 1 | 0 | 0 | 0 | 0 | 0 | 1 | 23 | 31 |  |  | PEAKS DB |
| total 1 peptides |  |  |  |  |  |  |  |  |  |  |  |  |  |  |  |  |  |  |  |  |  |  |  |  |  |  |  |  |  |
| --- | --- | --- | --- | --- | --- | --- | --- | --- | --- | --- | --- | --- | --- | --- | --- | --- | --- | --- | --- | --- | --- | --- | --- | --- | --- | --- | --- | --- | --- |

XP\_013920450.1

back to list

  

| Protein Coverage
| Supporting Peptides
|

Protein Coverage:

Supporting Peptides:

| Peptide | Uniq | -10lgP | Mass | Length | ppm | m/z | z | RT | Fraction | Scan | Source File | Area F2 | Area F1 | Area F3 | Area F4 | Area F5 | Area F6 | #Feature | #Feature F2 | #Feature F1 | #Feature F3 | #Feature F4 | #Feature F5 | #Feature F6 | Start | End | PTM | AScore | Found By |
| --- | --- | --- | --- | --- | --- | --- | --- | --- | --- | --- | --- | --- | --- | --- | --- | --- | --- | --- | --- | --- | --- | --- | --- | --- | --- | --- | --- | --- | --- |
| K.ELGVGFATR.K | Y | 43.57 | 948.5029 | 9 | -0.4 | 475.2585 | 2 | 36.46 | 6 | F6:16444 | 31122021\_RID\_2349\_6C.raw |  |  |  |  |  | 5.8274E4 | 1 | 0 | 0 | 0 | 0 | 0 | 1 | 23 | 31 |  |  | PEAKS DB |
| total 1 peptides |  |  |  |  |  |  |  |  |  |  |  |  |  |  |  |  |  |  |  |  |  |  |  |  |  |  |  |  |  |
| --- | --- | --- | --- | --- | --- | --- | --- | --- | --- | --- | --- | --- | --- | --- | --- | --- | --- | --- | --- | --- | --- | --- | --- | --- | --- | --- | --- | --- | --- |

XP\_026547098.1

back to list

  

| Protein Coverage
| Supporting Peptides
|

Protein Coverage:

Supporting Peptides:

| Peptide | Uniq | -10lgP | Mass | Length | ppm | m/z | z | RT | Fraction | Scan | Source File | Area F2 | Area F1 | Area F3 | Area F4 | Area F5 | Area F6 | #Feature | #Feature F2 | #Feature F1 | #Feature F3 | #Feature F4 | #Feature F5 | #Feature F6 | Start | End | PTM | AScore | Found By |
| --- | --- | --- | --- | --- | --- | --- | --- | --- | --- | --- | --- | --- | --- | --- | --- | --- | --- | --- | --- | --- | --- | --- | --- | --- | --- | --- | --- | --- | --- |
| K.ELGVGFATR.K | Y | 43.57 | 948.5029 | 9 | -0.4 | 475.2585 | 2 | 36.46 | 6 | F6:16444 | 31122021\_RID\_2349\_6C.raw |  |  |  |  |  | 5.8274E4 | 1 | 0 | 0 | 0 | 0 | 0 | 1 | 23 | 31 |  |  | PEAKS DB |
| total 1 peptides |  |  |  |  |  |  |  |  |  |  |  |  |  |  |  |  |  |  |  |  |  |  |  |  |  |  |  |  |  |
| --- | --- | --- | --- | --- | --- | --- | --- | --- | --- | --- | --- | --- | --- | --- | --- | --- | --- | --- | --- | --- | --- | --- | --- | --- | --- | --- | --- | --- | --- |

XP\_026561817.1

back to list

  

| Protein Coverage
| Supporting Peptides
|

Protein Coverage:

Supporting Peptides:

| Peptide | Uniq | -10lgP | Mass | Length | ppm | m/z | z | RT | Fraction | Scan | Source File | Area F2 | Area F1 | Area F3 | Area F4 | Area F5 | Area F6 | #Feature | #Feature F2 | #Feature F1 | #Feature F3 | #Feature F4 | #Feature F5 | #Feature F6 | Start | End | PTM | AScore | Found By |
| --- | --- | --- | --- | --- | --- | --- | --- | --- | --- | --- | --- | --- | --- | --- | --- | --- | --- | --- | --- | --- | --- | --- | --- | --- | --- | --- | --- | --- | --- |
| K.ELGVGFATR.K | Y | 43.57 | 948.5029 | 9 | -0.4 | 475.2585 | 2 | 36.46 | 6 | F6:16444 | 31122021\_RID\_2349\_6C.raw |  |  |  |  |  | 5.8274E4 | 1 | 0 | 0 | 0 | 0 | 0 | 1 | 23 | 31 |  |  | PEAKS DB |
| total 1 peptides |  |  |  |  |  |  |  |  |  |  |  |  |  |  |  |  |  |  |  |  |  |  |  |  |  |  |  |  |  |
| --- | --- | --- | --- | --- | --- | --- | --- | --- | --- | --- | --- | --- | --- | --- | --- | --- | --- | --- | --- | --- | --- | --- | --- | --- | --- | --- | --- | --- | --- |

XP\_007432588.1

back to list

  

| Protein Coverage
| Supporting Peptides
|

Protein Coverage:

Supporting Peptides:

| Peptide | Uniq | -10lgP | Mass | Length | ppm | m/z | z | RT | Fraction | Scan | Source File | Area F2 | Area F1 | Area F3 | Area F4 | Area F5 | Area F6 | #Feature | #Feature F2 | #Feature F1 | #Feature F3 | #Feature F4 | #Feature F5 | #Feature F6 | Start | End | PTM | AScore | Found By |
| --- | --- | --- | --- | --- | --- | --- | --- | --- | --- | --- | --- | --- | --- | --- | --- | --- | --- | --- | --- | --- | --- | --- | --- | --- | --- | --- | --- | --- | --- |
| K.ELGVGFATR.K | Y | 43.57 | 948.5029 | 9 | -0.4 | 475.2585 | 2 | 36.46 | 6 | F6:16444 | 31122021\_RID\_2349\_6C.raw |  |  |  |  |  | 5.8274E4 | 1 | 0 | 0 | 0 | 0 | 0 | 1 | 23 | 31 |  |  | PEAKS DB |
| total 1 peptides |  |  |  |  |  |  |  |  |  |  |  |  |  |  |  |  |  |  |  |  |  |  |  |  |  |  |  |  |  |
| --- | --- | --- | --- | --- | --- | --- | --- | --- | --- | --- | --- | --- | --- | --- | --- | --- | --- | --- | --- | --- | --- | --- | --- | --- | --- | --- | --- | --- | --- |

XP\_034278224.1

back to list

  

| Protein Coverage
| Supporting Peptides
|

Protein Coverage:

Supporting Peptides:

| Peptide | Uniq | -10lgP | Mass | Length | ppm | m/z | z | RT | Fraction | Scan | Source File | Area F2 | Area F1 | Area F3 | Area F4 | Area F5 | Area F6 | #Feature | #Feature F2 | #Feature F1 | #Feature F3 | #Feature F4 | #Feature F5 | #Feature F6 | Start | End | PTM | AScore | Found By |
| --- | --- | --- | --- | --- | --- | --- | --- | --- | --- | --- | --- | --- | --- | --- | --- | --- | --- | --- | --- | --- | --- | --- | --- | --- | --- | --- | --- | --- | --- |
| K.ELGVGFATR.K | Y | 43.57 | 948.5029 | 9 | -0.4 | 475.2585 | 2 | 36.46 | 6 | F6:16444 | 31122021\_RID\_2349\_6C.raw |  |  |  |  |  | 5.8274E4 | 1 | 0 | 0 | 0 | 0 | 0 | 1 | 23 | 31 |  |  | PEAKS DB |
| total 1 peptides |  |  |  |  |  |  |  |  |  |  |  |  |  |  |  |  |  |  |  |  |  |  |  |  |  |  |  |  |  |
| --- | --- | --- | --- | --- | --- | --- | --- | --- | --- | --- | --- | --- | --- | --- | --- | --- | --- | --- | --- | --- | --- | --- | --- | --- | --- | --- | --- | --- | --- |

XP\_015687553.1

back to list

  

| Protein Coverage
| Supporting Peptides
|

Protein Coverage:

Supporting Peptides:

| Peptide | Uniq | -10lgP | Mass | Length | ppm | m/z | z | RT | Fraction | Scan | Source File | Area F2 | Area F1 | Area F3 | Area F4 | Area F5 | Area F6 | #Feature | #Feature F2 | #Feature F1 | #Feature F3 | #Feature F4 | #Feature F5 | #Feature F6 | Start | End | PTM | AScore | Found By |
| --- | --- | --- | --- | --- | --- | --- | --- | --- | --- | --- | --- | --- | --- | --- | --- | --- | --- | --- | --- | --- | --- | --- | --- | --- | --- | --- | --- | --- | --- |
| K.ELGVGFATR.K | Y | 43.57 | 948.5029 | 9 | -0.4 | 475.2585 | 2 | 36.46 | 6 | F6:16444 | 31122021\_RID\_2349\_6C.raw |  |  |  |  |  | 5.8274E4 | 1 | 0 | 0 | 0 | 0 | 0 | 1 | 23 | 31 |  |  | PEAKS DB |
| total 1 peptides |  |  |  |  |  |  |  |  |  |  |  |  |  |  |  |  |  |  |  |  |  |  |  |  |  |  |  |  |  |
| --- | --- | --- | --- | --- | --- | --- | --- | --- | --- | --- | --- | --- | --- | --- | --- | --- | --- | --- | --- | --- | --- | --- | --- | --- | --- | --- | --- | --- | --- |

ETE71857.1

back to list

  

| Protein Coverage
| Supporting Peptides
|

Protein Coverage:

Supporting Peptides:

| Peptide | Uniq | -10lgP | Mass | Length | ppm | m/z | z | RT | Fraction | Scan | Source File | Area F2 | Area F1 | Area F3 | Area F4 | Area F5 | Area F6 | #Feature | #Feature F2 | #Feature F1 | #Feature F3 | #Feature F4 | #Feature F5 | #Feature F6 | Start | End | PTM | AScore | Found By |
| --- | --- | --- | --- | --- | --- | --- | --- | --- | --- | --- | --- | --- | --- | --- | --- | --- | --- | --- | --- | --- | --- | --- | --- | --- | --- | --- | --- | --- | --- |
| K.ELGVGFATR.K | Y | 43.57 | 948.5029 | 9 | -0.4 | 475.2585 | 2 | 36.46 | 6 | F6:16444 | 31122021\_RID\_2349\_6C.raw |  |  |  |  |  | 5.8274E4 | 1 | 0 | 0 | 0 | 0 | 0 | 1 | 23 | 31 |  |  | PEAKS DB |
| total 1 peptides |  |  |  |  |  |  |  |  |  |  |  |  |  |  |  |  |  |  |  |  |  |  |  |  |  |  |  |  |  |
| --- | --- | --- | --- | --- | --- | --- | --- | --- | --- | --- | --- | --- | --- | --- | --- | --- | --- | --- | --- | --- | --- | --- | --- | --- | --- | --- | --- | --- | --- |

P86802.1

back to list

  

| Protein Coverage
| Supporting Peptides
|

Protein Coverage:

Supporting Peptides:

| Peptide | Uniq | -10lgP | Mass | Length | ppm | m/z | z | RT | Fraction | Scan | Source File | Area F2 | Area F1 | Area F3 | Area F4 | Area F5 | Area F6 | #Feature | #Feature F2 | #Feature F1 | #Feature F3 | #Feature F4 | #Feature F5 | #Feature F6 | Start | End | PTM | AScore | Found By |
| --- | --- | --- | --- | --- | --- | --- | --- | --- | --- | --- | --- | --- | --- | --- | --- | --- | --- | --- | --- | --- | --- | --- | --- | --- | --- | --- | --- | --- | --- |
| V.GVTMAHELGHNLGMNHDEEK.K | Y | 43.00 | 2217.9895 | 20 | 0.4 | 740.3374 | 3 | 24.09 | 5 | F5:8477 | 31122021\_RID\_2349\_5C.raw |  |  |  |  | 2.5748E5 |  | 1 | 0 | 0 | 0 | 0 | 1 | 0 | 153 | 172 |  |  | PEAKS DB |
| total 1 peptides |  |  |  |  |  |  |  |  |  |  |  |  |  |  |  |  |  |  |  |  |  |  |  |  |  |  |  |  |  |
| --- | --- | --- | --- | --- | --- | --- | --- | --- | --- | --- | --- | --- | --- | --- | --- | --- | --- | --- | --- | --- | --- | --- | --- | --- | --- | --- | --- | --- | --- |

Peptide List

  
  

---

Prepared with PEAKS ™ (bioinfor.com)
